# Burden of 375 diseases and injuries, risk-attributable burden of 88 risk factors, and healthy life expectancy in 204 countries and territories, including 660 subnational locations, 1990–2023: a systematic analysis for the Global Burden of Disease Study 2023

**DOI:** 10.1016/S0140-6736(25)01637-X

**Published:** 2025-10-18

**Authors:** GBD 2023 Disease and Injury and Risk Factor Collaborators, Simon I Hay, Kanyin Liane Ong, Damian F Santomauro, Bhoomadevi A, Mohammad Amin Aalipour, Hasan Aalruz, Hazim S Ababneh, Ukachukwu O Abaraogu, Biruk Beletew Abate, Cristiana Abbafati, Nasir Abbas, Mitra Abbasifard, Mohsen Abbasi-Kangevari, Samar Abd ElHafeez, Ashraf Nabiel Abdalla, Mohammed Altigani Abdalla, Emad M Abdallah, Barkhad Aden Abdeeq, Nadin M I Abdel Razeq, Ahmed Abdelrahman Abdelgalil, Reda Abdel-Hameed, Michael Abdelmasseh, Mahmoud Abdelnabi, Wael M Abdel-Rahman, Sherief Abd-Elsalam, Sepideh Abdi, Mohammad Abdollahi, Meriem Abdoun, Arman Abdous, Jeza Muhamad Abdul Aziz, Deldar Morad Abdulah, Rizwan Suliankatchi Abdulkader, Adam Abdullahi, Auwal Abdullahi, Toufik Abdul-Rahman, Kulmira Abdykerimova, Habtamu Abebe Getahun, Aidin Abedi, Armita Abedi, Asrat Agalu Abejew, Roberto Ariel Abeldaño Zuñiga, E S Abhilash, Shehab Uddin Al Abid, Syed Hani Abidi, Alemwork Abie, Olugbenga Olusola Abiodun, Olumide Abiodun, Richard Gyan Aboagye, Shady Abohashem, Hassan Abolhassani, Ulric Sena Abonie, Nagah M Abourashed, Mohamed Abouzid, Dmitry Abramov, Lucas Guimarães Abreu, Dariush Abtahi, Rana Kamal Abu Farha, Fuad Hamdi A Abuadas, Aminu Kende Abubakar, Bilyaminu Abubakar, Eman Abu-Gharbieh, Sawsan Abuhammad, Ahmad Y Abuhelwa, Hana J Abukhadijah, Niveen ME Abu-Rmeileh, Salahdein Aburuz, Dina Abushanab, Raghu Ram Achar, Anirudh Balakrishna Acharya, Apurba Acharya, Ilana N Ackerman, Juan Manuel Acuna, Ousman Adal, Lisa C Adams, Lawan Hassan Adamu, Mesafint Molla Adane, Zenaw Debasu Addisu, Isaac Yeboah Addo, Oluwafemi Atanda Adeagbo, Tajudeen Adesanmi Adebisi, Isaac Akinkunmi Adedeji, David Adedia, Kamoru Ademola Adedokun, Rufus Adesoji Adedoyin, Oluwatobi E Adegbile, Oyelola A Adegboye, Nurudeen A Adegoke, Olumide Thomas Adeleke, Isaac Ayodeji Adesina, Miracle Ayomikun Adesina, Habeeb Omoponle Adewuyi, Temitayo Esther Adeyeoluwa, Olorunsola Israel Adeyomoye, Kishor Adhikari, Ripon Kumar Adhikary, Usha Adiga, Mohd Adnan, Qorinah Estiningtyas Sakilah Adnani, Prince Owusu Adoma, Leticia Akua Adzigbli, David Adzrago, Giuseppina Affinito, Ahmed M Afifi, Aanuoluwapo Adeyimika Afolabi, Rotimi Felix Afolabi, Saira Afzal, Gizachew Beykaso Agafari, Suneth Buddhika Agampodi, Temesgen Anjulo Ageru, Navidha Aggarwal, Mahdi Aghaalikhani, Sepehr Aghajanian, Seyed Mohammad Kazem Aghamir, César Agostinis Sobrinho, Anurag Agrawal, Williams Agyemang-Duah, Mahsa Ahadi, Bright Opoku Ahinkorah, Aqeel Ahmad, Danish Ahmad, Faisal Ahmad, Khabir Ahmad, Khurshid Ahmad, Muayyad M Ahmad, Noah Ahmad, Rabbiya Ahmad, Sajjad Ahmad, Tauseef Ahmad, Waqas Ahmad, Negar Sadat Ahmadi, Amir Mahmoud Ahmadzade, Mohadese Ahmadzade, Akeem Olayiwola Ahmed, Anisuddin Ahmed, Ayman Ahmed, Gasha Salih Ahmed, Haroon Ahmed, Junaid Ahmed, Luai A Ahmed, Mehrunnisha Sharif Ahmed, Meqdad Saleh Ahmed, Muktar Beshir Ahmed, Mushood Ahmed, Oli Ahmed, Shabbir Ahmed, Sindew Mahmud Ahmed, Gulzhanat Aimagambetova, Ahmed AJ Jabbar, Dolapo Emmanuel Ajala, Marjan Ajami, Azeezat Oluwafunmilayo Ajose, Hossein Akbarialiabad, Saeid Akbarifard, Oluwasefunmi Akeju, Roland Eghoghosoa Akhigbe, Olufemi Ambrose Akinkuotu, Karolina Akinosoglou, Mohammed Ahmed Akkaif, Sreelatha Akkala, Wole Akosile, Hammad Akram, Ashley E Akrami, Ralph Kwame Akyea, Alaa Al Amiry, Salah Al Awaidy, Syed Mahfuz Al Hasan, Omar Al Omari, Mohammad Al Qadire, Omar Al Ta'ani, Wasan A M Al Taie, Yazan Al Thaher, Omar Ali Mohammed Al Zaabi, Mohammad Ahmmad Mahmoud Al Zoubi, Mousa Ali Al-Abbadi, Yazan Al-Ajlouni, Tariq A Alalwan, Ziyad Al-Aly, Khurshid Alam, Manjurul Alam, Mohammad Khursheed Alam, Mostafa Alam, Rasmieh Mustafa Al-Amer, Abebaw Alamrew, Amani Alansari, Turki M Alanzi, Fahmi Y Al-Ashwal, Rahmeh Al-Asmar, Seyed Mohammad Amin Alavi, Mohammed Albashtawy, Astefanos Al-Dalakta, Khalifah A Aldawsari, Wafa A Aldhaleei, Mohammed S Aldossary, Robert W Aldridge, Raouf Alebshehy, Shereen M Aleidi, Bezawit Abeje Alemayehu, Tekletsadik Tekleslassie Alemayehu, Fentahun Alemnew, Melaku Birhanu Alemu, Ayman Al-Eyadhy, Ali M Alfalki, Fahad D Algahtani, Abdelazeem M Algammal, Mohammed Ridha Algethami, Adel Ali Saeed Al-Gheethi, Khairat Al-Habbal, Khalid F Alhabib, Nma Bida Alhaji, Samar Al-Hajj, Fadwa Naji Alhalaiqa, Mohammed Khaled Al-Hanawi, Aminu Alhassan Ibrahim, Ashraf Alhumaidi, Fahad A Alhumaydhi, Dari Alhuwail, Abid Ali, Haroon Muhammad Ali, Irfan Ali, Maratab Ali, Mohammad Daud Ali, Mohammed Usman Ali, Rafat Ali, Shahid Ali, Syed Shujait Ali, Syed Yusuf Ali, Waad Ali, Akram Al-Ibraheem, Gianfranco Alicandro, Montaha Al-Iede, Sheikh Mohammad Alif, Morteza Alipour, Samah W Al-Jabi, Mohammad A Aljasir, Mohamad Aljofan, Adel Al-Jumaily, Syed Mohamed Aljunid, Ahmad Alkhatib, Mayson H Alkhatib, Mustafa Alkhawam, Atefeh Allahbakhshian, Khaled S Allemailem, Mohammed Z Allouh, Wesam Taher Almagharbeh, Wael Almahmeed, Sabah Al-Marwani, Nihad A Almasri, Joseph Uy Almazan, Hesham M Al-Mekhlafi, Omar Almidani, Amr Almobayed, Khaldoon Aied Alnawafleh, Hasan Yaser Alniss, Margret Beaula Alocious Sukumar, Mahmoud A Alomari, Mohammad R Alosta, Jaber S Alqahtani, Saleh A Alqahtani, Mohammad R Alqudimat, Ahmad Rajeh Al-Qudimat, Ahmad Alrawashdeh, Intima Alrimawi, Sahel Majed Alrousan, Salman Khalifah Al-Sabah, Mohammed A Alsabri, Najim Z Alshahrani, Mansour Abdullah Alshehri, Zaid Altaany, Awais Altaf, Alaa B Al-Tammemi, Jaffar A Al-Tawfiq, Malik A Althobiani, Khalid A Altirkawi, Javier Alvarez-Galvez, Vera L Alves Carneiro, Nelson Alvis-Guzman, Nelson J Alvis-Zakzuk, Hassan Alwafi, Mohammad Al-Wardat, Yaser Mohammed Al-Worafi, Hany Aly, Mohammad Sharif Ibrahim Alyahya, Amal AlZahmi, Hosam Alzahrani, Karem H Alzoubi, Md Akib Al-Zubayer, Uchenna Anderson Amaechi, Ekiyor Joseph Amafah, Joy Amafah, Masoud Aman Mohammadi, Reza Amani-Beni, Adeladza Kofi Amegah, Faten Amer, Bardia Amidi, Amr Amin, Tarek Tawfik Amin, Alireza Amindarolzarbi, Saeed Amini, Ehsan Amini-Salehi, Nafiu Aminu, Majid Aminzare, Sohrab Amiri, Joanne O Amlag, Dickson A Amugsi, Jimoh Amzat, Filippos Anagnostakis, Roshan A Ananda, Robert Ancuceanu, Deanna Anderlini, David B Anderson, Jason A Anderson, Sofia Androudi, Susan C Anenberg, Song Peng Ang, Colin Angus, Nguyen Hoang Anh, Samuel Egyakwa Ankomah, Kabilan Annadurai, Amir Anoushiravani, Iman Ansari, Sumbul Ansari, Umair Ansari, Rahel Mulatie Anteneh, Josep M Antó, Catherine M Antony, Ernoiz Antriyandarti, Boluwatife Stephen Anuoluwa, Saleha Anwar, Sumadi Lukman Anwar, Razique Anwer, Shahnawaz Anwer, Anayochukwu Edward Anyasodor, Geminn Louis Carace Apostol, Juan Pablo Arab, Hossein Arabi, Jalal Arabloo, Mosab Arafat, Aleksandr Y Aravkin, Demelash Areda, Jorge Arias de la Torre, Hany Ariffin, Benedetta Armocida, Johan Ärnlöv, Jesu Arockiaraj, Mahwish Arooj, Anton A Artamonov, Kurnia Dwi Artanti, Raphael Taiwo Aruleba, Deepavalli Arumuganainar, Nurila Aryntayeva, Mahsa Asadi Anar, Muhammad Asaduzzaman, Syed Mohammed Basheeruddin Asdaq, Shewatatek Melaku Asefa, Mulu Tiruneh Asemu, Saeed Asgary, Mohammad Asghari-Jafarabadi, Charlie Ashbaugh, Syed Amir Ashraf, Tahira Ashraf, Mitra Ashrafi, Milad Ashrafizadeh, Bernard Kwadwo Yeboah Asiamah-Asare, Muhammad Shahzad Aslam, Saeed Aslani, Yuni Asri, Batyrbek Assembekov, Thomas Astell-Burt, Mahshid Ataei, Mirbahador Athari, Seyyed Shamsadin Athari, Maha Moh'd Wahbi Atout, Sachin R Atre, Alok Atreya, Julie Alaere Atta, Zeenah A Atwan, Zaure Maratovna Aumoldaeva, Marcel Ausloos, Abolfazl Avan, Núbia Carelli Pereira Avelar, Sana Javaid Awan, Babafela B Awosile, Adedapo Wasiu Awotidebe, Lemessa Assefa A Ayana, Seyyed HamidReza Ayatizadeh, Olatunde O Ayinde, Yusuf Oloruntoyin Ayipo, Seyed Mohammad Ayyoubzadeh, Davood Azadi, Sina Azadnajafabad, Alireza Azarboo, Ali Azargoonjahromi, Masood Azhar, Farya Azimi, Mohd Yusmaidie Aziz, Sadat Abdulla Aziz, Amin Azizan, Ahmed Y Azzam, Domenico Azzolino, Zaharaddeen Shuaibu Babandi, Rasha Babiker, Giridhara Rathnaiah Babu, Israel Tadesse Bacha, Muhammad Badar, Ashish D Badiye, Alaa Aboelnour Badran, Youngoh Bae, Arvind Bagga, Soroush Baghdadi, Nasser Bagheri, Sara Bagheri, Elahe Baghizadeh, Fereshteh Baghizadeh, Sana Baghizadeh, Khlood K Baghlaf, Najmeh Bahmanziari, Mohammad Amin Bahrami, Razieh Bahreini, Ruhai Bai, Atif Amin Baig, Vali Baigi, Shankar M Bakkannavar, Abdulaziz T Bako, Senthilkumar Balakrishnan, Wondu Feyisa Balcha, Maher Balkis, Jose Balmori-de-la-Miyar, Mohammadreza Balooch Hasankhani, Ovidiu Constantin Baltatu, Shatha Bamashmous, Maciej Banach, Morteza Banakar, Palash Chandra Banik, Rajon Banik, Shirin Barati, Noel C Barengo, Suzanne Lyn Barker-Collo, Hiba Jawdat Barqawi, Ismael A Barreras Beltran, Amadou Barrow, Sandra Barteit, Lingkan Barua, MD Abu Bashar, Zarrin Basharat, Shahid Bashir, Guido Basile, Pritish Baskaran, Rehana Basri, Quique Bassat, Mohammad-Mahdi Bastan, Sanjay Basu, Saurav Basu, Kavita Batra, Bernhard T Baune, Mahdis Bayat, Mohammad Amin Bayat Tork, Mulat Tirfie Bayih, Feyisa Shasho Bayisa, Nebiyou Simegnew Bayleyegn, Thomas Beaney, Neeraj Bedi, Narasimha M Beeraka, Priyamadhaba Behera, Jina Behjati, Babak Behnam, Amir Hossein Behnoush, Bezawit K Bekele, Asnake Gashaw Belayneh, Melesse Belayneh, Abel Cherkos Belete, Gokce Belge Bilgin, Michael Belingheri, Muhammad Bashir Bello, Olorunjuwon Omolaja Bello, Luis Belo, Apostolos Beloukas, Salaheddine Bendak, Riyad Bendardaf, Corina Benjet, Derrick A Bennett, Isabela M Bensenor, Samiun Nazrin Bente Kamal Tune, Habib Benzian, Zombor Berezvai, Maria Bergami, Alemshet Yirga Berhie, Abiye Assefa Berihun, Amiel Nazer C Bermudez, Eduardo Bernabe, Robert S Bernstein, Paulo J G Bettencourt, Ajeet Singh Bhadoria, Akshaya Srikanth Bhagavathula, Neeraj Bhala, Jeetendra Bhandari, Kayleigh Bhangdia, Ravi Bharadwaj, Sonu Bhaskar, Ajay Nagesh Bhat, Anup Bhat, Vivek Bhat, Priyadarshini Bhattacharjee, Shuvarthi Bhattacharjee, Gurjit Kaur Bhatti, Jasvinder Singh Bhatti, Manpreet Singh Bhatti, Rajbir Bhatti, Soumitra S Bhuyan, Sibhatu Kassa Biadgilign, Raluca Bievel-Radulescu, Can Bilgin, Cem Bilgin, Saeed Biroudian, Catherine Bisignano, Atanu Biswas, Bijit Biswas, Raaj Kishore Biswas, Ahmad Naoras Bitar, Molalegne Bitew, Bruno Bizzozero-Peroni, Espen Bjertness, Fiona M Blyth, Trupti Bodhare, Virginia Bodolica, Mahmut Bodur, Lucimere Bohn, Rachael Bokota, Obasanjo Afolabi Bolarinwa, Srinivasa Rao Bolla, Paria Bolourinejad, Aime Bonny, Sri Harsha Boppana, Berrak Bora Basara, Sanaz Bordbar, Hamed Borhany, Alejandro Botero Carvajal, Souad Bouaoud, Soufiane Boufous, Rupert R A Bourne, Christopher Boxe, Marija M Bozic, Jyoti Brahmaiah, Dejana Braithwaite, Nicholas J K Breitborde, Hermann Brenner, Edmond D Brewer, Gabrielle Britton, Julie Brown, Annie J Browne, Traolach Brugha, Claudia Buchweitz, Raffaele Bugiardini, Linh Phuong Bui, Norma B Bulamu, Tsion Samuel Bunare, Danilo Buonsenso, Asmat Burhan, Katrin Burkart, Richard A Burns, Felix Busch, Reinhard Busse, Yasser Bustanji, Zahid A Butt, Channa Buxbaum, Sanjay C J, Jack Cagney, Tianji Cai, Rose Cairns, Mehtap Çakmak Barsbay, Daniela Calina, Luis Alberto Cámera, Luciana Aparecida Campos, Ismael Campos-Nonato, Fan Cao, Yuchen Cao, Angelo Capodici, Rosario Cárdenas, Sinclair Carr, Giulia Carreras, Juan Jesus Carrero, Austin Carter, Andrea Carugno, Andre F Carvalho, Ana Paula Carvalho-e-Silva, Joao Mauricio Castaldelli-Maia, Carlos A Castañeda-Orjuela, Giulio Castelpietra, Alberico L Catapano, Maria Sofia Cattaruzza, Arthur Caye, Christopher R Cederroth, Luca Cegolon, Francieli Cembranel, Muthia Cenderadewi, Kelly M Cercy, Ester Cerin, Sonia Cerrai, Muge Cevik, Madhu Chakkere Shivamadhu, Chiranjib Chakraborty, Promit Ananyo Chakraborty, Sandip Chakraborty, Joht Singh Chandan, Rama Mohan Chandika, Miyuru Chandradasa, Eeshwar K Chandrasekar, Jung-Chen Chang, Vijay Kumar Chattu, Victoria Chatzimavridou-Grigoriadou, Lam Duc Chau, Sirshendu Chaudhuri, Akhilanand Chaurasia, Galmesa Bekana Chemeda, An-Tian Chen, Catherine S Chen, Guangjin Chen, Hana Chen, Haowei Chen, Hui Chen, Junhao Chen, Meng Xuan Chen, Shanquan Chen, Simiao Chen, Xiang Chen, Yifan Chen, Haojin Cheng, Ka Ching Cheung, Nicholas WS Chew, Gerald Chi, Ju-Huei Chien, Odgerel Chimed-Ochir, Patrick R Ching, Jesus Lorenzo Chirinos-Caceres, Clara G Chisari, William C S Cho, Bryan Chong, Yuen Yu Chong, Hou In Chou, Enayet Karim Chowdhury, Mohiuddin Ahsanul Kabir Chowdhury, Hanne Christensen, Steffan Wittrup McPhee Christensen, Dinh-Toi Chu, Isaac Sunday Chukwu, Eric Chung, Erin Chung, Sheng-Chia Chung, Sunghyun Chung, Muhammad Chutiyami, Arrigo Francesco Giuseppe Cicero, Liliana G Ciobanu, Rebecca M Cogen, Aaron J Cohen, Alyssa Columbus, Joao Conde, Stephen E Congly, Nathalie Conrad, Sara Conti, Mariana Oliveira Corda, Alexandru Corlateanu, Samuele Cortese, Paolo Angelo Cortesi, Claudia Cosma, Ewerton Cousin, Emma Johnson Cowart, Michael H Criqui, Andrew Crist, Jessica A Cruz, Natalia Cruz-Martins, Xiaolin Cui, Garland T Culbreth, Nour Dababo, Ali Dabbagh, Omid Dadras, Tukur Dahiru, Xiaochen Dai, Zhaoli Dai, Mayank Dalakoti, Koustuv Dalal, Gloria Dalla Costa, Giovanni Damiani, Emanuele D'Amico, Yohannes Tefera Damtew, Roy Arokiam Arokiam Daniel, Lucio D'Anna, Pojsakorn Danpanichkul, Samuel Demissie Darcho, Latefa Ali Dardas, Bahar Darouei, Reza Darvishi Cheshmeh Soltani, Anna Dastiridou, Gail Davey, Claudio Alberto Dávila-Cervantes, Nicole Davis Weaver, Dimash Davletov, Kairat Davletov, Elham Davoudi, Fernando Pio De la Hoz, Katie de Luca, Nicole K DeCleene, Edward Christopher Dee, Orla Deegan, Sindhura Deekonda, Amanda Deen, Louisa Degenhardt, Paria Dehesh, Lee Deitesfeld, Tadesse Asmamaw Dejenie, Pouria Delbari, Mohammad Delsoz, Dessalegn Demeke, Andreas K Demetriades, Desalegn Getnet Demsie, Edgar Denova-Gutiérrez, Tadios Niguss Derese, Ismail Dergaa, Hunegnaw Almaw Derseh, Emina Dervišević, Abraham Aregay Desta, Vinoth Gnana Chellaiyan Devanbu, Pradeep Kumar Devarakonda, Syed Masudur Rahman Dewan, Arkadeep Dhali, Kuldeep Dhama, Rajinder K Dhamija, Amol S Dhane, Narender K Dhania, Mandira Lamichhane Dhimal, Meghnath Dhimal, Sameer Dhingra, Bibha Dhungel, Marcello Di Pumpo, Diana Dias da Silva, Daniel Diaz, Luis Antonio Diaz, Kimia Didehvar, Lauren K Dillard, Adriana Dima, Xueting Ding, Temesgien Ergetie Dinkayehu, Huyen Phuc Do, Thao Huynh Phuong Do, Klara Georgieva Dokova, Christiane Dolecek, Regina-Mae Villanueva Dominguez, Francesco Dondi, Mario D'Oria, Fariba Dorostkar, Ojas Prakashbhai Doshi, Paulo Magno Martins Dourado, Robert Kokou Dowou, Menayit Tamrat Dresse, Tim Robert Driscoll, Ashel Chelsea Dsouza, Viola Savy Dsouza, Jiang Du, John Dube, Emeka W Dumbili, Samuel C Dumith, Jennifer Dunne, Andre Rodrigues Duraes, Senbagam Duraisamy, Oyewole Christopher Durojaiye, Ashit Kumar Dutta, Arkadiusz Marian Dziedzic, Abdel Rahman E'mar, Osamudiamen Ebohon, Ejemai Eboreime, Lamiaa Labieb Mahmoud Ebraheim, Alireza Ebrahimi, Mohammad Hossein Ebrahimi, Sara Ebrahimi, Abdelaziz Ed-Dra, Ekaette Godwin Edelduok, Kristina Edvardsson, Ferry Efendi, Behrad Eftekhari, Foolad Eghbali, Fatemeh Ehsani, Ashkan Eighaei Sedeh, Terje Andreas Eikemo, Ebrahim Eini, Michael Ekholuenetale, Temitope Cyrus Ekundayo, Rabie Adel El Arab, Abdelfatteh EL Omri, Maysaa El Sayed Zaki, Mohamed Ahmed Eladl, Reza Elahi, Said El-Ashker, Rana Elbeshbeishy, Noha Mousaad Elemam, Ghada Metwally Tawfik ElGohary, Muhammed Elhadi, Mohamed Elhoumed, Waseem El-Huneidi, Sherif Elkannishy, Omar Abdelsadek Abdou Elmeligy, Rami Elmorsi, Adel B Elmoselhi, Mohamed Hassan Elnaem, Gihan ELNahas, Mohammed Elshaer, Ibrahim Elsohaby, Abdelgawad Salah Abdelgawad Eltahawy, Tadele Emagneneh, Theophilus I Emeto, Victor Oghenekparobo Emojevwe, Destaw Endeshaw, Misganu Endriyas, Holly E Erskine, Christopher Imokhuede Esezobor, Derese Eshetu, Habitu Birhan Eshetu, Gilbert Eshun, Sharareh Eskandarieh, Majid Eslami, Rafaela Cavalheiro do Espírito Santo, Francesco Esposito, Kara Estep, Crystal Amiel M Estrada, Fahima Nasrin Eva, Elochukwu Ezenwankwo, Adewale Oluwaseun Fadaka, Heidar Fadavian, Adeniyi Francis Fagbamigbe, Ayesha Fahim, Ildar Ravisovich Fakhradiyev, Aliasghar Fakhri-Demeshghieh, Qiping Fan, Mohammad Farahmand, Emerito Jose Aquino Faraon, Mohammad Fareed, Zaki Farhana, Carla Sofia e Sá Farinha, MoezAlIslam Ezzat Mahmoud Faris, Andre Faro, Syed Muhammad Yousaf Farooq, Umar Farooque, Hossein Farrokhpour, Fatemeh Farshad, Farima Farsi, Md Omar Faruk, Folorunso Oludayo Fasina, Modupe Margaret Fasina, Emmanuel Toluwani Fasusi, Ali Fatehizadeh, Davood Fathi, Zareen Fatima, Mehdi Fazlzadeh, Li Fei, Valery L Feigin, Alireza Feizkhah, Ginenus Fekadu, Berhanu Elfu Feleke, Dechao Feng, Kaixin Feng, Xiaoqi Feng, Talukdar Raian Ferdous, Seyed-Mohammad Fereshtehnejad, Rodrigo Fernandez-Jimenez, Pietro Ferrara, Alize J Ferrari, André Ferreira, Nuno Ferreira, Natan Feter, Alexander Finnemore, Claudio Fiorilla, Florian Fischer, Ida Fitriana, Luisa S Flor, Federica Fogacci, Morenike Oluwatoyin Folayan, Marco Fonzo, Lisa M Force, Arianna Fornari, Carla Fornari, Ingeborg Forthun, Daniela Fortuna, Matteo Foschi, Maryam Fotouhi, Kayode Raphael Fowobaje, Juluis Visnel Foyet F, Richard Charles Franklin, Alberto Freitas, Jinming Fu, Nancy Fullman, Blima Fux, Sridevi G, Peter Andras Gaal, Dominic Dormenyo Gadeka, Márió Gajdács, Yaseen Galali, Silvano Gallus, Dhanraj Ganapathy, Shivaprakash Gangachannaiah, Mohd Ashraf Ganie, Dingwei Gao, Xiang Gao, Bashiru Garba, Fernando Barroga Garcia, Vanessa Garcia, Miguel Garcia-Argibay, David Garcia-Azorin, William M Gardner, Jacopo Garlasco, Zisis Gatzioufas, Prem Gautam, Rupesh K Gautam, Federica Gazzelloni, Feven Sahle Gebre, Miglas Welay Gebregergis, Haftay Gebremedhin Gebreslassie, Stefano Gelibter, Nsikakabasi Samuel George, Ali Gerami Matin, Genanew K Getahun, Kalab Yigermal Gete, Delaram J Ghadimi, Keyghobad Ghadiri, Fataneh Ghadirian, Amir Ghaffari Jolfayi, Seyyed-Hadi Ghamari, Arin Ghamkhar, Ali Ghandili, Moein Ghasemi, Mohammad-Reza Ghasemi, Shakiba Ghasemi Assl, Haniyeh Ghasrsaz, Ramy Mohamed Ghazy, Sailaja Ghimire, Nermin Ghith, Nasim Gholizadeh, Elena Ghotbi, Alessandro Gialluisi, Konstantinos Giannakis, Ruth Margaret Gibson, Artyom Urievich Gil, Gabriela Fernanda Gil, Syed Abdullah Gilani, Nora M Gilbertson, Tiffany K Gill, Themba G Ginindza, Bikash Ranjan Giri, Alem Abera Girmay, Alessandro Girombelli, Elena V Gnedovskaya, Laszlo Göbölös, Kimiya Gohari, Mahaveer Golechha, Pouya Goleij, Ali Golestani, Davide Golinelli, Melika Golmohammadi, Wenping Gong, Sameer Vali Gopalani, Yitayal Ayalew Goshu, Alessandra C Goulart, Aman Goyal, Ayman Grada, Simon Matthew Graham, Vittorio Grieco, Michal Grivna, Ashna Grover, Habtamu Alganeh Guadie, Shi-Yang Guan, Zhongyang Guan, Giovanni Guarducci, Mohammed Ibrahim Mohialdeen Gubari, Avirup Guha, Damitha Asanga Gunawardane, Xingzhi Guo, Zhaoyu Guo, Zheng Guo, Zhifeng Guo, Anish Kumar Gupta, Himanshu Gupta, Ishita Gupta, Lalit Gupta, Rajat Das Gupta, Rajeev Gupta, Sapna Gupta, Veer Bala Gupta, Vijai Kumar Gupta, Vipin Gupta, Vivek Kumar Gupta, Yonas Deressa Guracho, Lami Gurmessa, Reyna Alma Gutiérrez, Roberth Steven Gutiérrez-Murillo, Parishma Guttoo, Jose Guzman-Esquivel, Adrina Habibzadeh, Abrham Tesfaye Habteyes, Awoke Derbie Habteyohannes, Tesfahun Simon Hadaro, Najah R Hadi, Zahra Hadian, Abdul Hafiz, Faraidoon Haghdoost, Arian Haghtalab, Hailey Hagins, Demewoz Haile, Haimanot Ewnetu Hailu, Pritam Halder, Aram Halimi, Sebastian Haller, Kosar Hikmat Hama Aziz, Islam M Hamad, Randah R Hamadeh, Nadia M Hamdy, Sajid Hameed, Erin B Hamilton, Ahmad Hammoud, Mohammad Hamza, Umar Sabiu Hamza, Hannah Han, Didem Han Yekdeş, Asif Hanif, Nasrin Hanifi, Graeme J Hankey, Fahad Hanna, Ashanul Haque, Md Aminul Haque, Md Nuruzzaman Haque, Harapan Harapan, Hilda L Harb, Kassandra L Harding, Arief Hargono, Andy Martahan Andreas Hariandja, Josep Maria Haro, Ashley Ann Harris, Eka Mishbahatul Marah Has, Ahmed I Hasaballah, Faizul Hasan, Md Kamrul Hasan, Hamidreza Hasani, Ali Hasanpour- Dehkordi, Arezou Hashem Zadeh, Mohammad Hashem Hashempur, Nada Tawfig Hashim, Ammarah Hasnain, Amr Hassan, Ibrahim Nagmeldin Hassan, Ikrama Hassan, Nageeb Hassan, Mahgol Sadat Hassan Zadeh Tabatabaei, Shokoufeh Hassani, Mohammed Bheser Hassen, Lasanthi Wathsala Hathagoda, Rasmus J Havmoeller, Angie Hawat, Khezar Hayat, Youssef Hbid, Jiawei He, Jue He, Jeffrey J Hebert, Mohammad Heidari, Mehdi Hemmati, Claire A Henson, Molly E Herbert, Claudiu Herteliu, Austin Heuer, Sumudu Avanthi Hewage, Mojtaba Heydari, Zahra Heydarifard, Kamal Hezam, Yuta Hiraike, Ramesh Holla, Julia Hon, Alamgir Hossain, Lubna Hossain, Md Belal Hossain, Md Mahbub Hossain, Md Sabbir Hossain, Mohammad Bellal Hossain, Hassan Hosseinzadeh, Mehdi Hosseinzadeh, Mihaela Hostiuc, Sorin Hostiuc, Jada Averianna Houser, Mila Nu Nu Htay, Chengxi Hu, Yifei Hu, Junjie Huang, Weijun Huang, Yefei Huang, Mega Hasanul Huda, Atanesia Indriyani Human, Kyle Matthew Humphrey, Kiavash Hushmandi, Andreas Kattem Husøy, Javid Hussain, M Azhar Hussain, Salman Hussain, Dursa Hussein, Nawfal R Hussein, Mohamed Ibrahim Husseiny, Hong-Han Huynh, Bing-Fang Hwang, Luigi Francesco Iannone, Ahmed Ibrahim, Khalid S Ibrahim, Ramzi Ibrahim, Reem Ibrahim, Anel Ibrayeva, Francisco Javier Idalsoaga, Pulwasha Maria Iftikhar, Audrey L Ihler, Nayu Ikeda, Adalia Ikiroma, Jibran Ikram, Olayinka Stephen Ilesanmi, Irena M Ilic, Milena D Ilic, Muhammad Hamza Ilyas, Mohammad Tarique Imam, Masoud Imani, Mustapha Immurana, Lucius Chidiebere Imoh, Leeberk Raja Inbaraj, Arit Inok, Mujahid Iqbal, Lalu Muhammad Irham, Mustafa Alhaji Isa, Dr Md Shahinul Islam, Md Rabiul Islam, Md Shariful Islam, Farhad Islami, Faisal Ismail, Nahlah Elkudssiah Ismail, Yerlan Ismoldayev, Hiroyasu Iso, Gaetano Isola, Mosimah Charles Ituka, Masao Iwagami, Chinwe Juliana Iwu-Jaja, Ihoghosa Osamuyi Iyamu, Mahalaxmi Iyer, Veena J Iyer, Vinothini J, Jalil Jaafari, Louis Jacob, Kathryn H Jacobsen, Ali Jadidi, Farhad Jadidi-Niaragh, Morteza Jafarinia, Shabbar Jaffar, Haitham Jahrami, Ammar Abdulrahman Jairoun, Vikash Jaiswal, Mihajlo Jakovljevic, Ali Jaliliyan, Reza Jalilzadeh Yengejeh, Mohamed Jalloh, Armaan Jamal, Qazi Mohammad Sajid Jamal, Jazlan Jamaluddin, Jerin James, Tyler G James, Hasan Jamil, Safayet Jamil, Roland Dominic G Jamora, Masoud Jamshidi, Shaghayegh JamshidiRastabi, Rajiv Janardhanan, Esmaeil Jarrahi, Syed Sarmad Javaid, Anita Javanmardi, Javad Javidnia, Talha Jawaid, Qassim Jawell Odah Abed, Ruwan Duminda Jayasinghe, Yovanthi Anurangi Jayasinghe, Achala Upendra Jayatilleke, Kimia Jazi, Felix K Jebasingh, Sun Ha Jee, Jayakumar Jeganathan, Tadesse Hailu Jember, Belayneh Hamdela Jena, Diptismita Jena, Seogsong Jeong, Mahsa Jessri, Bijay Mukesh Jeswani, Vivekanand Jha, Zixiang Ji, Min Jiang, Weiqiu Jin, Wenyi Jin, Catherine O Johnson, Emily Katherine Johnson, Mohammad Jokar, Jost B Jonas, Tamas Joo, Abu Jor, Abel Joseph, Alex Joseph, Nitin Joseph, Charity Ehimwenma Joshua, Farahnaz Joukar, George Joy, Jacek Jerzy Jozwiak, Mikk Jürisson, Malik E Juweid, Madhanraj K, Billingsley Kaambwa, Zubair Kabir, Dler H Hussein Kadir, Ethan M Kahn, Ashish Kumar Kakkar, Leila R Kalankesh, Khalil Kalavani, Feroze Kaliyadan, Aidana Kaliyakparova, Sanjay Kalra, Md Moustafa Kamal, Mehnaz Kamal, Sivesh Kathir Kamarajah, Rajesh Kamath, Saltanat Kamenova, Arun Kamireddy, Ramat T Kamorudeen, Oleksandr Kamyshnyi, Haidong Kan, Mona Kanaan, Saddam Fuad Kanaan, Jiseung Kang, Samuel Berchi Kankam, Kehinde Kazeem Kanmodi, Sujitha Kannan, Suthanthira Kannan S, Rami S Kantar, Neeti Kapoor, Sujita Kumar Kar, Paschalis Karakasis, Reema A Karasneh, Hanie Karimi, Arman Karimi Behnagh, Samad Karkhah, Mohmed Isaqali Karobari, Tomasz M Karpiński, Manoj Kumar Kashyap, Abdene Weya Kaso, Hengameh Kasraei, Nigussie Assefa Kassaw, Adarsh Katamreddy, Patrick DMC Katoto, Joonas H Kauppila, Gbenga A Kayode, Nastaran Kazemi Rad, Mohammad-Hossein Keivanlou, Peter Njenga Keiyoro, Chukwudi Keke, John H Kempen, Salima Kerai, Vikash Ranjan Keshri, Kamyab Keshtkar, Emmanuelle Kesse-Guyot, Reza Khademi, Yousef Saleh Khader, Inn Kynn Khaing, Himanshu Khajuria, Sidra Khalid, Sumaira Khalid-Ariturk, Hazim O Khalifa, Anas Husam Khalifeh, Anees Ahmed Khalil, Mariam Khalil, Anita Khalili, Pantea Khalili, Ghazaleh Khalili-Tanha, Mohamed Khalis, Faham Khamesipour, Abdul Arif Khan, Ajmal Khan, Asaduzzaman Khan, Faiz Ullah Khan, Maseer Khan, Md Abdullah Saeed Khan, Mohammad Idreesh Khan, Mohammad Jobair Khan, Muhammad Hamza Khan, Muhammad Mueed Khan, Muhammad Umair Khan, Muhammad Umer Khan, Nusrat Khan, Ruby Khan, Salman Ali Khan, Serab Khan, Sumaiya Khan, Yusuf Saleem Khan, Zahid Khan, Srijana Khanal, Vishnu Khanal, Shaghayegh Khanmohammadi, Sameer Uttamaro Khasbage, Zenith Khashim, Khaled Khatab, Haitham Khatatbeh, Moawiah Mohammad Khatatbeh, Kavin Khatri, Hamid Reza Khayat Kashani, Afshin Khazaei, Peyman Kheirandish Zarandi, Sunil Kumar Khokhar, Mohammad Saeid Khonji, Najmaddin Salih Husen Khoshnaw, Atulya Aman Khosla, Farbod Khosravi, Mahmood Khosrowjerdi, P Ratan Khuman, Helda Khusun, Zemene Demelash Kifle, Hye Jun Kim, Jinho Kim, Min Seo Kim, Sungroul Kim, Ruth W Kimokoti, Yohannes Kinfu, Mary Kirk, Adnan Kisa, Sezer Kisa, Katarzyna Kissimova-Skarbek, Mika Kivimäki, Jessica Klusty, Abdul Basith KM, Shivakumar KM, Ann Kristin Skrindo Knudsen, Nazarii Kobyliak, Jonathan M Kocarnik, Sonali Kochhar, Michail Kokkorakis, Ali-Asghar Kolahi, Diana Gladys Kolieghu Tcheumeni, Farzad Kompani, Aida Kondybayeva, Anastasios Georgios Panagiotis Konstas, Isaac Koomson, Gerbrand Koren, Tapos Kormoker, Oleksii Korzh, Karel Kostev, Konstantinos Kotsis, Archana Koul, Parvaiz A Koul, Sindhura Lakshmi Koulmane Laxminarayana, Irene Akwo Kretchy, James-Paul Kretchy, Kewal Krishan, Chong-Han Kua, Ananya Kuanar, Barthelemy Kuate Defo, Raja Amir Hassan Kuchay, Burcu Kucuk Bicer, Mohammed Kuddus, Ilari Kuitunen, Omar Kujan, Anit Kujur, Mukhtar Kulimbet, Vishnutheertha Kulkarni, Shweta Kulshreshtha, Dewesh Kumar, Dhasarathi Kumar, Jogender Kumar, Manasi Kumar, Nitesh Kumar, Nithin Kumar, Rakesh Kumar, Sanjay Kirshan Kumar, Tushar Kumar, Vijay Kumar, Subramanian Kumaran, Jibin Kunjavara, Setor K Kunutsor, Almagul Kurmanova, Om P Kurmi, Maria Dyah Kurniasari, Krishna Prasad Kurpad, Pramod Kumar Kushawaha, Asep Kusnali, Christina Yeni Kustanti, Dian Kusuma, Tezer Kutluk, Assylkhan Kuttybayev, Michael Agyemang Kwarteng, Wai Hang Patrick Kwong, Evans F Kyei, Grace Kwakyewaa Kyei, Ville Kytö, Hmwe Hmwe Kyu, Pallavi L C, Adriano La Vecchia, Carlo La Vecchia, Muhammad Awwal Ladan, Lucie Laflamme, Chandrakant Lahariya, Daphne Teck Ching Lai, Anita Lakhani, Dharmesh Kumar Lal, Ratilal Lalloo, Tea Lallukka, Judit Lám, Iván Landires, Berthold Langguth, Ariane Laplante-Lévesque, Savita Lasrado, Kamaluddin Latief, Kenney Ki Lee Lau, Basira Kankia Lawal, Bilkisu Kankia Lawal, Saheed Akinmayowa Lawal, Aliyu Lawan, Harriet L S Lawford, Hilary R Lawlor, Dai Quang Le, Duc Huy Le, Huu-Hoai Le, Long Khanh Dao Le, Minh Huu Nhat Le, Nhi Huu Hanh Le, Thao Thi Thu Le, Trang Diep Thanh Le, Caterina Ledda, Hye Ah Lee, Seung Won Lee, Wei-Chen Lee, Yo Han Lee, James Leigh, Vasileios Leivaditis, Matthew J Lennon, Matilde Leonardi, Elvynna Leong, Janni Leung, Chengcheng Li, Haobo Li, Hui Li, Jianan Li, Jiaying Li, Jie Li, Jinbo Li, Ming-Chieh Li, Peng li, Shaojie Li, Wang-Zhong Li, Wei Li, Wei Li, Weilong Li, Wenjie Li, Xunliang Li, Yichong Li, Yongze Li, Zhengrui Li, Zhihui Li, Yanxue Lian, Xue-Zhen Liang, Stephen S Lim, Jialing Lin, Queran Lin, Ro-Ting Lin, Ya Lin, Daniel Lindholm, Christine Linehan, Yuewei Ling, Gang Liu, Haipeng Liu, Jue Liu, Xianliang Liu, Xiaofeng Liu, Xuefeng Liu, Yubo Liu, Yunfei Liu, Erand Llanaj, Michael J Loftus, Valerie Lohner, José Francisco López-Gil, Platon D Lopukhov, Stefan Lorkowski, Masoud Lotfizadeh, Shanjie Luan, Jailos Lubinda, Taraneh Lucas, Giancarlo Lucchetti, Alessandra Lugo, Raimundas Lunevicius, Peng Luo, Jay B Lusk, Angelina M Lutambi, Ricardo Lutzky Saute, Miltiadis D Lytras, Ellina Lytvyak, Hawraz Ibrahim M Amin, Kevin Sheng-Kai Ma, Zheng Feei Ma, Mahmoud Mabrok, Isis E Machado, Firoozeh Madadi, Seyed Ataollah Madinezad, Christian Madsen, Aurea Marilia Madureira-Carvalho, Mohammed Magdy Abd El Razek, Azzam A Maghazachi, D R Mahadeshwara Prasad, Sasikumar Mahalingam, Mehrdad Mahalleh, Nozad Hussein Mahmood, Alireza Mahmoudi, Farhad Mahmoudi, My Tra Mai, Rituparna Maiti, Azeem Majeed, Konstantinos Christos C Makris, Mohammad-Reza Malekpour, Reza Malekzadeh, Hardeep Singh Malhotra, Ahmad Azam Malik, Farihah Malik, Tabarak Malik, Deborah Carvalho Malta, Abdullah A Mamun, Mustapha Mangdow, Lokesh Manjani, Yosef Manla, Kamaruddeen Mannethodi, Farheen Mansoor, Marjan Mansourian, Mohammad Ali Mansournia, Ana M Mantilla Herrera, Lorenzo Giovanni Mantovani, Changkun Mao, Tahir Maqbool, Sajid Maqsood, Hamid Reza Marateb, Joemer C Maravilla, Konstantinos Margetis, Mirko Marino, Adilson Marques, Randall V Martin, Gabriel Martinez, Bernardo Alfonso Martinez-Guerra, Ramon Martinez-Piedra, Daniela Martini, Francisco Rogerlândio Martins-Melo, Miquel Martorell, Winfried März, Roy Rillera Marzo, Sammer Marzouk, Stefano Masi, Clara N Matei, Yasith Mathangasinghe, Stephanie Mathieson, Alexander G Mathioudakis, Manu Raj Mathur, Medha Mathur, Fernanda Penido Matozinhos, Rita Mattiello, Khurshid A Mattoo, Richard James Maude, Pallab K Maulik, Miranda L May, Mahsa Mayeli, Maryam Mazaheri, Antonio Mazzotti, Chioma Ngozichukwu Pauline Mbachu, Ikechukwu Innocent Mbachu, Martin McKee, Susan A McLaughlin, Steven M McPhail, Enkeleint A Mechili, Rishi P Mediratta, Jitendra Meena, Elahe Meftah, Medhin Mehari, Asim Mehmood, Man Mohan Mehndiratta, Entezar Mehrabi Nasab, Kala M Mehta, Vini Mehta, Subhash Mehto, Toni Meier, Tesfahun Mekene Meto, Hadush Negash Meles, Endalkachew Belayneh Melese, Satish Melwani, Aishe Memetova, Walter Mendoza, Godfred Antony Menezes, Ritesh G Menezes, Berihun Agegn Mengistie, Emiru Ayalew Mengistie, Sultan Ayoub Meo, Michelangelo Mercogliano, Atte Meretoja, Tuomo J Meretoja, Tomislav Mestrovic, Chamila Dinushi Kukulege Mettananda, Sachith Mettananda, Mohamed M M Metwally, Louise Mewton, Adquate Mhlanga, Andrea Michelerio, Ana Carolina Micheletti Gomide Nogueira de Sá, Hiwot Soboksa Mideksa, Paul Anthony Miller, Ted R Miller, Giuseppe Minervini, Wai-kit Ming, GK Mini, Mojgan Mirghafourvand, Erkin M Mirrakhimov, Seyed Ali Mirshahvalad, Mizan Kiros Mirutse, Yousef Mirzaei, Archana Mishra, Kumar Guru Mishra, Vinaytosh Mishra, Arup Kumar Misra, Philip B Mitchell, Prasanna Mithra, Sayan Mitra, Manasi Murthy Murthy Mittinty, Malihe Moazeni, Mohammadreza Mobayen, Madeline E Moberg, Shivani Modi, Ashraf Mohamadkhani, Jama Mohamed, Mona Gamal Mohamed, Nouh Saad Mohamed, Khabab Abbasher Hussien Mohamed Ahmed, Taj Mohammad, Sakineh Mohammad-Alizadeh-Charandabi, Abdolreza Mohammadi, Mohammad Reza Mohammadi, Seyed Omid Mohammadi, Abdollah Mohammadian-Hafshejani, Ibrahim Mohammadzadeh, Ramin Mohammadzadeh, Ammas Siraj Mohammed, Hussen Mohammed, Omer Mohammed, Shafiu Mohammed, Suleiman Mohammed, Yahaya Mohammed, Syam Mohan, Yugal Kishore Mohanta, Mohammad Mohseni, Amin Mokari-Yamchi, Ali H Mokdad, Alexandr Mokhirev, Peyman Mokhtarzadehazar, Sabrina Molinaro, Amirabbas Mollaei, Shaher Momani, Lorenzo Monasta, Amirabbas Monazzami, Himel Mondal, Stefania Mondello, Ahmed Al Montasir, Catrin E Moore, Yousef Moradi, Maziar Moradi-Lakeh, Paula Moraga, Lidia Morawska, Rafael Silveira Moreira, Brooks W Morgan, Negar Morovatdar, Mahdis Morovvati, Mahmoud M Morsy, Jakub Morze, Reza Mosaddeghi Heris, Jonathan F Mosser, Nogol Motamedgorji, Vincent Mougin, Simin Mouodi, Asma Mousavi, Seyede Zohre Mousavi, Amin Mousavi Khaneghah, Seyed Mohamad Sadegh Mousavi Kiasary, Mohamed Awad Abdalaziz Mousnad, Amanda Movo, Hagar Lotfy Mowafy, Kimia Mozahheb Yousefi, Matías Mrejen, Ahmed Msherghi, Rabia Mubarak, Sumaira Mubarik, Shiv K Mudgal, Syed Aun Muhammad, Muhammad Solihuddin Muhtar, Sukhes Mukherjee, Sumoni Mukherjee, Amartya Mukhopadhyay, Satinath Mukhopadhyay, M A Muktadir, Sileshi Mulatu, Francesk Mulita, Getaneh Baye Mulu, Chalie Mulugeta, Mulyadi Mulyadi, Muneeb Ahmad Muneer, Malaisamy Muniyandi, Kavita Munjal, Yanjinlkham Munkhsaikhan, Javier Muñoz Laguna, Anjana Munshi, Pradeep Manohar Muragundi, Michio Murakami, Yahye Hassan Muse, Ali Mushtaq, Ghulam Mustafa, Sherzad Ibrahim Mustafa, Mubarak Taiwo Mustapha, Sathish Muthu, Saravanan Muthupandian, Claude Mambo Muvunyi, Muhammad Muzaffar, Woojae Myung, Amin Nabavi, Fatemehzahra Naddafi, Ahamarshan Jayaraman Nagarajan, Shankar Prasad Nagaraju, Mohsen Naghavi, Pirouz Naghavi, Ganesh R Naik, Gurudatta Naik, Hiten Naik, Firzan Nainu, Sanjeev Nair, Soroush Najdaghi, Noureddin Nakhostin Ansari, Paul Nam, Vinay Nangia, Jobert Richie Nansseu, Ibrahim A Naqid, Shumaila Nargus, Delaram Narimani Davani, Yvonne Nartey, Bruno Ramos Nascimento, Gustavo G Nascimento, Abdallah Y Naser, Mohammad Naser, Abdulqadir J Nashwan, Hamide Nasiri, Mahmoud Nassar, Zuhair S Natto, Javaid Nauman, Zakira Naureen, Samidi Nirasha Kumari Navaratna, Anum Nawaz, M Omar Nawaz, Biswa Prakash Nayak, Shalini Ganesh Nayak, Javad Nazari, G Takop Nchanji, Rawlance Ndejjo, Anthony Wainaina Ndungu, Amanuel Tebabal Nega, Abigia Ashenafi Negash, Ionut Negoi, Ruxandra Irina Negoi, Alina Gabriela Negru, Jalil Nejati, Chakib Nejjari, Samata Nepal, Olivia D Nesbit, Henok Biresaw Netsere, Charles Richard James Newton, Marie Ng, Georges Nguefack-Tsague, Josephine W Ngunjiri, Anh Thy H Nguyen, Cuong Tat Nguyen, Huong Lan Thi Nguyen, Huong-Dung Thi Nguyen, Nghia Phu Nguyen, Phat Tuan Nguyen, The Phuong Nguyen, Trang Nguyen, Tu Anh Nguyen, Van Thanh Nguyen, Ambe Marius Ngwa, Robina Khan Niazi, Jing Nie, Luciano Nieddu, Yeshambel T Nigatu, Ali Nikoobar, Dina Nur Anggraini Ningrum, Vikram Niranjan, Abebe Melis Nisro, Jan Rene Nkeck, Princess Afia Nkrumah-Boateng, Chukwudi A Nnaji, Efaq Ali Noman, Shuhei Nomura, Syed Toukir Ahmed Noor, Mohammadamin Noorafrooz, Pardis Noormohammadpour, Mamoona Noreen, Masoud Noroozi, Jean Jacques Noubiap, Taylor Noyes, Valentine C Nriagu, Chisom Adaobi Nri-Ezedi, Jean Claude Nshimiyimana, Fred Nugen, Atoma Negera Nugusa, Mengistu H Nunemo, Aqsha Nur, Dieta Nurrika, Sylvester Dodzi Nyadanu, Felix Kwasi Nyande, Chimezie Igwegbe Nzoputam, Ogochukwu Janet Nzoputam, Bogdan Oancea, George Obaido, Erin M O'Connell, Adashi Margaret Odama, Ramez M Odat, Fabio Massimo Oddi, Ismail A Odetokun, Oluwakemi Ololade Odukoya, Joseph Kojo Oduro, Michael Safo Oduro, Onome Bright Oghenetega, Oluwaseun Adeolu Ogundijo, Abiola Ogunkoya, James Odhiambo Oguta, Doori Oh, Sarah Oh, Edel T O'Hagan, Hassan Okati-Aliabad, Sylvester Reuben Okeke, Deborah Oluwatosin Okeke-Obayemi, Akinkunmi Paul Okekunle, Olalekan John Okesanya, Onyedika A Okoli, Osaretin Christabel Okonji, John Olayemi Okunlola, Oluyemi Adewole Okunlola, Oluwaseyi Isaiah Olabisi, Andrew T Olagunju, Oladotun Victor Olalusi, Matthew Idowu Olatubi, Arão Belitardo Oliveira, Gláucia Maria Moraes Oliveira, Abdulhakeem Abayomi Olorukooba, Erik J Olson, Oluseye Olalekan Oludoye, Ronald Olum, Bolajoko Olubukunola Olusanya, Jacob Olusegun Olusanya, Oluwafemi G Oluwole, Folorunsho Bright Omage, Hany A Omar, Goran Latif Omer, Qi Chwen Ong, Sandersan Onie, Obinna E Onwujekwe, Franklyn Opara, Marcel Opitz, Michal Ordak, Verner N Orish, Raffaele Ornello, Atakan Orscelik, Alberto Ortiz, Esteban Ortiz-Prado, Augustus Osborne, Eric Osei, Samuel M Ostroff, John W Ostrominski, Uchechukwu Levi Osuagwu, Olayinka Osuolale, Godfred Otchere, Elham H Othman, Mostafa Monier Othman, Adrian Otoiu, Oche Joseph Otorkpa, Abdu Oumer, Jerry John Ouner, Amel Ouyahia, Guoqing Ouyang, Mayowa O Owolabi, Irene Amoakoh Owusu, Kolapo Oyebola, Tope Oyelade, Oyetunde T Oyeyemi, Ilker Ozsahin, Mahesh P A, Kevin Pacheco-Barrios, Inderbir Padda, Alicia Padron-Monedero, Jagadish Rao Padubidri, Anton Pak, Pramod Kumar Pal, Tamás Palicz, Raffaele Palladino, Raul Felipe Palma-Alvarez, Tejasri Paluvai, Feng Pan, Hai-Feng Pan, Parsa Panahi, Sujogya Kumar Panda, Songhomitra Panda-Jonas, Deepshikha Pande Katare, Ke Pang, Helena Ullyartha Pangaribuan, Georgios D Panos, Leonidas D Panos, Ioannis Pantazopoulos, Giovanni Paolino, Mario Virgilio Papa, Ilias Papadimopoulos, Paraskevi Papadopoulou, Utsav Parekh, Peyvand Parhizkar Roudsari, Amrita Parida, Chulwoo Park, Eun-Kee Park, Seoyeon Park, Arpit Parmar, Swapnil Parve, Ava Pashaei, Roberto Passera, Bhumi Hemal Patel, Hemal M Patel, Mitesh Patel, Neel Navinkumar Patel, Satyananda Patel, Ashlesh Patil, Shankargouda Patil, Dimitrios Patoulias, Apurba Patra, Mohammad Hridoy Patwary, Hilary Paul, Shrikant Pawar, Shubhadarshini Pawar, Hamidreza Pazoki Toroudi, Amy E Peden, Paolo Pedersini, Jarmila Pekarcikova, Veincent Christian Filipino Pepito, Prince Peprah, João Perdigão, Gavin Pereira, Maria Odete Pereira, Pablo Perez-Lopez, Norberto Perico, Simone Perna, Konrad Pesudovs, Pavlo Petakh, Olumuyiwa James Peter, Fanny Emily Petermann-Rocha, Hoang Nhat Pham, Nhat Truong Pham, Tung Thanh Pham, Anil K Philip, Michael R Phillips, Zayar Phyo, Brandon V Pickering, David M Pigott, Julian David Pillay, Luane Pinheiro Pinheiro Rocha, Zahra Zahid Piracha, Michael A Piradov, Edoardo Pirera, Enrico Pisoni, Dietrich Plass, Evgenii Plotnikov, Indrashis Podder, Dimitri Poddighe, Roman V Polibin, Peter Pollner, Ramesh Poluru, Arjun Pon Avudaiappan, Constance Dimity Pond, Ville T Ponkilainen, Ion Popa, Svetlana Popova, Djordje S Popovic, Maarten J Postma, Sajjad Pourasghary, Reza Pourbabaki, Farzad Pourghazi, Mohsen Poursadeqiyan, Naeimeh Pourtaheri, Attur Ravindra Prabhu, Sergio I Prada, Jalandhar Pradhan, Pranil Man Singh Pradhan, Rifky Octavia Pradipta, Peralam Yegneswaran Prakash, Chandra P Prasad, Akila Prashant, Elton Junio Sady Prates, Tina Priscilla, Natalie Pritchett, Harsh Priya, Hery Purnobasuki, Bharathi M Purohit, Jagadeesh Puvvula, Nameer Hashim Qasim, Xiang Qi, Zhipeng Qi, Jia-Yong Qiu, Zahiruddin Syed Quazi, Shahazad Niwazi Qurashi, Deepthi R, Navid Rabiee, Basuki Rachmat, Raghu Anekal Radhakrishnan, Venkatraman Radhakrishnan, Maja R Radojčić, Hadi Raeisi Shahraki, Ibrar Rafique, Pankaja Raghav, Pracheth Raghuveer, Leila Rahbarnia, Fakher Rahim, Hawbash Mohammed-Amin Rahim, Sajjad Rahimi, Afarin Rahimi-Movaghar, Vafa Rahimi-Movaghar, Fryad Majeed Rahman, Mahbubur Rahman, Mahfuzur Rahman, Md Mosfequr Rahman, Mohammad Hifz Ur Rahman, Mohammad Meshbahur Rahman, Mosiur Rahman, Saeed Rahmani, Masoud Rahmati, Ghasem Rahmatpour Rokni, Hakim Rahmoune, Pramila Rai, Diego Raimondo, Ivano Raimondo, Sunil Kumar Raina, Jeffrey Pradeep Raj, Adarsh Raja, Sandesh Raja, Sathish Rajaa, Erta Rajabi, Shahryar Rajai Firouzabadi, Gunaseelan Rajendran, Judah Rajendran, Vinoth Rajendran, Shaman Rajindrajith, Mohammad Amin Rajizadeh, Prashant Rajput, Mahmoud Mohammed Ramadan, Majed Ramadan, Kadar Ramadhan, Chitra Ramasamy, Shakthi Kumaran Ramasamy, Sheena Ramazanu, Zahra Ramezani, Marzieh Ramezani Farani, Pramod W Ramteke, Juwel Rana, Shailendra Singh Rana, Chhabi Lal Ranabhat, Nemanja Rancic, Smitha Rani, Fatemeh Ranjbar Noei, Chythra R Rao, Kumuda Rao, Mithun Rao, Sowmya J Rao, Davide Rasella, Vahid Rashedi, Mohammad-Mahdi Rashidi, Mohammad Aziz Rasouli, Ashkan Rasouli-Saravani, Prateek Rastogi, Azad Rasul, Devarajan Rathish, Abdur Rauf, Santosh Kumar Rauniyar, Ilari Rautalin, Ramin Ravangard, Dhwani Ravi, David Laith Rawaf, Salman Rawaf, Reza Rawassizadeh, Ramu Rawat, Ayita Ray, Mohammad Rayati, Iman Razeghian, Bahman Razi, Christian Razo, Filippo Recenti, Murali Mohan Rama Krishna Reddy, Elrashdy Redwan, Sanika Rege, Wajiha Rehman, Lennart Reifels, Giuseppe Remuzzi, Longbing Ren, Andre M N Renzaho, Serge Resnikoff, Luis Felipe Reyes, Mina Rezaei, Nazila Rezaei, Negar Rezaei, Nima Rezaei, Mohsen Rezaeian, Taeho Gregory Rhee, Mavra A Riaz, Antonio Luiz P Ribeiro, Jennifer Rickard, Moattar Raza Rizvi, Hannah Elizabeth Robinson-Oden, Hermano Alexandre Lima Rocha, João Rocha Rocha-Gomes, Mónica Rodrigues, Leonardo Roever, Peter Rohloff, Iftitakhur Rohmah, Susanne Röhr, David Rojas-Rueda, Megan L Rolfzen, Debby Syahru Romadlon, Michele Romoli, Marina Romozzi, Luca Ronfani, Jennifer Jacqueline Rosauer, Amirhossein Roshanshad, Morteza Rostamian, Gregory A Roth, Kunle Rotimi, Himanshu Sekhar Rout, Hanieh Rouzbahani, Reza Rouzbahani, Shiva Rouzbahani, Bedanta Roy, Nitai Roy, Parimal Roy, Poulami Roy, Priyanka Roy, Sharmistha Roy, Shubhanjali Roy, Susovan Roy Chowdhury, Parameswari Royapuram Parthasarathy, Enrico Rubagotti, Guilherme de Andrade Ruela, Susan Fred Rumisha, Michele Russo, Godfrey Mutashambara Rwegerera, Manjula S, Chandan S N, Aly M A Saad, Adnan Saad Eddin, Zahra Saadatian, Maha Mohamed Saber-Ayad, Cameron John Sabet, Siamak Sabour, Kabir P Sadarangani, Seyed Kiarash Sadat Rafiei, Basema Ahmad Saddik, Bashdar Abuzed Sadee, Tarannom Sadegh, Ehsan Sadeghi, Erfan Sadeghi, Fatemeh Sadeghi-Ghyassi, Mohd Saeed, Umar Saeed, Maryam Saeedi, Mehdi Safari, Sare Safi, Sher Zaman Safi, Rajesh Sagar, Mastooreh Sagharichi, Dominic Sagoe, Nondo Saha, Fatemeh Saheb Sharif-Askari, Narjes Saheb Sharif-Askari, Amirhossein Sahebkar, Pragyan Monalisa Sahoo, Kirti Sundar Sahu, Muhammad Soaib Said, Zahra Saif, S Mohammad Sajadi, Md Refat Uz Zaman Sajib, Mirza Rizwan Sajid, Morteza Saki, Nasir Salam, Payman Salamati, Luciane B Salaroli, Mohamed A Saleh, Leili Salehi, Mahdi Salehi, Marwa Rashad Salem, Mohammed Z Y Salem, Dauda Salihu, Sohrab Salimi, Malik Sallam, Giovanni A Salum, Sundeep Santosh Salvi, Hossein Samadi Kafil, Jayami Eshana Samaranayake, Waqas Sami, Yoseph Leonardo Samodra, Vijaya Paul Samuel, Abdallah M Samy, Sandeep G Sangle, Elaheh Sanjari, Sathish Sankar, Francesco Sanmarchi, Francesca Sanna, Lucas H C C Santos, Milena M Santric-Milicevic, Bruno Piassi Sao Jose, Krishna Prasad Sapkota, Sivan Yegnanarayana Iyer Saraswathy, Jacob Owusu Sarfo, Yaser Sarikhani, Hemen Sarma, Mohammad Sarmadi, Gargi Sachin Sarode, Sachin C Sarode, Satish Saroshe, Michele Sassano, Brijesh Sathian, Mukesh Kumar Sathya Narayanan, Paul A Saunders, Mehrdad Savabi Far, Monika Sawhney, Sangeeta Gopal Saxena, Ganesh Kumar Saya, Abu Sayeed, Mete Saylan, Christophe Schinckus, Ione Jayce Ceola Schneider, Rachel D Schneider, Art Schuermans, Austin E Schumacher, Ghil Schwarz, David C Schwebel, Falk Schwendicke, Catherine Schwinger, Amin Sedigh, Saravanan Sekaran, Mario Šekerija, Muthamizh Selvamani, Vimalraj Selvaraj, Yuliya Semenova, Mohammad H Semreen, Fikadu Waltengus Sendeku, Yigit Can Senol, Subramanian Senthilkumaran, Sadaf G Sepanlou, Andreea Claudia Serban, Edson Serván-Mori, Yashendra Sethi, Christian Sewor, Seyed Mohammad Seyed Alshohadaei, Allen Seylani, Matthew Seymour, Jamileh Shadid, Nilay S Shah, Sweni Shah, Shazlin Shaharudin, Muhammad Shahbaz, Samiah Shahid, Syed Ahsan Shahid, Wajeehah Shahid, Endrit Shahini, Fatemeh Shahrahmani, Hamid R Shahsavari, Moyad Jamal Shahwan, Masood Ali Shaikh, Alireza Shakeri, Ali Shakerimoghaddam, Ali S Shalash, Sunder Sham, Muhammad Aaqib Shamim, Farzane Shams, Mehran Shams-Beyranvand, Mohammad Ali Shamshirgaran, Anas Shamsi, Alfiya Shamsutdinova, Dan Shan, Abhishek Shankar, Mohammed Shannawaz, Xian Shao, Amin Sharifan, Javad Sharifi Rad, Avimanu Sharma, Bhoopesh Kumar Sharma, Bunty Sharma, Kamal Sharma, Manoj Sharma, Sourabh Sharma, Ujjawal Sharma, Vishal Sharma, Rajesh P Shastry, Shamee Shastry, Armin Shavandi, Ramzi Shawahna, Maryam Shayan, Babangida Shehu Bappah, Ali Sheidaei, Suchitra M Shenoy, Samendra P Sherchan, Suraj S Shetty, Fang Shi, Lin-Hong Shi, Mosa Shibani, Belayneh Fentahun Shibesh, Kenji Shibuya, Desalegn Shiferaw, Md Monir Hossain Shimul, Jae Il Shin, Min-Jeong Shin, Rahman Shiri, Reza Shirkoohi, Aminu Shittu, Abdul-karim Olayinka Shitu, Ivy Shiue, Velizar Shivarov, Nathan A Shlobin, Shayan Shojaei, Zahra Shokati Eshkiki, Azad Shokri, Sinegugu Nosipho Shongwe, Sina Shool, Seyed Afshin Shorofi, Gambhir Shrestha, Sunil Shrestha, Kerem Shuval, Nicole Remaliah Samantha Sibuyi, Emmanuel Edwar Siddig, Mohammad Sidiq, Martin Siegel, Diego Augusto Santos Silva, Gustavo Correia Basto da Silva, João Pedro Silva, Juan Carlos Silva, Luís Manuel Lopes Rodrigues Silva, Noah Joseph Bernard Silva de Leonardi, Padam Prasad Simkhada, Abhinav Singh, Akanksha Singh, Ambrish Singh, Baljinder Singh, Bhim Pratap Singh, Harmanjit Singh, Harpreet Singh, Jasbir Singh, Jasvinder A Singh, Kalpana Singh, Lucky Singh, Narinder Pal Singh, Paramdeep Singh, Poornima Suryanath Singh, Prashant Kumar Singh, Puneetpal Singh, Rakesh K Singh, Samer Singh, Satwinder Singh, Surendra Singh, Mukesh Kumar Sinha, Ratnesh Sinha, Robert Sinto, Sarah Brooke Sirota, Søren T Skou, David A Sleet, Erica Leigh N Slepak, Farrukh Sobia, MdSalman Sohel, Somaye Sohrabi, Balamrit Singh Sokhal, Ranjan Solanki, Solikhah Solikhah, Sameh S M Soliman, Weiyi Song, Younseong Song, Aayushi Sood, Prashant Sood, Soroush Soraneh, Reed J D Sorensen, Joan B Soriano, Michele Sorrentino, Fernando Sousa, Marco Aurelio Sousa, Ceren Soylu, Michael Spartalis, Sandra Spearman, Manraj Singh Sra, Chandrashekhar T Sreeramareddy, Bahadar S Srichawla, Suresh Kumar Srinivasamurthy, Shyamkumar Sriram, Lauryn K Stafford, Jeffrey D Stanaway, Antonina V Starodubova, Simona Cătălina Ştefan, Caroline Stein, Dan J Stein, Caitlyn Steiner, Timothy J Steiner, Jaimie D Steinmetz, Paschalis Steiropoulos, Aleksandar Stevanović, Leo Stockfelt, Lars Jacob Stovner, Kurt Straif, Peter Stubbs, Yu Su, Omer Subasi, Narayan Subedi, Claudia Kimie Suemoto, Alisha Suhag, Liang Sui, Thitiporn Sukaew, Surajo Kamilu Sulaiman, Auwal Garba Suleiman, Muritala Suleiman Odidi, Muhammad Suleman, Desy Sulistiyorini, Mark J M Sullman, Anusha Sultan Meo, Haitong Zhe Sun, Jing Sun, Mao-ling Sun, Xiaodong Sun, Zhong Sun, Zhuanlan Sun, Suraj Sundaragiri, Thanigaivel Sundaram, Johan Sundström, David Sunkersing, Sumam Sunny, Vinay Suresh, Chandan Kumar Swain, Vivianne M Swart, Dayinta Annisa Syaiful, Lukasz Szarpak, Mindy D Szeto, Sree Sudha T Y, Payam Tabaee Damavandi, Rafael Tabarés-Seisdedos, Seyed-Amir Tabatabaeizadeh, Shima Tabatabai, Celine Tabche, Ramin Tabibi, Mohammad Tabish, Jyothi Tadakamadla, Santosh Kumar Tadakamadla, Buhari Abdullahi Tafida, Farzad Taghizadeh-Hesary, Yasaman Taheri Abkenar, Moslem Taheri Soodejani, Amir Taherkhani, Jabeen Taiba, Shima Tajabadi, Iman M Talaat, Stella Talic, Byomkesh Talukder, Mircea Tampa, Jacques Lukenze Tamuzi, Jianye Tan, Ker-Kan Tan, Shynar Tanabayeva, Haosu Tang, Ekamol Tantisattamo, Ingan Ukur Tarigan, Mengistie Kassahun Tariku, Saba Tariq, Md Tariqujjaman, Nathan Y Tat, Razieh Tavakoli Oliaee, Rahele Tavakoly, Seyed Mohammad Tavangar, Mebrahtu G Tedla, Amare Teshome Tefera, Mojtaba Teimoori, Mohamad-Hani Temsah, Corey Teply, Masayuki Teramoto, Amensisa Hailu Tesfaye, Azimeraw Arega Tesfu, Jay Tewari, Alireza Teymouri, Omar Thaher, Pugazhenthan Thangaraju, Kavumpurathu Raman Thankappan, Rekha Thapar, Ismaeel Tharwat, Samar Tharwat, Hadiza Theyra-Enias, Mehakpreet Kaur Thind, Arun James Thirunavukarasu, Muthu Thiruvengadam, Rekha Thiruvengadam, Arulmani Thiyagarajan, Nihal Thomas, Geethika P Thota, Wei Tian, Jansje Henny Vera Ticoalu, Tenaw Yimer Tiruye, Madi Tleshev, Musliu Adetola Tolani, Sojit Tomo, Marcello Tonelli, Roman Topor-Madry, Ali Torkashvand, Mathilde Touvier, Marcos Roberto Tovani-Palone, Khaled Trabelsi, Eugenio Traini, Mai Thi Ngoc Tran, Nghia Minh Tran, Ngoc Ha Tran, Quynh Thuy Huong Tran, Tam Quoc Minh Tran, Thang Huu Tran, Nguyen Tran Minh Duc, Domenico Trico, Indang Trihandini, Samuel Joseph Tromans, Quynh Xuan Nguyen Truong, Thien Tan Tri Tai Truyen, Aristidis Tsatsakis, Gary Tse, Evangelia Eirini Tsermpini, Lorainne Tudor Car, Mike Tuffour Amirikah, Munkhtuya Tumurkhuu, Zhouting Tuo, Sok Cin Tye, Aniefiok John Udoakang, Atta Ullah, Himayat Ullah, Irfan Ullah, Saeed Ullah, Muhammad Umair, Krishna Kishore Umapathi, Lawan Umar, Muhammad Umar, Muhammad Umar, Shehu Salihu Umar, Andrew Underwood-Nakamura, Dinesh Upadhya, Era Upadhyay, Dipan Uppal, Daniele Urso, Jibrin Sammani Usman, Kelechi Julian Uzor, Dilber Uzun Ozsahin, Hande Uzunçıbuk, Pratyusha Vadagam, sara Vahdati, Asokan Govindaraj Vaithinathan, Omid Vakili, Alireza Vakilian, Pascual R Valdez, Mario Valenti, Gelareh Valizadeh, Jef Van den Eynde, Giloume Van Der Walt, Aaron van Donkelaar, Javad Varasteh, Ravi Prasad Varma, Priya Vart, Tommi Juhani Vasankari, Sampara Vasishta, Srivatsa Surya Vasudevan, Prabhakar Veginadu, Ashleigh S Vella, Balachandar Vellingiri, Narayanaswamy Venketasubramanian, Baskar Venkidasamy, Megan Verma, Poonam Verma, Massimiliano Veroux, Georgios-Ioannis Verras, Dominique Vervoort, Simone Vidale, Simone Villa, Jorge Hugo Villafañe, David Villarreal-Zegarra, Francesco S Violante, Sharath Chaitanya Vipparthy, Rachel Visontay, Luciano Magalhães Vitorino, Vasily Vlassov, Martin Vojtek, Stein Emil Vollset, Avina Vongpradith, Mehdi Vosoughi, Elpida Vounzoulaki, Hai Nam Vu, Linh Vu, Yasir Waheed, Mugi Wahidin, Megha Walia, Agnes Wamuyu Wamai, Jin-Yi Wan, Cong Wang, Fang Wang, Lei Wang, Liang Wang, Ruixuan Wang, Shaopan Wang, Shu Wang, Wei Wang, Xing Wang, Xuequan Wang, Yanzhong Wang, Yichen Wang, Yuan-Pang Wang, Zhihua Wang, Tanveer A Wani, Mary Njeri Wanjau, Ahmed Bilal Waqar, Muhammad Waqas, Paul Ward, Toyiba Hiyaru Wassie, Kosala Gayan Weerakoon, Ishanka Weerasekara, Fei-Long Wei, Xueying Wei, Robert G Weintraub, Daniel J Weiss, Eli J Weiss, Yi Feng Wen, Andrea Werdecker, Ronny Westerman, Joanna L Whisnant, Harvey A Whiteford, Taweewat Wiangkham, Yohanes Cakrapradipta Wibowo, Anggi Lukman Wicaksana, Dakshitha Praneeth Wickramasinghe, Nuwan Darshana Wickramasinghe, Samuel Wiebe, Angga Wilandika, Peter Willeit, Shadrach Wilson, Andrew Awuah Wireko, Gemechu Kumera Wirtu, Charles Shey Wiysonge, Abay Tadesse Woday, Marcin W Wojewodzic, Axel Walter Wolf, Tewodros Eshete Wonde, Yohannes Chemere Wondmeneh, Daniel Tarekegn Worede, Minichil Chanie Worku, Nigus Kassie Worku, Ai-Min Wu, Chenkai Wu, Felicia Wu, James Fan Wu, Jiayuan Wu, Jinyi Wu, Shi-Nan Wu, Zenghong Wu, Yihun Miskir Wubie, Yanjie Xia, Zhijia Xia, Guangqin Xiao, Hong Xiao, Na Xiao, Wanqing Xie, Hongquan Xing, Site Xu, Suowen Xu, Wanqing Xu, Xiang Xu, Xiaoyue Xu, Mukesh Kumar Yadav, Vikas Yadav, Mahnaz Yadollahi, Sajad Yaghoubi, Saba Yahoo (Syed), Galal Yahya, Kazumasa Yamagishi, Guangcan Yan, Haibo Yang, Weiguang Yang, Xinxin Yang, Yuichiro Yano, Haiqiang Yao, Laiang Yao, Amir Yarahmadi, Haya Yasin, Mohamed A Yassin, Yuichi Yasufuku, Sanni Yaya, Pengpeng Ye, Meghdad Yeganeh, Ali Cem Yekdeş, Mohammad Hossein YektaKooshali, Kuanysh A Yergaliyev, Renjulal Yesodharan, Subah Abderehim Yesuf, Saber Yezli, Siyan Yi, Muluken Yigezu, Zeamanuel Anteneh Yigzaw, Dehui Yin, Yulai Yin, Paul Yip, Malede Berihun Yismaw, Yazachew Engida Yismaw, Dong Keon Yon, Naohiro Yonemoto, Mustafa Z Younis, Abdilahi Yousuf, Chuanhua Yu, Jian Yu, Yong Yu, Faith H Yuh, Ghazala Yunus, Umar Yunusa, Aminu Abba Yusuf, Monal Yuwanati, Siddhesh Zadey, Vesna Zadnik, Mubashir Zafar, Manijeh Zaghampour, Emilia Zainal Abidin, Fathiah Zakham, Nazar Zaki, Giulia Zamagni, Burhan Abdullah Zaman, Sojib Bin Zaman, Abu Sarwar Zamani, Nelson Zamora, Aurora Zanghì, Heather J Zar, Kourosh Zarea, Mohammed Zawiah, Mohammed G M Zeariya, Abay Mulu Zenebe, Sebastian Zensen, Eyael M Zeru, Tiansong Zhan, Yongle Zhan, Beijian Zhang, Casper J P Zhang, Haijun Zhang, Jingya Zhang, Liqun Zhang, Meixin Zhang, Xiaoyi Zhang, Yunquan Zhang, Zhiqiang Zhang, Jianhui Zhao, Sheng Zhao, Zhongyi Zhao, Jinxin Zheng, Ming-Hua Zheng, Peng Zheng, Claire Chenwen Zhong, Jiayan Zhou, Juexiao Zhou, Maigeng Zhou, Bin Zhu, Zhengyang Zhu, Abzal Zhumagaliuly, Magdalena Zielińska, Liu Zihao, Ghazal Zoghi, Mohamed Ali Zoromba, Zhiyong Zou, Rafat Mohammad Zrieq, Liesl J Zuhlke, Lilik Zuhriyah, Alimuddin Zumla, Ahed H Zyoud, Sa'ed H Zyoud, Shaher H Zyoud, Michael Brauer, Theo Vos, Christopher J L Murray, Emmanuela Gakidou

**Affiliations:** A Institute for Health Metrics and Evaluation, University of Washington, Seattle, WA, USA; B Department of Health Metrics Sciences, School of Medicine, University of Washington, Seattle, WA, USA; C Queensland Centre for Mental Health Research, Wacol, QLD, Australia; D School of Public Health, The University of Queensland, Brisbane, QLD, Australia; E Amity Institute of Public Health, Amity University, Uttar Pradesh, India; F Shahid Beheshti University of Medical Sciences, Shahid Beheshti University of Medical Sciences, Tehran, Iran; G Department of Nursing, Al Zaytoonah University of Jordan, Amman, Jordan; H Department of Radiation Oncology, Massachusetts General Hospital, Boston, MA, USA; I School of Health & Life Sciences, University of the West of Scotland, Paisley, UK; J Department of Medical Rehabilitation, University of Nigeria Nsukka, Enugu, Nigeria; K Public Health, Curtin University, Perth, WA, Australia; L Department of Legal and Economic Studies, La Sapienza University, Rome, Italy; M Centre for Regenerative Medicine and Health, Hong Kong Institute of Science and Innovation, Chinese Academy of Sciences, Hong Kong, China; N Department of Internal Medicine, Rafsanjan University of Medical Sciences, Rafsanjan, Iran; O Clinical Research Development Unit, Rafsanjan University of Medical Sciences, Rafsanjan, Iran; P Non-communicable Diseases Research Center, Shahid Beheshti University of Medical Sciences, Tehran, Iran; Q Department of Epidemiology, Alexandria University, Alexandria, Egypt; R College of Pharmacy, Umm Al-qura University, Makkah, Saudi Arabia; S Hull York Medical School, University of Hull, Hull, UK; T Department of Biology, Qassim University, Buraydah, Saudi Arabia; U Department of Health and Nutrition, Save the Children, Hargeisa, Somalia; V School of Nursing, The University of Jordan, Amman, Jordan; W College of Pharmacy, King Saud University, Riyadh, Saudi Arabia; X Basic Science Department, University of Hail, Hail, Saudi Arabia; Y Chemistry Department, Al-Azhar University, Cairo, Egypt; Z Department of Surgery, Marshall University, Huntington, WV, USA; AA Department of Cardiovascular Medicine, Mayo Clinic, Phoenix, AZ, USA; AB Department of Medical Laboratory Science, University of Sharjah, Sharjah, United Arab Emirates; AC Department of Tropical Medicine and Infectious Diseases, Tanta University, Tanta, Egypt; AD Stanford Cancer Institute, Stanford University, Stanford, CA, USA; AE The Institute of Pharmaceutical Sciences (TIPS), Tehran University of Medical Sciences, Tehran, Iran; AF School of Pharmacy, Tehran University of Medical Sciences, Tehran, Iran; AG Department of Medicine, University of Setif Algeria, Sétif, Algeria; AH Department of Health, Sétif, Algeria; AI Faculty of Veterinary Medicine, Islamic Azad University, Karaj, Iran; AJ Komar University of Science and Technology, Sulaymaniyah, Iraq; AK Baxshin Hospital, Baxshin Research Center, Sulaymaniyah, Iraq; AL Community and Maternity Nursing Unit, University of Duhok, Duhok, Iraq; AM National Institute of Epidemiology, Indian Council of Medical Research, Chennai, India; AN Department of Population and Global Health, Harvard University, Boston, MA, USA; AO Department of Physiotherapy, Bayero University Kano, Kano, Nigeria; AP Department of Physiotherapy, Federal University Wukari, Wukari, Nigeria; AQ Department of Research, Toufik's World Medical Association, Antonova 10, Ukraine; AR Department of General Surgery and Clinical Anatomy, Kazakh National Medical University, Almaty, Kazakhstan; AS Department of Epidemiology and Biostatistics, University of Gondar, Gondar, Ethiopia; AT Department of Neurosurgery, University of Southern California, Los Angeles, CA, USA; AU Keck School of Medicine, University of Southern California, Los Angeles, CA, USA; AV Department of Emergency Medicine, Zanjan University of Medical Sciences, Zanjan, Iran; AW School of Pharmacy, Bahir Dar University, Bahir Dar, Ethiopia; AX Postgraduate Department, University of Sierra Sur, Miahuatlan de Porfirio Diaz, Mexico; AY Yhteiskuntadatatieteen keskus (Centre for Social Data Science), University of Helsinki, Helsinki, Finland; AZ Department of Botany, Sree Narayana Guru College Chelannur, Kozhikode, India; BA Nuffield Department of Population Health, University of Oxford, Oxford, UK; BB National Heart Foundation Hospital and Research Institute, Dhaka, Bangladesh; BC Department of Biomedical Sciences, Nazarbayev University School of Medicine, Astana, Kazakhstan; BD Department of Midwifery, Bahir Dar University, Bahir Dar, Ethiopia; BE Department of Internal Medicine, Federal Medical Centre, Abuja, Nigeria; BF Department of Community Medicine, Babcock University, Ilishan-Remo, Nigeria; BG Department of Family and Community Health, University of Health and Allied Sciences, Ho, Ghana; BH School of Population Health, University of New South Wales, Sydney, NSW, Australia; BI Cardiovascular Research Center, Massachusetts General Hospital, Boston, MA, USA; BJ Department of Radiology, Harvard University, Boston, MA, USA; BK Research Center for Immunodeficiencies, Tehran University of Medical Sciences, Tehran, Iran; BL Department of Medical Biochemistry and Biophysics, Karolinska Institute, Stockholm, Sweden; BM Department of Sport, Exercise and Rehabilitation, Northumbria University, Newcastle, UK; BN Basic Science Department, Preparatory Year, University of Hail, Hail, Saudi Arabia; BO Zoology Department, Benha University, Benha, Egypt; BP Department of Physical Pharmacy and Pharmacokinetics, Poznan University of Medical Sciences, Poznan, Poland; BQ Department of Cardiovascular Disease, Loma Linda University Medical Center, Loma Linda, CA, USA; BR Department of Pediatric Dentistry, Federal University of Minas Gerais, Belo Horizonte, Brazil; BS Department of Anesthesiology, Shahid Beheshti University of Medical Sciences, Tehran, Iran; BT Clinical Pharmacy and Therapeutics Department, Applied Science Private University, Amman, Jordan; BU Community Health Nursing Department, Jouf University, Sakaka, Saudi Arabia; BV Graduate School of Public Health, St. Luke's International University, Tokyo, Japan; BW Division of Population Data Science, National Cancer Center, Tokyo, Japan; BX Department of Pharmacology and Toxicology, Usmanu Danfodiyo University, Sokoto, Sokoto, Nigeria; BY Nigerian Institute of Medical Research, Lagos, Nigeria; BZ Clinical Sciences Department, University of Sharjah, Sharjah, United Arab Emirates; CA Department of Biopharmaceutics and Clinical Pharmacy, University of Jordan, Amman, Jordan; CB Department of Nursing, University of Sharjah, Sharjah, United Arab Emirates; CC Maternal and Child Health Nursing, Jordan University of Science and Technology, Irbid, Jordan; CD Department Pharmacy Practice and Pharmacotherapeutics, University of Sharjah, Sharjah, United Arab Emirates; CE Medical Research Center, Hamad Medical Corporation, Doha, Qatar; CF College of Health Sciences, Qatar University, Doha, Qatar; CG Birzeit University, Ramallah, Palestine; CH Department of Pharmacology and Therapeutics, United Arab Emirates University, Al Ain, United Arab Emirates; CI College of Pharmacy, University of Jordan, Amman, Jordan; CJ Department of Pharmacy, Hamad Medical Corporation, Doha, Qatar; CK Department of Biochemistry, Jagadguru Sri Shivarathreeswara University, Mysuru, India; CL Department of Restorative Dentistry, University of Sharjah, Sharjah, United Arab Emirates; CM Department of Forensic Medicine and Toxicology, Karnali Academy of Health Sciences, Jumla, Nepal; CN School of Public Health and Preventive Medicine, Monash University, Melbourne, VIC, Australia; CO Department of Clinical Medicine, American University of Antigua, Coolidge, Antigua and Barbuda; CP FIU Robert Stempel College of Public Health & Social Work, Florida International University, Miami, FL, USA; CQ Department of Emergency and Critical Care Nursing, Bahir Dar University, Bahir Dar, Ethiopia; CR Department of Diagnostic and Interventional Radiology, Technical University of Munich, Munich, Germany; CS Stanford University, Palo Alto, CA, USA; CT Department of Human Anatomy, Federal University Dutse, Dutse, Nigeria; CU Department of Anatomy, Bayero University Kano, Kano, Nigeria; CV College of Medicine and Health Sciences, Bahir Dar University, Bahir Dar, Ethiopia; CW Department of Clinical Pharmacy, Bahir Dar University, Bahir Dar, Ethiopia; CX School of Medicine, University of Sydney, Sydney, NSW, Australia; CY Centre for Social Research in Health, University of New South Wales, Sydney, NSW, Australia; CZ Department of Health Promotion, Education and Behavior, University of South Carolina, Columbia, SC, USA; DA Department of Public Health, University of KwaZulu-Natal, Durban, South Africa; DB Department of Microbiology, Ladoke Akintola University, Osogbo, Nigeria; DC NMC Healthcare, Independent Consultant, Sharjah, United Arab Emirates; DD Department of Sociology, Olabisi Onabanjo University, Ago-Iwoye, Nigeria; DE School of Basic and Biomedical Sciences, University of Health and Allied Sciences, Ho, Ghana; DF Department of Immunology, Roswell Park Comprehensive Cancer Center, Buffalo, NY, USA; DG Graduate Program Division, University at Buffalo, Buffalo, NY, USA; DH Department of Medical Rehabilitation, Obafemi Awolowo University, Ile-Ife, Nigeria; DI Department of Pediatrics, East Tennessee State University, Johnson City, TN, USA; DJ Center for Cardiovascular Risk Research, Center for Cardiovascular Risk Research, Johnson City, TN, USA; DK Menzies School of Health Research, Charles Darwin University, Darwin, NT, Australia; DL Translational Research Team, University of Sydney, Sydney, NSW, Australia; DM Melanoma Institute Australia, The University of Sydney, Sydney, NSW, Australia; DN Department of Family Medicine, Bowen University, Iwo, Nigeria; DO Department of Family Medicine, Bowen University Teaching Hospital, Ogbomoso, Nigeria; DP Department of Microbiology, University of Medical Sciences, Ondo, Ondo, Nigeria; DQ Slum and Rural Health Initiative Research Academy, Slum and Rural Health Initiative, Ibadan, Nigeria; DR Department of Physiotherapy, University of Ibadan, Ibadan, Nigeria; DS Department of Educational Counselling and Developmental Psychology, University of Ibadan, Ibadan, Nigeria; DT Department of Educational Psychology, University of Johannesburg, Johannesburg, South Africa; DU Department of Pharmacology and Therapeutics, University of Medical Sciences, Ondo, Ondo, Nigeria; DV Department of Veterinary Medicine, University of Ibadan, Ibadan, Nigeria; DW Department of Physiology, University of Medical Sciences, Ondo, Ondo, Nigeria; DX Department of Community Medicine, Tribhuvan University, Bharatpur, Nepal; DY Public Health Section, Himalayan Environment and Public Health Network (HEPHN), Chitwan, Nepal; DZ Department of Fisheries and Marine Bioscience, Jashore University of Science and Technology, Jashore, Bangladesh; EA Research School of Population Health, Australian National University, Canberra, ACT, Australia; EB Apollo Institute Of Medical Sciences & Research Chittoor, Apollo Hospital, Chittoor, India; EC Department of Biology, University of Hail, Hail, Saudi Arabia; ED Department of Public Health, Universitas Padjadjaran (Padjadjaran University), Bandung, Indonesia; EE Department of Health Administration and Education, University of Education Winneba, Winneba, Ghana; EF Department of Epidemiology and Biostatistics, University of Health and Allied Sciences, Ho, Ghana; EG National Institute on Minority Health and Health Disparities, National Institutes of Health, Bethesda, MD, USA; EH School of Public Health, University of Texas Health Science Center at Houston, Houston, TX, USA; EI Department of Public Health and Preventive Medicine, University of Naples “Federico II” Naples, Italy; EJ Department of Surgery, University of Toledo, Toledo, OH, USA; EK Technical Services Directorate, MSI Nigeria Reproductive Choices, Abuja, Nigeria; EL Department of Epidemiology and Medical Statistics, University of Ibadan, Ibadan, Nigeria; EM Department of Community Medicine, King Edward Memorial Hospital, Lahore, Pakistan; EN Department of Public Health, Public Health Institute, Lahore, Pakistan; EO Department of Public Health, Wachemo University, Hossana, Ethiopia; EP Department of New Initiatives, International Vaccine Institute, Seoul, South Korea; EQ College of Health Sciences and Medicine, Wolaita Sodo University, Wolaita Sodo, Ethiopia; ER MM College of Pharmacy, Maharishi Markandeshwar (Deemed to be University), Ambala, India; ES Department of Orthopedic Surgery and Sports Medicine, Boston Children's Hospital, Boston, MA, USA; ET Department of Neurosurgery, Alborz University of Medical Sciences, Karaj, Iran; EU Neuroscience Research Center, Iran University of Medical Sciences, Tehran, Iran; EV Urology Research Center, Tehran University of Medical Sciences, Tehran, Iran; EW Health Research and Innovation Sciences Center, Klaipeda University, Klaipeda, Lithuania; EX SPRINT Sport Physical Activity and Health Research & Innovation Center, Polytechnic Institute of Guarda, Guarda, Portugal; EY Trivedi School of Biosciences, Ashoka University, Sonipat, India; EZ Department of Public Health Sciences, Queen's University, Kingston, ON, Canada; FA Rajaie Trauma Research Center, Shiraz University of Medical Sciences, Shiraz, Iran; FB School of Public Health, University of Technology Sydney, Sydney, NSW, Australia; FC College of Medicine, Shaqra University, Shaqra, Saudi Arabia; FD School of Medicine and Psychology, Australian National University, Canberra, ACT, Australia; FE Health Research Institute, University of Canberra, Canberra, NSW, Australia; FF Biological Production Unit National Institute of Health Islamabad Pakistan, National Institute of Health, Islamabad, Pakistan; FG World Health Organisation, Islamabad, Pakistan; FH Department of Research, King Khaled Eye Specialist Hospital & Research Center, Riyadh, Saudi Arabia; FI Department of Health Informatics, Qassim University, Buraidha, Saudi Arabia; FJ School of Nursing, The University of Jordan, Amman, Jordan; FK Department of Clinical Pharmacy, Universiti Sains Malaysia, Penang, Malaysia; FL Department of Pharmacy Practice, The Islamia University of Bahawalpur, Bahawalpur, Pakistan; FM Department of Health and Biological Sciences, Abasyn University, Peshawar, Pakistan; FN Department of Natural Sciences, Lebanese American University, Beirut, Lebanon; FO School of Public Health, Zhejiang University, Hangzhou, China; FP Department of Community Health Sciences, Sohail University, Karachi, Pakistan; FQ College of Medicine, University of Cincinnati, Cincinnati, OH, USA; FR School of Medicine, Tehran University of Medical Sciences, Tehran, Iran; FS Department of Neuroscience, Mashhad University of Medical Sciences, Mashhad, Iran; FT Urology Department, Shahid Beheshti University of Medical Sciences, Tehran, Iran; FU Department of Veterinary Microbiology, University of Ilorin, Ilorin, Nigeria; FV Maternal and Child Health Division (MCHD), International Centre for Diarrhoeal Disease Research, Bangladesh, Dhaka, Bangladesh; FW Department of Women's and Children's Health, Uppsala University, Uppsala, Sweden; FX Institute of Endemic Diseases, University of Khartoum, Khartoum, Sudan; FY Swiss Tropical and Public Health Institute, University of Basel, Basel, Switzerland; FZ Medical Laboratory Science Department, University of Human Development, Sulaymaniyah, Iraq; GA Department of Biosciences, COMSATS Institute of Information Technology, Islamabad, Pakistan; GB Manipal College of Dental Sciences, Mangalore, Manipal Academy of Higher Education, Mangalore, India; GC Institute of Public Health, United Arab Emirates University, Al Ain, United Arab Emirates; GD College of Nursing, Majmaah University, Al Majmaah, Saudi Arabia; GE Department of Pathology and Microbiology, University of Duhok, Duhok, Iraq; GF College of Medicine and Public Health, Flinders University, Adelaide, SA, Australia; GG Faculty of Public Health, Jimma University, Jimma, Ethiopia; GH Department of Medicine, Rawalpindi Medical University, Rawalpindi, Pakistan; GI Department of Psychology, University of Chittagong, Chattogram, Bangladesh; GJ Department of Public Health Epidemiology, Debre Berhan University, Debre Berhan, Ethiopia; GK Menelik II Medical and Health Science College, EpiMetrics, Inc., Addis Ababa, Ethiopia; GL School of Medicine, Nazarbayev University, Astana, Kazakhstan; GM Clinical Academic Department of Women's Health, NU Medicine, Astana, Kazakhstan; GN Department of Medical Laboratory Technology, Erbil Polytechnic University, Erbil, Iraq; GO Bowen University Hospital, Bowen University, Iwo, Nigeria; GP National Nutrition and Food Technology Research Institute, Shahid Beheshti University of Medical Sciences, Tehran, Iran; GQ St George and Sutherland Clinical School, University of New South Wales, Sydney, NSW, Australia; GR Department of Water Engineering, Graduate University of Advanced Technology, Kerman, Iran; GS Oxford Vaccine Group, University of Oxford, Oxford, UK; GT Department of Physiology, Ladoke Akintola University, Ogbomoso, Nigeria; GU School of Veterinary Medicine, Texas Tech University, Amarillo, TX, USA; GV Department of Internal Medicine, University of Patras, Patras, Greece; GW Department of Internal Medicine and Infectious Diseases, University General Hospital of Patras, Patras, Greece; GX Department of Cardiology, Fudan University, Shanghai, China; GY Department of Management, Policy, and Community Health, University of Texas, Houston, TX, USA; GZ Faculty of Health and Behavioural Sciences, The University of Queensland, Brisbane, QLD, Australia; HA Department of Infection Prevention & Control, Baylor Scott & White Health, Frisco, TX, USA; HB Chicago College of Osteopathic Medicine, Midwestern University, Downers Grove, IL, USA; HC Feinberg School of Medicine, Northwestern University, Chicago, IL, USA; HD Centre for Academic Primary Care, University of Nottingham, Nottingham, UK; HE College of Pharmacy and Health Sciences, Ajman University, Ajman, United Arab Emirates; HF Center of Medical and Bio-allied Health Sciences Research, Ajman University, Ajman, United Arab Emirates; HG Department of Communicable Diseases, Ministry of Health, Muscat, Oman; HH Middle East, Eurasia, and Africa Influenza Stakeholders Network, Muscat, Oman; HI Division of Public Health Sciences, Washington University in St. Louis, St. Louis, MO, USA; HJ Fundamentals and Administration Department, Sultan Qaboos University, Muscat, Oman; HK Al Al-Bayt University, Mafraq, Jordan; HL Department of Internal Medicine, Allegheny Health Network, Pittsburgh, PA, USA; HM Department of General Education, Hamdan Bin Mohammed Smart University, Dubai, United Arab Emirates; HN Faculty of Pharmacy, Philadelphia University, Amman, Jordan; HO School of Pharmacy, Cardiff University, Cardiff, UK; HP Department of Adult Health and Critical Care, Sultan Qaboos University, Muscat, Oman; HQ School of Public Health, University of Texas, Houston, TX, USA; HR The University of Jordan School of Medicine, The University of Jordan, Amman, Jordan; HS Department of Rehabilitation, Montefiore Medical Center, Bronx, NY, USA; HT Department of Epidemiology, Columbia University, New York, NY, USA; HU Department of Biology, University of Bahrain, Zallaq, Bahrain; HV Department of Research and Development, Washington University in St. Louis, St. Louis, MO, USA; HW Clinical Epidemiology Center, US Department of Veterans Affairs (VA), St. Louis, MO, USA; HX Murdoch Business School, Murdoch University, Perth, WA, Australia; HY Department of Bioengineering, George Mason University, Fairfax, VA, USA; HZ Preventive Dentistry Department, Jouf University, Sakaka, Saudi Arabia; IA Department of Oral and Maxillofacial Surgery, Shahid Beheshti University of Medical Sciences, Tehran, Iran; IB School of Nursing, Yarmouk University, Irbid, Jordan; IC School of Nursing and Midwifery, Western Sydney University, Sydney, NSW, Australia; ID Department of Nursing and Midwifery, Woldia University, Woldia, Ethiopia; IE Department of Surgery, Hamad Medical Corporation, Doha, Qatar; IF Department of Health Information Management and Technology, Imam Abdulrahman Bin Faisal University, Dammam, Saudi Arabia; IG Department of Clinical Pharmacy, Al-Ayen Iraqi University, Thi-Qar, Iraq; IH Department of Clinical Pharmacy and Pharmacy Practice, University of Science and Technology, Sana'a, Yemen; II Faculty of Medicine, The University of Jordan, Amman, Jordan; IJ Faculty of Medicine, Ahvaz Jundishapur University of Medical Sciences, Ahvaz, Iran; IK Department of Community and Mental Health, Al Al-Bayt University, Mafraq, Jordan; IL Department of Internal Medicine, Cleveland Clinic, Cleveland, OH, USA; IM Division of Pediatric Cardiology, University of Colorado, Aurora, CO, USA; IN Heart Center, King Faisal Specialist Hospital & Research Center, Riyadh, Saudi Arabia; IO Division of Gastroenterology and Hepatology, Mayo Clinic, Rochester, MN, USA; IP General Directorate of Research and Studies, Ministry of Health, Riyadh, Saudi Arabia; IQ Institute of Health Informatics, University College London, London, UK; IR Department of Health, University of Bath, Bath, UK; IS College of Pharmacy, University of Sharjah, Sharjah, United Arab Emirates; IT School of Pharmacy, The University of Jordan, Amman, Jordan; IU Curtin School of Population Health, Curtin University, Perth, WA, Australia; IV Department of Health Systems and Policy, University of Gondar, Gondar, Ethiopia; IW Pediatric Intensive Care Unit, King Saud University, Riyadh, Saudi Arabia; IX Department of Epidemiology and Biostatistics, University of South Carolina, Columbia, SC, USA; IY Department of Public Health, University of Hail, Hail, Saudi Arabia; IZ Department of Bacteriology, Immunology, and Mycology, Suez Canal University, Ismailia, Egypt; JA Department of Family and Community Medicine, University of Jeddah, Jeddah, Saudi Arabia; JB Global Centre for Environmental Remediation, University of Newcastle, Newcastle, NSW, Australia; JC Cooperative Research Centre for Contamination Assessment and Remediation of the Environment, Newcastle, NSW, Australia; JD College of Medicine and Health Sciences, Khalifa University, Abu Dhabi, United Arab Emirates; JE Department of Cardiac Sciences, King Saud University, Riyadh, Saudi Arabia; JF Africa Center of Excellence for Mycotoxin and Food Safety, Minna, Nigeria; JG Epidemiology and Population Health Department, American University of Beirut, Beirut, Lebanon; JH British Columbia Injury Research Prevention Unit, British Columbia Children's Hospital Research Institute, Vancouver, BC, Canada; JI College of Nursing, Qatar University, Doha, Qatar; JJ Department of Health Services and Hospital Administration, King Abdulaziz University, Jeddah, Saudi Arabia; JK Health Economics Research Group, King Abdulaziz University, Jeddah, Saudi Arabia; JL Faculty of Applied Health Sciences (Physiotherapy), Tishk International University, Erbil, Iraq; JM Faculty of Dentistry, Ibn Al-Nafis University for Medical Sciences, Sana'a, Yemen; JN College of Applied Medical Sciences, Qassim University, Buraydah, Saudi Arabia; JO Information Science Department, Kuwait University, Kuwait, Kuwait; JP Health Informatics Unit and Geohealth Lab, Dasman Diabetes Institute, Dasman, Kuwait; JQ Department of Zoology, Abdul Wali Khan University Mardan, Mardan, Pakistan; JR Department of Biotechnology, University of Malakand, Chakdara, Pakistan; JS Department of Statistics and Operations Research, Aligarh Muslim University, Aligarh, India; JT School of Food and Agricultural Sciences, University of Management and Technology, Lahore, Pakistan; JU Department of Pharmacy, Mohammed Al-Mana College for Medical Sciences, Dammam, Saudi Arabia; JV Department of Medical Rehabilitation (Physiotherapy), University of Maiduguri, Maiduguri, Nigeria; JW The Nethersole School of Nursing, The Chinese University of Hong Kong, Hong Kong, China; JX Department of Biosciences, Jamia Millia Islamia, New Delhi, India; JY Centre for Biotechnology and Microbiology, University of Swat, Charbagh, Pakistan; JZ Center for Biotechnology and Microbiology, University of Swat, Swat, Pakistan; KA Biomedical Engineering Department, Johns Hopkins University, Baltimore, MD, USA; KB Department of Geography, Sultan Qaboos University, Muscat, Oman; KC Department of Nuclear Medicine, King Hussein Cancer Center, Amman, Jordan; KD Department of Diagnostic Radiology and Nuclear Medicine, The University of Jordan, Amman, Jordan; KE Department of Pathophysiology and Transplantation, Università degli Studi di Milano (University of Milan), Milan, Italy; KF Cystic Fibrosis Center, Fondazione IRCCS Ospedale Maggiore Policlinico, Milan, Italy; KG The School of Medicine, The University of Jordan, Amman, Jordan; KH Institute of Health and Wellbeing, Federation University Australia, Melbourne, VIC, Australia; KI Biomedical Physics Group, University of Hamburg, Hamburg, Germany; KJ Department of Clinical and Community Pharmacy, An-Najah National University, Nablus, Palestine; KK Department of Medical Laboratories, Qassim University, Buraydah, Saudi Arabia; KL Department of Biomedical Sciences, Nazarbayev University, Astana, Kazakhstan; KM School of Physics, Mathematics and Computing, The University of Western Australia, Perth, WA, Australia; KN Information and Communication Technology Research Pole (Lab-STICC), ENSTA Bretagne, Brest, France; KO Department of Public Health and Community Medicine, International Medical University, Kuala Lumpur, Malaysia; KP International Centre for Casemix and Clinical Coding, National University of Malaysia, Bandar Tun Razak, Malaysia; KQ College of Life Sciences, Birmingham City University, Birmingham, UK; KR Department of Biological Sciences and Chemistry (DBSC), University of Nizwa, Nizwa, Oman; KS Cardiovascular Division, University of Alabama, Birmingham, AL, USA; KT Tabriz University of Medical Sciences, Tabriz University of Medical Sciences, Tabriz, Iran; KU College of Medicine and Health Sciences, United Arab Emirates University, Al Ain, United Arab Emirates; KV Faculty of Medicine, Jordan University of Science and Technology, Irbid, Jordan; KW Faculty of Nursing, University of Tabuk, Tabuk, Saudi Arabia; KX Department of Cardiology, Heart, Vascular, and Thoracic Institute, Cleveland Clinic Abu Dhabi, Abu Dhabi, United Arab Emirates; KY College of Medicine and Health Sciences Academic Programs, Khalifa University, Abu Dhabi, United Arab Emirates; KZ Independent Consultant, Amman, Jordan; LA Rehabilitation Sciences Department, Qatar University, Doha, Qatar; LB Department of Medicine, Nazarbayev University, Astana, Kazakhstan; LC Department of Parasitology, University of Malaya, Kuala Lumpur, Malaysia; LD Department of Parasitology, Sana'a University, Sana'a, Yemen; LE Department of Urology, Cleveland Clinic Abu Dhabi, Abu Dhabi, United Arab Emirates; LF Nuffield Department of Surgical Sciences, University of Oxford, Oxford, UK; LG Ophthalmology Department, University of Miami, Miami, FL, USA; LH Nursing Faculty, University of Tabuk, Tabuk, Saudi Arabia; LI School of Public Health, SRM Institute of Science and Technology, Chennai, India; LJ Department of Physical Therapy and Rehabilitation Sciences, Jordan University of Science and Technology, Irbid, Jordan; LK Department of Rehabilitation Sciences and Physical Therapy, Jordan University of Science and Technology, Irbid, Jordan; LL Faculty of Nursing, Zarqa University, Zarqa, Jordan; LM Department of Respiratory Care, Prince Sultan Military College of Health Sciences, Dammam, Saudi Arabia; LN Liver, Digestive, and Lifestyle Health Research Section, King Faisal Specialist Hospital & Research Center, Riyadh, Saudi Arabia; LO Division of Gastroenterology and Hepatology, Weill Cornell Medicine, New York, NY, USA; LP American University of the Middle East, Egaila, Kuwait; LQ Surgical Research Section, Hamad Medical Corporation, Doha, Qatar; LR Department of Allied Medical Sciences, Jordan University of Science and Technology, Irbid, Jordan; LS Department of Nursing, Georgetown University, Washington, DC, USA; LT Macro-Fiscal Policy Department, Ministry of Finance, Dubai, United Arab Emirates; LU Department of Surgery, Kuwait University, Kuwait, Kuwait; LV Jaber Al Ahmad Al Sabah Hospital, Ministry of Health, Kuwait, Kuwait; LW Department of Emergency Medicine, Sana'a University, Sanaa, Yemen; LX Pediatric Emergency Medicine Department, Drexel University, Philadelphia, PA, USA; LY Department of Medical Rehabilitation Sciences, Umm Al-Qura University, Makkah, Saudi Arabia; LZ Department of Basic Sciences, Yarmouk University, Irbid, Jordan; MA Health Science Division, Sharjah, United Arab Emirates; MB Institute of Molecular Biology and Biotechnology, The University of Lahore, Lahore, Pakistan; MC Faculty of Health Sciences, Equator University of Science and Technology, Uganda, Masaka, Uganda; MD Research, Policy, and Training Directorate, Jordan Center for Disease Control, Amman, Jordan; ME Applied Science Research Center, Applied Science Private University, Amman, Jordan; MF Department of Specialty Internal Medicine, Johns Hopkins Aramco Healthcare, Dhahran, Saudi Arabia; MG Department of Medicine, Indiana University School of Medicine, Indianapolis, IN, USA; MH Department of Respiratory Therapy, King Abdulaziz University, Jeddah, Saudi Arabia; MI Respiratory Therapy Unit, King Abdulaziz University, Jeddah, Saudi Arabia; MJ University of Sharjah, Sharjah, United Arab Emirates; MK Faculty of Health Sciences, University of Cadiz, Cadiz, Spain; ML School of Sciences, University of Minho, Braga, Portugal; MM Research Group in Health Economics, Universidad de Cartagena (University of Cartagena), Cartagena, Colombia; MN Research Group in Hospital Management and Health Policies, Universidad de la Costa (University of the Coast), Barranquilla, Colombia; MO Department of Economic Sciences, Universidad de la Costa (University of the Coast), Barranquilla, Colombia; MP National Health Observatory, National Institute of Health, Bogota, Colombia; MQ Department of Clinical Pharmacology and Toxicology, Umm Al-Qura University, Makkah, Saudi Arabia; MR Department of Rehabilitation Sciences, Jordan University of Science and Technology, Irbid, Jordan; MS Department of Medical Sciences, Azal University for Human Development, Sana'a, Yemen; MT Department of Clinical Sciences, University of Science and Technology of Fujairah, Fujairah, United Arab Emirates; MU Department of Pediatrics, Cleveland Clinic, Cleveland, OH, USA; MV Institute of Public Health, United Arab Emirates University, Abu Dhabi, United Arab Emirates; MW Department of Physiotherapy, Taif University, Taif, Saudi Arabia; MX Department of Pharmaceutical Sciences, Qatar University, Doha, Qatar; MY Department of Clinical Pharmacy, Jordan University of Science and Technology, Irbid, Jordan; MZ Evaluation Unit, Global Alliance for Vaccines and Immunisations, Geneva, Switzerland; NA London School of Hygiene and Tropical Medicine, University of London, London, UK; NB Global Health Advocacy Incubator (GHAI), University of Central Nicaragua, Washington, DC, USA; NC Food and Beverages Safety Research Center, Urmia University of Medical Sciences, Urmia, Iran; ND Isfahan Cardiovascular Research Institute, Heart Failure Research Center., Isfahan University of Medical Sciences, Isfahan, Iran; NE Department of Biomedical Science, University of Cape Coast, Cape Coast, Ghana; NF Department of Pharmacy, An-Najah National University, Nablus, Palestine; NG Student Research Committee, Lorestan University of Medical Sciences, Khorramabad, Iran; NH Health Policy Research Center, Shiraz University of Medical Sciences, Shiraz, Iran; NI College of Medicine, University of Sharjah, Sharjah, United Arab Emirates; NJ Summer Program, University of Chicago, Chicago, IL, USA; NK Public Health and Community Medicine Department, Cairo University, Cairo, Egypt; NL Department of Radiology and Radiological Science, University of Maryland, Baltimore, MD, USA; NM Department of Health and Management Sciences, Khomein University of Medical Sciences, Khomein, Iran; NN Gastrointestinal and Liver Diseases Research Center, Guilan University of Medical Sciences, Rasht, Iran; NO Department of Pharmaceutics and Pharmaceutical Technology, Usmanu Danfodiyo University, Sokoto, Sokoto, Nigeria; NP School of Pharmacy, University of Botswana, Gaborone, Botswana; NQ Department of Food Safety and Hygiene, Zanjan University of Medical Sciences, Zanjan, Iran; NR Spiritual Health Research Center, Baqiyatallah University of Medical Sciences, Tehran, Iran; NS Department of Health and Wellbeing, African Population and Health Research Center, Nairobi, Kenya; NT Department of Sociology, Usmanu Danfodiyo University, Sokoto, Sokoto, Nigeria; NU Department of Sociology, University of Johannesburg, Johannesburg, South Africa; NV Center for Biomedical Image Computing & Analytics, University of Pennsylvania, Philadelphia, PA, USA; NW University of Bologna, Bologna, Italy; NX Department of General Medicine, Eastern Health, Box Hill, VIC, Australia; NY Faculty of Pharmacy, Carol Davila University of Medicine and Pharmacy, Bucharest, Romania; NZ Centre for Sensorimotor Performance, The University of Queensland, Brisbane, QLD, Australia; OA Neurology Department, Royal Brisbane and Women's Hospital, Brisbane, QLD, Australia; OB Faculty of Medicine and Health, University of Sydney, Sydney, NSW, Australia; OC Sydney Musculoskeletal Health, University of Sydney, Sydney, NSW, Australia; OD Department of Medicine, University of Thessaly, Volos, Greece; OE Department of Environmental and Occupational Health, George Washington University, Washington, DC, USA; OF Department of Internal Medicine, Rutgers University, Toms River, NJ, USA; OG Sarver Heart Center, University of Arizona, Tucson, AZ, USA; OH School of Health and Related Research, University of Sheffield, Sheffield, UK; OI Department of General Medicine, Thai Binh University of Medicine and Pharmacy in Vietnam, Thai Binh City, Vietnam; OJ Department of Management, University of Cape Coast, Cape Coast, Ghana; OK Department of Public Health, The Apollo University, Chittoor, India; OL Digestive Diseases Research Institute, Tehran University of Medical Sciences, Tehran, Iran; OM General Surgery Department, Shahid Beheshti University of Medical Sciences, Tehran, Iran; ON Department of Physiotherapy, Galgotias University, Greater Noida, India; OO Pharmacy Department, Critical Care, Cleveland Clinic Abu Dhabi, Abu Dhabi, United Arab Emirates; OP Department of Public Health, Debre Tabor University, Debre Tabor, Ethiopia; OQ Environment and Health over the Lifecourse Programme, Barcelona Institute for Global Health, Barcelona, Spain; OR Department of Experimental and Health Sciences, Pompeu Fabra University, Barcelona, Spain; OS Agribusiness Study Program, Sebelas Maret University, Surakarta, Indonesia; OT Department of Environmental and Occupational Health, University of Medical Sciences, Ondo, Ondo, Nigeria; OU Centre for Interdisciplinary Research in Basic Sciences (CIRBSc), Jamia Millia Islamia, New Delhi, India; OV School of Chemical and Life Sciences (SCLS), Jamia Hamdard, New Delhi, India; OW Department of Surgery, Gadjah Mada University, Yogyakarta, Indonesia; OX Department of Pathology, Imam Mohammad Ibn Saud Islamic University, Riyadh, Saudi Arabia; OY Department of Rehabilitation Sciences, Hong Kong Polytechnic University, Hong Kong, China; OZ Rural Health Research Institute, Charles Sturt University, Orange, NSW, Australia; PA School of Medicine and Public Health, Ateneo De Manila University, Pasig City, Philippines; PB Inter-Agency Committee on Environmental Health, Department of Health Philippines, Manila, Philippines; PC Division of Gastroenterology, Hepatology, and Nutrition, Virginia Commonwealth University, Richmond, VA, USA; PD Gastroenterology Department, Pontifical Catholic University of Chile, Santiago, Chile; PE Geneva University Hospital, University of Geneva, Geneva, Switzerland; PF Health Management and Economics Research Center, Iran University of Medical Sciences, Tehran, Iran; PG College of Pharmacy, Al Ain University, Abu Dhabi, United Arab Emirates; PH Department of Applied Mathematics, University of Washington, Seattle, WA, USA; PI College of Art and Science, Ottawa University, Surprise, AZ, USA; PJ School of Life Sciences, Arizona State University, Tempe, AZ, USA; PK Care in Long Term Conditions Research Division, King's College London, London, UK; PL CIBER Epidemiology and Public Health (CIBERESP), Madrid, Spain; PM Department of Paediatrics, University of Malaya, Kuala Lumpur, Malaysia; PN University of Malaya Medical Centre, University of Malaya, Kuala Lumpur, Malaysia; PO Department of Cardiovascular, Endocrine-Metabolic Diseases and Aging, Istituto Superiore di Sanità (ISS), Rome, Italy; PP Department of Neurobiology, Care Sciences and Society, Karolinska Institute, Stockholm, Sweden; PQ School of Health and Social Studies, Dalarna University, Falun, Sweden; PR Department of Biotechnology, Sri Ramaswamy Memorial Institute of Science and Technology, Kattankulathur, India; PS University College of Medicine & Dentistry, The University of Lahore, Lahore, Pakistan; PT Institute for Biomedical Problems, Russian Academy of Sciences, Moscow, Russia; PU Division of Epidemiology, Universitas Airlangga (Airlangga University), Surabaya, Indonesia; PV Wake Forest University, Winston Salem, NC, USA; PW Department of Periodontics, Saveetha University, Chennai, India; PX Department of Public Health, Kazakh National Medical University, Almaty, Kazakhstan; PY Department of Clinical Disciplines, Al Farabi Kazakh National University, Almaty, Kazakhstan; PZ School of Medicine, Shahid Beheshti University of Medical Sciences, Tehran, Iran; QA College of medicine, University of Arizona, Tucson, AZ, USA; QB Department of Community Medicine and Global Health, University of Oslo, Oslo, Norway; QC Department of Pharmacy Practice, AlMaarefa University, Riyadh, Saudi Arabia; QD School of Veterinary Medicine, Bahir Dar University, Bahir Dar, Ethiopia; QE Research Institute of Dental Sciences, Shahid Beheshti University of Medical Sciences, Tehran, Iran; QF National Agency for Strategic Research in Medical Education (NASRME), Ministry of Health and Medical Education, Tehran, Iran; QG Cabrini Research, Cabrini Health, Malvern, VIC, Australia; QH College of Applied Medical Science, University of Hail, Hail, Saudi Arabia; QI Pioneer Journal of Biostatistics and Medical Research (PJBMR), Pakistan, Pakistan; QJ School of Medicine, Zanjan University of Medical Sciences, Zanjan, Iran; QK Department of Radiation Oncology, Shandong University, Shandong, China; QL School of Health and Social Development, Deakin University, Melbourne, VIC, Australia; QM School of Traditional Chinese Medicine, Xiamen University Malaysia, Sepang, Malaysia; QN Faculty of Medicine, Nursing, and Health Sciences, Monash University, Melbourne, VIC, Australia; QO Nursing Department, Institute of Technology and Health Science RS dr Soepraoen, Malang, Indonesia; QP Faculty of Health Science, Institute of Technology and Health Science RS dr Soepraoen, Malang, Indonesia; QQ Atchabar Scientific Research Institute, Kazakh National Medical University, Almaty, Kazakhstan; QR School of Architecture, Design, and Planning, University of Sydney, Sydney, NSW, Australia; QS Department of Toxicology and Pharmacology, Tehran University of Medical Sciences, Tehran, Iran; QT Birjand University of Medical Sciences, Birjand, Iran; QU Department of Immunology, Zanjan University of Medical Sciences, Zanjan, Iran; QV Faculty of Nursing, Philadelphia University, Amman, Jordan; QW Hospital and Research Centre, Dr. D. Y. Patil Vidyapeeth Pune (Deemed to be University), Pune, India; QX Center for Clinical Global Health Education, Johns Hopkins University, Baltimore, MD, USA; QY Department of Forensic Medicine, Lumbini Medical College, Palpa, Nepal; QZ Management Policy and Community Health, University of Texas, Houston, TX, USA; RA College of Medicine, University of Basrah, Basrah, Iraq; RB кафедра, Al Farabi Kazakh National University, Almaty, Kazakhstan; RC School of Business, University of Leicester, Leicester, UK; RD Department of Statistics and Econometrics, Bucharest University of Economic Studies, Bucharest, Romania; RE Robarts Research Institute, The University of Western Ontario, London, ON, Canada; RF Department of Physiotherapy, Federal University of Santa Catarina, Araranguá, Brazil; RG School of Nursing and Public Health, University of KwaZulu-Natal, Durban, South Africa; RH Department of Public Health, Wollega University, Nekemte, Ethiopia; RI Department of Health Behavior and Society, Jimma University, Jimma, Ethiopia; RJ Trauma Research Center, Shiraz University of Medical Sciences, Shiraz, Iran; RK Psychiatry Department, University College Hospital, Ibadan, Ibadan, Nigeria; RL Medicinal Chemistry Unit, Kwara State University, Malete, Ilorin, Nigeria; RM Centre for Drug Research, Universiti Sains Malaysia, Pinang, Malaysia; RN Department of Health Information Management, Tehran University of Medical Sciences, Tehran, Iran; RO Laboratory Sciences Department, Arak University of Medical Sciences, Khomein, Iran; RP Department of Surgery, Washington University in St. Louis, St. Louis, MO, USA; RQ Department of Nursing, Shiraz University of Medical Sciences, Shiraz, Iran; RR Miami Cardiovascular Institute, Baptist Health South Florida, Inc, Miami, FL, USA; RS Department of Psychiatry, University of Social Welfare and Rehabilitation Sciences, Tehran, Iran; RT Advanced Medical & Dental Institute, Universiti Sains Malaysia, Penang, Malaysia; RU Department of Anesthesia, Cihan University -Sulaimaniya, Sulaymaniyah, Iraq; RV Department of Basic Sciences, University of Sulaimani, Sulaymaniyah, Iraq; RW Rheumatology Research Center, Tehran University of Medical Sciences, Tehran, Iran; RX Tehran University of Medical Sciences, Tehran, Iran; RY ASIDE Healthcare, Lewes, DE, USA; RZ Faculty of Medicine, October 6 University, 6th of October City, Egypt; SA Geriatric Unit, Fondazione IRCCS Ca' Granda Ospedale Maggiore Policlinico, Milan, Italy; SB Community Medicine Department, Ahmadu Bello University, Zaria, Nigeria; SC Physiology department, RAK Medical and Health Sciences University, Ras Alkhaimah, United Arab Emirates; SD Department of Population Medicine, Qatar University, Doha, Qatar; SE School of Public Health, Washington University in St. Louis, St. Louis, MO, USA; SF Directorate of Quality Assurance, Gomal University, Dera Ismail Khan, Pakistan; SG Department of Forensic Science, Government Institute of Forensic Science Nagpur, Nagpur, India; SH Rashtrasant Tukadoji Maharaj Nagpur University, Nagpur, India; SI Department of Clinical Pathology, Mansoura University, Mansoura, Egypt; SJ Microbiology Department, Horus University Egypt, Damietta, Egypt; SK Department of Precision Medicine, Sungkyunkwan University, Suwon, South Korea; SL Department of Pediatrics, All India Institute of Medical Sciences, New Delhi, India; SM Department of Orthopedics, University of California Los Angeles, Los Angeles, CA, USA; SN Orthopedic Institute for Children, Los Angeles, CA, USA; SO School of Medical Education and Learning Technologies, Shahid Beheshti University of Medical Sciences, Tehran, Iran; SP Shahid Rajii Hospital, Shahid Beheshti University of Medical Sciences, Tehran, Iran; SQ Taleghani Anesthesiologist, Shahid Beheshti University of Medical Sciences, Tehran, Iran; SR Dental Material Research Center, Islamic Azad University, Tehran, Iran; SS Pediatric Dentistry Department, King Abdulaziz University, Jeddah, Saudi Arabia; ST School of Public Health, Tehran University of Medical Sciences, Tehran, Iran; SU Healthcare Management Department, Shiraz University of Medical Sciences, Shiraz, Iran; SV College of Optometry, Pacific University, Forest Grove, OR, USA; SW Clinical Research Center, Nanjing Children's Hospital, Nanjing, China; SX International Medical School, Management and Science University, Alam, Malaysia; SY Sina Trauma and Surgery Research Center, Tehran University of Medical Sciences, Tehran, Iran; SZ Department of Epidemiology and Biostatistics, Tehran University of Medical Sciences, Tehran, Iran; TA Department of Forensic Medicine and Toxicology, Manipal Academy of Higher Education, Manipal, India; TB TIRR Memorial Hermann, Houston, TX, USA; TC Division of Biological Sciences, Tamil Nadu State Council for Science and Technology, Chennai, India; TD Lerner College of Medicine, Cleveland Clinic, Cleveland, OH, USA; TE Chen Senior Medical Center, Tamarac, FL, USA; TF Anahuac Business School, Universidad Anahuac Mexico, Mexico City, Mexico; TG Department of Epidemiology and Biostatistics, Kerman University of Medical Sciences, Kerman, Iran; TH College of Medicine, Alfaisal University, Riyadh, Saudi Arabia; TI Center of Innovation, Technology and Education (CITE), Anhembi Morumbi University, São José dos Campos, Brazil; TJ Periodontology, King Abdulaziz University, Jeddah, Saudi Arabia; TK Department of Hypertension, Medical University of Lodz, Lodz, Poland; TL Polish Mothers' Memorial Hospital Research Institute, Lodz, Poland; TM Dental Research Center, Tehran University of Medical Sciences, Tehran, Iran; TN Department of Non-communicable Diseases, Bangladesh University of Health Sciences, Dhaka, Bangladesh; TO Department of Anatomy, Saveh University of Medical Sciences, Saveh, Iran; TP Department of Translational Medicine, Florida International University, Miami, FL, USA; TQ School of Psychology, University of Auckland, Auckland, New Zealand; TR Department of Public and Environmental Health, University of The Gambia, Banjul, The Gambia; TS Department of Epidemiology, University of Florida, Gainesville, FL, USA; TT Heidelberg Institute of Global Health (HIGH), Heidelberg University, Heidelberg, Germany; TU Department of Community and Family Medicine, All India Institute of Medical Sciences, Gorakhpur, India; TV Alpha Genomics Private Limited, Islamabad, Pakistan; TW University Institute of Food Science and Technology, The University of Lahore, Lahore, Pakistan; TX Department of General Surgery and Medical-Surgical Specialties, University of Catania, Catania, Italy; TY Department of Community Medicine, Sri Manakula Vinayagar Medical College and Hospital, Puducherry, Puducherry, India; TZ College of Medicine, Jouf University, Sakaka, Saudi Arabia; UA ISGlobal Instituto de Salud Global de Barcelona (Barcelona Institute for Global Health), Universitat de Barcelona, Barcelona, Spain; UB Catalan Institution for Research and Advanced Studies (ICREA), Barcelona, Spain; UC Non-communicable Diseases Research Center, Tehran University of Medical Sciences, Tehran, Iran; UD School of Medicine, Iran University of Medical Sciences, Tehran, Iran; UE Center for Primary Care, Harvard University, Boston, MA, USA; UF School of Public Health, Imperial College London, London, UK; UG Department of Community Medicine, Employees State Insurance-Post Graduate Institute of Medical Sciences and Research, Kolkata, India; UH Department of Medical Education, University of Nevada Las Vegas, Las Vegas, NV, USA; UI Department of Psychiatry, University of Münster, Münster, Germany; UJ Department of Psychiatry, Melbourne Medical School, Melbourne, VIC, Australia; UK Cancer Research Center, Shahid Beheshti University of Medical Sciences, Tehran, Iran; UL Pastor Institute, Tehran University of Medical Sciences, Tehran, Iran; UM Biological Science Division, University of Chicago, Chicago, IL, USA; UN Department Nutrition and Dietetics, Bahir Dar University, Bahir Dar, Ethiopia; UO Epidemiology ang Biostatistics, Haramaya University, Harar, Ethiopia; UP Department of Surgery, Jimma University, Jimma, Ethiopia; UQ The George Institute for Global Health, Imperial College London, London, UK; UR School of Public Health, Dr. D. Y. Patil University, Mumbai, India; US Jazan University, Jazan, Saudi Arabia; UT Department of Human Anatomy and Histology, I.M. Sechenov First Moscow State Medical University, Moscow, Russia; UU Department of Community and Family Medicine, All India Institute of Medical Sciences, Bhubaneswar, India; UV Avicenna Biotech Research, Germantown, MD, USA; UW Department of Regulatory Affairs, Amarex Clinical Research, Germantown, MD, USA; UX Non-Communicable Diseases Research Center (NCDRC), Tehran, Iran; UY Milken Institute of Public Health, George Washington University, Washington, DC, USA; UZ Department of Public Health, Bahir Dar University, Bahir Dar, Ethiopia; VA Department of Public Health, University of South Africa, Pretoria, South Africa; VB Department of Midwifery, Arba Minch University, Arba Minch, Ethiopia; VC Department of Radiology, Mayo Clinic, Rochester, MN, USA; VD Department of Medicine and Surgery, University of Milan Bicocca, Milan, Italy; VE Direzione Sanitaria, Fondazione IRCCS San Gerardo dei Tintori, Monza, Italy; VF Infectious Disease Research Department, King Abdullah International Medical Research Center, Riyadh, Saudi Arabia; VG Department of Veterinary Microbiology, Usmanu Danfodiyo University, Sokoto, Sokoto, Nigeria; VH Department of Biological Sciences, University of Porto, Porto, Portugal; VI Research Unit on Applied Molecular Biosciences (UCIBIO), University of Porto, Porto, Portugal; VJ Department of Biomedical Sciences, University of West Attica, Athens, Greece; VK National AIDS Reference Center of Southern Greece, University of West Attica, Athens, Greece; VL Department of Industrial Engineering, American University of Sharjah, Sharjah, United Arab Emirates; VM Center of Excellence of Cancer Research, University of Sharjah, Sharjah, United Arab Emirates; VN Department of Epidemiology and Psychosocial Research, Ramón de la Fuente Muñiz National Institute of Psychiatry, Mexico City, Mexico; VO Department of Internal Medicine, University of São Paulo, São Paulo, Brazil; VP BRAC James P Grant School of Public Health, BRAC University, Dhaka, Bangladesh; VQ Department of Epidemiology and Health Promotion, New York University, New York, NY, USA; VR Institute of Marketing and Communication Sciences, Corvinus University of Budapest, Budapest, Hungary; VS Dipartimento di Scienze Mediche e Chirurgiche, University of Bologna, Bologna, Italy; VT Department of Nursing, Bahir Dar University, Bahir Dar, Ethiopia; VU School of Public Health, Johns Hopkins University, Baltimore, MD, USA; VV Department of Epidemiology and Biostatistics, University of the Philippines Manila, Manila, Philippines; VW Institute of Dentistry, Barts and the London School of Medicine and Dentistry, London, UK; VX Hubert Department of Global Health, Emory University, Atlanta, GA, USA; VY Department of Global Health, George Washington University, Washington, DC, USA; VZ Faculty of Medicine, Universidade Católica Portuguesa (Catholic University of Portugal), Sintra, Portugal; WA Center for Interdisciplinary Research in Health (CIIS), Universidade Católica Portuguesa (Catholic University of Portugal), Lisbon, Portugal; WB Department of Community and Family Medicine, All India Institute of Medical Sciences, Rishikesh, India; WC Community Health Department, University of South Wales, South Wales, UK; WD Department of Public Health, North Dakota State University, Fargo, ND, USA; WE Institute of Applied Health Research, University of Nottingham, Nottingham, UK; WF Institute of Applied Health Research, University of Birmingham, Birmingham, UK; WG Department of General Practice and Emergency Medicine, Karnali Academy of Health Sciences, Jumla, Nepal; WH Department of Medicine, University of Massachusetts Medical School, Worcester, MA, USA; WI Global Health Neurology Lab, NSW Brain Clot Bank, Sydney, NSW, Australia; WJ Division of Cerebrovascular Medicine and Neurology, National Cerebral and Cardiovascular Center, Suita, Japan; WK Department of General Medicine, Manipal Academy of Higher Education, Mangalore, India; WL Manipal College of Health Professions, Manipal Academy of Higher Education, Udupi, India; WM Department of Medicine, SUNY Upstate Medical University, Syracuse, NY, USA; WN Translational and Clinical Research Institute, Newcastle University, Newcastle upon Tyne, UK; WO School of Sport & Health Sciences, University of Brighton, Brighton, UK; WP Department of Public Health Research, Bengal Rural Welfare Service (BRWS), Kolkata, India; WQ Department of Medical Lab Technology, Chandigarh University, Mohali, India; WR Laboratory of Translational Medicine and Nanotherapeutics, Central University of Punjab, Bathinda, India; WS Department of Botanical and Environmental Sciences, Guru Nanak Dev University, Amritsar, India; WT Department of Pharmaceutical Sciences, Guru Nanak Dev University, Amritsar, India; WU Department of Health Administration, Rutgers University, New Brunswick, NJ, USA; WV Independent Consultant, Addis Ababa, Ethiopia; WW Fondazione Banca Degli Occhi Del Veneto, Carol Davila University of Medicine and Pharmacy, Venice, Italy; WX TAF Uludag Winter Training Center, Turkish Ministry of Defence, Bursa, Turkiye; WY Neurovascular Research Laboratory, Mayo Clinic College of Medicine, Rochester, MN, USA; WZ Medical Ethics Department, Iran University of Medical Sciences, Tehran, Iran; XA Department of Neurology, Institute of Post-Graduate Medical Education and Research and Seth Sukhlal Karnani Memorial Hospital, Kolkata, India; XB Department of Community and Family Medicine, All India Institute of Medical Sciences, Deoghar, India; XC Charles Perkins Centre, University of Sydney, Sydney, NSW, Australia; XD Clinical Research Centre, Sydney Local Health District, Sydney, NSW, Australia; XE Department of Clinical Pharmacy, Universiti Sultan Zainal Abidin, Besut, Malaysia; XF Health Biotechnology Directorate at Bio and Emerging Technology Institute, Addis Ababa University, Addis Ababa, Ethiopia; XG Department of Physical Education and Health, Universidad de la República, Rivera, Uruguay; XH School of Public Health, University of Sydney, Sydney, NSW, Australia; XI Department of Community and Family Medicine, All India Institute of Medical Sciences, Tamil Nadu, India; XJ School of Business Administration, American University of Sharjah, Sharjah, United Arab Emirates; XK Department of Nutrition and Dietetics, Ankara University, Ankara, Turkiye; XL Faculty of Psychology, Education, and Sport, University Lusofona, Porto, Portugal; XM Research Centre for Physical Activity, Health, and Leisure, University of Porto, Porto, Portugal; XN Global Healthcare Management, York University, London, UK; XO Demography and Population Studies, University of the Witwatersrand, Johannesburg, South Africa; XP Ophthalmology Department, Isfahan University of Medical Sciences, Isfahan, Iran; XQ Faculty of Medicine and Pharmaceutical Sciences, University of Douala, Douala, Cameroon; XR Department of Cardiology, Centre Hospitalier Montfermeil (Montfermeil Hospital Center), Montfermeil, France; XS Department of Anesthesia and Critical Care Medicine, Johns Hopkins University, Baltimore, MD, USA; XT General Directorate of Health Information Systems, Ministry of Health, Ankara, Turkiye; XU Department of Public Health, Tehran University of Medical Sciences, Tehran, Iran; XV Internal Medicine Department, Shahid Beheshti University of Medical Sciences, Tehran, Iran; XW Facultad de Salud (Faculty of Health), Universidad Santiago de Cali, Cali, Colombia; XX Department of Medicine, University Ferhat Abbas of Setif, Setif, Algeria; XY Department of Epidemiology and Preventive Medicine, University Hospital Saadna Abdenour, Setif, Algeria; XZ Transport and Road Safety (TARS) Research Centre, University of New South Wales, Sydney, NSW, Australia; YA Vision and Eye Research Institute, Anglia Ruskin University, Cambridge, UK; YB Department of Earth, Environment, and Equity, Howard University, Washington, DC, USA; YC Faculty of Medicine, University of Belgrade, Belgrade, Serbia; YD University Eye Hospital, Belgrade, Serbia; YE Department of Pathology, Apollo Institute of Medical Sciences & Research Chittoor, Chittoor, India; YF Cancer Population Sciences Program, University of Florida Health Cancer Center, Gainesville, FL, USA; YG Department of Psychiatry and Behavioral Health, Ohio State University, Columbus, OH, USA; YH Department of Psychology, Ohio State University, Columbus, OH, USA; YI Division of Clinical Epidemiology and Aging Research, German Cancer Research Center, Heidelberg, Germany; YJ CEVAXIN, Panama City, Panama; YK Institute for Scientific Research and High Technology Services, Panama City, Panama; YL Department of Injury, The George Institute for Global Health, Newtown, NSW, Australia; YM Faculty of Medicine, University of New South Wales, Sydney, NSW, Australia; YN The Malaria Atlas Project, Telethon Kids Institute, Perth, WA, Australia; YO Department of Health Sciences, University of Leicester, Leicester, UK; YP Hospital de Clínicas de Porto Alegre, Federal University of Rio Grande do Sul, Porto Alegre, Brazil; YQ Child & Adolescent Research Program, Programa de Depressão na Infância e na Adolescência, Porto Alegre, Brazil; YR Department of Medical and Surgical Sciences, University of Bologna, Bologna, Italy; YS College of Health Sciences, VinUniversity, Hanoi, Vietnam; YT Research Advancement Consortium in Health, Hanoi, Vietnam; YU Flinders Health and Medical Research Institute, Flinders University, Adelaide, SA, Australia; YV Department of Environmental Health, Bahir Dar University, Bahir Dar, Ethiopia; YW Department of Woman and Child Health and Public Health, Fondazione Policlinico Universitario A. Gemelli IRCCS (Agostino Gemelli University Polyclinic IRCCS), Rome, Italy; YX Global Health Research Institute, Università Cattolica del Sacro Cuore (Catholic University of Sacred Heart), Rome, Italy; YY School of Nursing, Universitas Harapan Bangsa (National Hope University), Banyumas, Indonesia; YZ National Centre for Epidemiology and Population Health, Australian National University, Canberra, ACT, Australia; ZA School of Medicine and Health, Technical University of Munich, Munich, Germany; ZB Department of Radiology, University of Cambridge, Cambridge, UK; ZC Department of Health Care Management, Technical University of Berlin, Berlin, Germany; ZD Department of Basic Biomedical Sciences, University of Sharjah, Sharjah, United Arab Emirates; ZE School of Public Health Sciences, University of Waterloo, Waterloo, ON, Canada; ZF Al Shifa School of Public Health, Al Shifa Trust Eye Hospital, Rawalpindi, Pakistan; ZG JSS Dental College & Hospital, Jagadguru Sri Shivarathreeswara University, Mysore, India; ZH Department of Sociology, University of Macau, Macau, China; ZI The Children's Hospital at Westmead, New South Wales Poisons Information Centre, Sydney, NSW, Australia; ZJ Faculty of Health Sciences Healthcare Management Department, Ankara University, Ankara, Turkiye; ZK Department of Clinical Pharmacy, University of Medicine and Pharmacy of Craiova, Craiova, Romania; ZL Department of Internal and Geriatric Medicine, Hospital Italiano de Buenos Aires (Italian Hospital of Buenos Aires), Buenos Aires, Argentina; ZM Board of Directors, Argentine Society of Medicine, Buenos Aires, Argentina; ZN Center of Innovation, Technology and Education (CITE), Anhembi Morumbi University, Sao Jose dos Campos, Brazil; ZO Center for Nutrition and Health Research, National Institute of Public Health, Cuernavaca, Mexico; ZP Department of Ophthalmology, Beijing Institute of Ophthalmology, Beijing, China; ZQ Department of Surgery, Chinese Academy of Medical Sciences, Beijing, China; ZR Unit of Hygiene and Public Health, Romagna Local Health Authority, Forlì-Cesena, Italy; ZS Interdisciplinary Research Center for Health Science, Sant'Anna School of Advanced Studies, Pisa, Italy; ZT Department of Health Care, Metropolitan Autonomous University, Mexico City, Mexico; ZU Department of Epidemiology, Harvard University, Boston, MA, USA; ZV Institute for Cancer Research, Prevention and Clinical Network, Florence, Italy; ZW Department of Medical Epidemiology and Biostatistics, Karolinska Institute, Stockholm, Sweden; ZX Department of Medicine and Surgery, University of Insubria, Varese, Italy; ZY IMPInstitute for Mental and Physical Health and Clinical Translation (IMPACT), Deakin University, Geelong, VIC, Australia; ZZ School of Health Science, University of Sydney, Sydney, NSW, Australia; AAA Education Center of Australia, Health Science College, Sydney, NSW, Australia; AAB Department of Psychiatry, University of São Paulo, São Paulo, Brazil; AAC Public Health Department, National University of Colombia, Bogota, Colombia; AAD Epidemiology and Public Health Evaluation Group, National University of Colombia, Bogota, Colombia; AAE Division of Country Health Policies and Systems (CPS), World Health Organisation, Trieste, Italy; AAF Mental Health Flagship, World Health Organization (WHO), Copenhagen, Denmark; AAG Department of Pharmacological and Biomolecular Sciences, University of Milan, Milan, Italy; AAH MultiMedica Sesto San Giovanni IRCCS, Sesto San Giovanni, Italy; AAI Department of Public Health and Infectious Diseases, La Sapienza University, Rome, Italy; AAJ Department of Psychiatry, Federal University of Rio Grande do Sul, Porto Alegre, Brazil; AAK Department of Otolaryngology, Head and Neck Surgery, University of Tübingen, Tübingen, Germany; AAL Department of Physiology and Pharmacology, Karolinska Institute, Stockholm, Sweden; AAM Department of Medical, Surgical, and Health Sciences, University of Trieste, Trieste, Italy; AAN Public Health Unit, University Health Agency Giuliano-Isontina (ASUGI), Trieste, Italy; AAO Department of Nutrition, Federal University of Santa Catarina, Florianópolis, Brazil; AAP College of Public Health, Medical, and Veterinary Sciences, James Cook University, Townsville, QLD, Australia; AAQ Department of Public Health, University of Mataram, Mataram, Indonesia; AAR Mary MacKillop Institute for Health Research, Australian Catholic University, Melbourne, VIC, Australia; AAS School of Public Health, University of Hong Kong, Hong Kong, China; AAT Institute of Clinical Physiology, Italian National Council of Research, Pisa, Italy; AAU Infection and Global Health Research, University of St Andrews, St Andrews, UK; AAV Regional Infectious Diseases Unit, NHS National Services Scotland, Edinburgh, UK; AAW Department of Biochemistry, Drexel University, Philadelphia, PA, USA; AAX Department of Biotechnology, Adamas University, Kolkata, India; AAY Institute for Skeletal Aging & Orthopedic Surgery, Hallym University, Chuncheon, South Korea; AAZ School of Population and Public Health, University of British Columbia, Vancouver, BC, Canada; ABA State Disease Investigation Laboratory, Animal Resources Development Department, Agartala, India; ABB Department of Applied Health Sciences, University of Birmingham, Birmingham, UK; ABC Clinical Nutrition Department, Jazan University, Jazan, Saudi Arabia; ABD Department of Psychiatry, University of Kelaniya, Ragama, Sri Lanka; ABE University Psychiatry Unit, Colombo North Teaching Hospital, Ragama, Sri Lanka; ABF Department of Anesthesiology and Perioperative Medicine, University of Rochester, Rochester, NY, USA; ABG College of Medicine, National Taiwan University, Taipei, Taiwan; ABH Department of Nursing, National Taiwan University Hospital, Taipei, Taiwan; ABI Department of Epidemiology and Biostatistics, Semey Medical University (SMU), Semey, Kazakhstan; ABJ Department of Community Medicine, Datta Meghe Institute of Medical Sciences, Sawangi, India; ABK Department of Endocrinology, University of Manchester, Manchester, UK; ABL Department of Endocrinology, Christie Hospital NHS Foundation Trust, Manchester, UK; ABM Department of Epidemiology, University of Texas, Houston, TX, USA; ABN Department of Public Health, Indian Institute of Public Health, Hyderabad, India; ABO Department of Oral Medicine and Radiology, King George's Medical University, Lucknow, India; ABP EPI, Oromia Health Bureau, Addis Ababa, Ethiopia; ABQ Peking Union Medical College Hospital, Chinese Academy of Medical Sciences, Beijing, China; ABR Hospital of Stomatology, Sun Yat-sen University, Guangzhou, China; ABS Faculty of Humanities and Health Sciences, Curtin University, Miri, Malaysia; ABT Clinical Research Center, Zhujiang Hospital of Southern Medical University, Guangzhou, China; ABU Science and Technology Department, Northern Jiangsu People's Hospital, Yangzhou, China; ABV Department of Urology, Academy of Medical Science, Kunming, China; ABW School of Dentistry, University of Michigan, Ann Arbor, MI, USA; ABX Faculty of Epidemiology and Population Health, London School of Hygiene & Tropical Medicine, London, UK; ABY Department of Computer, Electrical and Mathematical Sciences and Engineering, King Abdullah University of Science and Technology, Thuwal, Saudi Arabia; ABZ Department of Cardiology, Shanghai Jiao Tong University School of Medicine, Shanghai, China; ACA School of Chinese Medicine, Hong Kong Baptist University, Hong Kong, China; ACB Yong Loo Lin School of Medicine, National University of Singapore, Singapore, Singapore; ACC Division of Cardiovascular Medicine, Harvard University, Boston, MA, USA; ACD Department of Laboratory Medicine, Taichung Tzu-Chi Hospital Buddhist Tzu-Chi Medical Foundation, Tanzih, Taiwan; ACE Department of Medical Laboratory Science and Biotechnology, Central Taiwan University of Science and Technology, Taiwan; ACF Department of Public Health and Health Policy, Hiroshima University, Hiroshima, Japan; ACG Division of Infectious Diseases, Virginia Commonwealth University, Richmond, VA, USA; ACH Department of Public Health, Administration, and Social Sciences, Cayetano Heredia University, Lima, Peru; ACI Department of Medical and Surgical Sciences and Advanced Technologies “GF Ingrassia” University of Catania, Catania, Italy; ACJ Department of Clinical Oncology, Queen Elizabeth Hospital, Hong Kong, China; ACK Department of Medicine, National University of Singapore, Singapore, Singapore; ACL Department of Medicine, University of Hong Kong, Hong Kong, China; ACM School of Public Health, Curtin University, Perth, WA, Australia; ACN Department of Epidemiology and Preventative Medicine, Monash University, Melbourne, VIC, Australia; ACO Department of Public Health, Asian University for Women, Chittagong, Bangladesh; ACP Bispebjerg Hospital, University of Copenhagen, Copenhagen, Denmark; ACQ Department of Health Science and Technology, Aalborg University, Aalborg, Denmark; ACR Department of Physiotherapy, University College of Northern Denmark, Aalborg, Denmark; ACS The Interdisciplinary Research Group on Biomedicine and Health, VNU International School, Hanoi, Vietnam; ACT Faculty of Applied Sciences, VNU International School (VNUIS), Hanoi, Vietnam; ACU Department of Paediatric Surgery, Federal Medical Centre, Umuahia, Nigeria; ACV Department of Urology, The University of Queensland, Brisbane, QLD, Australia; ACW Department of AndroUrology, AndroUrology Centre, Brisbane, QLD, Australia; ACX Department of Pediatrics, University of Washington, Seattle, WA, USA; ACY Department of Health Informatics, University College London, London, UK; ACZ Health Data Research UK, London, UK; ADA Department of Health Behavior, Texas A&M University, College Station, TX, USA; ADB School of Nursing and Midwifery, University of Technology Sydney, Sydney, NSW, Australia; ADC Adelaide Medical School, University of Adelaide, Adelaide, SA, Australia; ADD School of Pharmacy and Medical Sciences, University of South Australia, Adelaide, SA, Australia; ADE Health Effects Institute, Boston, MA, USA; ADF Department of Biostatistics, Johns Hopkins University, Baltimore, MD, USA; ADG Nova Medical School, Nova University of Lisbon, Lisbon, Portugal; ADH Department of Medicine, University of Calgary, Calgary, AB, Canada; ADI Department of Cardiovascular Sciences, Katholieke Universiteit Leuven, Leuven, Belgium; ADJ General Administration, University of Milan Bicocca, Monza, Italy; ADK National School of Public Health, Nova University of Lisbon, Lisbon, Portugal; ADL Egas Moniz Center for Interdisciplinary Research, Egas Moniz School of Health and Science, Almada, Portugal; ADM Department of Respiratory Medicine and Allergology, Nicolae Testemitanu State University of Medicine and Pharmacy, Chisinau, Moldova; ADN School of Psychology, University of Southampton, Southampton, UK; ADO Department of Child and Adolescent Psychiatry, New York University, New York, NY, USA; ADP Research Center on Public Health (CESP), University of Milan Bicocca, Monza, Italy; ADQ Laboratory of Public Health, Instituto Auxologico Italiano IRCCS (Italian Auxological Institute), Milan, Italy; ADR Department of Health Sciences, University of Florence, Florence, Italy; ADS Department of Family Medicine and Public Health, University of California San Diego, La Jolla, CA, USA; ADT Life and Health Sciences Research Institute (ICVS), University of Minho, Braga, Portugal; ADU Institute for Research and Innovation in Health (i3S), University of Porto, Porto, Portugal; ADV School of Medicine, The Chinese University of Hong Kong, Shenzhen, Shenzhen, China; ADW Research Department, Cleveland Clinic Abu Dhabi, Abu Dhabi, United Arab Emirates; ADX Faculty of Medicine, University of Aleppo, Aleppo, Syria; ADY Department of Anesthesia, Critical Care and Pain Medicine, Shahid Beheshti University of Medical Sciences, Tehran, Iran; ADZ Research Center for Child Psychiatry, University of Turku, Turku, Finland; AEA Department of Health Statistics and Informatics, Northern Territory Government, Darwin, WA, Australia; AEB Department of Community Medicine, Ahmadu Bello University, Zaria, Nigeria; AEC School of Pharmacy and Charles Perkins Centre, University of Sydney, Sydney, NSW, Australia; AED Department of Public Health and Primary Care, University of Cambridge, Cambridge, UK; AEE Cardiovascular Metabolic Translational Research Program, National University of Singapore, Singapore, Singapore; AEF Institute for Health Sciences, Mid Sweden University, Sundsvall, Sweden; AEG Nutrition Department, Harvard University, Boston, MA, USA; AEH IRCCS Istituto Ortopedico Galeazzi (Galeazzi Orthopedic Institute IRCCS), University of Milan, Milan, Italy; AEI Department of Dermatology, Case Western Reserve University, Cleveland, OH, USA; AEJ The University of Adelaide, University of South Australia, Adelaide, SA, Australia; AEK Department of Community Medicine, Employees' State Insurance Model Hospital, Chennai, India; AEL Department of Brain Sciences, Imperial College London, London, UK; AEM Department of Internal Medicine, Texas Tech University, Lubbock, TX, USA; AEN Department of Public Health, Haramaya University, Harar, Ethiopia; AEO The University of Jordan, Amman, Jordan; AEP Heart Failure Research Center, Isfahan University of Medical Sciences, Isfahan, Iran; AEQ Department of Environmental Health, Arak University of Medical Sciences, Arak, Iran; AER 2nd University Ophthalmology Department, Aristotle University of Thessaloniki, Thessaloniki, Greece; AES Ophthalmology Department, University of Thessaly, Greece; AET Department of Global Health and Infection, Brighton and Sussex Medical School, Brighton, UK; AEU School of Public Health, Addis Ababa University, Addis Ababa, Ethiopia; AEV Department of Population and Development, Latin American Faculty of Social Sciences Mexico, Mexico City, Mexico; AEW Atchabarov Scientific-Research Institute of Fundamental and Applied Medicine, Kazakh National Medical University, Almaty, Kazakhstan; AEX Population Health Research Center, Kazakh National Medical University, Almaty, Kazakhstan; AEY biomedical and nutritional sciences, University of Massachusetts Lowell, lowell, MA, USA; AEZ Department of Public Health, National University of Colombia, Bogota, Colombia; AFA School of Health, Medical and Applied Sciences, CQUniversity, Brisbane, QLD, Australia; AFB Memorial Sloan Kettering Cancer Center, Memorial Sloan Kettering Cancer Center, New York, NY, USA; AFC Department of Rehabilitation Sciences, Qatar University, Doha, Qatar; AFD Department of Pediatrics, Brookdale University Hospital Medical Center, Brooklyn, NY, USA; AFE National Drug and Alcohol Research Centre, University of New South Wales, Sydney, NSW, Australia; AFF Department of Biostatistics and Epidemiology, Kerman University of Medical Sciences, Kerman, Iran; AFG Department of Medical Biochemistry, University of Gondar, Gondar, Ethiopia; AFH Department of Neurosurgery, Tehran University of Medical Sciences, Tehran, Iran; AFI Ophthalmology Department, University of Tennessee, Memphis, TN, USA; AFJ Department of Physiology, Bahir Dar University, Bahir Dar, Ethiopia; AFK Department of Neurosurgery, University of Edinburgh, Edinburgh, UK; AFL Department of Neurosurgery, National Health Service (NHS) Scotland, Edinburgh, UK; AFM Dirección de Nutrición, Salvador Zubiran National Institute of Medical Sciences and Nutrition, Mexico City, Mexico; AFN Research and Training Directorate, Eka Kotebe General Hospital, Addis Ababa, Ethiopia; AFO Department of Biological Sciences, University of Manouba, Manouba, Tunisia; AFP Department of Social Sciences, University of Jendouba, El Kef, Tunisia; AFQ Department of Nutrition and Dietetics, Bahir Dar University, Bahir Dar, Ethiopia; AFR Department of Forensic Medicine, University of Sarajevo, Sarajevo, Bosnia and Herzegovina; AFS Department of Statistics, Computer Science, Applications “G. Parenti” (DiSIA), University of Florence, Florence, Italy; AFT Chettinad Hospital & Research Institute, Chettinad Academy of Research and Education, Chennai, India; AFU Department of Cardiology, Icahn School of Medicine at Mount Sinai, New York, NY, USA; AFV Department of Pharmacy, United International University, Dhaka, Bangladesh; AFW Pharmacology Division, Center for Life Sciences Research Bangladesh, Dhaka, Bangladesh; AFX Sheffield Teaching Hospitals NHS Foundation Trust, Sheffield, UK; AFY Division of Pathology, ICAR-Indian Veterinary Research Institute, Bareilly, India; AFZ Neurology Department Institute of Human Behavior and Allied Sciences, University of Delhi, New Delhi, India; AGA Research and Development Cell, Dr. D. Y. Patil Vidyapeeth, Pune (Deemed to be University), Pune, India; AGB Department of Zoology, University of Delhi, Delhi, India; AGC Research Department, Planetary Health Research Centre, Kathmandu, Nepal; AGD Institute of Occupational, Social and Environmental Medicine, Goethe University, Frankfurt am Main, Germany; AGE Research Department, Nepal Health Research Council, Kathmandu, Nepal; AGF Department of Pharmacy Practice, National Institute of Pharmaceutical Education and Research Hajipur, Hajipur, India; AGG Population Interventions Unit, University of Melbourne, Melbourne, VIC, Australia; AGH Department of Life Science and Public Health, Università Cattolica del Sacro Cuore (Catholic University of the Sacred Heart), Rome, Italy; AGI Escola Superior de Saúde (Higher School of Health), Instituto Politécnico do Porto (Polytechnic Institute of Porto), Porto, Portugal; AGJ Public Health Intelligence Unit, National Institute of Public Health, Cuernavaca, Mexico; AGK Department of Gastroenterology, Pontifical Catholic University of Chile, Santiago, Chile; AGL University of California San Diego, La Jolla, CA, USA; AGM Department of Anesthesiology, Rutgers University, Newark, NJ, USA; AGN Department of Otolaryngology - Head and Neck Surgery, Medical University of South Carolina, Charleston, SC, USA; AGO Faculty of Management, Bucharest University of Economic Studies, Bucharest, Romania; AGP Joe C. Wen School of Population & Public Health, University of California Irvine, Irvine, CA, USA; AGQ Department of Psychiatry, Bahir Dar University, Bahir Dar, Ethiopia; AGR College of Health Sciences, VinUniversity, Ha Noi, Vietnam; AGS Institute of Health Economics and Technology (iHEAT), Hanoi, Vietnam; AGT Department of Medicine, Can Tho University of Medicine and Pharmacy, Can Tho, Vietnam; AGU Department of Social Medicine and Health Care Organisation, Medical University of Varna, Varna, Bulgaria; AGV Oxford Centre for Global Health Research, University of Oxford, Oxford, UK; AGW Mahidol Oxford Tropical Medicine Research Unit, Mahidol University, Bangkok, Thailand; AGX Nuclear Medicine Department, ASST Spedali Civili di Brescia and Università degli Studi di Brescia, Brescia, Italy; AGY Cardio-Thoraco-Vascular Department, Azienda Sanitaria Universitaria Giuliano Isontina, Trieste, Italy; AGZ Department of Medical Laboratory Sciences, Iran University of Medical Sciences, Tehran, Iran; AHA Independent Consultant, Bridgewater, NJ, USA; AHB Heart Institute, University of São Paulo, São Paulo, Brazil; AHC Clínica Pró Coração, São Paulo, Brazil; AHD Department of Epidemiology, University of Pittsburgh, Pittsburgh, PA, USA; AHE Department of Psychiatry, University of Pittsburgh Medical Center, Pittsburgh, PA, USA; AHF Department of Medicine, Bangalore Medical College and Research Institute, Bangalore, India; AHG Faculty of Health, Medicine and Life Sciences (FHML), Maastricht University, Maastricht, Netherlands; AHH Department of Pathology, China Medical University, Liaoning, China; AHI Office of Institutional Analysis, University of Windsor, Windsor, ON, Canada; AHJ School of Sociology, University College Dublin, Dublin, Ireland; AHK Postgraduate Program in Health Sciences, Federal University of Rio Grande do Sul, Rio Grande, Brazil; AHL School of Population Health, Curtin University, Perth, WA, Australia; AHM School of Medicine, Federal University of Bahia, Salvador, Brazil; AHN Department of Internal Medicine, Escola Bahiana de Medicina e Saúde Pública (Bahiana School of Medicine and Public Health), Salvador, Brazil; AHO Faculty of Science and Humanities, SRM Institute of Science and Technology, Kattankulathur, India; AHP Department of Infection and Tropical Medicine, University of Sheffield, Sheffield, UK; AHQ AlMaarefa University, Riyadh, Saudi Arabia; AHR Department of Conservative Dentistry with Endodontics, Medical University of Silesia, Katowice, Poland; AHS Department of Microbiology and Immunology, Northwestern University, Chicago, IL, USA; AHT Department of Biological and Chemical Sciences, Michael and Cecilia Ibru University, Delta State, Nigeria; AHU Department of Psychiatry, Dalhousie University, Halifax, NS, Canada; AHV Department of Psychiatry, University of Alberta, Edmonton, AB, Canada; AHW Histology Department, Zagazig University, Zagazig, Egypt; AHX Fred Hutchinson Cancer Research Center, Seattle, WA, USA; AHY Environmental and Occupational Health Research Center, Shahroud University of Medical Sciences, Shahroud, Iran; AHZ Higher School of Technology, Sultan Moulay Slimane University, Beni Mellal, Morocco; AIA Department of Zoology, University of Uyo, Ikot Akpaden, Nigeria; AIB School of Nursing and Midwifery, La Trobe University, Bundoora, VIC, Australia; AIC Advanced Nursing Department, Universitas Airlangga (Airlangga University), Surabaya, Indonesia; AID La Trobe University, Melbourne, VIC, Australia; AIE Gastrointestinal and Liver Disease Research Center, Guilan University of Medical Sciences, Rasht, Iran; AIF Iran University of Medical Sciences, Iran University of Medical Sciences, Tehran, Iran; AIG Semnan University of Medical Sciences and Health, Samara University, Semnan, Iran; AIH Isenberg School of Management, University of Massachusetts Amherst, Amherst, MA, USA; AII Massachusetts General Hospital, Boston, MA, USA; AIJ Centre for Global Health Inequalities Research (CHAIN), Norwegian University of Science and Technology, Trondheim, Norway; AIK Private Orthodontist, Ahvaz, Iran; AIL Faculty of Science and Health, University of Portsmouth, Hampshire, UK; AIM Almoosa College of Health Sciences, Al Ahsa, Saudi Arabia; AIN Clinical Pathology Department, Mansoura University, Mansoura, Egypt; AIO Department of Basic Medical Sciences, University of Sharjah, Sharjah, United Arab Emirates; AIP Department of Anatomy and Embryology, Mansoura University, Mansoura, Egypt; AIQ Department of Radiology, Tehran University of Medical Sciences, Tehran, Iran; AIR Deanship of Preparatory Year and Supporting Studies, Imam Abdulrahman Bin Faisal University, Dammam, Saudi Arabia; AIS College of Medicine, RAK Medical and Health Sciences University, Ras Al Khaimah, United Arab Emirates; AIT Faculty of Medicine, Ain Shams University, Cairo, Egypt; AIU Sharjah Institute for Medical Research, University of Sharjah, Sharjah, United Arab Emirates; AIV Department of Internal Medicine, Ain Shams University, Cairo, Egypt; AIW Section of Adult Hematology, King Saud University, Riyadh, Saudi Arabia; AIX College of Medicine, Korea University, Seoul, South Korea; AIY Houston Methodist Hospital, Houston, TX, USA; AIZ National Institute of Public Health Research, Ministry of Health, Nouakchott, Mauritania; AJA Department of Toxicology, Mansoura University, Mansoura, Egypt; AJB Faculty of Applied Health Science, Horus University, New Damietta, Egypt; AJC Pediatric Dentistry and Dental Public Health Department, Alexandria University, Alexandria, Egypt; AJD MD Anderson Cancer Center Department of Plastic Surgery, University of Texas, Houston, TX, USA; AJE Basic Medical Sciences Department, University of Sharjah, Sharjah, United Arab Emirates; AJF Research Institute of Medical & Health Sciences, University of Sharjah, Sharjah, United Arab Emirates; AJG School of Pharmacy and Pharmaceutical Sciences, Ulster University, Coleraine, UK; AJH Department of Neuropsychiatry, Ain Shams University, Cairo, Egypt; AJI Executive Committee, International Association for Women Mental Health, Potomac, MD, USA; AJJ Department of Infectious Diseases and Public Health, City University of Hong Kong, Hong Kong, China; AJK Department of Animal Medicine, Zagazig University, Zagazig, Egypt; AJL Faculty of Veterinary Medicine, Damanhour University, Damanhur, Egypt; AJM Department of Midwifery, Woldia University, Addis Ababa, Ethiopia; AJN Department of Public Health and Tropical Medicine, James Cook University, Townsville, QLD, Australia; AJO Department of Adult Health Nursing, Bahir Dar University, Bahir Dar, Ethiopia; AJP Health Research and Technology Transfer Directorate, South Ethiopia Region Public Health Institute, Jinka, Ethiopia; AJQ Department of Public Health, Hawassa University, Hawassa, Ethiopia; AJR Queensland Centre for Mental Health Research, Brisbane, QLD, Australia; AJS Department of Paediatrics, University of Lagos, Lagos, Nigeria; AJT Department of Paediatrics, Lagos University Teaching Hospital, Lagos, Nigeria; AJU Goba College of Medicine and Health Sciences, Madda Walabu University, Robe, Ethiopia; AJV Department of Health Promotion and Health Behavior, University of Gondar, Gondar, Ethiopia; AJW Wassa Amenfi East Municipal Health Directorate, Ghana Health Service, Wassa Akropong, Ghana; AJX Multiple Sclerosis Research Center, Tehran University of Medical Sciences, Tehran, Iran; AJY Department of Bacteriology and Virology, Semnan University of Medical Sciences, Semnan, Iran; AJZ Cancer Research Center, Semnan University of Medical Scinces, Semnan, Iran; AKA Health Research and Innovation Science Centre, Klaipeda University, Klaipeda, Lithuania; AKB Dipartimento di Scienze Biomediche e Neuromotorie (DIBINEM), University of Bologna, Bologna, Italy; AKC Department of Environmental and Occupational Health, University of the Philippines Manila, Manila, Philippines; AKD Department of Public Health, North South University, Dhaka, Bangladesh; AKE Drexel Dornsife School of Public Health, Drexel University, Philadelphia, PA, USA; AKF Department of Anesthesia, Cincinnati Children's Hospital Medical Center, Cincinnati, OH, USA; AKG Department of Biotechnology, University of the Western Cape, Cape Town, South Africa; AKH Department of Electrical and Computer Engineering, Tarbiat Modares University, Tehran, Iran; AKI Research Centre for Healthcare and Community, Coventry University, Coventry, UK; AKJ Department of Oral Biology, Riphah International University, Islamabad, Pakistan; AKK Director of the Scientific and Technological Park, Kazakh National Medical University, Almaty, Kazakhstan; AKL Department of Medicine, Korea University, Seoul, South Korea; AKM Department of Food Hygiene and Quality Control, University of Tehran, Tehran, Iran; AKN Department of Public Health Sciences, Clemson University, Clemson, SC, USA; AKO Pediatric Infectious Disease Research Center, Tehran University of Medical Sciences, Tehran, Iran; AKP Department of Health Policy and Administration, University of the Philippines Manila, Manila, Philippines; AKQ College of Medicine, AlMaarefa University, Riyadh, Saudi Arabia; AKR Saveetha Medical College and Hospital, Saveetha Institute of Medical and Technical Sciences (SIMATS), Chennai, India; AKS Division of Statistics, Bangladesh Bank, Sylhet, Bangladesh; AKT Environmental Statistics Unit, National Institute of Statistics, Lisbon, Portugal; AKU Ecological Economics and Environmental Management, NOVA University of Lisbon, Lisbon, Portugal; AKV Department of Clinical Nutrition and Dietetics, Applied Science Private University, Amman, Jordan; AKW Department of Psychology, Federal University of Sergipe, São Cristóvão, Brazil; AKX Department of Radiography and Imaging Technology, Green International University, Lahore, Pakistan; AKY Department of Family Medicine, Luton & Dunstable University Hospital, Luton, UK; AKZ Endocrinology and Metabolism Research Institute, Non-Communicable Diseases Research Center (NCDRC), Tehran, Iran; ALA Dentistry Research Institute, Tehran University of Medical Sciences, Tehran, Iran; ALB Obesity and Eating Habits Research Center, Tehran University of Medical Sciences, Tehran, Iran; ALC Department of Clinical Psychology, University of Dhaka, Dhaka, Bangladesh; ALD Community-based Inclusive Mental Health Department, Centre for Disability in Development (CDD), Dhaka, Bangladesh; ALE Department of Veterinary Tropical Diseases, University of Pretoria, Pretoria, South Africa; ALF Animal Production and Health Division (EMPRES), Food and Agriculture Organization of the United Nations, Rome, Italy; ALG Charité University Berlin, Charité Medical University Berlin (Charité Universitätsmedizin Berlin), Berlin, Germany; ALH Department of Chemistry and Biochemistry, University System of Georgia, Statesboro, GA, USA; ALI Pharmacy Department, University College Hospital, Ibadan, Ibadan, Nigeria; ALJ School of Engineering, Edith Cowan University, Joondalup, WA, Australia; ALK Department of Electrical and Computer Engineering (ECE), Tarbiat Modares University, Tehran, Iran; ALL University Institute of Radiological Sciences and Medical Imaging Technology, The University of Lahore, Lahore, Pakistan; ALM Department of Environmental Health Engineering, Ardabil University of Medical Science, Ardabil, Iran; ALN Department of Environmental Health Engineering, Tehran University of Medical Sciences, Tehran, Iran; ALO Department of Cardiovascular Surgery, Huazhong University of Science and Technology, Wuhan, China; ALP National Institute for Stroke and Applied Neurosciences, Auckland University of Technology, Auckland, New Zealand; ALQ Research Center of Neurology, Moscow, Russia; ALR Department of Social Medicine and Epidemiology, Guilan University of Medical Sciences, Rasht, Iran; ALS Department of Pharmacy, Wollega University, Nekemte, Ethiopia; ALT Department of Epidemiology and Biostatistics, Bahir Dar University, Bahir Dar, Ethiopia; ALU Department of Urology, The First Affiliated Hospital of Zhejiang Chinese Medical University, Hangzhou, China; ALV Division of Surgery & Interventional Science, University College London, London, UK; ALW School of Acupuncture-Tuina, Shandong University of Traditional Chinese Medicine, Jinan, China; ALX National Institute of Environmental Health, Chinese Center for Disease Control and Prevention, Beijing, China; ALY Department of Biomedical Engineering, University of Houston, Houston, TX, USA; ALZ Division of Neurology, University of Toronto, Toronto, ON, Canada; AMA Department of Neurobiology, Care Sciences, and Society, Karolinska Institute, Stockholm, Sweden; AMB Cardiovascular Health and Imaging Laboratory, Centro Nacional de Investigaciones Cardiovasculares (CNIC) (National Centre for Cardiovascular Disease Research), Madrid, Spain; AMC Department of Cardiology, Hospital Clinico San Carlos, IdISSC, Madrid, Spain; AMD Center for Public Health Research, University of Milan Bicocca, Monza, Italy; AME Laboratory of Public Health, IRCCS Istituto Auxologico Italiano, Milan, Italy; AMF Ophthalmology, Hospital Center of Porto, Porto, Portugal; AMG Department of Social Sciences, University of Nicosia, Nicosia, Cyprus; AMH Department of Biological Sciences, University of Southern California, Los Angeles, CA, USA; AMI School of Medicine, Federal University of Rio Grande do Sul, Porto Alegre, Brazil; AMJ Medical School, Universidad de Navarra, Pamplona, Spain; AMK Department of Public Health, University of Naples “Federico II” Naples, Italy; AML Institute of Public Health, Charité Medical University Berlin (Charité Universitätsmedizin Berlin), Berlin, Germany; AMM Department of Pharmacology, Gadjah Mada University, Yogyakarta, Indonesia; AMN Medical and Surgical Sciences Department, University of Bologna, Bologna, Italy; AMO Department of Child Dental Health, Obafemi Awolowo University, Ile-Ife, Nigeria; AMP Clinical Science Department, Nigerian Institute of Medical Research, Lagos, Nigeria; AMQ Department of Cardiac, Thoracic, Vascular Sciences and Public Health, University of Padova, Padova, Italy; AMR Division of Pediatric Hematology-Oncology, St. Jude Children's Research Hospital, Seattle, WA, USA; AMS Department of Neurology, Public Health and Disability, Fondazione IRCCS Istituto Neurologico Carlo Besta, Milan, Italy; AMT Department of Medicine and Surgery, University of Milan Bicocca, Monza, Italy; AMU Department of Disease Burden, Norwegian Institute of Public Health, Bergen, Norway; AMV Emilia-Romagna Region - Innovation in Healthcare and Social Services Department, Bologna, Italy; AMW Department of Neuroscience, Multiple Sclerosis Research Center, Ravenna, Italy; AMX Department of Biotechnological and Applied Clinical Sciences, University of L'Aquila, L'Aquila, Italy; AMY Department of Radiology, University of Southern California, Los Angeles, CA, USA; AMZ Department of Medicine, Iran University of Medical Sciences, Tehran, Iran; ANA Clinical Epidemiology Division (KEP), Karolinska Institute, Stockholm, Sweden; ANB Department of Microbiology and Parasitology, University of Buea, Buea, Cameroon; ANC College of Medicine, Dentistry and Public Health, James Cook University, Townsville, QLD, Australia; AND MEDCIDS, Faculty of Medicine of the University of Porto, University of Porto, Porto, Portugal; ANE Center for Health Technology and Services Research (CINTESIS), Porto, Portugal; ANF Department of Biostatistics, Xuzhou Medical University, Xuzhou, China; ANG Department of Pathology, Federal University of Espirito Santo, Vitória, Brazil; ANH Health Services Management Training Centre, Semmelweis University, Budapest, Hungary; ANI Department of Applied Social Sciences, Sapientia Hungarian University of Transylvania, Târgu-Mureş, Romania; ANJ School of Public Health, University of Ghana, Legon, Accra, Ghana; ANK Department of Public Health, University of Szeged, Szeged, Hungary; ANL Department of Food Technology, Salahaddin University-Erbil, Erbil, Iraq; ANM Department of Nutrition and Dietetics, Cihan University-Erbil, Erbil, Iraq; ANN Department of Medical Epidemiology, Mario Negri Institute for Pharmacological Research, Milan, Italy; ANO Department of Prosthodontics, Saveetha University, Chennai, India; ANP Department of Pharmacology, Manipal Academy of Higher Education, Manipal, India; ANQ Department of Endocrinology, Sheri Kashmir Institute of Medical Sciences, Srinagar, India; ANR Department of Endocrinology and Metabolism, All India Institute of Medical Sciences, Delhi, India; ANS Clinical Nuclear Medicine Center, Shanghai Tenth People's Hospital, Shanghai, China; ANT Department of Nuclear Medicine, Tongji University Tenth People's Hospital, Shanghai, China; ANU Key Lab of Environment and Health, Xuzhou Medical University, Xuzhou, China; ANV Department of Veterinary Public Health and Preventive Medicine, Usmanu Danfodiyo University, Sokoto, Sokoto, Nigeria; ANW Department of Public Health, SIMAD University Mogadishu. Somalia, Mogadishu, Somalia; ANX Centre for Innovation in Mental Health, University of Southampton, Southampton, UK; ANY School of Medicine, Orebro University, Orebro, Sweden; ANZ Department of Medicine, University of Valladolid, Valladolid, Spain; AOA Department of Neurology, Hospital Universitario Rio Hortega, Valladolid, Spain; AOB Infectious Diseases Unit, University of Verona, Verona, Italy; AOC Department of Ophthalmology, University of Basel, Basel, Switzerland; AOD Professional Services Division, Texas State Board of Pharmacy, Austin, TX, USA; AOE Department of Pharmacology, IES Institute of Pharmacy, Bhopal, India; AOF Independent Consultant, Rome, Italy; AOG College of Health Sciences, Addis Ababa University, Addis Ababa, Ethiopia; AOH Department of Midwifery, Adigrat University, Adigrat, Ethiopia; AOI School of Public Health, Adigrat University, Adigrat, Ethiopia; AOJ Department of Neurosciences, Neurology and Stroke Unit, ASST Grande Ospedale Metropolitano Niguarda, Milan, Italy; AOK Institute of Public Health, Jagiellonian University Medical College, Krakow, Poland; AOL School of Medicine and Population Health, University of Sheffield, Sheffield, UK; AOM School of Engineering and Applied Science, George Washington University, Washington, DC, USA; AON Department of Public Health, Menelik II Medical and Health Science College, Addis Ababa, Ethiopia; AOO Mayo Clinic, Rochester, MN, USA; AOP Infectious Disease Research Center, Kermanshah University of Medical Sciences, Kermanshah, Iran; AOQ Pediatric Department, Kermanshah University of Medical Sciences, Kermanshah, Iran; AOR School of Nursing and Midwifery, Shahid Beheshti University of Medical Sciences, Tehran, Iran; AOS Department of Cardiology, Iran University of Medical Sciences, Tehran, Iran; AOT Faculty of Medicine, Shahid Beheshti University of Medical Sciences, Tehran, Iran; AOU Social Determinants of Health Research Center, Shahid Beheshti University of Medical Sciences, Tehran, Iran; AOV Research Committee of Qom University of Medical Sciences, Qom University of Medical Sciences, Qom, Iran; AOW Department of Medical Biochemical Analysis, Cihan University-Erbil, Erbil, Iraq; AOX Neurology Department, Tehran University of Medical Sciences, Tehran, Iran; AOY Department of Medical Genetics, Shahid Beheshti University of Medical Sciences, Tehran, Iran; AOZ Center for Comprehensive Genetic Services, Shahid Beheshti University of Medical Sciences, Tehran, Iran; APA Department of Global Health Sciences, University of California San Francisco, San Francisco, CA, USA; APB School of Medicine, Mazandaran University of Medical Sciences, Mazandaran, Iran; APC Tropical Health Department, Alexandria University, Alexandria, Egypt; APD Family and Community Medicine Department, King Khalid University, Abha, Saudi Arabia; APE Research Group for Childhood Cancer, Danish Cancer Research Institute, Copenhagen, Denmark; APF Department of Dermatology, Mazandaran University of Medical Sciences, Sari, Iran; APG Obstetrics and Gynecology Department, Shahid Beheshti University of Medical Sciences, Tehran, Iran; APH Department of Epidemiology and Prevention, IRCCS Neuromed, Pozzilli, Italy; API GBD Collaborating Unit, Norwegian Institute of Public Health, Bergen, Norway; APJ Department of Medicine, Stanford University, Stanford, CA, USA; APK Country Office, World Health Organization (WHO), Astana, Kazakhstan; APL Discipline of Public Health Medicine, University of KwaZulu-Natal, Durban, South Africa; APM Department of Zoology, KKS Women's College, Balasore, India; APN Department of Nursing, Aksum University, Aksum, Ethiopia; APO Department of Anesthesiology and Critical Care Medicine, Ospedale SS Annunziata Savigliano, Savigliano, Italy; APP Third Department of Neurology, Research Center of Neurology, Moscow, Russia; APQ Department of Cardiac Surgery, Cleveland Clinic Abu Dhabi, Abu Dhabi, United Arab Emirates; APR Lerner College of Medicine, Case Western Reserve University, Cleveland, OH, USA; APS Department of Biostatistics, Tarbiat Modares University, Tehran, Iran; APT Quantitative Department, Non-Communicable Diseases Research Center (NCDRC), Tehran, Iran; APU Department of Health Systems and Policy Research, Indian Institute of Public Health, Gandhinagar, India; APV Department of Genetics, Sana Institute of Higher Education, Sari, Iran; APW Universal Scientific Education and Research Network (USERN), Kermanshah University of Medical Sciences, Kermanshah, Iran; APX Department of Life Sciences, Health and Healthcare Professions, Link Campus University, Rome, Italy; APY Health Services Research, Evaluation and Policy Unit, AUSL della Romagna, Ravenna, Italy; APZ Research Institute for Endocrine Sciences, Tehran, Iran; AQA Senior Department of Tuberculosis, The Eighth Medical Center of PLA General Hospital, Beijing, China; AQB College of Health Sciences, University of Sharjah, Sharjah, United Arab Emirates; AQC Midwifery Department, Debre Tabor University, Debre Tabor, Ethiopia; AQD Department of Epidemiology, Universidade de São Paulo (University of São Paulo), São Paulo, Brazil; AQE Department of Dermatology, Case Western Reserve University, Libertyville, IL, USA; AQF Nuffield Department of Orthopaedics, Rheumatology, and Musculoskeletal Sciences, University of Oxford, Oxford, UK; AQG Liverpool Orthopaedic and Trauma Service, University of Liverpool, Liverpool, UK; AQH Department of Public Health and Preventive Medicine, Charles University, Prague, Czech Republic; AQI National Institutes of Health, Bethesda, MD, USA; AQJ Department of Health Informatics, Bahir Dar University, Bahir Dar, Ethiopia; AQK Department of Epidemiology and Biostatistics, Anhui Medical University, Hefei, China; AQL Health Direction, Local Health Authority of Ferrara, Ferrara, Italy; AQM Department of Clinical Science, University Of Sulaimani, Sulaimani, Iraq; AQN Harrington Heart and Vascular Institute, Case Western Reserve University, Cleveland, OH, USA; AQO Division of Cardiovascular Medicine, Ohio State University, Columbus, OH, USA; AQP Department of Community Medicine, University of Peradeniya, Kandy, Sri Lanka; AQQ Department of Geriatric Neurology, Shaanxi Provincial People's Hospital, Xi'an, China; AQR Big Data Institute, University of Oxford, Oxford, UK; AQS Division of Epidemiology, Vanderbilt University Medical Center, Nashville, TN, USA; AQT Nanyang Maternal and Child Health Care Hospital, Nanyang Central Hospital, Nanyang, China; AQU Department of Nephrology, Max Super Specialty Hospital, New Delhi, India; AQV Non-communicable Diseases Division (NCD), Indian Council of Medical Research, New Delhi, India; AQW College of Medicine and Public Health, Flinders University, Adelaide, VIC, Australia; AQX Independent Consultant, Bharatpur, India; AQY Independent Consultant, Delhi, India; AQZ Department of Anaesthesia, Maulana Azad Medical College, New Delhi, India; ARA Centre for Noncommunicable Diseases and Nutrition, BRAC University, Dhaka, Bangladesh; ARB Department of Preventive Cardiology & Medicine, Eternal Heart Care Centre & Research Institute, Jaipur, India; ARC Department of Medicine, Mahatma Gandhi University Medical Sciences, Jaipur, India; ARD Department of Toxicology, Shriram Institute for Industrial Research, Delhi, India; ARE School of Medicine, Deakin University, Geelong, VIC, Australia; ARF School of Biotechnology, Dublin City University, Dublin, Ireland; ARG Department of Anthropology, Deemed University, Delhi, India; ARH Faculty of Health, Medicine and Life Sciences (FHML), Macquarie University, Sydney, NSW, Australia; ARI Department of Nursing, Wollega University, Nekemte, Ethiopia; ARJ Department of Epidemiology and Psychosocial Research, Ramón de la Fuente Muñiz National Institute of Psychiatry, Mexico City, Mexico; ARK Doctoral Program in Biomedical Gerontology, Pontifical Catholic University of Rio Grande do Sul, Porto Alegre, Brazil; ARL Department of Population and Quantitative Sciences, Case Western Reserve University, Cleveland, OH, USA; ARM Research Unit in Epidemiology Clinic, Mexican Institute of Social Segurity, Colima, Mexico; ARN Neurosurgery Department, Fasa University of Medical Sciences, Shiraz, Iran; ARO College of Health Science, Dilla University, Dilla, Ethiopia; ARP Department of Medical Microbiology, Bahir Dar University, Bahir Dar, Ethiopia; ARQ Department of Clinical Pharmacology and Medicine, University of Kufa, Najaf, Iraq; ARR Department of Microbiology and Parasitology, Umm Al-Qura University, Makkah, Saudi Arabia; ARS Epidemiology Programme, London School of Hygiene & Tropical Medicine, London, UK; ART The George Institute for Global health, University of New South Wales, Sydney, NSW, Australia; ARU School of Medicine, Urmia University of Medical Sciences, Urmia, Iran; ARV School of Medicine, Hamedan University of Medical Sciences, Hamedan, Iran; ARW Department of Public Health and Preventive Medicine, Monash University, Melbourne, VIC, Australia; ARX Department of Community Medicine, Post Graduate Institute of Medical Education and Research, Chandigarh, India; ARY Centre for Community Medicine, All India Institute of Medical Sciences, New Delhi, India; ARZ Research Center for Social Determinants of Health, Shahid Beheshti University of Medical Sciences, Tehran, Iran; ASA Department of Infectious Disease Epidemiology, Robert Koch Institute, Berlin, Germany; ASB Department of Public Health, Charité Insitute of Public Health, Berlin, Germany; ASC College of Science, University of Sulaimani, Sulaymaniyah, Iraq; ASD Department of Pharmacy, American University of Madaba, Amman, Jordan; ASE Department of Family and Community Medicine, Arabian Gulf University, Manama, Bahrain; ASF Biochemistry Department, Ain Shams University, Cairo, Egypt; ASG Department of Public Health, Green International University, Lahore, Pakistan; ASH Department of Medical and Technical Information Technology, Bauman Moscow State Technical University, Moscow, Russia; ASI Guthrie Medical Group, Guthrie Medical Group, Cortland, NY, USA; ASJ Department of Clinical Pharmacy and Pharmacy Practice, Bayero University Kano, Kano, Nigeria; ASK Edirne Public Health Center, Edirne Provincial Health Directorate, Edirne, Turkiye; ASL Sakarya University, Sakarya, Turkiye; ASM Department of Critical Care and Emergency Nursing, Zanjan University of Medical Sciences, Zanjan, Iran; ASN Centre for Neuromuscular and Neurological Disorders (Perron Institute), The University of Western Australia, Perth, WA, Australia; ASO Stroke Research Centre, Perron Institute for Neurological and Translational Science, Perth, WA, Australia; ASP Department of Health and Education, Torrens University Australia, Melbourne, VIC, Australia; ASQ Department of Chemistry, University of Hail, Hail, Saudi Arabia; ASR Pharmacy, BRAC University, Dhaka, Bangladesh; ASS Department of Population Science and Human Resource Development, University of Rajshahi, Rajshahi, Bangladesh; AST Medical Research Unit, Universitas Syiah Kuala (Syiah Kuala University), Banda Aceh, Indonesia; ASU Vital and Health Statistics, Ministry of Health, Beirut, Lebanon; ASV Department for Health, University of Bath, Bath, UK; ASW University of Nevada Reno, Reno, NV, USA; ASX Department of Epidemiology Population Biostatistics and Health Promotion, Universitas Airlangga (Airlangga University), Surabaya, Indonesia; ASY Directorate General of Health Human Resources, Ministry of Health, Jakarta, Indonesia; ASZ Research Unit, Parc Sanitari Sant Joan de Deu, Barcelona, Spain; ATA Department of Mental Health, Biomedical Research Networking Center for Mental Health Network (CiberSAM), Madrid, Spain; ATB Department of Advanced Nursing, Universitas Airlangga (Airlangga University), Surabaya, Indonesia; ATC School of Nursing and Midwifery, La Trobe University, Bundoora, VIC, Australia; ATD Department of Zoology and Entomology, Al-Azhar University, Cairo, Egypt; ATE Faculty of Nursing, Chulalongkorn University, Bangkok, Thailand; ATF Department of Health Research Methods, Evidence, and Impact, McMaster University, Hamilton, ON, Canada; ATG Department of Biochemistry and Molecular Biology, Tejgaon College, Dhaka, Bangladesh; ATH Department of Ophthalmology, Iran University of Medical Sciences, Tehran, Iran; ATI Department of Medical Surgical, Shahroud University of Medical Sciences, Shahrekord, Iran; ATJ Institute of Radiology and Radiological Sciences, Tehran University of Medical Sciences, Tehran, Iran; ATK institute of radiology and radiological sciences, Johns Hopkins University, Baltimore, MD, USA; ATL Research Center for Traditional Medicine and History of Medicine, Shiraz University of Medical Sciences, Shiraz, Iran; ATM Department of Periodontics, RAK Medical and Health Sciences University, Ras Al Khaimah, United Arab Emirates; ATN Department of Oral Rehabilitation, University of Khartoum, Khartoum, Sudan; ATO Department of Biotechnology, Lahore University of Biological and Applied Sciences, Lahore, Pakistan; ATP Department of Neurology, Cairo University, Cairo, Egypt; ATQ Department of Medicine, University of Khartoum Faculty of Medicine, Khartoum, Sudan; ATR Department of Community Medicine, Federal University Teaching Hospital, Lafia, Nigeria; ATS Department of Epidemiology and Community Medicine, Federal University of Lafia, Lafia, Nigeria; ATT Ajman University, Ajman, United Arab Emirates; ATU Department Health Metrics Science, University of Washington, Seattle, WA, USA; ATV National Data Management Center, Ethiopian Public Health Institute, Addis Ababa, Ethiopia; ATW Department of Paediatrics, University of Colombo, Colombo, Sri Lanka; ATX Paediatric Professorial Unit, Lady Ridgeway Hospital for Children, Colombo, Sri Lanka; ATY Skaane University Hospital, Skaane County Council, Malmö, Sweden; ATZ Faculty of Medicine, Damascus University, Damascus, Syria; AUA Institute of Pharmaceutical Sciences, University of Veterinary and Animal Sciences, Lahore, Pakistan; AUB Department of Pharmacy Administration and Clinical Pharmacy, Xian Jiaotong University, Xian, China; AUC UK Dementia Research Institute Care Research & Technology Centre, Imperial College London, London, UK; AUD Department of Neurosurgery, Beijing Fengtai Hospital, Beijing, China; AUE Faculty of Kinesiology, University of New Brunswick, Fredericton, NB, Canada; AUF School of Allied Health, Murdoch University, Murdoch, WA, Australia; AUG Community-Oriented Nursing Midwifery Research Center, Shahrekord University of Medical Sciences, Shahrekord, Iran; AUH Department of Medicine, MedStar Health, Washington, DC, USA; AUI Department of Medicine, Georgetown University, Washington, DC, USA; AUJ Babes-Bolyai University, Cluj-Napoca, Romania; AUK Australian Centre for Health Service Innovations, Queensland University of Technology, Brisbane, QLD, Australia; AUL Poostchi Ophthalmology Research Center, Shiraz University of Medical Sciences, Shiraz, Iran; AUM Department of Virology, Lorestan University of Medical Sciences, Khorramabad, Iran; AUN Department of Microbiology, Taiz University, Taiz, Yemen; AUO School of Medicine, Nankai University, Tianjin, China; AUP Graduate School of Medicine, University of Tokyo, Tokyo, Japan; AUQ Kasturba Medical College, Mangalore, Manipal Academy of Higher Education, Manipal, India; AUR Department of Physics, University of Rajshahi, Rajshahi, Bangladesh; AUS Centre for Advancing Health Outcomes, Vancouver, BC, Canada; AUT Department of Decision and Information Sciences, University of Houston, Houston, TX, USA; AUU Public Health Research Group, Nature Study Society of Bangladesh, Khulna, Bangladesh; AUV Department of Statistics, Shahjalal University of Science and Technology, Sylhet, Bangladesh; AUW Department of Population Sciences, University of Dhaka, Dhaka, Bangladesh; AUX School of Health and Society, University of Wollongong, Wollongong, NSW, Australia; AUY School of Engineering and Technology, Duy Tan University, Da Nang, Vietnam; AUZ Jadara Research Center, Jadara University, Irbid, Jordan; AVA Department of Internal Medicine, Carol Davila University of Medicine and Pharmacy, Bucharest, Romania; AVB Department of Legal Medicine and Bioethics, Carol Davila University of Medicine and Pharmacy, Bucharest, Romania; AVC Department of Clinical Legal Medicine, National Institute of Legal Medicine Mina Minovici, Bucharest, Romania; AVD Centre for Population Health (CePH), Department of Social and Preventive Medicine, Faculty of Medicine, University of Malaya, Kuala Lumpur, Malaysia; AVE Department of Community Medicine, Manipal University College Malaysia, Melaka, Malaysia; AVF Department of Psychological and Cognitive Sciences, Tsinghua University, Beijing, China; AVG Maternal Care and Child Health Department, Capital Medical University, Beijing, China; AVH Faculty of Medicine, The Chinese University of Hong Kong, Hong Kong, China; AVI Department of Otorhinolaryngology Head and Neck Surgery, Shanghai Jiao Tong University, Shanghai, China; AVJ School of Public Health, Xuzhou Medical University, Xuzhou, China; AVK Pediatric Nursing Department, University of Indonesia, Depok, Indonesia; AVL East Manggarai Regency Health Office, Ministry of Health, Borong, Indonesia; AVM Nephrology and Urology Research Center, Baqiyatallah University of Medical Sciences, Tehran, Iran; AVN Department of Neuromedicine and Movement Science, Norwegian University of Science and Technology, Trondheim, Norway; AVO Department of Finance and Economics, University of Sharjah, Sharjah, United Arab Emirates; AVP Department of Social Sciences and Business, Roskilde University, Roskilde, Denmark; AVQ Czech National Centre for Evidence-Based Healthcare and Knowledge Translation, Masaryk University, Brno, Czech Republic; AVR Institute of Biostatistics and Analyses, Masaryk University, Brno, Czech Republic; AVS Clinical Governance and Quality Improvement Head, Salale University, Gerba Guracha, Ethiopia; AVT Department of Biomolecular Sciences, University of Zakho, Zakho, Iraq; AVU Artur Riggs Diabetes & Metabolism Research Institute, Cancer Prevention and Research Institute, Duarte, CA, USA; AVV Zagazig University, Zagazig, Egypt; AVW International Master Program for Translational Science, Taipei Medical University, Taipei, Taiwan; AVX Department of Occupational Safety and Health, China Medical University, Taiwan, Taichung, Taiwan; AVY Department of Occupational Therapy, Asia University, Taiwan, Taichung, Taiwan; AVZ Department of Biomedical, Metabolic, and Neural Science, University of Modena and Reggio Emilia, Modena, Italy; AWA Gastroenterology and Hepatology, Alexandria University, Charleston, SC, USA; AWB Department of Biology, University of Zakho, Zakho, Iraq; AWC Genetics and Molecular Biology Department, Abu Dhabi University, Abu Dhabi, United Arab Emirates; AWD Science and Technology Park, Kazakh National Medical University, Almaty, Kazakhstan; AWE Department of Medicine, Western University, London, ON, Canada; AWF Departamento de Gastroenterologia (Department of Gastroenterology), Pontifical Catholic University of Chile, Santiago, Chile; AWG Health Policy and Management Department, City University of New York, New York, NY, USA; AWH Center for Nutritional Epidemiology and Policy Research, National Institutes of Biomedical Innovation, Health and Nutrition, Settsu, Japan; AWI Collaborative Alliance Research and Education (CARE) Programme, Episcope Research Service, Aberdeen, Scotland; AWJ Department of Cardiovascular Medicine, Cleveland Clinic, Cleveland, OH, USA; AWK West Africa RCC, Africa Centre for Disease Control and Prevention, Abuja, Nigeria; AWL Department of Community Medicine, University College Hospital, Ibadan, Ibadan, Nigeria; AWM Faculty of Medical Sciences, University of Kragujevac, Kragujevac, Serbia; AWN Department of Orthopaedic Surgery, Massachusetts General Hospital, Boston, MA, USA; AWO Department of Clinical Pharmacy, Prince Sattam bin Abdulaziz University, Al Kharj, Saudi Arabia; AWP Department of Biostatistics, Iran University of Medical Sciences, Tehran, Iran; AWQ Institute of Health Research, University of Health and Allied Sciences, Ho, Ghana; AWR Department of Chemical Pathology, University of Jos, Jos, Nigeria; AWS Department of Chemical Pathology, Jos University Teaching Hospital, Jos, Nigeria; AWT Department of Health Research, ICMR National Institute for Research in Tuberculosis, Chennai, India; AWU Faculty of Health and Life Sciences, University of Exeter, Exeter, UK; AWV Department of Psychology, Wuhan University, Wuhan, China; AWW Faculty of Pharmacy, Universitas Ahmad Dahlan, Yogyakarta, Indonesia; AWX Department of Microbiology, University of Maiduguri, Maiduguri, Nigeria; AWY Department of Biotechnology, Sharda University, Greater Noida, India; AWZ Journal of Biological Sciences and Public Health, Dhaka, Bangladesh; AXA School of Pharmacy, BRAC University, Dhaka, Bangladesh; AXB Department of Surveillance and Health Equity Science, American Cancer Society, Atlanta, GA, USA; AXC Clinical Laboratory Department, Tobruk University, Tobruk, Libya; AXD Department of Blood Transmitted Diseases, National Centre for Disease Control (NCDC), Tobruk, Libya; AXE Department of Clinical Pharmacy & Pharmacy Practice, Asian Institute of Medicine, Science and Technology, Bedong, Malaysia; AXF Malaysian Academy of Pharmacy, Puchong, Malaysia; AXG Department of Urology, Kazakh National Medical University, Almaty, Kazakhstan; AXH Public Health Department of Social Medicine, Osaka University, Suita, Japan; AXI Department of Medicine, University of Yaoundé I, Yaounde, Cameroon; AXJ Department of Health Services Research, University of Tsukuba, Tsukuba, Japan; AXK Department of Non-Communicable Disease Epidemiology, London School of Hygiene & Tropical Medicine, London, UK; AXL Department of Global Health, South African Medical Research Council, Cape Town, South Africa; AXM Department of Global Health, Stellenbosch University, Cape Town, South Africa; AXN Knowledge Translation Program, Centre for Health Evaluation and Outcome Sciences, Vancouver, BC, Canada; AXO Department of Biotechnology, Karpagam Academy of Higher Education, Coimbatore, India; AXP Indian Institute of Public Health, Public Health Foundation of India, Gandhinagar, India; AXQ Department of Environmental Health Engineering, Guilan University of Medical Sciences, Rasht, Iran; AXR Department of Physical Medicine and Rehabilitation, Université Paris Cité, Paris, France; AXS Research and Development Unit, Biomedical Research Networking Center for Mental Health Network (CiberSAM), Barcelona, Spain; AXT Department of Health Studies, University of Richmond, Richmond, VA, USA; AXU Department of Nursing, Arak University of Medical Sciences, Arak, Iran; AXV Department of Immunology, Tabriz University of Medical Sciences, Tabriz, Iran; AXW Shiraz Neuroscience Research Center, Shiraz University of Medical Sciences, Shiraz, Iran; AXX UCL Institute for Global Health, University of London, London, UK; AXY College of Medicine and Health Sciences, Arabian Gulf University, Manama, Bahrain; AXZ Government Hospitals, Manama, Bahrain; AYA Department of Health and Safety, Dubai Municipality, Dubai, United Arab Emirates; AYB Department of Research and Academic Affairs, Larkin Community Hospital, South Miami, FL, USA; AYC Department of Medicine, AMA School of Medicine, Makati, Philippines; AYD UNESCO-TWAS Section of Economic & Social Sciences, Humanities & Arts, The World Academy of Sciences UNESCO-TWAS, Trieste, Italy; AYE Shaanxi University of Technology, Hanzhong, China; AYF Department of Surgery, Iran University of Medical Sciences, Tehran, Iran; AYG Department of Environmental Engineering, Islamic Azad University, Ahvaz, Iran; AYH Department of Neurosurgery, Medical College of Wisconsin, Milwaukee, WI, USA; AYI Department of Public Health Sciences, University of Chicago, Chicago, IL, USA; AYJ Department of Health Informatics, Qassim University, Buraydah, Saudi Arabia; AYK Department of Primary Care Medicine, Universiti Malaya, Kuala Lumpur, Malaysia; AYL SRM Medical College Hospital and Research Centre, Sri Ramaswamy Memorial Institute of Science and Technology, Kattankulathur, India; AYM Department of Family Medicine, University of Michigan, Ann Arbor, MI, USA; AYN Division of Population Data Science, Institute for Cancer Control, National Cancer Center, Tokyo, Japan; AYO Department of Public Health, Daffodil International University, Dhaka, Bangladesh; AYP Department of Public and Community Health, Frontier University Garowe, Puntland, Somalia; AYQ Department of Neurosciences, University of the Philippines Manila, Manila, Philippines; AYR Institute for Neurosciences, St. Luke's Medical Center, Bonifacio Global City, Philippines; AYS Institute for Musculoskeletal Health, University of Sydney, Sydney, NSW, Australia; AYT Shahrekord University of Medical Sciences, Shahrekord, Iran; AYU Division of Medical Research, Sri Ramaswamy Memorial Institute of Science and Technology, Kattankulathur, India; AYV Department of Stem Cells and Developmental Biology, Royan Institution, Tehran, Iran; AYW Department of Medicine, University of Mississippi Medical Center, Jackson, MS, USA; AYX Department of Medicine, Jinnah Sindh Medical University, Karachi, Pakistan; AYY Karolinska Institutet Campus Solna, Karolinska Institute, Stockholm, Sweden; AYZ Invasive Fungi Research Center, Mazandaran University of Medical Sciences, Sari, Iran; AZA Department of Medical Mycology, Mazandaran University of Medical Sciences, Sari, Iran; AZB Department of Pharmacology, Imam Mohammad Ibn Saud Islamic University, Riyadh, Saudi Arabia; AZC Department of Nursing, Middle Technical University of Kut Technical Institute, Baghdad, Iraq; AZD Department of Oral Medicine and Periodontology, University of Peradeniya, Peradeniya, Sri Lanka; AZE Department of Oral Medicine and Periodontology, Saveetha University, Chennai, India; AZF Department of Research, University of Puthisastra, Phnom Penh, Cambodia; AZG Postgraduate Institute of Medicine, University of Colombo, Colombo, Sri Lanka; AZH Faculty of Graduate Studies, Institute for Violence and Injury Prevention, Colombo, Sri Lanka; AZI Iranian Tissue Bank and Research Center, Tehran University of Medical Sciences, Tehran, Iran; AZJ Department of Endocrinology, Diabetes and Metabolism, Christian Medical College and Hospital (CMC), Vellore, India; AZK Department of Medicine, University of Melbourne, Melbourne, VIC, Australia; AZL Department of Epidemiology and Health Promotion, Yonsei University, Seoul, South Korea; AZM Department of Epidemiology, Wachemo University, Hossana, Ethiopia; AZN Graphic Era (Deemed to be University), Dehradun, India; AZO Department of Food, Nutrition and Health, University of British Columbia, Vancouver, BC, Canada; AZP Department of Internal Medicine, GCS Medical College, Hospital & Research Centre, Ahmedabad, India; AZQ The George Institute for Global Health, New Delhi, India; AZR Manipal Academy of Higher Education, Manipal, India; AZS Department of Public Health, Tongji University, Shanghai, China; AZT Xuzhou Medical University, Xuzhou, China; AZU Department of Radiology, Zhongshan Hospital, Shanghai, China; AZV Department of Orthopedics, Wuhan University, Wuhan, China; AZW Department of Biomedical Sciences, City University of Hong Kong, Hong Kong, China; AZX Danish Center for Health Economics, University of Southern Denmark, Odense, Denmark; AZY Faculty of Veterinary Medicine, University of Calgary, Calgary, AB, Canada; AZZ Young Researchers and Elite Club, Islamic Azad University, Karaj, Iran; BAA Rothschild Foundation Hospital, Institut Français de Myopie, Paris, France; BAB Singapore Eye Research Institute, Singapore, Singapore; BAC Hungarian Health Management Association, Budapest, Hungary; BAD Department of Biomedical Engineering, Hong Kong Polytechnic University, Hong Kong, China; BAE Department of Gastroenterology and Hepatology, Stanford University, Stanford, CA, USA; BAF School of Public Health, Sri Ramaswamy Memorial Institute of Science and Technology (SRMIST), Chennai, India; BAG Department of Community Medicine, Manipal Academy of Higher Education, Mangalore, India; BAH Department of Economics, National Open University, Benin City, Nigeria; BAI Caspian Digestive Disease Research Center, Guilan University of Medical Sciences, Rasht, Iran; BAJ Nursing & Midwifery Research Department (NMRD), Hamad Medical Corporation, Doha, Qatar; BAK Department of Family Medicine and Public Health, University of Opole, Opole, Poland; BAL Institute of Family Medicine and Public Health, University of Tartu, Tartu, Estonia; BAM Department of Radiology and Nuclear Medicine, The University of Jordan, Amman, Jordan; BAN School of Public Health, National Institute of Epidemiology, Chennai, India; BAO Health Economics Unit, Flinders University, Adelaide, SA, Australia; BAP Research Department, TobaccoFree Research Institute Ireland, Dublin, Ireland; BAQ School of Public Health, University College Cork, Cork, Ireland; BAR Department of Statistics, Salahaddin University-Erbil, Erbil, Iraq; BAS Department of Business Administrations, Cihan University-Erbil, Erbil, Iraq; BAT Department of Pharmacology, Post Graduate Institute of Medical Education and Research, Chandigarh, India; BAU School of Management and Medical Informatics, Tabriz University of Medical Sciences, Tabriz, Iran; BAV Department of Health, Khoy Medical Sciences, Khoy, Iran; BAW Department of Dermatology, King Faisal University, Hofuf, Saudi Arabia; BAX Research Institute of Cardiology and Internal Medicine, Almaty, Almaty, Kazakhstan; BAY Department of Endocrinology, Bharti Hospital Karnal, Karnal, India; BAZ University Centre for Research and Development, Chandigarh University, Mohali, India; BBA Canberra Business School, University of Canberra, Hawker, ACT, Australia; BBB College of Pharmacy, Prince Sattam bin Abdulaziz University, Al Kharj, Saudi Arabia; BBC NIHR Global Health Research Unit on Global Surgery, University of Birmingham, Birmingham, UK; BBD Prasanna School of Public Health, Manipal Academy of Higher Education, Manipal, India; BBE Care and Public Health Research Institute (CAPHRI), Maastricht University, Maastricht, Netherlands; BBF Department of General Medical Practice No. 2, Kazakh National Medical University, Almaty, Kazakhstan; BBG Russell H. Morgan Department of Radiology and Radiological Science, Johns Hopkins University, Baltimore, MD, USA; BBH Department of Public Health, South Wales University, Treforest, UK; BBI Microbiology, Virology and Immunology Department, I. Horbachevsky Ternopil National Medical University, Ternopil, Ukraine; BBJ School of Public Health, Fudan University, Shanghai, China; BBK Department of Health Sciences, University of York, York, UK; BBL School of Health and Environmental Science, Korea University, Seoul, South Korea; BBM Department of Anesthesia, Critical Care and Pain Medicine, Massachusetts General Hospital, Boston, MA, USA; BBN T. H. Chan School of Public Health, Harvard University, Boston, MA, USA; BBO Office of the Executive Director, Cephas Health Research Initiative Inc, Ibadan, Nigeria; BBP MS Ramaiah Memorial Hospital, Ramaiah University of Applied Sciences, Banglore, India; BBQ Department of Community Medicine, ESIC Medical College and Hospital Chennai, Chennai, India; BBR The Hansjörg Wyss Department of Plastic and Reconstructive Surgery, NYU Langone Health, New York, NY, USA; BBS Cleft Lip and Palate Surgery Division, Global Smile Foundation, Norwood, MA, USA; BBT Department of Psychiatry, King George's Medical University, Lucknow, India; BBU 2nd Department of Cardiology, Aristotle University of Thessaloniki, Thessaloniki, Greece; BBV Department of Basic Medical Sciences, Yarmouk University, Irbid, Jordan; BBW Endocrine Research Center, Iran University of Medical Sciences, Tehran, Iran; BBX Department of Echocardiography, Iran University of Medical Sciences, Tehran, Iran; BBY Department of Medical–Surgical Nursing, Guilan University of Medical Sciences, Rasht, Iran; BBZ Saveetha Medical College and Hospital, Saveetha University, Chennai, India; BCA Chair and Department of Medical Microbiology, Poznan University of Medical Sciences, Poznan, Poland; BCB Amity Stem Cell Institute (ASCI), Amity University Haryana, Gurugram, India; BCC College of Health Science, Department of Public Health, Arsi University, Asella, Ethiopia; BCD Eye Research Center, Iran University of Medical Sciences, Tehran, Iran; BCE Department of Reproductive, Family and Population Health, Addis Ababa University, Addis Ababa, Ethiopia; BCF Department of Medicine, Jacobi Medical Center, New York, NY, USA; BCG Centre for Tropical Diseases and Global Health, Catholic University of Bukavu, Bukavu, Democratic Republic of the Congo; BCH Surgery Research Unit, University of Oulu, Oulu, Finland; BCI Department of Molecular Medicine and Surgery, Karolinska Institute, Stockholm, Sweden; BCJ International Research Center of Excellence, Institute of Human Virology Nigeria, Abuja, Nigeria; BCK Julius Centre for Health Sciences and Primary Care, Utrecht University, Utrecht, Netherlands; BCL Open, Distance and eLearning Campus, University of Nairobi, Nairobi, Kenya; BCM Center for Tobacco Research, Ohio State University, Columbus, OH, USA; BCN Department of Ophthalmology, Harvard University, Boston, MA, USA; BCO Eye Unit, MyungSung Medical College, Addis Ababa, Ethiopia; BCP Center of Global Child Health, The Hospital for Sick Children, Toronto, ON, Canada; BCQ Jindal School of Public Health and Human Development, O. P. Jindal Global University, Sonipat, Sonipat, India; BCR Department of Biomedical Informatics, Arizona State University, Phoenix, AZ, USA; BCS Department of Human Nutrition of INRAE, National Research Institute for Agriculture, Food and Environment, Paris, France; BCT Sorbonne Paris Nord University, Bobigny, France; BCU Faculty of Medicine, Mashhad University of Medical Sciences, Mashhad, Iran; BCV Health Policy Research Center, Shiraz University of Medical Sciences, Shiraz, Iran; BCW Department of Public Health, Jordan University of Science and Technology, Irbid, Jordan; BCX Amity Institute of Forensic Sciences, Amity University, Noida, India; BCY Lahore Medical Research Center, Lahore Medical Research Center, Lahore, Pakistan; BCZ College of Health Professions, Marshall University, Huntington, WV, USA; BDA Department of Veterinary Medicine, United Arab Emirates University, Al Ain, United Arab Emirates; BDB Faculty of Veterinary Medicine, Kafrelsheikh University, Kafrelsheikh, Egypt; BDC Department of Nursing, Zarqa University, Zarqa, Jordan; BDD University Institute of Diet and Nutritional Sciences, The University of Lahore, Lahore, Pakistan; BDE Department of Global Health, University of Washington, Seattle, WA, USA; BDF Department of Medicine, Guilan University of Medical Sciences, Rasht, Iran; BDG Department of Obstetrics & Gynecology, Iran University of Medical Sciences, Tehran, Iran; BDH Department of Medical Genetics and Molecular Medicine, Mashhad University of Medical Sciences, Mashhad, Iran; BDI Department of Public Health, Mohammed VI Center for Research and Innovation, Rabat, Morocco; BDJ Higher Institute of Nursing Professions and Health Techniques, Rabat, Morocco; BDK Food and Drug Research Center, Iran Food and Drug Administration, Tehran, Iran; BDL NITVAR, Indian Council of Medical Research, Pune, India; BDM Academy of Scientific and Innovative Research (AcSIR), India, Ghaziabad, India; BDN Natural and Medical Sciences Research Center, University of Nizwa, Nizwa, Oman; BDO School of Health and Rehabilitation Sciences, The University of Queensland, Brisbane, QLD, Australia; BDP Department of Pharmacy Administration and Clinical Pharmacy, Peking University, Beijing, China; BDQ Department of Pharmacy Administration and Clinical Pharmacy, The First Affiliated Hospital of Xi'an Jiaotong University, Xi'an, China; BDR Epidemiology Program, Jazan University, Jazan, Saudi Arabia; BDS Department of Community Medicine, National Institute of Preventive and Social Medicine, Dhaka, Bangladesh; BDT Department of Basic Health Sciences, Qassim University, Buraydah, Saudi Arabia; BDU BD Statistics Center for Research, Dhaka, Bangladesh; BDV Karachi Medical and Dental College, Karachi, Pakistan; BDW Center for Atmospheric Particle Studies (CAPS), Carnegie Mellon University, Pittsburgh, PA, USA; BDX Department of Mechanical Engineering (MechE), Carnegie Mellon University, Pittsburgh, PA, USA; BDY Aston Pharmacy School, College of Health and Life Sciences, Aston University, Birmingham, UK; BDZ Institute for Global Health, University College London, London, UK; BEA Silesian University of Technology, Gliwice, Poland; BEB Joint Doctoral School, Silesian University of Technology, Gliwice, Poland; BEC Dr. Panjwani Center for Molecular Medicine & Drug Research, University of Karachi, Karachi, Pakistan; BED International Center for Chemical and Biological Sciences, International Center for Chemical and Biological Sciences, Karachi, Pakistan; BEE College of Medicine, University of Hail, Hail, Saudi Arabia; BEF Department of Cardiology, University of South Wales, Treforest, UK; BEG Department of Cardiology, University of Buckingham, Buckingham, UK; BEH Central Department of zoology, Tribhuvan University, Kathmandu, Nepal; BEI Department of Health, Nepal Development Society, Chitwan, Nepal; BEJ Department of Preventable Non Communicable Disease, Menzies School of Health Research, Alice Springs, NT, Australia; BEK Department of Epidemiology, Non-Communicable Diseases Research Center (NCDRC), Tehran, Iran; BEL Department of Pharmacology, All India Institute of Medical Sciences, Raipur, India; BEM Department of Physiology and Biomedical Engineering, Mayo Clinic, Rochester, MN, USA; BEN College of Health, Wellbeing and Life Sciences, Sheffield Hallam University, Sheffield, UK; BEO College of Arts and Sciences, Ohio University, Zanesville, OH, USA; BEP Faculty of Nursing, Yarmouk University, Irbid, Jordan; BEQ Department of Orthopaedics, Postgraduate Medical Institute, Sangrur, India; BER Department of Neurosurgery, Shahid Beheshti University of Medical Sciences, Tehran, Iran; BES Asadabad School of Medical Sciences, Asadabad, Iran; BET Department of Biology, Science and Research Branch, Islamic Azad University, Tehran, Iran; BEU Penn Medicine, University of Pennsylvania, Philadelphia, PA, USA; BEV Bone and Joint Reconstruction Research Center, Iran University of Medical Sciences, Tehran, Iran; BEW University of Sulaimani College of Medicine, Sulaimani Polytechnic University, Sulaymaniyah, Iraq; BEX Department of Internal Medicine, Corewell Health East William Beaumont University Hospital, Royal Oak, MI, USA; BEY Department of Medical Oncology, Miami Cancer Institute, Miami, FL, USA; BEZ Department of Radiology, University of Washington, Seattle, WA, USA; BFA Cardiothoracic Imaging Section, University of Washington, Seattle, WA, USA; BFB Research Department, University of Inland Norway, Elverum, Norway; BFC Ashok & Rita Patel Institute of Physiotherapy, Charotar University of Science and Technology, Changa, Anand, India; BFD Faculty of Health Sciences, University of Muhammadiyah Prof. Dr. Hamka, Jakarta, Indonesia; BFE Program Division, SEAMEO Regional Center for Food and Nutrition, Jakarta, Indonesia; BFF Department of Pharmacology, University of Gondar, Gondar, Ethiopia; BFG Department of Biomedical Sciences, Seoul National University, Seoul, South Korea; BFH Department of Health Policy and Management, Korea University, Seoul, South Korea; BFI Cardiovascular Disease Initiative, Broad Institute of MIT and Harvard, Cambridge, MA, USA; BFJ Department of Environmental Health Sciences, Soonchunhyang University, Asan, South Korea; BFK Health and Healing Research, Education, and Service, Inc., Boston, MA, USA; BFL Millennium Prevention, Inc., Westwood, MA, USA; BFM The Pacific Community, Noumea, New Caledonia; BFN College of Medicine, Qatar University, Doha, Qatar; BFO School of Health Sciences, Kristiania University College, Oslo, Norway; BFP Department of International Health and Sustainable Development, Tulane University, New Orleans, LA, USA; BFQ Department of Nursing and Health Promotion, Oslo Metropolitan University, Oslo, Norway; BFR Department of Health Economics and Social Security, Jagiellonian University Medical College, Krakow, Poland; BFS Department of Brain Sciences, University College London, London, UK; BFT Department of Public Health, University of Helsinki, Helsinki, Finland; BFU Department of Public Health Dentistry, Krishna Vishwa Vidyapeeth (Deemed to be University), Karad, India; BFV Centre for Disease Burden, Norwegian Institute of Public Health, Bergen, Norway; BFW Endocrinology Department, Bogomolets National Medical University, Kyiv, Ukraine; BFX Scientific Department, Medical Laboratory CSD, Kyiv, Ukraine; BFY Global Healthcare Consulting, New Delhi, India; BFZ Department of Medicine, Harvard University, Boston, MA, USA; BGA Mycobacteriology Unit, Center for Health Promotion and Research, Bamenda, Cameroon; BGB Children's Medical Center, Tehran University of Medical Sciences, Tehran, Iran; BGC Scientific and Educational Center for Neurology and Applied Neuroscience, Kazakh National Medical University, Almaty, Kazakhstan; BGD Department of Ophthalmology, Aristotle University of Thessaloniki, Thessaloniki, Greece; BGE Centre for the Business and Economics of Health, The University of Queensland, Brisbane, QLD, Australia; BGF Copernicus Institute of Sustainable Development, Utrecht University, Utrecht, Netherlands; BGG Department of Science and Environmental Studies, The Education University of Hong Kong, Hong Kong, China; BGH Department of General Practice and Family Medicine, Kharkiv National Medical University, Kharkiv, Ukraine; BGI Department of Epidemiology, IQVIA, Frankfurt am Main, Germany; BGJ University Hospital Marburg, Marburg, Germany; BGK Department of Psychiatry, University of Ioannina, Ioannina, Greece; BGL Amity institute of Public Health and Hospital administration, Amity University Noida, Noida, India; BGM Department of Internal and Pulmonary Medicine, Sheri Kashmir Institute of Medical Sciences, Srinagar, India; BGN Kasturba Medical College, Manipal, Manipal Academy of Higher Education, Udupi, India; BGO School of Pharmacy, University of Ghana, Legon, Ghana; BGP Department of Public Health, Central University, Accra, Ghana; BGQ Central University, Accra, Ghana; BGR Department of Anthropology, Panjab University, Chandigarh, India; BGS School of Applied Science, Republic Polytechnic, Singapore, Singapore; BGT Centre for Biotechnology, Siksha ‘O’ Anusandhan Deemed to be University, Bhubaneswar, India; BGU Department of Demography, University of Montreal, Montreal, QC, Canada; BGV Department of Social and Preventive Medicine, University of Montreal, Montreal, QC, Canada; BGW Department of Biotechnology, Baba Ghulam Shah Badshah University, Jammu and Kashmir, India; BGX Department of Medical Education and Informatics, Gazi University Faculty of Medicine, Ankara, Turkiye; BGY Department of Biochemistry, University of Hail, Hail, Saudi Arabia; BGZ Department of Pediatrics, Kuopio University Hospital, Kuopio, Finland; BHA Institute of Clinical Medicine, University of Eastern Finland, Kuopio, Finland; BHB Dental School, The University of Western Australia, Perth, WA, Australia; BHC Rajendra Institute of Medical Sciences, Ministry of Health and Family Welfare, Ranchi, India; BHD Research and Publication Activity Division, Kazakh National Medical University, Almaty, Kazakhstan; BHE Center of Medicine and Public Health, Asfendiyarov Kazakh National Medical University, Almaty, Kazakhstan; BHF Department of Medicine, Queensland Health, Brisbane, QLD, Australia; BHG Amity Centre for Water Studies and Research, Amity University Rajasthan, Jaipur, India; BHH Department of Community Medicine, Rajendra Institute of Medical Sciences, Ranchi, India; BHI SRM Centre for Clinical Trials and Research (CCTR), Sri Ramaswamy Memorial Institute of Science and Technology, Chennai, India; BHJ Department of Pediatrics, Post Graduate Institute of Medical Education and Research, Chandigarh, India; BHK Institute for Excellence in Health Equity, New York University, New York, NY, USA; BHL Department of Psychiatry, University of Nairobi, Nairobi, Kenya; BHM Department of Pharmacology and Toxicology, National Institute of Pharmaceutical Education and Research, Hajipur, Hajipur, India; BHN College of Public Health & Health Informatics, University of Hail, Hail, Saudi Arabia; BHO Gastroenterology Department, Ahalia Hospital, Abu Dhabi, United Arab Emirates; BHP Allied Health Sciences, Bahria University Medical and Dental College, Karachi, Pakistan; BHQ Department of Anaesthesiology, Rajendra Institute of Medical Sciences, Ranchi, India; BHR Department of Economics, Manipal University, Jaipur, Jaipur, India; BHS IITM Pravartak Technologies Foundation, Chennai, India; BHT Section of Cardiology, University of Manitoba, Winnipeg, MB, Canada; BHU Department of Translational Health Sciences, University of Bristol, Bristol, UK; BHV Department of Clinical Subjects, Al Farabi Kazakh National University, Almaty, Kazakhstan; BHW Faculty of Health and Life Sciences, Coventry University, Coventry, UK; BHX Department of Medicine, McMaster University, Hamilton, ON, Canada; BHY Faculty of Medicine and Health Science, Universitas Kristen Satya Wacana (Satya Wacana Christian University), Salatiga, Indonesia; BHZ School of Nursing, Taipei Medical University, Taipei, Taiwan; BIA Division of Cardiology, University of Illinois, Champaign, IL, USA; BIB Department of Microbiology, Central University of Punjab, Bathinda, India; BIC National Research and Innovation Agency, Jakarta, Indonesia; BID Institute for Health Sciences, STIKES Bethesda Yakkum Yogyakarta Indonesia, Yogyakarta, Indonesia; BIE Department of Public Health and Epidemiology, Khalifa University of Science and Technology, Abu Dhabi, United Arab Emirates; BIF Faculty of Public Health, University of Indonesia, Depok, Indonesia; BIG Department of Pediatric Oncology, Medicana Health International, Istanbul, Turkiye; BIH Department of Pediatric Oncology, Hacettepe University, Ankara, Turkiye; BII Department of Clinical Surgical Sciences, University of the West Indies, St Augustine, Trinidad and Tobago; BIJ Department of Nursing, University of Massachusetts Boston, Boston, MA, USA; BIK Clinical Research Center, Turku University Hospital, Turku, Finland; BIL Heart Center, University of Turku, Turku, Finland; BIM Kasturba Medical College, Manipal, Manipal Academy of Higher Education, Manipal, India; BIN Pediatric Emergency Department, Fondazione IRCCS Ospedale Maggiore Policlinico, Milan, Italy; BIO Department of Clinical Sciences and Community Health, University of Milan, Milan, Italy; BIP Department of Nursing Science, Bayero University Kano, Kano, Nigeria; BIQ Department of Global Public Health, Karolinska Institute, Stockholm, Sweden; BIR Institute for Social and Health Sciences, University of South Africa, Pretoria, South Africa; BIS Division of Evidence Synthesis, Foundation for People-centric Health Systems, New Delhi, India; BIT Division of Lifestyle Medicine, Centre for Health: The Specialty Practice, New Delhi, India; BIU School of Digital Science, Universiti Brunei Darussalam (University of Brunei Darussalam), Bandar Seri Begawan, Brunei; BIV Institute of Applied Data Analytics, Universiti Brunei Darussalam (University of Brunei Darussalam), Bandar Seri Begawan, Brunei; BIW Department of Chemistry, Dayalbagh Educational Institute, Agra, India; BIX Indian Council of Medical Research, New Delhi, India; BIY School of Dentistry, The University of Queensland, Brisbane, QLD, Australia; BIZ NEVES Society for Patient Safety, Budapest, Hungary; BJA Unidad de Genética y Salud Pública, Instituto de Ciencias Médicas, Las Tablas, Panama; BJB Ministry of Health, Hospital Joaquín Pablo Franco Sayas, Las Tablas, Panama; BJC Department of Psychiatry and Psychotherapy, University of Regensburg, Regensburg, Germany; BJD Department of Behavioural Sciences and Learning, Linköping University, Linköping, Sweden; BJE Department of Otorhinolaryngology, Father Muller Medical College, Mangalore, India; BJF Centre for Family Welfare, University of Indonesia, Depok, Indonesia; BJG Department of Global Health and Health Security, Taipei Medical University, Taipei, Taiwan; BJH Department of Orthopaedics and Traumatology, The Chinese University of Hong Kong, Hong Kong, China; BJI Department of Clinical Pharmacy and Pharmacy Management, Kaduna State University, Kaduna, Nigeria; BJJ Department of Obstetrics and Gynaecology, University of Nottingham, Derby, UK; BJK Department of Obstetrics and Gynaecology, Ahmadu Bello University, Zaria, Nigeria; BJL Health Systems, Administration and Management, Babcock University, Sagamu, Nigeria; BJM Health Services Management Programme, Plasma University, Mogadishu, Somalia; BJN School of Physical Therapy, The University of Western Ontario, London, ON, Canada; BJO UQ Centre for Clinical Research, The University of Queensland, Brisbane, QLD, Australia; BJP Faculty of Medicine, Nam Can Tho University, Can Tho, Vietnam; BJQ PhD Program in Epidemiology, Vanderbilt University, Nashville, TN, USA; BJR Faculty of Medicine, University of Medicine and Pharmacy at Ho Chi Minh City, Ho Chi Minh City, Vietnam; BJS Department of Cardiovascular Research, Methodist Hospital, Merrillville, IN, USA; BJT Health Economics Division, Monash University, Burwood, VIC, Australia; BJU International Ph.D. Program in Medicine, Taipei Medical University, Taipei, Taiwan; BJV Research Center for Artificial Intelligence in Medicine, Taipei Medical University, Taipei, Taiwan; BJW University of Medicine and Pharmacy at Ho Chi Minh City, Ho Chi Minh City, Vietnam; BJX Independent Consultant, Ho Chi Minh City, Vietnam; BJY Department of Clinical and Experimental Medicine, University of Catania, Catania, Italy; BJZ Clinical Trial Center, Ewha Womans University, Seoul, South Korea; BKA Department of Family Medicine, University of Texas Medical Branch, Galveston, TX, USA; BKB Department of Preventive Medicine, Korea University, Seoul, South Korea; BKC Asbestos and Dust Diseases Research Institute, University of Sydney, Sydney, NSW, Australia; BKD Department of Cardiothoracic and Vascular Surgery, Westpfalz Klinikum, Kaiserslautern, Germany; BKE Department of Cardiothoracic Surgery, University of Patras, Patras, Greece; BKF Centre for Healthy Brain Ageing, University of New South Wales, Sydney, NSW, Australia; BKG SC Neurologia, Salute Pubblica e Disabilità (Neurology, Public Health, Disability Unit), Fondazione IRCCS Istituto Neurologico Carlo Besta, Milan, Italy; BKH Faculty of Science, (Universiti Brunei Darussalam) University of Brunei Darussalam, Bandar Seri Begawan, Brunei; BKI National Centre for Youth Substance Abuse Research, The University of Queensland, Brisbane, QLD, Australia; BKJ School of Public Health and Management, Guangzhou University of Chinese Medicine, Guangzhou, China; BKK Peking Union Medical College, Beijing, China; BKL Department of Rheumatology and Immunology, The People's Hospital of Baoan Shenzhen, Shenzhen, China; BKM Global Health Research Center, Guangdong Academy of Medical Sciences, Guangzhou, China; BKN Shanxi Medical University, Taiyuan, China; BKO Department of Health Promotion and Health Education, National Taiwan Normal University, Taipei, Taiwan; BKP School of Public Health, Peking University, Beijing, China; BKQ The First Affiliated Hospital of Guangzhou Medical University, Guangzhou Medical University, Guangzhou, China; BKR First Clinical Medicine, Shandong University of Traditional Chinese Medicine, Jinan, China; BKS Population Studies Center, University of Pennsylvania, Philadelphia, PA, USA; BKT Department of Radiation Oncology, Southern Medical University, Guangzhou, China; BKU Department of Nephrology, the Second Affiliated Hospital of Anhui Medical University, Hefei, China; BKV National Clinical Research Center for Cardiovascular Diseases, Chinese Academy of Medical Sciences, Shenzhen, China; BKW Department of Endocrinology and Metabolism, The First Hospital of China Medical University, Shenyang, China; BKX School of Medicine, Shanghai Jiao Tong University, Shanghai, China; BKY Tsinghua Vanke School of Public Health, Tsinghua University, Beijing, China; BKZ Department of Global Health and Population, Harvard University, Boston, MA, USA; BLA Discipline of Physiology, National University of Ireland, Galway, Galway, Ireland; BLB First Clinical Medical College, Shandong University of Chinese Medicine, Jinan, China; BLC International Centre for Future Health Systems, University of New South Wales, Sydney, NSW, Australia; BLD WHO Collaborating Centre for Public Health Education and Training, Imperial College London, London, UK; BLE Department of Food Science and Human Nutrition, Iowa State University, Ames, IA, USA; BLF College of Public Health, China Medical University, Taiwan, Taichung, Taiwan; BLG Asbestos Diseases Research Institute, Concord, NSW, Australia; BLH The First Affiliated Hospital of Wenzhou Medical University, Wenzhou Medical University, Wenzhou, China; BLI Department of Medical Sciences, Uppsala University, Uppsala, Sweden; BLJ Department of Medicine, Norrtälje Hospital (Tiohundra), Norrtälje, Sweden; BLK UCD Centre for Disability Studies, University College Dublin, Dublin, Ireland; BLL Management Science and Engineering, Stanford University, Stanford, CA, USA; BLM School of Life Sciences, University of Technology Sydney, Sydney, NSW, Australia; BLN Centre for Intelligent Healthcare, Coventry University, Coventry, UK; BLO Department of Epidemiology and Biostatistics, Peking University, Beijing, China; BLP School of Nursing and Health Sciences, Hong Kong Metropolitan University, Hong Kong, China; BLQ Department of Radiology and Biomedical Imaging, Yale University, New Haven, CT, USA; BLR Department of Radiology, Massachusetts General Hospital, Boston, MA, USA; BLS Lerner Research Institute, Cleveland Clinic, Cleveland, OH, USA; BLT Department of Quantitative Health Science, Case Western Reserve University, Cleveland, OH, USA; BLU Xiangya Hospital, Central South University, Changsha, China; BLV Institute of Child and Adolescent Health, Peking University, Beijing, China; BLW Department of Molecular Epidemiology, German Institute of Human Nutrition Potsdam-Rehbrücke, Potsdam, Germany; BLX German Center for Diabetes Research (DZD), München-Neuherberg, Germany; BLY Department of Infectious Diseases, Monash University, Melbourne, VIC, Australia; BLZ Department of Infectious Diseases, Alfred Health, Melbourne, VIC, Australia; BMA Department of Cardiology, University of Cologne, Cologne, Germany; BMB School of Medicine, Universidad Espíritu Santo, Samborondón, Ecuador; BMC Vicerrectoría de Investigación y Postgrado, Universidad de Los Lagos, Osorno, Chile; BMD Department of Epidemiology and Evidence-Based Medicine, I.M. Sechenov First Moscow State Medical University, Moscow, Russia; BME Institute of Nutritional Sciences, Friedrich Schiller University Jena, Jena, Germany; BMF Competence Cluster for Nutrition and Cardiovascular Health (nutriCARD), Jena, Germany; BMG Department of Community Health, Shahrekord University of Medical Sciences, Shahrekord, Iran; BMH Social Determinants of Health Research Center, Shahrekord University of Medical Sciences, Shahrekord, Iran; BMI Department of Spine Surgery, Qingdao Municipal Hospital Group, Qingdao, China; BMJ Geospatial Health and Development Team-Child Health Analytics, Telethon Kids Institute, Perth, WA, Australia; BMK Scientific Research and Surveillance Systems, Macha Research Trust, Choma, Zambia; BML Injury Prevention and Safety Promotion Research Center, Shahid Beheshti University of Medical Sciences, Tehran, Iran; BMM School of Medicine, Federal University of Juiz de Fora, Juiz de Fora, Brazil; BMN Department of Emergency General and Trauma Surgery, NHS University Hospitals of Liverpool Group, Aintree Hospital, Liverpool, UK; BMO Zhujiang Hospital of Southern Medical University, Southern Medical University, Guangzhou, China; BMP Department of Population Health Sciences, Duke University, Durham, NC, USA; BMQ Dodoma Medical Research Centre, National Institute for Medical Research in Tanzania, Dodoma, Tanzania; BMR Department of Neurosciences and Behavioral Sciences, University of São Paulo, Ribeirão Preto, Brazil; BMS College of Engineering, Effat University, Jeddah, Saudi Arabia; BMT Management of Information Systems Department, The American College of Greece, Aghia Paraskevi, Greece; BMU Department of Medicine, University of Alberta, Edmonton, AB, Canada; BMV Tulane University, New Orleans, LA, USA; BMW Department of Chemistry, Salahaddin University-Erbil, Erbil, Iraq; BMX Center for Global Health, University of Pennsylvania, Philadelphia, PA, USA; BMY Centre for Public Health and Wellbeing, University of the West of England, Bristol, UK; BMZ Faculty of Veterinary Medicine, Suez Canal University, Ismailia, Egypt; BNA Department of Microbiology and Parasitology, King Salman International University, South of Sinai, Egypt; BNB Department of Family Medicine, Mental and Public Health, Federal University of Minas Gerais, Ouro Preto, Brazil; BNC Anesthesiology Research Center, Shahid Beheshti University of Medical Sciences, Tehran, Iran; BND Associate Laboratory i4HB, University Institute of Health Sciences (CESPU), Gandra, Portugal; BNE UCIBIO Research Unit on Applied Molecular Biosciences, University Institute of Health Sciences, Gandra, Portugal; BNF Ophthalmology Department, Ministry of Health & Population, Aswan, Egypt; BNG Department of Forensic Medicine & Toxicology, Mysore Medical College & Research Institute, Mysooru, India; BNH Department of Health & Family Welfare, Government of Karnataka, Bangalore, India; BNI Department of Emergency Medicine, Sri Lakshmi Narayana Institute of Medical Science, Puducherry, Puducherry, India; BNJ Research Center, Cihan University-Sulaimaniya, Sulaymaniyah, Iraq; BNK Department of Ophthalmology, Tehran University of Medical Sciences, Tehran, Iran; BNL Neurology Department, University of Miami, Miami, FL, USA; BNM Institute of Health Science, Nam Can Tho University, Can Tho, Vietnam; BNN Department of Pharmacology, All India Institute of Medical Sciences, Bhubaneswar, India; BNO Department of Primary Care and Public Health, Imperial College London, London, UK; BNP Cyprus International Institute for Environmental and Public Health, Cyprus University of Technology, Limassol, Cyprus; BNQ Non-communicable Disease Research Center, Shiraz University of Medical Sciences, Shiraz, Iran; BNR Department of Neurology, King George's Medical University, Lucknow, India; BNS Rabigh Faculty of Medicine, King Abdulaziz University, Jeddah, Saudi Arabia; BNT University Institute of Public Health, The University of Lahore, Lahore, Pakistan; BNU Institute of Health, Jimma University, Jimma, Ethiopia; BNV Division of Research and Development, Lovely Professional University, Phagwara, India; BNW Department of Maternal-Child Nursing and Public Health, Federal University of Minas Gerais, Belo Horizonte, Brazil; BNX Poche Centre for Indigenous Health, The University of Queensland, Brisbane, QLD, Australia; BNY University of Kansas Medical Center, A.T. Still University, Kansas City, KS, USA; BNZ Internal Medicine Department, MedStar Health, Washington, DC, USA; BOA Internal Medicine Department, Eisenhower Health, Palm Desert, CA, USA; BOB Corporate Nursing and Midwifery Research Department, Hamad Medical Corporation, Doha, Qatar; BOC International Center for Chemical and Biological Sciences, University of Karachi, Karachi, Pakistan; BOD Department of Epidemiology and Biostatistics, Isfahan University of Medical Sciences, Isfahan, Iran; BOE Biomedical Engineering Research Center (CREB), Universitat Politècnica de Catalunya (Barcelona Tech - UPC), Barcelona, Spain; BOF West Moreton Hospital Health Services, Queensland Centre for Mental Health Research, Wacol, QLD, Australia; BOG School of Medicine and Surgery, University of Milan Bicocca, Monza, Italy; BOH Department of Urology, Anhui Medical University, Hefei, China; BOI Department of Food, Nutrition and Health, United Arab Emirates University, Al Ain, United Arab Emirates; BOJ Department of Biomedical Engineering, University of Isfahan, Isfahan, Iran; BOK Automatic Control Department, Universitat Politècnica de Catalunya (Barcelona Tech - UPC), Barcelona, Spain; BOL Far Eastern University, Manila, Philippines; BOM Department of Neurosurgery, Icahn School of Medicine at Mount Sinai, New York, NY, USA; BON Department of Food, Environmental and Nutritional Sciences, University of Milan, Milano, Italy; BOO Faculty of Human Kinetics, University of Lisbon, Lisbon, Portugal; BOP Department of Energy, Environmental, and Chemical Engineering, Washington University in St. Louis, St. Louis, MO, USA; BOQ Department of Physics and Atmospheric Science, Dalhousie University, Halifax, NS, Canada; BOR Department of Economics, Instituto Tecnologico Autonomo de Mexico (Autonomous Technology Institute of Mexico), Mexico City, Mexico; BOS Department of Infectious Diseases, Instituto Nacional de Nutrición Salvador Zubirán (Salvador Zubiran National Institute of Medical Sciences and Nutrition), Mexico City, Mexico; BOT Department of Non-communicable Diseases and Mental Health, Pan American Health Organization, Washington, DC, USA; BOU Department of Food, Environmental and Nutritional Sciences, University of Milan, Milan, Italy; BOV Campus Fortaleza, Federal Institute of Education, Science and Technology of Ceará, Fortaleza, Brazil; BOW Department of Nutrition and Dietetics, University of Concepción, Concepción, Chile; BOX Centre for Healthy Living, University of Concepción, Concepción, Chile; BOY Clinical Institute of Medical and Chemical Laboratory Diagnostics, Medical University of Graz, Graz, Austria; BOZ Medical Clinic V, Heidelberg University, Mannheim, Germany; BPA Faculty of Humanities and Health Sciences, Curtin University, Sarawak, Malaysia; BPB Jeffrey Cheah School of Medicine and Health Sciences, Monash University, Subang Jaya, Malaysia; BPC Medical Scientist Training Program, Northwestern University, Chicago, IL, USA; BPD Department of Clinical and Experimental Medicine, University of Pisa, Pisa, Italy; BPE Department of Dermatology, Carol Davila University of Medicine and Pharmacy, Bucharest, Romania; BPF Board of Directors, Association of Resident Physicians, Bucharest, Romania; BPG Department of Anatomy and Developmental Biology, Monash University, Clayton, VIC, Australia; BPH Department of Anatomy, Genetics and Biomedical Informatics, University of Colombo, Colombo, Sri Lanka; BPI University of Sydney, University of Sydney, Sydney, NSW, Australia; BPJ Division of Immunology, Immunity to Infection and Respiratory Medicine, University of Manchester, Manchester, UK; BPK North West Lung Centre, Manchester University NHS Foundation Trust, Manchester, UK; BPL Health Policy Research, Public Health Foundation of India, Gurugram, India; BPM Institute of Population Health Sciences, University of Liverpool, Liverpool, UK; BPN Department of Community Medicine, Geetanjali Medical College and Hospital in Udaipur India, Udaipur, India; BPO Department of Social Medicine, Federal University of Rio Grande do Sul, Porto Alegre, Brazil; BPP Department of Prosthetic Dental Sciences, Jazan University, Jazan, Saudi Arabia; BPQ Department of Epidemiology, Mahidol-Oxford Tropical Medicine Research Unit, Bangkok, Thailand; BPR Nuffield Department of Medicine, University of Oxford, Oxford, UK; BPS Research Division, The George Institute for Global Health, New Delhi, India; BPT School of Medicine, University of New South Wales, Sydney, NSW, Australia; BPU Department of Social Medicine and Family, Dezful University of Medical Sciences, Dezful, Iran; BPV Department of Biomedical and Neuromotor Sciences, University of Bologna, Bologna, Italy; BPW Orthopedic Trauma Pathology Department, IRCCS, Bologna, Italy; BPX Department of Paediatrics, Nnamdi Azikiwe University, Nnewi, Nigeria; BPY Department of Obstetrics and Gynaecology, Nnamdi Azikiwe University, Awka, Nigeria; BPZ Department of Health Services Research and Policy, London School of Hygiene & Tropical Medicine, London, UK; BQA Australian Centre for Health Services Innovation, Queensland University of Technology, Brisbane, QLD, Australia; BQB Digital Health and Informatics Directorate, Queensland Health, Brisbane, QLD, Australia; BQC Department of Healthcare, University of Vlora, Vlora City, Albania; BQD Clinic of Social and Family Medicine, University of Crete, Heraklion, Greece; BQE Division of Pediatric Hospital Medicine, Stanford University, Stanford, CA, USA; BQF Amir Oncology Hospital, Shiraz University of Medical Sciences, Shiraz, Iran; BQG Department of Epidemiology, Adigrat University, Adigrat, Ethiopia; BQH Department of Public Health, Jazan University, Jazan, Saudi Arabia; BQI Neurology Department, Janakpuri Super Specialty Hospital Society, New Delhi, India; BQJ Department of Neurology, Govind Ballabh Institute of Medical Education and Research, New Delhi, India; BQK Tehran Heart Center, Cardiovascular Diseases Research Institute, Tehran University of Medical Sciences, Tehran, Iran; BQL Department of Epidemiology and Biostatistics, University of California San Francisco, San Francisco, CA, USA; BQM Department of Dental Research Cell, Dr. D. Y. Patil University, Pune, India; BQN Department of Biosciences and Bioengineering, Indian Institute of Technology Dharwad, Dharwad, India; BQO Institute for Sustainable Agriculture and Food Economics (INL), Martin Luther University Halle-Wittenberg, Halle, Germany; BQP Office of Innovation, Competence Cluster for Nutrition and Cardiovascular Health (nutriCARD), Halle, Germany; BQQ Department of Public Health, Arba Minch University, Arba Minch, Ethiopia; BQR Department of Medical Laboratory Sciences, Adigrat University, Adigrat, Ethiopia; BQS Department of Internal Medicine, University of Gondar, Gondar, Ethiopia; BQT Johns Hopkins University, Baltimore, MD, USA; BQU Department of General Practice, Monash University, Melbourne, VIC, Australia; BQV Health Care Authority, Olympia, WA, USA; BQW Dirección General de Investigación, Desarrollo e Innovación (DGIDI), Universidad Científica del Sur (University of the South), Lima, Peru; BQX Department of Medical Microbiology and Immunology, Trinity Medical Sciences University, St. Vincent, Saint Vincent and the Grenadines; BQY Kasturba Medical College, Manipal Academy of Higher Education, Mangalore, India; BQZ Department of Pathology, Imam Abdulrahman Bin Faisal University, Dammam, Saudi Arabia; BRA School of Midwifery, University of Gondar, Gondar, Ethiopia; BRB Department of Physiology, King Saud University, Riyadh, Saudi Arabia; BRC Department of Public Health, University “Federico II” of Naples, Naples, Italy; BRD General Administration Department, Helsinki University Hospital, Helsinki, Finland; BRE School of Health Sciences, University of Melbourne, Melbourne, VIC, Australia; BRF Comprehensive Cancer Center, Helsinki University Hospital, Helsinki, Finland; BRG University of Helsinki, Helsinki, Finland; BRH University Centre Varazdin, University North, Varazdin, Croatia; BRI Department of Pharmacology, University of Kelaniya, Ragama, Sri Lanka; BRJ Clinical Medicine Department, Colombo North Teaching Hospital, Ragama, Sri Lanka; BRK Department of Paediatrics, University of Kelaniya, Ragama, Sri Lanka; BRL University Paediatrics Unit, Colombo North Teaching Hospital, Ragama, Sri Lanka; BRM Department of Pathology, Zagazig University, Zagazig, Egypt; BRN Stritch School of Medicine, Loyola University Chicago, Chicago, IL, USA; BRO Dermatology Unit, Fondazione IRCCS Policlinico San Matteo, Pavia, Italy; BRP Department of Oncology, Addis Ababa University, Addis Abeba, Ethiopia; BRQ Queensland Centre for Mental Health Research (QCMHR), The University of Queensland, Wacol, QLD, Australia; BRR College of Human Medicine, Michigan State University, Flint, MI, USA; BRS Multidisciplinary Department of Medical-Surgical and Dental Specialties, University of Campania Luigi Vanvitelli, Naples, Italy; BRT Saveetha Dental College and Hospitals, Saveetha University, Chennai, India; BRU Department of Public Health Dentistry, Saveetha Institute of Medical and Technical Sciences, Chennai, India; BRV Global Institute of Public Health, Ananthapuri Hospitals and Research Institute, Trivandrum, India; BRW Faculty of Nursing and Midwifery, Tabriz University of Medical Sciences, Tabriz, Iran; BRX Internal Medicine Programme, Kyrgyz State Medical Academy, Bishkek, Kyrgyzstan; BRY Department of Atherosclerosis and Coronary Heart Disease, National Center of Cardiology and Internal Disease, Bishkek, Kyrgyzstan; BRZ University Health Network, University of Toronto, Toronto, ON, Canada; BSA Department of Radiology, Health Sciences North, Sudbury, ON, Canada; BSB Bergen Center for Ethics and Priority Setting, University of Bergen, Bergen, Norway; BSC Department of Community Medicine, Apollo Institute of Medical Sciences and Research, Hyderabad, India; BSD Thumbay College of Management and AI in Healthcare, Gulf Medical University, Ajman, United Arab Emirates; BSE Research and Development Department, Panacea Institute of Interdisciplinary Research and Education, Varanasi, India; BSF Department of Pharmacology, All India Institute of Medical Sciences, Mangalagiri, India; BSG Discipline of Psychiatry and Mental Health, University of New South Wales, Sydney, NSW, Australia; BSH Central Clinical School, Faculty of Medicine and Health, University of Sydney, Sydney, NSW, Australia; BSI Melbourne School of Population and Global Health, University of Melbourne, Melbourne, VIC, Australia; BSJ Environment Research Center, Isfahan University of Medical Sciences, Isfahan, Iran; BSK Department of Environmental Health Engineering, Isfahan University of Medical Sciences, Isfahan, Iran; BSL Burn and Regenerative Medicine Research Center, Guilan University of Medical Sciences, Rasht, Iran; BSM Department of Internal Medicine, Albert Einstein Hospital, Philadelphia, PA, USA; BSN College of Applied and Natural Science, University of Hargeisa, Hargeisa, Somalia; BSO RAK College of Nursing, RAK Medical and Health Sciences University, Ras Alkhima, United Arab Emirates; BSP Nursing College, Sohag University, Sohag, Egypt; BSQ Molecular Biology Unit, Sirius Training and Research Centre, Khartoum, Sudan; BSR Bio-Statistical and Molecular Biology Department, Sirius Training and Research Centre, Khartoum, Sudan; BSS Faculty of Medicine, University of Khartoum, Khartoum, Sudan; BST Department of Biophysics, All India Institute of Medical Sciences, New Delhi, India; BSU Social Determinants of Health Research Center, Tabriz University of Medical Sciences, Tabriz, Iran; BSV Midwifery Department, Tabriz University of Medical Sciences, Tabriz, Iran; BSW Urology Department, Tehran University of Medical Sciences, Tehran, Iran; BSX Department of Bacteriology, Tarbiat Modares University, Tehran, Iran; BSY Department of Medicine, Anne Burnett Marion School of Medicine at Texas Christian University, Fort Worth, TX, USA; BSZ Modeling in Health Research Center, Shahrekord University of Medical Sciences, Shahrekord, Iran; BTA Skull Base Research Center, Shahid Beheshti University of Medical Sciences, Tehran, Iran; BTB Department of Pharmaceutics, Tabriz University of Medical Sciences, Tabriz, Iran; BTC School of Pharmacy, Haramaya University, Harar, Ethiopia; BTD Department of Public Health, Dire Dawa University, Dire Dawa, Ethiopia; BTE Department of Medicine, Government Medical College Kozhikode, Kozhikode, India; BTF Health Systems and Policy Research Unit, Ahmadu Bello University, Zaria, Nigeria; BTG Department of Health Sciences, Azare, National Institute for Research in Tribal Health, Bauchi, Nigeria; BTH Medical Microbiology Department, Usmanu Danfodiyo University, Sokoto, Sokoto, Nigeria; BTI Medical Microbiology Department, Usmanu Danfodiyo University Teaching Hospital, Sokoto, Nigeria; BTJ Department of Pharmacology, Dale View College of Pharmacy and Research Centre, Thruvananthapuram, India; BTK School of Health Sciences, University of Petroleum and Energy Studies, Dehradun, India; BTL Department of Applied Biology, University of Science and Technology Meghalaya, Ri-Bhoi, India; BTM Department of Health Services Management, Iran University of Medical Sciences, Iran, Iran; BTN Department of Health Services Management, Isfahan University of Medical Sciences, Isfahan, Iran; BTO Maternal and Childhood Obesity Research Center, Urmia University of Medical Sciences, Urmia, Iran; BTP Kazakh-Russian Medical University, Almaty, Kazakhstan; BTQ Institute of Clinical Physiology, National Research Council, Pisa, Italy; BTR Department Medical-Surgical Nursing, Golestan University of Medical Sciences, Gorgan, Iran; BTS Department of Mathematics, The University of Jordan, Amman, Jordan; BTT Nonlinear Dynamics Research Center (NDRC), Ajman University, Ajman, United Arab Emirates; BTU Clinical Epidemiology and Public Health Research Unit, Burlo Garofolo Institute for Maternal and Child Health, Trieste, Italy; BTV Department of Sport Physiology, Razi University, Kermanshah, Iran; BTW Department of Physiology, All India Institute of Medical Sciences, Deoghar, India; BTX Department of Biomedical and Dental Sciences and Morphofunctional Imaging, Messina University, Messina, Italy; BTY Department of Medicine, TMSS Medical College, Bogura, Bangladesh; BTZ Department of Medicine, Sofia Ismail Memorial Medical Centre, Bogura, Bangladesh; BUA Centre for Neonatal and Paediatric Infection, St. George's University of London, London, UK; BUB Department of Epidemiology and Biostatistics, Kurdistan University of Medical Sciences, Sanandaj, Iran; BUC Gastrointestinal and Liver Diseases Research Center, Iran University of Medical Sciences, Tehran, Iran; BUD Preventive Medicine and Public Health Research Center, Iran University of Medical Sciences, Tehran, Iran; BUE Computer, Electrical, and Mathematical Sciences and Engineering Division, King Abdullah University of Science and Technology, Thuwal, Saudi Arabia; BUF International Laboratory for Air Quality and Health, Queensland University of Technology, Brisbane, QLD, Australia; BUG Department of Public Health, Oswaldo Cruz Foundation, Recife, Brazil; BUH Department of Public Health, Federal University of Pernambuco, Recife, Brazil; BUI Clinical Research Development Unit, Mashhad University of Medical Sciences, Mashhad, Iran; BUJ Baan Clinic, Baan Clinic, Tehran, Iran; BUK Faculty of Medicine, October 6 University, Giza, Egypt; BUL Department of Biology and Biological Engineering, Chalmers University of Technology, Gothenburg, Sweden; BUM College of Medical Sciences, SGMK Copernicus University, Warsaw, Poland; BUN Neurosciences Research Center (NSRC), Tabriz University of Medical Sciences, Tabriz, Iran; BUO Student Research Committee, Tabriz University of Medical Sciences, Tabriz, Iran; BUP NHS National Services Scotland, Belfast, UK; BUQ Social Determinants of Health Research Center, Babol University of Medical Sciences, Babol, Iran; BUR Department of Audiology, School of Rehabilitation, Shahid Beheshti University of Medical Sciences, Tehran, Iran; BUS Faculty of Biotechnologies, ITMO University, Saint Petersburg, Russia; BUT Department of Physical and Environmental Sciences, Texas A&M University, Corpus Christi, TX, USA; BUU Shiraz University of Medical Sciences, Shiraz, Iran; BUV School of Pharmacy, Management and Science University (MSU), Shah Alam, Malaysia; BUW International University of Africa (IUA), Khartoum, Sudan; BUX Medical Microbiology and Immunology Department, Cairo University, Cairo, Egypt; BUY Antimicrobial Resistance Research Center, Iran University of Medical Sciences, Tehran, Iran; BUZ Hazrat-e Rasool General Hospital, Iran University of Medical Sciences, Tehran, Iran; BVA René Rachou Institute, Oswaldo Cruz Foundation, Belo Horizonte, Brazil; BVB Department of Radiology, University of Tripoli, Tripoli, Libya; BVC PMAS Arid Agriculture University Rawalpindi, Rawalpindi, Pakistan; BVD Unit of Pharmacotherapy, Epidemiology and Economics, University of Groningen (Rijksuniversiteit Groningen), Groningen, Netherlands; BVE Department of Epidemiology and Biostatistics, Wuhan University, Wuhan, China; BVF College of Nursing, All India Institute of Medical Sciences, Deoghar, India; BVG Institute of Molecular Biology and Biotechnology, Bahauddin Zakariya University Multan, Multan, Pakistan; BVH International Ph.D. Program in Biotech and Healthcare Management, Taipei Medical University, Taipei, Taiwan; BVI Department of Biochemistry, All India Institute of Medical Sciences, Bhopal, India; BVJ Knowledge Management Department, Prahlad Omkarwati Foundation (POF), Mumbai, India; BVK Independent Consultant, New Delhi, India; BVL Department of Medicine, National University Health System, Singapore, Singapore; BVM Department of Endocrinology & Metabolism, Institute of Post-Graduate Medical Education and Research and Seth Sukhlal Karnani Memorial Hospital, Kolkata, India; BVN Department of Mechanical Engineering, North Carolina Agricultural and Technical State University, Greensboro, NC, USA; BVO Department of Pediatrics and Child Health Nursing, Bahir Dar University, Bahir Dar, Ethiopia; BVP Department of Surgery, General University Hospital of Patras, Patras, Greece; BVQ Faculty of Medicine, University of Thessaly, Larissa, Greece; BVR Department of Pediatrics and Child Health Nursing, Debre Berhan University, Debre Berhan, Ethiopia; BVS College of Health Science, Woldia University, Woldia, Ethiopia; BVT Department of Nursing, Sam Ratulangi University, Manado, Indonesia; BVU Department of Medicine, Allama Iqbal Medical College, Lahore, Pakistan; BVV Department of Health Economics, National Institute for Research in Tuberculosis, Chennai, India; BVW Amity Institute of Pharmacy, Amity University, Noida, India; BVX Department of Community and Global Health, The University of Tokyo, Tokyo, Japan; BVY Epidemiology, Biostatistics and Prevention Institute (EBPI), University of Zürich, Zurich, Switzerland; BVZ Department of Human Genetics and Molecular Medicine, Central University of Punjab, Bathinda, India; BWA Manipal College of Pharmaceutical Sciences, Manipal Academy of Higher Education, Manipal, India; BWB Center for Infectious Disease Education and Research, The University of Osaka, Suita, Japan; BWC School of Postgraduate research and publications, Amoud University, Hargeisa, Somalia; BWD Department of Pediatrics & Pediatric Pulmonology, Institute of Mother & Child Care, Multan, Pakistan; BWE Department of Pathology and Microbiology, Duhok University, Duhok, Iraq; BWF Operational Research Center in Healthcare, Near East University, Nicosia, Cyprus; BWG Department of Research Methods, Orthopaedic Research Group, Coimbatore, India; BWH Central Research Laboratory, Meenakshi Medical College Hospital and Research Institute, Chennai, India; BWI University of Tabuk, Tabuk, Saudi Arabia; BWJ Prince Fahad bin Sultan Chair for Biomedical Research, University of Tabuk, Tabuk, Saudi Arabia; BWK Director General, Rwanda Biomedical Centre, Kigali, Rwanda; BWL College of Medicine and Health Sciences, University of Rwanda, Kigali, Rwanda; BWM Department of Technology, The University of Lahore, Lahore, Pakistan; BWN Research Centre for Health Sciences (RCHS), The University of Lahore, Lahore, Pakistan; BWO Department of Psychiatry, Seoul National University, Seoul, South Korea; BWP Department of Neuropsychiatry, Seoul National University Bundang Hospital, Seongnam, South Korea; BWQ Department of Ophthalmology, University of Tennessee, Memphis, TN, USA; BWR Department of Geriatric Health, Tabriz University of Medical Sciences, Tabriz, Iran; BWS Department of Health Education & Promotion, Gonabad University of Medical Sciences, Gonabad, Iran; BWT Research and Analytics Department, Initiative for Financing Health and Human Development, Chennai, India; BWU Department of Research and Analytics, Bioinsilico Technologies, Chennai, India; BWV Department of Nephrology, Manipal Academy of Higher Education, Manipal, India; BWW Department of Computer Science, University of Illinois, Champaign, IL, USA; BWX Department of Computer Science and IT, Torrens University, Adelaide, SA, Australia; BWY Department Health Services Research, University of Alabama at Birmingham, Birmingham, AL, USA; BWZ Department of Medicine, University of British Columbia, Vancouver, BC, Canada; BXA Faculty of Pharmacy, Hasanuddin University, Makassar, Indonesia; BXB Department of Pulmonary Medicine, Government Medical College, Thrissur, Thrissur, India; BXC Health Action by People, Trivandrum, India; BXD Neuroscience Research Center, Isfahan University of Medical Sciences, Isfahan, Iran; BXE Department of Physiotherapy, Tehran University of Medical Sciences, Tehran, Iran; BXF Research Center for War-affected People, Tehran University of Medical Sciences, Tehran, Iran; BXG Suraj Eye Institute, Nagpur, India; BXH Department for the Control of Disease, Epidemics, and Pandemics, Ministry of Public Health, Yaoundé, Cameroon; BXI Department of Public Heath, University of Yaoundé I, Yaoundé, Cameroon; BXJ Department of Biomedical Sciences, University of Zakho, Zakho, Iraq; BXK Bristol Medical School, University of Bristol, Bristol, UK; BXL Department of Clinical Medicine, Federal University of Minas Gerais, Belo Horizonte, Brazil; BXM Clinical Hospital, Federal University of Minas Gerais, Belo Horizonte, Brazil; BXN National Dental Research Institute Singapore, Duke-NUS Medical School, Singapore, Singapore; BXO Department of Applied Pharmaceutical Sciences and Clinical Pharmacy, Isra University, Amman, Jordan; BXP Critical Care Unit, Sheikh Shakhbout Medical City hospital, Abu Dhabi, United Arab Emirates; BXQ Division of Endocrinology and Diabetes, University of Vermont, South Burlington, VT, USA; BXR Department of Dental Public Health, King Abdulaziz University, Jeddah, Saudi Arabia; BXS Department of Health Policy and Oral Epidemiology, Harvard University, Boston, MA, USA; BXT Department of Circulation and Medical Imaging, Norwegian University of Science and Technology, Trondheim, Norway; BXU Department of Internal Medicine, Khyber Medical University, Islamabad, Pakistan; BXV School of Earth and Environmental Sciences, Cardiff University, Cardiff, UK; BXW Manipal College of Nursing, Manipal Academy of Higher Education, Manipal, India; BXX Department of Pediatrics, Arak University of Medical Sciences, Arak, Iran; BXY Department of Research, TroDDIVaT Initiative, Buea, Cameroon; BXZ Department of Disease Control and Environmental Health, Makerere University, Kampala, Uganda; BYA Department of Management Science and Project Planning, University of Nairobi, Nairobi, Kenya; BYB Department of Public Health, Johns Hopkins University, Baltimore, MD, USA; BYC MyungSung Medical College, Addis Ababa, Ethiopia; BYD Department of General Surgery, Carol Davila University of Medicine and Pharmacy, Bucharest, Romania; BYE Department of General Surgery, Emergency University Hospital Bucharest, Bucharest, Romania; BYF Department of Anatomy and Embryology, Carol Davila University of Medicine and Pharmacy, Bucharest, Romania; BYG Department of Cardiology, Cardio-Aid, Bucharest, Romania; BYH Department of Cardiology, University of Medicine and Pharmacy “Victor Babes” Timisoara, Romania; BYI Rocordis Heart Center, Cardiology and Cardiovascular Surgery Hospital, Timisoara, Romania; BYJ Health Promotion Research Center, Zahedan University of Medical Sciences, Zahedan, Iran; BYK Euromed Research Center, Euromed University of Fes, Fez, Morocco; BYL Faculty of Medicine, Pharmacy, and Dentistry, University Sidi Mohammed Ben Abdellah, Fez, Morocco; BYM Department of Community Medicine, Lumbini Medical College, Palpa, Nepal; BYN School of Nursing, University of Gondar, Gondar, Ethiopia; BYO Department of Psychiatry, University of Oxford, Oxford, UK; BYP Department of Neurosciences, Kenya Medical Research Institute/Wellcome Trust Research Programme, Kilifi, Kenya; BYQ Department of Public Health, University of Yaoundé I, Yaoundé, Cameroon; BYR Department of Biological Sciences, University of Embu, Embu, Kenya; BYS Institute for Global Health Innovations, Duy Tan University, Hanoi, Vietnam; BYT Faculty of Medicine, Nam Can Tho University, Can Tho, Vietnam; BYU International Medical Faculty, Nam Can Tho University, Can Tho, Vietnam; BYV Cardiovascular Research Department, Methodist Hospitals, Merrillville, IN, USA; BYW Department of Surgery, Danang Family Hospital, Danang, Vietnam; BYX Hitotsubashi Institute for Advanced Study (HIAS), Hitotsubashi University, Tokyo, Japan; BYY Institute for Cancer Control, National Cancer Center, Chuo-ku, Japan; BYZ Faculty of Public Health, VNU University of Medicine and Pharmacy, Hanoi, Vietnam; BZA Faculty of Medicine, Duy Tan University, Da Nang, Vietnam; BZB Department of Pediatrics, New York Medical College, New York, NY, USA; BZC Tuberculosis Group, Oxford University Clinical Research Unit, Vietnam, Ho Chi Minh City, Vietnam; BZD Department of General Medicine, University of Medicine and Pharmacy at Ho Chi Minh City, Ho Chi Minh City, Vietnam; BZE Department of Public Health, University of Bamenda, Bamenda, Cameroon; BZF International Islamic University Islamabad, Islamabad, Pakistan; BZG Population Research Institute, Nanjing University of Posts and Telecommunications, Nanjing, China; BZH Department of Humanities and Social Science, University for International Studies in Rome, Rome, Italy; BZI Institute for Mental Health Policy Research, Centre for Addiction and Mental Health, Toronto, ON, Canada; BZJ Public Health Department, Universitas Negeri Semarang (State University of Semarang & ReverseEquilibrium;), Kota Semarang, Indonesia; BZK Graduate Institute of Biomedical Informatics, Taipei Medical University, Taipei, Taiwan; BZL School of Medicine, University of Limerick, Limerick, Ireland; BZM Department of Public Health, UNICAF, Larnaca, Cyprus; BZN Department of Pathology, Hawassa University, Hawassa, Ethiopia; BZO Department of Internal Medicine and Specialties, University of Yaoundé I, Yaounde, Cameroon; BZP College of Health Sciences, University of Ghana, Accra, Ghana; BZQ Technical Department, University of Cape Town, Cape Town, South Africa; BZR School of Public Health and Family Medicine, University of Cape Town, Cape Town, South Africa; BZS School of Chemical & Biomolecular Engineering, University of Sydney, Sydney, NSW, Australia; BZT Faculty of Applied Sciences, Taiz University, Taiz, Yemen; BZU Global Research Institute, Keio University, Tokyo, Japan; BZV Family Health Research Institute, Tehran University of Medical Sciences, Tehran, Iran; BZW Canadian Institute for Health Information, Toronto, ON, Canada; BZX Department of Microbiology and Molecular Genetics, The Women University Multan, Multan, Pakistan; BZY School of Biomedical Engineering, Science and Health Systems, Drexel University, Philadelphia, PA, USA; BZZ Division of Cardiology, University of California San Francisco, San Francisco, CA, USA; CAA Internal Medicine Department, Maimonides Medical Center, Brooklyn, NY, USA; CAB Department of Paediatrics, Nnamdi Azikiwe University, Awka, Nigeria; CAC Global Health Department, Euclid University, Banqui, Central African Republic; CAD School of Information, University of California Berkeley, Berkeley, CA, USA; CAE Department of Public Health, Mattu University, Mattu, Ethiopia; CAF Department of Non-communicable Disease Epidemiology, London School of Hygiene & Tropical Medicine, London, UK; CAG Center for Health System and Strategy, Ministry of Health, Jakarta, Indonesia; CAH Department of Public Health, Banten School of Health Science, South Tangerang, Indonesia; CAI Ministry of Research, Technology and Higher Education, Higher Education Service Institutions (LL-DIKTI) Region IV, Bandung, Indonesia; CAJ Department of Nursing, University of Health and Allied Sciences, Ho, Ghana; CAK Center of Excellence in Reproductive Health Innovation (CERHI), University of Benin, Benin City, Nigeria; CAL Department of Physiology, University of Benin, Edo, Nigeria; CAM Department of Physiology, Benson Idahosa University, Benin City, Nigeria; CAN Department of Applied Economics and Quantitative Analysis, University of Bucharest, Bucharest, Romania; CAO Bioinformatics Department, National Institute of Research and Development for Biological Sciences, Bucharest, Romania; CAP College of Computing, Data Science and Society, University of California Berkeley, Berkeley, CA, USA; CAQ Brown School, Washington University in St. Louis, St. Louis, MO, USA; CAR Department of Biomedicine and Prevention, University of Rome “Tor Vergata” Rome, Italy; CAS Department of Veterinary Public Health and Preventive Medicine, University of Ilorin, Ilorin, Nigeria; CAT Department of Community Health and Primary Care, University of Lagos, Idi Araba, Nigeria; CAU Department of Family and Preventive Medicine, University of Utah, Salt Lake City, UT, USA; CAV Department of Population and Health, University of Cape Coast, Cape Coast, Ghana; CAW PSSM Data Sciences, Pfizer Research & Development, Pfizer Inc., Groton, CT, USA; CAX Department of Physiology, Adeleke University, Ede, Nigeria; CAY Department of Veterinary Public Health and Preventive Medicine, University of Ibadan, Ibadan, Nigeria; CAZ Department of Health Promotion and Education, University of Ibadan, Ibadan, Nigeria; CBA Sheffield Centre for Health and Related Research, University of Sheffield, Sheffield, UK; CBB Institute for Global Engagement & Empowerment, Yonsei University, Seoul, South Korea; CBC Westmead Applied Research Center, University of Sydney, Sydney, NSW, Australia; CBD University of Sydney, Sydney, NSW, Australia; CBE Counselling and Human Development Studies, University of Ibadan, Ibadan, Nigeria; CBF Department of Food and Nutrition, Seoul National University, Seoul, South Korea; CBG College of Medicine, University of Ibadan, Ibadan, Nigeria; CBH Faculty of Medicine, University of Thessaly, Volos, Greece; CBI Department of Medical Laboratory Science, Federal Neuropsychiatric Hospital, Abeokuta, Nigeria; CBJ Management Science and Healthcare Analytics, University of Michigan, Ann Arbor, MI, USA; CBK School of Pharmacy, University of the Western Cape, Cape Town, South Africa; CBL Department of Education Leadership and Management, University of Johannesburg, Johannesburg, South Africa; CBM Mathematical and Computer Sciences, University of Medical Sciences, Ondo, Ondo, Nigeria; CBN College of Health Sciences, Bowen University, Iwo, Nigeria; CBO Department of Psychiatry and Behavioural Neurosciences, McMaster University, Hamilton, ON, Canada; CBP Department of Psychiatry, University of Lagos, Lagos, Nigeria; CBQ Department of Neurology, University College Hospital, Ibadan, Ibadan, Nigeria; CBR Department of Medicine, University of Ibadan, Ibadan, Nigeria; CBS Department of Nursing Science, Bowen University, Iwo, Nigeria; CBT Cumming School of Medicine, University of Calgary, Calgary, AB, Canada; CBU Center for Clinical and Epidemiological Research, University of São Paulo, São Paulo, Brazil; CBV Associação Brasileira de Cefaleia em Salvas e Enxaqueca (ABRACES), São Paulo, Brazil; CBW Cardiology Department, Federal University of Rio de Janeiro, Rio de Janeiro, Brazil; CBX School of Health and Life Sciences, Teesside University, Middlesbrough, UK; CBY Department of Epidemiology, Johns Hopkins University, Baltimore, MD, USA; CBZ School of Public Health, Makerere University, Kampala, Uganda; CCA Centre for Healthy Start Initiative, Lagos, Nigeria; CCB Research Policy & Administration, Centre for Healthy Start Initiative, Lagos, Nigeria; CCC Department of Pharmacology and Therapeutics, Olabisi Onabanjo University, Sagamu, Nigeria; CCD Institute of Infectious Disease and Molecular Medicine, University of Cape Town, Cape Town, South Africa; CCE Institute of Chemistry, Universidade Estadual de Campinas (State University of Campinas), Campinas, Brazil; CCF Department of Computational Biology, Brazilian Agricultural Research Institute (EMBRAPA), Campinas, Brazil; CCG Department of Pharmacy Practice and Pharmacotherapeutics, University of Sharjah, Sharjah, United Arab Emirates; CCH Department of Pharmacology and Toxicology, Beni-Suef University, Beni-Suef, Egypt; CCI Surgery Department, Sulaimani University, Sulaimani, Iraq; CCJ ENT Department, Tor Vergata University of Rome, Rome, Italy; CCK Lee Kong Chian School of Medicine, Nanyang Technological University, Singapore, Singapore; CCL Department of Global Health and Social Medicine, Harvard University, Boston, MA, USA; CCM Wellspring Research, Wellspring Center Indonesia, Jakarta, Indonesia; CCN Department of Pharmacology and Therapeutics, University of Nigeria Nsukka, Enugu, Nigeria; CCO Department of Pharmaceutical Services, University College Hospital, Ibadan, Ibadan, Nigeria; CCP Institute of Diagnostic and Interventional Radiology and Neuroradiology, University Hospital Essen, Essen, Germany; CCQ Department of Pharmacotherapy and Pharmaceutical Care, Medical University of Warsaw, Warsaw, Poland; CCR Department of Microbiology and Immunology, University of Health and Allied Sciences, Ho, Ghana; CCS Sickle Cell Unit, Ho Teaching Hospital, Ho, Ghana; CCT Department of Biotechnological and Applied Clinical Sciences, University of L'Aquila, L'Aquila, Italy; CCU Department of Neurology, ASL Avezzano-Sulmona-L'Aquila, L'Aquila, Italy; CCV Department of Neurosurgery, University of California San Francisco, San Francisco, CA, USA; CCW Department of Nephrology and Hypertension, IIS-Fundacion Jimenez Diaz, Madrid, Spain; CCX Department of Medicine, Autonomous University of Madrid, Madrid, Spain; CCY One Health Global Research Group, Universidad de las Americas (University of the Americas), Quito, Ecuador; CCZ Department of Biological Sciences, Njala University, Freetown, Sierra Leone; CDA University of Health and Allied Sciences, University of Health and Allied Sciences, Ho, Ghana; CDB Henry M Jackson School of International Studies, University of Washington, Seattle, WA, USA; CDC Cardiovascular Division, Harvard University, Boston, MA, USA; CDD School of Medicine, Western Sydney University, Bathurst, NSW, Australia; CDE Department of Optometry and Vision Science, University of KwaZulu-Natal, KwaZulu-Natal, South Africa; CDF Department of Biological Sciences, Elizade University, Ilara-Mokin, Nigeria; CDG Department of Preventive and Social Medicine, University of Otago, Dunedin, New Zealand; CDH Faculty of Nursing, Applied Science Private University, Amman, Jordan; CDI Independent Consultant, Gothenburg, Sweden; CDJ School of Public Health, Texila American University, Georgetown, Guyana; CDK School of Public Health, Haramaya University, Harar, Ethiopia; CDL School of Nursing, University of California San Francisco, San Francisco, CA, USA; CDM Faculty of Medicine, University Ferhat Abbas of Setif, Setif, Algeria; CDN Division of Infectious Diseases, University Hospital of Setif, Setif, Algeria; CDO Department of General Surgery, Central South University, ChangSha, China; CDP Department of Medicine, University College Hospital, Ibadan, Ibadan, Nigeria; CDQ West African Center for Cell Biology of Infectious Pathogens, University of Ghana, Legon-Accra, Ghana; CDR Department of Biochemistry and Nutrition, Nigerian Institute of Medical Research, Lagos, Nigeria; CDS Division of Medicine, University College London, London, UK; CDT School of Medicine, Keele University, Keele, UK; CDU Department of Biosciences and Biotechnology, University of Medical Sciences, Ondo, Ondo, Nigeria; CDV Operational Research Center in Healthcare, Near East University, Nicosia, Turkiye; CDW Department of Mathematical Sciences, Saveetha School of Engineering, SIMATS, Chennai, India; CDX Department of Respiratory Medicine, Jagadguru Sri Shivarathreeswara University, Mysore, India; CDY Department of Physical Medicine and Rehabilitation, Harvard University, Boston, MA, USA; CDZ Universidad San Ignacio de Loyola, Lima, Peru; CEA Department of Medicine, Richmond University Medical Center, Staten Island, NY, USA; CEB National School of Public Health, Institute of Health Carlos III, Madrid, Spain; CEC Department of Forensic Medicine and Toxicology, Manipal Academy of Higher Education, Mangalore, India; CED Department of Neurology, National Institute of Mental Health and Neurosciences, Bangalore, India; CEE Department of Mental Health, Hospital Universitari Vall d'Hebron (CIBERSAM), Barcelona, Spain; CEF Biomedical Research Networking Center for Mental Health Network (CiberSAM), Barcelona, Spain; CEG Primary Health Center, Directorate of Public Health and Family Welfare, Eluru District, India; CEH Menzies Institute for Medical Research, University of Tasmania, Hobart, TAS, Australia; CEI Department of Ophthalmology, Heidelberg University, Heidelberg, Germany; CEJ Centre for Medical Biotechnology, Amity University Uttar Pradesh, Noida, India; CEK Department of Neurological Sciences, University of Nebraska Medical Center, Omaha, NE, USA; CEL Anesthesiology Department of The Third Xiangya Hospital, Central South University, Changsha, China; CEM National Institute of Health Research and Development, Ministry of Health Indonesia, Jakarta, Indonesia; CEN Division of Ophthalmology & Visual Sciences, University of Nottingham, Nottingham, UK; CEO First Department of Ophthalmology, Aristotle University of Thessaloniki, Thessaloniki, Greece; CEP Department of Neurology, University of Bern, Biel/Biene, Switzerland; CEQ Department of Neurology, University of Cyprus, Nicosia, Cyprus; CER Department of Emergency Medicine, University of Thessaly, Larissa, Greece; CES Department of Emergency Medicine, University of Bern, Bern, Switzerland; CET Unit of Dermatology, IRCCS Ospedale San Raffaele, Milan, Italy; CEU University of Padua, Padua, Italy; CEV Department of Medicine and Surgery, University of Bologna, Bologna, Italy; CEW Medical University of Vienna, Vienna, Austria; CEX Department of Science and Mathematics, Deree-The American College of Greece, Athens, Greece; CEY Department of Biophysics, University of Athens, Athens, Greece; CEZ Department of Forensic Medicine and Toxicology, All India Institute of Medical Sciences, Rajkot, India; CFA Digestive Diseases Research Center, Tehran University of Medical Sciences, Tehran, Iran; CFB Cardiac Research Center, Tehran University of Medical Sciences, Tehran, Iran; CFC Department of Sociology, Anthropology, and Public Health, University of Maryland, Baltimore, MD, USA; CFD Department of Medical Humanities and Social Medicine, Kosin University, Busan, South Korea; CFE Department of Biomedical Data Science, Stanford University, Stanford, CA, USA; CFF Department of Psychiatry, All India Institute of Medical Sciences, Bhubaneswar, India; CFG Department of Primary Care and General Practice, Kazan State Medical University, Kazan, Russia; CFH Department of Cardiology, Parve Nursing Home, Sindkhed Raja, India; CFI School of Nursing, University of British Columbia, Vancouver, BC, Canada; CFJ Department of Medical Sciences, University of Torino, Torino, Italy; CFK Department of Imaging, AOU Città della Salute e della Scienza di Torino (AOU City of Health and Science of Turin), Torino, Italy; CFL Institute of Physiotherapy, Ashok and Rita Patel Institute of Physiotherapy, Anand, India; CFM Department of Physiotherapy, Charotar University of Science and Technology, Anand, India; CFN Marwadi University Research and Development Cell, Marwadi University, Rajkot, India; CFO Department of Cardiovascular Medicine, University of Tennessee, Nashville, TN, USA; CFP Department of Physiology, All India Institute of Medical Sciences, Nagpur, India; CFQ College of Dental Medicine, Roseman University of Health Sciences, South Jordan, UT, USA; CFR Second Propedeutic Department of Internal Medicine, Aristotle University of Thessaloniki, Thessaloniki, Greece; CFS Department of Human Anatomy, All India Institute of Medical Sciences, Bathinda, India; CFT Department of Genetics, Yale University, New Haven, CT, USA; CFU Department of Interventional Cardiology, Cedars Sinai Medical Center, Los Angeles, CA, USA; CFV Physiology Research Center, Iran University of Medical Sciences, Tehran, Iran; CFW Department of Physiology, Iran University of Medical Sciences, Tehran, Iran; CFX IRCCS Fondazione Don Carlo Gnocchi, Milan, Italy; CFY Department of Clinical and Experimental Sciences, University of Brescia, Brescia, Italy; CFZ Department of Public Health, Trnava University, Trnava, Slovakia; CGA Center for Research and Innovation, Ateneo De Manila University, Pasig City, Philippines; CGB Australian Institute of Health Innovation, Macquarie University, Sydney, NSW, Australia; CGC Research Institute for Medicines, Universidade de Lisboa (University of Lisbon), Lisbon, Portugal; CGD Centre for Fertility and Health, Norwegian Institute of Public Health, Oslo, Norway; CGE Department of Applied Nursing, Federal University of Minas Gerais, Belo Horizonte, Brazil; CGF Faculty of Medicine, Autonomous University of Madrid, Madrid, Spain; CGG Mario Negri Institute for Pharmacological Research, Bergamo, Italy; CGH School of Optometry and Vision Science, University of New South Wales, Sydney, NSW, Australia; CGI Department of Biochemistry and Pharmacology, Uzhhorod National University, Uzhhorod, Ukraine; CGJ Facultad de Medicina (Faculty of Medicine), Universidad Diego Portales (Diego Portales University), Santiago, Chile; CGK School of Cardiovascular and Metabolic Health, University of Glasgow, Glasgow, UK; CGL Department of Internal Medicine, University of Arizona, Tucson, AZ, USA; CGM Department of Cardiovascular Medicine, Mayo Clinic, Rochester, MN, USA; CGN Department of Integrative Biotechnology, Sungkyunkwan University, Suwon, South Korea; CGO School of Pharmacy, University of Nizwa, Nizwa, Oman; CGP Shanghai Mental Health Center, Shanghai Jiao Tong University, Shanghai, China; CGQ Departments of Psychiatry and Epidemiology, Columbia University, New York, NY, USA; CGR Department of Epidemiology, Hiroshima University, Hiroshima, Japan; CGS Basic Medical Sciences Department, Durban University of Technology, Durban, South Africa; CGT Maternal and Child Nursing Department, Federal University of Minas Gerais, Belo Horizonte, Brazil; CGU International Center of Medical Sciences Research, Islamabad, Pakistan; CGV Riphah International University, Islamabad, Pakistan; CGW Department of Promoting Health, Maternal-Infant, Excellence and Internal and Specialized Medicine (PROMISE) G. D'Alessandro, University of Palermo, Palermo, Italy; CGX Air and Climate Unit, European Commission, Ispra, Italy; CGY Department of Environmental Hygiene, German Environment Agency, Berlin, Germany; CGZ Mental Health Research Institute, Tomsk National Research Medical Center, Tomsk, Russia; CHA Siberian State Medical University, Tomsk, Russia; CHB Department of Dermatology, Sagore Dutta Hospital, Kolkata, India; CHC Data Driven Health Division, Hungarian Healthcare Management Association, Budapest, Hungary; CHD Institute of Digital Health Sciences, Semmelweis University, Budapest, Hungary; CHE Department of Data Management and Analysis, The INCLEN Trust International, New Delhi, India; CHF Department of Medical and Surgical Oncology, Miami Cancer Institute, Miami, FL, USA; CHG Wicking Dementia Research and Teaching Centre, University of Newcastle, Hobart, TAS, Australia; CHH Department of Orthopedics and Traumatology, University of Tampere, Tampere, Finland; CHI Management Department, Bucharest University of Economic Studies, Bucharest, Romania; CHJ Academy of Romanian Scientists, Bucharest, Romania; CHK Dalla Lana School of Public Health, University of Toronto, Toronto, ON, Canada; CHL Department of Internal Medicine, University of Novi Sad, Novi Sad, Serbia; CHM Clinic for Endocrinology, Diabetes and Metabolic Disorders, Clinical Center of Vojvodina, Novi Sad, Serbia; CHN University Medical Center Groningen, University of Groningen, Groningen, Netherlands; CHO Center of Excellence in Higher Education for Pharmaceutical Care Innovation, Padjadjaran University, Bandung, Indonesia; CHP Department of Occupational Health and Safety Engineering, Shiraz University of Medical Sciences, Shiraz, Iran; CHQ Department of Occupational Health and Safety Engineering, Ardabil University of Medical Science, Ardabil, Iran; CHR Non-communicable Diseases Research Center, Bam University of Medical Sciences, Bam, Iran; CHS Centro de Investigaciones Clinicas (Clinical Research Center), Fundación Valle del Lili (Valle del Lili Foundation), Cali, Colombia; CHT Centro PROESA, Universidad ICESI, Cali, Colombia; CHU Department of Humanities and Social Sciences, National Institute of Technology Rourkela, Rourkela, India; CHV Department of Community Medicine and Public Health, Tribhuvan University, Kathmandu, Nepal; CHW T.H. Chan School of Public Health, Harvard University, Boston, MA, USA; CHX Department of Fundamental Nursing, Universitas Airlangga (Airlangga University), Surabaya, Indonesia; CHY Research Center in Advancing Community Healthcare, Surabaya, Indonesia; CHZ Department of Microbiology, Manipal Academy of Higher Education, Manipal, India; CIA Medical Oncology Lab, All India Institute of Medical Sciences, New Delhi, India; CIB Department of Biochemistry, JSS Academy of Higher Education and Research, Mysuru, India; CIC Department of Dermatology, Venereology and Leprosy-DVL, Apollo Institute of Medical Sciences and Research, Hyderabad, India; CID Centre for Dental Education and Research, All India Institute of Medical Sciences, New Delhi, India; CIE Department of Biology, Universitas Airlangga (Airlangga University), Surabaya, Indonesia; CIF Department of Biostatistics, Epidemiology, and Informatics, University of Pennsylvania, Philadelphia, PA, USA; CIG Department of Medical Instrumentation Techniques Engineering, Al-Rafidain University College, Baghdad, Iraq; CIH Department of Cybersecurity, Kyiv National University of Construction and Architecture, Kyiv, Ukraine; CII Rory Meyers College of Nursing, New York University, New York, NY, USA; CIJ Guangdong Cardiovascular Institute, Guangdong Academy of Medical Sciences, Guangzhou, China; CIK Department of Respiratory and Critical Care Medicine, Henan University of Science and Technology, Luoyang, China; CIL Global Consortium for Public Health and Research, Datta Meghe Institute of Higher Education and Research, Wardha, India; CIM College of Nursing and Health Sciences, Jazan University, Jazan, Saudi Arabia; CIN Department of Community Medicine, Rajiv Gandhi Institute of Medical Sciences, Bengaluru, India; CIO Department of Biomaterials, Saveetha Dental College and Hospitals, SIMATS, Saveetha University, Chennai, India; CIP Research Center for Public Health and Nutrition, National Research and Innovation Agency, Jakarta, Indonesia; CIQ Manipal College of Dental Sciences, Manipal Academy of Higher Education, Manipal, India; CIR Oman Dental College, Muscat, Oman; CIS Department of Medical Oncology, Cancer Institute (W.I.A), Chennai, India; CIT Division of Psychology and Mental Health, University of Manchester, Manchester, UK; CIU Department of Epidemiology and Biostatistics, Shahrekord University of Medical Sciences, Shahrekord, Iran; CIV Research and Development Coordination, National Institute of Health, Islamabad, Pakistan; CIW Department of Community and Family Medicine, All India Institute of Medical Sciences, Jodhpur, India; CIX Department of Epidemiology, National Institute of Mental Health and Neurosciences, Bengaluru, India; CIY Infectious and Tropical Diseases Research Center, Tabriz University of Medical Sciences, Tabriz, Iran; CIZ Osh State University, Osh, Kyrgyzstan; CJA Director of Central Asia Research Collaboration Group, Asfendiyarov Kazakh National Medical University, Almaty, Kazakhstan; CJB Department of Environmental Health Engineering, Torbat Heydariyeh University of Medical Sciences, Torbat Heydariyeh, Iran; CJC Health Science Research Centre, Torbat Heydariyeh University of Medical Sciences, Torbat Heydariyeh, Iran; CJD Iranian National Center for Addiction Studies, Tehran University of Medical Sciences, Tehran, Iran; CJE Faculty of Health Sciences, Qaiwan International University, Sulaymaniyah, Iraq; CJF Department of Epidemiology, Institute of Epidemiology, Disease Control and Research (IEDCR), Dhaka, Bangladesh; CJG Department of Pathobiology and Population Sciences (PPS), Royal Veterinary College (RVC), London, UK; CJH College of Medicine and Health Sciences, National University of Science and Technology, Sohar, Oman; CJI Department of Biostatistics, National Institute of Preventive and Social Medicine, Dhaka, Bangladesh; CJJ Health Service Research and Quality of Life Center (CEReSS), Aix-Marseille University, Marseille, France; CJK Faculty of Medicine, University of Setif Algeria, Setif, Algeria; CJL LIRSSEI Research Lab, University of Setif Algeria, Setif, Algeria; CJM Division of Gynecology and Human Reproduction Physiopathology, IRCCS Azienda Ospedaliero-Universitaria di Bologna, Bologna, Italy; CJN Department of Medical, Surgical and Experimental Sciences, University of Sassari, Sassari, Italy; CJO Gynecology and Breast Care Center, Mater Olbia Hospital, Olbia, Italy; CJP Dr. Rajendra Prasad Government Medical College, Tanda, Kangra, India; CJQ Department of Cardiology, Dow University of Health Sciences, Karachi, Pakistan; CJR Department of Medicine, Dow University of Health Sciences, Karachi, Pakistan; CJS Department of Infectious Diseases and Tropical Medicine, Tehran University of Medical Sciences, Tehran, Iran; CJT Emergency Medicine Department, Sri Manakula Vinayagar Medical College and Hospital, Puducherry, India; CJU Physiology Research Center, Kerman University of Medical Sciences, Kerman, Iran; CJV Centre for Chronic Disease Control, New Delhi, India; CJW Department of Clinical Sciences, University of Sharjah, Sharjah, United Arab Emirates; CJX Department of Cardiology, Mansoura University, Mansoura, Egypt; CJY Department of Population Health, King Saud bin Abdulaziz University for Health Sciences, Jeddah, Saudi Arabia; CJZ Department of Midwifery, Ministry of Health of the Republic of Indonesia, Palu, Indonesia; CKA Department of Anatomy, Govt. Siddhartha Medical College, Vijayawada, India; CKB Department of Radiology, Stanford University, Stanford, CA, USA; CKC Saw Swee Hock School of Public Health, National University of Singapore, Singapore, Singapore; CKD Department of Biological Science and Bioengineering, Inha University, Incheon, South Korea; CKE Department of Biotechnology, Hislop College, Nagpur, India; CKF Department of Molecular Biology & Genetic Engineering, RTM Nagpur University, Nagpur, India; CKG South Asian Institute for Social Transformation (SAIST), Dhaka, Bangladesh; CKH Department of Epidemiology, Biostatistics and Occupational Health, McGill University, Montreal, QC, Canada; CKI Department of Dentistry, All India Institute of Medical Sciences, Bathinda, India; CKJ Department of Research, Eastern Scientific LLC, Richmond, KY, USA; CKK Planetary Health Research Centre (PHRC), Kathmandu, Nepal; CKL Centre for Clinical Pharmacology, University of Defence in Belgrade, Belgrade, Serbia; CKM Centre for Clinical Pharmacology, Medical College of Georgia at Augusta University, Belgrade, Serbia; CKN Department of Forensic Medicine and Toxicology, Jagadguru Sri Shivarathreeswara University, Mysore, India; CKO Department of Nursing and Midwifery, Golestan University of Medical Sciences, Gorgan, Iran; CKP Department of Community Medicine, Manipal Academy of Higher Education, Manipal, India; CKQ Department of Oral Medicine and Radiology, Nitte University, Mangalore, India; CKR Department of Oral Pathology, Microbiology and Forensic Odontology, Sharavathi Dental College and Hospital, Shimogga, India; CKS Institute of Collective Health, Federal University of Bahia, Salvador, Brazil; CKT Barcelona Institute for Global Health, Barcelona, Spain; CKU Iranian Research Center on Aging, University of Social Welfare and Rehabilitation Sciences, Tehran, Iran; CKV Epidemiology and Biostatistics, Kurdistan University of Medical Sciences, Sanandaj, Iran; CKW Department of Immunology, Shahid Beheshti University of Medical Sciences, Tehran, Iran; CKX Department of Geography, Soran University, Soran, Iraq; CKY Department of Family Medicine, Rajarata University of Sri Lanka, Anuradhapura, Sri Lanka; CKZ University of Swabi, University of Swabi, Swabi, Pakistan; CLA Department of Global Health Policy, University of Tokyo, Tokyo, Japan; CLB Department of Neurosurgery, Helsinki University Hospital, Helsinki, Finland; CLC The National Institute for Stroke and Applied Neurosciences, Auckland University of Technology, Auckland, New Zealand; CLD Department of Health Services Management, Shiraz University of Medical Sciences, Shiraz, Iran; CLE Department of Psychiatry, St. John's National Academy of Health Sciences, Bangalore, India; CLF Inovus Medical, St Helens, UK; CLG Academic Public Health England, Public Health England, London, UK; CLH Department of Computer Science, Boston University, Boston, MA, USA; CLI Department of Mathematical Demography & Statistics, International Institute for Population Sciences, Mumbai, India; CLJ Interventional Cardiology Department, Saint Vincent Hospital, Worcester, MA, USA; CLK Gene Therapy Center, Tehran University of Medical Sciences, Tehran, Iran; CLL College of medicine, Shahid Beheshti University of Medical Sciences, Tehran, Iran; CLM Department of Hematology, North Khorasan University of Medical Sciences, Bojnurd, Iran; CLN Department of Hematology, Tarbiat Modares University, Tehran, Iran; CLO Clinical Epidemiology Unit, Lund University, Lund, Sweden; CLP Department of Neurosciences, Rehabilitation, Ophthalmology, Genetics, Maternal and Child Health, University of Genoa, Genoa, Italy; CLQ Department of Internal Medicine, Northwest Health, Porter, Valparaiso, IN, USA; CLR Department of Biological Sciences, King Abdulaziz University, Jeddah, Egypt; CLS Department of Protein Research, Research and Academic Institution, Alexandria, Egypt; CLT Institute for Health, Health Care Policy and Aging Research, Rutgers University, New Brunswick, NJ, USA; CLU The School of Pharmaceutical Sciences, University of Science Malaysia, Penang, Malaysia; CLV School of Public Health and China Center for Health Developments, Peking University, Beijing, China; CLW Division of Psychiatry, University College London, London, UK; CLX School of Medicine, Western Sydney University, Campbelltown, NSW, Australia; CLY Translational Health Research Institute, Western Sydney University, Campbelltown, NSW, Australia; CLZ Brien Holden Vision Institute, Sydney, NSW, Australia; CMA Unisabana Center for Translational Science, Universidad de La Sabana (Savannah University), Chia, Colombia; CMB Critical Care Department, Clinica Universidad De La Sabana (Savannah University Clinic), Chia, Colombia; CMC School of Environment, Tehran University, Tehran, Iran; CMD Endocrinology and Metabolism Research Institute, Tehran University of Medical Sciences, Tehran, Iran; CME Network of Immunity in Infection, Malignancy and Autoimmunity (NIIMA), Universal Scientific Education and Research Network (USERN), Tehran, Iran; CMF Department of Epidemiology and Biostatistics, Rafsanjan University of Medical Sciences, Rafsanjan, Iran; CMG Department of Public Health Sciences, University of Connecticut, Farmington, CT, USA; CMH Department of Psychiatry, Yale University, New Haven, CT, USA; CMI The University of Lahore, Lahore, Pakistan; CMJ Department of Internal Medicine, Federal University of Minas Gerais, Belo Horizonte, Brazil; CMK Centre of Telehealth, Federal University of Minas Gerais, Belo Horizonte, Brazil; CML Department of Surgery, University of Minnesota, Minneapolis, MN, USA; CMM Department of Surgery, University Teaching Hospital of Kigali, Kigali, Rwanda; CMN Department of Physiology and Physiotherapy, DIT University, Delhi, India; CMO Community Health Department, Federal University of Ceará, Fortaleza, Brazil; CMP Faculty of Medicine, University of Porto, Porto, Portugal; CMQ Department of Geography and Demography, University of Coimbra, Coimbra, Portugal; CMR Department of Clinical Research, University of Sao Paulo, Ribeirão Preto, Brazil; CMS Gilbert and Rose-Marie Chagoury School of Medicine, Lebanese American University, Beirut, Lebanon; CMT Division of Global Health Equity, Harvard University, Boston, MA, USA; CMU Center for Indigenous Health Research, Wuqu' Kawoq Maya Health Alliance, Tecpan, Guatemala; CMV Centre for Healthy Brain Ageing (CHeBA), University of New South Wales, Sydney, NSW, Australia; CMW Department of Environmental and Radiological Health Sciences, Colorado State University, Fort Collins, CO, USA; CMX Department of Anesthesiology, University of Nebraska Medical Center, Omaha, NE, USA; CMY Department of Neurosciences, Maurizio Bufalini Hospital, Cesena, Italy; CMZ Centre for Global Epilepsy, University of Oxford, Oxford, UK; CNA Fondazione Policlinico Universitario A. Gemelli, Cuore Università Cattolica del Sacro Cuore (Catholic University of Sacred Heart), Rome, Italy; CNB Department of Ophthalmology and Visual Sciences, University of Wisconsin-Madison, Madison, WI, USA; CNC School of Medicine, Gonabad University of Medical Sciences, Gonabad, Iran; CND Division of Cardiology, University of Washington, Seattle, WA, USA; CNE Department of Pharmacy Services, Alberta Health Services, Edmonton, AB, Canada; CNF West African Postgraduate College of Pharmacists, Lagos, Nigeria; CNG Department of Analytical and Applied Economics, Utkal University, Bhubaneswar, India; CNH RUSA Centre of Excellence in Public Policy and Governance, Utkal University, Bhubaneswar, India; CNI Isfahan University of Medical Sciences, Islamic Azad University, Isfahan, Iran; CNJ Family and Prevention Medicine, Isfahan University of Medical Sciences, Isfahan, Iran; CNK Department of Ophthalmology, University of Miami, Miami, FL, USA; CNL Faculty of Medicine, Quest International University Perak, Ipoh, Malaysia; CNM Department of Biochemistry and Food Analysis, Patuakhali Science and Technology University, Patuakhali, Bangladesh; CNN Department of Veterinary Microbiology, College of Veterinary Science and Animal Husbandry, Agartala, India; CNO Department of Medicine, North Bengal Medical College and Hospital, Siliguri, India; CNP Department of Labour, Government of West Bengal, Kolkata, India; CNQ Department of Public Health, New Mexico State University, Las Cruces, NM, USA; CNR Research Department, Indian Institute of Public Health, Delhi, India; CNS Department of Anesthesiology, Washington University in St. Louis, St. Louis, MO, USA; CNT Department of Biochemistry, Saveetha University, Chennai, India; CNU Departamento de Ciencias Básicas Médicas, Universidad ICESI, Cali, Colombia; CNV Advanced Campus Governador Valadares, Juiz de For a Federal University, Governador Valadares, Brazil; CNW Department of Health Statistics, National Institute for Medical Research, Dar es Salaam, Tanzania; CNX Department of Cardiology, SS. Annunziata Hospital - ASL2 Abruzzo, Chieti, Italy; CNY Department of Internal Medicine, Muhimbili University of Health and Allied Sciences, Dar es Salaam, Tanzania; CNZ Department of Internal Medicine, University of Botswana, Gaborone, Botswana; COA Department of Oral and Maxillofacial Surgery, Jagadguru Sri Shivarathreeswara University, Mysore, India; COB Cardiovascular Department, Zagazig University, Zagazig, Egypt; COC Department of Critical Care Anesthesiology, University of Alabama Birmingham, Birmingham, AL, USA; COD Faculty of Medicine, Gonabad University of Medical Sciences, Gonabad, Iran; COE Infectious Diseases Research Center, Gonabad University of Medical Sciences, Gonabad, Iran; COF Department of Medical Pharmacology, Cairo University, Giza, Egypt; COG Department of Epidemiology, Shahid Beheshti University of Medical Sciences, Tehran, Iran; COH Escuela de Kinesiología, Diego Portales University, Santiago de Chile, Chile; COI Universidad Autónoma de Chile, Santiago de Chile, Chile; COJ School of Public Health, Shahid Beheshti University of Medical Sciences, Tehran, Iran; COK Department of Public Health and Epidemiology, Khalifa University, Abu Dhabi, United Arab Emirates; COL Department of Computer, University of Science and Culture, Tehran, Iran; COM Research Center for Environmental Determinants of Health, Kermanshah University of Medical Sciences, Kermanshah, Iran; CON Department of Biostatistics, Shiraz University of Medical Sciences, Shiraz, Iran; COO Iranian Research Center for Evidence-based Medicine, Tabriz University of Medical Sciences, Tabriz, Iran; COP International Center of Medical Sciences Research, Islamabad, Pakistan; COQ Department of Nursing and Midwifery, Saveh University of Medical Sciences, Saveh, Iran; COR Department of Health, Shahid Beheshti University of Medical Sciences, Tehran, Iran; COS Ophthalmic Research Center, Shahid Beheshti University of Medical Sciences, Tehran, Iran; COT Faculty of Medicine, Bioscience and Nursing, MAHSA University, Selangor, Malaysia; COU Interdisciplinary Research Centre in Biomedical Materials (IRCBM), COMSATS Institute of Information Technology, Lahore, Pakistan; COV Department of Psychiatry, All India Institute of Medical Sciences, New Delhi, India; COW Department of Psychosocial Science, University of Bergen, Bergen, Norway; COX Sharjah Institute of Medical Sciences, University of Sharjah, Sharjah, United Arab Emirates; COY Center for Global Health Research, Saveetha University, Chennai, India; COZ Biotechnology Research Center, Mashhad University of Medical Sciences, Mashhad, Iran; CPA Canadian Red Cross, Red Cross, Ottawa, ON, Canada; CPB Department of Psychiatry, Ministry of Health, Manama, Bahrain; CPC College of Pharmacy, Al-Hadba University, Mosul, Iraq; CPD Department of Health and Kinesiology, University of Illinois, Champaign, IL, USA; CPE Department of Statistics, University of Gujrat, Gujrat, Pakistan; CPF Department of Microbiology, Ahvaz Jundishapur University of Medical Sciences, Ahvaz, Iran; CPG Department of Integrated Health Education, Federal University of Espirito Santo, Vitória, Brazil; CPH Faculty of Pharmacy, Mansoura University, Mansoura, Egypt; CPI Department of Health Education & Promotion, A.C.S. Medical College and Hospital, Karaj, Iran; CPJ Research Center for Health, Safety and Environment, Alborz University of Medical Sciences, Karaj, Iran; CPK Department of Endocrinology, Mayo Clinic, Rochester, MN, USA; CPL Student Research Committee, Kashan University of Medical Sciences, Kashan, Iran; CPM Public Health and Community Medicine Department, Cairo University, Giza, Egypt; CPN Technology Management Department, University College of Applied Sciences, Gaza, Palestine; CPO School of Economics and Management, University of Kassel, Kassel, Germany; CPP College of Nursing, Jouf University, Jouf, Saudi Arabia; CPQ Department of Pathology, Microbiology and Forensic Medicine, The University of Jordan, Amman, Jordan; CPR Department of Clinical Laboratories and Forensic Medicine, The University of Jordan, Amman, Jordan; CPS Department of Global Initiatives, Child Mind Institute, New York, NY, USA; CPT Department of Psychiatry and Legal Medicine, Federal University of Rio Grande do Sul, Porto Alegre, Brazil; CPU Clinical Research Division, Pulmocare Research and Education (PURE) Foundation, Pune, India; CPV Faculty of Health Sciences, Symbiosis International University, Pune, India; CPW Drug Applied Research Center, Tabriz University of Medical Sciences, Tabriz, Iran; CPX Surgical Department, North Colombo Teaching Hospital, Ragama, Sri Lanka; CPY Institute of Epidemiology and Preventive Medicine, National Taiwan University, Taipei, Taiwan; CPZ Benang Merah Research Center (BMRC), Minahasa Utara, Indonesia; CQA Department of Anatomy, Ras Al Khaimah Medical and Health Sciences University, Ras Al Khaimah, United Arab Emirates; CQB Department of Entomology, Ain Shams University, Cairo, Egypt; CQC Medical Ain Shams Research Institute (MASRI), Ain Shams University, Cairo, Egypt; CQD Department of Forensic Biology, Government Institute of Forensic Science Chhatrapati Sambhajinagar, Chhatrapati Sambhajinagar, India; CQE Epidemiology and Biostatistics, Tehran University of Medical Sciences, Tehran, Iran; CQF Department of Microbiology, Saveetha University, Chennai, India; CQG Primary Healthcare Department, Azienda USL di Bologna, Bologna, Italy; CQH Child Health Analytics Research Program, Telethon Kids Institute, Perth, WA, Australia; CQI University of São Paulo, São Paulo, Brazil; CQJ School of Public Health and Health Management, University of Belgrade, Belgrade, Serbia; CQK Department of Infectious Diseases and Tropical Medicine, Federal University of Minas Gerais, Belo Horizonte, Brazil; CQL Department of Sociology and Gerontology, Miami University, Oxford, OH, USA; CQM Independent Consultant, Thiruvananthapuram, India; CQN Department of Health, Physical Education and Recreation, University of Cape Coast, Cape Coast, Ghana; CQO Department of Public Health, Jahrom University of Medical Sciences, Jahrom, Iran; CQP Botany Department, Bodoland University, Kokrajhar, India; CQQ Faculty of Science, Queensland University of Technology, Brisbane, QLD, Australia; CQR Health Sciences Research Center, Torbat Heydariyeh University of Medical Sciences, Torbat Heydariyeh, Iran; CQS Department of Oral Pathology and Microbiology, Dr. D. Y. Patil Vidyapeeth, Pune (Deemed to be University), Pune, India; CQT Department of Community Medicine, Mahatma Gandhi Memorial Medical College, Indore, India; CQU Department of Geriatric and Long Term Care, Hamad Medical Corporation, Doha, Qatar; CQV Faculty of Health & Social Sciences, Bournemouth University, Bournemouth, UK; CQW Department of Epidemiology, National Institute for Research in Tuberculosis, Chennai, India; CQX Faculty of Science, Medicine and Health, University of Wollongong, Wollongong, NSW, Australia; CQY Precision Medicine Department, Università degli studi della Campania Luigi Vanvitelli (University of Campania Luigi Vanvitelli), Naples, Italy; CQZ Department of Public Health Sciences, University of North Carolina at Charlotte, Charlotte, NC, USA; CRA Department of Public Health Sciences, Coastal Carolina University, Conway, SC, USA; CRB Harvard Extension School, Harvard University, Cambridge, MA, USA; CRC Department of Preventive and Social Medicine, Jawaharlal Institute of Postgraduate Medical Education and Research, Puducherry, India; CRD Department of Post-Harvest Technology and Marketing, Patuakhali Science and Technology University, Patuakhali, Bangladesh; CRE Psychiatry Clinic, Holy Savior Armenian Hospital, Istanbul, Turkiye; CRF Faculty of Business and Computing, University of the Fraser Valley, Abbotsford, BC, Canada; CRG Graduate School of Business, ESAN University, Lima, Peru; CRH Faculty of Medicine, Katholieke Universiteit Leuven, Leuven, Belgium; CRI Stroke Unit and Neurology Unit, ASST Grande Ospedale Metropolitano Niguarda, Milan, Italy; CRJ Department of Psychology, University of Alabama at Birmingham, Birmingham, AL, USA; CRK Clinic for Conservative Dentistry and Periodontology, University Hospital of the Ludwig-Maximilians-University Munich, Munich, Germany; CRL Center for International Health, University of Bergen, Bergen, Norway; CRM Operating Room Department, Khomein University of Medical Sciences, Khomein, Iran; CRN Department of Medical Education, Tehran University of Medical Sciences, Tehran, Iran; CRO Department of Pharmacology, Saveetha University, Chennai, India; CRP Department of Medical Statistics, University of Zagreb, Zagreb, Croatia; CRQ Department of Epidemiology and Prevention of Chronic Noncommunicable Diseases, Croatian Institute of Public Health, Zagreb, Croatia; CRR Department of Applied Mechanics and Biomedical Engineering, Indian Institute of Technology Delhi, Chennai, India; CRS Emergency Department, Manian Medical Centre, Erode, India; CRT Department of Economics and Economic Policies, Bucharest University of Economic Studies, Bucharest, Romania; CRU Center for Health Systems Research, National Institute of Public Health, Cuernavaca, Mexico; CRV Department of Medicine, Swami Vivekanand Subharti University, Meerut, India; CRW National Heart, Lung, and Blood Institute, National Institutes of Health, Rockville, MD, USA; CRX Department of Medicine, Northwestern University, Chicago, IL, USA; CRY Rita A. Patel Institute of Physiotherapy, The Charutar Vidya Mandal (CVM) University, Anand, India; CRZ School of Health Sciences, Universiti Sains Malaysia, Kota Bharu, Malaysia; CSA H.E.J. Research Institute of Chemistry, University of Karachi, Karachi, Pakistan; CSB Department of Biotechnology, Quaid-i-Azam University Islamabad, Islamabad, Pakistan; CSC Department of Physics, The University of Lahore, Lahore, Pakistan; CSD Gastroenterology Unit, IRCCS, Bari, Italy; CSE Department of Chemistry, Institute for Advanced Studies in Basic Sciences (IASBS), Zanjan, Iran; CSF Center for Medical and Bio-Allied Health Sciences Research, Ajman University, Ajman, United Arab Emirates; CSG Independent Consultant, Karachi, Pakistan; CSH Noncommunicable Diseases Research Center, Neyshabur University of Medical Sciences, Neyshabur, Iran; CSI Neurology Department, Ain Shams University, Cairo, Egypt; CSJ Department of Pathology and Laboratory Medicine, Northwell Health, New York, NY, USA; CSK Department of Pharmacology, All India Institute of Medical Sciences, Jodhpur, India; CSL Division of Neurological Science, University of Bern, Bern, Switzerland; CSM School of Medicine, Alborz University of Medical Sciences, Karaj, Iran; CSN Department of Pathobiology, Shahid Bahonar University of Kerman, Kerman, Iran; CSO Science Department, Kazakh National Medical University, Almaty, Kazakhstan; CSP National University of Ireland, Galway, Galway, Ireland; CSQ Columbia University, New York, NY, USA; CSR Department of Radiation Oncology, All India Institute of Medical Sciences, New Delhi, India; CSS Amity Institute of Public Health, Amity University, Noida, India; CST Division of Nephrology, Southern Medical University, Guangzhou, China; CSU Department for Evidence-based Medicine and Evaluation, University for Continuing Education Krems, Krems, Austria; CSV Universidad Espíritu Santo, Samborondón, Ecuador; CSW Amity Institute of Biotechnology, Amity University Rajasthan, Rajasthan, India; CSX Department of Forensic Science, Shree Guru Gobind Singh Tricentenary University, Gurugram, India; CSY Department of Biotechnology, Graphic Era (Deemed to be University), Dehradun, India; CSZ UN Mehta Institute of Cardiology and Research Center, B.J. Medical College, Ahmedabad, India; CTA Department of Cardiology, Government Medical College, Ahmedabad, India; CTB Department of Social and Behavioral Health, University of Nevada Las Vegas, Las Vegas, NV, USA; CTC Department of Nephrology, Vardhman Mahavir Medical College, New Delhi, India; CTD Institute of Forensic Science & Criminology, Panjab University, Chandigarh, India; CTE Yenepoya Research Center, Yenepoya University, Mangalore, India; CTF Department of Engineering, Free University of Brussels, Brussels, Belgium; CTG Department of Physiology, Pharmacology, and Toxicology, An-Najah National University, Nablus, Palestine; CTH Ophthalmic Research Center (ORC), Shahid Beheshti University of Medical Sciences, Tehran, Iran; CTI Physiotherapy Department, Federal University of Health Sciences, Azare, Nigeria; CTJ Department of Microbiology, Kasturba Medical College, Mangalore, India; CTK Department of Biology, Morgan State University, Baltimore, MD, USA; CTL Department of Environmental Health Sciences, Tulane University, New Orleans, LA, USA; CTM KS Hegde Medical Academy, Nitte University, Mangalore, India; CTN Department of Epidemiology and Health Statistics, Wenzhou Medical University, Wenzhou, China; CTO The Department of Medicine & Therapeutics, The Chinese University of Hong Kong, Hong Kong, China; CTP General Surgery Department, Glasgow Royal Infirmary, Glasgow, UK; CTQ Department of HIV/AIDS Prevention and Control, Bahir Dar University, Bahir Dar, Ethiopia; CTR Tokyo Foundation for Policy Research, Tokyo, Japan; CTS Department of Public Health, Dambi Dollo University, Dembi Dollo, Ethiopia; CTT Department of Epidemiology, Jimma University, Jimma, Ethiopia; CTU Department of Pediatrics, Yonsei University, Seoul, South Korea; CTV Korea University, Seoul, South Korea; CTW Finnish Institute of Occupational Health, Helsinki, Finland; CTX Cancer Research Center, Tehran University of Medical Sciences, Tehran, Iran; CTY Cancer Biology Research Center, Tehran University of Medical Sciences, Tehran, Iran; CTZ Department of Medicine, Ladoke Akintola University, Ogbomoso, Nigeria; CUA Oulu Business School, University of Oulu, Oulu, Finland; CUB Martti Ahtisaari Institute, University of Oulu, Oulu, Finland; CUC Department of Experimental Research, Medical University Pleven, Pleven, Bulgaria; CUD Department of Genetics, Sofia University “St. Kliment Ohridiski” Sofia, Bulgaria; CUE Department of Neurosurgery, Columbia University Medical Center, New York, NY, USA; CUF Student Scientific Research Center, Tehran University of Medical Sciences, Tehran, Iran; CUG Alimentary Tract Research Center, Ahvaz Jundishapur University of Medical Sciences, Ahvaz, Iran; CUH Social Determinants of Health Research Center, Kurdistan University of Medical Sciences, Sanandaj, Iran; CUI Department of Public Health Medicine, University of KwaZulu-Natal, Durban, South Africa; CUJ Center for Technology and Innovation in Cardiovascular Informatics, Iran University of Medical Sciences, Tehran, Iran; CUK Department of Medical-Surgical Nursing, Mazandaran University of Medical Sciences, Sari, Iran; CUL Department of Nursing and Health Sciences, Flinders University, Adelaide, SA, Australia; CUM Department of Community Medicine and Public Health, Institute of Medicine, Kathmandu, Nepal; CUN Department of Research and Academics, Kathmandu Cancer Center, Bhaktapur, Nepal; CUO Person-Centered Research, Monash University, Melbourne, VIC, Australia; CUP Kenneth H. Cooper Institute, Texas Tech University Health Sciences Center, Dallas, TX, USA; CUQ Advanced Materials Division, Mintek, Randburg, South Africa; CUR Department of Biotechnology, University of the Western Cape, Bellville, South Africa; CUS Unit of Basic Medical Sciences, University of Khartoum, Khartoum, Sudan; CUT Department of Medical Microbiology and Infectious Diseases, Erasmus University, Rotterdam, Netherlands; CUU Galgotias Multidisciplinary Research & Development Cell, Galgotias University, Greater Noida, India; CUV Chair for General Economics, Health Economics and Econometrics, University of Greifswald, Greifswald, Germany; CUW Department of Physical Education, Federal University of Santa Catarina, Florianópolis, Brazil; CUX Faculty of Dentistry, Federal University of Minas Gerais, Belo Horizonte, Brazil; CUY Instituto Salud Publica, Pontifical Javeriana University, Bogota, Colombia; CUZ Sport Physical Activity and Health Research & Innovation Center (SPRINT), Polytechnic Institute of Guarda, Guarda, Portugal; CVA RISE Health, University of Beira Interior, Covilhã, Portugal; CVB School of Human and Health Sciences, University of Huddersfield, Huddersfield, UK; CVC Department of Dentistry, All India Institute of Medical Sciences, Bhopal, India; CVD Amity institute of Public Health and Hospital Administration, Amity University, Noida, India; CVE Department of Biochemistry, Central University of Punjab, Bathinda, India; CVF Department of Agriculture and Environmental Sceinces, National Institute of Food Technology Entrepreneurship and Management-Kundli (NIFTEM-K), Sonipat, India; CVG Department of Pharmacology, Government Medical College and Hospital, Chandigarh, India; CVH School of Pharmaceutical Sciences, IFTM University, Moradabad, India; CVI Department of Paediatrics, All India Institute of Medical Sciences, Bilaspur, India; CVJ School of Medicine, Baylor College of Medicine, Houston, TX, USA; CVK Department of Medicine Service, US Department of Veterans Affairs (VA), Houston, TX, USA; CVL Research Department, Hamad Medical Corporation, Doha, Qatar; CVM Faculty of Medicine and Health Sciences, Shree Guru Gobind Singh Tricentenary University, Gurugram, India; CVN Department of Radiodiagnosis, All India Institute of Medical Sciences, Bathinda, India; CVO Amity Institute of Public Health and Hospital Administration, Amity University, Noida, India; CVP National Institute of Cancer Prevention and Research, Noida, India; CVQ Department of Human Genetics, Punjabi University Patiala, Patiala, India; CVR Department of Biochemistry, Banaras Hindu University, Varanasi, India; CVS Institute of Medical Sciences, Banaras Hindu University, Varanasi, India; CVT Department of Computer Science & Engineering, Central University of Punjab, Bathinda, India; CVU Department of Community Medicine, Veer Chandra Singh Garhwali Government Institute of Medical Science and Research, Srinagar Garhwal, India; CVV Department of Physiotherapy, Manipal Academy of Higher Education, Manipal, India; CVW ESIC Medical College and Hospital, ESIC Medical College and Hospital, Ranchi, India; CVX Department of Internal Medicine, University of Indonesia, Jakarta, Indonesia; CVY Department of Internal Medicine, Dr. Cipto Mangunkusumo National Hospital, Jakarta Pusat, Indonesia; CVZ Department of Sports Science and Clinical Biomechanics, University of Southern Denmark, Odense, Denmark; CWA Department of Physiotherapy and Occupational Therapy, Næstved-Slagelse-Ringsted Hospitals, Slagelse, Denmark; CWB Division of Injury Prevention, The Bizzell Group, Atlanta, GA, USA; CWC Rollins School of Public Health, Emory University, Atlanta, GA, USA; CWD Department of Development Studies, Daffodil International University, Dhaka, Bangladesh; CWE School of Medicine, Keele University, Stoke-On-Trent, UK; CWF University Hospitals North Midlands, Stoke-on-Trent, UK; CWG Department of Systemic Pathology, Touro College of Osteopathic Medicine, Middletown, NY, USA; CWH Department of Pathology, American University of the Caribbean School of Medicine, Cupecoy, Saint Martin; CWI Faculty of Public Health, Universitas Ahmad Dahlan, Yogyakarta, Indonesia; CWJ Department of Medicinal Chemistry, University of Sharjah, Sharjah, United Arab Emirates; CWK Division of Engineering in Medicine, Harvard Medical School, Boston, MA, USA; CWL Department of Endocrinology, Case Western Reserve University, Cleveland, OH, USA; CWM School of Medicine, Babol University of Medical Sciences, Babol, Iran; CWN Hospital Universitario de La Princesa, Universidad Autónoma de Madrid (Autonomous University of Madrid), Madrid, Spain; CWO Centro de Investigación Biomédica en Red Enfermedades Respiratorias (CIBERES) (Center for Biomedical Research in Respiratory Diseases Network), Madrid, Spain; CWP Department of Public Health, Experimental and Forensic Medicine, University of Pavia, Pavia, Italy; CWQ School of Primary and Allied Health Care, Monash University, Melbourne, VIC, Australia; CWR Universidade Federal de Minas Gerais (Federal University of Minas Gerais), Belo Horizonte, Brazil; CWS Doheny Eye Institute, University of California Los Angeles, Pasadena, CA, USA; CWT 3rd Department of Cardiology, University of Athens, Athens, Greece; CWU Department of Hematology, Mayo Clinic, Rochester, MN, USA; CWV Medical and Diagnostic Research Centre, University of Hail, Hail, Saudi Arabia; CWW Department of Neurology, University of Massachusetts Medical School, Worcester, MA, USA; CWX Department of Pharmacology, RAK Medical and Health Sciences University, Ras Al Khaimah, United Arab Emirates; CWY College of Health and Public Service, University of North Texas, Denton, TX, USA; CWZ Nutrition and Dietetics Department, Federal Research Institute of Nutrition, Biotechnology and Food Safety, Moscow, Russia; CXA Department of Internal Disease, Pirogov Russian National Research Medical University, Moscow, Russia; CXB SAMRC Unit on Risk and Resilience in Mental Disorders, University of Cape Town, Cape Town, South Africa; CXC Department of Neurology, University of Copenhagen, Copenhagen, Denmark; CXD Department of Medicine, Democritus University of Thrace, Alexandroupolis, Greece; CXE Occupational and Environmental Medicine Department, University of Gothenburg, Gothenburg, Sweden; CXF Department of Neurology and Clinical Neurophysiology, Trondheim, Norway; CXG Global Observatory on Pollution and Health, Boston College, Chestnut Hill, MA, USA; CXH ISGlobal Instituto de Salud Global de Barcelona, Barcelona, Spain; CXI Discipline of Physiotherapy, University of Technology Sydney, Sydney, NSW, Australia; CXJ Department of Ophthalmology, Renmin Hospital of Wuhan University, Wuhan, China; CXK Department of Orthopaedics, Massachusetts General Hospital, Boston, MA, USA; CXL Department of Orthopaedics, Harvard University, Cambridge, MA, USA; CXM Research Department, Nepal Development Society, Kathmandu, Nepal; CXN School of Exercise and Nutrition Sciences, Deakin University, Melbourne, VIC, Australia; CXO Department of Geriatrics, University of São Paulo, Sao Paulo, Brazil; CXP Integrative Epidemiology Unit, University of Bristol, Bristol, UK; CXQ School of Acupuncture-Tuina, Shandong University of Traditional Chinese Medicine, Jinan, China; CXR Praboromarajchanok Institute, Ministry of Public Health, Nonthaburi, Thailand; CXS Department of Physiotherapy, Tishk International University, Erbil, Iraq; CXT Department of Human Anatomy, Federal University, Dutse, Dutse, Nigeria; CXU School of Life Sciences, Xiamen University, Xiamen, China; CXV Faculty of Health Science, Universitas Indonesia Maju, Jakarta, Indonesia; CXW Department of Life and Health Sciences, University of Nicosia, Nicosia, Cyprus; CXX School of Medicine, Medical Sciences and Nutrition, University of Aberdeen, Aberdeen, UK; CXY University Diabetes Center, King Saud University, Riyadh, Saudi Arabia; CXZ Yusuf Hamied Department of Chemistry, University of Cambridge, Cambridgeshire, UK; CYA Institute of Integrated Intelligence and Systems, Griffith University, Brisbane, QLD, Australia; CYB The First Hospital of China Medical University, China Medical University, Shenyang, China; CYC Department of Endocrinology and Metabolism, Affiliated Hospital of Shandong Second Medical University, Weifang, China; CYD Department of Biomedical Sciences, Universiti Putra Malaysia, Selangor, Malaysia; CYE High-Quality Development Evaluation Research Institute, Nanjing University of Posts and Telecommunications, Nanjing, China; CYF Gandhi Medical College, Kaloji Narayana Rao University of Health Sciences (KNRUHS), Secunderabad, India; CYG The George Institute for Global Health, Sydney, NSW, Australia; CYH Department of Population Health Sciences, University College London, London, UK; CYI Ashok & Rita Patel Institute of Physiotherapy, Charotar University of Science and Technology, Anand, India; CYJ Faculty of Public Health, Universitas Airlangga (Airlangga University), Surabaya, Indonesia; CYK Department of Clinical Research and Development, LUXMED Group, Warsaw, Poland; CYL Collegium Medicum, John Paul II Catholic University of Lublin, Lublin, Poland; CYM Northwestern University, Chicago, IL, USA; CYN Department of Pharmacology, All India Institute of Medical Sciences, Deoghar, India; CYO Department of Neurology, Neurocenter of Southern Switzerland (NSI), Lugano, Switzerland; CYP Department of Medicine, University of Valencia, Valencia, Spain; CYQ Carlos III Health Institute, Biomedical Research Networking Center for Mental Health Network (CiberSAM), Madrid, Spain; CYR Department of Basic Medical Sciences, Islamic Azad University, Mashhad, Iran; CYS Department of Internal Medicine, Islamic Azad University, Mashhad, Iran; CYT Department of Medical Education, Shahid Beheshti University of Medical Sciences, Tehran, Iran; CYU Department of Health, Safety, and Environmental Management, Abadan School of Medical Sciences, Abadan, Iran; CYV Saveetha Medical College and Hospital, Saveetha Institute of Medical and Technical Sciences, Chennai, India; CYW Department of Dentistry and Oral Health, La Trobe University, Bendigo, VIC, Australia; CYX School of Dentistry and Oral Health, Griffith University, Gold Coast, QLD, Australia; CYY Department of Physiotherapy, A.T. Still University, Azare, Nigeria; CYZ The Five Senses Health Institute, Iran University of Medical Sciences, Tehran, Iran; CZA Living Systems Institute, University of Exeter, Exeter, UK; CZB Department of Biostatistics and Epidemiology, Shahid Sadoughi University of Medical Sciences, Yazd, Iran; CZC Research Center for Molecular Medicine, Hamadan University of Medical Sciences, Hamadan, Iran; CZD Department of Environmental, Agricultural and Occupational Health, University of Nebraska Medical Center, Omaha, NE, USA; CZE Department of Pathology, Alexandria University, Alexandria, Egypt; CZF Department of Global Health, Florida International University, Miami, FL, USA; CZG Department of Dermato-Venereology, Dr. Victor Babes Clinical Hospital of Infectious Diseases and Tropical Diseases, Bucharest, Romania; CZH Department of Epidemiology, Stellenbosch University, Cape Town, South Africa; CZI Department of Medicine, Northlands Medical Group, Omuthiya, Namibia; CZJ Department of Joint and Orthopedics, Southern Medical University, Guangzhou, China; CZK Department of Orthopaedics, Chongqing Medical University, Chongqing, China; CZL Department of Surgery, National University of Singapore, Singapore, Singapore; CZM Department of Medicine, Kazakh National Medical University, Almaty, Kazakhstan; CZN State Key Laboratory of Numerical Modeling for Atmospheric Sciences and Geophysical Fluid Dynamics (LASG), Chinese Academy of Sciences, Beijing, China; CZO Department of Medicine, University of California Irvine, Orange, CA, USA; CZP Department of Public Health, Debre Markos University, Debre Markos, Ethiopia; CZQ Department of Pharmacology and Therapeutics, The University of Faisalabad, Faisalabad, Pakistan; CZR Department of Metabolism and Systems Science, University of Birmingham, Birmingham, UK; CZS Nutrition and Clinical Services Division, International Centre for Diarrhoeal Disease Research, Bangladesh, Dhaka, Bangladesh; CZT Taking Our Best Shot, Houston, TX, USA; CZU Department of Research and Innovation, Enventure Medical Innovation, Houston, TX, USA; CZV Basic Sciences in Infectious Diseases Research Center, Shiraz University of Medical Sciences, Shiraz, Iran; CZW Institute of General Practice and Interprofessional Care, University Hospital Tübingen, Tübingen, Germany; CZX Robert Bosch Center for Integrative Medicine and Health, Bosch Health Campus, Stuttgart, Germany; CZY Department of Pathology, Tehran University of Medical Sciences, Tehran, Iran; CZZ Indiana University School of Medicine, University of Missouri, Indianapolis, IN, USA; DAA University of Gondar, Ethiopian Medical Association, Gondar, Ethiopia; DAB Department of Urology, Sabzevar University of Medical Sciences, Sabzevar, Iran; DAC Research Chair for Evidence-Based Health Care and Knowledge Translation, King Saud University, Riyadh, Saudi Arabia; DAD Department of Preventive Medicine, Northwestern University, Chicago, IL, USA; DAE Department of Environmental and Occupational Health and Safety, University of Gondar, Gondar, Ethiopia; DAF Internal Medicine Department, King George's Medical University, Lucknow, India; DAG Department of Abdominal Surgery, Katholieke Universiteit Leuven, Leuven, Belgium; DAH Department of Surgery, University Hospital Bochum, Herne, Germany; DAI Amrita Vishwa Vidyapeetham, Amrita Institute of Medical Sciences, Kochi, India; DAJ Department of Economics, The American University in Cairo, Cairo, Egypt; DAK Rheumatology and Immunology Unit, Mansoura University, Mansoura, Egypt; DAL Department of Radiology, Kaduna State University, Kaduna, Nigeria; DAM Department of Gastroenterology, Hepatology, and Nutrition, The Hospital for Sick Children, Toronto, ON, Canada; DAN International Centre for Eye Health, London School of Hygiene & Tropical Medicine, London, UK; DAO Department of Applied Bioscience, Konkuk University, Seoul, South Korea; DAP Centre for Global Health Research, Saveetha University, Chennai, India; DAQ Clinical Epidemiology, Leibniz Institute for Prevention Research and Epidemiology, Bremen, Germany; DAR Department of Endocrinology, Diabetes and Metabolism, National Institutes of Health, Bethesda, MD, USA; DAS School of Public Health, Harbin Medical University, Harbin, China; DAT Faculty of Public Health, Universitas Sam Ratulangi (Sam Ratulangi University), Manado, Indonesia; DAU Department of Allied Health and Human Performance, University of South Australia, Adelaide, SA, Australia; DAV Public Health Department, Debre Markos University, Debre Markos, Ethiopia; DAW Department of Prosthetic Dentistry, Kazakh National Medical University, Almaty, Kazakhstan; DAX Department of Surgery, Ahmadu Bello University, Zaria, Nigeria; DAY Department of Biochemistry, All India Institute of Medical Sciences, Jodhpur, India; DAZ Department of Medicine, University of Calgary, Calgary, AB, Canada; DBA Interdisciplinary Health Data Center, Jagiellonian University Medical College, Kraków, Poland; DBB Merilyn and Glick Eye Institute, University of Indiana, Indianapolis, IN, USA; DBC Nutritional Epidemiology Research Team (EREN), National Institute for Health and Medical Research (INSERM), Paris, France; DBD Department of Health, Medicine and Human Biology, Sorbonne Paris Nord University, Bobigny, France; DBE High Institute of Sport and Physical Education of Sfax, University of Sfax, Sfax, Tunisia; DBF Department of Movement Sciences and Sports Training, The University of Jordan, Amman, Jordan; DBG Netherlands Organisation for Applied Scientific Research (TNO), Netherlands Organisation for Applied Scientific Research (TNO), Utrecht, Netherlands; DBH School of Medicine and Dentistry, Griffith University, Gold Coast, QLD, Australia; DBI Department of Health, Children's Hospital 1, Ho Chi Minh City, Vietnam; DBJ School of Biomedical Engineering, University of Technology Sydney, Sydney, NSW, Australia; DBK Second Department of Internal Medicine, Kansai Medical University, Hirakata, Japan; DBL John T. Milliken Department of Medicine, Washington University in St. Louis, St. Louis, MO, USA; DBM Department of Internal Medicine, University of Medicine and Pharmacy at Ho Chi Minh City, Ho Chi Minh City, Vietnam; DBN Department of Business Analytics, University of Massachusetts Dartmouth, Dartmouth, MA, USA; DBO Molecular Neuroscience Research Center, Shiga University of Medical Science, Shiga, Japan; DBP ALS Vietnam Research and Advocacy Initiative, ALS Vietnam, Quang Ngai, Vietnam; DBQ Adult Learning Disability Service, Leicestershire Partnership National Health Service Trust, Leicester, UK; DBR College of Health Science, Vin University, Hanoi, Vietnam; DBS Department of Medicine, University of Crete, Heraklion, Greece; DBT Department of Cardiology, Tianjin Medical University, Tianjin, China; DBU Kent and Medway Medical School, Kent and Medway Medical School, Canterbury, UK; DBV Department of Internal Medicine, Wake Forest University, Winston-Salem, NC, USA; DBW Department of Urology, The Second Hospital of Tianjin Medical University, Tianjin, China; DBX Joslin Diabetes Center, Harvard University, Boston, MA, USA; DBY Hayatabad Medical Complex, Postgraduate Medical Institute, Peshawar, Pakistan; DBZ Department of Allied Health Sciences, Iqra University Chak Shahzad Campus, Islamabad, Pakistan; DCA Department of Biology and Biochemistry, University of Houston, Houston, TX, USA; DCB Medical Genomics Research Department, King Abdullah International Medical Research Center, Riyadh, Saudi Arabia; DCC Department of Life Sciences, University of Management and Technology, Lahore, Pakistan; DCD Department of Pediatric Cardiology, Rush University, Chicago, IL, USA; DCE Department of Physiotherapy, Federal Ministry of Health, Azare, Nigeria; DCF Federal University of Health Sciences Teaching Hospital Azare, Azare, Nigeria; DCG Lahore Business School, The University of Lahore, Lahore, Pakistan; DCH Department of Medicine, Khairpur Medical College, Khairpur, Pakistan; DCI Department of Oncology, Federal Medical Centre, Gusau, Nigeria; DCJ Kasturba Medical College, Manipal Academy of Higher Education, Manipal, India; DCK Amity Institute of Biotechnology, Amity University Rajasthan, Jaipur, India; DCL Section of Advanced Heart Failure and Transplant, North Shore University Hospital, Manhasset, NY, USA; DCM Center for Neurodegenerative Diseases and the Aging Brain, University of Bari, Tricase, Italy; DCN Institute of Psychiatry, Psychology & Neuroscience, King's College London, London, UK; DCO Harvard Kennedy School, Harvard University, Cambridge, MA, USA; DCP Research Institute for Medical and Health Sciences, University of Sharjah, Sharjah, United Arab Emirates; DCQ Operational Research Center in Healthcare, Near East University, Mersin, Turkiye; DCR Department of Orthodontics, University of Trakya, Edirne, Turkiye; DCS Johnson & Johnson, Duquesne University, Pittsburgh, PA, USA; DCT Faculty of Medicine, Shiraz University of Medical Sciences, Shiraz, Iran; DCU College of Health and Sport Sciences, University of Bahrain, Zallaq, Bahrain; DCV Department of Clinical Biochemistry, Isfahan University of Medical Sciences, Isfahan, Iran; DCW Department of Neurology, Rafsanjan University of Medical Sciences, Rafsanjan, Iran; DCX Non-communicable Diseases Research Center, Rafsanjan University of Medical Sciences, Rafsanjan, Iran; DCY Sociedad Argentina de Medicina, Buenos Aires, Argentina; DCZ Hospital Vélez Sarsfield, Buenos Aires, Argentina; DDA Department of Biomedical Sciences, Humanitas University, Milan, Italy; DDB Dermatology Unit, IRCCS Humanitas Research Hospital, Milan, Italy; DDC Psychiatry and Mental Health, University of Cape Town, Cape Town, South Africa; DDD Faculty of Sciences, University of Guilan, Rasht, Iran; DDE Achutha Menon Centre for Health Science Studies, Sree Chitra Tirunal Institute for Medical Sciences and Technology, Thiruvananthapuram, India; DDF Department of Internal Medicine, University of Groningen, Groningen, Netherlands; DDG UKK Institute, Tampere, Finland; DDH Faculty of Medicine and Health Technology, Tampere University, Tampere, Finland; DDI Department of Biochemistry, Apollo Institute of Medical Sciences and Research Chittoor, Chittoor, India; DDJ Department of Otolaryngology Head and Neck Surgery, Louisiana State University Health Sciences Center, Shreveport, LA, USA; DDK School of Dentistry and Medical Sciences, Charles Sturt University, Wagga Wagga, NSW, Australia; DDL Population Oral Health, Sydney Dental School, University of Sydney, Sydney, NSW, Australia; DDM Department of Zoology, Central University of Punjab, Bathinda, India; DDN Department of Human Genetics & Molecular Biology, Bharathiar University, Coimbatore, India; DDO Raffles Neuroscience Centre, Raffles Hospital, Singapore, Singapore; DDP Centre for Biosciences and Biotechnology, Saveetha University, Chennai, India; DDQ Department of Surgery, University of Southampton, Southampton, UK; DDR College of Medicine and Veterinary Medicine, University of Edinburgh, Edinburgh, UK; DDS Department of Health Policy and Management, Johns Hopkins University, Baltimore, MD, USA; DDT Department of Neurology, Infermi Hospital, Rimini, Italy; DDU Department of Neurology & Stroke Unit, Sant'Anna Hospital, Como, Italy; DDV Department of Biomedical Sciences for Health, University of Milan, Milano, Italy; DDW Department of Physiotherapy, Universidad Europea de Madrid (European University of Madrid), Villaviciosa de Odón, Spain; DDX Digital Health Research Center, Instituto Peruano de Orientación Psicológica, Lima, Peru; DDY Department of Biomedical Informatics, University of Utah, Salt Lake City, UT, USA; DDZ Occupational Medicine Unit, Sant'Orsola Malpighi Hospital, Bologna, Italy; DEA Cardiac Electrophysiology, St Bernard's Medical Center, Jonesboro, AR, USA; DEB The Matilda Centre for Research in Mental Health and Substance Use, University of Sydney, Sydney, NSW, Australia; DEC Faculty of Medicine of Itajubá, Brazil, Itajubá, Brazil; DED Department of Health Care Administration and Economics, National Research University Higher School of Economics, Moscow, Russia; DEE Faculty of Pharmacy, University of Porto, Porto, Portugal; DEF Diabetes Research Centre, University of Leicester, Leicester, UK; DEG International Institute for Training and Research (INSTAR), VNU University of Medicine and Pharmacy, Hanoi, Vietnam; DEH NUST School of Health Sciences, National University of Science and Technology (NUST), Islamabad, Pakistan; DEI Széchenyi István University, Gyor, Hungary; DEJ Research Organization for Health, National Research and Innovation Agency, Bogor, Indonesia; DEK Department of Social Sciences, Chuka University, Kenya, Nairobi, Kenya; DEL Population Studies and Research Institute, University of Nairobi, Nairobi, Kenya; DEM School of Chinese Medicine, Beijing University of Chinese Medicine, Beijing, China; DEN University of Chicago, Chicago, IL, USA; DEO Brigham and Women's Hospital, Boston, MA, USA; DEP Department of Oncology, Xiang'an Hospital of Xiamen University, Xiamen, China; DEQ Department of Laboratory Medicine, Guangdong Provincial People's Hospital, Guangzhou, China; DER School of Biomedical Sciences, The University of Western Australia, Perth, WA, Australia; DES Department of Health Services Research, Management and Policy, University of Florida, Gainesville, FL, USA; DET Department of Artificial Intelligence, Xiamen University Malaysia, Xiamen, China; DEU Department of Neurosurgery, Capital Medical University, Beijing, China; DEV Department of Neurosurgery, Beijing Tiantan Hospital, Beijing, China; DEW College of Agriculture, Northwest A&F University, Xianyang City, China; DEX Enze Medical Health Academy, Taizhou Hospital of Zhejiang Province, Taizhou, China; DEY School of Life Course and Population Sciences, King's College London, London, UK; DEZ Division of Gastroenterology and Hepatology, Mayo Clinic, Jacksonville, FL, USA; DFA Division of Life Sciences and Medicine, University of Science and Technology of China, Heifei, China; DFB Department of Pharmaceutical Chemistry College of Pharmacy, King Saud University, Riyadh, Saudi Arabia; DFC School of Nursing Sciences, University of Nairobi, Nairobi, Kenya; DFD Faculty of Sciences, The University of Lahore, Lahore, Pakistan; DFE Key Laboratory of Computer-Aided Drug Design, Guangdong Medical University, Dongguan, China; DFF Department of Biotechnology and Genetic Engineering, Hazara University Mansehra, Mansehra, Pakistan; DFG Centre for Health Policy Research, Torrens University Australia, Adelaide, SA, Australia; DFH Department of Parasitology, Rajarata University of Sri Lanka, Anuradhapura, Sri Lanka; DFI Institute of Health and Wellbeing, Federation University, Melbourne, VIC, Australia; DFJ University of Adelaide, North Terrace, NSW, Australia; DFK Department of Orthopaedics, General Hospital of Central Theater Command, Wuhan, China; DFL Fourth Military Medical University, Xi'an, China; DFM Department of Geriatrics, The Eighth Affiliated Hospital of Sun Yat-sen University, Shenzhen, China; DFN Cardiology Department, Royal Children's Hospital, Melbourne, VIC, Australia; DFO Department of Critical Care and Neurosciences, Murdoch Children's Research Institute, Parkville, VIC, Australia; DFP Key Laboratory of Shaanxi Province for Craniofacial Precision Medicine Research, Stomatological Hospital (College) of Xi'an Jiaotong University, Xi'an, China; DFQ Demographic Change and Aging Research Area, Federal Institute for Population Research, Wiesbaden, Germany; DFR Competence Center of Mortality-Follow-Up of the German National Cohort, Federal Institute for Population Research, Wiesbaden, Germany; DFS Department of Physical Therapy, Naresuan University, Phitsanulok, Thailand; DFT Department of Experimental Pharmacology, Heidelberg University, Mannheim, Germany; DFU Department of Medical Surgical Nursing, Gadjah Mada University, Yogyakarta, Indonesia; DFV Department of Surgery, University of Colombo, Colombo, Sri Lanka; DFW Department of Community Medicine, Rajarata University of Sri Lanka, Anuradhapura, Sri Lanka; DFX Department of Clinical Neurosciences, University of Calgary, Calgary, AB, Canada; DFY Department of Community Health Sciences, University of Calgary, Calgary, AB, Canada; DFZ Department of Nursing, Universitas Aisyiyah Bandung, Bandung, Indonesia; DGA Institute of Clinical Epidemiology, Medical University Innsbruck, Innsbruck, Austria; DGB Research Organisation, Inter-Continental Omni-Research in Medicine Collaborative, Berlin, Germany; DGC School of Public Health, Debre Markos University, Debre Markos, Ethiopia; DGD Cochrane South Africa, South African Medical Research Council, Cape Town, South Africa; DGE Department of Public Health, Samara University, Samara, Ethiopia; DGF Department of Research, Cancer Registry of Norway, Oslo, Norway; DGG Department of Chemical Toxicology, Norwegian Institute of Public Health, Oslo, Norway; DGH Institute of Health and Care Sciences, University of Gothenburg, Gothenburg, Sweden; DGI Faculty of Health Sciences, Oslo Metropolitan University, Oslo, Norway; DGJ Faculty of Health, University of Technology Sydney, Sydney, NSW, Australia; DGK Brown School of Public Health, Washington University in St. Louis, St. Louis, MO, USA; DGL Department of Theory and Empiricism of Healthcare, Universität Kassel, Kassel, Germany; DGM Department of Pharmacy, University of Gondar, Gondar, Ethiopia; DGN The Second Affiliated Hospital, Wenzhou Medical University, Wenzhou, China; DGO Global Health Research Center, Duke Kunshan University, Kunshan, China; DGP Duke Global Health Institute, Duke University, Durham, NC, USA; DGQ Department of Food Science and Human Nutrition, Michigan State University, East Lansing, MI, USA; DGR Division of Hematology and Oncology, Medical College of Wisconsin, Milwaukee, WI, USA; DGS Affiliated Hospital of Guangdong Medical University, Guangdong Medical University, Zhanjiang, China; DGT Department of Public Health, Wuhan Fourth Hospital, Wuhan, China; DGU Eye Institute of Xiamen University, Xiamen University Malaysia, Xiamen, China; DGV Division of Gastroenterology, Huazhong University of Science and Technology, Wuhan, China; DGW Vanke School of Public Health, Tsinghua University, Beijing, China; DGX Western Institute of Digital-Intelligent Medicine, Chongqing Medical University, Chongqing, China; DGY Tongji Medical College, Huazhong University of Science and Technology, Wuhan, China; DGZ School of Public Health, Zhejiang University, Zhejiang, China; DHA Department of Public Health Science, Fred Hutchinson Cancer Research Center, Seattle, WA, USA; DHB School of Nursing and Rehabilitation, Shandong University, Jinan, China; DHC Department of Intelligent Medical Engineering, Anhui Medical University, Anhui, China; DHD Department of Surgery, The First Affiliated Hospital of Anhui Medical University, Hefei, Anhui, China; DHE Department of Respiratory and Critical Care Medicine, Nanchang University, Jiangxi, China; DHF Ruijin Hospital, Shanghai Jiao Tong University, Shanghai, China; DHG Department of Endocrinology, University of Science and Technology of China, Hefei, China; DHH School of Medicine, University of Rochester, Rochester, NY, USA; DHI Department of Nutrition, Tufts University, Boston, MA, USA; DHJ Department of Social and Behavioral Sciences, Harvard University, Boston, MA, USA; DHK Cardiovascular Program, The George Institute for Global Health, Sydney, NSW, Australia; DHL Department of Environmental Health and Epidemiology, National Institute for Research in Environmental Health, Bhopal, India; DHM Department of Basic Medical Sciences, Neyshabur University of Medical Sciences, Neyshabur, Iran; DHN Department of Community Medicine, Apollo Institute of Medical Sciences and Research, Hyderabad, India; DHO Department of Microbiology and Immunology, Zagazig University, Zagazig, Egypt; DHP Department of Cells and Tissues, Molecular Biology Institute of Barcelona, Barcelona, Spain; DHQ Department of Public Health, Juntendo University, Tokyo, Japan; DHR Department of Public Health Medicine, University of Tsukuba, Tsukuba, Japan; DHS Department of Public Health Administration, Linyi People's Hospital, Linyi, China; DHT Department of Cardiovascular Surgery, Tianjin Medical University General Hospital, Tian Jin, China; DHU Faculty of Medicine, Juntendo University, Tokyo, Japan; DHV School of Traditional Chinese Medicine, Beijing University of Chinese Medicine, Beijing, China; DHW Pritzker School of Medicine, University of Chicago, Chicago, IL, USA; DHX School of Public Health and Primary Care, The Chinese University of Hong Kong, Hong Kong, China; DHY Department of Medicine, Thomas Jefferson University, Philadelphia, PA, USA; DHZ Department of Medicine, Mashhad University of Medical Sciences, Mashhad, Iran; DIA College of pharmacy and Health Science, Ajman University, Ajman, United Arab Emirates; DIB Hematology Section, Hamad Medical Corporation, Doha, Qatar; DIC Department of Biostatistics and Data Science, The University of Osaka, Suita, Japan; DID National Center for Chronic and Noncommunicable Disease Control and Prevention, Chinese Center for Disease Control and Prevention, Beijing, China; DIE The George Institute for Global Health, University of New South Wales, Sydney, NSW, Australia; DIF School of Biotechnology, University of Tehran, Tehran, Iran; DIG Department of Public Health, Trakya University, Edirne, Turkiye; DIH Medical Biotechnology Research Center, Guilan University of Medical Sciences, Rasht, Iran; DII Manipal College of Nursing, Manipal Academy of Higher Education, Udupi, India; DIJ Department of Family Medicine, St. Paul's Hospital Millennium Medical College, Addis Ababa, Ethiopia; DIK Family Medicine Department, St. Peter's Specialized Hospital, Addis Ababa, Ethiopia; DIL Biostatics, Epidemiology, and Science Computing Department, King Faisal Specialist Hospital & Research Center, Riyadh, Saudi Arabia; DIM KHANA Center for Population Health Research, Phnom Penh, Cambodia; DIN Public Health Department, Dire Dawa University, Dire Dawa, Ethiopia; DIO Department of Health Promotion and Behavioral Science, Bahir Dar University, Bahir Dar, Ethiopia; DIP Department of Epidemiology, Xuzhou Medical University, Xuzhou, China; DIQ Nankai University, China Population and Development Research Center, Tianjin, China; DIR Centre for Suicide Research and Prevention, University of Hong Kong, Hong Kong, China; DIS Department of Social Work and Social Administration, University of Hong Kong, Hong Kong, China; DIT Department of Pharmacy, Bahir Dar University, Bahir Dar, Ethiopia; DIU Department of Pharmacology, Bahir Dar University, Bahir Dar, Ethiopia; DIV Pharmacy Department, Alkan Health Science, Business and Technology College, Bahir Dar, Ethiopia; DIW Department of Pediatrics, Kyung Hee University, Seoul, South Korea; DIX Department of Biostatistics, University of Toyama, Toyama, Japan; DIY Department of Health Policy and Management, Jackson State University, Jackson, MS, USA; DIZ School of Business & Economics, Universiti Putra Malaysia (University of Putra Malaysia), Kuala Lumpur, Malaysia; DJA Department of Public Health, Jigjiga University, Jigjiga, Ethiopia; DJB Department of Anesthesiology, Chongqing Medical University, Chongqing, China; DJC School of Medicine, Tongji University, Shanghai, China; DJD School of Public Health, Hubei University of Medicine, Shiyan, China; DJE Department of Basic Science, University of Hail, Hail, Saudi Arabia; DJF Faculty of Nursing, University of Alberta, Edmonton, AB, Canada; DJG Department of Haematology, Bayero University Kano, Kano, Nigeria; DJH Department of Haematology and Blood Transfusion, Aminu Kano Teaching Hospital, Kano, Nigeria; DJI Association for Socially Applicable Research (ASAR), Pune, India; DJJ Department of Emergency Medicine, Global Emergency Medicine Innovation and Implementation (GEMINI) Research Center, Durham, NC, USA; DJK Epidemiology and Cancer Registry Sector, Institute of Oncology Ljubljana, Ljubljana, Slovenia; DJL Family and Community Medicine Department, University of Hail, Hail, Saudi Arabia; DJM Islamic Azad University, Tehran, Iran; DJN Department of Environmental and Occupational Health, Universiti Putra Malaysia, Serdang, Malaysia; DJO Faculty of Medicine and Health Sciences, Hodeidah University, Hodeidah, Yemen; DJP Department of Computer Science and Software Engineering, United Arab Emirates University, Al Ain, United Arab Emirates; DJQ Basic Sciences Department, University of Duhok, Duhok, Iraq; DJR Department of Health Sciences, James Madison University, Harrisonburg, VA, USA; DJS Department of Computer and Self Development, Prince Sattam bin Abdulaziz University, Al Kharj, Saudi Arabia; DJT Health Investigation Center, Universidad Católica Boliviana San Pablo, Tarija, Bolivia; DJU San Pablo Catholic University Tarija Bolivia, Tarija, Bolivia; DJV Sant'Elia Hospital, University of Catania, Caltanissetta, Italy; DJW Department of Paediatrics and Child Health, University of Cape Town, Cape Town, South Africa; DJX Unit on Child & Adolescent Health, Medical Research Council South Africa, Cape Town, South Africa; DJY Nursing Care Research Center in Chronic Diseases, Ahvaz Jundishapur University of Medical Sciences, Ahvaz, Iran; DJZ Department of Clinical Practice, Northern Border University, Rafha, Saudi Arabia; DKA Institute of Diagnostic and Interventional Radiology and Neuroradiology, University of Duisburg-Essen, Essen, Germany; DKB Department of Psychiatry, Johns Hopkins University, Baltimore, MD, USA; DKC Department of Surgery, University of Hong Kong, Hong Kong, China; DKD Department of Cardiology, Zhongshan Hospital, Shanghai, China; DKE Department of International Health, Johns Hopkins University, Baltimore, MD, USA; DKF School of Public Policy and Administration, Xi ‘an Jiaotong University, Xi'an, China; DKG Medical Oncology Department of Gastrointestinal Cancer, Cancer Hospital of Dalian University of Technology, Shenyang, China; DKH School of Biomedical Engineering, Dalian University of Technology, Dalian, China; DKI Department of Internal Medicine, Jacobi Medical Center, Bronx, NY, USA; DKJ Department of Internal Medicine, Albert Einstein College of Medicine, Bronx, NY, USA; DKK School of Public Health, Wuhan University of Science and Technology, Wuhan, China; DKL Hubei Province Key Laboratory of Occupational Hazard Identification and Control, Wuhan University of Science and Technology, Wuhan, China; DKM Tianjin Medical University General Hospital, Tianjin Centers for Disease Control and Prevention, Tianjin, China; DKN Department of Epidemiology and Biostatistics, Zhejiang University, Hangzhou, China; DKO Fuwai Hospital, Chinese Academy of Medical Sciences, Beijing, China; DKP Department of Health Management, Shengjing Hospital of China Medical University, Shenyang, China; DKQ School of Global Health, Shanghai Jiao Tong University, Shanghai, China; DKR National Institute of Parasitic Diseases, Chinese Center for Disease Control and Prevention, Shanghai, China; DKS Department of Hepatology, Wenzhou Medical University, Wenzhou, China; DKT Jockey Club School of Public Health and Primary Care, The Chinese University of Hong Kong, Hong Kong, China; DKU School of Medicine, Stanford University, Stanford, CA, USA; DKV School of Data Science, The Chinese University of Hong Kong, Shenzhen, Shenzhen, China; DKW National Center for Chronic and Noncommunicable Disease Control China and Prevention, Beijing, China; DKX School of Public Health and Emergency Management, Southern University of Science and Technology, Shenzhen, China; DKY Adama Hospital Medical College, Adama Hospital Medical College, Adama, Ethiopia; DKZ Department of Biochemistry and Pharmacogenomics, Medical University of Warsaw, Warsaw, Poland; DLA School of Public Health, Bengbu Medical College, Bengbu, China; DLB Endocrinology and Metabolism Research Center, Hormozgan University of Medical Sciences, Bandar Abbas, Iran; DLC College of Nursing, Prince Sattam bin Abdulaziz University, Al Kharj, Saudi Arabia; DLD Faculty of Nursing, Mansoura University, Mansoura, Egypt; DLE Department of Medical-Surgical Nursing, University of Hail, Hail, Saudi Arabia; DLF Applied Science Research Centre, Applied Science Private University, Amman, Jordan; DLG Department of Medicine, University of Cape Town, Cape Town, South Africa; DLH Department of Public Health, Universitas Brawijaya, Malang, Indonesia; DLI Center for Clinical Microbiology, University College London, London, UK; DLJ NIHR-Biomedical Research Centre (NIHR-BRC), University College London Hospitals, London, UK; DLK Department of Chemistry, An-Najah National University, Nablus, Palestine; DLL Clinical Research Centre, An-Najah National University Hospital, Nablus, Palestine; DLM Department of Building Engineering and Environment, Palestine Technical University (Kadoorie), Tulkarem, Palestine; DLN Civil Engineering and Sustainable Structures, Palestine Technical University (Kadoorie), Tulkarem, Palestine

## Abstract

**Background:**

For more than three decades, the Global Burden of Diseases, Injuries, and Risk Factors Study (GBD) has provided a framework to quantify health loss due to diseases, injuries, and associated risk factors. This paper presents GBD 2023 findings on disease and injury burden and risk-attributable health loss, offering a global audit of the state of world health to inform public health priorities. This work captures the evolving landscape of health metrics across age groups, sexes, and locations, while reflecting on the remaining post-COVID-19 challenges to achieving our collective global health ambitions.

**Methods:**

The GBD 2023 combined analysis estimated years lived with disability (YLDs), years of life lost (YLLs), and disability-adjusted life-years (DALYs) for 375 diseases and injuries, and risk-attributable burden associated with 88 modifiable risk factors. Of the more than 310 000 total data sources used for all GBD 2023 (about 30% of which were new to this estimation round), more than 120 000 sources were used for estimation of disease and injury burden and 59 000 for risk factor estimation, and included vital registration systems, surveys, disease registries, and published scientific literature. Data were analysed using previously established modelling approaches, such as disease modelling meta-regression version 2.1 (DisMod-MR 2.1) and comparative risk assessment methods. Diseases and injuries were categorised into four levels on the basis of the established GBD cause hierarchy, as were risk factors using the GBD risk hierarchy. Estimates stratified by age, sex, location, and year from 1990 to 2023 were focused on disease-specific time trends over the 2010–23 period and presented as counts (to three significant figures) and age-standardised rates per 100 000 person-years (to one decimal place). For each measure, 95% uncertainty intervals [UIs] were calculated with the 2·5th and 97·5th percentile ordered values from a 250-draw distribution.

**Findings:**

Total numbers of global DALYs grew 6·1% (95% UI 4·0–8·1), from 2·64 billion (2·46–2·86) in 2010 to 2·80 billion (2·57–3·08) in 2023, but age-standardised DALY rates, which account for population growth and ageing, decreased by 12·6% (11·0–14·1), revealing large long-term health improvements. Non-communicable diseases (NCDs) contributed 1·45 billion (1·31–1·61) global DALYs in 2010, increasing to 1·80 billion (1·63–2·03) in 2023, alongside a concurrent 4·1% (1·9–6·3) reduction in age-standardised rates. Based on DALY counts, the leading level 3 NCDs in 2023 were ischaemic heart disease (193 million [176–209] DALYs), stroke (157 million [141–172]), and diabetes (90·2 million [75·2–107]), with the largest increases in age-standardised rates since 2010 occurring for anxiety disorders (62·8% [34·0–107·5]), depressive disorders (26·3% [11·6–42·9]), and diabetes (14·9% [7·5–25·6]). Remarkable health gains were made for communicable, maternal, neonatal, and nutritional (CMNN) diseases, with DALYs falling from 874 million (837–917) in 2010 to 681 million (642–736) in 2023, and a 25·8% (22·6–28·7) reduction in age-standardised DALY rates. During the COVID-19 pandemic, DALYs due to CMNN diseases rose but returned to pre-pandemic levels by 2023. From 2010 to 2023, decreases in age-standardised rates for CMNN diseases were led by rate decreases of 49·1% (32·7–61·0) for diarrhoeal diseases, 42·9% (38·0–48·0) for HIV/AIDS, and 42·2% (23·6–56·6) for tuberculosis. Neonatal disorders and lower respiratory infections remained the leading level 3 CMNN causes globally in 2023, although both showed notable rate decreases from 2010, declining by 16·5% (10·6–22·0) and 24·8% (7·4–36·7), respectively. Injury-related age-standardised DALY rates decreased by 15·6% (10·7–19·8) over the same period. Differences in burden due to NCDs, CMNN diseases, and injuries persisted across age, sex, time, and location. Based on our risk analysis, nearly 50% (1·27 billion [1·18–1·38]) of the roughly 2·80 billion total global DALYs in 2023 were attributable to the 88 risk factors analysed in GBD. Globally, the five level 3 risk factors contributing the highest proportion of risk-attributable DALYs were high systolic blood pressure (SBP), particulate matter pollution, high fasting plasma glucose (FPG), smoking, and low birthweight and short gestation—with high SBP accounting for 8·4% (6·9–10·0) of total DALYs. Of the three overarching level 1 GBD risk factor categories—behavioural, metabolic, and environmental and occupational—risk-attributable DALYs rose between 2010 and 2023 only for metabolic risks, increasing by 30·7% (24·8–37·3); however, age-standardised DALY rates attributable to metabolic risks decreased by 6·7% (2·0–11·0) over the same period. For all but three of the 25 leading level 3 risk factors, age-standardised rates dropped between 2010 and 2023—eg, declining by 54·4% (38·7–65·3) for unsafe sanitation, 50·5% (33·3–63·1) for unsafe water source, and 45·2% (25·6–72·0) for no access to handwashing facility, and by 44·9% (37·3–53·5) for child growth failure. The three leading level 3 risk factors for which age-standardised attributable DALY rates rose were high BMI (10·5% [0·1 to 20·9]), drug use (8·4% [2·6 to 15·3]), and high FPG (6·2% [–2·7 to 15·6]; non-significant).

**Interpretation:**

Our findings underscore the complex and dynamic nature of global health challenges. Since 2010, there have been large decreases in burden due to CMNN diseases and many environmental and behavioural risk factors, juxtaposed with sizeable increases in DALYs attributable to metabolic risk factors and NCDs in growing and ageing populations. This long-observed consequence of the global epidemiological transition was only temporarily interrupted by the COVID-19 pandemic. The substantially decreasing CMNN disease burden, despite the 2008 global financial crisis and pandemic-related disruptions, is one of the greatest collective public health successes known. However, these achievements are at risk of being reversed due to major cuts to development assistance for health globally, the effects of which will hit low-income countries with high burden the hardest. Without sustained investment in evidence-based interventions and policies, progress could stall or reverse, leading to widespread human costs and geopolitical instability. Moreover, the rising NCD burden necessitates intensified efforts to mitigate exposure to leading risk factors—eg, air pollution, smoking, and metabolic risks, such as high SBP, BMI, and FPG—including policies that promote food security, healthier diets, physical activity, and equitable and expanded access to potential treatments, such as GLP-1 receptor agonists. Decisive, coordinated action is needed to address long-standing yet growing health challenges, including depressive and anxiety disorders. Yet this can be only part of the solution. Our response to the NCD syndemic—the complex interaction of multiple health risks, social determinants, and systemic challenges—will define the future landscape of global health. To ensure human wellbeing, economic stability, and social equity, global action to sustain and advance health gains must prioritise reducing disparities by addressing socioeconomic and demographic determinants, ensuring equitable health-care access, tackling malnutrition, strengthening health systems, and improving vaccination coverage. We live in times of great opportunity.

**Funding:**

Gates Foundation and Bloomberg Philanthropies.

## Introduction

High-quality, comprehensive, mutually exclusive, and timely estimates of health and health loss produced through the Global Burden of Diseases, Injuries, and Risk Factors Study (GBD) are a valuable source of publicly accessible health data. For more than 30 years, GBD has equipped researchers, policy makers, and the public with evidence-based tools to better understand the global impact of diseases, injuries, and modifiable risk factors at the population level.^1^ GBD has enabled close, quantitative monitoring of progress towards international health targets, especially the UN Sustainable Development Goals (SDGs). An early innovation of GBD was the metric of disability-adjusted life-years (DALYs), which measures overall disease burden as the years of lost health and life combined, and has been adopted by global health institutions such as WHO. More recent work, including reports by WHO World Health Statistics^2^ the NCD Risk Factor Collaboration, and the Prospective Urban and Rural Epidemiological (PURE) study, provide estimates for specific diseases or risk factors; however, GBD is broader in scope.

Estimation of disease and injury burden in GBD has evolved since its inception and original publication, which established DALYs as the primary metric for burden analysis. GBD 2010 highlighted the rise of non-communicable diseases (NCDs), particularly mental disorders, musculoskeletal conditions, and cardiovascular diseases, while also accounting for persistent burden from communicable, maternal, neonatal, and nutritional (CMNN) diseases in low-income regions.^3^ GBD 2015 introduced improved geographical detail, allowing for more granular subnational assessments of disease burden.^4^ The 2015 iteration reinforced the ongoing epidemiological shift, showing reductions in infectious disease burden but increases in NCD-related DALYs, particularly due to metabolic risks. GBD 2017 expanded risk factor analysis and also revealed growing disparities in NCD burden across regions, with lower-income countries experiencing a dual burden of infectious and chronic diseases.^5^ GBD 2019 further refined cause-of-death modelling and highlighted increasing longevity alongside persistent morbidity, demonstrating that years lived with disability (YLDs) were rising faster than mortality reductions.^6^ GBD 2021 incorporated disruptions related to the COVID-19 pandemic and improved modelling of multimorbidity, emphasising the continued shift in burden towards NCDs, with metabolic and behavioural risk factors increasingly driving DALYs, even in regions with a historically high burden of infectious diseases.^7^ These advances underscore how the GBD framework has progressively illuminated the changing nature of global disease burden, reinforcing the need for targeted interventions to mitigate the growing impact of NCDs, while sustaining infectious disease control efforts. GBD 2023 shows disease burden trends amid a fundamentally altered and severely constrained global health financing system. As global budget cuts threaten progress towards the SDGs, future iterations of GBD must prioritise monitoring of the impact on populations globally. GBD 2023 can inform priorities for international health agendas, while anticipating demographic trends, the growing burden of NCDs, and other challenges.

**Research in context**

**Evidence before this study**

Since its inception in the early 1990s, the Global Burden of Diseases, Injuries, and Risk Factors Study (GBD) has systematically quantified health and health loss across time, age, sex, location, and sociodemographic groups. GBD introduced and uses disability-adjusted life-years (DALYs) as a measure of disease burden that captures disability and premature mortality. DALYs have been widely adopted by WHO, the UN, and public health agencies to measure overall disease burden in a population. Previous research efforts, including WHO World Health Statistics and initiatives such as the NCD Risk Factor Collaboration and the Prospective Urban and Rural Epidemiological (PURE) study, have advanced understanding of specific diseases or risk factors and, like GBD, are continuously updated, making it possible to track progress towards the UN Sustainable Development Goals. GBD stands out for its comprehensive scope, wealth of data, and rigorous methodological provenance and updates, as well as its global coverage and commitment to reporting scientific findings free of political bias and the influence of special interests.

**Added value of this study**

GBD 2023 analysed 375 diseases and injuries and 88 modifiable risk factors, providing updated estimates of prevalence, incidence, years lived with disability (YLDs), years of life lost (YLLs), DALYs, and risk-attributable DALYs for 204 countries and territories from 1990 to 2023. Our estimates of burden improved on those from GBD 2021 by the inclusion of data from more than 35 000 new sources, with particularly notable increases in data used to estimate the burden of diseases such as ischaemic heart disease, chronic obstructive pulmonary disease, and tuberculosis. Analyses were extended to five new causes: ulcerative colitis; Crohn's disease; thyroid diseases; other endocrine, metabolic, blood, and immune disorders; and electrocution. “Other pandemic-related outcomes” was removed as a cause. Additionally, we began to transition our primary tool to model prevalence from disease modelling meta-regression version 2.1 (DisMod-MR 2.1) to disease modelling age-time (DisMod-AT), which more effectively captures temporal trends in data. GBD 2023 advanced risk factor analyses from previous GBD cycles, strengthening attributable burden estimates by conducting 85 new or updated systematic reviews and incorporating additional data from more than 16 000 new sources, particularly for intimate partner violence, lead exposure, high BMI, and high fasting plasma glucose. Based on new evidence or further specification of outcomes or mediation factors, 50 new risk–outcome pairs, such as the relationship between particulate matter pollution and dementia, were analysed; two pairs were excluded (child wasting and malaria, and high alcohol use and nasopharynx cancer) for not meeting inclusion criteria or for overlapping with other outcomes. In total, 676 risk–outcome pairs were analysed for GBD 2023. Methods were updated for specific risk factors, notably regarding estimation of burden attributable to lead exposure and revision of the theoretical minimum risk exposure level for diet high in trans fatty acids.

**Implications of all the available evidence**

This study reaffirms that the global epidemiological transition has continued up to 2023. Although the COVID-19 pandemic temporarily disrupted health trends, the long-term decline in burden due to communicable, maternal, neonatal, and nutritional (CMNN) diseases has continued, whereas absolute burden of NCDs has risen sharply, largely due to demographic changes. It is an opportune time to revisit these two patterns at the highest policy levels. First, acknowledging and celebrating the staggering success of reducing the impact of CMNN diseases worldwide is important, alongside warnings that progress is fragile. The threats of stagnation or resurgence do not recede simply because our global policy focus might shift. Second, there is an opportunity to make substantial progress in reducing the burden of NCDs across sociodemographic strata. Since our present analyses show that almost half of total disease burden is attributable to specific modifiable risk factors—with increasing contributions, especially in ageing populations, of metabolic risks (eg, high systolic blood pressure, smoking, lead exposure, and ambient particulate matter air pollution)—considerable progress can be made by addressing risk-attributable burden, although successful mitigation varies substantially across risk factors. Equitable scaling of implementation remains a challenge, requiring coordinated policy efforts, targeted prevention strategies, and strengthened health-care systems to mitigate disparities and improve population health outcomes.

The comparative risk assessment framework of GBD has continually advanced in methodology and scope. Risk factor estimates have been a part of GBD since its inception. GBD 2010 refined a unified methodological foundation by systematically quantifying 67 risk factors across regions, highlighting a global epidemiological shift from CMNN disease-related to NCD-related risk factors, such as high systolic blood pressure (SBP), smoking, and poor diet.^8^ Subsequent updates in GBD 2015 enhanced risk exposure estimation, particularly for dietary and metabolic risks, and intensified policy attention on cardiovascular implications of air pollution.^9^ GBD 2016 further emphasised behavioural risks, including alcohol and drug use, and provided detailed subnational estimates that promoted localised policy responses.^10^ GBD 2017 refined mediation pathways and updated risk curves.^11^ GBD 2019 introduced methods to model non-linear exposure–response relationships and refined counterfactual analyses, shifting the policy discourse towards integrated, system-level interventions addressing complex interactions among risk factors.^12^ Most recently, GBD 2021 leveraged advanced Bayesian techniques and explicitly recognised the rising importance of climate-sensitive risks.^13^ An added dimension is the burden-of-proof methodology, which quantitatively evaluates the strength of evidence between risks and outcomes.^13^, ^14^, ^15^ The methodological innovations and policy messages contained in GBD 2021 underscored the transition from addressing individual health behaviours towards comprehensive systemic interventions to mitigate interconnected and emerging global health threats.

As part of *The Lancet*'s serialisation of GBD, GBD 2023 continues this theme by analysing the relationships between 375 diseases and injuries and 88 risk factors together in a single framework. GBD 2023 provides detailed and comprehensive estimates of health loss over the period of 1990–2023 to highlight major global trends that have preceded and persisted beyond the COVID-19 pandemic. GBD 2023 facilitates a deeper understanding of health disparities within and across populations, evaluates how differential health outcomes have changed over time, quantifies health improvements and gains, and serves as a valuable resource to help identify the specific policies and targeted interventions that will be most impactful. Here, the key GBD 2023 findings are presented for metrics quantifying health and health loss, including prevalence, incidence, YLDs, years of life lost (YLLs), and DALYs. Metrics used to measure the attributable burden of risk factors include relative risk and risk-attributable DALYs. The estimation of causes of deaths and YLLs for GBD 2023 is reported in a separate publication.^16^

This manuscript was produced with contributions from the GBD Collaborator Network and in accordance with the GBD Protocol.^17^

## Methods

### Overview

For each GBD round, newly available data and refined methods are used to update the complete time series of metrics from 1990 to the latest year of analysis. GBD 2023 results therefore supersede all previous estimates. GBD 2023 methods closely followed those used in GBD 2021.^7^, ^13^ A summary of the methods is given, with emphasis on any notable improvements; a more detailed description of all methods is available in appendix 1 and appendix 2. Based on review and approval by the GBD Scientific Council, which considers factors such as policy relevance, data availability, and epidemiological profile, we report here for the first time on health outcomes for five additional causes: ulcerative colitis; Crohn's disease; thyroid diseases; other endocrine, metabolic, blood, and immune disorders; and electrocution. The cause introduced in GBD 2021 titled “other pandemic-related outcomes” was removed because improved data availability since 2021 allowed for more precise assignment of pandemic-related outcomes to specific causes.^16^ These changes bring the total number of diseases and injuries reported in GBD 2023 to 375. We improved our burden estimates through the incorporation of data from more than 35 000 new sources. Moreover, we began transitioning from our principal tool to model prevalence—disease modelling meta-regression version 2.1 (DisMod-MR 2.1; appendix 1 section 2.6)—to an updated version, disease modelling age-time (DisMod-AT), with one of the main improvements being the ability to factor cohort effects over time and location-level covariates by age and sex (appendix 1 section 2.7). This allows us to model changes in prevalence or incidence among specific age cohorts as population segments grow older to more accurately reflect how disease patterns evolve over time, which is important for diseases with rapidly changing epidemiology, such as diabetes. Data availability by location and year for modelling of disease and injury burden is included in appendix 1 (figures S1, S2). Additionally, changes to the estimation of impairments and aetiologies have been made for GBD 2023 (appendix 1 sections 2.8, 6). Similarly, by adding more than 16 000 new data sources on risk factors, we improved estimates of risk-attributable burden (appendix 2 table S7). For GBD 2023, we conducted 85 new or updated systematic reviews of the literature on relative risk (appendix 2 section 2.1.3) and risk factor exposure (appendix 2 section 2.2.1). No new risk factors were added for GBD 2023; however, based on new evidence or further specification of outcomes, 50 new risk–outcome pairs were added, eight of which were based on further specification of mediation factors. Two pairs were removed from the analysis (appendix 2 table S3). Across all analytical components of the risk factor estimation process, 676 risk–outcome pairs were analysed for GBD 2023. Details of our standardised inclusion and exclusion criteria for GBD 2023 risk–outcome pairs are provided in appendix 2 (section 2.1.1). We also updated our methods for certain risk–outcome pairs, such as lead exposure and ischaemic heart disease (appendix 2 section 4). The inclusion of new data sources and methodological improvements for GBD 2023 contributed to improvements in internal consistency, trend stability, and cross-source harmonisation.

### Data sources and processing

Details for all data sources used for disease and injury burden and for risk factor estimation for GBD 2023 are available online via the GBD 2023 Sources Tool on the Global Health Data Exchange. All data sources underwent strict systematic quality assurance processes.^1^ Data sources ensure quality by applying data-vetting protocols to assess internal consistency, completeness, and plausibility; using tools, such as MR-BRT (meta-regression—Bayesian, regularised, trimmed), to adjust for known biases; and cross-validating new sources against existing datasets.

#### Burden of diseases and injuries

DALY calculations for GBD 2023 were based on more than 120 000 cause-related data sources, of which more than 35 000 were newly added between GBD 2021 and the current release. Cause-related sources included more than 98 000 total entries, distributed over 50 000 incidence-related and 25 000 prevalence-related sources, and a range of other sources necessary for tracking severity splits, duration, and similar characteristics. GBD 2023 included newly incorporated data sources on numerous causes, including cardiovascular diseases (eg, ischaemic heart disease and ischaemic stroke), chronic obstructive pulmonary disease, tuberculosis, asthma, and chronic kidney disease. Notable changes were most evident in chronic respiratory conditions and in specific regions where the inclusion of new data addressed gaps. Details on data sources for YLLs are documented in another publication^16^ and are available via the GBD 2023 Sources Tool. Estimates of burden reported here draw from a wide range of sources, including scientific literature, household surveys, disease registries, and clinical informatics, as detailed in appendix 1 (section 2.1). The process for conducting cause-specific literature reviews is detailed in appendix 1 (section 2.1.1). The search strategy covered online research databases, public governmental and international organisation websites, and published reports, as well as contributions of primary data from GBD collaborators. The methods and data sources for fatal estimates, such as vital registration systems, are discussed in a separate publication.^16^

Cause-related data with known biases, such as alternative case definitions or measurement methods, were adjusted using the meta-regression tool MR-BRT (appendix 1 section 2.5).^15^ The adjustment process involved analysing paired estimates based on reference and alternative case definitions for the same age, sex, location, and year. For data sources without sex-specific information, we applied a correction factor derived from the pooled, within-study sex ratios. Data missing both age and sex details were adjusted using a process (age-sex splitting) that leverages within-source sex ratios to adjust age-specific data from sources that reported by age and by sex separately. When data sources spanned wide age ranges (typically >25 years), we derived more granular age-specific estimates using age patterns based on other available data sources (appendix 1 section 2.3.5).

Data processing also extended to clinical data. The comprehensive series of data-processing steps are detailed in appendix 1 (section 2). We analysed data from several clinical settings, including inpatient hospital admissions, outpatient visits, and health insurance claims. For inpatient data reporting a single diagnosis, we adjusted data to account for factors such as re-admissions, non-primary diagnoses, and outpatient care. We made these adjustments by calculating age-sex-specific ratios by cause using RegMod, a new GBD regression modelling package, which was used in this instance to create correction factor models for clinical data (appendix 1 section 2.2.5). To ensure that estimates of inpatient data accurately reflected population data, inpatient sources were scaled using estimates of total inpatient admission rates per capita for each location-year-age-sex for which demographic data were incomplete.

Moreover, we adjusted inpatient sources to account for disparities in health-care access across all locations by scaling estimates using a scalar developed for the Healthcare Access and Quality Index (appendix 1 section 2.2.5).^17^ These adjustments produce standardised, population-level clinical estimates that represent both the incidence and prevalence of causes and mitigate the impact of known biases in the data.

#### Disease and injury burden attributable to risk factors

To estimate the burden of disease attributable to risk factors, we combined four inputs: exposure; relative risk of each health outcome associated with the risk factor; the theoretical minimum risk exposure level (TMREL); and deaths and burden for each of the health outcomes with which a risk factor is associated. In GBD 2023, we estimated the burden associated with 88 risk factors. The TMREL and deaths and burden for each health outcome associated with a risk factor are derived and did not require additional data, so data seeking for risk factor analyses focused on exposure and relative risks. The exposure estimation processes used more than 55 000 distinct data sources, about 16 000 of which were new for GBD 2023, related primarily to the incorporation of new data sources for various risk factors, such as sexual violence against children, intimate partner violence, bullying victimisation, high BMI, high fasting plasma glucose (FPG), and various dietary risk factors. These sources were identified through systematic reviews of risk factor exposure studies, in addition to other data that include household and health examination surveys and censuses, ground-sensing or remote-sensing data, and administrative records (appendix 2 section 2.2.1).

Relative risk estimates were derived from meta-analyses incorporating more than 3800 distinct data sources, more than 900 of which were new for GBD 2023. Data used to estimate relative risks were identified and extracted through systematic literature reviews of randomised controlled trials and prospective cohort studies reporting fatal and non-fatal health outcomes associated with risk factor exposures, and from studies underlying risk–outcome meta-analyses (appendix 2 section 2.1.3). Where data from randomised controlled trials or cohort studies were unavailable, odds ratios from case–control studies were potentially included in relative risk estimation (generally reflected by including a bias covariate in the burden-of-proof estimation framework). Across relative risk and exposure estimation processes, 85 new or updated systematic reviews were conducted. Decisions were made to undertake or prioritise reviews based on various circumstances, including the availability of literature providing new or more nuanced or detailed data, or newly available resources to support review of particular risk–outcome pairs. Appendix 2 includes PRISMA diagrams for each of the 85 systematic reviews and risk factor-specific strategies to maximise data collection, search procedures, and bias assessment (section 4), and systematic review and bias assessment guidelines (section 2.1.3). For risk factor exposure data, MR-BRT was used to adjust for bias and perform age-sex splitting; further details are provided in appendix 2 (section 2.2.2).

### Estimation methods

#### Burden of diseases and injuries

GBD 2023 estimated incidence, prevalence, YLDs, YLLs, and DALYs for 375 diseases and injuries: 371 with non-fatal outcomes and 292 with fatal outcomes. Specific diseases and injuries are organised within a four-level cause hierarchy. The broadest category—level 1—includes three large cause groupings of NCDs, CMNN diseases, and injuries. Level 2 categories are further disaggregated into specific subgroupings, such as cardiovascular diseases and transport injuries. Level 3 causes include specific causes (eg, stroke and road injuries). In some cases, level 3 causes are the most granular level of analysis; however, in other cases, causes are further disaggregated at level 4. Level 4 causes are the most specific (eg, ischaemic stroke and pedestrian road injuries). Detailed information on the GBD cause hierarchy is in appendix 1 (table S3).

The modelling of prevalence and incidence was mainly conducted using DisMod-MR 2.1, a Bayesian disease modelling meta-regression tool.^7^ A new tool, DisMod-AT, modelled prevalence and incidence for four causes: type 1 diabetes, major depressive disorder, anxiety disorders, and autism spectrum disorders (appendix 1 section 2.7). For certain diseases and injuries, the use of spatiotemporal Gaussian process regression (ST-GPR) models allowed for the analysis of data that are both heterogeneous and incomplete and which require statistical smoothing (appendix 1 section 2.4). The methodology for cause-specific estimations, including the calculation of sequela-specific prevalence, is described in appendix 1 (section 6).

To estimate YLDs, we calculated cause-age-sex-location-year-specific prevalence of sequelae (or duration of nature of injury) and then multiplied these prevalence values by their respective disability weights for each disease and injury. The process for estimating disability weights is detailed further in appendix 1 (section 2.9). YLDs were adjusted for comorbidity, assuming that a multiplicative function of disability weights accounts for the co-occurrence of non-fatal causes within individuals. YLLs were derived by multiplying the cause-age-sex-location-year-specific number of deaths by the standard life expectancy at the age of death for each cause, as detailed by the GBD 2023 Causes of Death Collaborators.^16^ DALYs were computed by summing YLDs and YLLs (appendix 1 section 4). A complementary measure to DALYs—healthy life expectancy (HALE), which measures a population's mean number of years of life spent in full health—was calculated using YLDs per capita and age-specific mortality rates by location, age, sex, year, and cause.^16^ This method was developed by Sullivan^19^ and described in appendix 1 (section 5). Both DALYs and HALE were estimated by location, age, sex, and year. More comprehensive details can be found in appendix 1 (sections 4, 5). Cause-specific disease and injury estimation methods were updated for GBD 2023 for several causes, including for rheumatic heart disease, autism spectrum disorders, and HIV/AIDS. Details on these and other cause-specific updates are in appendix 1 (section 6).

#### Disease and injury burden attributable to risk factors

Risk factor analysis was based on the comparative risk assessment framework, which is premised on a causal web of hierarchically organised, modifiable risk factors that affect health outcomes^20^, ^21^ (appendix 2 section 2, table S2). Risk factors were classified into a four-level hierarchy with the broadest categories—environmental and occupational, behavioural, and metabolic risks—at level 1. Level 1 categories were then further disaggregated, allowing for analysis focused both on risk groups at level 2 (eg, air pollution) and on increasingly granular risk factors at levels 3 (eg, particulate matter pollution) and 4 (eg, household air pollution from solid fuels). GBD 2021 and GBD 2023 included 88 total risk factors across hierarchy levels (appendix 2 table S1). Risk factor definitions and modelling details are in appendix 2 (section 4).

Described briefly are the methods for estimating each of the four inputs into assessing risk-attributable burden: exposure, relative risk of each health outcome associated with the risk factor, the TMREL, and deaths and burden for each of the health outcomes with which a risk factor is associated.

Methods to estimate mean levels of exposure to each risk factor by age-sex-location-year varied across risks. Data for most risks were extracted from household surveys and the scientific literature and were modelled using either ST-GPR or DisMod-MR 2.1.^7^, ^13^ Some risks (eg, ambient air pollution) required other approaches, such as satellite data and geospatial analysis for environmental exposures. For most risks, the distribution of exposure across individuals was estimated by modelling a measure of dispersion, usually the SD, and fitting an ensemble of parametric distributions to the predicted mean and SD (appendix 2 section 2.2.3 [step 2]). Summary exposure values (SEVs), reflecting both the prevalence of a given risk factor and the relative harm caused by that risk factor, were calculated from exposure estimates (appendix 2 section 2 [step 5]).

For the GBD 2023 risk factor analysis, we evaluated a total of 88 risk factors and 159 health outcomes, including four outcomes (bipolar disorder, bulimia nervosa, conduct disorder, and schizophrenia) that in previous iterations of the GBD had not been linked to any risk factors. At the most detailed risk and cause level, relative risks for a total of 676 risk–outcome pairs—including pairs in mediation pathways and pairs for which, by definition, a fixed percentage (often 100%) of the disease is attributed to the risk—were estimated. This included 50 new risk–outcome pairs, while two previously included pairs—child wasting and malaria, and high alcohol use and nasopharynx cancer—were excluded for not meeting inclusion criteria or for overlapping with other outcomes (appendix 2 table S3).

For 256 of the pairs for which standard effect size analyses were applicable to estimate the relative risk, we applied our burden-of-proof meta-regression approach.^13^, ^14^, ^15^ The burden-of-proof framework used a range of systematic strategies, including ensemble spline models to capture the potentially non-linear shape of the risk–outcome relationship, robust likelihood-based trimming of outliers, covariate selection and adjustment to account for known variation in input study design characteristics, and quantification and incorporation of remaining between-study heterogeneity into uncertainty. See appendix 2 (section 2.1.2–2.1.8 [step 1]) for details.

The burden-of-proof approach further generates a burden-of-proof risk function (BPRF), which is conservatively defined for harmful risks as the 5th and for protective risks as the 95th quantile relative risk curve, inclusive of between-study heterogeneity, closest to null. The BPRF extends relative risk estimates using the same data inputs and modelling processes to provide a conservative measure of both effect size and evidence strength that incorporates between-study heterogeneity to formally account for divergence or convergence in findings across input studies. For ease of interpretation and comparison, risk–outcome scores (ROSs) are calculated summarising average BPRFs across the data-dense range (15th to 85th percentile) of risk exposure levels reported in the input studies, and summary scores are mapped to a (one to five) star rating system, with higher positive ROS values and more stars corresponding to incrementally stronger evidence for the risk–outcome relationship (appendix 2 section 2.1.6, table S8). The uncertainty intervals (UIs) for relative risks estimated with burden-of-proof methods in this analysis include between-study heterogeneity for all risk factors except tobacco use, given concerns raised during GBD 2021 regarding the interpretation of the resulting wide UIs with respect to policy. Efforts to review and potentially revise the incorporation of unexplained between-study heterogeneity in UIs are part of regular GBD methodology updates. For the purposes of this combined GBD 2023 disease burden and risk factor analysis, we present BPRF-related metrics only in appendix 3 (table S18). More detailed BPRF results can be found in GBD 2021 Risk Factor Collaborators^13^ and other risk-specific papers,^22^, ^23^, ^24^, ^25^, ^26^, ^27^ and accessed through the Burden of Proof tool*.*

For each risk factor, the TMREL—the counterfactual level of exposure that is theoretically possible and would minimise health risks in exposed populations—was estimated either on the basis of epidemiological evidence quantifying risk–outcome relationships and the distribution of observed risk factor exposure (eg, ozone air pollution), or on the basis of risk factor definition (eg, smoking; appendix 2 section 2 [step 3], table S4). Differing TMRELs reflect the range of behavioural, metabolic, and environmental risk factors, including those for which zero exposure is theoretically achievable and those for which non-zero levels reflect minimum risk.

For each risk–outcome pair, estimates of exposure, TMREL, and relative risk were used to compute the population attributable fraction (PAF; the proportional difference between disease or injury burden at current levels of risk factor exposure and the burden that would have occurred had the population been exposed to the risk factor at the TMREL; appendix 2 section 2 [step 4]). For associations involving risk factors that act on outcomes via intermediate risks (ie, many risk factors, particularly dietary risks, are associated with disease outcomes mediated through metabolic risks, such as a relationship between diet high in sodium and hypertensive heart disease mediated through high SBP), PAFs were adjusted based on values estimated in the GBD 2023 mediation matrix (appendix 2 section 2 [step 6], table S5). Eight additional risk–outcome pairs were incorporated in the 2023 matrix, for a total of 165 mediated pairs (appendix 2 table S6).

To calculate measures of risk-attributable burden—ie, the disease burden (DALYs, deaths, YLLs, or YLDs) attributable to a particular risk factor or combination of risks—PAFs were multiplied by the estimated disease burden associated with particular outcomes (appendix 2 section 2 [step 7]). There have been several updates to the estimation of risk-attributable burden for GBD 2023, including for outcomes such as ischaemic heart disease, with prevalence and disease burden now modelled directly rather than on the basis of non-specific chest pain symptoms, and for risk factors such as lead exposure, with one of the important changes being that the effect of lead on ischaemic heart disease is now estimated directly, whereas this effect was previously exclusively mediated via high SBP. Additionally, names and case definitions were updated for some risk factors, such as sexual violence against children (previously childhood sexual abuse), and the TMREL was revised for one risk factor: diet high in trans fatty acids. See appendix 2 (section 4) for all GBD 2023 risk-specific methods.

For assessments of model robustness for the primary models used in estimating disease and injury burden and risk-attributable burden, refer to appendix 2 section 2.2.3 for ST-GPR, appendix 1 section 4.5 of GBD 2019 Diseases and Injuries Collaborators^6^ for DisMod-MR, appendix 1 section 6 for DisMod-AT, and Zheng and colleagues^14^ for burden-of-proof methods.

### GBD research and reporting practices

This research complies with the GATHER statement;^28^ a completed GATHER checklist is provided in appendix 1 (table S2). The University of Washington Institutional Review Board approved the GBD study (STUDY00009060) up to July 26, 2026. The software used for analyses included Python (version 3.10.4), Stata (version 13.1), and R (version 4.2.1). The statistical code used in GBD 2023 is publicly available online. An international network of collaborators helped to provide, review, and analyse the available data to generate health metrics; GBD 2023 drew on the expertise of more than 14 000 collaborators from more than 160 countries and territories.

All GBD 2023 estimates for diseases, injuries, and risk factors are reported by age, sex, location, and year for 25 age groups from early neonatal (0–6 days) to 95 years and older; for males, females, and all sexes combined; for every year from 1990 to 2023; and in 204 countries and territories grouped into 21 regions and seven super-regions. The super-regions are central Europe, eastern Europe, and central Asia; high income; Latin America and the Caribbean; north Africa and the Middle East; south Asia; southeast Asia, east Asia, and Oceania; and sub-Saharan Africa (appendix 1 section 1.1–1.2). GBD 2023 also produced estimates for 660 subnational locations in 20 countries (Brazil, China, Ethiopia, India, Indonesia, Italy, Iran, Japan, Kenya, Mexico, New Zealand, Nigeria, Norway, Pakistan, the Philippines, Poland, Russia, South Africa, the UK, and the USA). Results are also presented by Socio-demographic Index (SDI) quintile, which is a composite measure of lag-distributed income per capita, average years of education, and fertility rates among females younger than 25 years.^29^ Each location at the most specific level is assigned an SDI value ranging from 0 (lowest income and educational attainment, and highest fertility) to 100 and then grouped into quintiles from low SDI to high SDI (appendix 1 table S12).

Estimates are reported here as absolute counts and as rates per 100 000 person-years, with age-standardised rates calculated using the GBD 2023 world standard population^30^ to account for varying age structures across populations. Count data are presented to three significant figures, and rates are presented to one decimal place. Uncertainty was propagated throughout the estimation process. Mean estimates for all metrics reported represent the mean value across 250 draws from the estimate's distribution, with 95% UIs calculated as the 2·5th and 97·5th percentile values across the draws. To reduce computing power and time across the estimation process, the number of draws was reduced from 500 in GBD 2021 to 250 for GBD 2023. Simulations revealed that estimates and uncertainty were minimally affected by this reduction (see appendix 1 section 1.1 for more details).

### Role of the funding source

The funders of this study had no role in study design, data collection, data analysis, data interpretation, or the writing of the report.

## Results

### Overview

To capture worldwide long-term patterns of disease burden and changes in the global health outlook since the COVID-19 pandemic, we report estimated DALYs from 1990 to 2023, across level 1 causes, then primarily focus on results from 2010 to 2023, the most recent period of acute public health and policy interest. HALE results are presented in appendix 3 (table S9). For risk factor analyses, we present estimates of risk factor exposure in SEVs and risk-attributable burden in DALYs. More detailed estimates and metrics are presented in appendix 3. Comprehensive results can also be accessed through the GBD 2023 Results Tool and visualised via GBD Compare. Results specific to risk factor analyses can also be accessed through the Burden of Proof Tool.

### The changing landscape of global health across sociodemographic levels

**Figure 1:**
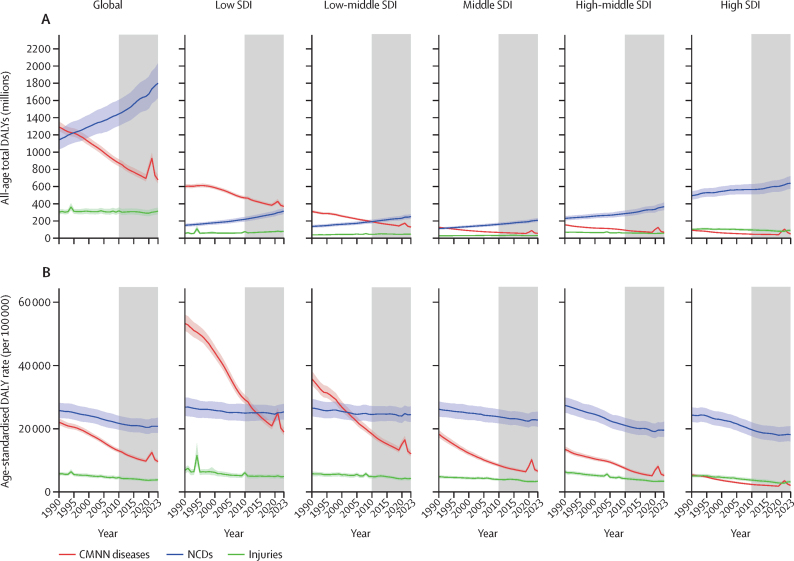
Trends of total DALYs (A) and age-standardised DALY rates (B) by GBD level 1 cause and by SDI quintile, 1990–2023 The grey shading indicates the 2010–23 period. The bump in injury-related DALYs that can be seen in the low SDI and global panels in 1994 is largely the result of the Rwanda genocide. The larger bump in CMNN diseases in almost all plots in 2021 and 2022 is the larger DALY effect of the COVID-19 pandemic. Shading around mean trend lines represents 95% uncertainty intervals. CMNN=communicable, maternal, neonatal, and nutritional. DALY=disability-adjusted life-year. GBD=Global Burden of Diseases, Injuries, and Risk Factors Study. NCDs=non-communicable diseases. SDI=Socio-demographic Index.

The number of global all-cause DALYs remained statistically stable between 1990 and 2023 (2·74 billion [95% UI 2·60–2·90] in 1990 and 2·80 billion [2·57–3·08] in 2023; appendix 3 table S10). This apparent stasis hides an epidemiological transition among level 1 causes that broadly reflects a decrease in burden due to CMNN diseases, a rise in NCD DALYs, and an unchanged level of injuries (figure 1A). Global age-standardised DALY rates—which account for variation in population structure—decreased for CMNN diseases, NCDs, and injuries, reflecting per-person improvements in burden between 1990 and 2023 (figure 1B), but these gains were not seen in DALY counts for NCDs or injuries because the global population is growing and ageing. The biggest deviation during the COVID-19 pandemic from these long-term favourable trajectories in DALY rates was for CMNN diseases, and this disturbance was more acute in countries in lower SDI quintiles (ie, the effect of the pandemic was greatest for countries with lower income per capita and educational attainment and higher fertility rates; figure 1B). A similar pandemic-related disruption was not evident for NCDs or injuries at the global level.

Declines in age-standardised DALY rates for CMNN diseases were steepest over time at lower SDI levels and more attenuated with higher SDI. In the high SDI quintile, total DALY counts and age-standardised rates for CMNN diseases were below those for injuries in all but the pandemic years (figure 1). Much less substantial decreases in age-standardised rates were seen for NCDs and injuries except in the high-middle and high SDI quintiles.

### Global trends in DALYs, 2010–23

Although the 2023 all-cause global DALYs rose 6·1% (95% UI 4·0–8·1) from 2·64 billion (2·46–2·86) in 2010 to 2·80 billion (2·57–3·08) in 2023, the global age-standardised DALY rate declined by 12·6% (11·0–14·1; appendix 3 table S10). Across level 1 causes, NCDs contributed the highest burden globally in 2023 and were the only disease group for which DALY counts increased between 2010 and 2023, from 1·45 billion (1·31–1·61) to 1·80 billion (1·63–2·03). However, age-standardised DALY rates for NCDs decreased during this period by 4·1% (1·9–6·3; appendix 3 table S10). DALY counts for CMNN diseases decreased from 874 million (837–917) in 2010 to 681 million (642–736) in 2023, and the age-standardised DALY rate decreased more markedly by 25·8% (22·6–28·7). DALY counts due to injuries also exhibited a decreasing but non-significant trend from 319 million (288–357) in 2010 to 316 million (280–356) in 2023. The age-standardised DALY rate due to injuries decreased by 15·6% (10·7–19·8) during the same period (appendix 3 table S10).

**Figure 2:**
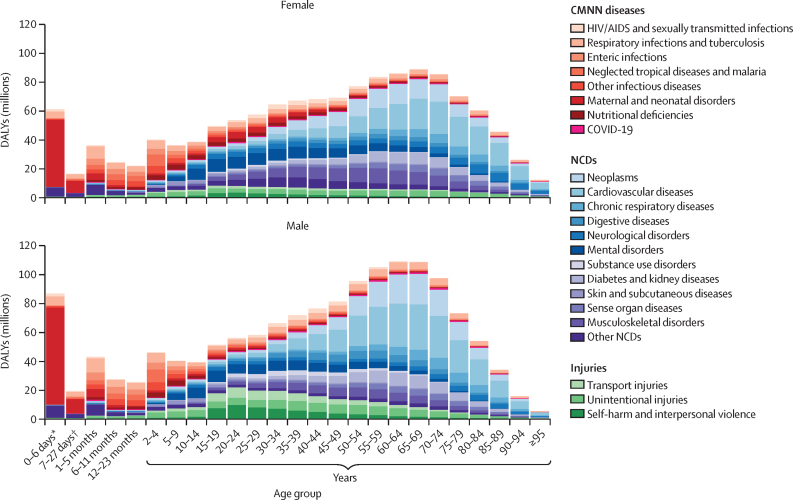
The distribution of global DALYs across age and sex for GBD level 2 causes in 2023 CMNN=communicable, maternal, neonatal, and nutritional. DALYs=disability-adjusted life-years. GBD=Global Burden of Diseases, Injuries, and Risk Factors Study. NCDs=non-communicable diseases. *Early neonatal. †Late neonatal.

In 2023, males accounted for 1·47 billion (95% UI 1·37–1·59) global all-cause DALYs and females for 1·33 billion (1·20–1·49; appendix 3 table S3; see figure 2 for age-cause-specific DALYs, by sex). For both sexes, ischaemic heart disease was the leading level 3 cause of DALY burden in 2023 (74·8 million [64·2–84·8] DALYs in females and 118 million [106–130] in males), followed by neonatal disorders (71·7 million [65·7–78·9] in females and 98·3 million [89·4–107] in males) and stroke (70·8 million [61·8–83·4] in females and 85·7 million [75·8–97·8] in males; appendix 3 table S3). By age group, CMNN diseases were leading causes of burden in children younger than 5 years, with maternal and neonatal disorders the greatest cause of DALYs in the neonatal phase (age <28 days), accounting for 72·2% (68·4–75·3) of 184 million (178–190) total DALYs in this age group in 2023 (figure 2; see the GBD 2023 Results Tool for total DALYs by age group). NCDs increasingly contributed to disease burden with ageing, accounting for 45·0% (40·8–49·8) of 152 million (130–180) total DALYs in individuals aged 5–14 years, 61·6% (58·6–64·1) of 882 million (778–1006) total DALYs for those aged 15–49 years, and 85·5% (84·6–86·3) of 1150 million (1060–1260) total DALYs for those aged 55 years and older. The burden of injuries was higher in males aged 10–54 years, accounting for 24·7% (22·4–27·0) of 590 million (529–662) total DALYs in this age group, compared with 11·1% (9·7–12·5) of 540 million (463–631) total DALYs in females of the same age, with the difference between sexes declining gradually for those 55 and older.

**Figure 3:**
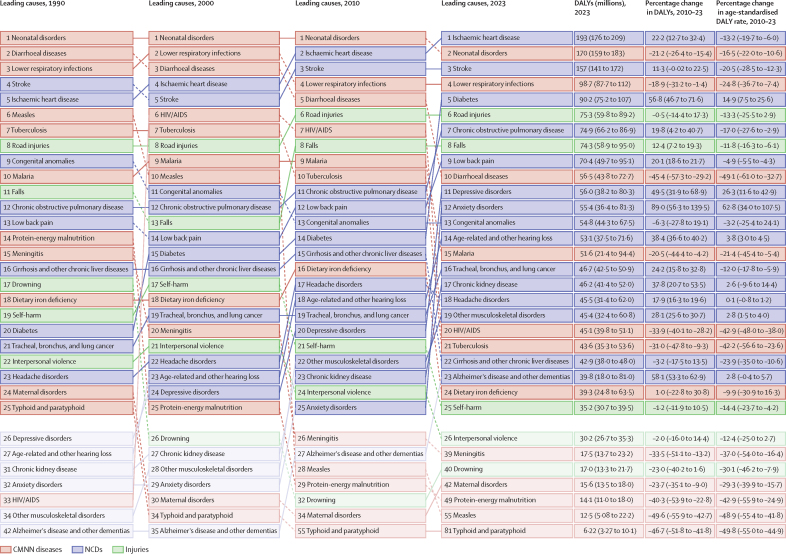
Leading 25 GBD level 3 causes of global DALYs in 1990, 2000, 2010, and 2023, for both sexes combined, and all ages Causes are connected by lines between time periods: solid lines represent an increase or no change in rank, and dashed lines represent a decrease in rank. Faded colours indicate that the cause is not within the top 25 causes of DALYs for that year. Data in parentheses are 95% uncertainty intervals. DALY=disability-adjusted life-year. GBD=Global Burden of Diseases, Injuries, and Risk Factors Study.

In total, 15 NCDs, seven CMNN diseases, and three types of injuries featured within the 25 leading level 3 causes of global DALYs in 2023 (figure 3). In 2010, neonatal disorders, ischaemic heart disease, and stroke were the leading causes of DALYs for all ages and sexes combined. In 2023, the top five causes were ischaemic heart disease (193 million [95% UI 176–209] DALYs), neonatal disorders (170 million [159–183]), stroke (157 million [141–172]), lower respiratory infections (98·7 million [87·7–112]), and diabetes (90·2 million [75·2–107]). Notable health gains among leading CMNN diseases included lower respiratory infections (with a decrease in the age-standardised DALY rate of 24·8% [7·4–36·7] between 2010 and 2023) and diarrhoeal diseases (decrease of 49·1% [32·7–61·0]). Rates also declined for HIV/AIDS (by 42·9% [38·0–48·0]), tuberculosis (42·2% [23·6–56·6]), and malaria (21·4% [5·4–45·4]). Another notable health gain among CMNN diseases was observed for neonatal disorders, which decreased in age-standardised DALY rate by 16·5% (10·6–22·0) and dropped from first ranking in 1990, 2000, and 2010 to second ranking globally in 2023.

Among the leading level 3 causes of DALYs in 2023, the largest health declines between 2010 and 2023—ie, increases in age-standardised DALY rates—were observed for NCD causes, including anxiety disorders with an increase of 62·8% (95% UI 34·0–107·5), depressive disorders (26·3% [11·6–42·9]), and diabetes (14·9% [7·5–25·6]; figure 3). Alzheimer's disease and other dementias also moved into the top 25 causes of DALYs for the first time (non-significant increase in age-standardised rate of 2·8% [–0·4 to 5·7]). With respect to level 3 categories of injury, age-standardised DALY rates did not show a statistical change for road injuries (non-significant decrease of 13·3% (–25·5 to 2·9), but declined for falls by 11·8% (6·1 to 16·3) and self-harm by 14·4% (4·2 to 23·7). DALY counts and age-standardised DALY rates by cause for 2010 and 2023 are presented in appendix 3 (tables S3, S4).

### Decomposition of DALYs into YLDs and YLLs

Global all-cause DALYs in 2023 were composed of 990 million (95% UI 756–1280) YLDs (equivalent to 35·4% [29·5–41·5] of 2·80 billion total DALYs; appendix 3 tables S5, S6) and 1·81 billion (1·78–1·84) YLLs (equivalent to 64·6% [59·5–69·5] of total DALYs; appendix 3 tables S7, S8). Global YLDs exhibited a non-significant increase from 786 million (597–1000) in 2010, and YLLs decreased significantly from 1·85 billion (1·84–1·86) in 2010. All-cause, age-standardised YLD rates remained statistically stable between 2010 and 2023, with a non-significant increase of 2·3% (–0·2 to 5·4; appendix 3 table S11). A total of 21 NCDs, three CMNN diseases, and one injury featured within the 25 leading level 3 causes of YLDs globally in 2023 (table 1). Low back pain, depressive disorders, and anxiety disorders were the top three causes of YLDs. Global YLLs decreased by 2·4% (0·9–3·9) between 2010 and 2023, and age-standardised YLL rates decreased considerably by 18·7% (17·5–19·9; appendix 3 table S12). Age-standardised YLD and YLL rates and the number of YLDs and YLLs by cause and sex for 2010, 2020, and 2023 are in appendix 3 (tables S5–S8).

**Table 1:** Top 25 leading level 3 causes of global YLDs in 2023 across all ages, for both sexes combined, YLD counts, age-standardised rates, and percentage change between 2010 and 2023

	**Percentage of all-cause YLDs**		**YLDs**			**Age-standardised rate of YLDs**		
	2010	2023	Counts (millions), 2010	Counts (millions), 2023	Percentage change, 2010–23	Per 100 000, 2010	Per 100 000, 2023	Percentage change, 2010–23
Low back pain	7·5% (6·4 to 8·7)	7·1% (6·1 to 8·3)	58·6 (41·4 to 78·9)	70·4 (49·7 to 95·1)	20·1% (18·6 to 21·7)	847·5 (600·4 to 1143·3)	805·9 (569·1 to 1084·1)	−4·9% (−5·5 to −4·3)
Depressive disorders	4·8% (3·8 to 6·1)	5·7% (4·5 to 7·4)	37·5 (25·7 to 51·0)	56·0 (38·2 to 80·3)	49·5% (31·9 to 68·9)	527·6 (361·4 to 717·2)	666·4 (454·6 to 952·5)	26·3% (11·6 to 42·9)
Anxiety disorders	3·7% (2·8 to 4·9)	5·6% (4·1 to 7·8)	29·3 (19·9 to 41·5)	55·4 (36·4 to 81·3)	89·0% (56·3 to 139·5)	415·6 (284·8 to 587·6)	676·6 (440·9 to 989·3)	62·8% (34·0 to 107·5)
Falls	6·2% (5·3 to 7·1)	5·4% (4·6 to 6·1)	48·6 (35·2 to 65·6)	53·1 (38·4 to 72·2)	9·4% (7·2 to 11·5)	708·2 (514·3 to 955·8)	606·7 (438·0 to 822·5)	−14·3% (−15·8 to −12·8)
Age-related and otherhearing loss	4·9% (4·1 to 5·8)	5·4% (4·5 to 6·4)	38·4 (27·0 to 51·5)	53·1 (37·5 to 71·6)	38·4% (36·6 to 40·2)	577·4 (409·7 to 775·1)	599·1 (424·5 to 804·9)	3·8% (3·0 to 4·5)
Headache disorders	4·9% (3·8 to 6·0)	4·6% (3·6 to 5·6)	38·6 (26·4 to 52·6)	45·5 (31·4 to 62·0)	17·9% (16·3 to 19·6)	541·3 (370·8 to 736·2)	542·0 (373·4 to 739·2)	0·1% (−0·8 to 1·2)
Diabetes	3·6% (3·2 to 4·1)	4·5% (3·9 to 5·0)	28·5 (20·0 to 38·1)	44·2 (31·0 to 59·7)	55·0% (52·6 to 58·0)	422·5 (296·5 to 565·2)	489·3 (343·3 to 658·9)	15·8% (14·0 to 17·6)
Other musculoskeletal disorders	4·3% (3·3 to 5·5)	4·4% (3·4 to 5·6)	33·6 (23·4 to 45·5)	43·2 (29·9 to 58·3)	28·4% (25·8 to 30·8)	479·1 (332·5 to 647·9)	494·1 (342·4 to 668·7)	3·1% (2·3 to 4·0)
Dietary iron deficiency	4·9% (3·6 to 6·4)	3·9% (2·8 to 5·3)	38·9 (25·0 to 59·0)	39·3 (24·8 to 63·5)	1·0% (−22·8 to 30·8)	565·2 (361·8 to 856·5)	509·4 (321·3 to 818·3)	−9·9% (−30·9 to 16·3)
Gynaecological diseases	2·9% (2·4 to 3·4)	2·9% (2·4 to 3·4)	22·8 (15·4 to 31·9)	28·6 (19·5 to 40·0)	25·6% (22·1 to 29·6)	315·6 (214·2 to 441·6)	341·4 (231·3 to 477·6)	8·2% (6·1 to 10·6)
Oral disorders	2·4% (1·6 to 3·2)	2·4% (1·7 to 3·3)	18·6 (11·2 to 27·4)	23·9 (14·5 to 35·1)	28·8% (24·4 to 33·0)	273·7 (166·0 to 401·4)	272·9 (164·4 to 402·4)	−0·3% (−3·3 to 2·8)
Blindness and vision loss	2·3% (2·0 to 2·8)	2·4% (1·9 to 2·9)	18·4 (12·9 to 26·0)	23·6 (16·3 to 33·5)	28·0% (25·5 to 30·6)	284·5 (198·8 to 399·5)	261·1 (180·3 to 368·9)	−8·2% (−10·4 to −6·1)
Neonatal disorders	2·4% (2·0 to 2·8)	2·4% (2·0 to 2·8)	18·5 (13·8 to 24·1)	23·6 (18·0 to 29·8)	27·8% (17·4 to 37·0)	264·8 (197·8 to 345·4)	304·5 (232·2 to 384·6)	15·0% (5·7 to 23·3)
Osteoarthritis	2·0% (1·2 to 3·8)	2·2% (1·3 to 4·3)	15·7 (7·47 to 33·7)	22·4 (10·7 to 48·2)	42·7% (41·0 to 44·4)	238·9 (114·1 to 513·1)	243·0 (115·8 to 523·5)	1·7% (0·7 to 2·8)
Neck pain	2·1% (1·6 to 2·7)	2·1% (1·6 to 2·6)	16·7 (11·1 to 24·5)	20·7 (13·8 to 30·1)	24·0% (20·9 to 27·3)	237·9 (158·5 to 345·3)	238·3 (158·6 to 346·3)	0·2% (−0·4 to 0·7)
Schizophrenia	1·8% (1·3 to 2·4)	1·7% (1·3 to 2·3)	14·0 (10·3 to 17·7)	17·0 (12·4 to 21·4)	21·1% (19·2 to 22·9)	196·2 (143·7 to 248·3)	197·4 (144·6 to 249·9)	0·6% (−0·1 to 1·3)
Stroke	1·6% (1·3 to 1·9)	1·7% (1·4 to 2·0)	12·6 (9·01 to 16·1)	16·7 (12·0 to 21·2)	31·9% (29·7 to 34·1)	192·8 (137·7 to 245·9)	186·0 (133·8 to 236·4)	−3·6% (−4·9 to −2·3)
Dermatitis	1·7% (1·1 to 2·5)	1·5% (1·0 to 2·2)	13·5 (7·88 to 21·8)	15·2 (8·9 to 24·2)	12·5% (11·0 to 14·4)	198·3 (115·7 to 321·5)	196·3 (114·7 to 316·7)	−1·0% (−1·7 to −0·5)
Chronic obstructivepulmonary disease	1·5% (1·2 to 1·9)	1·5% (1·2 to 1·9)	11·6 (9·61 to 13·3)	15·0 (12·6 to 17·6)	30·0% (26·1 to 33·5)	178·3 (148·7 to 206·1)	166·3 (139·1 to 194·8)	−6·7% (−9·4 to −3·7)
Asthma	1·5% (1·1 to 1·9)	1·4% (1·1 to 1·8)	11·5 (7·26 to 16·2)	14·1 (8·80 to 20·3)	22·6% (18·2 to 26·6)	167·3 (105·4 to 235·3)	173·7 (107·8 to 249·5)	3·8% (0·5 to 7·2)
COVID-19	NA	1·3% (0·6 to 2·5)	NA	12·5 (5·33 to 25·7)	NA	NA	152·0 (64·5 to 313·6)	NA
Alzheimer's disease andother dementias	1·0% (0·7 to 1·2)	1·2% (0·9 to 1·6)	7·69 (5·33 to 9·85)	12·2 (8·52 to 15·6)	58·3% (55·9 to 61·7)	131·6 (91·4 to 168·7)	137·4 (96·3 to 176·7)	4·4% (2·9 to 6·6)
Alcohol use disorders	1·3% (1·0 to 1·6)	1·2% (0·9 to 1·4)	10·3 (7·23 to 14·5)	11·6 (8·08 to 16·5)	12·6% (9·6 to 15·3)	143·4 (101·1 to 202·0)	136·4 (95·2 to 194·5)	−4·9% (−6·7 to −3·1)
Chronic kidney disease	1·0% (0·8 to 1·2)	1·0% (0·9 to 1·3)	7·48 (5·55 to 9·77)	10·3 (7·53 to 13·3)	37·0% (31·4 to 43·3)	114·9 (85·0 to 149·8)	114·5 (84·4 to 148·5)	−0·4% (−4·9 to 3·9)
Ischaemic heart disease	1·0% (0·9 to 1·2)	1·0% (0·9 to 1·1)	8·06 (5·82 to 10·8)	10·0 (7·26 to 13·5)	24·1% (20·8 to 27·4)	123·3 (89·3 to 165·2)	110·2 (80·1 to 147·9)	−10·6% (−12·6 to −9·0)

Data in parentheses are 95% uncertainty intervals. Causes are listed from first to last rank with respect to 2023 YLD counts and percentage of 2023 all-cause YLDs. Count data are presented to three significant figures, and rates are presented to one decimal place. GBD=Global Burden of Diseases, Injuries, and Risk Factors Study. NA=not applicable. YLDs=years lived with disability.

### Trends in DALYs by SDI, location, age, and sex

**Figure 4:**
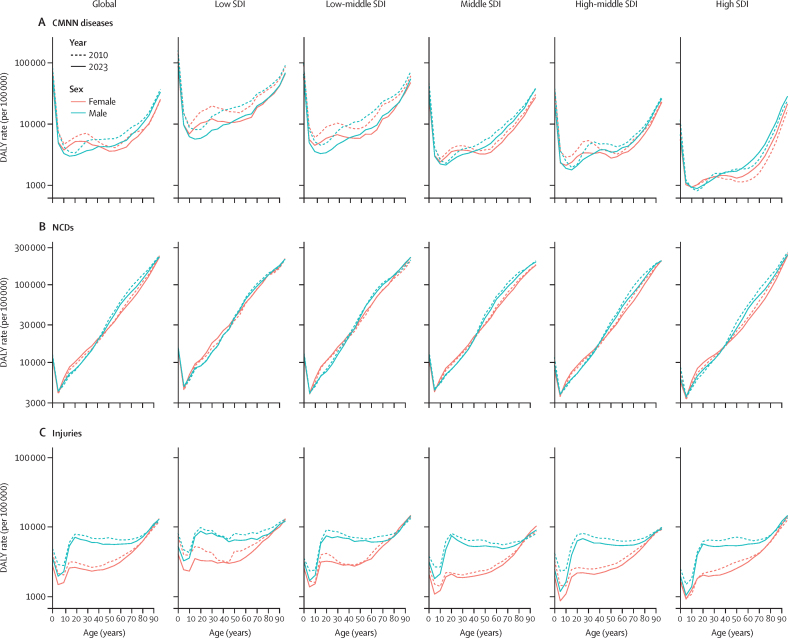
Age-specific DALY rates for CMNN diseases (A), NCDs (B), and injuries (C), by age, sex, year, and SDI quintile The y axis shows DALYs per 100 000 person-years on a logarithmic scale. CMNN=communicable, maternal, neonatal, and nutritional. DALY=disability-adjusted life-year. GBD=Global Burden of Diseases, Injuries, and Risk Factors Study. NCDs=non-communicable diseases. SDI=Socio-demographic Index.

Trends in DALYs at the global level were informed by complex patterns of cause-specific burden across location, age, and sex. Age-standardised DALY rates for NCDs in males in 2023 ranged from 19 519·6 (95% UI 17 667·9–21 818·2) per 100 000 in the high SDI quintile to 25 205·2 (23 054·5–27 396·0) per 100 000 in the low SDI quintile. In females, age-standardised DALY rates for NCDs ranged from 17 017·8 (14 711·8–20 005·1) per 100 000 in the high SDI quintile to 25 574·4 (22 701·9–28 560·9) per 100 000 in the low SDI quintile (GBD 2023 Results Tool and GBD Compare). Across all SDI quintiles, age-specific DALY rates from NCDs decreased with increasing age from 0–6 days to 5–9 years and then increased gradually with age (figure 4).

Age-standardised DALY rates for CMNN diseases in males in 2023 ranged from 2257·1 (95% UI 2033·9–2553·5) per 100 000 in the high SDI quintile to 19 212·4 (18 052·2–20 819·7) per 100 000 in the low SDI quintile. In females, they ranged from 1909·6 (1655·9–2271·7) per 100 000 in the high SDI quintile to 18 824·3 (17 538·6–20 419·6) per 100 000 in the low SDI quintile (GBD 2023 Results Tool and GBD Compare). Age-standardised DALY rates for CMNN diseases decreased between 2010 and 2023 in all SDI quintiles, ranging from a decrease of 34·9% (31·6–38·0) in the low SDI quintile to a decrease of 13·0% (7·2–17·8) in the high SDI quintile. Age-specific DALY rates for CMNN diseases were higher in females than in males in age groups younger than 45 years, and markedly higher among males than females in age groups 45 years and older (figure 4).

Age-standardised DALY rates for injuries in males in 2023 ranged from 4271·2 (95% UI 3792·2–4896·0) per 100 000 in the high SDI quintile to 6401·8 (5445·7–7262·9) per 100 000 in the low SDI quintile. In females, age-standardised DALY rates ranged from 2042·6 (1759·3–2331·2) per 100 000 in the middle SDI quintile to 3381·1 (2789·3–3977·4) per 100 000 in the low SDI quintile (GBD 2023 Results Tool and GBD Compare). Age-standardised DALY rates for injuries decreased between 2010 and 2023 in all SDI quintiles, ranging from a decrease of 13·5% (10·2–16·1) in the high SDI quintile to a decrease of 20·2% (15·7–23·6) in the high-middle SDI quintile. Across all SDI quintiles, DALY rates for injuries emerged 0–6 days after birth, declined between the age groups of 7–27 days after birth and 5–9 years, and increased with age thereafter. For age 15 years and older, DALY rates for injuries in males remained relatively stable with increasing age, except after about the age of 70 years, when it increased; for females, rates increased steadily with age after 40 years (figure 4).

**Figure 5:**
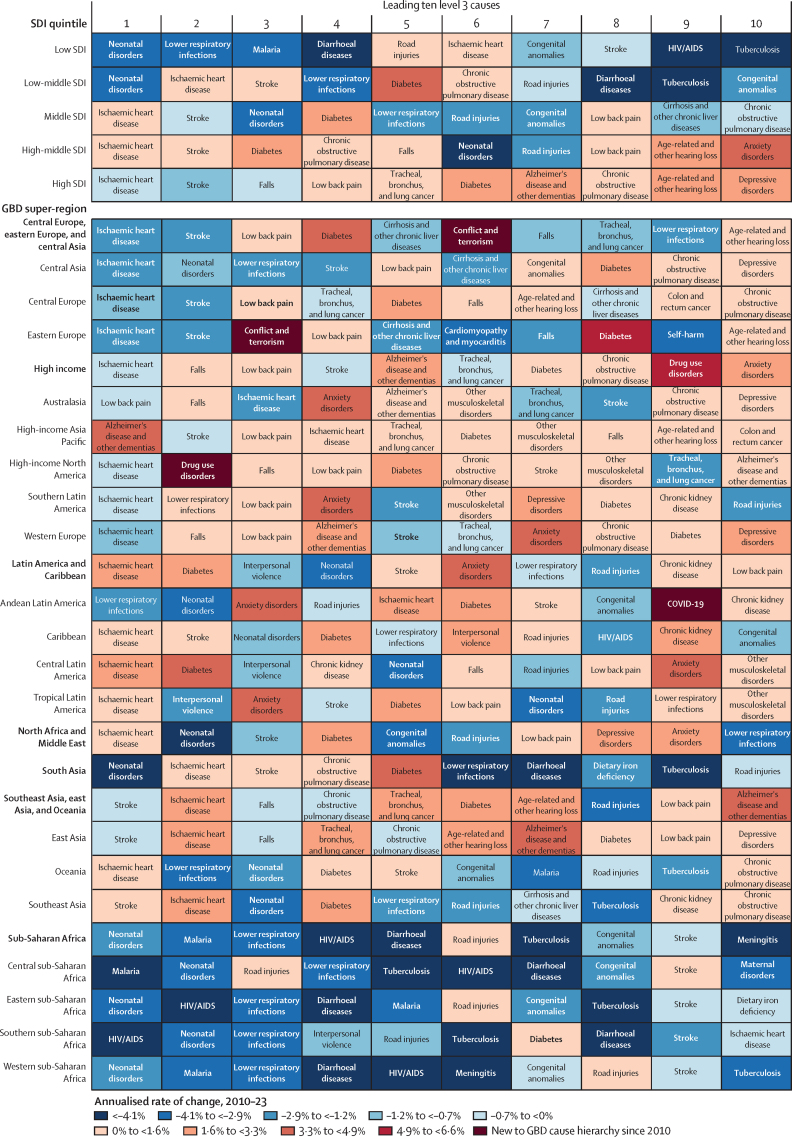
Leading ten GBD level 3 causes of 2023 DALYs by SDI quintile, GBD region and super-region, and annualised rate of change between 2010 and 2023 Level 3 causes are ranked by 2023 DALY counts from left (first) to right (tenth) for each GBD region and SDI quintile, with GBD super-regions in bold. DALY=disability-adjusted life-year. GBD=Global Burden of Diseases, Injuries, and Risk Factors Study. SDI=Socio-demographic Index.

Drivers of changes in DALYs by location are further illustrated in figure 5, which shows the ten leading level 3 causes of DALYs in 2023 and their annualised rate of change (ARC) between 2010 and 2023 by region, super-region, and SDI quintile. In the low SDI quintile, six of the ten leading level 3 causes of DALYs were CMNN diseases, led by neonatal disorders (92·6 million [95% UI 85·7–100] DALYs), lower respiratory infections (48·1 million [40·2–58·0]), and malaria (41·9 million [17·5–77·3]; GBD 2023 Results Tool). As SDI increased, more NCDs emerged in the top ten leading causes of DALYs (figure 5). In the high SDI quintile, nine of the ten leading level 3 causes of DALYs were NCDs, with the top three causes in total DALYs being ischaemic heart disease (71·0 million [65·7–74·8] DALYs), stroke (49·7 million [45·2–53·2]), and falls (33·1 million [25·6–43·2]). The largest changes in age-standardised DALY rates for ischaemic heart disease between 2010 and 2023 ranged from a non-significant increase of 7·7% (–13·7 to 30·3) in low SDI locations to a decrease of 25·5% (22·9 to 28·1) in high SDI locations (GBD 2023 Results Tool).

The leading level 3 causes of age-standardised DALY rates by location in 2023 are shown in appendix 3 (figure S1). Ischaemic heart disease was the leading cause of age-standardised DALY rates in 73 (35·8%) of 204 countries and territories. COVID-19 was not a leading cause of global DALYs in 2023. In sub-Saharan Africa, neonatal disorders, HIV/AIDS, malaria, and lower respiratory infections were the leading level 3 causes of burden in 27 countries in western and eastern sub-Saharan Africa, with HIV/AIDS leading in all continental countries in central sub-Saharan Africa, bar DR Congo and the Central African Republic. Ischaemic heart disease was the leading cause of burden in ten countries and territories in north Africa and the Middle East, five countries and territories in western Europe, and six countries and territories in central Asia. Stroke was the most burdensome cause in most locations in east Asia (appendix 3 figure S1).

### Trends in exposure to risk factors, 2010–23

At level 1 of the risk factor hierarchy (appendix 2 table S15), age-standardised SEVs grew between 2010 and 2023 only for metabolic risks, with an increasing mean ARC of 1·1% (95% UI 0·3–2·0; table 2). Conversely, there were small but significant decreases in SEVs for environmental and occupational risks and for behavioural risks, with ARC decreases of 0·8% (0·5–1·1) and 0·4% (0·2–0·7), respectively. Among specific level 2 risk factors, exposure increased significantly between 2010 and 2023 only for high BMI (ARC 1·3% [0·5–2·2]), kidney dysfunction (0·1% [0·1–0·2]), drug use (1·9% [0·5–2·6]), and low physical activity (0·5% [0·2–0·8]). Level 2 risk factors that showed significant declines in SEVs between 2010 and 2023 included unsafe water, sanitation, and handwashing, with a decreasing ARC of 1·4% (0·3–2·6), air pollution (decrease of 1·4% [1·2–1·7]), and tobacco use (decrease of 1·1% [0·5–1·6]). Results at more disaggregated levels of the risk factor hierarchy reveal that SEVs for some components of air pollution decreased, with household air pollution from solid fuels declining at an ARC of 3·2% (2·5–3·7), while exposure to ambient particulate matter and ambient ozone pollution increased, rising by 1·2% (0·9–1·5) and 1·3% (1·1–1·4), respectively. Age-standardised SEVs and percentage change over time by risk factor and sex are provided in appendix 3 (table S15).

**Table 2:** Global age-standardised SEVs in 1990, 2010, and 2023, and annualised rate of change over 1990–2023 and 2010–23, by GBD risk factor

			**SEV, 1990**	**SEV, 2010**	**SEV, 2023**	**Annualised rate of change, 1990–2023**	**Annualised rate of change, 2010–23**
All risk factors			24·5 (21·7 to 26·0)	24·0 (21·4 to 25·5)	23·6 (20·7 to 25·0)	−0·1% (−0·2 to 0·0)	−0·1% (−0·3 to 0·1)
Environmental or occupational risks			45·6 (40·2 to 49·1)	41·8 (36·0 to 46·3)	37·7 (32·1 to 42·3)	−0·6% (−0·8 to −0·4)	−0·8% (−1·1 to −0·5)
	Unsafe water, sanitation, and handwashing		44·4 (29·0 to 53·1)	33·7 (22·2 to 41·1)	28·1 (18·9 to 34·9)	−1·4% (−2·0 to −0·8)	−1·4% (−2·6 to −0·3)
		Unsafe water source	44·7 (33·1 to 56·6)	38·7 (25·3 to 52·2)	36·5 (22·4 to 51·3)	−0·6% (−1·4 to 0·1)	−0·5% (−1·7 to 0·7)
		Unsafe sanitation	56·9 (52·5 to 60·9)	40·7 (37·4 to 43·8)	30·8 (26·8 to 35·4)	−1·9% (−2·3 to −1·4)	−2·1% (−3·1 to −1·2)
		No access to handwashing facility	32·6 (15·4 to 43·8)	25·7 (11·6 to 35·0)	21·4 (10·0 to 29·2)	−1·3% (−2·4 to 0·0)	−1·4% (−3·7 to 0·7)
	Air pollution		51·5 (44·4 to 58·5)	45·4 (38·4 to 52·7)	37·6 (31·9 to 44·1)	−0·9% (−1·1 to −0·8)	−1·4% (−1·7 to −1·2)
		Particulate matter pollution	56·8 (51·2 to 62·5)	49·5 (44·4 to 54·8)	41·7 (37·0 to 47·0)	−0·9% (−1·1 to −0·8)	−1·3% (−1·5 to −1·1)
		Ambient particulate matter pollution	20·0 (16·0 to 25·3)	24·4 (20·1 to 29·2)	28·5 (23·6 to 33·8)	1·1% (0·5 to 1·7)	1·2% (0·9 to 1·5)
		Household air pollution from solid fuels	35·5 (29·7 to 41·9)	25·5 (21·1 to 30·4)	16·9 (13·2 to 21·2)	−2·2% (−2·8 to −1·7)	−3·2% (−3·7 to −2·5)
		Ambient ozone pollution	16·5 (14·3 to 20·1)	20·9 (18·1 to 25·2)	24·6 (21·6 to 28·8)	1·2% (1·1 to 1·3)	1·3% (1·1 to 1·4)
		Ambient nitrogen dioxide pollution	22·3 (0·0 to 50·6)	20·8 (0·0 to 48·3)	14·4 (0·0 to 40·7)	−1·3% (−2·5 to 0·0)	−2·9% (−5·6 to 0·0)
	Non-optimal temperature		28·5 (27·8 to 29·6)	33·9 (32·7 to 34·9)	32·2 (31·0 to 33·2)	0·4% (0·2 to 0·5)	−0·4% (−0·6 to −0·2)
		High temperature	30·9 (28·7 to 32·9)	40·5 (37·1 to 43·1)	40·4 (37·4 to 43·0)	0·8% (0·7 to 0·9)	0·0% (−0·1 to 0·1)
		Low temperature	25·6 (24·5 to 27·3)	26·5 (25·2 to 28·3)	24·2 (22·9 to 26·1)	−0·2% (−0·2 to −0·1)	−0·7% (−0·8 to −0·6)
	Other environmental risks		41·9 (14·3 to 51·2)	44·5 (14·0 to 52·4)	41·1 (12·8 to 48·3)	−0·1% (−0·4 to 0·4)	−0·6% (−1·0 to −0·3)
		Residential radon	25·7 (0·0 to 37·3)	25·2 (0·0 to 36·8)	25·0 (0·0 to 36·8)	−0·1% (−0·2 to 0·1)	0·0% (−0·2 to 0·1)
		Lead exposure	49·2 (8·1 to 60·7)	53·1 (8·4 to 62·1)	48·1 (7·3 to 56·4)	−0·1% (−0·5 to 0·4)	−0·8% (−1·2 to −0·4)
	Occupational risks		3·8 (2·4 to 8·2)	3·9 (2·6 to 8·3)	3·8 (2·5 to 8·3)	0·0% (−0·1 to 0·2)	−0·1% (−0·3 to 0·1)
		Occupational carcinogens	0·9 (0·7 to 1·6)	1·1 (0·8 to 1·8)	1·1 (0·8 to 1·9)	0·6% (0·4 to 0·7)	0·4% (0·2 to 0·5)
		Occupational exposure to asbestos	2·3 (2·2 to 2·4)	2·2 (2·1 to 2·3)	2·0 (1·9 to 2·2)	−0·4% (−0·7 to −0·2)	−0·8% (−1·1 to −0·5)
		Occupational exposure to arsenic	0·4 (0·1 to 0·8)	0·5 (0·1 to 0·9)	0·5 (0·2 to 0·9)	0·3% (0·1 to 0·8)	0·1% (−0·1 to 0·5)
		Occupational exposure to benzene	0·7 (0·3 to 1·7)	0·9 (0·4 to 2·0)	1·0 (0·5 to 2·1)	0·8% (0·6 to 1·1)	0·7% (0·5 to 0·9)
		Occupational exposure to beryllium	0·1 (0·1 to 0·1)	0·1 (0·1 to 0·1)	0·1 (0·1 to 0·1)	0·5% (0·4 to 0·5)	0·3% (0·2 to 0·4)
		Occupational exposure to cadmium	0·2 (0·2 to 0·2)	0·2 (0·2 to 0·2)	0·2 (0·2 to 0·2)	0·7% (0·6 to 0·8)	0·4% (0·2 to 0·6)
		Occupational exposure to chromium	0·4 (0·4 to 0·4)	0·5 (0·4 to 0·5)	0·5 (0·5 to 0·5)	1·0% (0·9 to 1·0)	0·6% (0·4 to 0·9)
		Occupational exposure to diesel engine exhaust	1·7 (1·7 to 1·7)	2·3 (2·2 to 2·3)	2·6 (2·6 to 2·7)	1·3% (1·2 to 1·3)	1·1% (0·9 to 1·2)
		Occupational exposure to formaldehyde	0·8 (0·7 to 0·8)	0·9 (0·9 to 1·0)	1·0 (0·9 to 1·0)	0·7% (0·6 to 0·8)	0·4% (0·1 to 0·6)
		Occupational exposure to nickel	0·4 (0·1 to 1·3)	0·5 (0·1 to 1·3)	0·5 (0·1 to 1·3)	0·2% (0·0 to 0·7)	0·1% (−0·2 to 0·5)
		Occupational exposure to polycyclic aromatic hydrocarbons	0·7 (0·7 to 0·7)	0·9 (0·9 to 1·0)	1·0 (1·0 to 1·0)	1·0% (0·9 to 1·0)	0·7% (0·4 to 0·9)
		Occupational exposure to silica	4·1 (1·7 to 9·8)	4·4 (2·1 to 10·0)	4·6 (2·2 to 10·3)	0·3% (0·1 to 0·7)	0·3% (0·1 to 0·5)
		Occupational exposure to sulphuric acid	1·0 (0·6 to 2·0)	1·0 (0·7 to 2·0)	1·0 (0·7 to 2·0)	0·2% (0·0 to 0·4)	0·0% (−0·2 to 0·2)
		Occupational exposure to trichloroethylene	0·2 (0·2 to 0·2)	0·3 (0·3 to 0·3)	0·3 (0·3 to 0·3)	1·0% (0·9 to 1·1)	0·7% (0·5 to 0·9)
		Occupational asthmagens	17·9 (15·4 to 20·9)	18·1 (15·6 to 21·1)	17·7 (15·5 to 20·6)	0·0% (−0·2 to 0·1)	−0·2% (−0·5 to 0·1)
		Occupational particulate matter, gases, and fumes	12·3 (0·0 to 73·5)	12·3 (0·0 to 72·0)	11·6 (0·0 to 69·0)	−0·2% (−0·3 to 0·0)	−0·4% (−0·6 to 0·0)
		Occupational noise	10·6 (10·2 to 11·2)	10·9 (10·5 to 11·4)	10·7 (10·3 to 11·2)	0·0% (0·0 to 0·0)	−0·2% (−0·2 to −0·1)
		Occupational injuries	NA	NA	NA	NA	NA
		Occupational ergonomic factors	40·2 (0·0 to 79·6)	39·8 (0·0 to 81·9)	38·2 (0·0 to 82·1)	−0·2% (−0·3 to 0·1)	−0·3% (−0·6 to 0·0)
Behavioural risks			20·2 (16·7 to 22·4)	18·9 (15·9 to 20·9)	17·8 (14·6 to 19·8)	−0·4% (−0·6 to −0·2)	−0·4% (−0·7 to −0·2)
	Child and maternal malnutrition		8·8 (4·5 to 14·7)	8·6 (4·5 to 14·3)	8·4 (4·2 to 13·8)	−0·1% (−0·4 to 0·2)	−0·2% (−0·6 to 0·2)
		Suboptimal breastfeeding	38·7 (35·3 to 42·9)	33·9 (30·8 to 37·8)	32·3 (29·3 to 35·7)	−0·6% (−0·6 to −0·5)	−0·4% (−0·5 to −0·2)
		Non-exclusive breastfeeding	43·7 (30·9 to 58·5)	38·8 (27·6 to 52·4)	35·7 (26·0 to 47·5)	−0·6% (−0·7 to −0·5)	−0·6% (−0·8 to −0·4)
		Discontinued breastfeeding	41·8 (40·5 to 43·1)	36·0 (35·1 to 37·0)	34·9 (33·7 to 36·1)	−0·5% (−0·6 to −0·5)	−0·2% (−0·4 to −0·1)
	Child growth failure		15·9 (10·1 to 23·0)	12·4 (7·9 to 19·0)	9·0 (5·2 to 14·9)	−1·7% (−2·6 to −1·3)	−2·5% (−4·1 to −1·6)
		Child underweight	21·6 (17·4 to 25·3)	17·4 (14·1 to 20·7)	13·6 (10·8 to 16·6)	−1·4% (−1·7 to −1·3)	−1·9% (−2·4 to −1·6)
		Child wasting	8·3 (5·6 to 10·5)	8·1 (5·5 to 10·1)	7·1 (4·8 to 9·2)	−0·5% (−0·9 to −0·1)	−1·0% (−2·0 to −0·3)
		Child stunting	26·7 (23·7 to 28·9)	21·3 (19·1 to 23·2)	16·6 (14·7 to 18·6)	−1·4% (−1·7 to −1·2)	−1·9% (−2·6 to −1·3)
	Low birthweight and short gestation		21·6 (19·0 to 24·4)	22·8 (20·1 to 25·8)	22·8 (20·2 to 25·8)	0·2% (0·1 to 0·2)	0·0% (−0·1 to 0·1)
		Short gestation	31·2 (27·2 to 35·2)	32·1 (28·2 to 36·6)	31·8 (27·9 to 36·2)	0·1% (0·0 to 0·1)	−0·1% (−0·2 to 0·0)
		Low birthweight	17·6 (16·0 to 19·2)	18·5 (16·9 to 20·3)	18·5 (16·9 to 20·2)	0·2% (0·1 to 0·2)	0·0% (−0·1 to 0·1)
	Iron deficiency		8·5 (5·9 to 11·5)	7·9 (6·0 to 10·2)	7·3 (5·6 to 9·5)	−0·5% (−1·4 to 0·6)	−0·6% (−1·9 to 0·8)
	Vitamin A deficiency		21·9 (0·0 to 38·6)	15·0 (0·0 to 22·9)	8·5 (0·0 to 13·7)	−2·9% (−3·9 to 0·0)	−4·4% (−5·5 to 0·0)
	Zinc deficiency		26·4 (0·0 to 41·4)	22·3 (0·0 to 35·2)	18·5 (0·0 to 29·9)	−1·1% (−1·7 to 0·0)	−1·4% (−2·5 to 0·0)
	Tobacco use		28·7 (26·8 to 30·8)	24·0 (22·7 to 25·4)	20·9 (19·6 to 22·6)	−1·0% (−1·2 to −0·6)	−1·1% (−1·6 to −0·5)
		Smoking	23·4 (20·8 to 26·1)	19·2 (17·6 to 20·8)	16·2 (14·9 to 17·7)	−1·1% (−1·5 to −0·7)	−1·3% (−2·1 to −0·5)
		Chewing tobacco	4·1 (2·3 to 6·8)	4·4 (3·3 to 5·9)	3·8 (2·5 to 5·8)	−0·3% (−2·1 to 1·7)	−1·1% (−4·1 to 1·9)
		Second-hand smoke	43·6 (40·1 to 47·1)	37·7 (34·8 to 40·7)	33·9 (30·5 to 37·7)	−0·8% (−1·2 to −0·3)	−0·8% (−1·5 to −0·1)
	High alcohol use		6·8 (4·9 to 9·5)	6·3 (4·7 to 8·6)	5·7 (4·3 to 7·8)	−0·5% (−1·0 to −0·1)	−0·8% (−1·3 to −0·2)
	Drug use		0·6 (0·5 to 0·7)	0·6 (0·4 to 0·8)	0·7 (0·4 to 1·2)	0·7% (−0·4 to 1·5)	1·9% (0·5 to 2·6)
	Dietary risks		39·9 (31·2 to 47·2)	38·6 (30·3 to 45·6)	38·4 (28·9 to 45·3)	−0·1% (−0·5 to 0·3)	0·0% (−0·7 to 0·6)
		Diet low in fruits	42·9 (34·2 to 49·5)	40·7 (32·9 to 45·0)	40·5 (33·7 to 46·6)	−0·2% (−0·7 to 0·5)	0·0% (−1·1 to 0·8)
		Diet low in vegetables	28·9 (17·0 to 36·5)	26·4 (16·0 to 31·4)	26·1 (15·2 to 31·9)	−0·3% (−1·1 to 0·4)	−0·1% (−1·5 to 1·2)
		Diet low in legumes	50·9 (0·0 to 67·0)	43·8 (0·0 to 58·7)	42·2 (0·0 to 58·7)	−0·6% (−1·5 to 0·4)	−0·3% (−2·3 to 1·7)
		Diet low in wholegrains	40·3 (31·2 to 50·6)	40·1 (31·9 to 49·9)	40·5 (30·9 to 51·4)	0·0% (−0·9 to 1·0)	0·1% (−1·7 to 1·6)
		Diet low in nuts and seeds	56·7 (47·3 to 67·3)	45·3 (37·6 to 52·1)	42·0 (31·5 to 53·0)	−0·9% (−1·9 to 0·0)	−0·6% (−2·4 to 1·0)
		Diet low in milk	63·1 (58·1 to 72·8)	65·0 (62·0 to 74·3)	65·1 (61·5 to 74·1)	0·1% (−0·2 to 0·3)	0·0% (−0·3 to 0·3)
		Diet high in red meat	26·1 (0·0 to 39·6)	26·3 (0·0 to 38·9)	26·7 (0·0 to 40·4)	0·1% (−0·7 to 1·1)	0·1% (−1·7 to 2·0)
		Diet high in processed meat	13·0 (9·1 to 17·6)	13·0 (10·5 to 15·7)	13·7 (10·8 to 17·1)	0·2% (−0·9 to 1·1)	0·4% (−1·9 to 2·4)
		Diet high in sugar-sweetened beverages	10·2 (4·3 to 18·3)	13·6 (10·3 to 17·7)	16·7 (11·9 to 23·4)	1·5% (−0·4 to 4·3)	1·6% (−1·4 to 5·0)
		Diet low in fibre	30·8 (16·7 to 47·5)	25·1 (14·0 to 41·1)	20·7 (9·3 to 35·2)	−1·2% (−3·7 to 1·1)	−1·5% (−5·3 to 1·7)
		Diet low in calcium	28·1 (24·1 to 38·1)	22·8 (19·4 to 29·3)	20·3 (17·1 to 26·8)	−1·0% (−1·6 to −0·4)	−0·9% (−1·9 to 0·1)
		Diet low in seafood omega-3 fatty acids	44·7 (31·8 to 56·1)	35·0 (21·6 to 46·9)	28·9 (15·4 to 42·9)	−1·3% (−2·9 to 0·1)	−1·5% (−5·2 to 1·1)
		Diet low in omega-6 polyunsaturated fatty acids	69·1 (41·2 to 82·8)	61·4 (35·7 to 75·9)	57·8 (34·3 to 73·4)	−0·5% (−1·0 to −0·1)	−0·5% (−1·5 to 0·4)
		Diet high in trans fatty acids	47·7 (32·6 to 61·1)	41·9 (29·3 to 54·4)	29·5 (19·8 to 40·2)	−1·5% (−2·8 to 0·0)	−2·7% (−5·6 to 0·6)
		Diet high in sodium	41·3 (18·2 to 66·5)	42·0 (19·3 to 69·2)	41·1 (16·6 to 67·2)	0·0% (−1·4 to 1·4)	−0·2% (−2·5 to 1·9)
	Intimate partner violence		18·3 (8·4 to 32·5)	17·6 (9·9 to 23·7)	17·4 (11·1 to 21·6)	−0·2% (−1·5 to 1·5)	−0·1% (−1·8 to 1·8)
	Sexual violence against children and bullying		12·2 (6·6 to 20·4)	12·6 (7·8 to 18·6)	12·2 (7·5 to 18·0)	0·0% (−1·4 to 1·4)	−0·2% (−2·2 to 1·7)
	Sexual violence against children		14·6 (6·9 to 25·0)	14·6 (8·0 to 22·5)	14·7 (8·6 to 22·4)	0·0% (−1·6 to 1·7)	0·0% (−2·2 to 2·4)
	Bullying victimisation		5·5 (2·5 to 10·5)	6·3 (3·0 to 11·9)	5·0 (2·5 to 9·2)	−0·3% (−0·7 to 0·2)	−1·9% (−2·6 to −1·0)
	Unsafe sex		NA	NA	NA	NA	NA
	Low physical activity		16·8 (14·0 to 19·5)	17·3 (14·5 to 20·3)	18·5 (15·6 to 21·5)	0·3% (0·1 to 0·5)	0·5% (0·2 to 0·8)
	Metabolic risks		14·3 (12·5 to 16·9)	17·5 (15·9 to 19·2)	20·3 (18·3 to 22·4)	1·0% (0·6 to 1·6)	1·1% (0·3 to 2·0)
	High fasting plasma glucose		14·4 (9·5 to 22·9)	15·3 (11·8 to 22·3)	17·6 (13·0 to 24·8)	0·6% (−0·9 to 2·2)	1·0% (−1·2 to 3·3)
	High LDL cholesterol		44·6 (35·9 to 54·4)	43·5 (35·8 to 53·2)	44·2 (35·8 to 54·4)	0·0% (−0·8 to 0·7)	0·1% (−1·0 to 1·3)
	High systolic blood pressure		27·3 (15·9 to 42·4)	28·4 (22·3 to 36·7)	30·3 (25·0 to 36·0)	0·3% (−1·0 to 1·7)	0·5% (−1·3 to 2·1)
	High BMI		13·2 (11·1 to 15·6)	17·2 (15·6 to 18·7)	20·4 (18·6 to 22·5)	1·3% (0·8 to 1·9)	1·3% (0·5 to 2·2)
	Low bone mineral density		22·7 (17·3 to 29·3)	21·6 (16·3 to 28·1)	21·1 (16·0 to 27·7)	−0·2% (−0·3 to −0·2)	−0·2% (−0·3 to 0·0)
	Kidney dysfunction		2·9 (1·9 to 4·9)	2·9 (1·9 to 4·8)	2·9 (2·0 to 4·9)	0·0% (−0·1 to 0·1)	0·1% (0·1 to 0·2)

Data in parentheses are 95% uncertainty intervals. NA is given to indicate risk factors for which SEVs are not calculated because a direct PAF approach is used—ie, PAFs are calculated directly from the disease rather than generated with the standard set of analytical processes. GBD=Global Burden of Diseases, Injuries, and Risk Factors Study. NA=not applicable. PAF=population attributable fraction. SEV=summary exposure value.

### Risk-attributable DALYs by age, sex, and location

**Figure 6:**
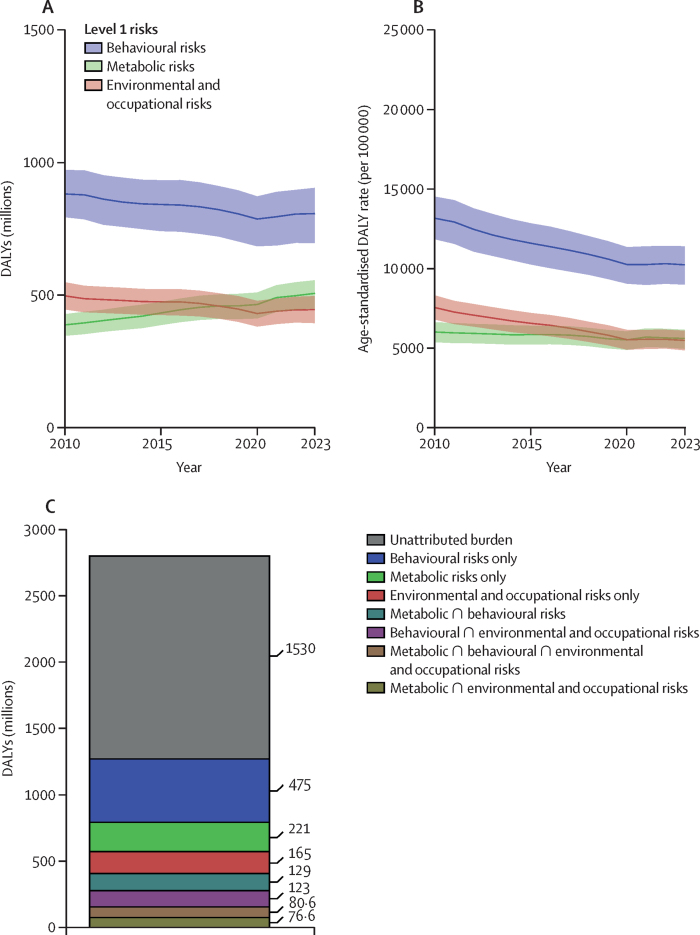
Global DALYs attributable to GBD level 1 risk factors (A) Global DALY counts attributable to level 1 risks, 2010–23. (B) Age-standardised DALY rates attributable to level 1 risks, 2010–23. (C) Global total DALY counts unattributed or attributable to level 1 risk factors, 2023. Mean estimates by level 1 risk factor in panels A and B are represented by coloured lines; the shading indicates 95% uncertainty intervals. For panel C, ∩ refers to a burden that is attributed to two or all three level 1 risk factors (ie, the intersecting set of DALYs that belong to both or all three risk factors). Mean estimates in panels A and B are aggregated to include all DALYs attributable exclusively to the specific level 1 risk factor plus those attributable to the intersection of that risk and one or both of the other level 1 risk factors (ie, for a single year, the DALY counts combined across the three lines sum to more than the total number of attributable DALYs for that year). In GBD 2023, 45·6% of total global DALYs were attributable to risk factors (appendix 3 table S13). DALY=disability-adjusted life-year. GBD=Global Burden of Diseases, Injuries, and Risk Factors Study.

**Figure 7:**
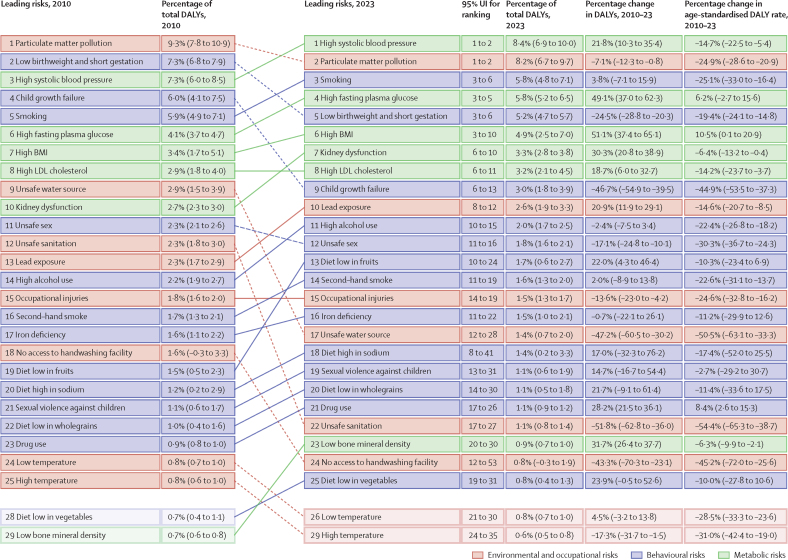
Leading 25 GBD level 3 risk factors by attributable DALYs as a percentage of total DALY counts (2010 and 2023), and percentage change in attributable DALY counts and age-standardised DALY rates from 2010 to 2023 Each column displays the top 25 risks in descending order for the specified year. Risk factors are connected by lines between time periods; solid lines represent an increase or lateral shift in ranking, and dashed lines represent a decrease in rank. Faded colours indicate that the cause is not within the top 25 causes of DALYs for that year. Data in parentheses are 95% UIs. DALY=disability-adjusted life-year. GBD=Global Burden of Diseases, Injuries, and Risk Factors Study. UI=uncertainty interval.

For level 1 risks, attributable global disease burden measured in DALY counts was highest in 2023 for behavioural risk factors (808 million [95% UI 697–905] attributable DALYs or 28·9% [25·1–32·0] of 2·80 billion total DALYs in 2023), followed by metabolic risks (507 million [449–556] or 18·1% [16·6–19·7]) and then environmental and occupational risks (445 million [395–496] or 16·0% [14·1–18·0]; figure 6). In aggregate, 1·27 billion (1·18–1·38) global DALYs (45·5% [43·4–47·8] of 2·80 billion total DALYs in 2023) were attributable to all 88 GBD 2023 risk factors combined (appendix 3 table S13). Further disaggregation of risk-attributable burden estimates showed that when ranked by percentage of total DALYs, high SBP was the leading level 3 risk globally in 2023 (8·4% [6·9–10·0] of total DALYs; figure 7; see appendix 3 table S13 for DALYs by outcome). Particulate matter pollution (encompassing both ambient and household air pollution) was the second leading risk (8·2% [6·7–9·7] of total DALYs), smoking ranked third (5·8% [4·8–7·1]), high FPG ranked fourth 5·8% [5·2–6·5]), and low birthweight and short gestation ranked fifth (5·2% [4·7–5·7]). Of the 25 leading level 3 risk factors in 2023, more than half (13) were behavioural risks.

The contribution of level 3 risk factors to global DALYs in 2023 varied by age (appendix 3 figure S2A–E) and sex (appendix 3 figure S2F–G). Among children younger than 5 years, risks related to child and maternal malnutrition, particulate matter pollution, and unsafe water, sanitation, and handwashing were leading level 3 risk factors, with no metabolic risks among the top ten. For children and adolescents aged 5–14 years, iron deficiency was the leading risk, followed by others related to unsafe water, sanitation, and handwashing, and child and maternal malnutrition. For the age group of 15–49 years, the top two risks were unsafe sex and occupational injuries. Metabolic risk factors gained prominence in this group, with high BMI and high SBP the third-ranked and fourth-ranked risks, followed by high alcohol use. For individuals aged 50–69 years, high SBP and smoking were the top risks, with metabolic risks such as high FPG, high BMI, high LDL cholesterol, and kidney dysfunction also prominent. A similar pattern was seen for individuals aged 70 years and older. The top ten risks for all-age females and males were similar, although smoking was the leading risk for males, but the 12th-ranked risk for females. Additionally, high alcohol use was the ninth-ranked risk factor among males, but for females was not among the top 25 risks. By contrast, unsafe sex and iron deficiency were the ninth-ranked and 11th-ranked risks, respectively, for females, but ranked 19th and 23rd for males.

**Figure 8:**
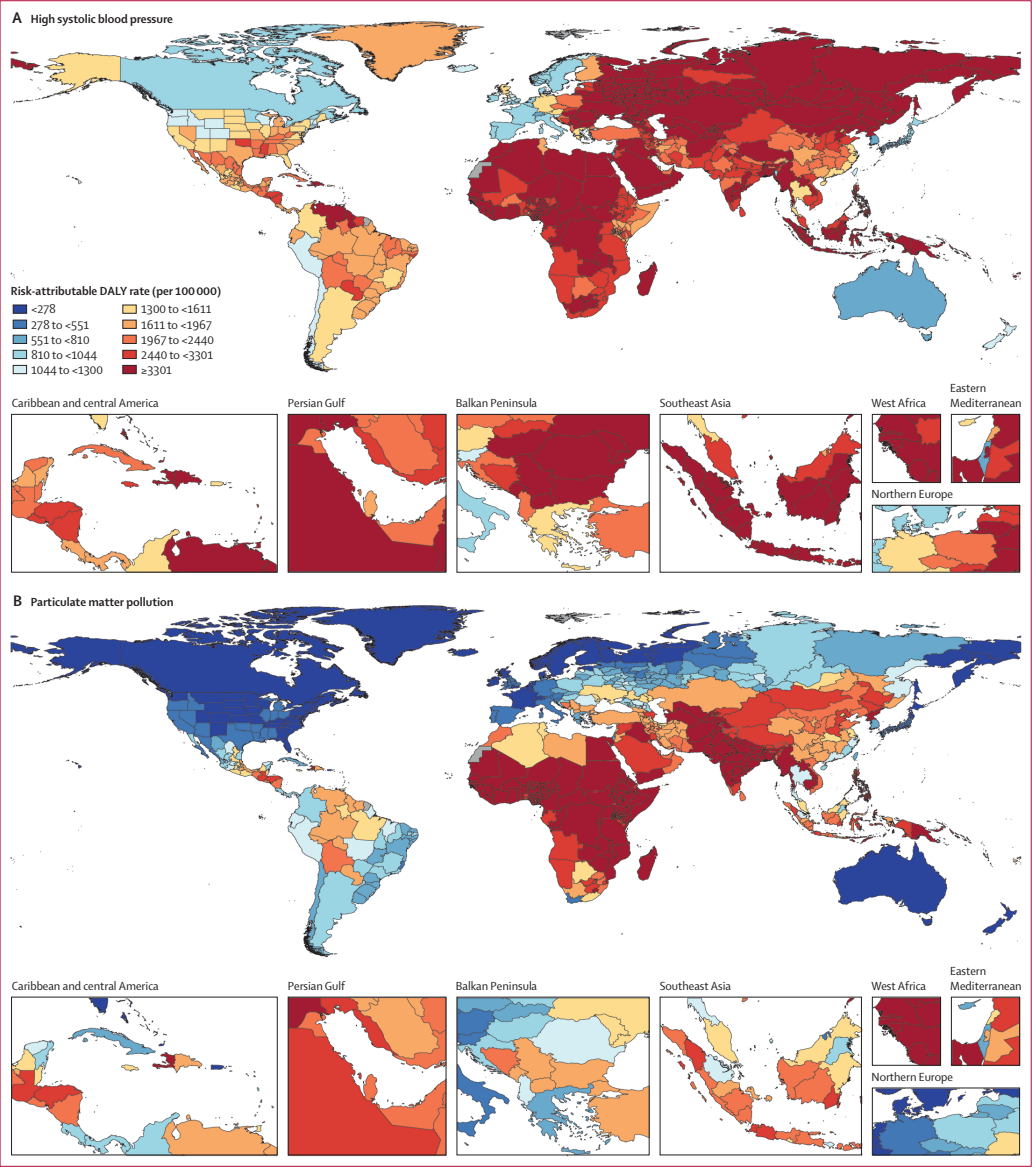

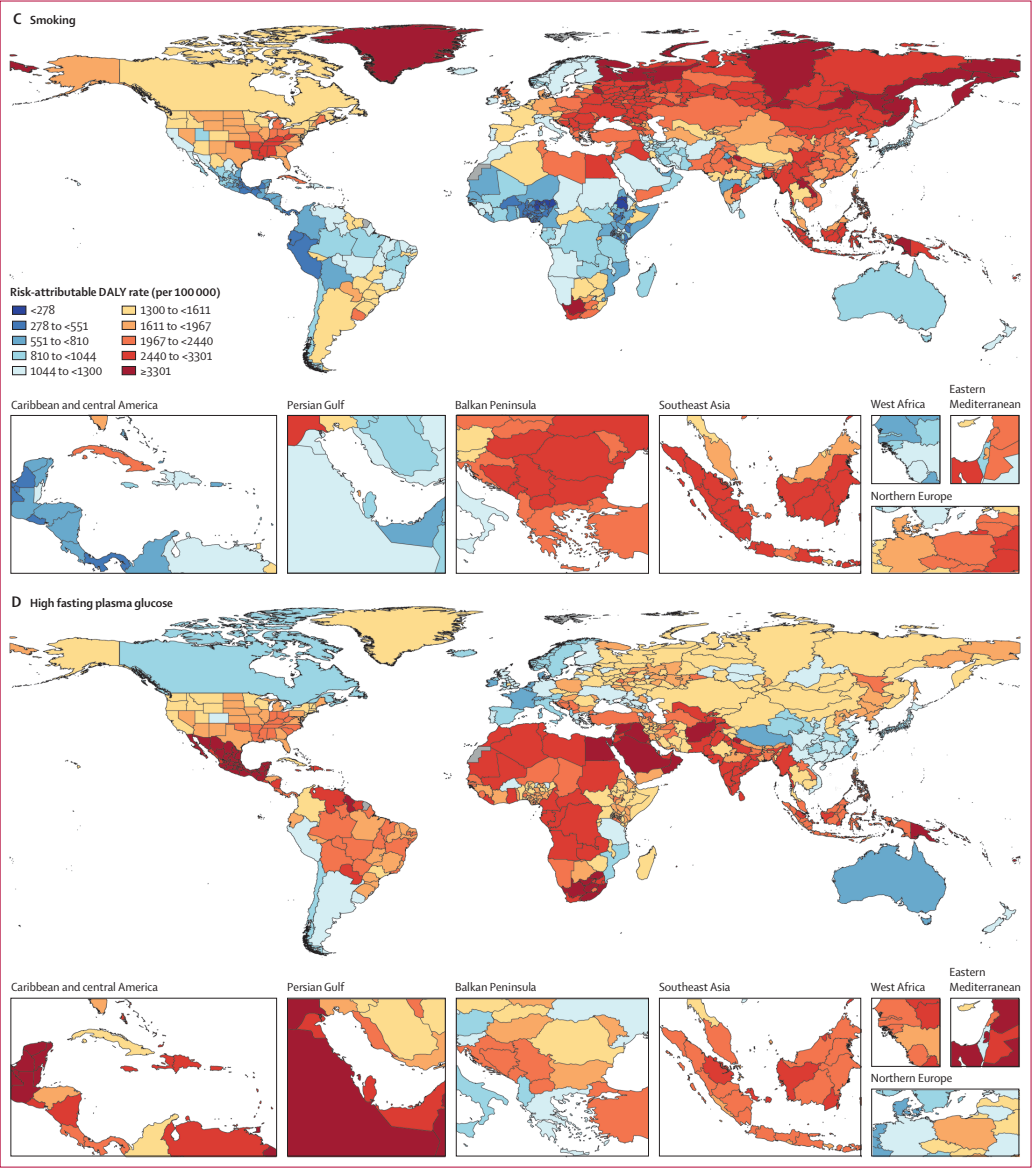

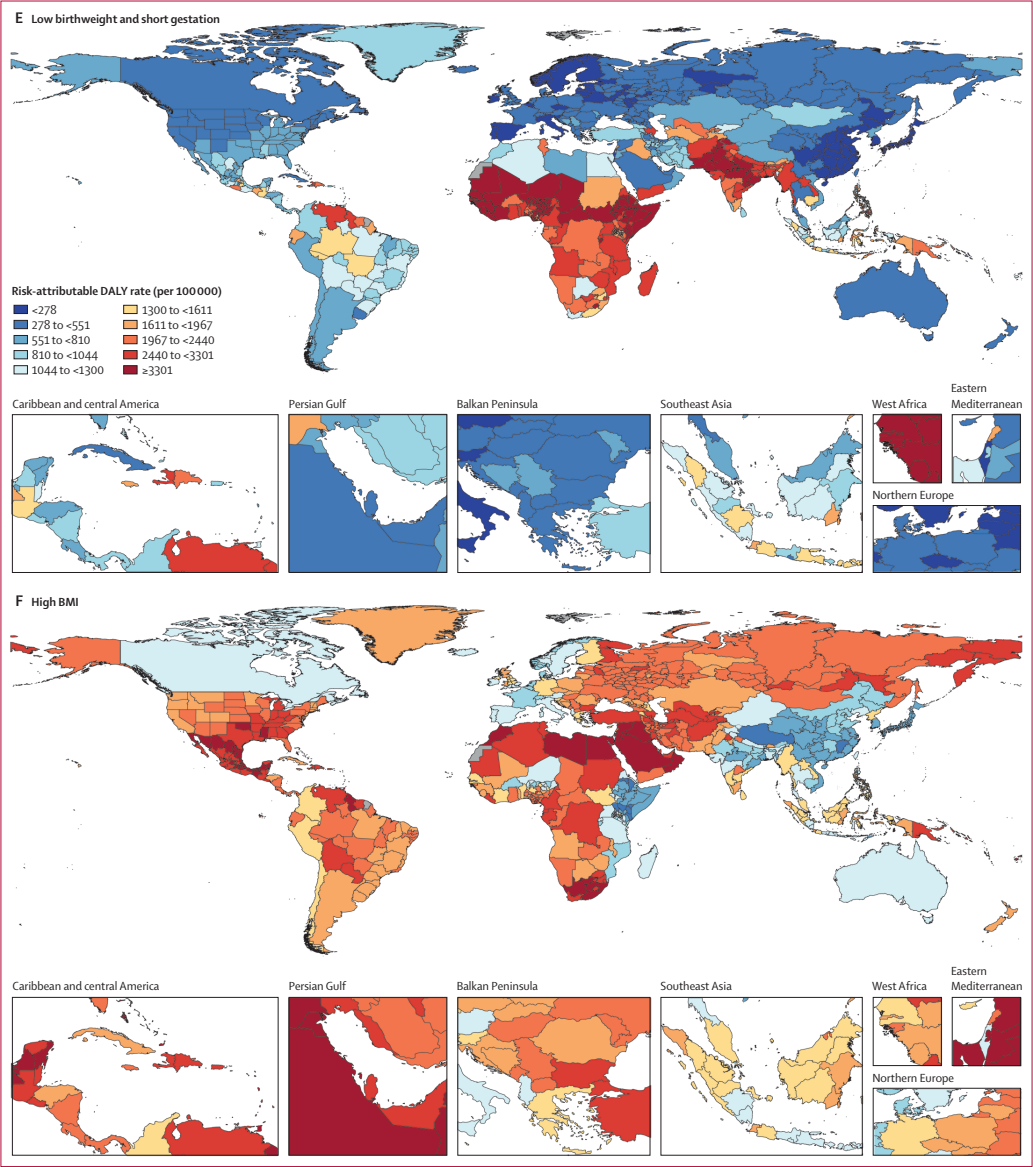

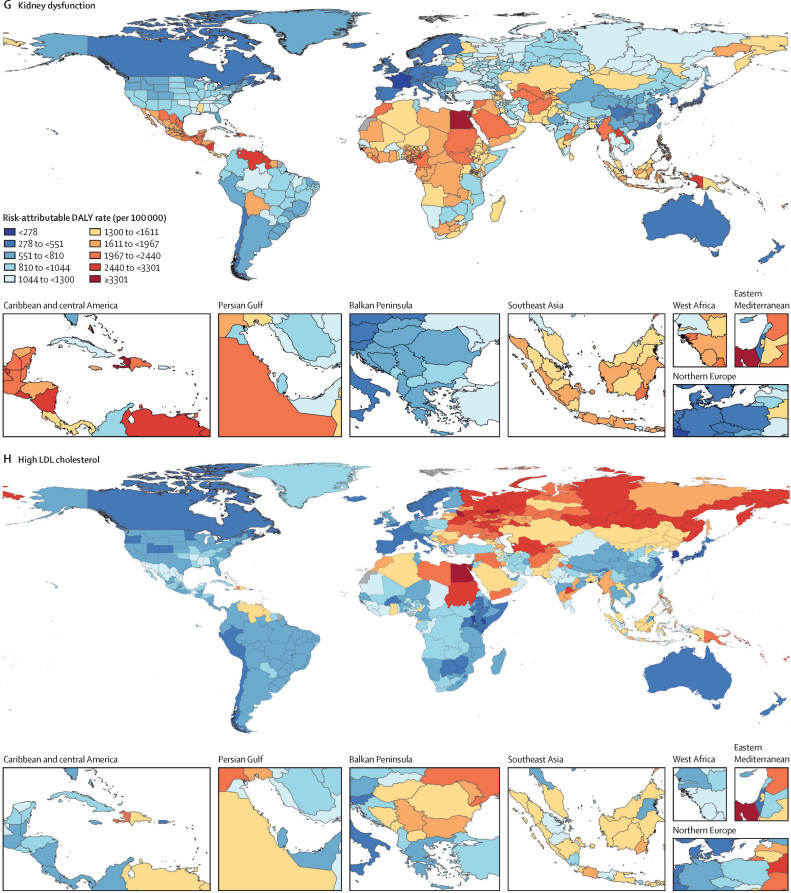

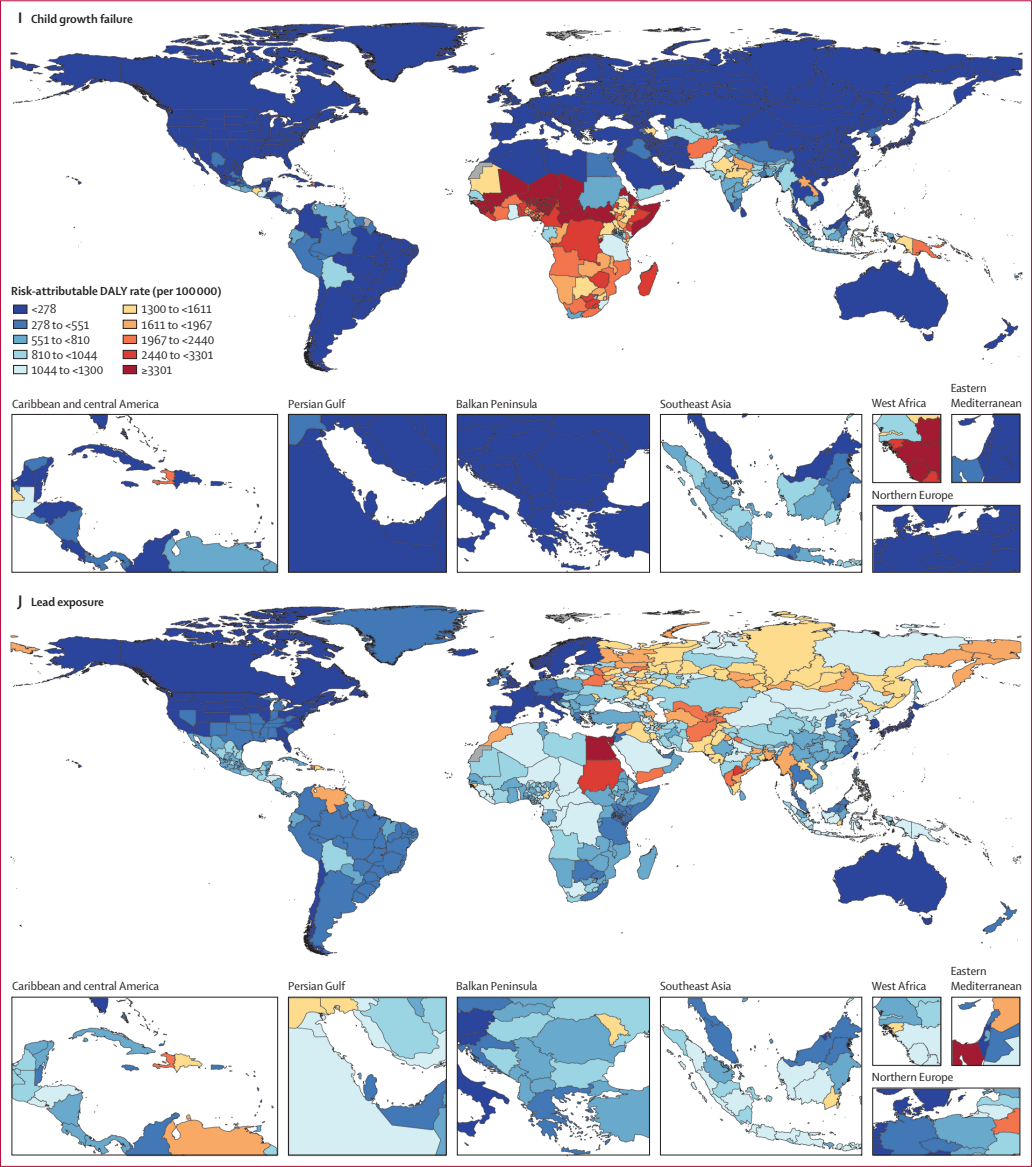
Age-standardised DALY rate attributable to the ten leading GBD level 3 risk factors, ranked by percentage of total DALY counts, by location, 2023 High systolic blood pressure (A), particulate matter pollution (B), smoking (C), high fasting plasma glucose (D), low birthweight and short gestation (E), high BMI (F), high LDL cholesterol (G), kidney dysfunction (H) child growth failure (I), and lead exposure (J). Dotted lines indicate disputed territories. DALY=disability-adjusted life-year. GBD=Global Burden of Diseases, Injuries, and Risk Factors Study.

**Figure 9:**
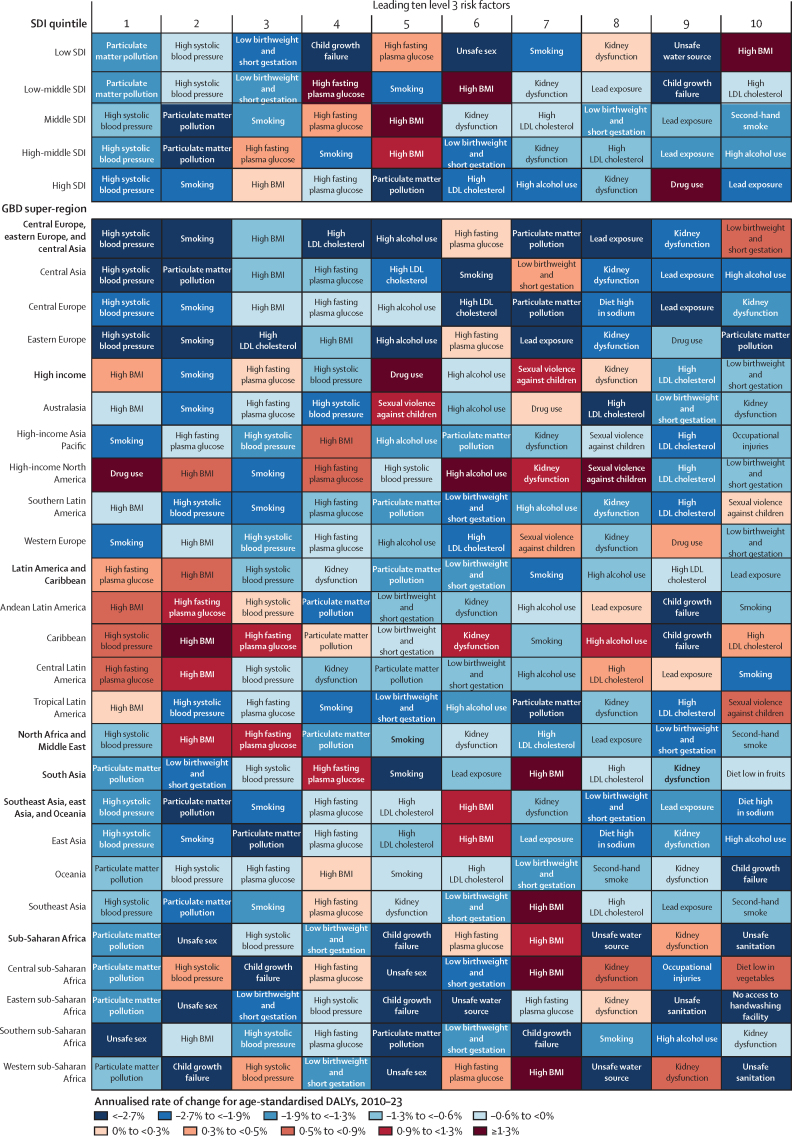
Leading ten GBD level 3 risk factors for 2023 attributable age-standardised DALY rates by SDI quintile, GBD region and super-region, and annualised rate of change between 2010 and 2023 For each region and super-region (in bold) and SDI quintile, level 3 risk factors are ranked by attributable age-standardised DALY rates from left (first) to right (tenth). DALY=disability-adjusted life-year. GBD=Global Burden of Diseases, Injuries, and Risk Factors Study. SDI=Socio-demographic Index.

The 2023 disease burden attributable to risk factors varied considerably by geography, as illustrated by the global distribution of age-standardised risk-attributable DALY rates for all GBD risk factors combined (appendix 3 figure S3) and maps of rates attributable to the ten leading global level 3 risks (figure 8; ranked according to percentage of total DALY counts). Age-standardised attributable DALY rates for high SBP, the leading level 3 risk factor in 2023, were highest in the super-regions of north Africa and the Middle East and central Europe, eastern Europe, and central Asia (figure 8A). At a regional level, high SBP was the leading contributor to burden in central Asia (highest in Tajikistan, at 7298·3 [95% UI 5930·8–8496·6] age-standardised DALYs per 100 000), Oceania (highest in Nauru, at 12 800·7 [10 101·2–15 840·3] age-standardised DALYs per 100 000), north Africa and the Middle East (highest in Egypt, at 8241·6 [6389·3–10 311·6] age-standardised DALYs per 100 000), eastern Europe (highest in Belarus, at 5604·4 [4514·0–6479·7] age-standardised DALYs per 100 000), southeast Asia (highest in Myanmar, at 6429·9 [4736·2–8134·3] age-standardised DALYs per 100 000), central sub-Saharan Africa (highest in the Central African Republic, at 4557·5 [3212·2–6066·3] age-standardised DALYs per 100 000), and western sub-Saharan Africa (highest in Guinea-Bissau, at 5541·2 [4302·6–6899·5] age-standardised DALYs per 100 000; figure 9; appendix 3 table S13). High SBP was the leading contributor to age-standardised burden in the middle, high-middle, and high SDI quintiles and the second-ranked contributor in the low and low-middle SDI groups (figure 9).

Attributable age-standardised 2023 DALY rates per 100 000 for the second leading risk, particulate matter pollution, were highest at the super-region level in south Asia, sub-Saharan Africa, and north Africa and the Middle East (figure 8B), and at the regional level in Oceania (highest in the Solomon Islands, at 10 226·8 [95% UI 8375·4–12 034·4]), south Asia (highest in Bangladesh, at 6095·0 [5340·3–6971·4]), and central, eastern, and western sub-Saharan Africa (highest in the Central African Republic, at 7933·3 [6052·0–9660·4], South Sudan, at 6524·5 [5025·2–7984·6], and Chad, at 7342·7 [5849·1–8652·9], respectively; figure 9; appendix 3 table S13). Particulate matter pollution was the leading level 3 risk factor in the low and low-middle SDI groups and the second leading risk factor in middle and high-middle SDI groups (figure 9). Smoking, the third leading level 3 contributor to global burden (as measured by percentage of total DALY counts), exhibited the highest age-standardised DALYs per 100 000 in central Europe, eastern Europe, and central Asia; southeast Asia, east Asia, and Oceania; north Africa and the Middle East; and south Asia super-regions (figure 8C). At a regional level, smoking was the leading risk in high-income Asia Pacific and western Europe (highest in Brunei, at 1581·3 [1123·5–2185·0] age-standardised DALYs per 100 000, and in Monaco, at 2204·1 [1637·0–2880·9] age-standardised DALYs per 100 000, respectively; figure 9; appendix 3 table S13). Smoking was the third leading risk in the middle SDI group (figure 9).

Global maps of 2023 age-standardised risk-attributable burden for the fourth to the tenth leading level 3 risk factors—high FPG, low birthweight and short gestation, high BMI, kidney dysfunction, high LDL cholesterol, child growth failure, and lead exposure—are shown in figure 8D–J. Of these risks, high FPG was the leading risk factor at a regional level in central Latin America, and high BMI was the top risk in Australasia, southern Latin America, Andean Latin America, and tropical Latin America (figure 9). Notably, in only two of 21 GBD regions were the leading risk factors not reflected in the top ten level 3 risks globally; these were high-income North America (and correspondingly the high SDI quintile), where drug use was the leading risk, and southern sub-Saharan Africa, where unsafe sex was the top risk (figure 9).

See appendix 3 for detailed estimates related to the attributable burden, including relative risks (table S17) and PAFs (tables S13, S14, S16) used to calculate attributable burden, and attributable burden measured in DALYs (table S13) and attributable deaths (table S14) presented for each risk factor and outcome, across geography and time.

### Trends in risk-attributable DALYs, 2010–23

Over the period 2010–23, all-age global DALY counts attributable to behavioural risks declined by 8·4% (95% UI 4·5–13·2), and those attributable to environmental and occupational risks declined by 10·4% (5·3–15·0). Conversely, global counts attributable to metabolic risks increased by 30·7% (24·8–37·3; figure 6; appendix 3 table S13). This seeming contradiction is due largely to the greater impact of metabolic risk factors on increasingly ageing populations, as evidenced by the decrease of 6·7% (2·0–11·0) seen in age-standardised global DALY rates attributable to metabolic risks over the same period. Notably, however, this decline in age-standardised DALY rates for metabolic risk factors was less pronounced than it was for behavioural risks (decline of 22·2% [19·2–25·4]) and environmental and occupational risks (decline of 27·3% [23·4–31·1]; figure 6, appendix 3 table S13). The smaller decline in age-standardised burden attributable to metabolic risks between 2010 and 2023 was due in part to a significant global increase in rates of burden attributable to high BMI, which rose by 10·5% (0·1 to 20·9), and a non-significant increase in high FPG, which rose by 6·2% (–2·7 to 15·6; figure 7). These increases stand in contrast to declining global age-standardised DALY rates over the same period for all other leading 25 level 3 risk factors except drug use, which rose by 8·4% (2·6–15·3; figure 7). The greatest decreases among the 22 other leading level 3 risk factors were for risks associated with unsafe water, sanitation, and handwashing (declines of 54·4% [38·7–65·3] for unsafe sanitation, 50·5% [33·3–63·1] for unsafe water source, and 45·2% [25·6–72·0] for no access to handwashing facility). Other notable declines in age-standardised DALY rates were seen for child growth failure (decrease of 44·9% [37·3–53·5]), unsafe sex (decrease of 30·3% [24·3–36·7]), smoking (decrease of 25·1% [16·4–33·0]), occupational injuries (decrease of 24·6% [16·2–32·8]), and particulate matter pollution (decrease of 24·9% [20·9–28·6]; figure 7; appendix 3 table S13).

### Trends in risk-attributable DALYs by SDI and location, 2010–23

**Figure 10:**
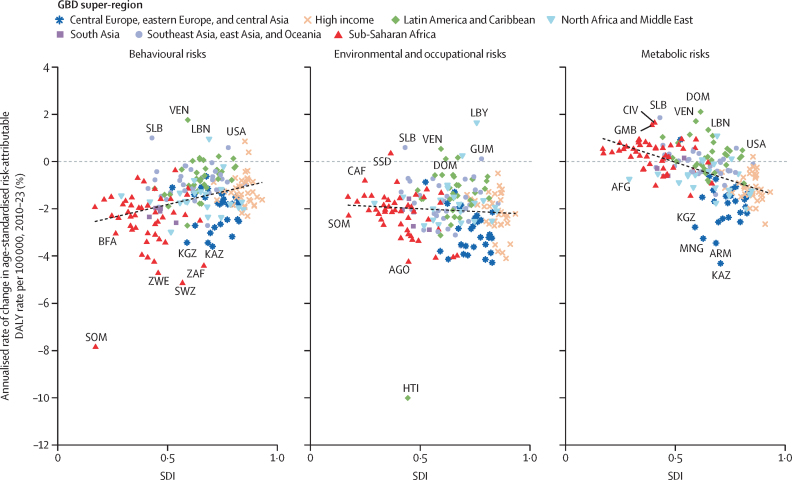
Annualised rate of change in age-standardised risk-attributable DALY rates by GBD level 1 risk, SDI quintile, and country or territory, 2010–23 The black dashed lines depict the linear regression line. Country and territory points are categorised by GBD super-region. Selected countries and territories are labelled by International Organization for Standardization 3 codes. AFG=Afghanistan. AGO=Angola. ARM=Armenia. BFA=Burkina Faso. CAF=Central African Republic. CAN=Canada. CIV=Côte D’Ivoire. DOM=Dominican Republic. GBD=Global Burden of Diseases, Injuries, and Risk Factors Study. GMB=The Gambia. GUM=Guam. HTI=Haiti. KAZ=Kazakhstan. KGZ=Kyrgyzstan. LBN=Lebanon. LBY=Libya. MNG=Mongolia. NER=Niger. SDI=Socio-demographic Index. SLB=Solomon Islands. SOM=Somalia. SSD=South Sudan. SWZ=Eswatini. VEN=Venezuela. ZAF=South Africa. ZWE=Zimbabwe.

Time trends in risk-attributable burden between 2010 and 2023 varied by both SDI level and location, as reflected in ARCs in age-standardised DALY rates attributable to overarching level 1 risk factors (figure 10). For behavioural risk factors, attributable burden generally declined over this period at a slower rate in higher than in lower SDI countries and territories. ARCs were negative in most countries, indicating a decline over time in burden attributable to behavioural risks; however, this burden increased over time in some countries, including Venezuela, the Solomon Islands, Lebanon, and the USA, where age-standardised DALYs attributable to behavioural risks rose by 25·9% (95% UI 15·1–36·1), 13·9% (3·8–25·5), 13·4% (2·9–23·0), and 11·9% (7·6–16·1), respectively (appendix 3 table S13). The rise in behavioural risk-attributable burden in the USA—the only country other than Canada in the high-income super-region that showed such an increase—was driven largely by a 124·2% (96·1 to 157·0) increase in age-standardised burden attributable to drug use, in addition to an increase in attributable burden for intimate partner violence (54·7% [5·8 to 99·2]) and non-significant rise in sexual violence against children (33·5% [–8·4 to 59·3]; appendix 3 table S13). In contrast to the overall pattern for behavioural risks, burden attributable to metabolic risks generally declined at a faster rate with increasing SDI. Approximately half as many countries and territories had positive ARCs (75)—indicating increasing burden attributable to metabolic risks—as negative ARCs (129), with age-standardised DALYs increasing in countries such as the Dominican Republic (31·2% [22·1–42·0]), the Solomon Islands (27·1% [13·6–43·2]), Venezuela (24·6% [18·8–31·8]), Côte d’Ivoire (23·5% [8·2–42·3]), and The Gambia (21·9% [6·7–40·7]; appendix 3 table S13). For environmental and occupational risks, there was minimal association between SDI and rate of change in attributable burden, and ARCs were generally negative.

Disaggregating to a more detailed level of the risk factor hierarchy, figure 9 presents ARCs between 2010 and 2023 for age-standardised DALYs attributable to the ten leading level 3 risk factors, stratified by SDI and GBD region. For countries in low and low-middle SDI quintiles, the greatest ARC declines over time were for child growth failure (in addition to unsafe sex and unsafe water source in the low SDI quintile), whereas the greatest increases were for metabolic risks: high BMI and high FPG. Middle, high-middle, and high SDI quintiles saw the greatest declines in burden attributable to particulate matter pollution, with decreasing burden attributable to smoking also common across these groups. As in the lower SDI regions, the greatest increases in attributable burden in middle and high-middle SDI quintiles were for high BMI and high FPG. In the high SDI quintile, the highest annualised rates of increase were for drug use, along with considerably lower rates of increase for high BMI.

With respect to the three leading 2023 level 3 risk factors globally—high SBP, particulate matter pollution, and smoking—annual rates of age-standardised burden attributable to high SBP declined between 2010 and 2023 in 17 of 21 GBD regions, with the highest rates of decrease in eastern Europe and central Asia (figure 9). Conversely, burden attributable to high SBP increased in the Caribbean and, to a lesser extent, in central and western sub-Saharan Africa. Of the regions in which particulate matter pollution was one of the ten leading risk factors, age-standardised DALYs attributable to particulate matter pollution decreased in nine regions, with the highest rates of decline in eastern Europe, east Asia, and central Europe. Notably, burden attributable to particulate matter pollution rose slightly in Australasia and the Caribbean. Smoking showed attributable burden decreases over time in all regions, with the highest rates of decline in central Asia, eastern Europe, tropical Latin America, and south Asia. Time trends in attributable burden for the third-ranking to the tenth-ranking level 3 risk factors, by region and by SDI, can also be seen in figure 9. Detailed estimates of change over time in attributable DALYs and deaths for each risk factor and outcome—by GBD super-region, region, and country—are available in appendix 3 (table S13).

## Discussion

The findings from GBD 2023 highlight the continuing epidemiological transition, with substantial reductions in CMNN disease burden contrasted by a rising burden of NCDs and metabolic risk factors, largely driven by ageing and population growth. Between 2010 and 2023, global age-standardised DALY rates decreased by nearly 13%, despite total DALY counts rising by about 6%. The reduction in CMNN diseases—particularly diarrhoeal diseases, HIV/AIDS, tuberculosis, and malaria, with approximate decreases in age-standardised DALY rates of 49%, 43%, 42%, and 21%, respectively—represents a major global health achievement up to 2023, yet neonatal disorders and lower respiratory infections remain leading causes of burden. NCDs now account for nearly two-thirds of global DALYs, with ischaemic heart disease, stroke, diabetes, and chronic respiratory diseases among the top contributors. Our analysis estimated that about 46% of total 2023 DALYs were attributable to the modifiable risk factors included in GBD 2023, particularly high SBP, particulate matter pollution, and smoking. Notably, age-standardised DALY rates attributable to high BMI, high FPG, and drug use increased, underscoring emerging global health challenges. Although substantial gains in health have been made, these results emphasise the need for risk-factor mitigation and targeted interventions to address the ever-rising burden of NCDs and sustain progress towards reducing the burden of CMNN diseases. Without advances in prevention, early diagnosis, and chronic disease management of NCDs, gains in longevity risk being offset by a rising burden of non-fatal diseases. This underscores the need for health systems and policy makers to prioritise healthy ageing, focusing not only on reducing mortality rates, but also on improving preventive care and disease management. Moreover, recent budgetary cuts to development assistance are re-ordering the global health system, posing a real and immediate threat to sustaining health gains.^31^, ^32^ The evolving situation demands not only targeted policy responses, but also rigorous, objective, ongoing monitoring.

A hallmark of GBD is the continued emphasis on data-driven estimation and ongoing collection and curation of health data. For this cycle of GBD, we added more than 34 000 new inputs of data for disease and injury burden estimation and about 16 000 new inputs for risk factor analysis. These additions represent surveys newly identified through active data seeking; collaborator feedback; expanded use of already-identified surveys and new phases of existing surveys, such as Multiple Indicator Cluster Surveys, available for the first time in this cycle; updates and revisions to reporting time series; and new systematic reviews to identify the latest data reported in the literature. Data updates not only focus on the new estimate years of 2022 and 2023, but also revisit the past, providing additional datapoints on causes and locations where past surveys are newly accessible, or filling in previous gaps in our database. Although differences remain among locations in the total amount of data accessible, we have successfully accessed data inputs from each of 204 countries and territories, including 660 subnational locations, from every year estimated, and for each cause and risk factor. Data inputs by cause and risk factor are detailed in the GBD 2023 Sources Tool, which allows users to explore the full array of data inputs by metric, by disease, injury, or risk, and by location.

We have seen the epidemiological transition continue despite a global financial crisis and the COVID-19 pandemic. Sociodemographic factors such as poverty, education, employment, and social inequalities continue to shape health outcomes by influencing access to health services, nutritional quality, and the ability to engage in preventive health behaviours. These determinants are particularly relevant with respect to the CMNN diseases, for which—despite progress in reducing mortality from infectious diseases and maternal and neonatal conditions—the burden remains disproportionately high in low and middle SDI countries due to persistent disparities in health-care access, vaccination coverage, and nutrition. This pattern can be seen at large geographical scales, as in the Sahel, the semi-arid expanse that includes countries within the GBD regions of central and western sub-Saharan Africa, and Eritrea. Malnutrition, both under-nutrition and the rising prevalence of obesity, represents a double burden of disease, demanding integrated strategies that address food security, health system strengthening, and social policies that promote health equity.

Addressing the global burden of disease requires focused action on key risk factors, particularly overweight and obesity, which have become major drivers of poor health outcomes worldwide. Although obesity rates vary across countries,^33^ they are rising in nearly all regions,^34^, ^35^ contributing to increased prevalence of diabetes^36^ and chronic kidney disease.^37^ Effective solutions must extend beyond individual choices to encompass structural determinants, including food availability and affordability, urban design, and public messaging on the health risks of high BMI. Despite no success in reversing obesity trends at the population level, governments and global health organisations must prioritise comprehensive strategies that promote healthier diets and increased physical activity, beginning with early-life interventions. Ischaemic heart disease, the leading cause of DALYs for both males and females globally, is another high-burden disease that requires a redoubling of efforts. As new approaches and innovative strategies for defining and treating coronary artery disease continue to evolve,^38^ health policy efforts must prioritise equitable access to prevention, detection, emergency services, and treatment, particularly in under-served and lower-resourced settings. Equitable access to evidence-based treatments should also be part of a broader effort to reduce weight-related disease burden and mortality. For example, a recent study showed that statin therapy was prescribed to less than 10% of eligible individuals for primary prevention of cardiovascular disease in many low-income and middle-income countries.^39^ Novel therapies, such as GLP-1 receptor agonists, which have demonstrated effectiveness in managing obesity, type 2 diabetes, and cardiovascular risk, remain largely inaccessible outside high-income countries.^40^ There is an urgent need to expand access to established essential medicines, while also improving clinical studies and population-level research for novel treatments globally.^41^, ^42^ Beyond obesity, tackling other major modifiable metabolic and behavioural risk factors—including high SBP, tobacco use, and substance use—is crucial. Although tobacco use has declined in high-income regions, it remains alarmingly high in others. Designed to align with the WHO Framework Convention on Tobacco Control, MPOWER measures^43^ provide a framework for enacting tobacco control, yet full implementation is needed to accelerate progress. Managing high blood pressure effectively requires widespread access to high-quality primary care, an area in which many health systems still fall short.

Despite substantial declines in exposure due to removal from motor vehicle fuels, lead exposure—recognised since the Roman Empire as a health risk factor^44^, ^45^—persists as an important contributor to cardiovascular disease burden, especially in central and eastern Europe and central Asia. Although these effects largely reflect accumulated bone lead concentrations driven by past exposure before removal of leaded gasoline, lead remains a ubiquitous environmental contaminant. Efforts to reduce exposure from paint in older houses, contaminated soil, drinking water, battery recycling, electronic waste, spices, cookware, and other consumer products, combined with surveillance to identify highly exposed populations, should be prioritised. Additionally, evidence continues to accumulate for the scope of NCDs affected by exposure to particulate matter air pollution (PM_2·5_), a risk factor for the eight leading causes of death globally, including dementia^46^ and type 2 diabetes,^47^ which have rapidly increasing mortality rates.^16^ Even low levels of PM_2·5_ have been associated with increased dementia risk,^48^ and more than a sixth of the global burden of type 2 diabetes was attributable to PM_2·5_ in 2023 (GBD 2023 Results Tool). Although the burden associated with one PM_2·5_ risk factor, household air pollution, has declined dramatically except in sub-Saharan Africa,^49^ ambient PM_2·5_ remains the leading global environmental risk factor. It is essential that policy makers align national standards with WHO guidelines and, crucially, develop implementation approaches to reduce exposures and consequent effects on health.^50^ The increasing evidence for the involvement of PM_2·5_ in major diseases suggests an opportunity for future research to help identify individuals at high risk and to inform potential prevention options.

GBD 2023 also highlights the staggering increase in the burden of mental disorders globally, the underlying causes, and even temporal trend, of which remain widely debated.^51^ There is convincing evidence that the COVID-19 pandemic resulted in secondary deterioration of mental health, leading to an increase in the prevalence of depressive and anxiety disorders.^52^ Notably, the largest increases in these disorders were estimated to have occurred following the onset of the COVID-19 pandemic. However, there is also convincing evidence that the prevalence of these disorders has been increasing steadily over the past two decades, especially for some locations within the high-income super-region.^7^ There are several competing and complementary theories for this increase, including increases in social media use, cyberbullying, child maltreatment, climate despair, and rising costs of living and income inequality,^51^, ^53^ with expanded mental health awareness and increased reporting further highlighting the problem. Meta-analyses suggest significant associations between social media use and symptoms of depression and anxiety, but further research is needed to explore the causal direction.^54^ However, the widespread use and influence of social media in many parts of the world might make it difficult to detect its effects at the individual level. Population-level studies are required to determine the relationship between social media use and mental disorders, as well as to design suitable interventions. For example, in Australia, the federal government recently passed a law effectively banning children younger than 16 years from accessing certain forms of social media. This presents a unique opportunity for researchers to further examine the effects of public health policy on social media and its effects on youth mental health. Focused efforts are needed to better understand these drivers and inform policies that can effectively address the growing mental health crisis.

GBD 2023 presents strong evidence for exposure to sexual abuse and intimate partner violence as additional preventable contributors to several mental disorders, notably major depressive disorder and anxiety disorders, as well as a large set of other conditions ranging from maternal disorders to asthma, as well as homicide and suicide (appendix 3 table S13).^27^ The highest rates of DALYs attributable to intimate partner violence and sexual violence against children were seen in sub-Saharan Africa, but high rates were also seen in high-income regions, demonstrating that the detrimental effects of sexual and intimate partner violence span across societies, regardless of socioeconomic status. Among reproductive-aged females, intimate partner violence ranked in the top five health risks, with an attributable DALY rate similar to that of iron deficiency, while the global DALY rate attributable to sexual violence against children was similar to that of unsafe sanitation (GBD Compare; appendix 3 table S13). Bullying victimisation also merits discussion as a modifiable risk factor, ranking sixth among the behavioural risk factors in attributable DALYs among young people aged 10–24 years, with highest rates observed in the north Africa and the Middle East and high-income super-regions (GBD Compare). Our estimates highlight specific health outcomes associated with exposure to violence—particularly gender-based violence—and quantify the health burden it engenders, adding further detail to the growing body of data illuminating the high prevalence of gender-based violence.^55^, ^56^ Together, these data are a call to action. Compared with other conditions with a similar magnitude of burden, efforts to prevent exposure to violence, as well as address the needs of survivors, have historically been under-prioritised. It is essential to better quantify and understand intimate partner violence and sexual violence against children, especially because both are often hidden and under-reported.

Overall, progress in CMNN diseases has been astounding over the period of study; despite profound setbacks in the form of the COVID-19 pandemic, this progress remains one of the shining achievements of global health. These gains are not unidirectional and are sustained through an imperfect constellation of national and international efforts in prevention, treatment, and cure. In an environment of reduced funds to combat the major sources of communicable disease burden,^57^ it is possible that we will see reversals in some of these trends. As we face these challenges and their effects, we believe that there has never been a time in which global health measurement is so important.

There are several limitations to the overall GBD enterprise that provide opportunities to refine and improve the quality and accuracy of the results. The iterative nature of GBD reflects the incorporation of new data sources, methodological improvements, and ongoing efforts to stabilise data and analytical processes. Despite these efforts, challenges persist due to variability in the availability and quality of input data. Inconsistent quality, flawed methodologies, and gaps in the collection of primary data make it difficult to accurately quantify the burden of disease without ongoing and thorough assessments of data quality. Additionally, lags in the availability of data for more recent years further contribute to these challenges. For example, because surveys were delayed due to COVID-19, just 19 STEPS surveys conducted since 2020 have been released to date, only five of which have the individual-level record data necessary to analyse some causes and risk factors. By contrast, the 4-year period before 2020 had more than 41 STEPS surveys. In time, more data will become available for this period, with additional details enriching summary reports, but the typical delays we see in the release of surveys and other datasets were compounded by COVID-19 physical distancing restrictions. To the extent possible, the GBD analytical framework—using a modelled statistical approach to synthesise all evidence available—is designed to account for issues of sparse or missing data and uncertainty arising from a multitude of sources, such as stochastic variation in input data, demographic adjustments, and bias due to input study characteristics. Input bias can be particularly impactful with respect to sex and age metadata related to summary statistics, as reporting of outcomes stratified by age and sex is often not available, requiring processing using age-splitting and sex-splitting algorithms to produce the more granular estimates presented in GBD. However, limitations associated with the quality and methods of primary data collection remain a recurring obstacle and highlight the need to strengthen data collection systems. Fully accounting for the range of uncertainties inherent in burden and risk factor estimation processes remains an ongoing challenge, and uncertainty and statistical variation cannot be eliminated.

There are also limitations specific to GBD disease burden measures. Time-varying differences in disease detection or reporting can bias estimates of prevalence or incidence, making it challenging to accurately quantify changing morbidity over time. Although we use crosswalking and MR-BRT adjustment tools to account for varying case definitions and data collection methods, and have further introduced an advanced DisMod-AT tool that will allow us to more accurately model temporal trends, we acknowledge that YLD trends over time might reflect both true morbidity and detection artifacts. More detailed and improved diagnostic data are therefore essential to more accurately capture changes in morbidity. Temporal trends in causes might also be attenuated due to limitations in the ability of DisMod-MR 2.1 to accurately estimate trends when data are sparse. For most causes that require the prevalence by severity to estimate YLDs, the estimated severity distribution is largely sourced from a small number of survey series conducted in Australia and the USA because of the scarcity of comprehensive data available in other countries. Without more data on severity across geography, there is a potential for bias in YLD estimation—particularly in settings in which access to care, diagnostic practices, and treatment availability differ considerably. However, work to address this concern is currently underway for some causes.^58^, ^59^ The quality and accuracy of comorbidity corrections, which are essential to ensure that estimated YLDs are unique to each cause and additive across causes, also require continuous improvement. For GBD 2023, we assumed independent comorbidity—ie, the chance of having a comorbid cause is equal to its prevalence. Assuming independent comorbidity can lead to underestimation of comorbidity, especially for causes such as mental disorders, which have substantial dependent comorbidity, and in turn might overestimate YLDs for some causes. However, in the context of sparse data on joint prevalence and functional health loss from comorbid states across all causes in GBD, the independence assumption is necessary to make estimation possible. Fortunately, simulation testing within epidemiological datasets has suggested accounting for dependent comorbidity has a minimal impact on the overall YLD counts (appendix 1 section 2.10). Limitations related to our estimation of YLLs are discussed in a parallel GBD 2023 publication.^16^

With respect to our risk factor analyses, it is unlikely that our present estimates capture all existing relationships between risk factors and health outcomes and all risk-attributable burden, although with every iteration of GBD we add new relationships and update evidence. Not only are additional risk factors and risk–outcome pairs considered in every round of GBD, so too are mediation relationships. With respect to mediation, we adjusted relative risk estimates for mediation based on the assumption that joint risks are multiplicative, but some combinations of risks might be super-multiplicative or sub-multiplicative. This issue might be particularly relevant to analyses of dietary risk factors that yield protective effects, such as fruit or wholegrain intake, or other coincident exposures, such as PM_2·5_ and high temperatures. More research is needed to better understand mediation effects to fully account for them to more accurately estimate attributable burden for inter-related risk factors; refining our mediation methods remains a continuing priority. With respect to TMRELs, we generally set them to equal to zero for those harmful risks where zero exposure is theoretically achievable or used the data to empirically derive non-zero levels reflecting minimum risk (or used clinical guidance where data are sparse or biological thresholds are well defined), with monotonically increasing risk functions. For protective risks, we generally set TMRELs at the 85th percentile of exposure in the available data to avoid extrapolating the risk function outside the data-rich range of the available literature, which could lead to exaggerated estimates of attributable burden and implausible levels of consumption. Although evidence suggests that these TMRELs yield accurate estimates of relative risk,^23^ further refinements might be needed.

In the present iteration of GBD, our burden-of-proof flexible meta-regression framework more accurately describes the true shape of the risk–outcome relationship rather than imposing log-linearity, systematically trims data outliers, tests and adjusts for bias in the input data, and formally quantifies and incorporates between-study heterogeneity unexplained by individual study design features into a measure of evidence strength that combines effect size and uncertainty accounting for this between-study heterogeneity. This analytical framework, however, has not yet been applied to all risk–outcome pairs because the work remains ongoing. Moreover, because the covariate selection and adjustment methods used to control for bias are data driven and rely on the high-level information that is available about input studies to meta-analyses (eg, which studies were gold standard *vs* not), they are unable to provide a more nuanced understanding of the impact of deviations from the gold standard, beyond testing and adjusting for systematic bias. A final limitation of the present risk analysis is that our assumption that the risk–outcome relationships assessed were constant across location and time (with the exception of relative risk functions involving temperature, which we varied according to annual mean temperature, and the relationship between high BMI and breast cancer, which has been shown to vary between Asian and non-Asian populations^60^) is unlikely to hold true for all cases. Burden-of-proof methods provide an analytical framework to identify variation in risk–outcome relationships by location or other population characteristics, but ultimately to do so will require additional primary studies systematically evaluating differences between population subgroups. We continue to evaluate the available evidence and will incorporate more location-specific or subgroup relative risk estimates as they are identified.

GBD evolves to ensure its scientific findings remain relevant, useful, and timely. With a strong commitment to exploring new scientific horizons and opening new opportunities for research, our goal is to update and publish the next iteration of GBD (GBD 2025) as soon as results are available. In addition to quantifying additional risk–outcome pairs and evaluating potential new metabolic, behavioural, and environmental risk factors, we aim to quantify the attributable burden of low educational attainment in GBD 2025, an effort we acknowledge will be challenging as the effects of education on disease burden are also mediated through numerous other risk factors. We hope to be able to expand to additional social determinants of health in future iterations of GBD. In addition to expanding the scope of GBD, efforts to continually improve the available estimates include incorporating additional health conditions and geographical areas, procuring and assimilating new data, improving methods to correct data discrepancies, and better representing uncertainty in our findings. Additionally, we are transitioning to DisMod-AT, an improved version of DisMod-MR, for most health outcomes. DisMod-AT is anticipated to improve the precision of age and time trends in our prevalence estimates, particularly by integrating the effects of population shocks. We will continue to test and validate DisMod-AT and will remain focused on ensuring harmonisation and consistency in estimates generated using this tool. For GBD 2025, we plan to integrate severity distributions according to levels of health-care access for several conditions. We also plan to account for changes to disease detection and diagnosis across time and location in future GBD iterations. Regarding adjustments to clinical data, future research could be enhanced by integrating alternative causal frameworks and using more granular data on health system usage. Last, there are efforts to improve the methods that account for comorbidities.

This GBD 2023 synthesis of estimates of disease and injury burden and risk-attributable burden provides policy makers, researchers, and public health practitioners with crucial insights to inform global health strategies. It shows a growing and ageing world where progress against CMNN diseases has been remarkable across all SDI levels and where continued progress against NCDs has been modest and outpaced by demographic changes. With focused efforts on prevention, risk mitigation, and more robust health systems, substantial strides can be made towards improving population health and achieving long-term sustainable development targets, as we move ever closer to consensus goals for health and wellbeing impact by 2030. The challenges remain considerable, however, with the potential for reversal of progress on CMNN diseases resulting from new and substantial reduction in funding for global health alongside rises in metabolic disease and risk factors.

### GBD 2023 Disease and Injury and Risk Factor Collaborators

### Affiliations

### Contributors

### Data sharing

For detailed information on data sources and estimates, please visit the Global Health Data Exchange GBD 2023 website at http://ghdx.healthdata.org/gbd-2023.

For the **NCD Risk Factor Collaboration** see https://www.ncdrisc.org/

For the **Prospective Urban and Rural Epidemiological (PURE) study** see https://www2.phri.ca/pure

For ***The Lancet*'s serialisation of GBD** see https://www.thelancet.com/gbd

For the **GBD 2023 Sources Tool** see https://ghdx.healthdata.org/gbd-2023/sources

For the **Global Health Data Exchange** see https://ghdx.healthdata.org/

For the **Burden of Proof tool** see https://vizhub.healthdata.org/burden-of-proof/

For the **statistical code** see https://ghdx.healthdata.org/gbd-2023/code

For the **GBD 2023 Results Tool** see https://vizhub.healthdata.org/gbd-results/

For **GBD Compare** see https://vizhub.healthdata.org/gbd-compare/

## Declaration of interests

D Abramov reports payment or honoraria for speakers bureaus from Bayer and AstraZeneca; participation on an Advisory Board with BridgeBio; receipt of equipment, materials, drugs, medical writing, gifts, or other services from Bayer in the form of medical writing assistance; all outside the submitted work. D Adzrago reports support for the present manuscript from the Intramural Research Program of the National Institutes of Health (NIH), and support for attending meetings and/or travel from the Intramural Research Program of the National Institutes of Health (NIH) outside the submitted work. The contributions of the NIH author(s) were made as part of their official duties as NIH federal employees, are in compliance with agency policy requirements, and are considered Works of the United States Government. However, the findings and conclusions presented in this paper are those of the author(s) and do not necessarily reflect the views of the NIH or the US Department of Health and Human Services. S Afzal reports support for the present manuscript from Institute of Public Health Lahore for study material, manuscripts, medical writings and library resources; grants or contracts from the Dean Institute of Public Health Lahore; payment or honoraria for lectures, presentations, speakers bureaus, manuscript writing or educational events from the Dean Institute of Public Health Lahore; support for attending meetings and/or travel from the Dean Institute of Public Health Lahore; participation on a Data Safety Monitoring Board or Advisory Board with Pakistan National Bioethics Committee as a Member, Institutional Review Board of Fatima Jinnah Medical University as a Member, Ethical Review Board and Data Monitoring Board Institute of Public Health Lahore Pakistan as a Member, Clinical Research Organization King Edward Medical University, Annals of King Edward Medical University Advisory Board as a Member; leadership or fiduciary roles in other board, society, committee or advocacy group, paid or unpaid, with Pakistan Higher Education Commission Research Committee as a Member, Pakistan Medical and Dental Commission Research and Journals Committee as a Member, Pakistan National Bioethics Committee as a Member, Pakistan Society of Internal Medicine as a Member, Pakistan Association of Medical Editors as a Member, Medical Microbiology and Infectious Diseases Society as a Member, Leads International as a Fellow, Faculty of Public Health UK as a Fellow, College of Physicians and Surgeons Pakistan as a Fellow; receipt of equipment, materials, drugs, medical writing, gifts or other services from Bergen University Norway; other financial or non-financial interests with Dean Institute of Public Health Birdwood Lahore; all outside the submitted work. C Agostinis Sobrinho reports grants or contracts from Fundação para a Ciência e Tecnologia (FCT) via grant CEECINST/00093/2021/CP2815/CT0001, outside the submitted work. A Amin reports the following patents pending: US20200253891A1; Method of Liver Cancer Treatment with Safranal-Based Formulations, US20200254049A1; Combination Therapy for Cancer, US20200253890A1; Suppression and Inhibition of CDC25B with Safranal-Based Formulations; all outside the submitted work. R Ancuceanu reports consulting fees from AbbVie and Merck Romania; payment or honoraria for lectures, presentations, speakers bureaus, manuscript writing or educational events from AbbVie, Laropharm, Reckitt, Merck Romania, and MagnaPharm; support for attending meetings and/or travel from Merck Romania and Reckitt; all outside the submitted work. J Ärnlöv reports payment or honoraria for lectures, presentations, speakers bureaus, manuscript writing or educational events from AstraZeneca, Boehringer Ingelheim, and Novartis; participation on a Data Safety Monitoring Board or Advisory Board with AstraZeneca, Boehringer Ingelheim, and Astella; all outside the submitted work. M S Aslam reports grants or contracts from Xiamen University Malaysia Research Fund (XMUMRF) for Grant No.: XMUMRF/2025-C15/ITCM/0006 Project title: Therapeutic and Toxicity Evaluation of Selected Medicinal Herbs for NAFLD: Exploring the Inter-Organelle Contact Sites Modulation Theory Role: Co-Investigator Dates: Jan 2025 - Dec 2027 (ongoing) and for Grant No.: XMUMRF/2023-C11/ISEM/0041 Project title: Children's Rights Education in the Early Years of Divorce: An Exploration of Adolescents’ Perspectives Role: Co-Investigator Dates: Jan 2023 - Dec 2025 (ongoing) – both internal XMUMRF research grants administered by Xiamen University Malaysia; funds disbursed to institutional research account only; no salary, honoraria, or personal payments to author; all outside the submitted work. O C Baltatu reports support for the present manuscript from the National Council for Scientific and Technological Development Fellowship (CNPq, 304224/2022-7), the Anima Institute (AI) Research Professor Fellowship, and Alfaisal University; leadership or fiduciary roles in other board, society, committee or advocacy group, paid or unpaid, with VividiWise Analytics as Managing Partner and São José dos Campos Tech Park - CITE as Biotech Advisory Board Member; all outside the submitted work. S Barteit reports grants or contracts from the Carl-Zeiss Foundation and the German Research Foundation (DFG); stock or stock options in CHEERS company, a for-profit company focusing on climate change and health evaluation and response systems; all outside the submitted work. A Beloukas reports grants or contracts from Gilead for a Research Grant and Sponsorship to the University of West Attica, and from GSK/ViiV for a Research Sponsorship to the University of West Attica; payment or honoraria for lectures, presentations, speakers bureaus, manuscript writing or educational events from Gilead and GSK paid to the University of West Attica; support for attending meetings and/or travel from Gilead and GSK paid to the University of West Attica; receipt of equipment, materials, drugs, medical writing, gifts or other services from Cepheid in the form of FOC reagents for a research project; all outside the submitted work. P J G Bettencourt reports the following patents planned, issued or pending: WO2020229805A1, BR112021022592A2, EP3965809A1, OA1202100511, US2023173050A1, EP4265271A2, EP4275700A2, EP4265271A3, EP4275700A3; all outside the submitted work. A S Bhagavathula reports support for attending meetings and/or travel from the North Dakota State University, American College of Epidemiology, and University of Virginia; leadership or fiduciary roles in other board, society, committee or advocacy group, paid or unpaid, with Board of Directors, American College of Epidemiology; Institute for Health Metrics and Evaluation as GBD Lead Collaborator; all outside the submitted work. S Bhaskar reports grants or contracts from Japan Society for the Promotion of Science (JSPS), Japanese Ministry of Education, Culture, Sports, Science and Technology (MEXT), Grant-in-Aid for Scientific Research (KAKENHI) (Grant ID: 23KF0126), JSPS and the Australian Academy of Science, JSPS International Fellowship (Grant ID: P23712); leadership or fiduciary roles in other board, society, committee or advocacy group, paid or unpaid, with Rotary District 9675, Sydney, Australia as District Chair, Diversity, Equity, Inclusion & Belonging, with Global Health & Migration Hub Community, Global Health Hub Germany, Berlin, Germany as Chair, Founding Member and Manager, with PLOS One, BMC Neurology, Frontiers in Neurology, Frontiers in Stroke, Frontiers in Public Health, Journal of Aging Research, Neurology International, Diagnostics, & BMC Medical Research Methodology as an Editorial Board Member; all outside the submitted work. A Biswas reports consulting fees from LUPIN Pharmaceuticals Ltd., INTAS Pharmaceuticals Ltd., Alkem Laboratories Ltd., and Torrent Pharmaceuticals Ltd.; all outside the submitted work. F M Blyth reports support for attending meetings and/or travel from International Association for the Study of Pain (IASP) as IASP Councilor; all outside the submitted work. R Cairns reports grants or contracts from Reckitt for an untied educational grant to study poisoning; payment or honoraria for lectures, presentations, speakers bureaus, manuscript writing or educational events from Pharmacy Guild of Australia and Reckitt; all outside the submitted work. A Caye reports consulting fees from Knight Therapeutics and EMS Pharmaceuticals; all outside the submitted work. H Christensen reports support for the present manuscript from Velux Foundation, Br Hartman Fonden, Lundbeck Foundation, Novo Foundation, Tvaersfonden; payment or honoraria for lectures, presentations, speakers bureaus, manuscript writing or educational events from Bayer A/S; participation on a Data Safety Monitoring Board or Advisory Board with Atricure (LEEAPS trial Data Safety Monitoring Board); leadership or fiduciary roles in other board, society, committee or advocacy group, paid or unpaid, with Action Plan for Stroke in Europe as Past Chair; all outside the submitted work. J Conde reports grants or contracts from OncoNanoAI: Artificial intelligence to discover the next generation of personalized nanoparticles for triple-negative breast cancer therapy (2025-2027) FCT Grant LISBOA2030-FEDER-00862500- 14998; patents planned, issued or pending: TRPV2 Antagonists. US Application No. US11273152B2, Surfactant-based cellulose hydrogel methods and uses thereof, PCT/IB2025/051694, 17/02/2025, Self-immolative micelle, methods and uses thereof, EP25165757, 24/03/2025; all outside the submitted work. S E Congly reports grants or contracts paid to their institution from AstraZeneca, Merck, Ipsen, Bausch Health, Oncoustics, Boehringer Ingelheim, and Gilead Sciences Canada; consulting fees paid to them from GSK and Boehringer Ingelheim; participation on a Data Safety Monitoring Board or Advisory Board with Boehringer Ingelheim, Gilead Sciences Canada, and AstraZeneca; leadership or fiduciary roles in other board, society, committee or advocacy group, paid or unpaid, with Canadian Association for the Study of the Liver as a Member of the Board of Directors and Alberta Society of Gastroenterology as Vice President; all outside the submitted work. N Conrad reports grants or contracts paid to their institution from the Wellcome Trust Career Development Award (grant number 318034/Z/24/Z), Research Foundation Flanders (grant number 12ZU922N), and KU Leuven (internal funding); all outside the submitted work. S Cortese reports grants or contracts from the National Institute for Health and Care Research (NIHR) and the European Research Agency; payment or honoraria for lectures, presentations, speakers bureaus, manuscript writing or educational events from the Association for Child and Adolescent Mental Health (ACAMH), the British Association of Psychopharmacology (BAP), Medice; support for attending meetings and/or travel from the Association for Child and Adolescent Mental Health (ACAMH), the British Association of Psychopharmacology (BAP), Medice; leadership or fiduciary roles in other board, society, committee or advocacy group, paid or unpaid, with the European ADHD Guideline Group (EAGG); all outside the submitted work. E C Dee reports support for the present manuscript from the US National Institutes of Health (NIH)/ National Cancer Institute (NCI) and the Prostate Cancer Foundation through the Prostate Cancer Foundation Young Investigator Award and through the Cancer Center Support Grant from the US NCI (P30 CA008748). A K Demetriades reports leadership or non-fiduciary roles in other board, society, committee or advocacy group, paid or unpaid, with EANS (European Association of Neurosurgical Societies) as a Board Member, AO SPIN as a Steering Committee Member for Knowledge Forum Degenerative, Global Neuro Foundation as a Board Member, AO Spine Foundation as a Steering Committee Member, Knowledge Forum Degenerative; all outside the submitted work. X Ding reports grants or contracts from the American Heart Association for a 2-year predoctoral fellowship (DOI: 10.58275/AHA.25PRE1373497.pc.gr.227106), quarterly payments made to their institution, outside the submitted work. L L M Ebraheim reports support for the present manuscript from the Gates Foundation (OPP1152504); royalties or licenses from the Institute for Health Metrics and Evaluation, outside the submitted work. A Faro reports support for the present manuscript from Brazilian National Council for Scientific and Technological Development (CNPq, Brazil), CNPq-funded researcher (PQ). L M Force reports support for the present manuscript from Gates Foundation, St. Jude Children's Research Hospital; grants or contracts from St. Baldrick's Foundation, Conquer Cancer Foundation, NIH Loan Repayment Program; leadership or fiduciary roles in other board, society, committee or advocacy group, unpaid, with Lancet Oncology International Advisory Board; all outside the submitted work. R C Franklin reports support for attending meetings and/or travel from Australasian College of Tropical Medicine (ACTM) - Annual Conference 2022-2024; leadership or fiduciary roles in other board, society, committee or advocacy group, paid or unpaid, with Australasian College of Tropical Medicine as President, Kidsafe Australia as President, Royal Life Saving Society Australia as a Board Member, and Auschem Training as a Board Member; all outside the submitted work. N Fullman reports grants or contracts from the Gates Foundation since March 2024 for work around childhood vaccination and drivers of non-vaccination in select countries; other financial or non-financial interests with Gates Ventures from June 2020 to June 2025, for work around childhood vaccination and vaccine delivery in low- and middle-income countries, and from Gates Foundation (July 2025 to present); all outside the submitted work. N M M Ghith reports support for attending meetings and/or travel from Danish Data Science Institute at the Technical University of Denmark, travel grant in 2023, outside the submitted work. Z Guan reports grants or contracts from Dementia Centre of Excellence and Curtin enAble Institute, Curtin University, outside the submitted work. A Guha reports grants or contracts from American Heart Association and US Department of Defense; leadership or fiduciary roles in other board, society, committee, or advocacy groups, paid or unpaid, with ZERO Prostate Cancer Health Equity Committee; all outside the submitted work. A A Harris reports grants support from Gates Foundation and Gavi, outside the submitted work. A Hassan reports consulting fees from Novartis, Sanofi Genzyme, Biologix, Astra Zeneca, Pfizer, Merz, Roche, Merck, Hikma Pharma, Janssen, Inspire Pharma, Future Pharma, and Elixir Pharma; payment or honoraria for lectures, presentations, speakers bureaus, manuscript writing or educational events from Novartis, Allergan, AbbVie, Merck, Biologix, Viatris, Pfizer, Eli Lilly, Janssen, Roche, Sanofi Genzyme, Bayer, Astrazeneca, Hikma Pharma, Al Andalus, Chemipharm, Lundbeck, Elixir, EvaPharma, Inspire Pharma, Future Pharma and Habib Scientific Office, and Everpharma; support for attending meetings and/or travel from Novartis, Allergan, Merz, Pfizer, Merck, Biologix, Roche, Sanofi Genzyme, Bayer, Hikma Pharma, Chemipharm, Al Andalus and Clavita Pharm; leadership or fiduciary roles in other board, society, committee or advocacy group, paid or unpaid, with MENA Headache Society as Vice President, Multiple Sclerosis Chapter of the Egyptian Society of Neurology as a Board Member, Headache Chapter of the Egyptian Society of Neurology as a Board Member, The International Headache Society (IHS) as a Member of the committee of education, the membership committee, and regional committee; all outside the submitted work. C Herteliu reports grants or contracts for the project “Analysis of the impact of Covid-19 on the main demographic indicators in Romania and the Republic of Moldova by using econometric modeling” code PN-IV-P8-8.3-ROMD-2023-0208 funded by the Romanian Ministry of Research, Innovation and Digitalization (MCID) through UEFISCDI, for a grant of the European Commission Horizon 4P-CAN (Personalised Cancer Primary Prevention Research through Citizen Participation and Digitally Enabled Social Innovation), for the project “Societal and Economic Resilience within multi-hazards environment in Romania” funded by European Union – NextgenerationEU and Romanian Government, under National Recovery and Resilience Plan for Romania, contract no.760050/ 23.05.2023, cod PNRR-C9-I8-CF 267/ 29.11.2022, through the Romanian Ministry of Research, Innovation and Digitalization, within Component 9, Investment I8, and for the project “A better understanding of socio-economic systems using quantitative methods from Physics” funded by European Union – NextgenerationEU and Romanian Government, under National Recovery and Resilience Plan for Romania, contract no.760034/ 23.05.2023, cod PNRR-C9-I8-CF 255/ 29.11.2022, through the Romanian Ministry of Research, Innovation and Digitalization, within Component 9, Investment I8; all outside the submitted work. A K Husøy reports payment or honoraria for lectures, presentations, speakers bureaus, manuscript writing or educational events from Teva Pharmaceuticals for a 45-minute lecture for nurses hosted by Teva in January 2025; leadership or fiduciary roles in other board, society, committee or advocacy group, unpaid, with Lifting The Burden (LTB), a UK-registered non-governmental organization, as Director and Trustee, and with The Journal of Headache and Pain (TJHP) as Editorial Board Member; all outside the submitted work. I M Ilic reports support for the present manuscript from Ministry of Science, Technological Development and Innovation of the Republic of Serbia, no. 451-03-137/2025-03/200110. M D Ilic reports support for the present manuscript from Ministry of Science, Technological Development and Innovation of the Republic of Serbia, no. 451-03-47/2023-01/200111. N E Ismail reports leadership or fiduciary roles in other board, society, committee, or advocacy group, unpaid, with Malaysian Academy of Pharmacy, Malaysia as the Bursar and Council Member and Malaysian Pharmacists Society Education Chapter Committee as a Committee Member; all outside the submitted work. I O Iyamu reports grants or contracts from Canadian Institutes for Health Research (CIHR) Health Systems Impact Fellowship (Funding Reference No. IF8-196153), Michael Smith Health Research BC Trainee Award (Award number - HSIF-2024-04465), and CIHR Canadian HIV Trials Network (CTN+) post-doctoral fellowship; consulting fees from Excellence Community Education Welfare Scheme; support for attending meetings and/or travel from Pacific Public Health Foundation; leadership or fiduciary roles in other board, society, committee or advocacy group, paid or unpaid, with Public Health Association of British Columbia as Vice President; all outside the submitted work. V Jha reports consulting fees/honoraria paid to the George Institute from Bayer, Astra Zeneca, Boehringer Ingelheim, Baxter, Vera, Visterra, Otsuka, Novartis, Astra Zeneca, Timberlyne, Biogen, Chinook, and Alpine; payment or honoraria for lectures, presentations, speakers bureaus, manuscript writing or educational events paid to the George Institute from Vera; all outside the submitted work. T Joo reports support for the present manuscript from EU4Health Programme 2021 - 2027 under Grant Agreement 101126953 (The Joint Action on CARdiovascular diseases and DIabetes – JACARDI. The views and opinions expressed are those of the author(s) only and do not necessarily reflect those of the European Union or the European Health and Digital Executive Agency (HaDEA). Neither the European Union nor the granting authority can be held responsible for them), and from National Research, Development and Innovation Office in Hungary (RRF- 2.3.1-21-2022-00006, Data-Driven Health Division of National Laboratory for Health Security. J J Jozwiak reports payment or honoraria for lectures, presentations, speakers bureaus, manuscript writing or educational events from Novartis, Adamed, Amgen, Boehringer Ingelheim, Servier, Novo Nordisk; all outside the submitted work. M K Kashyap reports grants or contracts from Indian Council of Medical Research (ICMR), New Delhi - Grant # 5/13/55/2020/NCD-III; patents planned, issued or pending: 202311003940 (Indian Patent-Pending), 202311058515 (Indian Patent-Pending); all outside the submitted work. J H Kempen reports salary support via institution for the present manuscript from Sight for Souls and Mass Eye and Ear Global Surgery Program; leadership or fiduciary roles in other board, society, committee or advocacy group, paid or unpaid, with Sight for Souls (a US 501c3 charity) as Board President; all outside the submitted work. M Kivimäki reports grants or contracts paid to their university from the Wellcome Trust (221854/Z/20/Z), Medical Research Council (MR/Y014154/1), and Research Council of Finland (350426), outside the submitted work. J M Kocarnik reports support for the present manuscript from Institute for Health Metrics and Evaluation as an employee, the Gates Foundation for funding to his institution, and American Lebanese Syrian Associated Charities for funding to his institution. A G Konstas reports grants or contracts from Thea Pharmaceuticals, Omni Vision, Vianex, Santen, Intermed; consulting fees from Thea Pharmaceuticals and Santen; payment or honoraria for lectures, presentations, speakers bureaus, manuscript writing or educational events from Thea Pharmaceuticals, Vianex, Intermed, Esteve Pharmaceuticals, Bayer; support for attending meetings and/or travel from Vianex, Thea Pharmaceuticals, Intermed, Santen; all outside the submitted work. K Krishan reports non-financial support from the UGC Centre of Advanced Study, CAS II, awarded to the Department of Anthropology, Panjab University, Chandigarh, India, outside the submitted work. T Lallukka reports support for the present manuscript from the Research Council of Finland (330527], paid to their institution. M-C Li reports grants or contracts from the National Science and Technology Council, Taiwan (NSTC 113-2314-B-003-002) and the “Higher Education Sprout Project” of National Taiwan Normal University.; leadership or fiduciary roles in other board, society, committee or advocacy group, paid or unpaid, with Journal of the American Heart Association as Technical Editor; all outside the submitted work. W-Z Li reports support for the present manuscript from the National Natural Science Foundation of China (82303338) and the Grant of State Key Laboratory of Respiratory Disease (SKLRD-Z- 202401). D Lindholm reports stock or stock options in AstraZeneca during time of employment (>2·5 years ago); other financial or non-financial interests with AstraZeneca as a former employee (>2·5 years ago); all outside the submitted work. H Liu reports other financial or non-financial interests as a mentor of the National Medical Research Association (NMRA, UK), a member of British Society for Cardiovascular Research (BSCR, UK), and a member of Cardiovascular Analytics Group (CVAG, HKSAR of China); all are non-profit academic associations; all outside the submitted work. J Liu reports support for the present manuscript from the National Natural Science Foundation (72474005) and Beijing Natural Science Foundation (L222027); royalties or licenses from the National Natural Science Foundation (72474005) and Beijing Natural Science Foundation (L222027); all outside the submitted work. V Lohner reports support for the present manuscript from Marga and Walter Boll Foundation, Kerpen, Germany. S Lorkowski reports grants or contracts paid to their institution from dsm-firmenich (formerly DSM Nutritional Products); consulting fees from Danone, Novartis Pharma, and Swedish Orphan Biovitrum (SOBI); payment or honoraria for lectures, presentations, speakers bureaus, manuscript writing or educational events from AMARIN Germany, Amedes Holding, AMGEN, Berlin-Chemie, Boehringer Ingelheim Pharma, Daiichi Sankyo Deutschland, Danone, Hubert Burda Media Holding, Janssen-Cilag, Lilly Deutschland, Novartis Pharma, Novo Nordisk Pharma, Roche Pharma, Sanofi-Aventis, Swedish Orphan Biovitrum (SOBI), SYNLAB Holding Deutschland; support for attending meetings and/or travel from AMGEN; participation on a Data Safety Monitoring Board or Advisory Board with AMGEN, Daiichi Sankyo Deutschland, Novartis Pharma, Sanofi-Aventis; all outside the submitted work. K S-K Ma reports grants or contracts from the International Team for Implantology, outside the submitted work. H R Marateb reports grants or contracts from Universitat Politècnica de Catalunya Barcelona Tech – UPC, outside the submitted work. S Masi reports grants or contracts from Servier via personal contracts for consulting activities, lectures, presentations, manuscript writing and educational events, Tuscany Region via grants for research projects in the field of arterial hypertension and management of SARS-CoV2 infection, and Italian Ministry of University and Research via grants for research projects in the field of heart failure; consulting fees from Servier; payment or honoraria for lectures, presentations, speakers bureaus, manuscript writing or educational events from Servier; support for attending meetings and/or travel from Servier; participation on a Data Safety Monitoring Board or Advisory Board with Servier; all outside the submitted work. R J Maude reports support for the present manuscript from Wellcome Trust. This research was supported in part by Wellcome Trust [Grant number 220211] as it provides core funding for Mahidol Oxford Tropical Medicine Research and contributes to his salary. He is required by Wellcome to acknowledge this grant in all publications. S A Meo reports grants or contracts from Ongoing Research Funding Program (ORF-2025-47), King Saud University, Riyadh, Saudi Arabia, outside the submitted work. T R Miller reports grants or contracts from AB InBev Foundation, National Institute of Mental Health (USA), Santa Clara County Public Health Department (California); payment for expert testimony from lawyers representing state & local plaintiffs in opioid litigation; all outside the submitted work. L Monasta reports support for the present manuscript from the Italian Ministry of Health (Ricerca Corrente 34/2017), payments made to the Institute for Maternal and Child Health IRCCS Burlo Garofolo. C E Moore reports participation on a Data Safety Monitoring Board or Advisory Board with Gwen Knight as Advisory Board member for her MRC grant on AMR, no payment made, with Leonid Chindelevitch as Advisory Board member for his UKRI grant on AMR, no payment made, with Wendy Thompson as Deputy Chair on the advisory board for the Tackling antimicrobial resistance across dentistry in Sub-Saharan Africa, travel claimed for meeting, and with Regional AMR Data Analysis for Advocacy, Response, and Policy (RADAAR) as Member of the Technical Advisory Group (TAG) of the IVI-led and Fleming Fund resourced project RADAAR Phase-2, no payment made; leadership or fiduciary roles in other board, society, committee or advocacy group, paid or unpaid, with Microbiology Society as Co-chair for the Impact and Influence committee and co-lead for the Knocking Out AMR project, travel claimed for meetings; all outside the submitted work. R d S Moreira reports grants or contracts from CNPq (National Council for Scientific and Technological Development) for the CNPq Research Productivity Scholarship (scholarship registration number 316607/2021-5), outside the submitted work. J F Mosser reports support for the present manuscript from the Gates Foundation via grant funding; grants or contracts from Gavi; payment or honoraria for lectures, presentations, speakers bureaus, manuscript writing or educational events from Providence Health & Services; support for attending meetings and/or travel from the Gates Foundation; all outside the submitted work. S Nomura reports support for the present manuscript from Ministry of Education, Culture, Sports, Science and Technology of Japan (24H00663) and Precursory Research for Embryonic Science and Technology from the Japan Science and Technology Agency (JPMJPR22R8). B Oancea reports support for the present manuscript from Ministry of Research, Innovation and Digitalization through the Core Program of the National Research, Development and Innovation Plan 2022- 2027, project no. PN 23-02-0101-Contract No. 7N/2023; PNRR/2022/C9/MCID/I8 project 760096. S Onie reports support for the present manuscript from National Health and Medical Research Council, Australia; consulting fees from World Health Organization for the amount of USD$9,000 from November 2023 to date; support for attending meetings and/or travel from Suicide Prevention Australia for travel and attendance fees for annual conference and International Association for Suicide Prevention for conference attendance fees; leadership or fiduciary roles in other board, society, committee or advocacy group, paid or unpaid, with International Association for Suicide Prevention as Vice President and Indonesian Association for Suicide Prevention as President; stock or stock options in Wellspring Indonesia, a local mental health clinic in Indonesia (not majority shareholder); all outside the submitted work. R Ornello reports consulting fees from Teva; payment or honoraria for lectures, presentations, speakers bureaus, manuscript writing or educational events from Novartis, Eli Lilly, Teva, AbbVie, Bayer, Pfizer, Lundbeck, Organon; support for attending meetings and/or travel from Teva and Novartis; participation on an Advisory Board with Eli Lilly and AbbVie; receipt of equipment, materials, drugs, medical writing, gifts or other services from Novartis; all outside the submitted work. A Ortiz reports grants or contracts from Sanofi paid to their institution The Fundación Jiménez Díaz Health Research Institute (IIS-FJD UAM) and as Director of the Catedra Astrazeneca-UAM of chronic kidney disease and electrolytes paid to their institution Universidad Autonoma de Madrid (UAM); consulting fees from Astellas, Astrazeneca, Bioporto, Boehringer Ingelheim, Fresenius Medical Care, GSK, Bayer, Sanofi- Genzyme, Lilly, Chiesi, Otsuka, Novo-Nordisk, and Sysmex; payment or honoraria for lectures, presentations, speakers bureaus, manuscript writing or educational events from Astellas, Astrazeneca, Bioporto, Boehringer Ingelheim, Fresenius Medical Care, GSK, Bayer, Sanofi- Genzyme, Sobi, Menarini, Lilly, Chiesi, Otsuka, Novo-Nordisk, Sysmex and Vifor Fresenius Medical Care Renal Pharma and Spafarma; support for attending meetings and/or travel from Astellas, Astrazeneca, Fresenius Medical Care, Boehringer- Ingelheim, Sanofi-Genzyme, Chiesi, Sobi, and Bayer; participation on a Data Safety Monitoring Board or Advisory Board with Astellas, Astrazeneca, Boehringer-Ingelheim, Fresenius Medical Care, Bayer, Sanofi-Genzyme, Chiesi, Otsuka, Novo Nordisk, and Sysmex; leadership or fiduciary roles in other board, society, committee or advocacy group, unpaid, with Council ERA. SOMANE; all outside the submitted work. P K Pal reports grants or contracts paid to their institution from Indian Council of Medical Research (ICMR), Department of Science & Technology (DST)-Science and Engineering Research Board, Department of Biotechnology (DBT), DST-Cognitive Science Research Initiative, Wellcome Trust UK-India Alliance DBT, PACE scheme of BIRAC, Michael J. Fox Foundation, SKAN (Scientific Knowledge for Ageing and Neurological ailments)-Research Trust; payment or honoraria for lectures, presentations, speakers bureaus, manuscript writing or educational events from the International Parkinson and Movement Disorder Society, and Movement Disorder Societies of Korea, Taiwan and Bangladesh, Japanese Society of Neurology, Teva Pharmaceutical Industries and Elsevier Inc (payment of one-thirds of the honorarium to their institute); support for attending meetings and/or travel from the National Institute of Mental Health and Neurosciences (NIMHANS), International Parkinson and Movement Disorder Society, and Movement Disorder Societies of Korea, Taiwan and Bangladesh, Japanese Society of Neurology and Asian Oceanian Congress of Neurology; leadership or fiduciary roles in other board, society, committee or advocacy group with Indian Academy of Neurology as Past President, Asian and Oceanian subsection of International Parkinson and Movement Disorder Society (MDS-AOS) as Past Secretary, Annals of Movement Disorders as Past Editor-in-Chief, the Parkinson Society of Karnataka as President, Infection Related Movement Disorders Study Group of MDS as Chair, Rare Movement Disorders Study Group of International Parkinson and Movement Disorder Society (IPMDS) as a Member, Education Committee of IAPRD as a Member, Rating Scales Education and Training Program Committee of IPMDS as a Member, Neurophysiology Study Group of IPMDS as a Member, Movement Disorders in Asia Study Group as a Member, Post-Stroke Movement Disorders as a Member, Ataxia Study Group of IPMDS as a Member, Ataxia Global Initiative as a Member, Movement Disorders Society of India as President, and the Education Committee of International Parkinson and Movement Disorder Society (IPMDS) as Chair—all unpaid posts except Annual Leadership stipend for 2023–2025, of which one-thirds to be paid to their institute; all outside the submitted work. R F Palma-Alvarez reports payment or honoraria for lectures, presentations, speakers bureaus, manuscript writing or educational events from Angelini, Casen Recordati, Lundbeck, Neuraxpharm, Rubió, Servier, and Takeda; support for attending meetings and/or travel from Angelini, Italfarmaco, Advanz Pharma, Takeda, and Lundbeck; all outside the submitted work. S K Panda reports support for the present manuscript from Siksha ‘O’ Anusandhan (Deemed to be University) via a salary; grants or contracts from File no. 17-59/2023-24/CCRH/Tech./Coll./ICMR- Diabetes/960] as co-investigator; all outside the submitted work. G D Panos reports support for attending meetings and/or travel (expenses covered without receiving direct payment) from Bayer Greece and Roche Hellas; all outside the submitted work. R Passera reports participation on a Data Safety Monitoring Board or Advisory Board with the Data Safety Monitoring Board dello studio “Consolidation with ADCT-402 (loncastuximab tesirine) after immunochemotherapy: a phase II study in BTKi- treated/ineligible Relapse/Refractory Mantle Cell Lymphoma (MCL) patients’ - FIL, Fondazione Italiana Linfomi, Alessandria (Italy), unpaid; leadership or fiduciary roles in other board, society, committee or advocacy group, paid or unpaid, with the EBMT Statistical Committee, European Society for Blood and Marrow Transplantation, Paris (France) as a member, and the IRB/IEC Comitato Etico AO SS. Antonio e Biagio Alessandria-ASL AL-VC (Italy) as a past Member (2020–2023); all outside the submitted work. A E Peden reports support for the present manuscript from the [Australian] National Health and Medical Research Council (Grant Number: APP2009306). V C F Pepito reports grants or contracts from Sanofi Consumer Healthcare to conduct studies on self-care in the Philippines, and Zuellig Family Foundation for writing manuscripts on health systems strengthening; all outside the submitted work. M A Piradov reports leadership or fiduciary roles in other board, society, committee, or advocacy group, paid or unpaid, with the Journal Annals of Clinical and Experimental Neurology as Editor-in-Chief, outside the submitted work. C D Pond reports grants or contracts paid to their university from Medical Research Futures Fund Australian Government (11 grants) and Department of Health and Ageing (1 grant); consulting fees from HNECC Primary Health Network for consulting on vertical integration project, Melbourne University for consulting on biomarkers project, Brain Health Collective for consulting on dementia, and Royal Australian College of General Practitioners for chairing research committee and related activities; payment or honoraria for lectures, presentations, speakers bureaus, manuscript writing or educational events from Dementia Training Australia and Melbourne University; support for attending meetings and/or travel from Royal Australian College of General Practitioners for travel related to role as chair of the Research Committee; leadership or fiduciary roles in other board, society, committee or advocacy group, paid or unpaid, with Research Foundation Board, RACGP as a Member; all outside the submitted work. S Rege reports leadership or fiduciary roles in other board, society, committee or advocacy group, paid or unpaid, with International Society for Pharmacoeconomics and Outcomes Research (ISPOR) Medication Adherence and Persistence (MAP) Special Interest Group (SIG) as Operational Lead, Editorial Board of Pharmacoepidemiology section within Frontiers in Pharmacology as Review Editor, PLOS ONE Editorial Board as Academic Editor, and Pain Management as Editorial Board Member; all outside the submitted work. L Ronfani reports support for the present manuscript from the Italian Ministry of Health (Ricerca Corrente 34/2017), payments made to the Institute for Maternal and Child Health IRCCS Burlo Garofolo. Y L Samodra reports grants or contracts from NSTC – Institute of Epidemiology and Preventive Medicine, NTU, Taiwan for a post-doctoral fellow contract; leadership or fiduciary roles in other board, society, committee or advocacy group, paid or unpaid, with Benang Merah Research Center, Indonesia as Co-Founder; other financial or non-financial interests with Jago Beasiswa (idebeasiswa.com) as a scholarship mentor; all outside the submitted work. V Sharma reports other financial or non-financial interests with DFSS (MHA)‘s research project (DFSS28(1)2019/EMR/6) at Institute of Forensic Science & Criminology, Panjab University, Chandigarh, India, outside the submitted work. J I Shin reports other financial or non-financial interests with Lee Youn Jae fellowship (JIS), outside the submitted work. V Shivarov reports patents planned, issued or pending with the Bulgarian Patent Office; other financial or non-financial interests with ICON plc in the form of a salary; all outside the submitted work. D D Silva reports grants or contracts from E2S|P.Porto, Porto, Portugal for contract as Adjunct Professor and CISA@LAQV|REQUIMTE for financial support as Integrated Researcher; payment or honoraria for lectures, presentations, speakers bureaus, manuscript writing or educational events from Faculty of Medicine of University of Porto, Portugal and Faculty of Pharmacy of University of Porto, Portugal; support for attending meetings and/or travel from E2S|P.Porto, Porto, Portugal and Erasmus+ Mobility; leadership or fiduciary roles in other board, society, committee or advocacy group, paid or unpaid, with the Portuguese Association of Forensic Sciences (APCF) as Directory Board Member; all outside the submitted work. J P Silva reports support for the present manuscript from Portuguese Foundation for Science and Technology for payment of a salary (contract with reference 2021.01789.CEECIND/CP1662/CT0014). L M L R Silva reports grants or contracts from SPRINT, Sport Physical Activity and Health Research e Innovation Center, Polytechnic of Guarda, 6300-559 6 Guarda, Portugal; and collaborate with RISE - UBI, Health Sciences Research Centre, University of Beira Interior, 6201-506 Covilhã, Portugal; all outside the submitted work. J A Singh reports consulting fees from ROMTech, Atheneum, Clearview Healthcare Partners, American College of Rheumatology, Yale, Hulio, Horizon Pharmaceuticals, DINORA, ANI/Exeltis, USA Inc., Frictionless Solutions, Schipher, Crealta/Horizon, Medisys, Fidia, PK Med, Two labs Inc., Adept Field Solutions, Clinical Care Options, Putnam Associates, FocusForward, Navigant Consulting, Spherix, MedIQ, Jupiter Life Science, UBM LLC, Trio Health, Medscape, WebMD, Practice Point Communications, and the National Institutes of Health; payment or honoraria for lectures, presentations, speakers bureaus, manuscript writing or educational events from Simply Speaking; support for attending meetings and/or travel from OMERACT, an international organization that develops measures for clinical trials and receives arm's length funding from 12 pharmaceutical companies, as past steering committee member to attend their meeting every 2 years; participation on a Data Safety Monitoring Board or Advisory Board with FDA Arthritis Advisory Committee (unpaid); leadership or fiduciary role in other board, society, committee or advocacy group, paid or unpaid as a past steering committee member of the OMERACT; stock or stock options in Atai Life Sciences, Kintara Therapeutics, Intelligent Biosolutions, Acumen Pharmaceutical, TPT Global Tech, Vaxart Pharmaceuticals, Atyu Biopharma, Adaptimmune Therapeutics, GeoVax Labs, Pieris Pharmaceuticals, Enzolytics Inc., Seres Therapeutics, Tonix Pharmaceuticals Holding Corp., Aebona Pharmaceuticals, and Charlotte's Web Holdings, Inc. and previously owned stock options in Amarin, Viking, and Moderna Pharmaceuticals; all outside the submitted work. S T Skou reports grants or contracts from European Union's Horizon 2020 research innovation program (payment to the hospital, grant agreement No 945377) and Region Zealand (payment to the hospital, program grant from Region Zealand (Exercise First)); royalties or licenses from Munksgaard for book chapters and TrustMe-Ed for online lecture; payment or honoraria for lectures, presentations, speakers bureaus, manuscript writing or educational events from Nestlé Health Science for presentation at webinar on osteoarthritis; other financial or non-financial interests as co-founder of GLA:D, ® a not-for profit initiative hosted at University of Southern Denmark aimed at implementing clinical guidelines for osteoarthritis in clinical practice; all outside the submitted work. J D Stanaway reports support for the present manuscript from Gates Foundation via grants to institution; grants or contracts paid to institution from Open Philanthropy and Novo Nordisk Foundation, outside the submitted work. D J Stein reports consultancy honoraria from Discovery Vitality, Kanna, L’Oreal, Lundbeck, Orion, Servier, Seaport Therapeutics, Takeda, and Wellcome, outside the submitted work. J Sundström reports direct or indirect stock ownership in companies (Anagram kommunikation AB, Sence Research AB, Symptoms Europe AB, MinForskning AB) providing services to companies and authorities in the health sector including Amgen, AstraZeneca, Bayer, Boehringer, Eli Lilly, Gilead, GSK, Göteborg University, Itrim, Ipsen, Janssen, Karolinska Institutet, LIF, Linköping University, Novo Nordisk, Parexel, Pfizer, Region Stockholm, Region Uppsala, Sanofi, STRAMA, Takeda, TLV, Uppsala University, Vifor Pharma, WeMind; all outside the submitted work. R Tabarés-Seisdedos reports grants or contracts from Valencian Regional Government's Ministry of Education (PROMETEO/CIPROM/2022/58) and the Spanish Ministry of Science, Innovation and Universities (PID2021-129099OB-I00). The funders were not involved in the design of the manuscript or decision to submit the manuscript for publication, nor will they be involved in any aspect of the study's conduct; all outside the submitted work. J H V Ticoalu reports leadership or fiduciary roles in other board, society, committee, or advocacy group, paid or unpaid, with Benang Merah Research Center, Indonesia as Co-Founder; all outside the submitted work. D Trico reports payment or honoraria for lectures, presentations, speakers bureaus, manuscript writing or educational events from AstraZeneca, Eli Lilly, and Novo Nordisk; patents planned, issued or pending with AstraZeneca; leadership or fiduciary roles in other board, society, committee or advocacy group, paid or unpaid, with EASD Early Career Academy and EASD Committee on Clinical Affairs; receipt of equipment, materials, drugs, medical writing, gifts or other services from Abbott and PharmaNutra; all outside the submitted work. S J Tromans reports grants or contracts paid to University of Leicester, their institution, as part of the 2023/4 Adult Psychiatric Morbidity Survey team, collecting epidemiological data on community-based adults living in England (a contracted study from NHS Digital, via the Department of Health and Social Care. Contributions on chapters of the 2023/4 Adult Psychiatric Morbidity Survey report), as lead on a study funded by the National Institute for Health and Care Research Clinical Research Network, on optimizing the survey design for people with learning disability and autistic people, as lead on a study from the National Institute for Health and Care Research related to reviewing a national training programme for health and social care professionals relating to learning disability and autism, and as co-applicant on study funded by the National Institute for Health and Care Research related to Identification, recording, and reasonable adjustments for people with a learning disability and autistic people in NHS electronic clinical record systems; support for attending meetings and/or travel from the Royal College of Psychiatrists for conference events due to their academic secretary role in the faculty of the Psychiatry of Intellectual Disability, and as event organizer and/or speaker; leadership or fiduciary roles in board, society, committee or advocacy groups, paid or unpaid as Academic Secretary for the Neurodevelopmental Psychiatry Special Interest Group and Psychiatry of Intellectual Disability Faculty at the Royal College of Psychiatrists, as Editorial Board Member for Progress in Neurology and Psychiatry, Advances in Mental Health and Intellectual Disability, Advances in Autism, BMC Psychiatry, and BJPsych Open, and as Editor of Psychiatry of Intellectual Disability Across Cultures (Oxford University Press) for which they received royalties; outside the submitted work. E Upadhyay reports patents planned, issued or pending for A system and method of reusable filters for anti-pollution mask (Published); a system and method for electricity generation through crop stubble by using microbial fuel cells (Published); A system for disposed personal protection equipment (PPE) into biofuel through pyrolysis and method (Published); A novel herbal pharmaceutical aid for formulation of gel and method thereof (Published); Herbal drug formulation for treating lung tissue degenerated by particulate matter exposure (Published); a method to transform cow dung into the wall paint by using natural materials and composition thereof (Filed); Biodegradable packaging composition and method of preparation thereof (Filed); Eco-friendly bio-shoe polish from banana and turmeric (Filed); Honey-based polyherbal syrup composition to treat air pollution-induced inflammation and preparation method thereof (Filed); Process for preparing a caffeine free, antioxidant and nutrient rich beverage (Filed); leadership or fiduciary roles in other board, society, committee or advocacy group, paid or unpaid, with Meteorological Society, Jaipur (India) as Executive Council Member, Indian Chapter and DSTPURSE Program as Member Secretary; all outside the submitted work. E Vounzoulaki reports grants or contracts from a National Institute for Health and Care Research (NIHR)(UK) Development and Skills Enhancement (DSE) Award until July 2026, outside the submitted work. P Willeit reports consulting fees from Novartis Pharmaceuticals, outside the submitted work. Y Yasufuku reports grants or contracts from Shionogi & Co., Ltd., paid from the joint research fund provided by this pharmaceutical company to The University of Osaka, outside the submitted work. S Zadey reports writing honoraria from Think Global Health and Hindu; leadership or fiduciary roles in other board, society, committee or advocacy group, paid or unpaid, with Association for Socially Applicable Research as Board Member, *Lancet* Citizens’ Commission on Reimagining India's Health System as Fellow, G4 Alliance Asia Working Group as Chair, Blood DESERT Coalition as Fellow, and Nivarana as Advisory Board Member; all outside the submitted work. J Zhao reports support for the present manuscript from Fundamental Research Funds for the Central Universities (2024BSSXM20). M Zielińska reports other financial or non-financial interests with Alexion, AstraZeneca Rare Disease as an employee, outside the submitted work. L J Zühlke reports grants or contracts from the Division of Research Capacity Development, Foreign Commonwealth and Development Office, UK, National Research Foundation of South Africa, South African Medical Research Council, (grant number Mid-Career Scientist Program, MR/S005242/1), and as Member of RHD Vaccine Advisory Committee of the LeDucq Foundation, outside the submitted work.

## References

[1] Murray CJL (2022). The Global Burden of Disease Study at 30 years. Nat Med.

[2] WHO (2025). World health statistics 2025: monitoring health for the SDGs, Sustainable Development Goals. https://iris.who.int/bitstream/handle/10665/381418/9789240110496-eng.pdf.

[3] Murray CJL, Vos T, Lozano R (2012). Disability-adjusted life years (DALYs) for 291 diseases and injuries in 21 regions, 1990–2010: a systematic analysis for the Global Burden of Disease Study 2010. Lancet.

[4] GBD 2015 DALYs and HALE Collaborators (2016). Global, regional, and national disability-adjusted life-years (DALYs) for 315 diseases and injuries and healthy life expectancy (HALE), 1990–2015: a systematic analysis for the Global Burden of Disease Study 2015. Lancet.

[5] GBD 2017 Disease and Injury Incidence and Prevalence Collaborators (2018). Global, regional, and national incidence, prevalence, and years lived with disability for 354 diseases and injuries for 195 countries and territories, 1990–2017: a systematic analysis for the Global Burden of Disease Study 2017. Lancet.

[6] GBD 2019 Diseases and Injuries Collaborators (2020). Global burden of 369 diseases and injuries in 204 countries and territories, 1990–2019: a systematic analysis for the Global Burden of Disease Study 2019. Lancet.

[7] GBD 2021 Diseases and Injuries Collaborators (2024). Global incidence, prevalence, years lived with disability (YLDs), disability-adjusted life-years (DALYs), and healthy life expectancy (HALE) for 371 diseases and injuries in 204 countries and territories and 811 subnational locations, 1990–2021: a systematic analysis for the Global Burden of Disease Study 2021. Lancet.

[8] Lim SS, Vos T, Flaxman AD (2012). A comparative risk assessment of burden of disease and injury attributable to 67 risk factors and risk factor clusters in 21 regions, 1990–2010: a systematic analysis for the Global Burden of Disease Study 2010. Lancet.

[9] GBD 2015 Risk Factors Collaborators (2016). Global, regional, and national comparative risk assessment of 79 behavioural, environmental and occupational, and metabolic risks or clusters of risks, 1990–2015: a systematic analysis for the Global Burden of Disease Study 2015. Lancet.

[10] GBD 2016 Risk Factors Collaborators (2017). Global, regional, and national comparative risk assessment of 84 behavioural, environmental and occupational, and metabolic risks or clusters of risks, 1990–2016: a systematic analysis for the Global Burden of Disease Study 2016. Lancet.

[11] GBD 2017 Risk Factor Collaborators (2018). Global, regional, and national comparative risk assessment of 84 behavioural, environmental and occupational, and metabolic risks or clusters of risks for 195 countries and territories, 1990–2017: a systematic analysis for the Global Burden of Disease Study 2017. Lancet.

[12] GBD 2019 Risk Factors Collaborators (2020). Global burden of 87 risk factors in 204 countries and territories, 1990–2019: a systematic analysis for the Global Burden of Disease Study 2019. Lancet.

[13] GBD 2021 Risk Factors Collaborators (2024). Global burden and strength of evidence for 88 risk factors in 204 countries and 811 subnational locations, 1990–2021: a systematic analysis for the Global Burden of Disease Study 2021. Lancet.

[14] Zheng P, Afshin A, Biryukov S (2022). The Burden of Proof studies: assessing the evidence of risk. Nat Med.

[15] Zheng P, Barber R, Sorensen RJD, Murray CJL, Aravkin AY (2021). Trimmed constrained mixed effects models: formulations and algorithms. J Comput Graph Stat.

[16] GBD 2023 Causes of Death Collaborators (2025). Global burden of 292 causes of death in 204 countries and territories and 660 subnational locations, 1990–2023: a systematic analysis for the Global Burden of Disease Study 2023. Lancet.

[17] Institute for Health Metrics and Evaluation (2024). Global Burden of Diseases, Injuries, and Risk Factors Study (GBD) Protocol. https://www.healthdata.org/sites/default/files/2024-06/GBD%20Protocol%20060424.pdf.

[19] Sullivan DF (1971). A single index of mortality and morbidity. HSMHA Health Rep.

[20] Murray CJ, Ezzati M, Lopez AD, Rodgers A, Vander Hoorn S (2003). Comparative quantification of health risks conceptual framework and methodological issues. Popul Health Metr.

[21] Murray CJ, Lopez AD (1997). Global mortality, disability, and the contribution of risk factors: Global Burden of Disease Study. Lancet.

[22] Dai X, Gil GF, Reitsma MB (2022). Health effects associated with smoking: a Burden of Proof study. Nat Med.

[23] Lescinsky H, Afshin A, Ashbaugh C (2022). Health effects associated with consumption of unprocessed red meat: a Burden of Proof study. Nat Med.

[24] Razo C, Welgan CA, Johnson CO (2022). Effects of elevated systolic blood pressure on ischemic heart disease: a Burden of Proof study. Nat Med.

[25] Stanaway JD, Afshin A, Ashbaugh C (2022). Health effects associated with vegetable consumption: a Burden of Proof study. Nat Med.

[26] Flor LS, Anderson JA, Ahmad N (2024). Health effects associated with exposure to secondhand smoke: a Burden of Proof study. Nat Med.

[27] Spencer CN, Khalil M, Herbert M (2023). Health effects associated with exposure to intimate partner violence against women and childhood sexual abuse: a burden of proof study. Nat Med.

[28] Stevens GA, Alkema L, Black RE (2016). Guidelines for Accurate and Transparent Health Estimates Reporting: the GATHER statement. Lancet.

[29] GBD 2021 Demographics Collaborators (2024). Global age-sex-specific mortality, life expectancy, and population estimates in 204 countries and territories and 811 subnational locations, 1950–2021, and the impact of the COVID-19 pandemic: a comprehensive demographic analysis for the Global Burden of Disease Study 2021. Lancet.

[30] GBD 2023 Demographics Collaborators (2025). Global age-sex-specific all-cause mortality and life expectancy estimates for 204 countries and territories and 660 subnational locations, 1950–2023: a demographic analysis for the Global Burden of Disease Study 2023. Lancet.

[31] Apeagyei AE, Bisignano C, Elliott H (2025). Tracking development assistance for health, 1990–2030: historical trends, recent cuts, and outlook. Lancet.

[32] Institute for Health Metrics and Evaluation (IHME) (2025).

[33] GBD 2021 US Obesity Forecasting Collaborators (2024). National-level and state-level prevalence of overweight and obesity among children, adolescents, and adults in the USA, 1990–2021, and forecasts up to 2050. Lancet.

[34] GBD 2021 Adult BMI Collaborators (2021). Global, regional, and national prevalence of adult overweight and obesity, 1990–2021, with forecasts to 2050: a forecasting study for the Global Burden of Disease Study. Lancet.

[35] GBD 2021 Adolescent BMI Collaborators (2025). Global, regional, and national prevalence of child and adolescent overweight and obesity, 1990–2021, with forecasts to 2050: a forecasting study for the Global Burden of Disease Study 2021. Lancet.

[36] Ong KL, Stafford LK, McLaughlin SA, the GBD 2021 Diabetes Collaborators (2023). Global, regional, and national burden of diabetes from 1990 to 2021, with projections of prevalence to 2050: a systematic analysis for the Global Burden of Disease Study 2021. Lancet.

[37] GBD 2023 Kidney Failure with Replacement Therapy Collaborators (2025). Global, regional, and national prevalence of kidney failure with replacement therapy and associated aetiologies, 1990–2023: a systematic analysis for the Global Burden of Disease Study 2023. Lancet Glob Health.

[38] Zaman S, Wasfy JH, Kapil V (2025). The *Lancet* Commission on rethinking coronary artery disease: moving from ischaemia to atheroma. Lancet.

[39] Marcus ME, Manne-Goehler J, Theilmann M (2022). Use of statins for the prevention of cardiovascular disease in 41 low-income and middle-income countries: a cross-sectional study of nationally representative, individual-level data. Lancet Glob Health.

[40] Roser P, Bajaj SS, Stanford FC (2022). International lack of equity in modern obesity therapy: the critical need for change in health policy. Int J Obes.

[41] Lincoff AM, Brown-Frandsen K, Colhoun HM (2023). Semaglutide and cardiovascular outcomes in obesity without diabetes. N Engl J Med.

[42] Jastreboff AM, Aronne LJ, Ahmad NN (2022). Tirzepatide once weekly for the treatment of obesity. N Engl J Med.

[43] WHO MPOWER. https://www.who.int/initiatives/mpower.

[44] Murphy T (March 11, 2004). Pliny the Elder's Natural History: the empire in the encyclopedia.

[45] McConnell JR, Chellman NJ, Plach A (2025). Pan-European atmospheric lead pollution, enhanced blood lead levels, and cognitive decline from Roman-era mining and smelting. Proc Natl Acad Sci USA.

[46] Huang X, Steinmetz J, Marsh EK (2025). A systematic review with a Burden of Proof meta-analysis of health effects of long-term ambient fine particulate matter (PM_2·5_) exposure on dementia. Nat Aging.

[47] GBD 2019 Diabetes and Air Pollution Collaborators (2022). Estimates, trends, and drivers of the global burden of type 2 diabetes attributable to PM_2·5_ air pollution, 1990–2019: an analysis of data from the Global Burden of Disease Study 2019. Lancet Planet Health.

[48] Semmens EO, Leary CS, Fitzpatrick AL (2023). Air pollution and dementia in older adults in the Ginkgo Evaluation of Memory Study. Alzheimers Dement.

[49] GBD 2021 HAP Collaborators (2025). Global, regional, and national burden of household air pollution, 1990–2021: a systematic analysis for the Global Burden of Disease Study 2021. Lancet.

[50] WHO (26 May 2025). Seventy-eighth World Health Assembly—daily update. https://www.who.int/news/item/26-05-2025-seventy-eighth-world-health-assembly—daily-update–26-may-2025.

[51] Blake JA, Sourander A, Kato A, Scott JG (2025). Will restricting the age of access to social media reduce mental illness in Australian youth?. Aust N Z J Psychiatry.

[52] Santomauro DF, Mantilla Herrera AM, Shadid J, the COVID-19 Mental Disorders Collaborators (2021). Global prevalence and burden of depressive and anxiety disorders in 204 countries and territories in 2020 due to the COVID-19 pandemic. Lancet.

[53] Haidt J (2024).

[54] Fassi L, Thomas K, Parry DA, Leyland-Craggs A, Ford TJ, Orben A (2024). Social media use and internalizing symptoms in clinical and community adolescent samples: a systematic review and meta-analysis. JAMA Pediatr.

[55] Women UN (Nov 25, 2024). Facts and figures: ending violence against women. https://www.unwomen.org/en/articles/facts-and-figures/facts-and-figures-ending-violence-against-women.

[56] Sardinha L, Maheu-Giroux M, Stöckl H, Meyer SR, García-Moreno C (2022). Global, regional, and national prevalence estimates of physical or sexual, or both, intimate partner violence against women in 2018. Lancet.

[57] The Lancet (2025). The demise of USAID: time to rethink foreign aid?. Lancet.

[58] Wu Y, Wulf Hanson S, Culbreth G (2024). Assessing the impact of health-care access on the severity of low back pain by country: a case study within the GBD framework. Lancet Rheumatol.

[59] Santomauro DF, Purcell C, Whiteford HA, Ferrari AJ, Vos T (2023). Grading disorder severity and averted burden by access to treatment within the GBD framework: a case study with anxiety disorders. Lancet Psychiatry.

[60] Yap Y-S, Lu Y-S, Tamura K (2019). Insights into breast cancer in the East *vs* the West: a review. JAMA Oncol.

