# Supplementary material for: Burden of 375 diseases and injuries, risk-attributable burden of 88 risk factors, and healthy life expectancy in 204 countries and territories, including 660 subnational locations, 1990–2023: a systematic analysis for the Global Burden of Disease Study 2023
Source: Lancet. 2025 Oct 18;406(10513):1873–922. doi: 10.1016/S0140-6736(25)01637-X (PMC12535840; doi:10.1016/S0140-6736(25)01637-X)
Supplement: Supplementary appendix 1 [file mmc1.pdf]

THE LANCET

Supplementary appendix 1

This appendix formed part of the original submission and has been peer reviewed. We post it as supplied by the authors.

Supplement to: GBD 2023 Disease and Injury and Risk Factor Collaborators. Burden of 375 diseases and injuries, risk-attributable burden of 88 risk factors, and healthy life expectancy in 204 countries and territories, including 660 subnational locations, 1990–2023: a systematic analysis for the Global Burden of Disease Study 2023. *Lancet* 2025; published online Oct 12. https://doi.org/10.1016/S0140-6736(25)01637-X.

Appendix 1: Non-fatal methods appendix to "Burden of 375 diseases and injuries, risk-attributable burden of 88 risk factors, and healthy life expectancy in 204 countries and territories, including 660 subnational locations, 1990 – 2023: a systematic analysis for the Global Burden of Disease Study 2023"

Preamble

This appendix provides further methodological detail for "Burden of 375 diseases and injuries, risk-attributable burden of 88 risk factors, and healthy life expectancy in 204 countries and territories, including 660 subnational locations, 1990 – 2023: a systematic analysis for the Global Burden of Disease Study 2023". This study complies with the Guidelines for Accurate and Transparent Health Estimates Reporting (GATHER) recommendations.[1] It includes detailed tables and information on data in an effort to maximise transparency in our estimation processes and provide a comprehensive description of analytical steps. We intend this appendix to be a living document, to be updated with each iteration of the Global Burden of Disease Study.

Portions of this appendix have been reproduced or adapted from appendices for Lim et al 2012,[2] GBD 2015 Disease and Injury Incidence and Prevalence Collaborators,[3] GBD 2016 Disease and Injury Incidence and Prevalence Collaborators,[4] GBD 2017 Disease and Injury Incidence and Prevalence Collaborators,[5] GBD 2019 Diseases and Injuries Collaborators,[6] and GBD 2021 Diseases and Injuries Collaborators.[7] References are provided for reproduced or adapted sections.

Table of Contents

Preamble **2**

Table of Contents **3**

List of methods appendix figures and tables **5**

Section 1: GBD Overview **6**

Section 1.1: Global Burden of Diseases, Injuries, and Risk Factors Study 2023 6

Section 1.2: Geographical locations of the analysis 8

Section 1.3: Statement of GATHER compliance 8

Section 1.4: GBD cause hierarchy 8

Section 1.5: Data input sources overview 9

Section 1.6: Funding sources 9

Section 1.7: Abbreviations 10

Section 2: Non-fatal outcome estimation **11**

Section 2.1: Data sources, identification, and extraction 11

Section 2.1.1: Systematic reviews 12

Section 2.1.2: Survey data preparation 12

Section 2.1.3: Disease registries 13

Section 2.1.4: Case notifications 13

Section 2.2: Clinical data sources and methods summary 13

Section 2.2.1: Mapping diagnoses to GBD diseases and injuries 13

Section 2.2.2 Individual-level clinical data sources with unique identifiers in GBD 2023 13

Section 2.2.3: Aggregate inpatient hospital admission sources used in GBD 2023 18

Section 2.2.4: Estimation of the inpatient utilization envelope 19

Section 2.2.5: Correction Factors 22

Section 2.2.6: Outpatient encounter data 28

Section 2.2.7: New data added for GBD 2023 29

Section 2.3 Data Adjustments 30

Section 2.3.1: Crosswalking 30

Section 2.3.4 Network Analysis 33

Section 2.3.5 Age sex splitting 34

Section 2.4: Spatiotemporal Gaussian process regression (ST-GPR) modelling 34

Section 2.4.1 Estimating mean functions 35

Section 2.4.1: Estimating error variance 37
Section 2.4.2: Estimating the covariance function 38
Section 2.4.3: Prediction using GPR 39
Section 2.4.4: Subnational scaling and aggregation 39
Section 2.5: MR-BRT meta-regression modelling 39
Section 2.5.1 MR-BRT Overview 39
Section 2.5.2 MR-BRT Formula 39
Section 2.5.3 MR-BRT Features 40
Section 2.6: DisMod-MR 2.1 estimation 41
Section 2.6.1: Estimation of sequelae and causes 41
Section 2.6.2: DisMod-MR 2.1 description 41
Section 2.6.3: DisMod-MR 2.1 likelihood estimation 46
Section 2.7: DisMod-AT estimation 47
Section 2.8: Impairment and underlying cause estimation 50
Section 2.8.1: Impairment squeeze 51
Section 2.9: Disability weights 53
Section 2.9.1: GBD 2010 Disability Weights Measurement Study 54
Section 2.9.2: GBD 2013 European disability weights measurement study 55
Section 2.10: Comorbidity correction (COMO) 56
Section 2.11: YLD computation, uncertainty, and residual YLDs 58
Section 2.11.1: Residual YLDs 58
Section 2.12: Birth prevalence 58
Section 3: SDI 59
Section 3.1: SDI definition 59
Section 3.2: Development of revised SDI indicator 59
Section 4: Estimation process for DALYs 61
Section 5: HALE 61
References 62
Section 6: Non-fatal cause-specific modelling descriptions 64
GBD 2023 non-fatal appendix write-ups in order 64

List of methods appendix figures and tables

All supplementary tables can be accessed here:
https://ghdx.healthdata.org/record/ihme-data/gbd-2023-yld-daly-hale-risk-1990-2023

Appendix Figures

Figure S1: Number of countries and territories with non-fatal data by disease group for a given year by GBD region

Figure S2: Number of countries and territories with non-fatal data for a given year by GBD region

Figure S3: Processing steps for individual-level clinical data sources with unique identifiers

Figure S4: Merative MarketScan Research Databases Enrollee Population by Year

Figure S5: Medicare Part A Enrollee Population by Year

Figure S6: Medicare Part A+B Enrollee Population by Year from 5% Enhanced Sample

Figure S7: Poland Enrollee Population by Year

Figure S8: Processing steps for aggregate inpatient hospital admission sources

Figure S9: Overview process of estimation of hospital utilization envelope

Figure S10: Overview of Correction Factor process

Figure S11: Readmissions correction model in linear space for acute encephalitis

Figure S12: Non-primary correction model in linear space for acute encephalitis

Figure S13: Non-primary and outpatient correction model in linear space for acute encephalitis

Figure S14: Processing steps for outpatient encounter data

Figure S15: System of differential equations that captures relationships between rates and compartments

Figure S16: DisMod-MR 2.1 sequence of estimation cascade

Figure S17: SF-12 composite scores and disability weights for 60 health states with fitted loess regression

Figure S18: DALY burden estimation for GBD 2023

Appendix Tables

Table S1: GBD 2023 location hierarchy with levels

Table S2: GATHER checklist of information that should be included in reports of global health estimates, with description of compliance and location of information for the Global Burden of Diseases, Injuries, and Risk Factors study 2023.

Table S3: GBD 2023 cause hierarchy with levels

Table S4: Clinical inpatient sources requiring the envelope adjustment

Table S5: Inclusion and exclusion criteria for data sources used in the inpatient utilization envelope model

Table S6a: Data aggregates that go into correction factor ratios with various data processing steps applied

Table S6b: Ratios for correction factor models

Table S7: How correction factors are applied to create various incidence and prevalence estimates

Table S8: Source-years of clinical data added for GBD 2023

Table S9: GBD 2023 sequelae, health states, health state lay descriptions, and disability weights

Table S10: GBD 2023 methods of estimating years lived with disability (YLDs) for 34 residual categories

Table S11: List of GBD 2023 non-fatal causes with prevalence at birth

Table S12: GBD 2023 Socio-Demographics Index groupings by location

Table S13: List of International Classification of Diseases (ICD) codes mapped to non-fatal causes and injuries in GBD 2023

Table S14: Source counts by cause for GBD 2023

Section 1: GBD Overview

Section 1.1: Global Burden of Diseases, Injuries, and Risk Factors Study 2023

The Global Burden of Diseases, Injuries, and Risk Factors Study (GBD) is a collaborative research effort aimed at estimating morbidity and mortality from a comprehensive set of diseases, injuries, and risk factors. The GBD Collaborator Network draws on the expertise of over 14,000 contributors from around the world. For this paper, we estimated numbers and rates of incidence, prevalence, years lived with disability (YLDs), and disability-adjusted life-years (DALYs) for the years 1990–2023; we estimated deaths and years of life lost (YLLs) for 1980–2023 by age, sex, and location.

To reduce computing power and time, we reduced the number of draws (or computations) per process to 250, from 500 in GBD 2021. Based on simulation testing, we determined that a change in the number of draws did not impact final mean estimates, nor lead to inappropriately narrow uncertainty estimates.

To help assess the impact of reduced draws, we conducted the following experiment. We first generated 1000 samples from a random variable $X \sim N(30, 10^2)$ to be used as our "true" distribution. For each *n* depicted in the plot below, we subset *n* samples with which we calculated the sample standard deviation. We then estimated a 95% uncertainty interval $30 \pm 1.96s$ and computed the percent of the "true" distribution covered by that 95% uncertainty interval. For each *n*, these steps were conducted 1000 times. The distribution of coverages from those 1000 simulations for each number of draws is shown below in green:

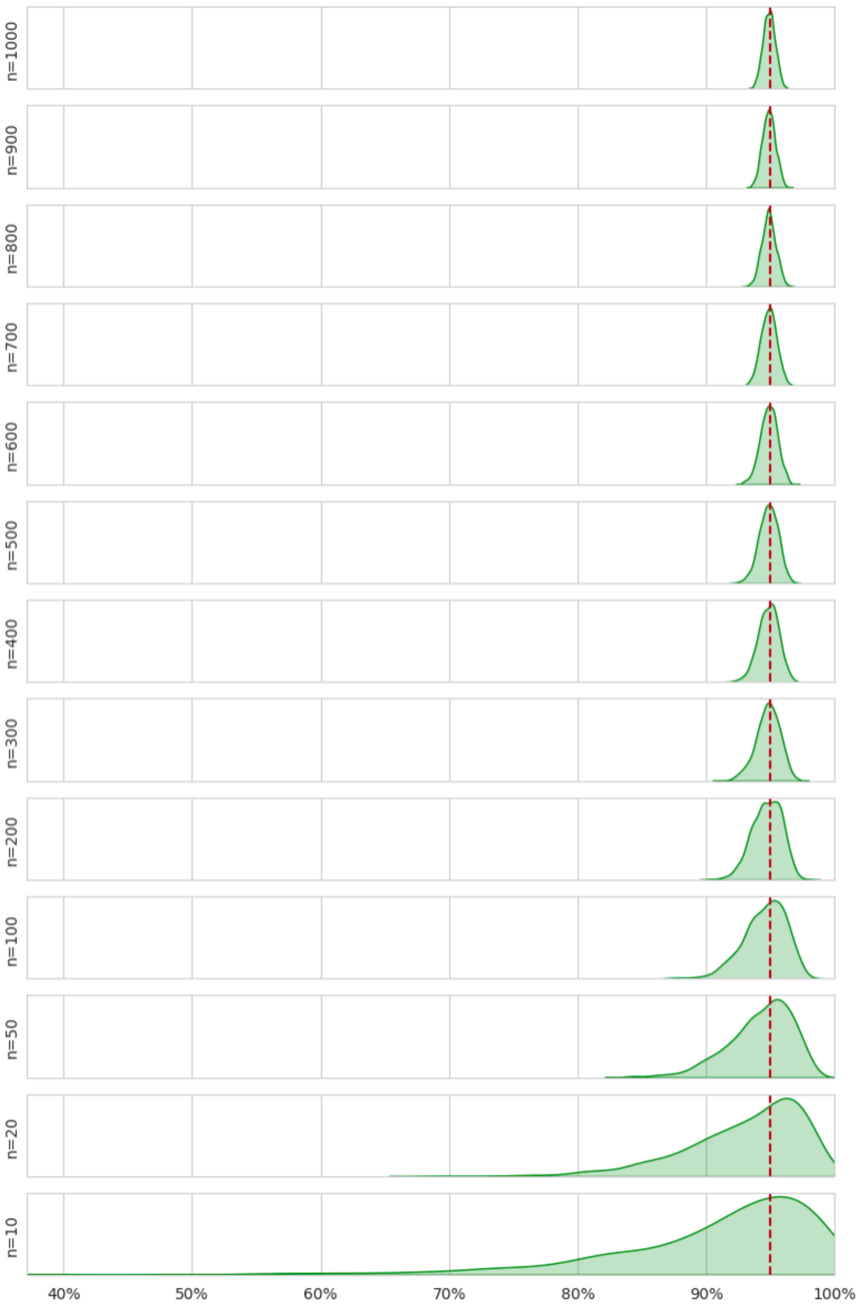

Section 1.2: Geographical locations of the analysis

We produced estimates for 204 countries and territories that were grouped into 21 regions and seven super regions (table 1). The seven super-regions are central Europe, eastern Europe, and central Asia; high income; Latin America and the Caribbean; north Africa and the Middle East; south Asia; southeast Asia, east Asia, and Oceania; and sub-Saharan Africa. In GBD 2023, we continue to analyse at subnational levels countries that were added in previous cycles, including Brazil, China, Ethiopia, India, Indonesia, Iran, Italy, Japan, Kenya, Mexico, New Zealand, Nigeria, Norway, Pakistan, Russia, the Philippines, Poland, South Africa, and the USA. All analyses are at the first level of administrative organisation within each country except for New Zealand (by Māori ethnicity), and the Philippines (by provinces). To meet data use requirements, in this publication we present subnational estimates for Brazil, India, Indonesia, Japan, Kenya, Mexico, and the USA; given space constraints, these results are presented in Appendix 3 instead of the main text. Subnational estimates for China are included in maps but are not reported in appendix tables. Subnational estimates for other countries will be released in separate publications, although please note that we only release estimates for a subset of these countries, per agreements with country partners.

At the most detailed spatial resolution, we generated estimates for 843 unique locations. As was done in GBD 2021, in GBD 2023, we continue to use the set of locations defined as standard locations and non-standard locations. Standard GBD locations are defined as the set of all subnationals belonging to countries where data quality is high and with populations over 200 million, in addition to all other countries. Standard locations include the subnationals for China, India, the USA, and Brazil, but not Indonesia; data for China, India, the USA, and Brazil are also included at the country level. All other countries with subnational estimates are defined as non-standard locations.

Section 1.3: Statement of GATHER compliance

This study complies with the Guidelines for Accurate and Transparent Health Estimates Reporting (GATHER) recommendations.[1] We have documented the steps involved in our analytical procedures and detailed the data sources used. See table 2 for the GATHER checklist. The GATHER recommendations may be found here: http://gather-statement.org/.

Section 1.4: GBD cause hierarchy

The GBD cause and sequelae list is organized hierarchically (see table 3) to accommodate different purposes and needs of various users.

The first two levels aggregate causes into general groupings. At Level 1 there are three cause groups: communicable, maternal, neonatal, and nutritional diseases (Group 1 diseases); non-communicable diseases (Group 2); and injuries (Group 3). These Level 1 aggregates are subdivided at Level 2 of the hierarchy into 22 cause groupings (e.g., neonatal disorders, neurological disorders, and transport injuries). The disaggregation into Levels 3 and 4 contains the finest level of detail for causes captured in GBD 2023. The greatest detail available for some causes, such as anxiety disorders or rheumatoid arthritis, is at Level 3 of the hierarchy, while other specific causes are at Level 4 of the hierarchy with an aggregate category at Level 3 (for example, depressive disorders at Level 3, which encompasses major depressive disorders and dysthymia at Level 4). Sequelae of diseases and injuries are organised at Levels

5 and 6 of the hierarchy. In GBD, sequelae are defined as distinct, mutually exclusive categories of health consequences that can be directly attributed to a cause. For example, both neuropathy and blindness due to diabetic retinopathy are sequelae of diabetes; stroke and ischaemic heart disease are not, as these consequences cannot be categorically ascribed to diabetes in an individual despite good evidence for increased risk of these outcomes. The finest detail for all sequelae estimated in GBD is at Level 6 and is aggregated into summary sequelae categories (Level 5) for causes with large numbers of sequelae. Examples include the grouping of the infectious disease episodes and long-term sequelae of meningitis. For GBD 2023 there are 3704 mutually exclusive and collectively exhaustive sequela, 2106 cause sequelae and 1598 injuries sequelae, and thus our YLD estimates at each level of the hierarchy sum to the total of the level above. Prevalence and incidence aggregation is estimated at the level of individuals who may have more than one sequela or disease and therefore are not additive.

The GBD cause list continues to evolve to reflect the policy relevance, and public health and medical care importance of the causes of major losses of health. The cause and sequelae list expanded based on input from the Scientific Council and GBD collaborator network. For GBD 2023, the causes of death cause list has increased to 292 causes, from the 288 causes in GBD 2021. The non-fatal cause list has expanded from 365 causes in GBD 2021 to 370 causes in GBD 2023. The total number of fatal and non-fatal causes combined for GBD 2023 is 375. As in GBD 2021, we made no estimates for YLDs for just five causes, either because no disability is possible (as is the case with sudden infant death syndrome); because disability may occur rarely but at levels too low for accurate estimation given the data (as for aortic aneurysm); or because the disability is captured by the complicating causes that led to that cause of death (as for indirect maternal deaths, late maternal deaths, and maternal deaths aggravated by HIV/AIDS).

Section 1.5: Data input sources overview

GBD 2023 synthesises a large and growing number of data input sources including surveys, censuses, vital statistics, and other health-related data sources. The data from these sources are used to estimate morbidity; illness, and injury; and attributable risk for 204 countries and territories from 1990 to 2023; mortality deaths are estimated from 1980 to 2023. The input sources are accessible through an interactive citation tool available in the GHDx.

Citations for specific GBD components, causes and risks, and locations can be found through the Data Input Sources Tool in GHDx: http://ghdx.healthdata.org/gbd/2020/data-input-sources. This tool allows users to view and access GHDx records for input sources and export a comma-separated value (CSV) file that includes metadata, citations, and information about where the data were used in GBD. As required by GATHER, additional metadata for input sources are available through the citation tool as well.

Section 1.6: Funding sources

This publication and the research it presents was funded by the Gates Foundation (award OPP1152504); Queensland Department of Health, Australia; the New Zealand Ministry of Health; the Norwegian Institute of Public Health; St. Jude Children's Research Hospital; UK Department of Health and Social Care; and Bloomberg Philanthropies. The funders of the study had no role in study design, data collection, data analysis, data interpretation, or writing of the report. All authors had full access to all data in the study and had final responsibility for the decision to submit for publication.

Section 1.7: Abbreviations

ARC	annualized rate of change
ASFR	age-specific fertility rate
ACMR	all-cause mortality rate
BMI	Body Mass Index
CMNN	Communicable, maternal, neonatal, and nutritional diseases
CoD	causes of death
CODEm	Cause of Death Ensemble modelling
COMO	comorbidity correction
COPD	Chronic obstructive pulmonary disease
CSMR	cause-specific mortality rates
CV	coefficient of variation
DALYs	disability-adjusted life-years
DisMod-AT	disease model-Bayesian age-time
DisMod-MR	disease model-Bayesian meta-regression
DW	disability weight
EDU15+	education for those 15 years old and older
EMR	excess mortality rate
GATHER	Guidelines for Accurate and Transparent Health Estimates Reporting
GBD	Global Burden of Diseases, Injuries, and Risk Factors Study
GHDx	Global Health Data Exchange
GPR	Gaussian process regression
HALE	healthy life expectancy
HAT	human African trypanosomiasis
ICD	International Classification of Diseases
ICG	ICD groups
IFD	in-facility delivery proportion
IHME	Institute for Health Metrics and Evaluation
LASSO	least absolute shrinkage and selection operator
LDI	lag-distributed income
LOESS	locally estimated scatterplot smoothing
MAD	median absolute deviation
MCCD	Medical Certification of Causes of Death
MEPS	Medical Expenditure Panel Survey
MICS	Multiple Indicators Survey
MR-BRT	Meta-regression—Bayesian, regularised, trimmed
NESARC	National Epidemiologic Survey on Alcohol and Related Conditions
NSMHWB	Australian National Survey of Mental Health and Wellbeing
NTDs	neglected tropical diseases
RSME	root mean square error
SARS-CoV 2	Severe acute respiratory syndrome coronavirus 2
SD	Standard deviation
SID HCUP	State Inpatient Database
SDI	Social Demographic Index
ST-GPR	spatiotemporal Gaussian process regression
TFR	total fertility rate
TFU25	younger than 25 years old (fertility rate)

UI uncertainty interval
UK United Kingdom
UI uncertainty interval
USA United States of America
WHO World Health Organization
YLDs years lived with disability
YLLs years of life lost

Section 2: Non-fatal outcome estimation

The GBD 2023 non-fatal estimation process describes the steps necessary to estimate incidence, prevalence, and YLDs for disease and injury sequelae in GBD 2023. Conceptually, the estimation effort is divided into eight major components: (1) compiling data sources through data identification and extraction; (2) data adjustments; (3) estimation of prevalence and incidence by cause and sequelae by using DisMod-MR 2.1, or alternative modelling strategies for select cause groups; (4) estimation by impairment; (5) severity distributions; (6) incorporation of disability weights (DWs); (7) comorbidity adjustment; and (8) the estimation of YLDs by sequelae and causes. Section 6 contains additional detail specific to each non-fatal disease, impairment, and injury, and their sequelae. Non-fatal modelling strategies vary significantly between causes.

Section 2.1: Data sources, identification, and extraction

Figure S1 is a map showing the number of countries and territories with non-fatal data for a given year, aggregated by GBD Region. We aggregate all “Communicable, maternal, neonatal, and nutritional diseases” in red, “Non-communicable diseases” in blue, and “Injuries” in green.

Figure S1. Number of countries and territories with non-fatal data by disease group for a given year by GBD region

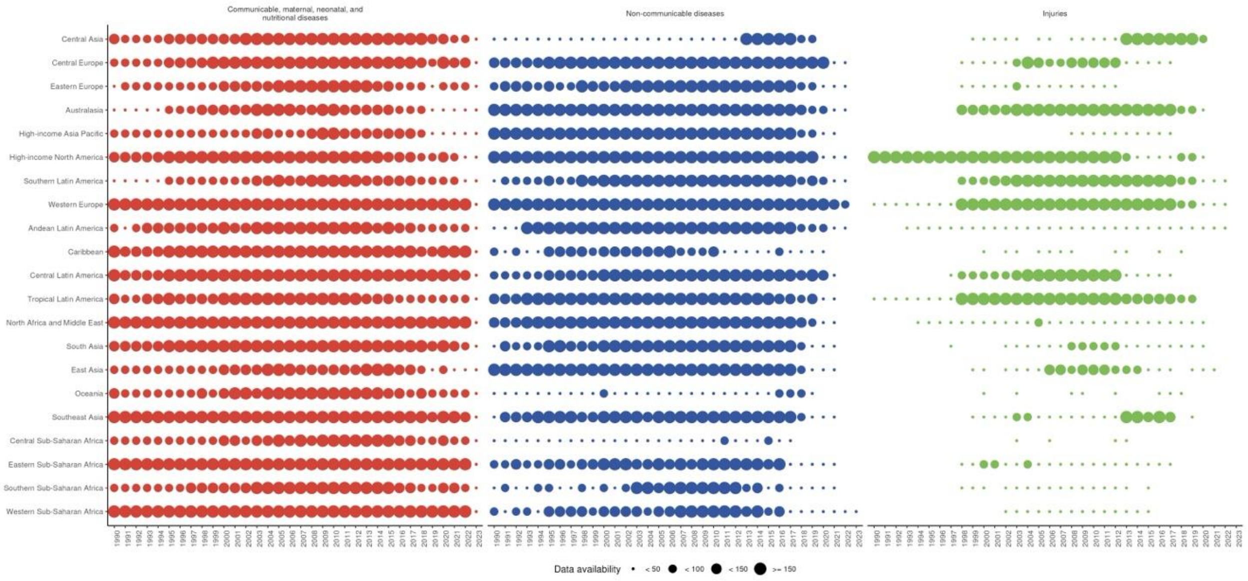

Figure S2 shows a map showing the number of countries and territories with non-fatal data for a given year, aggregated by GBD Region, across all the estimated causes in the GBD.

Figure S2. Number of countries and territories with non-fatal data for a given year by GBD region

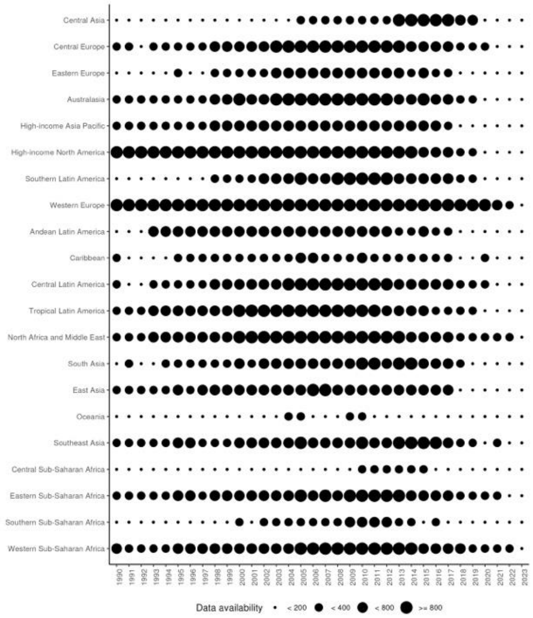

Section 2.1.1: Systematic reviews

For GBD 2023, updated systematic reviews were conducted for 122 causes and risk factors. For other disease sequelae, only a small fraction of the existing data appears in the published literature, and other sources predominate, such as survey data, disease registers, notification data, or hospital inpatient data. As was done in past rounds of GBD, data were systematically screened from household surveys archived in the GHDx (http://ghdx.healthdata.org/), including Demographic and Health Surveys, Multiple Indicator Cluster Surveys (MICS), Living Standards Measurement Surveys, and Reproductive Health Surveys. Other national health surveys were identified on the basis of survey series that had yielded usable data for past rounds of GBD, sources suggested to us by in-country GBD collaborators, and surveys identified in major multinational survey data catalogues such as the International Household Survey Network and the WHO Central Data Catalog, as well as through country Ministry of Health and Central Statistical Office websites. Citations for all data sources used for non-fatal estimation in GBD 2023 are provided in searchable form through a web tool (http://ghdx.healthdata.org/). A description of the search terms used for cause-specific systematic reviews are detailed by cause in Section 6.

Section 2.1.2: Survey data preparation

For GBD 2023, survey data for which we have access to the unit record data constitutes a substantial part of the underlying data used in the estimation process. During extraction, we concentrated on demographic variables (eg, location, sex, age), survey design variables (eg, sampling strategy and sampling weights), and the variables used to define the population estimate (eg, prevalence or a

proportion) and a measure of uncertainty (standard error, confidence interval or sample size, and number of cases).

Section 2.1.3: Disease registries

For GBD 2023 non-fatal estimation, disease registries were an important source for a select number of conditions such as cancers, end-stage renal disease, and congenital disorders.
Registry data is particularly key in the estimation of neoplasms when we consider the increasing attention to non-communicable diseases, particularly cancers, in low and middle-income areas of the world. The GHDx source tool (http://ghdx.healthdata.org/data-type/disease-registry) provides a comprehensive list of registry data used in GBD estimation processes.

Section 2.1.4: Case notifications

Case notifications, active screening, intervention coverage studies, and surveillance contributed to estimates of infectious diseases. If data were available, we extracted it from survey and administrative microdata; otherwise, data were extracted from published literature and reports. For many infectious diseases and neglected tropical diseases (NTDs), we used cases for which notification was made by countries to the WHO and other global monitoring entities. The causes for which we used WHO case notification data included tuberculosis, measles, yellow fever, rabies, dengue, cholera, whooping cough, human African trypanosomiasis (HAT), meningitis, and other infectious diseases and NTDs, such as Ebola.

Section 2.2: Clinical data sources and methods summary

Clinical data derived from electronic health records (EHRs) played a key role in the estimation of many non-fatal diseases, impairments, and injuries in GBD 2023. Data sources were heterogeneous in granularity, comprehensiveness, and level of detail, and the methods described below were used to transform data to be comparable and complete across locations, ages, sexes, years, and causes modelled in the GBD.

Section 2.2.1: Mapping diagnoses to GBD diseases and injuries

Most clinical sources are coded using the International Classification of Diseases (ICD) system that we map to GBD-defined diagnosis groups. ICD-9 and ICD-10 codes are mapped to what are termed "ICD code groups" (ICGs) with a many-to-one relationship, which simplifies the disease categorization and reduces complexity. ICGs are then mapped to a disease or injury modelling entity used by GBD modelers.

Mapping of ICD codes is not exhaustive as some causes in the GBD cause hierarchy do not use EHR data. We designate whether each modelling entity is processed in terms of incidence or prevalence, depending on the nature of the disease and the expected pattern of treatment. Table 13 shows the ICD codes used for non-fatal modelling by GBD cause, impairment, and injury.

Section 2.2.2 Individual-level clinical data sources with unique identifiers in GBD 2023

Figure S3. Processing steps for individual-level clinical data sources with unique identifiers

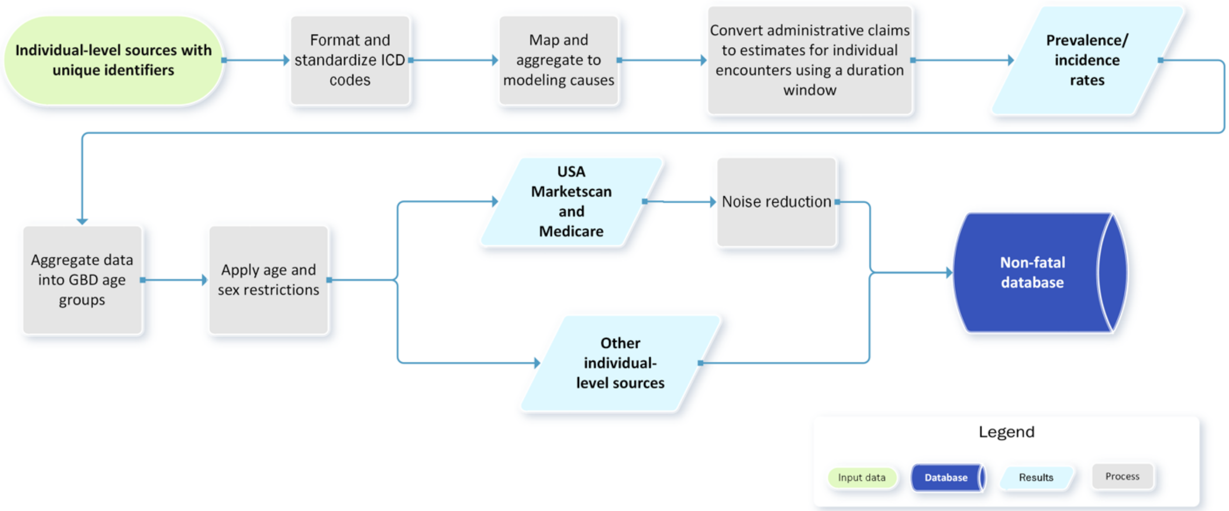

Poland National Health Fund Patient claims, United States Merative MarketScan Research Database claims, and United States Medicare claims were processed. These sources contain every health service encounter for the populations they covered, including hospital inpatient records, with same-day inpatient setting visits and overnight admissions, and outpatient, with general practitioner, emergency department (or urgent care), and specialty visits. We designate which health services each modelling entity requires data for, including, for example, inpatient only or inpatient *and* outpatient data. We mapped encounter-level data to ICD diagnoses to the diseases, impairments, and injuries modelled in the GBD. GBD causes are processed as "prevalence" or "incidence" based on the specification of the research team responsible for modelling the cause. Prevalent conditions are identified as any primary or non-primary diagnosis on any inpatient or outpatient claim within the year of interest. To reduce noise from spurious coding practices, a minimum of two outpatient claims for the same individual are required in a calendar year to count as a prevalent case. Incidence of a disease, impairment, or injury was calculated based on a duration window which varied by cause. Any individual who had multiple diagnoses for the same condition within the duration window is counted as a single incident case, and additional diagnoses outside of the duration window are treated as new incident cases.

After mapping to cause and identifying prevalent and incident cases by cause, for USA MarketScan and Medicare sources, we applied a noise reduction Poisson model to smooth trends over age and time and ensure compliance with data usage agreements by sharing modelled rates. More detail on the populations covered by each source is outlined below.

United States Merative MarketScan Research Database claims

For GBD 2023, we accessed data derived from the Truven database of USA private health insurance and Medicare private supplemental insurance for the years 2000 and 2010-2019.

Figure S4. Merative MarketScan Research Databases Enrollee Population by Year

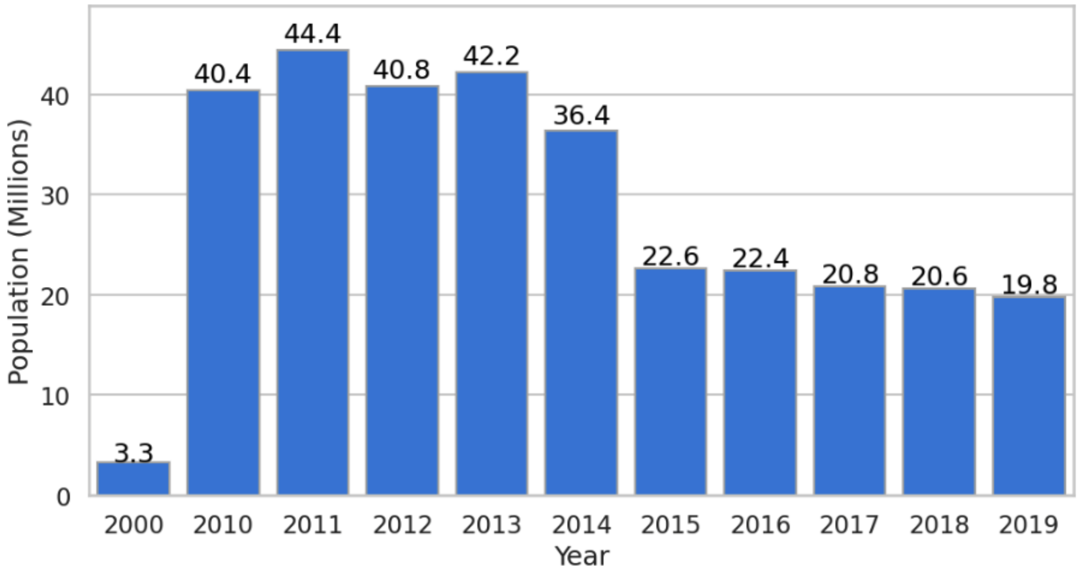

For each of these individuals, claims representing every health service encounter were used and all episodes of care were linked to individuals by unique identifiers. For the GBD, we subset the population in the MarketScan database to individuals with a full year of insurance coverage or those who were born or died in the year of interest, to ensure the sample includes all healthcare utilization for a given individual in that year.

United States Medicare claims

Medicare data from the Centers for Medicare and Medicaid Services (CMS), provided by the Research Data Assistance Center (ResDAC), was added for GBD 2023. Medicare is a federal health insurance program for people aged 65 and older, but also younger individuals with specific eligible conditions. Only data for enrollees 65 and older were processed for years 2000, 2010, and 2014-2016. Specific information on insurance eligibility by month allowed us to produce denominators of enrollees that can be a fraction of the year and calculated as enrollee-months. For GBD conditions that use inpatient-only data in their models, claims for Part A enrollees were used (figure S5) and notably does not include enrollees and their claims from Part C (also known as Medicare Advantage). For GBD causes that use data for both inpatient stays and outpatient encounters, a representative 5% sample of enrollees (figure S5) and their Part B claims from Medicare Carrier files were used, which include physician services and outpatient care. Claims for Part C enrollees were also not available for the 5% sample.

Figure S5. Medicare Part A Enrollee Population by Year

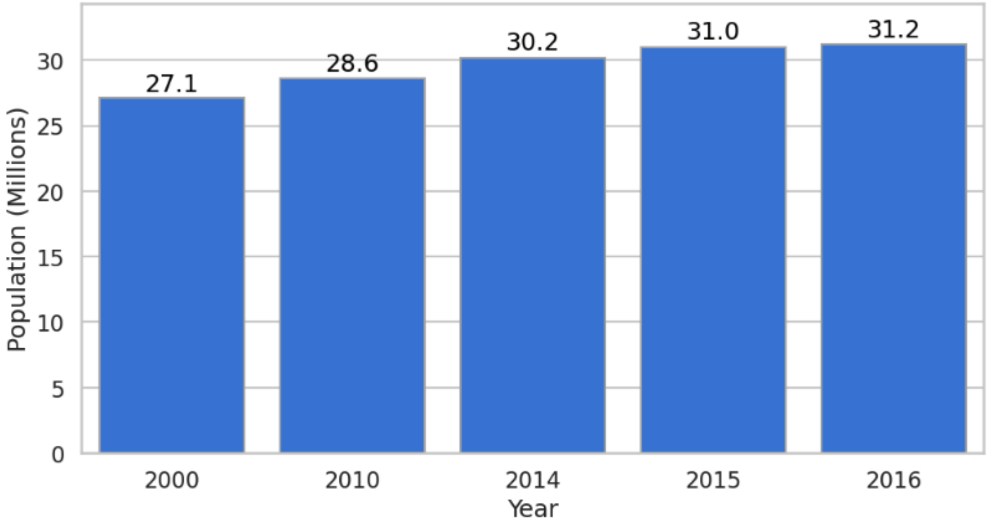

Figure S6. Medicare Part A+B Enrollee Population by Year from 5% Enhanced Sample

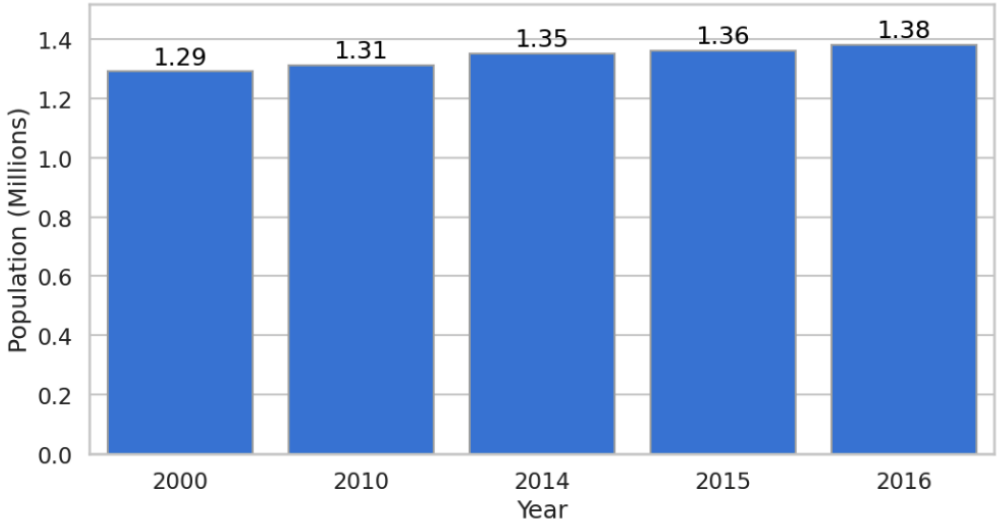

Poland National Health Fund Patient claims

Anonymized, individual-level claims data from Poland were accessed through an existing collaboration and institutional partnership with the Agency for Health Technology Assessment and Tariff System (AOTMiT). The data is derived from the National Health Fund (Narodowy Fundusz Zdrowia) database in Poland and is representative of every service encounter and episode of care in the public health care system (close to 92% population coverage) from 2015 to 2019, and for each region (voivodeship) in the country.

Figure S7. Poland Enrollee Population by Year

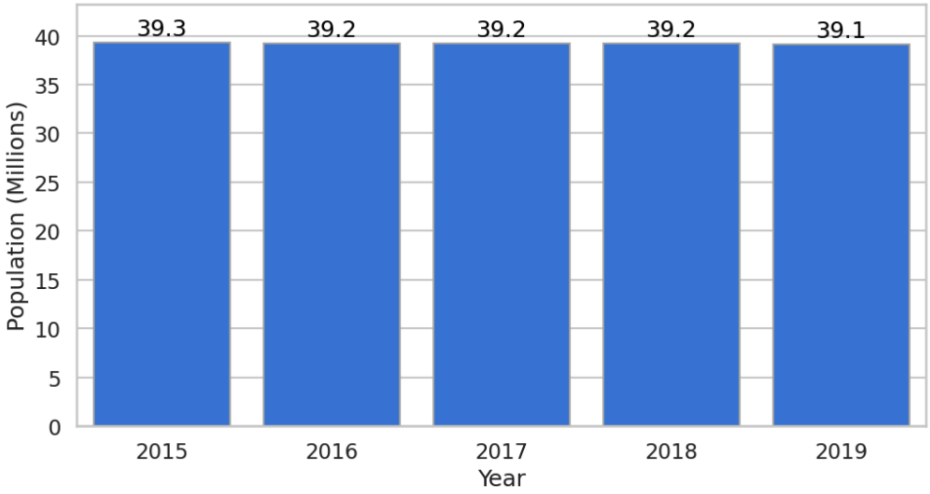

Other individual-level data sources, but without unique identifiers

Fee-for-service claims data from Singapore and Taiwan were also processed for GBD 2023. Tabulated inpatient-only claims data from Singapore for the years 1991-2019 were derived from the MediClaims Database and provided by the Ministry of Health of Singapore. The MediClaims data processed for the GBD is inclusive of all inpatient admissions in the country's public and private hospital facilities, and for all patients covered under MediShield Life, MediSafe, and MediFund, with admissions aggregated at the national level. Similarly, Taiwan National Health Insurance claims for the year 2016, derived from the National Health Insurance Research Database (NHIRD) and covering all residents in Taiwan under a universal single-payer health care system, was used. The NHIRD is representative of the whole population for Taiwan and covers both inpatient admissions and outpatient encounters.

Calculating uncertainty

Uncertainty in individual-level data sources was calculated using Wilson's approximation, utilizing sample size derived from enrollment data (i.e. MarketScan, Medicare) or GBD population estimates (i.e. Poland) depending on the source. Uncertainty in outpatient encounter data (Section 2.2.6) was also calculated using Wilson's approximation and GBD population. Uncertainty for aggregate inpatient data (Section 2.2.3) that are not complete for the population and use the inpatient utilization envelope came from multiplying 1000 sampled draws from the envelope and correction factor models and deriving uncertainty intervals from the upper and lower bounds. Inpatient sources that are complete for the population rely on creating 1000 draws from a Poisson distribution that are then multiplied by the correction factor draws to derive uncertainty.

Wilson’s approximation:

$$\sigma^2 = \frac{\frac{p(1-p)}{n} + \frac{1.96^2}{4n^2}}{\left(1 + \frac{1.96^2}{n}\right)^2}$$

where p is prevalence and n is the sample size

Section 2.2.3: Aggregate inpatient hospital admission sources used in GBD 2023

Figure S8. Processing steps for aggregate inpatient hospital admission sources

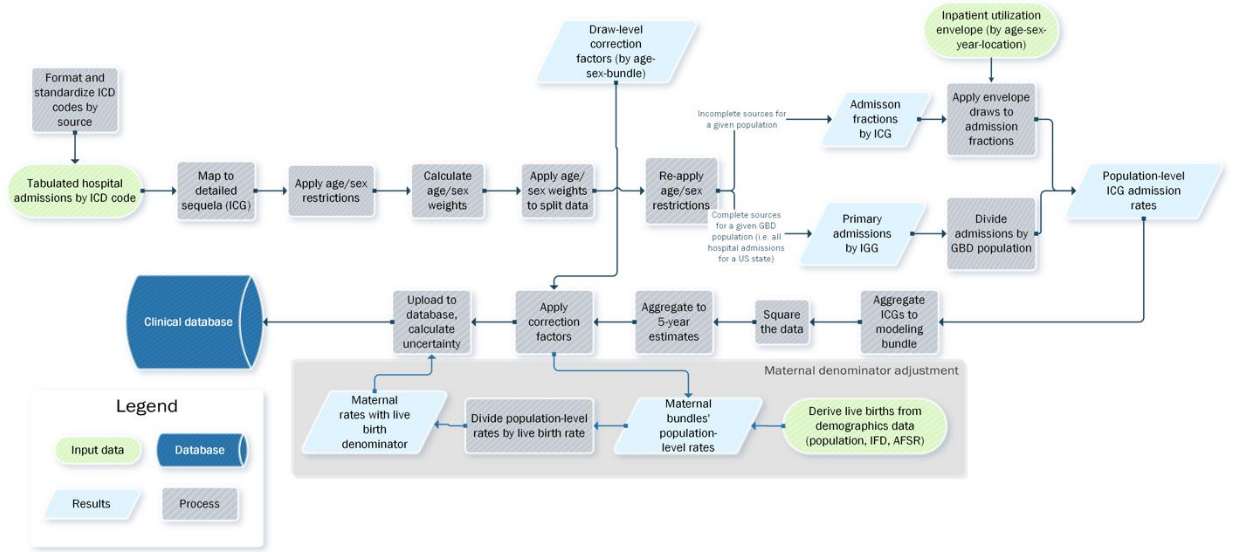

Hospital inpatient records data were extracted from 2817 location-years in 44 countries. For inpatient sources, a case of disease was defined as an overnight admission in a hospital or a stay of >24 hours, depending on how length of stay is represented in each source. ICD coding was standardized across sources and versions of ICD. Counts of admissions with a primary diagnosis of each cause were extracted from all sources and propagated through the aggregate hospital admissions data processing steps. For inpatient sources, a case of disease was defined as an overnight admission in a hospital or a stay of >24 hours, depending on how length of stay is represented in each source. We mapped each primary diagnosis code for a given admission to its modelling entity using the clinical ICD map. Non-primary diagnostic detail, where available, was included through the correction factor modelling process (Section 2.2.5). Admissions were then aggregated to create cause fractions, defined as the number of admissions for a given disease, impairment, or injury divided by total admissions for that age, sex, location, and year. In cases where a source is not complete for the country or subnational population, the inpatient utilization envelope was used and applied to the cause fractions (outlined in Section 2.2.4). For sources that are complete for the population, rates are created with numerators being primary admissions for a given condition and denominators being GBD population estimates.

Section 2.2.4: Estimation of the inpatient utilization envelope

The inpatient utilization envelope provided all-cause inpatient admission rate estimates from 1990 to 2019 for all age groups including <1 year detail, males and females, and 934 locations within the GBD 2023 framework. To inform nonfatal burden estimation, we applied those rates to upscale the number of cause-specific admission counts from several aggregate inpatient sources (table 4) that were not complete for the GBD location they represent and therefore did not capture all admissions in a given population. This approach enabled inclusion of these nonrepresentative sources covering 12 more countries.

Table 4. Clinical inpatient sources requiring the envelope adjustment

Location name	Subnational detail	Years of data
Argentina Public Hospital Discharge Statistics	No	2010, 2011
Armenia Hospital Data	No	2016
Botswana Health Management Data System (HMDS)	No	2007, 2008, 2009
Indonesia Integrated Hospital Data – Sistem Informasi Rumah Sakit (SIRS)	Yes	2013
Iran Hospital Data	No	2001-2010
Japan Diagnosis Procedure Combination Database	Yes	2010, 2011, 2012, 2013, 2014, 2015
Kenya National Inpatient Morbidity and Mortality Statistics	No	1999
Mexico Institutions of Health Sector Hospital Discharges	Yes	2004, 2018, 2019
Nepal Hospital Inpatient Discharges	No	2010, 2011, 2013, 2015
Portugal – European Hospital Morbidity Database	No	2015
Türkiye Diagnosis-Related Group Hospital Inpatient Database	No	2011, 2012
Viet Nam Hospital Data	No	2013

Case definition

We defined a hospital admission as hospitalization in a formal health care facility for, at least, an overnight stay. The inpatient utilization rate was the average number of admissions per person per year. We excluded inpatient admissions of healthy newborns and, wherever possible, excluded same-day discharges in accordance with the case definition.

Input data

We included sources either covering the complete population in a given location or using a nationally or subnationally representative sample of the population. We extracted the total inpatient admission

counts per year. The inputs comprised administrative data, national government reports, scientific literature, and surveys from 1990 to 2019.

Table 5. Inclusion and exclusion criteria for data sources used in the inpatient utilization envelope model

**Inclusion criteria**	**Exclusion criteria**
Case definitions: overnight stay, discharge	
Data types: Administrative data, Report, Scientific Literature, Survey	
Population coverage: complete in a location at the national or subnational level	Population coverage: incomplete for a given location at the national or subnational level
Time period covered: 1990 – 2019	
Recall: Annual admission counts / 12-month recall period	Recall: Less or over 12-month recall
Unit of measurement: total number of admissions for any cause excluding healthy newborns and day cases	Unit of measurement: total number of admitted persons.

The data search and extraction were completed in several steps. Firstly, we processed and included the available individual admission-level administrative inpatient data covering the entire populations in a country or a subnational location aggregating it by age group and sex. Secondly, we searched the GHDx for government reports concluding the search on December 12th, 2023. We restricted the search to the following GBD regions: Andean Latin America, the Caribbean, Central Asia, Central Latin America, Central Sub-Saharan Africa, Eastern Sub-Saharan Africa, Southern Sub-Saharan Africa, Western Sub-Saharan Africa, East Asia, Southeast Asia, Southern Latin America, Eastern Europe, High-income Asia Pacific, North Africa and the Middle East, Oceania, and South Asia. We applied no language restrictions and required all returned records to have tabulated reports. We screened the titles translated to English using a set of keywords, including "Health", "Statistical", "Yearbook", "Annual", "Report", "Statistics", "Bulletin", "Hospital", "Discharges", "Inpatient". Thirdly, we complemented the retrieved reports with external web searches on public government websites addressing missingness after the GHDx review, which resulted in the addition of government reports for 33 countries previously not catalogued in the GHDx. We also included three known multinational administrative data sources, including Eurostat, European Health for All database (HFA-DB), and the Organization for Economic Co-operation and Development (OECD) Health Statistics further expanding coverage. Finally, we used a subset of surveys used to produce utilization estimates in GBD 2021 reporting the number of admissions per person in the past 12 months. We applied no language restrictions to our search and required all returned records to contain either microdata or tabulated reports.

Data analysis

First, we split data points covering wider age bins and/or both sexes into GBD standard age/sex categories using inpatient utilization age patterns produced with a Bayesian spline cascade. We visually inspected the resulting age/sex specific data set to identify outliers. Finally, we modeled average annual per capita inpatient utilization rates for 934 locations, 25 age groups, and for males and females, from

1990 to 2019, where the 25 age categories were early neonatal (0-6 days), late neonatal (7-27 days), 1 to 5 months, 6-11 months, 12 to 23 months, 2 to 4 years, 5 to 9 years, followed by 5-year increments up to 90 to 94 years, and 95 years and older. We modelled per capita inpatient utilization rate with Spatiotemporal Gaussian Process Regression (ST-GPR) adjusting for total health expenditure per capita, log hospital beds per 1,000 population, and Sociodemographic Index (SDI).

Figure S9. Overview process of estimation of hospital utilization envelope

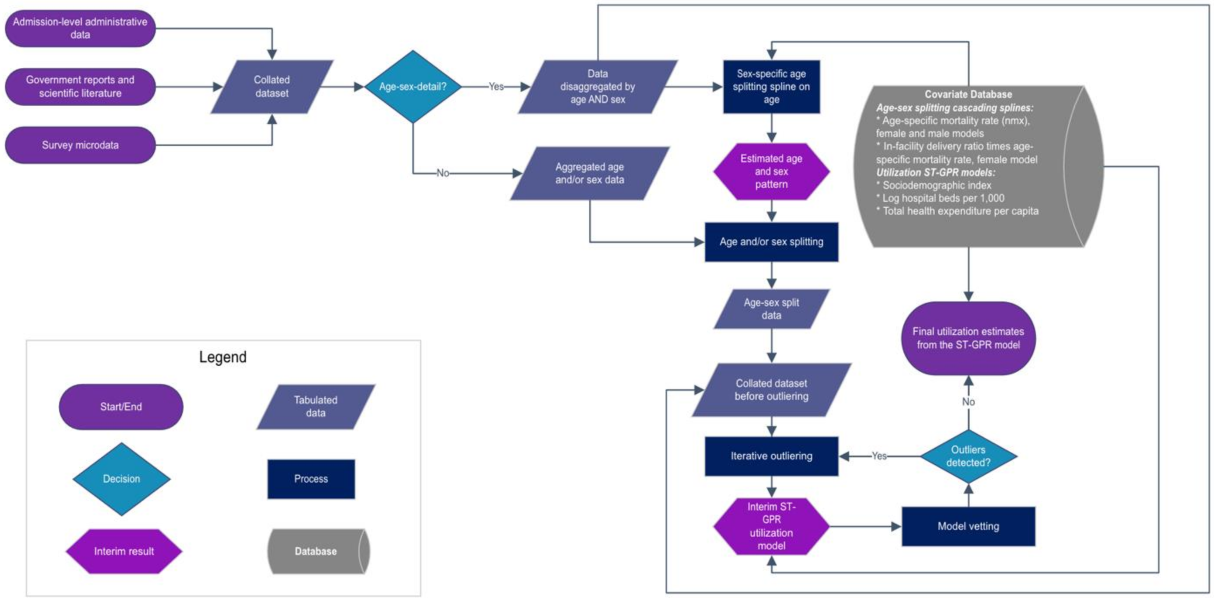

Changes from GBD 2021 to GBD 2023

This iteration of inpatient utilization models has seen several significant updates increasing the robustness and performance of the model. First, we newly incorporated over 2,000 location-years of government reports, adding on to and replacing lower-quality surveys. Second, we added over 300 location-years of aggregated individual-admission data with extensive age and sex detail where we could ensure adherence to the case definition of inpatient utilization, which could not always be readily confirmed in other data sources. Surveys that previously required crosswalking due to deviating case definition were excluded from the analysis in favor of widely available government reports. Third, we developed country-year-specific age patterns for each sex that we used to split aggregate data points, whereas the previous model relied on a global age pattern that could not capture variations across locations and years due to differences in population structure and health care systems. Fourth, we systematically excluded healthy newborns from the input data with <1 year age detail, which resulted in more reliable utilization estimates for infants. Fifth, we replaced the Healthcare Access and Quality Index (HAQi) indicator with Sociodemographic Index (SDI) to adjust the utilization model to remove potential co-linearity between HAQi and healthcare utilization and control for differences in SDI between countries. Finally, we eliminated spatial smoothing in the ST-GPR model due to the lack of evidence of any correlation in healthcare system specifications within the GBD location hierarchy.

Applying the inpatient utilization envelope to incomplete inpatient sources

We applied the inpatient utilization envelope to inpatient sources incomplete for the population they represent (table 4). To do so, we calculated age- and sex-specific cause fractions by dividing the number of cause-specific admissions by total inpatient admissions in the source. Then, we calculated the all-cause number of inpatient admissions by multiplying the total per capita inpatient utilization rate from the envelope by population of the respective demographic group. Finally, we multiplied the cause fractions by the total admission count, which amounted to the total number of cause-specific admissions in a given population.

We calculated total cause-specific admissions per age, sex, location, and year from every clinical source as follows:

$$\boldsymbol{CFr} = \frac{A_{cause}}{n}$$

$$\boldsymbol{A_{tot}} = AR * N$$

$$TA_{cause} = \boldsymbol{CFr} * \boldsymbol{A_{tot}}$$

where
CFr – cause fraction
A – admissions
n – sample size in a source
AR – estimated admission rate from the envelope
N – GBD population
TA – total admissions

per age, sex, location, and year.

Section 2.2.5: Correction Factors

We performed three corrections on the aggregate-level inpatient hospital admissions data sources to standardize all inpatient hospital admissions data to the same definition of care and to account for cases of disease that were not captured in these sources. These data adjustments include: deduplication of multiple admissions per individual for the same cause or condition, within one year (for prevalent conditions) and within a cause-specific duration (for incident causes), accounting for cases of disease that were non-primary diagnoses and not captured in the aggregate-level data, and accounting for outpatient admissions also not present in the aggregate-level inpatient admissions data. These corrections were applied to the aggregate-level inpatient admissions data as scalars, resulting in four incidence and prevalence estimates: (1) un-corrected inpatient admissions, primary diagnosis only; (2) inpatient admissions by individual encounter, deduplicated for readmissions , primary diagnosis only; (3) inpatient admissions by individual encounter, deduplicated for readmissions, accounting for all

diagnoses; and (4) inpatient admissions and outpatient encounters by individual, deduplicated for readmissions, accounting for all diagnoses. Estimate 4, where all three corrections are applied, was used for most causes, with exceptions based on the nature of the disease; in instances where outpatient care or non-primary diagnosis were not expected, estimates 3 or 2 were used instead.

Figure S10. Overview of Correction Factor process

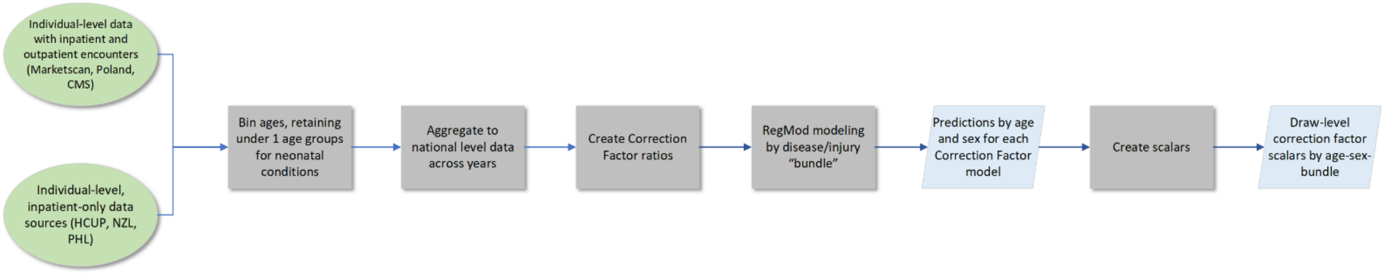

Correction Factor ratios for the estimates 2 and 3 above were calculated using all clinical sources that had individual-level data with unique patient identifiers and both primary and non-primary diagnosis positions. These sources include those described in "Section 2.2.2. Individual-level data sources with unique identifiers in GBD 2023": United States Merative MarketScan claims (2010-2019, in plots referenced as 'Marketscan'), Taiwan National Health Insurance claims (2016, 'TWN'), Poland National Health Fund Patient claims (2015-2019, 'POL'), United States Medicare claims (2000, 2010, 2014-2016, 'Medicare'), as well as inpatient admissions data from the Philippines Health Insurance PhilHealth(2013-2016, 2019, 'PHL'), New Zealand National Minimum Dataset (2000-2019, 'NZL'), and the United States HCUP State Inpatient Databases (2003-2009, 2016, 2018, 2019, 'HCUP'). Of these sources, only MarketScan, Medicare, Poland, and the Taiwan claims data included a link between inpatient and outpatient care and could be used in estimate 4 described above.

To create a correction for readmissions, to go from total admissions to single cases of disease, we calculate the ratio of the number of unique primary diagnoses among inpatient admissions for an individual to the total number of primary diagnoses among inpatient admissions for each cause by age, sex, and source. For a correction for missing non-primary diagnoses, we calculate the ratio of the number of deduplicated primary diagnoses among inpatient admissions to the number of deduplicated primary and non-primary diagnoses among inpatient admissions by age, sex, and source. To model a correction for both missing outpatient admissions and non-primary diagnoses, we calculate the ratio of the number of deduplicated primary diagnoses among inpatient admissions to the number of deduplicated primary and non-primary diagnoses among inpatient admissions and outpatient encounters by age, sex, and source. The data aggregates that make up these ratios can be found in Table 6a below and the ratios can be found in Table 6b. These three sets of ratios are always proportions between 0 and 1. The data that goes into each of the ratios is aggregated over year and subnational location and is specific by age group, sex, and source.

Table 6a. Data aggregates that go into correction factor ratios with various data processing steps applied

**Name**	**Description**	**D**	**N**	**O**
dno1	Deduplicated primary diagnoses, among inpatient admissions	X		
dno12	Deduplicated primary and non-primary diagnoses, among inpatient admissions	X	X	
dno123	Deduplicated primary and non-primary diagnoses, among inpatient admissions and outpatient encounters	X	X	X

Here, D means deduplication has been applied, N means non-primary diagnoses are included, and O means outpatient encounters are included.

Table 6b. Ratios for correction factor models

**Correction Factor**	**Corrects for**	**Ratio for modeling**
CF1	Readmissions	dno1/dno0
CF2	Non-primary Dx	dno1/dno12
CF3	Non-primary Dx and outpatient	dno1/dno123

For each GBD cause, each of the Correction Factor ratios were modeled in RegMod (see here for more information on RegMod) as a binomial model with a fixed effect on sex and a B-spline on age, as described by the equation below. For the spline on age, there were three quadratic knots spaced according to data density with linear tails. All models were conducted in logit-space in order to bound the results by 0 and 1. To get predictions from the models, 1000 draws were taken from the posterior distribution, with an uncertainty interval calculated from 0.025 and 0.975 quantiles in the draw-level results and point estimates calculated directly from model coefficients. Figures S11-13 show examples of the model results for acute encephalitis.

$$E[y|sex_i, age_i] = logit^{-1}(\beta_0 + \beta_1 sex_i + spline(age_i))$$

Figure S11. Readmissions correction model in linear space for acute encephalitis

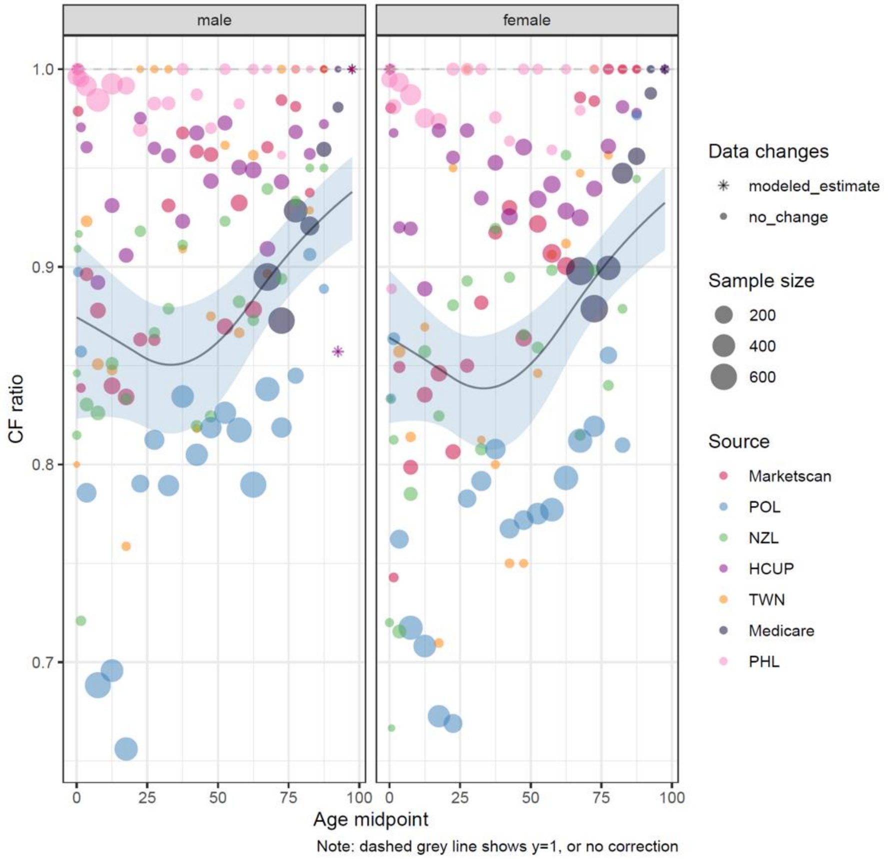

"Data changes" shows where the data has been modelled to be visualized according to data privacy restrictions. Note that these changes are not present when the model is fit, but only for visualization purposes.

Figure S12. Non-primary correction model in linear space for acute encephalitis

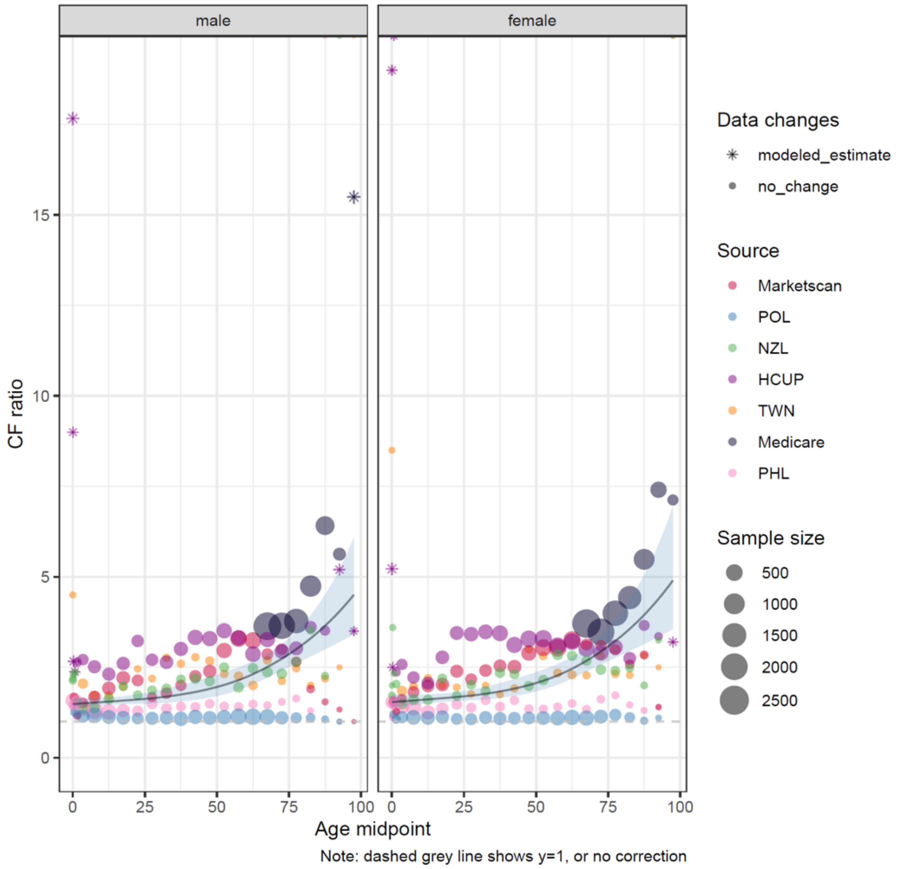

"Data changes" shows where the data has been modelled to be visualized according to data privacy restrictions. Note that these changes are not present when the model is fit, but only for visualization purposes. The data shown represents the inverse of the non-primary correction model to more clearly visualize the scalar applied to correct the inpatient data.

Figure S13. Non-primary and outpatient correction model in linear space for acute encephalitis

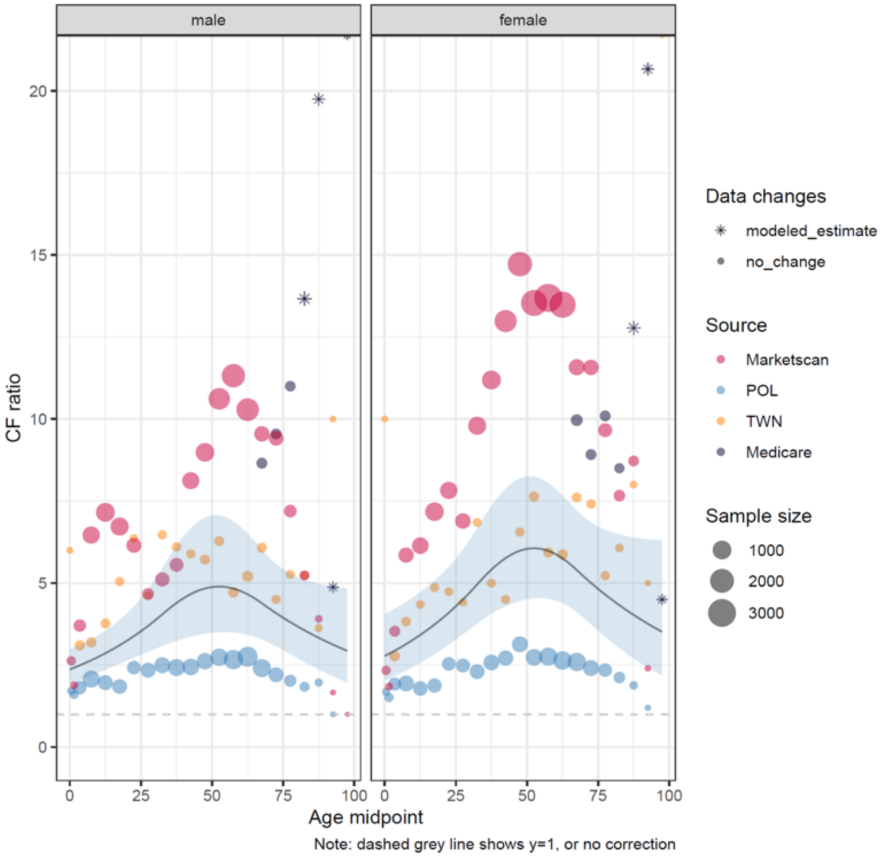

"Data changes" shows where the data has been modelled to be visualized according to data privacy restrictions. Note that these changes are not present when the model is fit, but only for visualization purposes. The data shown represents the inverse of the non-primary, outpatient correction model to more clearly visualize the scalar applied to correct the inpatient data.

For the readmissions correction, the model predictions were applied to inpatient data as a scalar, by age and sex. The readmissions correction is always a value between 0 and 1, so the effect was a decrease in inpatient admissions from total inpatient admissions to just incident encounters. For the non-primary diagnosis correction, the readmissions correction is first applied, followed by the inverse of the predictions from the non-primary correction model, increasing the estimate to include non-primary inpatient cases. For the outpatient and non-primary correction, the readmissions correction is also first applied and then followed by the inverse of the predictions from the outpatient and non-primary model, increasing the estimate to include both non-primary inpatient cases and outpatient cases. Table 7 outlines how the corrections are applied as the scalars described here to create different estimates. The corrections are applied at the draw-level over 1000 draws, the estimates are produced by taking the median of the draws, and the uncertainty intervals are produced by taking the 97.5 and 2.5 percentiles of the draws.

Table 7. How correction factors are applied to create various incidence and prevalence estimates

Estimate	Correction(s) applied	Scalar to apply	Scalar equation
Inpatient admissions by individual encounter, deduplicated for readmissions, primary diagnosis only	Readmissions	CF1	dno1/dno0
Inpatient admissions by individual encounter, deduplicated for readmissions, accounting for all diagnoses	Readmissions and non-primary Dx	CF1 * 1/CF2	dno1/dno0 * dno12/dno1
Inpatient admissions and outpatient encounters by individual, deduplicated for readmissions, accounting for all diagnoses	Readmissions, non-primary Dx, and outpatient	CF1 * 1/CF3	dno1/dno0 * dno123/dno1

Determination of maternal causes used separate cause fractions and a different scalar calculated from a maternal hospital admissions rate instead of the inpatient hospital envelope using the equation

$$\left(\frac{events}{\#\ of\ total\ hospital\ visits}\right) * \left(\frac{hospital\ visits}{live\ births}\right) * \left(\frac{births}{population}\right)$$

Determination of injuries used a separate correction factor from those described above which adjusted data that was only E-coded by data that contained E-codes and N-codes (nature of injury codes) with the following equation

$$\frac{1}{\frac{E\text{-}code\ primary\ dx}{E\text{-}code\ any\ dx + N\text{-}code\ any\ dx}}$$

A final adjustment was applied to each of the above estimates. The HAQ Index was used to account for differences in access and quality of health care across time and space. The HAQ Index adjustment was applied by dividing the above estimates by a scalar ranging from 0 to 100, where 0 represents the first percentile of observed access and quality and 100 the 99th percentile.

Section 2.2.6: Outpatient encounter data

Figure S14. Processing steps for outpatient encounter data

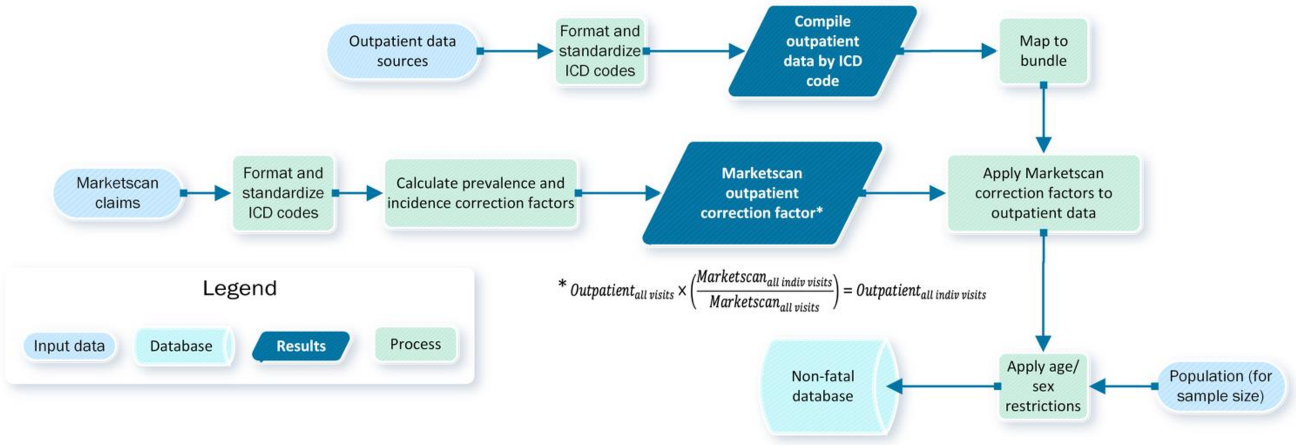

Outpatient encounter data that could not be linked to comprehensive inpatient records were available from the USA and Sweden for 109 location-years. No changes were made in the processing of outpatient data from GBD 2019, except for updates to the ICD mapping to GBD diseases and injuries. As with the aggregate inpatient sources, a scalar was calculated by using MarketScan claims data to adjust for multiple visits per individual within one year (for prevalent conditions) and within a cause-specific duration (for incident causes).

We have also processed and included electronic health records from general practices (GP data), provided by the Netherlands Institute for Health Services Research (Nivel), covering about 10% of the Dutch population, with approx. 1.75 million individuals registered with a GP each year. The data set includes incidence and prevalence numbers per 1,000 persons in the Netherlands, based on ICPC-1 coding system. From the ICPC-1 coding system, we were able to directly map over 100 codes to GBD disease and injury categories. The Dutch GP data were processed into 'illness episodes' by Nivel, using a three-year window: reporting calendar year + two previous calendar years. What this means is that the data received contains any outstanding episodes of care (for chronic conditions) *and* any new conditions recorded over a three-year period. For chronic conditions lasting one year or more, we have made a correction to the prevalence estimates for the GBD, subtracting out half of the incident cases in a year.

Section 2.2.7: New data added for GBD 2023

In addition to Medicare claims, several new sources were provided for non-fatal modelling as well as additional years of existing GBD clinical sources. Table 8 below summarizes the data added in this GBD round with relevant characteristics of the data source. Sources that include unique identifiers are either processed (A) at IHME in a secure environment, or (B) by collaborators before being shared for use in the GBD. Where available, 2020 and 2021 years of clinical data were processed and reviewed but not used directly in non-fatal models. Dramatic decreases in utilization during the COVID-19 pandemic were observed and further analyses are needed to disentangle the effects of care seeking behaviour on incidence and prevalence rates as well as true changes in rates for these conditions across the time series.

Table 8. Source-years of clinical data added for GBD 2023

Source	Years Added	Includes unique identifiers	Includes outpatient encounters	Complete for population
United States Merative MarketScan Research Database	2018, 2019	Yes	Yes	No
Poland National Health Fund Patient Claims	2019	Yes	Yes	Yes
Singapore MediClaims Database	2018, 2019	Yes	No	Yes
Mongolia H-Info Health Facility Data*	2019	No	Yes	Yes
Russia Statistical Yearbook	2018, 2019	No	Yes	Yes
South Korea Medical Services Claims Data*	2018, 2019	Yes	Yes	Yes
Austria Hospital Inpatient Discharges	2015-2018	No	No	Yes
Brazil Hospital Information System (SIH)	2017-2019	No	No	Yes
Chile Hospital Discharge Information System	2019	No	No	Yes
Georgia Hospital Data	2016, 2018, 2019	No	No	Yes
Germany Inpatient Admissions	2000-2019	No	No	Yes
Italy Hospital Inpatient Discharges	2019	No	No	Yes
Mexico Institutions of Health Sector Hospital Discharges*	2004-2019	No	No	No
Netherlands Nivel Primary Care Database	2011-2019	No	Yes – GP visits only	No
New Zealand National Minimum Dataset	2018, 2019	No	No	Yes
Philippines Health Insurance (PhilHealth) Data	2019	Yes	No	Yes
United States State Inpatient Databases (HCUP – US SID)	2016-2019^	Yes^	No	Yes

**New source for GBD 2023*

^Select states only

Section 2.3 Data Adjustments

Section 2.3.1: Crosswalking

Crosswalking refers to the process of adjusting data for known biases. An observation is considered biased if it differs in a systematic way from the standard GBD definition of the modeled parameter. Examples include self-reported rather than doctor-diagnosed measures of disease incidence, case ascertainment using disparate diagnostic tests, sampling from non-representative populations (e.g. blood donors), and observations derived from different types of surveillance systems, among others. If

the difference between an alternative measurement method and the GBD definition is systematic, we can model it as a function of covariates and use this model to predict the degree of adjustment needed for a given alternative or non-standard observation. The result of crosswalking is that GBD models can incorporate data from a wider range of sources.

Specifically, crosswalking involves:

1. Finding pairs of alternative and reference (e.g. self-reported and measured) observations that match on relevant criteria (e.g. age, sex, location and year);
2. Taking the difference between these observations in log or logit space, to ensure that the crosswalk adjustment remains bounded correctly;
3. Running a meta-regression model that estimates this difference potentially as a function of covariates;
4. Predicting how much each alternative data point in the original dataset should be adjusted; and
5. Applying the adjustment.

Section 2.3.2: Bias adjustment for alternative case definitions and study methods

In GBD 2023 we continued the practice started in GBD 2019 of crosswalking non-fatal and risk exposure data to account for alternative case definitions or study methods. The adjustments were applied prior to entering data into our main analytical tools of DisMod-MR 2.1 and ST-GPR, ensuring that all data inputs were expressed on a consistent scale. We also used this approach to convert data presented for both sexes to a male and female equivalent. The starting point was to explicitly state the reference case definition and study method and identify alternative definitions and study characteristics that fall within our inclusion criteria.

We compiled data from both within-study comparisons (ie, data that used alternative and reference definitions in the same population) and between-study comparisons (ie, data that used an alternative definition in one population and a reference definition in another population that overlap in location, time, age, and sex) of different case definitions. For between-study comparisons, we allowed a maximum calendar year difference between studies of five years. Where validation studies (ie, those carried out at the introduction of a new set of diagnostic criteria comparing to previous criteria) were available, we extracted data on the comparison of alternative to reference. For quantities of interest with multiple alternative definitions/methods we also looked for pairs comparing two alternatives.

If both between and within study pairs were available, we examined whether there was a systematic difference between these. If there was a significant difference, we made judgement call as to whether within-study or between study data comparisons were most appropriate. In general, this was the within-study data. However, there were important measurement or conceptual reasons for choosing between-study data. For example, for crosswalks between self-reported height and weight compared to measured height and weight, between-study comparisons may be preferable if respondents knew they would be measured and, therefore, were less likely to misreport their height and weight.

To quantify the degree of bias for an alternative data source, we calculated the difference between matched pairs of alternative and reference observations and used this quantity as the dependent variable in a mixed effect meta-regression model. The model could include any number of covariates to capture how bias might vary as a function of other variables, like age or sex. Predictions from the model were then used to convert alternative observations to their equivalent reference values. For GBD 2021 and later rounds, we developed an open-source Python package to facilitate the process of modeling and applying bias adjustments (ihmeuw-msca, 2023).

To choose covariates for the model, we examined whether there were systematic differences in the adjustments by key demographics (age, sex, geographic location, year) and other potential factors that may lead to variation in the degree of bias adjustment. We did this when there was a strong rationale, eg, biological plausibility, for variation by such characteristics. After fitting the model, for predicted adjustment factors that were not statistically significant, we still applied the adjustments if there was a conceptual reason to believe that the alternative definition is biased. This expands the variance of data points using a non-standard case definition or study method, effectively reducing their influence in subsequent modeling steps.

Section 2.3.3: Example bias adjustment calculation

As an example, we provide mathematical notation for a bias adjustment to a data source that measures prevalence using a non-standard case definition. We have pairs of alternative and reference observations (denoted i) that match on age, sex, location, and time period combination (denoted j). The degree of bias varies as a function of age and sex. Because the parameter of interest is prevalence, which is bounded by 0 and 1, we calculate the logit-scale difference between alternative and reference observations in a given matched pair:

$$y_{i,j} = logit\left(p_{i,j}^{alt}\right) - logit(p_{i,j}^{ref})$$

In preparing the data for this calculation, if the values of either the reference or alternative were zero, we aggregated values across age groups until both values had non-zero observations. We used the delta method to compute the standard error of the reference and alternative measures in logit space. The standard error of the logit-scale difference was computed as the square root of the sum of the variances of each data point in a pair.

If the parameter had instead been bounded by only 0, like incidence, we would have calculated the log-scale difference. From simulations we found that the two methods provide almost identical results for quantities that after adjustment do not exceed a value of 0.5 (eg, prevalence or proportion). The logit-scale difference method much better dealt with higher values and avoided prevalence or proportions to exceed one.

As a next step in this hypothetical example, we modeled the differences as the dependent variable in a mixed-effects meta-regression model with age and sex as covariates:

$$y_{i,j} = \beta_0 + \beta_1 \, age_i + \beta_2 \, sex_i + u_j + \epsilon_i$$
$$u_j \sim N(0, \gamma)$$

$$\epsilon_i \sim N(0, \sigma_i^2)$$

We then used the linear predictor of this model to predict the degree of bias adjustment needed for the various age and sex combinations among the alternative observations:

$$\hat{\delta}_{a,s} = f(age, sex) = \hat{\beta}_0 + \hat{\beta}_1\, age + \hat{\beta}_2\, sex$$

To adjust a particular alternative observation $p_{a,s}^{alt}$ we subtracted the adjustment factor in logit space, and the inverse logit transformation was applied to the result to convert back to natural units:

$$p_{a,s}^{adjusted} = logit^{-1}(logit(p_{a,s}^{alt}) - \hat{\delta}_{a,s})$$

The uncertainty for the adjusted logit-scale prevalence includes:

- uncertainty of the original observation in logit space,
- uncertainty from the posterior distribution of the predicted adjustment, and
- random intercepts in the meta-regression model (denoted γ above).

The variances from the three components were summed and then transformed into natural unit space using the delta method.

Section 2.3.4 Network Analysis

When there were multiple alternative case definitions or study methods, we used network analysis to leverage the additional information provided by indirect comparisons. For example, if A is the reference and B and C are two alternatives, the comparison of C versus A would be considered a direct comparison to the reference. This case was the subject of the previous section. In contrast, the combination of A versus B and B versus C provides an indirect comparison of the alternative C against the reference A. Or in other words, the inclusion of B-versus-C comparisons in the dataset provides additional information with which to estimate the difference between C and A.

Implementing a network analysis requires careful construction of the design matrix, or the dataset we pass to the mixed effects meta-regression model. Continuing the example with reference A and alternatives B and C, the design matrix for a network analysis with no covariates is created as follows:

- Create *k* dummy variables where *k* are all definitions/methods other than A (eg, *k* = B, C)
- Code dummy *k* as
    - 1 if the first term of the logit-scale difference is *k*;
    - -1 if *k* is second term of the logit-scale difference;
    - 0 otherwise

For example:

Study	Comparison	DummyB	DummyC
1	logit(B)-logit(A)	1	0
2	logit(B)-logit(A)	1	0
3	logit(C)-logit(A)	0	1

4	logit(C)-logit(A)	0	1
5	logit(C)-logit(B)	-1	1
6	logit(C)-logit(B)	-1	1

The coding structure outlined above assumes that all case definitions are mutually exclusive. In some cases, however, individual case definitions are composed of different sub-components or dimensions. For example, case definitions may vary by the type of symptoms that a respondent experiences as well as the recall period over which those symptoms are experienced. In the presence of sparse data, it may be difficult to find both direct and indirect comparisons of all individual case definitions. In these cases, an alternative approach is to assume different dimensions of case definitions have a multiplicative effect. In other words, the effect of recall period has the same relative effect across different categories of symptoms reported by respondents. To implement this coding scheme:

- Create *k* dummy variable columns for each case definition dimension.
- For each dummy variable k:
    - Add 1 if *k* is a component of the first term in the logit-scale difference.
    - Subtract 1 if *k* is a component of the second term in the logit-scale difference.

Network analysis is a feature of the open source Python package for conducting bias adjustments (ihmeuw-msca , 2023) mentioned earlier. The package abstracts away the need to create the design matrix manually as in this example and can incorporate an arbitrary number of alternative definitions and covariates.

Section 2.3.5 Age sex splitting

Before modelling, we ran a Dismod-MR 2.1 model with data disaggregated by age to estimate countries' age-pattern and then applied the estimated age-pattern to split aggregated all-age data into the 5-year age groups preferred for ST-GPR modelling. This procedure was done by calculating a constant, k, which was the ratio of the aggregated all-age data point, $\mu_{all\ age}$, to the all-age estimated utilisation rate from the DisMod-MR 2.1 model, $\widehat{\mu_d}$

$$k = \frac{\mu_{all\ age}}{\widehat{\mu_d}}$$

The constant, k, was then multiplied by age-specific utilisation rates from the DisMod-MR 2.1 model. Observation-specific uncertainty and uncertainty from the estimated age-pattern were both propagated into the uncertainty for a given post-splitting data point. The split data were then incorporated into the final DisMod-MR 2.1 model.

Section 2.4: Spatiotemporal Gaussian process regression (ST-GPR) modelling

The input data were modelled by using ST-GPR to allow for smoothing over age, time, and location in locations that were missing complete datasets. The flowchart showing the analytic steps can be found elsewhere.[6] The approach is a stochastic modelling technique that is designed to detect signals amidst noisy data. It also serves as a powerful tool for interpolating non-linear trends.[8,9] Unlike classical linear models that assume that the trend underlying data follows a definitive functional form, GPR assumes that the specific trend of interest follows a Gaussian process, which is defined by a mean function $m(\cdot)$

and a covariance function $Cov(\cdot)$. For example, let $p_{c,a,s,t}$ be the prevalence, in normal, log, or logit space, observed in country c, for age group a, and sex s at time t:

$$(p_{c,a,s,t}) = g_{c,a,s}(t) + \epsilon_{c,a,s,t}$$

where

$$\epsilon_{c,a,s,t} \sim Normal(0, \sigma_p^2)$$
$$g_{c,a,s}(t) \sim GP\left(m_{c,a,s}(t), Cov\left(g_{c,a,s}(t)\right)\right).$$

The derivation of the mean and covariance functions, $m_{c,a,s}(t)$ and $Cov\left(g_{c,a,s}(t)\right)$, along with a more detailed description of the error variance (σ_p^2), is described below.

Section 2.4.1 Estimating mean functions

We estimated mean functions by using a two-step approach. To be more specific, $m_{c,a,s}(t)$ can be expressed, depending on the prevalence transformation, as:

$$log(p_{c,a,s}(t)) = X_{c,a,s}\beta + h(r_{c,a,s,t})$$

$$logit(p_{c,a,s}(t)) = X_{c,a,s}\beta + h(r_{c,a,s,t})$$

$$p_{c,a,s}(t) = X_{c,a,s}\beta + h(r_{c,a,s,t})$$

where $X\beta$ is the summation of the components of a hierarchical mixed-effects linear regression, including the intercept and the product of covariates with their corresponding fixed-effect coefficients. Some models were run as hierarchical mixed-effects linear regressions with random effects on the levels of the location hierarchy. For most mixed-effects models, random effects were only used in the fit, not in the prediction. The second part of the equation, $h(r_{c,a,s,t})$, is a smoothing function for the residuals, $r_{c,a,s,t}$, derived from the linear model. Cause-specific methods details can be found in appendix section 6.

Although the linear component captures general trends over time, much of the data variability may still not be adequately accounted for. To address this, we fit a locally weighted polynomial regression (locally estimated scatterplot smoothing, or LOESS) function $h(r_{c,a,s,t})$ to systematically estimate this residual variability by borrowing strength across time, age, and space patterns (the spatiotemporal component of ST-GPR).[10,11] The time adjustment parameter, defined by λ, aims to borrow strength from neighboring time points (ie, the prevalence in this year is highly correlated with prevalence in the previous year but less so further back in time). The age-adjustment parameter, defined by ω, borrows strength from data in neighboring age groups. The space-adjustment parameter, defined by ξ, aims to borrow strength across the hierarchy of geographical locations. The spatial and temporal weights are combined into a single space-time weight to allow the amount of spatial weight given to a particular

point $r_{c,a,s,t}$ to fluctuate given the data availability at each time *t* and location-level *l* in the location hierarchy.

Let $w_{c,a,s,t}$ be the final weight assigned to observation $r_{c,a,s,t}$ with reference to a focal observation r_{c_0,a_0,s_0,t_0}. We first generated a temporal weight $t.\ w_{c,a,s,t}$ for smoothing over time, which was based on the scaled distance along the time dimension of the two observations (Ng M) :

$$t.w_{c,a,s,t} = \frac{1}{e^{\lambda|t-t_0|}}$$

Next, we generated a spatial weight to smooth over geography. Specifically, we defined a geospatial relationship by categorizing data based on the GBD location hierarchy (table 1). *zeta* acts as a scalar on a given datapoint given its proximity to the target location:

$$t.w_{c,a,s,t} = \zeta^{|c-c_0|}$$

For example, estimating a country, would use the following weighting scheme:

- Country data: ζ^0 = 1
- Regional data not from the country being estimated: ζ^1
- Data from other regions in the same super region: ξ^2
- Global data from other super regions: ζ^3

Under the spatial weighting specification, typical values of ζ range from [0.001, 0.2], where ζ can be interpreted as the amount to down weight regional datapoints compared to country datapoints for a given estimating country. For example, for a given datapoint $r_{c,a,s,t}$ and ζ = 0.01, a datapoint not within country *c* but within the same region *r* as $r_{c,a,s,t}$ would be assigned $\frac{1}{100}$ the weight of a datapoint within the country.

The spatial and temporal weights were then multiplied and summed across each level of the location hierarchy and normalised for each time period *t*. This procedure allowed the space-time weight to implicitly take into account the amount of data available at the country vs. region vs. super-region level and attribute spatial weight accordingly.
Given a normalisation constant,

$$K_i = \sum_{c\epsilon C} s.w_{c,t} * t.w_{c.t} + \sum_{c\epsilon R} s.w_{c,t} * t.w_{c.t} + \sum_{c\epsilon SR} s.w_{c,t} * t.w_{c.t}$$

the final space-time weight would then equal

$$w'_{c,a,s,t} = \frac{s.w_{c,t} * t.w_{c,t}}{K_i}$$

Finally, we calculated the weight $w''_{c,a,s,t}$ to smooth over age, which is based on a distance along the age dimension of two observations. For a point between the age a of the observation $r_{c,a,s,t}$ and a focal observation r_{c_0,a_0,s_0,t_0}, the weight is defined as follows:

$$w''_{c,a,s,t} = \frac{1}{e^{\omega|a-a_0|}}$$

The final weights were then computed by simply multiplying the space-time weights and age weights and normalising so all weights for a given time period *t* sum to 1. A full derivation of weights for each category, assuming the location being estimated was a country, follows:

1) If the observation $r_{c,t}$ belongs to the same country c_0 of the focal observation r_{c_0,t_0}:

$$w_{c,a,s,t} = \frac{(w'_{c,a,s,t}w''_{c,a,s,t})}{\sum_{c=c_0}(w'_{c,a,s,t}w''_{c,a,s,t})} \qquad \forall c = c_0$$

1) If the observation $r_{c,t}$ belongs to a different country than the focal observation r_{c_0,t_0}, but both belong to the same region *R*:

$$w_{c,a,s,t} = \frac{(w'_{c,a,s,t}w''_{c,a,s,t})}{\sum_{c\neq c_0}(w'_{c,a,s,t}w''_{c,a,s,t})} \qquad \forall c \neq c_0 \cap R[c] = R[c_0]$$

2) If the observation $r_{c,t}$ belongs to the same super region *SR* but to both a different country c_0 and a different region $R[c_0]$ than the focal observation r_{c_0,t_0}:

$$w_{c,a,s,t} = \frac{(w'_{c,a,s,t}w''_{c,a,s,t})}{\sum_{c\neq c_0}(w'_{c,a,s,t}w''_{c,a,s,t})} \qquad \forall c \neq c_0 \cap R[c] \neq R[c_0] \cap SR[c] = SR[c_0]$$

3) If the observation $r_{c,t}$ is from a different super region than the focal observation r_{c_0,t_0}(Ie, all other data currently not receiving a weight):

$$w_{c,a,s,t} = \frac{(w'_{c,a,s,t}w''_{c,a,s,t})}{\sum_{c\neq c_0}(w'_{c,a,s,t}w''_{c,a,s,t})} \qquad \forall c \neq c_0 \cap R[c] \neq R[c_0] \cap SR[c] \neq SR[c_0]$$

Observations could be down weighted by a factor of 0.1, usually because they were not geographically representative at the unit of estimation. Details of reasons for down weighting can be found in cause-specific modeling summaries. The final weights were then normalised such that the sum of weights across age, time, and geographic hierarchy for a reference group was 1.

Section 2.4.1: Estimating error variance

σ_p^2 represents the error variance in normal or transformed space including the sampling variance of the estimates and prediction error from any crosswalks performed. First, variance was systematically imputed if the data extraction did not include any measure of uncertainty. When some sample sizes for data were available, missing sample sizes were imputed as the 5th percentile of available sample sizes.

Missing variances were then calculated as $\sigma_p^2 = \frac{p*(1-p)}{n}$ for proportions or were predicted from the mean by using a regression for continuous values. When sample sizes were entirely missing and could not be imputed, the 95th percentile of available variances at the most granular geographic level (ie, first country, then region, etc.) were used to impute missing variances. For proportions where *p*n* or *(1-p)*n* is <20, variance was replaced by using the Wilson Interval Score method.

Next, if prevalence was modelled as a log transformation, the error variance was transformed into log-space by using the delta method approximation as follows:

$$\sigma_p^2 \cong \frac{\sigma_{p\prime}^2}{p_{c,a,s,t}^2}$$

where $\sigma_{p\prime}^2$ represents the error variance in normal space. If prevalence was modelled as a logit transformation, the error variance was transformed into logit-space by using the delta method approximation as follows:

$$\sigma_p^2 \cong \frac{\sigma_{p\prime}^2}{(p_{c,a,s,t} * (1 - p_{c,a,s,t}))^2}$$

Finally, prior to GPR, an approximation of non-sampling variance was added to the error variance. Calculations of non-sampling variance were done on normal-space variances. Non-sampling variance was calculated as the variance of inverse-variance weighted residuals from the space-time estimate at a given location-level hierarchy. If there were <10 data points at a given level of the location hierarchy, the non-sampling variance was replaced with that of the next highest geography level with >10 data points.

Section 2.4.2: Estimating the covariance function

The final input into GPR is the covariance function, which defines the shape and distribution of the trends. Here, we have chosen the Matern-Euclidian covariance function, which offers the flexibility to model a wide spectrum of trends with varying degrees of smoothness. The function is defined as follows:

$$M(t,t') = \sigma^2 \frac{2^{1-\nu}}{\Gamma(\nu)} \left(\frac{d(t,t')\sqrt{2\nu}}{l}\right)^{\nu} K_\nu \left(\frac{d(t,t')\sqrt{2\nu}}{l}\right)$$

where $d(\cdot)$ is a distance function; σ^2, ν, l, and K_ν are hyperparameters of the covariance function specifically σ^2 is the marginal variance, ν is the smoothness parameter that defines the differentiability of the function, l is the length scale, which roughly defines the distance between which two points become uncorrelated, and K_ν is the Bessel function. We approximated σ^2 by taking the normalised median absolute deviation $MADN(r_c')$ of the difference, which is the normalised absolute deviation of the difference of the first-stage linear regression estimate from the second-stage spatiotemporal smoothing step for each country. We then took the mean of these country-level MADN estimates for all countries with 10+ country-years of data to ensure that differences between first- and second-stage estimates had sufficient data to truly convey meaningful information on model uncertainty. We used the

parameter specification v=2 for all models. The scale parameter l used for each cause is reported in appendix sections 3.4 and 4.12.

Section 2.4.3: Prediction using GPR

We integrated over $g_{c,t}(t_*)$ to predict a full time series for country c, age a, sex s, and prediction time t_*as follows:

$$p_{c,a,s}(t_*) \sim N\left(m_{c,a,s,t}(t_*), \sigma_p^2 I + Cov\left(g_{c,a,s,t}(t_*)\right)\right)$$

Random draws of 1000 samples were obtained from the distributions above for every country for a given indicator. The final estimated mean for each country was the mean of the draws. In addition, 95% UIs were calculated by taking the 2.5 and 97.5 percentile of the sample distribution. The linear modelling process was implemented by using the lmer4 package in R, and the ST-GPR analysis was implemented through the PyMC2 package in Python.

Section 2.4.4: Subnational scaling and aggregation

To ensure internal consistency of the estimates between countries and their respective subnational locations, national estimates were either created by population-weighted aggregation or subnational estimates were adjusted by population-weighted scaling to the national estimates, depending on the data coverage of a given country compared to that of its subnational locations. For example, if data coverage was better at the national level than at its corresponding subnational locations for a given country and cause across age, sex, and time, estimates were rescaled to be consistent with the national level. Conversely, if data coverage was better at the subnational level, estimates for its parent country were generated through population-weighted aggregation of subnational estimates.
Estimates can also be scaled within logit space. Scaling in logit space ensures that subnational estimates of proportion models do not exceed one after being rescaled to the national estimate.

Section 2.5: MR-BRT meta-regression modelling

Section 2.5.1 MR-BRT Overview

MR-BRT is a meta-regression modeling tool developed at IHME. In contrast to other types of regression, meta-regression incorporates uncertainty in the dependent variable; each observation comes with its own standard error. This characteristic is important when the input data are results of scientific studies that are reported with uncertainty. Observations with greater uncertainty are given less weight in the model. To describe variation in the parameter of interest, MR-BRT can incorporate both fixed and random effects. Fixed effects include binary and continuous covariates as in a traditional regression model. Random effects describe group-level variation and are often used to characterize differences between studies beyond what is captured by measured covariates.

Section 2.5.2 MR-BRT Formula

Formally, a linear mixed effects meta-regression as implemented in MR-BRT can be described as:

$$y_{ij} = \beta_0 + \beta_1 x_1 + \cdots + \beta_n x_n + u_j + \epsilon_{ij}.$$

The variable y_{ij} refers to the value of observation i in study j; it is typically expressed in log or logit space to ensure that model predictions remain within logical constraints, for example that relative risks cannot be negative. The terms $\beta_0 + \beta_1 x_1 + \cdots + \beta_n x_n$ comprise the linear predictor, including both the intercept and the effects of any number of covariates. The term u_j is a random intercept corresponding to study j. The full set of random intercepts is assumed to follow a normal distribution where γ is the variance of between-study heterogeneity. Random effects can be estimated for continuous covariates as well, in which case they are called random slopes. The term ϵ_{ij} refers to the stochastic error corresponding to observation *i* in study *j*, and the set of values are assumed to follow a normal distribution in which observation-specific standard errors are known prior to modeling. This linear mixed effects formulation of the model covers most features MR-BRT. Features that involve nonlinear optimization techniques like the ratio model (described below) extend this framework and are described formally elsewhere.[12]

Section 2.5.3 MR-BRT Features

MR-BRT – as suggested by its full name "Meta-Regression with Bayesian priors, Regularization and Trimming" – comes equipped with several capabilities that expand upon the classical mixed effects meta-regression model:

Section 1: Bayesian priors can be applied to any estimated coefficient, enabling information from outside the dataset to be considered in the process of fitting the model. A Uniform prior sets hard bounds on the allowed values of an estimated coefficient. A Gaussian prior acts as a suggestion for the estimated value of a coefficient, with the standard deviation of the specified Gaussian distribution determining the strength of the prior.

Section 2: LASSO variable selection, also known as L1 regularization, can be implemented by specifying Laplace priors with mean 0 on the β coefficients. Similarly, ridge regression, also known as L2 regularization, can be implemented by specifying Gaussian priors with mean 0 on the β coefficients.

Section 3: Trimming is a method for identifying and removing the effects of outliers. Users define the proportion of points to be excluded and the algorithm determines which ones to exclude. Because the trimming algorithm is an integrated part of the model's likelihood function, MR-BRT identifies outliers and estimates the β coefficients simultaneously during the fitting process.

Section 4: A spline term may be used to describe the nonlinear effect of a covariate. MR-BRT implements a B-spline, or basis spline. Users have control over the flexibility of the estimated curve by specifying the number of knots, location of knots, spline degree (i.e. cubic or quadratic), linearity in the tail segments, convexity, concavity, or a monotonicity constraint requiring the spline to be non-decreasing or non-increasing.

Section 5: Pairs of exposure intervals may be used as an independent variable using a method known as the "ratio model". This feature is most often used when the epidemiological literature reports relative risks corresponding to a reference exposure range (e.g. BMI = [18,22)) and an alternative exposure range (e.g. BMI = [30,35)). It is usually used in conjunction with a spline to capture the nonlinear effect of the exposure. The ratio model works by integrating over the span of each interval and taking the ratio as part of the likelihood function.[12]

The source code for MR-BRT is publicly available on GitHub as the Python package mrtool (ihmeuw-msca, 2023). The mrtool package builds upon the open source mixed effects package LimeTr (https://github.com/zhengp0/limetr). For a full technical description of MR-BRT and the underlying mathematics.[12]

Section 2.6: DisMod-MR 2.1 estimation

Section 2.6.1: Estimation of sequelae and causes

The most extensively used estimation method is the Bayesian meta-regression method DisMod-MR 2.1. For some causes, such as HIV/AIDS or measles, disease-specific natural history models have been used for which the underlying three-state model in DisMod-MR 2.1 (susceptible, cases, dead) is insufficient to capture the complexity of a disease process. For some diseases with a range of sequelae differentiated by severity, such as COPD or diabetes mellitus, DisMod-MR 2.1 was used to meta-analyse the data on overall prevalence with separate DisMod-MR 2.1 models of the proportions of cases with different severity levels or sequelae. Likewise, DisMod-MR 2.1 was used to meta-analyse data on the proportions of liver cancer and cirrhosis due to underlying etiologies such as hepatitis B, hepatitis C, and alcohol use disorders.

Section 2.6.2: DisMod-MR 2.1 description

Until GBD 2010, non-fatal estimates in burden of disease assessments were based on a single data source on prevalence, incidence, remission, or a mortality risk selected by the researcher as most relevant to a particular location and time. For GBD 2010, we set a more ambitious goal: to evaluate all available information on a disease that passes a minimum quality standard. That required a different analytical tool that would be able to pool disparate information presented for varying age groupings and from data sources by using different case definitions. The DisMod-MR 1.0 tool used in GBD 2010 evaluated and pooled all available data, adjusted data for systematic bias associated with case ascertainment methods that varied from the reference and produced estimates by world regions with UIs by using Bayesian statistical methods. For GBD 2013, the improved DisMod-MR 2.0 increased computational speed, which allowed computations to be consistent between all disease parameters at the country rather than the region level. The hundred-fold increase in speed of DisMod-MR 2.0 was partly due to a more efficient rewrite of the code in C++, but also due to switching to a model specification of log rates rather than a negative binomial model used in DisMod-MR 1.0. In cross-validation tests, the log rates specification worked as well as or better than the negative binomial specification. In GBD 2015, we also improved how country covariates differentiate non-fatal estimates for diseases with sparse data. The coefficients for country covariates are re-estimated at each level of the cascade but with the default set to constraining estimates of covariates to the upper and lower bound of the global fit. For a given location, country coefficients are calculated by using both data and prior information available for that location. In the absence of data, the coefficient of its parent location is used to utilise the predictive power of our covariates in data-sparse situations.

For GBD 2016, the computational engine (DisMod-MR 2.1) remained substantively unchanged from GBD 2015. We updated the age prediction sets to include age groups 80–84 years, 85–89 years, 90–94 years, and 95 years and older to comply with changes across all functional areas of the GBD.

In GBD 2017, we continued to use DisMod-MR 2.1 because no substantial changes were made. Updates to computation include extending the terminal prediction year to 2017 and additional subnational units in Ethiopia, Iran, New Zealand, Norway, and the Russian Federation.

In GBD 2019, 2021 and 2023, no substantial changes were made to DisMod-MR 2.1, but we made more substantial changes to how we use the tool. First, we added the years 2019, 2020, and 2021, 2022 and 2023 as additional years of estimation. Second, we also included the option again to have random effects on cause-specific mortality rates (CSMR) and EMR. This functionality had been dropped a couple of GBD rounds earlier. Third, as we did all our adjustments for alternative case definitions and study methods as well as adjustments to combined-sex data points prior to entering data into DisMod-MR 2.1, we no longer used the functionality in DisMod-MR 2.1 to estimate coefficients for study and sex covariates and instead, processed data prior to entry in DisMod-MR 2.1 as described in the sections above on crosswalking and sex splitting. Fourth, based on simulation testing conducted in GBD 2019 we found that coverage improved, and errors reduced when passing down priors with a wider setting of minimum coefficient of variation (which determines the uncertainty around priors and hence how 'informative' the priors are) than had generally been used in past GBD iterations. We settled on a default value of 0.8 where in the past values of 0.4 or less had been more commonly used. We made some exceptions for highly prevalent conditions where a lower minimum coefficient of variation (CV) setting achieved the task of making priors less informative, but not completely uninformative.

DisMod-MR 2.1 simultaneously fits several epidemiologic measures using a Bayesian, nonlinear, mixed-effects regression model, which produces estimates of a consistent set of epidemiologic measures in a compartmental disease model, meaning that they are the solution to the set of differential equations that specify the model. The compartmental model assumes a steady state, and is often illustrated with the following figure S15, which we recreate here from Flaxman et a. (2015).[13] The system of differential equations that captures the relationships between rates and compartments follows the figure S15.

Figure S15. System of differential equations that captures relationships between rates and compartments

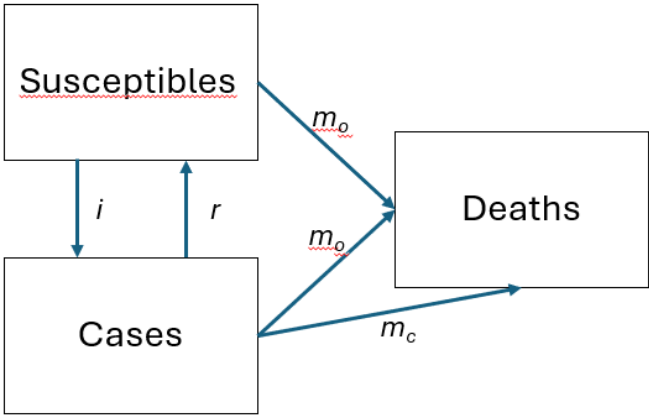

A three-compartment model of a disease in a population consisting of susceptibles, i.e. those in the population without disease, cases of the disease and deaths. Individuals move from susceptible to case by the incidence rate *i*, and can return to susceptible by remission rate *r*. Cases are susceptible to a mortality rate, m_i, that reflects the excess mortality in cases of the disease. Cases and susceptibles are subject to a mortality rate from other causes, m_o. Each quantity in figure S15 is a function of age and time, governed by the following differential equations:

$$\frac{d}{d\tau}S(a+\tau,t+\tau) = -(i+m_o)S + rC$$

$$\frac{d}{d\tau}C(a+\tau,t+\tau) = iS - (r+m_o+m_c)C$$

where:

S = $S(a,t)$ = stock of susceptibles

C = $C(a,t)$ = stock of cases, i.e. those in population with disease

i = $i(a,t)$ = incidence hazard for susceptibles S

r = $r(a,t)$ = remission ('cure') hazard for individuals with the disease C

m_o = $m_o(a,t)$ = without condition mortality hazard for susceptibles S

m_c = $m_c(a,t)$ = excess mortality hazard for individuals with the disease C

a and *t* denote age and time.

A Monte Carlo Markov Chain simulation starts with the prevalence at birth, and samples the posterior distribution integrating the observed values for all observations of all epidemiologic quantities, ensuring that susceptibles and cases sum to population size, and that the partial derivatives of each differential

equation in the system with respect to time zero. For most diseases the birth prevalence is set to zero. For disease that can be present at birth, a non-zero birth prevalence is specified. Population sizes for all combinations of year, age, sex, and location are input to DisMod-MR 2.1 from the latest GBD estimates, which are published iteratively and available by download from the GBD results tool.

The sequence of estimation occurs at five levels: global, super-region, region, country and, where applicable, subnational location. The super-region priors are generated at the global level with mixed-effects, non-linear regression by using all available data; the super-region fit, in turn, informs the region fit, and so on down the cascade. For some countries, additional models are fit for subnational locations. At each step in this cascade, only the data appropriate to that year, sex and geography are used to update the prior and yield the posterior. This is illustrated in figure S16 below.

Figure S16. Dismod-MR 2.1 sequence of estimation cascade

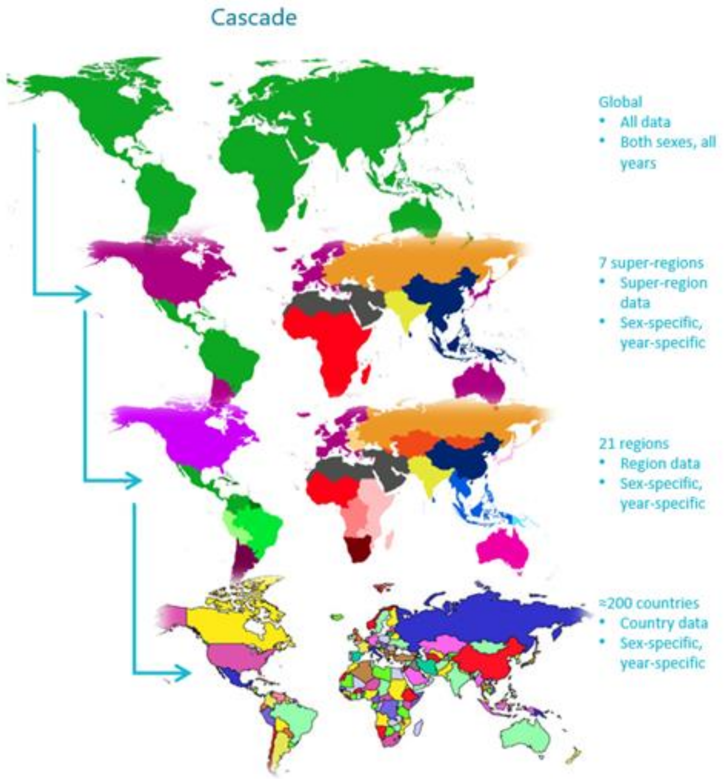

Once fitting is complete for the finest granularity, results are summed, at the draw level, weighting by population size, to produce final results for larger geographic units from the finest results.

Analysts can choose to branch the cascade in terms of time and sex at different levels depending on data density. The default used in most models is to branch by sex after the global fit but to retain all years of data until the lowest level in the cascade is reached.

The computational engine is limited to three levels of random effects; we differentiate estimates at the super-region, region, and country level. In GBD 2013, the subnational units of China, the United Kingdom and Mexico were treated as "countries" to enable a random effect to be estimated for every location with contributing data. However, the lack of a hierarchy between country and subnational units meant that the fit to country data contributed as much to the estimation of a subnational unit as the fits for all other countries in the region. We found inconsistency between the country fit and the aggregation of subnational estimates when the country's epidemiology varied from the average of the region. Adding an additional level of random effects required a prohibitively comprehensive rewrite of the underlying DisMod-MR engine. Instead, we added a fifth layer to the cascade, with subnational estimation informed by the country fit and country covariates, plus an adjustment based on the average of the residuals between the subnational location's available data and its prior. This technique mimicked the impact of a random effect on estimates among subnationals.

In GBD 2017 and 2019 GBD rounds we calculated priors on excess mortality and entered these as data points by matching sex-specific prevalence data with an age width of 20 or less with the corresponding CSMR for the same location and year. For stability, we excluded calculation of EMR for prevalence data points of less than 1 in a million. EMR is simply calculated as CSMR divided by prevalence. As with previous GBD years, for diseases with an average duration of less than a year (as indicated by a setting of remission greater than one), we ran an initial global model to get an equivalent prevalence and used the following formula to calculate EMR:

$$EMR = {}^{CSMR * (remission + (ACMR - CSMR) + EMR\_pred)}\!/_{incidence}$$

where,

ACMR is the all-cause mortality rate

EMR_pred is the EMR fit from an initial global DisMod model

Despite using the log of LDI or the HAQ Index as a covariate with a prior that the coefficient had to be negative, we found many disease models with an implausible distribution of mortality to prevalence (or incidence) ratios implying lower case fatality in locations with lower HAQ Index than in countries with higher HAQ Index. This likely signals an inconsistency between fatal and non-fatal data inputs. For GBD 2019, we decided to run regressions on EMR data (calculated as described above) first using MR-BRT with HAQ Index as a predictor. In general, we tend to think that CSMR estimates are more robust than non-fatal data because of much greater data availability and a lesser task in adjusting cause death data for garbage coding than the complex task of adjusting non-fatal data sources for alternative case definitions and study methods. To indicate that we would reduce the random effects on EMR and the minimum coefficient of variation for priors on EMR being created at each next level down the cascade.

Section 2.6.3: DisMod-MR 2.1 likelihood estimation

Analysts have the choice of using a Gaussian, log-Gaussian, Laplace, or Log-Laplace likelihood function in DisMod-MR 2.1. The default offset-log-Gaussian equation for the data likelihood is

$$-\log[p(y_j|\psi)] = \log(\sqrt{2\pi}) + \log(\delta_j + s_j) + \frac{1}{2}\left(\frac{\log(a_j+\eta_j) - \log(m_j+\eta_j)}{\delta_j + s_j}\right)^2$$

where,

y_{j} is the value of the point estimate of the epidemiologic measure (prevalence, incidence, remission, excess mortality rate, with-condition mortality rate, cause-specific mortality rate, relative risk or standardized mortality ratio) for observation j

Φ denotes all model random variables

η_j is the offset value, *eta*, for a particular "integrand" (prevalence, incidence, remission, excess mortality rate, with-condition mortality rate, cause-specific mortality rate, relative risk, or standardised mortality ratio)

a_j is the adjusted measurement for data point j, defined by

$$a_j = e^{(-u_j - c_j)} y_j$$

where:

u_j is the total "area effect" (i.e., the sum of the random effects at three levels of the cascade: super-region, region and country); random effects at the same level of geography are normally distributed with a group mean of zero; and

c_j is the total covariate effect (i.e., the mean combined fixed effects for sex, study level, and country level covariates), defined by

$$c_j = \sum_{k=0}^{K[I(j)\}-1} \beta_{I(j),k} \hat{\mathrm{X}}_{k,j}$$

with SD

$$s_j = \sum_{l=0}^{L[I(j)\}-1} \zeta_{I(j),l} \hat{\mathrm{Z}}_{k,j}$$

where:

k denotes the mean value of each data point in relation to a covariate (also called x-covariate)

I(j) denotes a data point for a particular integrand *j*

$\beta_{I(j),k}$ is the multiplier of kth covariate for the ith integrand

$\hat{X}_{k,j}$ is the value of covariate k corresponding to observation j

l denotes the SD of each data point in relation to a covariate (also called z-covariate)

$\zeta_{I(j),k}$ is the multiplier of the lth z-covariate for the jth integrand

δ_j is the standard deviation of the adjusted measurement for observation j, after adjustment of y_j for location random effects and fixed effect covariates relevant to that integrand

$$\delta_j = \log\left[y_j + e^{(-u_j - c_j)}\eta_j + c_j\right] - \log\left[y_j + e^{(-u_j - c_j)}\eta_j\right]$$

where:

m_j denotes the predicted value of the integrand corresponding to observation j based on underlying age-specific rates, without accounting for location random effects (u_j) or fixed effects for predictive covariates (c_j). That is to say, it is an integration of the fitted spline values for the age-range referred to by observation j, and defined by:

$$m_j = \frac{1}{B(j) - A(j)} \int_{A(j)}^{B(j)} I_j(a)da$$

where:

$A(j)$ is the lower bound of the age range for a data point

$B(j)$ is the upper bound of the age range for a data point

I_j denotes the function of age corresponding to the integrand for data point j

Section 2.7: DisMod-AT estimation

DisMod-AT was developed to meet the need for a modeling tool which can estimate disease progression over age and time simultaneously. This capability is crucial because many diseases exhibit age, time, and cohort dynamics, which cannot be modeled independently of each other.[14–16] By including both age and time in its model simultaneously, DisMod-AT is able to estimate dynamics for all three, producing much improved estimates of diseases for which they are strongly linked and advancing the state of the art for disease modeling.

The compartmental model of disease progression used in previous versions of DisMod (
Section 2.6: DisMod-MR 2.1 estimation) is also used by DisMod-AT, but with the addition of time t:

$$S'(a,t) = -\big(\iota(a,t) + \omega(a,t)\big)S(a,t) + \rho(a,t)C(a,t)$$

$$C'(a,t) = \iota(a,t)S(a,t) - \big(\rho(a,t) + \chi(a,t) + \omega(a,t)\big)C(a,t)$$

with the initial conditions $C(0,t) = P(0,t)$ and $S(0,t) = 1 - P(0,t)$, where:

$S(a,t)$ is the fraction of the population which is susceptible to the condition,

$C(a,t)$ is the fraction of the population with the condition,

$P(0,t)$ is birth prevalence rate; $\iota(a,t)$ is incidence rate,

$\rho(a,t)$ is remission rate; $\chi(a,t)$ is excess mortality rate, and

$\omega(a,t)$ is mortality rate from other causes.

DisMod-AT's key advance from DisMod-MR 2.1 is to explicitly consider the equations' age- and time-dependence simultaneously, whereas DisMod-MR 2.1 considered them time-independent. By taking time to be the time of birth for a population cohort, c, such that $f(a,t) = f(a, c+a) = f_c(a)$, these equations can be written:

$$S_c'(a) = -\left(\iota_c(a) + \omega_c(a)\right)S_c(a) + \rho_c(a)C_c(a)$$

$$C_c'(a) = \iota_c(a)S_c(a) - \left(\rho_c(a) + \chi_c(a) + \omega_c(a)\right)C_c(a)$$

DisMod-AT solves this system in age and time simultaneously by utilizing interior-point optimization methods rather than the Markov chain Monte Carlo method in DisMod-MR.[17–19] This advance is what allows DisMod-AT to estimate age and time dynamics in disease, which is impossible if the equations are considered independently of either age or time.

DisMod-AT uses the same geographical cascade structure as DisMod-MR, carrying estimation strength across geography using Bayesian priors (Section 1.2: Geographical locations of the analysis). First, DisMod-AT estimates disease at the global level drawing from all the world's data and passes that estimate to the seven super-regions as their prior after adjustment by the random effect for each super-region and the beta values of predictive covariates that were chosen of any of the primary rates (remission, incidence and excess mortality) in the model. Then, regions are fit using super-region estimates to inform their priors, followed by fits for country-level locations using region estimates to inform their priors, and concluding with fits for sub-national locations, where present, using country estimates to inform their priors.

The model for each estimated quantity, integrands in GBD publication nomenclature, is a likelihood function of the form:[20]

$$\mathcal{P}(y_i|u,\theta) = e^{D\left(y_i, A_i(u,\theta), \delta_i(\theta)\right)}$$

where y_i is the ith measurement (data) value, u are the random effects, θ are the fixed effects (i.e. of predictive covariates), D is the probability density function corresponding to y_i, specified as a configuration option by the analyst, $A_i(u,\theta)$ is the ith average integrand:[21]

$$A_i(u,\theta) = \frac{1}{b_i - a_i}\frac{1}{d_i - c_i}\int_{a(i)}^{b(i)}\int_{c(i)}^{d(i)}\frac{w_i(a,t)}{\overline{w}_i}I_i(a,t)e^{E_i(a,t)}\,\mathrm{d}t\,\mathrm{d}a$$

$I_i(a,t)$ is the ith integrand function, in terms of $\iota(a,t)$, $\rho(a,t)$, $\chi(a,t)$, $\omega(a,t)$, $S(a,t)$, and $C(a,t)$, $\overline{w}_i$ is the age-time weighting:

$$\overline{w}_i = \frac{1}{b_i - a_i} \frac{1}{d_i - c_i} \int_{a(i)}^{b(i)} \int_{c(i)}^{d(i)} w_i(a,t)\,\mathrm{d}t\,\mathrm{d}a$$

$E_i(a,t)$ is the effect for the ith measurement value, in terms of the fixed effects θ:

$$E_i(a,t) = \sum_{j \in K_i} x_{i,j} \left(\beta_j(a,t) + \Delta\beta_j(a,t) \right)$$

$\beta_j(a,t)$ is a piecewise linear function giving the effect for covariate $j \in K_i$, $x_{i,j}$ is the value of covariate $j \in K_i$, K_i are the set of covariates on the ith integrand; $\delta_i(\theta)$ is the transformed standard deviation corresponding to y_i:

$$\delta_i(\theta) = \begin{cases} \log\left(y_i + \eta_i + \sigma_i(\theta)\right) - \log(y_i + \eta_i) & \text{if log density} \\ \sigma_i(\theta) & \text{otherwise} \end{cases}$$

η_i is a small number which offsets log densities to prevent infinities, $\sigma_i(\theta)$ is the standard deviation corresponding to y_i:

$$\sigma_i(\theta) = \begin{cases} \Delta_i + \varepsilon_i(\theta) \\ \Delta_i\left(1 + \varepsilon_i(\theta)\right) \\ \sqrt{\Delta_i^2 + \varepsilon_i(\theta)} \\ \Delta_i\sqrt{1 + \varepsilon_i(\theta)} \end{cases}$$

Δ_i is a configurable option specifying a minimum coefficient of variation on the measurement, $\varepsilon_i(\theta)$ is the estimated average noise effect:

$$\varepsilon_i(\theta) = \frac{1}{\overline{w}_i} \int_{a(i)}^{b(i)} \int_{c(i)}^{d(i)} w_i(a,t) \sum_{j \in K_i} x_{i,j}\gamma_j(a,t)\,\mathrm{d}t\,\mathrm{d}a$$

and $\gamma_i(a,t)$ is a piecewise linear function for the covariate multiplier values.

Analysts specify the set of age points and of time points for which DisMod-AT estimates disease rates as configuration options. DisMod-AT interpolates between these points bilinearly when required. In tandem, analysts also specify Bayesian priors on the change in estimated rates between age or time points, configuring how much the model expects estimated rates to vary over each age or time increment. These priors can be specified for each rate in the compartmental model and may be specified over subsets of age or time points. Additionally, analysts have the option to specify Bayesian priors on covariate fixed effects, random effects, measurement value effects, and measurement noise effects.

We selected four causes—type 1 diabetes, major depressive disorder (MDD), anxiety disorders, and autism spectrum disorders—for our initial roll-out of DisMod-AT in GBD 2023 These causes were chosen over concerns that DisMod-MR 2.1 may not have been adequately capturing trends in the underlying data. For GBD 2021, the impact of the COVID-19 pandemic on MDD and anxiety disorders had to be quantified via a custom analysis after analysis in DisMod-MR 2.1. While we have not conducted a formal

comparison of results generated by DisMod-AT versus DisMod-MR for these conditions, DisMod-AT has improved our ability to model temporal trends and generate more robust estimates, especially where non-linear age or cohort effects are present. DisMod-AT has been more sensitive to the increase in prevalence of depressive and anxiety disorders and autism spectrum disorder which were evident in epidemiological data for some regions, and has allowed us to allowed us to more accurately model known adult epidemiology for type 1 diabetes, despite a paucity of data for adult age groups. DisMod-AT allowed the impact of the COVID-19 pandemic to directly influence the incidence of both MDD and anxiety disorders via a location-level covariate, avoiding the need for post-analysis adjustments required of DisMod-MR 2.1. The transition to DisMod-AT is ongoing, and its broader implementation across causes will be accompanied by systematic validation exercises.

Section 2.8: Impairment and underlying cause estimation

For GBD 2023, as in GBD 2021, GBD 2019, GBD 2017 and GBD 2016, we estimated the country-age-sex-year prevalence of nine impairments. Impairments in GBD are conditions or specific domains of functional health loss that are spread across many GBD causes as sequelae and for which there are better data to estimate the occurrence of the overall impairment than for each sequela based on the underlying cause. These impairments included anaemia, epilepsy, hearing loss, heart failure, intellectual disability, infertility, vision loss, Guillain-Barré syndrome, and pelvic inflammatory disease. Overall impairment prevalence was estimated by using DisMod-MR 2.1. We constrained cause-specific estimates of impairments, as in the 19 causes of blindness, to sum to the total prevalence estimated for that impairment. Anaemia, epilepsy, hearing loss, vision loss, heart failure, and intellectual disability were estimated at different levels of severity. Estimates were made separately for primary infertility (those unable to conceive), secondary infertility (those having trouble conceiving again), and whether the impairment affected men and/or women. In the case of epilepsy, we determined the proportions with idiopathic and secondary epilepsy as well as the proportions with severe and less severe epilepsy by using mixed effects regressions. The sparse data for the proportion of seizure-free, treated epilepsy were pooled in a random effects meta-analysis. DisMod-MR 2.1 models produced country-, age-, sex-, and year-specific severity levels of hearing loss and vision loss. Because of limited information on the severity levels of intellectual disability, we assumed a similar distribution of severity globally based on random effects meta-analysis of IQ-specific data for the overall impairment. This assumption was supplemented by cause-specific severity distributions for chromosomal causes and iodine deficiency; the severity of intellectual disability included in the long-term sequelae of causes including neonatal disorders, meningitis, encephalitis, neonatal tetanus, and malaria was estimated in combined health states of multiple impairments such as motor impairment, blindness, and/or seizures (R, 2015) .We changed the name of the intellectual disability impairment to specify that estimates reflect cases arising during the developmental period, which we have defined as ages under 20 years. The severity of heart failure was derived from our Medical Expenditure Panel Surveys (MEPS) analysis and therefore was not specific for country, year, age, or sex.

A detailed description of the methods of each impairment can be found in Section 6 of this appendix.

Section 2.8.1: Impairment squeeze

For the impairments epilepsy, intellectual disability, and blindness, mentioned above in Step 4, we often have better information regarding the total prevalence of the impairment rather than the prevalence of said impairment due to its various causes. For example, we have more data and a better idea of the total number of blind individuals (which we refer to herein as the blindness "envelope") in the world than we do the number of individuals who are blind due to a specific cause like retinopathy of prematurity or cataract. We achieve this consistency by either squeezing or inflating the individual sequela prevalence values so that their sums fit into each appropriate envelope. Blindness, epilepsy, and/or intellectual disability appear in various combinations with motor impairment levels as sequelae for a number of neonatal disorders and infectious diseases like malaria and neonatal tetanus ("Moderate motor impairment with blindness and epilepsy due to neonatal tetanus", for example). This presents an extra challenge because any squeeze or inflation of one of the impairments making up a sequela affects the others.

We set rules on how to do these adjustments sequentially. First, when the envelope of an impairment is smaller than the sum of all contributing causes, we redistribute the excess prevalent cases of combined impairment sequelae onto the sequelae that only have motor impairment (at a mild, moderate, or severe level) within the same cause grouping. Second, we apply the adjustments in a particular order such that we always fit at least one of the envelopes exactly where the other one or two envelopes may be exceeded by some amount. We first enforce a fit to the epilepsy impairment envelope, then intellectual disability, and last, blindness. Thus, the epilepsy envelope always matches exactly, whereas the intellectual disability and blindness envelopes may occasionally be exceeded on a draw-by-draw basis.

Sequelae were defined in terms of severity for 236 causes. We generally followed the same approach for estimating the distribution of severity we used in GBD 2021. In cases in which severity was related to a particular impairment, such as mild, moderate, and severe heart failure due to ischaemic heart disease or pulmonary arterial hypertension, the analysis was driven by impairment estimation methods. Severity levels for causes such as chronic kidney disease, epilepsy and COPD were modelled using DisMod-MR 2.1 or ST-GPR, whereas we performed meta-analyses to estimate the allocation of severity for causes such as rheumatoid arthritis, and multiple sclerosis. For dementia, we changed from using meta-analysis of three age categories to a more flexible model in MR-BRT using a spline on age. That allowed us to increase the number of studies informing severity from 7 to 67. For gallbladder and biliary diseases, we performed a meta-analysis of six community-based studies of the proportion of cases of gallbladder disease identified by ultrasonography who are symptomatic. In previous rounds, inpatient admission for gall bladder and biliary disease as a primary diagnosis were taken to represent symptomatic cases.
For many causes, we continue to have inadequate data on severity from surveys or the epidemiological literature. For those diseases, we made use of three population surveys: the MEPS (Medical Expenditure Panel Survey) 2000–2014, the [US] National Epidemiological Survey on Alcohol and Related Conditions (NESARC)[22] 2000–2001 and 2004–2005, and the Australian National Survey of Mental Health and Wellbeing of Adults (NSMHWB) 1997 (Mental Health and Wellbeing: Profile of Adults, Australia, 1998)[23]. Each dataset contained individual-level measurements of functional health status made by using the 12-Item Short Form Health Survey (SF-12) as well as diagnostic information on the causes affecting each individual.

To use the data collected by measuring the distribution of severity with the SF-12, the individual SF-12 summary scores were mapped to an equivalent DW. A convenience sample of respondents was asked to complete SF-12 for the hypothetical individual living in a health state described by using a selection of 60 of the 235 health states with their lay descriptions from the GBD DW surveys reflecting the full range of severity. Each of these health states has a measured DW associated with it on a zero to one scale. We collected 2783 usable responses in total.

The final relationship between SF-12 score and DW is depicted in Figure S17:

Figure S17. SF-12 composite scores and disability weights for 60 health states with fitted loess regression

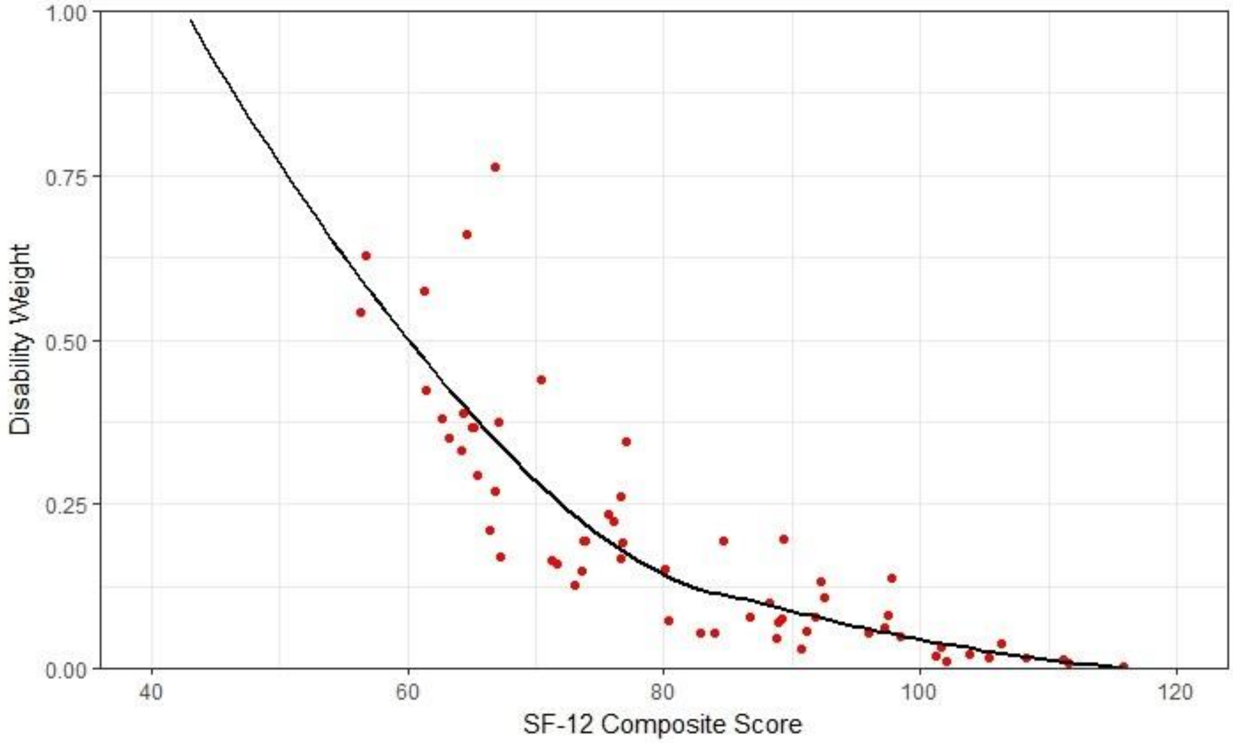

1.

To generate a smooth mapping from SF-12 combined scores to the GBD DW space, we used locally estimated scatterplot smoothing regression on the random effects for each health state. DWs were capped to remain between 0 and 1. All SF-12 survey data were thus transformed into DW space.
The second stage of the analysis was to build models predicting the transformed SF-12 scores as a function of the number of causes suffered by each individual. First, variable selection was performed by using least absolute shrinkage and selection operator (LASSO) regression to penalize the regression coefficients of highly correlated causes. The tuning parameter, λ, controls the strength of the least-squares penalty. When λ=0, LASSO regression returns the same results as ordinary least-squares regression. Higher values of λ impose a stronger penalty and constrain a greater number of model parameters to 0. A ten-fold cross-validation was used to find the value of the λ that minimized the mean cross-validated error. This process resulted in a λ value of 0.0013 and eliminated 10 causes from the

analysis. Transformed SF-12 scores into the DW scale for the remaining 190 causes were then modelled for each measure *m* of each individual *i* over *n* total causes in the survey as follows:

$$logit(DW)_{im} = \beta_0 + \beta_1 Condition1_{im} + \cdots + \beta_n Condition\ n_{im}$$

This equation effectively assumes that comorbid causes act to change SF-12 scores in a multiplicative fashion rather than an additive fashion.
To estimate the comorbidity-corrected effect of each cause (ie, in isolation) on total disability, we compared the predicted DW without the cause of interest (counterfactual DW) with the predicted DW including the cause of interest. Following the multiplicative comorbidity equation, the joint effect can be written

$$Condition\ specificDW = 1 - \frac{1 - predictedDW_m}{1 - counterfactualDW_m}$$

The mean of this cause-specific effect over all observations is the population marginal effect of a cause.

Using the model above, we estimate a counterfactual DW – the total individual DW excluding the effect of the cause of interest. We compared the observed distribution of functional health status with this counterfactual distribution to determine the marginal effect of the cause of interest. In other words, we estimated the health state for each individual and for each cause as the cumulative individual weight minus the effects of all comorbid causes.

$$Health\ state\ DW = 1 - \frac{1 - individual\ cumulativeDW_m}{1 - counterfactualDW_m}$$

The estimation strategy for health state-specific severity distributions for which there are multiple severity categories involved binning individuals' weights into severity cut-offs (eg, mild, moderate, and severe) for which DWs were derived. These bins were defined by using results from the GBD Disability Weights Studies[24] for causes that had multiple health states defined. Cut-offs for the severity group were the midpoints between DWs of the health state and cases distributed into severity bins accordingly. For example, individuals with a health state DW above the mid-point between the mild DW and the moderate DW for a particular condition would be assigned the moderate sequela. Cases were considered asymptomatic if the counterfactual weight was equal to or greater than the individual cumulative weight. The proportion of cases of a condition assigned to each level of severity for that condition was then used as the severity distribution of the condition for prevalence estimates to be apportioned accordingly into severity-specific prevalence estimates.

Section 2.9: Disability weights

To compute YLDs for a particular health outcome in a given population, the number of people living with that outcome is multiplied by a disability weight (DW) that represents the magnitude of health loss associated with the outcome. DWs are measured on a scale from 0 to 1; 0 implies a state equivalent to full health, and 1, a state equivalent to death.

Section 2.9.1: GBD 2010 Disability Weights Measurement Study

For GBD 2010, a primary data collection effort focused on measuring health loss rather than welfare loss by using a standardised approach of simple comparison questions directed to the general public across diverse communities.

Multi-country household surveys were conducted between Oct 28, 2009 and June 23, 2010 in five countries (Bangladesh, Indonesia, Peru, Tanzania, and the USA) selected to provide diversity across culture, language, and socioeconomic status.

Personal face-to-face computer-assisted interviews were conducted for all household surveys except for the survey in the US, which was conducted by computer-assisted telephone interview. Households were randomly selected by using a multistage stratified sampling design for which the probability of selection was proportional to the population size. In all cases, samples were designed to be representative of a given geographical area and, in the USA, to provide national representation.

For every contacted household, an adult respondent age 18 years or older was randomly selected by the survey program by means of the Kish approach. For face-to-face interviews, as many as three visits were made to selected households to establish contact. When a respondent was identified, as many as three return visits were made to do the survey at a time when the respondent was available. For the US telephone surveys, repeated calls were made up to seven times.

A web-based survey was posted at a dedicated URL between July 26, 2010 and May 16, 2011. The survey was initially available in English and subsequently available in Spanish and Mandarin. Recruitment of respondents occurred through several channels, such as news items and editorials in scientific journals, announcements at scientific meetings, postings on websites of institutions participating in the GBD, and social networking and communication mobilisation channels as well as direct contact with individuals and groups with known global health interests by tapping into the professional networks of the study investigators and their colleagues. Participants in the web-based survey were required to be ages 18 or older. Household surveys obtained oral informed consent from all participants; written informed consent was obtained from participants in the web survey. Ethical review board approval was obtained from each household survey site and the University of Washington, Seattle, WA.

Standardised survey instruments were developed to obtain comparative assessments of the full array of disease and injury sequelae, parsimoniously captured in 220 unique health states. Lay descriptions of health states formed the basis for all comparisons. These descriptions used simple, non-clinical vocabulary that emphasised the major functional consequences and symptoms associated with each health state. Development of these descriptions involved an iterative process of detailed consultation with experts participating in the GBD 2010 study; the goals was to capture the most relevant details of each health state while avoiding ambiguity and ensuring consistency. When possible, health states were grounded in standard clinical classifications systems. For example, the Canadian Cardiovascular Society grading scale was referenced for descriptions of stages of angina,[25] and the New York Heart Association

functional classification was referenced for severity of heart failure.[26] Pilot testing indicated that the lay descriptions in face-to-face interviews should not exceed 30 words.

A paired comparison question formed the basis of all surveys. The questions in the survey were framed with the following statement, "A person's health may limit how well parts of his body or mind work. As a result, some people are not able to do all of the things in life that others may do, and some people are more severely limited than others. I am going to ask you a series of questions about different health problems. In each question, I will describe two different people…" Descriptions of two hypothetical people, each with a particular health state, were presented to respondents who were then asked which person they regarded as healthier. Health pairs in all surveys were selected by a randomizing computer algorithm. In the five household surveys, paired comparisons were presented for a subset of 108 health states pertaining to chronic conditions. The framing of chronic and acute conditions is different as they were presented as causing life-long or temporary health loss. We chose to only field health states that could be framed as lasting a lifetime in the household surveys as we hypothesized that presenting differently framed comparisons would be difficult to convey in face-to-face interviews. In the web survey, we considered this more feasible because respondents could read and refer to the framing of the question for each pair-wise comparison. All 220 health states were thus evaluated in the web survey.

In addition, the web survey included questions relating to population health and health programs specifically—such as "Imagine two different health programs. The first program prevented 1000 people from getting an illness that causes rapid death. The second program prevented 2000 people from getting an illness that is not fatal but causes lifelong health problems resulting in moderate to severe disability. Which program would you say produced the greater overall health benefits?" This information was used to anchor the results from the pair-wise comparisons on the 0–1 DW scale.

Section 2.9.2: GBD 2013 European disability weights measurement study

The GBD 2010 DWs were critically dependent on the ways that outcomes were described to survey respondents. Descriptions for health states were designed to balance validity and parsimony, and this approach necessarily meant that some details of different health states had to be omitted. Because lay descriptions were developed collaboratively through individual expert groups organised around a particular set of health issues, some amount of variability in language and detail inevitably occurred. Criticisms and suggestions for improvement came from a number of commentators on the GBD 2010 DWs measurement study.[27–29]

GBD 2013 expanded the list of disease and injury causes and sequelae mapped to 235 unique health states. Additional data for the European Disability Weights Measurement Study were collected between September 23, 2013, and November 11, 2013, in Hungary, Italy, the Netherlands, and Sweden. The initiation of these surveys was connected to a project sponsored by the European Centre for Disease Prevention and Control.[30] The four selected countries were chosen to be representative of the four regions of Europe (east, south, middle, and north) in terms of age, sex, and education of the respondents. Respondents were recruited from standing internet panels in each country on the basis of quota sampling with reference to age, sex, and education in such a way as to maintain the population representativeness of these characteristics. Eligible participants were 18–65 years old and were

preselected in the Netherlands, where the age, sex, and education of respondents were already known, or in the other three countries, invited to participate via a web-link and then selected on the basis of their individual characteristics.

The protocol for the European DWs measurement study followed the protocol that was developed and implemented in the GBD 2010 DWs measurement study. Lay descriptions for some health states that lacked mention of an important symptom or for which consistency of wording across different levels of severity had been noted were reworded. The European DWs measurement study included 255 health states, of which 183 were used in the analyses of GBD 2013. Those 183 consisted of 135 of the 220 health states that were included in the European DWs measurement study with unmodified lay descriptions and 30 from GBD 2010 for which alternative lay descriptions were included. DWs were estimated for additional sequelae that were incorporated into GBD 2013 but had not been included in GBD 2010.

Finding high correlation in resulting DW values between the country surveys and the web survey, we analysed the results of all surveys together. We ran probit regression analyses on the answers to the pair-wise comparison questions by using dummies for each health state with a value of 1 for the first state in a pair, –1 for the second state in a pair, and 0 for all states other than the pair. This method formalizes the intuition that if two health states in a pair produce similar health loss, the answers are likely to be evenly split; a pair of health states with very different health loss get many more responses favouring one over the other. The statistical methods infer the distances between values attached to different health states based on the frequencies of responses to the paired comparisons.

A second analytic step is needed to anchor the resulting estimates onto the 0–1 DWs scale, where 0 equals no loss of health, with 1 meant to represent loss equivalent to death. We anchored results from the probit regression analysis onto the 0–1 scale by using population health equivalence data from the GBD 2010 web survey by using a linear regression of the probit coefficients from the analysis of paired comparisons on the logit-transformed DW estimates derived from interval regression of the population health equivalence responses. Using numerical integration, we then estimated mean values for DWs on the natural 0–1 scale. Uncertainty was estimated by bootstrapping with 1,000 samples. For a complete overview of disability weights applied to the Global Burden of Disease Study, see the GBD 2013 article by Salomon and colleagues.[24]

Section 2.10: Comorbidity correction (COMO)

The final stage in the estimation of YLDs is a micro-simulation, which adjusts for comorbidity. We refer to this micro-simulation process as “COMO” (for comorbidity correction). We estimated the co-occurrence of different diseases by simulating 20,000 individuals in each location-age-sex-year combination as exposed to the independent probability of having any of the sequelae included in GBD based on prevalence. We tested the contribution of dependent and independent comorbidity in the US MEPS data and found that independent comorbidity was the dominant factor even though well-known examples of dependent comorbidity exist, such as clustering of conditions like diabetes and stroke or anxiety and alcohol use disorders. Age was the main predictor of comorbidity such that age-specific micro-simulations accommodated most of the required comorbidity correction.[31]

The two components necessary for the computation of YLDs and are the two inputs into COMO: 1) prevalence of each disease sequela and 2) DWs. The prevalence values of causes are primarily produced by using DisMod-MR 2.1 and, for causes with multiple sequelae, subsequently apportioned into sequela-specific prevalence based on available estimates of the severity distribution. The estimation of DWs and severity distributions have been described earlier in this appendix.

The micro-simulation, as performed for each age-sex-location-year, can best be represented as a four-step process. First, simulated individuals (simulants) are exposed to independent probabilities of having each sequela, where the probability is equal to the prevalence estimate. For each simulant, the probability of having a disease sequela is equal to the estimated prevalence. Each simulant is determined to have or not have the disease sequelae based on a draw from a binomial distribution. From this simulation, simulants end up with any number of sequelae, from 0 up to the theoretical maximum given their demographics. Second, the DW for each simulant is estimated on the basis of the disease sequelae that they have acquired. The formula for the cumulative DW for a simulant is one minus the multiplicative sum of one minus each DW present

$$Simulant\ DW_l = 1 - \prod_{k=i}^{j}(1 - DW_k)$$

Where:

DW_k is the DW for the kth disease sequela that the simulant *l* has acquired.

Once the simulant DW is computed, the DW attributable to each sequela for the simulant is calculated by using the following formula:

$$ADW_{lk} = \frac{DW_k}{\sum_{k=i}^{k=j} DW_k} * Simulant\ DW_l$$

Where:

ADW_{lk} is the attributable DW for disease sequela *k* in simulant *l*,

DW_k is the DW for disease sequela *k*.

Simulant DW_l is the DW for simulant *l* from the combination of all sequelae that they have acquired. This formula apportions the overall simulant DW to each condition in proportion to the DW of each condition in isolation.

Finally, YLDs per capita in an age-sex-country-year are computed by taking the sum of the attributable DWs for a disease sequela across simulants.

$$YLD\ Rate_k = \frac{\sum_{l=1}^{n} ADW_{lk}}{n}$$

The actual number of YLDs from disease sequela *k* in an age-sex-location-year is then computed as the YLD rate *k* times the appropriate age-sex-location-year population.

By repeating the simulation process for each age-sex-country-year 250 times, the uncertainty in the prevalence of each disease sequela and the DW is propagated into the final comorbidity corrected YLD

results. We selected 20,000 simulants for each age-sex-location-year group on the basis of simulation testing, which has shown that results are stable for YLDs at this number of simulants even in the younger age groups when prevalence is relatively low. Mean results for YLDs that reflect 10 million simulants (20,000 simulants multiplied by 250 iterations to capture uncertainty) are very stable in each age-sex-location-year. For any given location-year-age-sex group, acause aggregate prevalence values were calculated as $1- \prod(1\text{-prevalence})$.

Section 2.11: YLD computation, uncertainty, and residual YLDs

We computed YLDs by sequela as prevalence multiplied by the DW for the health state associated with that sequela. The uncertainty ranges reported around YLDs incorporate uncertainty in prevalence and uncertainty in the DW. To do this, we take the 250 samples of comorbidity-corrected YLDs and 250 samples of the DW to generate 250 samples of the YLD distribution. We assume no correlation in the uncertainty in prevalence and DWs. The 95% uncertainty interval is reported as the 0.025 and 0.975 quantiles of the distribution. DW draws are not year specific; UIs for YLDs at different points in time for a given disease or sequela are correlated because of the shared uncertainty in the DWs. For this reason, changes in YLDs over time can be significant even if the UIs of the two estimates of YLDs largely overlap. Prevalence UIs are used to determine significance of change in YLDs over time since DW draws are year agnostic.

Section 2.11.1: Residual YLDs

Despite expanding our list of causes and sequelae in successive GBD iterations, many diseases remain for which we do not explicitly estimate disease prevalence and YLDs. Less common diseases and their sequelae were included in 34 residual categories (table 10). For 22 of these residual categories, epidemiological data on incidence or prevalence were available, so these were modelled accordingly. For 13 residual categories, epidemiological data on incidence and prevalence were not available, but sufficient CoD data allowed for CoD estimates. For these residual categories, we estimated YLDs by multiplying their YLL estimates by the ratio of YLDs to YLLs from the Level 3 causes in the same disease category that were explicitly modelled. This scaling was done for each country-sex-year. This approach made the simplifying assumption that the residual diseases caused disability proportionate to the ratio of disability to mortality in explicitly modelled diseases. We did not include causes with large disability but no or little mortality in estimating these ratios. For example, we estimated the YLDs from other neurological disorders from the YLD to YLL ratios for dementia, multiple sclerosis, and Parkinson's disease but did not include the YLDs from headaches and epilepsy in the ratio. Detailed information on how YLDs for residual causes were estimated are available in their respective cause writeups in section 6.

Section 2.12: Birth prevalence

A number of conditions are present at birth and quantifying them is important in fully describing the epidemiology of diseases within populations. These include many conditions included in the GBD cause group of neonatal disorders, infections that are transmitted from mother to child either transplacentally or during birth, and congenital birth defects arising either *de novo* or from maternal exposures. Although these conditions were included in the underlying models informing previous GBD iterations,

we developed a system for reporting them for the first time in GBD 2017; a list of these causes is reported in table 11.

Mathematically (ie, in the models), conditions present at birth are equivalent to “birth prevalence.” However, we report these as “incidence” in recognition of the way that GBD defines incidence as a new case of a disease or injury entering the population. To process these results for publication in GBD, we used a three-step process. First, the number of cases at birth was calculated as birth prevalence rate multiplied by number of live births for each location, sex, and year. Second, the number of cases present at birth were summed with incident cases during the early neonatal period (calculated as the 0-to-6-days incidence rate times the 0-to-6-days population), and the early neonatal incidence rate was recalculated by re-dividing by the 0-to-6-days population. Third, incidence rates for aggregate age groups were re-calculated by using the revised incidence figures for the early neonatal period.
Causes included in reporting are all of those for which birth prevalence has been estimated as part of existing modelling processes. Although extensive, this list should not be considered exhaustive of all of the conditions that can be present at birth.

Section 3: SDI

Section 3.1: SDI definition

The Socio-demographic Index (SDI) is a composite indicator of background social and economic conditions that influence health outcomes in each location. In short, it is the geometric mean of 0 to 1 indices of total fertility rate (TFR) for those younger than 25 years old (TFU25), mean education for those 15 years old and older (EDU15+), and lag-distributed income (LDI) per capita. After calculating SDI, values were multiplied by 100 for a scale of 0 to 100.

Section 3.2: Development of revised SDI indicator

SDI was originally constructed for GBD 2015 by using the Human Development Index (HDI) methodology, wherein a 0 to 1 index value was determined for each of the original three covariate inputs (TFR in ages 15 to 49 years, EDU15+, and LDI per capita) by using the observed minima and maxima over the estimation period to set the scales.[32]

In response to feedback from collaborators and the evolution of the GBD, we have refined the indicator with each GBD cycle. Beginning in GBD 2017, along with our expanded estimation of age-specific fertility, we replaced TFR with TFU25 as one of the three component indices. The TFU25 provides a better measure of women’s status in society because it focuses on ages at which childbearing disrupts the pursuit of education and entrance into the workforce. In addition, we observed that in highly developed countries, the TFU25 has tended to decline consistently over time despite rebounds in TFR driven by increasing fertility at older ages. The concordance correlation coefficient between SDI based on the GBD 2016 method and the updated method for GBD 2017 was 0.981.

During GBD 2016, we moved from using relative index scales to using absolute scales to enhance the stability of SDI interpretation over time because we noticed that the measure was highly sensitive to the addition of subnational units that tended to stretch the empirical minima and maxima.21 We selected the minima and maxima of the scales by examining the relationships each of the inputs had with life

expectancy at birth and under-5 mortality and by identifying points of limiting returns at both high and low values if they occurred before theoretical limits (eg, a TFU25 of 0) were reached.

Thus, for each covariate input, an index score of 0 represents the minimum level of each covariate input past which selected health outcomes can get no worse, and an index score of 1 represents the maximum level of each covariate input past which selected health outcomes cease to improve. As a composite, a location with an SDI of 0 would have a theoretical minimum level of sociodemographic development relevant to these health outcomes, and a location with an SDI of 1 (before multiplying by 100 for reporting) would have a theoretical maximum level of sociodemographic development relevant to these health outcomes.

We computed the index scores underlying SDI as follows:

$$I_{Cly} = \max\left(\frac{C_{ly} - C_{low}}{C_{high} - C_{low}}, 0.005\right)$$

Where:

> I_{cly} is the index for covariate *C*, location *l*, and year *y* and is equal to the difference between the value of that covariate in that location-year and the lower bound of the covariate divided by the difference between the upper and lower bounds for that covariate

If the values of input covariates fell outside the upper or lower bounds, they were mapped to the respective upper or lower bounds. We also note that the index value for TFU25 was computed as $1 - I_{TFU25ly}$ because lower TFU25s correspond to higher levels of development and thus higher index scores. For GBD 2023, we expanded the computation of SDI to 925 locations spanning the time period 1950–2023.

The composite SDI is the geometric mean of these three indices for a given location-year. The cut-off values used to determine quintiles for analysis were then computed by using country-level estimates of SDI for the year 2023, excluding countries with populations less than 1 million.

For GBD 2023, final SDI values were multiplied by 100 for reporting, in order to improve understanding of and broader engagement with the values. Final reporting values are on a 0 to 100 scale.

Example calculation

We present the equation used to calculate SDI for a hypothetical country in the year 2023:

$$TFU25 = 1.09;\ Mean\ educ\ yrs\ pc = 8.23;\ lnLDI = 9.60$$

$$I_{TFU25} = 1 - \frac{1.09 - 0}{3 - 0} = 0.637$$

$$I_{Educ} = \frac{8.23 - 0}{17 - 0} = 0.484$$

$$I_{\ln LDI} = \frac{9.60 - 5.52}{11.00 - 5.52} = 0.744$$

$$SDI = \sqrt[3]{I_{TFU25} \cdot I_{Educ} \cdot I_{\ln LDI}} = \sqrt[3]{.637 \cdot .484 \cdot .744} = 0.611$$

$$I_{lnLDI} = \frac{9.58 - 5.52}{11.00 - 5.52} = 0.741$$

$$SDI = \sqrt[3]{I_{TFU25} \cdot I_{Educ} \cdot I_{\ln LDI}} = \sqrt[3]{.855 \cdot .543 \cdot .741} = 0.701$$

$$\text{GBD 2023 reporting } SDI = 0.701 * 100 = 70.1$$

Section 4: Estimation process for DALYs

To estimate DALYs for GBD 2023, we started by estimating cause-specific mortality and non-fatal health loss. For each year for which YLDs have been estimated, we computed DALYs by adding YLLs and YLDs for each age-sex-location (Figure S18). Uncertainty in YLLs was assumed to be independent of uncertainty in YLDs. We calculated 250 draws for DALYs by summing the first draw of the 250 draws for YLLs and YLDs and then repeating for each subsequent draw. 95% UIs were computed by using the 25th and 975th ordered draw of the DALY uncertainty distribution. We calculated DALYs as the sum of YLLs and YLDs for each cause, location, age group, sex, and year.

Figure S18. DALY burden estimation for GBD 2023

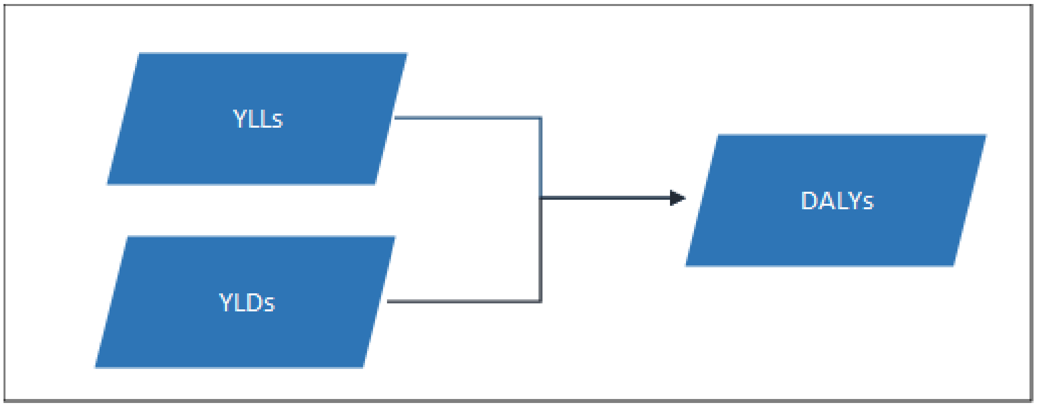

Section 5: HALE

The first step to calculating healthy life expectancy for a population (defined by sex, country, and year) was to compute average health of individuals for every age group in that population. We combined information about prevalences for all sequelae and their associated disability weights and accounted for comorbidity with a Monte Carlo simulation approach. We made the assumption that comorbidities were independent within each age group. We created simulations where individuals were exposed to each

sequela with a probability equal to the estimated prevalence of that sequela in each age group. This created a simulated population where the frequencies of many possible multi-morbidities were consistent with the underlying estimates of prevalence. We define 1 minus the disability weight as the positive health associated with each sequela. The combined health for a simulated individual was the product of these positive health values for all relevant sequelae in the presence of multiple sequelae.

Average health values are computed as 1 minus the YLD per person in a population, which are then used to compute health adjusted person years. We incorporated average health values into the life table using Sullivan's method. First, we multiplied values in the nLx (average person-years lived within an age interval starting at age x) column of the life table by the corresponding average health value in that interval. We recalculated the rest of the life table using the adjusted nLx values. Sullivan's method began with an adjusted estimate of health adjusted life years within the terminal age interval (equal to nLx multiplied by the average health value for the terminal age group) and subsequent calculations we produced estimates by iterating through younger age intervals, summing the health-adjusted person-years with all age intervals above the current age interval to generate health adjusted person years lived above a certain age (adjusted Tx) for each age group. After calculating adjusted Tx for all age groups, HALE was calculated by dividing the adjusted Tx for each age group by the proportion of hypothetical birth cohort still alive at age x.[33]

References

1 Stevens GA, Alkema L, Black RE, *et al.* Guidelines for Accurate and Transparent Health Estimates Reporting: the GATHER statement. *The Lancet* 2016; **388**: e19–23.

2 Lim SS, Vos T, Flaxman AD, *et al.* A comparative risk assessment of burden of disease and injury attributable to 67 risk factors and risk factor clusters in 21 regions, 1990-2010: a systematic analysis for the Global Burden of Disease Study 2010. *Lancet* 2012; **380**: 2224–60.

3 GBD 2015 Disease and Injury Incidence and Prevalence Collaborators. Global, regional, and national incidence, prevalence, and years lived with disability for 310 diseases and injuries, 1990-2015: a systematic analysis for the Global Burden of Disease Study 2015. *Lancet* 2016; **388**: 1545–602.

4 GBD 2016 Disease and Injury Incidence and Prevalence Collaborators. Global, regional, and national incidence, prevalence, and years lived with disability for 328 diseases and injuries for 195 countries, 1990-2016: a systematic analysis for the Global Burden of Disease Study 2016. *Lancet* 2017; **390**: 1211–59.

5 GBD 2017 Disease and Injury Incidence and Prevalence Collaborators. Global, regional, and national incidence, prevalence, and years lived with disability for 354 diseases and injuries for 195 countries and territories, 1990-2017: a systematic analysis for the Global Burden of Disease Study 2017. *Lancet* 2018; **392**: 1789–858.

6 GBD 2019 Diseases and Injuries Collaborators. Global burden of 369 diseases and injuries in 204 countries and territories, 1990-2019: a systematic analysis for the Global Burden of Disease Study 2019. *Lancet* 2020; **396**: 1204–22.

7 GBD 2021 Diseases and Injuries Collaborators. Global incidence, prevalence, years lived with disability (YLDs), disability-adjusted life-years (DALYs), and healthy life expectancy (HALE) for 371 diseases and

injuries in 204 countries and territories and 811 subnational locations, 1990-2021: a systematic analysis for the Global Burden of Disease Study 2021. *Lancet* 2024; **403**: 2133–61.

8 Vasudevan S, Ramos F, Nettleton E, Durrant-Whyte H. Gaussian process modeling of large-scale terrain. *Journal of Field Robotics* 2009; **26**: 812–40.

9 Rasmussen CE. Gaussian Processes in Machine Learning. In: Bousquet O, von Luxburg U, Rätsch G, eds. Advanced Lectures on Machine Learning: ML Summer Schools 2003, Canberra, Australia, February 2 - 14, 2003, Tübingen, Germany, August 4 - 16, 2003, Revised Lectures. Berlin, Heidelberg: Springer, 2004: 63–71.

10 Ng M, Fleming T, Robinson M, *et al.* Global, regional, and national prevalence of overweight and obesity in children and adults during 1980-2013: a systematic analysis for the Global Burden of Disease Study 2013. *Lancet* 2014; **384**: 766–81.

11 Ng M, Freeman MK, Fleming TD, *et al.* Smoking prevalence and cigarette consumption in 187 countries, 1980-2012. *JAMA* 2014; **311**: 183–92.

12 Zheng P, Barber R, Sorensen RJD, Murray CJL, Aravkin AY. Trimmed constrained mixed effects models: formulations and algorithms. *J Comput Graph Stat* 2021; **30**: 544–56.

13 Flaxman AD, Vos T, Murray CJL. An integrative metaregression framework for descriptive epidemiology. Seattle: University of Washington Press, 2015.

14 Bell A, Jones K. The impossibility of separating age, period and cohort effects. *Soc Sci Med* 2013; **93**: 163–5.

15 Glenn ND. Cohort analysts’ futile quest: statistical attempts to separate age, period and cohort effects. *American Sociological Review* 1976; **41**: 900–4.

16 Goldstein H. Age, period and cohort effects — a confounded confusion. *Journal of Applied Statistics* 1979; published online Jan 1. DOI:10.1080/02664767900000003.

17 Borwein J, Lewis A. Convex Analysis and Nonlinear Optimization. New York, NY: Springer, 2006 DOI:10.1007/978-0-387-31256-9.

18 More JJ, Sorensen DC. Newton’s method. Argonne National Lab. (ANL), Argonne, IL (United States), 1982 DOI:10.2172/5326201.

19 Wächter A, Biegler LT. On the implementation of an interior-point filter line-search algorithm for large-scale nonlinear programming. *Math Program* 2006; **106**: 25–57.

20 data_like - dismod_at documentation. https://dismod-at.readthedocs.io/latest/data_like.html (accessed March 11, 2025).

21 avg_integrand - dismod_at documentation. https://dismod-at.readthedocs.io/latest/avg_integrand.html (accessed March 11, 2025).

22 Hasin DS, Grant BF. The National Epidemiologic Survey on Alcohol and Related Conditions (NESARC) Waves 1 and 2: review and summary of findings. *Soc Psychiatry Psychiatr Epidemiol* 2015; **50**: 1609–40.

23 Henderson S, Andrews G, Hall W. Australia's Mental Health: An Overview of the General Population Survey*. *Aust N Z J Psychiatry* 2000; **34**: 197–205.

24 Salomon JA, Haagsma JA, Davis A, *et al.* Disability weights for the Global Burden of Disease 2013 study. *Lancet Glob Health* 2015; **3**: e712-723.

25 Campeau L. The Canadian Cardiovascular Society grading of angina pectoris revisited 30 years later. *Can J Cardiol* 2002; **18**: 371–9.

26 Dolgin M. Nomenclature and criteria for diagnosis of diseases of the heart and great vessels, 9th ed. Boston: Little, Brown, 1994.

27 Nord E. Disability weights in the Global Burden of Disease 2010: unclear meaning and overstatement of international agreement. *Health Policy* 2013; **111**: 99–104.

28 Taylor HR, Jonas JB, Keeffe J, *et al.* Disability weights for vision disorders in Global Burden of Disease study. *Lancet* 2013; **381**: 23.

29 Voigt K, King NB. Disability weights in the global burden of disease 2010 study: two steps forward, one step back? *Bull World Health Organ* 2014; **92**: 226–8.

30 Kretzschmar M, Mangen M-JJ, Pinheiro P, *et al.* New methodology for estimating the burden of infectious diseases in Europe. *PLOS Medicine* 2012; **9**: e1001205.

31 Vos T, Flaxman AD, Naghavi M, *et al.* Years lived with disability (YLDs) for 1160 sequelae of 289 diseases and injuries 1990-2010: a systematic analysis for the Global Burden of Disease Study 2010. *Lancet* 2012; **380**: 2163–96.

32 GBD 2015 Mortality and Causes of Death Collaborators. Global, regional, and national life expectancy, all-cause mortality, and cause-specific mortality for 249 causes of death, 1980-2015: a systematic analysis for the Global Burden of Disease Study 2015. *Lancet* 2016; **388**: 1459–544.

33 GBD 2017 DALYs and HALE Collaborators. Global, regional, and national disability-adjusted life-years (DALYs) for 359 diseases and injuries and healthy life expectancy (HALE) for 195 countries and territories, 1990-2017: a systematic analysis for the Global Burden of Disease Study 2017. *Lancet* 2018; **392**: 1859–922.

Section 6: Non-fatal cause-specific modelling descriptions

GBD 2023 non-fatal appendix write-ups in order

1. Acne vulgaris
2. Acute endocarditis

3. Acute glomerulonephritis
4. Acute hepatitis A, B, C, and E
5. Acute myocarditis
6. Alcohol use disorders
7. Alopecia areata
8. Alzheimer's disease and other dementias
9. Amphetamine use disorders
10. Anaemia
11. Anorexia nervosa
12. Anxiety disorders
13. Appendicitis
14. Ascariasis
15. Asthma
16. Atrial fibrillation and flutter
17. Attention-deficit/hyperactivity disorder
18. Autism spectrum disorders
19. Bipolar disorder
20. Blindness and vision loss
21. Bulimia nervosa
22. Cancers
23. Cannabis use disorders
24. Cellulitis
25. Chagas disease
26. Chronic kidney disease
27. Chronic obstructive pulmonary disease
28. Cirrhosis and other chronic liver diseases
29. Cocaine use disorders
30. Conduct disorder
31. Congenital birth defects
32. COVID-19
33. Cutaneous and mucocutaneous leishmaniasis
34. Cystic echinococcosis
35. Cysticercosis
36. Decubitus ulcer
37. Dengue
38. Dermatitis
39. Developmental intellectual disability
40. Diabetes mellitus
41. Diarrhoeal diseases
42. Diphtheria
43. Diverticular disease
44. Dracunculiasis (Guinea worm)
45. Dysthymia
46. Ebola virus disease
47. Encephalitis
48. Endocrine, metabolic, blood, and immune disorders
49. Epilepsy
50. Fistula

51. Foodborne trematodiases
52. Fungal skin diseases
53. Gallbladder and biliary diseases
54. Gastritis and duodenitis
55. Gastro-oesophageal reflux disease
56. Gout
57. Guillain-Barré syndrome
58. Gynaecological diseases
59. Haemoglobinopathies and haemolytic anaemias
60. Headache disorders
61. Hearing impairment
62. Heart failure
63. HIV/AIDS
64. Hookworm disease
65. Human African trypanosomiasis (HAT)
66. Infertility
67. Inflammatory bowel disease (IBD)
68. Inguinal, femoral, and abdominal hernia
69. Injuries
70. Interstitial lung disease and pulmonary sarcoidosis
71. Invasive non-typhoidal Salmonella (iNTS)
72. Ischaemic heart disease
73. Ischaemic stroke, intracerebral haemorrhage, and subarachnoid haemorrhage
74. Leprosy
75. Low back pain
76. Lower extremity peripheral arterial disease
77. Lower respiratory infections
78. Lymphatic filariasis
79. Major depressive disorder
80. Malaria
81. Maternal disorders
82. Measles
83. Meningitis
84. Motor neuron disease
85. Multiple sclerosis
86. Neck pain
87. Neonatal disorders
88. Non-alcoholic fatty liver diseases without cirrhosis
89. Non-rheumatic valvular heart disease
90. Nutritional deficiencies
91. Onchocerciasis
92. Opioid use disorders
93. Oral disorders
94. Osteoarthritis
95. Other cardiovascular diseases
96. Other chronic respiratory diseases
97. Other digestive diseases
98. Other drug use disorders

99. Other intestinal infectious diseases
100. Other mental disorders
101. Other musculoskeletal disorders
102. Other neglected tropical diseases
103. Other neurological disorders
104. Other sense organ diseases
105. Other skin and subcutaneous diseases
106. Other unspecified infectious diseases
107. Otitis media
108. Pancreatitis
109. Paralytic ileus and intestinal obstruction
110. Parkinson's disease
111. Pelvic inflammatory disease
112. Peptic ulcer disease
113. Pertussis (whooping cough)
114. Pneumoconiosis
115. Pruritus
116. Psoriasis
117. Pulmonary arterial hypertension
118. Pyoderma
119. Rabies
120. Rheumatic heart disease
121. Rheumatoid arthritis
122. Scabies
123. Schistosomiasis
124. Schizophrenia
125. Sexual violence
126. Sexually transmitted infections excluding HIV
127. Tetanus
128. Trichuriasis
129. Tuberculosis
130. Typhoid and paratyphoid fevers
131. Upper respiratory infections
132. Urinary diseases and male infertility
133. Urticaria
134. Varicella and herpes zoster
135. Vascular intestinal disorders
136. Viral skin diseases
137. Visceral leishmaniasis
138. Yellow fever
139. Zika virus disease

Acne vulgaris

Flowchart

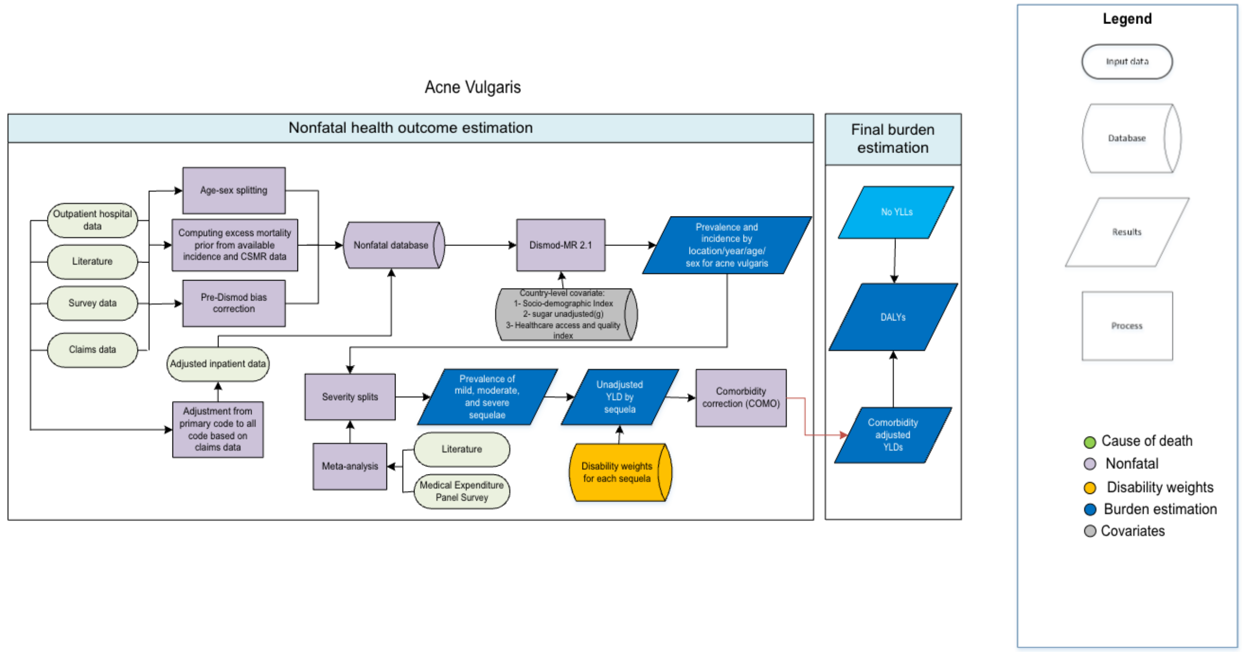

Input data and methodological summary for acne vulgaris

Case definition

Acne vulgaris is defined as a chronic inflammatory disease of the pilosebaceous unit associated with an increase in sebum secretion (ICD-10: L70, excluding L70.4). Acne vulgaris was included in the GBD 2021 cause group of skin and subcutaneous conditions.

Acne vulgaris

Quantity of interest	Reference or alternative	Definition
Acne vulgaris	Reference	Acne vulgaris as determined by Poland National Health Fund Patient Claims 2015-2019
Acne vulgaris	Alternative	All other data for acne vulgaris

Input data

In the GBD 2010 study, a systematic review of the literature was conducted using PubMed and Google Scholar to capture epidemiological data for acne vulgaris. The inclusion criteria stipulated that studies (1) must be published between 1980 and 2012; (2) must provide data on the incidence or prevalence of acne vulgaris; (3) must use samples representative of the general population (ie, samples derived from the experimental arm of clinical trials or based in dermatology clinics were excluded); (4) must use a sample size larger than 100; and (5) must provide sufficient information on study method and sample

characteristics to assess the quality of the study. For GBD 2016, the GBD 2010 search strategy was replicated in PubMed to capture epidemiological studies published between 2013 and 2016. An additional literature search was carried out for GBD 2017 for USA data to better inform the DisMod crosswalk from USA claims data to literature data and capture any studies missed in previous literature searches. This literature search also replicated the GBD 2010 search strategy and captured studies published between 1980 and 2017. Additional clinical data for GBD 2023 includes USA claims data 2010–2019 and Poland national claims 2015–2019 along with location-year of literature data. Our new sources of literature data are from Brazil, Bulgaria, Cameroon, Egypt, France, Germany, Ghana, New Zealand, Portugal, Saudi Arabia, Turkey, and Tanzania.

Data processing

For acne vulgaris, we crosswalked all data to the reference definition. We began by evaluating the number of observations of each alternate definition that matched with a corresponding observation from the reference definition. We considered "between" study matches, where the alternative was from the same GBD age group and sex, and the midpoint year of the study was within five years of the midpoint of the reference definition observation.

$$\log i\,t\left(y_i^{alt}\right) - \log i\,t\left(y_i^{ref}\right) = \beta_0 + \epsilon_i$$

Table 1: MR-BRT crosswalk adjustment factors for acne vulgaris

Data input	Reference or alternative case definition	Gamma	Beta coefficient, log/logit (95% UI)*	Adjustment factor**
Poland National Health Fund Outpatient Claims 2015–2019	Ref	0.1014	---	---
All other data	Alt		0.8362 (0.2122–1.4602)	1.3750 (1.2364–4.3070)

**MR-BRT crosswalk adjustments can be interpreted as the factor the alternative case definition is adjusted by to reflect what it would have been had it been measured using the reference case definition. If the log/logit beta coefficient is negative, then the alternative is adjusted up to the reference. If the log/logit beta coefficient is positive, then the alternative is adjusted down to the reference.*
***The adjustment factor column is the exponentiated beta coefficient. For log beta coefficients, this is the relative rate between the two case definitions. For logit beta coefficients, this is the relative odds between the two case definitions.*

Modelling strategy

In GBD 2023, we have made no substantive changes in the modelling strategy from GBD 2021.

DisMod-MR 2.1, a Bayesian meta-regression tool, was used to estimate prevalence by age, sex, year, and geography (subnational, country, region, super-region) for acne vulgaris. Since our available data only contained information on prevalence, we specified additional expert priors to further inform analyses. We assumed zero excess mortality and remission from 0.38 to 0.6, implying a duration of

approximately two to three years. This was in line with the available epidemiological data, expert opinion, and previous GBD work. A value prior of zero was set for incidence between the ages of 0 and 6, and 61 and 100. We used a time window of five years to determine which datapoints were used for a particular year of fit. The datasets for acne vulgaris were sufficiently large to make use of a relatively short time window of ten years to determine which datapoints were used for a particular year of fit. Socio-demographic Index, sugar unadjusted (g), and Healthcare Access and Quality Index were used as country-level covariates to guide estimates for countries with few or no data. Since GBD 2019, we have replaced our within-DisMod crosswalks with crosswalks completed using the MR-BRT modelling tool. We adjusted all our data toward the level of Poland National Health Fund Patient Claims 2015–2019 datapoints, which were more representative of the general population.

$$prevalence = incidence * duration$$

$$Remission = \frac{Cured\ cases}{person - year\ of follow - up\ in\ prevalent\ cases}$$

Table 2. Severity distribution, details on the severity levels for acne vulgaris and the associated disability weight (DW) with that severity.

Sequela	Severity level	Lay description	DW (95% CI)
Mild acne vulgaris	Disfigurement, level 1	The individual has a slight, visible physical deformity that is sometimes sore or itchy. Others notice deformity, which causes some worry and discomfort.	0.476 (0.381, 0.581)
Moderate acne vulgaris	Disfigurement, level 2	The individual has a visible physical deformity that is sore and itchy. Other people stare and comment, which causes the person to worry. The person has trouble sleeping and concentrating.	0.413 (0.32, 0.497)
Severe acne vulgaris	Disfigurement, level 3	The individual has an obvious physical deformity that is very painful and itchy. Physical deformity makes others uncomfortable, which causes the person to avoid social contact, feel worried, sleep poorly, and think about suicide.	0.111 (0.081, 0.149)

Table 3. Covariates. Summary of covariates used in the acne vulgaris DisMod-MR meta-regression model

Covariate	Type	Parameter	Value	Exponentiated beta (95% uncertainty interval)
Socio-demographic Index	Country-level	Prevalence	1.00 (0.99, 1.00)	2.71 (2.68, 2.72)

sugar unadjusted (g)	Country-level	Prevalence	0.0095 (–0.0053, 0.018)	1.01 (0.99, 1.02)
Healthcare Access and Quality Index	Country-level	Prevalence	0.035 (0.016, 0.050)	1.04 (1.02, 1.05)

Acute endocarditis

Flowchart

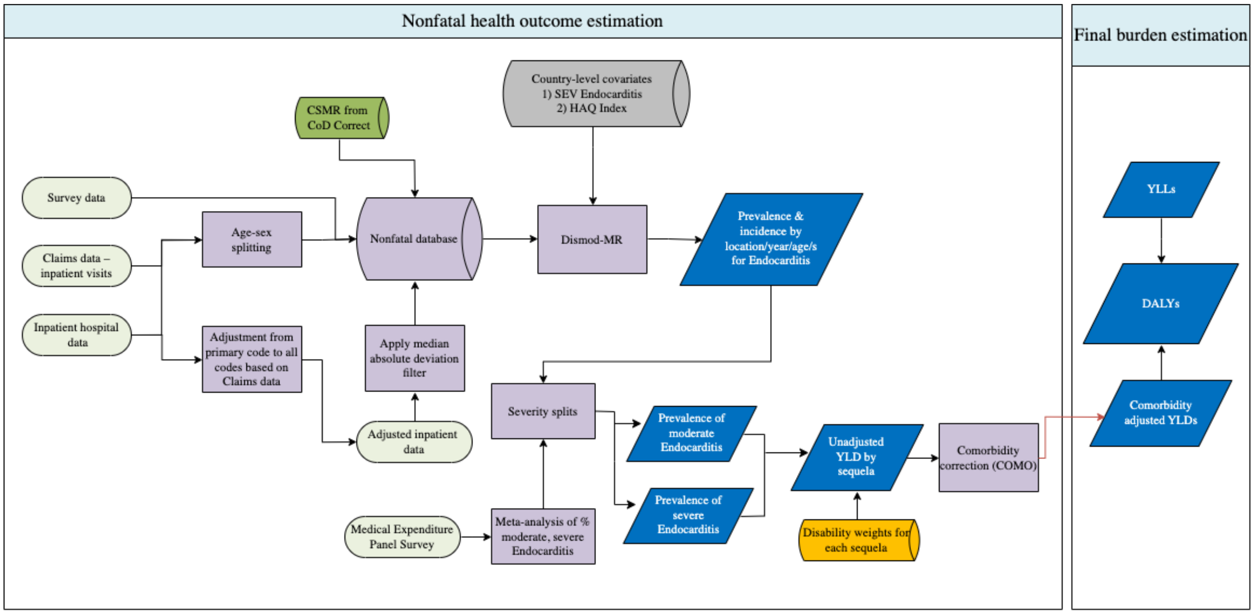

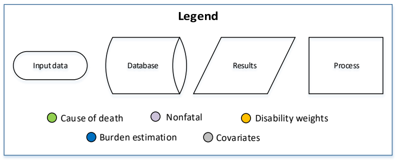

Input data and methodological summary for endocarditis

Case definition

Acute endocarditis is inflammation of the inner lining of the heart's chambers and valves, known as endocardium, caused by a bacterial or fungal infection of the heart, with a vegetation adherent to a heart valve or chordae. The standard for clinical diagnosis of infective endocarditis is through the Duke Criteria, which include confirmation through clinical criteria, specific blood tests, and cardiovascular imaging.

The GBD reference case definition is listed in Table 1, and the ICD codes used for inclusion are included in Table 2.

Table 1. Acute endocarditis reference definition

Quantity of interest	Reference or alternative	Definition
Acute endocarditis	Reference	The standard for clinical diagnosis of infective endocarditis is through the Duke Criteria, which include confirmation through various blood tests and cardiovascular imaging.
Acute endocarditis	Alternative	N/A

Table 2. ICD codes used for inclusion of endocarditis hospital and claims data

Cause	ICD-9	ICD-10
Endocarditis	421-421.9<br>424, 424.4-424.9<br>074.22, 112.81, 115-115.9	I33-I33.9, I38-I39.9<br>A32.82, B33.21, B37.6

Input data

We did not perform a systematic review for GBD 2023. A systematic review was last performed for GBD 2015. The following search terms were used: ((‘endocarditis’[MeSH Terms] OR ‘endocarditis’[All Fields]) AND ‘epidemiology’[Subheading]) OR ((‘endocarditis’[MeSH Terms] OR ‘endocarditis’[All Fields]) AND ((‘epidemiology’[Subheading] OR ‘epidemiology’[All Fields] OR ‘incidence’[All Fields] OR ‘incidence’[MeSH Terms]) OR (‘epidemiology’[Subheading] OR ‘epidemiology’[All Fields] OR ‘prevalence’[All Fields] OR ‘prevalence’[MeSH Terms]) OR ‘case fatality’[All Fields])) OR ((‘endocardium’[MeSH Terms] OR ‘endocardium’[All Fields]) AND inflammation[TIAB] AND ‘epidemiology’[Subheading]) OR ((‘endocardium’[MeSH Terms] OR ‘endocardium’[All Fields]) AND inflammation[TIAB] AND ((‘epidemiology’[Subheading] OR ‘epidemiology’[All Fields] OR ‘incidence’[All Fields] OR ‘incidence’[MeSH Terms]) OR (‘epidemiology’[Subheading] OR ‘epidemiology’[All Fields] OR ‘prevalence’[All Fields] OR ‘prevalence’[MeSH Terms]) OR ‘case fatality’[All Fields]))

- Dates included in search: 1/1/2013–3/16/2015
- Number of initial hits: 1246
- Number of sources included: 6

We did not include any non-literature-based data types, apart from the hospital and claims data described elsewhere. We excluded all outpatient data, as they were implausibly low when compared with inpatient data and claims data from the same locations. We used hospital data corrected for readmission (ie, excluding multiple admissions of a given patient for endocarditis within a year) and for non-primary diagnosis (ie, considering differently primary and secondary diagnosis of endocarditis in inpatient admission), based on the correction factors generated by the clinical informatics team. More information on how correction factors were made for this adjustment can be found in the “Claims data” section of the non-fatal appendix. Diagnosis of endocarditis requires a combination of information provided by imaging and blood cultures;[2] in low- and middle-income settings, this can pose a challenge in diagnosis, for example with regard to organism non-identification, resulting in unclear incidence of the disease.[3] For several locations with hospital data, there were either an implausible number of zero

counts of incident cases of endocarditis for all ages and sexes, or stochastic age patterns across the age range. To address this, we excluded any inpatient hospital datapoints which were at least two-fold higher or 0.5-fold lower than the age-specific median absolute deviation[1] value for regions identified with the highest-quality data – specifically, high-income North America, central Europe, western Europe, and high-income Asia Pacific. We based our exclusion criteria on national-year specific values for each study, age, and sex group adjusted by population size.

All data from Brazil, Mexico, Botswana, Iran, Nepal, Kenya, and Mongolia were excluded due to inconsistencies in coding practices or implausible values. In addition, subsets of data from Poland the USA were excluded due to concerns on the quality of the data sources.

Modelling strategy

For GBD 2023, we estimated the incidence and prevalence of acute endocarditis using a DisMod-MR Bayesian meta-regression model; the long-term prevalence of heart failure due to endocarditis is estimated as part of the heart failure modelling process. We set a minimum of 11 and maximum of 13 as value priors on remission to establish an average duration of one month. Country-level covariates used included the age-standardised endocarditis summary exposure variable scalar (SEV scalar) on incidence with a minimum of 0.75 and a maximum of 1.25 as value priors, and Healthcare Access and Quality (HAQ) Index on excess mortality with a minimum of –1 HAQ Index and a maximum of –0.1 HAQ Index as value priors. Table 3 gives the parameters, betas, and exponentiated betas for study-level and country-level covariates used in the model.

The proportion of moderate and severe for acute endocarditis were determined by the standard approach for severity splitting for GBD 2023 that used the Medical Expenditure Panel Survey (MEPS) to map endocarditis ICD codes (see Table 2) to quality-of-life metrics to quantify disability. Table 4 includes the severity level, lay descriptions, and disability weights (DWs) associated with acute endocarditis.

We evaluated models by comparing model fits with the data and with results from previous GBD estimation cycles. Apart from updates to the clinical informatics data included in the model, there have been no substantive updates for the acute endocarditis estimation process in the GBD 2023 round.

Table 3. Covariates. Summary of covariates used in the acute endocarditis DisMod-MR meta-regression model

Covariate	Parameter	Beta	Exponentiated beta (95% uncertainty interval)
Healthcare Access and Quality Index	Excess mortality rate	–0.1 (–0.1 to –0.1)	0.90 (0.90 to 0.90)

Table 4. Severity distribution. Details on the severity levels for acute endocarditis and the associated disability weight (DW) with that severity

Severity level	Lay description	DW (95% CI)
Moderate	Has a fever and aches, and feels weak, which causes some difficulty with daily activities.	0.051 (0.032–0.074)

Severe	Has a high fever and pain, and feels very weak, which causes great difficulty with daily activities.	0.133 (0.088–0.19)

References

[1] Huber, P.J. (2011). Robust Statistics. In: Lovric, M. (eds) *International Encyclopedia of Statistical Science*. Springer, Berlin, Heidelberg. https://doi.org/10.1007/978-3-642-04898-2_594

[2] Cuervo G, Escrihuela-Vidal F, Gudiol C, Carratalà J. Current Challenges in the Management of Infective Endocarditis. *Front Med* (Lausanne). 2021 Feb 22;8:641243. doi: 10.3389/fmed.2021.641243. PMID: 33693021; PMCID: PMC7937698.

[3] Njuguna B, Gardner A, Karwa R, Delahaye F. Infective Endocarditis in Low- and Middle-Income Countries. *Cardiol Clin*. 2017 Feb;35(1):153-163. doi: 10.1016/j.ccl.2016.08.011. PMID: 27886786.

Acute glomerulonephritis

Flowchart

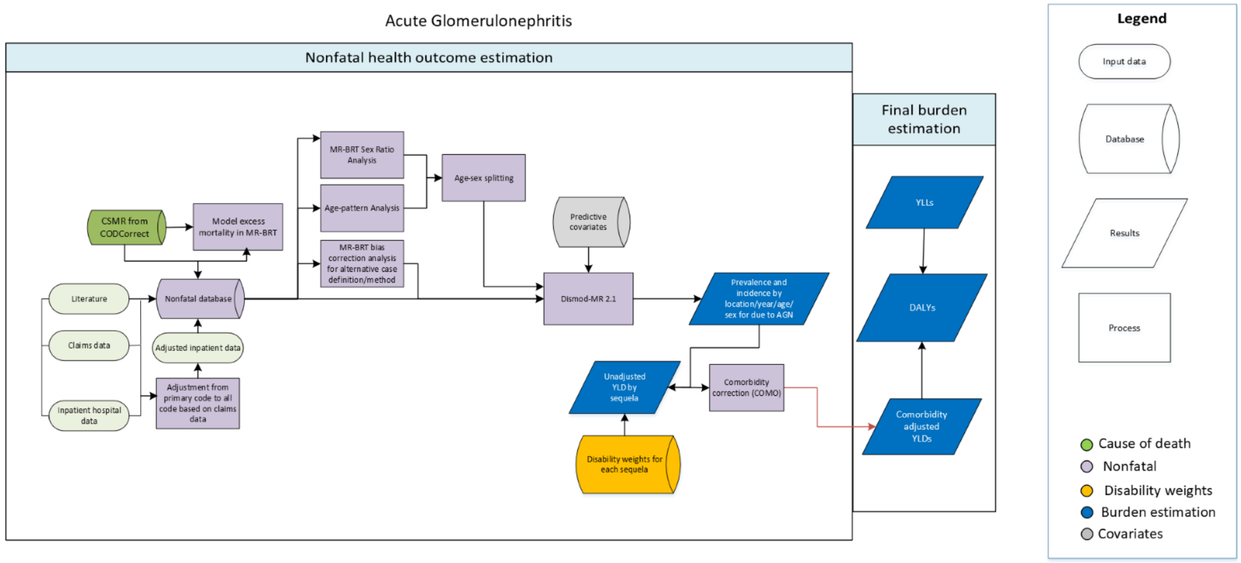

Input data and methodological summary for acute glomerulonephritis

Case definition

Acute glomerulonephritis (AGN) is an acute episode of glomerular injury accompanied by inflammation, generally presenting with haematuria, oedema, hypertension, and acute kidney injury. ICD codes for AGN include N00, N00.0, N00.1, N00.2, N00.3, N00.4, N00.5, N00.6, N00.7, N00.8, N00.9, N01, N01.1, N01.2, N01.3, N01.4, N01.5, N01.6, N01.7, N01.8, and N01.9.

In GBD 2017, our reference case definition for AGN was limited to post-infectious AGN; data sources that included other aetiologies of AGN were adjusted to this reference standard. Starting in GBD 2019, and continuing into GBD 2021 and GBD 2023, the reference case definition was based on ICD diagnosis in administrative data and thus was not specific to a single aetiology.

**Quantity of interest**	**Reference or alternative**	**Definition**
Incidence of acute glomerulonephritis	Reference	Cases of acute glomerulonephritis identified using ICD codes in administrative records that are considered population-representative.
Incidence of acute glomerulonephritis	Alternative	Cases identified from database of commercial claims from USA in 2010-2017 using ICD codes.
Incidence of acute glomerulonephritis	Alternative	Cases identified from database of commercial claims from USA in 2000 using ICD codes.

Input data

Input data

A systematic literature review was first conducted in 2010, and again in 2013, extracting a total of 14 articles. In addition, inputs for the non-fatal AGN model include incidence data extracted from the GBD library of hospital discharges and claims aggregated and processed by IHME. No additional data were added for GBD 2023.

Inputs to our non-fatal modelling also included cause-specific mortality rate (CSMR) estimates taken from our fatal modelling process (see CoD cause-specific modelling description for AGN in this appendix) and excess mortality rates (EMR) estimates modelled using MR-BRT (see the EMR data processing section below).

Incidence data processing

Hospital discharge data provide observations about encounters, generally with only the primary diagnostic code for the encounter. Claims data, on the other hand, link claims for all inpatient and outpatient encounters for a single individual and provide primary and secondary diagnoses for all encounters.

In GBD 2017, an individual was extracted from claims data as an incident case if that individual had one or more inpatient encounters with an appropriate ICD code as any diagnosis. Hospital discharges with an appropriate ICD code as primary diagnosis were extracted and adjusted to account for typical patterns of readmission.

Beginning in GBD 2019, however, we employed data processed with methods to capture cases that were diagnosed and/or treated in both inpatient and outpatient settings. Specifically, an individual was extracted from claims data as an incident case if that individual had at least one inpatient or outpatient encounter with an appropriate ICD code as any diagnosis; repeat encounters within 90 days, regardless of setting, were assumed to represent care for the same episode. Hospital discharge data were processed by extracting discharges with an appropriate ICD code as primary diagnosis and adjusting using correction factors derived from claims data. Specifically, we calculated the ratio of inpatient claims with AGN as primary diagnosis to total incident cases of AGN seen in claims data, and modelled these ratios as functions of age, sex, and Healthcare Access and Quality Index using MR-BRT (meta-regression—Bayesian, regularised, trimmed). Details of modelling and applying these ratios remained the same as in GBD 2019.

In addition to applying new ratios to hospital data for AGN, the methods for bias adjustment were updated in GBD 2019 to allow a more direct comparison between different case definitions and/or study designs. In past GBD cycles, we used data from published studies that employed rigorous case definitions for post-infectious AGN as our reference standard and adjusted clinical administrative data toward this reference standard by marking administrative data with binary covariates and estimating a fixed effect for this covariate in our DisMod-MR meta-regression modelling process. This amounts to adjusting data using an ecological comparison and is vulnerable to compositional bias; if data from different location-years were collected using different methods or case definitions, true spatiotemporal differences in epidemiology can be erroneously adjusted, and differences truly due to differences in methods can be erroneously estimated as differences in underlying epidemiology. Beginning in GBD 2019, we avoided this risk by making pre-modelling bias adjustments and dropping data types that could not be rigorously adjusted. This was done by conducting a meta-regression of the relationship between datapoints matched with regard to year, age, sex, and location, but differing with regard to one or more

study design characteristic. Data from studies that ascertained cases of post-infectious AGN based on serological, histological, and/or imaging findings were scarce, and we were not able to find overlapping datapoints from administrative data sources to estimate adjustment factors. As a result, these data were excluded and a new case definition was adopted: diagnosis of AGN of any aetiology as indicated by ICD code in a clinical encounter.

Sufficient matched data were identified, however, to develop an adjustment factor for USA claims data (extracted as described above) to account for selection bias due to commercial insurance. The table below shows these bias correction factors. Beta coefficients and adjustment factors incorporate study heterogeneity (gamma).

Table 1: MR-BRT crosswalk adjustment factors for acute glomerulonephritis

Data input	Reference or alternative case definition	Gamma	Beta coefficient, log (95% UI)*	Adjustment factor**
Hospital + non-USA claims	Ref	0.33	---	---
USA claims from year 2000	Alt		1.83 (–0.11 to 3.77)	6.21 (0.89–43.18)
USA claims from years 2010–2017	Alt		1.83 (0.96–2.70)	6.23 (2.61–14.89)

**MR-BRT crosswalk adjustments can be interpreted as the factor the alternative case definition is adjusted by to reflect what it would have been had it been measured using the reference case definition. If the log/logit beta coefficient is negative, then the alternative is adjusted up to the reference. If the log/logit beta coefficient is positive, then the alternative is adjusted down to the reference.*
***The adjustment factor column is the exponentiated beta coefficient. For log beta coefficients, this is the relative rate between the two case definitions. For logit beta coefficients, this is the relative odds between the two case definitions.*

Datapoints with an age-standardised incidence rate greater than 1.5 median absolute deviations from the median of the age-standardised incidence rate for all inpatient and non-USA claims data were marked as outliers and excluded from analysis. Hospital discharge data from Latvia, Meghalaya, Jordan, Qatar, Iran, and Turkey, and claims data from Poland were also marked as outliers because their estimates were implausibly high when compared to regional, super-regional, and global rates.

EMR input processing

In GBD 2017, EMR inputs were produced by fitting a preliminary compartment model to estimate prevalence from incidence data, matching preliminary prevalence resulting from each incidence datapoint with the corresponding CSMR value within the same age, sex, year, and location, and dividing CSMR by prevalence. This method of producing EMR inputs, however, demonstrated a rather unrealistic pattern of EMR compared to an expected pattern of decreasing EMR with greater access to quality health care. Such unexpected patterns often signal inconsistencies between CSMR estimates and data on prevalence and/or incidence. Thus, in an effort to provide greater guidance on the expected pattern of EMR, in GBD 2019, EMR estimates produced per above were used in a MR-BRT model with age, sex,

and Healthcare Access and Quality (HAQ) Index as predictors, with a prior on HAQ Index to have a negative coefficient. In GBD 2021, we employed the same MR-BRT method to predict EMR for each location, year, sex, and for ages 0, 10, 20….100; these predictions were used as inputs to our non-fatal models, in GBD 2021 and GBD 2023, with the latter described below.

Modelling strategy

DisMod-MR model

We have made no substantive changes in the modeling strategy from GBD 2021. Similar to previous rounds, we ran a DisMod-MR 2.1 model to produce estimates by year, age, sex, and location. Inputs to DisMod-MR for AGN include incidence, CSMR, and EMR inputs processed as described above. Prior settings in the DisMod-MR model included setting remission of three to four weeks. It was assumed that no one was born with AGN. The minimum coefficient of variation at the regional, super-regional, and global level was set at 0.8. The HAQ Index was included as a predictive covariate on EMR. The beta and exponentiated values of this predictive covariate (which can be interpreted as an odds ratio) are shown in the table below.

Table 2. Covariates. Summary of covariates used in the acute glomerulonephritis DisMod-MR meta-regression model

Covariate	Parameter	Exponentiated beta (95% uncertainty interval)
HAQ Index	Excess mortality rate	0.97 (0.97–0.97)

Severity split and disability weight

The basis of the GBD disability weight assessment is lay descriptions of sequelae highlighting major functional consequences and symptoms.[1] Disability weights (DW) for AGN are associated with systemic symptoms of fever, aches, weakness, and some difficulty with daily activities. The lay description and disability weight for acute glomerulonephritis are shown below.

Table 3. Severity distribution, details on the severity levels for acute glomerulonephritis and the associated disability weight (DW) with that severity.

Severity level	Lay description	DW (95% CI)
Acute glomerulonephritis	Has a fever and aches, and feels weak, which causes some difficulty with daily activities.	0.051 (0.032–0.074)

References

1. Salomon JA, Vos T, Hogan DR, et al. Common values in assessing health outcomes from disease and injury: disability weights measurement study for the Global Burden of Disease Study 2010. *The Lancet* 2012; 380: 2129–43.

Acute hepatitis: A, B, C, and E

Acute hepatitis A

Flowchart

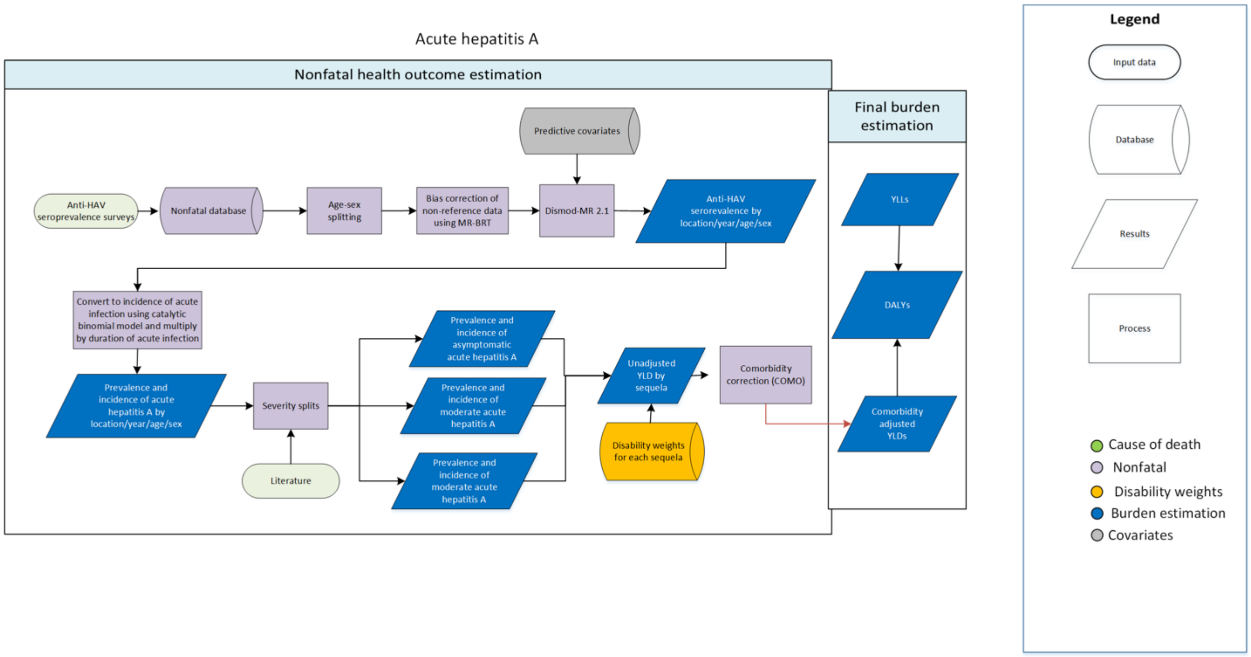

Input data and methodological summary for acute hepatitis A

Case definition

We define acute hepatitis A as an infection with the hepatitis A virus resulting in anti-HAV IgG seroconversion, regardless of symptoms.

Input data

Seroprevalence data inputs

We use anti-hepatitis A virus (HAV) seroprevalence data from population-based studies and surveys to estimate seroprevalence and seroincidence. The last systematic review was performed as part of GBD 2013. Additional data sources provided by collaborators were included in GBD 2019. No data changes were made as part of GBD 2023. A systematic review update is currently in progress for the next round of GBD.

Data processing

Because we produce sex-specific estimates, we adjusted data that reported on both sexes into male and female sex-specific estimates. We identified studies that reported sex-specific data and calculated the log ratio of female to male prevalence from studies that report sex-specific prevalence, modelling these log ratios in meta-regression—Bayesian, regularised, trimmed (MR-BRT), a regression tool developed at IHME. We then used the modelled sex ratio to adjust “both”-sex data values to expected “male” and “female” values. We calculated the male values as $val_{male} = val_{both} * \frac{pop_{both}}{(pop_{male}+ratio*pop_{female})}$. We calculated female values $val_{female} = ratio * val_{male}$.

Table 1: MR-BRT sex ratios for hepatitis A

Adjustment factor	Beta coefficient, log (95% UI)
Female to male ratio of anti-HAV seroprevalence	0.008 (–0.027 to 0.042)

**MR-BRT adjustments can be interpreted as the factor the alternative case definition is adjusted by to reflect what it would have been had it been measured using the reference case definition. If the log/logit beta coefficient is negative, then the alternative is adjusted up to the reference. If the log/logit beta coefficient is positive, then the alternative is adjusted down to the reference.*

We took as our reference population the general population of unvaccinated individuals living in a certain location in a certain year. Adjustment factors were estimated and applied prior to modelling to those prevalence data collected using non-reference study populations of pregnant women, blood donors, and a mix of vaccinated and unvaccinated individuals. Data were matched (by year, age, sex, location) for reference population and alternative populations, and their systematic differences were modelled using MR-BRT. Adjustment factors were estimated serially first for pregnant women and blood donors, then for a mix of vaccinated and unvaccinated individuals.

The process of adjusting for biases in non-reference data using MR-BRT with the logit-transformation method is described below:

1. Identify datapoints with overlapping year, age, sex, and location between reference and non-reference population data.
2. Logit transform overlapping datapoints of alternative and reference types.
3. Convert overlapping datapoints into a difference in logit space using the following equation: $logit(altnerative) - logit(reference)$
4. Use the delta method to compute standard errors of overlapping datapoints in logit space, then calculate standard error of logit difference using the following equation: $\sqrt{(variance\ of\ logit(alternative)) + (variance\ of\ logit(reference))}$
5. Using MR-BRT, conduct a random effects meta-regression to obtain the pooled logit difference of alternative to reference
6. Apply the pooled logit difference to all data points of non-reference types using the following equation:
$$new_{estimate} = inverse.logit((logit(alternative)) - (pooled\ logit\ difference))$$
7. Calculate new standard errors using the delta method, accounting for gamma (between-study heterogeneity)

The adjustment for pregnant women and blood donors was done separately from vaccinated population. Tables 2 and 3 show adjustment factors.

Table 2: MR-BRT crosswalk factors for anti-HAV seroprevalence non-representative populations

Data input	Reference or alternative case definition	Gamma	Beta coefficient, logit (95% CI)
General population	Reference	0.87	---
Blood donors	Alternative		0.85 (–0.95 to 2.58)
Pregnant women	Alternative		1.31 (–1.18 to 3.80)

**MR-BRT crosswalk adjustments can be interpreted as the factor the alternative case definition is adjusted by to reflect what it would have been had it been measured using the reference case definition. If the log/logit beta coefficient is negative, then the alternative is adjusted up to the reference. If the log/logit beta coefficient is positive, then the alternative is adjusted down to the reference.*

Table 3: MR-BRT crosswalk factors for anti-HAV seroprevalence vaccination status

Data input	Reference or alternative case definition	Gamma	Beta coefficient, logit (95% CI)
Unvaccinated study sample	Reference	1.01	---
Study sample included both vaccinated and unvaccinated individuals	Alternative		0.59 (–1.41 to 2.61)

**MR-BRT crosswalk adjustments can be interpreted as the factor the alternative case definition is adjusted by to reflect what it would have been had it been measured using the reference case definition. If the log/logit beta coefficient is negative, then the alternative is adjusted up to the reference. If the log/logit beta coefficient is positive, then the alternative is adjusted down to the reference.*

After sex-splitting and crosswalking, we adjusted broad age-group data (defined as age range was greater than 25 years) into five-year age bins using an estimated age pattern of seroprevalence model from the previous GBD round, a continued strategy from GBD 2019, assuming the age-distribution in the study sample was the same as the estimated population.

Modelling strategy

DisMod model of anti-HAV IgG seroprevalence

We model the seroprevalence of anti-HAV IgG using a DisMod-MR 2.1 model. Similar to previous GBD rounds, remission and excess mortality value priors of zero were used, and an incidence value prior range between 0 and 0.5 was used. Additionally, a relative-risk-weighted summary exposure variable for diarrhoea risk factors was included as a predictive covariate in the DisMod model to inform estimates for location-years with few or no primary data, with the coefficient in the fitted model shown below. In GBD 2023, we updated the minimum coefficient of variation at the regional, super-regional, and global level to 0.8 (previously 0.4).

Table 4. **Covariates.** Summary of country-level covariates used in the anti-HAV seroprevalence DisMod-MR 2.1 model

Covariate	Parameter	Exponentiated beta (95% CI)
Log-transformed age-standardised SEV* scalar: diarrhoea	Prevalence	1.31 (1.29–1.34)

*Summary exposure value

Acute hepatitis A incidence

As a log-normal model, DisMod-MR 2.1 performs poorly under modelling conditions for which prevalence approaches 100%. In view of the ubiquity of HAV infection and the reasonably stable force of infection among susceptible people across age groups, we used a catalytic binomial model to estimate the force of HAV infection on the basis of anti-HAV IgG seroprevalence. We derive incidence from the anti-HAV seroprevalence estimates using the following formula:

$$incidence = \frac{-\ln(1 - prevalence)}{age_{mid}} \times (1 - prevalence)$$

Severity splits and disability weights

We calculate acute symptomatic infections by multiplying incidence of acute infection by the probability of an acute infection being symptomatic. The probability of symptomatic infection comes from Armstrong and Bell[1] and is shown in the figure below (where probability of symptomatic infection is represented as “probability of jaundice”). The probability increases with age from ~1% in the first year of life to ~85% in adulthood.

The study used data from seven independent studies of community-wide hepatitis A outbreaks in different states in the USA. Each study included serological surveys of symptomatic and asymptomatic individuals. The functional form $P_j(a) = P_{jmax} * (1 - e^{(-r * a^s)})$ was derived from Edmunds et al,[3] the probability of jaundice in adults (P_{jmax}) was estimated from the pooled data of people 18 years of age and older. Data from people under 18 was then used to determine the model parameters r and s using maximum likelihood methods. The final probability model is:

$$Prob\ (symptomatic) = 0.852 * (1 - e^{-0.01244 * age^{1.903}})$$

Asymptomatic incidence is then derived by subtracting symptomatic incidence from total incidence. We then base severity splits for moderate and severe on expert opinion that the probability of severe infection follows a beta distribution with mean 0.6% (the below table reports percentiles of this distribution). We assume the rest of symptomatic infections are moderate.

Table 5. **Severity distribution,** details on the severity levels for acute hepatitis A

0th percentile	25th percentile	50th percentile	75th percentile	100th percentile
0.0024	0.0054	0.006	0.007	0.01

Prevalence of each sequela is calculated by multiplying by the disease duration of four weeks.

The table below illustrates the sequelae associated with acute hepatitis A, as well as the lay descriptions and associated disability weights.

Table 6. Disability weights, details on the associated disability weight (DW) for acute hepatitis A

Sequela	Description	Disability weight (95% CI)
Moderate	Has a fever and aches, and feels weak, which causes some difficulty with daily activities.	0.051 (0.032–0.074)
Severe	Has a high fever and pain, and feels very weak, which causes great difficulty with daily activities.	0.133 (0.088–0.19)
Asymptomatic	Infection with no apparent illness.	NA

Acute hepatitis B

Flowchart

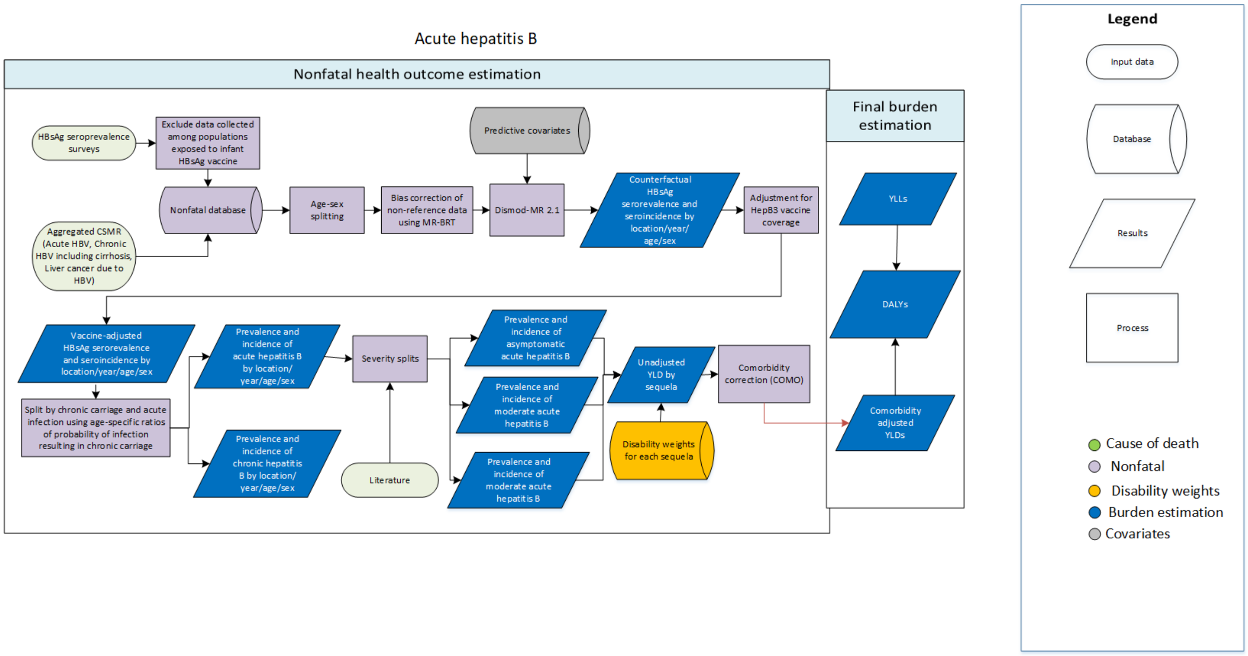

Input data and methodological summary for hepatitis B

Case definition

We define acute hepatitis B as the period corresponding to initial infection with the hepatitis B virus, regardless of symptoms.

Input data

Seroprevalence data inputs

We use hepatitis B surface antigen (HBsAg) seroprevalence data from population-based studies and surveys. The last systematic review conducted by IHME was performed as part of GBD 2013. This round, we completed an effort started in GBD 2019 to align data sources with those identified in Schweitzer and colleagues.[2]

Figure 1: PRISMA diagram of HBsAg sources

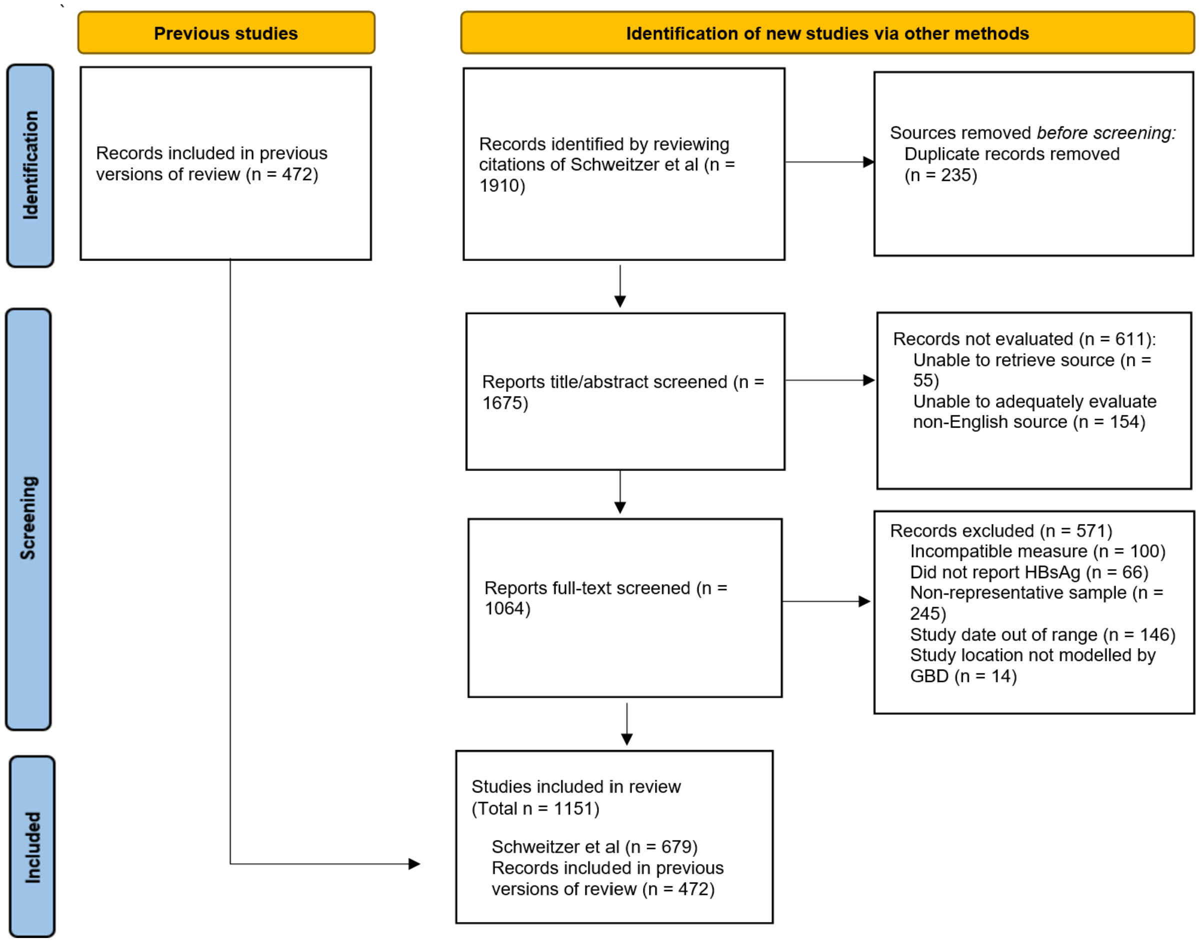

Cause-specific mortality rate (CSMR) inputs

We included CSMR inputs to our DisMod compartmental model for estimating seroprevalence of HBsAg. To obtain these, we summed CoDCorrected-CSMR estimates for acute hepatitis B, cirrhosis due to hepatitis B, and liver cancer due to hepatitis B from the GBD fatal estimation processes.

Overview of data processing and modelling

We modelled HBsAg seroprevalence using a multi-step approach. First, we subset our database to only data from unvaccinated populations and performed pre-modelling bias adjustments. Then, we fit a "counterfactual" HBsAg seroprevalence model, using only these data from unvaccinated populations in a DisMod-MR 2.1 compartmental model to obtain estimates of what the incidence and prevalence of chronic carriage would be in a steady state without vaccine intervention. Next, we modified those results using estimates of hepatitis B vaccine coverage and efficacy to obtain final estimates of incidence and prevalence of chronic hepatitis B carriage. Finally, we used natural history studies to infer what the total incidence of acute hepatitis B was from the incidence of chronic carriage. These processes are described in more detail below.

The rationale for this approach is as follows: prior to GBD 2019, the DisMod-MR 2.1 model of hepatitis B surface antigen positivity, using all available data, tended to follow the data from unvaccinated populations and poorly fit prevalence data from vaccinated populations at younger ages. DisMod-MR 2.1 assumes that diseases are steady state and employs data from a pre-set time window for the estimates for a given year. For example, estimates for the year 2000 can be set to utilise data from 1990 to 2010, 1995 to 2005, 1998 to 2002, and so on. Despite attempts to narrow the time window to between two- and five-year intervals, the model still did not capture rapid changes in seroprevalence that have resulted from vaccine uptake and cohort effects. In GBD 2019, we changed the modelling strategy to a counterfactual model to estimate what seroprevalence would be in the absence of vaccination efforts, and then adjusted by removing seroprevalent cases based on infant vaccine coverage and efficacy. The result of this process fit data from vaccinated cohorts better than DisMod models that were fit using all data, and so we continued this approach in GBD 2021 and GBD 2023.

Seroprevalence data processing

To prepare data for a counterfactual seroprevalence model, we started by excluding seroprevalence datapoints if all or at least 50% of study participants were born after the location-specific year of the hepatitis B three-dose vaccine introduction. Data collected from vaccinated populations were retained in the database to verify that subsequent modelling steps adequately accounted for the effect of vaccine programmes. Prior to modelling the data collected in samples of individuals not exposed to infant vaccination, we performed several data adjustments to correct for non-reference data-collection and reporting, including sex-splitting, crosswalking, and age-splitting.

Because we produce sex-specific estimates, we adjusted data that reported on both sexes into male and female sex-specific estimates. We identified studies that reported sex-specific data and calculated the log ratio of female to male prevalence from studies that report sex-specific prevalence, modelling these log ratios in meta-regression—Bayesian, regularised, trimmed (MR-BRT), a regression tool developed at IHME. We then used the modelled sex ratio to adjust “both”-sex data values to expected “male” and “female” values. We calculated the male values as $val_{male} = val_{both} * \frac{pop_{both}}{(pop_{male}+ratio*pop_{female})}$ We calculated female values $val_{female} = ratio * val_{male}$.

Table 1: MR-BRT sex ratios for HBsAg

Adjustment factor	Beta coefficient, log (95% CI)	Gamma
Female to male ratio of HBsAg seroprevalence	–0.359 (–0.383 to –0.335)	0.0013

**MR-BRT adjustments can be interpreted as the factor the alternative case definition is adjusted by to reflect what it would have been had it been measured using the reference case definition. If the log/logit beta coefficient is negative, then the alternative is adjusted up to the reference. If the log/logit beta coefficient is positive, then the alternative is adjusted down to the reference.*

Adjustment factors were estimated and applied prior to modelling to those prevalence data collected using non-reference study populations of pregnant women and blood donors. Data were matched (by year, age, sex, location) for general population and alternative populations, and their systematic differences were modelled using MR-BRT.

The process of adjusting for biases in non-reference data using MR-BRT with the logit-transformation method is described below:

1. Identify datapoints with overlapping year, age, sex, and location between reference and non-reference population data.
2. Logit transform overlapping datapoints of alternative and reference types.
3. Convert overlapping datapoints into a difference in logit space using the following equation: $logit(altnerative) - logit(reference)$
4. Use the delta method to compute standard errors of overlapping datapoints in logit space, then calculate standard error of logit difference using the following equation: $\sqrt{(variance\ of\ logit(alternative)) + (variance\ of\ logit(reference))}$
5. Using MR-BRT, conduct a random effects meta-regression to obtain the pooled logit difference of alternative to reference
6. Apply the pooled logit difference to all data points of non-reference types using the following equation:
$$new_{estimate} = inverse.logit((logit(alternative)) - (pooled\ logit\ difference))$$
7. Calculate new standard errors using the delta method, accounting for gamma (between-study heterogeneity)

Table 2: MR-BRT crosswalk factors for HBsAg seroprevalence non-representative populations

Data input	Reference or alternative population	Gamma	Beta coefficient, logit (95% CI)
General population	Reference	0.359	---
Blood donors	Alternative		–0.099 (–1.327 to 1.129)
Pregnant women	Alternative		0.0097 (–1.263 to 1.283)

**MR-BRT adjustments can be interpreted as the factor the alternative case definition is adjusted by to reflect what it would have been had it been measured using the reference case definition. If the log/logit beta coefficient is negative, then the alternative is adjusted up to the reference. If the log/logit beta coefficient is positive, then the alternative is adjusted down to the reference.*

After sex-splitting and crosswalking, we adjusted broad age-group data (defined as age range was greater than 25 years) into five-year age bins using an estimated age pattern of seroprevalence model from the previous GBD round, a continued strategy from GBD 2019, assuming the age-distribution in the study sample was the same as the estimated population.

Modelling strategy

Counterfactual seroprevalence model

We estimated HBsAg seroprevalence using DisMod-MR 2.1. We used the processed data described previously to generate location-age-sex-year-specific estimates. In addition to HBsAg seroprevalence and CSMR data, we included predictive covariates in the model to improve estimation of quantities of interest where data are absent or scarce. We included remission priors between 0 and 0.02, excess mortality prior between 0 and 0.1, and incidence priors between 0 and 0.05 for all ages. The summary of covariates using in the counterfactual HBsAg seroprevalence DisMod-MR 2.1 model are listed below.

Table 4. Covariates. Summary of predictive covariates used in the HBsAg seroprevalence DisMod-MR 2.1 model

Covariate	Parameter	Exponentiated beta (95% CI)
Log-transformed age-standardised SEV scalar: hepatitis B	Prevalence	1.02 (1.00–1.08)
Socio-demographic Index	Prevalence	0.14 (0.14–0.1)
Healthcare Access and Quality Index	Excess mortality rate	1.00 (1.00–1.00)

Adjustment for vaccination effects

After the completion of the counterfactual DisMod model, a post-hoc adjustment was performed to modify estimates of HBsAg seropositivity based on GBD-produced location-year-specific hepatitis B three-dose vaccine coverage and vaccine efficacy. For every age group and year, the coverage at that time those individuals were neonates was multiplied by the proportion of vaccine coverage by location and year and by vaccine efficacy of 95% to get the proportion of the population protected. Protected individuals were subtracted from the HBsAg cases estimated in the counterfactual DisMod model to get final estimates of HBsAg prevalence and incidence. An example of the counterfactual DisMod-MR 2.1 estimates and vaccine-adjusted estimates in comparison to included and excluded datapoints is shown in Figure 2.

Figure 2. Comparisons of counterfactual and vaccine-adjusted estimates to included and excluded datapoints

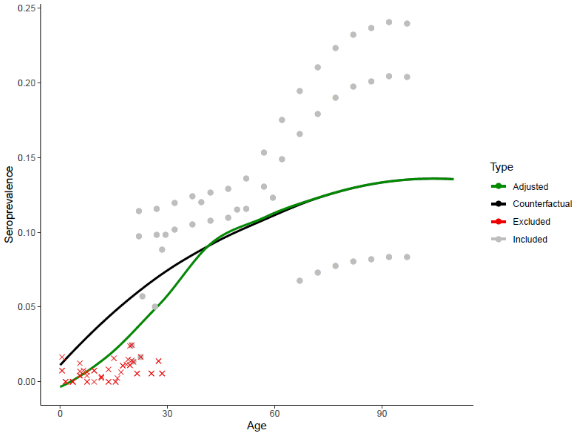

These final estimates of HBsAg seroprevalence serve as inputs to models for several entities, as described in the methods appendix sections on the estimation of the fatal and non-fatal burden of cirrhosis and other chronic liver diseases and liver cancer. The remainder of this section only discusses how HBsAg seroprevalence estimates are used to estimate acute hepatitis B infection.

Acute hepatitis B incidence and severity distribution estimation

The incidence obtained from the DisMod model of HBsAg is regarded as the incidence of chronic carriage. This is converted to the total incidence of hepatitis B infection by dividing age-specific estimates of the incidence of chronic carriage by age-specific estimates of the probability of infection resulting in carriage based on Edmunds and colleagues.[3] Edmunds et al only reports this probability for ages 0–25 years, so we assume the probability for ages 25+ is the same as estimates for age 25.

$$P(carrier \mid age \leq 6\ months) = 0.885$$
$$P(carrier \mid 6\ months \leq age < 25\ years) = e^{-0.645 \times age^{0.455}}$$
$$P(carrier \mid age \geq 25\ years) = e^{-0.645 \times 25^{0.455}} = 0.061$$

We then estimated the incidence of symptomatic acute infections by multiplying the incident infection rate calculated above by the probability of suffering from acute illness. We use estimates of this probability by age, both sexes, from McMahon et al 1985[4] to calculate the incident rate of acute

symptomatic infections. We assume perinatal cases are rare and use a probability of 1%, rather than McMahon's estimate for age 0.

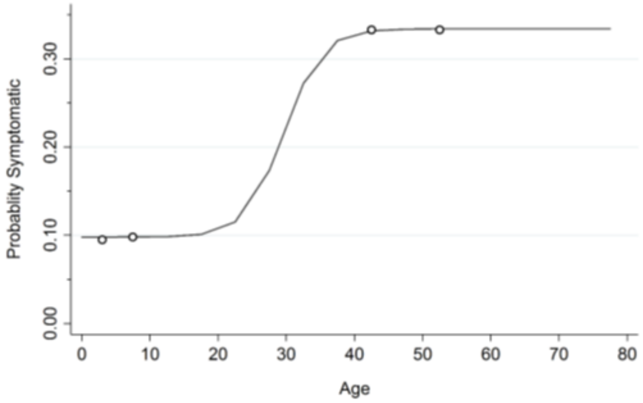

Asymptomatic incidence is then derived by subtracting symptomatic incidence from total incidence. Symptomatic cases were split into moderate (73%) and severe (27%) based on data from McMahon and colleagues. Prevalence of each sequela is calculated by multiplying by the disease duration of six weeks.

Health states and disability weights

The table below illustrates the sequelae associated with acute hepatitis B, as well as the lay descriptions and associated disability weights.

Table 5. Disability weights, details on the associated disability weight (DW) for acute hepatitis B

Sequela	Description	Disability weight
Moderate	Has a fever and aches, and feels weak, which causes some difficulty with daily activities.	0.051 (0.032–0.074)
Severe	Has a high fever and pain, and feels very weak, which causes great difficulty with daily activities.	0.133 (0.088–0.19)
Asymptomatic	Infection with no apparent illness.	NA

Acute hepatitis C

Flowchart

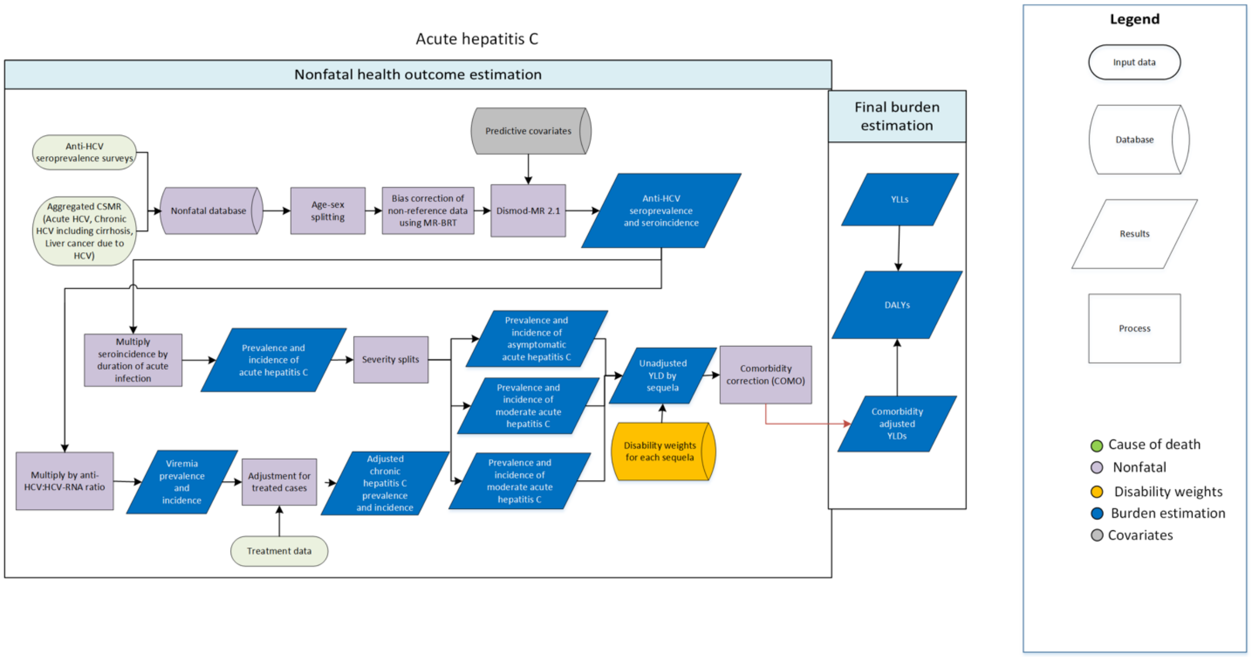

Input data and methodological summary for hepatitis C

Case definition

We define acute hepatitis C as the period corresponding to initial infection with the hepatitis C virus, resulting in anti-HCV IgG seroconversion, regardless of symptoms.

Input data

Seroprevalence data

To estimate morbidity for hepatitis C, we use anti-HCV seroprevalence data from population-based studies and surveys to estimate seroincidence and seroprevalence. The last systematic review performed by IHME was part of GBD 2013, we subsequently added sources collated by the Center for Disease Analysis and identified in the systematic review by Blach 2016,[5] as well as ongoing contributions from the GBD Collaborator Network.

CSMR inputs

We also use CSMR estimates from the GBD causes of death modelling process as inputs in our DisMod compartmental model of anti-HCV seropositivity, summing CSMR for acute hepatitis C, cirrhosis and other chronic liver diseases due to hepatitis C, and liver cancer due to hepatitis C.

Data on the ratio of anti-HCV seroprevalence to HCV viremia

In GBD 2019, we identified from our seroprevalence database 42 studies that reported on the prevalence of anti-HCV antibody and HCV-RNA in the same individuals, which we used as inputs to a meta-analysis of the ratio of seroprevalence to viraemia in untreated populations.

Treatment data

Additionally, we use hepatitis C treatment volumes to account for curative efforts. In GBD 2019, we included information on treatment for Egypt, Japan, and Australia. With collaboration from the Coalition for Global Hepatitis Elimination, we expanded our treatment database in GBD 2021. In GBD 2023, we used the same treatment data as used for GBD 2021. The data were from 34 countries: Egypt, Japan, Australia, Romania, Portugal, Latvia, Iceland, Slovenia, Spain, England, France, Bulgaria, Croatia, Hungary, Wales, Rwanda, South Africa, Argentina, Brazil, Morocco, Pakistan, Georgia, Ukraine, Indonesia, Mongolia, Canada, China, Ghana, India, Ireland, Malaysia, Mexico, and Russia. We extracted data on age, sex, and treatment type (ie, direct-acting antivirals, interferon, triple drug) when available.

Seroprevalence data processing

Because we produce sex-specific estimates, we adjusted data that reported on both sexes into male and female sex-specific estimates prior using them in regression models. To estimate an adjustment factor for this "sex-splitting" process, we identified studies that reported sex-specific prevalence, calculated the log ratio of female-to-male prevalence from studies, and pooled these log ratios using MR-BRT to account for inter-study heterogeneity. We then used the modelled sex ratio to adjust "both"-sex data values to expected "male" and "female" values. We calculated the male values as:

$$val_{male} = val_{both} * \frac{pop_{both}}{(pop_{male}+ratio*pop_{female})}.$$

We calculated female values as:

$$val_{female} = ratio * val_{male}.$$

Table 1: MR-BRT sex ratio for anti-HCV

Adjustment factor	Beta coefficient, log (95% CI)	Gamma
Female to male ratio of anti-HCV seroprevalence	–0.04306 (–0.376 to 0.29)	0.028

**MR-BRT adjustments can be interpreted as the factor the alternative case definition is adjusted by to reflect what it would have been had it been measured using the reference case definition. If the log/logit beta coefficient is negative, then the alternative is adjusted up to the reference. If the log/logit beta coefficient is positive, then the alternative is adjusted down to the reference.*

After sex-splitting, data collected using samples of non-reference populations were adjusted toward the levels expected if they had been conducted using a sample from the general (reference) population, a process referred to in GBD as "crosswalking". To estimate adjustment factors, we paired prevalence datapoints collected using samples of non-reference populations (specifically, samples of blood donors) to prevalence data collected using reference (general) populations, matching by year, age, sex, location, as reported in the original studies. Their systematic differences were modelled as a logit difference in prevalence using MR-BRT.

The process of adjusting for biases in non-reference data ("crosswalking") using MR-BRT with the logit-transformation method is described below:

1. Identify datapoints with overlapping year, age, sex, and location between reference and non-reference population data.
2. Logit transform overlapping datapoints of alternative and reference types.
3. Convert overlapping datapoints into a difference in logit space using the following equation: $logit(altnerative) - logit(reference)$
4. Use the delta method to compute standard errors of overlapping datapoints in logit space, then calculate standard error of logit difference using the following equation: $\sqrt{(variance\ of\ logit(alternative)) + (variance\ of\ logit(reference))}$
5. Using MR-BRT, conduct a random effects meta-regression to obtain the pooled logit difference of alternative to reference
6. Apply the pooled logit difference to all data points of non-reference types using the following equation:
$$new_{estimate} = inverse.logit((logit(alternative)) - (pooled\ logit\ difference))$$
7. Calculate new standard errors using the delta method, accounting for gamma (between-study heterogeneity)

Table 2: MR-BRT crosswalk factors for anti-HCV seroprevalence non-representative populations

Data input	Reference or alternative case definition	Gamma	Beta coefficient, logit (95% CI)
General population	Reference	0.187	---
Blood donors	Alternative		–0.603 (–1.52 to 0.315)

**MR-BRT adjustments can be interpreted as the factor the alternative case definition is adjusted by to reflect what it would have been had it been measured using the reference case definition. If the log/logit beta coefficient is negative, then the alternative is adjusted up to the reference. If the log/logit beta coefficient is positive, then the alternative is adjusted down to the reference.*

After sex-splitting and crosswalking, we adjusted broad age-group data (defined as age range was greater than 25 years) into five-year age bins using an estimated age pattern of seroprevalence from the previous GBD round, a continued strategy from GBD 2019, assuming the age-distribution in the study sample was the same as the estimated population.

Modelling strategy

DisMod model of anti-HCV seropositivity

We estimated anti-HCV seropositivity using DisMod-MR 2.1. We used the processed seroprevalence data described previously to generate location-age-sex-year-specific estimates. In addition to anti-HCV seroprevalence and CSMR data, we included predictive covariates in the model to improve estimation in quantities of interest where data are absent or scarce. We included remission priors of 0 for all ages and incidence priors maximum of 0.001 for 0 to 10 years of age. The summary of covariates used in the anti-HCV positivity DisMod-MR 2.1 model are listed below.

Table 3. Covariates. Summary of covariates used in the anti-HCV seroprevalence DisMod-MR 2.1 model

Covariate	Parameter	Exponentiated beta (95% CI)
Log-transformed age-standardised SEV scalar: hepatitis C	Prevalence	2.46 (2.46–2.46)
Socio-demographic Index	Prevalence	0.14 (0.14–0.14)

Estimating the incidence and point-prevalence of acute hepatitis C infection

The incidence of anti-HCV IgG seroconversion from DisMod-MR was treated as the incidence of acute hepatitis C infection. The incident infections were divided into asymptomatic (75%), moderate (24%), and severe (1%) states based on expert opinion and assigned the following health states and disability weights. We assumed duration of six weeks based on content expert consultation.

Table 4. Disability weights, details on the associated disability weight (DW) for acute hepatitis C

Sequela	Description	Disability weight
Moderate	Has a fever and aches, and feels weak, which causes some difficulty with daily activities.	0.051 (0.032–0.074)
Severe	Has a high fever and pain, and feels very weak, which causes great difficulty with daily activities.	0.133 (0.088–0.19)
Asymptomatic	Infection with no apparent illness.	NA

Estimating the prevalence of chronic hepatitis C (viraemia)

Beyond estimating burden due to acute hepatitis C, the DisMod model of anti-HCV seroprevalence was used to estimate prevalence of chronic infection (viraemia), which serves as an input to multiple estimation processes described in separate sections of this appendix (fatal and non-fatal burden of cirrhosis and other liver disease and liver cancer). This was done in three steps: first, we estimated the ratio of anti-HCV seroprevalence to viraemia; second, we converted seroprevalence estimates from DisMod to estimates of viraemia by multiplying by this ratio estimate at the draw level; and third, we reduced the number of prevalent cases based on the number of cases treated by national programmes. We used 42 studies that reported on the prevalence of anti-HCV antibody and HCV-RNA to produce a pooled estimate of proportion viraemic among the seropositive in untreated populations. This was used to correct outputs of our model of anti-HCV seropositivity to estimate viraemia. We examined the estimated coefficient based on super-region, particularly looking to see if there is a difference in the ratio of anti-HCV to HCV-RNA positivity in sub-Saharan Africa as suggested by expert collaborators. However, no difference was identified, and we used the same conversion factor globally. Below is a graph of the pooled estimated logit difference and logit difference and standard error of input studies.

Figure 1. Forest plot of studies that report both anti-HCV and HCV-RNA estimates

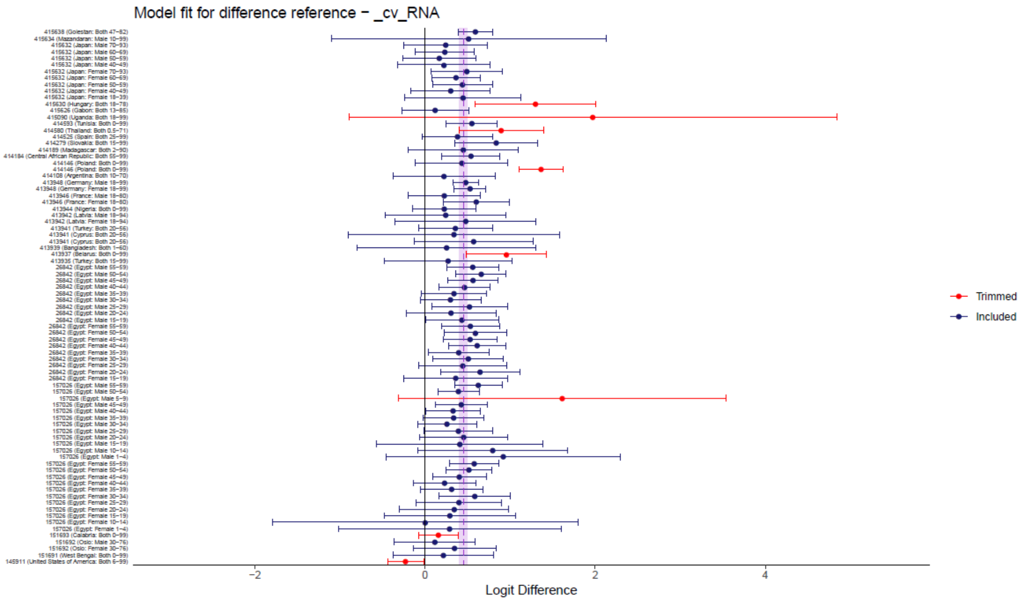

Treatment adjustment

We accounted for virus-clearing treatments of hepatitis C in locations where we had access to information on national treatment data. We processed treatment data by splitting both-sex data into sex-specific data and broad age-range data into five-year age bins using the age and sex distributions in our estimates of chronic HCV cases. (This implies equal access to treatment for all ages and both sexes whenever we have treatment data that do not report these characteristics.) We multiplied the number of cases treated by treatment efficacy (assumed 70% for pegylated interferon and 95% for direct-acting antiviral treatments). We subtracted the number of individuals treated in a year from the initial viraemic cases estimated in the steps above. The cumulative effect of treatment from year to year was the summation of treatment in the current year of treatment, as well as previous years and age groups, to ensure all years of treatment were reflected in final estimates. For locations where the treatment data did not include the final year of estimation, we assumed the proportion of cases treated in the last year of observed data was sustained until 2019 and assumed a decrease in treatment coverage in between 2020 and 2023, as described below.

We identified treatment data from seven locations (Australia, Brazil, Canada, England, Georgia, Mexico, and Rwanda) that provided information on the number of people treated in 2020, 2021, or both. We used treatment data from these locations to estimate service disruption due to COVID-19. From these, we calculated an average reduction in treatment services of 19.6% in 2020 compared to 2019 and of 20.1% in 2021 compared to 2020.

In the locations for which we had treatment data from 2020 and 2021, the number of people treated in the COVID-19 pandemic years was calculated using its own treatment data; for locations where we had treatment data for 2020 but not 2021, we assumed the proportion of cases treated in 2020 was sustained in 2021. For the countries for which we had some years of treatment data prior to 2020, but not in the COVID-19 years, we applied the average treatment reduction rates (%) between 2019–2020 and 2020–2021 that were calculated based on the seven locations, as described above. The same reduction from 2021 was applied to 2022 and 2023.

Acute hepatitis E
Flowchart

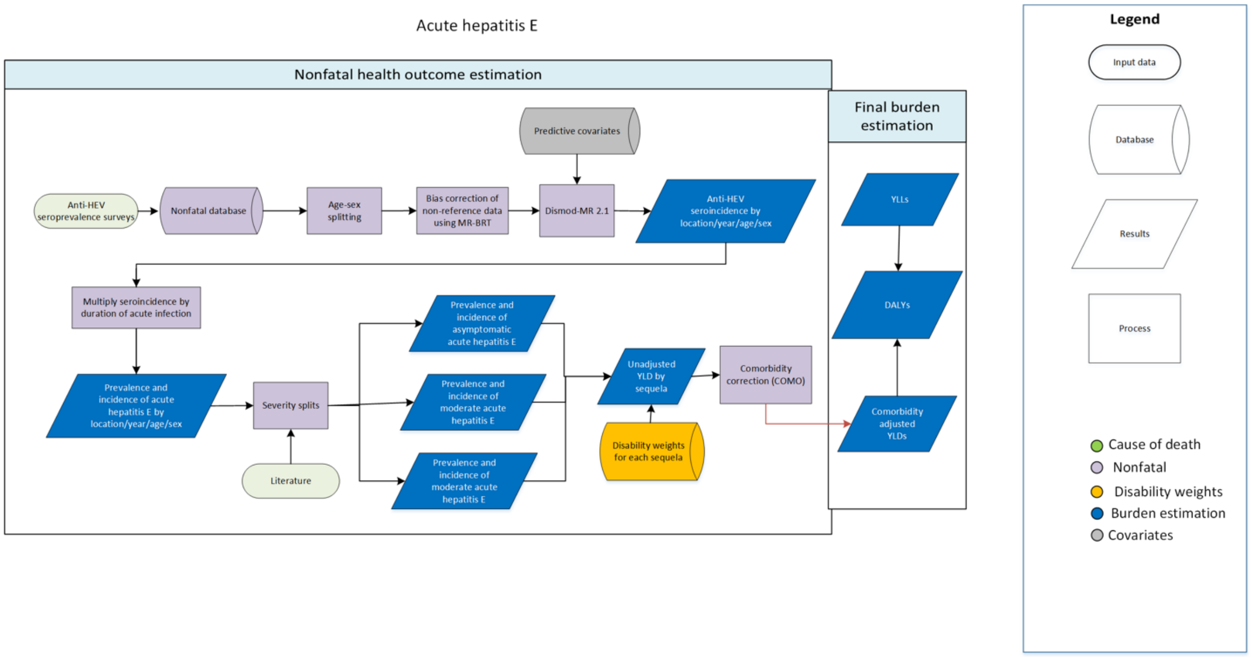

Input data and methodological summary for hepatitis E
Case definition

We define acute hepatitis E as an infection with the hepatitis E virus resulting in anti-HEV IgG seroconversion, regardless of symptoms.

Input data

We use anti-HEV seroprevalence data from population-based studies and surveys to estimate the incidence of infection. In GBD 2023, we began a systematic literature review using the following search string to identify recent seroprevalence data. Analogous search strings were used for other databases (Embase and LILACs):

(hepatitis e[mesh] OR hepatitis e virus[mesh] OR hepatitis e[tiab] OR anti-HEV[tiab] OR Enterically-Transmitted Non-A, Non-B Hepatitis[tiab] OR Epidemic Non-A, Non-B Hepatitis[tiab]) ***AND*** *(("Incidence"[Mesh] OR Inciden*[tiab] OR attack rate[tiab] OR force of infection[tiab])* ***OR*** *("Prevalence"[Mesh] OR Seroepidemiologic Studies[mesh] OR Prevalence[tiab] OR Seroprevalence[tiab] OR Seroposit*[tiab] OR Infect*[tiab] OR Epidemiol*[tiab] OR Epidemiology[subheading])* ***OR*** *("Seroconversion"[Mesh] OR Seroconver*[tiab]) OR (Seroreversion[tiab] OR Serolog*[tiab])* ***OR*** *("Immunity"[Mesh] OR Diagnosis [mesh] OR Immun*[tiab] OR Diagnos*[tiab] OR Duration[tiab] or natural history[tiab])* ***OR*** *("Disease"[Mesh] OR Risk factors[mesh] OR Diseas*[tiab] OR "Disease Attributes"[Mesh] OR diagnosis[subheading] OR Sever*[tiab] OR Critical[tiab])* ***OR*** *(Prognosis[mesh] OR Death*[tiab] OR Mortality [mesh] OR Mortalit*[tiab] OR case fatal*[tiab]))* ***NOT*** *(animals[mesh] NOT humans[mesh])*

The inclusion criteria included:

- Reports unadjusted prevalence or incidence of anti-HEV IgG
- Representative sample of the general population (~population-representative study)
- A convenience sample or other population group that is adequately representative.

- Subpopulations that we are interested in for which an adjustment can be made

The exclusion criteria included:
- Non-representative sampling or sampling of a specific population for which no valid adjustment can be made (eg, people receiving liver transplants, people with Guillain-Barré syndrome)
- Populations with comorbidities
- Subpopulations selected by race or ethnicity
- Unreliable data sources:
    - Self-report on HEV status
    - Studies with measurement issues (eg, studies based on blood donations that report "prevalence" among donated samples/sera instead of among individual donors.)
- Study designs such as systematic reviews, commentaries, editorials, replies, etc.

All pre-existing seroprevalence data used in GBD 2021 were identified using the search string from above. These sources were re-reviewed against the updated inclusion and exclusion criteria. Of the 93 sources from previous rounds of GBD, 54 were retained in the model.

Figure 1: PRISMA diagram of anti-HEV seroprevalence sources

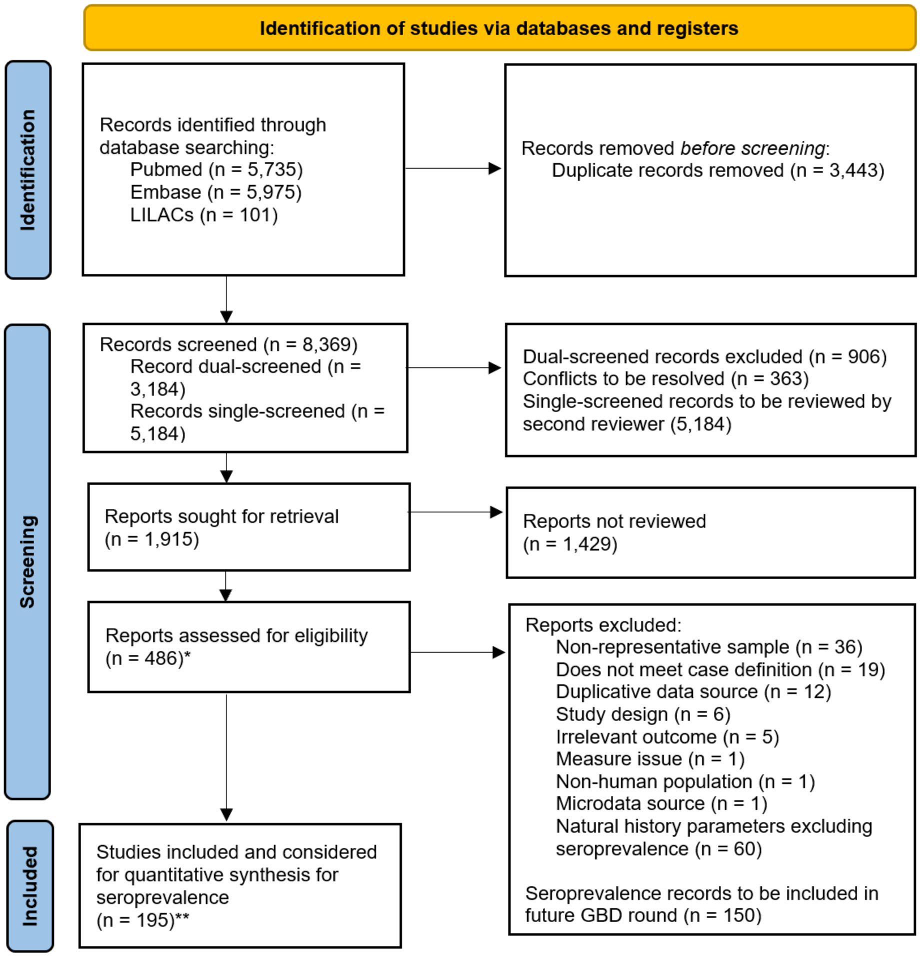

**93/486 reports identified and assessed for eligibility were pre-existing sources in the GBD database for HEV seroprevalence*

***67/195 studies were excluded in the final GBD 2023 seroprevalence model due to the inability to estimate reliable adjustment factors for aligning data from non-reference populations or those using alternative case definitions*

Data processing

Seroprevalence data processing

Because we produce sex-specific estimates, we adjusted data that reported on both sexes into male and female sex-specific estimates prior using them in regression models. To estimate an adjustment factor for this "sex-splitting" process, we identified studies that reported sex-specific prevalence, calculated the log ratio of female-to-male prevalence from studies, and pooled these log ratios using MR-BRT to account for inter-study heterogeneity. We then used the modelled sex ratio to adjust "both"-sex data values to expected "male" and "female" values. We calculated the male values as:

$$val_{male} = val_{both} * \frac{pop_{both}}{(pop_{male} + ratio * pop_{female})}$$

We calculated female values as: $val_{female} = ratio * val_{male}$.

Table 1: MR-BRT sex ratio for anti-HEV

Adjustment factor	Beta coefficient, log (95% CI)

Female to male ratio of anti-HEV seroprevalence	–0.182 (–0.434 to 0.406)

**MR-BRT adjustments can be interpreted as the factor the alternative case definition is adjusted by to reflect what it would have been had it been measured using the reference case definition. If the log/logit beta coefficient is negative, then the alternative is adjusted up to the reference. If the log/logit beta coefficient is positive, then the alternative is adjusted down to the reference.*

After sex-splitting, data collected using samples of non-reference populations were adjusted toward the levels expected if they had been conducted using a sample from the general (reference) population, a process referred to in GBD as "crosswalking". To estimate adjustment factors, we paired prevalence datapoints collected using samples of non-reference populations (specifically, samples of blood donors) to prevalence data collected using reference (general) populations, matching by year, age, sex, location, as reported in the original studies. Their systematic differences were modelled as a logit difference in prevalence using MR-BRT.

The process of adjusting for biases in non-reference data using MR-BRT with the logit-transformation method is described below:

1. Identify datapoints with overlapping year, age, sex, and location between reference and non-reference population data.
2. Logit transform overlapping datapoints of alternative and reference types.
3. Convert overlapping datapoints into a difference in logit space using the following equation: $logit(altnerative) - logit(reference)$
4. Use the delta method to compute standard errors of overlapping datapoints in logit space, then calculate standard error of logit difference using the following equation: $\sqrt{(variance\ of\ logit(alternative)) + (variance\ of\ logit(reference))}$
5. Using MR-BRT, conduct a random effects meta-regression to obtain the pooled logit difference of alternative to reference
6. Apply the pooled logit difference to all data points of non-reference types using the following equation:
$$new_{estimate} = inverse.logit((logit(alternative)) - (pooled\ logit\ difference))$$
7. Calculate new standard errors using the delta method, accounting for gamma (between-study heterogeneity)

Table 2: MR-BRT crosswalk factors for anti-HEV seroprevalence non-representative populations

Data input	Reference or alternative case definition	Gamma	Beta coefficient, logit (95% CI)
General population	Reference	0.43	---
Blood donors	Alternative		–0.06 (–1.55 to 1.43)

**MR-BRT adjustments can be interpreted as the factor the alternative case definition is adjusted by to reflect what it would have been had it been measured using the reference case definition. If the log/logit beta coefficient is negative, then the alternative is adjusted up to the reference. If the log/logit beta coefficient is positive, then the alternative is adjusted down to the reference.*

We adjusted broad age-group data into five-year age bins using an estimated regional age-pattern from age-specific data. Data in which the age range was greater than 25 years was categorised as broad age-range data.

Modelling strategy

DisMod model
We estimated anti-HEV seropositivity using DisMod-MR 2.1. We used the processed seroprevalence data described previously to generate location-age-sex-year-specific estimates. We included remission and excess mortality rate priors of 0 for all ages. In addition to anti-HEV seroprevalence data, we included predictive covariates in the model to improve estimation in quantities of interest where data are absent or scarce. The summary of covariates used in the anti-HEV positivity DisMod-MR 2.1 model is listed below.

Table 3. Covariates. Summary of covariates used in the anti-HEV seroprevalence DisMod-MR 2.1 model

Covariate	Parameter	Exponentiated beta (95% CI)
Proportion of the population living in the classic monsoon region (low-income countries)	Prevalence	2.05 (1.64–2.63)
Log-transformed SEV scalar: diarrhoea	Prevalence	1.04 (1.00–1.09)

Acute hepatitis E incidence and prevalence estimation

The seroincidence from DisMod-MR was treated as the incidence of acute hepatitis E infection. Prevalence of acute infection is calculated by multiplying age-sex-year-location-specific incidence of acute infection by the disease duration of four weeks.

Severity splits and disability weights

We calculate acute symptomatic infections by multiplying incidence of seroconversion by the probability of an acute infection being symptomatic. The probability of acute symptomatic infection was derived from total acute infection using the algorithm adapted from Edmonds and colleagues' 1993 publication.[3] Based on information published by Rein and colleagues,[7] we assume that the probability of symptomatic infection increases with age from ~1% in the first year of life to ~60% in adulthood.

Within symptomatic infections, we attribute an assumed proportion of symptomatic infections to a severe state, sampled from a beta distribution:

0th percentile	25th percentile	50th percentile	75th percentile	100th percentile
0.009	0.002	0.030	0.030	0.070

Symptomatic infections with moderate severity are estimated by subtracting severe infections from the total.

The table below illustrates the sequelae associated with acute hepatitis E, along with their descriptions and disability weights.

Table 4. Disability weights, details on the associated disability weight (DW) for acute hepatitis E

Sequela	Description	Disability weight
Moderate	Has a fever and aches, and feels weak, which causes some difficulty with daily activities.	0.051 (0.032–0.074)
Severe	Has a high fever and pain, and feels very weak, which causes great difficulty with daily activities.	0.133 (0.088–0.19)
Asymptomatic	Infection with no apparent illness.	NA

References

1. Armstrong GL, Bell BP. Hepatitis A Virus Infections in the United States: Model-Based Estimates and Implications for Childhood Immunization. *Pediatrics*. 2002 May 1;109(5):839–45.
2. Schweitzer, A., Horn, J., Mikolajczyk, R. T., Krause, G., & Ott, J. J. Estimations of worldwide prevalence of chronic hepatitis B virus infection: a systematic review of data published between 1965 and 2013. *Lancet*. 2015; 386(10003), 1546–1555
3. Edmunds WJ, Medley GF, Nokes DJ, Hall AJ, Whittle HC. The influence of age on the development of the hepatitis B carrier state. *Proc Biol Sci*. 1993 Aug 23;253(1337):197–201.
4. McMahon BJ, Alward WL, Hall DB, Heyward WL, Bender TR, Francis DP, et al. Acute hepatitis B virus infection: relation of age to the clinical expression of disease and subsequent development of the carrier state. *J Infect Dis*. 1985 Apr;151(4):599–603).
5. Blach S et al. Global prevalence and genotype distribution of hepatitis C virus infection in 2015: a modelling study. *The Lancet Gastroenterology & Hepatology*. 2017; 2(3):161-176.
6. Guadagnino, Vincenzo, et al. Prevalence, risk factors, and genotype distribution of hepatitis C virus infection in the general population: a community-based survey in southern Italy. *Hepatology* 26.4 (1997): 1006-1011.
7. Rein DB, Stevens GA, Theaker J, Wittenborn JS, Wiersma ST. The global burden of hepatitis E virus genotypes 1 and 2 in 2005. *Hepatology*. 2012 Apr 1;55(4):988–97.

Acute myocarditis

Flowchart

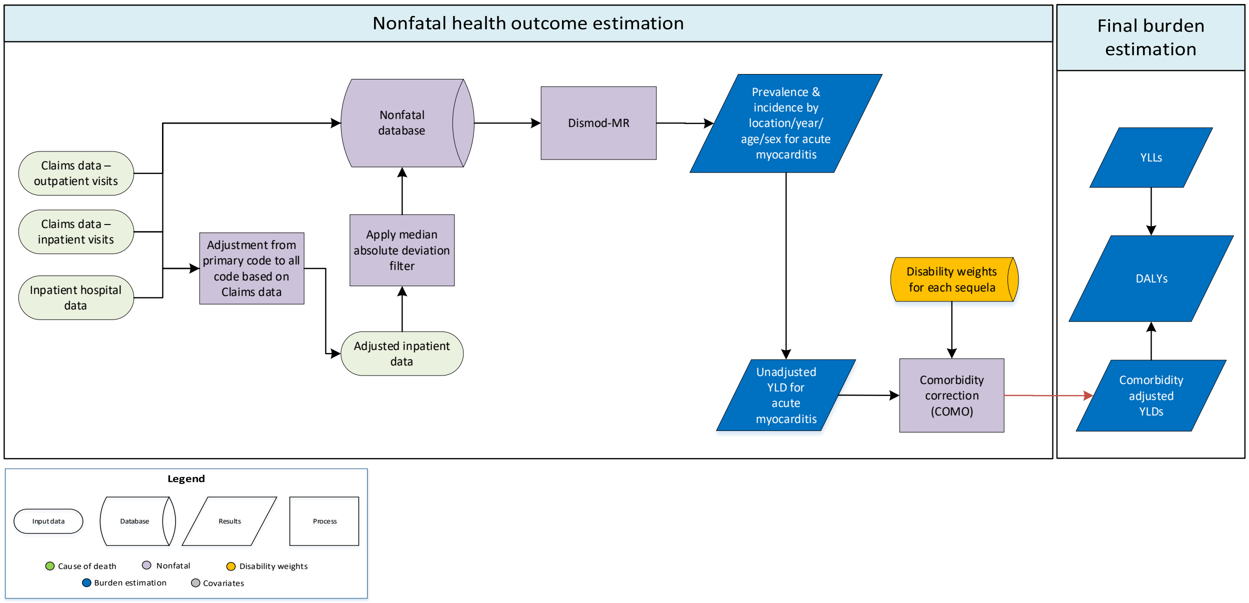

Input data and methodological summary for acute myocarditis

Case definition

Myocarditis is inflammation of the myocardium or middle layer of the heart wall muscles. It can be caused by viral infections, autoimmune conditions, and other non-ischaemic causes and can result in reduced ability of the heart to pump blood to the body. Acute myocarditis was defined for GBD as the acute and time-limited symptoms of myocarditis separate from its chronic heart-failure-related sequelae. Heart failure due to myocarditis is estimated separately in GBD (see methods for heart failure). Symptoms of acute myocarditis can be non-specific and include a flu-like or gastrointestinal syndrome, followed by anginal-type chest pain, arrhythmias, syncope, or heart failure.

The GBD reference case definition is listed in Table 1, and the ICD codes used for inclusion are included in Table 2.

Table 1. Myocarditis reference case definition

**Quantity of interest**	**Reference or Alternative**	**Definition**
Myocarditis	Reference	The physician standard for diagnosing acute myocarditis is endomyocardial biopsy. However, CMR imaging can serve as a non-invasive modality for diagnosis.
Myocarditis	Alternative	N/A

Table 2. ICD codes used for inclusion of myocarditis hospital and claims data

Cause	ICD-9	ICD-10
Acute myocarditis	422–422.9, 429, and 429.1	B33.2, I40–I41.8, I51.4, I51.5, I51.6

Input data

The preferred data sources for acute myocarditis were hospital admission data and other health facility data identifying cases of acute myocarditis. We have performed a systematic review of myocarditis in past cycles of GBD (GBD 2013 – see below) and found no sources that matched our criteria for modelling of the disease. As a result, we currently only use hospital admission incidence data to estimate acute myocarditis incidence and prevalence.

A systematic review was performed for GBD 2013 and updated for GBD 2015. A systematic review has not been completed since.

The GBD 2015 search terms included (cardiomyopathy AND epidemiology [MeSH Subheading]) OR (myocarditis AND epidemiology [MeSH Subheading]) OR (cardiomyopathy AND (incidence OR prevalence OR “case fatality”)) OR (myocarditis AND (incidence OR prevalence OR “case fatality”))

- Dates included in search: 1/1/2013–3/16/2015
- Number of initial hits: 3598
- Number of sources included: 0

The GBD 2013 search terms included: (hasabstract[text] AND Humans[MeSH] AND middle age[MeSH])) OR 21) AND ((cardiomyopathy/epidemiology[MeSH] OR cardiomyopathy/mortality[MeSH]) AND (prevalence[Title/Abstract] OR incidence[Title/Abstract]) AND ("2010"[Date - Publication] : "3000"[Date - Publication]) AND (hasabstract[text] AND Humans[MeSH] AND middle age[MeSH]))

We used inpatient hospital data adjusted for readmission, primary to any diagnosis, and inpatient to outpatient utilisation based on correction factors generated using USA claims data. More information on how correction factors were made for this adjustment can be found in the “Claims data” section of the non-fatal appendix. We excluded all outpatient data, as these were determined to be implausibly low when compared with inpatient data from the same locations and with claims data. We excluded any inpatient hospital datapoints that were at least two-fold higher or 0.5-fold lower than the age-sex-specific median absolute deviation value for regions identified with the highest-quality data – specifically, high-income North America, central Europe, western Europe, and high-income Asia Pacific. We based our exclusion criteria on national year-specific values for each study, age, and sex group adjusted by population size.

All data from Brazil, Mexico, Poland, Botswana, Nepal, Ecuador, Mongolia, Norway, and South Korea were excluded due to issues including inconsistent coding practices, implausible value time trends, or unresolved questions regarding the sources of these data. USA MarketScan Commercial Claims and Encounters Data from year 2000 were also excluded due to quality concerns.

Modelling strategy

The modelling strategy remained the same as for GBD 2021. Briefly, we estimated acute myocarditis using a DisMod-MR Bayesian meta-regression model, setting a minimum of 3 and maximum of 5 as value priors on remission to establish an average duration of three months. We set a value prior of zero for all ages on excess mortality. Table 3 provides the parameters, betas, and exponentiated betas for study-level and country-level covariates used in the model. Table 4 includes the severity level, lay descriptions, and disability weights (DWs) associated with acute endocarditis.

Table 3. Covariates. Summary of covariates used in the acute myocarditis DisMod-MR meta-regression model

Study covariate	Parameter	Beta	Exponentiated beta
HAQ Index	Excess mortality rate	–0.54 (–0.98 to –0.11)	0.58 (0.38–0.89)

Table 4. Severity distribution. Details on the severity levels for acute myocarditis and the associated disability weight (DW) with that severity

Severity level	Lay description	Disability weight (95% CI)
Acute myocarditis	Has a fever and aches, and feels weak, which causes some difficulty with daily activities.	0.051 (0.032–0.074)

References

[1] Huber, P.J. (2011). Robust Statistics. In: Lovric, M. (eds) International Encyclopedia of Statistical Science. Springer, Berlin, Heidelberg. https://doi.org/10.1007/978-3-642-04898-2_594

Alcohol use disorders

Flowchart

Alcohol use disorders

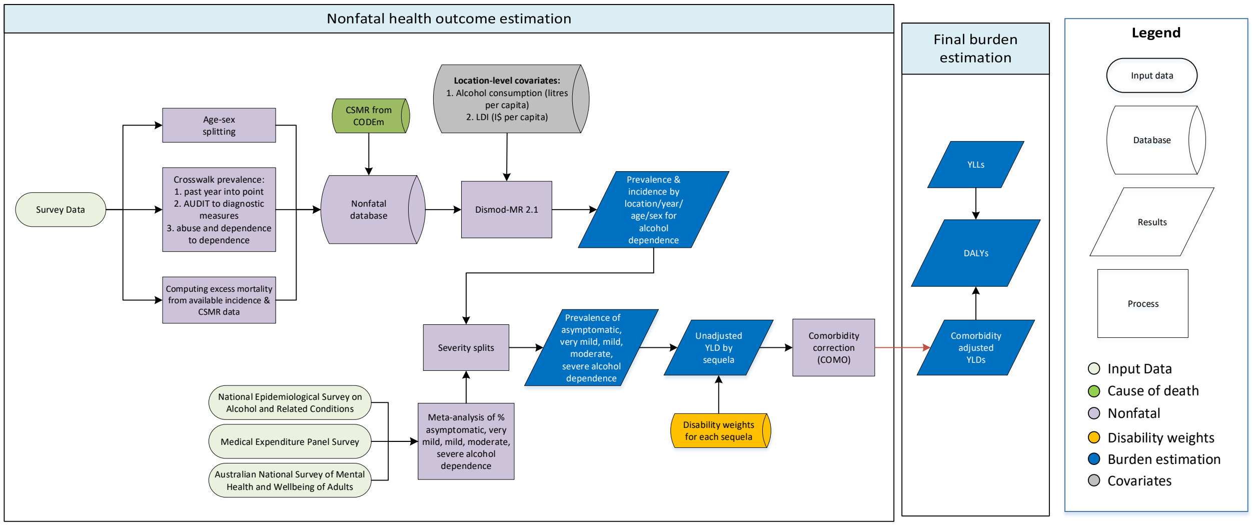

Input data and methodological summary for alcohol use disorders

Case definition

Alcohol use disorders case definition

Alcohol use disorder (AUD) is a medical condition marked by a problematic pattern of alcohol consumption, characterised by an inability to stop or control drinking despite negative social, occupational, or health consequences.[1] Within the Global Burden of Disease (GBD) study, alcohol use disorders include two related conditions: alcohol dependence and fetal alcohol syndrome (FAS), both of which are modelled to estimate non-fatal outcomes across the lifespan.

Transition in the literature from DSM-IV to DSM-5

The Global Burden of Disease (GBD) has historically relied on the DSM-IV framework for alcohol use disorders, which classified alcohol abuse and alcohol dependence as distinct conditions. Under DSM-IV guidelines, alcohol dependence and alcohol abuse were mutually exclusive diagnoses, distinguishing less severe harmful alcohol use (abuse) from more severe, chronic patterns of alcohol consumption (dependence).[2] Non-fatal estimates in the GBD primarily focus on alcohol dependence, capturing the more severe maladaptive patterns of alcohol use.

The DSM-5 later redefined alcohol use disorders by combining alcohol abuse and dependence into a single diagnosis: alcohol use disorder (AUD). This updated classification captures a spectrum of severity, ranging from mild to severe, providing a more nuanced understanding of problematic alcohol use.[2] Review of the literature indicates that there is substantial agreement between DSM-5 moderate to severe AUD and DSM-IV alcohol dependence.[3-5] Further consultation with experts has highlighted that

the DSM-5 has not yet achieved widespread global acceptance, particularly outside the Americas. As a result, GBD non-fatal estimates remain based on the DSM-IV classification of alcohol dependence.

Alcohol dependence case definition

Alcohol dependence is a substance-related disorder characterised by a severe maladaptive pattern of alcohol consumption. According to the Diagnostic and Statistical Manual of Mental Disorders (DSM-IV) criteria for alcohol dependence, at least three out of seven of the following criteria must be manifested during a 12-month period:

- Tolerance
- Withdrawal symptoms or clinically defined alcohol withdrawal syndrome
- Use in larger amounts or for longer periods than intended
- Persistent desire or unsuccessful efforts to cut down on alcohol use
- Time is spent obtaining alcohol or recovering from effects
- Social, occupational, and recreational pursuits are given up or reduced because of alcohol use
- Use is continued despite knowledge of alcohol-related harm (physical or psychological)

The DSM-IV code for alcohol dependence is 303.90, while the corresponding International Classification of Diseases (ICD) codes include ICD-9 codes 303, 305, 291, 357, 790, and E860, and ICD-10 codes F10.1 and F10.2.[6,7]

**Quantity of interest**	**Reference or alternative**	**Definition**
Alcohol dependence	Reference	Point prevalence of individuals diagnosed with alcohol dependence based on DSM or ICD criteria
Alcohol dependence	Alternative	Prevalence of individuals diagnosed with alcohol dependence using AUDIT, AUDADIS*, or CAGE* screening tools
Alcohol dependence	Alternative	Prevalence of individuals diagnosed with dependence and alcohol abuse (ie, excessive alcohol use without symptoms of dependence)
Alcohol dependence	Alternative	Prevalence of individuals diagnosed with alcohol dependence, measured at different durations: 1 month*, 6 months*, 12 months, and lifetime*

* *We do not have* crosswalks for these alternative definitions to the alcohol dependence reference definition due to insufficient matched data across age, sex, year, and location.

Input data

In GBD 2013 and GBD 2016, systematic literature reviews were conducted to capture studies on the prevalence, incidence, remission, duration, and excess mortality associated with alcohol dependence.

The reviews were carried out in three stages, which involved searching peer-reviewed literature databases such as Medline, Embase, and PubMed; exploring grey literature; and consulting experts.

The inclusion criteria required that (1) cases be defined by clinical thresholds established by the DSM and ICD; (2) sufficient information be provided on study methodology and sample characteristics to assess study quality; and (3) study samples must represent the general population. This led to the exclusion of inpatient or pharmacological treatment samples (except for mortality estimates), case studies, and samples focused on veterans or refugees.

Prevalence estimates were further refined by age and sex as necessary (Table 2). For studies reporting prevalence for both sexes, a global sex ratio estimated using MR-BRT (meta-regression with Bayesian priors, regularization, and trimming; see Appendix 1, Section 2) was applied to split the data. For studies reporting estimates across broad age groups spanning 20 years or more, these were divided into five-year age groups using the global age pattern estimated by DisMod-MR 2.1 (see Appendix 1, Section 2).

Table 2: MR-BRT age and sex splitting adjustment factors for alcohol dependence

Data input	Gamma	Beta coefficient, log (95% UI) *	Adjustment factor**
Female: Male	0.33	–0.69 (–1.35 to –0.04)	0.50
Age >20		0.12 (0.07 to 0.18)	1.13

**MR-BRT crosswalk adjustments can be interpreted as the factor the alternative case definition is adjusted by to reflect what it would have been had it been measured using the reference case definition. If the log/logit beta coefficient is negative, then the alternative is adjusted up to the reference. If the log/logit beta coefficient is positive, then the alternative is adjusted down to the reference.*

***The adjustment factor column is the exponentiated beta coefficient. For log beta coefficients, this is the relative rate between the two case definitions. For logit beta coefficients, this is the relative odds between the two case definitions.*

Key changes in GBD 2023

In GBD 2023, we re-evaluated the quality of data informing our estimates, re-extracted data from 30 unique country sources where means, case definitions, case counts, and sample sizes had been incorrectly extracted, and excluded 85 sources for various reasons. These exclusions included eight sources due to duplicative studies or samples, 12 that did not meet the case definition, 13 from literature reviews, 13 lacking relevant data, and 49 with unrepresentative samples, such as studies focusing on individuals in treatment, slum dwellers, or twins.

Bias correction

Due to insufficient data on alcohol dependence in some regions, three crosswalks were performed using MR-BRT to allow for the inclusion of data that did not meet our reference definitions in the epidemiological modelling of alcohol dependence. The first crosswalk converted estimates of alcohol

use disorders (alcohol abuse + alcohol dependence) to reflect what they would be if the data represented estimates of alcohol dependence. Similarly, the second crosswalk was performed using MR-BRT to adjust past-year prevalence estimates of alcohol dependence toward the level they would have been had the study measured point prevalence, as the latter is less susceptible to recall bias. The third crosswalk adjusted estimates of prevalence according to the Alcohol Use Disorder Identification Test (AUDIT) to what they would be had prevalence been determined based on diagnostic measures. For this final crosswalk, a systemic review was performed in GBD 2019 to identify AUDIT validation studies using the following search string:

(("audit"[tiab] AND "alcohol"[tiab]) OR "alcohol use disorders identification test"[tiab]) AND ("validation"[tiab] or "validity"[tiab]) NOT (animals[MeSH] NOT humans[MeSH])

Out of 303 total studies screened, 38 studies were found to report prevalence of alcohol dependence according to the AUDIT as well as according to physician diagnosis or reported specificity and sensitivity to allow for the calculation of prevalence. These studies were used to generate crosswalk parameters using MR-BRT (see appendix 1 section 2). All three crosswalks utilised a logit difference model, which has been described elsewhere. Briefly, alternative definition datapoints were logit transformed, and the MR-BRT beta was subtracted from them, after which they were transformed back into normal space.

Table 3: MR-BRT crosswalk adjustment factors for alcohol dependence

Data input	Reference or alternative case definition	Gamma	Beta coefficient, logit (95% UI)*	Adjustment factor**
Point prevalence	Ref	0.68	---	
Past- year prevalence	Alt		0.81 (–0.58 to 2.14)	2.25
Prevalence according to ICD and DSM diagnostic measures	Ref	0.76	---	
Prevalence according to AUDIT	Alt		1.09 (–0.40 to 2.63)	2.97
Alcohol dependence prevalence	Ref	0.57	---	
Alcohol dependence and abuse prevalence	Alt		1.04 (–0.03 to 2.19)	2.83

**MR-BRT crosswalk adjustments can be interpreted as the factor the alternative case definition is adjusted by to reflect what it would have been had it been measured using the reference case definition. If the log/logit beta coefficient is negative, then the alternative is adjusted up to the reference. If the log/logit beta coefficient is positive, then the alternative is adjusted down to the reference.*

***The adjustment factor column is the exponentiated beta coefficient. For log beta coefficients, this is the relative rate between the two case definitions. For logit beta coefficients, this is the relative odds between the two case definitions.*

Severity split inputs and disability weights

The basis of the GBD disability weight survey assessments are lay descriptions of sequelae highlighting major functional consequences and symptoms. The lay descriptions and disability weights for alcohol dependence severity levels are shown below.

Table 4. Severity distribution, details on the severity levels for alcohol dependence and the associated disability weight (DW) with that severity.

Severity level	Lay description	DW (95% CI)
Very mild	Drinks alcohol daily and has difficulty controlling the urge to drink. When sober, the person functions normally.	0.123 (0.082–0.177)
Mild	Drinks a lot of alcohol and sometimes has difficulty controlling the urge to drink. While intoxicated, the person has difficulty performing daily activities.	0.235 (0.16–0.327)
Moderate	Drinks a lot, gets drunk almost every week and has great difficulty controlling the urge to drink. Drinking and recovering cause great difficulty in daily activities, sleep loss, and fatigue.	0.373 (0.248–0.508)
Severe	Gets drunk almost every day and is unable to control the urge to drink. Drinking and recovering replace most daily activities. The person has difficulty thinking, remembering, and communicating, and feels constant pain and fatigue.	0.57 (0.396–0.732)

**Asymptomatic cases carried no disability weight*

Severity splits used in GBD 2023 were consistent with those used in GBD 2017. The United States' Medical Expenditure Panel Survey (MEPS, conducted in annual waves since 1996),[8] the US National Epidemiological Survey on Alcohol and Related Conditions (NESARC, conducted in two waves from 2001–2002 and 2004–2005),[9] and the Australian National Survey of Mental Health and Wellbeing of Adults (NSMHWB, conducted in 1997)[10] were used to estimate the proportion of alcohol dependence cases in the asymptomatic 40.9% (38.4–43.3); very mild 46.9% (43.7–50.0); mild 4.0% (1.8–5.8); moderate 3.4% (2.3–4.5); and severe 4.8% (3.0–7.0) disease categories.

Modelling strategy

The GBD epidemiological modelling strategy for alcohol dependence made use of DisMod-MR 2.1 to estimate prevalence by age, sex, year, and location. Standardised mortality ratio and relative risk data were excluded in the modelling process. Instead, we pulled in cause-specific mortality rate (CSMR) data from our CODEm and CoDCorrect analyses and matched it with prevalence datapoints for the same geography and study year to estimate priors on excess mortality rates (by dividing CSMR by prevalence). We assumed no incidence and mortality before age 10. An upper limit of 0.6 was placed on remission (in line with data from the US National Epidemiological Survey on Alcohol and Related Conditions (NESARC) as well as a declining trend with age to restrict DisMod-MR 2.1 from straying too far from the data inputs. Two country-level covariates were included in the DisMod-MR 2.1 model. The first covariate represents alcohol consumption, measured in litres of alcohol per capita. The second covariate, lag-distributed income per capita (LDI; I$ per capita), represents a moving average of gross domestic product (GDP) over time. LDI was also applied to excess mortality data, with a negative relationship assumed.

Table 5. Covariates. Summary of covariates used in the alcohol dependence DisMod-MR meta-regression model

Covariate	Type	Parameter	Exponentiated beta (95% uncertainty interval)
Litres of alcohol consumed per capita	Country	Prevalence	1.01 (1.00–1.03)
LDI (I$ per capita)	Country	Excess mortality rate	0.86 (0.82–0.90)

Fetal alcohol syndrome

Flowchart

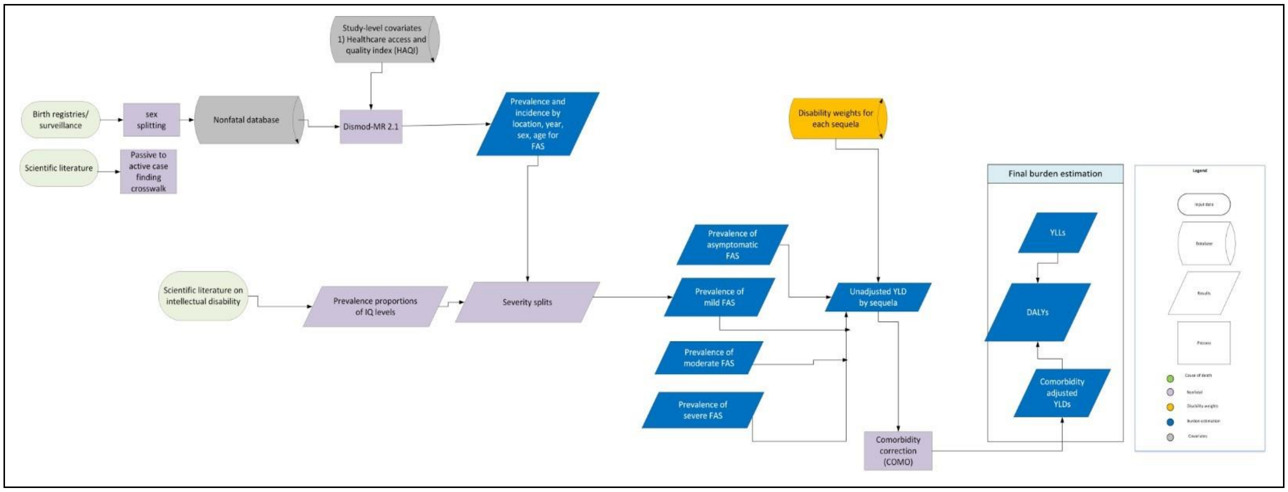

Input data and methodological summary for fetal alcohol syndrome

Case definition

Fetal alcohol syndrome (FAS; ICD 9: 760.71 and ICD-10: Q86.0 and P04.3)[11] is the most severe form of fetal alcohol spectrum disorder (FASD) and results from maternal alcohol consumption during pregnancy. In the Global Burden of Disease (GBD) model, only FAS cases were included, excluding other FASD manifestations such as partial fetal alcohol syndrome, alcohol-related neurodevelopmental disorder, and alcohol-related birth defects. FAS is characterised by maternal alcohol exposure, with or without documented prenatal exposure, leading to distinctive facial anomalies (eg, short palpebral fissures, flat upper lip, flattened philtrum, flat midface), growth delays (eg, height and weight ≤10th percentile on appropriate growth curves), and central nervous system abnormalities (eg, small cranial size at birth, ≤10th percentile, with cognitive impairments). Diagnoses were based on guidelines from the USA Institute of Medicine, the British Pediatric Association, and other recognised authorities (Table 1).

Table 1. Diagnostic guidelines for fetal alcohol spectrum disorders (FASDs), including fetal alcohol syndrome (FAS), partial fetal alcohol syndrome (PFAS), alcohol-related birth defects (ARBD), and alcohol-related neurodevelopmental disorder (ARND), according to the Institute of Medicine (IOM) as expounded upon by Hoyme and colleagues (2005)[12]

**Diagnostic criteria**	**Details**	**Condition**

A. Characteristic facial pattern	At least two of the following:<br>1. Short palpebral fissures (≤10th percentile).<br>2. Thin vermilion border of the upper lip (score 4 or 5 on the lip/philtrum guide).<br>3. Smooth philtrum (score 4 or 5 on the lip/philtrum guide).	
B. Growth retardation	Evidence of prenatal and/or postnatal growth retardation (height or weight ≤10th percentile).	
C. Brain abnormalities	Evidence of deficient brain growth or abnormal morphogenesis, including:<br>1. Structural brain abnormalities.<br>2. Head circumference ≤10th percentile.	
	Requires all features A–C or evidence of behavioral/cognitive abnormalities inconsistent with development level.	**Fetal alcohol syndrome (FAS)**
	Requires features A and B/C or evidence of behavioral/cognitive abnormalities inconsistent with development level.	Partial FAS (PFAS)
	Requires characteristic facial pattern (1A) and specific congenital structural defects (malformations/dysplasia) in at least one organ system.<br>If only minor anomalies are present, at least two must be identified.<br>Assumes normal growth and intellectual/behavioural characteristics.	Alcohol-related birth defects (ARBD)
	Assumes normal growth and structure but requires at least one of the following:<br>1. Deficient brain growth or abnormal morphogenesis, including:<br>a. Structural brain abnormalities.<br>b. Head circumference ≤10th percentile.<br>2. Behavioural/cognitive abnormalities inconsistent with development, unrelated to genetics, family, or environment.<br>a. Includes marked impairment in complex tasks (eg, problem-solving, planning, abstraction, metacognition, arithmetic), language deficits, or disordered behaviour.	Alcohol-related neurodevelopmental disorder (ARND)

Table 2. FAS case definitions in the GBD

**Quantity of interest**	**Reference or alternative**	**Definition**

Fetal alcohol syndrome	Reference	Prevalence of individuals diagnosed with fetal alcohol syndrome identified using active case finding methods
Fetal alcohol syndrome	Alternative	Prevalence of individuals diagnosed with fetal alcohol syndrome identified using passive case identification methods

Input data

A series of systematic literature reviews were last conducted in 2015 to capture studies reporting on the prevalence, incidence, remission, and excess mortality of FAS. These reviews included searches of peer-reviewed literature through electronic databases and consultations with experts. To qualify for inclusion, studies needed to use recognised classifications of FAS (eg, those from the USA Institute of Medicine, Table 1) and provide detailed methodology and sample characteristics for quality assessment, with no restrictions on publication language.

These reviews captured data from a range of studies, and in addition to these data, surveillance sources were also used. These included large, multi-year surveillance series such as the Western Australian Birth Defects Registry, South Australian Birth Defects Register, Brazilian Live Birth Information System (SINASC), Mexico Births Information Subsystem (SINAC), and newborn surveillance systems like the European Surveillance of Congenital Anomalies (EUROCAT) and WHO-SEAR Newborn and Birth Defects (NBBD) surveillance.

To ensure our models used the most granular data, data reported for both sexes were split using a global sex ratio estimated with MR-BRT (meta-regression—Bayesian, regularised, trimmed; see Appendix 1, Section 2) as shown in Table 3.

Table 3: MR-BRT sex splitting adjustment factors for fetal alcohol syndrome

Data input	Gamma	Beta coefficient, log (95% UI)*	Adjustment factor **
Female: Male	0	–0.28 (–0.67 to 0.11)	0.76

**MR-BRT crosswalk adjustments can be interpreted as the factor the alternative case definition is adjusted by to reflect what it would have been had it been measured using the reference case definition. If the log/logit beta coefficient is negative, then the alternative is adjusted up to the reference. If the log/logit beta coefficient is positive, then the alternative is adjusted down to the reference.*

***The adjustment factor column is the exponentiated beta coefficient. For log beta coefficients, this is the relative rate between the two case definitions. For logit beta coefficients, this is the relative odds between the two case definitions.*

Key changes in GBD 2023

In GBD 2023, we re-evaluated the quality of data informing our estimates, re-extracted data from 35 unique country sources where means, case definitions, case counts, and sample sizes had been incorrectly extracted, and excluded 36 sources for various reasons. These exclusions included four

sources that lacked details on ascertainment methods or only provided standardised data, one source with inaccessible full text, 11 that did not capture FAS, and 20 with unrepresentative samples, such as studies focused on Indigenous populations or groups with very high alcohol consumption prevalence in South Africa.

Bias correction

Prevalence data collected using both passive and active case finding methodologies was included in this model. As passive case finding methods are likely to underestimate the true prevalence of fetal alcohol syndrome, a crosswalk was applied to adjust those datapoints upwards. The expected difference in reported prevalence was modelled using MR-BRT. To adjust the passive case data, a logit difference model was used in which the beta coefficient was subtracted from the logit transformed prevalence data, the inverse logit of which was used in the model. Table 2 summarises the MR-BRT crosswalk coefficients.

Table 2: MR-BRT crosswalk adjustment factors for fetal alcohol syndrome

Data input	Reference or alternative case definition	Gamma	Beta coefficient, logit (95% UI)*	Adjustment factor**
Active case finding	Ref	1.87	---	
Passive case finding	Alt		–0.03 (–3.60 to 3.51)	0.9704

**MR-BRT crosswalk adjustments can be interpreted as the factor the alternative case definition is adjusted by to reflect what it would have been had it been measured using the reference case definition. If the log/logit beta coefficient is negative, then the alternative is adjusted up to the reference. If the log/logit beta coefficient is positive, then the alternative is adjusted down to the reference.*

***The adjustment factor column is the exponentiated beta coefficient. For log beta coefficients, this is the relative rate between the two case definitions. For logit beta coefficients, this is the relative odds between the two case definitions.*

Modelling strategy

We have made no substantive changes in the modelling strategy from GBD 2023. The GBD 2023 modelling strategy utilised DisMod-MR 2.1 (disease model—Bayesian meta-regression, described in appendix 1, section 2) to estimate prevalence by age, sex, year, and location. Prevalence was set to begin from birth. Incidence was set to zero given cases cannot manifest after birth (despite the fact they may not be diagnosed immediately at birth). Remission was also set to zero. Estimates from known high-drinking populations (eg, Indigenous populations) were not considered representative of the general population and were excluded. A country-level covariate was included representing the log proportion of pregnant women who drink during their pregnancy, estimated from a meta-analysis.[13] Table 3 below illustrates the covariate, parameter, beta and exponentiated beta values for the model.

Table 3. Covariates. Summary of covariates used in the fetal alcohol syndrome DisMod-MR meta-regression model

Covariate	Type	Parameter	Exponentiated beta (95% uncertainty interval)
Maternal drinking	Country	Prevalence	1.07 (1.00–1.22)

Severity split inputs and disability weights

There were no available data providing the prevalence of FAS by severity, likely because FAS represents the most severe form of fetal alcohol spectrum disorders (FASD) and requires a diagnosis only when specific and stringent criteria are met.[14] As a result, differentiating FAS by severity is uncommon. Consequently, an intellectual disability scale was used as a proxy for assessing FAS severity. A meta-analysis of studies reporting on intellectual disabilities in children with FAS was conducted to determine the proportion of children with FAS across various IQ levels. FAS severity categories were aligned with IQ levels for which prevalence data are available: severe FAS was matched to an IQ below 50, moderate FAS to an IQ between 50 and 69, mild FAS to an IQ between 74 and 84, and asymptomatic FAS to an IQ of 85 or higher. These prevalence data were then used to calculate severity distributions for FAS.

Table 4. Severity distribution, details on the severity levels for fetal alcohol syndrome in GBD 2 and the associated disability weight (DW) with that severity.

Severity level	Lay description	DW* (95% CI)
Mild	Is a little slow in developing physically and mentally, which causes some difficulty in learning but no other difficulties in daily activities.	0.016 (0.008–0.03)
Moderate	Is slow in developing physically and mentally, which causes some difficulty in daily activities.	0.056 (0.035–0.083)
Severe	Is very slow in developing physically and mentally, which causes great difficulty in daily activities.	0.179 (0.119–0.257)

**Asymptomatic cases carried no disability weight.*

References

1. Nehring SM, Chen RJ, Freeman AM. Alcohol Use Disorder. [Updated 2024 Mar 16]. In: StatPearls [Internet]. Treasure Island (FL): StatPearls Publishing; 2025 Jan. Available from: https://www.ncbi.nlm.nih.gov/books/NBK436003/
2. National Institute on Alcohol Abuse and Alcoholism (NIAAA). Alcohol use disorder: a comparison between DSM-IV and DSM-5. Accessed Jan 24, 2025.

https://www.niaaa.nih.gov/publications/brochures-and-fact-sheets/alcohol-use-disorder-comparison-between-dsm

3. Compton WM, Dawson DA, Goldstein RB, Grant BF. Crosswalk between DSM-IV dependence and DSM-5 substance use disorders for opioids, cannabis, cocaine, and alcohol. *Drug Alcohol Depend.* 2013;132(1-2):387–390. DOI:10.1016/j.drugalcdep.2013.02.036.
4. Dawson DA, Smith SM, Saha TD, Rubinsky AD, Grant BF. Comparative performance of the AUDIT-C in screening for DSM-IV and DSM-5 alcohol use disorders. *Drug Alcohol Depend.* 2012;126(3):384–388. doi:10.1016/j.drugalcdep.2012.05.029.
5. Agrawal A, Heath AC, Lynskey MT. DSM-IV to DSM-5: The impact of proposed revisions on diagnosis of alcohol use disorders. *Addiction.* 2011;106(11):1935–1943. doi:10.1111/j.1360-0443.2011.03517.x.
6. American Psychiatric Association. Diagnostic and Statistical Manual of Mental Disorders (DSM-IV-TR). 4th, Text Revision ed. Washington DC: American Psychiatric Association; 2000.
7. World Health Organization. The ICD-10 Classification of Mental and Behavioural Disorders. Clinical descriptions and diagnostic guidelines. Geneva: World Health Organization; 1992.
8. Agency for Healthcare Research and Quality. United States Medical Expenditure Panel Survey. Rockville, United States: Agency for Healthcare Research and Quality.
9. Introduction to the National Epidemiologic Survey on Alcohol and Related Conditions [http://pubs.niaaa.nih.gov/publications/arh29-2/74-78.htm]. Access date 1 December 2014.
10. Australian Bureau of Statistics. National Survey of Mental Health and Wellbeing of Adults 1997. Canberra: Australian Bureau of Statistics.
11. ICD-9-CM. 760.71 Alcohol affecting fetus or newborn via placenta or breast milk. Accessed Jan 24, 2025. http://www.icd9data.com/2015/Volume1/760-779/760-763/760/760.71.htm.
12. Hoyme HE, May PA, Kalberg WO, et al. A practical clinical approach to diagnosis of fetal alcohol spectrum disorders: clarification of the 1996 Institute of Medicine criteria. *Pediatrics.* 2005;115:39–47. https://pmc.ncbi.nlm.nih.gov/articles/PMC1380311/.
13. Stratton K, Howe C, Battaglia F, editors. Fetal alcohol syndrome. Diagnosis, epidemiology, prevention, and treatment. Washington DC: National Academy Press; 1996.
14. Popova S, Lange S, Probst C, Gmel G, Rehm J. Estimation of national, regional, and global prevalence of alcohol use during pregnancy and fetal alcohol syndrome: a systematic review and meta-analysis. *The Lancet Global Health* 2017.

Alopecia areata

Flowchart

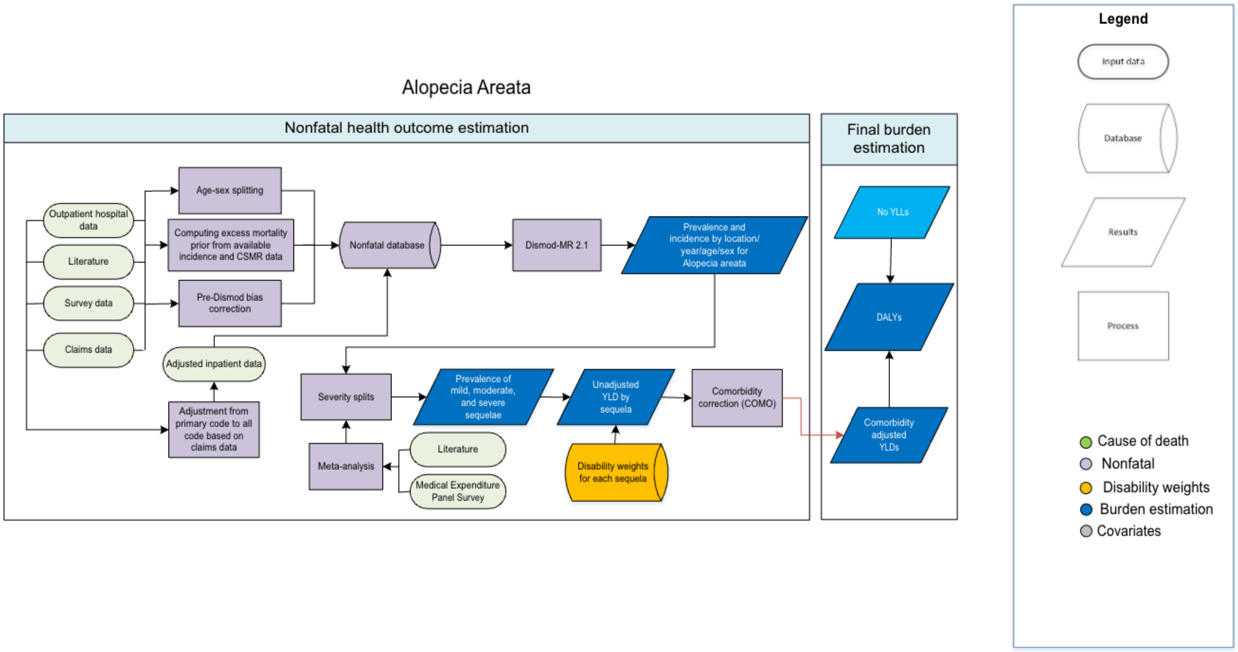

Input data and methodological summary for alopecia areata

Case definition

Alopecia areata is defined as a chronic inflammatory disease of the pilosebaceous unit associated with an increase in sebum secretion (ICD-10: L70, excluding L70.4). Alopecia areata was included in the GBD 2021 cause group of skin and subcutaneous conditions.

alopecia areata

Quantity of interest	Reference or alternative	Definition
Alopecia areata	Reference	Alopecia areata as determined by Poland National Health Fund Patient Claims
Alopecia areata	Alternative	All other data for alopecia areata

Input data

In the GBD 2016 study, a systematic review of the literature was conducted using PubMed to expand the GBD dataset (1980–2014) with new epidemiological data for alopecia areata between 2014 and 2016. The inclusion criteria stipulated that studies (1) must provide data on the incidence or prevalence of alopecia areata; (2) must use samples representative of the general population (ie, samples derived from the experimental arm of clinical trials or based in dermatology clinics were excluded); (3) must use a sample size larger than 100; and (4) must provide sufficient information on study method and sample characteristics to assess the quality of the study. Additional clinical data for GBD 2023 include more years of USA claims data, and Poland national claims along with location-year of literature data.

Data processing

For alopecia areata, we crosswalked all data to the reference definition. We began by evaluating the number of observations of each alternate definition that matched with a corresponding observation from the reference definition. We considered "between" study matches, where the alternative was from the same GBD age group, sex, and the midpoint year of the study was within five years of the midpoint of the reference definition observation.

$$\log it\left(y_i^{alt}\right) - \log it\left(y_i^{ref}\right) \;=\; \beta_0 + \epsilon_i$$

Table 1: MR-BRT crosswalk adjustment factors for alopecia areata

Data input	Reference or alternative case definition	Gamma	Beta coefficient, log/logit (95% UI)*	Adjustment factor**
Poland National Health Fund Patient Claims	Ref	0	---	---
All other data	Alt		1.2084 (1.2051–1.2116)	3.34798 (3.3369–3.3590)

**MR-BRT crosswalk adjustments can be interpreted as the factor the alternative case definition is adjusted by to reflect what it would have been had it been measured using the reference case definition. If the log/logit beta coefficient is negative, then the alternative is adjusted up to the reference. If the log/logit beta coefficient is positive, then the alternative is adjusted down to the reference.*
***The adjustment factor column is the exponentiated beta coefficient. For log beta coefficients, this is the relative rate between the two case definitions. For logit beta coefficients, this is the relative odds between the two case definitions.*

Modelling strategy

In GBD2023, we have made no substantive changes in the modelling strategy from GBD 2021.

DisMod-MR 2.1, a Bayesian meta-regression tool, was used to estimate prevalence by age, sex, year, and geography (subnational, country, region, super-region) for alopecia areata. Since our available data only contained information on prevalence, we specified additional expert priors to further inform analyses. We assumed zero excess mortality and remission from 1.7 to 1.8, implying a duration of approximately seven months. This was in line with the available epidemiological data, expert opinion, and previous GBD work. We used a time window of five years to determine which datapoints were used for a particular year of fit. The datasets for alopecia areata were sufficiently large to make use of a relatively short time window of ten years to determine which datapoints were used for a particular year of fit. Since GBD 2019, we have replaced our within-DisMod crosswalks with crosswalks completed using the MR-BRT modelling tool. We adjusted all our data toward the level of Poland National Health Fund Patient Claims datapoints, which were more representative of the general population.

$$prevalence = incidence * duration$$

$$Remission = \frac{Cured\ cases}{person - year\ of follow - up\ in\ prevalent\ cases}$$

Table 2. Severity distribution, details on the severity levels for alopecia areata and the associated disability weight (DW) with that severity

Sequela	Severity level	Lay description	DW (95% CI)
Mild alopecia areata	Disfigurement, level 1	The individual has a slight, visible physical deformity that others notice, which causes some worry and discomfort.	0.396 (0.299, 0.518)
Severe alopecia areata	Disfigurement, level 3	The individual has a visible physical deformity that causes others to stare and comment. As a result, the person is worried and has trouble sleeping and concentrating.	0.604 (0.482, 0.701)

Alzheimer’s disease and other dementias

Flowchart

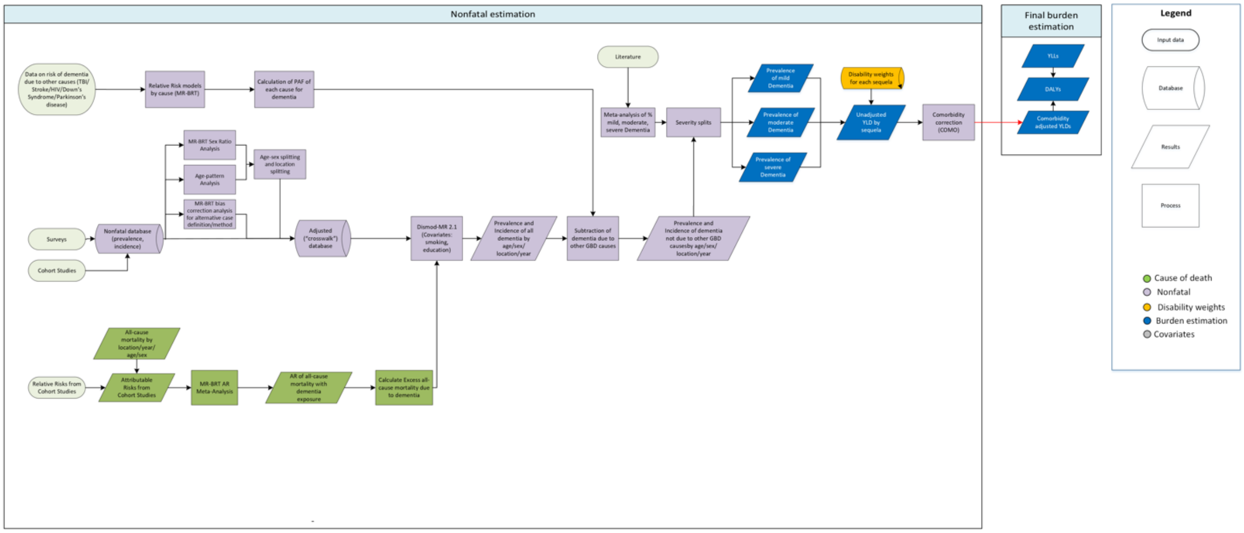

Input data and methodological summary

Case definition

Dementia is a progressive, degenerative, and chronic neurological disorder typified by memory impairment and other neurological dysfunctions. For the purposes of GBD 2023, we used the Diagnostic and Statistical Manual of Mental Disorders III, IV or V, or ICD case definitions as the reference. The DSM-IV definition is:

- Multiple cognitive deficits manifested by both memory impairment and one of the following: aphasia, apraxia, agnosia, disturbance in executive functioning
- Must cause significant impairment in occupational functioning and represent a significant decline
- Course is characterised by gradual onset and continuing cognitive decline
- Cognitive deficits are not due to other psychiatric conditions
- Deficits do not occur exclusively during the course of a delirium

The relevant ICD-10 codes for dementia are F00, F01, F02, F03, G30, and G31. The ICD-9 codes are 290, 291.2, 291.8, 294 and 331.

Alternative case definitions accepted for inclusion included (1) diagnosis based on the result of an algorithm, (2) diagnosis based on general practitioner data, (3) diagnosis based on clinical records, (4) diagnosis using 10/66 algorithm, and (5) diagnosis with NIA-AA (National Institute on Aging – Alzheimer’s Association) criteria instead of ICD or DSM.

A wide array of diagnostic and screening instruments exists, including Clinical Dementia Rating scale (CDR), Mini-Mental State Examination (MMSE), and the Geriatric Mental State (GMS). For severity rating purposes we use the CDR as the reference.

Unlike most causes in the Global Burden of Disease project, dementia mortality and morbidity estimates are modelled jointly. This is because of marked discrepancies between prevalence data and cause of death data. Specifically, age-standardised prevalence data suggest little to no variation over time (eg, 1990–2020), whereas age-standardised mortality rates in vital registrations in high-income countries have increased over this same period. Additionally, prevalence variation between countries is much smaller than the variation in death rates assigned to dementia in vital registration. We attribute these discrepancies to changing coding practices rather than epidemiological change.

Because of this joint procedure, descriptions of the mortality estimation process are included where relevant.

Input data

Model inputs

To inform our estimates of burden due to dementia, we use mortality data from relative risk studies and linked hospital to mortality data, as well as prevalence and incidence data from surveys and administrative data such as claims sources. For GBD 2023, we updated GBD 2021 estimates by including additional sex-specific, reference case definition data of dementia prevalence and incidence identified from a recent systematic review of studies up to February 2023 (further detailed below). We also added results from 18 studies that are part of the Cohort Studies of Memory in an International Consortium (COSMIC). We excluded all US MarketScan claims data, as done in GBD 2021, because of the paucity of data available to inform a reliable adjustment, and because the large amount of these data meant claims had an unduly large influence on the global fit of the dementia model.

The following search string was run through PubMed on February 3, 2023 to identify additional studies from prior GBD rounds:

> (((dementia[Title/Abstract] OR alzheimer[Title/Abstract] OR dementia[MeSH]) AND (incidence[Title/Abstract] OR prevalence[Title/Abstract] OR prevalent[Title/Abstract] OR incident[Title/Abstract])) AND ("2017/08/30"[Date - Publication] : "2023/02/03"[Date - Publication]) NOT (animals[MeSH] NOT humans[MeSH]) NOT (Review[Publication Type]))

We included cross-sectional or longitudinal studies that were population representative, based dementia diagnosis off cognition and functional tests, and reported relevant measures (prevalence, incidence, remission rate, excess mortality rate, relative risk of mortality, standardised mortality ratio, or with-condition mortality rate). We excluded case-control studies, randomised control trials, studies without clearly defined sample sizes, and studies where cognitive status was determined by biomarkers (eg, white matter, genetics, and neuroanatomy).

A total of 8196 records were identified from the initial search, from which 1146 full-text articles were assessed for eligibility. We extracted 235 studies that met our inclusion and exclusion criteria. For GBD

2023, we included only studies that report sex-specific reference case definition results. Processing of the remaining studies is underway for future GBD rounds.

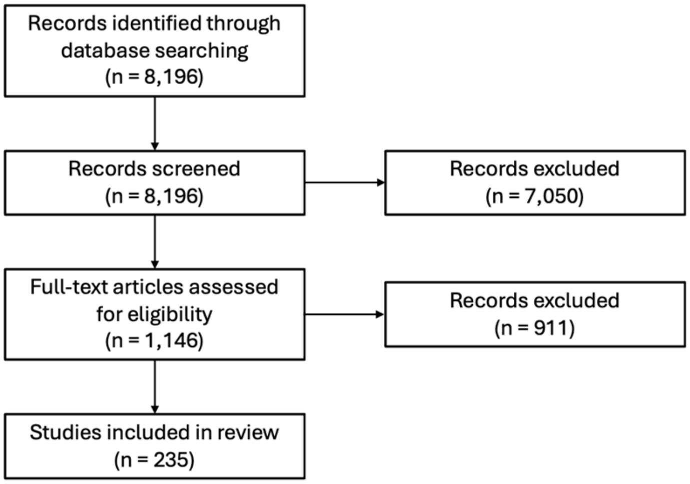

Severity splits

Our method of severity splits was redesigned for GBD 2019 and was not updated for GBD 2023. Briefly, a systematic review was conducted to collect information on the proportion of individuals in each dementia severity class out of the population of all individuals with dementia. There are a variety of commonly used methods for severity rating; we took the Clinical Dementia Rating (CDR) scale as our reference definition for severity classification, along with a doctor-given diagnosis according to DSM III, IV, V, or ICD case definitions as our reference definition for dementia.

However, as a neurodegenerative disorder with a wide range of categories in which symptoms manifest, there are an abundance of classification tools which discern between severity levels along different criteria. We accepted severities classified by:

- Clinical dementia rating sum-of-boxes (CSR-SB)
- Blessed test of information, memory, and concentration (BIMC)
- Global deterioration scale (GDS)
- Geriatric Mental State Examination (GMS)
- CAMDEX
- DSM-III-R
- Karasawa's

We excluded any studies which classified dementia severity according to scales that only evaluated cognitive function and memory, excluding activities of daily living (ADLs). The most prominent such scale is MMSE (Mini-Mental State Exam). The most recent systematic review covered literature published through August 2017.

The severity split analysis was conducted using the MR-BRT tool[1] (meta-regression—Bayesian, regularised, trimmed; additional information can be found in appendix 1, section 2). We multiplied

estimations of prevalence (country-year-sex-age-specific) by the fractions of mild, moderate, and severe dementia and estimated 95% uncertainty intervals at the 1000-draw level. The severity distributions over age for each sex are visualised below (figure 1), followed by a table describing each severity.

Figure 1. Severity ratios for each five-year age bin, by sex

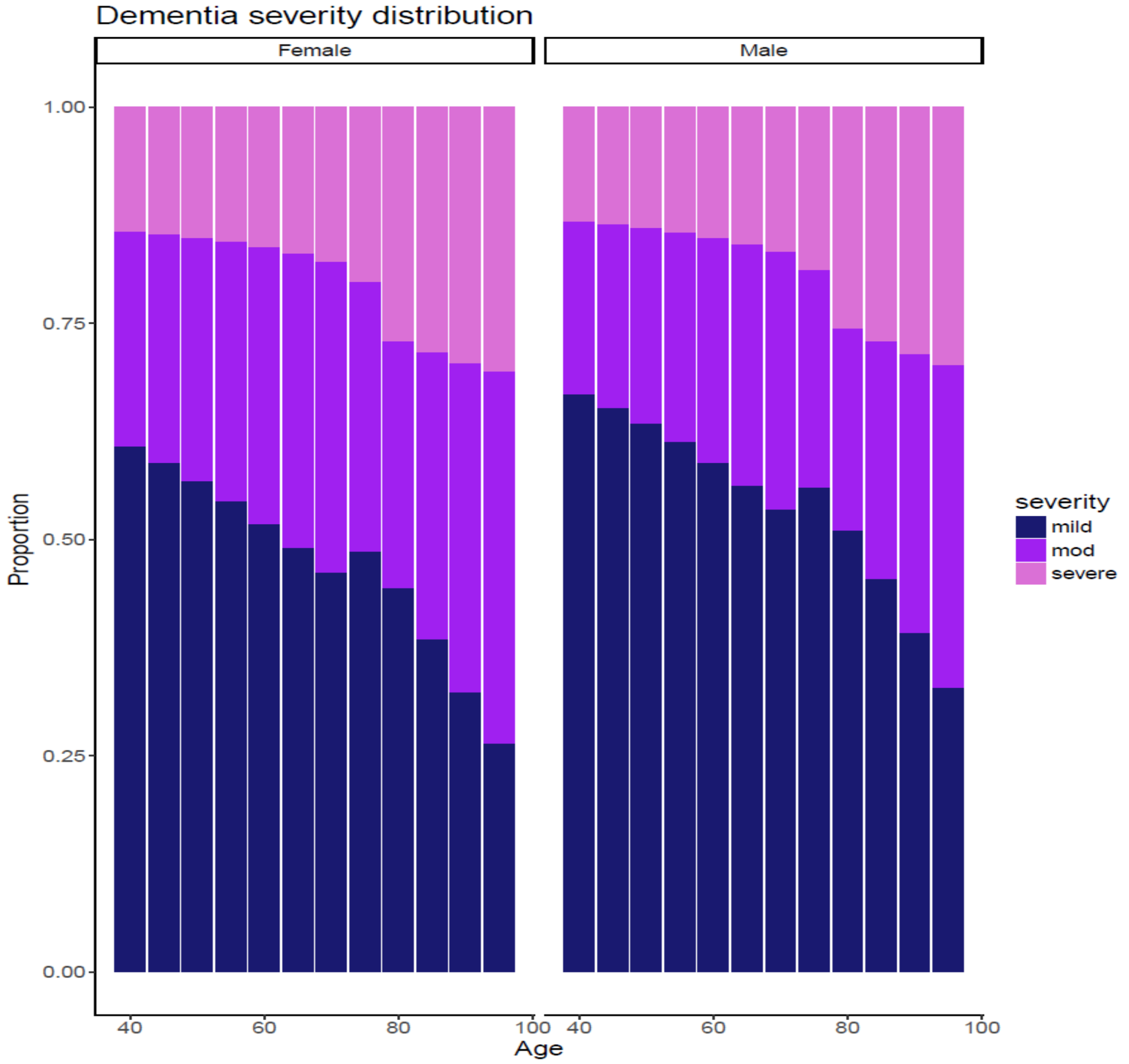

Table 1. Dementia severity levels

Severity level	Lay description
Mild	The person has some trouble remembering recent events and finds it hard to concentrate and make decisions and plans. They may have slight to moderate difficulty engaging in community affairs, complicated hobbies, and intellectual interests.
Moderate	The person retains highly learned material, but has severe memory problems, is disoriented with respect to time and sometimes place. They are severely impaired in their ability to handle problems and make social judgements. They require assistance with daily activities, and only retain simple chores and hobbies.

Severe	The person has complete memory loss, no longer recognises close family members, and requires help with all daily activities, including personal care.

Relative risk due to other causes

While the DSM definition excludes dementia cases where the syndrome is caused by other psychiatric disorders, it does not exclude dementia cases caused by other diseases not included in DSM. This includes stroke, Parkinson's disease, Down's syndrome, and traumatic brain injury (TBI), which are found elsewhere in the GBD cause list. To prevent double counting of prevalent cases, both under dementia and each of these other causes, we adjusted our dementia prevalence to exclude cases caused by these other conditions. To do so, in GBD 2019 we used data from the Aging, Demographics and Memory study (ADAMS), to estimate the relative risk of getting dementia for each condition included in the ADAMS dataset (stroke, Parkinson's disease, TBI). We then conducted more extensive systematic reviews on all of these conditions to model each separately (Table 2). Relative risk models were run using MR-BRT, and population attributable fractions (PAF) for each condition were calculated with the following equation, where exposure is defined as the prevalence of condition:

$$PAF = \frac{exposure * (RR - 1)}{[exposure * (RR - 1)] + 1}$$

Finally, attributable burden was calculated as the PAF multiplied by total burden (ie, dementia incidence/prevalence).

Table 2. Summary of each systematic review for relative risk due to other causes.

	**Stroke**	**Parkinson's disease**	**Down's syndrome**	**TBI**
**Data type**	Relative risks	Proportions and relative risks	Proportions	Relative risks
**Review hits**	504	1475	355	
**Accepted during title/abstract screening**	79	135	102	
**Accepted during full text**	35 (33 from systematic review and two from PubMed search)	56	26	45

Modelling strategy

For GBD 2023, we added sex-specific, reference case definition data to our prevalence and incidence data from GBD 2021. The processing steps for GBD 2021 are described below.

First, prevalence data were sex split, crosswalked, and age split. Studies with age and sex detail separately were split into age- and sex-specific datapoints. Data specified as "both"-sex data were split into male- and female-specific datapoints using MR-BRT to get a model ratio of female/male prevalence and then using the following equations:
Male prevalence:

$$prev_{male} = prev_{both} * \frac{pop_{both}}{(pop_{male} + ratio * pop_{female})}$$

Female prevalence:

$$prev_{female} = ratio * prev_{male}$$

We also split datapoints where the age range was greater than 25 years using the global age pattern.

Dementia studies are heterogeneous. Even with a smaller number of definitions (DSM/ICD), there are a large number of different ways to diagnose dementia. For example, out of 272 sources used in a previous round, GBD 2017, there were 263 different methods of diagnosing dementia (overlap was among those who used 10/66 protocol or AGECAT algorithm). Most use a two-step procedure, where you screen using a cognitive test and then only fully evaluate those that fall below a certain pre-defined threshold. We controlled for methods differences by adjusting alternative case definitions to reference, which we term "crosswalking". Study covariates are based on broad categories determined after going through the diagnostic heterogeneity, and there are some added for specific criteria that we know are biased. The same study-level covariates and models were used in GBD 2023 as in GBD 2021. Crosswalking was carried out using a logit difference network meta-regression analysis.

Table 4. MR-BRT crosswalk adjustment factors for dementia (network analysis)

Data input	Reference or alternative case definition	Gamma	Beta coefficient, logit (95% UI)*	Adjustment factor**
DSM or ICD case definition	Ref	0.34	---	---
Clinical records diagnosis criteria	Alt		–0.05 (–0.72 to 0.61)	0.95
Algorithm diagnosis criteria (AGECAT)	Alt		0.08 (–0.59 to 0.74)	1.08
NIA-AA diagnosis criteria	Alt		0.51 (–0.16 to 1.17)	1.67
10/66 algorithm diagnosis criteria	Alt		0.97 (0.30 to 1.64)	2.64
GP records used for diagnosis	Alt		–1.21 (–1.88 to –0.54)	0.30

**MR-BRT crosswalk adjustments can be interpreted as the factor the alternative case definition is adjusted by to reflect what it would have been had it been measured using the reference case definition. If the log/logit beta*

coefficient is negative, then the alternative is adjusted up to the reference. If the log/logit beta coefficient is positive, then the alternative is adjusted down to the reference.

***The adjustment factor column is the exponentiated beta coefficient. For log beta coefficients, this is the relative rate between the two case definitions. For logit beta coefficients, this is the relative odds between the two case definitions.*

Two country-level covariates were included in the initial DisMod-MR 2.1 model[1] (disease model—Bayesian meta-regression; details on this method can be found in appendix 1, section 2). Age-standardised education was used as a proxy for general brain health/use that may be protective of dementia – specifically Alzheimer's disease. Smoking prevalence (age-standardised, both sexes) was also used as a covariate to guide estimates, as the literature has shown a positive relationship between smoking and dementia.

Note that two DisMod-MR models were run with prevalence and incidence inputs – the first used prevalence, within-condition mortality rate, and relative risk data before mortality adjustments (DisMod-MR Model 1 in flowchart). The second used dementia prevalence and incidence data and cause-specific mortality rate inputs from final mortality modelling (DisMod-MR Model 2 in flowchart) which accounts for all dementia regardless of cause (this is the dementia impairment envelope). Tables 5 and 6 below summarise country-level covariates used in each of these DisMod-MR models.

Table 5. Summary of covariates used in the Alzheimer's disease DisMod-MR meta-regression model (adjusted prevalence, Model 1)

Covariate	Type	Parameter	Exponentiated beta (95% uncertainty interval)
Smoking prevalence (age-standardised)	Prevalence	1.00 (0.015 to 1.94)	2.72 (1.01 to 6.96)
Education (age-standardised)	Prevalence	–0.083 (–0.083 to –0.083)	0.92 (0.92 to 0.92)

Table 6. Summary of covariates used in the Alzheimer's disease DisMod-MR meta-regression model (unadjusted prevalence, Model 2)

Covariate	Type	Parameter	Exponentiated beta (95% uncertainty interval)
Smoking prevalence (age-standardised)	Incidence	1.35 (0.76 to 1.90)	3.86 (2.13 to 6.71)
Education (age-standardised)	Incidence	–0.012 (–0.037 to –0.0004)	0.99 (0.96 to 1.00)
Education (age-standardised)	Prevalence	–0.083 (–0.083 to –0.083)	0.92 (0.92 to 0.92)

As mentioned previously, the estimation of morbidity due to dementia occurs in conjunction with the mortality estimation. Additional details on this process can be found in the CoD capstone appendix (section 1, appendix 4).

We pulled the excess mortality rates from final fatal estimates into a final DisMod-MR model (Model 2). To prevent double counting of prevalent cases, both under dementia and under other causes that can lead to dementia, we adjusted our dementia prevalence to exclude cases caused by these other conditions, which include stroke, Parkinson's disease, traumatic brain injury, and Down's syndrome. To do so, we used data from the Aging, Demographics and Memory study (ADAMS) and new systematic reviews to estimate the relative risk of getting dementia for each condition included in the ADAMS dataset (stroke, Parkinson's disease, TBI). We first fit logistic regression models predicting the outcome of dementia given each exposure, with an additional covariate on age. We then used these models to predict the probability of dementia given each exposure at various ages and divided the probability of having dementia by the probability of not having dementia at each age to calculate relative risks. After calculating age-specific relative risks, we used these data and estimates of dementia prevalence from our DisMod-MR 2.1 model to calculate the population attributable fractions (PAFs) for each cause and age.

Finally, we multiplied the PAF by the total prevalence to get the amount of dementia prevalence that can be attributed to each cause and subtracted this from the total prevalence to get the prevalence of dementia that is not due to other GBD causes.

Amphetamine use disorders

Flowchart

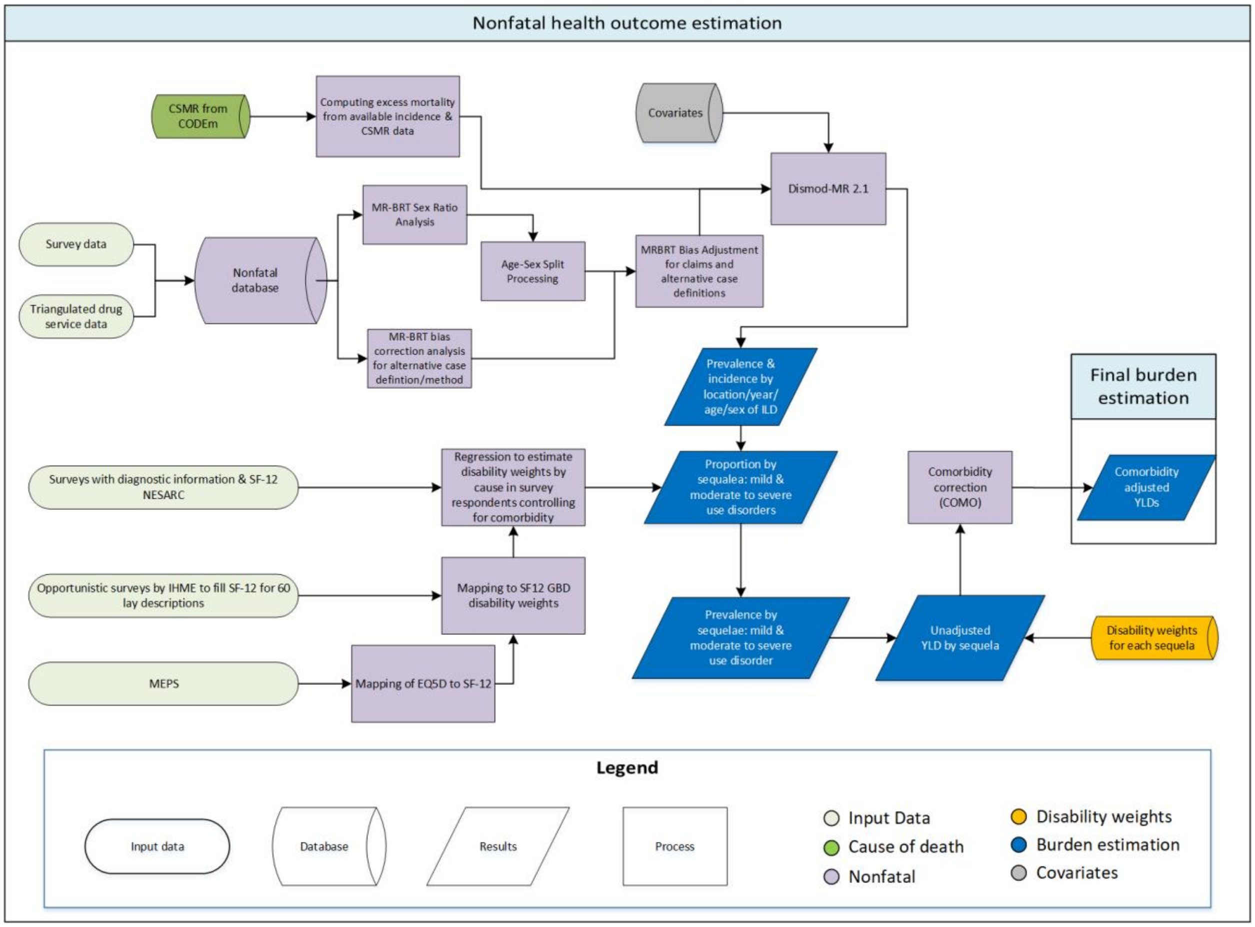

Input data and methodological summary for amphetamine use disorders

Case definition

Amphetamine dependence is a substance-related disorder involving a dysfunctional pattern of amphetamine use. Included in the Global Burden of Disease (GBD) disease modelling were cases meeting the Diagnostic and Statistical Manual of Mental Disorders (DSM-IV-TR) or the International Classification of Diseases (ICD-10) diagnostic criteria for amphetamine dependence (DSM: 304.40; ICD: F15.2), excluding those cases due to a general medical condition.[1,2] According to DSM-IV TR criteria, dependence involves a maladaptive pattern of substance use, leading to clinically significant impairment or distress. At least three of the following symptoms must be experienced within the same 12-month period:

- Tolerance, characterised by either
  - A need for increased amounts of the substance to achieve intoxication; or

    - Markedly diminished effect with continued use of the same amount of the substance;
- Withdrawal, characterised by either
    - Withdrawal symptoms characteristic to cannabis dependence; or
    - The same (or similar) substance is taken to avoid withdrawal symptoms;
- Substance taken in progressively larger amounts or for longer periods;
- Persistent desire or unsuccessful efforts to reduce substance use;
- Disproportionate time dedicated to obtaining the substance;
- Other important activities are given up because of the substance use; and
- Substance use is continued despite knowledge of physical or psychological problems occurring as a result of the substance.

**Quantity of interest**	**Reference or Alternative**	**Definition**
Amphetamine dependence prevalence	Reference	Amphetamine dependence measured using ICD or DSM diagnostic criteria
Amphetamine dependence prevalence	Alternative	A chronic condition in which a person craves amphetamines and is unable to control taking the drug. The person needs greater amounts to get the same effect and has withdrawal symptoms after stopping
Amphetamine dependence prevalence	Alternative	Amphetamine dependence measured using ICD or DSM diagnostic criteria in survey only.
Amphetamine dependence prevalence	Alternative	Regular (weekly) use of amphetamines by "indirect" method, i.e. using a multiplier from treatment data and surveys among drug users on prevalence of having accessed treatment over a recall period (mostly one year).
Amphetamine dependence prevalence	Alternative	Regular (weekly) use of amphetamines as measured in population surveys

Input data

In the GBD 2010 study, a systematic review was conducted to assess the prevalence, incidence, remission, and excess mortality associated with amphetamine dependence. This comprehensive review aimed to capture studies relevant to these aspects, employing a three-stage search strategy that included peer-reviewed literature (accessed through Medline, Embase, and PubMed), grey literature, and expert consultation. The methodology adopted for mental and substance use disorders involved conducting electronic database searches on a rolling basis to ensure thorough coverage.

The literature review for GBD 2010 was meticulously repeated for GBD 2013 to incorporate additional data published up to 2013. For GBD 2015, the second and third stages of the literature review were updated, and in GBD 2016, a focused search of peer-reviewed databases (Medline, Embase, and PsycINFO) was conducted to include studies published from 2013 to 2016. GBD 2017 expanded the scope further by incorporating additional sources identified by GBD experts and, where available, microdata. Notably, GBD 2017 also conducted two targeted systematic reviews to enhance the dataset significantly. The first review aimed to capture studies comparing Māori versus non-Māori populations within New Zealand, acknowledging the inclusion of these sub-groups. The second review leveraged the China National Knowledge Infrastructure database to identify studies not typically accessible through the main databases used in previous rounds.

The inclusion criteria for these systematic reviews were clearly defined: publications from 1980 onwards, diagnosis based on clinical thresholds as established by the DSM or ICD, provision of sufficient methodological and sample characteristic information to assess study quality, and the requirement for study samples to be representative of the general population. Studies focusing on inpatient or pharmacological treatment samples, case studies, veterans, or refugees were excluded, with no restrictions on the publication language. The methodologies employed for these systematic reviews have been detailed extensively in previous publications.

Age and sex splitting

Reported estimates of prevalence were split by age and sex where possible. First, if studies reported prevalence for broad age groups by sex (eg, prevalence in 15- to 65-year-old males and females separately), and also by specific age groups for both sexes combined (eg, prevalence in 15- to 30-year-olds, then in 31- to 65-year-olds, for males and females combined), age-specific estimates were split by sex using the reported sex ratio and bounds of uncertainty. Second, prevalence data for both sexes that could not be split using a within-study ratio were split using a sex ratio derived from a meta-analysis of existing sex-specific data using our meta-regression with Bayesian priors, regularization, and Trimming (Details on MR-BRT can be found in appendix 1, section 2).

The female to male ratio was 0.63 (0.44–0.92) for adults aged 20 and older and 0.60 (0.42–0.87) for youth under age 20. Finally, after the application of bias adjustments, where studies reported estimates across age groups spanning 20 years or more, these were split into five-year age groups using the super-region-specific prevalence age pattern estimated by our disease model—Bayesian meta-regression tool (DisMod-MR 2.1; see appendix 1, section 2) on all data prior to age-splitting.

Data adjustment

Due to insufficient data in the optimal case definition of amphetamine dependence, the prevalence dataset included datapoints of both use and dependence estimated using “direct” or “indirect” survey methods. “Direct” methods of measuring amphetamine dependence predominantly involve surveys of the general population that ask if respondents use or are dependent on amphetamine. Surveys tend to underestimate the prevalence of the most harmful and stigmatised forms of illicit drug use in ways that probably vary between countries and cultures.[3] “Indirect” methods are considered superior; they use different sources of data to indirectly estimate the total number of drug users (methods include

“multiplier methods,” back-projection and capture-recapture methods). Due to the lack of data available on amphetamine dependence from indirect survey methods (considered to be the gold standard for GBD purposes), estimates of use and/or estimates from direct survey methods were also included in the modelling. We marked studies reporting on the prevalence of amphetamine dependence obtained via direct methods as well as those reporting on the prevalence of amphetamine use obtained via direct methods and derived adjustment factors using MR-BRT. Due to limited overlapping data and roughly similar patterns of use, we combined amphetamine and cocaine data to derive a single adjustment factor. Beta coefficients in logit space are shown in the table below:

Table 1: MR-BRT crosswalk adjustment factors for amphetamine and cocaine use disorders

Data input	Reference or alternative case definition	Gamma	Beta coefficient, logit (95% UI)*	Adjustment factor **
Amphetamine dependence – indirect method	Ref	0.62	---	
Amphetamine use – indirect method	Alt		1.07 (–0.11 to 2.35)	2.92
Amphetamine dependence – direct method	Alt		–0.54 (–1.73 to 0.76)	0.58
Amphetamine use – direct method	Alt		0.54 (–0.65 to 1.81)	1.72

**MR-BRT crosswalk adjustments can be interpreted as the factor the alternative case definition is adjusted by to reflect what it would have been had it been measured using the reference case definition. If the log/logit beta coefficient is negative, then the alternative is adjusted up to the reference. If the log/logit beta coefficient is positive, then the alternative is adjusted down to the reference.*

***The adjustment factor column is the exponentiated beta coefficient. For log beta coefficients, this is the relative rate between the two case definitions. For logit beta coefficients, this is the relative odds between the two case definitions.*

Additionally, we adjusted for recall period to adjust from one-year recall to point-prevalence, again using combined cocaine and amphetamine data. Beta coefficients from MR-BRT are shown in the table below:

Table 2: MR-BRT crosswalk adjustment factors for amphetamine and cocaine use disorders

Data input	Reference or alternative case definition	Gamma	Beta coefficient, logit (95% UI)*	Adjustment factor**

Amphetamine dependence point prevalence	Ref	0	---	
Amphetamine dependence 1-year recall	Alt		0.71 (0.63–0.79)	2.03

**MR-BRT crosswalk adjustments can be interpreted as the factor the alternative case definition is adjusted by to reflect what it would have been had it been measured using the reference case definition. If the log/logit beta coefficient is negative, then the alternative is adjusted up to the reference. If the log/logit beta coefficient is positive, then the alternative is adjusted down to the reference.*

***The adjustment factor column is the exponentiated beta coefficient. For log beta coefficients, this is the relative rate between the two case definitions. For logit beta coefficients, this is the relative odds between the two case definitions.*

Modelling strategy

We have made no substantive changes in the modelling strategy from GBD 2021. Prior settings in DisMod included assuming no incidence, remission, and excess mortality before age 15, and an upper limit of 0.35 on remission. The minimum age of onset was corroborated with expert feedback and existing literature from various sources including the European Monitoring Centre for Drugs and Drug Addiction.[4] These settings were retained for GBD 2023.

Severity distribution

The basis of the GBD disability weight survey assessments are lay descriptions of sequelae highlighting major functional consequences and symptoms. The lay descriptions and disability weights for amphetamine dependence severity levels are shown below.

Table 3. Severity distribution, details on the severity levels for amphetamine use disorders in GBD 2021 and the associated disability weight (DW) with that severity.

Severity level	Lay description	DW (95% CI)
Mild	Uses stimulants (drugs) at least once a week and has some difficulty controlling the habit. When not using, the person functions normally.	0.079 (0.051–0.114)
Moderate to severe	Uses stimulants (drugs) and has difficulty controlling the habit. The person sometimes has depression, hallucinations, and mood swings, and has difficulty in daily activities.	0.486 (0.329–0.637)

**Asymptomatic cases carried no disability weight*

The proportion of people with amphetamine dependence within each of the severity levels was determined based on available data from US National Epidemiological Survey on Alcohol and Related Conditions (NESARC), conducted in two waves from 2001 to 2002 and 2004 to 2005.[5] NESARC is a direct

household survey. As such, it is expected to underestimate moderate to severe cases of drug dependence. The estimated distribution of amphetamine dependent cases by severity were asymptomatic (55%, 40–71), mild (19%, 12–27), and moderate/severe (26%, 16–35). As in GBD 2021, lag-distributed income (LDI) was included as a country covariate on EMR with bounds set at –1 and –0.1.

Table 4. Covariates. Summary of covariates used in the amphetamine use disorders DisMod-MR meta-regression model

Covariate	Parameter	Beta, log (95% uncertainty interval)	Exponentiated beta (95% uncertainty interval)
LDI ($ per capita)	Excess mortality rate	–0.1 (–0.1 to –0.1)	0.90 (0.90 to 0.90)

References

1. American Psychiatric Association. Diagnostic and Statistical Manual of Mental Disorders (DSM-IV-TR). 4th, Text Revision ed Washington DC: American Psychiatric Association. 2000.
2. World Health Organization. The ICD-10 Classification of Mental and Behavioural Disorders. Clinical descriptions and diagnostic guidelines Geneva: World Health Organization. 1992.
3. Reuter P, Trautmann F. A Report on Global Illicit Drugs Markets 1998-2007. Utrecht. 2009.
4. European Montioring Centre for Drugs and Drug Addiction. Lisbon, Portugal 2014.
5. Grant BF, Dawson DA. National Institute on Alcohol Abuse and Alcoholism. *Alcohol Health & Research World*. 2006; 29(2): p. 74.

Anaemia impairment (envelope and causal attribution)

Flowchart

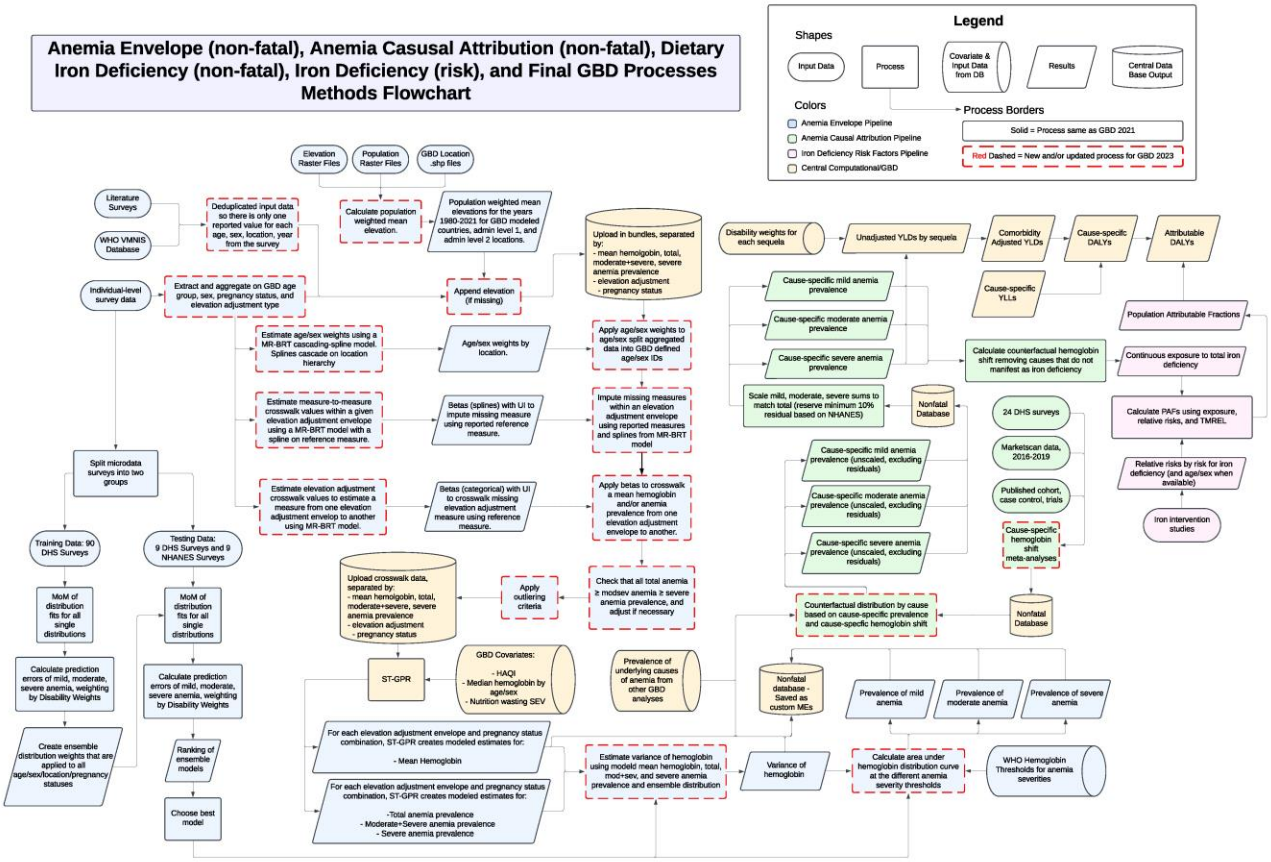

Input data and methodological summary for anaemia

Case definition

Anaemia is defined as decreased blood concentration of haemoglobin, irrespective of underlying cause, red blood cell morphology, or red blood cell function. Thresholds for defining individuals as being anaemic, as well as thresholds for anaemia severity, are based on the World Health Organization's (WHO) thresholds for haemoglobin in g/L.[1] There are not any international guidelines on appropriate thresholds for diagnosing anaemia in neonates. To estimate thresholds for the neonatal period that reflect the higher haemoglobin levels typically seen in newborns, we calculated the ratio of "normal" haemoglobin (defined as the 50th percentile of the global haemoglobin distribution) for the 0–6 days and 7–27 days periods over the 1–5-month age group by severity, and then multiplied the WHO 6–59-months thresholds by these ratios.

Table 1: Definitions of mild, moderate, and severe anaemia based on blood haemoglobin concentration

Sex	Age	Mild	Moderate	Severe
Both	0–6 days	145–159 g/L	100–144 g/L	<100 g/L
Both	7–27 days	120–134 g/L	85–119 g/L	<85 g/L
Both	1–5 months	100–109 g/L	70–99 g/L	<70 g/L
Both	5 months – 4 years	95–104 g/L	70–94 g/L	<70 g/L
Both	5 - 14 years	110–114 g/L	80–109 g/L	<80 g/L
Male	15+ years	110–129 g/L	80–109 g/L	<80 g/L
Female, non-pregnant	15+ years	110–119 g/L	80–109 g/L	<80 g/L
Female, pregnant	15+ years	100–109 g/L	70–99 g/L	<70 g/L

Overview of adjusting haemoglobin for elevation

Haemoglobin concentration increases with increasing elevation, a physiological response to lower ambient oxygen levels that aims to maintain oxygen delivery throughout the body. Due to this physiological response, WHO recommends adjusting haemoglobin to ensure all haemoglobin concentrations are assigned anaemia statuses using the same haemoglobin thresholds shown in Table 1. The amount at which haemoglobin is adjusted for elevation varies based on both the elevation at which the haemoglobin measurement was taken and the adjustment method used.

In 2023, WHO published a new elevation adjustment formula that differed from the method that was used to adjust haemoglobin for elevation in GBD 2021 and earlier,[1] as seen in Figure 1. The previous WHO elevation adjustment method was found to be under-adjusting haemoglobin for elevations below 3000 meters and over-adjusting haemoglobin for elevations above 3000 meters. The old WHO adjustment method was estimated using only data from small subset of demographics and locations and assumed a log-linear relationship between elevation and haemoglobin adjustment amount. The new WHO elevation-adjustment equation was developed by Biomarkers Reflecting Inflammation and Nutritional Determinants of Anaemia (BRINDA) using data from a larger subset of demographics from varying geographical locations.[2] Given this, BRINDA estimated a more robust and realistic biological relationship between elevation and haemoglobin adjustment amount and therefore is the elevation adjustment method used for GBD 2023.

Figure 1: Haemoglobin adjustment for elevation. The yellow curve is the WHO elevation adjustment method published in 2001, and the blue curve is the updated elevation adjustment method as proposed by WHO.

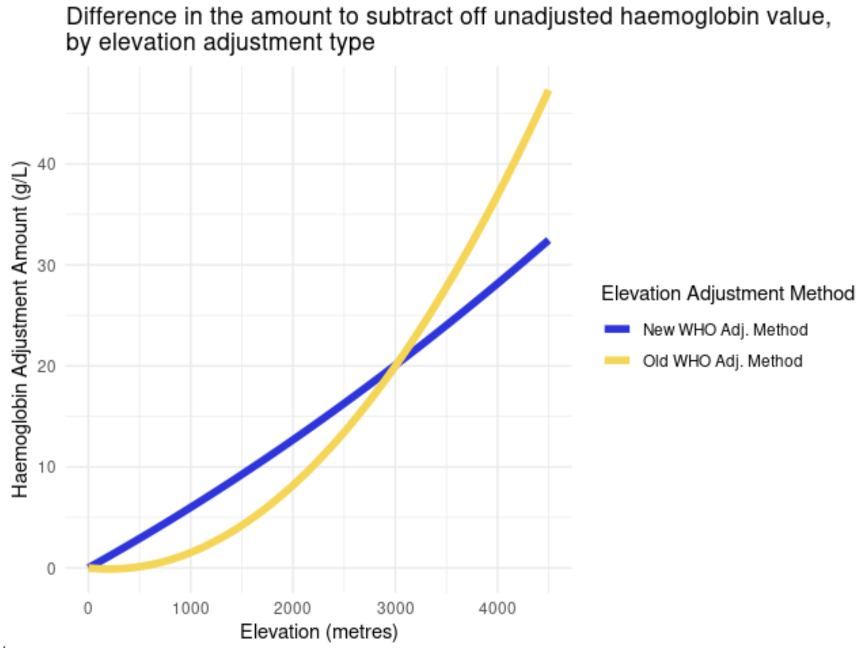

Overview of input data

Estimating total anaemia (called "the envelope") utilises data from a variety of sources. Inclusion criteria include quantitative measurement of haemoglobin in either a population-based sample or group judged to adequately represent the sex, age groups, and location of the study.

Population-based individual-level surveys (microdata) – including the Demographic and Health Survey (DHS), Multiple Indicator Cluster Survey (MICS) series, national micronutrient surveys, and other national and subnational nutrition surveys – comprised the bulk of input data. We supplemented these data with additional sources of mean haemoglobin and anaemia prevalence from the WHO Vitamin and Mineral Nutrition Information System[3] and other literature sources where available. An overview of the source-location-years available for both individual-level data and tabulated data can be seen in Figure 2.

Figure 2: Maps of all unique source-location-year counts of data used in the anaemia envelope, where a) shows the sources that supplied haemoglobin data, and b) shows sources that supplied any total, moderate + severe, and/or severe anaemia prevalence data. Countries that are grey did not supply any data for the given measure.

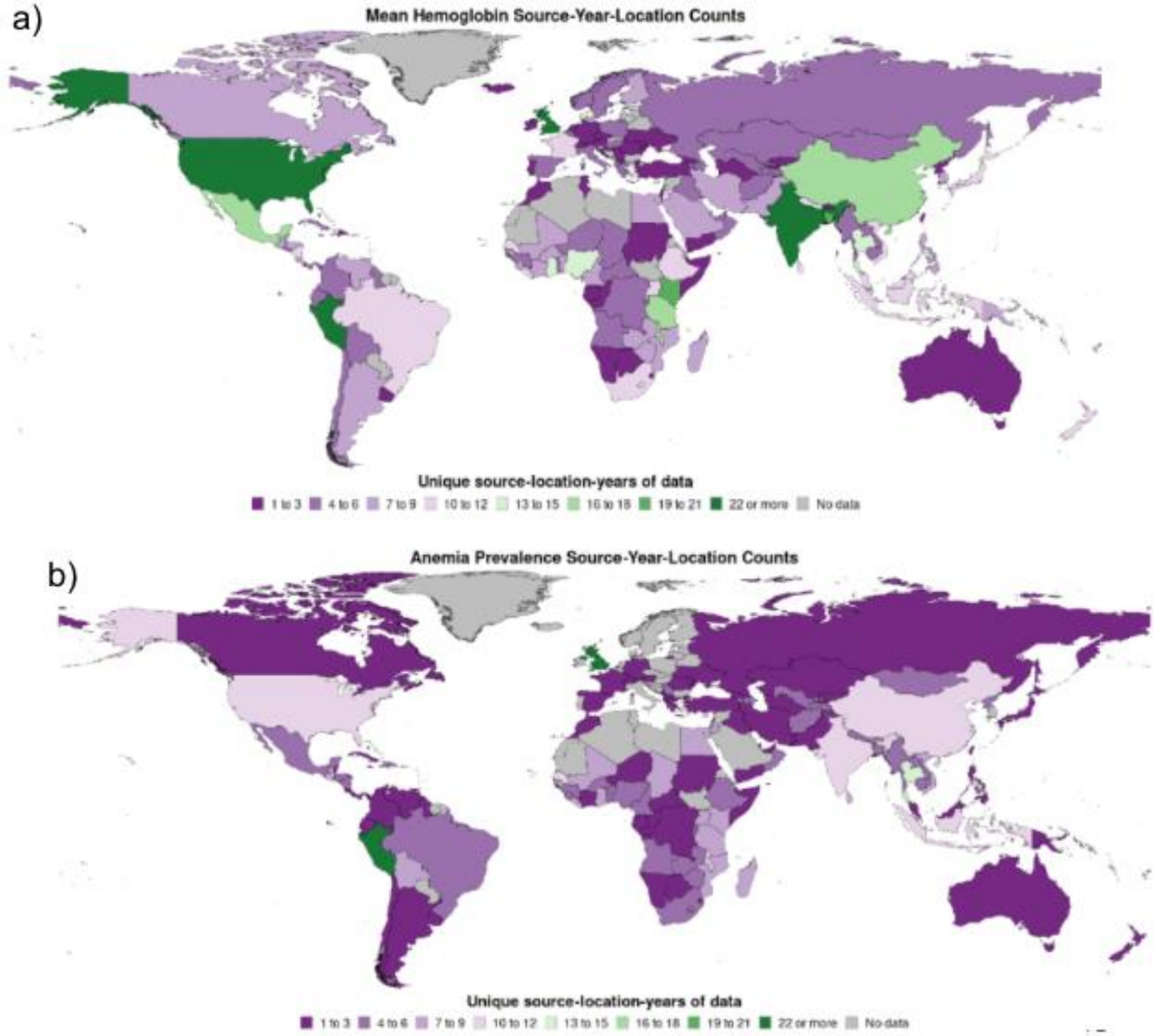

Data processing

For GBD 2023, we only reported final mean haemoglobin concentrations and anaemia prevalence values that were adjusted using the new WHO elevation-adjustment method. However, when processing all input data in the anaemia pipeline, we created three "anaemia envelopes" in parallel to be used to help inform the final new WHO elevation-adjusted dataset. The three envelopes consist of mean haemoglobin concentrations and anaemia prevalence values that were: 1) raw/unadjusted for elevation, 2) elevation-adjusted using the old WHO method, and 3) elevation-adjusted using the new WHO elevation adjustment method.

Step 1: Extracting, elevation adjusting, and assigning anaemia status to input data

In personal-level input data, we extracted haemoglobin concentrations, along with elevation (if present), age, sex, location, year, and pregnancy status. The haemoglobin concentrations were then flagged for the elevation-adjustment method used to report the haemoglobin concentrations in the survey. If the haemoglobin concentrations were adjusted using the old WHO elevation adjustment method and elevation was present (in meters), haemoglobin was first unadjusted for elevation. Next, all raw/unadjusted haemoglobin concentrations were adjusted using the new WHO elevation-adjustment method, again, granted elevation was present (in meters). For the surveys where elevation was not present, those haemoglobin concentrations were later adjusted for elevation in the subsequent steps below.

All personal-level haemoglobin concentrations were then assigned an anaemia status using the thresholds defined in Table 1. Next, the individual-level data were split by elevation-adjustment method used and aggregated into GBD age groups by sex and pregnancy status in six different formats: mean haemoglobin concentration, severe anaemia prevalence, moderate anaemia prevalence, moderate+severe anaemia prevalence, mild anaemia prevalence, and total anaemia prevalence. Tabulated data were also flagged for elevation-adjustment method used and appended to the appropriate anaemia envelope dataset.

Step 2: Creating and assigning elevation to surveys based on GBD locations

To properly apply elevation adjustments as described above, all input data needed to be assigned an elevation (in meters). If a survey provided elevation information, the reported elevation was used to elevation-adjust the raw/unadjusted haemoglobin concentration, as stated in Step 1.

If elevation was not provided, elevation needed to be assigned based on the most granular GBD location from which the survey originated from. We derived a dataset of "population-weighted mean elevations" for each GBD modelled country, admin level one, and admin level two locations, seen in Figure 3. Using the population-weighted approach allowed us to more accurately represent the average elevation at which an individual in the population resides. We created this dataset using an elevation raster file[4] and population raster files[5] from 1980–2021, where we take the mean elevation weighted by the population for each 5x5 kilometre grid within the polygon of the GBD location being aggregated. If a survey was published before 1980 or after 2021 and did not report elevation, it was assigned a population weighted mean elevation from 1980 and 2021, respectively.

Figure 3: Elevation raster file and the corresponding population weighted mean elevation output for all GBD modelled locations

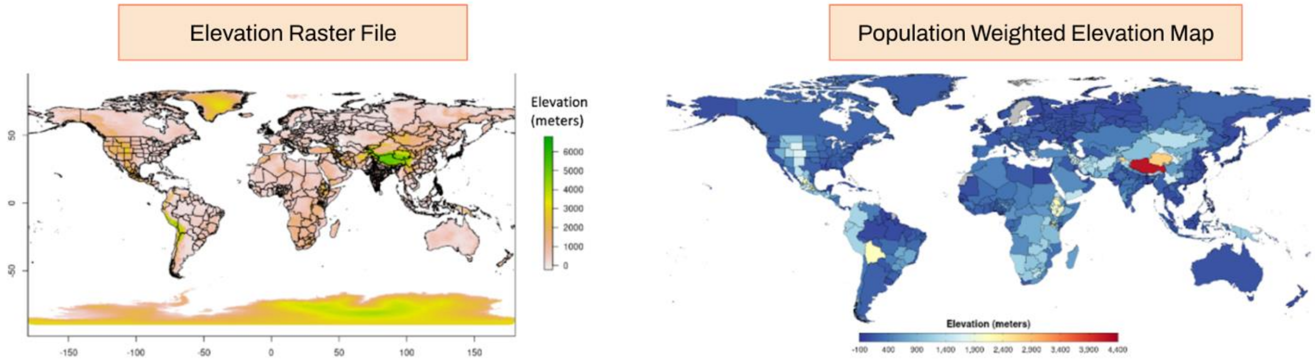

Step 3: Age-sex splitting literature and VMNIS data

Using collapsed individual-level survey data that had already been aggregated into GBD defined age-sex-pregnancy groups, we calculated age-sex patterns for mean haemoglobin, total anaemia, moderate+severe anaemia, and severe anaemia prevalence using the meta-regression—Bayesian, regularised, trimmed (MR-BRT) meta-analytic tool's cascading spline framework.[6] Males, non-pregnant females, and pregnant females are all modelled separately. Within those subgroups, we modelled 2+ year-olds and 0–2-year-olds separately, which allows us to isolate the varying age trends in the younger age groups. The MR-BRT model uses a quadratic spline on age and is cascaded on location hierarchy. Spline parameters used can be seen in Table 2. The cascading framework is shown in Table 3.

We then used the age-sex weight patterns from the cascading splines to split any data that spanned more than a single GBD age-sex group into the corresponding GBD age-sex groups it encapsulates. The GBD location associated with the age-sex weights to be used from the MR-BRT cascade model is assigned by the nearest location level hierarchy modelled in our MR-BRT cascading framework (ie, either using the same location, if that model is available, or using the model for a location hierarchy above). Figure 4 shows outputs from the cascade modelling pipeline, with examples from females 2 years and older from Hidalgo, Mexico (data-rich source) and males two years and older from Rwanda (data-sparse source). In each cascade model output, the splines on age follow the curvature of the spline of the location hierarchy above it as the location hierarchy being modelled becomes more granular.

Table 2: Age/sex weight MR-BRT settings and spline parameters used in each model for 3a) males and females 0–2 years old, 3b) Males and females 2–125 years old, 3c) pregnant females 10–54 years old.

Table 2a. Model parameters for 0–2-year-olds, both males and females

Measure	Measure transform	Knot locations (age in years)	Left tail linearity enforcement	Right tail linearity enforcement	Concavity or convexity enforcement
Haemoglobin (g/L)	Log	0.5, 1	False	False	Convex
Anaemia Prevalence	Logit	0.5, 1	False	False	None

Table 2b. Model parameters for 2–125-year-olds, both males and females

Measure	Measure transform	Knot locations (age in years)	Left tail linearity enforcement	Right tail linearity enforcement	Concavity or convexity enforcement
Haemoglobin (g/L)	Log	10, 15, 55, 70	False	True	Concave
Anaemia Prevalence	Logit	10, 15, 55, 70	False	True	Convex

Table 2c. Model parameters for pregnant women of reproductive age (10–54 years old)

Measure	Measure transform	Knot locations (age in years)	Left tail linearity enforcement	Right tail linearity enforcement	Concavity or convexity enforcement
Haemoglobin (g/L)	Log	25, 35	False	True	None
Anaemia Prevalence	Logit	25, 35	False	True	None

Table 3: Cascade *thetas* (weights) applied to each of the location hierarchies (the smaller the theta, the more the cascade model is influenced by the beta weights from its parent model).

Cascade location level	Theta weights
Global	---
Super-region	10
Region	8
Country	2
Admin Level 1	1
Admin Level 2	0.1

Figure 4: Example of cascade model outputs for a) females in Hidalgo (data-rich) and b) males in Rwanda (data-sparse). In each plot, the global, super-region, region, country, and admin level one (if applicable) estimated sex-specific age patterns are represented by the black, purple, yellow, red, and turquoise lines, respectively. The datapoints plotted are the input data used to inform the sex-specific age patterns.

Figure 4a. Females in Hidalgo (data-rich)

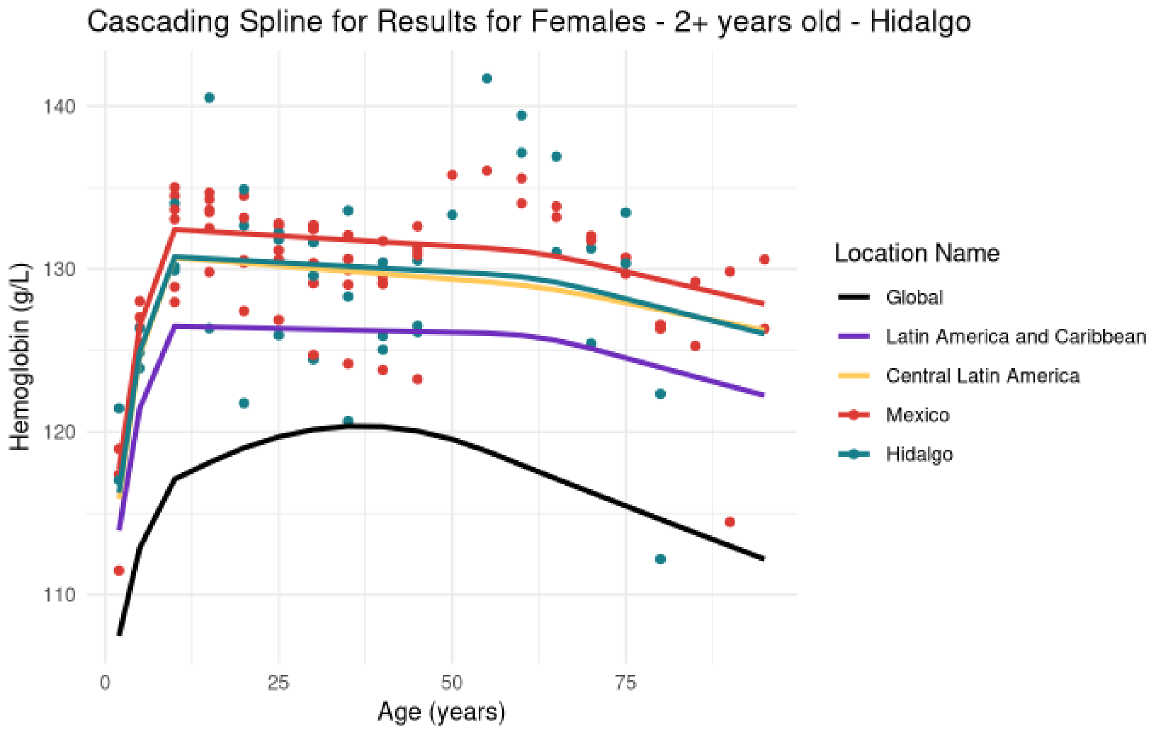

Figure 4b. Males in Rwanda (data-sparse)

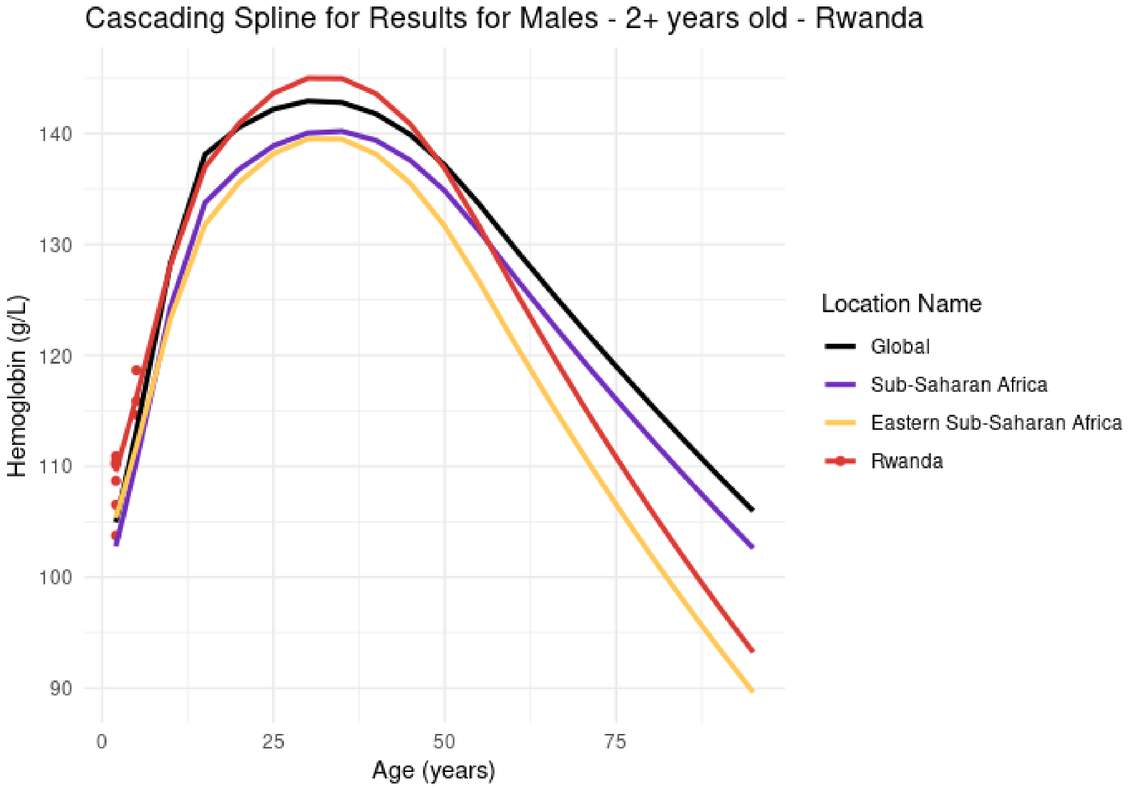

Step 4: Crosswalking by pregnancy status

Haemoglobin concentration varies systematically based on pregnancy status. Our models of mean haemoglobin and anaemia prevalence are based on these values among non-pregnant females, and these estimates are later adjusted to account for pregnancy prevalence for each location/year/age/sex as described in the "Fitting ensemble distributions with method of moments" methods section. Studies that only reported mean haemoglobin or anaemia prevalence among pregnant women were crosswalked to fit our model case definition of non-pregnant women. To estimate this crosswalk, we matched data sources containing age-specific estimates of mean haemoglobin or anaemia prevalence separately by pregnancy status and calculated the ratio of these values among pregnant females versus non-pregnant females within a single study. We log-transformed these ratios and calculated standard errors of the ratios using the delta method. We then meta-analysed these ratios using MR-BRT with a 10% trim setting. Although age was tested as a potential predictor in the model, we did not observe a significant age dependence of the ratios. The crosswalk effects are illustrated below.

Table 4: MR-BRT crosswalk values for adjusting mean haemoglobin by pregnancy status

Model	Data input	Reference or alternative case definition	Gamma	Beta coefficient, log (mean [Hb]) (95% UI)*	Adjustment factor**
**Mean haemoglobin**	Non-pregnant women	Ref	0.033	---	---
	Pregnant women	Alt		–0.08 (–0.15 to –0.02)	0.92 (0.86–0.98)

**MR-BRT crosswalk adjustments can be interpreted as the factor the alternative case definition is adjusted by to reflect what it would have been had it been measured using the reference case definition. If the log/logit beta coefficient is negative, then the alternative is adjusted up to the reference. If the log/logit beta coefficient is positive, then the alternative is adjusted down to the reference.*
***The adjustment factor column is the exponentiated beta coefficient. For log beta coefficients, this is the relative rate between the two case definitions. For logit beta coefficients, this is the relative odds between the two case definitions.*

Step 5: Measure-to-measure crosswalk to square the modelling dataset

To ensure internal consistency within all the reported mean haemoglobin and total, moderate+severe, and severe anaemia prevalence values, we implemented the measure-to-measure crosswalk (ie, squaring the dataset). The squaring process involves creating an input datapoint for each of the measures (mean haemoglobin, total anaemia, moderate+severe anaemia, severe anaemia prevalence) that are missing from all the age, sex, location, year, NID (survey) combinations present in the input data set for a given elevation adjustment envelope.

To perform this operation, we created MR-BRT models to estimate crosswalk values (betas) which use a reported reference measure to estimate a missing alternate measure. Using aggregated individual-level survey data, we first subset the data by the demographics (rows) shown in Table 1 so that the haemoglobin concentrations and anaemia thresholds assigned to the given demographic are consistent within the sample population being used in the MR-BRT model. After subsetting, we then create a dataset with one measure as the reference and a different measure as the alterative. The reference and alternative subsets are then matched by NID and GBD age, sex, location, and year. This is repeated for all demographics and combinations of the varying reference measures that can be used to impute a missing alternative measure, shown in Table 5.

Table 5: Reference measures used to impute alternative measures, and the transformation used on the alternative measures.

Reference measure	Alternative measure	Alternative measure transform
Mean haemoglobin	Total anaemia prevalence	Logit
Mean haemoglobin	Moderate+severe anaemia prevalence	Logit
Mean haemoglobin	Severe anaemia prevalence	Logit
Total anaemia prevalence	Mean haemoglobin	Log
Moderate+severe anaemia prevalence	Mean haemoglobin	Log

We modelled the alternative measure as a function of the reference measure. To account for the potential for non-linear relationships, we placed a continuous quadratic spline on the reference measure value with four internal frequency knots at the 10th, 30th, 70th, and 90th quantiles of the input data present along the exposure range. The spline was enforced to be monotonically decreasing. Linearity at the tails was not enforced, nor was convexity. No random effects were included in the model, and each of the models was run using a 5% trim setting. Figure 5 provides an example as to how the estimated betas were used to predict missing measures in our input data to complete the square by measure portion of the pipeline.

Figure 5: Example of using the betas estimated in the women of reproductive age MR-BRT model to predict moderate+severe anaemia using a reported mean haemoglobin value

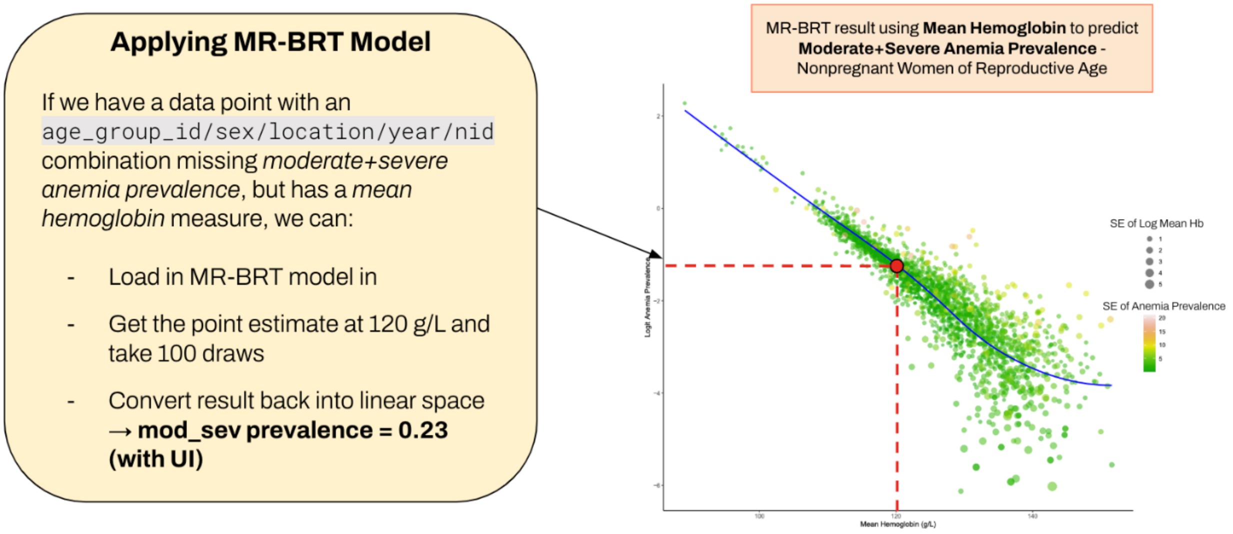

Step 6: Adjust input data for elevation

The final crosswalk step is to ensure all input data is adjusted using the new WHO elevation-adjustment method. If there were reported mean haemoglobin concentrations and anaemia prevalence values in either the unadjusted or old WHO elevation-adjusted envelopes for a given age, sex, location, year, NID combination that were not able to be elevation adjusted in Step 1, we needed to crosswalk those values into the new WHO elevation-adjusted envelope.

To adjust mean haemoglobin values to be representative of the new WHO elevation-adjustment method that were reported as unadjusted, we used the unadjusted mean haemoglobin concentration and population-weighted mean elevation and applied those values to the new WHO elevation-adjustment equation. To adjust mean haemoglobin values from the old WHO elevation-adjustment method to the new WHO elevation adjustment method, we used the population-weighted mean elevation to unadjust the mean haemoglobin, and then subsequently readjusted using the same method for unadjusted mean haemoglobin concentrations to new WHO elevation-adjusted values.

To crosswalk total anaemia, moderate+severe anaemia, severe anaemia prevalence values between elevation adjustment methods, we created a suite of MR-BRT models to estimate betas to perform the crosswalk. Using collapsed microdata where all elevation-adjustment methods are present, we subset the data by total, moderate+severe, and severe anaemia prevalence values. Using those subsets, we then create two data sets: 1) reported prevalence value from the unadjusted envelope as the reference and the reported new WHO elevation-adjusted reported value as the alternate, and 2) reported prevalence value from the old WHO elevation-adjusted envelope as the reference and the new WHO elevation-adjusted reported value as the alternate. The reference and alternative datasets are then matched on GBD age, sex, location, and year IDs along with the study's NID. The reference and alternative prevalence values were then logit-transformed and subtracted from each other, and we subsequently delta-transformed their standard errors. We then modelled the logit differences in anaemia prevalence using a MR-BRT model, with categorical covariates on elevation (in 400-metre bins), the non-transformed reference prevalence (in 20% increments), and created demographic categories based on the rows from Table 1. An example of the betas predicted for a given demographic can be seen in Figure 6. The result of this process gives us a mean haemoglobin concentration and total, moderate+severe, and severe anaemia prevalence values that were reported in any of the three anaemia envelopes to be all represented in the new WHO elevation-adjusted anaemia envelope.

Figure 6. Example of applying elevation crosswalk to estimate the new WHO elevation-adjusted total anaemia prevalence using an old WHO elevation-adjusted survey that reported total anaemia prevalence of 25% for non-pregnant WRA living at 1000 meters.

1. Load in the MR-BRT estimated betas for crosswalking total anaemia prevalence using the old WHO elevation-adjustment method to the new WHO elevation-adjustment method, shown in the table.

2. Identify the categories the data point to be crosswalked correlates to (highlighted in red).

3. Convert the reference total anaemia prevalence to logit space:

$$logit(0.250) = -1.099$$

4. Add the estimated betas to the value calculated in step 3.

$$logit(prev) + \beta_0 + \beta_{elevation} + \beta_{anemia} =$$

$$-1.099 + 0.156 + 0.337 - 0.108 = \mathbf{-0.713}$$

5. Convert back to linear space

$$inv\_logit(-0.713) = \mathbf{0.329}$$

Variable Type	Category	Beta
Intercept	---	0.156
Elevation Category	0 m - 400 m	REFERENCE
	400 m - 800 m	0.132
	800 m - 1200 m	0.337
	1200 m - 1600 m	0.516
	1600 m - 2000 m	0.510
	2000 m - 2400 m	0.461
	2400 m - 2800 m	0.413
	2800 m - 3200 m	0.209
	3200 m - 3600 m	-0.270
	3600 m - 4000 m	-0.259
	4000 m - 4400 m	-0.678
	4400 m - 4800 m	-0.877
	4800 m - 5200 m	-1.959
	5200 m - 5600 m	-2.624
Reference Anaemia Prevalence Category	0% to 20%	REFERENCE
	20% to 40%	-0.108
	40% to 60%	-0.164
	60% to 80%	-0.197
	80% to 100%	-0.128

Step 7: Ensuring total anaemia ≥ moderate+severe anaemia ≥ severe anaemia prevalence

Given the large amount of data processing and imputation, we implemented a procedure to ensure that total anaemia ≥ moderate+severe ≥ severe anaemia prevalence for each unique age, sex, location, year, NID combination in our dataset within each of the elevation adjustment envelopes. If these inequality constraints were not met for a given age, sex, year, location, NID combination, we invoked an adjustment step to re-impute the prevalence values that do not meet the inequalities shown above. This situation occurs when a survey only reports an anaemia prevalence for only one or two of the severity levels along with a mean haemoglobin, where we are then left to impute the remaining one or two missing anaemia prevalence values in our measure-to-measure imputation step. In some surveys, the reported anaemia prevalence value(s) is (are) far lower than what is estimated when using our measure-to-measure imputation step with the given reported mean haemoglobin concentration. In these cases, the measure-to-measure imputation step can potentially estimate a moderate+severe anaemia prevalence value that is larger than the reported total anaemia prevalence or estimate a severe anaemia prevalence that is larger than the reported moderate+severe anaemia prevalence.

To re-estimate anaemia prevalence values that violate the severity constraints of total anaemia ≥ moderate+severe ≥ severe anaemia prevalence, we utilised the MR-BRT models created in the measure-to-measure crosswalk step of the anaemia pipeline. This approach also requires that the mean haemoglobin measure be reported in the survey along with at least one of the anaemia prevalence values. If those two measures are present, then we first use the reported mean haemoglobin to estimate total anaemia, moderate+severe anaemia, and severe anaemia prevalence values for the given age, sex, location, year, NID combination using the MR-BRT measure-to-measure models. These

estimates are then used to create ratios of the imputed severe to moderate+severe anaemia prevalence estimates and imputed moderate+severe to total anaemia prevalence estimates. We then apply the ratios to the reported anaemia prevalence value to impute the missing anaemia prevalence value(s) that violated the inequality constraints.

If there are instances where anaemia prevalence values violate the anaemia prevalence inequalities and there is not enough information to implement this alternate imputation approach, the given age, sex, year, location, NID combination is outliered from all of the mean haemoglobin, total anaemia, moderate+severe anaemia, and severe anaemia datasets for the given elevation adjustment envelope.

Modelling strategy

Estimation of overall anaemia prevalence occurred in four steps: 1) ST-GPR models of mean haemoglobin and prevalence of total, moderate+severe, and severe anaemia, 2) calculation of ensemble weights, 3) generation of ensemble distributions, and 4) calculation of anaemia prevalence based on the ensemble distributions.

ST-GPR models of mean haemoglobin and anaemia prevalence

In previous GBD iterations, we modelled the mean and standard deviation of haemoglobin and used these as inputs to our ensemble model of haemoglobin concentration. To utilise additional data sources that report prevalence of anaemia directly, in GBD 2020 we amended our modelling process to include models of anaemia prevalence (total, moderate+severe, and severe) instead of modelling SD of haemoglobin directly.

We modelled 1) mean haemoglobin, 2) total anaemia prevalence, 3) moderate+severe anaemia prevalence, and 4) severe anaemia prevalence using spatiotemporal Gaussian process regression (ST-GPR), a three-step modelling process for generating estimates for every location, year, age, and sex in the GBD study. The first step of the ST-GPR process is to estimate a linear prior using predictive covariates to help guide the model's directionality and intercept. The following covariates were used in the stage-one linear prior step: summary exposure value (SEV) for child wasting, Healthcare Access and Quality (HAQ) Index, and 50th percentile of haemoglobin (pooled across all microdata sources). Random effects were not added to the linear stage-one step.

The second, spatiotemporal smoothing step of ST-GPR calculates the residual between our stage 1 regression estimate and each of our observed datapoints and then smooths this residual, drawing strength over space, age, and time and producing a revised stage 2 estimate for every location, year, age, and sex. The third step of ST-GPR is a Gaussian process regression, using the stage 2 estimates as a prior and the observed datapoints and their variance to 1) further smooth the residual between the stage 2 predictions and observed data and produce a final mean estimate for each location, year, age, and sex, and 2) estimate uncertainty around this mean estimate, quantified by taking 1000 draws from the posterior Gaussian process. More detailed information on the ST-GPR modelling process can be found in the main text methods appendix.

Ensemble distribution modelling

We modelled the full distribution of haemoglobin for each population (location/age/year/sex), from which we applied the WHO thresholds to calculate prevalence of anaemia by severity. In GBD 2015, a Weibull distribution was fit using shape and scale parameters estimated from mean haemoglobin. Since GBD 2016, we now combine multiple two-parameter distributions to create a more precise and unbiased ensemble distribution.

Generation of ensemble weights

First, we created a training and testing set of individual-level haemoglobin measurements. The training set consisted of 90 DHS surveys, providing 290 group-specific samples of microdata from children <5, males 15–45, pregnant females 15–45, and non-pregnant females 15–45 (not all groups were sampled in each DHS). A set of two-parameter distributions (gamma, mirrored gamma, Weibull, mirror lognormal, and mirrored Gumbel) were fit to the sample's haemoglobin mean and variance. These distributions were combined using weights optimised by a loss function of severity-specific prediction error weighted by the ratio of the severity's disability weight (DW) to mild anaemia DW. Weights were constrained to be positive and sum to 1, so that the resultant ensemble distribution is a proper probability density function. All permutations of the five distributions were tested (ie, we optimised weights for both a mix of all five distributions as well as a gamma-Weibull two-way combination).
The loss function is:

$$\sum_{i=i}^{n_{surveys}} \sum_{j=1}^{n_{demographics}} \sum_{k=1}^{n_{severities}} r_k \left| p_{ijk} - \widehat{p_{ijk}} \right|$$

such that:

$$\widehat{p_{ijk}} = \sum_{z=1}^{n_{distributions}} w_z \int_{t1_{jk}}^{t2_{jk}} PDF_{ijz}\, \mathrm{dt}\,,\ 0 \le \widehat{p_{ijk}} \le 1$$

where:

$i \in surveys : 1 \le i \le\ n_{surveys}$, the list of surveys (in either the training or testing set)

$j \in demographics : 1 \le j \le n_{demographics}$, the demographics (rows) found in Table 1

$k \in severities : 1 \le k \le n_{severities}$, the list of severities (mild, moderate, severe)

r is the ratio of the severity *k* disability weight to that of mild anaemia, r_{mild} = 1, $r_{moderate}$ = 13 and r_{severe} = 40

PDF is a probability density function fit to the sample mean and variance

t1 and t2 are the lower and upper bounds to the WHO anaemia definition for the group found in Table 1

w is the set of distribution weights s.t. $0 \leq w_z \leq 1$ and $\sum_{z=1}^{n_{distributions\ used}} w_z = 1$

$z \in distributions : 1 \leq z \leq n_{distributions}$, the list of distributions (gamma, mirror gamma, Weibull, mirror lognormal, and mirror gumbel)

The testing set consisted of nine National Health and Nutrition Examination Surveys (NHANES) and nine DHS surveys not included in the training data. Inclusion of NHANES as half the testing set ensured out-of-sample predictive validity by challenging the global weights, as it provided the ensemble distribution with high-income data (DHS is from LMICs) and data from adults >45 (DHS did not take blood tests from older populations). We selected the combination of distributions (including all individual component distributions) that minimised the loss function. For GBD 2023, this resulted in an ensemble distribution that was 40% gamma and 60% mirrored Gumbel.

Haemoglobin variance optimisation

To generate an ensemble distribution of haemoglobin concentration, we first required an estimate of the variance of the haemoglobin distribution. To generate an estimate of distribution variance, we used our ST-GPR estimates of mean haemoglobin and prevalence of total, moderate+severe, and severe anaemia prevalence in a variance-optimisation algorithm. For every location, year, age, and sex, we anchored the distributions at the estimated mean haemoglobin value and found the variance value that minimised the error between our estimates of severe, moderate+severe, and total anaemia and the corresponding values implied by a given mean haemoglobin and variance combination using the ensemble distribution of 40% gamma and 60% mirrored Gumbel and implementing the loss function shown above. We weighted the errors in the variance optimisation algorithm based on the severity-specific disability weights, such that the severe anaemia error was weighted the most and total anaemia was weighted the least.

Fitting ensemble distributions with method of moments

Using our global model weights and our estimates of mean haemoglobin and standard deviation of haemoglobin, we modelled the full distribution of haemoglobin for each location, year, age, and sex by fitting each component distribution on the modelled mean and standard deviation and weighting each individual distribution to create the ensemble distribution:

$$\sum_{z=1}^{n_{final\ distributions}} w_z * PDF_z$$

where:

$z \in \text{final } distributions : 1 \leq z \leq n_{final\ distributions}$, the list of chosen distributions (40% gamma and 60% mirrored Gumbel)

Because anaemia thresholds depend on pregnancy, we separately modelled the distribution of haemoglobin among pregnant and non-pregnant women. Our modelling process directly provided estimates of mean haemoglobin and variance of haemoglobin concentration among non-pregnant women; to estimate the mean haemoglobin among pregnant women, we shifted the non-pregnant mean haemoglobin estimate for each location, year, age, and sex by the crosswalk adjustment factor

calculated, as described in the data processing section. We assumed that the distribution variance was the same among pregnant and non-pregnant women.

We used these estimates of mean haemoglobin and SD of haemoglobin concentration by pregnancy status to produce separate weighted ensemble distributions. To combine these distributions to represent the entire population of females in a given location, year, and age, we used the proportion of pregnant women and weighted each of the haemoglobin distributions among pregnant and non-pregnant women accordingly to produce a final general population distribution.

Finding the area under the curve to calculate anaemia prevalence

Using the WHO anaemia thresholds shown in Table 1, we calculated the prevalence of mild, moderate, and severe anaemia for each location, year, age group, and sex as the area under the [Hb] concentration curve between the thresholds for each severity.

Input data and methodological summary for anaemia causal attribution

Causes and inputs

Anaemia can arise as a result of many different diseases. Each round of GBD, as evidence is identified and synthesised, additional GBD causes are added to the GBD anaemia causal attribution analysis. Additional changes for specific causes are incorporated to either improve efficiency or reflect changes to modelling approaches for underlying causes. For GBD 2023, these cause changes included adding explicit estimates for puerperal sepsis.

For each cause included in GBD anaemia causal attribution, two inputs are required. The first required input is cause-level prevalence generated from other cause-specific GBD estimates, which are estimated by various teams across IHME. The second required input is a cause-specific haemoglobin shift.

Haemoglobin shifts

Cause-specific haemoglobin shift refers to changes in Hb levels caused by a specific condition or disease. This shift can be measured as the difference in Hb levels between individuals with and without the condition or as changes in Hb levels after successfully treating an infection. Estimating the Hb shift for a specific cause of anaemia allows us to calculate the counterfactual Hb distribution, representing the impact of the anaemia cause on a population’s overall Hb levels. In GBD 2021, cause-specific Hb shifts were derived from published literature, most of which have remained unchanged since GBD 2010, and reported as single, non-age-specific scalar values without uncertainties. Recognising that the effects of a disease may vary by age, sex, and pregnancy status, GBD 2023 incorporated individual-level insurance-claims data from the MarketScan research database to estimate age-, sex-, and pregnancy-specific Hb shifts associated with sickle cell disease (stratified by genotypes: HbSS, HbSC, and HbS-beta thalassemia), sickle cell trait, HIV, postpartum haemorrhage, and puerperal sepsis.

The MarketScan research database includes patient-level Hb lab results and ICD-10-coded physician and hospital visit data for commercially insured individuals in the USA. For our analysis, we included approximately 2.7 million individuals with at least one Hb measurement and six or more months of continuous insurance enrollment between 2016 and 2019. The six-month minimum ensured sufficient

data for observing exposures and outcomes. Anaemia-causing conditions and pregnancy status were identified using ICD-10 codes. To minimise coding errors, chronic conditions required codes to appear in at least one inpatient visit or two outpatient visits on different dates, and acute conditions required codes to appear at least once within a clinically relevant time frame.[7] Pregnancy episodes were determined using a published algorithm based on likely pregnancy outcomes,[8] where cases potentially associated with atypical maternal Hb dynamics, such as multiple gestations, ectopic pregnancies, or pregnancies ending in abortion, were excluded.

In brief, for each anaemia cause, we conducted coarsened exact matching (CEM) to match exposed and non-exposed individuals by age, sex, pregnancy status, and US state of residence. Using CEM-derived weights, we then calculated Hb shift as the weighted difference in mean Hb levels between exposed and non-exposed groups for each unique combination of age, sex, pregnancy status, and location. These weighted mean differences, along with their uncertainties, were used as inputs for MR-BRT meta-regression models, where we included random effects for location (random slopes and intercepts) and assumed non-linear relationships across age groups using splines. For sickle cell disease, we used a cascading spline approach: first, we modelled Hb shifts associated with total sickle cell disease (stage-1 model), then applied these parameters to genotype-specific models (eg, HbSS, HbSC, and HbS-beta thalassemia), incorporating sparse data with a theta value of 0.1 to ensure the stage-1 model had greater influence. For HbSS, HbSC, and sickle cell trait, we supplemented the analysis with Hb shift data from children under 5 years old in the DHS Nigeria 2018 dataset. For HIV, DHS data from India, Cambodia, and 24 countries in sub-Saharan Africa were also included. For postpartum haemorrhage (PPH) and puerperal sepsis, Hb shifts did not vary by age but differed by postpartum days. Therefore, the MR-BRT models for these causes included a spline term for postpartum days, where the area under the curve represented the total Hb shift. For the total population, this Hb shift was applied to women of reproductive age and averaged over one year. For the pregnant population, it was applied to the 39-week pregnancy period. For each anaemia cause, we selected model parameters—including spline type (cubic or domain-based), frequency, and knot placement—to capture biologically plausible trends in Hb shifts across age, sex, and pregnancy status (Figure 7). Note that for GBD 2023, only non-pregnancy-specific results were used.

For anaemia causes that were not yet analysed or could not be analysed using MarketScan data, the same literature-based Hb shift values from GBD 2021 were applied. As in GBD 2021, Hb shifts for residual causes—primarily “other” causes in the GBD hierarchy that are known or suspected to contribute to anaemia—were not explicitly estimated. Instead, anaemia burden for each of these causes was assigned from the residual anaemia envelope in a manner analogous to fixed proportion redistribution used in the GBD cause-specific mortality analysis, while ensuring 80.5% of all residuals were attributed to dietary iron deficiency.[9] Our objective is to systematically include all anaemia causes as specific inputs to GBD anaemia causal attribution, ultimately eliminating the need for residual attribution. Additionally, wherever possible, we aim to estimate age-, sex-, and pregnancy-specific Hb shifts for all anaemia causes using MarketScan and other data sources. Table 6 summarises the cause-specific Hb shift values used in GBD 2023.

Table 6: Causes included in GBD anaemia causal attribution and associated haemoglobin shifts. If GBD 2023 haemoglobin shift column is left blank, then the haemoglobin shift from GBD 2021 was used in this

analysis. If GBD 2023 haemoglobin shift column is filled in, context on MR-BRT analysis in regards to the new shifts are present.

Causes with prevalence and haemoglobin shift inputs	Haemoglobin shift (g/L) – GBD 2021	Haemoglobin shift (g/L) – GBD 2023
HIV/AIDS	Females = –7.0 g/L<br>Males = –9.7 g/L	Average Hb shift (g/L) - MarketScan + DHS:<br>- Male: –3.3 (–3.8 to –2.8)<br>- Pregnant female: –2.2 (–2.9 to –1.6)<br>- Non-pregnant female: –5.2 (–5.7 to –4.7)
Malaria<br>*P falciparum* parasitaemia without clinical malaria<br>*P vivax* parasitaemia without clinical malaria<br>Clinical malaria	Both sexes = –8.37 g/L	
Malaria<br>*P vivax* parasitaemia without clinical malaria	Both sexes = –11.3 g/L	
Malaria<br>Clinical malaria	Both sexes = –20.0 g/L	
Schistosomiasis	Both sexes = –2.5 g/L	
Hookworm disease	Both sexes = –2.08 g/L	
Maternal haemorrhage	Females = –6.8 g/L	Average Hb shift (g/L)<br>- *MarketScan*:<br>o Total population: –2.8 (–9.1 to 3.5)<br>o Pregnant population: –1.5 (–2.4 to –0.5)
Maternal puerperal sepsis	*Not modelled*	Average Hb shift (g/L)<br>- *MarketScan*:<br>o Total population: –3.9 (–8.2 to 0.2)<br>o Pregnant population: –1.7 (–4.1 to 0.6)
Vitamin A deficiency (under 15 years only)	Both sexes = –3.6 g/L	
Cirrhosis and other chronic liver diseases, decompensated	Cirrhosis, both sexes = –18 g/L<br>Other, both sexes = –15 g/L	
Peptic ulcer disease	Both sexes = –15.11 g/L	
Gastritis	Both sexes = –15.11 g/L	
Ulcerative colitis	Both sexes = –20 g/L	
Crohn's disease	Both sexes = –23 g/L	
Chronic kidney disease<br>Stage III chronic kidney disease	CKD3, females = –16.34 g/L<br>CKD3, males = –11.84 g/L	
Chronic kidney disease<br>Stave IV chronic kidney disease	CKD4, females = –27.82 g/L<br>CKD4, males = –21.83 g/L	
Chronic kidney disease<br>Stage V chronic kidney disease	CKD5, females = –37.62 g/L<br>CKD5, males = –32.43 g/L	
Chronic kidney disease<br>End-stage renal disease	ESRD, both sexes = –30.7 g/L	

Uterine fibroids	Females = –13.7 g/L	
Menstrual disorders	Females = –3.0 g/L	
Thalassaemias<br>Beta-thalassaemia major	Both sexes = –70 g/L	
Thalassaemias<br>Haemoglobin E/beta-thalassaemia	Both sexes = –64 g/L	
Thalassaemias<br>Haemoglobin H disease	Both sexes = –56 g/L	
Thalasasemias trait<br>Beta-thalassaemia trait	Both sexes = –19 g/L	
Thalasasemias trait<br>Haemoglobin E trait	Both sexes = –6 g/L	
Sickle cell disorders<br>Homozygous sickle cell and severe sickle cell/beta-thalassaemia parent	Both sexes = –54 g/L	Average Hb shift (g/L)<br>- MarketScan + DHS:<br>○ Male: –31 (–34 to –28)<br>○ Pregnant female: –17 (–20 to –14)<br>○ Non-pregnant female: –26 (–28 to –23)
Sickle cell disorders<br>Haemoglobin SC disease	Both sexes = –36 g/L	Average Hb shift (g/L) - MarketScan + DHS:<br>○ Male: –22 (–25 to –20)<br>○ Pregnant female: –25 (–27 to –23)<br>○ Non-pregnant female: –15 (–21 to –10)
Sickle cell disorders<br>Mild sickle cell/beta-thalassaemia	Both sexes = –52 g/L	Average Hb shift (g/L) - MarketScan + DHS:<br>○ Male: –29 (–31 to –26)<br>○ Pregnant female: –21 (–24 to –18)<br>○ Non-pregnant female: –21 (–26 to –16)
Sickle cell trait	Both sexes = –5 g/L	Average Hb shift (g/L) - MarketScan + DHS:<br>○ Male: –9.2 (–12.5 to –7.4)<br>○ Non-preg female: –10.1 (–13.5 to –8.3)<br>○ Preg female: –6.2 (–9.1 to –3.8)
G6PD deficiency	Females = –0.074 g/L<br>Males = –0.152 g/L	
Hemizygous G6PD deficiency	Females = –0.016 g/L	
Thyroid diseases<br>Hyperthyroid disease	Both sexes = –4.2 g/L	
Thyroid diseases<br>Hypothyroid disease	Both sexes = –2.8 g/L	
**Estimated via fixed proportion redistribution methods* in GBD anaemia causal attribution**		
Other neglected tropical diseases	Both sexes = –3.0 g/L	

Other infectious diseases	Both sexes = –3.0 g/L	
Dietary iron-deficiency	Both sexes = –4.01 g/L	
Other haemoglobinopathies and haemolytic anaemias	Both sexes = –3.0 g/L	
Other endocrine, nutrition, blood, and immune disorders	Both sexes = –15 g/L	

**A minimum of 10% of all anaemia was assigned to residual categories based on analysis of NHANES-III data from the USA.*

Figure 7: MR-BRT outputs for haemoglobin shift analyses for a) sickle cell diseases by genotype, b) sickle cell trait, c) HIV, and d) postpartum haemorrhage and puerperal sepsis.

Figure 7a. Sickle cell diseases by genotype

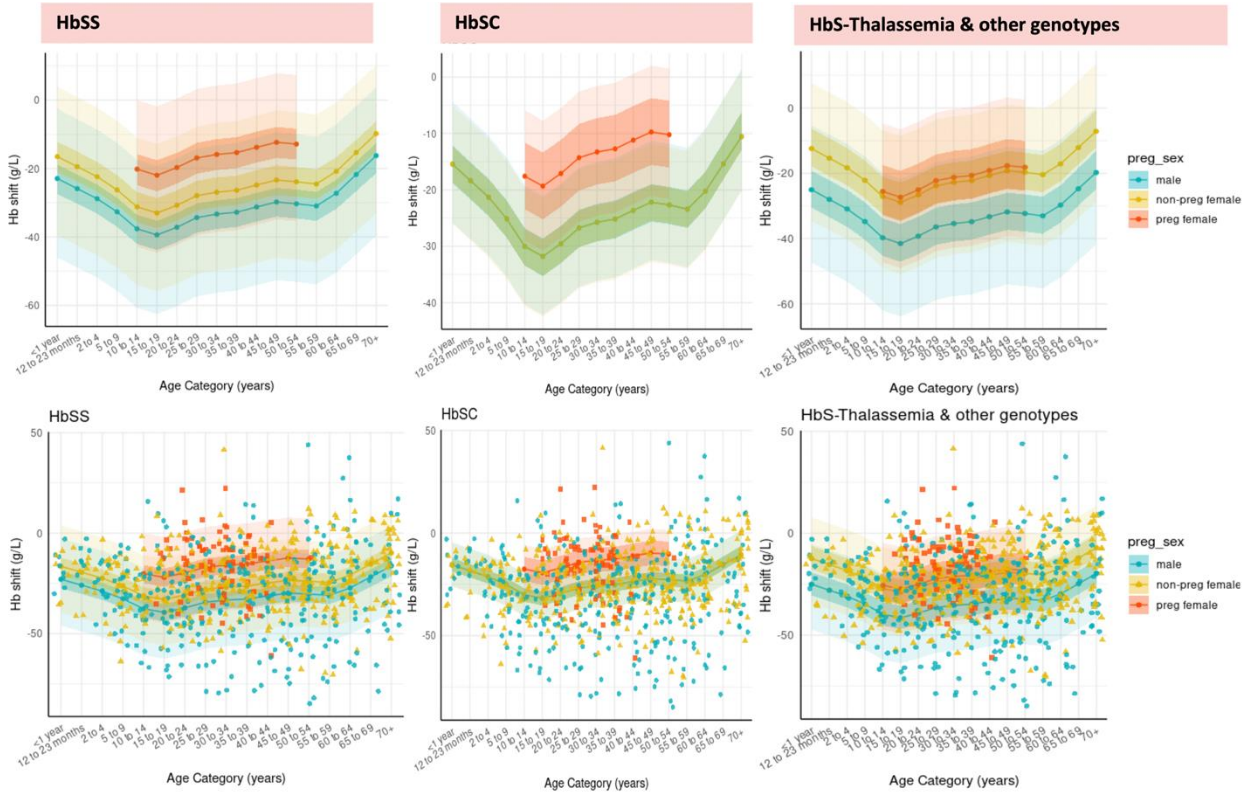

Figure 7b. Sickle cell trait

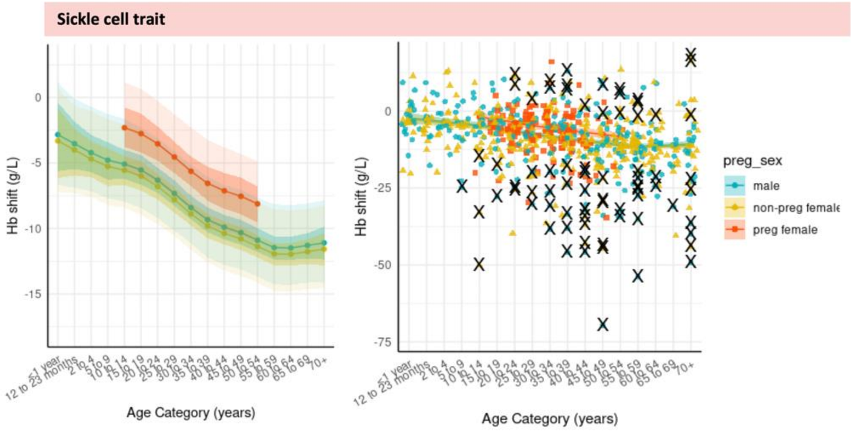

Figure 7c. HIV

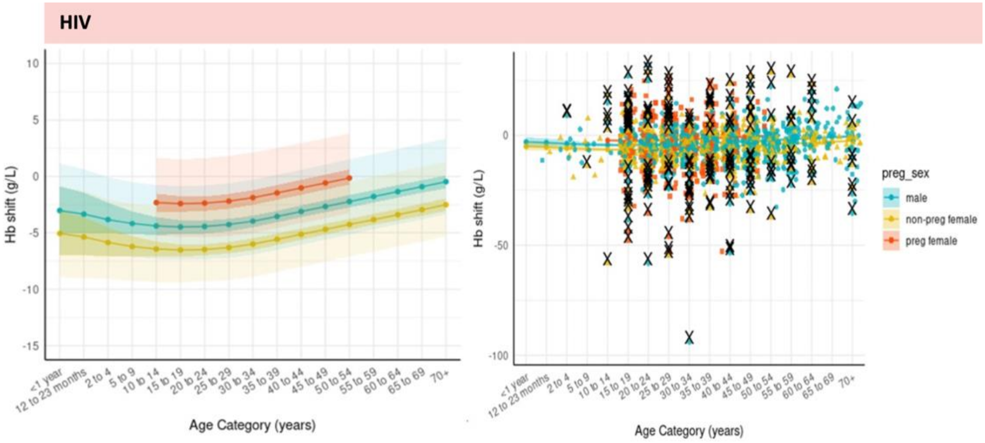

Figure 7d. Postpartum haemorrhage and puerperal sepsis

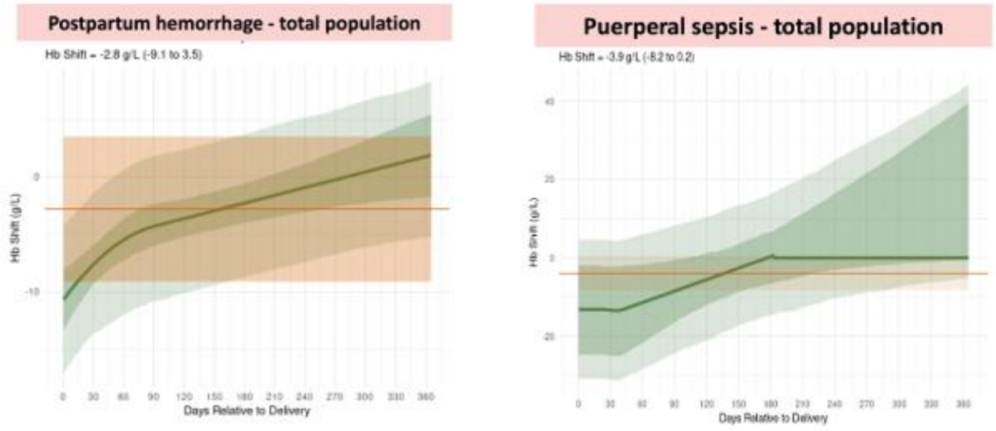

Modelling strategy

To estimate anaemia prevalence attributable to each cause, we used the modelled full distribution of haemoglobin for each location, year, age, and sex combined with the cause-specific prevalence and haemoglobin shift associated with each cause. We multiplied the haemoglobin shift by the estimated cause prevalence, returning a prevalence-weighted shift in the haemoglobin distribution specific to each cause, location, year, age, and sex. We then shifted our mean haemoglobin estimate by this cause prevalence-weighted haemoglobin shift and re-estimated the haemoglobin distribution, using the same distribution variance value. We calculated the difference in anaemia prevalence between our original distribution and this shifted counterfactual distribution for each anaemia severity, and this difference was the estimated prevalence of anaemia attributable to a given cause.

Whenever the sum of the cause-specific anaemia prevalences was greater than the initial estimated anaemia prevalence, we proportionally squeezed the estimated cause-specific anaemia prevalences to sum to 90% of the total anaemia prevalence envelope. As noted in Table 7, we reserved a minimum of 10% of all anaemia to be assigned to residual causes based on a review of findings from NHANES in the USA.[4,5] The residual envelope was distributed among the remaining four causes.

For all causes with population-specific prevalence estimates, we enforced a similar condition where the sum of mild, moderate, and severe cause-specific anaemia would not exceed the total cause prevalence within each population. We thus scaled the results to ensure the sum across anaemia severity matched total cause prevalence for each cause.

It is important to take note of the difference between "dietary iron deficiency" as a GBD cause and "iron deficiency" as a GBD risk. Many GBD causes lead to anaemia that clinically manifests as iron deficiency (or microcytosis), but where inadequate intake is not the underlying problem. Examples include neglected tropical diseases such as hookworm and schistosomiasis, malaria, gastrointestinal disorders, cirrhosis, maternal haemorrhage, menstrual disorders, uterine fibroids, and vitamin A deficiency. The name "dietary iron deficiency" is intended to differentiate, therefore, between inadequate intake and haemorrhagic or iron metabolism disorders. Additionally, because we have yet to include 100% of anaemia causes, estimates should be interpreted to also include some acute and chronic haemorrhagic states for which supplementation may be helpful but poor nutritional intake is not the only underlying problem. Examples include malabsorption syndromes, other micronutrient deficiencies besides vitamin A deficiency, and injuries with associated acute blood loss anaemia. "Iron deficiency" exposure as estimated for the GBD risk factors analysis, in contrast, includes a combined assessment of the magnitude of haematologic insult from all causes that manifest as iron deficiency. As mentioned above, our goal is to systematically add all causes of anaemia as specific inputs to GBD anaemia causal attribution, including inadequate iron intake, and eliminate the need for residual attribution.

The anaemia causal attribution process produces estimates for mild, moderate, and severe anaemia due to HIV. Using these estimates, we calculated proportions of HIV with mild, moderate, severe, and no anaemia for each demographic group. GBD produces estimates for seven HIV sub-causes: early HIV, symptomatic HIV, AIDS with antiretroviral treatment, AIDS without antiretroviral treatment, drug-sensitive HIV/AIDS–tuberculosis, multidrug-resistant HIV/AIDS–tuberculosis, and extensively drug-resistant HIV/AIDS–tuberculosis. We assumed the anaemia severity proportions were equivalent across

the seven sub-causes and estimated the anaemia severity levels for each by multiplying the HIV sub-causes by the anaemia proportions.

Changes from GBD 2021 to GBD 2023

The substantive changes to the modelling strategy for GBD 2023 include:

- Applied elevation-adjustments to haemoglobin using the new WHO elevation adjustment method published in 2023, which caused anaemia prevalence values to be higher for all modelled locations below 3000 meters for all estimated ages, sexes, and years.
- Calculated and assigned population-weighted mean elevations to surveys if elevation was not reported using the assigned GBD location for the given survey.
- Implemented new crosswalk steps to age-sex split means reported in tabulated sources that span multiple GBD age and/or sex categories and to impute missing measures among the various elevation-adjustment envelopes.
- Utilised the stage-1 linear prior functionality within ST-GPR and passed in only three of the 13 covariates used in GBD 2021 to model mean haemoglobin and the various severities of anaemia prevalence, which were 1) median haemoglobin by age and sex, 2) Healthcare Access and Quality Index by GBD location and year, and 3) summary exposure values for nutrition wasting in children under 5 years old.
- Updated haemoglobin shift values for a subset of the input causes that are used in the anaemia causal attribution pipeline.

References

1. Guideline on haemoglobin cutoffs to define anaemia in individuals and populations. Geneva: World Health Organization; 2024. Licence: CC BY-NC-SA 3.0 IGO.
2. Sharma, A.J., Addo, O.Y., Mei, Z. and Suchdev, P.S. (2019), Reexamination of hemoglobin adjustments to define anemia: altitude and smoking. *Ann. N.Y. Acad. Sci*., 1450: 190-203.
3. Bill and Melinda Gates Foundation (BMGF), Centers for Disease Control and Prevention (CDC), International Micronutrient Malnutrition Prevention and Control (IMMPaCt) Program, Nutrition International, World Health Organization (WHO). WHO Global Micronutrients Database . Geneva, Switzerland: World Health Organization (WHO).
4. Hastings, D. and P.K. Dunbar. National Geophysical Data Center, 1999. Global Land One-kilometer Base Elevation (GLOBE) v.1. National Geophysical Data Center, NOAA. doi:10.7289/V52R3PMS
5. Geography and Environmental Science, University of Southampton, WorldPop. Age and Sex Structures, Global Per Country 2000-2020. Southampton, United Kingdom: WorldPop, 2018.
6. Zheng, P., Barber, R., Sorensen, R. J. D., Murray, C. J. L., & Aravkin, A. Y. (2021). Trimmed Constrained Mixed Effects Models: Formulations and Algorithms. *Journal of Computational and Graphical Statistics*, 30(3), 544–556. https://doi.org/10.1080/10618600.2020.1868303
7. Johnson, K. M. et al. Development of a conceptual model for evaluating new non-curative and curative therapies for sickle cell disease. *PLOS ONE* 17, e0267448 (2022).
8. Sarayani, A. et al. Impact of the Transition from ICD–9–CM to ICD–10–CM on the Identification of Pregnancy Episodes in US Health Insurance Claims Data. *Clin Epidemiol* 12, 1129–1138 (2020).
9. Looker AC, Dallman PR, Carroll MD, Gunter EW, Johnson CL. Prevalence of iron deficiency in the United States. *JAMA* 1997; 277: 973–6.

Anorexia nervosa

Flowchart

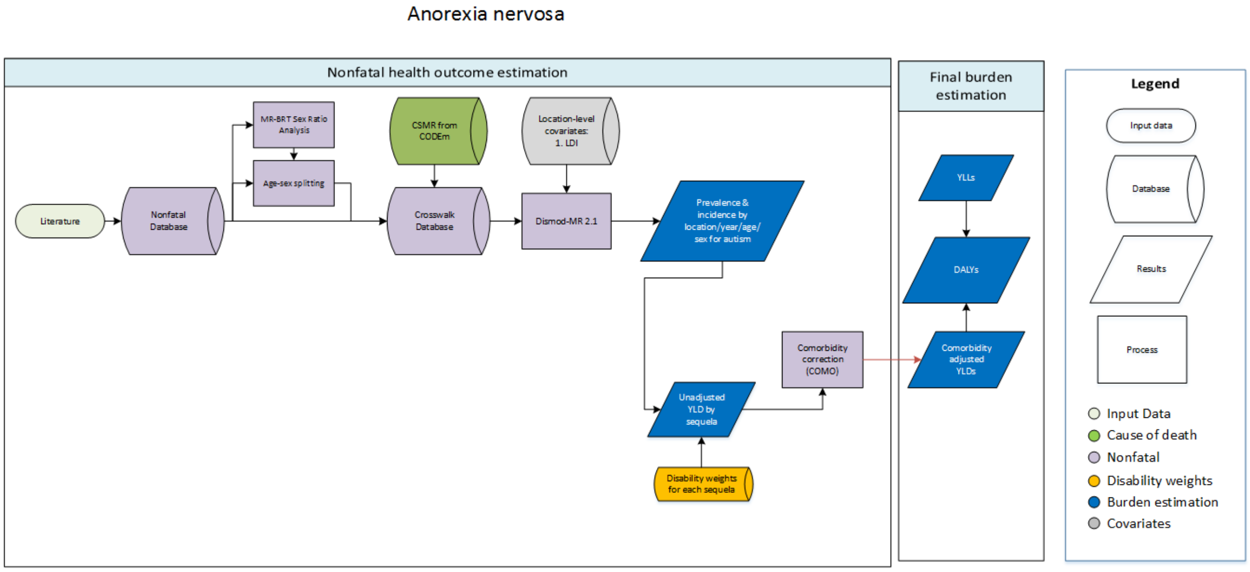

Input data and methodological summary for anorexia nervosa

Case definition

According to the Diagnostic and Statistical Manual of Mental Disorders fifth edition (DSM-5),[1] anorexia nervosa (AN) is an eating disorder characterised by:

a) Restriction of energy intake relative to requirements, leading to a significantly low body weight in the context of age, sex, developmental trajectory, and physical health. Significantly low weight is defined as a weight that is less than minimally normal or, for children and adolescents, less than that minimally expected.
b) Intense fear of gaining weight or becoming fat, or persistent behaviour that interferes with weight gain, even though at a significantly low weight.
c) Disturbance in the way in which one's body weight or shape is experienced, undue influence of body weight or shape on self-evaluation, or persistent lack of recognition of the seriousness of the current low body weight.

Included in the GBD study were cases meeting diagnostic criteria according to DSM[1] or the International Classification of Diseases (ICD; ICD-10 code F50.0).[2] Different versions of DSM (DSM-III, DSM-III-R, DSM-IV, DSM-IV-TR, DSM-5, and DSM-5-TR) and ICD (ICD-9, ICD-10 and ICD-11) were accepted.

Input data

Systematic literature reviews were conducted to capture studies reporting the prevalence, incidence, remission, and excess mortality of AN. The epidemiological systematic literature review for AN was conducted in three stages, involving electronic searches of the peer-reviewed literature (ie, via PsycInfo, Embase, and PubMed), the grey literature, and expert consultation. For mental disorders, we update our GBD electronic database searches on a rolling basis. A systematic review update was completed for GBD

2023 for eating disorders combined. Databases were searched on June 5, 2023, for publications after January 1, 2017. Below are the search terms used for each database:

PubMed: ("anorexia nervosa"[Title/Abstract] OR "bulimi* "[Title/Abstract] OR "Eating disorders"[Title/Abstract] OR "Eating disorder"[Title/Abstract] OR "Anorexic"[Title/Abstract] OR OSFED[Title/Abstract] OR “purging disorder”[Title/Abstract] OR "Feeding and Eating Disorders"[MeSH Terms:noexp] OR "anorexia nervosa"[MeSH Terms] OR "Binge-Eating Disorder"[MeSH Terms] OR "bulimia nervosa"[MeSH Terms]) AND (((((((((((((prevalen*[Title/Abstract]) OR (mortalit*[Title/Abstract])) OR (death*[Title/Abstract])) OR (inciden*[Title/Abstract])) OR (inciden*[Title/Abstract])) OR (remission[Title/Abstract])) OR (duration[Title/Abstract])) OR (remit*[Title/Abstract])) OR (epidemiolog*[Title/Abstract])) OR ("Prevalence"[Mesh])) OR ("Mortality"[Mesh])) OR ("Incidence"[Mesh])) OR ("Epidemiology"[Mesh:NoExp])) OR ("Morbidity"[Mesh:NoExp]))

Embase: ('anorexia nervosa':ab,ti OR bulimi*:ab,ti OR 'eating disorder':ab,ti OR 'eating disorders':ab,ti OR 'anorexic':ab,ti OR OSFED:ab,ti OR ‘purging disorder’:ab,ti OR 'eating disorder'/de OR 'anorexia nervosa'/exp OR 'binge eating disorder'/exp OR 'bulimia'/exp OR 'purging disorder'/exp) AND (prevalen*:ab,ti OR mortalit*:ab,ti OR death*:ab,ti OR inciden*:ab,ti OR remission:ab,ti OR duration:ab,ti OR remit*:ab,ti OR epidemiolog*:ab,ti OR 'prevalence'/exp OR 'epidemiology'/exp OR 'remission'/exp)

PsycInfo: (TI "anorexia nervosa" OR AB "anorexia nervosa" OR TI bulimi* OR AB bulimi* OR TI "eating disorders" OR AB "eating disorders" OR TI "eating disorder" OR AB "eating disorder" OR TI anorexic OR AB anorexic OR TI OSFED OR AB OSFED OR TI “purging disorder” OR AB “purging disorder” OR DE "Eating Disorders" OR DE "Anorexia Nervosa" OR DE "Binge Eating Disorder" OR DE "Bulimia" OR DE "Purging (Eating Disorders)") AND (TI prevalen* OR AB prevalen* OR TI mortalit* OR AB mortalit* OR TI death* OR AB death* OR TI inciden* OR AB inciden* OR TI remission OR AB remission OR TI duration OR AB duration OR TI remit* OR AB remit* OR TI epidemiolog* OR AB epidemiolog* OR DE "Mortality Rate" OR DE "Epidemiology" OR DE "Morbidity" OR DE "Remission (Disorders)")

In addition to the database search, a grey literature search and expert consultation were conducted. The systematic review update was conducted according to Preferred Reporting Items for Systematic Reviews and Meta-Analyses (PRISMA; see Figure 1).

Figure 1: PRISMA 2009 flow diagram

From: Moher D, Liberati A, Tetzlaff J, Altman DG, The PRISMA Group (2009). *P*referred *R*eporting *I*tems for *S*ystematic Reviews and *M*eta-*A*nalyses: The PRISMA Statement. PLoS Med 6(7): e1000097. doi:10.1371/journal.pmed1000097

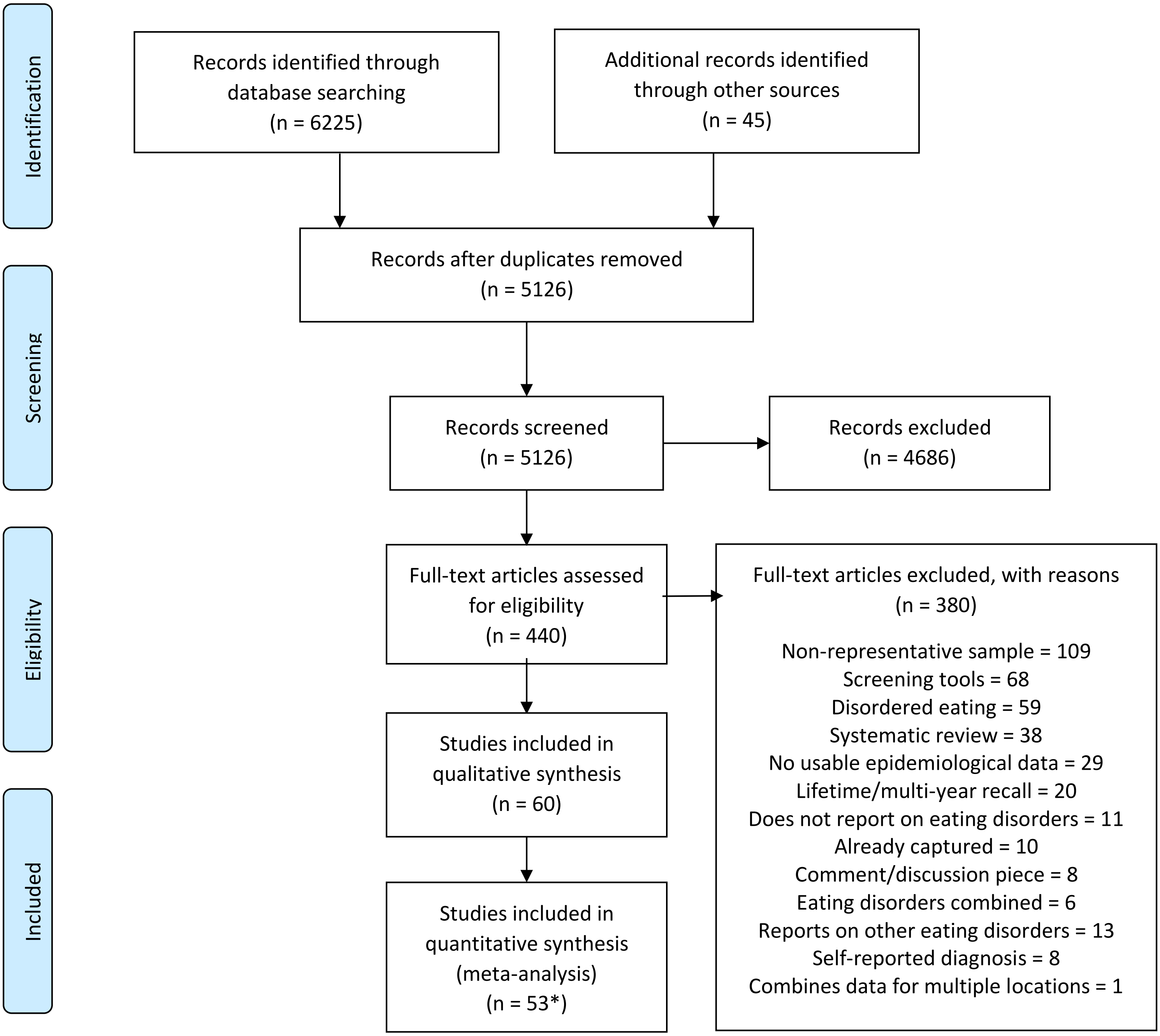

**The qualitative analysis led to the exclusion of additional studies with duplicative cohorts or methodological limitations impacting their eligibility and increasing measurement error within the data.*

The GBD inclusion criteria stipulated that: 1) the publication year must be from 1980 onward; 2) "caseness" must be based on clinical threshold as established by the DSM or ICD; 3) sufficient information must be provided on study method and sample characteristics to assess the quality of the study; and 4) study samples must be representative of the general population (ie, inpatient or pharmacological treatment samples, case studies, veterans, or refugee samples were excluded). No limitation was set on the language of publication. Methods used for this systematic review have been reported in greater detail elsewhere.[3]

Age-sex splitting

The extracted data underwent three types of age-sex splitting processes:

1. Where possible, estimates were further split by sex and age based on the available data. For instance, if studies reported prevalence for broad age groups by sex (eg, prevalence in 15–65-year-old males and females separately), and also by specific age groups but for both sexes combined (eg, prevalence in 15–30-year-olds, then in 31–65-year-olds, for males and females combined); age-specific estimates were split by sex using the reported sex-ratio and bounds of uncertainty.
1. A meta-regression—Bayesian, regularised, trimmed (MR-BRT) analysis was used to split the remaining both-sex estimates in the dataset. For each parameter, sex-specific estimates were matched by location, age, and year. A MR-BRT network meta-analysis was then used to estimate pooled sex ratios and bounds of uncertainty. These were then used to split the both-sex estimates in the dataset. The male-to-female prevalence ratio estimated was 0.29 (95% uncertainty interval [UI]: 0.16–0.54).
2. Studies reporting prevalence estimates across age groups spanning 25 years or more were split into five-year age groups using the prevalence age pattern estimated by DisMod-MR 2.1. The DisMod-MR model used to estimate the age pattern did not contain any previously age split data.

Bias corrections/crosswalks

We tested for a number of potential sources for bias in prevalence between studies, including use of ICD versus DSM diagnostic criteria, previous iterations of the DSM diagnostic criteria (eg, DSM-III, DSM-IV) versus new iterations of the DSM diagnostic criteria (ie, DSM-5), and past-year versus point recall. However, none of the crosswalks explored had a statistically significant impact on prevalence and therefore no bias corrections were applied to these estimates.

Modelling strategy

After the above data processes were applied, DisMod MR 2.1 was used to model the epidemiological data for AN. Adjustments to model priors or the dataset were made where appropriate. Where outliers were identified in the data, we reassessed the study's methodology and quality before a decision was made to exclude or include the data.

We assumed no incidence prior to age 5 or from 50 years onward. These settings are in line with those placed on the corresponding cause of death model for AN. A cap of 0.6 was placed on remission in order to obtain a more plausible fit of the model. For GBD 2023, we removed the cause-specific mortality rate (CSMR) data from our CODEm and CoDCorrect analysis from our DisMod-MR modelling and instead allowed DisMod-MR to follow other mortality data (standardised mortality ratios and relative risks)

sourced from the systematic literature reviews, which improved model fit for some locations. A country-level covariate, lagged distributed income (LDI), was included. This covariate represents a moving average of gross domestic product (GDP) over time. The limits placed on this covariate meant that prevalence was assumed to increase with rising GDP. LDI was also applied to excess mortality data in order to better inform regional distribution. A summary of location-level covariates and exponentiated values for AN are shown in Table 1.

Table 1: Summary of covariates used in the AN DisMod-MR meta-regression model

Covariate	Type	Parameter	Exponentiated beta (95% UI)
LDI ($ per capita)	Location-level	Prevalence	1.53 (1.39–1.64)
LDI ($ per capita)	Location-level	Excess mortality	0.74 (0.62–0.89)

Disability weight

The GBD disability weight survey assessments include lay descriptions of sequelae highlighting major functional consequences and symptoms. No severity splits were applied to AN. The lay description and disability weight for AN are shown in Table 2.

Table 2: Lay description for AN in GBD 2023 and the associated disability weight

Lay description	Disability weight (95% UI)
Feels an overwhelming need to starve and exercises excessively to lose weight. The person is very thin, weak, and anxious.	0.224 (0.150–0.312)

There were no significant changes in GBD 2023 results for AN compared to GBD 2021. While we continue to improve on the data and methods used to estimate the burden of mental disorders, some challenges need to be acknowledged. Firstly, we still have a large number of locations with no high-quality raw data available. Secondly, it is difficult to quantify and remove all variation due to measurement error in our epidemiological estimates. While we have improved the methodology used to account for known sources of bias in some cases, we still have very few datapoints to inform these adjustments. Thirdly, there is a paucity of research on the risk factors of mental disorders which can be used as predictive covariates in our epidemiological models.

References

1. American Psychiatric Association. Diagnostic and Statistical Manual of Mental Disorders, Fifth Edition. Arlington, VA: American Psychiatric Association; 2013.
2. World Health Organization. ICD-10 Classification of Mental and Behavioural Disorders: Clinical descriptions and diagnostic guidelines. Geneva: World Health Organization; 1992.
3. Ferrari AJ, Charlson FJ, Norman RE, et al. Burden of depressive disorders by country, sex, age, and year: findings from the global burden of disease study 2010. *PLoS Med* 2013; **10**(11): e1001547.

Anxiety disorders

Flowchart

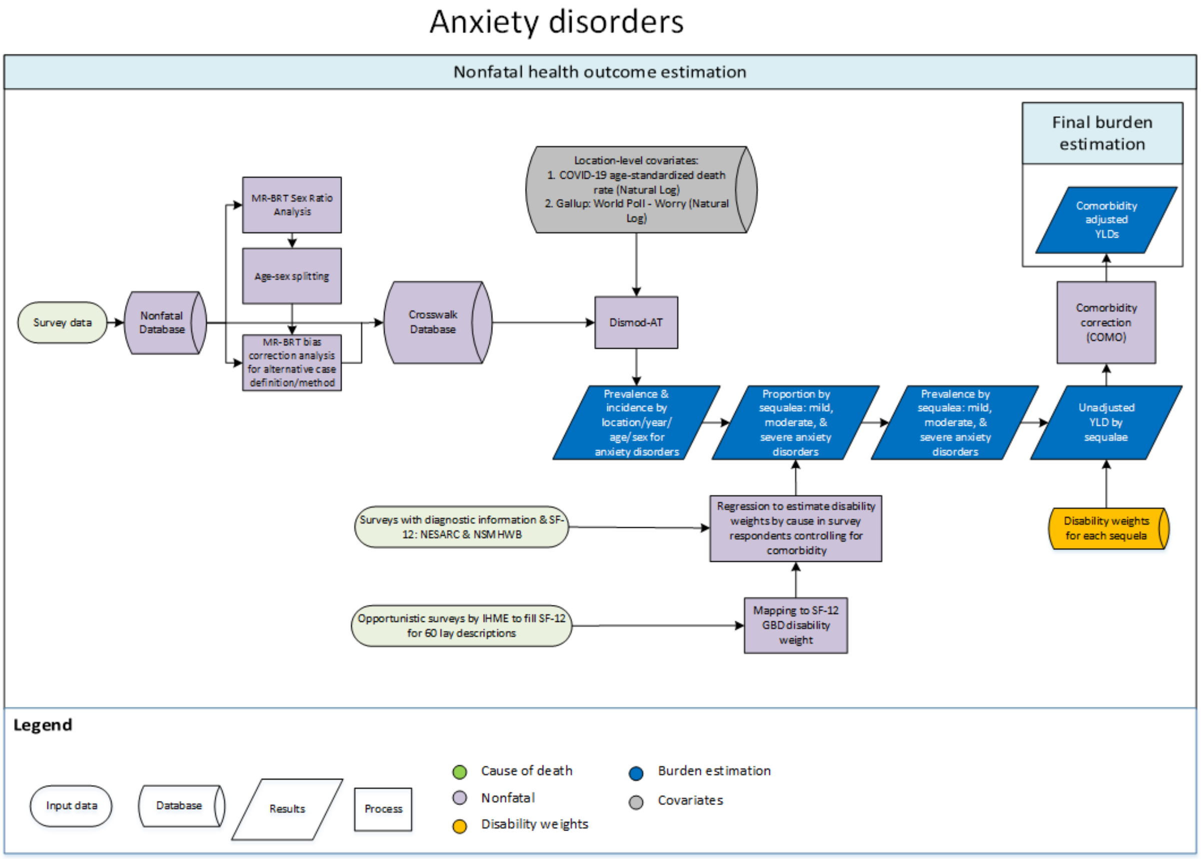

Input data and methodological summary for anxiety disorders

Case definition

Anxiety disorders are characterised by experiences of intense fear and distress, typically in combination with other physiological symptoms. We aimed to capture all cases of anxiety disorders reaching diagnostic threshold defined by the Diagnostic and Statistical Manual of Mental Disorders (DSM) or the World Health Organization (WHO) International Classification of Diseases (ICD).[1,2] The specific anxiety disorders included were panic disorder, agoraphobia, specific phobia, social phobia, obsessive-compulsive disorder (OCD), post-traumatic stress disorder (PTSD), generalised anxiety disorder (GAD) including overanxious disorder in childhood, separation anxiety disorder (SAD), and anxiety disorder "not otherwise specified" (NOS). These were identified by the following ICD-10 codes: F40-42, F43.0, F43.1, F93.0-93.2, F93.8. Excluded were anxiety disorders due to a general medical condition and substance-induced anxiety disorder. Different versions of DSM (DSM-III, DSM-III-R, DSM-IV, DSM-IV-TR, DSM-5, and DSM-5-TR) and ICD (ICD-9, ICD-10, and ICD-11) were accepted.

Anxiety disorders were modelled as a single cause for "any" anxiety disorder to avoid the double-counting of individuals meeting criteria for more than one anxiety disorder. Epidemiological estimates reporting an outcome for "any" or "total" anxiety disorders were included in analyses, if they reported on at least three anxiety disorders. This has been further explained in previous publications.[3,4]

Input data

The epidemiological systematic literature review for anxiety disorders was conducted in three stages involving electronic searches of the peer-reviewed literature (ie, via PsycInfo, Embase, and PubMed), the grey literature, and expert consultation. For mental disorders, we update our GBD electronic database searches on a rolling basis. A systematic review update for anxiety disorders was completed for GBD 2023. Databases were searched on March 17, 2021, for publications after May 18, 2018. Below are the search terms used for each database:

PubMed: (("panic disorder"[Title/Abstract] OR "panic disorders"[Title/Abstract] OR "social phobia"[Title/Abstract] OR "social phobias"[Title/Abstract] OR "specific phobia"[Title/Abstract] OR "specific phobias"[Title/Abstract] OR "agoraphobia"[Title/Abstract] OR "agoraphobias"[Title/Abstract] OR "GAD"[Title/Abstract] OR "selective mutism"[Title/Abstract] OR "Panic attack specifier"[Title/Abstract] OR "anxiety disorder"[Title/Abstract] OR "anxiety disorders"[Title/Abstract] OR "anxiety disorders"[MeSH Terms] OR "obsessive compulsive disorder"[Title/Abstract] OR "obsessive compulsive disorders"[Title/Abstract] OR "OCD"[Title/Abstract] OR "post traumatic stress disorder"[Title/Abstract] OR "post traumatic stress disorders"[Title/Abstract] OR "traumatic stress"[Title/Abstract] OR "PTSD"[Title/Abstract] OR "stress disorders, post traumatic"[MeSH Terms]) AND ("prevalen*"[Title/Abstract] OR "prevalence"[MeSH Terms] OR "inciden*"[Title/Abstract] OR "incidence"[MeSH Terms] OR "remit*"[Title/Abstract] OR "remission"[Title/Abstract] OR "recurrence"[MeSH Terms:noexp] OR "recurren*"[Title/Abstract] OR "duration"[Title/Abstract] OR "mortality"[Title/Abstract] OR "death*"[Title/Abstract] OR "mortality"[MeSH Terms] OR "epidemiolog*"[Title/Abstract] OR "Epidemiology"[MeSH Major Topic] OR "Epidemiology"[MeSH Terms:noexp] OR "Morbidity"[MeSH Terms:noexp]))

Embase: ('agoraphobia':ab,ti OR 'obsessive compulsive disorder':ab,ti OR 'post traumatic stress disorder':ab,ti OR 'anxiety disorder':ab,ti OR 'anxiety disorders':ab,ti OR 'anxiety disorder'/exp) AND (prevalen*:ab,ti OR mortalit*:ab,ti OR death*:ab,ti OR inciden*:ab,ti OR remission:ab,ti OR recurren*:ab,ti OR duration:ab,ti OR remit*:ab,ti OR epidemiolog*:ab,ti OR 'incidence'/exp OR 'mortality'/exp OR 'morbidity'/mj OR 'remission'/exp OR 'prevalence'/exp OR 'epidemiology'/exp)

PsycInfo: (TI panic disorder OR AB panic disorder) OR (TI post traumatic stress disorder OR AB post traumatic stress disorder) OR (TI posttraumatic stress disorder OR ptsd OR AB posttraumatic stress disorder or ptsd) OR (TI social phobia OR AB social phobia) OR (TI agoraphobia OR AB agoraphobia) OR (TI obsessive compulsive disorder OR ocd OR AB obsessive compulsive disorder OR ocd) OR (TI specific phobia OR AB specific phobia) OR (TI gad OR AB gad) OR (TI anxiety disorders OR AB anxiety disorders) AND (TI prevalen* OR AB prevalen*) OR (TI mortalit* OR AB mortalit*) OR (TI death* OR AB death*) OR (TI inciden* OR AB inciden*) OR (TI recurren* OR AB recurren*) OR (TI remission OR AB remission) OR (TI duration OR AB duration) OR (TI remit* OR AB remit*) OR (TI epidemiolog* OR AB epidemiolog*) OR (DE "Epidemiology")

In addition to the database search, a grey literature search and expert consultation were also conducted. The systematic review update was conducted according to Preferred Reporting Items for Systematic Reviews and Meta-Analyses (PRISMA; see Figure 1).

Figure 1: PRISMA flow diagram

From: Moher D, Liberati A, Tetzlaff J, Altman DG, The PRISMA Group (2009). *P*referred *R*eporting *I*tems for *S*ystematic Reviews and *M*eta-*A*nalyses: The PRISMA Statement. PLoS Med 6(7): e1000097. doi:10.1371/journal.pmed1000097

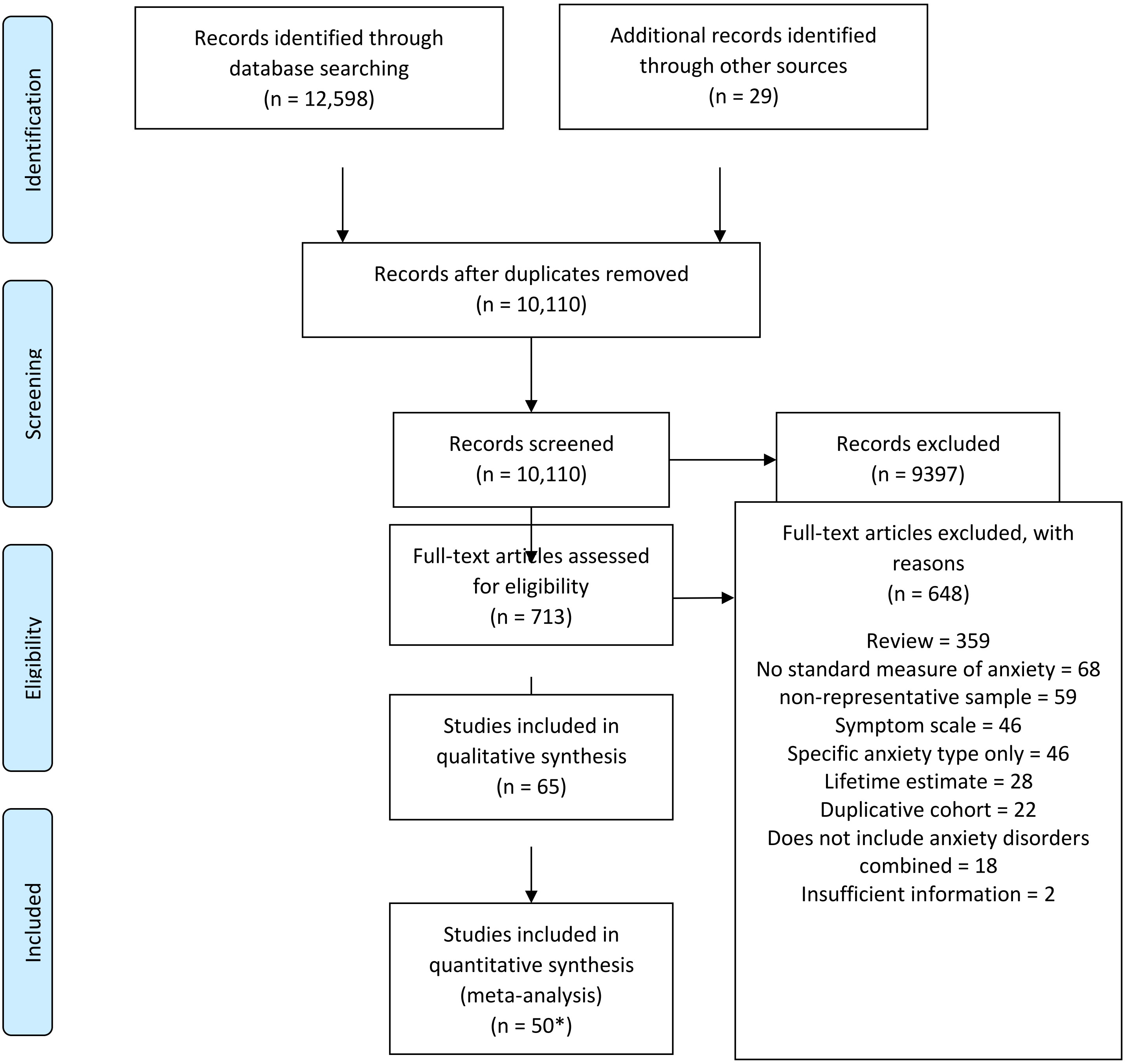

**The qualitative analysis led to the exclusion of additional studies with duplicative cohorts or methodological limitations impacting their eligibility and increasing measurement error within the data.*

The GBD inclusion criteria stipulated that: 1) the publication year must be from 1980 onward; 2) "caseness" must be based on clinical threshold as established by the DSM or ICD; 3) sufficient information must be provided on study method and sample characteristics to assess the quality of the study; 4) a minimum of three (or two if occurring during childhood) anxiety disorder subtypes must be included within the overall estimate; and 5) study sample must be representative of the

general population (ie, inpatient or pharmacological treatment samples, case studies, veterans, or refugee samples were excluded). No limitation was set on the language of publication. Methods used in this systematic review have been reported in greater detail elsewhere.[3,4]

Age-sex splitting

The extracted data underwent the following age-sex splitting processes:

1. Where possible, estimates were further split by sex and age based on the available data. For instance, if studies reported prevalence for broad age groups by sex (eg, prevalence in 15–65-year-old males and females separately), and also by specific age groups but for both sexes combined (eg, prevalence in 15–30-year-olds, then in 31–65-year-olds, for males and females combined); age-specific estimates were split by sex using the reported sex-ratio and bounds of uncertainty.
2. A meta-regression–Bayesian, regularised, trimmed (MR-BRT) analysis was used to split the remaining both-sex estimates in the dataset. For each parameter, sex-specific estimates were matched by location, age, and year. A MR-BRT network meta-analysis was then used to estimate pooled sex ratios and bounds of uncertainty. These were then used to split the both-sex estimates in the dataset. The male-to-female prevalence ratio estimated was 0.55 (95% uncertainty interval [UI]: 0.53–0.56).

Bias corrections/crosswalks

Estimates with known biases were adjusted/crosswalked accordingly prior to DisMod-AT. For each crosswalk of interest, pairs of the reference and the alternative estimates were matched by age, sex, location, and year. This was done for both within-study (where possible) and between-study pairs. These pairs were then used as inputs in a MR-BRT network meta-analysis. The MR-BRT analysis produced a pooled ratio between the reference estimates and alternative estimates, which was used to adjust all alternative estimates in the dataset. For anxiety disorders, a past-year recall ratio was used to adjust all past-year recall estimates towards the level they would have been if the estimate had capture point/past-month prevalence. The latter prevalence period is less affected by recall bias. See Table 1 for these adjustment factors used for anxiety disorders. The estimated UIs around the adjustment ratio incorporates gamma, which represents the between-study variance across all input data in the model. This added uncertainty widens the UIs for crosswalks with significant fixed effects. We also estimated an adjustment factor of 2.88 (2.39–3.47) to adjust estimates of anxiety disorder symptom prevalence derived from symptom scales down towards the level of anxiety disorder prevalence derived from diagnostic surveys. Because the former was most commonly used in surveys during the COVID-19 pandemic, we utilised this adjustment to ensure estimates used within our analysis was consistent to our definition of anxiety disorders. Due to a lack of symptom-scale to reference pairs of estimates available, we derived the adjustment factor by first estimating the symptom-scale prevalence from the sensitivity and specificity of nine validation studies[5-13] using representative samples and the estimated prevalence of anxiety disorders for the study population. The ratios between the anxiety disorder prevalence and symptom scale prevalence were then pooled across the studies via MR-BRT to quantify the adjustment factor.

Table 1: MR-BRT crosswalk adjustment factors for anxiety disorders

**Data input**	**Reference or alternative case definition**	**Gamma**	**Beta coefficient, logit (95% UI)***	**Adjustment factor****
Population survey	Reference: past-month or point prevalence	0.03		

Population survey	Alternative: past-year prevalence		0.46 (0.38–0.54)	1.58 (1.46–1.71)

**MR-BRT crosswalk adjustments can be interpreted as the factor the alternative case definition is adjusted by to reflect what it would have been had it been measured using the reference case definition. If the log/logit beta coefficient is negative, then the alternative is adjusted up to the reference. If the log/logit beta coefficient is positive, then the alternative is adjusted down to the reference.*
***The adjustment factor column is the exponentiated beta coefficient. For log beta coefficients, this is the relative rate between the two case definitions. For logit beta coefficients, this is the relative odds between the two case definitions.*

Modelling strategy

For GBD 2023, modelling transitioned from using DisMod-MR 2.1 to DisMod-AT. Bias and root mean square error (RMSE) are shown in Table 2 for each measure.

Table 2. Performance metrics for the anxiety disorders DisMod-AT model

**Measure**	**Bias**	**RMSE**
Prevalence	0.00532	0.0272
Remission	-0.0134	0.0596

Relative to DisMod-MR, DisMod-AT provides enhanced estimation machinery and modelling performance, enabling less data pre-processing and fewer assumptions to be made. As a result, the following methodological changes were made for GBD 2023:

i) For GBD 2021, studies reporting prevalence estimates across age groups spanning 25 years or more were split into five-year age groups based on the prevalence age pattern estimated by DisMod-MR. This process was not required for GBD 2023 due to the simultaneous modelling of age and time in DisMod-AT.

ii) For GBD 2021, the impact of the COVID-19 pandemic on anxiety disorders prevalence was estimated via a custom adjustment to baseline DisMod-MR 2.1 estimates extrapolated from a MR-BRT meta-regression on the association between change in prevalence and proxies of pandemic impact. This process was not required for GBD 2023 as the impact could be estimated within DisMod-AT using a location-level covariate.

When a base fit is completed in DisMod-AT, standardised residuals are produced for all datapoints. Any observation where the absolute value of the standardised residual is greater than 2 were considered model outliers and were flagged for additional review. We reassessed the study methodology and quality of estimates flagged in this way before a decision was made to exclude or include such data in final models.

Data across all epidemiological parameters were initially included in the modelling process. The incidence studies reported estimates which were very low relative to the prevalence data. As prevalence studies contributed much greater world coverage than incidence studies, we excluded the incidence data, relying instead on data from the other parameters. Within DisMod-AT, we assumed no incidence and prevalence before age 2. This minimum age of onset was corroborated with expert feedback and existing literature on anxiety disorders.

To enhance predictive accuracy, DisMod-AT models are refined through a covariate selection process, evaluating various location-level covariates for their effectiveness. This process begins with

an initial selection of covariates informed by existing evidence and expert consultations. Covariate selection is then refined using a forward stepwise method. In the initial step, each covariate is assessed against the base model. Following this, the best-performing covariate is retained, and the process repeats, evaluating the remaining covariates against the newly updated model. This stepwise selection continues until no further improvement in predictive power is noted, as measured by the performance of model covariates and the root mean square standardised residuals (RMSSR). The following location-level covariates were selected through this process and were used to inform estimation:

1. The Gallup World Poll – Worry item: The Gallup initiative conducts comprehensive and comparable national surveys across a wide range of countries worldwide.[14] This index asked respondents to report whether they experienced worry yesterday. The Gallup covariate was included as a means to test for a correlation between worry at a location level and anxiety disorder prevalence. Data from the Gallup worry item was modelled using the spatiotemporal Gaussian process regression (ST-GPR) to produce estimates for all years and locations required by DisMod-AT. The log of the modelled output was used as the covariate in DisMod-MR due to skewedness of the data. The relationship detected was in the expected direction (ie, the higher the proportion of individuals reporting worry in the previous day, the higher the prevalence rate).
2. The age-standardised death rate of COVID-19: This covariate identified, for each GBD location, the age-standardised, sex-specific death rate attributable to COVID-19. The inclusion of this covariate follows evidence of the relationship between COVID-19 and anxiety prevalence. To address the limited temporal range of available data and associated skewness, this covariate was log-transformed and data from 2021+ were linearly waned to reach zero by 2023.

A summary of covariates and exponentiated values for anxiety disorders are shown in Table 3.

Table 3. Summary of covariates used in the anxiety disorders DisMod-AT meta-regression model

**Covariate**	**Type**	**Parameter**	**Beta (95% UI)**	**Exponentiated beta (95% UI)**
Gallup World Poll – Worry	Location-level	Incidence	0.90 (0.78 to 1.01)	2.45 (2.18–2.74)
COVID-19 age-standardised death rate	Location-level	Incidence	0.11 (–0.01 to 0.24)	1.12 (0.99–1.28)

Note: DisMod-AT leverages on data from all parameters to estimate location-level-covariates for a specific parameter.

Severity splits and disability weights

The GBD disability weight survey assessments include lay descriptions of sequelae highlighting major functional consequences and symptoms. The lay descriptions and disability weights for anxiety disorder severity levels are shown in Table 4. To determine the proportion of people with anxiety disorders within each of the severity levels, we used data from the Australian National Survey of Mental Health and Wellbeing of Adults (NSMHWB, conducted in 1997).[15] The proportion of anxiety disorder cases falling within each level of severity was: asymptomatic 16.8% (14.2–19.5), mild 42.4% (32.9–50.2), moderate 24.8% (18.9–31.0), and severe 16.1% (10.2–22.9). The same severity distribution and disability weights were applied to the pre-COVID-19 and post-COVID-19 prevalent cases of anxiety disorders.

Table 4. Severity distribution, details on the severity levels for anxiety disorders, and the associated disability weight with that severity

**Severity level**	**Lay description**	**Disability weight (95% UI)**
Mild	Feels mildly anxious and worried, which makes it slightly difficult to concentrate, remember things, and sleep. The person tires easily but is able to perform daily activities.	0.03 (0.018–0.046)
Moderate	Feels anxious and worried, which makes it difficult to concentrate, remember things, and sleep. The person tires easily and finds it difficult to perform daily activities.	0.133 (0.091–0.186)
Severe	Constantly feels very anxious and worried, which makes it difficult to concentrate, remember things, and sleep. The person has lost pleasure in life and thinks about suicide.	0.523 (0.362–0.677)

Methodological changes have resulted in a small decrease globally in prevalence of anxiety disorders with large variation in changes across locations (some increasing and some decreasing) compared to GBD 2021. Our final prevalence estimates for anxiety disorders need to be considered along several limitations. Across our entire epidemiological modelling process, it is important to acknowledge that our case definition for anxiety disorders will need to be revised to better capture changes to the latest DSM/ICD criteria. Epidemiological estimates reporting an outcome for "any" or "total" anxiety disorders were included in GBD 2023 if they reported on at least three anxiety disorders. Future iterations of GBD will revisit the unique contribution of specific anxiety disorders. Secondly, we still have a large number of locations with no high-quality raw data available. Thirdly, it is difficult to quantify and remove all variation due to measurement error in our epidemiological estimates. While we have improved the methodology used to account for known sources of bias, in some cases, we still have very few datapoints to inform these adjustments. Fourthly, there is a paucity of research on the risk factors of mental disorders which can be used as predictive covariates in our epidemiological models.

References

1. American Psychiatric Association. Diagnostic and Statistical Manual of Mental Disorders (DSM-IV-TR). Fourth Edition, Text Revision ed. Washington DC: American Psychiatric Association; 2000.
2. World Health Organization. The ICD-10 Classification of Mental and Behavioural Disorders. Clinical descriptions and diagnostic guidelines. Geneva: World Health Organization; 1992.
3. Baxter AJ, Scott KM, Vos T, Whiteford HA. Global prevalence of anxiety disorders: a systematic review and meta-regression. *Psychological Medicine* 2013; **43**(05): 897-910.
4. Baxter AJ, Vos T, Scott KM, Ferrari AJ, Whiteford HA. The global burden of anxiety disorders in 2010. *Psychological Medicine* 2014; **44**(11): 2363-74.
5. Vrublevska J, Renemane L, Kivite-Urtane A, Rancans E. Validation of the generalized anxiety disorder scales (GAD-7 and GAD-2) in primary care settings in Latvia. *Front Psychiatry* 2022; **13**: 972628.
6. Spitzer RL, Kroenke K, Williams JBW, Löwe B. A Brief Measure for Assessing Generalized Anxiety Disorder: The GAD-7. *Archives of Internal Medicine* 2006; **166**(10): 1092-7.
7. Marlow M, Skeen S, Grieve CM, et al. Detecting Depression and Anxiety Among Adolescents in South Africa: Validity of the isiXhosa Patient Health Questionnaire-9 and Generalized Anxiety Disorder-7. *J Adolesc Health* 2023; **72**(1s): S52-s60.
8. Kroenke K SR, Williams JB, Monahan PO, Löwe B. Anxiety disorders in primary care: prevalence, impairment, comorbidity, and detection. *Ann Intern Med* 2007; **146**(5): 317-25.

9. Konkan R, Senormanci O, Guclu O, Aydin E, Sungur MZ. Validity and reliability study for the Turkish adaptation of the Generalized Anxiety Disorder-7 (GAD-7) scale/Yaygin Anksiyete Bozuklugu-7 (YAB-7) testi Turkce uyarlamasi, gecerlik ve guvenirligi. *Archives of Neuropsychiatry* 2013; **50**(1): 53-9.
10. Ip H, Suen YN, Hui CLM, et al. Assessing anxiety among adolescents in Hong Kong: psychometric properties and validity of the Generalised Anxiety Disorder-7 (GAD-7) in an epidemiological community sample. *BMC Psychiatry* 2022; **22**(1): 703.
11. García-Campayo J, Zamorano E, Ruiz MA, et al. Cultural adaptation into Spanish of the generalized anxiety disorder-7 (GAD-7) scale as a screening tool. *Health Qual Life Outcomes* 2010; **8**: 8.
12. Christensen H, Batterham PJ, Grant JB, Griffiths KM, Mackinnon AJ. A population study comparing screening performance of prototypes for depression and anxiety with standard scales. *BMC medical research methodology* 2011; **11**: 1-9.
13. Ahn J-K, Kim Y, Choi K-H. The Psychometric Properties and Clinical Utility of the Korean Version of GAD-7 and GAD-2. *Frontiers in Psychiatry* 2019; **10**.
14. Gallup G. The Gallup Poll: Public Opinion 2003: Rowman & Littlefield; 2004.
15. Australian Bureau of Statistics. National Survey of Mental Health and Wellbeing of Adults 1997. Canberra: Australian Bureau of Statistics.

Appendicitis

Flowchart

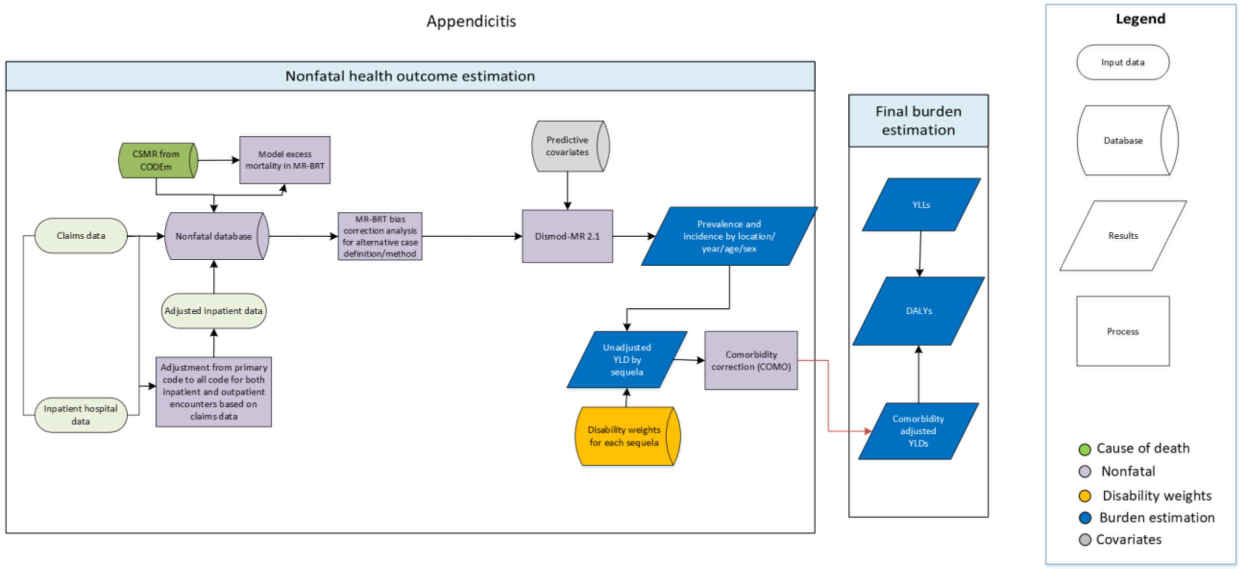

Input data and methodological summary for appendicitis

Case definition

Appendicitis is an inflammation of the appendix generally presenting with nausea, vomiting, and sharp pain in the right lower abdomen. Appendicitis carries risk of severe complications, including sepsis and death, and is usually treated surgically. ICD-10 codes included are K35-K35.3, K35.8, K35.80, K35.89, K35.9, K36, K36.0, K37, K37.0, K37.9, and K38.3.

Quantity of interest	Reference or Alternative	Definition
Incidence of appendicitis	Alternative	Cases identified from database of commercial claims from USA in 2010-2019 using ICD codes.
Incidence of appendicitis	Alternative	Cases identified from database of commercial claims from USA in 2000 using ICD codes.
Incidence of appendicitis	Reference	Cases of appendicitis identified using ICD codes in administrative records that are considered population-representative.

Input data

As in previous rounds of GBD, in GBD 2023 we modelled appendicitis using incidence data extracted from the IHME clinical administrative data library aggregated and processed for GBD as described in "Clinical input data and methods summary" section of this appendix. Notably, in GBD 2023, this library of hospital discharges and claims expanded with additional years of data from USA claims (years 2018, 2019), Poland claims (year 2019), new sources in Mongolia claims (year 2019), and South Korea (years 2018, 2019). Additional years of hospital discharges were also included in GBD 2023 from the USA (HCUP years 2016–2019), Austria, Brazil, Chile, Georgia, Italy, New Zealand, and the Philippines, as well

as the new sources of hospital discharges from Germany and Mexico and data from the US Medicare programme for age groups 65–69 and older, (years 2000, 2010, 2014-16). Appendicitis, specifically, also included the new administrative data from the Netherlands Nivel Primary Care database General Practitioner Register (years 2011–2022).

Prior to GBD 2023, given the heterogeneity within the clinical administrative data for this cause, we systematically excluded data series with an age-standardised incidence rate greater than two median absolute deviations from the median of the age-standardised incidence rate for all data. In GBD 2023, however, we explored this heterogeneity in greater detail and found that many extreme values were seen in inpatient data from sources that either do not cover the entire population or do not include a representative population sample, thus requiring extraction as discharge diagnosis cause fractions and conversion to incidence using GBD estimates of hospital admission rate per capita. (See the section of this appendix on the hospital utilisation estimates for information on this input.) In GBD 2023, we decided to exclude all inpatient sources that require this adjustment and only include the sources with full coverage of the target population. Lastly, we excluded US MarketScan data for individuals aged 65 and older, replacing them with CMS data, which are more representative of the USA population in these age groups.

Inputs to our non-fatal modelling also included cause-specific mortality rate (CSMR) estimates taken from our fatal modelling process (see CoD cause-specific modelling description for appendicitis in this appendix) and excess mortality rates (EMR) estimates modelled outside of DisMod (see the EMR data processing section below). We excluded the cause-specific mortality rate (CSMR) inputs for ages 1–10 in the Andean region and Zimbabwe (ZWE) due to inconsistencies in reported mortality trends.

Incidence data processing

Hospital discharge data provide observations about encounters, generally with only the primary diagnostic code for the encounter. Claims data, on the other hand, link claims for all inpatient and outpatient encounters for a single individual and provide primary and secondary diagnoses for all encounters.

In GBD 2017, an individual was extracted from claims data as an incident case if that individual had one or more inpatient encounters with an appropriate ICD code of appendicitis as any diagnosis. Hospital discharges with an appropriate ICD code as primary diagnosis were extracted and adjusted for readmission. In both GBD 2019 and GBD 2021, however, we employed data processing methods to capture cases that were diagnosed and/or treated in both inpatient and outpatient settings. Specifically, an individual was extracted from claims data as an incident case if that individual had at least one inpatient or outpatient encounter with an appropriate ICD code as any diagnosis within 28 days. Hospital discharge data were processed by extracting discharges with an appropriate ICD code as primary diagnosis and adjusting using correction factors derived from claims data. Specifically, we modelled the ratio of inpatient claims with appendicitis as primary diagnosis to total incident cases of appendicitis seen in claims data. In GBD 2021, we updated the method of estimating these correction factors by assigning three frequency-placed knots, instead of two, in the age-spline parameter of MR-BRT analysis. Other processing methods remained the same as in GBD 2019.

In GBD 2023, we refined our approach by updating the correction factor used for hospital discharge data adjustments. Instead of using correction factor 3, which was a single model of the ratio of inpatient claims with a primary diagnosis to total incident cases seen in claims data with complete information on

all diagnoses in all encounter types, we now apply correction factors 1 and 5 (CF1 and CF5). CF1 is defined as the ratio of deduplicated primary diagnoses among inpatient admissions to adjusted primary diagnoses among inpatient admissions, while CF5 is defined as the ratio of deduplicated primary and secondary diagnoses among inpatient encounters and outpatient encounters to deduplicated primary diagnoses among inpatient encounters. Like the previous approach, the approach this year accounts for non-primary inpatient diagnoses and outpatient cases and does so serially – to allow data-sources to contribute to estimation of de-duplication for readmission, multiple diagnosis in inpatient encounters, and/or outpatient care, depending on which of these relationships are captured in the dataset – rather than only employing sources that have complete information on all diagnoses and encounter types. This approach makes use of more diverse data inputs. This final scalar is applied to extracted primary diagnosis admission rate data as a product of CF1 and 1/CF5. See the section of this appendix on the processing of hospital data for more details.

As first done in GBD 2019, USA claims data (extracted and processed as described above) were adjusted to account for selection bias due to commercial insurance, using MR-BRT analysis. The USA MarketScan claims data from the year 2000 and from the years 2010–2019 were separately adjusted to account for selection bias due to commercial insurance, which was suspected to be differential over the years as coverage expanded. In contrast to GBD 2019, in GBD 2021 and GBD 2023, we used age as a covariate to estimate bias adjustment factors. (As mentioned, these data were excluded for individuals aged 65 years and older, due to the new availability of CMS data, but this adjustment was applied to data for younger age groups, which were retained.) The process of adjusting for biases in non-reference data using MR-BRT with the logit-transformation method is described below:

1. Identify datapoints with overlapping year, age, sex, and location between reference and non-reference population data.
2. Logit transform overlapping datapoints of alternative and reference types.
3. Convert overlapping datapoints into a difference in logit space using the following equation: $logit(altnerative) - logit(reference)$
4. Use the delta method to compute standard errors of overlapping datapoints in logit space, then calculate standard error of logit difference using the following equation: $\sqrt{(variance\ of\ logit(alternative)) + (variance\ of\ logit(reference))}$
5. Using MR-BRT, conduct a random effects meta-regression to obtain the pooled logit difference of alternative to reference
6. Apply the pooled logit difference to all data points of non-reference types using the following equation:
$$new_{estimate} = inverse.logit((logit(alternative)) - (pooled\ logit\ difference))$$
7. Calculate new standard errors using the delta method, accounting for gamma (between-study heterogeneity)

The table below shows bias correction factors estimated using MR-BRT.

Table 1: MR-BRT crosswalk adjustment factors for appendicitis

Data input	Reference or alternative data collection	Gamma	Covariate	Beta coefficient, logit (95% UI)*	Adjustment factor**

Hospital + non-USA claims	Ref	---	---	---	---
USA claims from year 2000	Alt	0	Age (continuous from 0 to 95+)	0.00024 (–0.0028 to 0.0033)	1.00 (0.997 to 1.003)
			Sex (female to male)	0.079 (–0.025 to 0.18)	1.08 (0.98 to 1.20)
			Intercept	–0.73 (–0.91 to –0.54)	0.48 (0.40 to 0.58)
USA claims from years 2010–2019	Alt	0.10	Age (continuous from 0 to 95+)	0.0028 (0.024 to 0.0031)	1.00 (0.82 to 1.22)
			Sex (female to male)	-0.022 (–0.033 to -0.0099)	0.98 (0.80 to 1.19)
			Intercept	–0.073 (-0.23 to 0.09)	0.93 (0.72 to 1.20)

**MR-BRT crosswalk adjustments can be interpreted as the factor the alternative case definition is adjusted by to reflect what it would have been had it been measured using the reference case definition. If the log/logit beta coefficient is negative, then the alternative is adjusted up to the reference. If the log/logit beta coefficient is positive, then the alternative is adjusted down to the reference.*
***The adjustment factor column is the exponentiated beta coefficient. For log beta coefficients, this is the relative rate between the two case definitions. For logit beta coefficients, this is the relative odds between the two case definitions.*

EMR processing

In GBD 2017, EMR inputs were produced by fitting a preliminary compartment model to estimate prevalence from incidence data, matching preliminary prevalence resulting from each incidence datapoint with the corresponding CSMR value within the same age, sex, year, and location, and dividing CSMR by prevalence. This method of producing EMR inputs, however, demonstrated a rather unrealistic pattern of EMR compared to an expected pattern of decreasing EMR with greater access to quality health care. Such unexpected patterns often signal inconsistencies between CSMR estimates and data on prevalence and/or incidence. Thus, in an effort to provide greater guidance on the expected pattern of EMR, in GBD 2019, EMR estimates produced per above were used in a MR-BRT model with age, sex, and Healthcare Access and Quality (HAQ) Index as predictors, with a prior on HAQ Index to have a negative coefficient. In GBD 2021, we employed the same MR-BRT method to predict EMR for each location, year, sex, and for ages 0, 10, 20….100. In GBD 2023, we added additional age point for prediction at 5. We increased the number of frequency-placed knots in the age spline parameter from three to five in MR-BRT, improving flexibility in capturing age-specific trends. Predictions from this model were used as inputs to our non-fatal model, described below.

Modelling strategy

DisMod model

Similar to previous rounds, we ran a DisMod-MR 2.1 model to produce estimates by age, sex, year, and location. Inputs to DisMod for appendicitis include incidence, CSMR, and EMR inputs processed as described above. A prior value was set on remission so that all cases remit within two weeks. Compared to previous rounds, we updated age-specific incidence priors to better align model estimates with observed data patterns across different age groups. The minimum coefficient of variation at the regional, super-regional, and global level remained set at 0.8. We included HAQ Index as a predictive covariate on EMR with a mean and standard deviation produced from the MR-BRT model described above. The fibre (g per day) consumption covariate was included as a predictive covariate on incidence. Betas and exponentiated values (which can be interpreted as odds ratios) of predictive covariates are shown in the table below.

Table 2. Covariates. Summary of covariates used in the appendicitis DisMod-MR meta-regression model

Covariate	Type	Parameter	Exponentiated beta (95% uncertainty interval)
Fibre, unadjusted (g)	Country-level	Incidence	1.00 (1.00–1.00)
Healthcare Access and Quality Index	Country-level	Excess mortality rate	0.96 (0.96–0.96)

Severity split and disability weight

The basis of the GBD disability weight survey assessments are lay descriptions of sequelae highlighting major functional consequences and symptoms. The lay descriptions and disability weights for appendicitis are shown below.

Table 3. Severity distribution, details on the severity levels for appendicitis and the associated disability weight (DW) with that severity.

Severity level	Lay description	DW (95% CI)
Severe	This person has severe pain in the belly and feels nauseated. The person is anxious and unable to carry out daily activities.	0.324 (0.219–0.442)

Ascariasis

Flowchart

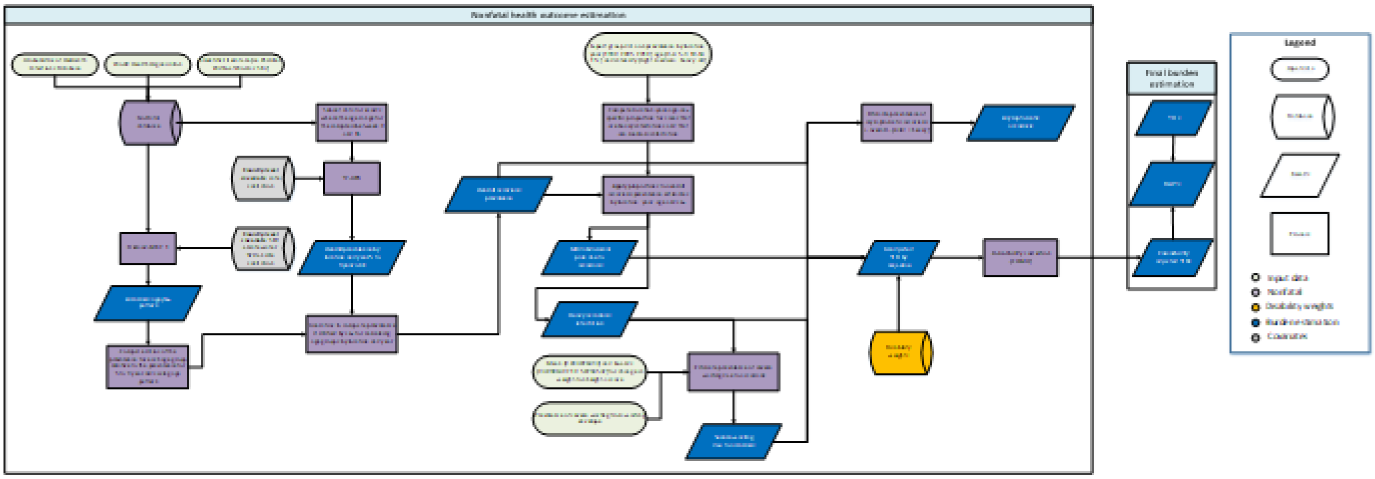

Input data and methodological summary for ascariasis

Case definition

Ascariasis is a helminthic disease caused by the parasitic roundworm *Ascaris lumbricoides*. It is one of the three intestinal nematode infections, or soil-transmitted helminthiasis (STH), modelled in GBD. Diagnosis is made by examination of stool by microscope or PCR, with or without concentration procedures. The ICD-10 codes for ascariasis are B77-B77.9. We used the following case definition for GBD 2023:

Quantity of interest	Reference or alternative	Definition
Ascariasis	Reference	Diagnosis made by examination of stool using Kato-Katz technique, resulting in positive for intestinal helminth eggs of type *A lumbricoides*.

Input data

The primary input data for this model was from the Global Atlas of Helminth Infections (GAHI) database and the Expanded Special Project for the Elimination of Neglected Tropical Diseases (ESPEN). The GAHI and ESPEN databases include surveys and studies conducted to measure the prevalence of STH.[1] Each record in the database contained metadata (ie, location, year, age range, sex) of each study sample and the prevalence of ascariasis in that sample.

We supplemented the GAHI and ESPEN data with survey-data collected in a literature review performed by Children Without Worms (CWW), which included countries outside of sub-Saharan Africa, a 2001–2004 China subnational survey to better inform our estimates in China, and additional data provided by the World Health Organization (WHO). For GBD 2023, we added data from systematic reviews (Figure

2a) and additional extracted data from the GAHI, CWW, and WHO datasets (Figure 2b). For all input data, we excluded datapoints where the age range of the sample was unknown and retained only those surveys utilizing the Kato-Katz diagnostic method.

Geographical restrictions

We conducted a literature review (last updated for GBD 2017) to determine the geographical extent of the disease and classify locations based on whether the disease was absent or present in each year. Locations that were geographically restricted in any given year did not have estimates made for them. Of note, we did not attempt a complete systematic review, since a single high-quality source could offer sufficient evidence of presence. Evidence of absence or presence was not available for every location for each year. Assumptions made for missing years took into consideration the epidemiological characteristics of the disease.

If evidence indicated disease presence for two non-consecutive years, we assumed presence for all years between the two. If evidence indicated disease absence for two non-consecutive years, we assumed absence for all years between the two. If evidence indicated a change in status (ie, from absent to present, or present to absent) between two non-consecutive years, then we conducted targeted searches to ascertain the relevant year of introduction or elimination for that location. In the cases where presence or absence information was missing for the start or end years of our study interval without evidence of any introduction or elimination events within the interval, we applied the status of the first and last presence/absence observations, respectively, to all years between the interval bound and the observation year. Table 1 shows the search strings and associated yield for each of the databases queried.

Table 1. Geographical restriction search strings

Database	Search string	Yield
PubMed	(Ascariasis[Title/Abstract] OR Ascaris[Title/Abstract] OR "A. lumbricoides"[Title/Abstract] OR Ascaris[MeSH] OR Trichuris[Title/Abstract] OR Trichuriasis[Title/Abstract] OR "Whip Worm"[Title/Abstract] OR "T. trichura"[Title/Abstract] OR Trichuris[MeSH] OR Hookworm[Title/Abstract] OR "A. duodenale"[Title/Abstract] OR "Ancylostoma duodenale"[Title/Abstract] OR ancylostomiasis[Title/Abstract] OR "N. americanus"[Title/Abstract] OR "Necator americanus"[Title/Abstract] OR necatoriasis[Title/Abstract] OR Ancylostoma [MeSH] OR Necator[MeSH]) AND (prevalence[Title/Abstract] OR incidence[Title/Abstract] OR epidemiology[Title/Abstract] OR surveillance[Title/Abstract]) NOT(Animals[MeSH] NOT Humans[MeSH])	2376
Web of Science	(Ascariasis OR Ascaris OR A. lumbricoides OR Trichuris OR Trichuriasis OR Whip Worm OR T. trichura OR Hookworm OR A. duodenale OR Ancylostoma duodenale OR anclyostomiasis OR N. americanus OR Necator americanus OR necatoriasis) AND TOPIC:(prevalence OR incidence OR epidemiology OR surveillance) NOTTOPIC: ((Animals NOT Humans)) Timespan: 1980-2016. Indexes: SCI-EXPANDED, SSCI, A&HCI, ESCI.	2266
SCOPUS	TITLE-ABS_KEY (ascariasis OR ascaris OR a. lumbricoides OR trichuris OR trichuriasis OR whip worm OR t. trichura OR hookworm OR a. duodenale OR	29

	ancylostoma duodenale OR anclyostomiasis OR n. americanus OR necator americanus OR necatoriasis) AND PUBYEAR>1979	

These papers classified location-years for all locations and years present in the literature. We only utilised papers that were explicitly concerned with ascariasis. Additionally, systematic literature reviews, meta-analyses, national health statistics publications, and collaborator input supported classification of location-years not present in the literature review wherever possible.

Modelling strategy

Prevalence model

In the estimation of overall morbidity due to ascariasis, we implemented a three-stage modelling framework. First, we utilised a spatiotemporal Gaussian process regression (ST-GPR) to generate a complete time series of estimates for each location where there are no geographical restrictions. ST-GPR attempts to model non-linear trends utilising a Gaussian process to fit a trend. We ran an age-restricted ST-GPR model, using all data with age bins between 0 and 16 because these data fall within the peak in prevalence across all age groups, the majority of data fall within these age ranges, and these data provide sufficient statistical power for our model. The following were the model specifications:

$$prevalence = sanitation + (1|level\ 1/level\ 2/level\ 3)$$

Levels 1, 2, and 3 refer to GBD location hierarchies, or nested random effects for super-region, region, and location. Covariate selection was based on directionality of the resulting beta values from the linear model, significant effect size, and subsequently out-of-sample testing for the resulting “best” combinations. The covariate used for the GBD 2023 model was proportion of population with access to sanitation, a change from the previous round which used Healthcare Access and Quality Index and safe water. The following hyperparameters were used: st_lambda = 0.25, st_omega = 2, st_zeta = 0.005, gpr_scale = 15. We selected these hyperparameters as they provided more weight to country-level data when estimating the prevalence for a given location-year, ensuring that the Gaussian process regressions follow country-specific data rather than region-specific data when estimating a time series for a location.

Table 2b. Covariates. Summary of covariates used in the ascariasis ST-GPR model

**Covariate**	**Beta coefficient, log (95% UI)**	**Standard error**	**Exponentiated beta (95% UI)**
Sanitation (proportion with access)	–2.614 (–3.765 to –1.463)	0.587	0.073 (0.023–0.232)

Age-pattern model

The next stage of the modelling process was using a DisMod Bayesian meta-regression model (DisMod-MR), to generate a global age-sex curve to disaggregate all-age, both-sex prevalence data. DisMod-MR is an integrated meta-regression framework that allows multiple datasets to be used within a singular

analysis regardless of age-binning, sources, and geographies. As a result, a variety of differently aggregated information combine to generate a consensus output. Our final model contained all processed GAHI data as input informed by two country-level covariates (ie, all risk factor summary exposure values (SEVs) for unsafe water and unsafe sanitation).

Table 2a. Covariates. Summary of covariates used in the ascariasis DisMod-MR model

**Covariate**	**Type**	**Parameter**	**Exponentiated beta (95% UI)**
SEV: unsafe water	Country-level	Proportion	4.43 (4.34–4.48)
SEV: unsafe sanitation	Country-level	Proportion	4.44 (4.38–4.48)

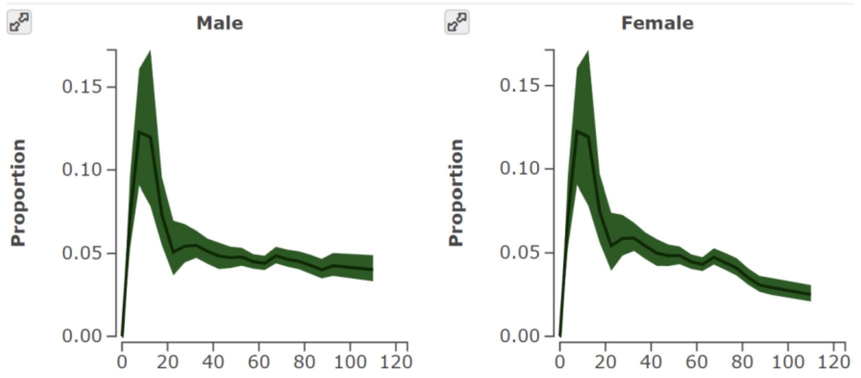

Figure 1: Global age-specific prevalence estimates for males (left) and females (right) for the year 2010. Proportion (prevalence) is on the Y-axis, and age in years on the X-axis. Screenshot from EpiViz tool.

Figure 1 shows the age-specific variation in the proportion of prevalence, differentiated by sex. When considered as a global aggregate, we see that reported male and female prevalence are very similar.

Imputations

The final stage of the overall prevalence modelling process is to impute the remaining age groups by borrowing information from the DisMod-MR global age-sex pattern and ST-GPR time series, by first assuming the estimates from ST-GPR are representative of the 5–9-year-old age group. Each additional age group is assigned a ratio representing how much larger or smaller the prevalence is compared to the prevalence of the reference group (5–9-year-olds) using the DisMod-MR global age-sex pattern. The following is the computation for each age group:

$$Ratio = \frac{prevalence_{[age\ start] to\ [age\ end]}}{prevalence_{5\ to\ 9}}$$

With a ratio for every age group by sex, we multiplied the ratio by the ST-GPR location-year estimates to impute estimates for the remaining age groups.

Health states/sequelae

The table below shows the list of sequelae due to ascariasis and the associated disability weights (DWs). Prevalence of medium infection and heavy infection are mapped to *mild abdominopelvic problems* and *heavy infestation of ascariasis,* respectively. Light infection or asymptomatic were not attributed any disability.

Table 3. Severity distribution, details on the severity levels for ascariasis and the associated disability weight (DW) with that severity

**Sequela**	**Lay description**	**DW (95% CI)**
Mild abdominopelvic problems	has some pain in the belly that causes nausea but does not interfere with daily activities	0.011 (0.005–0.021)
Heavy infestation	has cramping pain and a bloated feeling in the belly	0.027 (0.015–0.043)
Severe wasting	is extremely skinny and has no energy	0.128 (0.082–0.183)
Asymptomatic ascariasis	N/A	N/A

Following computations of location-year-age-sex-specific prevalence of ascariasis, we leverage information from the 2010 Expert Group (EG) data to conduct sequelae splits. The 2010 EG data provided estimates for heavy infestation, mild abdominopelvic problems, and asymptomatic ascariasis by location and for 1990, 2005, and 2010. These three values add up to *all cases* of ascariasis. Thus, for heavy infestation and mild abdominopelvic problems, we computed the proportion of cases that belong to our sequelae of interest over *all cases* of ascariasis. The following is the equation utilised to calculate heavy infestation and mild abdominopelvic problems:

$$Proportion_{sequelae} = \frac{prevalence_{sequelae}}{prevalence_{all\ cases}}$$

This calculates proportions for every location, year, and age group available. The EG data only had four age groups (0–4, 5–9, 10–14, 15+ years), so we applied the 15+ age group proportion for all remaining age groups. In addition, for the years 1995 and 2000, we applied the 1990 proportions, and for years 2015, 2019, and 2020–2021, we applied the 2010 proportions. Using these location-year-age-specific proportions, we multiplied the total ascariasis estimates to compute heavy infestation and mild abdominopelvic prevalence. To estimate the prevalence of asymptomatic ascariasis, prevalence of mild and heavy infestation were each subtracted from the overall ascariasis prevalence.

The final step in the modelling process was to estimate the prevalence of severe wasting due to ascariasis in age groups 1–5 months, 6–11 months, 12–23 months and 2–4 years. This was done separately using 1000 draws of prevalence of heavy infestation due to ascariasis and the wasting envelope prevalence. The initial step in determining prevalence of severe wasting due to ascariasis was generating 1000 draws of change in weight-for-height z-score per heavy prevalent case from a random normal distribution with mean = 0.493826493 and standard deviation = 0.04972834 (calculated from upper and lower bounds of the mean estimate). The mean, upper, and lower bounds were calculated

using findings from a meta-analysis[2] (last updated in GBD 2013). The prevalence of severe wasting due to ascariasis was then obtained as a function of change in weight-for-height z-score. The following are the computations:

$$Prevalence_{wasting\ due\ to\ ascariasis} = wasting - \Phi(\Phi^{-1}(\text{wasting}) - \text{z score} * \text{heavy infestation})$$

Where Φ is the standard normal cumulative distribution function and Φ^{-1} is the inverse standard normal cumulative distribution function.

Changes from GBD 2021 to GBD 2023

The major substantive change for GBD 2023 was the specification of new covariates for the ST-GPR global prevalence model.

There were also data changes between the rounds. New data inputs from scientific literature, ESPEN and expansion of age-specific data from the CWW and GAHI datasets were added to the model.

Limitations

As we attempt to improve the modelling processes for ascariasis, we recognise several limitations. We only include studies where Kato-Katz identifies infected individuals. Future updates to the model will include a systematic review for within-study comparisons of diagnostic performance to facilitate a diagnostic crosswalk model.

A secondary limitation to our data is that several included studies are not nationally representative, and therefore at a location level, the data are highly heterogeneous. Numerous studies within the database come from districts or villages, and in most cases, the studies were done in areas where prevalence is known to be high.

In addition, our current model does not include the impact of mass drug administration (MDA). In future rounds, we plan to integrate MDA coverage into the estimate of prevalence.

Furthermore, we made a large assumption that the global age-sex distributions were applicable to all locations. While we believe that prevalence should peak among adolescents and slowly decline afterward, there is likely variation across regions and locations. Given that our data are among children or all-age, it is very difficult to build an age trend at granular location levels. Thus, we allowed DisMod-MR to disaggregate our heterogeneous data in an effort to provide sensible age-sex curves.

We did not apply any adjustments for the COVID pandemic to ascariasis due to a lack of available data quantifying the impacts of the pandemic on NTD epidemiology.

We believe that more work will improve our sequelae split methods. Since the EG data do not provide all estimation years and age groups, several assumptions had to be made to estimate sequelae for all years, locations, and age groups in GBD. Further work will be required to gather additional data and improve these sequelae estimates.

References

1. London School of Hygiene and Tropical Medicine. Global Atlas of Helminth Infections – Soil Transmitted Helminths. London, United Kingdom: London School of Hygiene and Tropical Medicine.
2. Hall A, Hewitt G, Tuffrey V, de Silva N. A review and meta-analysis of the impact of intestinal worms on child growth and nutrition. *Maternal and Child Nutrition*. 2008. 4: 118-236.

Figure 2a. PRISMA 2023 flow diagram – systematic review of ascariasis prevalence literature (updates for GBD 2023)

Source: Page MJ, et al. BMJ 2021;372:n71. doi: 10.1136/bmj.n71. This work is licensed under CC BY 4.0. To view a copy of this license, visit https://creativecommons.org/licenses/by/4.0/

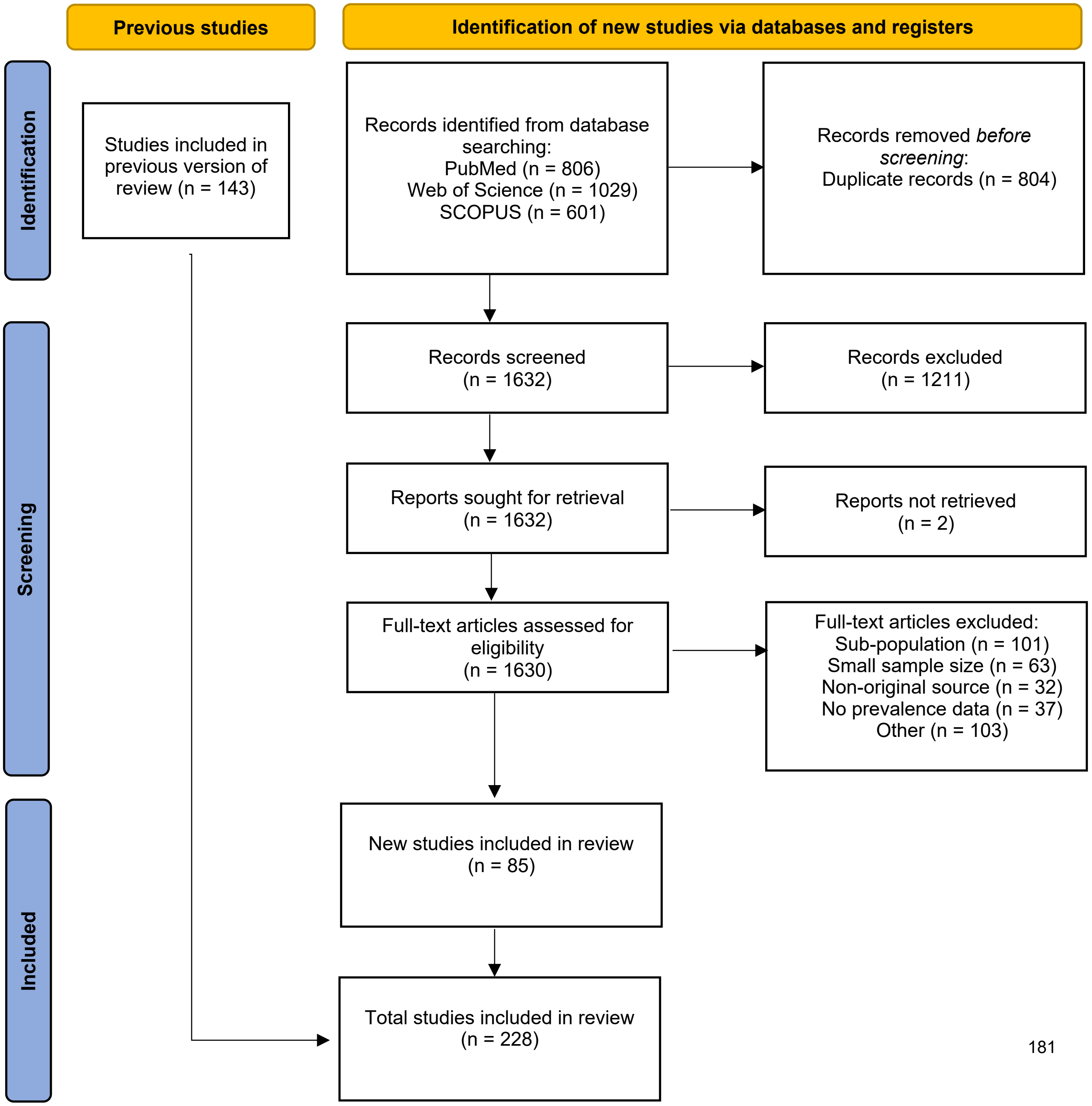

Figure 2b. PRISMA 2023 flow diagram – expansion of prevalence data from GAHI, CWW, and WHO databases (updates for GBD 2023)

Source: Page MJ, et al. BMJ 2021;372:n71. doi: 10.1136/bmj.n71. This work is licensed under CC BY 4.0. To view a copy of this license, visit https://creativecommons.org/licenses/by/4.0/

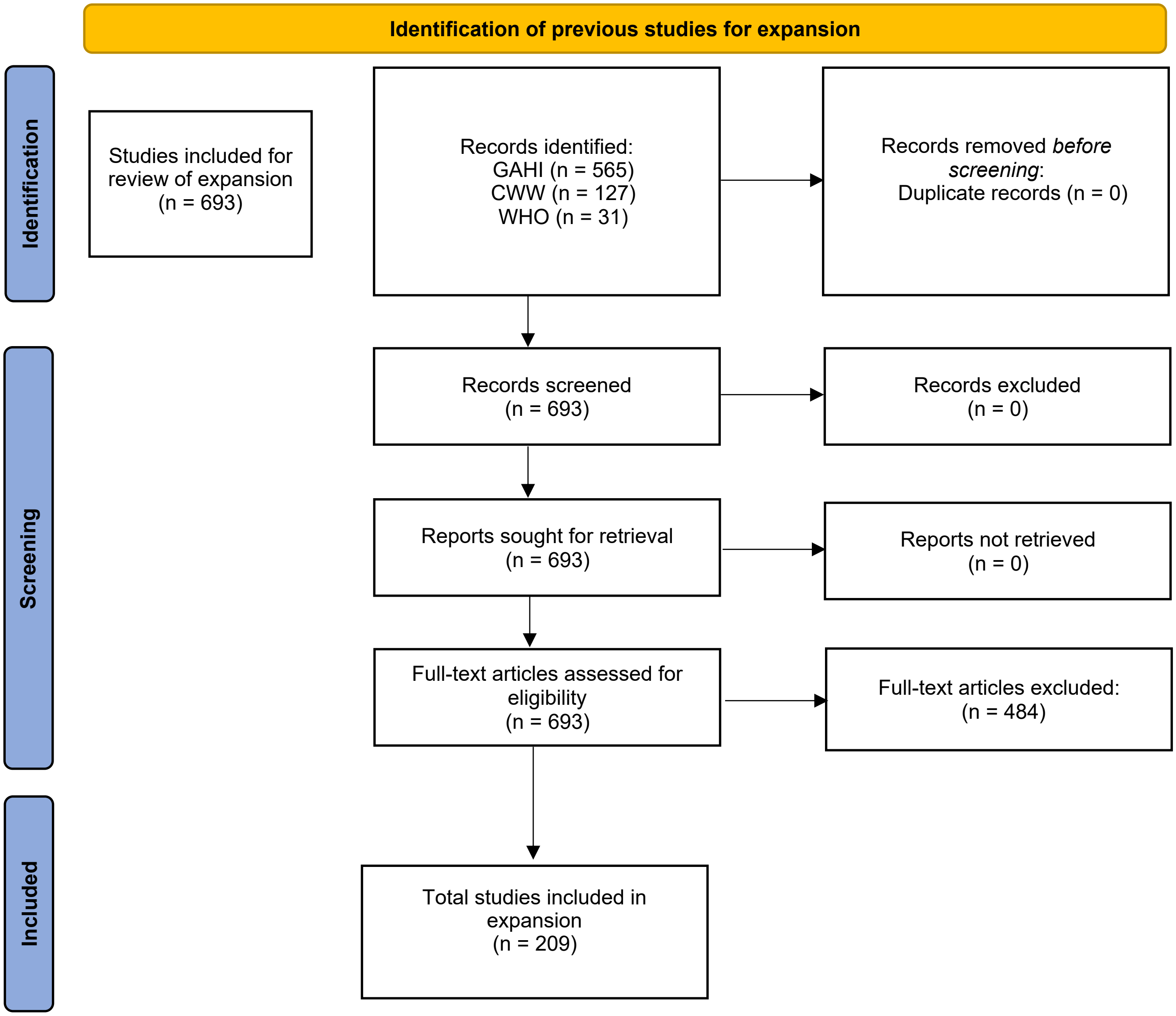

Asthma

Flowchart

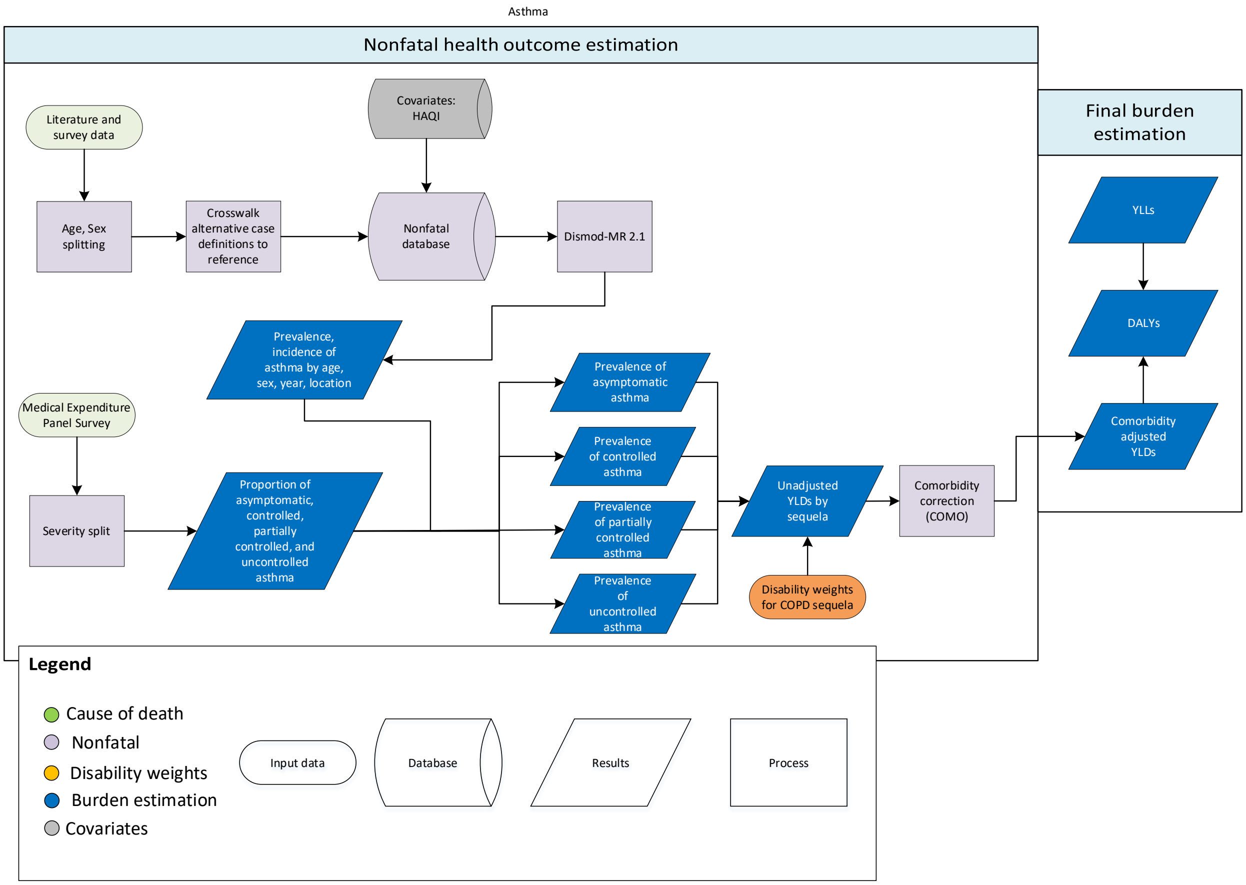

Input data and methodological summary for asthma

Case definition

Asthma is a chronic lung disease marked by spasms in the bronchi, usually resulting from an allergic reaction or hypersensitivity and causing difficulty in breathing. The relevant ICD-10 codes are J45 and J46. ICD-9 code is 493.

Table 1. Reference and alternative case definitions

Quantity of interest	Reference or alternative	Definition
Asthma	Reference	Self-report wheezing in the past 12 months and doctor's diagnosis of asthma
Asthma	Alternative	Self-report wheezing in the past 12 months
Asthma	Alternative	Self-report doctor's diagnosis of asthma
Asthma	Alternative	Self-report ever having asthma
Asthma	Alternative	Self-report currently having asthma

Input data

A systematic review for asthma was conducted in GBD 2023. The search strings below were used for PubMed and Web of Science between 01/01/2017 and 04/25/2023. A total of 980 unique records were identified, 506 were included in full-text screening, and 222 sources were extracted to be used in modelling for GBD 2023. Through the systematic review data were added for 81 new locations (four countries, 77 subnationals), and in 29 countries where the previous data source was from at least 15 years ago.

PubMed:
(Asthma*[TiAb] OR Asthma[MeSH]) AND (((prevalence[TiAb] OR prevalent[TiAb]) AND (cross-sectional OR "cross sectional" OR "disease frequency" OR "Cross-Sectional Studies"[MeSH Terms])) OR ((remission[TiAb] OR remit*[TiAb]) AND (cohort OR "Cohort Studies"[MeSH Terms]))) NOT (animals[MeSH] NOT humans[MeSH])

Web of Science:
((TI=Asthma* OR AB=Asthma*) OR ALL=Asthma)
AND ((((TI=prevalence OR AB=prevalence) OR (TI=prevalent OR AB=prevalent))
AND (ALL=cross-sectional OR ALL="cross sectional" OR All="disease frequency" OR ALL="Cross-Sectional Studies"))OR (((TI=remission OR AB=remission) OR (TI=remit* OR AB=remit*))
AND (All=cohort OR ALL="Cohort Studies"))) NOT (ALL=animals NOT ALL=humans)

In addition to the systematic review, data from the International Study of Asthma and Allergies in Children (ISAAC) and the Global Asthma Network (GAN) were updated. In total, for both survey series, new data were added for 83 locations compared to in GBD 2021.

For GBD 2023, only scientific literature or survey data that report prevalence, incidence, or remission were included. Previously, insurance claims data from USA, Poland, and Taiwan were included but dropped this round because after bias adjustment, the claims data still underestimated compared to literature data.

Figure 1: PRISMA flow diagram

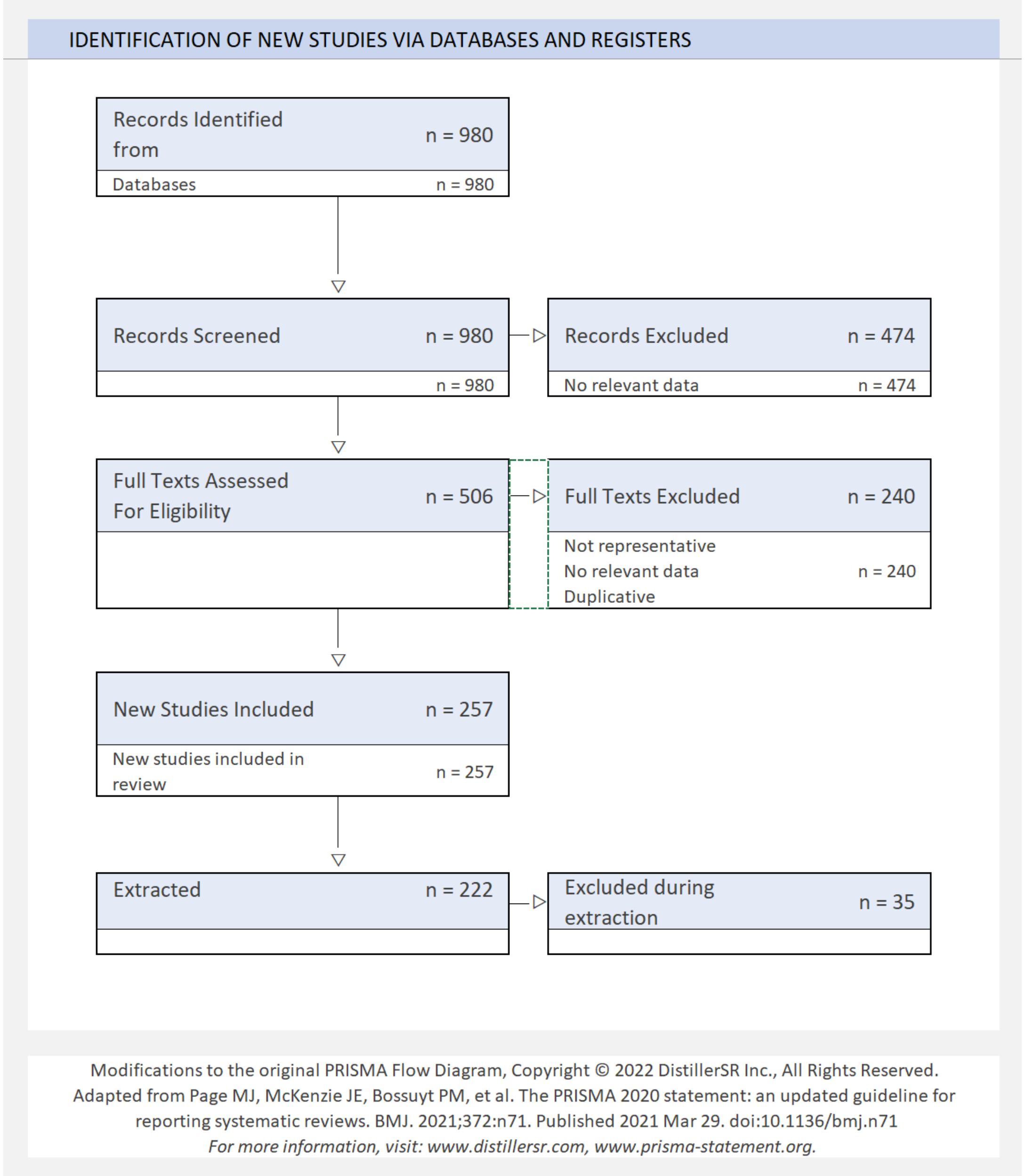

Data processing

Age and sex split
In some cases, data are reported by only age or only sex, but not both. For example, a study may have included the prevalence of males and females with asthma and then separately reported the prevalence of both sexes combined in smaller age bins (eg, 40–45 years, 46–50, etc.) that have asthma. In these cases, we perform an age-sex split by utilising proportions within the study to disaggregate the data.

When data are not disaggregated into male and female categories, we instead perform a sex-split on the data by applying sex proportions from all sex-specific input data. The sex split analysis was carried out using MR- BRT (meta-regression—Bayesian, regularised, trimmed; described in appendix 1, section 2 of the reference) and included a cubic spline with four interior knots on age to reflect the changing age and sex pattern in asthma. In early ages, male prevalence is higher; then, beginning in late adolescence, female prevalence increases relative to male prevalence through midage, and female prevalence decreases again in the oldest age groups.

Figure 2. MR-BRT input and output sex ratios for sex-splitting asthma prevalence

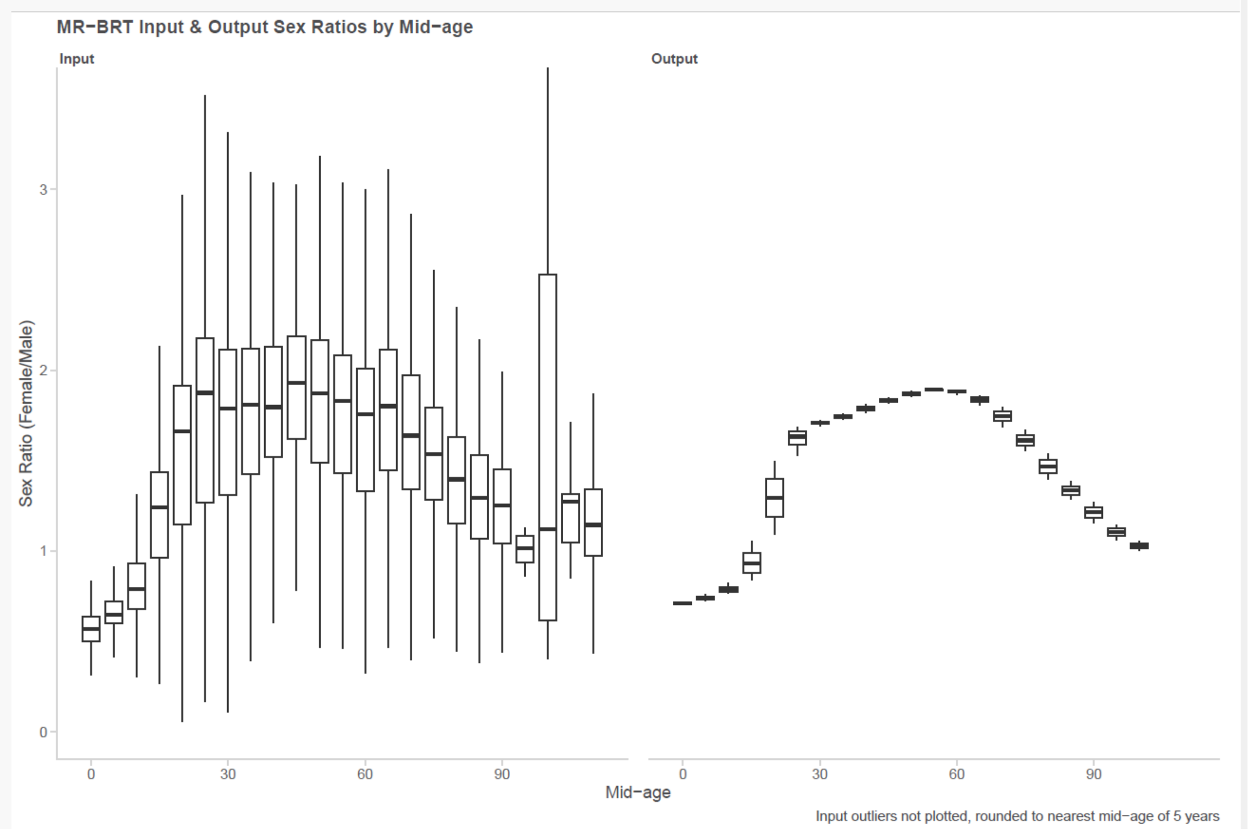

When data are aggregated into age categories larger than 25 years in adults or 10 years in adolescents, we split the data into smaller age bins based on the super-region age pattern from an initial DisMod model that only included input data with age ranges under 25 years.

Figure 3. Asthma prevalence age pattern by super-region

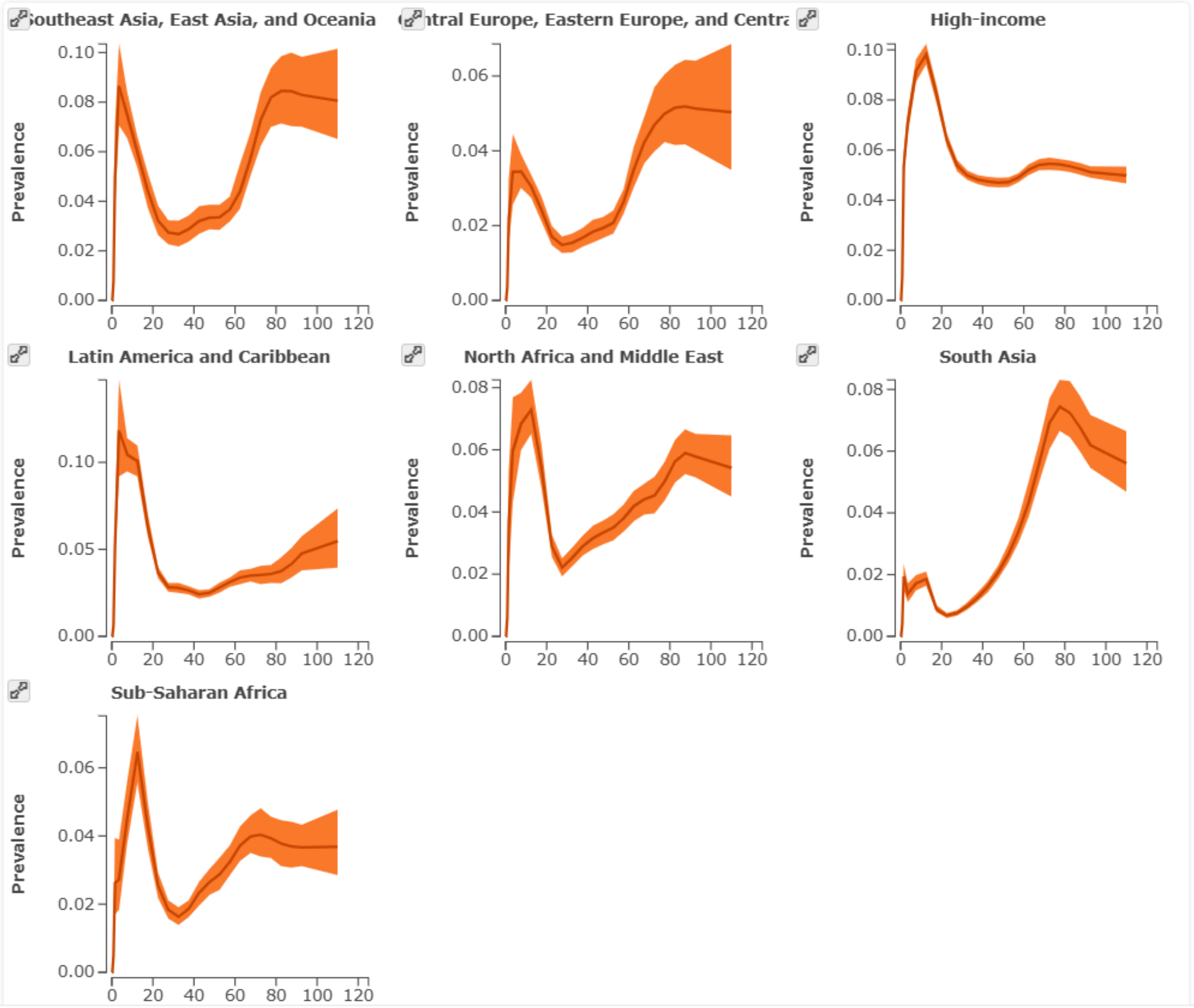

Bias adjustment

We made a series of adjustments to data that do not completely match our case definition, doctor's diagnosis and wheezing in the past year. The estimation of asthma in a population varies slightly by the case definition used (wheezing and diagnosis, only wheezing, etc.).

The adjustment is a logit-transformation method in MR-BRT. The general process is described below:

1. Identify datapoints with overlapping year, age, sex, and location between reference and alternative definitions.
2. Logit transform overlapping datapoints of alternative and reference case definitions.
3. Convert overlapping datapoints into a difference in logit space using the following equation:

$$logit(altnerative) - logit(reference)$$

4. Use the delta method to compute standard errors of overlapping datapoints in logit space, then calculate standard error of logit difference using the following equation:

$$\sqrt{(variance\ of\ alternative) + (variance\ of\ reference)}$$

5. Using MR-BRT, conduct a random effects meta-regression to obtain the pooled logit difference of alternative to reference.
6. Apply the pooled logit difference to all datapoints of alternative case definitions using the following equation:

$$new_{estimate} = inverse.logit((logit(alternative)) - (pooled\ logit\ difference))$$

7. Calculate new standard errors using the delta method, accounting for gamma (between-study heterogeneity).

The coefficients for bias adjustments are shown:

Table 2. MR-BRT crosswalk adjustment factors for asthma

Data input	Reference or alternative case definition	Gamma	Beta coefficient, logit (95% UI)*	Adjustment factor**
Wheezing in past year + doctor's diagnosis	Reference	0.38	---	---
Wheezing in past year	Alternative		0.52 (0.45–0.59)	1.68
Doctor's diagnosis	Alternative		0.14 (0.13–0.15)	1.15
Self-report ever asthma diagnosis	Alternative		0.28 (0.23–0.33)	1.32
Self-report current asthma diagnosis	Alternative		0.11 (0.03–0.18)	1.12

**MR-BRT crosswalk adjustments can be interpreted as the factor the alternative case definition is adjusted by to reflect what it would have been had it been measured using the reference case definition. If the log/logit beta coefficient is negative, then the alternative is adjusted up to the reference. If the log/logit beta coefficient is positive, then the alternative is adjusted down to the reference.*
***The adjustment factor column is the exponentiated beta coefficient. For log beta coefficients, this is the relative rate between the two case definitions. For logit beta coefficients, this is the relative odds between the two case definitions.*

Modelling strategy

A significant change to the modelling strategy for GBD 2023 was that we removed modelled cause-specific mortality rate (CSMR) as an input. The reason CSMR was excluded is due to incidence and prevalence estimates in early ages and older ages being inflated in Latin America and the Caribbean and south Asia super-regions and deviating from literature data in those locations.

We use DisMod-MR 2.1 (disease model—Bayesian meta-regression; described in appendix 1, section 2) as the main modelling tool for asthma. Prior settings include a maximum remission of 0.3 for ages 1 to 20, and maximum remission of 0.1 for ages 20 to 95+ (reflecting the upper bound of the highest observed data). Another prior setting restricts incidence or prevalence between the ages of 0 and 1 year, to avoid incorrectly counting pneumonia and other common respiratory illnesses with symptoms

of wheezing as asthma. Healthcare Access and Quality (HAQ) Index was added as a country-level covariate for excess mortality rate.

Table 3. Severity distribution, details on the severity levels for asthma and the associated disability weight (DW) with that severity.

Severity level	Lay description	DW (95% CI)	Severity distribution
Asymptomatic			0.362 (0.35–0.37)
Controlled	This person has wheezing and cough once a month, which does not cause difficulty with daily activities.	0.015 (0.007–0.026)	0.199 (0.136–0.278)
Partially controlled	This person has wheezing and cough once a week, which causes some difficulty with daily activities.	0.036 (0.022–0.055)	0.206 (0.151–0.258)
Uncontrolled	This person has wheezing, cough, and shortness of breath more than twice a week, which causes difficulty with daily activities and sometimes wakes the person at night.	0.133 (0.086–0.192)	0.233 (0.187–0.303)

Table 4. Covariates. Summary of covariates used in the asthma DisMod-MR meta-regression model

Covariate	Type	Parameter	Exponentiated beta (95% Uncertainty Interval)
Healthcare Access and Quality Index	Country-level	Excess mortality rate	0.99 (0.15–6.47)

Atrial fibrillation and flutter

Flowchart

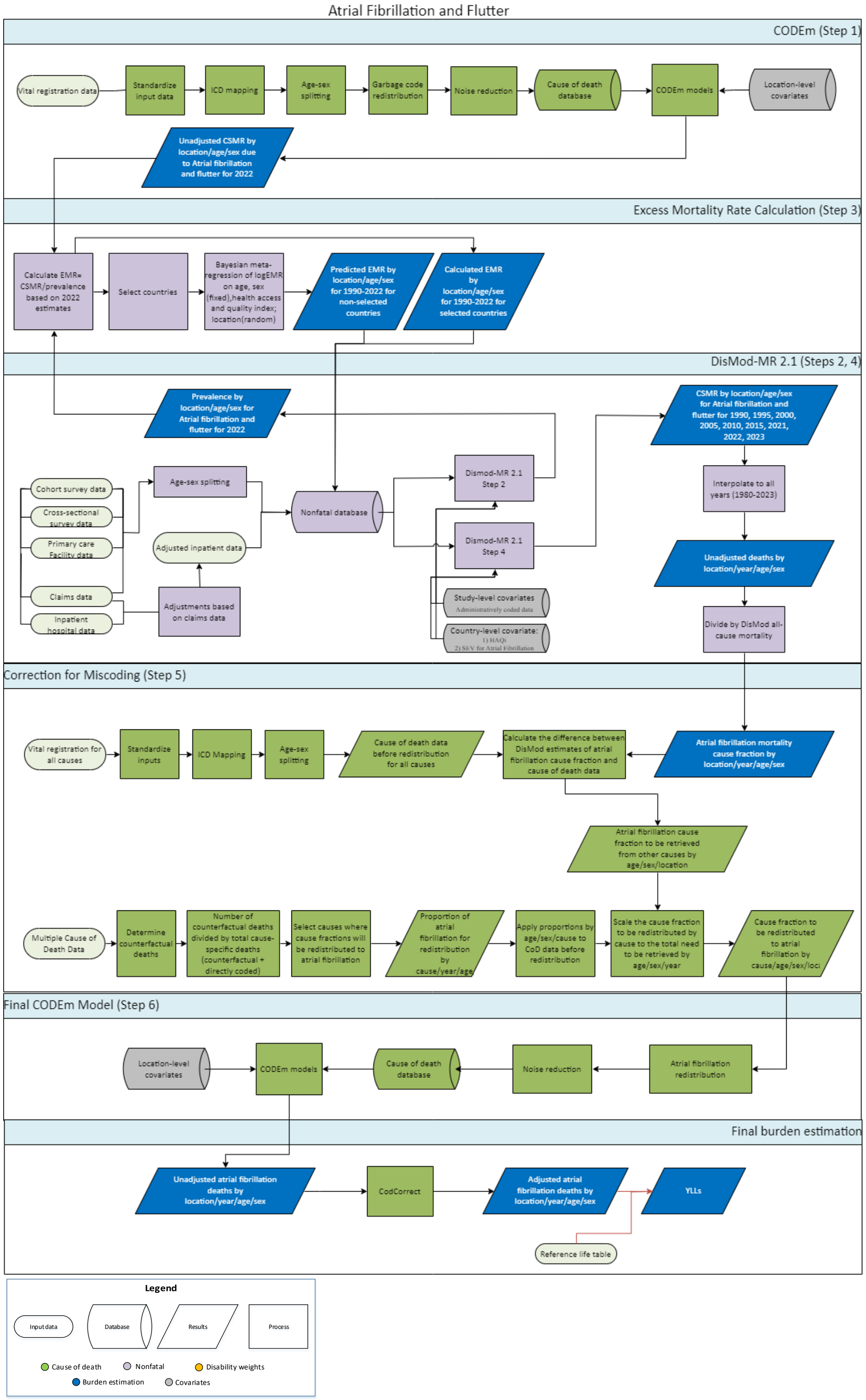

Input data and methodological summary for Atrial fibrillation and flutter

Case definition

Atrial fibrillation is a supraventricular arrhythmia due to disorganised depolarisation of the atrium. Atrial flutter is a macro-reentrant supraventricular arrhythmia, usually involving the cavo-tricuspid isthmus. Diagnosis requires an electrocardiogram (ECG) demonstrating 1) irregularly irregular RR intervals (in the absence of complete AV block); 2) no distinct P waves on the surface ECG, and 3) an atrial cycle length (when visible) that is usually variable and less than 200 milliseconds for atrial fibrillation or typical or atypical atrial flutter with a flutter wave and supraventricular tachycardia with either fixed or variable block.

The International Classification of Disease (ICD) codes used for inclusion of hospital and claims are I48-I48.9 for ICD-10 and 427.3 for ICD-9. Sources reporting ICD code identified atrial fibrillation and flutter was used as alternative case definitions, described in table 1 below.

Quantity of interest	Reference or alternative	Definition
Atrial fibrillation and flutter	Reference	Diagnosis requires an electrocardiogram (ECG) demonstrating 1) irregularly irregular RR intervals (in the absence of complete AV block); 2) no distinct P waves on the surface ECG, and 3) an atrial cycle length (when visible) that is usually variable and less than 200 milliseconds.
Atrial fibrillation and flutter	Alternative	Inpatient/outpatient hospital admission of atrial fibrillation and flutter ICD codes (listed above).
Atrial fibrillation and flutter	Alternative	Inpatient/outpatient hospital admission of atrial fibrillation and flutter ICD codes (listed above) from Truvan MarketScan database in the USA.
Atrial fibrillation and flutter	Alternative	Inpatient/outpatient hospital admission of atrial fibrillation and flutter ICD codes (listed above) from Center for Medicaid Services database in the USA.

Input data

Model inputs

We did not perform a systematic review for atrial fibrillation and flutter in GBD 2023; our last performed systematic review was in GBD 2021. In GBD 2021, we searched PubMed, Embase, and the virtual health library databases for sources. The dates of the search were 1/1/2015–12/31/2019. The search strings used in the systematic review are shown below:

PubMed: ("atrial fibrillation"[TIAB] OR "atrial flutter"[TIAB]) AND (incidence[TIAB] OR prevalence[TIAB] OR "case fatality"[TIAB] OR "excess mortality"[TIAB]) AND ("2015/01/01"[PDAT]: "2019/12/31"[PDAT]) NOT dog NOT animal NOT mice NOT goat NOT pig

Embase: ('atrial fibrillation':ab,ti OR 'atrial flutter':ab,ti) AND (incidence:ab,ti OR prevalence:ab,ti OR 'case fatality':ab,ti OR epidemiology:ab,ti OR 'excess mortality':ab,ti) NOT (rats:ab,ti OR mice:ab,ti OR dogs:ab,ti OR apes:ab,ti OR fish:ab,ti OR monkeys:ab,ti) AND [2015-2019]/py AND ('article'/it OR 'article in press'/it OR 'conference paper'/it OR 'review'/it)`

Virtual Health Library: (tw:("atrial fibrillation") OR tw:("atrial flutter")) AND (tw:(incidence) OR tw:(prevalence) OR tw:(mortality) OR tw:("case fatality") OR tw:(epidemiology)) AND NOT (tw:(rats) OR tw:(mice) OR tw:(dogs) OR tw:(apes) OR tw:(fish) OR tw:(monkeys)) AND (year_cluster:[2015 TO 2019])

Figure 1 shows a PRISMA diagram that displays the text review and extraction process.

Figure 1: PRISMA flow diagram

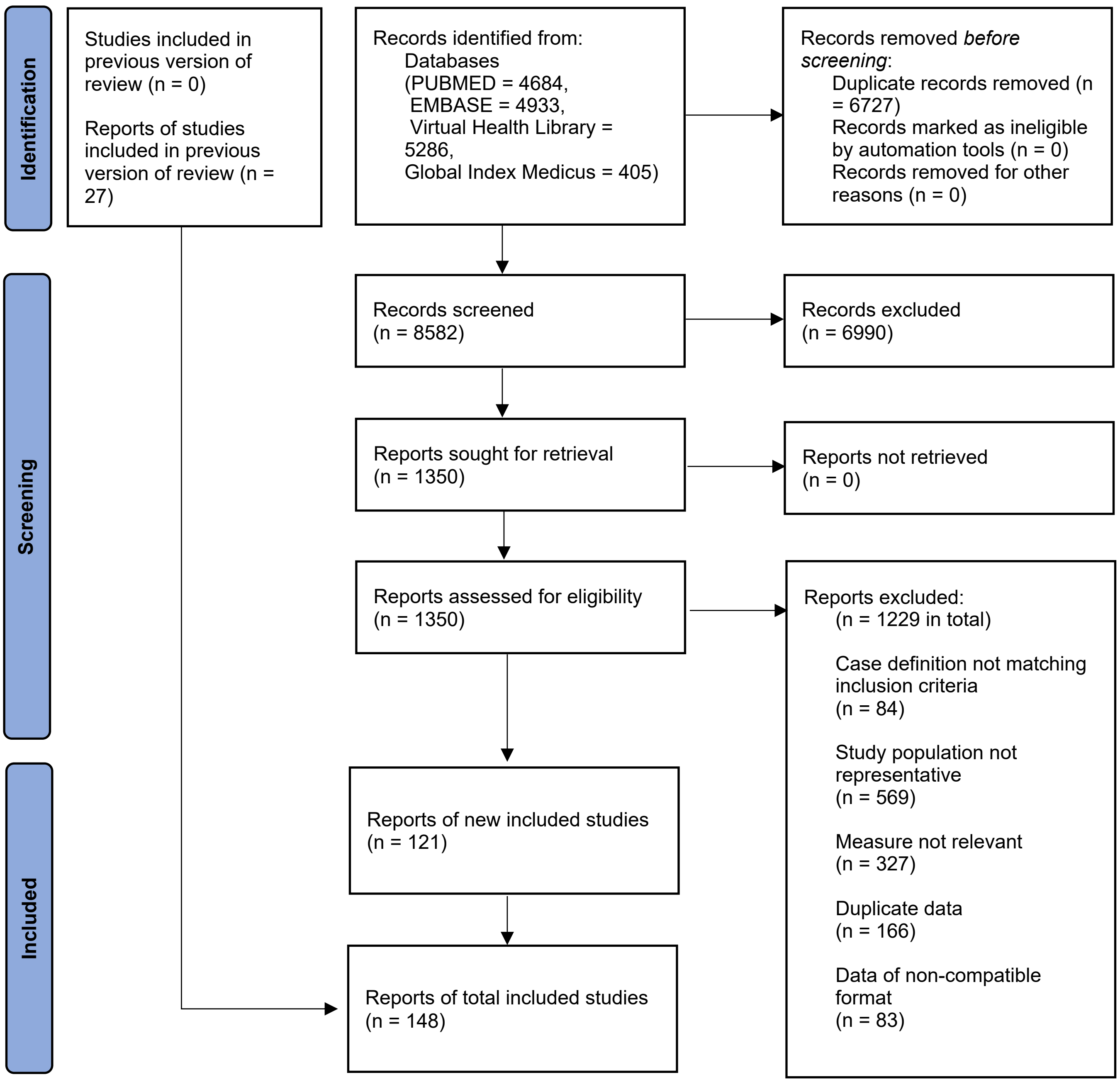

Apart from hospital and claims datapoints on prevalence, no non-literature-based data were included. We included hospital data corrected for readmission, primary to any diagnosis, and inpatient to outpatient utilisation ratios using adjustment factors calculated from USA claims data. We excluded hospital data in certain geographies (eg, the Philippines, China, India, Tibet, Kenya, Chile, Ecuador, Mexico, Botswana, Nepal, Brazil, and Japan) where the data were implausibly low. We also excluded all outpatient administrative data as the values for all locations were implausibly low, for example sources where the prevalence was consistently zero across all ages/sexes.

The reference case definition for atrial fibrillation was diagnosis based on an ECG reading. We adjusted datapoints which used an alternate definition, specifically claims and inpatient hospital data from outside the USA; claims and inpatient data obtained from the Truven MarketScan database; and claims and inpatient data obtained from the Centers for Medicare and Medicaid Services (CMS) database. We used MR-BRT crosswalking standard GBD procedures; more details can be found in the non-fatal appendix crosswalking section. Table 2 shows the adjustment factors produced by the crosswalking procedure. The coefficients in Table 2 below were used to calculate adjustment factors for alternative definitions. The formula for computing adjustment factors is given in equation 1 below. We also included a cubic spline on a standardised age variable (age-scaled) and a categorical sex variable to the crosswalking procedure to adjust for the possibly of bias. The cubic spline can be seen in figure 2 below.

We also split prevalence datapoints where the age range was greater than 25 years. Age splitting was based on the global sex-specific age pattern from a DisMod model that included only prevalence datapoints where the age range was less than 25 years. This was done to allow sources that had wider age ranges to be included in the analysis. These wide-age data sources previously caused issues in fitting known increasing prevalence for atrial fibrillation and flutter with age.

Equation 1: Calculation of adjustment factors:

$$Estimated\ Reference\ Def = invlogit(logit(Alternative\ Def) - [\sum_{s=0}^{b} Beta_{Alternative\ Def, spline\ basis_s} * Spline\ basis_s(age\_scaled)] - Beta * I(Sex))$$

$I(.)$ = Indicator function, b = Number of spline bases used

Age splines for adjustment factors:

We fit a cubic spline to the standardised age variable (named age_scaled, calculated as $age\ scaled = \frac{(mean\ age\ of\ study - mean(age\ of\ all\ studies))}{standard\ deviation(age\ of\ all\ studies)}$). We selected knots for the cubic spline on age scaled based on visual inspection of the spline fit to observed ratios used in computing adjustment factors. Figure 2 below shows the fit of this spline on the standardised age variable for males and females with the observed logit difference between alternative and reference definitions on the vertical axis.

Cause – covariate	Knot placement (age_scaled)	Knot placement (age in years)
Atrial fibrillation and flutter – age_scaled	–1.99, –0.65, 0.22, 0.51, 1.85	47.1, 63.4, 73.9, 77.4, 93.8

Table 2: MR-BRT crosswalk adjustment factors for atrial fibrillation and flutter

Data input	Reference or alternative case definition	Gamma	Beta coefficient, logit (95% CI)
Literature using ECG reading	Ref	0.01	-
Inpatient clinical informatics data, intercept	Alt		–0.51 (–0.53 to –0.49)
Inpatient clinical informatics data, spline_0	Alt		–0.11 (–0.12 to –0.10)
Inpatient clinical informatics data, spline_1	Alt		0.32 (0.27 to 0.36)
Inpatient clinical informatics data, spline_2	Alt		–0.22 (–0.26 to –0.19)
Inpatient clinical informatics data, spline_3	Alt		–0.24 (–0.27 to –0.21)
Inpatient clinical informatics data, male	Alt		–0.41 (–0.39 to –0.43)
MarketScan data, intercept	Alt		–0.28 (–0.29 to –0.26)
MarketScan data, spline_0	Alt		–0.02 (–0.03 to –0.02)
MarketScan data, spline_1	Alt		0.32 (0.29 to 0.35)
MarketScan data, spline_2	Alt		0.07 (0.04 to 0.11)
MarketScan data, spline_3	Alt		0.07 (0.03 to 0.10)
MarketScan data, male	Alt		0.01 (0 to 0.02)
USA claims data, intercept	Alt		0.01 (–0.01 to 0.03)
USA claims data, spline_0	Alt		–0.21 (–0.22 to –0.21)
USA claims data, spline_1	Alt		0.64 (0.60 to 0.67)
USA claims data, spline_2	Alt		0.04 (0.01 to 0.08)
USA claims data, spline_3	Alt		0.05 (0.02 to 0.08)
USA claims data, male	Alt		–0.06 (–0.07 to –0.06)

***The adjustment factor column is the exponentiated beta coefficient. For log beta coefficients, this is the relative rate between the two case definitions. For logit beta coefficients, this is the relative odds between the two case definitions.*

Figure 2a: Age-scaled spline for adjustment of inpatient clinical informatics prevalence data

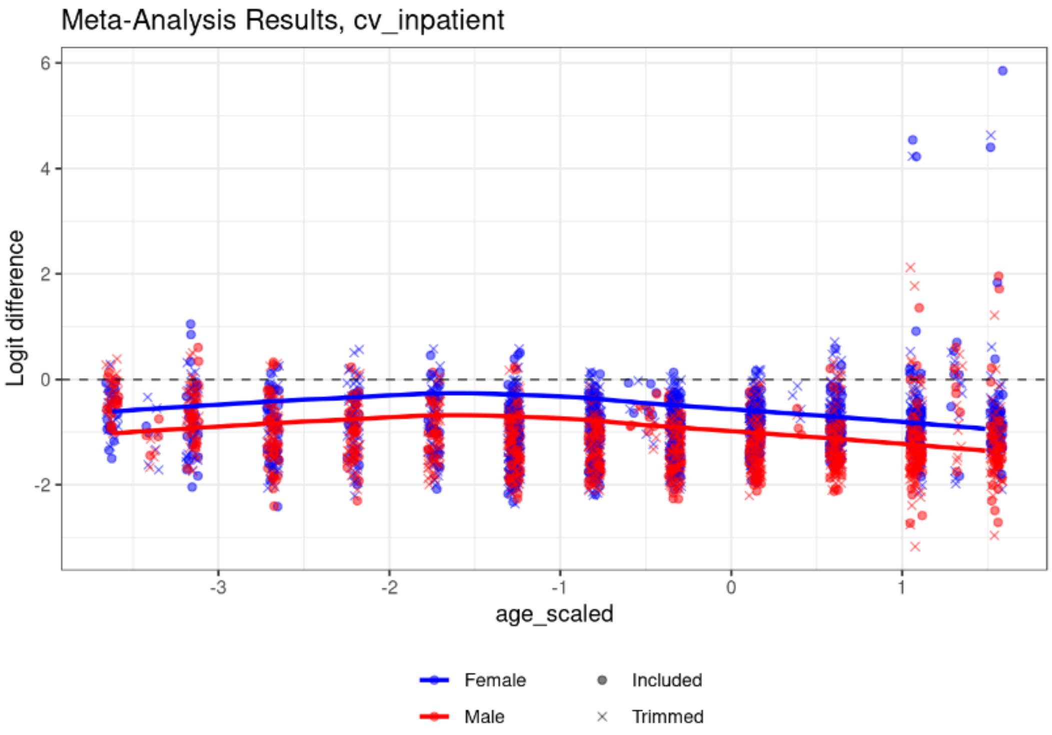

Figure 2b: Age-scaled spline for adjustment of MarketScan clinical informatics prevalence data

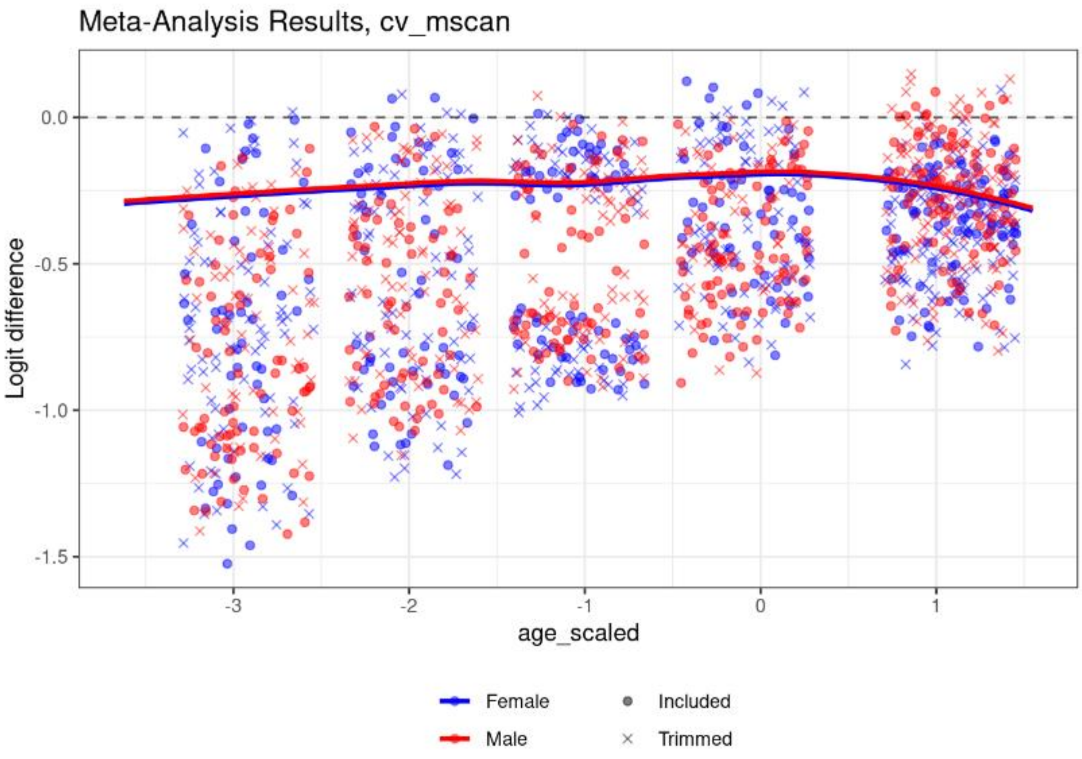

Figure 2c: Age-scaled spline for adjustment of CMS clinical informatics prevalence data

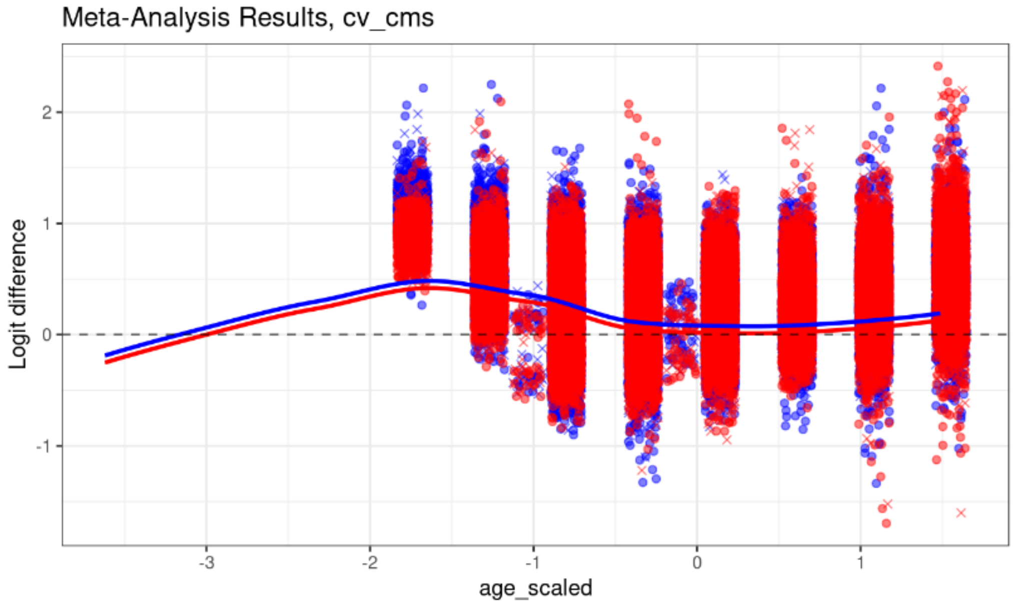

Modelling strategy

To address changes in coding practices for atrial fibrillation that resulted in an implausible trend of increasing death-certificate-based mortality rates, we used a prevalence-based modelling approach that combined DisMod-MR and CODEm models to generate estimates for atrial fibrillation and flutter. This approach, first used in GBD 2015, allowed us to generate more accurate estimates using observed prevalence and incidence rates along with modelled excess mortality rates generated from prevalence and cause-specific mortality estimates.

The modelling steps are illustrated in the above flowchart. Effect sizes for covariates included in both the DisMod-MR 2.1 and CODEm models can be found in the tables below.

In Step 1, we estimated deaths for atrial fibrillation using a standard CODEm approach. We used vital registration data (VR) for the CODEm models. We outliered ICD-8 and ICD-9 vital registration data that were discontinuous from other data in the time series and created an unlikely time trend. We also outliered datapoints that were implausibly low in multiple age groups. More details on this modelling strategy and the list of outliered locations can be seen in the appendix section regarding cause of death modelling of atrial fibrillation.

In Step 2, we estimated prevalence rates in DisMod-MR using data from published reports of cross-sectional and cohort surveys, as well as primary care facility data. We also used claims data covering inpatient and outpatient visits. For GBD 2023, inpatient hospital data were adjusted using age- and sex-specific information for: 1) readmission within one year; 2) primary diagnosis code to secondary codes; and 3) the ratio of inpatient to outpatient visits. These clinical informatics data were then further adjusted using MR-BRT to account for misclassification compared with reference data that used ECG to identify atrial fibrillation and flutter. We set priors of no remission and capped excess mortality at 0.4 for all ages. We included the Healthcare Access and Quality (HAQ) Index as a country-level, fixed-effect

covariate on excess mortality and the log-transformed, age-standardised summary exposure value (SEV) scalar for atrial fibrillation and flutter as a country-level, fixed-effect covariate on prevalence.

In Step 3, we calculated the excess mortality rate (EMR) for the year 2022 (defined as the cause-specific mortality rate [CSMR] estimated from CODEm divided by the prevalence rate from DisMod-MR). We then selected 21 countries based on four conditions: 1) ranking of 4 or 5 stars in the system for assessing the quality of VR data; 2) prevalence data available from the literature were included in the DisMod-MR estimation; 3) prevalence rate ≥0.005; and 4) CSMR ≥0.00002. Using information from these countries as input data, we ran a MR-BRT model of logEMR on sex, a cubic spline of age, and HAQ Index. Specifics on the MR-BRT framework can be found elsewhere in the appendix. We then predicted year-, age-, and sex-specific EMR using the results of this regression for all non-selected countries. Countries included in the regression were assigned their directly calculated values. These EMR datapoints were assigned to the time period 1990–2022 and uploaded into the non-fatal database in order to be used in modelling. Figures 3 illustrates the resulting estimated excess mortality rates described, and how estimates vary by levels of HAQ Index. Note that as access to quality health care decreases, the predicted excess mortality rate increases.

Figure 3: Modelled excess mortality rate relationship with age, sex, and Healthcare Access and Quality Index. *HAQ Index divided into tertiles.

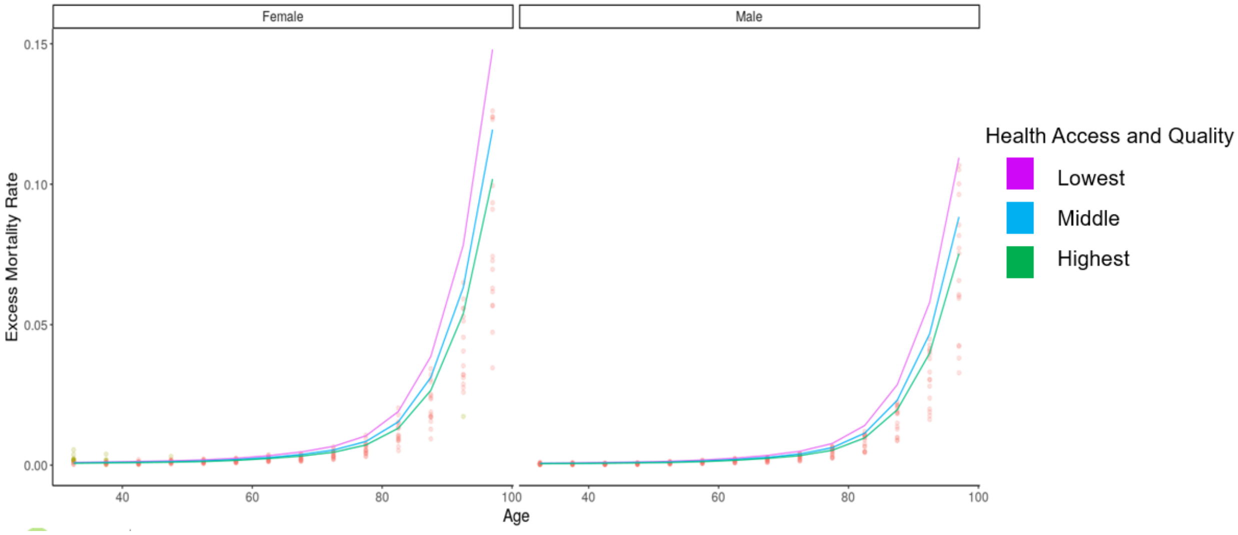

In Step 4, we reran DisMod-MR using the input data described in Step 2 along with the EMR estimated in Step 3. We included HAQ Index as a fixed-effect, country-level covariate on excess mortality and the log-transformed, age-standardised SEV scalar for atrial fibrillation and flutter as a fixed-effect, country-level covariate on prevalence. We included a value prior of zero for remission for all ages and set a value prior of zero for excess mortality and incidence for ages 0–30.

The prevalence from the DisMod-MR model in Step 4 was used as the finalised output for upload to the comorbidity adjustment and further processing into YLDs and DALYs.

The tables below include the study covariates, parameters, betas, and exponentiated betas.

Table 4a. Covariates. Summary of covariates used in the atrial fibrillation and flutter step 2 DisMod-MR meta-regression model

**Covariate**	**Parameter**	**Beta**	**Exponentiated beta**
Log-transformed age-standardised SEV scalar: a-fib	Prevalence	0.91 (0.86 to 0.95)	2.48 (2.36 to 2.60)
Healthcare Access and Quality Index	Excess mortality rate	–0.12 (–0.13 to –0.11)	0.89 (0.87 to 0.90)

Table 4b. Covariates. Summary of covariates used in the atrial fibrillation and flutter step 4 DisMod-MR meta-regression model

Covariate	Parameter	Beta	Exponentiated beta
Log-transformed age-standardised SEV scalar: a-fib	Prevalence	0.77 (0.75 to 0.80)	2.16 (2.12 to 2.21)
Healthcare Access and Quality Index	Excess mortality rate	–0.005 (–0.0051 to –0.0049)	1.00 (0.99 to 1.00)

Models were evaluated based on expert opinion, comparison with results from previous rounds of GBD, and model fit. No substantive changes were made to the modelling strategy for GBD 2021.

Severity splits and disability weights

Atrial fibrillation is split into symptomatic and asymptomatic based on standard GBD proportion information. For GBD 2023, we included heart failure due to atrial fibrillation and flutter sequela into the severity distribution. For details on the heart failure estimation process, see the heart failure section in the appendix. The table below includes lay descriptions and disability weights for the severity levels of atrial fibrillation:

Table 3. Severity distribution, details on the severity levels for atrial fibrillation and flutter in GBD 2023 and the associated disability weight (DW) with that severity

Severity level	Lay description	DW (95% CI)
Asymptomatic atrial fibrillation	No symptoms	N/A
Symptomatic atrial fibrillation, without heart failure	Has periods of rapid and irregular heartbeats and occasional fainting.	0.224 (0.151–0.312)
Symptomatic atrial fibrillation, with	Has periods of rapid and irregular heartbeats and occasional fainting, has been diagnosed with clinical heart failure, a chronic disease that requires medication	0.012 (0.006–0.023)

asymptomatic heart failure	every day and causes some worry but minimal interference with daily activities.	
Symptomatic atrial fibrillation, with mild heart failure	Has periods of rapid and irregular heartbeats and occasional fainting, is short of breath and easily tires with moderate physical activity, such as walking uphill or more than a quarter-mile on level ground. The person feels comfortable at rest or during activities requiring less effort.	0.042 (0.026–0.062)
Symptomatic atrial fibrillation, with moderate heart failure	Has periods of rapid and irregular heartbeats and occasional fainting, is short of breath and easily tires with minimal physical activity, such as walking only a short distance. The person feels comfortable at rest but avoids moderate activity.	0.072 (0.047–0.103)
Symptomatic atrial fibrillation, with severe heart failure	Has periods of rapid and irregular heartbeats and occasional fainting, is short of breath and feels tired when at rest. The person avoids any physical activity, for fear of worsening the breathing problems.	0.179 (0.122–0.312)

Attention-deficit (hyperactivity disorder)

Flowchart

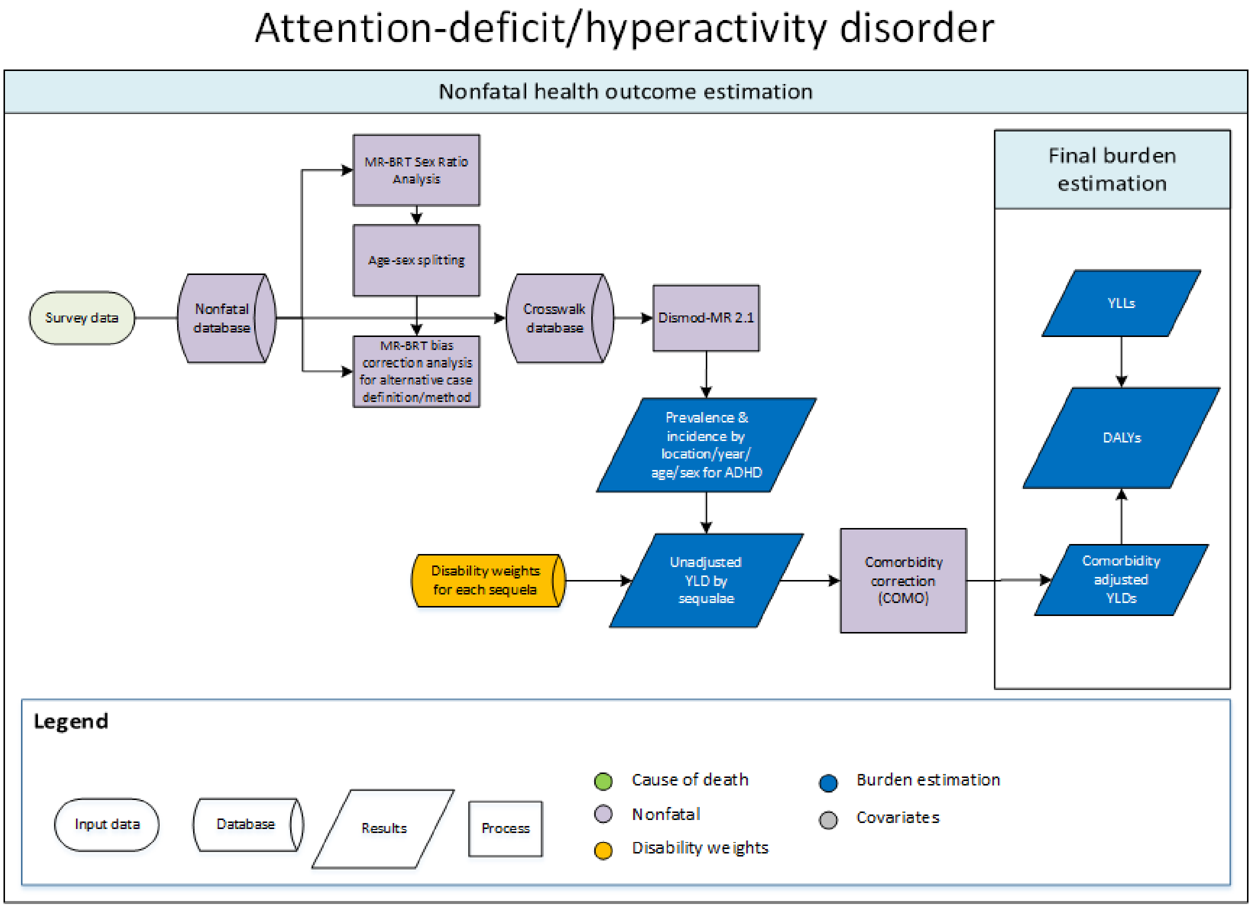

Input data and methodological summary for attention-deficit/hyperactivity disorder

Case definition

Attention-deficit/hyperactivity disorder (ADHD) is an externalising disorder characterised by persistent inattention and/or hyperactivity-impulsivity. As per criteria set by the Diagnostic and Statistical Manual of Mental Disorders Fourth Edition, Text Revision (DSM-IV-TR)[1] diagnosis requires six or more symptoms of inattention or hyperactivity-impulsivity to have persisted for at least six months in two or more settings causing significant impairment to functioning, with at least some impairing symptoms being present prior to 7 years of age (12 years of age in DSM-5).[2] Recognised symptoms include:

Inattention:

- often fails to give close attention to details or makes careless mistakes in schoolwork, work, or other activities

- often has difficulty sustaining attention in tasks or play activities
- often does not seem to listen when spoken to directly
- often does not follow through on instructions and fails to finish schoolwork, chores, or duties in the workplace (not due to oppositional behaviour or failure to understand instructions)
- often has difficulty organising tasks and activities
- often avoids, dislikes, or is reluctant to engage in tasks that require sustained mental effort (such as schoolwork or homework)
- often loses things necessary for tasks or activities (eg, toys, school assignments, pencils, books, or tools)
- is often easily distracted by extraneous stimuli
- is often forgetful in daily activities

Hyperactivity:

- often fidgets with hands or feet or squirms in seat
- often leaves seat in classroom or in other situations in which remaining seated is expected
- often runs about or climbs excessively in situations in which it is inappropriate (in adolescents or adults, may be limited to subjective feelings of restlessness)
- often has difficulty playing or engaging in leisure activities quietly
- is often "on the go" or often acts as if "driven by a motor"
- often talks excessively

Impulsivity:

- often blurts out answers before questions have been completed
- often has difficulty awaiting turn
- often interrupts or intrudes on others (eg, butts into conversations or games)

Included in the GBD study were cases meeting diagnostic criteria according to DSM[1] or the International Classification of Diseases (ICD)[3] (called "hyperkinetic disorders" in ICD). These were identified by the ICD-10 code: F90. Different versions of DSM (DSM-III, DSM-III-R, DSM-IV, DSM-IV-TR, DSM-5, and DSM-5-TR) and ICD (ICD-9, ICD-10, and ICD-11) were accepted.

Input data

The epidemiological systematic literature review for ADHD was conducted in three stages involving electronic searches of the peer-reviewed literature (ie, via PsycInfo, Embase, and PubMed), the grey literature, and expert consultation. For mental disorders, we update our GBD electronic database searches on a rolling basis. A systematic review update was completed for GBD 2023 for ADHD and conduct disorder combined. Databases were searched on September 4, 2023, for publications after September 2, 2018. Below is the search terms used for each database:

PubMed: ((((((("attention deficit and disruptive behavior disorders"[MeSH Terms]) OR ((attention*[Title/Abstract]) AND (disorder*[Title/Abstract]) AND (hyperactiv*[Title/Abstract]))) OR (hyperkinetic[Title/Abstract])) OR (adhd[Title/Abstract])) OR ("conduct disorder"[Title/Abstract])) OR ("conduct disorders"[Title/Abstract])) OR ("conduct disordered"[Title/Abstract])) OR (externali*[Title/Abstract])) OR ((disruptive[Title/Abstract]) AND (disorder[Title/Abstract])) AND ((((((((((prevalen*[Title/Abstract]) OR (mortalit*[Title/Abstract])) OR (death*[Title/Abstract])) OR (inciden*[Title/Abstract])) OR (remission[Title/Abstract])) OR (duration[Title/Abstract])) OR (remit*[Title/Abstract])) OR (epidemiolog*[Title/Abstract])) OR ("Prevalence"[MeSH])) OR

("Mortality"[MeSH])) OR ("Incidence"[MeSH])) OR ("Epidemiology"[MeSH:NoExp])) OR ("Morbidity"[MeSH:NoExp])

Embase: ('attention deficit disorder'/exp OR 'disruptive behavior'/exp OR 'conduct disorder'/exp OR 'disruptive behavior':ab,ti OR 'conduct disorder':ab,ti OR (attention:ab,ti AND disorder:ab,ti AND hyperactiv*:ab,ti) OR 'conduct disorders':ab,ti OR 'conduct disordered':ab,ti OR hyperkinetic:ab,ti OR adhd:ab,ti OR (disruptive:ab,ti AND disorder:ab,ti) OR externali*:ab,ti) AND ((prevalen*:ab,ti OR mortalit*:ab,ti OR death*:ab,ti OR inciden*:ab,ti OR remission:ab,ti OR duration:ab,ti OR remit*:ab,ti OR epidemiolog*:ab,ti OR 'prevalence'/exp OR 'epidemiology'/exp OR 'remission'/exp OR 'incidence'/exp OR 'mortality'/exp OR 'morbidity'/mj)

PsycInfo: ((title: (prevalen*)) OR (abstract: (prevalen*)) OR (title: (mortalit*)) OR (abstract: (mortalit*)) OR (title: (death*)) OR (abstract: (death*)) OR (title: (inciden*)) OR (abstract: (inciden*)) OR (title: (remission)) OR (abstract: (remission)) OR (title: (duration)) OR (abstract: (duration)) OR (title: (remit*)) OR (abstract: (remit*)) OR (title: (epidemiolog*)) OR (abstract: (epidemiolog*)) OR (Index Terms: ("Mortality Rate")) OR (Index Terms: ("Epidemiology")) OR (Index Terms: ("Morbidity")) OR (Index Terms: ("Remission (Disorders)"))) AND ((Index Terms: ("attention deficit disorder")) OR (Index Terms: ("conduct disorder")) OR (Index Terms: ("disruptive behavior")) OR (abstract: (adhd)) OR (title: (adhd)) OR (abstract: ("conduct disorder")) OR (title: ("conduct disorder")) OR (abstract: ("conduct disorders")) OR (title: ("conduct disorders")) OR (abstract: ("conduct disordered")) OR (title: ("conduct disordered")) OR (abstract: (externali*)) OR (title: (externali*)) OR ((abstract: (attention*) OR (title: (attention*))) AND (abstract: (disorder*) OR (title: (disorder*))) AND (abstract: (hyperactiv*) OR (title: (hyperactiv*)))) OR (abstract: (hyperkinetic)) OR (title: (hyperkinetic)) OR ((abstract: (disruptive) OR (title: (disruptive))) AND (abstract: (disorder) OR (title: (disorder)))))

In addition to the database search, a grey literature search and expert consultation were conducted. The systematic review update was conducted according to Preferred Reporting Items for Systematic Reviews and Meta-Analyses (PRISMA; see Figure 1).

Figure 1: PRISMA flow diagram

From: Moher D, Liberati A, Tetzlaff J, Altman DG, The PRISMA Group (2009). *P*referred *R*eporting *I*tems for *S*ystematic Reviews and *M*eta-*A*nalyses: The PRISMA Statement. PLoS Med 6(7): e1000097. doi:10.1371/journal.pmed1000097

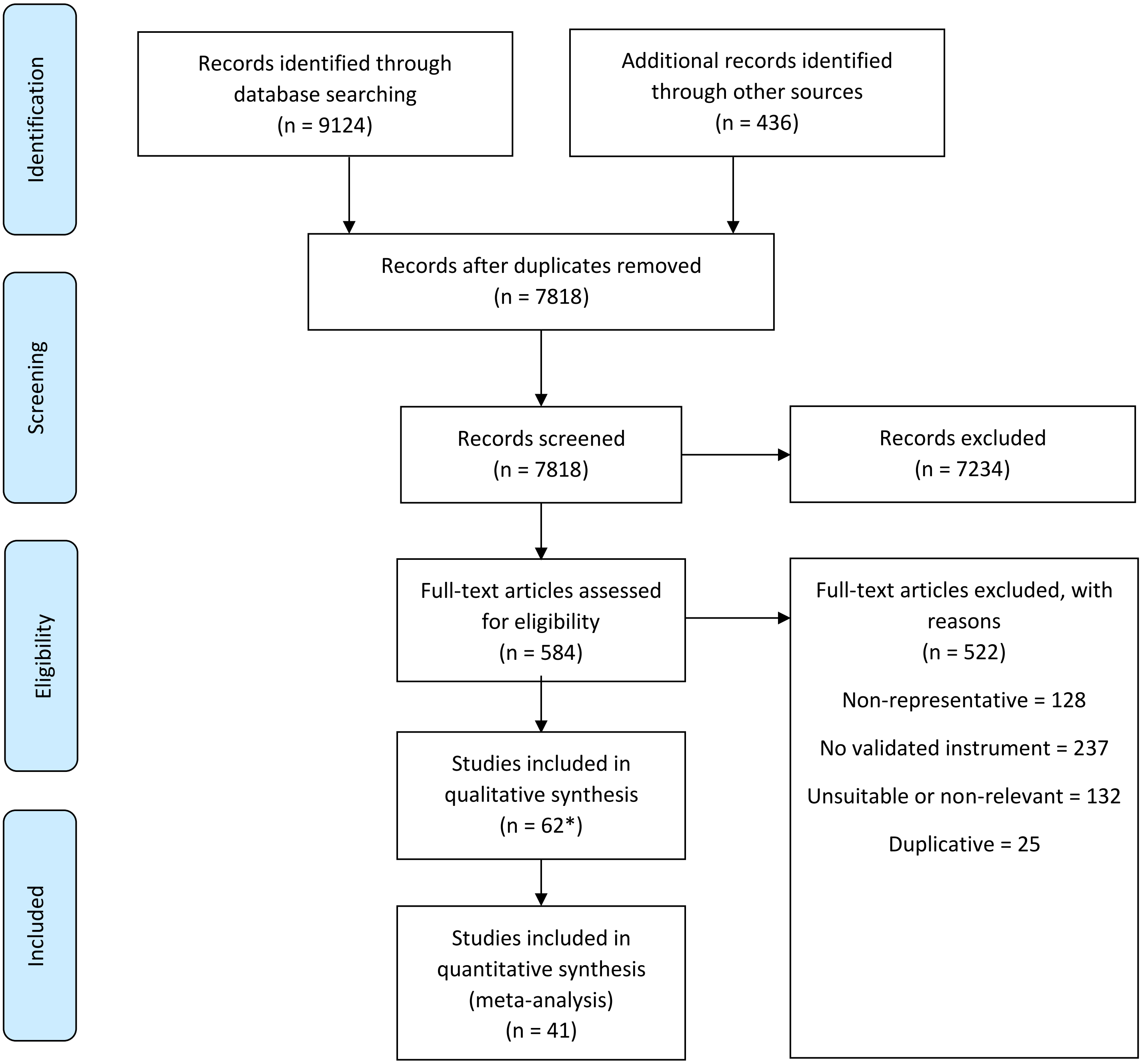

**The qualitative analysis led to the exclusion of additional studies with duplicative cohorts or methodological limitations impacting their eligibility and increasing measurement error within the data.*

The GBD inclusion criteria stipulated that: 1) the publication year must be from 1980 onward; 2) "caseness" must be based on clinical threshold as established by the DSM or ICD; 3) sufficient information must be provided on study method and sample characteristics to assess the quality of the study; and 4) study sample must be representative of the general population (ie, inpatient or pharmacological treatment samples, case studies, veterans, or refugee samples were excluded). No limitation was set on the language of publication. Methods used in previous systematic reviews have been reported in greater detail elsewhere.[4]

Age-sex splitting

The extracted data underwent two types of age-sex splitting processes:

1. Where possible, estimates were further split by sex and age based on the available data. For instance, if studies reported prevalence for broad age groups by sex (eg, prevalence in 15–65-year-old males and females separately), and also by specific age groups but for both sexes combined (eg, prevalence in 15–30-year-olds, then in 31–65-year-olds, for males and females combined); age-specific estimates were split by sex using the reported sex-ratio and bounds of uncertainty.
2. A meta-regression—Bayesian, regularised, trimmed (MR-BRT) analysis was used to split the remaining both-sex estimates in the dataset. For each parameter, sex-specific estimates were matched by location, age, and year. A MR-BRT network meta-analysis was then used to estimate pooled sex ratios and bounds of uncertainty. These were then used to split the both-sex estimates in the dataset. The male-to-female ratio was estimated as 2.51 (95% uncertainty interval [UI]: 2.39–2.64) for prevalence, 1.16 (0.55–2.42) for remission, and 2.42 (0.22–27.13) for incidence.

Bias corrections/crosswalks

Estimates with known biases were adjusted/crosswalked accordingly prior to DisMod-MR 2.1. For each crosswalk of interest, pairs of the reference and the alternative estimates were matched by age, sex, location, and year. This was done for both within-study (where possible) and between-study pairs. These pairs were then used as inputs in a MR-BRT network meta-analysis. The MR-BRT analysis produced a pooled ratio between the reference estimates and alternative estimates, which was used to adjust all alternative estimates in the dataset. Reference data informing the prevalence of ADHD consisted of estimates reporting past-month/point prevalence of ADHD that included impairment in their criterion for diagnosis. Two adjustment ratios were used for alternative data:

1. A past-year recall ratio adjusted all datapoints derived from past-year prevalence toward the level they would have been if the study had captured point/past-month prevalence. The latter prevalence period is less affected by recall bias.
2. A no-impairment ratio adjusted all datapoints that did not require cases to meet criteria for impairment toward the level they would have been if the study had required impairment for diagnosis.

See Table 1 for adjustment factors used for ADHD. The estimated UIs around the adjustment ratio incorporate gamma, which represents the between-study variance across all input data in the model. This added uncertainty widens the UIs for crosswalks with significant fixed effects.

Table 1: MR-BRT crosswalk adjustment factors for ADHD

Data input	Reference or alternative case definition	Gamma	Beta coefficient, log (95% UI)*	Adjustment factor**
Population survey	Reference: past-month/point prevalence, meeting the impairment criteria	0.05		
Population survey	Alternative: past-year prevalence		0.35 (0.19–0.52)	1.42 (1.21–1.67)
Population survey	Alternative: no impairment		0.42 (0.24–0.61)	1.53 (1.27–1.83)

**MR-BRT crosswalk adjustments can be interpreted as the factor the alternative case definition is adjusted by to reflect what it would have been had it been measured using the reference case definition. If the log/logit beta coefficient is negative, then the alternative is adjusted up to the reference. If the log/logit beta coefficient is positive, then the alternative is adjusted down to the reference.*

***The adjustment factor column is the exponentiated beta coefficient. For log beta coefficients, this is the relative rate between the two case definitions. For logit beta coefficients, this is the relative odds between the two case definitions.*

Modelling strategy

We have made no substantive changes in the modelling strategy from GBD 2021.

After the above data processes were applied, DisMod-MR 2.1 was used to model the epidemiological data for ADHD. Adjustments to model priors or to the dataset were made where appropriate. Where outliers were identified in the data, we re-assessed the study's methodology and quality before a decision was made to exclude or include the data.

Data across all epidemiological parameters were initially included in the modelling process. We assumed no incidence prior to 3 years of age or onward from 12 years of age. The minimum age of onset was set in consultation with experts and based on current literature, while the upper age limit on incidence was set in line with the latest DSM-5 criteria. Remission was set to zero prior to 12 years, in line with the restriction on incidence. Excess mortality was set to zero given only three estimates were found for this parameter and there were insufficient data to suggest an elevated risk of mortality in those with ADHD.

Severity splits and disability weight

The GBD disability weight survey assessments include lay descriptions of sequelae highlighting major functional consequences and symptoms. The lay description and disability weight for ADHD is shown in Table 2. The inclusion of an impairment crosswalk for ADHD means that outcomes represent cases of ADHD that are symptomatic and impaired. As modelled estimates do not include asymptomatic cases, no severity splits were required.

Table 2. Lay description for ADHD in GBD 2023 and the associated disability weight

Lay description	Disability weight (95% UI)
Is hyperactive and has difficulty concentrating, remembering things, and completing tasks.	0.045 (0.028–0.066)

Relative to GBD 2021, modelling of ADHD for GBD 2023 saw the addition of bias adjustments for recall and impairment, and a corresponding removal of the existing severity split between symptomatic and asymptomatic cases. While we continue to improve on the data and methods used in GBD, some challenges need to be acknowledged. Firstly, we still have a large number of locations with no high-quality raw data available. Secondly, it is difficult to quantify and remove all variations due to measurement error in our prevalence estimates. While we have improved the methodology used to account for known sources of bias (eg, survey methods or case definitions), we still have very few datapoints to inform such adjustments. Thirdly, there is a paucity of research on the risk factors of mental disorders which can be used as predictive covariates in our epidemiological models.

References

1. American Psychiatric Association. Diagnostic and Statistical Manual of Mental Disorders (DSM-IV-TR). 4th, Text Revision ed. Washington DC: American Psychiatric Association; 2000.
2. American Psychiatric Association. Diagnostic and Statistical Manual of Mental Disorders, Fifth Edition. Arlington, VA: American Psychiatric Association; 2013.
3. World Health ICD-10 Classification of Mental and Behavioural Disorders: Clinical descriptions and diagnostic guidelines. Geneva: World Health
4. Erskine HE, Ferrari AJ, Nelson P, et al. Research Review: Epidemiological modelling of attention-deficit/hyperactivity disorder and conduct disorder for the Global Burden of Disease Study 2010. *Journal of Child Psychology and Psychiatry* 2013; **54**(12): 1263-74.
5. Ezpeleta L, Keeler G, Erkanli A, Costello EJ, Angold A. Epidemiology of Psychiatric Disability in Childhood and Adolescence. *Journal of Child Psychology and Psychiatry and Allied Disciplines* 2001; **42**(7): 901-14.
6. Erskine HE, Ferrari AJ, Polanczyk GV, et al. The global burden of conduct disorder and attention-deficit/hyperactivity disorder in 2010. *Journal of Child Psychology and Psychiatry* 2014; **55**(4): 328-36.

Autism spectrum disorders

Flowchart

Autism Spectrum Disorders

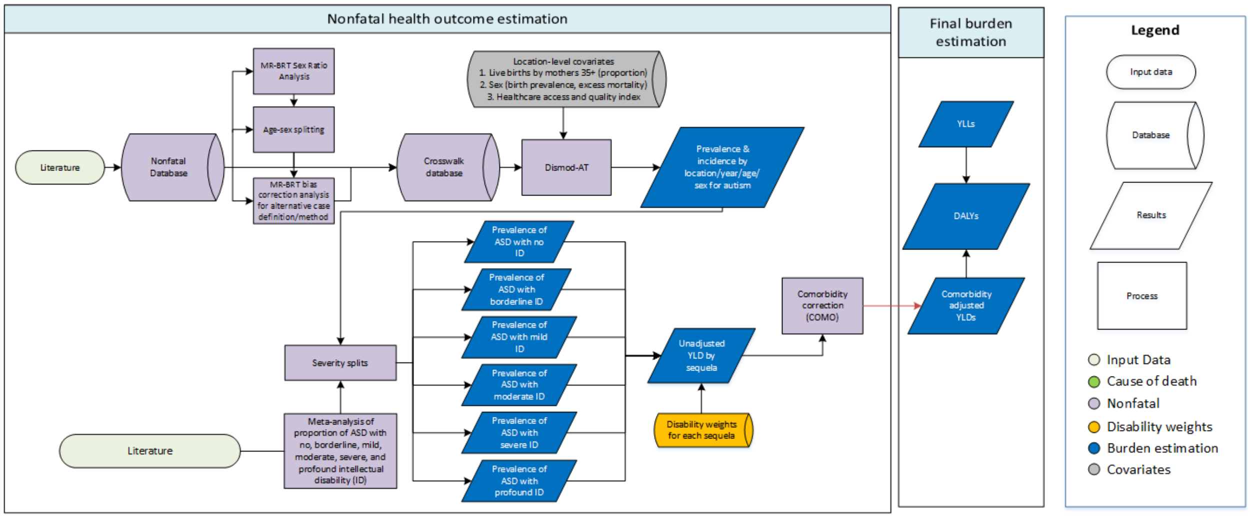

Input data and methodological summary for autism spectrum disorders

Case definition

Autism spectrum disorders (ASD) – also known as pervasive developmental disorders – are a group of neurodevelopmental disorders with onset occurring in early childhood. ASD is characterised by pervasive impairment in several areas of development, including social interaction and communication skills, along with restricted and repetitive patterns of behaviours and/or interests.

ASD was an umbrella for five sub-disorders according to the Diagnostic and Statistical Manual of Mental Disorders fourth edition, text revision (DSM-IV-TR):[1] autistic disorder, pervasive developmental disorder, pervasive developmental disorder not otherwise specified, Rett's disorder, Asperger's disorder, and childhood disintegrative disorder. ASD was also an umbrella for eight sub-disorders according to the International Statistical Classification of Diseases and Related Health Problems 10th Revision (ICD-10):[2] childhood autism (F84.0), atypical autism (F84.1), Rett's syndrome (F84.2), other childhood disintegrative disorder (F84.3), overactive disorder associated with mental retardation and stereotyped movements (F84.4), Asperger syndrome (F84.5), other pervasive developmental disorders (F84.8), and pervasive disorder unspecified (F84.9). However, it was amalgamated into a single disorder in the Diagnostic and Statistical Manual for Mental Disorders 5th edition (DSM-5).[3] A diagnosis of ASD according to the DSM-5 requires the following criteria to be met:

Persistent deficits in social communication and social interaction across multiple contexts, as manifested by all of the following, currently or by history:

1. *Deficits in social-emotional reciprocity, ranging, for example, from abnormal social approach and failure of normal back-and-forth conversation; to reduced sharing of interests, emotions, or affect; to failure to initiate or respond to social interactions.*
2. *Deficits in nonverbal communicative behaviours used for social interaction, ranging, for example, from poorly integrated verbal and non-verbal communication; to abnormalities in eye contact and body language or deficits in understanding and use of gestures; to a total lack of facial expressions and non-verbal communication.*
3. *Deficits in developing, maintaining, and understanding relationships, ranging, for example, from difficulties adjusting behaviour to suit various social contexts; to difficulties in sharing imaginative play or in making friends; to absence of interest in peers.*

Restricted, repetitive patterns of behaviour, interests, or activities, as manifested by at least two of the following, currently or by history:

1. *Stereotyped or repetitive motor movements, use of objects, or speech (eg, simple motor stereotypies, lining up toys or flipping objects, echolalia, idiosyncratic phrases).*
2. *Insistence on sameness, inflexible adherence to routines, or ritualised patterns of verbal or nonverbal behaviour (eg, extreme distress at small changes, difficulties with transitions, rigid thinking patterns, greeting rituals, need to take same route or eat same food every day).*
3. *Highly restricted, fixated interests that are abnormal in intensity or focus (eg, strong attachment to or preoccupation with unusual objects, excessively circumscribed or perseverative interests).*
4. *Hyper- or hyporeactivity to sensory input or unusual interest in sensory aspects of the environment (eg, apparent indifference to pain/temperature, adverse response to specific sounds or textures, excessive smelling or touching of objects, visual fascination with lights or movement).*

The symptoms must be present in the early developmental period, cause clinically significant impairment, and not be better explained by intellectual impairment or global developmental delay.

Different versions of DSM (DSM-III, DSM-III-R, DSM-IV, DSM-IV-TR, DSM-5, and DSM-5-TR) and ICD (ICD-9, ICD-10, and ICD-11) were accepted.

Input data

The epidemiological systematic literature review for ASD was first conducted for GBD 2017 and updated in April 2021 for GBD 2021. It was conducted in three stages involving electronic searches of the peer-reviewed literature (ie, via PsycInfo, Embase, and PubMed), the grey literature, and expert consultation. For mental disorders, we update our GBD electronic database searches on a rolling basis. An electronic search was not required for GBD 2023 and will be conducted in a subsequent round of GBD.

The GBD inclusion criteria stipulated that: 1) the diagnostic criteria must be from 1980 onward; 2) "caseness" must be based on clinical threshold as established by the DSM, ICD, Chinese Classification of Mental Disorders (CCMD), or diagnosed by a clinician using established tools; 3) sufficient information must be provided on study method and sample characteristics to assess the quality of the study; and 4) study samples must be representative of the general population (ie, case studies, veterans, or refugee samples were excluded). No limitation was set on the language of publication.

To improve data coverage for ASD, estimates of the prevalence of the DSM-IV-TR sub-disorder autistic disorder, ICD-10 childhood autism (F84.0), and their DSM-III, DSM-III-R, DSM-IV, ICD-9, and CCMD equivalents were also included with an adjustment so that they reflected what these estimates would be if the data represented ASD. Administrative prevalence estimates were included with bias corrections in the epidemiological modelling of ASD in GBD 2019. However, there were not enough data to explore the interaction between administrative estimates and time or health-care access quality. This potentially inflated prevalence in locations with good health-care access quality where the majority of ASD cases are diagnosed, and underestimated prevalence in locations where health-care access quality is poor and the majority of ASD cases are missed. Administrative prevalence estimates were therefore excluded in GBD 2021 and GBD 2023.

Age-sex splitting

The extracted data underwent two types of age-sex splitting processes:

1. Where possible, estimates were further split by sex and age based on the available data. For instance, if studies reported prevalence for broad age groups by sex (eg, prevalence in 15–65-year-old males and females separately), and also by specific age groups but for both sexes combined (eg, prevalence in 15–30-year-olds, then in 31–65-year-olds, for males and females combined); age-specific estimates were split by sex using the reported sex-ratio and bounds of uncertainty.
2. A meta-regression—Bayesian, regularised, trimmed (MR-BRT) analysis was used to split the remaining both-sex estimates in the dataset. For each parameter, sex-specific estimates were matched by location, age, and year, and a MR-BRT network meta-analysis was used to estimate pooled sex ratios and bounds of uncertainty. These were then used to split the both-sex estimates in the dataset. The male-to-female prevalence ratio was 2.40 (95% uncertainty interval [UI] 2.32–2.48). The male-to-female excess mortality rate ratio was 0.73 (0.62–0.86).

Bias corrections/crosswalks

Estimates with known biases were adjusted/crosswalked accordingly prior to DisMod-AT. Within the ASD epidemiological dataset, within-study estimates were paired by age, sex, location, and year, between the reference and alternative estimates. Pairs were also made between the different alternative estimates where applicable. The ratios between these estimates were then used as inputs in a MR-BRT network meta-analysis. This analysis produced pooled ratios between the reference estimates and alternative estimates. These ratios (see Table 1) were used to adjust all alternative estimates in the dataset. The estimated UIs around the adjustment ratio incorporate gamma, which represents the between-study variance across all input data in the model. This added uncertainty widens the UIs for crosswalks with significant fixed effects. ASD had two alternative definitions to crosswalk:

1. Estimates of autism (rather than of ASD).
2. General population survey without additional case-finding. These are studies that conduct household or school surveys but do not conduct additional active case-finding (such as reviewing special education records) to find cases likely to be missed by survey methodology.

Table 1: MR-BRT crosswalk adjustment factors for ASD

Data input	Reference or alternative case definition	Gamma	Beta coefficient, log (95% UI)*	Adjustment factor**
Population survey	Reference: estimate represents ASD from general population surveys, with additional case-finding or total population screening	0.008		
Population survey	Alternative: estimate represents autism (rather than ASD)		–0.81 (–0.93 to –0.69)	0.44 (0.39–0.50)
Population survey	Alternative: general population survey without additional case-finding		–0.35 (–0.50 to –0.20)	0.70 (0.61–0.82)

**MR-BRT crosswalk adjustments can be interpreted as the factor the alternative case definition is adjusted by to reflect what it would have been had it been measured using the reference case definition. If the log/logit beta coefficient is negative, then the alternative is adjusted up to the reference. If the log/logit beta coefficient is positive, then the alternative is adjusted down to the reference.*

***The adjustment factor column is the exponentiated beta coefficient. For log beta coefficients, this is the relative rate between the two case definitions. For logit beta coefficients, this is the relative odds between the two case definitions.*

Modelling strategy

For GBD 2023, modelling transitioned from using DisMod-MR 2.1 to DisMod-AT. Bias and root mean square error (RMSE) are shown in Table 2 for each measure. Relative to DisMod-MR, DisMod-AT provides enhanced estimation machinery and modelling performance, enabling less data pre-processing, and fewer assumptions to be made. As a result, the following methodological changes were made for GBD 2023. When a base fit is completed in DisMod-AT, standardised residuals are produced for all datapoints. Any observation where the absolute value of the standardised residual is greater than 2 were considered model outliers and were flagged for additional review. We reassessed the study methodology and quality of estimates flagged in this way before a decision was made to exclude or include such data in final models

We assumed all incidence of ASD occurred at birth and no remission across the lifespan. A maximum excess mortality rate of 0.3 was set to align with the largest observed confidence interval for an excess mortality rate estimate for ASD.

To enhance predictive accuracy, DisMod-AT models are refined through a covariate selection process, evaluating various location-level covariates for their effectiveness. This process begins with an initial selection of covariates (for incidence, birth prevalence, remission, and/or excess mortality) informed by existing evidence and expert consultations. Covariate selection is then refined using a forward stepwise method. In the initial step, each covariate is assessed against the base model. Following this, the best performing covariate is retained, and the process repeats, evaluating the remaining covariates against the newly updated model. This stepwise selection continues until no further improvement in predictive power is noted, as measured by the performance of model covariates and the root mean square standardised residuals (RMSSR). A summary of the location-level covariates and exponentiated values for ASD are shown in Table 3.

Table 2. Performance metrics for the ASD DisMod-AT model

Measure	Bias	RMSE
Prevalence	-0.00199	0.00592
Excess mortality	-0.00506	0.0185
Birth prevalence	0.0000627	0.00195

Table 3. Summary of covariates used in the ASD DisMod-AT meta-regression model

Covariate	Type	Parameter	Beta	Exponentiated beta
Percentage of births to women over 35	Location-level	Birth prevalence	0.69 (–0.43 to 1.81)	1.99 (0.65–6.13)
Sex	Location-level	Birth prevalence	0.94 (0.85 to 1.03)	2.56 (2.34–2.80)
Sex	Location-level	Excess mortality	–0.15 (–0.46 to 0.16)	0.86 (0.63–1.17)
Healthcare Access and Quality Index	Location-level	Excess mortality	–0.03 (–0.03 to –0.03)*	0.97 (0.97–0.97)*

Note: DisMod-AT leverages on data from all parameters to estimate location-level-covariates for a specific parameter.
**To achieve plausible variation of excess mortality rates across geography and time, the inclusion of HAQ Index on excess mortality was tested separately. Beta values between –0.01 to –0.09 were directly tested and –0.03 had the best out-of-sample predictive performance across the five random holdouts.*

Severity splits and disability weights

ASD is one of the causes that contribute to the intellectual disability (ID) envelope. As such, a gradation of ASD by level of intellectual disability was required. Meta-analyses were conducted using data from 19 studies that used reference sampling methodology and reported information on the IQ level of those with ASD in order to calculate the severity splits by six sequelae: ASD with 1) no ID, 2) borderline ID, 3) mild ID, 4) moderate ID, 5) severe ID, and 6) profound ID.

The disability weights for each sequela of ASD were calculated using the disability weights for the health states autism; Asperger's syndrome and other ASD; borderline ID; mild ID; moderate ID; severe ID; and profound ID. These disability weights and their lay descriptions are presented in Table 4.

Table 4: Severity distribution, details on the severity levels for ASD and the associated disability weight with that severity

Health state	Lay description	Disability weight (95% UI)

Autism	Has severe problems interacting with others and difficulty understanding simple questions or directions. The person has great difficulty with basic daily activities and becomes distressed by any change in routine.	0.262 (0.176–0.365)
Asperger's syndrome and other ASDs	Has difficulty interacting with other people and is slow to understand or respond to questions. The person is often preoccupied with one thing and has some difficulty with basic daily activities.	0.104 (0.071–0.147)
ID, borderline	Is slow in learning at school. As an adult, the person has some difficulty doing complex or unfamiliar tasks but otherwise functions independently.	0.011 (0.005–0.020)
ID, mild	Has low intelligence and is slow in learning at school. As an adult, the person can live independently, but often needs help to raise children and can only work at simple supervised jobs.	0.043 (0.026–0.064)
ID, moderate	Has low intelligence, and is slow in learning to speak and to do even simple tasks. As an adult, the person requires a lot of support to live independently and raise children. The person can only work at the simplest supervised jobs.	0.100 (0.066–0.142)
ID, severe	Has very low intelligence and cannot speak more than a few words, needs constant supervision and help with most daily activities, and can do only the simplest tasks.	0.160 (0.107–0.226)
ID, profound	Has very low intelligence, has almost no language, and does not understand even the most basic requests or instructions. The person requires constant supervision and help for all activities.	0.200 (0.133–0.283)

To estimate the disability weights for each sequela of ASD, the following steps were conducted, with each step pulling 1000 draws of each input:

1. A pooled disability weight for ASD was estimated:
$$DW_{ASD} = DW_{Autism} \times P_{Autism} + DW_{Asperger} \times (1 - P_{Autism})$$
Where *DW* is disability weight and *P* is the proportion of ASD cases estimated to meet DSM-IV criteria for the autism sub-type (derived from a meta-analysis of 19 studies, 0.41 [0.36–0.47]).
2. The disability weight for ASD and each remaining level of ID was estimated:
$$DW_{ASD+ID} = 1 - (1 - DW_{ASD}) \times (1 - DW_{ID})$$

The severity proportions from the meta-analysis used in the above process and the resulting disability weights for each sequela are presented in Table 5.

Table 5: Severity proportions and disability weights of ASD sequelae

**Sequela**	**Severity proportion (95% UI)**	**Disability weight (95% UI)**
ASD without ID	0.446 (0.395–0.496)	0.169 (0.114–0.236)

ASD with borderline ID	0.197 (0.159–0.235)	0.178 (0.123–0.244)
ASD with mild ID	0.149 (0.110–0.191)	0.205 (0.149–0.273)
ASD with moderate ID	0.139 (0.101–0.182)	0.252 (0.192–0.318)
ASD with severe ID	0.056 (0.034–0.084)	0.302 (0.236–0.373)
ASD with profound ID	0.014 (0.006–0.026)	0.336 (0.261–0.418)

Methodological changes have resulted in small decrease globally in prevalence of ASD with variation in changes across locations (some increasing and some decreasing) compared to GBD 2021. Our final prevalence estimates for ASD need to be considered along several limitations. While we continue to improve on the data and methods used to estimate the burden of mental disorders, other challenges need to be acknowledged. Firstly, we still have a large number of locations with no high-quality raw data available. Secondly, it is difficult to quantify and remove all variation due to measurement error in our epidemiological estimates. While we have attempted to account for known sources of bias, in some cases, we still have very few datapoints to inform these adjustments and to explore other interactions/bias adjustments. Thirdly, there is a paucity of research on the risk factors of mental disorders which can be used as predictive covariates in our epidemiological models.

References

1. American Psychiatric Association. Diagnostic and statistical manual of mental disorders : DSM-IV-TR. 4th ed., text revision. ed. Washington, DC: American Psychiatric Association; 2000.
2. World Health Organization. The ICD-10 classification of mental and behavioural disorders. Clinical descriptions and diagnostic guidelines. Geneva: World Health Organization; 1992.
3. American Psychiatric Association. Diagnostic and statistical manual of mental disorders: DSM-5. 4th Text Revision ed. Washington, DC: American Psychiatric Association Publishing; 2013.

Bipolar disorder

Flowchart

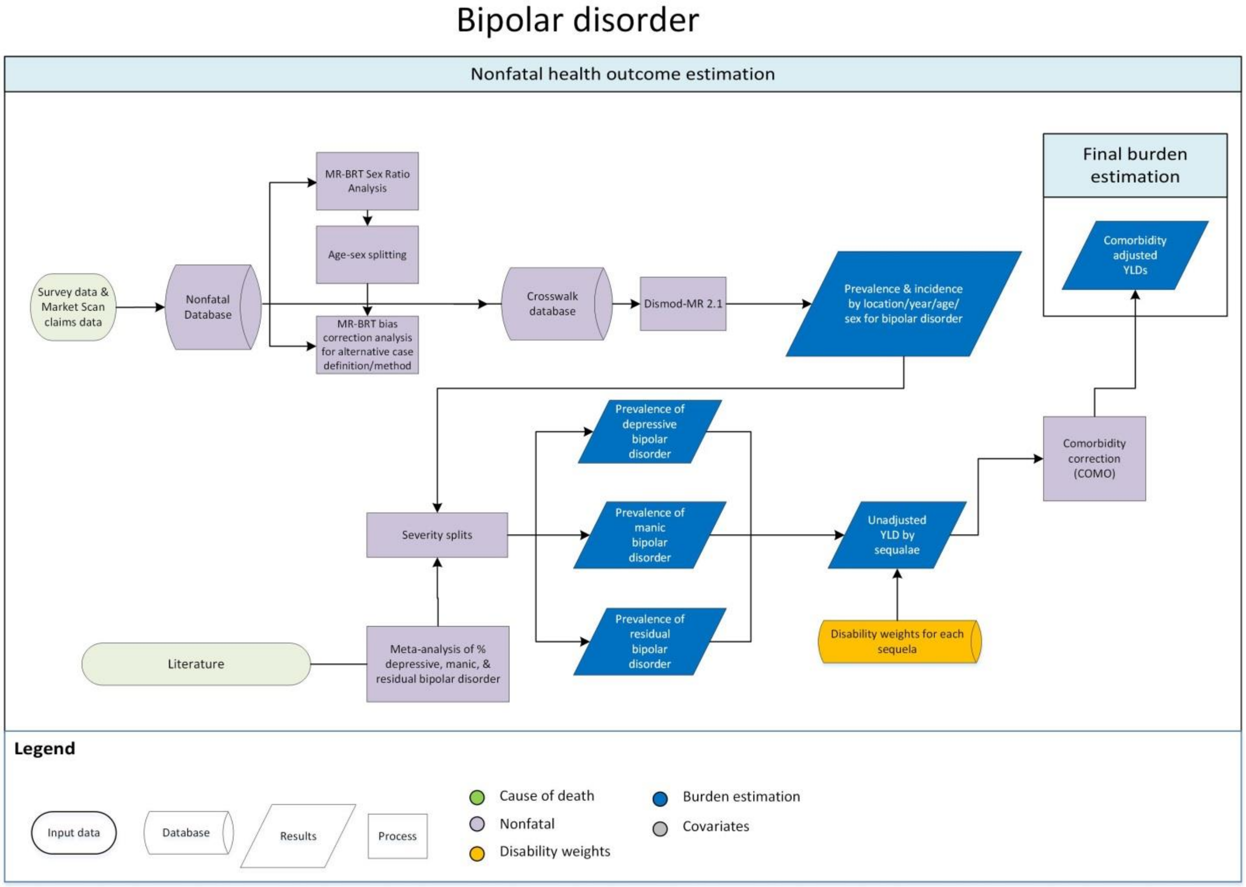

Input data and methodological summary for bipolar disorder

Case definition

Bipolar disorder is a serious mood disorder with little or no complete remission. Included in GBD disease modelling were cases meeting diagnostic criteria for bipolar disorder according to the Diagnostic and Statistical Manual of Mental Disorders (DSM), or the equivalent diagnosis in the International Classification of Diseases (ICD).[1,2] These are identified by the following codes: F30.0-F30.9, F31.0–F31.6, F31.8–F31.9, F34.0. Excluded were bipolar disorder due to a general medical condition or substance-induced cases. Different versions of DSM (DSM-III, DSM-III-R, DSM-IV, DSM-IV-TR, DSM-5, and DSM-5-TR) and ICD (ICD-9, ICD-10, and ICD-11) were accepted.

A diagnosis of bipolar disorder involves the experience of one or more manic, hypomanic, and/or major depressive episodes. According to DSM-IV-TR, a manic episode involves the experience of elevated, expansive, or irritable mood lasting for at least one week. During this period, at least three (or four if mood is only irritable) of the following symptoms must also be experienced: i) inflated self-esteem or grandiosity, ii) decreased need for sleep, iii) more talkative, iv) flight of ideas or experiences that

thoughts are racing, v) distractibility, vi) increase in goal-directed activity, and vii) excessive involvement in pleasurable activities with high potential for painful consequences.

A hypomanic episode involves the experience of elevated, expansive, or irritable mood lasting for at least four days. During this period, at least three (or four if mood is only irritable) of the symptoms previously listed for a manic episode must also be experienced.

A major depressive episode involves the experience of depressed mood almost all day, every day, for at least two weeks. A total of five out of nine criteria must be met to make a diagnosis and at least one of the five criteria should either be "depressed mood" for most of every day or "loss of interest in nearly all activities" for most of every day. The other seven criteria are: i) change in eating, appetite, or weight, ii) excessive sleeping or insomnia, iii) agitated or slow motor activity, iv) fatigue, v) feeling worthless or inappropriately guilty, vi) trouble concentrating, and vii) repeated thoughts about death.

Different subtypes of bipolar disorder can be diagnosed depending on the combination of symptoms experienced. Bipolar I disorder is characterised by at least one manic episode, which can also alternate with a major depressive episode. Bipolar II disorder is characterised by depressive and hypomanic episodes. Cyclothymia is characterised by subsyndromal hypomanic episodes alternating with major depressive episodes. Bipolar disorder not otherwise specified is characterised by clinically significant symptoms of bipolar disorder which do not meet criteria for the other diagnoses.[2,3] In GBD 2023 we estimated burden for the entire spectrum of bipolar disorder simultaneously, rather than individually for each subtype of the disorder. At a minimum, epidemiological studies needed to report on bipolar I and bipolar II to be included in analyses.

Input data

The epidemiological systematic literature review for bipolar disorder was conducted in three stages involving electronic searches of the peer-reviewed literature (ie, via PsycInfo, Embase, and PubMed), the grey literature, and expert consultation. For mental disorders, we update our GBD electronic database searches on a rolling basis. A systematic review update was completed for GBD 2023 for mood disorders together (depressive disorders and bipolar disorder). Databases were searched on March 23, 2021, for publications after April 1, 2018. Below are the search terms used for each database:

PubMed: ((((((((((((prevalen*[Title/Abstract]) OR (mortality[Title/Abstract])) OR (death*[Title/Abstract])) OR (inciden*[Title/Abstract])) OR (recurren*[Title/Abstract])) OR (remission[Title/Abstract])) OR (duration[Title/Abstract])) OR (remit*[Title/Abstract])) OR (epidemiolog*[Title/Abstract])) OR (prevalence[MeSH Terms])) OR (mortality[MeSH Terms])) OR (incidence[MeSH Terms])) OR (recurrence[MeSH Terms])) AND ((((((((((((depress*[Title/Abstract]) OR (dysthymi*[Title/Abstract])) OR (bipolar[Title/Abstract])) OR (manic[Title/Abstract])) OR (mania[Title/Abstract])) OR ("mood disorders"[Title/Abstract])) OR ("mood disorder"[Title/Abstract])) OR (mood disorders[MeSH Terms])) OR (depressive disorders[MeSH Terms])) OR (depressive disorders, major[MeSH Terms])) OR (bipolar disorders[MeSH Terms])) OR (dysthymic disorders[MeSH Terms]))

EMBASE: (depress*:ab,ti OR dysthymi*:ab,ti OR bipolar:ab,ti OR manic:ab,ti OR mania:ab,ti OR 'mood disorder':ab,ti OR 'mood disorders':ab,ti) AND (prevalen*:ab,ti OR mortality:ab,ti OR death*:ab,ti OR inciden*:ab,ti OR recurren*:ab,ti OR remission:ab,ti OR duration:ab,ti OR remit*:ab,ti OR epidemiolog*:ab,ti AND ([article]/lim OR [article in press]/lim) AND [humans]/lim AND [embase]/lim

PsycInfo: (title: depress* OR abstract: depress* OR title: dysthymi* OR abstract: dysthymi* OR title: bipolar OR abstract: bipolar OR title: manic OR abstract: manic OR title: mania OR abstract: mania OR title: "mood disorder" OR abstract: "mood disorder" OR title: "mood disorders" OR abstract: "mood disorders") AND (title: prevalen* OR abstract: prevalen* OR title: mortality* OR abstract: mortality* OR title: death* OR abstract: death* OR title: inciden* OR abstract: inciden* OR title: recurren* OR abstract: recurren* OR title: remission AND abstract: remission OR title: duration OR abstract: *duration OR title: remit* OR* abstract: remit* OR title: epidemiolog* OR abstract: epidemiolog*)

In addition to the database search, a grey literature search and expert consultation were also conducted. The systematic review update was conducted according to Preferred Reporting Items for Systematic Reviews and Meta-Analyses (PRISMA; see Figure 1).

Figure 1: PRISMA 2009 flow diagram

From: Moher D, Liberati A, Tetzlaff J, Altman DG, The PRISMA Group (2009). *P*referred *R*eporting *I*tems for *S*ystematic Reviews and *M*eta-*A*nalyses: The PRISMA Statement. PLoS Med 6(7): e1000097. doi:10.1371/journal.pmed1000097

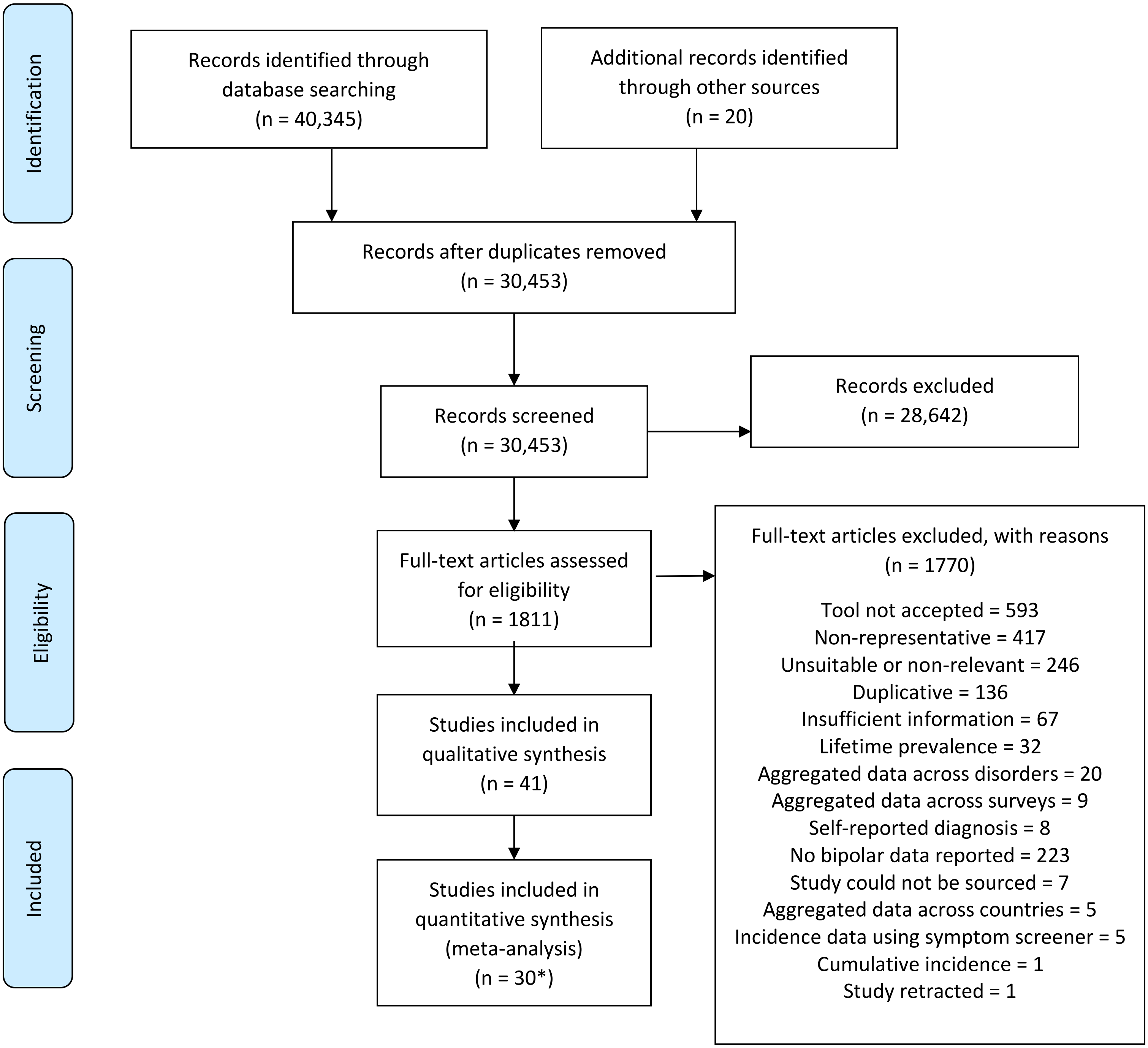

**The qualitative analysis led to the exclusion of additional studies with duplicative cohorts or methodological limitations impacting their eligibility and increasing measurement error within the data.*

The GBD inclusion criteria stipulated that: 1) the publication year must be from 1980 onward; 2) "caseness" must be based on clinical threshold as established by the DSM or ICD; 3) sufficient information must be provided on study method and sample characteristics to assess the quality of the study; 4) study samples must be representative of the general population (ie, inpatient or pharmacological treatment samples, case studies, veterans, or refugee samples were excluded); and 5) as previously explained, we estimated the burden for the entire spectrum of bipolar disorder rather than individually for each subtype of the disorder. Combined estimates of all subtypes of bipolar disorder were required. Studies reporting separate estimates for bipolar I, bipolar II, cyclothymia, and/or bipolar not otherwise specified were accepted if sufficient information was available to sum the disorder-specific estimates. No limitation was set on the language of publication.

Age-sex splitting

The extracted data underwent three types of age-sex splitting processes:

1. Where possible, estimates were further split by sex and age based on the available data. For instance, if studies reported prevalence for broad age groups by sex (eg, prevalence in 15–65-year-old males and females separately), and also by specific age groups but for both sexes combined (eg, prevalence in 15–30-year-olds, then in 31–65-year-olds, for males and females combined); age-specific estimates were split by sex using the reported sex ratio and bounds of uncertainty.
2. A meta-regression—Bayesian, regularised, trimmed (MR-BRT) analysis was used to split the remaining both-sex estimates in the dataset. For each parameter, sex-specific estimates were matched by location, age, and year, and a MR-BRT network meta-analysis was used to estimate pooled sex ratios and bounds of uncertainty. These were then used to split the both-sex estimates in the dataset. The male-to-female prevalence ratio estimated for prevalence, standardised mortality ratio, and relative risk estimates was 0.88 (95% uncertainty interval [UI]: 0.82–0.95), 0.96 (0.93–0.99), and 0.82 (0.72–0.92), respectively.
3. Studies reporting prevalence estimates across age groups spanning 25 years or more were split into five-year age groups using the prevalence age-pattern estimated by DisMod-MR 2.1. The DisMod-MR model used to estimate the age pattern did not contain any previously age-split data.

Bias corrections/crosswalks

Estimates with known biases were adjusted/crosswalked accordingly prior to DisMod-MR 2.1. For each crosswalk of interest, pairs of the reference and the alternative estimates were matched by age, sex, location, and year. This was done for both within-study (where possible) and between-study pairs. Pairs were also made between the different alternative estimates. The ratios between these estimates were then used as inputs in a MR-BRT network meta-analysis. This analysis produced pooled ratios between the reference estimates and alternative estimates, which were used to adjust all alternative estimates in the dataset. Two adjustment ratios were used for alternative data.

1. A point/past-month recall correction adjusted point/past-month prevalence estimates towards the level they would have been if the study had captured past-year prevalence. We set past-year prevalence as the desirable level due to the episodic nature of bipolar disorder. Estimates of point prevalence surveying symptoms experienced in the past 30 days or less may fail to diagnose cases of bipolar disorder in a residual state, thereby underestimating prevalence.

2. A lifetime recall correction adjusted all datapoints derived from lifetime prevalence towards the level they would have been if the study had captured past-year prevalence. Lifetime estimates were included as they are useful to capture potentially missed cases in the residual state.

See Table 1 for adjustment factors used for bipolar disorder. The estimated UIs around the adjustment factors incorporate gamma, which represents the between-study variance across all input data in the model. This added uncertainty widens the UIs for crosswalks with significant fixed effects.

Table 1: MR-BRT crosswalk adjustment factors for bipolar disorder

**Data input**	**Reference or alternative case definition**	**Gamma**	**Beta coefficient, log (95% UI)***	**Adjustment factor****
Population survey	Reference: past-year prevalence of bipolar disorder	0.09		
Population survey	Alternative: point or past-month prevalence		–0.46 (–0.61 to 0.30)	0.63 (0.54–0.74)
Population survey	Alternative: lifetime prevalence		0.42 (0.27 to 0.56)	1.52 (1.31–1.76)

**MR-BRT crosswalk adjustments can be interpreted as the factor the alternative case definition is adjusted by to reflect what it would have been had it been measured using the reference case definition. If the log/logit beta coefficient is negative, then the alternative is adjusted up to the reference. If the log/logit beta coefficient is positive, then the alternative is adjusted down to the reference.*
***The adjustment factor column is the exponentiated beta coefficient. For log beta coefficients, this is the relative rate between the two case definitions. For logit beta coefficients, this is the relative odds between the two case definitions.*

MarketScan data

We made use of USA MarketScan data in our prevalence dataset. These were prevalence data for bipolar disorder derived from claims information in a database of private and public insurance schemes. Given the sparseness of the bipolar disorder prevalence dataset, this allowed us to incorporate detailed prevalence estimates by state, sex, and age in our modelling. Evaluation of the age pattern of MarketScan data revealed that it was consistent to what can be observed in population-representative survey estimates; however, given that this data source only captures a subset of the population, the actual levels of prevalence, and the sex difference in prevalence, were not comparable and had to be adjusted accordingly.

We compared each year of MarketScan estimates against corresponding prevalence data from the National Comorbidity Survey Replication (NCS-R), a survey representative of the general USA population. The resulting prevalence ratios were used to adjust all MarketScan estimates before they were entered into the bipolar disorder model. The NCS-R: MarketScan ratios are presented in Table 2 below.

Table 2. MarketScan adjustment factors

**MarketScan year**	**Males (95% UI)**	**Females (95% UI)**

2000	3.32 (2.17–4.47)	2.49 (1.69–3.29)
2010	2.16 (1.42–2.91)	1.50 (1.02–1.98)
2011	2.11 (1.38–2.83)	1.49 (1.01–1.97)
2012	2.11 (1.38–2.84)	1.46 (0.99–1.92)
2013	2.07 (1.36–2.79)	1.45 (0.98–1.91)
2014	2.05 (1.35–2.76)	1.38 (0.94–1.82)
2015	1.76 (1.15–2.37)	1.19 (0.81–1.57)
2016	1.65 (1.08–2.21)	1.09 (0.74–1.43)
2017	1.66 (1.08–2.23)	1.05 (0.71–1.39)

Modelling strategy

We have made no substantive changes in the modelling strategy from GBD 2021.

After the above data processes were applied, DisMod MR 2.1 was used to model the epidemiological data for bipolar disorder. Adjustments to model priors or to the dataset were made where appropriate. Where outliers were identified in the data, we reassessed the study's methodology and quality before a decision was made to exclude or include the data.

Data across all epidemiological parameters were initially included in the modelling process. The three studies on incidence reported 0%, 0.1%, and 0.7% incidence of bipolar disorder and were low relative to the prevalence data. They were excluded from the final model where incidence was estimated using data from other parameters. We assumed no incidence and prevalence before age 10. This minimum age of onset was corroborated with expert feedback and was consistent with the available data. Remission was set to a maximum of 0.05 in agreement with literature and expert advice suggesting no or very little complete remission from bipolar disorder.[4,5]

Severity splits and disability weights

The GBD disability weight survey assessments include lay descriptions of sequelae highlighting major functional consequences and symptoms. The lay descriptions and disability weights for bipolar disorder severity levels are shown in Table 3. Information on the distribution of manic, depressive, and residual states of bipolar disorder was obtained from a systematic review of the literature[6] capturing data published between 1980 and 2012, and an update we conducted for GBD 2019 capturing data up to February 2018. Severity splits used in GBD 2023 were consistent with those used in GBD 2021.

Overall, 26 studies provided information on the proportion of bipolar disorder cases in a manic, depressive, or residual state. A MR-BRT analysis was used to explore between-study heterogeneity and to estimate the pooled proportion of cases falling within each bipolar health state. Two covariates were used in the analysis. The first was a sampling type covariate where the reference was population representative data or data from surveys of inpatients and outpatients combined. Alternatives for this covariate included data from inpatient-only samples, and outpatient-only samples. The second covariate was for bipolar subtypes where the reference was surveys screening for overall bipolar disorder (ie, bipolar I, bipolar II, and/or bipolar not otherwise specified) and the alternative included studies that reported data for bipolar I only (n=6). An income covariate was tested (ie, studies representative of high-income countries [n= 21] versus low- and middle-income countries [n=5]), but it was not statistically

significant and was not included in the final analysis. The proportion of bipolar disorder cases falling within each state were as follows: manic 18.7% (95% UI 9.1–30.7), depressive 31.7% (15.6–48.1), and residual 49.5% (24.9–74.1).

Table 3. Severity distribution, details on the severity levels for bipolar disorder and the associated disability weight with that severity

**Severity level**	**Lay description**	**DW (95% UI)**
Manic	Is hyperactive, hears and believes things that are not real, and engages in impulsive and aggressive behaviour that endangers the person and others.	0.492 (0.341–0.646)
Depressive*	Has constant sadness and has lost interest in usual activities. The person has some difficulty in daily life, sleeps badly, has trouble concentrating, and sometimes thinks about harming himself (or herself).	0.396 (0.267–0.531)
Residual	Has mild mood swings, irritability, and some difficulty with daily activities.	0.032 (0.018–0.051)

*Note. *Equivalent to the disability weight estimated for moderate major depressive disorder*

There were no significant changes in GBD 2023 results for bipolar disorder compared to GBD 2021. While we continue to improve on the data and methods used to estimate the burden of mental disorders, some challenges need to be acknowledged. Firstly, we still have a large number of locations with no high-quality raw data available. Secondly, it is difficult to quantify and remove all variation due to measurement error in our epidemiological estimates. While we have improved the methodology used to account for known sources of bias, in some cases we still have very few datapoints to inform these adjustments. Thirdly, there is a paucity of research on the risk factors of mental disorders which can be used as predictive covariates in our epidemiological models.

References

1. American Psychiatric Association. Diagnostic and Statistical Manual of Mental Disorders (DSM-IV-TR). Fourth Edition, Text Revision ed. Washington DC: American Psychiatric Association; 2000.
2. World Health Organization. The ICD-10 Classification of Mental and Behavioural Disorders. Clinical descriptions and diagnostic guidelines. Geneva: World Health Organization; 1992.
3. American Psychiatric Association. Diagnostic and Statistical Manual of Mental Disorders (DSM). Washington: American Psychiatric Association, 1952.
4. Angst J, Sellaro R. Historical perspectives and natural history of bipolar disorder. *Biological Psychiatry* 2000; **48**(6): 445–57.
5. Colom F, Vieta E. The road to DSM-V. Bipolar disorder episode and course specifiers. *Psychopathology* 2009; **42**(4): 209–18.
6. Ferrari AJ, Saha S, McGrath JJ, et al. Health states for schizophrenia and bipolar disorder within the Global Burden of Disease 2010 Study. *Population Health Metrics* 2012; **10**(1): 16.

Blindness and vision loss

Flowcharts

Vision loss

Nonfatal health outcome estimation

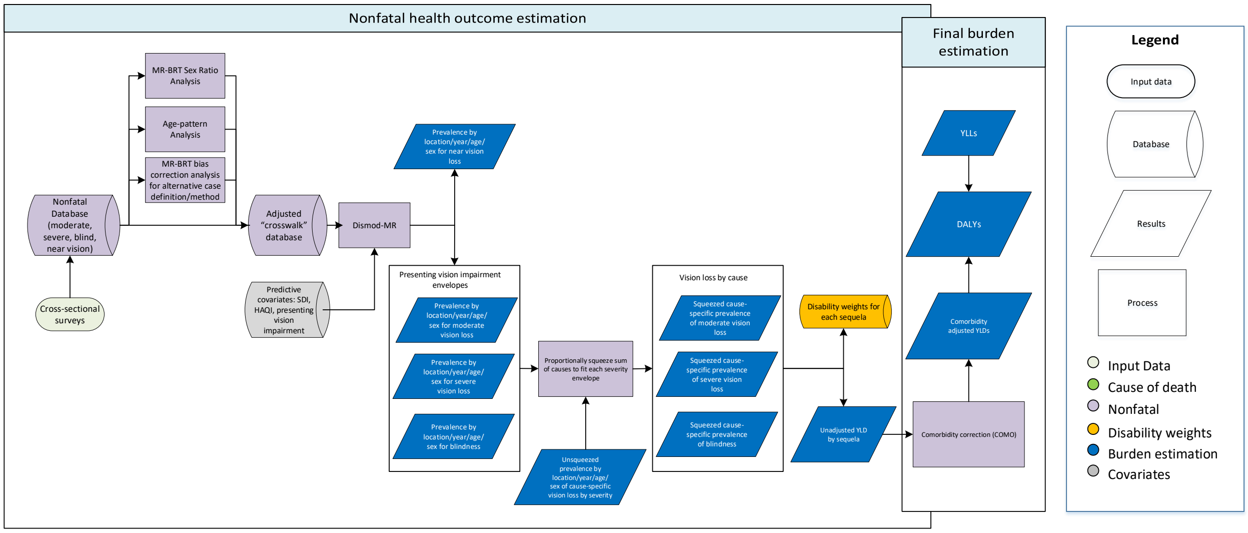

Cause-specific vision loss: cataract, glaucoma, macular degeneration, other vision loss

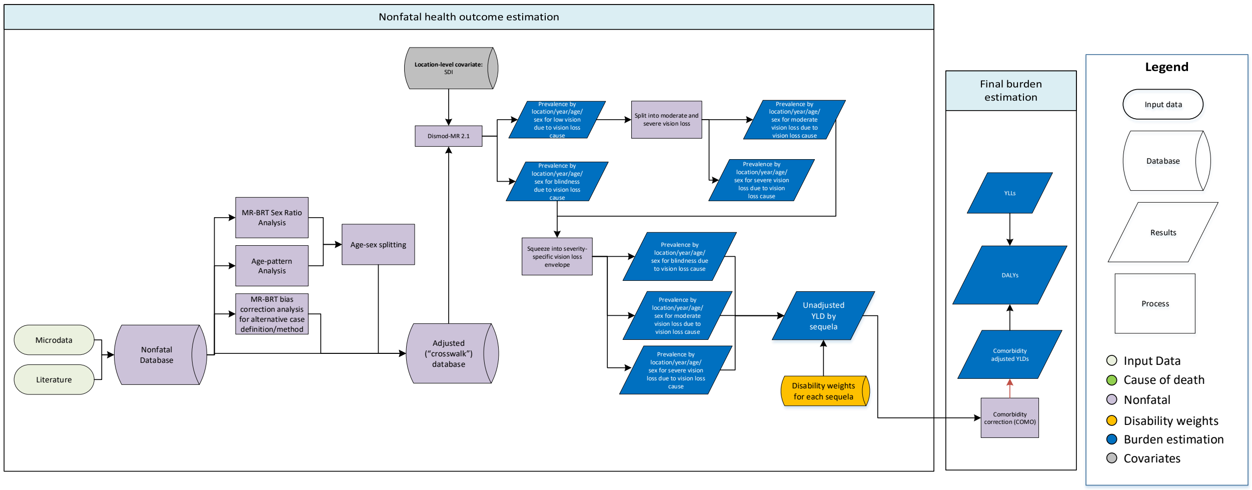

Cause-specific vision loss: diabetic retinopathy

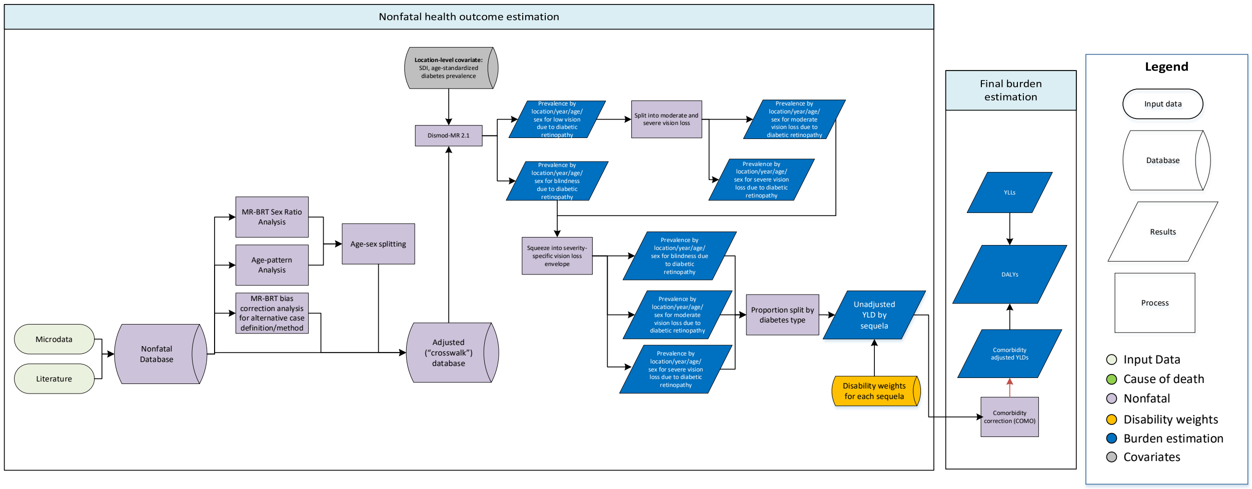

Cause-specific vision loss: trachoma

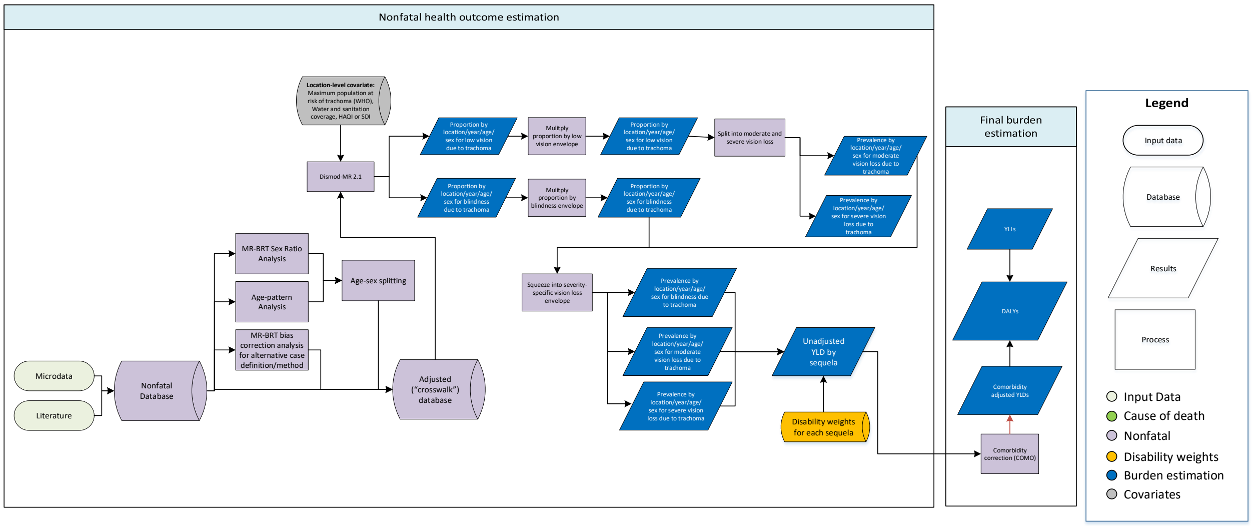

Case definition

Total vision loss and cause-specific vision loss are estimated for the following severities (Table 1). Severity of vision loss is determined using a measured visual acuity test such as a Snellen chart or LogMAR chart.

Table 1. Severity of vision loss assigned based on range of visual acuity

Vision loss severity	Case definition

Blindness	Distance visual acuity of <3/60 or <10% visual field around central fixation
Severe vision loss	Distance visual acuity of ≥3/60 and <6/60
Moderate vision loss	Distance visual acuity of ≥6/60 and <6/18
Near vision loss (uncorrected presbyopia)	Near visual acuity of <6/12 distance equivalent

Near vision loss describes the progressive inability to focus on near objects as individuals age (presbyopia). This impairs the ability to read. The majority of presbyopia can be corrected by the use of reading glasses, contact lenses, or refractive surgery.

We model vision loss due to the following causes: uncorrected refractive error, cataract, glaucoma, macular degeneration, diabetic retinopathy, trachoma, vitamin A deficiency, retinopathy of prematurity, meningitis, encephalitis, onchocerciasis, and a residual category of other vision loss. Vision loss due to vitamin A deficiency, retinopathy of prematurity, meningitis, encephalitis, and onchocerciasis are modelled as part of their underlying cause as described in their respective sections. (Table 2)

Table 2. Causes of vision loss

**Condition**	**Case definition**
Cataract	Clouding of the lens of the eye due to protein buildup that impairs vision. Cataracts can be addressed via surgical lens replacement.
Diabetic retinopathy	Damage to the retina caused by damaged blood vessels that can leak blood into the retina and cause scarring of the retina.
Glaucoma	A condition with increased intraocular pressure which can lead to damage of the optic nerve.
Age-related macular degeneration	Age-related deterioration of the macula, the part of the retina responsible for central vision, leading to central vision loss.
Uncorrected refractive error	Blurry vision due to the lens's inability to focus. The blurriness caused by refractive error can be addressed through the use of contact lenses, glasses, or refractive surgery. Uncorrected refractive error is the difference in acuity between presenting vision (whatever corrective lens the individual uses) and best-corrected vision.
Trachoma	Results from a conjunctival bacterial infection (*Chlamydia trachomatis*) that produces inflammation and inversion of the

	eyelids and eyelashes scratching and scarring the cornea, eventually leading to trichiasis and impaired vision from corneal scarring.

Input data

Model inputs

Data on overall vision loss come from surveys measuring visual acuity in representative population-based studies, either from publications in peer-reviewed and grey literature or surveys for which we had the unit record data. Data were excluded if no test was used of visual acuity that can be converted to the Snellen scale, and if a study did not assess "presenting" or "best-corrected" vision. Presenting vision is the visual acuity as measured with the glasses used by an individual. Best-corrected vision is with the best possible correction for refractive error, regardless of the strength of glasses used by an individual. A subset of these studies that reported vision loss by cause were used to estimate the prevalence of vision loss due to cataract, glaucoma, macular degeneration, diabetic retinopathy, and other causes.

No new systematic review was conducted for GBD 2023, but a data audit was conducted for all citations used in vision models. For GBD 2019 and GBD 2021, we added literature sources from a systematic review conducted by collaborators in the Vision Loss Expert Group (VLEG) where all screened abstracts were sent to regional expert groups to assess data quality for inclusion. This systematic review was conducted using the search engines MEDLINE, Embase, WHOLIS, SciELO, Open Grey, and other grey literature searches commissioned by VLEG from York Health Economics Consortium, UK, an organisation that has supported the VLEG by independently conducting these searches in the past. These searches covered the time period of 1980–2018. In total, since 2010, VLEG has provided data extracted from 137 studies, of which 67 came from the most recent systematic review update (2014–2018). Data from 95 of these literature sources that matched GBD inclusion criteria were newly added to vision models. The Vision Loss Expert Group also provided additional data provided by principal investigators for existing studies, 51 new RAAB surveys, and five-year disaggregated data for 151 RAAB surveys (previously only data for combined ages 50–99 were available), which better informed the age pattern for vision loss in this year's estimates.

Several adjustments were made to data extracted from the original data sources.

1) Where studies only reported "both"-sex data, a meta-regression in MR-BRT[1] (meta-regression—Bayesian, regularised, trimmed; additional information can be found in appendix 1, section 4.4.1 of the cited paper) was used to split these datapoints into sex-specific datapoints.
2) Where studies reported visual acuity spanning multiple thresholds (eg, <6/60, rather than separate severe and blind estimates), we applied a logit-difference adjustment meta-regression, using data from studies reporting vision loss by both severity levels.
3) Some studies reported best-corrected vision loss, but not presenting vision loss. We crosswalked these datapoints using a logit-difference meta-regression. This gave us predicted presenting vision loss datapoints for studies not explicitly reporting presenting vision loss.
4) Where datapoints spanned more than 25 years of age, we age-split using an algorithm that applies the age pattern of the super-region (from a DisMod-MR model that only contains data with age groups that span fewer than 25 years) to split the data to five-year age groups.

DisMod-MR 2.1 is the tool used to produce year-age-sex-location-specific prevalence estimates (disease model—Bayesian meta-regression; for details on this method see appendix 2, section 2)

Whereas other vision loss aetiologies are modelled based on prevalence data, vision loss due to trachoma is modelled as a proportion of the overall best-corrected vision loss envelope, a strategy that was chosen based on the nature of available data.

Modelling strategy

We modelled the prevalence of vision loss in two steps. In the first step, we estimated the total prevalence estimates of presenting vision loss: moderate vision loss, severe vision loss, blindness, and near vision loss (presbyopia). We directly derived prevalence of near vision loss from this step, whereas the remaining three models that reflect different severity levels of distance vision loss continued to the next step.

1) Estimate severity-specific vision loss (the "envelopes")

First, we ran five DisMod-MR 2.1 models to estimate the total prevalence estimates of vision loss: moderate presenting vision loss, severe presenting vision loss, presenting blindness, and uncorrected presbyopia. Non-reference case definitions were adjusted to reference (full visual acuity exam, presenting vision) using MR-BRT[1] (meta-regression—Bayesian, regularised, trimmed; additional information can be found in appendix 1, section 4.4.1 of the cited paper). Betas and exponentiated values, which can be interpreted as an odds ratio, are shown in the tables below for each adjustment for alternative case definitions. The best-corrected adjustment factor indicates whether the test measured visual acuity with the level of correction the patient presents with or the ophthalmologist provides additional correction via pinhole or lens correction. Rapid assessment corrects for potential biases in cause-specific vision loss from studies using expedited visual acuity measurement. The severity covariate splits mixed severity data (moderate/severe, severe/blindness) into severity-specific data. Gamma captures the between study heterogeneity.

Table 3. MR-BRT crosswalk adjustment factors for vision loss models

MR-BRT crosswalk adjustment factors for moderate vision loss envelope

Data input	Reference or alternative case definition	Gamma	Beta coefficient, logit (95% CI)*	Adjustment factor**
Presenting visual acuity, does not use rapid methodology	Ref	0.59	---	---
Best-corrected visual acuity	Alt		–1.11 (–2.27 to 0.06)	0.33
Uses rapid methodology	Alt		–0.06 (–1.23 to 1.11)	0.94

MR-BRT crosswalk adjustment factors for severe vision loss envelope

Data input	Reference or alternative case definition	Gamma	Beta coefficient, logit (95% CI)*	Adjustment factor**
Presenting visual acuity, does not use rapid methodology	Ref	0.69	---	---
Best-corrected visual acuity	Alt		–0.94 (–2.30 to 0.42)	0.39
Uses rapid methodology	Alt		0.11 (–1.25 to 1.48)	1.12

MR-BRT crosswalk adjustment factors for blindness envelope

Data input	Reference or alternative case definition	Gamma	Beta coefficient, logit (95% CI)*	Adjustment factor**
Presenting visual acuity, does not use rapid methodology	Ref	0.02	---	---
Best-corrected visual acuity	Alt		–0.15 (–0.19 to –0.15)	0.86
Uses rapid methodology	Alt		0.07 (–0.03 to 0.34)	1.07

MR-BRT crosswalk adjustment factors for cause-specific low vision models

Data input	Reference or alternative case definition	Gamma	Beta coefficient, logit (95% CI)*	Adjustment factor**
Does not use rapid methodology	Ref	0.70	---	---
Uses rapid methodology	Alt		0.12 (–0.03 to 0.34)	01.13

MR-BRT crosswalk adjustment factors for cause-specific blindness models

Data input	Reference or alternative case definition	Gamma	Beta coefficient, logit (95% CI)*	Adjustment factor**
Does not use rapid methodology	Ref	0.451	---	---
Uses rapid methodology	Alt		0.11 (–0.78 to 1.00)	01.11

**MR-BRT crosswalk adjustments can be interpreted as the factor the alternative case definition is adjusted by to reflect what it would have been had it been measured using the reference case definition. If the log/logit beta coefficient is negative, then the alternative is adjusted up to the reference. If the log/logit beta coefficient is positive, then the alternative is adjusted down to the reference.*

***The adjustment factor column is the exponentiated beta coefficient. For log beta coefficients, this is the relative rate between the two case definitions. For logit beta coefficients, this is the relative odds between the two case definitions.*

Mixed severity data (for example, mixed moderate and severe vision loss, or mixed severe vision loss and blindness) were split into severity-specific vision loss using a meta-regression in MR-BRT with a cubic spline on age.

Socio-demographic Index (SDI) and Healthcare Access and Quality (HAQ) Index were used as location covariates as a proxy measure of access to eye care such as cataract surgery. All predictors are listed below for each vision model. The exponentiated beta can be interpreted as an odds ratio. For example, in row 1 below, an exponentiated beta of 0.44 for SDI means that for every 1 unit change in SDI (measured on a scale from 0 to 1), moderate vision loss is lower by a factor of 0.44.

Table 4. Summary of predictive covariates used in vision DisMod-MR meta-regression models

Cause	Covariate	Type	Coefficient	Exponentiated beta (95% uncertainty interval)
Moderate vision loss envelope	Socio-demographic Index	Prevalence	–0.28 (–0.42 to –0.15)	0.75 (0.66 to 0.86)
Severe vision loss envelope	Socio-demographic Index	Prevalence	–1.97 (–2.0 to –1.91)	0.14 (0.14 to 0.15)
Blindness envelope	Socio-demographic Index	Prevalence	–1.2 (–1.57 to –0.82)	0.30 (0.21 to 0.44)
Blindness envelope	Healthcare Access and Quality Index	Prevalence	–0.016 (–0.021 to –0.012)	0.98 (0.98 to 0.99)
Moderate vision loss due to uncorrected refractive error	Socio-demographic Index	Prevalence	–0.62 (–0.85 to –0.38)	0.54 (0.43 to 0.68)
Severe vision loss due to uncorrected refractive error	Socio-demographic Index	Prevalence	–1.99 (–2.00 to –1.97)	0.14 (0.14 to 0.14)
Blindness due to uncorrected refractive error	Socio-demographic Index	Prevalence	–1.98 (–2.00 to –1.95)	0.14 (0.14 to 0.14)
Vision loss due to other vision loss	Socio-demographic Index	Prevalence	–0.99 (–1.0 to –0.99)	0.37 (0.37 to 0.37)
Blindness due to other vision loss	Socio-demographic Index	Prevalence	–0.001 (–0.002 to –0.000)	1.00 (1.00 to 1.00)
Vision loss due to macular degeneration	Socio-demographic Index	Prevalence	0.59 (0.23 to 0.93)	1.81 (1.26 to 2.53)
Blindness due to macular degeneration	Socio-demographic Index	Prevalence	–0.95 (–1.0 to –0.87)	0.39 (0.37 to 0.42)

Vision loss due to glaucoma	Socio-demographic Index	Prevalence	–0.86<br>(–0.99 to –0.71)	0.42<br>(0.37 to 0.49)
Blindness due to glaucoma	Socio-demographic Index	Prevalence	-1.99<br>(-2.0 to –1.98)	0.14<br>(0.14 to 0.14)
Vision loss due to cataract	Socio-demographic Index	Prevalence	–0.073<br>(–0.21 to -0.003)	0.93<br>(0.81 to 1.00)
Blindness due to cataract	Socio-demographic Index	Prevalence	–2.71<br>(–2.82 to –2.6)	0.066<br>(0.060 to 0.074)
Vision loss due to diabetes mellitus	Socio-demographic Index	Prevalence	–1.99<br>(–2.0 to –1.95)	0.14<br>(0.14 to 0.14)
Vision loss due to diabetes mellitus	Diabetes age-standard prevalence (proportion)	Prevalence	2.01<br>(1.81 to 2.23)	7.44<br>(6.10 to 9.32)
Blindness due to diabetes mellitus	Socio-demographic Index	Prevalence	–0.028<br>(–0.084 to –0.001)	0.97<br>(0.92 to 1.00)
Blindness due to diabetes mellitus	Diabetes age-standard prevalence (proportion)	Prevalence	3.99<br>(3.98 to 4.00)	54.30<br>(53.79 to 54.60)
Vision loss due to trachoma	Healthcare Access and Quality Index	Proportion	–4.00<br>(–4.00 to –3.99)	0.018<br>(0.018 to 0.019)
Vision loss due to trachoma	Max trachoma population at risk	Proportion	3.00<br>(2.99 to 3.00)	19.99<br>(19.81 to 20.09)
Vision loss due to trachoma	Improved water source (proportion access)	Proportion	–3.96<br>(–4.00 to –3.89)	0.019<br>(0.018 to 0.020)
Blindness due to trachoma	Healthcare Access and Quality Index	Proportion	–3.51<br>(–3.72 to –3.32)	0.030<br>(0.024 to 0.036)
Blindness due to trachoma	Max trachoma population at risk	Proportion	–0.34<br>(–0.82 to 0.051)	0.71<br>(0.44 to 1.05)
Blindness due to trachoma	Improved water source (proportion access)	Proportion	2.51<br>(2.10 to 2.92)	12.26<br>(8.17 to 18.49)

2) Estimate cause-specific vision loss

In the second step, we estimated the prevalence of vision loss due to multiple causes: refractive error, cataract, glaucoma, macular degeneration, diabetic retinopathy, retinopathy due to prematurity, trachoma, vitamin A deficiency, onchocerciasis, meningitis, and other causes not classified elsewhere (termed "other vision loss"). Vision loss due to retinopathy of prematurity, vitamin A deficiency, onchocerciasis, meningitis, tetanus, and neonatal conditions was modelled as part of these underlying causes; see their respective write-ups for more information.

For each of cataract, glaucoma, macular degeneration, diabetic retinopathy, and other vision loss, we ran two DisMod-MR 2.1 models: one for the combined category of moderate and severe vision loss due to the cause, and one for blindness due to the cause. Moderate and severe vision loss were modelled together because input data were mostly available for the aggregate. Refractive error was modelled using three models, one for each severity.

We used the following age restrictions based on input from the Vision Loss Expert Group:

Table 5. Age restriction in cause-specific DisMod-MR models.

Cause	Minimum age
Cataracts	20
Glaucoma	45
Macular degeneration	45
Diabetic retinopathy	20
Trachoma	15
Other vision loss	0

Vision loss due to trachoma was modelled as a proportion of the envelope, with separate proportion models for (severe and moderate) vision loss and blindness. We estimated the proportions of low vision and blindness due to trachoma using DisMod-MR 2.1 models. Our model included fixed effects on the maximum population at risk for trachoma (proportion) reported by WHO, the proportion of the population with access to sanitation, and HAQ Index. Finally, we applied geographical and age restrictions to ensure that we estimate zero proportions in non-endemic locations (see neglected tropical disease appendices for more information) and among those younger than 15 year of age (as scarring of the cornea due to trachoma takes decades to develop).

We split the moderate plus severe vision loss estimates for each cause into moderate and severe using the ratio of presenting moderate and severe vision loss envelopes. Onchocerciasis and retinopathy of prematurity are the two exceptions because moderate and severe vision loss are modelled as part of the estimation process of these causes.

We scaled the cause-specific vision loss prevalence to the total prevalence of the vision loss envelopes for each of the three severity levels. The final result is prevalence of vision loss due to each cause by severity.

Table 6. Health states and disability weights

Health state name	Health state description	Disability weight
Distance vision, moderate loss	This person has vision problems that make it difficult to recognise faces or objects across a room.	0.031 (0.019–0.049)
Distance vision, severe loss	This person has severe vision loss, which causes difficulty in daily activities, some emotional impact (for example, worry), and some difficulty going outside the home without assistance.	0.184 (0.125–0.259)
Distance vision, blindness	This person is completely blind, which causes great difficulty in some daily activities, worry and anxiety, and great difficulty going outside the home without assistance.	0.187 (0.124–0.26)
Near vision loss	This person has difficulty seeing things that are nearer than 3 feet if uncorrected by reading glasses, but has no difficulty with seeing things at a distance.	0.011 (0.005–0.02)

References

[1] Vos T, Lim SS, Abbafati C, *et al.* Global burden of 369 diseases and injuries in 204 countries and territories, 1990–2019: a systematic analysis for the Global Burden of Disease Study 2019. *The Lancet* 2020; **396**: 1204–22. doi: https://doi.org/10.1016/S0140-6736(20)30925-9

Bulimia nervosa

Flowchart

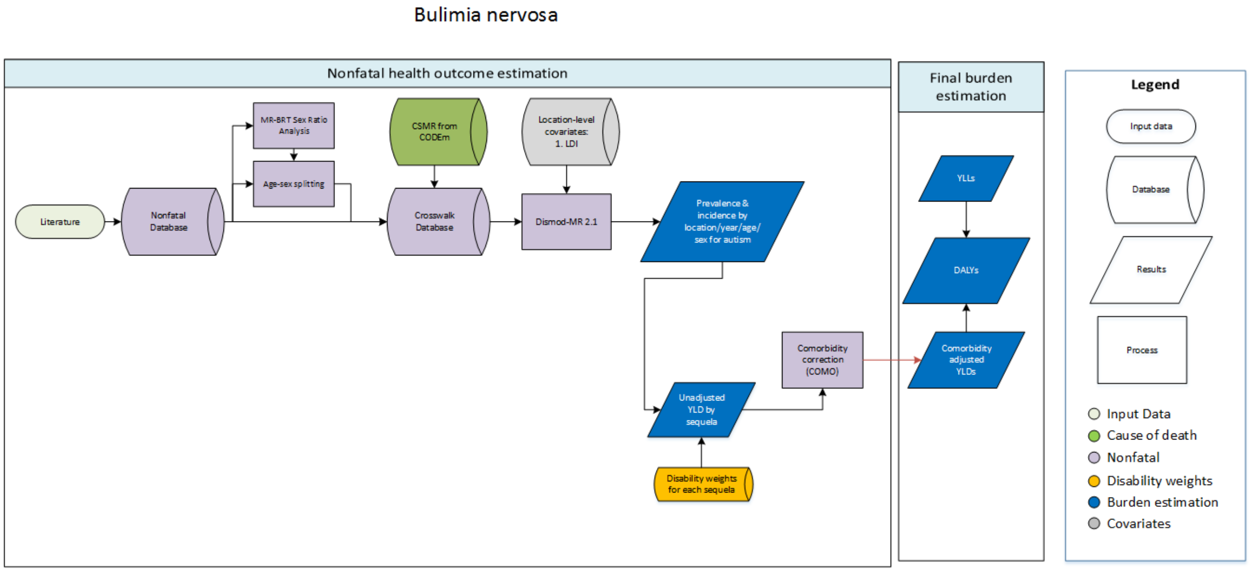

Input data and methodological summary for bulimia nervosa

Case definition

According to the Diagnostic and Statistical Manual of Mental Disorders fifth edition, text revision (DSM-5),[1] bulimia nervosa (BN) is an eating disorder characterised by:

a) Recurrent episodes of binge eating. An episode of binge eating is characterised by both of the following:
   1) eating, in a discrete period of time (eg, within any two-hour period), an amount of food that is definitely larger than most individuals would eat during a similar period of time and under similar circumstances.
   2) a sense of lack of control over eating during the episode (eg, a feeling that one cannot stop eating or control what or how much one is eating).

b) Recurrent inappropriate compensatory behaviour in order to prevent weight gain, such as self-induced vomiting; misuse of laxatives, diuretics, enemas, or other medications; fasting; or excessive exercise.

c) The binge eating and inappropriate compensatory behaviours both occur, on average, at least once a week for three months.

d) Self-evaluation is unduly influenced by body shape and weight.

e) The disturbance does not occur exclusively during episodes of anorexia nervosa.

Included in GBD were cases meeting diagnostic criteria according to DSM[1] or the International Classification of Diseases (ICD).[2] These were identified by the following code: F50.2. Different versions of DSM (DSM-III, DSM-III-R, DSM-IV, DSM-IV-TR, DSM-5, and DSM-5-TR) and ICD (ICD-9, ICD-10, and ICD-11) were accepted.

Input data

Systematic literature reviews were conducted to capture studies reporting the prevalence, incidence, remission, and excess mortality of BN. The epidemiological systematic literature review for BN was conducted in three stages involving electronic searches of the peer-reviewed literature (ie, via PsycInfo, Embase, and PubMed), the grey literature, and expert consultation. For mental disorders, we update our GBD electronic database searches on a rolling basis. A systematic review update was conducted for GBD 2023 for eating disorders combined. Databases were searched on June 5, 2023, for publications after January 1, 2017. Below are the search terms used for each database.

PubMed: ("anorexia nervosa"[Title/Abstract] OR "bulimi* "[Title/Abstract] OR "Eating disorders"[Title/Abstract] OR "Eating disorder"[Title/Abstract] OR "Anorexic"[Title/Abstract] OR OSFED[Title/Abstract] OR “purging disorder”[Title/Abstract] OR "Feeding and Eating Disorders"[MeSH Terms:noexp] OR "anorexia nervosa"[MeSH Terms] OR "Binge-Eating Disorder"[MeSH Terms] OR "bulimia nervosa"[MeSH Terms]) AND ((((((((((((prevalen*[Title/Abstract]) OR (mortalit*[Title/Abstract])) OR (death*[Title/Abstract])) OR (inciden*[Title/Abstract])) OR (inciden*[Title/Abstract])) OR (remission[Title/Abstract])) OR (duration[Title/Abstract])) OR (remit*[Title/Abstract])) OR (epidemiolog*[Title/Abstract])) OR ("Prevalence"[Mesh])) OR ("Mortality"[Mesh])) OR ("Incidence"[Mesh])) OR ("Epidemiology"[Mesh:NoExp])) OR ("Morbidity"[Mesh:NoExp]))

Embase: ('anorexia nervosa':ab,ti OR bulimi*:ab,ti OR 'eating disorder':ab,ti OR 'eating disorders':ab,ti OR 'anorexic':ab,ti OR OSFED:ab,ti OR ‘purging disorder’:ab,ti OR 'eating disorder'/de OR 'anorexia nervosa'/exp OR 'binge eating disorder'/exp OR 'bulimia'/exp OR 'purging disorder'/exp) AND (prevalen*:ab,ti OR mortalit*:ab,ti OR death*:ab,ti OR inciden*:ab,ti OR remission:ab,ti OR duration:ab,ti OR remit*:ab,ti OR epidemiolog*:ab,ti OR 'prevalence'/exp OR 'epidemiology'/exp OR 'remission'/exp)

PsycInfo: (TI "anorexia nervosa" OR AB "anorexia nervosa" OR TI bulimi* OR AB bulimi* OR TI "eating disorders" OR AB "eating disorders" OR TI "eating disorder" OR AB "eating disorder" OR TI anorexic OR AB anorexic OR TI OSFED OR AB OSFED OR TI “purging disorder” OR AB “purging disorder” OR DE "Eating Disorders" OR DE "Anorexia Nervosa" OR DE "Binge Eating Disorder" OR DE "Bulimia" OR DE "Purging (Eating Disorders)") AND (TI prevalen* OR AB prevalen* OR TI mortalit* OR AB mortalit* OR TI death* OR AB death* OR TI inciden* OR AB inciden* OR TI remission OR AB remission OR TI duration OR AB duration OR TI remit* OR AB remit* OR TI epidemiolog* OR AB epidemiolog* OR DE "Mortality Rate" OR DE "Epidemiology" OR DE "Morbidity" OR DE "Remission (Disorders)")

In addition to the database search, a grey literature search and expert consultation were also conducted. The systematic review update was conducted according to Preferred Reporting Items for Systematic Reviews and Meta-Analyses (PRISMA; see Figure 1).

Figure 1: PRISMA 2009 flow diagram

From: Moher D, Liberati A, Tetzlaff J, Altman DG, The PRISMA Group (2009). *P*referred *R*eporting *I*tems for *S*ystematic Reviews and *M*eta-*A*nalyses: The PRISMA Statement. PLoS Med 6(7): e1000097. doi:10.1371/journal.pmed1000097

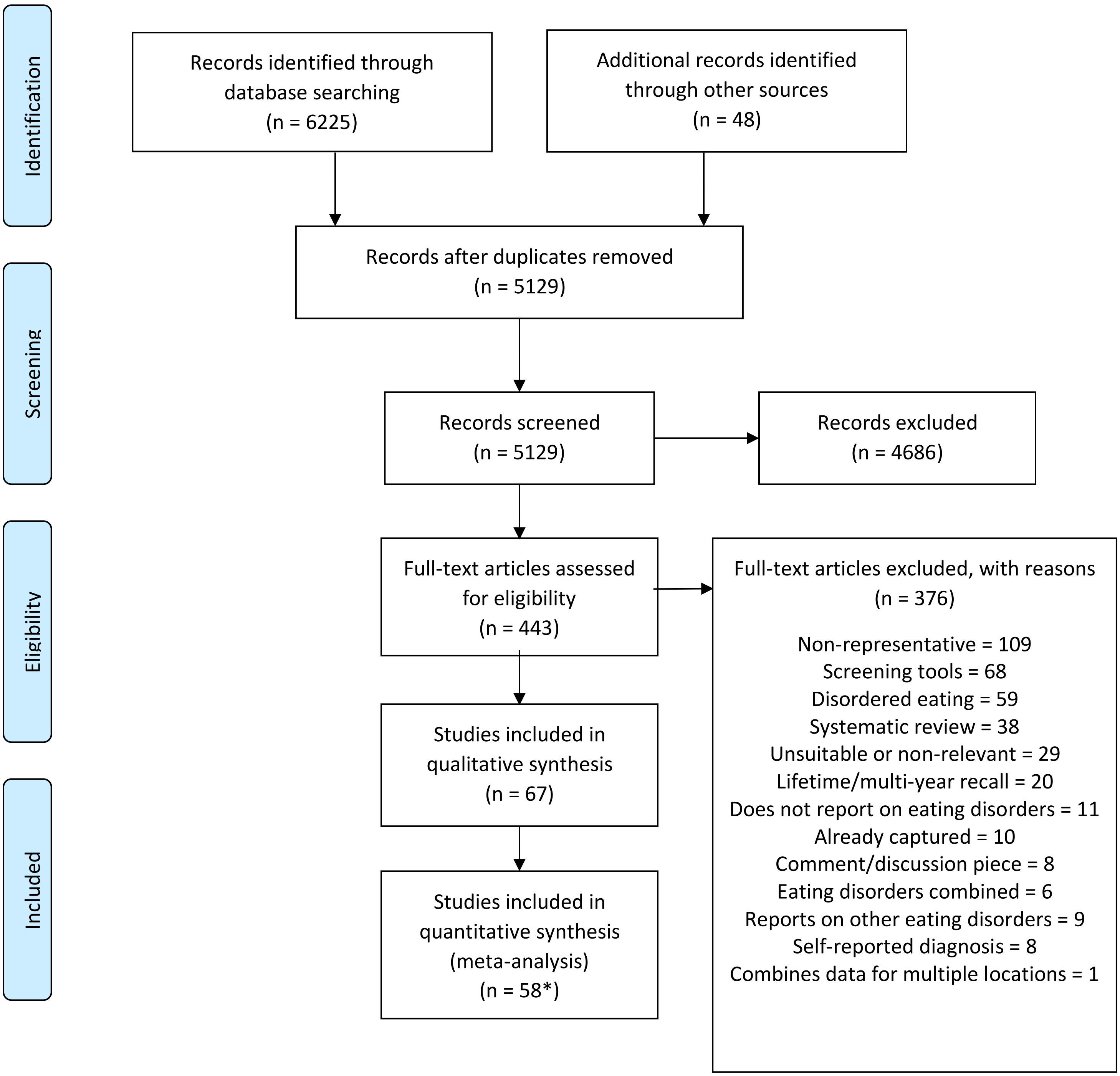

**The qualitative analysis led to the exclusion of additional studies with duplicative cohorts or methodological limitations impacting their eligibility and increasing measurement error within the data.*

The GBD inclusion criteria stipulated that: 1) the publication year must be from 1980 onward; 2) "caseness" must be based on clinical threshold as established by the DSM or ICD; 3) sufficient information must be provided on study method and sample characteristics to assess the quality of the study; and 4) study samples must be representative of the general population (ie, inpatient or pharmacological treatment samples, case studies, veterans, or refugee samples were excluded). No limitation was set on the language of publication. Methods used for this systematic review have been reported in greater detail elsewhere.[3]

Age-sex splitting

The extracted data underwent three types of age-sex splitting processes:

1. Where possible, estimates were further split by sex and age based on the available data. For instance, if studies reported prevalence for broad age groups by sex (eg, prevalence in 15–65-year-old males and females separately), and also by specific age groups but for both sexes combined (eg, prevalence in 15–30-year-olds, then in 31–65-year-olds, for males and females combined); age-specific estimates were split by sex using the reported sex ratio and bounds of uncertainty.
2. A meta-regression—Bayesian, regularised, trimmed (MR-BRT) analysis was used to split the remaining both-sex estimates in the dataset. For each parameter, sex-specific estimates were matched by location, age, and year. A MR-BRT network meta-analysis was then used to estimate pooled sex ratios and bounds of uncertainty. These were then used to split the both-sex estimates in the dataset. The male-to-female prevalence ratio estimated was 0.48 (95% uncertainty interval [UI] 0.39–0.60).

Bias corrections/crosswalks

Estimates with known biases were adjusted/crosswalked accordingly prior to DisMod-MR 2.1. For each crosswalk of interest, pairs of the reference and the alternative estimates were matched by age, sex, location, and year. This was done for both within-study (where possible) and between-study pairs. These pairs were then used as inputs in a MR-BRT network meta-analysis. The MR-BRT analysis produced a pooled ratio between the reference estimates and alternative estimates, which was used to adjust all alternative estimates in the dataset. Reference data informing the prevalence of BN consisted of estimates reporting past-month/point prevalence using a DSM-5 or ICD-11 diagnostic criteria. Two adjustment ratios were used for alternative data:

1. A past-year recall correction adjusted all datapoints derived from past-year prevalence towards the level they would have been if the study had captured point/past-month prevalence. The latter prevalence period is less affected by recall bias.
2. An old diagnostic criteria correction was used to adjust all prevalence estimates derived from previous iterations of the diagnostic criteria (ie, DSM-IV-TR, DSM-IV, DSM-III, ICD-10) towards the level they would have been if the estimate was derived from recent iterations of diagnostic criteria (ie, DSM-5, ICD-11).

See Table 1 for adjustment factors used for BN. The estimated UIs around the adjustment ratio incorporate gamma, which represents the between-study variance across all input data in the model. This added uncertainty widens the UIs for crosswalks with significant fixed effects.

Table 1: MR-BRT crosswalk adjustment factors for BN

Data input	Reference or alternative case definition	Gamma	Beta coefficient, log (95% UI)*	Adjustment factor**
Population survey	Reference: past-month/point prevalence following DSM-5 or ICD-11 diagnostic criteria	0.00		
Population survey	Alternative: past-year prevalence		0.45 (0.01 to 0.88)	1.57 (1.01–2.42)
Population survey	Alternative: old diagnostic criteria diagnosis (ie, DSM-III-R, DSM-IV, DSM-IV-TR, ICD-10)		–0.21 (–0.69 to 0.27)	0.81 (0.50–1.31)

Modelling strategy

After the above data processes were applied, DisMod MR 2.1 was used to model the epidemiological data for BN. Adjustments to model priors or the dataset were made where appropriate. Where outliers were identified in the data, we reassessed the study's methodology and quality before a decision was made to exclude or include the data.

We assumed no incidence prior to 10 years of age or onward from 40 years of age. In GBD 2021, a decision was made to remove BN as a cause of death due to the very limited data available to inform this model. There was also no clear epidemiological evidence from our systematic review of the literature to suggest that BN is associated with a statistically significant risk of death. Instead, excess mortality was set to zero in our analysis.

A country-level covariate, lagged distributed income (LDI), was also included. This covariate represents a moving average of gross domestic product (GDP) over time. The limits placed on this covariate meant that prevalence was assumed to increase with rising GDP. A summary of location-level covariates and exponentiated values for BN are shown in Table 2.

Table 2: Summary of covariates used in the BN DisMod-MR meta-regression model

Covariate	Type	Parameter	Exponentiated beta (95% UI)
LDI ($ per capita)	Location-level	Prevalence	1.50 (1.35–1.64)

Disability weight

The GBD disability weight survey assessments include lay descriptions of sequelae highlighting major functional consequences and symptoms. No severity splits were applied to BN. The lay description and disability weight for BN are shown in Table 3 below.

Table 3: Lay description for BN in GBD 2023 and the associated disability weight

Lay description	Disability weight (95% UI)
Has uncontrolled overeating followed by guilt, starving, and vomiting to lose weight.	0.223 (0.149–0.311)

In GBD 2023, the modelling strategy for BN was updated to include a past-year crosswalk and an old diagnostic criteria crosswalk, which had not been part of previous analyses. This methodological change was implemented to address heterogeneity in prevalence estimates and ensure alignment with data reflecting current episodes of BN according to the most up-to-date diagnostic criteria. The inclusion of the past-year crosswalk has led to a decrease in the estimated global prevalence of BN compared to GBD 2021.

While we continue to improve on the data and methods used to estimate the burden of mental disorders, some other challenges need to be acknowledged. Firstly, we still have a large number of locations with no high-quality raw data available. Secondly, it is difficult to quantify and remove all variation due to measurement error in our epidemiological estimates. While we have improved the methodology used to account for known sources of bias, in some cases we still have very few datapoints to inform these adjustments. Thirdly, there is a paucity of research on the risk factors of mental disorders which can be used as predictive covariates in our epidemiological models.

References

1. American Psychiatric Association. Diagnostic and Statistical Manual of Mental Disorders, Fifth Edition. Arlington, VA: American Psychiatric Association; 2013.
2. World Health Organization. ICD-11 for mortality and morbidity statistics. 2019. https://icd.who.int/en.
3. Ferrari AJ, Charlson FJ, Norman RE, et al. Burden of depressive disorders by country, sex, age, and year: findings from the global burden of disease study 2010. *PLoS Med* 2013; **10**(11): e1001547.

Neoplasms

This general framework for the GBD 2023 cancer estimation applies to all malignant neoplasms (ie, cancers) except for non-melanoma skin cancer (including basal cell carcinoma and squamous cell carcinoma); benign and in situ neoplasms (including intestinal; cervical and uterine; and other benign neoplasms); and myelodysplastic, myeloproliferative, and other haemopoietic neoplasms.

Flowchart

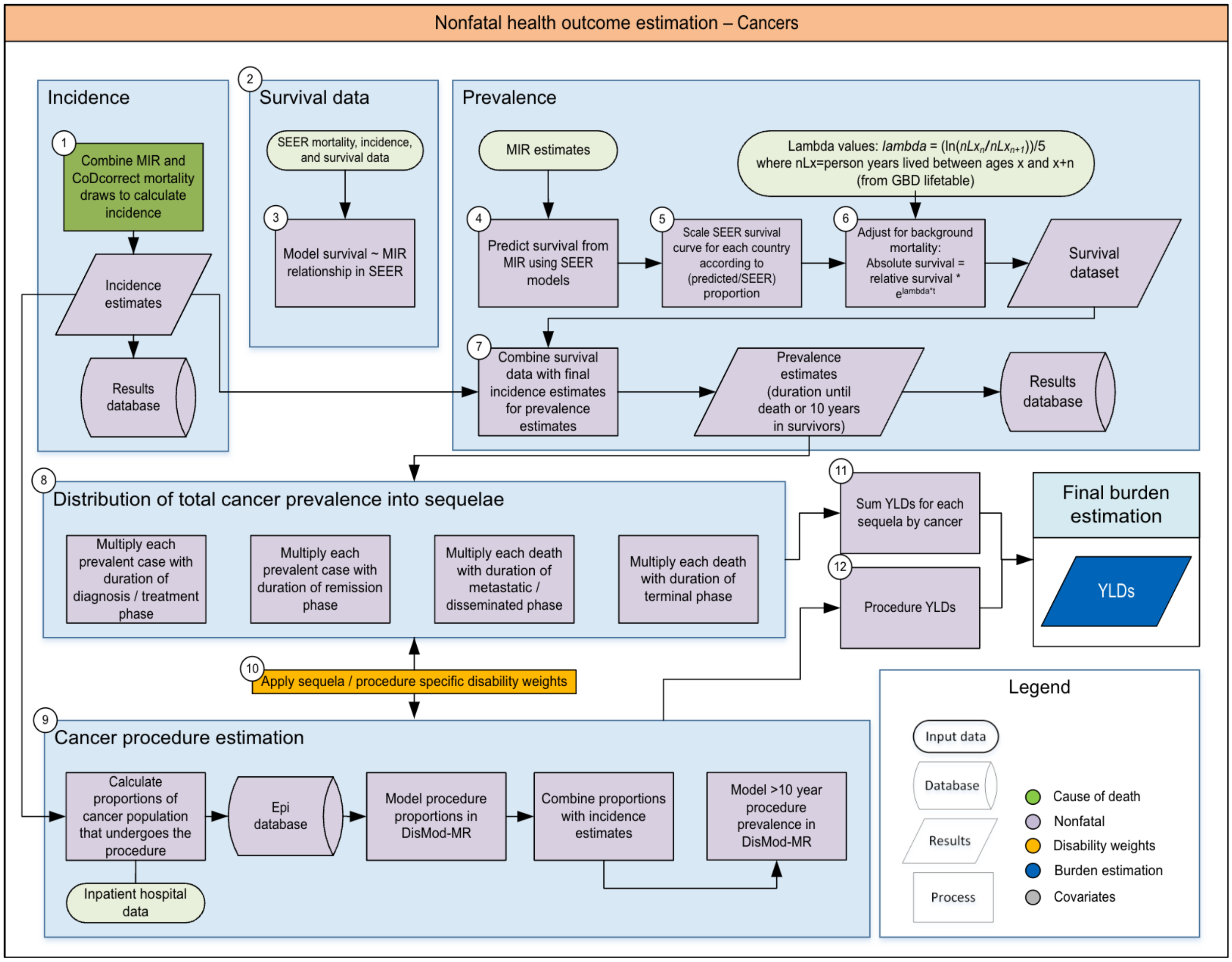

Figure 1. Flowchart of non-fatal estimation for all cancers except non-melanoma skin cancer, benign and in situ neoplasms, and myelodysplastic, myeloproliferative, and other haemopoietic neoplasms

Abbreviations: DisMod-MR, disease model – Bayesian meta-regression; GBD, Global Burden of Disease study; MIR, mortality-to-incidence ratio; SEER, Surveillance, Epidemiology, and End Results Program; YLDs, years lived with disability

Input data and methodological summary for all cancers except non-melanoma skin cancer, benign and in situ neoplasms, and myelodysplastic, myeloproliferative, and other haemopoietic neoplasms

Case definition

For GBD 2023, incidence, prevalence, and disability are estimated for all cancers and benign neoplasms as defined in ICD-10 (C00–D49). The associated ICD codes for neoplasms estimated for GBD 2023 are listed elsewhere in the GBD summary papers. Prevalence for cancers are estimated for a maximum of ten years after incidence, as in GBD 2013, GBD 2015, GBD 2016, GBD 2017, GBD 2019, and GBD 2021.[1–5] Prevalence extending beyond the ten-year period is only estimated for permanent sequelae resulting from five treatment-related surgical procedures (cystectomy, laryngectomy, mastectomy, prostatectomy, and stoma). To estimate disability for each cancer, the prevalence is split into four sequelae representing various phases of cancer, each with a corresponding disability weight. Additional disability is estimated for the five cancers with surgical procedures.

Table 1. Clinical case definition for all cancers estimated in GBD 2023

Quantity of interest	Clinical case definition
Acute lymphoid leukaemia	Malignant neoplasms of the blood and bone marrow (specifically acute lymphoid leukaemia); ICD-10 codes C91.0, C91.3, and C91.6.
Acute myeloid leukaemia	Malignant neoplasms of the blood and bone marrow (specifically acute myeloid leukaemia); ICD-10 codes C92.0, C92.3-C92.6, C93.0, C94.0, C94.2, and C94.4.
Bladder cancer	Malignant neoplasms of the bladder; ICD-10 code C67.
Brain and central nervous system cancer	Malignant neoplasms of the brain and central nervous system; ICD-10 codes C70-C72 and C75.1-C75.3.
Breast cancer	Malignant neoplasms of the breast; ICD-10 code C50.
Burkitt lymphoma	Malignant neoplasms of the lymph system (specifically Burkitt lymphoma); ICD-10 code C83.7.
Cervical cancer	Malignant neoplasms of the cervix; ICD-10 code C53.
Chronic lymphoid leukemia	Malignant neoplasms of the blood and bone marrow (specifically chronic lymphoid leukaemia); ICD-10 code C91.1.
Chronic myeloid leukaemia	Malignant neoplasms of the blood and bone marrow (specifically chronic myeloid leukaemia); ICD-10 codes C92.1-C92.2.
Colon and rectum cancer	Malignant neoplasms of the colon and rectum; ICD-10 codes C18-C21.
Esophageal cancer	Malignant neoplasms of the esophagus; ICD-10 code C15.
Eye cancer	Malignant neoplasms of the eye; ICD-10 code C69.
Gallbladder and biliary tract cancer	Malignant neoplasms of the gallbladder and biliary tract; ICD-10 codes C23-C24.
Hepatoblastoma	Malignant neoplasms of the liver (specifically hepatoblastoma); ICD-10 code C22.2.
Hodgkin lymphoma	Malignant neoplasms of the lymph system (specifically Hodgkin disease); ICD-10 code C81.
Kidney cancer	Malignant neoplasm of the kidneys; ICD-10 codes C64-C65.

Larynx cancer	Malignant neoplasms of the larynx; ICD-10 code C32.
Leukaemia	Malignant neoplasms of the blood and bone marrow (specifically leukaemia); ICD-10 codes C91-C95.
Lip and oral cavity cancer	Malignant neoplasms of the lips and oral cavity; ICD-10 codes C00-C08.
Liver cancer	Malignant neoplasms of the liver; ICD-10 codes C22.0-C22.8 and a proportion of C22.9 reflecting primary liver cancer.
Liver cancer due to alcohol use	Malignant neoplasms of the liver (specifically due to alcohol use); ICD-10 codes C22.0-C22.1, C22.3-C22.8, and a proportion of C22.9 reflecting primary liver cancer.
Liver cancer due to hepatitis B	Malignant neoplasms of the liver (specifically due to hepatitis B); ICD-10 codes C22.0-C22.1, C22.3-C22.8, and a proportion of C22.9 reflecting primary liver cancer.
Liver cancer due to hepatitis C	Malignant neoplasms of the liver (specifically due to hepatitis C); ICD-10 codes C22.0-C22.1, C22.3-C22.8, and a proportion of C22.9 reflecting primary liver cancer.
Liver cancer due to NASH	Malignant neoplasms of the liver (specifically due to NASH); ICD-10 codes C22.0-C22.1, C22.3-C22.8, and a proportion of C22.9 reflecting primary liver cancer.
Liver cancer due to other causes	Malignant neoplasms of the liver (specifically due to causes other than alcohol use, hepatitis B, hepatitis C, or NASH); ICD-10 codes C22.0-C22.1, C22.3-C22.8, and a proportion of C22.9 reflecting primary liver cancer.
Malignant neoplasm of the bone and articular cartilage	Malignant neoplasms of the bones and articular cartilage; ICD-10 codes C40-C41.
Malignant skin melanoma	Malignant neoplasms of the skin (specifically melanoma); ICD-10 code C43.
Mesothelioma	Malignant neoplasms of the mesothelium; ICD-10 code C45.
Multiple myeloma	Malignant neoplasms of plasma cells; ICD-10 codes C88 and C90.
Nasopharynx cancer	Malignant neoplasms of the nasopharynx; ICD-10 code C11.
Neuroblastoma and other peripheral nervous cell tumours	Malignant neoplasms of the nerve cells and other peripheral nervous cells (specifically neuroblastoma and other peripheral nervous cell malignancies); ICD-10 codes C47 and a proportion of C74 reflecting neuroblastomas of the adrenal gland.
Non-Hodgkin lymphoma	Malignant neoplasms of the lymph system (specifically non-Hodgkin lymphoma); ICD-10 codes C82-C86 and C96.
Other eye cancers	Malignant neoplasms of the eye (excluding retinoblastoma); ICD-10 codes C69.0-C69.1 and C69.3-C69.9.
Other leukaemia	Malignant neoplasms of the blood and bone marrow (specifically leukaemias not otherwise specified as ALL, AML, CLL, or CML), including ICD-10 codes C91.7, C92.7, C92.8, C93.1-C93.9, C94.3, C94.7, and C95.7.
Other malignant neoplasms	Malignant neoplasms not included in another GBD cancer cause, including ICD-10 codes such as C17, C30, C31, C48, C51, C52, C57, C60, C63, C66, and C68.

Other non-Hodgkin lymphoma	Malignant neoplasms of the lymph system (specifically non-Hodgkin lymphomas other than Burkitt lymphoma); ICD-10 codes C82-C86 (excluding codes mapped to Burkitt lymphoma) and C96.
Other pharynx cancer	Malignant neoplasms of the pharynx (specifically pharynx cancers which are not included in lip and oral cavity cancers or nasopharynx cancers); ICD-10 codes C09-C10 and C12-C13.
Ovarian cancer	Malignant neoplasms of the ovary; ICD-10 code C56.
Pancreatic cancer	Malignant neoplasms of the pancreas; ICD-10 code C25.
Prostate cancer	Malignant neoplasms of the prostate; ICD-10 code C61.
Retinoblastoma	Malignant neoplasms of the retina (specifically retinoblastoma); ICD-10 code C69.2.
Soft tissue and other extraosseous sarcomas	Malignant neoplasms of the soft tissues and other extraosseous sites (specifically sarcomas of these sites); ICD-10 code C49.
Stomach cancer	Malignant neoplasms of the stomach; ICD-10 code C16.
Testicular cancer	Malignant neoplasms of the testis; ICD-10 code C62.
Thyroid cancer	Malignant neoplasms of the thyroid; ICD-10 code C73.
Tracheal, bronchus, and lung cancer	Malignant neoplasms of the trachea, bronchus, and lungs; ICD-10 codes C33-C34.
Uterine cancer	Malignant neoplasms of the uterus; ICD-10 code C54.

Input data

In order to align estimates of incidence with estimates of mortality for each cancer site, ratios of mortality to incidence are modelled using matching cancer registry data. By multiplying these mortality-to-incidence ratios (MIRs) with incidence data from cancer registries where vital registration data is absent or of low quality, mortality data can be added to the cause of death modelling in CODEm. After the cause of death estimates have been finalised and passed through CoDCorrect, cancer incidence is calculated by dividing the cancer mortality estimates by the estimated MIRs. Data sources for cancer mortality are described elsewhere in the GBD summary articles. Data sources for cancer registry incidence data are limited to those that have matching mortality data or do not have overlapping vital registration data. Data sources used to estimate prevalence come from the USA SEER[6] (Surveillance, Epidemiology, and End Results Program) data for matching incidence, mortality, and survival. For a minority of cancer types not consistently captured by cancer registries, prevalence data from clinical informatics data sources are used. Data sources used to estimate the proportion of cancer patients undergoing surgical procedures and to determine sequelae duration will also be listed below.

Table 2a. Data inputs for neoplasms morbidity modelling by parameter

**Cause**	**Prevalence sources**	**Incidence sources**	**Deaths sources**	**All measures sources**
Neoplasms	375	3146	5293	9110
Lip and oral cavity cancer	3	817	4487	5307
Nasopharynx cancer	3	663	4067	4733
Other pharynx cancer	3	883	4388	5274
Oesophageal cancer	3	975	4854	5832

Stomach cancer	3	976	4794	5773
Colon and rectum cancer	3	1032	4932	5969
Liver cancer	3	1185	4444	5900
Hepatoblastoma	3	1185	4444	5900
Gallbladder and biliary tract cancer	3	907	4373	5283
Pancreatic cancer	3	1073	4557	5633
Larynx cancer	3	956	4747	5725
Tracheal, bronchus, and lung cancer	3	1104	4983	6090
Malignant neoplasm of bone and articular cartilage	3	791	3370	4146
Malignant skin melanoma	3	645	4111	4759
Non-melanoma skin cancer	3	1244	3984	5231
Mesothelioma	3	645	2389	3037
Neuroblastoma and other peripheral nervous cell tumours	3	682	3149	3834
Soft tissue and other extraosseous sarcomas	3	765	3133	3901
Breast cancer	3	1297	5008	6313
Cervical cancer	3	1052	4821	5876
Uterine cancer	3	1029	4853	5885
Ovarian cancer	3	1050	4581	5634
Prostate cancer	3	1098	4928	6034
Testicular cancer	3	639	3895	4537
Kidney cancer	3	1144	4577	5724
Bladder cancer	3	877	4441	5338
Eye cancer	3	763	3161	3927
Retinoblastoma	3	635	3113	3751
Other eye cancer	3	703	3142	3848
Brain and central nervous system cancer	3	1062	4513	5578
Thyroid cancer	3	948	4409	5360
Other malignant neoplasms	3	746	4115	4864
Hodgkin lymphoma	3	870	4086	4959
Non-Hodgkin lymphoma	3	1282	4590	5875
Burkitt lymphoma	3	920	81	1004
Other non-Hodgkin lymphoma	3	905	3167	4075
Multiple myeloma	3	783	3484	4270
Leukaemia	3	980	4381	5364
Other malignant neoplasms	3	746	4115	4738
Other neoplasms	375	0	3116	3491

As described elsewhere in the GBD summary articles, additional data from a literature review were utilised to proportionally split the age ≥10 years liver cancer mortality estimates (which excludes

hepatoblastoma) into the five aetiology groups included in the GBD. Table 2 lists the number of papers contributing to each of the liver cancer aetiology proportion models.

Table 2b. Data inputs for liver cancer subtypes morbidity modelling by parameter

Cause	Proportion data sources
Liver cancer	268
Liver cancer due to alcohol use	96
Liver cancer due to hepatitis B	267
Liver cancer due to hepatitis C	266
Liver cancer due to NASH	93
Liver cancer due to other causes	55

To standardise differences in reporting among these papers, we used the GBD meta-regression—Bayesian, regularised, trimmed (MR-BRT) method, which is detailed elsewhere in the GBD summary papers. Table 3 lists the MR-BRT crosswalk adjustment factors that were used to estimate sex-specific proportions from both-sex data, and to adjust for implicit versus explicit reporting of non-alcoholic steatohepatitis (NASH). Further details on the extraction, modelling, and application of these liver cancer aetiology splits are described elsewhere in the GBD summary articles.

Table 3. MR-BRT crosswalk adjustment factors for liver cancer aetiology proportion model inputs

Model	Crosswalk	Reference	Alternative	Gamma	Beta coefficient	Adjustment factor
Liver cancer due to alcohol	Sex split	male proportion	Both-sex proportion	0.00	–0.998	0.368
Liver cancer due to hepatitis B	Sex split	male proportion	Both-sex proportion	0.07	–0.421	0.656
Liver cancer due to hepatitis C	Sex split	male proportion	Both-sex proportion	0.42	0.299	1.349
Liver cancer due to other causes	Sex split	male proportion	Both-sex proportion	0.37	0.260	1.297
Liver cancer due to NASH	Sex split	male proportion	Both-sex proportion	0.00	0.090	1.095
Liver cancer due to NASH	NASH definition	Explicit NASH	Implicit NASH	0.91	–0.322	0.725

**MR-BRT crosswalk adjustments can be interpreted as the factor the alternative case definition is adjusted by to reflect what it would have been had it been measured using the reference case definition. If the beta coefficient is negative, then the alternative is adjusted up to the reference. If the beta coefficient is positive, then the alternative is adjusted down to the reference.*
***The adjustment factor column is the exponentiated beta coefficient.*

Modelling strategy

Estimation of cancer mortality and MIR estimation has been described elsewhere in the GBD summary articles. As both the fatal and non-fatal estimation processes utilise these same modelled MIR estimates, the MIR estimation process is detailed again below. To summarise, incidence and mortality data from cancer registries were matched by cancer, age, sex, year, and location to generate input MIRs, which

were then used to obtain MIR estimates using one of two MIR modelling approaches, depending on the cancer. In the non-fatal process, these estimated MIRs were then used to transform the final GBD cancer mortality estimates into GBD incidence estimates.

MIR data processing

For all causes that existed in GBD 2021, data-cleaning steps for MIR estimation were the same as for GBD 2021. For each cancer, MIRs from locations in Healthcare Access and Quality Index (HAQ Index) quintiles 1–4 were dropped if they were below the median of MIRs from locations in HAQ Index quintile 5. We also dropped MIRs from locations in HAQ Index quintiles 1–4 if the MIRs were above an outlier threshold calculated as the third quartile + 1.5 * IQR (inter-quartile range). We dropped all MIR data that were based on fewer than 15 incident cases to avoid excessive variation in the ratio due to small numbers. An exception to this threshold was made for mesothelioma, acute myeloid leukaemia, and new this round, acute lymphoid leukaemia, where instead we dropped MIRs that were based on fewer than ten cases because of lower data availability for these cancers. For the lower end of the age spectrum where cancers are generally rarer, we previously aggregated incidence and mortality to the youngest five-year age bin where SEER[7] reported at least 50 cases from 1990 to 2015, to avoid unstable MIR predictions in young age groups because of too few cases or deaths. For GBD 2023, instead of using the SEER-based age threshold, we allowed age groups with greater than ten observations and more than one unique location to retain their age-specific data and be modelled independently. As before, for age groups below this new threshold, the MIR estimates were then copied to all younger GBD age groups estimated for that cancer.

For the nine cancer causes largely affecting children and first estimated in GBD 2021, additional data processing steps were used to help stabilise the input data and MIR estimates. These additional steps were determined to be necessary due to the much smaller counts of cases and deaths in these causes. First, for retinoblastoma and hepatoblastoma, data were aggregated across sexes and across bins of ten calendar years (for example, 2000 to 2009). Data were then only excluded if there were less than 0.01 cases, since such small values were not directly reported but were a result of redistribution and were leading to implausibly high MIRs. For retinoblastoma, the cancer registry mortality data were too limited to generate sufficient MIR input data using only cancer registry data. To generate additional MIR input data for this cause, cancer registry incidence was matched with mortality from vital registration data by age-sex-year-location. These cancer registry–vital registration matched retinoblastoma MIR inputs were processed the same as the standard matched inputs.

Since MIRs can be above 1, especially in older age groups and for cancers with low cure rates, we used the 95th percentile (by age group) of the cleaned dataset (detailed above) to cap the MIR input data. These “upper cap” values were used to allow MIRs over 1 in some age groups but to constrain the MIRs to a maximum level. The addition of new data for GBD 2023 led to slightly different upper caps compared to GBD 2021 (see upper cap values for GBD 2023 below). For GBD 2023, we no longer set a minimum upper cap of 1 (regardless of the 95th percentile) for paediatric age groups (under 20 years).

**Age group (years)**	**0–4**	**5–9**	**10–14**	**15–19**	**20–24**	**25–29**	**30–34**	**35–39**	**40–44**	**45–49**
**Upper cap**	0.678	0.750	0.793	0.961	0.954	0.798	0.752	0.812	0.859	0.866

Age group (years)	50–54	55–59	60–64	65–69	70–74	75–79	80–84	85–89	90–94	95+
**Upper cap**	0.893	0.925	0.973	1.02	1.06	1.16	1.28	1.41	1.61	1.84

Any MIR values over this upper cap were winsorised to the cap value. To run the logit model in ST-GPR, the input data were first divided by the upper caps to get proportional data ranging from 0 to 1. Model predictions from ST-GPR were then rescaled back to MIRs by multiplying the scaled predictions by the upper caps. To constrain the MIRs at the lower end, we used the fifth percentile of the cancer and age-specific cleaned MIR input data to winsorise all model predictions below this lower cap.

MIR data modelling

As in previous GBD cycles, MIRs for most cancers were estimated with a three-step modelling approach using the general GBD spatiotemporal Gaussian process regression (ST-GPR) approach. The first step used a linear model for logit-transformed MIR as the outcome, with covariates for HAQ Index, sex, and categorical age group (number of categories depending on cancer type).[8] For the MIRs, this initial linear prior used the following general form:

$$\text{logit}\left(MIR_{c,a,s,t}\right) = \alpha + \beta_1 (HAQIndex)_{c,t} + \beta_2 \mathrm{I}_s + \sum_{a}^{A} \beta_a \mathrm{I}_a + \epsilon$$

MIR: mortality-to-incidence ratio
c: country (or subnational for subnationally modelled locations), a: age group, t: time (years); s: sex
HAQIndex: Healthcare Access and Quality Index
I: indicator variable
ϵ: error term

Results from the final linear model were used as input for spatiotemporal smoothing and a Gaussian process regression. The ST-GPR model has three main hyper-parameters that control for smoothing across time, age, and geography. These hyper-parameter values were iteratively evaluated in GBD 2019 for balancing model performance with local data availability and were unchanged for GBD 2023. The time adjustment parameter lambda (λ) aims to borrow strength from neighbouring time points (ie, the value in this year is highly correlated with the value in the previous year but less so further back in time) and was set to 0.05. The age adjustment parameter omega (ω) borrows strength from data in neighbouring age groups and was set to 0.5. The space adjustment parameter zeta (ζ) aims to borrow strength across the hierarchy of geographical locations and was set to 0.01. For the remaining parameters in the Gaussian process regression, we set amplitude to 1 (influences fluctuation from the mean function) and set the scale value to 10 (influences the time distance over which points are correlated). Additional details on ST-GPR are described elsewhere in the GBD summary papers. These models were used to obtain MIR estimates for all combinations of GBD age, sex, year, cause, and location. Datapoints were outliered manually if they clearly influenced the model in an unrealistic way. For example, a datapoint was marked as an outlier if it created a single-year, single age group spike in model predictions that was inconsistent with the trend suggested by surrounding datapoints.

For GBD 2021 the MIR modelling approach used for the nine newly estimated cancer causes differed by cause, depending on the data availability, the age distribution of cases and deaths, the reliability of the modelled estimates, and the GBD cause hierarchy. For GBD 2023, new data and processing improvements allowed most of these causes to now be modelled in ST-GPR, as detailed above. For two causes, retinoblastoma and hepatoblastoma, we continued to model MIRs using a negative binomial regression approach for GBD 2023. The negative binomial approach was used for these two cancer causes because it allows modelling of count data with overdispersion (meaning the mean and variance are allowed to differ in the underlying distribution), which was determined to be necessary due to the relatively rare deaths for these cancer causes and the age restrictions for these causes (age <10). MIRs were estimated for each age-sex-year-location using a negative binomial regression run in R (version 4.4.0) using glm.nb from the MASS package. Models included covariates for HAQ Index and categorical age groups and were offset by the logarithm of cases. For eye cancer, the MIRs were copied from the models for retinoblastoma (age 0-9) and "other eye cancer" (age 10+), the same as for GBD 2021.

Incidence estimation

For all cancers except retinoblastoma, the final GBD cancer mortality estimates (after CoDCorrect adjustment) were transformed to incidence estimates by using the MIRs specific to that cancer cause. Final mortality estimates at the draw-specific level were divided by the modelled MIR estimates (also at the draw-specific level) to generate a distribution of draws of incidence estimates (which provides an estimated mean incidence with 95% uncertainty interval). It was assumed that uncertainty in the MIR is independent of uncertainty in the estimated mortality.

For retinoblastoma, the incidence estimation approach above was used for all locations except those in the high-income super-region. For high-income countries, death from retinoblastoma is extremely rare, which can lead to estimated MIRs close to zero and underestimation of incidence with the above approach (due to a numerator close to zero). To address this, alternative MIRs specific to locations in the high-income super-region were estimated by matching CODEm mortality estimates with cancer registry incidence data for these locations. Incidence draws for these locations were then estimated as detailed above, dividing the CoDCorrect estimates by these alternative high-income-specific MIRs (rather than the globally informed MIRs). To avoid potential subsequent overestimation of incidence in these locations, the incidence rates were winsorised to the 2.5th and 97.5th percentiles of incidence rates across countries in the high-income super-region.

For neuroblastoma and other eye cancer, the MIR models produced a small subset of draws with values that were implausibly low in the high-income super-region, which initially led to an overestimation of incidence in this step. To account for this, we winsorised the high-income MIR draws to a floor based on the 5th percentile of all the high-income MIRs for neuroblastoma and all high-income MIRs excluding USA subnationals for other eye cancer.

Prevalence estimation

After transforming the final GBD cancer mortality estimates to incidence estimates (step 1 in the general cancer flowchart), incidence was combined with annual relative survival estimates from one to ten years after diagnosis (step 7 in the flowchart). Previous reports suggest that the value of (1 – MIR) may serve as a proxy for five-year relative survival, with the exact correlation varying slightly by cancer type.[9] Because this correlation varies, we trained cancer-specific prediction models to estimate five-year survival from MIRs, using data from SEER. We used SEER*Stat[10] to obtain mortality,[11] incidence,[12] and

relative survival[12] statistics from the nine SEER registries reporting from 1980 to 2014 (through 2014 so that all years have at least five years of follow-up time; step 2), by cancer type, sex, five-year time periods (eg, 1980–1984, 1985–1989, etc.), and five-year age groups (except combining 80+). For each cancer, we modelled five-year relative survival with MIRs calculated from SEER mortality and incidence, using a generalised linear model with a quasibinomial family and logit link, weighted by the number of index cases (step 3).

To reduce variability due to small samples, we only included MIRs based on at least 25 incident cases (except for the rarer cancers mesothelioma, acute myeloid leukaemia, and acute lymphoid leukaemia, where MIRs based on at least ten cases were included). These models were then applied to the GBD MIR estimates to predict an estimated five-year survival for each age, sex, year, and location combination (step 4). To prevent unrealistic values, predicted five-year survival values were winsorised to be between 0% and 100% survival.

To generate yearly survival estimates up to ten years, we downloaded SEER sex- and age-specific annual one- through ten-year relative survival data from persons diagnosed between 2001 and 2010 (2001 through 2010 so that all cases had at least five years of follow-up, with half having the full ten years of follow-up).[12] A proportional scalar was calculated as the predicted GBD five-year survival estimate divided by the SEER five-year survival statistic, and was then used to generate yearly survival estimates by scaling the one- to ten-year SEER curve to the GBD survival predictions under the proportional hazards assumption (step 5).

To facilitate the estimation of the total person-time survived, the estimated relative survival (the survival of cancer cases relative to those without cancer, given the expected background mortality) described above was next transformed into absolute survival estimates (the overall survival of cancer cases, including all causes of mortality; step 6 and 7 in the flowchart). To account for background mortality in the relative survival estimates, GBD 2023 lifetables were used to calculate lambda (λ) values:

$$\lambda = \frac{\ln\left(\frac{nLx_n}{nLx_{n+1}}\right)}{5}$$

nLx = person-years lived between ages x and x+n (from GBD lifetable).

GBD 2021 lifetables are described elsewhere in the GBD summary papers. Absolute survival was then calculated using an exponential survival function:

$$absolute\ survival = relative\ survival * e^{\lambda * t}$$

t = time (in years)

Absolute survival was combined with incidence to estimate the prevalence at each year after diagnosis.

Disability estimation

To estimate disability for each cancer, total prevalence is split into four sequelae: (1) diagnosis and primary therapy phase; (2) controlled phase; (3) metastatic phase; and (4) terminal phase (step 8 in the

flowchart). The diagnosis and primary therapy phase represents the time from the onset of symptoms to the end of initial treatment. The controlled phase represents the time between finishing primary treatment and the earliest of either cure (defined as recurrence- and progression-free survival after ten years); death from another cause; or progression to the metastatic phase. The metastatic phase represents the time period of intensive treatment for metastatic disease, as determined for each cancer by evaluating data from SEER[6] averages (Table 4). The terminal phase represents the one-month period prior to death. Each of these four sequelae has a separate disability weight, which are the same across cancer types (Table 6. Severity distribution). Because of the long-term disability associated with certain treatment-related procedures, additional disability beyond these four sequelae is estimated for five cancers: breast cancer (disability due to mastectomy), larynx cancer (disability due to laryngectomy), colon and rectum cancer (disability due to stoma), bladder cancer (disability due to incontinence from cystectomy), and prostate cancer (disability due to either incontinence or impotence from prostatectomy).

For the purposes of calculating disability due to cancer, survivors beyond ten years were considered cured. For this group, the survivor population prevalence person-time was divided into two sequelae: (1) diagnosis and primary therapy phase; and (2) controlled phase (or remission). For the population that did not survive beyond ten years, the yearly prevalence person-time was divided into four sequelae by assigning fixed durations for each of the: (4) terminal phase, (3) disseminated/metastatic phase, and (1) diagnosis and primary therapy phase, and assigning any remaining prevalence to the (2) controlled phase (step 8 in the flowchart). The duration of these four sequelae remained the same as for GBD 2013, GBD 2015, GBD 2016, GBD 2017, GBD 2019, and GBD 2021,[1–5] with the exception of updates to other malignant neoplasms (recalculated) and other non-Hodgkin lymphoma (set equal to non-Hodgkin lymphoma). Table 4 lists the duration of each, along with the sources used to determine their length. For the diagnosis and primary therapy phase, the duration was taken from primary literature or expert opinion. For the disseminated/metastatic phase, the duration was taken from primary literature, or as the median survival time reported by SEER for the persons described in the note column.

Table 4. Duration of four prevalence sequelae by cancer

Cause	Diagnosis and primary therapy phase (months)*	Controlled phase, or remission	Disseminated/ metastatic phase (months)*	Note for disseminated/ metastatic phase	Terminal phase (months)
Oesophageal cancer	5.0[13]	The remission phase duration is calculated based on the remaining time after attributing other sequelae durations.	4.6[14]	SEER Summary Stage 1977 (Distant site/node involved) 1995–2000	1.0
Stomach cancer	5.2[13]		3.9[14]	SEER Summary Stage 1977 (distant site/node involved) 1995–2000	1.0
Liver cancer	4.0		2.5[14]	SEER Summary Stage 1977 (distant site/node involved) 1995–2000	1.0
Hepatoblastoma	6.0		23.1[14]	SEER Summary Stage 1977 (distant site/node involved) 1995–2000	1.0
Larynx cancer	5.3[13]		8.8[14]	SEER Stage IVc	1.0

Tracheal, bronchus, and lung cancer	3.3[15]	The remission phase duration is calculated based on the remaining time after attributing other sequelae durations.	4.5[14]	SEER Summary Stage 1977 (distant site/node involved) 1995–2000	1.0
Breast cancer	3.0[15]		17.7[14]	SEER Summary Stage 1977 (distant site/node involved) 1995–2000	1.0
Cervical cancer	4.8[13]		9.2[14]	SEER Summary Stage 1977 (distant site/node involved) 1995–2000	1.0
Uterine cancer	4.6[13]		11.6[14]	SEER Summary Stage 1977 (distant site/node involved) 1995–2000	1.0
Prostate cancer	4.0[15]		30.4[14]	SEER Summary Stage 1977 (distant site/node involved) 1995–2000	1.0
Colon and rectum cancer	4.0[15]		9.7[14]	SEER Summary Stage 1977 (distant site/node involved) 1995–2000	1.0
Lip and oral cavity cancer	5.3[13]		9.3[14]	SEER Stage IVc	1.0
Nasopharynx cancer	5.3[13]		13.2[14]	SEER Stage IVc	1.0
Other pharynx cancer	5.3[13]		7.9[14]	SEER Stage IVc	1.0
Gallbladder and biliary tract cancer	4.0		3.5[14]	SEER Summary Stage 1977 (distant site/node involved) 1995–2000	1.0
Pancreatic cancer	4.1[13]		2.5[14]	SEER Summary Stage 1977 (distant site/node involved) 1995–2000	1.0
Malignant skin melanoma	2.9[16]		7.2[14]	SEER Summary Stage 1977 (distant site/node involved) 1995–2000	1.0
Ovarian cancer	3.2[15]		25.6[14]	SEER Summary Stage 1977 (distant site/node involved) 1995–2000	1.0
Testicular cancer	3.7[13]		19.5[14]	SEER Stage III	1.0
Kidney cancer	5.3[13]		5.4[14]	SEER Summary Stage 1977 (distant site/node involved) 1995–2000	1.0
Bladder cancer	5.1[13]		5.8[14]	SEER Summary Stage 1977 (distant site/node involved) 1995–2000	1.0
Brain and central nervous system cancer	5.0		6.9[14]	SEER median age-standardised survival all patients, all years	1.0
Thyroid cancer	3.0		19.4[14]	SEER Stage IVc	1.0
Mesothelioma	4.0		7.8[14]	SEER Summary Stage 1977 (distant site/node involved) 1995–2000	1.0
Hodgkin lymphoma	3.7[15]		26.0[17]	Literature	1.0

Non-Hodgkin lymphoma	3.7[15]		7.7[17]	Literature	1.0
Other non-Hodgkin lymphoma	3.7[15]		7.7[17]	Literature	1.0
Burkitt lymphoma	6.0		8.8[14]	SEER Summary Stage 1977 (distant site/node involved) 1995–2000	1.0
Multiple myeloma	7.0[13]		36.8[14]	SEER median age-standardised survival all patients, all years	1.0
Leukaemia	5.0[13]		43.7[14]	SEER median age-standardised survival all patients, all years	1.0
Acute lymphoid leukaemia	12.0		7.0[14]	SEER median age-standardised survival all patients, all years	1.0
Acute myeloid leukaemia	6.0		4.6[14]	SEER median age-standardised survival all patients, all years	1.0
Chronic lymphoid leukaemia	6.0		48[18]	SEER median age-standardised survival all patients, all years	1.0
Chronic myeloid leukaemia	6.0		4.6[14]	SEER median age-standardised survival for AML (patients with CML die in blast crisis, which is treated like AML) all patients, all years	1.0
Other leukaemia	6.0		48.0[18]	SEER median age-standardised survival all patients, all years	1.0
Other malignant neoplasms	4.9**		15.8[14]	SEER median age-standardised survival all patients, all years	1.0
Malignant neoplasm of bone and articular cartilage	10.0		19.8[14]	SEER Summary Stage 1977 (distant site/node involved) 1995–2000	1.0
Neuroblastoma and other peripheral nervous cell tumours	10.0	The remission phase duration is calculated based on the remaining time after attributing other sequelae durations.	47.4[14]	SEER Summary Stage 1977 (distant site/node involved) 1995–2000	1.0
Soft tissue and other extraosseous sarcomas	10.0		10.7[14]	SEER Summary Stage 1977 (distant site/node involved) 1995–2000	1.0
Eye cancer	2.9		16.0[14]	SEER median age-standardised survival all patients, all years	1.0
Other eye cancers	2.9		16.0[14]	SEER median age-standardised survival all patients, all years	1.0
Retinoblastoma	6.0		6.4[19]	Literature	1.0

* Superscripts refer to references used to inform these values. Durations without a superscript are based on expert opinion.

** "Other malignant neoplasms" value was calculated as the mean of all Level 3 causes (Level 4 causes were excluded from this calculation).

For cancer-specific procedure sequelae, hospital data were used to estimate the number of cancer patients undergoing mastectomy, laryngectomy, stoma, prostatectomy, or cystectomy (step 9 in the flowchart). Input data for these proportions came from clinical informatics sources of hospital data from the USA,[20,21] Canada,[22] and Mexico,[23] and remained the same as in GBD 2013, GBD 2015, GBD 2016, GBD 2017, GBD 2019, and GBD 2021.[1–5] Proportions for each procedure were generated by dividing the rate of the procedure (generated from the diagnostic codes in the hospital dataset) by the GBD age- and sex-specific disease incidence rates for that country. Diagnostic codes used are listed in Table 5:

Table 5. Procedure codes used to estimate cancer procedure proportions

Procedure	Cancer	Procedure code (ICD-9-CM[24])
Mastectomy	Breast cancer	854, 8541, 8542, 8543, 8544, 8545, 8546, 8547, 8548
Laryngectomy	Larynx cancer	301, 303, 304, 3029
Stoma	Colon and rectum cancer	461, 4610, 4611, 4613, 4862
Cystectomy	Bladder cancer	5771, 5779
Prostatectomy	Prostate	603, 604, 605, 606, 6062

To estimate procedure-related disability for each of these five cancers, the procedure proportions (proportion of each cancer population that undergo these procedures) were used as input for a proportion model in DisMod-MR 2.1 to estimate the proportions for all locations, by age, year, and by sex. Details of DisMod-MR 2.1 and clinical and claims data processing are available elsewhere in the GBD summary papers.

Since colostomy or ileostomy procedures are done for reasons other than cancer, a literature review was conducted for GBD 2013 to determine the proportion of ostomies due to colon and rectum cancer. Based on the results of the literature review that an average of 58% of ostomies are done for colon and rectum cancer, the “all cause” colostomy proportions were multiplied by 0.58.[25–27]

The final procedure proportions were applied to the incident cases of the respective cancers and multiplied with the proportion of the incident population surviving for ten years to determine the incident cases of the cancer population that underwent procedures and that survived beyond ten years. These estimates of survivors at ten years were then used as an input for DisMod-MR 2.1, with a remission specification of zero and an excess mortality rate prior of 0 to 0.1, as well as with increasing both the age of the population and the year by ten years to reflect prevalence after that population has survived ten years. The results from this model are incidence and lifetime prevalent cases of persons with these cancer-related sequelae who have survived beyond ten years.

Since disability associated with prostatectomy comes from impotence and incontinence, and not from the prostatectomy itself, 18% of the prostatectomy prevalence was assumed to have incontinence and 55% was assumed to have impotence, based on a literature review done for GBD 2013.[28–35] Cases were assigned disability for either impotence or incontinence, but no cases were assigned disability from both.

We assumed that for the population surviving up to ten years, only those in the remission phase experience additional disability due to procedures (eg, women suffering from metastatic breast cancer do not experience additional disability due to a mastectomy during this phase). To estimate the prevalence of the cancer population in remission during the first ten years after diagnosis with and

without procedure-related disability, we multiplied the prevalence of the population in the remission phase with the proportion of the population undergoing a procedure. This step allowed us to estimate disability during the remission phase for both the population experiencing disability due to the remission phase alone, as well as the population experiencing disability from both the remission phase and the additional procedure-related disability combined. This combined disability weight was calculated for each procedure using a multiplicative function to combine the remission phase disability weight with the procedure disability weight, as 1 - (1 - 'remission phase weight') * (1 - 'procedure weight'). For example, the combined disability weight for "remission phase, with mastectomy" was calculated as: 1 - (1 – 0.049) * (1 – 0.036) = 0.083.

YLD estimation

Lastly, the procedure sequelae prevalence and general sequelae prevalence were multiplied with their respective disability weights (Table 6) to obtain the number of YLDs for each sequela (steps 11 and 12 in the flowchart). The methods used to generate disability weights are described elsewhere in the GBD summary papers. In brief, disability weights were created from survey data to represent the magnitude of health loss associated with an outcome. These disability weights range from 0 (implying a state equivalent to full health) to 1 (a state equivalent to death). Summing these sequelae-specific YLDs then provides the total YLD estimate associated with each cancer cause.

Table 6. Severity distribution. Details on the severity levels for cancers and the associated disability weight with that severity.

**Health state**	**Lay description**	**Disability weight (95% uncertainty interval)**
Cancer, diagnosis and primary therapy<br>*All cancers except non-melanoma skin cancer*	This person has pain, nausea, fatigue, weight loss and high anxiety.	0.288<br>(0.193–0.399)
Cancer, controlled phase<br>*All cancers except non-melanoma skin cancer*	This person has a chronic disease that requires medication every day and causes some worry but minimal interference with daily activities.	0.049<br>(0.031–0.072)
Cancer, metastatic<br>*All cancers except non-melanoma skin cancer*	This person has severe pain, extreme fatigue, weight loss, and high anxiety.	0.451<br>(0.307–0.600)
Terminal phase, with medication<br>*All cancers except non-melanoma skin cancer*	This person has lost a lot of weight and regularly uses strong medication to avoid constant pain. The person has no appetite, feels nauseous, and needs to spend most of the day in bed.	0.540<br>(0.377–0.687)
Mastectomy<br>*Breast cancer*	This person had one of her breasts removed and sometimes has pain or swelling in the arms.	0.036<br>(0.020–0.057)
Stoma<br>*Colon and rectum cancer*	This person has a pouch attached to an opening in the belly to collect and empty stools.	0.095<br>(0.063–0.131)
Laryngectomy<br>*Larynx cancer*	This person has difficulty speaking, and others find it difficult to understand.	0.051<br>(0.032–0.078)
Urinary incontinence<br>*Bladder cancer; Prostate cancer*	This person cannot control urinating.	0.139<br>(0.094–0.198)

Impotence<br>*Prostate cancer*	This person has difficulty in obtaining or maintaining an erection.	0.017<br>(0.009–0.030)

Non-melanoma skin cancer (basal and squamous cell carcinoma)

Flowchart

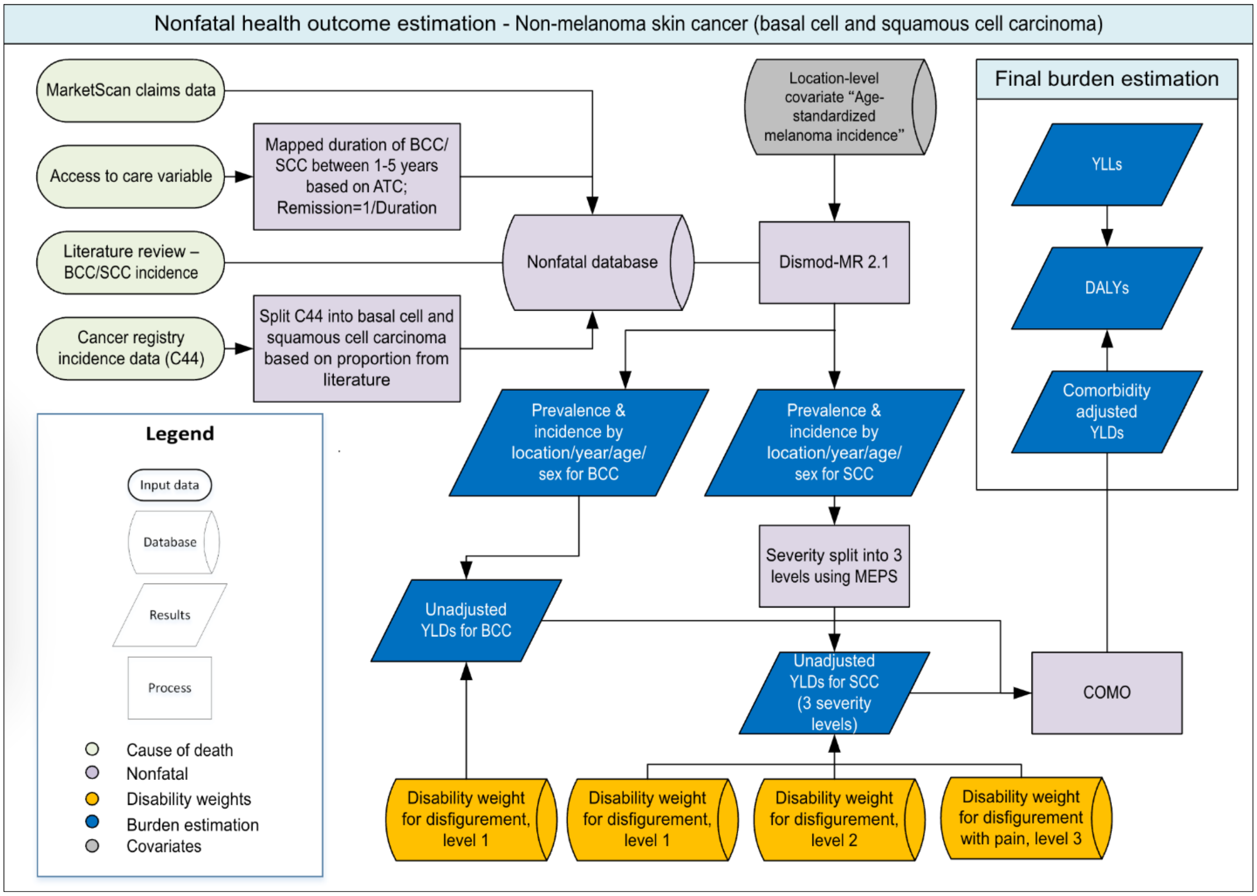

Figure 2. Flowchart of non-fatal estimation for non-melanoma skin cancer (basal and squamous cell carcinoma)

Abbreviations: BCC, basal cell carcinoma; COMO, comorbidity correction microsimulation; DALYs, disability-adjusted life-years; DisMod-MR, disease model – Bayesian meta-regression; MEPS, Medical Expenditure Panel Survey; SCC, squamous cell carcinoma; YLDs, years lived with disability; YLLs, years of life lost.

Input data and methodological summary for non-melanoma skin cancer (basal and squamous cell carcinoma)

Case definition

Non-melanoma skin cancer (NMSC) is defined as squamous cell carcinoma and basal cell carcinoma. NMSC does not include other types of skin cancer (eg, melanoma, Merkel cell carcinoma).

Table 7. Clinical case definition for non-melanoma skin cancer

Quantity of interest	Clinical case definition

Non-melanoma skin cancer	Malignant neoplasms of the skin, excluding melanoma; ICD-10 code C44. All deaths were attributed to squamous cell carcinoma.
Non-melanoma skin cancer (basal cell carcinoma)	Malignant neoplasms of the skin (specifically basal cell carcinoma). Incident cases coded to C44 but without morphology codes were assigned to basal or squamous cell carcinoma based on proportions informed by published literature. No mortality is estimated for this cause.
Non-melanoma skin cancer (squamous cell carcinoma)	Malignant neoplasms of the skin (specifically squamous cell carcinoma). Incident cases coded to C44 but without morphology codes were assigned to basal or squamous cell carcinoma based on proportions informed by published literature.

Input data

We estimated squamous cell carcinoma and basal cell carcinoma incidence by using data from cancer registries, primary literature, and insurance claims. Only cancer registries that were listed in Cancer Incidence in Five Continents (CI5)[36–46] as registering squamous cell carcinoma or basal cell carcinoma were included in the cancer registry incidence data.[47] In total, there were 1212 sources for basal cell carcinoma incidence data and 1417 sources for squamous cell carcinoma incidence data. Additional details on claims data processing are available elsewhere in the GBD summary papers.

Modelling strategy

For cancer registry data reported at the three-digit level (ie, C44: Other and unspecified malignant neoplasm of skin), fixed proportions reported in Karagas and colleagues were used to split C44 into squamous cell carcinoma and basal cell carcinoma.[48] These data, along with data from clinical and literature sources, were input into DisMod-MR 2.1 models to estimate incidence and prevalence for both squamous cell carcinoma and basal cell carcinoma. Prevalence was calculated as a function of two extreme scenarios (duration of one versus five years). Country-, age-, sex-, and year-specific duration was estimated using a country-age-sex-year-specific relative access-to-care score.

The access-to-care score was based on the melanoma mortality-to-incidence ratio:

$$Access\ to\ care = 1 - \frac{Age\ standardised\ MIR_{cys} - Age\ standardised\ MIR_{min}}{Age\ standardised\ MIR_{max} - Age\ standardised\ MIR_{min}}$$

c = country; y = year; s = sex
Age-standardised MIR_{min} = lowest MIR for all countries and years
Age standardised MIR_{max} = highest MIR for all countries and years

Remission was calculated as the inverse of these duration estimates and used as additional input for DisMod-MR 2.1. Country-level covariates of age-standardised melanoma incidence and lag-distributed income were included in the squamous cell carcinoma model while just age-standardised melanoma incidence was included in the basal cell carcinoma model.

To reflect differing degrees of disability due to squamous cell carcinoma we used three levels of severity that were derived from MEPS (Medical Expenditure Panel Survey),[49] resulting in fixed proportions of 80% mild disfigurement, 15% moderate, and 5% severe. For basal cell carcinoma, disability severity was split into 60% asymptomatic (without disability) and 40% with mild disfigurement. Prevalence was multiplied by distinct disability weights (Table 8) to generate YLDs.

Table 8. Severity distribution. Details on the severity levels for non-melanoma skin cancer (squamous and basal cell carcinoma) and the associated disability weight with that severity.

Cancer disability, severity	Health state	Lay description	Disability weight (95% uncertainty interval)
Cutaneous squamous cell carcinoma, mild	Disfigurement, level 1	This person has a slight, visible physical deformity that others notice, which causes some worry and discomfort.	0.011 (0.005–0.021)
Cutaneous squamous cell carcinoma, moderate	Disfigurement, level 2	This person has a visible physical deformity that causes others to stare and comment. As a result, the person is worried and has trouble sleeping and concentrating.	0.067 (0.044–0.096)
Cutaneous squamous cell carcinoma, severe	Disfigurement, level 3, with itch/pain	This person has an obvious physical deformity that is very painful and itchy. The physical deformity makes others uncomfortable, which causes the person to avoid social contact, feel worried, sleep poorly, and think about suicide.	0.576 (0.401–0.731)
Disfigurement due to basal cell carcinoma	Disfigurement, level 1	This person has a slight, visible physical deformity that others notice, which causes some worry and discomfort.	0.011 (0.005–0.021)

Table 9. Covariates. Summary of covariates used in non-melanoma skin cancer DisMod-MR meta-regression models

Cause	Covariate	Type	Measure	Exponentiated beta (95% uncertainty interval)
Squamous cell carcinoma	Age-standardised melanoma incidence	Country-level	Incidence hazard	1.77 (1.73–1.80)
Squamous cell carcinoma	LDI (I$ per capita)	Country-level	Excess mortality rate	0.82 (0.61–1.09)
Basal cell carcinoma	Age-standardised melanoma incidence	Country-level	Incidence hazard	6.58 (1.28–42.86)

Myelodysplastic, myeloproliferative, and other haemopoietic neoplasms

Flowchart

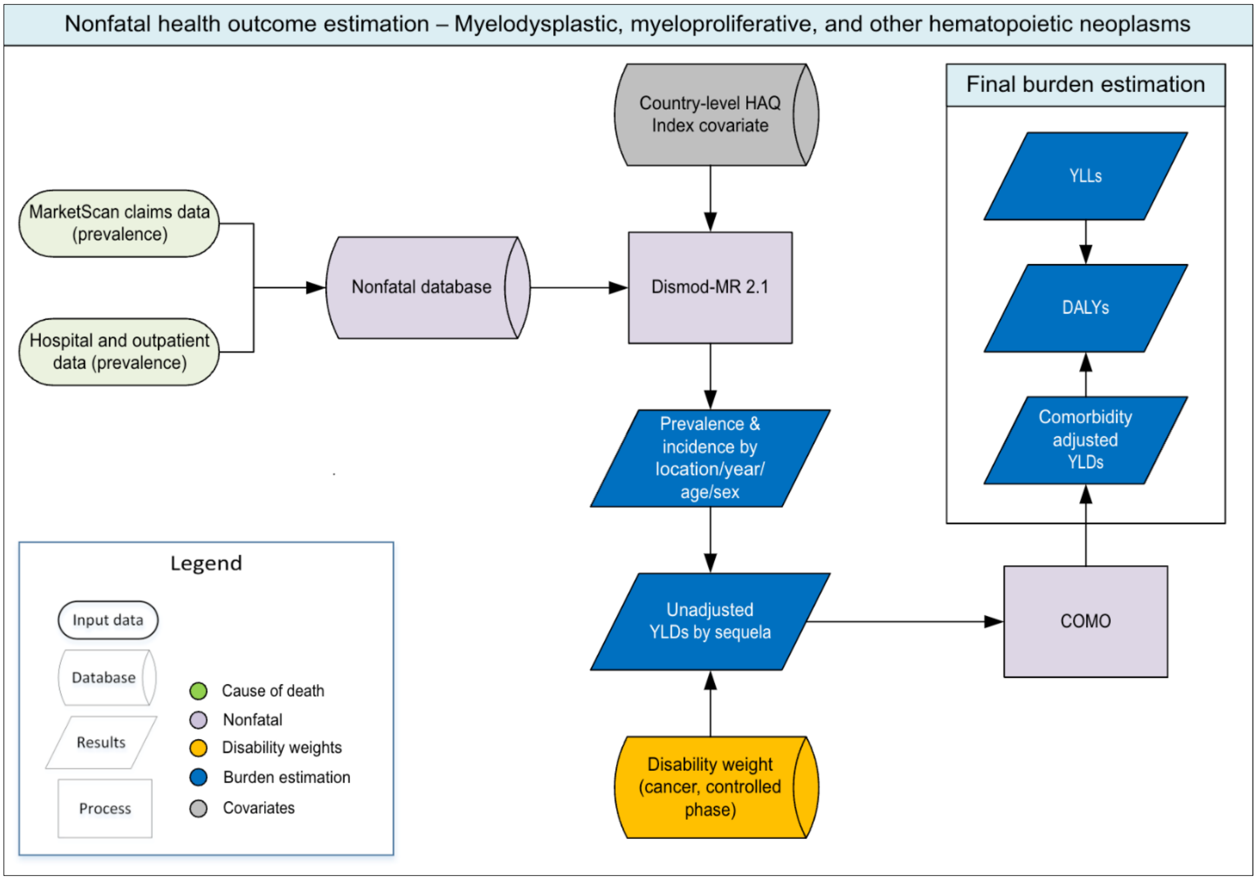

Figure 3. Flowchart of non-fatal estimation for myelodysplastic, myeloproliferative, and other haemopoietic neoplasms

Abbreviations: DisMod-MR, disease model – Bayesian meta-regression; COMO, comorbidity correction microsimulation; DALYs, disability-adjusted life-years; HAQ Index, Healthcare Access and Quality Index; YLDs, years lived with disability; YLLs, years of life lost

Input data and methodological summary for myelodysplastic, myeloproliferative, and other haemopoietic neoplasms

Case definition

Myelodysplastic, myeloproliferative, and other haemopoietic neoplasms (MDS/MPN) comprise a wide variety of diseases and outcomes, including ICD-10 codes D45 (polycythemia vera), D46 (myelodysplastic syndromes), and D47 (other neoplasms of uncertain behavior of lymphoid, hematopoietic, and related tissue). These were modelled together as a single group for GBD 2023 (the same as for GBD 2017, GBD 2019, and GBD 2021).

Table 10. Clinical case definition for myelodysplastic, myeloproliferative, and other haemopoietic neoplasms

Quantity of interest	Clinical case definition
Myelodysplastic, myeloproliferative, and other haemopoietic neoplasms	Neoplasms of the blood (specifically myelodysplastic, myeloproliferative, and other haemapoeitic neoplasms), including ICD-10 codes D45-D47.

Input data

We estimated MDS/MPN deaths using vital registration data (as outlined above). We did not use cancer registry data for these neoplasms, as it has only been reported within some cancer registries since 2001 and is recognised to be under-reported.[50] We estimated MDS/MPN incidence and prevalence using hospital and outpatient prevalence data from various health systems worldwide. As in GBD 2021, clinical data processing and adjustment methods based correction factors on hospital data from Poland,[51] using age splines with frequency-based knots. This reduced the size and uncertainty of the correction factors compared to GBD 2019, which had previously been based on claims data from MarketScan.[47] As in GBD 2019 and GBD 2021, these clinical data were adjusted for the HAQ Index of the location and inpatient data accounts for outpatient encounters. Additional details on clinical and claims data processing are available elsewhere in the GBD summary papers.

Modelling strategy

We modelled deaths for all locations and years, by age and by sex, using CODEm. As MDS/MPN can be a precursor to leukaemia, our MDS/MPN CODEm model used many of the same covariate priors as the CODEm model for acute myeloid leukaemia. CODEm methods, parameters, and covariates are described elsewhere in the GBD summary articles.

We modelled the incidence and prevalence of these diseases for all combinations of location, age, year, and sex using a prevalence model in DisMod-MR 2.1. For DisMod model specifications, cause-specific mortality rates came from the CODEm model, remission was specified to be zero, and the excess mortality rate was set to be inversely related to the HAQ Index covariate.

While this broad category of haemological neoplasms is heterogeneous in its components' severity or propensity for transformation to leukaemia, modelling the subtypes of MDS/MPN separately was not feasible for GBD 2023. This is a limitation and an area of desired future improvement as data availability improves. For GBD 2023, the "cancer, controlled phase" disability weight was assigned for all MDS/MPN cases (see Table 6).

Table 11. Covariates: summary of covariates used in the myelodysplastic, myeloproliferative, and other haemopoietic neoplasms DisMod-MR meta-regression model

Covariate	Type	Measure	Exponentiated beta (95% uncertainty interval)
Healthcare Access and Quality Index	Country-level	Excess mortality rate	0.98 (0.97–0.98)

Benign and in situ intestinal neoplasms; benign and in situ cervical and uterine neoplasms; other benign and in situ neoplasms

Flowchart

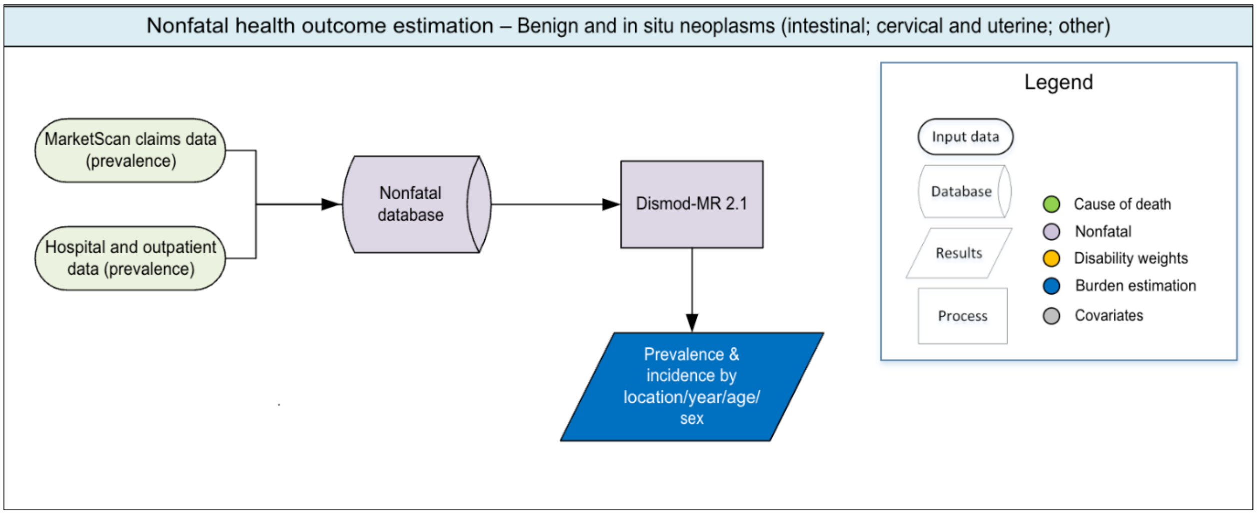

Figure 4. Flowchart of non-fatal estimation for benign and in situ intestinal neoplasms; benign and in situ cervical and uterine neoplasms; and other benign and in situ neoplasms

Abbreviations: DisMod-MR, disease model – Bayesian meta-regression.

Input data and methodological summary for benign and in situ intestinal neoplasms; benign and in situ cervical and uterine neoplasms; and other benign and in situ neoplasms

Case definition

For GBD 2023, we estimated three categories of benign and in situ neoplasms: (1) intestinal neoplasms; (2) cervical and uterine neoplasms; and (3) other benign and in situ neoplasms. Benign and in situ intestinal neoplasms were defined as any non-invasive intestinal growth of the digestive system beyond the stomach, from duodenum to anus. Benign and in situ cervical and uterine neoplasms were defined as any non-invasive cervical or uterine growth, except for uterine fibroids, which are modelled as a separate GBD cause. Other benign and in situ neoplasms were defined as any non-invasive neoplasms not covered by other GBD causes, such as lipomas, benign breast neoplasms, and non-melanoma skin neoplasms other than basal cell carcinoma or squamous cell carcinoma.

Table 12. Clinical case definition for benign and in situ neoplasms

Quantity of interest	Clinical case definition
Benign and in situ intestinal neoplasms	Benign and in situ neoplasms of the intestines, including ICD-10 codes D01, D12, D13.3, D13.9, D37.2–D37.5, K62.0–K62.1, and K63.5. No mortality is estimated for this cause.
Benign and in situ cervical and uterine neoplasms	Benign and in situ neoplasms of the cervix or uterus; ICD-10 codes D06, D07.0, D26, N84.0-N84.1, and N87. No mortality is estimated for this cause.
Other benign and in situ neoplasms	Benign and in situ neoplasms not specified as a part of another GBD neoplasm cause, including ICD-10 codes D00-D49 and N60.

Input data

To estimate the prevalence of each of these categories for all locations, by age, year, and sex, the prevalence of these neoplasms from clinical data was used as input for a prevalence model in DisMod-MR 2.1. We estimated incidence and prevalence for each of these benign neoplasms using hospital and outpatient prevalence data from various health systems worldwide. As in GBD 2021, clinical data processing and adjustment methods were based on correction factors on hospital data from Poland,[51] using age splines with frequency-based knots. This reduced the size and uncertainty of the correction factors compared to GBD 2019, which had previously been based on claims data from MarketScan.[47] As in GBD 2019 and GBD 2021, these clinical data were adjusted for the HAQ Index of the location and inpatient data accounts for outpatient encounters. Additional details on clinical and claims data processing are available elsewhere in the GBD capstones.

Of these three causes, we only estimated deaths for “other benign and in situ neoplasms”, as this cause includes neoplasms such as low-grade and other central nervous system neoplasms. Though non-malignant, these tumours can cause death from physically impairing vital nervous system functions. We estimated these deaths using only vital registration data (as outlined above), as these neoplasms are not consistently captured by cancer registries.

Modelling strategy

In the DisMod model for benign and in situ intestinal neoplasms, excess mortality rate was specified to be zero, and remission was allowed to vary from 0 to 1. In the DisMod model for benign and in situ cervical and uterine neoplasms, excess mortality rate was specified to be zero, and remission was allowed to vary from 0 to 0.75. In the DisMod model for other benign and in situ neoplasms, excess mortality rate was specified to be zero, and remission was allowed to vary from 0 to 1.

All three of these benign and in situ neoplasms are by definition benign and localised. As such, no deaths or disability were attributed to their occurrence in GBD 2023. The exception was for “other benign and in situ neoplasms”, where deaths were included for a subset of this broad category that includes neoplasms such as low-grade brain and central nervous system neoplasms. Deaths were modelled in CODEm, as described above. Because these are only a subset of the total neoplasms included in the cause, we did not assign any disability to these causes for GBD 2023. This is an area of desired future improvement as data availability improves.

References

1 Global Burden of Disease Cancer Collaboration. The Global Burden of Cancer 2013. *JAMA Oncology* 2015; **1**: 505–27.

2 Global Burden of Disease Cancer Collaboration. Global, Regional, and National Cancer Incidence, Mortality, Years of Life Lost, Years Lived With Disability, and Disability-Adjusted Life-years for 32 Cancer Groups, 1990 to 2015: A Systematic Analysis for the Global Burden of Disease Study. *JAMA Oncology* 2017; **3**: 524–48.

3 Global Burden of Disease Cancer Collaboration. Global, Regional, and National Cancer Incidence, Mortality, Years of Life Lost, Years Lived With Disability, and Disability-Adjusted Life-Years for 29 Cancer Groups, 1990 to 2016: A Systematic Analysis for the Global Burden of Disease Study. *JAMA Oncology* 2018; **4**: 1553–68.

4 Global Burden of Disease Cancer Collaboration. Global, Regional, and National Cancer Incidence, Mortality, Years of Life Lost, Years Lived With Disability, and Disability-Adjusted Life-Years for 29 Cancer Groups, 1990 to 2017: A Systematic Analysis for the Global Burden of Disease Study. *JAMA Oncology* 2019; **5**: 1749–68.

5 Global Burden of Disease Cancer Collaboration, Brian L. Gaw, Rixing Xu, *et al.* Cancer Incidence, Mortality, Years of Life Lost, Years Lived With Disability, and Disability-Adjusted Life Years for 29 Cancer Groups From 2010 to 2019: A Systematic Analysis for the Global Burden of Disease Study 2019. *JAMA Oncology* 2022; **8**: 420–44.

6 Surveillance, Epidemiology, and End Results (SEER) Program (www.Seer.Cancer.Gov) SEER*Stat Database: Incidence - SEER 18.

7 Surveillance, Epidemiology, and End Results (SEER) Program (www.seer.cancer.gov) SEER*Stat Database: Incidence - SEER 18 Regs Research Data + Hurricane Katrina Impacted Louisiana Cases, Nov 2017 Sub (1973-2015 varying) - Linked To County Attributes - Total U.S., 1969-2016 Counties, National Cancer Institute, DCCPS, Surveillance Research Program, released April 2018, based on the November 2017 submission.

8 Barber RM, Fullman N, Sorensen RJD, *et al.* Healthcare Access and Quality Index based on mortality from causes amenable to personal health care in 195 countries and territories, 1990–2015: a novel analysis from the Global Burden of Disease Study 2015. *The Lancet* 2017; **390**: 231–66.

9 Asadzadeh Vostakolaei F, Karim-Kos HE, Janssen-Heijnen MLG, Visser O, Verbeek ALM, Kiemeney LALM. The validity of the mortality to incidence ratio as a proxy for site-specific cancer survival. *Eur J Public Health* 2011; **21**: 573–7.

10 Surveillance Research Program, National Cancer Institute SEER*Stat software (seer.cancer.gov/seerstat) version 8.3.4.

11 Surveillance, Epidemiology, and End Results (SEER) Program (www.seer.cancer.gov) SEER*Stat Database: Mortality - All COD, Aggregated With State, Total U.S. (1969-2015) <Katrina/Rita Population Adjustment>, National Cancer Institute, DCCPS, Surveillance Research Program, released December 2017. Underlying mortality data provided by NCHS (www.cdc.gov/nchs).

12 Surveillance, Epidemiology, and End Results (SEER) Program (www.seer.cancer.gov) SEER*Stat Database: Incidence - SEER 9 Regs Research Data, Nov 2016 Sub (1973-2014) <Katrina/Rita Population Adjustment> - Linked To County Attributes - Total U.S., 1969-2015 Counties, National Cancer Institute, DCCPS, Surveillance Research Program, released April 2017, based on the November 2016 submission.

13 Neal RD, Din NU, Hamilton W, *et al.* Comparison of cancer diagnostic intervals before and after implementation of NICE guidelines: analysis of data from the UK General Practice Research Database. *Br J Cancer* 2014; **110**: 584–92.

14 Surveillance, Epidemiology, and End Results (SEER) Program (www.seer.cancer.gov) SEER*Stat Database: Incidence - SEER 18 Regs Research Data + Hurricane Katrina Impacted Louisiana Cases, Nov 2012 Sub (1973-2010 varying) - Linked To County Attributes - Total U.S., 1969-2011 Counties, National Cancer Institute, DCCPS, Surveillance Research Program, Surveillance Systems Branch, released April 2013, based on the November 2012 submission.

15 Allgar VL, Neal RD. Delays in the diagnosis of six cancers: analysis of data from the National Survey of NHS Patients: Cancer. *Br J Cancer* 2005; **92**: 1959–70.

16 Neal RD, Cannings-John R, Hood K, *et al.* Excision of malignant melanomas in North Wales: effect of location and surgeon on time to diagnosis and quality of excision. *Fam Pract* 2008; **25**: 221–7.

17 Kewalramani T, Nimer SD, Zelenetz AD, *et al.* Progressive disease following autologous transplantation in patients with chemosensitive relapsed or primary refractory Hodgkin's disease or aggressive non-Hodgkin's lymphoma. *Bone Marrow Transplant* 2003; **32**: 673–9.

18 Esteban D, Tovar N, Jiménez R, *et al.* Patients with relapsed/refractory chronic lymphocytic leukaemia may benefit from inclusion in clinical trials irrespective of the therapy received: a case-control retrospective analsysis. *Blood Cancer J* 2015; **5**: e356.

19 Kopelman JE, McLean IW, Rosenberg SH. Multivariate analysis of risk factors for metastasis in retinoblastoma treated by enucleation. *Ophthalmology* 1987; **94**: 371–7.

20 National Cancer Institute (United States). United States SEER Cancer Data 1973-2010. Bethesda, United States: National Cancer Institute (United States).

21 National Center for Health Statistics (NCHS), Centers for Disease Control and Prevention (CDC), US Census Bureau. United States National Hospital Discharge Survey. Hyattsville, United States: National Center for Health Statistics (NCHS), Centers for Disease Control and Prevention (CDC).

22 Canadian Institute for Health Information (CIHI). Canada Discharge Abstract Database 1994-2009. Ottawa, Canada: Canadian Institute for Health Information (CIHI).

23 Ministry of Health (Mexico). Mexico Ministry of Health Hospital Discharges 2000-2012. Mexico City, Mexico: Ministry of Health (Mexico).

24 ICD - ICD-9-CM - International Classification of Diseases, Ninth Revision, Clinical Modification. 2021; published online Nov 3. https://www.cdc.gov/nchs/icd/icd9cm.htm (accessed Dec 15, 2022).

25 Canova C, Giorato E, Roveron G, Turrini P, Zanotti R. Validation of a stoma-specific quality of life questionnaire in a sample of patients with colostomy or ileostomy. *Colorectal Dis* 2013; **15**: e692-698.

26 Caricato M, Ausania F, Ripetti V, Bartolozzi F, Campoli G, Coppola R. Retrospective analysis of long-term defunctioning stoma complications after colorectal surgery. *Colorectal Dis* 2007; **9**: 559–61.

27 Erwin-Toth P, Thompson SJ, Davis JS. Factors impacting the quality of life of people with an ostomy in North America: results from the Dialogue Study. *J Wound Ostomy Continence Nurs* 2012; **39**: 417–22; quiz 423–4.

28 Catalona WJ, Carvalhal GF, Mager DE, Smith DS. Potency, continence and complication rates in 1,870 consecutive radical retropubic prostatectomies. *J Urol* 1999; **162**: 433–8.

29 Donnellan SM, Duncan HJ, MacGregor RJ, Russell JM. Prospective assessment of incontinence after radical retropubic prostatectomy: objective and subjective analysis. *Urology* 1997; **49**: 225–30.

30 Eastham JA, Kattan MW, Rogers E, *et al.* Risk factors for urinary incontinence after radical prostatectomy. *J Urol* 1996; **156**: 1707–13.

31 Kundu SD, Roehl KA, Eggener SE, Antenor JAV, Han M, Catalona WJ. Potency, continence and complications in 3,477 consecutive radical retropubic prostatectomies. *J Urol* 2004; **172**: 2227–31.

32 Potosky AL, Davis WW, Hoffman RM, *et al.* Five-Year Outcomes After Prostatectomy or Radiotherapy for Prostate Cancer: The Prostate Cancer Outcomes Study. *JNCI Journal of the National Cancer Institute* 2004; **96**: 1358–67.

33 Sacco E, Prayer-Galetti T, Pinto F, *et al.* Urinary incontinence after radical prostatectomy: incidence by definition, risk factors and temporal trend in a large series with a long-term follow-up. *BJU Int* 2006; **97**: 1234–41.

34 Stanford JL, Feng Z, Hamilton AS, *et al.* Urinary and Sexual Function After Radical Prostatectomy for Clinically Localized Prostate CancerThe Prostate Cancer Outcomes Study. *JAMA* 2000; **283**: 354–60.

35 Walsh PC, Marschke P, Ricker D, Burnett AL. Patient-reported urinary continence and sexual function after anatomic radical prostatectomy. *Urology* 2000; **55**: 58–61.

36 Doll R, Payne P, Waterhouse J, editors. Cancer Incidence in Five Continents, Vol. I. Geneva: Union Internationale Contre le Cancer, 1966 https://publications.iarc.fr/Non-Series-Publications/Other-Non-Series-Publications/Cancer-Incidence-In-Five-Continents-Volume-I-1966 (accessed Feb 24, 2021).

37 Doll R, Muir CS, Waterhouse JA. Cancer Incidence in Five Continents, Vol. II. Geneva: Union Internationale Contre le Cancer, 1970.

38 Waterhouse J, Muir C, Correa P, Powell J. Cancer Incidence in Five Continents III. Lyon: IARC; 1976.

39 Waterhouse J, Muir C, Shanmugaratnam K, Powell J. Cancer Incidence in Five Continents IV. Lyon: IARC; 1982.

40 Muir C, Mack T, Powell J, Whelan S. Cancer Incidence in Five Continents V. Lyon: IARC; 1987.

41 Parkin D, Muir C, Whelan S, Gao Y, Ferlay J, Powell J. Cancer Incidence in Five Continents VI. Lyon: IARC; 1992.

42 Parkin D, Whelan S, Ferlay J, Raymond L, Young J. Cancer Incidence in Five Continents VII. Lyon: IARC; 1997.

43 Parkin D, Whelan S, Ferlay J, Teppo L, Thomas D. Cancer Incidence in Five Continents VIII. Lyon: IARC; 2002.

44 Curado M, Edwards B, Shin H, et al. Cancer Incidence in Five Continents IX. Lyon: IARC; 2007. http://www.iarc.fr/en/publications/pdfs-online/epi/sp160/CI5vol9-A.pdf.

45 Forman D, Bray F, Brewster D, et al. Cancer Incidence in Five Continents X. http://ci5.iarc.fr. Published 2013.

46 Bray F, Colombet M, Mery L, Piñeros M, Znaor A, Zanetti R and Ferlay J, editors (2017). Cancer Incidence in Five Continents, Vol. XI. Lyon: International Agency for Research on Cancer.

47 Truven Health Analytics. United States MarketScan Commercial Claims and Encounters Database. Ann Arbor, United States.

48 Karagas MR, Greenberg ER, Spencer SK, Stukel TA, Mott LA. Increase in incidence rates of basal cell and squamous cell skin cancer in New Hampshire, USA. *International Journal of Cancer* 1999; **81**: 555–9.

49 Medical Expenditure Panel Survey Home. Accessed November 15, 2019. https://meps.ahrq.gov/mepsweb/.

50 Cogle CR, Craig BM, Rollison DE, List AF. Incidence of the myelodysplastic syndromes using a novel claims-based algorithm: high number of uncaptured cases by cancer registries. *Blood* 2011; **117**: 7121–5.

51 National Health Fund (Poland). Poland National Health Fund Patient Claims 2016.

Cannabis use disorders

Flowchart

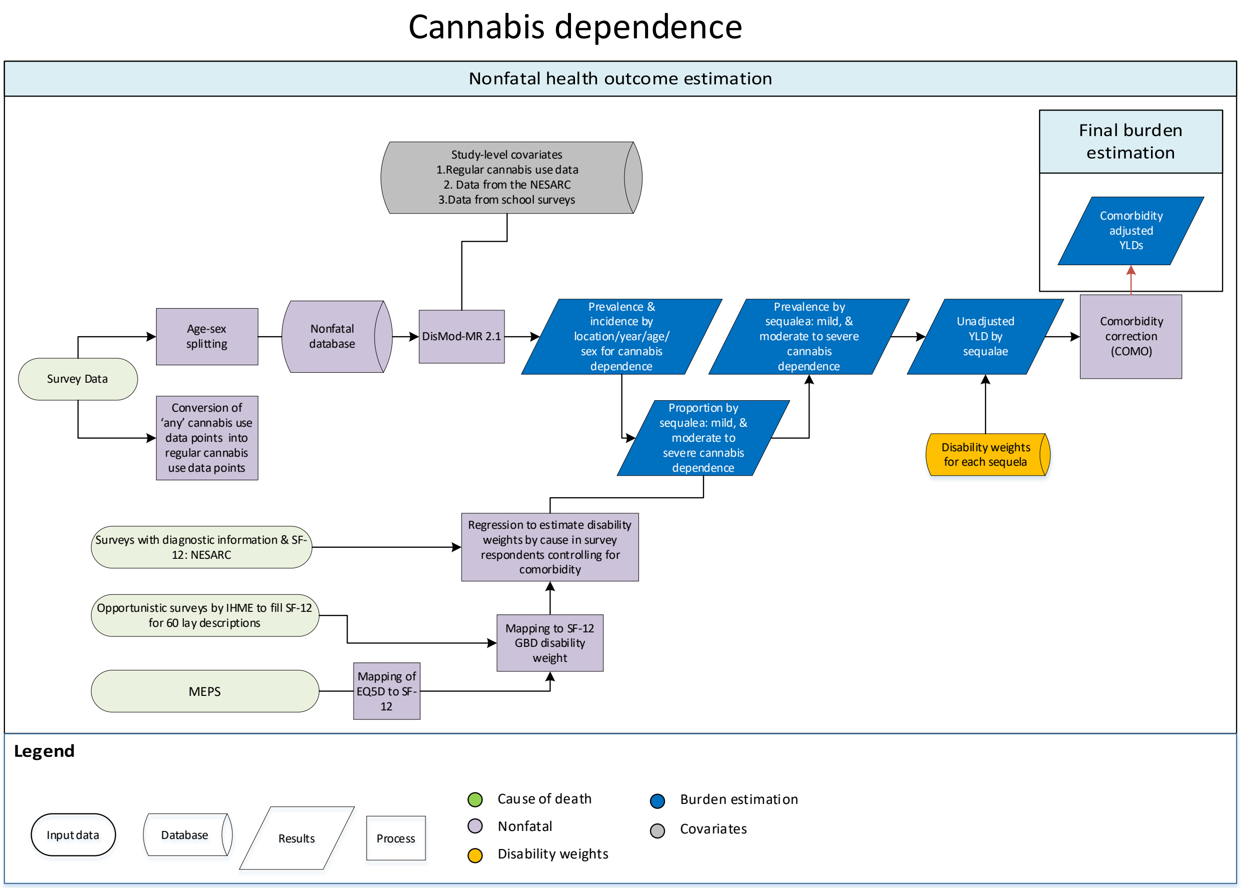

Input data and methodological summary for cannabis use disorders

Case definition

Cannabis use disorders are substance-related conditions characterised by a problematic pattern of cannabis use that disrupts functioning. The Global Burden of Disease (GBD) modelling included cases that met the diagnostic criteria for cannabis dependence based on the Diagnostic and Statistical Manual of Mental Disorders (DSM) or equivalent diagnoses in the International Classification of Diseases (ICD). These were identified using the following codes: DSM-IV: 304.30, ICD-9: 304.3 and 305.2, and ICD-10: F12.1, F12.2, and F12.9. Cases attributed to a general medical condition were excluded.[1,2]

According to DSM-IV-TR criteria, cannabis dependence involves a maladaptive pattern of cannabis use, leading to clinically significant impairment or distress. At least three of the following symptoms must be experienced within the same 12-month period:

- Tolerance, characterised by either
  - A need for increased amounts of the substance to achieve intoxication; or
  - Markedly diminished effect with continued use of the same amount of the substance;
- Withdrawal, characterised by either
  - Withdrawal symptoms characteristic to cannabis dependence; or
  - The same (or similar) substance is taken to avoid withdrawal symptoms;
- Substance taken in progressively larger amounts or for longer periods;
- Persistent desire or unsuccessful efforts to reduce substance use;
- Disproportionate time dedicated to obtaining the substance;
- Other important activities are given up because of the substance use; and
- Substance use is continued despite knowledge of physical or psychological problems occurring as a result of the substance.

The case definitions accepted for cannabis dependence are shown below.

Quantity of interest	Reference or alternative	Definition
Cannabis use disorders	Reference	Cannabis dependence as measured using ICD or DSM criteria
Cannabis use disorders	Alternative	Regular (weekly) use of cannabis
Cannabis use disorders	Alternative	Cannabis dependence, based on school survey
Cannabis use disorders	Alternative	Regular (weekly) use of cannabis based on household survey
Cannabis use disorders	Alternative	Regular (weekly) use of cannabis based on school survey

Input data

Systematic literature reviews were conducted to identify studies on the prevalence, incidence, duration, and excess mortality associated with cannabis dependence. The search process involved three stages: electronic searches of peer-reviewed literature through PsycInfo, Embase, and PubMed; searches of the grey literature; and expert consultations. This approach, established for mental and substance use disorders, involved conducting electronic database searches on a rolling basis.

All three stages of the literature review from GBD 2010 were repeated for GBD 2013 and GBD 2016, while only stages two and three were conducted for GBD 2017. To further enhance the cannabis dependence dataset, two targeted systematic reviews were performed.

The first review focused on cannabis dependence studies specific to Māori and non-Maori populations, reflecting their inclusion as distinct subgroups in GBD 2017. The second review explored studies on cannabis dependence in China, using the China National Knowledge Infrastructure database to capture research published in Chinese journals that may not be indexed in mainstream databases like PsycInfo, Embase, and PubMed.

The inclusion criteria stipulated that: (1) the publication year must be from 1980 onward; (2) "case" must be based on clinical threshold as established by the DSM or ICD; (3) sufficient information must be provided on study method and sample characteristics to assess the quality of the study; and (4) study samples must be representative of the general population (ie, inpatient or pharmacological treatment samples, case studies, and veterans or refugee samples were excluded). No limitation was set on the language of publication. Methods used for this systematic review have been reported in greater detail elsewhere.[3-6]

Key changes in GBD 2023

In GBD 2023, we re-evaluated data quality, re-extracted information from 36 country sources with incorrect data (means, case definitions, case counts, and sample sizes), and excluded eight sources: six for duplicative studies or samples, one for reporting only odds ratios, and one for having an unrepresentative convenience sample of 834 students.

Age and sex splitting

Reported estimates of prevalence were split by age and sex where possible. First, if studies reported prevalence for broad age groups by sex (eg, prevalence in 15- to 65-year-old males and females separately), and also by specific age groups for both sexes combined (eg, prevalence in 15- to 30-year-olds, then in 31- to 65-year-olds, for males and females combined), age-specific estimates were split by sex using the reported sex ratio and bounds of uncertainty. Second, prevalence data for both sexes that could not be split using a within-study ratio were split using a sex ratio derived from a meta-analysis of existing sex-specific data using our meta-regression—Bayesian, regularised, trimmed tool (MR-BRT). Details on MR-BRT can be found in appendix 1, section 2.

The female to male ratio was 0.49 (0.33–0.70) for ages 20 and above and 0.61 (0.42–0.88) for ages below 20. Finally, after the application of bias adjustments, where studies reported estimates across age groups spanning 25 years or more, these were split into five-year age groups using the super-region-specific prevalence age pattern estimated by our disease model—Bayesian meta-regression tool (DisMod-MR 2.1) on all data prior to age-splitting (see appendix 1, section 2).

Data adjustment

Due to limited data on the optimal case definition of cannabis dependence, the prevalence dataset included datapoints originally reporting any cannabis use, regular cannabis use (defined as weekly use), and cannabis dependence. Adjusting estimates of any cannabis use and regular cannabis use to cannabis dependence involved a two-step process.

In the first stage, estimates of any cannabis use were converted to regular cannabis use. Briefly, a ratio of any cannabis use to regular cannabis use was calculated by comparing similar estimates in the dataset, ensuring they matched in country, year, age group, sex, and prevalence type. Once these paired estimates were established, MetaXL, a meta-analysis add-in for Microsoft Excel, was used to calculate the ratio. Results indicated that estimates of any cannabis use were 2.9 times higher (95% CI: 2.5–3.3) than regular cannabis use estimates. This ratio was applied to adjust all any-use estimates downward to align with regular cannabis use levels.

The second stage, updated during GBD 2019, involved converting regular cannabis use estimates to cannabis dependence estimates using a logit-difference coefficient calculated through MR-BRT. This stage also accounted for bias in school-based surveys compared to household surveys among youth. An age-specific pattern emerged, prompting separate models for youth (under age 25) and adults (age 25 and older). A network analysis incorporating both direct and indirect comparisons was used to adjust youth data for two study-level covariates: regular use and school-based survey status. Consequently, two separate MR-BRT models were run—one for adults and one for youth.

Compared to GBD 2017, adjustments made using the logit-difference approach in MR-BRT resulted in slightly higher post-adjustment prevalence estimates among both youth and adults. No changes were made to these adjustment processes in GBD 2023.

Table 2: MR-BRT crosswalk adjustment factors for cannabis use disorder, youth

Data input	Reference or alternative case definition	Gamma	Beta coefficient, logit (95% UI)*	Adjustment factor**
Cannabis dependence, household-based	Ref	0.32	---	
Cannabis dependence, school-based	Alt		0.33 (–0.30 to 0.94)	1.39
Cannabis regular use, household-based	Alt		0.73 (0.12 to 1.34)	2.08
Cannabis regular use, school-based	Alt		1.08 (0.44 to 1.70)	2.94

**MR-BRT crosswalk adjustments can be interpreted as the factor the alternative case definition is adjusted by to reflect what it would have been had it been measured using the reference case definition. If the log/logit beta coefficient is negative, then the alternative is adjusted up to the reference. If the log/logit beta coefficient is positive, then the alternative is adjusted down to the reference.*

***The adjustment factor column is the exponentiated beta coefficient. For log beta coefficients, this is the relative rate between the two case definitions. For logit beta coefficients, this is the relative odds between the two case definitions.*

Table 3: MR-BRT crosswalk adjustment factors for cannabis use disorder, adults

Data input	Reference or alternative case definition	Gamma	Beta coefficient, logit (95% UI)*	Adjustment factor**
Cannabis dependence	Ref	0.28	---	
Cannabis regular use	Alt		1.31 (0.77–1.86)	3.71

**MR-BRT crosswalk adjustments can be interpreted as the factor the alternative case definition is adjusted by to reflect what it would have been had it been measured using the reference case definition. If the log/logit beta coefficient is negative, then the alternative is adjusted up to the reference. If the log/logit beta coefficient is positive, then the alternative is adjusted down to the reference.*

***The adjustment factor column is the exponentiated beta coefficient. For log beta coefficients, this is the relative rate between the two case definitions. For logit beta coefficients, this is the relative odds between the two case definitions.*

Modelling strategy

There have been no significant changes to the modelling strategy in GBD 2023.

Prior settings in DisMod included assuming no incidence before age 13. This minimum age of onset was corroborated with expert feedback and existing literature on cannabis dependence. We also assumed no incidence after age 70 as supported by data from various sources including the European Monitoring Centre for Drugs and Drug Addiction.[7] An upper limit of 0.25 was placed on remission, consistent with limits in the dataset. These settings were retained for GBD 2023. In GBD 2023, as in prior iterations, no country-level covariates were used in predictions.

Severity and disability

The basis of the GBD disability weight survey assessments are lay descriptions of health states highlighting major functional consequences and symptoms. The lay descriptions and disability weights for cannabis dependence severity levels are shown below.

Table 4. Severity distribution, details on the severity levels for cannabis use disorders in GBD 2023 and the associated disability weight (DW) with that severity.

Severity level	Lay description	DW (95% CI)
Mild	Uses marijuana at least once a week and has some difficulty controlling the habit. When not using, the person functions normally.	0.039 (0.024–0.06)

Moderate to severe	Uses marijuana daily and has difficulty controlling the habit. The person sometimes has mood swings, anxiety, and hallucinations, and has some difficulty in daily activities.	0.266 (0.178–0.364)

**Asymptomatic cases carried no disability weight*

The US National Epidemiological Survey on Alcohol and Related Conditions (NESARC, conducted in two waves from 2001 to 2002 and 2004 to 2005)[8] was used to estimate the proportion of cannabis dependence cases asymptomatic (58%, 51–63), mild (36%, 31–42), and moderate to severe (6%, 4–8). NESARC is a direct household survey. As such, it is expected to underestimate moderate to severe cases of drug dependence; however, there are very few sources of usable drug severity data.

References

1. American Psychiatric Association. Diagnostic and Statistical Manual of Mental Disorders (DSM-IV-TR). 4th, Text Revision ed. Washington DC: American Psychiatric Association; 2000.
2. World Health Organization. The ICD-10 Classification of Mental and Behavioural Disorders. Clinical descriptions and diagnostic guidelines. Geneva: World Health Organization; 1992.
3. Calabria B, Degenhardt L, Briegleb C, et al. Systematic review of prospective studies investigating "remission" from amphetamine, cannabis, cocaine or opioid dependence. Addictive Behaviors 2010; 35(8): 741-9.
4. Calabria B, Degenhardt L, Hall W, Lynskey M. Does cannabis use increase the risk of death? Systematic review of epidemiological evidence on adverse effects of cannabis use. Drug Alcohol Rev 2010; 29(3): 318-30.
5. Calabria B, Degenhardt L, Nelson P, et al. What do we know about the extent of cannabis use and dependence? Results of a global systematic review. Sydney: National Drug and Alcohol Research Centre, University of NSW, 2010.
6. Degenhardt L, Ferrari AJ, Calabria B, et al. The global epidemiology and contribution of cannabis use and dependence to the global burden of disease: results from the GBD 2010 study. PloS one 2013; 8(10): e76635.
7. European Monitoring Centre for Drugs and Drug Addiction. Lisbon, Portugal; 2014.
8. Introduction to the National Epidemiologic Survey on Alcohol and Related Conditions [http://pubs.niaaa.nih.gov/publications/arh29-2/74-78.htm]. Access date 1 December 2014.

Cellulitis

Flowchart

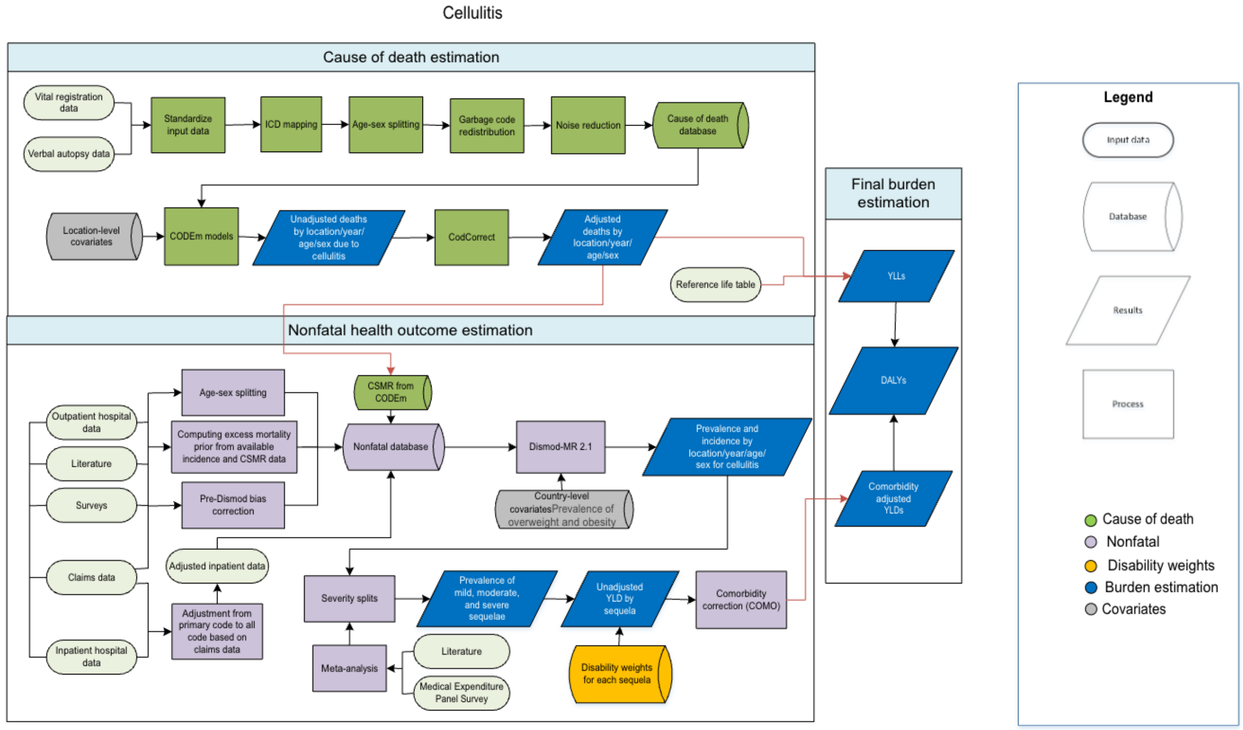

Case definition

Cellulitis was included in the GBD 2019 cause group of skin and subcutaneous conditions. Cellulitis is a skin disease marked by a bacterial infection that affects and spreads through the skin and soft tissues (ICD-10: L03).

Quantity of interest	Reference or alternative	Definition
Cellulitis	Reference	Cellulitis as determined by Poland's claims data 2015–2019
Cellulitis	Alternative	All other data for cellulitis

Input data

In the GBD 2010 study, a systematic review of the literature was conducted using PubMed and Google Scholar to capture epidemiological data for cellulitis. Due to lack of published data on the epidemiology of cellulitis, the literature search also included relevant incidence data from national inpatient or outpatient records in Europe, North America, and Latin America.

The inclusion criteria stipulated that studies (1) must be published between 1980 and 2012; (2) must provide data on the incidence or prevalence of cellulitis; (3) must use samples representative of the general population (ie, samples derived from the experimental arm of clinical trials or based in dermatology clinics were excluded); (4) must use a sample size larger than 100; and (5) must provide

sufficient information on study method and sample characteristics to assess the quality of the study. For GBD 2013, the GBD 2010 search strategy was replicated to capture epidemiological studies published between 2012 and 2013.
The final dataset also includes clinical data from USA, Brazil, England, Chile, New Zealand, Poland, South Korea, Mexico, and Germany. Cause-specific mortality rates for cellulitis estimated by CODEm.
Data were outliered or excluded if we found them unreasonable compared to regional, super-regional, and global rates.

Data processing

For Cellulitis, we crosswalked all data to the reference definition. We began by evaluating the number of observations of each alternate definition that matched with a corresponding observation from the reference definition. We considered "between" study matches, where the alternative was from the same GBD age group and sex, and the midpoint year of the study was within five years of the midpoint of the reference definition observation.

$$\log i\, t\left(y_i^{alt}\right) - \log i\, t\left(y_i^{ref}\right) \;=\; \beta_0 \;+ \epsilon_i$$

Table 1: MR-BRT crosswalk adjustment factors for cellulitis

Data input	Reference or alternative case definition	Gamma	Beta coefficient, logit (95% UI) *	Adjustment factor**
Cellulitis as defined by Poland National Health Fund Patient Claims	Ref	0	---	---
All other data	Alt		1.8917 (1.8908–1.8926)	6.6308 (6.6252–6.6365)

**MR-BRT crosswalk adjustments can be interpreted as the factor the alternative case definition is adjusted by to reflect what it would have been had it been measured using the reference case definition. If the log/logit beta coefficient is negative, then the alternative is adjusted up to the reference. If the log/logit beta coefficient is positive, then the alternative is adjusted down to the reference.*
***The adjustment factor column is the exponentiated beta coefficient. For log beta coefficients, this is the relative rate between the two case definitions. For logit beta coefficients, this is the relative odds between the two case definitions.*

Modelling strategy

In GBD 2023, we have made no substantive changes in the modelling strategy from GBD 2021. DisMod-MR 2.1 was used to estimate prevalence by age, sex, year, and geography (subnational, country, region, super-region) for cellulitis. Cellulitis was modelled with remission set between 12 and 30, implying a duration of 12 days to one month. This was in line with the available epidemiological data, expert opinion, and previous GBD work. The cellulitis dataset was sufficiently large to make use of a relatively short time window of five years to determine which datapoints were used for a particular year of fit.

In GBD 2023, we applied crosswalks using the MR-BRT modelling tool. We adjusted inpatient data along with USA MarketScan data and other data sources toward the level of Poland claims datapoints which were more representative of the general population.
In addition, prevalence of overweight and obesity was used as a country-level covariate to guide estimates for locations with few or no data. The covariate was restricted to a range of –0.5 to –0.1.

$$prevalence = incidence * duration$$

$$Remission = \frac{Cured\ cases}{person - yearoffollow - up\ in\ prevalent\ cases}$$

The tables below indicate the severity distribution of cellulitis, and covariates, parameters, and exponentiated beta values used in GBD 2023.

Table 2. Severity distribution, details on the severity levels for cellulitis and the associated disability weight (DW) with that severity.

Sequela	Severity level	Lay description	DW (95% CI)
Mild cellulitis	Infectious disease, acute episode, mild	This person has a low fever and mild discomfort, but no difficulty with daily activities.	0.241 (0.159–0.329)
Moderate cellulitis	Infectious disease, acute episode, moderate	This person has a fever and aches, and feels weak, which causes some difficulty with daily activities.	0.288 (0.222–0.352)
Severe cellulitis	Infectious disease, acute episode, severe	This person has a high fever and pain, and feels very weak, which causes great difficulty with daily activities.	0.471 (0.365–0.583)

Table 3. Covariates. Summary of covariates used in the Cellulitis DisMod-MR meta-regression model

Covariate	Type	Parameter	Exponentiated beta (95% uncertainty interval)
Prevalence of overweight and obesity	Country-level	Incidence hazard	0.74 (0.61–0.89)

Chagas disease

Flowchart

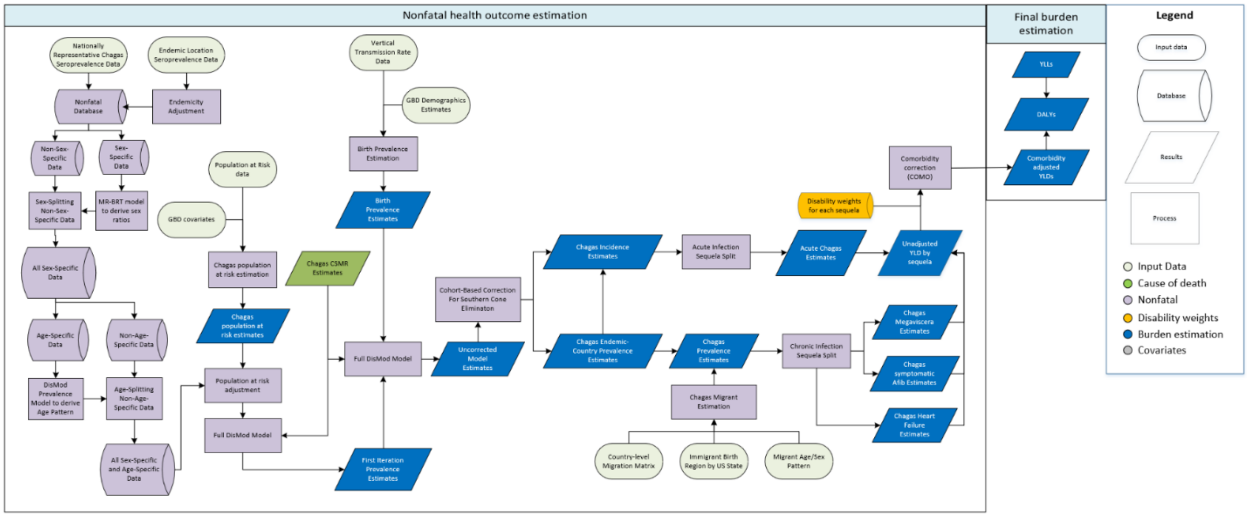

Input data and methodological summary for Chagas disease

Case definition

Chagas disease is a protozoan infection caused by *Trypanosoma cruzi*, transmitted primarily by triatomine insects. Acute infection can cause fever, rash, headache, swollen lymph nodes, and pain, while chronic infection can lead to cardiac and gastrointestinal disorders. It includes all ICD-10 codes under the heading B57 (Chagas disease), with codes B57.0-B75.1 corresponding to the acute phase, B57.2 corresponding to chronic cardiovascular sequelae, and B57.3 corresponding to chronic digestive sequelae.

Quantity of interest	Reference or alternative	Definition
Chagas disease	Reference	Prevalence determined through diagnosis of acute and chronic infections with the protozoa *Trypanosoma cruzi*. For diagnosis, two positive tests by different diagnostic methods are required, unless the diagnostic test was enzyme-linked immunosorbent assay (ELISA) or rapid immunochromatographic test (ICT). It includes all ICD-10 codes under the heading B57 (Chagas disease).

Input data

Model inputs

For GBD 2023, a systematic review of Chagas disease seroprevalence data was completed. We reviewed surveys from representative populations or censuses using two or more diagnostic methods (unless the

single method used was ELISA or ICT) that included Chagas disease seroprevalence data in humans in endemic locations for the years 1980–2021. We searched several academic search systems (PubMed, Web of Science, ScienceDirect, and SciELO) as well as reference lists of review papers (by hand and via online tools); we also asked our broad institutional network of collaborators for relevant sources (Figure 1). In our final analysis, we included a total of 145 sources (Figure 1). The systematic review was conducted in two stages: the initial protocol was published with the Open Science Framework (DOI 10.17605/OSF.IO/USWC3) and the updated protocol at PROSPERO (PROSPERO ID CRD42022368900).

We used a meta-regression—Bayesian, regularised, trimmed (MR-BRT) model with our sex-specific data to derive an estimate of the ratio of the male prevalence of Chagas disease to female prevalence of Chagas disease to split non-sex-specific data (Table 1). Then, a DisMod-MR 2.1 Bayesian meta-regression model fit to the input data with less than a 25-year age range was used to derive a global age pattern. This age pattern was then used to split the data that had an age range greater than 25 years (Figure 2). These estimates were then adjusted for population at risk as estimated by the Pan American Health Organization[1] (PAHO) (see section below).

Cause-specific mortality rates (CSMR) are also used in our modelling process, detailed in subsequent sections.

Table 1: MR-BRT crosswalk adjustment factors for Chagas disease

**Data input**	**Reference or alternative case definition**	**Gamma**	**Beta coefficient, log (95% CI)***	**Adjustment factor****
Female data	Ref	0	---	---
Male data	Alt		0.05 (0.006, 0.09)	1.05

**MR-BRT crosswalk adjustments can be interpreted as the factor the alternative case definition is adjusted by to reflect what it would have been had it been measured using the reference case definition. If the log/logit beta coefficient is negative, then the alternative is adjusted up to the reference. If the log/logit beta coefficient is positive, then the alternative is adjusted down to the reference.*

***The adjustment factor column is the exponentiated beta coefficient. For log beta coefficients, this is the relative rate between the two case definitions. For logit beta coefficients, this is the relative odds between the two case definitions.*

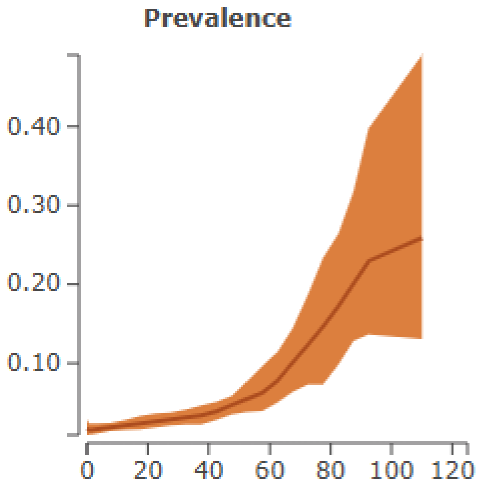

Figure 1: Latin America-specific age pattern for Chagas disease used to split all-age data into age-specific datapoints for further modelling

Population-at-risk adjustments

To convert seroprevalence data that are representative only of a population at risk to seroprevalence representative of the entire population at the GBD location level, we adjusted the age- and sex-split prevalence by the proportion of the population at risk for each location. First, we estimated the proportion of population at risk for years 1980–2023 by location by fitting a binomial generalised linear model with urbanicity, year, and location as covariates to estimates of population at risk from PAHO (for the years 2005 and 2010). For Mexico and Brazil, we estimated subnational proportions of population at risk by disaggregating national population at risk estimates to GBD subnational geographies using Chagas disease cause-specific mortality rate (CMSR) as a covariate. We assumed that the proportion at risk for years before 1980 was equal to the estimated proportions in 1980 for all locations.

Then, we applied the population-at-risk adjustment by multiplying the age-specific prevalence estimates by the average proportion of the population at risk during the life course for each birth cohort. For example, for individuals of age 50 in 2010, we estimate the average proportion of the population at risk from 1960 to 2010 and multiplied that value by the prevalence of Chagas disease. This approach, new for GBD 2023, aims to account for differences in cumulative exposure to Chagas disease over time by age cohort, particularly in locations where changes in Chagas risk factors and control measures have substantially decreased population-level exposure to Chagas disease over time.

Modelling strategy

We modelled Chagas disease using a full DisMod-MR 2.1 Bayesian meta-regression model incorporating seroprevalence data, as above, and CSMR estimates. We assume no remission. For Chile and Uruguay, where vector-based transmission has been interrupted, we eliminate all new infections except those via vertical transmission for the years after the interruption of vector-based transmission.[2-3] For non-endemic countries where ≥0.1% of the population are immigrants from endemic countries, we estimate the prevalence of imported chronic infections based on migration using migration data from the UN Population Division[4] and IPUMS.[5] For each non-endemic country, we estimate the total number of people infected with Chagas as the sum of the number of immigrants from each endemic country multiplied by the corresponding prevalence of Chagas in that endemic country.

We did not apply any adjustments for the COVID-19 pandemic to Chagas disease due to a lack of available data quantifying the impacts of the pandemic on NTD epidemiology.

Sequelae estimation

We estimate five sequelae: symptomatic acute infection from incidence, megaviscera, heart failure, atrial fibrillation and flutter, and chronic asymptomatic infection from prevalence. We assume that 5% of acute infections will be symptomatic.[6] The proportion of chronic infections resulting in a given sequela varies by sex and age (Figure 2). To estimate the prevalence of megaviscera among those infected with Chagas, we used age-specific probabilities from Coura and colleagues, 1995.[7] For the prevalence of atrial fibrillation and flutter attributable to Chagas we assume zero cases for individuals under the age of 10. We updated our approach to now represent only symptomatic atrial fibrillation and flutter, using proportions from Paixão and colleagues, 2020,[8] which are derived from the “global” atrial fibrillation symptomatic/asymptomatic proportions. To estimate the prevalence of heart failure attributable to Chagas among those who are infected, we used sex- and age-specific proportions from Sabino and colleagues, 2013[9] and assume zero cases for individuals under the age of 30. Please see Table 2 for a list of sequelae, their descriptions, and associated disability weights.

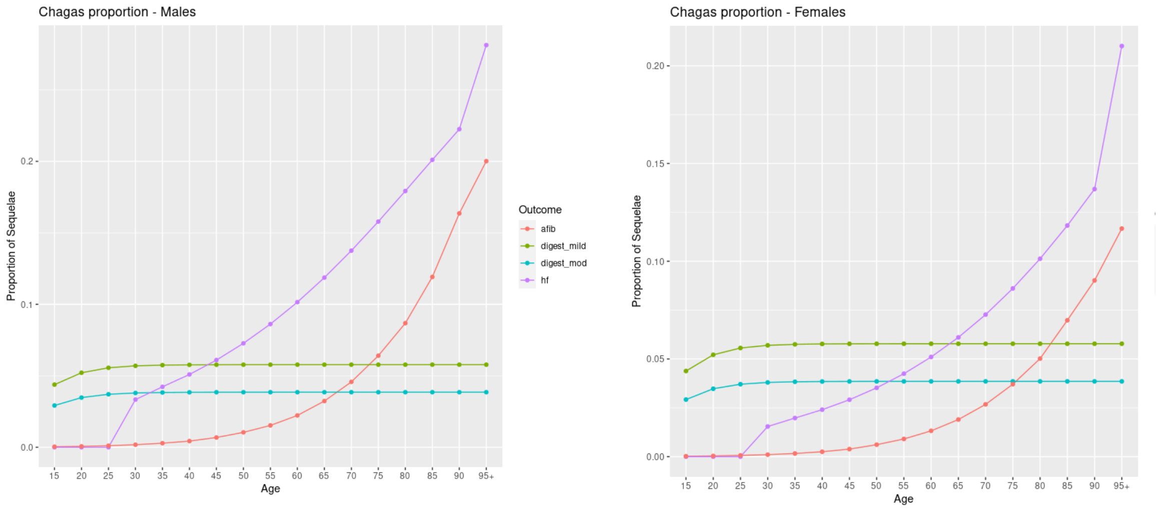

Figure 2. Chagas disease sequelas proportion by age and sex

Table 2. Sequelae, lay description, and disability weights

Sequelae	Description	Disability weight
Symptomatic atrial fibrillation and flutter due to Chagas disease	Has periods of rapid and irregular heartbeats and occasional fainting.	0.224 (0.151–0.312)
Mild heart failure due to Chagas disease	Is short of breath and easily tires with moderate physical activity, such as walking uphill or more than a quarter-mile on level ground. The person feels comfortable at rest or during activities requiring less effort.	0.041 (0.026–0.062)

Moderate heart failure due to Chagas disease	Is short of breath and easily tires with minimal physical activity, such as walking only a short distance. The person feels comfortable at rest but avoids moderate activity.	0.072 (0.047–0.103)
Severe heart failure due to Chagas disease	Is short of breath and feels tired when at rest. The person avoids any physical activity, for fear of worsening the breathing problems.	0.179 (0.122–0.251)
Mild chronic digestive disease due to Chagas disease	Has some pain in the belly that causes nausea but does not interfere with daily activities.	0.011 (0.005–0.021)
Moderate chronic digestive disease due to Chagas disease	Has pain in the belly and feels nauseated. The person has difficulties with daily activities.	0.114 (0.078–0.159)
Acute Chagas disease	Has a fever and aches, and feels weak, which causes some difficulty with daily activities.	0.051 (0.032–0.074)
Asymptomatic Chagas disease	Latent Chagas infection (ie, chronic infection with no apparent symptoms)	NA

Changes from GBD 2021 to GBD 2023

As detailed above, for GBD 2023 we implemented several data and model updates: we (1) conducted a systematic review of Chagas disease prevalence, (2) updated our population at risk estimation, and (3) made updates to the estimation of atrial fibrillation and flutter sequelae to capture only symptomatic atrial fibrillation and flutter with updated proportions.

References

1. Pan-American Health Organization (PAHO), World Health Organization (WHO). Quantitative Estimation of Chagas in the Americas
2. Abad-Franch F, Diotaiuti L, Gurgel-Gonçalves R, Gürtler RE. Certifying the interruption of Chagas disease transmission by native vectors: cui bono? Mem Inst Oswaldo Cruz 2013;108:251–4.
3. Coura JR. Chagas disease: control, elimination and eradication. Is it possible? Mem Inst Oswaldo Cruz 2013;108:962–7.
4. United Nations, Department of Economic and Social Affairs, Population Division (2013). Trends in International Migrant Stock: The 2013 Revision - Migrants by Destination and Origin. (United Nations database, POP/DB/MIG/Stock/Rev.2013/Origin).
5. IPUMS USA, University of Minnesota, www.ipums.org.
6. Teixeira AR, Nitz N, Guimaro MC, Gomes C, Santos-Buch CA. Chagas disease. Postgrad Med J 2006;82:788–98.
7. Coura JR, Naranjo MA, Willcox HP. Chagas' disease in the Brazilian Amazon: II. A serological survey. Rev Inst Med Trop São Paulo 1995; 37:103–7.
8. Paixão GMM, Silva LGS, Gomes PR, Lima EM, Ferreira MPF, Oliveira DM, Ribeiro MH, Ribeiro AH, Nascimento JS, Canazart JA, Ribeiro LB, Benjamin EJ, Macfarlane PW, Marcolino MS, Ribeiro AL. Evaluation of Mortality in Atrial Fibrillation: Clinical Outcomes in Digital Electrocardiography (CODE) Study. Glob Heart. 2020 Jul 28;15(1):48. doi: 10.5334/gh.772. PMID: 32923342; PMCID: PMC7413140.

9. Sabino EC, Ribeiro AL, Salemi VM, et al., for the National Heart, Lung, and Blood Institute Retrovirus Epidemiology Donor Study-II (REDS-II), International Component. Ten-year incidence of Chagas cardiomyopathy among asymptomatic Trypanosoma cruzi-seropositive former blood donors. Circulation 2013;127:1105–15.

Figure 3: PRISMA diagram for GBD 2023 systematic review of Chagas disease prevalence

Figure 3a. Initial systematic review for Chagas disease

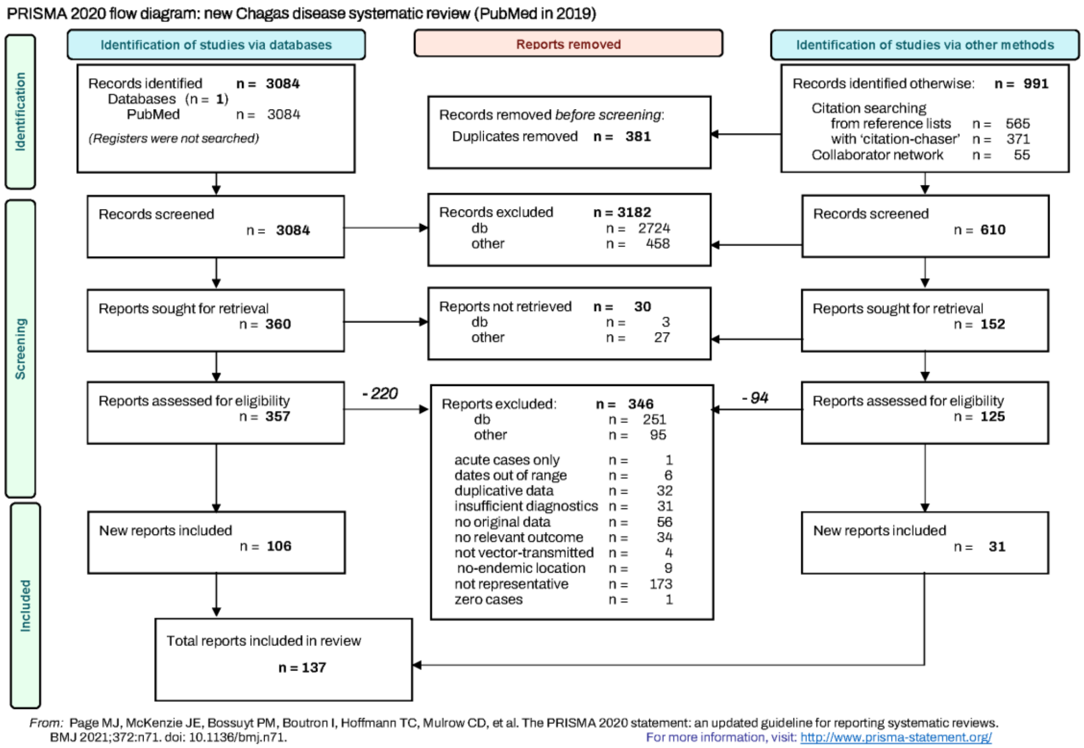

Figure 3b. Update to systematic review for Chagas disease, GBD 2023

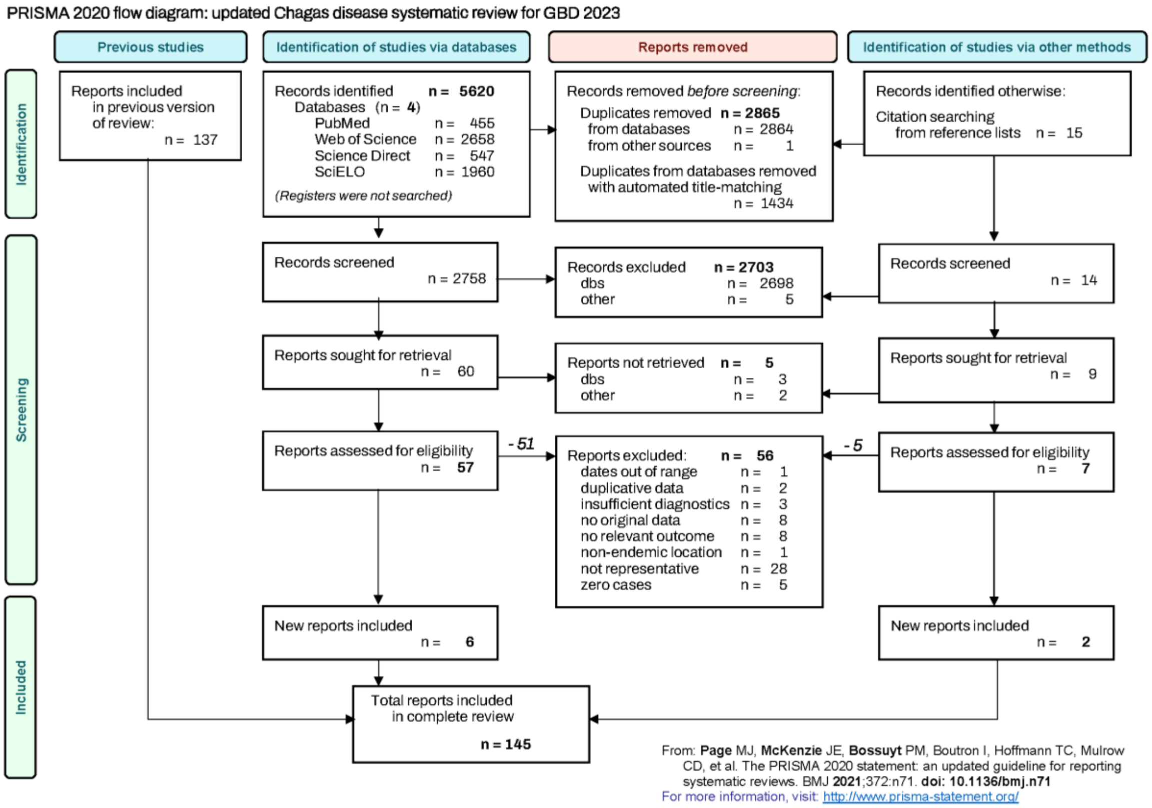

Chronic kidney disease

Flowchart

Chronic Kidney Disease (CKD)

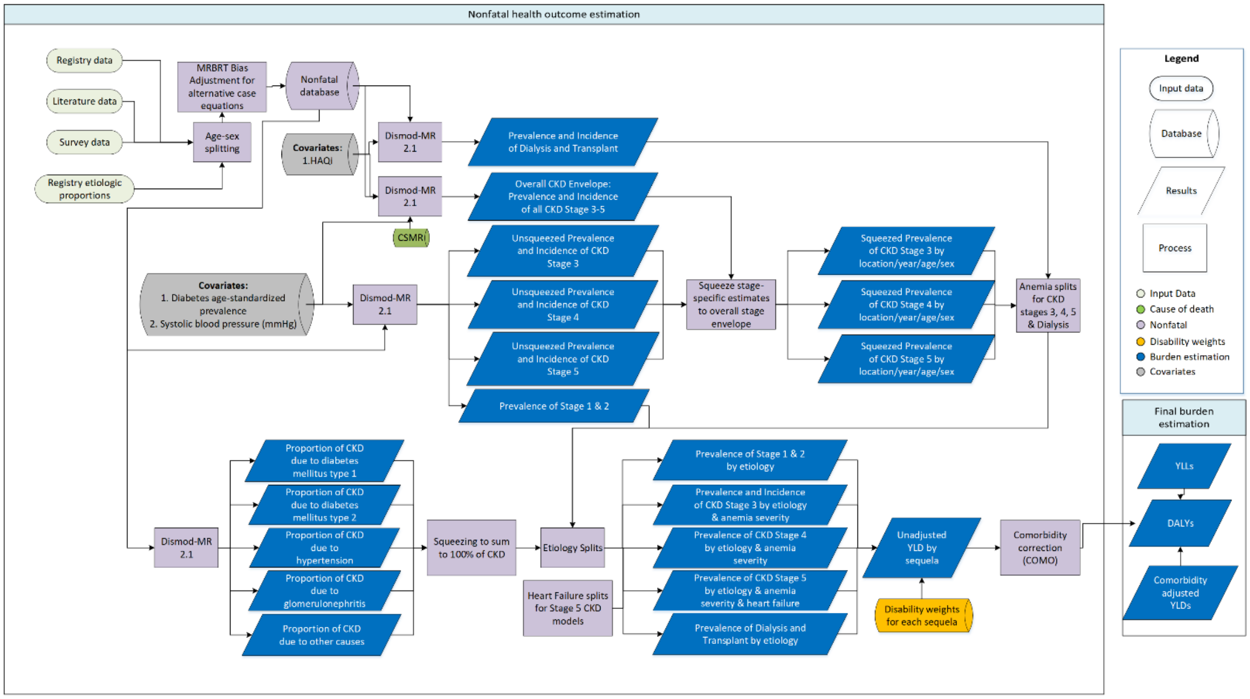

Input data and methodological summary for chronic kidney disease

Case definition

Chronic kidney disease (CKD) is defined as a permanent loss of kidney function as indicated by estimated glomerular filtration rate (eGFR) and urinary albumin to creatinine ratio (ACR). The CKD-EPI 2009 eGFR equation is considered our gold standard for those 18 years or older, and the Schwartz equation is our gold standard for those younger than 18. These equations can be found in Table 1. Alternative case definitions include the Cockcroft-Gault equation and the Modification of Diet in Renal Disease (MDRD) equation. Few data sources in our CKD models used the new CKD-EPI 2021 eGFR equation; therefore, this equation was also considered to be gold standard. The GBD study considers six stages of CKD as defined by degree of loss of kidney function or receipt of kidney replacement therapy. These definitions of the six stages can be found in Table 2. The ICD-9 codes associated with CKD include 585.1-585.9. The ICD-10 codes associated with CKD include N18.1-N18.9.

Table 1. CKD eGFR equations

Equation	Formula
CKD-EPI 2009	eGFR = $141 \times \min(S_{Cr}/\kappa, 1)^{\alpha} \times \max(S_{Cr}/\kappa, 1)^{-1.209} \times 0.993^{Age} \times 1.018$ [if female] $\times 1.159$ [if Black] κ is 0.7 for females and 0.9 for males α is –0.329 for females and –0.411 for males, where min indicates the minimum of Scr/κ or 1, and max indicates the maximum of Scr/κ or 1

Schwartz	eGFR = 0.413 x (height/Scr) if height is expressed in centimetres<br>OR<br>41.3 x (height/Scr) if height is expressed in metres

Table 2. GBD case definitions of CKD

Quantity of interest	Reference or alternative	Definition
Stages 1 & 2 chronic kidney disease	Reference	Albumin to creatinine ratio (ACR) of ≥30 mg/g and estimated glomerular filtration rate (eGFR) ≥ 60 mL/min/1.73m^2 as estimated using the CKD-EPI equation for individuals age ≥18 and the Schwartz equation for those <18.
Stages 1 & 2 chronic kidney disease	Alternative	Albumin to creatinine ratio (ACR) of ≥ a threshold other than 30 mg/g and estimated glomerular filtration rate (eGFR) ≥ 60 mL/min/1.73m^2 as estimated using the CKD-EPI equation for individuals age ≥18 and the Schwartz equation for those <18.
Stages 1 & 2 chronic kidney disease	Alternative	Albumin to creatinine ratio (ACR) of ≥30 mg/g and estimated glomerular filtration rate (eGFR) ≥ 60 mL/min/1.73m^2 as estimated using the MDRD equation (or modifications thereof) for individuals age ≥18.
Stages 1 & 2 chronic kidney disease	Alternative	Albumin to creatinine ratio (ACR) of ≥30 mg/g and estimated glomerular filtration rate (eGFR) ≥ 60 mL/min/1.73m^2 as estimated using the Cockcroft-Gault equation (standardised for body surface area) for individuals age ≥18.
Stages 1 & 2 chronic kidney disease	Alternative	Albumin to creatinine ratio (ACR) of ≥ a threshold other than 30 mg/g and estimated glomerular filtration rate (eGFR) ≥ 60 mL/min/1.73m^2 as estimated using the MDRD equation (or modifications thereof) for individuals age ≥18.
Stages 1 & 2 chronic kidney disease	Alternative	Albumin to creatinine ratio (ACR) of ≥ a threshold other than 30 mg/g and estimated glomerular filtration rate (eGFR) ≥ 60 mL/min/1.73m^2 as estimated using the Cockcroft-Gault equation (standardised for body surface area) for individuals age ≥18.
Stage 3 chronic kidney disease	Reference	Estimated glomerular filtration rate (eGFR) 30-59 mL/min/1.73m^2 as estimated using the CKD-EPI equation for individuals age ≥18 and the Schwartz equation for those <18 not on renal replacement therapy.
Stage 3 chronic kidney disease	Alternative	Estimated glomerular filtration rate (eGFR) 30-59 mL/min/1.73m^2 as estimated using the MDRD equation (or modifications thereof) for individuals age ≥18 not on renal replacement therapy.
Stage 3 chronic kidney disease	Alternative	Estimated glomerular filtration rate (eGFR) 30-59 mL/min/1.73m^2 as estimated using the Cockcroft-Gault equation (standardised for body surface area) for individuals age ≥18 not on renal replacement therapy.

Stage 4 chronic kidney disease	Reference	Estimated glomerular filtration rate (eGFR) 15-29 mL/min/1.73m^2 as estimated using the CKD-EPI equation for individuals age ≥18 and the Schwartz equation for those <18 not on renal replacement therapy.
Stage 4 chronic kidney disease	Alternative	Estimated glomerular filtration rate (eGFR) 15-29 mL/min/1.73m^2 as estimated using the MDRD equation (or modifications thereof) for individuals age ≥18 not on renal replacement therapy.
Stage 4 chronic kidney disease	Alternative	Estimated glomerular filtration rate (eGFR) 15-29 mL/min/1.73m^2 as estimated using the Cockcroft-Gault equation (standardised for body surface area) for individuals age ≥18 not on renal replacement therapy.
Stage 5 chronic kidney disease	Reference	Estimated glomerular filtration rate (eGFR) <15 mL/min/1.73m^2 as estimated using the CKD-EPI equation for individuals age ≥18 and the Schwartz equation for those <18 not on renal replacement therapy.
Stage 5 chronic kidney disease	Alternative	Estimated glomerular filtration rate (eGFR) <15 mL/min/1.73m^2 as estimated using the MDRD equation (or modifications thereof) for individuals age ≥18 not on renal replacement therapy.
Stage 5 chronic kidney disease	Alternative	Estimated glomerular filtration rate (eGFR) <15 mL/min/1.73m^2 as estimated using the Cockcroft-Gault equation (standardised for body surface area) for individuals age ≥18 not on renal replacement therapy.
End-stage renal disease after transplant	Reference	Ever received kidney transplant due to end-stage renal disease. Includes all kidney transplants due to ESRD, not just preemptive transplant.
End-stage renal disease on dialysis	Reference	On dialysis (haemodialysis or peritoneal dialysis) for >90 days.

Input data

Data seeking

A systematic review across three databases for CKD stages 1&2, 3, 4, and 5 was conducted for GBD 2023. Databases were searched on September 13, 2022, for publications after January 1, 2017. Below are the search terms for each database:

PubMed: (("chronic kidney disease"[Title/Abstract] OR "CKD"[Title/Abstract] OR "eGFR"[Title/Abstract]) AND ("prevalence"[Title/Abstract] OR "incidence"[Title/Abstract])) NOT ("animals"[MeSH Terms:noexp] OR "animal"[All Fields] OR "humans"[MeSH Terms])

Web of Science: ((AB=("chronic kidney disease" OR "CKD" or "eGFR")) AND TI=("prevalence" OR "incidence")) AND PY=(2017-2022)

ScienceDirect: Title contains (chronic kidney disease OR ckd OR egfr) AND Title contains (prevalence OR incidence) SCOPE: Library Resources / Articles, Books and More Material Type: Articles; Collection: ScienceDirect Journals (5 years ago present)

Below is the PRISMA diagram for the systematic review.

Figure 1: PRISMA diagram

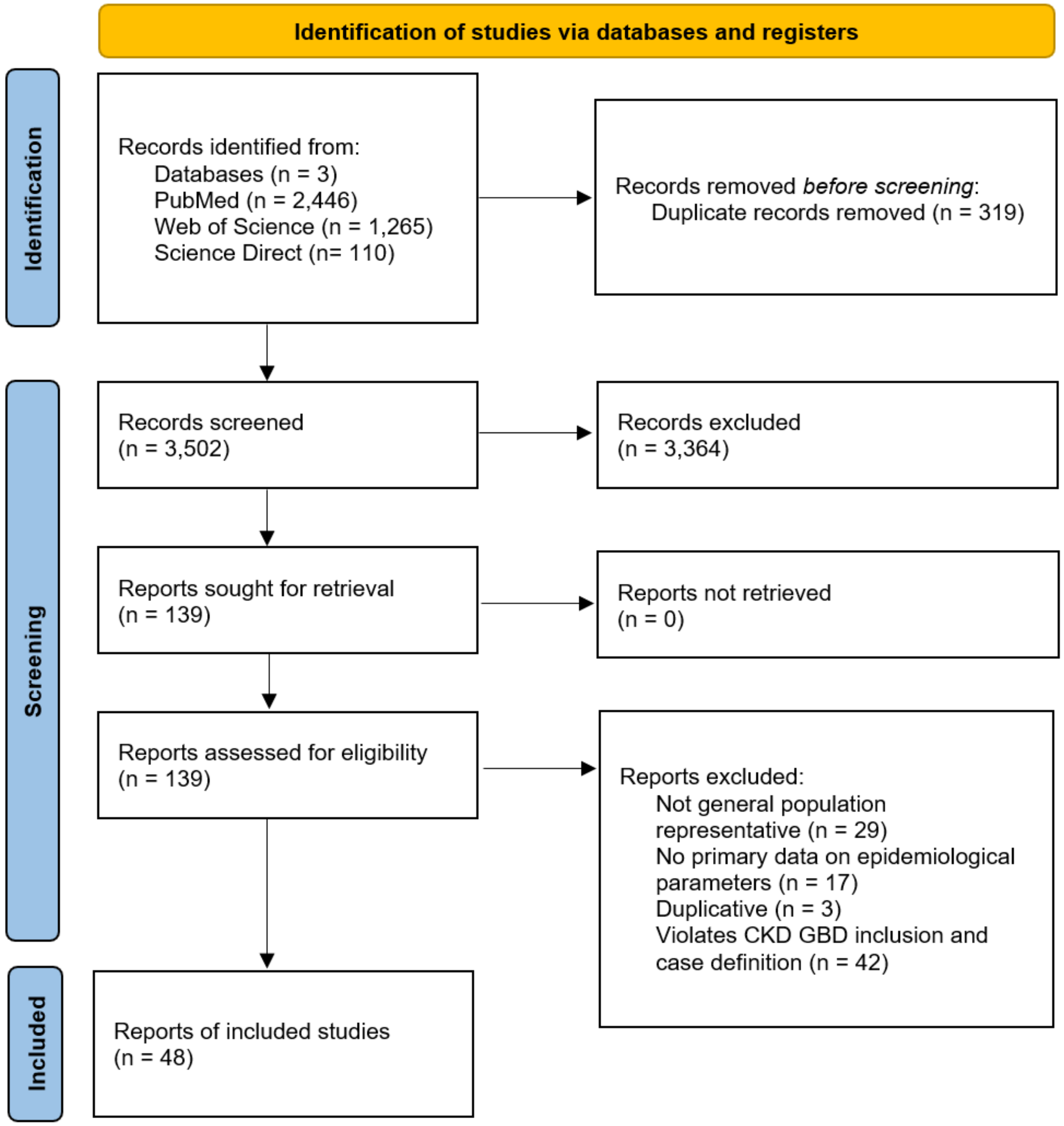

Data for end-stage renal disease on dialysis and end-stage renal disease after transplant mainly come from renal registries. All known renal registries used in GBD 2021 dialysis and transplant models were assessed for more recent years of data. We attempted to identify and access renal registries previously not extracted for models through a published systematic review of all renal registries. From this effort, we have newly included data from the Malaysian National Renal Registry and the Taiwan Renal Registry Data System into our models for GBD 2023.

Data processing

Age and sex split

In some cases, input data are reported for the same study that are age-specific or sex-specific, but not both. For example, a study may report the prevalences of males and females with stage 3 CKD and then separately report the prevalences of both sexes combined by smaller age bins (eg, 40–44, 45–49) that have stage 3 CKD. In these cases, we performed an age-sex split by utilising the sex ratios within the study to disaggregate the age-specific data into data that are both age- and sex-specific.

In cases where only data for both sexes combined are reported, we split these data into sex-specific data by applying a sex ratio outputted by our meta-regression—Bayesian, regularised, trimmed (MR-BRT)[1] tool (for methods details see appendix 1, section 2). The inputs into MR-BRT were sex ratios created from data that were already sex-specific for the given CKD stage.

In order to obtain an appropriate age pattern with which to split input data into smaller ages bins when necessary, we first ran a disease model—Bayesian meta-regression (DisMod-MR 2.1, for methods description see appendix 1, section 2) including all datapoints for a given CKD stage with an age range less than or equal to 25 years and a sample size greater than 50 persons. We then applied the age pattern by super-region to split input data, thereby allowing for variation in the age pattern by location.

Bias adjustments

For GBD 2023, we updated the adjustment factors applied to the alternative case definitions for CKD Stages 1&2, 3, 4, and 5.

We adjusted data using alternative case definitions with adjustment factors from a MR-BRT model to account for different estimates that result from these different equations. The adjustment is a logit-transformation method in MR-BRT. The general process is described below:

1. Using individual-level data accessible to us, calculate the prevalence of a given CKD stage according to each reference and alternative case definition detailed in table 2. Identify datapoints with overlapping year, age, sex, and location between reference and alternative definitions.
2. Logit-transform overlapping datapoints of alternative and reference case definitions.
3. Convert overlapping datapoints into a difference in logit space using the following equation:

$$logit(alternative) - logit(reference)$$

4. Use the delta method to compute standard errors of overlapping datapoints in logit space, then calculate standard error of logit difference using the following equation:

$$\sqrt{(variance\ of\ alternative) + (variance\ of\ reference)}$$

5. Using MR-BRT, conduct a random effects meta-regression to obtain the pooled logit difference of alternative to reference.
6. Apply the pooled logit difference to all datapoints of alternative case definitions using the following equation:

$$newestimate = inverse.logit(logit(alternative) - (pooled\ logit\ difference))$$

7. Calculate new standard errors using the delta method, accounting for gamma (between-study heterogeneity).

For the CKD stage 3 and CKD stages 3-5 envelope models, the MR-BRT models included a spline on age. Below are the dose-response curves for each alternative case definition that we adjusted to the

reference in GBD 2023. There was no spline used for CKD stages 1&2, 4, and 5. Table 3 below shows these adjustment factors used to adjust the data for GBD 2023.

Figure 2: Stage 3 MDRD equation

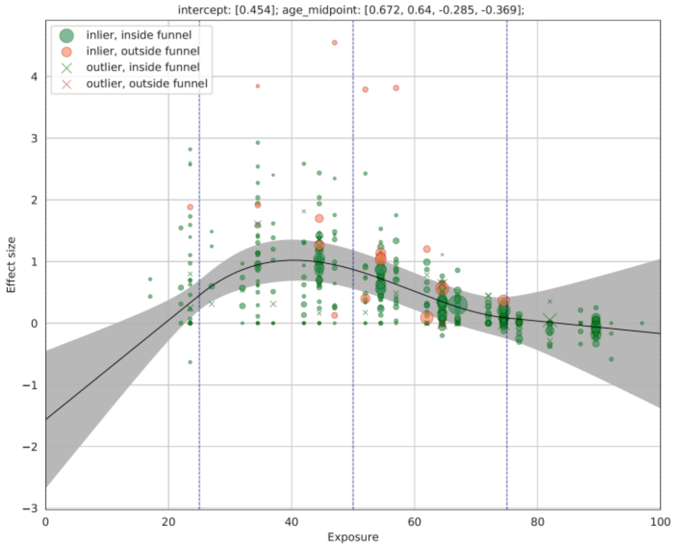

Figure 3: Stage 3 Cockcroft-Gault equation

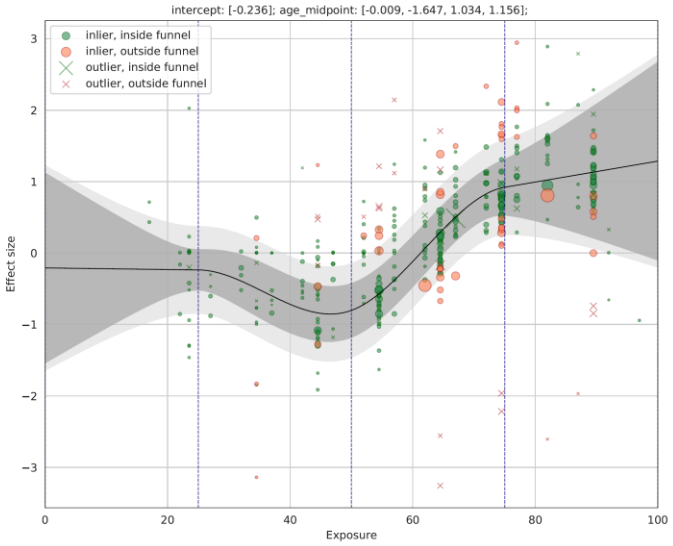

Figure 4: Stage 3-5 MDRD equation

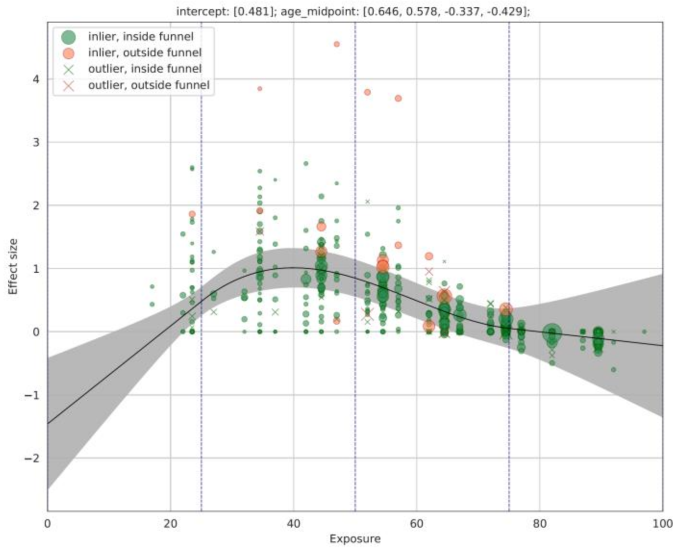

Figure 5: Stage 3-5 Cockcroft-Gault equation

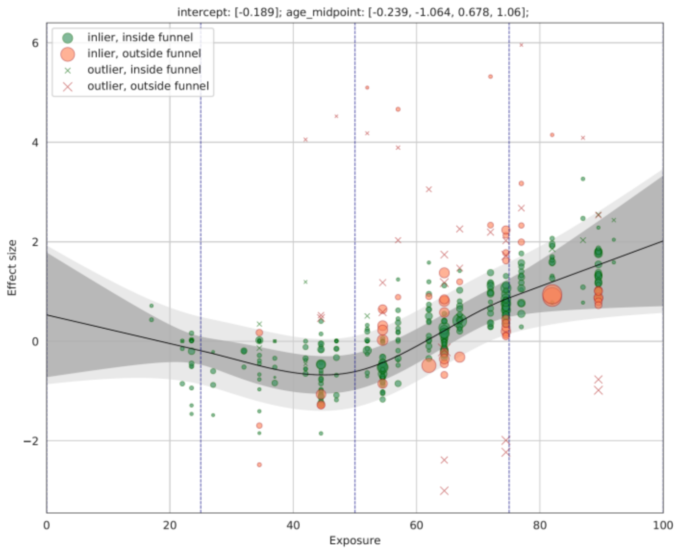

Table 3: MR-BRT crosswalk adjustment factors for CKD stages 1&2, 4, and 5

Data input	Reference or alternative case definition	Gamma	Beta coefficient, logit (SD)*	Adjustment factor**
Stage 1&2 ACR ≥30 mg/g and eGFR by CKD-EPI	Ref	---	---	---
Stage 1&2 ACR ≥17 mg/g and eGFR by Cockcroft-Gault	Alt	0.12	0.64 (0.03)	1.90

Stage 1&2 ACR ≥17 mg/g and eGFR by CKD-EPI	Alt	0.00	0.68 (0.02)	1.97
Stage 1&2 ACR ≥17 mg/g and eGFR by MDRD	Alt	0.00	0.63 (0.03)	1.88
Stage 1&2 ACR ≥20 mg/g and eGFR by Cockcroft-Gault	Alt	0.00	0.44 (0.02)	1.55
Stage 1&2 ACR ≥20 mg/g and eGFR by CKD-EPI	Alt	0.00	0.47 (0.02)	1.60
Stage 1&2 ACR ≥20 mg/g and eGFR by MDRD	Alt	0.01	0.44 (0.03)	1.55
Stage 1&2 ACR ≥25 mg/g and eGFR by Cockcroft-Gault	Alt	0.00	0.20 (0.02)	1.22
Stage 1&2 ACR ≥25 mg/g and eGFR by CKD-EPI	Alt	0.00	0.20 (0.02)	1.22
Stage 1&2 ACR ≥25 mg/g and eGFR by MDRD	Alt	0.00	0.18 (0.02)	1.20
Stage 1&2 ACR ≥30 mg/g and eGFR by Cockcroft-Gault	Alt	0.00	–0.01 (0.02)	0.99
Stage 1&2 ACR ≥30 mg/g and eGFR by MDRD	Alt	0.00	–0.02 (0.02)	0.98
Stage 4 CKD-EPI	Ref	---	---	---
Stage 4 Cockcroft-Gault	Alt	0.00	0.03 (0.06)	1.03
Stage 4 MDRD	Alt	0.00	–0.11 (0.05)	0.90
Stage 5 CKD-EPI	Ref	---	---	---
Stage 5 Cockcroft-Gault	Alt	0.00	–0.07 (0.10)	0.93
Stage 5 MDRD	Alt	0.00	–0.03 (0.08)	0.97

**MR-BRT crosswalk adjustments can be interpreted as the factor the alternative case definition is adjusted by to reflect what it would have been had it been measured using the reference case definition. If the logit beta coefficient is negative, then the alternative is adjusted up to the reference. If the logit beta coefficient is positive, then the alternative is adjusted down to the reference.*

***The adjustment factor column is the exponentiated beta coefficient. For logit beta coefficients, this is the relative odds between the two case definitions.*

Modelling strategy

CKD stage models

The modelling strategy for GBD 2023 is similar to GBD 2021.

We ran separate DisMod-MR 2.1 models for each stage of CKD and an aggregate CKD stage 3-5 model to produce estimates by age, sex, year, and country. Estimates from the CKD stage 3, 4, and 5 models were scaled by age, sex, year, and location to sum to the aggregate CKD stage 3-5 estimates.

Because DisMod-MR 2.1 does not incorporate disease progression in its compartmental model, we used "remission" as a proxy for progression, where a surviving prevalent case ceases to be a case in this stage. As CKD is a progressive disease, we assume there is no true remission, which allows us to apply this parameter substitution. To account for the progression of individuals from stage 3 to 4, from 4 to 5, and from dialysis to transplant, we back-calculated progression to later stages of CKD. This was done by calculating the ratio of the incidence of the next stage with the prevalence of the previous stage. For inclusion in the DisMod-MR 2.1 models, these custom input data were calculated as:

$$remission_s = \frac{incidence_{s+1}}{prevalence_s}, where\ s\ is\ stage$$

Furthermore, remission was set to zero for Stage 5, and the excess mortality parameter was used to account for progression to end-stage kidney disease and mortality due to CKD stage 5 collectively (even though "technically" this is not correct for those who go onto dialysis, this was a decision to facilitate modelling). Bounds on excess mortality were informed using a meta-analysis of survival analyses[2] of individuals with untreated CKD stage 5.

Table 4: Summary of covariates used in the CKD stage DisMod-MR meta-regression models

Model	Covariate	Type	Parameter	Exponentiated beta (95% uncertainty interval)
CKD Stage 1&2	Diabetes age-standardised prevalence	Country-level	Prevalence	1.03 (1.00–1.08)
	Mean systolic blood pressure	Country-level	Prevalence	2.96 (1.99–4.24)
CKD Stage 3	Diabetes age-standardised prevalence	Country-level	Prevalence	1.15 (1.07–1.23)
	Mean systolic blood pressure	Country-level	Prevalence	4.01 (3.51–4.45)
CKD Stage 4	Diabetes age-standardised prevalence	Country-level	Prevalence	1.03 (1.00–1.08)
	Mean systolic blood pressure	Country-level	Prevalence	2.46 (2.14–2.70)
CKD Stage 5	Diabetes age-standardised prevalence	Country-level	Prevalence	1.88 (1.73–2.05)

	Mean systolic blood pressure	Country-level	Prevalence	1.66 (1.03–2.90)
CKD Stage 3-5	Diabetes age-standardised prevalence	Country-level	Prevalence	1.02 (1.00–1.05)
	Mean systolic blood pressure	Country-level	Prevalence	2.34 (1.39–3.86)
	Healthcare Access and Quality Index	Country-level	Excess mortality rate	1.00 (1.00–1.00)
Dialysis	Healthcare Access and Quality Index	Country-level	Incidence	1.02 (1.02–1.02)
Transplant	Healthcare Access and Quality Index	Country-level	Incidence	1.02 (1.01–1.02)

CKD aetiology proportion models

To estimate aetiology proportions of CKD, we utilised three separate types of data.

First, we utilised data primarily from end-stage kidney registries that included CKD aetiologies to model ESRD aetiology proportions. Each aetiology was modelled with DisMod-MR 2.1 to obtain estimates of each by location, year, age, and sex. Data for CKD due to overall DM were more widely available than data by type of DM. To make use of all available data, we modelled the proportion of CKD due to overall DM with age-standardised diabetes prevalence as a county-level covariate. The proportions of CKD due to DM type 1 and DM type 2 were then scaled to sum to the proportion of overall DM by location, year, age, and sex. Mean systolic blood pressure (mmHg) was used as a country-level covariate in the CKD due to hypertension model. After modelling, the results from all five aetiology-specific models were adjusted proportionally so that estimates across the aetiologies sum to 100%. Then, the proportions were applied to prevalence estimates of dialysis and transplant to calculate aetiology-specific prevalence estimates.

Table 5. Summary of covariates used in the CKD aetiology DisMod-MR meta-regression models

**Model**	**Covariate**	**Type**	**Parameter**	**Exponentiated beta (95% uncertainty interval)**
CKD due to diabetes mellitus proportion	Age-standardised diabetes prevalence	Country-level	Proportion	2.71 (2.69–2.72)
CKD due to hypertension proportion	Mean systolic blood pressure	Country-level	Proportion	1.00 (1.00–1.01)

The second type of data comes from the Geisinger Health System in Pennsylvania.[3] These data contain age-sex-stage-specific aetiology proportions that allowed differential aetiologic composition of CKD across stages for disease progression. These data were used for CKD Stages 1&2, Stage 3, Stage 4, and

Stage 5. For everyone with CKD, we scanned their history of recorded International Classification of Diseases (ICD) codes to identify ICD codes for primary kidney diseases (see Table 6). We used this information to map individuals to GBD aetiologies by stage of CKD; individuals with CKD but with no history of a primary kidney disease ICD code were classified as having CKD of unknown aetiology. We ran a multinomial logistic regression including sex and a non-linear term for age to predict the probability of each aetiology by age and sex for each stage of CKD (1&2, 3, and 4&5 combined). For each stage, aetiology, age, and sex, we converted this probability into the proportion of CKD due to the given aetiology.

To maintain consistency between GBD estimates of type 1 diabetes prevalence and CKD due to type 1 diabetes prevalence and generalise the results of the Geisinger analysis to all locations, we performed a location-specific correction for the proportion of CKD due to type 1 and type 2 diabetes. For each diabetic subtype (e) for a given location (l), age (a), and sex (g) the ratio of subtype-specific diabetes prevalence to total diabetes prevalence (r) was calculated as:

$$r_{e,l,a,g} = \frac{prevalence_{e,l,a,g}}{prevalence_{dm1,l,a,g} + prevalence_{dm2,l,a,g}}$$

This ratio is used to adjust the proportion of CKD due to a given diabetic subtype (p) for a given CKD stage (s), location (l), age (a), and sex (g) by scaling the predicted proportion of CKD due to that subtype (k) by the ratio of total DM due to each diabetic subtype (e) in a location (l) to the ratio of total DM due to each diabetic subtype (e) in the USA.

$$p_{s,e,l,a,g} = k_{s,a,g} \times \frac{r_{e,l,a,s}}{r_{e,USA,a,s}}$$

Table 6. International Classification of Disease codes used for GBD aetiology mapping

CKD aetiology	ICD-9 codes	ICD-10 codes
Type 1 diabetes	250.41, 250.43	E10.2, E10.21, E10.22, E10.29
Type 2 diabetes	250.40, 250.42	E11.2, E11.21, E11.22, E11.29
Glomerulonephritis	581, 581.0, 581.1, 581.2, 581.3, 581.8, 581.81, 581.89, 581.9, 582, 582.0, 582.1, 582.2, 582.4, 582.8, 582.81, 582.89, 582.9, 583, 583.0, 583.1, 583.2, 583.4, 583.6, 583.7, 583.8, 583.81, 583.89, 583.9	N02, N02.0, N02.1, N02.2, N02.3, N02.4, N02.5, N02.6, N02.7, N02.8, N02.9, N03, N03.0, N03.1, N03.2, N03.3, N03.4, N03.5, N03.6, N03.7, N03.8, N03.9, N04, N04.0, N04.1, N04.2, N04.3, N04.4, N04.5, N04.6, N04.7, N04.8, N04.9, N05, N05.0, N05.1, N05.2, N05.3, N05.4, N05.5, N05.6, N05.7, N05.8, N05.9, N06, N06.0, N06.1, N06.2, N06.3, N06.4, N06.5, N06.6, N06.7, N06.8, N06.9
Hypertension	403, 403.0, 403.00, 403.01, 403.1, 403.10, 403.11, 403.6, 403.9, 403.90, 403.91, 404, 404.0, 404.00, 404.01, 404.02, 404.03, 404.1, 404.10, 404.11, 404.12, 404.13, 404.9, 404.90, 404.91, 404.92, 404.93	I12, I12.0, I12.1, I12.2, I12.9, I13, I13.0, I13.1, I13.10, I13.11, I13.2, I13.9

Other	589, 589.0, 589.1, 589.9, 753.0, 753.1, 753.10, 753.11, 753.12, 753.13, 753.14, 753.15, 753.16, 753.17, 753.19, 753.2, 753.20, 753.21, 753.22, 753.23, 753.29, 753.3, 283.11, 710.0, 753.0, 753.21, 753.22, 753.29	N07, N07.0, N07.1, N07.2, N07.3, N07.4, N07.5, N07.6, N07.7, N07.8, N07.9, N08, N08.0, N08.1, N08.2, N08.3, N08.4, N08.5, N08.8, N15.0, Q61, Q61.0, Q61.00, Q61.01, Q61.02, Q61.1, Q61.11, Q61.19, Q61.2, Q61.3, Q61.4, Q61.5, Q61.8, Q61.9, Q62, Q62.0, Q62.1, Q62.10, Q62.11, Q62.12, Q62.2, Q62.3, Q62.31, Q62.32, Q62.39, Q62.4, Q62.5, Q62.6, Q62.60, Q62.61, Q62.62, Q62.63, Q62.69, Q62.7, Q62.8, D59.3, M31.31, M32.14, M32.15, N11.9, N13.70, N13.8, Q60.2, Q63.8, N14.0, N14.1, N14.3, N25.89, N26.9, N28.0

The third type of data is from chronic kidney disease registries in India that have information on both stage (CKD Stages 1&2, Stage 3, Stage 4, and Stage 5) and aetiology. These data were modelled in separate stage- and aetiology-specific DisMod-MR 2.1 models to produce proportions for each combination by age, sex, and year for India. These proportions were combined with the Geisinger proportions described above using a weighted average, to account for different data source strengths across stage and age. These weighted averages by age, sex, stage, and aetiology were applied to prevalence estimates of CKD Stages 1&2, Stage 3, Stage 4, and Stage 5 for all locations. The lack of stage-aetiology information from areas in the world other than the USA and India is a key limitation in this analysis. Prior to GBD 2023, only stage-aetiology information from the Geisinger analysis was used.

Anaemia causal attribution

The age- and sex-specific anaemia prevalence for CKD was analysed as part of overall anaemia causal attribution for GBD 2023. The details of the anaemia analysis are described separately in the "anaemia impairment" section. Briefly, after estimating total anaemia, a series of counterfactual distributions were generated based on the age- and sex-specific prevalence of each anaemia-causing condition and the quantitative effect that the condition has on haemoglobin concentration in the blood, a so-called "haemoglobin shift," that was derived by meta-analysing cohort studies, observational studies, or trials comparing the haemotological status of those with as compared to without the disease. Due to limited data on haemoglogbin shift, all were assumed to be invariant over age, sex, location, and year.

Severity splits and disability weights

Estimates of prevalence and incidence are split using CKD aetiology proportion models, resulting in CKD estimates by stage and aetiology. Then a portion of each aetiology split for CKD stages 3, 4, and 5 is attributed a disability weight associated with mild, moderate, or severe anaemia. Then, each aetiology split for Stage 5 is attributed a disability weight associated with mild, moderate, or severe heart failure.

Table 7. Severity distribution, details on the severity levels for chronic kidney disease and the associated disability weight (DW) with that severity

Severity level	Lay description	DW (95% CI)

Albuminuria	Asymptomatic	--
CKD stage 3 without anaemia	Asymptomatic	--
CKD stage 3 with mild anaemia	Feels slightly tired and weak at times, but this does not interfere with normal daily activities.	0.004 (0.001–0.008)
CKD stage 3 with moderate anaemia	Feels moderate fatigue, weakness, and shortness of breath after exercise, making daily activities more difficult.	0.052 (0.034–0.076)
CKD stage 3 with severe anaemia	Feels very weak, tired, and short of breath, and has problems with activities that require physical effort or deep concentration.	0.149 (0.101–0.21)
CKD stage 4 without anaemia	Tires easily, has nausea, reduced appetite, and difficulty sleeping.	0.104 (0.07–0.147)
CKD stage 4 with mild anaemia	Combined disability weight	0.108 (0.072–0.151)
CKD stage 4 with moderate anaemia	Combined disability weight	0.15 (0.103–0.207)
CKD stage 4 with severe anaemia	Combined disability weight	0.237 (0.165–0.324)
CKD stage 5 without anaemia	Has lost a lot of weight and has constant pain. The person has no appetite, feels nauseated, and needs to spend most of the day in bed.	0.569 (0.389–0.727)
CKD stage 5 with mild anaemia	Combined disability weight	0.570 (0.391–0.727)
CKD stage 5 with moderate anaemia	Combined disability weight	0.591 (0.414–0.743)
CKD stage 5 with severe anaemia	Combined disability weight	0.631 (0.456–0.782)
End-stage kidney disease, on dialysis	Is tired and has itching, cramps, headache, joint pains, and shortness of breath. The person needs intensive medical care every other day lasting about half a day.	0.571 (0.397–0.725)
End-stage renal disease, on dialysis and mild anemia	Combined disability weight	0.573 (0.403–0.726)
End-stage renal disease, on dialysis and moderate anemia	Combined disability weight	0.593 (0.424–0.742)
End-stage renal disease, on dialysis and severe anemia	Combined disability weight	0.633 (0.462–0.781)
End-stage kidney disease, with kidney transplant	Sometimes feels tired and down, and has some difficulty with daily activities.	0.024 (0.014–0.039)
Stage 5 due to type 1 diabetes mellitus, with asymptomatic heart failure	Has lost a lot of weight and has constant pain. The person has no appetite, feels nauseous, and needs to spend most of the day in bed.	0.148 (0.100–0.205)
Stage 5 due to type 1 diabetes mellitus, with mild heart failure	Combined disability weight	0.141 (0.097–0.195)
Stage 5 due to type 1 diabetes mellitus, with moderate heart failure	Combined disability weight	0.168 (0.115–0.230)

Stage 5 due to type 1 diabetes mellitus, with severe heart failure	Combined disability weight	0.264 (0.186–0.358)
Stage 5 due to type 2 diabetes mellitus, with asymptomatic heart failure	Has lost a lot of weight and has constant pain. The person has no appetite, feels nauseous, and needs to spend most of the day in bed.	0.148 (0.100–0.205)
Stage 5 due to type 2 diabetes mellitus, with mild heart failure	Combined disability weight	0.141 (0.097–0.195)
Stage 5 due to type 2 diabetes mellitus, with moderate heart failure	Combined disability weight	0.168 (0.115–0.230)
Stage 5 due to type 2 diabetes mellitus, with severe heart failure	Combined disability weight	0.264 (0.186–0.358)
Stage 5 due to hypertension, with asymptomatic heart failure	Has lost a lot of weight and has constant pain. The person has no appetite, feels nauseous, and needs to spend most of the day in bed.	0.148 (0.1–0.205)
Stage 5 due to hypertension, with mild heart failure	Combined disability weight	0.141 (0.097–0.195)
Stage 5 due to hypertension, with moderate heart failure	Combined disability weight	0.168 (0.115–0.230)
Stage 5 due to hypertension, with severe heart failure	Combined disability weight	0.264 (0.186–0.358)
Stage 5 due to glomerulonephritis, with asymptomatic heart failure	Combined disability weight	0.148 (0.1–0.205)
Stage 5 due to glomerulonephritis, with mild heart failure	Combined disability weight	0.141 (0.097–0.195)
Stage 5 due to glomerulonephritis, with moderate heart failure	Combined disability weight	0.168 (0.115–0.230)
Stage 5 due to glomerulonephritis, with severe heart failure	Combined disability weight	0.264 (0.186–0.358)
Stage 5 due to other and unspecified causes, with asymptomatic heart failure	Has lost a lot of weight and has constant pain. The person has no appetite, feels nauseous, and needs to spend most of the day in bed.	0.148 (0.100–0.205)
Stage 5 due to other and unspecified causes, with mild heart failure	Combined disability weight	0.141 (0.097–0.195)
Stage 5 due to other and unspecified causes, with moderate heart failure	Combined disability weight	0.168 (0.115–0.230)

Stage 5 due to other and unspecified causes, with severe heart failure	Combined disability weight	0.264 (0.186–0.358)

Note: the DWs for CKD 4 and 5 stages with anaemia are derived from a multiplicative function combining the CKD stage DW and the corresponding severity of anaemia DW

References

1. Zheng, P., Barber, R., Sorensen, R. J., Murray, C. J., & Aravkin, A. Y. (2021). Trimmed constrained mixed effects models: formulations and algorithms. *Journal of Computational and Graphical Statistics*, 1-13.
2. Murtagh FEM, Marsh JE, Donohoe P, Ekbal NJ, Sheerin NS, Harris FE. Dialysis or not? A comparative survival study of patients over 75 years with chronic kidney disease stage 5. *Nephrol Dial Transplant*. 2007; 22(7): 1955–62.
3. Chang AR, Kirchner HL, Sang Y, Grams ME, Coresh J, Geisinger Health System, Johns Hopkins University (United States), Chronic Kidney Disease Prognosis Consortium (CKD-PC). United States - Pennsylvania Geisinger Health System Chronic Kidney Disease Etiology Proportions 1997-2017, Analyzed by CKD-PC. [Unpublished.]

Chronic obstructive pulmonary disease (COPD)

Flowchart

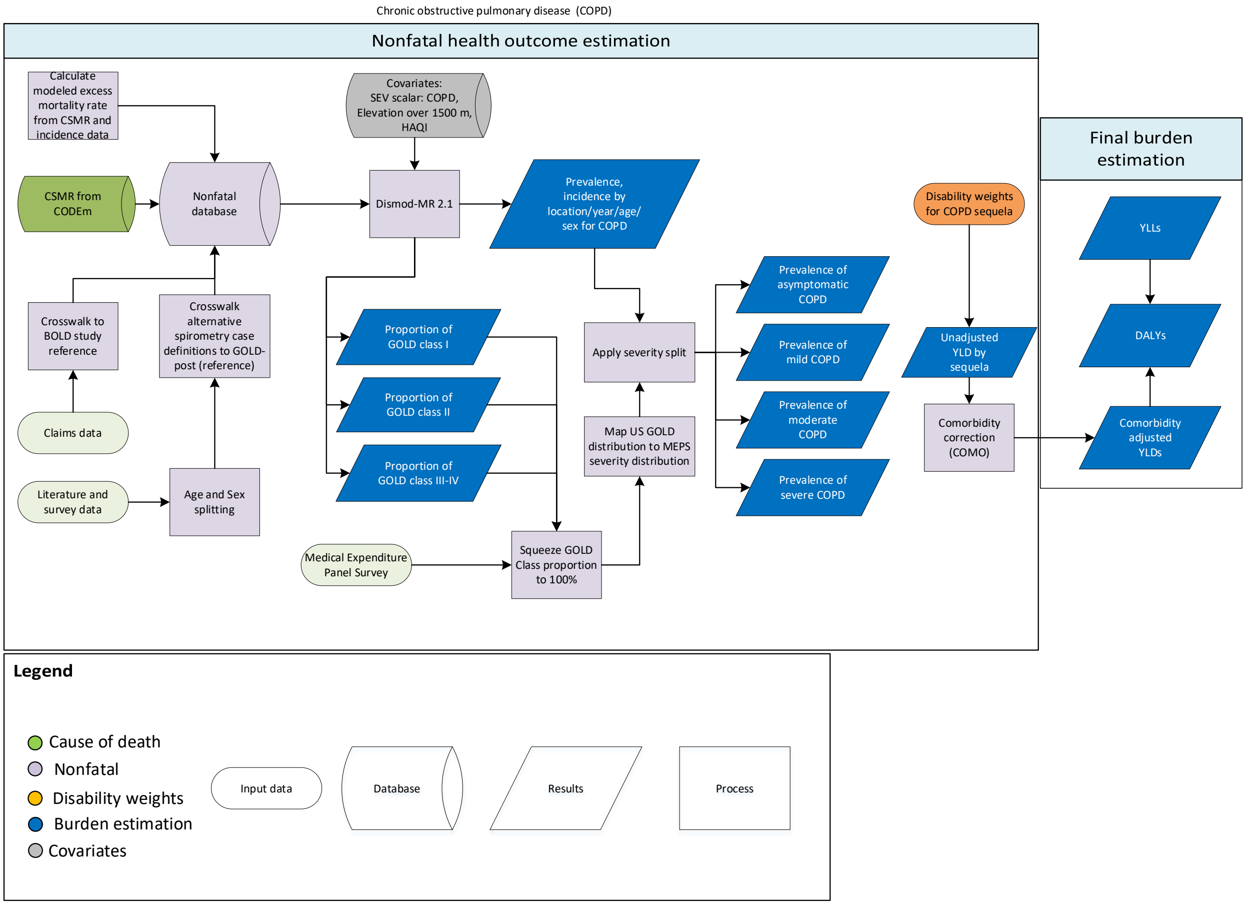

Input data and methodological summary for COPD

Case definition

Chronic inflammatory lung disease that causes obstructed airflow and breathing problems. It includes emphysema and chronic bronchitis. COPD is defined as in the Global Initiative for Chronic Obstructive Lung Disease (GOLD) classification: a measurement of <0.7 FEV_1/FVC (one second of forceful exhalation/total forced expiration) on spirometry after bronchodilation. ICD-10 codes associated with COPD include J41, J42, J43, J44, and J47. The corresponding ICD-9 codes are 491-492, and 496. J40, 490 and J47.

Table 1. Reference and alternative definitions for COPD and the GOLD Stage Proportion Models

**Quantity of interest**	**Reference or Alternative**	**Definition**
COPD prevalence	Reference	GOLD Post: GOLD standard after taking a bronchodilator. FEV_1/FVC <0.70

COPD prevalence	Alternative	GOLD Pre: GOLD standard without a bronchodilator.<br>FEV_1/FVC <0.70
COPD prevalence	Alternative	Lower limit of normal (LLN), post-bronchodilator.<br>FEV_1/FVC <5th percentile of predicted lung function (age, sex-specific)
COPD prevalence	Alternative	Lower limit of normal (LLN), pre-bronchodilator.<br>FEV_1/FVC <5th percentile of predicted lung function (age, sex-specific)
COPD prevalence	Alternative	European Respiratory Society or American Thoracic Society criteria.<br>FEV_1/FVC <5% predicted lung function and FEV_1 ≥70% of predicted lung function (age, sex-specific)
COPD GOLD stage 1	Reference	Mild. FEV_1 ≥ 80% of predicted lung function (age, sex-specific)
COPD GOLD stage 2	Reference	Moderate. FEV_1 is between 50% and 79% of normal lung function (age, sex-specific)
COPD GOLD stage 3-4	Reference	Severe. FEV_1 <50% of normal lung function (age, sex-specific)

Input data

COPD has the following data sources

- Prevalence, incidence, and remission data from literature
- Hospital claims data
- Proportion data of GOLD class severities from literature
- Burden of Obstructive Lung Disease (BOLD) Study data

Prevalence, incidence, and remission data relating to COPD are extracted from literature provided by collaborators or found via opportunistic review. All data must include spirometry-based measures. Other data come from hospital claims data for non-fatal estimation and vital registrations for cause of death.

The Burden of Obstructive Lung Disease (BOLD) data are specifically notable because of their standardised methodology across 17 countries with diverse social, economic, and environmental characteristics.

While no systematic review of the literature was completed for GBD 2023, additional data were included from GBD Collaborators, and an opportunistic search was done focused on adding new sources that report GOLD stage classifications. Notable data added this round include prevalence and incidence data from Netherlands Nivel Primary Care Database and Canadian Chronic Disease Surveillance System. GOLD stage proportion data was added in China, India, Sweden, Netherlands, Kyrgyzstan, and Portugal.

Table 2. MR-BRT crosswalk adjustment factors for COPD

Data input	Reference or alternative case definition	Gamma	Beta coefficient, log/logit (95% UI)*	Adjustment factor**
GOLD post	Ref	0.25	---	---
GOLD pre	Alt		0.50<br>(–0.02 to 1.07)	0.62<br>(0.49 to 0.74)
ERS	Alt		0.70	0.67

			(0.11 to 1.31)	(0.53 to 0.79)
LLN pre	Alt		0.10<br>(0.01 to 0.19)	0.52<br>(0.50 to 0.55)
LLN post	Alt		–0.34<br>(–0.50 to –0.19)	0.42<br>(0.38 to 0.45)
BOLD	Ref	0.19	---	---
MarketScan	Alt		–1.93<br>(–2.35 to –1.50)	0.13<br>(0.08 to 0.18)

**MR-BRT crosswalk adjustments can be interpreted as the factor the alternative case definition is adjusted by to reflect what it would have been had it been measured using the reference case definition. If the log/logit beta coefficient is negative, then the alternative is adjusted up to the reference. If the log/logit beta coefficient is positive, then the alternative is adjusted down to the reference.*
***The adjustment factor column is the exponentiated beta coefficient. For log beta coefficients, this is the relative rate between the two case definitions. For logit beta coefficients, this is the relative odds between the two case definitions.*

Data processing

Age and sex split
In some cases, data are reported by only age or only sex, but not both. For example, a study may have included the prevalence of males and females with COPD and then separately reported the prevalence for both sexes in smaller age bins (eg, ages 40–45, 46–50, etc.) that have COPD. In these cases, we perform an age-sex split by utilising proportions within the study to disaggregate the data.

When data are not disaggregated into male and female categories for a given data source, we instead perform a sex-split on the data by applying sex proportions from other studies that do have male- and female-specific data. Historically, male prevalence of COPD has been higher than female prevalence, but the sex gap has been shown to have narrowed in recent years. When data are aggregated into age categories larger than 25 years, we split into smaller age bins based on super-regional age patterns in the GBD 2021 COPD model.

Modelled excess mortality data

In GBD 2023, we continued modelling excess mortality rate (EMR) data outside of DisMod-MR 2.1 (disease model—Bayesian meta-regression; described in appendix 1, section 2).

Priors on EMR were estimated in DisMod by matching prevalence datapoints with their corresponding CSMR values within the same age, sex, year, location (by dividing CSMR by prevalence).

However, for many causes, DisMod estimated a rather unrealistic pattern of EMR compared to an expected pattern of decreasing EMR with greater access to quality health care. Such unexpected patterns often signal inconsistencies between CSMR estimates and the measures of prevalence and/or incidence.

In an effort to provide greater guidance to DisMod on the expected pattern of EMR, EMR data generated in the previous round were used as inputs for modelling in MR-BRT[2] (meta-regression—Bayesian, regularised, trimmed; described in appendix 1, section 2 of the reference) with age, sex, and

Healthcare Access and Quality (HAQ) Index included as covariates. Results from MR-BRT were then predicted for each location year, sex, and for ages 0, 10, 20 ….100.

This method led to improvements in the consistency of EMR relative to health-care access. We also included HAQ Index as a country-level covariate in DisMod to inform EMR with the mean and standard deviation produced from MR-BRT analysis.

Bias adjustments

In GBD 2023, we ran the same bias adjustment methods used in GBD 2021, by utilising a MR-BRT model outside of DisMod to allow a more direct comparison between different case definitions and/or study designs.

We made a series of adjustments to data that do not completely match our case definition. Different diagnosis criteria often lead to different estimates of COPD. For example, use of a bronchodilator is important to determine airway reversibility; testing spirometry without a bronchodilator may lead to higher likelihood of over-diagnosing COPD. Similarly, claims data are subject to biases. Claims data are often systematically lower than survey data, probably due to selection bias with regard to socioeconomic status. Adjustments are made to these data to correct these biases.

The adjustment is a logit-transformation method in MR-BRT. The general process is described below:

1. Identify datapoints with overlapping year, age, sex, and location between reference and alternative definitions.
2. Logit transform overlapping datapoints of alternative and reference case definitions.
3. Convert overlapping datapoints into a difference in logit space using the following equation:
    a. $logit(altnerative) - logit(reference)$
4. Use the delta method to compute standard errors of overlapping datapoints in logit space, then calculate standard error of logit difference using the following equation:
    a. $\sqrt{(variance\ of\ alternative) + (variance\ of\ reference)}$
5. Using MR-BRT, conduct a random effects meta-regression to obtain the pooled logit difference of alternative to reference.
6. Apply the pooled logit difference to all datapoints of alternative case definitions using the following equation:
    a. $new_{estimate} = inverse.logit((logit(alternative)) - (pooled\ logit\ difference))$
7. Calculate new standard errors using the delta method, accounting for gamma (between-study heterogeneity).

Data derived from claims from commercial health insurance in the USA were also adjusted using a factor estimated in MR-BRT. Claims data, notably USA MarketScan, were adjusted in relation to the BOLD study data. In this case, the BOLD data serve as the reference definition while the MarketScan data are the alternative definition.

Modelling strategy

The estimation of COPD burden has two distinct steps.

1. Estimate prevalence and incidence using a DisMod-MR 2.1 model.

2. Estimate proportion of COPD severities using GOLD class groupings in DisMod-MR 2.1.

After these two steps, the COPD prevalence and incidence are split by age, sex, and location for each severity level.

Step 1: Main COPD model – estimate prevalence and incidence using DisMod-MR 2.1

Model settings
We set remission to zero because individuals do not recover once they have COPD; the symptoms are only managed. Incidence ceiling is set at 0.0002 before age 15 and a ceiling at 0.0005 before age 30 to avoid a kick-up of estimates in age ranges with few or no primary data.

Each model includes a series of country-level covariates that describe spatiotemporal patterns.

- COPD standardised exposure variables (SEV) aggregates multiple risk factors into a single variable.
- Healthcare Access and Quality (HAQ) Index on EMR to capture country-level variation of EMR, assuming a negative coefficient (ie, lower mortality with rising GDP and HAQ). The priors of HAQ Index came from the EMR MR-BRT prediction.
- The proportion of elevation over 1500 m was included as a country-level covariate on EMR because of its significance in COPD cause of death models.

Step 2: GOLD class models to estimate proportions of severities
The GOLD class models use data from surveys that specified prevalence by GOLD class after expressing the values as a proportion of all COPD cases. We also restricted random effects to +/-0.5 to control implausible geographical variation.

Severity splits

The three GOLD class groupings reflect a grading based on a physiological measurement rather than a direct measurement of disease severity. To map the epidemiological findings by GOLD class into the three COPD health states for which we have disability weights (DW), we used the 2001–2011 Medical Expenditure Panel Survey (MEPS) data from the USA. Specifically, we convert the GOLD class designations estimated for the USA in 2005 (the midpoint of MEPS years of analyses) into GBD classifications of asymptomatic, mild, moderate, and severe COPD.

The table below shows the three health states of COPD and the corresponding lay descriptions and disability weights. The graph shows the average proportion by GOLD class (after scaling to 100%) across all ages for USA in 2005. We also show the proportion of MEPS respondents reporting any health service contact in the past year for COPD with a DW value attributable to COPD of 0, mild range (0 to midpoint between DWs for mild and moderate), moderate range (midpoint of DW values mild and moderate to midpoint of DW values for moderate and severe) and severe range (midpoint between DW values moderate and severe or higher). The DW value for COPD was derived from a regression with indicator variables for all health states reported by MEPS respondents and their reported overall level of disability derived from a conversion of 12-Item Short Form Surveys (SF-12) answers to GBD DW values. This

analysis gave the severity distribution for each GBD cause reported in MEPS after correcting for any comorbid causes individual respondents reported during a year.

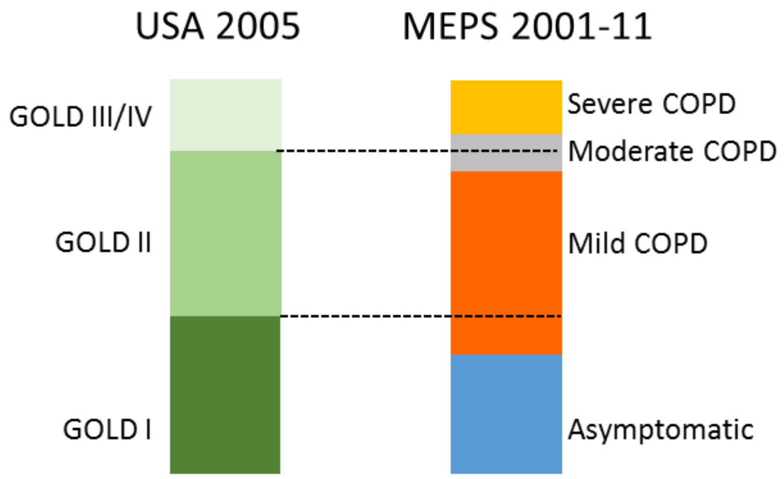

The algorithm to translate GOLD class to COPD DW categories first assigns GOLD III & IV to severe COPD and what remains to moderate. Next, GOLD class I is assigned to the asymptomatic category first and what remains goes to mild COPD. This algorithm is repeated for each age and sex category and for all 1000 draws from the DisMod models of GOLD classes and the MEPS analyses. We end up with proportions of each of the GOLD class categories that map onto GBD COPD health states with uncertainty bounds determined by the 25th and 975th values of the 1000 draws. These values are then applied to the estimates of the proportion of cases by GOLD class category, after scaling to 100%, by location, year, age, and sex. This assumes that the relationship between GOLD class and GBD COPD health states in the USA applies everywhere.

Table 3. Severity distribution, details on the severity levels for COPD and the associated disability weight (DW) with that severity.

Health state	Lay description	DW (95% CI)
Asymptomatic COPD	--	0.00
COPD and other chronic respiratory problems, mild	This person has cough and shortness of breath after heavy physical activity, but is able to walk long distances and climb stairs.	0.019 (0.011–0.033)
COPD and other chronic respiratory problems, moderate	This person has cough, wheezing, and shortness of breath, even after light physical activity. The person feels tired and can walk only short distances or climb only a few stairs.	0.225 (0.153–0.31)

COPD and other chronic respiratory problems, severe	This person has cough, wheezing, and shortness of breath all the time. The person has great difficulty walking even short distances or climbing any stairs, feels tired when at rest, and is anxious.	0.408 (0.273–0.556)

Table 4. Covariates. Summary of covariates used in the COPD DisMod-MR meta-regression model

Covariate	Type	Parameter	Exponentiated beta (95% uncertainty interval)
Log-transformed age-standardised SEV scalar: COPD	Country-level	Prevalence	2.47 (2.46–2.48)
Elevation over 1500 m (proportion)	Country-level	Excess mortality rate	1.14 (1.10–1.19)
Healthcare Access and Quality Index	Country-level	Excess mortality rate	0.98 (0.98–0.98)

Cirrhosis and other chronic liver diseases

Flowchart

Cirrhosis

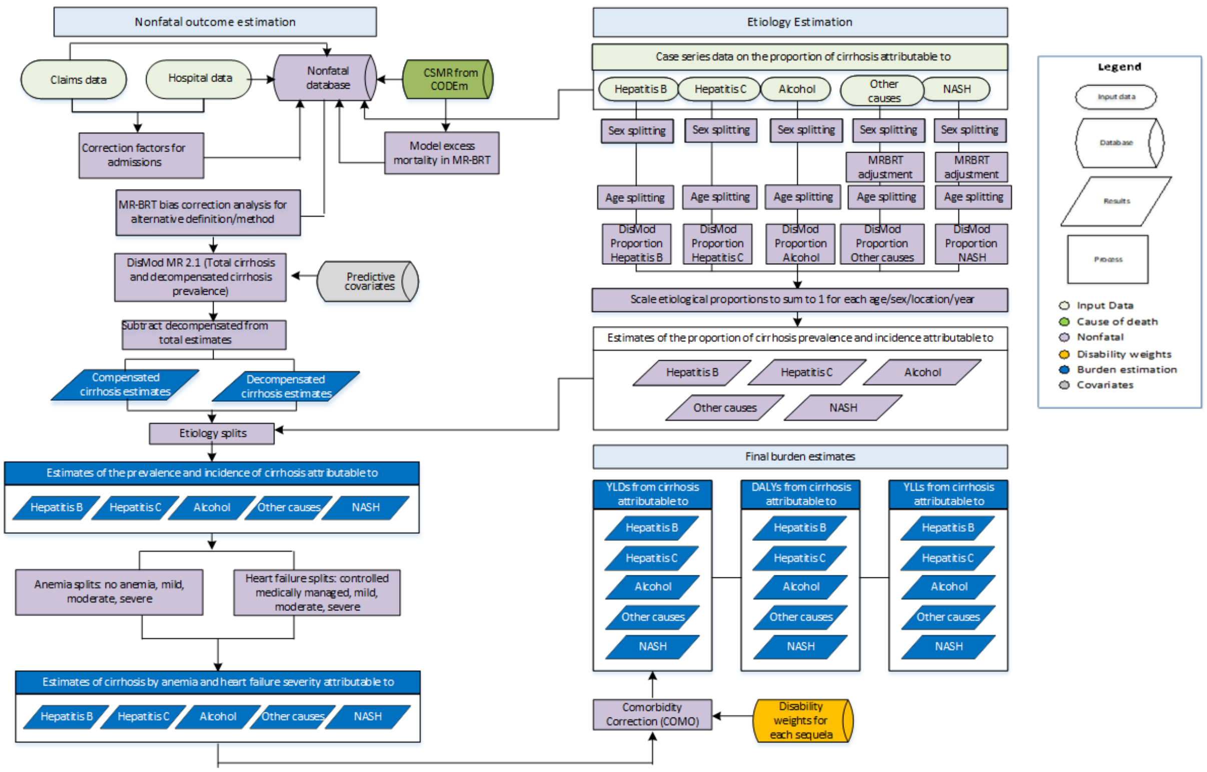

Input data and methodological summary for cirrhosis

Case definition

This cause encompasses both cirrhosis and a number of other chronic liver diseases that, left unchecked, may progress to cirrhosis. Cirrhosis is chronic, progressive replacement of healthy liver tissue by scarring, including cases where the liver is still able to functionally compensate for lost tissue, and decompensated cases, where liver function has become impaired, and complications develop (such as ascites, jaundice, gastrointestinal bleeding, renal failure, or encephalopathy). This cause also includes non-alcoholic fatty liver disease (without cirrhosis), chronic hepatitis B infection (without cirrhosis), and chronic hepatitis C infection (without cirrhosis).

This Level 3 cause includes five Level 4 causes:

- Cirrhosis and other chronic liver diseases due to alcohol use: cirrhosis (ie, scarring of liver) due to alcohol use, regardless of whether there is functional liver impairment and symptoms.
- Chronic hepatitis B including cirrhosis: Encompasses all chronic infection with hepatitis B virus, including cases that have developed cirrhosis (ie, scarring of the liver) and those that have not.
- Chronic hepatitis C including cirrhosis: Encompasses all chronic infection with hepatitis C virus, including cases that have developed cirrhosis (ie, scarring of the liver) and those that have not.

- Non-alcoholic fatty liver disease including cirrhosis: Encompasses all non-alcoholic fatty liver disease, including cases that have developed cirrhosis (ie, scarring of the liver) due to non-alcoholic steatohepatitis, and those that have not. (Non-alcoholic fatty liver disease and non-alcoholic steatohepatitis are older terms referring to progressive fat deposition with or without inflammation occurring in people with little or no alcohol consumption. This has been determined to result from metabolic derangements and to occur with or without concurrent alcohol consumption. The terms have been replaced by the terms metabolic-associated fatty liver disease and metabolic dysfunction-associated steatohepatitis, which will be employed in future rounds of GBD.)
- Cirrhosis and other chronic liver diseases due to other causes: Cirrhosis (ie, scarring of liver) due to other causes such as (but not limited to): Wilson's disease, PBC primary biliary cholangitis, hemochromatosis, and autoimmune disease, and cryptogenic cases, regardless of whether there is functional liver impairment and symptoms.

Input data

Input data for total cirrhosis and decompensated cirrhosis

As in previous rounds, in GBD 2023 we modelled the incidence and prevalence of total cirrhosis and of decompensated cirrhosis using data extracted from the IHME clinical administrative data library aggregated and processed for GBD as described in the "Clinical input data and methods summary" section of this appendix. Notably, in GBD 2023, this library of hospital discharges and claims expanded with additional years of data from USA claims (years 2018, 2019), Poland claims (year 2019), new sources in Mongolia claims (year 2019), and South Korea (years 2018, 2019). Additional years of hospital discharges were also included in GBD 2023 from the USA (HCUP years 2016–2019), Austria, Brazil, Chile, Georgia, Italy, New Zealand, and the Philippines, as well as the new sources of hospital discharges from Germany and Mexico and data from the US Medicare programme for age-groups 65–69 and older, (years 2000, 2010, 2014-16). The total cirrhosis model also included new administrative data from the Netherlands Nivel Primary Care Database General Practitioner Register (years 2011–2022).

Prior to GBD 2023, given the heterogeneity within the clinical administrative data for this cause, we systematically excluded data series with an age-standardised prevalence greater than either two or three median absolute deviations from the median of the age-standardised prevalence for all data. In GBD 2023, however, we explored this heterogeneity in greater detail and found that many extreme values were seen in inpatient data from sources that either do not cover the entire population or do not include a representative population sample, thus requiring extraction as discharge diagnosis cause fractions and conversion to prevalence using GBD estimates of hospital admission rate per capita. (See the section of this appendix on the hospital utilisation estimates for information on this input.) In GBD 2023, we decided to exclude all inpatient sources that require this adjustment and only include the sources with full coverage of the target population. Lastly, we excluded all USA MarketScan data from both total and decompensated cirrhosis models in GBD 2023. This decision was made because the ratio of individuals with cirrhosis seeking care in inpatient and outpatient settings differed significantly from the ratios observed in Poland's national claims data and USA Medicaid data, which cover the entire national population or primarily the elderly, respectively. This exclusion was made to account for potential selection bias associated with commercial insurance status.

Additional inputs to the non-fatal models of total and decompensated cirrhosis include cause-specific mortality rates (CSMR) produced for every year, age, sex, and location in the CoDCorrect process (please see the CoD cause-specific modelling description for cirrhosis in this appendix) and excess mortality rates (EMR) inputs generated using MR-BRT (see the EMR data processing section below).

Prevalence data processing for total cirrhosis and decompensated cirrhosis

Hospital discharge data provide observations about inpatient encounters, generally with only the primary diagnostic code for the encounter. Claims data, on the other hand, link claims for all inpatient and outpatient encounters for a single individual and provide primary and secondary diagnoses for all encounters.

The total cirrhosis model uses hospital discharge data adjusted to total cases diagnosed in inpatient and outpatient encounters, as well as claims data from Poland, Mongolia, and South Korea, where prevalent cases were identified directly from records for both inpatient and outpatient care. The decompensated model uses hospital discharge data adjusted only to account for readmissions and claims data from Poland and Mongolia where prevalent cases were identified only from inpatient records. (See sections of this appendix for details of hospital and claims data processing.)

A limitation of hospital discharge data is that individuals cannot be identified in the administrative records. As such, one person can have multiple hospital encounters for the same condition in a year, leading to overestimation of non-fatal burden. We resolved this issue using patient-level sources that do track individual hospital encounters for the same reason and correct for readmissions. Another concern is that hospital data do not reflect outpatient cases. We used Medicare data from the USA and national claims data from Poland, which contain both inpatient and outpatient data, to model ratios of inpatient primary admissions to total cases identified from inpatient and outpatient encounters using all encounter diagnoses, specific to sex, age group, and Healthcare Access and Quality Index, and then we applied these ratios to data sources that only reported inpatient hospital encounters with codes for primary diagnoses.

While the method of estimating the correction factor for deduplication of readmissions remained the same in GBD 2023 (the one used for decompensated cirrhosis), we refined the method of estimating the correction factors for outpatient encounters in GBD 2023 (the one used for total cirrhosis). Instead of using correction factor 3, which was a single model of the ratio of inpatient claims with a primary diagnosis to total prevalent cases seen in claims data with complete information on all diagnoses in all encounter types, we now apply correction factors 1 and 5 (CF1 and CF5). CF1 is defined as the ratio of deduplicated primary diagnoses among inpatient admissions to adjusted primary diagnoses among inpatient admissions, while CF5 is defined as the ratio of deduplicated primary and secondary diagnoses among inpatient encounters and outpatient encounters to deduplicated primary diagnoses among inpatient encounters. Like the previous approach, the approach this year accounts for non-primary inpatient diagnoses and outpatient cases, but does so serially, to allow data sources to contribute to estimation of de-duplication for readmission, multiple diagnosis in inpatient encounters, and/or outpatient care, depending on which of these relationships are captured in the dataset, rather than only employing sources that have complete information on all diagnoses and encounter types. This approach makes use of more diverse data inputs. This final scalar is applied to extracted primary diagnosis admission rate data as a product of CF1 and 1/CF5. See the section of this appendix on the processing of hospital data for more details.

Input data for cirrhosis aetiological proportions

To assign cirrhosis cases to specific aetiologies, we use data from cirrhosis case-series that report the proportion of cirrhosis cases attributed to alcohol, hepatitis B, hepatitis C, NASH, and other causes. A systematic review for these case-series data was conducted for GBD 2013 and updated with collaborator contributions in subsequent rounds. In GBD 2021, 11 new case-series studies were added from an ongoing literature review in PubMed using the search string below. Given time limitations, we expedited the search by looking for results that reported the terms "cirrhosis" and "cases" from the search hits. Studies that did not have these terms in the title/abstract are currently being screened for inclusion in the next round of GBD.

(((((((hepatitis b[Title/Abstract] OR "hepatitis b antibod*"[Title/Abstract] OR "hepatitis b antigens"[Title/Abstract] OR hbsag[Title/Abstract])) OR (hepatitis c[Title/Abstract] OR "hepatitis c antibod*"[Title/Abstract] OR "hepatitis c antigens"[Title/Abstract] OR "anti-hcv"[Title/Abstract] OR HCV-RNA[Title/Abstract]))))) AND (alcohol* OR "alcoholic disorders" OR cirrhosis))) AND (NAFLD OR "non-alcoholic fatty liver disease" OR NAFL)

The inclusion criteria for case-series data stipulated that:

- The publication year must be from 1980 onward.
- Sufficient information must be provided on study methods and sample characteristics to assess the quality of the study.
- The sample had to be a representative sample of those with decompensated cirrhosis without HCC, compensated cirrhosis without HCC, deaths due to cirrhosis without HCC (reference standard populations)*.
  ** Note: We included case-series that reported aetiologies for case-series in various stages of disease in our inclusion criteria but will disaggregate proportions in future rounds to identify variation in aetiologies by stage*.
- The cirrhosis cases should be identified by admin data, chart review, non-invasive test, liver biopsy, or other defined diagnostic criteria for which a valid adjustment can be made.
- The diagnosis of the aetiology can include the following reference definitions or an alternative definition that could produce a crosswalk.
  - Hepatitis B: confirmed via HBsAg
  - Hepatitis C: confirmed via anti-HCV OR HCV-RNA
  - Alcohol: reliable history of significant alcohol intake, clinical examination and laboratory features suggestive of significant alcohol intake
  - NAFLD: intake of less than 20 g of ethanol per day AND appropriate exclusion of other liver diseases

The exclusion criteria were as follows:

- If a study evaluated only a subset of aetiologies, the study must have exhaustively categorised all cirrhotic persons in the sample into a specific aetiology or an "other" or "unknown" category, but cannot have excluded cirrhotic persons from the denominator for not having one of the aetiologies under study.

- If the study reported on "multiple aetiologies" without specifying which aetiologies were concurrently present, the study can be included but those cases should be removed from both the numerator and the denominator in study extraction.
- Administrative records with no report on methods used to determine aetiology of cirrhosis were excluded.

Figure 1: Aetiological proportion data sources

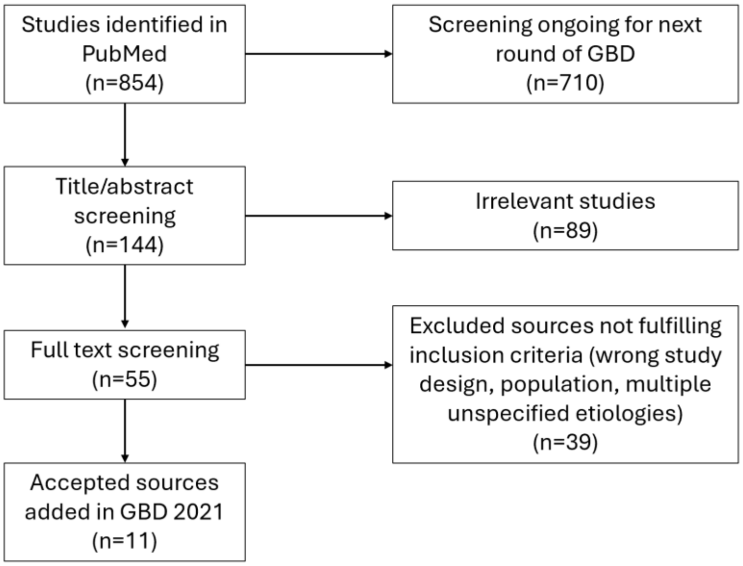

Input data counts

Details on source counts for clinical informatics data, including those used for total cirrhosis and decompensated cirrhosis models, are provided elsewhere in the methods appendix. The source counts used for cirrhosis aetiological proportion models are presented below.

Table 1: Data inputs for cirrhosis aetiological proportion modelling

Model	Measure	Total sources	Countries with data	Number of regions	Number of super-regions
Cirrhosis and other chronic liver diseases due to hepatitis B, proportion	Proportion	99	40	18	7
Cirrhosis and other chronic liver diseases due to hepatitis C, proportion	Proportion	98	40	17	7
Cirrhosis and other chronic liver diseases due to alcohol, proportion	Proportion	64	27	13	6
Proportion of cirrhosis due to other causes	Proportion	44	23	13	7
Proportion of cirrhosis due to NASH	Proportion	35	19	10	5

EMR input processing

In GBD 2017, EMR inputs were produced by matching prevalence datapoints with their corresponding CSMR values within the same age, sex, year, and location (by dividing CSMR by prevalence). For short-duration conditions (remission >1), the corresponding prevalence was derived by running an initial model and then applying the same CSMR/prevalence method. However, this method of producing EMR inputs demonstrated a rather unrealistic pattern of EMR compared to an expected pattern of decreasing EMR with greater access to quality health care. Such unexpected patterns often signal inconsistencies between CSMR estimates and the measures of prevalence and/or incidence. Thus, in an effort to provide greater guidance on the expected pattern of EMR, in GBD 2019, EMR data produced per above in GBD 2017 were modelled by age, sex, and Healthcare Access and Quality (HAQ) Index using MR-BRT, with a prior on HAQ Index having a negative coefficient. In GBD 2021, we employed the same MR-BRT method to predict EMR for each location, year, sex, and for ages 0, 10, 20….100; these predictions were used as inputs to our non-fatal model, below. The GBD 2021 method was also employed in GBD 2023.

Aetiological proportion input processing

Prior to modelling, we performed several adjustments to case-series data sources to correct for non-reference data collection methods, including "multi-aetiology splitting", "sex-splitting", and "age-splitting".

Some studies reported cases in which multiple risk factors of cirrhosis were identified. However, we do not have enough data on multiple aetiology data to estimate combinations of aetologies in distinct models. Instead, we reassigned these multi-aetiology cases to single aetiologies prior to modelling the five distinct aetologies. For this reassignment, we used proportions of single aetiologies observed in the same study. For example, if a study might reported 100 cases of cirrhosis total, of which 40 cases were due to hepatitis B, 20 due to alcohol, 30 due to NAFLD, and ten due to hepatitis B and alcohol, we would redistribute cases due to both hepatitis B and alcohol proportionately to cases of each aetiology. That is to say we would redistribute the ten cases of hepatitis B and alcohol by a ratio of 40:20, resulting in 47 cases of hepatitis B and 23 cases due to alcohol. The figure below shows the proportion of each aetiology before and after adjustment of multi-aetiology cases to single aetiologies.

Figure 2: Pre- and post-adjustment of multi-aetiology cases in a hypothetical case-series example

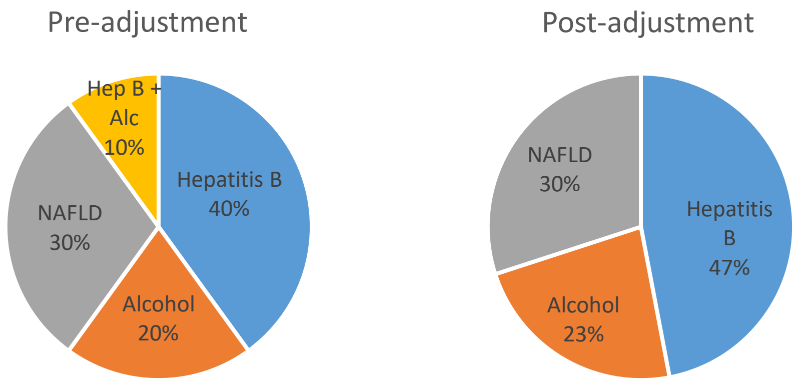

Because we produce sex-specific estimates, we adjusted data that reported on both sexes into male and female sex-specific estimates. We identified studies that reported proportions of cases due to specific aetiologies in a sex-specific fashion, and calculated the log ratios of the proportion due to a specific aetiology in females to the proportion due to the same aetiology in males, and we modelled these log ratios in MR-BRT. We then used the modelled sex-ratio to adjust "both"-sex data values to expected "male" and "female" values. We calculated the male values as:

$$val_{male} = val_{both} * \frac{pop_{both}}{(pop_{male} + ratio * pop_{female})}.$$

We calculated female values as:

$$val_{female} = ratio \cdot val_{male}.$$

The table below lists the estimated ratios of proportion of cases due to a given aetiology among females vs. the proportion of cases due to that aetiology in males.

Table 3: MR-BRT sex ratios for cirrhosis aetiology proportions

Cirrhosis etiology proportion	Beta coefficient, log (95% CI)*	Gamma	Interpretation
Cirrhosis due to hepatitis B	–0.26 (–0.39 to –0.13)	0.018	Higher for males
Cirrhosis due to hepatitis C	0.14 (–0.04 to 0.33)	0.089	Higher for females
Cirrhosis due to alcohol	–1.02 (–1.42 to –0.62)	0.312	Higher for males
Cirrhosis due to other causes	0.642 (0.41 to 0.87)	0.063	Higher for females
Cirrhosis due to NASH	0.80 (0.65 to 0.95)	0.005	Higher for females

**MR-BRT crosswalk adjustments can be interpreted as the factor the alternative case definition is adjusted by to reflect what it would have been had it been measured using the reference case definition. If the log/logit beta coefficient is negative, then the alternative is adjusted up to the reference. If the log/logit beta coefficient is positive, then the alternative is adjusted down to the reference.*

**MR-BRT adjustments can be interpreted as the factor the alternative case definition is adjusted by to reflect what it would have been had it been measured using the reference case definition. If the log/logit beta coefficient is negative, then the alternative is adjusted up to the reference. If the log/logit beta coefficient is positive, then the alternative is adjusted down to the reference.*

After extraction and sex-splitting, we then adjusted aetiological proportion data to account for the fact that older case-series data did not separately report the proportion of cirrhosis cases due to NASH. Epidemiological studies and hepatologists have indicated that cases labeled as "cryptogenic" in older studies may be un-identified cases of cirrhosis due to NASH. In GBD 2017, if case-series studies reported the proportion of cases that were "cryptogenic" without reporting a proportion due to NASH, they were all treated as cases of NASH. In GBD 2019, GBD 2021, and GBD 2023, however, we analysed case-series studies that reported both NASH and cryptogenic cases, modelling the proportion due to NASH out of the sum of NASH plus cryptogenic in MR-BRT. We then identified the case-series in our database that reported cryptogenic, but not NASH, as an aetiology of cirrhosis, and applied the modelled results to extract a proportion due to NASH and a proportion due to other causes.

Table 4: Cryptogenic-NASH adjustment factor in MR-BRT

Data input	Beta coefficient, logit (95% CI)	Gamma
Proportion of cryptogenic cases out of cryptogenic cases plus NASH cases reported in the same study	0.465 (0.231–0.698)	0.111

Data inputs for estimating the incidence and prevalence of chronic hepatitis B infection, the incidence and prevalence of chronic hepatitis C infection, and the incidence and prevalence of non-alcoholic fatty liver disease are described in the sections titled "Acute Hepatitis A, B, C and E" and "Nonalcoholic fatty liver disease without cirrhosis" of the "Non-fatal cause-specific modelling descriptions" section of this appendix.

Modelling strategy

We modelled the prevalence and incidence of total cirrhosis and the prevalence and incidence of decompensated cirrhosis using clinical informatics prevalence data, CSMR and EMR, processed as described above, assuming no remission, in full compartmental models in DisMod-MR 2.1. The summary of covariates and the exponentiated betas of the total cirrhosis and decompensated cirrhosis DisMod-MR 2.1 models are listed in Tables 5 and 6 below.

To estimate the prevalence of cirrhosis due to alcohol, hepatitis B, hepatitis C, NASH, and other causes, we developed five single-parameter models of five aetiological proportions using DisMod-MR 2.1 and used the results of these models to split the parent total cirrhosis and decompensated cirrhosis prevalence estimates.

Aetological proportion DisMod models

Data for aetiological proportion models are scant, and estimates are strengthened by using predictive covariates. As in previous rounds, DisMod models for the proportion of cirrhosis due to each aetiology

use the following predictive covariates: the prevalence of the precursor states that can give rise to each aetiology of cirrhosis (prevalence of hepatitis B, hepatitis C, alcohol consumption, etc.) and the most recent estimate of the proportion of liver cancer cases due to each aetiology, all with bounds limiting to positive associations. (See liver cancer appendix section for details on estimation of aetiological proportions for liver cancer.) The summary of covariates and the exponentiated betas of each aetiological proportion model are listed in tables below (Tables 7 to 11).

Table 5. Covariates. Summary of covariates used in the total cirrhosis DisMod-MR 2.1 model

Covariate	Parameter	Exponentiated beta (95% uncertainty interval)
Vaccine-adjusted HBsAg seroprevalence, age-standardised	Prevalence	51.49 (44.35–55.49)
Chronic hepatitis C, age-standardised	Prevalence	7.69 (1.69–34.64)
Alcohol drinker proportion, age-standardised	Prevalence	2.31 (2.11–2.57)
Litres of alcohol consumed per capita	Prevalence	1.00 (1.00–1.01)
Prevalence of obesity	Prevalence	1.01 (1.00–1.02)
Healthcare Access and Quality Index	Excess mortality rate	0.99 (0.99–0.99)

Table 6. Covariates. Summary of covariates used in the decompensated cirrhosis DisMod-MR 2.1 model

Covariate	Parameter	Exponentiated beta (95% uncertainty interval)
Vaccine-adjusted HBsAg seroprevalence, age-standardised	Prevalence	51.54 (44.84–54.49)
Chronic hepatitis C, age-standardised	Prevalence	1.42 (1.02–2.48)
Litres of alcohol consumed per capita	Prevalence	1.00 (1.00–1.00)
Alcohol drinker proportion, age-standardised	Prevalence	1.81 (1.65–1.99)
Prevalence of obesity	Prevalence	1.01 (1.00–1.12)
Healthcare Access and Quality Index	Excess mortality rate	0.99 (0.99–0.99)

Table 7. Covariates. Covariates used in the proportion of cirrhosis due to hepatitis B DisMod-MR meta-regression model

Covariate	Exponentiated beta (95% uncertainty interval)
Vaccine-adjusted HBsAg seroprevalence, age-standardised	1.64 (1.06–2.57)
Proportion of liver cancer due to hepatitis B, age-standardised	1.28 (1.02–1.72)
Hepatitis B vaccine coverage (proportion), aged through time	0.55 (0.38–0.88)
Proportion of cirrhosis due to alcohol	0.46 (0.37–0.65)
Proportion of cirrhosis due to hepatitis C	0.65 (0.45–0.93)
Proportion of cirrhosis due to other causes	0.68 (0.47–0.94)
Proportion of cirrhosis due to NASH	0.59 (0.39–0.94)

Table 8. Covariates. Covariates used in the proportion of cirrhosis due to hepatitis C DisMod-MR meta-regression model

Covariate	Exponentiated beta (95% uncertainty interval)
Chronic hepatitis C, age-standardised	1.79 (1.10–2.63)
Proportion of liver cancer due to hepatitis C, age-standardised	1.86 (1.19–2.63)
Proportion of cirrhosis due to alcohol	0.41 (0.37–0.50)
Proportion of cirrhosis due to hepatitis B	0.53 (0.38–0.80)
Proportion of cirrhosis due to other causes	0.94 (0.82–1.00)
Proportion of cirrhosis due to NASH	0.63 (0.41–0.94)

Table 9. Covariates. Covariates used in the proportion of cirrhosis due to alcohol DisMod-MR meta-regression model

Covariate	Exponentiated beta (95% uncertainty interval)
Litres of alcohol consumed per capita	1.01 (1.00–1.03)
Alcohol drinker proportion, age-standardised	1.58 (1.14–2.19)
Proportion of liver cancer due to alcohol, age-standardised	1.39 (1.02–2.17)

Table 10. Covariates. Covariates used in the proportion of cirrhosis due to other causes DisMod-MR meta-regression model

Covariate	Exponentiated beta (95% uncertainty interval)
Proportion of liver cancer due to other causes, age-standardised	1.91 (1.22–2.64)

Table 11. Covariates. Covariates used in the proportion of cirrhosis due to NASH DisMod-MR meta-regression model

Covariate	Exponentiated beta (95% uncertainty interval)
Mean BMI	1.27 (1.06–1.54)
Prevalence of obesity	2.19 (1.06–5.45)
NAFLD/NASH prevalence	3.22 (1.27–6.78)
Proportion of liver cancer due to NASH, age-standardised	2.30 (1.07–5.75)

Compensated cirrhosis prevalence estimation

Final decompensated cirrhosis prevalence estimates at the 1000-draw level were subtracted from the final total cirrhosis prevalence estimates at the 1000-draw level to generate 1000 draws of compensated cirrhosis prevalence estimates (which provides an estimated mean with 95% uncertainty interval).

Aetiology-specific cirrhosis prevalence estimation

We used the five aetiological proportion estimates to split the compensated and decompensated cirrhosis prevalence estimates. Proportions were rescaled to sum to one at the draw level and then multiplied by the estimates of the prevalence of decompensated cirrhosis and compensated cirrhosis.

Cause-level incidence estimation

In GBD, we consider cirrhosis to develop through one of five aetiological pathways: heavy alcohol use, chronic infection with hepatitis B or C, non-alcoholic steatohepatitis, and a residual category of multiple other causes. In order to develop cirrhosis, we assume that people must first have been at risk of developing cirrhosis through one of these five pathways. Nonetheless, the cirrhosis and other chronic liver disease estimates variably include precursor states depending on aetiology (see below), and incidence estimates reported in GBD reflect the incidence of the earliest stage of chronic liver disease estimated for that aetiology.

The incidence estimates for cirrhosis and chronic liver disease corresponding to each aetiology of cirrhosis were, therefore, calculated as follows:

- For alcohol use and other causes, cause-level incidence estimates are estimates of the incidence of compensated cirrhosis due to alcohol use and compensated cirrhosis due to other causes. Since all cases of cirrhosis must start as compensated and progress to decompensated, the incidence estimates from the DisMod-MR 2.1 compartmental model of total cirrhosis were treated as the incidence of compensated cirrhosis, and these were multiplied by the aetiological proportions for alcohol and for other causes at the draw level to estimate incidence of compensated cirrhosis due to alcohol use and compensated cirrhosis due to other causes.

- For cirrhosis and other chronic liver diseases due to chronic infection with hepatitis B, cause-level incidence estimates are estimates of the incidence of chronic hepatitis B infection (see “Acute Hepatitis A, B, C, and E” section of this appendix).

- For cirrhosis and other chronic liver diseases due to chronic infection with hepatitis C, cause-level incidence estimates are estimates of the incidence of chronic hepatitis C infection (see “Acute Hepatitis A, B, C, and E” section of this appendix).

- For cirrhosis and other chronic liver diseases due to non-alcoholic steatohepatitis, cause-level incidence estimates are the non-alcoholic fatty liver incidence estimates (see “Non-alcoholic fatty liver disease without cirrhosis” section of this appendix).

Sequelae and disability weights

We estimated the proportion of individuals with decompensated cirrhosis that had different severity levels of anaemia: no anaemia, mild anaemia, moderate anaemia, and severe anaemia. After estimation of decompensated cirrhosis due to each aetiology, we further split estimates to reflect these anaemia severity proportions. See the “Anaemia impairment (envelope and causal attribution)” section of this appendix for details. We also estimated the proportion of heart failure that was attributable to decompensated cirrhosis. The different severity levels of heart failure include controlled and medically

managed, mild, moderate, and severe. See the “Heart failure estimation” section of this appendix for details. Decompensated cirrhosis with and without anaemia and heart failure were assigned the following health states and disability weights.

Table 13: Disability weights for decompensated cirrhosis

Health state	Lay description	Disability weight (95% CI)
Decompensated cirrhosis of the liver	Has a swollen belly and swollen legs. The person feels weakness, fatigue and loss of appetite.	0.178 (0.113–0.243)
Decompensated cirrhosis of the liver and mild anaemia	Feels slightly tired and weak at times, but this does not interfere with normal daily activities.	0.181 (0.116–0.246)
Decompensated cirrhosis of the liver and moderate anaemia	Feels moderate fatigue, weakness, and shortness of breath after exercise, making daily activities more difficult.	0.220 (0.146–0.295)
Decompensated cirrhosis of the liver and severe anaemia	Feels very weak, tired and short of breath, and has problems with activities that require physical effort or deep concentration.	0.300 (0.202–0.397)
Decompensated cirrhosis of the liver, controlled, medically managed heart failure	Has been diagnosed with clinical heart failure, a chronic disease that requires medication every day and causes some worry but minimal interference with daily activities.	0.218 (0.154–0.298)
Decompensated cirrhosis of the liver, mild heart failure	Is short of breath and easily tires with moderate physical activity, such as walking uphill or more than a quarter-mile on level ground. The person feels comfortable at rest or during activities requiring less effort.	0.212 (0.150–0.290)
Decompensated cirrhosis of the liver, moderate heart failure	Is short of breath and easily tires with minimal physical activity, such as walking only a short distance. The person feels comfortable at rest but avoids moderate activity.	0.237 (0.167–0.320)
Decompensated cirrhosis of the liver, severe heart failure	Is short of breath and feels tired when at rest. The person avoids any physical activity, for fear of worsening the breathing problems.	0.324 (0.233–0.436)

Dividing the above symptomatic cirrhosis outcomes by aetiology, and combining with asymptomatic states, the total set of sequelae included in non-fatal estimation of cirrhosis and other chronic liver diseases are as shown in the table below.

Table 14: Comprehensive sequelae for non-fatal estimation of cirrhosis and other chronic liver diseases

Level	Cause name

Level 3	Cirrhosis and other chronic liver diseases
Level 4	Cirrhosis and other chronic liver diseases **due to alcohol***<br>- Compensated cirrhosis due to alcohol<br>- Decompensated cirrhosis due to alcohol (*3 levels of anaemia, 4 levels of heart failure, neither*)<br>- Decompensated cirrhosis due to alcohol, without anaemia or heart failure<br>- Decompensated cirrhosis due to alcohol, with mild anaemia<br>- Decompensated cirrhosis due to alcohol, with moderate anaemia<br>- Decompensated cirrhosis due to alcohol, with severe anaemia<br>- Decompensated cirrhosis due to alcohol, with medically managed heart failure<br>- Decompensated cirrhosis due to alcohol, with mild heart failure<br>- Decompensated cirrhosis due to alcohol, with moderate heart failure<br>- Decompensated cirrhosis due to alcohol, with severe heart failure
Level 4	Chronic **hepatitis B** including cirrhosis******<br>- Compensated cirrhosis due to hepatitis B<br>- Decompensated cirrhosis due to hepatitis B (*3 levels of anaemia, 4 levels of heart failure, neither*)<br>- Decompensated cirrhosis due to hepatitis B, without anaemia or heart failure<br>- Decompensated cirrhosis due to hepatitis B, with mild anaemia<br>- Decompensated cirrhosis due to hepatitis B, with moderate anaemia<br>- Decompensated cirrhosis due to hepatitis B, with severe anaemia<br>- Decompensated cirrhosis due to hepatitis B, with medically managed heart failure<br>- Decompensated cirrhosis due to hepatitis B, with mild heart failure<br>- Decompensated cirrhosis due to hepatitis B, with moderate heart failure<br>- Decompensated cirrhosis due to hepatitis B, with severe heart failure<br>- Chronic hepatitis B without cirrhosis
Level 4	Chronic **hepatitis C** including cirrhosis******<br>- Compensated cirrhosis due to hepatitis C<br>- Decompensated cirrhosis due to hepatitis C (*3 levels of anaemia, 4 levels of heart failure, neither*)<br>- Decompensated cirrhosis due to hepatitis C, without anaemia or heart failure<br>- Decompensated cirrhosis due to hepatitis C, with mild anaemia<br>- Decompensated cirrhosis due to hepatitis C, with moderate anaemia<br>- Decompensated cirrhosis due to hepatitis C, with severe anaemia<br>- Decompensated cirrhosis due to hepatitis C, with medically managed heart failure<br>- Decompensated cirrhosis due to hepatitis C, with mild heart failure<br>- Decompensated cirrhosis due to hepatitis C, with moderate heart failure<br>- Decompensated cirrhosis due to hepatitis C, with severe heart failure<br>- Chronic hepatitis C without cirrhosis
Level 4	**Non-alcoholic fatty liver disease** including cirrhosis******<br>- Compensated cirrhosis due to NASH<br>- Decompensated cirrhosis due to NASH (*3 levels of anaemia, 4 levels of heart failure, neither*)<br>- Decompensated cirrhosis due to NASH, without anaemia or heart failure<br>- Decompensated cirrhosis due to NASH, with mild anaemia<br>- Decompensated cirrhosis due to NASH, with moderate anaemia<br>- Decompensated cirrhosis due to NASH, with severe anaemia<br>- Decompensated cirrhosis due to NASH, with medically managed heart failure<br>- Decompensated cirrhosis due to NASH, with mild heart failure<br>- Decompensated cirrhosis due to NASH, with moderate heart failure<br>- Decompensated cirrhosis due to NASH, with severe heart failure<br>- NAFL/NASH (without cirrhosis)

Level 4	Cirrhosis and other chronic liver diseases **due to other causes***<br>- Compensated cirrhosis due to other causes<br>- Decompensated cirrhosis due to other causes (*3 levels of anaemia, 4 levels of heart failure, neither*)<br>- Decompensated cirrhosis due to other causes, without anaemia or heart failure<br>- Decompensated cirrhosis due to other causes, with mild anaemia<br>- Decompensated cirrhosis due to other causes, with moderate anaemia<br>- Decompensated cirrhosis due to other causes, with severe anaemia<br>- Decompensated cirrhosis due to other causes, with medically managed heart failure<br>- Decompensated cirrhosis due to other causes, with mild heart failure<br>- Decompensated cirrhosis due to other causes, with moderate heart failure<br>- Decompensated cirrhosis due to other causes, with severe heart failure

*Because these causes do not include estimates of pre-cirrhotic precursor states, they represent the prevalence and incidence of cirrhosis due to alcohol and cirrhosis due to other causes, respectively.
**Because these causes include estimates of pre-cirrhotic precursor states, they represent the prevalence and incidence of chronic hepatitis B infection, chronic hepatitis C infection, and NALFD, respectively, including those who have developed cirrhosis and those who have not.

Cocaine use disorders

Flowchart

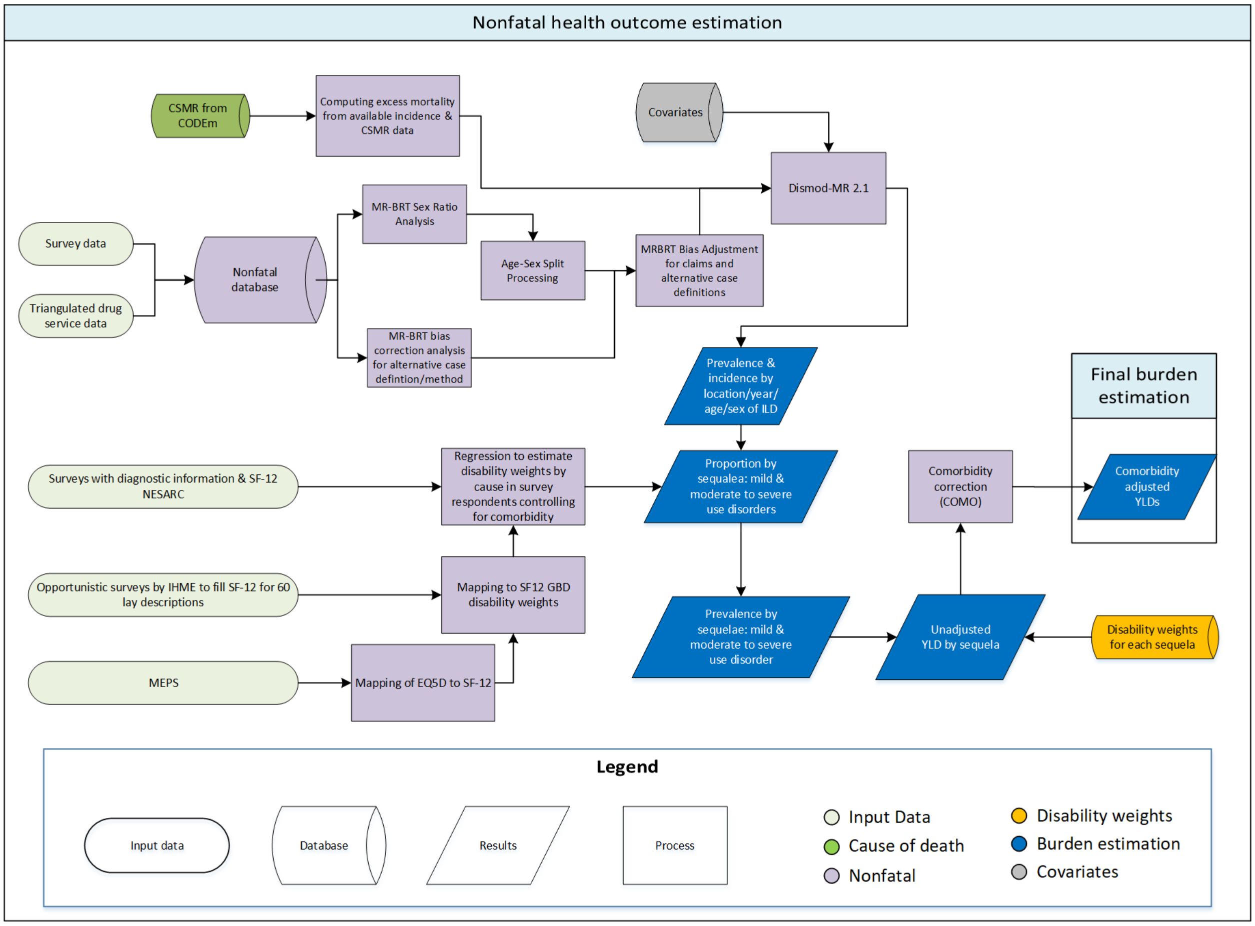

Input data and methodological summary for cocaine use disorders

Case definition

Cocaine dependence is a substance-related disorder involving a dysfunctional pattern of cocaine use. Included in the Global Burden of Disease (GBD) disease modelling were cases meeting the Diagnostic and Statistical Manual of Mental Disorders (DSM-IV-TR) or the International Classification of Diseases (ICD-10) diagnostic criteria for cocaine dependence (DSM: 304.20; ICD: F14.2), excluding those cases due to a general medical condition.[1,2] According to DSM-IV TR criteria, dependence involves a maladaptive pattern of substance use leading to clinically significant impairment or distress.

Quantity of interest	Reference or alternative	Definition
Cocaine dependence prevalence	Reference	Cocaine dependence by “indirect” method, ie, using a multiplier from treatment data and surveys among drug users on prevalence of having accessed treatment over a recall period (mostly one year).
Cocaine dependence prevalence	Alternative	A chronic condition in which a person craves cocaine and is unable to control taking the drug. The person needs greater amounts to get the same effect and has withdrawal symptoms after stopping
Cocaine dependence prevalence	Alternative	Cocaine dependence according to ICD or DSM criteria measured in population survey
Cocaine dependence prevalence	Alternative	Regular (weekly) use of cocaine as measured in population surveys
Cocaine dependence prevalence	Alternative	Cocaine dependence, 1 year recall

Input data

For the GBD 2023 round, there were no significant updates to the input data. The systematic review process for GBD 2010 involved a thorough search for studies on the prevalence, incidence, remission, and excess mortality associated with cocaine dependence. This process included searches of peer-reviewed literature (via Medline, Embase, and PubMed), grey literature, and expert consultations, adopting a rolling basis approach for electronic database searches for mental and substance use disorders. The methodology from GBD 2010 was replicated for GBD 2013 to include new data published up to 2013. For GBD 2015, the second and third stages of the literature review were updated, and for GBD 2016, a search of peer-reviewed databases (Medline, Embase, and PsycINFO) was conducted to include studies from 2013 to 2016. GBD 2017 expanded the search to include additional sources

identified by GBD experts and microdata where available. It also featured two targeted systematic reviews: one focusing on Maōri versus non-Maori populations in New Zealand and another using the China National Knowledge Infrastructure database to capture studies not typically included in the major databases. Methods used for this systematic review have been reported in greater detail elsewhere.[3,4]

Age and sex splitting

Reported estimates of prevalence were split by age and sex where possible. First, if studies reported prevalence for broad age groups by sex (eg, prevalence in 15- to 65-year-old males and females separately), and also by specific age groups for both sexes combined (eg, prevalence in 15- to 30-year-olds, then in 31- to 65-year-olds, for males and females combined), age-specific estimates were split by sex using the reported sex ratio and bounds of uncertainty. Second, prevalence data for both sexes that could not be split using a within-study ratio were split using a sex ratio derived from a meta-analysis of existing sex-specific data using the meta-regression with Bayesian priors, regularization, and trimming (MR-BRT) tool. Details on MR-BRT can be found in appendix 1 section 2.

The female to male ratio was 0.50 (0.39–0.66) for ages 20 and above, and 0.68 (0.51–0.89) for ages below 20. Finally, after the application of bias adjustments, where studies reported estimates across age groups spanning 25 years or more, these were split into five-year age groups using the super-region-specific prevalence age pattern estimated by our disease model—Bayesian meta-regression tool (DisMod-MR 2.1) on all data prior to age-splitting. Information on DisMod-MR 2.1 can be found in appendix 1 section 2.

Data adjustment

Due to insufficient data in the optimal case definition of cocaine dependence, the prevalence dataset included datapoints of both use and dependence estimated using “direct” or “indirect” survey methods. “Direct” methods of measuring cocaine dependence predominantly involve surveys of the general population that ask if respondents use or are dependent on cocaine. Surveys tend to underestimate the prevalence of the most harmful and stigmatised forms of illicit drug use in ways that probably vary between countries and cultures.[5] “Indirect” methods are considered superior; they use different sources of data to indirectly estimate the total number of drug users (methods include “multiplier methods,” back-projection, and capture-recapture methods). Due to the lack of data available on cocaine dependence from indirect survey methods (considered to be the gold standard for GBD purposes), estimates of use and/or estimates from direct survey methods were also included in the modelling. We marked studies reporting on the prevalence of cocaine dependence obtained via direct methods as well as those reporting on the prevalence of cocaine use obtained via direct methods and derived adjustment factors using MR-BRT. Due to limited overlapping data and roughly similar patterns of use, we combined amphetamine and cocaine data to derive a single adjustment factor. Betas coefficients, in logit space are shown in the table below:

Table 1: MR-BRT crosswalk adjustment factors for cocaine and amphetamine use disorders

Data input	Reference or alternative case definition	Gamma	Beta coefficient, logit (95% UI)*	Adjustment factor**
Cocaine dependence – indirect method	Ref	0.62	---	
Cocaine use – indirect method	Alt		1.07 (–0.11 to 2.35)	2.92
Cocaine dependence – direct method	Alt		–0.54 (–1.73 to 0.76)	0.58
Cocaine use – direct method	Alt		0.54 (–0.65 to 1.81)	1.72

**MR-BRT crosswalk adjustments can be interpreted as the factor the alternative case definition is adjusted by to reflect what it would have been had it been measured using the reference case definition. If the log/logit beta coefficient is negative, then the alternative is adjusted up to the reference. If the log/logit beta coefficient is positive, then the alternative is adjusted down to the reference.*

***The adjustment factor column is the exponentiated beta coefficient. For log beta coefficients, this is the relative rate between the two case definitions. For logit beta coefficients, this is the relative odds between the two case definitions.*

Subsequently, we adjusted for recall period to adjust from one-year recall to point prevalence, again using combined cocaine and amphetamine data. Beta coefficients from MR-BRT are shown in the table below:

Table 2: MR-BRT crosswalk adjustment factors for cocaine and amphetamine use disorders

Data input	Reference or alternative case definition	Gamma	Beta coefficient, logit (95% UI)*	Adjustment factor**
Cocaine dependence point prevalence	Ref	0	---	
Cocaine dependence 1-year recall	Alt		0.71 (0.63–0.79)	2.03

**MR-BRT crosswalk adjustments can be interpreted as the factor the alternative case definition is adjusted by to reflect what it would have been had it been measured using the reference case definition. If the log/logit beta coefficient is negative, then the alternative is adjusted up to the reference. If the log/logit beta coefficient is positive, then the alternative is adjusted down to the reference.*

***The adjustment factor column is the exponentiated beta coefficient. For log beta coefficients, this is the relative rate between the two case definitions. For logit beta coefficients, this is the relative odds between the two case definitions.*

Modelling strategy

We have made no substantive changes in the modelling strategy from GBD 2021. Prior settings in DisMod-MR 2.1 included assuming no incidence, remission, and excess mortality before age 15, and an upper limit of 0.2 on remission. The minimum age of onset was corroborated with expert feedback and existing literature from various sources including the European Monitoring Centre for Drugs and Drug Addiction.[6] These settings were retained for GBD 2023.

Severity and disability

The basis of the GBD disability weight survey assessments are lay descriptions of sequelae highlighting major functional consequences and symptoms. The lay descriptions and disability weights for cocaine dependence severity levels are shown below.

The proportion of people with cocaine dependence within each of the severity levels were determined based on available data from US National Epidemiological Survey on Alcohol and Related Conditions (NESARC), conducted in two waves from 2001 to 2002 and 2004 to 2005.[7] NESARC is a direct household survey. As such, it is expected to underestimate moderate to severe cases of drug dependence. The estimated distribution of cocaine dependent cases by severity were asymptomatic (50%, 37–64), mild (25%, 18–33), and moderate/severe (25%, 17–33).

Table 3. Severity distribution, details on the severity levels for cocaine use disorders in GBD 2023 and the associated disability weight (DW) with that severity.

Severity level	Lay description	DW (95% CI)
Mild	Uses cocaine at least once a week and has some difficulty controlling the habit. When not using it, the person functions normally.	0.116 (0.074–0.165)
Moderate to severe	Uses cocaine and has difficulty controlling the habit. The person sometimes has mood swings, anxiety, paranoia, hallucinations, and sleep problems, and has some difficulty in daily activities.	0.479 (0.324–0.634)

**Asymptomatic cases carried no disability weight.*

As in GBD 2021, lag-distributed income (LDI) was included as a country covariate on EMR with bounds set at –0.5 and –0.1.

Table 4. Covariates. Summary of covariates used in the cocaine use disorders DisMod-MR meta-regression model

**Covariate**	**Parameter**	**Beta, log (95% uncertainty interval)**	**Exponentiated beta (95% uncertainty interval)**
LDI ($ per capita)	Excess mortality rate	–0.1 (–0.1 to –0.1)	0.90 (0.90 to 0.90)

References

1. American Psychiatric Association. Diagnostic and Statistical Manual of Mental Disorders (DSM-IV-TR). 4th, Text Revision ed Washington DC: American Psychiatric Association. 2000.
2. World Health Organization. The ICD-10 Classification of Mental and Behavioural Disorders. Clinical descriptions and diagnostic guidelines Geneva: World Health Organization. 1992.
3. Degenhardt L, Bucello C, Calabria B, Nelson P, Roberts A, Hall W, et al. What data are available on the extent of illicit drug use and dependence globally? Results of four systematic reviews. Drug and alcohol dependence. 2011.
4. Calabria B, Degenhardt L, Briegleb C, Vos T, Hall W, Lynskey M, et al. Systematic review of prospective studies investigating “remission” from amphetamine, cannabis, cocaine or opioid dependence. Addictive Behaviors. 2010.
5. Reuter P, Trautmann F. A Report on Global Illicit Drugs Markets 1998-2007. Utrecht. 2009.
6. European Montioring Centre for Drugs and Drug Addiction. Lisbon, Portugal 2014.
7. Grant BF, Dawson DA. National Institute on Alcohol Abuse and Alcoholism. Alcohol Health & Research World. 2006; 29(2): p. 74.

Conduct disorder

Flowchart

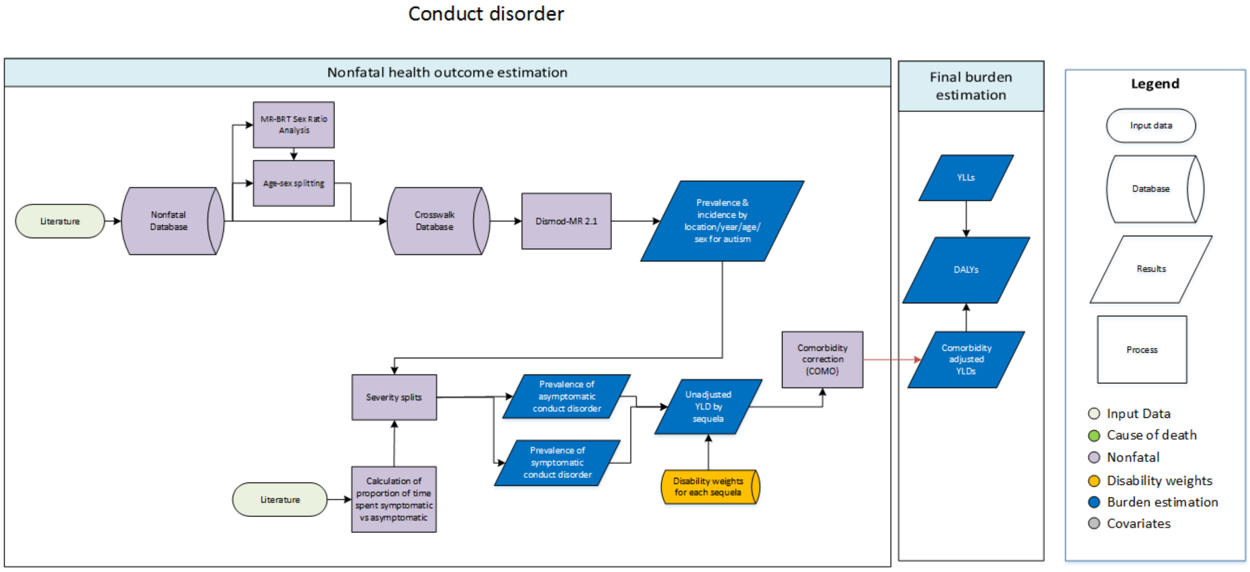

Input data and methodological summary for conduct disorder

Case definition

Conduct disorder (CD) is an externalising behaviour disorder characterised by a pattern of antisocial behaviour that violates the basic rights of others or major age-appropriate societal norms. As per criteria set by the Diagnostic and Statistical Manual of Mental Disorders fourth edition, text revision (DSM-IV-TR),[1] diagnosis requires three or more of the following symptoms to be present in the past 12 months (with at least one present in the last six months) and cause significant impairment in functioning. Symptoms include:

Aggression to people and animals

- often bullies, threatens, or intimidates others
- often initiates physical fights
- has used a weapon that can cause serious physical harm to others (eg, a bat, brick, broken bottle, knife, gun)
- has been physically cruel to people
- has been physically cruel to animals
- has stolen while confronting a victim (eg, mugging, purse snatching, extortion, armed robbery)
- has forced someone into sexual activity

Destruction of property

- has deliberately engaged in fire setting with the intention of causing serious damage
- has deliberately destroyed others' property (other than by fire setting)

Deceitfulness or theft

- has broken into someone else's house, building, or car
- often lies to obtain goods or favors or to avoid obligations (ie, "cons" others)

- has stolen items of nontrivial value without confronting a victim (eg, shoplifting, but without breaking and entering; forgery)

Serious violations of rules

- often stays out at night despite parental prohibitions, beginning before age 13 years
- has run away from home overnight at least twice while living in parental or parental surrogate home (or once without returning for a lengthy period)
- is often truant from school, beginning before age 13 years

CD is considered a disorder of childhood but can be diagnosed in adults who display such behaviours yet do not meet the criteria for antisocial personality disorder. However, there are almost no studies measuring adult CD as existing studies in this area tend to measure adult antisocial behaviour rather than adult CD.[2] As such, only childhood CD (ie, cases prior to 18 years of age) was modelled in GBD.

Included in GBD were cases meeting diagnostic criteria according to DSM[1] or the International Classification of Diseases (ICD).[3] These were identified by the following code: F91. Different versions of DSM (DSM-III, DSM-III-R, DSM-IV, DSM-IV-TR, DSM-5, and DSM-5-TR) and ICD (ICD-9, ICD-10, and ICD-11) were accepted.

Input data

The epidemiological systematic literature review for CD was conducted in three stages involving electronic searches of the peer-reviewed literature (ie, via PsycInfo, Embase, and PubMed), the grey literature, and expert consultation. For mental disorders, we update our GBD electronic database searches on a rolling basis. A systematic review update was completed for GBD 2023 for attention-deficit/hyperactivity disorder and CD together. Databases were searched on September 4, 2023, for publications after September 2, 2018. Below are the search terms used for each database:

PubMed: ((((((("attention deficit and disruptive behavior disorders"[MeSH Terms]) OR ((attention*[Title/Abstract]) AND (disorder*[Title/Abstract]) AND (hyperactiv*[Title/Abstract]))) OR (hyperkinetic[Title/Abstract])) OR (adhd[Title/Abstract])) OR ("conduct disorder"[Title/Abstract])) OR ("conduct disorders"[Title/Abstract])) OR ("conduct disordered"[Title/Abstract])) OR (externali*[Title/Abstract])) OR ((disruptive[Title/Abstract]) AND (disorder[Title/Abstract])) AND (((((((((((prevalen*[Title/Abstract]) OR (mortalit*[Title/Abstract])) OR (death*[Title/Abstract])) OR (inciden*[Title/Abstract])) OR (remission[Title/Abstract])) OR (duration[Title/Abstract])) OR (remit*[Title/Abstract])) OR (epidemiolog*[Title/Abstract])) OR ("Prevalence"[MeSH])) OR ("Mortality"[MeSH])) OR ("Incidence"[MeSH])) OR ("Epidemiology"[MeSH:NoExp])) OR ("Morbidity"[MeSH:NoExp])

Embase: ('attention deficit disorder'/exp OR 'disruptive behavior'/exp OR 'conduct disorder'/exp OR 'disruptive behavior':ab,ti OR 'conduct disorder':ab,ti OR (attention:ab,ti AND disorder:ab,ti AND hyperactiv*:ab,ti) OR 'conduct disorders':ab,ti OR 'conduct disordered':ab,ti OR hyperkinetic:ab,ti OR adhd:ab,ti OR (disruptive:ab,ti AND disorder:ab,ti) OR externali*:ab,ti) AND ((prevalen*:ab,ti OR mortalit*:ab,ti OR death*:ab,ti OR inciden*:ab,ti OR remission:ab,ti OR duration:ab,ti OR remit*:ab,ti OR epidemiolog*:ab,ti OR 'prevalence'/exp OR 'epidemiology'/exp OR 'remission'/exp OR 'incidence'/exp OR 'mortality'/exp OR 'morbidity'/mj)

PsycInfo: ((title: (prevalen*)) OR (abstract: (prevalen*)) OR (title: (mortalit*)) OR (abstract: (mortalit*)) OR (title: (death*)) OR (abstract: (death*)) OR (title: (inciden*)) OR (abstract: (inciden*)) OR (title: (remission)) OR (abstract: (remission)) OR (title: (duration)) OR (abstract: (duration)) OR (title: (remit*)) OR (abstract: (remit*)) OR (title: (epidemiolog*)) OR (abstract: (epidemiolog*)) OR (Index Terms: ("Mortality Rate")) OR (Index Terms: ("Epidemiology")) OR (Index Terms: ("Morbidity")) OR (Index Terms: ("Remission (Disorders)"))) AND ((Index Terms: ("attention deficit disorder")) OR (Index Terms: ("conduct disorder")) OR (Index Terms: ("disruptive behavior")) OR (abstract: (adhd)) OR (title: (adhd)) OR (abstract: ("conduct disorder")) OR (title: ("conduct disorder")) OR (abstract: ("conduct disorders")) OR (title: ("conduct disorders")) OR (abstract: ("conduct disordered")) OR (title: ("conduct disordered")) OR (abstract: (externali*)) OR (title: (externali*)) OR ((abstract: (attention*) OR (title: (attention*))) AND (abstract: (disorder*) OR (title: (disorder*))) AND (abstract: (hyperactiv*) OR (title: (hyperactiv*)))) OR (abstract: (hyperkinetic)) OR (title: (hyperkinetic)) OR ((abstract: (disruptive) OR (title: (disruptive))) AND (abstract: (disorder) OR (title: (disorder)))))

In addition to the database search, a grey literature search and expert consultation were conducted. The systematic review update was conducted according to Preferred Reporting Items for Systematic Reviews and Meta-Analyses (PRISMA; see Figure 1).

Figure 1: PRISMA 2009 flow diagram

From: Moher D, Liberati A, Tetzlaff J, Altman DG, The PRISMA Group (2009). *P*referred *R*eporting *I*tems for *S*ystematic Reviews and *M*eta-*A*nalyses: The PRISMA Statement. PLoS Med 6(7): e1000097. doi:10.1371/journal.pmed1000097

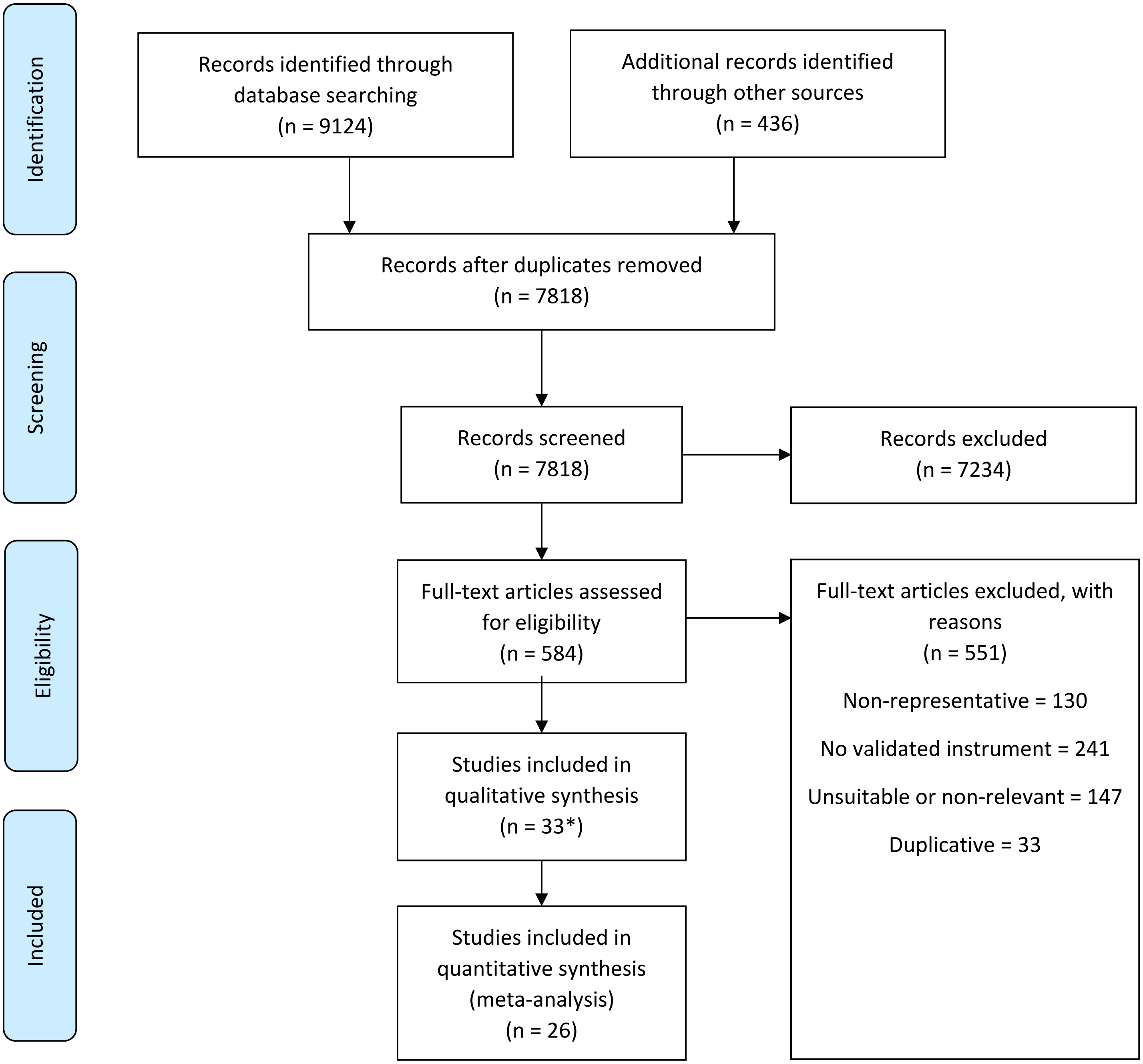

**The qualitative analysis led to the exclusion of additional studies with duplicative cohorts or methodological limitations impacting their eligibility and increasing measurement error within the data.*

The GBD inclusion criteria stipulated that: 1) the publication year must be from 1980 onward; 2) "caseness" must be based on clinical threshold as established by the DSM or ICD; 3) sufficient information must be provided on study method and sample characteristics to assess the quality of the study; and 4) study sample must be representative of the general population (ie, inpatient or pharmacological treatment samples, case studies, veterans, or refugee samples were excluded). No limitation was set on the language of publications. Methods used in previous systematic reviews have been reported in greater detail elsewhere.[2]

Age-sex splitting

The extracted data underwent two types of age-sex splitting processes:

1. Where possible, estimates were further split by sex and age based on the available data. For instance, if studies reported prevalence for broad age groups by sex (eg, prevalence in 15–65-year-old males and females separately), and also by specific age groups but for both sexes combined (eg, prevalence in 15–30-year-olds, then in 31–65-year-olds, for males and females combined); age-specific estimates were split by sex using the reported sex-ratio and bounds of uncertainty.
2. A meta-regression—Bayesian, regularised, trimmed (MR-BRT) analysis was used to split the remaining both-sex estimates in the dataset. For each parameter, sex-specific estimates were matched by location, age, and year. A MR-BRT network meta-analysis was then used to estimate pooled sex ratios and bounds of uncertainty. The male-to-female prevalence ratio estimated was 2.49 (95% uncertainty interval [UI]: 2.13–2.91).

Bias corrections/crosswalks

No crosswalks were applied to the estimates for CD.

Modelling strategy

We have made no substantive changes in the modelling strategy from GBD 2021.

After the above data processes were applied, DisMod MR 2.1 was used to model the epidemiological data for CD. Adjustments to model priors or the dataset were made where appropriate. Where outliers were identified in the data, we reassessed the study's methodology and quality before a decision was made to exclude or include the data.

Data across all epidemiological parameters were initially included in the modelling process. We assumed no incidence or prevalence prior to 5 years of age or after 18 years of age. The minimum age of onset was set in consultation with experts, while the upper age limit was set in line with DSM criteria. Excess mortality was set to zero given the absence of data demonstrating an association between CD and an increased risk of death. Remission and incidence were capped between ages 4 and 17 years in order to gain a more plausible output.

Severity splits and disability weight

The GBD disability weight survey assessments include lay descriptions of sequelae highlighting major functional consequences and symptoms. The lay description and disability weight for CD are shown in

Table 1. A severity split for the proportion of time spent symptomatic versus asymptomatic was based on data from the Great Smoky Mountains Study, which assessed the levels of disability found in children and adolescents with mental disorders.[4] Of those with CD, 72% reported disability, while 20% of individuals with no diagnosis reported disability at the time of survey. Using these as estimates of the proportion of time with disability in the "average case", the proportion of disability in children without a diagnosis was subtracted from the proportion with disability for CD, giving an adjusted proportion of 52%. Detailed descriptions of this methodology have been published elsewhere.[5]

Table 1. Lay description for CD in GBD 2023 and the associated disability weight

**Lay description**	**Disability weight (95% UI)**
Has frequent behaviour problems, which are sometimes violent. The person often has difficulty interacting with other people and feels irritable.	0.241 (0.159–0.341)

There were no significant changes in GBD 2023 results for conduct disorder compared to GBD 2021. While we continue to improve on the data and methods used to estimate the burden of mental disorders, some challenges need to be acknowledged. Firstly, we still have a large number of locations with no high-quality raw data available. Secondly, it is difficult to quantify and remove all variation due to measurement error in our epidemiological estimates. While we have improved the methodology used to account for known sources of bias, in some cases we still have very few datapoints to inform these adjustments. Thirdly, there is a paucity of research on the risk factors of mental disorders which can be used as predictive covariates in our epidemiological models.

References

1. American Psychiatric Association. Diagnostic and Statistical Manual of Mental Disorders (DSM-IV-TR). 4th, Text Revision ed. Washington DC: American Psychiatric Association; 2000.
2. Erskine HE, Ferrari AJ, Nelson P, et al. Research Review: Epidemiological modelling of attention-deficit/hyperactivity disorder and conduct disorder for the Global Burden of Disease Study 2010. *Journal of Child Psychology and Psychiatry* 2013; **54**(12): 1263-74.
3. World Health Organization. ICD-10 Classification of Mental and Behavioural Disorders: Clinical descriptions and diagnostic guidelines. Geneva: World Health Organization; 1992.
4. Ezpeleta L, Keeler G, Erkanli A, Costello EJ, Angold A. Epidemiology of Psychiatric Disability in Childhood and Adolescence. *J Child Psychol Psychiatry* 2001; **42**(7): 901-14.
5. Erskine HE, Ferrari AJ, Polanczyk GV, et al. The global burden of conduct disorder and attention-deficit/hyperactivity disorder in 2010. *Journal of Child Psychology and Psychiatry* 2014; **55**(4): 328-36.

Congenital birth defects

Flowchart

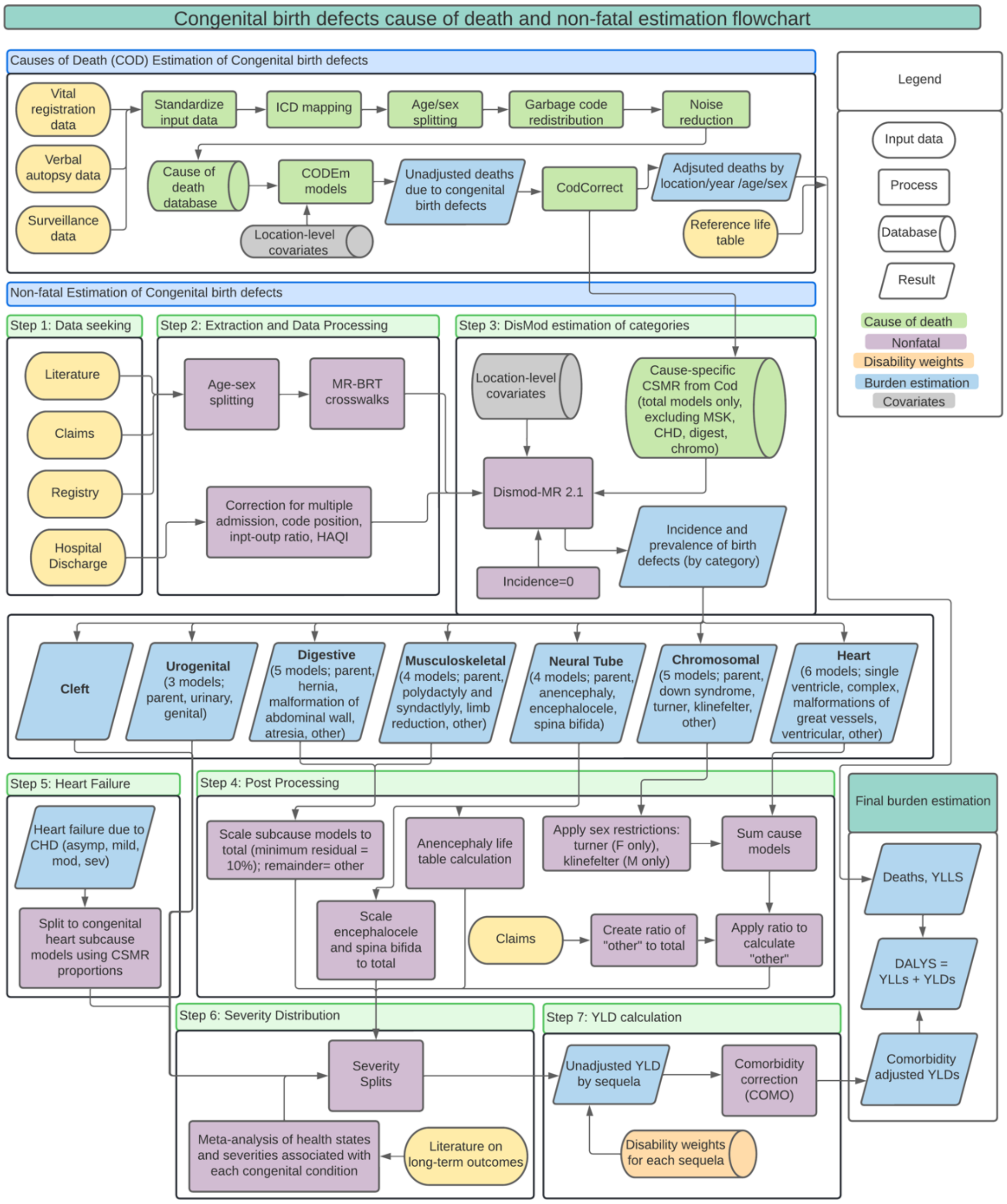

Registry flow chart

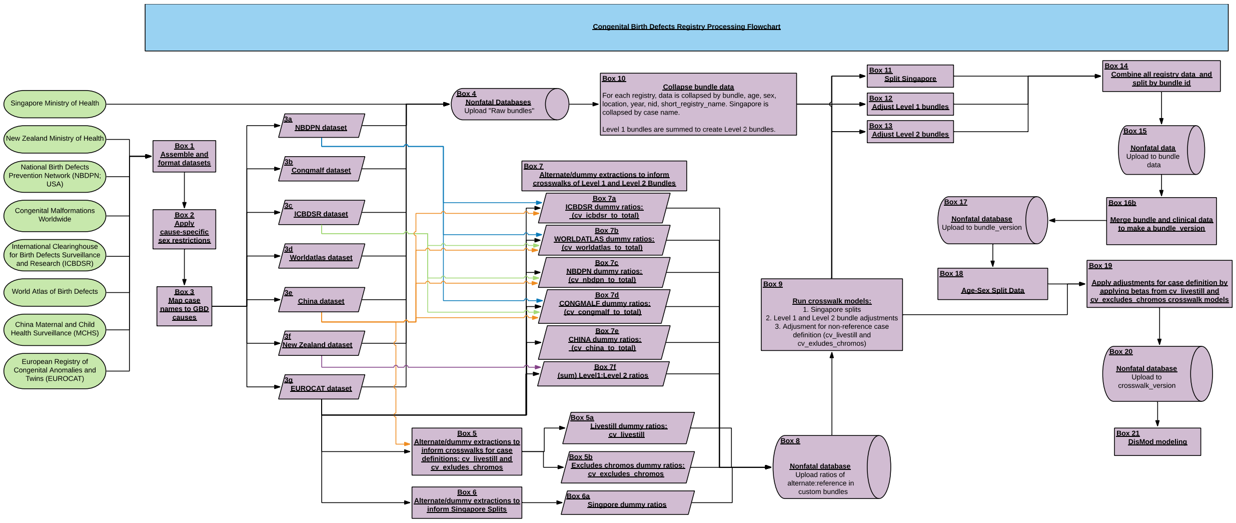

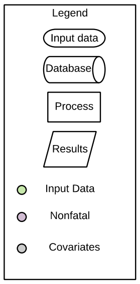

Level 1 (subcause) and Level 2 (parent) bundle list

Congenital muskuloskeletal anomalies
Level 2 bundle:
- 602 - Total muscoskeletal congenital anomalies

Level 1 bundles:
- 604 - Congenital msk - subcause - Limb reduction deficits
- 606 - Congenital msk - subcause - Polydactyly and syndactyly
- 3776 - Congenital msk - subcause - clubfoot
- 3779 - Congenital msk - subcause - hip dysplasia
- 2972 - Other musculoskeletal congenital anomalies

Neural tube defects
Level 2 bundle:
- 608 - Total neural tube defects

Level 1 bundles:
- 610 - Congenital neural - subcause - Anencephaly
- 612 - Congenital neural - subcause - Encephalocele
- 614 - Congenital neural - subcause - Spina Bifida

Congenital muskuloskeletal anomalies
Level 2 bundle:
- 620 - Total congenital digestive anomalies

Level 1 bundles:
- 622 - Congenital digestive - subcause - Congenital diaphragmatic hernia
- 624 - Congenital digestive - subcause - Congenital malformations of the abdominal wall
- 626 - Congenital digestive - subcause - Congenital atresia and/or stenosis of the digestive tract
- 2975 - Other digestive congenital anomalies

Congenital heart disease
Level 2 bundle:
- 628 - Total congenital heart defects

Level 1 bundles:
- 630 - Congenital heart - subcause - Single ventricle and single ventricle pathway heart defects
- 632 - Congenital heart - subcause - Critical malformations of great vessels, congenital valvular heart disease and patent ductus arteriosis
- 634 - Congenital heart - subcause - Ventricular septal defect and atrial septal defect
- 636 - Congenital heart - subcause - Severe congenital heart defects excluding single ventricle and single ventricle pathway
- 2978 - Other congenital cardiovascular defects

Congenital chromosomal anomalies
Level 2 bundle:
- 3029 - Total chromosomal congenital anomalies

Level 1 bundles:
- 436 - Congenital chromo -subcause - Down syndrome
- 437 - Congenital chromo - subcause - Turner syndrome
- 438 - Congenital chromo - subcause - Klinefelter syndrome
- 638 - Congenital chromo - subcause - Edward Syndrome and Patau Syndrome
- 439 - Other chromosomal anomalies, genetic syndrome and micro-deletions

Congenital urogenital anomalies
Level 1 bundles:
616 - Congenital urinary anomalies
618 - Congenital genital anomalies

Orofacial clefts
Level 1 bundle:
435 - Orofacial clefts

Other Level 1 Bundles not currently directly used in congenital birth defects modeling:
3188 - Other congenital birth defects
248 - Congenitlal cataract
3065 - Congenital hearing loss

Input Data and Methodological Summary for Congenital birth defects

Case definition

The GBD case definition of congenital birth defects, also referred to as congenital anomalies, includes any condition present at birth that is a result of abnormalities of embryonic development, excluding those that are directly the result of infections or substance abuse (eg, fetal alcohol syndrome, congenital syphilis), modelled elsewhere in GBD, and excludes minor anomalies as they are defined by European Surveillance of Congenital Anomalies (EUROCAT). We have estimated the prevalence and associated disability for the following categories of congenital birth defects (those in bold are GBD causes):

1. Neural tube defects
    a. Anencephaly
    b. Encephalocele
    c. Spina bifida
2. Congenital heart anomalies
    a. Single ventricle and single ventricle pathway defects
    b. Complex congenital heart defects excluding single ventricle and single ventricle pathway defects
    c. Malformations of great vessels, congenital valvular heart disease, and patent ductus arteriosus
    d. Ventricular septal defect and atrial septal defect
    e. Other congenital cardiovascular anomalies
3. Orofacial clefts: Cleft lip and cleft palate
4. Total chromosomal congenital birth defects
    a. Down syndrome
    b. Turner syndrome
    c. Klinefelter syndrome
    d. Other chromosomal abnormalities
        i. Edwards syndrome and Patau syndrome
        ii. Other chromosomal abnormalities, genetic syndromes, and micro-deletions
5. Urogenital congenital anomalies
    a. Congenital urinary anomalies
    b. Congenital genital anomalies
6. Digestive congenital anomalies
    a. Congenital diaphragmatic hernia
    b. Congenital malformations of the abdominal wall
    c. Congenital atresia and/or stenosis of the gastrointestinal tract
    d. Other congenital malformations of the digestive tract
7. Congenital musculoskeletal and limb anomalies
    a. Polydactyly and syndactyly
    b. Limb reduction defects
    c. Other musculoskeletal congenital anomalies
8. Other congenital birth defects: all birth defects (excluding minor anomalies) not contained in the other categories

Input data

Several types of data sources are used in the estimation of congenital anomalies: literature prevalence; with-condition mortality and excess mortality data; birth prevalence and neonatal with-condition mortality data from a number of international birth defects registries and surveillance systems; inpatient hospital and MarketScan claims data (a trusted data source) prepared internally by the GBD research team; and cause-specific mortality estimates produced by the causes of death analysis.

First, we extracted data from a number of international birth defects registries. The International Clearinghouse for Birth Defects Surveillance and Research (ICBDSR) reports birth prevalence from a number of international member registries. The World Atlas Report also published birth prevalence estimates from these international registries prior to the publication of ICBDSR reports. EUROCAT reports the birth prevalence of anomalies for a variety of locations in western Europe as reported by participating member registries. China's Maternal and Child Health Surveillance survey (MCHS) reports

birth prevalence and early neonatal mortality data for all subnational locations of China. The National Birth Defects Prevention Network (NBDPN) reports birth prevalence estimates as compiled by a number of subnational registries within the USA. The Birth Defects Registry of India (BDRI) reports congenital anomalies from participating hospitals within India.

Second, we used inpatient hospital and claims data (from the USA, Taiwan, and Singapore) for all congenital anomalies causes and sub-cause models. These data were prepared centrally by the Clinical Informatics research team and are described in detail in the Clinical Informatics section of this appendix. Four rounds of data bias correction were employed in the processing of clinical data. This included 1) adjustment for readmission, 2) correction of primary diagnoses to all diagnoses, 3) adjustment for inpatient-to-outpatient ratio, and 4) adjustment based on Healthcare Access and Quality Index. Of note, in GBD 2017 we used congenital birth defects data only using the first two corrections but changed starting in GBD 2019 to use clinical data that had all four corrections applied. This change was facilitated by improvements in analysis of corrections by the Clinical Informatics team and was a change made across GBD. Of note, we also changed the mapping of club foot and hip dysplasia starting in GBD 2019: previously they were mapped to "limb reduction defects" but were included only in the total for musculoskeletal birth defects with the plan to disaggregate models in the future.

Third, we included data from a systematic review of the available literature for all types of congenital birth defects that was completed in GBD 2015 by constructing search strings designed to capture information on the prevalence, associated mortality, and long-term health outcomes associated with each sub-category of congenital anomalies. All results were screened – first abstracts, then full-text screenings – to ensure the availability of required information and the representativeness of the reported population, and the exclusion of duplicate data also reported as part of the birth registry data inputs.

Data processing

Age-sex splitting

Starting with GBD 2019, any data that were not sex-specific or did not fit entirely within GBD age groups were age- and sex-split to fit these groups prior to modelling using empirical age- and sex-patterns derived from previous DisMod-MR 2.1 models of the same condition. This is described further below.

Crosswalks in MR-BRT

A number of the input data sources used for the estimation of congenital birth defects are known to have biases leading to under-reporting or over-reporting relative to the true prevalence of congenital anomalies among livebirths and all subsequent age groups. We used meta-regression—Bayesian, regularised, trimmed (MR-BRT)—to develop statistical models that were used to adjust non-reference data. The alternate definitions that were crosswalked are described below. The specifics of each MR-BRT crosswalk are described below (for "registry to total" crosswalks) and in the corresponding cause-specific sections (for live/stillbirth and exclusion of chromosomal conditions crosswalks).

Live/stillbirths: Where necessary, we used a crosswalk to adjust for the inclusion of stillbirths in the reported birth prevalence estimates in literature and registry data sources, as stillbirths are not included in our case definition of prevalence among livebirths. Each of these crosswalks used a log-transformed neonatal mortality rate as a dose–response (spline/linear) covariate in the crosswalks.

Exclusion of chromosomal conditions: Some sources report birth defects in isolation (ie, excluding any persons who have a coexisting genetic or chromosomal disorder). Our reference definition is the inclusion of chromosomal diagnoses. These splines did not consider any additional covariates.

Registry to total: For a subset of congenital causes, particularly congenital heart anomalies, we noted substantial differences in the lists of case definitions being reported to the various congenital registries. For each type of congenital birth defects, registries with the most complete list of reported case definitions – ie, the highest case ascertainment – were used as reference registries and were considered the gold standard for that type of congenital birth defect, or modellable entity. For each modellable entity, we used registry-specific crosswalks to adjust non-gold-standard registries to match the case ascertainment seen in the gold-standard registry. No splines were used in these crosswalks.

Table 1: Crosswalks for data from Congenital Malformations Worldwide[1]

Reference registry	Modellable entity name	Gamma	Beta coefficient, log (95% CI)	Adjustment factor*
EUROCAT, China	Total musculoskeletal congenital anomalies	0.043446	–1.659 (–1.752 to –1.567)	0.1902 (0.1734 to 0.2088)
EUROCAT, China	Total neural tube defects	0	–8.862e-05 (–0.0175 to 0.01737)	0.9999 (0.9826 to 1.018)
New Zealand, ICBDMS, EUROCAT	Congenital urinary anomalies	0.410011	–0.8497 (–0.9172 to –0.7822)	0.4275 (0.3996 to 0.4574)
EUROCAT, ICBDMS, NBDPN	Total congenital digestive anomalies	0.007148	–0.1384 (–0.1517 to –0.1252)	0.8707 (0.8593 to 0.8823)
EUROCAT, ICBDMS	Congenital malformations of the abdominal wall	0.00356	–0.06254 (–0.0806 to –0.04448)	0.9394 (0.9226 to 0.9565)
NBDPN	Congenital atresia and/or stenosis of the digestive tract	0	–6.641e-21 (–0.01697 to 0.01697)	1 (0.9832 to 1.017)
EUROCAT	Total congenital heart defects	0.043003	–1.716 (–1.806 to –1.626)	0.1798 (0.1643 to 0.1968)
EUROCAT, NBDPN	Single ventricle and single ventricle pathway heart defects	0.039983	–0.4149 (–0.4979 to –0.3318)	0.6604 (0.6078 to 0.7176)
EUROCAT, NBDPN	Severe congenital heart defects excluding single ventricle and single ventricle pathway	0.011641	–0.6259 (–0.6711 to –0.5807)	0.5348 (0.5112 to 0.5595)
EUROCAT, China	Total chromosomal congenital anomalies	0.046543	–0.6162 (–0.7063 to –0.5262)	0.54 (0.4935 to 0.5909)

Table 2: Crosswalks for data from *International Clearinghouse for Birth Defects Monitoring System (ICBDMS)*[2]

Reference registry	Modellable entity name	Gamma	Beta coefficient, log (95% CI)	Adjustment factor*
EUROCAT, China	Total musculoskeletal congenital anomalies	0.076511	–1.393 (–1.506 to –1.279)	0.2484 (0.2217 to 0.2784)
EUROCAT, New Zealand, China	Polydactyly and syndactyly	0.008397	–0.3163 (–0.3577 to –0.2749)	0.7288 (0.6993 to 0.7596)
EUROCAT, China	Total neural tube defects	0	–8.862e-05 (–0.01755 to 0.01737)	0.9999 (0.9826 to 1.018)
EUROCAT	Total congenital heart defects	0.03719	–1.426 (–1.51 to –1.342)	0.2402 (0.2209 to 0.2612)
EUROCAT, NBDPN	Single ventricle and single ventricle pathway heart defects	0.039983	–0.4149 (–0.4979 to –0.3318)	0.6604 (0.6078 to 0.7176)
EUROCAT, NBDPN	Critical malformations of great vessels, congenital valvular heart disease, and patent ductus arteriosus	0.028993	–0.5232 (–0.5936 to –0.4529)	0.5926 (0.5523 to 0.6358)
EUROCAT, NBDPN	Severe congenital heart defects excluding single ventricle and single ventricle pathway	0.025623	–0.3316 (–0.3952 to –0.268)	0.7178 (0.6735 to 0.7649)
EUROCAT, China	Total chromosomal congenital anomalies	0.046543	–0.6162 (–0.7063 to –0.5262)	0.54 (0.4935 to 0.5909)

Table 3: Crosswalks for data from *NBDPN*[3]

Reference registry	Modellable entity name	Gamma	Beta coefficient, log (95% CI)	Adjustment factor*
EUROCAT, China	Total musculoskeletal congenital anomalies	0.018925	–1.008 (–1.065 to –0.9506)	0.3649 (0.3446 to 0.3865)
EUROCAT, China	Total neural tube defects	0	–8.862e-05 (–0.01755 to 0.01737)	0.9999 (0.9826 to 1.018)
EUROCAT, ICBDMS	Congenital malformations of the abdominal wall	0.00356	–0.06254 (–0.0806 to –0.04448)	0.9394 (0.9226 to 0.9565)
EUROCAT	Total congenital heart defects	0.000931	0.07634 (0.06148 to 0.09121)	1.079 (1.063 to 1.096)
EUROCAT, China	Total chromosomal congenital anomalies	0.046895	–0.5862 (–0.6766 to –0.4957)	0.5565 (0.5083 to 0.6091)

Table 4: Crosswalks for data from *New Zealand Birth Defects Registry*

Reference registry	Modellable entity name	Gamma	Beta coefficient, log (95% CI)	Adjustment factor*
EUROCAT, China	Total musculoskeletal congenital anomalies	0.023344	–0.7577 (–0.815 to –0.7003)	0.4688 (0.4426 to 0.4964)
EUROCAT, China	Total neural tube defects	0.020533	–0.7704 (–0.8242 to –0.7165)	0.4628 (0.4386 to 0.4884)
EUROCAT	Total congenital heart defects	0.010986	–0.5204 (–0.5605 to –0.4803)	0.5943 (0.5709 to 0.6186)
EUROCAT, NBDPN	Single ventricle and single ventricle pathway heart defects	0.010111	–0.682 (–0.7232 to –0.6407)	0.5056 (0.4852 to 0.5269)
EUROCAT, NBDPN	Critical malformations of great vessels, congenital valvular heart disease, and patent ductus arteriosus	0.01217	–0.7667 (–0.8119 to –0.7215)	0.4645 (0.444 to 0.486)
EUROCAT, NBDPN	Severe congenital heart defects excluding single ventricle and single ventricle pathway	0.01263	–0.9006 (–0.9483 to –0.853)	0.4063 (0.3874 to 0.4261)
EUROCAT, China	Total chromosomal congenital anomalies	0.030019	–0.6302 (–0.6956 to –0.5648)	0.5325 (0.4988 to 0.5685)

Table 5: Crosswalks for data from *Singapore Birth Defects Registry*

Reference registry	Modellable entity name	Gamma	Beta coefficient, log (95% CI)	Adjustment factor*
EUROCAT	Down syndrome	0.043064	0.6031 (0.5145 to 0.6918)	1.828 (1.673 to 1.997)
EUROCAT	Total musculoskeletal congenital anomalies	0.000336	0.05078 (0.04121 to 0.06034)	1.052 (1.042 to 1.062)
EUROCAT	Limb reduction deficits	0.03955	1.609 (1.518 to 1.7)	4.999 (4.565 to 5.475)
EUROCAT	Polydactyly and syndactyly	0.014893	1.282 (1.229 to 1.334)	3.603 (3.419 to 3.796)
EUROCAT	Total neural tube defects	0.032992	0.9013 (0.8212 to 0.9813)	2.463 (2.273 to 2.668)
EUROCAT	Anencephaly	0.045988	1.471 (1.377 to 1.565)	4.354 (3.964 to 4.782)
EUROCAT	Encephalocele	0.040592	1.506 (1.417 to 1.594)	4.508 (4.126 to 4.925)
EUROCAT	Spina bifida	0.025375	1.068 (0.9968 to 1.14)	2.911 (2.71 to 3.127)
EUROCAT	Congenital diaphragmatic hernia	0.047244	2.253 (2.16 to 2.345)	9.512 (8.675 to 10.43)
EUROCAT	Congenital malformations of the abdominal wall	0.006371	1.001 (0.9622 to 1.039)	2.72 (2.618 to 2.827)

EUROCAT	Congenital atresia and/or stenosis of the digestive tract	0.009226	0.5386 (0.4939 to 0.5833)	1.714 (1.639 to 1.792)
EUROCAT	Single ventricle and single ventricle pathway heart defects	0.045698	2.017 (1.924 to 2.11)	7.519 (6.852 to 8.252)
EUROCAT	Critical malformations of great vessels, congenital valvular heart disease, and patent ductus arteriosus	0.022149	1.243 (1.178 to 1.308)	3.466 (3.247 to 3.699)
EUROCAT	Ventricular septal defect and atrial septal defect	0.002317	0.1981 (0.1757 to 0.2205)	1.219 (1.192 to 1.247)
EUROCAT	Severe congenital heart defects excluding single ventricle and single ventricle pathway	0.036296	1.417 (1.334 to 1.5)	4.125 (3.797 to 4.482)
EUROCAT	Edward syndrome and Patau syndrome	0.046072	1.525 (1.434 to 1.617)	4.597 (4.193 to 5.04)

Table 6: Crosswalks for data from *World Atlas of Birth Defects*[6]

Reference registry	Modellable entity name	Gamma	Beta coefficient, log (95% CI)	Adjustment factor*
EUROCAT	Total musculoskeletal congenital anomalies	0.02394	–1.394 (–1.461 to –1.328)	0.248 (0.2321 to 0.265)
EUROCAT, China	Total neural tube defects	0	–0.1143 (–0.1327 to –0.0959)	0.892 (0.8758 to 0.9086)
New Zealand, ICBDMS, EUROCAT	Congenital urinary anomalies	0.142379	–0.1339 (–0.1754 to –0.09251)	0.8746 (0.8391 to 0.9116)
EUROCAT, ICBDMS	Congenital malformations of the abdominal wall	0.00356	–0.06254 (–0.0806 to –0.04448)	0.9394 (0.9226 to 0.9565)
EUROCAT	Total congenital heart defects	0.03719	–1.426 (–1.51 to –1.342)	0.2402 (0.2209 to 0.2612)
EUROCAT, NBDPN	Single ventricle and single ventricle pathway heart defects	0.039983	–0.4149 (–0.4979 to –0.3318)	0.6604 (0.6078 to 0.7176)
EUROCAT, NBDPN	Critical malformations of great vessels, congenital valvular heart disease, and patent ductus arteriosus	0.028993	–0.5232 (–0.5936 to –0.4529)	0.5926 (0.5523 to 0.6358)
EUROCAT, NBDPN	Severe congenital heart defects excluding single ventricle and single ventricle pathway	0.006351	–0.4172 (–0.4526 to –0.3818)	0.6589 (0.636 to 0.6826)
EUROCAT, China	Total chromosomal congenital anomalies	0.046543	–0.6162 (–0.7063 to –0.5262)	0.54 (0.4935 to 0.5909)

Determining outliers and data thresholds

Under-reporting of congenital birth defects is common and can vary by source, location, year, sex, and age. In order to have an empirical, systematic approach to outliering of data, we adapted the non-zero floor approach used by the GBD cause-specific mortality analysis. After all age-sex splitting and crosswalking was complete, the first step was to calculate median absolute deviation (MAD) for the age group of birth, where registry and literature data were combined with all clinical data for the early neonatal age group (0–6 days). The thresholds chosen were –0.5 MAD and +3 MAD, with any data outside of these bounds being identified as outliers. This was determined based on the right-skewed distribution observed in most of the congenital data and the expert prior that under-reporting is far more prevalent than over-reporting – and therefore the bias is asymmetric. In any case where the lower MAD bound was negative, we used a threshold of 0.

For most models, we calculated the MADs using only the EUROCAT data, which we found to be the most reliable source for prevalence of congenital disorders. Exceptions were neural tube defects (all data sources), urinary birth defects (EUROCAT and USA claims data), musculoskeletal defects (only USA claims data), and chromosomal anomalies, which differed by condition given the high volume of zeroes in the data. For Down syndrome, we used all data. For Edward syndrome and Patau syndrome, we used all non-zero EUROCAT data. For Turner and Klinefelter syndromes, we used EUROCAT data and logged mean absolute deviation and exponentiated this to determine bounds for these data.

To evaluate data for older age groups, we employed two approaches. First, we outliered data from any location-year-source that was outliered for the first stage MAD algorithm. Second, using all clinical and literature data, we developed a model with fixed effects by age to estimate implied MAD bounds for each non-zero age group and again applied the same thresholds of –0.5 MAD and +3 MAD.

Modelling strategy

Overview

We have made no substantive changes in the modeling strategy from GBD 2021. All available input data were utilised in a series of DisMod-MR 2.1 models to estimate the prevalence of each category of congenital anomalies across the full life course for each location/age/sex combination. Incidence was set to zero for all congenital models, as congenital conditions occur at the time of birth and by GBD case definition, congenital cases do not occur after birth. Remission was allowed only in the models of a select subset of causes for which surgical intervention or spontaneous remission can completely eliminate the disability due to that congenital condition. Cause-specific priors and slope priors were used to guide biologically plausible DisMod-MR 2.1 estimates of excess mortality and remission where applicable.

For most of the congenital birth defects causes, we ran DisMod-MR 2.1 models of all defects combined (termed “parent” models). This allowed us to use data on all anomalies within each cause as well as to leverage CSMR results from the GBD cause of death (CoD) analysis. When CSMR data were used as an input, DisMod-MR 2.1 pairs each CSMR datum with a matching prevalence datapoint by age, sex, location, and year. After matching, CSMR is divided by prevalence to calculate an implied excess mortality rate (EMR) datum. All EMR data are then used in driving the model. Of note, EMR data are not calculated when prevalence data are of broader than GBD age groups or are for both sexes combined.

We used CSMR as input to all of the models except congenital heart anomalies, chromosomal abnormalities, digestive congenital anomalies, congenital musculoskeletal and limb anomalies, and urogenital congenital anomalies. For congenital heart anomalies, the reason is that excess mortality would be underestimated in older ages if CSMR results were used because despite continuing higher rates of mortality through adolescence and adulthood, many of these deaths are not coded as being due to congenital heart disease. Similarly, congenital musculoskeletal and digestive anomalies estimates for CSMR in older children, adolescents, and adults are much lower than would be suggested by cohort and cross-sectional studies of survival, as few of these deaths are coded as being due to the congenital birth defect present. Finally, for urogenital congenital anomalies, in addition to our modelling urinary and genital anomalies separately, the mechanism of death in older ages will typically be via development of chronic kidney disease, and these deaths are classified in GBD as being due to chronic kidney disease due to other conditions. Details are in each cause-specific section below.

Location-level covariates

Location-level covariates were used in each of the congenital DisMod-MR 2.1 models based on published information about the risk factors for these birth defects. Folic acid availability was used as a covariate on prevalence for all neural tube defects models and a subset of the congenital

musculoskeletal anomalies models. A folic acid fortification covariate was used in the neural tube defects and cleft models, which was modelled based on data from the Global Fortification Data Exchange. The legality of abortion was used as a covariate on prevalence for conditions in which prenatal diagnosis is commonly available and the prognosis is severe enough to cause a high rate of termination of pregnancy following prenatal diagnosis: these include all chromosomal conditions and a subset of the congenital heart anomalies. Maternal consumption of alcohol during pregnancy as a proportion of all pregnancies was used as a covariate on prevalence for all congenital heart defects. The proportion of livebirths by mothers age 35+ was used as a covariate on all chromosomal models. Across many of the congenital models, the Healthcare Access and Quality Index covariate was used to guide the global pattern of with-condition mortality and excess mortality, as was the natural log of the lag-distributed income per capita (LN-LDI). For most of the severe congenital conditions, the mortality associated with the condition is highly dependent on access to adequate surgical interventions and other medical care during the first hours, weeks, and years of life.

Post-model processing

For those causes with a parent model (neural tube defects), we then squeezed the sum of the specific sub-cause prevalence estimates to these total prevalence estimates to ensure internal consistency of our cause-level and sub-cause estimates. The prevalence of other heart, musculoskeletal, and digestive anomalies was derived by reducing the total envelope model for each cause by its sub-causes to derive the difference that was attributable to other anomalies in that category.

Assigning health states and sequelae for long-term outcomes

To determine the distribution of health outcomes associated with the congenital causes, we performed a review of available literature on the long-term health outcomes of survivors in cohorts born with each type of congenital malformation. For conditions requiring surgical intervention shortly after birth to ensure survival, the health states included in the disability weight calculations correspond to the post-surgery outcomes reported in cohorts of individuals born with these life-threatening congenital conditions. Where data were available from multiple cohorts, we pooled these cohorts together to calculate the proportion of individuals with each health state. Where data on the joint distribution of the long-term health outcomes were not available, we assumed independence of each long-term health outcome. Combined disability weights were calculated for all necessary combinations of existing disability weights.

Neural tube defects

To ensure internal consistency of the estimates of each sub-type of neural tube defects, we developed a model of the total prevalence of neural tube defects and used these location-, year-, sex-, and age-specific prevalence estimates to scale the estimates of anencephaly, encephalocele, and spina bifida prevalence. This modelling strategy allowed us to incorporate the cause-specific mortality estimates from the GBD CoD analysis and allowed us to use literature data where the prevalence and mortality estimates were reported for the total of all neural tube defects only.

Crosswalks

The MR-BRT crosswalk results are shown below.

Table 7: MR-BRT crosswalk betas for alternate definitions (reference = livebirths including those with chromosomal anomalies)

Crosswalk	Beta	Standard error
Excluding chromosomal diagnoses adjustment	–0.038	0.028

Figure 1: MR-BRT crosswalk of alternate definition (livebirths and stillbirths included) with spline on log-transformed neonatal mortality rate

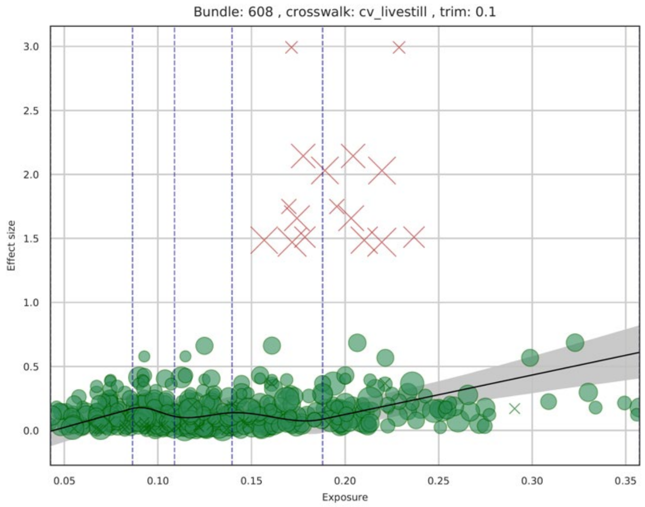

Modelling strategy

The DisMod-MR 2.1 model of total neural tube defects used cause-specific mortality (CSMR) estimates from the GBD CoD analysis for neural tube defects. This model had a minimum excess mortality of 0.5 for the first week of age and a minimum excess mortality of 0.0003 for ages 1–100 years as the risk of death due to neural tube defects is greatest shortly after birth. The model also used an increased smoothness (maximum xi=3) on EMR in order to allow high excess mortality in the early neonatal age group. Random effects on prevalence were limited to 0–0.75 to limit geographical variation in the estimated birth prevalence, and all min coefficient of variation (cv) settings were 0.8.

Table 8: Location-level covariate effects

Covariate name	Measure	Beta value	Exponentiated value
Folic acid, unadjusted (µg)	Prevalence	–0.0005 (–0.00064 to –0.00036)	1.00 (1.00–1.00)
Composite fortification standard and folic acid inclusion	Prevalence	–0.31 (–0.35 to –0.27)	0.73 (0.70–0.76)
Healthcare Access and Quality Index	EMR	–0.041 (–0.041 to –0.04)	0.96 (0.96–0.96)

Anencephaly

Case definition and associated health states

Anencephaly is the absence of a major portion of the brain, skull, and scalp. Anencephaly corresponds to the ICD-10 codes Q00.0 and Q00.2. All infants with anencephaly are assigned the health state of severe motor and cognitive impairment.

Crosswalks

The MR-BRT crosswalk results are shown below.

Table 9: MR-BRT crosswalk betas for alternate definitions (reference = livebirths including those with chromosomal anomalies)

Crosswalk	Beta	Standard error
Excluding chromosomal diagnoses adjustment	–0.030	0.163

Figure 2: MR-BRT crosswalk of alternate definition (livebirths and stillbirths included) with spline on log-transformed neonatal mortality rate

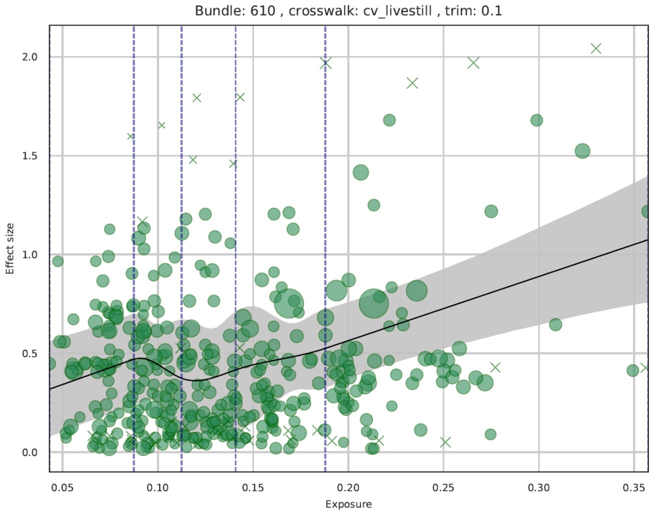

Modelling strategy

The life expectancy for infants born with anencephaly is on the order of hours or days; none of these infants survives past the neonatal age period. Because of the extremely high excess mortality associated with this condition and the short age range over which the prevalence varies, we used a custom modelling process to estimate the prevalence of anencephaly. We first used DisMod-MR 2.1 to model the prevalence of anencephaly at birth for every location, year, age, and sex combination. We then used literature data on outcomes from the largest available cohort of infants born with anencephaly,[7,8] using the precise time of death information from this cohort to create a life table that applied the high EMRs to all cases of anencephaly at birth.

We applied these mortality rates to both sexes and all locations, generating the time lived by infants with anencephaly during the early and late neonatal age groups by location, year, and sex. We then used GBD 2019 mortality estimates to calculate the time lived by all infants during the early and late neonatal age groups by location, year, and sex, and used these two values to calculate the prevalence of anencephaly in the early and late neonatal age groups; after one month of age, all available literature indicates that no infants born with anencephaly are still alive.

The DisMod-MR 2.1 model for the birth prevalence of anencephaly has random effects on prevalence limited to ±0.5. As this model was designed to estimate only the prevalence at birth, incidence, remission, and excess mortality were set to zero for all ages, and the only age mesh points were 0 and 100 years of age.

Table 10: Location-level covariate effects

Covariate name	Measure	Beta value	Exponentiated value
Legality of abortion	Prevalence	–0.0006 (–0.0016 to –0.00003)	1.00 (1.00–1.00)
Folic acid, unadjusted (μg)	Prevalence	–0.000096 (–0.00035 to –0.0000042)	1.00 (1.00–1.00)
Composite fortification standard and folic acid inclusion	Prevalence	–0.42 (–0.5 to –0.34)	0.66 (0.61–0.71)

Encephalocele

Case definition and associated health states

Encephalocele is characterised by sac-like protrusions of the brain and meninges through openings in the skull. Encephalocele corresponds to the ICD-10 codes Q01.2, Q01.8, and Q01.9. Our case definitions of spina bifida and encephalocele do not consider surgical intervention for either condition as remission.

Cases of spina bifida and encephalocele are split into every combination of mild, moderate, and severe motor impairment, all severities of intellectual disability, and urinary incontinence. These proportions were calculated using a pooled analysis of available literature on the long-term outcomes in cohorts of individuals born with each subtype of neural tube defects. The distribution of health states associated with encephalocele[9–11] was derived separately from the distribution of health states associated with spina bifida,[12,13] although these two categories of neural tube defects are associated with the same list of long-term outcome sequelae.

Crosswalks

The MR-BRT crosswalk results are shown below.

Table 11: MR-BRT crosswalk betas for alternate definitions (reference = livebirths including those with chromosomal anomalies)

Crosswalk	Beta	Standard error
Excluding chromosomal diagnoses adjustment	–0.068	0.074

Figure 3: MR-BRT crosswalk of alternate definition (livebirths and stillbirths included) with spline on log-transformed neonatal mortality rate

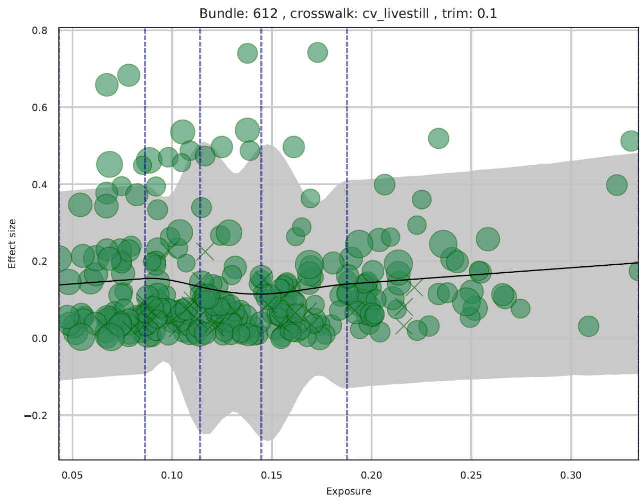

Modelling strategy

The DisMod-MR 2.1 model for encephalocele had a minimum excess mortality prior of 0.2 for the first week of age and a minimum excess mortality prior of 0.0003 for ages 1–54. Excess mortality was restricted to 0–0.1 thereafter, as we believe that those with encephalocele would no longer be dying of this condition past age 55. The model also used an increased smoothness on EMR (maximum xi=3). Random effects on prevalence were limited to ±0.5, as we expect limited geographical variation in the birth prevalence of encephalocele.

Table 12: Location-level covariate effects

Covariate name	Measure	Beta value	Exponentiated value
Legality of abortion	Prevalence	–0.004 (–0.0053 to –0.0027)	1.00 (0.99–1.00)
Folic acid, unadjusted (μg)	Prevalence	–0.00054 (–0.00091 to –0.00016)	1.00 (1.00–1.00)
Composite fortification standard and folic acid inclusion	Prevalence	–0.29 (–0.34 to –0.25)	0.75 (0.71–0.78)
Healthcare Access and Quality Index	EMR	–0.025 (–0.049 to –0.001)	0.98 (0.95–1.00)

Spina bifida

Case definition and associated health states

Spina bifida occurs when part of the spinal cord and/or meninges are uncovered by skin. Spina bifida occulta, a much less severe form of spina bifida, in which the defect in vertebral column remains covered by skin, is excluded from the GBD case definition of spina bifida. Spina bifida corresponds to the ICD-10 codes Q05.0, Q05.4, Q05.6, Q05.7, Q05.8, and Q05.9. Our case definitions of spina bifida and encephalocele do not consider surgical intervention for either condition as remission.

Cases of spina bifida and encephalocele are split into every combination of mild, moderate, and severe motor impairment, all severities of intellectual disability, and urinary incontinence. These proportions were calculated using a pooled analysis of available literature on the long-term outcomes in cohorts of individuals born with each subtype of neural tube defects. The distribution of health states associated with encephalocele[9–11] was derived separately from the distribution of health states associated with

spina bifida,[12,13] although these two categories of neural tube defects are associated with the same list of long-term outcome sequelae.

Crosswalks

The MR-BRT crosswalk results are shown below.

Table 13: MR-BRT crosswalk betas for alternate definitions (reference = livebirths including those with chromosomal anomalies)

Crosswalk	Beta	Standard error
Excluding chromosomal diagnoses adjustment	–0.0386	0.034

Figure 4: MR-BRT crosswalk of alternate definition (livebirths and stillbirths included) with spline on log-transformed neonatal mortality rate

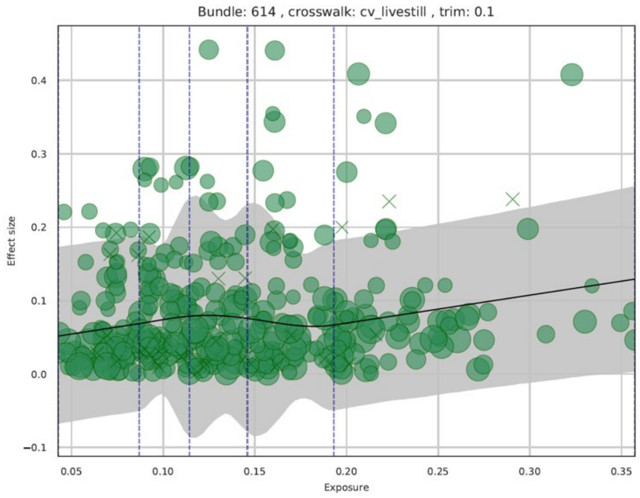

Modelling strategy

The DisMod-MR 2.1 model for spina bifida had a minimum excess mortality of 0.2 for the first week of age, and a minimum of 0.0002 for ages 1+ years, and a maximum smoothness on EMR of xi=3. Random effects on prevalence were also limited to ±0.5.

Table 14: Location-level covariate effects

Covariate name	Measure	Beta value	Exponentiated value
Legality of abortion	Prevalence	–0.0078 (–0.0087 to –0.0069)	0.99 (0.99–0.99)
Folic acid, unadjusted (μg)	Prevalence	–0.00017 (–0.00045 to –0.0000069)	1.00 (1.00–1.00)
Composite fortification standard and folic acid inclusion	Prevalence	–0.054 (–0.098 to –0.013)	0.95 (0.91–0.99)
Healthcare Access and Quality Index	EMR	–0.025 (–0.049 to –0.00086)	0.98 (0.95–1.00)
Healthcare Access and Quality Index	With-condition mortality rate	–0.052 (–0.064 to –0.041)	0.95 (0.94–0.96)

Post-model processing

Prevalence of spina bifida and encephalocele were summed and scaled to match the total for neural tube defects parent model by location, age group, sex, and year. Age-specific anencephaly prevalence was calculated separately as described above.

Congenital heart anomalies

Summary and associated health states

There are many distinct types of congenital heart anomalies with a range of anatomical patterns, severities, and requirements for medical treatment. For the purpose of estimating non-fatal outcomes, in GBD 2017, congenital heart anomalies were split into five sub-categories based on both the anatomical characteristics and the treatment requirements of each condition:

1. Single ventricle and single ventricle pathway defects
2. Complex congenital heart defects excluding single ventricle and single ventricle pathway defects
3. Malformations of great vessels, congenital valvular heart disease, and patent ductus arteriosus
4. Ventricular septal defect and atrial septal defect
5. Other congenital cardiovascular anomalies

To estimate the burden of total congenital heart anomalies, we used claims data to calculate a ratio of other-to-total, and this was applied to the sum of the other four sub-causes for each location, age group, sex, and year.

Every case of congenital heart anomalies was associated with a health state of congenital heart disease, except for a proportion of ventricular and atrial septal defects (VSD/ASD) that are considered asymptomatic. All congenital heart anomalies cases were split into a proportion without intellectual disability and a proportion with every severity from borderline to profound intellectual disability. The proportion of congenital heart anomalies cases experiencing each severity of intellectual disability were calculated using available literature sources on the prevalence and severity of intellectual disability in congenital heart anomalies populations.[14–16] The proportion of VSD/ASD cases attributed to the asymptomatic category was derived from literature sources on the long-term outcomes of patients

diagnosed with septal defects at birth.[17–19] GBD estimates of congenital heart failure were assigned to the congenital heart anomalies categories according to the proportion of total congenital heart cause-specific mortality assigned to each category of congenital heart anomalies.

Total congenital heart anomalies

Crosswalks

The MR-BRT crosswalk results are shown below.

Table 15: MR-BRT crosswalk betas for alternate definitions (reference = livebirths including those with chromosomal anomalies)

Crosswalk	Beta	Standard error
Excluding chromosomal diagnoses adjustment	–0.099	0.0010

Figure 5: Funnel plot of MR-BRT crosswalk betas for alternate definitions (reference = livebirths including those with chromosomal anomalies)

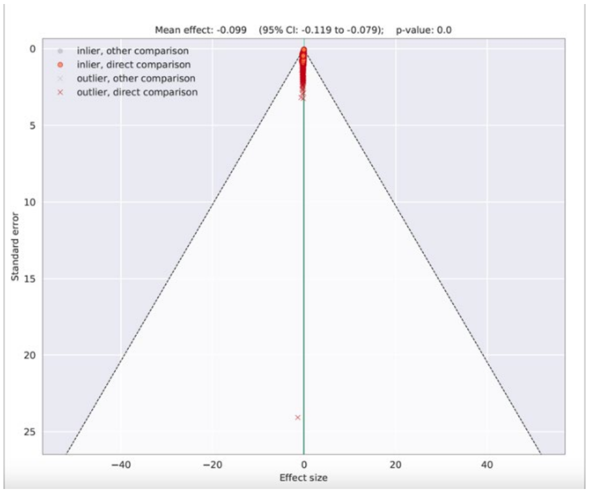

Figure 6: MR-BRT crosswalk of alternate definition (livebirths and stillbirths included) with the log-transformed neonatal mortality rate

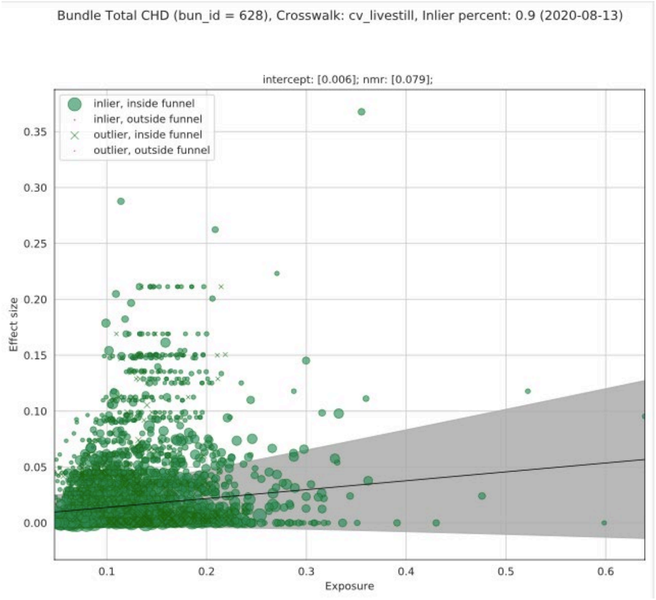

Modelling strategy

In the DisMod-MR 2.1 model of total congenital heart anomalies, random effects on prevalence were limited to ±0.5 to limit geographical variation in the estimates of birth prevalence. The minimum EMR for the neonatal age range was set to 0.1. The smoothness on EMR was increased to xi=3.0 to allow high excess mortality in the neonatal age groups and lower EMRs in older ages.

Table 16: Location-level covariate effects

Covariate name	Measure	Beta value	Exponentiated value
Maternal alcohol consumption during pregnancy (proportion)	Prevalence	0.011 (0.00036 to 0.040)	1.01 (1.00–1.04)
Healthcare Access and Quality Index	Prevalence	0.00020 (0.000047 to 0.00035)	1.00 (1.00–1.00)
Healthcare Access and Quality Index	EMR	–0.025 (–0.048 to –0.0013)	0.98 (0.95–1.00)

Single ventricle and single ventricle pathway defects

Case definition

Single ventricle and single ventricle pathway defects include tricuspid atresia, hypoplastic left heart syndrome, mitral valve atresia, single left ventricle, double outlet right ventricle, and pulmonary atresia; the corresponding ICD-10 codes are Q20.1, Q20.2, Q20.4, Q22.4, Q22.6, and Q23.4. Each of the single ventricle and single ventricle pathway conditions requires surgical intervention shortly after birth to ensure infant survival.

Crosswalks

The MR-BRT crosswalk results are shown below.

Table 17: MR-BRT crosswalk betas for alternate definitions (reference = livebirths including those with chromosomal anomalies)

Crosswalk	Beta	Standard error
Excluding chromosomal diagnoses adjustment	–0.071	0.023

Figure 7: Funnel plot of MR-BRT crosswalk betas for alternate definitions (reference = livebirths including those with chromosomal anomalies)

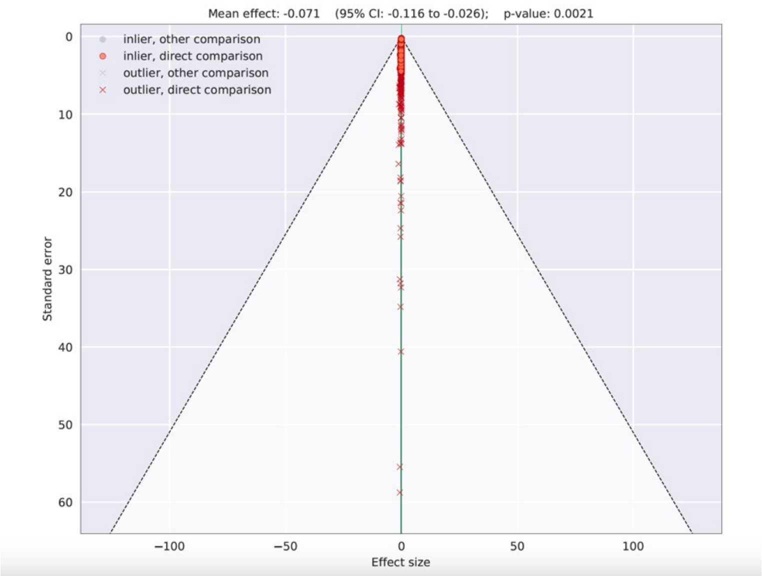

Figure 8: MR-BRT crosswalk of alternate definition (livebirths and stillbirths included) with the log-transformed neonatal mortality rate

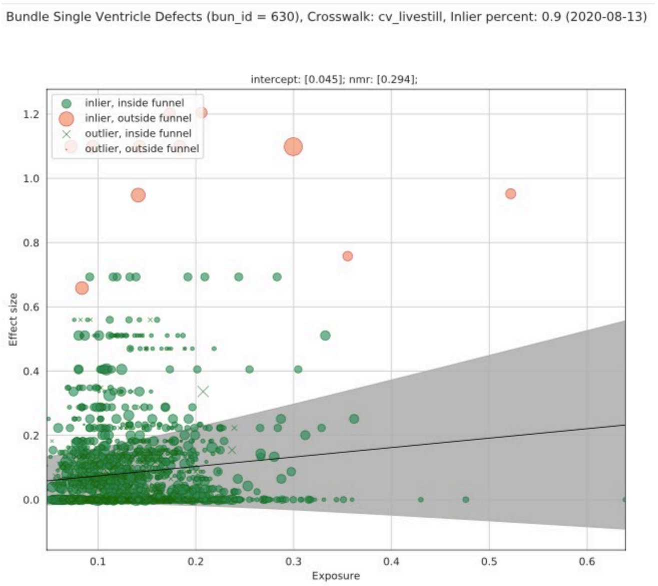

Modelling strategy

In the DisMod-MR 2.1 model of single ventricle and single ventricle pathway heart defects, random effects on prevalence were limited to ±0.5 to limit the estimated geographical variation in birth prevalence. A minimum EMR of 8 was set for the early neonatal period to capture the high mortality risk, based on expert priors and a review of available literature on the mortality risk among infants born with single ventricle and single ventricle pathway heart defects. The smoothness on EMR was set to 5.0

in order to fit steep changes in the EMR during the first weeks of life, as the risk of death due to these congenital heart anomalies is greatest shortly after birth and diminishes over the life course.

Table 18: Location-level covariate effects

Covariate name	Measure	Beta value	Exponentiated value
Maternal alcohol consumption during pregnancy (proportion)	Prevalence	0.061 (0.0038 to 0.14)	1.06 (1.00–1.15)
Healthcare Access and Quality Index	EMR	–0.05 (–0.098 to –0.0024)	0.95 (0.91–1.00)

Complex congenital heart defects excluding single ventricle and single ventricle pathway defects

Case definition

Complex congenital heart defects excluding single ventricle and single ventricle pathway defects include common arterial trunk, common truncus, discordant ventriculo-arterial connection, transposition of great vessels, atrioventricular septal defect, endocardial cushion defect, tetralogy of Fallot, aortopulmonary septal defect, pulmonary valve atresia, congenital stenosis of aortic valve, and total anomalous pulmonary venous connection. This category of severe congenital heart defects includes ICD-10 codes Q20.0, Q20.3, Q21.2, Q21.3, Q21.4, Q22.0, Q23.0, and Q26.2.

Crosswalks

The MR-BRT crosswalk results are shown below.

Table 19: MR-BRT crosswalk betas for alternate definitions (reference = livebirths including those with chromosomal anomalies)

Crosswalk	Beta	Standard error
Excluding chromosomal diagnoses adjustment	–0.191	0.017

Figure 9: Funnel plot of MR-BRT crosswalk betas for alternate definitions (reference = livebirths including those with chromosomal anomalies)

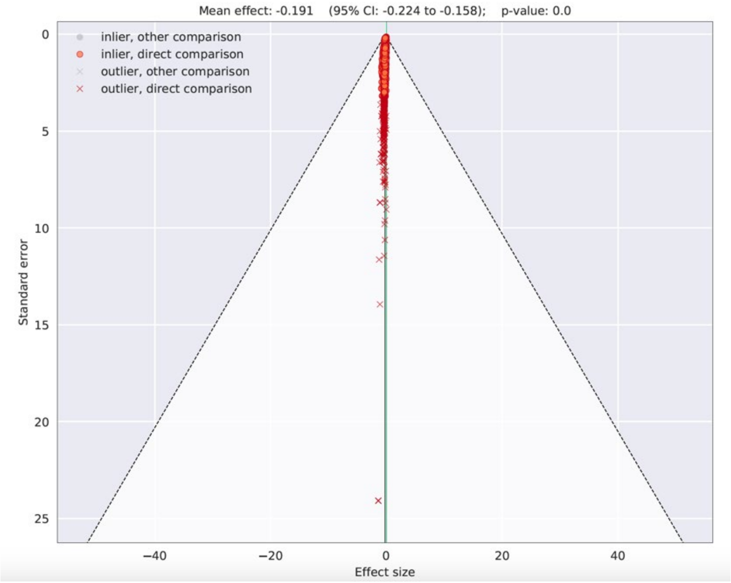

Figure 10: MR-BRT crosswalk of alternate definition (livebirths and stillbirths included) with the log-transformed neonatal mortality rate

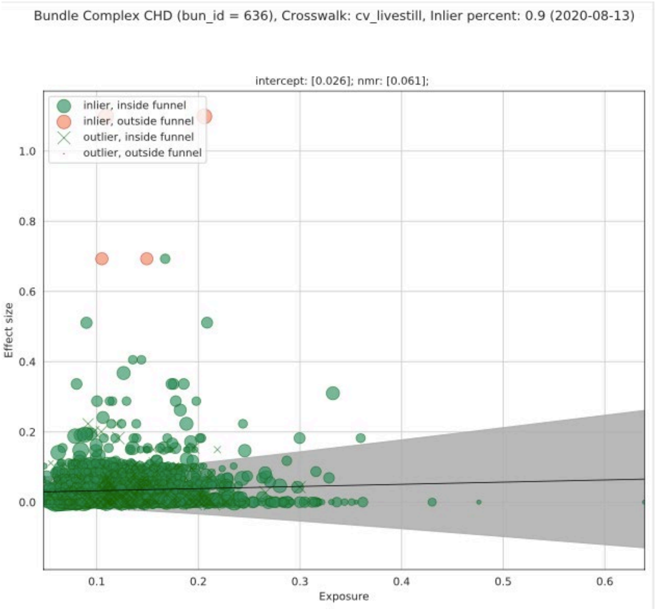

Modelling strategy

In the DisMod-MR 2.1 model of congenital heart defects excluding single ventricle and single ventricle pathway defects, random effects on prevalence were limited to ±0.5. A minimum EMR of 1.0 for the early neonatal period was enforced to capture the high risk of mortality associated with these conditions. The smoothness on EMR was set to xi = 3.0 to allow the model to fit steep changes in the mortality rate of these conditions in the neonatal age period.

Table 20: Location-level covariate effects

Covariate name	Measure	Beta value	Exponentiated value
Maternal alcohol consumption during pregnancy (proportion)	Prevalence	0.25 (0.032 to 0.49)	1.29 (1.03–1.63)
Healthcare Access and Quality Index	EMR	–0.025 (–0.049 to –0.0011)	0.97 (0.95–1.00)

Malformations of great vessels, congenital valvular heart disease, and patent ductus arteriosus

Case definition

The malformations of vessels and valves in this sub-cause category include Ebstein’s anomaly, congenital pulmonary valve stenosis, pulmonary valve insufficiency, other malformations of the pulmonary valve, malformations of the tricuspid valve, tricuspid atresia or stenosis, insufficiency of the aortic valve, mitral stenosis or insufficiency, and other malformations of aortic and mitral valves. Patent ductus arteriosus cases are only included among infants of >37 weeks gestational age, as premature infants often have minor patent ductus arteriosus that closes shortly after birth. The ICD-10 codes corresponding to the critical malformations of great vessels category include Q22.1, Q22.2, Q22.3, Q22.5, Q22.8, Q22.9, Q23.1, Q23.2, Q23.3, Q23.8, Q23, Q25.1, Q25.2, Q25.3, Q25.4, Q25.5, and Q25.0. The majority of these conditions require medical attention within the first few weeks of life.

Crosswalks

The MR-BRT crosswalk results are shown below.

Table 21: MR-BRT crosswalk betas for alternate definitions (reference = livebirths including those with chromosomal anomalies)

Crosswalk	Beta	Standard error
Excluding chromosomal diagnoses adjustment	–0.074	0.0096

Figure 11: Funnel plot of MR-BRT crosswalk betas for alternate definitions (reference = livebirths including those with chromosomal anomalies)

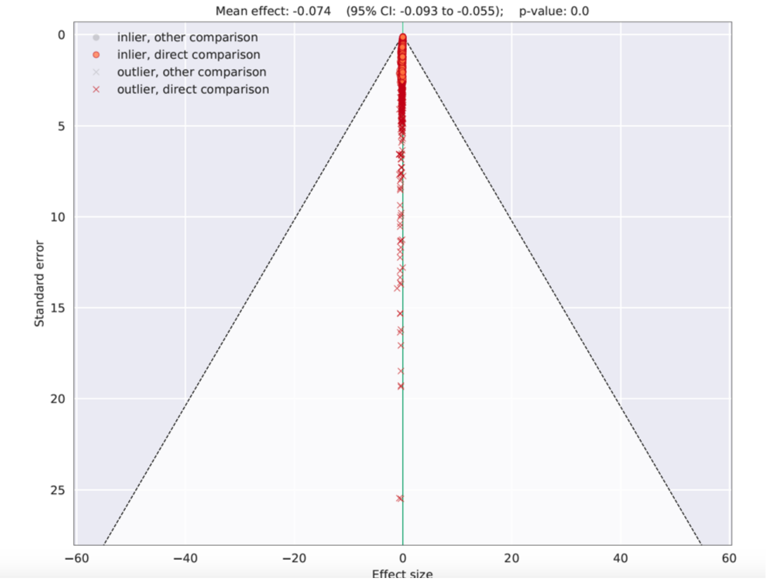

Figure 12: MR-BRT crosswalk of alternate definition (livebirths and stillbirths included) with the log-transformed neonatal mortality rate

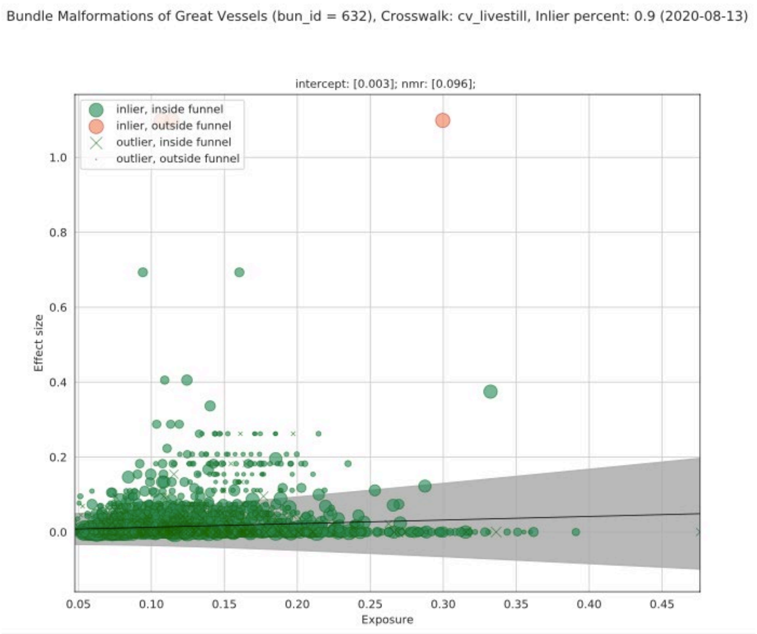

Modelling strategy

In the DisMod-MR 2.1 model of critical malformations of great vessels, congenital valvular heart disease, and patent ductus arteriosus, random effects on prevalence were limited to 0–0.5. A minimum EMR of 1.0 was set for the early neonatal period to capture the high mortality risk associated with these conditions. The smoothness on excess mortality was increased to xi=3.0 to fit steep changes in the mortality associated with these conditions during and after the neonatal period, as the risk of death due to congenital heart anomalies is highest shortly after birth.

Table 22: Location-level covariate effects

Covariate name	Measure	Beta value	Exponentiated value
Maternal alcohol consumption during pregnancy (proportion)	Prevalence	0.091 (0.0027 to 0.23)	1.10 (1.00–1.26)
Healthcare Access and Quality Index	EMR	–0.025 (–0.049 to –0.001)	0.98 (0.95–1.00)

Ventricular septal defects and atrial septal defects

Case definition

Ventricular septal defects and atrial septal defects includes holes in the walls separating the chambers of the heart. Many of these septal defects close spontaneously, while other require surgical care. The ICD-10 codes corresponding to ventricular septal defect and atrial septal defect are Q21.0 and Q21.1, respectively.

Crosswalks

The MR-BRT crosswalk results are shown below.

Table 23: MR-BRT crosswalk betas for alternate definitions (reference = livebirths including those with chromosomal anomalies)

Crosswalk	Beta	Standard error

Excluding chromosomal diagnoses adjustment	–0.089	0.0093

Figure 13: Funnel plot of MR-BRT crosswalk betas for alternate definitions (reference = livebirths including those with chromosomal anomalies)

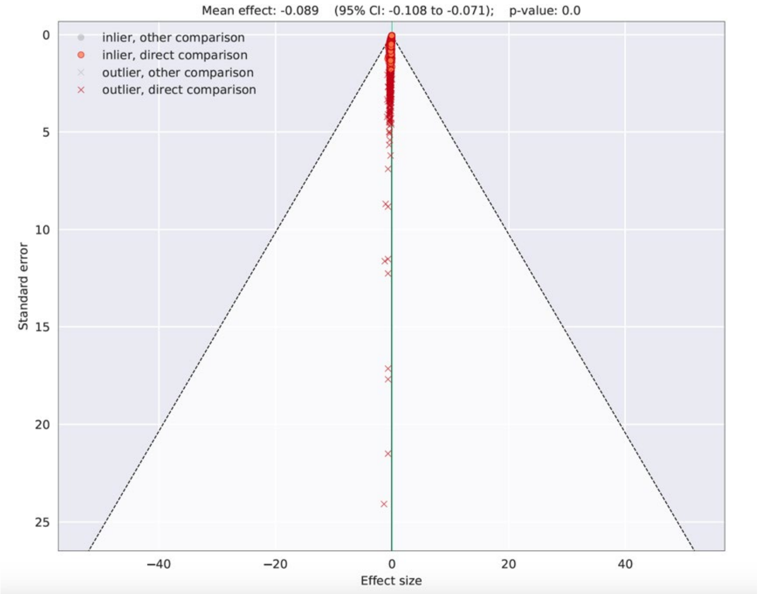

Figure 14: MR-BRT crosswalk of alternate definition (livebirths and stillbirths included) with the log-transformed neonatal mortality rate

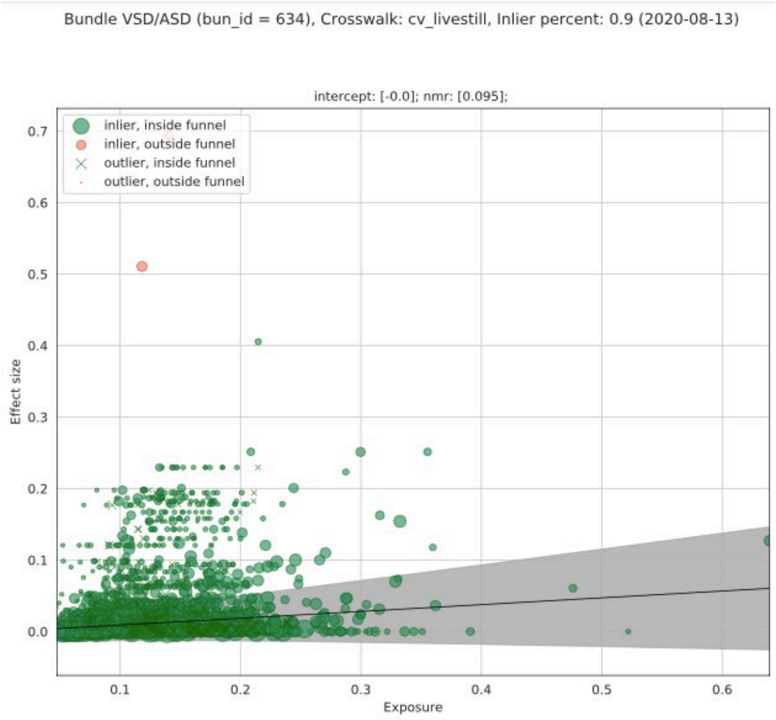

Modelling strategy

In the DisMod-MR 2.1 model of ventricular septal defects and atrial septal defects (VSD/ASD), remission was set to 0.05–0.2 for ages 0–5 years, 0–0.05 for ages 5–10 years, and zero for all subsequent ages. Random effects on prevalence were limited to ±0.3 to limit the random geographical variation in the estimated birth prevalence. No minimum EMR was set in this model, as VSD/ASD cases are not associated with EMRs as high as the other sub-types of congenital heart defects. The smoothness on EMR was set to xi=3.0, and a decreasing slope prior was set on remission for all ages, with remission set to zero past age 10 years.

Table 24: Location-level covariate effects

Covariate name	Measure	Beta value	Exponentiated value
Maternal alcohol consumption during pregnancy (proportion)	Prevalence	0.0040 (0.00013 to 0.013)	1.00 (1.00–1.01)
Healthcare Access and Quality Index	EMR	–0.026 (–0.05 to –0.002)	0.97 (0.95–1.00)

Other congenital cardiovascular anomalies

Case definition

The fifth and final sub-cause category of congenital heart anomalies is other congenital cardiovascular anomalies, which correspond to ICD-10 codes Q27, Q27.1, Q27.2, Q27.3, Q27.30, Q27.31, Q27.32, Q27.33, Q27.34, Q27.39, Q27.4, Q27.8, Q27.9, Q28, Q28.0, Q28.1, Q28.2, Q28.3, Q28.8, and Q28.9.

Modelling strategy

Other congenital cardiovascular anomalies are modelled by applying the ratio of other congenital heart anomalies to total congenital heart anomalies as it is reflected in MarketScan data, to the sum of the sub-causes of congenital cardiovascular anomalies. The result is prevalence of other congenital cardiovascular anomalies by age/year/sex/location. Specifically, we use claims data to calculate the proportion of cases that are due to the other causes. To do that, we sum the cases for the specified congenital sub-causes and the other category sub-causes. We divide the number of other sub-cause cases by the total number of cases to obtain the proportion. In order to have a valid proportion, we only use datapoints for which we have the combination of age, sex, location, and year for all sub-causes. We then calculate the prevalence of other: p_other = (p_sum_subcauses / 1-prop_other) – p_sub_subcauses.

Orofacial clefts

Case definition and associated health states

Orofacial clefts include isolated cleft lip, isolated cleft palate, and combined cleft lip and cleft palate. Cleft lip is an opening in the upper lip that may extend into the nose, and with cleft palate, the roof of the mouth contains an opening into the nose. Both conditions are the result of the tissues of the face not joining properly during development. The GBD case definition of orofacial clefts includes isolated cleft palate, which corresponds to ICD-10 codes Q35.2, Q35.3, Q35.5, Q35.6, Q35.7, Q35.8, and Q35.9,

and cleft palate with or without cleft lip, which corresponds to ICD-10 codes Q36.0, Q36.1, Q36.9, Q37.1, Q37.5, Q37.8, and Q37.9. Craniofacial clefts that do not include the oropharynx are excluded.

These conditions can be successfully treated by surgery, which is typically done during the first few months or years of life but may occasionally be completed later in life. The sequelae associated with orofacial clefts are disfigurement level 1, disfigurement level 2, and disfigurement level 2 with speech problems. Additionally, a proportion of the population with orofacial clefts is considered to be asymptomatic. In the absence of data, we assumed the proportion of each is equal.

Crosswalks

The MR-BRT crosswalk results are shown below.

Table 25: MR-BRT crosswalk betas for alternate definitions (reference = livebirths including those with chromosomal anomalies)

Crosswalk	Beta	Standard error
Excluding chromosomal diagnoses adjustment	–0.055	0.012

Figure 15: MR-BRT crosswalk of alternate definition (livebirths and stillbirths included) with spline on log-transformed neonatal mortality rate

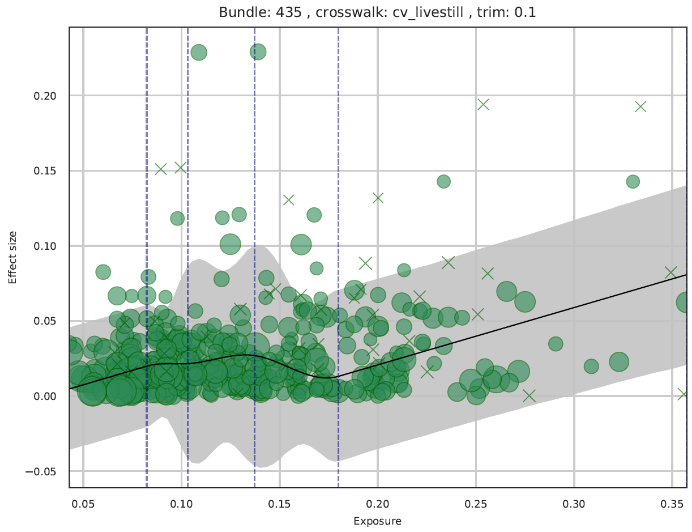

Modelling strategy

The DisMod-MR 2.1 model of orofacial clefts had random effects on prevalence limited to ±0.8, as we expected limited variation in birth prevalence of orofacial clefts. The model settings allow increased smoothness on both EMR and remission (maximum xi=5.0) to fit steep changes in the rates mortality and remission during the first few years of life.

Incidence was set to zero for all ages. Remission was set to zero for the first three months of life, as cleft lip and/or palate are rarely corrected in the first few months of life. A maximum remission of 0.8 was set for ages 3 months to 2 years, the age range in which cleft repair is most commonly performed, allowing up to 75% of cleft cases to be repaired between 3 months and 2 years of age. Remission was bounded from 0 to 0.07 for ages 2–5 years, 0–0.004 for ages 5–20 years, then bounded from 0–0.002 for ages 20–50 years, and set at zero for ages 50+ years. These limits on remission reflect our priors that up to 20% of remaining cleft cases are repaired between 2 and 5 years of age, another 5% may be repaired

between 5 and 20 years of age, and a maximum 5% of remaining cases are surgically repaired between ages 20 and 50 years.

Priors on EMR were set at a maximum of 2.5 for the early neonatal period, 0.01 for ages 5–10, and 0.000001 for ages 10+. These limits on excess mortality reflect our priors that up to 5% of individuals with orofacial clefts die in the first week of life, up to 5% die in the following three weeks, up to 20% die in the next 11 months, another maximum of 20% before 5 years of ages, and a maximum of 5% of the remaining individuals die between ages 5 and 10 years.

Table 26: Location-level covariate effects

Covariate name	Measure	Beta value	Exponentiated value
Healthcare Access and Quality Index	Prevalence	–0.000097 (–0.00019 to –0.000015)	1.00 (1.00–1.00)
Folic acid, unadjusted (μg)	Prevalence	–0.00016 (–0.00026 to –0.000071)	1.00 (1.00–1.00)
Composite fortification standard and folic acid inclusion	Prevalence	–0.0077 (–0.014 to –0.0015)	0.99 (0.99–1.00)
LN-LDI (I$ per capita)	EMR	–0.75 (–0.75 to –0.75)	0.47 (0.47–0.47)

Chromosomal anomalies

In addition to Down syndrome, Turner syndrome, and Klinefelter syndrome, hundreds of different types of chromosomal abnormalities and other genetic syndromes have been identified, described, and categorised. Commonalties between genetic syndromes include the predisposition of affected persons to have dysmorphic body features, congenital heart disease, endocrine problems, and neurodevelopmental abnormalities that can lead to intellectual disability. Many of those with chromosomal abnormalities can be readily recognised or suspected by such features. While each has hallmark physical features and diagnostic criteria, most also require sophisticated laboratory facilities to confirm diagnosis; therefore, especially in lower-resource settings, a large number of cases are diagnosed as having "unspecified chromosomal abnormalities" – an ICD code that corresponds to the GBD cause of "other chromosomal abnormalities." Additionally, most congenital birth defects registries have only limited scope as they only track a subset of genetic syndromes.

Total chromosomal anomalies

Crosswalks

The MR-BRT crosswalk results are shown below.

Figure 16: MR-BRT crosswalk of alternate definition (livebirths and stillbirths included) with spline on log-transformed neonatal mortality rate

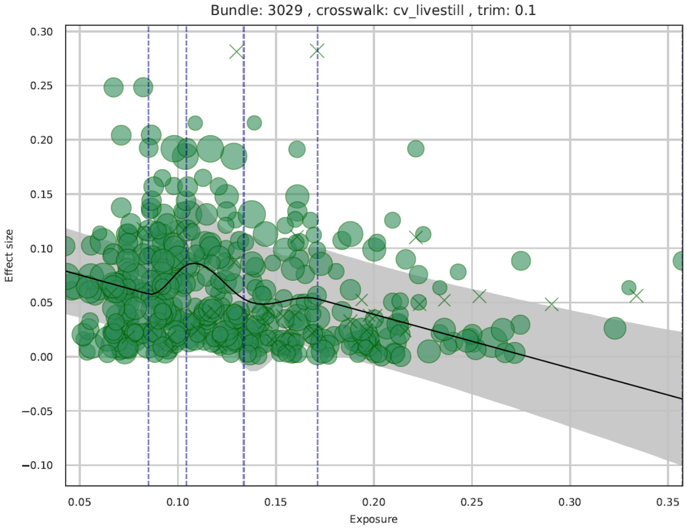

Modelling strategy

To maximise the data basis for estimating chromosomal abnormalities and genetic syndromes, we completed an analysis of all chromosomal abnormalities together, leveraging cause-specific mortality results from the GBD CoD analysis (for Down syndrome plus "other chromosomal abnormalities"), all prevalence data from registries, and clinical administrative data (hospital and claims). This model estimates total chromosomal abnormalities in DisMod-MR 2.1 and served as the basis for scaling the remaining specific causes (Down, Klinefelter, Turner, Edwards, Patau) and estimating the remainder.

Table 27: Location-level covariate effects

Covariate name	Measure	Beta value	Exponentiated value
Legality of abortion	Prevalence	–0.0054 (–0.007 to –0.004)	0.99 (0.99–1.00)
Livebirths 35+ (proportion)	Prevalence	0.24 (0.22 to 0.26)	1.27 (1.24–1.30)
Healthcare Access and Quality Index	EMR	–0.02 (–0.022 to –0.018)	0.98 (0.98–0.98)

Down syndrome

Case definition and associated health states

Down syndrome, also known as Trisomy 21, is the presence of a third copy of chromosome 21, typically caused by non-disjunction during the production of gametes. Down syndrome is associated with several specific physical characteristics, including decreased muscle tone, flat facial features, an upward slant to the eyes, abnormally shaped ears, a single deep crease across the centre of the palm, folded skin on the inner corners of the eyes, and ability to extend joints beyond the usual, among others. The GBD case definition of Down syndrome includes ICD-10 codes Q90.0, Q90.1, Q90.2, and Q90.9.

Individuals with Down syndrome may have several combinations of sequelae. Those included in the GBD sequelae list are intellectual disability, congenital heart disease, and dementia. The joint distribution of intellectual disability, congenital heart disease, and dementia associated with cases of Down syndrome was derived from a review of literature on long-term outcomes in cohorts of Down syndrome individuals. To calculate the severity distribution of intellectual disability due to Down syndrome, we used literature values for the IQ distribution of individuals with Down syndrome[20]and calculated the area under the curve. We obtained age-specific proportions of individuals with Down syndrome and dementia, and thus global age patterns were modelled to calculate the proportion of the population with each combination of sequelae for each of the following age ranges: 0–44 years, 45–49 years, 50–54 years, 55–69 years, 70–79 years, and 80+ years.

Crosswalks

The MR-BRT crosswalk results are shown below.

Figure 17: MR-BRT crosswalk of alternate definition (livebirths and stillbirths included) with spline on log-transformed neonatal mortality rate

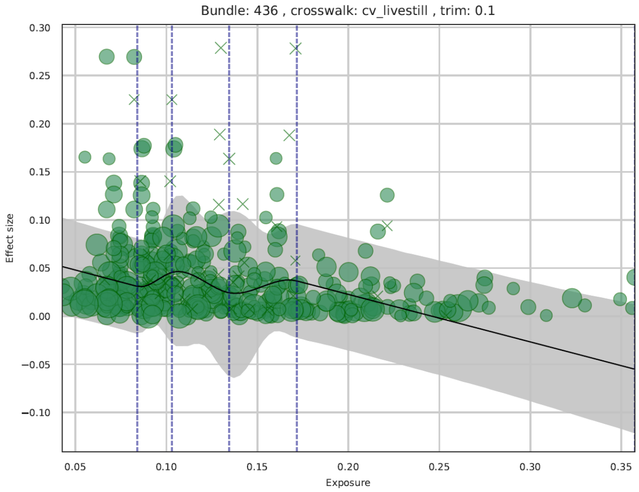

Modelling strategy

The DisMod-MR 2.1 model of Down syndrome excluded all data with a prevalence of zero as outliers, as we expect that these low values are indicative of under-reporting in the data sources. The DisMod-MR 2.1 model used CSMR data from the corresponding Down syndrome model in the GBD CoD analysis, and converted these data to EMR estimates where matching prevalence data are available. Random effects EMR were limited to ±0.1, and on prevalence to ±0.2, to limit the geographical variation in birth prevalence allowed in the model. The maximum smoothness on EMR was increased to xi=3.0 to fit the observed steep decline in the mortality risk associated with Down syndrome after the neonatal age range.

Table 28: Location-level covariate effects

Covariate name	Measure	Beta value	Exponentiated value
Legality of abortion	Prevalence	–0.0047 (–0.0055 to –0.004)	1.00 (0.99–1.00)
Livebirths 35+ (proportion)	Prevalence	0.0014 (0.000039 to 0.0040)	1.00 (1.00–1.00)
Healthcare Access and Quality Index	EMR	–0.043 (–0.044 to –0.043)	0.96 (0.96–0.96)

Turner syndrome

Case definitions and associated health states

Turner syndrome, also known as 45 XO, is a condition in which a female is partly or completely missing an X chromosome. Turner syndrome can lead to a variety of medical and developmental problems, including short height, failure to commence puberty, infertility, heart defects, learning disabilities, and difficulty with social adjustment. The GBD case definition of Turner syndrome includes ICD-10 codes Q96.0, Q96.3, and Q96.9. The sequelae associated with Turner syndrome are congenital heart disease,

infertility, and the combination of both congenital heart disease and infertility; additionally, a subset of individuals with Turner syndrome are asymptomatic. The distribution of these sequelae was determined by a review of existing literature on the long-term health consequences of Turner syndrome.[21–23]

Crosswalks

The MR-BRT crosswalk results are shown below.

Figure 18: MR-BRT crosswalk of alternate definition (livebirths and stillbirths included) with spline on log-transformed neonatal mortality rate

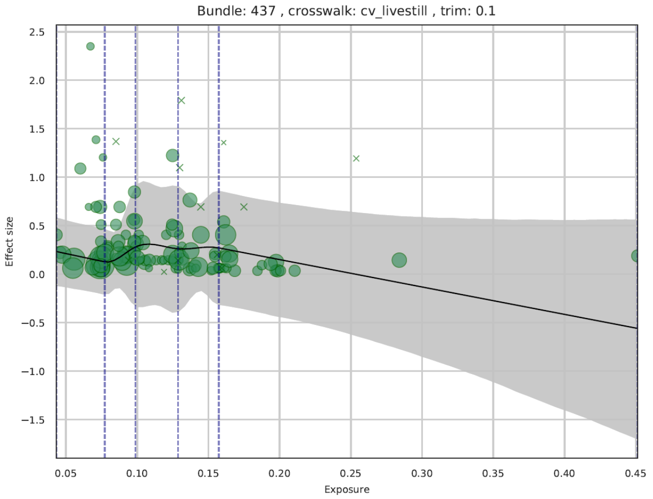

Modelling strategy

One of the known limitations to the use of birth prevalence data on Turner syndrome is that individuals with Turner syndrome are commonly diagnosed later in life rather than prenatally or at birth. Thus, we implemented a correction factor to account for under-diagnosis in all birth registry data sources, using available literature on the trends in age pattern of Turner syndrome diagnosis over time.[23] Although improvements in diagnosis have occurred over time, only between 15% and 30% of all diagnosed Turner syndrome cases are diagnosed before 1 year of age. Additionally, the reported denominators from all birth registries – the number of livebirths in each registry catchment area – were adjusted to include only female births using the GBD fertility estimates of the age, year, and location-specific proportion of total livebirths that are female. Furthermore, all prevalence data with values of zero were excluded as outliers, as these low values indicate severe under-reporting in the input data. These modelling strategy changes address known causes of under-reporting of Turner syndrome in the previous iterations of the GBD and led to higher estimates than reported previously.

The DisMod-MR 2.1 model of Turner syndrome had an EMR capped at 0.1 (slightly higher than the highest available literature estimate of EMR). The model did not have a slope prior set on EMR as the risk of mortality associated with Turner syndrome is not specific to the neonatal ages. This model also allows an increased maximum smoothness on EMR (maximum xi=3.0) and random effects on prevalence limited to ±0.5 to limit random geographical variation in the estimated birth prevalence of Turner syndrome.

Table 29: Location-level covariate effects

Covariate name	Measure	Beta value	Exponentiated value
Livebirths 35+ (proportion)	Prevalence	–0.15 (–0.28 to –0.02)	0.96 (0.76–0.98)
Healthcare Access and Quality Index	EMR	–0.025 (–0.049 to 0)	0.98 (0.95–1.00)

Klinefelter syndrome

Case definitions and associated health states

Klinefelter syndrome, also known as 47 XXY, is a condition in which a male is born with an extra X chromosome in all or some of his cells. We also include other genotypes with supernumerary X chromosomes, eg, XXXY, XXXXY, etc. The primary feature of Klinefelter syndrome is sterility, but it can cause a variety of other conditions, including weaker muscles, increased height, poor coordination abilities, smaller genitals, breast growth, and reduced sexual drive as a result of lower testosterone levels. The GBD case definition of Klinefelter syndrome includes ICD-10 codes Q98.0, Q98.5, and Q99.8. The sequelae associated with Klinefelter syndrome are borderline intellectual disability, mild intellectual disability, primary infertility, the combination of borderline intellectual disability and infertility, and the combination of mild intellectual disability and infertility. In addition, a subset of individuals with Klinefelter syndrome are asymptomatic. The distribution of these sequelae was determined by a review of existing literature on the long-term health consequences of Klinefelter syndrome.[24–26]

Crosswalks

The MR-BRT crosswalk results are shown below.

Figure 19: MR-BRT crosswalk of alternate definition (livebirths and stillbirths included) with spline on log-transformed neonatal mortality rate

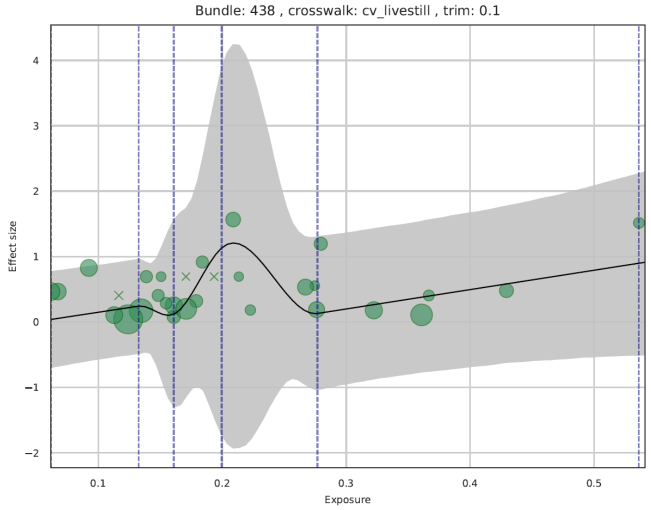

Modelling strategy

As discussed above for Turner syndrome, one limitation to the use of birth registry data for the estimation of Klinefelter syndrome is that many individuals with Klinefelter syndrome are not diagnosed prenatally or at birth. To correct this systematic under-reporting in the birth registry data, we applied a correction factor to all birth registry input data using available literature on the age pattern of Klinefelter syndrome diagnosis.[26] We also adjusted the both-sex livebirth denominators provided in registry data using location-, age-, and year-specific proportions of all livebirths that were male. Furthermore, all prevalence data with values of zero were excluded as outliers, as these low values indicate severe under-reporting in the input data.

The DisMod-MR 2.1 model of Klinefelter syndrome had an EMR maximum limit of 0.015, allowing the model to fit estimates of excess mortality up to slightly higher than the highest reported literature values. The model did not have a slope prior set on excess mortality and allowed an increased smoothness on EMR.

Table 30: Location-level covariate effects

Covariate name	Measure	Beta value	Exponentiated value
Legality of abortion	Prevalence	–0.00027 (–0.00092 to –0.000004)	1.00 (1.00–1.00)
Livebirths 35+ (proportion)	Prevalence	0.23 (0.13 to 0.30)	1.26 (1.14–1.35)
Healthcare Access and Quality Index	EMR	–0.025 (–0.049 to –0.0019)	0.98 (0.95–1.00)

Edwards and Patau syndromes

Case definitions and associated health states

Edwards syndrome, also known as Trisomy 18, is the condition in which infants are born with a third copy of chromosome 18. Patau syndrome, also known as Trisomy 13, is the condition in which infants are born with a third copy of chromosome 13. The GBD estimates the combined prevalence of these two conditions in a single model as they present similarly and are associated with similar rates of excess mortality. Infants with Edwards syndrome typically have low birthweights and a range of associated

conditions including a small head and jaw, limb abnormalities, and severe intellectual disability. Infants with Patau syndrome have a range of associated defects including musculoskeletal anomalies, developmental abnormalities of the nervous system such as microcephaly, congenital heart defects, and severe intellectual disability. The ICD-10 code for Edwards syndrome is Q91.3, and the ICD-10 code for Patau syndrome is Q91.7. All cases of Edwards and Patau syndrome were assigned the sequela of severe motor and cognitive impairment, and a proportion of these cases are also associated with congenital heart disease. The proportion of cases with associated congenital heart disease was 0.775, derived by pooling estimates from available literature as of GBD 2021 on the health states associated with the two trisomies.[27,28]

Crosswalks

The MR-BRT crosswalk results are shown below.

Figure 20: MR-BRT crosswalk of alternate definition (livebirths and stillbirths included) with spline on log-transformed neonatal mortality rate

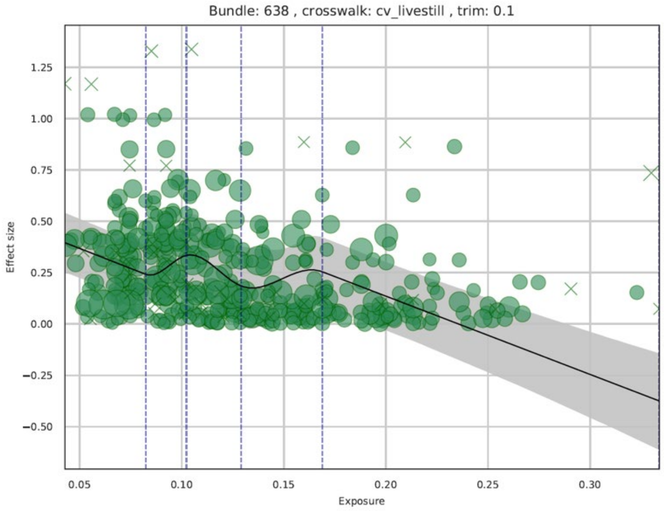

Modelling strategy

In the DisMod-MR 2.1 model of Edwards syndrome and Patau syndrome, random effects on prevalence were limited to ±0.5, reflecting the expectation of limited geographical variation in the birth prevalence of Edwards syndrome and Patau syndrome. A decreasing slope prior was set on EMR for ages 0–1 year, and an increasing slope prior was set on EMR for all ages 1+ years, as individuals with these trisomies generally die within the first few years of life. The model allowed a maximum smoothness of xi=3.0 in order to fit high excess mortality in the early age groups.

Table 31: Location-level covariate effects

Covariate name	Measure	Beta value	Exponentiated value
Legality of abortion	Prevalence	–0.004 (–0.0058 to –0.0022)	1.00 (0.99–1.00)
Livebirths 35+ (proportion)	Prevalence	0.034 (0.0013 to 0.091)	1.03 (1.00–1.10)
Healthcare Access and Quality Index	EMR	–0.025 (–0.049 to –0.0023)	0.98 (0.95–1.00)

All input data with birth prevalence values of zero were excluded as outliers, as these values represent under-reporting and low case ascertainment in the input data rather than a true lack of these chromosomal conditions in the corresponding locations.

Other chromosomal abnormalities, genetic syndromes, and micro-deletions

Case definitions and associated health states

Unbalanced chromosomal rearrangements are genetic anomalies that typically occur due to meiotic non-disjunction, when homologous chromosomes do not separate normally in nuclear division during gamete formation. The GBD case definition of other chromosomal abnormalities includes unbalanced chromosomal rearrangements such as 47, XXX (Triple X syndrome), other meiotic nondisjunction events, and other female sex chromosome abnormalities, in addition to other unspecified chromosomal abnormalities. The GBD case definition corresponds to the ICD-10 codes Q92.0, Q97.0, Q97.8, and Q99.9. Excluded from this definition are the chromosomal abnormalities of Down syndrome, Turner syndrome, Klinefelter syndrome, Edwards syndrome, and Patau syndrome, which are each modelled separately. The sequelae associated with other chromosomal anomalies include intellectual disability, intellectual disability with dementia, intellectual disability with congenital heart disease and dementia, and intellectual disability with congenital heart disease. Additionally, a proportion of the individuals with unbalanced chromosomal anomalies are asymptomatic. In the absence of available literature on the long-term health outcomes among individuals with other chromosomal conditions, the severity distributions associated with Down syndrome were used for the sequelae associated with other chromosomal anomalies.

Post-model processing

Other chromosomal anomalies were calculated based on reducing the model of total chromosomal anomalies by each of the chromosomal sub-causes, and the remaining prevalence was attributed to other chromosomal anomalies. Specifically, we use claims data to calculate the proportion of cases that are due to the other causes. To do that, we sum the cases for the specific sub-causes and the other sub-causes cases. We divide the number of other sub-cause cases by the total number of cases to obtain the proportion. In order to have a valid proportion, we only use datapoints for which we have the combination of age, sex, location, and year for all sub-causes. We then calculate the prevalence of other: p_other = (p_sum_subcauses / 1-prop_other) – p_sub_subcauses.

Congenital musculoskeletal and limb anomalies

The GBD definition of congenital musculoskeletal and limb anomalies includes any anomalies of the muscles or skeletal system present at birth that are not caused by a defined chromosomal syndrome. Within the range of congenital musculoskeletal anomalies, we explicitly model three sub-categories: polydactyly and syndactyly, limb reduction defects, and all other congenital musculoskeletal anomalies.

Total musculoskeletal birth defects

Crosswalks

The MR-BRT crosswalk results are shown below.

Table 32: MR-BRT crosswalk betas for alternate definitions (reference = livebirths including those with chromosomal anomalies)

Crosswalk	Beta	Standard error
Excluding chromosomal diagnoses adjustment	–0.053	0.007

Figure 21: MR-BRT crosswalk of alternate definition (livebirths and stillbirths included) with spline on log-transformed neonatal mortality rate

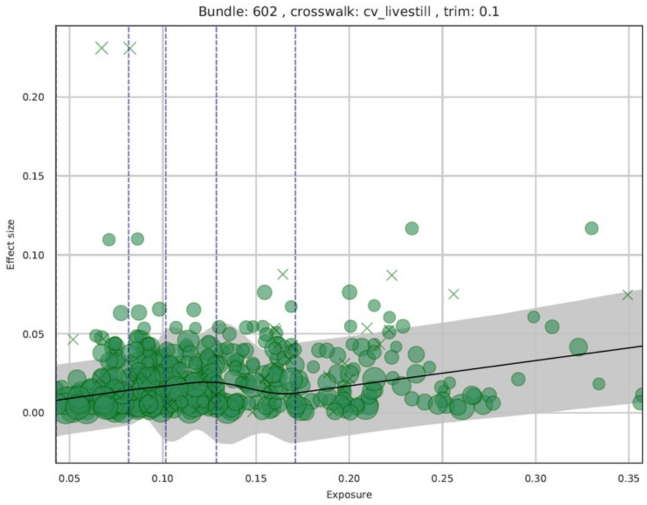

Modelling strategy

The DisMod-MR 2.1 model of total congenital musculoskeletal anomalies used cause-specific mortality estimates from the corresponding model in the GBD CoD analysis, and converted these data to excess mortality estimates where corresponding prevalence data were available. Random effects on prevalence were limited to ±1.0 to limit geographical variation in the birth prevalence of congenital musculoskeletal anomalies. Smoothness on EMR was increased to xi=3.0 to allow the model to fit a steep decrease in EMR after the earliest age groups.

Table 33: Location-level covariate effects

Covariate name	Measure	Beta value	Exponentiated value
Legality of abortion	Prevalence	–0.00012 (–0.00032 to –0.0000069)	1.00 (1.00–1.00)
Healthcare Access and Quality Index	EMR	–0.074 (–0.15 to –0.0042)	0.93 (0.86–1.00)
Age-standardised summary exposure value (SEV) for household air pollution	Prevalence	0.0063 (0.00033 to 0.016)	1.01 (1.00–1.02)
Age-standardised SEV for smoking	Prevalence	0.024 (0.0013 to 0.063)	1.02 (1.00–1.07)

Limb reduction defects

Case definitions and associated health states

Limb reduction defects are the condition where a part or all of the arm or limb of a fetus fails to form during development, so that the limb is either reduced from its normal size or missing entirely. The GBD case definition of limb reduction defects corresponds with ICD-10 codes Q71 (all three-digit codes under Q71), Q72 (all three-digit codes), Q73.0, Q73.1, and Q73.8. Of note, club foot and hip dysplasia are no longer included in this category as of GBD 2019.

All cases of limb reduction defects are associated with level 2 disfigurement. A proportion of limb reduction defect cases are associated with no motor impairment, mild motor impairment with and without pain, and moderate motor impairment with and without pain. The distribution of health states associated with congenital limb reduction was derived from an analysis of available literature on the long-term outcomes among individuals with congenital limb reductions.[29,30]

Crosswalks

The MR-BRT crosswalk results are shown below.

Table 34: MR-BRT crosswalk betas for alternate definitions (reference = livebirths including those with chromosomal anomalies)

Crosswalk	Beta	Standard error
Excluding chromosomal diagnoses adjustment	–0.042	0.034

Figure 22: MR-BRT crosswalk of alternate definition (livebirths and stillbirths included) with spline on log-transformed neonatal mortality rate

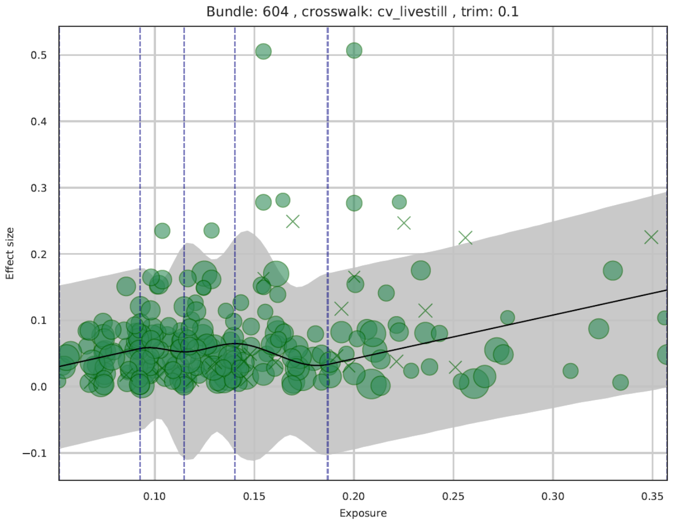

Modelling strategy

In the DisMod-MR 2.1 model of limb reduction defects, random effects on prevalence were limited to ±0.75 to limit geographical variation in the estimated birth prevalence. The EMR was set to a maximum of 0.02 for all ages to reflect the relatively low mortality risk of congenital limb anomalies. Remission for

the first 3 months of life was restricted to 0–0.02, while for 3 months to 2 years it was allowed to go up to a maximum of 1. From ages 2–5 years, remission was restricted to 0–0.1, and for all ages after 5 years old, remission was bound between 0–0.004.

Table 35: Location-level covariate effects

Covariate name	Measure	Beta value	Exponentiated value
Legality of abortion	Prevalence	–0.00039 (–0.00094 to –0.000029)	1.00 (1.00–1.00)
Healthcare Access and Quality Index	EMR	–0.025 (–0.049 to –0.00051)	0.98 (0.95–1.00)
Age-standardised SEV for household air pollution	Prevalence	1.39 (1.25 to 1.52)	4.00 (3.50–4.57)

Polydactyly and syndactyly

Case definitions and associated health states

Polydactyly is the condition of being born with at least one extra digit on either the hand or the foot, while syndactyly is absence of at least one digit. Our case definition of polydactyly corresponds to ICD-10 code Q69, and syndactyly corresponds to Q70. The sequela and health state associated with all cases of polydactyly and syndactyly is level 1 disfigurement. Remission is allowed in the model of polydactyly and syndactyly, as individuals born with these conditions may have them surgically corrected and are then no longer considered within our case definition.

Crosswalks

The MR-BRT crosswalk results are shown below.

Table 36: MR-BRT crosswalk betas for alternate definitions (reference = livebirths including those with chromosomal anomalies)

Crosswalk	Beta	Standard error
Excluding chromosomal diagnoses adjustment	–0.05	0.011

Figure 23: MR-BRT crosswalk of alternate definition (livebirths and stillbirths included) with spline on log-transformed neonatal mortality rate

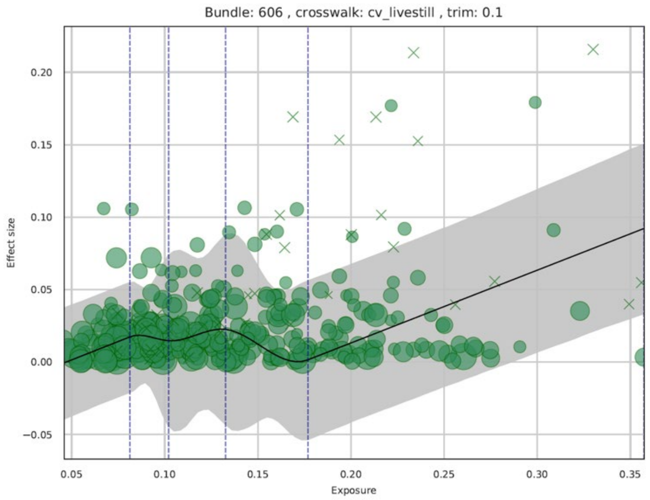

Modelling strategy

The DisMod-MR 2.1 model of polydactyly and syndactyly limited random effects on prevalence to ±0.75, as we expected limited geographical variation in the birth prevalence estimates. Excess mortality priors were set to 0 for ages 0–54 years and had a max of 0.1 for ages 55+ years, as it is not expected that someone would die of these conditions at an early age. The remission rate was bounded from 0 to 0.02 for the first 3 months of life, as surgical correction of polydactyly or syndactyly rarely occurs in the first few months of life. Remission was bounded between 0 and 5 for ages 3 months to 2 years, and between 0 and 0.5 for ages 2–5 years, the ages during which surgical correction is most likely to occur, then set to a maximum of 0.02 after 5 years of age. The smoothness on remission was set to xi=1.5 in order to facilitate steep changes in remission rates during the first few years of life.

Table 37: Location-level covariate effects

Covariate name	Measure	Beta value	Exponentiated value
LDI (I$ per capita)	Remission	1.01 (0.54–1.47)	2.74 (1.72–4.36)

Other congenital musculoskeletal anomalies

Case definitions and associated health states

The other congenital musculoskeletal anomalies included within the total estimate of congenital musculoskeletal anomalies includes clubfoot, skeletal dysplasias, congenital deformities of the spine, congenital dysplasia of the hip, and other congenital musculoskeletal anomalies. This “other” category corresponds to ICD-10 codes Q65, Q65.0, Q65.00, Q65.01, Q65.02, Q65.1, Q65.2, Q65.8, Q65.81, Q65.82, Q65.89, Q65.9, Q66, Q66.0, Q66.1, Q68, Q68.1, Q68.2, Q68.6, Q68.8, Q74, Q74.1, Q74.2, Q74.3, Q74.9, Q75, Q75.0, Q75.5, Q75.9, Q79.8, Q79.9, Q76, Q76.1, Q76.2, Q76.3, Q76.4, Q76.41, Q76.411, Q76.412, Q76.413, Q76.414, Q76.415, Q76.419, Q76.42, Q76.425, Q76.426, Q76.427, Q76.428, Q76.429, Q76.49, Q76.8, Q76.9, Q77, Q77.0, Q77.1, Q77.2, Q77.3, Q77.4, Q77.5, Q77.6, Q77.7, Q77.8, Q77.9, Q78, Q78.0, Q78.1, Q78.2, Q78.3, Q78.4, Q78.5, Q78.6, Q78.8, and Q78.9.

In the absence of comprehensive literature on the long-term outcomes associated with the category of other congenital musculoskeletal anomalies, prevalence estimates of other congenital musculoskeletal anomalies were assigned health states using the proportions derived for limb reduction defects.

Post-model processing

Other congenital musculoskeletal anomalies are modelled by applying the ratio of other congenital digestive anomalies to total congenital digestive anomalies as it is reflected in MarketScan data, to the sum of the sub-causes of congenital musculoskeletal anomalies. The result is prevalence of other congenital musculoskeletal anomalies by age/year/sex/location. Specifically, we use claims data to calculate the proportion of cases that are due to the other causes. To do that, we sum the cases for the specific sub-causes and the other sub-cause cases. We divide the number of other sub-cause cases by total number of cases to obtain the proportion. In order to have a valid proportion, we only use datapoints for which we have the combination of age, sex, location, and year for all sub-causes. We then calculate the prevalence of other: p_other = (p_sum_subcauses / 1-prop_other) – p_sub_subcauses.

Urogenital congenital anomalies

The GBD case definition of urogenital congenital anomalies includes anomalies of the genitals and the urinary system that are present at birth. While some types of urogenital congenital anomalies encompass both the urinary and genital systems, we have assigned each congenital condition as a malformation of either the urinary or the genital system in a mutually exclusive fashion and model anomalies of the urinary and genital systems separately with distinct model specifications.

Congenital urinary anomalies

Case definitions and associated health states

Congenital urinary anomalies include congenital malformation of the collecting system, ureter, bladder, and kidney, as well as bladder exstrophy and epispadias. The ICD-10 codes included in the category of urinary anomalies are Q64.0, Q64.1, Q60-Q61, and Q62-Q63.

The total prevalence of congenital urinary anomalies was split into proportions with and without each of the following health states: urinary incontinence, impotence, recurrent urinary tract infections and other recurring abdominal issues, and atypical genitalia (corresponding to level 1 disfigurement in the GBD Disability Weights Study). The distribution of these long-term outcomes was derived from a review of available literature on the long-term outcomes experienced by cohorts of individuals born with a range of congenital urogenital anomalies.[31–36]

Crosswalks

The MR-BRT crosswalk results are shown below.

Table 38: MR-BRT crosswalk betas for alternate definitions (reference = livebirths including those with chromosomal anomalies)

Crosswalk	Beta	Standard error
Excluding chromosomal diagnoses adjustment	–0.032	0.008

Figure 24: MR-BRT crosswalk of alternate definition (livebirths and stillbirths included) with spline on log-transformed neonatal mortality rate

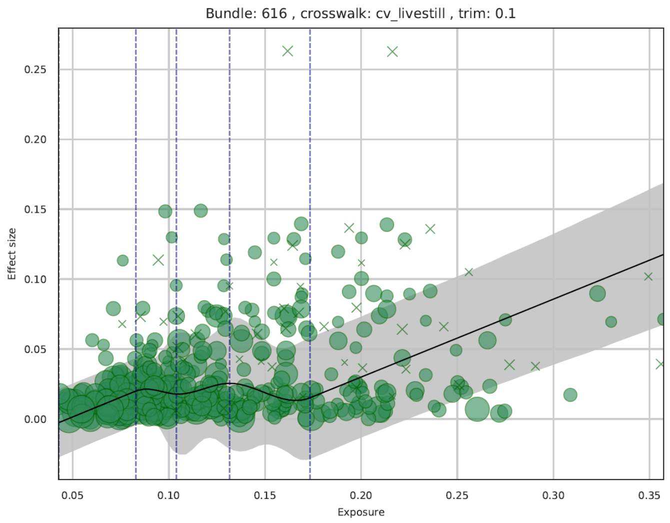

Modelling strategy

In the DisMod-MR 2.1 model of congenital urinary anomalies, random effects on prevalence were limited to ±0.5, and random effects on with-condition mortality were limited to ±1.0. The maximum EMR was set to 0.1 for all ages. The smoothness on EMR was set to xi=3 to fit changes in the EMR during the neonatal period. CSMR was also pulled in from our CoD model of congenital urogenital anomalies. As we assume no death due to congenital genital anomalies, this model represents deaths associated with exclusively congenital urinary anomalies.

Table 39: Location-level covariate effects

Covariate name	Measure	Beta value	Exponentiated value
Age-standardised SEV for ambient particulate matter	Prevalence	0.013 (0.00036 to 0.046)	1.01 (1.00–1.05)
Age-standardised SEV for high fasting plasma glucose	Prevalence	2.80 (2.61 to 2.97)	16.39 (13.61–19.57)
Healthcare Access and Quality Index	EMR	–0.029 (–0.03 to –0.027)	0.97 (0.97–0.97)

Congenital genital anomalies

Case definitions and associated health states

Congenital genital anomalies include hypospadias, ambiguous or indeterminate sex, other congenital abnormalities of the male genitalia, and a variety of female genital malformations. ICD-10 codes Q50–Q52, Q54, Q56, and Q55 (excluding Q55.20–Q55.21) are included in the case definition of congenital genital anomalies. Undescended testicles are excluded from the case definition of genital anomalies, as this is not considered a severe condition.

Cases of congenital genital anomalies was split into proportions with and without primary infertility, impotence, recurrent urinary tract infections and other recurring abdominal issues, and atypical genitalia. Estimates were produced for the prevalence of every possible combination of those long-term

sequelae, assuming independence between the outcomes. The distribution of these long-term outcomes was derived from a review of available literature on the long-term outcomes experienced cohorts of individuals born with a range of congenital urogenital anomalies.[31–36]

Crosswalks

The MR-BRT crosswalk results are shown below.

Table 40: MR-BRT crosswalk betas for alternate definitions (reference = livebirths including those with chromosomal anomalies)

Crosswalk	Beta	Standard error
Excluding chromosomal diagnoses adjustment	–0.019	0.011

Figure 25: MR-BRT crosswalk of alternate definition (livebirths and stillbirths included) with spline on log-transformed neonatal mortality rate

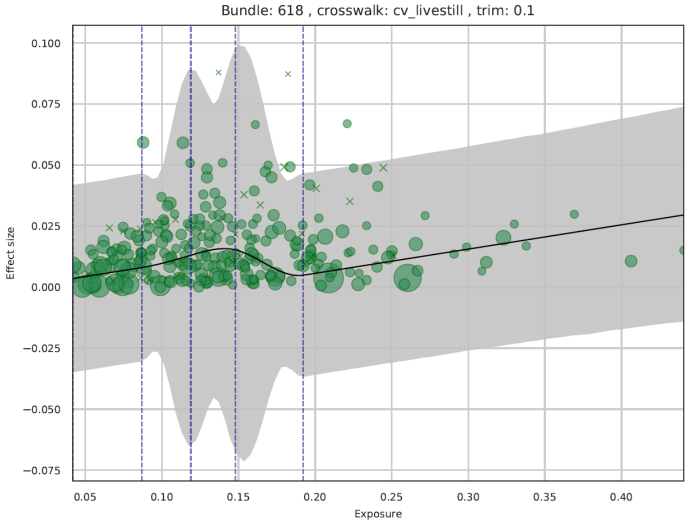

Modelling strategy

In the DisMod-MR 2.1 model of congenital genital anomalies, random effects on prevalence were limited to ±0.75 to limit random geographical variation in the estimates of birth prevalence. Excess mortality was set to zero for all ages, as we do not believe that individuals are dying due to genital anomalies. This is consistent with our CoD analysis, in which the only causes reflected in our urogenital mortality estimates are congenital urinary conditions.

Table 41. Location-level covariate effects

Covariate name	Measure	Beta value	Exponentiated value
Age-standardised SEV for ambient particulates	Prevalence	0.050 (0.0018–0.14)	1.05 (1.00–1.15)
Age-standardised SEV for high fasting plasma glucose	Prevalence	0.25 (0.042–0.48)	1.29 (1.04–1.62)

Digestive congenital anomalies

Case definitions

Digestive congenital anomalies include any anomalies of the gastrointestinal tract present at birth as the result of abnormal embryonic development. The parent cause of congenital digestive anomalies is split into four sub-cause categories.

Total digestive congenital anomalies

To ensure internal consistency in the estimates of each sub-type of congenital digestive anomalies, we generated a model to estimate the total prevalence and associated mortality due to all congenital digestive anomalies, then fit the estimates of each sub-type of congenital digestive anomalies proportionally to the envelope of this total model. The prevalence estimates of other congenital digestive anomalies were derived by reducing the total envelope model for each cause by its sub-causes to derive the difference that was attributable to other anomalies in that category. This modelling strategy allowed us to utilise the GBD CoD estimates as input to the total congenital digestive anomalies estimates and allowed us to incorporate literature data that reported only the total prevalence of all digestive anomalies.

Crosswalks

The MR-BRT crosswalk results are shown below.

Table 42: MR-BRT crosswalk betas for alternate definitions (reference = livebirths including those with chromosomal anomalies)

Crosswalk	Beta	Standard error
Excluding chromosomal diagnoses adjustment	–0.078	0.011

Figure 26: MR-BRT crosswalk of alternate definition (livebirths and stillbirths included) with spline on log-transformed neonatal mortality rate

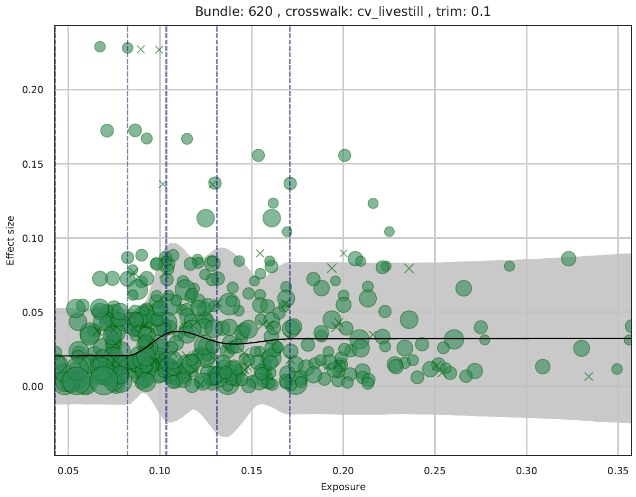

Modelling strategy

The DisMod-MR 2.1 model of total congenital digestive anomalies used cause-specific mortality estimates from the corresponding GBD CoD model of congenital digestive anomalies, and these data were converted to excess mortality estimates where corresponding cause-specific mortality estimates were available. The model had random effects on prevalence limited to ±0.5 and random effects on excess mortality limited to ±0.1. The model also had a slope prior on remission to decrease with age and have an overall all-ages maximum of 1.0. The smoothness on EMR was increased to xi=3.0 in order to fit steep changes in EMR during the neonatal age period.

Table 43: Location-level covariate effects

Covariate name	Measure	Beta value	Exponentiated value
Age-standardised SEV for smoking	Prevalence	0.052 (0.0021 to 0.20)	1.05 (1.00–1.22)
Age-standardised SEV for high BMI	Prevalence	0.99 (0.96 to 1.00)	2.69 (2.62–2.72)
Litres of alcohol consumed per capita	Prevalence	0.0031 (0.00013 to 0.0080)	1.00 (1.00–1.01)
Healthcare Access and Quality Index	EMR	–0.027 (–0.028 to –0.026)	0.97 (0.97–0.97)

Congenital diaphragmatic hernia

Case definitions and associated health states

Congenital diaphragmatic hernia, a life-threatening malformation of the diaphragm that allows the abdominal organs to push into the chest cavity and obstructs proper formation of the lungs, is modelled separately from all other congenital malformations of the digestive system. Congenital diaphragmatic hernia corresponds to ICD-10 code Q79.0.

The health outcomes associated with congenital diaphragmatic hernia include every combination of disfigurement, chronic abdominal pain, mild chronic respiratory problems, breathlessness, mild intellectual disability, and a proportion of patients who are asymptomatic. The distribution of these long-term health outcomes was derived from a pooled analysis of available literature on the long-term outcomes in surviving patients born with congenital diaphragmatic hernias.[37–39]

Crosswalks

The MR-BRT crosswalk results are shown below.

Table 44: MR-BRT crosswalk betas for alternate definitions (reference = livebirths including those with chromosomal anomalies)

Crosswalk	Beta	Standard error
Excluding chromosomal diagnoses adjustment	–0.063	0.035

Figure 27: MR-BRT crosswalk of alternate definition (livebirths and stillbirths included) with spline on log-transformed neonatal mortality rate

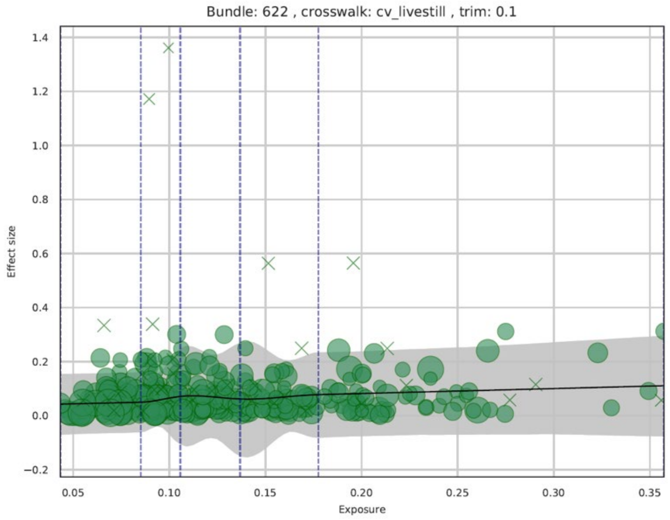

Modelling strategy

In the DisMod-MR 2.1 model of congenital diaphragmatic hernia, random effects on prevalence were set to ±0.5. The maximum excess mortality for the early neonatal age period was set to 10.0, and to 0.05 for all subsequent ages. A decreasing slope prior on remission rate was set for all ages and smoothness on EMR was increased to xi=3.0 in order to fit steep changes in EMR during the first weeks of life.

Table 45: Location-level covariate effects

Covariate name	Measure	Beta value	Exponentiated value
Age-standardised SEV for smoking	Prevalence	0.48 (0.17 to 0.79)	1.62 (1.18–2.21)
Age-standardised SEV for high BMI	Prevalence	0.065 (0.0047 to 0.15)	1.07 (1.00–1.16)
Litres of alcohol consumed per capita	Prevalence	0.00057 (0.000023 to 0.0016)	1.00 (1.00–1.00)
Healthcare Access and Quality Index	EMR	–0.025 (–0.049 to –0.0012)	0.98 (0.95–1.00)

Congenital malformations of the abdominal wall

Case definitions and associated health states

All congenital malformations of the abdominal wall are modelled together as a distinct sub-category. The primary diagnoses in this category are gastroschisis, omphalocele, and prune belly syndrome, corresponding to ICD-10 codes Q79.3, Q79.2, and Q79.4, respectively.

The health outcomes associated with congenital malformations of the abdominal wall include every combination of constipation, chronic abdominal pain, and disfigurement and concern about scars. The distribution of these outcomes was calculated from a pooled analysis of literature sources on the long-term outcomes among surviving individuals born with congenital malformations of the abdominal wall.[40,41]

Crosswalks

The MR-BRT crosswalk results are shown below.

Table 46: MR-BRT crosswalk betas for alternate definitions (reference = livebirths including those with chromosomal anomalies)

Crosswalk	Beta	Standard error
Excluding chromosomal diagnoses adjustment	–0.069	0.025

Figure 28: MR-BRT crosswalk of alternate definition (livebirths and stillbirths included) with spline on log-transformed neonatal mortality rate

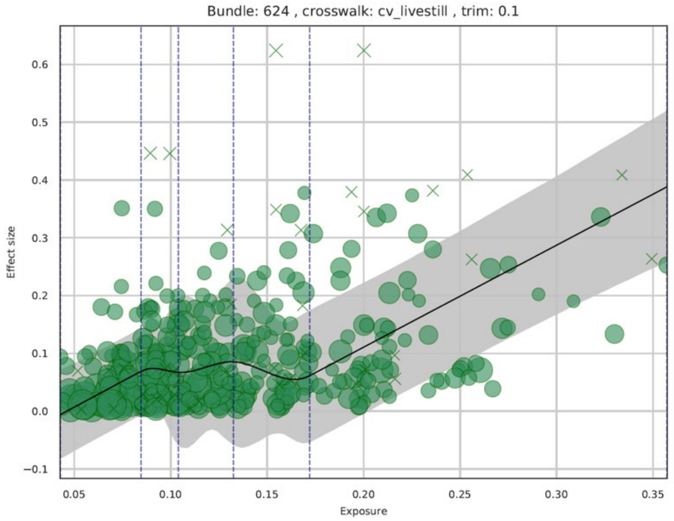

Modelling strategy

The DisMod-MR 2.1 model of congenital malformations of the abdominal wall had random effects on prevalence limited to ±0.5. The minimum EMR was set to 0.5 with a maximum EMR of 10.0, for the early neonatal period. For ages 0.5–100 years, excess mortality max was set to 0.05. A decreasing slope prior on remission was set for all ages, and the smoothness on EMR was set to xi=3.0, allowing the model to fit a steep decrease in the EMR after the neonatal age period.

Table 47: Location-level covariate effects

Covariate name	Measure	Beta value	Exponentiated value
Age-standardised SEV for smoking	Prevalence	0.047 (0.0023 to 0.13)	1.05 (1.00–1.13)
Age-standardised SEV for high BMI	Prevalence	0.038 (0.0018 to 0.10)	1.04 (1.00–1.11)
Litres of alcohol consumed per capita	Prevalence	0.00064 (0.000018 to 0.0017)	1.00 (1.00–1.00)
Healthcare Access and Quality Index	EMR	–0.025 (–0.049 to 0)	0.98 (0.95–1.00)

Congenital atresia and/or stenosis of the digestive tract

Case definitions and associated health states

All variations of congenital atresia and/or stenosis of the digestive tract are modelled together as the third distinct sub-category of digestive congenital anomalies. This includes biliary atresia, oesophageal atresia and/or stenosis with and without trachea-oesophageal fistula, and atresia and stenosis of the small intestine, large intestine, rectum, and anus. The ICD-10 codes included in the digestive tract atresia and stenosis sub-cause category are Q42.0, Q42.1, Q42.2, Q42.3, Q42.4, Q42.8, Q42.9, Q42.8, Q42.9, Q42.0, Q42.1, Q42.2, Q42.3, Q42.4, Q41 (Q41.0, Q41.1, Q41.2, Q41.8, Q41.9), Q44.2, Q39.0, Q39.1, and Q39.2.

The outcomes associated with congenital atresia and/or stenosis of the abdominal tract include every combination of dysphagia, acid reflux, chronic abdominal pain and/or nausea, and chronic respiratory problems; the distribution of these long-term outcomes was also derived from available long-term follow-up studies.[42,43]

Crosswalks

The MR-BRT crosswalk results are shown below.

Table 48: MR-BRT crosswalk betas for alternate definitions (reference = livebirths including those with chromosomal anomalies)

Crosswalk	Beta	Standard error
Excluding chromosomal diagnoses adjustment	–0.093	0.016

Figure 29: MR-BRT crosswalk of alternate definition (livebirths and stillbirths included) with spline on log-transformed neonatal mortality rate

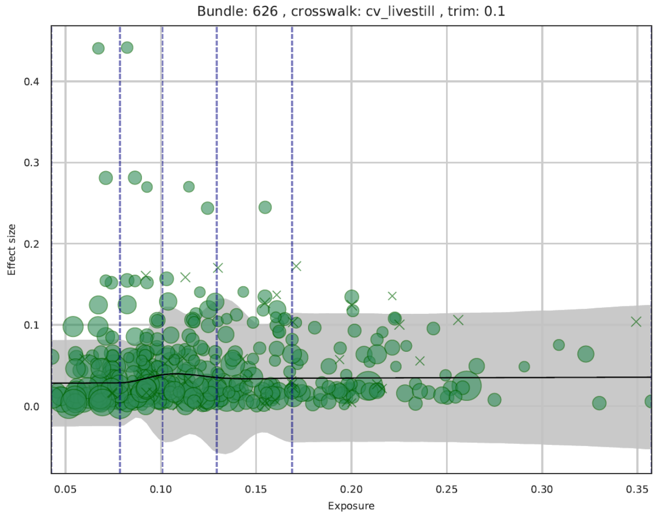

Modelling strategy

In the DisMod-MR 2.1 model of congenital atresia and/or stenosis of the digestive tract, random effects on prevalence were set to ±0.5, and random effects on with-condition mortality were set to ±1.0. A decreasing slope prior on remission was set for all ages, as remission is most likely just after birth. The smoothness on EMR was increased to xi=3.0 in order to fit steep changes in EMR during the first weeks of life, with value priors set to 2–15 for the early neonatal period and 0 for ages 70–100 years.

Table 49: Location-level covariate effects

Covariate name	Measure	Beta value	Exponentiated value
Age-standardised SEV for smoking	Prevalence	0.076 (0.0039 to 0.19)	1.08 (1.00–1.21)
Age-standardised SEV for high BMI	Prevalence	0.99 (0.96 to 1.00)	2.68 (2.62–2.72)
Litres of alcohol consumed per capita	Prevalence	0.0028 (0.00029 to 0.0059)	1.00 (1.00–1.01)
Healthcare Access and Quality Index	EMR	–0.025 (–0.049 to –0.00079)	0.98 (0.95–1.00)

Other congenital malformations of the digestive tract

Case definitions and associated health states

Other congenital malformations of the digestive tract includes ICD-10 codes Q38 (Q38.0, Q38.3, Q38.4, Q38.6, Q38.7, Q38.8), Q39 (Q39.3, Q39.4, Q39.5, Q39.6, Q39.8, Q39.9), Q40 (Q40.0, Q40.1, Q40.2, Q40.3, Q40.8, Q40.9), Q43 (Q43.1, Q43.2, Q43.3, Q43.4, Q43.5, Q43.6, Q43.7, Q43.8, Q43.9), Q44 (Q44.0, Q44.1, Q44.3, Q44.4, Q44.5, Q44.6, Q44.7), Q45 (Q45.0, Q45.1, Q45.2, Q45.3, Q45.8, Q45.9),

Q79.1, and Q79.5 (Q79.51, Q79.59). Inguinal hernias present at birth are excluded from the case definition of gastrointestinal congenital anomalies and are modelled separately as part of the estimation of inguinal hernias.

The distribution of health outcomes associated with other congenital digestive anomalies was considered to be the same as the health outcomes associated with atresia and/or stenosis of the abdominal tract.

Post-model processing

Other congenital digestive anomalies are calculated by summing all of the sub-causes of congenital digestive anomalies and subtracting this sum from the total congenital digestive model (by age/sex/year/location). This residual is the prevalence of other congenital digestive anomalies. If this residual is less than 10% of the total congenital digestive anomalies model, the other sub-causes are squeezed down and other congenital digestive anomalies becomes 10% of the total congenital digestive anomalies model.

Other congenital birth defects

In addition, of the specific types of congenital anomalies outlined in the preceding pages, there are a number of other types of defects that may be present at birth. These other congenital defects include anomalies of the ears, eyes, face, and neck; respiratory malformation and diseases; skin disorders; phakomatoses; and other neurological disorders that are not included in the case definition of neural tube defects. Estimates of the YLDs attributable to these other congenital anomalies are derived from a YLL:YLD ratio. This ratio was calculated for all congenital birth defects combined, but excluding congenital heart defects, as the location-age-sex-year-specific ratio of YLLs from the CoD estimates to YLDs from the non-fatal analyses described above. This ratio was then applied to the YLLs estimates for other congenital anomalies to derive estimated YLDs for other congenital anomalies.

References

1. Congenital Malformations Worldwide: A Report from the International Clearinghouse for Birth Defects Monitoring Systems | GHDx. http://internal-ghdx.healthdata.org/record/congenital-malformations-worldwide-report-international-clearinghouse-birth-defects (accessed April 21, 2021).

2. International Clearinghouse for Birth Defects Surveillance and Research | GHDx. http://internal-ghdx.healthdata.org/series/international-clearinghouse-birth-defects-surveillance-and-research (accessed April 21, 2021).

3. United States National Birth Defects Prevention Network (NBDPN) | GHDx. http://internal-ghdx.healthdata.org/series/united-states-national-birth-defects-prevention-network-nbdpn (accessed April 21, 2021).

4. New Zealand Birth Defects Registry (NZBDR) | GHDx. http://internal-ghdx.healthdata.org/series/new-zealand-birth-defects-registry-nzbdr (accessed April 21, 2021).

5. Immigration and Checkpoints Authority (ICA) (Singapore). Singapore Registry of Births and Deaths - Live Births By Birth Order. Singapore, Singapore: Singapore Department of Statistics.

6. European Surveillance of Congenital Anomalies (EUROCAT), International Centre on Birth Defects, World Health Organization (WHO). World Atlas of Birth Defects. 2nd ed. Geneva, Switzerland: World Health Organization (WHO), 2003.

7. Jaquier M. Anencephaly Online Survey. Anencephaly.info [Internet]. 2006.

8. Jaquier M, Klein A, Boltshauser E. Spontaneous pregnancy outcome after prenatal diagnosis of anencephaly. *BJOG*. 2006; 113(8): 951-3.

9. Lanton AP. The Characteristics of Patients with Encephaloceles. *Z Kinderchir*. 1990; 45(Suppl 1): 18-9.

10. Da Silva SL, Jeelani Y, Dang H, Krieger MD, McComb JG. Risk factors for hydrocephalus and neurological deficit in children born with an encephalocele. *J Neurosurg Pediatr*. 2015; 15(4): 392–8.

11. Lo BWY, Kulkarni AV, Rutka JT, Jea A, Drake JM, Lamberti-Pasculli M, Dirks PB, Thabane L. Clinical predictors of developmental outcome in patients with cephaloceles. *J Neurosurg Pediatr*. 2008; 2(4): 254–7.

12. Moeini Naghani I, Hashemi Zonouz T, Shahjouei S, Homayoun AA, Nejat F, El Khashab M. Congenital cardiac anomalies in myelomeningocele patients. *Acta Med Acad*. 2014; 43(2): 160–4.

13. Oakeshott P, Hunt GM, Poulton A, Reid F. Open spina bifida: birth findings predict long-term outcome. *Arch Dis Child.* 2012; 97(5): 474–6.

14. Riehle-Colarusso T, Autry A, Razzaghi H, Boyle CA, Mahle WT, Van Naarden Braun K, Correa A. Congenital Heart Defects and Receipt of Special Education Services. *Pediatrics*. 2015; 136(3): 496–504.

15. Menting ME, Cuypers JAAE, Opić P, Utens EMWJ, Witsenburg M, van den Bosch AE, van Domburg RT, Meijboom FJ, Boersma E, Bogers AJJC, Roos-Hesselink JW. The unnatural history of the ventricular septal defect: outcome up to 40 years after surgical closure. *J Am Coll Cardiol*. 2015; 65(18): 1941–51.

16. Gaynor JW, Stopp C, Wypij D, Andropoulos DB, Atallah J, Atz AM, Beca J, Donofrio MT, Duncan K, Ghanayem NS, Goldberg CS, Hövels-Gürich H, Ichida F, Jacobs JP, Justo R, Latal B, Li JS, Mahle WT, McQuillen PS, Menon SC, Pemberton VL, Pike NA, Pizarro C, Shekerdemian LS, Synnes A, Williams I, Bellinger DC, Newburger JW, International Cardiac Collaborative on Neurodevelopment (ICCON) Investigators. Neurodevelopmental outcomes after cardiac surgery in infancy. *Pediatrics*. 2015; 135(5): 816–25.

17. Wren C, O'Sullivan JJ. Survival with congenital heart disease and need for follow up in adult life. *Heart*. 2001; 85(4): 438–43.

18. Gabriel HM, Heger M, Innerhofer P, Zehetgruber M, Mundigler G, Wimmer M, Maurer G, Baumgartner H. Long-term outcome of patients with ventricular septal defect considered not to require surgical closure during childhood. *J Am Coll Cardiol*. 2002; 39(6): 1066–71.

19. Neumayer U, Stone S, Somerville J. Small ventricular septal defects in adults. *Eur Heart J*. 1998; 19(10): 1573–82.

20. Epstein CJ. Down syndrome (trisomy 21). In: Scriver CR, Beaudet AL, Sly WS, Valle D, eds. The Metabolic Basis of Inherited Disease. 7th ed. New York, United States: McGraw Hill Inc., 1995.

21. Stochholm K, Juul S, Juel K, Naeraa RW, Gravholt CH. Prevalence, incidence, diagnostic delay, and mortality in Turner syndrome. *J Clin Endocrinol Metab*. 2006; 91(10): 3897–902.

22. Price WH, Clayton JF, Collyer S, De Mey R, Wilson J. Mortality ratios, life expectancy, and causes of death in patients with Turner's syndrome. *J Epidemiol Community Health*. 1986; 40(2): 97-102.

23. Massa G, Verlinde F, De Schepper J, Thomas M, Bourguignon JP, Craen M, de Zegher F, Francois I, Du Caju M, Maes M, Heinrichs C, in collaboration with the Belgian Study Group for Paediatric Endocrinology. Trends in age at diagnosis of Turner syndrome. *Arch Dis Child*. 2005; 90(3): 267-8.

24. Abramsky L, Chapple J. 47,XXY (Klinefelter syndrome) and 47,XYY: estimated rates of and indication for postnatal diagnosis with implications for prenatal counselling. *Prenat Diagn*. 1997; 17(4): 363–8.

25. Nahata L, Rosoklija I, Yu RN, Cohen LE. Klinefelter Syndrome: Are We Missing Opportunities for Early Detection?. *Clin Pediatr*. 2013; 52(10): 936-41.

26. Bojesen A, Juul S, Gravholt CH. Prenatal and Postnatal Prevalence of Klinefelter Syndrome: A National Registry Study. *J Clin Endocrinol Metab*. 2003; 88(2): 622-6.

27. Petry P, Polli JB, Mattos VF, Rosa RCM, Zen PRG, Graziadio C, Paskulin GA, Rosa RFM. Clinical features and prognosis of a sample of patients with trisomy 13 (Patau syndrome) from Brazil. *Am J Med Genet A*. 2013; 161A(6): 1278–83.

28. Polli JB, Groff D de P, Petry P, Mattos VF, Rosa RCM, Zen PRG, Graziadio C, Paskulin GA, Rosa RFM. Trisomy 13 (Patau syndrome) and congenital heart defects. *Am J Med Genet A*. 2014; 164A(1): 272–5.

29. Johansen H, Østlie K, Andersen LØ, Rand-Hendriksen S. Adults with congenital limb deficiency in Norway: demographic and clinical features, pain and the use of health care and welfare services. A cross-sectional study. *Disabil Rehabil*. 2015; 37(22): 2076–82.

30. Johansen H, Dammann B, Oinæs Andersen L, Andresen I-L. Children with congenital limb deficiency in Norway: issues related to school life and health-related quality of life. A cross-sectional study. *Disabil Rehabil*. 2016; 38(18): 1803–10.

31. Ching CB, Wood HM, Ross JH, Gao T, Angermeier KW. The Cleveland Clinic experience with adult hypospadias patients undergoing repair: their presentation and a new classification system. *BJU Int*. 2011; 107(7): 1142–6.

32. Davies MC, Liao L-M, Wilcox DT, Woodhouse CRJ, Creighton SM. Anorectal malformations: what happens in adulthood?. *BJU Int*. 2010; 106(3): 398–404.

33. Rintala RJ. Congenital cloaca: Long-term follow-up results with emphasis on outcomes beyond childhood. *Semin Pediatr Surg.* 2016; 25(2): 112–6.

34. Sircili MHP, e Silva FA de Q, Costa EMF, Brito VN, Arnhold IJP, Dénes FT, Inacio M, de Mendonca BB. Long-term surgical outcome of masculinizing genitoplasty in large cohort of patients with disorders of sex development. J *Urol*. 2010; 184(3): 1122–7.

35. van der Zwan YG, Callens N, van Kuppenveld J, Kwak K, Drop SLS, Kortmann B, Dessens AB, Wolffenbuttel KP, Dutch Study Group on DSD. Long-term outcomes in males with disorders of sex development. *J Urol*. 2013; 190(3): 1038–42.

36. Warne SA, Wilcox DT, Creighton S, Ransley PG. Long-term gynecological outcome of patients with persistent cloaca. *J Urol*. 2003; 170(4 Pt 2): 1493–6.

37. Crankson SJ, Al Jadaan SA, Namshan MA, Al-Rabeeah AA, Oda O. The immediate and long-term outcomes of newborns with congenital diaphragmatic hernia. *Pediatr Surg Int*. 2006; 22(4): 335-40.

38. Öst E, Joelsson MÖ, Burgos CM, Frenckner B. Self-assessed physical health among children with congenital diaphragmatic hernia. *Pediatr Surg Int*. 2016; 32(5): 493–503.

39. Rocha GM, Bianchi RF, Severo M, Rodrigues MM, Baptista MJ, Correia-Pinto J, Guimarães HA. Congenital Diaphragmatic Hernia. The Post-neonatal period. Part II. *Eur J Pediatr Surg*. 2008; 18(5): 307–12.

40. van Eijck FC, Wijnen RMH, van Goor H. The incidence and morbidity of adhesions after treatment of neonates with gastroschisis and omphalocele: a 30-year review. *J Pediatr Surg*. 2008; 43(3): 479–83.

41. Harris EL, Minutillo C, Hart S, Warner TM, Ravikumara M, Nathan EA, Dickinson JE. The long term physical consequences of gastroschisis. *J Pediatr Surg*. 2014; 49(10): 1466–70.

42. Dingemann C, Meyer A, Kircher G, Boemers TM, Vaske B, Till H, Ure BM. Long-term health-related quality of life after complex and/or complicated esophageal atresia in adults and children registered in a German patient support group. *J Pediatr Surg*. 2014; 49(4): 631–8.

43. Lilja HE, Wester T. Outcome in neonates with esophageal atresia treated over the last 20 years. *Pediatr Surg Int.* 2008; 24(5): 531–6.

COVID-19

Flowcharts

Cases of SARS-CoV-2 infection from the IHME COVID infections model

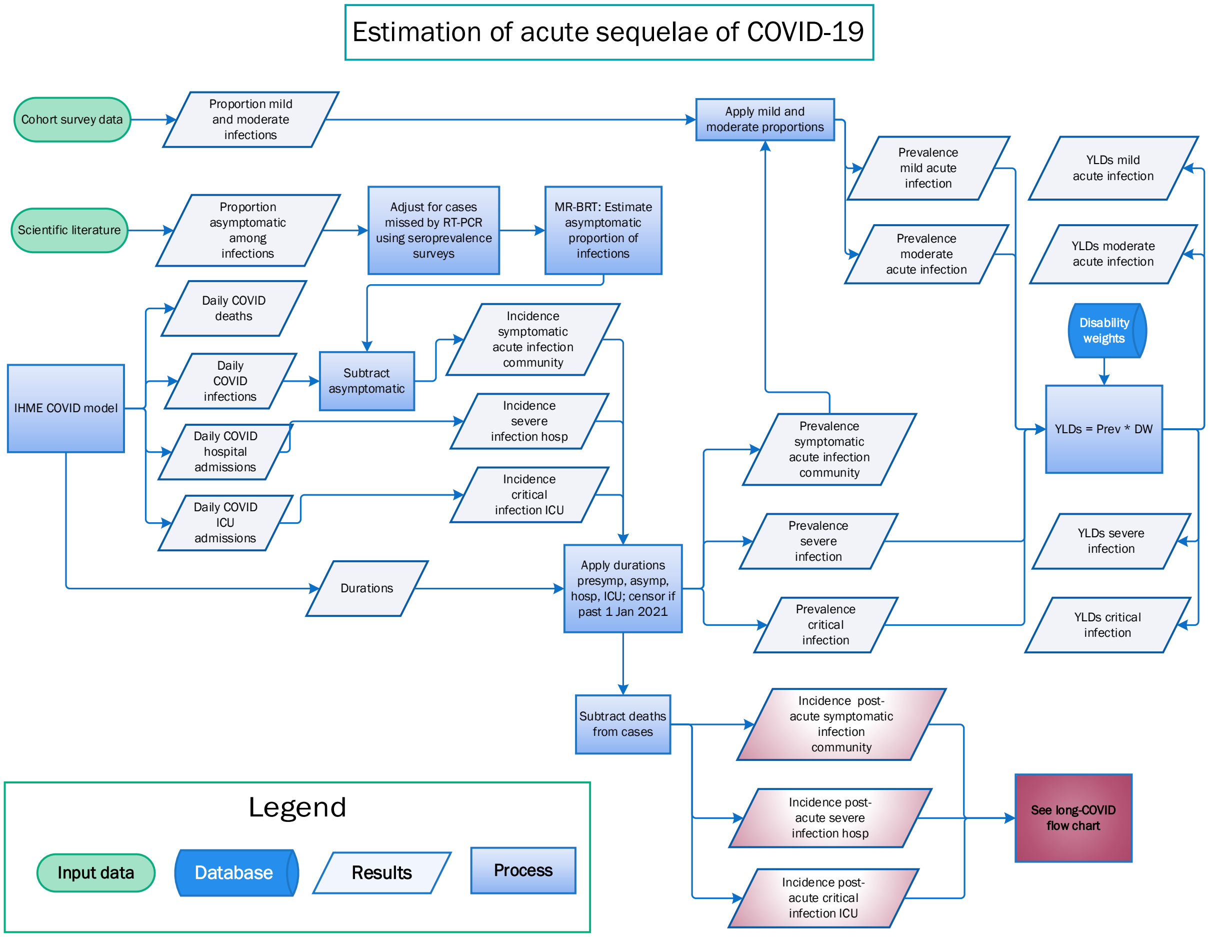

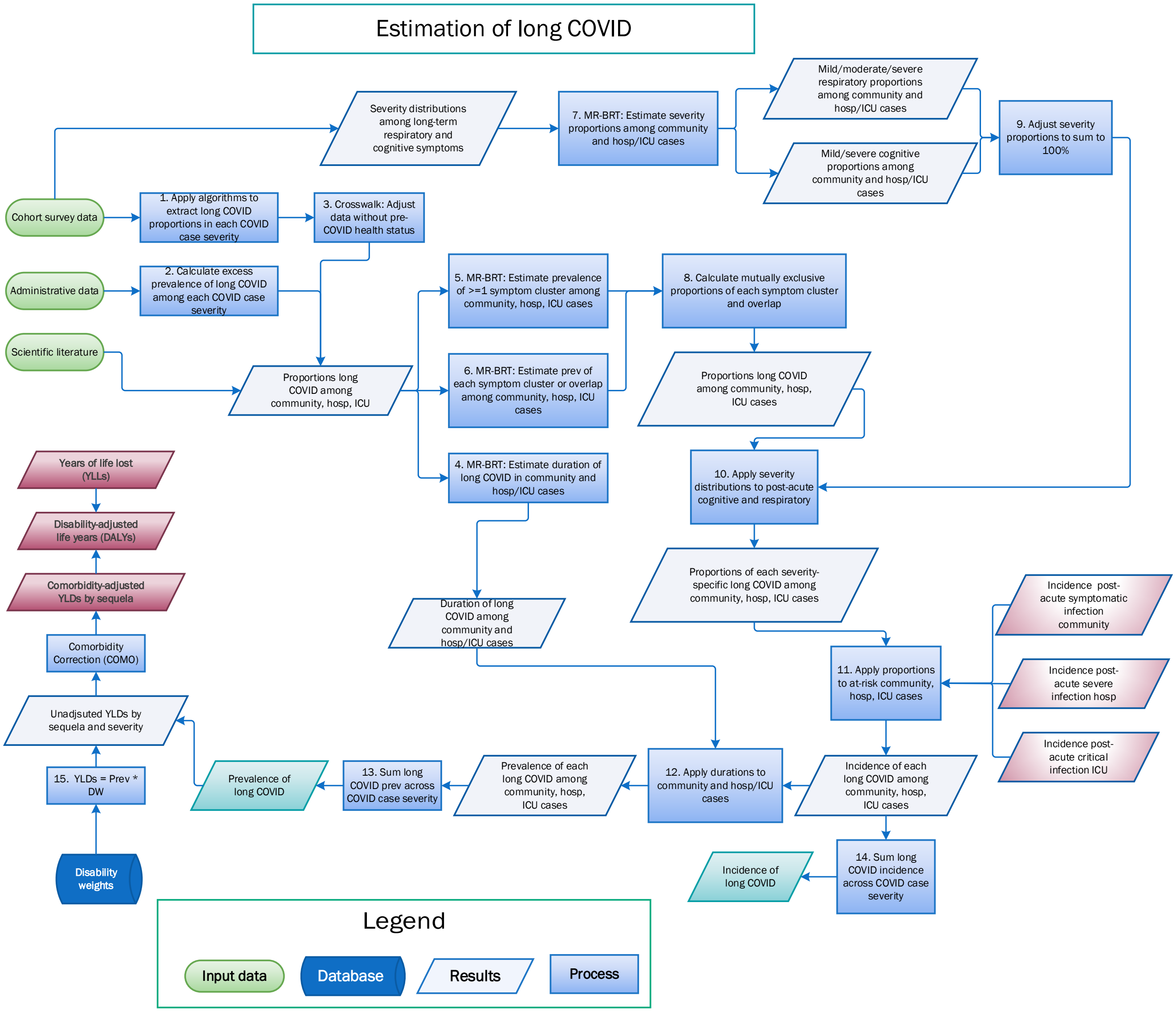

Estimation of long COVID
Severity distributions among long-term respiratory and cognitive symptoms
7. MR-BRT: Estimate severity proportions among community and hosp/ICU cases
Mild/moderate/severe respiratory proportions among community and hosp/ICU cases
Mild/severe cognitive proportions among community and hosp/ICU cases
9. Adjust severity proportions to sum to 100%
Cohort survey data
1. Apply algorithms to extract long COVID proportions in each COVID case severity
3. Crosswalk: Adjust data without pre-COVID health status
Administrative data
2. Calculate excess prevalence of long COVID among each COVID case severity
Scientific literature
Proportions long COVID among community, hosp, ICU
5. MR-BRT: Estimate prevalence of >=1 symptom cluster among community, hosp, ICU cases
6. MR-BRT: Estimate prev of each symptom cluster or overlap among community, hosp, ICU cases
4. MR-BRT: Estimate duration of long COVID in community and hosp/ICU cases
8. Calculate mutually exclusive proportions of each symptom cluster and overlap
Proportions long COVID among community, hosp, ICU cases
10. Apply severity distributions to post-acute cognitive and respiratory
Proportions of each severity-specific long COVID among community, hosp, ICU cases
Years of life lost (YLLs)
Disability-adjusted life years (DALYs)
Comorbidity-adjusted YLDs by sequela
Comorbidity Correction (COMO)
Unadjsuted YLDs by sequela and severity
15. YLDs = Prev * DW
Disability weights
Duration of long COVID among community and hosp/ICU cases
Incidence post-acute symptomatic infection community
Incidence post-acute severe infection hosp
Incidence post-acute critical infection ICU
11. Apply proportions to at-risk community, hosp, ICU cases
Incidence of each long COVID among community, hosp, ICU cases
12. Apply durations to community and hosp/ICU cases
Prevalence of each long COVID among community, hosp, ICU cases
13. Sum long COVID prev across COVID case severity
Prevalence of long COVID
14. Sum long COVID incidence across COVID case severity
Incidence of long COVID
Legend
Input data
Database
Results
Process

Input data and methodological summary for long COVID

Case definitions

Infections: People infected with the severe respiratory syndrome coronavirus 2 (SARS-CoV-2), including both asymptomatic and symptomatic cases and regardless of testing availability, quality, or hospital utilisation.

Need for hospital admissions: Symptomatic SARS-CoV-2 cases severe enough to warrant hospitalisation, regardless of access or utilisation.

Need for ICU admissions: Critical symptomatic SARS-CoV-2 cases that required ICU care, regardless of access or utilisation.

Deaths: Deaths due to SARS-CoV-2 infections.

Table 1. List of case definitions for the non-fatal outcomes due to SARS-CoV-2 infection

Quantity of interest	Reference, alternative, or clinical	Definition
Post-acute symptom cluster: fatigue, bodily pain and/or mood swings	Clinical	Fatigue with bodily pain and/or mood swings after an acute episode of COVID-19 which affect daily functioning and were not preexisting symptoms before SARS-CoV-2 infection.
Post-acute symptom cluster: fatigue, bodily pain and/or mood swings	Reference	Self-report case with new or worse symptoms of fatigue with bodily pain and/or mood swings, after an acute episode of COVID-19 which affect daily functioning.
Post-acute symptom cluster: fatigue, bodily pain and/or mood swings	Alternative	Self-report case with symptoms of fatigue, with bodily pain and/or mood swings, after an acute episode of COVID-19 which affect daily functioning but without comparison to pre-COVID health.
Post-acute symptom cluster: fatigue, bodily pain and/or mood swings	Alternative	Case with fatigue derived from medical record databases, matched with COVID-negative people.
Post-acute symptom cluster: fatigue, bodily pain and/or mood swings	Alternative	Self-reported case of fatigue after an acute episode of COVID-19, from published studies.
Post-acute symptom cluster: respiratory symptoms	Clinical	Shortness of breath and other respiratory symptoms such as cough after an acute episode of COVID-19 which affect daily functioning and were not preexisting symptoms before SARS-CoV-2 infection.

Post-acute symptom cluster: respiratory symptoms	Reference	Self-reported case of newly developed or worsened shortness of breath and other respiratory symptoms such as cough after an acute episode of COVID-19 which affect daily functioning.
Post-acute symptom cluster: respiratory symptoms	Alternative	Self-reported case of shortness of breath and other respiratory symptoms such as cough after an acute episode of COVID-19 which affect daily functioning but without comparison to pre-COVID health.
Post-acute symptom cluster: respiratory symptoms	Alternative	Case of shortness of breath derived from medical record databases, matched with COVID-negative people.
Post-acute symptom cluster: respiratory symptoms	Alternative	Self-reported case of shortness of breath after an acute episode of COVID-19, from published studies.
Post-acute symptom cluster: cognitive symptoms	Clinical	Memory problems or poor concentration after an acute episode of COVID-19 which affect daily functioning and were not preexisting symptoms before SARS-CoV-2 infection.
Post-acute symptom cluster: cognitive symptoms	Reference	Self-reported case of new onset or worsened memory problems or poor concentration after an acute episode of COVID-19 which affect daily functioning.
Post-acute symptom cluster: cognitive symptoms	Alternative	Self-reported case of memory problems or poor concentration after an acute episode of COVID-19 which affect daily functioning but without comparison to pre-COVID health.
Post-acute symptom cluster: cognitive symptoms	Alternative	Case with cognitive symptoms derived from medical record databases, matched with COVID-negative people.
Post-acute symptom cluster: cognitive symptoms	Alternative	Self-reported case of memory problems or poor concentration after an acute episode of COVID-19, from published studies.
SARS-CoV-2 infection	Reference	People infected with the virus SARS-CoV-2, including both asymptomatic and symptomatic cases.
Asymptomatic SARS-CoV-2 infection	Reference	Proportion of SARS-CoV-2 infections that exhibit no clinical symptoms due to infection.
Mild COVID-19 acute episode	Reference	A case of COVID-19 with mild symptoms corresponding to the GBD health state of mild infectious disease episode.
Moderate COVID-19 acute episode	Reference	A case of COVID-19 with moderate symptoms corresponding to the GBD health state of moderate infectious disease episode.
Severe COVID-19 acute episode	Reference	A case of COVID-19 needing hospitalisation due to severe symptoms corresponding to the GBD health state of severe infectious disease episode.

Critical COVID-19 acute episode	Reference	A case of COVID-19 needing ICU care due to critical symptoms corresponding to the GBD health state of ICU admission.
Mild respiratory symptom cluster	Reference	A case with mild respiratory symptoms defined by the GBD health state of mild COPD (has cough or shortness of breath after heavy physical activity but is able to walk long distances and climb stairs).
Moderate respiratory symptom cluster	Reference	A case with moderate respiratory symptoms defined by the GBD health state of moderate COPD (has cough, wheezing, and shortness of breath, even after light physical activity).
Severe respiratory symptom cluster	Reference	A case with severe respiratory symptoms defined by the GBD health state of severe COPD (has cough, wheezing, and shortness of breath all the time and great difficulty walking even short distances or climbing any stairs).
Mild cognitive symptom cluster	Reference	A case with mild cognitive symptoms defined by the GBD health state of mild dementia (having some trouble remembering recent events and finding it hard to concentrate and make decisions and plans).
Severe cognitive symptom cluster	Reference	A case with severe cognitive symptoms defined by the GBD health state of moderate dementia (having memory problems and confusion, feeling disoriented, and needing help with some daily activities).

Infections

Estimates of SARS-CoV-2 infections were obtained from the IHME COVID-19 Forecasting Team.[1]

Systematic literature review

Methods

The design and dissemination of findings for this systematic literature review and meta-analysis followed the Preferred Reporting Items for Systematic Review and Meta-Analysis (PRISMA) statement (see Figure 1).[2] The study protocol implemented in GBD 2021 was documented in the International Prospective Register of Systematic Reviews (PROSPERO), Registration Number: CRD42020210101.[3]

Information sources and search

For the initial analysis, we had search terms for the study developed by co-authors at Duke University in consultation with a medical librarian who specialises in systematic literature reviews. We expanded upon the initial search strategy, adding new search strings to account for any additional non-fatal outcomes due to COVID-19. Additionally, we expanded the study publication dates to account for any studies or analyses on long COVID published between 2020 and 2023. Search terms were used to identify articles describing non-fatal, clinical outcomes in patients with confirmed COVID-19. The search strategy was reviewed and refined by the core team of experts at IHME, as well as the core group of expert collaborators working on long COVID. We used the following databases as part of our systematic

literature review: PubMed, Embase, CINAHL, Global Health, Web of Science, and WHO Covid-19 Research Database.

Eligibility criteria

As in GBD 2021, we included studies of people with SARS-CoV-2 confirmed by RT-PCR test with clinical outcomes caused by COVID-19 and diagnosed by health professionals. We excluded studies among populations with pre-existing conditions and where COVID-19 was self-reported or suspected. We excluded papers that only reported imaging (ie, CT images) and/or laboratory tests alone without reporting non-fatal clinical outcomes. We also excluded the following study types: case reports with a sample size of 20 or less, editorials, commentaries, scientific or conference abstracts, and protocol papers without primary data. For GBD 2023, we included only published studies that reported on symptoms at multiple follow-up times since infection rather than a single time point.

Study selection and data extraction

Studies identified in each database were imported into DistillerSR, a systematic review software, and duplicates were removed. Two reviewers independently screened in pairs at the title/abstract and full-text levels against the inclusion and exclusion criteria. The extracted variables included geographical location, sample characteristics, COVID case definition, clinical outcomes, and length of follow-up. We extracted the most detailed data reported by age and sex. For clinical outcomes, we extracted proportions and uncertainty values reported by the authors.

Published literature data

For GBD 2023, we conducted a systematic literature review and added a significant portion of papers on the non-fatal outcomes of COVID-19, published between 2020 and 2023. For the systematic literature review, we used web-based software DistillerSR to track and screen sources for inclusion in the final analysis.

Databases used: PubMed, Embase, CINAHL, Global Health, Web of Science, WHO Covid-19 Research Database.

Dates & total sources queried:

PubMed	Embase	CINAHL	Global Health	Web of Science	WHO Covid-19 Research Database
9/21/2023	9/21/2023	9/21/2023	9/21/2023	9/21/2023	9/21/2023
2860	3140	678	1141	3397	3044

Total sources queried: 14,260
Total number of duplicate sources removed: 6958
Total number of sources meeting the inclusion criteria, with the extracted data: 155

Concordance between the Data Extraction Analyst (DEA) and Lead Research Scientist was established by each TA reviewing 10% of the initial query (7302 unique hits) from PubMed to ensure that all eligible sources were included and agreed upon. This concordant set was used to train Distiller AI functionality, which generated a score probability of inclusion for all hits. All sources with a score greater than 0.9 were included for full text review (291 sources), and all sources with score 0.7–0.9 were manually screened by the Data Extraction Analyst (1389) to result in a total of 837 sources for full text review.

Figure 1. PRISMA flow diagram of systematic literature review for long COVID

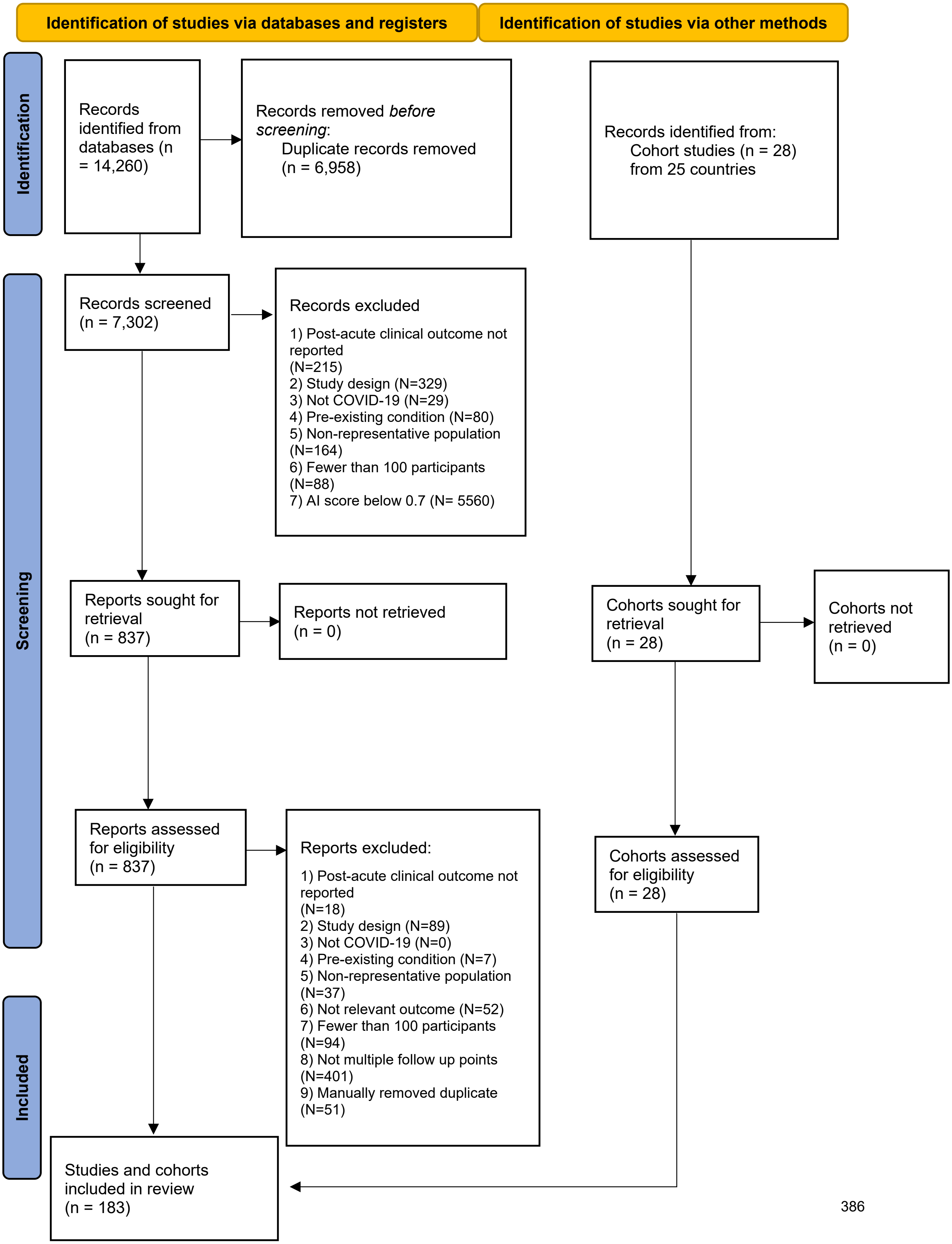

Cohort studies with individual-level data

We included a total of 28 cohorts with individual-level data in GBD 2023: 13 cohorts were following community infections, while 15 cohorts were following cases that were hospitalised due to COVID-19. Compared to our initial analysis, where the longest follow-up of patients was 12 months, in our updated analysis for GBD 2023, we included two studies where the longest patient follow-up was greater than 36 months. Additionally, 12 of the cohort studies had a follow-up of patients greater than 24 months, while 18 studies had a follow-up greater than 12 months. All cohort study data were provided to IHME by the core network of experts and GBD Collaborators working on the long COVID analysis in their home countries or regions.

Methods overview

Our long COVID analysis is split into two large components: estimating the acute sequelae of COVID-19 and estimating the post-acute sequelae among survivors of COVID-19.

First, infections were multiplied by the pooled estimate of the proportion of infections without symptoms, and deaths were subtracted from the estimate of symptomatic cases and to get estimates by age, sex, year, and country of symptomatic survivors of COVID-19 infection. Then, infections were followed through the disease course to obtain surviving cases of mild/moderate non-hospitalised, severe hospitalised, and critical ICU cases of COVID-19 at risk for post-acute symptoms.

Second, post-acute sequelae were estimated similar to GBD 2021. The proportions of symptomatic survivors with one or more of the three symptom clusters of long COVID (with fatigue, cognitive problems, and shortness of breath as the key symptoms) were extracted from 28 international, individual-level cohort studies and two (2) USA medical record databases. Data from cohort studies with individual case records available that did not report on excess risk of long COVID symptom clusters in comparison to controls or self-reported health status prior to COVID-19 were adjusted by the ratio of excess to total symptoms from studies that reported both. Then, the proportions with long COVID symptom clusters by follow-up time since the end of the acute infection were estimated using a Bayesian meta-regression tool, separately for hospitalised and non-hospitalised cases. Subsequently, estimates from studies providing distributions of symptom cluster overlap and severity gradients of cognitive and respiratory problems were pooled.

GBD 2023 includes one major methodological change to this process. The relationship between risk of long COVID and variant was estimated by modelling studies that followed samples of people with COVID-19 from different times in the pandemic, with each study using the same process and survey instrument for the different samples.

Finally, the global estimates of symptomatic COVID-19 survivors were multiplied by the proportions experiencing one or more of the symptom clusters at three months post infection, after accounting for differential risk by variant.

Acute sequelae of SARS-CoV-2 infection

Asymptomatic cases

Case definitions

An asymptomatic case is defined as a person infected with detectable viral load of SARS-CoV-2 but without symptoms. Data and methods to estimate the proportion asymptomatic that amount to SARS-CoV-2 infections has not changed since GBD 2021.

Data

Data sources were obtained from a published systematic literature review which contains the proportion of confirmed positive COVID cases through antibody testing that were asymptomatic, from studies across the world.[4]

We have two primary inclusion criteria: 1) antibody screening studies; and 2) randomly selected sample to increase representativeness. Of the 18 antibody screening studies included in the review, six met our inclusion and exclusion criteria (Table 2).

Table 2. Input data of the proportion of asymptomatic cases among SARS-CoV-2 infections

**Author**	**Location**	**Sample**
Ward et al.[5]	China	17 576
Pollán et al.[6]	Hubei	3053
Da Silva et al.[7]	Shandong	1167
Feehan et al.[8]	Bahrain	311
Hippich et al.[9]	Hubei	47
Mahajan et al.[10]	Guangdong	23

[Step 1]

The standard error of each datapoint was calculated using the following equation for a binomial distribution.

$$Standard\ error = \sqrt{\frac{proportion_{asymp} * (1 - proportion_{asymp})}{sample\ size}}$$

[Step 2]

Methods

First, we pooled the studies using a simple random effects model with the MR-BRT tool in logit space to constrain the estimate between 0 and 1 (Figure 2). The delta method was used to convert the standard error into logit space for the meta-analysis.

Figure 2. Pooled estimate of proportion asymptomatic among SARS-CoV-2 infections

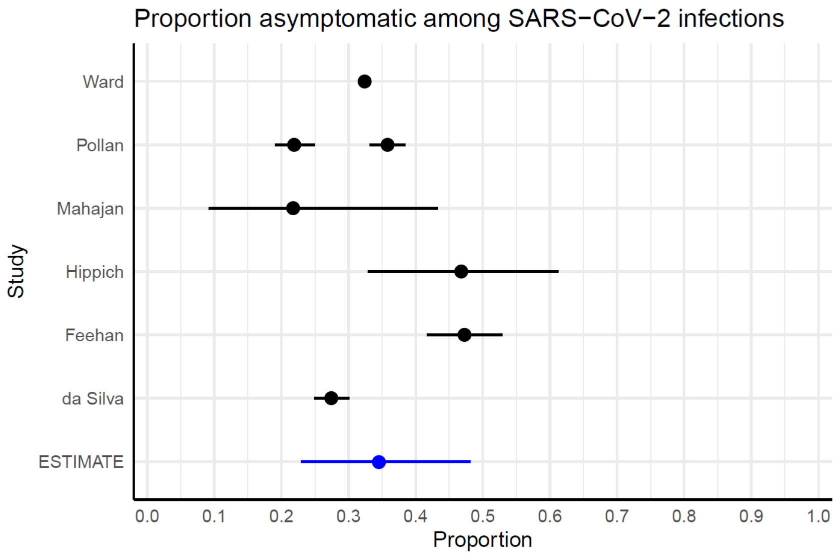

The data are high-quality but heterogeneous in the observed proportions asymptomatic, ranging from 22% to 47% asymptomatic. This could be due to differential rather than consistent antibody testing capture of SARS-CoV-2 infections in different settings, true variation in the proportion asymptomatic due to different underlying risk factors in the study populations, or differential symptom recall by the patients in these studies.

As in GBD 2021, asymptomatic cases are assumed to not be at risk for long COVID, due to lack of data.

Community cases

Case definition

Community cases of COVID-19 are defined as symptomatic, non-hospitalised, mild/moderate cases of COVID-19.

$$inc_{comm} = infections * (1 - prop\ asymp) - hosp\ admissions$$

where $hosp\ admissions$ represents the need for hospital admissions, corresponding to infections multiplied by the age-specific hospitalisation-infection ratio estimated in GBD 2021. In GBD 2023, we removed the paradoxical imposition that some deaths occur without needing hospitalisation.

Hospitalised and ICU cases

Case definition

Hospitalised cases of COVID-19 at risk for long COVID are defined as cases of COVID-19 needing hospitalisation but not ICU care, regardless of access to or utilisation of care, who survive the acute episode. Similarly, ICU cases at risk for long COVID are defined as cases of COVID-19 needing ICU care due to critical acute symptoms, regardless of access/utilisation, who survive the acute episode.

$$inc_{hosp\ at\ risk} = infections * HIR(1 - CFR_{hosp}) - inc_{icu}$$

$$inc_{icu\ at\ risk} = infections * HIR * IHR * (1 - CFR_{icu})$$

Where HIR=hospital-infection ratio; IHR=ICU-hospital ratio; CFR=case-fatality ratio

Proportion of deaths among hospitalised and ICU cases

Case fatality among hospitalised and ICU patients was estimated in GBD 2021.

Data

Age-specific data on COVID deaths among hospitalised and/or ICU patients proved extremely difficult to find, and we found only one comprehensive source with this level of detail from the Netherlands COVID-19 ICU online dashboard.[11]

Methods

Case fatality among hospitalised and ICU patients was extracted and fit with a sixth-order polynomial to most closely follow the curves of the data so that case fatality estimates could be extracted for every five-year age group (Figure 3). The value for case fatality for age group 5–9 was extrapolated back to age 0 due to lack of data at the very young ages.

Figure 3. Case fatality ratios among hospitalised and ICU COVID-19 patients by age

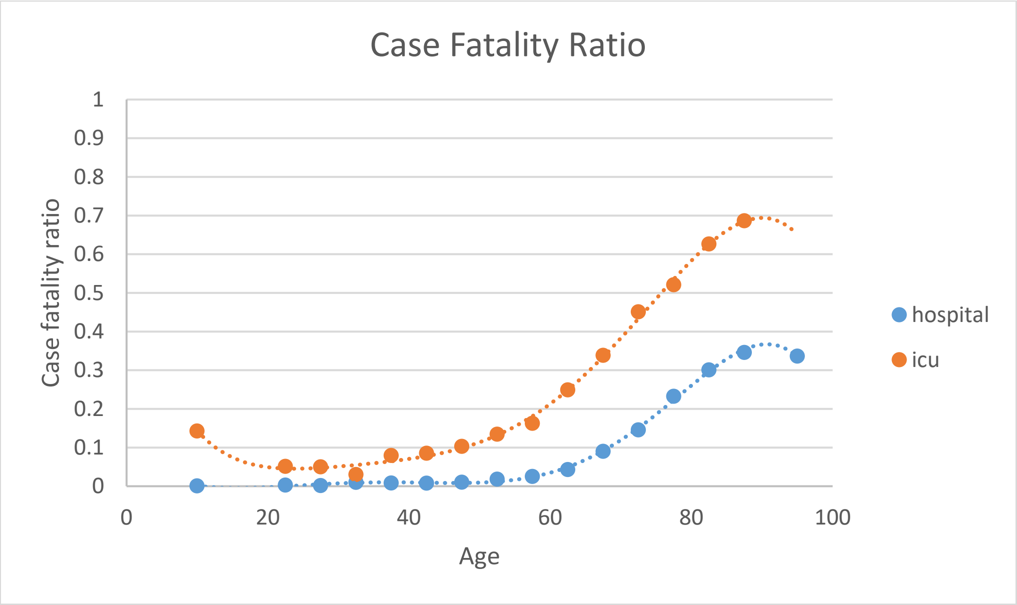

Post-acute sequelae of SARS-CoV-2 infection

Case definition

The case definition remains the same as in GBD 2021.

On October 6, 2021, the World Health Organization published a clinical case definition of post-COVID-19 condition developed by Delphi consensus.[12] During the Delphi consensus process, the following items attained the pre-defined threshold for consensus (70% of answers in range of 7–9 on Likert scale:[13]

1. A history of SARS-CoV-19 infection,
2. Three symptoms: cognitive dysfunction/brain fog, fatigue, and shortness of breath,
3. Importance of including "persistent" as descriptor of the nature of symptoms in case definition
4. Post COVID-19 is to be considered a diagnosis of exclusion determined by a health provider when symptoms cannot be explained by an alternative diagnosis,
5. That symptoms have an impact on everyday functioning,
6. Importance to include a separate case definition for post-COVID-19 condition for children.

All other items did not reach the threshold for consensus and should be labelled "partial consensus". In terms of Delphi methodology, therefore, they should not have appeared in the case definition. The authors of the WHO Post COVID-19 clinical case definition state that they also included additional items that "reached borderline significance" without defining the threshold.

In our analysis, we focus on those items listed above that have reached the threshold for consensus:

1. A SARS-CoV-19 infection is our starting point,
2. The three symptoms mentioned are the three key symptoms of the three symptom clusters we defined, and our algorithms for the ten cohort studies required mention of impact on everyday functioning (most commonly, at least a score of 2 on the usual activities question of EQ5D-5L),
4. Item 4 above pertains to a clinical case definition, rather than a case definition in a research setting; the equivalence in research would be exclusion of those who reported the same or worse symptoms prior to COVID-19. This has been built into our definition;
6. Lastly, we found that we could apply the same case definitions to children and adults; we did find that the cognition symptom cluster was less commonly reported by children (or their parents/caregivers).

With regard to the minimum duration included in the WHO case definition, all of the options from 2 weeks to 6 months were in the range of "partial consensus" with small differences in the proportions mentioning a value between 1 and 3 months. There was no option given to respondents to choose one particular duration only. Similarly, the "minimum period from onset of COVID-19 to presence of symptoms" items had answers for all options between 1 and 6 months, as well as "no time period" within the range of "partial consensus". For this paper, we chose to make three months from the acute infection symptom onset the starting point of long COVID.

For the purposes of quantifying all health loss due to COVID-19 in the Global Burden of Disease study, we also quantify the health loss during the acute infection phase and that experienced by cases of long COVID prior to meeting the criterion of a minimum duration of three months after infection.

[Step 1]

Cohort data

Information sources and search

Instead of relying on published reports only, we also contacted authors of published studies and ongoing COVID-19 follow-up studies that were registered at the ISRCTN registry, which prospectively registers clinical trials and observational studies.[14] With researchers from each of these follow-up studies, we developed algorithms to select cases of any of the three symptom clusters by severity level by choosing symptom questions and measures employed in each study that would most closely match the wording of the health state descriptions that were presented to respondents of the GBD DW surveys to derive disability weights (**Table 3**). We utilised the cohort data with explicit questions comparing current symptoms to those pre-COVID to adjust the remainder of the cohort data lacking pre-COVID comparisons.

Table 3. Health states, lay descriptions, and disability weights used for the quantification of YLDs due to COVID-19 infection and long COVID

**Outcome**	**Health State**	**Lay description**	**DW (95% UI)**
Acute infection			
Mild (ie, does not need hospitalisation)	Mild infection	has a low fever and mild discomfort, but no difficulty with daily activities.	0.006 (0.002–0.012)
Moderate (ie, does not need hospitalisation)	Moderate infection	has a fever and aches, and feels weak, which causes some difficulty with daily activities.	0.051 (0.032–0.074)
Severe (ie, needs hospitalisation)	Severe infection	has a high fever and pain, and feels very weak, which causes great difficulty with daily activities.	0.133 (0.088–0.190)
Critical (ie, needs ICU care)	ICU admission	is very ill and often asleep or unconscious; when awake cannot move in bed, cannot speak, is completely dependent on others and is anxious.	0.743 (0.556–0.878)
Long COVID sequelae			
Mild respiratory symptoms	Chronic respiratory problems, mild	has cough and shortness of breath after heavy physical activity but is able to walk long distances and climb stairs.	0.019 (0.011–0.039)
Moderate respiratory symptoms	Chronic respiratory problems, moderate	has cough, wheezing, and shortness of breath, even after light physical activity. The person feels tired and can walk only short distances or climb only a few stairs.	0.225 (0.153–0.310)
Severe respiratory symptoms	Chronic respiratory problems, severe	has cough, wheezing, and shortness of breath all the time. The person has great difficulty walking even short distances or climbing any stairs, feels tired when at rest, and is anxious.	0.408 (0.273–0.556)

Mild cognitive symptoms	Cognitive problems, mild	has some trouble remembering recent events and finds it hard to concentrate and make decisions and plans.	0.069 (0.046–0.099)
Severe cognitive symptoms	Cognitive problems, moderate	has memory problems and confusion, feels disoriented, at times hears voices that are not real, and needs help with some daily activities.	0.377 (0.252–0.508)
Fatigue syndrome	Infectious disease, post-acute consequences	is always tired and easily upset. The person feels pain all over the body and is depressed.	0.219 (0.148–0.308)

GBD disability weights (DWs) quantify the health loss as a fraction of time lived within a health state. For severe cognitive symptoms of long COVID we use the health state of moderate cognitive problems that is also used in GBD for moderate dementia.

Table 4. Follow-up studies of long COVID, age and sex distributions, their inclusion of community and/or hospitalised cases, sample sizes, follow-up period, comparison method, and reported symptoms or symptom clusters.

**Follow-up study**	**Sample size**	**Follow-up since end of acute episode (months)**	**Outcomes**
StopCOVID Cohort (Russia)[15,16]	3445 adults, 1072 children	7 months, 12 months	Fatigue cluster, respiratory cluster by severity, cognitive cluster, Fatigue sub-threshold cluster, alopecia, sleep problems, taste smell loss, dizziness, headache, anxiety, depression
Isfahan COVID Cohort (Iran)[17]	2287 adults, 82 children	6 months, 12 months, 24 months	Fatigue cluster, respiratory cluster, cognitive cluster, Fatigue sub-threshold cluster, alopecia, sleep problems, taste smell loss, headache, anxiety, depression
Zürich SARS-CoV-2 Cohort (Switzerland)[18]	Prosp: 1106 adults; Retro: 437 adults	Prospective: 1/3/6/9/12/18/24/30/36 months; Retrospective: 6/9/12/18/24/30/36/42 months	Fatigue cluster, respiratory cluster by severity, cognitive by severity cluster, Fatigue sub-threshold cluster, alopecia, sleep problems, taste smell loss, headache, anxiety, depression
Zürich SARS-CoV-2 Vaccination Cohort (Switzerland)	575 adults	Up to 30 months from the date of Omicron infection	Fatigue cluster, respiratory cluster by severity, cognitive cluster by severity, Fatigue sub-threshold cluster, alopecia, sleep problems,

Follow-up study	Sample size	Follow-up since end of acute episode (months)	Outcomes
			taste smell loss, headache, anxiety, depression
Corona Immunitas (Switzerland)	Zurich: 1044 adults<br>Ticino: 851 adults<br>Vaud: 850 adults	Up to 1 year from the date of Omicron infection	Fatigue cluster, respiratory cluster by severity, cognitive cluster, Fatigue sub-threshold cluster, alopecia, sleep problems, taste smell loss, headache, anxiety, depression
Cohort of Positive Patients for COVID-19 (Luxembourg)	685 all ages	12 months, 15 months, 24 months	Fatigue cluster, respiratory cluster, cognitive cluster
Bogota Sabana University Clinic Long Covid Cohort Study (Colombia)	292 adults	3 months, 6 months, 12 months	Fatigue cluster, respiratory cluster by severity, cognitive cluster by severity, Fatigue sub-threshold cluster, sleep problems, taste smell loss, joint/muscle pain, dizziness, anxiety, depression
Amazonas COVACManaus Long Covid Follow-up Survey (Brazil)	991 adults	1st follow-up: median 288 days<br>2nd follow-up: median 961 days	Fatigue cluster, respiratory cluster by severity, cognitive cluster by severity
West Bank Long Covid Project (Palestine)	5628 adults	1st follow-up: median 277 days<br>2nd follow-up: median 599 days	Fatigue cluster, respiratory cluster, cognitive cluster by severity
UK Virus Watch (England & Wales)	58,620 adults	Consists of 6 follow-up surveys (Feb 2021, May 2021, Mar 2022, Feb 2023, Oct 2023, Apr 2024) with follow-up responses between 1 month & 2 years	Fatigue cluster, respiratory cluster by severity, cognitive cluster by severity
CO-FLOW (Netherlands)[19]	285 adults	3 months, 6 months (up to 2 years)	Fatigue cluster, respiratory cluster by severity, cognitive cluster

Follow-up study	Sample size	Follow-up since end of acute episode (months)	Outcomes
Rome ISARIC (Italy)[20]	52 adults<br>82 children	Median 2 months (adults), Median less than 2 months (children)	Fatigue cluster, respiratory cluster, cognitive cluster
Helbok et al. (Austria)[21]	85 adults	3 months	Fatigue cluster, cognitive cluster
Faroe Islands[22]	204 all ages	1 month,<br>2 months,<br>3 months	Fatigue cluster, respiratory cluster, cognitive cluster
US Longitudinal COVID-19 Cohort HAARVI (USA)[23]	177 adults	Median<br>6 months	Fatigue cluster, respiratory cluster, cognitive cluster
pa-COVID (Germany)[24,25]	145 adults	1 month,<br>3 months	Fatigue cluster, respiratory cluster by severity, cognitive cluster
PronMed ICU (Sweden)[26,27]	158 adults	4–7 months	Fatigue cluster, respiratory cluster, cognitive cluster
PRA administrative data (USA)[28]	1,009,885 adults	Median 6–7 months (195 days)	ICD codes for fatigue cluster, respiratory cluster, cognitive cluster
Veterans Affairs administrative data (USA)[29,30]	51,376 COVID+<br>5,051,832 non-COVID	6 months	ICD codes for fatigue cluster, respiratory cluster, cognitive cluster

[a] Data from studies with individual record data that did not report on the differences in pre-COVID health and health at follow-up were adjusted based on ratios of excess to total reported symptom clusters from cohorts that did contain this information.

[b] The two US administrative databases allowed the identification of controls matched to those with a positive PCR test for COVID-19 based on a range of demographics and comorbid conditions. We took the difference between cases and controls as the proportion of symptoms attributable to COVID-19.

[Step 2]

Administrative data

Information sources and search

In addition, analyses were received in GBD 2021 from collaborators at two US administrative databases—Veterans Affairs Health Administration and Pharmaceutical Research Associates (PRA) Health Sciences, a data collection of private health insurance plans—based on the International Classification of Diseases, Tenth Revision, Clinical Modification (ICD-10-CM) codes for the primary symptoms belonging to the three symptom clusters of interest among cases with COVID-19 compared to non-COVID-19 cases matched on demographic characteristics and pre-existing common health problems (Appendix 2 Data Inputs).[28–30] ICD codes are provided in 5.

Veterans Affairs COVID cases were matched to 4,990,835 controls using the procedure outlined in Al-Aly et al.[29]

PRA Health Services COVID cases were matched 1:1 to 1,009,885 controls by month of diagnosis, ten-year age group, sex, race, and previously diagnosed diabetes, heart failure, cancer, and stroke. COVID patients were included if their initial diagnosis was between March and October 2020 and they also had at least one outpatient or inpatient visit between November 2020 and January 2021. Controls were eligible for matching if they had no COVID diagnosis prior to January 2021, had at least two visits between March and October 2020, and had at least one outpatient or inpatient visit between November 2020 and January 2021.

After matching, the excess rate of symptoms associated with COVID-19 diagnosis was defined as the difference in the reported symptom ICD codes between cases and controls.

Table 5. ICD-10-CM codes used to extract administrative data for cognitive symptoms, fatigue, and respiratory symptoms

ICD-10-CM CODE	ICD-10-CM CODE DESCRIPTION	Symptom cluster
'R404'	Transient alteration of awareness	Cognitive
'R410'	Disorientation, unspecified	Cognitive
'R411'	Anterograde amnesia	Cognitive
'R412'	Retrograde amnesia	Cognitive
'R413'	Other amnesia	Cognitive
'R4182'	Altered mental status, unspecified	Cognitive
'R41840'	Attention and concentration deficit	Cognitive
'R41841'	Cognitive communication deficit	Cognitive
'R4189'	Other symptoms and signs involving cognitive functions and awareness	Cognitive
'R419'	Unspecified symptoms and signs involving cognitive functions and awareness	Cognitive
'R531'	Weakness	Fatigue
'R5381'	Other malaise	Fatigue
'R5382'	Chronic fatigue, unspecified	Fatigue
'R5383'	Other fatigue	Fatigue

'J9610'	Chronic respiratory failure, unspecified whether with hypoxia or hypercapnia	Respiratory
'J9611'	Chronic respiratory failure with hypoxia	Respiratory
'J9612'	Chronic respiratory failure with hypercapnia	Respiratory
'J9620'	Acute and chronic respiratory failure, unspecified whether with hypoxia or hypercapnia	Respiratory
'J9621'	Acute and chronic respiratory failure with hypoxia	Respiratory
'J9622'	Acute and chronic respiratory failure with hypercapnia	Respiratory
'J9690'	Respiratory failure, unspecified, unspecified whether with hypoxia or hypercapnia	Respiratory
'J9691'	Respiratory failure, unspecified with hypoxia	Respiratory
'J9692'	Respiratory failure, unspecified with hypercapnia	Respiratory
'J988'	Other specified respiratory disorders	Respiratory
'J989'	Respiratory disorder, unspecified	Respiratory
'J99'	Respiratory disorders in diseases classified elsewhere	Respiratory
'R05'	Cough	Respiratory
'R0600'	Dyspnea, unspecified	Respiratory
'R0602'	Shortness of breath	Respiratory
'R0603'	Acute respiratory distress	Respiratory
'R0609'	Other forms of dyspnea	Respiratory
'R071'	Chest pain on breathing	Respiratory

Data adjustments

[Step 3]

Adjust for underlying rates of symptom clusters

To maintain our case definition of symptom clusters due directly to COVID-19, the proportions of patients with each symptom cluster needed to account for pre-existing symptoms. For cohorts with questions about pre-COVID-19 health status, this excess risk of each symptom cluster could be directly calculated. Some cohorts, however, lacked such questions in the survey instruments and thus reported inflated counts of symptoms among COVID-19 patients. We adjusted the proportion data from these latter cohorts using the observed adjustment among cohorts with pre-COVID-19 health status.

First, data were re-extracted from the cohort studies with individual record data that reported information on pre-COVID health status, with adapted algorithms to exclude the information on pre-COVID health status to make these data comparable to the cohort studies that lack this information. The logit differences between data with and without pre-COVID health status for these six cohorts were pooled in a meta-analysis with a study-level random effect, separately by symptom cluster to estimate four overall adjustment factors (Table 6). These coefficients were then applied to corresponding symptom cluster data for the other cohorts with individual record data to adjust their data for pre-COVID health status.

Table 6. Model coefficients for crosswalk adjustment to account for underlying rates of symptom clusters

Symptom cluster	Adjustment beta coefficient, logit (SD)
Any long COVID	0.500 (0.233)
Post-acute fatigue syndrome	0.452 (0.273)
Respiratory symptoms	0.545 (0.125)
Cognitive symptoms	0.191 (0.019)

Adjust for reporting individual symptoms, age, and administrative data

We accounted for other sources of bias within the MR-BRT models described below by including indicator variables for bias characteristics and estimating a correction factor within the models. For data that reported individual symptoms rather than symptom clusters (fatigue, shortness of breath, and single cognitive issues) or reported overall long COVID proportions from a longer symptom list than our three symptom clusters, we estimated correction factors within each model (Tables 7-10). Also, given that administrative data likely under-estimate true disease rates, we adjusted VA and PRA data sources where possible using an indicator variable within the MR-BRT models (Tables 9 and 10). Age was incorporated as a binary variable for data among children and young people under age 20, so that predictions could be made separately for ages under 20 and 20 and older.

Adjust follow-up time

The value of each follow-up time was shifted to be relative to the end of the acute episode, as defined by the acute disease course described above. For example, hospital data measured since hospital discharge are shifted ten days to represent follow-up since ten days after hospital discharge, which marks the assumed end of the acute symptomatic phase.

[Step 4]

Duration estimates

All symptom cluster models were logit-linear regressions, in order to constrain the outcome proportions between zero and one, and were conducted in MRTool 0.0.2.[31]

We estimated the rate of recovery among COVID patients with long COVID with a logit-linear regression of the logit-transformed prevalence of any symptom cluster on follow-up time of cohort data. Given the scarcity of data (in the hospital model in particular), we assumed the same recovery rate applies to all symptom clusters.

Separate models were run for symptomatic non-hospitalised cases and for hospital/ICU cases, described as the “Overall long COVID” models below, and we calculated distributions of durations integrating the area below the fitted curve using the following equation.

$$Duration = \frac{\int_{F=0}^{F_{end}} \frac{e^{\beta_0} * e^{\beta_1 * F}}{1 + e^{\beta_0} * e^{\beta_1 * F}} dF}{prop_{start}}$$

where F represents follow-up day, β_0 is the intercept of the model, β_1 is the slope on follow-up day, and F_{end} represents the follow-up day when the proportion of cases with long COVID drops below 0.001, a threshold selected as the end of the recovery curve. F_{end} is calculated as

$$F_{end} = \frac{\log\left(\frac{0.001}{1-0.001}\right) - \beta_0}{\beta_1}$$

Evaluating the above integral gives

$$Duration = \frac{\frac{1}{\beta_1} * \log\left(abs\left(1+e^{\beta_0}\right) * e^{\beta_1 * F_{end}}\right) - \frac{1}{\beta_1} * \log\left(abs\left(1+e^{\beta_0}\right)\right)}{prop_{start}}$$

where $prop_{start}$ is the intercept in normal space as

$$prop_{start} = \frac{e^{\beta_0}}{1+e^{\beta_0}}$$

We sampled the parameters of each model 1000 times to evaluate the above equations 1000 times and to calculate uncertainty around the overall duration estimates that we report.

For each date of infection in each estimation year, we estimated the proportion who developed long COVID after three months. Prevalent cases were truncated at the end of either year, so that time spent with long COVID contributed to the prevalent cases in the appropriate year. For instance, for the year 2020, day-specific duration was calculated as

$$Duration_{day} = \frac{\int_{F=0}^{Dec\ 31,2020-day} \frac{e^{\beta_0} * e^{\beta_1 * F}}{1+e^{\beta_0} * e^{\beta_1 * F}} dF}{prop_{start}}$$

such that the integral is evaluated from onset of long COVID symptoms until the end of 2020 to obtain the day-specific average duration experienced within 2020 for incident long COVID cases of each day.

Prevalence estimates

[Step 5]

Overall long COVID

Prevalence of overall long COVID was defined as having at least one of the three symptom clusters when extracted from the individual-level cohort data. First, we modelled this prevalence of overall long COVID. Estimates of individual symptom clusters and overlaps between clusters were adjusted to sum to overall long COVID.

For the overall long COVID models among symptomatic non-hospitalised cases and hospital/ICU cases, we included cohort data from which we were able to extract the number of patients with at least one of the three symptom clusters. For symptomatic non-hospitalised cases, the MRTool regression had a random effect on study, and fixed effects on whether the study used a more comprehensive symptom list (as in the recovery pattern model above), whether the data were among females only or males only, whether the data were among individuals <20 years, and on follow-up time (Table 7). The hospital/ICU regression also had a random effect on study and fixed effects

on whether the data were among ICU patients, whether the data were among females only or males only, and on follow-up time (Table 8). We modelled hospital and ICU data together because there were insufficient data on ICU admissions to support a separate model. To obtain estimates among ICU cases, we simply included the beta coefficient on ICU in the posterior estimates, and we excluded it for estimates among hospital cases.

MRTool trimmed 10% of the datapoints in order to make the estimates more robust. Trimming observations according to the likelihood is a method from the field of robust statistics.[32,33] We used a least trimmed squares (LTS) estimator that seeks to fit the specified majority of the most self-coherent data, giving an understanding of the overall relationship in the face of outlying observations as described in Zheng et al.[31]

Table 7. Model parameters for community overall long COVID

**Fixed effect**	**Prior (SD)**	**Source of prior**	**Final estimated beta coefficient, logit (SD)**
Female (ref: Both sexes)	0.223 (0.048)	Simple random effect meta-analysis of only sources with sex-specific and both-sex data	0.177 (0.350)
Male (ref: Both sexes)	–0.241 (0.050)	Simple random effect meta-analysis of only sources with sex-specific and both-sex data	–0.230 (0.158)
Follow-up time	n/a		–0.00243 (0.00116)
Under age 20 (ref: Over age 20)	–0.786 (0.111)	Simple random effect meta-analysis of only sources with child and adult data	–0.744 (0.953)
Uses publication-specific long list of symptoms to define "any long COVID symptom"	n/a		1.51 (6.15)

Table 8. Model parameters for hospital/ICU overall long COVID

**Fixed effect**	**Prior (SD)**	**Source of prior**	**Final estimated beta coefficient, logit (SD)**
ICU	0.575 (0.184)	Simple random effect meta-analysis of only VA and PRA hospital and ICU data	0.579 (0.204)
Female (ref: Both sexes)	0.223 (0.048)	Simple random effect meta-analysis of only sources with sex-specific and both-sex data	0.213 (0.152)
Male (ref: Both sexes)	-0.241 (0.050)	Simple random effect meta-analysis of only sources with sex-specific and both-sex data	–0.240 (0.070)
Follow-up time	n/a		–0.00366 (0.000442)
Uses publication-specific long list of symptoms to define "any long COVID symptom"	n/a		1.49 (0.630)

[Step 6]

Individual symptom clusters

To model individual symptom clusters, we ran MRTool meta-regression models on all data of each symptom cluster, including administrative data and published sources that reported symptoms we mapped to symptom clusters, such as cough mapping to respiratory symptoms. MRTool trimmed 10% of the datapoints in order to make the estimates more robust. There were too few datapoints to run separate models for ICU-admitted cases; in the hospital models for each symptom cluster, an indicator variable was used for those admitted to ICU in order to predict their proportions, with the coefficient informed by the observed relationship between ICU and general hospital ward data.[28,30] In addition, indicator variables were added for sex, whether the data were from an administrative source, and for individual symptoms reported in the published articles (fatigue, cognitive dysfunction, shortness of breath). Table 9 and Table 10 display the fixed effects included in the non-hospitalised and hospital/ICU models, respectively, and each model also had a random effect on study.

Table 9. Model parameters for each symptom cluster model among community cases. Sources of the priors are the same as in the overall long COVID models.

Fixed effect	Fatigue Prior (SD)	Fatigue Beta Coefficient, Logit (SD)	Respiratory Prior (SD)	Respiratory Beta Coefficient, Logit (SD)	Cognitive Prior (SD)	Cognitive Beta Coefficient, Logit (SD)
**Female (ref: Both sexes)**	0.299 (0.046)	0.297 (0.675)	0.296 (0.0567)	0.262 (0.0541)	0.0890 (0.0373)	0.0267 (0.442)
**Male (ref: Both sexes)**	-0.209 (0.052)	-0.204 (0.672)	-0.207 (0.0624)	-0.135 (0.0572)	-0.126 (0.0399)	-0.0485 (0.736)
**Follow-up time**	-0.00243 (1.15)	-0.00207 (0.00467)	-0.00243 (1.15)	-0.00231 (0.000155)	-0.00243 (1.15)	-0.0008 (0.00424)
**Alternative outcome definitions from publications (fatigue, memory problems, cough, shortness of breath)**	n/a	Fatigue 1.33 (3.98)	n/a	Shortness of breath 1.54 (0.206)	n/a	Memory problems 0.793 (6.40)
**Under age 20 (ref: Over age 20)**	-1.10 (0.112)	-1.12 (2.77)	-1.46 (0.396)	-1.01 (0.284)	-2.04 (0.379)	-1.63 (8.35)

Table 10. Model parameters for each symptom cluster model among hospital/ICU cases. Sources of the priors are the same as in the overall long COVID models.

Fixed effect	Fatigue Prior (SD)	Fatigue Beta Coefficient, Logit (SD)	Respiratory Prior (SD)	Respiratory Beta Coefficient, Logit (SD)	Cognitive Prior (SD)	Cognitive Beta Coefficient, Logit (SD)
**ICU**	0.450 (0.187)	0.564 (0.0813)	1.17 (0.169)	0.923 (0.0933)	1.27 (0.610)	0.503 (0.223)
**Female (ref: Both sexes)**	0.299 (0.0461)	0.272 (0.0498)	0.296 (0.0567)	0.231 (0.452)	0.0890 (0.0373)	0.181 (0.0969)
**Male (ref: Both sexes)**	-0.209 (0.0515)	-0.198 (0.0564)	-0.207 (0.0624)	-0.143 (0.0857)	-0.126 (0.0399)	-0.229 (0.0563)
**Follow-up time**	-0.00366 (0.00382)	-0.00206 (0.000230)	-0.00366 (0.00382)	-0.00340 (0.00115)	-0.00366 (0.00382)	-0.00188 (0.000269)
**Alternative outcome definitions from publications (fatigue, memory problems, cough, shortness of breath)**	n/a	Fatigue 1.57 (0.167)	n/a	Shortness of breath 0.739 (0.411)	n/a	Memory problems 1.75 (0.459)

[Step 6]

Overlap of symptom clusters

To model the overlap of symptom clusters, we ran MRTool meta-analysis models on available cohort data of each overlap of symptom clusters among long COVID patients, rather than among all COVID patients above, because the proportions are small. Table 11 displays the fixed effects included in the models, and each model also had a random effect on study. Also, due to sparse data, we modelled non-hospitalised data and hospitalised data together with a fixed effect on the latter, and no data were trimmed.

Table 11. Model parameters for each overlap of symptom clusters model among long COVID cases.

Fixed effect	Fatigue and Respiratory Beta Coefficient, Logit (SD)	Fatigue and Cognitive Beta Coefficient, Logit (SD)	Respiratory and Cognitive Beta Coefficient, Logit (SD)	Fatigue, Respiratory, and Cognitive Beta Coefficient, Logit (SD)
Hospital/ICU	0.339 (0.184)	0.160 (0.185)	0.130 (0.173)	-0.0268 (0.209)

[Step 7]

Severity distributions

We also modelled severity distributions of cognitive and respiratory symptoms using MRTool with available cohort data of each severity among all cognitive or respiratory cases. Each severity-specific model had a random effect on study and a fixed effect on whether the data were among hospitalised patients (Table 12 and Table 13). There were insufficient severity-specific data to model these proportions by follow-up time, and no data were trimmed.

Table 12. Model parameters for severity-specific cognitive symptom models

Fixed effect	Mild cognitive Beta Coefficient, Logit (SD)	Severe cognitive Beta Coefficient, Logit (SD)
Hospital/ICU	-0.632 (0.246)	1.16 (0.335)

Table 131. Model parameters for severity-specific respiratory symptom models

Fixed effect	Mild respiratory Beta Coefficient, Logit (SD)	Moderate respiratory Beta Coefficient, Logit (SD)	Severe respiratory Beta Coefficient, Logit (SD)
Hospital/ICU	-0.468 (0.310)	0.100 (0.491)	0.732 (0.168)

Severity-specific estimates were adjusted to sum to 100% before being used to split the overall cognitive and respiratory results by severity.

Declining risk by variant

For GBD 2023, there was sufficient evidence to estimate the variable risk of long COVID by variant. From the systematic literature review described above, in addition to targeted data seeking, we identified eight studies that sampled people with COVID-19 from different times in the pandemic and followed them for ongoing symptoms using the same process and survey instruments within each study. We anchored each sample by the number of months since January 2020 and estimated the relationship between risk of ongoing symptoms and the number of months since January 2020 with a random effect on study, in a MRTool meta-analysis model with no data trimming. Table 14 and Figure 4 display this estimated relationship.

Table 14. Model parameter for risk of long COVID by calendar time

Fixed effect	Beta Coefficient, Logit (SD)
Months since January 2020	-0.125 (0.00207)

Figure 4. Risk of long COVID by calendar time, (a) unadjusted and (b) adjusted for study-level random effect

(a)

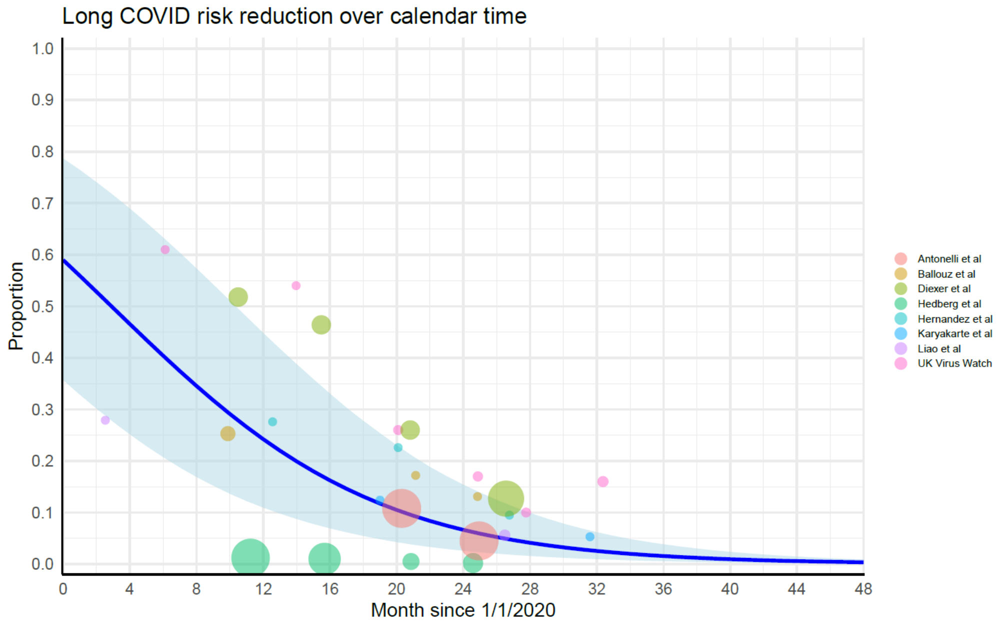

(b)

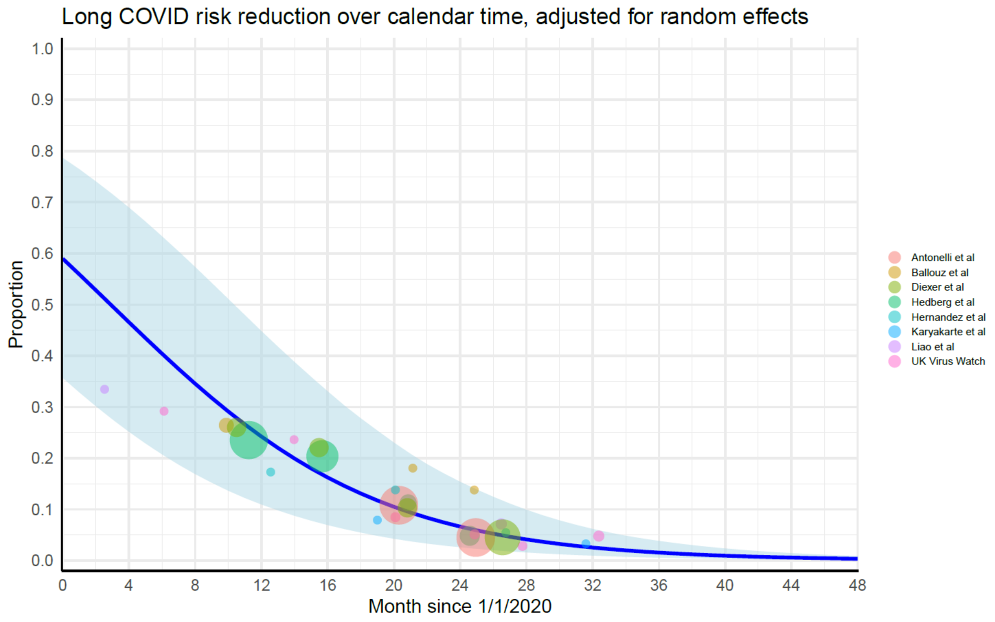

The proportions of COVID-19 cases with each symptom cluster were then generated for every month since January 2020 using this estimated relationship, to account for declining risk by variant.

Incidence and prevalence estimates

[Steps 8, 9, 10, 11, 12]

Incidence of long COVID symptom clusters and overlaps was calculated by multiplying surviving symptomatic COVID cases (community, hospitalised, and ICU cases who recovered from the acute infection) by the proportions of symptom clusters that were adjusted to sum to the overall long COVID estimate. These cases were then multiplied by day-specific durations to obtain prevalence of each symptom cluster and overlap in each estimation year. All calculations were conducted using 1000 draws of each quantity to propagate uncertainty through each analytical step.

[Steps 13, 14]

Incidence and prevalence estimates were then summed across the COVID case severities: among community mild/moderate cases, cases needing hospitalisation, and cases needing ICU care.

YLD estimates

[Step 15]

We calculated YLDs by multiplying the prevalence of each symptom cluster and overlap by the corresponding disability weights (Table 3). For overlap clusters, the combined disability weight was calculated using a multiplicative equation:

$$combined\ DW = 1 - (1 - DW_1) * (1 - DW_2)$$

Or

$$combined\ DW = 1 - (1 - DW_1) * (1 - DW_2) * (1 - DW_3)$$

References

1 Team C-19 F, Reiner RC, Collins JK, Murray CJL. Forecasting the trajectory of the COVID-19 pandemic into 2023 under plausible variant and intervention scenarios: a global modelling study. 2023; : 2023.03.07.23286952.

2 Page MJ, McKenzie JE, Bossuyt PM, *et al.* The PRISMA 2020 statement: an updated guideline for reporting systematic reviews. *BMJ* 2021; **372**: n71.

3 Mao W, Vos T, Wulf Hanson S, *et al.* Long-term and short-term disease burden of COVID-19: a systematic review and meta-analysis. *PROSPERO 2020 CRD42020210101* https://www.crd.york.ac.uk/prospero/display_record.php?RecordID=210101.

4 Oran DP, Topol EJ. Prevalence of Asymptomatic SARS-CoV-2 Infection. *Ann Intern Med* 2020; M20-3012.

5 Ward H, Cooke G, Atchison C, *et al.* Declining prevalence of antibody positivity to SARS-CoV-2: a community study of 365,000 adults. 2020 http://spiral.imperial.ac.uk/handle/10044/1/83634 (accessed Sept 27, 2021).

6 Pollán M, Pérez-Gómez B, Pastor-Barriuso R, *et al.* Prevalence of SARS-CoV-2 in Spain (ENE-COVID): a nationwide, population-based seroepidemiological study. *Lancet* 2020; **396**: 535–44.

7 Silva AAM da, Lima-Neto LG, Azevedo C de MPES de, *et al.* Population-based seroprevalence of SARS-CoV-2 and the herd immunity threshold in Maranhão. *Rev Saude Publica* 2020; **54**: 131.

8 Feehan AK, Fort D, Velasco C, *et al.* The importance of anosmia, ageusia and age in community presentation of symptomatic and asymptomatic SARS-CoV-2 infection in Louisiana, USA; a cross-sectional prevalence study. *Clin Microbiol Infect* 2021; **27**: 633.e9-633.e16.

9 Hippich M, Holthaus L, Assfalg R, *et al.* A Public Health Antibody Screening Indicates a 6-Fold Higher SARS-CoV-2 Exposure Rate than Reported Cases in Children. *Med (N Y)* 2021; **2**: 149-163.e4.

10 Mahajan S, Srinivasan R, Redlich CA, *et al.* Seroprevalence of SARS-CoV-2-Specific IgG Antibodies Among Adults Living in Connecticut: Post-Infection Prevalence (PIP) Study. *Am J Med* 2021; **134**: 526-534.e11.

11 ESRI Nederland. Coronavirus IC en verpleegafdeling-opnamen (Stichting NICE). https://experience.arcgis.com/experience/e58fd5e1779b4cdd9d81e44b9b1032d0 (accessed May 4, 2021).

12 A clinical case definition of post COVID-19 condition by a Delphi consensus, 6 October 2021. https://www.who.int/publications-detail-redirect/WHO-2019-nCoV-Post_COVID-19_condition-Clinical_case_definition-2021.1 (accessed Nov 5, 2021).

13 Brown BB. Delphi Process: A Methodology Used for the Elicitation of Opinions of Experts. RAND Corporation, 1968 https://www.rand.org/pubs/papers/P3925.html (accessed Nov 9, 2021).

14 ISRCTN Registry. https://www.isrctn.com/ (accessed June 15, 2021).

15 Osmanov IM, Spiridonova E, Bobkova P, *et al.* Risk factors for long covid in previously hospitalised children using the ISARIC Global follow-up protocol: A prospective cohort study. *medRxiv* 2021; :2021.04.26.21256110.

16 Munblit D, Bobkova P, Spiridonova E, *et al.* Asthma is associated with Long Covid in previously hospitalised adults: StopCOVID cohort study. *Clinical and Experimental Allergy* in review.

17 Sarrafzadegan N, Mohammadifard N, Haghjooy Javanmard S, *et al.* Isfahan COVID cohort study: Rationale, methodology, and initial results. *J Res Med Sci* 2021; **27**. https://www.jmsjournal.net/text.asp?2022/27/1/65/355060.

18 Puhan M, Fehr J, Abela I, *et al.* Zurich SARS-CoV-2 Cohort: Towards a long-term control of SARS-CoV-2 transmission - Identifying the epidemiological, immunological and viral genetic drivers of SARS-CoV-2 transmission and pathogenesis in a representative population-based cohort. https://www.isrctn.com/ISRCTN14990068 (accessed June 11, 2021).

19 Bek LM, Berentschot JC, Hellemons ME, *et al.* CO-FLOW: COvid-19 Follow-up care paths and Long-term Outcomes Within the Dutch health care system: study protocol of a multicenter prospective cohort study following patients 2 years after hospital discharge. *BMC Health Services Research* 2021; **21**: 847. https://www.trialregister.nl/trial/8710.

20 Buonsenso D, Munblit D, De Rose C, *et al.* Preliminary evidence on long COVID in children. *Acta Paediatr* 2021; **110**: 2208–11.

21 Rass V, Beer R, Schiefecker AJ, *et al.* Neurological outcome and quality of life 3 months after COVID-19: A prospective observational cohort study. *Eur J Neurol* 2021; published online March 7. DOI:10.1111/ene.14803. https://clinicaltrials.gov/ct2/show/NCT04416100.

22 Petersen MS, Kristiansen MF, Hanusson KD, *et al.* Long COVID in the Faroe Islands - a longitudinal study among non-hospitalized patients. *Clin Infect Dis* 2020; ciaa1792.

23 Logue JK, Franko NM, McCulloch DJ, *et al.* Sequelae in Adults at 6 Months After COVID-19 Infection. *JAMA Netw Open* 2021; **4**: e210830.

24 Kurth F, Roennefarth M, Thibeault C, *et al.* Studying the pathophysiology of coronavirus disease 2019: a protocol for the Berlin prospective COVID-19 patient cohort (Pa-COVID-19). *Infection* 2020; **48**: 619-626. https://www.drks.de/drks_web/navigate.do?navigationId=trial.HTML & TRIAL_ID=DRKS00021688.

25 Thibeault C, Mühlemann B, Helbig ET, *et al.* Clinical and virological characteristics of hospitalised COVID-19 patients in a German tertiary care centre during the first wave of the SARS-CoV-2 pandemic: a prospective observational study. *Infection* 2021; published online April 22. DOI:10.1007/s15010-021-01594-w.

26 Hultström M, Frithiof R, Lipcsey M. PRONMED Uppsala COVID-19 ICU Biobank. 2021; published online March 18. https://clinicaltrials.gov/ct2/show/NCT04474249.

27 Ekbom E, Frithiof R, Öl E, *et al.* Impaired diffusing capacity for carbon monoxide is common in critically ill Covid-19 patients at four months post-discharge. *Respiratory Medicine* 2021; **182**: 106394.

28 A global healthcare intelligence partner. PRA Health Sciences. https://prahs.com/ (accessed June 15, 2021).

29 Al-Aly Z, Xie Y, Bowe B. High-dimensional characterization of post-acute sequelae of COVID-19. *Nature* 2021; **594**: 259–64.

30 VA research during the COVID-19 pandemic. https://www.research.va.gov/covid-19.cfm (accessed June 15, 2021).

31 Zheng P, Barber R, Sorensen RJD, Murray CJL, Aravkin AY. Trimmed Constrained Mixed Effects Models: Formulations and Algorithms. *arXiv:190910700 [math, stat]* 2020; published online Oct 27. http://arxiv.org/abs/1909.10700 (accessed June 15, 2021).

32 Huber P. Robust Statistics. John Wiley & Sons, 2004.

33 Rousseeuw P, Leroy A. Robust regression and outlier detection. John Wiley & Sons, 2005.

Cutaneous and mucocutaneous leishmaniasis

Flowchart

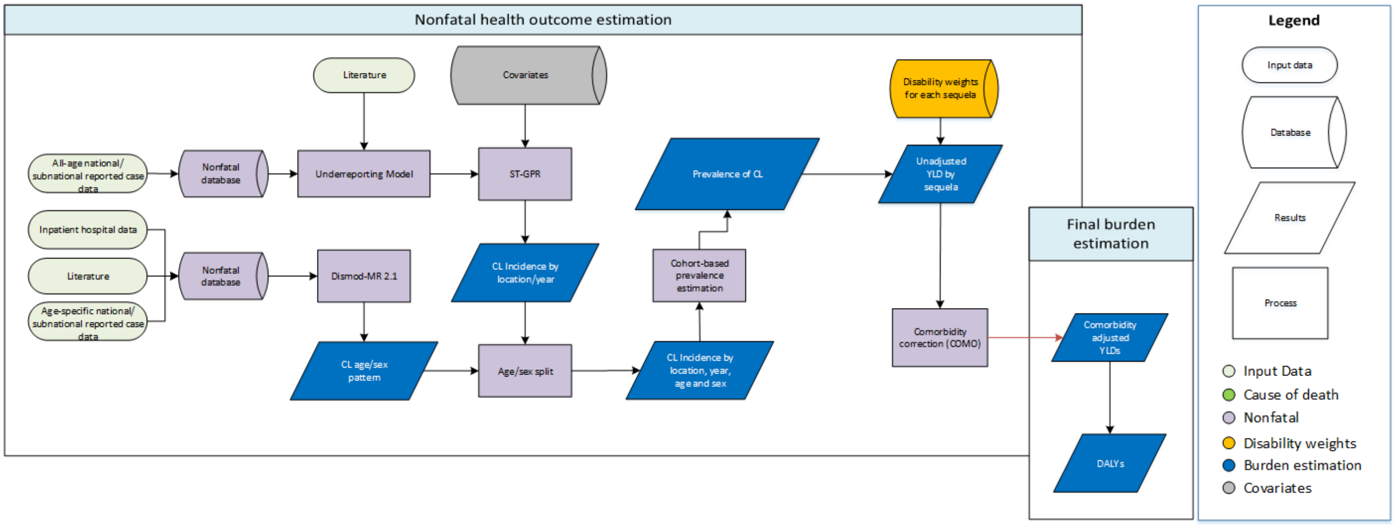

Input data and methodological summary for cutaneous leishmaniasis

Case definition

Cutaneous and mucocutaneous leishmaniasis (CL) is a group of syndromes caused by the *Leishmania* parasite, transmitted through the bite of phlebotomine sandflies. The most common form is cutaneous leishmaniasis, and those infected typically present with a well-demarcated skin lesion at the site of the sandfly bite. This initial lesion is usually painless and may enlarge and ulcerate, developing a scab or crust. Mucocutaneous leishmaniasis is less common and begins with a primary cutaneous ulcer progressing to partial or complete destruction of nasopharyngeal tissues. The ICD-9 codes related to CL are 085.4 and 085.5, and the ICD-10 codes are B55.1 and B55.2. We used the following case definition for GBD 2023:

Quantity of interest	Reference or alternative	Definition
Cutaneous and mucocutaneous leishmaniasis	Reference	A person identified as a case through a case notification system or by clinical diagnosis of skin lesions, with or without laboratory confirmation.

Input data

Incidence

Current estimation for the all-age, both-sex incidence envelope is based upon location-representative information rather than site-specific epidemiological measures due to the absence of global foci maps allowing for upscaling of geographically precise information. The primary input data are case notification time-series reported by national control programmes, ministries of health, and the World Health

Organization (WHO). This is supplemented by systematic literature review (last updated for GBD 2015) to identify alternate sources of data for years missing information. For countries with subnational estimates, in-country collaborators have compiled information for respective programmes or identified key resources, again supplemented by literature reviews. Where possible, information disaggregating location-level statistics by age and sex were extracted.

Geographical restrictions

There are strong climatic and biogeographical constraints on the geographical distribution of CL resulting in a focal rather than global distribution. As a result, it is necessary to identify locations burdened by the disease through space and time as distinct from countries where CL is absent. Tags were assigned to each location-year based upon the outcome of a search of IHME databases, as well as location-specific searches of PubMed. Each location-year is tagged as follows:

- Present – where a specific citation of either an autochthonous laboratory-confirmed case (ie, a case with PCR, serological, or parasitological diagnosis), reported case (ie, a case noted as CL, but with no supporting diagnostic), or supporting evidence (ie, confirmed infection in animal reservoirs or sandfly vectors).
- Protocol Present – for a given location-year, where no specific citation is used, but is present for another year in the same location, it is assumed that CL is present given that eradication of the pathogen has not been achieved.
- Absent – where PubMed location-specific searches returned zero relevant results, in locations scoring –25 or lower as evaluated by Pigott and colleagues[2] (the threshold for “absence” in that study), locations were tagged as Absent.
- Protocol Absent – as with Absent, locations with zero relevant PubMed results, but with greater than –25 as evaluated by Pigott and colleagues,[2] were tagged as Protocol Absent.

Cutaneous Leishmaniasis Geographic Restrictions: 2010 (Endemic: 188)

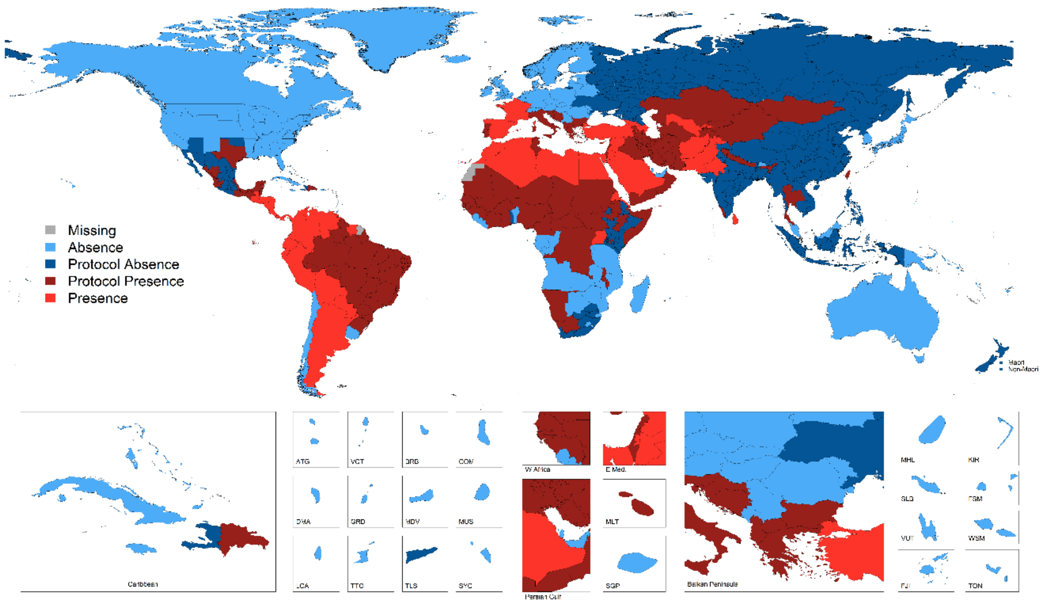

Figure 1: Cutaneous leishmaniasis geographical restrictions for the year 2010. GBD locations tagged as present are coloured in red; dark red represents protocol presence, dark blue represents protocol absence, and absence is represented by light blue. Locations missing tags are presented in grey.

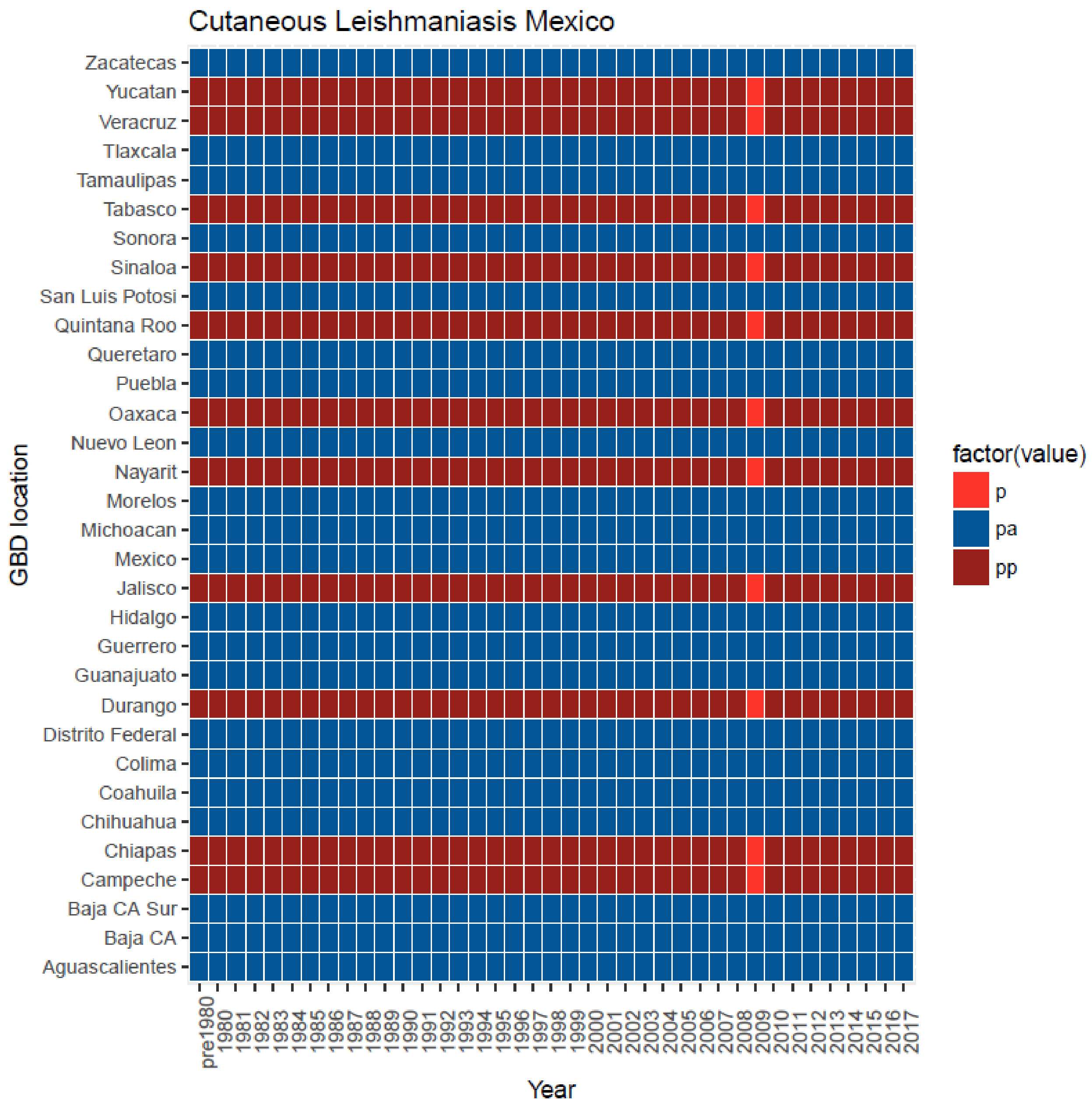

Figure 2: Cutaneous leishmaniasis geographical restrictions for Mexican subnationals. Locations tagged as present are coloured in red; dark red represents protocol presence, and dark blue represents protocol absence.

Modelling strategy

Under-reporting adjustment

Reported case count data were translated into estimates of true case counts by using published under-reporting scalars derived from a study.[1] This analysis provided estimates of plausible incidence ranges of CL based on published data and expert judgment of the magnitude of under-reporting. The incidence

ranges were determined at the country and/or region level and were based on reported estimates multiplied by probable under-reporting factors.

All-age, both-sex incidence modelling

The under-reporting-adjusted, all-age, both-sex incidence data were modelled using spatiotemporal Gaussian process regression (ST-GPR) to produce a complete time series of estimates for each GBD location. The GBD 2021 model was run using a linear model with two covariates, leishmaniasis endemicity and proportion of population with access to sanitation, with random effects by country, region, and super-region. For GBD 2023, the linear regression phase of ST-GPR was updated to a custom negative binomial model in order to better account for zero and near-zero case counts. Separate linear models were run with all combinations of seven selected covariates with random intercepts by country and region, resulting in a total of 128 models. Only models that met prior assumptions for covariate directionality (see Table 1) were kept. The 22 remaining models were then weighted by first calculating the out-of-sample root-mean-square deviation (OOS RMSE) using five-fold cross-validation, which was used to create a weight for each model as a proportion of the total OOS RMSE. These weights were multiplied by the in-sample predictions for each model and then summed, in order to create an ensemble model used as the first stage for the final ST-GPR model. The final ST-GPR model was run using the following hyperparameters: st_lambda = 0.5, st_omega = 1, st_zeta = 0.01, gpr_scale = 1.

Table 1: Negative binomial model covariates and directionality priors for ensemble

**Covariate**	**Directionality**
Healthcare Access and Quality Index	–1
Urbanicity	–1
Population density (under 150 ppl/km^2)	+1
Improved water source (proportion with access)	–1
Universal health coverage	–1
Sanitation (proportion with access)	–1
Socio-demographic Index	–1

Age-sex pattern

The next stage of the modelling process used a DisMod Bayesian meta-regression (DisMod-MR) model to generate country-specific age-sex curves to disaggregate all-age, both-sex incidence estimates. DisMod-MR is an integrated meta-regression framework that allows multiple datasets to be used within a singular analysis regardless of age-binning, sources, and geographies. As a result, a variety of differently aggregated information is combined to generate a consensus output. We ran a single-parameter DisMod-MR model using age- and sex-specific case notification data informed by two country-level covariates (see Table 2).

Table 2: Covariates. Summary of covariates used in the CL DisMod-MR model

**Covariate**	**Type**	**Parameter**	**Exponentiated beta (95% UI)**
Socio-demographic Index	Country-level	Incidence	0.18 (0.15–0.20)

Healthcare Access and Quality Index	Country-level	Incidence	2.46 (2.16–2.80)

Prevalence modelling

Following standard GBD estimation protocols, incidence estimates were used to calculate disease prevalence (by multiplication with duration). The duration of initial acute infection was set to six months.[3]

Prevalence of chronic infection was estimated assuming that a fixed proportion of cases would result in facial scarring, using a cohort-based estimation process. The average proportion of sores that occurred on the face was calculated based upon a sample-weighted average of the proportion from four studies conducted in north Africa and the Middle East.[4-7] This proportion was 0.476. We additionally assumed that only those who did not have appropriate access to health care would be assigned long-term sequelae, which was estimated as a proportion using the universal health coverage (UHC) covariate. CL incidence estimates were then multiplied by the average proportion of facial sores and the proportion of people without adequate health-care access in each location-year, in order to estimate the incidence of people with long-term sequelae for each year:

$$chronic_{l,y,s,a} = chronic_{l,y-1,s,a-1} * \left(1 - uhc_{l,y,s,a}\right) * 0.476$$

In previous rounds, this cohort estimation process began in 1980. For GBD 2023, the cohort model was run starting in 1890, in order to generate an improved steady-state estimate of prevalence in 1980 while accounting for cases occurring in earlier years. The model was run with single-year age bins to accumulate chronic cases across ages from year to year. We made additional assumptions for treatment, population, and incidence. This included no treatment prior to 1940 and backfilling years without data with the earliest available value (see Table 3).

Table 3: Assumptions for long-term sequela model

**Treatment**	**Population**	**Incidence**
<1940 no treatment	<1950 backfill	<1980 backfill
1940 treatment begins and remains constant; assume UHC 1990 estimate	1950–1990 varies by year	1980–1990 varies by year

Health states/sequelae

One health state is assigned to CL (Table 4).

Table 4. Severity distribution, details on the severity levels for CL and the associated disability weight (DW) with that severity

**Sequela**	**Health state lay description**	**DW (95% CI)**	**Duration**
Cutaneous and mucocutaneous leishmaniasis	"has a slight, visible physical deformity that others notice, which causes some worry and discomfort"	0.011 (0.005–0.021)	6 months<br>(46.7% *UHC)<br>Lifelong

Changes from GBD 2021 to GBD 2023

The first stage of the ST-GPR model previously used a linear regression of log-transformed rates to calculate an all-age-sex time series for incidence. This approach required an offset for zero case counts, which overestimated incidence in locations with small case counts. The GBD 2023 model used a negative binomial regression to better account for low and zero case counts, thereby providing more plausible estimates in locations where incidence is low. This methodological update also allowed the inclusion of data with case counts of zero, which had previously been excluded from the model. Also, incidence data from the Brazil Information System for Notifiable Diseases (SINAN) was added to Brazil subnationals, in addition to country-level data from the WHO Global Health Observatory.

The cohort-based modelling of the prevalence of long-term sequelae began in 1980 in previous rounds. For GBD 2023, this cohort model was updated to start in 1890 to better account for accumulation of historical cases, resulting in higher predictions at the global level throughout the time series.

Limitations

Given the focus on location-representative estimates, the existing model is focused on national case counts. This excludes a large resource of published literature and grey literature focused on site-specific surveillance or surveys. While some pathogens have integrated subnational approaches as a building block for national estimates (eg, schistosomiasis), this has yet to be implemented for CL. Regardless of contribution to the global incidence model, these data could be used in future rounds to inform age-sex splits, as well as a variety of other key parameters, particularly duration parameters, which are currently lacking uncertainty.

We did not apply any adjustments for the COVID-19 pandemic to CL due to a lack of available data quantifying the impacts of the pandemic on NTD epidemiology.

References

1. Alvar J, Vélez ID, Bern C, *et al.* Leishmaniasis Worldwide and Global Estimates of Its Incidence. *PLoS One* 2012; 7.
2. Pigott DM, Bhatt S, Golding N, *et al.* Global distribution maps of the leishmaniases. *eLife* 2014; 3. DOI:10.7554/eLife.02851
3. Reithinger R, Dujardin J-C, Louzir H, Pirmez C, Alexander B, Brooker S. Cutaneous leishmaniasis. *The Lancet Infectious Diseases.* 2007; 7:581–96.
4. Momeni AZ, Aminjavaheri M. Clinical picture of cutaneous leishmaniasis in Isfahan, Iran. *Int J Dermatol.* 1994; 33:260–5.
5. Gurel MS, Ulukanligil M, Ozbilge H. Cutaneous leishmaniasis in Sanliurfa: epidemiologic and clinical features of the last four years (1997-2000). *Int J Dermatol.* 2002; 41:32–7.
6. Sharifi I, Fekri AR, Aflatonian MR, Nadim A, Nikian Y, Kamesipour A. Cutaneous leishmaniasis in primary school children in the south-eastern Iranian city of Bam, 1994-95. *Bull World Health Organ.* 1998; 76:289–93.
7. Mujtaba G, Khalid M. Cutaneous leishmaniasis in Multan, Pakistan. *Int J Dermatol.* 1998; 37:843–5.

Cystic echinococcosis

Flowchart

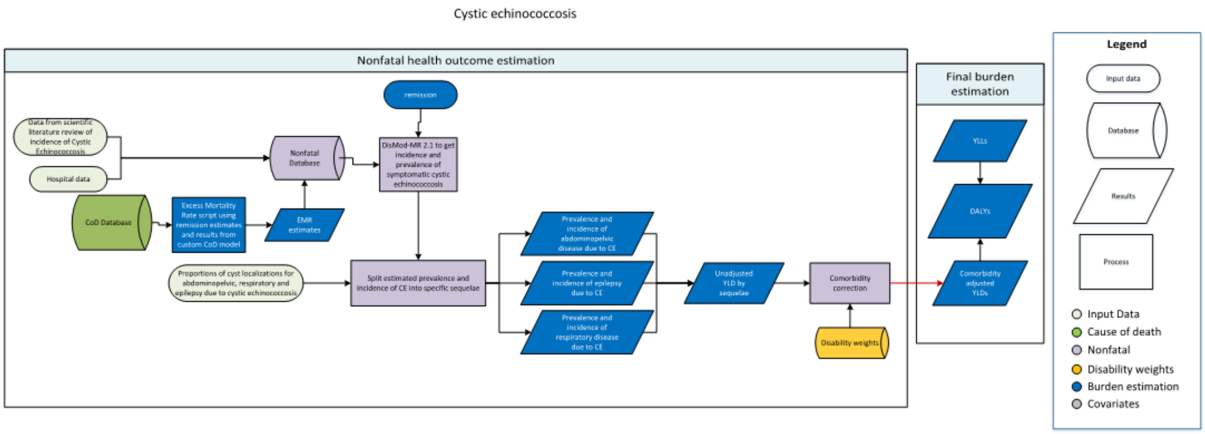

Input data and methodological summary for cystic echinococcosis

Case definition

Cystic echinococcosis (CE) is an infection with *Echinococcus* tapeworms, which are transmitted primarily via consumption of food, water, or soil contaminated with animal feces. Larval growth causes cyst formation in different parts of the body, especially the liver, lungs, and central nervous system; symptoms include abdominal pain, respiratory distress, and neurological symptoms including seizures. Diagnosis is made by clinical findings, imaging, serology, and tissue pathology. The ICD-9 and ICD-10 codes for echinococcosis are 122.0-122.9 and B67-B67.9, respectively. We used the following case definition for GBD 2023:

**Quantity of interest**	**Reference or alternative**	**Definition**
Cystic echinococcosis	Reference	Diagnosis of cystic echinococcosis based on imaging findings or clinical findings, based on ICD-9 codes 122.0-122.9 or ICD-10 codes B67-B67.9.

Systematic literature review

The non-fatal estimation for CE focused on estimating incidence and prevalence of CE and its sequelae. A systematic review of literature was conducted in PubMed for GBD 2015 using the following search string:

> ("echinococcosis"[Title/Abstract] OR "hydatid disease"[Title/Abstract] OR "hydatidosis"[Title/Abstract] OR "echinococcal disease"[Title/Abstract] OR "Echinococcus granulosus infection"[Title/Abstract]) AND (“1990”[Date – Publication] : “2015”[Date – Publication]) AND (epidemiology OR incidence OR prevalence).

This yielded 1619 studies, of which 279 were included during the title/abstract screening. Following the full-text screening, 77 studies (32 incidence, 43 prevalence, and two both) were included and extracted – studies were excluded because of one or more of the following reasons:

1. study not population-based
2. study does not have primary data on prevalence and/or incidence
3. study not in humans
4. study on sub-populations
5. review study

Since we were interested in modelling symptomatic CE cases, we only used data on incidence of patients diagnosed by imaging techniques (mainly ultrasonography). Therefore, we excluded prevalence data, which were mostly from serological studies. Data from these extracted studies were combined with data from studies extracted during GBD 2013.

Hospital data

Hospital data prepared by the GBD team were used as additional input into our models. These data were adjusted to account for multiple hospital episodes of a single case and non-primary diagnoses.

Geographical restrictions

For CE, we performed targeted searches to classify location-years in PubMed and Google Scholar. Geographical restrictions were populated by reviewing sources referenced by Deplazes and colleagues[1] along with ad hoc searches in PubMed for evidence of active transmission of CE in respective countries.

Modelling strategy

The morbidity model for CE involved a multi-step process. First, incidence data reported for both sexes were split into sex-specific inputs. To sex-split our both-sex datapoints, we used sex-specific inputs in a meta-regression—Bayesian, regularised, trimmed (MR-BRT) model to derive a ratio of female CE incidence to both-sex incidence (from scientific literature data). The resultant log ratio was applied to both-sex datapoints to calculate out females, and males were calculated via subtraction. The beta coefficients of the adjustment are presented in Table 1.

Table 1: MR-BRT crosswalk adjustment factors for cystic echinococcosis

**Data input**	**Reference or alternative case definition**	**Gamma**	**Beta coefficient, log (95% UI)***	**Adjustment factor****
Females	Ref	0.32	---	---
Both sex	Alt		0.15 (–1.03; 1.33)	1.16

**MR-BRT crosswalk adjustments can be interpreted as the factor the alternative case definition is adjusted by to reflect what it would have been had it been measured using the reference case definition. If the log/logit beta coefficient is negative, then the alternative is adjusted up to the reference. If the log/logit beta coefficient is positive, then the alternative is adjusted down to the reference.*

***The adjustment factor column is the exponentiated beta coefficient. For log beta coefficients, this is the relative rate between the two case definitions. For logit beta coefficients, this is the relative odds between the two case definitions.*

We then split all-age case data into age-specific observations using a global age pattern derived from a DisMod Bayesian meta-regression (DisMod-MR) model. The age pattern was developed using a single-parameter incidence model in DisMod-MR. Uncertainty was propagated throughout the sex- and age-splitting processes, such that final sex- and age-specific incidence estimates reflect the uncertainty of the original data.

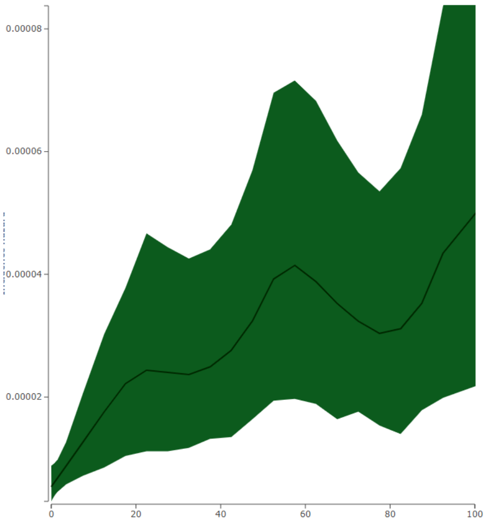

Figure 1. Global age pattern of cystic echinococcosis incidence produced by DisMod-MR

Then, DisMod-MR was used to model incidence and prevalence of symptomatic CE using incidence data from systematic reviews in GBD 2013 and 2015 and hospital data, excess mortality rate (EMR) estimates, and an assumed remission of 0.15–0.25 per case per year (duration 2–6.7 years, average 5 years). We used urbanicity, echinococcosis endemicity, and proportion of population involved in agricultural activities as country-level covariates. To estimate the EMR used in the final DisMod-MR model, we first fit an initial DisMod-MR model using the input data, covariates (excluding EMR), and allowing remission to range from 0 to 1. We then obtained predictions of EMR from this initial DisMod-MR model, which were used in turn as input data for a MR-BRT model. The MR-BRT model estimated EMR for all national-level locations, using the Healthcare Access and Quality (HAQ) Index, age, and sex as covariates. This approach produced predictions of remission and EMR that were 3.5 times higher than values predicted from previous models at the global level. We therefore adjusted all predicted EMR values, dividing by 3.5 in order to return the overall scale of EMR that was predicted by previous models, while also leveraging the relationships between HAQ Index and EMR estimated by the MR-BRT model. Last, the predicted EMR values from the MR-BRT model, after rescaling, were used as inputs into our final DisMod-MR model. This approach helps to ensure that the excess mortality trends implied by the final DisMod-MR model better match expected patterns across different levels of HAQ Index.

Geographical restrictions were applied to set incidence and prevalence to zero in location-years where the disease was not endemic.

Table 2. DisMod-MR model covariates

Covariate	Type	Parameter	Exponentiated beta
Sex	Study-level	Incidence	0.96 (0.92–1.01)
Urbanicity	Country-level	Incidence	1.00 (0.99–1.00)
Echinococcosis endemicity	Country-level	Incidence	20.06 (20.05–20.09)
Proportion of population involved in agricultural activities	Country-level	Incidence	1.00 (1.00–1.00)
Sex	Study-level	Excess mortality rate	1.21 (0.96–1.49)

After producing all-case prevalence draws, 1000 draws of proportions for abdominal, respiratory, and epileptic symptoms among echinococcosis cases adding up to 1 were generated. Uncertainty in the splitting proportions was captured by drawing them from a Dirichlet distribution, informed by published data on cysts localization.[2] On average, the proportions of abdominal, respiratory, and epileptic symptoms due to echinococcosis were 0.5, 0.47, and 0.03, respectively. These proportions were used to split the prevalence and incidence from DisMod-MR into the three sequelae.

Model evaluation was done by separately assessing the fit of the DisMod-MR model and checking the estimates produced after estimating incidence and prevalence of sequelae due to CE. Plots of time trends of incidence and prevalence across locations and age were used to evaluate the results. In addition, maps of the global distribution of incidence and prevalence were assessed across time.

We did not apply any adjustments for the COVID pandemic to cystic echinococcosis due to a lack of available data quantifying the impacts of the pandemic on NTD epidemiology.

Sequelae due to cystic echinococcosis

The table below shows the sequelae due to echinococcosis and their associated disability weights.

Table 3. Sequelae, lay descriptions, and disability weights (DWs)

Sequela	Lay description	DW (95% CI)
Chronic respiratory disease	"has cough and shortness of breath after heavy physical activity, but is able to walk long distances and climb stairs."	0.019 (0.011–0.033)
Abdominal problems	"has pain in the belly and feels nauseated. The person has difficulties with daily activities."	0.114 (0.078–0.159)
Epilepsy	(Combined DW)	NA

Changes from GBD 2021 to GBD 2023

There were no substantive changes to the modelling strategy for GBD 2023.

References

1. Deplazes P, Rinaldi L, Alvarez Rojas CA, Torgerson PR, Harandi MF, Romig T, Antolova D, Schrurer JM, Lahmar S, Cringoli G, Magambo J, Thompson RC, Jenkins EJ. Global Distribution of Alveolar and Cystic Echinococcosis. *Advanced Parasitology*. 2017. 95: 315-493.
2. Raether W, Hänel H. Epidemiology, clinical manifestations and diagnosis of zoonotic cestode infections: an update. *Parasitology Research*. 2003. 91:412-438.

Cysticercosis

Flowchart

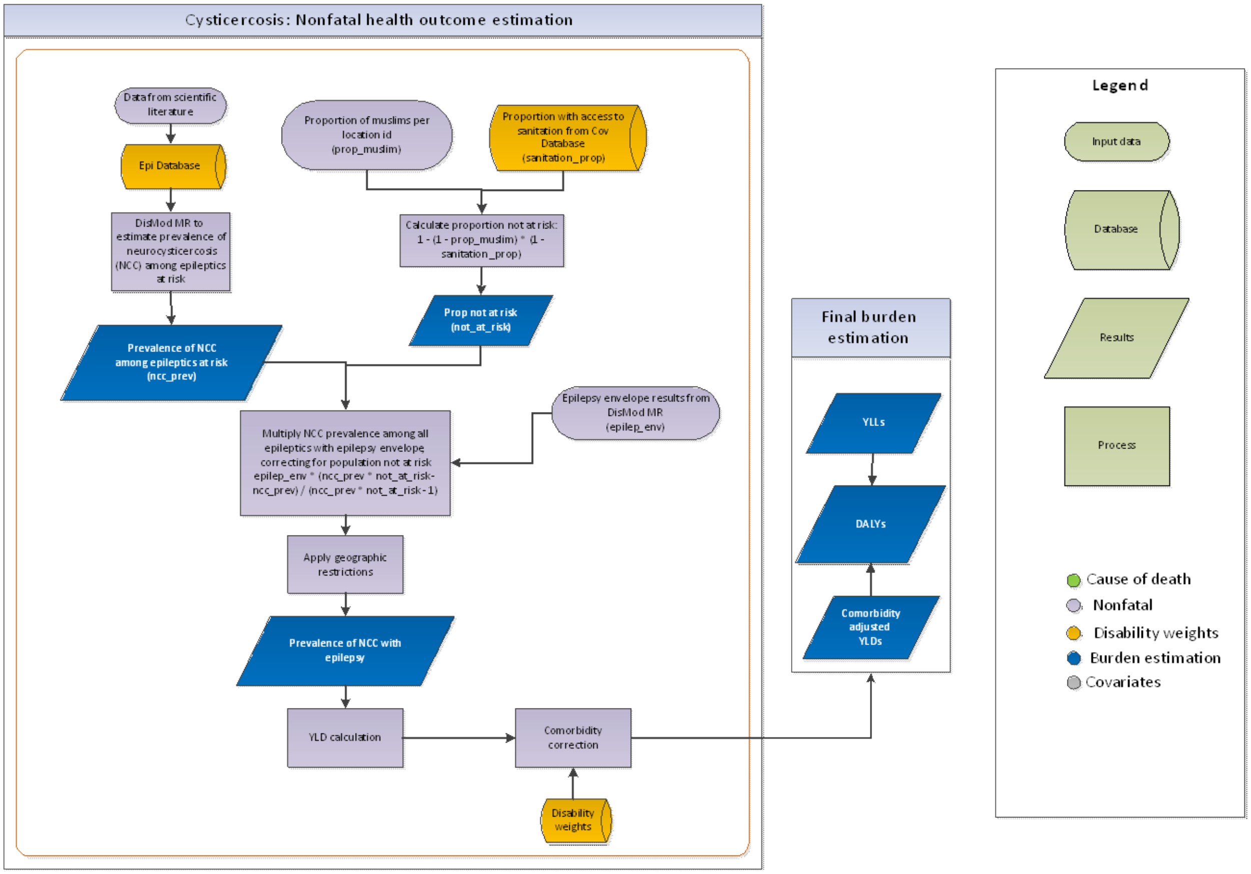

Input data and methodological summary for cysticercosis

Case definition

Cysticercosis is a parasitic disease caused by the pig tapeworm *Taenia solium*, transmitted via ingestion of contaminated food or water. Neurocysticercosis (NCC) is caused by parasites traveling into the central nervous system. NCC can result in epilepsy and other neurological symptoms. Diagnosis is made by positively identifying cysts in an epilepsy patient. The ICD-10 codes for cysticercosis are B69-B69.9.

Quantity of interest	Reference or alternative	Definition
Cysticercosis	Reference	An epilepsy patient with either (a) *T Solium* identified in excised cysticerci from tissues by microscopic examination, or (b) identification of cysticerci by CT scan, MRI, or X-ray and positive result on CDC immunoblot assay.
Cysticercosis	Alternative	An epilepsy patient with calcified cystic lesions in the brain identified by CT scan, MRI, or X-ray; or positive result on CDC immunoblot assay. [A "probable" case.]

Input data

Systematic literature review

Non-fatal cysticercosis estimates are based upon the estimated prevalence of NCC among at-risk epileptics, as well as the prevalence of NCC with epilepsy. A systematic review of the scientific literature in the PubMed database was conducted for GBD 2015 using the following search string:

> *"cysticercosis"[Title/Abstract] OR "neurocysticercosis"[Title/Abstract] OR "cysticerciasis"[Title/Abstract] OR "Taenia solium"[Title/Abstract]) AND ("1990"[Date – Publication] : "2015"[Date – Publication]) AND (epidemiology OR prevalence)*

This search string returned 1038 studies. Of those studies, 166 were selected for inclusion during the title/abstract screening. Following the full-text screening, 17 of the 166 studies were included for extraction. The studies that were excluded were dropped because of one or more of the following reasons:

1. The study was not of epileptics
2. The study was not population-based
3. The study did not have primary data on prevalence of NCC among epileptics at risk
4. The study was of non-human animals
5. The study was on comorbidities with NCC other than epilepsy
6. The study was of a sub-population, (eg, patients with neurological disorders)
7. It was a review study

Data processing

Input data observations were classified as having either a probable or a definite diagnosis. We used crosswalking methods to calibrate the observations classified as "probable" using the observations classified as "definite". Sixteen within-study comparisons were identified to crosswalk the data using definite diagnosis as a reference using meta-regression—Bayesian, regularised, trimmed (MR-BRT) [Table 1].

Table 1. MR-BRT crosswalk adjustment factors

Data input	Reference or alternative case definition	Gamma	Beta coefficient, log (95% CI)*	Adjustment factor**
**Definite**	Ref	0.62	---	---
**Probable**	Alt		0.59 (0.22, 0.96)	0.55

**MR-BRT crosswalk adjustments can be interpreted as the factor that the alternative diagnostic is adjusted by to reflect what it would have been had it been measured using the reference diagnostic. If the log/logit beta coefficient is negative, then the alternative is adjusted up to the reference. If the log/logit beta coefficient is positive, then the alternative is adjusted down to the reference.*
***The adjustment factor column is the exponentiated beta coefficient. For log beta coefficients, this is the relative rate between the two diagnostics. For logit beta coefficients, this is the relative odds between the two diagnostics.*

Covariates

Data on the proportion of the population that is Muslim were obtained from the PEW Research Center.[1] This proportion was incorporated as a continuous covariate with a range between 0 and 1.

Epilepsy envelope

The modelling pipeline incorporates 1000 draws of epilepsy prevalence estimates from the epilepsy DisMod Bayesian meta-regression (DisMod-MR) model for GBD 2023 – details on this modelling process can be found elsewhere.

Modelling strategy

DisMod-MR was used to model the prevalence of NCC among at-risk epileptics. In the model, Socio-Demographic Index (SDI), religion (binary, >50% Muslim), and the per capita pigs raised within extensive agricultural systems, were used as country-level covariates (Table 2).

Table 2. DisMod-MR model covariates

**Covariate**	**Type**	**Parameter**	**Exponentiated beta**
Religion (binary, >50% Muslim)	Country-level	Prevalence	0.22 (0.16, 0.33)
Socio-demographic Index	Country-level	Prevalence	0.14 (0.14, 0.16)
Pigs raised in extensive agricultural systems per capita	Country-level	Prevalence	3.07 (1.34, 6.38)

After running DisMod-MR, we adjusted the fraction of people with epilepsy attributable to cysticercosis in endemic countries for the population at risk based on the proportion of the population without access to sanitation and the proportion of the population that is Muslim. The following equation is used for estimating NCC prevalence among epileptics at risk:

$$Prevalence_{NCC\ prevalence} = Prevalence_{epilepsy} * \frac{NM - N}{NM - 1}$$

Where *prevalence* = prevalence of all-cause epilepsy in total population, *N* = proportion of NCC among epileptics at risk (non-Muslims without access to sanitation), and *M* = proportion of population not at risk of contracting NCC. It was assumed that the prevalence of epilepsy due to causes other than NCC is the same regardless of whether a population is at risk or not. It was also assumed that Muslims and non-Muslims have equal access to sanitation. Non-endemic administration units were automatically given an NCC prevalence estimate of zero.

Model evaluation was done by assessing the fit of the DisMod-MR model and checking the estimates produced after estimating the prevalence of NCC. Time-series prevalence plots across locations and ages were used to evaluate the results. In addition, maps of the global distribution of prevalence of NCC among epileptics at risk and the prevalence of NCC with epilepsy were also assessed across time.

We did not apply any adjustments for the COVID-19 pandemic to cysticercosis due to a lack of available data quantifying the impacts of the pandemic on NTD epidemiology.

Changes from GBD 2021 to GBD 2023

There were no substantive changes to the modelling strategy for GBD 2023.

References

1. “Table: Muslim Population by Country Pew Research Center, Washington, D.C.” (July 7, 2017). http://www.pewforum.org/2011/01/27/table-muslim-population-by-country/

Decubitus ulcer

Flowchart

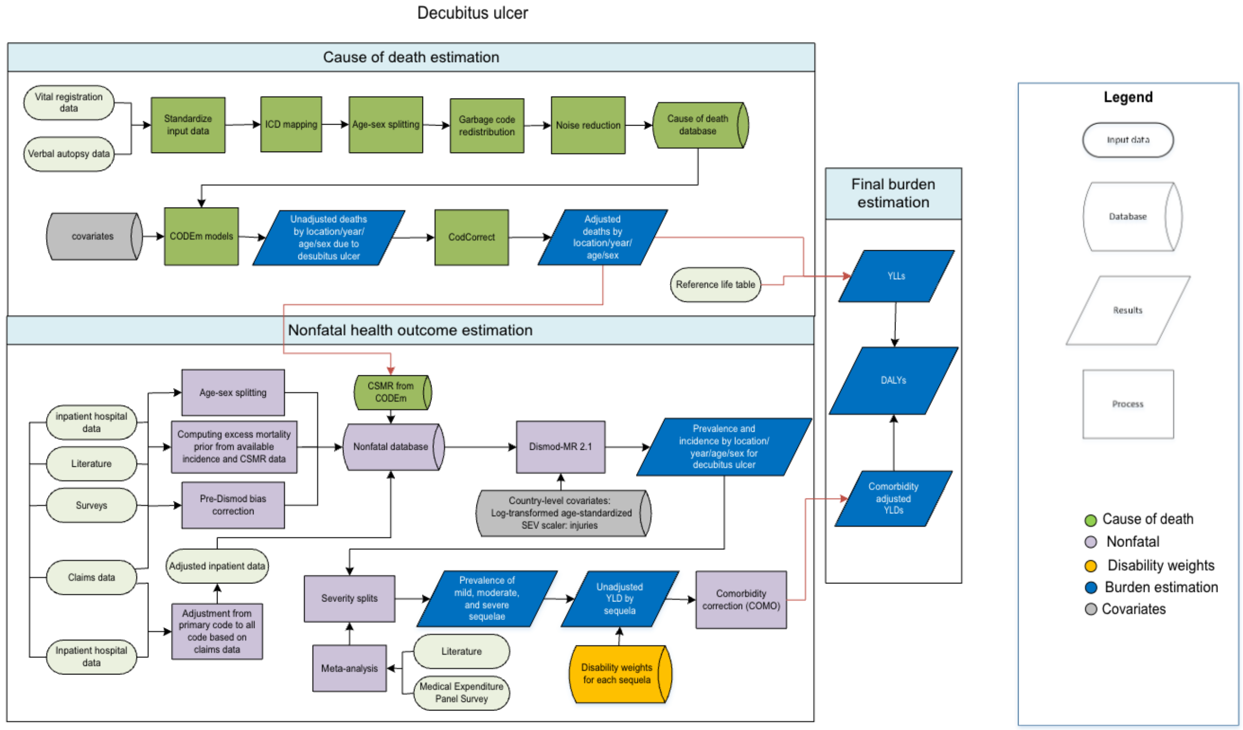

Case definition

Decubitus ulcer is defined as an injury to the skin and underlying tissue resulting from an obstruction of blood flow due to pressure on the skin. Also known as pressure ulcer/sore (ICD-10: L89). Hospital admission for decubitus (ICD-10: L89) (from the table below).

Quantity of interest	Reference or alternative	Definition
Decubitus ulcer	Reference	Decubitus ulcer as determined by Poland's claims data
Decubitus ulcer	Alternative	All other data for decubitus ulcer

Input data

In the GBD 2010 study, a systematic review of the literature was conducted using PubMed and Google Scholar to capture epidemiological data for decubitus ulcer. The inclusion criteria stipulated that studies (1) must be published between 1980 and 2012; (2) must provide data on the incidence or prevalence of decubitus ulcer; (3) must use samples representative of the general population (ie, samples derived from the experimental arm of clinical trials or based in dermatology clinics were excluded); (4) must use a sample size larger than 100; and (5) must provide sufficient information on study method and sample characteristics to assess the quality of the study. The data from literature were sparse but contained both prevalence and incidence estimates. For GBD 2013, the GBD 2010 search strategy was replicated to capture epidemiological studies published between 2012 and 2013. The available data were from high-

income countries. Hospital inpatient, USA claims data from 2000 and 2010–2016, Taiwan (province of China) claims data for 2016, and Poland claims data for 2015–2017 were also used for GBD 2021. For GBD 2023, we have added year-locations of inpatient and outpatient clinical data. The new data sources include Poland National Health Fund Patient Claims from 2015 to 2019, USA MarketScan Commercial Claims from 2010 to 2019, and South Korea Medical Services Claims Data 2018 and 2019. We have also included location-years of inpatient data, mainly from Mexico, Germany, the USA, Chile, Austria, the UK, Brazil, New Zealand, etc. We also have survey data from the UK added for this round.

The final dataset also included cause-specific mortality rates for decubitus ulcer estimated by CODEm. Data were outliered or excluded if we found them unreasonable compared to regional, super-regional, and global rates.

Data processing

For decubitus ulcer, we crosswalked all data to the reference definition. We began by evaluating the number of observations of each alternate definition that matched with a corresponding observation from the reference definition. We considered "between" study matches, where the alternative was from the same GBD age group and sex, and the midpoint year of the study was within five years of the midpoint of the reference definition observation.

$$\log i\, t\left(y_i^{alt}\right) - \log i\, t\left(y_i^{ref}\right) = \beta_0 + \epsilon_i$$

Table 1: MR-BRT crosswalk adjustment factors for decubitus ulcer

Data input	Reference or alternative case definition	Gamma	Beta coefficient, logit (95% UI)*	Adjustment factor**
Decubitus ulcer as defined by Poland National Health Fund Patient Claims	Ref	0.10136	---	---
All other data	Alt		0.0001145 (–0.6239 to 0.6241)	1.0001145 (0.5358 to 1.8666)

**MR-BRT crosswalk adjustments can be interpreted as the factor the alternative case definition is adjusted by to reflect what it would have been had it been measured using the reference case definition. If the log/logit beta coefficient is negative, then the alternative is adjusted up to the reference. If the log/logit beta coefficient is positive, then the alternative is adjusted down to the reference.*

***The adjustment factor column is the exponentiated beta coefficient. For log beta coefficients, this is the relative rate between the two case definitions. For logit beta coefficients, this is the relative odds between the two case definitions.*

Modelling strategy

In GBD 2023, we have made no substantive changes in the modelling strategy from GBD 2021. DisMod-MR 2.1 was used to estimate prevalence by age, sex, year, and geography (subnational, country, region, super-region) for decubitus ulcer. Per expert advice, remission was set from 3 to 4, implying a duration of three to four months. This was based on the assumption that remission does not change

with treatment. These values were also in line with the available epidemiological data, expert opinion, and previous GBD work. The decubitus ulcer dataset was sufficiently large to make use of a relatively short time window of five years to determine which datapoints were used for a particular year of fit.
In GBD 2023, we applied crosswalks using the MR-BRT modelling tool. We adjusted inpatient data along with USA MarketScan data and other data sources toward the level of Poland claims datapoints, which were more representative of the general population. In addition, log-transformed age-standardized SEV scaler: injuries was used as a country-level covariate to guide estimates for locations with few or no data. The covariate was restricted to a range of 0.1 to 0.5. For incidence hazard, we restricted location random effects to (0.0009–0.0015) in Nepal, Iran, and Botswana.

$$prevalence = incidence * duration$$

$$Remission = \frac{Cured\ cases}{person - year of follow - up\ in\ prevalent\ cases}$$

Table 2. Severity distribution, details on the severity levels for decubitus ulcer and the associated disability weight (DW) with that severity.

Severity level	Lay description	DW (95% CI)
Mild decubitus ulcer	The person has a slight, visible physical deformity that is sometimes sore or itchy. Others notice deformity, which causes some worry and discomfort.	0.43 (0.319–0.55)
Moderate decubitus ulcer	The person has a visible physical deformity that is sore and itchy. Other people stare and comment, which causes the person to worry. The person has trouble sleeping and concentrating.	0.326 (0.248–0.394)
Severe decubitus ulcer	The person has an obvious physical deformity that is very painful and itchy. Physical deformity makes others uncomfortable, which causes the person to avoid social contact, feel worried, sleep poorly, and think about suicide.	0.245 (0.179–0.318)

Table 3. Covariates. Summary of covariates used in the decubitus ulcer DisMod-MR meta-regression model

Covariate	Type	Parameter	Exponentiated beta (95% uncertainty interval)
Log-transformed age-standardised SEV scaler: injuries	Country-level	prevalence	1.35 (1.11–1.64)

Dengue

Flowchart

Dengue – GBD 2023

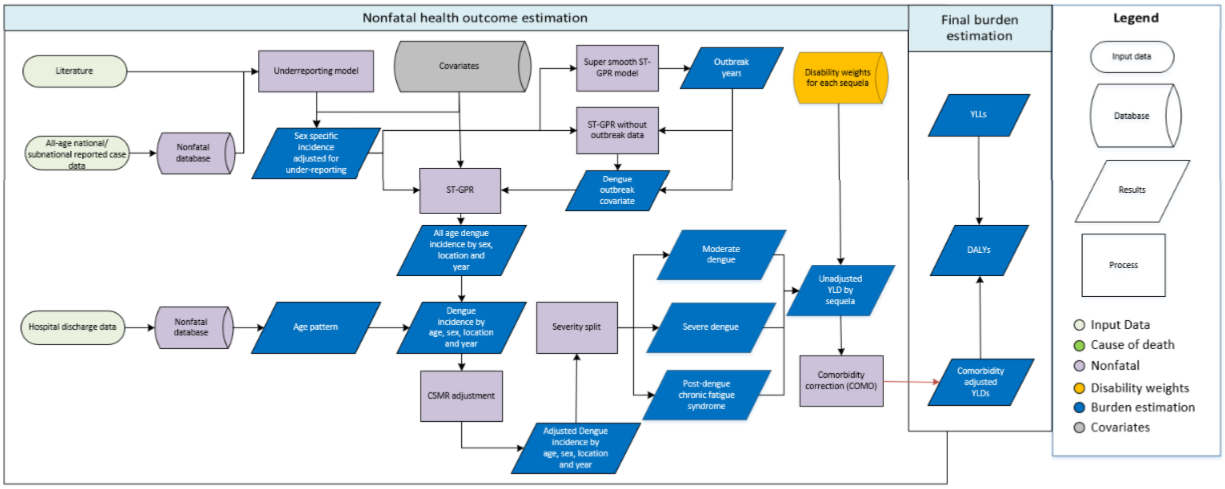

Input data and methodological summary for dengue

Case definition

Dengue is mosquito-borne viral infection that causes febrile illness and, in severe cases, jaundice, haemorrhage, and death. It includes all ICD-10 codes under the heading A90 (Dengue fever [classical dengue]) and A91 (Dengue haemorrhagic fever). We used the following case definition for GBD 2023:

Quantity of interest	Reference or alternative	Definition
Dengue	Reference	Case of dengue confirmed by any of: virus isolation in cell culture; detection of viral nucleic acid by PCR; NS1 antigen detection by ELISA or rapid test; serological detection of IgM or IgG antibodies by ELISA or rapid test or haemagglutination inhibition.[1]
Dengue	Reference	Cases of dengue notified to public health agencies.
Dengue	Reference	WHO definition of a probable case of dengue through clinical diagnosis based on combination of (a) residency in or travel to dengue-endemic area and (b) fever and (c) two of the following criteria: nausea or vomiting; rash; aches and pains; tourniquet test positive; leukopenia; any warning sign requiring strict observation and medical intervention, including abdominal pain or tenderness, persistent vomiting, fluid accumulation, mucosal bleed, lethargy or restlessness, liver enlargement >2 cm; increase in HCT concurrent with rapid decrease in platelet count.[1]

Input data

Model inputs

For GBD 2023, we modelled dengue incidence based on reported cases, which were updated to include data sources through 2023, including data from the OpenDengue project.[2] Age-specific data were collated separately to enable disaggregation of all-age and both-sex case data into age- and sex-specific inputs prior to modelling. Studies that compared incidence of dengue among passive and active case (including enhanced surveillance) detection systems, identified via a systematic literature review conducted in GBD 2019, were used to estimate a correction factor to adjust for under-reporting. Scientific literature sources were used for assumptions related to severity (see section on severity splits and disability weights below).

Data processing: correction for under-reporting

Since dengue disease is often under-reported due to health system capacity or misdiagnosis as other febrile illnesses, a systematic literature review was conducted in GBD 2019 to identify sources that compared incidence rates reported via active versus passive surveillance. We searched PubMed for dengue under-reporting using the following search terms:

> (("active"[Title/Abstract] AND "passive"[Title/Abstract]) OR "case detection"[Text Word] OR "under reporting"[Text Word] OR "coverage"[Text Word]) AND dengue[MeSH Terms]

In our final analysis, we included a total of 17 comparisons, from seven sources using enhanced surveillance compared to passive surveillance, to generate an adjustment factor to correct for under-reporting. The under-reporting adjustment factors were estimated using meta-regression—Bayesian, regularised, trimmed (MR-BRT). Prior to GBD 2023, population density was included in the MR-BRT model as a covariate. However, this covariate produced implausibly large adjustments in several locations with high population density; for GBD 2023, we have switched to using the Socio-demographic Index (SDI) instead. The uncertainty from the MR-BRT meta-regression was applied to the adjustment. Table 1 presents the correction factors for under-reporting.

To ensure that the estimated number of deaths would not exceed the number of severe cases, following the under-reporting adjustment we also applied a theoretical minimum incidence (TMI) adjustment using the cause-specific mortality rate (CSMR). The mortality-to-incidence ratio was checked and input data adjusted in cases where the mortality-to-incidence ratio exceeded the fraction of cases that we assumed are severe (5.5%) based on the literature (see more information in sections below).

Table 1: MR-BRT crosswalk adjustment factors for under-reporting due to dengue

**Data input**	**Gamma**	**Beta coefficient***, log (95% CI)	**Adjustment factor****
Intercept	0.24	–3.21 (–5.53; –0.89)	0.04
Socio-demographic Index		2.14 (–0.90; 5.19)	8.50

**MR-BRT crosswalk adjustments can be interpreted as the factor the alternative case definition is adjusted by to reflect what it would have been had it been measured using the reference case definition. If the log/logit beta coefficient is negative, then the alternative is adjusted up to the reference. If the log/logit beta coefficient is positive, then the alternative is adjusted down to the reference.*

***The adjustment factor column is the exponentiated beta coefficient. For log beta coefficients, this is the relative rate between the two case definitions. For logit beta coefficients, this is the relative odds between the two case definitions.*

Dengue incidence data reported for both sexes were first split into sex-specific inputs. To sex-split our both-sex datapoints, we used sex-specific inputs in a MR-BRT model to derive a ratio of female dengue incidence to both-sex incidence (scientific literature data). The resultant log ratio was applied to both-sex datapoints to calculate out females, and males were calculated via subtraction. The beta coefficients of the adjustment are presented in Table 2.

Table 2. Ratio of males:females estimated using MR-BRT

**Data input**	**Reference or alternative case definition**	**Gamma**	**Beta coefficient, log (variance)**	**Adjustment factor**
Intercept	Females (ref)	0.0051	–0.068 (0.0027)	1.005

**MR-BRT crosswalk adjustments can be interpreted as the factor the alternative case definition is adjusted by to reflect what it would have been had it been measured using the reference case definition. If the log/logit beta coefficient is negative, then the alternative is adjusted up to the reference. If the log/logit beta coefficient is positive, then the alternative is adjusted down to the reference.*

***The adjustment factor column is the exponentiated beta coefficient. For log beta coefficients, this is the relative rate between the two case definitions. For logit beta coefficients, this is the relative odds between the two case definitions.*

Modelling strategy

For GBD 2023 we updated our modelling strategy from using a “custom hybrid” approach, described in previous GBD publications, in favor of a new modelling approach using multiple spatiotemporal Gaussian process regression (ST-GPR) models to generate incidence estimates. In brief, we first identify potential outbreak years for each location, develop an outbreak covariate that reflects the difference between reported data and counterfactual “outbreak-free” incidence in these location-years, then fit final ST-GPR models that allow for discontinuities in the time series of dengue incidence in locations where sufficient data are available to estimate the presence or absence of outbreaks. In general, compared to previous GBD estimates, this strategy produces incidence time-series with more year-to-year variation in incidence for most countries and aims to more closely reflect the known epidemiology of dengue.

ST-GPR

First, we fit an initial ST-GPR model to all data using hyperparameters designed to produce a large degree of spatial and temporal smoothing. In the absence of comprehensive data identifying all historical dengue outbreaks, we used this model to identify potential outbreaks by location and year, here defined as incidence datapoints higher than the upper 95% confidence interval bound from the GPR phase of the ST-GPR model. Second, we fit an ST-GPR model that excluded all data from these potential outbreak years, producing estimates of “outbreak-free” dengue incidence. We then created a covariate to represent the magnitude of the outbreak for each potential outbreak year by taking the difference between the logit of incidence from the original under-reporting-adjusted datapoint and the

logit of incidence from this outbreak-free dengue ST-GPR model. Third, we fit two final ST-GPR models with location random effects using all of the available data, including those from outbreak years: one model using the outbreak covariate, and one without the outbreak covariate. For locations with six or more available incidence datapoints, we used the results from the model with the outbreak covariate included. For locations with fewer than six datapoints or no data, there were insufficient data to reasonably estimate whether each datapoint represented an outbreak or not; for these locations, the model without the outbreak covariate was used. For subnational locations, if either the subnational location or the national location had at least six datapoints, the results from the outbreak-covariate-inclusive ST-GPR model were used.

All four ST-GPR models were run using all-age incidence and the settings listed in Table 3. The covariates used were the population-weighted probability of dengue infection,[3] GBD-location level CSMR, population density, proportion of the population living between 0 and 15 absolute degrees latitude, Healthcare Access and Quality (HAQ) Index, and a dengue outbreak covariate generated from the first two ST-GPR models (used in the final outbreak-inclusive model only). For all models, we excluded data for location-years with zero cases reported (under the assumption that in dengue-endemic settings, reports of zero cases are more likely to be due to under-reporting than true zeros).

Table 3. ST-GPR model settings

	**ST-GPR model**			
**Parameter**	**Model 1 (super smooth model)**	**Model 2 (no outbreak data)**	**Model 3 (all data plus outbreak covariate)**	**Model 4 (all data, no outbreak covariate)**
**Lambda**	0.01	0.1	0.05	0.05
**Omega**	2	1	1	1
**Zeta**	0.05	0.01	0.01	0.01
**Scale**	1	1	1	1
**Amplitude**	1	1	1	1

Age splitting

Initial model testing showed that inclusion of data from the 2009 Cabo Verde dengue outbreak resulted in implausibly high values for West African locations, largely due to the limited number of data inputs for this modelling region (34 total inputs). The rest of the data from Cabo Verde were additionally excluded – the values in the underlying data suggested substantial under-reporting and, in initial testing, led the model to predict no cases. The all-age estimates were then disaggregated by age using an overall age pattern derived from the age-specific hospital discharge data inputs. This age pattern was modelled using a negative binomial regression with cubic spline variables for age group.

Theoretical minimum incidence adjustment

Similar to the TMI adjustment applied to the input data, to ensure that there weren't more deaths than severe cases, we made a final adjustment to incidence results (prior to age-splitting) using the CSMR. The mortality-to-incidence ratio was checked and incidence results adjusted in cases where the mortality-to-incidence ratio exceeded the fraction of cases that we assumed are severe (5.5%) based on the literature (see more information in the next section).

Severity splits and disability weights

The resulting incidence estimates were then split into moderate (94.5%) and severe (5.5%) sequelae, based on the proportion of reported cases that were severe using data from WHO and PAHO reports and scientific literature.[4-10] Prevalence of moderate dengue was calculated assuming a duration of six days and prevalence of severe dengue estimated using an assumption of duration of 14 days based on Whitehead and colleagues, 2007.[11] We assume that 8.4% of symptomatic infections will produce post-acute chronic fatigue lasting an average of six months based on Teixeira and colleagues, 2010.[12] Disability weights are presented in Table 4.

Table 4. Severity distribution, details on the severity levels for dengue and the associated disability weight (DW) with that severity

Severity level	Lay description	DW (95% CI)
Moderate	Has a fever and aches, and feels weak, which causes some difficulty with daily activities.	0.051 (0.032–0.074)
Severe	Has a high fever and pain, and feels very weak, which causes great difficulty with daily activities.	0.133 (0.088–0.19)
Post-dengue chronic fatigue syndrome	Is always tired and easily upset. The person feels pain all over the body and is depressed.	0.219 (0.148–0.308)

We did not apply any adjustments for the COVID-19 pandemic to dengue due to a lack of available data quantifying the impacts of the pandemic on NTD epidemiology.

Changes from GBD 2021 to GBD 2023

For GBD 2023 we implemented several methodological updates. We (1) updated data inputs through the year 2023, including OpenDengue[2]; (2) used SDI instead of population density in the under-reporting model; and (3) shifted from a custom hybrid approach to a new multi-step modelling process, as described above.

References

1. "Dengue Outbreak Toolbox." https://www.who.int/emergencies/outbreak-toolkit/disease-outbreak-toolboxes/dengue-outbreak-toolbox.
2. Clarke J, Lim A, Gupte P, Pigott DM, van Panhuis WG, Brady OJ. A global dataset of publicly available dengue case count data. *Sci Data*. 2024 Mar 14;11(1):296.
3. Messina JP, Brady OJ, Golding N, Kraemer MU, Wint GW, Ray SE, Pigott DM, Shearer FM, Johnson K, Earl L, Marczak LB. The current and future global distribution and population at risk of dengue. *Nature Microbiology*. 2019 Sep;4(9):1508-15.

4. World Health Organization (WHO). WHO DengueNet. Geneva, Switzerland: World Health Organization.
5. Pan American Health Organization (PAHO). Number of Reported Cases of Dengue and Dengue Hemorrhagic Fever (DHF) in the Americas, by Country 2003. Washington, D.C., United States: Pan American Health Organization (PAHO), 2005.
6. Pan American Health Organization (PAHO). Number of Reported Cases of Dengue and Dengue Hemorrhagic Fever (DHF) in the Americas, by Country 2004. Washington, D.C., United States: Pan American Health Organization (PAHO), 2005.
7. Pan American Health Organization (PAHO). Number of Reported Cases of Dengue and Dengue Hemorrhagic Fever (DHF) in the Americas, by Country 2005. Washington, D.C., United States: Pan American Health Organization (PAHO), 2006.
8. Pan American Health Organization (PAHO). Number of Reported Cases of Dengue and Dengue Hemorrhagic Fever (DHF) in the Americas, by Country 2006. Washington, D.C., United States: Pan American Health Organization (PAHO), 2007.
9. Pan American Health Organization (PAHO). Number of Reported Cases of Dengue and Dengue Hemorrhagic Fever (DHF) in the Americas, by Country 2007. Washington, D.C., United States: Pan American Health Organization (PAHO), 2008.
10. Lin C-C, Huang Y-H, Shu P-Y, Wu H-S, Lin Y-S, Yeh T-M, Liu H-S, Liu C-C, Lei H-Y. Characteristic of dengue disease in Taiwan: 2002-2007. *Am J Trop Med Hyg*. 2010; 82(4): 731-9.
11. Whitehead SS, Blaney JE, Durbin AP, Murphy BR. Prospects for a dengue virus vaccine. *Nature Reviews Microbiology*. 2007 Jul;5(7):518-28.
12. Teixeira L de AS, Lopes JSM, Martins AG da C, Campos FAB, Miranzi S de SC, Nascentes GAN. Persistence of dengue symptoms in patients in Uberaba, Minas Gerais State, Brazil. *Cad Saúde Pública* 2010; **26**: 624–30.

Dermatitis

Flowchart

Atopic Dermatitis

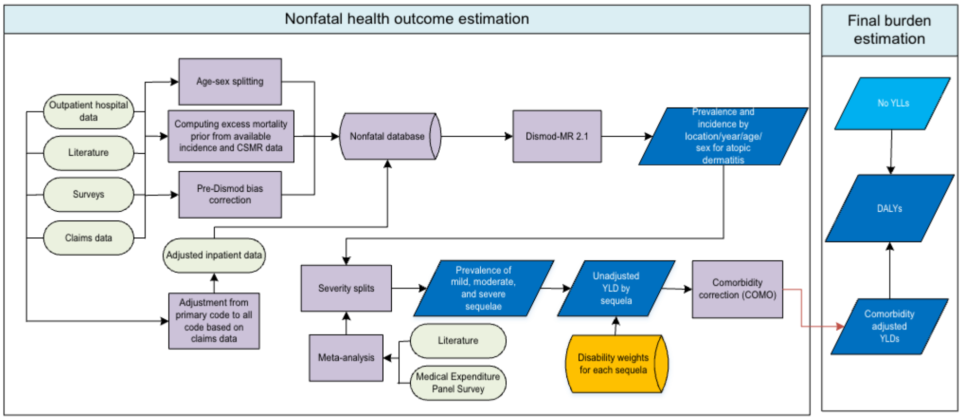

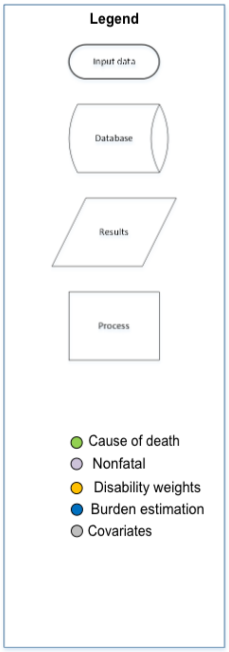

Contact Dermatitis

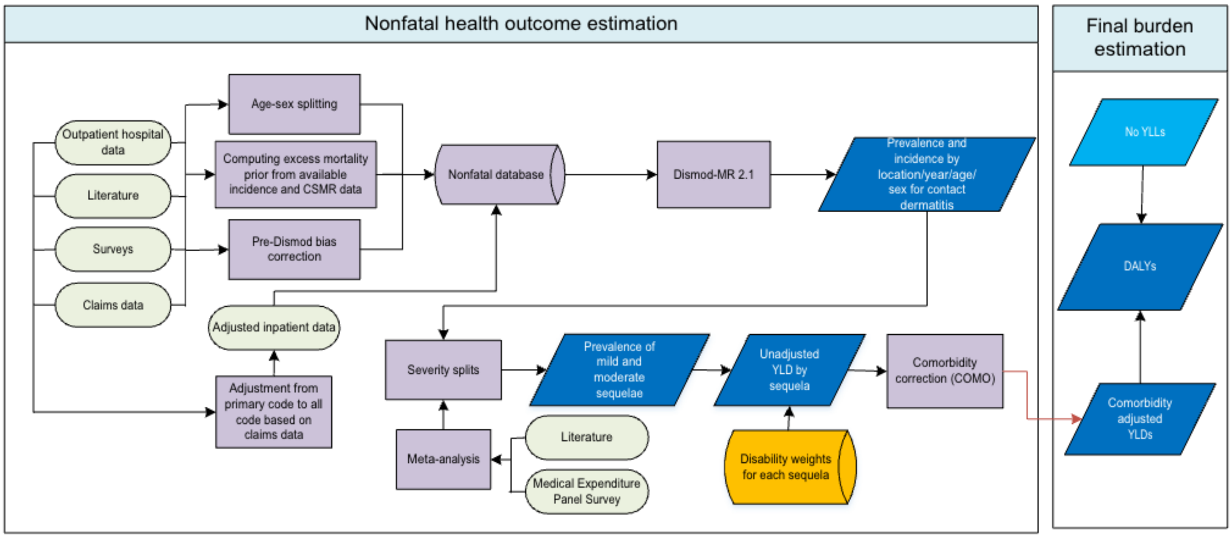

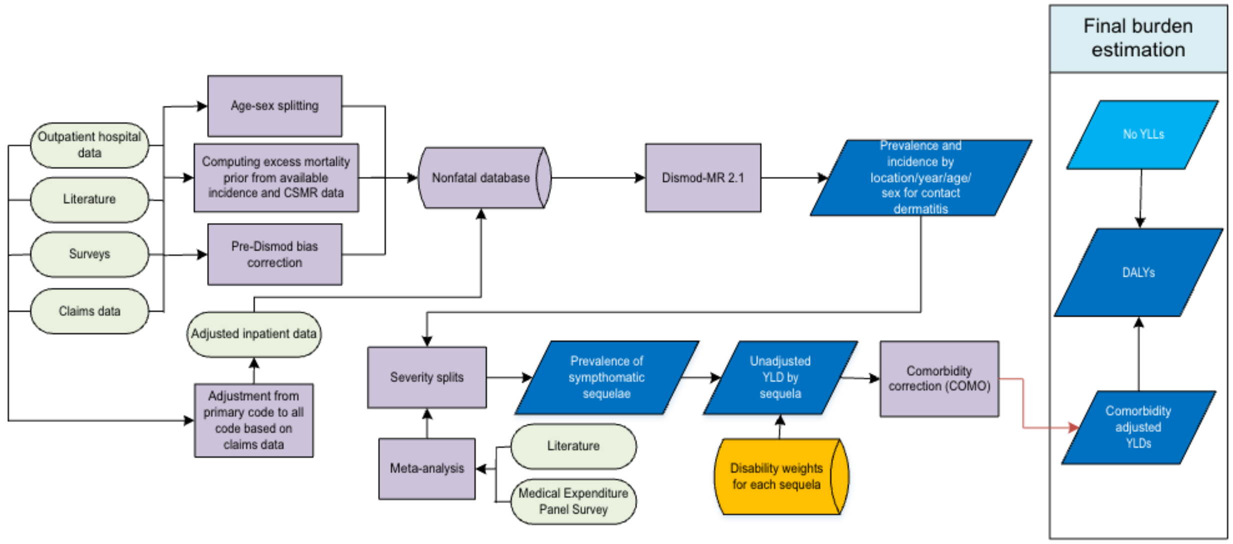

Input data and methodological summary for dermatitis

Case definition

Dermatitis, or eczema, refers to inflammation of the dermal layer of the skin, with disruption of the epidermal barrier. This inflammation leads to rashes that are commonly red, scaly, or flaky. Atopic dermatitis is defined as relapsing inflammation of the dermal layer of the skin with disruption of the epidermal barrier (dermatitis). Associated with elevated serum immunoglobulin E and some degree of immune dysregulation, it can be localised or widespread (ICD-10: L20). Contact dermatitis is defined as localised inflammation of the dermal layer of skin with disruption of the epidermal barrier (dermatitis) caused by direct contact with allergens or irritants (ICD: 10: L22-26). Seborrheic dermatitis is defined as inflammation of the dermal layer of the skin with disruption of the epidermal barrier (dermatitis) affecting the sebaceous-gland-rich areas of skin (ICD-10: L21).

Dermatitis:

**Quantity of interest**	**Reference or alternative**	**Definition**
Atopic dermatitis	Reference	Atopic dermatitis as determined by Poland National Health Fund Patient Claims 2015-2019
	Alternative	All other atopic dermatitis data sources
Contact dermatitis	Reference	Contact dermatitis as determined by Poland National Health Fund Patient Claims 2015-2019
	Alternative	All other contact dermatitis data sources
Seborrheic dermatitis	Reference	Seborrheic dermatitis as determined by Poland National Health Fund Patient Claims 2015-2019
	Alternative	All other seborrheic dermatitis data sources

Input data

Data for dermatitis came from scientific literature and claims submitted for individuals to USA commercial insurance and Poland. A literature review was conducted in GBD 2016 for studies of the incidence and prevalence of dermatitis, the details of which are described in the appendix to GBD 2016, and the results of this review were used in GBD 2021. Inpatient data were regarded as inappropriate for this chronic, non-fatal condition that is primarily cared for in non-acute settings. The data for dermatitis were expanded based on recommendations of research articles and reviews by the skin expert group.

For GBD 2023, clinical data from USA MarketScan Claims and Medicare Data 2015 to2019 and Poland National Health Fund Patient Claims 2015 to 2019 have been added for all dermatitis causes. There are also expert-approved year-locations of literature data from the USA, Italy, Nepal, Germany, Tanzania, Türkiye, Singapore, Iceland, Estonia, Cameroon, the UK, the Netherlands, Sweden, Latvia, Jamaica, Mexico, Norway, China, and France have been added for atopic dermatitis.

Data were marked as outliers and excluded if we found them unreasonable when compared to regional, super-regional, and global rates. See descriptions of individual modelling approaches for more information.

Data processing

For all dermatitis causes, we crosswalked all data to the reference definition. We began by evaluating the number of observations of each alternate definition that matched with a corresponding observation from the reference definition. We considered "between" study matches, where the alternative was from the same GBD age group and sex, and the midpoint year of the study was within five years of the midpoint of the reference definition observation.

$$\log i\, t\left(y_i^{alt}\right) - \log i\, t\left(y_i^{ref}\right) \; = \; \beta_0 \; + \epsilon_i$$

Table 1: MR-BRT crosswalk adjustment factors for dermatitis

Data input	Reference or alternative case definition	Gamma	Beta coefficient, log/logit (95% UI)*	Adjustment factor**
Atopic dermatitis	Ref	0.1013	---	---
	Alt		–0.1010 (–0.7246, 0.5227)	0.9039 (0.4844, 1.6866)
Contact dermatitis	Ref	0.8782	---	---
	Alt		0.8025 (–1.0342, 2.6392)	2.2311 (0.3555, 14.0023)
Seborrheic dermatitis	Ref	0	---	---
	Alt		0.9868 (0.9852, 0.9883)	2.6825 (2.6785, 2.6868)

**MR-BRT crosswalk adjustments can be interpreted as the factor the alternative case definition is adjusted by to reflect what it would have been had it been measured using the reference case definition. If the log/logit beta coefficient is negative, then the alternative is adjusted up to the reference. If the log/logit beta coefficient is positive, then the alternative is adjusted down to the reference.*

***The adjustment factor column is the exponentiated beta coefficient. For log beta coefficients, this is the relative rate between the two case definitions. For logit beta coefficients, this is the relative odds between the two case definitions.*

Modelling strategy

DisMod-MR 2.1 was used to estimate prevalence by age, sex, year, and geography (subnational, country, region, super-region) for atopic dermatitis, contact dermatitis, and seborrheic dermatitis. Separate models were run for each cause. We have made no substantive changes in the modelling strategy from GBD 2021.

Model parameters

Atopic dermatitis

Since our available data mostly contained information on prevalence, we specified additional expert priors to further inform analyses. The prior value on excess mortality was set to zero, and the prior value on remission was set to 0–0.2 (equivalent to five years to lifetime duration). Since GBD 2019, we replaced our within-DisMod crosswalks with crosswalks completed using the MR-BRT modelling tool. We adjusted administrative data, along with data from the Medical Expenditure Panel Survey, USA MarketScan 2015 to 2019 data, and our other data sources toward the level of Poland National Health Fund Patient Claims datapoints, which were more representative of the general population. A time window of ten years was used to determine which datapoints were used for a particular year of fit.

Contact dermatitis

Similar to atopic dermatitis, most prevalence data were available for contact dermatitis. Per expert advice, the remission parameter was set from 0.1 to 4, excess mortality was set to zero, and incidence was set to zero prior to age 6. Since GBD 2019, we replaced our within-DisMod crosswalks with crosswalks completed using the MR-BRT modelling tool. We adjusted USA MarketScan 2015–2019 data and the rest of our data sources toward the level of Poland National Health Fund Patient Claims datapoints which were more representative of the general population.

Seborrheic dermatitis

As with contact dermatitis, the available data were mostly prevalence estimates. Per expert advice, settings were placed on incidence for 0–4 years, and 60–100 years to 0–0.01. Excess mortality was set to zero while a setting of 0.1–12 was placed on remission, implying a duration of one month to ten years. Since GBD 2019, we replaced our within-DisMod crosswalks with crosswalks completed using the MR-BRT modelling tool. We adjusted USA MarketScan 2015–2019 data, and along with our other data sources toward the level of Poland National Health Fund Patient Claims datapoints which were more representative of the general population.

Table 2. Severity distribution, details on the severity levels for dermatitis and the associated disability weight (DW) with that severity.

Sequela	Severity level	Lay description	DW (95% CI)
Mild atopic dermatitis	Disfigurement, level 1 with itch/pain	The person has a slight, visible physical deformity that is sometimes sore or itchy. Others notice deformity, which causes some worry and discomfort.	0.672 (0.548–0.768)

Moderate atopic dermatitis	Disfigurement, level 2, with itch/pain	The person has a visible physical deformity that is sore and itchy. Other people stare and comment, which causes the person to worry. The person has trouble sleeping and concentrating.	0.217 (0.144–0.309)
Severe atopic dermatitis	Disfigurement, level 3, with itch/pain	The person has an obvious physical deformity that is very painful and itchy. Physical deformity makes others uncomfortable, which causes the person to avoid social contact, feel worried, sleep poorly, and think about suicide.	0.112 (0.083–0.156)
Mild contact dermatitis	Disfigurement, level 1 with itch/pain	The person has a slight, visible physical deformity that is sometimes sore or itchy. Others notice deformity, which causes some worry and discomfort.	0.272 (0.233–0.308)
Moderate contact dermatitis	Disfigurement, level 2, with itch/pain	The person has a visible physical deformity that is sore and itchy. Other people stare and comment, which causes the person to worry. The person has trouble sleeping and concentrating.	0.105 (0.07–0.144)
Symptomatic seborrheic dermatitis	Disfigurement, level 1 with itch/pain	The person has a slight, visible physical deformity that is sometimes sore or itchy. Others notice deformity, which causes some worry and discomfort.	0.443 (0.435–0.455)

Developmental intellectual disability

Flowchart

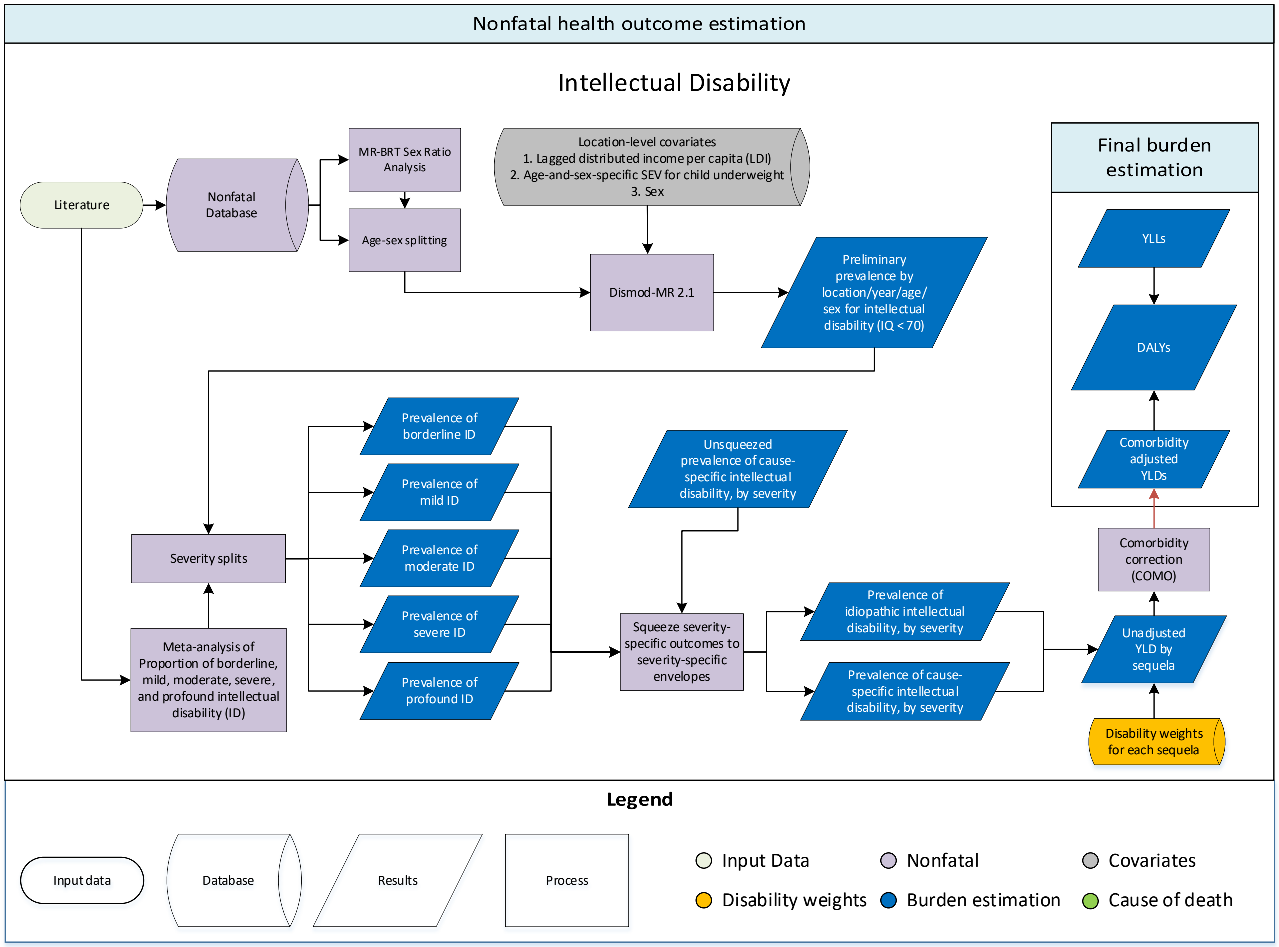

Case definition

Developmental intellectual disability (ID) is a condition of below-average mental ability. Consistent with the American Association on Intellectual and Developmental Disabilities, we define developmental intellectual disability as a condition originating before age 18 (as such, it does not include impairment due to stroke, Alzheimer's disease, or other conditions that affect older populations). We model the severities shown in Table 1, as measured by score on intelligence quotient (IQ) tests, which are standardised to have a mean of 100.

Table 1. ID severity definitions

**Severity of intellectual disability**	**IQ score**
Profound	0 to 19
Severe	20 to 34
Moderate	35 to 49
Mild	50 to 69
Borderline	70 to 85

Input data

Model inputs

The epidemiological systematic literature review for intellectual disability (IQ score <70) was first conducted for GBD 2016 in three stages involving electronic searches of the peer-reviewed literature (ie, via PsycInfo, Embase, and PubMed), the grey literature, and expert consultation. A systematic review update was completed for GBD 2023. Databases were searched on September 7, 2023, for publications after January 1, 2015. Below are the search terms used for each database.

PubMed: ("mentally retarded"[Title/Abstract] OR "mental retardation"[Title/Abstract] OR "Intellectual Disability"[Title/Abstract] OR "Intellectual Disabilities"[Title/Abstract] OR "intellectually disabled"[Title/Abstract] OR "intellectual impairment"[Title/Abstract] OR "intellectually impaired"[Title/Abstract] OR "intellectual and developmental disabilities"[Title/Abstract] OR "intellectual and developmental disability"[Title/Abstract] OR "intellectual or developmental disabilities"[Title/Abstract] OR "intellectual or developmental disability"[Title/Abstract] OR "intellectual development disorder"[Title/Abstract] OR "intellectual development disorders"[Title/Abstract] OR "intellectual developmental delay"[Title/Abstract] OR "intellectual developmental delays"[Title/Abstract] OR "intellectual developmental disability"[Title/Abstract] OR "intellectual developmental disabilities"[Title/Abstract] OR "intellectual dysfunction"[Title/Abstract] OR "mental handicap"[Title/Abstract] OR "mentally handicapped"[Title/Abstract] OR "mental deficiency"[Title/Abstract] OR "mental deficiencies"[Title/Abstract] OR "mentally deficient"[Title/Abstract] OR "mental disability"[Title/Abstract] OR "mental disabilities"[Title/Abstract] OR "mentally disabled"[Title/Abstract] OR "mental delay"[Title/Abstract] OR "mental delays"[Title/Abstract] OR "mental subnormality"[Title/Abstract] OR "mentally subnormal"[Title/Abstract] OR "Intellectual Disability"[MeSH Major Topic]) AND ("prevalen*"[Title/Abstract] OR "mortalit*"[Title/Abstract] OR "death*"[Title/Abstract] OR "inciden*"[Title/Abstract] OR "remission"[Title/Abstract] OR "duration"[Title/Abstract] OR "remit*"[Title/Abstract] OR "epidemiolog*"[Title/Abstract] OR "prevalence"[MeSH Terms] OR "mortality"[MeSH Terms] OR "incidence"[MeSH Terms] OR "Epidemiology"[MeSH Terms:noexp] OR "Morbidity"[MeSH Terms:noexp])

Embase: ('intellectual impairment'/de OR (((mental* OR intellectual*) NEXT/1 (retard* OR delay* OR deficien* OR handic* OR subnormal* OR impair* OR disab* OR dysfunction*)):ab,ti) OR ((intellectual NEXT/1 developmental NEXT/1 (disab* OR disorder* OR delay*)):ab,ti)) AND ((prevalen*:ab,ti OR mortalit*:ab,ti OR death*:ab,ti OR inciden*:ab,ti OR remission:ab,ti OR duration:ab,ti OR remit*:ab,ti OR epidemiolog*:ab,ti OR 'incidence'/exp OR 'mortality'/exp OR 'morbidity'/mj OR 'remission'/exp OR 'prevalence'/exp OR 'epidemiology'/exp))

PsycInfo: (DE intellectual disability OR (TI (intellectual OR intellectually OR mental OR mentally) AND TI (disabled OR disability OR disabilities OR impaired OR impairment OR retardation OR retarded OR delay OR delays OR delayed OR handicap OR handiccaped OR deficient OR deficiencies OR deficiency OR subnormal OR subnormality OR dysfunction) OR AB (intellectual OR intellectually OR mental OR mentally) AND AB (disabled OR disability OR disabilities OR impaired OR impairment OR retardation OR retarded OR delay OR delays OR delayed OR handicap OR handiccaped OR deficient OR deficiencies OR deficiency OR subnormal OR subnormality OR dysfunction)) OR (TI intellectual development disorder

OR TI intellectual developmental disability OR AB intellectual development disorder OR AB intellectual developmental disability OR TI intellectual developmental disabilities OR AB intellectual developmental disabilities OR TI intellectual development disorders OR AB intellectual development disorders)) AND ((DE remission (disorders) OR DE epidemiology OR DE morbidity OR DE mortality rate OR DE prevalence OR DE incidence) OR ((TI prevalen* OR AB prevalen*) OR (TI mortalit* OR AB mortalit*) OR (TI death* OR AB death*) OR (TI inciden* OR AB inciden*) OR (TI remission OR AB remission) OR (TI duration OR AB duration) OR (TI remit* OR AB remit*) OR (TI epidemiolog* OR AB epidemiolog*))

In addition to the database search, a grey literature search and expert consultation were also conducted. The systematic review update was conducted according to Preferred Reporting Items for Systematic Reviews and Meta-Analyses (PRISMA; see Figure 1).

Figure 1: PRISMA 2009 flow diagra

From: Moher D, Liberati A, Tetzlaff J, Altman DG, The PRISMA Group (2009). *P*referred *R*eporting *I*tems for *S*ystematic Reviews and *M*eta-*A*nalyses: The PRISMA Statement. PLoS Med 6(7): e1000097. doi:10.1371/journal.pmed1000097

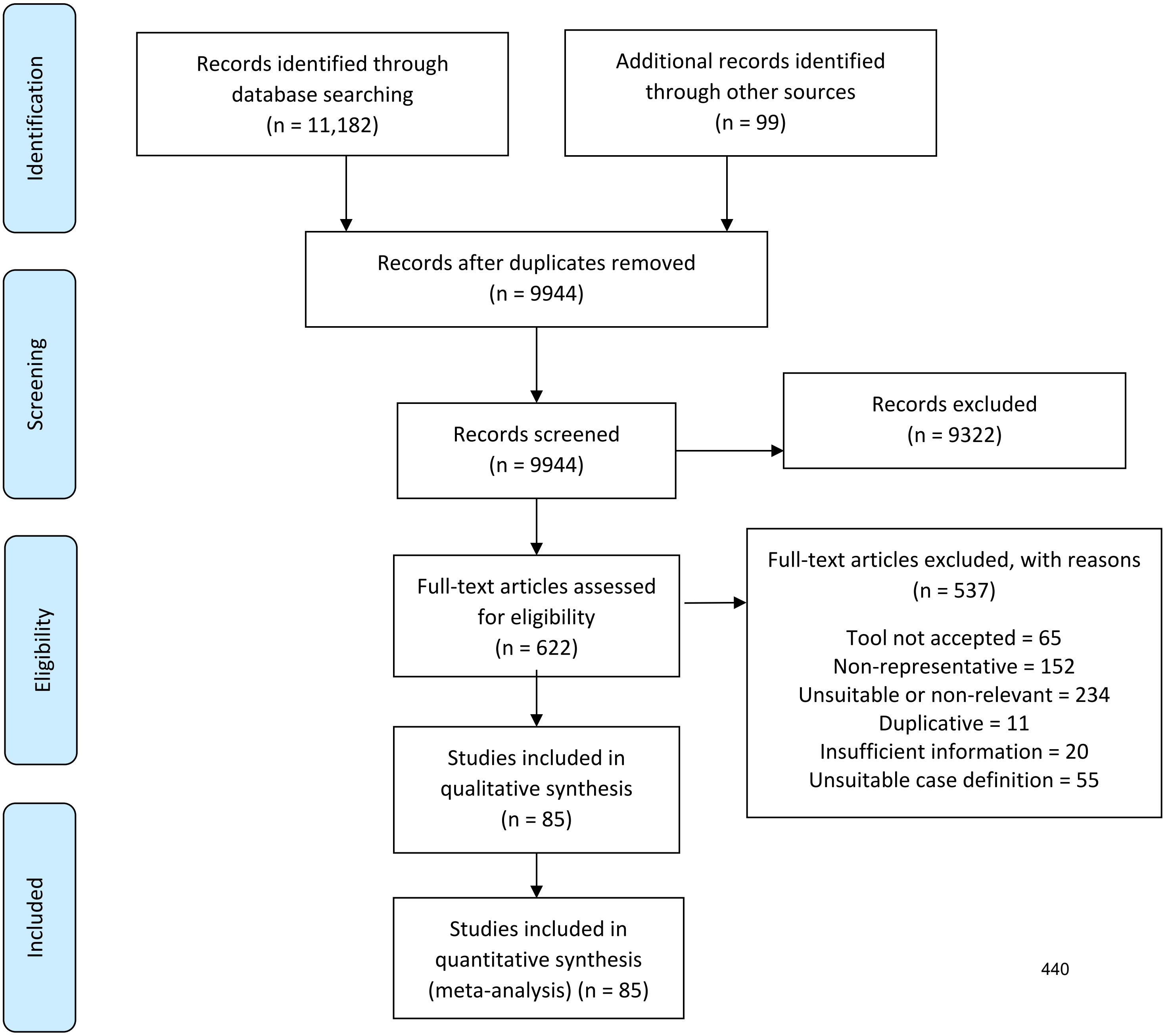

We included studies that estimated the general population prevalence of intellectual disability. We excluded studies that did not use a case definition based on intelligence quotient (IQ) and studies that investigated non-representative groups, such as hospital patients or people of a specific ethnicity.

Data processing

Age-sex splitting

The extracted data underwent three types of age-sex splitting processes:

1. Where possible, estimates were further split by sex and age based on the available data. For instance, if studies reported prevalence for broad age groups by sex (eg, prevalence in 15–65 year-old-males and females separately), and also by specific age groups but for both sexes combined (eg, prevalence in 15–30-year-olds, then in 31–65-year-olds, for males and females combined); age-specific estimates were split by sex using the reported sex-ratio and bounds of uncertainty.
2. A meta-regression—Bayesian, regularised, trimmed (MR-BRT) analysis was used to split the remaining both-sex estimates in the dataset. For each parameter, sex-specific estimates were matched by location, age, and year. A MR-BRT network meta-analysis was then used to estimate pooled sex ratios and bounds of uncertainty. These were then used to split the both-sex estimates in the dataset. The male-to-female prevalence ratio estimated was 1.35 (95% uncertainty interval [UI] 1.35–1.36).

Severity splits – disability weights

The GBD disability weight survey assessments include lay descriptions of sequelae highlighting major functional consequences and symptoms. The lay descriptions and disability weights for ID are shown in Table 2.

Table 2. Intellectual disability severity disability weights

**Health state**	**Lay description**	**Disability weight (95% UI)**
Borderline intellectual functioning	This person is slow in learning at school. As an adult, the person has some difficulty doing complex or unfamiliar tasks but otherwise functions independently.	0.011 (0.005–0.02)
Intellectual disability/mental retardation, mild	This person has low intelligence and is slow in learning at school. As an adult, the person can live independently but often needs help to raise children and can only work at simple supervised jobs.	0.043 (0.026–0.064)
Intellectual disability/mental retardation, moderate	This person has low intelligence and is slow in learning to speak and to do even simple tasks. As an adult, the person requires a lot of support to live independently and raise children. The person can only work at the simplest supervised jobs.	0.1 (0.066–0.142)
Intellectual disability/mental retardation, severe	This person has very low intelligence and cannot speak more than a few words, needs constant supervision and help with most daily activities, and can do only the simplest tasks.	0.16 (0.107–0.226)

Intellectual disability/mental retardation, profound	This person has very low intelligence, has almost no language, and does not understand even the most basic requests or instructions. The person requires constant supervision and help for all activities.	0.2 (0.133–0.283)

Modelling strategy

We modelled the prevalence of ID, both aetiology-specific ID and idiopathic ID, over multiple steps.

First, we ran a DisMod-MR 2.1 model to estimate the total prevalence of intellectual disability of level IQ <70. We included lagged distributed income and child underweight summary exposure value (SEV) in the model as predictive covariates. Table 3 shows raw and exponentiated model coefficients for the covariates used in the estimation process for the DisMod model.

Table 3. Model coefficient values (raw and exponentiated)

Covariate	Parameter	Coefficient (95% UI)	Exponentiated coefficient (95% UI)
Lagged distributed income (LDI) per capita	Prevalence	–0.03 (–0.08 to 0.00)	0.97 (0.92–1)
Age- and sex-specific SEV for child underweight	Prevalence	1.24 (0.25 to 2.32)	3.47 (1.29–10.14)
Sex	Prevalence	0.31 (0.25 to 0.37)	1.36 (1.29–1.44)

Second, we split the total prevalence of idiopathic ID into four severity levels: mild (IQ 50–69), moderate (IQ 35–49), severe (IQ 20–34), and profound (IQ below 20). We pooled a subset of studies that distinguished intellectual disability by these severity levels. We used cumulative severity levels (ie, IQ <50, IQ <35, and IQ <20) to maximise the number of sources. We estimated these cumulative severities' proportion of the <70 envelope via random effects meta-analyses. These proportions were used to estimate discrete severities from the overall intellectual disability (IQ <70) prevalence. We estimated the final severity level, borderline disability (IQ 70–84), via another random-effects meta-analysis of the ratio of IQ 70–84 to IQ <70. The uncertainty of the pooled fractions and ratios was propagated throughout our calculations using 1000 draws from a normal distribution with mean and standard error estimated by the meta-analysis. The results of the meta-analysis are shown in Table 4.

Table 4. Proportion of intellectual disability cases by severity

Severity	Mean	Standard error
Borderline*	0.779	0.232
Mild	0.513	0.029
Moderate	0.270	0.017
Severe	0.130	0.012
Profound	0.086	0.010

**Borderline intellectual disability reported as a proportion of all non-borderline intellectual disability cases.*

Third, we estimated prevalence of each aetiology-specific intellectual disability using models of the following parent causes. Since we model only developmental intellectual disability, causes that affect older populations such as stroke and Alzheimer's disease are not included in the causal attribution process.

Parent causes included in causal attribution:

- Neonatal preterm birth complications (<28w, 28-32w, 32-36w)
- Neonatal encephalopathy due to birth asphyxia and trauma
- Congenital birth defects (diaphragmatic hernia, cardiovascular anomalies)
- Haemolytic disease and other neonatal jaundice
- Meningitis (pneumococcal, *H influenzaea* type B, meningococcal, other bacterial)
- Encephalitis
- Malaria
- Neonatal tetanus
- Neonatal sepsis and other neonatal infections
- Iodine deficiency
- African trypanosomiasis
- Down syndrome
- Klinefelter syndrome
- Chromosomal abnormalities (unbalanced rearrangements, Down syndrome, Edwards syndrome, Patau syndrome, other chromosomal abnormalities)
- Neural tube defects (eg, spina bifida, encephalocele)
- Hypertensive disorders of pregnancy (eclampsia, preeclampsia)
- Autism spectrum disorders (ASD)
- Fetal alcohol syndrome

We calculated the prevalence of idiopathic ID by subtracting all severity- and aetiology-specific ID from the severity-specific envelope assuming the residuals to represent idiopathic disability. If the residual was less than 5% of the severity-specific envelope, the prevalence of all aetiology-specific ID was proportionally squeezed to fit within 95% of the envelope, leaving 5% for idiopathic ID. As we estimated the prevalence of individual aetiology-specific ID by models from the respective parent causes, the squeezing may have resulted in a distorted balance of prevalence estimates within their parent causes. Unique processes for specific parent causes are described in their respective cause write-ups. For GBD 2023, all aetiology-specific models were squeezed into their respective discrete severity envelopes.

Diabetes mellitus

Diabetes mellitus prevalence is estimated for overall diabetes mellitus, diabetes mellitus type 1, and diabetes mellitus type 2 in GBD 2023.

Flowchart

Figure 1: Calculating prevalence of diabetes mellitus (total, type 1, and type 2)

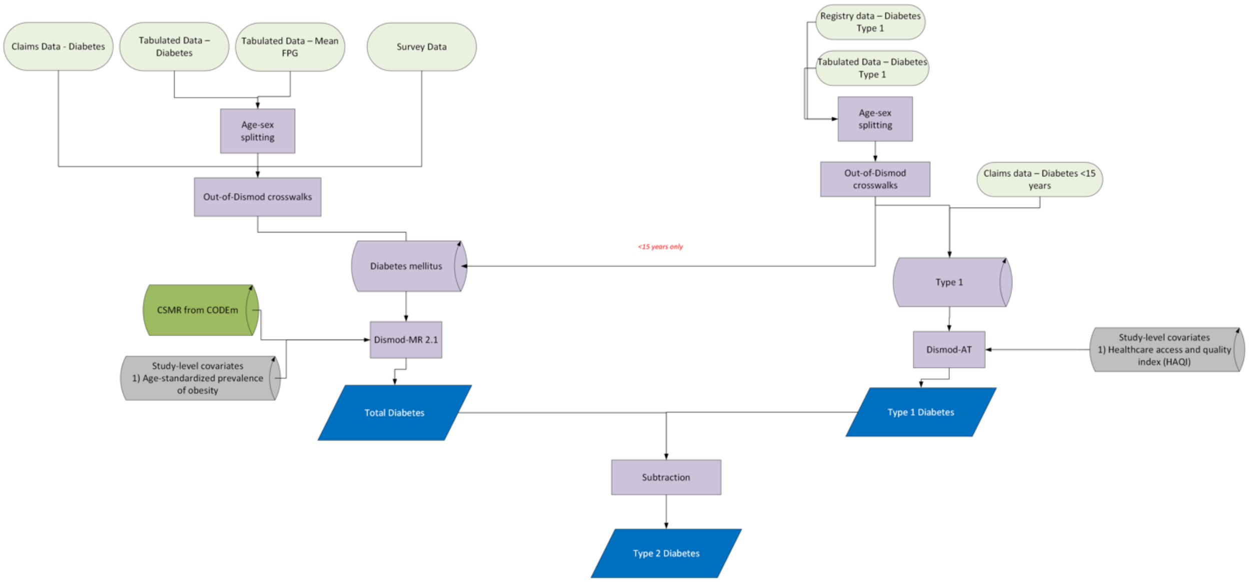

Figure 2: Calculating type-specific diabetes sequelae

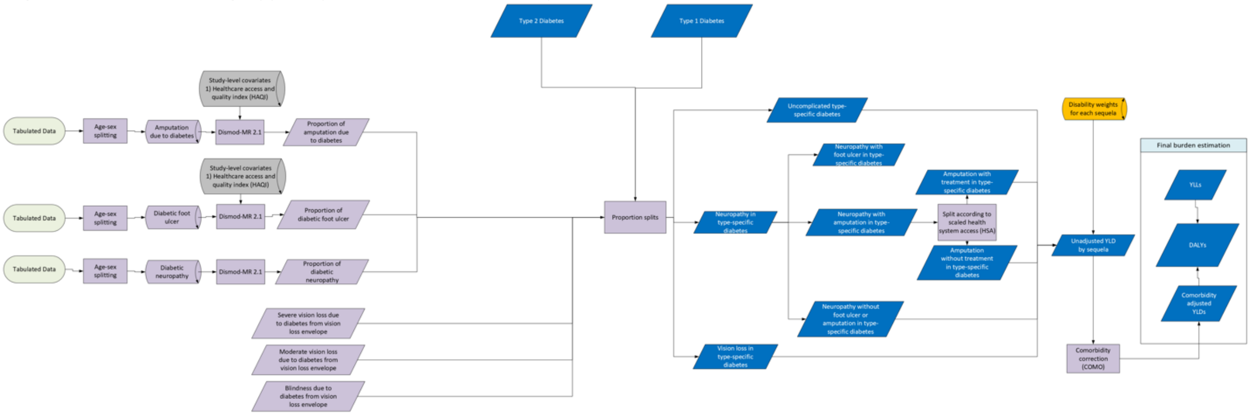

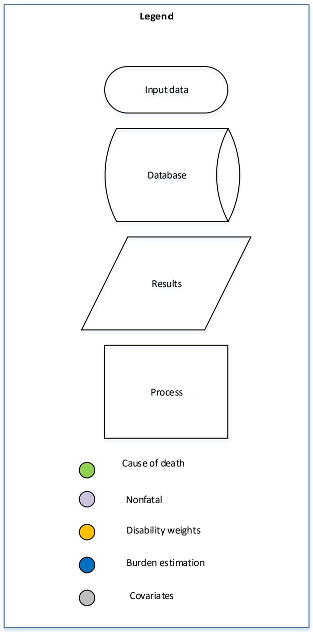

Case definitions

Total diabetes clinical case definition

A metabolic disorder in which the body does not produce enough or does not respond normally to insulin, causing chronic high blood sugar (glucose) levels which over time leads to serious damage to the heart, blood vessels, eyes, kidneys, and nerves.

Type 1 diabetes clinical case definition

A metabolic disorder in which the body produces little to no insulin due to autoimmune destruction of pancreatic β-cells, causing chronic high blood sugar (glucose) levels which over time leads to serious damage to the heart, blood vessels, eyes, kidneys, and nerves.

Type 2 diabetes clinical case definition

A metabolic disorder in which the body does not respond normally to insulin, causing chronic high blood sugar (glucose) levels which over time leads to serious damage to the heart, blood vessels, eyes, kidneys, and nerves.

Diabetes operational case definition

Reference and alternative case definitions and diagnostic criteria are presented in the table below. The below quantities of interest and case definitions are limited to those for which we model. For example, diabetes mellitus type 2 is not included in the table below because it is not modelled; rather, it is calculated by subtracting estimated diabetes mellitus type 1 from estimated overall diabetes mellitus for each sex, age, location, and year.

In GBD 2023, for type 1 diabetes, a singular reference case definition is used, no alternative case definitions were identified in the data upon review.

Table 1: Case definitions for diabetes mellitus

Quantity of interest	Reference or alternative	Definition
Diabetes mellitus	Reference	Fasting plasma glucose (FPG) ≥126 mg/dl (7 mmol/L) or current treatment (insulin or drugs)
Diabetes mellitus	Alternative	FPG greater than a threshold not equal to 126 mg/dl (7mmol/L) or current treatment (insulin or drugs)
Diabetes mellitus	Alternative	Blood sugar tests measured from glycated haemoglobin (HbA1c) or current treatment (insulin or drugs)
Diabetes mellitus	Alternative	Blood sugar tests measured from oral glucose tolerance test (OGTT) or current treatment (insulin or drugs)
Diabetes mellitus	Alternative	Blood sugar tests measured from post-prandial glucose test (PPG) or current treatment (insulin or drugs)
Diabetes mellitus	Alternative	Combination of non-FPG blood sugar test(s) and FPG or current treatment (insulin or drugs)
Diabetes mellitus	Alternative	Blood sugar tests measured from FPG, HbA1c, OGTT, or PPG (no treatment)
Diabetes mellitus type 1	Reference	Cases of physician-diagnosed type 1 diabetes, or type 1 diabetes cases in a diabetic registry or hospital, or any case of diabetes in persons <15 years who are on insulin
Neuropathy	Reference	People with diabetes mellitus who have diabetic neuropathy determined by microfilament test
Neuropathy	Alternative	People with diabetes mellitus who have diabetic neuropathy determined by a test that is not a microfilament test
Diabetic foot	Reference	People with diabetes mellitus who have diabetic foot, which is a poorly healing ulcer
Amputations due to diabetes mellitus	Reference	People with diabetes mellitus who have a lower limb amputation
Amputations due to diabetes mellitus	Alternative	People with diabetes mellitus who have a specific part of the lower limb amputated (eg, toes only, feet only, below ankle only)
Low vision due to diabetic retinopathy	Reference	Low vision (presenting visual acuity of <6/18 ≥3/60 in the better eye using the Snellen chart) from damage to the retina caused by damaged blood vessels due to diabetes. Presenting vision is measured using any corrective lenses currently in use.
Low vision due to diabetic retinopathy	Alternative	Low vision (presenting visual acuity of <6/18 ≥3/60 in the better eye using the Snellen chart) from damage to the retina caused by damaged blood vessels due to diabetes, as measured by Rapid Assessment of Avoidable Blindness (RAAB) surveys.
Blindness due to diabetic retinopathy	Reference	Blindness (acuity in the better eye of <3/60 or <10% visual field around central fixation point) from damage to the retina caused by damaged blood vessels that can leak blood into the retina and cause scarring. Presenting vision is measured using any corrective lenses currently in use.
Blindness due to diabetic retinopathy	Alternative	Blindness (acuity in the better eye of <3/60 or <10% visual field around central fixation point) from damage to the retina caused by damaged blood vessels that can leak blood into the retina and cause scarring as measured by Rapid Assessment of Avoidable Blindness (RAAB) surveys.

Diabetes mellitus, diabetes mellitus type 1, diabetes mellitus type 2

Key updates in GBD 2023:

- Additional data sources have been identified via collaborator network, GHDx, opportunistic reviews and clinical data.
- An updated systematic review was completed to include published literature through February 2022.

Data seeking

A total of 96 new sources were included in the overall diabetes mellitus model for GBD 2023. Of these new sources, 52 were also included in the diabetes mellitus type 1 model for GBD 2023 as they were specific to type 1 diabetes mellitus.

Collaborator-provided sources that were either shared directly with us or were identified through searching the Global Health Data Exchange (GHDx) were reviewed for inclusion.

An opportunistic search was conducted to identify scientific literature that reported on mortality-related measures, including relative risk of mortality, standard mortality ratio and with-condition mortality rate for overall diabetes or type 1 diabetes. A total of 57 sources were identified, of which, 39 sources were used in the overall diabetes model and 30 sources were used in the type 1 diabetes model.

Additionally, clinical data prepared by the clinical informatics team that are representative of the sum of inpatient and outpatient visits from USA, Poland, South Korea, and Mongolia were included in overall diabetes and type 1 diabetes models. Data from USA Medicare, Poland patient claims data, Korea national health insurance services data, and Mongolia H-info health systems data are all new additions to GBD 2023.

Systematic review

An updated systematic review was conducted for GBD 2023, with the goal of covering overall diabetes mellitus model, Type 1 diabetes and high fasting plasma glucose exposure for GBD 2023.

A combined search terms for diabetes (parent and type 1) and mean FPG was used, based on the GBD 2019 search terms, The search took place for the following dates: 10/17/2018-2/11/2022:

(((diabetes[TI] AND (prevalence[TIAB] OR incidence[TIAB])) OR ('Diabetes Mellitus'[MeSH Terms] AND 'epidemiology'[MeSH Terms]) OR (diabetes[TI] AND 'epidemiology'[MeSH Terms]) NOT gestational[All Fields] NOT ('neoplasms'[MeSH Terms] OR 'neoplasms'[All Fields] OR 'cancer'[All Fields]) NOT ('mice'[MeSH Terms] OR 'mice'[All Fields]) NOT ('schizophrenia'[MeSH Terms] OR 'schizophrenia'[All Fields]) NOT ('emigrants and immigrants'[MeSH Terms] OR ('emigrants'[All Fields] AND 'immigrants'[All Fields]) OR 'emigrants and immigrants'[All Fields] OR 'immigrants'[All Fields]) NOT ('pregnancy'[MeSH Terms] OR 'pregnancy'[All Fields] OR 'gestation'[All Fields]) NOT ('rats'[MeSH Terms] OR 'rats'[All Fields] OR 'rat'[All Fields]) NOT ('kidney'[MeSH Terms] OR 'kidney'[All Fields]) NOT renal[All Fields] NOT ('vitamins'[Pharmacological Action] OR 'vitamins'[MeSH Terms] OR 'vitamins'[All Fields] OR 'vitamin'[All Fields])) OR (((“glucose”[Mesh] OR “hyperglycemia”[Mesh] OR “prediabetic state”[Mesh]) AND "Geographic Locations"[Mesh] NOT "United States"[Mesh]) AND ("humans"[Mesh] AND "adult"[MeSH]) AND ("Data Collection"[Mesh] OR "Health Services Research"[Mesh] OR "Population Surveillance"[Mesh] OR "Vital statistics"[Mesh] OR

"Population"[Mesh] OR "Epidemiology"[Mesh] OR surve*[TiAb]) NOT Comment[ptyp] NOT Case Reports[ptyp] NOT "hospital"[TiAb])) AND (("2018/10/17"[Date - Publication] : "3000"[Date - Publication]))

Figure 1: Prisma flow diagram for diabetes mellitus models GBD 2023 systematic review

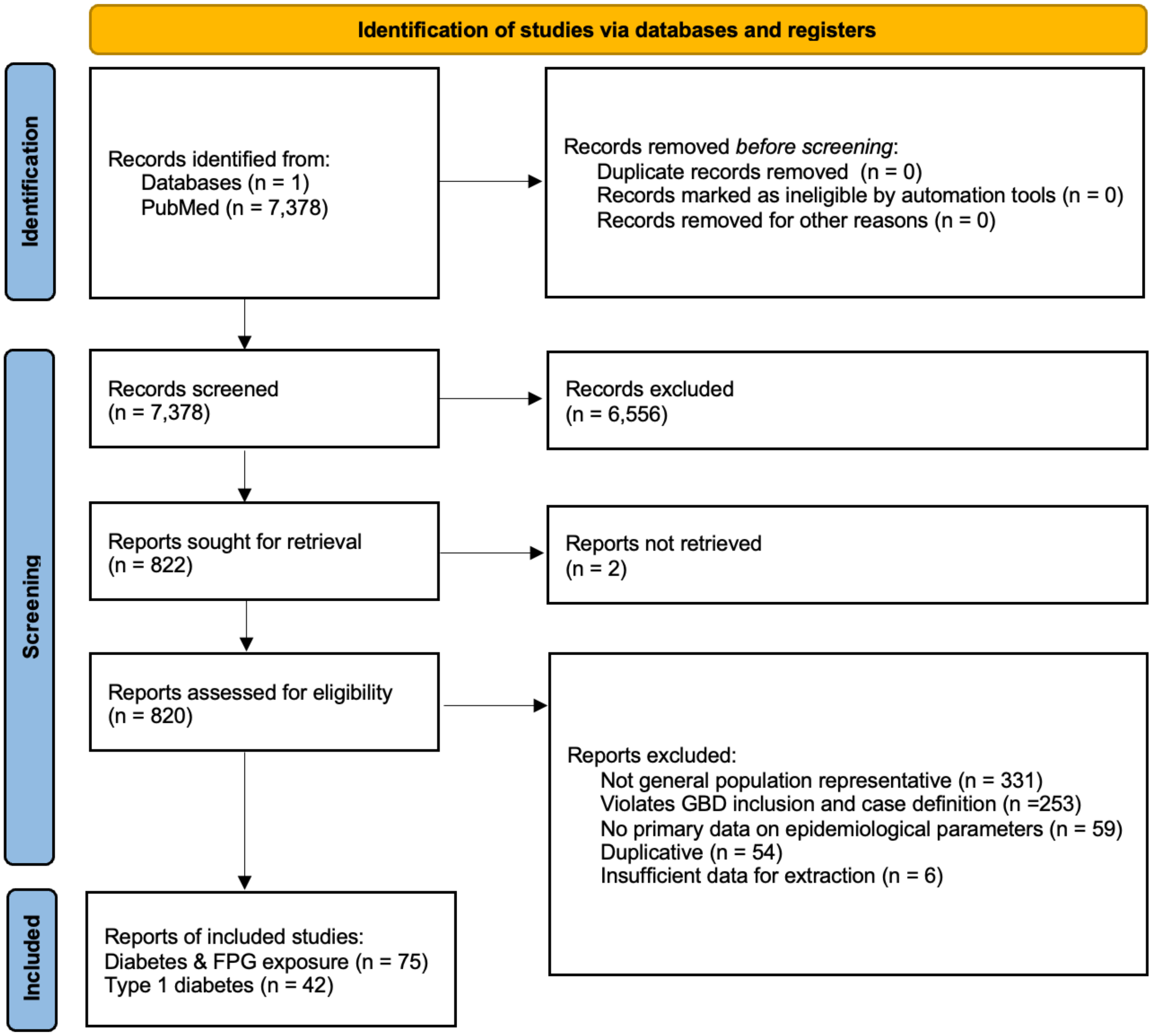

Main exclusion reasons include duplicative studies, not population representative, not one of the GBD reference or alternative case definitions, no primary data on epidemiological parameters, and insufficient data for extraction (ie, sample size, cases, uncertainty etc.).

Data inputs

Overall diabetes mellitus

Purpose

To incorporate all available population-representative data of diabetes, we accepted other measures of blood sugar (glycated haemoglobin A1c, oral glucose tolerance test, post-prandial glucose test) in addition to fasting plasma glucose to define diabetes. Studies that used random plasma glucose to define diabetes or self-reporting of diabetes status were not accepted.

Data

Data inputs came from four types of sources:

- Estimates of diabetes in a representative population
- Estimates of mean FPG in a representative population
- Individual-level data of blood sugar from surveys
- Clinical data inclusive of inpatient and outpatient data from USA, Poland, South Korea, and Mongolia

When a study reported both mean FPG and prevalence of diabetes, we used the prevalence of diabetes. Where possible, individual-level data from a cohort superseded any data described in a published paper. Individual-level data were collapsed and aggregated to produce estimates for each age group, sex, location, and year a survey was conducted.

Diabetes mellitus type 1

Purpose

To incorporate all available population-representative data of diabetes type 1, we accepted data that reported diabetes type 1, juvenile-onset diabetes, and insulin-dependent diabetes among children.

Data

Data inputs came from two types of sources:

- Published estimates of type 1 diabetes mellitus in a representative population
- Diabetic registries
- Clinical data inclusive of inpatient and outpatient data from USA, Poland, South Korea, and Mongolia in ages below 15

There were two studies, one by Rogers and colleagues in the USA[1] and the other by Thunander[2] and colleagues in Sweden that reported bimodal incidence age patterns where there was high incidence in the young ages and a second peak between 50 and 80. This bimodal pattern was not otherwise seen in other sources or type 1 diabetes registries, and after discussion with diabetes expert collaborators, it was decided to remove these two studies given plausibility of misdiagnosis in the older age groups.

Diabetes mellitus type 2

We found that the diagnostic criteria in the methodological sections of papers that report estimates of type 2 diabetes mellitus are not sufficiently specific for GBD. Thus, we calculated estimates of diabetes mellitus type 2 by subtracting the estimates of diabetes mellitus type 1 from estimates of overall diabetes mellitus for each age, sex, and location from 1990 to 2023.

Data processing

Overall diabetes mellitus

Key updates in GBD 2023:

- Age splitting was no longer applied to data with broad age bins as it had limited impact on model estimates overall and to avoid introducing age pattern assumptions based on global patterns.
- Updated sex ratios to age specific sex ratios for prevalence data and produced separate all-age sex ratios for incidence data.
- Bias adjustments were expanded to additional alternative case definitions, and adjustment factors were updated with new microdata.
- Bias adjustment for clinical data were updated and applied to USA MarketScan data and Poland claims data.
- Any clinical data in ages 85 and above were not included as input data to the model given concerns with reliable bias adjustment and population representativeness.

We performed several processing steps to the data to address sampling and measurement inconsistencies that will ensure the data are comparable across data sources and between high fasting plasma glucose modelling efforts.

1. *Small sample size and null prevalence*: Data with a sample size of ten or less or if the source's mean prevalence was reported to be zero were outliered prior to modelling.
2. *Mean FPG processing*: We used an ensemble distribution to estimate the prevalence of diabetes based on mean FPG for sources where data on prevalence of diabetes were not available but there was data on mean FPG. Essentially, we constructed a distribution based on unit-level data available in 31 different countries. Then we predicted out the prevalence of diabetes by age and sex. This provides the conversion of mean FPG to prevalence of diabetes defined as FPG greater than or equal to 126 mg/dL (7 mmol/L). Because this definition is not consistent with our reference case definition (which also includes those on treatment), we then adjust these datapoints to the reference case definition. For information on how these adjustments are made, please see the section, "Age splitting and bias adjustments" below.
3. *Sex splitting*: In GBD 2023, reported estimates of prevalence were split by sex and where possible. Studies with broad age groups estimates were no longer age-split given minimal impact on age-specific model estimates after testing models with and without age-splitting. First, if studies reported prevalence for broad age groups by sex and by specific age groups but for both sexes combined, age-specific estimates were split by sex using the sex ratio from within the study. Then, input data reporting prevalence for both sexes that could not be split using a within-study ratio were split using a sex ratio derived from a meta-analysis of existing sex-specific data (excluding studies already split using within-study sex ratios) using meta-regression—Bayesian, regularised, trimmed (MR-BRT). Age-specific female to male ratio for diabetes prevalence is shown in figure 2. Additionally, incidence data for diabetes were separately split using an all-age female to male ratio of 0.68 (95% CI: 0.60–0.77).

Figure 2. Prevalence of diabetes female to male sex ratio

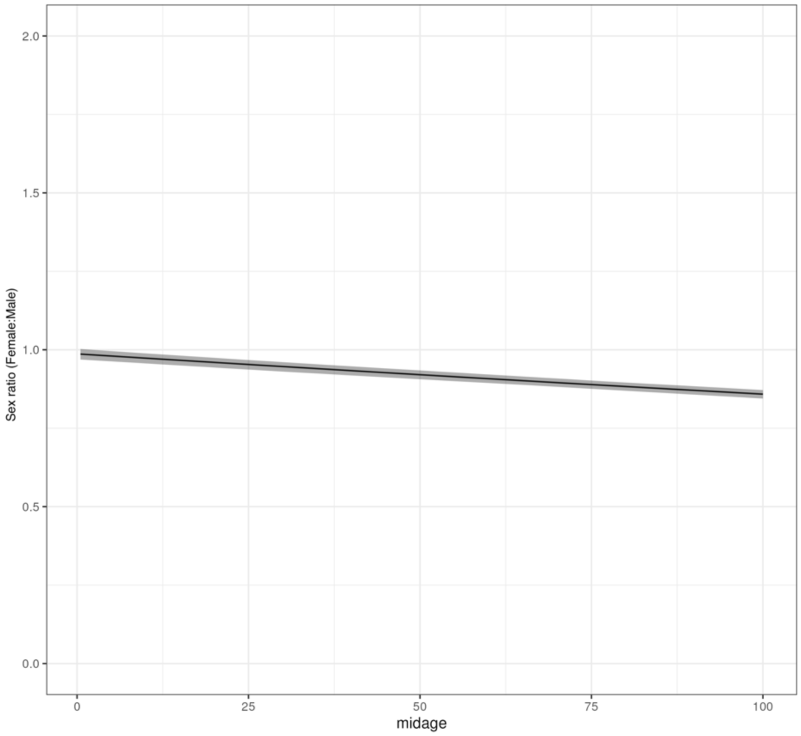

4. *Alternative definition bias adjustments:* We adjusted estimates from alternative case definitions to the reference case definition. Ratios were constructed between alternative case definitions and the reference case definition using microdata from surveys that measured glucose level based on different glucose tests on a single. We used MR-BRT analysis and performed this analysis in logit space due to the high prevalence of diabetes to ensure post-adjustment prevalence values do not exceed 1. Detailed steps are outlined below and adjustment factors for each alternative definition are outlined in table 2.

5. *Clinical data bias adjustments:* Clinical data often underestimate prevalence due to not being able to capture individuals who do not seek care and/or are undiagnosed. We examined and adjusted for clinical data bias to ensure population representation by comparing these data to population representative population surveys from the same country. However, we assume that claims data in persons <15 years are type 1 diabetes and that 100% of people with diabetes are captured in this age group. Thus, we only consider adjustments of the claims data for reported estimates in persons >15 years. When we compared USA Medicare data to USA NHANES data and South Korea clinical data to Korea NHANES data, we found that estimates were similar; therefore, these two sources did not undergo any bias adjustment. We were not able to include Mongolia claims data for ages >15 this round. This is because there were not sufficient reference data to produce a reliable adjustment factor, and we believe the claims data are heavily biased if no adjustments were applied. For Poland claims data, a sex covariate was included to account for differences in the adjustment factor by sex upon observing differences by sex. There were a total of five years of Poland claims data from the years 2015-2019; all five surveys used the Poland lipidogram study[3] from 2015–2016 as reference given the rather stable estimates of claims data over the five-year period. Detailed steps are outlined below, and adjustment factors for each clinical data source are outlined in table 3. Additionally, given small sample sizes and minimal representation in population surveys of people over the age of 85, we were not able to confidently apply bias adjustment factors for clinical data from this age group and therefore did not include clinical data in ages 85 and above in the model.

The process of adjusting for non-reference or clinical data using MR-BRT with the logit-transformation method is described below:

1. Identify datapoints with overlapping year, age, sex, and location between alternative case definition and reference case definition.
2. Logit transform overlapping datapoints of alternative and reference case definitions.
3. Convert overlapping datapoints into a difference in logit space using the following equation: logit(alternative) – logit(reference).
4. Use the delta method to compute standard errors of overlapping datapoints in logit space, then calculate standard error of logit difference using the following equation: √((variance of alternative) + (variance of reference)).
5. Using MR-BRT, conduct a random effects meta-regression to obtain the pooled logit difference of alternative to reference.
6. Apply the pooled logit difference to all datapoints of alternative case definitions using the following equation: New estimate = inverse.logit((logit(alternative)) – (pooled logit difference)).
7. Calculate new standard errors using the delta method, accounting for gamma (between-study heterogeneity).

Table 2: MR-BRT crosswalk adjustment factors for overall diabetes mellitus alternative case definitions

**Case definition** (fpg = fasting plasma glucose mmol/L; ogtt = oral glucose tolerance test mmol/L; hbA1c= hemoglobin A1c %)	**Reference or alternative case definition**	**Gamma**	**Beta coefficient log/logit (95% UI) ***	**Adjustment factor ** **
fpg ≥7mmol/L or on treatment	Ref	---	---	---
fpg ≥5.5 or on treatment	Alt	0.27	1.62 (1.6 to 1.65)	5.05 (4.95 to 5.21)
fpg ≥5.6	Alt	0.25	1.34 (1.32 to 1.37)	3.82 (3.74 to 3.94)
fpg ≥5.6 ogtt ≥11.1 or on treatment	Alt	0.63	1.88 (1.75 to 2.02)	6.55 (5.75 to 7.54)
fpg ≥5.6 or on treatment	Alt	0.2	1.4 (1.38 to 1.42)	4.06 (3.97 to 4.14)
fpg ≥6.1	Alt	0.06	0.59 (0.57 to 0.6)	1.8 (1.77 to 1.82)
fpg ≥6.1 or ogtt ≥11.1 or on treatment	Alt	0.25	0.89 (0.79 to 0.99)	2.44 (2.2 to 2.69)
fpg ≥6.1 or ogtt ≥7.8 or on treatment	Alt	0.1	1.63 (1.55 to 1.71)	5.1 (4.71 to 5.53)
fpg ≥6.1 or on treatment	Alt	0.03	0.69 (0.67 to 0.7)	1.99 (1.95 to 2.01)
fpg ≥6.7	Alt	0	-0.01 (-0.02 to 0)	0.99 (0.98 to 1.00)
fpg ≥6.7 or ogtt ≥10	Alt	0.3	0.5 (0.39 to 0.61)	1.65 (1.48 to 1.84)
fpg ≥6.7 or ogtt ≥11.1	Alt	0.36	0.27 (0.15 to 0.38)	1.31 (1.16 to 1.46)
fpg ≥6.9 or ogtt ≥11.1 or on treatment	Alt	0.08	0.09 (0.01 to 0.17)	1.09 (1.01 to 1.19)
fpg ≥7	Alt	0	-0.22 (-0.23 to -0.21)	0.8 (0.79 to 0.81)
fpg ≥7 or Hba1c ≥6.4 or on treatment	Alt	0.03	0.27 (0.24 to 0.31)	1.31 (1.27 to 1.36)
fpg ≥7 or Hba1c ≥6.5 or on treatment	Alt	0.03	0.22 (0.18 to 0.25)	1.25 (1.2 to 1.28)
fpg ≥7 or ogtt ≥11 or Hba1c ≥6.5 or on treatment	Alt	0	-0.19 (-0.29 to -0.09)	0.83 (0.75 to 0.91)
fpg ≥7 or ogtt ≥11 or on treatment	Alt	0.06	0.06 (-0.02 to 0.14)	1.06 (0.98 to 1.15)

fpg ≥7 or ogtt ≥11.1	Alt	0.08	0.02 (-0.06 to 0.1)	1.02 (0.94 to 1.11)
fpg ≥7 or ogtt ≥11.1 or Hba1c ≥6.5	Alt	0	-0.21 (-0.3 to -0.11)	0.81 (0.74 to 0.90)
fpg ≥7 or ogtt ≥11.1 or Hba1c ≥6.5 or on treatment	Alt	0	-0.21 (-0.31 to -0.11)	0.81 (0.73 to 0.90)
fpg ≥7 or ogtt ≥11.1 or Hba1c ≥6.9 or on treatment	Alt	0	-0.31 (-0.41 to -0.21)	0.73 (0.66 to 0.81)
fpg ≥7 or ogtt ≥11.1 or on treatment	Alt	0.07	0.04 (-0.04 to 0.12)	1.04 (0.96 to 1.13)
fpg ≥7 or ogtt ≥12.2	Alt	0.11	-0.18 (-0.27 to -0.09)	0.84 (0.76 to 0.91)
fpg ≥7 or ogtt ≥12.2 or on treatment	Alt	0.1	-0.15 (-0.24 to -0.06)	0.86 (0.79 to 0.94)
fpg ≥7.8	Alt	0.01	-0.66 (-0.68 to -0.65)	0.52 (0.51 to 0.52)
fpg ≥7.8 or ogtt ≥11.1	Alt	0.03	-0.22 (-0.3 to -0.15)	0.8 (0.74 to 0.86)
fpg ≥7.8 or ogtt ≥11.1 or on treatment	Alt	0.01	-0.2 (-0.27 to -0.13)	0.82 (0.76 to 0.88)
fpg ≥7.8 or on treatment	Alt	0	-0.29 (-0.3 to -0.27)	0.75 (0.74 to 0.76)
fpg ≥8 or ogtt ≥11.1	Alt	0.03	-0.24 (-0.31 to -0.16)	0.79 (0.73 to 0.85)
fpg ≥8 or ogtt ≥11.1 or on treatment	Alt	0.01	-0.21 (-0.28 to -0.13)	0.81 (0.76 to 0.88)
fpg ≥9.2 or on treatment	Alt	0.01	-0.48 (-0.5 to -0.47)	0.62 (0.61 to 0.63)
fpg ≥10 or ogtt ≥11.1 or on treatment	Alt	0.01	-0.27 (-0.34 to -0.19)	0.76 (0.71 to 0.83)
fpg ≥11.1 or ogtt ≥11.1	Alt	0.01	-0.3 (-0.38 to -0.22)	0.74 (0.68 to 0.8)
fpg ≥11.1 or ogtt ≥11.1 or on treatment	Alt	0.01	-0.27 (-0.35 to -0.2)	0.76 (0.7 to 0.82)
Hba1c ≥6 or on treatment	Alt	0.11	0.74 (0.7 to 0.78)	2.1 (2.01 to 2.18)
Hba1c ≥6.5	Alt	0.11	-0.36 (-0.4 to -0.32)	0.7 (0.67 to 0.73)
Hba1c ≥6.5 or on treatment	Alt	0.09	-0.1 (-0.13 to -0.07)	0.9 (0.88 to 0.93)
ogtt ≥10	Alt	0	0.51 (0.31 to 0.7)	1.67 (1.36 to 2.01)
ogtt ≥10 or on treatment	Alt	0	0.54 (0.35 to 0.74)	1.72 (1.42 to 2.1)
ogtt ≥11.1	Alt	0	0.06 (-0.16 to 0.27)	1.06 (0.85 to 1.31)
ogtt ≥11.1 or on treatment	Alt	0	0.11 (-0.1 to 0.32)	1.12 (0.9 to 1.38)

Table 3: MR-BRT crosswalk adjustment factors for overall diabetes mellitus clinical data

**Data input**	**Reference or alternative case definition**	**Gamma**	**Beta coefficient log/logit (95% UI) ***	**Adjustment factor ****
US NHANES 2001-2002	Ref	0	---	---
US MarketScan 2000	Alt		-0.71 (-0.9 to -0.51)	0.49 (0.41 to 0.6)
US NHANES 2009-2010	Ref	0	---	---
US MarketScan 2010	Alt		-0.48 (-0.65 to -0.31)	0.62 (0.52 to 0.73)
US NHANES 2011-2012	Ref	0		
US MarketScan 2011	Alt		-0.47 (-0.65 to -0.29)	0.63 (0.52 to 0.75)
US NHANES 2011-2012	Ref	0		
US MarketScan 2012	Alt		-0.46 (-0.65 to -0.28)	0.63 (0.52 to 0.76)
US NHANES 2013-2014	Ref	0	---	---
US MarketScan 2013	Alt		-0.48 (-0.65 to -0.31)	0.62 (0.52 to 0.73)
US NHANES 2013-2014	Ref	0	---	---

US MarketScan 2014	Alt		-0.44 (-0.61 to -0.27)	0.64 (0.54 to 0.76)
US NHANES 2015-2016	Ref	0	---	---
US MarketScan 2015	Alt		-0.48 (-0.65 to -0.3)	0.62 (0.52 to 0.74)
US NHANES 2015-2016	Ref	0	---	---
US MarketScan 2016	Alt		-0.49 (-0.66 to -0.32)	0.61 (0.52 to 0.73)
US NHANES 2017-2020	Ref	0	---	---
US MarketScan 2017	Alt		-0.59 (-0.75 to -0.43)	0.55 (0.47 to 0.65)
US NHANES 2017-2020	Ref	0	---	---
US MarketScan 2018	Alt		-0.67 (-0.82 to -0.51)	0.51 (0.44 to 0.6)
US NHANES 2017-2020	Ref	0	---	---
US MarketScan 2019	Alt		-0.64 (-0.8 to -0.48)	0.53 (0.45 to 0.62)
Poland Lipidogram Study 2015-2016	Ref	0.05		
Poland Patient Claims Data 2015-2019 - Male	Alt		-1.00 (-1.15 to -0.84)	0.37 (0.32 to 0.43)
Poland Patient Claims Data 2015-2019 - Female	Alt		-0.89 (-1.15 to -0.63)	0.41 (0.32 to 0.53)

***The adjustment factor column is the exponentiated beta coefficient. For log beta coefficients, this is the relative rate between the two case definitions. For logit beta coefficients, this is the relative odds between the two case definitions.*

Diabetes mellitus type 1

Key updates in GBD 2023:

- Alternative case definitions were removed due to lack of data.
- Sex ratios were updated to reflect age-specific sex ratios.
- In GBD 2021 imputed incidence type 1 adult data were used to improve data coverage on adult population; however, this method was foregone given new modelling strategy. See modelling strategy for details.

All of the data from scientific literature are from a diabetic registry, hospital discharge data review, physician interview, or insulin use. We assumed that there is no systematic bias between these sources and consider sources identified through these methods as reference. The majority of the data are incidence data.

Based on the assumption that the sum of inpatient and outpatient clinical in persons <15 years are type 1 diabetes and that 100% of people with diabetes are captured in this age group, we make no adjustments to data in these ages. Clinical data are received and processed by the clinical informatics team, details of methods of clinical data preparation and processing are outlined in detail elsewhere. Inpatient and outpatient visits are deduplicated and processed to produce one year prevalence of diabetes.

First, if studies from the literature reported incidence for broad age groups by sex and by specific age groups but for both sexes combined, age-specific estimates were split by sex using the sex ratio from within the study. Input data reporting data for both sexes that could not be split using a within-study ratio were split using a sex ratio derived from a meta-analysis of existing sex-specific data (excluding

studies already split using within-study sex ratios) using MR-BRT. Age-specific female to male ratios for prevalence of diabetes are shown in figure 3.

Figure 3. Type 1 diabetes incidence female to male sex **ratio**

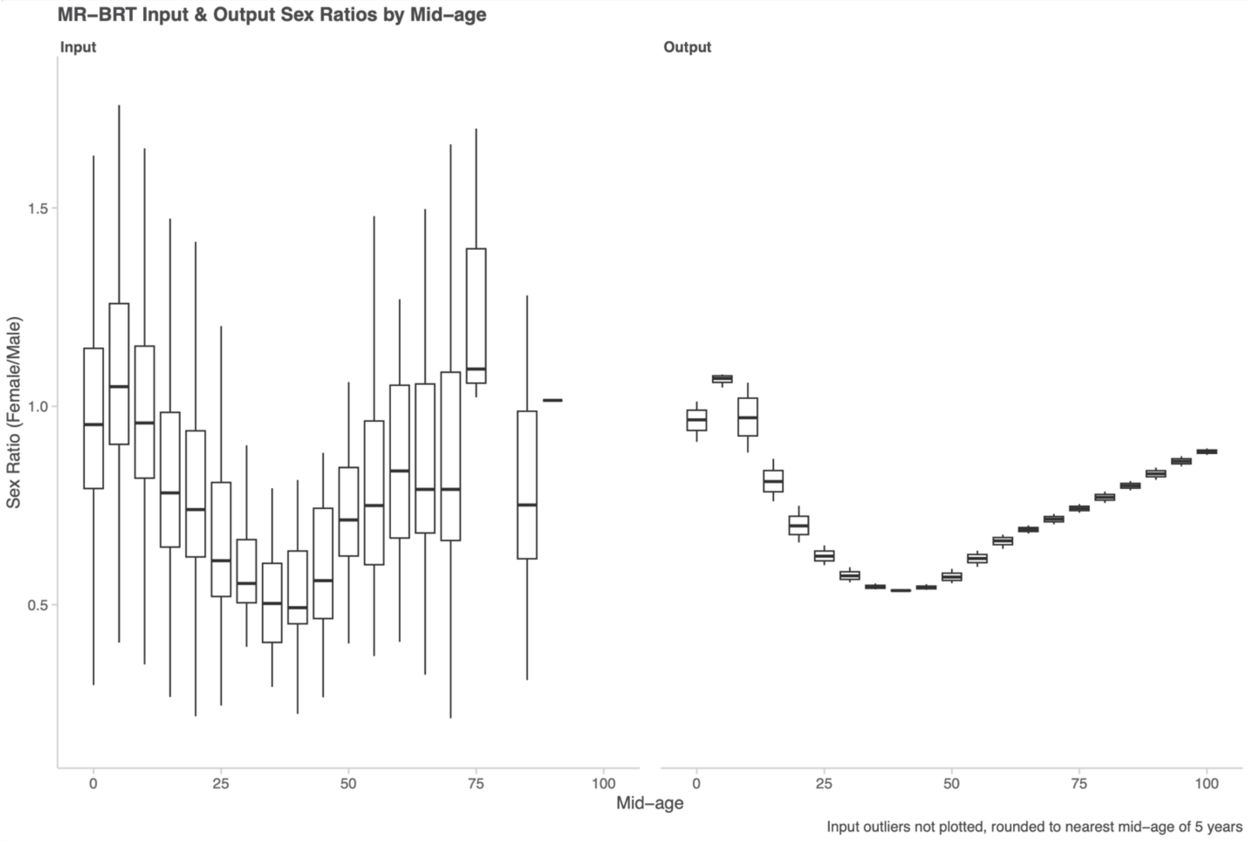

Modelling strategy

Overall diabetes mellitus

Key updates in GBD 2023:
The modelling strategy in GBD 2023 for overall diabetes remained the same compared to GBD 2021.

We estimated diabetes mellitus using DisMod-MR 2.1, which produces estimates of the prevalence of diabetes for each age, sex, geographical location, and year. We used data that reported prevalence and incidence for diabetes mellitus. After modelling, we replaced the total diabetes estimates for less than 15 years with the estimates from the type 1 diabetes mellitus model for each age, sex, location, and year for this age range. This was to ensure that the <15 years estimates for total diabetes mellitus and type 1 diabetes mellitus were equivalent, because we assume type 2 diabetes mellitus cannot occur before 15 years of age.

<u>Model parameters and estimates</u>

- We set a value prior of 0 for remission for ages 0 to 14.
- We set a value prior of a maximum value of 0.01 for remission for ages 15 to 100.
- We set a value prior of a maximum value of 0.15 for excess mortality for all ages.

- We set a value prior of 0 for incidence for ages 0 to 1.
- We set a value prior of a maximum value of 0.0008 for incidence for ages 1 to 15.
- We set a value prior of a maximum value of 0.1 for incidence for ages 15 to 100.

Table 6: Summary of covariates used in the diabetes mellitus DisMod-MR model

Covariate	Type	Parameter	Exponentiated beta (95% uncertainty interval)
Prevalence of obesity (age-standardised)	Country-level	Prevalence	10.81 (10.20–11.62)
Year	Country-level	Prevalence	1.01 (1.01–1.01)

Diabetes mellitus type 1

Key updates in GBD 2023:

- In GBD 2023, Type 1 diabetes was modelled using DisMod-AT instead of DisMod-MR 2.1. This is the first round of GBD where DisMod-AT has been used.
- Previously included covariates on incidence for the proportion of livebirths in women aged 35+ and maternal education were removed.
- The majority of value priors for incidence were removed except at birth.

For GBD 2023, we estimated type 1 diabetes mellitus using DisMod-AT. Bias and root mean square error (RMSE) are shown in Table 7 for each measure. More details about DisMod-AT can be found in the non-fatal section of appendix 1 section 2. We used data that reported incidence, prevalence data, standard mortality ratio, and with-condition mortality rate for type 1 diabetes. We decided to not include reported type 1 diabetes prevalence in non-claims sources because we found that other estimates of prevalence were inconsistent with corresponding incidence data. Similarly, we did not include prevalence of diabetes type 1 in people >15 years from claims sources because of poor reporting on type of diabetes. We made an exception for prevalence data from the Norway Adult Diabetes Registry for the entire age range after confirmation from collaborators regarding its reliability.

Table 7. Performance metrics for the type 1 diabetes DisMod-AT model

**Measure**	**Bias**	**RMSE**
S incidence	0.0000331	0.000109
Prevalence	0.000383	0.000672
With-condition mortality rate	0.00222	0.00570
Standardized mortality ratio	0.955	2.69

<u>Model parameters and estimates</u>

- We set a value prior of near 0 (1.0x10^-6) at age 0 for incidence to restrict incidence in neonates.
- We set a loose value prior of Ln(0.0001,1) for ages above 0 for incidence.
- We set a loose value prior of Ln(0.01,1) for excess mortality.

Five different covariates were tested for inclusion as part of the DisMod-AT covariate selection process. They are maternal education, proportion of livebirths in women aged 35+, proportion of livebirths in women aged 40+, HAQ Index, and LDI (I$ per capita). While LDI, HAQ Index and maternal education were selected, the inclusion of LDI and maternal education produced implausible geographical and time trends for incidence and were ultimately removed from the model. HAQ Index, applied to excess mortality rate, was the only covariate included in the type 1 diabetes model.

Table 8: Summary of covariates used in the diabetes mellitus type 1 DisMod-AT model

Covariate	Type	Parameter	Exponentiated beta (95% uncertainty interval)
Healthcare Access and Quality Index	Country-level	Excess mortality rate	0.998 (0.996-0.999)

Diabetes outcomes

Data seeking

No systematic review was conducted for the diabetes mellitus outcomes for GBD 2023. Previous systematic reviews for diabetic neuropathy, diabetic foot ulcer, and amputation due to diabetes mellitus were undertaken for GBD 2017.

Data inputs

Diabetic neuropathy

Purpose

Data

1. Data inputs came from
   - Estimates of neuropathy in a representative population of people with diabetes
2. Covariates
   - None

Diabetic foot ulcer

1. Data inputs came from
   - Estimates of foot ulcer in a representative population of people with diabetes
2. Covariates
   - Healthcare Access and Quality Index

Amputation due to diabetes

1. Data inputs came from
   - Estimates of amputation in a representative population of people with diabetes
2. Covariates
   - Healthcare Access and Quality Index

Data processing

Diabetic neuropathy, diabetic foot ulcer, and amputation due to diabetes

All input data and sources were reviewed for GBD 2019. We found that nearly all sources reported estimates in age ranges that exceed 50 years. We identified a single study for each outcome that reported estimates in age bins of <25 years. We applied this age pattern to the remaining datapoints. Due to a lack of data in the diabetic outcome models, no adjustments were undertaken for alternative case definitions, and therefore all case definitions were treated as reference.

Modelling strategy

For GBD 2023, there were no substantial updates to modelling strategy compared to GBD 2021

We estimated amputation due to diabetes mellitus, diabetic neuropathy, and diabetic foot for diabetes mellitus type 1 and diabetes mellitus type 2 using DisMod-MR 2.1. We then multiply all proportion draws from neuropathy/foot/amputation models by the parent diabetes model so that all estimates are in the same population-space.

We use the estimates of blindness, moderate, and severe vision loss due to diabetes from GBD vision loss results to estimate the proportion of the population with diabetes who have these conditions. The vision loss estimates are derived as part of the vision loss impairment analyses based on data ascribing vision loss to underlying causes in population-based surveys. Further details on these analyses can be found in the appendix section for vision loss estimation. The diabetes process takes these estimates into account when estimating diabetic outcomes.
First, we ensure that the sum of the prevalence for neuropathy due to diabetes mellitus, moderate vision loss due to diabetes mellitus, severe vision loss due to diabetes mellitus, and blindness due to diabetes mellitus does not exceed 90% of the prevalence of all diabetes mellitus. If the sum exceeds 90%, then we rescale the individual outcomes to 90%. This treats vision loss and neuropathy as mutually exclusive categories by assuming a patient will not have both simultaneously. From here, we calculate uncomplicated diabetes as the remainder of diabetes cases exclusive of neuropathy and vision loss.

We perform the same check to ensure that the prevalence of amputation due to diabetes mellitus and prevalence of foot ulcer due to diabetes mellitus does not exceed 90% of the prevalence of neuropathy due to diabetes mellitus. This treats foot ulcer and amputation as mutually exclusive categories by assuming a patient will not have both simultaneously.
In addition, we estimate the prevalence of amputation due to diabetes by splitting into with and without treatment using scaled health systems access (HSA) values. For diabetic amputation, we calculated a distribution of treated versus untreated amputation, defined as receiving a prosthesis or not. We first rescaled the IHME estimates to be between 0 and 0.9, under the assumption that 10% of amputees will not receive a prosthetic, even in high-income countries. We based this assumption on the retrospective study by Moore and colleagues, which found that about 80% of patients following major lower extremity amputation were fitted with prostheses in the authors' institutions from 1978 to 1986 in the USA.[5] We then performed a population-weighted average of this country-specific value to obtain a proxy for the proportion of amputees that receive a prosthetic by super-region. Because these are rough estimates based on large assumptions, we applied confidence intervals of +/- 50% of the value to reflect our uncertainty.

Model parameters and estimates

Diabetic neuropathy

- We set a value prior on the proportion of 0 from ages 0 to 1.

Diabetic foot ulcer

- We set a value prior on the proportion of 0 from ages 0 to 10.

Amputation due to diabetes

- We set a value prior of 0 for incidence for ages 0 to 15.
- We set a value prior of 0 for remission for all ages.

Table 10: Summary of covariates used in the diabetic foot ulcer DisMod-MR model

Covariate	Type	Parameter	Exponentiated beta (95% uncertainty interval)
Healthcare Access and Quality Index	Country-level	Proportion	0.99 (0.99–1.00)

Table 11: Summary of covariates used in the amputations due to diabetes DisMod-MR model

Covariate	Type	Parameter	Exponentiated beta (95% uncertainty interval)
Healthcare Access and Quality Index	Country-level	Prevalence	0.99 (0.98–1.01)

Severity distributions

We derived the disability weights for each sequela from the GBD disability weight survey. The table below illustrates the severity levels, lay descriptions, and associated disability weights applicable for outcomes related to diabetes mellitus type 1 and diabetes mellitus type 2:

Table 12: Details on the severity levels for diabetes mellitus and the associated disability weight (DW) with that severity.

Severity level	Lay description	DW (95% CI)
Uncomplicated diabetes mellitus (type 1 or type 2)	Has a chronic disease that requires medication every day and causes some worry, but minimal interference with daily activities	0.049 (0.031–0.072)
Diabetic neuropathy without diabetic foot or amputation	Has pain, tingling, and numbness in the arms, legs, hands, and feet. The person sometimes gets cramps and muscle weakness.	0.133 (0.089–0.187)

Diabetic neuropathy with diabetic foot	Has a sore on the foot that is swollen and causes some difficulty in walking.	0.150 (0.103–0.208)[a]
Diabetic neuropathy with treated amputation	Has lost part of one leg, leaving pain and tingling in the stump. The person has an artificial leg that helps in moving around.	0.1667 (0.114–0.229)[a]
Diabetic neuropathy with untreated amputation	Has lost part of one leg, leaving pain and tingling in the stump. The person does not have an artificial leg, has frequent sores, and uses crutches.	0.282 (0.198–0.379)[a]
Moderate vision loss due to diabetes mellitus	Has vision problems that make it difficult to recognise faces or objects across a room.	0.031 (0.019–0.049)
Severe vision loss due to diabetes mellitus	Has severe vision loss, which causes difficulty in daily activities, some emotional impact (for example worry), and some difficulty going outside the home without assistance.	0.184 (0.125–0.259)
Blindness due to diabetes mellitus	Is completely blind, which causes great difficulty in some daily activities, worry and anxiety, and great difficulty going outside the home without assistance.	0.187 (0.124–0.26)

[a] The disability weights are produced from a combination of two health states: neuropathy and diabetic foot/amputation

Comparison to other published estimates

Overall diabetes

We identified two groups who also make global estimates of diabetes, the International Diabetes Federation (IDF) and the NCD Risk Factor Collaboration (NCD-RisC). The IDF publishes annual updates to their estimates, with the most recent estimates published in the 10th atlas (https://www.diabetesatlas.org/en/), and NCD-Risc published estimates in the paper "Worldwide trends in diabetes prevalence and treatment from 1990 to 2022: a pooled analysis of 1108 population-representative studies with 141 million participants."
Below is a table comparing the global number of diabetes reported by GBD 2021, IDF 10th Atlas, and NCD-Risc for the closest years that align with 1990, 2010, and 2021.

Organisation	Source	1990	2010	2021
IHME	GBD 2023	188 million	372 million	534 million
International Diabetes Federation	IDF 10th Atlas	151 million (2000)	285 million	537 million
NCD Risk collaboration	Figure 7	198 million	350 million	828 million (2022)

There are several methodological and analytical differences between each group's approach, which explains differences in the number of cases. The table below summarises the main differences.

Organisation	Age	Case definition	Analysis
IHME	All ages	FPG ≥7 mmol/L (126 mg/dL) or currently on treatment (insulin or drugs)	Bayesian hierarchical meta-regression
International diabetes federation	20–79 years	FPG ≥7 mmol/L (126 mg/dL) or OGTT ≥11.1 mmol/L (200 mg/dL) or HbA1c ≥6.5% or random plasma glucose ≥11.1 mmol/L (200 mg/dL) or self-report diabetes status	Generalised linear regression model
NCD Risk collaboration	≥18 years	FPG ≥7 mmol/L (126 mg/dL) or HbA1c >=6.5%	Bayesian hierarchical model

Type 1 diabetes

We also compared our new Type 1 diabetes estimates for year 2021 to global paper published titled "Global incidence, prevalence, and mortality of type 1 diabetes in 2021 with projection to 2040: a modelling study" by Gregory & Robinson and colleagues.

Global	Gregory & Robinson and colleagues	GBD 2023 (using DisMod-AT)	GBD 2021 (using DisMod-MR)
Global total (0-99)	8.4million	8.3 million	19.6 million
Global <20 years	1.5million	1.3 million	2.8 million
Global 20+	6.9million	7.0 mil	16.9 million

References

[1]Rogers, M.A.M., Kim, C., Banerjee, T. *et al.* Fluctuations in the incidence of type 1 diabetes in the United States from 2001 to 2015: a longitudinal study. *BMC Med* **15**, 199 (2017).
https://doi.org/10.1186/s12916-017-0958-6

[2]M. Thunander, C. Petersson, K. Jonzon, J. Fornander, B. Ossiansson, C. Torn, S. Edvardsson, M. Landin-Olsson, Incidence of type 1 and type 2 diabetes in adults and children in Kronoberg, Sweden, *Diabetes Research and Clinical Practice*, Volume 82, Issue 2, 2008, Pages 247-255, ISSN 0168-8227. https://doi.org/10.1016/j.diabres.2008.07.022.

[3] College of Family Physicians in Poland (CFPiP), Częstochowa University of Technology, Polish Lipid Association, Silesian Analytical Laboratories. Poland LIPIDOGRAM 2015-2016 Study.

[4]Moore TJ, Barron J, Hutchinson F 3rd, Golden C, Ellis C, Humphries D. Prosthetic Usage following major lower extremity amputation. *Clin Orthop Relat Res.* 1989 Jan;(238):219-24.

Diarrhoeal diseases

Flowchart

Diarrheal diseases

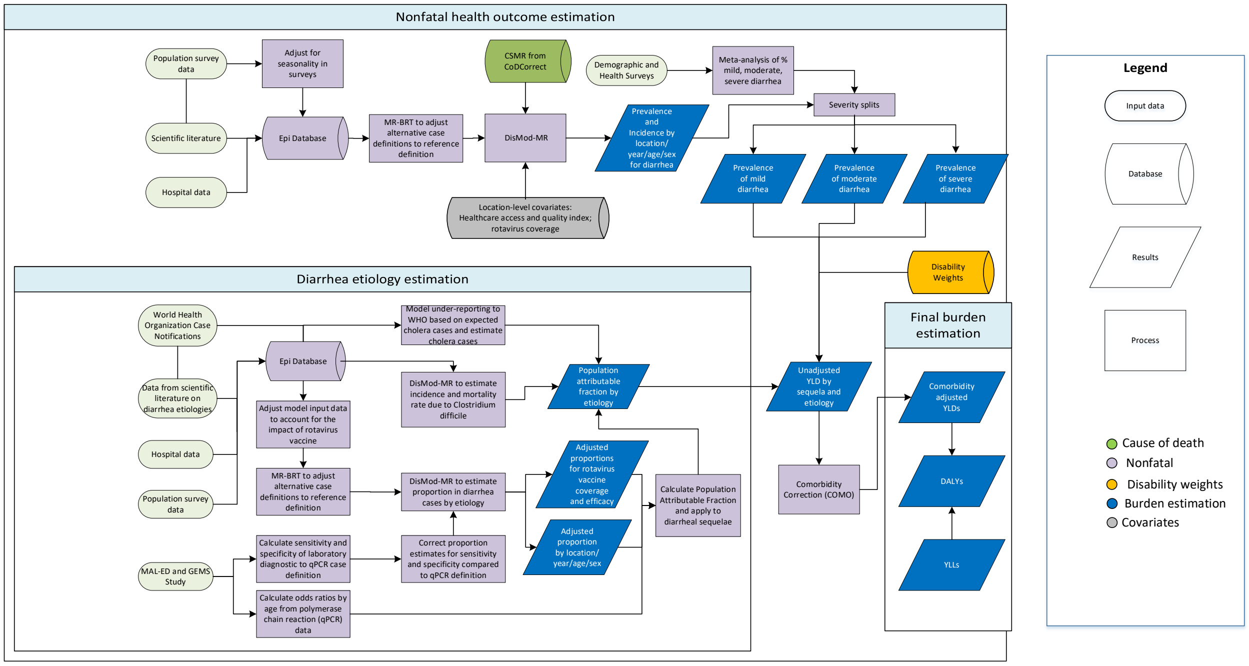

Input data and methodological summary for diarrhoeal diseases

Case definition

We defined diarrhoeal disease episodes as three or more loose stools in a 24-hour period. In the diarrhoea models, self-reported prevalence is the reference category for all data adjustments. Hospital input data use ICD-9 codes 001-009.9 and ICD-10 codes A00-A09.

The case definitions accepted for diarrhoea are shown below.

**Quantity of interest**	**Reference or alternative**	**Definition**
Incidence or prevalence of diarrhoea	Reference	Three or more abnormally loose stools in a 24-hour period. Self-reported or parental report for children.
Incidence of inpatient diarrhoea episodes	Alternative	Incidence of diarrhoea episodes that become inpatients reported in health care data.
Incidence of diarrhoea episodes in clinical claims data	Alternative	Incidence of diarrhoea episodes reported in claims data.

Input data

Model inputs

We used three main types of data in the diarrhoea non-fatal burden estimation: hospital data, population-based surveys, and data from scientific literature.

The first type of data is the incidence of diarrhoea in hospital settings including inpatient and claims data. These data were identified using the ICD-9 codes 001-009.9 and ICD-10 codes A00-A09 and were adjusted prior to modelling for multiple admissions and multiple diagnoses. To be consistent with the population-based survey data, adjusted hospital data were transformed from incidence to prevalence using the following equation:

$$Prevalence = Incidence * \frac{duration(days)}{365}$$

The second type of data are from population-representative surveys, such as the Demographic and Health Surveys and the Multiple Indicator Cluster Surveys. We converted the prevalence of maternal-reported two-week period from surveys to point prevalence in one-year age groups using this equation:

$$Point\ Prevalence = Period\ Prevalence * \frac{Duration}{(Recall\ Period + Duration - 1)}$$

Where the mean duration was the duration in days, an average of 4.3 days (4.2–4.4) in both equations.[1]

Survey data were adjusted for seasonality. Surveys are frequently conducted over several months. To account for seasonal variation in diarrhoea prevalence, we fit a mixed-effects generalised additive model for each GBD region with a forced periodicity and a random intercept by country. The ratio between the monthly model-fit diarrhoea prevalence and the corresponding regional diarrhoea prevalence is a scalar to adjust survey data by month and geography.

The third type of data are from scientific literature. Inclusion criteria include diarrhoea as the case definition, studies with a sample size of at least 100, and a study duration of at least one year to avoid bias in the seasonal timing of diarrhoea. We excluded studies that reported on diarrhoeal outbreaks exclusively and studies that combined acute gastroenteritis with and without diarrhoea. We included all literature data sources used in GBD 2021 and conducted an updated review of literature for GBD 2023 covering the period 12/01/2020 to 1/09/2024 for diarrhoea prevalence, incidence, and all diarrhoea aetiologies.

Aetiologies

We extracted data on all aetiologies from scientific literature that reported the proportion of diarrhoea cases that tested positive for each pathogen. For *C difficile*, we extracted incidence and prevalence. We applied the same inclusion and exclusion criteria described above for diarrhoea.

We searched articles using a PubMed search term that combined non-specific and aetiology-specific diarrhoea using the following search string:

(diarrhoea*[title/abstract] OR "Diarrhea"[Mesh] OR diarrhea*[title/abstract]) AND

(2020/12/01:2024/01/09[PDat]) AND (inciden*[title/abstract] OR incidence[Mesh] OR prevalent*[title/abstract] OR cases[title/abstract] OR prevalence[Mesh] OR epidemiology[title/abstract] OR epidemiology[Mesh] OR salmonella[title/abstract] OR salmonella[Mesh] OR aeromona*[title/abstract] OR aeromonas[Mesh] OR shigell*[title/abstract] OR shigella[Mesh] OR enteropathogenic[title/abstract] OR enterotoxigenic[title/abstract] OR "Enterotoxigenic Escherichia coli"[Mesh] OR "Enteropathogenic Escherichia coli"[Mesh] OR *coli[title/abstract] OR campylobacter[title/abstract] OR campylobacter[Mesh] OR amoebiasis[title/abstract] OR "Amebiasis"[Mesh] OR entamoeb*[title/abstract] OR cryptosporid*[title/abstract] OR "Cryptosporidiosis"[Mesh] OR rotavir*[title/abstract] OR rotavirus[Mesh] OR norovirus[title/abstract] norovirus[Mesh] OR adenovirus[title/abstract] OR "Adenovirus Infections, Human"[Mesh] OR clostridium*[title/abstract] OR Clostridioides difficile[title/abstract] OR "Clostridioides difficile"[Mesh] OR c. diff*[title/abstract] OR “astrovir*”[Mesh] OR “astrovir*”[title/abstract] OR “sapovir*”[Mesh] OR “sapovir*”[title/abstract] OR etiolog*[title/abstract] OR aetiolog*[title/abstract] OR pathogen*[title/abstract]) NOT

(appendicitis[title/abstract] OR esophag*[title/abstract] OR gastritis[title/abstract] OR liver[title/abstract] OR case report[title/abstract] OR case-report[title/abstract] OR "Case Reports" [Publication Type] OR therapy[title] OR Crohn[title/abstract] OR "inflammatory bowel"[title/abstract] OR irritable[title/abstract] OR travel*[title] OR Outbreak[title] OR Review[ptyp]) NOT

(animals[MeSH] NOT humans[MeSH])

We identified 2504 studies, of which 88 met our inclusion criteria. We extracted data for location, sex, year, and age.

Figure 1. Diarrhoeal disease systematic review PRISMA diagram

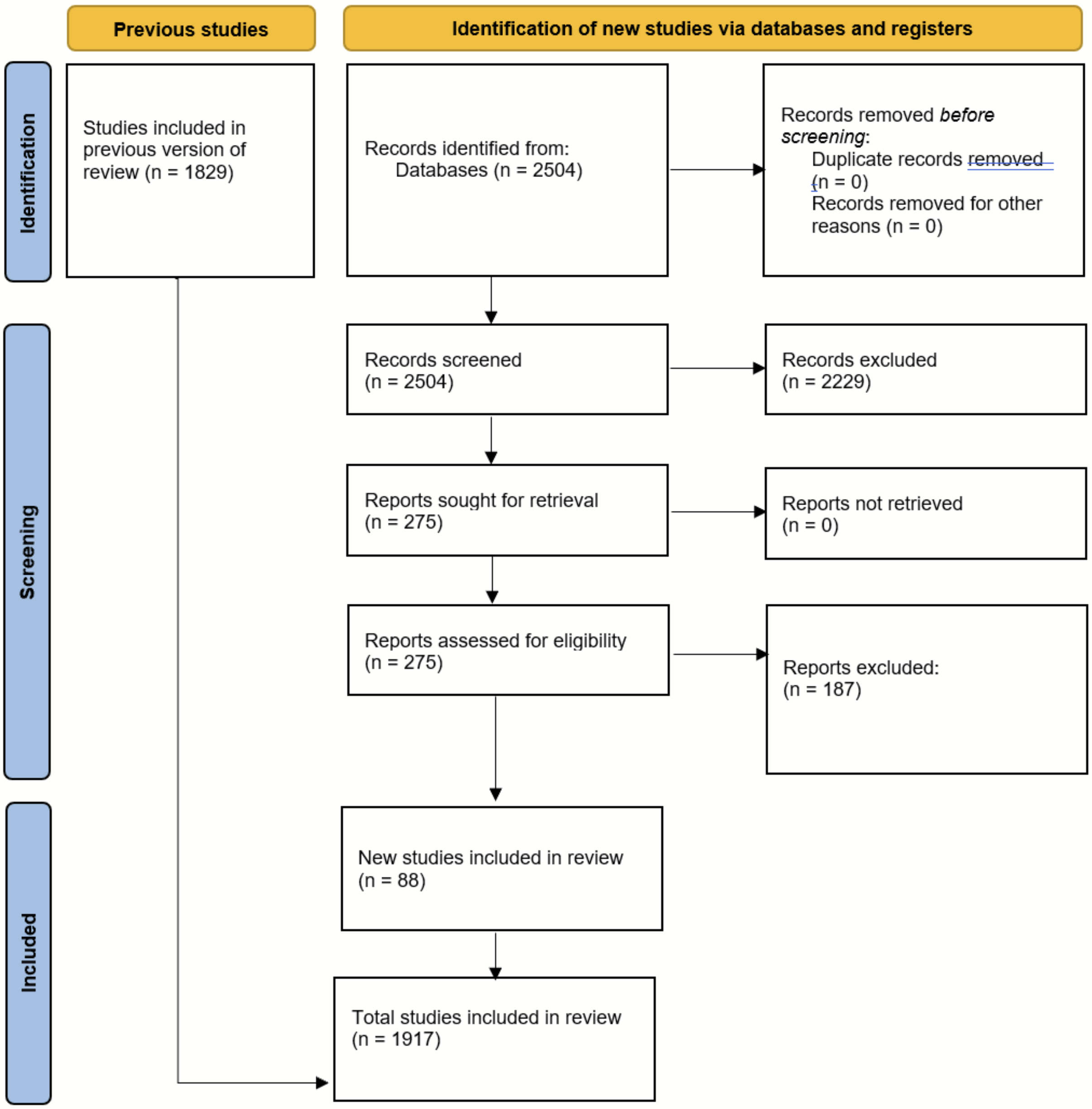

Additionally, we searched specifically for data sources detailing rotavirus coverage and vaccine efficacy using the following search string:

(((rotavirus[title/abstract] AND vaccine[title/abstract] AND (efficacy[title/abstract] OR effectiveness[title/abstract]) AND (2020/12/31[PDAT] : 2024/10/22[PDAT])))) NOT Review[Publication Type] NOT (animals[MeSH] NOT humans[MeSH])

We identified 185 studies from this search string along with 108 additional studies identified by reviewing meta-analyses captured by the search string. Of these 293 sources, 14 met our inclusion criteria.

Figure 2. Rotavirus vaccine efficacy systematic review PRISMA diagram

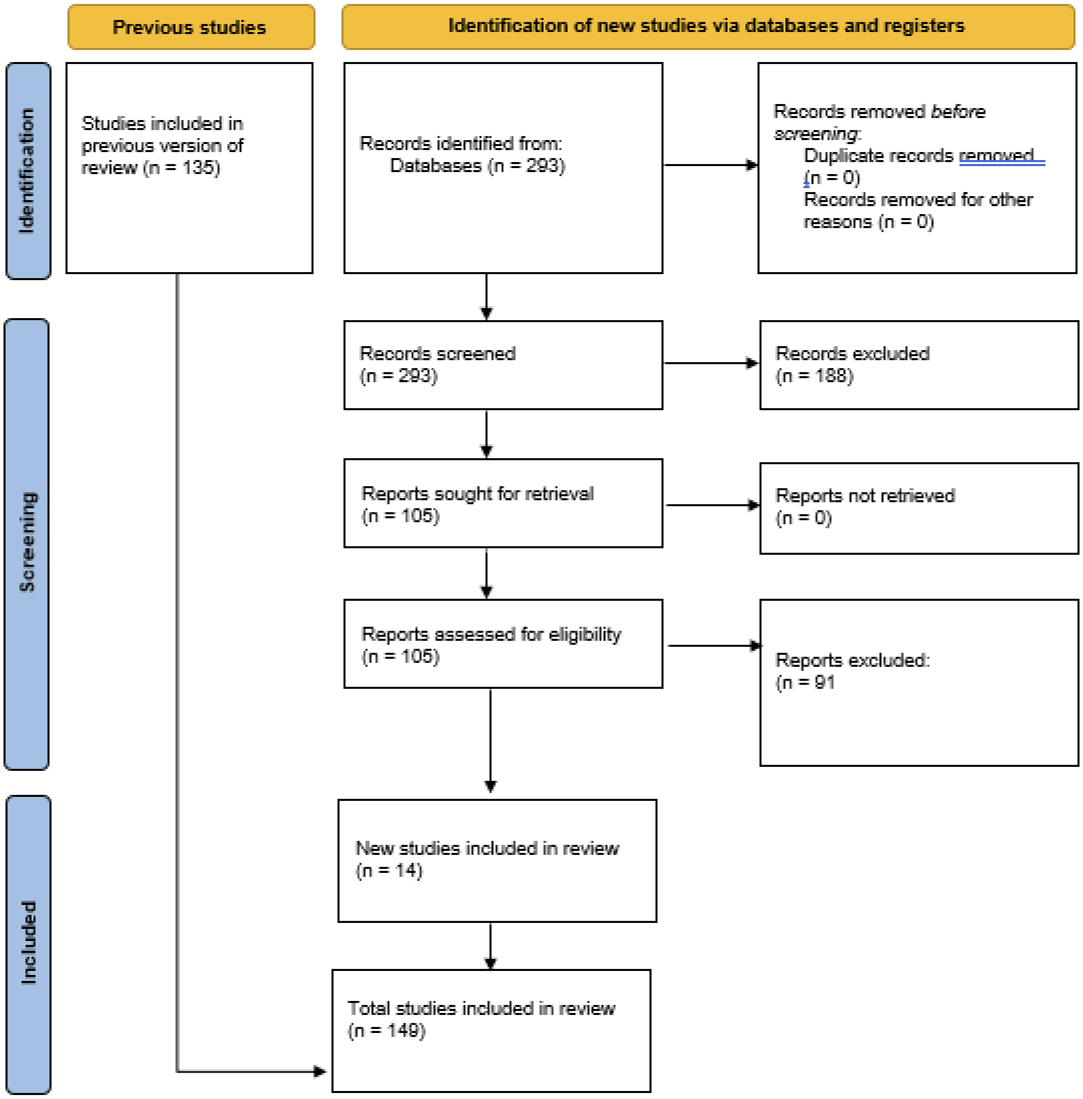

To update our cholera proportion estimates, we used an additional PubMed search string spanning from January 2019 to December 2023:

(cholera*[title/abstract] OR cholera[Mesh]) AND (diarrhea*[title/abstract] OR diarrhoea*[title/abstract] OR diarrhea[Mesh]) AND (epidemiolog*[title/abstract] OR epidemiology[Mesh] OR inciden*[title/abstract] OR incidence[Mesh] OR prevalent*[title/abstract] OR prevalence[Mesh] OR death*[title/abstract] OR mortality[title/abstract] OR mortality[Mesh] OR fatal*[title/abstract] OR case*[title/abstract] OR proportion*[title/abstract]) NOT ("case report"[title/abstract] OR "case reports"[Publication Type] OR review[Publication Type]) AND (("2019/01/01"[PDat] : "2023/12/21"[PDat])) NOT (animals[MeSH] NOT humans[MeSH])

We identified 244 studies, of which seven met our inclusion criteria.

CFR data for cholera were extracted from an updated systematic review with sources identified using the following PubMed search string capturing sources from January 2016 to October 2024. We identified 354 sources, of which 39 met our inclusion criteria. Additionally, two surveillance data series were updated for India and Malawi.

(Cholera*[TiAb] OR cholera [MeSH] OR "vibrio cholerae" [MeSH]) AND ("case fatality"[TiAb] OR "mortality"[TiAb] OR "cfr"[TiAb]) NOT (animals[MESH] NOT humans[MESH]) ("2016/01/01"[PDAT] : "2024/10/10"[PDAT])

Figure 3. Cholera proportion systematic review PRISMA diagram

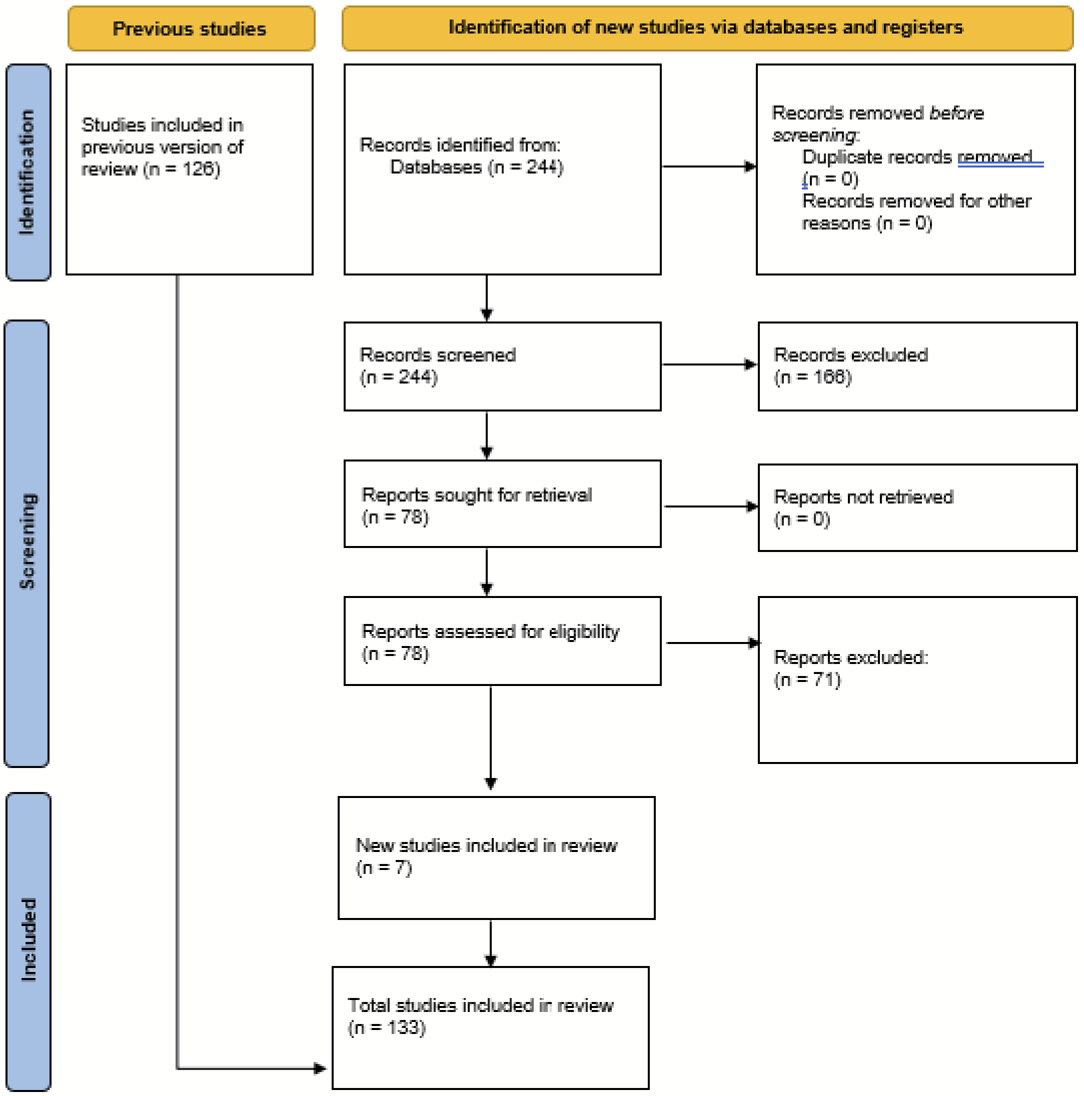

Figure 4. Cholera CFR systematic review PRISMA diagram

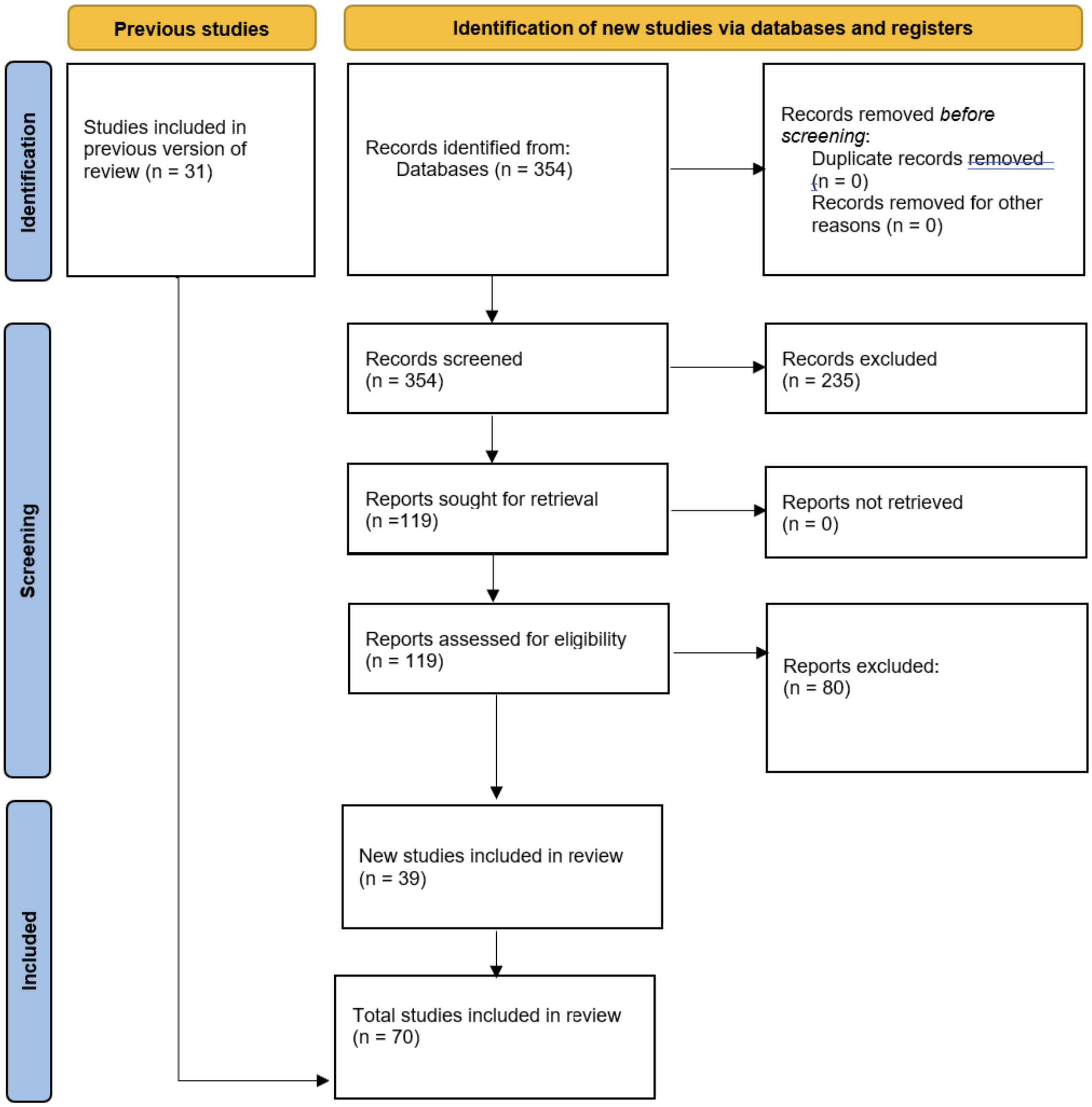

We used the Global Enteric Multicenter Study (GEMS), a seven-site, case-control study of moderate-to-severe diarrhoea in children under 5 years,[2] and the MAL-ED study,[3] a multi-site birth cohort, to calculate odds ratios for the diarrhoeal pathogens. We analysed raw data for a systematic reanalysis, representative of the distribution of cases and controls by age and site that were tested for the presence of pathogen using quantitative polymerase chain reaction (qPCR).[4]

Data that did not use qPCR for detection were adjusted for sensitivity and specificity prior to modelling in order to standardise data regardless of detection method. Adjusting these data prior to modelling allowed us to adjust only data that did not use qPCR, as well as better control for values at extreme bounds and capture uncertainty in modelling.

Case fatality rate (CFR) data for *Clostridium difficile* were collected from ICD-coded hospital records from Austria, Brazil, Canada, Italy, Mexico, New Zealand, and the USA. ICD codes A04.7 (ICD-10) and 008.45 (ICD-9) were used to identify intestinal infections with *Clostridium difficile*. Supplemental data for Romania collected as part of the International Nosocomial Infection Control Consortium (DOI: 10.1016/j.ajic.2016.01.005) were also included. We standardised age and sex across all datasets to the following most-detailed groups using the GBD causes of death age-sex splitting algorithm for age: 0–6, 7–27, and 28–364 days, and 1–4, 5–9, 10–14, 15–19, 20–24, 25–29, 30–34, 35–39, 40–44, 45–49, 50–54, 55–59, 60–64, 65–69, 70–74, 75–79, 80–84, 85–89, 90–94, 95+ years; and sex: male and female. This algorithm is based on the assumption that the age-sex pattern of the death or case rate for a given infectious syndrome or pathogen is inherent to the pathology of the disease and is therefore constant across location and year. Crude case-fatality rates were then estimated for the input data for each GBD age group and sex.

Data crosswalks

One of the GBD core principles is to use all available data to inform our estimates. To account for differences between studies, we conducted a meta-regression of the ratio of reference to non-reference data using the meta-regression—Bayesian, regularised, trimmed (MR-BRT) tool. When possible, crosswalks based on data matched within studies on age, sex, and location are used. When not possible, ratios between alternative and reference case definitions/methods were based on data matched between studies, nearby in age, year, with exact matches on sex and location. We adjusted inpatient data and claims data up to the level of self-reported data (our reference case definition) (table 1).

Table 1. Diarrhoeal disease crosswalk coefficients

Data input	Reference or alternative case definition	Gamma	Beta coefficient, logit (95% UI)*	Adjustment factor (95% UI)**
Self-reported diarrhoea	Ref	--	--	
Clinical and literature, inpatient	Alt	0	–5.96 (–5.99 to –5.92)	0.003 (0.003 to 0.003)
Claims, MarketScan	Alt	0	0.02 (–0.13 to 0.16)	1.02 (0.88 to 1.17)

**MR-BRT crosswalk adjustments can be interpreted as the factor the alternative case definition is adjusted by to reflect what it would have been had it been measured using the reference case definition. If the log/logit beta coefficient is negative, then the alternative is adjusted up to the reference. If the log/logit beta coefficient is positive, then the alternative is adjusted down to the reference.*
***The adjustment factor column is the exponentiated beta coefficient. For log beta coefficients, this is the relative rate between the two case definitions. For logit beta coefficients, this is the relative odds between the two case definitions.*

Age-sex splits

Data were age- and sex-split based on population and a modelled age-curve generated using age-specific data as inputs in MR-BRT to better estimate the distribution of non-age-specific data.

Severity split inputs

Diarrhoeal diseases have three severity levels: mild, moderate, and severe (Table 2). The proportion of diarrhoea cases that are assigned to each comes from a systematic review of diarrhoea severity.[1] Mild cases are the proportion of diarrhoea cases that did not seek medical care (64.8%); moderate cases are the proportion that sought medical care but did not have severe dehydration or bloody stool (28.9%); and severe cases are the proportion that sought medical care with severe dehydration or bloody stool (6.9%). These proportions are based on the frequency of dehydration and bloody stool among community-based studies reported in the systematic review.

Table 2. Severity distribution, details on the severity levels for diarrhoea and the associated disability weight (DW) with that severity.

Severity level	Lay description	Disability weight (95% CI)	Proportion
Mild	Has diarrhoea defined as 3 or more loose stools in a 24-hour period with no dehydration.	0.074 (0.049–0.104)	64.8%
Moderate	Has diarrhoea defined as 3 or more loose stools in a 24-hour period with painful cramps and feeling thirsty and any dehydration.	0.188 (0.125–0.264)	28.9%
Severe	Has diarrhoea defined as 3 or more loose stools in a 24-hour period with painful cramps and is very thirsty or feels nauseated or tired and/or severely dehydrated.	0.247 (0.164–0.348)	6.9%

Modelling strategy

Diarrhoea incidence and prevalence

The non-fatal diarrhoeal disease burden is modelled in DisMod-MR 2.1, a Bayesian meta-regression modelling framework. DisMod-MR produces estimates of the incidence, prevalence, and remission of diarrhoea for each age, sex, geographical location, and year. We defined remission, or the time to recovery, as five days average. The reference category for our input data is community-based diarrhoea episodes such as data from population-representative surveys or community cohorts. As described in the data crosswalks section above, input data that are from a different population, such as hospital inpatient groups, are adjusted before modelling by determining a meta-regression ratio of non-reference to reference data values, so that they are consistent with the reference category. Country-level covariates are used to inform the model (Table 3).

Table 3. Covariates. Summary of covariates used in the diarrhoea DisMod-MR meta-regression model

Covariate	Type	Parameter	Exponentiated beta (95% uncertainty interval)
RotaC vaccine lagged five-year coverage	Country-level	Prevalence	0.51 (0.50–0.52)

Socio-demographic Index	Country-level	Prevalence	0.095 (0.089–0.10)
Sex	Study-level	Prevalence	0.97 (0.97–0.98)
Healthcare Access and Quality Index	Country-level	Excess mortality rate	0.94 (0.94–0.94)
Sex	Study-level	Excess mortality rate	1.33 (1.32–1.34)

Aetiologies

We estimated diarrhoeal disease aetiologies independently from overall diarrhoea envelope using a counterfactual strategy for enteric adenovirus, *Aeromonas*, *Entamoeba histolytica* (amoebiasis), *Campylobacter*, *Cryptosporidium*, enteropathogenic *Escherichia coli* (EPEC), enterotoxigenic *Escherichia coli* (ETEC), norovirus, non-typhoidal *Salmonella* infections, rotavirus, *Shigella*, astrovirus, and sapovirus. *Vibrio cholerae* and *C difficile* were modelled separately (Table 4).

Table 4. Inpatient to community crosswalk coefficients for diarrhoeal disease aetiologies, not including *Vibrio cholerae* or *C difficile*

Aetiology	Data input	Reference or alternative case definition	Gamma	Beta coefficient, logit (95% UI)*	Adjustment factor (95% UI)**
All	Community-based samples	Ref	--	--	--
Adenovirus	Clinical, inpatient	Alt	0.09	0.25 (0.06 to 0.43)	1.28 (1.06 to 1.54)
Aeromonas	Clinical, inpatient	Alt	0.07	0.07 (–0.17 to 0.3)	1.07 (0.85 to 1.35)
Amoebiasis	Clinical, inpatient	Alt	0.82	0.17 (–0.44 to 0.78)	1.18 (0.64 to 2.18)
Astrovirus	Clinical, inpatient	Alt	0	0.15 (0.03 to 0.28)	1.17 (1.03 to 1.32)
Campylobacter	Clinical, inpatient	Alt	0.25	–0.09 (–0.32 to 0.13)	0.91 (0.73 to 1.14)
Cryptosporidium	Clinical, inpatient	Alt	0.05	0.25 (0.11 to 0.38)	1.28 (1.12 to 1.46)
EPEC	Clinical, inpatient	Alt	0.02	0.03 (–0.09 to 0.16)	1.03 (0.91 to 1.17)

ETEC	Clinical, inpatient	Alt	0.00	0.11 (0 to 0.21)	1.11 (1 to 1.24)
Norovirus	Clinical, inpatient	Alt	0.05	0.05 (–0.08 to 0.17)	1.05 (0.92 to 1.19)
Rotavirus	Clinical, inpatient	Alt	0.37	0.67 (0.49 to 0.85)	1.96 (1.64 to 2.34)
Salmonella	Clinical, inpatient	Alt	0.15	0.49 (0.21 to 0.78)	1.64 (1.23 to 2.17)
Sapovirus	Clinical, inpatient	Alt	0.01	–0.01 (–0.13 to 0.12)	0.99 (0.87 to 1.13)
Shigellosis	Clinical, inpatient	Alt	0.37	0.29 (0.03 to 0.56)	1.34 (1.03 to 1.75)

**MR-BRT crosswalk adjustments can be interpreted as the factor the alternative case definition is adjusted by to reflect what it would have been had it been measured using the reference case definition. If the log/logit beta coefficient is negative, then the alternative is adjusted up to the reference. If the log/logit beta coefficient is positive, then the alternative is adjusted down to the reference.*

***The adjustment factor column is the exponentiated beta coefficient. For log beta coefficients, this is the relative rate between the two case definitions. For logit beta coefficients, this is the relative odds between the two case definitions.*

Diarrhoeal aetiologies are attributed to diarrhoeal cases using a counterfactual approach. We calculated a population attributable fraction (PAF) from the proportion of diarrhoea cases that are positive for each aetiology. The PAF represents the relative reduction in diarrhoea burden if there was no exposure to a given aetiology. As diarrhoea can be caused by multiple pathogens and the pathogens may co-infect, PAFs can overlap and are not scaled to sum to 100%. We calculated the PAF from the proportion of diarrhoea cases that are positive for each aetiology. We used the following formula to estimate PAF:[5]

$$PAF = Proportion * (1 - \frac{1}{OR})$$

Where *Proportion* is the proportion of diarrhoea cases positive for an aetiology and *OR* is the odds ratio of diarrhoea given the presence of the pathogen.

We dichotomised the continuous qPCR test result using the value of the cycle threshold (Ct) that most accurately discriminated between cases and controls. The Ct values range from 0 to 35 cycles representing the relative concentration of the target gene in the stool sample. A low value indicates a higher concentration of the pathogen, while a value of 35 indicates the absence of the target in the sample. We used the lower Ct value when we had multiple Ct values for the cut-point. The case definition for each pathogen is a Ct value that is below the established cutoff point (Table 5).

Table 5. Single to multi-pathogen study crosswalk coefficients for diarrhoeal disease aetiologies, not including *Vibrio cholerae* or *C difficile*

Aetiology	Data input	Reference or alternative case definition	Gamma	Beta coefficient, logit (95% UI)*	Adjustment factor (95% UI)**
All	Multi-pathogen studies	Ref	--	--	
Adenovirus	Single pathogen	Alt	0.88	0.08 (–0.99 to 1.14)	1.08 (0.37 to 3.13)
Aeromonas	Single pathogen	Alt	N/A	N/A	NA
Amoebiasis	Single pathogen	Alt	N/A	N/A	NA
Astrovirus	Single pathogen	Alt	0	1.11 (0.66 to 1.56)	3.04 (1.93 to 4.78)
Campylobacter	Single pathogen	Alt	N/A	N/A	N/A
Cryptosporidium	Single pathogen	Alt	0	1.46 (0.88 to 2.04)	4.32 (2.42 to 7.71)
EPEC	Single pathogen	Alt	N/A	N/A	N/A
ETEC	Single pathogen	Alt	0.00	0.15 (0.02 to 0.27)	1.16 (1.02 to 1.31)
Norovirus	Single pathogen	Alt	0.56	0.15 (–0.52 to 0.83)	1.17 (0.59 to 2.29)
Rotavirus	Single pathogen	Alt	0.62	0.15 (–0.08 to 0.37)	1.16 (0.92 to 1.45)
Salmonella	Single pathogen	Alt	0.00	0.32 (0.07 to 0.56)	1.37 (1.07 to 1.76)
Sapovirus	Single pathogen	Alt	0.00	0.18 (–0.24 to 0.61)	1.2 (0.78 to 1.85)
Shigellosis	Single pathogen	Alt	0.00	0.42 (0.33 to 0.52)	1.53 (1.39 to 1.68)

**MR-BRT crosswalk adjustments can be interpreted as the factor the alternative case definition is adjusted by to reflect what it would have been had it been measured using the reference case definition. If the log/logit beta*

coefficient is negative, then the alternative is adjusted up to the reference. If the log/logit beta coefficient is positive, then the alternative is adjusted down to the reference.
***The adjustment factor column is the exponentiated beta coefficient. For log beta coefficients, this is the relative rate between the two case definitions. For logit beta coefficients, this is the relative odds between the two case definitions.*

We used a generalised linear mixed effects logistic regression model to calculate the odds ratio for under 1 year and 1–2 years old for each of our pathogens from the MAL-ED study. The MAL-ED study was used exclusively because the samples tested from that study are community-based samples, which we determined were more representative of non-fatal diarrhoea than the GEMS samples, which tested only moderate-to-severe diarrhoea. The odds ratio for 1–2 years was applied to all GBD age groups over 5 years. There were three pathogen-age odds ratios that were not statistically significant: *Aeromonas* and amoebiasis in under 1 year and *Campylobacter* in 1–2 years. If the odds ratio was not statistically significant, we transformed the odds ratios only for those aetiologies in log-space such that exponentiated values could not be below 1. The transformation was:

$$Odds\ ratio = exp(log(OR) - 1)) + 1$$

We modelled the proportion data using the Bayesian meta-regression tool DisMod-MR to estimate the proportion of positive diarrhoea cases for each separate aetiology by location/year/age/sex and to adjust for the covariates. We used the estimated sensitivity and specificity of the original laboratory diagnostic test results from the pooled GEMS and MAL-ED qPCR stool samples compared to the qPCR test result to adjust our proportion before we modelled the proportions:[6]

$$Proportion_{True} = \frac{(Proportion_{Observed} + Specificity - 1)}{(Sensitivity + Specificity - 1)}$$

We used this correction to account for the fact that the proportions we used are based on a new test that is not consistent with the laboratory-based case definition (qPCR versus GEMS conventional laboratory testing for pathogens).[7] Because of differences in the type of PCR used in the original (non-reference qPCR diagnostic) between GEMS and MAL-ED in detecting norovirus, we combined the sensitivity and specificity results for norovirus such that 50% of the draws were coming from GEMS test results exclusively, and 50% of the draws were coming from MAL-ED test results exclusively. Additionally, because the original laboratory diagnostic technique used for *Campylobacter* in MAL-ED was one not commonly used, we only used GEMS to determine the sensitivity and specificity of bacterial culture compared to qPCR in detecting *Campylobacter*.[8]

In order to be consistent with the odds ratios that we obtained, we adjusted our proportion estimates of any EPEC to typical EPEC only. This adjustment was informed by all available data that reported both atypical and typical EPEC. We applied the same approach to differentiate between heat-stable toxin (ST) and heat-labile toxin-producing (LT) ETEC. For the first time, GBD 2019 split these serotypes so that estimates from GBD 2019 onward represent the diarrhoeal disease burden attributable to ST-ETEC. This was based on work showing that ST-ETEC was much more pathogenic than LT-ETEC. As some of our proportion data were extracted for any ETEC, we determined a proportion of all ETEC that produced ST from all available data reporting both ETEC and ST-ETEC and applied that ratio to data representing all

ETEC so that they represented ST-ETEC only. For cholera, we used the literature review to estimate the expected number of cholera cases for each country-year using the incidence of diarrhoea (estimated using DisMod-MR) and the proportion of diarrhoea cases that are positive for cholera. We assigned cholera PAF using odds ratios from the qPCR results to estimate a number of cholera-attributable cases. We compared this expected number of cholera cases to the number reported to WHO at the country-year level.[9] We modelled the under-reporting fraction to correct the cholera case notification data for all countries using health system access and the diarrhoea SEV scalar to predict total cholera cases. We used the age-specific proportion of positive cholera samples in DisMod-MR and our incidence estimates to predict the number of cholera cases for each age/sex/year/location. Finally, we modelled the case-fatality ratio of cholera using DisMod-MR to estimate the number of cholera deaths.

For *C difficile*, we modelled incidence data identified via systematic review and excess mortality estimates in DisMod-MR 2.1. Excess mortality rates (EMRs) were computed based on case-fatality rates by age from hospital data and duration using the following equation:

$$EMR = -\ln(1 - CFR)/duration$$

Duration was assumed to be 1.0 month (0.3–1.7).

C difficile CFRs were calculated using MR-BRT, a meta-analytic mixed effects structure. The main model can be specified as follows:

$$logit(y_i) = X_i\beta + u_i 1 + \epsilon_i, \qquad \epsilon_i \sim N(0, \Sigma_i), \quad u_i \sim N(0, \gamma)$$

where

- y_i contains CFRs for data source i
- Design matrix X_i contains as columns the following covariates
  - HAQ Index
  - dummy-coded indicator for age group
    - neonatal–5 years, 5–50 years, 50–70 years, and 70 years and older
  - dummy-coded ICU indicator for data source
    - 1 if data source only compiles information on ICU patients, 0 if a mix between ICU/non-ICU patients
  - dummy-coded indicator for pathogen
- β are fixed effect multipliers
- ϵ_i are observation error terms with known variances
- u_i are data source-specific random intercepts with unknown covariance γ

We also implemented a prior on γ, the data source random effect. Many input data sources cover only a single country, leading to low variability in HAQ Index within each data source. Such collinearity adversely influenced the accuracy of the estimated effect of HAQ Index, which was instrumental in extrapolating trends from the input data to global results. To emphasise the contribution of HAQ Index over data source in the modelled estimates, we implemented a strong Gaussian prior (mean 0, standard error 0.001) on γ. Predictions for *Clostridium difficile* CFRs were generated for each country and age group as a function of each country's HAQ Index, assuming mixed ICU/non-ICU patients.

For rotavirus, we explicitly accounted for rotavirus vaccine efficacy when estimating attributable fraction. The impact of the rotavirus vaccine is dependent on modelled vaccine coverage for a location-year and on the rotavirus vaccine efficacy (VE). Numerous studies demonstrate a difference in VE by national income and development.[10] We used a meta-regression with SDI as covariate to predict the rotavirus VE by location and year.

Starting from GBD 2019, we explicitly incorporated the results from our analysis of VE to produce more robust estimates of the proportion of diarrhoea that has rotavirus over time and space. We assumed that the impact of the vaccine can be represented as 1 minus the product of the estimated vaccine coverage and VE.

$$Vaccine\ impact = 1 - vaccine\ coverage * vaccine\ efficacy$$

Both of these values vary in time and space but not by age. To avoid discontinuities in our DisMod model, we adjusted the input proportion data to remove the impact of the rotavirus vaccine by dividing the observed proportion by the vaccine impact.

$$Rotavirus\ proportion_{Adjusted} = \frac{Rotavirus\ proportion}{1 - Cov_{RotaV} * VE_{Modeled}}$$

The result from DisMod is the modelled proportion of diarrhoea positive for rotavirus in the absence of the vaccine. This modelled value is then multiplied by the impact of the rotavirus vaccine to determine the estimated proportion of diarrhoea positive for rotavirus in the presence of the vaccine. Our modified attributable fraction is then:

$$DisModPAF = Modeled\ Proportion\ (from\ DisMod) * \left(1 - \frac{1}{OR}\right)$$

The last step is to account for the expected impact of the rotavirus vaccine. We do this using the equation below:

$$PAF_{Rota} = DisModPAF * \frac{(1 - Cov_{RotaV} * VE_{Modeled})}{(1 - DisModPAF * Cov_{RotaV} * VE_{Modeled})}$$

Where the final attributable fraction for rotavirus is the product of the PAF estimated in DisMod-MR and the expected reduction in that PAF given modelled vaccine coverage and modelled VE by location-year, and this value is only applied to children 28 days to 5 years old. The product of the rotavirus attributable fraction and the number of deaths or cases of diarrhoea is the number of deaths and cases caused by rotavirus.

References

1 Lamberti LM, Fischer Walker CL, Black RE. Systematic review of diarrhea duration and severity in children and adults in low- and middle-income countries. *BMC Public Health* 2012; **12**: 276.

2 Kotloff KL, Nataro JP, Blackwelder WC, *et al.* Burden and aetiology of diarrhoeal disease in infants and young children in developing countries (the Global Enteric Multicenter Study, GEMS): a prospective, case-control study. *Lancet* 2013; **382**: 209–22.

3 Platts-Mills JA, Liu J, Rogawski ET, *et al.* Use of quantitative molecular diagnostic methods to assess the aetiology, burden, and clinical characteristics of diarrhoea in children in low-resource settings: a reanalysis of the MAL-ED cohort study. *Lancet Glob Health* 2018; **6**: e1309–18.

4 Liu J, Gratz J, Amour C, *et al.* A Laboratory-Developed TaqMan Array Card for Simultaneous Detection of 19 Enteropathogens. *J Clin Microbiol* 2013; **51**: 472–80.

5 Miettinen OS. Proportion of disease caused or prevented by a given exposure, trait or intervention. *Am J Epidemiol* 1974; **99**: 325–32.

6 Reiczigel J, Földi J, Ozsvári L. Exact confidence limits for prevalence of a disease with an imperfect diagnostic test. *Epidemiol Infect* 2010; **138**: 1674–8.

7 Platts-Mills JA, Operario DJ, Houpt ER. Molecular diagnosis of diarrhea: current status and future potential. *Curr Infect Dis Rep* 2012; **14**: 41–6.

8 Platts-Mills JA, Liu J, Gratz J, *et al.* Detection of Campylobacter in stool and determination of significance by culture, enzyme immunoassay, and PCR in developing countries. *J Clin Microbiol* 2014; **52**: 1074–80.

9 World Health Organization. Global Health Observatory data repository: Cholera. 2016. http://apps.who.int/gho/data/node.main.174?lang=en (accessed Aug 25, 2016).

10 Lamberti LM, Ashraf S, Walker CLF, Black RE. A Systematic Review of the Effect of Rotavirus Vaccination on Diarrhea Outcomes Among Children Younger Than 5 Years. *Pediatr Infect Dis J* 2016; **35**: 992–8.

Diphtheria

Flowchart

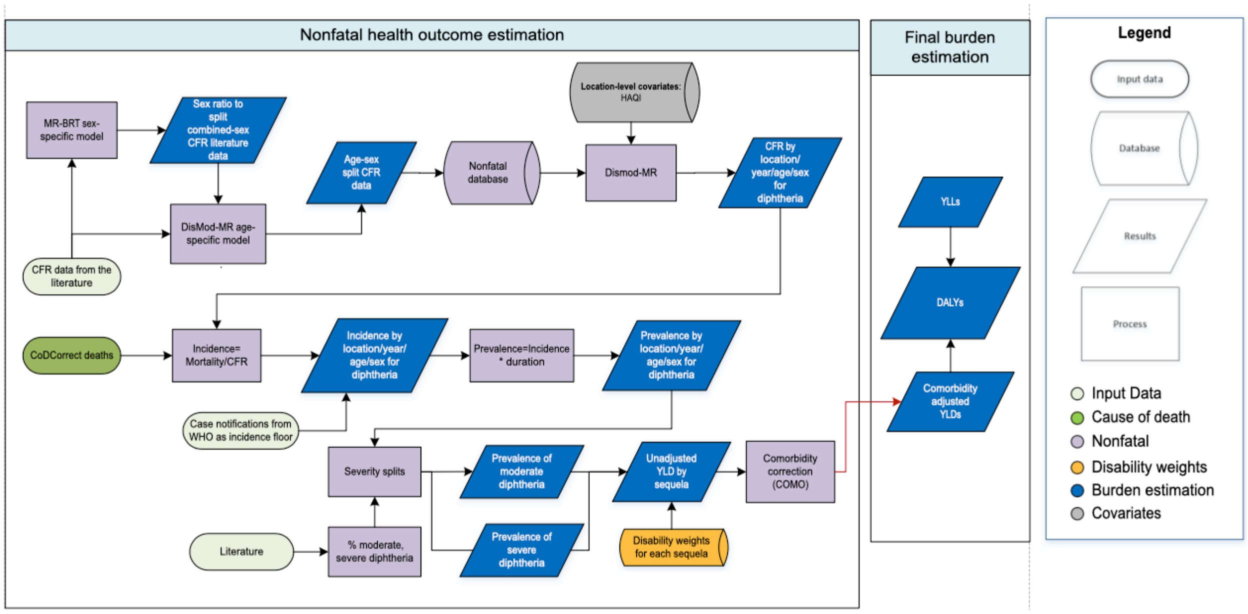

Input data and methodological summary for diphtheria

Case definition

Diphtheria is a disease of the respiratory tract caused by the bacterial pathogen *Corynebacterium diphtheriae*. Typical manifestations include fever, purulent pharyngeal discharge, and pseudomembrane formation along the respiratory tract. Toxigenic strains of *C diphtheriae* may lead to cardiac and neurological complications associated with an elevated risk of death. For diphtheria, ICD-10 codes are A36-A36.9, Z22.2, Z23.6, and ICD-9 codes are 032-032.9, V02.4, V03.5, and V74.3.

**Quantity of interest**	**Reference or alternative**	**Definition**
Diphtheria case-fatality ratio	Reference	Ratio of fatal cases of diphtheria to total confirmed cases of diphtheria in the sample

Input data

Model inputs

The non-fatal diphtheria model has two primary inputs. The first is data obtained from systematic reviews of the diphtheria case-fatality ratio (CFR) literature. The second is GBD mortality estimates of diphtheria, calculated per country by either Cause of Death Ensemble modelling (CODEm) or a negative binomial regression model. New in GBD 2023, we used case notifications from the World Health Organization (WHO) Joint Reporting Form (JRF) to create a case notification floor such that our non-fatal estimates always equal or exceed notified cases.

The diphtheria CFR systematic review was updated in GBD 2023. New data were added to existing sources from earlier systematic reviews, conducted approximately every three years. For GBD 2023, the search terms used in PubMed were: (diphtheria [MeSH Terms] OR diphtheria [TiAb]) AND (mortality[MeSH Terms] OR mortality [TiAb] OR "case fatality rate" [TiAb] OR "case fatality ratio" [TiAb] OR "CFR" [TiAb] OR death*[TiAb] OR died [TiAb] OR die [TiAb]) AND (1980[PDAT]: 3000[PDAT]*)*. Data were excluded if they were missing information about diphtheria cases and deaths, included data from non-representative populations, reported on non-respiratory diphtheria, or were limited to specific clinical manifestations or severities. New in GBD 2023, we assessed all citations for the completeness of diphtheria case ascertainment and determined the cases and deaths reporting sources. Studies were only included in the model if they met our criteria for complete case ascertainment or used population-representative reporting of cases and deaths.

Input data processing

Scant sex- and age-specific diphtheria CFR data are currently available, precluding the estimation of location- or year-specific sex and age patterns. Instead, a global sex ratio and age pattern were generated using all available sex- and age-specific diphtheria CFR data. The global sex ratio and age pattern were then used to split non-sex- or -age-specific CFR data prior to inclusion in the final CFR model. Uncertainty was propagated in the splitting process.

The sex ratio was modelled using meta-regression—Bayesian, regularised, trimmed (MR-BRT) a Bayesian meta-regression tool. For studies that included CFR data that were separate sex- and age-specific data, the within-study sex ratio was used to split the age-specific data rather than the global sex ratio. The female/male sex adjustment factor calculated for use in GBD 2023 modelling was 1.38 (CI 1.15–1.66), compared to 0.849 in GBD 2021. The change in the female to male ratio was driven primarily by changes to inclusion criteria in the CFR review, as discussed above.

Table 1: MR-BRT sex-splitting adjustment factor for diphtheria CFR

**Data input**	**Reference or alternative case definition**	**Beta coefficient, log (95% CI)**	**Adjustment factor* (95 % CI)**
Sex (female/male)	N/A	0.324 (0.506, 0.141)	1.382 (1.151, 1.658)

**MR-BRT crosswalk adjustments can be interpreted as the factor the alternative case definition is adjusted by to reflect what it would have been had it been measured using the reference case definition. If the log/logit beta coefficient is negative, then the alternative is adjusted up to the reference. If the log/logit beta coefficient is positive, then the alternative is adjusted down to the reference.*

***The adjustment factor column is the exponentiated beta coefficient. For log beta coefficients, this is the relative rate between the two case definitions. For logit beta coefficients, this is the relative odds between the two case definitions.*

To generate a global age pattern, diphtheria CFR data representing age range widths of ten years or less were used to fit a DisMod-MR model. The Healthcare Access and Quality (HAQ) Index was included as a

location-level covariate. The final global age pattern – produced by DisMod for each sex and age group from early neonatal to 95+ years – was used to split the remaining data.

Figure 1. CFR global age pattern for diphtheria CFR (L: male, R: female)

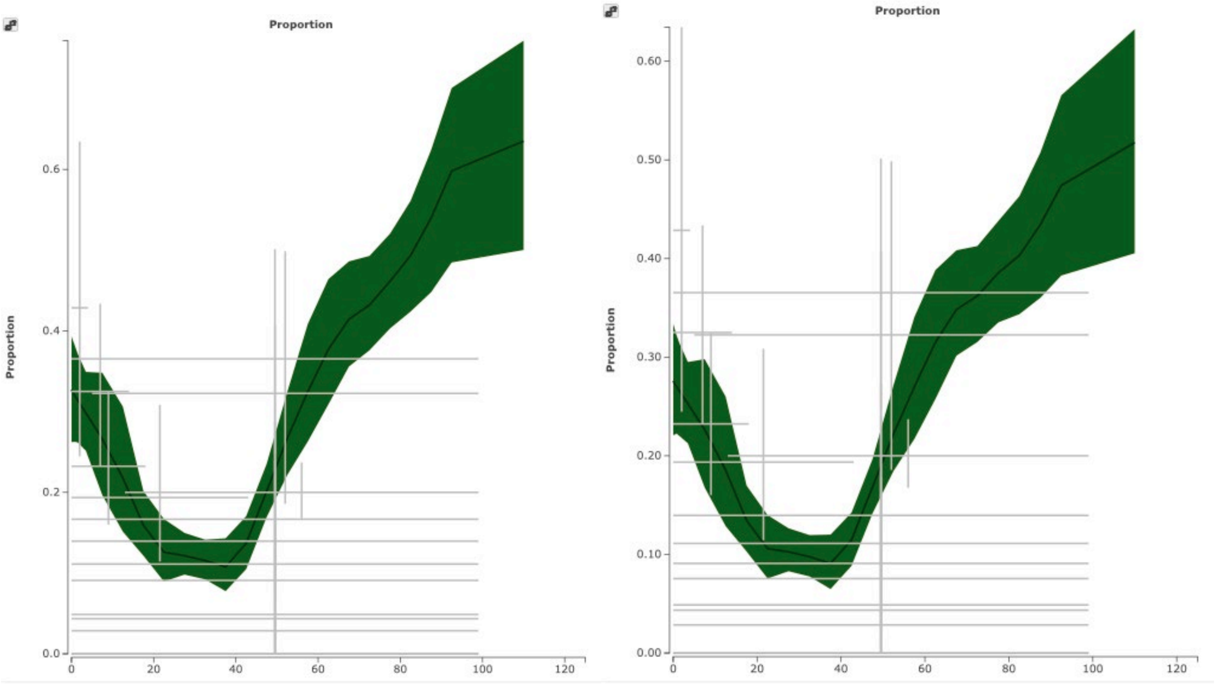

Modelling strategy

We used DisMod-MR to produce location-, year-, age-, and sex-specific diphtheria CFR estimates from our sex- and age-specific input data. We used the HAQ Index as a location-level covariate, enforcing a directional prior so locations with higher HAQ Index are predicted to have lower CFR.

Table 3 displays the raw and exponentiated magnitudes of covariate influence, which can be interpreted as odds ratios. In GBD 2021, the exponentiated beta coefficient for HAQ Index was 0.95; in GBD 2023, the exponentiated coefficient was 0.45, suggesting a much greater effect of HAQ Index on CFR patterns. In previous GBD rounds, CFR estimates were primarily driven by super-regional effects; with the addition of new data and the exclusion of non-representative studies, the GBD 2023 model estimates much greater within-region variability.

Incidence was calculated as the mortality rate divided by the case-fatality ratio. The diphtheria mortality rate was modelled using either CODEm or a negative binomial regression (see diphtheria in Cause of Death appendix). For location-years where modelled incidence was lower than JRF case notifications, we replaced modelled estimates with case notifications and calculated uncertainty intervals by sampling

from a posterior distribution centered around this mean. Prevalence was calculated as the product of incidence and case duration, with an estimated mean of 27.5 days.

For all countries, we produced estimates for all age groups between post-neonatal and 59 years. Calculations with 1000 samples (draws) were taken from the posterior distribution to propagate uncertainty throughout the modelling process. Draw-level estimates were then summarised as means and 95% uncertainty intervals (2.5th and 97.5th percentiles of all draws).

Severity split and disability weights

Non-fatal diphtheria case estimates were split by severity according to distributions derived from literature reviews. 70% (95% CI: 66.5–73.5) of cases are presumed moderate, and the remaining 30% (95% CI: 26.5–33.5) severe. Table 2 provides severity level descriptions in addition to these weights.

Table 2. Severity distribution, details on the severity levels for diphtheria in GBD 2023 and the associated disability weight (DW) with that severity

**Severity level**	**Lay description**	**DW (95% CI)**
Moderate diphtheria	Has a fever and aches, and feels weak, which causes some difficulty with daily activities.	0.051 (0.032–0.074)
Severe diphtheria	Has a high fever and pain, and feels very weak, which causes great difficulty with daily activities.	0.133 (0.088–0.19)

Table 3. Covariates. Summary of covariates used in the diphtheria CFR DisMod-MR meta-regression model

**Covariate**	**Type**	**Parameter**	**Exponentiated beta (95% uncertainty interval)**
Healthcare Access and Quality (HAQ) Index	Country-level	Case fatality ratio	0.41 (0.33–0.49)

Changes from GBD 2021 to GBD 2023

In GBD 2023, two major modelling changes were implemented since the previous round. First, our CFR model only included data from population-level studies and those determined to have complete case ascertainment. This led to far lower CFR estimates compared to previous rounds. Second, we used WHO case notification data as a floor on incidence estimates, assuring that incidence predictions always equal or exceed notified cases.

Figure 4: PRISMA 2020 flow diagram for diphtheria CFR

From: Page MJ, McKenzie JE, Bossuyt PM, et al. The PRISMA 2020 statement: an updated guideline for reporting systematic reviews. *BMJ*. 2021;372:n71. Published 2021 Mar 29. doi:10.1136/bmj.n71

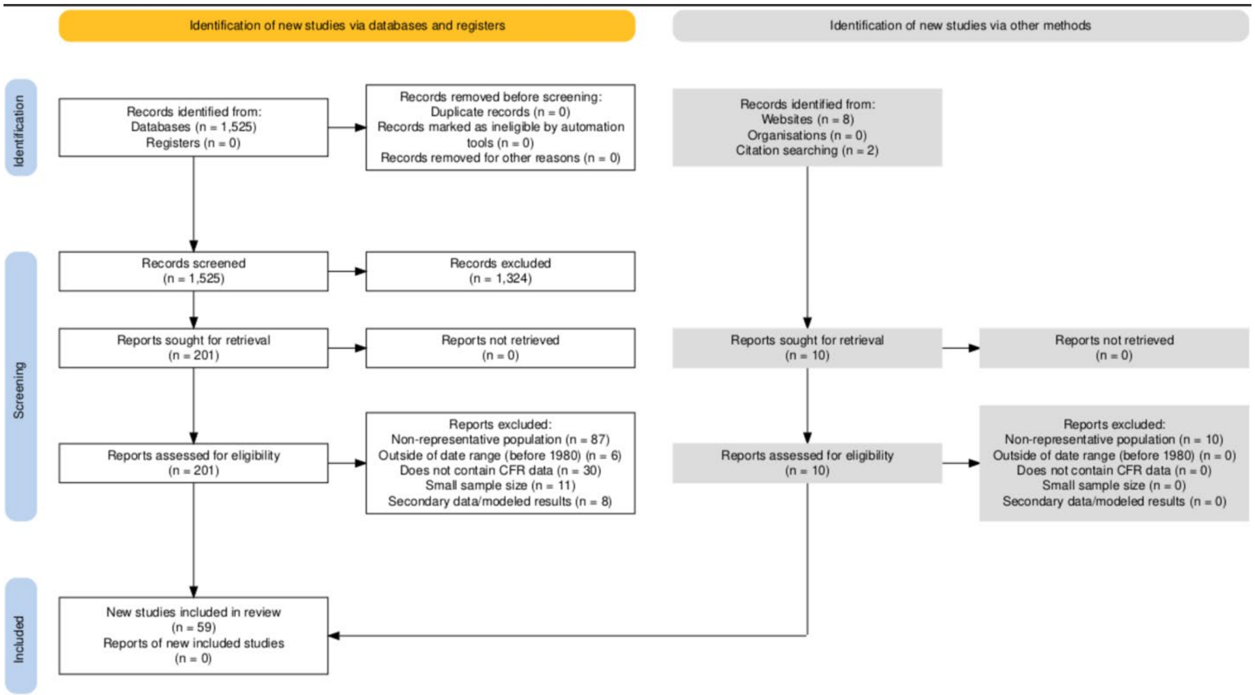

Diverticular disease

Flowchart

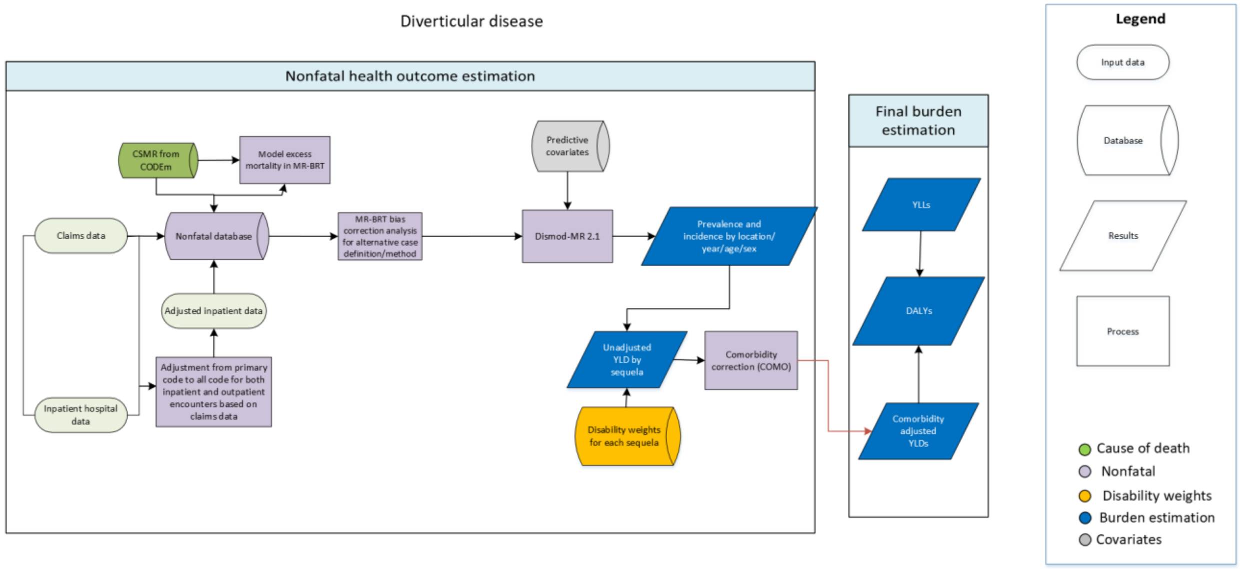

Input data and methodological summary for diverticular disease

Case definition

Diverticular disease encompasses a spectrum of conditions related to colonic diverticula, ranging from asymptomatic diverticulosis to symptomatic diverticulitis and its complications. Clinically significant cases typically present with abdominal pain, gastrointestinal bleeding, or sepsis, with radiographic or colonoscopic evidence of colonic diverticulosis or diverticulitis as the underlying source. In general population studies, diverticulosis is often identified incidentally through radiographic, colonoscopic, or post-mortem examination, even in asymptomatic individuals. In administrative data, diverticular disease is primarily captured through ICD codes. The ICD-10 codes included for diverticular disease are K57.10-K57.13, K57.2, K57.20, K57.21, K57.3, K57.30-K57.33, K57.4, K57.40-K57.41, K57.5, K57.50-K57.53, K57.8, K57.80-K57.81, K57.9, K57.90-K57.93, covering both diverticulosis and diverticulitis with or without complications.

**Quantity of interest**	**Reference or alternative**	**Definition**
Prevalence of diverticular disease	Alternative	Cases identified from database of commercial claims from USA in 2010-2019 using ICD codes.
Prevalence of diverticular disease	Alternative	Cases identified from database of commercial claims from USA in 2000 using ICD codes.
Prevalence of diverticular disease	Reference	Cases of appendicitis identified using ICD codes in administrative records that are considered population-representative.

Input data

Inputs

The model included prevalence data from hospital discharges and claims. See the section of this appendix on hospital and claims data for details of the IHME database and extraction and processing methods. For this cause, in particular, we excluded from our analysis any hospital inpatient datasets that did not cover the entire target population, thus requiring extraction as discharge diagnosis cause fractions and conversion to total prevalence of the disease using estimates of hospital admission rate per capita. We also excluded US MarketScan data for individuals aged 65 and older, retaining USA CMS data as the sole source for the USA in these age groups.

Inputs to our non-fatal modelling also included cause-specific mortality rate (CSMR) estimates taken from our fatal modelling process (see CoD cause-specific modelling description for diverticular disease in this appendix).

Prevalence input processing

Claims data generally link claims for all inpatient and outpatient encounters for a single individual and provide primary and secondary diagnoses for all encounters. Hospital discharge data, on the other hand, provide observations about encounters, generally with only the primary diagnostic code for the encounter.

Individuals were extracted from claims sources as prevalent cases if they had at least one inpatient or two outpatient encounters with an appropriate ICD code. Enrollees in the same year and location who were of the same age group and sex were counted in the prevalence denominator.

For hospital data, a discharge was extracted if an appropriate ICD code appeared as the primary discharge diagnosis, and the population of the catchment area was applied to generate a primary discharge rate.

We then applied correction factors 1 and 5 (CF1 and CF5). CF1 is defined as the ratio of deduplicated primary diagnoses among inpatient admissions to adjusted primary diagnoses among inpatient admissions, while CF5 is defined as the ratio of deduplicated primary and secondary diagnoses among inpatient encounters and outpatient encounters to deduplicated primary diagnoses among inpatient encounters. This approach accounts for non-primary inpatient diagnoses and outpatient cases, and does so serially to allow data sources to contribute to estimation of de-duplication for readmission, multiple diagnoses in inpatient encounters, and/or outpatient care, depending on which of these relationships are captured in the dataset, rather than only employing sources that have complete information on all diagnoses and encounter types. This approach makes use of more diverse data inputs. This final scalar is applied to extracted primary diagnosis admission rate data as a product of CF1 and 1/CF5. See the section of this appendix on the processing of hospital data for more details.

USA MarketScan data (extracted and processed as claims data per above) were adjusted to account for selection bias due to commercial insurance using MR-BRT analysis. We used age as an additional covariate to estimate bias adjustment factors. (Note that per above, MarketScan data were only employed for individuals under age 65 years; CMS claims were used for those 65 years and older and did not require adjustment for selection bias.)

The process of adjusting for biases in non-reference data using MR-BRT with the logit-transformation method is described below:

1. Identify datapoints with overlapping year, age, sex, and location between reference and non-reference population data.
2. Logit transform overlapping datapoints of alternative and reference types.
3. Convert overlapping datapoints into a difference in logit space using the following equation: $logit(altnerative) - logit(reference)$
4. Use the delta method to compute standard errors of overlapping datapoints in logit space, then calculate standard error of logit difference using the following equation: $\sqrt{(variance\ of\ logit(alternative)) + (variance\ of\ logit(reference))}$
5. Using MR-BRT, conduct a random effects meta-regression to obtain the pooled logit difference of alternative to reference
6. Apply the pooled logit difference to all data points of non-reference types using the following equation:
$$new_{estimate} = inverse.logit((logit(alternative)) - (pooled\ logit\ difference))$$
7. Calculate new standard errors using the delta method, accounting for gamma (between-study heterogeneity)

Table 1: MR-BRT crosswalk adjustment factors for diverticular disease

Data input	Reference or alternative data collection	Gamma	Covariate	Beta coefficient, logit (95% UI)*	Adjustment factor**
Hospital + non-USA claims	Ref	---	---	---	---
USA claims from year 2000	Alt	0	Age (continuous from 0 to 95+)	0.03 (0.024 to 0.038)	1.031 (1.024to 1.038)
			Sex (female to male)	0.05 (–0.056 to 0.16)	1.052 (0.946 to 1.171)
			Intercept	–2.09 (–2.47 to –1.71)	0.124 (0.084 to 0.181)
USA claims from years 2010–2019	Alt	0.015	Age (continuous from 0 to 95+)	0.005 (0.0039 to 0.0059)	1.0005 (0.976 to 1.035)
			Sex (female to male)	0.13(0.11 to 0.15)	1.140 (1.101 to 1.181)
			Intercept	–0.20 (-0.28 to –0.12)	0.820 (0.751 to 0.896)

**MR-BRT crosswalk adjustments can be interpreted as the factor the alternative case definition is adjusted by to reflect what it would have been had it been measured using the reference case definition. If the log/logit beta*

coefficient is negative, then the alternative is adjusted up to the reference. If the log/logit beta coefficient is positive, then the alternative is adjusted down to the reference.
***The adjustment factor column is the exponentiated beta coefficient. For log beta coefficients, this is the relative rate between the two case definitions. For logit beta coefficients, this is the relative odds between the two case definitions.*

Modelling strategy

DisMod model

We ran a DisMod-MR 2.1 model to produce estimates by age, sex, year, and location. Inputs to DisMod for diverticular disease include prevalence, CSMR, and EMR inputs processed as described above. A prior value was set on remission so that all cases remit within two weeks. The minimum coefficient of variation at the regional, super-regional, and global level was set at 0.8. No predictive covariates were employed.

Severity split and disability weight

The basis of the GBD disability weight survey assessments are lay descriptions of sequelae highlighting major functional consequences and symptoms.[1] The lay descriptions and disability weights for diverticular disease are shown below. The method for determining how many prevalent cases of diverticular disease experience each severity level at a given moment in time was the SF-12-based condition-specific marginal disability weight distribution method,[2] which is described elsewhere in this appendix.

Table 2. Severity distribution, details on the severity levels for diverticular disease and the associated disability weight (DW) with that severity

Severity level	Lay description	DW (95% CI)	Distribution
Asymptomatic	--	0	0.559 (0.556–0.563)
Abdominopelvic problem, mild	has some pain in the belly that causes nausea but does not interfere with daily activities.	0.011 (0.005–0.021)	0.160 (0.128–0.198)
Abdominopelvic problem, moderate	has pain in the belly and feels nauseous. The person has difficulties with daily activities.	0.114 (0.078–0.159)	0.145 (0.121–0.162)
Abdominopelvic problem, severe	has severe pain in the belly and feels nauseous. The person is anxious and unable to carry out daily activities.	0.324 (0.220–0.442)	0.136 (0.108–0.166)

References

1. Salomon JA, Vos T, Hogan DR, et al. Common values in assessing health outcomes from disease and injury: disability weights measurement study for the Global Burden of Disease Study 2010. *The Lancet*. 2012;380(9859):2129-2143. doi:10.1016/S0140-6736(12)61680-8

2. Burstein R, Fleming T, Haagsma J, Salomon JA, Vos T, Murray CJL. Estimating distributions of health state severity for the global burden of disease study. *Popul Health Metr*. 2015;13(1):31. doi:10.1186/s12963-015-0064-y

Dracunculiasis (Guinea worm)

Flowchart

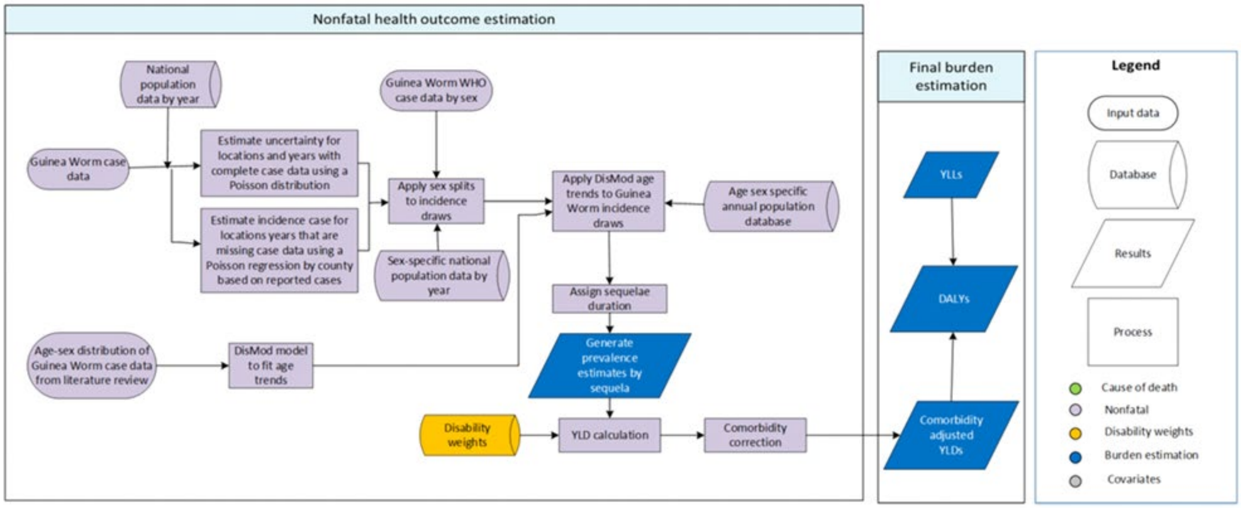

Input data and methodological summary for Guinea worm

Background

Guinea worm disease is caused by the parasitic worm *Dracunculus medinensis*. The transmission cycle begins when Guinea worm larvae are released in stagnant water (eg, ponds, lakes, open wells), where they are ingested by freshwater copepods (small crustaceans sometimes called water fleas) of the genus *Cyclops.*[1] When a person consumes water containing *Cyclops*, the copepods are dissolved by gastric acids and intestinal enzymes and the larvae are released. Larvae then migrate through the intestinal wall and travel to the connective tissues. The larvae mature and mate 60–90 days after infection; shortly thereafter, the male dies and the fertilized female worm continues to move through the victim's connective tissues. Approximately 10–14 months post-infection, the adult worm creates a painful burning blister on the skin that develops and enlarges over several days, usually from the feet or lower limbs. Blister formation may be preceded by a slight fever, itchy rash, nausea, vomiting, and diarrhoea. To relieve the pain associated with the worm's emergence, infected persons immerse the infected part of their body in local stagnant water sources, such as ponds. Upon entering the water, the female worm will expel her larvae and the cycle can begin again.[1-4]

The global campaign to eradicate Guinea worm began in 1980, when the US Centers for Disease Control and Prevention (CDC) suggested that Guinea worm eradication would be an ideal indicator of the success of the International Drinking Water Supply and Sanitation Decade of 1981–1990; in 1981, Guinea worm eradication was adopted as a sub-goal of this United Nations advocacy effort.[1,5] In 1986, the World Health Assembly adopted a resolution to eliminate Guinea worm disease, and since then, the Carter Center has led a coalition that includes ministries of health of endemic countries, CDC, the World Health Organization (WHO), the United Nations Children's Fund (UNICEF), thousands of village volunteers, and supervisory staff, supported by numerous donors.[5]

To break the cycle of transmission, ministries of health in endemic countries implement a suite of interventions: case detection and containment, provision of safe water sources, distribution of filter cloths and pipe filters, water source treatment with Abate® (a larvacide), and health education.

By design, the Guinea worm eradication programmatic infrastructure covers the entire at-risk population in endemic countries. Since case containment[6] is a key intervention designed to not only interrupt transmission but also monitor progress toward eradication, incident cases of Guinea worm disease are nationally representative. To implement case containment as an intervention, all cases of Guinea worm disease are identified. Containment is defined as detection within 24 hours of the worm's emergence; the patient did not contaminate any water source; the patient received proper wound care and health education on not entering any water source; a supervisor verified the case as dracunculiasis within seven days; and Abate® is used if there is any uncertainty about contamination of water sources or known contamination of water sources.[7] Case reporting occurs at the village level on a monthly basis; case data are then aggregated within the national Guinea Worm Eradication Program and reported to WHO. In settings where annual case reports are low (suggesting no transmission) or transmission has been interrupted, cash rewards are promoted to enhance surveillance activities.

Case definition

A Guinea worm case is defined as an individual with Guinea worm disease. A person is counted as a case only once in a calendar year, ie, when the first Guinea worm emerged from that person, although an individual may have more than one worm emerge at a time and/or more than one worm emerge during the year. These cases are confirmed through the Guinea Worm Eradication Program infrastructure by clinical exam and verification by local supervisors. All specimens from case-patients are sent to the CDC for laboratory evaluation and confirmation.[7] We used the following case definition for GBD 2023:

Quantity of interest	Reference or alternative	Definition
Guinea worm	Reference	An incident case is defined as a person exhibiting a skin lesion with emergence of a Guinea worm at least once in a calendar year. National disease programmes confirm number of reported cases by clinical exam and verification by local supervisors. In recent years, specimens from case-patients are sent to the US Centers for Disease Control and Prevention (CDC) for laboratory evaluation and confirmation.

Input data

Data sources

1) Case data by geography, by year
2) Literature review of age/sex distribution
3) Literature review for sequelae (type, duration, and proportion)

Case data: Annual case data were reported by WHO in the Weekly Epidemiological Record (WER) for the period 1990–2017. For years or geographies for which WHO WER reports were not published, the following sources were also used to extract case counts:

1) CDC's Morbidity and Mortality Weekly Reports

2) 1990–1999 total country reports[8]
3) India subnational estimates: India MOH report (1984–1999)
4) The Carter Center's Guinea worm wrap-up: disaggregation of case totals for Sudan and South Sudan pre-2011 (independence) to ensure case totals from 1990–2010 are consistent with current national boundaries; 2018 thru 2023 provisional case data.

Subnational data

India: Subnational data for India were obtained from the Ministry of Health (MOH) for the period 1984–1999; cases were reported by year and state.

Kenya: Subnational data from Kenya were requested from the MOH but not obtained. To split cases by subnational unit, the Carter Center Guinea worm wrap-up was reviewed to identify districts with endemic villages. A national survey conducted in 1993/1994 found cases in Turkana and West Pokot counties, but case totals were not reported by county. Indigenous transmission was interrupted in 1995, with imported cases reported until 2005. WER reports from 1999 to 2006 document that all imported cases from 1998 to 2005 occurred in Turkana County. All cases in Kenya are currently analysed in GBD as occurring in Turkana County as we are unable to disaggregate the data.

Ethiopia: Subnational data for the Ethiopian state of Gambella were obtained from country reports and WHO reports covering the period of 1990–2019[8] and Carter Center Guinea worm wrap-up for remaining years.

Pakistan: Subnational data for endemic Pakistan provinces were obtained from country reports for the period 1988–1994; cases were reported by year and state.[8]

Nigeria: Subnational data for Nigeria were obtained from the Carter Center for the period 1987–2008; cases were reported by year and state.

Geographical restrictions

Only the following countries were identified as Guinea-worm endemic as of 1990[8]: Benin, Burkina Faso, Cameroon, Central African Republic, Chad, Côte d'Ivoire, Ethiopia, Ghana, India, Kenya, Mali, Mauritania, Niger, Nigeria, Pakistan, Senegal, Sudan, South Sudan, Togo, Uganda, and Yemen. Any country not reporting Guinea worm as of 1990 was not included in the GBD model.

Geographical restrictions by year were also implemented to account for the period post-transmission to reflect the accomplishments of the Guinea worm eradication campaign. Geographical restrictions for countries that were endemic in 1990 were defined based on data reported post-interruption of transmission. In the GBD analysis, Guinea worm disease was no longer modelled for the year that followed the last reported case (imported or indigenous) provided that the subsequent years through 2023 also had no case reports. To ensure that cases were attributed to burden in the country in which the case was detected, both indigenous and imported cases were included. For example, Kenya reported its last (imported) case in 2005, and as no other cases were reported through 2013, incidence from 2006 onward is zero. For Chad, there were no cases reported in 2001–2009; however, all other years had at least one case reported, and thus the model reflects the entire period of 1990–2023.

Accounting for possible under-reporting

Once national eradication programmes were initiated, national case searches were conducted to improve the accuracy of national case estimates. These searches were designed to enumerate prevalent Guinea worm disease cases and identify endemic villages to direct intervention and surveillance activities. For the majority of years included in the GBD analysis, the total number of Guinea worm cases reported is equivalent to a national census, as all cases are identified and reported. Nevertheless, not all endemic countries were able to initiate full national surveillance as of 1990.

The model does not account for the possibility that cases occurred in communities that were not included in routine surveillance or did not achieve 100% reporting coverage over time. However, any cases that may have been undetected would likely not have been a significant increase over annual totals given the comprehensive nature of Guinea worm disease surveillance activities. Nevertheless, there are years for which the annual case data are inconsistent with preceding/following annual case totals and could not be accounted for in our model. For example, Niger reported 500 cases in 1992, despite reporting 32,829 cases in 1991 and 25,346 cases in 1993. In those instances, the following datapoints were identified as outliers and excluded from analysis as follows:

Table 1. List of reported case data outliered in the analysis to account for possible under-reporting

Country	Year	Reported cases
Central African Republic	1996	9
Central African Republic	1997	5
Ethiopia	1992	303
Kenya (Turkana County)	1990	6
Uganda	1990	4704
Uganda*	1992	126,369
Benin	1991	4006
Benin	1992	4315
Chad	1992	156
Côte d'Ivoire	1990	1360
Mali	1990	884
Mauritania	1992	1557
Niger	1992	500
Senegal	1990	38
Togo	1990	3042
Togo	1991	5118
South Sudan*	1996	116,844
Sudan	1994	132

**For these two datapoints, we do not dispute that over 100,000 cases of Guinea worm likely occurred. However, given the amount of missing data in the early time series for these two countries, inclusion of these resulted in implausibly high case predictions (over 1 million cases in Uganda in 1990 and over 1.5 million for South Sudan from 1990 to 1995).*

Age/sex distribution

Generally, the risk of Guinea worm infection varies according to sex- or age-specific differences in access to safe drinking water. A study in Ethiopia found women were more likely to experience Guinea worm disease than men; in India, men experienced greater risk of infection.[1] Exposure to unsafe water sources varies largely on mobility patterns and type of water sources: communities in which infected water is carried in for consumption are more likely to see more Guinea worm disease in children and older adults.[9] Once interventions to control the spread of Guinea worm infection are implemented, the age and sex distribution likely changes to reflect variation in coverage and uptake of eradication interventions, such as larvicide of water sources and case-containment rates; age/sex case data are currently not available.

The evidence base available to describe risk of infection by age is as follows:

1) Studies from Nigeria:
    a. Guinea worm disease not common among children <1 year of age; increase in risk by age.[10]
    b. More boys ages 5–9 years than girls were infected (11.9% versus 6.8%); women ages 20–29 years had higher prevalence of infection than men (13.4% versus 4.7%); overall, the prevalence in both men and women was highest in ages 10–14 years and 30 years or older.[11]
    c. The mean age of cases was 25.8 years (95% CI: 23.9, 27.7) for males and 26.9 years for females (95% CI: 23.7, 30.1).[12]
2) Other countries:
    a. Sudan: No significant age trend among lower-endemicity villages; higher-endemicity villages (n=4) had higher prevalence in children and older adults. This study[13] attributes the difference in age trends to community-level water source.
    b. Ghana: The trend in age of first infection reported was similar for males and females, with more females experiencing first infection between 15 and 19 years and males between 20 and 24 years of age. The proportion of men with Guinea worm disease was much higher than among women 25–54 years of age. Adults >15 years of age were more likely to be infected than children.[14]

The evidence base available to describe the risk of infection by gender is as follows:

1) Studies from Nigeria:
    a. No difference among males and females.[10]
    b. No overall gender difference comparing total males infected to total females infected, although gender differences for certain age groups[11] (see notes above).
    c. Two-thirds of cases reported among 47 villages from 1971 to 1974 were male.[12]

WHO Weekly Epidemiological Record (WER) age reports: Age and sex data were reported by country for 2009 onward; these data capture the age distribution for Chad, Ethiopia, Ghana, Mali, and South Sudan. We excluded these data as the age/sex distribution is only described for children <15 years or adults, which does not permit fitting an age trend across multiple categories.

WER sex-specific data: Sex-specific differences in the burden of Guinea worm disease could reflect differing levels of access to eradication program interventions, in addition to risk factors associated with

local transmission dynamics. Since the data reported from 2009 to 2015 are the only available nationally representative data, we used the overall sex difference to generate sex-specific incidence and prevalence, with females experiencing a slightly higher risk (53%) compared to males (47%):

Table 2. WHO Weekly Epidemiological Record total worm burden by gender, by year

Year	Female	Male	Total	% Female	% Male
**2009**	1699	1490	3189	53%	47%
**2010**	976	821	1797	54%	46%
**2011**	524	534	1058	50%	50%
**2012**	273	269	542	50%	50%
**2013**	79	69	148	53%	47%
**2014**	63	63	126	50%	50%
**2015**	9	13	22	41%	59%
**Total**	3623	3259	6882	53%	47%

There is limited evidence to suggest that risk varies jointly by sex and age; however, evidence for this modification also suggests that such age- and sex-specific risks may vary by endemic community within a given geography (in some settings, women at higher risk, in others men, but not for all age strata). Without additional data sources in which cases are disaggregated by age and sex, this joint relationship is not modelled.

To model age-specific variation, we used data from seven studies with age-specific case data to generate an age trend in a DisMod model. We further assumed no Guinea worm disease occurred in infants less than 1 year of age.

Severity splits/sequelae

Sequelae associated with Guinea worm relate to the wound at the site of the worm's emergence, which can include abscesses and chronic ulcerations. Joint and tissue damage can occur, as well as secondary infection in connective tissues.[15] During the worm's emergence, which takes approximately one month to exit the body, the ulcer is painful and itchy.[1] The wound is subject to secondary infection and scarring. Possible long-term consequences of Guinea worm infection include arthritis or other permanent damage to connective tissues; however, data on this are limited. In the Greenwood study,[12] 41.7% of all cases experienced infection at the site of emergence, and the annual proportion of cases with definite arthritis ranged from 1.6% to 7.3% of all cases.

While an individual experiences Guinea worm disease, they are generally unable to work and have limited mobility at the time prior and during emergence and in the subsequent period in which they are healing. Although most worms emerge in the feet and lower legs, there are reports of worms exiting at other sites,[15] which could cause other disability not accounted for here. A study in Nigeria found that 98% of worms emerged in the lower limbs.[16] The Greenwood study[12] also observed that 88.4% emerged in the lower limbs. Therefore, for the purposes of estimating the burden of Guinea worm disease in GBD, all disability associated with Guinea worm disease is attributed to lower limb conditions, pain, and

lack of mobility. Due to limited data, we cannot account for differential disability based on number of worms emerging at the same time.

The following evidence base was reviewed to determine the proportion of cases attributed to each sequela, as well as duration of sequelae.

Duration of disability and type of disability:

Studies from Nigeria:

1) 93.4% incapacitated for an average of 26 days.[10]
2) Average disability duration 12.7 weeks; 58% unable to leave the home for a mean duration of 4.2 weeks; duration of disability greater among those older than 50 years compared to those younger than 50 years.[17]
3) 21% of cases were totally incapacitated due to their infection (not permanently disabled).[16]
4) A survey of 17 villages from 1971 to 1975 found that duration of disability was approximately 100 days.[11]
5) Weekly visits to 47 villages from 1971 to 1974 reported mean duration of illness ranging from 4.2 weeks to 7.2 weeks. 17.4% of cases had an active infection which persisted for 10 weeks or more.[12]

Studies from other countries:

6) Benin: From two villages in highly endemic areas, estimated 39–59 days of disability experienced after worm emergence.[18]
7) Ghana: 28.2% experienced pain 12–18 months post-emergence; 5% unable to carry out at least one daily activity; 0.5% permanently impaired (ligament damage to thumb).[19]
8) Ghana: Complete disability experienced among males with Guinea worm disease lasted approximately 5 weeks among those untreated. Among cases provided supportive care (wound management), the duration of disability was 2.5 weeks.[14]

Modelling strategy

Incidence

The incidence of Guinea worm disease is modelled in GBD using two approaches: for years and locations for which case data were reported, 1000 draws of incidence were estimated using a beta distribution of cases and total population minus cases; for years and locations for which case data were missing (largely the early 1990s), a Poisson regression of all case data was implemented per country, using the total population as the offset. The predicted incidence and standard error were used to generate a random distribution of 1000 incidence draws. Incidence was multiplied by duration of sequelae to calculate prevalence.

Sex-specific incidence

To account for the proportion of cases in females compared to males (53% to 47%), the incidence draws were multiplied by the sex proportion and the total population (to estimate number of cases by sex), then divided by the sex-specific total population for that year to calculate sex-specific incidence.

Age-specific incidence

In order to generate age-specific incidence, a literature search was conducted to identify national and subnational data sources in which age-specific prevalence was reported. The only nationally representative data available were WER reports from 2009 onward; however, age was only reported as less than 15 years of age or 15 years of age and older. In order to generate a trend over the life course, eight subnational data sources were identified. The prevalence of Guinea worm disease was extracted by age category reported in the original paper. An age trend was fit using a DisMod Bayesian meta-regression (DisMod-MR) model to generate a global age-sex curve to disaggregate all-age, both-sex incidence data. Under the assumption that Guinea worm disease occurs approximately one year post-infection, incidence among children aged less than 1 year was set to zero.

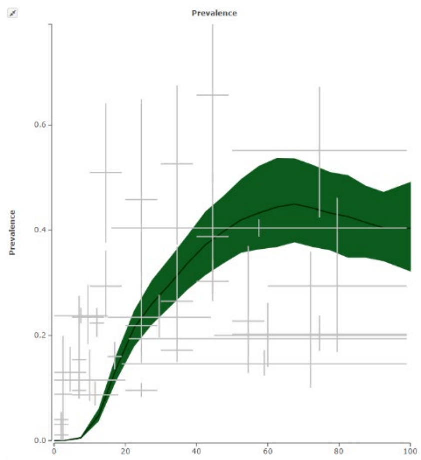

Figure 1. Age-specific prevalence model generated by DisMod-MR

Sequelae splits

For all cases, we assume each experiences pain and disfigurement (Level 2), and musculoskeletal problems, lower limb (moderate) for a period of one month, followed by two months of pain and disfigurement (mild). We then assume that 30% of all cases will then experience disfigurement Level 1 with itch/pain for an additional nine months (approximately a year of disability) to account for longer-term disability associated with recovery.

Prevalence of the sequelae listed in Table 3 was calculated by multiplying the age- and sex-specific incidence draw by the duration of the health state (in years).

1) Guinea worm pain associated with worm emergence (Level 2): all cases, 1 month
2) Guinea worm pain associated with worm emergence (Level 1): all cases, 2 months plus 30% of cases for an additional 9 months
3) Lower limb musculoskeletal problems: all cases, 1 month

Table 3. Severity distribution, details on the severity levels for Guinea worm and the associated disability weight (DW) with that severity

**Sequela**	**Lay description**	**DW (95% CI)**

Disfigurement, Level 2, with itch/pain	Has a visible physical deformity that is sore and itchy. Other people stare and comment, which causes the person to worry. The person has trouble sleeping and concentrating.	0.188 (0.125–0.267)
Disfigurement, Level 1, with itch/pain	Has a slight, visible physical deformity that is sometimes sore or itchy. Others notice the deformity, which causes some worry and discomfort.	0.027 (0.015–0.042)
Musculoskeletal problems, lower limbs, moderate	Has moderate pain in the leg, which makes the person limp, and causes some difficulty walking, standing, lifting and carrying heavy things, getting up and down and sleeping.	0.079 (0.054–0.11)

We did not apply any adjustments for the COVID-19 pandemic to EVD due to a lack of available data quantifying the impacts of the pandemic on NTD epidemiology.

Changes from GBD 2021 to GBD 2023

There were no substantive changes to the modelling strategy for GBD 2023.

References

1. Cairncross S, Muller R, Zagaria N. Dracunculiasis (Guinea worm disease) and the eradication initiative. *Clin Microbiol Rev* 2002; **15**: 223–46.
2. Biswas G, Sankara DP, Agua-Agum J, Maiga A. Dracunculiasis (guinea worm disease): eradication without a drug or a vaccine. *Philos Trans R Soc Lond B Biol Sci* 2013; **368**: 20120146.
3. Ruiz-Tiben E, Hopkins DR. Dracunculiasis (Guinea worm disease) eradication. *Adv Parasitol* 2006; **61**: 275–309.
4. Greenaway C. Dracunculiasis (guinea worm disease). *CMAJ* 2004; **170**: 495–500.
5. Hopkins DR, Ruiz-Tiben E, Downs P, Withers PC, Roy S. Dracunculiasis eradication: neglected no longer. *Am J Trop Med Hyg* 2008; **79**: 474–9.
6. Kappus KD, Hopkins DR, Ruiz-Tiben E, *et al.* A strategy to speed the eradication of dracunculiasis. *World Health Forum* 1991; **12**: 220–5.
7. Prevention CfDCa. Guinea worm wrap-up Atlanta, GA: WHO Collborating center for Research, Training and Eradication of Dracunculiasis, CDC; 2015.
8. Hopkins DR, Ruiz-Tiben E, Diallo N, Withers PC, Jr., Maguire JH. Dracunculiasis eradication: and now, South Sudan. *The American journal of tropical medicine and hygiene* 2013; **89**.
9. Watts SJ, Brieger WR, Yacoob M. Guinea worm: an in-depth study of what happens to mothers, families and communities. *Soc Sci Med* 1989; **29**: 1043–9.
10. Adeyeba OA, Kale OO. Epidemiology of dracunculiasis and its socio-economic impact in a village in south-west Nigeria. *West Afr J Med* 1991; **10**: 208–15.
11. Kale OO. The clinico-epidemiological profile of guinea worm in the Ibadan district of Nigeria. *Am J Trop Med Hyg* 1977; **26**: 208–14.
12. Greenwood B, Greenwood A, Bradley A. Guinea worm infection in northern Nigeria: reflections on a disease approaching eradication. *Trop Med Int Health* 2017; **22**: 558–66.
13. Tayeh A, Cairncross S. The impact of dracunculiasis on the nutritional status of children in South Kordofan, Sudan. *Ann Trop Paediatr*. 1996; **16**:221-6.
14. Belcher DW, Wurapa FK, Ward WB, Lourie IM. Guinea worm in southern Ghana: its epidemiology and impact on agricultural productivity. *Am J Trop Med Hyg* 1975; **24**: 243–9.

15. Muller R. Guinea worm disease: epidemiology, control, and treatment. *Bull World Health Organ* 1979; **57**: 683–9.
16. Okoye SN, Onwuliri CO, Anosike JC. A survey of predilection sites and degree of disability associated with guineaworm (Dracunculus medinensis). *Int J Parasitol.* 1995; **25**:1127-9.
17. Smith GS, Blum D, Huttly SR, Okeke N, Kirkwood BR, Feachem RG. Disability from dracunculiasis: effect on mobility. *Ann Trop Med Parasitol* 1989; **83**: 151–8.
18. Chippaux JP, Banzou A, Agbede K. [Social and economic impact of dracunculosis: a longitudinal study carried out in 2 villages in Benin]. *Bull World Health Organ* 1992; **70**: 73–8.
19. Hours M, Cairncross S. Long-term disability due to guinea worm disease. *Trans R Soc Trop Med Hyg* 1994; **88**: 559–60.

Dysthymia

Flowchart

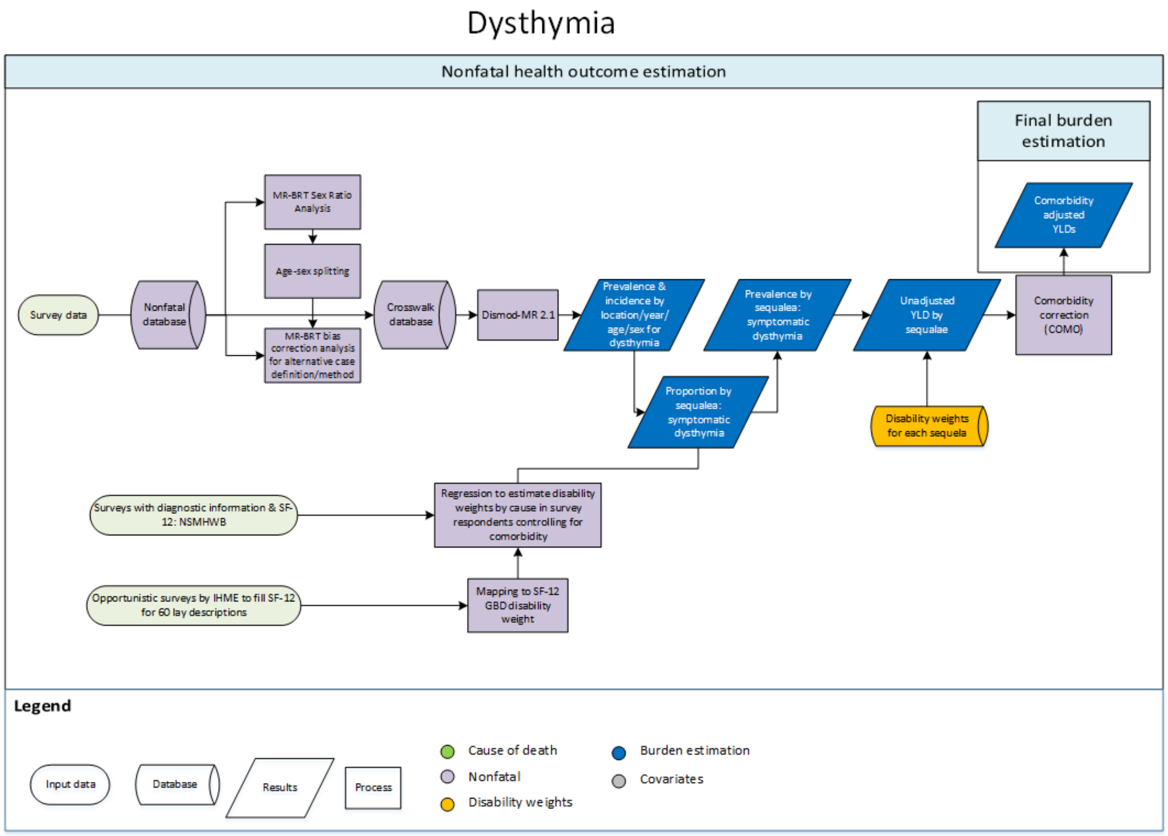

Input data and methodological summary for dysthymia

Case definition

Dysthymia is a mood disorder consisting of chronic depression, demonstrating less severe but longer-lasting symptoms than major depressive disorder. Included in GBD disease modelling were cases meeting diagnostic criteria for dysthymia according to the Diagnostic and Statistical Manual of Mental Disorders (DSM), or the equivalent diagnosis in the International Classification of Diseases (ICD).[1,2] These were identified by the code F34.1, excluding those cases due to a general medical condition or substance-induced cases.[1,2] Different versions of DSM (DSM-III, DSM-III-R, DSM-IV, DSM-IV-TR, DSM-5, and DSM-5-TR) and ICD (ICD-9, ICD-10, and ICD-11) were accepted.

According to DSM-IV TR criteria, dysthymia involves the experience of chronically depressed mood for most of the day, more days than not, for at least two years (or at least one year in children and adolescents). During this period, at least two of the following symptoms must also be experienced:

- poor appetite or overeating
- insomnia or hypersomnia
- low energy or fatigue
- low self-esteem
- poor concentration or indecisiveness

- feelings of hopelessness

Input data

The epidemiological systematic literature review for dysthymia was conducted in three stages involving electronic searches of the peer-reviewed literature (ie, via PsycInfo, Embase, and PubMed), the grey literature, and expert consultation. For mental disorders, we update our GBD electronic database searches on a rolling basis. A systematic review update was completed for GBD 2023 for mood disorders together (depressive disorders and bipolar disorder). Databases were searched on March 23, 2021, for publications after April 1, 2018. Below are the search terms used for each database:

PubMed: ((((((((((((prevalen*[Title/Abstract]) OR (mortality[Title/Abstract])) OR (death*[Title/Abstract])) OR (inciden*[Title/Abstract])) OR (recurren*[Title/Abstract])) OR (remission[Title/Abstract])) OR (duration[Title/Abstract])) OR (remit*[Title/Abstract])) OR (epidemiolog*[Title/Abstract])) OR (prevalence[MeSH Terms])) OR (mortality[MeSH Terms])) OR (incidence[MeSH Terms])) OR (recurrence[MeSH Terms])) AND (((((((((((depress*[Title/Abstract]) OR (dysthymi*[Title/Abstract])) OR (bipolar[Title/Abstract])) OR (manic[Title/Abstract])) OR (mania[Title/Abstract])) OR ("mood disorders"[Title/Abstract])) OR ("mood disorder"[Title/Abstract])) OR (mood disorders[MeSH Terms])) OR (depressive disorders[MeSH Terms])) OR (depressive disorders, major[MeSH Terms])) OR (bipolar disorders[MeSH Terms])) OR (dysthymic disorders[MeSH Terms]))

Embase: (depress*:ab,ti OR dysthymi*:ab,ti OR bipolar:ab,ti OR manic:ab,ti OR mania:ab,ti OR 'mood disorder':ab,ti OR 'mood disorders':ab,ti) AND (prevalen*:ab,ti OR mortality:ab,ti OR death*:ab,ti OR inciden*:ab,ti OR recurren*:ab,ti OR remission:ab,ti OR duration:ab,ti OR remit*:ab,ti OR epidemiolog*:ab,ti)

PsycInfo: (title: depress* OR abstract: depress* OR title: dysthymi* OR abstract: dysthymi* OR title: bipolar OR abstract: bipolar OR title: manic OR abstract: manic OR title: mania OR abstract: mania OR title: "mood disorder" OR abstract: "mood disorder" OR title: "mood disorders" OR abstract: "mood disorders") AND (title: prevalen* OR abstract: prevalen* OR title: mortality* OR abstract: mortality* OR title: death* OR abstract: death* OR title: inciden* OR abstract: inciden* OR title: recurren* OR abstract: recurren* OR title: remission AND abstract: remission OR title: duration OR abstract: *duration OR title: remit* OR* abstract: remit* OR title: epidemiolog* OR abstract: epidemiolog*)

In addition to the database search, a grey literature search and expert consultation were also conducted. The systematic review update was conducted according to Preferred Reporting Items for Systematic Reviews and Meta-Analyses (PRISMA; see Figure 1).

Figure 1: PRISMA 2009 flow diagram

From: Moher D, Liberati A, Tetzlaff J, Altman DG, The PRISMA Group (2009). *P*referred *R*eporting *I*tems for *S*ystematic Reviews and *M*eta-*A*nalyses: The PRISMA Statement. PLoS Med 6(7): e1000097. doi:10.1371/journal.pmed1000097

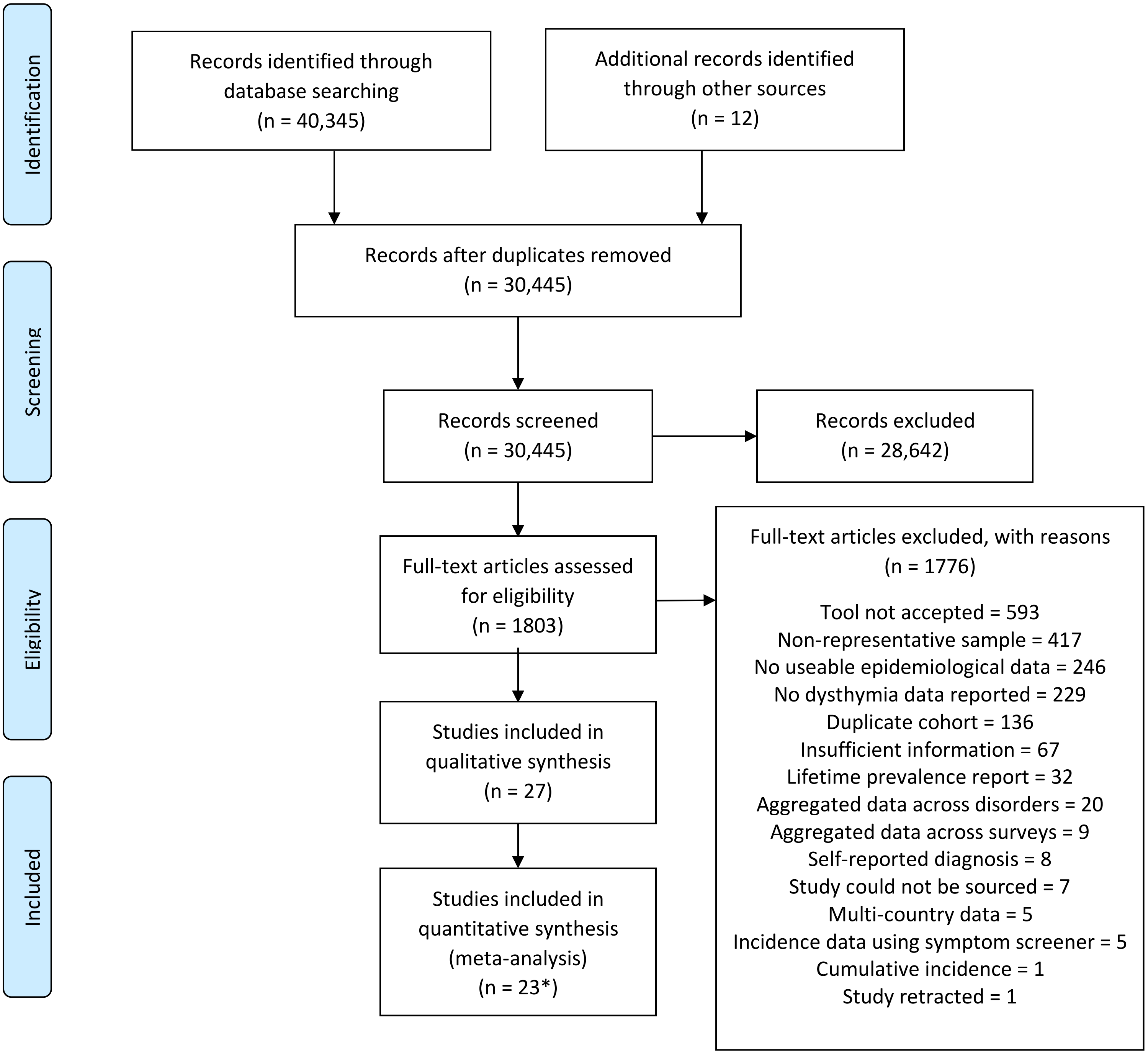

**The qualitative analysis led to the exclusion of additional studies with duplicative cohorts or methodological limitations impacting their eligibility and increasing measurement error within the data.*

The GBD inclusion criteria stipulated that 1) the publication year must be from 1980 onward; 2) "caseness" must be based on clinical threshold as established by the DSM or ICD; 3) sufficient information must be provided on study method and sample characteristics to assess the quality of the study; and 4) study samples must be representative of the general population (eg, inpatient or pharmacological treatment samples, case studies, veterans, or refugee samples were excluded). No limitation was set on the language of publication. Methods used for this systematic review have been reported in greater detail elsewhere.[3,4]

Age-sex splitting

The extracted data underwent three types of age-sex splitting processes:

1. Where possible, estimates were further split by sex and age based on the available data. For instance, if studies reported prevalence for broad age groups by sex (eg, prevalence in 15–65-year-old males and females separately), and also by specific age groups but for both sexes combined (eg, prevalence in 15–30-year-olds, then in 31–65-year-olds, for males and females combined); age-specific estimates were split by sex using the reported sex-ratio and bounds of uncertainty.
2. A meta-regression—Bayesian, regularised, trimmed (MR-BRT) analysis was used to split the remaining both-sex estimates in the dataset. For each parameter, sex-specific estimates were matched by location, mid-age, and year. A MR-BRT network meta-analysis was then used to estimate pooled sex ratios. Given evidence to suggest that the sex ratio in depressive disorders varies with age,[5-7] we also tested for an age interaction in the model but it was not significant. The global sex ratio for remission was estimated as 0.69 (95% UI 0.32–1.46).
3. Studies reporting prevalence estimates across age groups spanning 25 years or more were split into five-year age groups using the prevalence age pattern estimated by DisMod-MR 2.1. The DisMod-MR model used to estimate the age pattern did not contain any previously age-split data.

Bias corrections/crosswalks

Estimates with known biases were adjusted/crosswalked accordingly prior to DisMod-MR 2.1. For each crosswalk of interest, pairs of the reference and the alternative estimates were matched by age, sex, location, and year. This was done for both within-study (where possible) and between-study pairs. These pairs were then used as inputs in a MR-BRT network meta-analysis. The MR-BRT analysis produced a pooled ratio between the reference estimates and alternative estimates, which was used to adjust all alternative estimates in the dataset. Reference data informing the prevalence of dysthymia consisted of estimates reporting past-month/point prevalence of dysthymia using a diagnostic tool that was administered by a clinician. Two adjustment ratios were used for alternative data:

1. A lay-interviewer correction was used to adjust all prevalence estimates derived from trained lay interviewers towards the level they would have been if the estimate was derived from clinically trained interviewers (ie, psychologist or psychiatrist). We consider interviews conducted by clinicians to be more sensitive to detecting cases of dysthymia.
2. A past-year recall correction adjusted all datapoints derived from past-year prevalence towards the level they would have been if the study had captured point/past-month prevalence. The latter prevalence period is less affected by recall bias.

See Table 1 for adjustment factors using for dysthymia. The estimated uncertainty intervals (UIs) around the adjustment factor incorporate gamma, which represents the between-study variance across all input data in the model. This added uncertainty widens the UIs for crosswalks with significant fixed effects.

Table 1: MR-BRT crosswalk adjustment factors for dysthymia

Data input	Reference or alternative case definition	Gamma	Beta coefficient, log (95% UI)*	Adjustment factor**
Population survey	Reference: past month/point clinical diagnosis			
Population survey	Alternative: past-year prevalence	0.008	0.40 (0.28 to 0.51)	1.48 (1.32–1.67)
Population survey	Alternative: lay-interviewer diagnosis	0.11***	–0.21 (–1.07 to 0.62)	0.81 (0.34–1.86)

**MR-BRT crosswalk adjustments can be interpreted as the factor the alternative case definition is adjusted by to reflect what it would have been had it been measured using the reference case definition. If the log/logit beta coefficient is negative, then the alternative is adjusted up to the reference. If the log/logit beta coefficient is positive, then the alternative is adjusted down to the reference.*

***The adjustment factor column is the exponentiated beta coefficient. For log beta coefficients, this is the relative rate between the two case definitions. For logit beta coefficients, this is the relative odds between the two case definitions.*

****The adjustment factor for lay-interviewer data has a different Gamma value because this adjustment is derived from major depressive disorder prevalence data. This approach was necessary due to the lack of sufficient dysthymia data to directly inform the adjustment.*

Modelling strategy

After the above data processes were applied, DisMod MR 2.1 was used to model the epidemiological data for dysthymia. Adjustments to model priors or the dataset were made where appropriate. Where outliers were identified in the data, we reassessed the study's methodology and quality before a decision was made to exclude or include the data.

Data across all epidemiological parameters were initially included in the modelling process. The incidence studies reported estimates which were very low relative to the prevalence data. As prevalence studies contributed much greater world coverage than incidence studies, we excluded the incidence data, relying instead on data from the other parameters. We assumed no incidence and prevalence before age 3. This minimum age of onset was corroborated with expert feedback and was consistent with the available data. Excess mortality was set to zero as there is no epidemiological evidence to suggest that dysthymia is associated with a statistically significant risk of mortality.[3,4]

Severity splits and disability weights

The GBD disability weight survey assessments include lay descriptions of sequelae highlighting major functional consequences and symptoms. The lay description and disability weight for a symptomatic state of dysthymia is shown in Table 2. Given the milder and more stable presentation of dysthymia, it was assigned the same disability weight as that for mild major depressive disorder. To determine the proportion of people with symptomatic and asymptomatic dysthymia, the US National Epidemiological Survey on Alcohol and Related Conditions (NESARC, conducted in two waves from 2001–2002 and 2004–2005)[8] and the Australian National Survey of Mental Health and Wellbeing of Adults (NSMHWB,

conducted in 1997)[9] were used. The proportion of dysthymia cases falling within each severity level were as follows: asymptomatic 29% (23–36), and symptomatic 71% (64–77).

Table 2. Lay description for dysthymia in GBD 2023 and the associated disability weight

**Severity level**	**Lay description**	**Disability weight (95% UI)**
Symptomatic dysthymia	Feels persistent sadness and has lost interest in usual activities. The person sometimes sleeps badly, feels tired, or has trouble concentrating but still manages to function in daily life with extra effort.	0.145 (0.099–0.209)

In GBD 2023, the modelling strategy for dysthymia was updated to include a past-year crosswalk, which had not been part of previous analyses. This methodological change was implemented to address heterogeneity in prevalence estimates and ensure alignment with data reflecting current episodes of dysthymia. The inclusion of this crosswalk led to a decrease in the estimated global prevalence of dysthymia compared to GBD 2021.

While we continue to improve on the data and methods used to estimate the burden of mental disorders, some challenges need to be acknowledged. Firstly, we still have a large number of locations with no high-quality raw data available. Secondly, it is difficult to quantify and remove all variation due to measurement error in our epidemiological estimates. While we have improved the methodology used to account for known sources of bias, in some cases we still have very few datapoints to inform these adjustments. Thirdly, there is a paucity of research on the risk factors of mental disorders which can be used as predictive covariates in our epidemiological models.

References

1. American Psychiatric Association. Diagnostic and Statistical Manual of Mental Disorders (DSM). Washington: American Psychiatric Association, 1952.
2. World Health Organization. The ICD-10 Classification of Mental and Behavioural Disorders. Clinical descriptions and diagnostic guidelines. Geneva: World Health Organization; 1992.
3. Charlson FJ, Ferrari AJ, Flaxman AD, Whiteford HA. The epidemiological modelling of dysthymia: application for the Global Burden of Disease Study 2010. *J Affect Disord* 2013; **151**(1): 111-20.
4. Ferrari AJ, Charlson FJ, Norman RE, et al. Burden of depressive disorders by country, sex, age, and year: findings from the global burden of disease study 2010. *PLoS Med* 2013; **10**(11): e1001547.
5. Patten SB, Wang JL, Williams JV, et al. Descriptive epidemiology of major depression in Canada. *The Canadian Journal of Psychiatry* 2006; **51**(2): 84-90.
6. Patten SB, Williams JV, Lavorato DH, Wang JL, Bulloch AG, Sajobi T. The association between major depression prevalence and sex becomes weaker with age. *Social psychiatry and psychiatric epidemiology* 2016; **51**(2): 203-10.
7. Salk RH, Hyde JS, Abramson LY. Gender differences in depression in representative national samples: meta-analyses of diagnoses and symptoms. *Psychological bulletin* 2017; **143**(8): 783.
8. Introduction to the National Epidemiologic Survey on Alcohol and Related Conditions [http://pubs.niaaa.nih.gov/publications/arh29-2/74-78.htm]. Access date 1 December 2014.
9. Australian Bureau of Statistics. National Survey of Mental Health and Wellbeing of Adults 1997. Canberra: Australian Bureau of Statistics.

Ebola virus disease

Flowchart

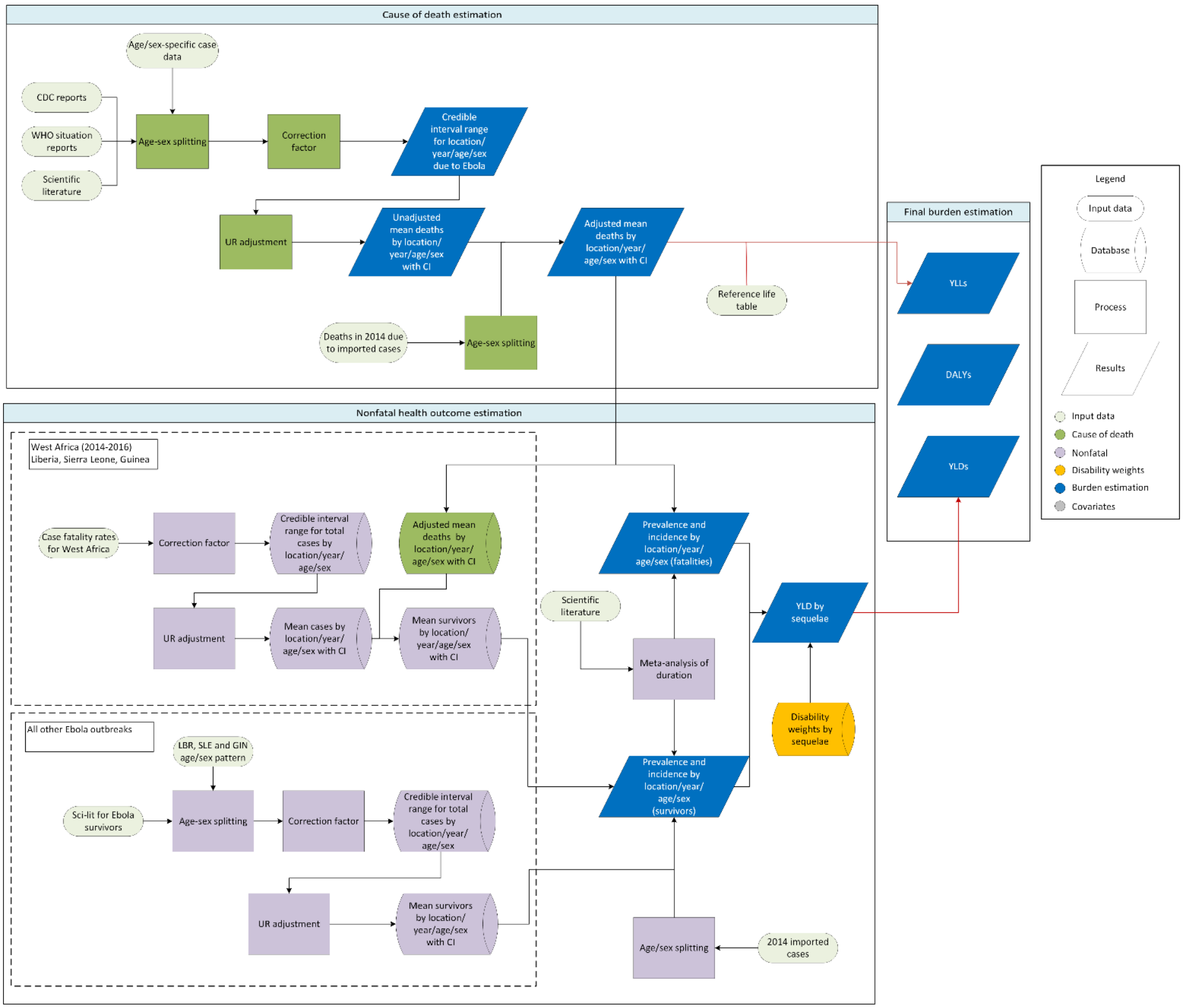

Input data and methodological summary for Ebola virus disease

Case definition

Ebola virus disease (EVD) is a relatively rare viral pathogen linked with high case-fatality rates in both humans and non-human primates. The disease is zoonotic, and while bats have been implicated as reservoirs, definitive host species are yet to be identified. Once a human becomes infected after viral transmission from animal sources either directly or indirectly, secondary human-to-human transmission is possible, primarily through exchange of infectious bodily fluids and secretions. Clinical cases typically

present initially as a febrile illness, similar to a number of different pathogens, which can subsequently be followed by haemorrhagic complications and death. Historically there have been a number of outbreaks, usually no more than a few hundred cases, typically constrained to one country, focused in Central Africa. The West African outbreak, however, which started in Guinea in 2013, claimed more lives than all previous outbreaks combined and spread across the region seeding additional outbreaks. The ICD code for Ebola is A98.4, but no data used in the modelling reference this code (ie, all the data are from literature extractions). Data for EVD were only included if the case was identified as either "probable" or "confirmed" as per World Health Organization (WHO) definitions. We used the following case definition for GBD 2023:

Quantity of interest	Reference or alternative	Definition
Ebola	Reference	Prevalence determined using cases identified as either "probable" or "confirmed". A confirmed case is any suspected or probable case with a positive laboratory result through either detection of virus RNA via reverse transcriptase-polymerase chain reaction, or by detection of IgM antibodies directed against Ebola. A probable case is any suspected case evaluated by a clinician or any deceased suspected case with an epidemiological link to a confirmed case.

Input data

Two distinct sequelae were assigned to EVD to be incorporated into the YLD estimation process: (i) sequela associated with the initial symptomatic phase of the infection (associated with all cases of EVD) and (ii) sequela characterising the long-term post-EVD consequences of infection. As such, data were required both to ascertain the number of deaths as well as those surviving from each outbreak.
Data on fatal cases inherited from the GBD 2017 mortality estimation process were converted into incidence of cases of Ebola (with fatal outcomes) by cross-referencing locational annualised population estimates.

In order to calculate the numbers of survivors from each outbreak, two data sources were referenced, one based upon modelled estimates of the main three countries in the West African Ebola outbreak (namely Sierra Leone, Liberia, and Guinea), supplemented by WHO Situation Reports covering the clusters of 2016 cases and literature references covering all other subsequent outbreaks.

Age-sex patterns derived from the age- and sex-specific input data were applied to total envelope estimates as reported by WHO and Centers for Disease Control and Prevention (CDC). Raw numbers of survivors were estimated by subtracting total deaths from total cases.

For all other outbreaks, numbers of survivors were directly evaluated based upon numbers published in a previous review[1,2] and consulting original documents describing these outbreaks. This initial review was also updated to include the outbreaks that occurred in the Democratic Republic of the Congo (DRC) in 2014,[3] cases in 2016 and 2017, the 2018 DRC Equateur Province outbreak,[4] the 2018–2020 DRC Ituri, North Kivu, and South Kivu Provinces outbreak,[5,6,7] including cases in Uganda,[8] the outbreaks in 2020 in the DRC Equateur Province,[9] outbreaks in 2021 in Guinea and DRC North Kivu Province,[10] outbreaks in

2022 in DRC Equateur[11] and North Kivu Provinces,[12] and also the Mubende District[13] of Uganda. This resulted in datasets describing each outbreak with variable degrees of detail: some fully describing the age and sex breakdown of all survivors (eg, Rosello and colleagues[14]) and others simply providing the final total. Only confirmed or probable cases were included, as per the case definition. Outbreaks that spanned multiple years, in the absence of sufficient data providing an accurate breakdown, were apportioned between the years by evenly assigning a uniform number of survivors to each month of the outbreak's duration. An additional search was conducted to identify imported cases from the West African outbreak during 2014 and 2015.

Modelling strategy

Data on cases (both survivors and fatalities) resulting from imported cases from 2014 and 2015 were used as specific count data as it was assumed to be an accurate representation of the cases and outbreaks in these countries, all of which were on high alert for importation of cases.[17,18]

All other input data were processed prior to inclusion in GBD to account for any potential under-reporting of deaths. A meta-analysis of existing under-reporting studies from the literature was performed using a random effects model with a DerSimonian-Laird estimator. A variety of sources were included, capturing a number of different estimation processes, all identified by literature review. The figure below shows the different effect sizes of the different studies, as well as the resulting GBD 2016 (used in GBD 2023) correction factor, with the GBD 2015 correction factor for reference. The correction factor ranged from 1.5147 to 2.5720, with a mean of 2.0433.

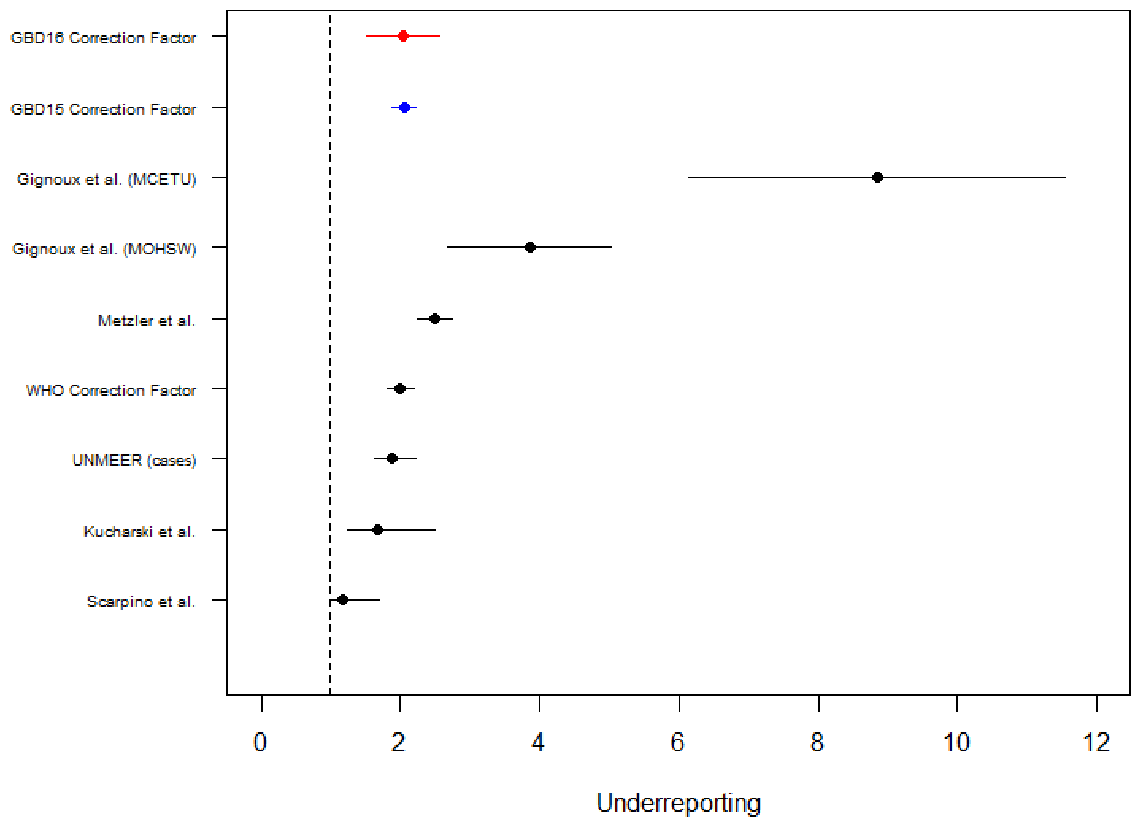

Figure 1. Under-reporting of Ebola case data

In order to capture this potential variation, all input data were multiplied by the lower and upper limit of this estimated correction factor; these numbers then provided the lower and upper bounds from which draw values were taken. For outbreaks where no data were supplied for age and/or sex, the pattern observed in the West African outbreak (for which there were the most comprehensive data) was used to apportion these total values.

1000 draws were taken from a normal distribution fitted between these lower and upper bound values, which generated mean estimates stratified by age, sex, location, and year along with credible intervals for these numbers. For the West African outbreak, this generated total case numbers, from which the estimated number of deaths was subtracted to provide an estimate for the total number of survivors. For all other outbreaks, this data processing directly estimated the total number of survivors from each outbreak. These count data were converted into prevalence estimates by cross-referencing estimates of population size.

To estimate the duration of the sequelae categories, previous modelled assessments of the West African outbreak were consulted.[1,2] The duration of initial infection for patients was calculated as the total time period between onset of symptoms to death or to discharge from hospital (8.2 days [7.9–8.4] and 15.1 days [14.6–15.6], respectively). These time periods were assumed to be appropriate for characterising all other outbreaks. This time period was then assigned a disability weight corresponding to "infectious disease, acute episode, severe."

For long-term sequelae estimation, the proportion of survivors still suffering post-acute consequences was modelled using an exponential function with proportions of survivors still reporting poor health states (derived from a number of survivor studies[19-29]) reported over different time periods. The average duration of post-Ebola sequelae was then calculated as 0.9042 years (0.3673–1.4268).

The final combination of YLDs associated with prevalent initial onset of disease and prevalent post-EVD consequences was then calculated to provide an overall YLD estimate stratified by age, sex, location, and year.

Health states/sequelae

The table below shows the list of sequelae due to EVD and the associated disability weights. It was not possible to create bespoke disability weights for the more specific sequelae often associated with EVD (eg, haemorrhaging or ocular complications in survivors), and thus existing disability weights were co-opted. General high fevers and weakness characterise the majority of presenting cases,[15] with long-term complications generally related to weakness and arthralgia.[16]

Table 1. Severity distribution, details on the severity levels for EVD and the associated disability weight (DW) with that severity

Sequelae	Description	DW (95% CI)

Infectious disease, acute episode, severe	Has a high fever and pain and feels very weak, which causes great difficulty with daily activities	0.133 (0.088–0.19)
Infectious disease, post-acute consequences (fatigue, emotional lability, insomnia)	Is always tired and easily upset. The person feels pain all over the body and is depressed	0.219 (0.148–0.308)

Changes from GBD 2021 to GBD 2023

There were no substantive changes to the modelling strategy for GBD 2023.

Limitations

Data on Ebola outbreaks prior to 2014 are sparse, and as a result, many values derived from the West African outbreak were assumed to be valid for historical outbreaks as well. This may mask significant differences that exist between these outbreaks, some of which were caused by different species of Ebola virus. In order to minimise this problem, we chose to implement a data-driven approach – for those outbreaks where sufficiently detailed historical data could be obtained, this was used in preference to any assumed age-sex breakdown.

Haemorrhagic manifestations are currently not considered as an explicit health state for disability weighting, and as a result, the current classification (of infectious disease, acute episode, severe) may be an underestimate. In contrast, the post-Ebola disease sequelae disability weighting may overestimate this burden, particularly when applied over a long period of time. In both instances, however, these disability weightings represent the most relevant linkages in the absence of bespoke values being generated.

Due to so few historical survivors of EVD, only a handful of studies have tracked the long-term sequelae among cohorts of survivors beyond a two-year period. Given the large number of survivors from the West African outbreak, it is likely that future parameterisation of this component will become much better data-driven. The current log-linear regression model extends for a period of 20 years and therefore could prove to be an overestimate of duration. In addition, ocular manifestations are not currently considered within the sequelae envelope – future iterations will consider health states identified by ongoing cohort analyses of Ebola survivors. Comments from collaborators in previous cycles have highlighted ocular conditions for inclusion; however, definitive evidence of a linkage with Ebola remains inconclusive. A study (conducted in West Africa) comparing Ebola survivors with background prevalence rates of many of the symptoms reported in survivors (eg, uveitis), suggested no difference in rates of these ophthalmic complications.[30] Understanding which of the many observed clinical outcomes in patients are caused by the virus, as opposed to incidentally co-morbid, is a necessary prerequisite for inclusion in the GBD.

We did not apply any adjustments for the COVID-19 pandemic to EVD due to a lack of available data quantifying the impacts of the pandemic on NTD epidemiology.

References

1. Pigott DM, Golding N, Mylne A, *et al.* Mapping the zoonotic niche of Ebola virus disease in Africa. *Elife* 2014; **3**:e04395.
2. Mylne A, Brady OJ, Huang Z, *et al.* A comprehensive database of the geographic spread of past human Ebola outbreaks. *Sci Data* 2014; **1**: 140042.
3. Maganga GD, Kapetshi J, Berthet N, *et al.* Ebola virus disease in the Democratic Republic of Congo. *N Engl J Med* 2014; **371**: 2083–91.
4. World Health Organization (WHO). WHO Ebola Situation Report 2018 - Number 17. 2018.
5. World Health Organization (WHO). WHO Ebola Situation Report 2019 - Number 69. 2019.
6. World Health Organization (WHO). WHO Ebola Situation Report 2019 - Number 73. 2019.
7. World Health Organization (WHO). WHO Ebola Situation Report 2019 - Number 98. 2020.
8. World Health Organization (WHO). WHO Ebola Situation Report 2019 - Number 45. 2019.
9. 2020 Democratic Republic of the Congo, Equateur Province | Democratic Republic of Congo | Outbreaks | Ebola (Ebola Virus Disease) | CDC. 2020; published online Nov 19. https://www-cdc-gov.offcampus.lib.washington.edu/vhf/ebola/outbreaks/drc/2020-june.html (accessed January 11, 2021).
10. Centers for Disease Control and Prevention (CDC). Ebola (Ebola Virus Disease). 2021 Democratic Republic of the Congo, North Kivu Province & Guinea, N'Zérékoré prefecture. 2021. https://www.cdc.gov/vhf/ebola/history/chronology.html (accessed July 27, 2022).
11. World Health Organization (WHO). WHO Outbreak News Bulletin. 2022 Democratic Republic of the Congo, Equateur Province. 2022. https://www.who.int/emergencies/disease-outbreak-news/item/2022-DON398 (accessed July 4, 2022).
12. World Health Organization (WHO). WHO Weekly Bulletin on Outbreaks and other Emergencies 2022 - Number 40. 2022.
13. World Health Organization (WHO). WHO Weekly Bulletin on Outbreaks and other Emergencies 2023 - Number 1. 2023.
14. Rosello A, Mossoko M, Flasche S, *et al.* Ebola virus disease in the Democratic Republic of the Congo, 1976-2014. *Elife* 2015; **4**. DOI:10.7554/eLife.09015.
15. Schieffelin JS, Shaffer JG, Goba A, *et al.* Clinical Illness and Outcomes in Patients with Ebola in Sierra Leone. *N Engl J Med* 2014; **371**:2092–100.
16. Tiffany A, Vetter P, Mattia J, *et al.* Ebola Virus Disease Complications as Experienced by Survivors in Sierra Leone. *Clin Infect Dis* 2016; **62**:1360–6.
17. Fasina FO, Shittu A, Lazarus D, *et al.* Transmission dynamics and control of Ebola virus disease outbreak in Nigeria, July to September 2014. *Euro Surveill* 2014; **19**:20920.
18. Althaus CL, Low N, Musa EO, Shuaib F, Gsteiger S. Ebola virus disease outbreak in Nigeria: Transmission dynamics and rapid control. *Epidemics* 2015; **11**:80–4.
19. UNMEER. Sierra Leone: Ebola emergency Weekly Situation Report No. 7. 2014 https://www.humanitarianresponse.info/system/files/documents/files/UNMEER_NERC_SitRep_07Dec.pdf.
20. Clark D V, Kibuuka H, Millard M, *et al.* Long-term sequelae after Ebola virus disease in Bundibugyo, Uganda: a retrospective cohort study. *Lancet Infect Dis* 2015; **15**:905–12.
21. Qureshi AI, Chughtai M, Loua TO, *et al.* Study of Ebola Virus Disease Survivors in Guinea. *Clin Infect Dis* 2015; **61**:1035–42.
22. Rowe AK, Bertolli J, Khan AS, *et al.* Clinical, virologic, and immunologic follow-up of convalescent Ebola hemorrhagic fever patients and their household contacts, Kikwit, Democratic Republic of the Congo. Commission de Lutte contre les Epidémies à Kikwit. *J Infect Dis* 1999; **179 Suppl**:S28-35.
23. Bwaka MA, Bonnet MJ, Calain P, *et al.* Ebola hemorrhagic fever in Kikwit, Democratic Republic of the Congo: clinical observations in 103 patients. *J Infect Dis* 1999; **179 Suppl**:S1-7.

24. Mohammed H, Vandy AO, Stretch R, *et al.* Sequelae and Other Conditions in Ebola Virus Disease Survivors, Sierra Leone, 2015. *Emerg Infect Dis* 2017; **23**:66–73.
25. Nanyonga M, Saidu J, Ramsay A, Shindo N, Bausch DG. Sequelae of Ebola Virus Disease, Kenema District, Sierra Leone. *Clin Infect Dis* 2016; **62**:125–6.
26. Mattia JG, Vandy MJ, Chang JC, *et al.* Early clinical sequelae of Ebola virus disease in Sierra Leone: a cross-sectional study. *Lancet Infect Dis* 2016; **16**:331–8.
27. Epstein L, Wong KK, Kallen AJ, Uyeki TM. Post-Ebola Signs and Symptoms in U.S. Survivors. *N Engl J Med* 2015; **373**:2484–6.
28. Etard J-F, Sow MS, Leroy S, *et al.* Multidisciplinary assessment of post-Ebola sequelae in Guinea (Postebogui): an observational cohort study. *Lancet Infect Dis* 2017. DOI:10.1016/S1473-3099(16)30516-3.
29. Scott JT, Sesay FR, Massaquoi TA, Idriss BR, Sahr F, Semple MG. Post-Ebola Syndrome, Sierra Leone. *Emerg Infect Dis* 2016; **22**:641–6.
30. Steptoe, PJ, Scott JT, Baxter, JM, *et al.* Novel retinal lesion in Ebola survivors, Sierra Leone, 2016. *Emerg Infect Dis* 2017; **23**:1102-9.

Encephalitis

Flowchart

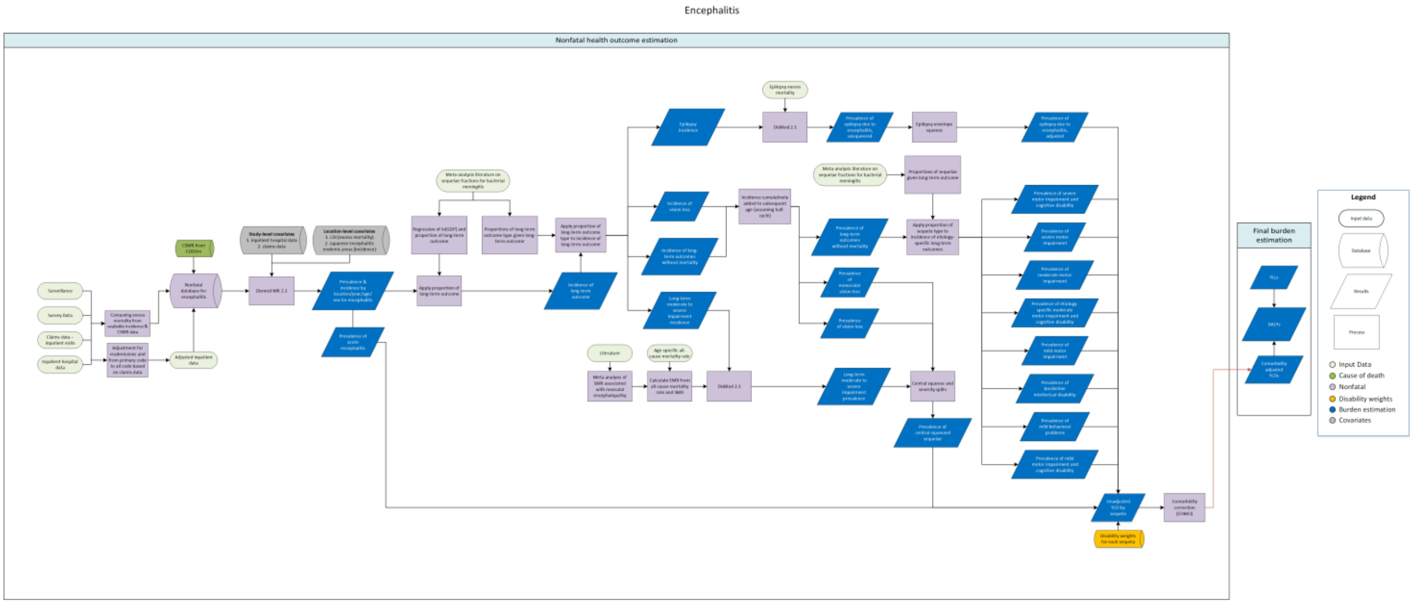

Input data and methodological summary for encephalitis

Case definition

Encephalitis is a disease caused by an acute inflammation of the brain. Symptoms of encephalitis can include flu-like symptoms like headache, fever, drowsiness, and fatigue, and at times, seizures, hallucinations, or stroke. Included in the GBD modelling were cases meeting ICD-10 diagnostic criteria for encephalitis (A83-A86.4, B94.1, F07.1, G04-G05.8).

The case definitions accepted for encephalitis are shown below.

Quantity of interest	Reference or alternative	Definition
Incidence of encephalitis	Reference	Encephalitis from inpatient data.
Incidence of encephalitis	Alternative	Encephalitis from USA private claims data.
Incidence of encephalitis	Alternative	Cases detected by epidemiological surveillance.

Input data

In the GBD 2023 study, the only data input updated was cause-specific mortality rate (CSMR), which leveraged final mortality rates from GBD 2021. We used the same input data from GBD 2021 including clinical informatics and the literature data identified from our systematic review in GBD 2021 as well as literature data from earlier GBD rounds.[1]

Bias corrections

Hospital data were flagged with a covariate for inpatient hospital data and were used as the reference category. Claims data were flagged with year-specific covariates. Surveillance data were flagged with covariates specific to the type of surveillance (eg, active versus passive and sentinel-based versus population-based). Both claims and surveillance data were crosswalked up to the reference category.

Table 2: MR-BRT crosswalk adjustment factors for encephalitis

Data input	Reference or alternative case definition	Gamma	Basis function on age midpoint	B-spline coefficient, logit (95% UI)*	Adjustment factor **
Inpatient hospital (CF2)	Ref		---	---	---
Claims, inpatient only	Alt	0.00	age_mid_0	2.54 (1.94 to 3.15)	12.70
			age_mid_1	2.83 (2.45 to 3.20)	16.83
			age_mid_2	–0.10 (–0.69 to 0.49)	0.90
			age_mid_3	1.50 (1.12 to 1.88)	4.50
			age_mid_4	1.12 (0.93 to 1.31)	3.07
Claims, inpatient only, year 2000	Alt	0.00	age_mid_0	1.65 (–3.67 to 6.98)	5.23
			age_mid_1	1.71 (–1.46 to 4.88)	5.54
			age_mid_2	0.36 (–4.06 to 4.78)	1.43
			age_mid_3	0.49 (–2.02 to 3.00)	1.63
			age_mid_4	1.01 (0.05 to 1.96)	2.73
Surveillance	Alt	0.77	---	–4.00 (–5.71 to –2.28)	0.02

**MR-BRT crosswalk adjustments can be interpreted as the factor the alternative case definition is adjusted by to reflect what it would have been had it been measured using the reference case definition. If the log/logit beta coefficient is negative, then the alternative is adjusted up to the reference. If the log/logit beta coefficient is positive, then the alternative is adjusted down to the reference.*

***The adjustment factor column is the exponentiated beta coefficient. For log beta coefficients, this is the relative rate between the two case definitions. For logit beta coefficients, this is the relative odds between the two case definitions.*

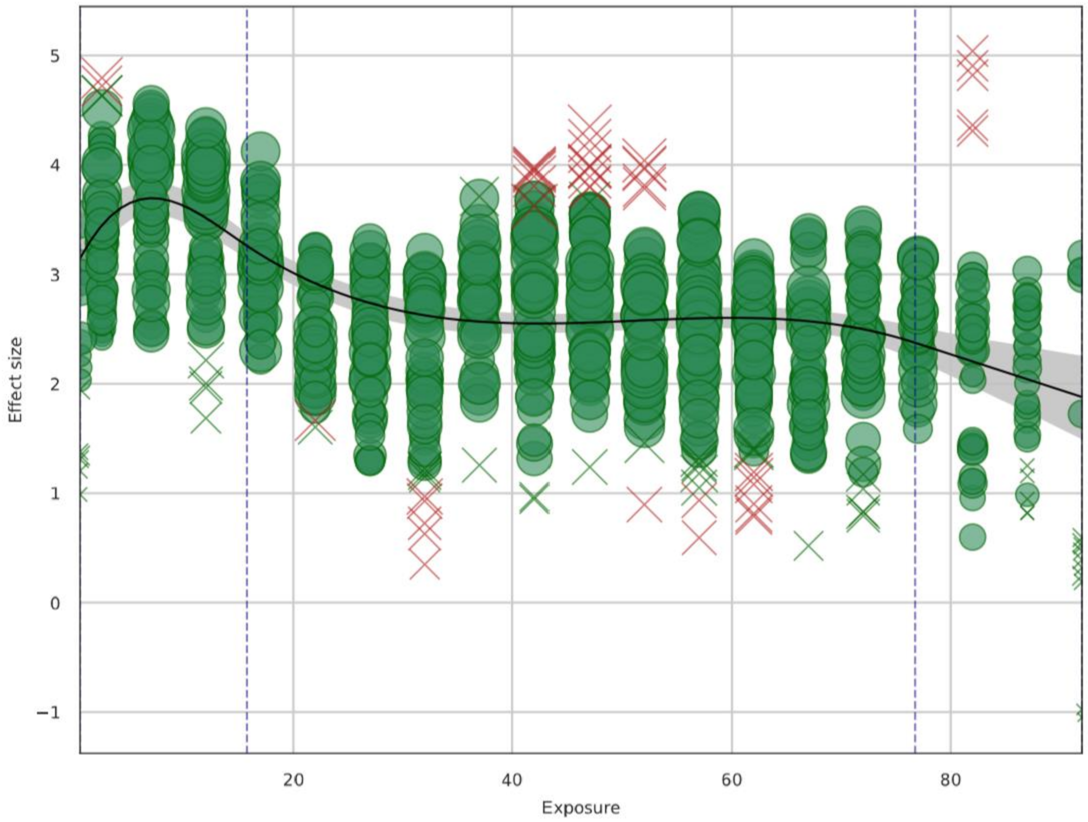

Figure 2a. Cubic spline on age midpoint for MarketScan claims crosswalk (exposure is age midpoint, effect size is the adjustment factor in logit space)

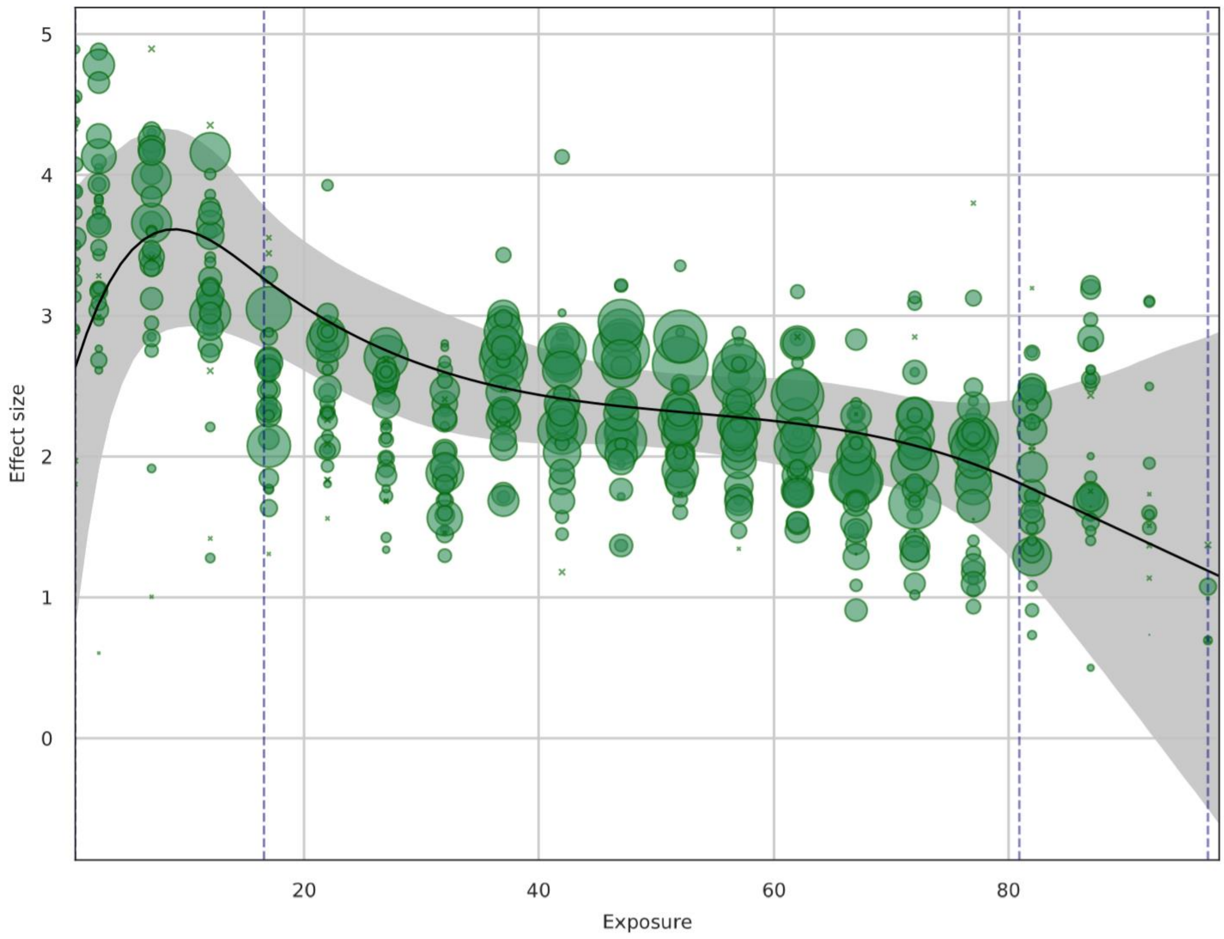

Figure 2b. Cubic spline on age midpoint for MarketScan 2000 claims crosswalk (exposure is age midpoint, effect size is the adjustment factor in logit space)

Modelling strategy

Non-fatal outcomes were modelled using a combination of custom models and DisMod-MR 2.1. First, the overall incidence and prevalence of encephalitis were modelled to estimate the short-term morbidity due to acute infection. This DisMod model had a set duration (1/remission) of three weeks. We also imposed caps on excess mortality for ages 10–50. USA claims data were grouped into year-specific covariates based on quality and were crosswalked to the reference data, which we extracted from literature and inpatient hospital data. We used the function in DisMod-MR 2.1 to pull in cause-specific mortality rate (CSMR) data from our CODEm and CoDCorrect analyses and match with incidence datapoints for the same geography. We calculated excess mortality rate to estimate priors for EMR by dividing CSMR by prevalence, calculated from remission and incidence. To help inform trends where we lack data, we applied a binary country-level covariate at the subnational and country level that indicates if the location is in a Japanese encephalitis-endemic area.[2] We also applied a lag-distributed income covariate to excess mortality. Betas and exponentiated values (which can be interpreted as an odds ratio)

are shown in the tables below for study-level covariates and country-level covariates. We outliered incidence input datapoints with zero cases that were dragging down final estimates. We also improved our time efficiency and estimation accuracy by using an ordinary differential equations solver (ODE solver) in place of traditional DisMod-MR.

Table 3. Covariates. Summary of covariates used in the encephalitis DisMod-MR meta-regression model

Covariate	Type	Parameter	Exponentiated beta (95% uncertainty interval)
Japanese encephalitis endemic area	Country-level covariate	Incidence	1.10 (1.10–1.11)
LDI (log transformed)	Country-level covariate	Excess mortality	1.00 (1.00–1.00)

In addition to short-term sequelae as a result of acute encephalitis, we also modelled the long-term outcomes from encephalitis.

Sequelae splits

We first split the long-term sequelae among survivors of acute infection. We calculated the acute phase survivors by applying the excess mortality (calculated by the acute encephalitis DisMod model) to the incidence of each aetiology (excess mortality was converted to case fatality rate by $e^{(-\text{excess mortality} \times 1/(\text{excess mortality} + \text{remission}))}$. The survivors were then subject to long-term sequelae by applying the post-discharge proportions of health consequences calculated by a meta-analysis by Edmond and colleagues.[3] We calculated the ratio of acute encephalitis cases that result in a major long-term impairment, and the ratio of minor impairments to major impairments, based off a regression of log-transformed GDP and ratio values from Edmond and colleagues. This regression was done differently from last year when we used GNI. The regression is shown below: $y = -0.33590 \ln(\text{GDP}) + 1.15230$

We assumed a similar pattern of health outcomes for encephalitis infection survivors as with other bacterial meningitis survivors (except hearing loss, as we could not find evidence of hearing loss as a consequence of encephalitis infection). We used these two ratios to calculate the proportions of survivors who contract a long-term minor impairment and those who contract a long-term major impairment. The proportion with major impairments were further split (again using pooled proportions from Edmond and colleagues) into specific major impairments, which were grouped into vision loss, moderate to severe cognitive impairments, and epilepsy.

The calculated incidence of long-term sequelae was then converted to prevalence by two different approaches. For the sequelae not associated with excess mortality, which were vision loss, intellectual disability, motor impairment, and behavioural problems, the incidence of each age was cumulatively added up to the subsequent age (assuming half-cycle) to construct prevalence at each age. If the sequela is associated with excess mortality (epilepsy and moderate-to-severe cognitive impairments), the calculated incidence was used as an input to the ODE solver, together with the corresponding mortality parameters (excess mortality data from the epilepsy envelope DisMod model, and standardised mortality ratio data from a neonatal encephalopathy meta-analysis, converted to excess mortality using

all-cause mortality estimates) to estimate the prevalence. Vision loss and epilepsy estimates were squeezed and severity split centrally.

Disability weights

The basis of the GBD disability weight survey assessments is lay descriptions of sequelae highlighting major functional consequences and symptoms. The lay descriptions and disability weights for sequelae associated with encephalitis are shown below.

Table 4. Severity distribution, details on the severity levels for encephalitis in GBD 2023 and the associated disability weight (DW) with that severity

Severity split	Lay description	DW (95% CI)
Mild behaviour problems	This person is hyperactive and has difficulty concentrating, remembering things, and completing tasks.	0.045 (0.028–0.066)
Moderate motor impairment	This person has some difficulty in moving around, and difficulty in lifting and holding objects, dressing, and sitting upright, but is able to walk without help.	0.061 (0.040–0.089)
Moderate motor plus cognitive impairments	This person has some difficulty in moving around, holding objects, dressing, and sitting upright, but can walk without help. This person has low intelligence and is slow in learning to speak and to do simple tasks.	0.20 (0.13–0.29)
Long-term mild motor impairment	This person has some difficulty in moving around but is able to walk without help.	0.01 (0.005–0.019)
Borderline intellectual disability	This person is slow in learning at school. As an adult, the person has some difficulty doing complex or unfamiliar tasks but otherwise functions independently.	0.011 (0.005–0.020)
Severe motor impairment	This person is unable to move around without help, and is not able to lift or hold objects, get dressed, or sit upright.	0.40 (0.27–0.55)
Epilepsy	(combined DW)	NA
Blindness	Is completely blind, which causes great difficulty in some daily activities, worry and anxiety, and great difficulty going outside the home without assistance.	0.19 (0.12–0.26)
Acute encephalitis	This person has a high fever and pain, and feels very weak, which causes great difficulty with daily activities.	0.13 (0.088–0.19)
Mild intellectual disability	This person has low intelligence and is slow in learning at school. As an adult, the person can live independently but often needs help to raise children and can only work at simple supervised jobs.	0.043 (0.026–0.064)
Monocular distance vision loss	This person is blind in one eye and has difficulty judging distances.	0.017 (0.009–0.029)
Mild motor plus cognitive impairments	This person has some difficulty in moving around but is able to walk without help. The person is slow in	0.031 (0.018–0.050)

	learning at school. As an adult, the person has some difficulty doing complex or unfamiliar tasks but otherwise functions independently.	
Severe motor plus cognitive impairments	This person cannot move around without help, and cannot lift or hold objects, get dressed, or sit upright. The person also has very low intelligence, speaks few words, and needs constant supervision and help with all daily activities.	0.54 (0.37–0.70)
Moderate vision impairment due to encephalitis	This person has vision problems that make it difficult to recognize faces or objects across a room.	0.031 (0.019–0.049)
Severe vision impairment due to encephalitis	This person has severe vision loss, which causes difficulty in daily activities, some emotional impact (for example worry), and some difficulty going outside the home without assistance.	0.18 (0.13–0.26)

References

1. Ferrari AJ, Santomauro DF, Aali A, Abate YH, Abbafati C, Abbastabar H, et al. Global incidence, prevalence, years lived with disability (YLDs), disability-adjusted life-years (DALYs), and healthy life expectancy (HALE) for 371 diseases and injuries in 204 countries and territories and 811 subnational locations, 1990–2021: a systematic analysis for the Global Burden of Disease Study 2021. The Lancet. 2024 May 18;403(10440):2133–61.
2. Centers for Disease Control and Prevention (CDC). CDC Yellow Book 2016 Health Information for International Travel. Oxford University Press USA; 2016.
3. Edmond K, Clark A, Korczak VS, Sanderson C, Griffiths UK, Rudan I. Global and regional risk of disabling sequelae from bacterial meningitis: a systematic review and meta-analysis. Lancet Infect Dis. 2010 May;10(5):317–28.

Endocrine, metabolic, blood, and immune disorders (EMBID)

Flowchart

Endocrine, Metabolic, Blood, Immune Disorders (EMBID)

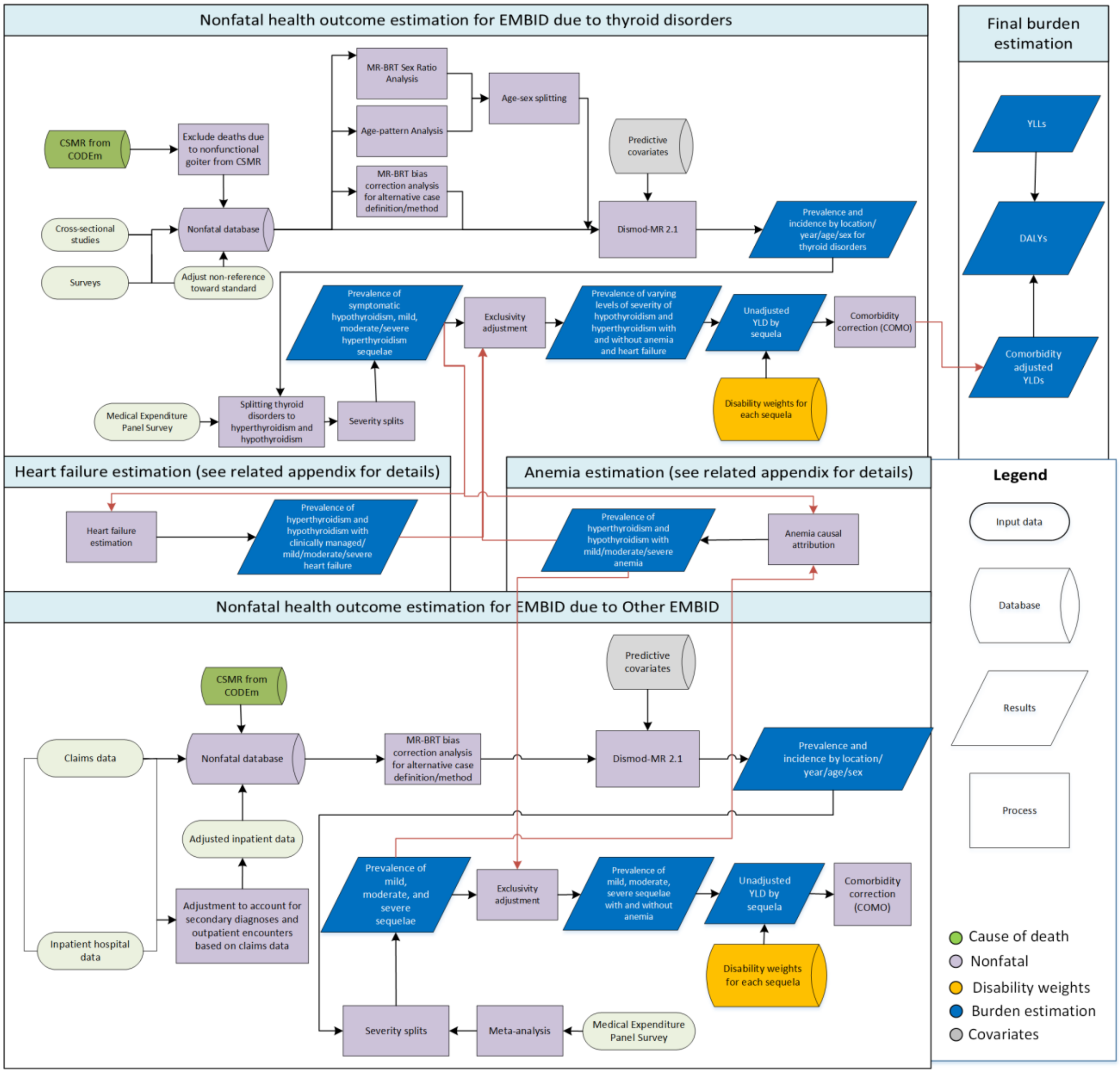

Input data and methodological summary for endocrine, metabolic, blood, and immune disorders

Case definitions

Endocrine, metabolic, blood, and immune disorders (EMBID) is a Level 3 residual cause consisting of conditions that are not related to nutritional deficiencies (eg, iodine deficiency) and do not map to other causes within the diabetes, urogenital, blood, and endocrine disease hierarchy. It is referred to in this appendix section as Level 3 EMBID. Within Level 3 EMBID, there are two Level 4 causes: 1) thyroid disorders and 2) other EMBID excluding thyroid disorders (referred to as "other EMBID" throughout this report).

Thyroid disorders are defined as acquired thyroid disorders with abnormal thyroid function, evidenced by abnormal levels of thyroid-stimulating hormone and thyroxine, or by being on treatment for thyroid dysfunction. Specifically, abnormal levels of thyroid-stimulating hormone and thyroxine are defined as:

- Overproduction of thyroid hormones: serum thyroid-stimulating hormone (TSH) concentration of 0.5 mIU/L or less; confirmed with high serum free T4 result (by laboratory standard)
- Underproduction of thyroid hormones: serum TSH concentration of 5 mIU/L or greater; confirmed with low serum free T4 result (by laboratory standard)

This Level 4 cause excludes goiter, as it is captured in a separate cause within GBD specific to iodine deficiency, where goiter serves as a proxy for iodine deficiency.

Other EMBID includes other endocrine, metabolic, immune, or blood disorders. In terms of ICD codes, GBD's definition of other EMBID excludes the codes for nutritional deficiencies, diabetes, anaemia, haemoglobinopathies, haemolytic disorders, and iron deficiency, which are modelled as separate causes or impairments; as well as those for obesity and hypercholesterolaemia, which are modelled as risk factors.

ICD-10 codes for other EMBID include: D64.4, D64.8, D68-D68.6, D68.8-D68.9, D69.6, D73- D73.5, D73.8-D73.9, D74.0, D74.8-D74.9, D75-D75.2, D75.8-D75.9, D76-D76.3, D80-D80.9, D81-D81.9, D82-D82.4, D82.8-D82.9, D83-D83.2, D83.8-D83.9, D84-D84.1, D84.8-D84.9, D86.8, D89-D89.2, D89.8-D89.9, E04-E04.2, E04.8-E04.9, E16.. 1-E16.4, E16.8-E16.9, E20-E20.1, E20.8-E20.9, E21-E21.5, E22-E22.2, E22.8-E22.9, E23.0, E23.2-E23.3, E23.6-E23.7, E24-E24.1, E24.3, E24.9, E25.0, E25.8-E25.9, E26-, E26.8-E26.9, E27-E27.2, E27.4-E27.5, E27.8-E27.9, E28-E28.1, E28.3, E28.8-E28.9, E29-E29.1, E29.8-E29.9, E30-E30.1, E30.8-E30.9, E31-E31.2, E31.8-E31.9, E32-E32.1, E32.8-E32.9, E34-E34.5, E34.8-E34.9, E67-E67.3, E67.8, E70-E70.5, E70.8-E70.9, E71-E71.5, E72-E72.5, E72.8-E72.9, E73-E73.1, E73.8-E73.9, E74-E74.4, E74.8-E74.9, E75-E75.6, E76-E76.3, E76.8-E76.9, E77-E77.1, E77.8-E77.9, E79-E79.2, E79.8-E79.9, E80-E80.7, E84-E84.9, and E88-E88.9.

The above codes are identical in GBD 2023 as in GBD 2021. In GBD 2021, we removed D70-D70.4, D70.8-D70.9, D72-D72.1, D72.8-D72.9, D75.1, E26.1 E83-E83.9, E85-E85.9, E88.3, E71.43, D69-D69.4, and D69-D69.8 from Level 3 EMBID and its child causes, although those codes were mapped to Level 3 EMBID in earlier rounds of GBD.

Overall strategy

We utilised two databases for Level 3 EMBID as inputs to two separate, complete compartmental DisMod-MR models: thyroid disorders and other EMBID. The model outputs of thyroid disorders and other EMBID were separately split into relevant severity levels and sequelae, combined with disability

weights to produce unadjusted YLDs, and adjusted for comorbidity. Final estimates of incidence, prevalence, and comorbidity-adjusted YLDs were then summed to produce the final non-fatal estimates of Level 3 EMBID.

All input data used for thyroid disorders and other EMBID are summarised below.

Thyroid disorders

Input data

For thyroid disorders, we extracted prevalence and incidence data from peer-reviewed publications identified via systematic literature reviews conducted by Madariaga and colleagues in 2014[1] and Taylor and colleagues in 2018.[2] We also included microdata from the USA National Health and Nutrition Examination Survey (NHANES) from years 2001–2002, 2007–2008, and 2009–2010.

The non-fatal model of thyroid disorders also included cause-specific mortality rate (CSMR) estimates taken from our fatal modelling process (see CoD cause-specific modelling description for thyroid disorders in this appendix). Prior to using these CSMR estimates in the non-fatal model, we conducted an adjustment to remove deaths due to non-functional goitres (see the CSMR data processing section below).

Prevalence and incidence data processing

Because we produce sex-specific estimates, we adjusted data that reported on both sexes into male and female sex-specific estimates. We identified studies that reported sex-specific data and calculated the log ratio of female to male prevalence from studies that report sex-specific prevalence, modelling these log ratios in meta-regression—Bayesian, regularised, trimmed (MR-BRT), a regression tool developed at IHME. We then used the modelled sex ratio to adjust "both"-sex data values to expected "male" and "female" values. We calculated the male values as

$$val_{male} = val_{both} * \frac{pop_{both}}{(pop_{male} + ratio * pop_{female})}.$$

We calculated female values as $val_{female} = ratio * val_{male}$.

This sex-splitting process was done separately for prevalence and incidence. For prevalence, the ratio of female to male cases derived from MR-BRT analysis was 2.71 (95% UI 1.88–3.91). For incidence, we only had sex-specific datapoints and thus did not require a separate sex-splitting data process.

We then adjusted data from the studies that used non-reference TSH cut-offs to diagnose thyroid disorders. To do this, we leveraged TSH distributions found in the National Health Surveys from Chile and the USA. Specifically, we calculated the prevalence of hyperthyroidism and hypothyroidism

[1]Garmendia Madariaga A, Santos Palacios S, Guillén-Grima F, Galofré JC. The incidence and prevalence of thyroid dysfunction in Europe: a meta-analysis. *J Clin Endocrinol Metab*. 2014 Mar;99(3):923-31. doi: 10.1210/jc.2013-2409. Epub 2014 Jan 1. PMID: 24423323.
[2]Taylor PN, Albrecht D, Scholz A, Gutierrez-Buey G, Lazarus JH, Dayan CM, Okosieme OE. Global epidemiology of hyperthyroidism and hypothyroidism. *Nat Rev Endocrinol*. 2018 May;14(5):301-316. doi: 10.1038/nrendo.2018.18. Epub 2018 Mar 23. PMID: 29569622.

produced using both alternative (non-reference) TSH cut-offs and reference TSH cut-offs. Then we calculated the logit difference of the non-reference prevalence estimates and the reference prevalence estimates. These logit differences were used as an input to MR-BRT (meta-regression—Bayesian, regularised, trimmed) to estimate the adjustment factors, which were then applied to all prevalence datapoints extracted from studies that used alternative TSH cut-offs.

We also made a systematic bias adjustment to prevalence datapoints from studies that only included previously undiagnosed individuals in the study sample (ie, studies where the numerator of prevalent cases was newly diagnosed as part of the study and neither the numerator nor the denominator included individuals who were previously diagnosed with thyroid disorders and/or on treatment). For this, we first identified studies that reported prevalence for both reference (ie, studies where both numerator and denominator included those who were previously diagnosed with thyroid disorders and/or on treatment) and non-reference (ie, studies where both numerator and denominator excluded those who were previously diagnosed with thyroid disorders and/or on treatment) (Figure 1). We then calculated the logit difference between the two datapoints for each study and used it as an input to MR-BRT the same manner described above. The adjustment factor was then applied to all datapoints that only reported the prevalence of previously undiagnosed thyroid disorders among those not already on treatment.

The process of adjusting for non-reference data using MR-BRT with the logit-transformation method is described below:

1. Identify datapoints with overlapping year, age, sex, and location between reference and non-reference population data.
2. Logit transform overlapping datapoints of alternative and reference types.
3. Convert overlapping datapoints into a difference in logit space using the following equation: $logit(altnerative) - logit(reference)$
4. Use the delta method to compute standard errors of overlapping datapoints in logit space, then calculate standard error of logit difference using the following equation: $\sqrt{(variance\ of\ logit(alternative)) + (variance\ of\ logit(reference))}$
5. Using MR-BRT, conduct a random effects meta-regression to obtain the pooled logit difference of alternative to reference
6. Apply the pooled logit difference to all data points of non-reference types using the following equation:
$$new_{estimate} = inverse.logit((logit(alternative)) - (pooled\ logit\ difference))$$
7. Calculate new standard errors using the delta method, accounting for gamma (between-study heterogeneity)

Figure 1. Studies reported both reference and non-reference (alternative) data; used in MR-BRT

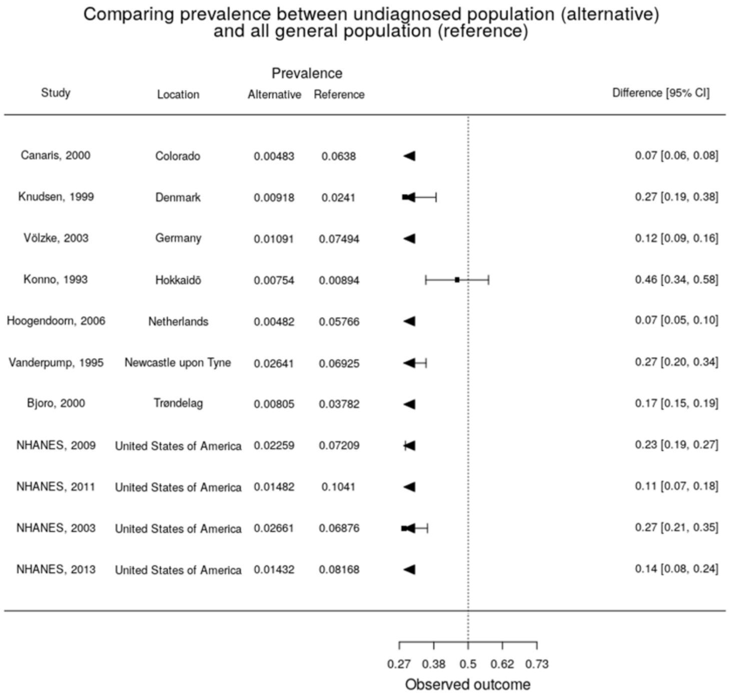

Table 2. MR-BRT crosswalk adjustment factors for thyroid disorders

Data input	Reference or alternative case definition	Gamma	Beta coefficient, logit (95% CI)	Adjustment factor*
General population	Ref	0	--	--
Excluding those currently on treatment	Alt		–1.45 (–1.78 to –1.12)	0.234 (0.17 to 0.33)

**MR-BRT crosswalk adjustments can be interpreted as the factor the alternative case definition is adjusted by to reflect what it would have been had it been measured using the reference case definition. If the log/logit beta coefficient is negative, then the alternative is adjusted up to the reference. If the log/logit beta coefficient is positive, then the alternative is adjusted down to the reference.*

***The adjustment factor column is the exponentiated beta coefficient. For log beta coefficients, this is the relative rate between the two case definitions. For logit beta coefficients, this is the relative odds between the two case definitions.*

After adjustments for case definitions (alternative TSH thresholds) and populations (general population or only the population not already on treatment), we also split datapoints where the age range was greater than 25 years using the global age pattern informed by the datapoints with fine age groups (ie, ages 5–9, 10–14, and 15–20…).

CSMR data processing

Unlike the fatal model, our non-fatal model excludes non-functional goitres. To maintain consistency between the fatal and non-fatal input data, we conducted a custom modelling process to estimate CSMR of thyroid disorders excluding non-functional goitre. Specifically, this was done by calculating the proportion of nonfunctional goitre deaths from the total thyroid disorders deaths by year and super-

region using cause of death data extracted from data-rich locations (see CoD cause-specific modelling description in this appendix) and applying these proportions to final CoDCorrected CSMR for thyroid diseases.

Modelling strategy

DisMod-MR model

We made no changes to the modelling strategy from GBD 2021. We ran a DisMod-MR 2.1 model to produce estimates by age, sex, year, and country. Inputs to DisMod-MR were prevalence, incidence, and CSMR estimates described above. We set a maximum disease duration of two years and assumed that no one was born with these thyroid disorders. The minimum coefficient of variation at the regional, super-regional, and global level was 0.8.

We included Healthcare Access and Quality Index and proportion of households using iodised salt (adjusted) as predictive covariates to inform excess mortality and incidence. The beta and exponentiated values of these covariates (which can be interpreted as an odds ratio) are shown in the table below.

Table 3. Covariates. Summary of covariates used in the thyroid disorders DisMod-MR meta-regression model

Covariate	Parameter	Exponentiated beta (95% uncertainty interval)
Healthcare Access and Quality Index	Excess mortality rate	1.00 (1.00–1.00)
Proportion of households using iodised salt (adjusted)	Incidence	0.14 (0.14–0.16)

Severity split and disability weight

The nature and severity of symptoms and functional impairment suffered due to thyroid disorders vary between hyperthyroidism and hypothyroidism. Individuals with thyroid disorders may move between hyper- and hypothyroid states over the course of their illness. To estimate a snapshot of the relative prevalence of each, we used data from the Medical Expenditure Panel Survey (MEPS) to split the estimates of thyroid disorders into hyperthyroidism and hypothyroidism. MEPS is an overlapping panel survey of the non-institutionalised USA population that collects data on respondents' health service interactions. Panels are initiated every year. Each panel is two years long and consists of five rounds.

Disease	ICD-9 codes used in MEPS	Frequency	Proportion
Hyperthyroidism	242, 245	1138	9.8%
Hypothyroidism	243, 244, 246	10,417	90.2%

Severity split & disability weight

The distribution of cases among differing severities of hypothyroidism and hyperthyroidism were derived from analysis of MEPS. In 2000, MEPS began using 12-Item Short Form Surveys (SF-12) to collect data on functional health status. The SF-12 survey is administered twice per panel (about once per year). To translate SF-12 scores into GBD disability weights, 62 lay descriptions for health states representing the full range of disability weight values (from most mild to most severe) were selected. A convenience sample of respondents was then asked to complete an SF-12 form for an individual with the health state described in the lay descriptions of these conditions. Composite mental and physical SF-12 score was regressed on GBD disability weight to derive the relationship between disability weight and SF-12 score. Individual respondent scores were then regressed on reported conditions to predict cumulative disability from the individuals' diagnoses, and a counterfactual cumulative disability excluding a single condition was predicted to obtain a comorbidity-corrected condition-specific disability weights for all individual with that single condition. The distribution of these condition-specific disability weights was used to derive the proportion of individuals with the condition that fall within each GBD severity category.

The basis of the GBD disability weight survey assessments is lay descriptions of sequelae highlighting major functional consequences and symptoms. Hyperthyroidism and hypothyroidism are assigned different lay descriptions associated with different disability weights. Specifically, hypothyroidism is split into asymptomatic and symptomatic categories. Hyperthyroidism is split into asymptomatic, mild, and moderate/severe categories. The lay descriptions and disability weights for thyroid disorders are shown below.

Table 4. Severity distribution, details on the severity levels for thyroid disorders and the associated disability weight (DW) with that severity

Disease	Severity level	Lay description	DW (95% CI)	Distribution (95% CI)
Hypothyroidism	Asymptomatic	--	--	0.436 (0.432–0.441)
	Symptomatic	Has low energy and feels cold	0.019 (0.010–0.032)	0.564 (0.559–0.568)
Hyperthyroidism	Asymptomatic	--	--	0.417 (0.410–0.423)
	Mild	Has a chronic disease that requires medication every day and causes some worry but minimal interference with daily activities	0.049 (0.031–0.072)	0.418 (0.360–0.447)
	Moderate/severe	Feels nervous, has palpitations, sweats a lot, and has difficulty sleeping	0.145 (0.095–0.202)	0.165 (0.138–0.222)

Other endocrine, metabolic, blood, and immune disorders

Input data

The “other EMBD” model used prevalence data from the IHME clinical administrative data library aggregated and processed for GBD as described in “Clinical Input data and methods summary” section of this appendix. Notably, in GBD 2023, this library was expanded with additional years of data from USA claims (years 2018 and 2019), Poland claims (year 2019), as well as hospital discharges in Austria, Brazil, Chile, Georgia, Germany, Italy, Mexico, New Zealand, the Philippines, and the USA. We also added for the first time Medicare data in the USA for adults aged 65 years and above.

We explored the heterogeneity within the clinical administrative data for this cause and found that many extreme values were seen in inpatient data from sources that either do not cover the entire population or do not include a representative population sample, thus requiring extraction as discharge diagnosis cause fractions and conversion to prevalence using GBD estimates of hospital admission rate per capita. (See the section of this appendix on the hospital utilisation estimates for information on this input.) In GBD 2023, we decided to exclude all inpatient sources that require this adjustment and only include the sources with full coverage of the target population.

Inputs to our non-fatal modelling also included cause-specific mortality rate (CSMR) estimates taken from our fatal modelling process (see CoD cause-specific modelling description for appendicitis in this appendix) and excess mortality rate (EMR) estimates modelled outside of DisMod (see the EMR data processing section below).

Prevalence data processing

Hospital discharge data provide observations about encounters, generally with only the primary diagnostic code for the encounter. Claims data, on the other hand, link claims for all inpatient and outpatient encounters for a single individual and provide primary and secondary diagnoses for all encounters.

An individual was extracted from claims data as a prevalent case if that individual had at least one inpatient or two outpatient encounters with an appropriate ICD code as any diagnosis within one year. Hospital discharge data were processed by extracting discharges with an appropriate ICD code as primary diagnosis and adjusting using correction factors derived from claims data. Specifically, we apply correction factors 1 and 5 (CF1 and CF5). CF1 is defined as the ratio of deduplicated primary diagnoses among inpatient admissions, to adjusted primary diagnoses among inpatient admissions, while CF5 is defined as the ratio of deduplicated primary and secondary diagnoses among inpatient encounters and outpatient encounters to deduplicated primary diagnoses among inpatient encounters. This approach accounts for non-primary inpatient diagnoses and outpatient cases serially, to allow data sources to contribute to estimation of de-duplication for readmission, multiple diagnoses in inpatient encounters, and/or outpatient care, depending on which of these relationships are captured in the dataset, rather than only employing sources that have complete information on all diagnoses and encounter types. This approach makes use of more diverse data inputs. This final scalar is applied to extracted primary diagnosis admission rate data as a product of CF1 and 1/CF5. See the section of this appendix on the processing of hospital data for more details.

The USA claims data (extracted and processed as described above) were adjusted to account for selection bias due to commercial insurance, using the same MR-BRT analysis described above. The USA

MarketScan claims data from the year 2000 and from the years 2010–2019 were separately adjusted to account for selection bias due to commercial insurance, which was suspected to be differential over the years as coverage expanded.

The process of adjusting for biases in non-reference data using MR-BRT with the logit-transformation method is described below:

1. Identify datapoints with overlapping year, age, sex, and location between reference and non-reference population data.
2. Logit transform overlapping datapoints of alternative and reference types.
3. Convert overlapping datapoints into a difference in logit space using the following equation: $logit(altnerative) - logit(reference)$
4. Use the delta method to compute standard errors of overlapping datapoints in logit space, then calculate standard error of logit difference using the following equation: $\sqrt{(variance\ of\ logit(alternative)) + (variance\ of\ logit(reference))}$
5. Using MR-BRT, conduct a random effects meta-regression to obtain the pooled logit difference of alternative to reference
6. Apply the pooled logit difference to all data points of non-reference types using the following equation:
$$new_{estimate} = inverse.logit((logit(alternative)) - (pooled\ logit\ difference))$$
7. Calculate new standard errors using the delta method, accounting for gamma (between-study heterogeneity)

Table 5. MR-BRT crosswalk adjustment factors for other endocrine, metabolic, blood, and immune disorders

Data input	Reference or alternative data collection	Gamma	Covariate	Beta coefficient, logit (95% CI)	Adjustment factor*
Hospital + non-USA claims	Ref	0.085		---	---
USA claims from year 2000	Alt		Age (continuous from 0 to 95+)	–0.024 (–0.026 to –0.022)	0.494 (0.493 to 0.495)
			Sex (female to male)	0.660 (0.569 to 0.751)	0.659 (0.639 to 0.679)
			Intercept	–0.232 (–0.405 to –0.059)	0.442 (0.400 to 0.485)
USA claims from years 2010–2019	Alt		Age (continuous from 0 to 95+)	–0.015 (–0.586 to –0.557)	0.496 (0.358 to 0.636)
			Sex (female to male)	0.380 (–0.192 to 0.951)	0.594 (0.452 to 0.721)
			Intercept	1.270 (0.679 to 1.862)	0.781 (0.663 to 0.866)

**MR-BRT crosswalk adjustments can be interpreted as the factor the alternative case definition is adjusted by to reflect what it would have been had it been measured using the reference case definition. If the log/logit beta*

coefficient is negative, then the alternative is adjusted up to the reference. If the log/logit beta coefficient is positive, then the alternative is adjusted down to the reference.
***The adjustment factor column is the exponentiated beta coefficient. For log beta coefficients, this is the relative rate between the two case definitions. For logit beta coefficients, this is the relative odds between the two case definitions.*

Modelling strategy

DisMod-MR model

We fit a DisMod-MR 2.1 compartmental model to produce estimates by age, sex, year, and country. Input data were prevalence and CSMR estimates described above. Prior settings in the DisMod-MR model included setting maximum remission of four years. We also assumed that no one was born with other EMBID. The minimum coefficient of variation at the regional, super-regional, and global level was set at 0.8.

We included lagged distributed income (LDI) as a predictive covariate to inform excess mortality, with a lower bound of –0.5 and an upper bound of –0.1. The beta and exponentiated values of this covariate (which can be interpreted as an odds ratio) are shown in the table below.

Table 6. Covariates. Summary of covariates used in the other endocrine, metabolic, blood, and immune disorders DisMod-MR meta-regression model

Covariate	Parameter	Exponentiated beta (95% uncertainty interval)
LDI (I$ per capita)	Excess mortality rate	0.90<br>(0.90–0.90)

Severity split & disability weight

Other EMBID is split into four levels of severity: asymptomatic, mild, moderate, and severe. The lay descriptions, disability weights, and severity distribution for other EMBID are shown below. The severity distributions of other EMBID were derived from analysis of the Medical Expenditure Panel Surveys (MEPS) in the same manner as hyperthyroidism and hypothyroidism as described above.

Table 7. Severity distribution, details on the severity levels for endocrine, metabolic, blood, and immune disorders and the associated disability weight (DW) with that severity.

Disease	Severity level	Lay description	DW<br>(95% CI)	Distribution<br>(95% CI)
Other EMBID	Asymptomatic	--	--	0.431<br>(0.427–0.436)
	Mild	Feels slightly tired and weak at times, but this does not interfere with normal daily activities	0.004<br>(0.001–0.008)	0.202<br>(0.148–0.262)
	Moderate	Has a chronic disease that requires medication every day and causes	0.049<br>(0.031–0.072)	0.189<br>(0.149–0.226)

		some worry but minimal interference with daily activities		
	Severe	Easily bruises and sometimes bleeds from the gums and nose; feels weak and has some difficulty with daily activities	0.159 (0.106–0.226)	0.178 (0.148–0.220)

Epilepsy impairment envelope

Flowchart

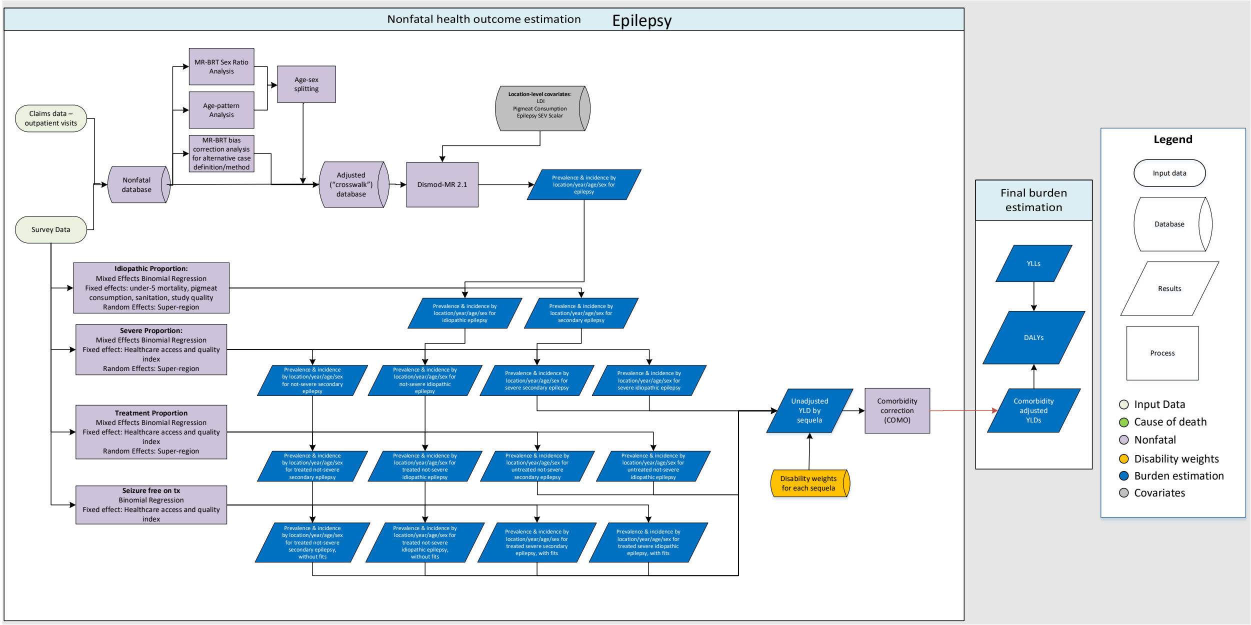

Case definition

Since GBD 2013, we have used the following definitions from the “Guidelines for Epidemiologic Studies on Epilepsy”: 1) Epilepsy: a condition characterised by recurrent (two or more) epileptic seizures, unprovoked by any immediate identified cause, and 2) “Active” epilepsy: a prevalent case of active epilepsy is defined as a person with epilepsy who has had at least one epileptic seizure in the previous five years, regardless of antiepileptic drug (AED) treatment. We also use the following ICD-10 codes for epilepsy: G40 (Neuro, epilepsy, total) and G41 (Neuro, epilepsy, status epilepticus). We define severe epilepsy as having seizures one or more times per month.

Input data and processing

Data inputs

The primary data inputs for the epilepsy modelling strategy were measurements of prevalence, incidence, remission rate, excess mortality rate, relative risk of mortality, standardised mortality ratio, or with-condition mortality rate for all epilepsy, regardless of cause, severity or treatment status.

For GBD 2021, we conducted a systematic review covering 01/10/2016 to 01/28/2020 using the following search string:

(2016/10/01[PDAT] : 3000[PDAT]) AND ("epilepsy"[MeSH Terms] OR "epilepsy, partial, motor"[MeSH Terms] OR "epilepsy, benign neonatal"[MeSH Terms] OR "epilepsy, reflex"[MeSH Terms] OR "myoclonic epilepsy, juvenile"[MeSH Terms] OR "epilepsy, frontal lobe"[MeSH Terms] OR "epilepsy, complex partial"[MeSH Terms] OR "epilepsy, post-traumatic"[MeSH Terms] OR "epilepsy, temporal lobe"[MeSH

Terms] OR "epilepsy, absence"[MeSH Terms] OR "epilepsy, tonic-clonic"[MeSH Terms] OR "epilepsies, myoclonic"[MeSH Terms] OR "epilepsies, partial"[MeSH Terms] OR epilep*[Title/Abstract]) AND (inciden*[Title/Abstract] OR prevalen*[Title/Abstract]) NOT (animals[MeSH] NOT umans[MeSH])

We included representative, population-based surveys that reported on prevalence, incidence, remission rate, excess mortality rate, relative risk of mortality, standardised mortality ratio, or with-condition mortality rate. We excluded studies with no clearly defined sample (eg, among clinic attenders or patient organisation members with non-specific or non-representative catchment area).

PRISMA 2020 Flow Diagram

From: Moher D, Liberati A, Tetzlaff J, Altman DG, The PRISMA Group (2009). *Preferred Reporting Items for Systematic Reviews and Meta-Analyses*: The PRISMA Statement. PLoS Med 6(7): e1000097. doi:10.1371/journal.pmed1000097

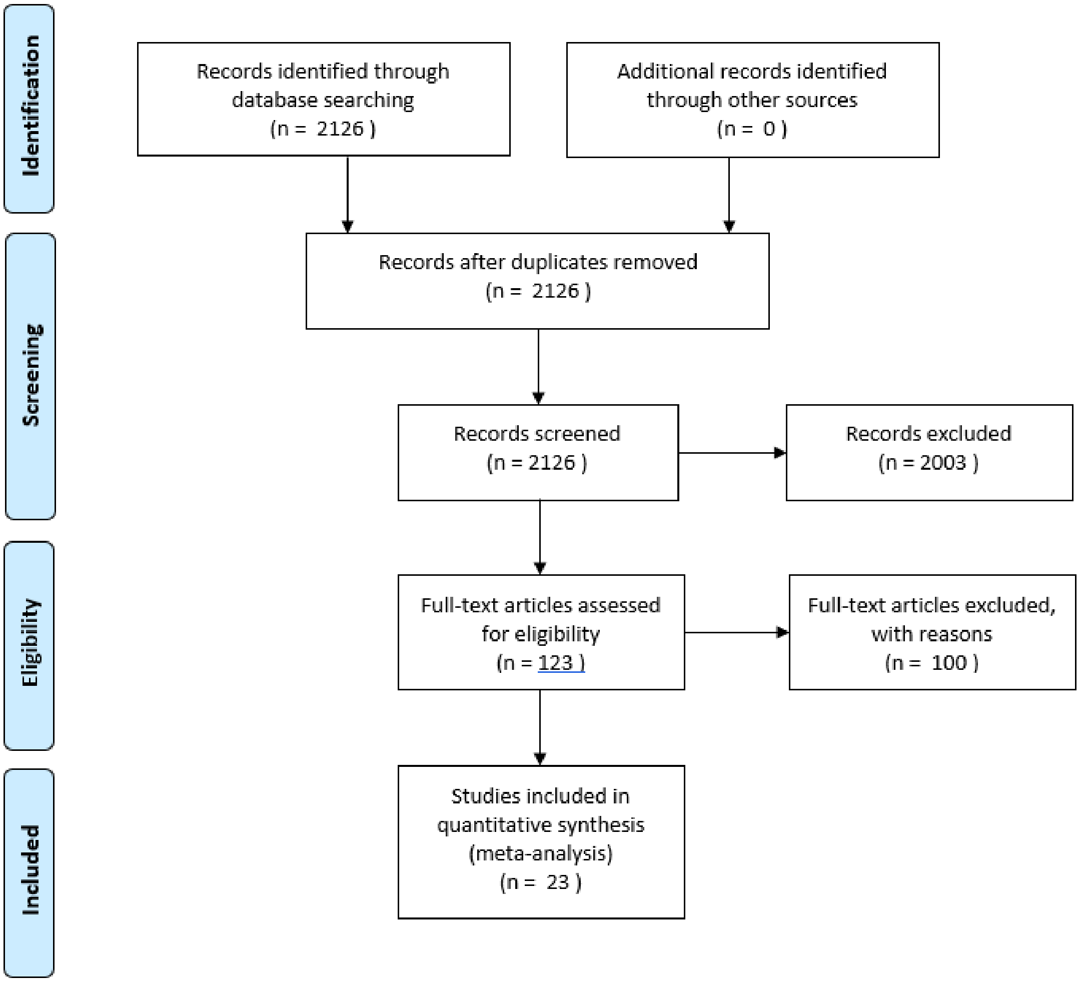

Although epilepsy was not originally scheduled for revision in GBD 2023, key adjustments were implemented to enhance the model. Active recall data from the Canadian Chronic Disease Surveillance System (CCDSS) covering 2005–2022 were extracted and incorporated into the model. Additionally, 30 data sources from WHO's Global Epilepsy Report 2019 not yet included in our database were screened, leading to the inclusion of 12 eligible sources—four with incidence data and ten with prevalence data. Four sources requiring adjustment from lifetime to active epilepsy were deferred for future inclusion.

For epilepsy modelling in GBD 2023, we use the following clinical data sources: Poland claims data from 2018, and Taiwan claims data from 2016. While we have previously used US MarketScan claims data from the years 2000 and 2010–2017, with the addition of the US MarketScan claims data for 2018 we found that there were so much MarketScan claims data in comparison to the smaller population studies that MarketScan was having an unduly large impact on the model. As such we decided to drop all US MarketScan claims data as we trust the smaller population studies more.

Additional data inputs include data on the proportion of epilepsy that is primary or idiopathic, the proportion of epilepsy that is severe (one or more fits per month), the proportion of epilepsy that is untreated (the treatment gap), and the proportion of treated epilepsy that is treated without fits (no fits reported in the preceding year).

Data processing

For GBD 2023, raw data with large age ranges were split into five-year age groups using the age pattern generated from a DisMod 2.1 model (details on this method can be found in appendix 1, section 2) with input data of only less than 25 years age range. Standard GBD sex-splitting methods were used for studies with only "both"-sex datapoints. We modelled the ratio of female/male prevalence in MR-BRT and calculated male prevalence:

$$prev_{male} = prev_{both} * \frac{pop_{both}}{(pop_{male} + ratio * pop_{female})}$$

And then calculated female prevalence:

$$prev_{female} = ratio * prev_{male}$$

For epilepsy, the modelled female/male ratio demonstrated a higher prevalence in males and was used to proportionally split "both"-sex datapoints into male and female datapoints (as seen in the figure below).

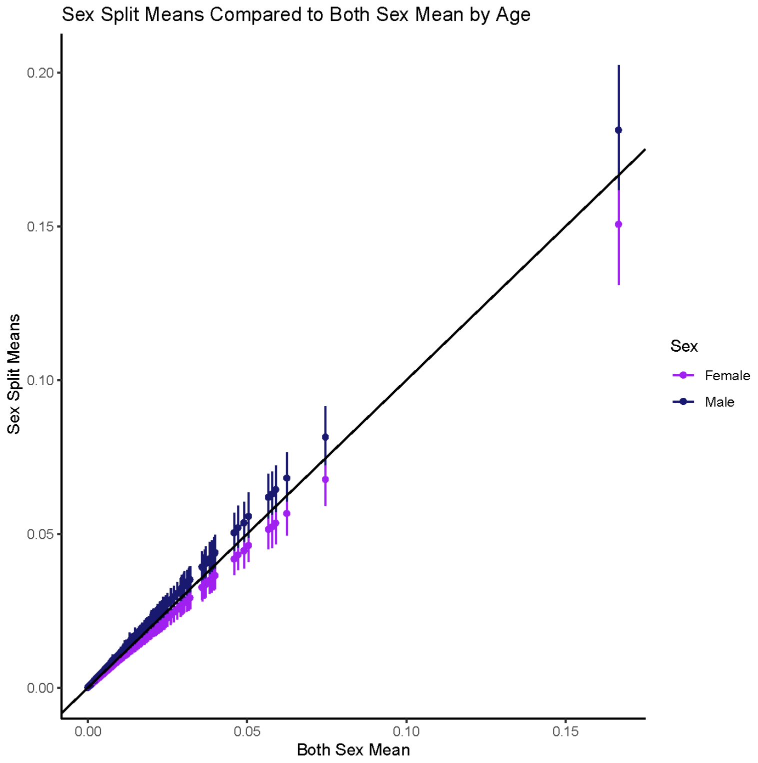

For GBD 2023, adjustment factors for all study-level covariates were determined using matched data (by year, age, sex, location) for reference and alternative case definitions in a logit ratio meta-regression. Studies that asked for lifetime recall were crosswalked to the reference definition for epilepsy (see case definition).

The table below shows adjustment factors estimated using MR-BRT.

MR-BRT crosswalk adjustment factors for epilepsy impairment envelope

Data input	Reference or alternative case definition	Gamma	Beta coefficient, log (95% UI)*	Adjustment factor**
	Ref	N/A	N/A	N/A
Recall lifetime	Alt	0.39	0.26 (–0.75 to 1.27)	1.3

**MR-BRT crosswalk adjustments can be interpreted as the factor the alternative case definition is adjusted by to reflect what it would have been had it been measured using the reference case definition. If the log/logit beta coefficient is negative, then the alternative is adjusted up to the reference. If the log/logit beta coefficient is positive, then the alternative is adjusted down to the reference.*

***The adjustment factor column is the exponentiated beta coefficient. For log beta coefficients, this is the relative rate between the two case definitions. For logit beta coefficients, this is the relative odds between the two case definitions.*

Modelling strategy

We modelled the prevalence of epilepsy in two steps: first, we created an epilepsy impairment envelope. Second, we split the envelope into primary (or idiopathic) and secondary epilepsies. Each of these was subdivided into "severe" (on average one or more fits per month) and "non-severe." Non-severe cases were subdivided into "treated" and "un-treated." Finally, "treated" cases were divided into "treated cases with fits" (between one and 11 fits on average in the preceding year) and "treated cases without fits" (no fits reported in the preceding year).

In the first step, we used DisMod-MR 2.1 for the epilepsy impairment envelope to model a consistent fit between incidence, prevalence, remission, and fatal data.

We also included the SEV epilepsy scalar, which summarises the epilepsy risk exposure level from all epilepsy risk factors for each country, as a predictive covariate on prevalence. We included cause-specific mortality rate (CSMR) estimates from the epilepsy mortality model as input data to the DisMod model. Where age-specific prevalence data were available, we calculated excess mortality rate (EMR) from prevalence and CSMR. We included the log of the lag-distributed income (LDI) as a covariate on EMR to account for lower mortality in developed countries. We included Bayesian priors on remission to account for the scarcity of remission data, setting bounds on remission from 0 to 0.25 for ages 0–60 and 0 to 0.05 for ages 61–100. The table below indicates the covariates used in the estimation process, as well as parameters, betas, and exponentiated betas.

Covariates. Summary of covariates used in the epilepsy impairment envelope DisMod-MR model.

Covariate	Type	Beta coefficient, log difference (95% UI)*	Adjustment factor**
Log-transformed age-standardised SEV scalar: idiopathic epilepsy	Prevalence	0.98 (0.82 to 1.14)	2.67 (2.27 to 3.14)
LDI ($ per capita)	Excess mortality rate	–0.55(–0.97 to –0.12)	0.58 (0.38 to 0.88)

In the second step, we used mixed-effects generalised linear models (binomial family) run in GBD 2023 to predict the proportion of idiopathic epilepsy, the proportion of severe epilepsy, the proportion of treated epilepsy, and the proportion of epilepsy that is treated without fits.

Because not all of the data on the proportion of idiopathic epilepsy used optimal case-finding methods (using CT scans or MRIs in addition to EEGs in order to diagnose secondary epilepsy), we first ran an initial linear regression model with a covariate on study quality. We then used the beta from this model to crosswalk studies with non-optimal case finding methods to those with adequate methods. The adjusted data were then used in the regression for the proportion of epilepsy that is idiopathic, with a fixed effect on SDI as well as a random effect on super-region.

We used similar models to predict the proportion of severe epilepsy and treatment gap based on the reported proportions extracted from the systematic review. To predict the proportion of severe epilepsy

and the treatment gap, we used mixed-effects models with a fixed effect on the log of HAQ Index and a random effect on super-region.

For the regression to determine the proportion of treated epilepsy cases that had not had a fit in the last year, there was a much smaller dataset, and therefore we could not use a random effect in the model. Therefore, we used generalised linear model (binomial family) to generate predictions for the proportion of treated epilepsy that was seizure-free with a fixed effect on the log of HAQ Index.

Severity splits and disability weights
The table below illustrates the severity levels, descriptions, and disability weights associated with epilepsy. These are calculated using regressions from literature (ie, frequency of seizures).

Severity level	Lay description	Disability weights (95% CI)
severe (seizures ≥ once per month)	This person has sudden seizures one or more times each month, with violent muscle contractions and stiffness, loss of consciousness, and loss of urine or bowel control. Between seizures, the person has memory loss and difficulty concentrating.	0.552 (0.375–0.71)
less severe (seizures < once per month)	This person has sudden seizures two to five times a year, with violent muscle contractions and stiffness, loss of consciousness, and loss of urine or bowel control.	0.263 (0.173–0.367)
Treated without fits	This person has a chronic disease that requires medication every day and causes some worry but minimal interference with daily activities.	0.49 (0.031–0.072)

Foodborne trematodiases

Flowcharts

Clonorchiasis

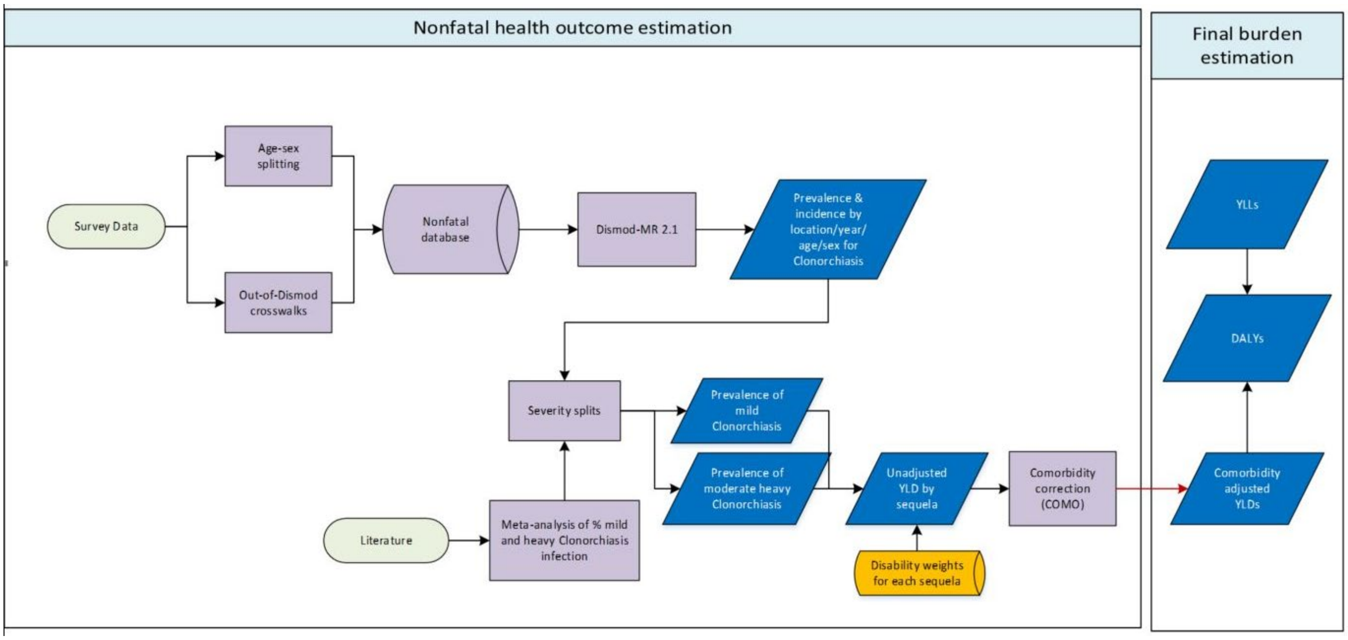

Fascioliasis

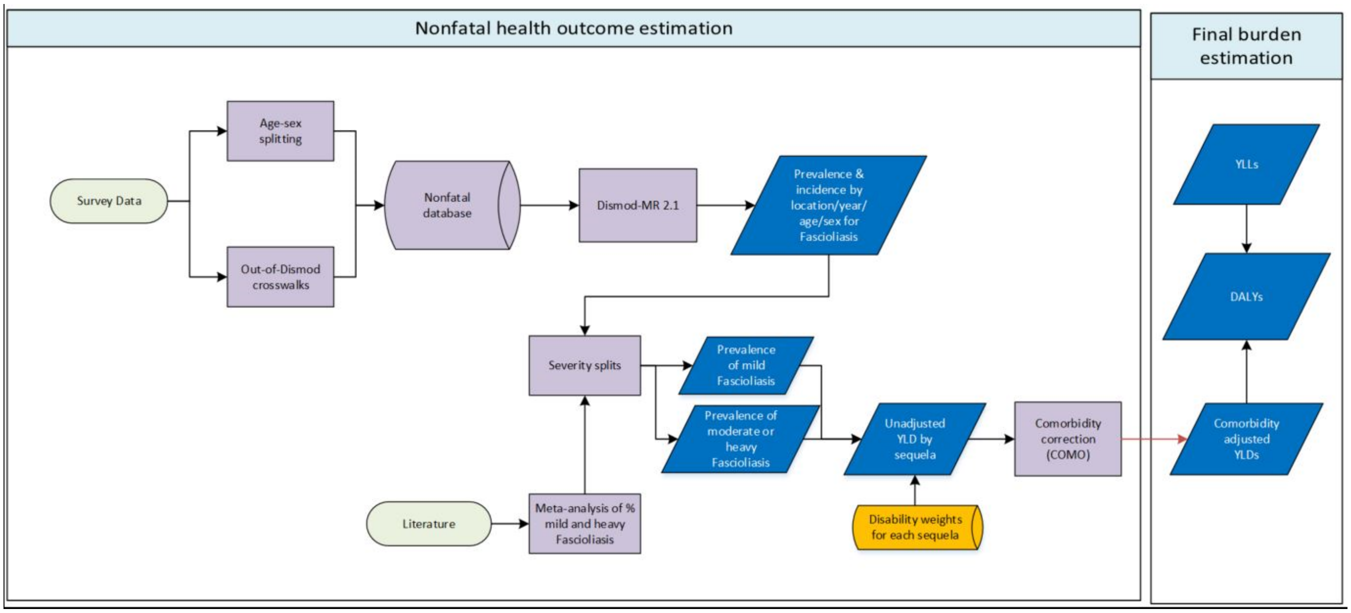

Intestinal fluke

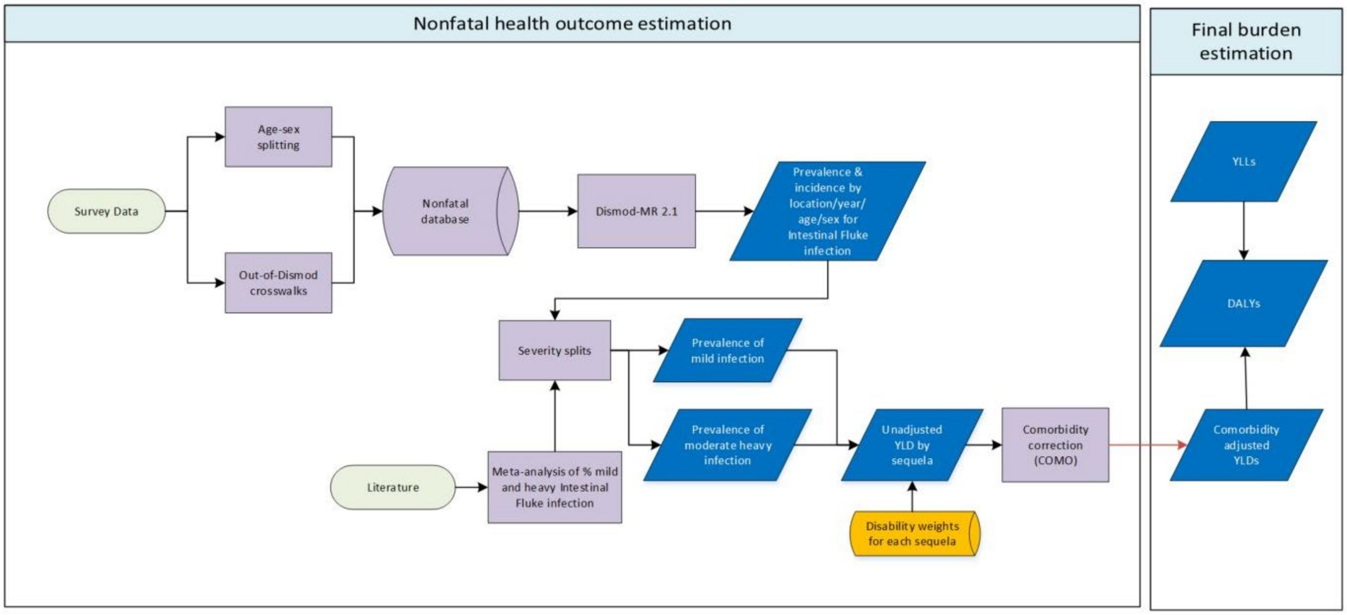

Opisthorchiasis

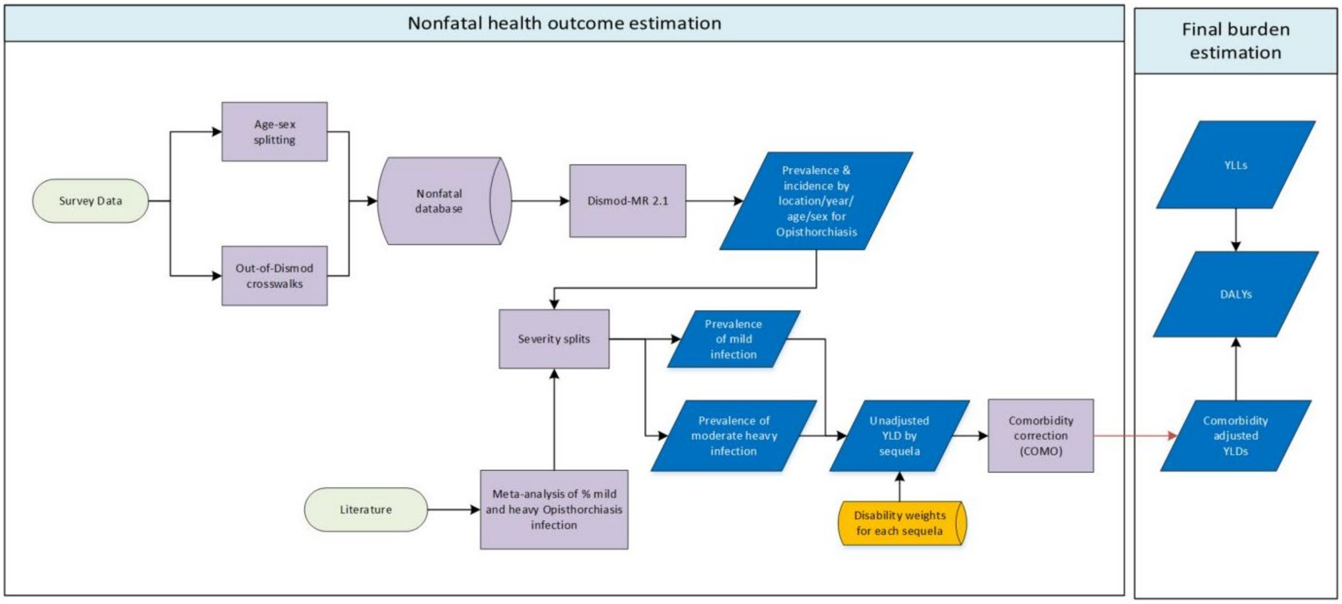

Paragonimiasis

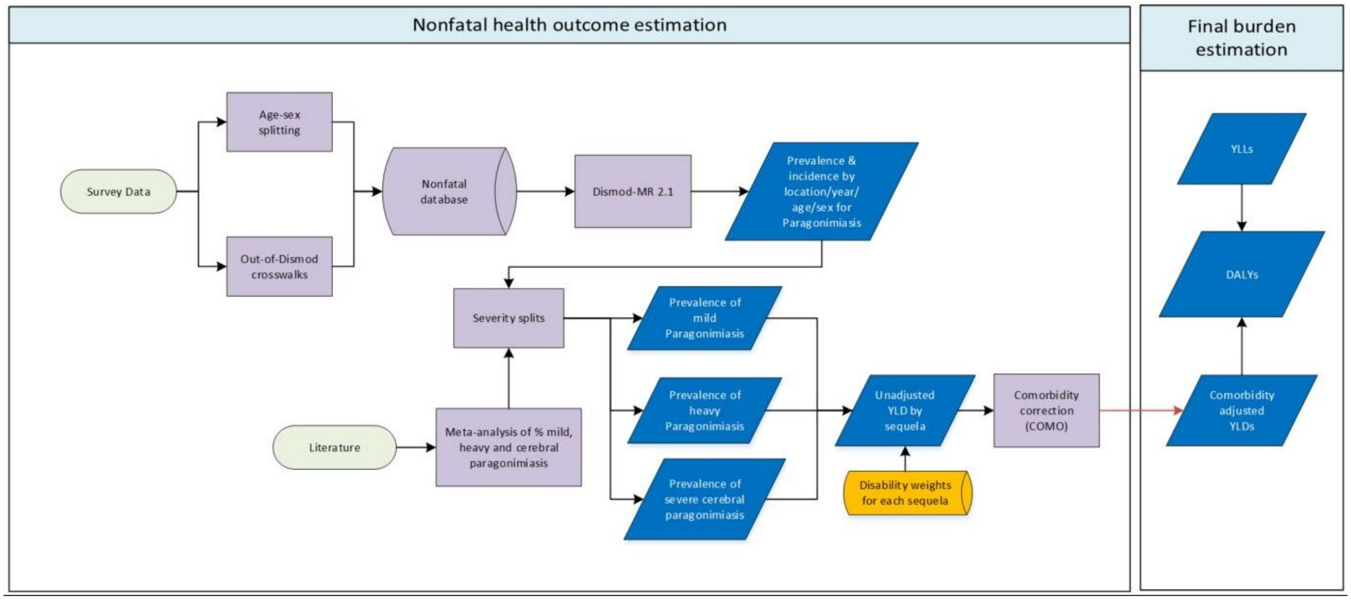

Input data and methodological summary for foodborne trematodiases

Case definition

The foodborne trematodiases (FBT) are a group of diseases that result from infection with parasitic worms of the class Trematoda, also known as flukes, via consumption of contaminated food. Infection of the liver, gallbladder, lungs, or brain can result in abdominal pain, chronic respiratory symptoms, neurological symptoms including epilepsy, and cholangiocarcinoma (bile duct cancer). In ICD-10, FBT are listed under code B66.[1]
FBT is subdivided into six types of FBT (see Table 1):

- Clonorchiasis is a parasitic disease that results from infection with the liver fluke *Clonorchis sinensis*, transmitted primarily via consumption of raw or undercooked fish. In addition to acute infectious symptoms, longer-term complications can result from inflammation of the liver, inflammation of the gallbladder, inflammation of the pancreas, and biliary obstruction. Clinical manifestations include abdominal pain, nausea, vomiting, weight loss, fatigue, jaundice, and cholangiocarcinoma (bile duct cancer).
- Fascioliasis is a parasitic disease that results from infection with the liver flukes *Fasciola hepatica* or *Fasciola gigantica*, transmitted primarily via consumption of contaminated raw water plants such as watercress. Acute clinical manifestations include abdominal pain, nausea, vomiting, fever, and rash, while chronic manifestations include jaundice, hepatomegaly, and weight loss, along with inflammation of the liver, gallbladder, and/or pancreas.
- Intestinal flukes are a diverse set of parasites including *Fasciolopsis buski*, *Metagonimus yokogawai*, *Heterophyes heterophyes*, *Echinostoma* species, and others, which can cause disease after infection of the intestinal tract. They are most commonly acquired via consumption of water plants, fish, and/or crustaceans. Many infected individuals are asymptomatic, but clinical manifestations can include abdominal pain, diarrhea, vomiting, or weight loss.

- Opisthorchiasis is a parasitic disease that results from infection with the liver flukes *Opisthorchis felineus* or *Opisthorchis viverrini*, transmitted primarily via consumption of raw or undercooked fish. In addition to acute infectious symptoms, longer-term complications can result from inflammation of the liver, inflammation of the gallbladder, inflammation of the pancreas, and biliary obstruction. Clinical manifestations include abdominal pain, nausea, vomiting, weight loss, fatigue, jaundice, and cholangiocarcinoma (bile duct cancer).
- Paragonimiasis (normal and cerebral infections) is a parasitic disease that results from infection with lung flukes of the genus *Paragonimus*, most commonly *Paragonimus westermani*, transmitted via consumption of contaminated food—most commonly raw or undercooked crabs, crayfish, or snails. Acute infection can result in fever, abdominal pain, rash, chest pain, and cough; late infection can cause hemoptysis (cough with bloody sputum). Less common clinical manifestations result from spread of the parasite outside of the lungs, including to the central nervous system (causing meningitis or encephalitis, resulting in headache, fever, vomiting, and/or seizures), the intestines (causing nausea, vomiting and/or diarrhea), the kidneys (causing bloody urine), or the skin (causing skin nodules).

Table 1. Subtypes of FBT

	**Species of FBT**	**Category**	**Carcinogen**
1	Clonorchiasis	Liver fluke	Associated with cholangiocarcinoma
2	Opisthorchiasis<br>(*O. viverrini* & *O. felineus*)	Liver fluke	Associated with cholangiocarcinoma<br>(*O. viverrini*)
3	Fascioliasis	Liver fluke	No available evidence
4	Intestinal flukes (*Fasciolopsis buski, Metagonimus yokogawai, Heterophyes heterophyes, Echinostoma species,* and others)	Intestinal fluke	No available evidence
5	Paragonimiasis	Lung fluke	

Table 2. Case definitions used for estimation of non-fatal burden of FBTs

**Quantity of interest**	**Reference or alternative**	**Definition**
Clonorchiasis	Reference	Prevalence of Clonorchiasis fluke infections identified by the presence of eggs in microscopic examination of stool or serological tests.
Opisthorchiasis	Reference	Prevalence of Opisthorchiasis fluke infections identified by the presence of eggs in microscopic examination of stool.
Fascioliasis	Reference	Prevalence of Fascioliasis fluke infections identified by the presence of eggs in microscopic examination of stool or serological tests.
Intestinal fluke	Reference	Prevalence of intestinal fluke infections identified by the presence of either adult worms or eggs in the examination of stool.
Normal and cerebral Paragonimiasis	Reference	Prevalence of Paragonimiasis fluke infections identified by the presence of eggs in microscopic examination of stool or serological tests.

Thresholds for heavy infection and duration by species of FBT

The majority of people infected with FBTs are asymptomatic. When symptoms do occur, they are often non-specific. Among the clinical symptomatic group, severity is associated with worm burden, typically measured by faecal egg counts, and the duration of infection. The thresholds for heavy infection and duration by species of FBT are shown in Table 3. The clinical presentation of FBT depends on the target organs (liver, lung, or

intestines). Clonorchiasis and opisthorchiasis patients may suffer from loss of appetite, fullness, indigestion, diarrhoea, pain in the right upper quadrant, lassitude, weight loss, ascites, and oedema.[2,3] Cholangitis, obstructive jaundice, intra-abdominal mass, cholecystitis, and gallbladder or intrahepatic stones may occur as complications.[3,4]

Table 3. Thresholds for heavy infection and duration by species of FBT

	**Species of FBT**	**Case thresholds for heavy infection**	**Duration**
1	Clonorchiasis	10,000 eggs per g of faeces	lifelong
2	Opisthorchiasis	10,000 eggs per g of faeces	lifelong
3	Fascioliasis	1,000 eggs per g of faeces	lifelong
4	Intestinal fluke	1,000 eggs per g of faeces	lifelong
5	Paragonimiasis	100 eggs per 5 ml sputum	lifelong
6	Cerebral paragonimiasis	Any infection of the brain with flukes and/or eggs of *Paragonimus* spp.	lifelong

Input data

Model inputs

For GBD 2010, the data came from an expert group analysis, which used the results of a systematic literature review performed by Furst and colleagues as a starting point.[5] Furst and colleagues searched PubMed, WHOLIS, FAOBIB, Embase, CAB Abstracts, Literatura Latino Americana e do Caribe em Ciências de Saùde (LILACS), ISI Web of Science, BIOSIS preview, Science Direct, African Journals OnLine (AJOL), and the System for Information on Grey Literature in Europe (SIGLE), period Jan 1, 1980, to Dec 31, 2008. The initial number of studies identified through the literature review was ~34,000 references. The literature review included extracted data from 181 studies. For GBD 2013 and GBD 2015, the search strategy was replicated to capture epidemiological studies published between 2008 and 2015.

Input data for the assessment of the total national number of infected people

Only studies that used countrywide surveys to estimate the national prevalence rates were included (or for China, province-wide surveys). We included only national studies because FBT shows a highly focal spatial distribution, and local cross-sectional surveys would profoundly underestimate or overestimate true national prevalence. Infection is highly related to food habits, and there are greatly varying differences between national and subnational prevalence rates. This search was last updated for GBD 2015; the final dataset contained 29 prevalence studies from 17 countries. We used raw data from the selected studies as input for DisMod Bayesian meta-regression (DisMod-MR).

Prevalence of intestinal fluke infection

Intestinal fluke infections can be caused by several different pathogens, such as *Metagonimus* spp., *Echinostoma* spp., and *Neodiplostomatidae.*[6] When assessing the prevalence of intestinal fluke infection, we added the identified prevalence for each parasite species in order to obtain the overall prevalence of intestinal fluke infections. This approach may lead to a certain overestimation of the true prevalence, because people may be co-infected with more than one intestinal fluke species. There is not sufficient evidence about the proportion of co-infections to effectively account for this in our modelling process, but the resulting overestimation of the true prevalence may be offset by the assumptions made in our modelling approach and the many challenges in

generating the underlying epidemiological parameters (eg, diagnostic inaccuracy in the detection of infections with the more than 50 intestinal fluke species). Also of note, the transmission sources of intestinal fluke infections are species-specific and therefore vary. For instance, *Fasciolopsis buski* is usually transmitted by eating raw water plants with the infective parasite stage attached to the water plants, whereas *Neodiplostomatidae* are transmitted by eating undercooked and infested frogs, snakes, and tadpoles. Because of these different transmission pathways, the rate of co-infection might in fact be smaller than expected.

Input data to differentiate between asymptomatic and heavy infections

We estimated the proportion of heavily infected among all infected in all available national and regional cross-sectional surveys. It is expected that heavy infection increases with age, and there are data available on heavy infection by age group. We therefore decided to include age-dependent rates of heavy infection for clonorchiasis, opisthorchiasis, and intestinal fluke infection. For (cerebral) paragonimiasis and fascioliasis there were not sufficient age-dependent data on high-intensity FBT infection for this approach, and we therefore used an estimate of the rate of heavy infection that did not vary by age.

Data pre-processing

We used a meta-regression—Bayesian, regularised, trimmed (MR-BRT) model with our sex-specific data to derive an estimate of the ratio of the male prevalence of all-species FBT infection to female prevalence of all-species FBT infection to split non-sex-specific data. Then, a DisMod-MR 2.1 Bayesian meta-regression model using the age-specific input data was run to derive an age pattern to apply to split the all-age data.

Table 4: MR-BRT crosswalk adjustment factors for all-species FBT infection

**Data input**	**Reference or alternative case definition**	**Gamma**	**Beta coefficient, log (95% CI)***	**Adjustment factor****
Female data	Ref	0.82	---	---
Male data	Alt		0.48 (–1.16 to 2.12)	1.62

**MR-BRT crosswalk adjustments can be interpreted as the factor the alternative case definition is adjusted by to reflect what it would have been had it been measured using the reference case definition. If the log/logit beta coefficient is negative, then the alternative is adjusted up to the reference. If the log/logit beta coefficient is positive, then the alternative is adjusted down to the reference.*

***The adjustment factor column is the exponentiated beta coefficient. For log beta coefficients, this is the relative rate between the two case definitions. For logit beta coefficients, this is the relative odds between the two case definitions.*

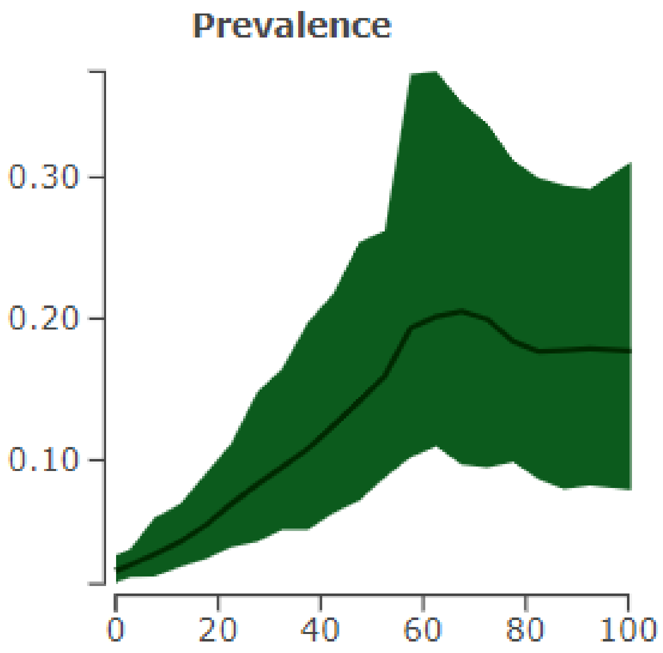

Figure 1: Global age pattern for all-species FBT infection used to split all-age data into age-specific datapoints for further modelling

Modelling strategy

We used a three-step process for the disease modelling of FBT. In the first step, we used DisMod-MR 2.1 to estimate the prevalence of FBT by age, sex, year, and country. In the second we differentiated between asymptomatic and heavy infections. MetaXL (a meta-analysis add-in for Microsoft Excel) was used to estimate the proportion of heavily infected among all infected by age group for clonorchiasis, opisthorchiasis, and intestinal fluke infection (see Tables 5 and 6). These proportions were used to estimate the prevalence of heavy FBT infection. The third step consisted of deselecting countries that have no autochthonous case reports of FBTs.

Table 5. Percentage of high-intensity infection by age group and type of FBT (based on eight FBT prevalence studies)

Age category	Clonorchiasis			Opisthorchiasis			Intestinal fluke infection		
	**Mean**	**Low**	**High**	**Mean**	**Low**	**High**	**Mean**	**Low**	**High**
**0-9**	30%	17%	44%	10%	0%	29%	8%	3%	14%
**10-19**	15%	0%	43%	15%	0%	69%	11%	8%	14%
**20-29**	18%	10%	29%	16%	0%	52%	18%	15%	21%
**30-39**	17%	5%	34%	21%	0%	56%	22%	17%	28%
**40-49**	22%	13%	32%	28%	1%	68%	22%	13%	32%
**50-59**	18%	0%	49%	29%	0%	75%	17%	9%	28%
**60+**	32%	18%	47%	25%	0%	64%	15%	8%	23%

Table 6. Percentage of high-intensity infection by type of FBT (based on four FBT prevalence studies)

Type of FBT	Mean	Low	High
**Paragonimiasis**	23%	0%	59%
**Fascioliasis**	19%	3%	41%

Cerebral paragonimiasis

It was assumed that 0.8% of paragonimiasis cases have cerebral involvement. This proportion was used to estimate the prevalence of cerebral paragonimiasis. This proportion is based on one study. The data are from Oh SJ. The rate of cerebral involvement in paragonimiasis: an epidemiologic study. *Jpn J Parasitol* 1969; 18:211-14. The study was performed in Paju, South Korea. This is an area with 6738 inhabitants, and according to the survey, it was estimated that 29.6% of all individuals would react to intradermal test (an immunological reaction indicating previous or current contact with the parasite). 25% of all "positive reactors" may have eggs in their sputum (active infection with the parasite currently present in the human host). If these rates are applied to the community, the number of patients with active paragonimiasis would be at least 498 (=6,738*0.296*0.250). Furthermore, four cases of cerebral paragonimiasis were found in this community. Therefore, four out of 498 individuals with active paragonimus infection suffered from cerebral infection (=0.80%; 95% confidence interval 0.019–1.587).

Severity splits and disability weights

For GBD 2023, FBT was not split into health states with different severities, with the exception of paragonimiasis. The table below shows the disability weights that were used to calculate the burden of FBT in years lived with disability (YLDs) for GBD 2023.

Table 7. Disability weights that were used to calculate FBT YLDs

Sequelae	Severity description	Health state name	Disability weight
**Asymptomatic clonorchiasis**	Clonorchiasis, currently without symptoms	N/A	0.000 (0.000–0.000)
**Heavy clonorchiasis**	Abdominal pain and nausea reported as moderate	Abdominopelvic problem, moderate	0.114 (0.078–0.159)
**Asymptomatic opisthorchiasis**	Opisthorchiasis, currently without symptoms	N/A	0.000 (0.000–0.000)
**Heavy opisthorchiasis**	Abdominal pain and nausea reported as moderate	Abdominopelvic problem, moderate	0.114 (0.078–0.159)
**Asymptomatic fascioliasis**	Fascioliasis, currently without symptoms	N/A	0.000 (0.000–0.000)
**Heavy fascioliasis**	Abdominal pain and nausea reported as moderate	Abdominopelvic problem, moderate	0.114 (0.078–0.159)
**Asymptomatic intestinal fluke infection**	Intestinal fluke infection, currently without symptoms	N/A	0.000 (0.000–0.000)

**Heavy intestinal fluke infection**	Abdominal pain and nausea reported as moderate	Abdominopelvic problem, moderate	0.114 (0.078–0.159)
**Mild paragonimiasis due to foodborne trematodiases**	COPD and other chronic respiratory problems, mild	Has cough and shortness of breath after heavy physical activity, but is able to walk long distances and climb stairs.	0.019 (0.011-0.033)
**Moderate paragonimiasis due to foodborne trematodiases**	COPD and other chronic respiratory problems, moderate	Has cough, wheezing and shortness of breath, even after light physical activity. The person feels tired and can walk only short distances or climb only a few stairs.	0.225 (0.153-0.31)
**Severe paragonimiasis due to foodborne trematodiases**	COPD and other chronic respiratory problems, severe	Has cough, wheezing and shortness of breath all the time. The person has great difficulty walking even short distances or climbing any stairs, feels tired when at rest, and is anxious.	0.408 (0.273-0.556)
**Cerebral paragonimiasis**	Epilepsy	(combined DW)	--

Note. N/A: not applicable

We did not apply any adjustments for the COVID-19 pandemic to foodborne trematodiases due to a lack of available data quantifying the impacts of the pandemic on NTD epidemiology.

Changes from GBD 2021 to GBD 2023

There were no substantive changes to the modelling strategy for GBD 2023.

References

1. WHO. *International Statistical Classification of Diseases and Related Health Problems. 10th Revision. Version for 2007.* 2007 [cited 2009 October 14, 2009]; Available from: http://apps.who.int/classifications/apps/icd/icd10online/.
2. Rim, H.J., *Clonorchiasis: an update.* J Helminthol, 2005. **79**(3): p. 269-81.
3. Pungpak, S., et al., *Clinical features in severe opisthorchiasis viverrini.* Southeast Asian J Trop Med Public Health, 1985. **16**(3): p. 405-9.
4. Rim, H.J., *The current pathobiology and chemotherapy of clonorchiasis.* Korean J Parasitol, 1986. **24**(Suppl.): p. 1-141.
5. Furst, T., J. Keiser, and J. Utzinger, *Global burden of human food-borne trematodiasis: a systematic review and meta-analysis.* Lancet Infect Dis, 2012. **12**(3): p. 210-21.
6. Furst, T., et al., *Manifestation, diagnosis, and management of foodborne trematodiasis.* BMJ, 2012. **344**: p. e4093.

Fungal skin diseases

Flowchart

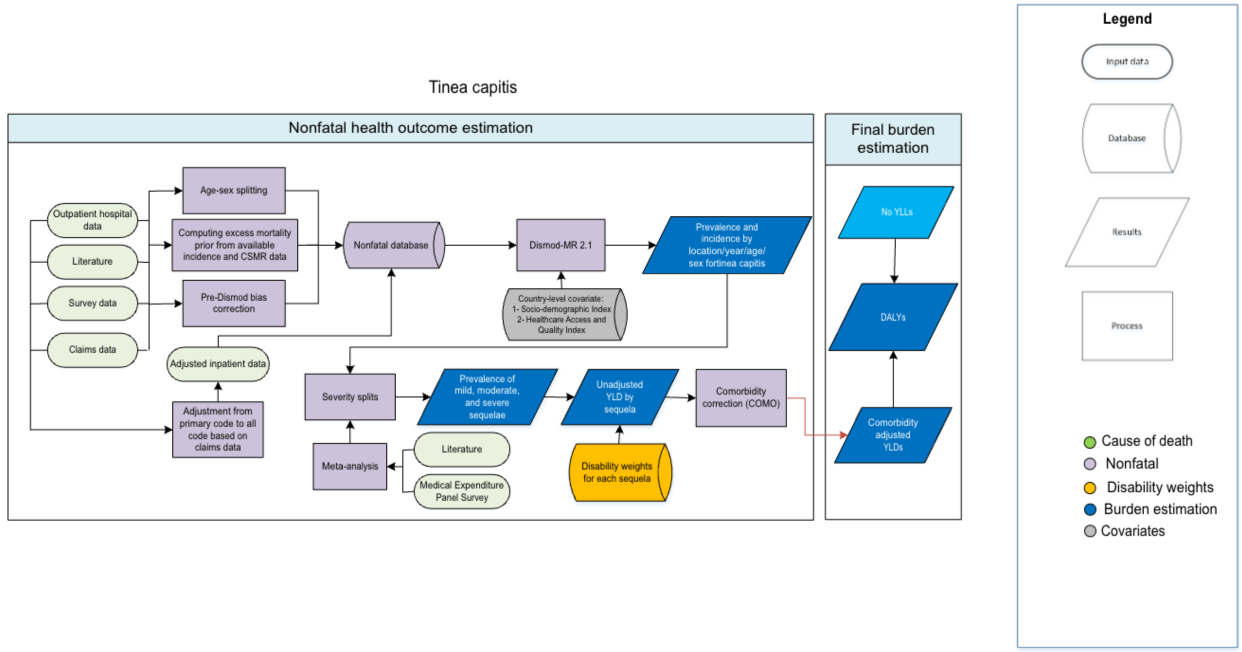

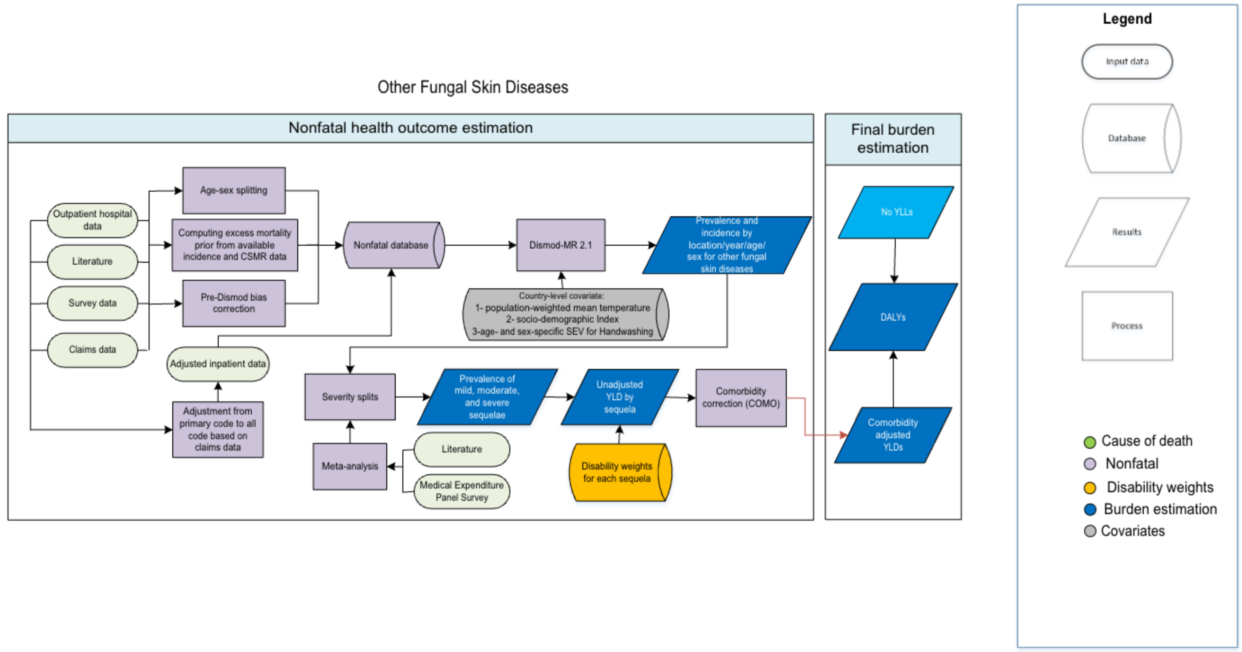

Input data and methodological summary for fungal skin diseases

Case definition

Fungal diseases were included in the GBD 2020 cause group of skin and subcutaneous conditions and consisted of tinea capitis and a residual group of “any” other fungal disease. Similar to GBD 2017, tinea

capitis was modelled separately from the other fungal skin diseases. This was done to better accommodate differences in burden between tinea capitis and other subtypes of fungal skin diseases.

The residual group of "any" other fungal skin disease included any fungal skin disease that was specifically not tinea capitis or onychomycosis (ie, fungal nail infection). The ICD-10 (1) list of other fungal skin diseases includes tinea manuum (ICD-10: B35.2), or hand ringworm; tinea pedis (ICD-10: B35.3), or athlete's foot; tinea corporis (ICD-10:B35.4), or ringworm of the body; tinea imbricata (ICD-10:B35.5), a superficial fungal infection limited to parts of Asia and Central America; tinea cruris (ICD-10:B35.6), also known as dhobi itch, groin ringworm, or jock itch. In GBD 2016, we added dermatophytosis (ICD10:B35.9).

Fungal skin diseases

Quantity of interest	Reference or alternative	Definition
Tinea capitis	Reference	Tinea capitis as diagnosed by dermatologists
	Alternative	Other data inputs for tinea capitis
Other fungal skin diseases	Reference	Other fungal diseases as determined by Poland National Health Fund Patient Claims 2015-2019
	Alternative	Other data inputs for tinea capitis

Input data

For GBD 2010, a systematic review of the literature using PubMed and Google Scholar was conducted to capture epidemiological data for fungal skin diseases. The literature search also included any relevant data from the Medical Expenditure Panel Survey (MEPS) in the USA in 2000–2009. The inclusion criteria stipulated that studies (1) must be published between 1980 and 2012; (2) must provide data on the incidence or prevalence of fungal skin diseases; (3) must use samples representative of the general population (ie, samples derived from the experimental arm of clinical trials or based in dermatology clinics were excluded); (4) must use a sample size larger than 100; and (5) must provide sufficient information on study method and sample characteristics to assess the quality of the study. For GBD 2013, the GBD 2010 search strategy was replicated to capture epidemiological studies published between 2012 and 2013. For GBD 2017, the GBD 2010 search strategy was replicated in PubMed to capture epidemiological studies published between 2013 and 2017. For GBD 2023, we have added year-locations of outpatient clinical data. The new data sources for tinea capitis include the USA, Mongolia, Poland, South Korea, and Sweden. For other fungal skin diseases, the new sources for clinical data are from the USA, Poland, and South Korea. Data were outliered or excluded if we found them unreasonable when compared to regional, super-regional, and global rates.

Data processing

For fungal skin diseases, we crosswalked all data to the reference definition. We began by evaluating the number of observations of each alternate definition that matched with a corresponding observation from the reference definition. We considered "between" study matches, where the alternative was from the same GBD age group and sex, and the midpoint year of the study was within five years of the midpoint of the reference definition observation.

$$\log i\,t\left(y_i^{alt}\right) - \log i\,t\left(y_i^{ref}\right) \;=\; \beta_0 \;+ \epsilon_i$$

Table 1: MR-BRT crosswalk adjustment factors for fungal skin diseases

Data input	Reference or alternative case definition	Gamma	Beta coefficient, log/logit (95% UI)*	Adjustment factor**
Tinea capitis	Ref	0	---	---
	Alt		–4.2753 (–4.2851 to –4.2655)	0.0139 (0.0138, 0.0140)
Other fungal skin diseases	Ref	0.1014	---	---
	Alt		1.4922 (0.8682 to 2.1162)	4.4468 (2.3825, 8.2994)

**MR-BRT crosswalk adjustments can be interpreted as the factor the alternative case definition is adjusted by to reflect what it would have been had it been measured using the reference case definition. If the log/logit beta coefficient is negative, then the alternative is adjusted up to the reference. If the log/logit beta coefficient is positive, then the alternative is adjusted down to the reference.*

***The adjustment factor column is the exponentiated beta coefficient. For log beta coefficients, this is the relative rate between the two case definitions. For logit beta coefficients, this is the relative odds between the two case definitions.*

Modelling strategy

DisMod-MR 2.1, a Bayesian meta-regression tool, was used to estimate tinea capitis and other fungal skin diseases prevalence by age, sex, year, and geography (subnational, country, region, super-region). Separate models were run for tinea capitis and other fungal skin diseases.

Tinea capitis

To help inform the distribution of tinea capitis across the lifespan, excess mortality was set at zero, remission was set at 0.5 to 4, and incidence was set at 0 to 0.02 between 20 and 100 years. This was in agreement with the available prevalence data and expert advice. We made use of a relatively long time window of 20 years to determine which datapoints were used for a particular year of fit. This means that for the year 2000, for instance, DisMod-MR 2.1 incorporated all datapoints ranging from 1980 to present to estimate prevalence. Since GBD 2019, we replaced our within-DisMod crosswalks with crosswalks completed using the MR-BRT modelling tool. We adjusted all our datapoints toward the level of dermatologist-diagnosed datapoints, which were more representative of the general population. We limited random effects for sub-Saharan Africa (–1,1), north Africa and the Middle East (–1, 1), southeast Asia, east Asia, and Oceania (–1, 1), and western Europe (–0.1, 1) to improve model estimates. In addition, the Socio-demographic Index and the Healthcare Access and Quality Index were used as country-level covariates to guide estimates for countries with few or no data.

Other fungal skin diseases

For other fungal skin diseases, excess mortality rate was set at zero, and remission set between 0.33 and 4. In GBD 2023, we have crosswalks completed using the MR-BRT modelling tool. We adjusted all clinical and literature data sources toward the level of Poland claims datapoints which were more representative of the general population. We limited random effects for Nigeria (–0.5, 0.5) and Ethiopia (–0.5, 0.5) to improve model estimates. In addition, population-weighted mean temperature, Socio-demographic Index, and age- and sex-specific SEV for handwashing were used as country-level

covariates to guide estimates for countries with few or no data. We have made no substantive changes in the modelling strategy from GBD 2019.

$$prevalence = incidence * duration$$

$$Remission = \frac{Cured\ cases}{person - yearoffollow - up\ in\ prevalent\ cases}$$

Table 2. Severity distribution, details on the severity levels for [insert cause name] and the associated disability weight (DW) with that severity.

Severity level	Lay description	DW (95% CI)
Infectious disease, acute episode, mild	The person has a low fever and mild discomfort but no difficulty with daily activities.	0.006 (0.002–0.012)

Table 3. Covariates. Summary of covariates used in the fungal skin disease DisMod-MR meta-regression model

	Covariate	Type	parameter	value	Exponentiated beta (95% uncertainty interval)
Tinea capitis	Socio-demographic Index	Country-level	prevalence	–1.09 (–1.89, 0.043)	0.33 (0.15–1.04)
	Healthcare Access and Quality Index	Country-level	prevalence	–0.031 (–0.04, –0.021)	0.97 (0.96–0.98)
Other fungal skin diseases	Population-weighted mean temperature	Country-level	prevalence	0.022 (0.019, 0.024)	1.02 (1.02–1.02)
	Socio-demographic Index	Country-level	prevalence	2.77 (0.73, 4.07)	15.95 (2.07–58.26)
	Age- and sex-specific SEV for handwashing	Country-level	prevalence	4.16 (4.11, 4.22)	64.20 (60.95–67.76)

Gallbladder and biliary diseases

Flowchart

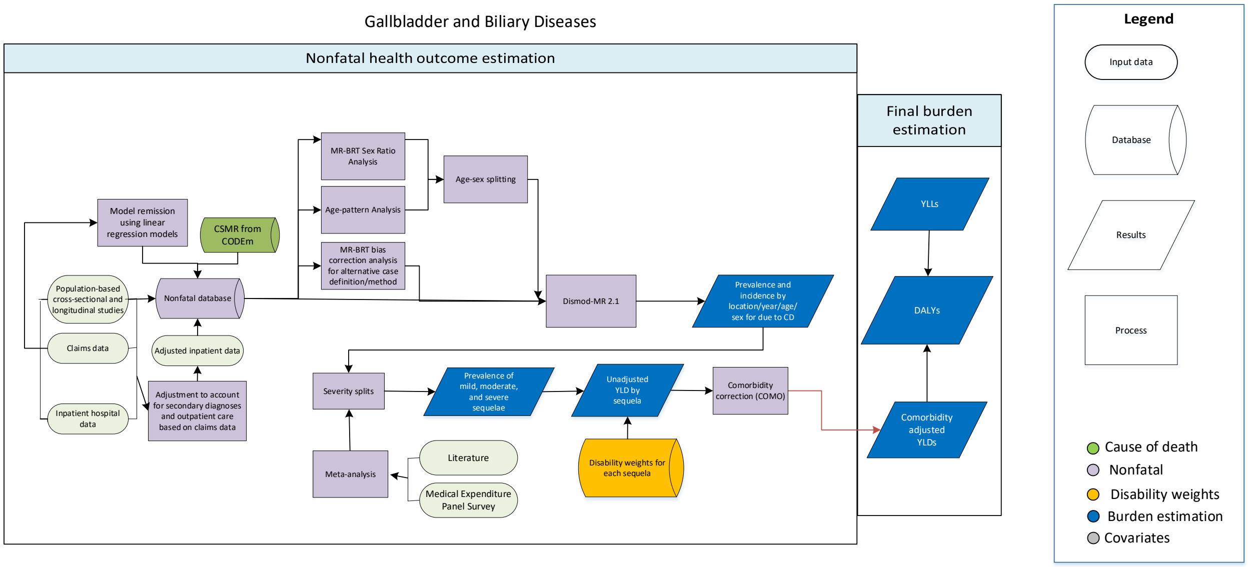

Input data and methodological summary for gallbladder and biliary diseases

Case definition

Gallbladder and biliary diseases encompass gallstones, cholecystitis, cholangitis, and other non-cancer diseases of the gallbladder and biliary tract, including those with and without symptoms. Gallstones are crystalline masses formed abnormally in the gallbladder or bile ducts from bile pigments, cholesterol, or calcium salts that can cause abdominal pain. Cholecystitis is an inflammation of the gallbladder, and cholangitis is an inflammation of the bile duct, which can result from obstruction by gallstones and cause severe abdominal pain, nausea, vomiting, fever, and jaundice.

ICD-10 codes for gallstone and biliary diseases included in GBD are K80, K81, K82, and K83. The procedure codes used to identify remission of gallbladder and biliary diseases are 47400-47480, 47490-47544, 47550-47556, 47562-47579, 47600-47715, 47720-47900, 47999-47999.

Quantity of interest	Reference or alternative	Definition
Prevalence of total biliary diseases	Alternative	Cases of gallbladder and biliary diseases identified using ICD codes in administrative records that are considered population-representative.
Prevalence of total biliary diseases	Alternative	Cases identified from database of commercial claims from USA in 2010-2017 using ICD codes.
Prevalence of total biliary diseases	Alternative	Cases identified from database of commercial claims from USA in 2000 using ICD codes.

Prevalence of total biliary diseases	Reference	Gallstones identified by ultrasound, regardless of symptoms, in population-based surveys.

Input data

Inputs

A systematic review was conducted to identify data on the prevalence of total gallbladder and biliary disease for GBD 2016. The search string used was ((gall bladder disease[Title/Abstract] OR cholecyst*[Title/Abstract] AND prevalence[Title/Abstract] AND ("2010/01/01"[Date - Publication] : "2016/11/01"[Date - Publication])) NOT(animals[MeSH] NOT humans[MeSH])). We excluded studies that were not representative of a general population described by year, age, sex, and location (ie, studies of clinically defined subpopulations such as *H pylori* cohorts or patients presenting with pain) and without sufficient information on study and sampling methods. We also excluded reviews.

In addition to data from the search-string-based review, input data for the total model included clinical administrative data from the GBD clinical informatics datasets, which were extracted as prevalence. No new data were added in GBD 2023. The input data and processing steps for modelling in GBD 2023 were the same as in GBD 2021. Inputs to our non-fatal modelling also included cause-specific mortality rate (CSMR) estimates taken from our fatal modelling process (see CoD cause-specific modelling description for gallbladder and biliary diseases in this appendix) and remission estimates modelled outside of DisMod-MR (see the remission data processing section below).

Prevalence input processing

Hospital discharge data provide observations about encounters, generally with only the primary diagnostic code for the encounter. Claims data, on the other hand, link claims for all inpatient and outpatient encounters for a single individual and provide primary and secondary diagnoses for all encounters.

We extracted prevalent cases for the total gallbladder and biliary disease database from claims data as individuals that had one inpatient or two outpatient encounters with a gallbladder and biliary disease ICD code as any diagnosis. Hospital discharge data were processed by extracting discharges with an appropriate ICD code as primary diagnosis and adjusting using correction factors (ie, correction factor 3) derived from claims data. Specifically, we modelled from the ratio of inpatient claims with gallbladder and biliary diseases as primary diagnosis to total prevalent cases of gallbladder and biliary diseases seen in claims data.

As for data from the literature sources, we started by splitting data reported in the literature for both sexes combined using the pooled sex-ratio estimated from studies that reported prevalence in males and females separately. The ratio of female to male cases derived from MR-BRT analysis was 1.69 (95% UI 1.07–2.68).

In GBD 2023, we carried forward the practice from GBD 2019 and GBD 2021 of employing data from studies in which general population samples were screened for both symptomatic and asymptomatic cases of gallbladder and biliary diseases using ultrasonography, treating these as the reference data type to which other types must be adjusted. (Earlier rounds of GBD used ICD-based clinical administrative

data as reference.) Claims and hospital discharge data were adjusted toward this reference standard to account for systematic differences prior to modelling in DisMod-MR. The USA MarketScan claims data from the year 2000 and from the years 2010–2017 were separately adjusted to account for selection bias due to commercial insurance, which was suspected to be differential over the years as coverage expanded.

The process of adjusting for biases in non-reference data using MR-BRT (meta-regression—Bayesian, regularised, trimmed) with the logit-transformation method is described below:

1. Identify datapoints with overlapping year, age, sex, and location between reference and non-reference population data.
2. Logit transform overlapping datapoints of alternative and reference types.
3. Convert overlapping datapoints into a difference in logit space using the following equation: $logit(altnerative) - logit(reference)$
4. Use the delta method to compute standard errors of overlapping datapoints in logit space, then calculate standard error of logit difference using the following equation: $\sqrt{(variance\ of\ logit(alternative)) + (variance\ of\ logit(reference))}$
5. Using MR-BRT, conduct a random effects meta-regression to obtain the pooled logit difference of alternative to reference
6. Apply the pooled logit difference to all data points of non-reference types using the following equation:
$$new_{estimate} = inverse.logit((logit(alternative)) - (pooled\ logit\ difference))$$
7. Calculate new standard errors using the delta method, accounting for gamma (between-study heterogeneity)

The table below shows bias correction factors estimated using MR-BRT.

Table 1. MR-BRT crosswalk adjustment factors for gallbladder and biliary diseases

Data input	Reference or alternative case definition	Gamma	Beta coefficient, logit (95% CI)	Adjustment factor*
Ultrasound-based diagnosis	Ref	0.008	---	---
Hospital + non-USA claims	Alt		–3.01 (–3.11 to –2.92)	0.05 (0.045 to 0.054)
USA claims from year 2000	Alt		–2.07 (–2.27 to –1.87)	0.13 (0.10 to 0.16)
USA claims from years 2010-2017	Alt		–2.40 (–2.61 to 2.20)	0.09 (0.07 to 0.11)

**MR-BRT crosswalk adjustments can be interpreted as the factor the alternative case definition is adjusted by to reflect what it would have been had it been measured using the reference case definition. If the log/logit beta coefficient is negative, then the alternative is adjusted up to the reference. If the log/logit beta coefficient is positive, then the alternative is adjusted down to the reference.*

***The adjustment factor column is the exponentiated beta coefficient. For log beta coefficients, this is the relative rate between the two case definitions. For logit beta coefficients, this is the relative odds between the two case definitions.*

Finally, we split datapoints where the age range was greater than 25 years using the global age pattern informed by the datapoints with fine age groups (ie, ages 5–9, 10–14, and 15–20…). Datapoints with an age-standardised prevalence greater than two median absolute deviations from the median of the age-standardised prevalence for all inpatient and non-USA claims data were marked as outliers and excluded from analysis.

Remission processing

As first done in GBD 2019, we generated remission inputs from USA claims data, defined as a number of people with procedure codes to permanently correct gallbladder and biliary disease (eg, cholecystectomy) among all people with diagnosis of gallbladder and biliary diseases, and regressed against Healthcare Access and Quality (HAQ) Index and sex. This was to better inform DisMod-MR on the increasing pattern of remission with greater access to quality health care. In GBD 2021, we updated this by including an additional covariate, age, to capture age variations in remission. The results from the regression model were then used to predict remission estimates for each location, year, and sex for ages 0, 10, 20…100, and in GBD 2023 we continued with these inputs.

Figure 1. Predicted remission in function of age, sex, and HAQ Index

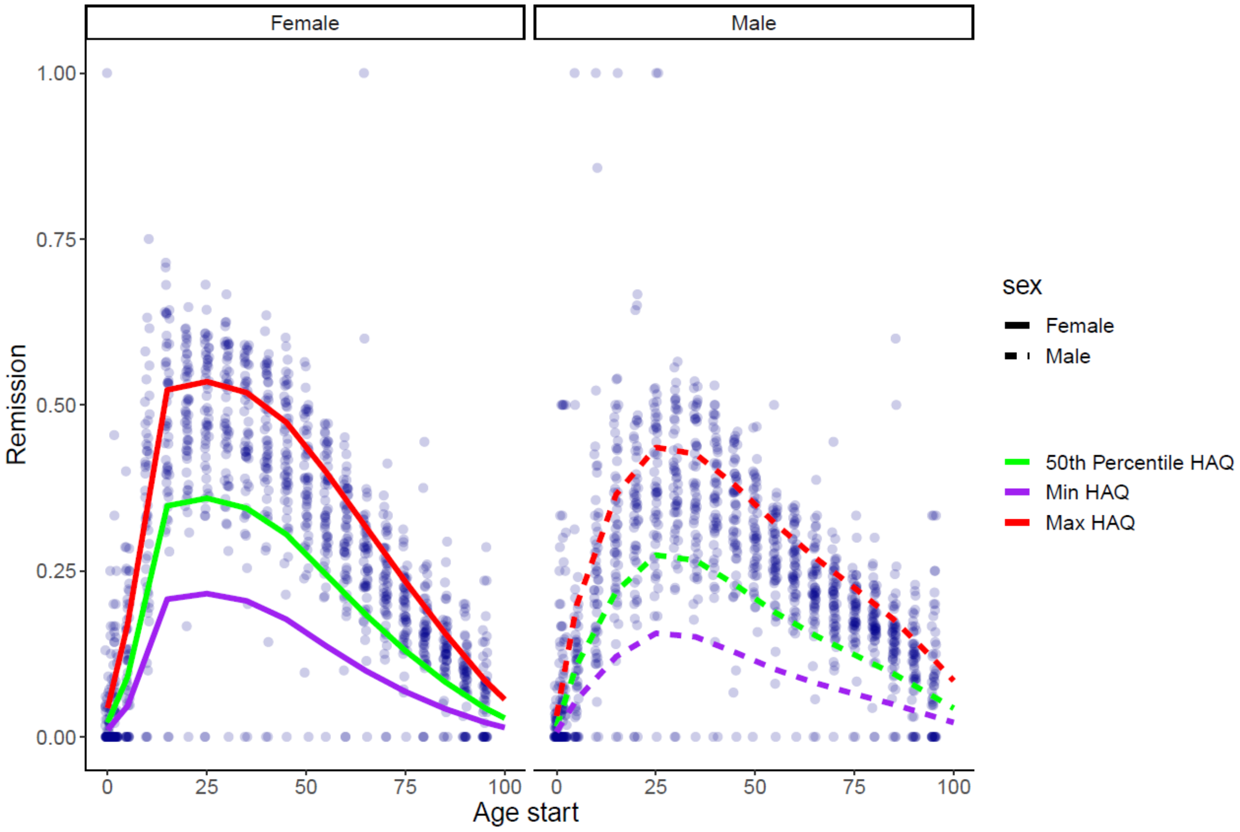

EMR processing

Similar to previous rounds, EMR inputs were produced inside DisMod-MR by matching prevalence datapoints with their corresponding CSMR values within the same age, sex, year, and location (by dividing CSMR by prevalence).

Modelling strategy

Modelling

We ran a DisMod-MR 2.1 model to produce estimates by age, sex, year, and country. Inputs to DisMod-MR for total gallbladder and biliary diseases include incidence, remission, and CSMR and EMR inputs processed as described above. The minimum coefficient of variation at the regional, super-regional, and global level was set at 0.8. No predictive covariates were employed.

Severity split and disability weight

The basis of the GBD disability weight survey assessments are lay descriptions of sequelae highlighting major functional consequences and symptoms. As has been done since GBD 2019, cases from the total model were divided into asymptomatic and symptomatic groups using proportions found in a review of six studies of the natural history of gallbladder and biliary diseases and modelled in MR-BRT. Symptomatic cases of gallbladder and biliary diseases were then divided into mild, moderate, and severe sequelae according to severity distributions derived from data from the Medical Expenditure Panel Survey (MEPS). MEPS is an overlapping panel survey of the non-institutionalised USA population that collects data on respondents' health service interactions. Panels are initiated every year. Each panel is two years long and consists of five rounds. In 2000, MEPS began using 12-Item Short Form Surveys (SF-12) to collect data on functional health status. The SF-12 survey is administered twice per panel (about once per year)[1]. In order to translate SF-12 scores into GBD disability weights, 62 lay descriptions for conditions representing the full range of disability weight values (from mildest to most severe) were selected. A convenience sample of respondents was then asked to complete an SF-12 form for an individual with the health state described in the lay descriptions of these conditions. Composite mental and physical SF-12 score was regressed on GBD disability weight to derive the relationship between disability weight and SF-12 score. Individual respondent scores were then regressed on reported conditions to predict cumulative disability from the individuals' diagnoses, and a counterfactual cumulative disability excluding a single condition was predicted to obtain a comorbidity-corrected condition-specific disability weights for all individual with that single condition. The distribution of these condition-specific disability weights was used to derive the proportion of individuals with the conditions that fall within each GBD severity category. Asymptomatic cases were assigned no disability. The lay descriptions and disability weights for gallbladder and biliary diseases are shown below.

Table 2. Severity distribution, details on the severity levels for gallbladder and biliary diseases in GBD 2023 and the associated disability weight (DW) with that severity

Severity level	Lay description	DW (95% CI)	Distribution (95% CI)
Asymptomatic	--	0	0.739 (0.566–0.966)
Mild	This person has some pain in the belly that causes nausea but does not interfere with daily activities.	0.011 (0.005–0.021)	0.153 (0.122–0.183)
Moderate	This person has pain in the belly and feels nauseous. The person	0.114 (0.080–0.159)	0.065 (0.042–0.086)

	has difficulties with daily activities.		
Severe	This person has severe pain in the belly and feels nauseated. The person is anxious and unable to carry out daily activities.	0.324 (0.219–0.442)	0.043 (0.031–0.054)

The severity distribution of gallbladder and biliary disease was derived from analysis of the MEPS. MEPS is an overlapping panel survey of the non-institutionalised USA population that collects data on respondents' health service interactions. Panels are initiated every year. Each panel is two years long and consists of five rounds. In 2000, MEPS began using 12-Item Short Form Surveys (SF-12) to collect data on functional health status. The SF-12 survey is administered twice per panel (about once per year). This survey has been employed to estimate distributions of severity levels for diverse GBD diseases as previously described and summarised below.[1]

To translate SF-12 scores into GBD disability weights, 62 lay descriptions for conditions representing the full range of disability weight values (from mildest to most severe) were selected. A convenience sample of respondents was then asked to complete an SF-12 form for an individual with the health state described in the lay descriptions of these conditions. Composite mental and physical SF-12 score was regressed on GBD disability weight to derive the relationship between disability weight and SF-12 score. Individual respondent scores were then regressed on reported conditions to predict cumulative disability from the individuals' diagnoses, and a counterfactual cumulative disability excluding a single condition was predicted to obtain a comorbidity-corrected condition-specific disability weights for all individual with that single condition. The distribution of these condition-specific disability weights was used to derive the proportion of individuals with the conditions that fall within each GBD severity category.

References

1. Burstein R, Fleming T, Haagsma J, Salomon JA, Vos T, Murray CJL. Estimating distributions of health state severity for the global burden of disease study. *Popul Health Metr*. 2015;13(1):31. doi:10.1186/s12963-015-0064-y

Gastritis and duodenitis

Flowchart

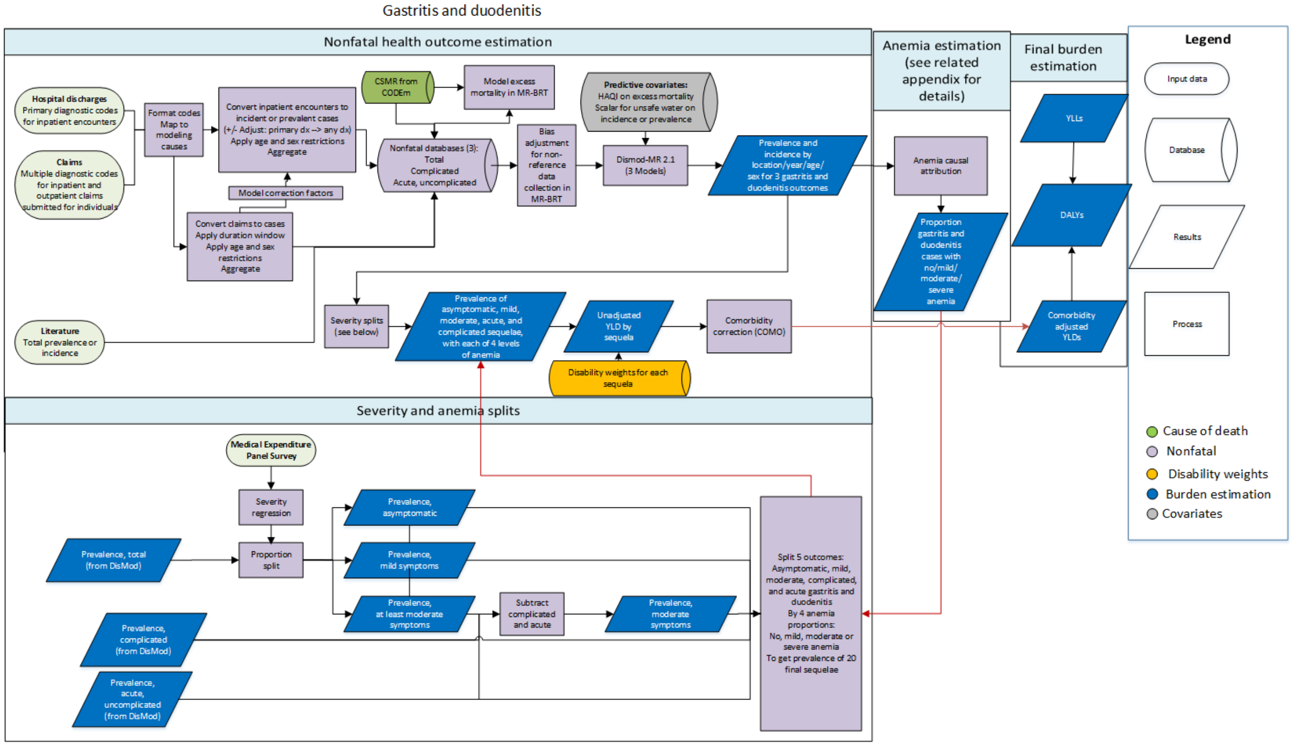

Input data and methodological summary for gastritis and duodenitis

Case definition

Although gastritis and duodenitis refer to inflammation of the mucosal lining of the stomach and duodenum, respectively, we adopt the common practice of using these terms to describe gastropathy and duodenopathy, meaning any form of injury to the mucosal lining of the stomach and duodenum, be it inflammatory (such as due to infection or autoimmune disease) or otherwise (such as due to nonsteroidal anti-inflammatory medications), regardless of symptoms.

In practice and in the GBD, both inflammatory and non-inflammatory mucosal damage are classified together as gastritis and duodenitis. These entities exclude cases of damage that extends through the muscularis mucosa, which defines peptic ulcer disease, which is estimated and described separately.

Gold-standard diagnosis of gastritis and duodenitis is by biopsy, although endoscopic visualisation and a number of biochemical and microbiological tests have good predictive value. Gastritis and duodenitis can acutely produce severe symptoms or have a subtle onset and evolve into a chronic illness characterised by asymptomatic periods and periods of abdominal pain, bloating, nausea, and early satiety. Complications such as haemorrhage may develop. Chronic gastritis is associated with elevated risk of gastric cancer.

In GBD 2023, gastritis and duodenitis were defined by diagnostic codes as described below. The ICD-10 code for gastritis and duodenitis is K29. ICD-10 codes for complicated gastritis and duodenitis are

K29.01, K29.21, K29.31, K29.41, K29.51, K29.61, K29.71, K29.81, K29.91. ICD-10 codes for acute gastritis are K29.0, K 29.00, K29.1, K29.2, and K29.20. Equivalent ICD-9 codes were used where appropriate.

Quantity of interest	Reference or Alternative	Definition
Incidence of complicated gastritis/duodenitis	Alternative	Cases identified from database of commercial claims from USA in 2000 using ICD codes.
Incidence of complicated gastritis/duodenitis	Alternative	Cases identified from database of commercial claims from USA in 2010-2016 using ICD codes.
Incidence of complicated gastritis/duodenitis	Reference	Cases of complicated gastritis/duodenitis identified using ICD codes in administrative records that are considered population-representative.
Incidence of severe, acute, uncomplicated gastritis/duodenitis	Alternative	Cases identified from database of commercial claims from USA in 2000 using ICD codes.
Incidence of severe, acute, uncomplicated gastritis/duodenitis	Alternative	Cases identified from database of commercial claims from USA in 2010-2016 using ICD codes.
Incidence of severe, acute, uncomplicated gastritis/duodenitis	Reference	Cases of severe, acute, uncomplicated gastritis/duodenitis identified using ICD codes in administrative records that are considered population-representative.
Prevalence of total gastritis and duodenitis	Alternative	Cases identified from database of commercial claims from USA in 2000 using ICD codes.
Prevalence of total gastritis and duodenitis	Alternative	Cases identified from database of commercial claims from USA in 2010-2016 using ICD codes.
Prevalencetotal gastritis and duodenitis	Reference	Cases of gastritis and duodenitis identified using ICD codes in administrative records that are considered population-representative.

Overall strategy

As in GBD 2017, GBD 2019 and GBD 2021, the GBD 2023 non-fatal estimation strategy for gastritis and duodenitis consisted of:

- Estimating the prevalence of total gastritis and duodenitis
- Dividing the total prevalent cases into asymptomatic, mild, and at least moderate severity levels
- Separately estimating the prevalence of gastritis and duodenitis with complication
- Separately estimating the prevalence of gastritis and duodenitis, acute, without complication (but with sufficient severity to require hospitalisation)
- Subtracting prevalent cases of gastritis and duodenitis with complication and gastritis and duodenitis, acute, without complication (but with sufficient severity to require hospitalisation) from prevalent cases of gastritis and duodenitis of at least moderate severity

Input data

Data sources

As in previous rounds, our GBD 2023 gastritis and duodenitis models relied primarily on data from hospital discharges and claims, assembled by IHME and described elsewhere in this appendix. These data are supplemented by data from peer-reviewed publications identified via systematic reviews of the literature conducted using PubMed and Embase for previous rounds of GBD, most recently GBD 2016. In brief, to be included, studies from all sources needed to:

> 1) Report a standard epidemiological measure (incidence, prevalence, case-fatality ratio, standardised mortality rate, etc.) of gastritis, duodenitis, or both.
> 2) Provide sufficient information on study methods and sample characteristics to assess its quality and make appropriate adjustments.
> 3) Use a gold-standard case definition based on endoscopy and biopsy, or use a well-defined alternative case definition that could be adjusted toward a reference standard.
> 4) Be conducted in a representative sample of a general population defined only by year, age, sex, and location, or be conducted in a representative sample of a well-defined sub-population for which valid adjustments could be made, or ascertain all cases for a defined catchment area for which GBD population estimates are available.

As in GBD 2021, the GBD 2023 gastritis and duodenitis modelling strategy used three separate databases: total gastritis and duodenitis, gastritis and duodenitis with complication (such as haemorrhage), and gastritis and duodenitis, acute, without complication (but with sufficient severity to require hospitalisation). The total gastritis and duodenitis dataset included data from hospital discharges and claims coded with any gastritis or duodenitis ICD code, as well as data from peer-reviewed publications. The gastritis and duodenitis with complication dataset included hospital discharges and inpatient claims with ICD codes specifying the occurrence of complications. The gastritis and duodenitis, uncomplicated, acute dataset included only hospital discharges and inpatient claims with ICD codes specifying that a complication did not occur.

No new prevalence or incidence data were added in GBD 2023.

Inputs to our non-fatal modelling also included cause-specific mortality rate (CSMR) estimates taken from our fatal modelling process (see CoD cause-specific modelling description for appendicitis in this appendix) and excess mortality rates (EMR) estimates modelled outside of DisMod (see the EMR data processing section below).

Prevalence and incidence data processing

The extraction and processing of prevalence and incidence data for gastritis and duodenitis were identical in GBD 2023, GBD 2021, and GBD 2019. The preponderance of these data came from claims and hospital discharges. Hospital discharge data provide observations about encounters, generally with only the primary diagnostic code for the encounter. Claims data, on the other hand, link claims for all inpatient and outpatient encounters for a single individual and provide primary and secondary diagnoses for all encounters.

For the total gastritis and duodenitis database, an individual was extracted from claims data as a prevalent case if they had any gastritis and duodenitis ICD code as any diagnosis in one or more inpatient encounters or two or more outpatient encounters. Hospital discharges were extracted if an

appropriate ICD code appeared as the primary discharge diagnosis, and the discharges were then adjusted using a correction factor estimated from claims data. Specifically, the correction factor (known as CF3) was modelled as the ratio of inpatient claims with an appropriate primary diagnostic code to all prevalent cases (inpatient and outpatient) in claims data, using MR-BRT.

For the gastritis and duodenitis with complication dataset and the gastritis and duodenitis, uncomplicated, acute dataset, individuals were extracted from claims as incident cases if they had an inpatient claim with an appropriate ICD code as any diagnosis. These incident cases were extracted linking multiple encounters for an individual and assuming multiple claims within a 60-day window represented multiple encounters for care of a single incident episode, and multiple claims separated by more than 60 days represented separate episodes of illness and, thus, additional incident cases. Hospital discharges were extracted if an appropriate ICD code appeared as the primary diagnosis, and the discharges were then adjusted using a correction factor estimated from claims data. Specifically, the correction factor (known as CF2), was modelled as the ratio of inpatient claims with an appropriate primary diagnostic code to all incident (inpatient) cases in claims data, using MR-BRT.

Details of the extraction, utilisation envelope, and correction factor models used to process hospital discharge and claims data for all GBD causes (including gastritis and duodenitis) are found in a separate section of this appendix on claims, inpatient hospital, and outpatient data.

Epidemiological measurements from peer-reviewed publications were manually extracted for the most granular age-sex groups reported, with a measure of uncertainty and information on the study design. Extracted measures were marked with dichotomous variables to indicate alternative (non-reference) case definitions, study populations, or other study design features. Where a single study reported measures using more than one case definition, multiple measurements were extracted to create paired data for modelling adjustment factors.

For total gastritis and duodenitis, we sought to use a gold-standard case definition of endoscopy without clinical indication, and to develop adjustments for alternative case definitions of endoscopy with clinical indication, serology (pepsinogen), diagnostic code in administrative data (such as hospital discharges or claims), and self-reported diagnosis (current or with 12-month recall). Unfortunately, only a single study in our database used endoscopy to survey for gastritis in a general population selected without regard to symptoms, two used endoscopy performed only in symptomatic persons, eight used serology, and four used self-report; among these, a total of three matches in year, age, sex, and location were observed between the studies, and no matches were observed between any of these data types and data from administrative sources. Thus, valid adjustments toward the gold-standard definition could not be estimated, we dropped the endoscopy-based data, and we adopted diagnostic code in administrative data as our reference case definition.

A pre-modelling adjustment was made to account for the fact that claims data from the USA only cover a commercially insured sub-population. Commercial claims data were available for all 51 USA subnational locations, and matched hospital discharge data covering the general population for one or more years were available for 24 USA subnational locations. Thus, 24 sets of paired data were used as inputs to a model of the difference in logit prevalence between alternative (commercially biased) and reference (general population) data in MR-BRT. The estimated mean logit differences were applied to USA claims data as bias correction prior to modelling in DisMod-MR 2.1 (below).

The process of adjusting alternative data types using MR-BRT with the logit-transformation method is described below:

1. Identify datapoints with overlapping year, age, sex, and location between reference and non-reference population data.
2. Logit transform overlapping datapoints of alternative and reference types.
3. Convert overlapping datapoints into a difference in logit space using the following equation: $logit(altnerative) - logit(reference)$
4. Use the delta method to compute standard errors of overlapping datapoints in logit space, then calculate standard error of logit difference using the following equation: $\sqrt{(variance\ of\ logit(alternative)) + (variance\ of\ logit(reference))}$
5. Using MR-BRT, conduct a random effects meta-regression to obtain the pooled logit difference of alternative to reference
6. Apply the pooled logit difference to all data points of non-reference types using the following equation:
$$new_{estimate} = inverse.logit((logit(alternative)) - (pooled\ logit\ difference))$$
7. Calculate new standard errors using the delta method, accounting for gamma (between-study heterogeneity)

The table below shows bias correction factors estimated using MR-BRT.

Table 1.1: MR-BRT crosswalk adjustment factors for total gastritis and duodenitis

Data input	Reference or alternative data collection	Gamma	Beta coefficient, logit difference (95% CI)	Adjustment factor*
Hospital + non-USA claims	Reference	0.83	---	---
USA claims from year 2000	Alternative		–0.44 (–2.7 to 1.9)	0.39 (0.066 to 0.87)
USA claims from years 2010–2016	Alternative		–0.030 (–1.7 to 1.7)	0.49 (0.15 to 0.85)

For gastritis and duodenitis with complication, and gastritis and duodenitis, uncomplicated, acute, only administrative data were available. Pre-modelling adjustments were made to data from commercial claims, using an approach similar to that described above for total peptic ulcer disease data.

Table 1.2: MR-BRT crosswalk adjustment factors for gastritis and duodenitis with complication

Data input	Reference or alternative data collection	Gamma	Beta coefficient, logit difference (95% CI)	Adjustment factor*
Hospital + non-USA claims	Reference	0.16	---	---
USA claims from year 2000	Alternative		–0.42 (–0.89 to 0.054)	0.40 (0.29 to 0.51)
USA claims from years 2010–2016	Alternative		–0.24 (–0.57 to 0.093)	0.44 (0.36 to 0.52)

Table 1.3: MR-BRT crosswalk adjustment factors for gastritis and duodenitis, uncomplicated, acute

Data input	Reference or alternative data collection	Gamma	Beta coefficient, logit difference (95% CI)	Adjustment factor*
Hospital + non-USA claims	Reference	0.21	---	---
USA claims from year 2000	Alternative		0.29 (–0.29 to 0.87)	0.57 (0.43 to 0.71)
USA claims from years 2010–2016	Alternative		–0.072 (–0.51 to 0.36)	0.48 (0.37 to 0.59)

**Adjustment factor is the inverse-logit-transformed beta coefficient; <0.5 represents that alternative is adjusted upward; >0.5 represents that alternative is adjusted downward.*

After adjustment, for each source-location-year-sex combination, the age-standardised mean was calculated, and the data series was excluded if this was zero or was greater than two times the median absolute deviation above or below the median for the database.

EMR processing

EMR inputs have evolved in serial rounds of GBD. In GBD 2017, EMR inputs were produced by matching total gastritis and duodenitis prevalence datapoints with their corresponding CSMR values within the same age, sex, year, and location (by dividing CSMR by prevalence). However, this method of producing EMR inputs demonstrated a rather unrealistic pattern of EMR compared to an expected pattern of decreasing EMR with greater access to quality health care. (Such unexpected patterns often signal inconsistencies between CSMR estimates and the measures of prevalence and/or incidence.) Thus, in an effort to provide greater guidance on the expected pattern of EMR, in GBD 2019, EMR data produced as above in GBD 2017 were modelled by age, sex, and Healthcare Access and Quality (HAQ) Index using MR-BRT, with a prior on HAQ Index having a negative coefficient. We then predicted EMR for each country, year, sex, and for ages 0, 10, 20….100. These predictions were used as inputs to our total gastritis and duodenitis DisMod model in GBD 2019, GBD 2021, and GBD 2023.

Modelling strategy

Total gastritis and duodenitis, symptomatic and asymptomatic

Similar to previous rounds, we ran a DisMod-MR 2.1 model to produce estimates by age, sex, year, and location. Inputs to DisMod for total gastritis and duodenitis included prevalence, CSMR, and EMR inputs processed as described above, and expert priors for other epidemiological measures. Prior value of remission was bounded from 0 to 1 (a minimum duration of one year). The minimum coefficient of variation at the regional, super-regional, and global level was set at 0.8, and the time window of data to include for fitting was five years. We included HAQ Index as a predictive covariate on EMR with a mean and standard deviation produced from the MR-BRT model described above. Predictive covariates for alcohol consumption and access to safe water were applied to prevalence, which we forced positive with a lower bound of zero on the priors. Betas and exponentiated values (which can be interpreted as an odds ratio) are shown in the tables below for all predictive covariates in the DisMod model.

Table 2.1: DisMod-MR 2.1 model covariates for total gastritis and duodenitis

**Covariate**	**Parameter**	**Exponentiated beta**

Litres of alcohol per capita	Prevalence	1.00 (1.00–1.00)
Scaled exposure variable for unsafe water	Prevalence	2.12 (2.12–2.13)
Healthcare Access and Quality Index	Excess mortality	0.97 (0.97–0.97)

Complicated gastritis and duodenitis

The DisMod model for complicated gastritis and duodenitis included incidence data as described above. The prior value of incidence was bounded to 0 to 0.3, the prior value of EMR was bounded to 0.1 to 10, and the prior value of remission was bounded to 6 to 13 cases of remission per person-year (disease duration 4 to 8.7 weeks). A location-level covariate for HAQ Index was applied to EMR, and location-level covariates for the log-transformed age-standardised death rate due to gastritis and duodenitis and unsafe water access were applied to incidence. Random effects for all super-regions except for the high-income super-region were bounded to –0.25 to 0.25. Betas and exponentiated values (which can be interpreted as odds ratios) are shown in the table below for all covariates.

Table 2.2: DisMod-MR 2.1 model covariates for gastritis and duodenitis with complication

**Covariate**	**Parameter**	**Exponentiated beta**
Natural log of age-standardised death rate	Incidence	1.00 (1.00–1.00)
Scaled exposure variable for unsafe water access	Incidence	1.00 (1.00–1.00)
Healthcare Access and Quality Index	Excess mortality rate	0.61 (0.37–0.98)

Acute gastritis and duodenitis, without complication

The DisMod model for acute, uncomplicated gastritis and duodenitis included incidence data as described above. The prior value on incidence was set to 0 through age 5 years, the range of prior values on EMR was bounded to 0 to 0.1, and the range of prior values on remission was bounded to 6 to 13 cases per person-year. Location-level covariates were applied for log-transformed, lag-distributed income (on excess mortality rate), log-transformed age-standardised death rate due to gastritis and duodenitis (on incidence), and per capita alcohol consumption (on incidence). Betas and exponentiated values (which can be interpreted as odds ratios) are shown for these covariates in the tables below.

Table 2.3: DisMod-MR 2.1 model covariates for gastritis and duodenitis, uncomplicated, acute

**Covariate**	**Parameter**	**Exponentiated beta**
Log-transformed lag-distributed income	Excess mortality rate	0.61 (0.38–1.00)
Natural log of age-standardised death rate	Incidence	1.00 (1.00–1.00)

Severity split & disability weight

The basis of the GBD disability weight survey assessments are lay descriptions of health states highlighting major functional consequences and symptoms.

Prevalence draws from the total gastritis and duodenitis model were divided into asymptomatic, mild, and at least moderate severity levels using proportions derived from the Medical Expenditure Panel Survey (MEPS). It must be noted that the MEPS analysis uses quality-of-life data from individuals who had a health care encounter for gastritis and duodenitis within the preceding 12 months and were interviewed about their quality of life in the preceding four weeks, so the asymptomatic proportion represents those with diagnosed disease who were asymptomatic in a given period of time, not those always asymptomatic who may have gastritis and duodenitis on lab tests or endoscopy if examined for study or screening purposes. After dividing the total prevalence draws by these three proportions, the complicated and uncomplicated acute prevalence draws were subtracted from the at least moderate draws.

Table 3.1: The asymptomatic, mild, and remaining moderate prevalent cases were then assigned the following lay descriptions and disability weights.

Severity level	Lay description	DW (95% CI)
Diagnosed gastritis and duodenitis, not in a symptomatic episode	--	0
Mild gastritis and duodenitis episode	This person has some pain in the belly that causes nausea but does not interfere with daily activities.	0.011 (0.005–0.021)
Moderate gastritis and duodenitis episode	This person has pain in the belly and feels nauseous. The person has difficulties with daily activities.	0.114 (0.080–0.159)

Table 3.2: Gastritis and duodenitis, with complication, and gastritis and duodenitis, uncomplicated, acute, were then assigned the following lay descriptions and disability weights.

Severity level	Lay description	DW (95% CI)
Gastritis and duodenitis, with complication	This person vomits blood and feels nauseous.	0.325 (0.209–0.462)
Gastritis and duodenitis, acute, uncomplicated	This person has severe pain in the belly and feels nauseous. The person is anxious and unable to carry out daily activities.	0.324 (0.220–0.442)

These five final health states were then combined with health states for anaemia. Methods for causal attribution of anaemia due to gastritis and duodenitis can be found in the “Impairment and underlying cause estimation” and the “Non-fatal cause-specific modelling description” titled “Anaemia”.

Gastro-oesophageal reflux disease

Flowchart

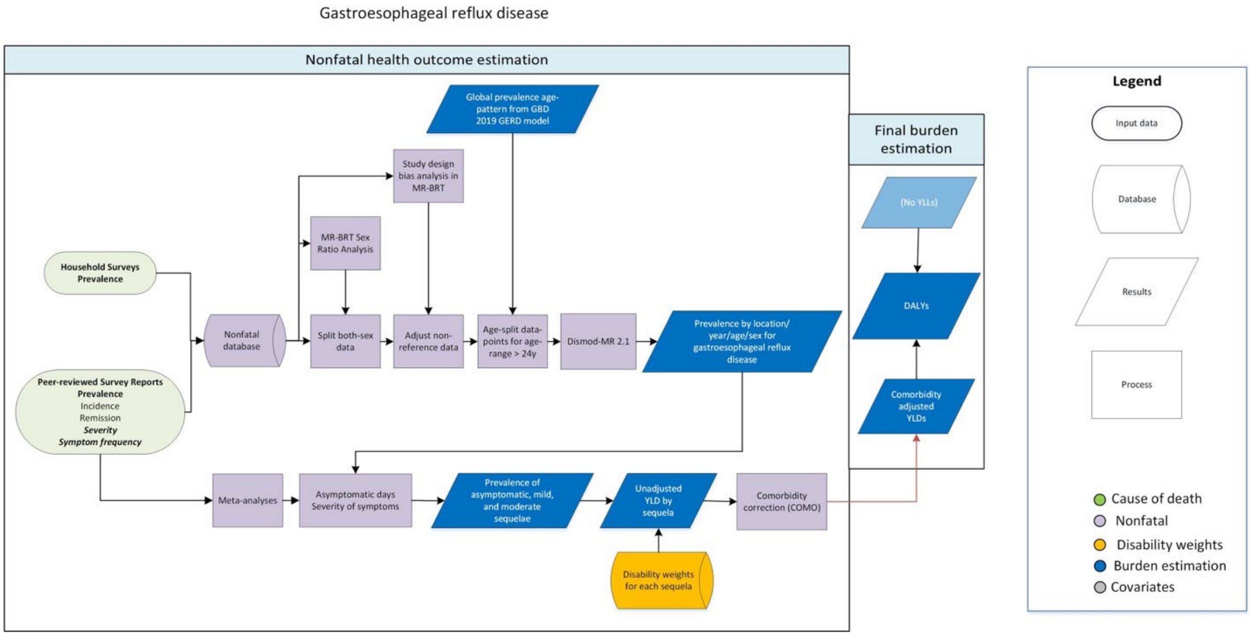

Input data and methodological summary for gastro-oesophageal reflux disease

Case definition

Gastro-oesophageal reflux disease (GORD) is a chronic condition that results when the reflux of stomach contents causes troublesome symptoms, complications, or both. The cardinal symptoms of typical GORD are heartburn (a burning feeling behind the breastbone) and regurgitation (the unpleasant sensation of material moving upward from the stomach toward the mouth).

In GBD, the occurrence of heartburn, regurgitation, or both, at least once weekly over a 12-month recall period is employed as the reference case definition.

Individuals who experience oesophageal complications (ulceration, metaplasia, etc.) without symptoms, whose sole symptom of gastro-oesophageal reflux is chest pain without typical reflux symptoms, or who experience reflux primarily as a trigger or exacerbating factor in respiratory or head and neck diseases (chronic cough, dental erosion, etc.) were not included. This strategy avoids double-counting disability already attributed to other underlying diseases modelled in GBD. Likewise, we regarded newborn reflux as a separate disease, which is modelled elsewhere and excluded from this analysis.

**Quantity of interest**	**Reference or Alternative**	**Definition**
Prevalence of total gastro-oesophageal reflux disease	Alternative	Self-reported heartburn assessed using one of several structured questionnaires; differs from reference case definition by asking about heartburn but NOT asking about regurgitation.

Prevalence of total gastro-oesophageal reflux disease	Alternative	Self-reported regurgitation assessed using one of several structured questionnaires; differs from reference case definition by asking about regurgitation but NOT asking about heartburn.
Prevalence of total gastro-oesophageal reflux disease	Alternative	Diagnosis made using one of several structured symptom questionnaires; differs from reference case definition by asking about and requiring the presence of symptoms beyond just heartburn and regurgitation.
Prevalence of total gastro-oesophageal reflux disease	Alternative	Diagnosis made using one of several structured symptom questionnaires; differs from reference case definition by asking about and counting as positive symptoms beyond just heartburn and regurgitation.
Prevalence of total gastro-oesophageal reflux disease	Alternative	Diagnosis made using one of several structured symptom questionnaires; differs from reference case definition by using a recall period shorter than 12 months.
Prevalence of total gastro-oesophageal reflux disease	Alternative	Diagnosis made using one of several structured symptom questionnaires; differs from reference case definition by using a minimum symptom frequency greater than just weekly.
Prevalence of total gastro-oesophageal reflux disease	Alternative	Diagnosis made using one of several structured symptom questionnaires; differs from reference case definition by using a minimum symptom frequency threshold that was less than weekly (at least every 2 weeks, at least monthly, etc.).
Prevalence of total gastro-oesophageal reflux disease	Alternative	Diagnosis made using one of several structured symptom questionnaires; differs from reference case definition by integrating information about symptom nature, frequency, and severity into a diagnostic score, rather than a simple cut-off of at least weekly heartburn and/or regurgitation.
Prevalence of total gastro-oesophageal reflux disease	Alternative	Cases that are identified using ICD codes in administrative records that are considered population-representative.
Prevalence of total gastro-oesophageal reflux disease	Reference	Self-reported symptoms of heartburn and/or regurgitation at least weekly for 12 months, assessed using one of several structured questionnaires, administered to a population-representative sample of survey respondents.

Input data

Data inputs and processing for GORD in GBD 2023 are unchanged from GBD 2021.

Data inputs

Data inputs for estimating the prevalence of GORD in GBD 2023 came from a systematic review conducted for GBD 2017. In brief, peer-reviewed publications reporting epidemiological measures of GORD were identified via a search-string-based review in PubMed, citations of those articles identified by search-string, and suggestions from the GBD Collaborator Network. Two household surveys – the USA National Health Interview Surveys in 2007 and 2012 – were identified from the Global Health Data

Exchange as asking participants about the occurrence of typical reflux symptoms and were also included. In brief, data from all sources had to:

1) Report a standard epidemiological measure (incidence, prevalence, case–fatality ratio, standardised mortality rate, etc.) of GORD or provide individual-level data from which one could be calculated.
2) Provide sufficient information on study methods and sample characteristics to assess its quality and make appropriate adjustments.
3) Use our reference case-definition, or use a well-defined alternative case-definition that could be adjusted toward our reference standard.
4) Be conducted in a representative sample of a general population defined only by year, age, sex, and location, or be conducted in a representative sample of a well-defined sub-population for which valid adjustments could be made, or ascertain all cases for a defined catchment area for which GBD population estimates are available.
5) Provide information on uncertainty (sample size, standard deviation, or confidence interval) and follow-up time.
6) Be written in a language that the modelling team could read (English, French, Portuguese, or Spanish).

In our search, all studies reporting incidence or remission of GORD provided insufficient information on person-time of observation and were excluded, so only prevalence data were included. Data from claims data extracted and prepared for GBD as described elsewhere in this appendix were used to develop adjustment factors for published studies from the search-string-based review that ascertained cases based on diagnostic codes in administrative data but were not used in the primary analysis of GORD prevalence.

Prevalence measurements from peer-reviewed publications were manually extracted for the most granular age-sex groups reported, with a measure of uncertainty and information on the study design. Prevalence estimates were extracted from individual-level data from two household surveys using questionnaire text, skip-pattern, and weights for complex sampling strategies provided in the documentation from original study investigators. Extracted measurements were marked with dichotomous variables to indicate alternative (non-reference) case definitions, study populations, or other study design features. Where a single study measured using more than one case definition, multiple measurements were extracted to create paired data for modelling adjustment factors.

Data processing

For studies that reported prevalence by age for both sexes combined, and prevalence by sex for all ages combined, we calculated the sex-ratio of cases in that study and applied it to the age-specific prevalence measures to estimate age-sex-specific prevalence.

To estimate sex-specific prevalence from studies that reported prevalence only for both sexes combined, we modelled the log sex ratio in MR-BRT using all sex-specific prevalence measurements from all other studies in the database: 0.24 (–0.23 to 0.70) and combined this with the GBD sex-specific population estimates for the relevant age group. These were applied by calculating male prevalence:

$$prev_{male} = prev_{both} * \frac{pop_{both}}{(pop_{male} + ratio * pop_{female})}$$

and then calculating female prevalence: $prev_{female} = ratio * prev_{male}$

For GORD, 27 studies used our reference case definition. The remaining studies had one or more non-reference study design feature thought to systematically bias prevalence measurements: questionnaire only asked subjects about heartburn, questionnaire only asked subjects about regurgitation, case definition required subjects to have additional symptoms to qualify as having GORD (such as sleep disruption or sour taste in mouth), case definition allowed subjects to qualify as having GORD due to having symptoms other than heartburn and regurgitation, recall period was less than 12 months, case definition required more than weekly symptoms, case definition included those with less than weekly symptoms, case definition used a scoring system that integrated information on number, frequency, and duration of symptoms, or cases were identified based on diagnostic code in administrative data. These were modelled as independent effects in a network meta-analysis in MR-BRT, using 82 studies. Adjustments were modelled as difference in logit prevalence between alternative and reference data. The estimated mean logit differences were applied to non-reference data types as bias correction prior to modelling in DisMod-MR 2.1 (below).

The process of adjusting non-reference data using MR-BRT with the logit-transformation method is described below:

1. Identify datapoints with overlapping year, age, sex, and location between reference and non-reference population data.
2. Logit transform overlapping datapoints of alternative and reference types.
3. Convert overlapping datapoints into a difference in logit space using the following equation: $logit(altnerative) - logit(reference)$
4. Use the delta method to compute standard errors of overlapping datapoints in logit space, then calculate standard error of logit difference using the following equation: $\sqrt{(variance\ of\ logit(alternative)) + (variance\ of\ logit(reference))}$
5. Using MR-BRT, conduct a random effects meta-regression to obtain the pooled logit difference of alternative to reference
6. Apply the pooled logit difference to all data points of non-reference types using the following equation:
$$new_{estimate} = inverse.logit((logit(alternative)) - (pooled\ logit\ difference))$$
7. Calculate new standard errors using the delta method, accounting for gamma (between-study heterogeneity)

The table below shows bias correction factors for study design characteristics estimated using MR-BRT.

Table 1: MR-BRT crosswalk adjustment factors for gastro-oesophageal reflux disease

Data input	Reference or alternative data collection	Gamma	Beta coefficient, logit difference (95% CI)	Adjustment factor*
Heartburn and/or regurgitation at least weekly for 12 months	Reference	0.61	---	---
Only asked about heartburn	Alternative		–0.61 (–2.1 to 0.92)	0.35 (0.11 to 0.72)
Only asked about regurgitation	Alternative		–0.26 (–1.8 to 1.3)	0.43 (0.14 to 0.78)
Required additional symptoms to meet case definition	Alternative		0.25 (–1.3 to 1.8)	0.56 (0.23 to 0.86)
Could meet case definition with other symptom options	Alternative		0.58 (–0.96 to 2.1)	0.64 (0.28 to 0.89)
Shorter recall period	Alternative		0.26 (–1.3 to 1.8)	0.56 (0.22 to 0.86)
Required greater minimum symptom frequency to meet case definition	Alternative		–1.2 (–2.7 to 0.35)	0.23 (0.063 to 0.59)
Had lower symptom frequency requirement to	Alternative		0.89 (–0.63 to 2.4)	0.71 (0.35 to 0.92)

meet case definition				
Used diagnostic score integrating multiple domains	Alternative		–0.027 (–1.6 to 1.5)	0.49 (0.17 to 0.82)
Diagnostic code in administrative data	Alternative		–1.7 (–3.2 to –0.13)	0.16 (0.039 to 0.47)

**Adjustment factor is the inverse-logit-transformed beta coefficient; <0.5 represents that alternative is adjusted upward; >0.5 represents that alternative is adjusted downward.*

Data sources that used non-reference study designs were dropped for which valid adjustments could not be developed: sampling of populations defined by profession (4), convenience sampling from waiting rooms (3), case definition limited to endoscopically confirmed erosive oesophagitis (1), and self-reported diagnosis without symptom-based questions (1).

Subsequently, datapoints for samples spanning 25 years of age or more were disaggregated by applying the age-pattern observed in the global fit for the GBD 2017 GORD model.

Specific datapoints from some sources from subnational locations were excluded if relatively high values in young age groups led to overestimation of the entire age range.

Modelling strategy

Compartmental DisMod model

A full compartmental model of GORD epidemiology was run using DisMod-MR 2.1. Adjusted prevalence data as processed for GBD 2023 and described above were the inputs. Parameter settings were unchanged from GBD 2021. Excess mortality rate was assumed a priori to be 0, and remission prior was set to 0.2 to 0.5 cases per person-year. Incidence was forced to 0 from birth to age 5 years, and after this age, prior was set to 0 to 0.2 cases per person-year. In previous rounds, we trialed covariates for mean body-mass index, prevalence of obesity, and per capita alcohol consumption, but these were not predictive so were removed from the model.

Severity split & disability weight

Severity distributions and disability weights for GORD are unchanged from GBD 2021.

Throughout the literature, the severity of GORD is often divided into three or four categories, using definitions such as those in the table below. In GBD 2017, we reviewed the studies in our prevalence database, above, and, if provided, extracted counts of cases of each severity as reported. These cases were then mapped to one of two GBD GORD severities (also shown in the table below). These categories were mapped to GBD health states, which are associated with disability weights. The basis of the GBD disability weight survey assessments are lay descriptions of health states highlighting major functional consequences and symptoms, also shown below.

Table 2: Sample mapping of reported GORD severity levels to GBD GORD severity levels

Literature severity levels	GBD severity level	Lay description

Mild: can be ignored	Mild/moderate	Often has a burning sensation in the back of the chest after eating
Moderate: cannot be ignored but does not affect lifestyle	Mild/moderate	Often has a burning sensation in the back of the chest after eating
Severe: affects lifestyle	Severe (abdom_mod)	Has pain in the belly* and feels nauseous. Has difficulty with daily activities.
Very severe: has marked effect on lifestyle	Severe (abdom_mod)	Has pain in the belly* and feels nauseous. Has difficulty with daily activities.

*We acknowledge that gastro-oesophageal reflux symptoms are felt in the chest, not the belly, but opine that a health state that incorporates other gastrointestinal symptoms and indicates interference with daily activities, such as difficulty eating and sleeping, better represents more severe gastro-oesophageal reflux disease than a health state that describes only post-prandial heartburn.

The proportion of cases in each of the GBD GORD severities was then estimated using the metafor package (version 2.0-0) in R (version 3.4). Inputs to this meta-analysis are shown below. In GBD 2017, all studies with severity information for sample of cases defined by at least weekly symptoms were included, whether the defining symptoms were heartburn, regurgitation, either, or both, and regardless of recall period or duration; thus 15 studies were included. In GBD 2019, we limited the severity meta-analysis to only those studies that used the reference case definition of heartburn and/or regurgitation at least weekly for 12 months; thus, only four studies were included. The GBD 2019 proportions were applied in GBD 2021 and GBD 2023.

Meta-analysis of proportion severe/very severe for GORD

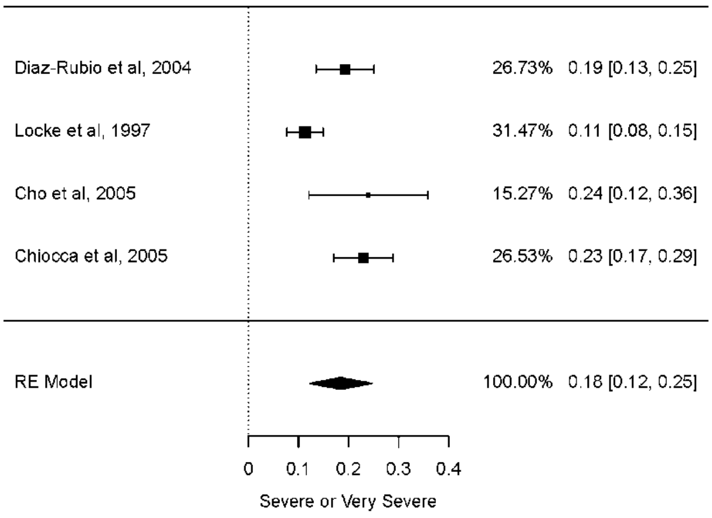

Many studies in the literature also report the frequency of GORD symptoms as the proportions of cases in each of a set of mutually exclusive and collectively exhaustive frequency categories. Examples include: 1–6 days/week and daily; 1 day/week, 2–6 days/week and daily; 1–3 days/week, 4–6 days/week, and daily; etc. For each study, for each frequency category, 1000 proportion draws were generated using a beta distribution with case counts in and out of the frequency category as shape parameters. We then assume that the number of days symptomatic within a category are uniformly distributed. We combine proportion draws and this assumption about mean days symptomatic in each category to produce draws

of the mean number of days/week symptomatic across all cases in a study. Means and standard deviations of these draws are displayed in the forest plot below.

Meta-analysis of days/week spent symptomatic for GORD

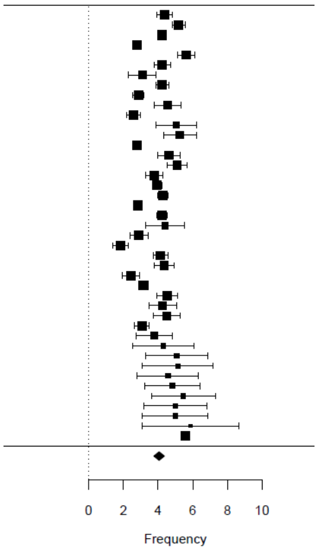

These inputs were then combined in a meta-analysis, and final mean and standard deviation were divided by seven to estimate the proportion of cases symptomatic on a given day, with uncertainty.

Table 3: Severity and frequency categories were combined to generate four categories, as shown

GBD severity-frequency category	Proportion that experience this severity of symptoms when symptomatic	Proportion that are symptomatic on any given day	DW (95% CI)
Mild/moderate GORD, asymptomatic days	0.72 (0.71–0.74)	0.42 (0.38–0.46)	None
Mild/moderate GORD, symptomatic days		0.58 (0.54–0.62)	0.027 (0.015–0.046)
Severe GORD, asymptomatic days	0.28 (0.26–0.29)	0.42 (0.38–0.46)	None
Severe GORD, symptomatic days		0.58 (0.54–0.62)	0.114 (0.080–0.159)

Gout

Flowchart

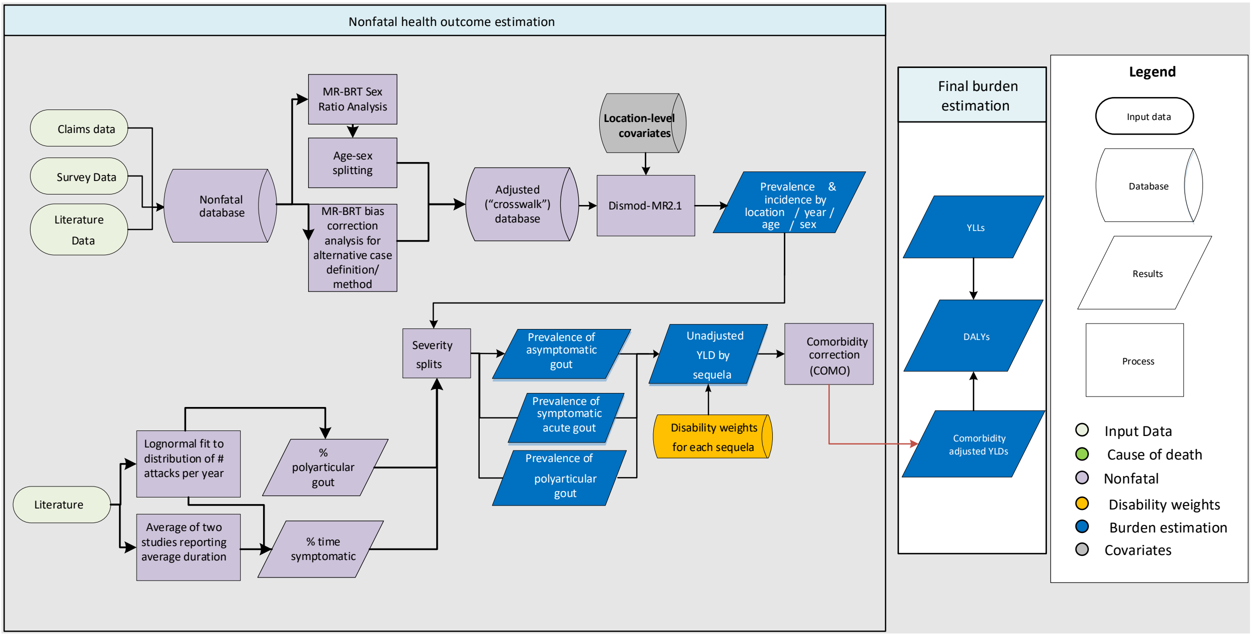

Input data and methodological summary for gout

Case definition

Gout is a rheumatic disease that is characterised by deposition of monosodium urate (MSU) crystals in the synovial fluid of joints and in other tissues, causing inflammation. The crystal formation is caused by elevated urate levels in extracellular fluids. Case definitions are found in Table 1. The ICD-10 code for gout is M10, and the ICD-9 code is 274.

Table 1. Case definitions for gout modelling

Quantity of interest	Reference or alternative	Definition
Gout	Reference	American College of Rheumatology 1977 (ARA 1977 or Wallace Criteria) survey criteria requiring the presence of MSU crystals in joint fluid or the presence of a tophus proven to contain MSU crystals and at least six of 12 gout symptoms or findings (>1 attack of acute arthritis, development of maximal inflammation within a day, attack of monarticular arthritis, observation of joint erythema, pain or swelling in the first MTP joint, unilateral attack involving the first MTP joint, unilateral attack involving tarsal joint, suspected tophus, hyperuricemia, asymmetrical swelling within a joint on X-ray and negative culture of joint fluid for

		microorganisms during attack of joint inflammation) to make a diagnosis
Gout	Alternative	Self-reported diagnosis of gout
Gout	Alternative	Gout reported in USA claims data based on ICD codes.
Gout	Alternative	Gout reported in Taiwan claims data based on ICD codes.
Gout	Alternative	Physician diagnosis of gout, criteria unspecified
Gout	Alternative	Physician diagnosis of gout using diagnostic criteria other than the ARA 1977

Input data

The most recent systematic review for gout was conducted in GBD 2013 for studies published between 1980 and 2009 using the following search terms on MEDLINE, EMBASE, CINAHL, CAB Abstracts, WHO Library (WHOLIS), and OpenSIGLE. For prevalence and incidence, the following search terms were used: (gout* OR hyperuricemia) AND (prevalen* OR inciden* OR cross-sectional OR cross sectional OR epidemiol* OR survey OR population-based OR population based OR population study OR population sample OR cohort OR follow-up OR follow up OR longitudinal OR regist*) AND (list of names of all GBD countries).

Exclusion criteria were:

- Sub-populations clearly not representative of the national population
- Not a population-based study
- Low sample size (less than 150)
- Review rather than original studies

For GBD 2019, 14 additional studies shared through the GBD Collaborator Network were added. In addition, data from USA claims for 2000 and 2010–2014 by state, and Taiwan claims data from 2016 were included.

Age and sex splitting

Reported estimates of prevalence were split by age and sex where possible. First, if studies reported prevalence for broad age groups by sex (eg, prevalence in 15- to 65-year-old males and females separately), and also by specific age groups for both sexes combined (eg, prevalence in 15- to 30-year-olds, then in 31- to 65-year-olds, for males and females combined), age-specific estimates were split by sex using the reported sex ratio and bounds of uncertainty. Second, prevalence data for both sexes that could not be split using a within-study ratio were split using a sex ratio derived from a meta-analysis of existing sex-specific data using MR-BRT (Meta-regression—Bayesian, regularised, trimmed, described in appendix 1 section 2). The female to male ratio was 0.33 (0.33–0.34). Finally, after the application of bias adjustments, where studies reported estimates across age groups spanning 25 years or more, these

were split into five-year age groups using the prevalence age pattern estimated by DisMod-MR 2.1 (disease model—Bayesian meta-regression, see appendix 1 section 2).

Data adjustment

We used study covariates for studies relying on self-reported diagnoses and those identifying sources through a diagnostic code in administrative data, which include gout ICD codes as well as read codes used in the UK health system. We used MR-BRT to adjust alternative case definition and claims data in the USA from the year 2000 and from 2010 onward and for Taiwan claims data to the reference case definition. Matched data were based off age, sex, year, and location. The mean and standard error for the coefficients were calculated using the MR-BRT crosswalk adjustment method. Betas and exponentiated values (which can be interpreted as an odds ratio) for these covariates are shown in the table below:

Table 3: MR-BRT crosswalk adjustment factors for gout

Data input	Reference or alternative case definition	Gamma	Beta coefficient, log (95% CI)*	Adjustment factor**
Physician-diagnosed gout	Ref	0.55	---	---
Self-reported gout	Alt		0.33 (0.05 to 0.60)	1.39 (1.05 to 1.83)
Gout identified with administrative data	Alt		0.29 (0.29 to 0.30)	1.34 (1.34 to 1.35)
USA claims data – 2000	Alt		–1.88 (–2.84 to –0.92)	0.15 (0.058 to 0.40)
USA claims data – 2010–2016	Alt		–1.55 (–2.00 to –1.09)	0.22 (0.13 to 0.34)
Taiwan claims data – 2016	Alt		0.30 (0.27 to 0.33)	1.35 (1.31 to 1.40)

**MR-BRT crosswalk adjustments can be interpreted as the factor the alternative case definition is adjusted by to reflect what it would have been had it been measured using the reference case definition. If the log/logit beta coefficient is negative, then the alternative is adjusted up to the reference. If the log/logit beta coefficient is positive, then the alternative is adjusted down to the reference.*

***The adjustment factor column is the exponentiated beta coefficient. For log beta coefficients, this is the relative rate between the two case definitions. For logit beta coefficients, this is the relative odds between the two case definitions.*

Modelling strategy

There have been no significant changes to the modeling strategy in GBD 2023.

Prior settings included assuming the excess mortality rate and remission of gout did not exceed 0.01 and 0.2, respectively, and that there was no incidence or prevalence of gout before the age of 15 years. We

included the summary exposure variable (SEV) scalar for gout, which summarises exposure to risks estimated in GBD to impinge on gout, ie, low glomerular filtration rate, as a country covariate. We set bounds of 0.75 to 1.25 as the SEV is constructed in a way that if our risk estimates are accurate the value should be 1.

Table 4. Covariates. Summary of covariates used in the gout DisMod-MR meta-regression model

**Covariate**	**Type**	**Parameter**	**Beta (95% uncertainty interval)**	**Exponentiated beta (95% uncertainty interval)**
Log-transformed age-standardised SEV scalar: Gout	Country-level	Prevalence	1.25 (1.24–1.25)	3.48 (3.45–3.49)

Severity and disability

The basis of the GBD disability weight (DW) survey assessments are lay descriptions of sequelae highlighting major functional consequences and symptoms. The lay descriptions and disability weights for gout severity levels are shown below.

Table 5. Severity distribution, details on the severity levels for gout in GBD 2023 and the associated disability weight (DW) with that severity.

Severity level	Lay description	DW (95% CI)
Gout, acute	This person has severe pain and swelling in the leg, making it very difficult to get up and down, stand, walk, lift, and carry heavy things. The person has trouble sleeping because of the pain.	0.295 (0.196–0.409)
Polyarticular gout (same as for severe RA)	This person has severe, constant pain and deformity in most joints, causing difficulty moving around, getting up and down, eating, dressing, lifting, carrying, and using the hands. The person often feels sadness, anxiety, and extreme fatigue.	0.581 (0.403–0.739)
Asymptomatic gout	This person has a diagnosis of gout without pain or functional difficulties.	0

To calculate the severity distribution of gout, we used three studies on the distribution of the number of gout attacks per year and fitted a lognormal curve using a least squared differences method.[1,2,3] In the absence of data on the proportion of gout cases who have chronic polyarticular gout, we assumed the proportion was equal to those who would have 52 attacks a year (ie, weekly) or more as implied by the lognormal curve.

The average number of attacks was estimated from the lognormal fit: 5.66 (5.14–6.18). From two studies, we derived an average duration of attacks of 6.1 days (5.4–6.8) by simple averaging. The resulting proportion of time symptomatic for acute gout was taken as the multiplication of these two estimates divided by the number of days in a year: 9.4% (8.0–10.9).

Figure 1: Distribution of cases by frequency

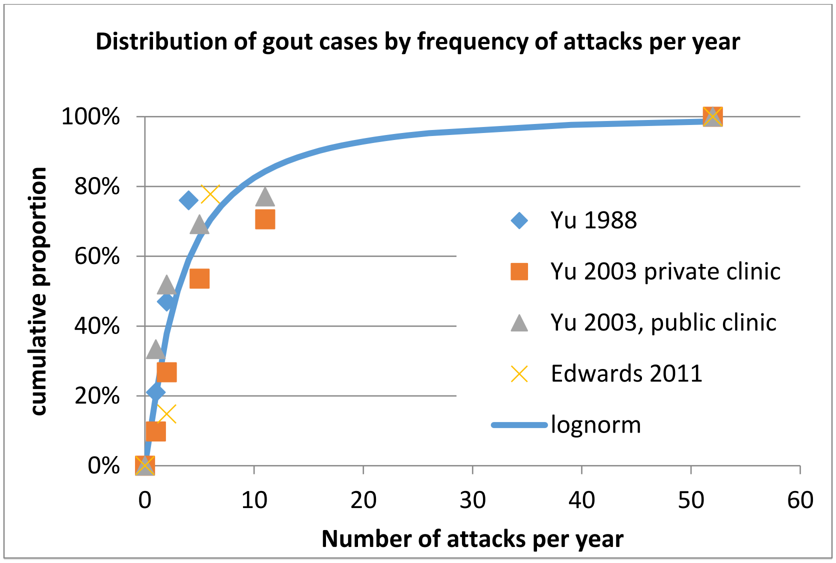

References

1. Edwards NL, Sundy JS, Forsythe A, Blume S, Pan F, Becker MA. Work productivity loss due to flares in patients with chronic gout refractory to conventional therapy. *Journal of Medical Economics*. 2011; 14(1).
2. Yu, KH, Luo SF, et al. Younger age of onset of gout in Taiwan. *Rheumatology*. 2003; 42(1): p. 166-170.
3. Yu TF, et al. Diversity of clinical features in gouty arthritis. *Seminars in Arthritis and Rheumatism*. 1984; 13(4): p. 360-368.

Guillain-Barré syndrome (GBS) impairment

Flowchart

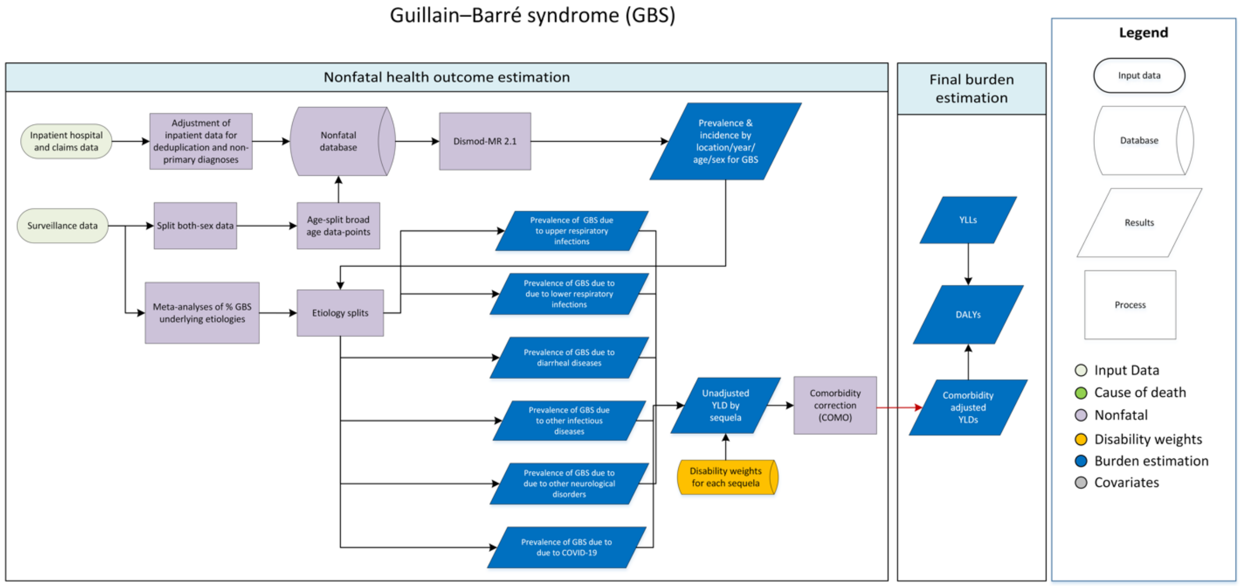

Case definition

Guillain-Barré syndrome (GBS) is a rare condition that usually occurs as a complication of a preceding infectious disease, such as a respiratory or gastrointestinal infection. It is considered an immune-mediated nerve dysfunction with rapid onset of weakness in the feet and legs, and sometimes the arms, which then progresses toward the trunk. In the acute phase, about 25% of cases required mechanical ventilation for survival. The majority of cases fully recover within months to a year. The following ICD-10 and ICD-9 codes are used: G61.0 (GBS) and 357.0 (acute infective polyneuritis). Literature studies are accepted if they are population-based and include physician-diagnosed GBS.

Input data

Morbidity model inputs

An updated systematic review was done for GBD 2023 from September 2017 to April 2023 using PubMed with the search string (((((("guillain barre syndrome"[MeSH Terms] OR ("guillain"[Title/Abstract] AND ("barre"[Title/Abstract] OR "barre"[Title/Abstract]) AND Title/Abstract[All Fields] AND "syndrome"[Title/Abstract])) OR "guillain-barre syndrome"[Title/Abstract]) OR "guillain-barre syndrome"[Title/Abstract]) OR "polyradiculoneuropathy"[Title/Abstract]) OR "Guillain-Barre syndrome"[Title/Abstract]) AND ("prevalence"[Title/Abstract] OR "incidence"[Title/Abstract] OR "epidemiology"[Title/Abstract] OR "remission"[Title/Abstract]))) AND ("2017/09/26"[Date - Publication] : "2017/09/26"[Date - Publication]). This search yielded 610 hits with 41 sources marked for extraction. A flowchart documenting this review is displayed below.

Figure 1. PRISMA diagram of GBD 2023 systematic review of scientific literature data

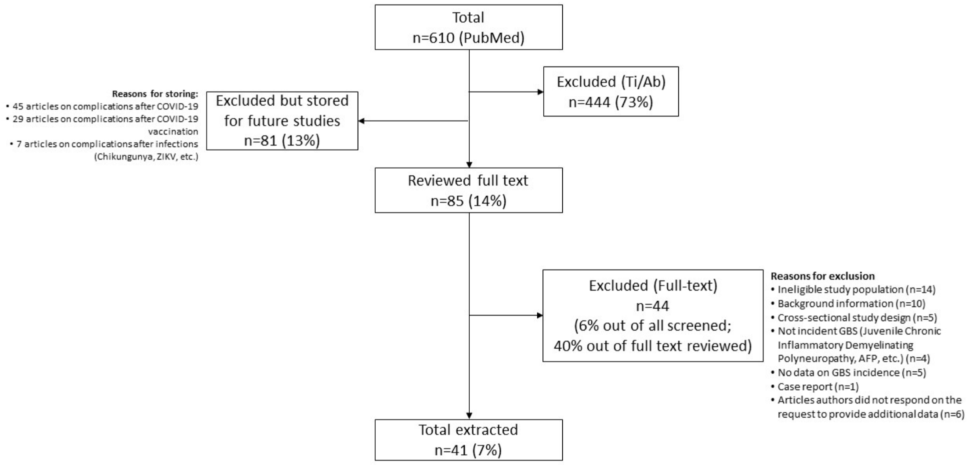

In order to incorporate GBS due to COVID-19 into the model's aetiology split, an additional search was undertaken using this larger systematic review to identify GBS data showing the proportion of cases associated with a preceding COVID-19 infection. From the 610 hits yielded in the original systematic review, 80 studies were flagged for potential association with COVID-19 and 13 studies (three identified from the systematic review and ten identified independently) contained relevant data on the proportion of GBS attributed to COVID-19.

Figure 2. PRISMA diagram of GBD 2023 systematic review of COVID-19/GBS proportion data

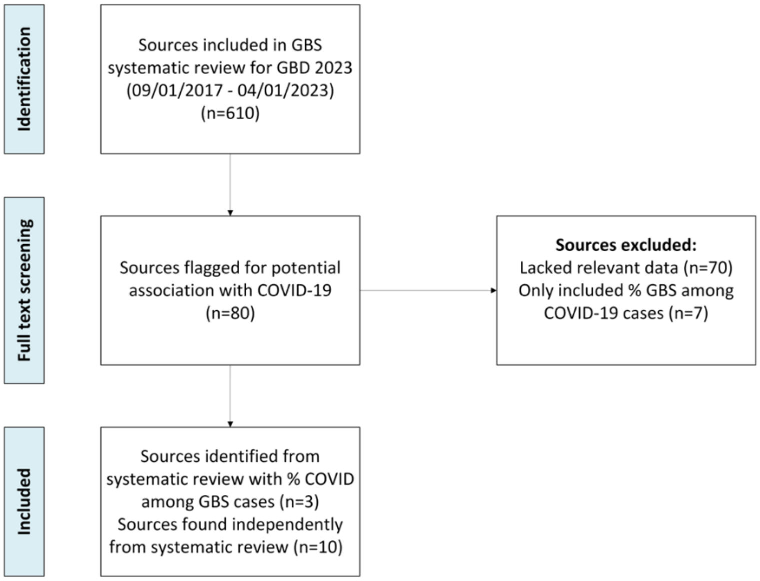

Inpatient hospital and claims incidence data were included using the ICD codes listed above. For GBD 2023, it was determined that, given the severity of most GBS cases, only inpatient clinical cases should be considered. Using select inpatient sources with unique patient identifiers and data on both primary and non-primary diagnoses, de-identified inpatient data with only primary diagnoses were adjusted

downward to remove duplicate diagnoses for the same individual and upward to account for non-primary diagnoses. Clinical data were evaluated and some sources (ie, Armenia, Botswana, and certain years of Austria inpatient data) were outliered. This was primarily due to limited sample size, leading to inflated estimates compared to estimates from sources with larger sample sizes.

For GBD 2023, a comprehensive data audit was conducted on all previously extracted data informing the model for total GBS. This process included removing duplicate rows from clinical data sources, re-extracting preprocessed data (eg, age-splitting, sex-splitting) from 88 sources to capture them in their raw form, converting 32 sources from cumulative incidence to incidence rates, and reviewing the extraction quality of the remaining data.

Aetiology data inputs

Information on aetiology splits comes from systematic reviews of the literature completed for GBD rounds 2010, 2017, and 2023. These reviews searched for articles providing information on the proportion of GBS cases with any described infectious cause, the proportion of GBS cases attributed to influenza, the proportion of GBS cases attributed to upper respiratory infection, the proportion of GBS cases attributed to diarrhoeal disease, the proportion of GBS attributed to other infection, and (most recently) the proportion of GBS attributed to COVID-19 infection.

Data processing

Data extracted from published surveys, disease registries, surveillance studies, and medical facilities were sometimes reported for both sexes or broadly defined age-groups in aggregate. In these cases, data were sex split and/or age split. Standard GBD sex-splitting methods were used for studies with only "both" sex datapoints. We modelled the ratio of female/male prevalence with a MR-BRT (meta-regression—Bayesian, regularised, trimmed) model (Additional information can be found in appendix 1, section 2) and calculated male prevalence:

$$prev_{male} = prev_{both} * \frac{pop_{both}}{(pop_{male} + ratio * pop_{female})}$$

And then calculated female prevalence:

$$prev_{female} = ratio * prev_{male}$$

For GBS, the modelled female/male ratio demonstrated a higher prevalence in males and was used to proportionally split "both"-sex datapoints into male and female datapoints.

For GBD 2023, raw data with large age ranges were split into five-year age bins using a global age pattern generated from a DisMod-MR 2.1 (disease model—Bayesian meta-regression, details on this method can be found in appendix 1, section 2) model that only included input data with less than a 25-year age range.

Modelling strategy

Estimating excess mortality

DisMod-MR 2.1 was used to estimate the prevalence of GBS for every location, year, age, and sex. For GBD 2023, in order to account for the survival rate, a random effects meta-analysis of survival rates following the acute phase of GBS was conducted. This generated a survival rate of 94% (95% UI 91–97).

Figure 3. Random-effects meta-analysis of survival rates following the acute phase of GBS

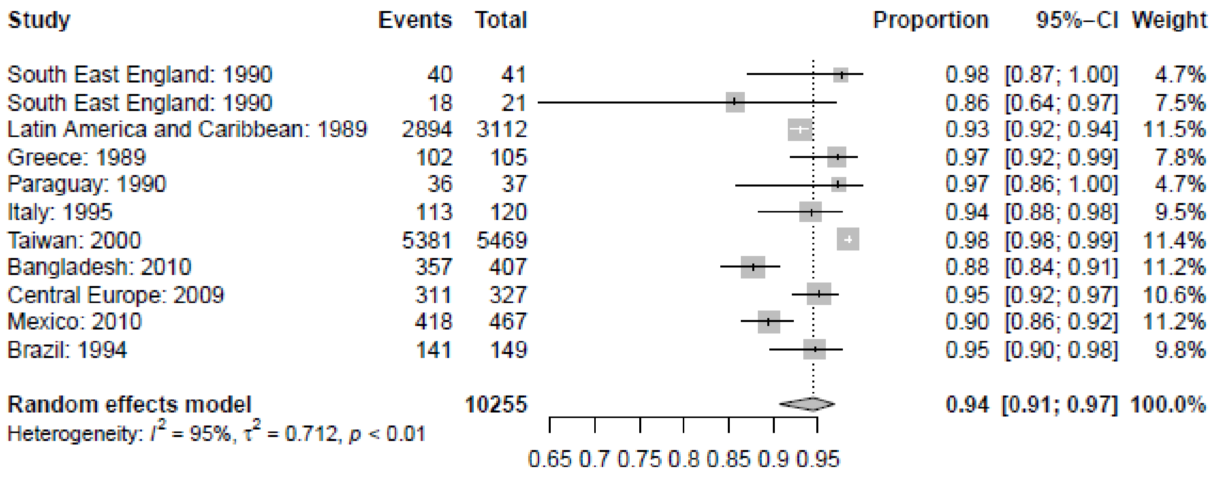

This survival rate was then used to calculate excess mortality rate based on the following equation:

$$-\ln(survival\%)\ /remission$$

The result was established as a prior on excess mortality rate equaling 0.12 (max/min thresholds 0.06–0.19).

Our methodology for GBD 2023 differs from that employed in GBD 2021, which used the pooled survival rate to adjust the incidence of people surviving after the acute phase of GBS. This method was updated for GBD 2023 in order to better retain incident cases in the estimated total of GBS cases.

In DisMod-MR 2.1, remission equals 1/duration of illness in years. A duration of 6 months was assumed, based on literature indicating that the majority of people are able to walk independently 6 months after disease onset, and a remission of 2. Consequently, a prior range of 1–3 for remission was established.

Aetiology split

To divide overall prevalence of GBS by underlying aetiology, a random-effects meta-analysis was conducted to pool the proportions of GBS cases attributable to each specific cause, including influenza, upper respiratory infections, diarrhoeal diseases, COVID-19, and other infectious diseases. These proportions were then “squeezed”, or scaled relative to one another, to ensure they summed to the total proportion of GBS attributed to any known infectious aetiology. Finally, the proportion that remained after accounting for GBS cases due to known infectious aetiology was assigned to the “GBS due to neurological disease” category.

For GBD 2023, the aetiology modelling strategy was updated to incorporate GBS due to COVID-19 infection. For years prior to 2020, this includes all aetiologies other than COVID-19. For 2020 onward, the pooled proportion of GBS due to COVID-19 infection is first applied, then the proportions for the rest of the other splits are squeezed to fit the remainder of the total after the proportion of COVID-19-related GBS has been removed. This was because GBS due to COVID-19 was captured most comprehensively by literature during 2020 onward, making us want to prioritise this split before incorporating the rest.

The results of these aetiology splits for years prior to 2020 and for 2020 onward are shown below:

Table 1. GBS aetiology split for years prior to 2020

**Aetiology**	**Mean**	**Lower**	**Upper**
Other neurological disorders	0.377	0.315	0.685
Influenza	0.119	0.078	0.177
Upper respiratory infections	0.327	0.269	0.39
Diarrhoeal diseases	0.108	0.085	0.137
Other infectious diseases	0.068	0.051	0.091

Table 2. GBS aetiology split for years 2020 onward

**Aetiology**	**Mean**	**Lower**	**Upper**
Other neurological disorders	0.254	0.212	0.298
Influenza	0.080	0.053	0.119
Upper respiratory infections	0.221	0.181	0.263
Diarrhoeal diseases	0.073	0.057	0.92
Other infectious diseases	0.046	0.034	0.061
COVID infection	0.326	0.221	0.453

Disability weights

The health state for paraplegia was used for all Guillain-Barré cases. It is described as “paralysed from the waist down, cannot feel or move the legs, and has difficulties with urine and bowel control. The person uses a wheelchair to move around”. The disability weight is 0.296 (0.198–0.414).

Gynaecological diseases

Uterine fibroids

Flowchart

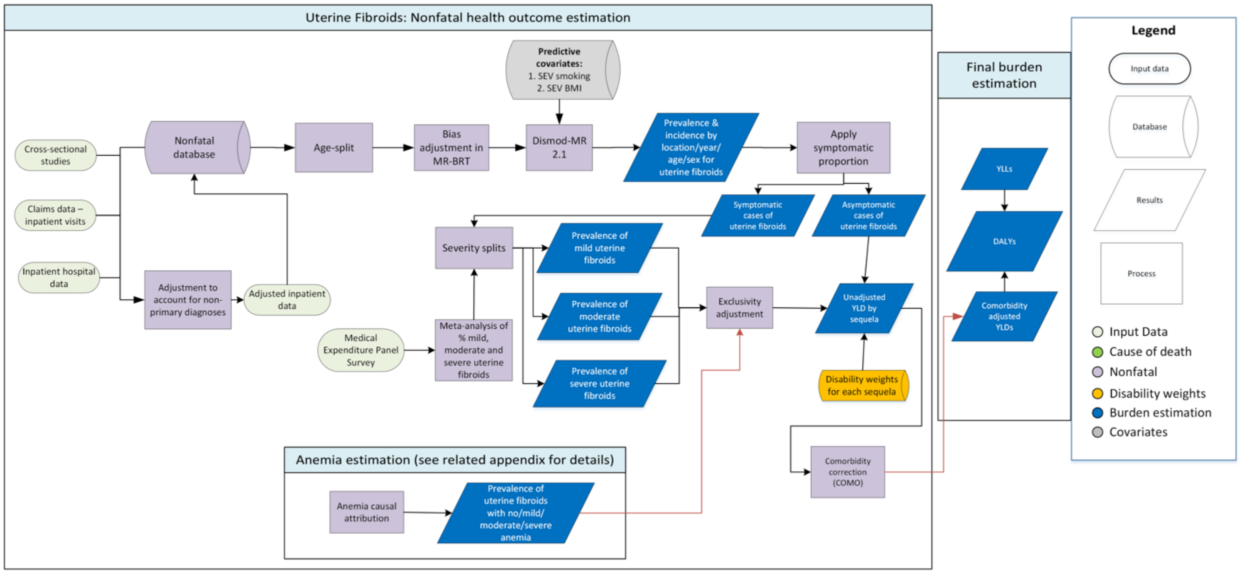

Input data and methodological summary for uterine fibroids

Case definition

Uterine fibroids, also called uterine myomas or leiomyomas, are non-cancerous tumours that develop from the muscle tissue of the uterus, which sometimes cause symptoms but can be asymptomatic and only get detected incidentally when imaging studies are conducted for unrelated reasons. When present, symptoms include abdominopelvic pain, painful intercourse, infertility, and heavy vaginal bleeding, which can lead to anaemia. Some fibroids can be detected by signs and symptoms identified during a clinical interview and pelvic exam, but the diagnosis should be confirmed by ultrasonography, hysterectomy, hysterosalpingography, sonohysterography, laparoscopy, or imaging tests such as MRI or CT scan.

For GBD 2023, we used the definition proposed by the American College of Obstetricians and Gynecologists (ACOG) as the reference case definition, that is, cases of uterine fibroids diagnosed by pelvic exam followed by ultrasonography, hysteroscopy, hysterosalpingography, sonohysterography, or laparoscopy.[1] We also incorporated studies that ascertained cases by self-report, pelvic exam only, or via diagnostic codes in administrative data, by adjusting data toward our reference case definition, as described below.

Quantity of interest	Reference or alternative	Definition

Prevalence of uterine fibroids	Reference clinical data type	Cases identified from database of commercial claims from USA using ICD codes.
Prevalence of uterine fibroids	Alternative clinical data type	Cases of uterine fibroids identified using ICD codes in administrative records that are considered population-representative.
Prevalence of uterine fibroids	Reference	ACOG definition: Diagnosis of uterine fibroids by pelvic exam followed by pelvic ultrasonography, hysteroscopy, hysterosalpingography (X-ray test), sonohysterography, or laparoscopy.
Prevalence of uterine fibroids	Alternative	Cases of uterine fibroids identified via self-report by respondents in longitudinal or cross-sectional surveys, or in questionnaires inquiring about diagnosis of uterine fibroids in the past months, years, or lifetime.
Prevalence of uterine fibroids	Alternative	Symptomatic cases of uterine fibroids identified via pelvic exam or ultrasonography, often as identified by respondents in retrospective cross-sectional studies asked about previous diagnosis of uterine fibroids at any point in their lifetime.
Prevalence of uterine fibroids	Alternative	Cases identified from administrative records using ICD codes, after commercial claims and population-representative sources have been crosswalked to a common standard of reference clinical data type.

Input data

Input data

The initial GBD systematic review for uterine fibroids was done in GBD 2010, when Ovid MEDLINE, EMBASE, CINAHL, CAB abstracts, WHOLIS, and ISGLE database were searched. The search strings used in the initial search were as follows:

> **PUBMED**: (“Leiomyoma”[Mesh] OR fibroid OR fibroids OR leiomyoma OR leiomyomas OR leimyoma OR leimyomas OR leyomyoma OR leyomyomas OR fibromyoma OR fibromyomas OR fibroma OR fibromas OR myoma OR myomas) AND (“Genitalia, Female”[Mesh] OR “Gynecology”[Mesh] OR “Uterus”[Mesh] OR genital OR genitals OR genitalia OR gynecology OR gynaecology OR gynecologic OR gynecological OR gynaecologic OR gynaecological OR uterine OR uterus OR hysterectomy) AND (“Prevalence”[Mesh] OR prevalence OR prevalences)
>
> **EMBASE**: (‘uterus myoma’/exp OR fibroid OR fibroids OR leiomyoma OR leiomyomas OR leimyoma OR leimyomas OR leyomyoma OR leyomyomas OR fibromyoma OR fibromyomas OR fibroma OR fibromas OR myoma OR myomas) AND (‘uterus’/exp OR ‘gynecology’/exp OR ‘female genital system’/exp OR genital OR genitals OR genitalia OR gynecology OR gynaecology OR gynecologic OR gynecological OR gynaecologic OR gynaecological OR uterine OR uterus OR hysterectomy) AND (prevalence/exp OR prevalence OR prevalences)

Published studies were subsequently augmented with contributions from the GBD Collaborator Network in later rounds of GBD.

Exclusion criteria were reviews, studies that did not provide primary data on epidemiological parameters (eg, commentary), and clearly non-representative studies (eg, of only high-risk pregnant women).

In addition to data from peer-reviewed publications, inputs for the uterine fibroid model include prevalence data extracted from the GBD library of hospital discharges and claims aggregated and processed by IHME. With changes to the hospital and claims administrative data-processing algorithms implemented since GBD 2017, most notably the addition of a requirement that two outpatient visits coded to a cause are required for a person to count as “a case” of a given disease, the inpatient-to-outpatient corrected administrative data became much more variable. This is hypothesised to be due to differences in care-seeking and health-care provision patterns for women with uterine fibroids, including differences between countries in whether women who have procedures for fibroids are categorised as inpatients or outpatients. We therefore used only inpatient hospital and claims data.

In claims data, an individual was extracted as a prevalent case if they had at least one inpatient encounter with an appropriate ICD code in any diagnostic position, and the denominator was enrollees in the same age group and location that year. Hospital discharges were extracted if an appropriate code appeared as a discharge diagnosis. Correction factors from claims data were then applied to the hospital discharges to estimate the number of cases represented by the encounters, adjusting for the fact that some facilities only provide the primary discharge diagnosis. Discharges with an appropriate ICD code in any diagnostic field were extracted as encounters and adjusted using a correction factor from claims data to estimate the number of incident cases, and another to account for some sources only providing primary diagnoses (see the section on non-fatal data sources, identification, and extraction for a description of GBD modelling of hospital utilisation and processing of inpatient and claims data in this appendix).

Data processing

The first step of data processing was age-splitting. For any datum that did not entirely fit within a GBD age group, the observation was split to be multiple age-specific datapoints based on the age pattern predicted by GBD 2017 DisMod-MR 2.1 models.

As mentioned before, the ACOG case definition of uterine fibroids was set as the reference case definition; this definition encompasses both symptomatic and asymptomatic cases that can be detected by pelvic exam or ultrasonography, sonohysterography, laparoscopy, or imaging tests such as MRI and CT scans. We consider clinical diagnoses indicated by ICD codes in administrative data (inpatient hospital and claims only), self-report, and symptomatic-only cases to be alternative case definitions.

To make data comparable, we began by evaluating the number of observations of each alternate definition that matched with a corresponding observation from the reference definition. Due to data scarcity, we only found “between-study” matches. That means we matched observations from different studies by age group, location (at the regional level), and whether the midpoints of the study periods were within five years of each other. All observations that matched were paired with one another and logit-transformed, and the difference of the logit mean values were calculated. The standard error of the logit difference was calculated using the delta method. We then modelled the logit difference using meta-regression—Bayesian, regularised, trimmed (MR-BRT), a meta-analytic tool developed for the Global Burden of Disease study. The process of adjusting for biases in non-reference data using MR-BRT with logit-transformation method is described below:

1. Identify datapoints with overlapping year, age, sex, and location between reference and non-reference population data.
2. Logit transform overlapping datapoints of alternative and reference types.
3. Convert overlapping datapoints into a difference in logit space using the following equation: $logit(altnerative) - logit(reference)$
4. Use the delta method to compute standard errors of overlapping datapoints in logit space, then calculate standard error of logit difference using the following equation: $\sqrt{(variance\ of\ logit(alternative)) + (variance\ of\ logit(reference))}$
5. Using MR-BRT, conduct a random effects meta-regression to obtain the pooled logit difference of alternative to reference
6. Apply the pooled logit difference to all data points of non-reference types using the following equation:
$$new_{estimate} = inverse.logit((logit(alternative)) - (pooled\ logit\ difference))$$
7. Calculate new standard errors using the delta method, accounting for gamma (between-study heterogeneity)

Adjustment of data inputs for uterine fibroids was conducted in two steps, using the above method serially. In the first step, we adjusted only clinical data to a common standard, by using claims data as the reference definition and inpatient hospital data as the alternative definition. In this model, we trimmed 10% of the data and added a quadratic spline on age, assuming non-linear tails. Our final model results for this crosswalk process are illustrated below.

Uterine fibroids MR-BRT crosswalk adjustments factors by age for hospital (alternate) to claims (reference) data

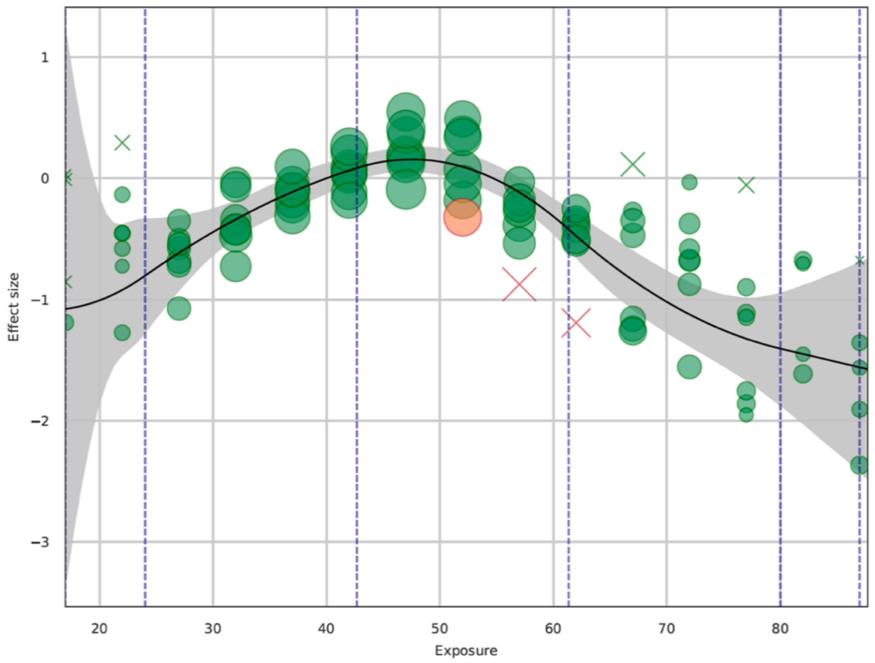

**Exposure on the x-axis is GBD age group and effect size is the logit-transformed difference of inpatient to claims data.*

According to this model, hospital data underestimated the number of uterine fibroid cases for most age groups. With ages 40–55 years, the inverse relationship is true.

Once the clinical data were adjusted, we performed a network MR-BRT considering the ACOG definition as the reference and clinical data (inpatient hospital and claims, adjusted per above), self-report, and symptomatic-only cases as alternative definitions. The adjustment factors for each of the included covariates in the models are summarised in the following table.

Table 1: MR-BRT crosswalk adjustment factors for uterine fibroids

Data input	Reference or alternative case definition	Gamma	Beta coefficient, logit (95% UI)*	Adjustment factor**
ACOG definition	Ref	1.13	---	---
Self-report	Alt		−2.99 (−3.41, −2.54)	0.05 (0.03, 0.07)
Symptomatic cases	Alt		−3.56	0.03

			(–5.22 to –1.83)	(0.005, 0.134)
Clinical data	Alt		–1.82<br>(–2.18, –1.47)	0.01<br>(0.10, 0.02)

**MR-BRT crosswalk adjustments can be interpreted as the factor the alternative case definition is adjusted by to reflect what it would have been had it been measured using the reference case definition. If the log/logit beta coefficient is negative, then the alternative is adjusted up to the reference. If the log/logit beta coefficient is positive, then the alternative is adjusted down to the reference.*

***The adjustment factor column is the exponentiated beta coefficient. For log beta coefficients, this is the relative rate between the two case definitions. For logit beta coefficients, this is the relative odds between the two case definitions.*

Modelling strategy

After standardisation of prevalence data to the ACOG definition, we modelled incidence, prevalence, remission, and excess mortality due to uterine fibroids in DisMod-MR 2.1.

As in previous GBD iterations, incidence was set to zero prior to 10 years of age, and we assumed no excess mortality. We set the minimum coefficient of variation to 0.8 and the priors on the location random effects to +/- 0.5.

In GBD 2019, a large number of potentially predictive covariates were selected *a priori* based on a non-systematic literature review, including summary exposure value (SEV) for smoking, body-mass index, systolic blood pressure, physical activity, alcohol consumption, the age-standardised death rate (lnASDR) of sexually transmitted infections (STIs) from GBD 2019 CoD analyses, prevalence of pelvic inflammatory disease from GBD 2019 non-fatal analyses, prevalence of contraception, and total fertility rate, and tested in preliminary DisMod models. From this list, two covariates were selected and used in the final GBD 2019 DisMod model. These same two covariates were used in GBD 2021 and GBD 2023, as shown in the following table:

Table 2. Covariates. Summary of covariates used in the uterine fibroids DisMod-MR meta-regression model

Covariate	Parameter	Exponentiated beta (95% uncertainty interval)
Age-standardised SEV for high body-mass index	Prevalence	1.15<br>(1.08–1.22)
Age-standardised SEV for smoking	Prevalence	1.09<br>(1.02–1.19)

The above modelling strategy is consistent with that employed beginning in GBD 2019 but is a change from previous GBD cycles when only symptomatic fibroids were modelled in DisMod, using clinical data that included only inpatient encounters. The assumption at that time was that all inpatient admissions represented fibroids that were symptomatic enough to warrant medical care. Total fibroids in previous cycles were then calculated based on a single study that reported 50% of the total cases of uterine fibroids to be symptomatic.[2] Beginning in GBD 2019 and continuing in GBD 2021 and GBD 2023, the use of MR-BRT analysis allowed us to quantify the relationship between the prevalence of total uterine

fibroids versus only symptomatic uterine fibroid cases. This allowed us adjust data toward the reference case definition prior to DisMod modelling, and thus to model total fibroids cases directly.

Severity splits and disability weights

We split total cases of uterine fibroids into symptomatic and asymptomatic cases of fibroids using the beta coefficient obtained in the crosswalk during data processing. The coefficient suggests that most uterine fibroids cases (97%) are asymptomatic. This proportion seem to be consistent with other studies that suggest that the majority of women with uterine fibroids do not experience symptoms[3,4] but is a notably significant departure from the proportion identified prior to GBD 2019. The remaining symptomatic cases were all assumed to have symptoms such as abdominal discomfort, bleeding, and consequently, anaemia due to fibroids.

The age-specific anaemia prevalence for symptomatic cases of uterine fibroids was analysed as part of overall anaemia causal attribution for GBD 2023. The details of the anaemia analysis are described separately in the “anaemia impairment” section of this appendix. Briefly, after estimating total anaemia, a series of counterfactual distributions are generated based on the age- and sex-specific prevalence of each anaemia-causing condition and the quantitative effect that the condition has on haemoglobin concentration in the blood, a so-called “haemoglobin shift,” that was derived by meta-analysing cohort studies, observational studies, or trials comparing the haematological status of those with as compared to without the disease. Due to limited data on haemoglobin shift, all were assumed to be invariant over age, location, and year. It should be noted that anaemia alone is not ascribed to fibroids, but only in conjunction with mild abdominal pain with the assumption that more severe, symptomatic cases would be more likely to cause anaemia. Disability weights for each sequela are listed below for reference.

Table 3. Severity distribution, details on the severity levels for uterine fibroids and the associated disability weight (DW) with that severity

Severity level	Lay description	DW (95% CI)
Asymptomatic		--
Uterine fibroids, symptomatic, without anaemia	Has some pain in the belly that causes nausea but does not interfere with daily activities.	0.011 (0.005, 0.021)
Uterine fibroids, symptomatic, with mild anaemia	Has some pain in the belly that causes nausea but does not interfere with daily activities, and feels slightly tired and weak at times, but this does not interfere with normal daily activities.	0.015 (0.062, 0.040)
Uterine fibroids, symptomatic, with moderate anaemia	Has some pain in the belly that causes nausea but does not interfere with daily activities, and feels moderate fatigue, weakness, and shortness of breath after exercise, making daily activities more difficult.	0.062 (0.040, 0.093)
Uterine fibroids, symptomatic, with severe anaemia	Has some pain in the belly that causes nausea but does not interfere with daily activities, and feels very weak, tired, and short of breath, and has problems with activities that require physical effort or deep concentration.	0.158 (0.109, 0.219)

Polycystic ovarian syndrome

Flowchart

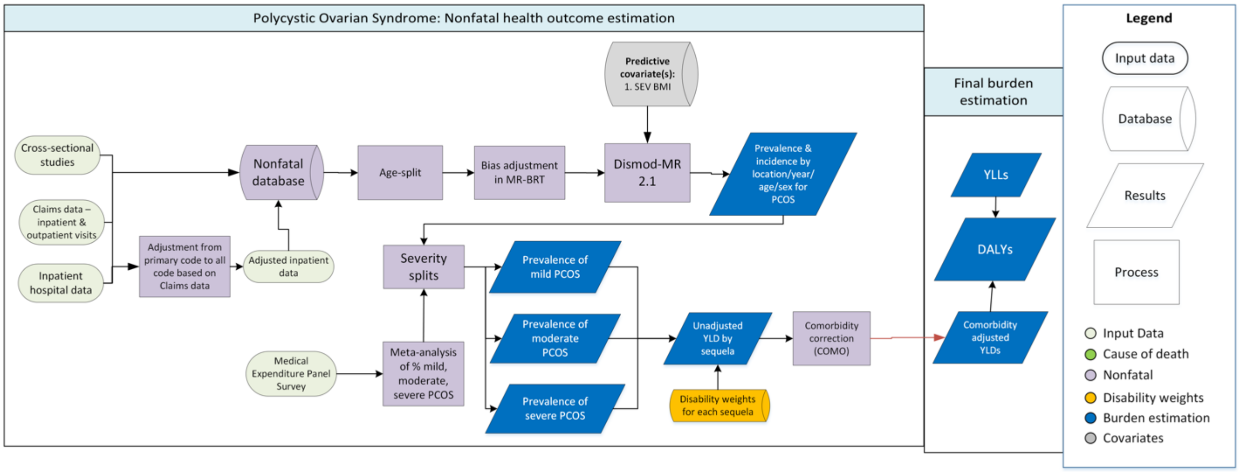

Input data and methodological summary for polycystic ovarian syndrome

Case definition

Polycystic ovarian syndrome (PCOS) is an endocrinopathy characterised by hyperandrogenism, ovulatory dysfunction, and polycystic ovaries which can lead to infrequent menstruation, excess hair growth (hirsutism), acne, obesity, and infertility.[5,13]

There is no universally accepted definition of PCOS.[6] Expert-generated diagnostic criteria include the National Institutes of Health (NIH) diagnostic,[7] the Rotterdam criteria,[8] and the Androgen Excess Society (AES) definition.[9] All diagnostic approaches require the presence of more than one sign or symptom and recommend that other causes of hyperandrogenism and oligomenorrhoea or amenorrhoea (such as congenital adrenal hyperplasia, hyperprolactinaemia, and androgen-secreting neoplasms) should be excluded.

In GBD 2019, we standardised the reference definition of all gynaecological diseases, including PCOS, to the ACOG definitions. According to ACOG, however, PCOS diagnosis can be accomplished using any of the three diagnostic approaches mentioned previously (NIH, Rotterdam, or AES).[5] As the Rotterdam and AES definitions have been criticised for including more mild phenotypes,[9] we continued using the NIH definition, which noted the disorder as having 1) hyperandrogenism and/or hyperandrogenemia, 2) oligo-ovulation, and 3) exclusion of known disorders, as our reference definition.[6]

Quantity of interest	Reference or Alternative	Definition
Prevalence of PCOS	Reference clinical data type	Cases identified from database of commercial claims from USA using ICD codes.
Prevalence of PCOS	Alternative clinical data type	Cases of polycystic ovarian syndrome identified using ICD codes in administrative records that are considered population-representative.
Prevalence of PCOS	Reference	Cases of polycystic ovarian syndrome identified using NIH/NICHD criteria for which three signs must be present: clinical or biochemical evidence of

		hyperandrogenism, oligomenorrhoea, and the exclusion of other disorders in a general population.
Prevalence of PCOS	Alternative	Cases of polycystic ovarian syndrome identified using Rotterdam criteria for which 2/3 hyperandrogenism, oligoamenorhhea, and/or polycystic ovaries by ultrasound diagnosis criteria for which three signs must be present: clinical or biochemical evidence of hyperandrogenism, oligomenorrhoea, and the exclusion of other disorders records that are considered in the general population.
Prevalence of PCOS	Alternative	Cases of polycystic ovarian syndrome identified using AES criteria for which hyperandrogenism plus one out of remaining two are required for diagnosis: oligoamenorhhea and/or polycystic ovaries by ultrasound diagnosis in a general population.
Prevalence of PCOS	Alternative	Cases of polycystic ovarian syndrome identified via self-report by respondents in longitudinal or cross-sectional surveys or questionnaires inquiring about self-reported diagnosis of polycystic ovarian syndrome in the past months, years, or lifetime.
Prevalence of PCOS	Alternative	Cases identified from administrative records using ICD codes, after commercial claims and population-representative sources have been crosswalked to a common standard of reference clinical data type.

Input data

Input data

For GBD 2010, a systematic review of PCOS throughout the world was conducted. Ovid MEDLINE, EMBASE, CINAHL, CAB abstracts, WHOLIS, ISGLE, and PUBMED databases were searched. Search strings were as follows:

> **PUBMED:** (“Polycystic Ovary Syndrome”[Mesh] OR “Polycystic Ovary Syndrome” OR “Sclerocystic Ovary Syndrome” OR “Sclerocystic Ovarian Degeneration” OR “Stein-Leventhal Syndrome” OR “Stein Leventhal Syndrome” OR “Sclerocystic Ovaries” OR “Sclerocystic Ovary”) AND (“Incidence”[Mesh] OR Incidence OR Incidences OR “Prevalence”[Mesh] OR Prevalence OR Prevalences)
> **EMBASE:** (“ovary polycystic disease”/exp OR “cystic ovary” OR “micropolycystic ovary” OR “multiple follicle cyst” OR “ovary polycystic syndrome” OR “ovary, micropolycystic” OR “ovary, polycystic” OR “polycystic ovarian disease” OR “polycystic ovary” OR “polycystic ovary disease” OR “polycystic ovary syndrome”) AND (‘incidence’/exp OR incidence OR incidences OR ‘prevalence’/exp OR prevalence OR prevalences)

We excluded reviews and studies that did not provide primary data on epidemiological parameters (eg, commentary) and clearly non-representative studies (eg, of only high-risk pregnant women).

In subsequent rounds of GBD, data from the GBD 2010 systematic review were serially augmented by data from claims and hospital discharges aggregated, extracted, and processed by IHME as described elsewhere in this appendix. In claims data, individuals were extracted as prevalent cases if they had at least one inpatient encounter or two outpatient encounters with an appropriate ICD code in a given year, and the denominator applied was all enrollees that year in the same age group and location. Hospital discharges were extracted if they had an appropriate ICD code as primary diagnosis and adjusted using a correction factor. Specifically, we used claims data to model the ratio of inpatient claims with PCOS as primary diagnosis to total prevalent cases of PCOS seen in both inpatient and outpatient settings (see the section on non-fatal data sources, identification, and extraction for a description of GBD modelling of hospital utilisation and processing of inpatient and claims data in this appendix), and this ratio was applied to hospital discharge data.

Data processing

Prior to modelling, we performed age-splitting to ensure all data fit into specific GBD standard age groups. Briefly, the age-splitting algorithm uses population weights that are determined by dividing the result predicted by GBD 2017 DisMod-MR 2.1 models for a specific age group by the result for the aggregate age specified in a given input datapoint. Age-specific values were then calculated by multiplying the aggregate input datapoint by these age specific weights.

Because prevalence and incidence of PCOS among reproductive-aged women vary according to the diagnostic criteria, we used the NIH case definition as the reference definition and adjusted the data from alternative definitions using two MR-BRT models. Acceptable alternate definitions included the Rotterdam definition, AES definition, self-report, and clinical data. We started by evaluating the number of observations of each alternate definition that matched with a corresponding observation from the reference definition. Due to data scarcity, we only found “between-study” matches. That means we matched observations from different studies by age group, location, and whether the midpoints of the study periods were within five years of each other.

To perform the crosswalk, all observations that matched were paired with one another and logit-transformed and the difference of the logit mean values was calculated. The standard error of the logit difference was calculated using the delta method. Adjustment of data inputs for PCOS was conducted in two steps. In the first step, we adjusted only clinical data to a common standard, by using claims data as the reference definition and inpatient hospital data as the alternative definition, entering the logit-transformed mean difference and standard error as inputs to run a MR-BRT model, trimming 10% of the data and assuming linear tails. Our final model results for this crosswalk process are illustrated in the next figure and table.

Table 1: MR-BRT crosswalk adjustment factors for polycystic ovarian syndrome to standardise between different clinical administrative data types

Data input	Reference or alternative case definition	Gamma	Beta coefficient, logit (95% UI)*	Adjustment factor**
Claims data	Ref	0	---	---
Inpatient data	Alt		–1.52 (–2.05, –0.95)	0.18 (0.11, 0.28)

**MR-BRT crosswalk adjustments can be interpreted as the factor the alternative case definition is adjusted by to reflect what it would have been had it been measured using the reference case definition. If the log/logit beta coefficient is negative, then the alternative is adjusted up to the reference. If the log/logit beta coefficient is positive, then the alternative is adjusted down to the reference.*

***The adjustment factor column is the exponentiated beta coefficient. For log beta coefficients, this is the relative rate between the two case definitions. For logit beta coefficients, this is the relative odds between the two case definitions.*

For the second MR-BRT model, we used a network analysis to crosswalk the different diagnostic criteria including the NIH definition as the reference and the Rotterdam diagnostic criteria, the AES definition, self-report, and clinical data as alternative definitions. The adjustment factors for each of the included covariates in the models are summarised in the following table.

Table 2: MR-BRT crosswalk adjustment factors for polycystic ovarian syndrome to standardise between different diagnostic criteria

Data input	Reference or alternative case definition	Gamma	Beta coefficient, logit (95% UI)*	Adjustment factor**
NIH definition	Ref	0.43	---	---
Rotterdam definition	Alt		0.22 (0.12, 0.32)	0.55 (0.47, 0.58)
AES definition	Alt		–0.006 (–0.10, 0.09)	0.50 (0.47, 0.52)
Self-report cases	Alt		–0.60 (–0.69, –0.52)	0.35 (0.33, 0.37)
Clinical data	Alt		–3.88 (–5.48, –2.33)	0.02 (0.004, 0.09)

**MR-BRT crosswalk adjustments can be interpreted as the factor the alternative case definition is adjusted by to reflect what it would have been had it been measured using the reference case definition. If the log/logit beta coefficient is negative, then the alternative is adjusted up to the reference. If the log/logit beta coefficient is positive, then the alternative is adjusted down to the reference.*

***The adjustment factor column is the exponentiated beta coefficient. For log beta coefficients, this is the relative rate between the two case definitions. For logit beta coefficients, this is the relative odds between the two case definitions.*

Modelling strategy

There were no changes to the modelling strategy and settings between GBD 2021 and GBD 2023. We modelled prevalence, incidence, and remission of PCOS using DisMod-MR 2.1. Incidence was set to zero prior to 10 years of age and after 55 years of age to reflect that women are only susceptible between menarche and menopause. Remission until age 54 was bounded to have a maximum value of 1 per 10 person-years. After age 55, no priors for remission were set. PCOS is not considered a cause of death, and therefore, excess mortality rate was set to zero. We set the minimum coefficient of variation (which helps determine the influence of Bayesian priors from the geographical cascade relative to local data) to 0.8 and set the parameter xi (which controls age smoothing) to have a maximum value of 3. In addition, a decreasing slope prior for incidence starting at age 16 was used to help the model to match the highest incidence observed in the data among younger ages (13–20 years). The time span of data used to fit for a particular year was set to five years.

In GBD 2019, a set of potentially associated factors were selected based on a non-systematic literature review and evaluated for predictive power in a series of test models; these included the summary exposure values (SEV) for smoking, high body-mass index, low physical activity, and alcohol consumption; the age-standardised death rate (lnASDR) of sexually transmitted infections (STIs) from the previous round's CoD analyses; prevalence of pelvic inflammatory disease from the previous round's non-fatal analyses; prevalence of contraception; total fertility rate; and the Socio-demographic Index (SDI). Most of the covariates from this list were not associated with the prevalence of PCOS in test models. Because obesity plays an important role in the aetiology of the syndrome, we include the relative risk-weighted prevalence of high body-mass index as a predictive covariate to help drive the magnitude of prevalence estimates in areas of sparse or absent data (the coefficient for the fitted model in this round is shown in the following table).

Table 3. **Covariates.** Summary of covariates used in the polycystic ovarian syndrome DisMod-MR meta-regression model

Covariate	Parameter	Exponentiated beta (95% uncertainty interval)
Age-standardised SEV for high body-mass index	Prevalence	1.91 (1.67–2.15)

Severity splits and disability weights

The basis of the GBD disability weight (DW) survey assessment are lay descriptions of sequelae highlighting major functional consequences and symptoms. Unfortunately, no health states specific to PCOS were included in the GBD disability weights survey. The main sequelae of PCOS are infertility and hyperandrogenism/hirsutism, the latter of which was approximated with the health state of "disfigurement, level 1." The NIH definition, which we designated as the reference case definition, considers that most cases of PCOS have hyperandrogenism and hirsutism, and therefore we assumed that a majority of PCOS would experience this sequela. Disability weights for each sequela are listed below for reference.

Table 4. **Severity distribution,** details on the severity levels for polycystic ovarian syndrome and the associated disability weight (DW) with that severity

Severity level	Lay description	DW (95% CI)
Asymptomatic		--
Hirsutism	Has a slight, visible physical deformity that others notice, which causes some worry and discomfort.	0.011 (0.005, 0.021)
Infertility, primary	Wants to have a child and has a fertile partner, but the couple cannot conceive.	0.008 (0.003, 0.015)
Infertility, secondary	Has at least one child and wants to have more children. The person has a fertile partner, but the couple cannot conceive.	0.005 (0.002, 0.011)
Hirsutism and primary infertility	Has a slight, visible physical deformity that others notice, which causes some worry and discomfort, and wants to have a child and has a fertile partner, but the couple cannot conceive.	0.018 (0.009, 0.035)
Hirsutism and primary infertility	Has a slight, visible physical deformity that others notice, which causes some worry and discomfort, and has at least one child and wants to have more children. The person has a fertile partner, but the couple cannot conceive.	0.016 (0.007, 0.031)

Endometriosis

Flowchart

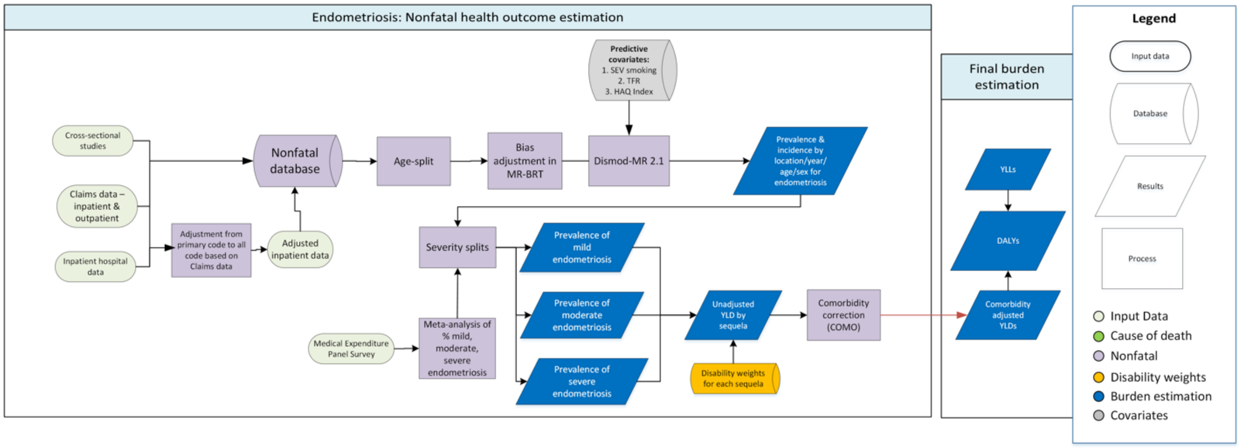

Input data and methodological summary for endometriosis

Case definition

Endometriosis is a gynaecological condition defined as growth of endometrial tissue outside the uterus regardless of symptoms. Common symptoms include chronic abdominal and pelvic pain, especially before and during a menstrual period and during sexual intercourse. Endometriosis can also lead to infertility. For GBD 2023, we define endometriosis cases according to the ACOG guidelines as cases diagnosed by pelvic exam confirmed by laparoscopy or laparotomy.[9]

Quantity of interest	Reference or alternative	Definition
Prevalence of endometriosis	Reference clinical data type	Cases identified from database of commercial claims from USA using ICD codes.
Prevalence of endometriosis	Alternative clinical data type	Cases of endometriosis identified using ICD codes in administrative records that are considered population-representative.
Prevalence of endometriosis	Reference	ACOG definition: Diagnosis by pelvic exam confirmed by laparoscopy or pathology.
Prevalence of endometriosis	Alternative	Cases of endometriosis identified via self-report by respondents in longitudinal or cross-sectional surveys or questionnaires inquiring about self-reported diagnosis of endometriosis in the past months, years, or lifetime
Prevalence of endometriosis	Alternative	Cases identified from administrative records using ICD codes, after commercial claims and population-representative sources have been crosswalked to a common standard clinical data type.

Input data

Input data

A systematic review of endometriosis prevalence was conducted for GBD 2010. The review consisted of a PubMed search and a systematic review of endometriosis throughout the world. Ovid MEDLINE,

EMBASE, CINAHL, CAB abstracts, WHOLIS, and ISGLE database were searched. The search strings for PubMed and EMBASE were as follows:

PUBMED: ("Endometriosis"[Mesh] OR Endometriosis OR Endometrioses OR Endometrioma OR Endometriomas OR Adenomyosis) AND ("Incidence"[Mesh] OR Incidence OR Incidences OR "Prevalence"[Mesh] OR Prevalence OR Prevalences)

EMBASE: ('endometriosis'/exp OR endometriosis OR endometrioses OR endometrioma OR endometriomas OR adenomyosis) AND ('incidence'/exp OR incidence OR incidences OR 'prevalence'/exp OR prevalence OR prevalences)

Exclusion criteria for the initial systematic review were reviews, studies that did not provide primary data on epidemiological parameters (eg, commentary), and clearly non-representative studies (eg, of only high-risk pregnant women).

In subsequent rounds of GBD, data from the GBD 2010 systematic review were serially augmented by data from claims and hospital discharges aggregated, extracted, and processed by IHME as described elsewhere in this appendix. In claims data, individuals were extracted as prevalent cases if they had at least one inpatient encounter or two outpatient encounters with an appropriate ICD code in a given year, and the denominator applied was all enrollees that year in the same age group and location. Hospital discharges were processed by extracting discharges with an appropriate ICD code as primary diagnosis and adjusting using a correction factor. Specifically, we used claims data to model the ratio of inpatient claims with endometriosis as primary diagnosis to total prevalent cases of endometriosis seen in both inpatient and outpatient settings and this ratio was applied to hospital discharge data. (See the section on non-fatal data sources, identification, and extraction for a description of GBD modelling of hospital utilisation and processing of inpatient and claims data in this appendix.)

Data processing

Any datum referring to a sample of women with an age range that did not entirely fit within a GBD age group was split into age-specific datapoints based on the age pattern predicted by GBD 2017 DisMod-MR 2.1 models.

Once the data were age-split, we adjusted data collected using non-reference case definitions, study populations, or other data collection methods using an MR-BRT analysis. To do this, first we counted the number of observations of each alternate definition that matched with a corresponding observation from the reference definition. We matched observations by age group and location (at the region level) and when the midpoint of the study was within five years of the midpoint of the reference definition observation.

All matched observations were paired with one another and logit-transformed, and the difference of the mean values was calculated in logit space. The standard error of the difference in logits was calculated using the delta method. As for uterine fibroids and PCOS, adjustment of data inputs for endometriosis was conducted in two steps, using MR-BRT and following the general steps to GBD crosswalking described above. In the first step, we adjusted only clinical data to a common standard, by using claims data as the reference definition and inpatient hospital data as the alternative definition; for this, we used logit-transformed mean difference and standard errors for matched claims and hospital data as inputs to run a MR-BRT model with a cubic spline on age and four knots, trimming 10% of the data and assuming linear tails. Our final model results for this crosswalk process are illustrated in the next figure.

Endometriosis MR-BRT crosswalk adjustment factors by age for hospital (alternate) to claims (reference) data.

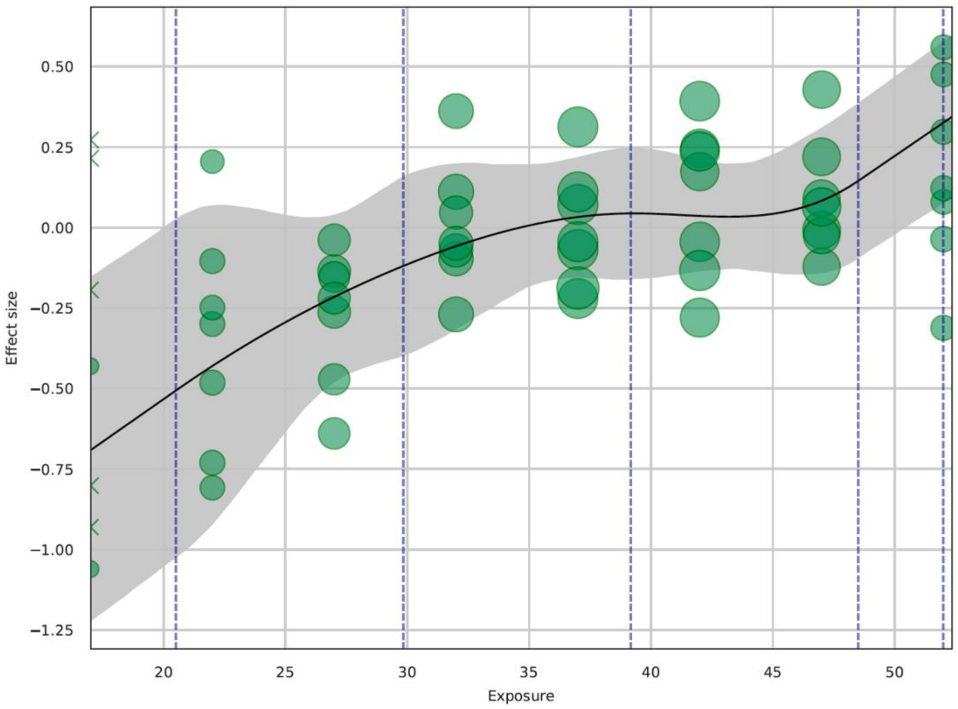

**Exposure on the x-axis is GBD age group and effect size is the logit-transformed difference of inpatient to claims data.*

According to this model, hospital data underestimated the number of endometriosis cases for the earlier age groups. After age 35, the inverse relationship is true.

After the first crosswalk, we performed a network MR-BRT analysis to adjust the data sources that use alternative definitions (clinical data and self-report endometriosis cases) considering the ACOG definition as the reference. The adjustment factors for each of the covariates included in the model are summarised in the following table.

Table 1: MR-BRT crosswalk adjustment factors for endometriosis

Data input	Reference or alternative case definition	Gamma	Beta coefficient, logit (95% UI)*	Adjustment factor**
ACOG definition	Ref	1.13	---	---
Self-report	Alt		0.15 (0.13, 0.17)	0.54 (0.53, 0.55)
Clinical data	Alt		–0.22 (–0.23, –0.21)	0.44 (0.43, 0.45)

**MR-BRT crosswalk adjustments can be interpreted as the factor the alternative case definition is adjusted by to reflect what it would have been had it been measured using the reference case definition. If the log/logit beta coefficient is negative, then the alternative is adjusted up to the reference. If the log/logit beta coefficient is positive, then the alternative is adjusted down to the reference.*
***The adjustment factor column is the exponentiated beta coefficient. For log beta coefficients, this is the relative rate between the two case definitions. For logit beta coefficients, this is the relative odds between the two case definitions.*

Modelling strategy

We have made no substantive changes in the modelling strategy from GBD 2021. We used DisMod-MR 2.1, a Bayesian meta-regression epidemiological tool, to generate incidence, prevalence, and remission estimates for endometriosis by age, sex, year, and location.

As in previous GBD iterations, incidence was assumed to be zero except between the ages of 15 and 50 years. This is because a woman must enter puberty before she can get endometriosis, and the condition remits spontaneously after the onset of menopause. The Bayesian prior on remission was bounded from 0 to 0.2 before the age of 50 years and was set to be equal to 0.2 (1/remission = duration = 5 years) from the age of 51 years through the end of life. We also bound the excess mortality rate among the prevalent cases to a maximum of 3 deaths per 10,000 person-years and used the Healthcare Access and Quality (HAQ) Index as the lone predictive covariate on this parameter.

Prior to GBD 2019, no covariates were used to inform the prevalence estimates of endometriosis. For GBD 2019, a non-systematic literature review was conducted to identify possible predictive covariates, which identified the following: the summary exposure values (SEV) for smoking, high body-mass index, low physical activity, and alcohol consumption; the age-standardised death rate (lnASDR) of sexually transmitted infections (STIs); the prevalence of pelvic inflammatory diseases; prevalence of contraception; and total fertility rate (TFR). The covariates were tested in preliminary models in GBD 2019, and TFR and the risk-weighted prevalence of smoking were selected as covariates in the final model. These covariates were used again in GBD 2023, with corresponding beta coefficients and exponentiated values as shown in the following:

Table 2. Covariates. Summary of covariates used in the endometriosis DisMod-MR meta-regression model

Covariate	Parameter	Exponentiated beta (95% uncertainty interval)
Total fertility rate	Prevalence	1.26 (1.22–1.31)
Age-standardised SEV for smoking	Prevalence	1.23 (1.15–1.31)
Healthcare Access and Quality Index	Excess mortality rate	0.99 (0.98–1.00)

Severity splits and disability weights

The basis of the GBD disability weight (DW) survey assessment are lay descriptions of sequelae highlighting major functional consequences and symptoms. The GBD 2010 systematic literature review identified three studies that were combined to inform the severity distribution of those with endometriosis. Only one study reported on the proportion of endometriosis cases with chronic abdominal pain,[11] and another was found to contain data on the distribution of pain severity.[12] Data from each study were combined to calculate a pooled proportion of 69.4% (95% CI 66.5–72.4) of women with endometriosis who have abdominal pain and, of those who suffer pain, 8.2% (7.3–9.1) with mild pain; 75.1% (73.6–76.5) with moderate pain; and 16.8% (15.5–18.0) with severe pain. No information was available on the proportion of time spent with pain. From the Australian Longitudinal Women's Health Study (ALWHS), we were able to derive an estimate of the proportion of women who have

endometriosis and long-term infertility.[13] The excess risk of being permanently infertile with endometriosis (relative to no endometriosis) was calculated as the difference in risk of being infertile with and without endometriosis. This excess risk was 6.2% (4.3–8.3). Disability weights for each sequela are listed below for reference.

Table 3. Severity distribution, details on the severity levels for endometriosis and the associated disability weight (DW) with that severity

Severity level	Lay description	DW (95% CI)
Asymptomatic		--
Mild abdominal pain due to endometriosis	Has some pain in the belly that causes nausea but does not interfere with daily activities.	0.011 (0.005, 0.021)
Moderate abdominal pain due to endometriosis	Has pain in the belly and feels nauseous. The person has difficulties with daily activities.	0.114 (0.078, 0.159)
Severe abdominal pain due to endometriosis	Has severe pain in the belly and feels nauseous. The person is anxious and unable to carry out daily activities.	0.324 (0.219, 0.442)
Primary infertility due to endometriosis	Wants to have a child and has a fertile partner, but the couple cannot conceive.	0.008 (0.003, 0.015)
Secondary infertility due to endometriosis	Has at least one child and wants to have more children. The person has a fertile partner, but the couple cannot conceive.	0.005 (0.002, 0.011)
Mild abdominal pain and primary infertility due to endometriosis	Has some pain in the belly that causes nausea but does not interfere with daily activities, and wants to have a child and has a fertile partner, but the couple cannot conceive.	0.018 (0.009, 0.036)
Moderate abdominal pain and primary infertility due to endometriosis	Has pain in the belly and feels nauseous. The person has difficulties with daily activities, and wants to have a child and has a fertile partner, but the couple cannot conceive.	0.121 (0.083, 0.168)
Severe abdominal pain and primary infertility due to endometriosis	Has severe pain in the belly and feels nauseous. The person is anxious and unable to carry out daily activities, and wants to have a child and has a fertile partner, but the couple cannot conceive.	0.329 (0.227, 0.445)
Mild abdominal pain and secondary infertility due to endometriosis	Has some pain in the belly that causes nausea but does not interfere with daily activities, and has at least one child and wants to have more children. The person has a fertile partner, but the couple cannot conceive.	0.016 (0.007, 0.031)
Moderate abdominal pain and secondary infertility due to endometriosis	Has pain in the belly and feels nauseous. The person has difficulties with daily activities, and has at least one child and wants to have more children. The person has a fertile partner, but the couple cannot conceive.	0.119 (0.081, 0.164)
Severe abdominal pain and secondary infertility due to endometriosis	Has severe pain in the belly and feels nauseous. The person is anxious and unable to carry out daily activities, and has at least one child and wants to have more children. The person has a fertile partner, but the couple cannot conceive.	0.328 (0.225, 0.444)

Genital prolapse

Flowchart

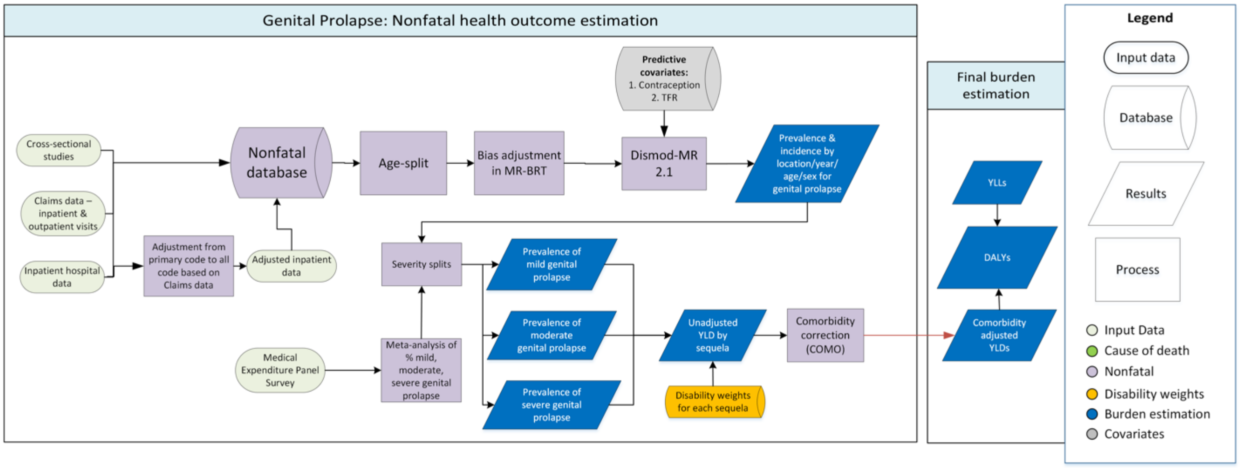

Input data and methodological summary for genital prolapse

Case definition

As defined by ACOG, genital prolapse, also called pelvic organ prolapse, is the clinically relevant descent of one or more of the female pelvic structures, including the uterus, bladder, rectum, small or large bowel, or vagina.[15] Risk of prolapse increases with age and can be exacerbated by vaginal childbirth or physical strain. The ICD-10 code associated with genital prolapse is N81. In an effort to standardise the case definitions of all gynaecological diseases, in GBD 2019, we started using the ACOG definition of genital prolapse as the reference definition.[14] The ACOG definition states that mild descent of the pelvic organs should not be considered pathological unless women experience symptoms such as pressure with or without a bulge, sexual dysfunction, or if it is disrupting normal lower urinary tract or bowel function.[14]

Quantity of interest	Reference or alternative	Definition
Prevalence of genital prolapse	Reference clinical data type	Cases identified from database of commercial claims from USA using ICD codes.
Prevalence of genital prolapse	Alternative clinical data type	Cases of genital prolapse identified using ICD codes in administrative records that are considered population-representative.
Prevalence of genital prolapse	Reference	ACOG definition: Clinically relevant descent of one or more of the pelvic structures, including the anterior and posterior vagina walls, uterus (cervix), apex of the vagina, bladder, rectum, small or large bowel or vagina.
Prevalence of genital prolapse	Alternative	Cases of genital prolapse identified via self-report by respondents in longitudinal or cross-sectional surveys or questionnaires inquiring about self-reported diagnosis of genital prolapse in the past months, years, or lifetime.
Prevalence of genital prolapse	Alternative	Cases of genital prolapse from cross-sectional studies in published literature where the case definition required that cases experience symptoms.
Prevalence of genital prolapse	Alternative	Cases identified from administrative records using ICD codes, after commercial claims and population-representative sources have been crosswalked to a common standard clinical data type.

Input data

Input data

Data sources used to inform the genital prolapse non-fatal estimates include data from peer-reviewed literature identified in a previous systematic review (mainly from population-level and community prevalence surveys), claims data, and hospital administrative data. The last comprehensive literature review was completed in GBD 2010, where we identified data on prevalence of genital prolapse using the following search strings:

> **PUBMED:** (("genital prolapse" OR "genital prolapses" OR "vaginal prolapse" OR "vaginal prolapses" OR "uterine prolapse" OR "uterine prolapses" OR "uterovaginal prolapse" OR "uterus prolapse" OR "pelvic organ prolapse" OR "urogenital prolapse" OR "vaginal vault prolapse" OR cystocele OR cystoceles OR "Vaginal enterocele" OR "urethrocele" OR "urethroceles") AND (prevalence OR prevalences OR epidemiology OR incidence OR incidences)) OR (("Uterine prolapse"[MeSH] OR "Pelvic organ prolapse"[MeSH] OR "cystocele"[MeSH]) AND ("Prevalence"[MeSH] OR "Epidemiology"[MeSH])
>
> **EMBASE:** (("genital prolapse" OR "genital prolapses" OR "vaginal prolapse" OR "vaginal prolapses" OR "uterine prolapse" OR "uterine prolapses" OR "uterovaginal prolapse" OR "uterus prolapse" OR "pelvic organ prolapse" OR "urogenital prolapse" OR "vaginal vault prolapse" OR cystocele OR cystoceles OR "Vaginal enterocele" OR "urethrocele" OR "urethroceles") AND ('incidence'/exp OR incidence OR incidences OR 'prevalence'/exp OR prevalence OR prevalences)) OR (('Uterus prolapse'/exp, 'Pelvic organ prolapse'/exp, 'Cystocele'/exp, 'Enterocele'/exp) AND ('incidence'/exp OR incidence OR incidences OR 'prevalence'/exp OR prevalence OR prevalences))

We excluded studies that did not provide primary data on epidemiological parameters (eg, reviews, commentary) and clearly non-representative studies.

In subsequent rounds of GBD, data from the GBD 2010 systematic review were serially augmented by data from claims and hospital discharges aggregated, extracted, and processed by IHME as described elsewhere in this appendix. In claims data, individuals were extracted as prevalent cases if they had at least one inpatient encounter or two outpatient encounters with an appropriate ICD code in a given year, and the denominator applied was all enrollees that year in the same age-group and location. Hospital discharges were processed by extracting discharges with an appropriate ICD code as primary diagnosis and adjusting using a correction factor. Specifically, we used claims data to model the ratio of inpatient claims with genital prolapse as primary diagnosis to total prevalent cases of genital prolapse seen in both inpatient and outpatient settings and this ratio was applied to hospital discharge data. (See the section on non-fatal data sources, identification, and extraction for a description of GBD modelling of hospital utilisation and processing of inpatient and claims data in this appendix.)

Data processing

The first step to process the data was age-sex splitting. For any datum that did not entirely fit within a GBD age group, the observation was split to be multiple age-specific datapoints based on the age pattern predicted by GBD 2017 DisMod-MR 2.1 models.

As the prevalence estimates on self-reported symptoms were markedly lower than the prevalence identified by medical examination, we used MR-BRT models to crosswalk the data collected from non-reference definitions including symptomatic cases, self-reported cases, and clinical data to the reference definition (cases of genital prolapse diagnosed by medical examination).

To perform the crosswalk, all observations that matched were paired with one another and logit-transformed, and the difference of the logit mean values was calculated. The standard error of the logit difference was calculated using the delta method. Adjustment of data inputs for genital prolapse was conducted in two steps, using the MR-BRT steps described in the section above on uterine fibroids. In

the first step, we adjusted only clinical data to a common standard, by using claims data as the reference definition and inpatient hospital data as the alternative definition. We then used the logit-transformed mean difference and standard error as inputs to run a MR-BRT model with a cubic spline on age and four knots, trimming 10% of the data and assuming linear tails. Our final model results for this crosswalk process are illustrated in the next figure.

Genital prolapse MR-BRT crosswalk adjustments factors by age for hospital (alternate) and claims (reference) data.

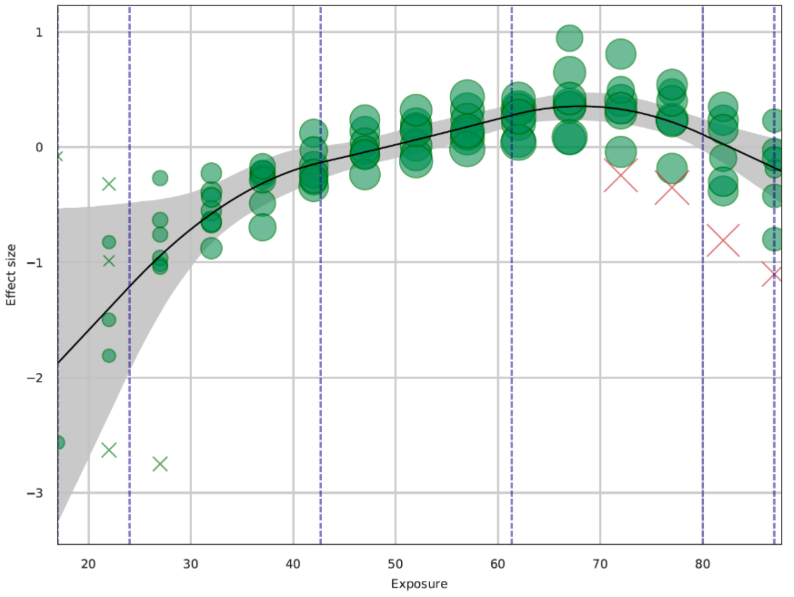

**Exposure on the x-axis is GBD age group and effect size is the logit-transformed difference of inpatient to claims data. MR-BRT model ran with a quadratic spline on age, linear tails, and trimming 10% of the data.*

According to this model, hospital data underestimated the number of genital prolapse cases for younger ages and up to age 50 and after age 80. Between ages 50 and 80, the inverse relationship is true.

In the second step, clinical data (as adjusted in the first step) were included as an alternative definition along with symptomatic and self-reported cases in a network MR-BRT model, where the reference definition was cases diagnosed using the ACOG definition. The adjustment factors for each of the covariates included in the model are summarised in the following table.

Table 1: MR-BRT crosswalk adjustment factors for genital prolapse

Data input	Reference or alternative case definition	Gamma	Beta coefficient, logit (95% UI)*	Adjustment factor**
ACOG definition	Ref	0.51	---	---
Self-report	Alt		−3.48 (−4.55, −2.43)	0.03 (0.01, 0.08)
Symptomatic cases	Alt		−2.24 (−3.33, −1.13)	0.10 (0.03, 0.24)
Clinical data	Alt		−5.58 (−5.77, −5.38)	0.004 (0.003, 0.005)

**MR-BRT crosswalk adjustments can be interpreted as the factor the alternative case definition is adjusted by to reflect what it would have been had it been measured using the reference case definition. If the log/logit beta coefficient is negative, then the alternative is adjusted up to the reference. If the log/logit beta coefficient is positive, then the alternative is adjusted down to the reference.*
***The adjustment factor column is the exponentiated beta coefficient. For log beta coefficients, this is the relative rate between the two case definitions. For logit beta coefficients, this is the relative odds between the two case definitions.*

Modelling strategy

There were no changes to the modelling strategy and settings between GBD 2021 and GBD 2023. We used DisMod-MR 2.1 to estimate the prevalence, incidence, and remission of genital prolapse. As in previous GBD iterations, incidence was set to zero prior to 15 years of age. Excess mortality rate was set to zero for all ages. The Bayesian prior on remission was bounded from 0 to 0.1 (1/remission = duration = 10 years) for all ages. We set the minimum coefficient of variation to 0.8 and the time span of data used to fit for a particular year to five years. To ensure that the age pattern of the estimates was consistent with the age pattern observed in the literature and because it is highly unlikely that young women would experience genital prolapse, we marked as outliers and excluded all data that reported prevalence values higher than 5% for women under 25 years.

In GBD 2019, we also conducted a non-systematic literature review to find the main predictors of genital prolapse that could inform DisMod-MR 2.1 estimates. We tested the association between the prevalence of genital prolapse and the summary exposure values (SEV) for smoking, high body-mass index, and low physical activity; the prevalence of contraception; and total fertility rate (TFR). No significant statistical association was found between the prevalence of prolapse and most of the aforementioned covariates. Since GBD 2019, we have used log-transformed total fertility rate and the prevalence of contraception as predictive covariates as multiparity is a recognised risk factor for prolapse. The following table illustrates covariates, measures, parameters, beta, and exponentiated beta values of the final model that was selected based on a combination of qualitative and quantitative goodness of fit to input data, plausibility of geographical and temporal trends, and consistency of age pattern.

Table 2. Covariates. Summary of covariates used in the genital prolapse DisMod-MR meta-regression model

Covariate	Parameter	Exponentiated beta (95% uncertainty interval)
Contraception (modern) prevalence (proportion by age)	Prevalence	7.53 (4.50–16.19)
Total fertility rate	Prevalence	2.29 (2.15–2.46)

Severity splits and disability weights

The basis of the GBD disability weight (DW) survey assessment are lay descriptions of sequelae highlighting major functional consequences and symptoms. To determine the proportion of people

within each domain of disability, several studies from the systematic review were identified to contain information on the proportion of women with symptoms. These data were pooled and applied to prevalence estimates. This included two studies with information on the proportion of women with prolapse who experience a bulging sensation (pooled proportion = 11.7% [95% CI 6.8–19.4]),[16,17] three that reported on the proportion with stress incontinence (pooled proportion = 52.8% [40.1–65.1]),[18–20] and one that reported on the frequency (measured as proportion of the year) of incontinence symptoms (pooled proportion = 7.9% [4.6–13.6]).[20] Percentages were combined to calculate the proportion of women who fall into both stress incontinence and bulging sensation categories at a given moment in time. The lay descriptions and disability weights for genital prolapse are shown below.

Table 3. Severity distribution, details on the severity levels for genital prolapse and the associated disability weight (DW) with that severity

Severity level	Lay description	DW (95% CI)	Distribution (95% CI)
Asymptomatic		--	0.807 (0.704–0.910)
Abdominal pain due to genital prolapse	Has some pain in the belly that causes nausea but does not interfere with daily activities.	0.011 (0.005–0.021)	0.072 (0.000–0.162)
Stress incontinence due to genital prolapse	Loses small amounts of urine without meaning to when coughing, sneezing, laughing, or during physical exercise.	0.020 (0.011–0.035)	0.111 (0.059–0.162)
Abdominal pain and stress incontinence due to genital prolapse	Loses small amounts of urine without meaning to when coughing, sneezing, laughing, or during physical exercise, and has some pain in the belly that causes nausea but does not interfere with daily activities.	0.031 (0.016–0.054)	0.010 (0.000–0.021)

Premenstrual syndrome

Flowchart

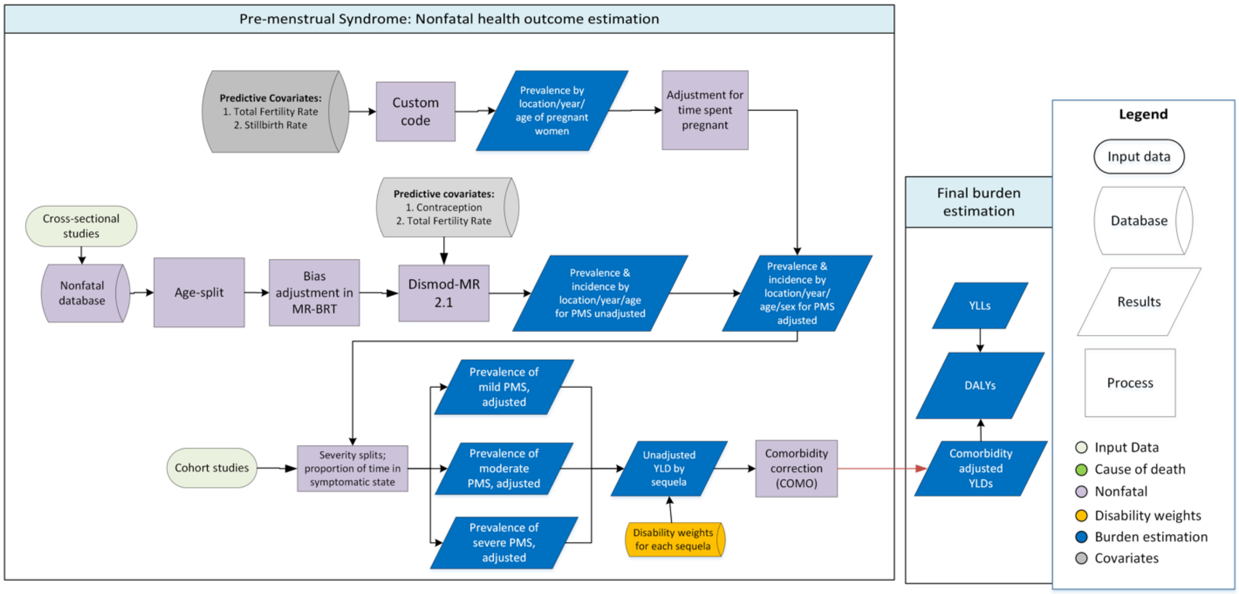

Input data and methodological summary for premenstrual syndrome

Case definition

Premenstrual syndrome (PMS) refers to psychological and physical symptoms that occur during the luteal phase of the menstrual cycle. Symptoms vary in nature and severity, but include tenderness, bloating, irritability, fatigue, abdominal pain, and altered mental states. PMS ceases when a woman is pregnant and when she reaches menopause. Lacking definitive and universally accepted diagnostic criteria for PMS, in GBD 2019, we started using the diagnostic criteria proposed by the American College of Obstetricians and Gynecologists (ACOG) as the reference definition. The ACOG definition of PMS requires at least one emotional or physical symptom to be experienced by women during the five days before menses and remit within four days of onset of menses, with no recurrence at least until day 13 of the cycle, in each of three prior menstrual cycles. Additionally, identifiable dysfunction in social or economic performance and prospective confirmation for two cycles are required.

**Quantity of interest**	**Reference or alternative**	**Definition**
Prevalence of PMS	Reference	ACOG definition: at least one of a list of emotional and physical symptoms to be experienced by women during the five days before menses and remit within four days of onset of menses, with no recurrence at least until day 13 of the cycle, in each of three prior menstrual cycles. Identifiable dysfunction in social or economic performance and prospective confirmation for two cycles are required from a non-pregnant population via questionnaire
Prevalence of PMS	Alternative	ICD definition: Cases of PMS identified using ICD codes in administrative records that are considered population-representative.

Prevalence of PMS	Alternative	Premenstrual Symptoms Screening Tool (PSST) definition: Premenstrual symptoms must occur in the last week before menses and remit within a few days of onset of follicular phase, and they must reach a level of severity that interferes with functioning in work, family, and social relationships. At least five symptoms (including at least one major dysphoric symptom) out of a list of 11 symptoms must have been present in the majority of cycles in the preceding 12 months. Symptoms must be confirmed prospectively by daily monitoring for at least two consecutive symptomatic menstrual cycles and cannot be merely an exacerbation of another disorder.
Prevalence of PMS	Alternative	Moderate or severe cases definition: Capture the women's subjective evaluation of their premenstrual symptoms and the impact of the symptoms on their daily life. Women had to report the presence of all six emotional and physical symptoms defined by the study, and these symptoms must have caused significant disturbance in their daily functioning.
Prevalence of PMS	Alternative	Other definitions

Input data

Input data

A comprehensive literature review was completed in GBD 2010, where we identified data on prevalence of PMS using the following search strings:

> **PUBMED:** "Premenstrual Syndrome"[Mesh] OR (premenstrual AND syndrome) OR (premenstrual AND syndrome) OR (premenstrual AND tension) OR (premenstrual AND tensions) OR (premenstrual AND stress) OR "premenstrual dysphoric disorder" OR "premenstrual dysphoric disorders" OR (menstrual AND distress) AND (("Incidence"[Mesh] OR incidence OR incidences OR onset OR occurrence) OR ("Prevalence"[Mesh] OR prevalence OR prevalences)
>
> **EMBASE:** 'premenstrual syndrome'/exp OR 'premenstrual dysphoric disorder'/exp OR (premenstrual AND syndrome) OR (premenstrual AND syndromes) OR (premenstrual AND tension) OR (premenstrual AND tensions) OR (premenstrual AND stress) OR "premenstrual dysphoric disorder" OR "premenstrual dysphoric disorders" OR (menstrual AND distress) AND (('incidence'/exp OR incidence OR incidences OR onset OR occurrence) OR (prevalence/exp OR prevalence OR prevalences)

Exclusion criteria for the initial systematic review were studies that did not provide primary data on epidemiological parameters (eg, commentary) and reviews.

Administrative data (claims and hospital discharge data aggregated and processed for GBD and coded with ICD-10 code N94.3) were considered for inclusion in modelling PMS but were ultimately not incorporated, as we believe that the likelihood that women with PMS would seek care in the medical system would be more variable across time and space than the true epidemiological variation.

Data processing

We performed age-splitting to ensure all data fit into GBD standard age groups. In other words, for any datum that did not entirely fit within a GBD age group, the observation was split to be multiple age-specific datapoints based on the age pattern predicted by GBD 2017 DisMod-MR 2.1 models.

Case definitions for PMS vary widely, including varying constellations of symptoms and varying requirements for when symptoms occur relative to menses and over how many cycles; ascertainment methods and recall periods also vary in published studies. We use as our reference definition the ACOG criteria, which state that the patient reports at least one of each of the following affective and somatic symptoms during the five days before their menses and these appear in three consecutive cycles: depression, angry outbursts, irritability, anxiety, confusion, social withdrawal; breast tenderness,

abdominal bloating, headache, or swelling of extremities. Alternative ("non-reference") data include those that use the WHO/ICD-10 definition of having at least one premenstrual symptom during the period of assessment, those that use the Premenstrual Symptoms Screening Tool (PSST) definition, those that limit their measurement to cases of premenstrual syndrome described as "moderate or severe", those that employ other definitions of PMS that are not frequently used, and those that report period-prevalence.

To adjust the non-reference data, we followed the general steps to crosswalking in GBD, as described above. Specifically, we first evaluated the number of observations of each alternate type that matched with a corresponding observation of the reference type. Due to data scarcity, we found only "between-study" matches. That means we matched observations of different studies from the same region where the midpoint of the age range for the observation was within 20 years of the midpoint of the reference definition observation. Using the same logic, we found all the matches among all possible combinations of alternative data types. All observations that matched were paired with one another and logit transformed, and the difference of the mean values was calculated in logit space. The standard error of the logit difference was calculated using the delta method, and these were entered into a meta-regression—Bayesian, regularised, trimmed (MR-BRT) network model, trimming 10% of the data. The adjustment factors for each of the included non-reference data characteristics in the models are summarised in the following table.

Table 1: MR-BRT crosswalk adjustment factors for premenstrual syndrome

Data input	Reference or alternative case definition	Gamma	Beta coefficient, logit (95% UI)*	Adjustment factor**
ACOG definition	Ref	1.03	---	---
WHO/ICD-10 definition	Alt		2.08 (1.99, 2.17)	0.89 (0.87, 0.90)
Premenstrual syndrome screening tool	Alt		–1.47 (–1.32, –1.17)	0.19 (0.21, 0.24)
Other definitions	Alt		–0.42 (–0.33, –0.05)	0.39 (0.41, 0.49)
Moderate and severe cases only	Alt		–0.38 (–0.45, –0.30)	0.41 (0.39, 0.42)
Period prevalence studies	Alt		–0.60 (–0.67, –0.52)	0.35 (0.33, 0.37)

**MR-BRT crosswalk adjustments can be interpreted as the factor the alternative case definition is adjusted by to reflect what it would have been had it been measured using the reference case definition. If the log/logit beta coefficient is negative, then the alternative is adjusted up to the reference. If the log/logit beta coefficient is positive, then the alternative is adjusted down to the reference.*

***The adjustment factor column is the exponentiated beta coefficient. For log beta coefficients, this is the relative rate between the two case definitions. For logit beta coefficients, this is the relative odds between the two case definitions.*

Modelling strategy

There were no changes to the modelling strategy and settings between GBD 2021 and GBD 2023. After the data adjustments, we used DisMod-MR-2.1 to estimate the prevalence, incidence, and remission of PMS. As in previous GBD iterations, incidence was set to zero prior to 10 years of age and after 49 years. This is because a woman is by definition only susceptible between menarche and menopause. We assumed no excess mortality from PMS and further assumed that the duration of the condition is between 3.3 and 5 years (remission rate = 0.2–0.3 per person-year). The minimum coefficient of variation was 0.8, and the time span of data used to fit a particular year was 5 years.

In GBD 2019, potential predictive covariates for PMS were selected a priori based on a non-systematic literature review and tested in preliminary models; these included the summary exposure values (SEV) for smoking, high body-mass index, high sodium intake, alcohol consumption, and low physical activity. Beginning in GBD 2019, the model has included risk-weighted prevalence of BMI, which was selected based on a combination of qualitative and quantitative goodness of fit to input data, plausibility of geographical and temporal trends, and consistency of age pattern. The following table shows the coefficients for the covariates used in the PMS model.

Table 2. Covariates. Summary of covariates used in the premenstrual syndrome DisMod-MR meta-regression model

Covariate	Parameter	Exponentiated beta (95% uncertainty interval)
Age-standardised SEV for high body-mass index	Prevalence	0.83<br>(0.58–1.11)

Post-modelling adjustment

Studies on the prevalence of PMS consistently excluded women who were not regularly menstruating. To re-parameterise our estimates to reflect the prevalence in the entire population of women aged 10–54 years, we divided DisMod estimates of PMS by the prevalence of pregnancy; beginning in GBD 2019, the prevalence of pregnancy estimated for this purpose combined GBD estimates of age-specific fertility rate (ASFR) and the stillbirth ratio (SBR). The equation used to compute the prevalence of pregnancy was as follows:

$$\textit{Prevalence of pregnancy} = (ASFR + (SBR * ASFR)) * 46/52$$

Where ASFR is the age-specific fertility rate, SBR is the stillbirth ratio (stillbirths per livebirth), and 46/52 is the proportion of the year spent pregnant (40 weeks) and postpartum (6 weeks). This is in contrast to GBD 2017 and earlier, when prevalence of pregnancy was estimated via a DisMod model using the UNPOP fertility estimates as input data.

Severity splits and disability weights

The basis of the GBD disability weight (DW) survey assessment are lay descriptions of sequelae highlighting major functional consequences and symptoms. Unfortunately, no specific disability weights for PMS were estimated during the GBD Disability Weight Measurement Survey. Instead, we identified two health states – abdominopelvic problem (mild) and major depression (mild) – as the closest

approximations of the symptoms associated with PMS. To determine the proportion of people within each of these severity levels, five studies were consulted. Three of the prevalence studies in the systematic review provided information on the proportion of PMS cases who feel depressed.[21,23] The pooled proportion was 74.2% (95% CI 69.6–78.3). Two other studies addressed the proportion of women with PMS who experience abdominal pain.[24,25] The pooled proportion was 41.1% (95% CI 31.7–51.3).

Since PMS symptoms do not occur for the entire year, we also weighted these proportions by the time spent symptomatic. Two studies estimated the average duration of PMS, and the pooled duration was 5.0 (95% CI 4.8–5.2) days.[26,27] One of these also determined the number of cycles for which symptoms were experienced in a year, from which we estimated an average of 9.6 symptomatic cycles per year.[27] Time spent symptomatic was then calculated as the average duration of symptomatic PMS times the number of cycles that were symptomatic, for an average time symptomatic of 13.1% (95% CI 12.3–13.9) of the year.

The lay descriptions and disability weights for premenstrual syndrome are shown below.

Table 3. Severity distribution, details on the severity levels for premenstrual syndrome and the associated disability weight (DW) with that severity

Severity level	Lay description	DW (95% CI)	Distribution (95% CI)
Asymptomatic		--	0.889 (0.868–0.909)
Depression due to premenstrual syndrome	Feels persistent sadness and has lost interest in usual activities. The person sometimes sleeps badly, feels tired, or has trouble concentrating but still manages to function in daily life with extra effort.	0.145 (0.099–0.209)	0.014 (0.001–0.026)
Abdominal pain due to premenstrual syndrome	Has some pain in the belly that causes nausea but does not interfere with daily activities.	0.011 (0.005–0.021)	0.057 (0.038–0.076)
Abdominal pain and depression due to premenstrual syndrome	Feels persistent sadness and has lost interest in usual activities. The person sometimes sleeps badly, feels tired, or has trouble concentrating but still manages to function in daily life with extra effort, and has some pain in the belly that causes nausea but does not interfere with daily activities.	0.155 (0.107–0.220)	0.040 (0.029–0.052)

Other gynaecological diseases – non-menstrual disorders

Flowchart

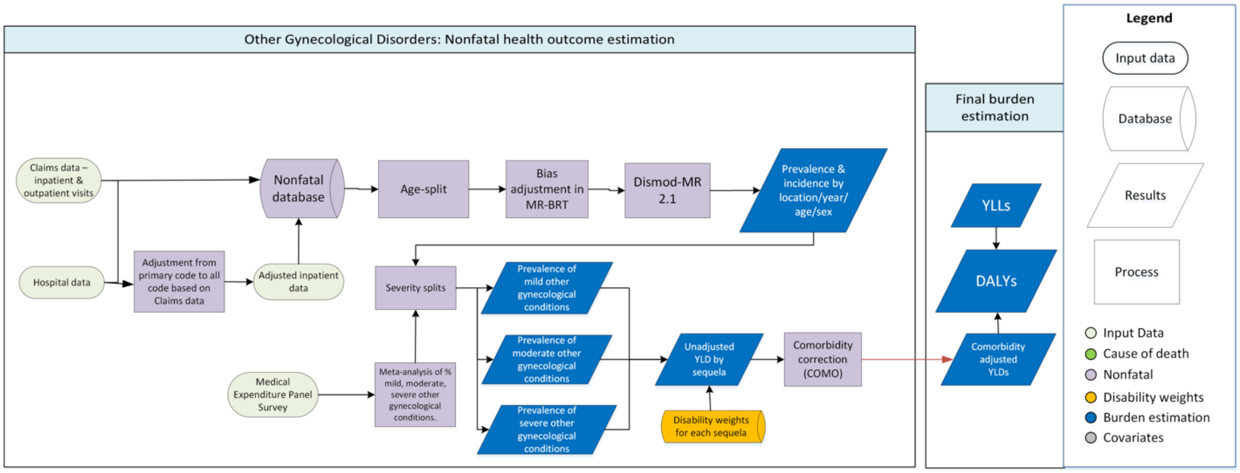

Input data and methodological summary for other gynaecological diseases – non-menstrual disorders

Case definition

Other gynaecological diseases encompass all gynaecological disorders that are not menstruation- or bleeding-related and do not fall under the heading of any of the other gynaecological causes in the GBD. Specifically, other gynaecological disorders include breast disorders; inflammatory disease of cervix uteri; diseases of Bartholin's gland; other inflammation of vagina and vulva; vulvovaginal ulceration and inflammation in diseases classified elsewhere; non-inflammatory disorders of ovary, fallopian tube, and broad ligament; other non-inflammatory disorders of the uterus, cervix, vagina, vulva, and perineum; and menopausal and other perimenopausal disorders.

The ICD-10 codes used for this cause are B37.3–B37.49, N61–N64.9, N72, N75–N77.8, N83–N86, and N88–N90.9.

Quantity of interest	Reference or alternative	Definition
Prevalence of other gynaecological diseases	Reference	Cases identified from database of commercial claims from USA using ICD codes.
Prevalence of other gynaecological diseases	Alternative	Cases identified using ICD codes in administrative records that are considered population-representative.

Input data

Input data

Data inputs come from claims and hospital discharges aggregated, extracted, and processed by IHME as described elsewhere in this appendix. In claims data, individuals were extracted as prevalent cases if they had at least one inpatient encounter or two outpatient encounters with an appropriate ICD code in

a given year, and the denominator applied was all enrollees that year in the same age group and location. Hospital discharges were processed by extracting discharges with an appropriate ICD code as primary diagnosis and adjusting using a correction factor. Specifically, we used claims data to model the ratio of inpatient claims with other gynaecologic disorders as primary diagnosis to total prevalent cases seen in both inpatient and outpatient settings, and this ratio was applied to hospital discharge data. (See the section on non-fatal data sources, identification, and extraction for a description of GBD modelling of hospital utilisation and processing of inpatient and claims data in this appendix.)

Data processing

A detailed explanation of the clinical data processing is described elsewhere in the appendix. In accordance with principles for data processing, to make hospital inpatient data and claims data comparable, we began by evaluating the number of observations from hospital inpatient data (alternate definition) that matched with a corresponding observation from claims data (reference definition). We matched the observations by age group and location. All observations that matched were paired with one another and logit transformed, and the difference of the mean logit values of each was calculated. The standard error of the logit difference was calculated using the delta method. To perform the crosswalk, we used a meta-regression—Bayesian, regularised, trimmed (MR-BRT). In this model we trimmed 10% of the data and added a cubic spline on age, assuming linear tails. Our final model results for this crosswalk process are illustrated in the following figure.

MR-BRT crosswalk adjustments factors by age for hospital (alternate) and claims (reference) data.

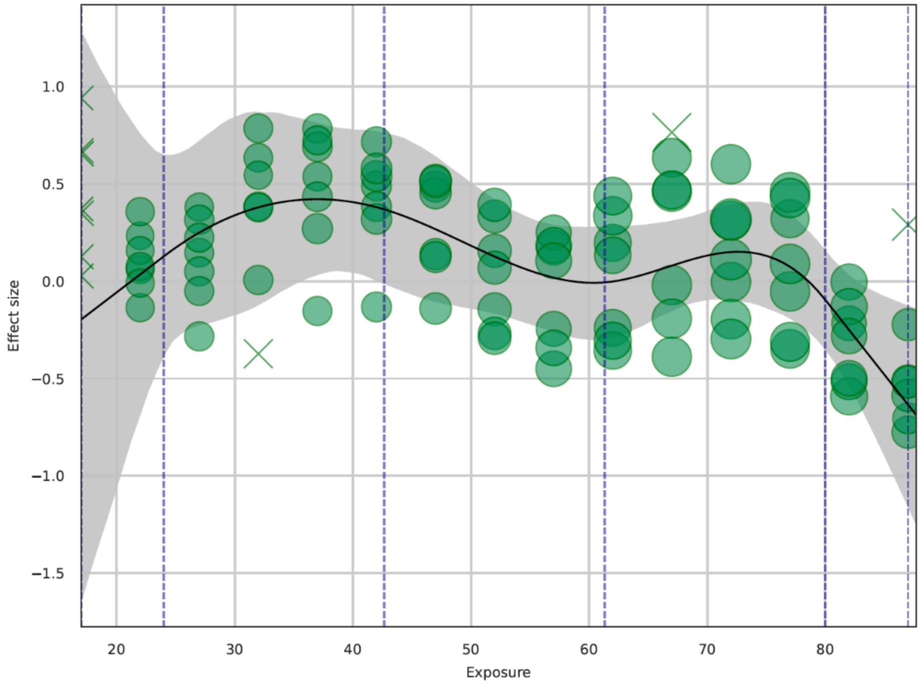

**Exposure on the x-axis is GBD age group and effect size is the logit-transformed difference of inpatient to claims data.*

According to this model, hospital data overestimated the number of other gynaecological diseases for most of the age groups. Before age 20 and after age 75, the inverse relationship is true.

Modelling strategy

There were no changes to the modelling strategy and settings between GBD 2021 and GBD 2023. We used DisMod-MR 2.1 to estimate the burden of other gynaecological diseases. Incidence was set to zero prior to 15 years of age, and we assumed no excess mortality from other gynaecological conditions over the same age range. We set the minimum coefficient of variation to 0.8 and the time span of data used to fit a particular year to 5 years. We do not use any predictive covariates in this model.

Severity splits and disability weights

The basis of the GBD disability weight (DW) survey assessment are lay descriptions of sequelae highlighting major functional consequences and symptoms.[28] To determine the proportion of women with other gynaecological conditions who fall into each severity level of abdominopelvic problem, data from the Medical Expenditure Panel Survey (MEPS) were used. MEPS is an overlapping panel survey of the non-institutionalised USA population that collects data on respondents' health service interactions. Panels are initiated every year. Each panel is two years long and consists of five rounds. In 2000, MEPS began using 12-Item Short Form Surveys (SF-12) to collect data on functional health status. The SF-12 survey is administered twice per panel (about once per year). This survey has been employed to estimate distributions of severity levels for diverse GBD diseases as previously described and summarised below.[29]

In order to translate SF-12 scores into GBD disability weights, 62 lay descriptions for conditions representing the full range of disability weight values (from most mild to most severe) were selected. A convenience sample of respondents was then asked to complete an SF-12 form for an individual with the health state described in the lay descriptions of these conditions. Composite mental and physical SF-12 score was regressed on GBD disability weight to derive the relationship between disability weight and SF-12 score. Individual respondent scores were then regressed on reported conditions to predict cumulative disability from the individuals' diagnoses, and a counterfactual cumulative disability excluding a single condition was predicted to obtain a comorbidity-corrected condition-specific disability weights for all individual with that single condition. The distribution of these condition-specific disability weights was used to derive the proportion of individuals with the conditions that fall within each GBD severity category.

The lay descriptions and disability weights for other gynaecological conditions are shown in the following table.

Table 1. Severity distribution, details on the severity levels for other gynaecological diseases – non-menstrual disorders and the associated disability weight (DW) with that severity

Severity level	Lay description	DW (95% CI)	Distribution (95% CI)
Asymptomatic		--	0.398<br><br>(0.385–0.409)
Mild other gynaecological disorders	Has some pain in the belly that causes nausea but does not interfere with daily activities.	0.011<br>(0.005–0.021)	0.419<br><br>(0.348–0.478)

Moderate other gynaecological disorders	Has pain in the belly and feels nauseous. The person has difficulties with daily activities.	0.114 (0.078–0.159)	0.120 (0.076–0.174)
Severe other gynaecological disorders	Has severe pain in the belly and feels nauseous. The person is anxious and unable to carry out daily activities.	0.324 (0.219–0.442)	0.063 (0.044–0.083)

Other gynaecological diseases – menstrual disorders

Flowchart

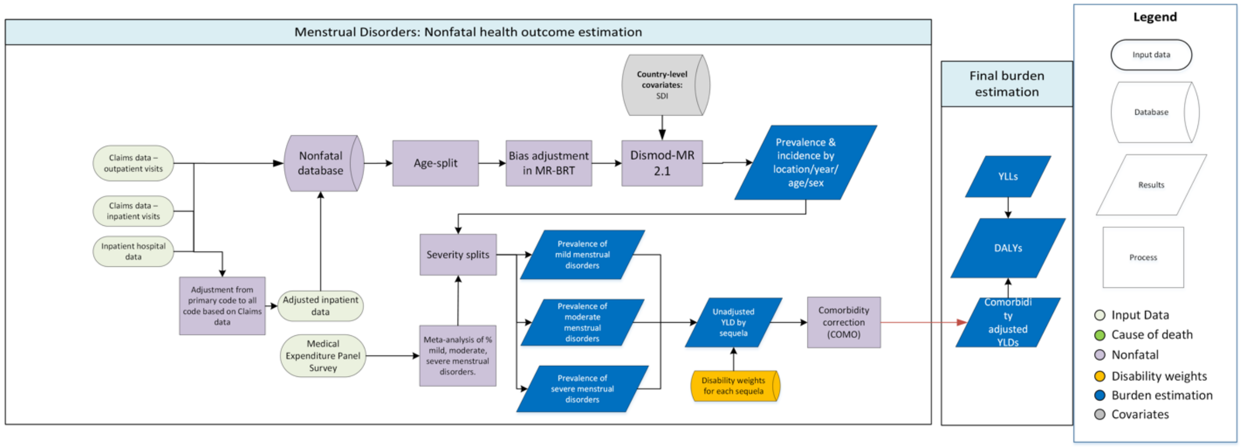

Input data and methodological summary for other gynaecological diseases – menstrual disorders

Case definition

Menstrual disorders encompasses all gynaecological disorders that are menstruation- or bleeding-related that do not fall under the heading of any of the other named gynaecological causes in the GBD. Specifically, menstrual disorders include absent, scanty, and rare menstruation, pain, and other conditions associated with female genital organs and menstrual cycle as defined by the ICD.

The ICD-10 codes used for this cause are N91-N95.9.

**Quantity of interest**	**Reference or alternative**	**Definition**
Prevalence of menstrual disorders	Reference	Cases identified from database of commercial claims from USA using ICD codes.
Prevalence of menstrual disorders	Alternative	Cases identified using ICD codes in administrative records that are considered population-representative.

Input data

Input data

Input data sources for other gynaecological diseases – menstrual disorders were the same as for other gynaecological diseases – non-menstrual disorders, and they were processed in the same way, with the only exception that the relevant ICD codes for each dataset differed, as described in the case definition section for each model.

Data processing

To make hospital inpatient data and claims data comparable, we began by evaluating the number of observations from hospital inpatient data (alternate definition) that matched with a corresponding observation from claims data (reference definition). We matched the observations by age group, location, and when the midpoint of the study was within five years of the midpoint of the reference definition observation. All observations that matched were paired with one another and logit transformed, and the difference of the logit mean values was calculated. The standard error of the logit difference was calculated using the delta method. To perform the crosswalk, we used a meta-regression—Bayesian, regularised, trimmed (MR-BRT). In this model we trimmed 10% of the data and added a cubic spline on age, assuming linear tails. Our final model results for this crosswalk process are illustrated in the following figure.

MR-BRT crosswalk adjustments factors by age for hospital (alternate) and claims (reference) data

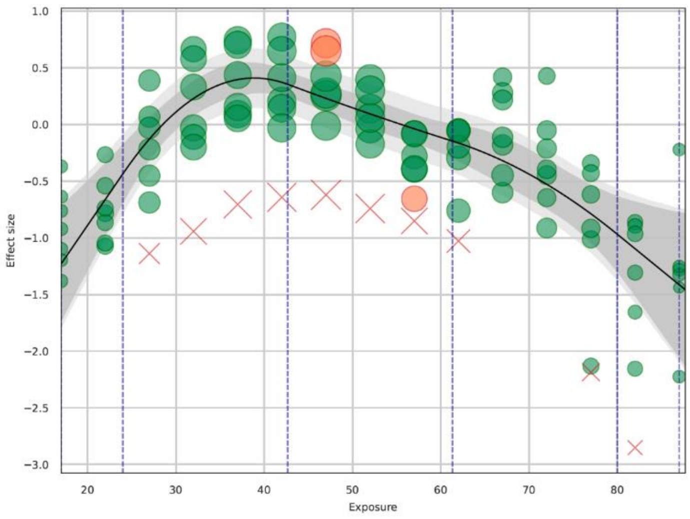

**Exposure on the x-axis is GBD age group and effect size is the logit-transformed difference of inpatient to claims data.*

According to this model, hospital data underestimated the number of menstrual gynaecological disorders for ages 15 to 30 years. Between ages 30 and 54 years, the inverse relationship is true.

Modelling strategy

We used DisMod-MR 2.1 to estimate the burden of menstrual disorders. Incidence was set to zero prior to 10 years of age and after 55 years. We assume no excess mortality from menstrual disorders. We set the minimum coefficient of variation to 0.8 and the time span of data used to fit a particular year to 5 years.

In GBD 2019, we evaluated the association between the prevalence of these conditions and potential predictive covariates including the summary exposure values (SEV) for smoking, high body-mass index, sodium intake, alcohol consumption, and low physical activity, along with Socio-demographic Index (SDI), total fertility rate, use of contraception, the prevalence of pelvic inflammatory diseases, and the age-standardised rate of sexually transmitted infections. However, none of the prior mentioned variables, except SDI, were associated with the prevalence of these conditions. From the list of covariates, we included the prevalence of pelvic inflammatory disease (PID) and the summary exposure value for high body-mass index as prevalence predictors in the final model, which was selected based on

a combination of qualitative and quantitative goodness of fit to input data, plausibility of geographical and temporal trends, and consistency of age pattern. These covariates were applied in GBD 2021 and 2023 as well.

Table 1. Covariates. Summary of covariates used in the other gynaecological diseases – menstrual disorders DisMod-MR meta-regression model

Covariate	Parameter	Exponentiated beta (95% uncertainty interval)
Pelvic inflammatory disease age-standardised prevalence	Prevalence	1.66<br>(1.08–2.54)
Age-standardised SEV for high body-mass index	Prevalence	0.98<br>(0.82–1.25)

Severity splits and disability weights

Anaemia causal attribution analysis used prevalence of menstrual disorders and information on the quantitative effect of menstrual disorders on haemoglobin levels to estimate the proportion of overall anaemia by severity that is due to menstrual disorders. The details of the anaemia analysis are described separately in the "Anaemia impairment" section. Briefly, after estimating total anaemia, a series of counterfactual distributions are generated based on the age- and sex-specific prevalence of each anaemia-causing condition and the quantitative effect that the condition has on haemglobin concentration in the blood, a so-called "haemoglobin shift," that was derived by meta-analysing cohort studies, observational studies, or trials comparing the haematological status of those with as compared to without the disease. Due to limited data on haemologbin shift, all were assumed to be invariant over age, sex, location, and year.

Table 2. Severity distribution, details on the severity levels for other gynecological diseases – menstrual disorders and the associated disability weight (DW) with that severity

Severity level	Lay description	DW (95% CI)
Menstrual disorders, without anaemia		--
Menstrual disorders, with mild anaemia	Feels slightly tired and weak at times, but this does not interfere with normal daily activities.	0.004<br>(0.001, 0.008)
Menstrual disorders, with moderate anaemia	Feels moderate fatigue, weakness, and shortness of breath after exercise, making daily activities more difficult.	0.052<br>(0.034, 0.076)
Menstrual disorders, with severe anaemia	Feels very weak, tired, and short of breath, and has problems with activities that require physical effort or deep concentration.	0.149<br>(0.10, 0.21)

References

1 Uterine Fibroids - ACOG. https://www.acog.org/Patients/FAQs/Uterine-Fibroids?IsMobileSet=false (accessed Oct 18, 2019).

2 Divakar H. Asymptomatic uterine fibroids. *Best Pract Res Clin Obstet Gynaecol* 2008; 22: 643–54.

3 Okolo S. Incidence, aetiology and epidemiology of uterine fibroids. *Best Pract Res Clin Obstet Gynaecol* 2008; 22: 571–88.

4 Schwartz SM, Marshall LM, Baird DD. Epidemiologic contributions to understanding the etiology of uterine leiomyomata. *Environ Health Perspect* 2000; 108 Suppl 5: 821–7.

5 Polycystic Ovary Syndrome: ACOG Practice Bulletin, Number 194. *Obstet Gynecol* 2018; 131: e157.

6 Franks S. Controversy in clinical endocrinology: diagnosis of polycystic ovarian syndrome: in defense of the Rotterdam criteria. *J Clin Endocrinol Metab* 2006; 91: 786–9.

7 Dunaif A, Givens JR, Haseltine FP, Merriam GR, eds. Polycystic ovary syndrome. Oxford, UK:Blackwell; 59 – 69

8 Rotterdam ESHRE/ASRM-Sponsored PCOS Consensus Workshop Group. Revised 2003 consensus on diagnostic criteria and long-term health risks related to polycystic ovary syndrome. *Fertil Steril* 2004; 81: 19–25.

9 Azziz R, Carmina E, Dewailly D, et al. The Androgen Excess and PCOS Society criteria for the polycystic ovary syndrome: the complete task force report. Fertil Steril 2009; 91: 456–88.

10 Endometriosis - ACOG. https://www.acog.org/Patients/FAQs/Endometriosis?IsMobileSet=false (accessed Oct 21, 2019).

11 Sinaii N, Plumb K, Cotton L, et al. Differences in characteristics among 1,000 women with endometriosis based on extent of disease. *Fertil Steril* 2008; 89: 538–45.

12 Sinaii N, Cleary SD, Ballweg ML, Nieman LK, Stratton P. High rates of autoimmune and endocrine disorders, fibromyalgia, chronic fatigue syndrome and atopic diseases among women with endometriosis: a survey analysis. *Hum Reprod* Oxf Engl 2002; 17: 2715–24.

13 Loxton D, Dobson A, Byles J, Tooth L. Australian Longitudinal Study on Women's Health (ALSWH). http://www.alswh.org.au/.

14 Committee on Practice Bulletins-Gynecology, American Urogynecologic Society. Practice Bulletin No. 185: Pelvic Organ Prolapse. *Obstet Gynecol* 2017; 130: e234–50.

15 Slieker-ten Hove MCP, Pool-Goudzwaard AL, Eijkemans MJC, Steegers-Theunissen RPM, Burger CW, Vierhout ME. Symptomatic pelvic organ prolapse and possible risk factors in a general population. *Am J Obstet Gynecol* 2009; 200: 184.e1-7.

16 Scherf C, Morison L, Fiander A, Ekpo G, Walraven G. Epidemiology of pelvic organ prolapse in rural Gambia, West Africa. *BJOG Int J Obstet Gynaecol* 2002; 109: 431–6.

17 Lawrence JM, Lukacz ES, Nager CW, Hsu J-WY, Luber KM. Prevalence and co-occurrence of pelvic floor disorders in community-dwelling women. *Obstet Gynecol* 2008; 111: 678–85.

18 Gomman HM, Nossier SA, Fotohi EM, Kholeif AE. Prevalence and factors associated with genital prolapse: a hospital-based study in Alexandria (Part I). *J Egypt Public Health Assoc* 2001; 76: 313–35.

19 Chuenchompoonut V, Bunyavejchevin S, Wisawasukmongchol W, Taechakraichana N. Prevalence of genital prolapse in Thai menopausal women (using new standardization classification). J Med Assoc Thail Chotmaihet Thangphaet 2005; 88: 1–4.

20 Townsend MK, Danforth KN, Lifford KL, et al. Incidence and remission of urinary incontinence in middle-aged women. *Am J Obstet Gynecol* 2007; 197: 167.e1-5.

21 Nisar N, Zehra N, Haider G, Munir AA, Sohoo NA. Frequency, intensity and impact of premenstrual syndrome in medical students. *J Coll Physicians Surg*--Pak JCPSP 2008; 18: 481–4.

22 Tabassum S, Afridi B, Aman Z, Tabassum W, Durrani R. Premenstrual syndrome: frequency and severity in young college girls. *JPMA J Pak Med Assoc* 2005; 55: 546–9.

23 Steiner M, Macdougall M, Brown E. The premenstrual symptoms screening tool (PSST) for clinicians. *Arch Womens Ment Health* 2003; 6: 203–9.

24 Choi D, Lee D-Y, Lehert P, Lee IS, Kim SH, Dennerstein L. The impact of premenstrual symptoms on activities of daily life in Korean women. *J Psychosom Obstet Gynaecol* 2010; 31: 10–5.

25 Deuster PA, Adera T, South-Paul J. Biological, social, and behavioral factors associated with premenstrual syndrome. *Arch Fam Med* 1999; 8: 122–8.

26 Hylan TR, Sundell K, Judge R. The impact of premenstrual symptomatology on functioning and treatment-seeking behavior: experience from the United States, United Kingdom, and France. *J Womens Health Gend Based Med*. 1999; 8(8): 1043-52.

27 Robinson RL, Swindle RW. Premenstrual symptom severity: impact on social functioning and treatment-seeking behaviors. *J Womens Health Gend Based Med*. 2000; 9(7): 757-68.

28 Salomon JA, Vos T, Hogan DR, et al. Common values in assessing health outcomes from disease and injury: disability weights measurement study for the Global Burden of Disease Study 2010. *Lancet* 2012; 380: 2129–43.

29 Burstein R, Fleming T, Haagsma J, Salomon JA, Vos T, Murray CJL. Estimating distributions of health state severity for the global burden of disease study. *Popul Health Metr* 2015; 13: 31.

Haemoglobinopathies and haemolytic anaemias

This document describes the non-fatal disease burden modelling process for GBD 2023 for each of sickle cell disorders, thalassaemias, glucose-6-phosphate dehydrogenase (G6PD) deficiency, sickle cell trait, thalassaemia trait, hemizygous G6PD deficiency, and other haemoglobinopathies and haemolytic anaemias.

Flowchart

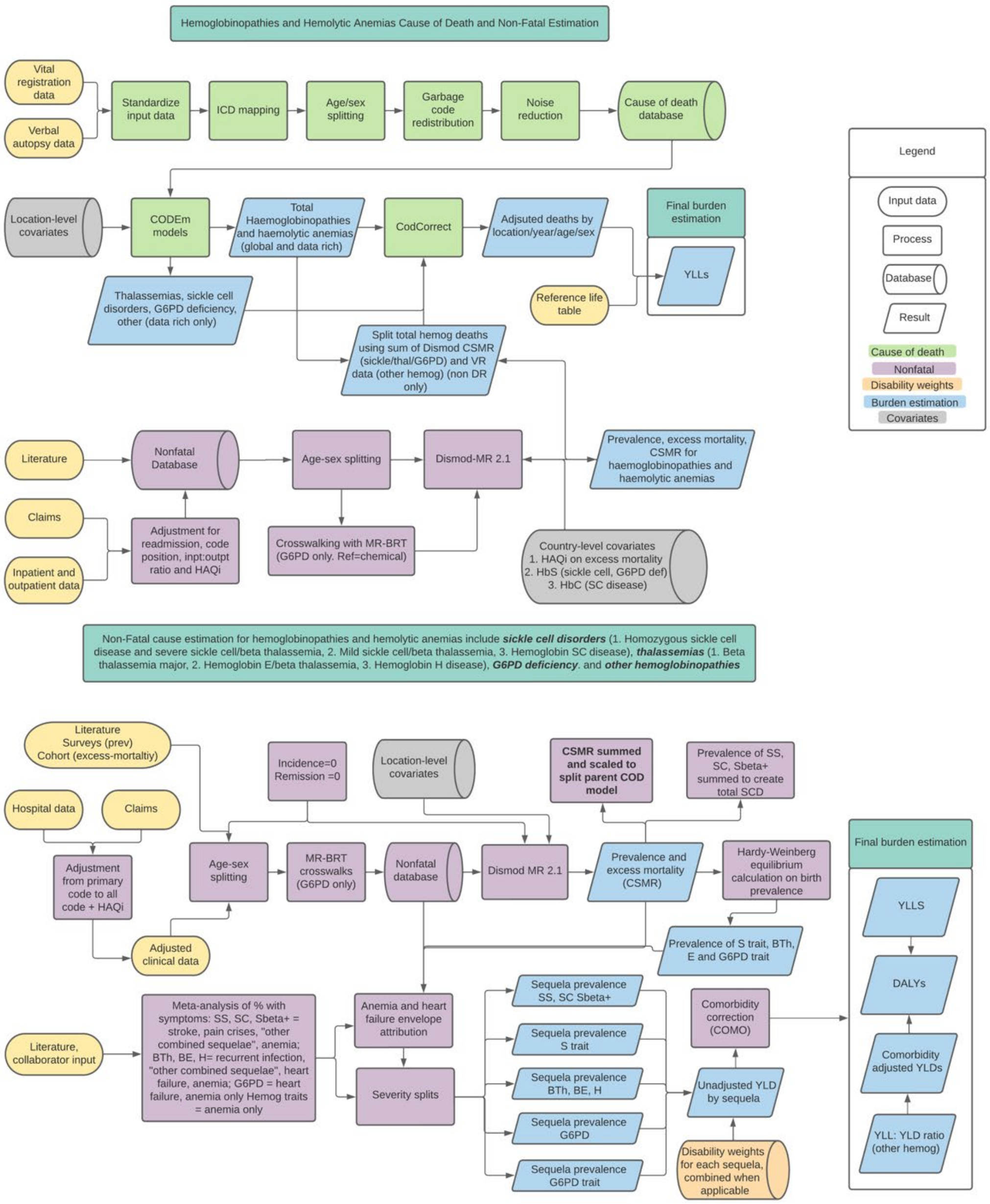

Input data and methodological summary for haemoglobinopathies and haemolytic anaemias

Case definition

Haemoglobinopathies and haemolytic anaemias span four GBD causes: thalassaemias, sickle cell disorders, G6PD deficiency, and other haemoglobinopathies and haemolytic anaemias. Case definitions for each of the types of thalassaemias and sickle cell were based on genotype. G6PD deficiency is an X-linked recessive genetic disease, and our reference definition was based on quantitative decline in G6PD activity during reagent (ie, chemical) testing; genotype or other testing was an acceptable alternate definition and adjusted as described below. Sickle cell trait, thalassaemia trait, and hemizygous G6PD deficiency were all similarly defined by genotype. They were estimated from the component disease models' estimates of birth prevalence assuming Hardy-Weinberg equilibrium. YLDs due to other haemoglobinopathies and haemolytic anaemias were estimated assuming the YLD-to-YLL ratio for each age, sex, location, and year was similar to that of the aggregate of sickle cell, thalassaemias, and G6PD deficiency. Most conditions in this group are aplastic anaemias.

Several unique combinations of genetic mutations lead to distinct phenotypes with different natural history, which has led us to estimate several distinct subtypes of thalassaemias and sickle cell disorders. The three thalassaemia models included 1) beta-thalassaemia major, 2) haemoglobin E/beta-thalassaemia, and 3) haemoglobin H disease (genotype = - - / - alpha). Sickle cell models included 1) homozygous sickle cell and severe sickle cell/beta-thalassaemia where the latter genotype had either a severe version of the sickle gene (assumed to always be the case if unspecified and west of the Arabian peninsula) or a nonsense (as opposed to reduced activity) mutation at the other beta haemoglobin gene locus; 2) haemoglobin sickle cell disease; and 3) "mild" sickle cell-beta-thalassaemia. G6PD deficiency was estimated in a single model.

Input data

Three sources of data were used for DisMod-MR 2.1 models: literature (generally from community prevalence surveys, birth screening, and cohort studies), claims data, and ICD-9 and ICD-10 hospital discharge data that were adjusted for ICD code position, readmission, inpatient-to-outpatient ratio, and location-specific Healthcare Access and Quality (HAQ) Index. Of note, there were no hospital data available for haemoglobin E/beta-thalassaemia, haemoglobin H disease, or G6PD deficiency. Our last comprehensive literature review was completed in GBD 2017, where we identified data on prevalence, excess mortality rate, or with-condition mortality rate. Age-specific survival probabilities from cohort studies were converted to corresponding with-condition mortality rates.

The systematic literature review used the following search strings in PubMed:

> (G6PD[Title/Abstract] OR G6PD deficiency[Title/Abstract] OR glucose-6 phosphate dehydrogenase[Title/Abstract] OR glucose-6-phosphate dehydrogenase deficiency[Title/Abstract] AND (survival[Title/Abstract] OR mortality[Title/Abstract] OR prevalence[Title/Abstract] OR incidence[Title/Abstract]) AND (2013/01/01[PDat] : 2016/12/31[PDat])) AND "humans"[MeSH Terms]

(sickle cell[Title/Abstract] AND (mortality[Title/Abstract] OR survival[Title/Abstract] OR prevalence[Title/Abstract] OR incidence[Title/Abstract]) AND (2013/04/01[PDat] : 2016/12/31[PDat])) AND "humans"[MeSH Terms]

(thalassemias [Title/Abstract] AND (prevalence[Title/Abstract] OR incidence[Title/Abstract] OR survival[Title/Abstract] OR mortality[Title/Abstract]) AND (2013/01/01[PDat] : 2016/12/31[PDat])) AND "humans"[MeSH Terms]

The search was completed on July 5, 2016, and supplemented similar searches that were completed for GBD 2010 and GBD 2013. The G6PD deficiency search yielded 120 results, of which 57 were selected for full text review and 32 were extracted. The sickle cell search yielded 488 results, of which 49 were selected for full text review and 22 were extracted. The thalassaemias search yielded 27 results; ten had full text review, and four were extracted.

We extracted prevalence data from population-level and community surveys as well as with-condition mortality and excess mortality data from cohort studies. Age-specific survival proportions were converted to with-condition mortality rates as needed. We also included data from hospital and claims data for a subset of haemoglobinopathy models, including beta-thalassaemia major, haemoglobin E/beta-thalassaemia, homozygous sickle cell and severe sickle cell/beta-thalassaemia, haemoglobin SC disease, and mild sickle cell/beta-thalassaemia.

Processing of clinical administrative data (ie, hospital and claims) were based on ICD-9 and ICD-10 codes as listed in Table 1. The extraction and processing of hospital and claims data is described separately.

Figure 1. PRISMA diagram of GBD 2016 literature review

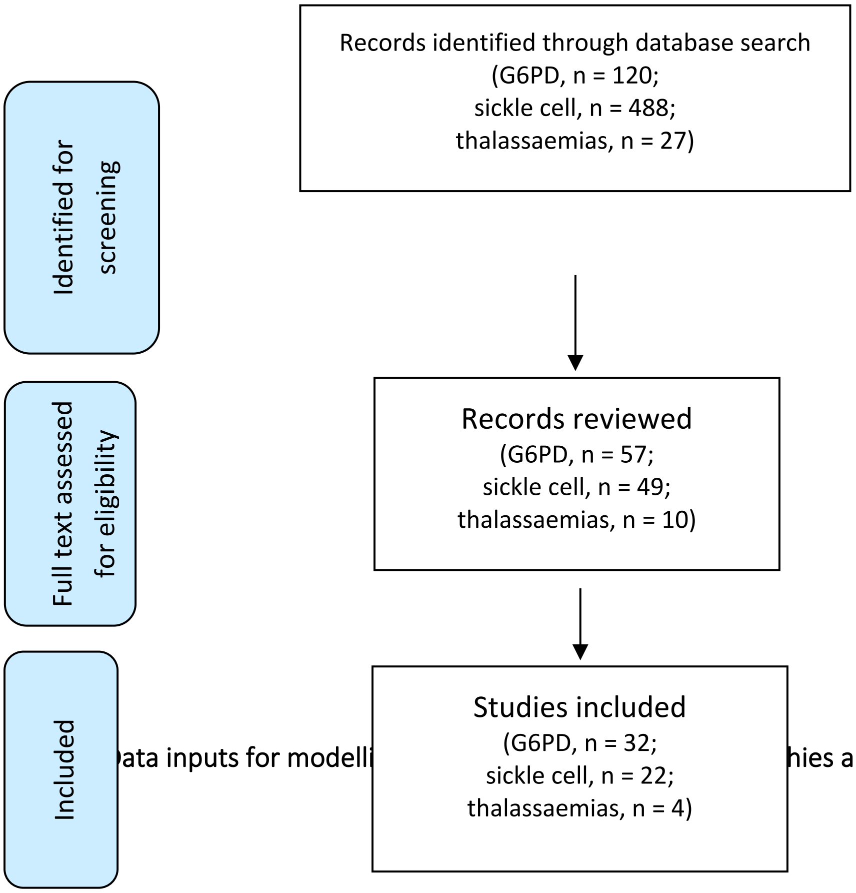

[illegible]ata inputs for modelli[illegible]hies and haemolytic anaemias

Condition	New sources	Total sources	Countries with data
Haemoglobinopathies and haemolytic anaemias (all measures)	94	790	138
Prevalence	66	752	138
Excess mortality rate	20	25	13
With-condition mortality rate	11	16	11
Other	0	9	6
Thalassaemias (all measures)	69	354	94
Prevalence	63	348	94
Excess mortality rate	5	6	6
With-condition mortality rate	1	1	1
Sickle cell disorders (all measures)	88	556	117
Prevalence	66	533	117
Excess mortality rate	15	19	9
With-condition mortality rate	10	15	10
G6PD deficiency (all measures)	0	185	69
Prevalence	0	176	68
Other	0	9	6

Table 2. International classification of diseases codes for haemoglobinopathies and haemolytic anaemias in GBD 2023 cause of death analysis

Condition	ICD-10 code	ICD-9 code
Thalassaemias	D56	282.4
Sickle cell disorders	D57	282.5–282.6
G6PD deficiency	D55	282.2–282.3
Other haemoglobinopathies and haemolytic anaemias	D58–D64.8	282.0–282.1, 282.7–285.8

Data processing

Data processing strategies did not change from GBD 2021 such that we conducted age-sex splitting and crosswalking in the same methods detailed as follows.

The first step of the process was age-sex splitting. For any datum that did not entirely fit within a GBD age group or was for both sexes combined, the observation was split to be multiple age-sex-specific datapoints based on the age and sex pattern predicted by GBD 2019 DisMod-MR 2.1 models. For thalassaemias and sickle cell disorders, this was the only processing completed.

For G6PD deficiency, we crosswalked all data to the reference definition of chemical test. We began by evaluating the number of observations of each alternate definition that matched with a corresponding observation from the reference definition. A match was considered "within" study if it was from the same data source and an exact match for age, sex, location, and year. A match was considered

"between" study if it was from the same GBD location, GBD age group and sex, and the midpoint of the study was within five years of the midpoint of the reference definition observation. Because the prevalence of G6PD deficiency itself can vary between studies, and the difference between reagent and chemical testing is expected to be a largely constant phenomenon, we restricted the crosswalk only to be based on within-study matches. There were no matches for diagnostics that were not based on either genetic or reagent testing. All of these data were therefore dropped from the model. The total number of datapoints and matches is shown in the table below.

Table 3. Datapoints and matches between alternate and reference definitions

	**Reference (cv_dx_chemical)**	**Alternate #1 (cv_dx_genetic)**	**Alternate #2 (cv_dx_other)**
Number of datapoints	6370	2578	9
Within-study matches to reference	--	397	0

The ratio of prevalence from alternate:reference was calculated, log-transformed, the standard error of the ratio calculated using the delta method, and all were analysed using meta-regression—Bayesian, regularised, trimmed (MR-BRT) a meta-regression tool developed for GBD 2019. We tested the relationships as a function of sex, age, and the variability as a function of location (grouped into super-regions). Only sex remained a significant predictor, so it was the only additional factor included in the final crosswalk model. We trimmed 10% of the data from the MR-BRT model. Our covariate betas for each of the included covariates in the model are summarised in the table below.

Table 4. MR-BRT crosswalk adjustment factors for haemoglobinopathies and haemolytic anaemias

**Data input**	**Reference or alternative case definition**	**Gamma**	**Beta coefficient, log (95% CI)**	**Adjustment factor***
Chemical test	Reference	0.06	---	---
Genetic test	Alternative		0.291 (–0.175 to 0.755)	1.33 (0.84–2.13)
Sex	Alternative		–0.027	

**MR-BRT crosswalk adjustments can be interpreted as the factor the alternative case definition is adjusted by to reflect what it would have been had it been measured using the reference case definition. If the log beta coefficient is negative, then the alternative is adjusted up to the reference. If the log beta coefficient is positive, then the alternative is adjusted down to the reference. The adjustment factor column is the exponentiated beta coefficient and can be interpreted as the relative rate between the two case definitions.*

Modelling strategy

The three sickle cell prevalence sub-causes were summed to create a fourth, total sickle cell disorders model. Covariates for the prevalence of haemoglobin S (HbS) and haemoglobin C (HbC) to the sickle cell and G6PD deficiency models bS and HbC rasters were summarised into GBD geographies from Malaria Atlas Project publications on them and assumed to be invariant over time and age. We estimated the non-fatal burden of haemoglobinopathies in four parts.

DisMod-MR 2.1 modelling of disease

First, we used the datasets described above to estimate prevalence for each age-sex-location-year using DisMod-MR 2.1. Natural-log-transformed lag-distributed income per capita was used as a covariate on excess mortality for most models. HbS and HbC allele frequency covariates were used as covariates on prevalence for each of the subtypes of sickle cell disorders and for G6PD deficiency, where the effect size and predictive power were expectedly much smaller. HAQ Index was also used as a covariate for excess mortality rate in the homozygous sickle cell and severe sickle cell/beta-thalassaemia model. A full table of all the location-level covariates and their effect sizes is shown below.

Final models were selected on a combination of qualitative and quantitative goodness of fit to input data, plausibility of geographical and temporal trends, consistency of age pattern, and, when available, comparison with other published studies on haemoglobinopathy epidemiology. Directionality, magnitude, and plausibility of study-level and country-level covariates were also considered in the process of model development. Of note, due to the nature of statistical modelling, final results do not always cover the values reported in input data.

Table 5. Covariate, parameter, beta, and exponentiated beta values for each model

Model	Covariate	Parameter	Beta	Exponentiated beta
Beta-thalassaemia major	UHC	EMR	–0.026 (–0.047 to –0.0037)	0.97 (0.95–1.00)
Haemoglobin E/beta-thalassaemia	UHC	EMR	–0.025 (–0.05 to –0.0024)	0.98 (0.95–1.00)
Haemoglobin E/beta-thalassaemia	Year	Prev	0.020 (0.018 to 0.020)	1.02 (1.02 to 1.02)
Haemoglobin H disease	Year	Prev	–0.018 (–0.019 to –0.017)	0.98 (0.98 to 0.98)
Homozygous sickle cell and severe sickle cell/beta-thal	(HbS)^2	Prev	49.94 (49.90 to 50.00)	5.02e+21 (4.69e+21 to 5.18e+21)
Homozygous sickle cell and severe sickle cell/beta-thal	UHC	EMR	–0.028 (–0.048 to –0.0036)	0.97 (0.95 to 1.00)
Haemoglobin SC disease	HbS	Prev	19.99 (19.98 to 20.00)	4.82e+8 (4.76e+8 to 4.85e+8)
Haemoglobin SC disease	HbC	Prev	10.00 (9.99 to 10.00)	2.19e+4 (2.18e+4 to 2.20e+4)
Haemoglobin SC disease	UHC	EMR	–0.024 (–0.046 to –0.0038)	0.98 (0.96 to 1.00)
Mild sickle cell/beta-thalassaemia	HbS	Prev	19.99 (19.97 to 20.00)	4.80e+8 (4.71e+8 to 4.85e+8)
Mild sickle cell/beta-thalassaemia	UHC	EMR	–0.025	0.98 (0.95 to 1.00)

			(−0.048 to −0.0029)	
G6PD deficiency	|Latitude|	Prev	−0.003 (−0.0045 to −0.0016)	1.00 (1.00 to 1.00)
G6PD deficiency	HbC	Prev	0.068 (0.0031 to 0.17)	1.07 (1.00 to 1.19)
G6PD deficiency	HbS	Prev	0.12 (0.0043 to 0.40)	1.13 (1.00 to 1.50)

Abbreviations: UHC=universal health coverage. EMR=excess mortality rate. Prev=prevalence. HbS=haemoglobin S trait prevalence. HbC=haemoglobin C trait prevalence.

Hardy-Weinberg equilibrium to estimate carrier prevalence

Second, we calculated prevalence of haemoglobinopathy traits (sickle cell trait, haemoglobin E trait, haemoglobin beta trait, hemizygous G6PD) by back-calculating from birth prevalence estimates from corresponding DisMod-MR 2.1 models, assuming Hardy-Weinberg equilibrium and no excess mortality. Because G6PD deficiency is an X-linked disease, hemizygous G6PD can only occur in females.

Severity distributions and sequelae of disease

With the exception of anaemia, only homozygous individuals were considered to experience disability. Estimated sequelae of thalassaemias included anaemia (described separately), heart failure (described separately), and periodic severe infection. Another series of common, but not universal, sequelae also occur in those with thalassaemias, including splenomegaly, skeletal deformity, delayed growth/puberty, diabetes, hypothyroidism, and leg ulcers. Given sparse data on the occurrence of these sequelae, they were approximated with a health state named "other combined sequelae of thalassaemia," for which we used the disability weight (DW) corresponding to a health state of "generic uncomplicated disease, anxiety about diagnosis and daily medication" which, of note, was also used to approximate the disability for those with cancer in remission. For sickle cell disorders, we similarly estimated YLDs for anaemia (described separately), stroke, and pain crises separately and approximated the myriad additional complications of sickle cell disease with the health state "other combined sequelae of sickle cell disease." The only sequelae estimated for G6PD deficiency were anaemia (described separately) and heart failure (described separately). Notably, however, G6PD deficiency is considered to be asymptomatic for a vast majority of those with the condition, with only a very small subset of around 1 in 1 million having chronic haemolysis (Class I disease) and approximately 1% having periodic haemolytic episodes (Class II disease) with exposure to environmental, pharmaceutical, or food products. Females heterozygous for G6PD deficiency exhibit chimerism, as one X chromosome becomes dominant in each of the red blood cells, so we estimated half as many heterozygous females will be symptomatic as homozygous females. Table 6 has all the disabling health states that were included in calculation of YLDs for haemoglobinopathies and haemolytic anaemias.

Anaemia causal attribution

The age- and sex-specific anaemia prevalence for each of the haemoglobinopathies, as well as the estimates of anaemia due to carrier/trait state, were analysed as part of overall anaemia causal attribution for GBD 2023. The details of the anaemia analysis are described separately in the "anaemia impairment" section. Briefly, after estimating total anaemia, a series of counterfactual distributions are

generated based on the prevalence of each anaemia-causing condition and the quantitative effect that the condition has on haemoglobin concentration in the blood, a so-called "haemoglobin shift," that was derived by meta-analysing cohort studies, observational studies, or trials comparing the haemotological status of those with as compared to without the disease. Due to limited data on haemoglobin shift, all were assumed to be invariant over age, sex, location, and year.

YLL:YLD ratio for other haemoglobinopathies and haemolytic anaemias

Finally, we calculated the YLD-to-YLL ratio for all haemoglobinopathies and then applied it to YLLs estimated for other haemoglobinopathies and haemolytic anaemias in our cause-specific mortality analysis. Quantitative crosswalk results for each model are shown below.

Table 6. Health states for haemoglobinopathies and haemolytic anaemias

Severity level	Lay description	DW (95% CI)	Cause
Mild anaemia	Feels slightly tired and weak at times, but this does not interfere with normal daily activities.	0.004 (0.001–0.008)	All
Moderate anaemia	Feels moderate fatigue, weakness, and shortness of breath after exercise, making daily activities more difficult.	0.052 (0.034–0.076)	All
Severe anaemia	Feels very weak, tired, and short of breath, and has problems with activities that require physical effort or deep concentration.	0.149 (0.101–0.209)	All
Severe abdominopelvic problem (proxy for vaso-occlusive crisis)	Has severe pain in the belly and feels nauseous. The person is anxious and unable to carry out daily activities.	0.324 (0.22–0.442)	Sickle cell disorders
Stroke, long-term consequences, moderate plus cognition problems	Has some difficulty in moving around, in using the hands for lifting and holding things, dressing and grooming, and in speaking. The person is often forgetful and confused.	0.316 (0.206–0.437)	Sickle cell disorders
Combined sequelae of disease (approximation of all other sequelae)	Has a chronic disease that requires medication every day and causes some worry but minimal interference with daily activities.	0.012 (0.006-0.023)	Sickle cell disorders
Controlled, medically managed heart failure	has a chronic disease that requires medication every day and causes some worry but minimal interference with daily activities.	0.049 (0.031-0.072)	Thalassaemias
Mild heart failure	Is short of breath and easily tires with moderate physical activity, such as walking uphill or more than a quarter-mile on level ground. The person feels comfortable at rest or during activities requiring less effort.	0.041 (0.026–0.062)	Thalassaemias
Moderate heart failure	Is short of breath and easily tires with minimal physical activity, such as walking only a short distance. The person feels comfortable at rest but avoids moderate activity.	0.072 (0.047–0.103)	Thalassaemias

Severe heart failure	Is short of breath and feels tired when at rest. The person avoids any physical activity for fear of worsening the breathing problems.	0.179 (0.122–0.251)	Thalassaemias
Severe infection	Has a high fever and pain, and feels very weak, which causes great difficulty with daily activities.	0.133 (0.088–0.19)	Thalassaemias

Changes from GBD 2021 to GBD 2023

There were no substantive changes to the modelling strategy for GBD 2023.

Headaches

Flowchart

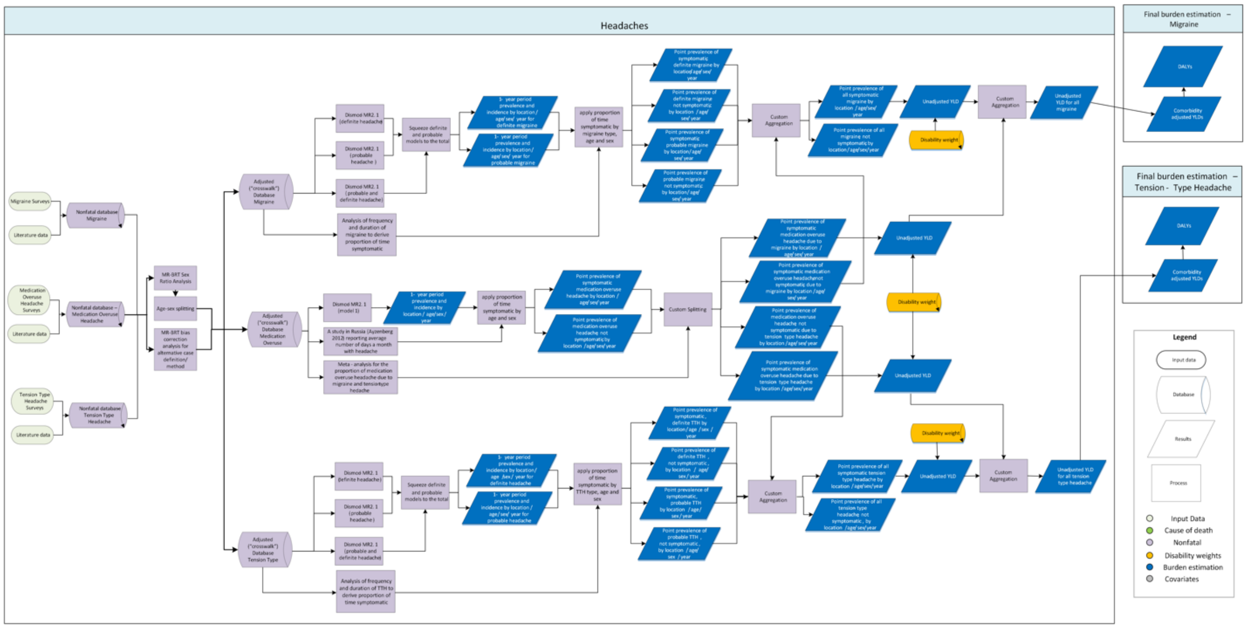

Input data and methodological summary for headaches

In GBD 2023, we report estimates for migraine and tension-type headaches (TTH). However, in our modelling process, we collect data on more granular information pertaining to migraine and TTH where we include definite and probable diagnosis types for each headache. Additionally, we also collect data for medication overuse headaches which inform estimates for migraine and TTH.

Case definition

Migraine

Migraine is a disabling primary headache disorder, typically characterised by recurrent moderate or severe unilateral pulsatile headaches. The two major types are migraine without aura and migraine with aura (transient neurological symptoms). In GBD, we do not distinguish between migraine with and without aura as most epidemiological studies report on overall migraine only. The reference diagnostic criteria for migraine are adapted from the International Classification of Headache Disorders (ICHD)-3, where at least one headache in the past year needs to fulfill the below criteria:

- Headache attacks lasting 4–72 hours (untreated or unsuccessfully treated)
- Headache has at least two of the following four characteristics:
    - Unilateral location
    - Pulsating quality
    - Moderate or severe pain intensity
    - Aggravation by or causing avoidance of routine physical activity
- During headache at least one of the following:
    - Nausea and/or vomiting

- Photophobia and phonophobia
- Not better accounted for by another ICHD-3 diagnosis

The migraine category includes both definite migraine cases and probable migraine cases. Definite migraine is a headache that satisfies all the criteria outlined above, while probable migraine satisfies all but one of the above criteria. Studies that have looked at the reasons for cases with probable headache not fulfilling criteria for definite diagnosis have suggested that most often it is the duration criterion that is left unfilled.[1,2,3,4,5]

Tension-type headache

Tension-type headache (TTH) is characterised by a dull, non-pulsatile, diffuse, band-like (or vice-like) pain of mild to moderate intensity in the head or neck. The reference diagnostic criteria for tension-type headache are adapted from the ICHD-3, in which at least one headache in the past year needs to fulfill the below criteria:

1. Lasting from 30 minutes to 7 days
2. At least two of the following four characteristics:
    a. Bilateral location
    b. Pressing or tightening (non-pulsating) quality
    c. Mild or moderate intensity
    d. Not aggravated by routine physical activity such as walking or climbing stairs
3. Both of the following:
    a. No nausea or vomiting
    b. No more than one of photophobia or phonophobia
4. Not better accounted for by another ICHD-3 diagnosis

TTH includes both definite TTH cases and probable TTH cases. Definite TTH is a headache that satisfies all criteria outlined above, while probable TTH satisfies all but one of the above criteria.

Medication overuse headache

Both migraine and tension-type headache can give rise to medication overuse headache (MOH), with the following International Classification of Headache Disorders (ICHD-3) diagnostic criteria:

1. Headache occurring ≥15 days/month in a patient with a pre-existing headache disorder
2. Regular overuse for >3 months of one or more drugs that can be taken for acute and/or symptomatic treatment of headache
3. Not better accounted for by another ICHD-3 diagnosis

ICHD-3 explicitly states that, when a person fulfils criteria for both migraine and MOH, both diagnoses should be given. However, our GBD headache collaborators indicated that in population survey practice, a screening question on chronic headache is used first, followed by questions to determine if medication overuse is present. This means the diagnoses of migraine and MOH become mutually exclusive (obviating any potential problem of double-counting).

Input data

There are no updates to input data for GBD 2023. This is a major limitation, and caution is warranted when interpreting estimates and trends in estimated years post 2017. A systematic review is underway for the next GBD round.

Migraine

We last conducted a systematic review of migraine for GBD 2017, which covered papers published through September 2017. The search string for this review was (((((("migraine disorders"[MeSH Terms] OR migraine[All Fields]) AND ((prevalence[Title/Abstract] OR incidence[Title/Abstract] OR remission[Title/Abstract] OR epidemiology[Title/Abstract])))))).

Inclusion criteria of the systematic reviews were:

- Representative, population-based surveys
- Reporting of prevalence of migraine headache

In GBD 2017, we decided to exclude medical claims data as the adjustment needed to make the claims data comparable to population representative surveys was unstable.

Tension-type headache

We last conducted a systematic review of TTH for GBD 2017, which covered papers published through September 2017. The search string for this review was ((((("headache"[MeSH Terms]) OR ("headache"[Title/Abstract] AND "tension"[Title/Abstract])) AND ("epidemiology"[Title/Abstract] OR "prevalence"[Title/Abstract] OR "incidence"[Title/Abstract] OR "remission"[Title/Abstract])))).

Inclusion criteria of the systematic reviews were:

- Representative, population-based surveys
- Reporting of prevalence of TTH headache

In GBD 2017, we decided to exclude medical claims data, as the adjustment needed to make the claims data comparable to population representative surveys was unstable.

Medication overuse headache

We last conducted a systematic review of MOH for GBD 2017, which covered papers published through September 2017. The search string for this review was (("headache"[MeSH Terms] OR "headache"[Title/Abstract]) AND ("pharmaceutical preparations"[MeSH Terms] OR "pharmaceutical preparations"[Title/Abstract] OR "medication"[Title/Abstract]) AND ("epidemiology"[Title/Abstract] OR "prevalence"[Title/Abstract] OR "incidence"[Title/Abstract] OR "remission"[Title/Abstract])).

Inclusion criteria of the systematic reviews were:

- Representative, population-based surveys
- Reporting of prevalence of MOH headache

Study bias adjustment

We used a list of binary adjustment criteria which are a modified version of quality indicators of epidemiological studies on headache[7] and are shown in the table below.

Table 1: Study covariates

Study covariate	Notation	
	**Less desirable (1)**	**Reference (zero)**
**Other than one-year recall period**	Point prevalence	One-year prevalence
**Not representative**	Selected population	General population or community-based sample from whole country OR general population or community-based sample from defined region within a country, or school-based (for children)
**Low-quality sampling method**	Not stated OR no (or failed) attempt to secure representativeness	Total defined population, or random sample corrected for population demographics OR random sample uncorrected for population demographics
**Poor response**	Not stated, or <70%	70–100%
**Low-quality survey method and type of interviewer**	Not stated OR self-administered (unsupervised) questionnaire OR telephone or face-to-face interview by untrained or unspecified interviewer(s)	Face-to-face interview with headache expert or trained interviewer
**Low-quality validation of diagnostic instrument**	Instrument not specified or not validated OR validated, but sensitivity and/or specificity <70% OR validated only in screen-positive sub-sample, or in clinic or unspecified sample, but sensitivity and specificity <70%	Validated in target population or similar, and sensitivity and specificity >70%, or all diagnoses made in face-to-face or telephone interviews by headache expert
**Low-quality diagnostic criteria**	Not stated OR stated, other than ICHD OR ICHD (or reasonable modification)	ICHD (or reasonable modification)
**Headache type assumed**	Probable/definite headache has been assumed based on descriptions and not stated explicitly	Didn't have to assume headache type

Studies based on lifetime recall of headaches were not included because of the concern of significant recall bias. For migraine and tension-type headache, we additionally tagged studies where the type of headache (probable/definite) was not explicitly mentioned in the report but the type was determined based on the diagnostic criteria stated to the best of our understanding. This covariate is called "Headache type assumed".

The mean and standard error for the coefficients were calculated using the meta-regression—Bayesian, regularised, trimmed (MR-BRT) adjustment method. All study covariates were initially evaluated independently for each of the three types of headache. However, covariate values varied not only in magnitude but in direction across the three headache types. Because we assume that the same study covariate should adjust data at least in the same direction for all headache types, the final study covariates were evaluated taking all migraine, tension-type, and medication overuse headache data into

account. Studies conducted in a school setting were not adjusted for, as we were unable to find matches to inform a reliable crosswalk. These studies were not excluded because the headache models are relatively data-sparse. Betas and inverse-logit values for these covariates are shown in the table below:

Table 2: MR-BRT crosswalk adjustment factors for headaches

Data input	Reference or alternative case definition	Gamma	Beta coefficient, logit (95% UI)*	Adjustment factor**
Other than one-year recall	Alt	1.20	–0.89 (–0.97 to –0.80)	0.30 (0.28 to 0.31)
Not representative	Alt		–0.39 (–0.45 to –0.33)	0.40 (0.39 to 0.42)
Low-quality sampling method	Alt		0.73 (0.66 to 0.79)	0.67 (0.66 to 0.69)
Poor response	Alt		–0.45 (–0.53 to –0.36)	0.40 (0.37 to 0.41)
Low-quality survey method	Alt		–0.22 (–0.31 to –0.13)	0.45 (0.42 to 0.47)
Low-quality diagnostic instrument	Alt		0.15 (0.13 to 0.19)	0.54 (0.53 to 0.55)
Low-quality diagnostic criteria	Alt		–0.37 (–0.43 to –0.32)	0.41 (0.39 to 0.42)
Headache type assumed	Alt		0.37 (0.33 to 0.42)	0.59 (0.58 to 0.60)

**MR-BRT crosswalk adjustments can be interpreted as the factor the alternative case definition is adjusted by to reflect what it would have been had it been measured using the reference case definition. If the log/logit beta coefficient is negative, then the alternative is adjusted up to the reference. If the log/logit beta coefficient is positive, then the alternative is adjusted down to the reference.*

***The adjustment factor column is the exponentiated beta coefficient. For log beta coefficients, this is the relative rate between the two case definitions. For logit beta coefficients, this is the relative odds between the two case definitions.*

Total headache model data adjustment

Because some data sources, especially earlier data from before ICHD became the standard (the initial criteria were published in 1988), largely report on definite migraine or TTH, we adjusted studies that reported only on definite headache type to the total headache type in order to better inform the total headache model. All data that reported on both definite and total migraine/TTH were used in regression models in order to derive an age- and sex-specific adjustment. This approach is consistent with GBD 2019 and 2021. The adjustment is shown in the graphs below.

Figure 1. Definite migraine to total migraine adjustment

Male Female

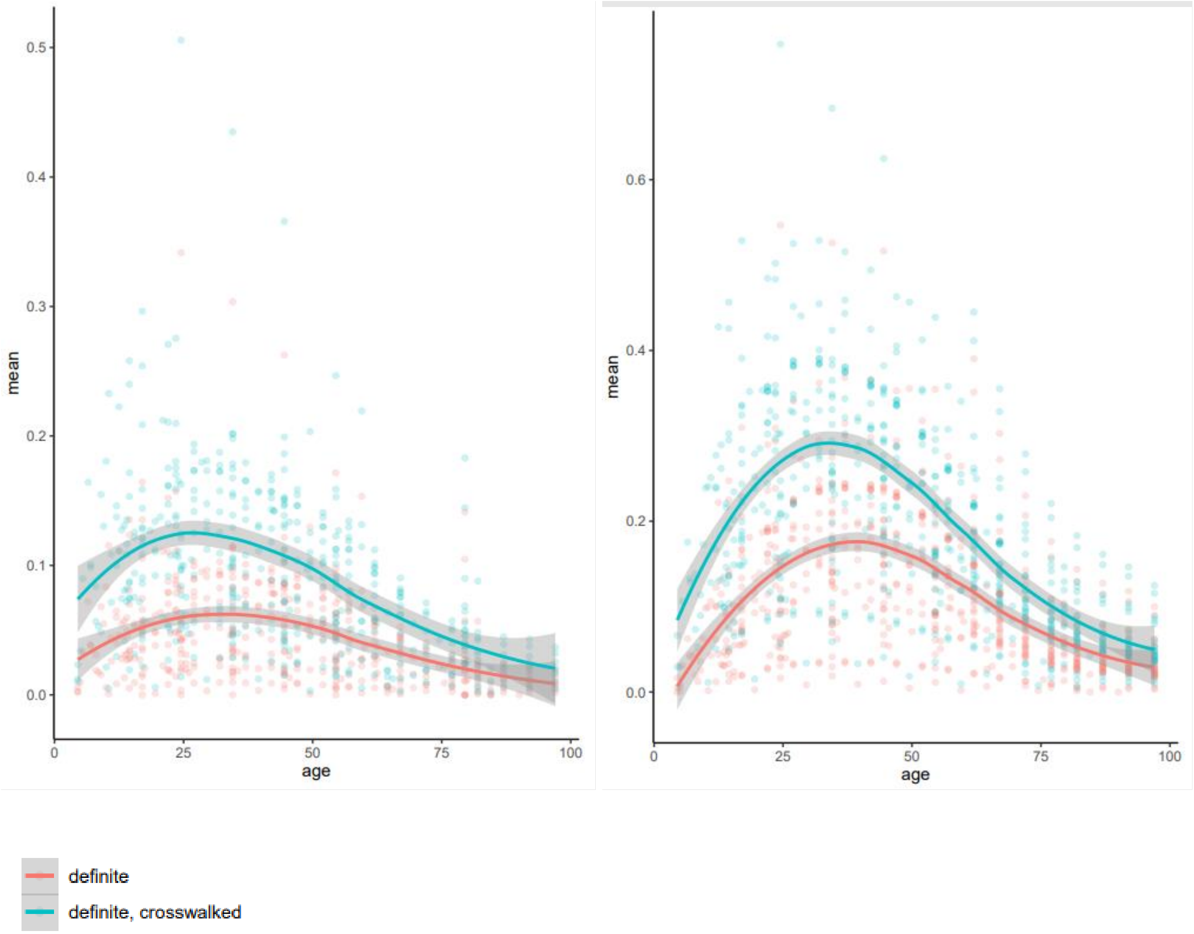

0.5
0.4
0.3
0.2
0.1
0.0
mean
0
25
50
75
100
age
0.6
0.4
0.2
0.0
mean
0
25
50
75
100
age
definite
definite, crosswalked

Figure 2. Definite TTH to total TTH adjustment

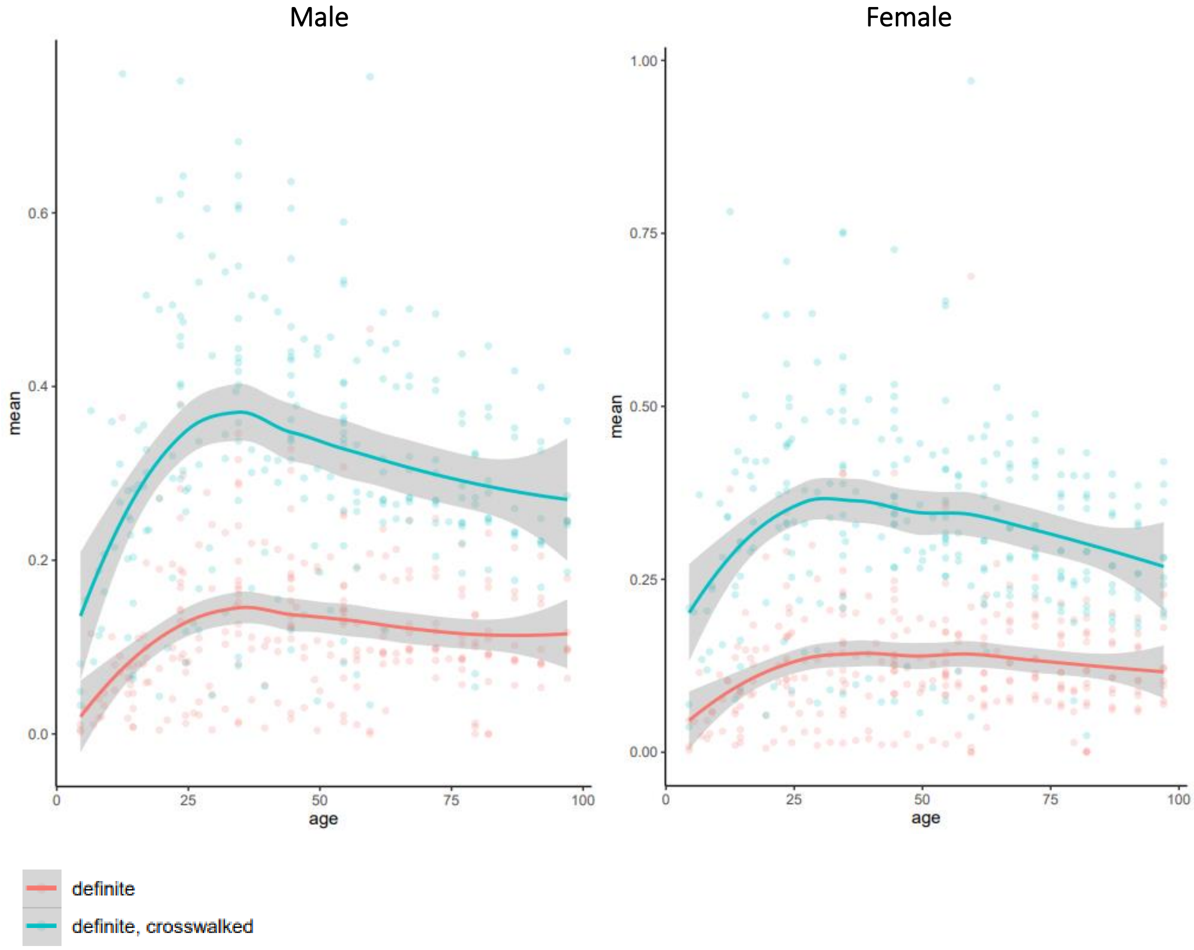

Modelling strategy

In GBD 2023, we made one significant update to the time-symptomatic analysis to better capture differences in headache frequency and duration by age and sex (described below). This is a substantial update compared to previous GBD rounds. However, the remainder of the modelling strategy has largely remained the same compared to GBD 2019 and 2021, where standard DisMod-MR settings across all headache models included setting excess mortality to zero, and assuming that there was no incidence or prevalence before the age of 5 years. No covariates are used in any of the DisMod-MR models.

Migraine

We made no substantive changes in the modelling strategy of migraine from GBD 2019 and 2021. We continue to run separate DisMod-MR models for definite migraine, probable migraine, and the total migraine category and set an upper bound on remission of 0.1 across all models. After running the separate models, we then scaled the results of probable and definite headache to the total migraine envelope to ensure consistency.

Tension-type headache

We made no substantive changes in the modelling strategy of TTH from GBD 2019 and 2021. We ran separate DisMod-MR models for definite TTH, probable TTH, and the total TTH category, setting an upper bound on remission of 0.5 across all models. After running the separate models, we then scaled the results of probable and definite headache to the total headache envelope to ensure consistency.

Medication overuse headache

We made no substantive changes in the modelling strategy since GBD 2017. Prior settings in the DisMod-MR model included an upper bound on remission of 0.4.

As medication overuse headache can develop from migraine or tension-type headache, we split medication overuse into sequelae of both primary headache disorders, specifically medication overuse headache due to migraine and medication overuse headache due to TTH. Based on a 2017 meta-analysis of three sources, 73.2% (63.7–81.0) of medication overuse headache is assigned to medication overuse headache due to migraine. The forest plot is shown below.

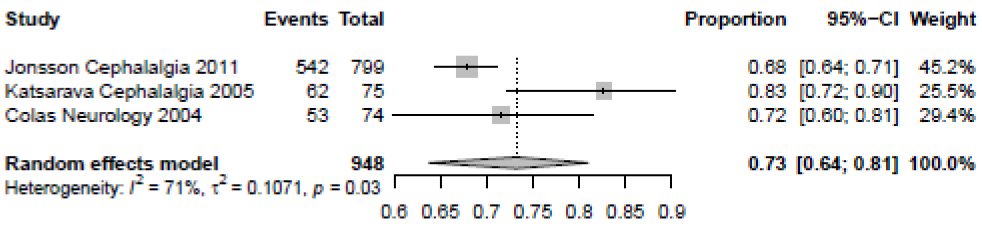

Time-symptomatic analysis

Starting from GBD 2023, we have updated our time-symptomatic analysis for migraine and TTH using multi-country survey unit-record data from 24 countries, with data from at least one country in each GBD super-region, in the Lift the Burden survey series provided by our collaborators. Prior to GBD 2023, only 19 country sources were used for this analysis to produce proportion of time symptomatic estimates for definite and migraine and TTH. Previously these estimates were not age- or sex-specific. In this updated analysis, we pooled observations from all 24 countries which resulted in 45,574 individual records and calculated the mean frequency and duration by sex and by three age groups (5–35, 35–49, and 50 and above). Proportion time-symptomatic values can be interpreted as the average percentage of time in a year a person is actively experiencing a given headache based on the case definitions. The updated proportion time-symptomatic are as below.

Table 3. Proportion of time-symptomatic by age, sex and headache type

	Definite migraine (SD)	Probable migraine (SD)	Definite TTH (SD)	Probable TTH (SD)
Male				
5–35 years	0.0634 (0.0044)	0.0360 (0.0034)	0.0261 (0.0016)	0.0139 (0.0024)

35–49 years	0.0786 (0.0057)	0.0336 (0.0030)	0.0248 (0.0018)	0.0099 (0.0012)
50 and above	0.0867 (0.0087)	0.0604 (0.0078)	0.0355 (0.0029)	0.0244 (0.0058)
Female				
5–35 years	0.0930 (0.0039)	0.0565 (0.0035)	0.0398 (0.0021)	0.0188 (0.0023)
35–49 years	0.1013 (0.0043)	0.0519 (0.0033)	0.0359 (0.0022)	0.0215 (0.0037)
50 and above	0.1277 (0.0071)	0.0716 (0.0056)	0.0456 (0.0033)	0.0356 (0.0085)

For medication overuse headache, no updates have been made this round. We continue to use proportion of time symptomatic of 0.532, across age and sex, based on meta-analysed results from two available studies on frequency and used the one available study on duration.

Proportions are then applied to prevalence of each headache respectively to produce symptomatic prevalence of each headache type.

Headache severity distribution

Table 4. Severity distribution, details on the severity levels for headaches and the associated disability weight (DW) with that severity.

**Severity level**	**Lay description**	**DW (95% CI)**
Symptomatic probable migraine	has severe, throbbing head pain and nausea that cause great difficulty in daily activities and sometimes confine the person to bed. Moving around, light, and noise make it worse.	0.441 (0.294–0.588)
Symptomatic definite migraine	has severe, throbbing head pain and nausea that cause great difficulty in daily activities and sometimes confine the person to bed. Moving around, light, and noise make it worse.	0.441 (0.294–0.588)
Symptomatic medication overuse headache due to migraine	has daily headaches, felt as dull pain and often lasting all day, with poor sleep, nausea and fatigue. The person takes medicine for the headaches, which provides little relief but is needed to avoid having worse symptoms.	0.223 (0.146–0.313)
Symptomatic probable tension-type headache	has a moderate headache that also affects the neck, which causes difficulty in daily activities.	0.037 (0.022–0.057)
Symptomatic definite tension-type headache	has a moderate headache that also affects the neck, which causes difficulty in daily activities.	0.037 (0.022–0.057)
Symptomatic medication overuse headache due to tension-type headache	has daily headaches, felt as dull pain and often lasting all day, with poor sleep, nausea and fatigue. The person takes medicine for the headaches, which provides little relief but is needed to avoid having worse symptoms.	0.223 (0.146–0.313)

References

[1]Kim B-K, Chung YK, Kim J-M, Lee K-S, Chu MK. Prevalence, clinical characteristics and disability of migraine and probable migraine: A nationwide population-based survey in Korea. *Cephalalgia* 2013; **33**: 1106–16. https://doi.org/10.1016/S0140-6736(20)30925-9[1]

[2] Lantéri-Minet M, Valade D, Géraud G, Chautard M, Lucas C. Migraine and probable migraine – results of FRAMIG 3, a French nationwide survey carried out according to the 2004 IHS classification. *Cephalalgia*; **25**: 1146–58.

[3] Pfaffenrath V, Fendrich K, Vennemann M, *et al.* Regional variations in the prevalence of migraine and tension-type headache applying the new IHS criteria: the German DMKG Headache Study. *Cephalalgia*; **29**: 48–57.

[4] Rasmussen BK, Jensen R, Olesen J. A Population-Based Analysis of the Diagnostic Criteria of the International Headache Society. *Cephalalgia* 1991; **11**: 129–34.

[5] Fendrich K, Vennemann M, Pfaffenrath V, *et al.* Headache Prevalence Among Adolescents — The German DMKG Headache Study. *Cephalalgia* 2007; **27**: 347–54.

[6] Vos T, Lim SS, Abbafati C, *et al.* Global burden of 369 diseases and injuries in 204 countries and territories, 1990–2019: a systematic analysis for the Global Burden of Disease Study 2019. *The Lancet* 2020; **396**: 1204–22. https://doi.org/10.1016/S0140-6736(20)30925-9

[7] Steiner TJ, Stovner LJ et al [2013]. Improving quality in population surveys of headache prevalence, burden, and cost: key methodological considerations. *J Headache Pain*, 14: 87.

Hearing impairment

Flowchart

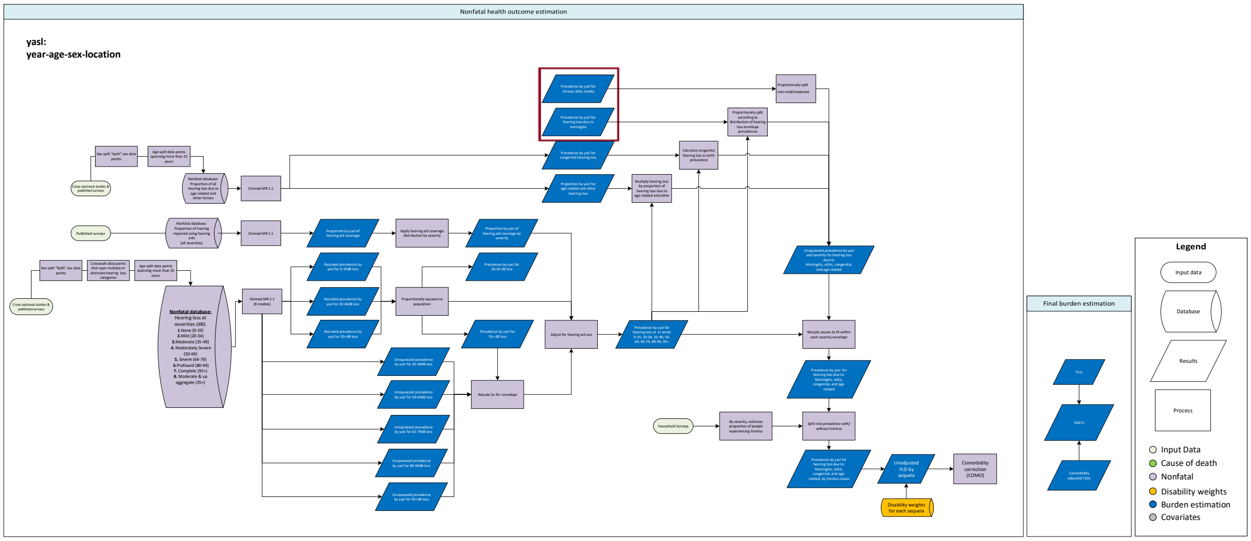

Case definition

Hearing impairment is defined as the biologically irreversible loss of the ability to perceive externally produced sounds, either through damage to the middle or inner ear or to neural circuitry underlying hearing. Hearing loss can be caused by genetics, altered neonatal development, loss of inner ear hair cells, or infection, and is common during ageing. Hearing impairment is an estimation of the prevalence of hearing loss at a range of severities, as measured by the softest sound that an individual can hear in their better ear, taken as the average across frequencies from 500 to 4000 hertz.

Hearing impairment is modelled for every year, age, sex, and location in the following severity categories:

Table 1: Severity thresholds of hearing loss

Severity thresholds of interest for hearing loss	
**Severity**	**Threshold (in decibels)**
None	0–19
Mild	20–34
Moderate	35–49
Moderately severe	50–64
Severe	65–79
Profound	80–94
Complete	95+

For data with other decibel ranges, we adjust to the ranges in Table 1. We modelled the following causes of hearing loss: congenital, meningitis, otitis, and age-related, and other. Congenital hearing loss is defined as hearing loss present at birth. Age-related and other hearing loss includes causes not identified as meningitis, otitis, or congenital. This includes presbycusis, the gradual loss of hearing with age, caused by breakdown of sensory receptors in the inner ear. For all causes, we estimate hearing loss with and without tinnitus, the perception of noise or ringing in the ears.

Unadjusted estimates of the prevalence of hearing loss due to meningitis and chronic otitis media are produced separately as part of each underlying cause's modelling process, as described in their respective sections. Along with the congenital and age-related aetiologies, these unadjusted estimates are incorporated into the overall hearing loss model, as detailed below.

Input data and processing

Studies on hearing loss typically report the prevalence of hearing loss by severity, in categories that are mutually exclusive and exhaustive. The severity grouping that an individual is put into depends on the softest decibel level at which they can hear a sound. However, these severity groupings are not standardised across literature. For example, one study may report the prevalence of mild, moderate, and severe hearing loss across the range of decibels. Another study may simply report the prevalence of the study population with no hearing loss and those who have hearing loss, regardless of range. To standardise severity groupings, we established seven mutually exclusive and exhaustive categories that the GBD would use to model and report the severity of hearing loss. These are referred to as "severity-specific envelopes". The range of decibel values applicable to each severity category can be seen in table 1.

For the estimation of severity-specific envelopes, we used prevalence measurements and individual-level data extracted from published surveys identified in a series of systematic reviews, or from sources provided by the GBD Collaborator Network.

Data sources up to 2008 were identified by a published systematic review (http://www.ncbi.nlm.nih.gov/pubmed/19444763). For GBD 2013, we conducted a systematic review covering 2008–2013 with the following search terms:

> (hearing impairment[Title/Abstract] OR deafness[Title/Abstract] OR hearing loss[Title/Abstract]) AND (prevalence[Title/Abstract]) AND ("2008"[PDAT] : "3000"[PDAT]) AND (cross sectional OR survey)

For GBD 2016, we conducted an additional systematic review using the following search terms:

(hearing impairment[Title/Abstract] OR deafness[Title/Abstract] OR hearing loss[Title/Abstract] OR audiometry[Title/Abstract]) AND (prevalence[Title/Abstract]) AND ("2008/11/26"[PDAT]: "3000"[PDAT]) AND (cross sectional OR survey)

This was conducted on November 30, 2016, and returned 239 results, of which 17 were accepted.

For GBD 2021, we conducted an additional systematic review using the following search terms:

hearing imp*[Title/Abstract] OR deaf*[Title/Abstract] OR hearing loss[Title/Abstract] OR audio*[Title/Abstract]) AND (prevalen*[Title/Abstract]) AND ("2016/11/16"[PDAT] : "3000"[PDAT]) AND (cross sectional OR survey)

This was conducted on November 5, 2019, and returned 431 results, of which 17 were accepted. The PRISMA diagram for this systematic review is shown below.

Figure 1: PRISMA flow diagram

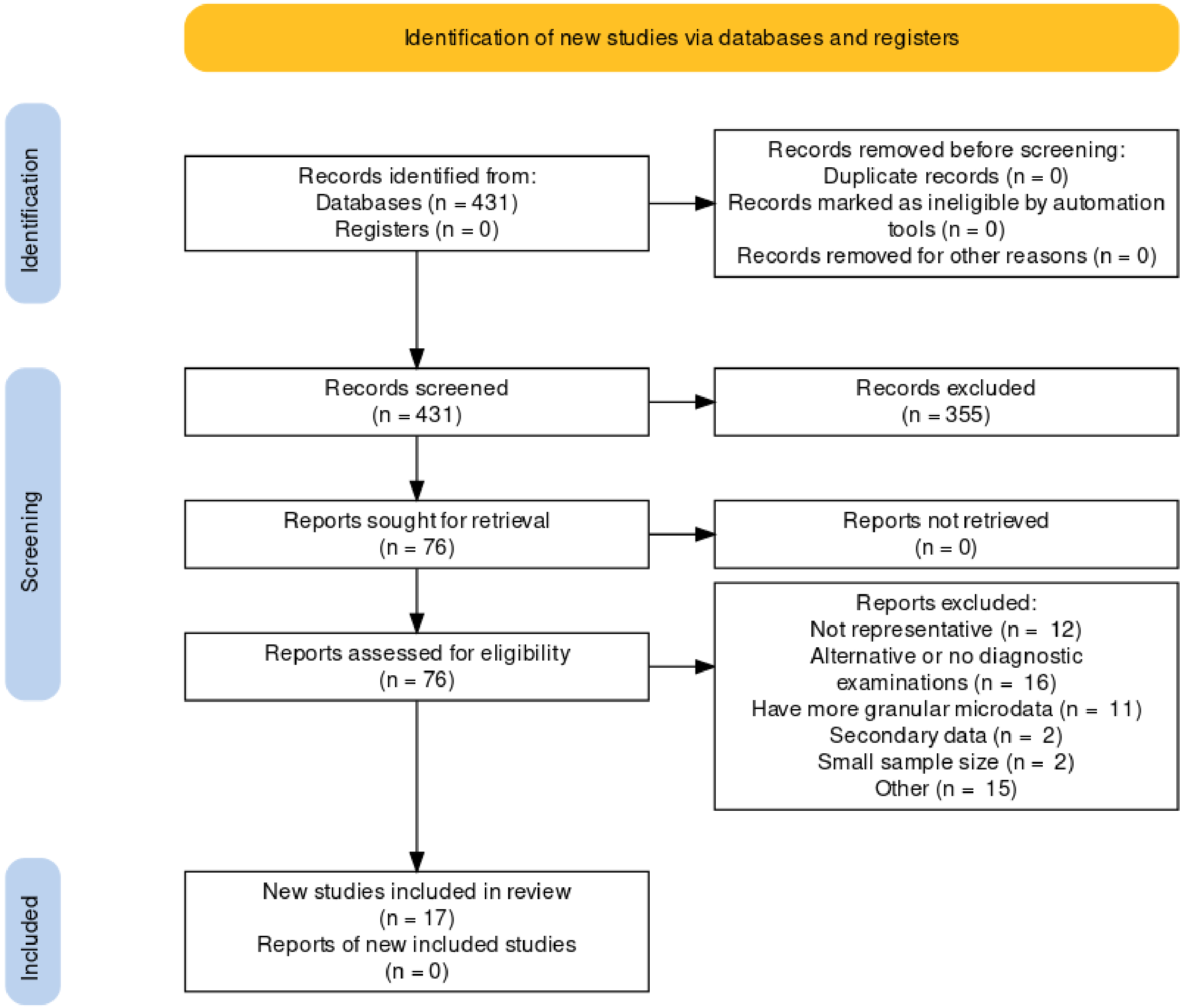

In addition to the search-string hits above, we identified household surveys that measured hearing loss – the United States National Health and Examination Surveys (NHANES) and the Health Survey for England (HSE) – and extracted prevalence measurements from individual-level data.

Self-reported hearing loss data were excluded. This includes censuses in the Integrated Public Use Microdata Series (IPUMS), the WHO Studies on Global Ageing and Adult Health (SAGE), and the WHO Multi-Country Survey Study on Health and Responsiveness (MCSS). Self-reported use of hearing aids (such as in MCSS, SAGE, and NHANES), however, was used to estimate hearing aid coverage.

For studies that did not report prevalence by sex, datapoints were split by sex by running a regression on the log ratio of female/male prevalence within each severity-specific dataset using MR-BRT (meta-regression—Bayesian, regularised, trimmed; for methods details, see appendix 1, section 2), then applying this ratio to non-specific data (Table 2). Datapoints that also reported data in wide age groups (>25 years) were split into five-year age bins by applying the age pattern of the best severity-specific GBD 2019 model.

Table 2. Sex-split coefficients for hearing loss models, with 95% UIs, log-space

**Model**	**Sex split model coefficient, log-space (95% UI)**
Hearing loss, 0–19 dB	0.0171 (–0.0163 to 0.0505)
Hearing loss, 20–34 dB	–0.211 (–0.583 to 0.161)
Hearing loss, 35–49 dB	–0.240 (–0.511 to 0.031)
Hearing loss, 35+ dB	–0.139 (–0.48 to 0.202)
Hearing loss, 50–64 dB	–0.468 (–0.629 to –0.307)
Hearing loss, 65–79 dB	–0.383 (–0.536 to –0.230)
Hearing loss, 80–94 dB	–0.0738 (–0.562 to 0.414)
Hearing loss, 95+ dB	–0.149 (–0.317 to 0.019)

Where studies reported hearing loss spanning multiple thresholds (eg, 80+, rather than 80–94 and 95+) or severity categories that did not align with GBD thresholds, we crosswalked data with the MR-BRT methodology to the appropriate GBD severity categories.

To create adjustment factors between alternate and reference threshold categories, we used microdata extracted from NHANES surveys. These data reported the exact decibel at which each person experienced hearing loss. We estimated the prevalence of each alternate and reference severity category by aggregating microdata into groups specific to age and sex. The prevalent population for each alternate or reference category was composed of every individual that fell within the range of decibels for a given severity. Adjustment factors were estimated using univariate analysis as the logit difference between the prevalence of an alternate category and the prevalence of its corresponding reference category. A table of each adjustment factor can be found below.

Table 3: MR-BRT crosswalk adjustment factors

Reference Category (dB)	Alternate Category (dB)	Gamma	Beta coefficient, logit (95% UI)*	Adjustment factor**
0-19	0-24	0	0.60 (0.54 to 0.67)	1.82 (1.72 to 1.95)
	0-25	0	0.70 (0.64 to 0.77)	2.01 (1.90 to 2.16)
	0-29	0.23	1.13 (0.68 to 1.59)	3.10 (1.97 to 4.90)
	0-30	0.21	1.24 (0.83 to 1.68)	3.46 (2.29 to 5.37)
	0-39	0.91	1.67 (–0.04 to 3.58)	5.31 (0.96 to 35.87)
	0-40	0.96	1.71 (–0.05 to 3.53)	5.53 (0.95 to 34.12)
0-19	10-25	2.06	–1.34 (–1.40 to –1.36)	0.26 (0.25 to 0.26)
20-34	0-24	2.50	3.40 (–1.46 to 8.28)	29.96 (0.23 to 3944.19)
	0-25	2.45	3.49 (–1.53 to 8.29)	32.79 (0.22 to 3983.83)
	0-29	2.30	3.82 (–0.85 to 8.29)	45.60 (0.43 to 3983.83)

	0-30	2.27	3.89 (–0.24 to 8.42)	48.91 (0.79 to 4536.90)
	0-39	1.95	4.48 (0.61 to 8.55)	88.23 (1.84 to 5166.75)
	0-40	1.91	4.50 (0.86 to 8.14)	90.02 (2.36 to 3428.92)
	15-24	.42	0.28 (0.28 to 0.29)	1.32 (1.32 to 1.34)
	20-39	0.13	0.27 (0.02 to 0.52)	1.31 (1.02 to 1.68)
	20-40	0.15	0.29 (0.003 to 0.59)	1.34 (1.00 to 1.80)
	20-200	0.41	0.52 (–0.35 to 1.32)	1.68 (0.70 to 3.74)
	21-39	0.20	0.12 (–0.29 to 0.52)	1.13 (0.75 to 1.68)
	25-39	0.35	–0.39 (–1.04 to 0.34)	0.68 (0.35 to 1.40)
	26-30	0	–1.42 (–1.42 to –1.42)	0.24 (0.24 to 0.24)
	26-40	0.43	–0.50 (–1.36 to 0.28)	0.61 (0.26 to 1.32)
	26-99	0.84	–0.03 (–1.65 to 1.73)	0.97 (0.19 to 5.64)
	26-200	0.84	–0.03 (–1.74 to 1.54)	0.97 (0.18 to 4.66)
	30-40	0.56	–1.06 (–2.24 to 0.007)	0.35 (0.11 to 1.01)
	30-200	0.96	–0.37 (–2.12 to 1.43)	0.69 (0.12 to 4.18)
35-49	0-39	2.45	5.18 (0.16 to 10.08)	177.68 (1.17 to 23 860.99)
	0-40	2.42	5.24 (0.41 to 10.17)	188.67 (1.51 to 26 108.08)
	20-39	0.71	1.45 (0.04 to 2.85)	4.26 (1.04 to 17.29)
	20-40	0.69	1.49 (0.10 to 2.88)	4.44 (1.11 to 17.81)
	21-39	0.66	1.31 (0.02 to 2.67)	3.71 (1.02 to 14.44)
	25-39	0.54	0.76 (–0.27 to 1.93)	2.14 (0.76 to 6.89)
	26-40	0.51	0.67 (–0.30 to 1.75)	1.95 (0.74 to 5.75)
	30-40	0.47	0.09 (-0.89 to 1.05)	1.09 (0.41 to 2.86)
	31-50	0.52	0.10 (0.29 to 0.74)	1.11 (1.34 to 2.10)
	35-60	0	0.38 (0.38 to 0.39)	1.46 (1.46 to 1.48)
	40-64	0.37	–0.10 (–0.85 to 0.61)	0.90 (0.43 to 1.84)
	40-69	0.40	–0.04 (–0.82 to 0.811)	0.96 (0.44 to 2.25)
	41-55	0.32	–0.45 (–1.06 to 0.23)	0.64 (0.35 to 1.26)
	41-60	0.35	–0.29 (–0.99 to 0.37)	0.75 (0.37 to 1.45)
	41-70	0.44	–0.12 (–1.06 to 0.76)	0.89 (0.35 to 2.14)
50-64	31-60	0	0.82 (0.82 to 0.82)	2.27 (2.27 to 2.27)
50-64	35-60	0.03	1.52 (1.51 to 1.53)	4.57 (4.53 to 4.62)
50-64	40-64	0.27	1.13 (0.58 to 1.68)	3.10 (1.79 to 5.37)
	40-69	0.29	1.22 (0.64 to 1.80)	3.39 (1.90 to 6.05)
	41-55	0.4	0.72 (–0.09 to 1.53)	2.05 (0.91 to 4.62)
	41-60	0.31	0.92 (0.30 to 1.55)	2.51 (1.35 to 4.71)
	41-70	0.32	1.13 (0.49 to 1.77)	3.10 (1.63 to 5.87)
	41-80	0	1.27 (1.26 to 1.28)	3.56 (3.53 to 3.60)

	51-70	0.18	0.06 (–0.31 to 0.42)	1.06 (0.73 to 1.52)
	55-69	0.29	–0.42 (–1.00 to 0.15)	0.66 (0.37 to 1.16)
	56-70	0.33	–0.43 (–1.10 to 0.24)	0.65 (0.33 to 1.27)
65-79	40-69	0.77	2.44 (0.92 to 3.99)	11.47 (2.51 to 54.05)
	41-80	0	2.61 (2.59 to 2.63)	13.60 (13.33 to 13.87)
	51-70	0.67	1.35 (0.01 to 2.68)	3.86 (1.01 to 14.59)
	55-69	0.69	0.86 (–0.53 to 2.24)	2.36 (0.59 to 9.39)
	56-70	0.66	0.84 (–0.47 to 2.16)	2.32 (0.63 to 8.67)
	61-80	0.19	0.35 (–0.04 to 0.72)	1.42 (0.96 to 2.05)
	61-99	0.14	0.46 (0.17 to 0.75)	1.58 (1.19 to 2.12)
	65-84	0.02	0.03 (–0.01 to 0.08)	1.03 (0.99 to 1.08)
	70-89	0.21	–0.20 (–0.63 to 0.22)	0.82 (0.53 to 1.25)
	70-94	0.21	–0.20 (–0.62 to 0.24)	0.82 (0.54 to 1.27)
	70-95	0.21	–0.20 (–0.63 to 0.23)	0.82 (0.53 to 1.26)
	71-90	0.3	–0.26 (–0.86 to 0.34)	0.77 (0.42 to 1.40)
	71-99	0.3	–0.16 (–0.75 to 0.44)	0.85 (0.47 to 1.55)
	71-200	0.31	–0.19 (–0.81 to 0.42)	0.83 (0.44 to 1.52)
80-94	61-99	1.01	1.58 (–0.42 to 3.58)	4.85 (0.66 to 35.87)
	65-84	0.91	0.92 (–0.89 to 2.73)	2.51 (0.41 to 15.33)
	70-89	0.81	0.54 (–1.06 to 2.14)	1.72 (0.35 to 8.50)
	70-94	0.73	0.44 (–1.01 to 1.88)	1.55 (0.36 to 6.55)
	70-95	0.73	0.44 (–1.00 to 1.89)	1.55 (0.37 to 6.62)
	71-90	0.61	0.25 (–0.96 to 1.45)	1.28 (0.38 to 4.26)
	71-99	0.61	0.37 (–0.83 to 1.58)	1.45 (0.44 to 4.85)
	71-200	0.66	0.41 (–0.88 to 1.71)	1.51 (0.41 to 5.53)
	80-200	0	0.00 (–0.04 to 0.04)	1.00 (0.96 to 1.04)
	81-90	0	–0.027(–0.32 to 0.26)	0.97 (0.72 to 1.30)
	81-99	0	$–3.92e^{-16}$ (–0.04 to 0.03)	1.00 (0.96 to 1.03)
	81-200	0	0.00 (–0.04 to 0.04)	1.00 (0.96 to 1.04)
	85-200	0	$–4.37e^{-24}$ (–0.04 to 0.04)	1.00 (0.96 to 1.04)
	90-99	0	0.00 (–0.03 to 0.03)	1.00 (0.97 to 1.03)
	90-200	0	0.00 (–0.03 to 0.03)	1.00 (0.97 to 1.03)
35-200	15-200	0	2.70 (2.69 to 2.70)	14.88 (14.73 to 14.88)
35-200	20-200	0.15	1.79 (1.48 to 2.10)	5.99 (4.39 to 8.17)
	25-200	0	1.17 (1.16 to 1.17)	3.22 (3.19 to 3.22)

	26-200	0.14	1.02 (0.73 to 1.31)	2.77 (2.08 to 3.71)
	26-99	0.14	1.02 (0.73 to 1.31)	2.77 (2.08 to 3.71)
	30-200	0.07	0.55 (0.40 to 0.70)	1.73 (1.49 to 2.01)
	31-200	0.05	0.43 (0.33 to 0.54)	1.54 (1.39 to 1.72)
	31-99	0.04	0.44 (0.34 to 0.54)	1.55 (1.40 to 1.72)
	40-200	0.04	–0.49 (–0.58 to –0.39)	0.61 (0.56 to 0.68)
	40-99	0.05	–0.48 (–0.59 to –0.38)	0.62 (0.55 to 0.68)
	41-200	0.09	–0.59 (–0.78 to –0.39)	0.55 (0.46 to 0.68)
	41-99	0.10	–0.58 (–0.78 to –0.39)	0.56 (0.46 to 0.68)
95-2000	61-99	0.80	2.42 (0.84 to 4.03)	11.25 (2.32 to 56.26)
	71-99	0.90	0.65 (–1.14 to 2.43)	1.92 (0.32 to 11.36)
	71-200	0.88	0.60 (–1.13 to 2.33)	1.82 (0.32 to 10.28)
	80-200	0.22	0.08 (–0.34 to 0.52)	1.08 (0.71 to 1.68)
	81-99	0.21	0.08 (–0.35 to 0.50)	1.08 (0.70 to 1.65)
	81-200	0.18	0.05 (–0.30 to 0.41)	1.05 (0.74 to 1.51)
	85-200	0	0.00 (–0.04 to 0.04)	1.00 (0.96 to 1.04)
	90-99	0	0.00 (–0.02 to 0.02)	1.00 (0.98 to 1.02)
	90-200	0	0.00 (–0.02 to 0.02)	1.00 (0.98 to 1.02)
	91-99	0	0.00 (–0.03 to 0.02)	1.00 (0.97 to 1.02)
	91-200	0	0.00 (–0.02 to 0.02)	1.00 (0.98 to 1.02)
	95-99	0	0.00 (–0.02 to 0.02)	1.00 (0.98 to 1.02)
	96-99	0	0.00 (–0.02 to 0.02)	1.00 (0.98 to 1.02)

**MR-BRT crosswalk adjustments can be interpreted as the factor the alternative case definition is adjusted by to reflect what it would have been had it been measured using the reference case definition. If the log/logit beta coefficient is negative, then the alternative is adjusted up to the reference. If the log/logit beta coefficient is positive, then the alternative is adjusted down to the reference.*

***The adjustment factor column is the exponentiated beta coefficient. For log beta coefficients, this is the relative rate between the two case definitions. For logit beta coefficients, this is the relative odds between the two case definitions.*

Modelling strategy

We modelled the prevalence of hearing loss over five steps. First, we ran three DisMod-MR 2.1 (disease model—Bayesian meta-regression; for methods description, see appendix 1, section 2) models to estimate the total prevalence of the following levels of hearing by y-a-s-l: normal hearing (0–19 dB), mild hearing loss (20–34 dB), and moderate hearing loss and above (35+ dB). For normal hearing loss (0–19 dB), DisMod-MR 2.1 had trouble fitting prevalence values close to 100% in very young ages. Initial models attempted to follow lower prevalence datapoints in teen and middle-aged populations, and resulting estimates of the prevalence of normal hearing in infants were implausible in the face of the data. As a solution, we modelled all data adjusted to the normal hearing loss category as 1 – prevalence, to accommodate for the fact that DisMod-MR 2.1 interacts better with datapoints at lower values. We then took the complement of the fitted model at the draw level to obtain normal hearing prevalence estimates. Next, we rescaled the prevalence estimates from the three models (0–19, 20–34, 35+) to sum to 1 for every year, age, sex, and location. We estimated prevalence of normal hearing for the purpose of correctly scaling the other two models only, and hence it did not form part of further analysis.

These three models used Socio-demographic Index (SDI) as a covariate. The estimated betas are shown in the table below.

Table 4: Covariates

Model	Covariate name	Measure	Beta value	Exponentiated value
Hearing loss impairment at 0-19 dB	Socio-demographic Index	Prevalence	0.0035 (0.00018 to 0.0096)	1.00 (1.00 to 1.01)
Hearing loss impairment at 35+ dB	Socio-demographic Index	Prevalence	–0.21 (–0.53 to –0.013)	0.81 (0.59 to 0.99)
Hearing loss impairment at 95+ dB	Socio-demographic Index	Prevalence	–1.9 (–2.0 to –1.72)	0.15 (0.14 to 0.18)

Second, we ran five additional DisMod-MR 2.1 models for each severity level of hearing loss greater than mild hearing loss: moderate (35–49 dB), moderately severe (50–64 dB), severe (65–79 dB), profound (80–94 dB), and complete (95+ dB). We then rescaled the prevalence estimates from these models to fit within the prevalence estimated for 35+ dB in the first step. By the end of the second step, we had estimated prevalence of six severity levels of hearing loss, including mild (20–34 dB).

Third, we ran two additional DisMod-MR 2.1 models to (1) estimate the proportion of the hearing impaired who use a hearing aid, deemed "hearing aid coverage", and (2) estimate the proportion of hearing loss across all severities that is attributable to age-related and other factors.

Fourth, we adjusted the prevalence of each of the six hearing loss severity levels estimated in steps one and two to account for hearing aid use. To do this, we made the assumption that the use of a hearing aid reduces the severity of impairment by one category. The model used to estimate hearing aid

coverage represents *all* severity categories. To estimate the proportion of hearing aid coverage for *each* severity category, we used data obtained from the Nord-Trondelag study and NHANES surveys. These two sources provided detailed information on hearing aid coverage among the impaired by age, sex, and most important, severity. We ran a logistic regression on age with binary indicators for severity levels and sex. Outputs of this regression were the proportion of individuals at every severity of hearing impairment that used a hearing aid. We assumed that 0% of people in the completely deaf category (95+) used a hearing aid. We then took estimates of hearing aid coverage that were produced in step 3 and scaled the estimate by dividing the value produced in each location by the value produced for Norway. This was to correct for any bias created by using adjustment factors calculated primarily with data from Norway. From there, we multiplied the scaled value of hearing aid coverage for each location by each of the six proportions of severity-specific coverage. This gave us the proportion of individuals in each severity category that use a hearing aid. Then, we shifted the identified fraction of people in each severity category that used a hearing aid to the category directly below. This provided the adjusted prevalence of six severity levels of all-cause hearing loss.

Fifth, we estimated the prevalence of hearing loss due to multiple causes: otitis media, congenital, meningitis, and age-related and other causes not classified elsewhere. In GBD 2017, we estimated the prevalence of hearing loss for each subtype of meningitis (pneumococcal, *H influenzae* type B meningitis, meningococcal, and other bacterial), but from GBD 2019 onward, we estimated the prevalence of hearing loss for meningitis as a whole. See the meningitis cause write-up for further details. For congenital hearing loss, we assumed that all hearing loss occurring at the time of birth is of congenital nature. We also assumed that all hearing loss due to otitis media is at the mild or moderate level. Because data on the aetiology of hearing loss are more stable in younger ages, up to the age of 20, we implemented proportional squeezes to scale cause-specific hearing loss prevalence to the total prevalence of each severity level. Above age 20, we subtracted the prevalence of congenital hearing loss, meningitis, and otitis from the total and called any remainder age-related and other hearing loss. Since we ensured that congenital prevalence was constant in each age group for every location, year, and sex combination after conducting the proportional squeeze, the sum of the prevalence of all hearing loss aetiologies sometimes exceeded the total prevalence of some severity levels.

Finally, we estimated the percentage of people experiencing tinnitus. We determined the proportion of people suffering from tinnitus using data from NHANES years that asked about the frequency each survey respondent heard ringing, roaring, and/or buzzing (1999, 2001, 2003, and 2011–2012). We labelled anyone with mild hearing loss and ringing, roaring, or buzzing "at least once a month" as a mild hearing loss with tinnitus case. Anyone with moderate hearing through to severe hearing loss and ringing, roaring, or buzzing "at least once a day" was labelled as a moderate hearing loss with tinnitus case. Anyone with complete hearing loss who responded that they "almost always" had ringing or buzzing was labelled as a complete hearing loss with tinnitus case. Using the data from NHANES, we calculated confidence intervals assuming a binomial distribution. We assumed the same distribution of tinnitus across all aetiologies of hearing loss. This is the same strategy used in previous GBD cycles.

Table 5: Health states and disability weights

Health state name	Health state description	Disability weight
Hearing loss, mild	has great difficulty hearing and understanding another person talking in a noisy place (for example, on an urban street).	0.010 (0.004–0.019)

Hearing loss, mild, with ringing	has great difficulty hearing and understanding another person talking in a noisy place (for example, on an urban street), and sometimes has annoying ringing in the ears.	0.021 (0.012–0.036)
Hearing loss, moderate	is unable to hear and understand another person talking in a noisy place (for example, on an urban street), and has difficulty hearing another person talking even in a quiet place or on the phone.	0.027 (0.015–0.042)
Hearing loss, moderate, with ringing	is unable to hear and understand another person talking in a noisy place (for example, on an urban street), and has difficulty hearing another person talking even in a quiet place or on the phone, and has annoying ringing in the ears for more than 5 minutes at a time, almost every day.	0.074 (0.048–0.107)
Hearing loss, moderately severe	(custom DW from hearing loss impairment envelope)	0.092 (0.064–0.129)
Hearing loss, moderately severe, with ringing	(custom DW from hearing loss impairment envelope)	0.167 (0.114–0.231)
Hearing loss, severe	is unable to hear and understand another person talking, even in a quiet place, and unable to take part in a phone conversation. Difficulties with communicating and relating to others cause emotional impact at times (for example worry or depression).	0.158 (0.104–0.227)
Hearing loss, severe, with ringing	is unable to hear and understand another person talking, even in a quiet place, and unable to take part in a phone conversation, and has annoying ringing in the ears for more than 5 minutes at a time, almost every day. Difficulties with communicating and relating to others cause emotional impact at times (for example worry or depression).	0.261 (0.174–0.361)
Hearing loss, profound	is unable to hear and understand another person talking, even in a quiet place, is unable to take part in a phone conversation, and has great difficulty hearing anything in any other situation. Difficulties with communicating and relating to others often cause worry, depression, and loneliness.	0.204 (0.134–0.288)
Hearing loss, profound, with ringing	is unable to hear and understand another person talking, even in a quiet place, is unable to take part in a phone conversation, has great difficulty hearing anything in any other situation, and has annoying ringing in the ears for more than 5 minutes at a time, several times a day. Difficulties with communicating and relating to others often cause worry, depression, or loneliness.	0.277 (0.182–0.388)
Hearing loss, complete	cannot hear at all in any situation, including even the loudest sounds, and cannot communicate verbally or use a phone. Difficulties with communicating and relating to others often cause worry, depression, or loneliness.	0.215 (0.143–0.307)
Hearing loss, complete, with ringing	cannot hear at all in any situation, including even the loudest sounds, and cannot communicate verbally or use a phone, and has very annoying ringing in the ears for more than half of the day. Difficulties with communicating and relating to others often cause worry, depression, or loneliness.	0.316 (0.211–0.436)

Heart failure impairment

Flowcharts

Overall modelling strategy

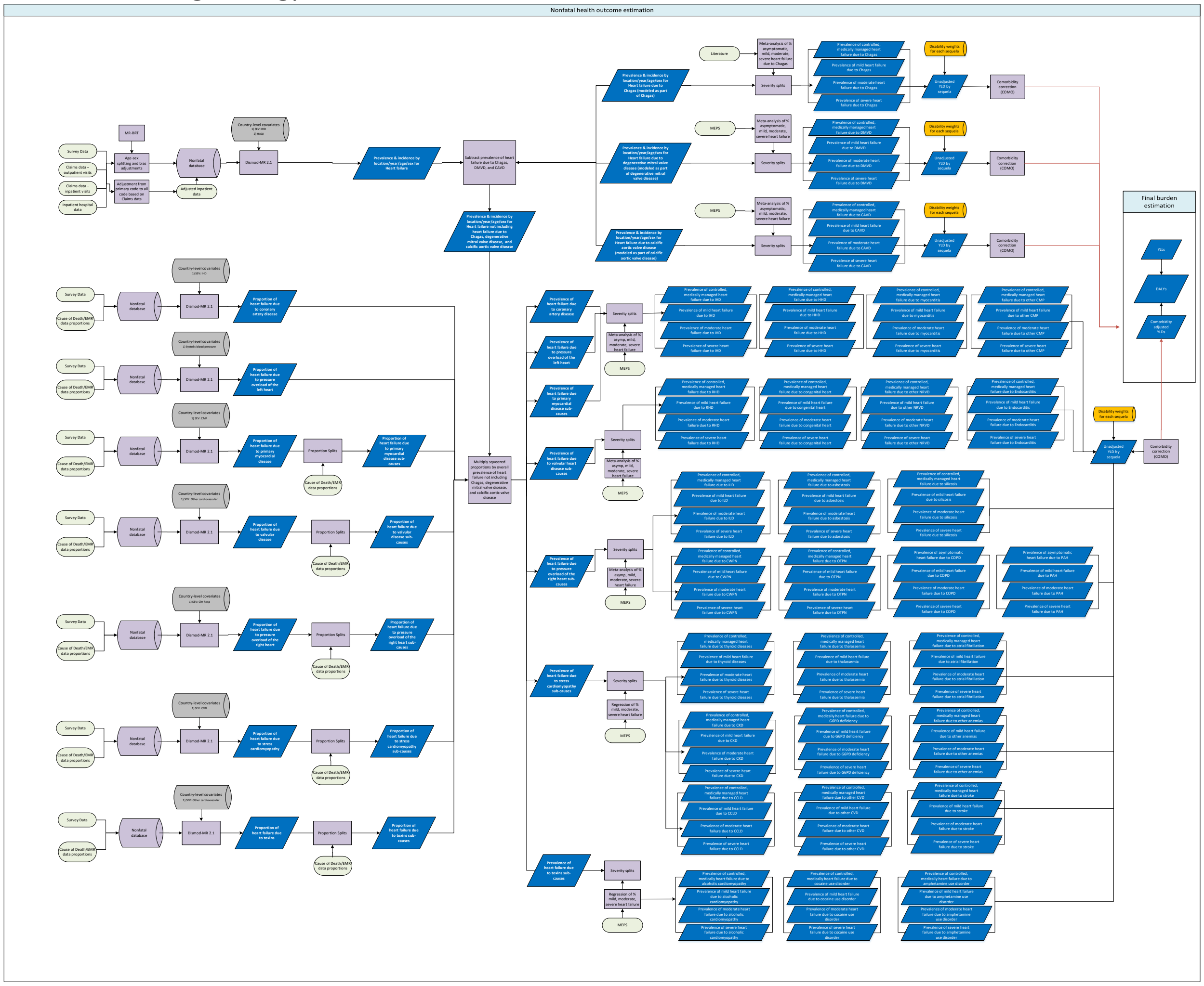

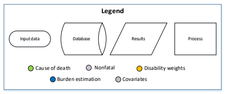

Abbreviations

DMVD: degenerative mitral valve disease; CAVD: calcific aortic valve disease; IHD: ischaemic heart disease; CMP: cardiomyopathy and myocarditis; HHD: hypertensive heart disease; ILD: interstitial lung disease; CWPN: coal workers pneumoconiosis; OTPN: other pneumoconiosis; COPD: chronic obstructive pulmonary disease; RHD: rheumatic heart disease; CVD: cardiovascular disease; NRVD: non-rheumatic valve disease; PAH: pulmonary arterial hypertension; CKD: chronic kidney disease; CCLD: cirrhosis and other chronic liver diseases.

Proportion splits and correction factor estimation

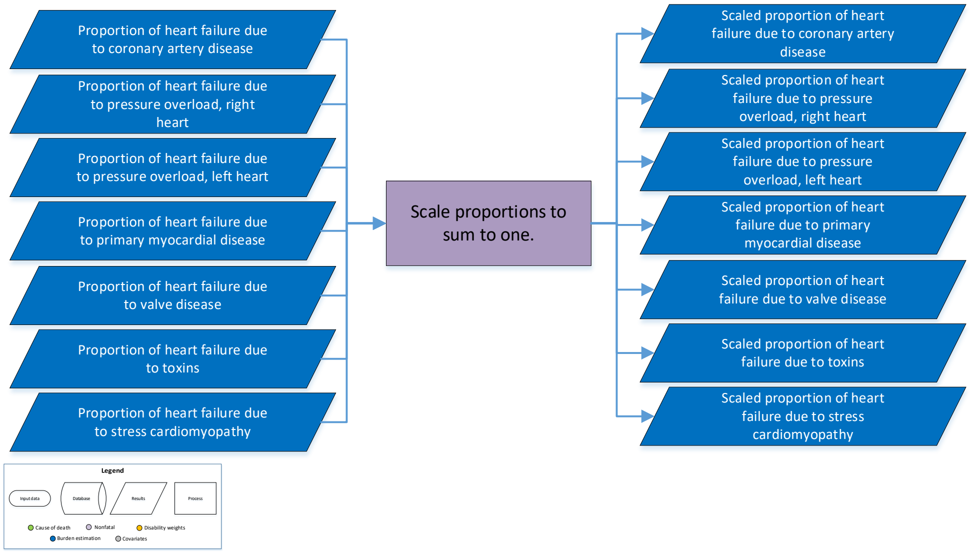

Case definition

Prevalent heart failure (HF) was defined in GBD according to the Universal Definition of Heart Failure as a clinical syndrome with signs or symptoms due to structural and/or functional cardiac abnormality and corroborated by elevated natriuretic peptide levels, objective evidence of pulmonary or systemic congestion, or echocardiographic studies.[1] This is inclusive of structural heart disease with current or previous symptoms of HF (ACC/AHA Stage C and D).[2] It does not capture cases with evidence of structural heart disease, abnormal cardiac function, or elevated natriuretic peptide levels but who have not yet experienced clinical signs and symptoms (ACC/AHA Stage B). Reference and alternative case definitions are shown in Table 1.

Table 1: Reference and alternative case definitions for heart failure

**Condition**	**Reference or Alternative**	**Definition**
Heart failure	Reference	Heart failure as diagnosed by clinicians using structured criteria such as the Framingham[3] or European Society of Cardiology[4] criteria. Prevalent heart failure was defined as structural heart disease with current or previous symptoms of HF or marked HF symptoms that interfere with daily life and with recurrent hospitalisations despite attempts to optimise treatment.
Heart failure	Alternative	Heart failure prevalence as coded in administrative health facility data.

Input data

Data acquisition

A systematic review was performed GBD 2016 and updated in GBD 2021, along with an unstructured review in 2019. In 2016, the search terms used were: "heart failure"[TIAB] AND (epidemiology[MeSH Terms] OR prevalence[TIAB] OR incidence[TIAB] OR mortality[TIAB]) AND ("1990/01/01"[PDAT] : "2016/09/02"[PDAT]) NOT “animal model” NOT rat NOT mice NOT diabetes[TIAB] NOT “renal transplant”[TIAB]. The dates of the search were 01/01/1990 through 09/02/2016. 37,891 initial hits

were returned, and 57 sources were added. An unstructured review yielded an additional 30 sources, of which six were extracted. In 2019, a review of 8 systematic review articles yielded 519 sources to review, of which 14 were extracted. In 2020, the search terms were: ("heart failure"[TIAB] OR “cardiac failure”[TIAB]) AND (epidemiology[MeSH Terms] OR prevalence[TIAB] OR incidence[TIAB] OR “excess mortality”[TIAB] OR “case fatality”[TIAB]) AND ("2016/01/01"[PDAT] : "2020/1/2"[PDAT]) NOT “animal model” NOT rat NOT mice NOT diabetes[TIAB] NOT “renal transplant”[TIAB]. 4469 initial hits were returned, and 27 sources were added.

Figure 1: PRISMA 2020 flow diagram

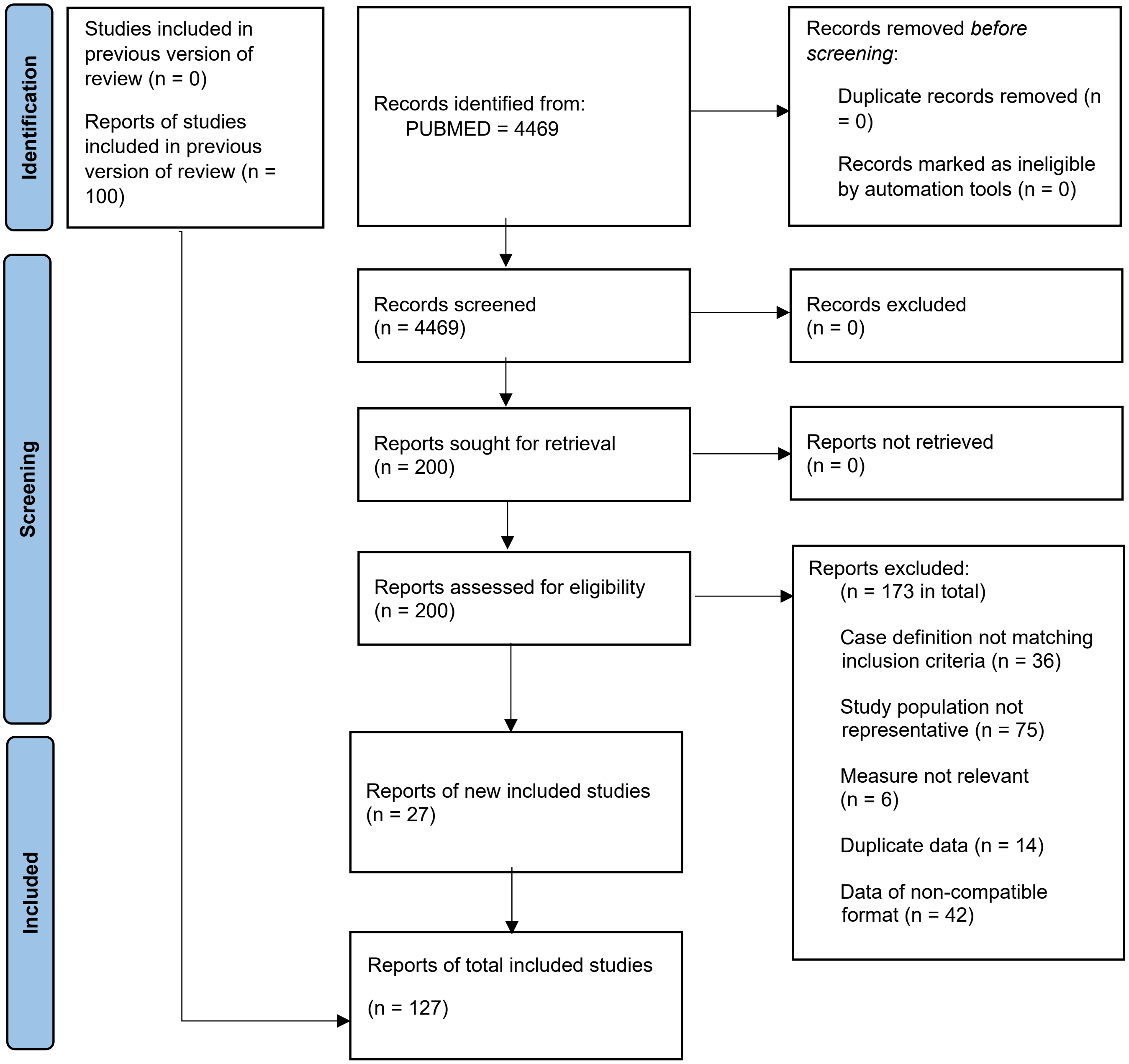

The final dataset also included ICD-coded administrative health facility data. Inpatient hospital data were corrected for readmission, primary diagnosis to any diagnosis ratios, and inpatient to outpatient utilisation ratios using adjustment factors calculated from individual-level claims data. Descriptions of search strategies and the methodology used to process these data are described in Appendix 1, Section 2 of this manuscript. Administrative health facility data were excluded if the facilities were not representative of the national population.

Additionally, we used the following data sources to estimate the proportion of heart failure attributable to each aetiology: 1) vital registration (VR) data from Mexico, Brazil, Taiwan, Colombia, and the USA; and 2) linked inpatient admissions and VR data from Friuli Venezia, Italy.

Data processing
We used the modelling software meta-regression—Bayesian, regularised, trimmed (MR-BRT) to process data in preparation for modelling, including 1) bias adjustment for datapoints using alternative case definitions, 2) splitting both-sex datapoints into sex-specific estimates, and 3) splitting datapoints with an age range of greater than 25 years. Details of MR-BRT and the adjustment processes can be found in Appendix 1, Section 2. Table 2 shows MR-BRT crosswalk adjustment factors; the formula for computing adjustment factors for prevalence is given in Equation 1. Age-splitting was based on the global sex-specific age pattern obtained from a single-parameter disease model—Bayesian meta-regression (DisMod-MR 2.1) model computed using prevalence data with age ranges of less than 25 years.

Equation 1: Calculation of adjustment factors**:**

$$Estimated\ Reference\ Def\ = invlogit(logit(Alternative\ Def) - Beta_{Alternative\ Def} - Beta_{Age_{scaled}} * Age\ Scaled)$$

Table 2: MR-BRT crosswalk adjustment factors for heart failure prevalence

Data input	Reference or alternative case definition	Gamma	Beta coefficient, logit (95% CI)
Structured criteria	Reference	0.04	---
Inpatient data	Alternate		–0.249 (–0.441, –0.057)
Age, scaled			0.093 (0.081, 0.105)

Modelling strategy

Overview
To estimate the burden of heart failure due to each of 27 underlying causes (Table 3), we first estimated the overall prevalence of heart failure and then the proportion of heart failure each cause was responsible for. The latter process includes an initial assessment of the fraction of heart failure cases attributable to each of seven high-level parent cause groupings, followed by further division into the detailed causes within each of these groupings. The selection for aetiological causes was based on a review of the literature, a quantitative analysis of the causes most commonly co-occurring with HF in 93 location-years of death certificates and inpatient records, and expert opinion regarding diseases that lead to clinical heart failure. In our review of the literature, we identified diseases whose biological mechanisms are proven to cause heart failure. In our quantitative analysis, we assessed death certificate records with both underlying and contributing causes of death, and hospital records with primary and non-primary diagnoses recorded. With these data, we restricted to patients with heart failure diagnoses and identified all other diseases recorded in each record. For each country, we tallied the number of times each disease was diagnosed alongside heart failure and identified the 20 most common diseases. Figure 2 is an illustrative example, showing the 20 most commonly co-occurring causes in Italian hospital

data. In our expert consultations, we asked disease experts to review and augment our aetiology list. Though hundreds of diseases can result in heart failure, the 27 causes included represent common, clinically relevant, and quantifiable entities that can be stably estimated, and include an "other" category to ensure estimates are collectively exhaustive and mutually exclusive.

Table 3: Heart failure aetiologies and proportion models

Proportion model	Aetiology
**Coronary artery disease**	Ischaemic heart disease
**Pressure overload, left heart**	Hypertensive heart disease
**Pressure overload, right heart**	COPD<br>Interstitial lung disease and pulmonary sarcoidosis<br>Silicosis<br>Asbestosis<br>Coal workers pneumoconiosis<br>Other pneumoconiosis<br>Pulmonary arterial hypertension
**Valve diseases**	Rheumatic heart disease<br>Congenital heart abnormalities<br>Other non-rheumatic valvular diseases<br>Endocarditis<br>Degenerative mitral disease<br>Calcific aortic disease
**Primary myocardial disease**	Myocarditis<br>Other cardiomyopathy
**Toxins**	Alcoholic cardiomyopathy<br>Cocaine use disorders<br>Amphetamine use disorders<br>Chagas
**Stress cardiomyopathies**	Thyroid disease<br>Thalassaemias<br>G6PD deficiency<br>Other haemoglobinopathies and haemolytic anaemias<br>Other cardiovascular and circulatory disorders<br>Atrial fibrillation<br>Chronic kidney disease<br>Cirrhosis and other chronic liver diseases<br>Acute stroke (subarachnoid haemorrhage, intracerebral haemorrhage, ischaemic)<br>Chronic stroke (subarachnoid haemorrhage, intracerebral haemorrhage, ischaemic)

Figure 2: 20 most commonly co-occurring diseases with heart failure in Italy hospital data

YLL cause	count
Garbage Code	184505
Hypertensive heart disease	40884
Atrial fibrillation and flutter	30721
Ischemic heart disease	28023
Diabetes mellitus type 2	16934
Chronic obstructive pulmonary disease	14967
Chronic kidney disease	12909
Ischemic stroke	8267
Alzheimer's disease and other dementias	4785
Non-rheumatic calcific aortic valve disease	4460
Urinary tract infections	4138
Other cardiovascular and circulatory diseases	3605
Cirrhosis and other chronic liver diseases	3322
Non-rheumatic degenerative mitral valve disease	3266
Lipoprotein metabolism and other lipidaemias disorders	3201
Obesity	3118
Other lower respiratory infections	3048
Rheumatic heart disease	2664
Peripheral artery disease	2252
Other endocrine, metabolic, blood, and immune disorders	2057

Prevalence estimation

Overall prevalence of heart failure was estimated in DisMod-MR 2.1 using literature data and administrative health facility data. We also included modelled excess mortality data as described below. We set a prior of no remission for all ages. All data adjustments were done outside of DisMod-MR 2.1, described above. Coefficients of covariates included in the DisMod-MR 2.1 model of heart failure prevalence are listed in Table 4.

Case-fatality (CFR) data extracted from a review of published literature were transformed into excess mortality rate (EMR), under the assumption that deaths among those with a diagnosis of heart failure were caused by heart failure. The transformation between CFR and EMR was performed using Equation 2.

Equation 2:

$$EMR = -(\ln{(1 - CFR)}/time(years)$$

We then used the transformed EMR data described above as inputs to a MR-BRT model (Figure 3). We modelled ln(EMR) on sex and a cubic spline for age. We included Healthcare Access and Quality (HAQ) Index as a covariate and specified a prior of a negative coefficient on the association between HAQ Index and EMR. Results from this model were then predicted for each location year, sex, and ten-year age groups. We included HAQ Index as a country-level covariate in DisMod-MR 2.1 to inform EMR with a mean and standard deviation produced from MR-BRT.

Figure 3: MR-BRT model predictions for excess mortality rate (EMR)

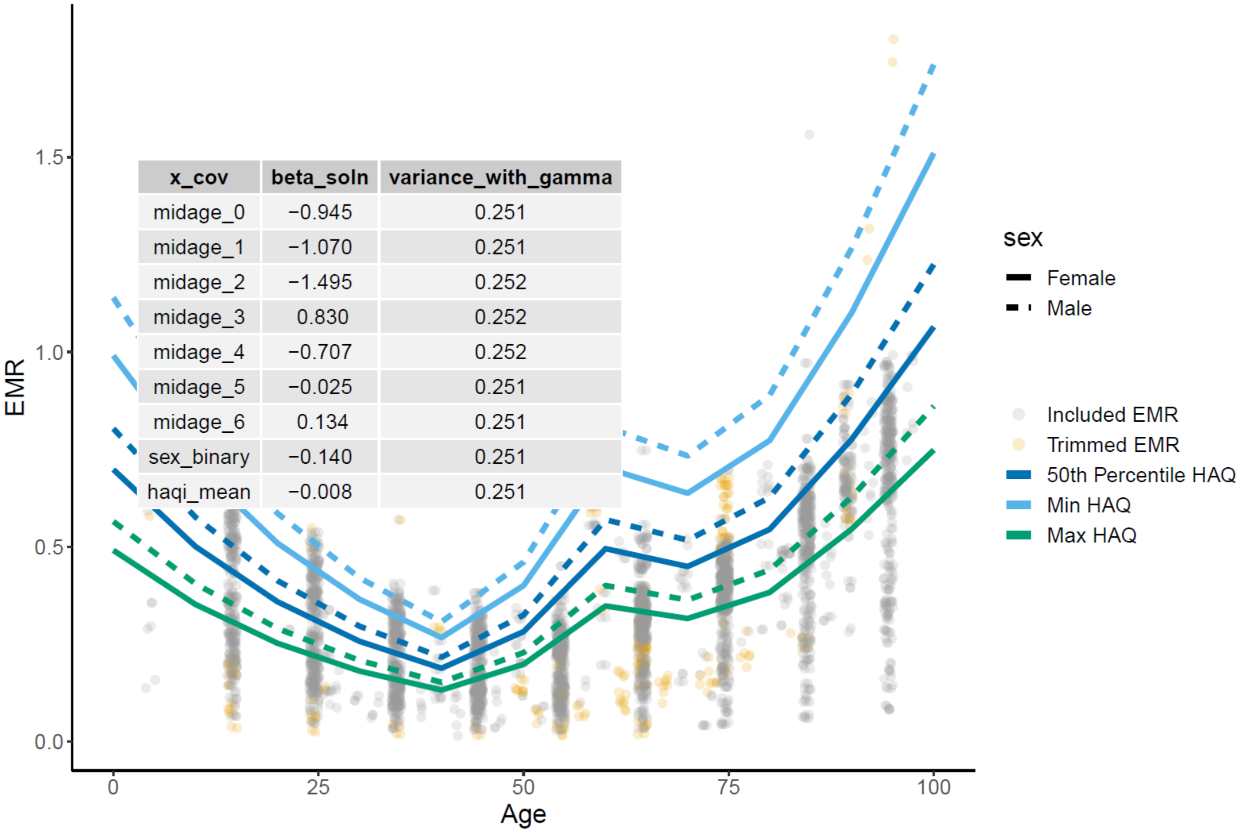

Table 4: Coefficients for covariates included in the DisMod-MR model of the overall prevalence of heart failure

Study covariate	Parameter	Beta	Exponentiated beta
Healthcare Access and Quality Index	Excess mortality rate	–0.008 (–0.008 to –0.008)	0.99 (0.99 to 0.99)

Estimates for the prevalence of heart failure due to Chagas, degenerative mitral valve disease (DMVD), calcific aortic valve disease (CAVD), and pulmonary arterial hypertension (PAH) were generated separately because the methods for those diseases explicitly estimate the burden of heart failure due to those causes. The methods appendix sections for each of these diseases contain additional details. Due to sparse input data for overall heart failure in locations where Chagas is endemic, our models of overall heart failure in those locations were likely underestimates of true prevalence. We thus added the estimates of heart failure due to Chagas to the estimates of overall prevalence. We subtracted the prevalence of heart failure due to DMVD, CAVD, and PAH from the overall heart failure estimates to give an adjusted prevalence of heart failure due to all remining aetiologies.

GBD estimates separately the prevalence of acute stroke (defined as 30 days or less after the initial stroke) and chronic stroke (defined as 30 days or more after the initial stroke). To estimate the burden of heart failure due to stroke, we similarly needed to provide acute and chronic estimates. Due to the temporal aspect of acute stroke, we estimated the prevalence of heart failure due to this cause

separately from others. We reviewed the literature and assumed that 5% of acute strokes resulted in heart failure.[5] Similar to DMVD, CAVD, and PAH, we subtracted the prevalence of heart failure due to acute stroke from the overall heart failure estimates to give an adjusted prevalence of heart failure due to all other aetiologies. Heart failure due to chronic stroke was estimated using the same methodology as other aetiologies.

Aetiological fraction estimation
To estimate the proportion of heart failure attributable to each cause, we utilised the epidemiological relationship between prevalence, cause-specific mortality, and excess mortality, as described in Equation 3.

Equation 3:

$$Prevalence_{HF\ due\ to\ aetiology} = \frac{Cause\ Specific\ Mortality\ Rate_{HF\ due\ to\ aetiology}}{Excess\ Mortality\ Rate_{HF\ due\ to\ aetiology}}$$

First, we calculated the cause-specific mortality rate (CSMR) for heart failure due to each aetiology. We used age-, sex-, and location-specific CSMR (post CoDCorrect) for each aetiology, multiplied by the fraction of deaths that also involved heart failure as described in Equation 4.

Equation 4:

$$CSMR_{HF\ due\ to\ aetiology} = CSMR_{aetiology} * Proportion\ deaths\ with\ HF_{aetiology}$$

This fraction was a modelled quantity, informed by person-level VR data from the USA, Mexico, Brazil, Taiwan, and Colombia, which contained the underlying cause of death as well as all codes in the causal chain. From these sources, we calculated the fraction of underlying deaths from each aetiology in which heart failure was coded in the causal chain. These data were modelled in MR-BRT to generate age- and sex-specific estimates of this proportion. For hypertensive heart disease, alcoholic cardiomyopathy, and other cardiomyopathy, we set the proportion to be 1, as all deaths due to these causes involve heart failure.

Next, we estimated the excess mortality rate (EMR) for heart failure due to each aetiology. We used uniquely identified person-level hospital discharge data for the entire Italian region of Friuli Venezia Giulia linked to all death records from the region. This is the only location available to GBD with population-level linked data of this kind. Inpatient data contained all primary and non-primary diagnoses associated with the visit, and mortality data contained the underlying cause of death as well as all codes in the causal chain. We identified patients with heart failure due to each aetiology as individuals with hospital-coded heart failure concurrent with or subsequent to an admission with a hospital code for the aetiology. Excess mortality rate for heart failure due to each aetiology was calculated by subtracting the background mortality rate from the mortality rate of persons with heart failure due to that aetiology. We modelled this quantity in MR-BRT to generate age- and sex-specific estimates of this value (Figure 4a and 4b). Due to the small number of deaths in younger ages, we assumed equal EMR across aetiologies for ages under 45.

Figure 4a: Modelled EMR for ischaemic heart disease

Figure 4b: Modelled EMR for chronic obstructive pulmonary disease

The aetiology-specific prevalence values calculated using Equation 1 were translated to proportions by dividing aetiology-specific prevalence by overall HF prevalence for each age, sex, year, and location stratum. These were aggregated to the seven broadest and mutually exclusive and collectively exhaustive cause groupings: coronary artery disease, pressure overload of the left heart, pressure overload of the right heart, valve diseases, primary myocardial diseases, toxins, and stress cardiomyopathies (Table 3).

These proportion data, along with literature data on aetiological attribution of heart failure, were used to inform DisMod-MR 2.1 models. An exception to this approach was made for sub-Saharan Africa, where we excluded the proportion estimates generated from death data, relying instead on published literature to determine the proportions of heart failure aetiologies. This decision was based on expert

opinion and evidence from THESUS-HF study, a large-scale, prospective, echocardiographic study of heart failure aetiologies in multiple African countries, that aetiological patterns differed significantly from what would have been determined from death data.[6] Table 7 shows the coefficients for the covariates included in the DisMod models for the seven main sub-cause proportion envelopes.

Table 5: Coefficients for covariates in DisMod-MR 2.1 for the seven main sub-cause proportion models

Sub-cause	Covariate	Parameter	Beta	Exponentiated beta
Heart failure due to coronary artery disease	Log-transformed, age-standardised SEV scalar: IHD	Proportion	0.75 (0.75–0.75)	2.12 (2.12–2.12)
Heart failure due to pressure overload of the left heart	Systolic blood pressure (mmHg)	Proportion	1.5E-5 (9.2E-7 – 3.6E-5)	1.00 (1.00–1.00)
Heart failure due to pressure overload of the right heart	Log-transformed, age-standardised SEV scalar: COPD	Proportion	0.75 (0.75–0.75)	2.12 (2.12–2.12)
Heart failure due to primary myocardial disease	Log-transformed, age-standardised SEV scalar: CMP	Proportion	0.75 (0.75–0.75)	2.12 (2.12–2.12)
Heart failure due to valve diseases	Log-transformed, age-standardized SEV scalar: CVD	Proportion	0.75 (0.75–0.75)	2.12 (2.12–2.12)
Heart failure due to stress cardiomyopathies	Log-transformed age-standardised SEV scalar: CVD	Proportion	0.75 (0.75–0.75)	2.12 (2.12–2.12)
Heart failure due to toxins	Age-standardised SEV for alcohol use	Proportion	1.25 (1.25–1.25)	3.49 (3.49–3.49)

After scaling the results from the seven proportion models to sum to one, we scaled each aetiology within its respective proportion model. For all aetiologies estimated via this process, we then multiplied the aetiological proportion by overall heart failure prevalence for each age, sex, location, and year stratum. Figure 6 shows the results of this process.

Figure 6: Global, aetiology-, age-, and sex-specific burden of heart failure

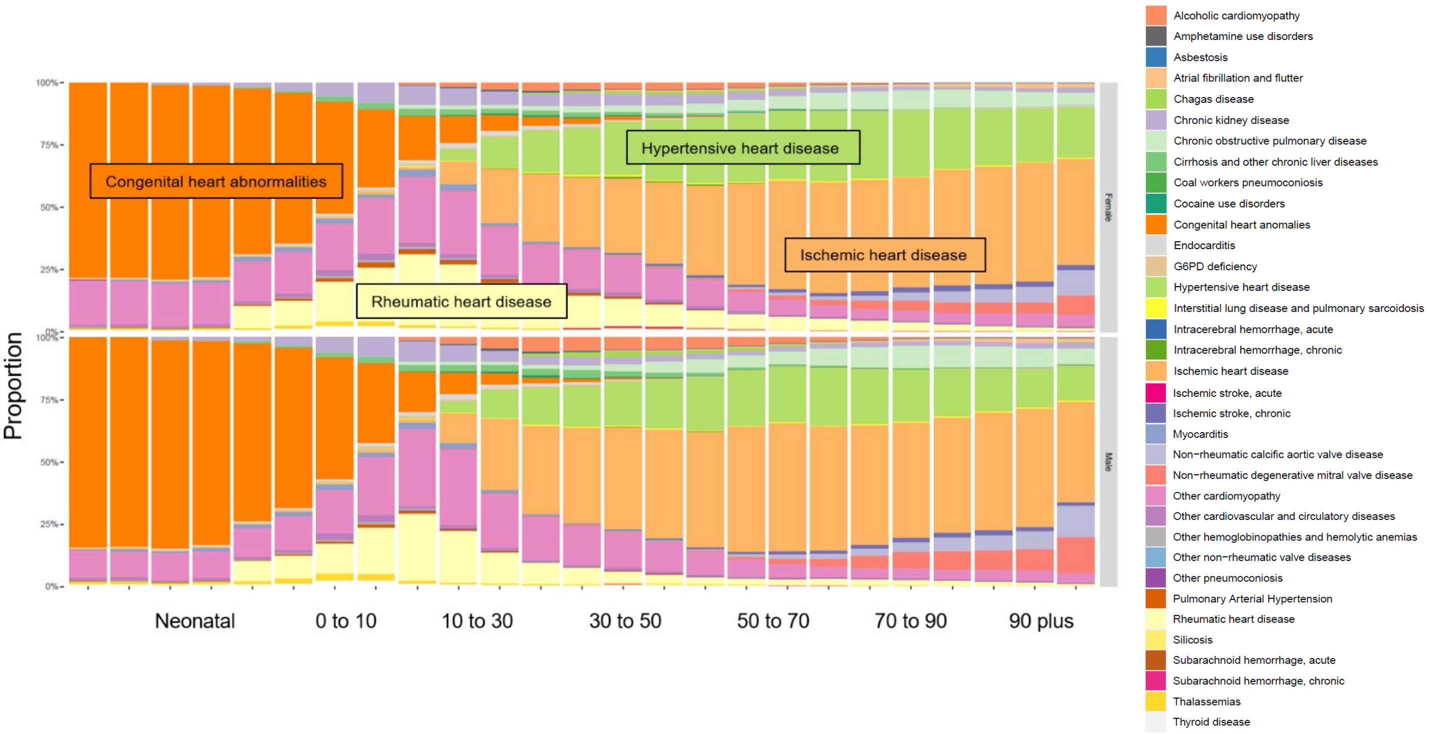

Models were evaluated based on expert opinion, comparison of results with other rounds of GBD, and model fit.

Severity split inputs

Aetiology-specific estimates of HF were split into asymptomatic, mild, moderate, and severe heart failure based on an analysis of MEPS data, except for Chagas disease. MEPS is the only available population-based source that links EQ5D to ICD codes, allowing the application of standard GBD disability methods. For Chagas, which is not adequately represented in MEPS, we based the severity splits on a meta-analysis of NYHA class among persons diagnosed with heart failure due to Chagas disease in areas where Chagas is endemic.[7–10] Disability weights were established for these severities using the standard approach for GBD 2023.

Table 4. Severity distribution, details on the severity levels for heart failure in GBD 2023 and the associated disability weight (DW) with that severity

Severity level	Lay description	DW (95% CI)
Controlled, medically managed	Has been diagnosed with clinical heart failure, a chronic disease that requires medication every day and causes some worry but minimal interference with daily activities.	0.049 (0.031–0.072)

Mild	Is short of breath and easily tires with moderate physical activity, such as walking uphill or more than a quarter-mile on level ground. The person feels comfortable at rest or during activities requiring less effort.	0.041 (0.026–0.062)
Moderate	Is short of breath and easily tires with minimal physical activity, such as walking only a short distance. The person feels comfortable at rest but avoids moderate activity.	0.072 (0.047–0.103)
Severe	Is short of breath and feels tired when at rest. The person avoids any physical activity, for fear of worsening the breathing problems.	0.179 (0.122–0.251)

References

1 Bozkurt B, Coats AJ, Tsutsui H, *et al.* Universal Definition and Classification of Heart Failure: A Report of the Heart Failure Society of America, Heart Failure Association of the European Society of Cardiology, Japanese Heart Failure Society and Writing Committee of the Universal Definition of Heart Failure. *J Card Fail* 2021; : S1071-9164(21)00050-6.

2 Maddox TM, Januzzi JL, Allen LA, *et al.* 2024 ACC Expert Consensus Decision Pathway for Treatment of Heart Failure With Reduced Ejection Fraction. *Journal of the American College of Cardiology* 2024; **83**: 1444–88.

3 McKee PA, Castelli WP, McNamara PM, Kannel WB. The natural history of congestive heart failure: the Framingham study. *N Engl J Med* 1971; **285**: 1441–6.

4 Ponikowski P, Voors AA, Anker SD, *et al.* 2016 ESC Guidelines for the diagnosis and treatment of acute and chronic heart failure: The Task Force for the diagnosis and treatment of acute and chronic heart failure of the European Society of Cardiology (ESC)Developed with the special contribution of the Heart Failure Association (HFA) of the ESC. *Eur Heart J* 2016; **37**: 2129–200.

5 Norberg E, Odenstedt-Herges H, Rydenhag B, Oras J. Impact of Acute Cardiac Complications After Subarachnoid Hemorrhage on Long-Term Mortality and Cardiovascular Events. *Neurocrit Care* 2018; **29**: 404–12.

6 Damasceno A, Mayosi BM, Sani M, *et al.* The causes, treatment, and outcome of acute heart failure in 1006 Africans from 9 countries. *Arch Intern Med* 2012; **172**: 1386–94.

7 Sabino EC, Ribeiro AL, Salemi VMC, *et al.* Ten-Year Incidence of Chagas Cardiomyopathy Among Asymptomatic Trypanosoma cruzi–Seropositive Former Blood Donors. *Circulation* 2013; **127**: 1105–15.

8 Ribeiro AL. Cohort of patients attending the Chagas disease outpatient clinic of Evandro Chagas Hospital.

9 Ribeiro AL. NYHA Class Information in a Cohort of Chagas dilated cardiomyopathy; Hospital das Clínicas da UFMG.

10 Ribeiro ALP, dos Reis AM, Barros MVL, *et al.* Brain natriuretic peptide and left ventricular dysfunction in Chagas' disease. *Lancet* 2002; **360**: 461–2.

HIV/AIDS

Flowchart

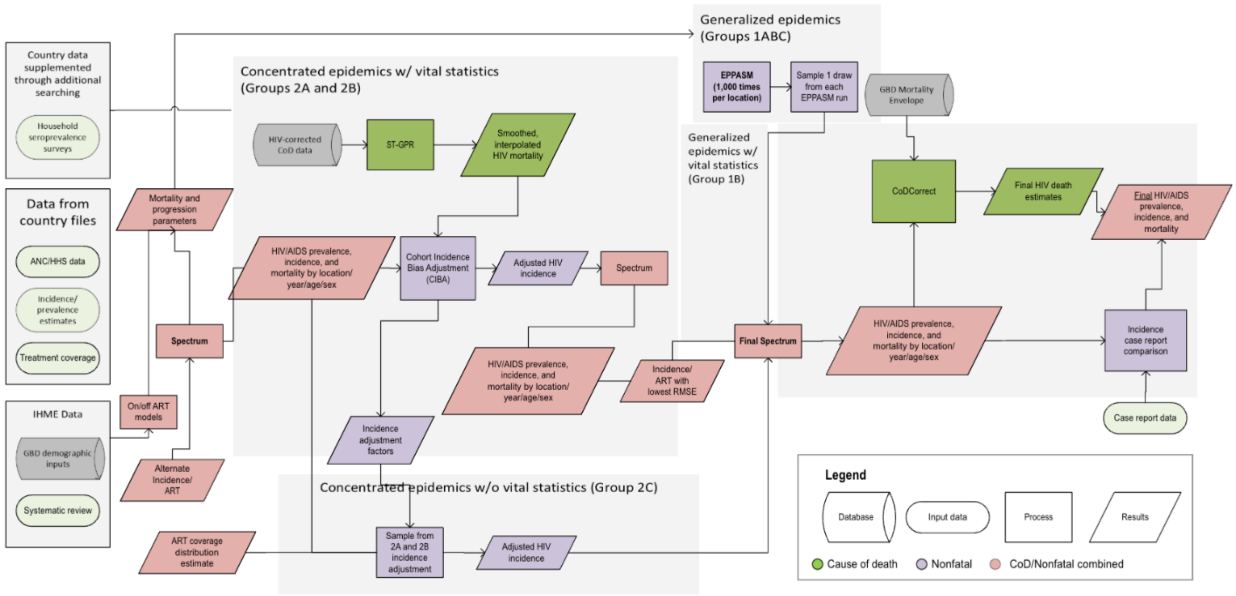

Input data and methodological summary for HIV/AIDS

Input data

Prevalence data

Household seroprevalence surveys
Geographically representative HIV seroprevalence survey results were used as inputs to the model for countries with generalised HIV epidemics where available. From these surveys, we used age- and sex-specific prevalence data.

GBD demographic inputs

Location-specific population, births, fertility, migration, and HIV-free survival rates from GBD 2021 were used as inputs in modelling all locations.

Data from countries

The files compiled by UNAIDS for their HIV/AIDS estimation process were our main source of data for producing estimates of HIV burden. The files are often built by within-country experts with the support of UNAIDS, which publishes estimates annually on behalf of countries and only shares their files when permission is granted. The files contain the HIV-specific information which is needed to run the Spectrum,[4] and Estimation and Projection Package-Age Sex (EPP-ASM)[5] models. Spectrum and EPP-ASM require the following input data: AIDS mortality among people living with HIV with and without ART,

CD4 progression among people living with HIV not on ART, ART coverage among adults and children, cotrimoxazole coverage among children, coverage of breastfeeding among women living with HIV, prevention of mother-to-child transmission (PMTCT) coverage, and CD4 thresholds for treatment eligibility. EPP-ASM additionally uses HIV prevalence data from surveillance sites and representative surveys. Antenatal care clinic (ANC) and treatment coverage data from UNAIDS were used in modelling Group 1 countries. We extracted all of these data from the proprietary format used by UNAIDS.

The EPP-ASM and Spectrum models used for GBD estimation vary slightly from those used by UNAIDS, with details on this variation included below. On top of the differences in model structure, we integrate our estimates of input model parameters, including new transition parameters and demographic rates. The differences between our estimates and UNAIDS' estimates reflect differences in model structure, model parameters, and the location-specific data used to calibrate our models.

Vital registration data

We used all available sources of cause of death data and sample registration data from the GBD Causes of Death database after garbage code redistribution and HIV/AIDS mis-coding correction in Group 2 countries and India.[3,7] There are two different cause of death data sources for HIV/AIDS in China: the Disease Surveillance Points (DSP) system and the Notifiable Infectious Disease Reporting (NIDR) system. Both systems are administered by the Chinese Center for Disease Control and Prevention, but the reported number of deaths due to HIV is significantly lower in DSP. Therefore, we have used the provincial-level ratio of deaths due to HIV/AIDS from NIDR to those from DSP, choosing the larger ratio between years 2013 and 2014, and scaled the reported deaths in the DSP system, which is in turn used in the spatiotemporal Gaussian process regression (ST-GPR).

On-ART mortality literature data

GBD 2023 used the same set of literature data extracted for GBD 2021.[1]

To be included, studies must include only HIV-positive people over the age of 15 who receive antiretroviral therapy (ART) but who were ART-naïve prior to the study. In addition, studies must report either a duration-specific (time since initiation of ART) mortality proportion or a hazard ratio across age or sex, and must not include children.

For duration-specific survival data, studies must report uncertainty on mortality estimates or provide stratum-specific sample sizes and must include duration-specific data to allow for calculation of 0–6, 7–12, or 13–24-month conditional mortality. In addition, studies must either report separate mortality and loss-to-follow-up (LTFU) curves, be corrected for LTFU using vital registration data or double sampling, or be conducted in a high-income setting. Finally, studies must report the percentage of participants who are male and the median age of participants.

Hazard ratio data for ages or sexes can only be used if the hazard ratios are controlled for other variables of interest (age, sex, and CD4 category). In GBD 2021, we included 61 studies, 13 of which were new this cycle. Of these studies, we added ten to inform the estimation age-sex hazard ratios, and three studies informed LFTU curves.

Off-ART mortality and CD4 progression literature data

In GBD 2013, we systematically reviewed the literature on mortality without ART to characterise uncertainty in the progression and death rates. We searched terms related to pre-ART or ART-naïve survival since seroconversion.8 After screening, we identified 13 cohort studies that included the cohorts used by UNAIDS, from which we extracted survival at each one-year point after infection. Screening for additional, recently published studies in GBD 2015, GBD 2016, and GBD 2017 identified no new cohort studies for inclusion in this analysis. We did not search for new studies in GBD 2019 or GBD 2021.

Modelling strategy

Case definition

Infection with the human immunodeficiency virus (HIV) causes influenza-like symptoms during the acute period following infection and can lead to acquired immunodeficiency syndrome (AIDS) if untreated. HIV attacks the immune system of its host, leaving infected individuals more susceptible to opportunistic infections like tuberculosis. Although there are two different subtypes of HIV, HIV-1 and HIV-2, no distinction is made in our estimation process or presentation of results. For HIV, ICD-10 codes are B20-B24, C46-C469, D84.9; ICD-9 codes are 042-044, 112-118 (after 1980), 130 (after 1980), 136.3-136.8 (after 1980), 176.0-176.9 (after 1980), 279 (after 1980); and ICD-9 BTL codes are B184-B185.

Country groupings

Countries were divided into groups: Groups 1A, 1B, and 2A, 2B, and 2C. Group 1 includes countries with HIV prevalence data from antenatal care clinics or nationally or subnationally representative population-based seroprevalence surveys. Group 1A included countries with a peak of at least 0.5% prevalence, and Group 1B includes countries with a peak of at least 0.25% prevalence and vital registration completeness less than 65%.

The remaining countries made up Group 2, which are further subdivided in Group 2A, 2B, and 2C based on availability of vital registration data. Group 2A consisted of countries with high-quality vital registration data; Group 2B consisted of countries with available cause of death data that is not high-quality; and Group 2C countries were those without any vital registration data. Quality was measured based on a star rating system as described elsewhere.[3]

Both groups of countries relied on the same approach to modelling on- and off-antiretroviral therapy (ART) mortality, as described below.

Results were aggregated by super-region as defined by the Global Burden of Disease study. These super-regions are depicted in figure S2.

Figure 1. HIV-specific country groupings based on data availability

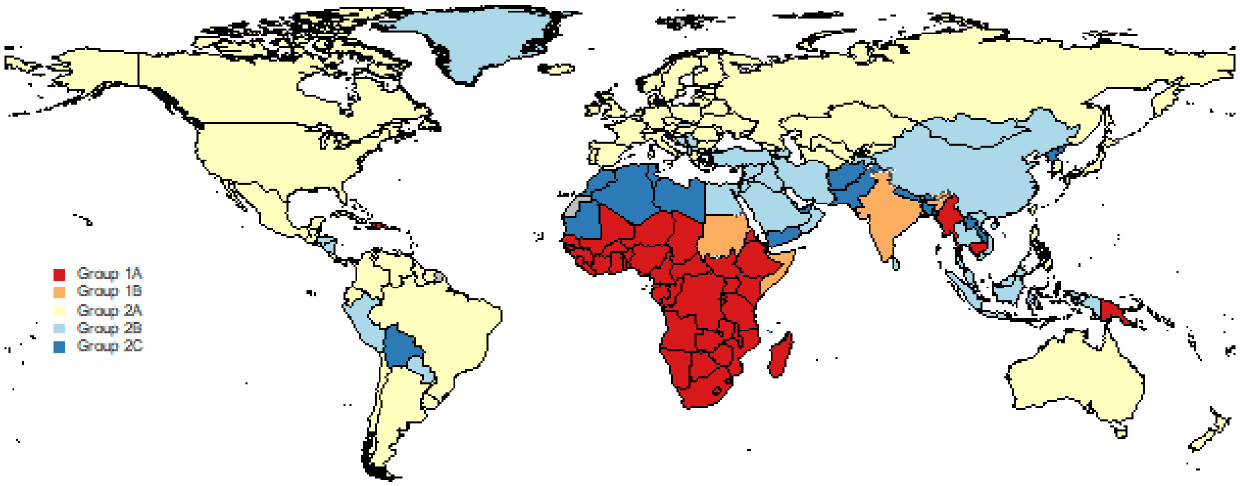

Figure 2. Global Burden of Disease 2021 super-regions

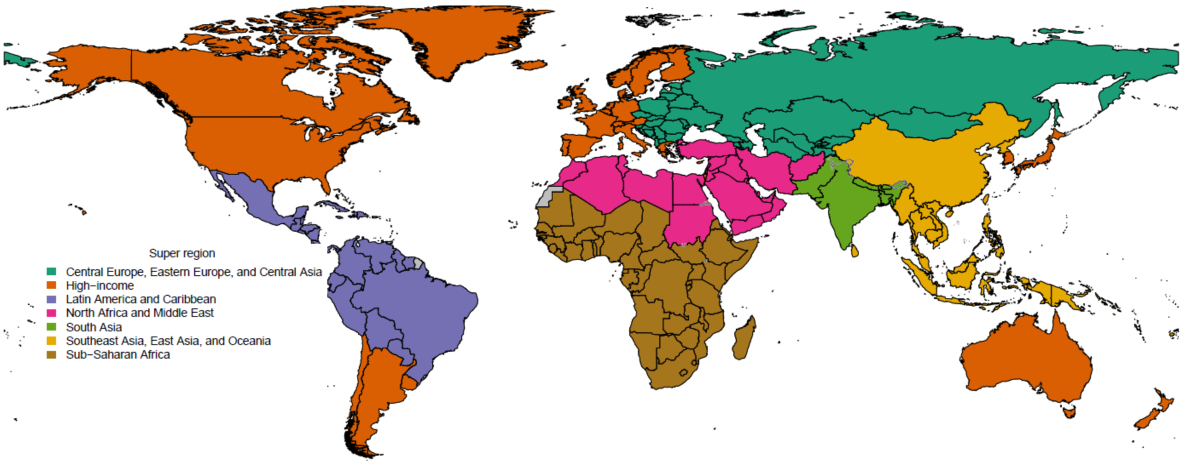

On-ART, off-ART mortality and CD4 progression parameters

On-ART mortality

We employ the meta-regression tool meta-regression with Bayesian priors, regularisation, and Trimming (MR-BRT), developed for the Global Burden of Disease (GBD) study 2019 for modelling. This tool combines the Bayesian framework with mixed-effects models to incorporate prior knowledge in the form of regularisation or constraints in the optimisation. This approach addresses some of the limitations of traditional meta-analyses by exploring between-study heterogeneity; accounting for small numbers of studies; allowing for nonlinear measurements, priors, constraints, and observation measurement errors. Specifically, we modelled the non-linear relationship between baseline CD4 and mortality, weighted each observation by the inverse of its reported variance, captured between-study

heterogeneity through a random effects model, and applied a monotonic decreasing prior on baseline CD4 levels.

The modelling has two stages. In the first stage, CD4 is modelled as a continuous variable. The outcome (logit of conditional probability of mortality after subtracting the background mortality) is exclusively regressed against the spline of the measures of CD4 to ensure the non-linear dynamics are captured by aggregating data from all studies. In this model, a spline degree of 2 is employed with a total of 4 knots, and 5% of the datapoints are trimmed. In the second stage, the other covariates are integrated into a mixed-effects model along with the fitted values from the first stage: age (15–24, 25–34, 35–44, 44–54, 55–100), sex (male, female), time since initiation of ART (0–6, 7–12, 13–24 months). The modelling is implemented in three regions, and we model each region separately: sub-Saharan Africa, high-income, other countries.

We corrected reported probabilities of death for loss to follow-up using an approach developed by Verguet and colleagues.[2] Verguet and colleagues used tracing and follow-up studies to empirically estimate the relationship between death in LTFU and the rate of LTFU.

To create estimates of sex-specific hazard ratios, we use the *metan* function in Stata to create estimates of relative risks separately by region, using female age groups as the reference group within each age strata. The age and sex hazard ratios were applied to the study-level mortality rates, accounting for the distribution of ages and sexes in the mortality data. We then subtracted HIV-free mortality from the model life table process to calculate study-level age-sex HIV-specific mortality.

We then used MR-BRT to synthesise the age-sex-split study-level data into estimates of conditional probability of death over initial CD4 count. We replaced our on-ART mortality rates with those estimated off treatment if they were higher. Prior to analysing the data, we apply a logit transformation of the outcome variable, which is the conditional probability of mortality after individuals initiate treatment with ART. This transformation, along with the corresponding variance transformation, enables the data to be analysed using a powerful Gaussian mixed effects model (MR-BRT), with random (Gaussian) effects by study. The random errors in our model are assumed to follow a normal distribution. The link function used in our meta-regression model is the logit function. The random-effect term in our model accounts for between-study variability. This term is assumed to follow a normal distribution, capturing the heterogeneity across different studies included in the meta-analysis.

We estimate mortality for each region in its own DisMod model based on data from the IeDEA cohort collaboration[3] and include a covariate for year as mortality among the LFTU has been found to decline in recent years.[4] Finally, in cases where on-ART rates were higher, we replaced our estimated on-ART mortality rates by rates off ART to account for progression to lower CD4 categories. This ensured individuals would not experience higher mortality when they entered treatment in Spectrum or EPP-ASM.

Off-ART mortality and CD4 progression

Following UNAIDS assumptions, off-ART mortality and CD4 progression is modelled as shown in the figure below.

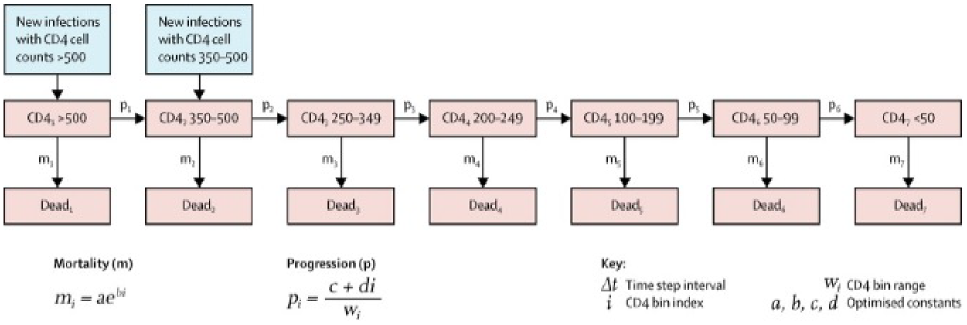

The death and progression rates between CD4 categories vary by age according to four age groups: 15–24 years, 25–34 years, 35–44 years, and 45 years or older. We modelled the logit of the conditional probability of death between years in these studies using the following formula:

$$logit(m_{ijk}) = \beta_0 + \sum_{i=1}^{4} \beta_{1i} a_i + \sum_{j=1}^{12} \beta_{2j} t_j + u_k + \epsilon_{ijk}$$

In the formula, *m* is conditional probability of death from year t_j to t_{j+1}, a_i is an indicator variable for age group at seroconversion (15–24 years, 25–34 years, 35–44 years, and 45 years or older), t_j is an indicator variable of year since seroconversion, and u_k is a study-level random effect. The categories of the variable t_j are one-year intervals up to 12 years since seroconversion, after which data availability was sparse. The baseline level was the lowest category for each indicator, so 15–24 years for age at seroconversion and 0 years for years since seroconversion. The model assumed a multivariate normal distribution for the study-level random effects, u_k, and the error term, ε, after the logit transformation of the conditional probabilities of death.

By sampling the multivariate normal distribution represented by the fitted mean and variance-covariance matrix, we generated 1000 survival curves for each age group that capture the systematic variation in survival across the available studies. For each of the 1000 survival curves, we used a framework modelled after the UNAIDS optimisation framework in which we find a set of progression and death rates that minimises the sum of the squared errors for the fit to the survival curve.[5,6]

Disease model

We used two different components to derive year-, age-, and sex-specific estimates of HIV incidence, prevalence, and mortality depending on locations' availability of data and extent of HIV burden, as described below:

1. EPP-ASM was used to estimate incidence, prevalence, and mortality that are consistent with serosurveillance data from antenatal care clinics and/or prevalence surveys.
2. Spectrum is a compartmental HIV progression model used to generate age-sex-specific incidence, prevalence, and death rates from input incidence and prevalence curves and assumptions about intervention scale-up and local variation in epidemiology. This model was used in conjunction with EPP-ASM for India and for all Group 2 countries.

Group 1: EPP–ASM

53 countries – as well as subnational locations in India, Kenya, Ethiopia, Nigeria, and South Africa – were included in Group 1 with available ANC data and/or at least one geographically representative HIV seroprevalence survey. For all these locations we used EPP-ASM, which was updated to incorporate the new ANC bias adjustment.

In EPP-ASM,[7] the transmission rate, r(t), is a simple transmission model applied at each time step (1/10 of a year) to the population. 'r' represents the number of new cases expected to emanate from a single case. Over 3000 iterations, a new r(t) is drawn, the full epidemic is determined and compared to the observed prevalence data to determine its likelihood. Beyond the end of the data, a prior distribution on r(t) helps to determine how we should expect the epidemic to behave. This assumption was different in EPP-ASM versus EPP. In EPP-ASM in most countries, we extended a random walk into the future based on the 'r-hybrid' r(t). The r-hybrid assumes a logistic decay until the year 2003, a linear interpolation until year 2008, and a random walk form after this.

For GBD 2023, we continued to use our modified version of EPP-ASM both to improve the fit to data and to generate paediatric estimates. We built a paediatric module in EPP-ASM that mirrored early updates to the paediatric module in Spectrum.[8] This child module included CD4 progression and CD4-specific mortality rates taken from a model fit to survival data from IeDEA and child initiation of ART based on ART distribution data from IeDEA. Perinatal and breastfeeding transmission was calculated as a function of prevalence among pregnant women and PMTCT programme data. We were thus able to utilise EPP-ASM to produce HIV incidence, prevalence, and mortality estimates for all ages. Additionally, we improved fit to prevalence data through allowing flexibility in the age distribution of incidence over time. We parameterised the ratio of incidence among ages 15–24:25+ as a constant before year 2000 and a linear regression thereafter. This allowed for the shifts in the age distribution of incidence observed over the course of the HIV epidemic to be reflected in our results. Finally, we utilised GBD demographic inputs and substituted in our own assumptions about HIV progression rates and on/off-ART mortality.

To incorporate uncertainty in our demographic and progression parameters, we ran EPP-ASM with separate draws of CD4 progression, on- and off-ART mortality rates, fertility, and HIV-free mortality. This process produced 1000 posterior distributions for each of the locations that make up Group 1. For every location in the group, we sampled one draw from each of the sets of EPP-ASM results to create a final distribution. By sampling one draw from each set, we ensured that the distribution of mortality parameters dictating the relationship between incidence and prevalence aligned with those used in the GBD demographics estimates.

We also continued to use the approach implemented in GBD 2019[9] to address selection bias resulting from temporal and geographical variation in ANC reporting. The ANC data which EPP-ASM uses cannot be assumed as representative of HIV prevalence in the full population. This is especially the case when there are minimal or no nationally representative prevalence surveys to anchor estimates, as in the early epidemic.[10]

EPP-ASM has embedded approaches to adjust for the bias associated with using prevalence among ANC-site-attending pregnant women to estimate prevalence among the both-sexes population. For the bias between pregnant women and the national both-sexes population, it makes assumptions around the difference in total fertility rate among HIV-positive and HIV-negative women, and the difference in prevalence between men and women. For the bias associated with the data coming from ANC sites, the specification of the likelihood of observed ANC data includes random intercepts for each clinic. The random intercepts allow each site's baseline prevalence to vary randomly around the overall mean prevalence. In other words, factors that could drive differences between sites' HIV prevalence levels are "adjusted" for.

However, the embedded approach does not explicitly account for the fact that the location of the clinic in space may also drive its HIV prevalence level. For example, we might expect rural sites to be more correlated than urban sites. Thus, to further adjust for this bias, we used an offset term that represents the difference in the prevalence among the national, both-sexes population and the prevalence among

the female, pregnant population associated with an ANC site location. The offset term was derived for each location as the difference between the adjusted prevalence in a given site-year and the adjusted national prevalence in that year. These estimates are adjusted for covariates that are thought to influence prevalence, for example, access to health-care facilities, malaria incidence, and male circumcision.

Thus, our final strategy for estimating the likelihood of the observed ANC data was:

$$\boldsymbol{W_{st} = \varphi^{-1}(\rho_t) + \vartheta_{st} + u_s + e_{st}}$$

$$\boldsymbol{e_{st} \sim N(0, \sigma_{st}^2)}$$

$$\boldsymbol{u_s \sim N(0, \sigma_s^2)}$$

Where:
W_{st} = the probit transformed prevalence at site *s* and time *t*
ρ_t= The national prevalence adjusted to represent prevalence among pregnant women from the model simulation
ϑ_{st} = The offset term representing the difference between the adjusted prevalence in a given site-year and the adjusted national prevalence in that year
φ^{-1} = probit transformation
e_{st} = Site-specific error term
u_s = Site-specific intercept

Group 2: Spectrum

For GBD 2013, we created an exact replica of Spectrum in Python. This enabled us to run thousands of iterations of the model at once on our computing cluster and allowed for more flexible input data structures. Additionally, we scaled all input values by a uniformly sampled factor between 0.9 and 1.1 to generate estimates with realistic ranges of uncertainty. For example, if treatment retention rates across CD4 categories were 0.906, 0.759, 0.787, 0.795, 0.785, 0.756, 0.813, and 0.700, we multiplied each number by an array of equivalent size that contained factors ranging from 0.9 to 1.1. At each draw, the array would contain different, randomly selected factors in the same range. Further, we previously improved our sex-specific modelling strategy in Spectrum by sex-splitting incidence based on a model fit to the sex ratio of prevalence observed in countries with representative surveys and updated the Spectrum paediatric module to reflect changes made by UNAIDS.[13] Our child module was revised to include CD4 progression and CD4-specific mortality rates taken from a model fit to survival data from IeDEA. Finally, we updated child initiation of ART to include data on ART distribution from IeDEA. These changes were retained in GBD 2021.

ART coverage distribution
Spectrum determines the number of people initiating ART treatment across each CD4 category based on eligibility criteria, and the number of expected deaths and untreated people. In other words, groups with a large proportion of people living with HIV and high numbers of expected deaths initiated the most individuals into treatment.

We improved the basis for this distribution using survey microdata and country-level wealth information. Three relevant surveys were available: Uganda AIS 2011 and Kenya AIS 2007 and 2012. These surveys conducted CD4 count measurements and include a question regarding the amount of time that an individual receiving ART had been enrolled in treatment. Survey data provide cross-

sectional CD4 count information; however, the Spectrum modelling framework tracks individuals by categorical CD4 count at the initiation of treatment. To crosswalk the cross-sectional survey data into estimates of CD4 count at treatment initiation, we built a model using relevant cohort data which tracked changes in CD4 count after initiation of treatment to translate an individual's current CD4 count and duration on treatment into CD4 count at initiation of treatment. The functional form for changes in CD4 count as a function of duration on treatment was a natural spline on duration with knots at 3, 12, 24, and 36 months, and an interaction between initial CD4 count and duration.

After crosswalking, we predicted the probability of being on treatment as a function of individual income (measured through an asset-based index), stratified by CD4 count, age, and sex. The results of this prediction were translated into country-specific age-sex-year-CD4 count probabilities of coverage using a conversion factor between individual income and lag-distributed GDP per capita. We used stochastic frontier analysis to constrain the maximum possible coverage for a given degree of income and CD4 count.

Predicted probabilities of coverage were input to Spectrum to inform the distribution, and not the overall level, of ART treatment by CD4 count. Within Spectrum, the probabilities of coverage are converted to counts of expected individuals on treatment in each CD4 count group. These are scaled to the distribution across CD4 count groups to match the input data on the number of people on ART coming from UNAIDS country files. In cases where the predicted number of individuals initiating treatment exceeds the total number of untreated individuals in a CD4 count group, we reallocate treatment evenly to other CD4 count groups.

Group 2: Countries without survey data and vital registration data

33 countries had neither geographically representative seroprevalence surveys nor reliable vital registration systems, these make up group 2C locations. To produce estimates of HIV burden in these countries, we used Spectrum to produce estimates of burden. As above, the estimates of incidence, prevalence, and mortality were incorporated into the rest of the machinery via the reckoning process.

Spatiotemporal Gaussian process regression (ST-GPR)

Countries with vital registration data (Groups 2A and 2B)
Vital registration is one of the highest-quality sources of data on HIV burden in many countries, so generating estimates that are consistent with these data with necessary adjustment to account for any potential under-reporting is critical. We identified 121 countries – as well as 760 subnational locations from China, Japan, Indonesia, India, Mexico, Sweden, the Philippines, Poland, Italy, the UK, Ukraine, Russia, New Zealand, Iran, Norway, and the USA – with usable points of vital registration data, verbal autopsy (VA) data, or sample registration system (SRS) data. In India, Vietnam, and Indonesia, we used SRS and VA data, respectively, as input mortality for CIBA. For India, we extracted the resulting age-sex distribution of incidence but scaled the level to match the adult incidence rate estimated from EPP for each state.

We estimated full time series for HIV deaths using ST-GPR fit to available cause of death data. We analysed mortality trends using ST-GPR starting in 1981, the year that HIV was first identified in the USA.[11] For ST-GPR, we adjusted the lambda (time weight) and GPR scale according to the completeness of vital registration data, with 4- and 5-star quality vital registration using parameters designed to follow the data more closely. We produced separate splines by country/age group, up to the peak year of death rate. We then ran a linear regression with fixed effects on region, age, and sex. Following this, we ran space-time residual smoothing, in which time, age, and space weights are used to inform smoothing

of the residuals between datapoints and the linear regression estimate. From this process, we generated space-time estimates with the applied weights, along with the median absolute deviation (MAD) of the space-time estimates from the data. The MAD was calculated at various levels of the geographical hierarchy (eg, subnational and national), and was added into the data variance term. The data variance and space-time estimates were then analysed using Gaussian process regression to return a final estimate of mortality along with uncertainty. ST-GPR deaths were used as final deaths in group 2A and group 2B.

Although Spectrum produces HIV mortality estimates that are within the realm of possibility in most countries using the incidence curves provided in the UNAIDS country files, it is a deterministic model that has not yet been integrated into an optimisable framework. Therefore, in order to "fit" it to vital registration data, we need to adjust input incidence. In contrast to GBD 2019 and previous cycles, in addition to adjusting input incidence, we determined the most plausible best treatment input based on fit to vital registration as well.

Additional adjustments

Additional adjustments enabled us to use case surveillance data and HIV mortality estimated as part of the GBD all-cause mortality life table process. In countries and territories with high-quality case notification data, we scaled incidence results to align with case reports after accounting for an assumed average of five years' lag to diagnosis. In previous GBD cycles, HIV mortality went through a "reckoning process" that was intended to be a method of reconciling separate estimates of HIV mortality (and its resulting effect on estimates of HIV-free and all-cause mortality) in Group 1 countries by averaging estimates of HIV mortality from the model life table process and EPP-ASM. Additional details on the reckoning can be found elsewhere.[12] In GBD 2023, this processed was removed and HIV deaths instead went through the standard GBD 2023 CoDCorrect process.[13]

Changes for GBD 2023

In GBD 2023, we ran three individual MR-BRT models to produce on-ART mortality rates for input in our disease models. This represented a shift away from 90 different models used in GBD 2021. The new MR-BRT modelling approach for on-ART mortality is implemented in three regions, which are modelled separately as sub-Saharan Africa, high-income, and other countries.

For GBD 2023, ST-GPR was run using a generalised set of hyperparameters, rather than the country-specific hyperparameters in previous cycles. This was done to ensure a consistent method for estimating mortality for countries with vital registration data. These generalised hyperparameters result in trends that better incorporate regional estimates with country-specific mortality data to give country-level estimates with greater spatial smoothing than GBD 2021 and previous rounds.

In previous GBD cycles, HIV incidence for Group 1 was "reconciled" by averaging estimates of HIV mortality from the model life table process and EPP-ASM.[1] In GBD 2023, we no longer averaged the two sets of estimates, and instead HIV mortality estimates were processed in the standard GBD CoDCorrection process, which is described in further detail in the CoDCorrect section of this appendix.

References

1 Carter A, Zhang M, Tram KH, *et al.* Global, regional, and national burden of HIV/AIDS, 1990–2021, and forecasts to 2050, for 204 countries and territories: the Global Burden of Disease Study 2021. *The Lancet HIV* 2024; **11**: e807–22.

2 Verguet S, Lim SS, Murray CJL, Gakidou E, Salomon JA. Incorporating Loss to Follow-up in Estimates of Survival Among HIV-Infected Individuals in Sub-Saharan Africa Enrolled in Antiretroviral Therapy Programs. *Journal of Infectious Diseases* 2013; **207**: 72–9.

3 Tarwater PM, Margolick JB, Jin J, *et al.* Increase and plateau of CD4 T-cell counts in the 3 1/2 years after initiation of potent antiretroviral therapy. *Journal of Acquired Immune Deficiency Syndromes* 2001; **27**: 168–75.

4 Zhou J, Kumarasamy N, Ditangco R, *et al.* The TREAT Asia HIV Observational Database. *J Acquir Immune Defic Syndr* 2005; **38**: 174–9.

5 Ghys PD, Żaba B, Prins M. Survival and mortality of people infected with HIV in low and middle income countries: results from the extended ALPHA network. *AIDS* 2007; **21**: S1–4.

6 Hallett TB, Zaba B, Todd J, *et al.* Estimating Incidence from Prevalence in Generalised HIV Epidemics: Methods and Validation. *PLoS Med* 2008; **5**: e80.

7 Eaton JW, Brown T, Puckett R, *et al.* The Estimation and Projection Package Age-Sex Model and the r-hybrid model: new tools for estimating HIV incidence trends in sub-Saharan Africa. *AIDS* 2019; **33**: S235–44.

8 Stover J, Glaubius R, Mofenson L, *et al.* Updates to the Spectrum/AIM model for estimating key HIV indicators at national and subnational levels. *AIDS* 2019; **33**: S227–34.

9 Jahagirdar D, Walters MK, Novotney A, *et al.* Global, regional, and national sex-specific burden and control of the HIV epidemic, 1990–2019, for 204 countries and territories: the Global Burden of Diseases Study 2019. *The Lancet HIV* 2021; **8**: e633–51.

10 Ng M, Gakidou E, Murray CJ, Lim SS. A comparison of missing data procedures for addressing selection bias in HIV sentinel surveillance data. *Popul Health Metr* 2013; **11**: 12.

11 Pneumocystis Pneumonia --- Los Angeles. https://www.cdc.gov/mmwr/preview/mmwrhtml/june_5.htm (accessed Feb 12, 2024).

12 Vos T, Lim SS, Abbafati C, *et al.* Global burden of 369 diseases and injuries in 204 countries and territories, 1990–2019: a systematic analysis for the Global Burden of Disease Study 2019. *The Lancet* 2020; **396**: 1204–22.

13 GBD 2021 Causes of Death Collaborators. Global burden of 288 causes of death and life expectancy decomposition in 204 countries and territories and 811 subnational locations, 1990-2021: a systematic analysis for the Global Burden of Disease Study 2021. *Lancet* 2024; **403**: 2100–32.

Hookworm disease

Flowchart

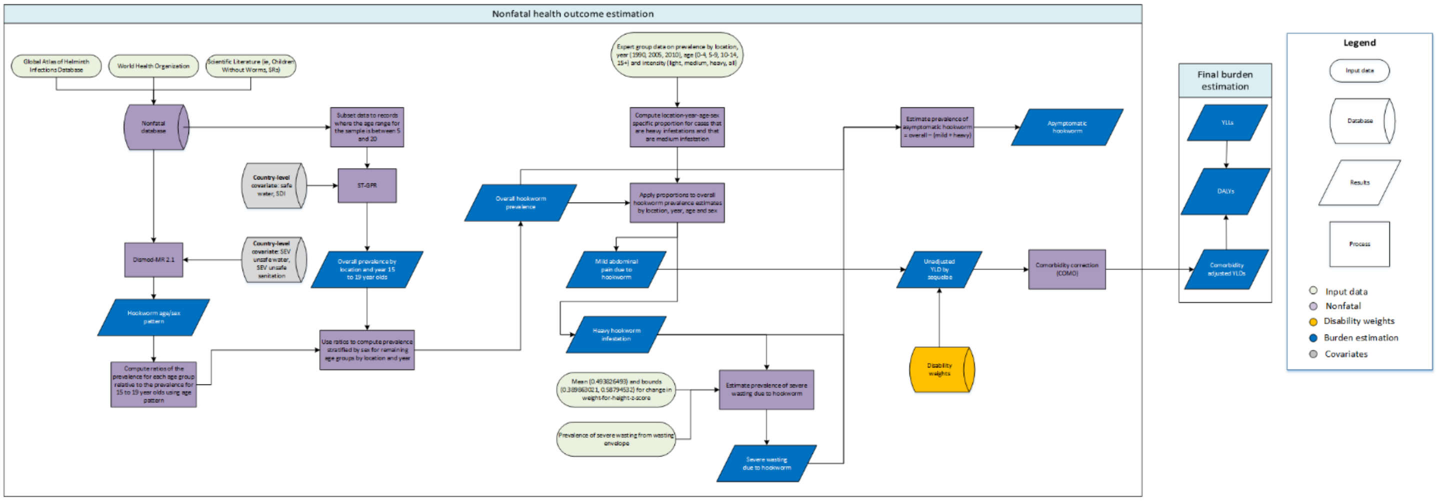

Input data and methodological summary for hookworm disease

Case definition

Hookworm disease is a helminthic disease caused by intestinal parasites in the roundworm group, *Ancylostoma duodenale* and *Necator americanus*. It is one of the three intestinal nematode infections, or soil-transmitted helminthiasis (STH), that we model in GBD. Diagnosis is made by examination of stool by microscope or PCR, with or without concentration procedures. The ICD-10 codes for hookworm disease are B76-B76.9. We used the following case definition for GBD 2023:

Quantity of interest	Reference or alternative	Definition
Hookworm	Reference	Diagnosis made by examination of stool using Kato-Katz technique, resulting in positive for intestinal helminth eggs of type hookworm.

Input data

The primary input data for this model were from the Global Atlas of Helminth Infections (GAHI) database and the Expanded Special Project for the Elimination of Neglected Tropical Diseases (ESPEN). The GAHI and ESPEN databases include surveys and studies conducted to measure the prevalence of STH.[1] Each record in the database contained metadata (ie, location, year, age range, sex) of each study sample and the prevalence of hookworm in that sample.

We supplemented the GAHI and ESPEN data with survey data collected in a literature review performed by Children Without Worms (CWW), which included countries outside of sub-Saharan Africa; a 2001–2004 China subnational survey to better inform our estimates in China; and additional data provided by the World Health Organization (WHO). For GBD 2023, we added data from systematic reviews (Figure

2a) and additional extracted data from the GAHI, CWW, and WHO datasets (Figure 2b). For all input data, we excluded datapoints where the age range of the sample was unknown and retained only those surveys utilising the Kato-Katz diagnostic method.

Geographical restrictions

We conducted a literature review (last updated for GBD 2017) to determine the geographical extent of the disease and classify locations based on whether the disease was absent or present in each year. Locations that were geographically restricted in any given year did not have estimates made for them. Of note, we did not attempt a complete systematic review, since a single high-quality source could offer sufficient evidence of presence. Evidence of absence or presence was not available for every location for each year. Assumptions made for missing years took into consideration the epidemiological characteristics of the disease.

If evidence indicated disease presence for two non-consecutive years, we assumed presence for all years between the two. If evidence indicated disease absence for two non-consecutive years, we assumed absence for all years between the two. If evidence indicated a change in status (ie, from absent to present, or present to absent) between two non-consecutive years, then we conducted targeted searches to ascertain the relevant year of introduction or elimination for that location. In the cases where presence or absence information was missing for the start or end years of our study interval without evidence of any introduction or elimination events within the interval, we applied the status of the first and last presence/absence observations, respectively, to all years between the interval bound and the observation year. Table 1 shows the search strings and associated yield for each of the databases queried.

Table 1. Geographical restriction search strings

**Database**	**Search String**	**Yield**
PubMed	(Ascariasis[Title/Abstract] OR Ascaris[Title/Abstract] OR "A. lumbricoides"[Title/Abstract] OR Ascaris[MeSH] OR Trichuris[Title/Abstract] OR Trichuriasis[Title/Abstract] OR "Whip Worm"[Title/Abstract] OR "T. trichura"[Title/Abstract] OR Trichuris[MeSH] OR Hookworm[Title/Abstract] OR "A. duodenale"[Title/Abstract] OR "Ancylostoma duodenale"[Title/Abstract] OR ancylostomiasis[Title/Abstract] OR "N. americanus"[Title/Abstract] OR "Necator americanus"[Title/Abstract] OR necatoriasis[Title/Abstract] OR Ancylostoma [MeSH] OR Necator[MeSH]) AND (prevalence[Title/Abstract] OR incidence[Title/Abstract] OR epidemiology[Title/Abstract] OR surveillance[Title/Abstract]) NOT(Animals[MeSH] NOT Humans[MeSH])	2376
Web of Science	(Ascariasis OR Ascaris OR A. lumbricoides OR Trichuris OR Trichuriasis OR Whip Worm OR T. trichura OR Hookworm OR A. duodenale OR Ancylostoma duodenale OR anclyostomiasis OR N. americanus OR Necator americanus OR necatoriasis) AND TOPIC:(prevalence OR incidence OR epidemiology OR surveillance) NOTTOPIC: ((Animals NOT Humans)) Timespan: 1980-2016. Indexes: SCI-EXPANDED, SSCI, A&HCI, ESCI.	2266
SCOPUS	TITLE-ABS_KEY (ascariasis OR ascaris OR a. lumbricoides OR trichuris OR trichuriasis OR whip worm OR t. trichura OR hookworm OR a. duodenale	29

	OR ancylostoma duodenale OR anclyostomiasis OR n. americanus OR necator americanus OR necatoriasis) AND PUBYEAR>1979	

These papers classified location-years for all locations and years present in the literature. We only utilised papers that are explicitly concerned with hookworm disease. Additionally, systematic literature reviews, meta-analyses, national health statistics publications, and collaborator input supported classification of location-years not present in the literature review wherever possible.

Modelling strategy

Prevalence model

In the estimation of overall morbidity due to hookworm disease, we implemented a three-stage modelling framework. We first utilised a spatiotemporal Gaussian process regression (ST-GPR) to generate a complete time series of estimates for each location where there are no geographical restrictions. ST-GPR attempts to model non-linear trends utilising a Gaussian process to fit a trend. We ran an age-restricted ST-GPR model, using all data with age bins between 5 and 20 because these data fall within the peak in prevalence across all age groups, the majority of data fall within these age ranges, and these data provide sufficient statistical power for our model. The following were the model specifications:

$$prevalence = sdi + improved\ water\ source + (1|level\ 1/level\ 2/level\ 3)$$

Levels 1, 2, and 3 refer to GBD location hierarchies, or nested random effects for super-region, region, and location. Covariate selection was based on directionality of the resulting beta values from the linear model, significant effect size, and subsequently out-of-sample testing for the resulting "best" combinations. The covariates used for the GBD 2023 model were Socio-demographic Index (SDI) and proportion of population with access to improved water sources. Improved toilet types and improved water sources are defined by the Joint Monitoring Programme.[4] The following hyperparameters were used: st-lambda = 0.25, st-omega = 2, st-zeta = 0.005, gpr-scale = 15. We selected these hyperparameters as they provided more weight to country-level data rather than region-level data when estimating the prevalence for a given location-year.

Table 2b. Covariates. Summary of covariates used in the hookworm ST-GPR model

**Covariate**	**Beta coefficient, log (95% UI)**	**Standard error**	**Exponentiated beta (95% UI)**
Improved water	–4.036 (–6.139 to –1.933)	1.073	0.018 (0.002 to 0.145)
Socio-demographic Index	–7.970 (–11.634 to –4.315)	1.864	$3.45*10^{-4}$ ($8.86*10^{-6}$ to 0.133)

Age pattern model

The next stage of the modelling process used a DisMod Bayesian meta-regression (DisMod-MR) model, to generate a global age-sex curve to disaggregate all-age, both-sex prevalence data. DisMod-MR is an

integrated meta-regression framework that allows multiple datasets to be used within a singular analysis regardless of age-binning, sources, and geographies. As a result, a variety of differently aggregated information is combined to generate a consensus output. Our final model contained all processed GAHI data as input informed by two country-level covariates (ie, all risk factors summary exposure values [SEV] for unsafe water and unsafe sanitation).

Table 2a. **Covariates.** Summary of covariates used in the hookworm DisMod-MR model

**Covariate**	**Type**	**Parameter**	**Exponentiated beta (95% UI)**
SEV unsafe water	Country-level	Proportion	4.43 (4.34–4.48)
SEV unsafe sanitation	Country-level	Proportion	4.44 (4.36–4.48)

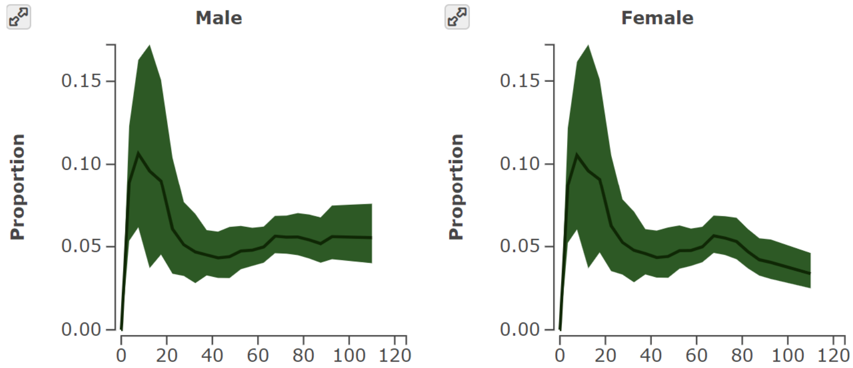

Figure 1: Global age-specific prevalence estimates for males (left) and females (right) for the year 2010. Proportion (prevalence) is on the Y-axis, and age in years on the X-axis. Screenshot from EpiViz tool.

Figure 1 shows the age-specific variation in prevalence rates, differentiated by sex. When considered as a global aggregate, we see that reported male and female prevalence are very similar. Prevalence peaks among young adults, followed by a decline and then stabilising during adulthood. These age-sex curves are similar to what has been reported in the literature.[2,3]

Imputations

The final stage of the overall prevalence modelling process is to impute the remaining age groups by borrowing information from the DisMod-MR global age-sex pattern and ST-GPR time series, by first assuming that the estimates from ST-GPR are representative of the 15–19-year-old age group. Each additional age group is assigned a ratio representing how much larger or smaller the prevalence is compared to the prevalence of the reference group (15–19-year-olds) using the DisMod-MR global age-sex pattern. The following is the computation for each age group:

$$Ratio = \frac{prevalence_{[age\ start]to\ [age\ end]}}{prevalence_{15\ to\ 19}}$$

With a ratio for every age group by sex, we multiplied the ratio by the ST-GPR location-year estimates to impute estimates for the remaining age groups.

Health states/sequelae

The table below shows the list of sequelae due to hookworm and the associated disability weights (DWs). Prevalence of medium infection and heavy infection were mapped to *mild abdominopelvic problems* and *heavy infestation of hookworm,* respectively. Light infection or asymptomatic were not attributed any disability.

Table 3. Severity distribution, details on the severity levels of hookworm and the associated disability weight (DW) with that severity

**Sequela**	**Lay description**	**DW (95% CI)**
Mild abdominopelvic problems	Has some pain in the belly that causes nausea but does not interfere with daily activities	0.011 (0.005–0.021)
Heavy infestation	Has cramping pain and a bloated feeling in the belly	0.027 (0.015–0.044)
Severe wasting	Is extremely skinny and has no energy	0.128 (0.082–0.183)
Asymptomatic hookworm disease	N/A	N/A
Mild anaemia	Feels slightly tired and weak at times, but this does not interfere with normal daily activities	0.004 (0.001–0.008)
Moderate anaemia	Feels moderate fatigue, weakness, and shortness of breath after exercise, making daily activities more difficult	0.052 (0.034–0.076)
Severe anaemia	Feels very weak, tired and short of breath, and has problems with activities that require physical effort or deep concentration	0.149 (0.101–0.210)

Following computations of location-year-age-sex-specific prevalence of hookworm, we leveraged information from the 2010 Expert Group (EG) data to conduct sequelae splits. The 2010 EG data provided estimates for heavy infestation, mild abdominopelvic problems, and asymptomatic hookworm by location and for 1990, 2005, and 2010. These three values add up to *all cases* of hookworm. Thus, for heavy infestation and mild abdominopelvic problems, we computed the proportion of cases that belong to our sequelae of interest over *all cases* of hookworm. More specifically, the following is the computation by heavy infestation and mild abdominopelvic problems:

$$Proportion_{sequelae} = \frac{prevalence_{sequelae}}{prevalence_{all\ cases}}$$

This calculates proportions for every location, year, and age group available. The EG data only had four age groups (0–4, 5–9, 10–14, 15+ years), so we applied the 15+ age group proportion for all remaining age groups. In addition, for the years 1995 and 2000, we applied the 1990 proportions, and for years 2015, 2019, and 2020–2021, we applied the 2010 proportions. Using these location-year-age-specific proportions, we multiplied the total hookworm estimates to compute heavy infestation and mild abdominopelvic prevalence. To estimate the prevalence of asymptomatic hookworm, prevalence of mild and heavy infestation were each subtracted from the overall hookworm prevalence.

The final step in the modelling process was to estimate the prevalence of severe wasting due to hookworm in age groups 1–5 months, 6–11 months, 12–23 months, and 2–4 years. This was done separately using 1000 draws of prevalence of heavy infestation due to hookworm and the wasting envelope prevalence. The initial step in determining prevalence of severe wasting due to hookworm was generating 1000 draws of change in weight-for-height z-score per heavy prevalent case from a random normal distribution with mean = 0.493826493 and standard deviation = 0.04972834 (calculated from upper and lower bounds of the mean estimate). The mean, upper, and lower bounds were based on a published article.[5] The prevalence of severe wasting due to hookworm was then obtained as a function of change in weight-for-height z-score. The following are the computations:

$$Prevalence_{wasting\ due\ to\ hookworm} = wasting - \Phi(\Phi^{-1}(\text{wasting}) - \text{z score} * \text{heavy infestation})$$

Where Φ is the standard normal cumulative distribution function and Φ^{-1} is the inverse standard normal cumulative distribution function. Finally, the age- and sex-specific anaemia prevalence for hookworm was analysed as part of overall anaemia causal attribution for GBD 2023. The description of the details of the anaemia analysis are in the "Anaemia impairment" section. Briefly, after estimating total anaemia, a series of counterfactual distributions of anaemia severity are generated based on the age- and sex-specific prevalence of each anaemia-causing condition, and the quantitative effect that the condition has on haemoglobin concentration in the blood, a "haemoglobin shift." Haemoglobin shifts due to hookworm disease were derived by meta-analysing cohort studies, observational studies, or trials comparing the haematological status of those with as compared to without the disease, which due to limited data, was assumed to be invariant over age, sex, location, and year. The generated counterfactual distributions of anaemia severity inform the estimated prevalence of anaemia attributable to hookworm disease.

Changes from GBD 2021 to GBD 2023

The major change from GBD 2021 was the addition of new data between the rounds. New data inputs from scientific literature, ESPEN, and expansion of age-specific data from the CWW and GAHI datasets were added to the model.

Limitations

As we attempt to improve the modelling processes for hookworm, we recognise that there are several limitations. We only include studies where Kato-Katz identifies infected individuals. Future updates to the model will include a systematic review for within-study comparisons of diagnostic performance to facilitate a crosswalk model.

A secondary limitation to our data is that several included studies are not nationally representative, and therefore at a location level, the data are highly heterogeneous. Numerous studies within the database come from districts or villages, and in most cases, the studies were done in areas where prevalence is known to be high.

In addition, our current model does not include the impact of mass drug administration (MDA). In future rounds, we plan to integrate MDA coverage into the estimate of prevalence.

Furthermore, we made a large assumption that the global age-sex distributions were applicable to all locations. While we believe that prevalence should peak among adolescents and slowly decline afterward, there is likely variation across regions and locations. Given that our data are among children or all-age, it is very difficult to build an age trend at granular location levels. Thus, we allowed DisMod-MR to disaggregate our heterogeneous data in an effort to provide sensible age-sex curves.

We did not apply any adjustments for the COVID-19 pandemic to hookworm disease due to a lack of available data quantifying the impacts of the pandemic on NTD epidemiology.

We believe that more work will improve our sequelae split methods. Since the EG data do not provide all estimation years and age groups, several assumptions had to be made to estimate sequelae for all years, locations, and age groups in GBD. Further work will be required to gather additional data and improve these sequelae estimates.

References

1. London School of Hygiene and Tropical Medicine. Global Atlas of Helminth Infections – Soil Transmitted Helminths. London, United Kingdom: London School of Hygiene and Tropical Medicine.
2. Riess H, Clowes P, Kroidl, Kowuor D, Nsojo A, Mangu C, Schule S, Mansmann U, Geldmacher C, Mhina S, Maboko L, Hoelscher M, Saathoff E. Hookworm Infection and Environmental Factors in Mbeya Region, Tanzania: A Cross-Sectional, Population-Based Study. *PLoS Neglected Tropical Diseases*. 2013. 7. e2408.
3. Pullan R, Kabatereine N, Quinnell R, Brooker S. Spatial and Genetic Epidemiology of Hookworm in a Rural Community in Uganda. *PLoS Neglected Tropical Diseases*. 2010. 4. e713.
4. "Improved and Unimproved Water Sources and Sanitation Facilities." *WHO / UNICEF Joint Monitoring Programme: Wat/san Categories*. The WHO/UNICEF, n.d. Web. 08 June 2016
5. Hall A, Hewitt G, Tuffrey V, de Silva N. A review and meta-analysis of the impact of intestinal worms on child growth and nutrition. *Maternal and Child Nutrition*. 2008. 4. 118-236.

Figure 2a. PRISMA 2023 flow diagram – systematic review of hookworm prevalence literature (updates for GBD 2023)

Source: Page MJ, et al. BMJ 2021;372:n71. doi: 10.1136/bmj.n71. This work is licensed under CC BY 4.0. To view a copy of this license, visit https://creativecommons.org/licenses/by/4.0/

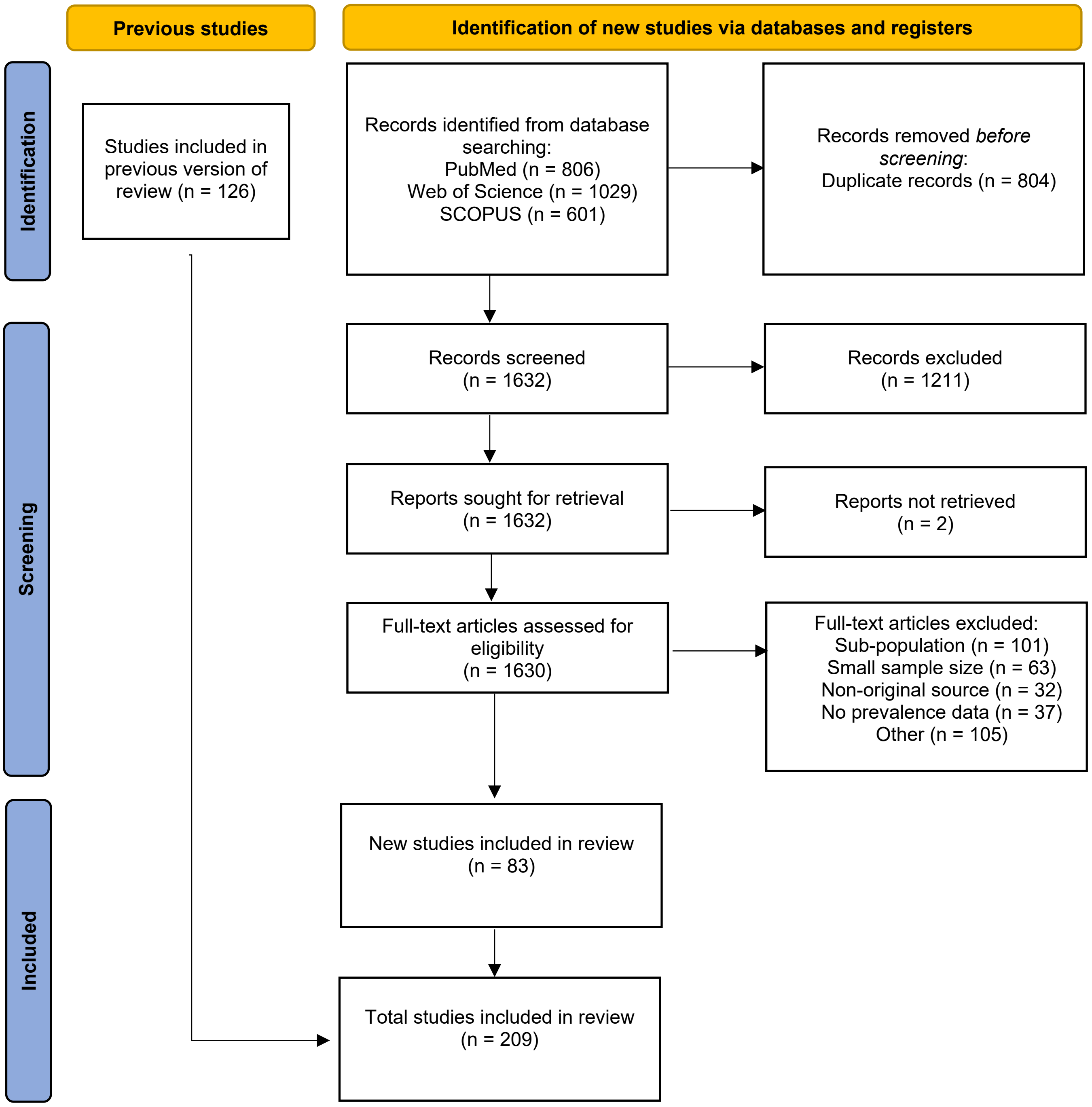

Figure 2b. PRISMA 2023 flow diagram – expansion of prevalence data from GAHI, CWW, and WHO databases (updates for GBD 2023)

Source: Page MJ, et al. BMJ 2021;372:n71. doi: 10.1136/bmj.n71. This work is licensed under CC BY 4.0. To view a copy of this license, visit https://creativecommons.org/licenses/by/4.0/

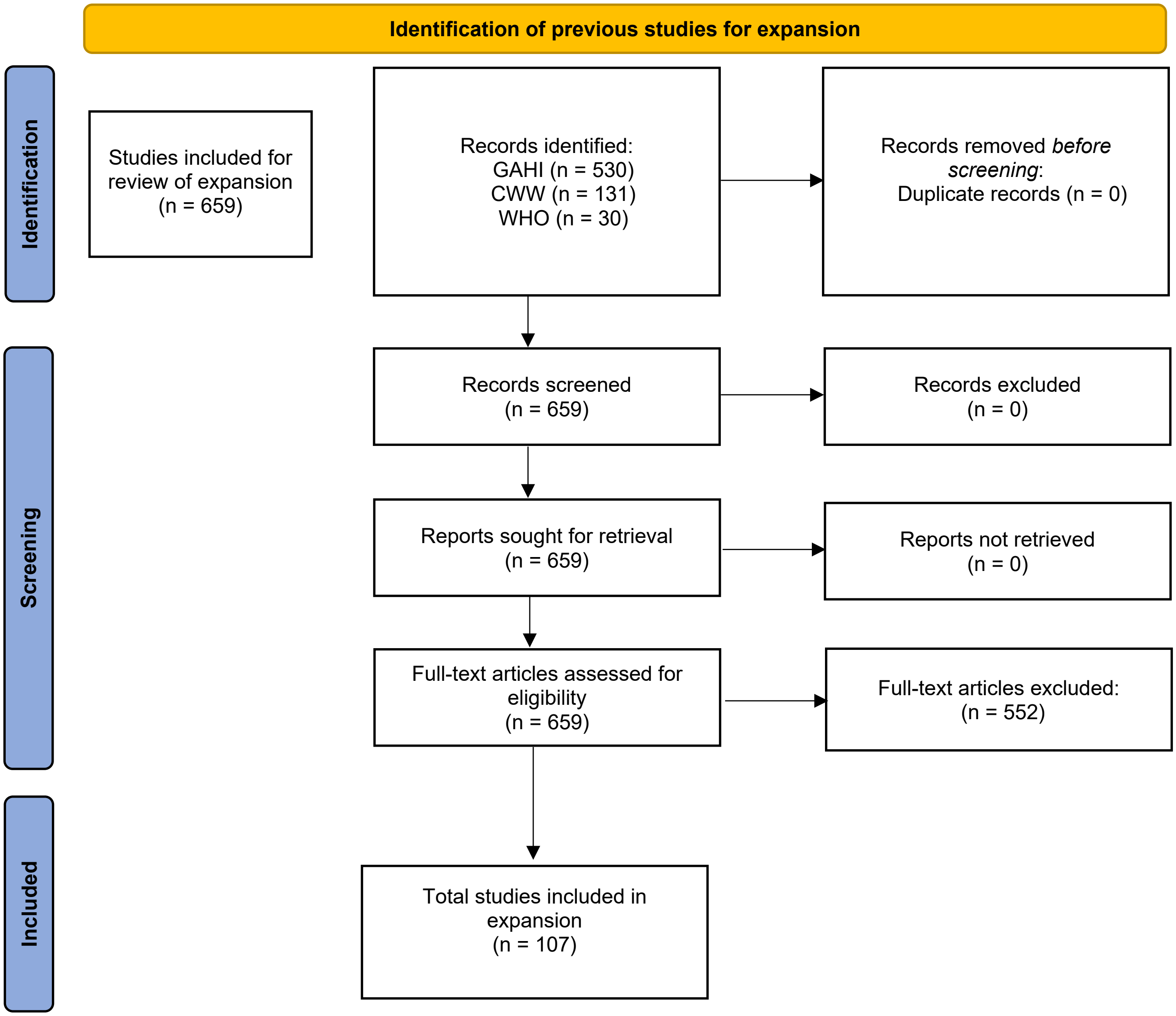

Human African trypanosomiasis (HAT)

Flowchart

African trypanosomiasis

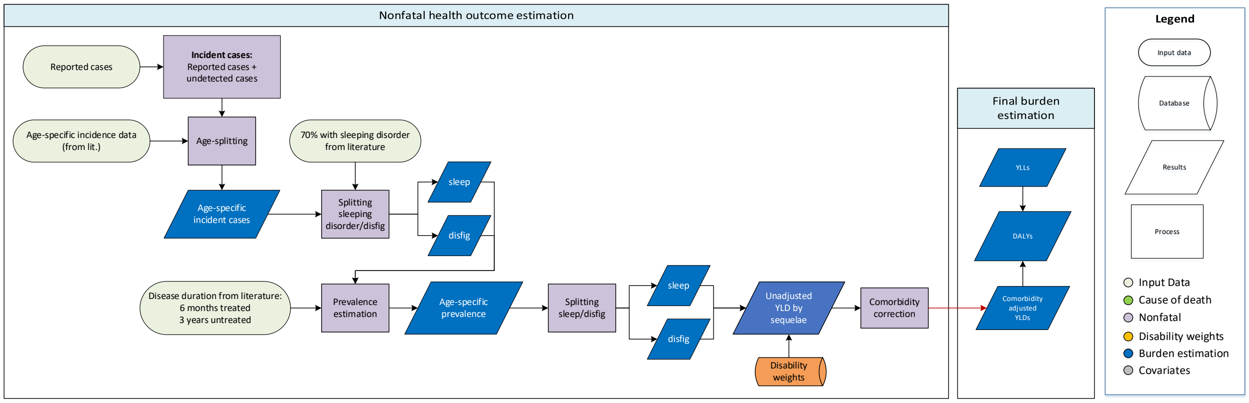

Input data and methodological summary for human African Trypanosomiasis

Case definition

Human African trypanosomiasis (HAT), also referred to as “sleeping sickness”, is a protozoan infection transmitted by tsetse flies that causes headache, fever, and joint pain, then progresses to neurological involvement including sleep, movement, speech, and psychiatric disorders; seizures; coma; and (if untreated) death. It is caused by the parasite *Trypanosoma brucei* with two subspecies, namely *T.b. rhodesience* and *T.b. gambiense*. Cases are diagnosed through laboratory methods which rest on finding the parasite in body fluid or tissue by microscopy. In highly endemic or epidemic areas where the likelihood of false positives in serological tests is deemed lower, a seropositive individual is considered affected even in the absence of parasitological confirmation. The ICD-10 codes for HAT are B56.0, B56.1, and B56.9. We used the following case definition for GBD 2023:

Quantity of interest	Reference or alternative	Definition
Human African trypanosomiasis (incidence)	Reference	Parasitological confirmation via microscopy. In highly endemic or epidemic areas, seropositive cases in absence of parasitological confirmation are included.
Human African trypanosomiasis (prevalence)	Reference	Parasitological confirmation via laboratory methods (microscopy). A seropositive individual is considered affected in absence of parasitological confirmation in highly endemic or epidemic areas.

Input data

Model inputs

Data sources for GBD 2023:

1) Annual case totals 1980–2022: National-level annual case totals from 1990–2022 were obtained from WHO’s publicly available dataset.[1]

Subnational data:

Kenya: Kenyan subnational estimates are attributed to Busia County. Identification of subnational locations for Kenyan case data were obtained via studies published in the peer-reviewed literature[2] and review of maps published from via the WHO HAT Atlas.[3]

2) Age/sex data: Data on the age and sex distribution of HAT were last updated for GBD 2017. Cases were extracted from the peer-reviewed literature via a systematic review of sources identified in PubMed using the following search string:

   ((African trypanosomiasis[Title/Abstract] AND (incidence[Title/Abstract] OR burden[Title/Abstract] OR prevalence[Title/Abstract] OR community[Title/Abstract])) AND ("1990"[Date – Publication] : "2017"[Date – Publication]))

   This yielded 219 studies, of which only three met the inclusion criteria and were extracted.[4-6] The inclusion criteria were:

   1. Studies representative of the national population
   2. Population-based studies
   3. Studies with primary data on incidence
   4. Studies of human African trypanosomiasis (excluded studies on animal African trypanosomiasis)

3) Population at risk estimates: 1980–2015 population at risk estimates from GBD 2010 ArcGIS analysis using geocoded case notifications for 2000 to 2009[7] and population Count Grid estimates from Gridded Population of the World 3.[8]

4) Screening coverage: Data on active versus passive screening coverage were obtained from a Weekly Epidemiological Report[9] identifying the population screened from 1997 to 2004 at the national level.

5) Geographical restrictions: Data file of all GBD locations, defining location as either endemic or non-endemic for HAT. Estimates are not produced for non-endemic countries, nor are they generated for countries with a history of HAT transmission but no data reported by WHO from 1990 to 2022.

Modelling strategy

Geographical restrictions

For countries historically considered endemic for HAT, but which have no reported case data or estimate of the population at risk, estimates are not produced. These countries include Botswana, Ethiopia, Guinea-Bissau, and Rwanda.

Among countries where population at-risk data are available, if no cases were reported to WHO, we assume the incidence of HAT is zero for those years and generate model estimates accordingly.

Modelling steps

Non-fatal estimates for HAT were generated as follows:

1. The incidence of reported HAT cases among the population at risk was calculated as the total number of reported cases divided by the population at risk estimates generated by the GBD working group for the period 1980–2015. Population at risk estimates for 2016–2023 were generated by assuming an annual 2% rate of population growth.

2. To estimate the number of cases that were likely undetected by country and year, a multi-level mixed-effects linear regression of log-transformed incidence rate (ratio of reported HAT cases to population at risk) on log-transformed screening coverage (ratio of number screened for HAT to population at risk), with country random effects, was performed. Gaps were then filled using interpolation between years and extrapolation from 2021 to 2023 for reported cases. This model generates a beta-coefficient which is used to estimate the case detection rate (see step 4).

   For countries with particularly sparse data on screening coverage, we used alternative approaches to avoid excessive extrapolation. Among countries with data reported only for years 1997–2004, the proportion of the at-risk population screened from 1997 was used retrospectively for the period 1980–1996, and the screening coverage from 2004 was carried forward from 2005 to 2023. For countries with no screening data reported, we used the mean screening coverage for the region.

3. Assuming the same proportion in treated (reported) and untreated (undetected) cases, the incidence estimates were then split into the two sequelae, skin disfigurement and sleeping disorder. This was done by generating 1000 draws of the splitting proportion for the sequelae (70%–74% with sleeping disorder) based on a study that reported presence of symptoms at admission of patients in treatment centres.[10] Draws were generated from a beta distribution with alpha parameter = 1884 and beta parameter = 649.

4. To compute prevalence of HAT, 1000 draws of total duration of symptoms in untreated cases were generated from a normal distribution with mean = [ln(3) – 0.5 * sigma^2] and standard deviation = sigma, where sigma = [ln(4.39)-ln(1.92))/(invnormal(0.975)*2)]: these parameters were based on a study of *T.b. gambiense*[10] which estimated an average duration of three years to untreated cases. An estimated duration of six months was applied to cases that received treatment, based on findings from a paper about *T.b. rhodesiense* in Uganda.[11]

5. Prevalence was then estimated from the incident cases before applying age pattern. Prevalence of treated and untreated cases were summed up, assuming that untreated cases have been prevalent up to their death for a certain duration.[12] For untreated cases, it was assumed that half the duration is spent with sleeping disorder (severe motor and cognitive impairment) and disfigurement.[10] Treated (ie, reported) cases are assumed to have been prevalent for 0.5 years, and for the fraction of treated cases that present with sleeping disorder, it was assumed that this is present for half the total duration and that the rest of the duration is spent suffering from disfiguring skin disease. Among reported cases assumed to be detected prior to stage 2 infection, we do not attribute any of the duration of morbidity to sleeping disorder.

6. Finally, an age pattern was applied to the prevalence estimates using the incidence studies from Sudan,[6] DRC,[4] and Uganda.[5] The age pattern employed a cubic spline to account for the higher risk of infection among working-age adults.

Severity splits/sequelae

The basis of the GBD disability weight (DW) survey assessments are lay descriptions of sequelae highlighting major functional consequences and symptoms. The lay descriptions and disability weights for HAT sequelae due to HAT are shown below in Table 1.

Table 1. Health states for human African trypanosomiasis

**Sequela**	**Lay description**	**DW (95% CI)**
Skin disfigurement, level 1	Has a slight, visible physical deformity that is sometimes sore or itchy. Others notice the deformity, which causes some worry and discomfort.	0.027 (0.015–0.042)
Motor plus cognitive impairments, severe	Cannot move around without help, and cannot lift or hold objects, get dressed, or sit upright. The person also has very low intelligence, speaks few words, and needs constant supervision and help with all daily activities	0.542 (0.37–0.702)

We did not apply any adjustments for the COVID-19 pandemic to human African trypanosomiasis due to a lack of available data quantifying the impacts of the pandemic on NTD epidemiology.

Changes from GBD 2021 to GBD 2023

There were no substantive changes to the modelling strategy for GBD 2023.

References

1. The Global Health Observatory, WHO. “Human African Trypanosomiasis (Sleeping Sickness).” https://www.who.int/data/gho/data/themes/topics/human-african-trypanosomiasis.
2. Rutto JJ, Osano O, Thuranira EG, Kurgat RK, Odenyo VA. Socio-economic and cultural determinants of human african trypanosomiasis at the Kenya - Uganda transboundary. *PLoS Negl Trop Dis* 2013; **7**(4): e2186.
3. Simarro PP, Cecchi G, Paone M, et al. The Atlas of human African trypanosomiasis: a contribution to global mapping of neglected tropical diseases. *Int J Health Geogr* 2010; **9**: 57.
4. Lutumba P, Makieya E, Shaw A, Meheus F, Boelaert M. Human African trypanosomiasis in a rural community, Democratic Republic of Congo. *Emerg Infect Dis* 2007; **13**(2): 248-54.
5. Fevre EM, Odiit M, Coleman PG, Woolhouse ME, Welburn SC. Estimating the burden of rhodesiense sleeping sickness during an outbreak in Serere, eastern Uganda. *BMC Public Health* 2008; **8**: 96.
6. Moore A, Richer M, Enrile M, Losio E, Roberts J, Levy D. Resurgence of sleeping sickness in Tambura County, Sudan. *Am J Trop Med Hyg* 1999; **61**(2): 315-8.
7. Mapping the distribution of human African trypanosomiasis, WHO. “The Atlas of Human African Trypanosomiasis (2000-2018): Kenya.” http://quarry.essi.upc.edu:8080/who/Distribution/HAT_Kenya_2000-2018.jpg.

8. Earth Science Data Systems, NASA. “Gridded Population of the World, Version 3 (GPWv3): Population Density Grid | NASA Earthdata.” Earth Science Data Systems, NASA. https://www.earthdata.nasa.gov/data/catalog/sedac-ciesin-sedac-gpwv3-popdens-3.00.
9. World Health Organization. Human African trypanosomiasis (sleeping sickness): epidemiological update. *Weekly epidemiological record* 2006; **February 24**(8): 69-80.
10. Blum J, Schmid C, Burri C. Clinical aspects of 2541 patients with second stage human African trypanosomiasis. *Acta Trop* 2006; **97**(1): 55-64.
11. Odiit M, Kansiime F, Enyaru JC. Duration of symptoms and case fatality of sleeping sickness caused by Trypanosoma brucei rhodesiense in Tororo, Uganda. *East Afr Med J* 1997; **74**(12): 792-5.
12. Checchi F, Filipe JA, Haydon DT, Chandramohan D, Chappuis F. Estimates of the duration of the early and late stage of gambiense sleeping sickness. *BMC Infect Dis* 2008; **8**: 16.

Infertility (impairment)

Flowchart

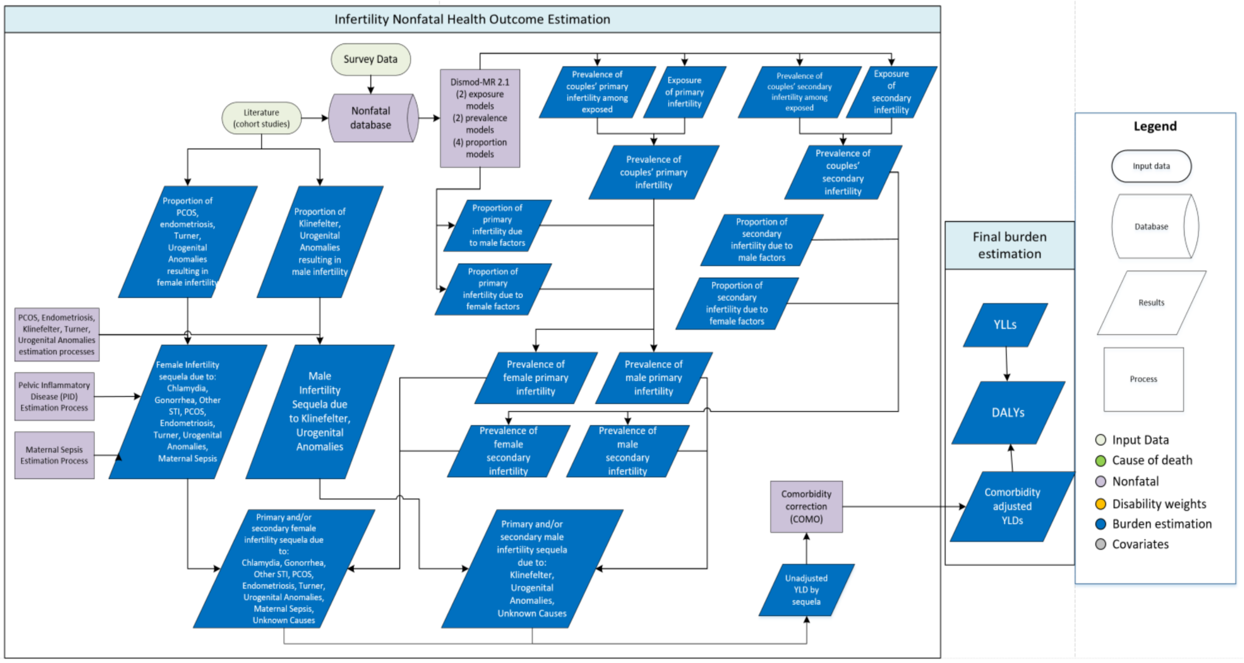

Input data and methodological summary for infertility impairment

Case definition

Clinically, infertility is a diagnosis made in couples and encompasses both primary infertility and secondary infertility, which are defined as inability to conceive a pregnancy after 12 months of vaginal intercourse without using contraceptives (primary: without any prior birth, secondary: after at least one prior birth).

In GBD, we employ the following case definitions for measuring the prevalence of infertility using household survey data:

- Primary infertility is diagnosed in a couple who have not had a livebirth, desire a child, have been in a union for five years or longer, and are not using contraceptives at the time of the survey.
- Secondary infertility is diagnosed in a couple who last had a livebirth more than five years ago, desire a child, have been in a union for five years or longer, and are not using contraceptives at the time of the survey.

These case definitions agree with definitions of infertility for use with household survey data that were proposed and described in detail by Mascarenhas and colleagues, which are operationalised to separately estimate the prevalence of primary infertility among couples at risk, the prevalence of couples at risk for primary infertility, the prevalence of secondary infertility among couples at risk, and the prevalence of couples at risk for secondary infertility.[1] For GBD purposes, however, we extend this

analysis to then estimate the proportion of primary and secondary infertility in couples that is attributable to female factors and male factors, and express prevalence of primary and secondary infertility out of all females and males of reproductive age in the general population. We also execute a "causal attribution" process to assign cases of primary female infertility, primary male infertility, secondary female infertility, and secondary male infertility to likely underlying causes and classify the remainder as idiopathic (ie, unknown causes).

Infertility envelopes and exposures

**Quantity of interest**	**Reference or Alternative**	**Definition**
Primary infertility envelope	Reference	Couple who are together for 5+ years, are not using contraception, want a child, and has not had a livebirth among those couples and couples who have been together for 5+ years and has had a livebirth as measured based on the responses of women ages 15–49 years old in general household surveys.
Primary infertility exposure	Reference	Married, not using contraception, and wants a child OR married and has had a livebirth among all women surveyed as measure based on the responses of women ages 15–49 years old in general household surveys.
Secondary infertility envelope	Reference	Married 5+ years, had a livebirth more than five years ago but not in the last five years, not using contraception, and wants children among them and married 5+ years, who have had a livebirth more than five years ago and had a livebirth within the last five years as measured based on the responses of women ages 15–49 years old in general household surveys.
Secondary infertility exposure	Reference	Married, has had a livebirth, not using contraception, and wants a child OR married, had a birth in the last five years, and has had more than one livebirth among all women surveyed as measured based on the responses of women ages 15–49 years old in general household surveys.

Female and male primary and secondary infertility

**Quantity of interest**	**Reference or alternative**	**Definition**
Female primary infertility	Reference	Case series of females in couples with primary infertility identified from infertility clinics. Couples with primary infertility are those who have not had a livebirth, who wish to have a child, and have been in a union without using contraceptives but have not had a pregnancy in 12 months.
Female secondary infertility	Reference	Case series of females in couples with secondary infertility identified from infertility clinics. Couples with secondary infertility are those who wish to have a child and have been in a union without using contraceptives since the last livebirth but have not had a pregnancy in 12 months.
Male primary infertility	Reference	Case series of males in couples with primary infertility identified from infertility clinics. Couples with primary infertility are those who have not had a livebirth, who wish to have a child, and have been in a union

		without using contraceptives, but have not had a pregnancy in 12 months.
Male secondary infertility	Reference	Case series of males in couples with secondary infertility identified from infertility clinics. Couples with secondary infertility are those who wish to have a child and have been in a union without using contraceptives since the last livebirth but have not had a pregnancy in 12 months.

Input data

Our primary data sources for estimating the prevalence of infertility are population-based household surveys. No new surveys were added in GBD 2023.

Data were extracted for female respondents in five-year age groups between 15 and 49 from population-based surveys including the Demographic and Health Surveys (DHS), World Fertility Surveys (WFS), Reproductive Health Surveys (RHS), Family and Fertility Survey (FFS), and others (EUR, NSF, PCD, PFM). Such surveys only ask fertility-related questions to women in a marriage or union. Even though only women are interviewed, we treated the responses as a proxy for the infertility of couples in unions because the questions are not structured in a way that it is possible to determine which partner is the cause of the couples' inability to conceive a child.

The combination of variables in surveys that were used to construct datasets for each of four models (primary "impairment" and "exposure" and secondary "impairment" and "exposure") are illustrated in the table below. As described below, prevalence of primary and secondary infertility in the population is estimated by multiplying prevalence among those with the "impairment" of infertility by the prevalence of the "exposure".

Table 1: Data extraction definitions used in estimation of infertility

Model name	Indicator	Numerator	Denominator
**Primary infertility (exposure)**	At risk for primary infertility among all women	Married, not using contraception and wants a child OR married and has had a livebirth (A+B+C)	All women surveyed
**Primary infertility (impairment)**	Primary infertility among married women	Married 5+ years, not using contraception, wants a child, and has not had a livebirth (A)	Numerator + Married 5+ years and has had a livebirth (A+B)
**Secondary infertility (exposure)**	At risk for secondary infertility among all women	Married, has had a livebirth, not using contraception, and wants a child, OR married, had a birth in the last 5 years, and has had more than one livebirth (A+B+C)	All women surveyed
**Secondary infertility (impairment)**	Secondary infertility among married women	Married 5+ years, had a livebirth more than five years ago but not in the last five years, not using contraception, and wants a child (A)	Numerator + Married 5+ years, had a livebirth more than 5 years ago and had a live birth within the last 5 years (A+B)

A second dataset informed estimates of which component of primary and secondary infertility was due to female and male factors, respectively. To obtain data on the sex breakdown for infertility, we used data from a systematic review of the literature conducted for GBD 2010 which used the following search string:

Causes[Title/abstract] AND infertility[Title] NOT mouse NOT murine NOT rat NOT rodent

This produced 626 hits from PubMed and studies were excluded according to the following criteria:

1. studies not representative of the national population;
2. studies that provide no raw data,
3. studies that provide only estimates;
4. studies performed before 1970;
5. case studies or studies with sample size less than 50;
6. studies that provide no data on the sex of the partner responsible for infertility among couples.

The majority of excluded studies were excluded because of the latter criterion. In total, 15 studies were included in our analysis for the sex breakdown among infertile couples. Infertility among couples was reported as due to one of the following causes: male factor, female factor, both, or unknown. Data reporting couples' infertility due to both partners were allocated to both male factor and female factor, and data reporting couples with infertility of unknown cause were allocated to male and female factors based on the proportion observed in other couples in the study.

Klinefelter's and Turner's syndrome cases were all considered to be infertile, but for all other causes of infertility, we required data inputs for estimating the prevalence of infertility due to each disease. The proportion of reproductive-age adults with urogenital anomalies with infertility was taken from Ching and colleagues 2011 and Davies and colleagues 2010[2,3] (see also "Congenital birth defects" section in this appendix). The proportions of endometriosis and polycystic ovarian syndrome cases with infertility were taken from the Australian Longitudinal Women's Health Study (ALWHS)[4] (see also the "Gynaecological diseases" section of this appendix). For STIs and maternal sepsis, the proportion of incident cases that go on to develop infertility were obtained from studies by Weström and colleagues[5,6] and were used to determine the proportion of PID cases that are caused by STIs and go on to develop infertility and puerperal sepsis that go on to develop infertility, respectively (see also the "Maternal disorders" sections in this appendix). The application of these proportions is described further in the "Causal attribution" section of this write-up.

Data sources specific to the infertility impairment estimation process did not change between GBD 2021 and GBD 2023, although data sources may have changed for estimating the prevalence of one or more disease for which infertility is a sequela, leading to shifts in the attribution of infertility to different GBD diseases. See the "Congenital birth defects," "Gynaecological diseases," "Pelvic inflammatory disease," and "Maternal disorders" portions of the "Non-fatal modelling methods" section in this appendix for updates on data sources for GBD causes that result in infertility.

Data processing

The processing of infertility data was unchanged in GBD 2023 from GBD 2021, and consisted of age-splitting only. Individual-level data from surveys were extracted and aggregated into GBD age groups, but for any summary measure extracted from published reports that referred to an age range that did not entirely fit within a GBD age group, the observation was split to be multiple age-specific datapoints

based on the age pattern predicted by DisMod-MR 2.1 models from the previous round. All extracted indicators were sex-specific and used similar case definitions and data collection methods, so sex-splitting and crosswalking were not required.

Modelling strategy

We estimated the prevalence of primary and secondary infertility by sex and cause in three steps: 1) estimation of couples' infertility [four DisMod-MR 2.1 models], 2) estimation of infertility by sex [four DisMod-MR 2.1 models], and 3) causal attribution of infertility. All DisMod-MR 2.1 models were run as single parameter models (either prevalence or proportion). We assumed zero infertility prior to age 15 or after age 50 years.

In previous rounds, we tested the predictive value of several covariates in all DisMod models: the prevalence of pelvic inflammatory disease, the risk-weighted prevalence of smoking, obesity, and alcohol use as measured by the summary exposure values (SEV) for smoking, body-mass index, and alcohol consumption (%), and the age-standardised death rate (lnASDR) of sexually transmitted infections (STIs) as country-level covariates. Predictive covariates by model are summarised below.

Estimation of couples' infertility

To estimate the prevalence of primary and secondary infertility among couples, we first ran four DisMod-MR 2.1 models to estimate the four parameters detailed above: (1) prevalence of primary infertility exposure, (2) proportion of primary infertility among the exposed, (3) prevalence of secondary infertility exposure, and (4) proportion of secondary infertility among the exposed.

Table 2. Covariates. Summary of covariates used in the prevalence of primary infertility exposure DisMod-MR model

Covariate	Parameter	Exponentiated beta (95% uncertainty interval)
Age-standardised death rate (lnASDR) of sexually transmitted infections excluding HIV	Prevalence	1.00 (1.00–1.00)

Table 3. Covariates. Summary of covariates used in the prevalence of primary infertility among the exposed DisMod-MR model

Covariate	Parameter	Exponentiated beta (95% uncertainty interval)
Age-standardised death rate (lnASDR) of sexually transmitted infections excluding HIV	Prevalence	1.00 (0.98–1.02)

Table 4. Covariates. Summary of covariates used in the prevalence of secondary infertility exposure DisMod-MR model

Covariate	Parameter	Exponentiated beta (95% uncertainty interval)
Age-standardised death rate (lnASDR) of sexually transmitted infections excluding HIV	Prevalence	0.99 (0.99–0.99)

Table 5. Covariates. Summary of covariates used in the prevalence of secondary infertility among the exposed DisMod-MR model

Covariate	Parameter	Exponentiated beta (95% uncertainty interval)
Age-standardised death rate (lnASDR) of sexually transmitted infections excluding HIV	Prevalence	1.00 (1.00–1.00)

We then multiplied estimates of the proportion of infertility among the exposed by the prevalence of exposure to obtain the prevalence of primary infertility among couples and the prevalence of secondary infertility among couples.

Estimation of infertility by sex

Next, we ran four DisMod-MR 2.1 models to estimate the prevalence of primary and secondary infertility by sex: (1) proportion of primary infertility attributable to female factor, (2) proportion of secondary infertility attributable to female factor, (3) proportion of primary infertility attributable to male factor, and (4) proportion of secondary infertility attributable to male factor. The time window of fit on each of these models was 10 years.

Table 6. Covariates. Summary of covariates used in the proportion of secondary infertility attributable to female factor DisMod-MR model

Covariate	Parameter	Exponentiated beta (95% uncertainty interval)
Age-standardised death rate (lnASDR) of sexually transmitted infections excluding HIV	Proportion	0.47 (0.32–0.64)

Because infertility in some couples is attributable to both partners rather than just one, the sum of the proportions due to each partner can exceed one. When the sum of the proportions estimated by DisMod is lower than one, however, we re-scale them to sum to one. We multiplied the prevalence of primary and secondary infertility derived in step 1 by the proportion due to male and female factors to estimate the following: (1) Female primary infertility (prevalence), (2) Male primary infertility (prevalence), (3) Female secondary infertility (prevalence), (4) Male secondary infertility (prevalence).

Causal attribution of infertility

There are nine identified causes of infertility in the GBD 2023 cause list: pelvic inflammatory disease (PID) due to sexually transmitted infections (STIs), including chlamydial infection, gonococcal infection, and other STIs; maternal sepsis; polycystic ovarian syndrome (PCOS); endometriosis; congenital Klinefelter syndrome; congenital Turner syndrome; and congenital urogenital anomalies. For each disease, we first determine the total prevalence of infertility due to that disease for each year-age-sex-location combination, and then we assign those to primary and secondary infertility, by sex, according to their natural history, as described below.

Total infertility by cause

For Klinefelter syndrome and Turner syndrome, all prevalent cases of these diseases were considered to be infertile throughout the reproductive years.

For urogenital anomalies, endometriosis, and PCOS, the prevalence of infertility among prevalent disease cases (either alone or in combination with another sequela) were derived from the published sources described above. We applied the proportion of prevalent urogenital anomalies, endometriosis, and PCOS cases with each infertility-related sequela to the prevalence of each disease estimated in DisMod-MR 2.1. This resulted in the prevalence of each infertility-related sequela due to each disease, as listed in the table below. See the "Congenital birth defects" and "Gynaecological diseases" sections in the "Non-fatal modelling methods" portion of this appendix for details on the DisMod models.

For STIs and maternal sepsis, we estimated the prevalence of infertility due to these causes by first estimating incidence of these diseases, applying a published estimate of the proportion of incident cases that go on to develop infertility, and then using these incidence estimates as inputs to an additional DisMod-MR 2.1 compartmental model to estimate the prevalence in the relevant age groups over time. For STIs, we started with four custom models output from the PID estimation process: the prevalence and incidence of PID, the proportion of PID due to chlamydial infection, the proportion of PID due to gonococcal infection, and the proportion of PID due to other STIs. See the pelvic inflammatory diseases section in the non-fatal "Modelling methods" portion of this appendix for details about the data inputs and modelling strategy. We then multiplied each proportion model by the PID envelope model to get the following outputs: the prevalence and incidence of PID due to chlamydial infection, the prevalence and incidence of PID due to gonococcal infection, and the prevalence and incidence of PID due to other STIs. Next, we took an estimate of the proportion of incident PID cases that go on to experience infertility from the analysis of Weström and colleagues,[5] applied it to the incident cases of PID due to each STI, and used DisMod-MR 2.1 to calculate the corresponding prevalence for each subsequent age group through their reproductive years, assuming zero remission or excess mortality. This process produced the following models: the prevalence of infertility due to chlamydial infection, the prevalence of infertility due to gonococcal infection, and the prevalence of infertility due to other STIs.

For maternal sepsis, estimates of puerperal sepsis incidence were taken directly from DisMod (see the "Non-fatal modelling methods: Maternal disorders" section of this appendix for details of DisMod maternal sepsis modelling). These were multiplied by an estimate of the proportion of incident cases that go on to develop infertility, also taken from Westrom,[6] and the product was input into a subsequent DisMod compartmental model to estimate prevalence of infertility due to maternal sepsis over age and time.

Table 7: Infertility sequela by cause

Cause Group	Cause	Sequelae*
Sexually transmitted infections (STIs)	PID due to chlamydial infection	• Infertility due to chlamydial infection
	PID due to gonococcal infection	• Infertility due to gonococcal infection
	PID due to other STI	• Infertility due to other STIs

Gynaecological diseases	Endometriosis	• Mild abdominal pain and infertility due to endometriosis<br>• Moderate abdominal pain and infertility due to endometriosis<br>• Severe abdominal pain and infertility due to endometriosis<br>• Infertility due to endometriosis
	Polycystic ovarian syndrome	• Infertility due to PCOS<br>• Hirsutism & infertility due to PCOS
Maternal disorders	Maternal sepsis	• Infertility due to puerperal sepsis
Congenital birth defects	Congenital Turner	• Heart diseases with infertility due to Turner syndrome<br>• Infertility due to Turner syndrome
	Congenital urogenital anomalies	• Infertility due to genital anomalies<br>• Atypical genitalia and infertility due to genital anomalies<br>• Infertility and recurrent urinary tract infections or other abdominal issues due to genital anomalies<br>• Infertility and impotence due to genital anomalies<br>• Atypical genitalia, recurrent urinary tract infections or other abdominal issues, and infertility due to genital anomalies<br>• Atypical genitalia, infertility, and impotence due to genital anomalies<br>• Infertility, impotence, and recurrent urinary tract infections or other abdominal issues and impotence due to genital anomalies<br>• Atypical genitalia, infertility, impotence, and recurrent urinary tract infections or other abdominal issues and impotence due to genital anomalies
	Congenital Klinefelter	• Borderline intellectual disability with infertility due to Klinefelter syndrome<br>• Mild intellectual disability with infertility due to Klinefelter syndrome<br>• Infertility due to Klinefelter syndrome

**Each of these diseases has other sequela that are not related to infertility that are not listed in this table.*

Sex- and stage-specific attribution of infertility

Once the infertility-related sequelae for each disease are estimated, the models are split into primary or secondary infertility for females or males.

Infertility by stage – female

The following GBD diseases can cause infertility in females: chlamydial infection, gonococcal infection, other STIs, endometriosis, PCOS, maternal sepsis, congenital Turner syndrome, and congenital urogenital anomalies.

To split the female infertility sequela into primary or secondary infertility, we aggregate the primary and secondary female impairment envelopes and then calculate the proportion of the aggregate envelope that is primary infertility and the proportion that is secondary infertility. We multiply the primary and secondary proportions by the prevalence of each female infertility sequela for PCOS, endometriosis, gonococcal infection, chlamydial infection, and other STIs to split each sequela into primary or secondary sequela. All female infertility sequelae from congenital Turner and congenital urogenital syndromes are assigned to female primary infertility. The infertility sequela from maternal sepsis is assigned to female secondary infertility.

Next, we estimate the portion of female infertility that is due to unknown causes, by stage. To do this, we sum the prevalence of all female primary infertility sequelae for comparison to 95% of the female primary infertility impairment envelope. If the prevalence of the summed female primary sequelae is less than 95% of the female primary envelope, then the difference between the prevalence of the summed sequelae and the envelope is assigned to "primary female infertility due to other causes," also known as idiopathic primary female infertility. If the sum of the female primary infertility sequelae is greater than 95% of the envelope, each individual sequela is scaled to 95% of its prevalence estimates, then the scaled sequelae are aggregated. We assign the difference between the rescaled, aggregated female primary infertility sequela and the female primary infertility impairment envelope to idiopathic primary female infertility. The same process is used to estimate idiopathic secondary female infertility, substituting the female primary sequelae and envelope for their secondary counterparts.

Finally, we determine the cases of endometriosis and PCOS sequelae that were misallocated to sequelae with infertility. We aggregate the primary and secondary sequelae for each disease and compare to the prevalence of the sequelae prior to being split into primary and secondary. If the prevalence of the original sequelae is greater than the prevalence of the aggregated type-specific sequelae, we assign the excess cases back to the disease, and not to the infertility-related sequela due to the disease. The excess cases are not included in years lived with disability (YLDs) or disability-adjusted life-years (DALYs) estimates for infertility but are included in disease-specific YLDs and DALYs for PCOS or endometriosis.

Infertility by stage – male

The following diseases can cause infertility in males: congenital Klinefelter syndrome and congenital urogenital anomalies.

To estimate male primary infertility due to unknown causes, we aggregate the prevalence of all Klinefelter and urogenital anomalies sequelae and subtract the summed value from the male primary infertility impairment envelope. We assign the remaining prevalence to idiopathic male primary infertility impairment. All Klinefelter and urogenital anomalies sequelae are assigned to primary infertility.

There are specific causes of secondary infertility in males in the GBD framework, so we assigned the entire secondary male infertility impairment envelope to idiopathic secondary male infertility impairment.

Table 8: Infertility sequelae by sex, stage, and cause

<table>
<tr><th>Cause</th><th>Female</th><th>Male</th></tr>
<tr><td>[PID due to] chlamydial infection</td><td>Primary/secondary infertility due to chlamydial infection</td><td>NA</td></tr>
<tr><td>[PID due to] gonococcal infection</td><td>Primary/secondary infertility due to gonococcal infection</td><td>NA</td></tr>
<tr><td>[PID due to] other STIs</td><td>Primary/secondary infertility due to other STIs</td><td>NA</td></tr>
<tr><td>Endometriosis</td><td>Mild abdominal pain and primary/secondary infertility due to endometriosis
Moderate abdominal pain and primary/secondary infertility due to endometriosis
Severe abdominal pain and primary/secondary infertility due to endometriosis
Primary/secondary Infertility due to endometriosis</td><td>NA</td></tr>
<tr><td>Polycystic ovarian syndrome</td><td>Primary/secondary infertility due to PCOS
Hirsutism and primary/secondary infertility due to PCOS</td><td>NA</td></tr>
<tr><td>Maternal sepsis</td><td>Secondary infertility due to puerperal sepsis</td><td>NA</td></tr>
<tr><td>Congenital Turner</td><td>Heart diseases with primary infertility due to Turner syndrome
Primary infertility due to Turner syndrome</td><td>NA</td></tr>
<tr><td>Congenital Klinefelter</td><td>NA</td><td>Borderline intellectual disability with primary infertility due to Klinefelter syndrome
Mild intellectual disability with primary infertility due to Klinefelter syndrome
Primary infertility due to Klinefelter syndrome</td></tr>
<tr><td>Congenital urogenital anomalies</td><td colspan="2">Primary infertility due to genital anomalies
Atypical genitalia and primary infertility due to genital anomalies
Primary infertility and recurrent urinary tract infections or other abdominal issues due to genital anomalies
Primary infertility and impotence due to genital anomalies</td></tr>
</table>

	• Atypical genitalia, recurrent urinary tract infections or other abdominal issues, and primary infertility due to genital anomalies<br>• Atypical genitalia, primary infertility, and impotence due to genital anomalies<br>• Primary infertility, impotence, and recurrent urinary tract infections or other abdominal issues and impotence due to genital anomalies<br>• Atypical genitalia, primary infertility, impotence, and recurrent urinary tract infections or other abdominal issues and impotence due to genital anomalies
Unknown	• Idiopathic primary and secondary infertility

Sequelae and disability weights

Every person with infertility is assumed to experience the health state as determined from the GBD disability weights survey.[7] The lay descriptions of primary and secondary infertility are below.

Table 9. Severity distribution, details on the severity levels for infertility impairment and the associated disability weight (DW) with that severity.

Severity level	Lay description	DW (95% CI)
Infertility, primary	This person wants to have a child and has a fertile partner, but the couple cannot conceive.	0.008 (0.003–0.015)
Infertility, secondary	This person has at least one child and wants to have more children. The person has a fertile partner, but the couple cannot conceive.	0.005 (0.002–0.011)

References

1. Mascarenhas, Maya N., et al. National, regional, and global trends in infertility prevalence since 1990: a systematic analysis of 277 health surveys. *PLoS medicine* 9.12 (2012): e1001356.
2. Ching CB, Wood HM, Ross JH, Gao T, Angermeier KW. The Cleveland Clinic experience with adult hypospadias patients undergoing repair: their presentation and a new classification system. *BJU Int*. 2011; 107(7): 1142–6.
3. Davies MC, Liao L-M, Wilcox DT, Woodhouse CRJ, Creighton SM. Anorectal malformations: what happens in adulthood?. *BJU Int*. 2010; 106(3): 398–404.
4. Loxton D, Dobson A, Byles J, Tooth L. Australian Longitudinal Study on Women's Health (ALSWH). http://www.alswh.org.au/.
5. Weström L, Joesoef R, Reynolds G, Hagdu A, Thompson SE. Pelvic inflammatory disease and fertility. A cohort study of 1,844 women with laparoscopically verified disease and 657 control women with normal laparoscopic results. *Sex Transm Dis* 1992; 19: 185–92.
6. Weström LV. Chlamydia and its effect on reproduction. *J Br Fer Soc* 1996; 1: 23–30.
7. Salomon JA, Vos T, Hogan DR, et al. Common values in assessing health outcomes from disease and injury: disability weights measurement study for the Global Burden of Disease Study 2010. *Lancet* 2012; 380: 2129–43.

Inflammatory bowel disease

Flowchart

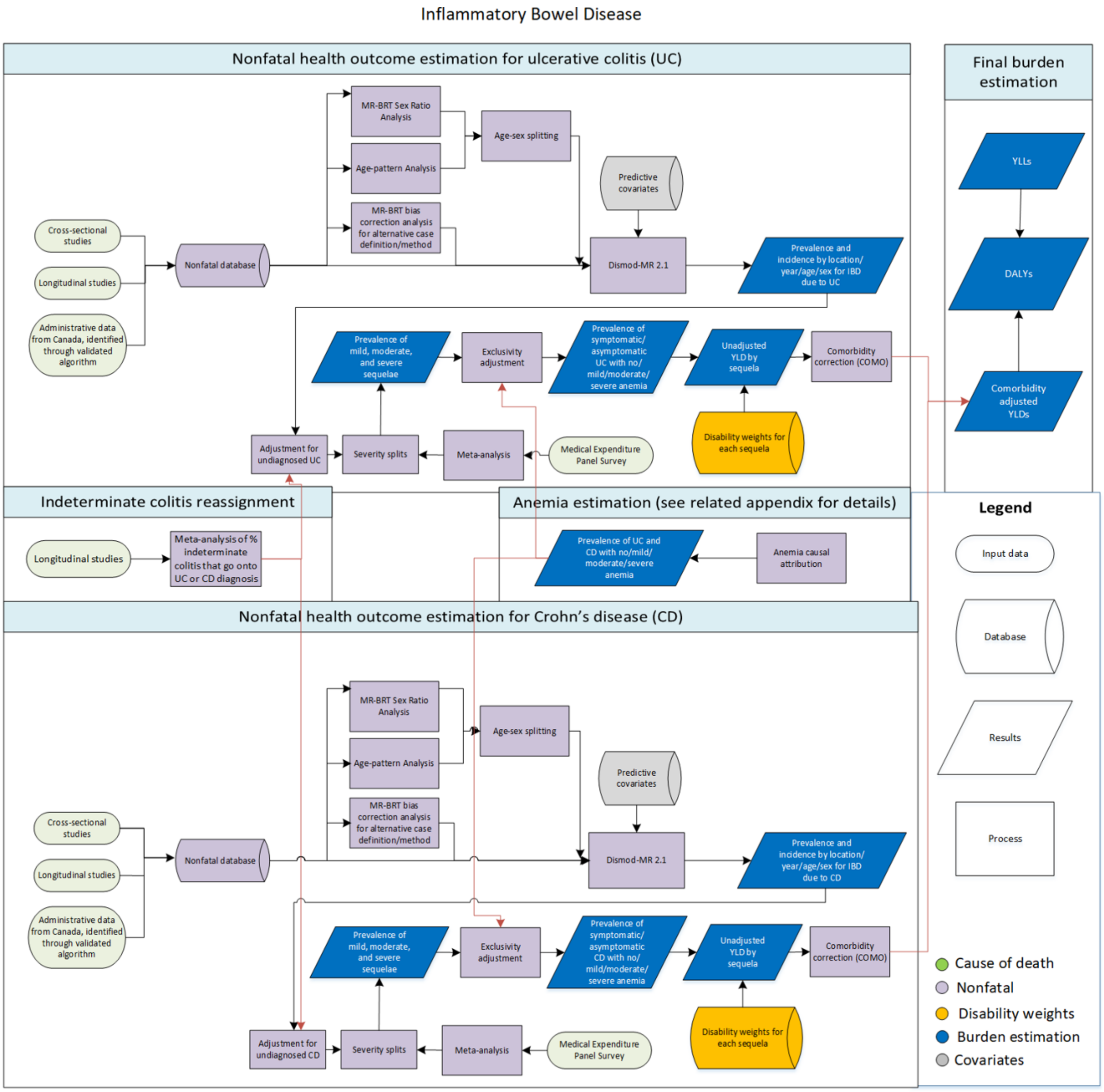

Input data and methodological summary for inflammatory bowel disease

Case definition

Inflammatory bowel disease is a group of chronic diseases resulting from non-infectious inflammation of the colon and gastrointestinal tract. These include Crohn's disease (inflammation throughout the gastrointestinal tract, but predominantly the small and large intestine) and ulcerative colitis (inflammation of the colon and rectum). These diseases result in episodes of abdominal pain,

gastrointestinal bleeding, diarrhoea, and potential additional complications, with many patients also experiencing intervening asymptomatic periods.

For both Crohn's disease and ulcerative colitis, we treat two kinds of case definitions as equivalent reference standards: cases that are diagnosed by endoscopy, imaging studies, or biopsy in a patient with appropriate clinical signs and symptoms, or cases that are identified through a patient database (including both inpatient and outpatient care) using an algorithm for identification of cases with International Classification of Diseases (ICD) codes that is validated through detailed chart review. Data collected by identifying cases from a patient database with ICD codes but not using a validated algorithm are included in our inputs but treated as non-reference.

In some cases of inflammatory bowel disease, neither Crohn's disease nor ulcerative colitis can be definitively diagnosed, and a diagnosis of indeterminate colitis is applied, indefinitely, or until definitive features of Crohn's or ulcerative colitis declare themselves.

ICD codes are K50 for Crohn's disease, K51 for ulcerative colitis, and K52 for indeterminate colitis.

Quantity of interest	Reference or alternative	Definition
Incidence of Crohn's disease	Alternative	Crohn's disease cases identified through a patient database using a unvalidated ICD code-based case-identification algorithm
Incidence of Crohn's disease	Reference	Crohn's disease diagnosed based on endoscopy, imaging studies, and biopsy in a patient with appropriate clinical signs and symptoms from a representative sample of the general population (either by study personnel, or in clinical care as confirmed by study personnel chart review), or cases that are identified through via a patient database (including both inpatient and outpatient care) using a validated ICD code-based case-identification algorithm
Incidence of ulcerative colitis	Alternative	Ulcerative colitis cases identified through a patient database using a unvalidated ICD code-based case-identification algorithm
Incidence of ulcerative colitis	Reference	Ulcerative colitis diagnosed based on endoscopy, imaging studies, and biopsy in a patient with appropriate clinical signs and symptoms from a representative sample of the general population (either by study personnel, or in clinical care as confirmed by study personnel chart review), or cases that are identified via a patient database (including both inpatient and outpatient care) using a validated ICD code-based case-identification algorithm
Prevalence of Crohn's disease	Alternative	Crohn's disease cases identified through a patient database using a unvalidated ICD code-based case-identification algorithm
Prevalence of Crohn's disease	Reference	Crohn's disease diagnosed based on endoscopy, imaging studies, and biopsy in a patient with appropriate clinical signs and symptoms from a representative sample of the general population (either by study personnel, or in clinical care as confirmed by study personnel chart review), or cases that are identified through via a patient database (including both inpatient and outpatient care) using a validated ICD code-based case-identification algorithm
Prevalence of ulcerative colitis	Alternative	Ulcerative colitis cases identified through a patient database using a unvalidated ICD code-based case-identification algorithm
Prevalence of ulcerative colitis	Reference	Ulcerative colitis diagnosed based on endoscopy, imaging studies, and biopsy in a patient with appropriate clinical signs and symptoms from a

		representative sample of the general population (either by study personnel, or in clinical care as confirmed by study personnel chart review), or cases that are identified via a patient database (including both inpatient and outpatient care) using a validated ICD code-based case-identification algorithm

Overall strategy

As in GBD 2019 and GBD 2021, in GBD 2023 we utilised two databases for inflammatory bowel disease as inputs to two separate, complete compartmental DisMod models: ulcerative colitis and Crohn's disease. We then adjusted both for the proportion of indeterminate colitis cases thought to represent undiagnosed ulcerative colitis and Crohn's and then applied distributions of the frequency of symptoms.

Input data and data processing

Input data

For GBD 2016, a systematic literature review was conducted to capture studies of prevalence and incidence for all inflammatory bowel diseases. A PubMed search was conducted using the following search string: (("crohn disease"[MeSH Terms] OR ("crohn"[All Fields] AND "disease"[All Fields]) OR "crohn disease"[All Fields] OR ("crohn's"[All Fields] AND "disease"[All Fields]) OR "crohn's disease"[All Fields]) OR ("colitis, ulcerative"[MeSH Terms] OR ("colitis"[All Fields] AND "ulcerative"[All Fields]) OR "ulcerative colitis"[All Fields] OR ("ulcerative"[All Fields] AND "colitis"[All Fields])) OR (Inflammatory[All Fields] AND Bowl[All Fields]) OR (("irritable bowel syndrome"[MeSH Terms] OR ("irritable"[All Fields] AND "bowel"[All Fields] AND "syndrome"[All Fields]) OR "irritable bowel syndrome"[All Fields]) AND ("diarrhoea"[All Fields] OR "diarrhea"[MeSH Terms] OR "diarrhea"[All Fields]))) AND ("epidemiology"[Subheading] OR "epidemiology"[All Fields] OR "epidemiology"[MeSH Terms]) AND (("2016"[PDAT]) NOT (animals[MeSH] NOT humans[MeSH])).

The exclusion criteria were:

1. Studies clearly not representative of a geographically defined general population.
2. Studies that did not provide primary data on epidemiological parameters, eg, a commentary piece.

In GBD 2019, we re-reviewed and excluded studies that included diagnosis by self-report or did not provide sufficient information about the study population to derive crude prevalence or incidence estimates.

For GBD 2021, we added additional data from peer-reviewed publications identified via a systematic review that was conducted by Ng and colleagues in 2017.[1] We also added administrative data from Canada that were extracted using a validated algorithm.[2]

[1]Ng SC, Shi HY, Hamidi N, Underwood FE, Tang W, Benchimol EI, et al. Worldwide incidence and prevalence of inflammatory bowel disease in the 21st century: a systematic review of population-based studies. *The Lancet*. 2018 23;390(10114):2769–78.

[2]Coward S, Clement F, Benchimol EI, Bernstein CN, Avina-Zubieta JA, Bitton A, Carroll MW, Hazlewood G, Jacobson K, Jelinski S, Deardon R, Jones JL, Kuenzig ME, Leddin D, McBrien KA, Murthy SK, Nguyen GC, Otley AR, Panaccione R, Rezaie A, Rosenfeld G, Peña-Sánchez JN, Singh H,

In addition to the abovementioned prevalence and incidence data in previous rounds of GBD we used prevalence inputs extracted from hospital inpatient discharge and claims data processed by IHME, as described elsewhere in this appendix. As a chronic disorder that is primarily treated in outpatient settings, IBD cases are poorly captured in inpatient discharge data. To address this, IHME has historically modelled correction factors (ie, ratios of inpatient admissions to total cases identified in both inpatient and outpatient care) using MarketScan insurance claims data from the USA. Despite this adjustment, however, we noted a large discrepancy between the adjusted inpatient discharge data and the reference data in locations where we had both data sources, suggesting a systematic bias in the ratio of inpatient admissions to total cases observed in the commercially insured population. In GBD 2021, we attempted to improve the correction factors by modelling them using multiple claims sources – from Poland and USA MarketScan data. The correction factors and age-sex patterns, however, were so different between the two data sources that correction factor models using both of them would not converge. From this, we inferred that there were substantial differences in coding practices and sites of care across countries. Until we have more data to derive more robust correction factors, we decided to exclude clinical informatics data in GBD 2021, a decision we carried forward in GBD 2023.

No new data were added in GBD 2023. The input data and processing steps for modelling in GBD 2023 were the same as in GBD 2021.

Data processing

The first step processing extracted data was to split data reported for both sexes using the pooled sex ratio estimated from studies that reported prevalence or incidence in males and females separately. For prevalence, the ratios of female to male cases derived from MR-BRT analysis were 0.91 (95% UI 0.60–1.38) and 1.13 (95% UI 0.36–3.53) for ulcerative colitis and Crohn's disease, respectively. For incidence, the ratios of female to male cases derived from MR-BRT analysis were 0.92 (0.65–1.30) and 1.20 (0.59–2.45) for ulcerative colitis and Crohn's disease, respectively.

Next, for both ulcerative colitis and Crohn's disease models, the non-reference (or "alternative") datapoints were adjusted toward the reference, which we refer to as "stringent criteria" in shorthand, using a meta-regression approach called meta-regression—Bayesian, regularised, trimmed (MR-BRT) analysis. Specifically, we identified studies that reported data using both reference and non-reference methods (Figure 1). We then calculated the logit difference between the two datapoints for each study and used these calculated logit-differences as inputs to MR-BRT to estimate pooled logit-difference. This constituted an adjustment factor which was applied to all datapoints collected with non-reference methods. The process of adjusting for non-reference data using MR-BRT with the logit-transformation method is described below:

Targownik LE, Kaplan GG. Past and Future Burden of Inflammatory Bowel Diseases Based on Modeling of Population-Based Data. *Gastroenterology*. 2019 Apr;156(5):1345-1353.e4.

1. Identify datapoints with overlapping year, age, sex, and location between reference and non-reference population data.
2. Logit transform overlapping datapoints of alternative and reference types.
3. Convert overlapping datapoints into a difference in logit space using the following equation: $logit(altnerative) - logit(reference)$
4. Use the delta method to compute standard errors of overlapping datapoints in logit space, then calculate standard error of logit difference using the following equation: $\sqrt{(variance\ of\ logit(alternative)) + (variance\ of\ logit(reference))}$
5. Using MR-BRT, conduct a random effects meta-regression to obtain the pooled logit difference of alternative to reference
6. Apply the pooled logit difference to all data points of non-reference types using the following equation:
$$new_{estimate} = inverse.logit((logit(alternative)) - (pooled\ logit\ difference))$$
7. Calculate new standard errors using the delta method, accounting for gamma (between-study heterogeneity)

Figure 1. Studies reported both reference and non-reference (alternative) data used in MR-BRT

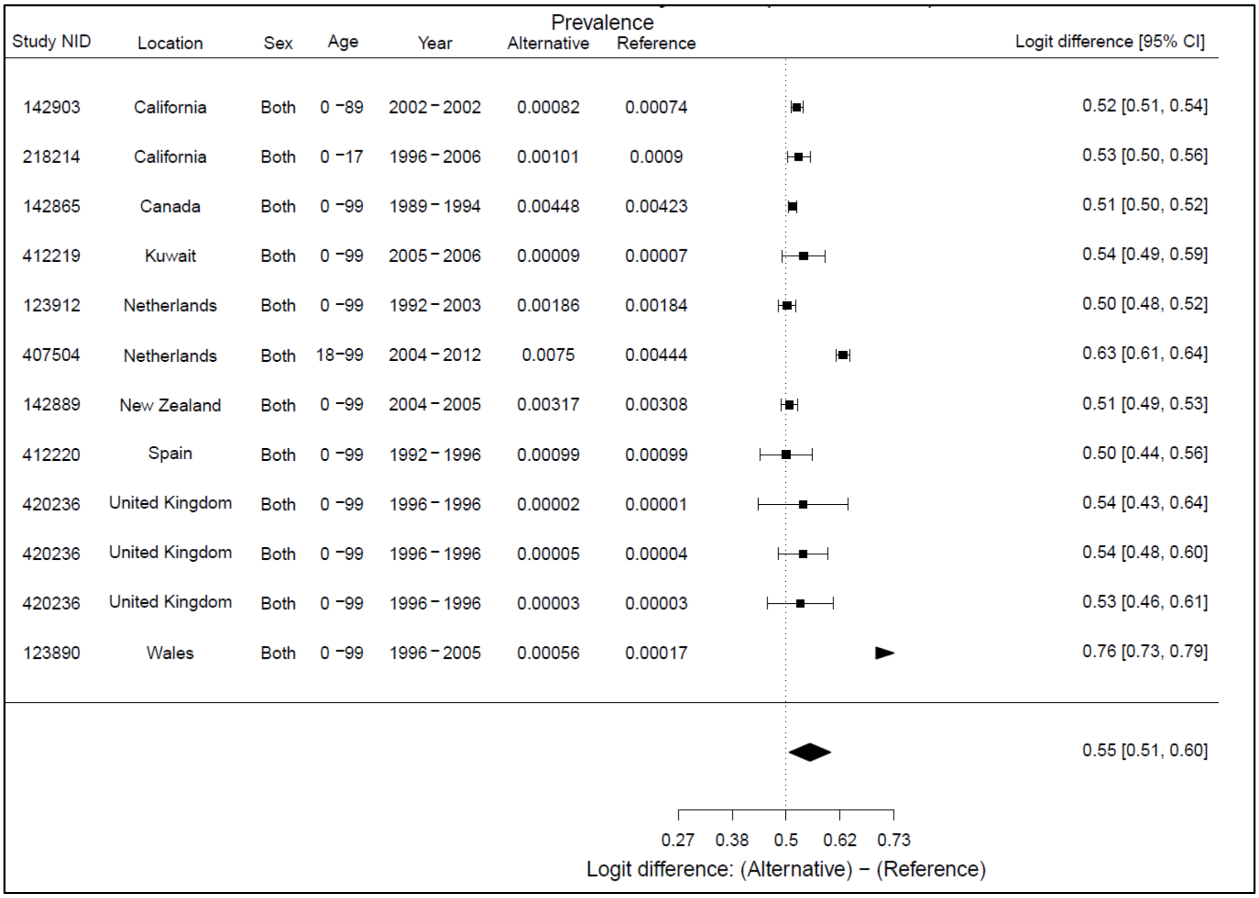

Study NID	Location	Sex	Age	Year	Prevalence Alternative	Prevalence Reference	Logit difference [95% CI]
142903	California	Both	0 −89	2002 − 2002	0.00082	0.00074	0.52 [0.51, 0.54]
218214	California	Both	0 −17	1996 − 2006	0.00101	0.0009	0.53 [0.50, 0.56]
142865	Canada	Both	0 −99	1989 − 1994	0.00448	0.00423	0.51 [0.50, 0.52]
412219	Kuwait	Both	0 −99	2005 − 2006	0.00009	0.00007	0.54 [0.49, 0.59]
123912	Netherlands	Both	0 −99	1992 − 2003	0.00186	0.00184	0.50 [0.48, 0.52]
407504	Netherlands	Both	18−99	2004 − 2012	0.0075	0.00444	0.63 [0.61, 0.64]
142889	New Zealand	Both	0 −99	2004 − 2005	0.00317	0.00308	0.51 [0.49, 0.53]
412220	Spain	Both	0 −99	1992 − 1996	0.00099	0.00099	0.50 [0.44, 0.56]
420236	United Kingdom	Both	0 −99	1996 − 1996	0.00002	0.00001	0.54 [0.43, 0.64]
420236	United Kingdom	Both	0 −99	1996 − 1996	0.00005	0.00004	0.54 [0.48, 0.60]
420236	United Kingdom	Both	0 −99	1996 − 1996	0.00003	0.00003	0.53 [0.46, 0.61]
123890	Wales	Both	0 −99	1996 − 2005	0.00056	0.00017	0.76 [0.73, 0.79]
							0.55 [0.51, 0.60]

This bias adjustment method allows a more direct comparison between the reference and non-reference data. Prior to GBD 2019, we adjusted alternative case definitions or study design characteristics to the reference standard by creating binary covariates for these alternative groups and estimating a fixed effect for these covariates in our DisMod meta-regression modelling process. This

amounts to adjusting data using an ecological comparison and is vulnerable to compositional bias; if data from different location-years were collected using different methods or case definitions, true spatiotemporal differences in epidemiology can be erroneously adjusted, and differences truly due to differences in methods can be erroneously estimated as differences in underlying epidemiology. By using pairing measurements made on the same sample using reference and non-reference methods, we were able to directly estimate the influence of the methods and adjust non-reference datapoints more accurately.

The table below shows bias correction factors estimated using MR-BRT.

Table 1. MR-BRT crosswalk adjustment factors for inflammatory bowel disease

Data input	Reference or alternative case definition	Gamma	Beta coefficient, logit (95% CI)	Adjustment factor*
Stringent criteria	Ref	0	--	--
ICD-code based administrative data	Alt		0.06 (0.04–0.09)	1.07 (1.04–1.10)

**MR-BRT crosswalk adjustments can be interpreted as the factor the alternative case definition is adjusted by to reflect what it would have been had it been measured using the reference case definition. If the log/logit beta coefficient is negative, then the alternative is adjusted up to the reference. If the log/logit beta coefficient is positive, then the alternative is adjusted down to the reference.*
***The adjustment factor column is the exponentiated beta coefficient. For log beta coefficients, this is the relative rate between the two case definitions. For logit beta coefficients, this is the relative odds between the two case definitions.*

After adjustment of non-reference data, we split datapoints where the age range was greater than 25 years using the super-region age patterns informed by the datapoints with fine age groups (ie, ages 5–9, 10–14, and 15–20…).

We excluded any data for subnational locations under the age of 20 years that had excessive influence on the estimation of pseudo-random effects and the subnational prior distribution and led the model to ignore more abundant data in older age groups; this occurred in some subnational locations in Japan and the USA.

Modelling strategy

Modelling

The modelling strategy for all inflammatory bowel disease encompasses two separate DisMod models for ulcerative colitis and Crohn's disease, which are then adjusted to account for inflammatory bowel disease due to indeterminate colitis.

Non-infective inflammatory bowel disease due to ulcerative colitis, pre-adjustment (for indeterminate colitis)

Similar to GBD 2021, we ran a DisMod-MR 2.1 model to produce estimates by age, sex, year, and country. Prior settings included setting incidence to zero for ages 0 to 2 and 0.00025 for ages 80 to 100, remission to zero for all ages, and excess mortality rate (EMR) to 0.2 for all ages. The minimum

coefficient of variation at the regional, super-regional, and global level was set at 0.8. Predictive covariates included Socio-demographic Index on incidence and Healthcare Access and Quality Index on EMR.

Non-infective inflammatory bowel disease due to Crohn's disease, pre-adjustment (for indeterminate colitis)

Similar to GBD 2021, we ran a DisMod-MR 2.1 model to produce estimates by age, sex, year, and country. Prior settings included setting incidence to zero for ages 0 to 2 and 0.00025 for ages 80 to 100, remission to zero for all ages, and excess mortality rate (EMR) to 0.2 for all ages. The minimum coefficient of variation at the regional, super-regional, and global level was set at 0.8. Predictive covariates included Socio-demographic Index on incidence and Healthcare Access and Quality Index on EMR.

Betas and exponentiated values for predictive covariates (which can be interpreted as an odds ratio) are shown in the table below.

Table 2. Covariates. Summary of covariates used in the inflammatory bowel disease DisMod-MR meta-regression models

Ulcerative colitis

Covariate	Parameter	Exponentiated beta (95% uncertainty interval)
Socio-demographic Index	Incidence	5.94 (5.25–6.83)
Healthcare Access and Quality Index	Excess mortality rate	0.60 (0.37–0.97)

Crohn's disease

Covariate	Parameter	Exponentiated beta (95% uncertainty interval)
Socio-demographic Index	Incidence	7.31 (7.11–7.39)
Healthcare Access and Quality Index	Excess mortality rate	0.61 (0.38–0.96)

Indeterminate colitis adjustment

After running DisMod-MR, the model outputs of ulcerative colitis and Crohn's disease were adjusted to account for indeterminate colitis. This approach assumed that all indeterminate colitis cases would be ultimately confirmed as either ulcerative colitis or Crohn's disease. Specifically, we identified studies that reported the proportion of indeterminate colitis cases in the total number of IBD cases. Then, we ran a meta-regression model in R to find a pooled proportion of indeterminate colitis attributable to the burden of ulcerative colitis and Crohn's disease. Both ulcerative colitis and Crohn's disease model

outputs were adjusted using the pooled proportion at the draw level. The adjusted estimates were then combined to estimate the total burden of IBD.

Severity split & disability weight

The basis of the GBD disability weight survey assessments are lay descriptions of sequelae highlighting major functional consequences and symptoms. For GBD 2023, we used the Medical Expenditure Panel Survey to find the proportion of ulcerative colitis and Crohn's disease asymptomatic versus symptomatic during a given four-week period. The lay descriptions and disability weights for sequelae associated with inflammatory bowel disease are shown below.

Table 3. Severity distribution, details on the severity levels for inflammatory bowel disease in GBD 2023 and the associated disability weight (DW) with that severity

Severity split	Lay description	DW (95% CI)
Crohn's disease, currently asymptomatic	--	0
Crohn's disease, symptomatic	This person has cramping abdominal pain, has diarrhoea several times a day, and feels very tired for two months every year. When the person does not have symptoms, there is anxiety about them returning.	0.231 (0.156–0.32)
Ulcerative colitis, currently asymptomatic	--	0
Ulcerative colitis, symptomatic	This person has cramping abdominal pain, has diarrhoea several times a day, and feels very tired for two months every year. When the person does not have symptoms, there is anxiety about them returning.	0.231 (0.156–0.32)

The age-specific anaemia prevalence for symptomatic cases of ulcerative colitis and Crohn's disease was analysed as part of overall anaemia causal attribution for GBD 2023. The details of the anaemia analysis are described separately in the "Anaemia impairment" section. Briefly, after estimating total anaemia, a series of counterfactual distributions are generated based on the age- and sex-specific prevalence of each anaemia-causing condition and the quantitative effect that the condition has on haemoglobin concentration in the blood, a so-called "haemoglobin shift," that was derived by meta-analysing cohort studies, observational studies, or trials comparing the haematological status of those with as compared to without the disease. Due to limited data on haemoglobin shift, all were assumed invariant over age, sex, location, and year. Disability due to anaemia resulting from ulcerative colitis and Crohn's disease was combined with disability for the health states in the table above in producing final estimates of disability for these diseases.

Inguinal, femoral, and abdominal hernia

Flowchart

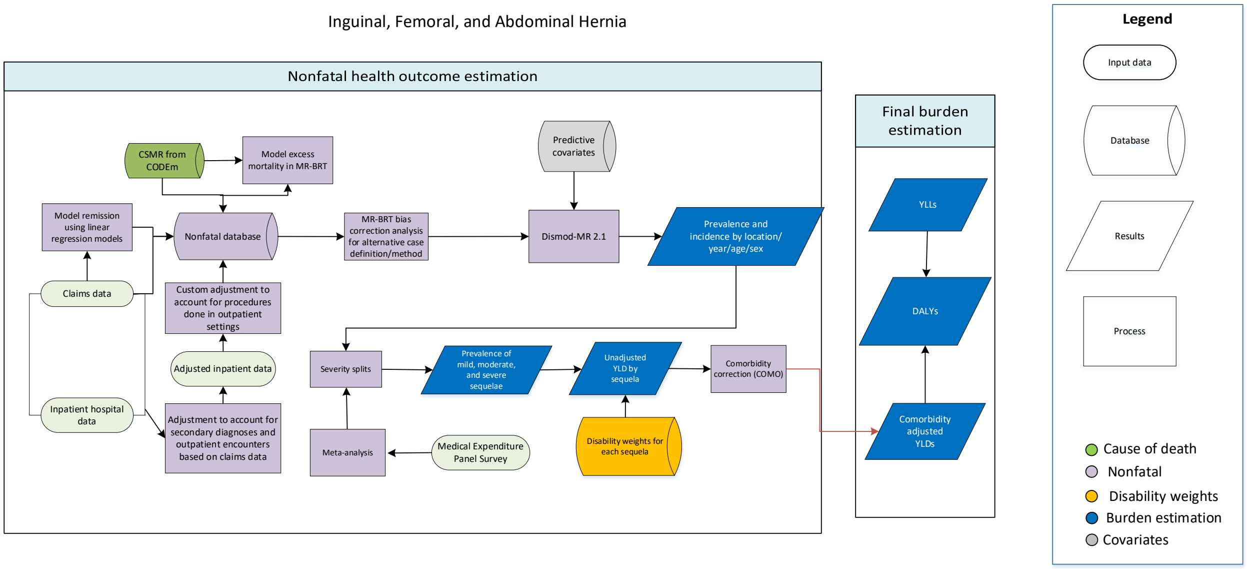

Input data and methodological summary for inguinal, femoral, and abdominal hernia

Case definition

Hernia refers to the protrusion of an abdominal internal organ through an opening in the tissue that holds it in place, regardless of symptoms. Inguinal, femoral, and abdominal hernia comprises the disorders in which portions of the digestive tract protrude through defects in the walls of the abdominal cavity. These occasionally lead to life-threatening acute complications, but more commonly are asymptomatic or cause chronic or intermittent pain. Symptomatic hernia is surgically repaired.

ICD-10 codes are K40, K41, K42, K44, K45, and K46 and all their 4-digit and 5-digit constituents. The ICD-9 codes are 550, 551, 552, 553 and their constituents, with the exceptions of 551.1-3, 552.1-3, and 553.1-3. The procedure codes for hernia repair are 43336-43337, 44050, 49491-49492, 49495-49496, 49500-49501, 49505, 49507, 49525, 49540, 49550, 49553, 49555, 49557, 49560-49561, 49565-49566, 49568, 49570, 49572, 49585, 49587, 49590, 49650-49653, and 54640.

Quantity of interest	Reference or Alternative	Definition
Prevalence of both symptomatic and asymptomatic cases (total)	Alternative	Cases identified from database of commercial claims from USA in 2010-2017 using ICD codes.
Prevalence of both symptomatic and asymptomatic cases (total)	Alternative	Cases identified from database of commercial claims from USA in 2000 using ICD codes.

Prevalence of both symptomatic and asymptomatic cases (total)	Reference	Cases of inguinal, femoral, and abdominal hernia identified using ICD codes in administrative records that are considered population-representative.

Overall strategy

In GBD 2017, two databases were developed for inguinal, femoral, and abdominal hernia to separately model total (symptomatic + asymptomatic cases) and symptomatic cases. In GBD 2019 and GBD 2021, the DisMod model for symptomatic cases was dropped, and we only modelled total cases of hernia in DisMod; an updated severity distribution was then applied as described below. This GBD 2021 approach was carried forward in GBD 2023.

Input data

Inputs

Like GBD 2021, the model included prevalence data from the IHME database of hospital discharges and claims, as described elsewhere in this appendix. No new data were added in GBD 2023. The input data and processing steps for modelling in GBD 2023 were the same as the GBD 2021.

Inputs to our non-fatal modelling also included cause-specific mortality rate (CSMR) estimates taken from our fatal modelling process (see CoD cause-specific modelling description for hernia in this appendix) and remission and excess mortality rates (EMR) estimates modelled outside of DisMod (see the remission and EMR data processing sections below).

Prevalence data processing

Data for GBD 2023 were processed by the same methods as in GBD 2021, which are broadly similar to approaches used in GBD 2019. Specifically, for claims data, we extracted prevalent cases of hernia for the total hernia database if an individual had one inpatient or two outpatient encounters with a hernia ICD code as any diagnosis. Hospital discharge data are indexed at the encounter level and generally only include an ICD code for the primary discharge diagnosis, so encounters with a hernia ICD code were extracted and then multiplied by a correction factor, described in the next paragraph, to estimate how many total prevalent cases gave rise to those hospital discharges.

We assumed that individuals in our database from USA claims with either an inpatient encounter with a hernia ICD code or an outpatient encounter with both hernia ICD code and procedural code for hernia repair was symptomatic, but that globally, most symptomatic cases of hernia were treated in an inpatient setting. Consequently, we summed the inpatient and outpatient encounters with procedures in the USA claims data, estimated the ratio of this sum to all encounters with hernia ICD codes, and applied this ratio to international hospital discharge data to estimate total hernia cases for populations for which individual-level claims data were not available.

Although better able to capture the relationship between inpatient and outpatient care, USA claims data were still regarded as suffering from selection bias due to commercial health insurance status. Thus, total hernia prevalence data extracted from USA claims from the year 2000 and from the years 2010–2017 were adjusted to total hernia prevalence estimated from hospital discharges after application of

the correction factor. This was done in MR-BRT using the logit-transformation method as described below:

1. Identify datapoints with overlapping year, age, sex, and location between reference and non-reference population data.
2. Logit transform overlapping datapoints of alternative and reference types.
3. Convert overlapping datapoints into a difference in logit space using the following equation: $logit(altnerative) - logit(reference)$
4. Use the delta method to compute standard errors of overlapping datapoints in logit space, then calculate standard error of logit difference using the following equation: $\sqrt{(variance\ of\ logit(alternative)) + (variance\ of\ logit(reference))}$
5. Using MR-BRT, conduct a random effects meta-regression to obtain the pooled logit difference of alternative to reference
6. Apply the pooled logit difference to all data points of non-reference types using the following equation:
$$new_{estimate} = inverse.logit((logit(alternative)) - (pooled\ logit\ difference))$$
7. Calculate new standard errors using the delta method, accounting for gamma (between-study heterogeneity)

The table below shows bias correction factors of alternative case definitions using MR-BRT.

Table 1. MR-BRT crosswalk adjustment factors for inguinal, femoral, and abdominal hernia

Data input	Reference or alternative case definition	Gamma	Covariate	Beta coefficient, logit (95% CI)	Adjustment factor*
Hospital + non-USA claims	Ref	0.001		---	---
USA claims from year 2000	Alt		Age (continuous from 0 to 95+)	–0.03 (–0.033 to –0.027)	0.97 (0.968 to 0.973)
			Intercept	4.04 (4.01 to 4.08)	57.00 (55.10 to 58.95)
USA claims from years 2010–2017	Alt		Age (continuous from 0 to 95+)	–0.02 (–0.026 to –0.022)	0.976 (0.974 to 0.978)
			Intercept	4.47 (4.37 to 4.57)	87 (70.17 to 96.31)

**MR-BRT crosswalk adjustments can be interpreted as the factor the alternative case definition is adjusted by to reflect what it would have been had it been measured using the reference case definition. If the log/logit beta coefficient is negative, then the alternative is adjusted up to the reference. If the log/logit beta coefficient is positive, then the alternative is adjusted down to the reference.*

***The adjustment factor column is the exponentiated beta coefficient. For log beta coefficients, this is the relative rate between the two case definitions. For logit beta coefficients, this is the relative odds between the two case definitions.*

Datapoints with an age-standardised prevalence greater than two median absolute deviations from the median of the age-standardised prevalence for all inpatient and non-USA claims data were marked as outliers and excluded from analysis.

Remission processing

In several rounds prior to GBD 2019, we used remission estimates derived from a single, large study of mean wait times for elective surgical repair in OECD countries conducted by Siciliani and colleagues. Starting in GBD 2019, we aimed to better inform DisMod on the increasing pattern of remission with greater access to quality health care. To do so, we used remission data from the USA claims, defined as the number of people with a hernia repair procedure code among all people with hernia diagnosis, and regressed against Healthcare Access and Quality (HAQ) Index and sex with an assumption that hernia does not resolve on its own without a surgical repair, so remission is zero at a theoretical HAQ Index value of zero. In GBD 2021, we updated this by including an additional covariate, age, to capture age variations in remission. The results from the regression model were then used to predict remission estimates for each location, year, and sex for ages 0, 10, 20…100. This latter remission processing method was employed in GBD 2023.

Figure 1. Predicted remission in function of age, sex, and HAQ Index

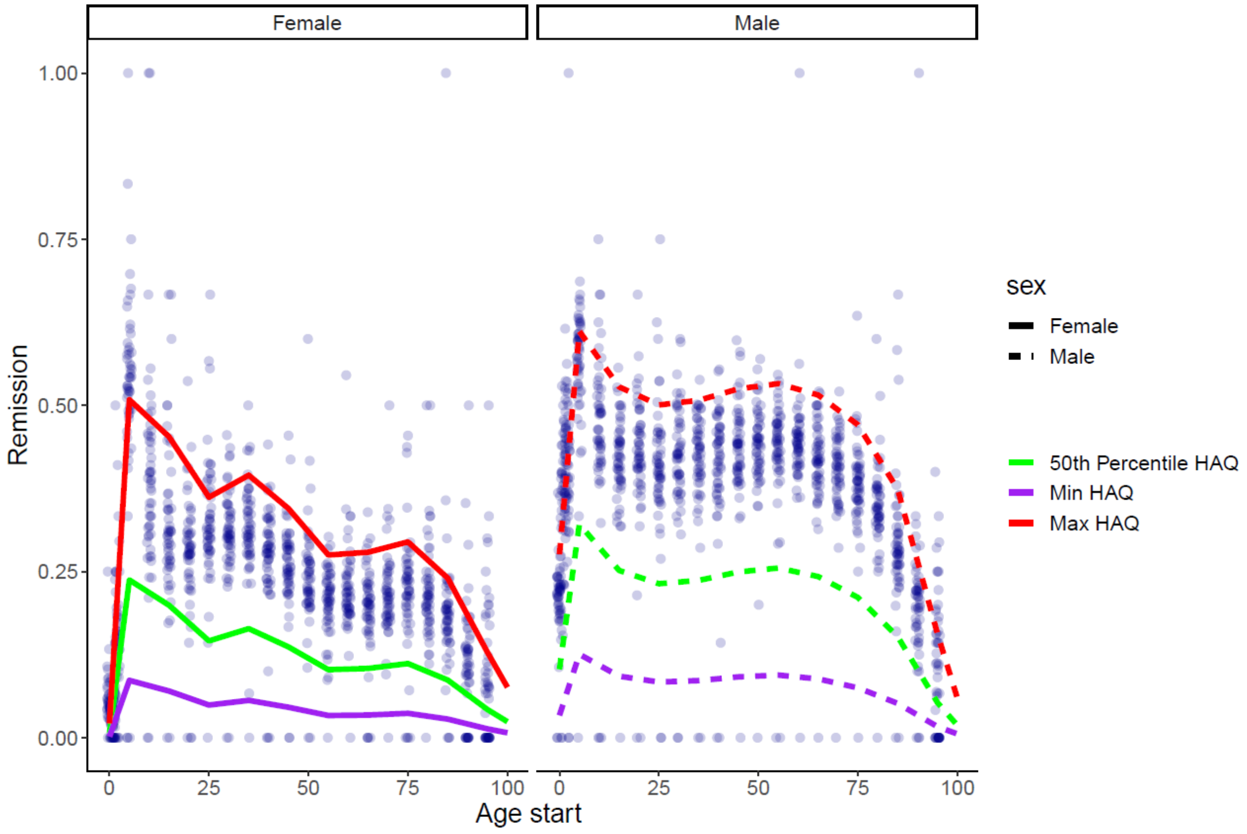

EMR processing

In GBD 2017, EMR inputs were produced by matching prevalence datapoints with their corresponding CSMR values within the same age, sex, year, and location (by dividing CSMR by prevalence). However, this method of producing EMR inputs demonstrated a rather unrealistic pattern of EMR compared to an expected pattern of decreasing EMR with greater access to quality health care. Such unexpected patterns often signal inconsistencies between CSMR estimates and the measures of prevalence and/or incidence. Thus, in an effort to provide greater guidance on the expected pattern of EMR, in GBD 2019, EMR data produced per above in GBD 2017 were modelled by age, sex, and HAQ Index using MR-BRT, with a prior on HAQ Index having a negative coefficient. In GBD 2021, we employed the same MR-BRT method to predict EMR for each location, year, sex, and for ages 0, 10, 20….100, and these predictions were used as inputs to our non-fatal model, below. EMR processing has not changed in GBD 2023.

Modelling strategy

Modelling

Similar to previous rounds, we ran a DisMod-MR 2.1 model to produce estimates by age, sex, year, and location. Inputs to DisMod for hernia include prevalence, remission, CSMR, and EMR inputs processed as described above. We changed several DisMod settings in GBD 2021. First, we removed the prior on EMR, which had a bound of 0 to 0.00002 between ages 0 and 15. We also removed an upper bound of incidence rate at 0.01 between ages 0 and 20. This was to capture high incident cases of hernia in children. The minimum coefficient of variation at the regional, super-regional, and global level was set at 0.8. In contrast to GBD 2019, we allowed birth prevalence of hernia. We used smoking prevalence and mean BMI as predictive covariates for prevalence. The HAQ Index and lag-distributed income (log transformed) covariates were applied to EMR and remission, respectively. Betas and exponentiated values for these predictive covariates (which can be interpreted as an odds ratio) are shown in the table below. Model settings did not change further in GBD 2023.

Table 2. Covariates. Summary of predictive covariates used in the total inguinal, femoral, and abdominal hernia DisMod-MR meta-regression model

Covariate	Parameter	Exponentiated beta (95% uncertainty interval)
Smoking prevalence	Prevalence	7.28 (7.13–7.38)
Mean BMI	Prevalence	0.86 (0.85–0.87)
Healthcare Access and Quality Index	Excess mortality rate	0.99 (0.99–0.99)
LDI (I$ per capita)	Remission	1.65 (1.65–1.65)

Severity split and disability weight

The DisMod model of symptomatic hernia used in GBD 2017 was dropped in GBD 2019, and symptom occurrence and severity distribution were estimated from Medical Expenditure Panel Survey (MEPS) data using standard GBD methodology. MEPS is an overlapping panel survey of the non-institutionalised USA population that collects data on respondents' health service interactions. Panels are initiated every year. Each panel is two years long and consists of five rounds. In 2000, MEPS began using 12-Item Short Form Surveys (SF-12) to collect data on functional health status. The SF-12 survey is administered twice per panel (about once per year). This survey has been employed to estimate distributions of severity levels for diverse GBD diseases as previously described and summarised below.[1]

In order to translate SF-12 scores into GBD disability weights, 62 lay descriptions for conditions representing the full range of disability weight values (from most mild to most severe) were selected. A convenience sample of respondents was then asked to complete an SF-12 form for an individual with the health state described in the lay descriptions of these conditions. Composite mental and physical SF-12 score was regressed on GBD disability weight to derive the relationship between disability weight and SF-12 score. Individual respondent scores were then regressed on reported conditions to predict cumulative disability from the individuals' diagnoses, and a counterfactual cumulative disability excluding a single condition was predicted to obtain a comorbidity-corrected condition-specific disability weights for all individual with that single condition. The distribution of these condition-specific disability weights was used to derive the proportion of individuals with the conditions that fall within each GBD severity category.

The basis of the GBD disability weight survey assessments are lay descriptions of sequelae highlighting major functional consequences and symptoms. Prevalent cases of symptomatic hernia were divided according to severity distributions derived from data from the MEPS to assign them to mild, moderate, and severe sequelae. Asymptomatic cases were assigned no disability. The lay descriptions and disability weights for inguinal, abdominal, and femoral hernia are shown below.

Table 3. Severity distribution, details on the severity levels for Inguinal, femoral, and abdominal hernia in GBD 2023 and the associated disability weight (DW) with that severity

Severity level	Lay description	DW (95% CI)	Proportion of total prevalent cases
Asymptomatic	--	0	0.356 (0.351–0.362)
Mild	This person has some pain in the belly that causes nausea but does not interfere with daily activities.	0.011 (0.005–0.021)	0.326 (0.252–0.390)
Moderate	This person has pain in the belly and feels nauseous. The person has difficulties with daily activities.	0.114 (0.080–0.159)	0.160 (0.117–0.208)
Severe	This person has severe pain in the belly and feels nauseated. The person is anxious and unable to carry out daily activities.	0.324 (0.219–0.442)	0.158 (0.123–0.193)

References

1. Burstein R, Fleming T, Haagsma J, Salomon JA, Vos T, Murray CJL. Estimating distributions of health state severity for the global burden of disease study. *Popul Health Metr*. 2015;13(1):31. doi:10.1186/s12963-015-0064-y

Injuries

Flowchart

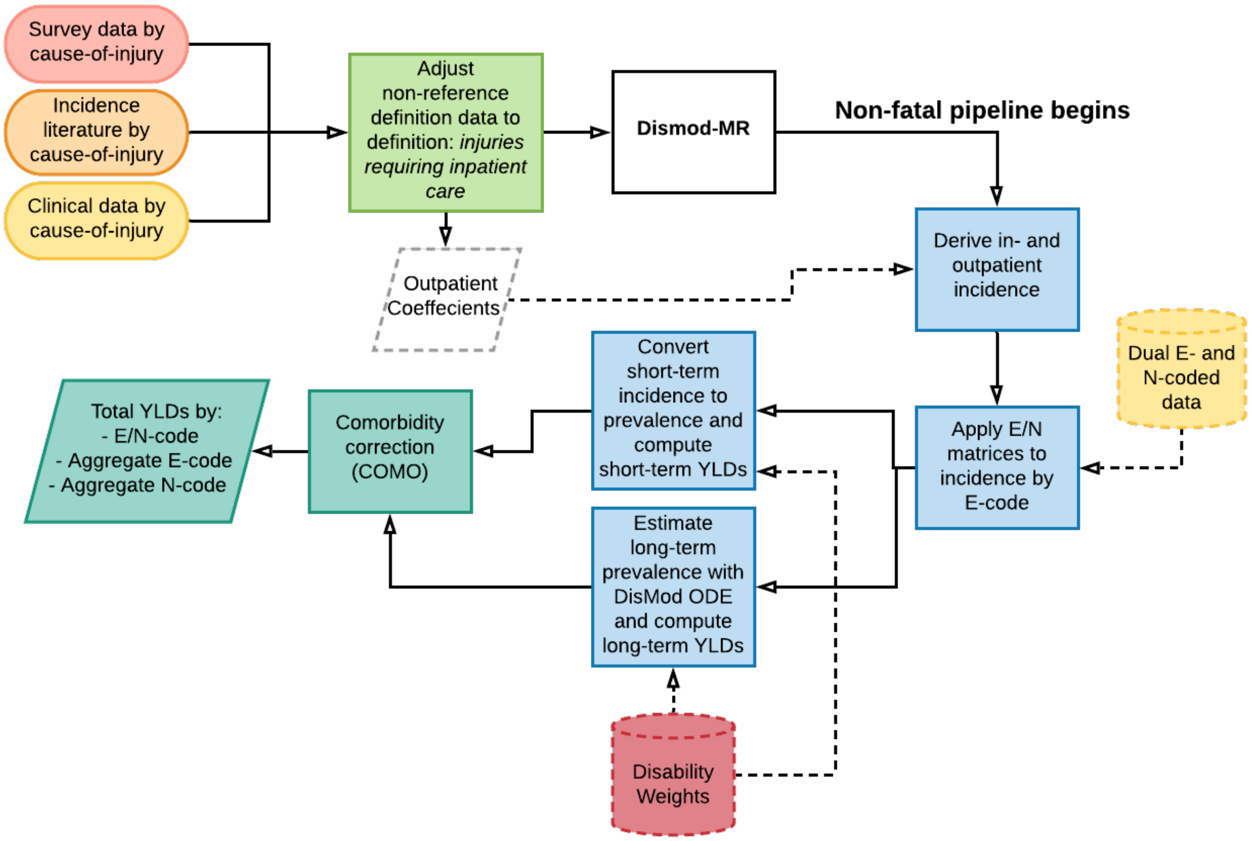

Case definition

For GBD 2023, the Injuries estimation process for non-fatal health outcomes encompasses a range of 37 causes, including transport injuries, falls, drowning, animal contact, self-harm, and interpersonal violence (excluding sexual violence, as described in a separate Appendix section). Injury incidence is defined using ICD-9 codes E000-E999 and ICD-10 chapters V to Y. Chapters S and T in ICD-10 and codes 800-999 in ICD-9 are used to estimate morbidity. Fatal discontinuities, defined as changes in deaths due to sudden, unexpected spikes in mortality that depart from the underlying mortality trend, are also included in the injuries framework and include the following causes: police conflict and executions ("state actor violence"), conflict and terrorism, and exposure to forces of nature. Specific non-fatal methods for these causes are outlined further below.

Each of these 37 causes of injury can result in a variety of physical injury sequelae (eg, traumatic brain injury), which we call the "nature of injury." Although the initial models are at the "cause of injury" level (eg, drowning), each cause of injury is distributed into cause-nature pairs to capture the disability that develops from the resulting nature of injury. We report incidence, prevalence, and years lived with disability (YLDs) due to injuries at the cause-nature pair level.

We make additional distinctions between inpatient and outpatient injuries and between short-term and long-term injuries. Inpatient injuries are defined as injuries that lead to overnight hospitalisation, whereas outpatient injuries are defined as ones treated in outpatient settings or emergency care. We define short-term injuries as injuries lasting less than one year and long-term injuries as those lasting longer than one year, at which point we assume lifelong disability.

Input data

Model inputs

To estimate morbidity from injuries, we use data from hospital records, emergency department records, insurance claims, and population-representative surveys to produce YLDs by country, year, sex, age, external cause-of-injury, and nature-of-injury category. Many countries report hospital data using a mix of cause-of-injury and nature-of-injury codes. To retain as much of the data as possible, we include all datasets that had at least 15% of cases coded to the cause of injury. In GBD 2015, we chose 45% as the threshold but have since lowered the threshold to 15%. We made this distinction after assessing the proportions of major injury causes (road injuries and falls) in each of the data sources. We concluded that there were no obvious differences between country data with 15%–45% coverage of external cause codes and those with more than 45% coverage. Below the 15% threshold, the external cause of injury coding became more disproportionate when compared to sources with higher external cause of injury coding. We assessed the raw hospital data to make sure that there was no disproportionate coding to certain causes in the 15%–45% cause-of-injury coding range. We increased the cause-specific injury cases from these datasets proportionately to sum to the total number of injury cases.

Conflict, war and executions, and police conflict data are obtained from the Uppsala Conflict Data Program [1], the International Institute for Strategic Studies [2], the Armed Conflict Location and Event Dataset [3], the Social Conflict Analysis Database [4], the Global Terrorism Database [5] and vital registration systems. Disaster data are obtained from the International Disaster Database from the Center for Research on the Epidemiology of Disasters [6]. Supplemental sources, such as collaborator accounts and news reports, are also used as sources for these causes.

Data searches

GBD 2023 utilised the same data as GBD 2021, with some updates to existing data and additions of new data. For GBD 2023, hospital and emergency department records were supplemented with more recent and available site-years (*Clinical data processing and estimation is described in detail in a separate section of this Appendix)*. Infrequently, datapoints were marked as outliers when a datapoint did not follow the age or time pattern as expected based on subject-matter or in-country experts and/or if the incidence rate of people sustaining an injury from a certain cause of injury was not plausible based on subject-matter or in-country experts.

Modelling strategy

As in previous GBD iterations, two categories of injury severity were separately modelled for each injury: injuries warranting inpatient care (inpatient) and injuries warranting other health care (outpatient). Injuries warranting inpatient care refer to injury cases of sufficient severity to require inpatient care if there are no restrictions in access to health care. Injuries warranting other health care refer to injury cases of sufficient severity to require health care attention but not hospitalisation. This category includes emergency department visits. To best measure the burden of injuries, GBD 2023 estimates excluded trivial injuries by restricting morbidity analysis to cases warranting some form of health care in a system with full access to health care. We intended to include cases with injuries that did not receive care in areas with restricted access to health care but that would have warranted some type of health care in a system with full access to health care. In some surveys, after asking about recall of injuries in the past month or year, respondents were further probed on whether they sought care and why they did not. This allowed us to include cases who cited financial or geographical barriers as reasons for not seeking care.

Cause-of-injury incidence

The list of unique (ie, not counting aggregate categories like road injuries or interpersonal violence) cause-of-injury categories was expanded compared to GBD 2021 as electrocution was modelled separately for the first time in GBD 2023. We treat police conflict and executions ("state actor violence") as a typical cause of injury rather than as a fatal discontinuity; however, the cause is modelled using the fatal discontinuity estimation strategy using incidence-to-mortality ratios because we do not have incidence data.

The majority of data that exist for external causes-of-injury estimation are incidence data. Incidence for cause-of-injury categories was modelled using Bayesian meta-regression method DisMod-MR 2.1 *(See appendix 1 section 2)*. Multiple datasets from hospital and emergency/outpatient departments, insurance claims, and surveys were fed into these incidence models. We separately estimated two categories of injury severity: inpatient and outpatient injuries. Coefficients for country-level covariates used in each cause-specific DisMod-MR model are shown in Table 1.

Excess mortality modelling (EMR)

In previous rounds, priors on excess mortality rate (EMR) were estimated in DisMod-MR 2.1 by matching prevalence datapoints with their corresponding cause-specific mortality rate (CSMR) values within the same age, sex, year, location (by dividing CSMR by prevalence). For many injuries, DisMod-MR 2.1 estimated a rather unrealistic pattern of EMR since we expect a pattern of decreasing EMR with greater access to quality health care. An example of this is shown in Figure 1 below. Such unexpected patterns often signal inconsistencies between CSMR estimates and the measures of prevalence and/or incidence. For injuries with large fatal and non-fatal inconsistencies, we implemented a new EMR modelling method described in the following section. These injuries included drowning, falls, poisoning by carbon

monoxide, interpersonal violence, assault by firearm, assault by sharp object, assault by other means, self-harm, self-harm by firearm, other transport injuries, and all road injuries.

To provide greater guidance to DisMod-MR 2.1 on the expected pattern of EMR, EMR data generated through a previous iteration was modelled using the meta-regression—Bayesian, regularised, trimmed (MR-BRT) tool with age, sex, and HAQ Index as predictors (See appendix 1 section 2). An upper bound of zero was included for HAQ Index based on the a priori assumption that greater access to health care leads to decreased mortality. For violence by firearm, sharp object, and other means, the mortality rate of homicide by firearm was also included as a covariate, since we assume that country-years with higher rates of violence by firearm are prone generally to more fatal violence. Results from MR-BRT were then used to predict EMR for each location-year, sex, and for ages 0-100 in ten-year increments. For the 16 injuries using EMR inputs modelled from MR-BRT, we set the trimming parameter to trim 10% of the datapoints, added a cubic-spline on age with knots set by data density, and a fixed effect on sex. The final MR-BRT predictions were then uploaded as EMR input data to DisMod-MR 2.1.

Figure 1. EMR estimates versus HAQ Index by location, for female road injuries DisMod-MR 2.1 results.

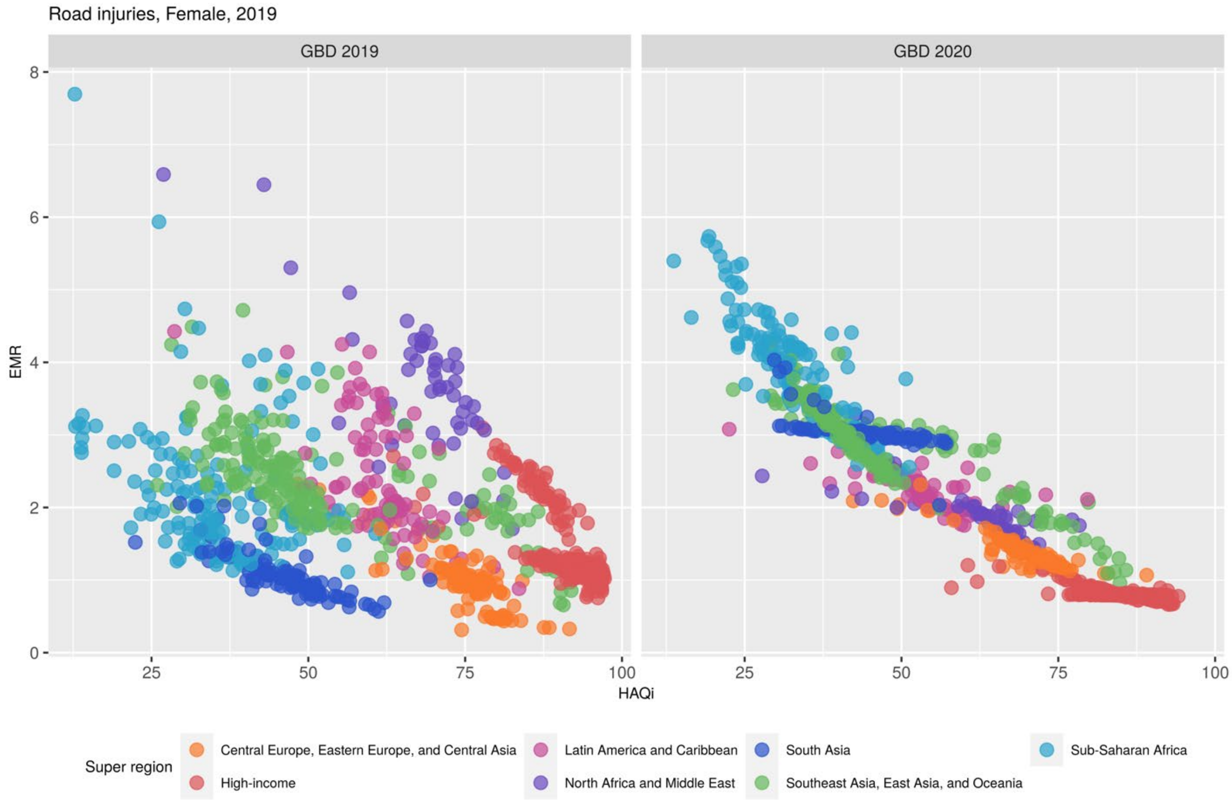

The left plot shows the EMR estimates from DisMod-MR in GBD 2019, while the right plot shows the EMR estimates from DisMod-MR in GBD 2021 after implementing the new EMR method. On the left, south Asia is an example of a region in which locations are not following an expected pattern of health access versus EMR, in that it has lower estimates of EMR than some high-income locations. On the right, a more visible trend is shown between EMR and HAQ Index across all locations.

Adjusting data

For GBD 2023, we derived coefficients to adjust type of care (inpatient or outpatient only) and presence of care-seeking behaviour (care versus no care) to maximise data included in inpatient-only and outpatient-only models for every injury. This was performed out of DisMod-MR 2.1. First, ST-GPR was used to estimate the proportion of people who were able to receive care for their injuries using the ratio of individuals who received inpatient or outpatient care to all injured individuals who did or did not receive care, based on World Health Survey data on road injuries spanning over 50 countries. These proportions allowed us to adjust data to the definition "injuries that received inpatient or outpatient care." Then, MR-BRT was used to adjust "received care" incidence and outpatient incidence both to inpatient incidence using inpatient versus outpatient incidence comparisons from the United States National Hospital Ambulatory Medical Care Survey. This process is summarised in Figure 2, and an example of a MR-BRT output can be seen in Figure 3.

Figure 2. Overview of data adjustment process using road injuries data from World Health Survey data

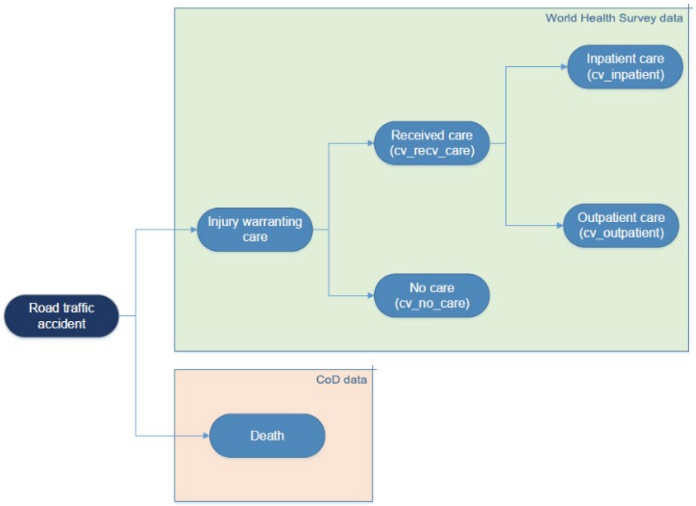

Figure 3. MR-BRT model for road injuries by age.

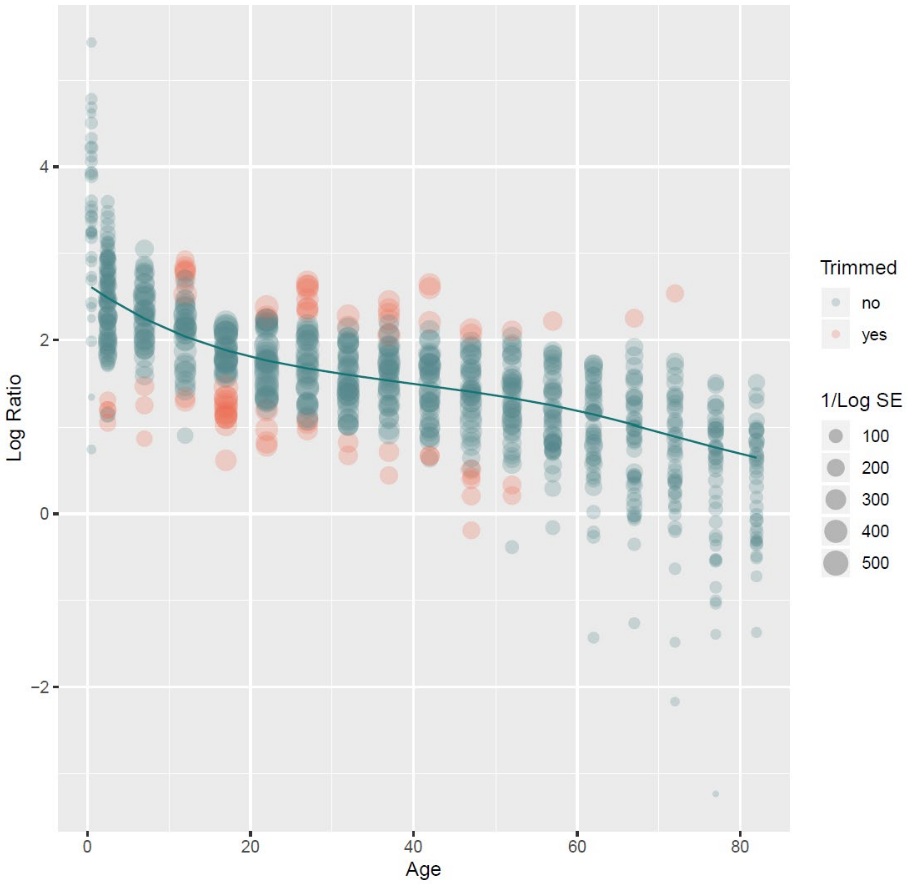

The y-axis shows the log of the ratio of outpatient cases to inpatient cases for each age along the x-axis. This shows how outpatient or ED visits without admission are more probable per inpatient admission in younger ages, while in the oldest ages, it is less likely for a road injury case to be seen only as an outpatient relative to each observed inpatient admission. The red datapoints show data that were trimmed by MR-BRT. See Figures 5–18 for MR-BRT plots for other injuries.

Table 1. Country-level covariates for DisMod-MR 2.1 incidence models for injuries

**Model**	**Covariate**	**Exponentiated value**
Road injuries (EMR)	Log-transformed age-standardised SEV scalar: Road Inj	3.48 (3.46–3.49)
	Vehicles - 2+4 wheels (per capita)	1.25 (1.23–1.27)
Pedestrian road injuries by road vehicle (EMR)	Log-transformed age-standardised SEV scalar: Pedest	3.06 (2.66–3.45)
Cyclist road injuries (EMR)	Log-transformed age-standardised SEV scalar: Cyclist	3.39 (3.16–3.49)
	Vehicles - 2+4 wheels (per capita)	1.25 (1.22–1.27)
Motorcyclist road injuries (EMR)	Log-transformed age-standardised SEV scalar: Mot Cyc	3.33 (3.06–3.48)
	Vehicles - 2 wheels (per capita)	1.72 (1.67–1.76)

Motor vehicle road injuries (EMR)	Log-transformed age-standardised SEV scalar: Mot Veh	2.19 (2.12–2.31)
	Vehicles - 4 wheels (per capita)	1.13 (1.10–1.15)
Other road injuries (EMR)	Log-transformed age-standardised SEV scalar: Oth Road	2.17 (2.12–2.25)
Other transport injuries (EMR)	Log-transformed age-standardised SEV scalar: Oth Trans	3.26 (2.84–3.48)
Falls	Log-transformed age-standardised SEV scalar: Falls	3.39 (3.29–3.48)
	Healthcare Access and Quality Index	1.05 (1.05–1.05)
Drowning (EMR)	Log-transformed age-standardised SEV scalar: Drown	3.23 (2.83–3.48)
	Coastal population within 10 km (proportion)	1.37 (1.25–1.50)
Fire, heat, and hot substances	Log-transformed age-standardised SEV scalar: Fire	3.43 (3.35–3.49)
	Indoor air pollution (all cooking fuels)	0.51 (0.48–0.55)
Poisonings	Log-transformed age-standardised SEV scalar: Poison	3.38 (3.22–3.48)
Poisoning by carbon monoxide (EMR)	Log-transformed SEV scalar: Poison	2.74 (2.22–3.34)
Poisoning by other means	Log-transformed SEV scalar: Poison	3.21 (2.78–3.48)
Exposure to mechanical forces	Log-transformed age-standardised SEV scalar: Mech	3.48 (3.47–3.49)
Unintentional firearm injuries	Log-transformed age-standardised SEV scalar: Mech Gun	3.12 (2.66–3.47)
Other exposure to mechanical forces	Log-transformed age-standardised SEV scalar: Oth Mech	3.45 (3.39–3.49)
Adverse effects of medical treatment	Socio-demographic Index	1.65 (1.65–1.65)
Animal contact	Log-transformed age-standardised SEV scalar: Animal	3.46 (3.41–3.49)
	LDI (I$ per capita)	0.74 (0.74–0.74)
Venomous animal contact	Log-transformed age-standardised SEV scalar: Venom	2.17 (2.12–2.27)
Non-venomous animal contact	Log-transformed age-standardised SEV scalar: Non Ven	3.47 (3.43–3.49)
Pulmonary aspiration and foreign body in airway	Log-transformed age-standardised SEV scalar: F Body Asp	3.16 (2.64–3.48)
Foreign body in eyes	—	—
Foreign body in other body part	Log-transformed SEV scalar: Oth F Body	2.74 (2.25–3.27)
Environmental heat and cold exposure	Population-weighted mean temperature	1.00 (1.00–1.01)
	90th percentile climatic temperature in the given country-year.	1.02 (1.00–1.05)
	LDI (I$ per capita)	0.74 (0.74–0.74)
Other unintentional injuries	Log-transformed age-standardised SEV scalar: Oth Unint	3.41 (3.27–3.49)

Electrocution	Healthcare Access and Quality Index	1.01 (1.01–1.02)
Self-harm (EMR)	Log-transformed age-standardised SEV scalar: Self Harm	3.20 (2.85–3.47)
	Major depressive disorder (age-standardised)	6.36 (4.87–7.34)
Self-harm by firearm (EMR)	Log-transformed age-standardised SEV scalar: Self Other	3.43 (3.38–3.48)
Self-harm by other specified means	Log-transformed age-standardised SEV scalar: Self Harm	3.32 (3.14–3.47)
Interpersonal violence (EMR)	Log-transformed age-standardised SEV scalar: Violence	3.17 (2.94–3.39)
	Socio-demographic Index	0.14 (0.14–0.14)
Assault by firearm (EMR)	Log-transformed age-standardised SEV scalar: Viol Gun	2.64 (2.40–2.91)
	Socio-demographic Index	6.91 (6.61–7.21)
Assault by sharp object (EMR)	Log-transformed age-standardised SEV scalar: Viol Knife	2.26 (2.14–2.40)
	Healthcare Access and Quality Index	0.97 (0.97–0.97)
Assault by other means (EMR)	Log-transformed age-standardised SEV scalar: Oth Viol	3.43 (3.34–3.49)
	Socio-demographic Index	0.78 (0.66–0.93)

Incidence of non-fatal injury discontinuities
Due to the sporadic nature of the incidence of injuries and a lack of time trend that results from injury discontinuities, DisMod-MR 2.1 was not used to model incidence due to injury discontinuities, exposure to forces of nature (ie, natural disaster), conflict and terrorism, and state actor violence. Instead, incidence-to-mortality ratios were averaged over super-region, year, and sex to limit the variability in the ratios applied to fatal discontinuities. For disaster incidence, the incidence-to-mortality ratio was calculated as an average of road injuries and drowning if there was a water-related natural disaster in that specific country-year noted in the International Disaster Database (if no water-related disaster, the incidence-to-mortality ratio from road injuries alone was used) [6]. For conflict and terrorism, the incidence-to-mortality ratio was calculated as an average of the road injuries and interpersonal violence causes. We treated executions and police conflict as similar to the fatal discontinuities in that we imputed the incidence using the incidence-to-mortality ratio of interpersonal violence. These incidence-to-mortality ratios were applied to mortality estimates from shock events from the Cause of Death database and shocks modelling process to calculate non-fatal discontinuity injuries incidence.

Analysis to inform nature-of-injury category hierarchy and long-term probability of injuries
Similar to GBD 2021, we used follow-up data obtained from a pooled dataset of six follow-up studies from China, the Netherlands, and the USA (see Table 2). These studies followed patients for at least one year after the injury. We also used the Medical Expenditure Panel Survey (MEPS) [7]. MEPS is a large-scale overlapping continuous panel survey of the USA non-institutionalised population that collects information on use and cost of health care and SF-12 responses. SF-12 responses are elicited twice over the two-year period that any individual is part of the study. Thus, MEPS offered the benefit of including health state measures of non-injured and destined to be injured and the benefit of having pre-injury and post-injury SF-12 responses. We pooled all available MEPS data over a 19-year span.

The follow-up studies used different patient-reported outcome measures to assess health status, namely the SF-36, Version 1 SF-12, and the EQ-5D. To enable comparison across the six datasets, it was necessary to analyse the data in a standardised patient-reported outcome measure. First, we mapped all patient-reported outcome measures to Version 2 SF-12 (SF-12v2). Second, we normalised the health status measurements by mapping the SF-12 scores to a corresponding disability weight based on several opportunistic surveys asking respondents to score SF-12 based on the lay descriptions for a selection of 60 GBD health states. We ran a regression of logit-transformed disability weight on nature-of-injury category and age group and never-injured status. The pooled dataset informed both the nature-of-injury category hierarchy and the long-term probability of injuries, discussed below.

Table 2. Details of injury follow-up surveys used in GBD 2023

**Dataset**	**Year**	**Type of data collected**	**Type of patients**	**Setting**	**Sample size* and response**
Guangdong follow-up survey, China[8]	2006–2007	Follow-up survey among sample of ISS patients	Patients (15+ years) who were hospitalised that had been injured by road traffic injury, fall, blunt or penetrating trauma	Based on three national injury surveillance hospitals in Zhuhai, Guangdong Province in China	998 (response 87%)
LIS follow-up survey, Netherlands[9]	2001–2002	Follow-up survey among stratified sample of ISS patients (oversampling less common, severe injuries)	Patients (15+ years) who visited the emergency department of a hospital and were discharged to the home environment and patients who were admitted to hospital	Based on 17 public hospitals in the Netherlands	8564 (response 37%)
LIS follow-up survey, Netherlands[10]	2007–2008	Follow-up survey among stratified sample	Patients (15+ years) who visited the emergency	Based on 15 public hospitals	8057 (response 36%)

		of ISS patients (oversampling less common, severe injuries)	department of a hospital and were discharged to the home environment and patients who were admitted to hospital	in the Netherlands	
NSCOT – National study on Costs and Outcomes of Trauma, USA[11]	2001–2002	A prospective cohort study was conducted among a sample of adult trauma patients treated at Level I trauma centres and non-trauma centre hospitals	Patients treated for a moderate to severe injury (as defined by at least one injury of an Abbreviated Injury Scale (AIS) score of 3 or greater	Based on 69 hospitals in 12 states in the USA	5191 (response 61%)
SCTBIFR – South Carolina Traumatic Brain injury Follow-up Registry, USA[12]	1999–2002	A prospective cohort study was conducted among injured in-patients with a traumatic brain injury-related injury	Patients (15+ years) who were admitted to hospitals and met the CDC case definition of TBI – trauma to the head associated with altered consciousness, amnesia, neurological abnormalities, skull fracture, intracranial lesion, or death	Discharged from all nonfederal in-state acute care hospitals	7613 (response 28%)
Burns outcome study, Netherlands[13]	2003–2006	A multicentre prospective cohort was conducted among adult	Injury patients who sustained severe burns	Three public hospitals with specialised burn units.	311 (response 78%)

		(severe) burn patients			

*number of patients that met the inclusion criteria; response rate = percentage of patients who responded to the follow-up survey (in case of multiple follow-up times the response rate of the first follow-up moment is reported).

<u>Nature-of-injury category hierarchy</u>

Multiple injuries can occur in one individual. Similar to GBD 2021, a nature-of-injuries severity hierarchy was used to establish a one-to-one relationship between cause-of-injury and nature-of-injury category (Table 3). This means that in the case of multiple injuries the nature-of-injury category that was likely to be responsible for the largest burden was selected. To construct the hierarchy, we used data from the pooled dataset of follow-up studies [8-13]. The output of the regression of logit-transformed disability weight on nature-of-injury category and individual characteristics of the follow-up studies were used to calculate the mean long-term disability attributable to each nature-of-injury category. The ranking of nature-of-injury categories by their long-term disability weights formed the basis of our severity hierarchy. Hierarchies were developed separately, for injuries warranting inpatient care and injuries warranting other health care.

Table 3. Nature-of-injury hierarchies: combination of empirical hierarchies estimated from pooled follow-up studies and expert adjustments, for inpatient and outpatient injuries

Rank	Inpatient hierarchy	Outpatient hierarchy
1	Spinal cord lesion below neck level	Fracture of pelvis
2	Amputation of lower limbs, bilateral	Fracture of patella, tibia or fibula, or ankle
3	Amputation of upper limbs, bilateral	Fracture of hip
4	Spinal cord lesion at neck level	Fracture of skull
5	Fracture of hip	Amputation of thumb
6	Fracture of femur, other than femoral neck	Fracture of vertebral column
7	Amputation of upper limb, unilateral	Multiple fractures, dislocations, crashes, wounds, sprains, and strains
8	Amputation of lower limb, unilateral	Internal haemorrhage in abdomen and
9	Multiple fractures, dislocations, crashes, wounds, sprains, and strains	Fracture of femur, other than femoral neck
10	Effect of different environmental factors	Dislocation of hip
11	Fracture of patella, tibia or fibula, or ankle	Amputation of toe/toes
12	Moderate-Severe traumatic brain injury	Fracture of hand (wrist and other distal part
13	Fracture of foot bones except ankle	Amputation of fingers (excluding thumb)
14	Internal haemorrhage in abdomen and pelvis	Burns, <20% of total burned surface area without lower airway burns
15	Crush injury	Dislocation of knee
16	Minor traumatic brain injury	Contusion in any part of the body
17	Fracture of pelvis	Minor traumatic brain injury
18	Nerve injury	Foreign body in respiratory system
19	Severe chest injury	Severe chest injury
20	Dislocation of hip	Drowning and non-fatal submersion
21	Burns, ≥20% total burned surface area or ≥10% burned surface are if head/neck or hands/wrist involved w/o lower airway burns	Asphyxiation

22	Lower airway burns	Poisoning requiring urgent care
23	Fracture of skull	Effect of different environmental factors
24	Amputation of thumb	Foreign body in GI and urogenital system
25	Fracture of hand (wrist and other distal part of [illegible]	Fracture of sternum and/or fracture of one or more ribs
26	Fracture of vertebral column	Nerve injury
27	Contusion in any part of the body	Fracture of face bones
28	Open wound(s)	Dislocation of shoulder
29	Amputation of toe/toes	Injury to eyes
30	Dislocation of knee	Fracture of clavicle, scapula, or humerus
31	Amputation of fingers (excluding thumb)	Fracture of radius and/or ulna
32	Drowning and non-fatal submersion	Fracture of foot bones except ankle
33	Asphyxiation	Foreign body in ear
34	Burns, <20% total burned surface area without lower airway burns	Muscle and tendon injuries, including sprains and strains lesser dislocations
35	Muscle and tendon injuries, including sprains and strains lesser dislocations	Superficial injury of any part of the body
36	Fracture of face bones	Open wound(s)
37	Foreign body in respiratory system	Complications following therapeutic
38	Poisoning requiring urgent care	
39	Foreign body in GI and urogenital system	
40	Fracture of sternum and/or fracture of one or	
41	Dislocation of shoulder	
42	Injury to eyes	
43	Fracture of clavicle, scapula, or humerus	
44	Fracture of radius and/or ulna	
45	Foreign body in ear	
46	Superficial injury of any part of the body	
47	Complications following therapeutic	

Cause-nature matrices
Because injury disability is linked more to the nature of injury than to the cause of injury, matrices were generated to map the proportion of each cause-of-injury category that results in a particular nature-of-injury category. These matrices are based on a collection of dual-coded (ie, both cause-of-injury and nature-of-injury coded) hospital and emergency department datasets. The data for this step came from inpatient, outpatient, and emergency room discharge data from Argentina, Brazil, Bulgaria, China, Chile, Colombia, Cyprus, Czech Republic, Denmark, Egypt, Estonia, Georgia, Great Britain, Hungary, Iceland, Iran, Italy, India, Kyrgyzstan, Latvia, Malta, Mauritius, Mexico, Mongolia, Mozambique, the Netherlands, New Zealand, Norway, the Philippines, Portugal, Slovenia, Spain, Sweden, Macedonia, Uganda, the USA, and Zambia. We applied our nature-of-injury severity hierarchy above to assert that every observation had one cause of injury and one nature of injury.

Dirichlet models were used to estimate all the nature-of-injury category proportions for one cause of injury simultaneously. These models allow for consistent borrowing of information across age, sex, inpatient/outpatient, and high/low-income countries and assert that the nature-of-injury proportions within a cause-of-injury category must add up to 1. One cause-nature matrix was created for each combination of injury warranting hospital admission versus injury warranting other health care,

high/low-income countries (a binary variable based on GBD super-region), male/female, and age category. Applying these matrices to our cause-of-injury incidence from DisMod-MR 2.1, we produced cases of injury warranting hospital admission and incidence of injury warranting other health care by cause and nature of injury. For causes that are subsets of other causes (child and parent causes), the cause-nature matrix was applied directly to the child causes. Afterward, the incidences of the child cause-nature combinations were scaled to sum to the incidence of the parent cause.

Probability of permanent health loss

Disability due to injury was assumed to affect all cases in the short-term with a proportion having long-term (permanent) outcomes. The probability of long-term outcomes was needed to estimate the incidence and subsequently the prevalence of cases with permanent health loss. In our conceptual model, individuals who suffer from a non-fatal injury will, in the long term, return to either full or partial health. If one-year post-injury patients return to a health status with more disability than their pre-injury health status, injury patients are assumed to have permanent disability from their injury. The difference between the pre-injury health states and health status one year after injury is assumed to be their permanent level of injury-related disability. We assessed the probability of developing permanent health loss using the pooled dataset of follow-up studies [8-13] and the MEPS [7] that were also used to generate the nature-of-injury hierarchy. To assess the probability of permanent health loss, we estimated the effects using a logit-linear mixed effects regression:

$$\begin{aligned} Logit(DW)_{im} = {} & \alpha + \beta(injuries_{im}) + \beta(never\ injured_i) + \beta(never\ injured_i * age_i) \\ & + \beta(fracture\ of\ pelvis_i) + \beta(fracture\ of\ pelvis_i * age_i) + \beta(poisoning_i * age_i) \\ & + \beta(moderate\ to\ severe\ TBI_i * age_i) + RE_c + RE_i \end{aligned}$$

where we included dummies for all the nature-of-injury categories $(injuries_{im})$, with the reference category being no injury (from MEPS dataset). We also included a dummy for never injured prior to the current injury, age, interactions between age and never-injured status, and interactions with three long-term nature-of-injury categories that were found to significantly vary with age: pelvis fractures, poisonings, and moderate/severe traumatic brain injuries. In notation, subscript m refers to patient-reported outcome measure, i refers to individual, and c refers to country. Random effects (RE) were included to control for variation between countries and individuals.

After predicting overall disability at one-year follow-up, we estimated a counterfactual by setting all observations to “no injury,” the reference group for $\beta(injuries_{im})$ in our model. The disability attributable to the nature of injury at one year was assumed to be the difference between our counterfactual of no injury and predicted disability with injury. The probability of treated long-term outcomes was estimated via the ratio of this attributable disability relative to the long-term disability weight for that injury.

$$Probability\ of\ long-term\ disability = \frac{with\ injury\ disability_{im} - counterfactual\ disability_{im}}{DW_m}$$

We developed estimates of the probability of permanent health loss by nature-of-injury category, injury severity level (injuries warranting inpatient admission and injuries warranting other health care), and age. These probabilities are shown in Figure 4 for three selected age groups (25-30, 50-55, 75-80) and selected nature-of-injury categories by inpatient and outpatient. Moderate-severe TBI and spinal cord

lesions only have inpatient injury long-term probabilities, and nerve injury, open wounds, and severe chest injury have long-term probabilities of zero for outpatient cases.

Figure 4. Long-term probabilities derived from the MEPS data for selected nature of injuries and age groups

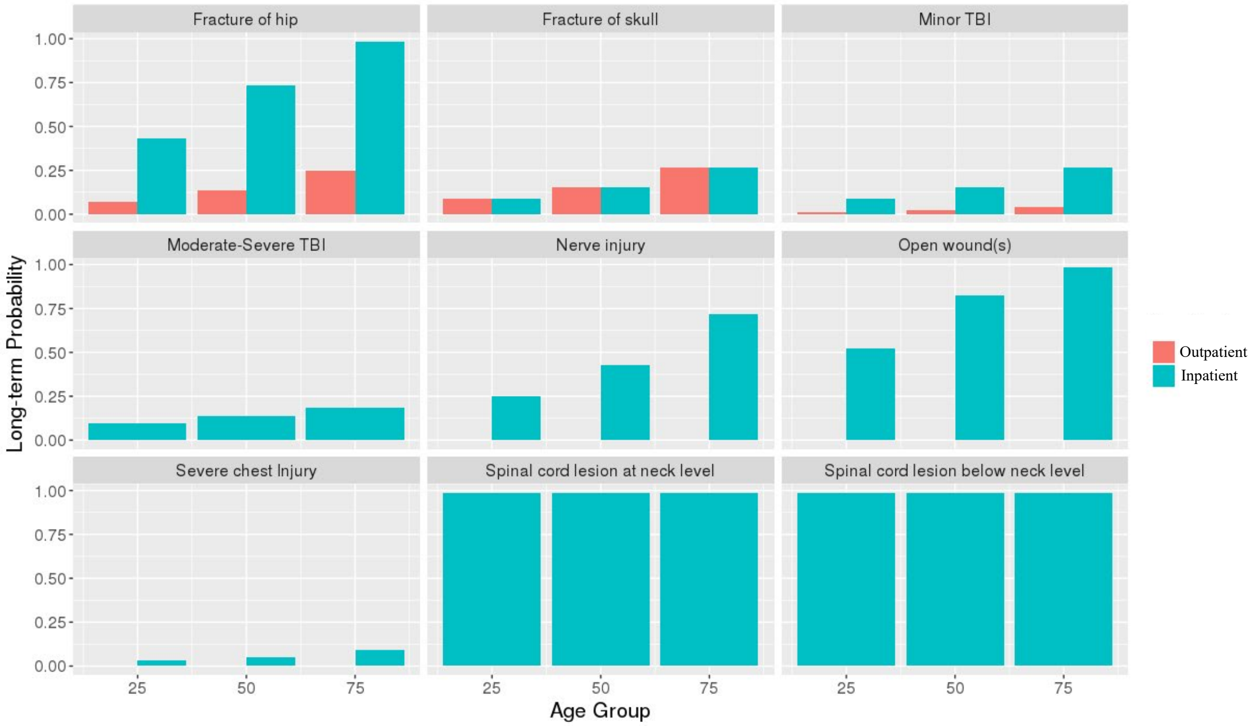

Disability associated with treated and untreated cases

For many nature-of-injury categories, a separate disability weight is used for treated versus untreated cases. To estimate the percentage treated for injuries in a given location-year, we used the Healthcare Access and Quality (HAQ) Index with the same strategy described for the probability of permanent health loss. We chose a reasonable cutoff for the HAQ Index (75 on a scale of 0–100) as the threshold at and above which 100% of injuries were treated. This value captured most OECD countries for all years back to 1980. We then scaled all remaining location-years between 10% and 100% treated based on their HAQ Index value and used that as the percentage treated in a given location-year. This was done at the draw level to propagate uncertainty. We made the decision to ignore any long-term disability from injuries with implausibly high estimates of long-term disability.

Custom disability weight adjustments

Traumatic brain injury (TBI)

An analysis was performed to create two custom combined disability weights (DWs) for long-term traumatic brain injury (TBI) from the GBD 2013 minor, moderate, and severe long-term TBI DWs [14]. Minor long-term TBI is defined as episodes of headaches, memory problems, and difficulty concentrating, moderate TBI also includes dizziness and anxiety, and severe TBI includes dependence on others. Custom weights were computed for two reasons: First, while mild TBI can be isolated using ICD codes, there was no meaningful way to distinguish between moderate and severe TBI within ICD codes,

which would have been necessary for the E-N matrix of the injuries pipeline. Second, the severity of an incident case of TBI might not necessarily align with the severity of the long-term outcome. For instance, a case of TBI categorised as minor after the incident could lead to moderate or severe long-term outcomes. Data from a follow-up study of TBI patients [15] detailed by severity of TBI incident as well as severity of long-term outcomes were used to inform logit models that estimated the proportion of minor incident TBI (N27) and moderate/severe incident TBI (N28) cases that resulted in minor, moderate, and severe long-term outcomes. The logit models' distributions of outcome severity of the initial TBI incident were then used to create new weighted combinations of the minor, moderate, and severe long-term TBI DWs, producing two custom DWs for the minor TBI n-code (N27) and moderate/severe TBI n-code (N28), shown in Table 4: Disability weights for long-term TBI, before and after custom adjustment, below. These custom DWs were only applied to the proportion of TBI cases estimated to have long-term outcomes.

For example, from the described analysis we found that out of all minor TBI incident cases with long-term outcomes, approximately 77% of those long-term outcomes were minor, approximately 21% were moderate, and approximately 2% were severe. So, the combined DW for minor TBI (N27) would be weighted as 77% of the original minor long-term TBI DW, 21% of the original moderate long-term TBI DW, and 2% of the original severe long-term TBI DW.

Table 4. Disability weights for long-term TBI, before and after custom adjustment

	Minor TBI, long-term	Moderate TBI, long-term	Severe TBI, long-term
**Original DW**	0.094 (0.063–0.133)	0.231 (0.156–0.324)	0.637 (0.462–0.786)
**Combined DW**	0.132 (0.090–0.182)	0.164 (0.112–0.226)	

Spinal cord injury (SCI)

Spinal cord lesions are grouped into two nature of injuries (n-codes): lesions at the neck level (N33), and lesions below the neck level (N34), where neck level is defined as at the level of the cervical spinal cord. To determine the disability weight of each of these n-codes, different levels of severity and their frequency were accounted for (Table 5). Data were used from a study [16] that reported on the distribution of spinal cord injuries by their severity after 1 year of recovery, with severity graded according to the American Spinal Injury Association (ASIA) Impairment Scale score. The frequency of each grade of severity after 1 year was calculated. Each grade of severity was assigned two corresponding disability weight, one for a treated injury and one for an untreated injury. A grade of E was treated as having full health (a disability weight of 0).

Table 5. ASIA Impairment Scale score and proportions mapped to GBD health state descriptions for long-term treated spinal cord injuries

ASIA Impairment Scale Score after 1 year	Proportion after 1 year	GBD health state lay description (at neck level, treated)	GBD health state lay description (below neck level, treated)
A	50.2%	is paralyzed from the neck down, with no feeling or control over any part of the body below the neck, and no urine or bowel control.	is paralyzed from the waist down, cannot feel or move the legs and has difficulties with urine and bowel

			control. The person uses a wheelchair to move around.
B	7.4%	is paralyzed from the neck down and cannot feel or move the arms and legs.	is paralyzed from the waist down and cannot feel or move the legs. The person uses a lightweight and comfortable wheelchair to move around.
C	14.0%	is paralyzed from the neck down and cannot feel or move the arms and legs.	is paralyzed from the waist down and cannot feel or move the legs. The person uses a lightweight and comfortable wheelchair to move around.
D	27.1%	has some difficulty in moving around, and difficulty in lifting and holding objects, dressing and sitting upright, but is able to walk without help	has some difficulty in moving around, and difficulty in lifting and holding objects, dressing and sitting upright, but is able to walk without help.
E	1.3%	N/A	N/A

Table 7. ASIA Impairment Scale score and proportions mapped to GBD health state descriptions for long-term untreated spinal cord injuries

ASIA Impairment Scale Score after 1 year	Proportion after 1 year	GBD health state lay description (at neck level, untreated)	GBD health state lay description (below neck level, untreated)
A	50.2%	is paralyzed from the neck down, with no feeling or control over any part of the body below the neck, and no urine or bowel control. Arms and legs are in fixed, bent positions, and the person gets frequent infections and pressure sores.	is paralyzed from the waist down, cannot feel or move the legs and has difficulties with urine and bowel control. Legs are in fixed, bent positions, and the person gets frequent infections and pressure sores.
B	7.4%	is paralyzed from the neck down and cannot feel or move the arms and legs. Arms and legs are in fixed, bent positions, and the person gets frequent infections and pressure sores.	is paralyzed from the waist down and cannot feel or move the legs. Legs are in fixed, bent positions, and the person gets frequent infections and pressure sores.
C	14.0%	is paralyzed from the neck down and cannot feel or move the arms and legs. Arms and legs are in fixed, bent positions, and the person gets frequent infections and pressure sores.	is paralyzed from the waist down and cannot feel or move the legs. Legs are in fixed, bent positions, and the person gets frequent infections and pressure sores.
D	27.1%	has some difficulty in moving around, and difficulty in lifting and holding objects, dressing and sitting upright, but is able to walk without help	has some difficulty in moving around, and difficulty in lifting and holding objects, dressing and sitting upright, but is able to walk without help.
E	1.3%	N/A	N/A

Afterward, a weighted average of these disability weights was calculated based on the frequency of each grade of severity and used as the final disability weight. This process for calculating a final disability weight was conducted separately for lesions at versus below neck level.

Table 8. Disability weights associated with long-term SCI

Health state	Disability weight
Spinal cord lesion at neck level (treated)	0.589 (0.415–0.748)
Spinal cord lesion at neck level (untreated)	0.732 (0.544–0.871)
Spinal cord lesion below neck level (treated)	0.296 (0.198–0.414)
Spinal cord lesion below neck level (untreated)	0.623 (0.434–0.777)

Duration of short-term health loss

To determine the duration for treated cases of short-term injury, we analysed patient responses from two Dutch Injury Surveillance System follow-up studies conducted from 2001–2003 and 2007–2009 [17]. These studies collected data at 2.5, 5, 9, and 24 months post-injury to determine whether injury patients were still experiencing problems due to their injury. If not, the patients were asked how many days they had experienced problems. The injury patients that still reported having problems one year after the injury were assumed to be captured in our analysis of permanent disability. The duration for treated cases of short-term injury was estimated for injuries warranting inpatient admission and injuries warranting other health care separately. The estimates were supplemented by expert-driven estimates of short-term duration for nature-of-injury categories that did not appear in the Dutch dataset and untreated injuries.

Calculation of prevalence from incidence data – short-term injury

For short-term injury outcomes, which were assumed to be less than one year in duration, the prevalence for each cause-of-injury/nature-of-injury/severity-level grouping was approximated by the incidence for that grouping multiplied by the associated nature-of-injury/severity-level-specific duration.

Calculation of prevalence from incidence data – permanent health loss

For permanent health loss, we assumed no remission and thus integrated incidence over time to arrive at prevalence estimates. We used DisMod-MR ODE (ie, the "engine" of DisMod-MR 2.1) to carry out this integration for each combination of cause of injury and nature of injury by country, year, and sex. For this step we used random effects meta-analysis to pool data on standardised mortality ratios derived from literature reviews for spinal cord injury, burns covering more than 20% of the body, moderate to severe traumatic brain injury, hip fracture, and multiple significant injuries [18-29]. In Figure 5 we include examples of these meta-analyses: hip fractures and traumatic brain injuries.

For all other nature-of-injury categories, we assumed no long-term excess mortality. For the incidence estimates derived from injury discontinuities – "exposure to forces of nature" and "conflict and terrorism" – we did not use DisMod-MR 2.1 as discontinuities by definition violate the assumption of a steady state in DisMod-MR 2.1 to estimate prevalence from incidence. For these two cause-of-injury categories, we coded the differential equations from DisMod ODE that determine the relationship between incidence, remission, mortality risk, and prevalence into Python and streamed out the prevalence from the incidence in the years of war or disaster by integrating over one year at a time.

Figure 5. Meta-analyses of standardised mortality ratios derived from literature reviews: hip fractures and traumatic brain injury

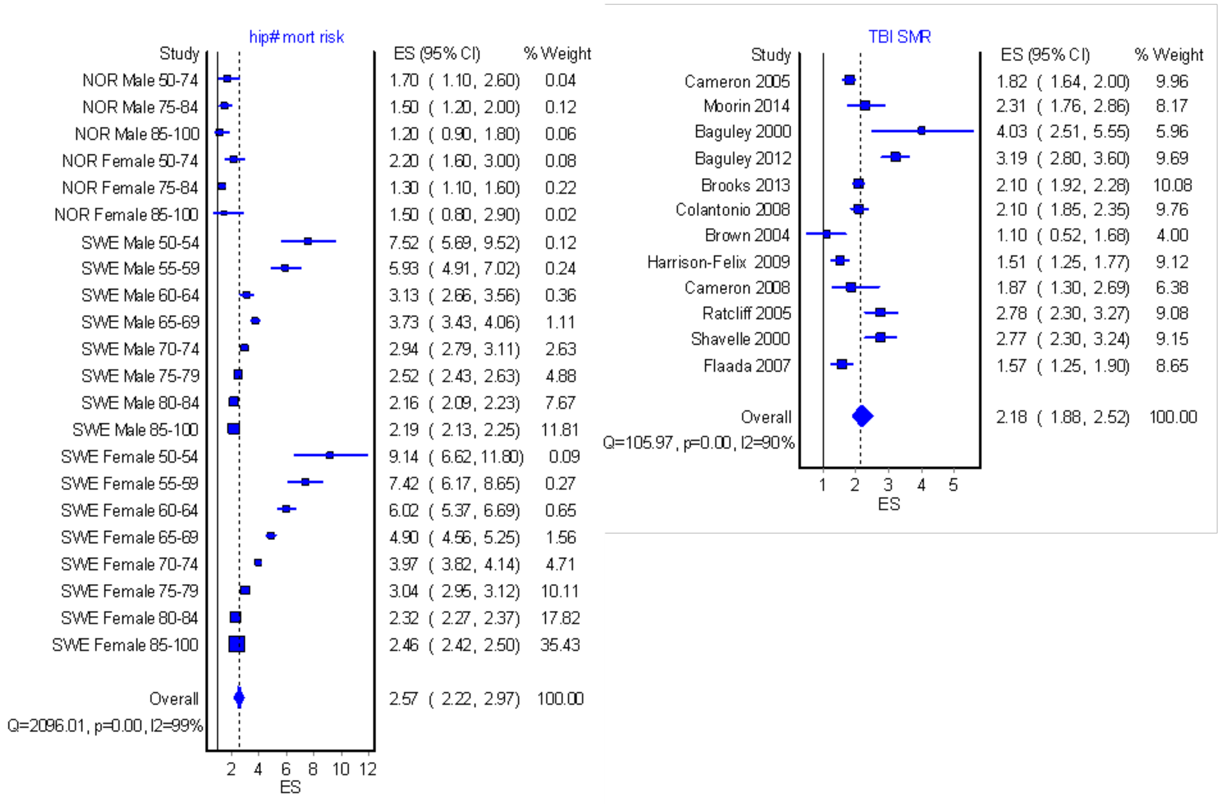

MR-BRT models (continued)

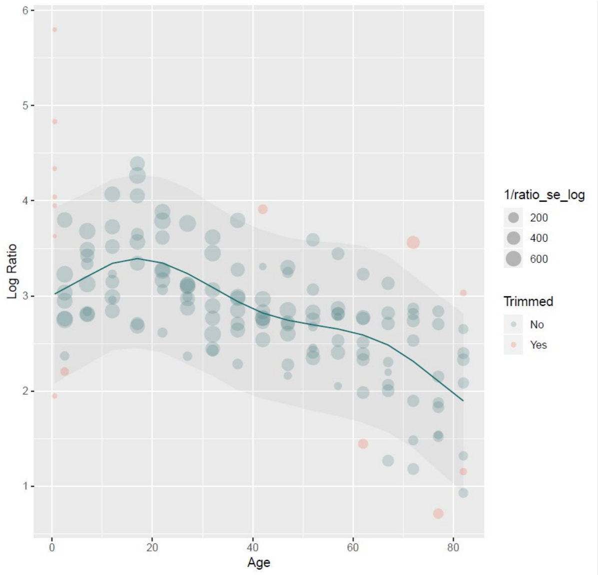

Figure 6. MR-BRT model for animal contact

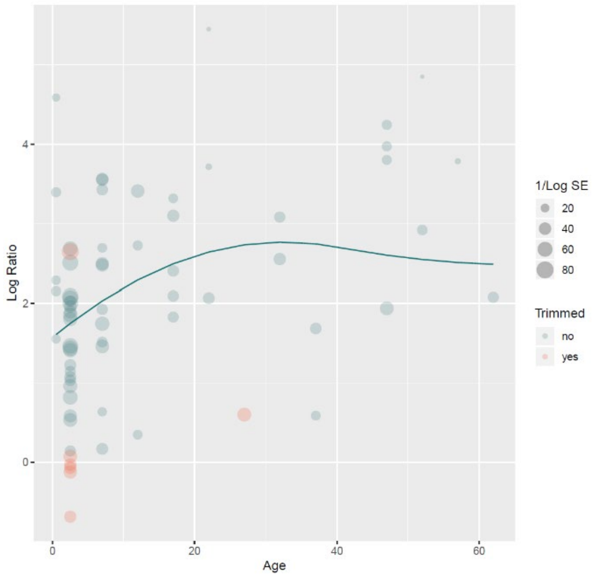

Figure 7. MR-BRT model for drowning

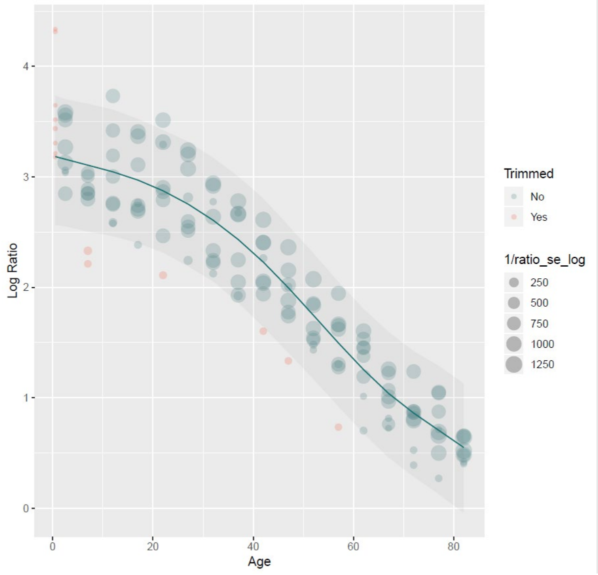

Figure 8. MR-BRT model for falls

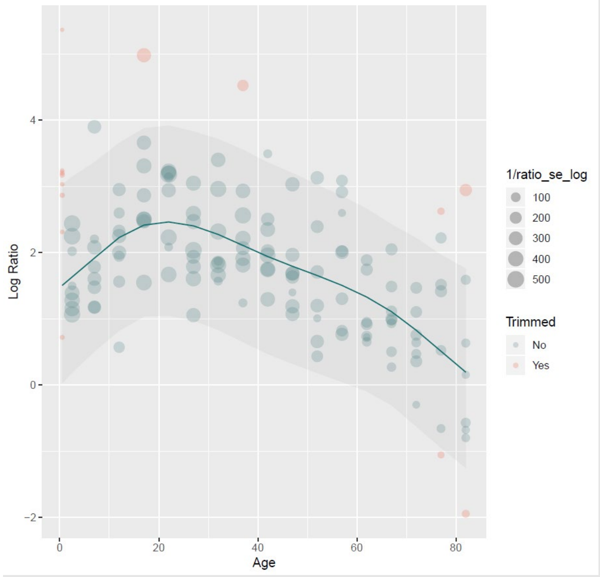

Figure 9. MR-BRT model for fire, heat, and hot substances

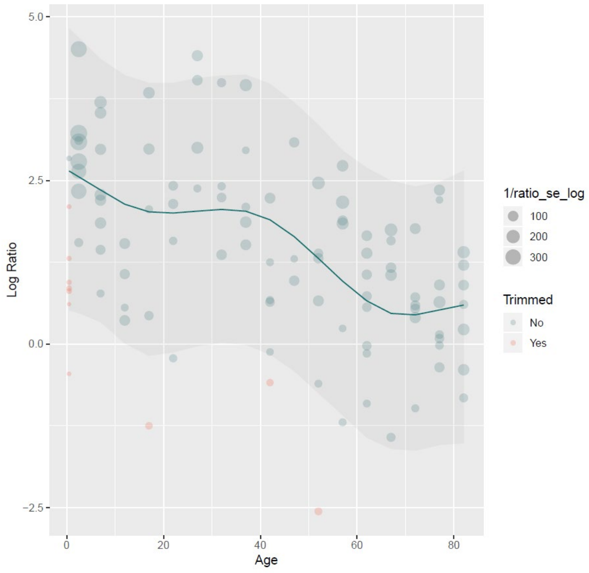

Figure 10. MR-BRT model for pulmonary aspiration and foreign body in airway

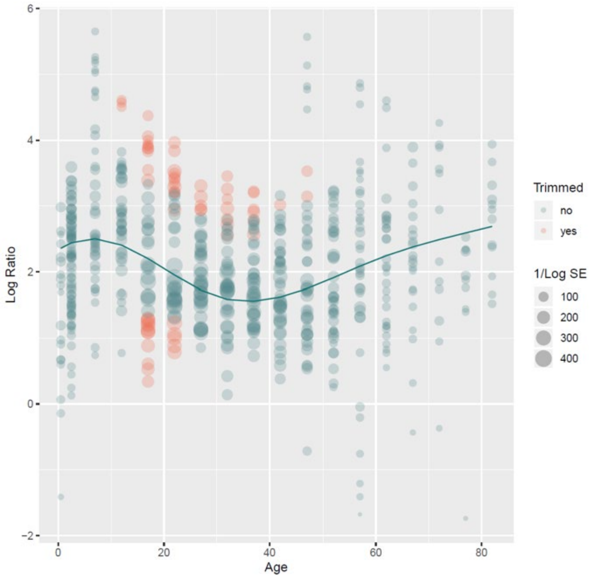

Figure 11. MR-BRT model for interpersonal violence

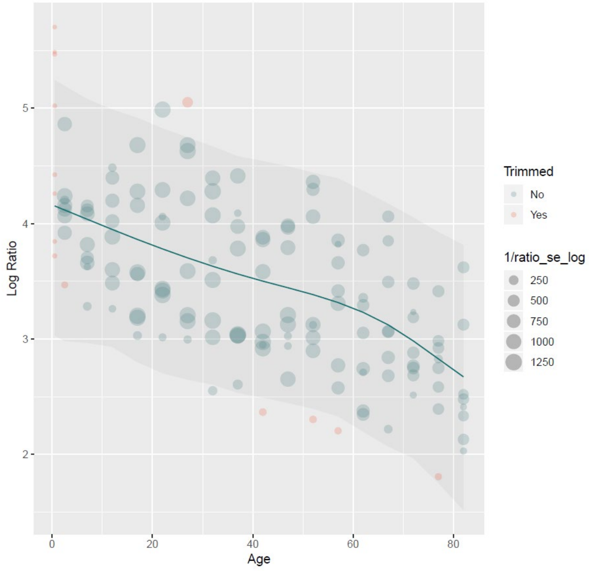

Figure 12. MR-BRT model for exposure to mechanical forces

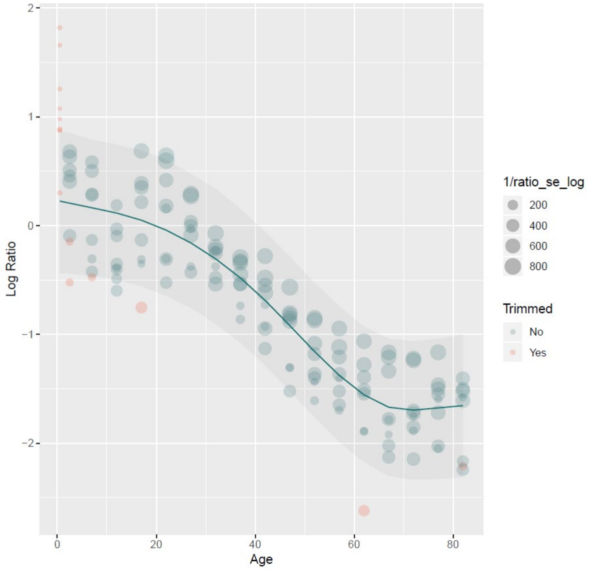

Figure 13. MR-BRT model for adverse effects of medical treatment

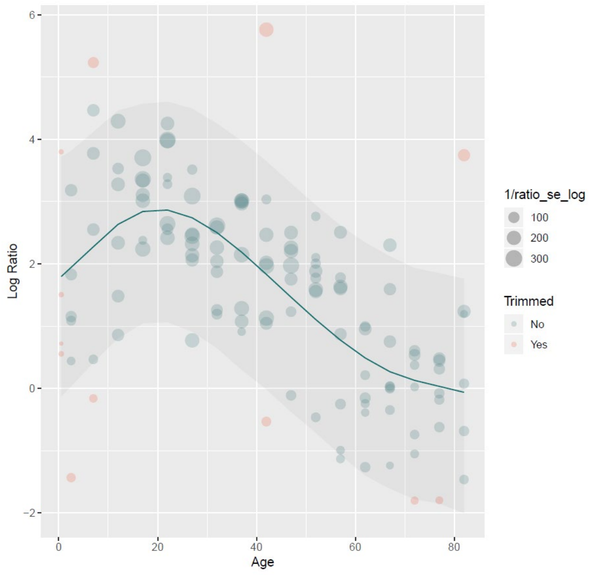

Figure 14. MR-BRT model for exposure to forces of nature

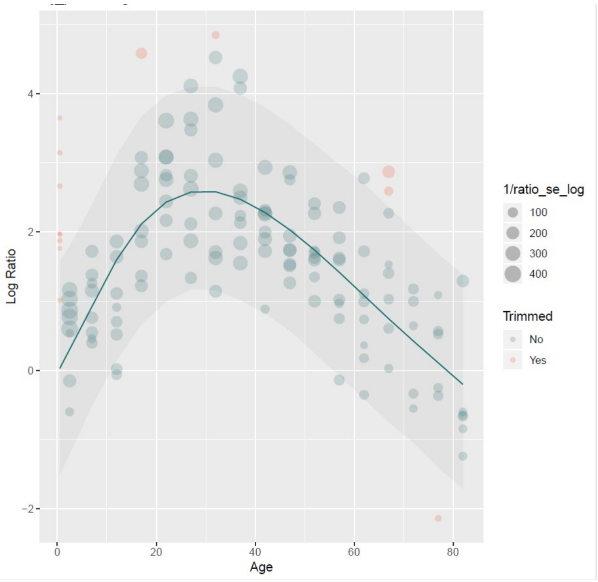

Figure 15. MR-BRT model for poisonings

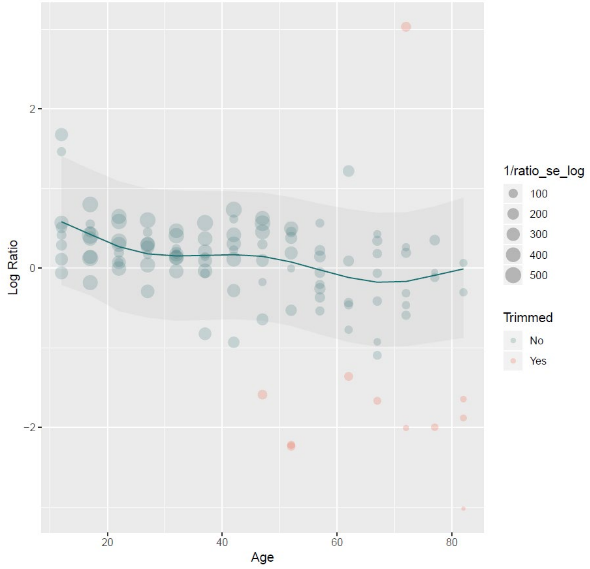

Figure 16. MR-BRT model for self-harm

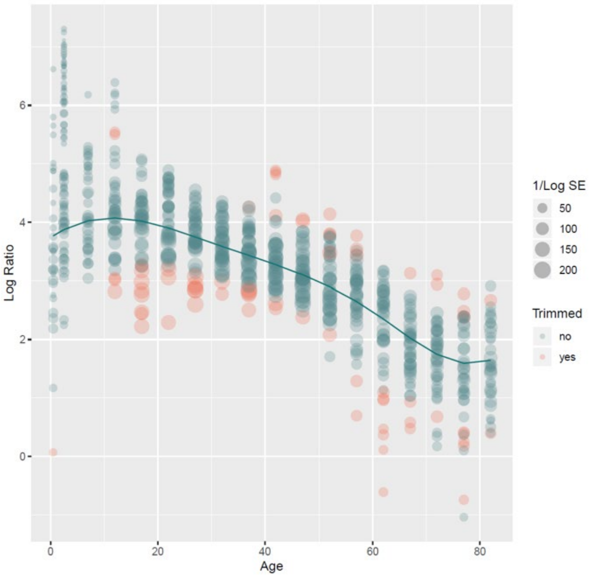

Figure 17. MR-BRT model for other unintentional injuries

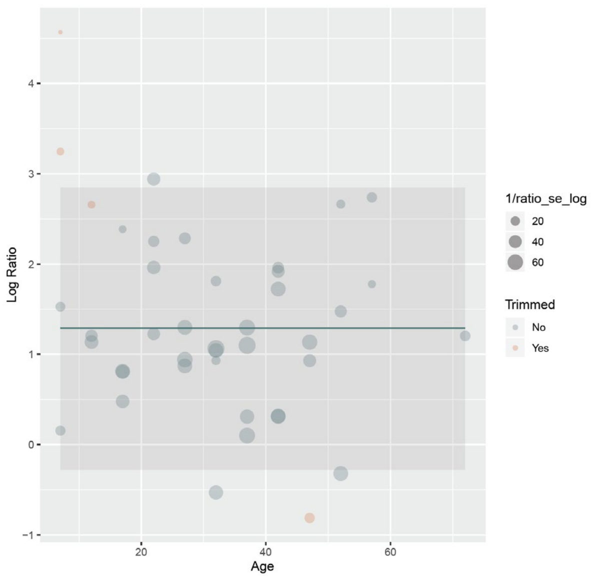

Figure 18. MR-BRT model for electrocution

References

1. Department of Peace and Conflict Research, Uppsala University. UCDP Nonstate Conflict Dataset. Uppsala, Sweden: Department of Peace and Conflict Research, Uppsala University.

2. International Institute for Strategic Studies. International Institute for Strategic Studies Armed Conflict Database. London, United Kingdom: International Institute for Strategic Studies.

3. Climate Change and African Political Stability Project (CCAPS). Armed Conflict Location and Event Dataset, Realtime - Robert S. Strauss Center as referenced in Raleigh, Clionadh, Andrew Linke, Havard Hegre and Joakim Karlsen. 2010. Introducing ACLED-Armed Conflict Location and Event Data. Journal of Peace Research 47(5), 1-10.

4. Salehyan I, Hendrix CS, Hamner J, Case C, Linebarger C, Stull E, Williams J, Robert S. Strauss Center for International Security and Law. Social Conflict in Africa: A New Database. *Int Interact*. 2012; 38(4): 503-511.

5. National Consortium for the Study of Terrorism and Responses to Terrorism (START). Global Terrorism Database. College Park, MD, United States of America: University of Maryland, 2018.

6. Centre for Research on the Epidemiology of Disasters (CRED). EM-DAT: The OFDA/CRED International Disaster Database. Brussels, Belgium: Catholic University of Leuven.

7. Agency for Healthcare Research and Quality. United States Medical Expenditure Panel Survey. Rockville, United States: Agency for Healthcare Research and Quality.

8. Chinese Center for Disease Control and Prevention (CCDC). China Zhuhai Study 2006-2007 - China CDC.

9. Consumer Safety Institute (Netherlands). Netherlands Injury Surveillance System 2002.

10. Consumer Safety Institute (Netherlands). Netherlands Injury Surveillance System 2008.

11. Mackenzie EJ, Rivara FP, Jurkovich GJ, et al. The National Study on Costs and Outcomes of Trauma. *J Trauma* 2007; 63: S54-67; discussion S81-86.

12. CDC, Medical University of South Carolina, South Carolina Department of Disabilities and Special Needs, South Carolina Department of Health and Environmental Control. South Carolina Traumatic Brain Injury Follow-up Registry 1999-2013. USA.

13. van Loey NE, van Beeck EF, Faber BW, van de Schoot R, Bremer M. Health-Related Quality of Life After Burns: A Prospective Multicentre Cohort Study With 18 Months Follow-Up. *J Trauma*. 2011; 72(2): 513-520.

14. Salomon JA, Haagsma JA, Davis A, Noordhout CM de, Polinder S, Havelaar AH, et al. Disability weights for the Global Burden of Disease 2013 study. *The Lancet Global Health*. 2015 Nov 1;3(11):e712–23.

15. Radboud University Medical Center. Netherlands Radboud University Brain Injury Cohort Study 1998-2011.

16. Marino RJ, Ditunno JF, Donovan WH, Maynard F. Neurological Recovery After Traumatic Spinal Cord Injury: Data From the Model Spinal Cord Injury Systems. *Arch Phys Med Rehabil* 1999;80:1391-6.

17. Polinder S, van Beeck EF, Essink-Bot ML, Toet H, Looman CW, Mulder S, Meerding WJ. Functional outcome at 2.5, 5, 9, and 24 months after injury in the Netherlands. *J Trauma*. 2007; 62(1): 133-41.

18. Strauss D, Shavelle R, DeVivo MJ, Day S. An analytic method for longitudinal mortality studies. *J Insur Med* 2000; 32: 217–25.

19. Shavelle R, Strauss D. Comparative mortality of adults with traumatic brain injury in California, 1988–97. *J Insur Med* 2000; 32: 163–6.

20. Baguley IJ, Nott MT, Howle AA, et al. Late mortality after severe traumatic brain injury in New South Wales: a multicentre study. *Med J Aust* 2012; 196: 40–5.

21. Middleton JW, Dayton A, Walsh J, Rutkowski SB, Leong G, Duong S. Life expectancy after spinal cord injury: a 50-year study. *Spinal Cord* 2012; 50: 803–11.

22. Brooks JC, Strauss DJ, Shavelle RM, Paculdo DR, Hammond FM, Harrison-Felix CL. Long-term disability and survival in traumatic brain injury: results from the National Institute on Disability and Rehabilitation Research Model Systems. *Arch Phys Med Rehabil* 2013; 94: 2203–9.

23. Baguley I, Slewa-Younan S, Lazarus R, Green A. Long-term mortality trends in patients with traumatic brain injury. *Brain Inj* 2000; 14: 505–12.

24. Ratcliff G, Colantonio A, Escobar M, Chase S, Vernich L. Long-term survival following traumatic brain injury. *Disabil Rehabil* 2005; 27: 305–14.

25. Frankel HL, Coll JR, Charlifue SW, et al. Long-term survival in spinal cord injury: a fifty year investigation. *Spinal Cord* 1998; 36: 266–74.

26. Harrison-Felix CL, Whiteneck GG, Jha A, DeVivo MJ, Hammond FM, Hart DM. Mortality over four decades after traumatic brain injury rehabilitation: a retrospective cohort study. *Arch Phys Med Rehabil* 2009; 90: 1506–13.

27. Moorin R, Miller TR, Hendrie D. Population-based incidence and 5-year survival for hospital-admitted traumatic brain and spinal cord injury, Western Australia, 2003-2008. *J Neurol* 2014; 261: 1726–34.

28. Colantonio A, Escobar MD, Chipman M, et al. Predictors of postacute mortality following traumatic brain injury in a seriously injured population. *J Trauma* 2008; 64: 876–82.

29. Flaada JT, Leibson CL, Mandrekar JN, et al. Relative risk of mortality after traumatic brain injury: a population-based study of the role of age and injury severity. *J Neurotrauma* 2007; 24: 435–45.

Interstitial lung disease and pulmonary sarcoidosis (ILD)

Flowchart

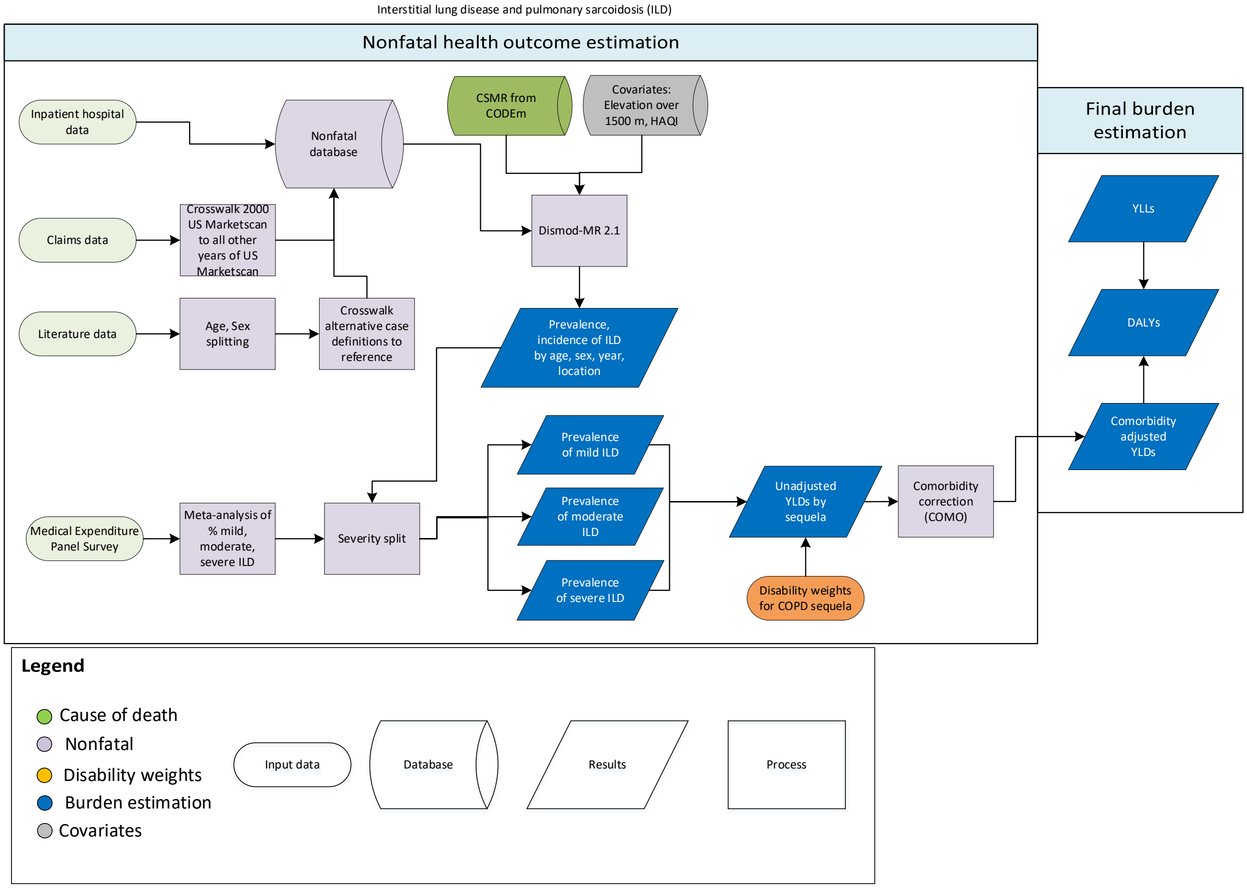

Input data and methodological summary for ILD

Case definition

Interstitial lung diseases and pulmonary sarcoidosis are a collection of chronic respiratory diseases that impair lung function and oxygen uptake through scarring and/or inflammation. The relevant ICD codes are D86 and J84. For interstitial lung disease, we use the American Thoracic Society as the gold standard definition.

Table 1. Case definitions for ILD

Quantity of interest	Reference or Alternative	Definition
All ILD (includes interstitial pulmonary fibrosis [IPF] and sarcoidosis)	Reference	Captures inclusively both forms of ILD we accept, IPF and sarcoidosis. Can report either with a “broad” or “narrow” definition. A broad definition is cases being confirmed through ICD coding exclusively. A narrow definition includes additional medical tests (ie, chest CT, biopsy) or physician review of medical history to confirm diagnosis of ILD.

IPF only	Alternative	Reports either a “broad” or “narrow” definition of only IPF
Sarcoidosis only	Alternate	Reports either a “broad” or “narrow” definition of only pulmonary sarcoidosis
USA MarketScan claims, all years except 2000	Reference	USA MarketScan private insurance claims data for all years except 2000
USA MarketScan 2000	Alternative	USA MarketScan private insurance claims data for 2000

Input data

A systematic review of ILD was conducted for GBD 2023. The search string utilised was the following:

("lung diseases, interstitial"[MeSH Terms] OR "interstitial lung disease" OR "interstitial lung diseases" OR "ILD" OR "sarcoidosis, pulmonary"[MeSH Terms] OR "pulmonary sarcoidosis" OR "idiopathic pulmonary fibrosis"[MeSH Terms] OR "pulmonary fibrosis" OR "IPF")
AND (prevalence[TiAb] OR prevalent[TiAb] OR incidence[TiAb] OR incident[TiAb] OR remission[TiAb])
NOT (animals[MeSH] NOT humans[MeSH])
AND (2017/09/01[PDAT] : 2021/08/11[PDAT])

Of the 1666 results generated from the search string, 73 were included in full-text screening, and 18 sources were extracted to be included in modelling. The primary reasons for exclusion were non-representative populations and no reported prevalence or incidence measures included in the study (see PRISMA diagram for more details). Data were added through the systematic review in the UK, Japan, Italy, Canada, and Finland.

Data used to make estimates of ILD are from three sources. The first is literature data from previous systematic reviews – usually from smaller-scale studies of prevalence or incidence. The second data type is claims data for the USA (MarketScan), Poland, and Taiwan (province of China). The sources and preparation of these data are described elsewhere in this paper. The third data type is adjusted hospital inpatient records. We correct for hospital readmission and deduplication from primary and secondary diagnoses in inpatient and outpatient encounters.

Roughly a quarter of the total input data come from the literature data, the rest is claims and inpatient hospital data. For literature data, there are a number of case definitions or subsets of this category that get identified and reported on.

Currently, we categorise the following subsets of ILD:

- Idiopathic pulmonary fibrosis (IPF), scarring of the lung tissue of unknown origin.
- Pulmonary sarcoidosis: immune cells gather in clumps (granulomas), which can build up in lungs and cause scarring. Autoimmune disorder.

Certain literature sources require histopathological confirmation of ILD, either through a chest CT, additional physician review of medical records, or biopsy. Other data sources rely entirely on ICD coding to capture ICD cases. Often, in literature, this is categorised by “narrow” and “broad” definitions, where the “narrow” definition corresponds to some sort of additional medical imaging, and the “broad” definition generally refers to a classification based only on ICD coding.

Bias adjustments
To adjust for cases that do not match our reference definition, we utilise a MR-BRT model outside of DisMod to allow a more direct comparison between different case definitions.

We made a series of adjustments to data that don't completely match our case definition. Data that only report idiopathic pulmonary fibrosis (IPF) or sarcoidosis under-report estimates of ILD in a population. USA claims data from 2000 tend to differ from other years of USA claims data. We make adjustments to these data to reflect these possible variations. The adjustment is a logit-transformation method in MR-BRT. The general process is described below:

1. Identify datapoints with overlapping year, age, sex, and location between reference and alternative definitions.
2. Logit transform overlapping datapoints of alternative and reference case definitions.
3. Convert overlapping datapoints into a difference in logit space using the following equation:
$$logit(altnerative) - logit(reference)$$
4. Use the delta method to compute standard errors of overlapping datapoints in logit space, then calculate standard error of logit difference using the following equation:
$$\sqrt{(variance\ of\ alternative) + (variance\ of\ reference)}$$
5. Using MR-BRT, conduct a random effects meta-regression to obtain the pooled logit difference of alternative to reference
6. Apply the pooled logit difference to all datapoints of alternative case definitions using the following equation:
$$new_{estimate} = inverse.logit((logit(alternative)) - (pooled\ logit\ difference))$$
7. Calculate new standard errors using the delta method, accounting for gamma (between-study heterogeneity)

Table 2. MR-BRT crosswalk adjustment factors for ILD

Data input	Reference or alternative case definition	Gamma	Beta coefficient, logit (95% UI)*	Adjustment factor**
All ILD	Ref	0.23	---	---
Only IPF	Alt		–1.46<br>(–2.09 to –0.79)	0.23
Only sarcoidosis	Alt		–1.07<br>(–1.71 to –0.40)	0.34
USA MarketScan (all other years)	Ref	---	---	---
USA MarketScan 2000	Alt	0.0	–0.31<br>(–0.32 to –0.29)	0.73

**MR-BRT crosswalk adjustments can be interpreted as the factor the alternative case definition is adjusted by to reflect what it would have been had it been measured using the reference case definition. If the log/logit beta coefficient is negative, then the alternative is adjusted up to the reference. If the log/logit beta coefficient is positive, then the alternative is adjusted down to the reference.*
***The adjustment factor column is the exponentiated beta coefficient. For log beta coefficients, this is the relative rate between the two case definitions. For logit beta coefficients, this is the relative odds between the two case definitions.*

Modelling strategy

For GBD 2023, there were no substantial changes to the modelling strategy for ILD. Estimates for ILD are produced using a standard DisMod-MR 2.1 approach. We use prior settings of zero remission, and we constrain the super-region random effects to –0.5 to 0.5 to ensure model stability.

We used two covariates, proportion of population living in elevation over 1500 m and the Healthcare Access and Quality (HAQ) Index on excess mortality rate to improve estimations in data-sparse locations.

Table 3. Severity distribution, details on the severity levels for ILD and the associated disability weight (DW) with that severity.

Severity level	Lay description	DW (95% CI)
Asymptomatic	--	0.00
Mild	Has cough and shortness of breath after heavy physical activity, but is able to walk long distances and climb stairs.	0.019 (0.011–0.033)
Moderate	Has cough, wheezing, and shortness of breath, even after light physical activity. The person feels tired and can walk only short distances or climb only a few stairs.	0.225 (0.153–0.312)
Severe	Has cough, wheezing, and shortness of breath all the time. The person has great difficulty walking even short distances or climbing any stairs, feels tired when at rest, and is anxious.	0.408 (0.273–0.556)

Table 4. Covariates. Summary of covariates used in the ILD DisMod-MR meta-regression model

Covariate	Type	Parameter	Exponentiated beta (95% uncertainty interval)
Elevation over 1500 m (proportion)	Country-level	Excess mortality rate	1.04 (1.00–1.12)
Healthcare Access and Quality Index	Country-level	Excess mortality rate	0.98 (0.98–0.98)

Figure 1: PRISMA 2020 flow diagram

Haddaway, N. R., Page, M. J., Pritchard, C. C., & McGuinness, L. A. (2022). PRISMA2020: An R package and Shiny app for producing PRISMA 2020-compliant flow diagrams, with interactivity for optimised digital transparency and Open Synthesis Campbell Systematic Reviews, 18, e1230. https://doi.org/10.1002/cl2.1230

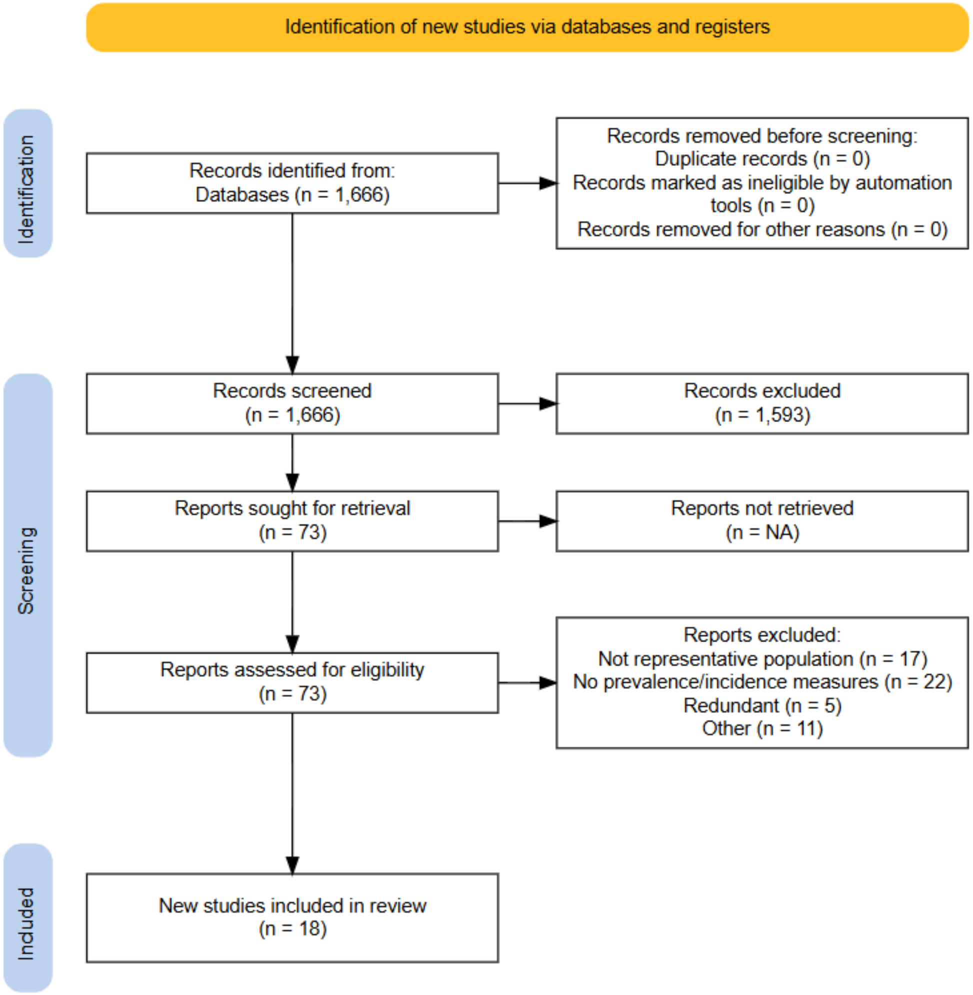
Identification of new studies via databases and registers
Identification
Records identified from:
Databases (n = 1,666)
Records removed before screening:
Duplicate records (n = 0)
Records marked as ineligible by automation tools (n = 0)
Records removed for other reasons (n = 0)
Screening
Records screened
(n = 1,666)
Records excluded
(n = 1,593)
Reports sought for retrieval
(n = 73)
Reports not retrieved
(n = NA)
Reports assessed for eligibility
(n = 73)
Reports excluded:
Not representative population (n = 17)
No prevalence/incidence measures (n = 22)
Redundant (n = 5)
Other (n = 11)
Included
New studies included in review
(n = 18)

Invasive non-typhoidal salmonella (iNTS)

Flowchart

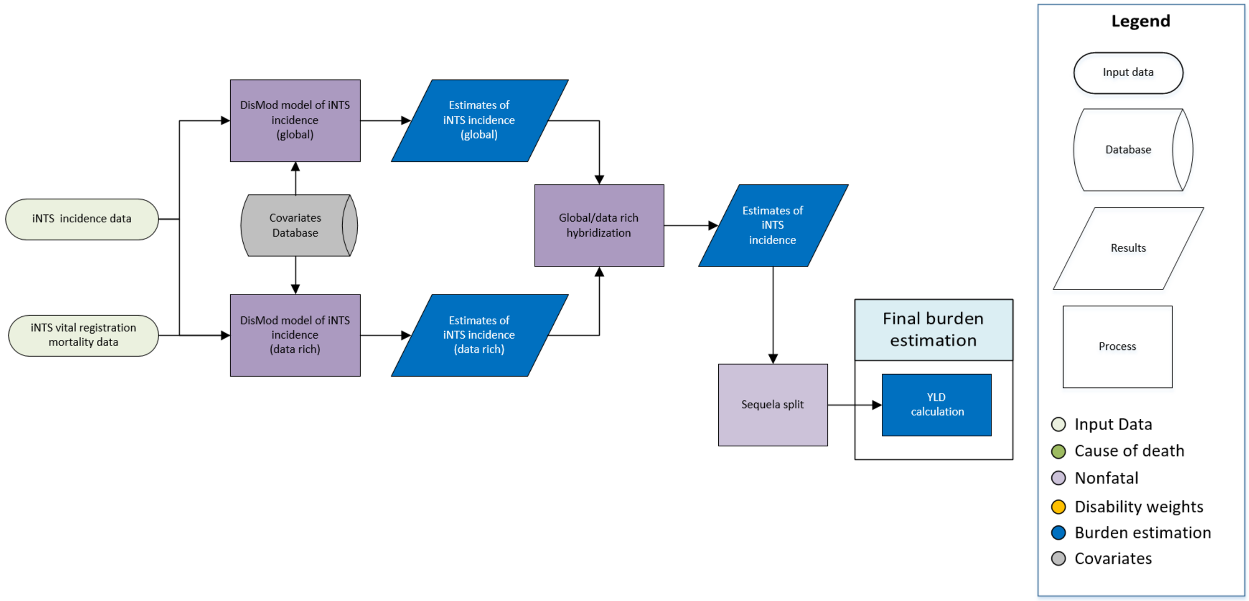

Input Data and Methodological Summary for Invasive non-typhoidal salmonella (iNTS)

Case definition

Non-typhoidal *Salmonella* infections are typically associated with diarrhoea. When these bacteria invade a typically sterile site like blood, they produce invasive non-typhoidal *Salmonella* (iNTS) disease. Whereas non-typhoidal *Salmonella* infections typically produce diarrhoeal illness, iNTS is typically febrile and can manifest in diverse symptoms that vary with severity and the exact site of the infection. Blood culture is the standard diagnostic for iNTS and has good sensitivity and specificity. We thus define a case of iNTS as any blood-culture-confirmed non-typhoidal *Salmonella* infection.

Input data

Model inputs

We conducted a systematic review for studies of iNTS incidence for GBD 2017, including sources that provided iNTS incidence rates derived from either active surveillance or, more commonly, hospital- or clinic-based surveillance with adjustments for health care utilisation. Studies of special populations (eg, people living with HIV/AIDS) were excluded. We updated this systematic review for GBD 2023 (Figure 1).

Severity splits

Given the typical severity of iNTS and the breadth of potential symptoms and manifestations, we assign all cases to the severe acute infectious disease episode health state, with a disability weight of 0.133 (0.088–0.19)

Table 1: Severity distribution for invasive non-typhoidal *Salmonella*

Sequela	Description	Disability weight
Severe acute infectious disease episode	Has a high fever and pain, and feels very weak, which causes great difficulty with daily activities.	0.133 (0.088–0.19)

Modelling strategy

We modelled incidence using two DisMod models: 1) a model that includes only incidence data, used to produce estimates for moderate and high burden regions; and 2) a model that includes additional incidence estimates derived from vital registration data from data-rich counties, used to produce estimates for low-burden regions. Both DisMod models used HIV mortality rate, malaria incidence adjusted for antimalarial coverage and drug effectiveness, and the summary exposure values (SEV) for sanitation as country-level covariates. We used no study-level covariates in the models.

We estimated prevalence as the product of incidence times duration. We estimated the duration of iNTS based on duration parameters reported in the scientific literature, with reported duration parameters including mean, median, range, standard deviation, and interquartile range. Because studies differed in how they reported duration, we were unable to use a simple meta-analysis approach. To leverage information on duration from all studies, we used approximate Bayesian computation (ABC). ABC employs a simple grid search in which we assumed that iNTS duration, in days, follows a negative binomial distribution with a one-day offset such that the resulting distribution had a minimum possible value of one day. We used a random negative binomial generator that took three inputs: the length of the randomly generated vector, N, the number of trials, n, and the probability of success in each trial, p. We trialed combinations of values of n and p using a simple grid search. For each combination, and for each duration data point, we generated 10,000 vectors from an offset random negative binomial distribution, where the length of each vector equaled the sample size of the study. Thus, each vector represented a random realisation of a possible distribution of durations for a given study. We estimated deviations between these realisations and the corresponding input data using an empirical cumulative distribution, and selected the best combination of values for n and p based on the root mean squared error. We estimated a mean duration of seven days (95% CI: 1–24).

Changes from GBD 2021 to GBD 2023

We updated our systematic review for GBD 2023 (Figure 1) and updated the code pipeline to improve robustness and efficiency, but made no substantive changes to our modelling strategy for iNTS between GBD 2021 and GBD 2023.

Figure 1: PRISMA flow diagram

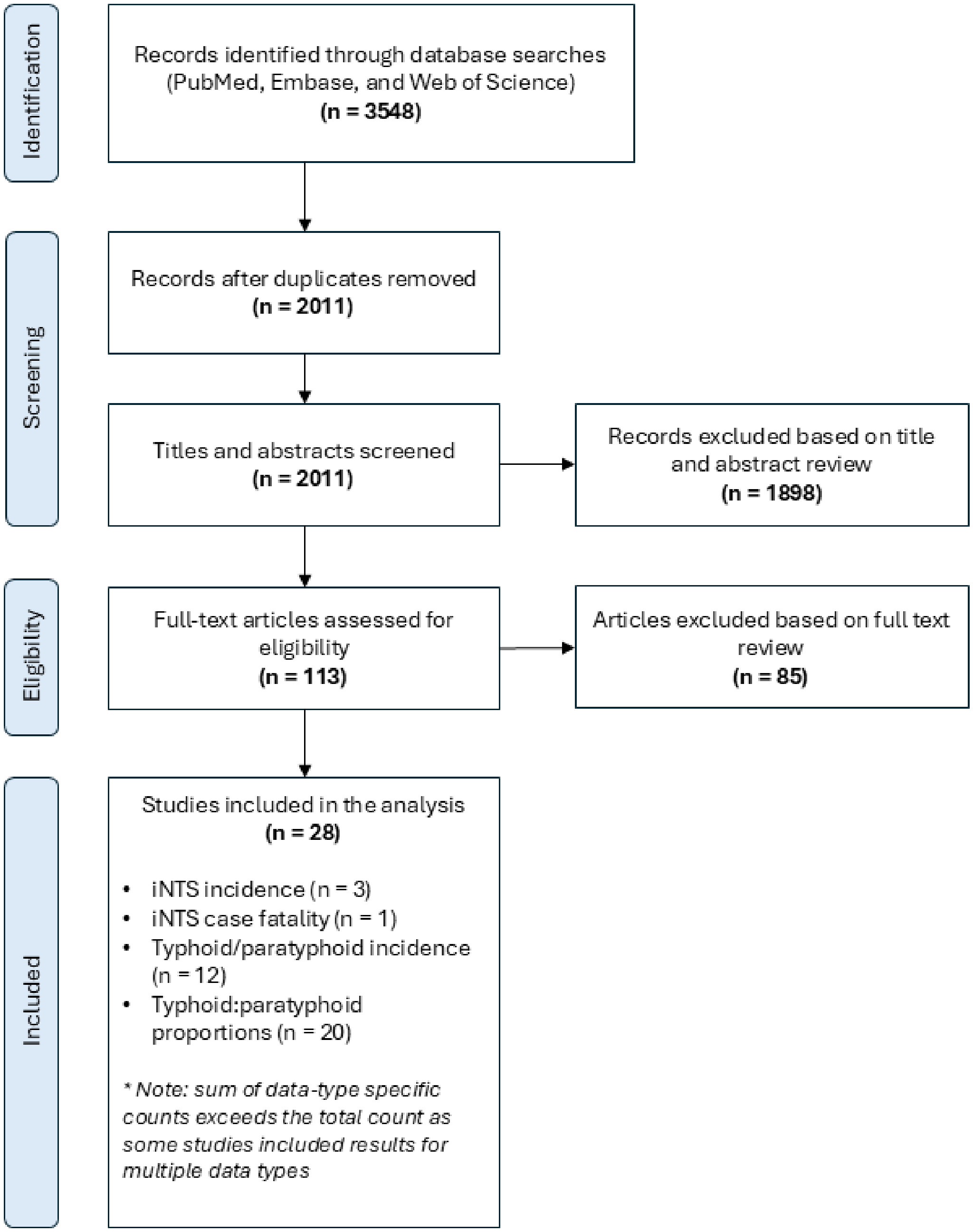

Ischaemic heart disease

Flowchart

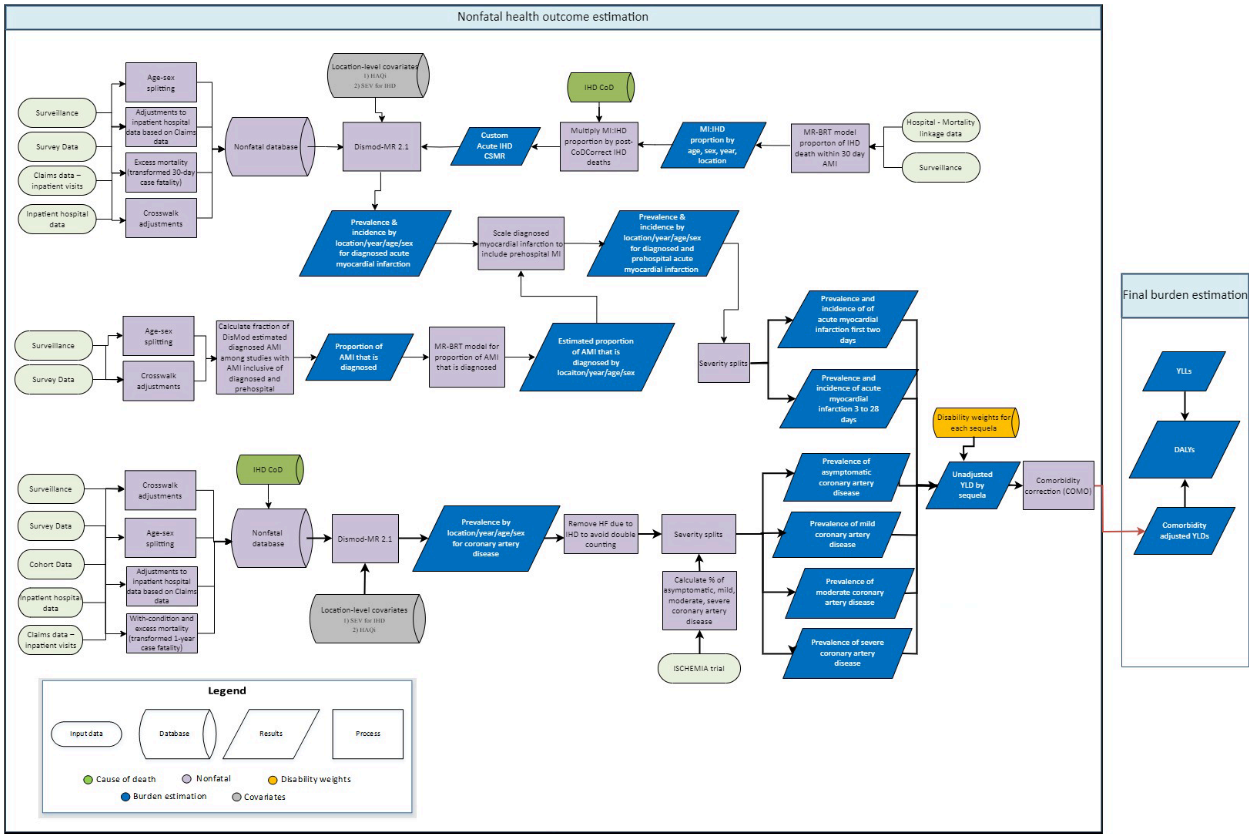

Input data and methodological summary for ischaemic heart disease

Case definition

Ischaemic heart disease (IHD) is a disease that limits the supply of blood to the heart. IHD is typically due to the narrowing of the coronary arteries, usually due to atherosclerosis, which limits blood flow. GBD estimates IHD as the aggregate of discrete sequelae, consisting of myocardial infarction (heart attacks), stable coronary artery disease, and heart failure due to IHD (ischaemic cardiomyopathy).

This appendix describes the input data and modelling processes for acute myocardial infarction (MI) and stable coronary artery disease as implemented for JACC 2022. Details of the heart failure due to IHD estimation process can be found in the heart failure appendix.

<u>Case definitions:</u>

1) Acute myocardial infarction (MI): Definite and possible MI according to the fourth universal definition of myocardial infarction:[4]
    a. When there is clinical evidence of myocardial necrosis in a clinical setting consistent with myocardial ischaemia or
    b. Detection of a rise and/or fall of cardiac biomarker values and with at least one of the following: i) symptoms of ischaemia, ii) new or presumed new ST-segment-T wave changes or new left bundle branch block, iii) development of pathological Q waves in the ECG, iv) imaging evidence of new loss of viable myocardium or new regional wall motion abnormality, or v) identification of an intracoronary thrombus by angiography or autopsy.
    c. Sudden (abrupt) unexplained cardiac death, involving cardiac arrest or no evidence of a non-coronary cause of death.

The prevalence of MI is considered from the onset of the event to 28 days after the event and is divided into an acute phase (0-2 days) and subacute phase (3-28 days). We also included unstable angina when reported separately as specified in the fourth universal definition.

2) Chronic IHD: Consists of stable coronary artery disease and ischaemic cardiomyopathy.
    a. Stable coronary artery disease was characterised by any of the following criteria:
        i. Greater than 50% epicardial coronary stenosis as determined by coronary angiography, computed tomography or invasive, in one or more of the coronary arteries.[3]
        ii. Current or past evidence of myocardial infarction or inducible myocardial ischaemia determined by non-invasive diagnostic tests such as the Bruce-protocol exercise treadmill test (ECG evidence of horizontal or down-sloping ST depression of ≥1mm at 80ms from J point), stress echocardiography test identification of regional wall function abnormalities using standard procedures, or history of coronary revascularisation procedure.[5,6]

    b. Heart failure due to IHD: Clinical diagnosis of heart failure using structured criteria such as the Framingham or European Society of Cardiology criteria, and heart failure is of ischaemic aetiology.[7,8]

Reference and alternate definitions of acute myocardial infarction and stable coronary artery disease are shown in Tables 1a and 1b, respectively. International classification of disease (ICD) codes mapped to acute myocardial infarction and stable coronary artery disease are listed in Tables 2a and 2b.

Table 1a: Reference and alternate definitions for acute myocardial infarction

Quantity of interest	Reference or alternate	Definition
Acute myocardial infarction (MI) incidence and excess mortality	Reference	Definite and possible MI according to the fourth universal definition of myocardial infarction; includes recurrent cases and cases who died before reaching medical care
Acute myocardial infarction (MI) incidence and excess mortality	Alternate	Cases diagnosed prior to regular use of troponin as part of the standard case definition
Acute myocardial infarction (MI) incidence and excess mortality	Alternate	First-ever cases only
Acute myocardial infarction (MI) incidence and excess mortality	Alternate	Only includes persons who survived to the hospital

Table 1b: Reference and alternate definitions for stable coronary artery disease

Quantity of interest	Reference or alternate	Definition
Stable coronary artery disease (prevalence)	Reference	Greater than 50% epicardial coronary stenosis as determined by coronary angiography, computed tomography or invasive, in one or more of the coronary arteries. Current or past evidence of myocardial infarction or inducible myocardial ischaemia determined by non-invasive diagnostic tests or procedure history.
Stable coronary artery disease (prevalence)	Alternate	Self-reported history of diagnosed coronary artery disease “Has a doctor ever told you that you had {coronary artery disease, acute myocardial infarction, heart attack, angina}?”
Stable coronary artery disease (prevalence)	Alternate	Administrative medical record history of coronary artery disease including any inpatient/outpatient hospitalisation (see ICD list in table 2b) or history of prescribed medications for coronary artery disease including nitroglycerin combined with anticoagulant, acetylsalicylic acid, beta-blocker, or statin.

Stable coronary artery disease (prevalence)	Alternate	Definite Q-wave diagnosis of prior myocardial infarction on resting 12-lead ECG specified by Minnesota codes 1-1-x, 1-2-x, 1-3-x.
Stable coronary artery disease (prevalence)	Alternate	Inpatient or outpatient admission for ischaemic heart disease. ICD-9 code 410-414, ICD-10 code I20-I25 extracted from tabulated reports.
Stable coronary artery disease (prevalence)	Alternative	Inpatient or outpatient admission for ischaemic heart disease. ICD-9 code 410-414, ICD-10 code I20-I25 from tabulated reports, individual-level hospital admissions data inclusive of inpatient/outpatient

Table 2a: ICD codes mapped to acute myocardial infarction

ICD code	Description
410	Acute myocardial infarction
411.0	Post-myocardial infarction syndrome
412	Old myocardial infarction
I21	Acute myocardial infarction
I22	Subsequent ST elevation (STEMI) and non-ST elevation (NSTEMI) myocardial infarction
I23	Certain current complications following ST elevation (STEMI) and non-ST elevation (NSTEMI) myocardial infarction (within the 28-day period)
I24.1	Dressler's syndrome

Table 2b: ICD codes mapped to coronary artery disease

ICD code	Description
410	Acute myocardial infarction
411	Other acute and subacute forms of ischaemic heart disease
412	Old myocardial infarction
413	Angina pectoris
414	Atherosclerotic heart disease of native coronary artery without angina pectoris
I20	Angina pectoris
I21	Acute myocardial infarction
I22	Subsequent myocardial infarction
I23	Complications following myocardial infarction
I24	Other acute ischaemic heart disease
I25	Chronic ischaemic heart disease

Input data

Myocardial infarction

For GBD 2023, a systematic review was done for myocardial infarction. The following databases were searched: EMBASE, PubMed, and Virtual Health Library (VHL). The dates of the search were 01/01/2020– 12/31/2022.

The search strings used were (("myocardial infarction"[tiab] AND (incidence OR "case fatality" OR "excess mortality")) OR ("acute coronary syndrome"[tiab] AND (incidence OR "case fatality" OR "excess

mortality")) OR (angina[tiab] AND (incidence OR prevalence OR "case fatality" OR "excess mortality"))) AND ("2020/12/31"[PDAT] : "2022/12/31"[PDAT]) NOT rat[tiab] NOT mice[tiab] NOT monkey[tiab] NOT pig[tiab] NOT animals[tiab].

The findings were reported according to the PRISMA (Preferred Reporting Items for Systematic reviews and Meta-Analyses) statement and registered with PROSPERO (PROSPERO record CRD42022323863). Figure 1 shows the PRISMA diagram for the systematic review. In the diagram, screening refers to reviewing of the title and abstract of an article for relevant information, not screening of the entire article. Reasons for exclusion include non-representativeness, use of different case definitions, studies reporting age-standardised data and data reported in a non-compatible format.

In total, 14 400 studies were returned after deduplication, (1348 from EMBASE, 12 554 from PubMed, and 498 from VHL). 127 articles were extracted.

Figure 1: PRISMA 2020 flow diagram – acute myocardial infarction[19]

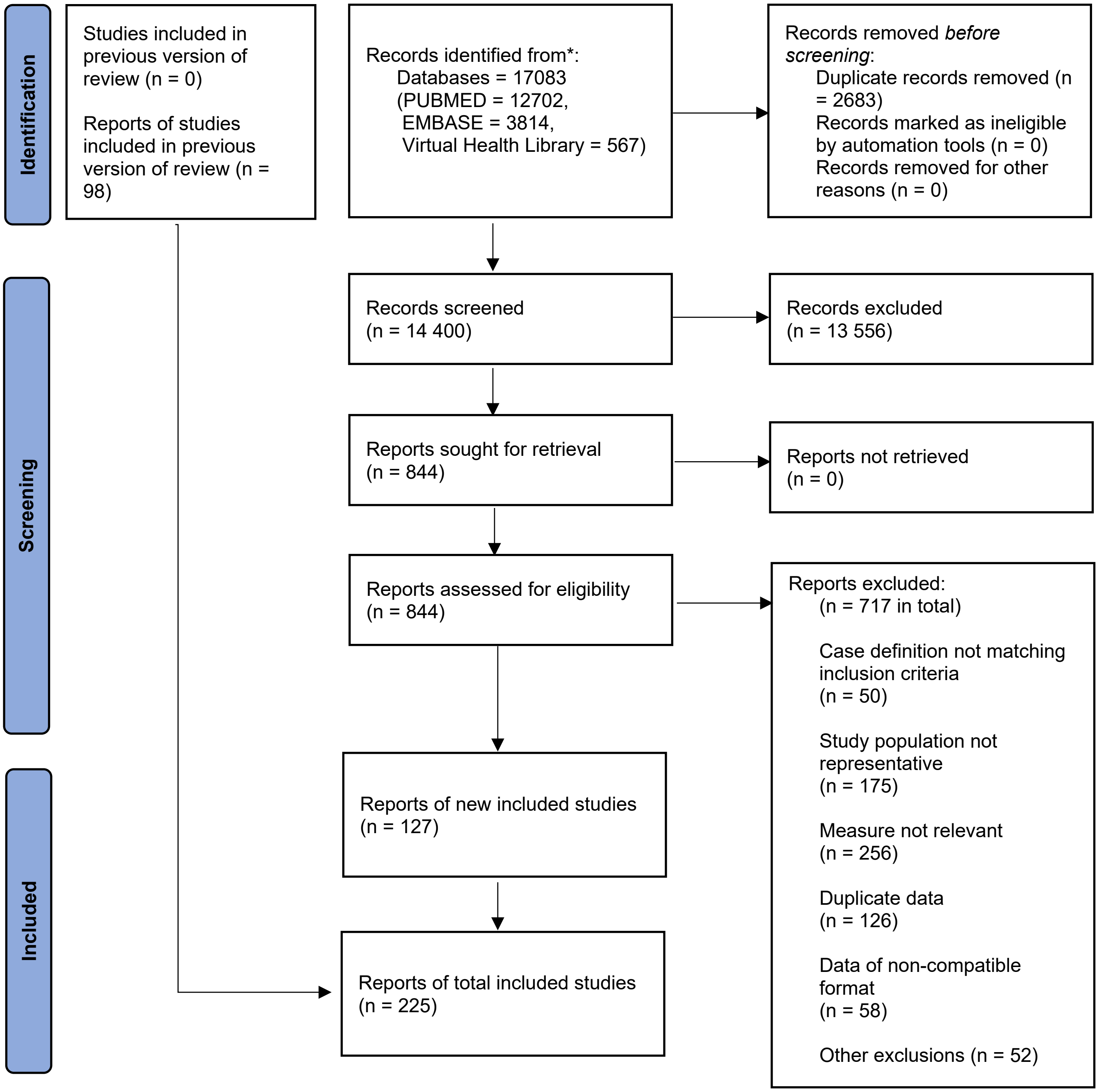

We also performed searches for GBD 2013, 2015, 2019, and 2021. Search terms, parameters, and results returned will be provided on request.

For myocardial infarction, we included administrative health facility data from inpatient hospital visits or related claims data. We adjusted sources that only reported tabulated hospital admissions of primary diagnosis to include primary and secondary diagnosis of myocardial infarction and account for

readmission within a one-month time frame. We outliered administrative health facility data from China, Brazil, Indonesia, Mongolia, Norway, Georgia, and Armenia that were inconsistent with published cohorts in the respective regions in which crosswalking could not be used to address the inconsistency. We also accepted results on population-level disease rates from studies identified in the literature review.

Data transformations and adjustments
The 30-day case fatality proportion for acute myocardial infarction was extracted from published cohort studies and myocardial infarction registries. We transformed 30-day case fatality data into excess mortality using the equation:

$$excess\ mortality\ rate = \frac{-\log\left(1 - case\ fatality\ proportion\right)}{30/365}$$

Case fatality proportion was expressed as excess mortality rate under the assumption that death within 30 days of an acute myocardial infarction event would be due to the event.

We adjusted incidence measurements for myocardial infarction found in published studies when they used alternative definitions so they would agree with our case reference definition using MR-BRT (meta-regression—Bayesian, regularised, trimmed) modelling tool. MR-BRT and the process of data adjustment are discussed elsewhere in the appendix. For myocardial infarction, we adjusted using multiple different covariates: a covariate to capture only first-ever MI, using studies where all events were included as the reference; a covariate to adjust estimates from studies that included fatal cases that did not survive the event in time to reach health care, using sources that included non-sudden fatal and non-fatal cases as reference; and a covariate to adjust for studies that did not use troponin measurements in their case diagnosis, using sources that did include troponin measurements in their diagnostic method. The coefficients for myocardial infarction reported in Table 4a can be used to calculate adjustment factors for alternative definitions. The formula for computing adjustment factors is given in Equation 1 below. We also included a standardised age variable (age-scaled) and a sex variable in the regression to adjust for the possibly of bias. Splines were not used in this model.

Equation 1: Calculation of adjustment factors without splines:

$$Estimated\ Reference\ Def = invlogit(logit(Alternative\ Def) - [\sum_{a=0}^{b} Beta_{Alternative\ Def_a} * I(Alternate\ Def_a)] - Beta_{sex} * I(Sex) - Beta_{age} * age_{scaled})$$

$I(.)$ = Indicator function, b = Number of alternate definitions used

Table 4a: MR-BRT crosswalk adjustment factors for diagnosed myocardial infarction

Data input	Measure	Reference or alternative case definition	Gamma	Beta coefficient, logit (95% CI) *	Adjustment factor**
Any event, non-sudden fatal and non-fatal events, used troponin	Incidence	Ref	---	---	

First-ever MI, intercept	Incidence	Alt	0.155	–0.20 (–0.55 to 0.16)	0.82
First-ever MI, age-scaled	Incidence	Alt		0.09 (–0.21 to 0.39)	1.09
First-ever MI, male	Incidence	Alt		0.01 (–0.30 to 0.30)	1.00
Non-fatal MI, intercept	Incidence	Alt		0.46 (0.16 to 0.77)	1.59
Non-fatal MI, age-scaled	Incidence	Alt		–0.14 (–0.44 to 0.17)	0.87
Non-fatal MI, male	Incidence	Alt		–0.10 (–0.40 to 0.21)	0.91
Troponin not used as part of definition, intercept	Incidence	Alt		–0.20 (–0.51 to 0.10)	0.82
Troponin not used as part of definition; age-scaled	Incidence	Alt		0.11 (–0.19 to 0.42)	1.12
Troponin not used as part of definition, sex (male)	Incidence	Alt		0.07 (–0.23 to 0.37)	1.07
Non-fatal MI, intercept	Excess mortality	Alt	0.09	0.84 (–0.16 to 0.21)	2.33
Non-fatal MI, age-scaled	Excess mortality	Alt		–0.27 (0.09 to –0.46)	0.76
Non-fatal MI, male	Excess mortality	Alt		0.03 (–0.16 to 0.21)	1.03

**MR-BRT crosswalk adjustments can be interpreted as the factor the alternative case definition is adjusted by to reflect what it would have been had it been measured using the reference case definition. If the log/logit beta coefficient is negative, then the alternative is adjusted up to the reference. If the log/logit beta coefficient is positive, then the alternative is adjusted down to the reference.*
***The adjustment factor column is the exponentiated beta coefficient. For log beta coefficients, this is the relative rate between the two case definitions. For logit beta coefficients, this is the relative odds between the two case definitions.*

Once the crosswalk was performed, we split incidence and excess mortality datapoints where the age range was greater than 25 years. Age splitting was based on the global sex-specific age pattern from a DisMod model that only used as input data incidence or excess mortality information from scientific literature with less than a 25-year age range. Datapoints included in the model used to generate the age pattern could use either the reference or alternate case definition.

Separately, we crosswalked incidence data where incident myocardial infarction events included both diagnosed and prehospital cases of myocardial infarction to be consistent with the fourth universal

definition. These data was used to estimate the prehospital myocardial infarction adjustment scalar after the diagnosed myocardial infarction DisMod estimates were produced. We adjusted for studies that reported for first-ever diagnosis and use of troponins for diagnosis as described earlier. Studies that included prehospital myocardial infarction cases but did not meet our gold-standard critiera included three types of studies:

1. Studies that used MONICA-style ascertainment of prehospital events by categorising into “definite”, “possible”, and “unclassifiable” events.
2. Studies that used vital registration death records to identify prehospital events with no history of diagnosis for myocardial infarction.
3. Studies that reported sudden cardiac death where the death occurred within 1 hour of symptom onset or within 24 hours of last time witnessed alive and the cause of death was determined to be cardiovascular.

We crosswalked these data to our gold-standard data meeting the fourth universal definition of myocardial infarction using network meta-analysis in MR-BRT (meta-regression—Bayesian, regularised, trimmed) modelling tool. MR-BRT and the process of data adjustment are discussed elsewhere in the appendix. The coefficients for prehospital myocardial infarction reported in Table 4b can be used to calculate adjustment factors for alternative definitions. The formula for computing adjustment factors is given in Equation 2. We also included a standardised age variable (age-scaled) and a sex variable in the regression to adjust for the possibly of bias. Splines were not used in this model.

Table 4b: MR-BRT crosswalk adjustment factors for studies including prehospital myocardial infarction

Data input	Measure	Reference or alternative case definition	Gamma	Beta coefficient, logit (95% CI) *	Adjustment factor**
Any event including non-fatal events, fatal non-sudden events, and fatal sudden events	Incidence	Ref	---	---	
First-ever MI, intercept	Incidence	Alt		–0.05 (–0.17 to 0.06)	0.95
First-ever MI, age-scaled	Incidence	Alt		0.10 (0.07 to 0.13)	1.10
First-ever MI, male	Incidence	Alt		–0.01 (–0.06 to 0.06)	1.00
Troponin not used as part of definition, intercept	Incidence	Alt		–0.04 (–0.11 to 0.04)	0.97
Troponin not used as part of definition; age-scaled	Incidence	Alt		–0.05 (–0.06 to –0.04)	0.95

Troponin not used as part of definition, sex (male)	Incidence	Alt		0<br>(–0.01 to 0.01)	1.0
Prehospital-MONICA style (intercept)	Incidence	Alt		–0.02<br>(–0.5 to 0.49)	0.98
Prehospital-MONICA style; age-scaled	Incidence	Alt		–0.01<br>(–0.41 to 0.40)	1.0
Prehospital-MONICA style (male)	Incidence	Alt		–0.08<br>(–0.12 to –0.03)	0.93
Prehospital – vital registration (intercept)	Incidence	Alt		0.13<br>(0.12 to 0.14)	1.14
Prehospital – vital registration; (age-scaled)	Incidence	Alt		0.09<br>(0.08 to 0.09)	1.09
Prehospital – vital registration (male)	Incidence	Alt		0<br>(–0.01 to 0.01)	1.0
Prehospital – sudden cardiac death (intercept)	Incidence	Alt		0.13<br>(–0.15 to 0.41)	1.14
Prehospital – sudden cardiac death; (age-scaled)	Incidence	Alt		–0.12<br>(–0.30 to 0.06)	0.09
Prehospital sudden cardiac death (male)	Incidence	Alt		0.07<br>(–0.20 to 0.34)	1.07

**MR-BRT crosswalk adjustments can be interpreted as the factor the alternative case definition is adjusted by to reflect what it would have been had it been measured using the reference case definition. If the log/logit beta coefficient is negative, then the alternative is adjusted up to the reference. If the log/logit beta coefficient is positive, then the alternative is adjusted down to the reference.*
***The adjustment factor column is the exponentiated beta coefficient. For log beta coefficients, this is the relative rate between the two case definitions. For logit beta coefficients, this is the relative odds between the two case definitions.*

Once the crosswalk was done, we split incidence into five-year age bins. Age splitting was based on the global sex-specific age pattern from a DisMod model that only used as input data incidence information from scientific literature with less than a 25-year age range. Datapoints included in the model used to generate the age pattern could use either the reference or alternate case definition.

We used the crosswalked and age-split studies that included prehospital myocardial infarction cases and the estimated incidence of diagnosed myocardial infarction to calculate the proportion of myocardial infarction cases that were diagnosed by location, age, sex, and year. The equation for calculating the proportion is shown below in equation 3.

Equation 3: Calculation for proportion of myocardial infarction that is diagnosed

$$Proportion\ diagnosed\ MI = \frac{Estimated\ incidence\ diagnosed\ MI\ from\ DisMod\ model}{Crosswalked\ and\ age\ split\ incident\ MI\ from\ studies\ including\ diagnosed\ and\ prehospital\ cases}$$

This proportion data was used to model a scaling factor to capture all cases of myocardial infarction inclusive of cases that happened prehospitalisation, the modelling of this scaling factor is described in the modelling section below.

Stable coronary artery disease

A systematic review specific to coronary artery disease was performed for GBD 2023. We searched the PubMed database over the dates 01/01/1980 through 12/31/2022. The search string terms used were: ("coronary heart disease"[ti] AND (prevalence OR incidence OR "case fatality" OR "excess mortality")) OR ("ischaemic"[ti] AND (prevalence OR incidence OR "case fatality" OR "excess mortality")) OR ("CHD"[ti] AND (prevalence OR incidence OR "case fatality" OR "excess mortality")) OR ("CAD"[ti] AND (prevalence OR incidence OR "case fatality" OR "excess mortality")) OR ("IHD"[ti] AND (prevalence OR incidence OR "case fatality" OR "excess mortality")) OR ("ischemic heart disease"[ti] AND (prevalence OR incidence OR "case fatality" OR "excess mortality")) OR ("coronary artery disease"[ti] AND (prevalence OR incidence OR "case fatality" OR "excess mortality")) OR (angina[ti] AND (incidence OR prevalence OR "case fatality" OR "excess mortality")) OR (("cardiovascular disease"[ti] AND ("coronary heart disease"[tiab] OR "coronary artery disease"[tiab] OR "ischemic heart disease"[tiab] OR "CAD"[tiab] OR "IHD"[tiab] OR "angina"[tiab])) AND (incidence OR prevalence OR "case fatality" OR "excess mortality")) AND ("1980/01/01"[PDAT] : "2023/12/31"[PDAT]) NOT rat[ti] NOT mice[ti] NOT monkey[ti] NOT pig[ti] NOT animals[ti] NOT rats[ti] NOT mice[ti] NOT monkeys[ti] NOT review[pt] NOT "case reports"[pt] NOT editorial[pt] NOT "congenital"[ti].

The findings were reported according to the PRISMA (Preferred Reporting Items for Systematic reviews and Meta-Analyses) statement and registered with PROSPERO (PROSPERO record CRD42022323863). A total 27 107 results were returned, and 452 sources were extracted. Details can be found in the PRISMA diagram below.

Figure 2: PRISMA 2020 flow diagram – coronary artery disease[19]

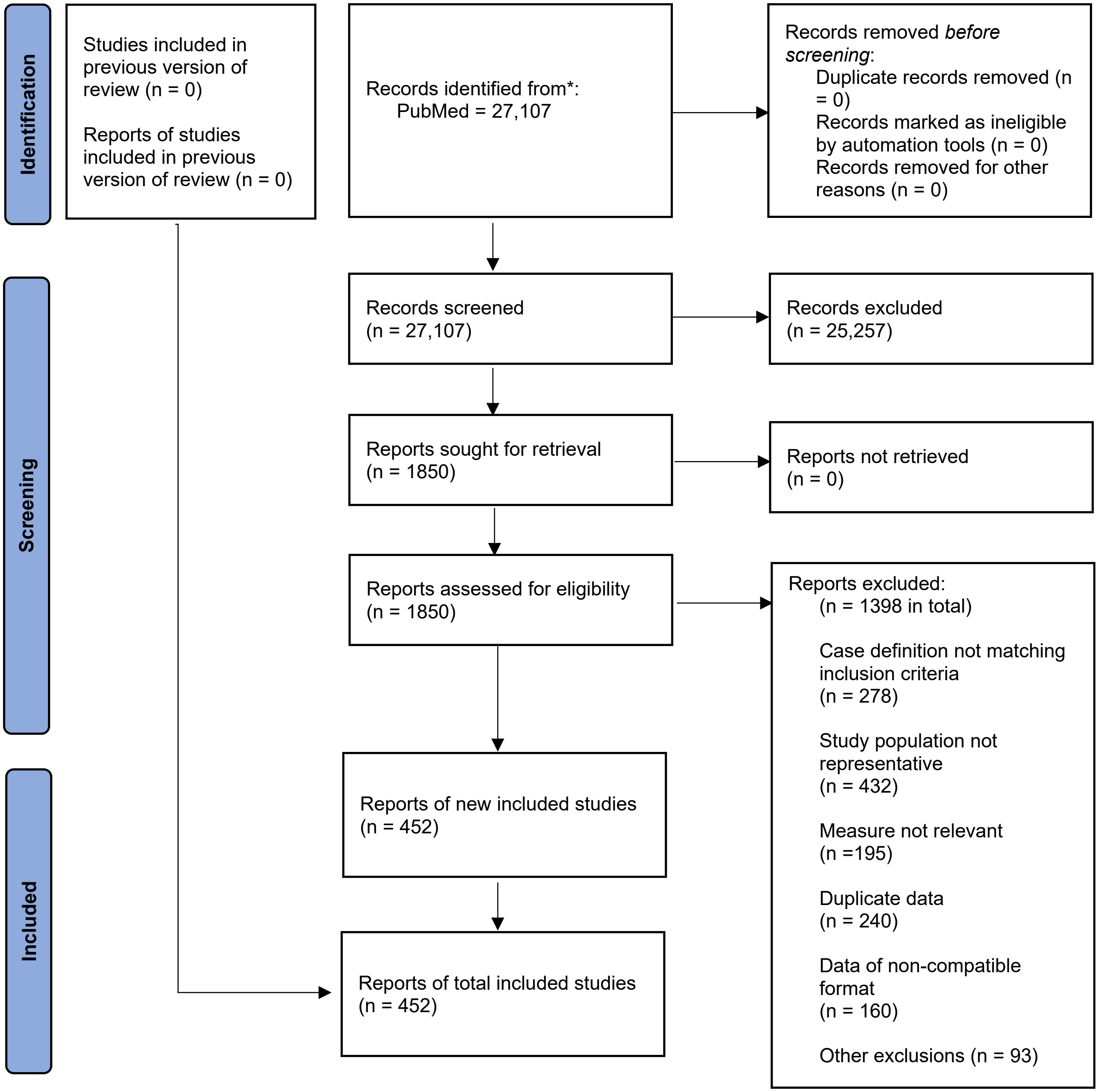

Data transformations and adjustments

The case-fatality proportion of coronary artery disease was extracted from the literature collected in systematic review as the number of fatal cases of coronary artery disease among prevalent cases. We also extracted the time of follow-up in years for each study that reported case-fatality proportion so we could calculate a rate. We expressed case-fatality proportion as a rate using the rate equation:

$$rate = \frac{-\log(1 - case\ fatality\ proportion)}{time}$$

We expressed case-fatality proportion as either excess mortality rate or with-condition mortality rate depending on the endpoint of the study. When the endpoint was death from ischaemic heart disease, myocardial infarction, or any other cardiovascular disease, we expressed case-fatality rate as excess mortality rate. When the endpoint of the study was all-cause mortality from any condition, we expressed case-fatality rate as with-condition mortality rate. Both excess mortality rate and with-condition mortality rate were used in DisMod modelling.

We included survey data of prevalence of self-reported history of a health professional diagnosis of coronary artery disease, coronary heart disease, myocardial infarction, or angina from national health examination surveys. We indexed the Global Health Data Exchange (https://ghdx.healthdata.org/global-health-data-exchange) to find all surveys with keyword "ischaemic heart disease". There were 2604 returned results; each result was assessed for inclusion of the self-reported history of coronary artery disease question, and survey methods were reviewed to determine population representation. We excluded any studies that were not representative of the population. We extracted 143 surveys.

We adjusted prevalence data obtained from the systematic review that reported alternate case definitions of coronary artery disease including the prevalence of self-reported history of diagnosis of coronary artery disease or myocardial infarction, sources that reported prevalent inpatient and outpatient admissions for coronary artery disease using ICD codes (table 2b) extracted from published studies or processed by IHME Clinical Informatics, and sources that reported prevalence of Q-wave evidence of prior myocardial infarction from community health examinations. Sources that reported the aggregated prevalence of >50% epicardial coronary artery stenosis, evidence of myocardial ischemia on diagnostic testing, and history of coronary revascularisation were considered the reference. Specifics on the crosswalking process are discussed elsewhere in the GBD methods appendix. Table 5 shows the coefficients adjustments made to the alternative definition.

We split coronary artery disease prevalence datapoints where the age range was greater than 25 years. Age-splitting was based on the global sex-specific age pattern from a DisMod model that only used prevalence input data from scientific literature with less than a 25-year age range and administrative claims data. We excluded prevalence data with broad age ranges where it was impossible to age-split due to small sample size, as these data caused the known age pattern for increased risk of coronary artery disease to be masked in the estimates generated from DisMod.

Equation 2: Calculation of adjustment factors with splines:

$$Estimated\ Reference\ Def = invlogit(logit(Alternative\ Def) - [\sum_{s=0}^{b} Beta_{Alternative\ Def, spline\ basis_s} * Spline\ basis_s(age\_scaled)] - Beta * I(Sex))$$

$I(.)$ = Indicator function, b = Number of spline bases used

Table 5: MR-BRT crosswalk adjustment factors for coronary artery disease prevalence

Data input	Measure	Reference or alternate case definition	Gamma	Beta coefficient, log/logit (95% UI) *	Adjustment factor**
Stable coronary artery disease defined by >50% coronary stenosis or current or past evidence of myocardial ischaemia/coronary revascularisation	Prevalence	Reference	0.29	---	---
Administrative coded, intercept	Prevalence	Alt		–0.51 (–1.13 to 0.09)	0.60
Administrative coded, age_scaled	Prevalence	Alt		0.10 (–0.49 to 0.68)	1.10
Administrative coded, male	Prevalence	Alt		–0.09 (–0.67 to 0.49)	0.91
Self-reported coronary artery disease, intercept	Prevalence	Alt		–0.27 (–0.86 to 0.33)	0.77
Self-reported coronary artery disease, age-scaled	Prevalence	Alt		–0.09 (–0.66 to 0.49)	0.92
Self-reported coronary artery disease, male	Prevalence	Alt		0.10 (-0.68 to 0.48)	0.91
Self-reported history of myocardial infarction, intercept	Prevalence	Alt		–1.40 (–2.0 to –0.80)	0.25
Self-reported history of myocardial infarction, male	Prevalence	Alt		0.15 (–0.44 to 0.73)	1.16
Self-reported history of myocardial infarction, male	Prevalence	Alt		0.08 (–0.40 to 0.66)	1.08
Q-wave on resting ECG, intercept	Prevalence	Alt		–1.12 (–1.73 to –0.52)	0.33
Q-wave on resting ECG, age_scaled	Prevalence	Alt		–0.25 (–0.84 to 0.34)	0.78
Q-wave on resting ECG, male	Prevalence	Alt		–0.14 (–0.72 to 0.45)	0.87
Administrative coded and self-reported, intercept	Prevalence	Alt		0.23 (–0.39 to 0.85)	1.26

Administrative coded and self-reported, age_scaled	Prevalence	Alt		–0.65 (–1.38 to 0.08)	0.52
Administrative coded and self-reported, male	Prevalence	Alt		–0.61 (–1.21 to –0.01)	0.55
Administrative coded and Q-wave on resting ECG, intercept	Prevalence	Alt		–1.10 (–1.75 to –0.46)	0.33
Administrative coded and Q-wave on resting ECG, age_scaled	Prevalence	Alt		0.27 (–0.39 to 0.92)	1.31
Administrative coded and Q-wave on resting ECG, male	Prevalence	Alt		0.56 (–0.05 to 1.16)	1.75
Administrative health facility data – IHME, intercept	Prevalence	Alt		–0.89 (–1.49 to –0.28)	0.41
Administrative health facility data – IHME, age-scaled	Prevalence	Alt		0.33 (–0.25 to 0.90)	1.39
Administrative health facility data – IHME, male	Prevalence	Alt		0.13 (–0.45 to 0.70)	1.13

**MR-BRT crosswalk adjustments can be interpreted as the factor the alternative case definition is adjusted by to reflect what it would have been had it been measured using the reference case definition. If the log/logit beta coefficient is negative, then the alternative is adjusted up to the reference. If the log/logit beta coefficient is positive, then the alternative is adjusted down to the reference.*

***The adjustment factor column is the exponentiated beta coefficient. For log beta coefficients, this is the relative rate between the two case definitions. For logit beta coefficients, this is the relative odds between the two case definitions.*

Modelling strategy

Myocardial infarction

We first estimated myocardial infarction incidence and prevalence of cases reaching health care (diagnosed myocardial infarction) using a DisMod-MR 2.1 model and separately modelled a scaling factor to adjusted estimated diagnosed myocardial infarction incidence and prevalence to capture prehospital myocardial infarction cases using a custom MR-BRT model. The product of the diagnosed myocardial infarction incidence and prevalence with the prehospital myocardial infarction scalar was the final incidence and prevalence of myocardial infarction. We included data on incidence, excess mortality, and cause-specific mortality in a DisMod-MR 2.1 model. We set a value prior of one month for remission (11/13). We also set a value prior for the maximum excess mortality rate of 10 for all ages.

Predictive covariates included the Healthcare Access and Quality (HAQ) Index as a fixed-effect country-level covariate on excess mortality, forcing an inverse relationship. We also included the log-transformed age-standardised SEV scalar for IHD as a fixed-effect country-level covariate for incidence. Covariates and associated coefficients can be found in Table 7.

To generate MI-specific cause-specific mortality, we split post-CoDCorrect IHD deaths into the proportion of IHD deaths with a confirmed diagnosis of myocardial infarction. Using both individual-level and tabulated sources linking hospitalisation events to subsequent mortality records (Table 6), we used the meta-analytic Bayesian modelling tool MR-BRT to estimate the proportion of IHD deaths associated with a diagnosis of myocardial infarction within 30 days of death with age, sex, year, and the Healthcare Access and Quality (HAQ) Index as linear covariates in the model. Models were validated by selecting the set of covariates and model specifications that minimised the root mean squared error. The result of the model is an estimated proportion of IHD deaths with a diagnosis of myocardial infarction within 30 days of death. We then apply this proportion to the post-CoDCorrect IHD deaths to get the cause-specific mortality rate of acute myocardial infarction. The form of the regression is given in equation 3 below. The estimated proportion of IHD deaths allocated to diagnosed myocardial infarction is shown in figure 1 stratified by SDI quintiles.

Equation 3: MR-BRT model form for proportion of IHD deaths with confirmed myocardial infarction diagnosis

$$logit(proportion) = Spline(age) + \beta_o sex + \beta_1 HAQi + \beta_2 AgeSexInteration + \beta_{3,\dots,8} year + \beta_9 intercept$$

Table 6. Previously collected sources reporting proportion of IHD deaths with diagnosed myocardial infarction within 30 days of death

Source information	Location	Type	Years
New Zealand Linked National Minimum Dataset to Mortality Collection Data[10]	New Zealand	Individual linked dataset	1999-2018
Friuli-Venezia Linked Dataset of hospitalization to mortality[11]	Italy	Individual linked dataset	2005-2015
Trends in out-of-hospital deaths due to coronary heart disease in Sweden (1991 to 2006)[12]	Sweden	Literature, population	1991-2006

National Board of Health and Welfare (Sweden). Sweden Cause of Death Register 1993-2018[13]	Sweden	Online database	1993-2018
Acute myocardial infarction hospital admissions and deaths in England: a national follow-back and follow-forward record-linkage study[14]	England	Literature, population	2006-2010
Acute Myocardial Infarction Population Incidence and Mortality Rates, and 28-day Case-fatality in Older Adults. The REGICOR Study[15]	Spain	Literature, population	1996-2008

Figure 1. Modelled proportion of IHD deaths allocated to diagnosed myocardial infarction, with associated covariates and coefficients

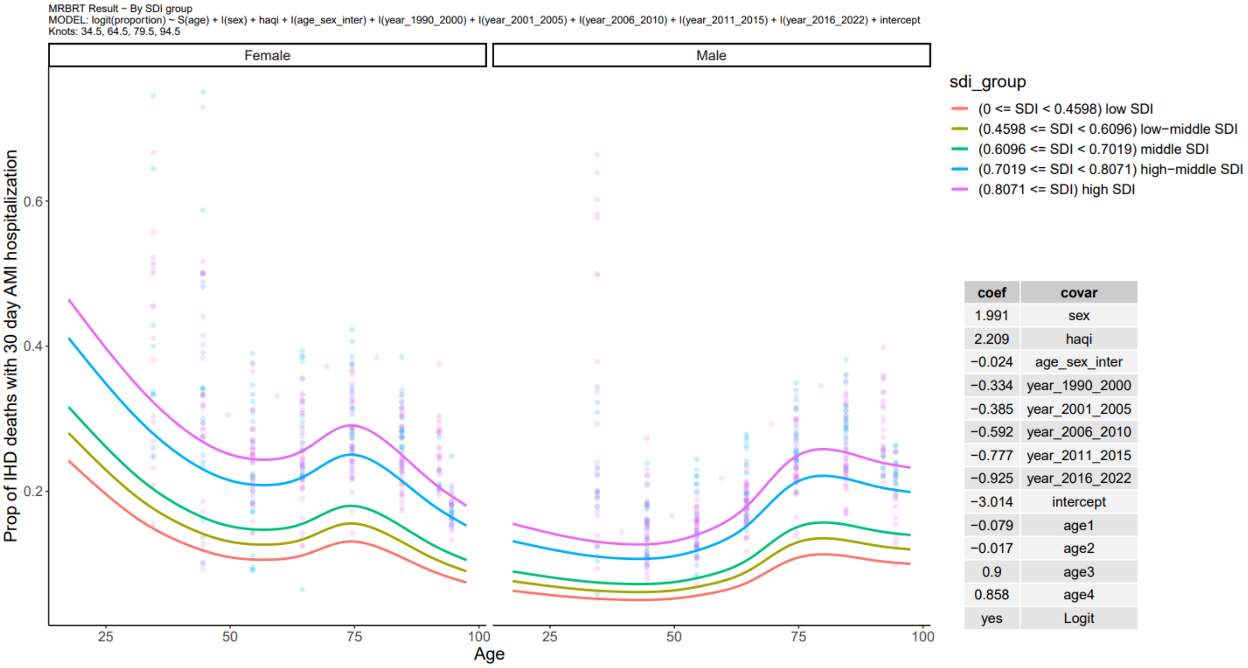

Table 7. Covariates: summary of covariates used in the myocardial infarction DisMod-MR meta-regression model

Covariate	Parameter	Beta	Exponentiated beta
Healthcare Access and Quality (HAQ) Index	Excess mortality rate	–0.051 (–0.052 to –0.051)	0.95 (0.95 to 0.95)
Log-transformed age-standardised SEV scalar: IHD	Incidence	0.91 (0.88 to 0.94)	2.48 (2.40 to 2.56)

With estimates of diagnosed myocardial infarction incidence and prevalence produced from the DisMod-MR 2.1 model, we then ran a separate MR-BRT model to estimate a scalar for the additional number of prehospital myocardial infarction incident and prevalent cases. The input data to this model are the proportion of incident myocardial infarction that is diagnosed as described in equation 3 above. The MR-BRT model included covariates for age, sex, HAQ Indes, and interaction term between age and HAQ Index, the IHD SEV scalar, and an intercept. We used a spline with 4 internal knots on the interaction term between age and HAQ Index to capture different age patterns by HAQ Index. The MR-BRT model was run in logit space to constrain the proportion between 0 and 1. The form of the regression model is shown in equation 4.

Equation 4. MR-BRT model form of proportion of incident myocardial infarction that is diagnosed

$$logit(proportion) = \beta_0 intercept + \beta_1 age + \beta_2 sex + \beta_3 HAQi + Spline(ageHAQiInteraction) + \beta_4 IHDSEV$$

The resulting proportion of incident diagnosed myocardial infarction was then predicted for all age, sex, year, locations using location-specific covariates. The resulting proportion estimates are visualised below by HAQ Index quintile in figure 2. We inverted the estimated proportions to produce the scaling factor applied to estimates of diagnosed myocardial infarction to produce estimates of incidence and prevalence of both diagnosed and prehospital myocardial infarction combined. The scaling factor is shown in figure 3 by HAQ Index quintile.

Figure 2: Modeled proportion of incident diagnosed myocardial infarction by HAQ Index quintile

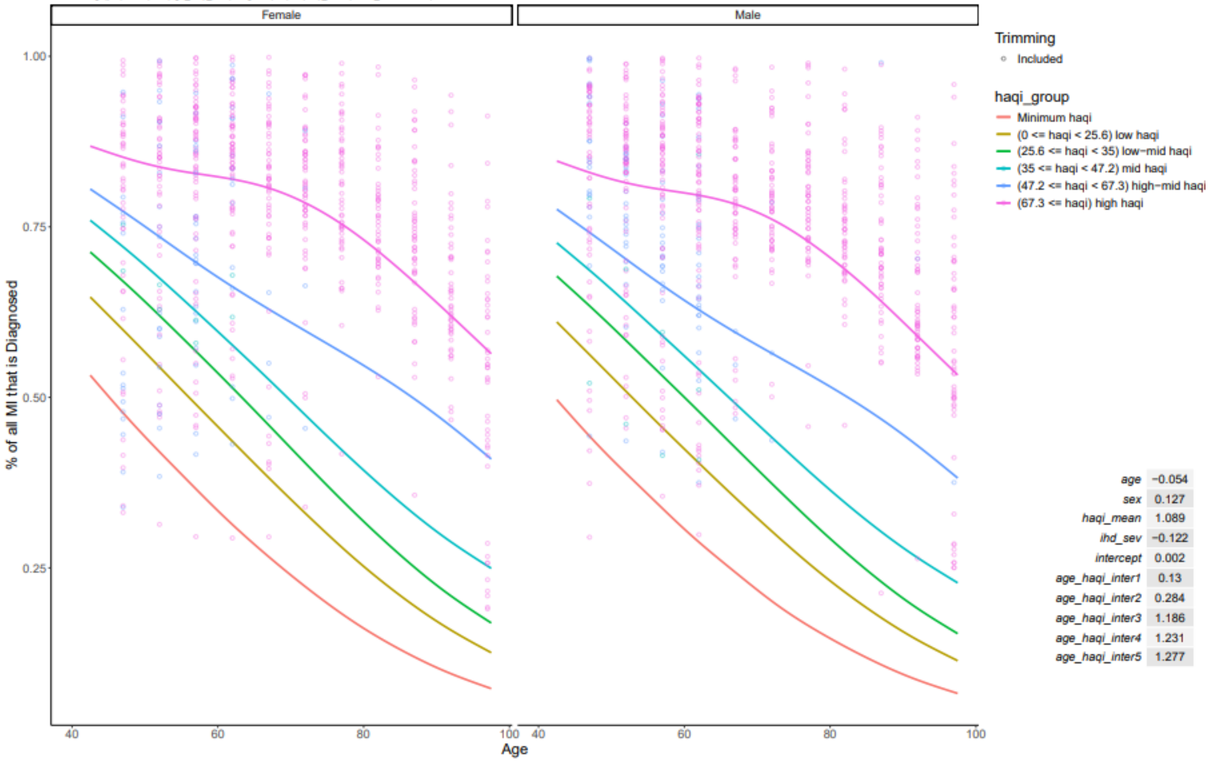

Figure 3: Modelled scaling factor of incident diagnosed myocardial infarction to include prehospital myocardial infarction cases by HAQ Index quintile

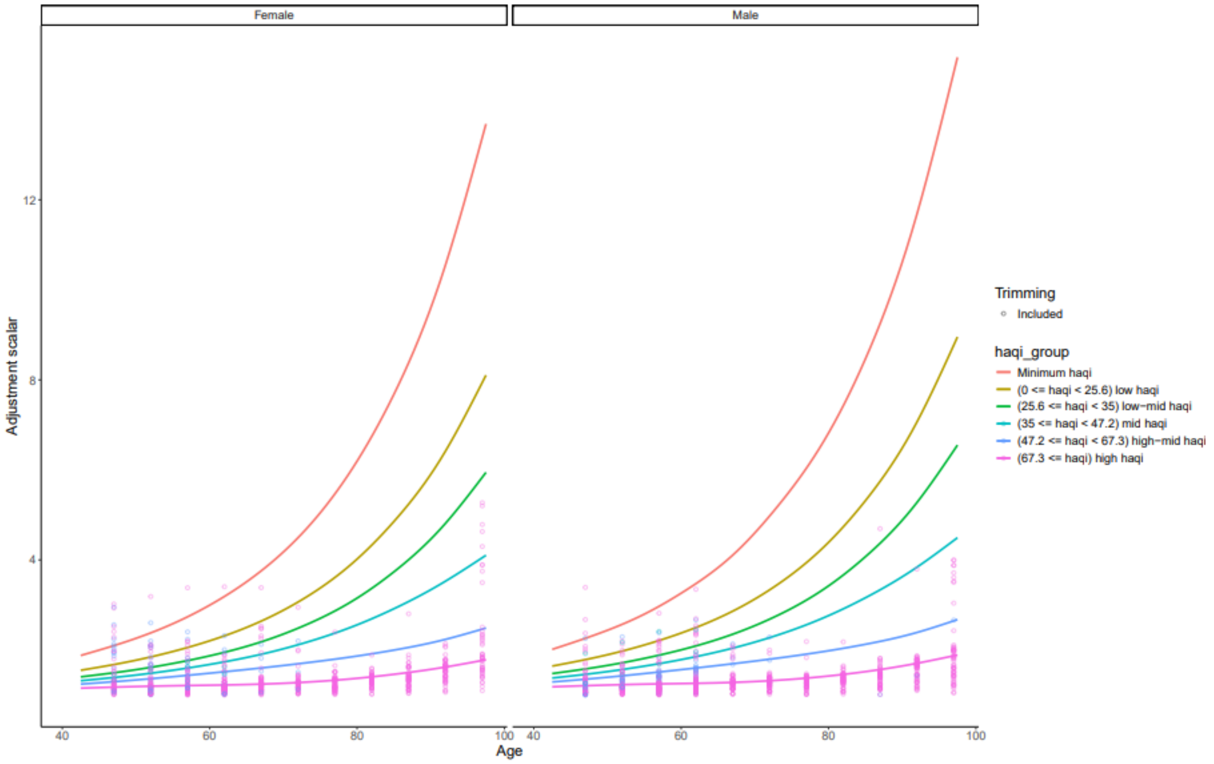

The adjustment scalar was applied to diagnosed myocardial infarction estimates to produce an estimate of the combined incidence and prevalence of diagnosed and prehospital myocardial infarction, becoming the acute myocardial infarction estimates.

Severity split inputs

Acute myocardial infarction was split into two severity levels by length of time since the event – days 1 and 2 versus days 3 through 28. Disability weights were established for these two severities using the standard approach for GBD 2021.

Table 8. Severity distribution, details on the severity levels for myocardial infarction in GBD 2021 and the associated disability weight (DW) with that severity

Severity level	Lay description	DW (95% CI)
Acute myocardial infarction, days 1–2	Has severe chest pain that becomes worse with any physical activity. The person feels nauseated, short of breath, and very anxious.	0.432 (0.288–0.579)
Acute myocardial infarction, days 3–28	Gets short of breath after heavy physical activity, and tires easily, but has no problems when at rest. The person has to take medication every day and has some anxiety.	0.074 (0.049–0.105)

Stable coronary artery disease

We used prevalence data from published scientific literature and population-representative survey series, along with data on excess mortality, with-condition mortality, and cause-specific mortality for IHD, to estimate the prevalence of coronary artery disease for all locations.

We included a value prior of zero for remission for all ages. We also included a value prior of 1 for excess mortality for all ages.

We also included the log-transformed, age-standardised SEV scalar for IHD as a fixed effect, country-level covariate on prevalence and Healthcare Access and Quality (HAQ) Index as a fixed effect, country-level covariate on excess mortality, forcing an inverse relationship with HAQ Index. Covariates and associated coefficients can be found in Table 9.

Table 9. Covariates. Summary of covariates used in the stable coronary artery disease DisMod-MR meta-regression model

Covariate	Parameter	Beta	Exponentiated beta
Log-transformed age-standardised SEV scalar: IHD	Prevalence	0.91<br>(0.89 to 0.94)	2.49<br>(2.53 to 2.55)
Healthcare Access and Quality (HAQ) Index	Excess mortality rate	–0.01<br>(–0.013 to –0.007)	0.99<br>(0.99 to 0.99)

Post-DisMod exclusivity adjustments

Estimates of prevalent coronary artery disease included subjects with heart failure due to IHD; to avoid double counting, we removed the estimates of prevalent heart failure due to IHD from the prevalence of coronary artery disease and reported sequelae of heart failure due to ischaemic heart disease separately. More details on the prevalence of heart failure due to ischaemic heart disease can be found in the heart failure appendix.

Severity split inputs

The proportion of asymptomatic, mild, moderate, and severe coronary artery disease was determined utilising findings from the Initial Invasive or Conservative Strategy for Stable Coronary Disease (ISCHEMIA) trial. This study reported summary metrics for patient quality of life using the Seattle Angina Questionnaire (SAQ) instrument for an international population of those with obstructive coronary artery disease.[16,17] The proportion of asymptomatic, mild, moderate, and severe cases of coronary artery disease were determined by set cut-offs of the SAQ overall summary health score, an aggregated metric of participants' responses across multiple dimensions and individual responses to a question regarding angina frequency. SAQ summary scores range from 0 to 100, where higher scores indicate lower disability. We defined asymptomatic coronary artery disease as cases who experienced zero angina symptoms within the past month or those with a SAQ summary score of 100. Mild coronary artery disease was defined as those with a summary SAQ score of greater than 75 but less than 100. Moderate coronary artery disease was defined as those with a summary SAQ score greater than 50 but less than 75. Severe coronary artery disease was defined as those with a summary SAQ score less than or equal to 50. These SAQ summary score cut-offs were determined by expert opinion. Disability weights were established for these severities using the standard approach for GBD 2021 and are shown in Table 10.

Table 10. Severity distribution, details on the severity levels for coronary artery disease in GBD 2019 and the associated disability weight (DW) with that severity

Severity level	Lay description	DW (95% CI)

Asymptomatic coronary artery disease		N/A
Mild coronary artery disease	Has chest pain that occurs with strenuous physical activity, such as running or lifting heavy objects. After a brief rest, the pain goes away.	0.033 (0.02–0.052)
Moderate coronary artery disease	Has chest pain that occurs with moderate physical activity, such as walking uphill or more than half a kilometer (around a quarter-mile) on level ground. After a brief rest, the pain goes away.	0.08 (0.052–0.113)
Severe coronary artery disease	Has chest pain that occurs with minimal physical activity, such as walking only a short distance. After a brief rest, the pain goes away. The person avoids most physical activities because of the pain.	0.167 (0.11–0.24)

Citations

[1] Hamilton Health Sciences, McMaster University (Canada), Population Health Research Institute (PHRI).

[2] Lawlor DA, Adamson J, Ebrahim S. Performance of the WHO Rose angina questionnaire in post-menopausal women: Are all of the questions necessary? *Journal of Epidemiology & Community Health* 2003;57:538-541.

[3] Leipsic J, Abbara S, Achenbach S, Cury R, Earls JP, Mancini GJ, Nieman K, Pontone G, Raff GL. SCCT guidelines for the interpretation and reporting of coronary CT angiography: a report of the Society of Cardiovascular Computed Tomography Guidelines Committee. *J Cardiovasc Comput Tomogr*. 2014 Sep-Oct;8(5):342-58. doi: 10.1016/j.jcct.2014.07.003. Epub 2014 Jul 24. PMID: 25301040.

[4] Thygesen K, Alpert JS, Jaffe AS, Chaitman BR, Bax JJ, Morrow DA, White HD; Executive Group on behalf of the Joint European Society of Cardiology (ESC)/American College of Cardiology (ACC)/American Heart Association (AHA)/World Heart Federation (WHF) Task Force for the Universal Definition of Myocardial Infarction. Fourth Universal Definition of Myocardial Infarction (2018). *J Am Coll Cardiol*. 2018 Oct 30;72(18):2231-2264. doi: 10.1016/j.jacc.2018.08.1038. Epub 2018 Aug 25. PMID: 30153967.

[5] Bruce RA. Exercise testing of patients with coronary heart disease. Principles and normal standards for evaluation. *Ann Clin Res*. 1971 Dec;3(6):323-32. PMID: 5156892.

[6] Sicari R, Nihoyannopoulos P, Evangelista A, Kasprzak J, Lancellotti P, Poldermans D, Voigt JU, Zamorano JL; European Association of Echocardiography. Stress Echocardiography Expert Consensus Statement--Executive Summary: European Association of Echocardiography (EAE) (a registered branch of the ESC). *Eur Heart J*. 2009 Feb;30(3):278-89. doi: 10.1093/eurheartj/ehn492. Epub 2008 Nov 11. PMID: 19001473.

[7] http://www.framinghamheartstudy.org/share/protocols/soe0_03s_protocol.pdf

[8] 2016 ESC Guidelines for the diagnosis and treatment of acute and chronic heart failure: The Task Force for the diagnosis and treatment of acute and chronic heart failure of the European Society of

Cardiology (ESC) Developed with the special contribution of the Heart Failure Association (HFA) of the ESC. Eur Heart J 2016; 37(27): 2129-2200.

[9] Timonin S, Shkolnikov VM, Andreev E, Magnus P, Leon DA. Evidence of large systematic differences between countries in assigning ischaemic heart disease deaths to myocardial infarction: the contrasting examples of Russia and Norway. *Int J Epidemiol*. 2022 Jan 6;50(6):2082-2090. doi: 10.1093/ije/dyab188. Epub 2021 Sep 11. PMID: 34999891; PMCID: PMC8743129.

[10] Ministry of Health (New Zealand). New Zealand National Minimum Dataset 2016. Wellington, New Zealand: Ministry of Health (New Zealand).

[11] Institute for Maternal and Child Health - IRCCS "Burlo Garofolo", Italian National Institute of Statistics. Italy - Friuli-Venezia Giulia Multiple Causes of Death Data 2007.

[12] Dudas Kerstin, Lappas Georg, Stewart Simon, Rosengren Annika. Trends in Out-of-Hospital Deaths Due to Coronary Heart Disease in Sweden (1991 to 2006). *Circulation*. 2011; 123(1): 46-52.

[13] National Board of Health and Welfare (Sweden). Sweden Cause of Death Register.

[14] Asaria P, Elliott P, Douglass M, Obermeyer Z, Soljak M, Majeed A, Ezzati M. Acute myocardial infarction hospital admissions and deaths in England: a national follow-back and follow-forward record-linkage study. *Lancet Public Health*. 2017 Apr;2(4):e191-e201. doi: 10.1016/S2468-2667(17)30032-4. Epub 2017 Mar 1. PMID: 29253451; PMCID: PMC6196770.

[15] Vázquez-Oliva G, Zamora A, Ramos R, Marti R, Subirana I, Grau M, Dégano IR, Marrugat J, Elosua R. Acute Myocardial Infarction Population Incidence and Mortality Rates, and 28-day Case-fatality in Older Adults. The REGICOR Study. *Rev Esp Cardiol* (Engl Ed). 2018 Sep;71(9):718-725. English, Spanish. doi: 10.1016/j.rec.2017.10.019. Epub 2017 Nov 22. PMID: 29174866.

[16] Spertus, A John et al. "Development and evaluation of the Seattle Angina questionnaire: A new functional status measure for coronary artery disease". *Journal of the American College of Cardiology*, Vol 25, Issue 2, February 1995, pages 333-341.

[17] Spertus JA, Jones PG, Maron DJ, O'Brien SM, Reynolds HR, Rosenberg Y, Stone GW, Harrell FE Jr, Boden WE, Weintraub WS, Baloch K, Mavromatis K, Diaz A, Gosselin G, Newman JD, Mavromichalis S, Alexander KP, Cohen DJ, Bangalore S, Hochman JS, Mark DB; ISCHEMIA Research Group. Health-Status Outcomes with Invasive or Conservative Care in Coronary Disease. *N Engl J Med*. 2020 Apr 9;382(15):1408-1419. doi: 10.1056/NEJMoa1916370. Epub 2020 Mar 30. PMID: 32227753; PMCID: PMC7261489.

[18] Ikemura N, Spertus J, Nguyen D, et al. INTERNATIONAL COMPARISONS OF HEALTH STATUS OUTCOMES IN PATIENTS UNDERGOING INITIAL INVASIVE VERSUS CONSERVATIVE MANAGEMENT FOR CHRONIC CORONARY DISEASE: INSIGHTS FROM THE ISCHEMIA TRIAL. *J Am Coll Cardiol*. 2023 Mar, 81 (8_Supplement) 1111.
https://doi.org/10.1016/S0735-1097(23)01555-3

[19] Page MJ, McKenzie JE, Bossuyt PM, Boutron I, Hoffmann TC, Mulrow CD, et al. The PRISMA 2020 statement: an updated guideline for reporting systematic reviews. *BMJ* 2021;372:n71. doi: 10.1136/bmj.n71

Ischaemic stroke, intracerebral haemorrhage, and subarachnoid haemorrhage

Flowcharts

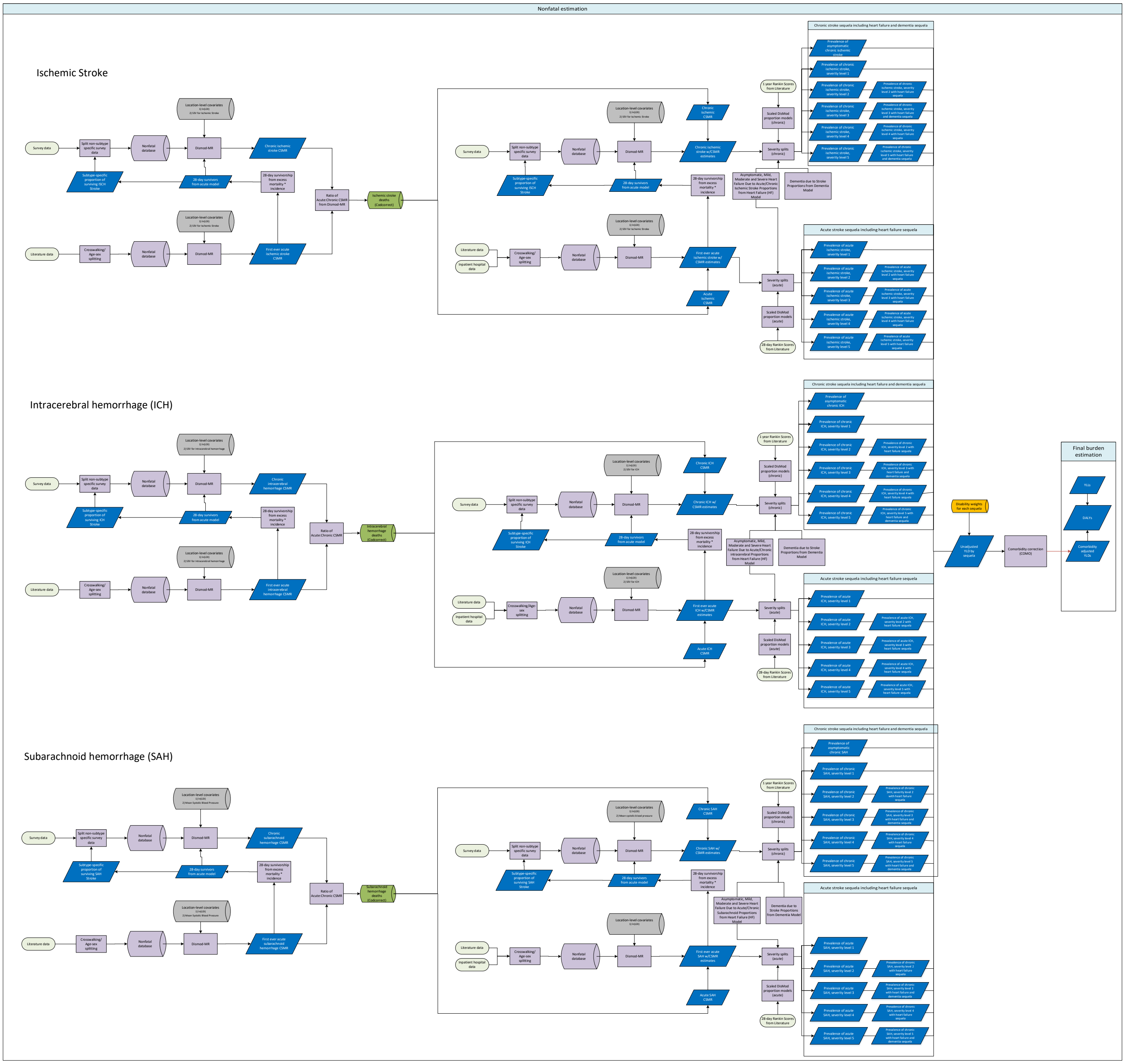

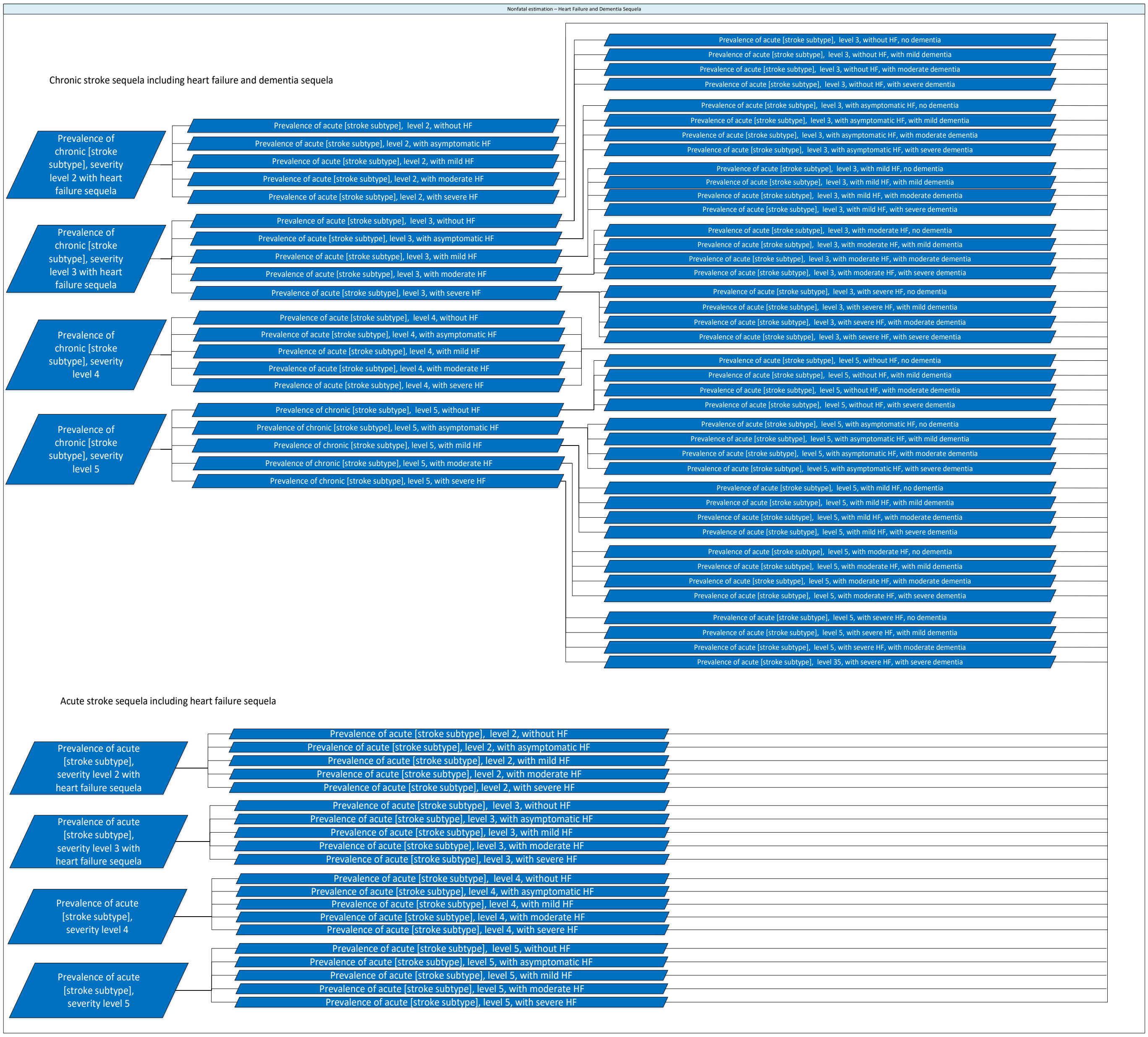
Nonfatal estimation – Heart Failure and Dementia Sequela
Chronic stroke sequela including heart failure and dementia sequela
Prevalence of chronic [stroke subtype], severity level 2 with heart failure sequela
Prevalence of acute [stroke subtype], level 2, without HF
Prevalence of acute [stroke subtype], level 2, with asymptomatic HF
Prevalence of acute [stroke subtype], level 2, with mild HF
Prevalence of acute [stroke subtype], level 2, with moderate HF
Prevalence of acute [stroke subtype], level 2, with severe HF
Prevalence of chronic [stroke subtype], severity level 3 with heart failure sequela
Prevalence of acute [stroke subtype], level 3, without HF
Prevalence of acute [stroke subtype], level 3, with asymptomatic HF
Prevalence of acute [stroke subtype], level 3, with mild HF
Prevalence of acute [stroke subtype], level 3, with moderate HF
Prevalence of acute [stroke subtype], level 3, with severe HF
Prevalence of chronic [stroke subtype], severity level 4
Prevalence of acute [stroke subtype], level 4, without HF
Prevalence of acute [stroke subtype], level 4, with asymptomatic HF
Prevalence of acute [stroke subtype], level 4, with mild HF
Prevalence of acute [stroke subtype], level 4, with moderate HF
Prevalence of acute [stroke subtype], level 4, with severe HF
Prevalence of chronic [stroke subtype], severity level 5
Prevalence of chronic [stroke subtype], level 5, without HF
Prevalence of chronic [stroke subtype], level 5, with asymptomatic HF
Prevalence of chronic [stroke subtype], level 5, with mild HF
Prevalence of chronic [stroke subtype], level 5, with moderate HF
Prevalence of chronic [stroke subtype], level 5, with severe HF
Prevalence of acute [stroke subtype], level 3, without HF, no dementia
Prevalence of acute [stroke subtype], level 3, without HF, with mild dementia
Prevalence of acute [stroke subtype], level 3, without HF, with moderate dementia
Prevalence of acute [stroke subtype], level 3, without HF, with severe dementia
Prevalence of acute [stroke subtype], level 3, with asymptomatic HF, no dementia
Prevalence of acute [stroke subtype], level 3, with asymptomatic HF, with mild dementia
Prevalence of acute [stroke subtype], level 3, with asymptomatic HF, with moderate dementia
Prevalence of acute [stroke subtype], level 3, with asymptomatic HF, with severe dementia
Prevalence of acute [stroke subtype], level 3, with mild HF, no dementia
Prevalence of acute [stroke subtype], level 3, with mild HF, with mild dementia
Prevalence of acute [stroke subtype], level 3, with mild HF, with moderate dementia
Prevalence of acute [stroke subtype], level 3, with mild HF, with severe dementia
Prevalence of acute [stroke subtype], level 3, with moderate HF, no dementia
Prevalence of acute [stroke subtype], level 3, with moderate HF, with mild dementia
Prevalence of acute [stroke subtype], level 3, with moderate HF, with moderate dementia
Prevalence of acute [stroke subtype], level 3, with moderate HF, with severe dementia
Prevalence of acute [stroke subtype], level 3, with severe HF, no dementia
Prevalence of acute [stroke subtype], level 3, with severe HF, with mild dementia
Prevalence of acute [stroke subtype], level 3, with severe HF, with moderate dementia
Prevalence of acute [stroke subtype], level 3, with severe HF, with severe dementia
Prevalence of acute [stroke subtype], level 5, without HF, no dementia
Prevalence of acute [stroke subtype], level 5, without HF, with mild dementia
Prevalence of acute [stroke subtype], level 5, without HF, with moderate dementia
Prevalence of acute [stroke subtype], level 5, without HF, with severe dementia
Prevalence of acute [stroke subtype], level 5, with asymptomatic HF, no dementia
Prevalence of acute [stroke subtype], level 5, with asymptomatic HF, with mild dementia
Prevalence of acute [stroke subtype], level 5, with asymptomatic HF, with moderate dementia
Prevalence of acute [stroke subtype], level 5, with asymptomatic HF, with severe dementia
Prevalence of acute [stroke subtype], level 5, with mild HF, no dementia
Prevalence of acute [stroke subtype], level 5, with mild HF, with mild dementia
Prevalence of acute [stroke subtype], level 5, with mild HF, with moderate dementia
Prevalence of acute [stroke subtype], level 5, with mild HF, with severe dementia
Prevalence of acute [stroke subtype], level 5, with moderate HF, no dementia
Prevalence of acute [stroke subtype], level 5, with moderate HF, with mild dementia
Prevalence of acute [stroke subtype], level 5, with moderate HF, with moderate dementia
Prevalence of acute [stroke subtype], level 5, with moderate HF, with severe dementia
Prevalence of acute [stroke subtype], level 5, with severe HF, no dementia
Prevalence of acute [stroke subtype], level 5, with severe HF, with mild dementia
Prevalence of acute [stroke subtype], level 5, with severe HF, with moderate dementia
Prevalence of acute [stroke subtype], level 35, with severe HF, with severe dementia
Acute stroke sequela including heart failure sequela
Prevalence of acute [stroke subtype], severity level 2 with heart failure sequela
Prevalence of acute [stroke subtype], level 2, without HF
Prevalence of acute [stroke subtype], level 2, with asymptomatic HF
Prevalence of acute [stroke subtype], level 2, with mild HF
Prevalence of acute [stroke subtype], level 2, with moderate HF
Prevalence of acute [stroke subtype], level 2, with severe HF
Prevalence of acute [stroke subtype], severity level 3 with heart failure sequela
Prevalence of acute [stroke subtype], level 3, without HF
Prevalence of acute [stroke subtype], level 3, with asymptomatic HF
Prevalence of acute [stroke subtype], level 3, with mild HF
Prevalence of acute [stroke subtype], level 3, with moderate HF
Prevalence of acute [stroke subtype], level 3, with severe HF
Prevalence of acute [stroke subtype], severity level 4
Prevalence of acute [stroke subtype], level 4, without HF
Prevalence of acute [stroke subtype], level 4, with asymptomatic HF
Prevalence of acute [stroke subtype], level 4, with mild HF
Prevalence of acute [stroke subtype], level 4, with moderate HF
Prevalence of acute [stroke subtype], level 4, with severe HF
Prevalence of acute [stroke subtype], severity level 5
Prevalence of acute [stroke subtype], level 5, without HF
Prevalence of acute [stroke subtype], level 5, with asymptomatic HF
Prevalence of acute [stroke subtype], level 5, with mild HF
Prevalence of acute [stroke subtype], level 5, with moderate HF
Prevalence of acute [stroke subtype], level 5, with severe HF

Input data and methodological summary

Case definition

Stroke was defined according to WHO criteria as rapidly developing clinical signs of focal (or less commonly global) disturbance of cerebral function lasting more than 24 hours or leading to death with no apparent cause other than that of vascular origin (1). Cases of transient ischaemic attack (TIA) were not included.

Acute stroke: Stroke cases are considered acute from the day of incidence of a first-ever stroke through day 28 following the event.

Chronic stroke: Stroke cases are considered chronic beginning 28 days following the occurrence of an event. Chronic stroke includes the late sequelae of an acute stroke and all recurrent stroke events. GBD 2015 adopted this broader definition of chronic stroke than what was used in prior iterations to model acute strokes using only first-ever incident events.

Ischaemic stroke: Ischaemic strokes are characterized by occlusion of blood flow to part of the brain due to hypoperfusion, most commonly due to a thrombus or embolism. It is defined as an episode of neurological dysfunction caused by focal cerebral, spinal, or retinal infarction

Intracerebral haemorrhage: Intracerebral haemorrhage is characterised by the rupture of a blood vessel resulting in bleeding into the intracerebral part of the brain. It is defined as focal collection of blood within the brain parenchyma or ventricular system that is not caused by trauma and results in a clinical stroke.

Subarachnoid haemorrhage: Subarachnoid haemorrhage is defined as bleeding into the subarachnoid space (the space between the arachnoid membrane and the pia mater of the brain or spinal cord) resulting in a clinical stroke.

The reference definitions for ischaemic stroke and intracerebral haemorrhage were first-ever, subtype-specific stroke, which included subjects who did not survive to hospital admission. For these two subtypes we included, after adjustment, sources which used the following alternate definitions: 1) sources which included first and recurrent strokes; 2) sources which reported only estimates for all subtypes combined; and 3) sources which included only stroke cases which survived to hospital admission; 4) sources that were ascertained from administrative inpatient hospital data 5) sources that were ascertained from administrative hospital data in the USA.

The reference definition for subarachnoid haemorrhage was first-ever, subtype-specific stroke, with aneurysmal and non-aneurysmal events combined, which included subjects who did not survive to hospital admissions. For subarachnoid haemorrhage, we included, after adjustment for bias, sources which used the following alternate definitions: 1) sources which included first and recurrent strokes; 2) sources which reported only estimates for aneurysmal subarachnoid haemorrhage; and 3) sources which included only stroke cases which survived to hospital admission.

A summary of reference and alternate case definitions by stroke subtype is given in table 1. ICD codes used to identify stroke subtypes are given in table 2.

Table 1: Reference and alternate case definitions by stroke subtype

**Quantity of interest**	**Reference or alternative**	**Definition**
Ischaemic stroke	Reference	WHO definition for ischaemic stroke
Ischaemic stroke	Alternative	First-ever and recurrent stroke
Ischaemic stroke	Alternative	All stroke subtypes combined
Ischaemic stroke	Alternative	Stroke cases that survived to hospital admission only
Ischaemic stroke	Alternative	Stroke cases that were ascertained from inpatient admission ICD codes
Ischaemic stroke	Alternative	Stroke cases that were ascertained from inpatient admission ICD codes in the USA
Intracerebral haemorrhage	Reference	WHO definition for intracerebral haemorrhage
Intracerebral haemorrhage	Alternative	First-ever and recurrent stroke
Intracerebral haemorrhage	Alternative	All stroke subtypes combined
Intracerebral haemorrhage	Alternative	Stroke cases that survived to hospital admission only
Intracerebral haemorrhage	Alternative	Stroke cases that were ascertained from inpatient admission ICD codes
Subarachnoid haemorrhage	Reference	WHO definition for subarachnoid haemorrhage
Subarachnoid haemorrhage	Alternative	First-ever and recurrent stroke
Subarachnoid haemorrhage	Alternative	Stroke cases that survived to hospital admission only
Subarachnoid haemorrhage	Alternative	Aneurysmal subarachnoid haemorrhage reported only

Table 2: ICD codes used for inclusion of hospital and claims data

Stroke subtype	ICD-9	ICD-10
Ischaemic stroke	433-435.9, 437.0-437.1, 437.5-437.8	I63-I63.9, I65-I66.9, I67.2-I67.3, I67.5-I67.6, I69.3
Intracerebral haemorrhage	431-432.9, 437.2	I61-I62, I62.1-I62.9, I68.1-I68.2, I69.1-I69.2
Subarachnoid haemorrhage	430-430.9	I60-I60.9, I62.0, I67.0-I67.1, I69.0

Input data

A systematic review was not performed for stroke in GBD 2023. The last systematic review was performed for stroke models in GBD 2021 in accordance with PRISMA systematic review guidelines. We searched PubMed for our systematic review; the search strings we used and a PRISMA diagram displaying the text review and extraction process are found below:

PubMed: ("stroke"[TIAB] OR "ischemic stroke"[TIAB] OR "ischaemic stroke"[TIAB] OR "cerebral infarction"[TIAB] OR "intracerebral hemorrhage"[TIAB] OR "intracerebral haemorrhage"[TIAB] OR "subarachnoid hemorrhage"[TIAB] OR "subarachnoid haemorrhage"[TIAB]) AND (incidence[TIAB] OR prevalence[TIAB] OR "excess mortality"[TIAB] OR "case fatality"[TIAB] OR "mortality ratio"[TIAB]) AND ("2017/09/01"[PDAT] : "2020/02/25"[PDAT])

Figure 1: PRISMA 2020 flow diagram

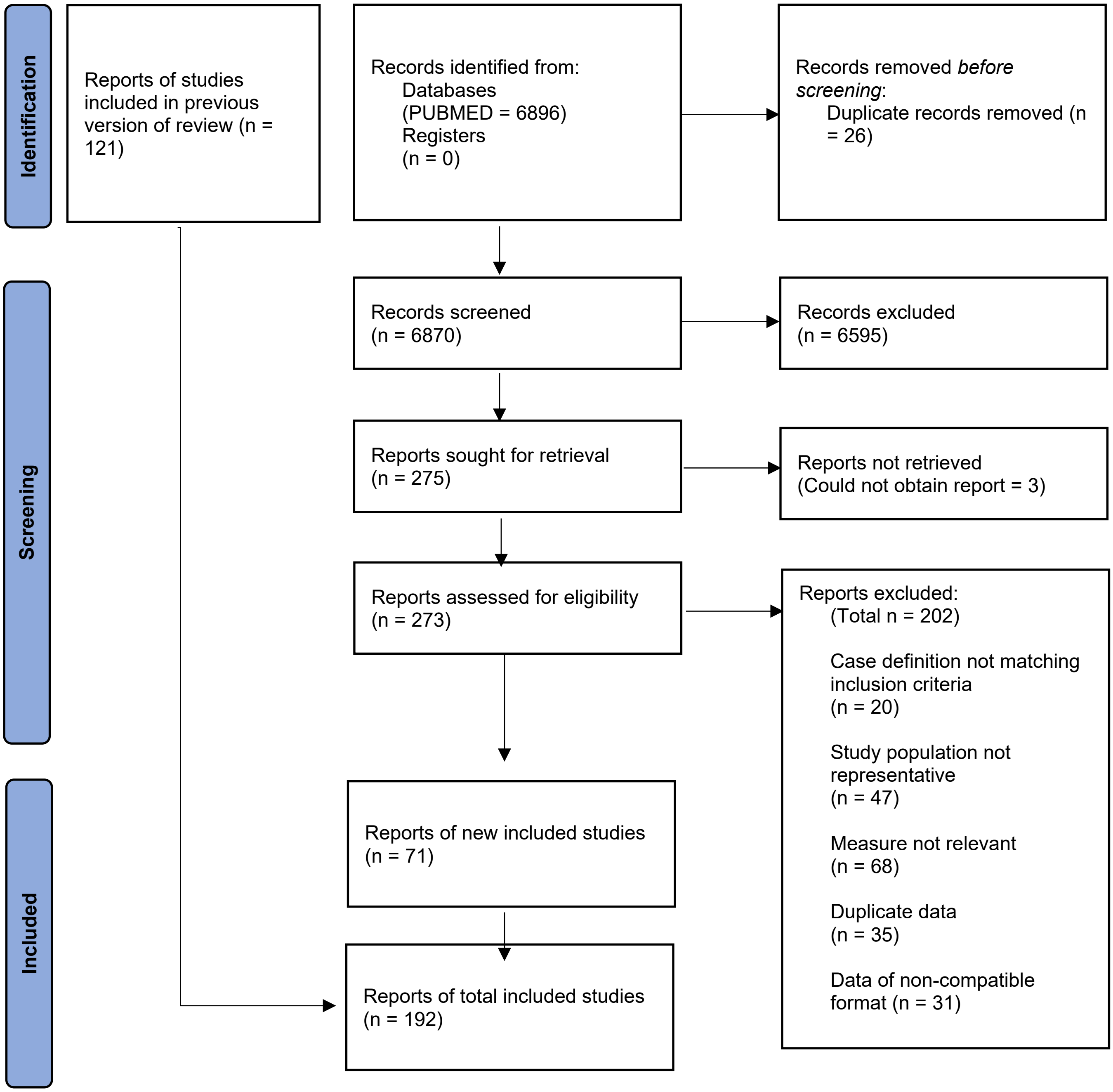

In addition to incidence data obtained from the literature reviews for acute stroke, we included inpatient hospital data, adjusted for readmission and primary to any diagnosis using correction factors estimated from claims data in the USA, Poland, Taiwan (province of China), and New Zealand. We excluded data for locations where the datapoints were implausibly low (Viet Nam, the Philippines, India, Nepal, China, Tibet, Kenya, Kyrgyzstan, Chile, Botswana, England, Brazil, Mexico, and Indonesia). For GBD 2023, we split incident unspecified strokes (ICD-10 I64) reported in the hospital data into ischaemic stroke, intracerebral haemorrhage, and subarachnoid haemorrhage according to the proportions of

subtype-specific coded strokes in the inpatient hospital data by source. We also split ICD-10 I62, other and unspecified non-traumatic intracranial haemorrhage, into intracerebral haemorrhage and subarachnoid haemorrhage using the same approach. In addition, we included unpublished stroke registry data for acute ischaemic stroke, acute intracerebral haemorrhage, and acute subarachnoid haemorrhage.

The 30-day case-fatality proportion of acute strokes were extracted from the literature and unpublished stroke registries. We expressed 30-day case fatality proportion as a rate (excess mortality rate) using the rate equation $excess\ mortality\ rate = \frac{-\log(1-case\ fatality\ proportion)}{30/365}$. Case-fatality proportion was expressed as excess mortality rate under the assumption that death within 30 days of an acute stroke event would be due to the stroke event.[1]

For the chronic stroke models, we included survey data for prevalent stroke. These surveys were identified based on expert opinion and review of major survey series focused on world health that included questions regarding self-reported history of stroke. These surveys reported on the prevalence of all strokes, and we therefore split the prevalence into estimates of subtype-specific stroke prevalence using a custom method described in the modelling strategy section below. We indexed all available surveys on the Global Health Data Exchange as of 06/20/2023. This returned 1842 new potential sources for our most recent update, of which we extracted microdata for 182.

Case-fatality proportions of chronic stroke were extracted from the literature and unpublished stroke registries. We expressed case-fatality proportion of beyond 30-day acute events as with-condition mortality rate using rate equation $with\ condition\ mortality\ rate = \frac{-\log(1-case\ fatality\ proportion)}{335/365}$ under the assumption that death beyond 30 days may be due to other causes than the index stroke event.[2]

As with many models in GBD, the diversity of data sources available means that we needed to adjust available data to our reference case definition. We thus crosswalked incidence and excess mortality data used in the acute models that did not meet our reference case definitions using MR- BRT, a Bayesian meta-regression tool develop for the GBD. More information on MR-BRT can be found elsewhere in the appendix.

We adjusted datapoints for first and recurrent strokes combined, using data for first strokes only as reference. For ischaemic stroke and intracerebral haemorrhage, we adjusted datapoints that reported all stroke subtypes combined, using as reference studies with subtype-specific information. We also adjusted data which included only persons who survived to hospital admission, using as reference data on both fatal and nonfatal strokes. In addition, we adjusted subtype-specific, inpatient clinical informatics data using subtype-specific literature estimates as a reference. These adjustments can be examined more closely in Table 4. The coefficients in Tables 4a, 4b, and 4c below can be used to calculate adjustment factors for alternative definitions. The formula for computing adjustment factors is given in equation 1 below. We included a cubic spline constructed on a standardised age variable (age scaled) and a sex variable to the crosswalking procedure to adjust for variation by age and sex. With the inclusion of the spline covariate on age, calculating adjustment factors is dependent on what segment of the age spline an adjustment is made, this is shown in tables 3a, 3b, and 3c below.

We split incidence datapoints where the age range was greater than 25 years for all stroke subtypes. Age splitting was based on the global sex-specific age pattern from a single-parameter DisMod model that only included incidence datapoints with less than a 25-year age range.

Equation 1: Calculation of adjustment factors:

$$Estimated\ Reference\ Def = invlogit(logit(Alternative\ Def) - [\sum_{s=0}^{b} Beta_{Alternative\ Def, spline\ basis_s} * Spline\ basis_s(age\_scaled)] - Beta * I(Sex))$$

$I(.)$ = Indicator function, b = Number of spline bases used

No data adjustments were necessary for the chronic stroke models.

Age splines for adjustment factors:
We fit a cubic spline to the standardised age variable (named age_scaled), calculated as:

$$age\ scaled = \frac{(mean\ age\ of\ study - mean(age\ of\ all\ studies))}{standard\ deviation(age\ of\ all\ studies)})$$

We selected knots for the cubic spline on age based on visual inspection of the spline fit to the observed ratios used in computing adjustment factors; see Figures 2a, 2b, and 2c below as examples. The knot placements for the age splines are listed in Table 3. We did not use a spline on age to adjust alternate definitions for subarachnoid haemorrhage incidence or excess mortality rate data for any stroke subtype. The fit of these splines versus the standardised age variable for males and females with the observed logit difference between alternative and reference definitions on the vertical axis are shown in Figures 2a through 2f.

Table 3: Knot placement for age splines

**Stroke subtype**	**Knot placement (age_scaled)**	**Knot placement (age in years)**
Ischaemic stroke	–1.25, –1, –0.75, 0.75, 1, 1.25	49.9, 53.9, 58.1, 82.6, 86.6, 90.7
Intracerebral haemorrhage	–2.95, –1.00, –0.06, 0.85, 1.77	22.1, 54.0, 69.3, 84.2, 99.2

Table 4a: MR-BRT crosswalk adjustment factors for ischaemic stroke

Data input	Measure	Reference or alternate case definition	Gamma	Beta coefficient, logit (95% CI)	**Adjustment factor

First-ever, subtype-specific, fatal and non-fatal events	Incidence	Ref	---	---	
Acute first-ever stroke, intercept	Incidence	Alt	0.063	0·32 (0·21 to 0·43)	1.38
Acute first-ever stroke, spline_0	Incidence	Alt	0.063	–0·76 (–0·8 to –0·73)	0·47
Acute first-ever stroke, spline_1	Incidence	Alt	0.063	–0·17 (–0·21 to –0·14)	0.84
Acute first-ever stroke, ages spline_2	Incidence	Alt	0.063	–0·36 (–0·38 to –0·33)	0.70
Acute first-ever stroke, ages spline_3	Incidence	Alt	0.063	0 (–0·03 to 0·03)	1.0
Acute first-ever stroke, ages spline_4	Incidence	Alt	0.063	–0·03 (–0·05 to 0)	0.97
Acute first-ever stroke, ages spline_5	Incidence	Alt	0.063	0·29 (0·26 to 0·32)	1.33
Acute first-ever stroke, ages spline_6	Incidence	Alt	0.063	0·19 (0·18 to 0·21)	1.21
Acute first-ever stroke, male	Incidence	Alt	0.063	0·08 (0·07 to 0·08)	1.08
All stroke, intercept	Incidence	Alt	0.063	0·38 (0·29 to 0·48)	1.47
All stroke, spline_0	Incidence	Alt	0.063	0·37 (0·24 to 0·50)	1.45
All stroke, spline_1	Incidence	Alt	0.063	0·57 (0·46 to 0·68)	1.76
All stroke, ages spline_2	Incidence	Alt	0.063	–0·43 (–0·52 to –0·33)	0.65
All stroke, ages spline_3	Incidence	Alt	0.063	0·07 (–0·03 to 0·17)	1.07
All stroke, ages spline_4	Incidence	Alt	0.063	–0·1 (–0·19 to 0)	0.91
All stroke, ages spline_5	Incidence	Alt	0.063	–0·5 (–0·62 to –0·38)	0.61
All stroke, ages spline_6	Incidence	Alt	0.063	–0·39 (–0·48 to –0·31)	0.67
All stroke, male	Incidence	Alt	0.063	–0·13	0.87

				(–0·15 to –0·12)	
Hospital, intercept	Incidence	Alt		–0·21 (–0·33 to –0·09)	0.81
Hospital, spline_0	Incidence	Alt		0·62 (0·58 to 0·66)	1.86
Hospital, spline_1	Incidence	Alt		0·58 (0·54 to 0·62)	1.79
Hospital, ages spline_2	Incidence	Alt		0·4 (0·37 to 0·43)	1.49
Hospital, ages spline_3	Incidence	Alt		0·31 (0·28 to 0·34)	1.36
Hospital, ages spline_4	Incidence	Alt		–0·04 (–0·07 to –0·01)	0.96
Hospital, ages spline_5	Incidence	Alt		–0·27 (–0·3 to –0·23)	0.77
Hospital, ages spline_6	Incidence	Alt		–0·08 (–0·11 to –0·06)	0.92
Hospital, male	Incidence	Alt		–0·19 (–0·2 to –0·19)	0.82
Inpatient clinical informatics, intercept	Incidence	Alt		0·44 (0·34 to 0·54)	1.55
Inpatient clinical informatics, spline_0	Incidence	Alt		0·47 (0·44 to 0·5)	1.60
Inpatient clinical informatics, spline_1	Incidence	Alt		0·51 (0·48 to 0·54)	1.67
Inpatient clinical informatics, ages spline_2	Incidence	Alt		0·18 (0·16 to 0·2)	1.20
Inpatient clinical informatics, ages spline_3	Incidence	Alt		0·36 (0·34 to 0·39)	1.43
Inpatient clinical informatics, ages spline_4	Incidence	Alt		–0·07 (–0·09 to –0·05)	0.93
Inpatient clinical informatics, ages spline_5	Incidence	Alt		0·03 (0 to 0·06)	1.03
Inpatient clinical informatics, ages spline_6	Incidence	Alt		0·06 (0·04 to 0·08)	1.07

Inpatient clinical informatics, male	Incidence	Alt		–0·19 (–0·2 to –0·19)	0.88
US claims data, intercept	Incidence	Alt		0·89 (0·76 to 1·02)	2.43
US claims data, spline_0	Incidence	Alt		0·2 (0·16 to 0·23)	1.22
US claims data, spline_1	Incidence	Alt		0·29 (0·26 to 0·33)	1.34
US claims data, ages spline_2	Incidence	Alt		–0·08 (–0·1 to –0·05)	0.93
US claims data, ages spline_3	Incidence	Alt		0·33 (0·31 to 0·36)	1.39
US claims data, ages spline_4	Incidence	Alt		–0·03 (–0·06 to –0·01)	0.97
US claims data, ages spline_5	Incidence	Alt		0·04 (0·01 to 0·07)	1.04
US claims data, ages spline_6	Incidence	Alt		0·08 (0·06 to 0·1)	1.09
US claims data, male	Incidence	Alt		–0·17 (–0·17 to –0·16)	0.85
Acute first-ever stroke, intercept	Excess mortality rate	Alt	0.21	0·14 (–0·08 to 0·37)	1.15
Acute first-ever stroke, age_scaled	Excess mortality rate	Alt		–0·31 (–0·33 to –0·29)	0.73
Acute first-ever stroke, male	Excess mortality rate	Alt		0·23 (0·21 to 0·25)	1.26
All stroke, intercept	Excess mortality rate	Alt		0·37 (0·12 to 0·62)	1·45
All stroke, age_scaled	Excess mortality rate	Alt		–0·09 (–0·15 to –0·03)	0·91
All stroke, male	Excess mortality rate	Alt		0·23 (0·18 to 0·28)	1·26
Hospital, intercept	Excess mortality rate	Alt		–0·36 (–0·72 to 0)	0·70

Hospital, age_scaled	Excess mortality rate	Alt		0·13 (–0·03 to 0·28)	1·14
Hospital, male	Excess mortality rate	Alt		–0·05 (–0·26 to 0·17)	0·95

Figure 2a: Age-scaled spline for adjustment of all stroke to ischaemic stroke incidence data

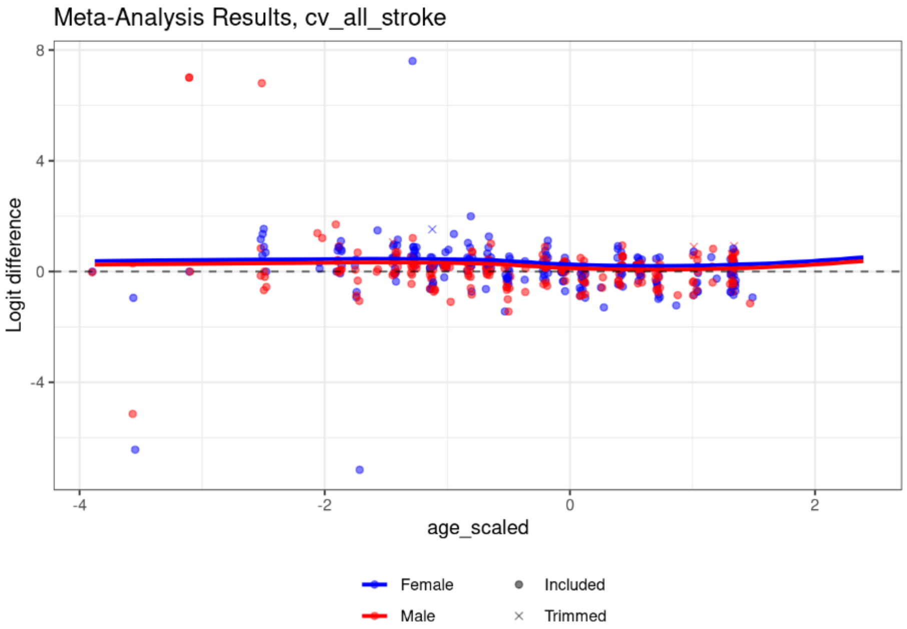

Figure 2b: Age-scaled spline for adjustment of hospital-only ischaemic stroke incidence data

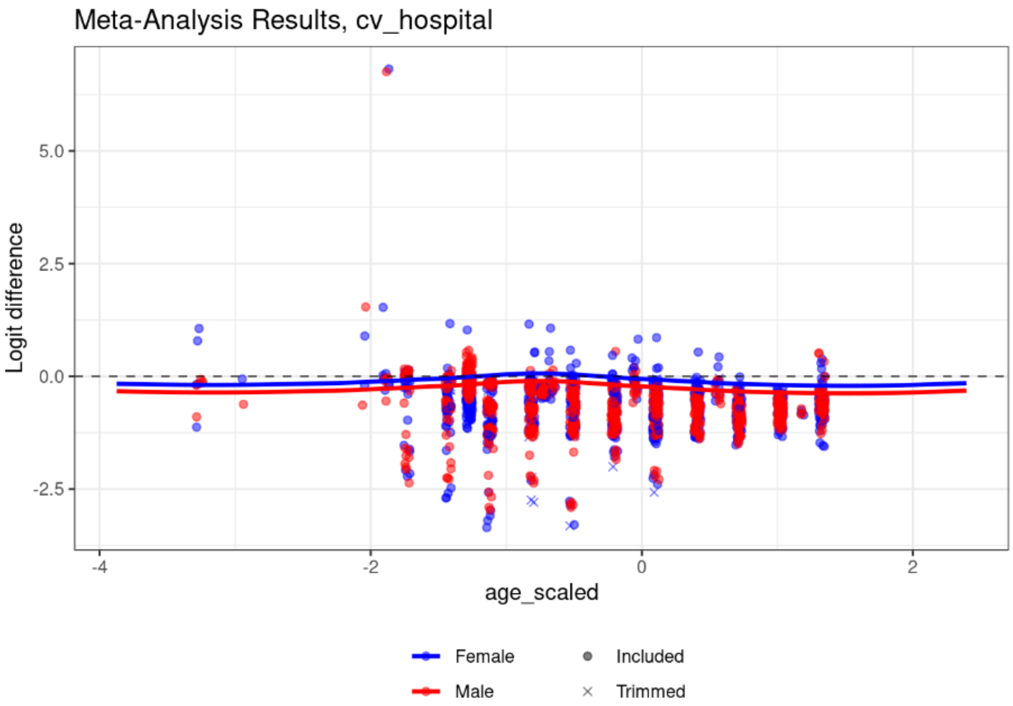

Figure 2c: Age-scaled spline for adjustment of inpatient clinical informatics ischaemic stroke incidence data

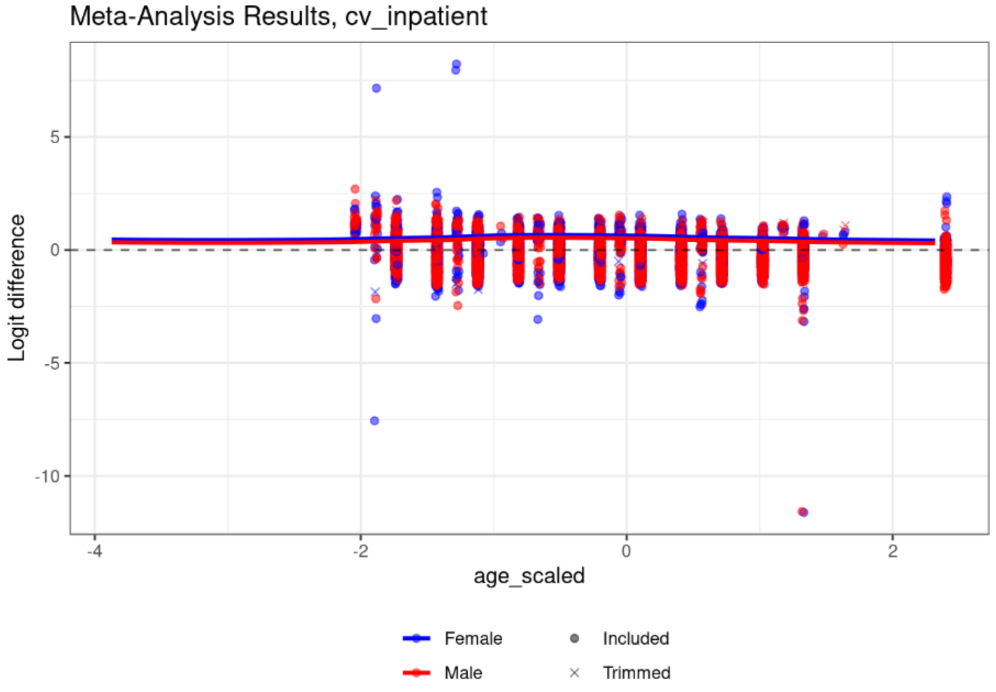

Figure 2d: Age-scaled spline for adjustment of first-ever and subsequent ischaemic stroke incidence data

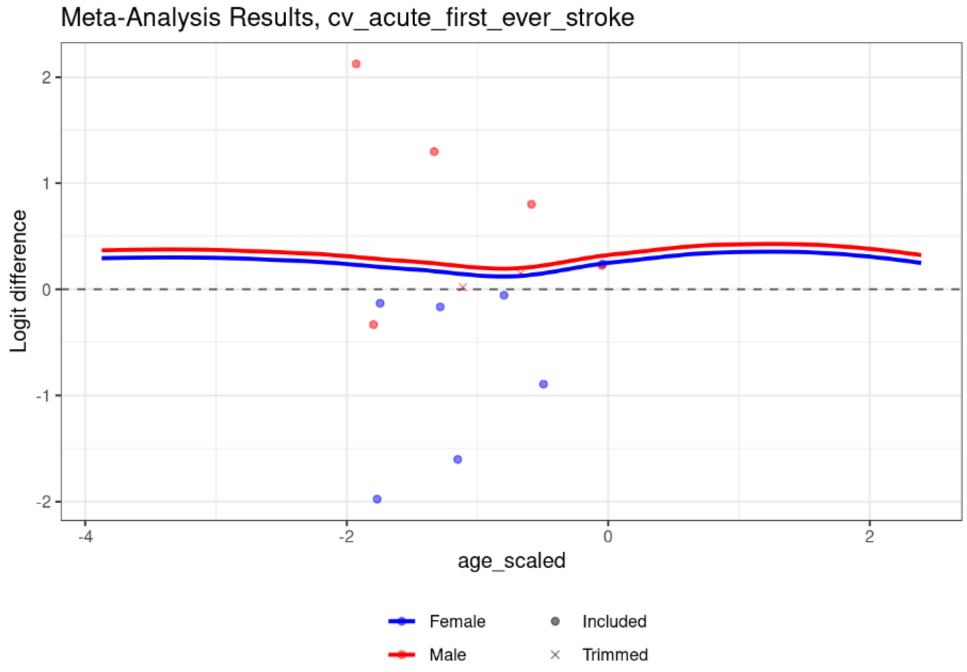

Table 4b: MR-BRT crosswalk adjustment factors for intracerebral haemorrhage

Data input	Measure	Reference or alternate case definition	Gamma	Beta coefficient, logit (95% CI)	**Adjustment factor
First-ever, subtype-specific, fatal and nonfatal events	Incidence	Ref	---	---	---
Acute first-ever stroke, intercept	Incidence	Alt	0.12	–0·03 (–0·22 to 0·16)	0.97
Acute first-ever stroke, spline_0	Incidence	Alt		0·04 (0 to 0·08)	1.04
Acute first-ever stroke, spline_1	Incidence	Alt		0·02 (–0·1 to 0·14)	1.02
Acute first-ever stroke, ages spline_2	Incidence	Alt		0·18 (0·12 to 0·24)	1.20
Acute first-ever stroke, ages spline_3	Incidence	Alt		0·13 (0·08 to 0·18)	1.14
Acute first-ever stroke, Male	Incidence	Alt		0·1 (0·07 to 0·14)	1.11
All stroke, intercept	Incidence	Alt		1·96 (1·81 to 2·11)	7·12
All stroke, spline_0	Incidence	Alt		0·05 (–0·01 to 0·1)	1.05
All stroke, spline_1	Incidence	Alt		0·11 (–0·05 to 0·27)	1.12
All stroke, ages spline_2	Incidence	Alt		0·03 (–0·05 to 0·12)	1.03
All stroke, ages spline_3	Incidence	Alt		0·15 (0·08 to 0·22)	1.16
All stroke, male	Incidence	Alt		–0·24 (–0·28 to –0·21)	0.78
Hospital, intercept	Incidence	Alt		–0·08 (–0·29 to 0·13)	0.93
Hospital, spline_0	Incidence	Alt		–0·09 (–0·13 to –0·05)	0.91
Hospital, spline_1	Incidence	Alt		0·19 (0·1 to 0·29)	1.21
Hospital, ages spline_2	Incidence	Alt		–0·01 (–0·06 to 0·04)	0.99
Hospital, ages spline_3	Incidence	Alt		–0·04 (–0·08 to 0)	0.96
Hospital, male	Incidence	Alt		0·01 (–0·02 to 0·04)	1.01
Inpatient clinical informatics, intercept	Incidence	Alt		0·94 (0·8 to 1·08)	2.56

Inpatient clinical informatics, spline_0	Incidence	Alt		–0·04 (–0·06 to –0·02)	0.96
Inpatient clinical informatics, spline_1	Incidence	Alt		0·08 (0·02 to 0·14)	1.08
Inpatient clinical informatics, ages spline_2	Incidence	Alt		–0·07 (–0·1 to –0·04)	0.93
Inpatient clinical informatics, ages spline_3	Incidence	Alt		–0·15 (–0·18 to –0·13)	0.86
Inpatient clinical informatics, male	Incidence	Alt		0 (–0·01 to 0·02)	1.0
USA claims data, male	Incidence	Alt		–0·01 (–0·03 to 0·01)	0.99
USA claims data, spline_0	Incidence	Alt		–0·11 (–0·13 to –0·08)	0.90
USA claims data, spline_1	Incidence	Alt		0·35 (0·27 to 0·43)	1.42
USA claims data, ages spline_2	Incidence	Alt		0·14 (0·1 to 0·18)	1.15
USA claims data, ages spline_3	Incidence	Alt		0·05 (0·01 to 0·08)	1.05
USA claims data, intercept	Incidence	Alt		1·63 (1·44 to 1·82)	5.10
Acute first-ever stroke, intercept	Excess mortality rate	Alt		0·33 (0·11 to 0·55)	1.39
Acute first-ever stroke, age_scaled	Excess mortality rate	Alt		–0·36 (–0·38 to –0·33)	0.70
Acute first-ever stroke, male	Excess mortality rate	Alt		–0·05 (–0·08 to –0·03)	0.95
All stroke, intercept	Excess mortality rate	Alt		–0·52 (–0·75 to –0·29)	0.59
All stroke, age_scaled	Excess mortality rate	Alt		0·22 (0·19 to 0·26)	1.25
All stroke, male	Excess mortality rate	Alt		–0·11 (–0·14 to –0·07)	0.90

Figure 2e: Age-scaled spline for adjustment of all stroke to intracerebral haemorrhage incidence data

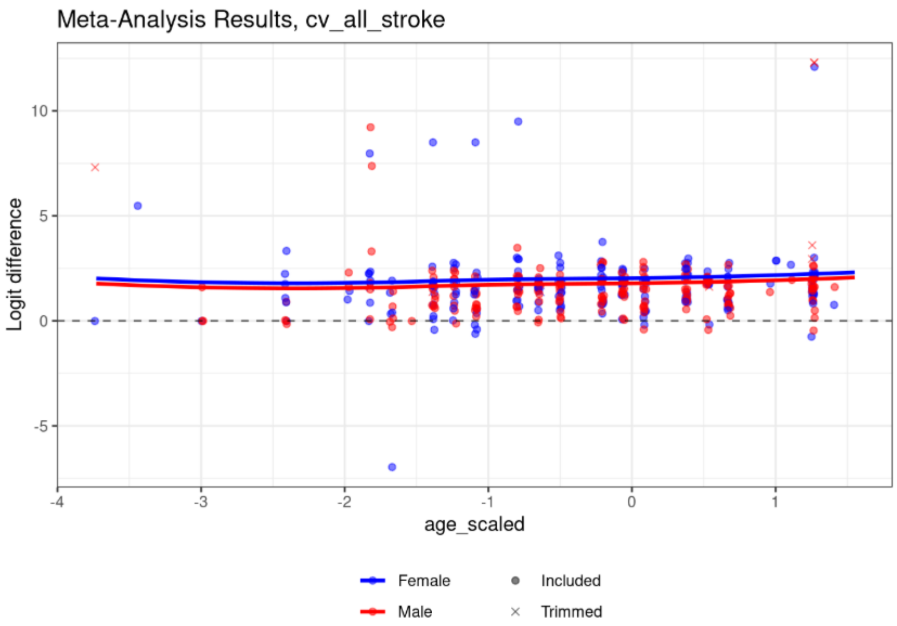

Figure 2f: Age-scaled spline for adjustment of hospital-only intracerebral haemorrhage incidence data

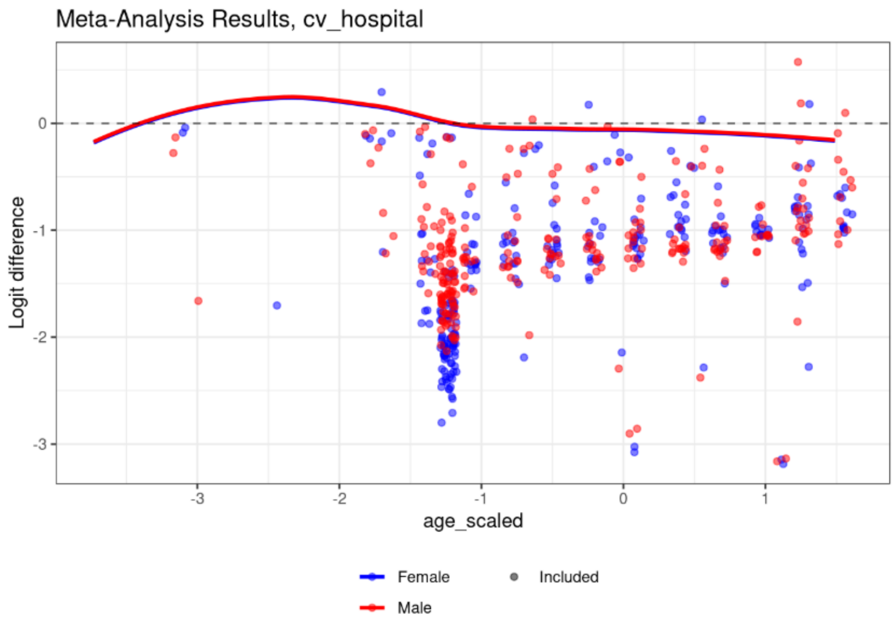

Figure 2g: Age-scaled spline for adjustment of inpatient clinical informatics intracerebral haemorrhage incidence data

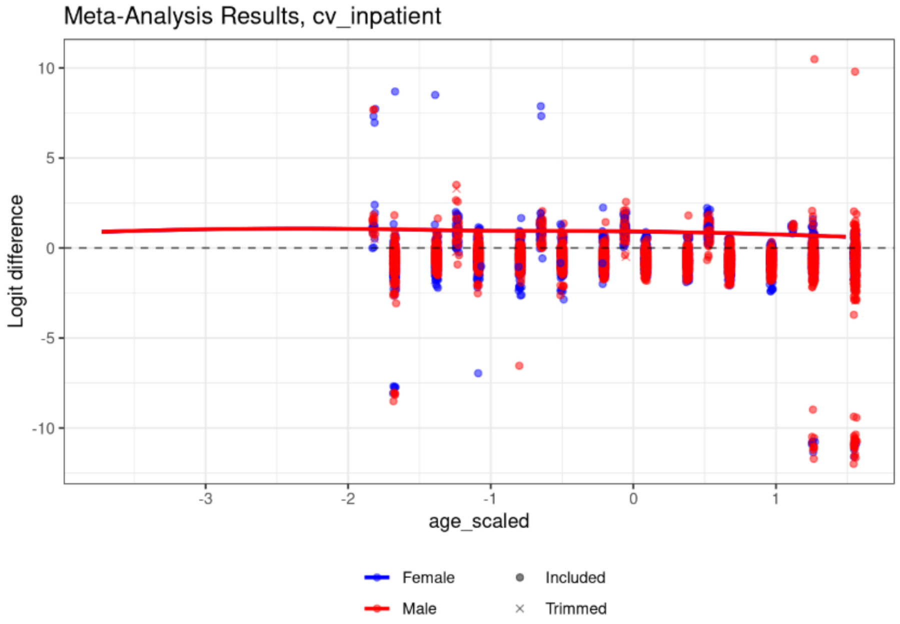

Figure 2h: Age-scaled spline for adjustment of first-ever and subsequent intracerebral haemorrhage incidence data

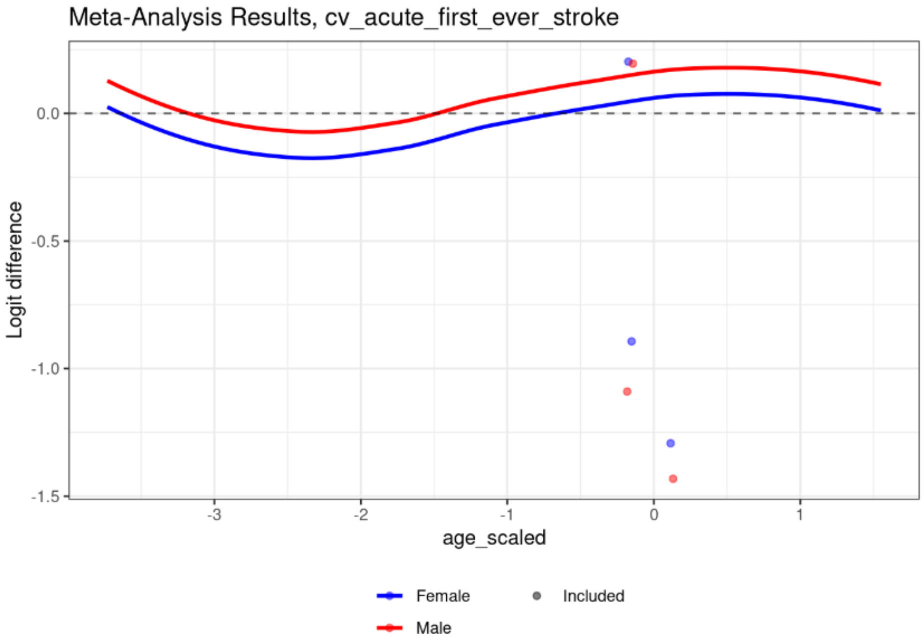

Table 4c: MR-BRT crosswalk adjustment factors for subarachnoid haemorrhage

Data input	Measure	Reference or alternate case definition	Gamma	Beta coefficient, logit (95% CI)	**Adjustment factor
First-ever, subtype-specific, fatal and nonfatal events	Incidence	Ref	---	---	
Aneurysmal subarachnoid haemorrhage only, intercept	Incidence	Alt	0.10	–0·12 (–0·22 to –0·01)	0.89
Aneurysmal subarachnoid haemorrhage only, age_scaled	Incidence	Alt		–0·09 (–0·11 to –0·07)	0.92
Aneurysmal subarachnoid haemorrhage only, male	Incidence	Alt		–0·07 (–0·1 to –0·04)	0.93
Acute first-ever stroke, intercept	Incidence	Alt		0·04 (–0·1 to 0·18)	1.04
Acute first-ever stroke, age_scaled	Incidence	Alt		0·05 (0·02 to 0·07)	1.05
Acute first-ever stroke, male	Incidence	Alt		0·02 (–0·01 to 0·06)	1.02
Inpatient clinical informatics, intercept	Incidence	Alt		1·24 (1·11 to 1·37)	3.46
Inpatient clinical informatics, age_scaled	Incidence	Alt		0·05 (0·05 to 0·06)	1.06
Inpatient clinical informatics, male	Incidence	Alt		0 (–0·02 to 0·01)	1.00
Hospital, intercept	Incidence	Alt		–0·14 (–0·28 to –0·01)	0.87
Hospital, age_scaled	Incidence	Alt		–0·11 (–0·12 to –0·1)	0.90
Hospital, male	Incidence	Alt		0·01 (–0·01 to 0·02)	1.01
USA Claims data, intercept	Incidence	Alt		2·25 (2·1 to 2·4)	9.47
USA Claims data, age_scaled	Incidence	Alt		0·61 (0·6 to 0·62)	1.83
USA Claims data, male	Incidence	Alt		0·19 (0·18 to 0·21)	1.22

Severity split inputs

The table below illustrates the severity level, lay description, and disability weights for GBD 2023. In previous iterations of GBD, severity splits for stroke were based on the standard approach described elsewhere (3). For GBD 2016, we undertook a review to identify epidemiological literature which reported the degree of disability at 28 days (for acute stroke) or one year (for chronic stroke) using the

modified Rankin scale (mRS) and the Mini-Mental State Examination (MMSE) or the Montreal Cognitive Assessment (MoCA). The mRS assesses functional capabilities, while the MMSE and MoCA tests provide evaluations of cognitive functioning. We then mapped these measures to the existing GBD categories as indicated in table 5a below. This approach allowed us to include location-specific information and can be updated as more data on functional or cognitive status become available.

We used established cut-offs[3] of the mRS to determine three levels of physical limitation; asymptomatic stroke corresponds to an mRS of 0, mild physical limitations correspond to an mRS of 1 or 2, moderate physical limitations correspond to an mRS of 3, and severe physical limitations correspond to an mRS of 4 or 5. Within the moderate and severe levels of physical limitations we included categories for those with and without cognitive impairment. This was defined by a MMSE score of less than 24 or an MoCA score of less than 26. In total, this creates six groups of stroke severity (asymptomatic, mild, moderate without cognitive impairment, moderate with cognitive impairment, severe without cognitive impairment, and severe with cognitive impairment). Within these six groups of severity, we further accounted for heart failure due to stroke (stress cardiomyopathy due to acute stroke) and dementia due to stroke as described below.

For GBD 2023, we updated the severity splits for both acute and chronic stroke subtypes to include sequelae leading to controlled, medically managed, mild, moderate, and severe heart failure. The process of estimating heart failure is described elsewhere in the appendix. We also included updates to our chronic stroke model with cognitive impairment to add sequelae of mild, moderate, and severe dementia. The process for estimating dementia is described elsewhere in the appendix. The GBD methods for estimating burden for heart failure and dementia produce estimates of their respective disease burden due to stroke.

For cases of heart failure due to stroke, this involves first estimating the amount of heart failure due to acute stroke and the amount persisting as chronic stroke subtypes. We then split the heart failure due to stroke cases into the stroke severity levels dependent on the mRS and MMSE and MoCA exams as shown in Table 5a and 5b below. We next split heart failure due to each stroke severity level into the four severity levels for heart failure (controlled, medically managed; mild; moderate; severe).

A similar process accounts for dementia due to stroke cases. In accounting for dementia due to stroke, we assume that dementia due to stroke is a subset of the moderate stroke with cognitive impairment and severe stroke with cognitive impairment and adjust only these categories. Unlike heart failure, the GBD estimation process does not produce dementia due to stroke by subtype. We first split the dementia due to stroke cases into stroke subtypes proportionally by the number of estimated chronic stroke subtype cases by age, sex, year, and location. We then assign the dementia due to stroke subtype cases to stroke severity levels 3 and 5 (moderate with cognitive impairment and severe with cognitive impairment, respectively) proportionally, with at least 10% of dementia due to stroke cases included in stroke severity level 3 and the remaining 90% of cases included in stroke severity level 5. The proportions of dementia due to stroke severity levels 3 and 5 were determined by expert opinion. If the cases arose that we had more estimated dementia due to severe stroke with cognitive impairment than we did severe stroke with cognitive impairment cases, the differential number of dementia due to severe stroke cases were added to the moderate stroke with cognitive impairment cases. Finally, the severe and moderate cases of stroke with dementia were further split into severity levels of dementia according to the severity splits described in the appendix section for dementia. Combined disability weights were then assigned to the severity levels of stroke with heart failure and dementia using the standard GBD method for combining disability weights; these are shown in tables 5a and 5b below.

Acute stroke severity splits

Table 5a. Severity distribution, details on the severity levels for acute stroke in GBD 2020 and the associated disability weight (DW) with that severity.

Severity level	Lay description	Modified Rankin score	Cognitive status	DW (95% CI)
Stroke, mild	Has some difficulty in moving around and some weakness in one hand but is able to walk without help.	1	N/A	0.019 (0.010 to 0.032)
Stroke, moderate, with no heart failure	Has some difficulty in moving around, and in using the hands for lifting and holding things, dressing, and grooming.	2, 3	MoCA ≥26 or MMSE ≥24	0.070 (0.046 to 0.099)
Stroke, moderate, with controlled, medically managed heart failure	Has some difficulty in moving around, and in using the hands for lifting and holding things, dressing, and grooming. Has been diagnosed with clinical heart failure, a chronic disease that requires medication every day and causes some worry but minimal interference with daily activities.	2, 3	MoCA ≥26 or MMSE ≥24	0.116 (0.076 to 0.164)
Stroke, moderate, with mild heart failure	Has some difficulty in moving around, and in using the hands for lifting and holding things, dressing, and grooming. Is short of breath and easily tires with moderate physical activity, such as walking uphill or more than a quarter-mile on level ground. The person feels comfortable at rest or during activities requiring less effort.	2, 3	MoCA ≥26 or MMSE ≥24	0.109 (0.074 to 0.154)
Stroke, moderate, with moderate heart failure	Has some difficulty in moving around, and in using the hands for lifting and holding things, dressing, and grooming. Is short of breath and easily tires with minimal physical activity, such as walking only a short distance. The person feels comfortable at rest but avoids moderate activity.	2, 3	MoCA ≥26 or MMSE ≥24	0.137 (0.091 to 0.191)
Stroke, moderate, with severe heart failure	Has some difficulty in moving around, and in using the hands for lifting and holding things, dressing, and	2, 3	MoCA ≥26 or MMSE ≥24	0.236 (0.165 to 0.319)

	grooming. Is short of breath and feels tired when at rest. The person avoids any physical activity, for fear of worsening the breathing problems.			
Stroke, moderate plus cognition problems, with no heart failure	Has some difficulty in moving around, in using the hands for lifting and holding things, dressing and grooming, and in speaking. The person is often forgetful and confused.	2, 3	MoCA <26 or MMSE <24	0.316 (0.206 to 0.437)
Stroke, moderate plus cognition problems, with controlled, medically managed heart failure	Has some difficulty in moving around, in using the hands for lifting and holding things, dressing and grooming, and in speaking. The person is often forgetful and confused. Has been diagnosed with clinical heart failure, a chronic disease that requires medication every day and causes some worry but minimal interference with daily activities.	2, 3	MoCA <26 or MMSE <24	0.349 (0.241 to 0.470)
Stroke, moderate plus cognition problems, with mild heart failure	Has some difficulty in moving around, in using the hands for lifting and holding things, dressing and grooming, and in speaking. The person is often forgetful and confused. Is short of breath and easily tires with moderate physical activity, such as walking uphill or more than a quarter-mile on level ground. The person feels comfortable at rest or during activities requiring less effort.	2, 3	MoCA <26 or MMSE <24	0.344 (0.237 to 0.464)
Stroke, moderate plus cognition problems, with moderate heart failure	Has some difficulty in moving around, in using the hands for lifting and holding things, dressing and grooming, and in speaking. The person is often forgetful and confused. Is short of breath and easily tires with minimal physical activity, such as walking only a short distance. The person feels comfortable at rest but avoids moderate activity.	2, 3	MoCA <26 or MMSE <24	0.365 (0.253 to 0.487)
Stroke, moderate plus cognition problems, with	Has some difficulty in moving around, in using the hands for lifting and holding things, dressing and	2, 3	MoCA <26 or MMSE <24	0.437 (0.308 to 0.575

severe heart failure	grooming, and in speaking. The person is often forgetful and confused. Is short of breath and feels tired when at rest. The person avoids any physical activity, for fear of worsening the breathing problems.			
Stroke, severe, with no heart failure	Is confined to bed or a wheelchair, has difficulty speaking, and depends on others for feeding, toileting, and dressing.	4, 5	MoCA ≥26 or MMSE ≥24	0.552 (0.377 to 0.707)
Stroke, severe, with controlled, medically managed heart failure	Is confined to bed or a wheelchair, has difficulty speaking, and depends on others for feeding, toileting, and dressing. Has been diagnosed with clinical heart failure, a chronic disease that requires medication every day and causes some worry but minimal interference with daily activities.	4, 5	MoCA ≥26 or MMSE ≥24	0.573 (0.408 to 0.720)
Stroke, severe, with mild heart failure	Is confined to bed or a wheelchair, has difficulty speaking, and depends on others for feeding, toileting, and dressing. Is short of breath and easily tires with moderate physical activity, such as walking uphill or more than a quarter-mile on level ground. The person feels comfortable at rest or during activities requiring less effort.	4, 5	MoCA ≥26 or MMSE ≥24	0.570 (0.404 to 0.720)
Stroke, severe, with moderate heart failure	Is confined to bed or a wheelchair, has difficulty speaking, and depends on others for feeding, toileting, and dressing. Is short of breath and easily tires with minimal physical activity, such as walking only a short distance. The person feels comfortable at rest but avoids moderate activity.	4, 5	MoCA ≥26 or MMSE ≥24	0.584 (0.417 to 0.732)
Stroke, severe, with severe heart failure	Is confined to bed or a wheelchair, has difficulty speaking, and depends on others for feeding, toileting, and dressing. Is short of breath and feels tired when at rest. The person avoids any physical activity, for fear of worsening the breathing problems.	4, 5	MoCA ≥26 or MMSE ≥24	0.630 (0.458 to 0.777)
Stroke, severe plus cognition problems, no heart failure	Is confined to bed or a wheelchair, depends on others for feeding, toileting, and dressing, and has	4,5	MoCA <26 or MMSE <24	0.588 (0.411 to 0.744)

	difficulty speaking, thinking clearly, and remembering things.			
Stroke, severe plus cognition problems, controlled, medically managed heart failure	Is confined to bed or a wheelchair, depends on others for feeding, toileting, and dressing, and has difficulty speaking, thinking clearly, and remembering things. Has been diagnosed with clinical heart failure, a chronic disease that requires medication every day and causes some worry but minimal interference with daily activities.	4,5	MoCA <26 or MMSE <24	0.608 (0.438 to 0.759)
Stroke, severe plus cognition problems, mild heart failure	Is confined to bed or a wheelchair, depends on others for feeding, toileting, and dressing, and has difficulty speaking, thinking clearly, and remembering things. Is short of breath and easily tires with moderate physical activity, such as walking uphill or more than a quarter-mile on level ground. The person feels comfortable at rest or during activities requiring less effort.	4,5	MoCA <26 or MMSE <24	0.604 (0.436 to 0.758)
Stroke, severe plus cognition problems, moderate heart failure	Is confined to bed or a wheelchair, depends on others for feeding, toileting, and dressing, and has difficulty speaking, thinking clearly, and remembering things. Is short of breath and easily tires with minimal physical activity, such as walking only a short distance. The person feels comfortable at rest but avoids moderate activity.	4,5	MoCA <26 or MMSE <24	0.617 (0.448 to 0.768)
Stroke, severe plus cognition problems, severe heart failure	Is confined to bed or a wheelchair, depends on others for feeding, toileting, and dressing, and has difficulty speaking, thinking clearly, and remembering things. Is short of breath and feels tired when at rest. The person avoids any physical activity, for fear of worsening the breathing problems.	4,5	MoCA <26 or MMSE <24	0.659 (0.488 to 0.808)

Chronic stroke severity splits

Table 5b. Severity distribution, details on the severity levels for chronic stroke in GBD 2020 and the associated disability weight (DW) with that severity

Severity level	Lay description	Modified Rankin score	Cognitive status	DW (95% CI)
Stroke, asymptomatic		0	N/A	N/A
Stroke, mild	Has some difficulty in moving around and some weakness in one hand but is able to walk without help.	1	N/A	0.019 (0.010 to 0.032
Stroke, moderate, with no heart failure	Has some difficulty in moving around, and in using the hands for lifting and holding things, dressing, and grooming.	2, 3	MoCA ≥26 or MMSE ≥24	0.070 (0.046 to 0.099
Stroke, moderate, with controlled, medically managed heart failure	Has some difficulty in moving around, and in using the hands for lifting and holding things, dressing, and grooming. Has been diagnosed with clinical heart failure, a chronic disease that requires medication every day and causes some worry but minimal interference with daily activities.	2, 3	MoCA ≥26 or MMSE ≥24	0.082 (0.053 to 0.118
Stroke, moderate, with mild heart failure	Has some difficulty in moving around, and in using the hands for lifting and holding things, dressing, and grooming. Is short of breath and easily tires with moderate physical activity, such as walking uphill or more than a quarter-mile on level ground. The person feels comfortable at rest or during activities requiring less effort.	2, 3	MoCA ≥26 or MMSE ≥24	0.108 (0.074 to 0.154
Stroke, moderate, with moderate heart failure	Has some difficulty in moving around, and in using the hands for lifting and holding things, dressing, and grooming. Is short of breath and easily tires with minimal physical activity, such as walking only a short distance. The person feels comfortable at rest but avoids moderate activity.	2, 3	MoCA ≥26 or MMSE ≥24	0.137 (0.091 to 0.191
Stroke, moderate, with severe heart failure	Has some difficulty in moving around, and in using the hands for lifting and holding things, dressing, and grooming. Is short of breath and feels tired when at rest. The person avoids any physical activity, for fear of worsening the breathing problems.	2, 3	MoCA ≥26 or MMSE ≥24	0.236 (0.165 to 0.319
Stroke, moderate plus cognition problems, with no	Has some difficulty in moving around, in using the hands for lifting and holding things, dressing and grooming,	2, 3	MoCA <26 or MMSE <24	0.316 (0.206 to 0.437

heart failure, with no dementia	and in speaking. The person is often forgetful and confused.			
Stroke, moderate plus cognition problems, with no heart failure, with mild dementia	Has some difficulty in moving around, in using the hands for lifting and holding things, dressing and grooming, and in speaking. The person is often forgetful and confused. The person has some trouble remembering recent events and finds it hard to concentrate and make decisions and plans. They may have slight to moderate difficulty engaging in community affairs, complicated hobbies, and intellectual interests.	2, 3	MoCA <26 or MMSE <24	0.134 (0.091 to 0.187
Stroke, moderate plus cognition problems, with no heart failure, with moderate dementia	Has some difficulty in moving around, in using the hands for lifting and holding things, dressing and grooming, and in speaking. The person is often forgetful and confused. The person retains highly learned material, but has severe memory problems, is disoriented with respect to time and sometimes place. They are severely impaired in their ability to handle problems and make social judgements. They require assistance with daily activities and only retain simple chores and hobbies.	2, 3	MoCA <26 or MMSE <24	0.420 (0.295 to 0.555
Stroke, moderate plus cognition problems, with no heart failure, with severe dementia	Has some difficulty in moving around, in using the hands for lifting and holding things, dressing and grooming, and in speaking. The person is often forgetful and confused. The person has complete memory loss, no longer recognizes close family members, and requires help with all daily activities, including personal care.	2, 3	MoCA <26 or MMSE <24	0.487 (0.345 to 0.628
Stroke, moderate plus cognition problems, with controlled, medically managed heart failure, with no dementia	Has some difficulty in moving around, in using the hands for lifting and holding things, dressing and grooming, and in speaking. The person is often forgetful and confused. Has been diagnosed with clinical heart failure, a chronic disease that requires medication every day and causes some worry but minimal interference with daily activities.	2, 3	MoCA <26 or MMSE <24	0.325 (0.219 to 0.443

Stroke, moderate plus cognition problems, with controlled, medically managed heart failure, with mild dementia	Has some difficulty in moving around, in using the hands for lifting and holding things, dressing and grooming, and in speaking. The person is often forgetful and confused. Has been diagnosed with clinical heart failure, a chronic disease that requires medication every day and causes some worry but minimal interference with daily activities. The person has some trouble remembering recent events and finds it hard to concentrate and make decisions and plans. They may have slight to moderate difficulty engaging in community affairs, complicated hobbies, and intellectual interests.	2, 3	MoCA <26 or MMSE <24	0.145 (0.098 to 0.207)
Stroke, moderate plus cognition problems, with asymptomatic heart failure, with moderate dementia	Has some difficulty in moving around, in using the hands for lifting and holding things, dressing and grooming, and in speaking. The person is often forgetful and confused. Has been diagnosed with clinical heart failure, a chronic disease that requires medication every day and causes some worry but minimal interference with daily activities. The person retains highly learned material, but has severe memory problems, is disoriented with respect to time and sometimes place. They are severely impaired in their ability to handle problems and make social judgements. They require assistance with daily activities and only retain simple chores and hobbies.	2, 3	MoCA <26 or MMSE <24	0.427 (0.305 to 0.561)
Stroke, moderate plus cognition problems, with asymptomatic heart failure, with severe dementia	Has some difficulty in moving around, in using the hands for lifting and holding things, dressing and grooming, and in speaking. The person is often forgetful and confused. Has been diagnosed with clinical heart failure, a chronic disease that requires medication every day and causes some worry but minimal interference with daily activities. The person has complete memory loss, no longer recognizes close family members, and requires help with all daily activities, including personal care.	2, 3	MoCA <26 or MMSE <24	0.493 (0.354 to 0.633)

Stroke, moderate plus cognition problems, with mild heart failure, with no dementia	Has some difficulty in moving around, in using the hands for lifting and holding things, dressing and grooming, and in speaking. The person is often forgetful and confused.	2, 3	MoCA <26 or MMSE <24	0.344 (0.237 to 0.464
Stroke, moderate plus cognition problems, with mild heart failure, with mild dementia	Has some difficulty in moving around, in using the hands for lifting and holding things, dressing and grooming, and in speaking. The person is often forgetful and confused. The person has some trouble remembering recent events and finds it hard to concentrate and make decisions and plans. They may have slight to moderate difficulty engaging in community affairs, complicated hobbies, and intellectual interests.	2, 3	MoCA <26 or MMSE <24	0.170 (0.117 to 0.238)
Stroke, moderate plus cognition problems, with mild heart failure, with moderate dementia	Has some difficulty in moving around, in using the hands for lifting and holding things, dressing and grooming, and in speaking. The person is often forgetful and confused. The person retains highly learned material, but has severe memory problems, is disoriented with respect to time and sometimes place. They are severely impaired in their ability to handle problems and make social judgements. They require assistance with daily activities and only retain simple chores and hobbies.	2, 3	MoCA <26 or MMSE <24	0.444 (0.320 to 0.577)
Stroke, moderate plus cognition problems, with mild heart failure, with severe dementia	Has some difficulty in moving around, in using the hands for lifting and holding things, dressing and grooming, and in speaking. The person is often forgetful and confused. The person has complete memory loss, no longer recognizes close family members, and requires help with all daily activities, including personal care.	2, 3	MoCA <26 or MMSE <24	0.508 (0.368 to 0.647)
Stroke, moderate plus cognition problems, with moderate heart failure, with no dementia	Has some difficulty in moving around, in using the hands for lifting and holding things, dressing and grooming, and in speaking. The person is often forgetful and confused. Is short of breath and easily tires with minimal physical activity, such as walking only a short distance. The person feels	2, 3	MoCA <26 or MMSE <24	0.365 (0.253 to 0.487

	comfortable at rest but avoids moderate activity.			
Stroke, moderate plus cognition problems, with moderate heart failure, with mild dementia	Has some difficulty in moving around, in using the hands for lifting and holding things, dressing and grooming, and in speaking. The person is often forgetful and confused. Is short of breath and easily tires with minimal physical activity, such as walking only a short distance. The person feels comfortable at rest but avoids moderate activity. The person has some trouble remembering recent events and finds it hard to concentrate and make decisions and plans. They may have slight to moderate difficulty engaging in community affairs, complicated hobbies, and intellectual interests.	2, 3	MoCA <26 or MMSE <24	0.196 (0.134 to 0.270)
Stroke, moderate plus cognition problems, with moderate heart failure, with moderate dementia	Has some difficulty in moving around, in using the hands for lifting and holding things, dressing and grooming, and in speaking. The person is often forgetful and confused. Is short of breath and easily tires with minimal physical activity, such as walking only a short distance. The person feels comfortable at rest but avoids moderate activity. The person retains highly learned material, but has severe memory problems, is disoriented with respect to time and sometimes place. They are severely impaired in their ability to handle problems and make social judgements. They require assistance with daily activities and only retain simple chores and hobbies.	2, 3	MoCA <26 or MMSE <24	0.461 (0.334 to 0.596)
Stroke, moderate plus cognition problems, with moderate heart failure, with severe dementia	Has some difficulty in moving around, in using the hands for lifting and holding things, dressing and grooming, and in speaking. The person is often forgetful and confused. Is short of breath and easily tires with minimal physical activity, such as walking only a short distance. The person feels comfortable at rest but avoids moderate activity. The person has complete memory loss, no longer recognizes close family members, and	2, 3	MoCA <26 or MMSE <24	0.523 (0.381 to 0.663)

	requires help with all daily activities, including personal care.			
Stroke, moderate plus cognition problems, with severe heart failure, with no dementia	Has some difficulty in moving around, in using the hands for lifting and holding things, dressing and grooming, and in speaking. The person is often forgetful and confused. Is short of breath and feels tired when at rest. The person avoids any physical activity, for fear of worsening the breathing problems.	2, 3	MoCA <26 or MMSE <24	0.437 (0.308 to 0.575
Stroke, moderate plus cognition problems, with severe heart failure, with mild dementia	Has some difficulty in moving around, in using the hands for lifting and holding things, dressing and grooming, and in speaking. The person is often forgetful and confused. Is short of breath and feels tired when at rest. The person avoids any physical activity, for fear of worsening the breathing problems. The person has some trouble remembering recent events and finds it hard to concentrate and make decisions and plans. They may have slight to moderate difficulty engaging in community affairs, complicated hobbies, and intellectual interests.	2, 3	MoCA <26 or MMSE <24	0.289 (0.206 to 0.381)
Stroke, moderate plus cognition problems, with severe heart failure, with moderate dementia	Has some difficulty in moving around, in using the hands for lifting and holding things, dressing and grooming, and in speaking. The person is often forgetful and confused. Is short of breath and feels tired when at rest. The person avoids any physical activity, for fear of worsening the breathing problems. The person retains highly learned material, but has severe memory problems, is disoriented with respect to time and sometimes place. They are severely impaired in their ability to handle problems and make social judgements. They require assistance with daily activities and only retain simple chores and hobbies.	2, 3	MoCA <26 or MMSE <24	0.522 (0.385 to 0.665)
Stroke, moderate plus cognition problems, with severe heart	Has some difficulty in moving around, in using the hands for lifting and holding things, dressing and grooming, and in speaking. The person is often forgetful and confused. Is short of	2, 3	MoCA <26 or MMSE <24	0.576 (0.428 to 0.721)

failure, with severe dementia	breath and feels tired when at rest. The person avoids any physical activity, for fear of worsening the breathing problems. The person has complete memory loss, no longer recognizes close family members, and requires help with all daily activities, including personal care.			
Stroke, severe, with no heart failure,	Is confined to bed or a wheelchair, has difficulty speaking, and depends on others for feeding, toileting, and dressing.	4, 5	MoCA ≥26 or MMSE ≥24	0.552 (0.377 to 0.707
Stroke, severe, with asymptomatic heart failure	Is confined to bed or a wheelchair, has difficulty speaking, and depends on others for feeding, toileting, and dressing. Has been diagnosed with clinical heart failure, a chronic disease that requires medication every day and causes some worry but minimal interference with daily activities.	4, 5	MoCA ≥26 or MMSE ≥24	0.558 (0.389 to 0.711
Stroke, severe, with mild heart failure	Is confined to bed or a wheelchair, has difficulty speaking, and depends on others for feeding, toileting, and dressing. Is short of breath and easily tires with moderate physical activity, such as walking uphill or more than a quarter-mile on level ground. The person feels comfortable at rest or during activities requiring less effort.	4, 5	MoCA ≥26 or MMSE ≥24	0.570 (0.403 to 0.72
Stroke, severe, with moderate heart failure	Is confined to bed or a wheelchair, has difficulty speaking, and depends on others for feeding, toileting, and dressing. Is short of breath and easily tires with minimal physical activity, such as walking only a short distance. The person feels comfortable at rest but avoids moderate activity.	4, 5	MoCA ≥26 or MMSE ≥24	0.584 (0.417 to 0.732
Stroke, severe, with severe heart failure	Is confined to bed or a wheelchair, has difficulty speaking, and depends on others for feeding, toileting, and dressing. Is short of breath and feels tired when at rest. The person avoids any physical activity, for fear of worsening the breathing problems.	4, 5	MoCA ≥26 or MMSE ≥24	0.630 (0.458 to 0.777
Stroke, severe plus cognition problems, no	Is confined to bed or a wheelchair, depends on others for feeding, toileting, and dressing, and has	4,5	MoCA <26 or MMSE <24	0.588 (0.411 to 0.744

heart failure, with no dementia.	difficulty speaking, thinking clearly, and remembering things.			
Stroke, severe plus cognition problems, no heart failure, with mild dementia.	Is confined to bed or a wheelchair, depends on others for feeding, toileting, and dressing, and has difficulty speaking, thinking clearly, and remembering things. The person has some trouble remembering recent events and finds it hard to concentrate and make decisions and plans. They may have slight to moderate difficulty engaging in community affairs, complicated hobbies, and intellectual interests.	4,5	MoCA <26 or MMSE <24	0.134 (0.091 to 0.187
Stroke, severe plus cognition problems, no heart failure, with moderate dementia.	Is confined to bed or a wheelchair, depends on others for feeding, toileting, and dressing, and has difficulty speaking, thinking clearly, and remembering things. The person retains highly learned material, but has severe memory problems, is disoriented with respect to time and sometimes place. They are severely impaired in their ability to handle problems and make social judgements. They require assistance with daily activities and only retain simple chores and hobbies.	4,5	MoCA <26 or MMSE <24	0.420 (0.295 to 0.555
Stroke, severe plus cognition problems, no heart failure, with severe dementia.	Is confined to bed or a wheelchair, depends on others for feeding, toileting, and dressing, and has difficulty speaking, thinking clearly, and remembering things. The person has complete memory loss, no longer recognizes close family members, and requires help with all daily activities, including personal care.	4,5	MoCA <26 or MMSE <24	0.487 (0.345 to 0.628
Stroke, severe plus cognition problems, asymptomatic heart failure, with no dementia	Is confined to bed or a wheelchair, depends on others for feeding, toileting, and dressing, and has difficulty speaking, thinking clearly, and remembering things. Has been diagnosed with clinical heart failure, a chronic disease that requires medication every day and causes some worry but minimal interference with daily activities.	4,5	MoCA <26 or MMSE <24	0.593 (0.421 to 0.747

Stroke, severe plus cognition problems, asymptomatic heart failure, with mild dementia	Is confined to bed or a wheelchair, depends on others for feeding, toileting, and dressing, and has difficulty speaking, thinking clearly, and remembering things. Has been diagnosed with clinical heart failure, a chronic disease that requires medication every day and causes some worry but minimal interference with daily activities. The person has some trouble remembering recent events and finds it hard to concentrate and make decisions and plans. They may have slight to moderate difficulty engaging in community affairs, complicated hobbies, and intellectual interests.	4,5	MoCA <26 or MMSE <24	0.588 (0.425 to 0.734)
Stroke, severe plus cognition problems, asymptomatic heart failure, with moderate dementia	Is confined to bed or a wheelchair, depends on others for feeding, toileting, and dressing, and has difficulty speaking, thinking clearly, and remembering things. Has been diagnosed with clinical heart failure, a chronic disease that requires medication every day and causes some worry but minimal interference with daily activities. The person retains highly learned material, but has severe memory problems, is disoriented with respect to time and sometimes place. They are severely impaired in their ability to handle problems and make social judgements. They require assistance with daily activities and only retain simple chores and hobbies.	4,5	MoCA <26 or MMSE <24	0.719 (0.540 to 0.856)
Stroke, severe plus cognition problems, asymptomatic heart failure, with severe dementia	Is confined to bed or a wheelchair, depends on others for feeding, toileting, and dressing, and has difficulty speaking, thinking clearly, and remembering things. Has been diagnosed with clinical heart failure, a chronic disease that requires medication every day and causes some worry but minimal interference with daily activities. The person has complete memory loss, no longer recognizes close family members, and requires help with all daily activities, including personal care.	4,5	MoCA <26 or MMSE <24	0.750 (0.578 to 0.882)

Stroke, severe plus cognition problems, mild heart failure, with no dementia	Is confined to bed or a wheelchair, depends on others for feeding, toileting, and dressing, and has difficulty speaking, thinking clearly, and remembering things. Is short of breath and easily tires with moderate physical activity, such as walking uphill or more than a quarter-mile on level ground. The person feels comfortable at rest or during activities requiring less effort.	4,5	MoCA <26 or MMSE <24	0.605 (0.436 to 0.758
Stroke, severe plus cognition problems, mild heart failure, with mild dementia	Is confined to bed or a wheelchair, depends on others for feeding, toileting, and dressing, and has difficulty speaking, thinking clearly, and remembering things. Is short of breath and easily tires with moderate physical activity, such as walking uphill or more than a quarter-mile on level ground. The person feels comfortable at rest or during activities requiring less effort. The person has some trouble remembering recent events and finds it hard to concentrate and make decisions and plans. They may have slight to moderate difficulty engaging in community affairs, complicated hobbies, and intellectual interests.	4,5	MoCA <26 or MMSE <24	0.600 (0.439 to 0.745)
Stroke, severe plus cognition problems, mild heart failure, with moderate dementia	Is confined to bed or a wheelchair, depends on others for feeding, toileting, and dressing, and has difficulty speaking, thinking clearly, and remembering things. Is short of breath and easily tires with moderate physical activity, such as walking uphill or more than a quarter-mile on level ground. The person feels comfortable at rest or during activities requiring less effort. The person retains highly learned material, but has severe memory problems, is disoriented with respect to time and sometimes place. They are severely impaired in their ability to handle problems and make social judgements. They require assistance with daily activities and only retain simple chores and hobbies.	4,5	MoCA <26 or MMSE <24	0.727 (0.553 to 0.861)

Stroke, severe plus cognition problems, mild heart failure, with severe dementia	Is confined to bed or a wheelchair, depends on others for feeding, toileting, and dressing, and has difficulty speaking, thinking clearly, and remembering things. Is short of breath and easily tires with moderate physical activity, such as walking uphill or more than a quarter-mile on level ground. The person feels comfortable at rest or during activities requiring less effort. The person has complete memory loss, no longer recognizes close family members, and requires help with all daily activities, including personal care.	4,5	MoCA <26 or MMSE <24	0.757 (0.589 to 0.886)
Stroke, severe plus cognition problems, moderate heart failure, with no dementia	Is confined to bed or a wheelchair, depends on others for feeding, toileting, and dressing, and has difficulty speaking, thinking clearly, and remembering things. Is short of breath and easily tires with minimal physical activity, such as walking only a short distance. The person feels comfortable at rest but avoids moderate activity.	4,5	MoCA <26 or MMSE <24	0.617 (0.448 to 0.768
Stroke, severe plus cognition problems, moderate heart failure, with mild dementia	Is confined to bed or a wheelchair, depends on others for feeding, toileting, and dressing, and has difficulty speaking, thinking clearly, and remembering things. Is short of breath and easily tires with minimal physical activity, such as walking only a short distance. The person feels comfortable at rest but avoids moderate activity. The person has some trouble remembering recent events and finds it hard to concentrate and make decisions and plans. They may have slight to moderate difficulty engaging in community affairs, complicated hobbies, and intellectual interests.	4,5	MoCA <26 or MMSE <24	0.612 (0.450 to 0.756)
Stroke, severe plus cognition problems, moderate heart failure, with moderate dementia	Is confined to bed or a wheelchair, depends on others for feeding, toileting, and dressing, and has difficulty speaking, thinking clearly, and remembering things. Is short of breath and easily tires with minimal physical activity, such as walking only a short distance. The person feels comfortable at rest but avoids moderate activity.	4,5	MoCA <26 or MMSE <24	0.735 (0.562 to 0.868)

	The person retains highly learned material, but has severe memory problems, is disoriented with respect to time and sometimes place. They are severely impaired in their ability to handle problems and make social judgements. They require assistance with daily activities and only retain simple chores and hobbies.			
Stroke, severe plus cognition problems, moderate heart failure, with severe dementia	Is confined to bed or a wheelchair, depends on others for feeding, toileting, and dressing, and has difficulty speaking, thinking clearly, and remembering things. Is short of breath and easily tires with minimal physical activity, such as walking only a short distance. The person feels comfortable at rest but avoids moderate activity. The person has complete memory loss, no longer recognizes close family members, and requires help with all daily activities, including personal care.	4,5	MoCA <26 or MMSE <24	0.764 (0.596 to 0.892)
Stroke, severe plus cognition problems, severe heart failure, with no dementia	Is confined to bed or a wheelchair, depends on others for feeding, toileting, and dressing, and has difficulty speaking, thinking clearly, and remembering things. Is short of breath and feels tired when at rest. The person avoids any physical activity, for fear of worsening the breathing problems.	4,5	MoCA <26 or MMSE <24	0.659 (0.489 to 0.808
Stroke, severe plus cognition problems, severe heart failure, with mild dementia	Is confined to bed or a wheelchair, depends on others for feeding, toileting, and dressing, and has difficulty speaking, thinking clearly, and remembering things. Is short of breath and feels tired when at rest. The person avoids any physical activity, for fear of worsening the breathing problems. The person has some trouble remembering recent events and finds it hard to concentrate and make decisions and plans. They may have slight to moderate difficulty engaging in community affairs, complicated hobbies, and intellectual interests.	4,5	MoCA <26 or MMSE <24	0.655 (0.489 to 0.794)
Stroke, severe plus cognition problems, severe	Is confined to bed or a wheelchair, depends on others for feeding, toileting, and dressing, and has	4,5	MoCA <26 or MMSE <24	0.764 (0.593 to 0.890)

heart failure, with moderate dementia	difficulty speaking, thinking clearly, and remembering things. Is short of breath and feels tired when at rest. The person avoids any physical activity, for fear of worsening the breathing problems. The person retains highly learned material, but has severe memory problems, is disoriented with respect to time and sometimes place. They are severely impaired in their ability to handle problems and make social judgements. They require assistance with daily activities and only retain simple chores and hobbies.			
Stroke, severe plus cognition problems, severe heart failure, with severe dementia	Is confined to bed or a wheelchair, depends on others for feeding, toileting, and dressing, and has difficulty speaking, thinking clearly, and remembering things. Is short of breath and feels tired when at rest. The person avoids any physical activity, for fear of worsening the breathing problems. The person has complete memory loss, no longer recognizes close family members, and requires help with all daily activities, including personal care.	4,5	MoCA <26 or MMSE <24	0.790 (0.626 to 0.910)

Table 6: Data input counts for the estimation process for the custom severity splits.

	**Acute proportion**	**Chronic proportion**
**Site-years (total)**	9	16
**Number of countries with data**	6	13
**Number of GBD regions with data (out of 21 regions)**	6	7
**Number of GBD super-regions with data (out of 7 super-regions)**	4	5

The model to split stroke into the six initial severity splits was last updated in GBD 2017. We used DisMod-MR, a Bayesian meta-regression tool, to model the six severity levels, with an independent proportion model for each. The data we used to inform these splits is summarised in table 6. Reports which grouped mRS scores differently than our mapping (eg, 0-2) were adjusted in DisMod by estimating the association between these alternate groupings and our preferred mappings. These statistical associations were used to adjust datapoints to the referent category as necessary. The six models were scaled such that the sum of the proportions for all levels equaled 1.

Modelling strategy

The general approach employed for all the components of the stroke modelling process is detailed in the bullet points below.

- Datapoints were adjusted from alternative to reference case definitions using estimates from statistical models generated by MR-BRT (discussed elsewhere in the appendix) for the acute models. Coefficients for these crosswalks can be found in Table 2a, 2b, and 2c.

- The GBD summary exposure values (SEV), which are the relative risk-weighted prevalence of exposure, were included as covariates for the ischaemic stroke or intracerebral haemorrhage models as appropriate, and a covariate for country income was used as a country-level covariate for both models (4). Subarachnoid haemorrhage did not include an SEV covariate, but did include a covariate for country income for excess mortality. Coefficients for these covariates can be found in Table 7a, 7b, 7c for fixed effects located below.

- We used the ratio of acute:chronic cause-specific mortality estimated in DisMod-MR models without cause-specific mortality rate to provide estimates to divide GBD 2023 CoDCorrected stroke deaths into acute and chronic stroke deaths, using the global average for the proportion of acute:chronic stroke mortality. The acute and chronic models were then run using the same incidence, prevalence, and case-fatality data as well as the custom cause-specific mortality rates as input data.

- We ran the first-ever acute subtype-specific models with CSMR as derived from CoDCorrect and epidemiological data as described above using DisMod-MR.

- We then calculated the rate of surviving until 28 days after an acute event for all three subtypes using the modelled estimates of excess mortality and incidence from the acute stroke models by age group, sex, year, and location. We then calculated the proportion of subtype-specific stroke survivors by age, sex, year, and location. These proportions were used to split the survey series input data on all stroke prevalence into the three subtypes to enable their use as input data into the chronic stroke DisMod models; Figure 3 shows an example for the United States National Health Interview Survey 2000-2001.

Figure 3: Example of all-stroke prevalence survey split into subtype-specific proportions using proportion of 28-day subtype-specific surviving cases

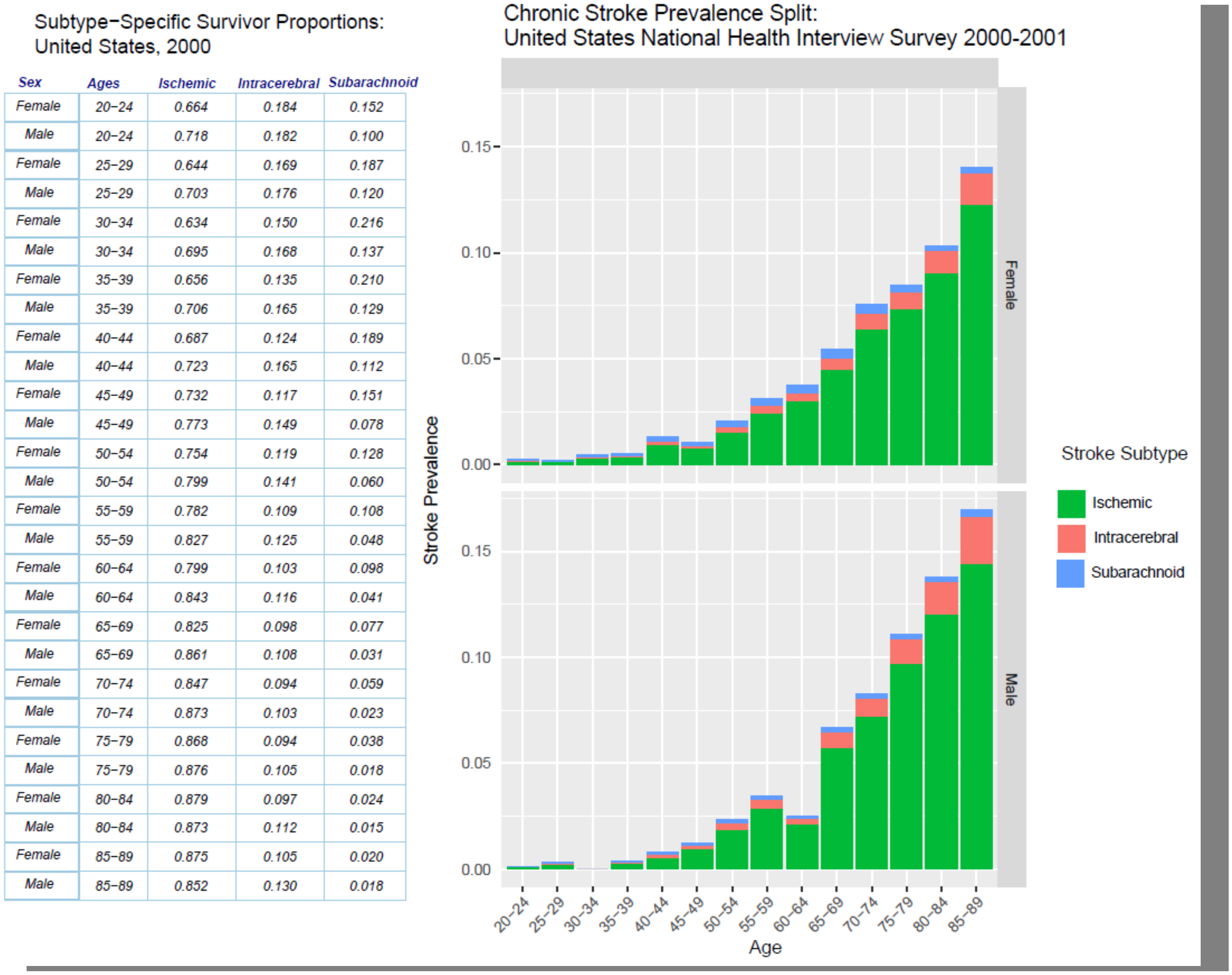

Subtype−Specific Survivor Proportions:
United States, 2000

Sex	Ages	Ischemic	Intracerebral	Subarachnoid
Female	20–24	0.664	0.184	0.152
Male	20–24	0.718	0.182	0.100
Female	25–29	0.644	0.169	0.187
Male	25–29	0.703	0.176	0.120
Female	30–34	0.634	0.150	0.216
Male	30–34	0.695	0.168	0.137
Female	35–39	0.656	0.135	0.210
Male	35–39	0.706	0.165	0.129
Female	40–44	0.687	0.124	0.189
Male	40–44	0.723	0.165	0.112
Female	45–49	0.732	0.117	0.151
Male	45–49	0.773	0.149	0.078
Female	50–54	0.754	0.119	0.128
Male	50–54	0.799	0.141	0.060
Female	55–59	0.782	0.109	0.108
Male	55–59	0.827	0.125	0.048
Female	60–64	0.799	0.103	0.098
Male	60–64	0.843	0.116	0.041
Female	65–69	0.825	0.098	0.077
Male	65–69	0.861	0.108	0.031
Female	70–74	0.847	0.094	0.059
Male	70–74	0.873	0.103	0.023
Female	75–79	0.868	0.094	0.038
Male	75–79	0.876	0.105	0.018
Female	80–84	0.879	0.097	0.024
Male	80–84	0.873	0.112	0.015
Female	85–89	0.875	0.105	0.020
Male	85–89	0.852	0.130	0.018

- Twenty-eight-day survivorship data and the post-split prevalence surveys were uploaded into the chronic subtype-specific with CSMR models. These chronic models also use CSMR as derived from CoDCorrect and epidemiological data as described above. Models were evaluated based on expert opinion, comparison with previous iterations, and model fit.

Tables 7a, 7b, 7c below indicate the covariates used by cause in the estimation process, as well as the beta and exponentiated beta values.

Table 7a: Coefficients for covariates used in the acute and chronic ischaemic stroke DisMod-MR models

Model	Variable name	Measure	Beta	Exponentiated beta
First-ever acute ischaemic stroke without CSMR	Log-transformed age-standardised SEV scalar: Ischaemic stroke	Incidence	0.78 (0.75 to 0.82)	2.18 (2.12 to 2.27)

First-ever acute ischaemic stroke without CSMR	LDI (I$ per capita)	Excess mortality rate	–0.44 (–0.48 to –0.40)	0.65 (0.62 to 0.67)
Chronic ischaemic stroke without CSMR	Log-transformed SEV scalar: Ischaemic stroke	Prevalence	0.76 (0.75 to 0.77)	2.13 (2.12 to 2.16)
Chronic ischaemic stroke without CSMR	LDI (I$ per capita)	Excess mortality rate	–0.13 (–0.17 to –0.1)	0.88 (0.84 to 0.90)
First-ever acute ischaemic stroke with CSMR	Log-transformed age-standardised SEV scalar: Ischaemic stroke	Incidence	0.87 (0.84 to 0.91)	2.40 (2.32 to 2.48)
First-ever acute ischaemic stroke with CSMR	Healthcare Access and Quality Index	Excess mortality rate	–0.015 (–0.016 to –0.014)	0.95 (0.93 to 0.96)
Chronic ischaemic stroke with CSMR	Log-transformed SEV scalar: Ischaemic stroke	Prevalence	0.76 (0.75 to 0.77)	2.13 (2.12 to 2.16)
Chronic ischaemic stroke with CSMR	Healthcare Access and Quality Index	Excess mortality rate	–0.23 (–0.31 to –0.11)	0.80 (0.73 to 0.90)

Table 7b: Coefficients for covariates used in the acute and chronic intracerebral haemorrhage DisMod-MR models

Model	Variable name	Measure	Beta	Exponentiated beta
First-ever acute intracerebral haemorrhage without CSMR	Log-transformed SEV scalar: Intracerebral Haemorrhage	Incidence	0.76 (0.75 to 0.79)	2.15 (2.12 to 2.21)
First-ever acute intracerebral haemorrhage without CSMR	LDI (I$ per capita)	Excess mortality rate	–0.46 (–0.50 to –0.42)	0.63 (0.61 to 0.66)
Chronic intracerebral haemorrhage without CSMR	Log-transformed SEV scalar: Intracerebral haemorrhage	Prevalence	0.77 (0.75 to 0.80)	2.16 (2.12 to 2.22)
Chronic intracerebral haemorrhage without CSMR	LDI (I$ per capita)	Excess mortality rate	–0.13 (–0.18 to –0.10)	0.88 (0.83 to 0.90)
First-ever acute intracerebral haemorrhage with CSMR	Log-transformed SEV scalar: Intracerebral Haemorrhage	Incidence	0.76 (0.75 to 0.77)	2.13 (2.12 to 2.15)
First-ever acute intracerebral haemorrhage with CSMR	Healthcare Access and Quality Index	Excess mortality rate	–0.77 (–0.83 to –0.71)	0.46 (0.44 to 0.49)
Chronic intracerebral haemorrhage with CSMR	Log-transformed SEV scalar: Intracerebral haemorrhage	Prevalence	0.75 (0.75 to 0.75)	2.12 (2.12 to 2.12)

Chronic intracerebral haemorrhage with CSMR	Healthcare Access and Quality Index	Excess mortality rate	–0.87 (–0.96 to –0.8)	0.42 (0.38 to 0.45)

Table 7c: Coefficients for covariates used in the acute and chronic subarachnoid DisMod-MR models

Model	Variable name	Measure	Beta	Exponentiated beta
First-ever acute subarachnoid haemorrhage without CSMR	Systolic blood pressure (mmHg)	Incidence	0.03 (0.02 to 0.03)	1.03 (1.02 to 1.03)
First-ever acute subarachnoid haemorrhage without CSMR	LDI (I$ per capita)	Excess mortality rate	–0.47 (–0.50 to –0.42)	0.63 (0.61 to 0.66)
Chronic subarachnoid haemorrhage without CSMR	LDI (I$ per capita)	Excess mortality rate	–0.14 (–0.21 to –0.1)	0.87 (0.81 to 0.90)
First-ever acute subarachnoid haemorrhage with CSMR	Log-transformed SEV scalar: Subarachnoid haemorrhage	Incidence	0.75 (0.75 to 0.75)	2.12 (2.12 to 2.12)
First-ever acute subarachnoid haemorrhage with CSMR	Healthcare Access and Quality Index	Excess mortality rate	–0.95 (–1 to –0.86)	0.39 (0.37 to 0.42)
Chronic subarachnoid haemorrhage with CSMR	Log-transformed SEV scalar: Subarachnoid haemorrhage	Prevalence	0.75 (0.75 to 0.75)	2.12 (2.12 to 2.12)
Chronic subarachnoid haemorrhage with CSMR	Healthcare Access and Quality Index	Excess mortality rate	–1 (–1 to –0.99)	0.37 (0.37 to 0.37)

References

[1] Viitanen M, Winblad B, Asplund K. Autopsy-verified causes of death after stroke. *Acta Med Scand*. 1987;**222**(5):401-8. doi: 10.1111/j.0954-6820.1987.tb10956.x. PMID: 3425392.

[2] Brønnum-Hansen H, Davidsen M, Thorvaldsen P; Danish MONICA Study Group. Long-term survival and causes of death after stroke. *Stroke*. 2001 Sep;32(9):2131-6. doi: 10.1161/hs0901.094253. PMID: 11546907.

[3] Saver JL, Chaisinanunkul N, Campbell BCV, Grotta JC, Hill MD, Khatri P, Landen J, Lansberg MG, Venkatasubramanian C, Albers GW; XIth Stroke Treatment Academic Industry Roundtable. Standardized Nomenclature for Modified Rankin Scale Global Disability Outcomes: Consensus Recommendations From Stroke Therapy Academic Industry Roundtable XI. *Stroke*. 2021 Aug;52(9):3054-3062. doi: 10.1161/STROKEAHA.121.034480. Epub 2021 Jul 29. PMID: 34320814.

Leprosy

Flowchart

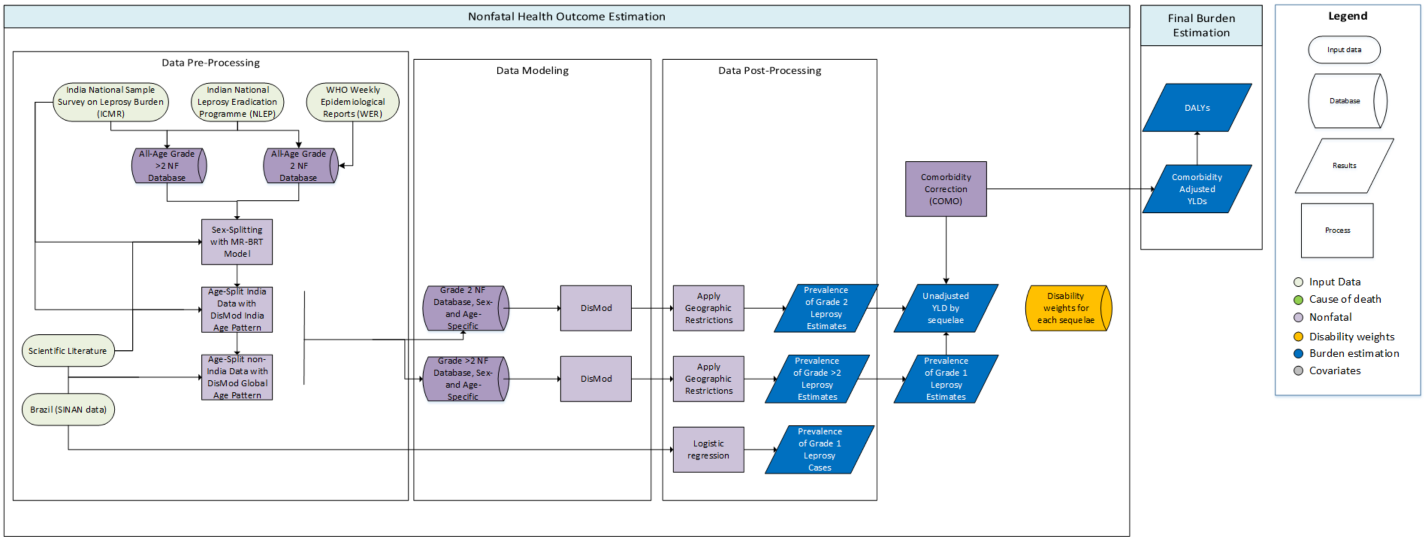

Input data and methodological summary for leprosy

Case definition

Leprosy is a chronic bacterial infection caused by *Mycobacterium leprae*, primarily affecting the nervous system, skin, respiratory tract, and eyes. Transmission occurs via contact with fluid from the nose and mouth of an infected individual. Leprosy can be diagnosed based on clinical manifestations, such as hypopigmented or reddish skin lesions with loss of sensations or thickening of the peripheral nerves accompanied by loss of sensation, and/or a positive skin smear for acid-fast bacilli. The ICD-10 code for leprosy is A30.9. We used the following case definition for GBD 2023:

Quantity of interest	Reference or alternative	Definition
Leprosy	Reference	An incident case of leprosy is defined as one identified through case notification or surveillance systems or via clinical diagnosis. Clinical diagnosis of leprosy can be based on clinical manifestations, such as hypopigmented or reddish skin lesion with loss of sensation, with or without involvement of peripheral nerves, and/or confirmation involving a skin-smear or biopsy.

Input data

We used five data sources inform estimates of leprosy incidence and prevalence by grade-classification:

(i) WHO Weekly Epidemiological Record reports disaggregated by Grade 2 and less than Grade 2 disability from 2000 to 2018. Data from 1990–2000 were not disaggregated by grade, and we hope to split them to use in future cycles.

(ii) Indian National Leprosy Eradication Programme subnational incidence data were used from 2010–2017.

(iii) The 2010–2011 India National Sample Survey on Leprosy Burden from the Indian Council of Medical Research (ICMR) prevalence data were used to estimate prevalence in India subnationals and to inform sex- and age-split models.

(iv) Data from Brazil's Information System for Notifiable Diseases (SINAN) informed the age-split models and the severity split model to disaggregate less than Grade 2 estimates into Grade 1 and Grade 0 estimates. These data were not used in the main prevalence models due to concerns that hospital-based reporting might over-represent prevalence at the subnational and national level.

(v) Relevant scientific literature was used to inform the sex- and age-split models.

Modelling strategy

Incidence

The non-fatal estimation process for leprosy began with nationally representative case notification data published by the World Health Organization (WHO) or Ministries of Health. The analysis was implemented in three steps: (1) data pre-processing, (2) data modelling, and (3) post-processing, which included applying geographical restrictions and quantification of sequela.

Sex-splitting

First, data reported in both sexes were split into male and female incidence inputs. To sex-split our both-sex datapoints, we used sex-specific inputs in a Bayesian meta-regression (MR-BRT) model to derive a ratio of female leprosy incidence to both leprosy incidence (using scientific literature data). The resultant log ratio was applied to both-sex datapoints to calculate out females, and males were calculated via subtraction. The result was a higher prevalence of leprosy among males as opposed to females and is consistent with published gender disparity in leprosy cases.[1-3]

Table 1: MR-BRT crosswalk adjustment factors for leprosy

Data input	Reference or alternative case definition	Gamma	Beta coefficient, log ratio (95% CI)*	Adjustment factor**
Female data	Ref	0.126	---	---
Both data	Alt		0.257 (–0.53 to 1.05)	1.28

**MR-BRT crosswalk adjustments can be interpreted as the factor the alternative case definition is adjusted by to reflect what it would have been had it been measured using the reference case definition. If the log/logit beta coefficient is negative, then the alternative is adjusted up to the reference. If the log/logit beta coefficient is positive, then the alternative is adjusted down to the reference.*

***The adjustment factor column is the exponentiated beta coefficient. For log beta coefficients, this is the relative rate between the two case definitions. For logit beta coefficients, this is the relative odds between the two case definitions.*

Age splitting

We then split all-age case data into age-specific observations using two age patterns derived from a DisMod Bayesian meta-regression model (DisMod-MR), one specific for India (derived using ICMR and Indian scientific literature) and another global age pattern for non-India locations (derived using SINAN and non-Indian scientific literature). Both age patterns were developed using single-parameter incidence models in DisMod-MR. Uncertainty is propagated throughout the sex- and age-splitting processes, such that final sex- and age-specific incidence estimates reflect uncertainty of the original data.

The age patterns can be found below:

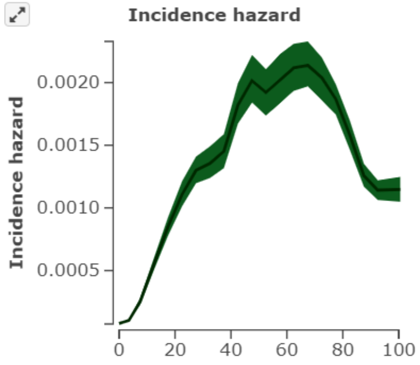

Figure 1a. Global age pattern for leprosy used to split non-India all-age data into age-specific datapoints for further modelling.

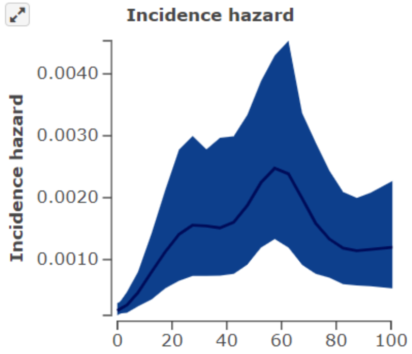

Figure 1b. India age pattern for leprosy used to split India all-age data into age-specific datapoints for further modelling.

Prevalence

We used a compartmental model in DisMod-MR to derive prevalence of leprosy from incident case reports. The reported case data were grade-specific, allowing us to implement two models, Grade <2 and Grade 2. In the Grade <2 leprosy model, we assumed no incident cases among children less than 2 years old and an average duration of 2 years, to account for the broad spectrum of disability associated with Grade 1 and the availability of treatment. In the Grade 2 model, we assumed no incident cases occurring among children less than 5 years old and assume no remission, as Grade 2 leprosy causes permanent disfigurement and/or disability.

Estimates of Grade <2 leprosy were disaggregated into Grade 1 and Grade 0 estimates using age- and sex-specific data reported by Brazil. Using these data, proportions of cases of Grade 1 and Grade 0 were calculated via logistic regression using a generalised estimating equation to account for repeated measures among the subjects in that cohort.

Table 2a. Covariates. Summary of covariates used in the leprosy DisMod-MR less than Grade 2 model

**Covariate**	**Type**	**Parameter**	**Exponentiated beta (95% UI)**
Latitude 15 to 30 (proportion)	Country-level	Prevalence	0.68 (0.64–0.71)
Healthcare Access and Quality Index	Country-level	Prevalence	0.08 (0.076–0.085)

Table 2b. Covariates. Summary of covariates used in the leprosy DisMod-MR Grade 2 model

**Covariate**	**Type**	**Parameter**	**Exponentiated beta (95% UI)**
Latitude 15 to 30 (proportion)	Country-level	Prevalence	2.80 (1.53–12.13)
Healthcare Access and Quality Index	Country-level	Prevalence	0.048 (0.011–0.067)

Geographical restrictions

Geographical restrictions were applied to generate zero estimates in countries for which transmission is not considered endemic. We do not account for imported cases of leprosy.

Table 3. Severity distribution, details on the severity levels for leprosy and the associated disability weight (DW) with that severity

**Severity level**	**Lay description**	**DW (95% CI)**
Disfigurement level 1 due to leprosy	Has a slight, visible physical deformity that others notice, which causes some worry and discomfort.	0.011 (0.005–0.021)
Disfigurement level 2 due to leprosy	Has a visible physical deformity that causes others to stare and comment. As a result, the person is worried and has trouble sleeping and concentrating.	0.067 (0.044–0.096)

We did not apply any adjustments for the COVID-19 pandemic to leprosy due to a lack of available data quantifying the impacts of the pandemic on NTD epidemiology.

Changes from GBD 2021 to GBD 2023

There were no substantive changes to the modelling strategy for GBD 2023.

References

1. Kumar R, Singhasivanon P, Sherchand JB, *et al.* Gender difference in socio-epidemiological factors for leprosy in the most hyper-endemic district of Nepal. *Nepal Med Coll J* 2004; 6: 98–105.
2. Peters ES, Eshiet AL. Male-female (sex) differences in leprosy patients in south eastern Nigeria: females present late for diagnosis and treatment and have higher rates of deformity. *Lepr Rev* 2002; 73: 262–7.
3. Ramos JM, Martínez-Martín M, Reyes F, Lemma D, Belinchón I, Gutiérrez F. Gender differential on characteristics and outcome of leprosy patients admitted to a long-term care rural hospital in South-Eastern Ethiopia. *Int J Equity Health* 2012; 11: 56.

Low back pain

Flowchart

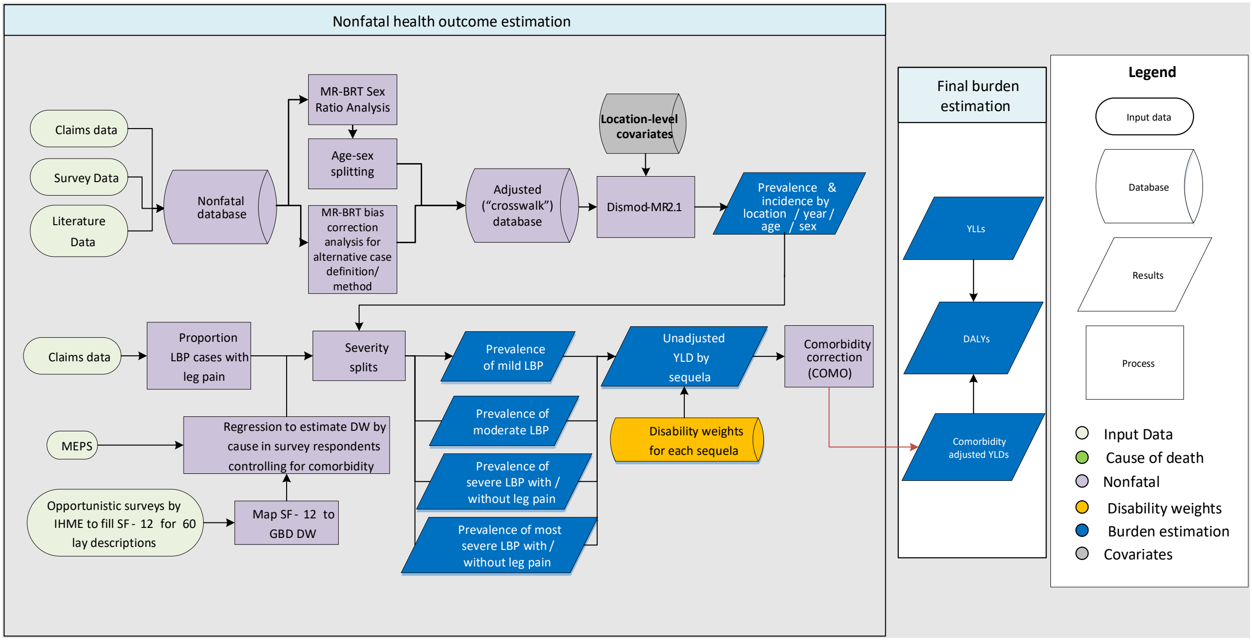

Input data and methodological summary for low back pain

Case definition

Low back pain (LBP) is defined as low back pain (with or without pain referred into one or both lower limbs) that lasts for at least one day. The "low back" is defined as the area on the posterior aspect of the body from the lower margin of the twelfth ribs to the lower gluteal folds.

The case definitions accepted for low back pain are shown below.

Reference or alternative	Definition
Reference	Self-reported current low back pain (with or without pain referred to one or both lower limbs) that lasts for at least one day. The "low back" is defined as the area on the posterior aspect of the body from the lower margin of the twelfth ribs to the lower gluteal folds. Low back pain identified in Taiwan claims is also currently treated as the reference.
Alternative	Current low back pain that lasts for at least 3 months (chronic)
Alternative	Low back pain that lasts for at least one day within the last 1 week to 1 month
Alternative	Low back pain that lasts for at least one day within the last 2 months to 1 year
Alternative	Current low back pain in a study population of schoolchildren that lasts for at least one day

Alternative	Current low back pain that lasts for at least one day and is activity-limiting
Alternative	Includes a broader anatomical region (eg, low back and upper back, low back and neck, etc.)
Alternative	Low back pain identified in USA claims data 2000 based on ICD codes below.
Alternative	Low back pain identified in USA claims data 2010–2012, 2014 based on ICD codes below.

ICD-10 codes for LBP are M54.3, M54.4 and M54.5. The ICD-9 code is 724.

Input data

The last systematic review was conducted for GBD 2021 utilising the following search string:

> (((Back Pain[MeSH] OR "back pain"[TiAb]) AND (prevalen*[TiAb] OR inciden*[TiAb]) AND (2017/09/01[PDAT] : 3000[PDAT])) OR(((prevalen* OR inciden*) AND ("neck pain" OR "neck ache" OR "neckache" OR "cervical pain" OR Neck Pain[Mesh])) AND (2017/12/20[PDAT] : 3000[PDAT]))) NOT (animals[MeSH] NOT humans[MeSH])

This returned 1346 entries, of which 35 low back pain sources were extracted.

Exclusion criteria were:

1. Sub-populations clearly not representative of the national population
2. Not a population-based study
3. Low sample size (less than 150)
4. Review rather than original studies

Figure 2: PRISMA diagram of low back pain systematic review from 2021

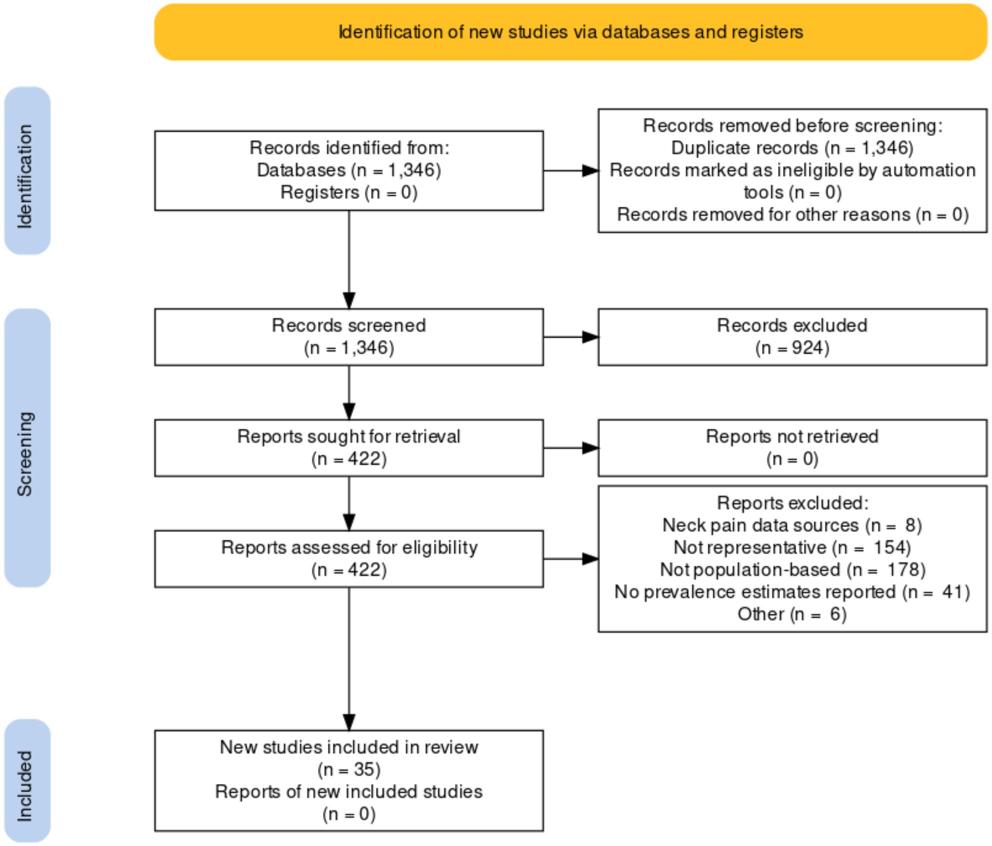

Additional information was derived from unit record data of surveys in the GHDx, GBD's repository of population health data including the World Health surveys and national health surveys. Opportunistically, additional studies encountered during data review were added. In addition, data from USA claims data for 2000, 2010–2012, and 2014–2016 by state were included.

Age and sex splitting

Reported estimates of prevalence were split by age and sex where possible. First, if studies reported prevalence for broad age groups by sex (eg, prevalence in 15- to 65-year-old males and females separately), and also by specific age groups for both sexes combined (eg, prevalence in 15- to 30-year-olds, then in 31- to 65-year-olds, for males and females combined), age-specific estimates were split by sex using the reported sex ratio and bounds of uncertainty. Second, prevalence data for both sexes that could not be split using a within-study ratio were split using a sex ratio derived from a meta-analysis of existing sex-specific data using MR-BRT (meta-regression—Bayesian, regularised, trimmed; for more information on MR-BRT, see appendix 1 section 2). The female to male ratio was 1.19 (1.03–1.40). Finally, after the application of bias adjustments, where studies reported estimates across age groups spanning 25 years or more, these were split into five-year age groups using the prevalence age pattern estimated by DisMod-MR 2.1 (disease model—Bayesian meta-regression, described in appendix 1 section 2).

Data adjustment

We attempted to correct for bias among studies that defined low back pain with too broad an anatomical region, episode duration of greater than three months, recall periods of one week to one month, recall periods between two months and one year, or as activity-limiting LBP, as well as studies conducted among schoolchildren. We added three additional covariates for claims data in the USA from the year 2000 and from 2010 onward and for Taiwan claims data. These adjustment factors were estimated as the logit difference between the prevalence of alternate case definition data and that of the reference definition for comparable age, sex, year, and location calculated using the MR-BRT network crosswalk adjustment method. Unadjusted low back pain prevalence data are often already close to 1, especially for older age groups, and a logit difference strategy ensures that any prevalence data requiring adjustment to a higher value do not exceed 1. Data on chronic low back pain (duration greater than three months) were not included in the final model, as we were unable to find matches to inform a reliable crosswalk. Moreover, data on low back pain in schoolchildren and studies reporting back pain in a broad anatomical region were not adjusted, as we could not find matches to inform a crosswalk. Betas and exponentiated values for these covariates are shown in the table below:

Table 2: MR-BRT crosswalk adjustment factors for LBP

Data input	Reference or alternative case definition	Gamma	Beta coefficient, logit (95% CI)*	Adjustment factor**
Point prevalence	Ref	0.59	---	---
Recall periods of 1 week to 1 month	Alt		0.03 (–0.03 to 0.09)	1.03 (0.97 to 1.092)
Recall periods between 2 months and one year	Alt		0.73 (0.68 to 0.78)	2.08 (1.97 to 02.18)
Activity-limiting LBP	Alt		–1.65 (–1.66 to –1.63)	0.19 (0.19 to 0.20)
USA claims data – 2000	Alt		–1.28 (–1.59 to –0.97)	0.28 (0.20 to 0.37)
USA claims data – 2010–2012, 2014–2017	Alt		–0.66 (–0.81 to –0.51)	0.52 (0.44 to 0.60)

**MR-BRT crosswalk adjustments can be interpreted as the factor the alternative case definition is adjusted by to reflect what it would have been had it been measured using the reference case definition. If the log/logit beta coefficient is negative, then the alternative is adjusted up to the reference. If the log/logit beta coefficient is positive, then the alternative is adjusted down to the reference.*

***The adjustment factor column is the exponentiated beta coefficient. For log beta coefficients, this is the relative rate between the two case definitions. For logit beta coefficients, this is the relative odds between the two case definitions.*

After adjusting data for case definition, we outliered data with a median absolute deviation of 1.5 or more above the age-standardised mean. This was done in a systematic way to cull data that were implausibly high.

Modelling strategy

There have been no significant changes to the modeling strategy in GBD 2023.

Prior settings in the DisMod-MR model included setting excess mortality to zero, and it was assumed that there was no incidence or prevalence of low back pain before the age of 5 years. We included the SEV scalar for low back pain as a country covariate. This combines the exposure measures for risks estimated to impinge on LBP in GBD: occupational ergonomic exposure and increased BMI. We set bounds of 0.75 to 1.25 as the SEV is constructed in a way that if our risk estimates are accurate the value should be 1.

Table 2. Covariates. Summary of covariates used in the LBP DisMod-MR meta-regression model

Covariate	Type	Parameter	Log beta (95% uncertainty interval)	Exponentiated beta (95% uncertainty interval)
Log-transformed age-standardised SEV scalar: Back pain	Country-level	Prevalence	0.75 (0.75–0.76)	2.12 (2.12–2.14)

Severity and disability

The basis of the GBD disability weight survey assessments are lay descriptions of sequelae highlighting major functional consequences and symptoms. The lay descriptions and disability weights for LBP severity levels are shown below.

Table 3. Severity distribution, details on the severity levels for LBP and the associated disability weight (DW) with that severity.

Severity level	Lay description	DW (95% CI)
Low back pain, mild	This person has mild back pain, which causes some difficulty dressing, standing, and lifting things.	0.020 (0.011–0.035)
Low back pain, moderate	This person has moderate back pain, which causes difficulty dressing, sitting, standing, walking, and lifting things.	0.054 (0.035–0.079)
Low back pain, severe without leg pain	This person has severe back pain, which causes difficulty dressing, sitting, standing, walking, and lifting things. The person sleeps poorly and feels worried.	0.272 (0.182–0.373)
Low back pain, severe with leg pain	This person has severe back and leg pain, which causes difficulty dressing, sitting, standing, walking, and lifting things. The person sleeps poorly and feels worried.	0.325 (0.219–0.446)

Low back pain, most severe without leg pain	This person has constant back pain, which causes difficulty dressing, sitting, standing, walking, and lifting things. The person sleeps poorly, is worried, and has lost some enjoyment in life.	0.372 (0.250–0.506)
Low back pain, most severe with leg pain	This person has constant back and leg pain, which causes difficulty dressing, sitting, standing, walking, and lifting things. The person sleeps poorly, is worried, and has lost some enjoyment in life.	0.384 (0.256–0.518)

The severity distributions are derived from an analysis of the Medical Expenditure Panel Surveys (MEPS) in the USA. MEPS is an overlapping continuous panel survey of the USA non-institutionalised population whose primary purpose is to collect information on the use and cost of health care. Panels are two years long and are conducted in five rounds, which are conducted every five to six months. A new panel begins annually, while the last panel is in its second year. Each panel typically contains about 30,000 to 35,000 individual respondents.

MEPS was initiated in 1996 but only began collecting health status data in the form of SF-12 responses in 2000. We used data from 2000–2014 in our analysis. Respondents self-administer the SF-12 twice per panel, at rounds 2 and 4, typically about a year apart. Only adults 18 years and older completed the SF-12. MEPS also usually collects information on diagnoses based on self-report of reasons for encounters with health services. In addition, diagnoses are derived through additional questions on "problems that bother you" or conditions that led to "disability days," ie, days out of role due to illness. Professional coders translate the verbatim text into three-digit ICD-9 codes. The main reason for LBP being measured in MEPS relates to health care contact. From MEPS, the severity distribution for LBP without leg pain and with leg pain were derived as shown in the below table.

Table 4. Severity distribution, details on the distribution of severity splits for LBP with and without leg pain

Severity level	Distribution without leg pain	Distribution with leg pain
Low back pain, mild	0.41 (0.31–0.53)	0.27 (0.19–0.37)
Low back pain, moderate	0.35 (0.25–0.44)	0.36 (0.28–0.43)
Low back pain, severe	0.10 (0.08–0.12)	0.14 (0.10–0.16)
Low back pain, most severe	0.14 (0.09–0.20)	0.23 (0.15–0.32)

We used USA claims data (2012) to derive the proportion of cases with low back pain who report leg pain. The proportions were different by age group as shown in Figure 2. The proportion in each severity level in each age group is calculated by multiplying the proportion in the severity level and the proportion with or without leg pain.

Figure 2: Proportion of LBP with leg pain

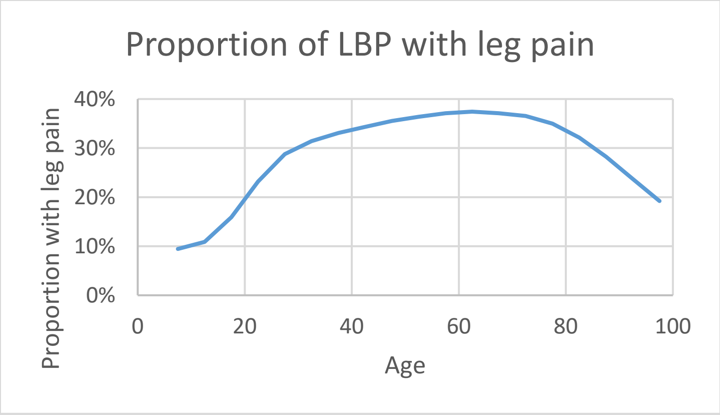

Table 6. Proportion of LBP with leg pain

Age (years)	Proportion with leg pain
5–9	9.4% (9.1–9.8)
10–14	10.9% (10.7–11.1)
15–19	15.9% (15.8–16.1)
20–24	23.2% (23.0–23.4)
25–29	28.8% (28.6–28.9)
30–34	31.4% (31.3–31.6)
35–39	33.1% (32.9–33.2)
40–44	34.3% (34.2–34.4)
45–49	35.5% (35.4–35.6)
50–54	36.4% (36.3–36.5)
55–59	37.1% (37.0–37.2)
60–64	37.4% (37.3–37.5)
65–69	37.1% (36.9–37.3)
70–74	36.5% (36.4–36.7)
75–79	35.0% (34.8–35.2)
80–84	32.1% (31.9–32.4)
85–89	28.3% (28.0–28.5)
90–94	23.7% (23.2–24.2)
95–100	19.2% (18.2–20.2)

References

MEPS-HC Panel Design and Collection Process, Agency for Healthcare Research and Quality, Rockville, Md. https://meps.ahrq.gov/mepsweb/survey_comp/hc_data_collection.jsp

Lower extremity peripheral arterial disease

Flowchart

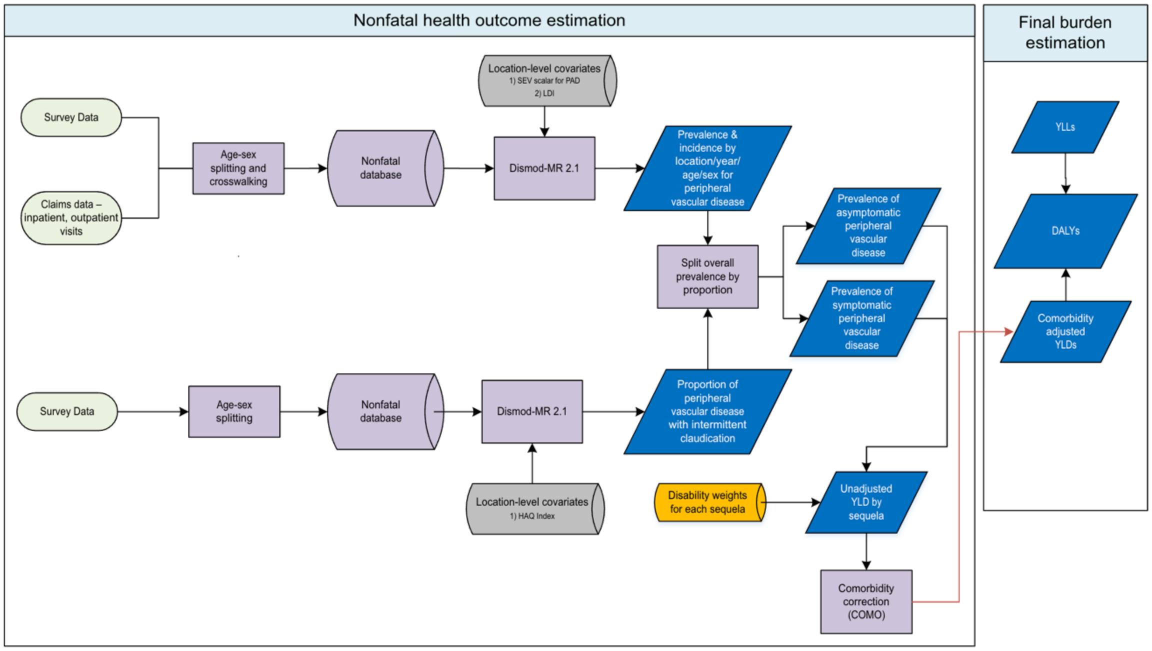

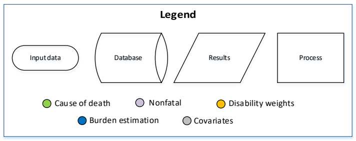

Input data and methodological summary for Lower exremity peripheral arterial disease

Case definition

For GBD 2023, lower extremity peripheral arterial disease (PAD) was defined as having an ankle-brachial index (ABI) ≤0.9. Intermittent claudication was defined clinically as leg pain on exertion among those with an ABI below that threshold.

Table 1: Reference and alternate definitions of lower extremity peripheral arterial disease

Quantity of interest	Reference or alternate	Definition
Prevalence of lower extremity peripheral arterial disease	Reference	Persons with an ankle brachial index (ABI) ≤0.9. ABI is the ratio of systolic blood pressure measured at the ankle and the arm
Prevalence of lower extremity peripheral	Alternate	Lower extremity peripheral arterial disease as identified in administrative claims,

arterial disease		outpatient, or primary care data
Proportion of patients with lower extremity peripheral arterial disease and intermittent claudication	Reference	Persons with an ankle brachial index (ABI) <0.9 who report pain due to claudication

Table 2: ICD-10 codes for claims data included in GBD 2023 mapped to lower extremity peripheral arterial disease.

ICD-10 code	ICD-10 cause name
440.20, 440.21, 440.22, 440.23, 440.24, 440.29, 440.4, 440.8, 440.9	Atherosclerosis of native arteries of the extremities
443, 443.1, 443.2, 443.8, 443.81, 443.82, 443.89,443.9	Other peripheral vascular disease
I70.2	Atherosclerosis of native arteries of the extremities
I70.20, I70.201, I70.202, I70.203, I70.208, I70.209	Unspecified atherosclerosis of native arteries of extremities
I70.21, I70.211, I70.212, I70.213, I70.218, I70.219	Atherosclerosis of native arteries of extremities with intermittent claudication
I70.22, I70.221, I70.222, I70.223, I70.228, I70.229	Atherosclerosis of native arteries of extremities with rest pain
I70.23, I70.231, I70.232, I70.233, I70.234, I70.235, I70.238, I70.239	Atherosclerosis of native arteries of right leg with ulceration
I70.24, I70.241, I70.242, I70.243, I70.244, I70.245, I70.248, I70.249	Atherosclerosis of native arteries of left leg with ulceration
I70.25	
I70.26, 70.261, I70.262, I70.263,	Atherosclerosis of native arteries of other extremities with

I70.268, I70.269<br><br><br>I70.29, I70.291, I70.292, I70.293, I70.298, I70.299	ulceration<br><br><br>Atherosclerosis of native arteries of extremities with gangrene<br><br><br>Other atherosclerosis of native arteries of extremities
I73, I73.1, I73.8, I73.81, I73.89, I73.9	Other peripheral vascular diseases

Input data

A systematic review was last performed for PAD and intermittent claudication for GBD 2023. We searched PubMed, Embase, and the Virtual Health Library databases. Search strings for each database are given below. Prior to the current review, the last review was completed in GBD 2016.

PubMed:
('peripheral vascular disease'[TIAB] AND 'epidemiology'[Subheading]) OR ('peripheral arterial disease'[TIAB] AND 'epidemiology'[Subheading]) OR ('peripheral artery disease'[TIAB] AND 'epidemiology'[Subheading]) OR ('intermittent claudication'[TIAB] AND 'epidemiology'[Subheading]) OR ('ankle-brachial index'[TIAB] AND 'epidemiology'[Subheading]) OR ('ankle brachial index'[TIAB] AND 'epidemiology'[Subheading]) OR ('peripheral artery occlusive disease'[TIAB] AND 'epidemiology'[Subheading]) OR ('peripheral obliterative arteriopathy'[TIAB] AND 'epidemiology'[Subheading]) OR ('peripheral vascular disease'[TIAB] AND 'prevalence'[MeSH Terms]) OR ('peripheral vascular disease'[TIAB] AND 'incidence'[MeSH Terms]) OR ('peripheral vascular disease'[TIAB] AND 'case fatality'[All Fields]) OR ('symptomatic claudication'[TIAB] AND (proportion[All Fields] OR percent[All Fields])) OR ('intermittent claudication'[TIAB] AND (proportion[All Fields] OR percent[All Fields])) AND ("Geographic Locations"[Mesh] OR "Population"[Mesh] OR "population*"[TiAb] OR "nation*"[TiAb] OR "communit*"[TiAb] OR "reside*"[TiAb] OR "residing"[TiAb] OR "inhabitants"[TiAb] OR "living in"[TiAb]) NOT (Comment[pt] OR Case Reports[pt] OR Review[pt] OR Editorial[pt] OR Clinical Trial[pt] OR News[pt]) NOT ("Animal Experimentation"[Mesh] OR "Models, Animal"[Mesh] OR "mouse"[tiab] OR "mice"[tiab] OR "rat"[tiab] OR "rats"[tiab] OR "dog"[tiab] OR "dogs"[tiab] OR "cat"[tiab] OR "cats"[tiab]) NOT "tissue culture" [TIAB] AND 2015/01/01:2023/12/31[dp]

Embase:
('peripheral vascular disease':ti,ab,kw AND 'epidemiology' OR ('peripheral arterial disease':ti,ab,kw AND 'epidemiology') OR ('peripheral artery disease':ti,ab,kw AND 'epidemiology') OR ('intermittent claudication':ti,ab,kw AND 'epidemiology') OR ('ankle-brachial index':ti,ab,kw AND 'epidemiology') OR ('ankle brachial index':ti,ab,kw AND 'epidemiology') OR ('peripheral artery occlusive disease':ti,ab,kw AND 'epidemiology') OR ('peripheral obliterative arteriopathy':ti,ab,kw AND 'epidemiology') OR ('peripheral vascular disease':ti,ab,kw AND 'prevalence'/exp) OR ('peripheral vascular disease':ti,ab,kw AND 'incidence'/exp) OR ('peripheral vascular disease':ti,ab,kw AND 'case fatality') OR ('symptomatic

claudication':ti,ab,kw AND ('proportion' OR 'percent')) OR ('intermittent claudication':ti,ab,kw AND ('proportion' OR 'percent'))) AND ('geography'/exp OR 'population'/exp OR 'population*':ti,ab,kw OR 'nation*':ti,ab,kw OR 'communit*':ti,ab,kw OR 'reside*':ti,ab,kw OR 'residing':ti,ab,kw OR 'inhabitants':ti,ab,kw OR 'living in':ti,ab,kw) NOT ('comment':it OR 'case reports':it OR 'review':it OR 'editorial':it OR 'clinical trial':it OR 'news':it) NOT ('animal experiment'/exp OR 'animal model'/exp OR 'mouse':ti,ab,kw OR 'mice':ti,ab,kw OR 'rat':ti,ab,kw OR 'rats':ti,ab,kw OR 'dog':ti,ab,kw OR 'dogs':ti,ab,kw OR 'cat':ti,ab,kw OR 'cats':ti,ab,kw) NOT 'tissue culture':ti,ab,kw AND [2015-01-01 to 2023-12-31]/pd

Virtual Health Library:
(mh:("peripheral vascular disease" OR "peripheral arterial disease" OR "peripheral artery disease" OR "intermittent claudication" OR "ankle-brachial index" OR "ankle brachial index" OR "peripheral artery occlusive disease" OR "peripheral obliterative arteriopathy")) AND (mh:("epidemiology" OR "prevalence" OR "incidence" OR "case fatality") AND (mh:"Geographic Locations" OR mh:"Population" OR "population*" OR "nation*" OR "communit*" OR "reside*" OR "residing" OR "inhabitants" OR "living in")) AND NOT (pt:"Comment" OR pt:"Case Reports" OR pt:"Review" OR pt:"Editorial" OR pt:"Clinical Trial" OR pt:"News") AND NOT (mh:"Animal Experimentation" OR mh:"Models, Animal" OR "mouse" OR "mice" OR "rat" OR "rats" OR "dog" OR "dogs" OR "cat" OR "cats") AND NOT "tissue culture" AND (entry_date:2015* OR entry_date:2016* OR entry_date:2017* OR entry_date:2018* OR entry_date:2019* OR entry_date:2020* OR entry_date:2021* OR entry_date:2022* OR entry_date:2023*)

The search was conducted from 1/1/2015 to 12/31/2023. The PRISMA diagram in figure 1 shows the relevant search information.

We also used claims data in the USA. Besides the claims data from the USA, which include the Truven database of private health insurance and subset of public insurance schemes of Medicaid and Medicare (CMS), we did not include any non-literature-based data types. We did not use inpatient hospital data, since PAD are expected to be rare in inpatient data but common in outpatient data as it is a condition usually managed on an outpatient basis, except for specific surgical interventions. This discrepancy leads to implausible correction factors based on inpatient/outpatient information from claims data (~150X); thus, adjusted inpatient data cannot be used. Including unadjusted inpatient data in the model is likely to lead to incorrect estimates as hospitalisation and procedure rates vary by geography based on access to and patterns of care.

Figure 1: PRISMA flow diagram

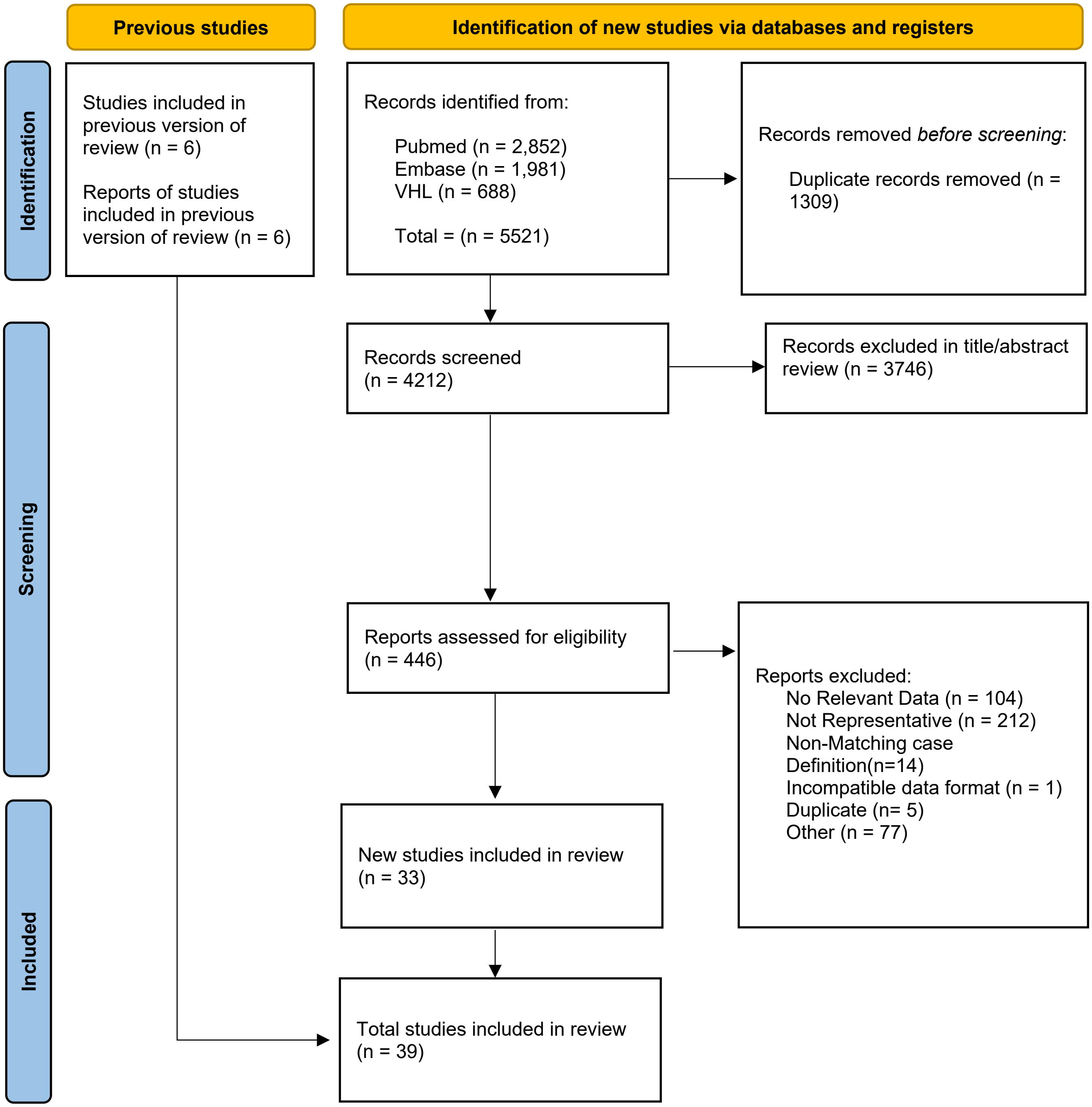

From: Page MJ, McKenzie JE, Bossuyt PM, Boutron I, Hoffmann TC, Mulrow CD, et al. The PRISMA 2020 statement: an updated guideline for reporting systematic reviews. BMJ 2021;372:n71. doi: 10.1136/bmj.n71

For GBD 2023, we adjusted prevalence data from claims using the MR-BRT data adjustment procedure described else where the appendix. Our reference data were prevalence of PAD based on directly measured ABI values. Alternate definitions were drawn from the Truven database of private health insurance and from CMS. We incorporated a quadratic spline, constructed on a standardised age variable (age scaled) and a sex variable into the crosswalking procedure to adjust for variation by age and sex. The selection of the knots for the spline on age was based on visual inspection of the spline fit to the observed ratios between alternate and reference definitions, which were used in computing adjustment factors. The knots were strategically place at values of the variable age scaled –2, –0.75, 0, 0.75. The coefficients in Table 4 below can be used to calculate adjustment factors for the alternative definition prevalence values. The formula for computing adjustment factors for prevalence is given in equation 1 below. Proportion data were not adjusted.

Table 4: MR-BRT crosswalk adjustment factors for lower extremity peripheral arterial disease

Data input	Measure	Reference or alternative case definition	Gamma	Beta coefficient, logit (95% UI)	Adjustment factor**
Measured ABI less than or equal to 0.90	Prevalence	Ref	0.001	---	---
Claims data, intercept	Prevalence	Alt		–1.80 (–1.82 to –1.78)	0.17
Claims data, age scaled 0	Prevalence	Alt		0.028 (0.020 to 0.035)	1.03
Claims data, age scaled 1	Prevalence	Alt		0.48 (0.45 to 0.53)	1.63
Claims data, age scaled 2	Prevalence	Alt		0.40 (0.37 to 0.43)	1.49
Claims data, sex (male)	Prevalence	Alt		0.35 (0.33 to 0.37)	1.42
CMS data, intercept	Prevalence	Alt		–1.20 (–1.22 to –1.18)	0.30
CMS data, age scaled 0	Prevalence	Alt		0.043 (0.035 to 0.051)	1.04
CMS data, age scaled	Prevalence	Alt		0.31 (0.27 to 0.35)	1.37

1					
CMS data, age scaled 2	Prevalence	Alt		0.24<br>(0.21 to 0.27)	1.27
CMS data, sex (male)	Prevalence	Alt		0.30<br>0.28 to 0.31)	1.34

Claims data: Truven database of private health insurance.
CMS data: public insurance schemes of Medicaid and Medicare.
***The adjustment factor column is the exponentiated beta coefficient. For log beta coefficients, this is the relative rate between the two case definitions. For logit beta coefficients, this is the relative odds between the two case definitions.*

Equation 1: Calculation of adjustment factors:

$$Estimated\ Reference\ Def = invlogit(logit(Alternative\ Def) - [\sum_{s=0}^{b} Beta_{Alternative\ Def, spline\ basis_s} * Spline\ basis_s(age\_scaled)] - Beta * I(Sex))$$

MR-BRT crosswalk adjustments in table 4 can be interpreted as the factor the alternative case definition is adjusted by to reflect what it would have been had it been measured using the reference case definition. If the log/logit beta coefficient is negative, then the alternative is adjusted up to the reference. If the log/logit beta coefficient is positive, then the alternative is adjusted down to the reference.

Severity splits and disability weights
We used the proportion of intermittent claudication to split the overall prevalence of PAD into symptomatic and asymptomatic PAD. Table 5 shows the severity levels and associated disability weights (DWs).

Table 5: Severity levels for lower extremity peripheral arterial disease in GBD 2023 and associated disability weights

Severity level	Lay description	DW (95% CI)
Asymptomatic	No symptoms	No DW assigned
Symptomatic	Has cramping pains in the legs after walking a medium distance. The pain goes away after a short rest.	0.014 (0.007–0.025)

Modelling strategy

Prevalence of lower extremity peripheral arterial disease
For GBD 2023, we used DisMod MR 2.1 to model the overall prevalence of PAD using prevalence data from literature studies and adjusted claims data. Further statistical details regarding DisMod MR 2.1 can be found in a separate section of this appendix.

We included the log-transformed, age-standardised SEV scalar for PAD and log-transformed LDI as fixed-effect, country-level covariates. We set value priors of zero for incidence from ages 0 to 30. We also set a value prior to 0 for remission for all ages. Additionally, we set a value prior to zero for excess mortality in between ages 0 and 30 as well as a value prior between 0 and 0.055 for excess mortality in between ages 30 and 100.

The table below illustrates the beta values and exponentiated beta values for the covariates chosen for the overall PAD model.

Table 6: Summary of covariates used in the lower extremity peripheral arterial disease DisMod-MR meta-regression model

Covariate	Parameter	Beta	Exponentiated beta
Log-transformed age-standardised SEV scalar: PAD	Prevalence	0.86<br>(0.79 to 0.95)	2.37<br>(2.19 to 2.60)
LDI (I$ per capita)	Excess mortality rate	–0.3<br>(–0.49 to –0.12)	0.74<br>(0.61 to 0.89)

Proportion of lower extremity peripheral arterial disease with intermittent claudication
We used DisMod-MR to model the proportion of PAD with intermittent claudication. We set a value prior to zero for the proportion for ages 0 to 40. We included the Healthcare Access and Quality Index score as a country-level covariate for excess mortality.

The table below illustrates the study covariates, parameters, beta, and exponentiated beta values for the proportion model for intermittent claudication.

Table 7: Summary of covariates used in the intermittent claudication DisMod-MR meta-regression (proportion) model.

Covariate	Parameter	Beta	Exponentiated beta
Healthcare Access and Quality Index	Proportion	–0.0055<br>(–0.012 to –0.00056)	0.99<br>(0.99 to 1.00)

Estimation of asymptomatic and symptomatic sequelae
To obtain final estimates for the sequelae of interest, we multiplied the prevalence estimates from the overall PAD model by the proportion estimated as having symptomatic disease from the intermittent claudication model at the draw level by age, sex, year, and location to generate the prevalence of symptomatic and asymptomatic PAD for each demographic grouping.

Models were evaluated based on expert review, comparisons with estimates from prior rounds of GBD, and assessing model fit.

There have been no substantive changes from GBD 2021 in terms of modelling strategy for PAD.

Lower respiratory infections (LRI)

Flowchart

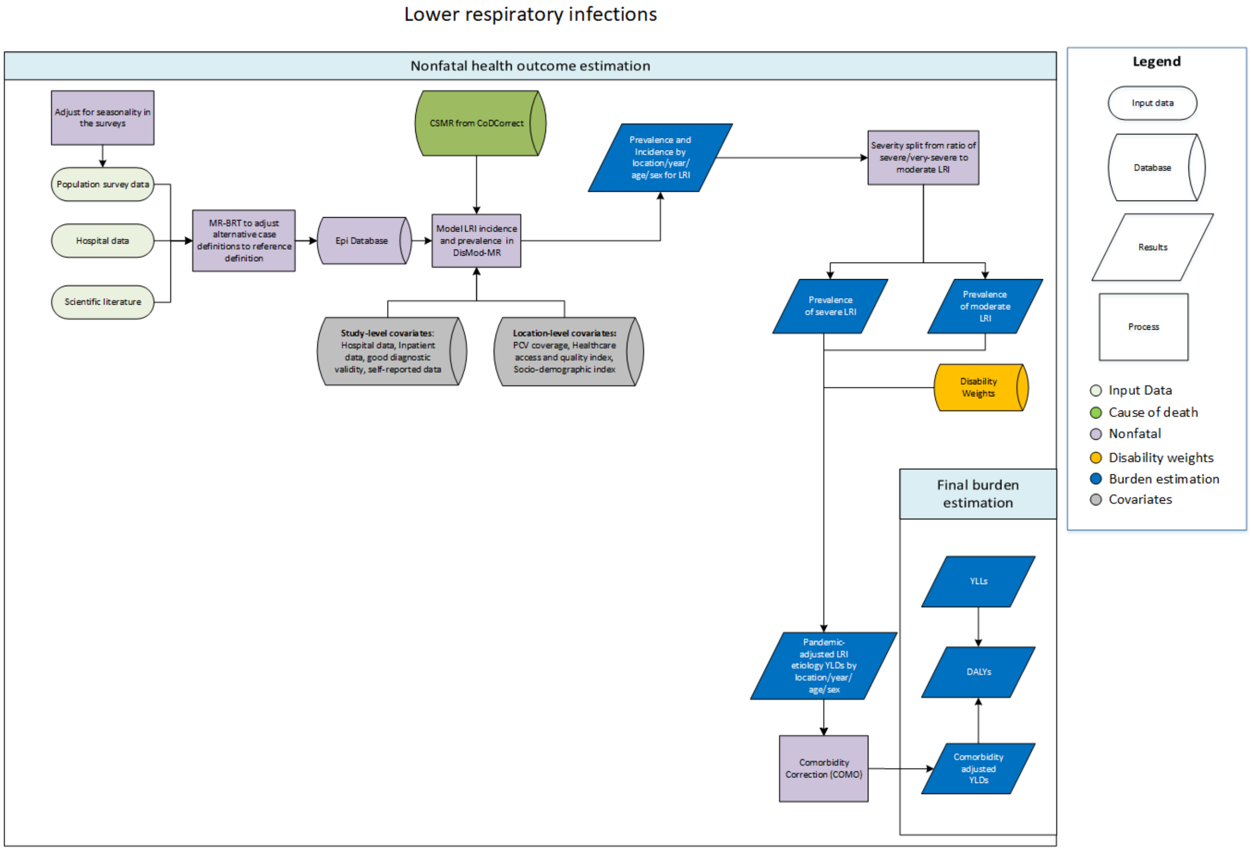

Input data and methodological summary for lower respiratory infections

Case definition

Lower respiratory infections (LRI) are defined by the GBD study as pneumonia or bronchiolitis. Symptoms include cough, fever, and shortness of breath. Included in the GBD modelling were cases meeting ICD-9 diagnostic criteria for LRI (079.82, 466-469, 470.0, 480-481.9, 482.0-482.89, 483.0-483.9, 484.1-484.2, 484.6-484.7, 487-490.9, 510-511.9, 513.0-513.9) and ICD-10 diagnostic criteria for LRI (A48.1, A70, B96.0-96.1, B97.21, B97.4-B97.6, J09-J11.89, J12-J13.9, J14-J14.0, J15-J15.8, J20-J21.9, J85.1, J91.0, P23.0-P23.4, U04-U04.9). In addition, the following garbage codes were redistributed entirely to LRI in ICD-9 (482, 482.9-483, 484, 484.3-484.5, 484.8-486.9, 770.0, V12.61) and ICD-10 (J15.9, J1-J19.6, J22-J22.9, P23, P23.5-P23.9). The GBD case definition of LRI does not include tuberculosis or COVID-19; although these pathogens can infect the lower respiratory tract, they are modelled separately due to their individual public health significance.

Table 1: Case definitions accepted for lower respiratory infections

**Quantity of interest**	**Reference or alternative**	**Definition**
Incidence or prevalence of lower respiratory infections	Reference	Clinician-diagnosed episode of pneumonia or bronchiolitis
Incidence or prevalence of lower respiratory infections	Alternative	Hospitalised episodes of lower respiratory infection (ICD-9 codes 073.0-073.6, 079.82, 466-469, 480-489, 513.0, and 770.0 and ICD-10 codes A48.1, J09-J22, J85.1, P23-P23.9, and U04)
Prevalence of lower respiratory infections	Alternative	Maternal-reported symptoms of under-5 acute lower respiratory infections including cough with difficulty breathing, fever, and symptoms in the chest.

Input data

Overall LRI

Input data included all data used in GBD 2021 and new data identified in our updated systematic review, newly acquired surveys, and new claims and inpatient data. These data measure lower respiratory infection incidence and prevalence. They come from a systematic literature review, hospital inpatient and outpatient data, claims data from the USA, and population-representative surveys.

The PubMed search string below was used to look for the incidence or prevalence of LRI.

((“lower respiratory”[title] OR pneumonia[title]) AND (2021/01/01[PDat] : 2023/12/31[PDat]) AND ((incidence OR prevalence OR epidemiology) OR (etiolog*[title/abstract] OR influenza[title/abstract] OR “respiratory syncytial virus”[title/abstract])) AND Humans[MeSH Terms]) NOT(autoimmune[title/abstract] OR COPD [title/abstract] OR “cystic fibrosis”[title/abstract] OR Review[ptyp]) NOT (animals[MeSH] NOT humans[MeSH])

Figure 1: PRISMA flow diagram

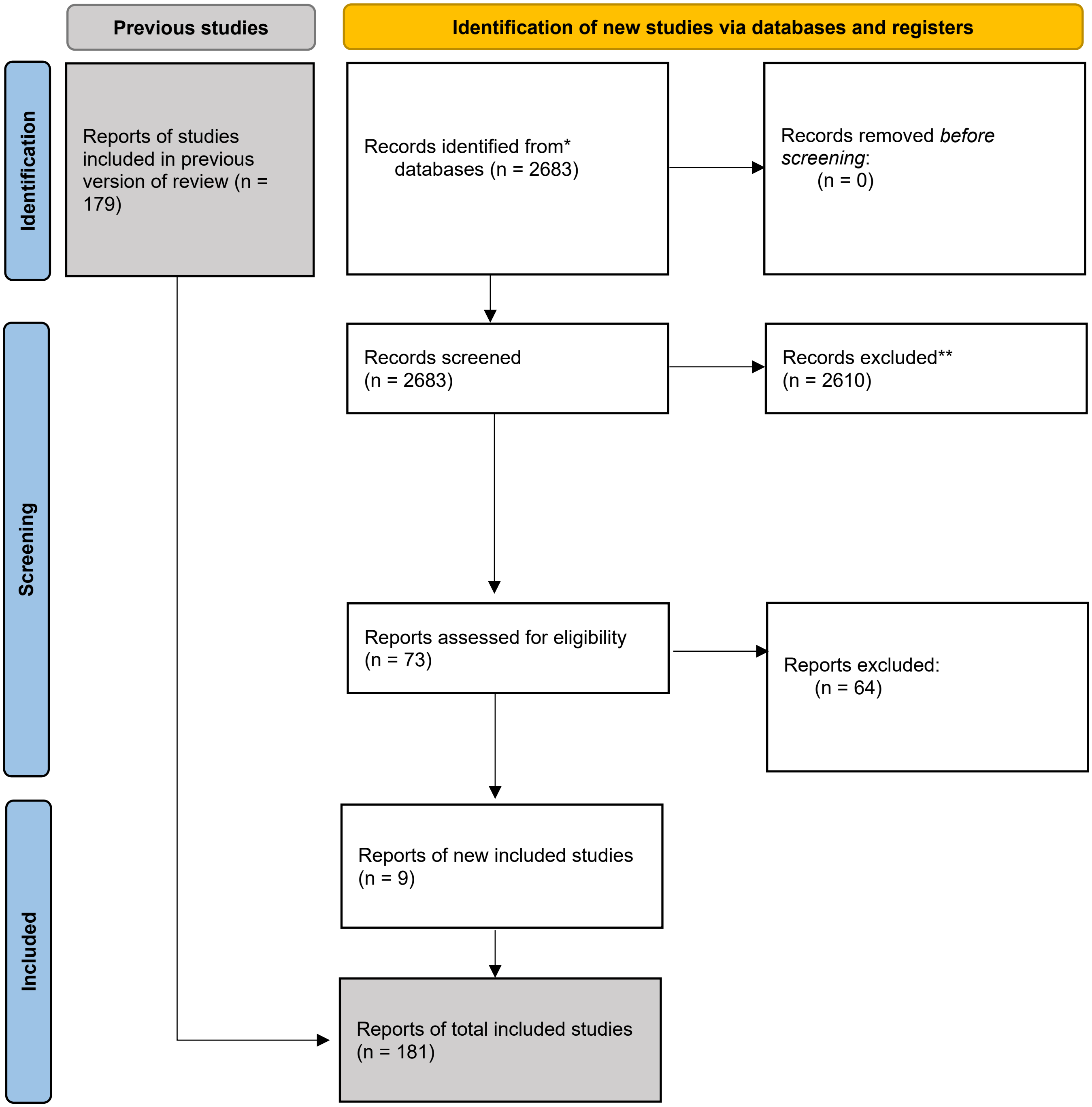

Data were outliered or excluded if we found them unreasonable when compared to regional, super-regional, and global rates.

To estimate the non-fatal burden of LRI, we also used self-reported prevalence of LRI symptoms from population-representative surveys, such as the Demographic and Health Survey and the Multiple

Indicator Cluster Survey. When possible, we extracted survey data by one-year age group and by sex. We converted these data from two-week period prevalence to point prevalence. The equation for this adjustment is:

1) $$Point\ Prevalence = \frac{Period\ Prevalence * Duration}{(Recall\ Period + Duration - 1)}$$

We accepted four survey definitions for the prevalence of symptoms of LRI: 1) Cough with difficulty breathing with symptoms in the chest with a fever was our gold standard, but we also accepted 2) Cough with difficulty breathing with symptoms in the chest *without* fever, 3) Cough with difficulty breathing with fever, and 4) Cough with difficulty breathing *without* fever. To make these definitions comparable, we identified the surveys that met the best case definition (definition 1). Within these surveys, we calculated the ratio of the prevalence of the best case definition to the prevalence of the alternate definitions. This ratio was used as the dependent variable in a meta-regression. The results from that meta-regression were used to adjust the prevalence and uncertainty for all the surveys that reported alternate case definitions **(Table 2a)**.

Table 2a: MR-BRT crosswalk adjustment factors for lower respiratory infections, surveys

Data input	Reference or alternative case definition	Gamma	Crosswalk covariate	Beta coefficient, log (95% UI)	Adjustment Factor**
Cough, with difficulty breathing and fever	ref	--	--	--	--
Survey, chest without fever	alt	0.03	intercept	–0.47 (–0.49 to –0.45)	0.63
Survey, difficulty breathing without fever	alt	0.13	intercept	–1.20 (–1.25 to –1.15)	0.30
Survey, difficulty breathing with fever	alt	0.05	intercept	–0.65 (–0.68 to –0.62)	0.52

***The adjustment factor column is the exponentiated beta coefficient. For log beta coefficients, this is the relative rate between the two case definitions. For logit beta coefficients, this is the relative odds between the two case definitions.*

Survey data were adjusted for seasonality. An inclusion criterion for scientific literature is a study duration longer than one year to avoid bias in the seasonal timing of LRI. Surveys are frequently conducted over several months. To account for seasonal variation in LRI symptom prevalence, we fit a generalised additive model with a forced periodicity for each GBD region. The model is mixed-effects with random effects on each country. The model accounts for the year of the survey and the case definition used. The percentage difference between the monthly model-fit LRI prevalence and the mean fitted LRI prevalence is a scalar to adjust survey data by month and geography.

In addition to survey data, hospital inpatient and USA inpatient claims data were included in the LRI modelling. These data are adjusted prior to modelling for readmissions and multiple diagnoses. To make the data more consistent in the modelling process, we converted all incidence data to prevalence. We found the ratio of the prevalence of LRI in hospitalisation records to the prevalence of LRI in our case definition (clinician-diagnosed pneumonia or bronchiolitis) for locations that contained data on both these prevalence values. We then regressed this ratio in a meta-regression to predict the adjustment factor for hospitalisation data to make them compatible with the reference case definition for our modelling. This meta-regression considered the Socio-demographic Index (SDI) as a predictor of this ratio for inpatient data, assuming that location-years with higher values of SDI are more likely to have access to health care, making this ratio smaller in those location-years **(Table 2b)**.

Claims data for GBD 2023 include MarketScan (USA), and data from Taiwan (province of China), Poland, and Russia. MarketScan data are retrieved by IHME's Clinical Informatics Team. As with inpatient clinical data, these data are converted first to prevalence, then compared to the reference definition for LRI using a meta-regression model **(Table 2b)**. Taiwan claims data were dropped as there were no reference data to match with and because the values there were systematically different from those in the USA.

Table 2b: MR-BRT crosswalk adjustment factors for lower respiratory infections: clinical inpatient, hospital-based studies, and inpatient claims to reference

Data input	Reference or alternative case definition	Gamma	Crosswalk covariate	Beta coefficient, log or logit (95% UI)	Adjustment factor**
Clinician-diagnosed pneumonia or bronchiolitis	ref		--	--	--
Clinical, inpatient	alt	0.32	Intercept	Logit 1.43 (0.96 to 1.98)	4.32
Clinical, inpatient	alt		sdi_0	Logit –0.95 (–1.51 to –0.39)	0.40
Clinical, inpatient	alt		sdi_1	Logit –1.19 (–1.93 to –0.58)	0.32
Clinical, inpatient	alt		sdi_2	Logit –1.24 (–2.08 to –0.59)	0.31
Literature, hospital-based	alt	0.15	Intercept	Log 0.80 (0.46 to 1.12)	2.25
Self-report	alt	0.32	Intercept	Log –1.03 (–1.35 to –0.71)	0.36

Claims, MarketScan	alt	0.32	Intercept	Logit 0.89 (0.29 to 1.49)	2.54

**MR-BRT crosswalk adjustments can be interpreted as the factor the alternative case definition is adjusted by to reflect what it would have been had it been measured using the reference case definition. If the log/logit beta coefficient is negative, then the alternative is adjusted up to the reference. If the log/logit beta coefficient is positive, then the alternative is adjusted down to the reference.*

***The adjustment factor column is the exponentiated beta coefficient. For log beta coefficients, this is the relative rate between the two case definitions. For logit beta coefficients, this is the relative odds between the two case definitions.*

We performed a systematic review of the duration of symptoms of LRI. We sought consistency with our case definition of LRI and defined our duration as the time between the onset of symptoms to the resolution of increased work of breathing. Although crucial, there were very limited data on spatial, temporal, or age-specific duration, which may vary based on severity, aetiology, and treatment. We identified 485 titles from PubMed and extracted six studies which were used in a meta-analysis (mean duration 7.79 days [6.2–9.64]). We used this as the duration of LRI in our conversions from period to point prevalence and for the conversion between incidence and prevalence.

Severity splits

The distribution of moderate (85%) and severe (15%) lower respiratory infections is determined by a meta-analysis of the ratio of severe to all LRI from studies that report the incidence of moderate and severe lower respiratory infections.

We used the health states of acute infectious disease episode, moderate and severe, with the lay descriptions and disability weight values shown in **Table 3** below:

Table 3. Severity distribution, details on the severity levels for lower respiratory infections and the associated disability weight (DW) with that severity.

Severity level	Lay description	DW (95% CI)
Moderate	Has a fever and aches and feels weak, which causes some difficulty with daily activities.	0.051 (0.032–0.074)
Severe	Has a high fever and pain and feels very weak, which causes great difficulty with daily activities.	0.133 (0.088–0.19)

Modelling strategy

Overall LRI

The non-fatal lower respiratory infection burden is modelled in DisMod-MR, a Bayesian meta-regression modelling framework. DisMod-MR produces estimates of the incidence, prevalence, and remission of LRI for each age, sex, geographical location, and year. The models are informed by country-level covariates (**Table 6**).

Table 6. Covariates used in the lower respiratory infections DisMod-MR meta-regression model

Covariate	Type	Parameter	Exponentiated beta (95% uncertainty interval)
Socio-demographic Index	Country-level	Prevalence	0.14 (0.14–0.14)
Healthcare Access and Quality Index	Country-level	Excess mortality	0.37 (0.15–0.96)

The methods used for estimating LRI aetiologies have been published elsewhere.[1]

References

1. Naghavi M, Vollset SE, Ikuta KS, Swetschinski LR, Gray AP, Wool EE, et al. Global burden of bacterial antimicrobial resistance 1990–2021: a systematic analysis with forecasts to 2050. *The Lancet*. 2024 Sep 28;404(10459):1199–226.

Lymphatic filariasis

Flowchart

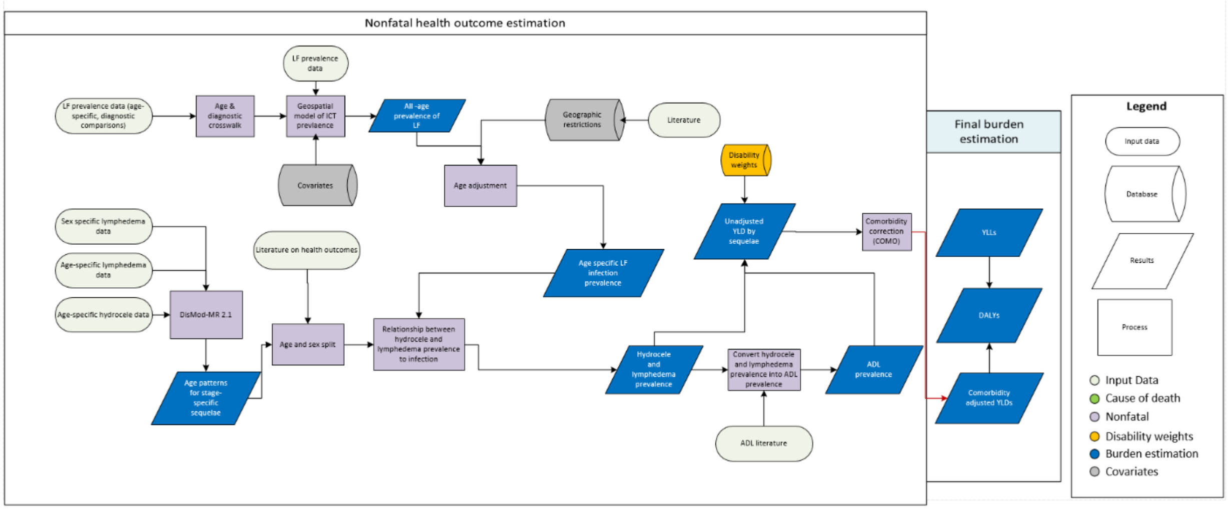

Input data and methodological summary for lymphatic filariasis

Case definition

Lymphatic filariasis (LF) is a neglected tropical disease in which threadlike nematodes invade the lymphatic system. The worms responsible – *Wuchereria bancrofti*, *Brugia malayi,* and *Brugia timori* – are spread from human to human via mosquitoes. Chronic infection can lead to lymphoedema (a swelling of the legs, also known in its more extreme manifestation as elephantiasis), hydrocele (a collection of fluid in the sac around the testicles), as well as recurrent debilitating episodes of acute adenolymphangitis. We used the following case definition for GBD 2023:

Quantity of interest	Reference or alternative	Definition
Lymphatic filariasis	Reference	Prevalent cases of lymphatic filariasis as confirmed through antigenaemia (ICT) diagnostic testing.
Lymphatic filariasis	Alternative	Prevalent cases of lymphatic filariasis as confirmed through microfilariaemia (MF) diagnostic testing.

Input data

In October 2016, a systematic review of the scientific literature was conducted in the PubMed database. Literature relevant to LF prevalence or incidence was identified using the search string “(Lymphatic filariasis AND prevalence) OR (Lymphatic filariasis AND (prevalence OR incidence OR "mass drug administration" OR MDA OR coverage)) OR (Lymphoedema, hydrocele) OR (Transmission Assessment Survey (TAS)) OR (Lymphatic filariasis AND mapping)”. Additional data on LF infection prevalence collected under the Global Programme for the Elimination of Lymphatic Filariasis were obtained through

the Expanded Special Project for Elimination of Neglected Tropical Diseases (ESPEN) and the World Health Organization (WHO).

In May 2019, a second systematic review of the scientific literature was conducted to identify new potential data sources. The review used the same search string as was used in 2016, again within the PubMed database. Updated data were also obtained from both ESPEN and WHO.

In 2023, updated data was obtained from both ESPEN and WHO, and a third systematic review of the scientific literature was conducted to identify new potential data sources specific to India. This review used the same search string as in 2016 and was conducted within the PubMed database. This India-focused effort was a part of a larger collaborative project with external partners in India.

Modelling strategy

We first estimate the prevalence of LF infection using a geospatial model, as described by Cromwell and colleagues (2020).[1] Time-series models are used to estimate LF infection prevalence in areas not appropriate for geospatial modelling. We then use the LF infection prevalence estimates to estimate the prevalence of LF sequelae: hydrocele, lymphoedema, and acute adenolymphangitis (ADL).

Infection prevalence modelling

Covariates

The geospatial model relies on covariates at the 5 × 5-km grid-cell resolution to represent environmental factors associated with LF transmission, including elevation, precipitation, vegetation, and temperature, as well as socioeconomic measures potentially associated with vector-borne disease burden. Geospatial estimates of population coverage with insecticide-treated bed nets (ITN), indoor residual spraying, and LF MDA (of any drug regimen) were included to account for interventions known to reduce transmission. Malaria (*Plasmodium falciparum* and *Plasmodium vivax*) prevalence and incidence was included as proxies for exposure to vector-borne disease. Variance inflation factor (VIF) analysis was performed for each modelling region to select the final set of covariates used for modelling. The final selections included 23 covariates for modelling regions within Africa, 22 covariates for south Asia, 21 covariates for southeast Asia and 21 covariates for Hispaniola. Covariate selection and VIF analysis were last conducted for GBD 2019. The resulting covariate selections were carried over to GBD 2023. The final covariate selections, by region, are detailed in Table 1.

Table 1: Final covariate selection, by modelling region

**Modelling region**	**Selected covariates**
Africa (central, northern & eastern, and western)	Travel time to nearest settlement; wet day frequency; distance from rivers 25 m wide or more; elevation; vegetation index; urbanicity; land equipped for irrigation; MDA for lymphatic filariasis; malaria incidence; precipitation; slope; stunting; tasseled cap brightness; population; wasting; Human development Index (HDI); nighttime lights; years of education; access to antimalarial drugs; indoor residual spraying for mosquito control; proportion of individuals who slept under an insecticide-treated map; wasting in under 5-year-olds; mortality in under 5-year-olds; stunting in under 5-year-olds

South Asia	Travel time to nearest settlement; aridity index; average daily mean temperature; distance from rivers; distance from rivers 25 m wide or more; distance from rivers or lakes of 50 sq km or more; nighttime lights; elevation; vegetation index; urbanicity; growing season length; land equipped for irrigation; MDA for lymphatic filariasis; diurnal difference in land surface temperature; malaria prevalence; malaria incidence; precipitation; slope; tasseled cap brightness; population; wasting in under 5-year-olds; mortality in under 5-year-olds
Southeast Asia	Travel time to nearest settlement; aridity index; average daily mean temperature; distance from rivers; distance from rivers 25 m wide or more; distance from rivers or lakes of 50 sq km or more; nighttime lights; elevation; vegetation index; urbanicity; growing season length; land equipped for irrigation; MDA for lymphatic filariasis; diurnal difference in land surface temperature; malaria prevalence; malaria incidence; precipitation; slope; tasseled cap brightness; population; Human development Index (HDI)
Hispaniola	Travel time to nearest settlement; aridity index; average daily mean temperature; distance from rivers; distance from rivers 25 m wide or more; distance from rivers or lakes of 50 sq km or more; nighttime lights; elevation; vegetation index; urbanicity; growing season length; land equipped for irrigation; MDA for lymphatic filariasis; diurnal difference in land surface temperature; malaria prevalence; precipitation; slope; stunting in under 5-year-olds; tasseled cap brightness; population; wasting in under 5-year-olds

Input data adjustments

To derive global estimates of all-age infection prevalence using data that were reported across different age and diagnostic categories, we used age and diagnostic crosswalk models to adjust the input data prior to modelling. Due to the introduction and the rapid adoption of ICT card tests in the mid-2000s and their higher sensitivity compared to blood smear microscopy, data derived from diagnosis by blood microscopy were first adjusted to be comparable to diagnosis with ICT. We identified peer-reviewed published surveys that reported prevalence in at least two age groups from within the same study population. The non-linear age-dependent relationship between MF and ICT prevalence was then calculated using surveys that reported both measures by fitting a logistic regression model with a basis spline on the ratio of ICT to MF prevalence by age. Infection prevalence measured and reported from a single age group, typically adults in baseline surveys or children in Transmission Assessment Surveys (TAS), was then adjusted to reflect all-age infection prevalence. The age crosswalk model was similarly structured and fit using surveys reporting ICT prevalence for multiple age groups.

Geostatistical modelling

Bayesian geostatistical models were fit separately for each of the following modelling regions based on a review of LF endemicity: (1) eastern sub-Saharan Africa, with Zimbabwe, Sudan, and Egypt; (2) western sub-Saharan Africa; (3) central sub-Saharan Africa; (4) south Asia; (5) southeast Asia and Oceania, with Brunei; and (6) Hispaniola. We employed an ensemble method to select covariates, capture possible non-linear effects, and account for the complex interactions among them. For each modelling region, three sub-models were fit to predict the prevalence of LF for geo-referenced datapoints, with cross validation. The three sub-models included a generalised additive model (GAM), a generalised boosted model (GBM), and a lasso regression model. All sub-models included country-level fixed effects. We modelled LF infection prevalence using a spatially and temporally explicit generalised linear mixed effects model via the integrated nested Laplace approximation (INLA) method. The spatiotemporal variation beyond that described by the included covariates was modelled as a Gaussian process with covariance as a Kronecker product of the spatial and temporal error processes. Spatial covariance was modelled using a Matérn function, and the temporal covariance was modelled using a first- or second-order

autoregressive function. Prevalence estimates were generated using the in-sample sub-model predictions as covariates and summarising 1000 samples from the posterior distribution as the mean; 95% uncertainty intervals (UIs) were generated from the 2·5th and 97·5th percentiles. This model was fit in R-INLA using stochastic partial differential equations (SPDE) to model the spatiotemporal processes. This process resulted in 1000 prevalence estimates for each 5 × 5-km grid-cell within the modelling space, repeated for each year being modelled. These 5 × 5-km grid-cells were then aggregated to their respective GBD administrative units, resulting in 1000 mean prevalence estimates for each GBD administrative unit within the modelling space for each year being modelled.

Time-series modelling

Geostatistical methods were not practical for estimating LF infection prevalence for the following locations due to their small geographical areas (<25 km^2), missing covariate data, or limited geo-referenced data: American Samoa, Brazil, Cook Islands, Fiji, French Polynesia, Guyana, Kiribati, Maldives, Marshall Islands, New Caledonia, Niue, Palau, Samoa, Tonga, Tuvalu, Vanuatu, and Wallis and Futuna. Instead, Bayesian time-series models for endemic UIs were fit to estimate annual national prevalence (Cromwell and colleagues (2020), Appendix 2).[1]

Estimate adjustments

The geostatistical models and the non-geostatistical time-series models all resulted in all-age and all-sex estimates of LF infection prevalence. Using an age-pattern generated from crosswalking methods, we converted the all-age and all-sex estimates into age-specific and sex-specific estimates of LF infection prevalence for each GBD administrative unit and year.

In the absence of data for 2023, prevalence was projected using an optimised exponential weighting model. For all pairs of consecutive years t and $t-1$ between 1990 and 2022, we calculated the difference d in the logit of prevalence p for each location i (using a logit transformation to ensure that all prevalence values were bounded between zero and one):

$$d_{i,t,t-1} = logit(p_t) - logit(p_{t-1})$$

We then calculated the weighted average of these logit differences using an exponential weighting scheme. In this approach, more recent trends were given more weight in calculating the average difference in the logit of prevalence, with a parameter ω determining the relative influence of more recent years compared to earlier years. For a given difference in the logit of prevalence between years t and $t-1$, the weight w_j depends upon ω and the number of years between year t and the last year of prevalence estimates, k:

$$w_j = e^{-\omega\times(k-t)}$$

For each location i, these weights were then used to calculate a weighted average of differences in the logit of prevalence:

$$\bar{d_\iota} = \frac{\sum_{t=1991}^{k} w_t d_{i,t,t-1}}{\sum_{t=1991}^{k} w_t}$$

Finally, the weighted average of differences was used to estimate prevalence in year $k+1$:

$$p_{i,k+1} = logit^{-1}(logit(p_{i,k}) + \bar{d}_i)$$

To optimise the one-year-ahead predictive performance of this projection method, we evaluated a range of possible values of ω between 0 and 1. For each candidate value of ω, we withheld data from 2022, then projected prevalence in 2022 for each location using only the data from preceding years. We repeated this process for all years between 1990 and 2022, then compared these projections to the withheld prevalence values, calculating root-mean-squared error across all locations and years. We then selected the value of ω that minimised out-of-sample root-mean-squared-error (ω = 0.084).

Sequelae modelling

Lymphoedema and hydrocele modelling

For GBD 2019, we reviewed published studies on the prevalence of hydrocele due to LF and lymphoedema due to LF, as well as programme monitoring data for which LF infection and hydrocele or lymphoedema prevalence were reported from within the same study population.

For GBD 2019, we adjusted non-sex-specific data on lymphoedema due to LF to be sex-specific. Hydrocele data are not reported or modelled for females. We then adjusted any non-age-specific lymphoedema data and non-age-specific hydrocele data to be age-specific according to five-year age groups using age patterns modelled from age-specific data in DisMod-MR 2.1.

Two separate disability models were implemented, one for lymphoedema and one for hydrocele – the process essentially the same. The age patterns can be found in Figures 1a and Figure 1b, below. The community-level prevalence reported in studies for which hydrocele or lymphoedema were also reported was used as a covariate (adjusted to represent ICT prevalence) to predict the prevalence of hydrocele and lymphoedema.

For GBD 2023, the prevalence of hydrocele due to LF and lymphoedema due to LF were estimated by applying the age-pattern generated for GBD 2019, outlined above, to the age-specific and sex-specific LF infection prevalence estimates from the geostatistical and time-series models, also outlined above.

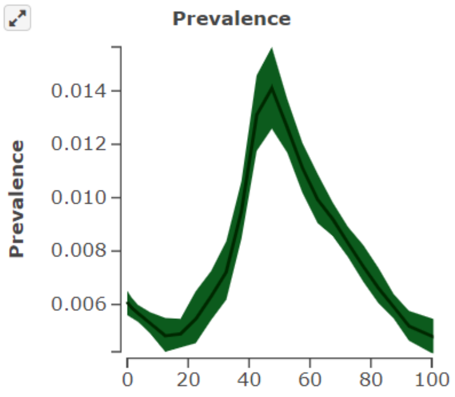

Figure 1a. Global age-pattern for lymphoedema due to LF used to split all-age data into age-specific datapoints for further modelling

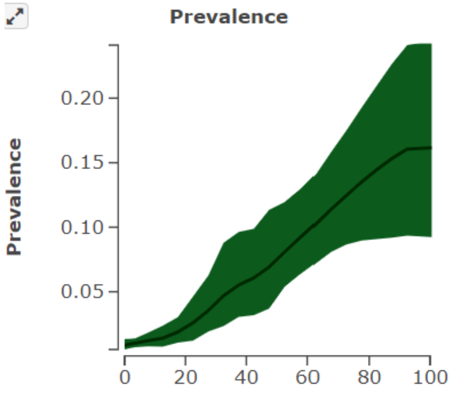

Figure 1b. Global age-pattern for hydrocele due to LF used to split all-age data into age-specific datapoints for further modelling

ADL prevalence estimates

Lastly, the third and final sequela for LF was estimated, acute ADL. After prevalence of lymphoedema and hydrocele were estimated, we assumed the following for prevalent lymphoedema cases: 95% experience a total of four ADL episodes per year, with an average duration of seven days. For prevalent hydrocele, we assume 70% of cases experience a total of two episodes per year, with an average duration of seven days. These assumptions were used to estimate the annual prevalence of acute ADL episodes.

Disability weights

The table below shows the list of sequelae due to LF and the associated disability weights.

Table 2. Severity distribution, details on the severity levels for lymphatic filariasis and the associated disability weight (DW) with that severity

**Sequela**	**Lay description**	**DW (95% CI)**
Lymphoedema	Has swollen legs with hard and thick skin, which causes difficulty in moving around	0.109 (0.073–0.154)
Hydrocele	Has swelling and tenderness in the testicles and pain during urination	0.128 (0.086–0.18)
Acute adenolymphangitis due to lymphatic filariasis	Has a fever and aches and feels weak, which causes some difficulty with daily activities	0.051 (0.032–0.074)

We did not apply any adjustments for the COVID-19 pandemic to lymphatic filariasis due to a lack of available data quantifying the impacts of the pandemic on NTD epidemiology.

Changes from GBD 2021 to GBD 2023

There were no substantive changes to the modelling strategy for GBD 2023.

References

1. Cromwell EA, Schmidt CA, Kwong KT, *et al.* The global distribution of lymphatic filariasis, 2000–18: a geospatial analysis. *The Lancet Global Health* 2020; **8**: e1186–94.

Major depressive disorder

Flowchart

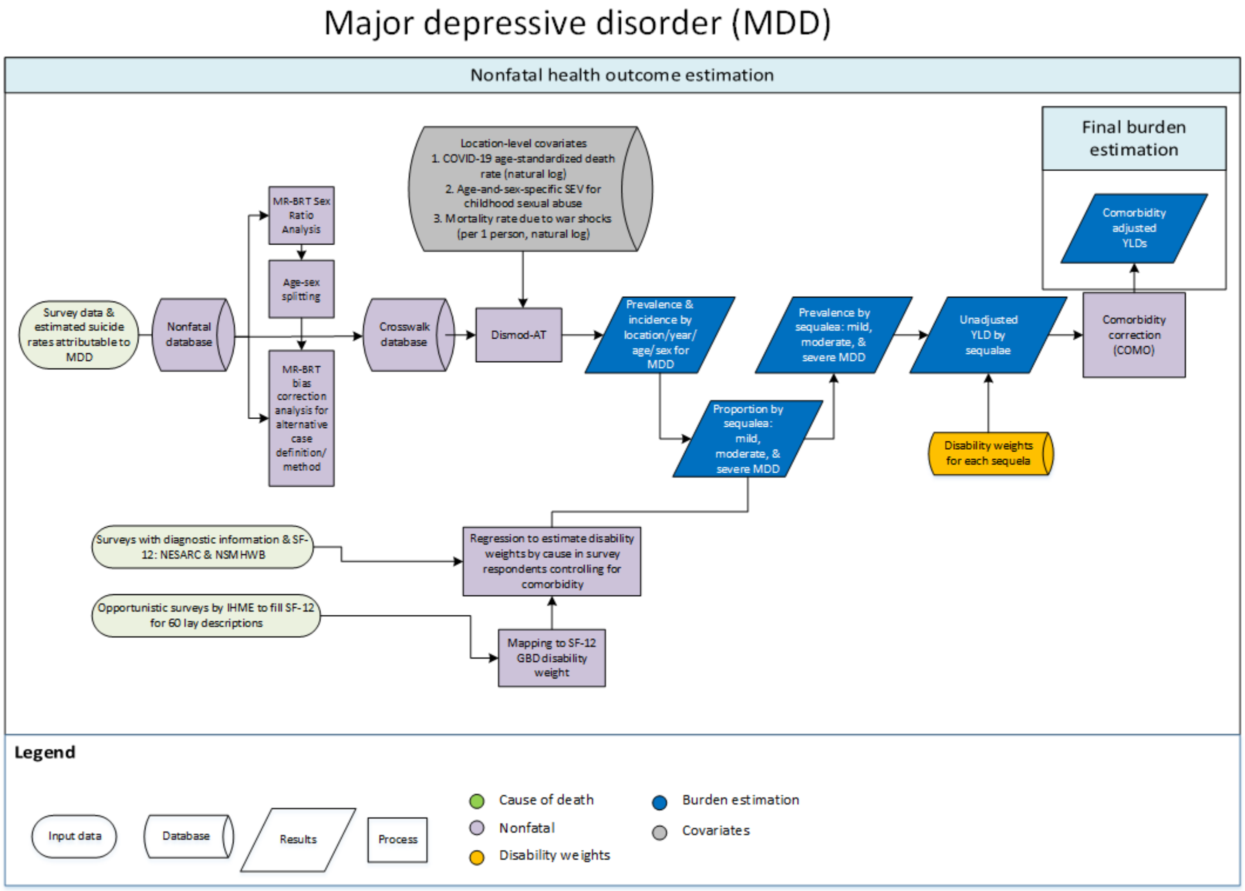

Input data and methodological summary for major depressive disorder

Case definition

Major depressive disorder (MDD) is an episodic mood disorder involving the experience of one or more major depressive episode(s). Included in the GBD disease modelling were cases meeting diagnostic criteria for MDD according to the Diagnostic and Statistical Manual of Mental Disorders (DSM) or the equivalent diagnosis of recurrent depression in the International Classification of Diseases (ICD).[1,2] These were identified by the following ICD-10 codes: F32.0–9, F33.0–9; excluding those cases due to a general medical condition or substance induced cases.[1,2] Different versions of DSM (DSM-III, DSM-III-R, DSM-IV, DSM-IV-TR, DSM-5, and DSM-5-TR) and ICD (ICD-9, ICD-10, and ICD-11) were accepted.

According to DSM-IV-TR criteria, MDD involves the presence of at least one major depressive episode, which is the experience of either depressed mood or loss of interest/pleasure, for most of every day, for at least two weeks. This must represent a change from the person's baseline and impaired functioning observed across social, occupational, and educational domains.

In addition to one of the two symptoms above, four out of the following seven criteria must also be met to make a diagnosis:

- change in eating, appetite, or weight
- excessive sleeping or insomnia
- agitated or slow motor activity
- fatigue
- feeling worthless or inappropriately guilty
- trouble concentrating
- repeated thoughts about death

MDD was modelled as an episodic disorder with the average length of a major depressive episode (ie, duration) specified. This method has been discussed in greater detail in previous publications.[3,4]

Input data

The epidemiological systematic literature review for MDD was conducted in three stages involving electronic searches of the peer-reviewed literature (ie, via PsycInfo, Embase, and PubMed), the grey literature, and expert consultation. For mental disorders, we update our GBD electronic database searches on a rolling basis. A systematic review update was completed for GBD 2023 for mood disorders together (depressive disorders and bipolar disorder). Databases were searched on March 23, 2021, for publications after April 1, 2018. Below are the search terms used for each database:

PubMed: ((((((((((((prevalen*[Title/Abstract]) OR (mortality[Title/Abstract])) OR (death*[Title/Abstract])) OR (inciden*[Title/Abstract])) OR (recurren*[Title/Abstract])) OR (remission[Title/Abstract])) OR (duration[Title/Abstract])) OR (remit*[Title/Abstract])) OR (epidemiolog*[Title/Abstract])) OR (prevalence[MeSH Terms])) OR (mortality[MeSH Terms])) OR (incidence[MeSH Terms])) OR (recurrence[MeSH Terms])) AND (((((((((((depress*[Title/Abstract]) OR (dysthymi*[Title/Abstract])) OR (bipolar[Title/Abstract])) OR (manic[Title/Abstract])) OR (mania[Title/Abstract])) OR ("mood disorders"[Title/Abstract])) OR ("mood disorder"[Title/Abstract])) OR (mood disorders[MeSH Terms])) OR (depressive disorders[MeSH Terms])) OR (depressive disorders, major[MeSH Terms])) OR (bipolar disorders[MeSH Terms])) OR (dysthymic disorders[MeSH Terms]))

EMBASE: (depress*:ab,ti OR dysthymi*:ab,ti OR bipolar:ab,ti OR manic:ab,ti OR mania:ab,ti OR 'mood disorder':ab,ti OR 'mood disorders':ab,ti) AND (prevalen*:ab,ti OR mortality:ab,ti OR death*:ab,ti OR inciden*:ab,ti OR recurren*:ab,ti OR remission:ab,ti OR duration:ab,ti OR remit*:ab,ti OR epidemiolog*:ab,ti

PsycInfo: (title: depress* OR abstract: depress* OR title: dysthymi* OR abstract: dysthymi* OR title: bipolar OR abstract: bipolar OR title: manic OR abstract: manic OR title: mania OR abstract: mania OR title: "mood disorder" OR abstract: "mood disorder" OR title: "mood disorders" OR abstract: "mood disorders") AND (title: prevalen* OR abstract: prevalen* OR title: mortality* OR abstract: mortality* OR title: death* OR abstract: death* OR title: inciden* OR abstract: inciden* OR title: recurren* OR abstract: recurren* OR title: remission AND abstract: remission OR title: duration OR abstract: *duration OR title: remit* OR* abstract: remit* OR title: epidemiolog* OR abstract: epidemiolog*)

In addition to the database search, a grey literature search and expert consultation were also conducted. The systematic review update was conducted according to Preferred Reporting Items for Systematic Reviews and Meta-Analyses (PRISMA; see Figure 1).

Figure 1: PRISMA 2009 flow diagram

From: Moher D, Liberati A, Tetzlaff J, Altman DG, The PRISMA Group (2009). *P*referred *R*eporting *I*tems for *S*ystematic Reviews and *M*eta-*A*nalyses: The PRISMA Statement. PLoS Med 6(7): e1000097. doi:10.1371/journal.pmed1000097

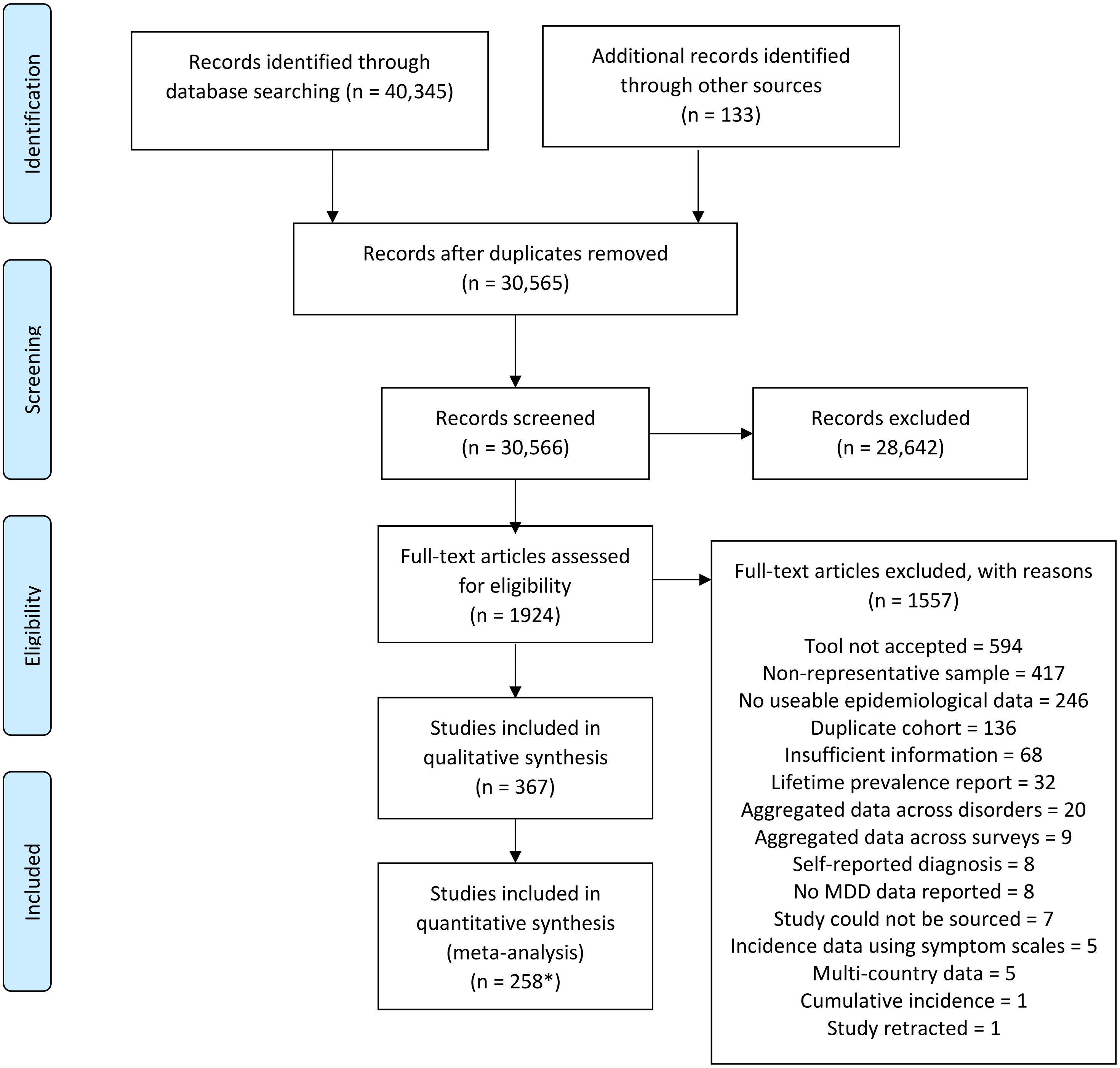

**The qualitative analysis led to the exclusion of additional studies with duplicative cohorts or methodological limitations impacting their eligibility and increasing measurement error within the data.* The GBD inclusion criteria stipulated that: 1) the publication year must be from 1980 onward; 2) “caseness” must be based on clinical threshold as established by the DSM or ICD; 3) sufficient information must be provided on study method and sample characteristics to assess the quality of the study; and 4) study samples must be representative of the general population (ie, inpatient or pharmacological treatment samples, case studies, veterans, or refugee samples were excluded). No limitation was set on the language of publication. Methods used for this systematic review have been reported in greater detail elsewhere.[3]

Age-sex splitting

The extracted data underwent three types of age-sex splitting processes:

1. Where possible, estimates were further split by sex and age based on the available data. For instance, if studies reported prevalence for broad age groups by sex (eg, prevalence in 15–65-year-old males and females separately), and also by specific age groups but for both sexes combined (eg, prevalence in 15–30-year-olds, then in 31–65-year-olds, for males and females combined); age-specific estimates were split by sex using the reported sex ratio and bounds of uncertainty.

2. A meta-regression—Bayesian, regularised, trimmed (MR-BRT) analysis was used to split the remaining both-sex estimates in the dataset. For each parameter, sex-specific estimates were matched by location, mid-age, and year. A MR-BRT network meta-analysis was then used to estimate pooled sex ratios. Given evidence to suggest that the sex ratio in depression varies with age,[5-7] we also tested for an age interaction in the model. We found that the sex difference in MDD decreased significantly with age, ie, prevalence in males (compared to females) increased significantly with increasing age. The global sex ratio (at the mean mid-age of data informing the sex-ratio model) was estimated as 0.55 (95% uncertainty interval [UI] 0.55–0.56) while Figure 2 shows the estimated male-to-female prevalence ratio by age. Age-specific sex ratios were used to split both-sex estimates in the dataset.

Figure 2. Sex ratios by age for MDD

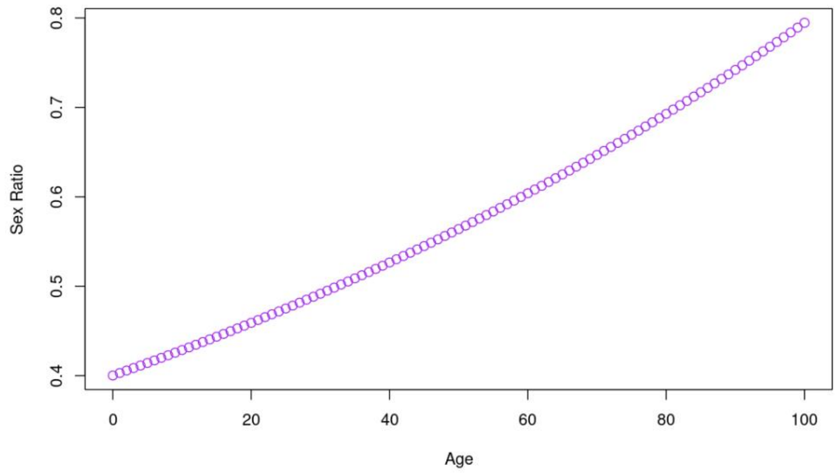

Bias corrections/crosswalks

Estimates with known biases were adjusted/crosswalked accordingly prior to DisMod-AT. For each crosswalk of interest, pairs of the reference and the alternative estimates were matched by age, sex, location, and year. This was done for both within-study (where possible) and between-study pairs. These pairs were then used as inputs in a MR-BRT network meta-analysis. The MR-BRT analysis produced a pooled ratio between the reference estimates and alternative estimates, which was used to adjust all alternative estimates in the dataset. Reference data informing the prevalence of MDD consisted of estimates reporting past-month/point prevalence of MDD using a diagnostic tool that was administered by a clinician. Four adjustment ratios were used for alternative data:

1. A past-year recall ratio adjusted all datapoints derived from past-year prevalence towards the level they would have been if the study had captured point/past-month prevalence. The latter prevalence period is less affected by recall bias.
2. A symptom scale ratio adjusted all datapoints derived using a symptom scale toward the level they would have been if the scale had strictly adhered to DSM or ICD thresholds for MDD.
3. A World Health Survey ratio adjusted all World Health Survey data downwards towards the level they would have been had the study strictly adhered to DSM or ICD thresholds for MDD. The World Health Surveys are surveys conducted by the World Health Organization in close to 70 countries. While these surveys capture useful information on the prevalence of depression, they make use of a symptom scale which does not fully meet DSM and ICD criteria for MDD. This adjustment works essentially in the same way as the previous symptom scale adjustment.
4. A lay-interviewer ratio was used to adjust all prevalence estimates derived from trained lay interviewers towards the level they would have been if the estimate was derived from clinically trained interviewers (eg, psychologist or psychiatrist). We consider interviews conducted by clinicians to be more sensitive to detecting cases of MDD, particularly in locations where western-based mental health case definitions and instruments are yet to be fully validated.
5. A DSM-5 ratio was used to adjust all prevalence estimates derived from DSM-5 diagnostic criteria towards the level they would have been if the estimate was derived from previously published diagnostic criteria (ie, DSM-IV-TR, DSM-IV, DSM-III, ICD-10). It was decided that DSM-

5 data would be adjusted to levels of previous DSM iterations, rather than older iterations of DSM being adjusted to reflect DSM-5 criteria. This is because during the crosswalk estimation process for GBD 2023 there were only 14 studies reporting on prevalence using DSM-5 criteria and these reflected nine locations, largely weighted towards western locations, with the exception of West Java, Nepal, Taiwan, and Rio De Grande do Sul. Out of these four locations, data for three were reported on youth and would not be representative. Based on this it was concluded that there are insufficient data using DSM-5 across both LMICs and HICs across the lifespan to inform a crosswalk. In addition, if a DSM-IV to DSM-5 crosswalk was calculated, it would be informed by data from three locations only; USA, Rio Grande do Sul, and Sweden.

See Table 1 for adjustment factors used for MDD. The estimated UIs around the adjustment ratio incorporate gamma, which represents the between-study variance across all input data in the model. This added uncertainty widens the UIs for crosswalks with significant fixed effects.

Table 1: MR-BRT crosswalk adjustment factors for MDD

**Data input**	**Reference or alternative case definition**	**Gamma**	**Beta coefficient, log (95% UI)***	**Adjustment factor****
Population survey	Reference: past-month/point prevalence, from a diagnostic tool following DSM-III, DSM-IV, or ICD criteria, administered by a clinician	0.11		
Population survey	Alternative: past-year prevalence		0.73 (0.61 to 0.84)	2.07 (1.85–2.32)
Population survey	Alternative: symptom scale		1.03 (0.98 to 1.08)	2.80 (2.67–2.94)
Population survey	Alternative: World Health Survey data		0.96 (0.83 to 1.10)	2.62 (2.30–3.00)
Population survey	Alternative: lay-interviewer diagnosis		–0.26 (–0.46 to –0.05)	0.77 (0.63–0.95)
Population survey	Alternative: DSM-5 criteria diagnosis		0.32 (0.21 to 0.44)	1.38 (1.23–1.55)

**MR-BRT crosswalk adjustments can be interpreted as the factor the alternative case definition is adjusted by to reflect what it would have been had it been measured using the reference case definition. If the log/logit beta coefficient is negative, then the alternative is adjusted up to the reference. If the log/logit beta coefficient is positive, then the alternative is adjusted down to the reference.*

***The adjustment factor column is the exponentiated beta coefficient. For log beta coefficients, this is the relative rate between the two case definitions. For logit beta coefficients, this is the relative odds between the two case definitions.*

Modelling strategy

For GBD 2023, modelling of MDD transitioned from using DisMod-MR 2.1 to DisMod-AT. Bias and root mean square error (RMSE) are shown in Table 2 for each measure.

Table 2. Performance metrics for the MDD DisMod-AT model

**Measure**	**Bias**	**RMSE**

Prevalence	-0.00642	0.0206
Excess mortality	-0.0897	0.0897
Relative risk	0.380	0.7781
Standardized mortality ratio	1.39	1.74

Relative to DisMod-MR, DisMod-AT provides enhanced estimation machinery and modelling performance, enabling less data pre-processing and fewer assumptions to be made. As a result, the following methodological changes were made for GBD 2023.

i) For GBD 2021, studies reporting prevalence estimates across age groups spanning 25 years or more were split into five-year age groups based on the prevalence age pattern estimated by DisMod-MR. This process was not required for GBD 2023 due to the simultaneous modelling of age and time in DisMod-AT.

ii) For GBD 2021, excess mortality data available in the dataset were supplemented by estimated suicide rates (by age, sex, year, and location) attributable to MDD. These methods were introduced to address machinery limitations of DisMod-MR and were no longer necessary in GBD 2023 with the use of DisMod-AT.

iii) For GBD 2021, the impact of the COVID-19 pandemic on MDD prevalence was estimated via a custom adjustment to baseline DisMod-MR 2.1 estimates extrapolated from a MR-BRT meta-regression on the association between change in prevalence and proxies of pandemic impact. This process was not required for GBD 2023 as the impact could be estimated within DisMod-AT using a location-level covariate.

When a base fit is completed in DisMod-AT, standardised residuals are produced for all datapoints. Any observations where the absolute value of the standardised residual is greater than 2 were considered model outliers and were flagged for additional review. We reassessed the study methodology and quality of estimates flagged in this way before a decision was made to exclude or include such data in final models.

Data across all epidemiological parameters were initially included in the modelling process. However, given that the few incidence datapoints available typically excluded cases of MDD at baseline, new major depressive episodes in people with previous episodes were not counted and incidence was underestimated. For this reason, we chose to exclude all raw incidence data in the final model and instead allowed DisMod-AT to calculate incidence based on data from other parameters. We assumed no incidence and prevalence before age 3. This minimum age of onset was corroborated with expert feedback and existing MDD literature.[3] An average remission rate for a major depressive episode of 1.45 (1.3–1.6) was used. This was derived from the five longitudinal studies[8-12] fitting a lognormal curve with least squared differences to data on the proportion of incident cases still fulfilling the case definition for MDD at intervals over a one-year period (See Figure 3). As data were only available for a follow-up of one year, a decision had to be made about the maximum allowable duration of an episode. Setting this at 40 years, the average duration implied by the lognormal fit was 0.65 (0.59–0.70) of a year.[13]

Figure 3. Time to recovery in episodes of MDD from five studies

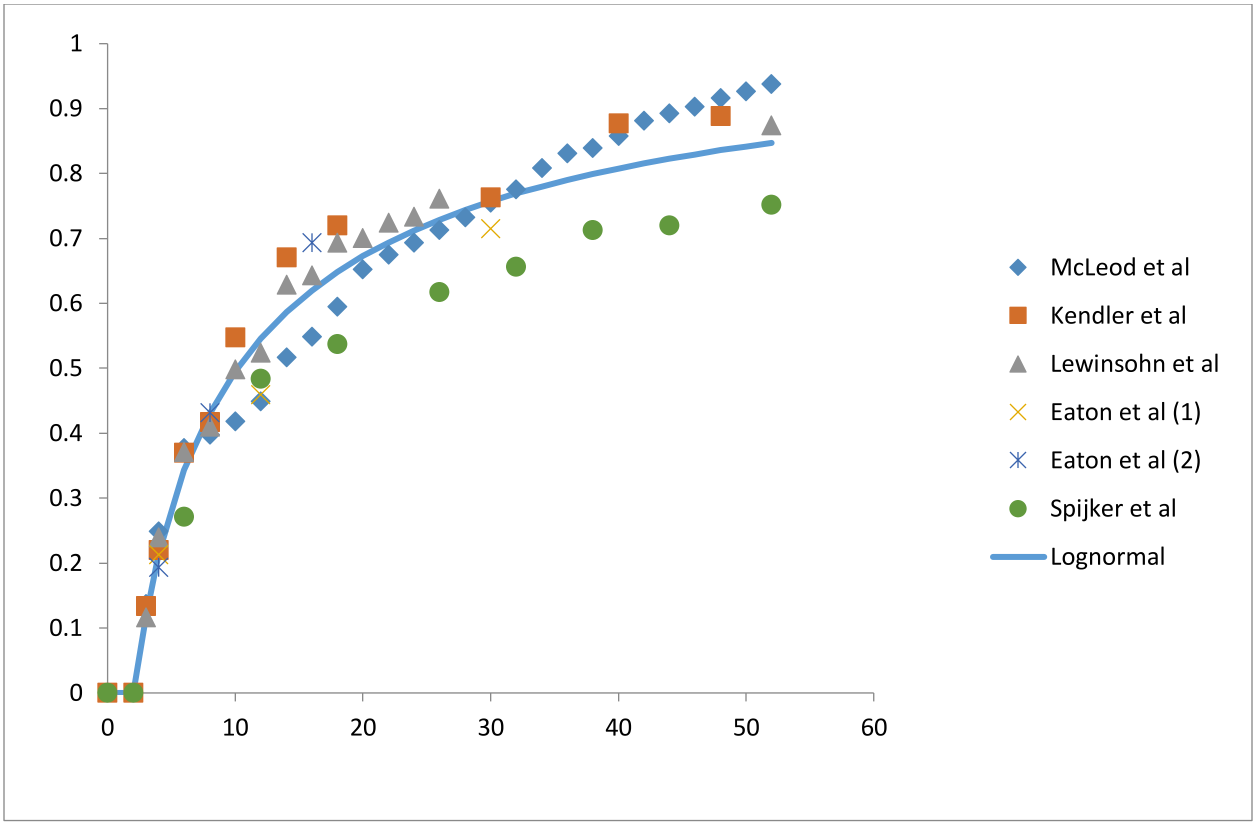

In addition to these changes, for GBD 2023 we removed an adjustment added to estimate the impact of the COVID-19 pandemic. Data informing this adjustment were sourced from a COVID-19 specific systematic review conducted in 2021. For GBD 2023, these data were integrated into the primary MDD dataset where they met inclusion criteria, alongside new COVID-19 relevant data found as part of the updated systematic review. For GBD 2023, the impact of COVID-19 was integrated into DisMod-AT modelling using a location-level covariate, representing the location specific age-standardised death rate attributable to COVID-19.

To enhance predictive accuracy, DisMod-AT models are refined through a covariate selection process, evaluating various location-level covariates for their effectiveness. This process begins with an initial selection of covariates informed by existing evidence and expert consultations. Covariate selection is then refined using a forward stepwise method. In the initial step, each covariate is assessed against the base model. Following this, the best-performing covariate is retained, and the process repeats, evaluating the remaining covariates against the newly updated model. This stepwise selection continues until no further improvement in predictive power is noted, as measured by the performance of model covariates and the root mean square standardised residuals (RMSSR). The following location-level covariates were selected through this process, and were used to inform estimation:

1. The age-standardised death rate of COVID-19: This covariate identified, for each GBD location, the age-standardised, sex-specific death rate attributable to COVID-19. The inclusion of this covariate follows evidence of the relationship between COVID-19 and MDD prevalence. To address the limited temporal range of available data and associated skewness, this covariate was log-transformed, and data from 2021+ were linearly waned to reach 0 by 2023.

2. An age- and sex-specific summary exposure value (SEV) scalar: This made use of the fraction of MDD burden caused by childhood sexual abuse (a risk factor of MDD included in the GBD study) to inform the estimation of incidence.
3. The per person mortality rate due to war shocks: This covariate identified, for each GBD location year, the mortality rate due to war and terrorism per one person. It was used given the existing evidence to show a positive association between conflict status and the prevalence of MDD.[16,17] The log of the modelled output was used as the covariate in DisMod AT due to skewness of the data.

A summary of covariates and exponentiated values for MDD are shown in Table 3.

Table 3. Summary of covariates used in the major depressive disorder DisMod-AT meta-regression model

**Covariate**	**Type**	**Parameter**	**Beta (95% UI)**	**Exponentiated beta (95% UI)**
COVID-19 age-standardised death rate	Location-level	Incidence	0.06 (0.04–0.07)	1.06 (1.05–1.07)
Age- and sex-specific SEV for childhood sexual abuse	Location-level	Incidence	1.91 (1.41–2.41)	6.73 (4.08–11.10)
Mortality rate due to war shocks (per 1 person)	Location-level	Incidence	0.01 (–0.02 to 0.05)	1.01 (0.98–1.05)

Note: DisMod-AT leverages on data from all parameters to estimate location-level-covariates for a specific parameter.

Severity splits and disability weights

The GBD disability weight survey assessments include lay descriptions of sequelae highlighting major functional consequences and symptoms. The lay descriptions and disability weights for MDD severity levels are shown in Table 4. To determine the proportion of people with MDD within each of the severity levels, estimates from the Australian National Survey of Mental Health and Wellbeing of Adults (NSMHWB, conducted in 1997)[14] were used. The proportion of MDD cases falling within each severity level were as follows: asymptomatic 13% (10–17), mild 59% (49–69), moderate 17% (13–22), and severe 10% (3–20). The same severity distribution and disability weights were applied to the pre-COVID-19 and post-COVID-19 prevalent cases of MDD.

Table 4. Severity distribution, details on the severity levels for major depressive disorder and the associated disability weight (DW) with that severity.

**Severity level**	**Lay description**	**DW (95% UI)**
Mild	Feels persistent sadness and has lost interest in usual activities. The person sometimes sleeps badly, feels tired, or has trouble concentrating but still manages to function in daily life with extra effort.	0.145 (0.099–0.209)

Moderate	Has constant sadness and has lost interest in usual activities. The person has some difficulty in daily life, sleeps badly, has trouble concentrating, and sometimes thinks about harming himself (or herself).	0.396 (0.267–0.531)
Severe	Has overwhelming, constant sadness and cannot function in daily life. The person sometimes loses touch with reality and wants to harm or kill himself (or herself).	0.658 (0.477–0.807)

Methodological changes have resulted in a small decrease globally in prevalence of MDD with large variation in changes across locations (some increasing and some decreasing) compared to GBD 2021. Our final prevalence estimates for MDD need to be considered along several limitations. Across our entire epidemiological modelling process, we still have a large number of locations with no high-quality raw data available. It is also difficult to quantify and remove all variation due to measurement error in our epidemiological estimates. While we have improved the methodology used to account for known sources of bias, in some cases we still have very few datapoints to inform these adjustments. Finally, there is a paucity of research on the risk factors of mental disorders which can be used as predictive covariates in our epidemiological models.

References

1. American Psychiatric Association. Diagnostic and Statistical Manual of Mental Disorders (DSM). Washington: American Psychiatric Association, 1952.
2. World Health Organization. The ICD-10 Classification of Mental and Behavioural Disorders. Clinical descriptions and diagnostic guidelines. Geneva: World Health Organization; 1992.
3. Ferrari AJ, Charlson FJ, Norman RE, et al. Burden of depressive disorders by country, sex, age, and year: findings from the global burden of disease study 2010. *PLoS Med* 2013; **10**(11): e1001547.
4. Ferrari AJ, Charlson FJ, Norman RE, et al. The epidemiological modelling of major depressive disorder: application for the Global Burden of Disease Study 2010. *PloS one* 2013; **8**(7).
5. Salk RH, Hyde JS, Abramson LY. Gender differences in depression in representative national samples: meta-analyses of diagnoses and symptoms. *Psychological Bulletin* 2017; **143**(8): 783.
6. Patten SB, Williams JV, Lavorato DH, Wang JL, Bulloch AG, Sajobi T. The association between major depression prevalence and sex becomes weaker with age. *Social Psychiatry and Psychiatric Epidemiology* 2016; **51**(2): 203-10.
7. Patten SB, Wang JL, Williams JV, et al. Descriptive epidemiology of major depression in Canada. *The Canadian Journal of Psychiatry* 2006; **51**(2): 84-90.
8. Eaton WW, Anthony JC, Gallo J, et al. Natural history of Diagnostic Interview Schedule/DSM-IV major depression. The Baltimore Epidemiologic Catchment Area follow-up. *Arch Gen Psychiatry* 1997; **54**(11): 993-9.
9. Kendler KS, Walters EE, Kessler RC. The prediction of length of major depressive episodes: results from an epidemiological sample of female twins. *Psychol Med* 1997; **27**(1): 107-17.
10. Lewinsohn PM, Clarke GN, Seeley JR, Rohde P. Major depression in community adolescents: age at onset, episode duration, and time to recurrence. *J Am Acad Child Adolesc Psychiatry* 1994; **33**(6): 809-18.
11. McLeod JD, Kessler RC, Landis KR. Speed of recovery from major depressive episodes in a community sample of married men and women. *J Abnorm Psychol* 1992; **101**(2): 277-86.

12. Spijker J, de Graaf R, Bijl RV, Beekman AT, Ormel J, Nolen WA. Duration of major depressive episodes in the general population: results from The Netherlands Mental Health Survey and Incidence Study (NEMESIS). *Br J Psychiatry* 2002; **181**: 208-13.
13. Vos T, Haby MM, Barendregt JJ, Kruijshaar M, Corry J, Andrews G. The burden of major depression avoidable by longer-term treatment strategies. *Archives of General Psychiatry* 2004; **61**(11): 1097-103.
14. Australian Bureau of Statistics. National Survey of Mental Health and Wellbeing of Adults 1997. Canberra: Australian Bureau of Statistics.

Malaria

Flowchart

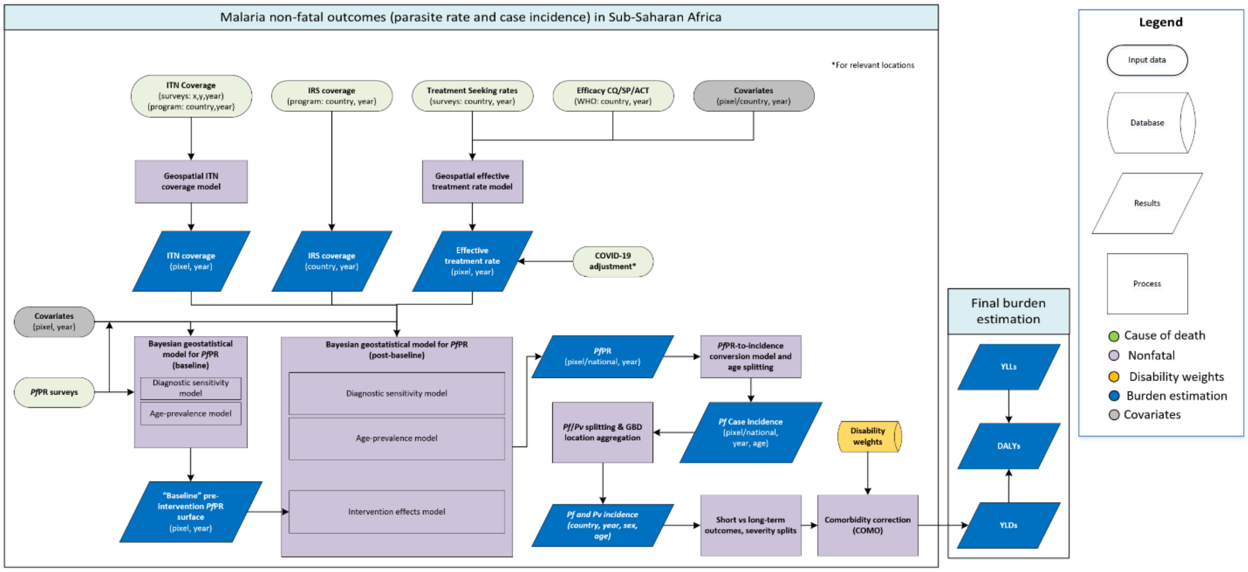

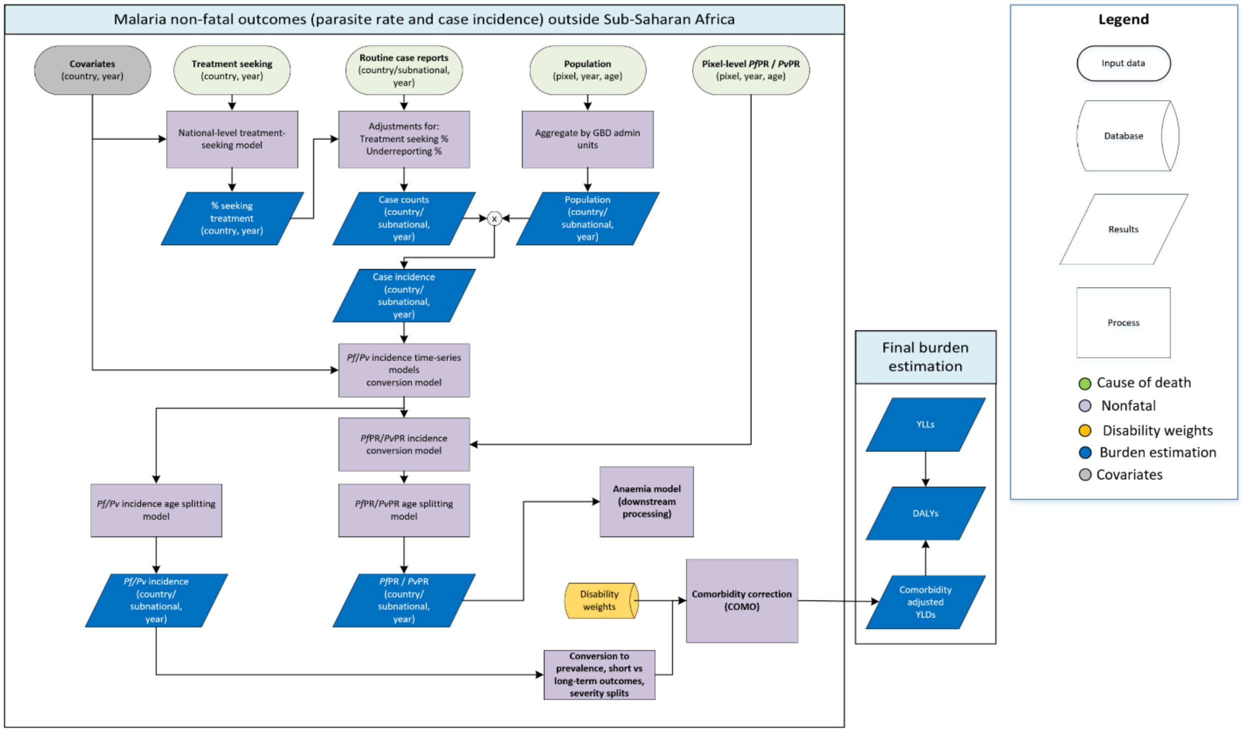

Input data and methodological summary for malaria

Case definition

Malaria is an acute parasitic mosquito-borne disease. An individual with uncomplicated malaria experiences one to two weeks of persistent fever, chills/shivering, sweating, joint pains, and headache. The individual will likely be lethargic and feverish, with loss of daily function during the attack. Individuals with an untreated *P falciparum* infection may develop severe malaria, which includes the symptoms of uncomplicated malaria but may also involve swelling, difficulty breathing, unconsciousness, and potentially death. Microscopy is considered the gold-standard diagnostic approach for the purposes of GBD. The relevant ICD-10 codes are B50-B54.

**Quantity of interest**	**Reference or alternative**	**Definition**
Malaria	Reference	Prevalence of people with detectable *P falciparum* or *P vivax* parasites through microscopy and/or rapid diagnostic tests and clinical symptoms for malaria (any of fever, diarrhoea, vomiting). This definition includes ICD-10 codes B50-B54.
Malaria	Alternative	Prevalence of people in malaria-endemic locations with clinical symptoms (any of fever, diarrhoea, vomiting) for whom diagnostic testing was either inconclusive or unavailable.
Disability due to cerebral malaria in children under 5	Reference	Proportion of children under 5 with cerebral malaria who go on to have long-term disability (motor impairment, intellectual disability, seizures, and blindness).

Input data

Primary data inputs were:

(i) Routine malaria case reports from national routine surveillance systems. These were obtained at the national level from the WHO World Malaria Report and at the subnational administrative level, wherever possible, via an exhaustive search of published and grey literature sources along with online data portals hosted by national ministries of health. Each retained record consisted of an annual count of malaria cases along with a distinction between confirmed and unconfirmed diagnoses, and differentiation by malaria parasite species.

(ii) Cross-sectional, geolocated, and community-representative observations of infection prevalence for *Plasmodium falciparum* (referred to hereafter as *P falciparum* parasite rate, *Pf*PR).

(iii) For GBD 2023, data on *Plasmodium knowlesi* (*P knowlesi)* have been included for Malaysia. This was done to better account for malaria burden in Malaysia, where *P knowlesi* is the primary strain of malaria impacting the country, as well as to increase alignment with World Malaria Report results. Although the strain does exist elsewhere in the region, prevalence is still comparatively low relative to other *Plasmodium species* and specific data on *P knowlesi* are often lacking.[1] The addition of *P knowlesi* to estimates of malarial burden was therefore limited to Malaysia for GBD 2023 and included with *P falciparum* during processing.

(iv) The input data for estimation of treatment seeking was expanded for GBD 2023 to include additional Multiple Indicator Cluster Survey (MICS) sources, as well as additional data on

treatment seeking for fever (from both Demographic and Health Survey [DHS] and MICS) for countries non-endemic for malaria.

These malaria epidemiological metrics were augmented in the modelling by:

(v) Malaria Atlas Project (MAP) modelled estimates of malaria control intervention population coverage (insecticide-treated bednets [ITNs], indoor residual spraying [IRS], and effective treatment with an antimalarial drug) resolved to 5 km x 5 km pixel-year level (for sub-Saharan Africa) and country-year level (outside sub-Saharan Africa).

(vi) A large suite of environmental, sociodemographic, and economic covariates resolved to 5 km x 5 km pixel-year level (for sub-Saharan Africa) and country-year level (outside sub-Saharan Africa).

Modelling strategy

The suitability, availability, and quality of *Pf*PR and routine case reporting data, as well as detailed intervention coverage information, differ markedly inside versus outside sub-Saharan Africa. As such, we developed separate modelling strategies for countries inside sub-Saharan Africa versus those outside. The exceptions were Botswana, Cabo Verde, Comoros, Djibouti, Eritrea, Ethiopia, Mauritania, Mauritius, Namibia, São Tomé and Príncipe, Senegal, South Africa, and Swaziland. Despite being part of Africa, these countries exhibit epidemiological trends and have data availability/quality more akin to non-African settings.

PfPR and case incidence modelling: Africa

Modelling was conducted in the following steps:

(i) The large assembly of geolocated *Pf*PR surveys maintained by MAP was used in a Bayesian spatiotemporal geostatistical model to predict *Pf*PR for every pixel-year in sub-Saharan Africa, representing an update to earlier work.[2-3] The model considered (i) *Pf*PR survey participant age ranges and diagnostic types; (ii) coverage of ITNs, IRS, and effective antimalarial drug coverage, and how these metrics changed through time at each date and prediction location; (iii) environmental conditions at each date and prediction location (including density of vegetation, temperature, humidity, rainfall, elevation, and proximity to populated areas). The outcome was a predicted space-time "cube" of *Pf*PR, standardised to the 2–10 age range, for each year 1980–2023.

(ii) The *Pf*PR cube was then converted into an equivalent cube of the predicted incidence rate of clinical malaria. This conversion was achieved using an established model[4] taking seasonality, treatment level, and recent prevalence trends at the location level into account and provided estimates stratified first into three broad age bins (0–5; 5–15; <15) and then into the final 23 GBD 2023 age bins.

PfPR and case incidence modelling: outside Africa

Malaria-endemic countries outside Africa tend to have less *Pf*PR data than those inside, in part because prevalence is generally lower. Furthermore, *Pf*PR surveys are rare in areas of lower prevalence, and thus this metric becomes an inefficient way to measure malaria risk. In contrast, routine surveillance systems outside Africa are generally stronger, meaning that reports of malaria cases from health systems are more reliable and provide some insight into the total malaria burden in the community.

Modelling outside Africa was carried out in the following steps:

(i) National and subnational case reports were first subject to adjustments to identify and minimise bias. Bias in reported case numbers arises from various sources. First, a fraction of cases in the community will fail to seek treatment or will attend a private or informal health care provider that will not provide a record of that case to the routine surveillance system. We adjusted for these factors by modelling the fraction of cases seeking care from different provider categories based on data from nationally representative cross-sectional household surveys (primarily from DHS program and MICS).[5-6] Another factor for which we must adjust is cases reaching formal clinics that may not be subject to a confirmatory diagnostic test. We adjusted for this by assuming the fraction of unconfirmed cases that were truly malaria would equal the fraction of positives among all those tested. A final factor we adjust for is incomplete data as many routine surveillance systems fail to capture all case reports, with facilities/regions missing from the national totals in a given year. We adjusted for this based on reporting completeness statistics published nationally by WHO.
   a. For GBD 2023, several updates were made to the treatment-seeking estimation process. The modelling process was split into two parts: a national-level model and a subnational-level model, with the subnational-level model values then being adjusted to align with national-level results. Please see Nguyen and colleagues, 2023[6] for further details regarding updates to the treatment-seeking modelling process.

(ii) These adjusted routine case reports were georeferenced using digitised administrative boundary data using a spatial database of such boundaries collated and maintained by MAP.

(iii) Each case report was converted into an estimate of clinical incidence rate by dividing it by the estimated population in each unit, with the latter quantity derived by combining high-resolution gridded population data and the aforementioned administrative boundaries.

(iv) Bayesian time-series models were then applied to the case reports for each country to impute incidence rates for years with missing data. The results from this analysis, in conjunction with the adjusted case reports, constitute the incidence values delivered for GBD 2023.

(v) The incidence rate for each country-year was then converted to an inferred *Pf*PR value using the same model described earlier.[4] This allowed us to utilise these polygon-level surveillance data and the *Pf*PR point-level data (where present) within the same modelling framework.

(vi) The combined *Pf*PR survey point data and (pseudo) *Pf*PR administrative unit data were then used in a Bayesian spatiotemporal geostatistical model to predict *Pf*PR at pixel-year level across all countries. As for the Africa model, *Pf*PR was standardised by age and diagnostic type and informed by a wide suite of covariates. An additional mechanism was developed to allow polygon (ie, administrative unit) and point (ie, survey) data to be used jointly to infer the predicted space-time surfaces.

(vii) The predicted *Pf*PR cube was then adjusted to ensure that, after conversion to pixel-level incidence, the incidence counts per country-year would precisely match the incidence results from step (iv). The summarised *Pf*PR values (ie, population-weighted and tallied for each country-year) from the adjusted *Pf*PR cube constitute the *Pf*PR values delivered for GBD 2023.

Total malaria cases by country, year, sex

The pixel-level predictions of clinical incidence rate (both inside and outside of Africa) were combined with high-resolution gridded population data to estimate total cases per pixel-year. These were then

aggregated to GBD national/subnational areas. Inside sub-Saharan Africa, for countries endemic for *P vivax* and *P falciparum*, we calculated the number of cases due to *P vivax* by applying the fraction of *P vivax* and *P falciparum* obtained from WHO and a literature review.[7-8] Outside sub-Saharan Africa, we followed the identical procedure for *P vivax* and *P falciparum.* Final age-splitting was accomplished using age-versus-incidence rate relationships gleaned from the paper by Cameron and colleaguges, 2015.[4]

Determining YLDs for malaria

As in GBD 2021, we used a two-step process for determining malaria severity. For acute cases, severity splits for mild, moderate, and severe malaria were produced by analysis of Medical Expenditure Panel Survey (MEPS) data. These sequelae and their associated disability weights are presented below.

Table 1. Severity distribution, details on the severity levels for malaria and the associated disability weight (DW) with that severity

**Severity level**	**Lay description**	**DW (95% CI)**
Mild malaria	Has a low fever and mild discomfort but no difficulty with daily activities.	0.006 (0.002–0.012)
Moderate malaria	Has a fever and aches and feels weak, which causes some difficulty with daily activities.	0.051 (0.032–0.074)
Severe malaria	Has a high fever and pain and feels very weak, which causes great difficulty with daily activities.	0.133 (0.088–0.19)

To determine long-term neurological burden due to malaria, we used the work by Roca-Felter and colleagues (2008)[9] that examined the number of uncomplicated cases that led to longer-term impairment. Analytically, this means multiplying incidence estimates (described in the section above) for persons under 20 by 0.00029 (0.000077–0.00057). This adjusted case estimate is then combined with excess mortality rates derived from all-cause mortality and standardised mortality ratios for neonatal encephalopathy (NE) in a DisMod model to produce prevalence estimates of long-term sequelae for all estimation years. Implicit in this process is an assumption that the disability and trend of impairment due to severe malaria follow NE. The subsequent severity splitting follows NE as well.

To determine the burden of acute (short-term) malaria, the incidence estimation results are combined and converted to prevalence by matching each draw with a draw of duration of clinical illness. Consistent with GBD 2021, we used a uniform distribution between 14 and 28 days for duration.

Malaria COVID-19 adjustment

A COVID-19 adjustment was applied to the years 2020–2022 utilising PULSE surveys from WHO, conducted by country government officials on health-care service disruption.[10-13] This adjustment was applied to antimalarial effective treatment rates, which are used in the prevalence estimation process, and subsequently incidence for only the pandemic-impacted years 2020–2022. Please see Dzianach and colleagues, 2023[14] for further details on how the adjustments were derived. Currently these adjustments have only been applied to 26 countries located in Africa (see full list below) due to the lack of a complementary approach to introducing the adjustments for countries outside of Africa. For countries in which a national survey was conducted after the onset of the pandemic, the new empirical

data on treatment seeking for fever superseded the need to model these disruptions, and no adjustment was applied for the study years. At the time our estimates were generated, these were the best data available to help account for the impact of COVID-19 on malaria. As new data become available, we will continue to refine our adjustment approach.

COVID-19 adjustments were applied to the following countries: Angola, Burundi, Benin, Central African Republic, Côte d'Ivoire, Cameroon, Democratic Republic of the Congo, Congo, Gabon, Ghana, Guinea-Bissau, Equatorial Guinea, Kenya (and subnationals), Liberia, Mozambique, Malawi, Nigeria (and subnationals), Rwanda, Sudan, Sierra Leone, Somalia, South Sudan, Chad, Togo, Uganda, and Zambia.

Changes from GBD 2021 to GBD 2023

As mentioned above, for GBD 2023 there were two major data updates included in addition to routine annual data updates: (1) including data on *P knowlesi* for Malaysia, and (2) expanding data inputs for treatment-seeking estimation.

For modelling, the primary update included for GBD 2023 was to the treatment-seeking estimation process, detailed above. Further details regarding the updates to treatment seeking can be found in Nguyen and colleagues, 2023.[6]

References

1. Lee, WC., Cheong, F.W., Amir, A. *et al. Plasmodium knowlesi*: the game changer for malaria eradication. *Malar J* **21**, 140 (2022).
2. Bhatt, S. et al. The effect of malaria control on *Plasmodium falciparum* in Africa between 2000 and 2015. *Nature* (2015).
3. Gething, P. W. et al. Mapping *Plasmodium falciparum* Mortality in Africa between 1990 and 2015. *New England Journal of Medicine* 375, 2435-2445 (2016).
4. Cameron, E. et al. Defining the relationship between infection prevalence and clinical incidence of *Plasmodium falciparum* malaria. *Nature Communications* 6:8170 (2015).
5. Battle, K.E. et al. Treatment-seeking rates in malaria endemic countries. *Malar J* **15**, 20 (2016).
6. Nguyen M, Dzianach PA, Castle PECW, Rumisha SF, Rozier JA, Harris JR, et al. (2023) Trends in treatment-seeking for fever in children under five years old in 151 countries from 1990 to 2020. *PLOS Glob Public Health* 3(8): e0002134. https://doi.org/10.1371/journal.pgph.0002134
7. Battle, K.E. et al. Mapping the global endemicity and clinical burden of *Plasmodium vivax*, 2000-17: a spatial and temporal modelling study. *The Lancet*, doi:10.1016/S0140-6736(19)31096-7 (2019).
8. Weiss, D. J. et al*.* Mapping the global prevalence, incidence, and mortality of *Plasmodium falciparum*, 2000-17: a spatial and temporal modelling study. *The Lancet*, doi:10.1016/S0140-6736(19)31097-9 (2019).
9. Roca-Feltrer, A. et al. Estimates of the burden of malaria morbidity in Africa in children under the age of 5 years. *Tropical Medicine & International Health*, doi: 10.1111/j.1365-3156.2008.02076(13)771-83 (2008).

10. Pulse survey on continuity of essential health services during the COVID-19 pandemic: interim report, 27 August 2020. Geneva: World Health Organization; 2020 (https://www.who.int/publications/i/item/WHO-2019-nCoV-EHS_continuity-survey-2020.1).
11. Second round of the national pulse survey on continuity of essential health services during the COVID-19 pandemic: January−March 2021. Geneva: World Health Organization; 2021 (https://apps.who.int/iris/bitstream/handle/10665/340937/WHO-2019-nCoV-EHScontinuity-survey-2021.1-eng.pdf?sequence=1&isAllowed=y).
12. Third round of the global pulse survey on continuity of essential health services during the COVID-19 pandemic. Geneva: World Health Organization; 2022 (https://www.who.int/publications/i/item/WHO-2019-nCoV-EHS_continuity-survey-2022.1).
13. Fourth round of the global pulse survey on continuity of essential health services during the COVID-19 pandemic. Geneva: World Health Organization; 2023 (https://www.who.int/publications/i/item/WHO-2019-nCoV-EHS_continuity-survey-2023.1).
14. Dzianach PA, Rumisha SF, Lubinda J, Saddler A, van den Berg M, Gelaw YA, Harris JR, Browne AJ, Sanna F, Rozier JA, et al. Evaluating COVID-19-Related Disruptions to Effective Malaria Case Management in 2020–2021 and Its Potential Effects on Malaria Burden in Sub-Saharan Africa. *Tropical Medicine and Infectious Disease*. 2023; 8(4):216.
15. Page MJ, McKenzie JE, Bossuyt PM, Boutron I, Hoffmann TC, Mulrow CD, Shamseer L, Tetzlaff JM, Akl EA, Brennan SE, Chou R. *Int J Surg*. 2021 Apr 1;88:105906.

Figure 1: GBD 2023 PRISMA diagram for PR and API literature review[15]

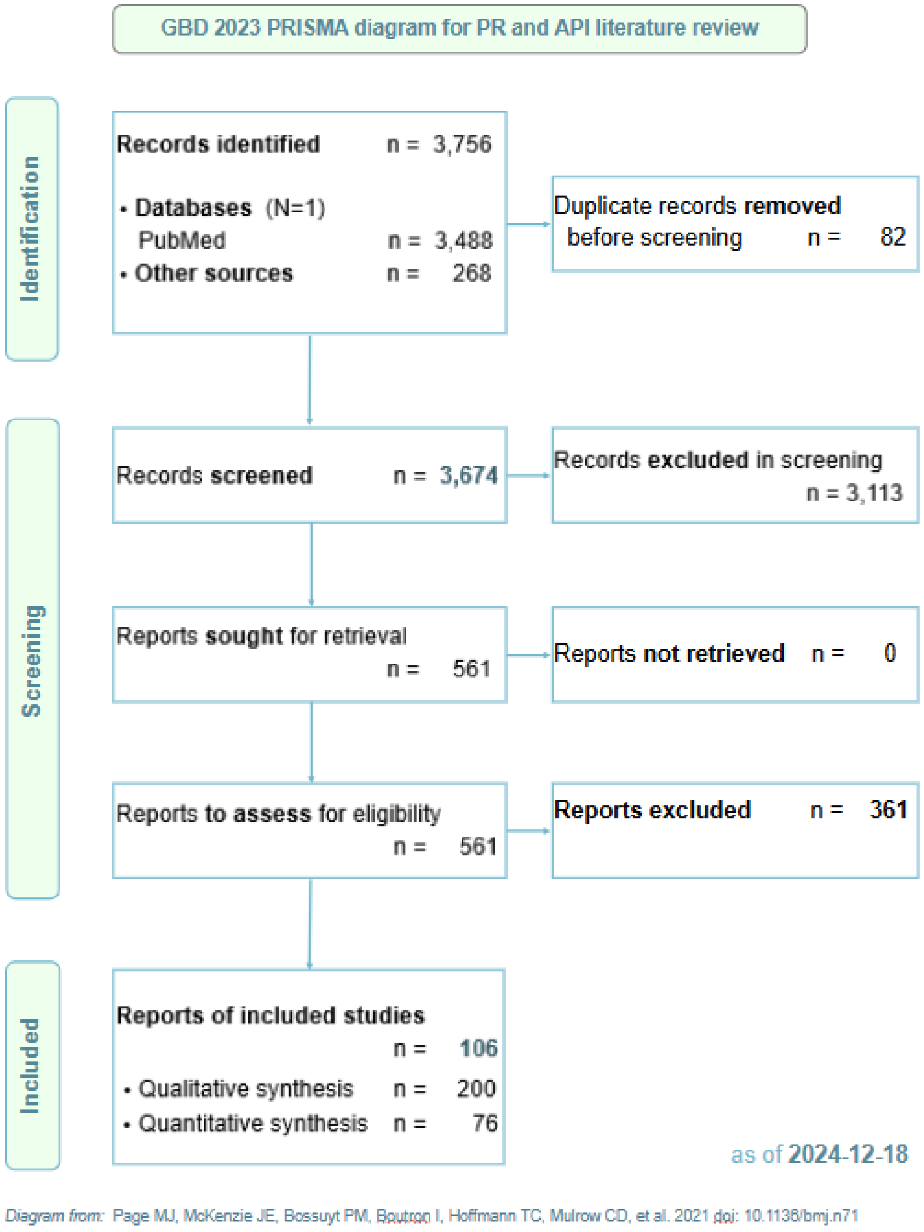

Maternal disorders

Non-fatal burden estimation for maternal disorders includes burden resulting from seven direct obstetric events or conditions, or groups of events and conditions: 1) abortion and miscarriage; 2) ectopic pregnancy; 3) obstructed labour and uterine rupture; 4) maternal haemorrhage; 5) maternal sepsis and other maternal infections; 6) maternal hypertensive disorders; and 7) other [direct] maternal disorders. These correspond to seven of nine subcauses of maternal death for which we estimate fatal burden. We do not estimate non-fatal burden related to the diseases and injuries underlying indirect maternal deaths and maternal deaths aggravated by HIV/AIDS, based on the premise that non-fatal burden associated with these diseases and injuries is captured in the respective underlying GBD cause.

Flowchart

Figure 1. Analytical flowchart for estimation of nonfatal burden of maternal disorders[1] for GBD 2023

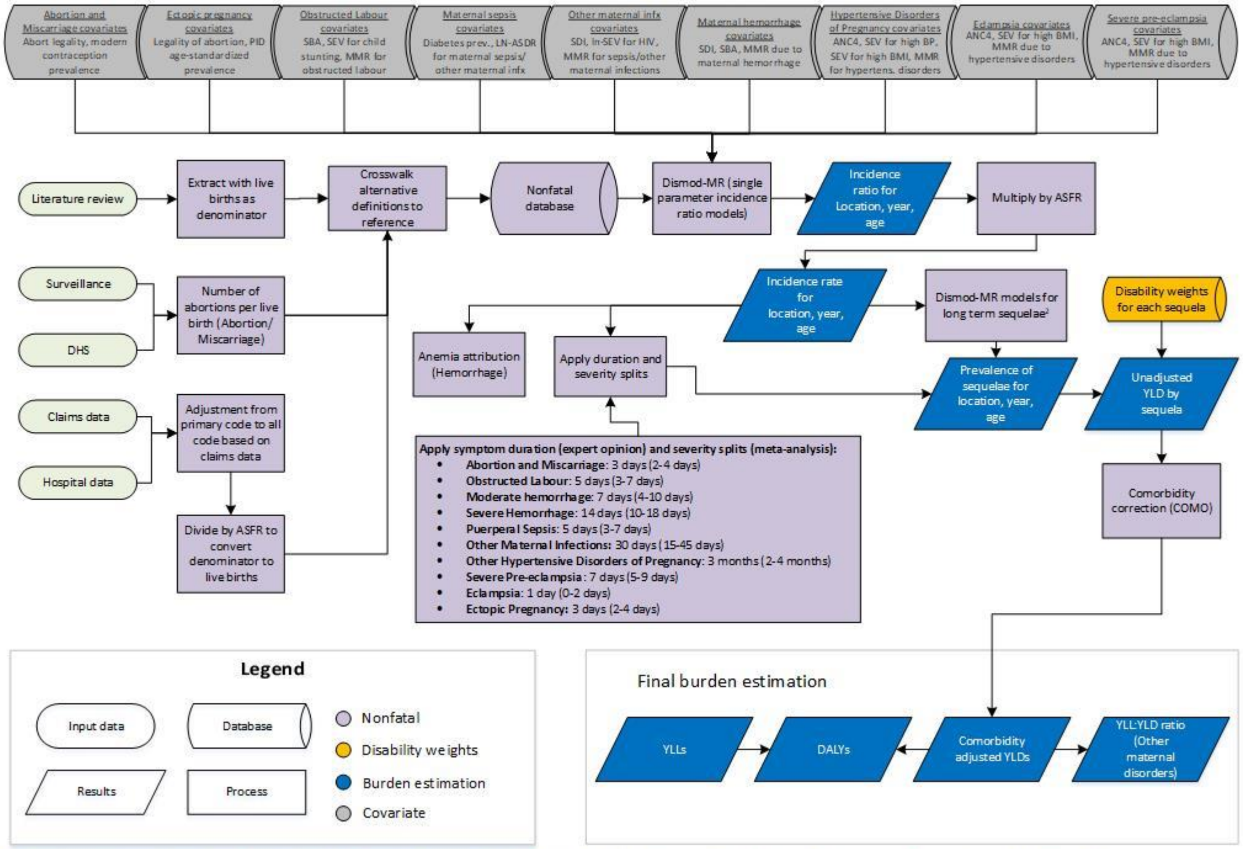

[1]Maternal disorders estimated for GBD 2023 include: 1) Abortion and miscarriage; 2) Obstructed labor and uterine rupture; 3) Maternal hemorrhage; 4) Maternal sepsis and other maternal infections; 5) Maternal hypertensive disorders; 6) Ectopic pregnancy 7) Other maternal disorders

[2]Long term sequelae include long term sequelae of severe pre-eclampsia, long term sequelae of eclampsia, and infertility due to puerperal sepsis.

Input data and methodological summary

Case definition

GBD non-fatal maternal disorders are direct obstetric complications of pregnancy, childbirth, and the postpartum period:

1) **Abortion and miscarriage** comprise a single disorder in the GBD. **Miscarriage,** also known as spontaneous abortion, is defined as spontaneous loss of pregnancy before 24 weeks of gestation and is measured on GBD as those that are clinically apparent and require medical care. **Abortion**, also known as intended abortion, is defined as elective or medically indicated termination of pregnancy at any gestational age, regardless of symptoms or complications.

2) **Ectopic pregnancy** is pregnancy that implants outside of the uterine cavity. It is ideally diagnosed by visualisation at surgery with histological confirmation following resection of ectopic pregnancy tissue, visualisation of an extrauterine gestational sac with a yolk sac or embryo (with or without a heartbeat) on transvaginal ultrasound (TVUS), or a combination of >2000 IU/mL serum human chorionic gonadotropin (hCG) and no evidence of an intrauterine pregnancy on TVUS.

3) **Maternal obstructed labor and uterine rupture** is defined as encompassing acute events of **obstructed labour** (arrest in the first or second stage of active labour despite strong uterine contractions including shoulder dystocia) and **uterine rupture during labour** (non-surgical breakdown of uterine wall during labour and delivery. Obstructed labour may be due to malpresentation, malposition, or cephalopelvic disproportion, but the presence of these factors alone is not sufficient to diagnose obstructed labour. (*Estimation of the incidence and short-term disability due to these acute events is described in this appendix section*.)

4) **Maternal haemorrhage** includes both postpartum haemorrhage and antepartum haemorrhage. It also includes placental disorders with haemorrhage regardless of blood volume lost or timing of bleeding event. Placental disorders without haemorrhage are not included in maternal haemorrhage but are included as other direct maternal disorders, described below.

    a. **Antepartum haemorrhage** is defined as any vaginal bleeding from any cause at or beyond 20 weeks of gestation and prior to onset of labour, except for post-coital spotting from cervical friability or bloody show late in third trimester just before onset of labor.
    b. **Postpartum haemorrhage** is defined as heavier than expected postpartum bleeding, >500 ml following vaginal delivery or >1000 ml after cesarean delivery.

5) **Maternal sepsis and other maternal infections** encompass the following:

    a. **Maternal sepsis** is sepsis during pregnancy, labor, and delivery, or postpartum defined by an identified source of infection, clinical signs of infection (vital sign abnormalities), and evidence of organ dysfunction.
    b. **Other maternal infections** are defined as other infections (without sepsis) believed to have a close epidemiological relationship with pregnancy, including genitourinary tract infections (excluding sexually transmitted diseases), obstetrical wound infections, and breast infections related to childbirth and lactation.

    (Some GBD visualisations may refer to "puerperal sepsis", which is outdated terminology; data and models so labelled represent the maternal sepsis definition provided above.)

6) **Hypertensive disorders of pregnancy** include **gestational hypertension** (new onset of hypertension in a pregnant person after 20 weeks of gestation as defined by having a blood pressure measured >140/90 on more than one occasion), **pre-eclampsia** (hypertension [>140/90 on more than one occasion] with proteinuria [≥0.3 g/L] or signs of end-organ damage), **severe**

pre-eclampsia (preeclampsia with severe hypertension [>160/100] or signs of end organ damage [liver: low platelets, elevated liver enzymes, coagulation issues; kidney: elevated creatinine; central nervous system: headaches or visual disturbances]), and **eclampsia** (defined as hypertension and seizures, with or without proteinuria). This definition excludes chronic hypertension in a pregnant person (hypertension present prior to 20 weeks of gestation), unless superimposed preeclampsia develops.

7) **Other [direct] maternal disorders** include a variety of different obstetric complications, the most common of which in ICD-10 coded VR mortality data include O88 (obstetric embolism), O26 (maternal care for other conditions predominantly related to pregnancy), O90 (complications of the puerperium, not elsewhere classified), O75 (other complications of labor and delivery, not elsewhere classified), C58 (malignant neoplasm of placenta), and O36 (maternal care for other fetal problems).

Input data

Non-fatal burden estimation utilises data on the incidence of maternal disorders extracted from previously published studies and from clinical administrative data processed by IHME. Datasets as-extracted and as-processed can be downloaded for each incidence model from the GBD online visualisation tools. Non-fatal burden estimation also makes use of demographic quantities (population, fertility) estimated by GBD, some empirical inputs on the severity of certain maternal disorders, and clinical expert opinion on the severity and duration of several maternal disorders.

Incidence data from published studies

A systematic review seeking data on cause-specific mortality and incidence of maternal disorders is conducted with each round of GBD. We most recently updated our search on February 2, 2022, using the following search string:

(((("Postpartum Hemorrhage" OR "Uterine Hemorrhage") OR (maternal[Title/Abstract] OR pregnan*[Title/Abstract] OR mothers) AND (haemorrhag*[Title/Abstract] OR hemorrhag*[Title/Abstract]) NOT "case report"[All fields]) OR (("induced abortion" OR "Therapeutic abortion" OR "legal Abortion" OR "medical abortion" OR "miscarriage" OR "Abortion, Induced"[Mesh] OR "Abortion, Therapeutic"[Mesh] OR "Abortion, Legal"[Mesh] OR "ectopic Pregnancy") NOT ("case report"[Title/Abstract] OR "birth defect"[Title/Abstract] OR congenital[Title/Abstract])) OR ("obstructed labour" OR "obstructed labor" OR "labour dystocia" OR "labor dystocia" OR dystocia OR "cephalopelvic disproportion" OR "cephalo-pelvic disproportion") OR (("obstetric fistula" OR "vesicovaginal fistula") OR "rectovaginal fistula") OR (("Puerperal Infection"[Mesh] OR "Puerperal Infection" OR ((maternal[Title/Abstract] OR pregnan*[Title/Abstract]) AND (Sepsis OR infection[Title/Abstract]))) NOT "case report") OR ((pre-eclampsia[Title/Abstract] OR preeclampsia[Title/Abstract] OR eclampsia[Title/Abstract] OR Pre-Eclampsia[Mesh] OR Eclampsia[Mesh] OR "Hypertension, Pregnancy-Induced"[Mesh] OR "pregnancy induced hypertension"[Title/Abstract] OR "gestational hypertension"[Title/Abstract] OR "Hypertensive disorders of pregnancy"[Title/Abstract]) NOT ("case report" OR "kidney donor"[Title/Abstract] OR "kidney donors"[Title/Abstract] OR polymorphism*[Title/Abstract] OR endotheli*[Title/Abstract]))) OR ((("maternal mortality"[Title/Abstract] OR "maternal death"[Title/Abstract] OR "maternal deaths"[Title/Abstract] OR "MM"[Title/Abstract] OR "confidential enquiry"[Title/Abstract] OR "confidential inquiry"[Title/Abstract] OR ((obstetric[Title/Abstract] OR pregnan*[Title/Abstract]) AND (etiology[Title/Abstract] OR cause[Title/Abstract] OR pattern[Title/Abstract]) AND (death[Title/Abstract] OR mortality[Title/Abstract]))) NOT (fetal[Title/Abstract] OR newborn*[Title/Abstract] OR neonatal[Title/Abstract] OR "case report" [Title/Abstract] OR "case study" [Title/Abstract] OR pathogenesis[Title/Abstract] OR thromboprophylaxis[Title/Abstract])) OR ((("maternal mortality"[Title/Abstract] OR "maternal death"[Title/Abstract] OR "maternal deaths"[Title/Abstract] OR "MMR"[Title/Abstract]) AND ("Afghanistan"[Title/Abstract] OR "Albania"[Title/Abstract] OR "Algeria"[Title/Abstract] OR "Andorra"[Title/Abstract] OR "Angola"[Title/Abstract] OR "Antigua and Barbuda"[Title/Abstract] OR "Argentina"[Title/Abstract] OR "Armenia"[Title/Abstract] OR "Azerbaijan"[Title/Abstract] OR "Bahrain"[Title/Abstract] OR "Bangladesh"[Title/Abstract] OR "Barbados"[Title/Abstract] OR "Belarus"[Title/Abstract] OR "Belize"[Title/Abstract] OR "Benin"[Title/Abstract] OR "Bhutan"[Title/Abstract] OR "Bolivia"[Title/Abstract] OR "Bosnia and Herzegovina"[Title/Abstract] OR "Botswana"[Title/Abstract] OR "Brazil"[Title/Abstract] OR "Brunei"[Title/Abstract] OR "Bulgaria"[Title/Abstract] OR "Burkina Faso"[Title/Abstract] OR "Burundi"[Title/Abstract] OR "Cambodia"[Title/Abstract] OR "Cameroon"[Title/Abstract] OR "Cape Verde"[Title/Abstract] OR "Central African Republic"[Title/Abstract] OR "Chad"[Title/Abstract] OR "China"[Title/Abstract] OR "Colombia"[Title/Abstract] OR "Comoros"[Title/Abstract] OR "Congo"[Title/Abstract] OR "Costa Rica"[Title/Abstract] OR "Croatia"[Title/Abstract] OR "Cuba"[Title/Abstract] OR "Cyprus"[Title/Abstract] OR "Côte d'Ivoire"[Title/Abstract] OR "Democratic Republic of the Congo"[Title/Abstract] OR "Djibouti"[Title/Abstract] OR "Dominica"[Title/Abstract] OR "Dominican Republic"[Title/Abstract] OR "Ecuador"[Title/Abstract] OR "Egypt"[Title/Abstract] OR "El Salvador"[Title/Abstract] OR "Equatorial Guinea"[Title/Abstract] OR "Eritrea"[Title/Abstract] OR "Ethiopia"[Title/Abstract] OR "Federated States of Micronesia"[Title/Abstract] OR "Fiji"[Title/Abstract] OR "Gabon"[Title/Abstract] OR "Georgia"[Title/Abstract] OR "Ghana"[Title/Abstract] OR "Grenada"[Title/Abstract] OR "Guatemala"[Title/Abstract] OR "Guinea"[Title/Abstract] OR "Guinea-Bissau"[Title/Abstract] OR "Guyana"[Title/Abstract] OR "Haiti"[Title/Abstract] OR "Honduras"[Title/Abstract] OR "India"[Title/Abstract] OR "Indonesia"[Title/Abstract] OR "Iran"[Title/Abstract] OR "Iraq"[Title/Abstract] OR "Jamaica"[Title/Abstract] OR "Jordan"[Title/Abstract] OR "Kazakhstan"[Title/Abstract] OR "Kenya"[Title/Abstract] OR "Kiribati"[Title/Abstract] OR "Kuwait"[Title/Abstract] OR "Kyrgyzstan"[Title/Abstract] OR "Laos"[Title/Abstract] OR "Latvia"[Title/Abstract] OR "Lebanon"[Title/Abstract] OR "Lesotho"[Title/Abstract] OR

"Liberia"[Title/Abstract] OR "Libya"[Title/Abstract] OR "Lithuania"[Title/Abstract] OR "Macedonia"[Title/Abstract] OR "Madagascar"[Title/Abstract] OR "Malawi"[Title/Abstract] OR "Malaysia"[Title/Abstract] OR "Maldives"[Title/Abstract] OR "Mali"[Title/Abstract] OR "Malta"[Title/Abstract] OR "Marshall Islands"[Title/Abstract] OR "Mauritania"[Title/Abstract] OR "Mauritius"[Title/Abstract] OR "Moldova"[Title/Abstract] OR "Mongolia"[Title/Abstract] OR "Montenegro"[Title/Abstract] OR "Morocco"[Title/Abstract] OR "Mozambique"[Title/Abstract] OR "Myanmar"[Title/Abstract] OR "Namibia"[Title/Abstract] OR "Nepal"[Title/Abstract] OR "Nicaragua"[Title/Abstract] OR "Niger"[Title/Abstract] OR "Nigeria"[Title/Abstract] OR "North Korea"[Title/Abstract] OR "Oman"[Title/Abstract] OR "Pakistan"[Title/Abstract] OR "Palestine"[Title/Abstract] OR "Panama"[Title/Abstract] OR "Papua New Guinea"[Title/Abstract] OR "Paraguay"[Title/Abstract] OR "Peru"[Title/Abstract] OR "Philippines"[Title/Abstract] OR "Qatar"[Title/Abstract] OR "Romania"[Title/Abstract] OR "Russia"[Title/Abstract] OR "Rwanda"[Title/Abstract] OR "Saint Lucia"[Title/Abstract] OR "Saint Vincent and the Grenadines"[Title/Abstract] OR "Samoa"[Title/Abstract] OR "Saudi Arabia"[Title/Abstract] OR "Senegal"[Title/Abstract] OR "Serbia"[Title/Abstract] OR "Seychelles"[Title/Abstract] OR "Sierra Leone"[Title/Abstract] OR "Singapore"[Title/Abstract] OR "Solomon Islands"[Title/Abstract] OR "Somalia"[Title/Abstract] OR "South Africa"[Title/Abstract] OR "South Sudan"[Title/Abstract] OR "Sri Lanka"[Title/Abstract] OR "Sudan"[Title/Abstract] OR "Suriname"[Title/Abstract] OR "Swaziland"[Title/Abstract] OR "Syria"[Title/Abstract] OR "São Tomé and Principe"[Title/Abstract] OR "Taiwan"[Title/Abstract] OR "Tajikistan"[Title/Abstract] OR "Tanzania"[Title/Abstract] OR "Thailand"[Title/Abstract] OR "The Bahamas"[Title/Abstract] OR "The Gambia"[Title/Abstract] OR "Timor-Leste"[Title/Abstract] OR "Togo"[Title/Abstract] OR "Tonga"[Title/Abstract] OR "Trinidad and Tobago"[Title/Abstract] OR "Tunisia"[Title/Abstract] OR "Turkmenistan"[Title/Abstract] OR "Uganda"[Title/Abstract] OR "Ukraine"[Title/Abstract] OR "United Arab Emirates"[Title/Abstract] OR "Uruguay"[Title/Abstract] OR "Uzbekistan"[Title/Abstract] OR "Vanuatu"[Title/Abstract] OR "Venezuela"[Title/Abstract] OR "Vietnam"[Title/Abstract] OR "Yemen"[Title/Abstract] OR "Zambia"[Title/Abstract] OR "Zimbabwe"[Title/Abstract])) NOT ("demographic and health survey"[Title/Abstract] OR "demographic and health surveys "[Title/Abstract] OR DHS[Title/Abstract] OR "reproductive health survey"[Title/Abstract] OR "reproductive health surveys"[Title/Abstract] OR RHS[Title/Abstract])) OR ((HIV[Title/Abstract] OR "Acquired Immunodeficiency Syndrome"[Title/Abstract] OR AIDS[Title/Abstract]) AND (pregnan*[Title/Abstract] OR "postpartum"[Title/Abstract] OR "post partum"[Title/Abstract]) AND ("mortality"[Title/Abstract] OR "death"[Title/Abstract]) NOT "case report")) AND (2019/05/10[PDat] : 3000[PDat]) NOT (animals[MeSH] NOT humans[MeSH]))

For GBD 2023, a total of 10,435 new, deduplicated published reports were identified and had their title and abstract reviewed. Of these, 499 sources were selected for full text review, and 163 met criteria for inclusion for one or more indicator of maternal disorder burden.

Figure 2: PRISMA 2020 flow diagram

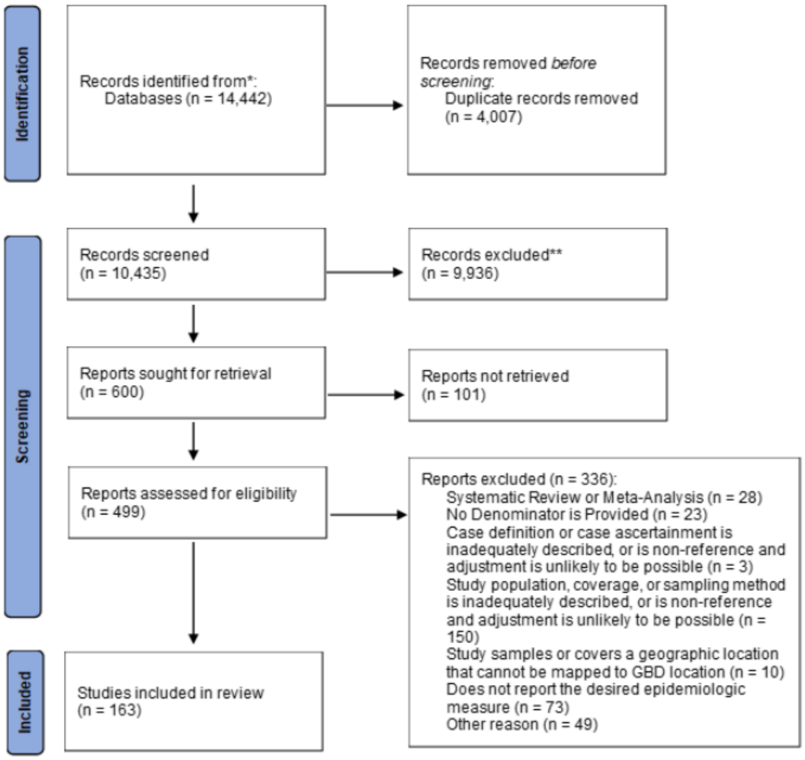

The number of these 163 sources that were specifically extracted for estimating the incidence of each maternal disorder can be found in the appendix section on GBD non-fatal source counts. All data on the frequency of maternal disorders were preferentially extracted as incidence ratios, that is, incident cases of the maternal disorder (regardless of how the pregnancy ended) per 100,000 livebirths, which was the most commonly reported parameter in the literature, and one which approximates the size of the

population at risk. If data were only available as incident cases per birth (including both stillbirths and livebirths in the denominator), incident cases per female population, or some other metric, they were converted to incidence ratio using GBD 2023 demographic estimates (fertility rate, stillbirth ratio, etc.) specific to the year, age group, and location where the datum was collected. Published data sources used a variety of case definitions and ascertainment methods, and sometimes reported the incidence of multiple disorders or the incidence of both more broadly or narrowly defined conditions in the list provided in the preceding section. Each extracted measurement was mapped to its most closely matching GBD case definition, as listed above, and may have been adjusted in the crosswalking process, described below.

Incidence from clinical administrative data

In addition to data sources identified as part of our systematic reviews, clinical administrative data processed by IHME were used for incidence estimation. Available clinical administrative datasets include hospital discharge data from 45 countries and claims data from Poland, Singapore, and the USA. Hospital discharge and claims data were extracted as described in the appendix section on hospital and claims data.

In brief, claims data are indexed by individual and year, and incident cases of maternal disorders were identified by the presence of an appropriate diagnostic code in a primary or secondary position during any encounter, with the first encounter in the year being counted and subsequent encounters occurring within 365 days that also carried an ICD code for the same maternal disorder being treated as additional care for the same incident event. The denominator of individuals at risk were females in the same age group that were enrolled the entire calendar year, and data were extracted only for five-year age groups from 10–14 through 50–54. If ICD codes for maternal disorders were seen associated with encounters for individuals outside this age range, they were regarded as either misclassification or as such rare events that incidence measurements extracted from these datasets were too imprecise to provide useful information for modelling.

Hospital discharge data are indexed by encounter and may provide only the primary discharge diagnosis code, or primary code and one or more secondary diagnoses. Incident cases of maternal disorders were identified from hospital discharge data by first extracting encounters with an appropriate ICD code as primary diagnosis, then multiplying this by ratios modelled from claims data: how often ICD codes for that maternal disorder appear as primary versus any discharge diagnosis in inpatient encounters and how many inpatient encounters for this maternal disorder appear per total incident cases. Data were extracted in five-year age groups, and modelled ratios were also specific to age group and modelled as a function of the Healthcare Access and Quality Index, as described elsewhere in this appendix. After incident cases were extracted and transformed with these ratios, they were combined with one of two population denominators. For hospital data from institutions or systems that provide all hospital care for a geographically defined population, incident cases were combined with female population estimates from GBD. For hospital data from institutions or systems with less than complete population coverage, incident cases were combined with a count of all admissions from the same dataset for the same year, age, and sex to produce hospitalisation “cause fractions”, and multiplied by an estimate of population-level year-age-sex-location-specific all-cause hospital utilisation modelled by GBD and described elsewhere in this appendix.

The ICD-9 and ICD-10 codes that mapped to each maternal disorder for processing of clinical administrative data are listed in the following table.

Table 1. Maternal disorder ICD codes

Non-fatal model	ICD-10 code(s)	ICD-9 code(s)
Maternal abortive outcome	N96, O01-O08	630-632, 634-639
Ectopic pregnancy	O00, O367	633
Obstructed labour and uterine rupture	O64-O66, O71, O83-O84	652.7, 653, 660, 665, 669.5
Maternal haemorrhage	O20, O44-O46, O67, O72	640-641, 661.2, 666
Maternal sepsis	O85	659.3, 670
Hypertensive disorders of pregnancy	O11, O13-16	642 (excluding 642.0-642.2)
Severe pre-eclampsia	O14.1	642.5
Eclampsia	O15	642.6
Other maternal infections	O23, O41.1, O42.1, O75.2-O75.3, O86, O91	646.5-646.6, 658.4, 659.2, 672, 674-675

The above extraction and processing steps for clinical administrative data provided estimates of incident cases per female population for the relevant years, age groups and locations. These were further transformed by dividing by GBD estimates of age-specific fertility rate and proportion of deliveries occurring in facilities to obtain estimates of incident cases per livebirth, to combine with data from the literature and use in incidence models.

Prior to GBD 2023, claims data from MarketScan, a database of commercial insurance claims in the USA, were used as inputs to estimate maternal disorder incidence, as well as for estimating ratios for correcting hospital discharge data from other sources. We noted, however, that incidence estimates from MarketScan differed considerably from incidence estimates from the Healthcare Cost and Utilization Project (HCUP), which provides more population-representative data on hospital discharges. We hypothesised that this could be due to differences in coding practices by clinicians providing care for patients with commercial health insurance or due to socioeconomic differences between pregnant women with commercial insurance and the general population of pregnant women in the USA. After extensive efforts to develop adjustment factors for the commercial bias in MarketScan data using standard GBD “crosswalk” methods (described elsewhere in this appendix), we determined that these adjustments were insufficient to overcome that bias, and a large volume of these heavily processed data had excessive influence on final incidence estimates at the global and local levels. Thus, we excluded MarketScan data from the maternal disorder incidence models, although they were employed in estimating primary-to-any diagnosis and inpatient-discharge-to-total case ratios for transforming hospital discharge datasets.

Additionally, we identified that hospital discharge data with less than complete coverage often had implausible extreme values, and hypothesised that estimates of all-cause hospital utilisation, even after adjustment for age-specific fertility and in-facility delivery, poorly captured the differential use of hospitals for delivery care versus other forms of health care. We thus excluded hospital discharge data with less than complete coverage from maternal disorder incidence models and retained only those with complete coverage.

Crosswalking and age-splitting

After extracting and processing incidence data from our systematic review and clinical administrative sources, as described above, we developed and applied adjustment factors to correct for systematic

biases in data sources that use non-reference case definitions or otherwise systematically differ from our reference source types; this process is referred to as "crosswalking". The GBD methods for crosswalking non-reference data towards a reference standard is described in detail in a separate section of this appendix. For maternal disorders with crosswalks based on data source type (for example, surveillance versus hospital discharge data), we chose as reference the data source with the most plausible values or that were the most conceptually representative of overall incidence. For maternal disorders with case definition crosswalks, we crosswalked alternative case definitions to our GBD reference definition. We subsequently split observations for broad age groups into observations for standard five-year GBD age groups. In GBD 2021, we age-split data prior to crosswalking, but in GBD 2023, we conducted crosswalks first, so crosswalk adjustments were based on the actual data extracted from primary sources.

To crosswalk data, we had to identify pairs of observations where incidence of a given maternal disorder was measured in the same population (defined by year, age, and location) using the alternative case definition or source type matched to an observation made using the reference definition or type. We preferentially used within-study matches, which is to say, we preferentially employed pairs of observations where both an alternative and reference case definition were applied to the same sample of individuals in the same study. If there were fewer than 15 matches available within sources, however, we allowed for between-study matches as well, which is to say, we identified observations from different sources where the study samples could be regarded as independent samples of the same underlying population.

For all maternal disorders, the difference between the two matched observations was calculated in log space. We calculated both the log-transformed means and standard deviations of each observation using the "linear_to_log" utility from the central "crosswalk" package, then calculated the difference (and standard deviation of the difference) between the log-transformed means using the delta method. Log differences were modeled using meta-regression—Bayesian, regularised, trimmed (MR-BRT), and the modelled adjustment factors were applied to all datapoints in the original dataset using that alternative definition using the "adjust_orig_vals" central function. All code employed for GBD 2023 estimation is available for review in our repository.

Following crosswalking, any datapoints that were not in standard five-year GBD age groups were age-split using the age pattern from a preliminary GBD 2023 model, which used the bested crosswalk version from GBD 2021 launched in the GBD 2023 environment to ensure that all GBD 2023-estimated geographical locations were present.

The results of the crosswalks are shown in the section below. In each graph, the effect size is the log of the adjustment coefficient estimated by the model, the x-axis is the age, and the points are the input ratios. If the adjustment factor is negative, then the alternative is underestimating the incidence of the specific disorder, relative to the reference, whereas when it is positive, the inverse is true.

Abortion and miscarriage

Surveillance data are considered the reference standard for abortion and miscarriage. We crosswalked all clinical administrative data to surveillance data. We used between-study matches given that the crosswalk for this cause was by data type, and we allowed for an age difference of up to five years between matches. We used 3265 matched pairs in the MR-BRT model. Results of the crosswalk, below, show that clinical administrative data underestimate the number of abortions and miscarriages

compared to surveillance across all ages, with the correction highest among age groups younger than 20.

Figure 3. Clinical to surveillance crosswalk, by age, for abortion and miscarriage

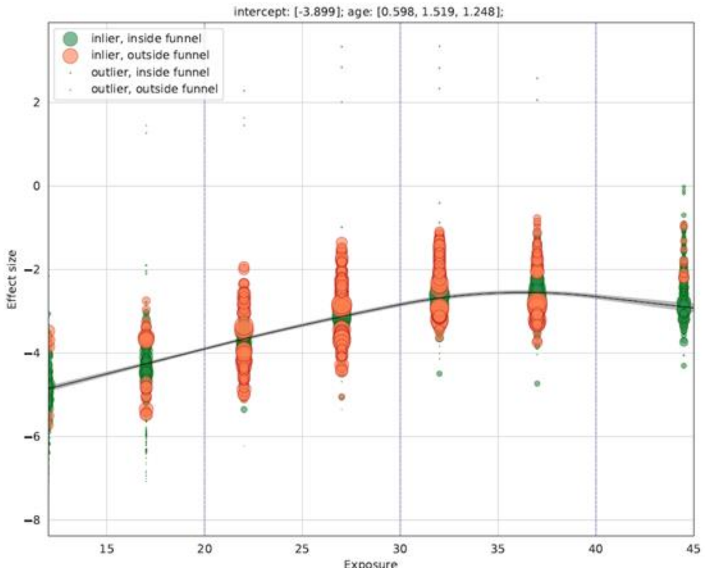

Ectopic pregnancy

For ectopic pregnancy, we explored a crosswalk to adjust MarketScan data toward the HCUP standard but found that data were implausible even after adjustment and excluded them. All other data were included without crosswalking.

Maternal haemorrhage

Maternal haemorrhage used a case definition crosswalk, where the reference definition included both postpartum (PPH) and antepartum (APH) haemorrhage. Data sources that included only APH or PPH were crosswalked to total haemorrhage by age. We used within-study matches because several sources included data for overall haemorrhage as well as one or both specific timings. Thus, matched observations referred to the same sample of individuals. For the postpartum haemorrhage to total haemorrhage crosswalk, we used 80 matched pairs in the MR-BRT model; for the antepartum haemorrhage to total haemorrhage crosswalk, we used 47 matches. Results of the two crosswalks, below, show the log-ratio of antepartum or postpartum haemorrhage only to total haemorrhage, by age, which is the factor to be employed in transforming observations from studies that only measured antepartum or postpartum haemorrhage, in order to incorporate these studies into models of total maternal haemorrhage. Data for most studies were centered around age 35, which is the midpoint of studies reporting on aggregate age groups of either 15–49 or 10–54. These plots demonstrate that the ratio of postpartum to total haemorrhage is larger than the ratio of antepartum to total haemorrhage,

which is expected, and that the ratio of antepartum to total haemorrhage is lower in younger age groups.

Figure 4. Postpartum to total haemorrhage crosswalk, left, and antepartum to total haemorrhage crosswalk, right

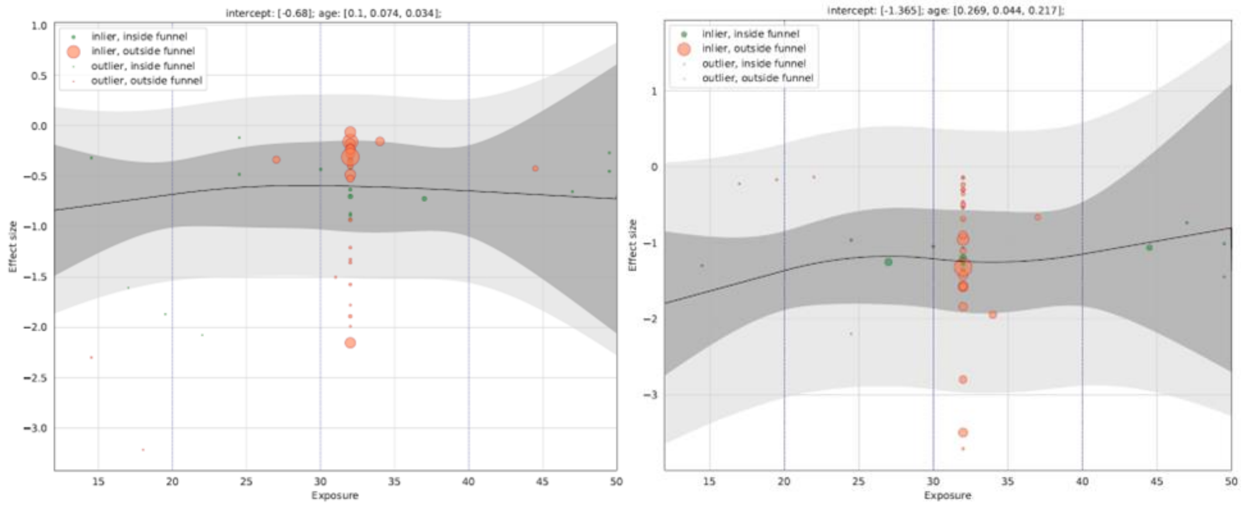

Maternal sepsis

For maternal sepsis, our reference data type was focused studies extracted from the scientific literature that employed a case definition matched to our GBD case definition. Clinical administrative data processed by IHME were crosswalked to this reference standard. To estimate the crosswalk, we used between-study matches and allowed a difference of up to five years for matches, which yielded 54 matches. Results of the crosswalk, below, show that clinical administrative data overestimate the incidence of maternal sepsis relative to measurements in the literature across all age groups, with overestimation most notable in the upper and lower extremities of age. As a result of this crosswalk, all clinical data were adjusted downward.

Figure 5. Clinical administrative data to literature crosswalk

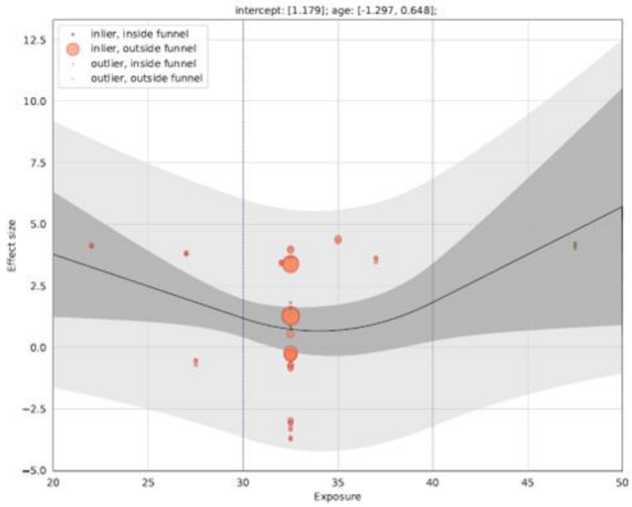

For maternal sepsis, we also explored a crosswalk to adjust MarketScan data toward the HCUP standard but found that data were implausible even after adjustment and excluded them.

Other maternal infections

For other maternal infections, we explored a crosswalk to adjust MarketScan data toward the HCUP standard but found that data were implausible even after adjustment and excluded them. All other data were included without crosswalking.

Hypertensive disorders of pregnancy

For hypertensive disorders of pregnancy (HDoP), our reference case definition was total HDoP. We crosswalked two alternative case definitions separately to our reference, sources that reported only on pre-eclampsia (PE) but did not include gestational hypertension and sources that reported only on gestational hypertension. We extracted and sought matched data to develop a crosswalk for studies that included chronic hypertension without superimposed preeclampsia in their total HDoP case definition but found insufficient matches for modelling a crosswalk factor.

Given that many sources reported on both total HDoP and one or both of the two narrower definitions (preeclampsia only and gestational hypertension only), we used within-study matched observations for modelling crosswalks. For the preeclampsia to total HDoP crosswalk, we included 20 matches; for the gestational hypertension to total HDoP crosswalk, we included 18 matches. We attempted to run crosswalks with age splines, but the majority (18/20 for preeclampsia, 16/18 for gestational hypertension) were observations reported for an aggregate age group, so we did not have sufficient information to inform a reasonable age pattern. For that reason, we applied the same crosswalk across all ages for each crosswalk. In both cases, the alternative case definition represented a subset of total HDoP, so alternative definitions were adjusted upward.

Figure 6. Funnel plot for gestational hypertension to hypertensive disorders of pregnancy case definition crosswalk

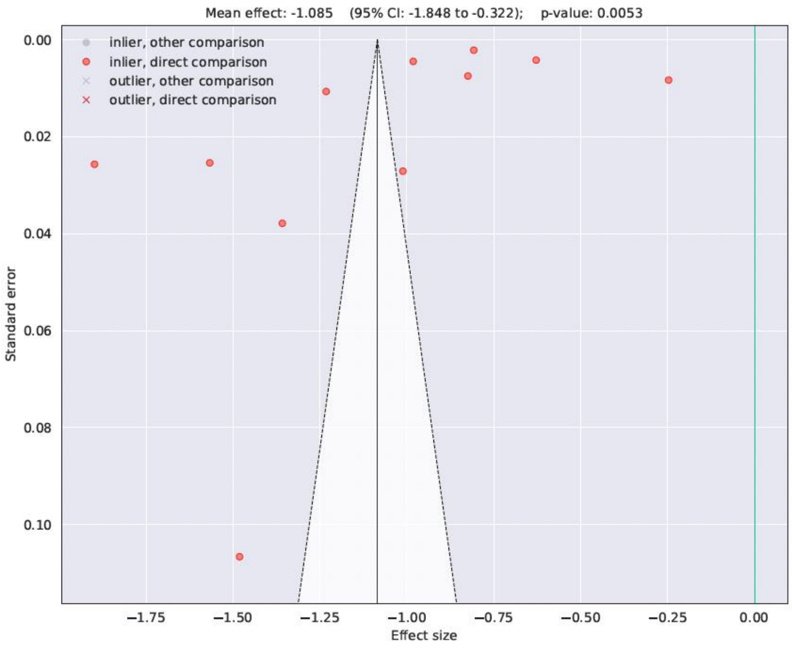

Figure 7. Funnel plot for preeclampsia to hypertensive disorders of pregnancy case definition crosswalk

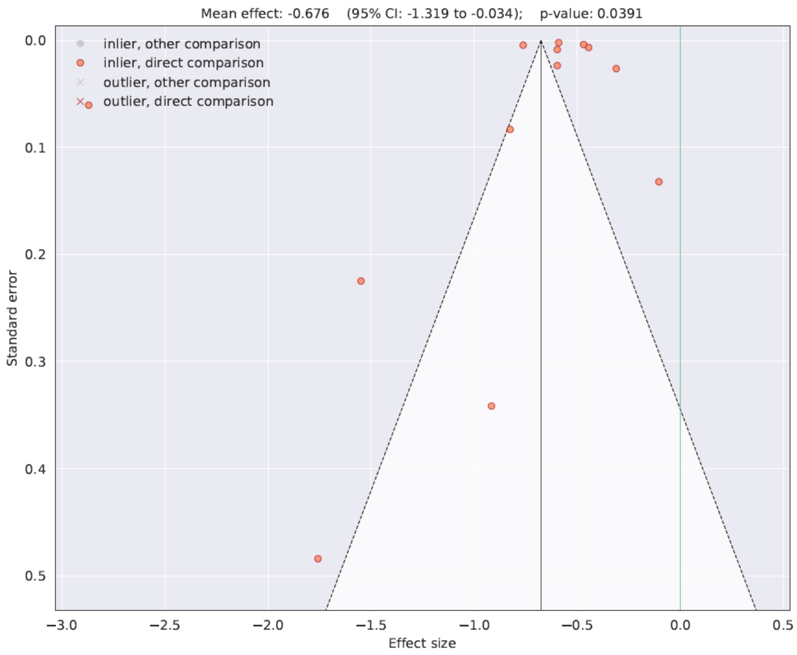

Severe pre-eclampsia

For severe pre-eclampsia, we explored a crosswalk to adjust MarketScan data toward the HCUP standard but found that data were implausible even after adjustment and excluded them. All other data were included without crosswalking.

Eclampsia

For eclampsia, our reference data type was literature studies that matched our GBD case definition, and we crosswalked all clinical administrative data to these reference literature data by age. We used between-study matches and allowed a year difference of up to five years for matches, which resulted in 153 matches. Results of the crosswalk, shown in the figure below, demonstrate that clinical administrative data approximate the literature case definition closely in all age groups. We applied the clinical to literature crosswalk, although data were only minimally adjusted.

Figure 8. Clinical administrative to literature crosswalk

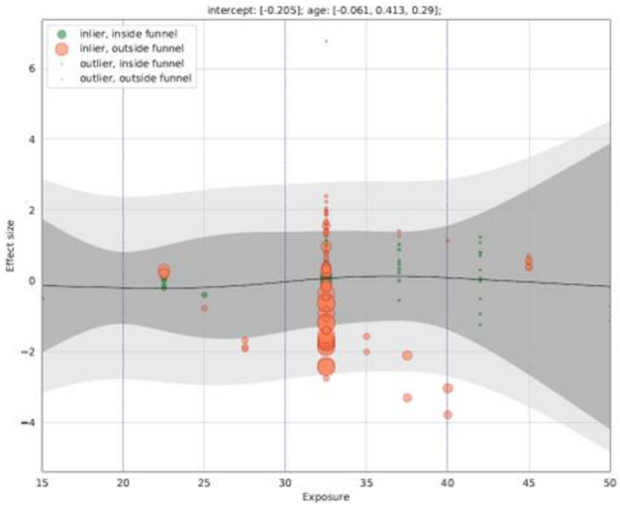

For eclampsia, we also explored a crosswalk to adjust MarketScan data toward the HCUP standard but found that data were implausible even after adjustment and excluded them.

Other inputs

Beyond the above-described inputs for estimating maternal disorder incidence, several additional inputs are employed in converting incidence estimates to estimates of YLDs. We used age-specific fertility rates from GBD 2023 to convert incidence ratio to incidence rate. We used published data to estimate the proportion of maternal haemorrhage that is non-severe versus severe, the proportion of incident eclampsia and severe pre-eclampsia cases that go on to develop long-term sequelae, and the proportion of puerperal sepsis cases that go on to develop secondary infertility; these are included in the reference list of this appendix section, below. For GBD 2023, we relied on expert opinion to determine the duration of short-term symptoms and functional impairment for each of the maternal disorders and their key subtypes, a quantity that is required to calculate annualised point-prevalence for YLD calculation; in future rounds we plan to incorporate measured symptom durations reported in scientific literature or inferred from administrative data.

Modelling strategy

Modelling incidence ratios

We used the datasets described above to estimate incidence ratio for each specified pregnancy complication for each year-age-location combination in the GBD 2023 estimation framework using DisMod-MR 2.1. A series of country covariates were chosen to help drive the magnitude of estimates in areas of sparse or absent data. We included the respective log-transformed maternal mortality ratio (MMR) for each maternal disorder that was estimated in GBD 2023 as a predictive covariate for several models. Maternal sepsis and ectopic pregnancy used the log-transformed age-standardised death rate (LN-ASDR) as a covariate rather than MMR. The coefficients of the covariates in each model are shown below. No specific age or slope priors were used. All models were run with a time window of five years; haemorrhage, hypertensive disorders of pregnancy, abortion and miscarriage, and other maternal infections were run with MinCV=0.4; the rest used the GBD default of 0.8. The detailed settings for final GBD 2023 DisMod-MR 2.1 models can be viewed in EpiViz, but a summary of the predictive covariates employed and their estimated beta coefficients is provided below.

Table 2: Covariate coefficients from DisMod models for incidence ratio of maternal disorders

Model	Covariate name	Beta value	Exponentiated beta value
Abortion and miscarriage	Legality of abortion	1.8e-6 (9.5e-8 to 5.2e-6)	1.0 (1.0 to 1.0)
	Contraception (modern) prevalence	-5.1e-4 (-1.5e-3 to -2.2e-5)	1.0 (1.0 to 1.0)
Ectopic pregnancy	Age-standardised prevalence of pelvic inflammatory disease	0.5 (0.1 to 1.0)	1.7 (1.1 to 2.6)
	lnASDR of ectopic pregnancy	8.1e-4 (2.8e-5 to 2.5e-3)	1.0 (1.0 to 1.0)
Maternal haemorrhage	Skilled birth attendance proportion	-0.7 (-0.9 to -0.4)	0.5 (0.4 to 0.7)
	Socio-demographic Index	-0.4 (-0.5 to -0.3)	0.7 (0.6 to 0.7)
	MMR due to maternal haemorrhage	1.0 (0.1 to 2.0)	2.7 (1.1 to 7.0)
Obstructed labour	Skilled birth attendance proportion	-3.5e-3 (-1.1e-2 to 1.3e-4)	1.0 (1.0 to 1.0)
	Age-standardised SEV for child stunting	6.7e-3 (2.7e-4 to 2.0e-2)	1.0 (1.0 to 1.0)
	MMR due to obstructed labour	1.0 (0.0 to 2.0)	2.7 (1.0 to 7.3)
Maternal sepsis	Age-standardised diabetes prevalence proportion	1.0 (0.2 to 1.9)	2.9 (1.2 to 6.7)
	lnASDR for maternal sepsis and other maternal infections	0.2 (0.1 to 0.2)	1.2 (1.2 to 1.2)
Other maternal infections	Socio-demographic Index	-9.3e-3 (-2.6e-2 to -4.5e-4)	1.0 (1.0 to 1.0)
	Log-transformed age-standardised SEV for HIV	0.9 (1.8 to 1.0)	2.4 (2.2 to 2.7)
	MMR due to sepsis and other maternal infections	1.0 (0.0 to 1.9)	2.7 (1.0 to 6.9)
Maternal hypertensive disorders	Antenatal care (4 visits) coverage proportion	-9.4e-5 (-2.7e-4 to –3.7e-6)	1.0 (1.0 to 1.0)
	Age-standardised SEV for high blood pressure	2.0 (2.0 to 2.0)	7.4 (7.4 to 7.4)
	Age-standardised SEV for high body-mass index	2.0 (2.0 to 2.0)	7.4 (7.4 to 7.4)
Severe pre-eclampsia	Antenatal care (4 visits) coverage proportion	-1.0e-2 (-2.8e-2 to -3.5e-4)	1.0 (1.0 to 1.0)
	MMR due to maternal hypertensive disorders	1.0 (0.0 to 2.0)	2.7 (1.0 to 7.2)
	Age-standardised SEV for high body-mass index	2.0 (1.9 to 2.0)	7.2 (6.8 to 7.4)
Eclampsia	Antenatal care (4 visits) coverage proportion	-2.0 (-2.0 to -2.0)	0.1 (0.1 to 0.1)
	MMR due to maternal hypertensive disorders	1.0 (0.1 to 2.0)	2.8 (1.1 to 7.1)
	Age-standardised SEV for high body-mass index	1.3e-3 (8.0e-5 to 3.3e-3)	1.0 (1.0 to 1.0)

If the exponentiated beta coefficient is smaller than 1 then the covariate is negatively associated with the outcome; if it is greater than 1 then the inverse is true.

Estimating incidence rates, prevalence, and YLDs

After completion of DisMod-MR 2.1 models, all age-specific incidence ratios were then converted to incidence rates by multiplying by ASFR and then to prevalence by applying globally assumed durations of short-term disability, with or without intervening steps to account for different levels of severity.

For abortion and miscarriage, incidence ratios from DisMod-MR 2.1 were multiplied by ASFR, and then prevalence was calculated assuming incident cases all have the same severity of acute disability (abdominopelvic pain, moderate) and it persists for an average of three days (+/-1). The same approach

and duration were applied to ectopic pregnancy, although a more severe disability was applied (abdominopelvic pain, severe).

Maternal haemorrhage incidence ratios were multiplied by ASFR, and the resulting incidence rates were split between moderate (500 to <1000 mL blood loss) and severe (≥1000 mL blood loss) based on a meta-analysis of 19 studies.[1] For the average duration of acute symptoms, we consulted with clinician collaborators and assigned a duration of seven days (+/-3) for moderate haemorrhage and 14 days (+/-4) for severe haemorrhage, and the assigned disabilities were for abdominopelvic pain, moderate and abdominopelvic pain, severe. The age-specific anaemia prevalence following maternal haemorrhage was estimated as part of overall anaemia causal attribution for GBD 2023. The details of the anaemia analysis are described separately in the "Anaemia Impairment" section. Disability weights for anaemia sequelae refer to symptoms of anaemia such as fatigue and decreased exercise tolerance and anaemia-related disability persists after resolution of the acute symptoms of haemorrhage.

Obstructed labour incidence ratios were multiplied by ASFR and then by an assigned duration of five days (+/-2), based on clinical expert determination.

Maternal sepsis and other maternal infections were estimated using multiple DisMod-MR 2.1 models. Maternal sepsis incidence ratios were estimated in a DisMod-MR 2.1 single-parameter regression model, then were multiplied by ASFR and an assigned a duration of five days (+/-2) to estimate short-term disability, which was based on the disability weight for infectious disease, acute episode, severe. Furthermore, based on a single longitudinal study of infertility,[5] we estimated that 9% (95% UI 7.7–10) of incident cases of maternal sepsis continue on to have secondary infertility due to maternal sepsis. We apply this proportion to the incidence rates calculated for maternal sepsis and use the product as input data for a compartmental model in DisMod-MR 2.1 in order to obtain estimates of the point-prevalence of secondary infertility due to maternal sepsis in the general female population across all years. Incidence ratios for other maternal infections were estimated in their own DisMod-MR 2.1 single-parameter model, multiplied by ASFR, and then assigned a wide potential duration of 15 to 45 days (mean 30) and a disability weight for infectious disease, acute episode, moderate.

Hypertensive disorders of pregnancy (HDoP) was also estimated using multiple DisMod-MR 2.1 models. The overall incidence of HDoP was estimated in one DisMod-MR 2.1 model, as was the incidence of severe pre-eclampsia, and the results of the latter were subtracted from the former. The difference was treated as a combination of gestational hypertension and non-severe pre-eclampsia, termed "other HDoP" and multiplied by ASFR, then a duration of 3 months (95% UI 2–4), and a disability weight for chronic disease and daily medication. Incidence ratios for severe pre-eclampsia were multiplied by ASFR, then by a duration of 7 days (+/-2) and assigned a disability weight associated with a combination of moderate abdominopelvic pain, tension-type headache, and mild motor and cognitive impairment. Eclampsia was estimated in a separate DisMod-MR 2.1 model, multiplied by ASFR, and assigned a duration of one day (+/-1) and a disability weight for moderate abdominopelvic pain and severe epilepsy. We further estimated that those with severe pre-eclampsia and eclampsia go on to have long-term sequelae, based on previously published longitudinal studies.[2-4] We estimated these long-term sequelae by using the incidence rate results of severe pre-eclampsia and eclampsia as input data for two full-compartment DisMod-MR 2.1 models. 62% (57–67) of the severe pre-eclampsia cases are estimated to go on to long-term sequelae. For eclampsia, we estimate that 6.5% (6.1–6.9) of the cases continue on to long-term sequelae in data-rich locations, compared to 11% (10.8–12) in non-data-rich locations. We

apply these percentages to the incidence rate–converted outputs of the severe pre-eclampsia and eclampsia DisMod-MR 2.1 models and use the resulting dataset as the input for the long-term sequelae models.

The sequelae, health states, lay descriptions, and disability weights for each maternal disorder are listed in the table below. Disability weights in GBD were calculated from two large surveys carried out in 2010 and 2013 as described in the disability weight section of the appendix. We assigned abdominopelvic pain of varying severity to approximate the disability from maternal haemorrhage, obstructed labour, ectopic pregnancy, and abortion and miscarriage. We used two health states to estimate the disability weight due to eclampsia (moderate abdominal pain and severe epilepsy). Tension-type headaches and mild motor plus cognitive impairment were used for severe pre-eclampsia. When two or more health states were combined for one sequela, the disability weight was calculated as described in the disability weights section of the Disease and Injuries Appendix.

Table 3. Health states and disability weights for each of the non-fatal maternal disorders

Sequela	Health state name	Health state description	Disability weight
Maternal abortive outcome	Abdominopelvic problem, moderate	Has pain in the belly and feels nauseous. The person has difficulties with daily activities.	0.114 (0.078–0.159)
Ectopic Pregnancy	Abdominopelvic problem, moderate	Has pain in the belly and feels nauseous. The person has difficulties with daily activities.	0.114 (0.078–0.159)
Maternal haemorrhage (<1 L blood lost)	Abdominopelvic problem, moderate	Has pain in the belly and feels nauseous. The person has difficulties with daily activities.	0.114 (0.078–0.159)
Maternal haemorrhage (>1 L blood lost)	Abdominopelvic problem, severe	Has severe pain in the belly and feels nauseous. The person is anxious and unable to carry out daily activities.	0.324 (0.22–0.442)
Mild anaemia due to maternal haemorrhage	Anaemia, mild	Feels slightly tired and weak at times, but this does not interfere with normal daily activities.	0.004 (0.001–0.008)
Moderate anaemia due to maternal haemorrhage	Anaemia, moderate	Feels moderate fatigue, weakness, and shortness of breath after exercise, making daily activities more difficult.	0.052 (0.034–0.076)
Severe anaemia due to maternal haemorrhage	Anaemia, severe	Feels very weak, tired and short of breath, and has problems with activities that require physical effort or deep concentration.	0.149 (0.101–0.209)
Obstructed labour, acute event	Abdominopelvic problem, severe	Has severe pain in the belly and feels nauseous. The person is anxious and unable to carry out daily activities.	0.324 (0.22–0.442)
Puerperal sepsis	Infectious disease, acute episode, severe	Has a high fever and pain, and feels very weak, which causes great difficulty with daily activities.	0.133 (0.088–0.19)
Infertility due to puerperal sepsis	Infertility, secondary	Has at least one child and wants to have more children. The person has a fertile partner, but the couple cannot conceive.	0.005 (0.002–0.011)
Other maternal infections	Infectious disease, acute episode, moderate	Has a fever and aches, and feels weak, which causes some difficulty with daily activities.	0.051 (0.032–0.074)
Severe pre-eclampsia	Moderate abdominal pain, tension-type headaches, mild motor plus cognitive impairment	Has pain in the belly and feels nauseous. The person has difficulties with daily activities. Has a moderate headache that also affects the neck, which causes difficulty in daily activities. Has some difficulty in moving around but is able to walk without help. The person is slow in learning at school. As an adult, the person has	0.174 (0.120–0.239)

		some difficulty doing complex or unfamiliar tasks but otherwise functions independently.	
Eclampsia	Moderate abdominal pain and severe epilepsy	Has pain in the belly and feels nauseous. The person has difficulties with daily activities. Has sudden seizures with violent muscle contractions and stiffness, loss of consciousness, and loss of urine or bowel control. Between seizures the person has memory loss and difficulty concentrating.	0.602 (0.427–0.753)
Long term sequelae of severe pre-eclampsia	Tension-type headaches, mild motor plus cognitive impairment	Has a moderate headache that also affects the neck, which causes difficulty in daily activities. Has some difficulty in moving around but is able to walk without help. The person is slow in learning at school. As an adult, the person has some difficulty doing complex or unfamiliar tasks but otherwise functions independently.	0.067 (0.041–0.103)
Long term sequelae of eclampsia	Tension-type headaches, mild motor plus cognitive impairment	Has a moderate headache that also affects the neck, which causes difficulty in daily activities. Has some difficulty in moving around but is able to walk without help. The person is slow in learning at school. As an adult, the person has some difficulty doing complex or unfamiliar tasks but otherwise functions independently.	0.067 (0.041–0.103)
Other hypertensive disorders of pregnancy	Generic uncomplicated disease: worry and daily medication	Has a chronic disease that requires medication every day and causes some worry but minimal interference with daily activities.	0.049 (0.031–0.072)

Uncertainty and model selection

For all explicitly modelled maternal disorders, uncertainty bounds include uncertainty due to input data, crosswalks of non-reference data, uncertainty in numerical solutions (posteriors) of each DisMod-MR 2.1 model, duration of symptoms, and proportion of all persons with each type of symptom.

In consultation with GBD researchers and collaborators, final models were selected on a combination of qualitative and quantitative goodness of fit to input data, plausibility of geographical and temporal trends, consistency of age pattern, and, when available, comparison with other published studies on the epidemiology of pregnancy complications. Directionality, magnitude, and plausibility of adjustment factors and predictive covariates were also considered in the process of model development. Of note, due to the nature of statistical modelling, final results do not always cover the values reported in input data.

Other direct and indirect maternal causes

We estimated YLDs for other [direct] maternal disorders using the YLD-to-YLL ratio approach. This is to say, the ratios of YLD:YLL were pooled for all the explicitly modelled maternal disorders described above and multiplied by the YLL for other [direct] maternal disorders. For other subcauses of maternal disorders, including late maternal death, indirect maternal disorders, and maternal death complicated by HIV/AIDS, we did not estimate any non-fatal burden based on the premise that the associated disability is captured in other causes.

References

1 Sloan N, Durocher J, Aldrich T, Blum J, Winikoff B. What measured blood loss tells us about postpartum bleeding: a systematic review. *BJOG* 2010; **117**: 788–800.

2 Roes EM, Raijmakers MT, Schoonenberg M, Wanner N, Peters WH, Steegers EA. Physical well-being in women with a history of severe preeclampsia. *J Matern Fetal Neonatal Med* 2005; **18**: 39–45.

3 Okanloma KA, Moodley J. Neurological complications associated with the pre-eclampsia/eclampsia syndrome. *Int J Gynaecol Obstet* 2000; **71**: 223–5.

4 Usta IM, Sibai BM. Emergent management of puerperal eclampsia. *Obstet Gynecol Clin North Am* 1995; **22**: 315–35.

5 Westrom LV. Chlamydia and its effect on reproduction. *J Br Fer Soc* 1996; **1**: 23–30.

Measles

Flowchart

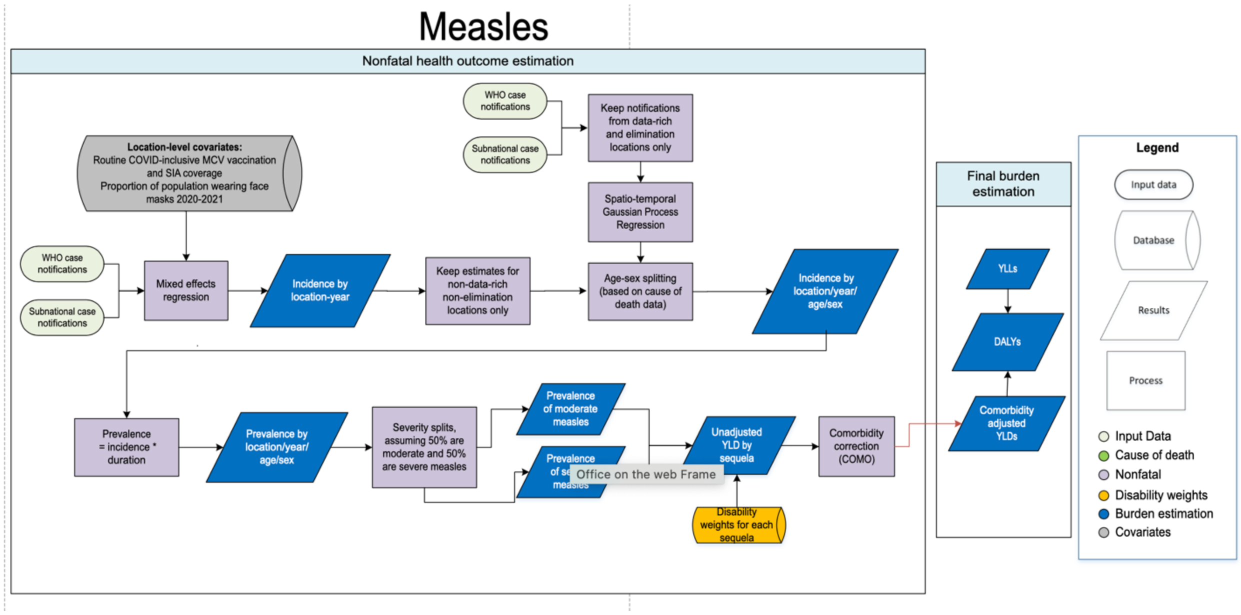

Input data and methodological summary for measles

Case definition

Measles is a systemic illness caused by infection with the highly contagious measles virus. It is characterised by fever, cough, conjunctivitis, rhinitis, and a diffuse maculopapular rash. Infection in healthy children is often benign and self-limited, though acute complications include otitis media, diarrhoea, pneumonia, and encephalitis. In severe cases, measles complications can lead to long-term disability and death. For measles, ICD-10 codes are B05-B05.9, Z24.4, and ICD-9 codes are 055-055.9, 484.0, V04.2, V73.2.

Quantity of interest	Reference or alternative	Definition
Measles case fatality rate	Reference	Ratio of deaths from measles to cases of measles in a sample
Measles incidence	Reference	Cases reported by national measles surveillance systems to WHO or subnational case notifications reported by national surveillance system. Cases may be diagnosed via laboratory confirmation (including IgM or PCR) or by clinical diagnosis following presentation with a febrile rash associated with cough, runny nose, fever, and conjunctivitis.

Input data

Model inputs

We fit a custom measles incidence model to the following data: measles case notifications released annually by the World Health Organization (WHO) in its Joint Reporting Form (JRF); annualised monthlyWHO case reports for 2023; and subnational case notifications from the USA and Brazil. As in GBD 2021, we used modelled estimates of measles-containing-vaccine (MCV) vaccination coverage proportions for first and second doses, and supplementary immunisation campaign (SIA) coverage as predictors of measles incidence. In GBD 2023, we used COVID-inclusive MCV coverage estimates and a masking covariate to account for the impact of COVID-19 on measles incidence. This approach differs from the COVID adjustment used in GBD 2021, wherein COVID-free counterfactual estimates of measles incidence were modelled followed by a post-hoc adjustment for the impact of the pandemic.

In high-income, central Europe/eastern Europe/central Asia, and Latin America and the Caribbean super-regions, as well as in WHO-verified measles elimination locations outside of these super-regions, modelled estimates of measles incidence were replaced by case notifications reported to WHO in years for which case notification data are available. This approach implies complete case reporting in these locations. In China and Jordan, modelled estimates of measles incidence are replaced by case notifications because of the strength of surveillance systems in these locations. In all locations mentioned above, missing data were imputed using results from a spatiotemporal Gaussian process regression (ST-GPR) model fit to WHO JRF annual measles case notifications for all countries and subnational data from Brazil, Japan, Great Britain, Indonesia, Italy, Poland, South Africa, and the USA. New in GBD 2023, in locations without trusted case notification systems or where measles has not been eliminated, we replaced modelled estimates with WHO case notifications when our model predicted lower incidence than notified cases. Finally, in locations with known outbreaks in 2022–2023, we used additional WHO and national surveillance system data to replace annual JRF notifications where we considered measles outbreak data were considered more accurate than the JRF.

Modelling strategy

The general modelling approach used for GBD 2023 is similar to that used in GBD 2021 with the exception of methods used for handling COVID-19-related impacts. First, we estimate measles cases in every location, using case notification inputs and a mixed-effects linear regression model. This model uses measles incidence rate as the dependent variable and estimates of five-year rolling mean routine COVID-inclusive measles vaccination rates (first- and second-dose measles-containing vaccines), and five-year lagged coverage of SIAs as predictors. We used rolling means of MCV coverage calculated over the preceding five-year interval to better capture population-level vaccine-derived immunity among under 5-year-olds. The five-year duration of the lag on SIAs was chosen from models tested with five-, seven-, and ten-year lags because it had the best out-of-sample performance in a five-fold cross validation framework. New in GBD 2023, we included a masking covariate as an additional predictor in the model for calendar years 2020 and 2021 to account for the impact of COVID mitigation strategies on the measles transmission.

In further detail, log-transformed incidence rates were regressed on the log of the proportion unvaccinated with first- and second-dose COVID-inclusive measles-containing vaccine (calculated using five-year rolling mean coverage), and additional SIA coverage lagged by one, two, three, four, and five years, the log of 1 – (proportion population reporting masking outside the home), with super-region, region, and country-level random effects:

$$Y_{ij} = \beta_0 + \beta_1 (1\text{-}MCV1_{ij}) + \beta_2 (1\text{-}MCV2_{ij}) + \beta_{a3}\, SIA_{aij} + \beta_4 ln(1\text{-}masking) + u_j + e_{ij}$$

In the equation above, Y_{ij} is the natural log of measles incidence rate per 100,000 people; β_0 is the fixed-effect intercept; β_1 is the fixed-effects slope on the log-transformed proportion unvaccinated with first-dose measles vaccine (calculated using rolling mean coverage over the preceding five years); β_2 is the fixed-effects slope on the log-transformed proportion unvaccinated with second-dose measles vaccine coverage (similarly calculated using rolling mean five-year coverage); β_{a3} is the fixed-effects slope on supplementary measles immunisation campaign coverage (administered doses over the target population of all under-15s) lagged by a=1-5 years; β_4 is the fixed effects slope on the log proportion of population who do not report masking outside the home; u_j is the location-level random effects; e_{ij} is the residual; i is the year; and j is the location. To adjust for underreporting, we assume a 95% attack rate in under 5-year-olds in the absence of vaccination, by generating a standard random effect consistent with this assumption and then applying that random effect in all years and locations when predicting from the model. From the fitted model, 1000 incidence predictions (draws) were generated for all ages, sexes, locations, and years using the estimated variance-covariance matrix. For locations that use case notifications directly, 1000 incidence draws were generated from a binomial distribution.

Our both-sex/all-age measles case estimates were split into age- and sex-specific cases by utilising age-sex distributions obtained from cause of death modelling in CODEm. For all countries, we produced estimates for all age groups between six months and 64 years, under the assumption that all individuals born before 1957 are immune to measles. In under-5 age groups, incidence predictions exceeded 0.9 on rare occasions; these draws were capped at an incidence of 0.9. Prevalence rates were calculated by multiplying case predictions by an average case duration of ten days and dividing by GBD-estimated population in each location; incidence rates were computed by dividing estimated cases by population in each location. This procedure was repeated for 1000 samples (draws) from the posterior distribution and summarised by the mean of the draws with 95% uncertainty intervals (2.5th and 97.5th percentiles of all draws).

Severity splits

We assume 50% of measles cases were acute episodes of moderate infectious disease and 50% were acute episodes of severe infectious disease. The lay descriptions and disability weights for measles severity levels derived from the GBD disability weights study are shown in Table 1.

Table 1. Severity distribution, details on the severity levels for measles in GBD 2021 and the associated disability weight (DW) with that severity

**Severity level**	**Lay description**	**DW (95% CI)**
Moderate	Has a fever and aches, and feels weak, which causes some difficulty with daily activities.	0.051 (0.032–0.074)
Severe	Has a high fever and pain, and feels very weak, which causes great difficulty with daily activities.	0.133 (0.088–0.19)

Changes from GBD 2021 to GBD 2023

The major substantive change to our modelling strategy for GBD 2023 was the handling of COVID-19 related impacts on measles incidence. In GBD 2023, we used five-year rolling mean routine COVID-inclusive measles vaccination rates, and a masking covariate as an additional predictor in the model for calendar years 2020 and 2021, to account for the impact of COVID mitigation strategies on the measles transmission.

Meningitis

Flowchart

Meningitis

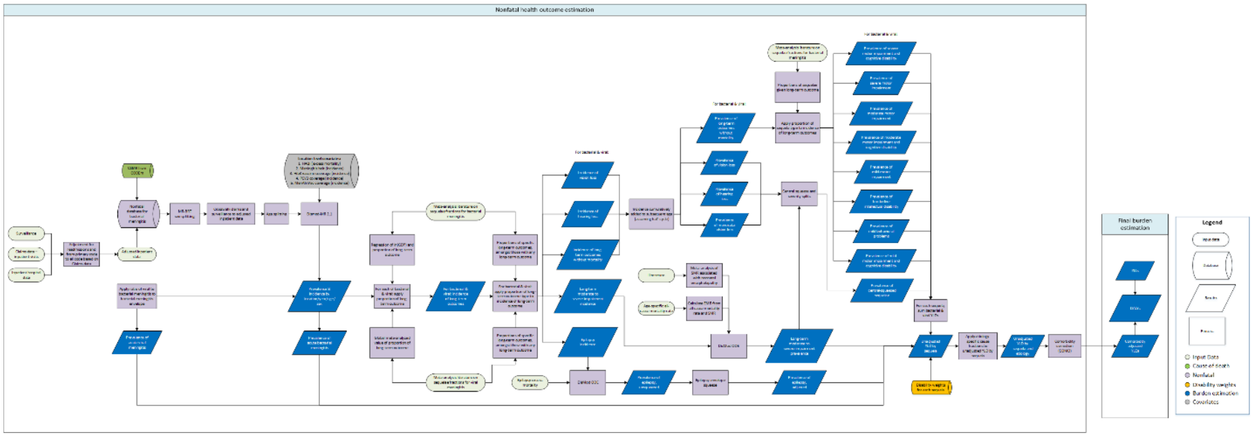

Input data and methodological summary for meningitis

Case definition

Meningitis is a disease caused by inflammation of the meninges, the protective membrane surrounding the brain and spinal cord, and is typically caused by an infection in the cerebrospinal fluid. Symptoms include headache, fever, stiff neck, and sometimes seizures. Included in the GBD modelling were cases meeting ICD-10 diagnostic criteria for meningitis due to bacteria or viruses (A39-A39.9, A87-A87.9, G00.0-G00.8, and G00-G03.0).

The case definitions accepted for meningitis are shown below.

**Quantity of interest**	**Reference or alternative**	**Definition**
Incidence of meningitis	Reference	Meningitis from inpatient hospital in clinical data or from literature, where cases are diagnosed by antigen test, blood test, cerebrospinal fluid test, polymerase chain reaction test, or latex agglutination test.
Incidence of meningitis	Alternative	Meningitis from private claims data.
Incidence of meningitis	Alternative	Cases detected by epidemiological surveillance.

Input data

Input data included all data previously used from GBD 2019, new data identified in our updated systematic review, newly acquired surveys, and new claims and inpatient data. Meningitis incidence data come from a systematic literature review, hospital inpatient and outpatient data, claims data from the USA, and surveillance data. In addition, sequelae and severity splits for bacterial meningitis were informed by a meta-analysis from Edmond and colleagues,[1] while sequelae and severity splits for viral meningitis were informed by a meta-analysis from Hudson and colleagues.[2]

The PubMed search string below was used to look for the incidence or prevalence of meningitis.

("meningitis"[MeSH Terms] OR "meningitis"[Title/Abstract]) AND ("prevalen*"[Title/Abstract] OR "inciden*"[Title/Abstract] OR "case*"[Title/Abstract]) NOT ("case report"[Title/Abstract] OR "case-report"[Title/Abstract])) AND (2020/3/11[DP] : 3000[DP]) NOT ("animals"[MeSH Terms] NOT "humans"[MeSH Terms])

The inclusion criteria stipulated that: (1) the publication year must be between 1980 and the present year; (2) "caseness" was based on presence of bacterial pathogens in blood (with additional clinical presentation of meningitis) or cerebrospinal fluid, as diagnosed by culture, antigen test, polymerase chain reaction test, latex agglutination test, or Gram staining; (3) sufficient information must be provided on study method and sample characteristics to assess the quality of the study; and (4) study samples must be representative of the general population. No limitation was set on the language of publication. We identified 265 studies after title-abstract screening, of which 133 met our inclusion criteria and were extracted. We excluded studies that were unrepresentative of the general population, studies that used animals as subjects, and studies (for incidence) with study population under 100.

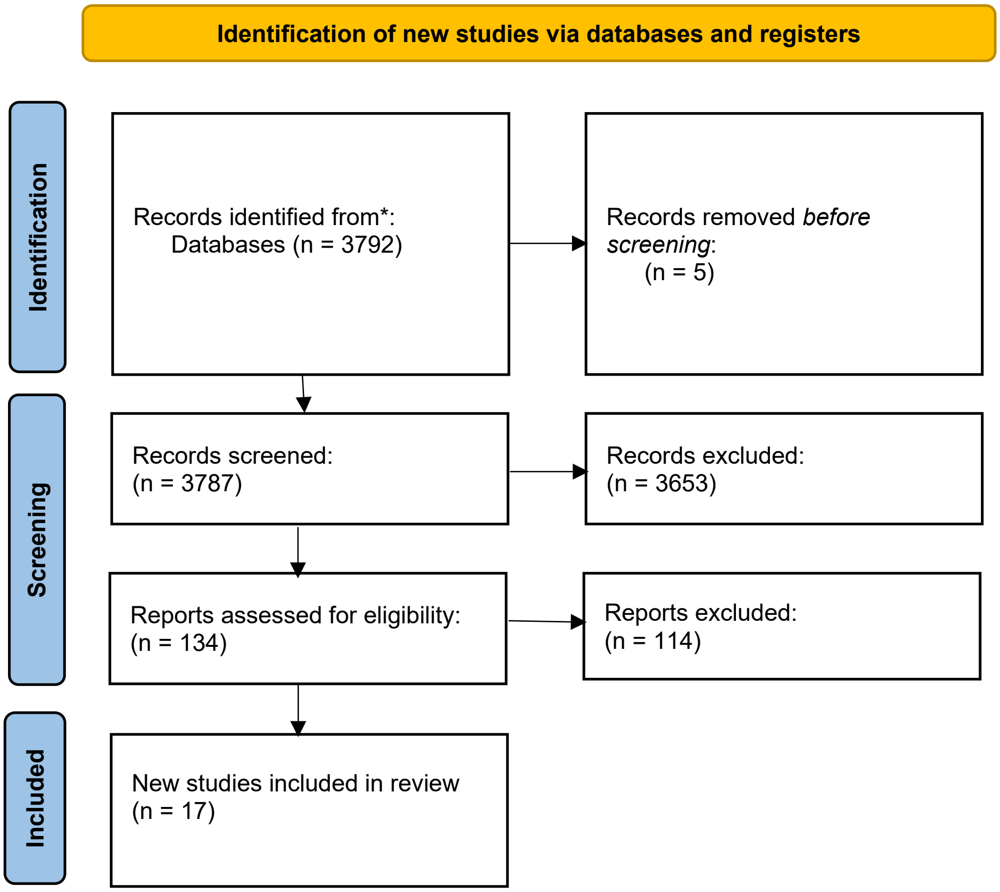

Figure 1: PRISMA diagram for meningitis 2023 systematic review for incidence

Bias corrections – incidence data

Hospital data were flagged with a covariate for inpatient hospital data and were used as the reference category. Claims data were flagged with year-specific covariates. Surveillance data were flagged with a covariate. For GBD 2021, an additional covariate was added to note surveillance with a broad definition, including suspected or viral meningitis. As described later, in non-fatal data modelling, we estimate bacterial meningitis first and add viral at the very end. Therefore, in our initial non-fatal data processing, we aim to include bacterial meningitis only to avoid double-counting viral cases. This "broadly defined" category was applied only to certain surveillance sources. The crosswalk allowed for composite definitions, such that a source that is both a surveillance source and broadly defined would have both adjustments additively applied. Both claims and surveillance data were crosswalked to the reference category.

Table 2a: MR-BRT crosswalk adjustment factors for meningitis incidence

Data input	Reference or alternative case definition	Gamma	Basis function	B-spline coefficient, logit (95% UI)*	Adjustment factor **
Inpatient hospital (CF2)	Ref		---	---	---
Claims, inpatient only	Alt	0.00	intercept	0.60 (0.47 to 0.73)	1.82
			age_mid_0	–0.09 (–0.17 to –0.01)	0.92
			age_mid_1	0.33 (–0.02 to 0.69)	1.40
			age_mid_2	–0.24 (–0.39 to –0.09)	0.79
			age_mid_3	–0.26 (–0.40 to –0.13)	0.77
Claims, inpatient only, year 2000	Alt	0.00	intercept	0.26 (–0.47 to 0.99)	1.30
			age_mid_0	–0.25 (–0.72 to 0.21)	0.78
			age_mid_1	0.74 (–1.40 to 2.87)	2.09
			age_mid_2	–0.97 (–1.92 to –0.03)	0.38
			age_mid_3	–0.57 (–1.33 to 0.20)	0.57
Surveillance	Alt	0.00	intercept	–3.60 (–5.31 to –1.90)	0.03
			HAQ Index	0.04 (0.03 to 0.04)	1.04
Broadly defined	Alt	2.21	intercept	4.45 (1.77 to 7.12)	85.44
			HAQ Index	–0.04 (–0.05 to –0.04)	0.96

**MR-BRT crosswalk adjustments can be interpreted as the factor the alternative case definition is adjusted by to reflect what it would have been had it been measured using the reference case definition. If the log/logit beta*

coefficient is negative, then the alternative is adjusted up to the reference. If the log/logit beta coefficient is positive, then the alternative is adjusted down to the reference.
***The adjustment factor column is the exponentiated beta coefficient. For log beta coefficients, this is the relative rate between the two case definitions. For logit beta coefficients, this is the relative odds between the two case definitions.*

Modelling strategy

Non-fatal outcomes were modelled using a combination of custom models and DisMod-MR 2.1. First, the overall incidence and prevalence of bacterial meningitis were modelled to estimate the short-term morbidity due to acute infection. This DisMod model had a set duration (1/remission) of four weeks with a range ±2 weeks. We also imposed caps on excess mortality for neonates and elders based on the highest excess mortality estimates from GBD 2019. We used the function in DisMod-MR 2.1 to pull in cause-specific mortality rate (CSMR) data from our CODEm and CoDCorrect analyses. We calculated excess mortality rate to estimate priors by dividing CSMR by prevalence, calculated from remission and incidence. To help inform trends where we lack data, we applied country-level covariates for proportion of the population at the subnational and country levels that lives within the meningitis belt in sub-Saharan Africa,[3] coverage of MenAfriVac vaccine initiative, coverage of Hib3, and coverage of PCV3. Betas and exponentiated values (which can be interpreted as an odds ratio) are shown in the tables below country-level covariates. After bacterial meningitis was modelled using DisMod, viral meningitis incidence was calculated using the age-sex-location-year-specific ratios of bacterial:viral meningitis from estimates of viral meningitis.

Disability weights

The basis of the GBD disability weight survey assessments is descriptions of sequelae highlighting major functional consequences and symptoms. The lay descriptions and disability weights for sequelae associated with meningitis are shown below.

Table 3. Severity distribution, details on the severity levels for meningitis and the associated disability weight (DW) with that severity

Severity split	Lay description	DW (95% CI)
Acute meningitis	This person has a high fever and pain, and feels very weak, which causes great difficulty with daily activities.	0.133 (0.088–0.19)
Acute viral meningitis	This person has a high fever and pain, and feels very weak, which causes great difficulty with daily activities.	0.133 (0.088–0.19)
Mild behaviour problems	This person is hyperactive and has difficulty concentrating, remembering things, and completing tasks.	0.045 (0.028–0.066)
Mild hearing loss	This person has great difficulty hearing and understanding another person talking in a noisy place (for example, on an urban street).	0.01 (0.004–0.019)
Mild hearing loss with ringing	This person has great difficulty hearing and understanding another person talking in a noisy	0.021 (0.012–0.036)

	place (for example, on an urban street), and sometimes has annoying ringing in the ears.	
Moderate hearing loss	This person is unable to hear and understand another person talking in a noisy place (for example, on an urban street), and has difficulty hearing another person talking even in a quiet place or on the phone.	0.027 (0.015–0.042)
Moderate hearing loss with ringing	This person is unable to hear and understand another person talking in a noisy place (for example, on an urban street), and has difficulty hearing another person talking even in a quiet place or on the phone and has annoying ringing in the ears for more than 5 minutes at a time, almost every day.	0.074 (0.048–0.107)
Moderately severe hearing loss	(custom DW from hearing loss impairment envelope)	
Moderately severe hearing loss with ringing	(custom DW from hearing loss impairment envelope)	
Severe hearing loss	This person is unable to hear and understand another person talking, even in a quiet place, and unable to take part in a phone conversation. Difficulties with communicating and relating to others cause emotional impact at times (for example worry or depression).	0.158 (0.105–0.227)
Profound hearing loss	This person is unable to hear and understand another person talking, even in a quiet place, is unable to take part in a phone conversation, and has great difficulty hearing anything in any other situation. Difficulties with communicating and relating to others often cause worry, depression, or loneliness.	0.204 (0.134–0.288)
Complete hearing loss	This person cannot hear at all in any situation, including even the loudest sounds, and cannot communicate verbally or use a phone. Difficulties with communicating and relating to others often cause worry, depression, or loneliness.	0.215 (0.144–0.307)
Severe hearing loss with ringing	This person is unable to hear and understand another person talking, even in a quiet place, is unable to take part in a phone conversation, and has annoying ringing in the ears for more than 5 minutes at a time, almost every day. Difficulties with communicating and relating to others cause emotional impact at times (for example, worry or depression).	0.261 (0.175–0.36)
Profound hearing loss with ringing	This person is unable to hear and understand another person, even in a quiet place, is unable to take part in a phone conversation, has great difficulty hearing anything in any other situation,	0.277 (0.182–0.387)

	and has annoying ringing in the ears for more than 5 minutes at a time, several times a day. Difficulties with communicating and relating to others often cause worry, depression, or loneliness.	
Complete hearing loss with ringing	This person cannot hear at all in any situation, including even the loudest sounds, and cannot communicate verbally or use a phone, and has very annoying ringing in the ears for more than half of the day. Difficulties with communicating and relating to others often cause worry, depression, or loneliness.	0.316 (0.212–0.435)
Moderate motor impairment	This person has some difficulty in moving around, and difficulty in lifting and holding objects, dressing, and sitting upright, but is able to walk without help.	0.061 (0.04–0.089)
Moderate motor plus cognitive impairments	This person has some difficulty in moving around, holding objects, dressing, and sitting upright, but can walk without help. This person has low intelligence and is slow in learning to speak and to do simple tasks.	0.203 (0.134–0.29)
Long-term mild motor impairment	This person has some difficulty in moving around but is able to walk without help.	0.01 (0.005–0.02)
Borderline intellectual disability	This person is slow in learning at school. As an adult, the person has some difficulty doing complex or unfamiliar tasks but otherwise functions independently.	0.011 (0.005–0.02)
Severe motor impairment	This person is unable to move around without help, and is not able to lift or hold objects, get dressed, or sit upright.	0.402 (0.268–0.545)
Epilepsy	(combined DW)	NA
Blindness	Is completely blind, which causes great difficulty in some daily activities, worry and anxiety, and great difficulty going outside the home without assistance.	0.187 (0.124–0.26)
Mild intellectual disability	This person has low intelligence and is slow in learning at school. As an adult, the person can live independently but often needs help to raise children and can only work at simple, supervised jobs.	0.043 (0.026–0.065)
Monocular distance vision loss	This person is blind in one eye and has difficulty judging distances.	0.017 (0.009–0.029)
Mild motor plus cognitive impairments	This person has some difficulty in moving around but is able to walk without help. The person is slow in learning at school. As an adult, the person has some difficulty doing complex or unfamiliar tasks but otherwise functions independently.	0.031 (0.018–0.05)
Severe motor plus cognitive impairments	This person cannot move around without help, and cannot lift or hold objects, get dressed, or sit upright. The person also has very low intelligence,	0.542 (0.37–0.702)

	speaks few words, and needs constant supervision and help with all daily activities.	
Moderate vision impairment	The person has vision problems that make it difficult to recognise faces or objects across a room.	0.031 (0.019–0.049)
Severe vision impairment	The person has severe vision loss, which causes difficulty in daily activities, some emotional impact (for example, worry), and some difficulty going outside the home without assistance.	0.184 (0.125–0.258)

Table 4a. Covariates. Summary of covariates used in the meningitis DisMod-MR meta-regression model

Covariate	Type	Parameter	Exponentiated beta (95% uncertainty interval)
Hib3 vaccine coverage (proportion)	Country-level	Incidence	0.64 (0.63–0.65)
PCV3 coverage (proportion)	Country-level	Incidence	0.62 (0.61–0.63)
Meningitis belt (proportion)	Country-level	Incidence	1.19 (1.06–1.35)
Proportion of total population covered by MenAfriVac initiative (meningitis meningococcal type A vaccine)	Country-level	Incidence	0.99 (0.99–1.00)
Healthcare Access and Quality Index	Country-level	Excess mortality	1.00 (0.99–1.00)

Modelling strategy: long-term sequelae estimation

We split the long-term sequelae among survivors of acute infection. We calculated the acute-phase survival proportion by applying the excess mortality (estimated by the acute meningitis DisMod model) to incidence; excess mortality was converted to case-fatality rate by $e^{(\text{-excess mortality x 1/(excess mortality + remission)})}$. We multiplied this proportion times the bacterial and viral incidence to get the incidence of meningitis-with-survival for bacterial and viral meningitis, respectively.

The survivors were then subject for long-term sequelae by applying the post-discharge proportions of health consequences calculated by a meta-analysis by Edmond and colleagues for bacterial meningitis, and Hudson and colleagues for viral meningitis.[Error! Bookmark not defined.] We calculated the proportion of acute meningitis survivors who experience major or minor long-term impairments for all aetiologies. This proportion was based off a regression of log-transformed GDP and ratio values from Edmonds and colleagues. The regression is shown below:

$$y = -0.347\ln(GDP) + 1.28$$

For viral meningitis, we used a single global, meta-analysed proportion value for viral meningitis: because viral is not treatable by antibiotics, we assume that the proportion with sequelae does not vary greatly by country. The proportion with any impairments was further split into specific impairments according to the proportions from the aetiology-relevant systematic review, which were grouped into vision loss, hearing loss, motor/cognitive impairments, and epilepsy.

All subsequently described steps were performed in parallel for bacterial and viral meningitis. The calculated incidence of long-term sequelae was then converted to prevalence by two different approaches. For the sequelae not associated with excess mortality, which were vision loss, hearing loss, intellectual disability, motor impairment, and behavioural problems, the incidence of each age was cumulatively added up to the subsequent age (assuming half-cycle) to construct prevalence at each age. If the sequela is associated with excess mortality (epilepsy and moderate-to-severe cognitive impairments), the calculated incidence was used as in input to the ODE solver together with the corresponding mortality parameters (excess mortality data from the epilepsy envelope DisMod model, and standardised mortality ratio data from a neonatal encephalopathy meta-analysis, converted to excess mortality using all-cause mortality estimates) to estimate the prevalence. Vision loss, hearing loss, and epilepsy estimates were squeezed and severity split centrally.

To calculate YLDs attributable to all meningitis, bacterial and viral attributable YLDs were ultimately summed. To calculate proportion of YLDs attributable to viral meningitis, the viral meningitis YLDs were divided by the summed YLDs. The methods used for estimating LRI aetiologies have been published elsewhere.[4]

References

1. Edmond K, Clark A, Korczak VS, Sanderson C, Griffiths UK, Rudan I. Global and regional risk of disabling sequelae from bacterial meningitis: a systematic review and meta-analysis. *Lancet Infect Dis.* 2010 May;10(5):317–28.

2. Hudson JA, Broad J, Martin NG, Sadarangani M, Galal U, Kelly DF, et al. Outcomes beyond hospital discharge in infants and children with viral meningitis: A systematic review. *Reviews in Medical Virology*. 2020 Mar;30(2):e2083.

3. Centers for Disease Control and Prevention (CDC). CDC Yellow Book 2016 Health Information for International Travel. Oxford University Press USA; 2016.

4. Naghavi M, Vollset SE, Ikuta KS, Swetschinski LR, Gray AP, Wool EE, et al. Global burden of bacterial antimicrobial resistance 1990–2021: a systematic analysis with forecasts to 2050. *The Lancet*. 2024 Sep 28;404(10459):1199–226.

Motor neuron diseases

Flowchart

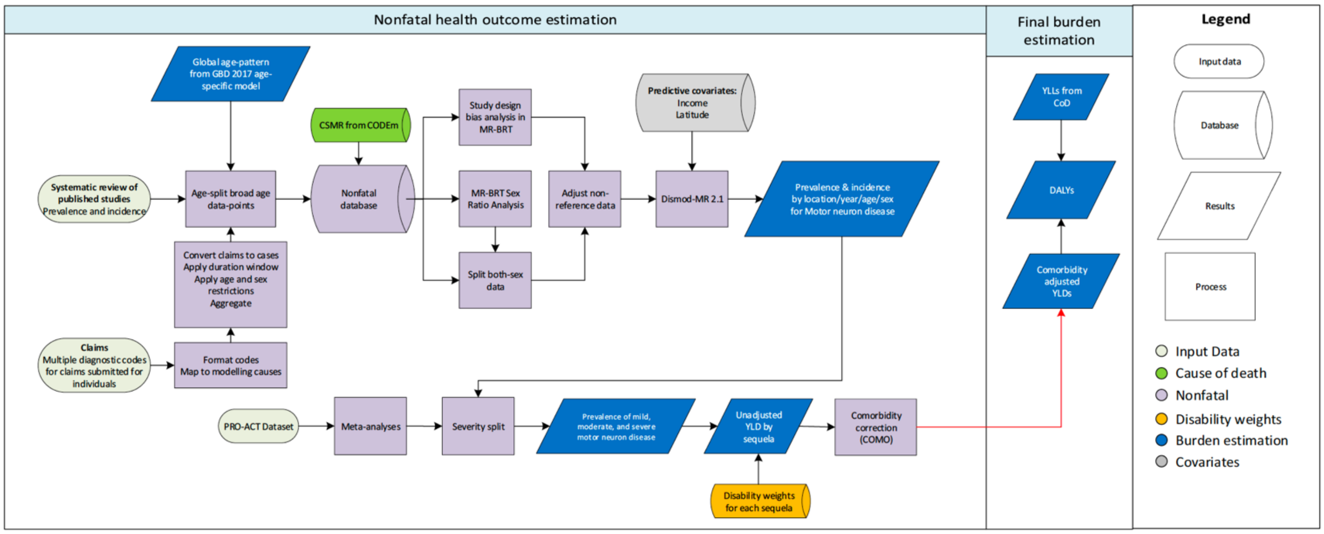

Input data and methodological summary for motor neuron diseases

Case definition

Motor neuron diseases (MND) are a set of chronic, degenerative, and progressive neurological conditions typified by the destruction of upper and/or lower motor neurons and the subsequent deterioration of voluntary muscle activity. The most common MND is amyotrophic lateral sclerosis (ALS). The El Escorial Criteria are the gold standard diagnostic criteria. The ICD-10 code corresponding to motor neuron diseases is G12.

Input data and data processing

Data inputs

A full systematic review was last conducted for GBD 2015. The following search string guided our search, which resulted in 3146 hits with 58 sources meeting extraction criteria: (1) the study is a representative population-based study with well-defined sample, (2) reports on prevalence, incidence, remission, excess mortality, relative risk of mortality, standardised mortality ratio, or with-condition mortality rate for motor neuron diseases in aggregate or a specified motor neuron disease.

(('motor neuron disease'[MeSH Terms] OR ('motor'[All Fields] AND 'neuron'[All Fields] AND 'disease'[All Fields]) OR 'motor neuron disease'[All Fields] OR ('motor'[All Fields] AND 'neuron'[All Fields] AND 'diseases'[All Fields]) OR 'motor neuron diseases'[All Fields]) OR ('amyotrophic lateral sclerosis'[MeSH Terms] OR ('amyotrophic'[All Fields] AND 'lateral'[All Fields] AND 'sclerosis'[All Fields]) OR 'amyotrophic lateral sclerosis'[All Fields]) OR ALS[All Fields] OR ('motor neuron disease'[MeSH Terms] OR ('motor'[All Fields] AND 'neuron'[All Fields] AND 'disease'[All Fields]) OR 'motor neuron disease'[All Fields] OR ('primary'[All Fields] AND 'lateral'[All Fields] AND 'sclerosis'[All Fields]) OR 'primary lateral sclerosis'[All Fields]) OR ('Politics Life Sci'[Journal] OR 'pls'[All Fields]) OR ('muscular atrophy, spinal'[MeSH Terms] OR

('muscular'[All Fields] AND 'atrophy'[All Fields] AND 'spinal'[All Fields]) OR 'spinal muscular atrophy'[All Fields] OR ('progressive'[All Fields] AND 'muscular'[All Fields] AND 'atrophy'[All Fields]) OR 'progressive muscular atrophy'[All Fields]) OR PBP[All Fields] OR ('pseudobulbar palsy'[MeSH Terms] OR ('pseudobulbar'[All Fields] AND 'palsy'[All Fields]) OR 'pseudobulbar palsy'[All Fields])) AND (('epidemiology'[Subheading] OR 'epidemiology'[All Fields] OR 'epidemiology'[MeSH Terms]) OR population-based[All Fields])

Data from the systematic review were manually extracted for GBD 2015. For GBD 2021, a data audit was performed on all previously extracted data. This process included the removal of duplicate rows, re-extraction of preprocessed data (eg, age-splitting and sex-splitting) to ensure they were captured in their raw form, and a comprehensive review of extraction quality for the remaining data.

Beyond systematic review data, as in previous rounds of GBD, we also made use of claims data as obtained and processed by the GBD Clinical Informatics team and described in a separate section of this appendix. These data link claims for all inpatient and outpatient encounters for a single individual and provide primary and secondary diagnoses for all encounters. An individual was extracted from claims data as a prevalent case if they had any MND code as any diagnosis in one or more inpatient encounters or two or more outpatient encounters.

Data processing

For GBD 2023, as in GBD 2021, all sex-specific data were used to estimate a pooled sex-ratio using a MR-BRT (metaregression—Bayesian, regularised, trimmed) model (Additional information can be found in appendix 1, section 2). This ratio was combined with sex-specific population estimates for the year-age-location combinations corresponding to each datapoint reported for both sexes combined, to estimate sex-specific datapoints prior to modelling. These were applied by calculating male prevalence:

$$prev_{male} = prev_{both} * \frac{pop_{both}}{(pop_{male} + ratio * pop_{female})}$$

and then calculating female prevalence:

$$prev_{female} = ratio * prev_{male}$$

(Or the equivalent equations for incidence or other epidemiological measure.)

Datapoints for samples spanning 25 years of age or more were disaggregated into five-year age groups according to the global age pattern estimated in a DisMod-MR 2.1 model that used only datapoints with an age range less than or equal to 25 years.

For GBD 2019, all previously extracted studies were reviewed and assigned a design variable to indicate if the case definition was limited to ALS only or encompassed all MND. Two pre-modelling adjustments were then made to adjust for systematic biases in some data sources: data reporting on ALS only and data from USA claims in the year 2000. The latter includes MarketScan 2000 data, which has lower-coverage data compared to later years of this source and therefore requires appropriate adjustment. Two studies of ALS only were found to be closely matched in year, age, sex, and time with three studies of MND more broadly, and the log ratios for all matched pairs were entered into a MR-BRT meta-analysis. Commercial claims data from the USA in 2000 were matched to USA claims data from later

years with more complete coverage of the population, and these log-ratios were entered into a separate MR-BRT model. This was maintained for GBD 2023.

MR-BRT crosswalk adjustment factors

Data input	Reference or alternative case definition	Beta coefficient, log (95% CI)*	Adjustment factor**
Surveys of all MND using combined clinical, imaging, electrophysiology, and imaging criteria<br>OR<br>Claims data from location-years other than USA 2000	Ref	---	---
USA claims from year 2000	Alt	–0.026 (–1.2 to 1.1)	0.97 (0.31 to 3.1)
Surveys limited to ALS only	Alt	–0.13 (–0.23 to –0.029)	0.88 (0.79 to 0.97)

**MR-BRT crosswalk adjustments can be interpreted as the factor the alternative case definition is adjusted by to reflect what it would have been had it been measured using the reference case definition. If the log/logit beta coefficient is negative, then the alternative is adjusted up to the reference. If the log/logit beta coefficient is positive, then the alternative is adjusted down to the reference.*
***The adjustment factor column is the exponentiated beta coefficient. For log beta coefficients, this is the relative rate between the two case definitions. For logit beta coefficients, this is the relative odds between the two case definitions.*

After extraction and processing, some studies were marked as outliers and excluded on a case-by-case basis if they were inconsistent with established regional or temporal trends or if concerns about study quality were identified during extraction and processing.

Modelling strategy

We use DisMod-MR 2.1 (disease model—Bayesian meta-regression; details on this method can be found in appendix 1, section 2) as the main analytical tool for MND estimation. Inputs included prevalence and incidence data, as described above, as well as the cause-specific mortality rate (CSMR) estimated in the GBD causes of death analysis, and excess mortality rate (EMR) obtained by dividing CSMR by prevalence datapoints. Prior settings are limited to 0 remission at all ages and maximum incidence of 0.0004. We also constrain the super-region random effects for prevalence and incidence to –0.5 and 0.5 to account for spurious inflation of regional differences.

We employed the following covariates to improve model predictions:

Covariate	Measure	Beta coefficient (95% CI)	Exponentiated
Absolute value of average latitude	Prevalence	0.037 (0.036 to 0.038)	1.04 (1.04 to 1.04)
LDI (I$ per capita)	Excess mortality rate	–0.5 (–0.5 to –0.5)	0.61 (0.61 to 0.61)

Although there are no known cures for MND, we expect disease management to differ globally – largely as a function of available resources. To capture this, we use the natural log of lagged distributed income per capita as a proxy to capture this relationship in the estimation of excess mortality.

As described in the literature, extreme latitude may be associated with higher prevalence and incidence of motor neuron disease, although the pathway to explain the association is not understood. Our operationalisation of latitude is created by taking the absolute value of a population-weighted average of latitude by country. The underlying population distribution rasters are part of the Gridded Population of the World dataset.

Severity splits

To calculate severity and disability due to MND, we analysed a dataset from Pooled Resource Openaccess ALS Clinical Trials (PRO-ACT). PRO-ACT is a large ALS clinical trials dataset, with a total of 8635 ALS patient records from multiple completed clinical trials. Among these, we conducted the final analysis with n=4838 (56%) of the patients with complete ALS Function Rating Score (ALSFRS) with average follow-up time of 184 days (min: –22, max: 648), in which 2999 (62%) received experimental (medication) treatments and 1301 (27%) received placebo (in these trials, the medications tested were found to be no better than placebo with respect to their effects on ALS progression).

The ALSFRS is an instrument for evaluating the functional status of patients with amyotrophic lateral sclerosis. It can be used to monitor functional changes in a patient over time. It measures (1) speech, (2) salivation, (3) swallowing, (4) handwriting, (5) cutting food and handling utensils (with or without gastrostomy), (6) dressing and hygiene, (7) turning in bed and adjusting bed clothes, (8) walking, (9) climbing stairs, and (10) breathing. Each task is rated on a 5-point scale from 0 = can't do, to 4 = normal ability. Individual item scores are summed to produce a reported total score of between 0 and 40 (worst to best). ALSFRS has been revised to ALSFRS-R, which includes 12 questions (ALSFRS Q10 changes to (10) dyspnoea, (11) orthopnea, and (12) respiratory insufficiency), with individual item scores summed to a score between 0 and 48.

To eliminate any bias from the treatment effects on the ALSFRS, only the first observation at the time of trial is selected. If the first observation is missing at the time of trial (or prior), the next non-missing observation is selected to be included in the final analysis.

We subsequently mapped ALSFRS scores into GBD severities, and sequelae into different combinations of speech problems, chronic obstructive pulmonary disease, and motor impairment using the following logic:

Motor impairment

The ALSFRS assesses motor function of the legs through questions on walking (Q8) and stair climbing (Q9).

Combined score	Severity level
8	None
5-7	Mild
2-4	Moderate
0-1	Severe

The ALSFRS also assesses motor impairment through questions on handwriting (Q4), cutting food and handling utensils (Q5), and dressing and hygiene (Q6).

Combined score	Severity level
12	None
9-11	Mild
3-8	Moderate
0-2	Severe

After determining case severity on these two separate metrics, we aggregate by taking the most severe ranking (eg, severe + mild = a severe case).

Respiratory problems

Question 10 of the ALSFRS describes breathing difficulty as a function of MND.

ALSFRS score	Description	Severity level
4	Normal	None
3	Shortness of breath with minimal exertion	Mild
2	Shortness of breath at rest	Moderate
0-1	Intermittent ventilator assistance required/ventilator-dependent	Severe

Speech problems

Speech impairment due to MND is derived from ALSFRS question 1, which describes speech impediments. A score of 4 on this question denotes no impairment, while all other values suggest some impairment.

Creating sequelae

After determining the severity status of each case for the three symptom umbrellas, we subsequently estimated the relative proportion of each combination of symptom class and their respective severities. Those without any symptoms (eg, no severity) were categorised as having worry about the diagnosis for disability estimation. The following table displays the various sequelae and their associated proportions.

Sequela	Proportion (mean)	Proportion (lower)	Proportion (upper)
Mild motor impairment, mild respiratory problems and speech problems due to motor neuron disease	0.01779	0.01658	0.01909
Mild motor impairment, moderate respiratory problems and speech problems due to motor neuron disease	0.00270	0.00225	0.00324

Mild motor impairment, severe respiratory problems and speech problems due to motor neuron disease	0.00082	0.00059	0.00113
Mild motor impairment, and speech problems due to motor neuron disease	0.02052	0.01922	0.02190
Mild motor impairment, mild respiratory problems, and speech problems due to motor neuron disease	0.03377	0.03210	0.03552
Moderate motor impairment, moderate respiratory problems, and speech problems due to motor neuron disease	0.00715	0.00640	0.00799
Moderate motor impairment, severe respiratory problems, and speech problems due to motor neuron disease	0.00286	0.00240	0.00342
Moderate motor impairment, and speech problems due to motor neuron disease	0.03041	0.02883	0.03208
Severe motor impairment, moderate respiratory problems, and speech problems due to motor neuron disease	0.02247	0.02111	0.02392
Severe motor impairment, severe respiratory problems, and speech problems due to motor neuron disease	0.01365	0.01259	0.01479
Severe motor impairment and speech problems due to motor neuron disease	0.04765	0.04567	0.04970
Mild respiratory problems and speech problems due to motor neuron disease	0.01157	0.01060	0.01263
Moderate respiratory problems and speech problems due to motor neuron disease	0.00142	0.00111	0.00182
Severe respiratory problems and speech problems due to motor neuron disease	0.00023	0.00013	0.00043
Speech problems due to motor neuron disease	0.02457	0.02315	0.02608
Mild motor impairment and mild respiratory problems due to motor neuron disease	0.02245	0.02109	0.02389
Mild motor impairment and moderate respiratory problems due to motor neuron disease	0.00275	0.00230	0.00329
Mild motor impairment and severe respiratory problems due to motor neuron disease	0.00068	0.00047	0.00097
Mild motor impairment due to motor neuron disease	0.10388	0.10103	0.10681
Moderate motor impairment and mild respiratory problems due to motor neuron disease	0.06744	0.06511	0.06985
Mild motor impairment and mild respiratory problems due to motor neuron disease	0.02245	0.02109	0.02389
Mild motor impairment and moderate respiratory problems due to motor neuron disease	0.00275	0.00230	0.00329
Mild motor impairment and severe respiratory problems due to motor neuron disease	0.00068	0.00047	0.00097
Mild motor impairment due to motor neuron disease	0.10388	0.10103	0.10681
Moderate motor impairment and mild respiratory problems due to motor neuron disease	0.06744	0.06511	0.06985

Moderate motor impairment and moderate respiratory problems due to motor neuron disease	0.01302	0.01199	0.01413
Moderate motor impairment and severe respiratory problems due to motor neuron disease	0.00412	0.00356	0.00477
Moderate motor impairment due to motor neuron disease	0.20136	0.19760	0.20518
Severe motor impairment and mild respiratory problems due to motor neuron disease	0.06902	0.06666	0.07146
Severe motor impairment and moderate respiratory problems due to motor neuron disease	0.02000	0.01872	0.02137
Severe motor impairment and severe respiratory problems due to motor neuron disease	0.01062	0.00969	0.01163
Severe motor impairment due to motor neuron disease	0.15037	0.14702	0.15378
Mild respiratory problems due to motor neuron disease	0.00643	0.00571	0.00723
Moderate respiratory problems due to motor neuron disease	0.00044	0.00028	0.00069
Severe respiratory problems due to motor neuron disease	0.00005	0.00001	0.00017
Asymptomatic, but worry about diagnosis due to motor neuron disease	0.03738	0.03562	0.03921

To determine disability due to these sequelae, we use the standard multiplicative aggregation formula as described in the main text. The following table provides description and disability weight assigned to the sequelae as appropriate.

Symptom group	Severity level	Lay description	DW (95%)
Respiratory problems	Asymptomatic		
Respiratory problems	Mild	Has cough and shortness of breath after heavy physical activity, but is able to walk long distances and climb stairs.	0.019 (0.011–0.033)
Respiratory problems	Moderate	Has cough, wheezing, and shortness of breath, even after light physical activity. The person feels tired and can walk only short distances or climb only a few stairs.	0.225 (0.153–0.31)
Respiratory problems	Severe	Has cough, wheezing, and shortness of breath all the time. The person has great difficulty walking even short distances or climbing any stairs, feels tired when at rest, and is anxious.	0.408 (0.273–0.556)
Motor impairment	Asymptomatic		

Motor impairment	Mild	Has some difficulty in moving around but is able to walk without help.	0.01 (0.005–0.019)
Motor impairment	Moderate	Has some difficulty in moving around and difficulty in lifting and holding objects, dressing, and sitting upright, but is able to walk without help.	0.061 (0.04–0.089)
Motor impairment	Severe	Is unable to move around without help, and is not able to lift or hold objects, get dressed, or sit upright.	0.402 (0.268–0.545)
Speech problems	No		
Speech problems	Yes	Has difficulty speaking, and others find it difficult to understand.	0.051 (0.032–0.078)
Asymptomatic, but worry	Yes	Has a disease diagnosis that causes some worry but minimal interference with daily activities.	0.012 (0.006–0.023)

Multiple sclerosis (MS)

Flowchart

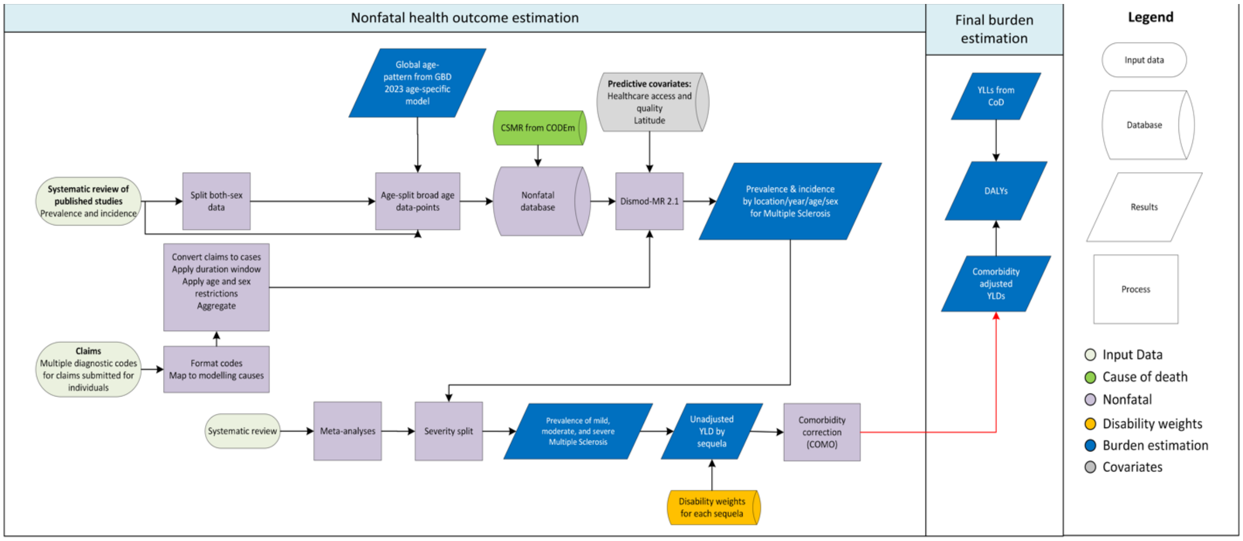

Input data and methodological summary for Multiple sclerosis

Case definition

Multiple sclerosis is a chronic, degenerative, and progressive neurological condition typified by the damaging of the myelin sheaths. McDonald's criteria for diagnosis are considered the contemporary gold standard. For GBD 2023, as for previous rounds, diagnosis by McDonald's criteria, other published criteria (such as Poser, Schumacher, or McAllen criteria), and clinical neurological exam are all considered acceptable case definitions. The ICD-10 code for MS is G35.

Input data and processing

The data underpinning estimates of burden due to MS are generally of two types. The first are representative, population-based, cross-sectional or longitudinal studies reported in peer-reviewed journals and identified via a search-string-based review. This was updated for GBD 2023 with a search that yielded 1408 hits, including 31 additional systematic reviews screened for further sources and 123 full texts selected for extraction. Estimates of epidemiological measures (eg, prevalence, incidence) were manually extracted from these publications. A flowchart documenting this review is displayed below.

Figure 1. PRISMA diagram of GBD 2021 systematic review of scientific literature data for MS

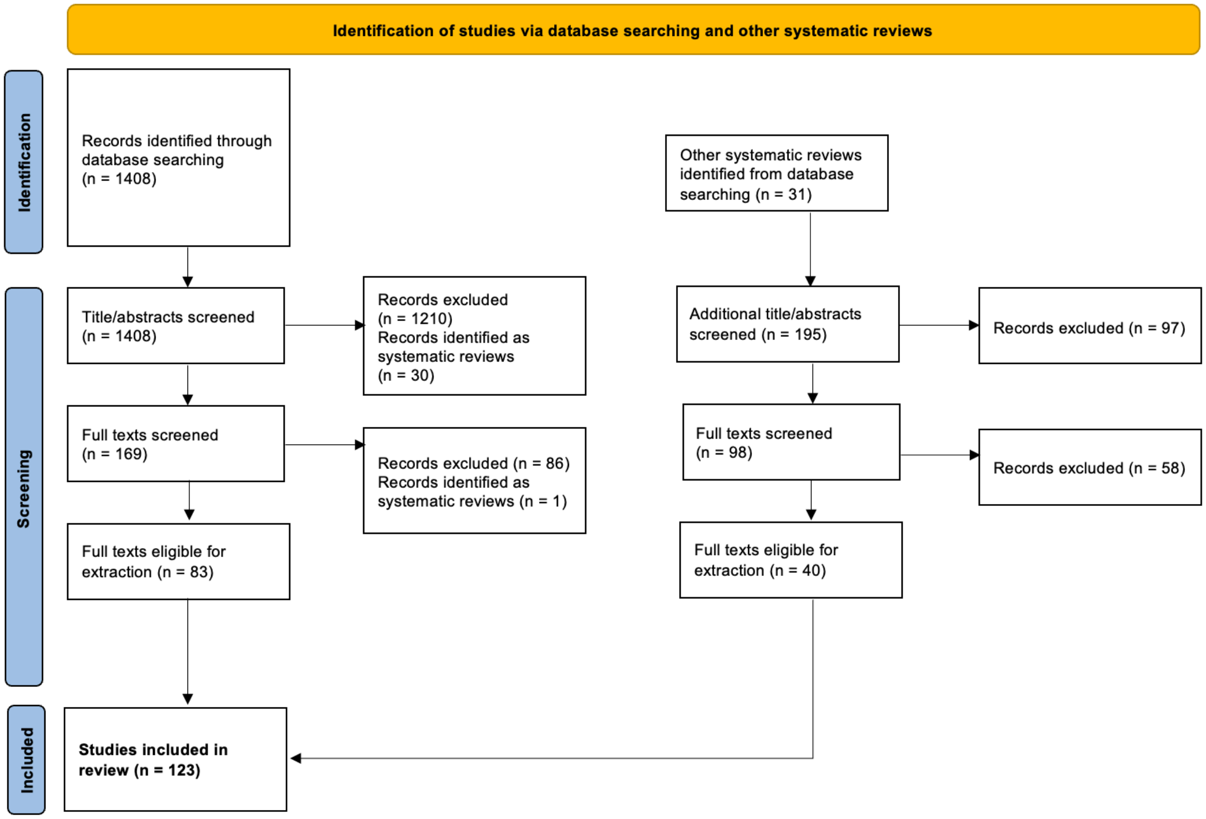

From: Page MJ, McKenzie JE, Bossuyt PM, Boutron I, Hoffmann TC, Mulrow CD, et al. The PRISMA 2020 statement: an updated guideline for reporting systematic reviews. BMJ 2021;372:n71. doi: 10.1136/bmj.n71

The second type of data informing MS burden is claims data, as obtained and processed by the GBD Clinical Informatics team and described in a separate section of this appendix. These data link claims from all inpatient and outpatient encounters for an individual and include both primary and secondary diagnoses for each encounter. Claims sources from Poland, Taiwan, and the USA were used in the MS non-fatal model for GBD 2023.

For studies that reported epidemiological measures (generally prevalence or incidence) by age for both sexes combined, as well as by sex for all ages combined, we calculated the sex-ratio of cases in that study and applied it to the age-specific measures to estimate age-sex-specific measures.

For studies that reported epidemiological measures for both sexes combined only, all sex-specific data were used to estimate a pooled sex-ratio using a MR-BRT (metaregression—Bayesian, regularised, trimmed) model (additional information can be found in appendix 1, section 2). This ratio was combined with sex-specific population estimates for the year-age-location combinations corresponding to each datapoint reported for both sexes combined, to estimate sex-specific datapoints prior to modelling. These were applied by calculating male prevalence:

$$prev_{male} = prev_{both} * \frac{pop_{both}}{(pop_{male} + ratio * pop_{female})}$$

and then calculating female prevalence:

$$prev_{female} = ratio * prev_{male}$$

(Equivalent equations were used for incidence.)

Subsequently, datapoints for samples spanning 25 years of age or more were disaggregated into five-year age groups by applying the age pattern observed in the global fit of a DisMod-MR 2.1 model that used only datapoints with an age range less than or equal to 25 years.

No crosswalking was done to adjust for differences in diagnostic criteria, as it would be challenging to account for variations in diagnostic categories (eg, possible MS, probable MS) as well as variation in false positive and negative rates. However, there is interest in further exploring this in the future.

In previous GBD rounds, USA MarketScan data from the year 2000 were crosswalked using all other data with a validated diagnostic criterion (eg, McDonald's; Poser) in order to account for providing lower-coverage data compared to later years of MarketScan. However, for GBD 2023, we decided to exclude MarketScan 2000 altogether. Additional information on crosswalking can be found in appendix 1, section 2.

After extraction and processing, some studies were marked as outliers and excluded on a case-by-case basis if they were inconsistent with established regional or temporal trends or if concerns about study quality were identified during extraction and processing.

Modelling strategy

Compartmental model

We used DisMod-MR 2.1 as the main analytical tool for the MS estimation process (additional information can be found in appendix 1, section 2). Inputs included prevalence and incidence data, as described above, as well as the cause-specific mortality rate (CSMR) estimated in the GBD causes of death analysis, and excess mortality rate (EMR) obtained by dividing CSMR by prevalence datapoints. Prior settings included zero remission for all ages, due to the incurable nature of MS, and zero incidence or excess mortality for persons under 5 years old, due to the rarity of MS in this age group. We also constrained the random effects for prevalence and incidence to a minimum of –1 and a maximum of 1 for all locations.

We employed the following covariates to improve model predictions:

**Covariate**	**Measure**	**Beta coefficient, log (95% CI)**	**Adjustment factor****

Absolute value of average latitude	incidence	0.021 (0.017 to 0.024)	1.02 (1.02 to 1.02)
Healthcare Access and Quality index	excess mortality rate	–0.036 (–0.075 to –0.024)	0.97 (0.93 to 0.98)

As described in the literature, extreme latitude is associated with higher incidence and prevalence of MS, although the pathway to explain the association is not fully understood. Our operationalisation of latitude is created by a population-weighted average of latitude by country and taking the absolute value. The underlying population distribution rasters are part of the Gridded Population of the World dataset. In GBD 2023, unlike previous rounds, we used latitude as a covariate only for MS incidence and not for prevalence. This is because including latitude as a covariate for both measures resulted in unrealistically high estimates in high-latitude, data-sparse locations (eg, Greenland) compared to more data-rich locations within the same region.

Although there are no known cures for MS, we expect disease management to differ globally – largely as a function of available resources. To capture this, we use the Healthcare Access and Quality Index covariate to capture this relationship in the estimation of excess mortality.

Severity splits

As we have done since GBD 2013, we used Kurtzke's Expanded Disability Status Scale (EDSS) to determine severity splits for MS. The EDSS scores corresponding to each severity are as follows:

Asymptomatic: EDSS = 0
Mild: 0 < EDSS ≤ 3.5
Moderate: 3.5 < EDSS ≤ 6.5
Severe: 6.5 < EDSS ≤ 9.5

The table below illustrates severity levels, lay descriptions, and DWs.

**Severity level**	**Lay description**	**DW (95% CI)**
Asymptomatic	-	0 (0-0)
Mild	Has mild loss of feeling in one hand, is a little unsteady while walking, has slight loss of vision in one eye, and often needs to urinate urgently.	0.183 (0.124–0.253)
Moderate	Needs help walking, has difficulty with writing and arm coordination, has loss of vision in one eye and cannot control urinating.	0.463 (0.313–0.613)
Severe	Has slurred speech and difficulty swallowing. The person has weak arms and hands, very limited and stiff leg movement, has loss of vision in both eyes and cannot control urinating.	0.719 (0.534–0.858)

Because not all sources had information on the number of cases with EDSS stage 0, instead of reporting on a mild category, we implemented a two-step meta-analysis strategy. First, we subsetted the studies to those that reported on the number of cases with EDSS stage 0 and did meta-analyses on the proportion of asymptomatic and mild cases. Then, we conducted meta-analyses on the full dataset to get the proportion mild, moderate, and severe, and we squeezed the asymptomatic and mild categories from the previous meta-analyses into the mild category established by the meta-analysis on the full dataset.

The following figures provide the result of the first meta-analysis on the asymptomatic and mild categories.

Figure 1. Asymptomatic cases of MS

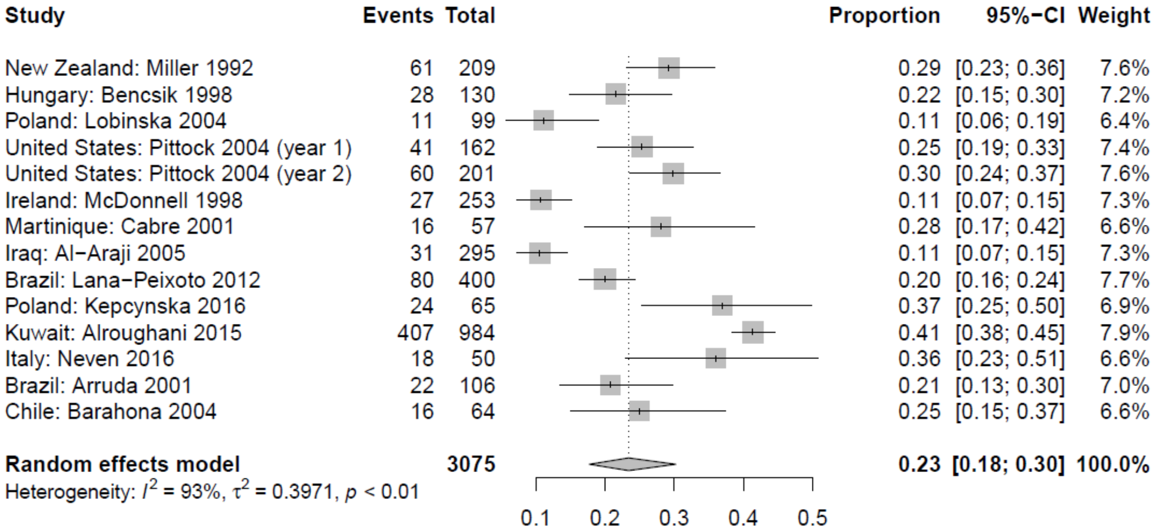

Figure 2. Mild cases of MS

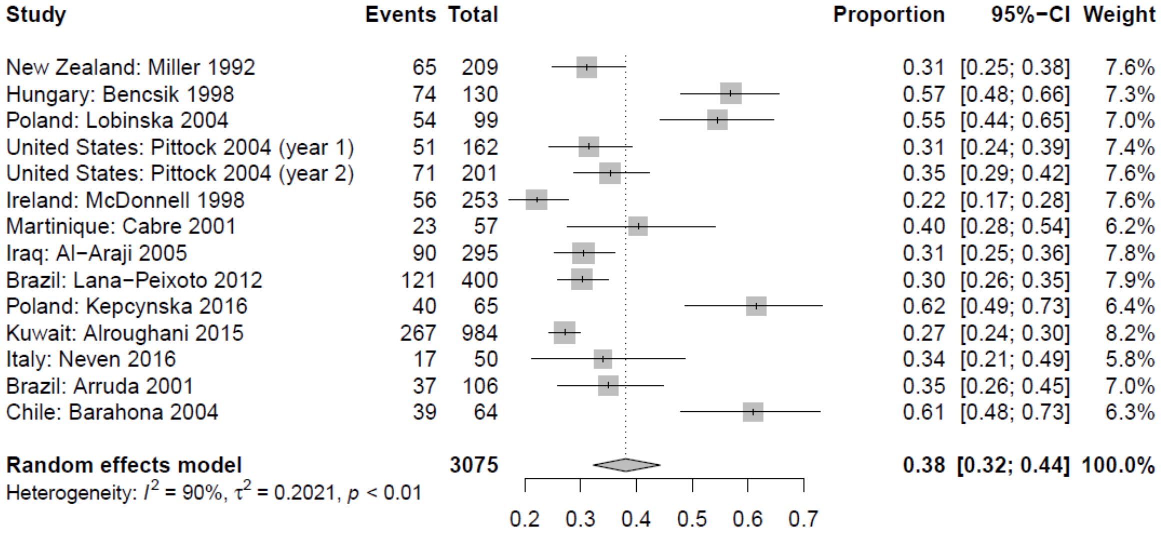

The following figures provide the results of the second meta-analysis on the mild, moderate, and severe categories.

Figure 3. Mild cases of MS (including both asymptomatic and mild categories)

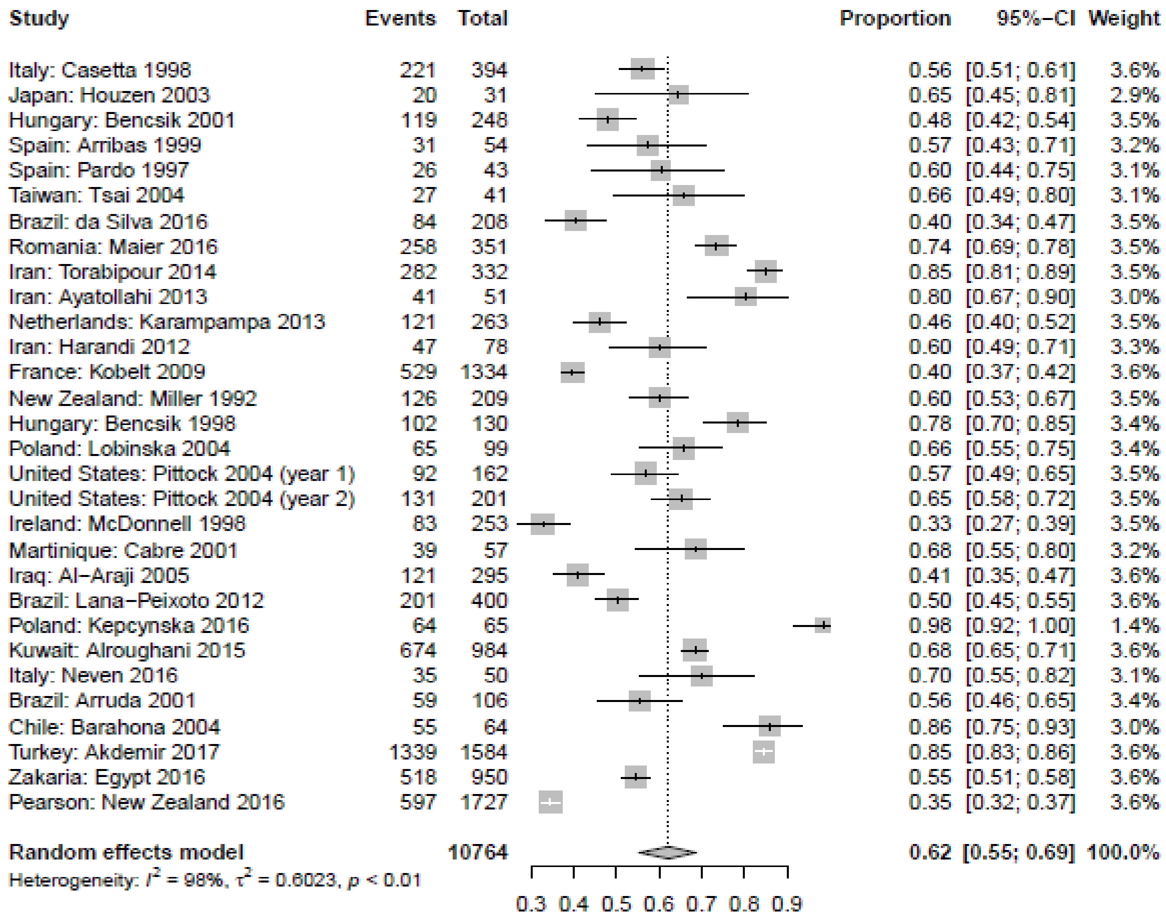

Figure 4. Moderate cases of MS

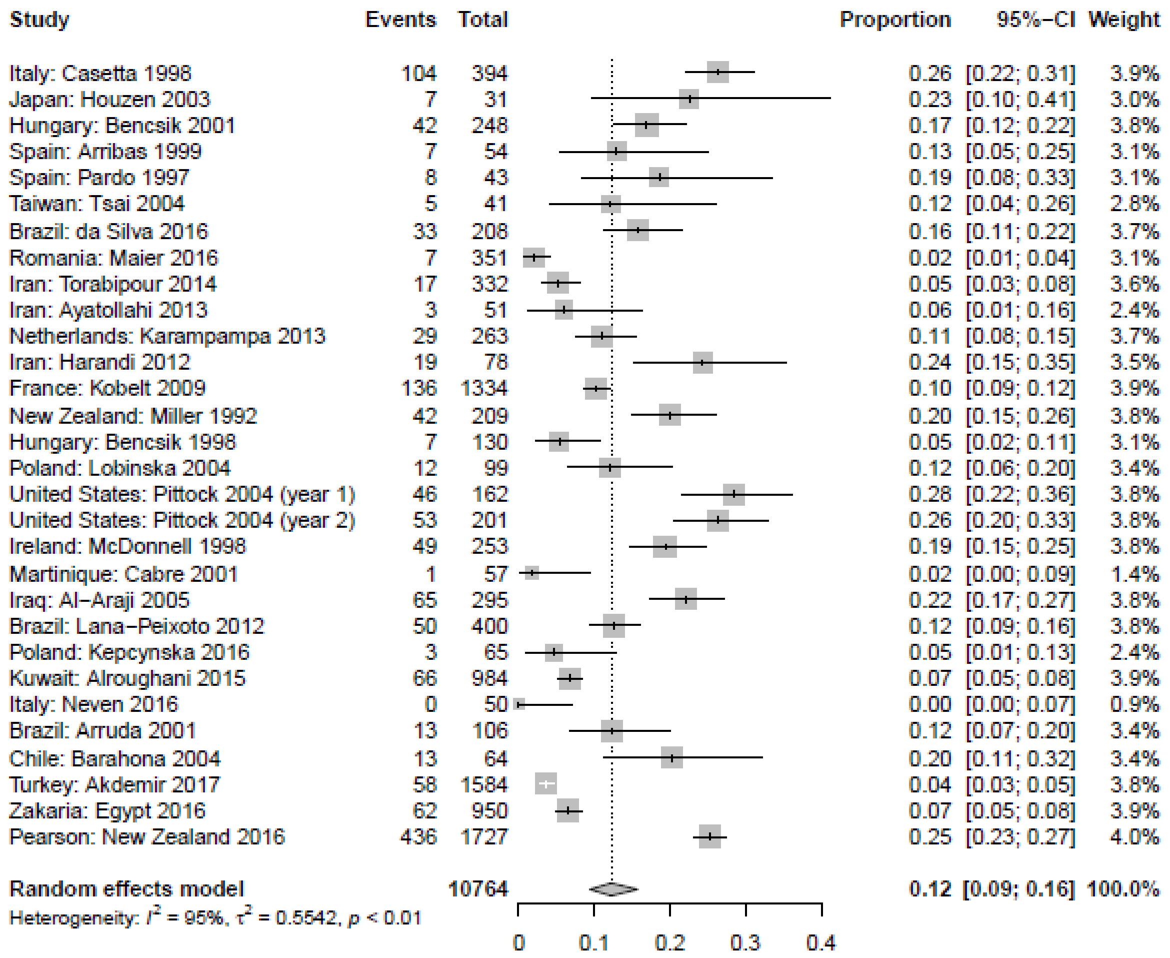

Figure 5. Severe cases of MS

Study	Events	Total	Proportion	95%−CI	Weight
Italy: Casetta 1998	69	394	0.18	[0.14; 0.22]	3.6%
Japan: Houzen 2003	4	31	0.13	[0.04; 0.30]	2.1%
Hungary: Bencsik 2001	87	248	0.35	[0.29; 0.41]	3.6%
Spain: Arribas 1999	16	54	0.30	[0.18; 0.44]	3.0%
Spain: Pardo 1997	9	43	0.21	[0.10; 0.36]	2.7%
Taiwan: Tsai 2004	10	41	0.24	[0.12; 0.40]	2.8%
Brazil: da Silva 2016	91	208	0.44	[0.37; 0.51]	3.6%
Romania: Maier 2016	86	351	0.25	[0.20; 0.29]	3.6%
Iran: Torabipour 2014	33	332	0.10	[0.07; 0.14]	3.4%
Iran: Ayatollahi 2013	7	51	0.14	[0.06; 0.26]	2.6%
Netherlands: Karampampa 2013	113	263	0.43	[0.37; 0.49]	3.6%
Iran: Harandi 2012	12	78	0.15	[0.08; 0.25]	3.0%
France: Kobelt 2009	669	1334	0.50	[0.47; 0.53]	3.7%
New Zealand: Miller 1992	41	209	0.20	[0.14; 0.26]	3.5%
Hungary: Bencsik 1998	21	130	0.16	[0.10; 0.24]	3.3%
Poland: Lobinska 2004	22	99	0.22	[0.14; 0.32]	3.3%
United States: Pittock 2004 (year 1)	54	162	0.33	[0.26; 0.41]	3.5%
United States: Pittock 2004 (year 2)	56	201	0.28	[0.22; 0.35]	3.5%
Ireland: McDonnell 1998	121	253	0.48	[0.42; 0.54]	3.6%
Martinique: Cabre 2001	18	57	0.32	[0.20; 0.45]	3.1%
Iraq: Al-Araji 2005	110	295	0.37	[0.32; 0.43]	3.6%
Brazil: Lana−Peixoto 2012	150	400	0.38	[0.33; 0.42]	3.7%
Poland: Kepcynska 2016	22	65	0.34	[0.23; 0.47]	3.2%
Kuwait: Alroughani 2015	244	984	0.25	[0.22; 0.28]	3.7%
Italy: Neven 2016	15	50	0.30	[0.18; 0.45]	3.0%
Brazil: Arruda 2001	34	106	0.32	[0.23; 0.42]	3.4%
Chile: Barahona 2004	33	64	0.52	[0.39; 0.64]	3.2%
Turkey: Akdemir 2017	187	1584	0.12	[0.10; 0.13]	3.7%
Zakaria: Egypt 2016	370	950	0.39	[0.36; 0.42]	3.7%
Pearson: New Zealand 2016	625	1727	0.36	[0.34; 0.39]	3.7%
**Random effects model**		**10764**	**0.28**	**[0.24; 0.33]**	**100.0%**

Heterogeneity: $I^2 = 96\%$, $\tau^2 = 0.3752$, $p < 0.01$

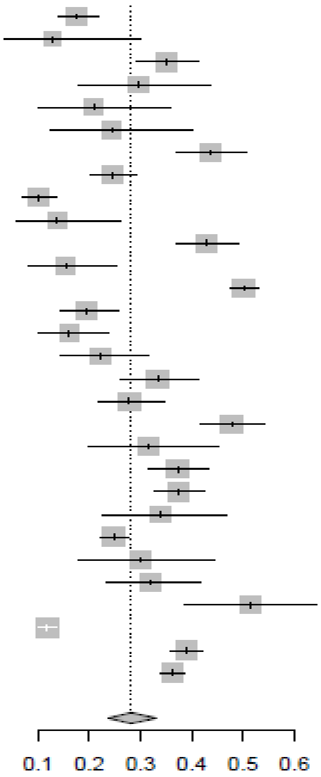

Neck pain

Flowchart

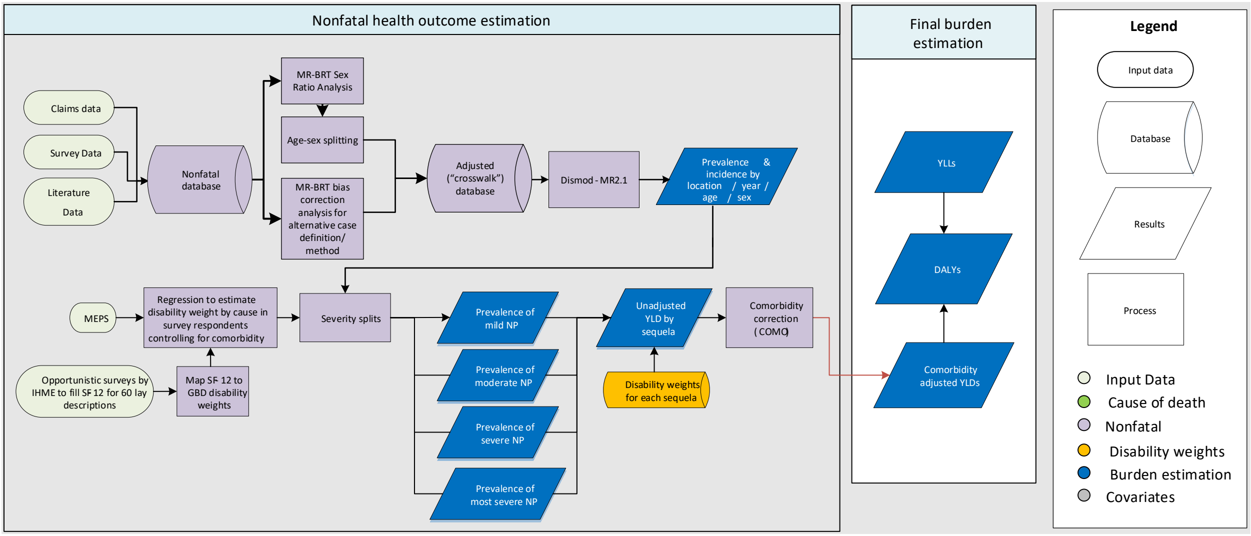

Input data and methodological summary for neck pain

Case definition

The list of accepted neck pain definitions is found below.

Reference or alternative	Case definition
Reference	Current neck pain (+/- pain referred into the upper limb(s)) that lasts for at least one day. This includes Taiwan claims data, which align with the reference definition
Alternative	Current pain (+/- pain referred into the upper limb(s)) that includes the neck in addition to a broader anatomical region (eg, neck and back) that lasts for at least one day
Alternative	Current neck pain (+/- pain referred into the upper limb(s)) that lasts for at least 3 months (chronic)
Alternative	Current neck pain (+/- pain referred into the upper limb(s)) that lasts for at least one day in the last 1 week to 1 month
Alternative	Current neck pain (+/- pain referred into the upper limb(s)) that lasts for at least one day in the last 2 months to 1 year
Alternative	Current neck pain (+/- pain referred into the upper limb(s)) among a study population of schoolchildren that lasts for at least one day

Alternative	Current neck pain (+/- pain referred into the upper limb(s)) that lasts for at least one day and is activity-limiting
Alternative	Current neck pain (with or without reported pain into the upper limb(s)) captured in USA claims data based on the ICD codes below.

The ICD-10 code for neck pain is M54.2. The ICD-9 code is 723.1.

Input data

Ovid MEDLINE, EMBASE, CINAHL, CAB abstracts, WHOLIS, and SIGLE databases were searched for GBD 2010, and PUBMED was searched through October 2017 for GBD 2017. There were no age, sex, or language restrictions. The terms neck pain, neck ache, neckache, and cervical pain individually and combined with each of the following terms: prevalen*, inciden*, cross-sectional, cross sectional, epidemiol*, survey, population-based, population based, population study, population sample.

Exclusion criteria were:

1. Sub-populations clearly not representative of the national population
2. Not a population-based study
3. Studies on a specific type of neck pain (eg, following neck fracture)
4. Low sample size (less than 150)
5. Review rather than original studies

An update systematic review was conducted for neck pain and low back pain simultaneously in GBD 2021, using the following search string:

(((Back Pain[MeSH] OR "back pain"[TiAb]) AND (prevalen*[TiAb] OR inciden*[TiAb]) AND (2017/09/01[PDAT] : 3000[PDAT])) OR(((prevalen* OR inciden*) AND ("neck pain" OR "neck ache" OR "neckache" OR "cervical pain" OR Neck Pain[Mesh])) AND (2017/12/20[PDAT] : 3000[PDAT]))) NOT (animals[MeSH] NOT humans[MeSH])

This returned 1346 entries, of which eight neck pain sources were extracted.

Figure 2: PRISMA diagram of neck pain systematic review from 2021

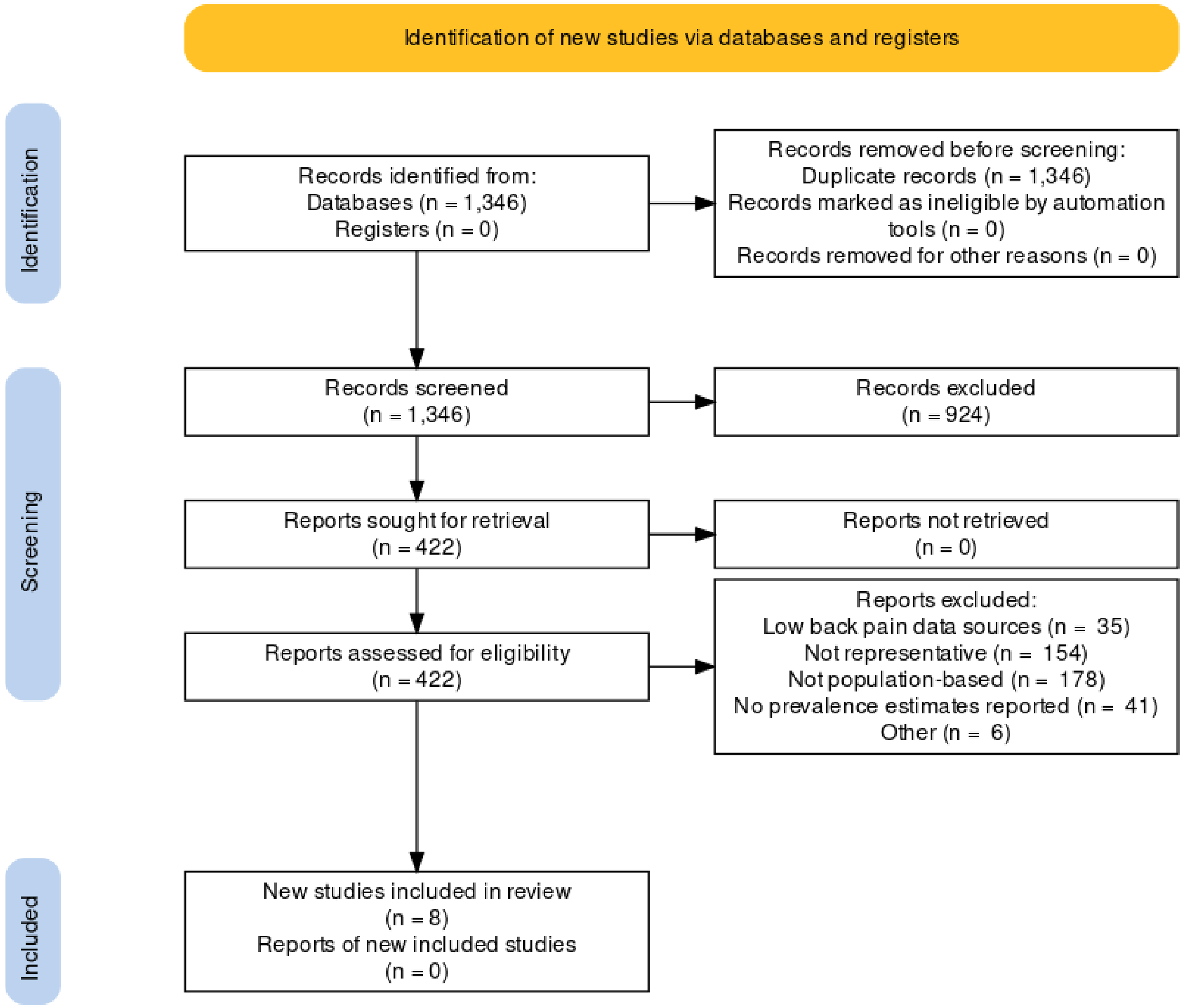

Additional information was derived from unit record data of surveys in the GHDx, GBD's repository of population health data including National Health and Nutrition Examination Survey (NHANES) and National Health Interview Survey (NHIS) in the USA. Opportunistically, additional studies encountered during data review were added for GBD 2019. In addition, data from USA claims data for 2000 and 2010–2015 by state and Taiwan claims data from 2016 were included.

Age and sex splitting

Reported estimates of prevalence were split by age and sex where possible. First, if studies reported prevalence for broad age groups by sex (eg, prevalence in 15- to 65-year-old males and females separately), and also by specific age groups for both sexes combined (eg, prevalence in 15- to 30-year-olds, then in 31- to 65-year-olds, for males and females combined), age-specific estimates were split by sex using the reported sex ratio and bounds of uncertainty. Second, prevalence data for both sexes that could not be split using a within-study ratio were split using a sex ratio derived from a meta-analysis of existing sex-specific data using MR-BRT (meta-regression—Bayesian, regularised, trimmed; for more

information, see appendix 1 section 2). The female to male ratio was 1.47 (1.19–1.83). Finally, after the application of bias adjustments, where studies reported estimates across age groups spanning 25 years or more, these were split into five-year age groups using the prevalence age pattern estimated by DisMod-MR 2.1 (disease model—Bayesian meta-regression, described in appendix 1 section 2).

Data adjustment

We used MR-BRT to calculate adjustment factors to correct for biases introduced by alternative case definitions. These alternative case definitions were studies that reported too broad an anatomical region, episode duration of greater than three months, recall periods of one week to one month, recall periods between two months and one year, activity-limiting neck pain, and studies conducted among schoolchildren. We added three additional covariates for claims data in the USA from the year 2000 and from 2010 onward and for Taiwan claims data. The mean and standard error for the coefficients were calculated using a two-step MR-BRT network crosswalk adjustment method. The covariate for claims data from Taiwan was not included in the final adjustments, as we were unable to find matches to inform a reliable crosswalk. MarketScan claims data were not included in the final model, as fluctuations in ICD coding prevented the construction of a reliable crosswalk. Betas and exponentiated values (which can be interpreted as an odds ratio) for these two covariates are shown in the table below:

Table 2: MR-BRT crosswalk adjustment factors for neck pain

Data input	Reference or alternative case definition	Gamma	Beta coefficient, logit (95% CI)*	Adjustment factor**
Point prevalence	Ref		---	---
Anatomical region too broad	Alt	0.12	0.97 (0.76 to 1.18)	2.63 (2.13 to 3.25)
Episode duration ≥3 months	Alt	0.12	–0.78 (–0.91 to –0.65)	0.46 (0.40 to 0.52)
Recall periods of 1 week to 1 month	Alt	0	1.13 (1.08 to 1.19)	3.10 (2.94 to 3.29)
Recall periods between 2 months and one year	Alt	0	1.68 (1.63 to 1.73)	5.37 (5.10 to 5.64)
Studies among schoolchildren	Alt	0.12	1.07 (0.78 to 1.36)	2.92 (2.18 to 3.90)
Activity-limiting neck pain	Alt	0	–1.13 (–1.14 to –1.12)	0.32 (0.32 to 0.33)

**MR-BRT crosswalk adjustments can be interpreted as the factor the alternative case definition is adjusted by to reflect what it would have been had it been measured using the reference case definition. If the log/logit beta coefficient is negative, then the alternative is adjusted up to the reference. If the log/logit beta coefficient is positive, then the alternative is adjusted down to the reference.*

***The adjustment factor column is the exponentiated beta coefficient. For log beta coefficients, this is the relative rate between the two case definitions. For logit beta coefficients, this is the relative odds between the two case definitions.*

After adjusting data for case definition, we outliered data with a median absolute deviation of 2 or more above or below the age-standardised mean. This was done in a systematic way to identify and remove data that were implausibly high or low.

Modelling strategy

There have been no significant changes to the modelling strategy in GBD 2023.

Prior settings in the DisMod-MR model included setting excess mortality to zero, and it was assumed that there was no incidence or prevalence of neck pain before the age of 5 years.

Severity and disability

The basis of the GBD disability weight survey assessments are lay descriptions of health states highlighting major functional consequences and symptoms. The lay descriptions and disability weights for neck pain severity levels are shown below.

Table 3. Severity distribution, details on the severity levels for NP in GBD 2021 and the associated disability weight (DW) with that severity.

Severity level	Lay description	DW (95% CI)	Proportions
Neck pain, mild	This person has neck pain, and has difficulty turning the head and lifting things	0.052 (0.036–0.074)	0.67 (0.57–0.75)
Neck pain, moderate	This person has constant neck pain, and has difficulty turning the head, holding arms up, and lifting things	0.112 (0.079–0.162)	0.12 (0.08–0.19)
Neck pain, severe	This person has severe neck pain, and difficulty turning the head and lifting things. The person gets headaches and arm pain, sleeps poorly, and feels tired and worried	0.226 (0.147–0.323)	0.06 (0.05–0.07)
Neck pain, most severe	This person has constant neck pain and arm pain, and difficulty turning the head, holding arms up, and lifting things. The person gets headaches, sleeps poorly, and feels tired and worried	0.300 0.199–0.434)	0.15 (0.11–0.20)

The severity distributions are derived from an analysis of the Medical Expenditure Panel Surveys (MEPS) in the USA. MEPS is an overlapping continuous panel survey of the United States non-institutionalised population whose primary purpose is to collect information on the use and cost of health care. Panels are two years long and are conducted in five rounds, which are conducted every five to six months. A new panel begins annually, while the last panel is in its second year. Each panel typically contains about 30,000 to 35,000 individual respondents

MEPS was initiated in 1996 but only began collecting health status data in the form of SF-12 responses in 2000. We used data from 2000–2014 for our analysis, which was last updated in GBD 2019. Respondents self-administer the SF-12 twice per panel, at rounds two and four, typically about a year apart. Only adults 18 years and older completed the SF-12. MEPS also usually collects information on diagnoses based on self-report of reasons for encounters with health services. In addition, diagnoses are derived through additional questions on “problems that bother you” or conditions that led to “disability days,”

ie, days out of role due to illness. Professional coders translate the verbatim text into three-digit ICD-9 codes. The main reason for neck pain being measured in MEPS relates to health-care contact.

To derive a crosswalk of SF-12 values into a scale comparable with that used by the GBD disability weights, small studies on convenience samples were conducted asking respondents to fill in SF-12 to reflect 62 lay descriptions of diverse severity that were used to derive the GBD disability weights. From these responses a relationship between SF-12 summary score and the GBD DWs was derived. With regression methods, average disability weights were calculated for each of 156 conditions for which there were corresponding diagnoses in MEPS, while controlling for any comorbid other condition by adding dummy variables for each condition. As our case definition is for point prevalence of neck pain, we ignored the proportion of MEPS respondents with a neck pain diagnosis for whom in our regression we found no disability attributable to neck pain. For the remaining cases we binned the amount of DW attributed to neck pain across the four health states assuming thresholds at the midpoints between DW values.

References

MEPS-HC Panel Design and Collection Process, Agency for Healthcare Research and Quality, Rockville, Md. https://meps.ahrq.gov/mepsweb/survey_comp/hc_data_collection.jsp

Neonatal disorders

Morbidity due to neonatal disorders is modelled as five individual causes: neonatal preterm birth, neonatal encephalopathy due to birth asphyxia and trauma, neonatal sepsis and other neonatal infections, haemolytic disease and other neonatal jaundice, and other neonatal disorders. Each cause is modelled separately due to differences in data availability and pathology, though many input data types and modelling approaches are shared across the causes. The process for each cause is documented below.

Neonatal preterm birth

Flowchart

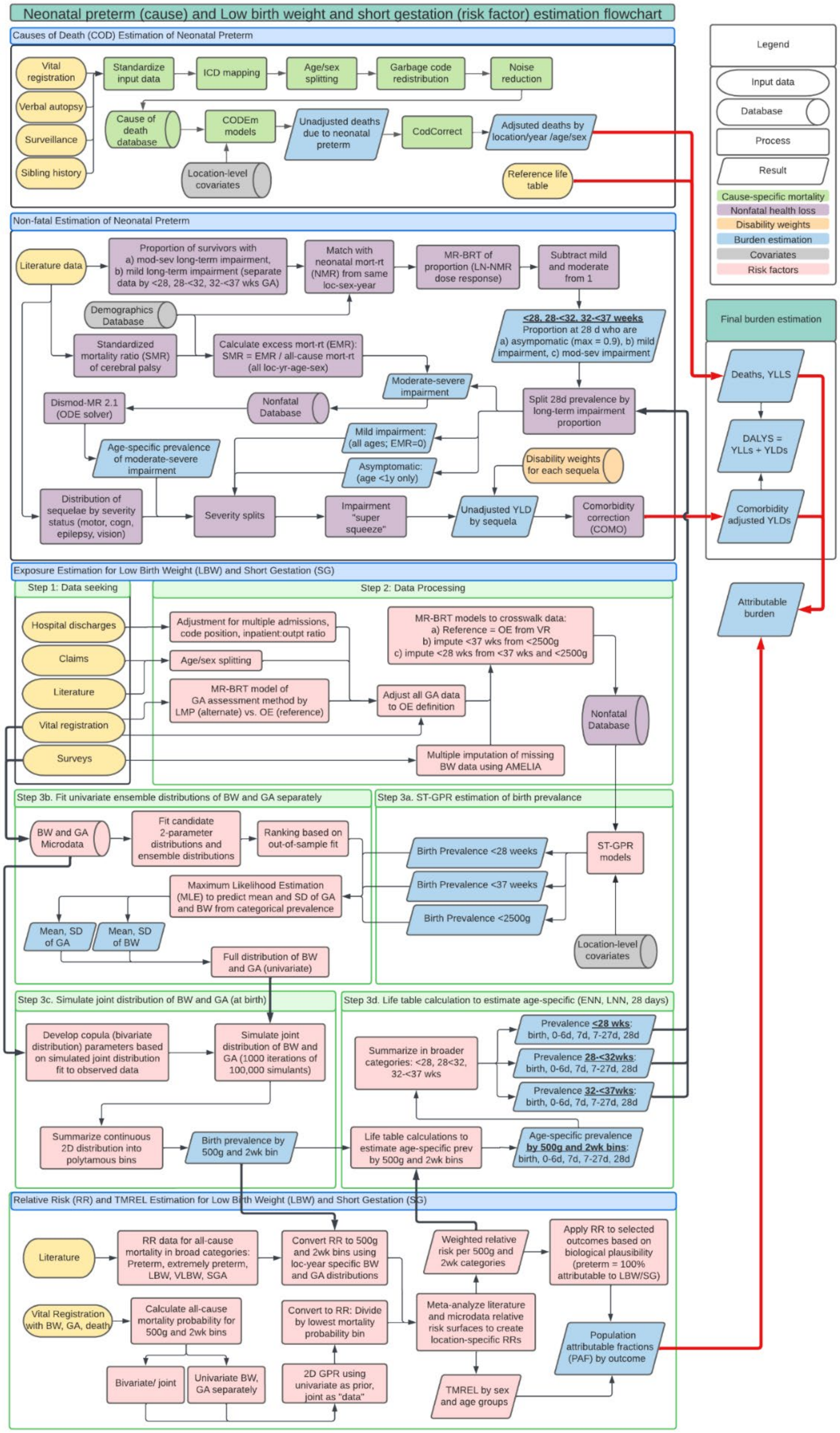

Case definition

Preterm birth is defined as livebirth before 37 completed weeks of gestation. Three categories of preterm birth, based on WHO definitions of prematurity, are presented in GBD estimates: extremely preterm birth (<28 weeks), very preterm birth (28 to <32 weeks), and moderate-to-late preterm birth (32 to <37 weeks).

Modelling summary

We model the non-fatal burden of neonatal preterm birth in five main steps (Table 1). To estimate non-fatal burden due to neonatal preterm birth, the distribution of gestational age at birth is modelled for every location/year/sex. Models of all-cause mortality rates by gestational age are used to estimate the gestational age distribution of surviving neonates from birth until 28 days (Step 1). The proportion of extremely preterm, very preterm, and moderate-to-late preterm neonates who experience long-term impairment are modelled in three severity categories: no impairment (asymptomatic cases), mild impairment, and moderate-to-severe impairment (Step 2). The impairment proportions are applied to estimates of all survivors of preterm birth from birth to 95+ years (the terminal age group in modelled GBD) in order to estimate the prevalence of impairment due to preterm birth by severity category, at all ages. Disability due to asymptomatic preterm birth is estimated until the first year of life, after which no impairment is assumed. Mild and moderate-to-severe impairment is assumed to persist until death, with all excess mortality due to preterm birth attributed to moderate-to-severe impairment (Step 3). Mild and moderate-to-severe impairment are further split into estimates of sequela (Step 4) and then disability weights are applied (Step 5).

Table 1. Analytic steps in estimation of YLDs due to preterm birth

Step	Summary of modeling strategy
1	A. Model univariate distributions at birth<br>B. Model joint distributions at birth<br>C. Model joint distributions from birth to 28 days
2	Model proportion of neonates born preterm who will go on to experience mild, moderate-to-severe, or no long-term impairment, by gestational age category
3	Model all survivors of preterm birth, by severity category, at all ages
4	Model sequela due to preterm birth
5	Apply disability weights to each sequela to calculate YLDs

The strategy to model gestational age distributions from birth until 28 days is the same for both the estimation of non-fatal burden due to preterm birth, described in this appendix, and the estimation of the exposure due to the risk factors "Low birthweight and short gestation" (LBWSG). Estimates of non-fatal burden due to preterm birth require only the modelled gestational age distributions as inputs; however, LBWSG exposure requires the joint distribution of gestational age and birthweight. Because the non-fatal burden due to preterm birth and LBWSG exposure share the same process, the joint estimation of gestational age and birthweight distributions is described in this appendix, even though only gestational age distributions are used in this analysis.

LBWSG risk factor methodological summary

Short gestational age and low birthweight are highly correlated risk factors associated with poor child health outcomes. The "low birthweight and short gestation" (LBWSG) risk factor quantifies the burden of disease attributable to increased risk of death and disability due to 1) less than ideal birthweight ("low birthweight") and 2) shorter than ideal length of gestation ("short gestation").

Within GBD, attributable burden is generally estimated separately for each individual risk factor, but the combined burden attributable to multiple risk factors is of general interest. In GBD, attributable burden due to multiple risk factors is typically estimated through a "mediation analysis" that is applied after independent estimation of each risk factor's exposure, relative risk, theoretical minimum risk exposure level (TMREL), and population attributable fraction (PAF). In the mediation analysis, a "mediation factor" adjusts the PAF of each risk factor by the amount of attributable burden mediated through the other GBD risk factors. While mediation may be common, direct quantification of the joint exposure, relative risk, and PAF of the combined risk factors is conceptually more straightforward.

In GBD 2016, LBWSG became the first group of GBD risk factors in which combined attributable burden is quantified by direct estimation of the joint exposure, relative risk, TMREL, and PAF of multiple risk factors. After first directly estimating the joint exposure, relative risk, TMREL, and PAF of birthweight and gestational age together, we then separate out the independent PAFs due to birthweight only or gestational age only. Because of this modelling strategy, the joint GBD risk factor quantifying the burden of disease due to both less than ideal birthweight ("low birthweight") and shorter than ideal gestational age ("short gestation") is grouped into a single "parent" risk factor termed "low birthweight and short gestation". LBWSG is disaggregated into two "child" risk factors: "low birthweight for gestation" and "short gestation for birthweight". Low birthweight for gestation quantifies the burden of disease attributable to less than ideal birthweight, after adjusting for the influence of gestational age. Likewise, short gestation for birthweight quantifies the burden of disease attributable to shortened gestational age, after adjusting for the influence of birthweight.

Ideally, the model for joint exposure and joint relative risk would be fully continuous. To simplify the computation for the analysis, a grid of 500-gram and two-week units ("bins") is used as the LBWSG dimensions and to approximate a fully continuous joint distribution model (see Figure 1).

Figure 1. Fully continuous analysis of joint gestational age and birthweight (left) is approximated with a grid of birthweight and gestational age with 500-gram and two-week "bins" (right)

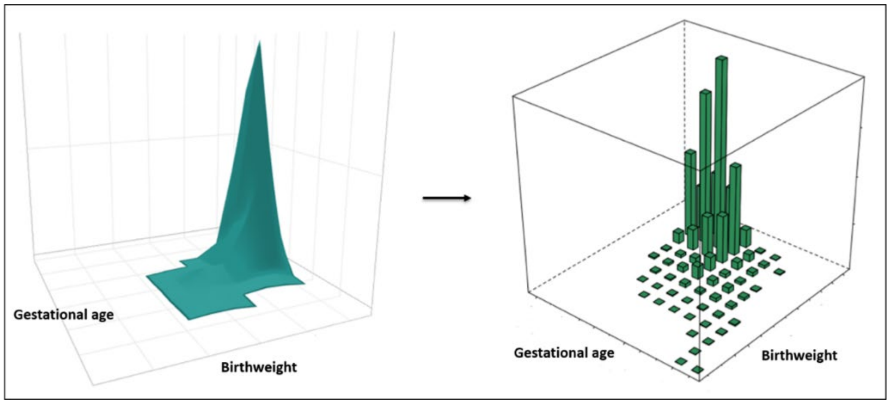

LBWSG case definition

"Low birthweight" has historically referred to any birthweight less than 2500 grams, dichotomising birthweight into two categories: "normal" and "low". In the context of the GBD LBWSG risk factor, low

birthweight refers to any birthweight less than the birthweight TMREL (the birthweight that minimises risk at the population level). Because LBWSG is estimated in a grid of 500-gram and 2-week bins, any 500-gram birthweight unit less than the TMREL, which was determined as [38, 40) weeks and [3500, 4000) g for the LBWSG parent risk factor, is considered "low birthweight". This includes, for example, birthweight of [2500, 3000) grams, which the traditional, dichotomous definition of "low birthweight" would not include.

Like birthweight, gestational age is typically classified into broad categories. "Preterm" is used to describe any newborn baby born less than 37 completed weeks of gestation. In the GBD context, "short gestation" is used to refer to all gestational ages below the gestational age TMREL.

Exposure modelling strategy

In LBWSG, exposure refers to the portion of the joint distribution of gestational age and birthweight less than the TMREL, by location/year/sex (l/y/s), from birth to the end of the neonatal period. Modelling LBWSG exposure can be summarised in three steps:

A. Model univariate gestational age and birthweight distributions at birth, by l/y/s
B. Model joint distributions of gestational age and birthweight at birth, by l/y/s
C. Model joint distributions from birth to the end of the neonatal period, by l/y/s

Input data and data processing

Input data needed to model univariate gestational age and birthweight distributions at birth (Step A):

- Prevalence of preterm birth (<37 weeks), by l/y/s
- Prevalence of preterm birth (<28 weeks), by l/y/s
- Mean gestational age, by l/y/s
- Gestational age microdata
- Prevalence of low birthweight (<2500 grams), by l/y/s
- Mean birthweight, by l/y/s
- Birthweight microdata

To model joint distributions of gestational age and birthweight (Step B), joint microdata of gestational age and birthweight are also required. Additional inputs to modelling joint distributions from birth to 28 days (Step C) are all-cause mortality by l/y/s and joint birthweight and gestational age microdata linked to mortality outcomes.

Prevalence of extremely preterm birth (<28 weeks) and preterm birth (<37 weeks) were modelled using vital registration, survey, and clinical data. For the preterm models, only inpatient and insurance claims data were included from clinical informatics datasets; outpatient data were excluded because they were more likely to capture repeated visits by the same child rather than unique visits. Prevalence of low birthweight (<2500 grams) was modelled using only vital registration and survey data.

Literature review

Before GBD 2016, available preterm birth data were sourced by a technical working group. In GBD 2016 and GBD 2017, we conducted systematic reviews to identify additional sources beyond the data already used in the models. The PubMed database was searched using the following search string:

> ((("Infant, Premature"[Mesh] OR ("infant"[All Fields] AND "premature"[All Fields]) OR "premature infant"[All Fields] OR ("preterm"[All Fields] AND "infant"[All Fields]) OR "preterm infant"[All Fields] OR ("infant, newborn"[MeSH Terms] OR ("infant"[All Fields] AND "newborn"[All Fields]) OR "newborn infant"[All Fields] OR ("newborn"[All Fields] AND "infant"[All Fields])) AND (premature[All Fields] OR

preterm[All Fields]) OR "premature birth"[MeSH Terms] OR ("premature"[All Fields] AND "birth"[All Fields]) OR "premature birth"[All Fields] OR ("preterm"[All Fields] AND "birth"[All Fields]) OR "preterm birth"[All Fields]) ((("Infant, Premature"[Mesh] OR ("infant"[All Fields] AND "premature"[All Fields]) OR "premature infant"[All Fields] OR ("preterm"[All Fields] AND "infant"[All Fields]) OR "preterm infant"[All Fields] OR ("infant, newborn"[MeSH Terms] OR ("infant"[All Fields] AND "newborn"[All Fields]) OR "newborn infant"[All Fields] OR ("newborn"[All Fields] AND "infant"[All Fields])) AND (premature[All Fields] OR preterm[All Fields]) OR "premature birth"[MeSH Terms] OR ("premature"[All Fields] AND "birth"[All Fields]) OR "premature birth"[All Fields] OR ("preterm"[All Fields] AND "birth"[All Fields]) OR "preterm birth"[All Fields]) AND ("1985"[PDAT] : "3000"[PDAT]) AND "humans"[MeSH Terms].

The exclusion criteria were studies that did not provide primary data on epidemiological parameters, non-representative studies (eg, only high-risk pregnancies), and reviews. Table 2 shows the search hits, number of full-texts reviewed, and number of extracted sources.

Table 2. LBWSG search hits, full-text review, extracted sources

Search	Hits	Full-text review	Extracted	Search date
GBD 2017	16 174	2200	154	6/6/2017

Data processing

Any data that didn't fit a GBD age group were split into age groups using a model that was run using only age-specific data. Starting in GBD 2019, as was the case with all other non-fatal analyses, we applied empirical age and sex ratios from previous models to disaggregate observations that did not entirely fit in one GBD age category or sex. Ratios were determined by dividing the result for a specific age and sex by the result for the aggregate age and sex specified in a given observation.

Low birthweight (<2500 grams) data were extracted from literature, vital registration systems, and surveys. Survey data (most commonly from DHS and MICS) were observed to have high missingness of birthweight responses. We evaluated the patterns of missingness and found a number of distinct patterns that suggested non-random omission of birthweight observations. We therefore imputed missing birthweight values using the Amelia II (Version 1.7.6) package in R. Birthweight was predicted using the following variables also in the DHS surveys: urbanicity, sex, birthweight recorded on card, birth order, maternal education, paternal education, child age, child weight, child height, mother's age at birth, mother's weight, shared toilet facility, and household water treated.

After imputation, we completed a number of additional steps to standardise the dataset by applying a series of crosswalks. "Crosswalking" is a process of reducing non-random bias by adjusting non-standard data to the likely value had the data been collected using a reference definition, technique, or sample. Three crosswalks were applied for birthweight and gestational age data, all of the statistical models for which were developed using meta-regression—Bayesian, regularised, trimmed (MR-BRT).

First was a crosswalk for method of gestational age assessment that included three separate models. All microdata that reported GA and both obstetric estimate (OE) and last menstrual period were crosswalked to OE using the relationship derived from USA GA microdata (Figure 2). This crosswalk was developed with a spline on LMP to reliably match on the data that needed to be crosswalked.

Next, for all data that were only categorical, we adjusted all gestational age data to a reference definition of obstetric estimate (OE), which also included tabulations of the crosswalked microdata above. Two alternate definitions regularly appeared, and both were crosswalked separately. These were last menstrual period (LMP) for each of <37 weeks' and <28 weeks' gestation (Tables 3 and 4) and other measure of gestation age (Tables 5 and 6).

The second set of crosswalks adjusted data derived from clinical administrative sources (ie, hospital discharges and insurance claims) to matched vital registration data using OE (Tables 7 and 8).

The third set of crosswalks served to "square the input dataset" to ensure that every location-year with data had an observation for each of <2500 g (birthweight), <37 weeks, and <28 weeks. This process used relationships between input data types to maximise the volume of data later input to models. Low birthweight data (<2500 g) were crosswalked to preterm (<37 weeks) data (Table 9), preterm to extremely preterm (Table 10), and extremely preterm to preterm (Table 11).

Figure 2. MR-BRT OE-LMP crosswalk adjustment factor by LMP-reported gestational age

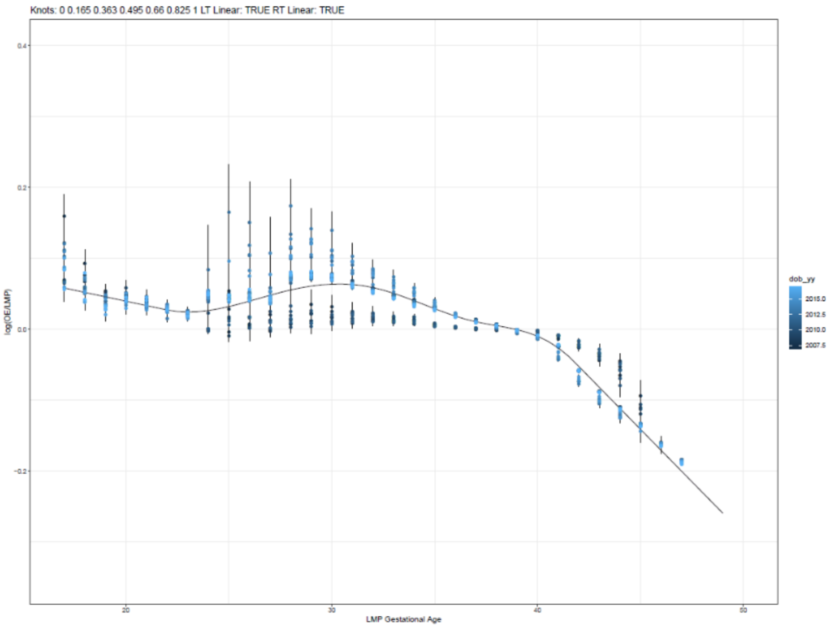

Table 3. MR-BRT OE-LMP crosswalk adjustment factor for preterm birth (<37 weeks' gestation)

Data input	Reference or alternative case definition	Gamma	Beta coefficient, log (95% CI)	Adjustment factor*
Obstetric estimate	Reference	0.01	---	---
Last menstrual period	Alt		0.187 (0.142, 0.231)	1.205 (1.153, 1.260)

Table 4. MR-BRT OE-LMP crosswalk adjustment factor for extremely preterm (<28 weeks' gestation)

Data input	Reference or alternative case definition	Gamma	Beta coefficient, log (95% CI)	Adjustment factor*
Obstetric estimate	Reference	0.00	---	---
Last menstrual period	Alt		0.0284 (0.268, 0.300)	1.328 (1.308, 1.349)

Table 5. MR-BRT OE-other measure crosswalk adjustment factor for preterm birth (<37 weeks' gestation)

Data input	Reference or alternative case definition	Gamma	Beta coefficient, log (95% CI)	Adjustment factor*
Obstetric estimate	Reference	0.10	---	---
Other measurement	Alt		–0.243 (–0.494, 0.009)	0.785 (0.610, 1.01)

Table 6. MR-BRT OE-other measure crosswalk adjustment factor for extremely preterm birth (<28 weeks' gestation)

Data input	Reference or alternative case definition	Gamma	Beta coefficient, log (95% CI)	Adjustment factor*
Obstetric estimate	Reference	0.37	---	---
Other measurement	Alt		0.154 (–0.486, 0.793)	1.166 (0.615, 2.210)

Table 7. MR-BRT VR-claims crosswalk adjustment factor for preterm birth (<37 weeks' gestation)

Data input	Reference or alternative case definition	Gamma	Beta coefficient, log (95% CI)	Adjustment factor*
Vital registration	Reference	0.07	---	---
Insurance claims	Alt		–0.712 (–0.909, –0.515)	0.491 (0.403, 0.597)

Table 8. MR-BRT VR-insurance claims crosswalk adjustment factor for extremely preterm birth (<28 weeks' gestation)

Data input	Reference or alternative case definition	Gamma	Beta coefficient, log (95% CI)	Adjustment factor*
Vital registration	Reference	0.02	---	---
Insurance claims	Alt		–1.258 (–1.447, –1.07)	0.284 (0.235, 0.344)

Table 9. MR-BRT low birthweight to preterm birth (<37 weeks' gestation)

Data input	Reference or alternative case definition	Gamma	Beta coefficient, log (95% CI)	Adjustment factor*
Preterm birth	Reference	0.08	---	---
Low birthweight	Alt		–0.479 (–0.518, –0.440)	0.620 (0.596, 0.644)

Table 10. MR-BRT preterm (<37 weeks' gestation) to extremely preterm (<28 weeks' gestation)

Data input	Reference or alternative case definition	Gamma	Beta coefficient, log (95% CI)	Adjustment factor*
28 weeks	Reference	0.06	---	---
37 weeks	Alt		3.221 (3.161, 3.281)	25.053 (23.600, 26.604)

Table 11. MR-BRT extremely preterm (<28 weeks' gestation) to preterm (<37 weeks' gestation)

Data input	Reference or alternative case definition	Gamma	Beta coefficient, log (95% CI)	Adjustment factor*
37 weeks	Reference	0.05	---	---
28 weeks	Alt		–3.208 (–3.266, –3.150)	0.0404 (0.0381, 0.0428)

**MR-BRT crosswalk adjustments can be interpreted as the factor the alternative case definition is adjusted by to reflect what it would have been had it been measured using the reference case definition. If the log/logit beta coefficient is negative, then the alternative is adjusted up to the reference. If the log/logit beta coefficient is positive, then the alternative is adjusted down to the reference.*

***The adjustment factor column is the exponentiated beta coefficient. For log beta coefficients, this is the relative rate between the two case definitions. For logit beta coefficients, this is the relative odds between the two case definitions.*

These data adjustments had the effect of dramatically increasing the size of each of the modelling datasets and are primarily responsible for most changes in preterm estimates between GBD 2019 and subsequent rounds. After all crosswalks, we performed a deduplication step on GA models. Namely, if low birthweight data in countries that were 1) categorised as "data-rich" locations in cause of death modelling or had at least ten consecutive years of vital registration data recording gestational age, and 2) had both preterm birth and low birthweight data, crosswalked low birthweight data were outliered so that the model was informed only by the gestational age data.

Modelling strategy

We have made no substantive changes in the modeling strategy from GBD 2021.

Step 1A: Model univariate birthweight and gestational age distributions at birth, by l/y/s

Microdata are the ideal data source for modelling distributions; however, microdata are not widely available for birthweight and are scarcer for gestational age. Categorical prevalence data are more readily available from a wider range of locations and years for low birthweight (<2500 g), extremely preterm (<28 weeks of gestation), and preterm birth (<37 weeks of gestation). Because categorical prevalence has wider availability than microdata, we use prevalence data to assist in modelling birthweight and gestational age ensemble distributions.

Ensemble distribution models can be constructed with three pieces of information: mean of the distribution, variance of the distribution, and the weights of the distributions being used in the ensemble. To model mean and variance for all l/y/s for birthweight and gestational age, we first used spatiotemporal Gaussian process regression (ST-GPR) models to model prevalence of low birthweight, extremely preterm and preterm birth for all l/y/s at birth. To model mean birthweight for all l/y/s, OLS

linear regression was used to regress mean birthweight on log-transformed low birthweight prevalence. This model was then used to predict mean birthweight for all l/y/s, using the prevalence of low birthweight (<2500 g) modelled for all l/y/s in ST-GPR. Similarly, to model gestational age mean for all l/y/s, OLS linear regression model was used to regress mean gestational age on log-transformed preterm prevalence. Mean gestational age for all l/y/s was predicted using the preterm birth (<37 weeks) estimate modelled in ST-GPR.

Global ensemble weights for gestational age were derived by using all available gestational age and birthweight microdata in Table 12 to select the ensemble weights. The distribution families included in the optimisation process were exponential, gamma, gumbel, Weibull, log-normal, normal, mirrored gamma, and mirrored gumbel. As an advancement starting in GBD 2021, ensemble weights were fit that specifically targeted the fit at 28 weeks and 37 weeks for gestational age and 1500 grams and 2500 grams for low birthweight. Prior to GBD 2021, the fit of these models had been optimised to reduce error across the entire distribution. Additionally, as an improvement starting in GBD 2021, this ensemble weight fitting strategy optimised on all microdata sources simultaneously, as opposed to separately.

For each l/y/s, given the mean and ensemble weights, the variance was optimised to minimise error on the prevalence of preterm birth (<37 weeks) for the gestational age distribution and prevalence of low birthweight (<2500 grams) for the birthweight distribution.

Step 1B: Model joint birthweight and gestational age distributions at birth, by l/y/s

To model the joint distribution of gestational age and birthweight from separate distributions, information was needed about the correlation between the two distributions. Distributions of gestational age and birthweight are not independent; the Spearman correlation for each country where joint microdata were available (Table 12), pooling across all years of data available, ranged from 0.25 to 0.49. The overall Spearman correlation was 0.38, pooling across all countries in the dataset.

Table 12. Summary of microdata inputs

Location	Years of data	Total births*	Format of data	Spearman correlation	Used in ensemble weight selection	Used in copula parameter selection	Used in relative risk models
BRA	2016	2,854,380	Microdata	0.37	Yes	Yes	No
ECU	2003–2015	2,473,039	Microdata	0.34	Yes	Yes	No
ESP	1990–2014	8,537,220	Microdata	0.42	Yes	Yes	No
JPN	1995–2015	23,644,506	Tabulations	0.41	No	No	Yes
MEX	2008–2012	10,256,117	Microdata	0.35	Yes	Yes	No
NOR	1990–2014	1,489,210	Microdata	0.44	Yes	Yes	Yes
NZL	1990–2016	1,600,501	Microdata	0.25	Yes	Yes	Yes
SGP	1993–2015	972,775	Tabulations	0.41	No	No	Yes
TWN	1998–2002	1,331,760	Tabulations	0.38	No	No	Yes
URY	1996–2014	698,622	Microdata	0.49	Yes	Yes	No
USA	1990–2014	81,929,879	Microdata	0.38	Yes	Yes	Yes

** Pooled across all years and sexes, excluding data missing year of birth, gestational age, or birthweight*

Joint distributions between the birthweight and gestational age marginal distributions were modelled with copulae. The Copula and VineCopula packages in R were used to select the optimal copula family and copula parameters to model the joint distribution, using joint microdata from the country-years in Table 12. The copula family selected from the microdata was "Survival BB8", with theta parameter set to 1.75 and delta parameter set to 1.

The joint distribution of birthweight and gestational age per location-year-sex was modelled using the global copula family and parameters selected and the location-year-sex gestational age and birthweight

distributions. The joint distribution was simulated 100 times to capture uncertainty. Each simulation consisted of 10,000 simulated joint birthweight and gestational age datapoints. Each joint distribution was divided into 500 g by two-week bins to match the categorical bins of the relative risk surface. Birth prevalence was then calculated for each 500 g by two-week bin.

Step 1C: Model joint distributions from birth to the end of the neonatal period, by l/y/s

Early neonatal prevalence and late neonatal prevalence were estimated using life table approaches for each 500 g and two-week bin. Using the all-cause early neonatal mortality rate for each location-year-sex, births per location-year-sex-bin, and the relative risks for each location-year-sex-bin in the early neonatal period, the all-cause early neonatal mortality rate was calculated for each location-year-sex-bin. The early neonatal mortality rate per bin was used to calculate the number of survivors at seven days and prevalence in the early neonatal period. Using the same process, the all-cause late neonatal mortality rate for each location-year-sex was paired with the number of survivors at seven days and late neonatal relative risks per bin to calculate late neonatal prevalence and survivors at 28 days.

Step 2: Model proportion of neonates born preterm who will go on to experience mild, moderate-to-severe, or no long-term impairment, by gestational age category

Using mild impairment proportion and moderate-to-severe impairment proportion data, we ran a single mixed-effects linear regression model, regressing on Healthcare Access and Quality (HAQ) Index and with a dummy variable on each gestational age and proportion type, to generate country-year-sex-specific estimates of both parameters for each gestational age (Figure 3). The remainder of 1 – (mild proportion + moderate-severe proportion) was assigned to asymptomatic proportion, by gestational age. The maximum sum of the mild and moderate-severe proportions was capped at 90%.

Figure 3. Preterm birth mild, moderate-severe impairment regression on HAQ Index (log), by gestational age

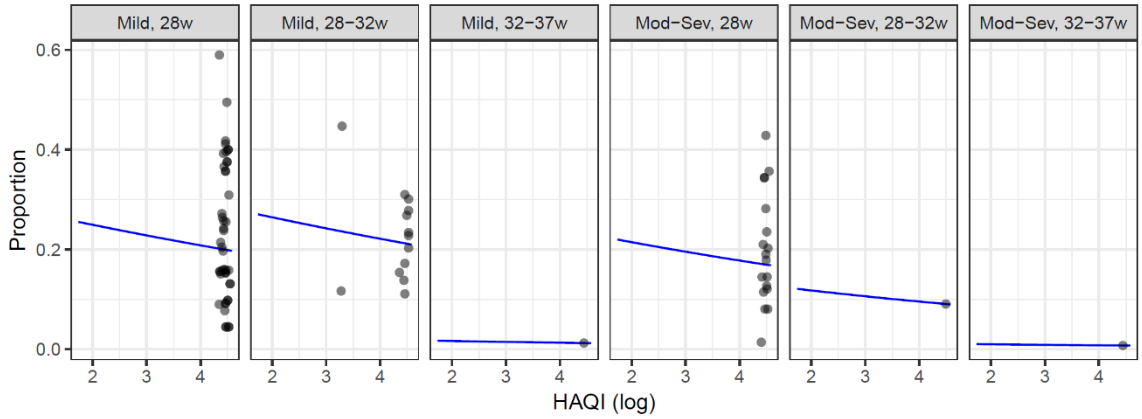

Step 3: Model all survivors of preterm birth, by severity category, at all ages

Asymptomatic, mild, and moderate-severe impairment proportions at 28 days, modelled in Step 2, were applied to prevalence at 28 days. Prevalence of survivors of extremely preterm birth, very preterm birth, and moderate-to-late preterm birth to 28 days was estimated in the modelling step described in Step 1C. Asymptomatic prevalence was assumed to be the same from birth to one year as at 28 days. Asymptomatic prevalence was set to zero after one year, as no burden is assumed after the first year of life. Mild prevalence was assumed to be the same at all GBD age groups as at 28 days. This was both a pragmatic decision in terms of reducing complexity of subsequent modelling steps, but also reflects a lack of data and therefore an assumption of no excess mortality among those born preterm who develop mild impairment.

The sum of asymptomatic and mild impairment in the early and late neonatal periods was subtracted from the neonatal preterm birth envelope estimates for each gestational age in the early and late neonatal periods, respectively, to estimate moderate-severe impairment.

Standardised mortality ratios of cerebral palsy were used as input data to model the prevalence of prematurity for ages older than the neonatal period based on the assumption that complications of prematurity are one of the reasons that young children go on to develop cerebral palsy. These data were used across all four neonatal causes and for many other causes in the GBD study. We ran a meta-analysis for a 0–19 age group and a 20–99 age group, and the SMR values were converted to EMR using the formula:

$$EMR = (\text{location-sex-age-specific } \textit{all-cause mortality rate}) * (\text{age-specific } SMR - 1)$$

To model moderate-severe prevalence at older ages, a DisMod-MR 2.1 model was run on the existing moderate-severe prevalence estimate (eg, prevalence in the early neonatal period), and on excess mortality estimates derived from the standard mortality ratios (SMR) of cerebral palsy. Remission and incidence were set to zero. The input dataset was entirely complete as every location had an input datum for early neonatal prevalence as well as specific values for EMR at every age-location-sex-year, so we did not specify location-level covariates and the model was set to not pass any priors for any parameter during the estimation cascade, functionally meaning the final estimate age-location-sex-year was not informed by any adjacent locations or years.

Step 4: Model sequelae due to preterm birth

Asymptomatic cases were by definition assigned no disability weight and therefore no YLDs. Mild impairment and moderate-severe impairment due to neonatal preterm birth are split into the sequelae listed in Table 13. The proportion for mild sequelae was split equally between motor and motor plus cognitive impairment. The proportions for each moderate/severe sequela were extracted from a study by Badawi and colleagues and are listed in Table 13. The proportions were the same across gestational age categories.

Table 13. Proportion of each sequela of neonatal preterm birth

Sequelae of neonatal preterm birth	Mild	Moderate-severe
Mild motor impairment	0.500	
Mild motor plus cognitive impairment	0.500	
Moderate motor only		0.173
Moderate motor impairment + epilepsy		0.100
Moderate motor impairment + blindness		0.018
Moderate motor impairment + blindness + epilepsy		0.009
Moderate motor impairment + blindness + cognitive impairment		0.032
Moderate motor impairment + epilepsy + cognitive impairment		0.183
Moderate motor impairment + blindness + epilepsy + cognitive impairment		0.017
Severe motor only		0.152
Severe motor impairment + epilepsy		0.033
Severe motor impairment + blindness		0.006
Severe motor impairment + blindness + epilepsy		0.003
Severe motor impairment + blindness + cognitive impairment		0.038
Severe motor impairment + epilepsy + cognitive impairment		0.216
Severe motor impairment + blindness + epilepsy + cognitive impairment		0.020
Mild retinopathy of prematurity	0.07	0.07
Moderate retinopathy of prematurity	0.19	0.19

Severe retinopathy of prematurity	0.13	0.13
Retinopathy of prematurity with blindness	0.26	0.26

Prematurity was additionally assessed to be a cause of vision loss via development of retinopathy of prematurity. The proportion of infants born with prematurity and surviving to the end of the neonatal period who go onto develop retinopathy of prematurity is applied to prevalence of preterm birth at 28 days. Proportional splits were estimated by regressing proportion of ROP among preterm infants on natural-log-transformed neonatal mortality rate from 55 studies in 19 countries. The prevalence of infants with ROP is then split into five vision sequelae of varying severity: asymptomatic, mild, moderate, severe, and complete vision loss (blindness). The proportional splits of retinopathy of prematurity by severity are also listed in Table 13 and are the same across gestational age categories. The mild impairment estimates are split into two sequelae, and the moderate-to-severe impairment estimates are split into 14 sequelae. The mild sequelae were derived by splitting the mild prevalence equally. The proportions for each moderate/severe sequela were extracted from a study by Badawi and colleagues[1] and are listed in Table 13 in descending order. These proportions were also used to split impairments into sequelae across the other neonatal causes.

Step 5: Use disability weights to each sequela to calculate YLDs

Each sequela is associated with a health state, which is used to calculate YLDs. The disability weights for all the health states of all the neonatal disorders are listed in the table below. Some health states are combined using a multiplicative approach to calculate the disability of certain sequelae.

Table 14. Disability weights and lay descriptions by health state

Health state	Description	Disability weight
Motor impairment, mild	Has some difficulty in moving around but is able to walk without help.	0.01 (0.005–0.019)
Motor impairment, moderate	Has some difficulty in moving around, and difficulty in lifting and holding objects, dressing and sitting upright, but is able to walk without help.	0.061 (0.040–0.089)
Motor impairment, severe	Is unable to move around without help, and is not able to lift or hold objects, get dressed or sit upright.	0.402 (0.268–0.545)
Motor plus cognitive impairments, mild	Has some difficulty moving around but is able to walk without help. The person is slow in learning at school. As an adult, the person has some difficulty doing complex or unfamiliar tasks but otherwise functions independently.	0.031 (0.018–0.050)
Motor plus cognitive impairments, moderate	Has some difficulty in moving around, holding objects, dressing, and sitting upright, but can walk without help. The person has low intelligence and is slow in learning to speak and to do simple tasks.	0.203 (0.134–0.290)
Motor plus cognitive impairments, severe	Cannot move around without help, and cannot lift or hold objects, get dressed, or sit upright. The person also has very low intelligence, speaks few words, and needs constant supervision and help with all daily activities.	0.542 (0.374–0.702)
Distance vision blindness	Is completely blind, which causes great difficulty in some daily activities, worry and anxiety, and great difficulty going outside the home without assistance.	0.187 (0.124–0.260)
Epilepsy, less severe (seizures < once per month)	Has sudden seizures two to five times a year, with violent muscle contractions and stiffness, loss of consciousness, and loss of urine or bowel control.	0.263 (0.173–0.367)
Epilepsy, severe (seizures ≥ once per month)	Has sudden seizures one or more times each month, with violent muscle contractions and stiffness, loss of consciousness, and loss of urine or bowel control. Between seizures the person has memory loss and difficulty concentrating.	0.552 (0.375–0.71)
Abdominopelvic problem, severe (proxy for EHB without kernicterus)	Has severe pain in the belly and feels nauseous. The person is anxious and unable to carry out daily activities.	0.324 (0.220–0.442)

Neonatal encephalopathy due to birth asphyxia and trauma

Flowchart

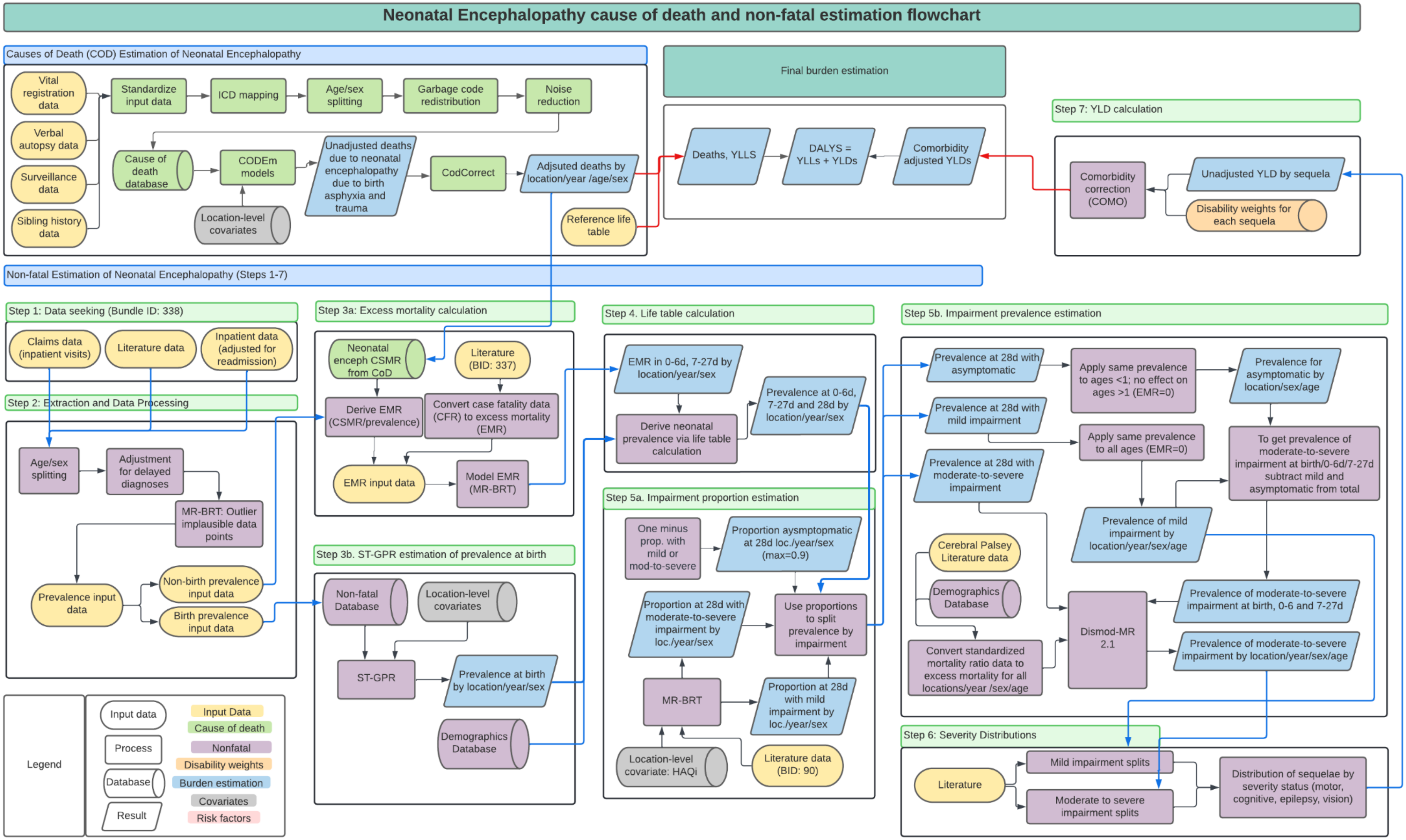

Case definition

Neonatal encephalopathy (NE) due to birth asphyxia and birth trauma is defined in the GBD non-fatal analyses as injury to the brain in the first few moments or days of life in an infant born at term. The case definition does not include trauma that is not associated with brain injury due to inconsistent coding in clinical administrative datasets. NE is often used interchangeably with the term hypoxic-ischaemic encephalopathy (HIE), but the terms are not strictly synonymous because it is believed that only a subset of NE cases are actually triggered by a hypoxic or ischaemic event. NE has multiple aetiologies and is defined by its symptoms – abnormal neurological function, including reduced level of consciousness, seizures, depression of tone and reflexes, or difficulty maintaining respiration.

Modelling strategy

We have made no substantive changes in the modeling strategy from GBD 2021. Modelling the non-fatal burden of neonatal encephalopathy occurs in six main steps.

Table 15. Analytical steps in estimation of YLDs due to neonatal encephalopathy due to birth asphyxia and trauma

Step	Summary of modelling strategy
1	Model NE prevalence at birth using ST-GPR
2	Estimate NE prevalence in the early neonatal period, late neonatal period, and at exactly 28 days using a life table algorithm
3	Model case-fatality ratio and asymptomatic, mild, and moderate-severe impairment proportions at 28 days using mixed effect regressions, then split prevalence at 28 days by severity of impairment
4	Model impairment prevalence at younger and older ages based on 28-day impairment prevalence
5	Split mild and moderate/severe impairment prevalence into sequelae
6	Apply disability weights to each sequela to calculate YLDs

Step 1: Model NE prevalence at birth using ST-GPR

Our modelling process used spatiotemporal Gaussian process regression (ST-GPR) to model the prevalence of neonatal encephalopathy at birth and produced estimates in the early and late neonatal periods using a life table algorithm.

Input data and data processing

Prevalence

We sourced data on prevalence of neonatal encephalopathy at birth from literature and clinical informatics data.

A systematic review for NE was last completed for GBD 2015. The PubMed database was searched using the following search string:

> ((("infant"[Title/Abstract] OR "newborn"[Title/Abstract] OR "newborn infant"[Title/Abstract]) AND ("encephalopathy"[Title/Abstract] OR "neonatal encephalopathy"[Title/Abstract] OR "perinatal asphyxia"[Title/Abstract] OR "asphyxia neonatorum"[Title/Abstract] OR "newborn encephalopathy"[Title/Abstract] OR "hypoxic ischaemic encephalopathy"[Title/Abstract] OR ("birth trauma"[Title/Abstract] AND "birth asphyxia"[Title/Abstract]))) AND ("2012"[PDAT] : "3000"[PDAT]) AND "humans"[MeSH Terms])

The exclusion criteria were studies that did not provide primary data on epidemiological parameters, non-representative studies (eg, only high-risk pregnancies), and reviews. We extracted 60 studies from this review.

Clinical informatics data (hospital and claims) formed the bulk of the input data for the neonatal encephalopathy birth prevalence model, including inpatient hospital and inpatient claims data. Using

the meta-regression—Bayesian, regularised, trimmed (MR-BRT) tool, we modelled and applied a correction factor to account for multiple hospital admissions for a single case of neonatal encephalopathy. We did not include outpatient data in the model because we do not believe it to be representative of the true prevalence of neonatal encephalopathy. This is because neonates with neonatal encephalopathy in the countries where hospital data were available are almost sure to be admitted to the hospital, whereas outpatient data are more likely to capture repeated visits by the same child as they grow.

In GBD 2019, we had standardised data processing to be the same across all sources of clinical informatics data (inpatient hospital and claims data) by ensuring the codes included in claims data matched the codes included in hospital data. This approach standardised the clinical data, but we still observed substantial heterogeneity between clinical and literature data. Investigation of the root cause of the heterogeneity led to the exclusion of those with a solitary discharge diagnosis of P20 (intrauterine hypoxia) from being counted as cases of NE.

In GBD 2021, clinical informatics data were processed to reflect the discrete under-5 age groups within the GBD study. Because many sources are not linked across years, these splits led to implausible age patterns and an under-ascertainment of cases at birth. Based on the assumption that all cases present in older age groups in the <1-year GBD age groups would necessarily have been present at birth, we adjusted the under-1 inpatient data to back add cases that first "appeared" at older ages to the numerator of the prevalence in younger ages.

Both of these changes technically create a mismatch between GBD mapping of ICD codes for neonatal encephalopathy for non-fatal versus mortality analyses, but we believe this is likely a more accurate representation of how the codes are used. These changes in clinical mapping and processing eliminated the need for crosswalking between clinical informatics source types, but also had the consequence of limiting the size of the dataset because not all sources contained the necessary level of detail to make the necessary distinctions. Significant heterogeneity in NE data from clinical sources remains and is a priority research area going forward in GBD.

We applied empirical sex ratios derived from clinical informatics data to disaggregate literature observations that were sex-aggregated. We calculated these ratios as the pooled sex distribution of birth prevalence from all clinical informatics data sources, which are disaggregated by sex. It is our intention to update this splitting process annually.

Lastly, because of significant residual heterogeneity in input data, especially from clinical administrative sources, we used an MR-BRT model to identify outliers in the birth prevalence data. We logit transformed our prevalence data and calculated standard errors using the delta method and fit a cubic spline on the Healthcare Access and Quality (HAQ) Index with fixed effects on sex and age group with a 40% trimming parameter. All trimmed data were marked as outliers in the model. Additionally, we outliered all inpatient hospital data from the USA as they were implausibly low compared to both USA claims data and clinical data from similar countries.

Modelling strategy

Starting with GBD 2021, we implemented a demographic life table modelling approach to produce prevalence estimates for the early neonatal, late neonatal, and 28-day age groups. We first modelled the prevalence of neonatal encephalopathy at birth using spatiotemporal Gaussian process regression (ST-GPR), a three-step modelling procedure for generating estimates for every location, year, age, and sex in the GBD study. The first step of the ST-GPR process is an ensemble linear mixed-effects regression of our data on a set of potentially predictive covariates taken from the GBD study covariates database.

We tested every combination of these covariates in individual, sex-specific mixed-effects linear regressions with nested random effects at the super-region, region, and location levels. We then evaluated and ranked each of these sub-models by their out-of-sample root-mean-squared error (RMSE). Finally, to produce initial estimates for every location, year, age, and sex in the analysis, we averaged the 50 top-performing models where the estimated coefficients were 1) statistically significant at $p < 0.05$, and 2) in the expected direction. We tested the following covariates in the ensemble prior: antenatal care coverage (1+ visits), in-facility delivery, lag-distributed income per capita, livebirths among women aged 35+ years, total fertility rate, maternal care and immunisation, Socio-demographic Index, Healthcare Access and Quality Index, maternal education (6+ years and 12+ years), ambient particulate matter summary exposure value (SEV), household air pollution SEV, low birthweight SEV, short gestation SEV, and smoking SEV. The covariates selected in each ensemble prior, including their frequency and relative influence (indicated by the standardised beta coefficients for each covariate), are displayed in Figure 4.

Figure 4. Standardised betas from ST-GPR ensemble stage 1 prior

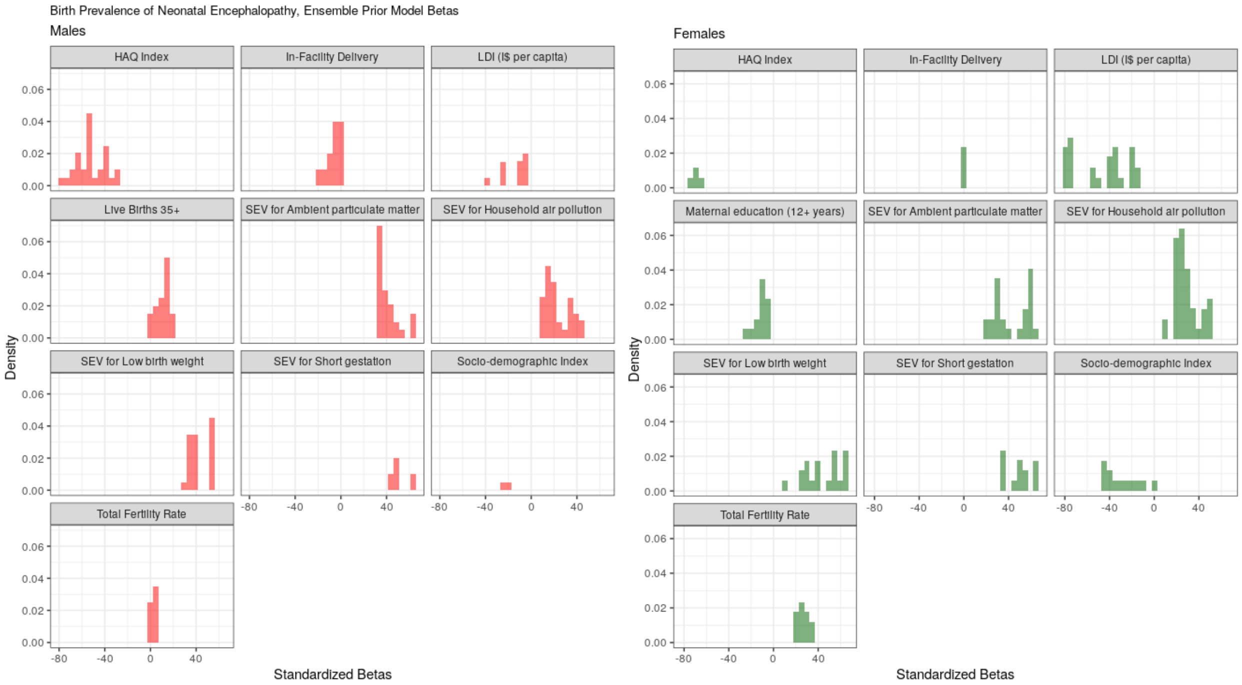

The second, spatiotemporal smoothing step of ST-GPR calculates the residual between our stage 1 regression estimate and each of our observed datapoints and then smooths this residual, drawing strength over space and time and producing a revised stage 2 estimate of birth prevalence for every location, year, and sex. The third step of ST-GPR is a Gaussian process regression, using the stage 2 estimates as a prior and the observed datapoints and their variance to 1) further smooth the residual between the stage 2 predictions and observed data and produce a final mean estimate for each location, year, and sex, and 2) estimate uncertainty around this mean estimate, quantified by taking 1000 draws from the posterior Gaussian process. More detailed information on the ST-GPR modelling process can be found in the main text methods appendix.

Step 2: Estimate NE prevalence in the early neonatal, late neonatal, and 28-day periods using a life table algorithm

Excess mortality data modelling

Our life table algorithm requires excess mortality estimates for every location, year, age, and sex. To generate these estimates, we modelled excess mortality in MR-BRT, using excess mortality data calculated from case-fatality ratio data as well as derived excess mortality from our prevalence data and modelled cause-specific mortality rates.

We extracted case-fatality ratio (CFR) data from literature as the proportion of deaths in the neonatal period (<28 days of life) among cases of neonatal encephalopathy. We did not conduct a separate literature review for this CFR data; rather, it was extracted whenever identified from the search described above. In order to use this CFR data for the life table algorithm detailed below, CFR was transformed into an excess mortality rate (EMR) using the formula:

$$EMR = -\frac{\ln(1 - CFR)}{\frac{days\ of\ observation\ period}{365}}$$

This is analogous to the transformation of cumulative incidence (proportion) to an incidence rate (person-year denominator). The denominator in this equation is the number of days in the observation period for the datapoint – for example, data that followed newborns with neonatal encephalopathy for one year would have a denominator of 1.

Additionally, we calculated excess mortality data wherever we have prevalence data by taking the cause-specific mortality rate estimates from the GBD cause of death analysis divided by the corresponding prevalence datapoint in a given location, year, age, and sex.

For our MR-BRT model, we log-transformed all EMR data and calculated standard errors using the delta method. We fit a cubic spline on the HAQ Index with fixed effects on age group and sex. From this, we generated 1000 draws of estimated EMR for every location, year, age, and sex in the analysis.

Life table algorithm

The next step in our modelling process is a life table algorithm that uses our estimates of birth prevalence of neonatal encephalopathy, excess mortality data, and mortality data from the GBD mortality analysis to generate prevalence estimates in the early and late neonatal age groups and at exactly 28 days for every location, year, and sex.

We began with cases at birth derived from our birth prevalence model and calculated cases at the end of the early neonatal period using the equation:

$$n_7 = n_0\, e^{-EMR_{0-7} t_7}$$

Where n is the number of cases at age 0 or 7 days, EMR_{0-7} is the modelled excess mortality rate for the early neonatal period, and t_7 is the duration of the early neonatal period in years. We repeated this calculation to get cases at the end of the late neonatal period. Prevalence at exactly 28 was a straightforward calculation of cases at 28 days divided by population at 28 days.

To calculate the period prevalence in the early and late neonatal periods, we first summed the person-years of cases who lived to the end of the period and the person-years of the cases who died during the period. We then divided that sum by the sum of the person-years of the general population who

lived to the end of the period and the person-years of the general population who died during the period, as follows:

$$p_{0-7} = \frac{n_7 t_{0-7} + n_0(1 - e^{-EMR_{0-7}t_7})a_{0-7}}{N_7 t_{0-7} + N_0\, ACMR_{0-7}t_{0-7}a_{0-7}}$$

In this equation, a_{0-7} represents the average years lived in the age interval $[0,7)$ by persons who die in this interval, and $ACMR_{0-7}$ represents the all-cause mortality ratio in that interval.

We computed the population (N_7) at the end of the early and late neonatal periods by using the all-cause mortality rate as follows:

$$N_7 = N_0\,(1 - ACMR_{0-7}t_{0-7})$$

In total, we estimated prevalence in the early and late neonatal periods and at exactly 28 days for every location, year, and sex through this method. This method incorporated uncertainty from the initial estimates of birth prevalence and excess mortality but did not include uncertainty from population estimates.

Step 3: Model impairment proportions at 28 days, then split prevalence at 28 days by severity of impairment

Infants who survive neonatal encephalopathy may go on to experience long-term disability or impairment. We categorised impairment for neonatal encephalopathy into three severities: asymptomatic, mild, and moderate-to-severe impairment.

Input data

Data on the proportion of cases of neonatal encephalopathy that go on to develop mild impairment and moderate-to-severe impairment were extracted from a systematic literature review that was last completed in GBD 2013 and updated in GBD 2015. The same search string described above was used to identify impairment data. In GBD 2021, we identified and extracted data from two additional studies to inform our estimates of mild and moderate-to-severe impairment proportion.

Modelling strategy

In GBD 2019, we modelled the proportion of mild impairment and moderate-severe impairment using a linear mixed-effects regression of log impairment proportion on HAQ Index. In GBD 2021, we updated the model to use a more appropriate logit transformation of the proportion data and changed the method from a linear regression to a MR-BRT model, which allowed us to fit the data using a more-flexible quadratic, monotonically increasing spline on HAQ Index and to weight data by their standard error. These changes led to an increase in the moderate-severe proportion at HAQ Index below approximately 80 and to an increase in the mild proportion at every value of HAQ Index. Figure 5 demonstrates the impact of these changes by showing the predicted impairment proportion values for each severity by level of HAQ Index for GBD 2019 (red series) and GBD 2021 (blue series).

Figure 5. MR-BRT model of impairment proportions

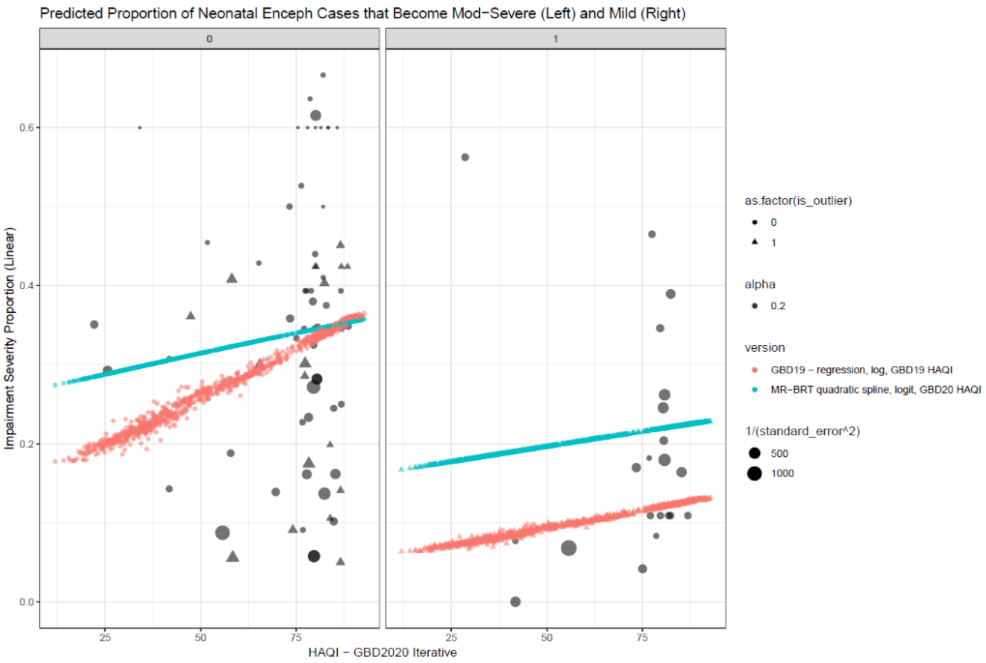

From this MR-BRT model, we generated 1000 draws of estimates of proportion mild and moderate-to-severe impairment for every unique location-year combination. We checked that every location, year, draw pairing of mild and moderate-to-severe impairment proportion never summed to greater than 90%, reserving at least 10% of long-term impairment as asymptomatic. However, no draw pairings summed to greater than 90% and so no draws were adjusted. For every location, year, draw we assigned the remainder proportion, calculated as 1 – (mild impairment proportion + moderate-to-severe impairment proportion), as the proportion with asymptomatic impairment.

We multiplied our estimated impairment proportions by the prevalence at 28 days calculated from our life table algorithm in Step 2 to generate impairment-specific prevalence estimates. Asymptomatic prevalence was extended to other ages based on the assumption that asymptomatic prevalence at 28 days is the same as at early neonatal, late neonatal, and post-neonatal, and that there is no burden and therefore no asymptomatic prevalence after 1 year of age. Mild prevalence was extended to other ages based on the assumption that the mild prevalence at 28 days is the same as the mild prevalence at all other ages. This assumption is grounded in the lack of excess mortality and remission among those born with mild neonatal encephalopathy (ie, no one can develop the disease after birth, no one dies from it, and no one recovers from it, so the number of cases is constant across age).

Step 4: Model long-term impairment prevalence at all ages

Input data

Standardised mortality ratios of cerebral palsy were used as input data to model the prevalence of neonatal encephalopathy for ages older than the neonatal period based on the assumption that brain damage in the neonatal period is one of the reasons that young children go on to develop cerebral palsy. These data were used across all four neonatal causes and for many other causes in the GBD study. We ran a meta-analysis for a 0–19 age group and a 20–99 age group, and the SMR values were converted to EMR using the formula:

$$EMR = (\text{location-sex-age-specific } \textit{all-cause mortality rate}) * (\text{age-specific } SMR - 1)$$

Modelling strategy

To estimate the prevalence of moderate-severe impairment at other ages, we needed to account for excess mortality. Because there is excess mortality, the number of cases of moderate-severe impairment declines with age. The sum of asymptomatic and mild impairment prevalence in the early and late neonatal periods was subtracted from the neonatal encephalopathy envelope prevalence estimates (Step 1) to estimate moderate-severe impairment prevalence. This reflects the assumption that all deaths in the early and late neonatal period were among those with moderate-severe impairment, and all newborns who developed asymptomatic or mild neonatal sepsis did not experience excess mortality.

To model moderate-severe prevalence at older ages, a DisMod-MR 2.1 model was run on the existing moderate-severe prevalence estimate (eg, prevalence in the early neonatal period), and on excess mortality estimates derived from the standard mortality ratios (SMR) of cerebral palsy. Remission and incidence were set to zero. The input dataset was entirely complete as every location had an input datum for early neonatal prevalence as well as specific values for EMR at every age-location-sex-year, so we did not specify location-level covariates and the model was set to not pass any priors for any parameter during the estimation cascade, functionally meaning the final estimate age-location-sex-year was not informed by any adjacent locations or years.

Step 5: Split mild and moderate-to-severe prevalence into sequelae

The mild impairment estimates are split into two sequelae, and the moderate-to-severe impairment estimates are split into 14 sequelae. The mild sequelae were derived by splitting the mild prevalence equally. The proportions for each moderate/severe sequela were extracted from a study by Badawi and colleagues[1] and are listed in the table below in descending order. These proportions were also used to split impairments into sequelae across the other neonatal causes.

Table 16. Health states by severity

Sequelae of neonatal encephalopathy	Mild	Moderate-severe
Mild motor impairment	0.500	
Mild motor plus cognitive impairment	0.500	
Moderate motor only		0.173
Moderate motor impairment + epilepsy		0.100
Moderate motor impairment + blindness		0.018
Moderate motor impairment + blindness + epilepsy		0.009
Moderate motor impairment + blindness + cognitive impairment		0.032
Moderate motor impairment + epilepsy + cognitive impairment		0.183
Moderate motor impairment + blindness + epilepsy + cognitive impairment		0.017
Severe motor only		0.152
Severe motor impairment + epilepsy		0.033
Severe motor impairment + blindness		0.006
Severe motor impairment + blindness + epilepsy		0.003
Severe motor impairment + blindness + cognitive impairment		0.038
Severe motor impairment + epilepsy + cognitive impairment		0.216
Severe motor impairment + blindness + epilepsy + cognitive impairment		0.020

Step 6: Use disability weights to calculate YLDs

Each sequela is associated with a health state, which is used to calculate YLDs. The health states used for neonatal encephalopathy are largely the same as the health states for other neonatal causes (see table below). Some health states were combined to calculate the burden of certain sequelae.

Table 17. Disability weights and lay descriptions by health state

Health state	Description	Disability weight
Motor impairment, mild	Has some difficulty in moving around but is able to walk without help.	0.01 (0.005–0.019)
Motor impairment, moderate	Has some difficulty in moving around, and difficulty in lifting and holding objects, dressing and sitting upright, but is able to walk without help.	0.061 (0.040–0.089)
Motor impairment, severe	Is unable to move around without help, and is not able to lift or hold objects, get dressed or sit upright.	0.402 (0.268–0.545)
Motor plus cognitive impairments, mild	Has some difficulty moving around but is able to walk without help. The person is slow in learning at school. As an adult, the person has some difficulty doing complex or unfamiliar tasks but otherwise functions independently.	0.031 (0.018–0.050)
Motor plus cognitive impairments, moderate	Has some difficulty in moving around, holding objects, dressing, and sitting upright, but can walk without help. The person has low intelligence and is slow in learning to speak and to do simple tasks.	0.203 (0.134–0.290)
Motor plus cognitive impairments, severe	Cannot move around without help, and cannot lift or hold objects, get dressed or sit upright. The person also has very low intelligence, speaks few words, and needs constant supervision and help with all daily activities.	0.542 (0.374–0.702)
Distance vision blindness	Is completely blind, which causes great difficulty in some daily activities, worry and anxiety, and great difficulty going outside the home without assistance.	0.187 (0.124–0.260)
Epilepsy, less severe (seizures < once per month)	Has sudden seizures two to five times a year, with violent muscle contractions and stiffness, loss of consciousness, and loss of urine or bowel control.	0.263 (0.173–0.367)
Epilepsy, severe (seizures ≥ once per month)	Has sudden seizures one or more times each month, with violent muscle contractions and stiffness, loss of consciousness, and loss of urine or bowel control. Between seizures the person has memory loss and difficulty concentrating.	0.552 (0.375–0.71)
Abdominopelvic problem, severe (proxy for EHB without kernicterus)	Has severe pain in the belly and feels nauseous. The person is anxious and unable to carry out daily activities.	0.324 (0.220–0.442)

Haemolytic disease and other neonatal jaundice

Flowchart

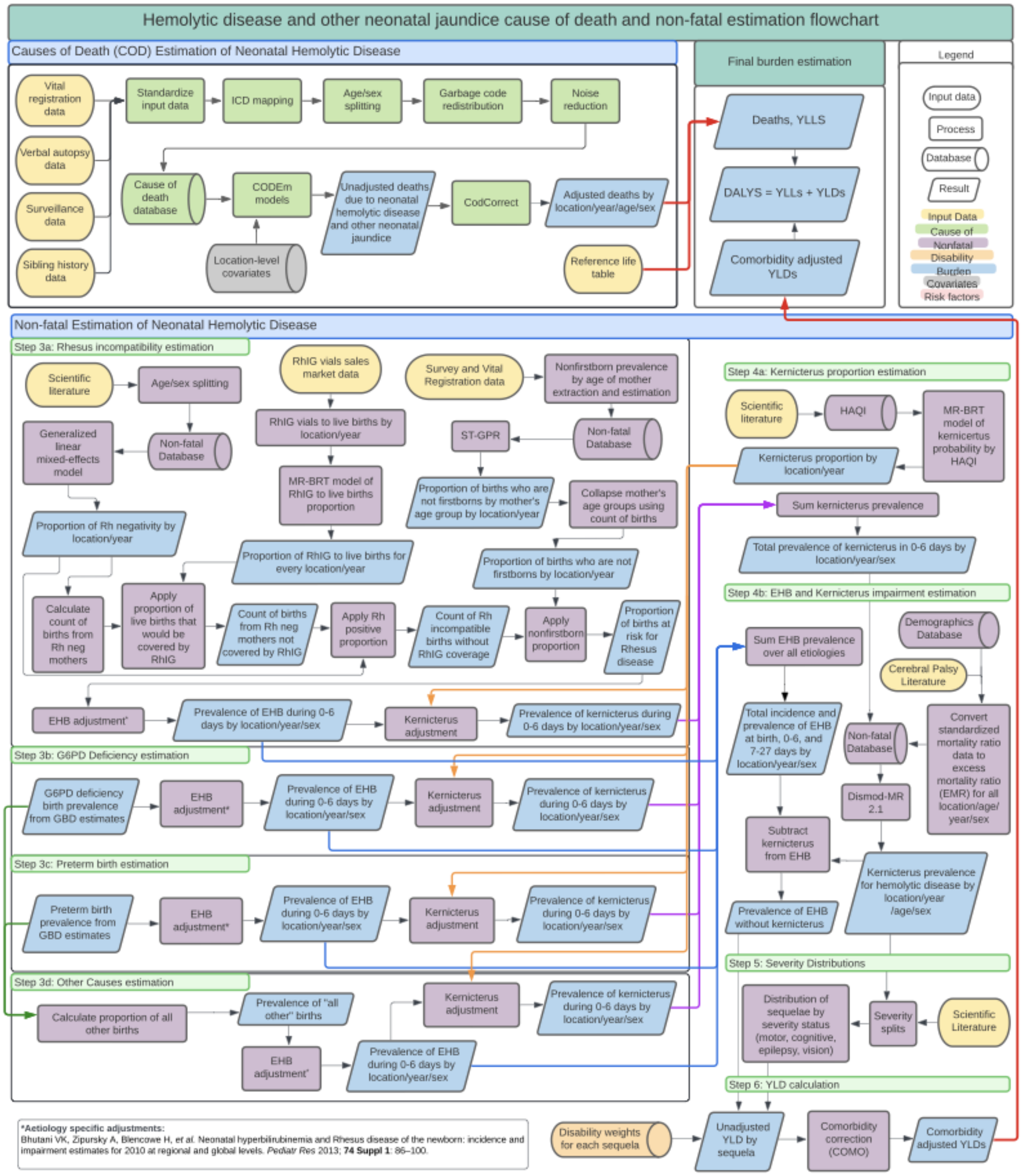

Case definition

Haemolytic disease of the newborn and other neonatal jaundice refers to several aetiologies by which an infant develops extreme hyperbilirubinaemia (EHB) and can then go on to develop kernicterus. We define jaundice as total serum bilirubin (TSB) >5 mg/dL and EHB as TSB >25 mg/dL in the neonatal period. Kernicterus is defined as bilirubin-induced brain injury following an EHB episode and is a clinical diagnosis. GBD estimates are limited to incidence, prevalence, and YLDs due to EHB and kernicterus. We

classify EHB that does not progress to kernicterus as mild impairment and kernicterus as moderate/severe impairment. The aetiologies that inform our estimates for EHB and kernicterus are Rhesus (Rh) disease, preterm birth, glucose-6-phosphate dehydrogenase deficiency (G6PD), and other causes.

Modelling strategy

Modelling the non-fatal burden of haemolytic disease occurs in seven main steps.

Table 18. Analytical steps in estimation of YLDs due to haemolytic disease and other neonatal jaundice

Step	Summary of modelling approach
1	Prevalence of EHB due to Rh disease:<br>a. Rh-negativity prevalence<br>b. Rho(D) immune globulin vials to live births proportion<br>c. Non-firstborn prevalence
2	Prevalence of EHB due to G6PD deficiency, preterm birth, and other causes
3	Proportion of EHB due to Rh disease, G6PD deficiency, preterm birth, and other causes who develop kernicterus
4	Prevalence (all ages) of kernicterus (accounting for increased long-term mortality)
5	Prevalence (in neonates only) of EHB without kernicterus
6	Split moderate/severe impairment (kernicterus) prevalence into sequelae
7	Apply disability weights to each sequela to calculate YLDs

USA claims data and hospital data were not included in the haemolytic disease modelling process because they are not coded separately by aetiology. We are working to develop an analytical framework whereby these data could be incorporated into GBD estimates.

Step 1: EHB due to Rh disease

Birth prevalence of EHB due to Rh disease is estimated using the following equation:

$$\text{EHB Prevalence} = (\text{Rh negativity prevalence} * (1 - \text{Rho(D) immune globulin vials to live births proportion})) * (1 - \text{Rh negativity prevalence}) * (\text{non-firstborn prevalence}) * 0.15$$

The three components included in the above equation are Rh negativity prevalence, doses of Rho(D) immune globulin (RhIG) (a prophylactic medication to prevent RhD isoimmunisation in mothers), and non-firstborn birth prevalence. Rh negativity prevalence represented estimates of the prevalence of pregnancies from Rh-negative blood type mothers. Market data on RhIG distribution to countries around the world was used to estimate the proportion of Rh-negative mothers who are protected by the RhIG. Non-firstborn birth prevalence was used to further quantify births who are at risk of Rhesus disease as RhD isoimmunisation in mothers affects births after the firstborn. The inputs and analytical approach that inform each of these components are described below.

For this GBD round, we updated our estimation to assume that every Rh-negative mother would receive one dose of RhIG, if available. Previously, our estimation assumed that only Rh-incompatible pregnancies received RhIG, which is the practice of only a small subset of countries. We were also able to remove the assumption that locations with a neonatal mortality rate (NMR) of less than five had full coverage of RhIG for Rh-incompatible pregnancies; this was due to a significant increase in our data coverage of RhIG sales across the globe and across years. See Step 1b for further details.

Step 1a: Rh negativity prevalence

Rh negativity prevalence data were extracted from literature based on the following search, first completed as a systematic review for GBD 2010. The systematic review was last updated in GBD 2019 to include additional years since GBD 2010. The PubMed database was searched using the search string below on February 7, 2019, and returned 466 results. 39 results were screened for full-text review, and eight were ultimately extracted. The exclusion criteria were studies that did not provide primary data on epidemiological parameters, non-representative studies (eg, only high-risk pregnancies), and reviews.

> ((newborn[Title/Abstract] OR neonat*[Title/Abstract]) AND (haemolytic[Title/Abstract] OR hemolytic[Title/Abstract] OR hyperbilirubin*[Title/Abstract] OR jaundice[Title/Abstract] OR "glucose-6"[Title/Abstract] OR G6PD[Title/Abstract] OR EHB[Title/Abstract] OR phototherapy[Title/Abstract] OR "ABO incompatibility"[Title/Abstract] OR "RH incompatibility"[Title/Abstract] OR "rh blood group system"[Title/Abstract] OR Rhesus[Title/Abstract] OR "erythroblastosis fetalis"[Title/Abstract] OR kernicterus[Title/Abstract]) AND (prevalen*[Title/Abstract] OR inciden*[Title/Abstract] OR mortality[Title/Abstract] OR severity[Title/Abstract] OR "long term"[Title/Abstract])) AND (2015/05/01[PDAT] : 3000[PDAT]) NOT "Case Reports"[PT]

Figure 6. PRISMA flow diagram for the GBD 2019 Rh-negativity prevalence systematic review

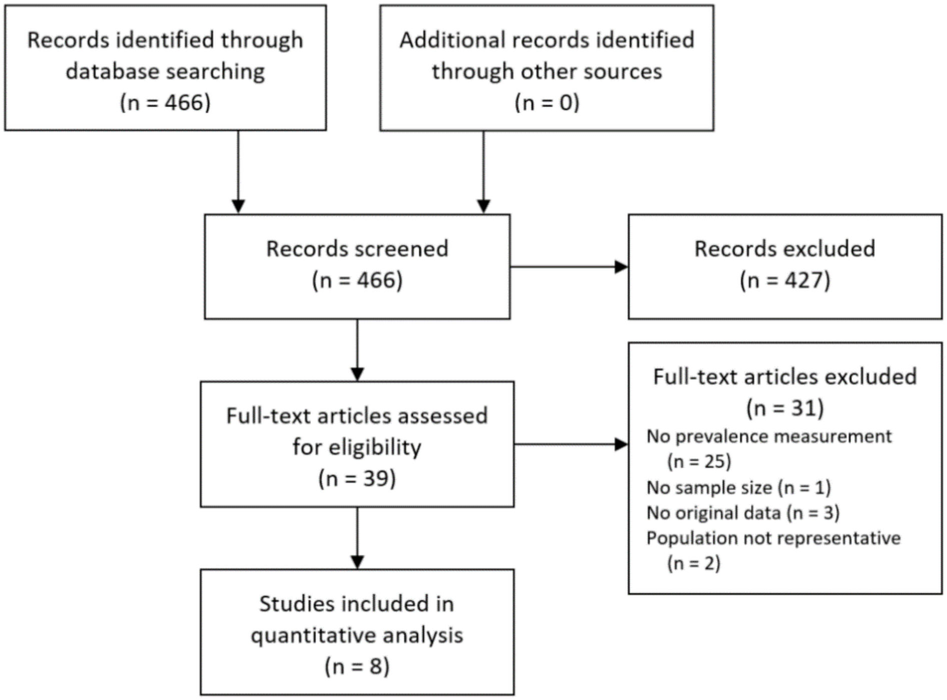

The literature included for quantitative analysis were studies on blood group typing and distribution, blood donors, Rh factor distribution, RhD blood antigens, blood group alloimmunisation, blood transfusion, and efficacy of antibody screening. We extracted data on Rh negativity prevalence covering a total of 49 countries and three subnational locations.

For this round we included additional sources covering 27 countries on Rh-negative population proportions of Rh negativity as reported by blood banks, national blood services, and government statistics. We also extracted Rh negative blood type proportions from literature sources, where we conducted a targeted search for the regions of Oceania, central sub-Saharan Africa, and Andean Latin America to have full coverage of representation across the GBD regions. We ran a binomial generalised linear mixed-effects model (GLMM) using glmer from the lme4 package provided by the R statistical software, to estimate the population proportion of the Rh negative blood type for every GBD location.

Our specifications for the binomial model included a fixed effect on the global intercept of the Rh-negative proportion and nested random effects using the GBD location hierarchy. We custom estimated the Rh-negative proportion for the New Zealand Māori population with the model fit from the Oceania region as they are an indigenous Polynesian population. In our estimation for pregnancies from Rh-negative mothers, we assumed that Rh negativity prevalence did not change over time and that it did not vary by age or sex, so we acknowledge a limitation in capturing any gender-specific or time series trends on Rh negativity. In previous rounds, we had run a single-parameter DisMod-MR 2.1 model on Rh negativity prevalence but without assumptions of age, sex, or time-varying effects, we updated this model to a simple GLMM model as the more properly fitted method.

Step 1b: Rho(D) immune globulin prophylaxis doses

Previously, we used data from the Marketing Research Bureau (MRB) on the distribution of RhIG vials as cited by Bhutani and colleagues,[2] for which we had a single year's worth of data (2010) for 138 countries with non-zero dose values reported for only 30 countries; the proportion of RhIG doses distributed in 2010 for Rh-incompatible pregnancies was used as a constant over time for our estimation of the prevalence of babies who are not protected by RhIG. For this GBD round, we used an expanded report by MRB that included 30 years' worth of data covering RhIG distribution to 96 countries, representing all GBD super-regions and regions except eastern and western sub-Saharan Africa. Due to the addition of these data, we removed the assumption around full RhIG coverage of Rh-incompatible pregnancies based on a country's NMR.

We used a MR-BRT model with the new RhIG distribution data to estimate distributed RhIG doses to livebirths ratios, including the HAQ Index as a covariate and a cascading spline based on the GBD location hierarchy. In applying the model results against Rh negativity prevalence, we assumed that one dose of RhIG (300 mcg or 1500 IU) was used for the Rh negative mother, as was assumed in Bhutani and colleagues[2] and would be compatible with recommendations from most obstetric guidelines at the international and country levels. In doing so, we were able to estimate the proportion of pregnancies for every location-year that were not covered by RhIG prophylaxis.

Step 1c: Non-firstborn birth prevalence

Our input data for modeling non-firstborn birth prevalence included the Demographic Health Surveys (DHS) program's population-representative child and birth history modules, vital registration data from the United Nations (UN) Demographic Yearbook, UNICEF Multiple Indicator Cluster Surveys (MICS), and vital registration birth data for subnational locations in Brazil and, new for this round, for subnational locations in Mexico and the Philippines. We expanded global coverage of birth order data by 53 locations and additionally extracted the age of the mother at the time of birth to enhance our accuracy in modeling non-firstborn proportion estimates. Our estimation process calculated non-firstborn prevalence by dividing the number of non-first births over the total births of that location-year, stratified by the mother's age group (the reproductive ages of 10 to 54 split into five-year bins). We then ran an ST-GPR model on non-firstborn prevalence with an age-specific cohort cumulative fertility (CCF) covariate replacing the total fertility rate (TFR) covariate used in previous rounds.

Extreme hyperbilirubinemia (EHB) probability

The 0.15 multiplier used in the EHB prevalence formula, also cited in Bhutani and colleagues,[2] was derived from Zipursky and colleagues,[3] which cited the Clark[4] study on trials for anti-D gamma globulin before widespread availability of RhIG; this multiplier was used to represent the proportion of babies at risk for Rh disease who go on to develop EHB. We do not have corresponding information on the

proportion of babies at risk for Rh disease who only develop jaundice (and not EHB), which prevents our being able to estimate overall jaundice.

Step 2: EHB due to G6PD deficiency, neonatal preterm birth, and other causes

Input data

The data used to estimate EHB due to non-Rh disease were prevalence of G6PD deficiency and prevalence of neonatal preterm birth, both of which came from corresponding GBD models, and the proportion of cases who develop EHB. Birth prevalence estimates for G6PD deficiency are described in the appendix section on "Haemoglobinopathies and haemolytic anaemias", and neonatal preterm birth is described in the first neonatal section above. The proportion of cases that develop EHB for each of these causes, also cited in Bhutani and colleagues,[2] was derived from combined Canada and Denmark population studies[9-15] that specified causes for EHB (not including Rh disease because of effective national Rh prophylaxis programs) and unpublished data that were further provided by study authors Sgro and Ebbesen. These aetiology-specific EHB proportions are listed in the table below.

Table 19. Proportion of cases of G6PD deficiency, preterm birth, and other causes that develop EHB

Aetiology	EHB proportion
G6PD deficiency	0.0013<br>(0.00085, 0.002)
Neonatal preterm birth	0.00045<br>(0.00029, 0.0007)
All other births	0.00038<br>(0.00033, 0.00163)

Modelling strategy

To model the prevalence of EHB due to G6PD deficiency, preterm birth, and other causes, we started with birth prevalence results for these three conditions. Birth prevalence estimates for G6PD deficiency and neonatal preterm birth came from the corresponding GBD models of those two conditions. The birth prevalence of other causes was based on the assumption that all babies who do not have any of the three modelled conditions (Rh disease, G6PD deficiency, and preterm birth) still have some probability of developing EHB. We therefore summed the birth prevalence of Rh disease, G6PD deficiency, and preterm births (as calculated in previous steps), and subtracted this from 1 to get the birth prevalence of all other causes as follows:

$$\text{"Other" birth prevalence} =$$

$$1 - (\text{Rh disease birth prevalence} + \text{G6PD deficiency birth prevalence} + \text{preterm birth prevalence})$$

We calculated prevalence of EHB by multiplying each birth prevalence estimate by the aetiology-specific scalar from the table above, representing the proportion of children who are expected to develop EHB.

Step 3: Kernicterus prevalence for Rhesus disease, G6PD deficiency, neonatal preterm birth, and other causes

Input data

Data on the probability of kernicterus were extracted from literature based on the following search, first completed as a systematic review for GBD 2019. This search was also designed to identify data on the probability of EHB and prevalence of neonatal jaundice as a whole. The PubMed database was searched using the search string below on April 25, 2019, returning 2212 results. 151 results were screened for full-text review, and 36 were extracted.

((newborn[Title/Abstract] OR neonat*[Title/Abstract]) AND (haemolytic[Title/Abstract] OR hemolytic[Title/Abstract] OR hyperbilirubin*[Title/Abstract] OR jaundice[Title/Abstract] OR icter*[Title/Abstract] OR "exchange transfusion"[Title/Abstract] OR “acute bilirubin encephalopathy” [Title/Abstract] OR EHB[Title/Abstract] OR phototherapy[Title/Abstract] OR kernicterus[Title/Abstract]) AND (prevalen*[Title/Abstract] OR inciden*[Title/Abstract] OR mortality[Title/Abstract] OR severity[Title/Abstract] OR "long term"[Title/Abstract])) AND (1980[PDAT] : 3000[PDAT]) NOT "Case Reports"[PT]

We included data in our model of kernicterus probability if the total serum bilirubin level in study participants was directly specified or could be reasonably inferred, and if the outcome matched our case definition of kernicterus (bilirubin-induced brain dysfunction). The exclusion criteria were studies that did not provide primary data on epidemiological parameters, non-representative studies (eg, only high-risk pregnancies), and reviews. In GBD 2021, we identified and extracted five new literature sources on the probability of kernicterus given an initial level of total serum bilirubin and included them in the meta-analysis.

Figure 7. PRISMA flow diagram for the GBD 2019 kernicterus proportion systematic review

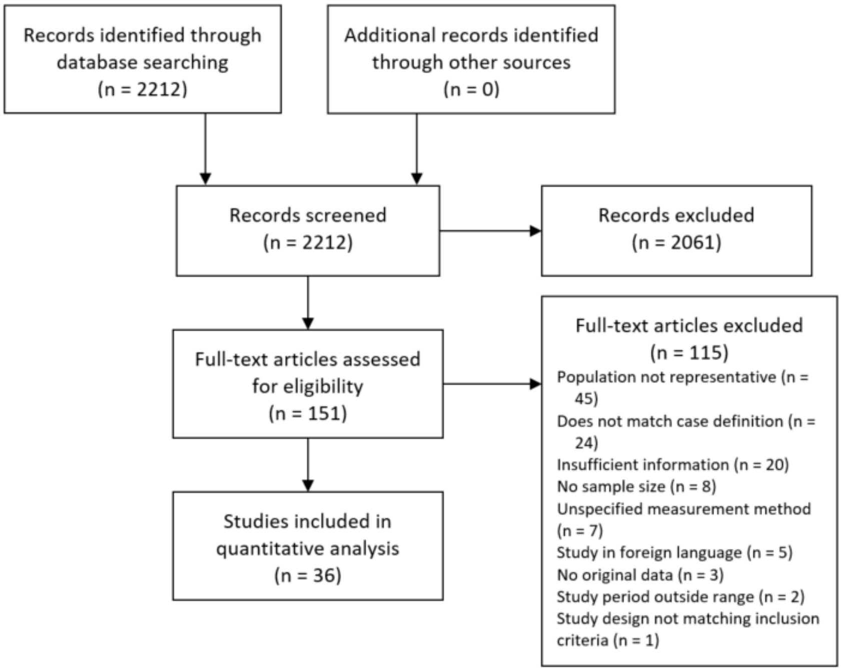

Modelling strategy

In GBD 2017, we had calculated kernicterus prevalence with the same approach used to calculate EHB prevalence – in this case by multiplying EHB prevalence by literature-derived scalars representing the proportion of EHB cases that develop kernicterus. Starting in GBD 2019, using the data that were extracted from our systematic reviews described above, we instead modelled kernicterus probability as a function of HAQ Index and initial total serum bilirubin (TSB) level, and generated location-year-specific kernicterus proportions. However, for kernicterus prevalence from EHB from Rhesus disease we had continued to use a pooled value of 0.072 (0.038, 0.112)[5-8] from literature derived from the USA and UK until this round, when we modelled this aetiology-specific kernicterus prevalence to provide a wider representation of outcomes.

To go into more detail about the modelling approach to estimate these new location-year-specific kernicterus proportions, we used the extracted data to develop a monotonic cubic spline model in MR-

BRT, with 10% trimming and covariates for the HAQ Index and TSB (three threshold levels: 15, 25, and 35 mg/dL), with a spline on the HAQ Index. The model results are shown in Figure 8 below, the first figure (panel A) in logit space and the second figure (panel B) in linear space. For this GBD round, we removed the uncertainty on gamma in our model. We used from this model the probability of kernicterus when initial TSB is 25 mg/dL, which is the minimum EHB threshold, to represent the probability of kernicterus among those with EHB, pairing with location-year-specific HAQ Index values.

Figure 8. Predicted kernicterus proportion for total serum bilirubin levels as a function of HAQ Index, (A) logarithmic scale vs (B) linear scale

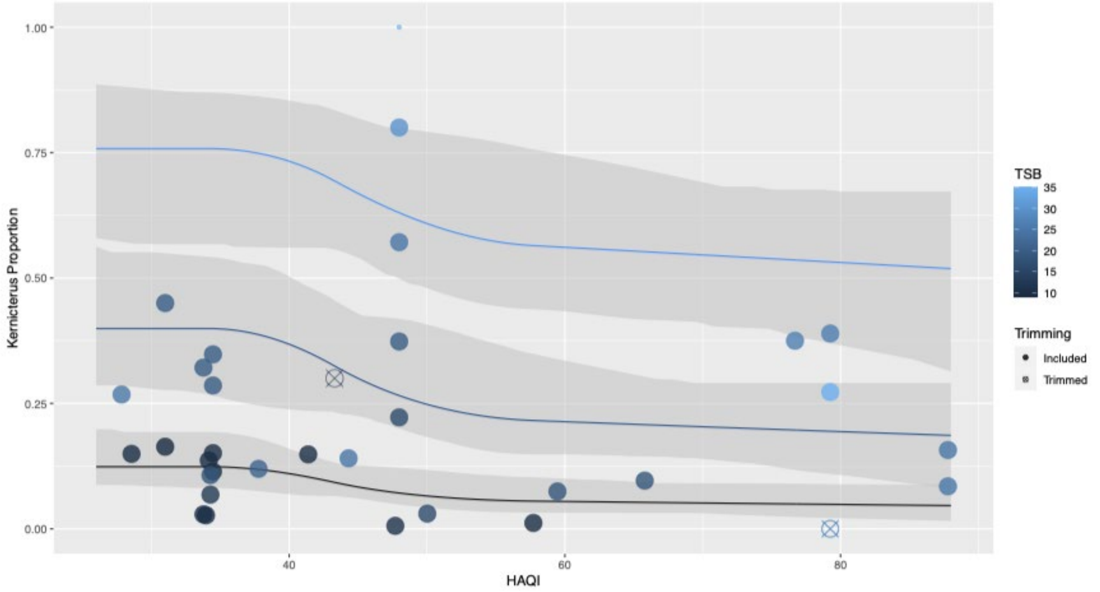

A.

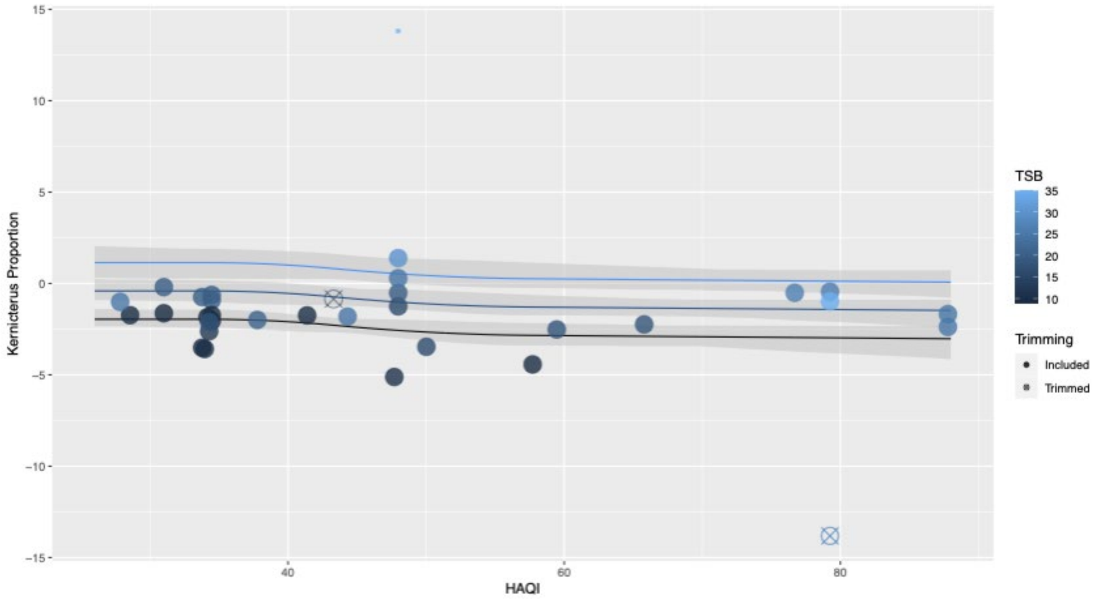

B.

Finally, we calculated total kernicterus prevalence across aetiologies in the 0–6-day period by summing kernicterus prevalence from its four aetiologies: Rh disease, G6PD deficiency, preterm birth, and other causes.

$$
\begin{aligned}
\text{Total kernicterus prevalence} \\
&= (\text{Kernicterus prevalence due to Rh disease}) \\
&+ (\text{Kernicterus prevalence due to G6PD deficiency}) \\
&+ (\text{Kernicterus prevalence due to preterm birth complications}) \\
&+ (\text{Kernicterus prevalence due to other causes})
\end{aligned}
$$

Step 4: Kernicterus prevalence at all ages (moderate/severe impairment)

Input data

Standardised mortality ratios of cerebral palsy were used as input data to model the prevalence of kernicterus for ages older than the neonatal period based on the assumption that acute bilirubin encephalopathy (ie, kernicterus) is one of the reasons that young children go on to develop cerebral palsy. These data were used across all four neonatal causes and for many other causes in the GBD study. We ran a meta-analysis for a 0–19 age group and a 20–99 age group, and the SMR values were converted to EMR using the formula:

$$EMR = (\text{location-sex-age-specific } \textit{all-cause mortality rate}) * (\text{age-specific } SMR - 1)$$

Modelling strategy

To model moderate-severe (kernicterus) prevalence at older ages, a DisMod-MR 2.1 model was run on the existing moderate-severe prevalence estimate (eg, prevalence in the early neonatal period), and on excess mortality estimates derived from the standard mortality ratios (SMR) of cerebral palsy. Remission and incidence were set to zero. The input dataset was entirely complete as every location had an input datum for early neonatal prevalence as well as specific values for EMR at every age-location-sex-year, so we did not specify location-level covariates and the model was set to not pass any priors for any parameter during the estimation cascade, functionally meaning the final estimate age-location-sex-year was not informed by any adjacent locations or years.

Step 5: EHB without kernicterus (mild impairment)

We represent mild impairment as impairment due to having EHB alone (no progression to kernicterus). To estimate this, we summed EHB prevalence across all four aetiologies and then subtracted the summed kernicterus prevalence across the four aetiologies. This was estimated for the 0–6-day and 7–27-day age groups. Prevalence of EHB without kernicterus from the post-neonatal period onward was assumed to be zero.

Step 6: Split into health states and pair with disability weights to calculate YLDs

The kernicterus estimates were split into 14 sequelae corresponding to moderate and severe disability, and the EHB without kernicterus estimate was associated with one sequela with mild disability. The proportions for each moderate/severe sequela were extracted from a study by Badawi and colleagues[1] and are listed in the table below in descending order. These proportions were also used to split impairments into sequelae across the other neonatal causes.

Table 20. Health states of haemolytic disease and other neonatal jaundice by severity

Sequelae of neonatal haemolytic disease and other neonatal jaundice	EHB	Kernicterus
Severe abdominopelvic problem	1.000	
Moderate motor impairment only		0.173
Moderate motor impairment + epilepsy		0.100
Moderate motor impairment + blindness		0.018
Moderate motor impairment + blindness + epilepsy		0.009
Moderate motor impairment + blindness + cognitive impairment		0.032

Moderate motor impairment + epilepsy + cognitive impairment		0.183
Moderate motor impairment + blindness + epilepsy + cognitive impairment		0.017
Severe motor impairment only		0.152
Severe motor impairment + epilepsy		0.033
Severe motor impairment + blindness		0.006
Severe motor impairment + blindness + epilepsy		0.003
Severe motor impairment + blindness + cognitive impairment		0.038
Severe motor impairment + epilepsy + cognitive impairment		0.216
Severe motor impairment + blindness + epilepsy + cognitive impairment		0.020

Step 7: Split into health states and pair with disability weights to calculate YLDs

Each sequela was associated with a unique GBD health state and paired with corresponding disability weights to calculate YLDs. The health states that were used for neonatal haemolytic disease and other neonatal jaundice are largely the same as the health states for other neonatal causes (see table below). Some health states were combined to calculate the burden of certain sequelae.

Table 21. Disability weights and lay descriptions by health state

Health state	Description	Disability weight
Motor impairment, mild	Has some difficulty in moving around but is able to walk without help.	0.01 (0.005–0.019)
Motor impairment, moderate	Has some difficulty in moving around, and difficulty in lifting and holding objects, dressing and sitting upright, but is able to walk without help.	0.061 (0.040–0.089)
Motor impairment, severe	Is unable to move around without help, and is not able to lift or hold objects, get dressed or sit upright.	0.402 (0.268–0.545)
Motor plus cognitive impairments, mild	Has some difficulty moving around but is able to walk without help. The person is slow in learning at school. As an adult, the person has some difficulty doing complex or unfamiliar tasks but otherwise functions independently.	0.031 (0.018–0.050)
Motor plus cognitive impairments, moderate	Has some difficulty in moving around, holding objects, dressing, and sitting upright, but can walk without help. The person has low intelligence and is slow in learning to speak and to do simple tasks.	0.203 (0.134–0.290)
Motor plus cognitive impairments, severe	Cannot move around without help, and cannot lift or hold objects, get dressed or sit upright. The person also has very low intelligence, speaks few words, and needs constant supervision and help with all daily activities.	0.542 (0.374–0.702)
Distance vision blindness	Is completely blind, which causes great difficulty in some daily activities, worry and anxiety, and great difficulty going outside the home without assistance.	0.187 (0.124–0.260)
Epilepsy, less severe (seizures < once per month)	Has sudden seizures two to five times a year, with violent muscle contractions and stiffness, loss of consciousness, and loss of urine or bowel control.	0.263 (0.173–0.367)
Epilepsy, severe (seizures ≥ once per month)	Has sudden seizures one or more times each month, with violent muscle contractions and stiffness, loss of consciousness, and loss of urine or bowel control. Between seizures the person has memory loss and difficulty concentrating.	0.552 (0.375–0.71)
Abdominopelvic problem, severe (proxy for EHB without kernicterus)	Has severe pain in the belly and feels nauseous. The person is anxious and unable to carry out daily activities.	0.324 (0.220–0.442)

Neonatal sepsis and other neonatal infections

Flowchart

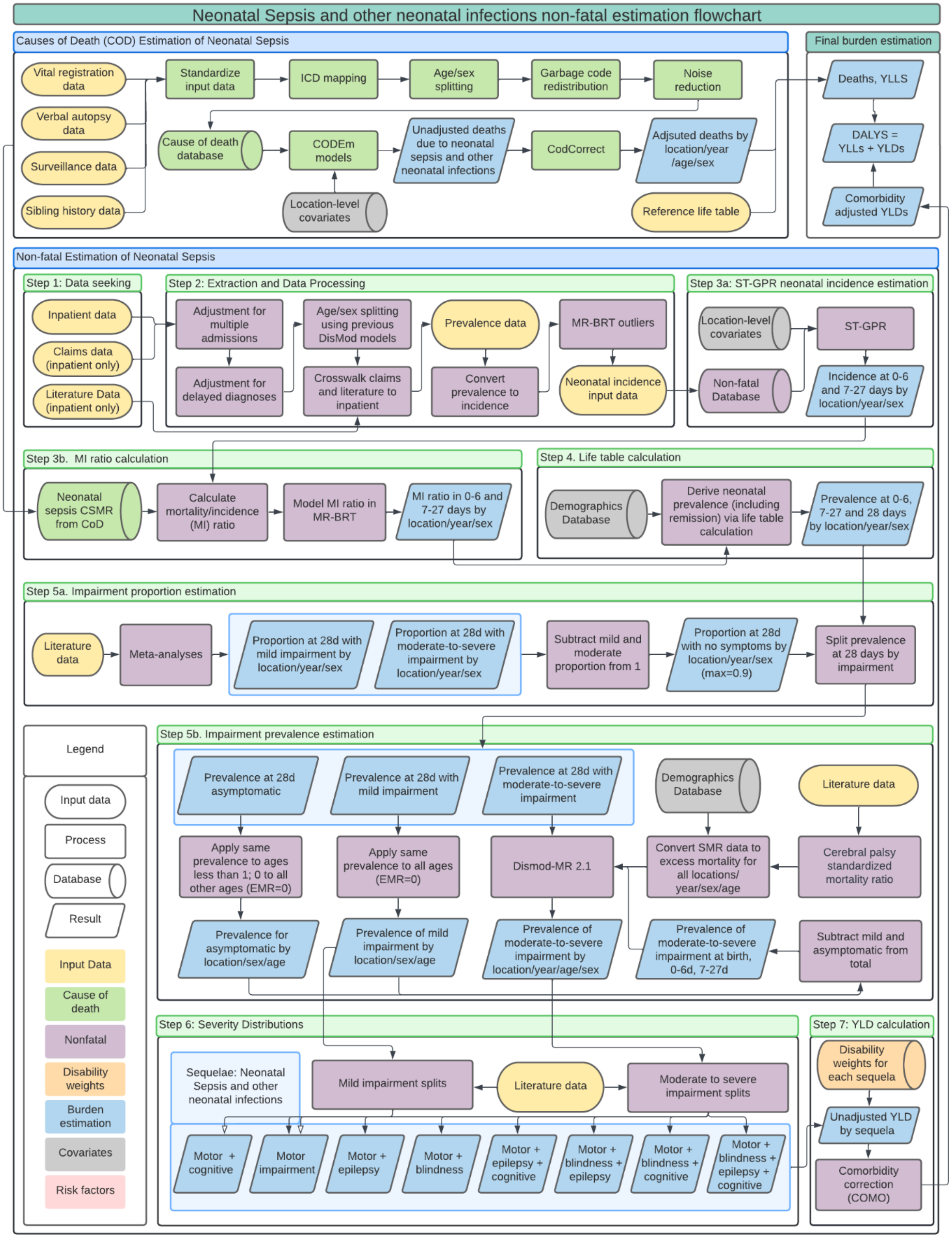

Case definition

Neonatal sepsis and other neonatal infections are infections during the neonatal period that advance to a systemic bloodstream infection (sepsis) and infections that occur during the neonatal period that are not already modelled separately in the GBD.

Modelling strategy

We have made no substantive changes in the modeling strategy from GBD 2021. Modelling the non-fatal burden of neonatal sepsis and other neonatal infections occurs in six main steps:

Table 22. Analytical steps in estimation of YLDs due to neonatal sepsis and other neonatal infections

Step	Summary of modelling strategy
1	Model neonatal sepsis incidence in the early and late neonatal periods in ST-GPR
2	Estimate neonatal sepsis prevalence in the early neonatal period, late neonatal period, and at exactly 28 days using a life table algorithm
3	Meta-analyse asymptomatic, mild, and moderate-severe impairment proportions at 28 days, then split prevalence at 28 days by severity of impairment
4	Model impairment prevalence at younger and older ages based on 28-day impairment prevalence
5	Split mild and moderate-severe impairment prevalence into sequelae
6	Apply disability weights to each sequela to calculate YLDs

Step 1: Model neonatal sepsis incidence in the early and late neonatal periods in ST-GPR

In previous GBD rounds we had used DisMod MR 2.1 to model neonatal sepsis prevalence in the early and late neonatal periods. From GBD 2021, we used spatiotemporal Gaussian process regression (ST-GPR) to model the incidence of neonatal sepsis in these age groups. We then used these as inputs in a life table algorithm to produce estimates of neonatal sepsis prevalence in the early neonatal period, late neonatal period, and at 28 days.

Input data

We extracted data on prevalence and incidence of neonatal sepsis and other neonatal infections from literature and clinical informatics data. All prevalence data were then converted to incidence before being input to ST-GPR.

A systematic literature review for neonatal sepsis was last completed for GBD 2015. The PubMed database was searched using the following search string:

> (("infant"[Title/Abstract] OR "newborn"[Title/Abstract] OR "newborn infant"[Title/Abstract]) AND ("neonatal sepsis"[All Fields] OR "neonatal septicaemia"[All Fields] OR "neonatal meningitis"[All Fields] OR "early sepsis"[All Fields] OR "early septicaemia"[All Fields] OR "tetanus"[All Fields] OR "meningitis"[All Fields] OR "sepsis"[All Fields])) AND ("2012"[PDAT] : "3000"[PDAT]) AND "humans"[MeSH Terms]

To be included, published data sources had to report on specific infections, or groups of infections, and provide diagnostic criteria for how cases were identified. The exclusion criteria were studies that did not provide primary data on epidemiological parameters (eg, a commentary piece), non-representative studies (eg, only high-risk pregnancies, nosocomial infection rates, preterm infants, ICU populations), and review articles. We did not find any studies that reported on all neonatal infections, only sepsis.

Clinical informatics data (hospital and claims) formed the bulk of the input data for the neonatal sepsis envelope model. Only inpatient data were included from these datasets because we believe they are more representative of the true prevalence of neonatal sepsis than outpatient data; infants with neonatal sepsis in the countries from which hospital data were available are almost sure to be admitted

to the hospital, whereas outpatient data are more likely to capture repeated visits by the same child as they grow. Clinical data processing is described separately.

Data processing

Starting with GBD 2021, clinical informatics data were processed to reflect the discrete under-5 age groups within the GBD study. Because many sources are not linked across years, these splits led to implausible age patterns and an under-ascertainment of cases at birth. Based on the assumption that all cases present in older age groups in the <1-year GBD age groups would necessarily have been present at birth, we adjusted the under-1 inpatient data to back-add cases that first "appeared" at older ages to the numerator of the prevalence in younger ages.

We applied empirical age ratios from previous DisMod-MR 2.1 models to disaggregate observations that did not entirely fit in one GBD age category. We calculated these ratios by dividing the result for a specific age and sex by the result for the aggregate age and sex specified in a given observation.

Our reference case definition for neonatal sepsis data was inpatient hospital data. We crosswalked data from inpatient claims and literature data to the reference definition using MR-BRT before modelling in ST-GPR. The adjustment factors applied were as follows:

Table 23. MR-BRT crosswalk adjustment factors for neonatal sepsis and other neonatal infections

Data input	Reference or alternative case definition	Gamma	Beta coefficient, log (95% CI)*	Adjustment factor**
Hospital data	Reference	--	--	--
Claims data	Alternate	0.74	0.25 (–0.44 to 0.94)	1.28 (0.64–2.56)
Literature data	Alternate	5.83	–1.64 (–4.99 to 1.71)	0.19 (0.01–5.53)

**MR-BRT crosswalk adjustments can be interpreted as the factor the alternative case definition is adjusted by to reflect what it would have been had it been measured using the reference case definition. If the log/logit beta coefficient is negative, then the alternative is adjusted up to the reference. If the log/logit beta coefficient is positive, then the alternative is adjusted down to the reference.*
***The adjustment factor column is the exponentiated beta coefficient. For log beta coefficients, this is the relative rate between the two case definitions. For logit beta coefficients, this is the relative odds between the two case definitions.*

Lastly, because of significant residual heterogeneity in input data, especially from clinical administrative sources, we used an MR-BRT model to identify outliers in the data. We log-transformed our incidence data and calculated standard errors using the delta method and fit a cubic spline on Healthcare Access and Quality (HAQ) Index with fixed effects on age group, a prior of decreasing monotonicity, and a 30% trimming parameter. All trimmed data were marked as outliers in the model.

Modelling strategy

Due to inconsistencies in estimates of neonatal sepsis prevalence between GBD rounds when modelling in DisMod MR-2.1, we significantly amended our modelling approach for GBD 2021. In previous GBD rounds, we ran a DisMod MR-2.1 model to estimate prevalence of neonatal sepsis in the early and late neonatal periods directly. We then interpolated these results to estimate the prevalence at 28 days. For GBD 2021, we amended this process to model the incidence of neonatal sepsis in the early and late neonatal periods and used a life table algorithm to calculate prevalence in these age groups and at 28 days. Unlike other neonatal cause models, we did not estimate birth prevalence for neonatal sepsis and other neonatal infections.

We first modelled the incidence of neonatal sepsis and other neonatal infections in the early and late neonatal periods using spatiotemporal Gaussian process regression (ST-GPR), a three-step modelling procedure for generating estimates for every location, year, age, and sex in the GBD study. The first step of the ST-GPR process is an ensemble linear mixed-effects regression of our data on a set of potentially predictive covariates taken from the GBD study covariates database. We tested every combination of these covariates in individual, sex-specific mixed-effects linear regressions with nested random effects at the super-region, region, and location levels. We then evaluated and ranked each of these sub-models by their out-of-sample root-mean-squared error (RMSE). Finally, to produce initial estimates for every location, year, age, and sex in the analysis, we averaged the 50 top-performing models where the estimated coefficients were 1) statistically significant at $p < 0.05$, and 2) in the expected direction. We tested the following covariates in the ensemble prior: lag-distributed income per capita, Socio-demographic Index, Healthcare Access and Quality Index, unsafe water SEV, unsafe sanitation SEV, maternal care and immunisation index, livebirths among women aged 35+ years, preterm birth SEV, low birthweight SEV, short gestation SEV, smoking SEV, mortality due to war and conflict, and neonatal CSMR.

The second, spatiotemporal smoothing step of ST-GPR calculates the residual between our stage 1 regression estimate and each of our observed datapoints and then smooths this residual, drawing strength over space, age, and time and producing a revised stage 2 estimate for every location, year, age, and sex. The third step of ST-GPR is a Gaussian process regression, using the stage 2 estimates as a prior and the observed datapoints and their variance to 1) further smooth the residual between the stage 2 predictions and observed data and produce a final mean estimate for each location, year, age, and sex, and 2) estimate uncertainty around this mean estimate, quantified by taking 1000 draws from the posterior Gaussian process. More detailed information on the ST-GPR modelling process can be found in the main text methods appendix.

Step 2: Estimate neonatal sepsis prevalence in the early neonatal period, late neonatal period, and at exactly 28 days using a life table algorithm

Mortality/incidence ratio modelling

Our life table algorithm requires mortality-to-incidence (MI) ratio estimates for every location, year, age, and sex. To generate these estimates, we modelled the MI ratio in MR-BRT, using MI ratio data derived from our incidence estimates and modelled cause-specific mortality rates. We log-transformed all MI ratio data and calculated standard errors using the delta method. We fit a monotonically decreasing cubic spline on the Healthcare Access and Quality Index with fixed effects on age and sex and a 20% trimming parameter. From this model we generated 1000 draws of estimated MI ratio for every location, year, age, and sex included in the analysis.

Life table algorithm

The next step in our modelling process is a life table algorithm that uses estimates of population at birth and in the early and late neonatal periods, incidence of neonatal sepsis in the early and late neonatal periods, mortality-to-incidence ratios, remission estimates, and mortality data from the GBD mortality analysis to generate prevalence estimates in the early and late neonatal age groups and at exactly 28 days for every location, year, and sex.

Early neonatal age group calculations

We first calculated incident cases of neonatal sepsis in the early neonatal period using the equation:

$$cases_{enn} = births * (1 - e^{-Inc_{enn} * t_{0-7}})$$

where Inc_{Enn} is the modelled incidence rate in the early neonatal period and $t_{0\text{-}7}$ is the number of days in the early neonatal period. We then calculated remitted cases in the early neonatal period as

$$remitted\ cases_{enn} = cases_{enn} * (1 - e^{-Remrate*t_{0-6}})$$

where *Remrate* is the remission rate. We generated 1000 draws of the remission rate from a normal distribution with mean 40 and standard deviation of 5.1, approximating a mean remission rate of 40 with confidence interval (30–50). Next, we calculated deaths among cases in the early neonatal period as

$$deaths_{enn} = cases_{enn} * MIratio_{enn}$$

Where $MIratio_{enn}$ is the modelled MI ratio for a given location, year, age, and sex. Finally, we calculated the population vulnerable to infection after the neonatal period as the population at birth minus all-cause deaths in the early neonatal period and the surviving cases of neonatal sepsis from the early neonatal period (which we assume cannot be re-infected):

$$population_{7days} = births - (all\ cause\ mortality_{enn} + survivors_{enn})$$

Late neonatal age group calculations

The equations for the late neonatal period calculations mirror those used in the early neonatal period, beginning with the calculated population at 7 days described above. The equations follow the same order as in the early neonatal period:

$$cases_{lnn} = population_{7days} * (1 - e^{-Inc_{lnn}*t_{7-27}})$$
$$remitted\ cases_{lnn} = cases_{lnn} * (1 - e^{-Remrate*t_{7-27}})$$
$$deaths_{lnn} = cases_{lnn} * MIratio_{lnn}$$

28 days calculations

Finally, to estimate prevalence at exactly 28 days, we first calculated the population at 28 days as:

$$population_{28days} = population_{7days} + survivors_{enn} - all\ cause\ mortality_{lnn}$$

Where $population_{7days}$ is the calculated population at the end of the early neonatal period, $survivors_{enn}$ are the surviving early neonatal cases of sepsis (which are not included in the $population_{7days}$ estimate), and $all\ cause\ mortality_{lnn}$ is the total all-cause deaths in the late neonatal period. We then calculated prevalence at 28 days as:

$$prevalence_{28days} = \frac{survivors_{enn} + survivors_{lnn}}{population_{28days}}$$

Step 3: Model impairment proportions at 28 days, then split prevalence at 28 days by severity of impairment

Infants who survive neonatal sepsis may go on to experience long-term disability or impairment. We categorised impairment for neonatal sepsis and other neonatal infections into three severities: asymptomatic, mild, and moderate-to-severe impairment.

Input data

Data on the proportion of cases of neonatal sepsis that go on to develop mild impairment and moderate-to-severe impairment were extracted from a systematic literature review that was

last completed in GBD 2013 and updated in GBD 2015. The same search string described above in Step 1 was used to identify impairment data.

Modelling strategy

Using mild impairment proportion and moderate-to-severe impairment proportion data, we ran separate meta-analyses to generate estimates of both parameters. The remainder of 1 – (mild proportion + moderate-severe proportion) was assigned as the asymptomatic proportion.

Table 24. Proportion of mild and moderate-to-severe impairment of neonatal sepsis and other neonatal infections at 28 days

Parameter	Estimate (95% UI)
Mild impairment proportion	10.2% (7.2–12.9)
Moderate-to-severe impairment proportion	4.3% (2.5–6.0)

Figure 9. Mild impairment meta-analysis

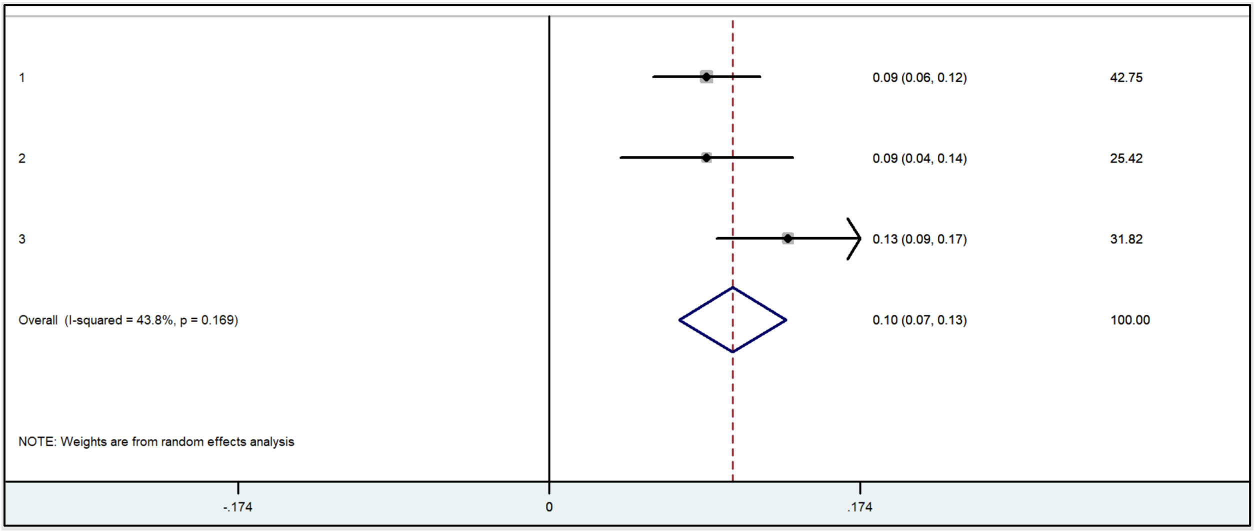

Figure 10. Moderate-to-severe impairment meta-analysis

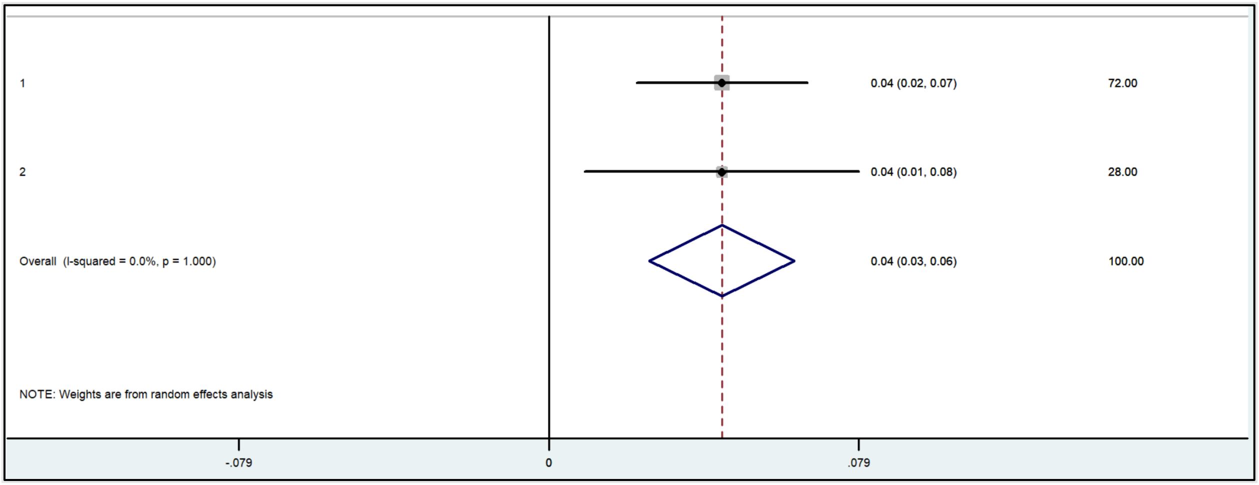

We multiplied our estimated impairment proportions by the prevalence at 28 days calculated from our life table algorithm in Step 2 to generate impairment-specific prevalence estimates. Asymptomatic

prevalence was extended to other ages based on the assumption that asymptomatic prevalence at 28 days is the same as at early neonatal, late neonatal, and post-neonatal, and that there is no burden and therefore no asymptomatic prevalence after 1 year of age. Mild prevalence was extended to other ages based on the assumption that the mild prevalence at 28 days is the same as the mild prevalence at all other ages. This assumption is grounded in the lack of excess mortality and remission among those who develop mild neonatal sepsis (ie, no one can develop the disease after the neonatal period, no one dies from it, and no one recovers from it, so the number of cases is constant across age).

Step 4: Model long-term impairment prevalence at all ages

Input data

Standardised mortality ratios of cerebral palsy were used as input data to model the prevalence of neonatal sepsis for ages older than the neonatal period based on the assumption that severe sepsis is one of the reasons that young children go on to develop cerebral palsy. These data were used across all four neonatal causes and for many other causes in the GBD study. We ran a meta-analysis for a 0–19 age group and a 20–99 age group, and the SMR values were converted to EMR using the formula:

$$EMR = (location\text{-}sex\text{-}age\text{-}specific\, all\text{-}cause\ mortality\ rate) * (age\text{-}specific\, SMR - 1)$$

Modelling strategy

To estimate the prevalence of moderate-severe impairment at other ages, we needed to account for excess mortality. Because there is excess mortality, the number of cases of moderate-severe impairment declines with age. The sum of asymptomatic and mild impairment in the early and late neonatal periods was subtracted from the neonatal sepsis envelope prevalence estimates (Step 1) in the early and late neonatal periods to estimate moderate-severe impairment. This reflects the assumption that all deaths in the early and late neonatal period were among those with moderate-severe impairment, and all newborns who developed asymptomatic or mild neonatal sepsis did not experience excess mortality.

To model moderate-severe prevalence at older ages, a DisMod-MR 2.1 model was run on the existing moderate-severe prevalence estimate (eg, prevalence in the early neonatal period), and on excess mortality estimates derived from the standard mortality ratios (SMR) of cerebral palsy. Remission and incidence were set to zero. The input dataset was entirely complete as every location had an input datum for early neonatal prevalence as well as specific values for EMR at every age-location-sex-year, so we did not specify location-level covariates and the model was set to not pass any priors for any parameter during the estimation cascade, functionally meaning the final estimate age-location-sex-year was not informed by any adjacent locations or years.

Step 5: Split mild and moderate-severe impairment prevalence into sequelae

The mild impairment estimates are split into two sequelae, and the moderate-to-severe impairment estimates are split into 14 sequelae. The mild sequelae were derived by splitting the mild prevalence equally. The proportions for each moderate/severe sequela were extracted from a study by Badawi and colleagues[1] and are listed in the table below in descending order. These proportions were also used to split impairments into sequelae across the other neonatal causes.

Table 25. Health states by severity

Sequelae of neonatal sepsis and other neonatal infections	Mild	Moderate-severe
Mild motor impairment	0.500	
Mild motor plus cognitive impairment	0.500	
Moderate motor only		0.173

Moderate motor impairment + epilepsy		0.100
Moderate motor impairment + blindness		0.018
Moderate motor impairment + blindness + epilepsy		0.009
Moderate motor impairment + blindness + cognitive impairment		0.032
Moderate motor impairment + epilepsy + cognitive impairment		0.183
Moderate motor impairment + blindness + epilepsy + cognitive impairment		0.017
Severe motor only		0.152
Severe motor impairment + epilepsy		0.033
Severe motor impairment + blindness		0.006
Severe motor impairment + blindness + epilepsy		0.003
Severe motor impairment + blindness + cognitive impairment		0.038
Severe motor impairment + epilepsy + cognitive impairment		0.216
Severe motor impairment + blindness + epilepsy + cognitive impairment		0.020

Step 6: Use disability weights to calculate YLDs

Each sequela is associated with a health state, which is used to calculate YLDs. The health states used for neonatal sepsis are largely the same as the health states for other neonatal causes (see table below). Some health states were combined to calculate the burden of certain sequelae.

Table 26. Disability weights and lay descriptions by health state

Health state	Description	Disability weight
Motor impairment, mild	Has some difficulty in moving around but is able to walk without help.	0.01 (0.005–0.019)
Motor impairment, moderate	Has some difficulty in moving around, and difficulty in lifting and holding objects, dressing, and sitting upright, but is able to walk without help.	0.061 (0.040–0.089)
Motor impairment, severe	Is unable to move around without help, and is not able to lift or hold objects, get dressed or sit upright.	0.402 (0.268–0.545)
Motor plus cognitive impairments, mild	Has some difficulty moving around but is able to walk without help. The person is slow in learning at school. As an adult, the person has some difficulty doing complex or unfamiliar tasks but otherwise functions independently.	0.031 (0.018–0.050)
Motor plus cognitive impairments, moderate	Has some difficulty in moving around, holding objects, dressing, and sitting upright, but can walk without help. The person has low intelligence and is slow in learning to speak and to do simple tasks.	0.203 (0.134–0.290)
Motor plus cognitive impairments, severe	Cannot move around without help, and cannot lift or hold objects, get dressed or sit upright. The person also has very low intelligence, speaks few words, and needs constant supervision and help with all daily activities.	0.542 (0.374–0.702)
Distance vision blindness	Is completely blind, which causes great difficulty in some daily activities, worry and anxiety, and great difficulty going outside the home without assistance.	0.187 (0.124–0.260)
Epilepsy, less severe (seizures < once per month)	Has sudden seizures two to five times a year, with violent muscle contractions and stiffness, loss of consciousness, and loss of urine or bowel control.	0.263 (0.173–0.367)
Epilepsy, severe (seizures ≥ once per month)	Has sudden seizures one or more times each month, with violent muscle contractions and stiffness, loss of consciousness, and loss of urine or bowel control. Between seizures the person has memory loss and difficulty concentrating.	0.552 (0.375–0.71)
Abdominopelvic problem, severe (proxy for EHB without kernicterus)	Has severe pain in the belly and feels nauseous. The person is anxious and unable to carry out daily activities.	0.324 (0.220–0.442)

Other neonatal disorders

In addition to the neonatal disorders described above, there are many diverse types of neonatal disorders with a range of severities and associated sequelae. Because these other neonatal disorders are diverse in their underlying causes and risk factors as well as in their associated health outcomes, modelling them together in a DisMod-MR 2.1 model would not produce reliable estimates of prevalence or excess mortality. Instead, we calculated the YLDs caused by other neonatal disorders directly using a YLD/YLL ratio.

We calculated the ratio of YLDs to YLLs across the specified neonatal disorders for which non-fatal outcomes were modelled, using YLL estimates from the GBD cause of death (CoD) analysis. We then multiplied this YLD/YLL ratio by the YLL estimate for other neonatal disorders from the GBD CoD analysis, providing us with an estimate of the YLDs associated with other neonatal disorders.

A full list of the ICD codes classified as other neonatal disorders in the mortality analysis is provided below. The codes that made up the largest proportion of deaths were P29: cardiovascular disorders originating in the perinatal period and P00: newborn (suspected to be) affected by maternal conditions that may be unrelated to present pregnancy.

ICD-9 codes:

> 760, 760.0-760.64, 760.8-760.9, 761, 761.2-761.6, 766, 770, 772, 772.0, 775.0, 776, 776.0-776.5, 776.7-776.9, 777, 778, 779

ICD-10 codes:

> P00, P01, P01.2-01.6, P01.8-01.9, P04, P04.2, P04.5-04.6, P04.8-04.9, P08, P29, P50, P51, P53, P54, P61, P61.0-61.1, P61.3-61.6, P61.8-61.9, P70, P70.1, P70.4, P70.8-70.9, P71, P72, P72.0, P72.2, P72.8-9, P76, P83, P84, P94, P94.1-2, P94.8-9, P96, P96.3, P96.8

References

1 Badawi N, Felix JF, Kurinczuk JJ, *et al.* Cerebral palsy following term newborn encephalopathy: a population-based study. *Dev Med Child Neurol* 2005; **47**: 293–8.

2 Bhutani VK, Zipursky A, Blencowe H, *et al.* Neonatal hyperbilirubinemia and Rhesus disease of the newborn: incidence and impairment estimates for 2010 at regional and global levels. *Pediatr Res* 2013; **74 Suppl 1**: 86–100.

3 Zipursky A. The universal prevention of Rh immunization. *Clin Obstet Gynecol* 1971;14:869–84.

4 Clarke CA: Prevention of Rhesus iso-immunization. *Seminars in Hematology* 6:201, 1969.

5 Walker W. Haemolytic Disease of the Newborn. In: *Recent Advances in Paediatrics*, 4th edn. London, UK: JA Churchill, 1970.

6 Vaughan VC. Kernicterus in erythroblastosis fetalis. *J Pediatr* 1946; **29**: 462–73.

7 Mollison PL, Cutbush M. Exchange transfusion in haemolytic disease of the newborn. *Lancet* 1948; **2**: 522–7.

8 Hsia DY, Allen FH Jr, Gellis SS, Diamond LK. Erythroblastosis fetalis. VIII. Studies of serum bilirubin in relation to kernicterus. *N Engl J Med* 1952;247:668–71.

9 Ebbesen F. Recurrence of kernicterus in term and near-term infants in Denmark. *Acta Paediatr* 2000;89:1213–7.

10 Ebbesen F, Andersson C, Verder H, et al. Extreme hyperbilirubinaemia in term and near-term infants in Denmark. *Acta Paediatr* 2005;94:59–64.

11 Ebbesen F, Bjerre JV, Vandborg PK. Relation between serum bilirubin levels =450 µmol/L and bilirubin encephalopathy; a Danish population-based study. *Acta Paediatr* 2012;101:384–9.

12 Sgro M, Campbell D, Barozzino T, Shah V. Acute neurological findings in a national cohort of neonates with severe neonatal hyperbilirubinemia. *J Perinatol* 2011;31:392–6.

13 Sgro M, Campbell D, Shah V. Incidence and causes of severe neonatal hyperbilirubinemia in Canada. *CMAJ* 2006;175:587–90.

14 Sgro M, Campbell DM, Kandasamy S, Shah V. Incidence of chronic bilirubin encephalopathy in Canada, 2007-2008. *Pediatrics* 2012;130: e886–90.

15 Vandborg PK, Hansen BM, Greisen G, Jepsen M, Ebbesen F. Follow-up of neonates with total serum bilirubin levels = 25mg/dL: a Danish population-based study. *Pediatrics* 2012;130:61–6.

Non-alcoholic fatty liver disease without cirrhosis

Flowchart

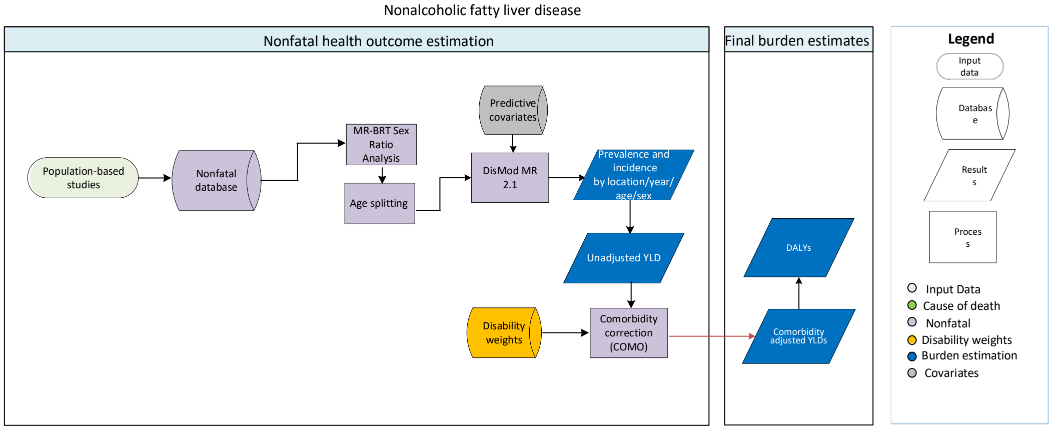

Input data and methodological summary for non-alcoholic fatty liver disease

Case definition

Non-alcoholic fatty liver disease (NAFLD) encompasses the spectrum of non-alcoholic fatty liver disease including fat deposition, inflammation, scarring, and fibrosis. The term, non-alcoholic steatohepatitis (NASH) appears in other places within GBD, which refers to the inflammatory stage in this spectrum. NAFLD without cirrhosis includes all degrees of NAFLD that have not progressed to cirrhosis, although we refer to it simply as "NAFLD" in this appendix section. Modelling details of cirrhosis due to NASH, a more severe form of NAFLD that accompanies scarring of the liver, can be found in the "Cirrhosis" section of this appendix.

A consensus statement was published by experts in the field of hepatology in June 2023, revising the nomenclature for this cause of disease to metabolic dysfunction-associated steatosis of the liver (MASLD).[1] This consensus was adopted after the GBD 2021 analysis concluded, and we continue to refer to this cause of disease as "NAFLD" for the GBD 2021 study report. The updated name will be proposed to the GBD Scientific Council to consider for adoption in the next GBD iteration.

Input data

We use population-based studies that report the prevalence of NAFLD. The following inclusion criteria were used:

[1] Rinella ME, Lazarus JV, Ratziu V, Francque SM, Sanyal AJ, Kanwal F, Romero D, Abdelmalek MF, Anstee QM, Arab JP, Arrese M, Bataller R, Beuers U, Boursier J, Bugianesi E, Byrne C, Castro Narro GE, Chowdhury A, Cortez-Pinto H, Cryer D, Cusi K, El-Kassas M, Klein S, Eskridge W, Fan J, Gawrieh S, Guy CD, Harrison SA, Kim SU, Koot B, Korenjak M, Kowdley K, Lacaille F, Loomba R, Mitchell-Thain R, Morgan TR, Powell E, Roden M, Romero-Gómez M, Silva M, Singh SP, Sookoian SC, Spearman CW, Tiniakos D, Valenti L, Vos MB, Wong VW, Xanthakos S, Yilmaz Y, Younossi Z, Hobbs A, Villota-Rivas M, Newsome PN; NAFLD Nomenclature consensus group. A multi-society Delphi consensus statement on new fatty liver disease nomenclature. *Hepatology*. 2023 Jun 24. doi: 10.1097/HEP.0000000000000520. Epub ahead of print. PMID: 37363821.

(1) Sample size greater than 100.
(2) Sample representative of general population for location.
(3) Sufficient description of methods to assess study quality.
(4) Does not exclude comorbidities.
(5) NAFLD diagnosed by ultrasound (USS) or other diagnostic imaging modality.

The last systematic review was performed for GBD 2017, using the search string below.

("Steatohepatitides"[Title/Abstract]) OR ("NAFLD"[Title/Abstract] OR "NAFL"[Title/Abstract] OR "NASH"[Title/Abstract] OR)) AND ("prevalence"[Title/Abstract] OR "incidence"[Title/Abstract) AND ("1990/01/01"[PDAT] : "2017/07/26"[PDAT]) NOT (animals[MeSH] NOT humans[MeSH]))

Although biopsy provides the gold-standard clinical case definition, this invasive procedure is not typically employed in population-based surveys or screening programmes. In consultation with GI experts, we thus chose ultrasound or other imaging study as our reference case diagnostics. We excluded any studies using serum diagnostics or fatty liver indexes and scores to diagnose NAFLD. Studies were excluded if they ascertained cases only among patients presenting for GI complaints or in specialty outpatient clinics, or if they excluded patients with comorbidities.

Since the majority of NAFLD cases are asymptomatic, we generally preferred studies with active case-finding methods and did not make use of administrative data from hospitals or claims, which severely underestimate NAFLD prevalence. An exception to this is that we accepted Asian studies pooling data from general checkups, where participation in checkups is high and ultrasound is a part of the checkup regimen (eg, South Korea, Japan, and some parts of China).

Data were marked as outliers and excluded if we found they differed substantially when compared to regional, super-regional, and global rates.

Data processing

Because we produce sex-specific estimates, we adjusted data that reported on both sexes into male and female sex-specific estimates. We identified studies that reported sex-specific prevalence and calculated the log ratio of female to male prevalence, then modelled these log ratios in a meta-regression tool, meta-regression—Bayesian, regularised, trimmed (MR-BRT). We then used the modelled sex ratio to adjust "both"-sex data values to expected "male" and "female" values. We calculated the male values as $val_{male} = val_{both} * \frac{pop_{both}}{(pop_{male}+ratio*pop_{female})}$. We calculated female values $val_{female} = ratio * val_{male}$.
After sex-splitting, we adjusted broad age-group data (defined as age range was greater than 25 years) into five-year age bins using an estimated age pattern of seroprevalence model from the previous GBD round, assuming the age-distribution in the study sample was the same as the estimated population.

Modelling strategy

DisMod model

We modelled prevalence and incidence of NAFLD using DisMod-MR 2.1. Prior settings include zero incidence from age 0 to 5, excess mortality and remission bound between 0 and 0.01 for all ages.

Several factors known to be associated with NAFLD prevalence in prior studies, for which we have prevalence estimates available for all GBD year-age-sex-location combinations, were employed as predictive covariates. Associations between predictive covariates and NAFLD prevalence for year-age-sex-location combinations with NAFLD prevalence data are used to help predict NAFLD prevalence for year-age-sex-location combinations with few or no data. A table of predictive covariates and their coefficients is shown below.

Table 1. Covariates. Summary of covariates used in the NAFLD DisMod-MR meta-regression model

Covariate	Parameter	Exponentiated beta (95% uncertainty interval)
Mean BMI	Prevalence	1.13 (1.08–1.17)
Prevalence of obesity	Prevalence	3.65 (1.47–7.12)
Age-standardised SEV* for high fasting plasma glucose	Prevalence	2.74 (2.50–2.98)

*Estimation of scaled exposure variables (SEVs) is described in a separate appendix section

Adjustment

We performed a post-hoc adjustment of estimates from DisMod to account for the fact that most studies of NAFLD historically excluded individuals with high alcohol consumption. Prevalence from such studies reflect the prevalence in low- or non-consumers of alcohol, but GBD burden estimation requires estimates of prevalence in the general population. We multiplied location-year-sex-age-specific prevalence estimates from the NAFLD DisMod model by the proportion of the general population that consumes <70 g (female) and <140 g (male) of alcohol per week to approximate data for the general population. This proportion is estimated by the alcohol risk factor team and is year-, age-, sex-, and location-specific.

Disability weights

Cases of NAFLD without cirrhosis are asymptomatic and assigned a disability weight of zero.

Non-rheumatic valvular heart diseases
Calcific aortic valve disease
Degenerative mitral valve disease
Other non-rheumatic valve diseases

Flowchart
Calcific aortic valve and degenerative mitral valve disease

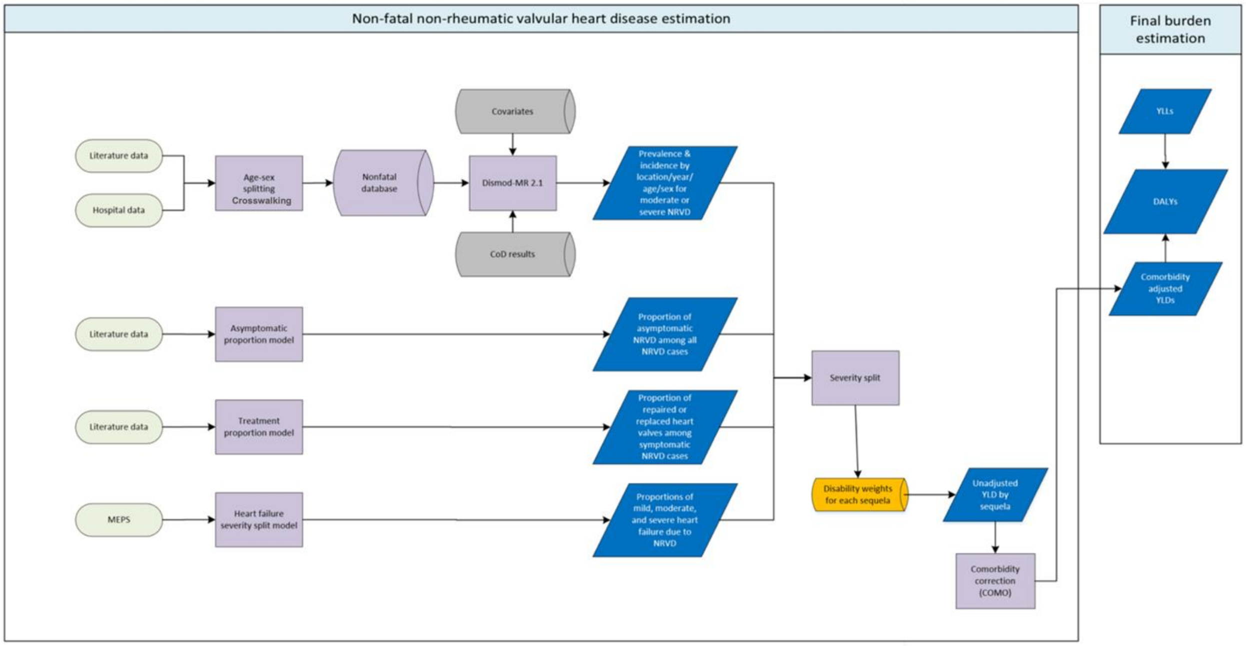

Input data and methodological appendix for non-rheumatic valve diseases

Case definitions
Non-rheumatic valvular heart disease
The non-rheumatic valve diseases (NRVD) are a group of cardiac conditions characterised by damage to at least one of the four heart valves. These conditions include calcific aortic valvular heart disease, degenerative mitral valvular heart disease, and other non-rheumatic valvular heart diseases. Estimates of NRVD in the GBD do not include valve disease with an aetiology that was congenital, rheumatic, or infectious. Valve disease due to these aetiologies is modelled as part of other causes in the GBD. All NRVD models were restricted to persons at or above the age of 15 to exclude congenital valve disorders. This age restriction is consistent with other progressive cardiovascular diseases modelled in the GBD.

Calcific aortic valve disease
Non-rheumatic calcific aortic valve disease (CAVD) is a condition where the aortic valve in the heart becomes stiff and hard due to the buildup of calcium deposits. CAVD was defined as physician diagnosis based on echocardiographic findings of haemodynamically moderate or severe aortic stenosis or regurgitation according to criteria from the American Heart Association and American College of Cardiology (AHA/ACC) (Table 1). CAVD did not include disease with an aetiology that was congenital, rheumatic, or infectious. Mild haemodynamic aortic stenosis or regurgitation was not included in our

case definition because mildly abnormal haemodynamic parameters are difficult to differentiate from non-pathological stenosis and/or regurgitation and are generally not reported in population-based studies. Table 3 shows the reference and alternative case definitions possible for data included in CAVD burden estimation.

Table 1: AHA/ACC definitions of moderate/severe aortic stenosis and regurgitation

Stenosis	Maximum jet velocity ≥3 m/s<br>Mean pressure gradient ≥20 mmHg
Regurgitation	Central jet mitral regurgitation ≥25% of the left ventricular outflow tract<br>Vena contracta ≥0.3 cm<br>Regurgitant volume ≥30 mL/beat<br>Regurgitant fraction ≥30%<br>Angiography grade ≥2+

Degenerative mitral valve disease
Non-rheumatic degenerative mitral valve disease (DMVD) is a condition where the mitral valve, which separates the two left chambers of the heart, becomes damaged due to weakening of the valve tissue, leading to leakage of blood across the valve. DMVD was defined as physician diagnosis based on echocardiographic findings of haemodynamically moderate or severe mitral regurgitation according to criteria from the AHA/ACC Cardiology (Table 2). DMVD did not include disease with an aetiology that was congenital, rheumatic, infectious, traumatic, carcinoid, or functional (ie, secondary to left ventricular remodelling due to heart failure from another cause). Mitral valve stenosis was considered to have rheumatic aetiology and therefore was not included. Haemodynamically mild mitral regurgitation was not included in our case definition because mild disease is challenging to differentiate from non-pathological regurgitation and is generally not reported in population-based studies. Table 3 shows the reference and alternative case definitions possible for data included in CAVD burden estimation.

Table 2: AHA/ACC definitions of moderate/severe mitral regurgitation

Regurgitation	Central jet mitral regurgitation >20% of the left atrium<br>Vena contracta ≥0.7 cm<br>Regurgitant volume ≥60 mL/beat<br>Regurgitant fraction ≥50%<br>Effective regurgitant orifice ≥0.4 cm^2<br>Angiography grade ≥2+

Table 3: Reference and alternative case definitions for CAVD and DMVD

**Quantity of interest**	**Reference or alternative**	**Definition**
CAVD	Reference	*Reference:* Clinical diagnosis of aortic valve stenosis or regurgitation due to progressive calcification of the aortic valve or annulus leading to hemodynamically moderate or severe aortic stenosis or

		regurgitation. Cases are determined by echocardiography by a physician.
CAVD	Alternative	Prevalence of CAVD as identified in administrative clinical informatics data.
DMVD	Reference	Degenerative mitral valve disease is defined as myxomatous degeneration of the mitral valve leading to regurgitation or prolapse. Cases are determined by echocardiography by a physician.
DMVD	Alternative	Prevalence of DMVD as identified in administrative clinical informatics data.

Other non-rheumatic valve disease
Other non-rheumatic valve disease (other NRVD) is a residual category that captures non-rheumatic, non-congenital valve disorders of the tricuspid and pulmonary valves. This includes tricuspid regurgitation, tricuspid stenosis, pulmonary regurgitation, and pulmonary stenosis. Other non-rheumatic valve diseases did not include tricuspid or pulmonary valve disease with an aetiology that was congenital, rheumatic, infectious, traumatic, carcinoid, or functional (ie, secondary to heart failure due to another cause). All estimates for other NRVD are produced as part of the heart failure estimation process; details are presented in the heart failure section of this appendix.

Input data

Data acquisition
Systematic reviews for CAVD and DMVD were last performed for GBD 2017. We searched PubMed using the following search strings:

CAVD: ("aortic stenosis"[Title/Abstract] OR "aortic regurgitation"[Title/Abstract]) NOT ("Transcatheter Aortic Valve Replacement"[MeSH] OR "Transcatheter aortic valve implantation"[KEYWORD]) AND (epidemiology[MeSH Major Topic] OR epidemiology[Subheading] OR epidemiology[MeSH Terms] OR prevalence[Title/Abstract] OR mortality[Title/Abstract]) NOT (animals[MeSH] NOT humans[MeSH]) AND ("1980/1/01"[PDAT] : "2017/12/31"[PDAT]) NOT Comment[ptyp] NOT Case Reports[ptyp]

DMVD: ("mitral stenosis"[Title/Abstract] OR "mitral regurgitation"[Title/Abstract]) AND ("epidemiology"[MeSH Major Topic] OR "epidemiology"[Subheading] OR "epidemiology"[MeSH Terms] OR prevalence [Title/Abstract] OR mortality[Title/Abstract]) NOT (animals[MeSH] NOT humans[MeSH]) AND ("1980/1/01"[PDAT] : "2017/12/31"[PDAT]) NOT Comment[ptyp] NOT Case Reports[ptyp]

We also included administrative inpatient hospital and claims data for both CAVD and DMVD. Descriptions of search strategies and the methodology used to process these data are described in Appendix 1, Section 2. Inpatient data were adjusted to reflect multiple visits in the same year, non-primary diagnoses, and inpatient to outpatient utilisation ratios. All administrative data were excluded below age 30 for both CAVD and DMVD.

Data on treatment, haemodynamic severity, and symptomatic status of CAVD and DMVD were collected from a subset of the studies reporting prevalence obtained from the systematic review described above.

Data processing
We used the modelling software meta-regression—Bayesian, regularised, trimmed (MR-BRT) to process data in preparation for modelling, including 1) bias adjustment for datapoints using alternative case

definitions, 2) splitting both-sex datapoints into sex-specific estimates, and 3) splitting datapoints with an age range of greater than 25 years. Details of MR-BRT and the adjustment processes can be found in Appendix 1, Section 2. Tables 4a and 4b show MR-BRT crosswalk adjustment factors for CAVD and DMVD, respectively. The formula for computing adjustment factors for prevalence is given in Equation 1 below. Age-splitting was based on the global sex-specific age pattern obtained from a single-parameter disease model—Bayesian meta-regression (DisMod-MR 2.1) model computed using prevalence data with age ranges of less than 25 years.

Equation 1: Calculation of adjustment factors**:**

$$Estimated\ Reference\ Def = invlogit(logit(Alternative\ Def) - Beta_{Alternative\ Def} - Beta_{Age_{scaled}} * Age\ Scaled)$$

Table 4a: MR-BRT adjustment factors for CAVD

Data input	Reference or alternative case definition	Gamma	Beta coefficient, logit (95% CI)
Literature	Reference	0.33	---
Inpatient, intercept	Alternative		–1.54 (–2.39 to –0.69)
Inpatient, male	Alternative		0.42 (0.2 to 0.64)
Inpatient, age spline 0	Alternative		–0.85 (–1.09 to –0.61)
Inpatient, age spline 1	Alternative		–0.89 (–1.1 to –0.69)
Claims, intercept	Alternative		–0.17 (–0.44 to 0.11)
Claims, male	Alternative		0.32 (0.25 to 0.4)
Claims, age spline 0	Alternative		–0.16 (–0.2 to –0.11)
Claims, age spline 1	Alternative		–0.43 (–0.47 to –0.39)

Table 4b: MR-BRT adjustment factors for DMVD

Data input	Reference or alternative case definition	Gamma	Beta coefficient, logit (95% CI)
Literature	Reference	0.03	---
Inpatient, intercept	Alternative		–2.02 (–2.85 to –1.19)
Inpatient, age scaled	Alternative		–0.88 (–1.01 to –0.75)
Claims, intercept	Alternative		–0.75 (–0.97 to –0.53)
Claims, age scaled	Alternative		–0.56 (–0.59 to –0.53)

MR-BRT crosswalk adjustments can be interpreted as the factor the alternative case definition is adjusted by to reflect what it would have been had it been measured using the reference case definition. If the logit beta coefficient is negative, then the alternative is adjusted up to the reference. If the logit beta coefficient is positive, then the alternative is adjusted down to the reference. The adjustment factor column is the exponentiated beta coefficient. For logit beta coefficients, this is the relative odds between the two case definitions.

Modelling strategy

Overview

We used parallel modelling strategies to model CAVD and DMVD. As mentioned above, estimates of morbidity for other NRVD were generated as part of the heart failure estimation process.

Figure 1 visualises the framework used. We first ran cause-specific models to estimate the prevalence and incidence of combined haemodynamically moderate and severe CAVD and DMVD. We then estimated the proportion of those with prevalent disease who were haemodynamically moderate. We next estimated the proportion of those with haemodynamically severe disease who were treated with valve repair or replacement procedures. The remaining proportion – those with untreated symptomatic disease – was split into four proportions: 1) controlled, medically managed heart failure; 2) mild failure; 3) moderate failure; and 4) severe heart failure. All proportions were calculated and converted to population prevalence at the draw level, thus propagating uncertainty from each step through to all subsequent steps. Additional details for each step follow.

Figure 1: Modelling framework for calcific aortic valve disease and degenerative mitral valve disease

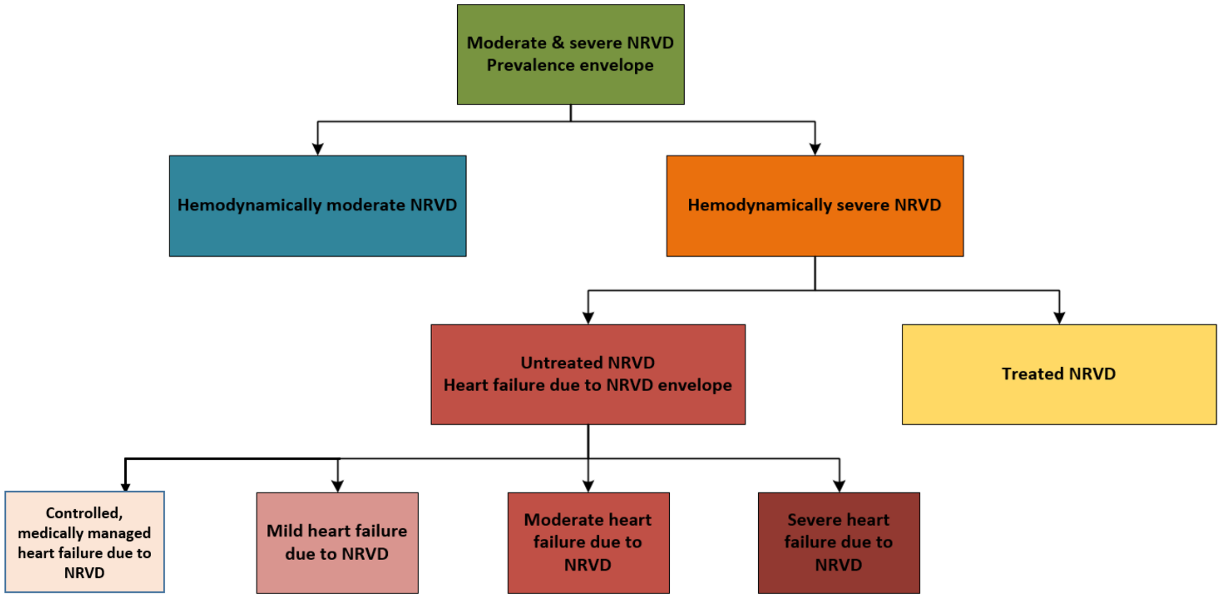

The health states, lay descriptions, and associated disability weights for the NRVD sequelae are shown in Table 5.

Table 5: Sequelae, health state lay descriptions, and associated disability weights

Sequela	Health state lay description	Disability weight

Asymptomatic non-rheumatic valve disease	--	0
Non-rheumatic valve disease after treatment	Has a chronic disease that requires medication every day and causes some worry but minimal interference with daily activities.	0.049 (0.031–0.072)
Controlled, medically managed heart failure due to non-rheumatic valve disease	Has been diagnosed with clinical heart failure, a chronic disease that requires medication every day and causes some worry but minimal interference with daily activities.	0.049 (0.031–0.072)
Mild heart failure due to non-rheumatic valve disease	Is short of breath and easily tires with moderate physical activity, such as walking uphill or more than a quarter mile on level ground. The person feels comfortable at rest or during activities requiring less effort.	0.041 (0.026–0.062)
Moderate heart failure due to non-rheumatic valve disease	Is short of breath and easily tires with minimal physical activity, such as walking only a short distance. The person feels comfortable at rest but avoids moderate activity.	0.072 (0.047–0.103)
Severe heart failure due to non-rheumatic valve disease	Is short of breath and feels tired when at rest. The person avoids any physical activity, for fear of worsening the breathing problems.	0.179 (0.122–0.251)

Overall prevalence and incidence estimation

We first estimated overall prevalence and incidence by age, sex, location, and year using DisMod-MR 2.1. Separate models were run for CAVD and DMVD. Full details of the DisMod-MR 2.1 modelling software can be found in Appendix 1, Section 2. In addition to the data described above, we also used cause-specific mortality rates from the fatal modelling process as inputs. Covariates included in the DisMod-MR 2.1 models for CAVD and DMVD in Tables 6a and 6b. For both models, we set value priors of zero for incidence from ages 0 to 15 and value priors of zero for remission for all ages.

Table 6a: Covariates and coefficients for CAVD DisMod-MR 2.1 model

Covariate	Parameter	Coefficients	Exponentiated coefficients
Log-transformed, age-standardised CVD SEV scalar	Prevalence	0.75 (0.75 to 0.75)	2.12 (2.12 to 2.12)
Healthcare Access and Quality Index	Excess mortality rate	–0.023 (–0.027 to –0.021)	0.97 (0.97 to 0.98)

Table 6b: Covariates and coefficients for DMVD DisMod-MR 2.1 model.

Covariate	Integrand	Coefficients	Exponentiated coefficients
Healthcare Access and Quality Index	Excess mortality rate	–0.1 (–0.1 to –0.1)	0.90 (0.90 to 0.90)

Haemodynamically moderate proportion estimation

We estimated the proportion of individuals with overall valve disease who were haemodynamically moderate. There was a total of five data sources (Belgium, Iceland, the Netherlands, Spain, and USA) that reported the proportion of individuals who were haemodynamically moderate. Due to the sparsity of the data, we were not able to generate separate estimates of this proportion for CAVD and DMVD.

We modelled a proportion with uncertainty that varied by age with the following regression:

$$logit(y) = \beta_0 + \beta_1 age + \gamma$$

where y is the proportion of haemodynamically moderate disease, *age* is the midpoint age for each datapoint, and γ is a random effect for each data source. The regression coefficients are reported in Table 7.

Table 7: Haemodynamically moderate NRVD regression coefficients.

**Covariate**	**Coefficients**	**Transformed coefficients**
Intercept (β_0)	6.6 (4.9 to 8.4)	0.998 (0.992 to 0.999)
Age (β_1)	–0.07 (–0.093 to –0.047)	0.932 (0.911 to 0.954)

Figure 2: Results of model for haemodynamically moderate NRVD

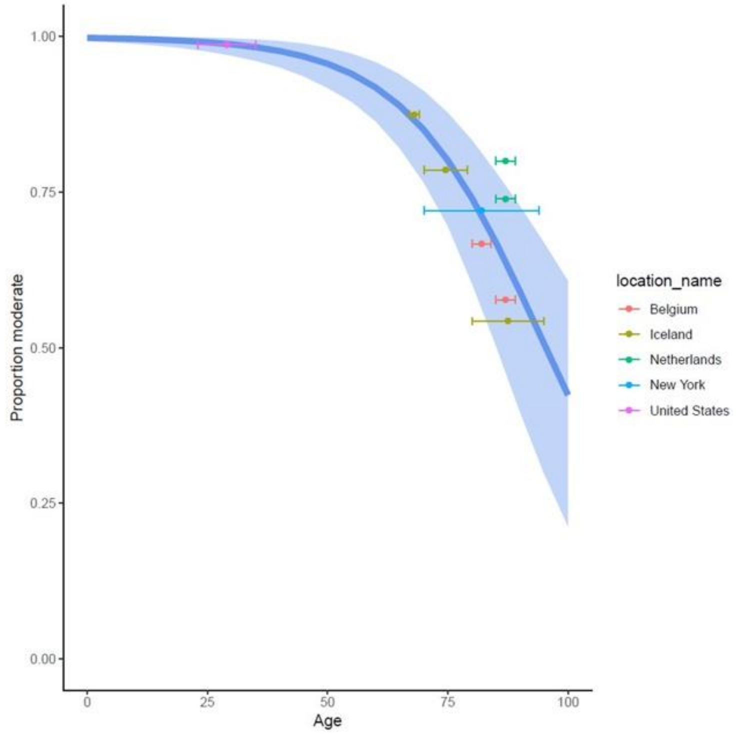

The prevalence of haemodynamically moderate valve disease and haemodynamically severe disease were then calculated by multiplying the prevalence envelope by the proportion of those with haemodynamically moderate disease by age, sex, location, and year.

Treated proportion

We next estimated the proportion of individuals with haemodynamically severe disease who had been treated with valve replacement or repair. We assumed that these procedures were not performed on any individuals with haemodynamically moderate disease.

The input data available were all from relatively high-income geographies, yet it is important that we capture the difference in treatment probability based on the likelihood of access to care. Because of this challenge, we ran a regression using the Healthcare Access and Quality (HAQ) Index predicting the level of treatment and set a prior that the proportion of individuals with a valve replacement or repair was zero where HAQ Index was equal to zero. This assumption allowed us to estimate an increasing relationship between HAQ Index and proportion treated, where the estimated proportion treated was based on data where HAQ Index was high.

We used the regression equation:

$$logit(y) = \alpha + \beta_1 * haqi + \beta_2 * age + \beta_3 * severity$$

where y is the proportion of individuals with haemodynamically severe disease who had a valve replacement or repair, $haqi$ is the Healthcare Access and Quality Index, age is the midpoint of the age range for a given datapoint, and $severity$ is an indicator variable to adjust for datapoints where the denominator of the proportion treated included both haemodynamically moderate and haemodynamically severe individuals.

The prevalence of those with treated valve disease and the prevalence of those with untreated haemodynamically severe disease were calculated by multiplying the prevalence of haemodynamically severe disease by the proportion of those with treated valve disease by age, sex, location, and year. The results of this regression are reported in Table 8 and plotted for three ages in Figure 3.

Table 8: Treated calcific aortic valve and degenerative mitral valve disease regression coefficients

**Covariate**	**Coefficients**	**Transformed coefficients**
Intercept (β_0)	–4.69 (–5.90 to –3.43)	0.009 (0.003 to 0.032)
HAQI (β_1)	0.080 (0.070 to 0.089)	1.083 (1.073 to 1.093)
Age (β_2)	–0.029 (–0.04 to –0.015)	0.971 (0.957 to 0.985)
Severity (β_3)	–0.947 (–1.40 to –0.54)	0.377 (0.246 to 0.578)

Figure 3: Results of treatment model for three ages

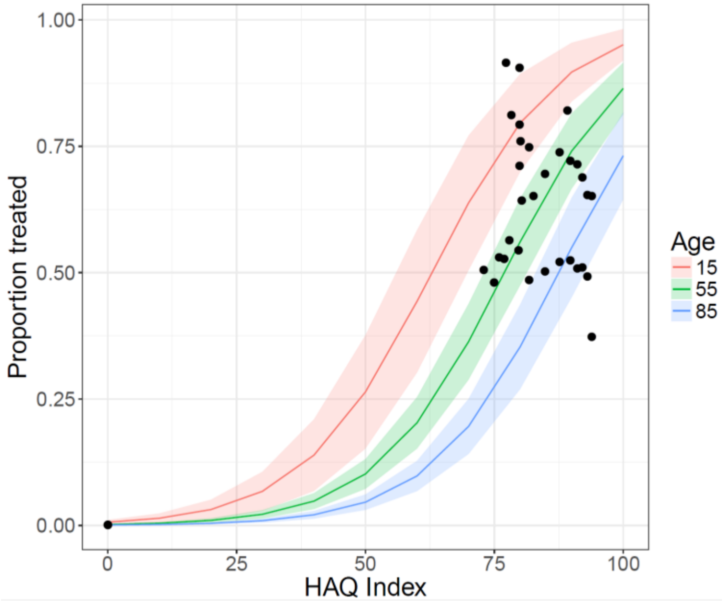

We assumed that all untreated haemodynamically severe disease would result in heart failure. The proportions of 1) controlled, medically managed heart failure, 2) mild heart failure, 3) moderate heart failure, and 4) severe heart failure due to valve disease were estimated using the approach described in the heart failure section of the appendix. These proportions are based on an analysis of MEPS, a population-based survey that links EQ5D to ICD codes, allowing the application of GBD's standard disability methods.

Nutritional deficiencies

Nutritional deficiencies is a parent cause for the non-fatal estimation of the following sub-causes:

1. Vitamin A deficiency
2. Iodine deficiency
3. Dietary iron deficiency
4. Protein-energy-malnutrition
5. Other nutritional deficiencies

Since these five subcauses are modelled separately, each with its own case definition, input data, modeling strategy, and severity distribution analysis, we present each subcause sequentially.

Vitamin A deficiency

Flowchart

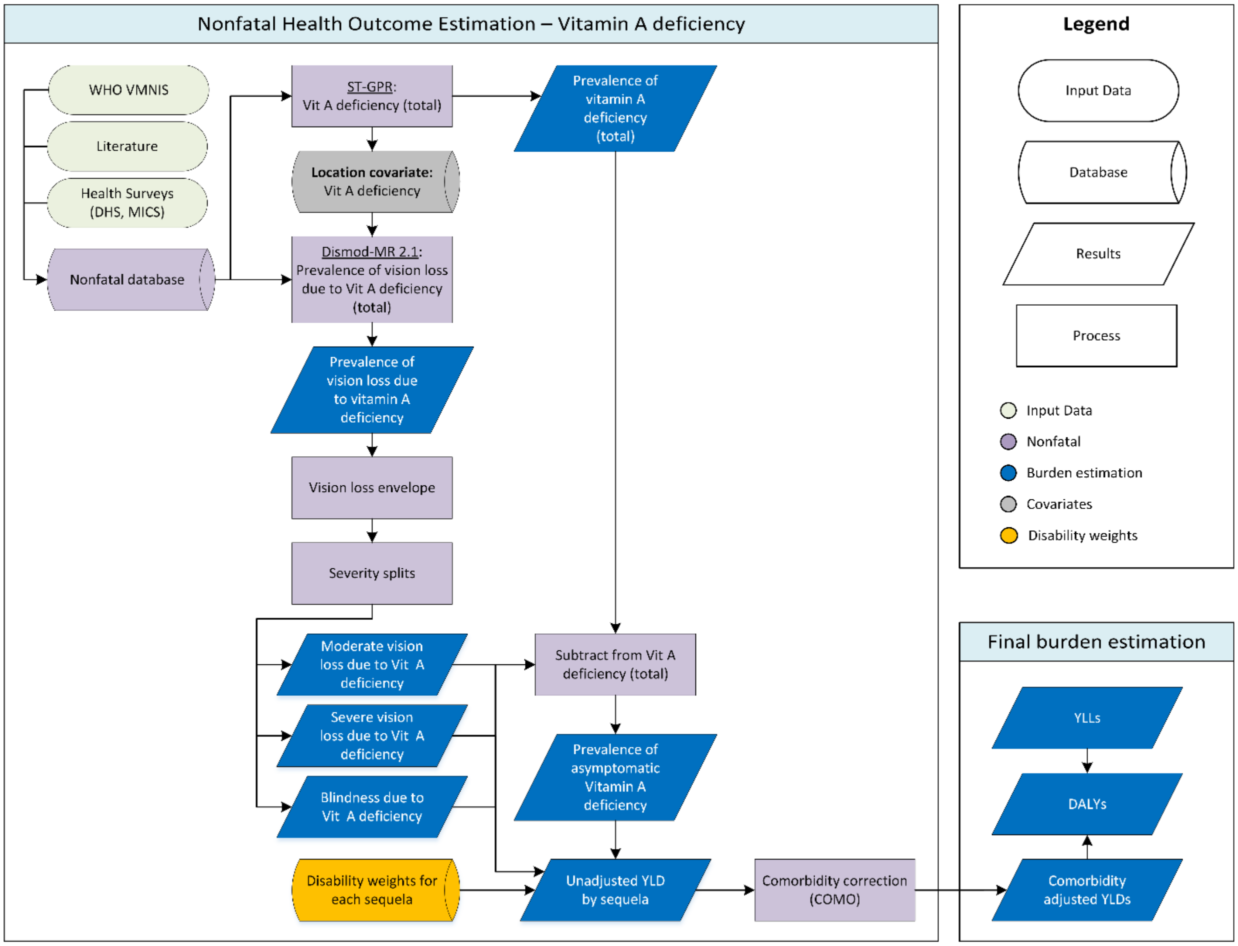

Case definition

Vitamin A deficiency is a condition due to low dietary intake or bioavailability of vitamin A that is inadequate to satisfy physiological needs, which is characterised by low serum or/and breast milk retinol or/and retinol binding concentration, or/and clinical symptoms such as night blindness, xerophthalmia. In GBD 2023, the case definition of vitamin A deficiency is the prevalence of serum retinol concentration <0.7 µmol/L.

In GBD 2023, the assessment of vitamin A deficiency burden involves the quantification of total vitamin A deficiency, anemia due to vitamin A deficiency, as well as blindness and vision loss due to vitamin A deficiency, which are associated with corneal ulcerations and corneal scars.

Input data

For GBD 2021, we used data from the WHO Vitamin and Mineral Nutrition Information System (WHO VMNIS), health surveys such as DHS and MICS, and studies identified through literature review for the vitamin A deficiency model. We outliered small-scale studies that are not population representative. We used data from the WHO Vitamin and Mineral Nutrition Information System for the vision loss model. A systematic review was last conducted for GBD 2013. The PubMed search terms were: ((vitamin A deficiency [Title/Abstract] AND prevalence[Title/Abstract]) AND ("2009"[Date – Publication] : "2013"[Date – Publication])). Exclusion criteria were:

1. Studies that were not population-based, eg, hospital or clinic-based studies
2. Studies that did not provide primary data on epidemiological parameters, eg, commentaries
3. Review articles
4. Case series
5. Self-reported cases

Modelling strategy

No major changes to the modelling strategy for vitamin A deficiency were made in GBD 2023 as compared to GBD 2021. We updated a sex-ratio model using MR-BRT (meta-regression—Bayesian, regularised, trimmed) based on updated vitamin A deficiency data. The sex ratio model was used to split prevalence data reported for both sexes into male and female prevalences. To derive the age pattern, we used a cascading spline model in which "super-region" and "country" serve as the hierarchical levels of location used as cascading layers. In the cascading spline approach, the age pattern estimation for a country is partially informed by the model fitted to the data within its super-region and all the global data, in addition to the data specific to the country. In the absence of data for both country and super-region, the age pattern estimation is informed by the data from all countries (global data). The age pattern is used to conduct age splitting for the studies that report vitamin A deficiency for wide non-GBD age groups. We used a new age splitting package called "PyDisagg" to conduct the age splitting. PyDisagg[1] disaggregates proportions, rate, and count observations based on a proportionality assumption. We seek to disaggregate a user-provided datum D into subcomponents D_i, guaranteeing that:

$$sum(D_i) = D$$

$$D_i = g_i p_i$$

where p_i is population weight and g_i a transform of the global rate (f_i).

$$g_i = T^{-1}(\beta + T(f_i))$$

The basic use case has T = ln, guaranteeing that the recovered rates are proportional to the global. In addition, PyDisagg allows splitting of bounded quantities using the appropriate logit transform for T. PyDisagg version 0.6.0 disaggregates observations of both continuous quantities (eg, age-splitting of age-aggregated observations) as well as discrete quantities (including sex and multiple categories). Uncertainty for split datapoints is obtained using asymptotic statistics. Specifically, we use uncertainty of the inputs (ie, uncertainty of the aggregate and uncertainty of the age pattern) together with a generalised delta method that effectively computes a linearised map from the inputs to the outputs and uses that to obtain posterior uncertainty intervals for the split datapoints.

We applied the spatiotemporal Gaussian process regression (ST-GPR) framework to estimate the mean prevalence of vitamin A deficiency from 1980 to 2024 for national and subnational locations, sexes, and for all GBD age groups. The vitamin A deficiency ST-GPR model utilised three location-level covariates as a fixed effect: age-standardised summary exposure values (SEV) for stunting (logit scale), Socio-demographic Index (logit scale), and the availability of vitamin A (µg RAE) (log scale). Since GBD 2021, vitamin A supplementation was omitted as a covariate in the vitamin A deficiency model due to its lack of statistical significance in the ST-GPR model. We also observed that when the coverage of vitamin A supplementation was included as a covariate in the vitamin A deficiency ST-GPR model, it resulted in an implausible temporal trend. To account for the effect of location levels, the ST-GPR model incorporated these levels (region/super-region/country) as a random effect. Since GBD 2019, we have introduced the assumption that the duration of vitamin A deficiency is one year, which implies that prevalence and incidence are equal. The process of vetting and validating models was accomplished primarily through an examination of ST-GPR scatterplots by GBD 2023 locations from 1990 to 2024. Any poorly fitting datapoints were re-inspected for error at the level of extraction. If errors in data extraction were found, re-extraction was done.

The vision loss due to vitamin A deficiency model was run as a single-parameter meta-regression on prevalence in DisMod-MR with vitamin A deficiency prevalence as a location-level covariate. The case definition for vision loss due to vitamin A deficiency is aligned with the WHO Vitamin and Mineral Nutrition Information System database's definition of a corneal scar. In GBD 2019, we modelled the sex ratio for vision loss due to vitamin A deficiency outside of DisMod-MR using MR-BRT (meta-regression—Bayesian, regularised, trimmed) and applied this ratio to split both-sex data prior to DisMod-MR modelling.

Table 1 Covariates. Summary of covariates used in the vitamin A deficiency models (GBD 2023)

**Vitamin A model**	**Modelling strategy**	**Covariate**	**Type**	**Parameter**
Vitamin A deficiency	ST-GPR	Vitamin A availability unadjusted (µg RAE)(log)	Country-level	Prevalence and incidence
	ST-GPR	Age-standardised SEV for stunting (logit)	Country-level	Prevalence and incidence
	ST-GPR	SDI (logit)	Country-level	Prevalence and incidence
Vision loss	DisMod-MR	Vitamin A deficiency (age-standardised)	Country-level	Prevalence

Our GBD results include explicit estimates of total vitamin A deficiency, although those without vision loss are assumed to be asymptomatic. Description of how our estimates of total vision loss described above are parsed into moderate vision loss, severe vision loss, and blindness can be found in the modelling description for the "vision loss impairment". Sequelae and corresponding disability weights for each of the health states associated with vitamin A deficiency are shown in Table 2.

Table 2. Severity, lay description, and disability weight (DW)

Sequela	Health state name	Lay description	Disability weight
Asymptomatic vitamin A deficiency	Asymptomatic	--	--
Moderate vision impairment due to vitamin A deficiency	Distance vision, moderate impairment	Has vision problems that make it difficult to recognise faces or objects across a room.	0.031 (0.019–0.049)
Severe vision impairment due to vitamin A deficiency	Distance vision, severe impairment	Has severe vision loss, which causes difficulty in daily activities, some emotional impact (for example worry), and some difficulty going outside the home without assistance.	0.184 (0.125–0.258)
Blindness due to vitamin A deficiency	Distance vision blindness	Is completely blind, which causes great difficulty in some daily activities, worry and anxiety, and great difficulty going outside the home without assistance.	0.187 (0.124–0.26)
Mild anaemia due to vitamin A deficiency	Mild anaemia	Feels slightly tired and weak at times, but this does not interfere with normal daily activities.	0.004 (0.001–0.008)
Moderate anaemia due to vitamin A deficiency	Moderate anaemia	Feels moderate fatigue, weakness, and shortness of breath after exercise, making daily activities more difficult.	0.052 (0.034–0.076)
Severe anaemia due to vitamin A deficiency	Severe anaemia	Feels very weak, tired and short of breath, and has problems with activities that require physical effort or deep concentration.	0.149 (0.101–0.209)

Iodine deficiency

Flowchart

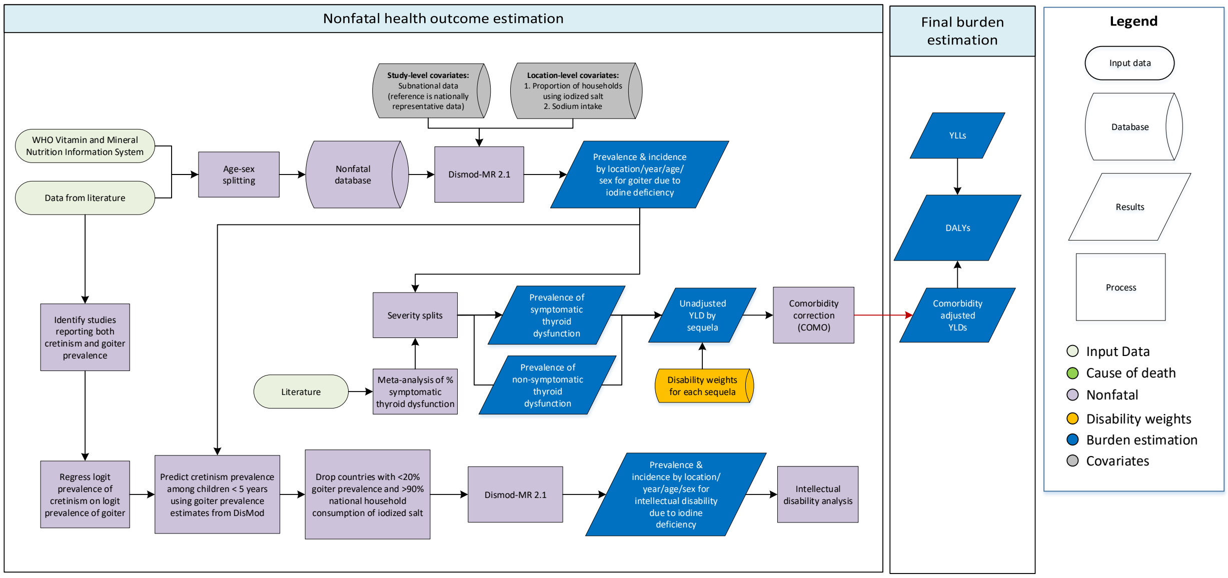

Case definition

Iodine deficiency is a condition characterised by impaired production of thyroid hormones due to insufficient iodine intake which results in adverse health ranging from thyroid gland enlargement (goitre) to severe physical and mental retardation. Our assessment of the non-fatal burden of iodine deficiency includes estimates of only the subset of iodine deficiency associated with visible goitre (grade 2) and its associated sequelae, including thyroid dysfunction, heart failure, and intellectual disability (historically referred to as "cretinism"). It does not include estimates of subclinical iodine deficiency or non-visible goitre (grade 1) induced by iodine deficiency.

Input data

For GBD 2023, data from the WHO Vitamin and Mineral Nutrition Information System and published studies were used for the visible goitre model. The extraction and accompanying systematic review were last conducted for GBD 2013. The PubMed search terms were: ((iodine deficiency [Title/Abstract] AND prevalence [Title/Abstract]) AND ("2009"[Date – Publication] : "2013"[Date – Publication]))

The exclusion criteria were:

1. Studies that were not population-based, eg, hospital or clinic-based studies
2. Studies that did not provide primary data on epidemiological parameters, eg, commentaries

3. Review articles
4. Case series
5. Self-reported cases

All input data for iodine deficiency are already in our gold-standard case definition (prevalence of visible goitre and prevalence of intellectual disability due to iodine deficiency), so no bias corrections are needed.

Modelling strategy

The iodine deficiency modelling strategy includes iodine deficiency and associated sequelae heart failure, thyroid dysfunction, and intellectual disability. The process is composed of two models for visible goitre and intellectual disability due to iodine deficiency and severity splits for the other sequela. No changes to the modelling strategy were made in GBD 2023.

In GBD 2019, we implemented a two-step process to estimate the prevalence of grade 2 goitre, which we carried into GBD 2021 and GBD 2023. We first used all available data to construct an age pattern model that captured the prevalence age-trend in the data, which was used to split data spanning an age range greater than 25 years into narrower age bins. Then we modelled the prevalence of visible goitre using the new age-split data. In this model, we introduced several new assumptions: visible goitre incidence can be non-decreasing across age (ie, we removed a decreasing slope prior), a small amount of remission is possible, and birth prevalence is not possible. These assumptions were based on evidence in the literature showing that the highest levels of visible goitre are in middle-aged people and were prompted by observing that the previously strict parameters were limiting the predictive power of the model. We also continued to use proportion of households using iodised salt and sodium intake as country-level covariates. The coefficients for these covariates are in the table below.

Table 1. Visible goitre covariates. Summary of covariates used in the visible goitre DisMod-MR meta-regression model

Covariate	Type	Parameter	Exponentiated beta (95% UI)
Proportion of households using iodised salt	Country-level	Prevalence	0.0028 (0.0024–0.0034)
Sodium intake	Country-level	Prevalence	1.11 (1.08–1.13)

For GBD 2023, no changes were made to the strategy for the intellectual disability model compared to GBD 2021. Consistent with GBD 2021 approach, we estimated the prevalence of intellectual disability due to iodine deficiency (cretinism) by regressing datapoints from studies reporting both cretinism and goitre prevalence in the same population. To do so, we first transformed cretinism prevalence and goitre prevalence into logit space, regressed the logit prevalence of cretinism on the logit prevalence of goitre, and predicted for all national locations using the goitre estimates from the DisMod-MR 2.1 model above. We dropped locations with total goitre prevalence less than 20% and locations with household iodised salt consumption greater than 90%. We kept observations in children younger than 5 years and used these data as incidence input in a second DisMod-MR 2.1 to generate location-year-age-sex-specific estimates. We modelled with zero remission, zero incidence after age 5, and proportion of

households using iodised salt as a covariate on incidence (Table 2). We repeated the dropout criteria of total goitre prevalence and iodised salt consumption on the DisMod-MR 2.1 output.

Table 2. Intellectual disability due to iodine deficiency covariates. Summary of covariates used in the intellectual disability due to iodine deficiency DisMod-MR meta-regression model.

Covariate	Type	Parameter	Exponentiated beta (95% UI)
Proportion of households using iodised salt	Country-level	Incidence	0.14 (0.14–0.14)

The severity split distribution did not change for GBD 2023. Initial severity proportions are visible goitre without symptoms of thyroid dysfunction (proportion=0.915, 95% confidence interval (CI): 0.904–0.926); goitre with symptoms of thyroid dysfunction (proportion=0.085, 95% confidence interval (CI): 0.084–0.086). Additionally, we split the intellectual disability due to iodine deficiency model into severe and profound ID using ID proportion assumptions. Everyone with ID is assumed to have thyroid dysfunction, while heart failure is assumed to only occur in people with profound intellectual disability (which we split into mild, moderate, and severe heart failure). Heart failure attributable to iodine deficiency was modelled separately, and the methods for this outcome are presented separately in the section for heart failure and its aetiologies. Table 3 provides details on the severity states downstream of iodine deficiency.

Table 3. Severity distribution, details on the severity levels for iodine deficiency and the associated disability weight (DW) with that severity.

Sequela	**Health state name**	**Lay description**	**Disability weight**
Visible goitre without symptoms	Disfigurement, level 1	Has a slight, visible physical deformity that others notice, which causes some worry and discomfort.	0.011 (0.005–0.021)
Visible goitre with symptoms without intellectual disability or heart failure	Iodine-deficiency goitre	Has a large mass in the front of the neck. The person sometimes has weakness and fatigue, constipation, and weight gain.	0.199 (0.133–0.276)
*Visible goitre with **severe** intellectual disability due to iodine deficiency*	Intellectual disability / mental retardation, severe	Has very low intelligence and cannot speak more than a few words, needs constant supervision and help with most daily activities, and can do only the simplest tasks.	0.326 (0.233–0.438)*
	Iodine-deficiency goitre	(see above)	
*Visible goitre with **profound** intellectual disability due to iodine deficiency*	Intellectual disability / mental retardation, profound	Has very low intelligence, has almost no language, and does not understand even the most basic requests or instructions. The person requires constant supervision and help for all activities.	0.358 (0.252–0.475)*

	Iodine-deficiency goitre	(see above)	
*Visible goitre with profound intellectual disability and **mild** heart failure due to iodine deficiency*	Intellectual disability / mental retardation, profound	(see above)	0.384 (0.276–0.502)*
	Iodine-deficiency goitre	(see above)	
	Heart failure, mild	Is short of breath and easily tires with moderate physical activity, such as walking uphill or more than a quarter-mile on level ground. The person feels comfortable at rest or during activities requiring less effort.	
*Visible goitre with profound intellectual disability and **moderate** heart failure due to iodine deficiency*	Intellectual disability / mental retardation, profound	(see above)	0.403 (0.293–0.524)*
	Iodine-deficiency goitre	(see above)	
	Heart failure, moderate	Is short of breath and easily tires with minimal physical activity, such as walking only a short distance. The person feels comfortable at rest but avoids moderate activity.	
*Visible goitre with profound intellectual disability with **severe** heart failure due to iodine deficiency*	Intellectual disability / mental retardation, profound	(see above)	0.471 (0.344–0.602)*
	Iodine-deficiency goitre	(see above)	
	Heart failure, severe	Is short of breath and feels tired when at rest. The person avoids any physical activity, for fear of worsening the breathing problems.	

Dietary iron deficiency

Flowchart

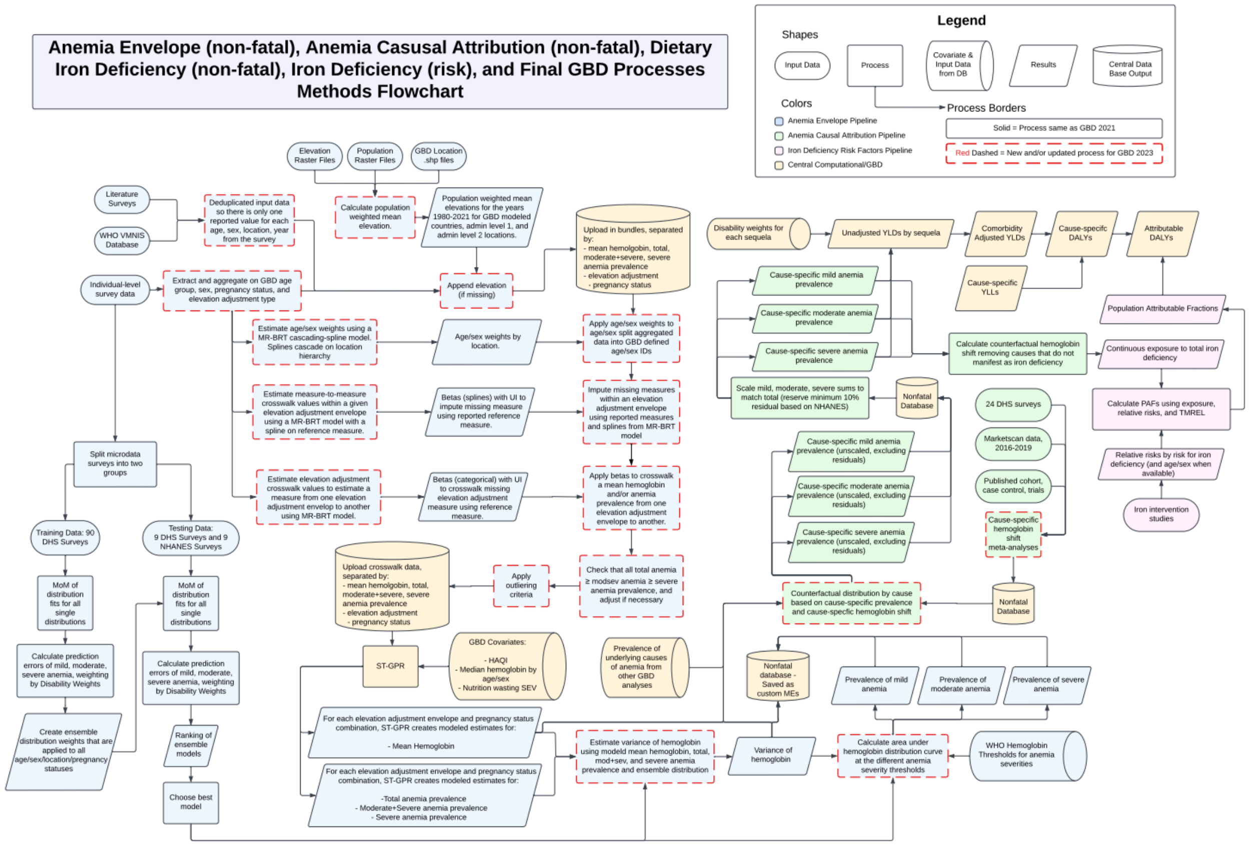

Case definition

Dietary iron deficiency in the GBD cause analysis is defined as mild, moderate, or severe anaemia that is the result of inadequate dietary intake of iron, but not due to other causes of inadequate absolute or functional iron availability to meet the body's needs.

Methodological summary

Dietary iron deficiency was quantified as an output of the GBD Anaemia Causal Attribution framework. The GBD anaemia model has two main steps – estimation of the anaemia envelope and causal attribution – both of which inherently impact estimates of iron deficiency. See the methodological description of "Anaemia (impairment)" for detailed description of the analytic approach and inputs.

Briefly, the first step is estimating anaemia envelope – the prevalence of mild, moderate, and severe anaemia prevalence for each GBD location, age group, sex, and year. The inputs to the envelope model are mean and standard deviation (SD) of haemoglobin [Hb] concentration. Mean haemoglobin is modelled directly in ST-GPR, and standard deviation is estimated using a variance optimisation algorithm that takes as inputs the modelled mean haemoglobin estimates and estimates of the prevalence of severe, moderate+severe, and total anaemia (also modelled in ST-GPR). For every location, year, age,

and sex, we anchor the distributions at the estimated mean [Hb] value and find the variance value that minimises the error between our ST-GPR estimates of severe, moderate+severe, and total anaemia and the corresponding values implied by a given mean and variance [Hb] combination.

Individual-level data sources are then used to develop a set of ensemble distribution weights using method of moments, which are then paired with mean and SD results to produce estimates of the entire distribution of haemoglobin for each population group. A population group is a specific geography, sex, age group, and year combination. The second step is anaemia causal attribution, which generates counterfactual haemoglobin distributions for each cause of anaemia based on the cause-level prevalence (or incidence, in the case of maternal haemorrhage) estimates from the respective GBD analyses and cause-specific haemoglobin shifts that were determined via meta-analysis of data from the literature, population-based surveys, and insurance claims databases for each cause. The counterfactual distribution methods used the same ensemble distribution weights as the overall anaemia envelope because there are inadequate data to guide alternate distributions for each subcause. Mild, moderate, and severe anaemia were assigned to each cause based on the difference between the counterfactual and observed haemoglobin distributions in each population group. The sum of severity-specific prevalence was then summed to match the total, with a minimum residual of 10%,[2,3] and then the remainder was distributed between five GBD causes using fixed proportion redistribution methods: 1) dietary iron deficiency (GBD cause), 2) other haemoglobinopathies and haemolytic anaemias, 3) other infectious diseases, 4) other neglected tropical diseases, and 5) endocrine, metabolic, blood, and immune disorders.

It is important to take note of the difference between “dietary iron deficiency” as a GBD cause and “iron deficiency” as a GBD risk. Many GBD causes lead to anaemia that clinically manifests as iron deficiency (or microcytosis), but where inadequate intake is not the underlying problem. Examples include neglected tropical diseases such as hookworm, malaria, and schistosomiasis, gastrointestinal disorders, cirrhosis, maternal haemorrhage, menstrual disorders, uterine fibroids, and vitamin A deficiency. The name “dietary iron deficiency” is intended to differentiate, therefore, between inadequate dietary intake of iron and haemorrhagic or disorders of iron metabolism. Additionally, because we have yet to include 100% of anaemia causes, estimates should be interpreted to also include some acute and chronic haemorrhagic states for which supplementation may be helpful, but poor nutritional intake is not the only underlying problem. Examples include malabsorption syndromes, other micronutrient deficiencies besides vitamin A deficiency, and injuries with associated acute blood loss anaemia. “Iron deficiency” exposure as estimated for the GBD risk factors analysis, in contrast, includes a combined assessment of the magnitude of haemological insult from all causes that manifest as iron deficiency. Our goal is to systematically add all causes of anaemia as specific inputs to GBD Anaemia Causal Attribution, including inadequate iron intake, and eliminate the need for residual attribution.

Protein-energy malnutrition

Flowchart

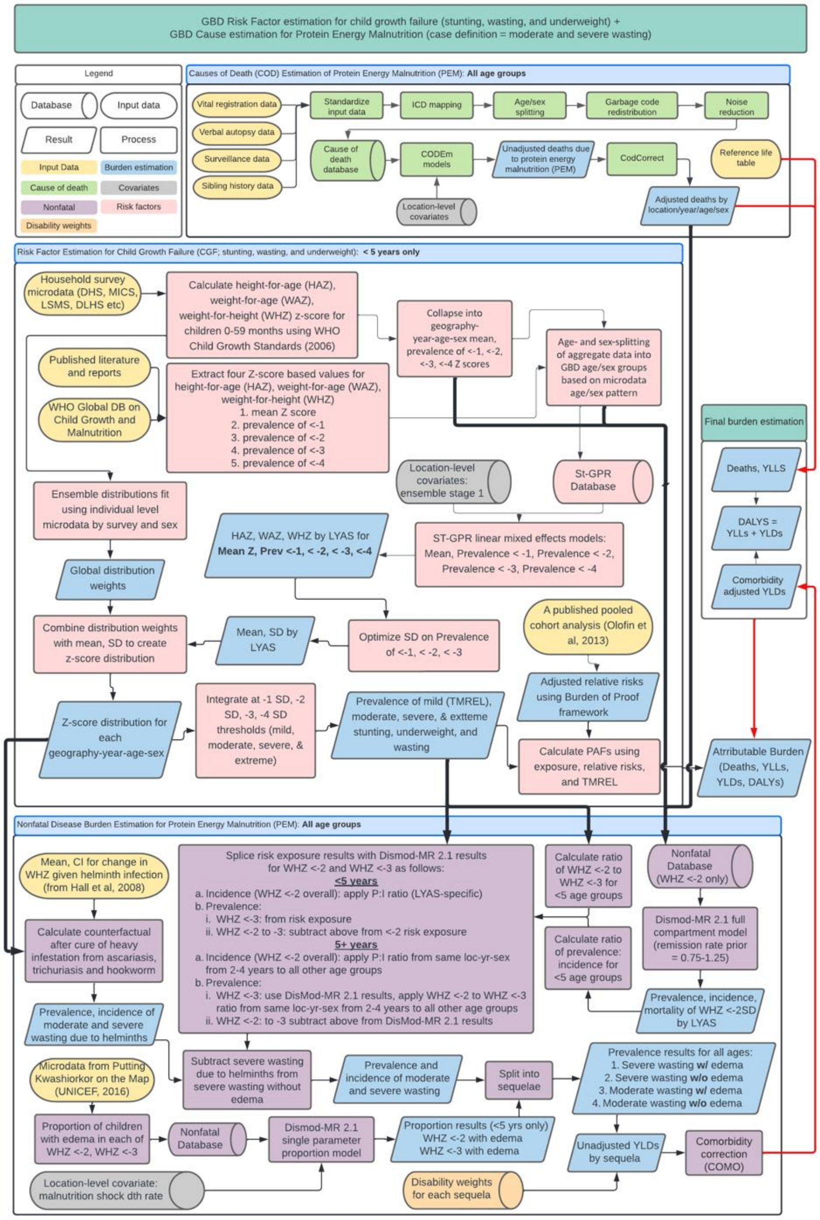

Case definition

Protein-energy malnutrition (PEM) includes moderate and severe acute malnutrition, commonly referred to as "wasting", and was defined in terms of weight-for-height Z-scores (WHZ) on the WHO 2006 growth standard for children. We quantified non-fatal PEM burden in four mutually exclusive and collectively exhaustive categories, reflecting distinct gradations of disability that can occur: moderate wasting without oedema (WHZ < –2SD to < –3 SD), moderate wasting with oedema (WHZ < –2SD to < –3 SD), severe wasting without oedema (WHZ < –3SD), and severe wasting with oedema (WHZ < –3SD). The

aggregate of categories that include "oedema" can be considered equivalent to the disease state commonly referred to as "kwashiorkor", and severe wasting can likewise be considered equivalent to "marasmus." For PEM, ICD 10 codes are E40-E46.9, E64.0, and ICD 9 codes are 260-263.9. This classification reflects a moderate shift from GBD 2015, when moderate wasting without oedema was not included in our non-fatal estimates, and by definition is associated with higher prevalence estimates than previously published by GBD. The other GBD 2015 categories – kwashiorkor, marasmus, and severe wasting – have unchanged case definitions, but have been renamed for clarity and consistency. This revised GBD 2016 case definition more closely aligns with others and allows for better application to the international nutrition community's programming and estimates related to non-fatal PEM. This change continued into GBD 2023.

Input data and data processing

The input data for this model come in two primary streams. First, we used individual-level and tabulated child anthropometry data from health surveys, literature, and national reports, and centralised them to inform the prevalence of WHZ decrement in each category corresponding to our case definitions. For details on estimation of wasting (WHZ < –2 and WHZ < –3) data identification and processing, see the methodological description of "Child growth failure" in the GBD 2023 Risk Factors appendix. Second, to inform the proportion of children under 5 years who have signs of organ failure manifested as oedema (ie, kwashiorkor), we used a compiled dataset of surveys conducted using Standardised Monitoring and Assessment of Relief and Transitions (SMART) methods. All data were extracted with the most detailed standard demographic identifiers available, including age, sex, country, year, and subnational location if available. No alternate case identifications were identified for oedema data, so no crosswalks were required or performed.

Modelling strategy

We used five parallel models to inform our estimates, all of which produced age-sex-specific results: 1) Prevalence of WHZ < –2 in children under 5 years in ST-GPR, 2) Prevalence of WHZ < –3 in children under 5 years in ST-GPR, 3) Proportion of those with WHZ < –2 who have oedema in under 5 years in DisMod-MR 2.1, 4) Proportion of those with WHZ < –3 who have oedema in under 5 years in DisMod-MR 2.1, and 5) Prevalence, incidence, and excess mortality of WHZ < –2 in all ages in DisMod-MR 2.1.

Using available information from scientific publications, which suggest the mean duration of illness is nine months, and conversations with collaborators and nutrition experts, we applied what we consider a plausible set of remission rate bounds of 0.25–1.25 (# of remitted cases of PEM per person-year of illness) to the final of the five models. These bounds allowed DisMod-MR to mathematically derive an internally consistent solution for incidence, prevalence, remission, excess mortality, and cause-specific mortality using all available data. This could only be done for the aggregate PEM definition (prevalence of WHZ < –2) to ensure that the case definition for prevalence matched that of the mortality results. The incidence-to-prevalence ratio derived from the final model was applied equally across all the categories of non-fatal PEM. Future work in systematically evaluating longitudinal datasets on nutrition and growth failure will allow us to improve the empirical basis for PEM incidence estimates, including improved resolution for the component categories.

For details on estimation of wasting (WHZ < –2 and WHZ < –3) estimation, see the methodological description of "Child Growth Failure" in the GBD 2023 Risk Factors appendix. Location-level covariate

effects for each of the three DisMod-MR 2.1 models are shown in the tables below. The two DisMod-MR models shown below for proportion of oedema among total and severe wasting were most recently run in GBD 2019.

Table 2a: Location-level covariate effects for proportion of oedema among total wasting

Measure	Covariate	Beta value	Exponentiated
Proportion	Energy unadjusted (kcal)	–1 (–1 to –1)	0.37 (0.37–0.37)
Proportion	Malnutrition shock log-transformed mortality rate	1 (1 to 1)	2.72 (2.72–2.72)

Table 2b. Location-level covariate effects for proportion of oedema among severe wasting

Measure	Covariate	Beta value	Exponentiated
Proportion	Energy unadjusted (kcal)	–1 (–1 to –1)	0.37 (0.37–0.37)
Proportion	Malnutrition shock log-transformed mortality rate	1 (1 to 1)	2.72 (2.72–2.72)

Table 2c. Location-level covariate effects for total wasting (moderate + severe, with and without oedema)

Measure	Covariate	Beta value	Exponentiated
Prevalence	Sanitation (prop access)	–0.00044 (–0.0016 to –0.000015)	1.00 (1.00–1.00)
Prevalence	Socio-demographic Index	–0.87 (–0.92 to –0.80)	0.42 (0.40–0.45)
Prevalence	Malnutrition shock, log-trans mortality rate	0.0028 (0.00042 to 0.0055)	1.00 (1.00–1.01)
Excess mortality rate	Healthcare Access and Quality Index	–0.064 (–0.064 to –0.063)	0.94 (0.94–0.94)

The results of the first four models were used for children under 5 years. Arithmetic transformations were performed to ensure that the final results fit into the mutually exclusive, collectively exhaustive categories of moderate and severe wasting, with and without oedema. We assumed zero prevalence of oedema in people over 5 years old. The results of the final model were used for all age groups 5 years and older, and the proportion of moderate versus severe wasting in each of those age groups was derived from the first set of models.

We applied disability weights from the GBD disability weight survey to the prevalence of the above sequelae according to their corresponding health state and severity level. The sequelae, along with their lay descriptions and disability weights for health states derived from the GBD disability weights study, are shown below. We assumed that those with moderate wasting, but no oedema, did not have any direct disability due to this condition.

Table 3. Sequelae, severity, lay description, and disability weights (DWs)

Sequela	Health state name	Lay description	DW (95% CI)
Moderate wasting without oedema	Asymptomatic	--	--
Moderate wasting with oedema	Kwashiorkor	Is very tired and irritable and has diarrhoea.	0.051 (0.031–0.079)
Severe wasting without oedema	Severe wasting	Is extremely skinny and has no energy.	0.128 (0.082–0.183)

Severe wasting with oedema	Kwashiorkor + severe wasting	Is very tired and irritable and has diarrhoea.	0.051 (0.031–0.079)
		Is extremely skinny and has no energy.	0.128 (0.082–0.183)

Following the assignment of disability weights to the various sequelae, the resulting years lived with disability (YLDs) go through the comorbidity simulator, which accounts for any comorbidity and corrects accordingly. The final outputs are comorbidity-adjusted YLDs, which are combined with years of life lost (YLLs) for final disability-adjusted life years (DALYs).

Other nutritional deficiencies

Other nutritional deficiencies encompass a wide variety of causes of morbidity, ranging from vitamin deficiencies to other nutritional anaemias. In GBD 2023, as done previously, we treat these causes as a single category, given their relatively limited burden, diversity in underlying causes and risk factors, and data availability. Instead of modelling them in a traditional modelling format, we calculate the YLDs associated with other nutritional deficiencies using a YLD/YLL ratio.

The first input for this non-fatal portion of other nutritional deficiencies burden is the YLL estimates from the GBD 2019 causes of death (CoD) analysis. The causes and their associated ICD-10 codes that constitute other nutritional deficiencies for CoD are listed below. Additionally, CoD includes specific models for protein-energy malnutrition, another nutritional cause of morbidity and mortality; as protein-energy malnutrition has a specific non-fatal model that results in YLDs, we can calculate the YLD/YLL ratio for protein-energy malnutrition. We multiply the YLL estimates for other nutritional deficiencies from CoD by the YLD/YLL ratio for PEM, providing us with an estimate of the YLDs associated with other nutritional deficiencies. There were no changes in modelling strategy for other nutritional deficiencies from GBD 2021.

Table 1. Definitions, ICD-10 codes and descriptions included in the other nutritional deficiencies model

GBD cause	ICD-10 code
Other nutritional deficiencies	D51-D52.0 (vitamin B12 deficiency anaemia and folate deficiency anaemia)
Other nutritional deficiencies	D52.8-D53.9 (other nutritional anaemias)
Other nutritional deficiencies	D64.3 (other sideroblastic anaemias)
Other nutritional deficiencies	E51-E61.9 (thiamine, niacin, other B group vitamins, ascorbic acid, vitamin D, other vitamin, dietary calcium, dietary selenium, dietary zinc, and other nutrient element deficiencies)
Other nutritional deficiencies	E63-E64.0 (other nutritional deficiencies and sequelae of protein-calorie malnutrition)
Other nutritional deficiencies	E64.2-E64.9 (sequelae of vitamin C deficiency, rickets, other nutritional deficiencies, and unspecified nutritional deficiencies)
Other nutritional deficiencies	M12.1-M12.19 (Kaschin-Beck disease)

References

1 Alexander Hsu, Peng Zheng, Kelsey Maass, Aleksandr Aravkin, Sameer Ali. pyDisagg: Dissaggregation under Generalized Proportionality Assumptions. 2025; published online Jan 13. DOI:10.5281/ZENODO.14641582.

2 Centers for Disease Control and Prevention (CDC). Iron deficiency--United States, 1999-2000. *MMWR Morb Mortal Wkly Rep* 2002; **51**: 897–9.

3 Looker AC, Dallman PR, Carroll MD, Gunter EW, Johnson CL. PRevalence of iron deficiency in the united states. *JAMA* 1997; **277**: 973–6.

Onchocerciasis

Flowchart

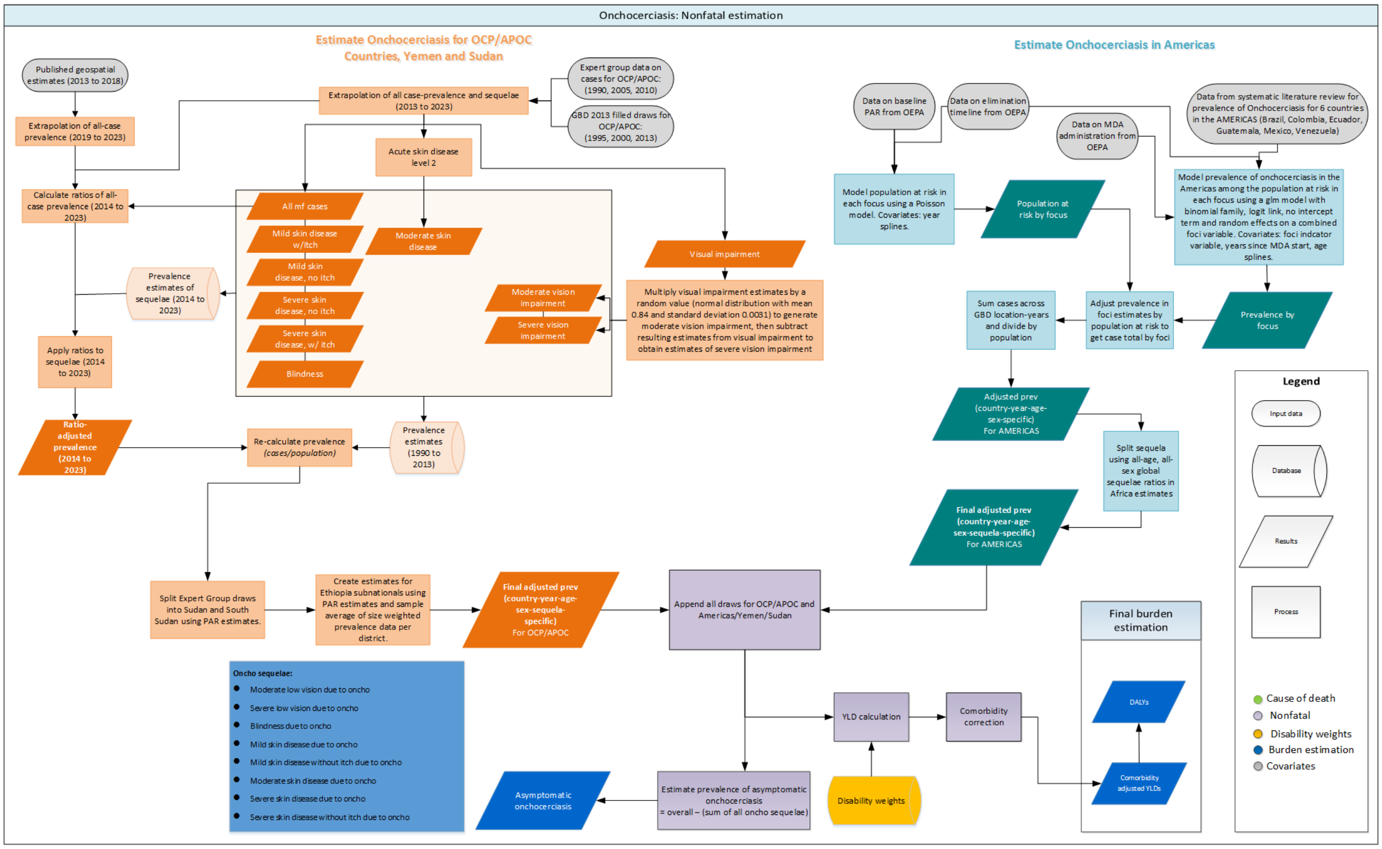

Input data and methodological summary for onchocerciasis

Case definition

Onchocerciasis, also known as river blindness, is a parasitic disease caused by *Onchocerca volvulus*. It is transmitted via the bite of one of several species of *Similium* blackflies that have historically bred in fast-moving freshwater rivers and tributaries throughout sub-Saharan Africa, Central America, and South America. Diagnosis can be made by skin snip biopsy to identify larvae, surgical removal of nodules and exam for adult worms, slit lamp exam of anterior part of the eye where larvae or lesions caused by them are visible, and antibody tests (mostly useful to visitors to areas with parasites). The ICD-10 code for onchocerciasis is B73. We used the following case definition for GBD 2023:

Quantity of interest	Reference or alternative	Definition
Onchocerciasis	Reference	Presence of *O volvulus* microfilariae in a skin snip under microscopy.

Input data

Sub-Saharan Africa, 1990–2013

Prevalence data prepared by the GBD 2010 and 2013 expert groups (EG) were used for modelling the non-fatal outcomes resulting from onchocerciasis in Africa. This included 1000 draws of infection and morbidity (visual impairment, blindness, and skin conditions) cases with confidence intervals categorised by country, age, and sex for years 1990, 1995, 2000, 2005, 2010, and 2013. Details about the materials and methods used by the EG to generate these draws can be found elsewhere.[1-5] These data represented all African countries included in the African Programme for Onchocerciasis Control (APOC) and the Onchocerciasis Control Programme (OCP) for which initial Rapid Epidemiological Mapping of Onchocerciasis (REMO) assessments demonstrated a need for Community-Directed Treatment with Ivermectin (CDTI) (defined as having a prevalence of skin nodules greater than 20%). Four countries – Rwanda, Mozambique, Kenya, and Gabon – were designated as hypo-endemic countries after initial REMO assessments and not included due to sparsity of cases and paucity of data. Estimates for Sudan from GBD 2010 were reassigned to South Sudan in GBD 2013 after its independence in 2011 since REMO assessments indicated that the vast majority of cases occurred in that area of the former Sudan. The tables below show the countries included in each programme and the number of corresponding GBD locations they represent.

Table 1. APOC and OCP countries

	**APOC Countries**	**OCP Countries**
Countries included	Angola, Burundi, Cameroon, Central African Republic, Chad, Congo, Democratic Republic of Congo, Ethiopia, Equatorial Guinea, Liberia, Malawi, Nigeria, South Sudan, Tanzania, and Uganda	Benin, Burkina Faso, Côte d'Ivoire, Ghana, Guinea Bissau, Guinea, Mali, Niger, Senegal, Sierra Leone, and Togo
Hypo-endemic countries not included	Rwanda, Mozambique, Kenya, Gabon, Sudan	
GBD countries & subnationals provided by EG	15	11
GBD world regions	3	1

Sub-Saharan Africa, 2015–2023

For GBD 2023, estimates from a published geospatial analysis[1] were utilised to project the EG estimates for years post-2015.

Americas

Prevalence data for modelling non-fatal outcomes resulting from onchocerciasis in the Americas were extracted via a systematic literature review (last updated for GBD 2019). Web of Science, Scopus, and PubMed were searched with the following search strings:

Table 2. Search strings for systematic review of onchocerciasis in the Americas

Database	Search string	Yield
PubMed	(oncho*[Title/Abstract] OR "river blindness"[Title/Abstract] OR "O. volvulus"[Title/Abstract] OR "robles disease"[Title/Abstract] OR "blinding filariasis"[Title/Abstract] OR "coast erysipelas"[Title/Abstract] OR “sowda” [Title/Abstract] OR “nodding syndrome”[Title/Abstract]) AND (“1980”[Date – Publication] : “2016”[Date – Publication]) AND (epidemiology[Title/Abstract] OR prevalence[Title/Abstract] OR incidence[Title/Abstract] OR surveillance[Title/Abstract] OR”MDA”[Title/Abstract] OR “Mass Drug Administration”[Title/Abstract] OR “Community-directed treatment with ivermectin”[Title/Abstract] OR “CDTI”[Title/Abstract] OR “mass treatment”[Title/Abstract] OR “multiple ivermectin treatments”[Title/Abstract] OR “monthly doses of ivermectin”[Title/Abstract] OR “large scale treatment”[Title/Abstract] OR REMO[Title/Abstract] OR “Rapid epidemiological mapping of onchocerciasis”[Title/Abstract] OR APOC[Title/Abstract] OR “African Programme for Onchocerciasis Control”[Title/Abstract] OR OCP[Title/Abstract] OR “Onchocerciasis Control Programme”[Title/Abstract]) NOT(Animals[MeSH] NOT Humans[MeSH])	986
Web of Science	TS=(oncho* OR "river blindness" OR "O. volvulus" OR "robles disease" OR "blinding filariasis" OR "coast erysipelas" OR sowda OR “nodding syndrome”) AND TS=(epidemiology OR prevalence OR incidence OR surveillance OR MDA OR “Mass Drug Administration” OR “Community-directed treatment with ivermectin” OR CDTI OR “mass treatment” OR “multiple ivermectin treatments” OR “monthly doses of ivermectin” OR “large scale treatment” OR REMO OR “Rapid epidemiological mapping of onchocerciasis” OR APOC OR “African Programme for Onchocerciasis Control” OR OCP OR “Onchocerciasis Control Programme”) NOT TS=((Animals NOT Humans))	1144
SCOPUS	(TITLE-ABS-KEY(oncho* OR "river blindness" OR "O. volvulus" OR "robles disease" OR "blinding filariasis" OR "coast erysipelas")) AND TITLE-ABS-KEY(epidemiology OR prevalence OR incidence OR surveillance OR MDA OR "Mass Drug Administration" OR "Community-directed treatment with ivermectin" OR CDTI OR "mass treatment" OR "multiple ivermectin treatments" OR "monthly doses of ivermectin" OR "large scale treatment" OR REMO OR "Rapid epidemiological mapping of onchocerciasis" OR APOC OR "African Programme for Onchocerciasis Control" OR OCP OR "Onchocerciasis Control Programme") AND NOT KEY(Animals NOT Humans) AND PUBYEAR > 1979	2000

This yielded 4130 results in total, which was reduced to 2502 after removing duplicates. The titles and abstracts were screened for inclusion or exclusion with the following criteria:

Exclusion criteria:

- Pre-1980
- Non-original source
- Non-representative population
  - Vulnerable populations (eg, slum-dwellers, prisoners, orphans, high-risk jobs, etc.)
  - Hospital-based samples (including saved stool samples)
  - Non-native peoples (eg, migrants, expats, nomads, etc.)
  - Immunosuppression/illness (eg, HIV, TB, CA, RA, asthma, malaria, handicap, etc.)
- Non-human population
- Does not meet case definition
- Case-control study

61 articles were identified for full text screening and extraction from the historically endemic American countries: Guatemala, Brazil, Ecuador, Venezuela, Mexico, and Colombia.

Modelling strategy

Sub-Saharan Africa, 1990–2013

In the first step of the non-fatal modelling for onchocerciasis, EG estimates of prevalence were exponentially extrapolated to obtain estimates post-2013. Acute skin disease level 2 was mapped to the moderate skin disease sequela. Uncertainty was quantified and provided by the EG for all estimates except OCP cases of visual impairment and blindness. Uncertainty was added during the splitting process of visual impairment into moderate and severe vision impairment sequelae. The process includes multiplying visual impairment estimates by a random value from a normal distribution with mean 0.84 and standard deviation 0.0031 to generate moderate visual impairment (details of calculation described elsewhere[2]). The resulting estimates are subtracted from visual impairment to generate severe vision impairment. Prevalence of sequelae was calculated by dividing the cases by the population.

Sub-Saharan Africa, 2015–2023

In previous GBD rounds, no further adjustments were applied to the extrapolated estimates. For GBD 2023, the rates of change from published geospatial estimates[1] were used to adjust the time trends for total prevalence of onchocerciasis to better reflect epidemiological trends. Using geospatial predictions from 2013–2018, a linear model was fit to predict estimates through 2023. The estimates were then adjusted using the log difference of the geospatial and EG estimates in the year 2013. Ratios between the EG and the adjusted geospatial estimates were then calculated for each year post-2014 and subsequently applied to each of the sequelae from the prior EG extrapolation.

Subnationals

The next step in modelling morbidity begins with the process of estimating the prevalence of onchocerciasis in GBD subnational locations. In Nigeria, we assume no subnational prevalence variation, and thus subnational prevalence estimates are set equal to national estimates.

Prevalence for the Ethiopia subnationals was estimated separately and appended to the Africa model. Subnational draws were split proportionally based on sample size weighted prevalence from prevalence data, using population at risk (PAR) estimates derived from digitising a map of onchocerciasis-endemic districts in 2015 from Meribo and colleagues to convert into case space using GBD populations.[3] In prior rounds, WorldPop estimates from 2015 were used for population. A proportion of cases falling into each subnational was then used to split national case numbers provided by EG draws into each subnational. The cases were further split by age-sex proportions within each subnational. This is a change from the previous round, where the same split occurred across each age-sex within a subnational.

Splits for Sudan/South Sudan

Since EG draws were provided before the independence of South Sudan in 2011, Sudan estimates from the EG were partitioned between Sudan and South Sudan. PAR estimates pre- and post-Abu Hamed foci elimination in 2015 in Sudan were used to proportionally split cases between the two countries.[4] REMO maps showing definite needs for CDTI were digitised and overlaid with population per pixel rasters to

produce estimates of PAR pre-Abu Hamed elimination. Post-Abu Hamed elimination in 2015, REMO maps were edited to remove the foci as definite CDTI areas and estimates were reproduced.

Yemen

Prevalence of onchocerciasis in Yemen was modelled separately and combined with the Africa model. Due to limited data, this was done utilising one datapoint from the Ministry of Health published in 1991 only accounting for population change.[23] Furthermore, the global age-sex trend was imposed to produce age-sex-specific estimates. The clinical manifestations of onchocerciasis in Yemen differ from those observed in other regions, almost exclusively consisting of onchodermatitis without ocular involvement.[24] All cases of onchocerciasis in Yemen were mapped to mild skin disease due to onchocerciasis without itch, though future efforts will seek to identify additional data to better inform severity distributions in Yemen.

Americas

In the next step, prevalence of onchocerciasis in the Americas was modelled separately and combined with the Africa and Yemen models. For the GBD estimation period, onchocerciasis is known to have occurred in six countries of Central and Southern America: Mexico, Guatemala, Colombia, Ecuador, Brazil, and Venezuela. The epidemiology of onchocerciasis is very different in these countries than in Africa because it has only occurred in relatively small, well-defined foci. These foci have been mapped and thoroughly monitored since the early 1990s with the formation of the Onchocerciasis Elimination Program of the Americas (OEPA) and all of the prevalence surveys conducted are only representative of these areas. Additionally, certain foci are geographically continuous across national boundaries. Therefore, we modelled onchocerciasis in these countries at the focus level among the PAR in each focus instead of at the national level.

PAR for each focus was modelled using data from OEPA on baseline population at risk[7] and data from OEPA and peer-reviewed studies on dates of elimination in each focus.[7-22] This was done with a Poisson model using year splines as a covariate, and 1000 draws of the population at risk were drawn from the predicted mean and standard error. The prevalence of disease among the population at risk was subsequently modelled using a generalised linear model with a binomial family, logit link, no intercept term, and random effects on a combined-foci variable created by grouping foci by geographical contiguity and nearness when data were sparse. Covariates included an indicator term on the foci, the number of years since MDA began, and splines on age. 1000 draws of prevalence were calculated from 1000 draws of beta values from the variance-covariance matrix and adjusted by the estimated population at risk in each focus-year to determine the number of cases. The cases were then summed by GBD geography and year and divided by national population to find the national prevalence. While the model predicted case values very close to zero in the countries where elimination has occurred, these were overwritten to zero values for all years after certified elimination. The ratio of global all-age, all-sex prevalence of each sequela to the all-cases prevalence from the Africa estimates was applied to all-cases prevalence from the Americas to calculate prevalence of each sequela.

Severity splits/sequelae

To estimate the prevalence of asymptomatic onchocerciasis, the prevalence of morbidity (vision loss, blindness, and skin conditions) was subtracted from the overall onchocerciasis prevalence. Moderate vision impairment, severe vision impairment, and blindness estimates were each multiplied by a factor of 8/33 (details of calculation described elsewhere[2]) before subtraction to account for cases that have concurring symptoms.

The table below shows the list of common clinical manifestations of onchocerciasis and the sequelae to which they have been mapped along with the lay description and the associated disability weight (DW) of each sequela.

Table 3: Severity distribution, details on severity levels for onchocerciasis and the associated disability weight (DW) with that severity

**Clinical manifestation**	**Sequela name**	**Lay description**	**DW (95% UI)**
Uveitis; Punctate keratitis; Optic neuritis; Torpid Iritis; Onchochorioretinitis	Moderate vision impairment	Has vision problems that make it difficult to recognise faces or objects across a room.	0.031 (0.019–0.049)
Sclerosing keratitis; Optic neuropathy; Optic atrophy; Choroidoretinopathy; Cataracts	Severe vision impairment	Has severe vision loss, which causes difficulty in daily activities, some emotional impact (for example worry), and some difficulty going outside the home without assistance.	0.184 (0.125–0.258)
Blindness	Blindness	Is completely blind, which causes great difficulty in some daily activities, worry and anxiety, and great difficulty going outside the home without assistance.	0.187 (0.124–0.260)
Acute papular onchodermatitis; Onchocercomata (subcutaneous nodules)	Mild skin disease	Has a slight, visible physical deformity that is sometimes sore or itchy. Others notice the deformity, which causes some worry and discomfort.	0.027 (0.015–0.042)
Chronic papular onchodermatitis; Lichenified onchodermatitis (“sowda”); Lymphadenopathy	Mild skin disease without itch	Has a slight, visible physical deformity that others notice, which causes some worry and discomfort.	0.011 (0.005–0.021)
Skin atrophy; Depigmentation (“leopard skin”)	Moderate skin disease	Has a visible physical deformity that is sore and itchy. Other people stare and comment, which causes the person to worry. The person has trouble sleeping and concentrating.	0.188 (0.124–0.267)
Hanging groin; Lymphoedema	Severe skin disease without itch	Has an obvious physical deformity that makes others uncomfortable, which causes the person to avoid social	0.405 (0.275–0.546)

		contact, feel worried, sleep poorly, and think about suicide.	
NA	Asymptomatic onchocerciasis	NA	NA

Changes from GBD 2021 to GBD 2023

We updated the estimation of prevalence for onchocerciasis for the later time series, 2014–2023, using a ratio comparison derived from previously published geospatial estimates (methods described above), resulting in improved time trends for recent years.

In addition, the process used to estimate PAR in Ethiopia subnationals was updated.

Limitations

As we attempt to improve the modelling processes for onchocerciasis, we recognise limitations. While the inclusion of incorporating geospatial estimates improved the time trends for later years, we recognise that not all sequelae have the same rate of change. In future rounds, we will investigate a transition to a full geospatial model to better account for variability across sequelae, location and time.

We did not apply any adjustments for the COVID-19 pandemic to onchocerciasis due to a lack of available data quantifying the impacts of the pandemic on NTD epidemiology.

References

1. Schmidt CA, Cromwell EA, Hill E, Donkers KM, Schipp MF, Johnson KB, Pigott DM; LBD 2019 Neglected Tropical Diseases Collaborators; Hay SI. The prevalence of onchocerciasis in Africa and Yemen, 2000-2018: a geospatial analysis. *BMC Med*. 2022 Sep 7;20(1):293.
2. Causes of blindness and vision impairment in 2020 and trends over 30 years, and prevalence of avoidable blindness in relation to VISION 2020: the Right to Sight: an analysis for the Global Burden of Disease Study. *Lancet Glob Health* 2020; **9**: e144–60.
3. Meribo K, Kebede B, Feleke SM, *et al.* Review of Ethiopian Onchocerciasis Elimination Programme. *Ethiop Med J* 2017; **55**: 55–63.
4. Zarroug IMA, Hashim K, ElMubark WA, *et al.* The First Confirmed Elimination of an Onchocerciasis Focus in Africa: Abu Hamed, Sudan. *Am J Trop Med Hyg* 2016; **95**: 1037–40.
5. Noma M, Zouré HGM, Tekle AH, Enyong PAI, Nwoke BEB, Remme JHF. The geographic distribution of onchocerciasis in the 20 participating countries of the African Programme for Onchocerciasis Control: (1) priority areas for ivermectin treatment. *Parasit Vectors* 2014; **7**: 325.
6. Coffeng LE, Stolk WA, Hoerauf A, *et al.* Elimination of African onchocerciasis: modeling the impact of increasing the frequency of ivermectin mass treatment. *PLoS One* 2014; **9**: e115886.
7. Murdoch ME, Asuzu MC, Hagan M, Makunde WH, Ngoumou P, Ogbuagu KF, Okello D, Ozoh G, Remme J. Onchocerciasis: the clinical and epidemiological burden of skin disease in Africa. Ann Trop Med Parasitol. 2002; 96(3): 283-296.
8. Brieger WR, Awedoba AK, Eneanya CI, Hagan M, Ogbuagu KF, Okello DO, Ososanya OO, Ovuga EB, Noma M, Kale OO, Burnham GM, Remme JH. The effects of ivermectin on onchocercal skin

disease and severe itching: results of a multicentre trial. Trop Med Int Health. 1998; 3(12): 951-61.
9. México. http://www.oepa.net/Mexico.htm (accessed July 7, 2017).
10. Guatemala. http://www.oepa.net/guatemala.html (accessed July 7, 2017).
11. Venezuela. http://www.oepa.net/venezuela.html (accessed July 7, 2017).
12. Colombia. http://www.oepa.net/colombia.html (accessed July 7, 2017).
13. Ecuador. http://www.oepa.net/ecuador.html (accessed July 7, 2017).
14. Rodríguez-Pérez MA, Unnasch TR, Domínguez-Vázquez A, *et al.* Lack of Active Onchocerca volvulus Transmission in the Northern Chiapas Focus of Mexico. *The American Journal of Tropical Medicine and Hygiene* 2010; **83**: 15–20.
15. Rodríguez-Pérez MA, Domínguez-Vázquez A, Unnasch TR, *et al.* Interruption of Transmission of Onchocerca volvulus in the Southern Chiapas Focus, México. *PLOS Neglected Tropical Diseases* 2013; **7**: e2133.
16. Rodríguez-Pérez MA, Unnasch TR, Domínguez-Vázquez A, *et al.* Interruption of Transmission of Onchocerca volvulus in the Oaxaca Focus, Mexico. *The American Journal of Tropical Medicine and Hygiene* 2010; **83**: 21–7.
17. Cruz-Ortiz N, Gonzalez RJ, Lindblade KA, *et al.* Elimination of Onchocerca volvulus Transmission in the Huehuetenango Focus of Guatemala. Journal of Parasitology Research. 2012. https://www.hindawi.com/journals/jpr/2012/638429/abs/ (accessed July 7, 2017).
18. Jr FR, Rizzo N, Espinoza CED, *et al.* One Hundred Years After Its Discovery in Guatemala by Rodolfo Robles, Onchocerca volvulus Transmission Has Been Eliminated from the Central Endemic Zone. *The American Journal of Tropical Medicine and Hygiene* 2015; **93**: 1295–304.
19. Gonzalez RJ, Cruz-Ortiz N, Rizzo N, *et al.* Successful interruption of transmission of Onchocerca volvulus in the Escuintla-Guatemala focus, Guatemala. *PLoS Negl Trop Dis* 2009; **3**: e404.
20. Lindblade KA, Arana B, Zea-Flores G, *et al.* Elimination of Onchocercia volvulus transmission in the Santa Rosa focus of Guatemala. *Am J Trop Med Hyg* 2007; **77**: 334–41.
21. Convit J, Schuler H, Borges R, *et al.* Interruption of Onchocerca volvulus transmission in Northern Venezuela. *Parasites & Vectors* 2013; **6**: 289.
22. WHO | WHO declares Ecuador free of onchocerciasis (river blindness). WHO. http://www.who.int/neglected_diseases/ecuador_free_from_onchocerciasis/en/ (accessed July 7, 2017).
23. WHO Expert Committee on Onchocerciasis Control (1993 : Geneva, Switzerland) & World Health Organization. (1995). Onchocerciasis and its control : report of a WHO Expert Committee on Onchocerciasis Control. World Health Organization. https://iris.who.int/handle/10665/37346
24. Al-Kubati A-S, Mackenzie CD, Boakye D, *et al.* Onchocerciasis in Yemen: moving forward towards an elimination program. *International Health* 2018; **10**: i89–96.

Opioid use disorders

Flowchart

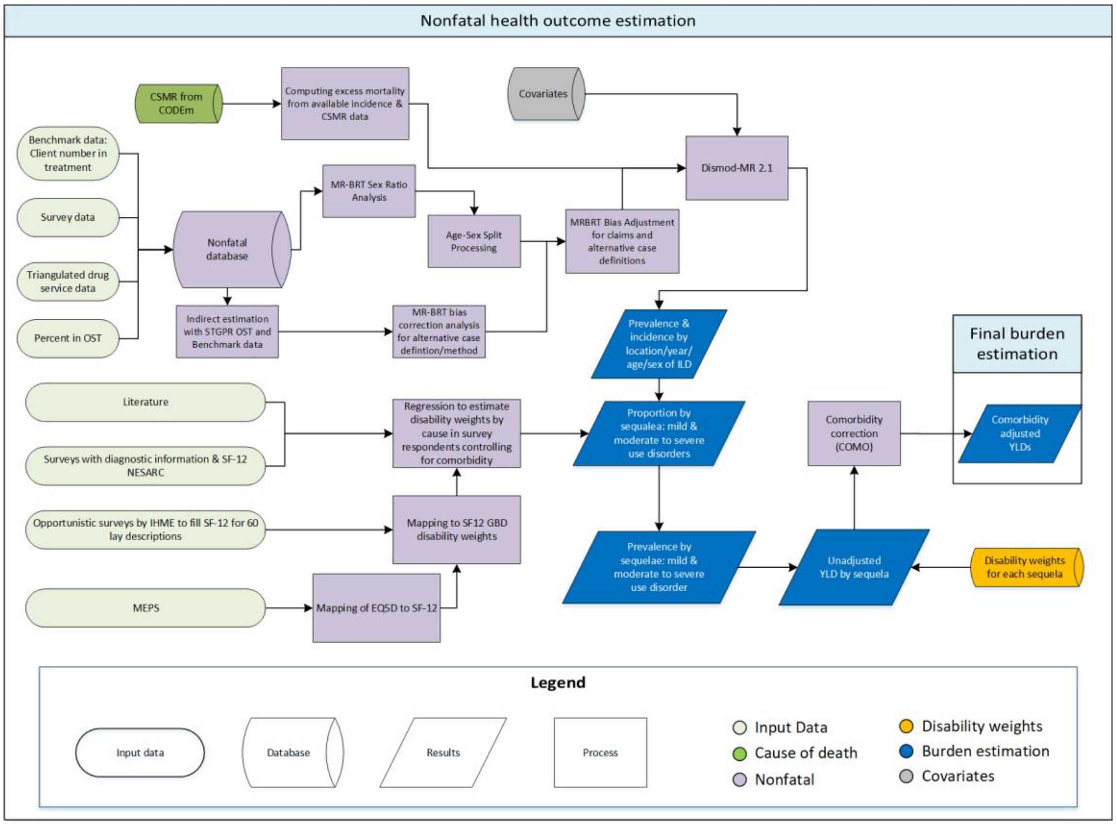

Input data and methodological summary for opioid use disorders

Case definition

We define opioid use disorders as "a maladaptive pattern of opioid abuse, leading to clinically significant impairment or distress that includes symptoms of dependence, such as withdrawal symptoms or progressive tolerance." Opioid dependence is a substance-related disorder involving a dysfunctional pattern of opioid use. The GBD disease modelling included cases that met the diagnostic criteria for opioid dependence as defined by the Diagnostic and Statistical Manual of Mental Disorders (DSM-IV-TR) and the International Classification of Diseases (ICD). These criteria encompass the following codes from DSM-IV and ICD-9 (304.0, 305.5, 965.0, 970.1, E850.0, E935, E940.1) and ICD-10 (F11.2, T14.0, Z79.8), excluding cases attributed to a general medical condition [1,2,3]. To meet the DSM-IV TR criteria, at least three of the following symptoms must be experienced within the same 12-month period:

- Tolerance, characterised by either
    - A need for increased amounts of the substance to achieve intoxication; or

- Markedly diminished effect with continued use of the same amount of the substance;

- Withdrawal, characterised by either
    - Withdrawal symptoms characteristic to dependence; or
    - The same (or similar) substance is taken to avoid withdrawal symptoms;
- Substance taken in progressively larger amounts or for longer period;
- Persistent desire or unsuccessful efforts to reduce substance use;
- Disproportionate time dedicated to obtaining the substance;
- Other important activities are given up because of the substance use; and
- Substance use is continued despite knowledge of physical or psychological problems occurring as a result of the substance.

**Quantity of interest**	**Reference or alternative**	**Definition**
Opioid use disorders	Reference	Opioid dependence derived from using an "indirect method", ie, using a multiplier from treatment data and surveys among drug users on prevalence of having accessed treatment over a recall period (mostly one year); or using a multiplier from notified cases of HIV deemed to have contracted the disease through injecting opioids and a survey of seroprevalence of HIV among those with opioid dependence
Opioid use disorders	Alternative	Opioid dependence derived from using "direct method", ie, in a population survey
Opioid use disorders	Alternative	A chronic condition in which a person craves opioids and is unable to control taking the drug. The person needs greater amounts to get the same effect and has withdrawal symptoms after stopping

Input data

Systematic reviews were conducted in GBD 2010 and GBD 2017. The first review was repeated in GBD 2013 and GBD 2016 to update literature sources. The GBD 2017 review was targeted towards Māori and non-Māori populations in New Zealand, and cases in China, using primarily the China National Knowledge Infrastructure database. The inclusion criteria stipulated that 1) the publication year must be from 1980 onward; 2) "cases" must be based on clinical threshold as established by the DSM or ICD; 3) sufficient information must be provided on study method and sample characteristics to assess the quality of the study; and (4) study samples must be representative of the general population (ie, inpatient or pharmacological treatment samples, case studies, and veterans or refugee samples were excluded). No limitation was set on the language of publication. Methods used for this systematic review have been reported in greater detail elsewhere.[4,5]

In GBD 2021, we included data utilising sources of opioid users in substitution therapy and literature surveys of percentage of drug users in substitution therapy. Inclusion criteria for these sources were 1) currently in substitution treatment; 2) primary drug of use disorder was opioids, including synthetic

opiates. We used these data to construct indirect estimates of prevalence of opioid use disorder. For details on these data and process, please see the IHME-indirect data creation.

Age and sex splitting

Reported estimates of prevalence were split by age and sex where possible. First, if studies reported prevalence for broad age groups by sex (eg, prevalence in 15- to 65-year-old males and females separately), and also by specific age groups for both sexes combined (eg, prevalence in 15- to 30-year-olds, then in 31- to 65-year-olds, for males and females combined), age-specific estimates were split by sex using the reported sex ratio and bounds of uncertainty. Second, prevalence data for both sexes that could not be split using a within-study ratio were split using a sex ratio derived from a meta-analysis of existing sex-specific data using MR-BRT (meta-regression—Bayesian, regularised, trimmed; described in appendix 1, section 2). The female to male ratio was 0.61 (0.51–0.73) for ages 20 and above and 1.12 (0.92–1.35) for ages below 20. Finally, after the application of bias adjustments, where studies reported estimates across age groups spanning 25 years or more, these were split into five-year age groups using the prevalence age pattern estimated by DisMod-MR 2.1 (disease model—Bayesian meta-regression tool) on all data prior to age-splitting. More details on DisMod-MR 2.1 can be found in appendix 1 section 2.

IHME-indirect data creation

Prevalence data include data created by IHME using an indirect multiplier method. This is a method of matching two datasets with partial information about opioid users to estimate the total population. This is already the primary type of data in previous iterations of opioid use disorder modelling.

The first source of data comes primarily from government records of the number of people with opioid dependence in substitution therapy. The second source of data comes from literature sources that describe the percentage of people with opioid dependence in treatment.

We run the data on percentage of dependents in treatment through our spatiotemporal Gaussian process regression tool (ST-GPR, details in appendix 1, section 2) model to get coverage estimates by every year, location, and sex. We assume treatment percentage doesn't differ by age due to data constraints.

We then calculate the following to get the total population of people with opioid dependence:

Opioid population = Number in treatment / ST-GPR estimated coverage; year, sex, location

This opioid population estimate is divided by the total population in each location-year-sex grouping to get an indirect estimate of opioid prevalence.

Data adjustment

The prevalence dataset included datapoints of both use and dependence estimated using "direct" or "indirect" survey methods. "Direct" methods of measuring opioid dependence predominantly involve surveys of the general population that ask if respondents use or are dependent on opioids. Surveys tend to underestimate the prevalence of the most harmful and stigmatised forms of illicit drug use in ways that probably vary between countries and cultures.[6] "Indirect" methods are considered superior, but they use different sources of data to indirectly estimate the total number of drug users (methods

include “multiplier methods,” back-projection and capture-recapture methods) that are often poorly documented. In GBD 2019, direct surveys of opioid dependence were adjusted by a factor derived from MR-BRT by the logit differences to indirect literature data. In GBD 2021 and onward, we modified this approach to use the IHME-indirect data instead of the indirect literature to create the adjustment factor instead. We updated our data adjustment process by matching direct surveys to the IHME-indirect created data to increase the amount of information available for creating adjustment factors using MR-BRT. The beta and exponentiated value for this covariate are shown in the table below:

Table 2: MR-BRT crosswalk adjustment factors for opioid use disorder

Data input	Status	Gamma	Beta coefficient, logit*	Adjustment factor**
IHME-indirect created opioid dependence data	Ref	0.24	---	---
Opioid dependence – direct method	Alt		–1.07	0.25

**MR-BRT crosswalk adjustments can be interpreted as the factor the alternative case definition is adjusted by to reflect what it would have been had it been measured using the reference case definition. If the log/logit beta coefficient is negative, then the alternative is adjusted up to the reference. If the log/logit beta coefficient is positive, then the alternative is adjusted down to the reference.*

***The adjustment factor column is the exponentiated beta coefficient. For log beta coefficients, this is the relative rate between the two case definitions. For logit beta coefficients, this is the relative odds between the two case definitions.*

Modelling strategy

Prior settings in DisMod included assuming no incidence and excess mortality before age 15. This minimum age of onset was corroborated with expert feedback and existing literature on opioid dependence. We also assumed no incidence after age 64 as supported by data from various sources including the European Monitoring Centre for Drugs and Drug Addiction.[7] An upper limit of 0.2 was placed on remission consistent with limits in the dataset. These settings were retained for GBD 2023.

As in GBD 2021, age-standardised prevalence of intravenous drug use and log-transformed estimates of defined daily doses for statistical purposes (SDDD; consumption per day per million population) of prescribed opioid analgesics were included as country-level covariates. SDDD were modelled in GBD 2017 via spatiotemporal Gaussian process regression (ST-GPR) using data supplied by the International Narcotics Control Board (INCB). These 2017 estimates were carried forward into 2023. Subnational estimates for the USA were estimated by crosswalking national estimates with the state/national ratios of opioid prescriptions per 100 persons supplied by the Centers for Disease Control and Prevention.

We continued to generate excess mortality rate (EMR) data using the MR-BRT method, stratified by age and sex, with a prior assumption that the Healthcare Access and Quality (HAQ) Index has a negative association with EMR. The MR-BRT results were then used to predict EMR for each location, year, sex, and for ages 0, 10, 20, ..., 100. The HAQ Index was included as a country-level covariate to inform EMR estimates, with the mean and standard deviation derived from MR-BRT outputs.

In previous modelling rounds, EMR priors were estimated in DisMod by matching prevalence datapoints with their corresponding cause-specific mortality rates (CSMR) within the same age, sex, year, and location (by dividing CSMR by prevalence). However, for many causes, DisMod produced unrealistic EMR patterns which did not align with the expected trend of decreasing EMR with improved health-care access and quality. These discrepancies often indicated inconsistencies between CSMR estimates and measures of prevalence and/or incidence.

For opioid use disorder, the MR-BRT analysis did not find evidence to support the assumed negative relationship between the HAQ Index and EMR, indicating that the HAQ Index did not significantly impact EMR. As a result, EMR predictions were consistent across locations with both high and low HAQ Index values, suggesting that the data did not align with the expectation set by the negative prior. This outcome was primarily driven by data from high-income regions and the central Europe, eastern Europe, and central Asia super-regions, where the majority of the data originate. Consequently, this approach produced prevalence estimates that closely aligned with cause-of-death estimates.

Estimates for Afghanistan and Iran, two countries within the generally low-prevalence north Africa and Middle East super-region, were significantly constrained despite having some of the highest prevalence input data globally. These constraints were due to low values for the intravenous drug use and prescription opioid covariates in both countries, which resulted in country priors much lower than their reported prevalence data. Additionally, intravenous drug use was included as a country-level covariate for EMR, with its influence restricted to a range between 0 and 2.

Table 3. Covariates. Summary of covariates used in the opioid use disorders DisMod-MR meta-regression model

**Covariate**	**Parameter**	**Beta, log (95% uncertainty interval)**	**Exponentiated beta (95% uncertainty interval)**
Intravenous drug use (age-standardised proportion)	Prevalence	0.26 (0.036–0.47)	1.29 (1.04–1.60)
Opioids per million population per day (10-year lag)	Prevalence	0.097 (0.084–0.11)	1.10 (1.09–1.12)
Intravenous drug use (age-standardised proportion)	Excess mortality rate	1.92 (1.81–2.00)	6.84 (6.12–7.36)

Note, a bound was set on the coefficient for opioids per million per day in an effort to make the model follow the high prevalence data in Iran and Afghanistan more closely.

Severity and disability

The basis of the GBD disability weight survey assessments are lay descriptions of health states highlighting major functional consequences and symptoms. The lay descriptions and disability weights for opioid dependence severity levels are shown below.

Table 4. Severity distribution, details on the severity levels for opioid use disorders in GBD 2023 and the associated disability weight (DW) with that severity

Severity level	Lay description	DW (95% CI)
Mild	Uses heroin (or methadone) daily and has difficulty controlling the habit. When not using, the person functions normally.	0.335 (0.221–0.473)
Moderate to severe	Uses heroin daily and has difficulty controlling the habit. When the effects wear off, the person feels severe nausea, agitation, vomiting, and fever. The person has a lot of difficulty in daily activities.	0.697 (0.510–0.843)

**Asymptomatic cases carried no disability weight.*

The proportion of people with opioid dependence within each of the severity levels was determined based on available data from US National Epidemiological Survey on Alcohol and Related Conditions (NESARC), conducted in two waves from 2001–2002 and 2004–2005,[8] and the Comorbidity and Trauma study conducted in 2005–2008.[9] NESARC is a direct household survey. As such, it is expected to underestimate moderate to severe cases of drug dependence. The estimated distribution of opioid dependent cases by severity were asymptomatic (16%, 13–19), mild (37%, 20–55), and moderate/severe (47%, 29–64).

References

1. American Psychiatric Association. Diagnostic and Statistical Manual of Mental Disorders (DSM-IV-TR). 4th, Text Revision ed Washington DC: American Psychiatric Association; 2000.
2. World Health Organization. The ICD-10 Classification of Mental and Behavioural Disorders. Clinical descriptions and diagnostic guidelines Geneva: World Health Organization; 1992.
3. National Institute on Drug Abuse (US). Opioid Use Disorder. In: Medications for Opioid Use Disorder Save Lives. Washington (DC): National Academies Press (US); 2019. Table 7, Opioid Use Disorder Codes (ICD-9 and ICD-10). Available from: https://www.ncbi.nlm.nih.gov/books/NBK557173/table/sb256.tab7/

4. Degenhardt L, Bucello C, Calabria B, Nelson P, Roberts A, Hall W, et al. What data are available on the extent of illicit drug use and dependence globally? Results of four systematic reviews. *Drug and alcohol dependence*. 2011.
5. Calabria B, Degenhardt L, Briegleb C, Vos T, Hall W, Lynskey M, et al. Systematic review of prospective studies investigating "remission" from amphetamine, cannabis, cocaine or opioid dependence. *Addictive Behaviors*. 2010.
6. Shand FL, Degenhardt L, Slade T, Nelson EC. Sex differences amongst dependent heroin users: Histories, clinical characteristics and predictors of other substance dependence. *Addictive behaviors*. 2011; 36(1): p. 27-36.
7. European Monitoring Centre for Drugs and Drug Addiction. Lisbon, Portugal; 2014.
8. Grant BF, Dawson DA. National Institute on Alcohol Abuse and Alcoholism. *Alcohol Health & Research World*. 2006; 29(2): p. 74.
9. Shand FL, Slade T, Degenhardt L, Baillie A, Nelson EC. Opioid dependence latent structure: two classes with differing severity? *Addiction*. 2011; 106(3): p. 590-8.

Oral disorders

This document describes the non-fatal disease burden modelling process for GBD 2023 for each of edentulism, caries of deciduous teeth, caries of permanent teeth, chronic periodontal disease, and other oral disorders.

Input data and methodological summary for oral disorders

Input data

Data seeking and systematic literature reviews were completed for all oral disorders together, given the overlap in data types and data sources that inform the models. An initial literature review was done by the Expert Group for GBD 2010 in PubMed, Embase, Latin American and Caribbean Health Sciences (LILACS), and Scientific Electronic Library Online (SciELO), including published articles as well as the results of national and subnational reports. An updated systematic review was last completed on February 11, 2018, for GBD 2017 in PubMed and Embase. The search strings used are below:

> **PubMed:** ((((Deciduous caries[Title/Abstract]) OR (milk caries[Title/Abstract]) OR (baby caries[Title/Abstract]) OR (caries[Title/Abstract]) OR (dental health[Title/Abstract]) OR (oral health[Title/Abstract])) OR ((Permanent caries[Title/Abstract]) OR (caries prevalence[Title/Abstract]) OR (dental health[Title/Abstract]) OR (oral health[Title/Abstract])) OR ((Periodontal disease[Title/Abstract]) OR (periodontitis[Title/Abstract]) OR (periodontal[Title/Abstract])) OR ((Edentulism[Title/Abstract]) OR (edentulous[Title/Abstract]) OR (endentulousness[Title/Abstract]) OR (severe tooth loss[Title/Abstract]) OR (total tooth loss[Title/Abstract]) OR (complete tooth loss[Title/Abstract]))) AND ((prevalence[Title/Abstract]) OR (incidence[Title/Abstract])) AND (2013/06/01[PDat] : 2016/12/31[PDat]))

> **Embase:** 'deciduous caries':ab,ti OR 'milk caries':ab,ti OR 'baby caries':ab,ti OR caries:ab,ti OR 'permanent caries':ab,ti OR 'caries prevalence':ab,ti OR 'dental health':ab,ti OR 'oral health':ab,ti OR 'periodontal disease':ab,ti OR periodontitis:ab,ti OR periodontal:ab,ti OR edentulism:ab,ti OR edentulous:ab,ti OR endentulousness:ab,ti OR 'severe tooth loss':ab,ti OR 'total tooth loss':ab,ti OR 'complete tooth loss':ab,ti AND (prevalence:ab,ti OR incidence :ab,ti) AND [2008-2016]/py AND [humans]/lim AND [embase]/lim NOT [medline]/lim

For GBD 2019, we completed a targeted systematic review of LILACS and SciELO, focusing first on articles from the most recent period, from 2014 to 2018, which were subject to full-text screening. The search used for LILACS and SciELO was the same:

> **LILACS/SciELO:** "(deciduous caries OR milk caries OR baby caries OR caries OR dental health OR oral health OR permanent caries OR caries prevalence OR periodontal disease OR periodontitis OR periodontal OR edentulism OR edentulous OR edentulousness OR complete tooth loss OR tooth loss OR toothloss OR number of teeth OR dentate OR edentate) AND (prevalence OR incidence OR survey OR epidemiology)".

A total of 1696 citations were identified after deduplication, 147 were selected for full text review, and 77 new sources were extracted from the following countries: Argentina (1), Brazil (47), Chile (5), Colombia (5), Cuba (5), Ecuador (1), El Salvador (1), Honduras (1), Mexico (5), Peru (5), and Venezuela (1).

For GBD 2021, we reviewed the Global Health Data Exchange (https://ghdx.healthdata.org) for oral health surveys and national reports with oral epidemiology data. A total of 105 new sources were identified and extracted for inclusion in GBD 2021 models. We eliminated many datapoints to avoid repetition in the dataset, while striving to maintain as much data detail as possible. Redundancy tended to arise in three data descriptors: age, gender, and urbanicity. Our order of preference for maintaining detail was age, followed by gender, then urbanicity. Additionally, many of the studies presented dmft or DMFT (decayed-missing-filled teeth) scores, which represent lifetime prevalence and were often described as “caries experience”. For the purposes of measuring the burden of disability from dental caries, we considered only data on current prevalence to be relevant, and thus converted lifetime prevalence data to current prevalence and incidence where possible. There were no new data sources added for GBD 2023.

Edentulism

Flowchart

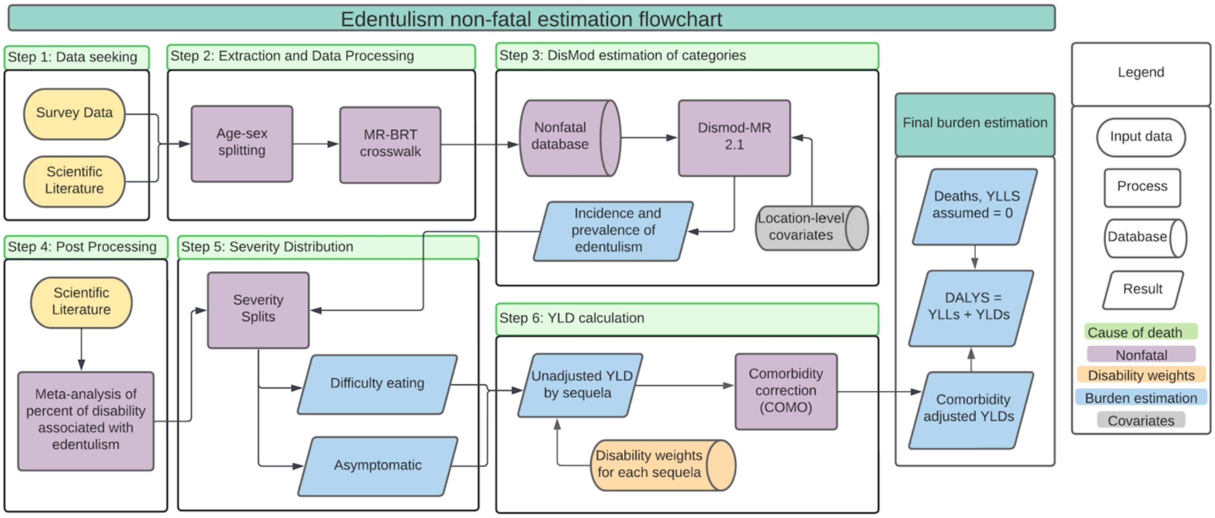

Case definition

The case definition of edentulism includes any individual with zero remaining permanent teeth; toothlessness of infancy is not included. The assessment of this disease includes quantification of the prevalence of the disease as well as estimation of the major sequelae: asymptomatic toothlessness and symptomatic toothlessness leading to “great difficulty in eating meat, fruits, and vegetables”. A small body of evidence has begun to emerge that implicates edentulousness as predisposing individuals to increased risk for ischaemic cardiovascular events, including myocardial infarction and stroke. These data are sparse but have been included in models estimating the excess mortality of those with complete tooth loss. Given that the association is believed to be ecological rather than causal, however, edentulism has not been estimated as an underlying cause of death, and it is not included in the risk factor analysis for cardiovascular diseases.

Input data and data processing

Details of the systematic literature reviews appear earlier in this write-up. In addition to published studies, we also utilised self-report data on toothlessness from World Health Survey (WHS) for 47 countries as well as a number of national oral health surveys identified through the Global Health Data Exchange.

Age-sex splitting

The first step of data processing was age splitting. For any datum that did not entirely fit within a GBD sex or age group, the observation was split to be multiple age-sex-specific datapoints based on the age and sex pattern predicted by previous DisMod-MR 2.1 models.

Crosswalks in meta-regression—Bayesian, regularised, trimmed (MR-BRT)

Prior to GBD 2021, we crosswalked self-reported (ie, WHS) data on toothlessness to the reference definition of oral examination; but at present, we no longer found a statistically significant difference between self-report and oral examination, so no adjustment of self-report data was completed.

Figure 1: Funnel plot showing logit-transformed ratio of edentulism prevalence for alternate (self-report) versus reference (oral examination)

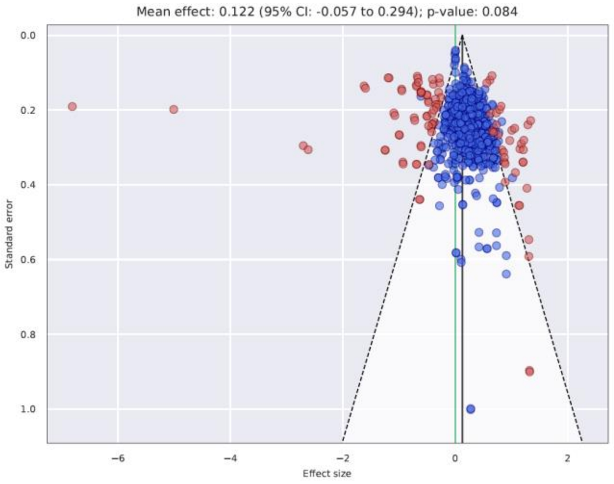

.

Modelling strategy

Estimates for the prevalence of edentulism were calculated for each location/year/sex/age using DisMod-MR 2.1. As would be expected for an irreversible condition, remission was fixed at zero for all ages. Mortality and relative risk were both fixed at zero before age 30, as any excess cardiovascular events resulting from severe tooth loss would not be expected at younger ages. We also assigned incidence and prevalence to be zero during childhood. Incidence was allowed to rise beginning at age 15, which was chosen based on the age at which the permanent dentition is expected to have fully formed in all individuals. The random effect limits for all locations were bounded at +/- 1.

As mentioned above, the criteria for diagnosis of edentulism are straightforward, and bias in the dataset was considered negligible. Thus, no study-level covariates were used in modelling the prevalence of edentulism. We included two location-level covariates in the model: 1) log-transformed lag-distributed

income (LDI) with a minimum beta value of 0.02, and 2) log-transformed age-standardised summary exposure value (SEV) scalar of cardiovascular disease (CVD) in recognition of the common risk factors between CVD and tooth loss.

Table 4: Covariate, parameter, beta, and exponentiated beta values for edentulism

Covariate name	Measure	Beta (UI)	Exponentiated beta (UI)
LDI (I$ per capita)	Prevalence	–0.217 (–0.225 to –0.209)	0.805 (0.798–0.811)
Age- and sex-specific SEV for smoking	Prevalence	0.048 (0.002 to 0.123)	1.05 (1.002–1.131)
Age- and sex-specific SEV for high fasting plasma glucose	Prevalence	0.263 (0.068 to 0.466)	1.301 (1.07–1.594)

Models were vetted based on the plausibility of the results, the extent to which estimates fit the data, and the plausibility of the range of estimates across location hierarchies.

Severity distributions and disability weights

The disability weight used for symptomatic toothlessness leading to "great difficulty in eating meats, fruits, and vegetables" is 0.067 (0.045–0.095) as determined by the GBD disability survey. We considered all those with severe tooth loss and no access to dentures to experience this disability. However, the proportion of those with edentulism and severe tooth loss who have dentures has not been studied extensively.

In order to estimate the proportion of edentulous individuals with no access to dentures, we completed a supplemental literature review of dentures prevalence for GBD 2010. Only six systematic surveys of dentures prevalence were identified, all in high- and middle-income countries. All were completed since 2000. After extracting the data from the studies, we performed linear regressions of denture presence and denture absence against health system access (HSA), a standardised covariate of treatment availability used in many disease estimation models. From the results of the regression, the prevalence of no dentures was calculated for all super-regions. We then completed a population-weighted average of all countries in the super-region based on 2003 populations, the average year of the dentures studies. Uncertainties for the prevalence of dentures were calculated by finding the standard deviation and standard error of the calculated prevalence values.

The estimated prevalence of dentures in each location was used to calculate the proportion of individuals with asymptomatic edentulism and severe tooth loss (ie, those who have access to dentures) and difficulty eating due to edentulism and severe tooth loss (ie, those without access to dentures). This latter sequela was included as a cause of years lost due to disability (YLDs).

Caries of permanent teeth and caries of deciduous teeth

Separate estimates of caries of deciduous teeth and caries of permanent teeth

The natural histories of deciduous and permanent caries share many similarities, but they also share some important differences. Age patterns of decay in permanent and deciduous dentition are distinct, and duration of a carious lesion in deciduous teeth also tends to be shorter than an untreated episode of permanent caries. Sugar consumption and feeding with formula are both associated with development of deciduous caries, while sugar consumption is associated with the development of caries

of permanent teeth. Finally, it is unclear whether the gender patterns and regional differences are the same for both deciduous and permanent caries. For all of these reasons, we elected to model deciduous caries and permanent caries as separate entities and then add the estimates together for an overall estimation of the global burden of dental caries. This is the modelling approach which has been taken in each iteration since GBD 2010.

Flowchart

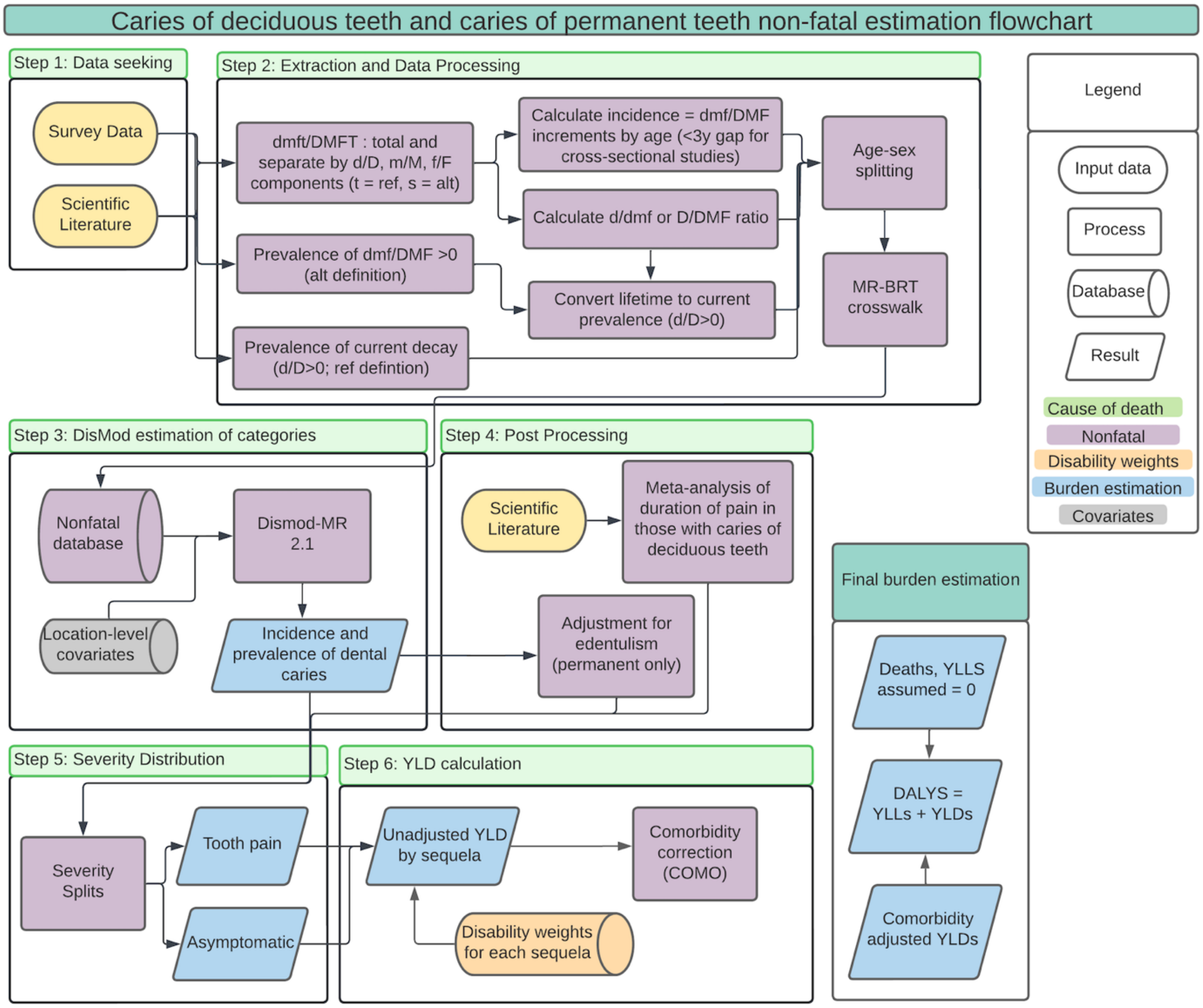

Case definition

The case definition for dental caries is "teeth with unmistakable coronal cavity at dentin level, root cavity in cementum that feel soft or leathery to probing, temporary or permanent restorations, or missing teeth extracted due to a caries lesion". Excluded definitions include crowns with isolated cosmetic defects, stained enamel pits, or fissures without visible cavitation or softening, fluorosis, and abrasion lesions. This definition corresponds to an ICD-9 code of 521.0 and an ICD-10 code of K02.3–K02.9. Most caries are subclinical in the sense that they do not cause symptoms a majority of the time. Once a carious lesion develops, it will occasionally recede without intervention, but often it worsens with time and eventually requires either filling or extraction.

Public health dentists commonly measure dental caries using the dmft/DMFT index, which is an incremental measure of the proportion of unhealthy teeth and is also a measure of an individual's lifetime prevalence of caries. Lowercase letters (dmft) are used for deciduous dentition and uppercase letters (DMFT) for permanent dentition. D is for decayed, M for missing, F for filled, and T for teeth. The maximum dmft score is 20 and the maximum DMFT score is 32. Furthermore, some dentists prefer to measure dental caries in terms of tooth surfaces, rather than number of teeth, and report their results using an analogous decay-missing-filled surfaces (dmfs/DMFS) index. The maximum dmfs score is 88, and the maximum DMFS score is 128 or 148 depending on whether the third molars are counted.

The DMFT index is easy to measure, and inter-rater reliability is high. However, the primary shortcoming of the DMFT is that it does not discriminate well between current and past caries. Strategies we employed to maximally utilise dmf/DMF data for estimating the prevalence of burden due to permanent caries are described below.

Input data and data processing

The approach for systematic literature review is described above. The reference definition for this model was presence of one or more teeth with current decay (for prevalence), whereas each additional carious tooth was counted as a separate incident event.

Converting lifetime to current prevalence

Many of the studies presented dmft or DMFT scores, which represent lifetime prevalence and were often described as "caries experience". For the purposes of measuring the burden of disability from dental caries, we converted lifetime prevalence data to current prevalence for individuals aged 20 years and under. We did this by multiplying the observed lifetime prevalence by the ratio of d/D to dmf/DMF. When d/dmf or D/DMF information was available from the same study, this ratio was applied. When not available from the same study, the pooled ratio from the closest matching GBD geography was used for the multiplication (country, region, super-region, global).

Calculation of incidence from dmft/DMFT increment

Whereas in the deciduous dentition, a vast majority of the dmf index is accounted for by caries, tooth loss is a major contributor to the DMF index for the permanent dentition. Caries of permanent teeth may not necessarily be the primary driver of this tooth loss, as other factors such as periodontal disease and trauma may contribute significantly. Thus, we performed the conversions of incremental dmf/DMF scores to incidence values for permanent caries only in individuals aged 20 years or less and for all ages in the case of deciduous caries. For longitudinal studies, the difference between the dmf/DMF score in the initial versus subsequent examination was taken to be equivalent to the number of incident caries over that time period. This assumes a negligible proportion of dmf/DMF increment is due to trauma in children. For cross-sectional studies examining children of different ages, we only calculated incidence when the gap in age was ≤3 years given the propensity for strong cohort effects in caries epidemiology.

Age and sex splitting

For any datum that did not entirely fit within a GBD sex or age group, the observation was split to be multiple age-sex-specific datapoints based on the age and sex pattern predicted by previous DisMod-MR 2.1 models. It is our intention to update this with each cycle of GBD.

Crosswalks in MR-BRT

We then crosswalked alternative to reference definitions. To make data comparable, we began by evaluating the number of observations of each alternate definition that matched with a corresponding observation from the reference definition. Owing to the significant heterogeneity in data on caries incidence and prevalence, we limited the comparisons to only "within"-study matches, where a match was defined as both methods of ascertainment being performed in the identical study population. The ratio of alternative to reference was calculated and logit-transformed. Standard error of the ratio was calculated using the delta method. Sex was included as a fixed effect and, for prevalence only, midpoint of age as a spline. The adjustment factors and spline plots for the crosswalks are shown below.

The funnel plot is for demonstration only; the final crosswalk was derived from the MR-BRT model represented by the spline plot in Figure 1. For deciduous caries, we also adjusted data that were DMFS-derived (ie, calculated from d/dmfs as opposed to d/dmft), even though data are comparatively sparse, because there is strong suggestion of age-specific relationship in these two measures. This sub-analysis would be strengthened by additional data. We elected to adjust rather than drop the alternate data because the age groups involved are comparatively data-sparse. We intend to focus on identifying additional data for early childhood caries for the next GBD systematic data extraction effort.

Figure 1: Funnel plot (left) and spline plot by age (right) showing logit-transformed ratio of deciduous caries prevalence for alternate (converted from d/dmf) versus reference (d>0)

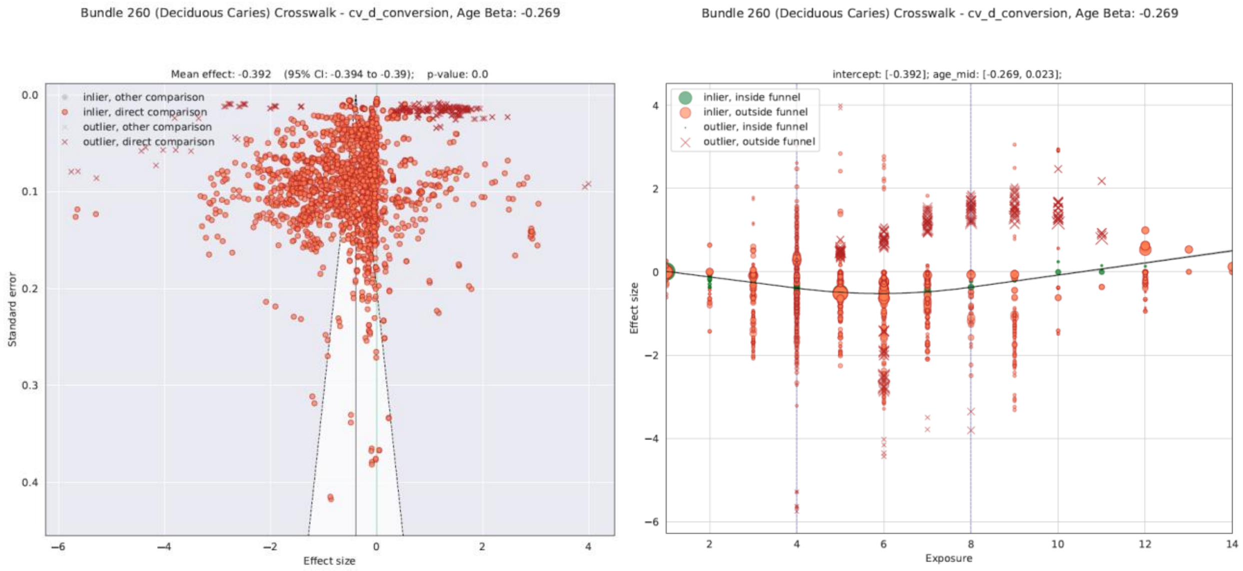

Figure 2: Funnel plot (left) and spline plot by age (right) showing logit-transformed ratio of deciduous caries incidence for alternate (dmfs measurement) versus reference (dmft measurement)

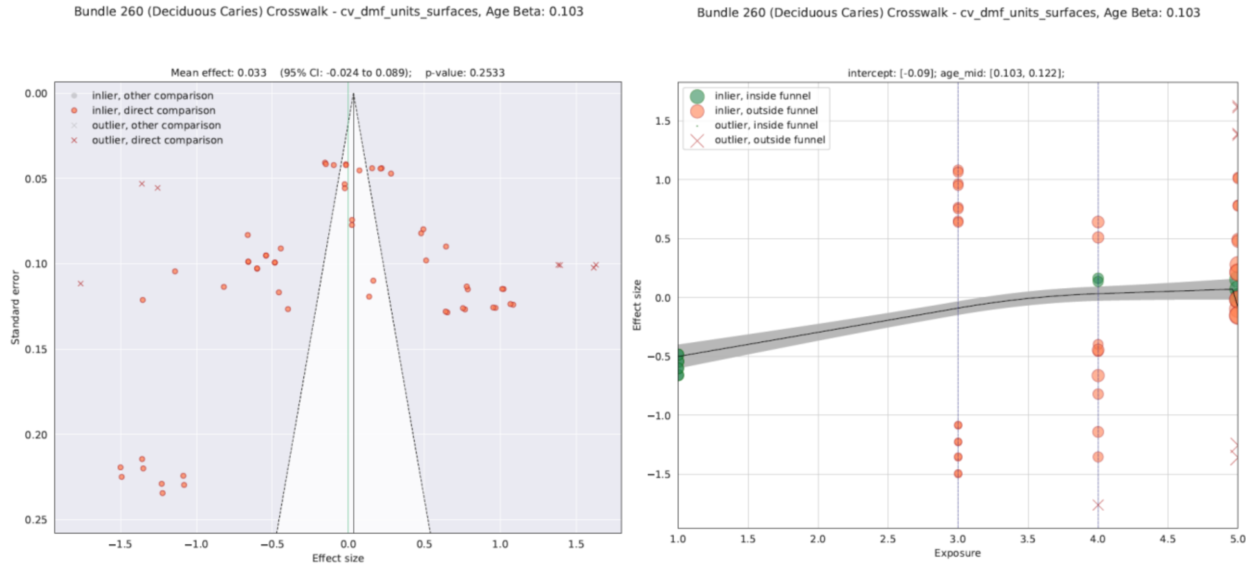

The same crosswalks were evaluated for data on caries of permanent teeth. The results of those crosswalks are shown in Figures 3 and 4, respectively.

Figure 3: Funnel plot (left) and spline plot by age (right) showing logit-transformed ratio of caries of permanent teeth prevalence for alternate (converted from D/DMF) versus reference (D>0)

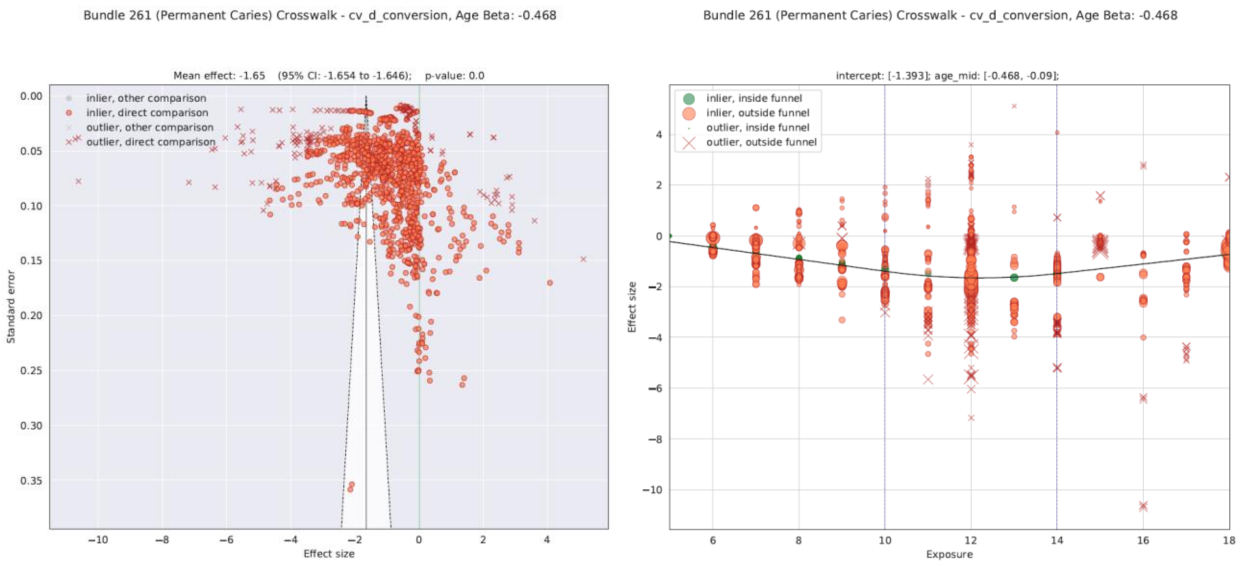

Figure 4: Funnel plot (left) and spline plot by age (right) showing logit-transformed ratio of caries of permanent teeth prevalence for alternate (DMFS measurement) versus reference (DMFT measurement)

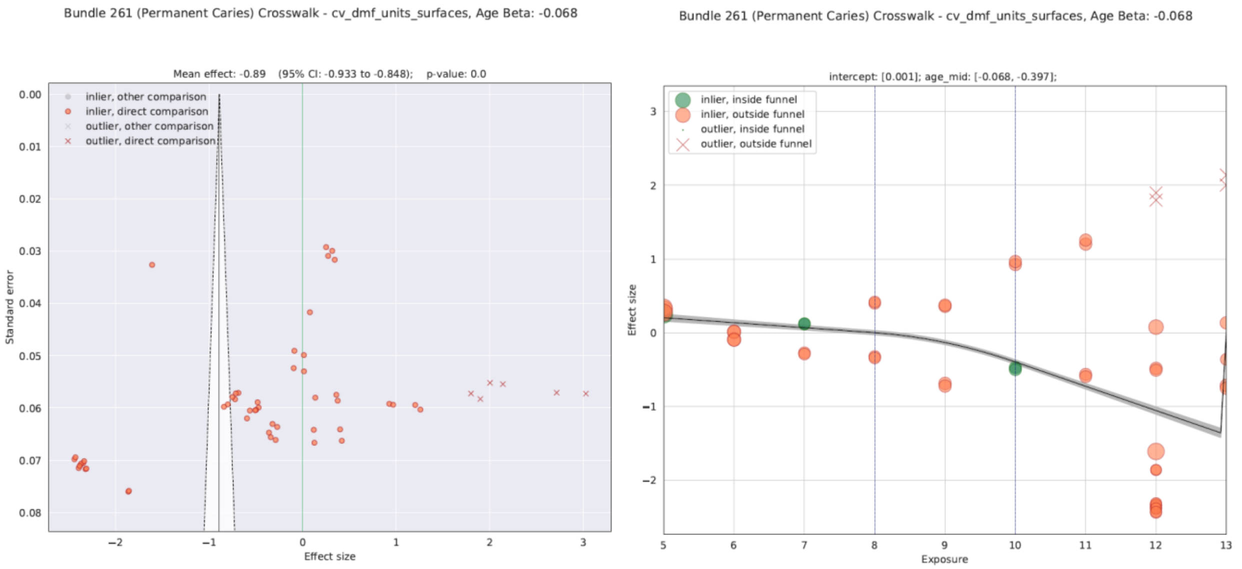

For prevalence data on caries of permanent teeth, there was a significant and age-dependent difference in prevalence data derived from measurement of surfaces as compared to teeth, so DMFS-derived prevalence data were adjusted to the reference of teeth as shown in Figure 4. As described above, the D/DMF conversion was only completed for data from children under 13 years of age because these were the only age groups from which we felt confident in deriving accurate measures of relationships between D/DMFS and D/DMFT. There were insufficient data to inform an assessment of whether or not there is a difference between cohort-based caries incidence and incidence derived from DMF increment.

Modelling strategy

DisMod model development

Serious health consequences of caries were also assumed to be uncommon and death very rare. We therefore assigned excess mortality to be zero from age 0 to 100. For both types of caries, most of the model settings were similar. The primary difference was in value priors. We assumed zero incident caries in infants under 1 year old and similarly zero incident deciduous caries from age 11 onward. For permanent caries, we assumed zero incident cases in children under 5. Location-level covariates were assigned separately on prevalence and incidence. Sugar availability in food from the GBD diet analysis was used as a covariate on incidence with a positive beta, while prevalence was assigned log-transformed LDI with a negative beta to reflect the association with access to dental care.

Table 3: Covariate, parameter, beta, and exponentiated beta values for dental caries of deciduous teeth

Covariate name	Measure	Beta (UI)	Exponentiated beta (UI)
LDI (I$ per capita)	Prevalence	–0.116 (–0.134 to –0.095)	0.89 (0.875–0.909)
Age- and sex-specific SEV for high sweetened beverages	Incidence	0.37 (0.022 to 0.878)	1.448 (1.023–2.407)

Table 4: Covariate, parameter, beta, and exponentiated beta values for dental caries of permanent teeth

Covariate name	Measure	Beta (UI)	Exponentiated beta (UI)
LDI (I$ per capita)	Prevalence	–0.171 (–0.216 to –0.131)	0.843 (0.806–0.877)
Age- and sex-specific SEV for high sweetened beverages	Incidence	0.3 (0.024 to 0.697)	1.35 (1.024–2.008)

Although studies were screened carefully during data extraction to ensure that they specified whether they were measuring permanent or deciduous caries, some datapoints were marked as outliers during modelling due to their high prevalence values in young ages, as it was deemed likely that some of these studies were reporting deciduous in addition to permanent caries. As with deciduous caries, models for permanent caries were vetted based on the plausibility of the results, the extent to which estimates fit the data, and the plausibility of the range of estimates across location hierarchies.

Correction for edentulism

One systematic source of bias in the literature was the exclusion of edentate individuals from the study populations, which leads to systematic overestimation of caries prevalence when modelled over the entire population. To account for this bias, we used our GBD estimates of edentulism prevalence to adjust YLD estimates for caries of permanent teeth. Final DisMod-MR 2.1 estimates of edentulism prevalence were paired with the corresponding results for caries of permanent teeth by age group, sex, location, and year to adjust for the proportion of the population that was excluded from the denominator of permanent caries models. No adjustment was made to the estimates of caries of deciduous teeth.

Severity distributions and disability weights

As described above, the GBD definition of disability associated with symptomatic dental caries is "this person has a toothache, which causes some difficulty eating". The disability weight associated with this condition is 0.01 (0.005–0.019), as derived from the GBD disability weights study.

Not all those with dental caries experience this disability all the time. We considered only those with active dentinal decay to experience symptomatic tooth pain. Those with deciduous caries who had undergone exfoliation or had their cavities filled were considered to have no disability. Likewise, those with permanent caries who had received fillings, had their cavities extracted, or lost a carious tooth altogether were considered to have no disability. Thus, two additional pieces of information are required to complete the calculation of YLDs: proportion with symptoms and duration of disability.

To determine which segment of the population has ongoing tooth pain and the proportion of time spent with tooth pain, we considered several different options. First, we examined the data on dental caries symptoms and disability from the Medical Expenditure Panel Survey (MEPS) conducted by the USA Department of Health and Human Services in 2000–2009. MEPS data were widely used in GBD 2010 analyses. Respondents to the survey are asked about all medical conditions. Conditions for which provider care was sought are reported by the respondents at every round, and respondents also report problems for which they did not see a provider if the symptoms were "bothering" them. Conditions can be added to the condition roster if 1) they are reported as a reason for a medical event, 2) the condition was reported as the reason for one or more disability days, or 3) the condition was "bothering" the person during the reference period. Conditions are then recorded as verbatim text and coded to ICD-9CM 3rd digit codes by professional medical coders. These ICD-9 codes were mapped to GBD causes, including dental caries. From the MEPS, symptomatic caries in the previous year were reported by 48.4% (44.3–52.9) of the respondents. This number is in agreement with our DisMod-MR 2.1 estimates of 1–2 years' duration in high-income North America for permanent caries if we consider people to only have symptoms at the end of a course of caries. The two primary shortcomings of using this approach are 1) it does not provide enough detail to differentiate between the experiences of those with deciduous versus permanent caries, and 2) it indicates the proportion of those with caries who were symptomatic during the previous year, but it does not provide information on the amount of time during that year spent with symptoms (ie, one day versus 12 months). The approach described below addresses both issues.

To determine duration, we adapted the method employed by the Australian Burden of Disease (AusBoD) Study in 1996. For total duration, we used the posterior estimates of duration from final DisMod-MR 2.1 models. For those with symptoms, we split this total duration into two distinct phases of caries disability. The "initial" phase is characterised by *periodic* pain that we assigned to occur an average of one hour per day. The "terminal" phase is a period of *constant* symptoms at the end of an episode. The length of the terminal phase was determined by literature review as described by the AusBoD group. For deciduous caries, we used a study by Mason and colleagues of children in the UK presenting to a casualty ward with tooth pain.[1] The length of time each child had been experiencing tooth pain was recorded. Based on the distribution of time courses, a log-normal distribution was plotted that approximated the average duration of *constant* symptoms at 27.6 days leading up to seeking care. For permanent caries, a similar study of the tooth pain experience of adults in New Zealand who presented to hospital dental departments and an emergency clinic[2] resulted in an estimated 55.2 days spent in the terminal phase of caries. For those with severe disease, the length of time spent in the terminal phase was subtracted from the total duration to determine the amount of time spent in the initial phase. For

those with mild disease, we considered the entire duration to be spent in the initial phase. These calculations were last completed as part of the GBD 2013 analysis.

To determine proportion with symptoms, we completed a supplemental literature review of tooth pain and caries. We identified a total of 21 studies with data about the prevalence of pain. The studies were grouped according to the type of dentition studied (deciduous or permanent) and the location of the study group (high-income or low- and middle-income countries). We extracted data on the proportion in each group who described symptoms of pain related to their caries as well as a subset who described their symptoms as being severe. The proportions in each group were weighted according to sample size to give estimates of the relative sizes of three groups: asymptomatic, mild, and severe. The results of this meta-analysis are illustrated in the table below.

We considered asymptomatic individuals to experience no disability. Those with mild disease spent the entire duration in the initial phase of disease (one hour of pain per day). Those with severe disease spent a majority of the duration in the initial phase followed by a period of time in the terminal phase (constant pain). YLDs were calculated by multiplying the prevalence, duration, proportion, and disability weight for each age, country, sex, and year.

Table 7: Duration and distribution of severity for tooth pain due to caries of deciduous and permanent teeth

	# of studies	% symptomatic of total	% severe among symptomatic	% mild of total	% severe of total	% asymptomatic of total
Deciduous caries						
Data-rich	5	0.35	0.257	0.26	0.09	0.65
All others	4	0.555	0.438	0.312	0.243	0.445
Permanent caries						
Data-rich	6	0.602	0.315	0.412	0.189	0.398
All others	6	0.954	0.548	0.432	0.521	0.046
Duration of phases						
Initial phase				1 hour per day		
Terminal phase (deciduous caries)				27.6 days		
Terminal phase (permanent caries)				55.2 days		

Chronic periodontal disease

Flowchart

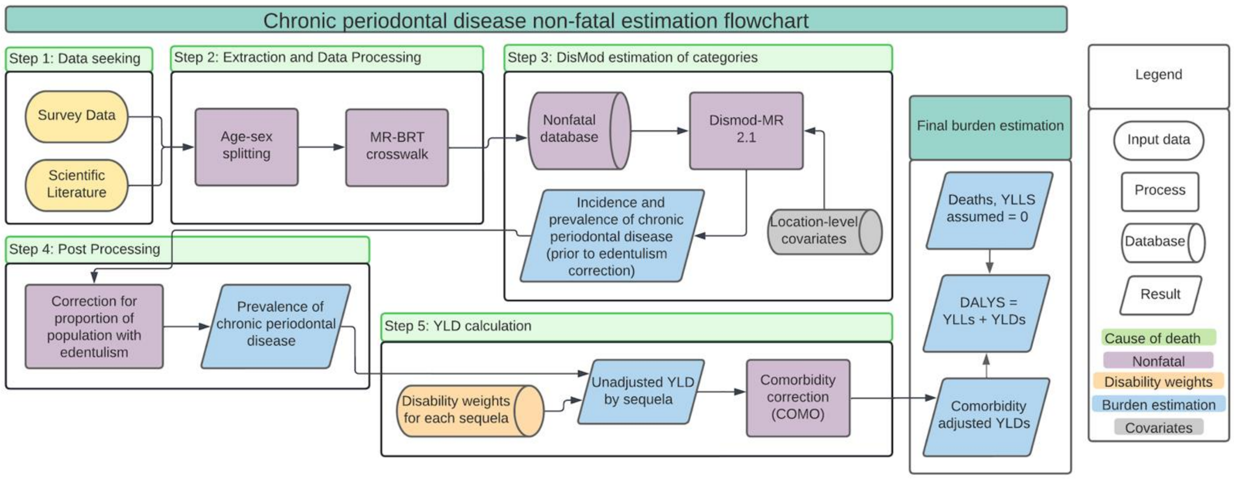

Case definition

Chronic periodontal disease is caused by chronic bacterial infection around the teeth. Symptoms of gingivitis, the mildest form of the disease, include swelling, redness, and propensity of the gums to bleed when perturbed. If the infection is not treated appropriately, it will eventually spread below the gum line, leading to a chronic inflammatory state of the periodontal tissues. Over time, there will be loss of gingival tissue and alveolar bone destruction. Teeth will become loose and may need to be extracted.

The GBD definition of disability associated with symptomatic severe periodontal disease is "bad breath, a bad taste in the mouth, and gums that bleed a little from time to time, but which does not interfere with daily activities". The ICD-10 codes for periodontal disease are K05.0–K05.6, and the ICD-9 codes are 523.0–523.9.

Defining periodontal disease in a meaningful, reproducible manner has been an ongoing challenge for public health dentists. Attachment loss (AL) and pocket depth (PD) have emerged as the most common metrics of periodontal health measurement. AL is measured as the difference between the distance from the gingival margin to the bottom of the pocket and the distance from the cemento-enamel junction to the bottom of the pocket.

The Community Periodontal Index (CPI) is a classification system that was developed by WHO as a standardised method of periodontal health measurement. CPI classification is based on the examination of all teeth present in the mouth for absence or presence of gingival bleeding and absence or presence of periodontal pockets. A standard-sized probe is used, with depth markings from 0.5 to 11.5 mm. The probe is inserted into the sulcus between a tooth and the gingiva until it meets resistance. The surrounding area is then explored with the probe to determine the maximum depth of the pocket. Multiple areas around each tooth are probed. Pocket scores range from 0 to 2 in order of increasing severity. When the CPI method was employed, we considered those with Class 2 only (pocket of 6 mm or more). Additionally, loss of attachment may be collected for specific index teeth by dividing the mouth in sextants. The two molars in each posterior sextant are paired for recording and, if one is

missing, there is no replacement. If no index tooth is present in a sextant qualifying for examination, all the teeth that are present in that sextant are examined and the highest score is recorded as the score for the sextant. We excluded studies in which the study population was reported as the number of sextants rather than the number of individuals surveyed. CPI is a modification of Community Periodontal Index of Treatment Needs (CPITN) that does not include the assessment of periodontal treatment needs. Also, Class 2 of CPI is equivalent Class 4 of CPITN.

In 2007, a new CDC proposal for gold-standard diagnosis of severe, chronic periodontitis was published.[1] This standard specified that a stricter definition of the condition should be implemented. This more exclusive definition of chronic periodontal disease includes ≥2 interproximal sites with AL ≥6 mm ***AND*** ≥1 interproximal site with PD ≥5 mm. This definition has not been adopted by GBD.

A small body of evidence has begun to emerge that implicates chronic periodontal disease as predisposing individuals to increased risk for ischaemic cardiovascular events including myocardial infarction and stroke. These data are sparse but have been included in models estimating the excess mortality of those with chronic periodontal disease. Given that the association is believed to be ecological rather than causal, however, periodontal disease has not been estimated as an underlying cause of death and it is not included in the risk factor analysis for cardiovascular diseases.

Input data

Details of the systematic review are provided above. We implemented a hierarchical preference for case definitions. We included the following definitions of severe periodontal disease commonly found in the literature:

1. CPITN – Class 4 only
2. CPI – Class 3 only
3. Clinical AL >6 mm
4. Clinical AL >5 mm
5. Clinical AL >4 mm
6. Gingival PD >5 mm

If more than one type of data was included in a study, our first preference was for CPITN = 4, followed by AL >6 mm, and PD >5. All were considered equivalently as reference definitions with no additional crosswalking performed. For those sources that did not provide data on any of the components of CPITN Class 4, but did provide data on CPITN Class 3, AL >5 mm, or AL >4 mm, we utilised these data after crosswalking in MR-BRT as described below.

Age-sex splitting

For any datum that did not entirely fit within a GBD sex or age group, the observation was split to be multiple age-sex-specific datapoints based on the age and sex pattern predicted by previous DisMod-MR 2.1 models

Crosswalks in MR-BRT

We then crosswalked alternative to reference definitions. To make data comparable, we began by evaluating the number of observations of each alternate definition that matched with a corresponding observation from the reference definition. All alternative definitions were mutually exclusive with one

another, so three separate crosswalks were performed using only within-study matches, defined as both methods of ascertainment being performed in the identical study population. The ratio of alternative to reference was calculated and logit-transformed. Standard error of the ratio was calculated using the delta method. Sex was included as a fixed effect and, for prevalence only, midpoint of age as a spline. The total number of matches, the adjustment factors, and the spline plots for periodontal disease crosswalks are shown below.

Figure 1: Funnel plot (left) and spline plot by age (right) showing logit-transformed ratio of chronic periodontal diseases prevalence as measured with alternate (top = CPITN 3; middle = attachment loss >4 mm; bottom = attachment loss >5 mm) versus reference (CPITN 4, AL =>6 mm)

Bundle 262 (Periodontal Disease) Crosswalk - cv_cpiclass3, Age Beta: 0.65

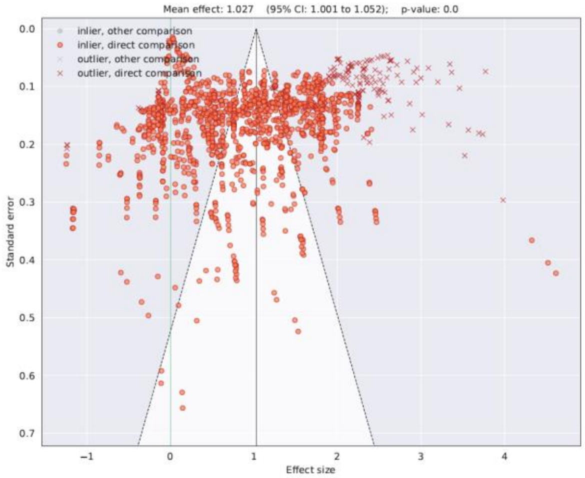

Bundle 262 (Periodontal Disease) Crosswalk - cv_cpiclass3, Age Beta: 0.65

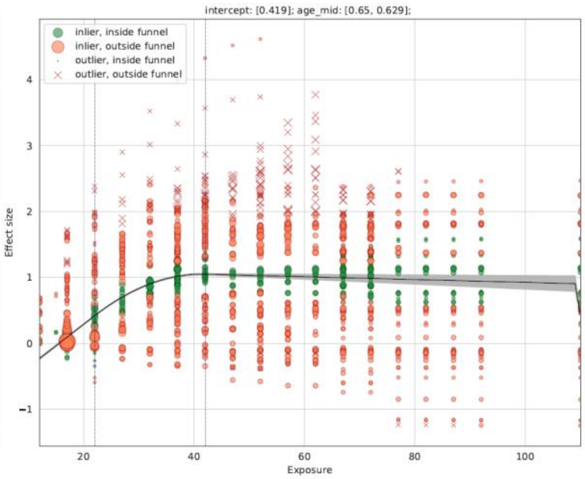

Bundle 262 (Periodontal Disease) Crosswalk - cv_atchloss4ormore, Age Beta: 0.793

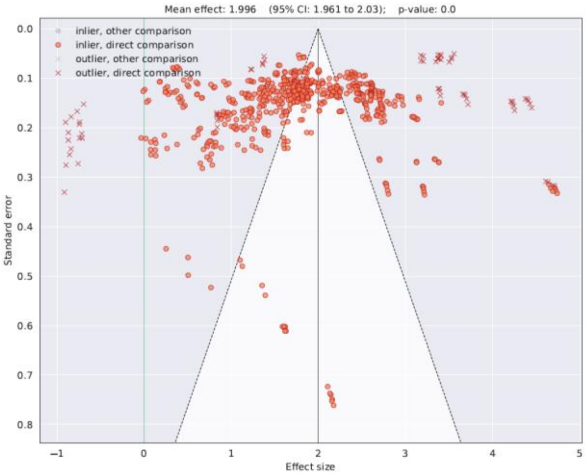

Bundle 262 (Periodontal Disease) Crosswalk - cv_atchloss4ormore, Age Beta: 0.793

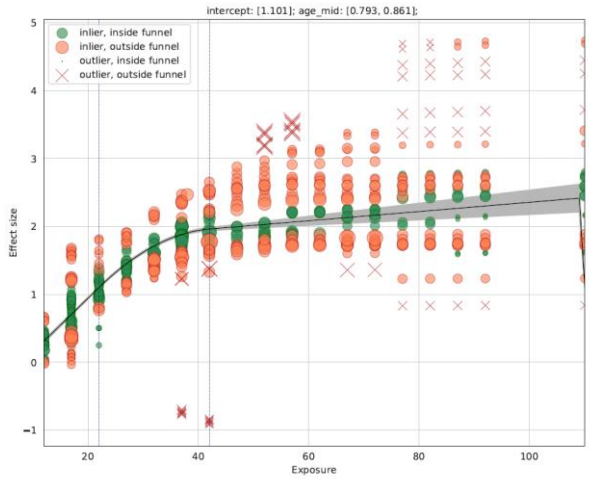

Bundle 262 (Periodontal Disease) Crosswalk - cv_atchloss5ormore, Age Beta: 0.629

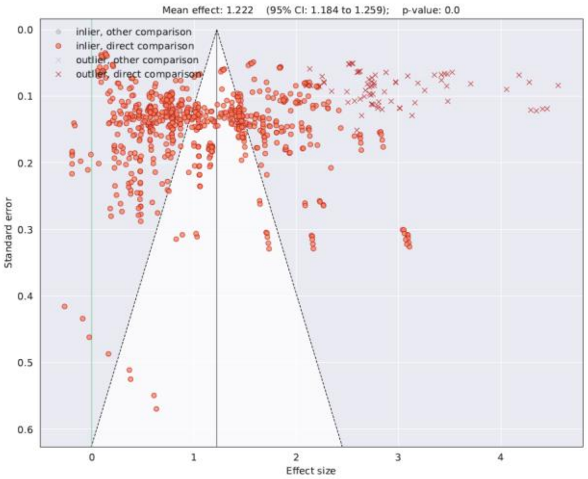

Bundle 262 (Periodontal Disease) Crosswalk - cv_atchloss5ormore, Age Beta: 0.629

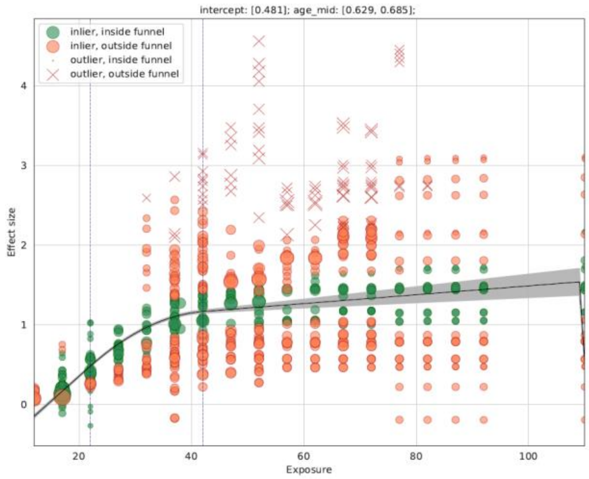

Modelling strategy

First, estimates for the prevalence of chronic periodontal disease were generated for each location/year/sex/age using DisMod-MR 2.1. Mortality was fixed to zero, and relative risk was fixed to 1.0 before age 30, as any excess cardiovascular events that occur in those with severe tooth loss would not be expected at young ages. Incidence and prevalence were assigned to be zero until age 8, as periodontal disease is largely considered to be a disease of adulthood. Incidence was allowed to rise beginning at age 9, based on the youngest age at which there was a non-zero point estimate for prevalence in the dataset. Additional bounds were assigned for incidence, remission, and excess mortality to improve plausibility in the DisMod estimates. Remission was bounded from 0 to 0.05, excess mortality rate from 0 to 0.0001, and incidence from 0 to 0.05. We considered these bounds to reasonably reflect the natural history of the disease. Three location-level covariates were used as shown in the table below.

Table 4: Covariate, parameter, beta, and exponentiated beta values for chronic periodontal diseases

Covariate name	Measure	Beta (UI)	Exponentiated beta (UI)
LDI (I$ per capita)	Prevalence	0.21 (0.156–0.261)	1.234 (1.169–1.298)
Age-standardised SEV for smoking	Prevalence	0.171 (0.01–0.464)	1.187 (1.01–1.591)
Age-standardised SEV for high fasting plasma glucose	Prevalence	0.39 (0.032–0.985)	1.476 (1.032–2.678)

Models were vetted based on the plausibility of the results, the extent to which estimates fit the data, and the plausibility of the range of estimates across location hierarchies.

Correction for edentulism

One systematic source of bias in the literature was the exclusion of edentate individuals from the study populations, which leads to systematic overestimation of periodontal disease prevalence when modelled over the entire population. To account for this bias, we used our GBD estimates of edentulism prevalence to adjust YLD estimates for chronic periodontal disease. Final DisMod-MR 2.1 estimates of edentulism prevalence were paired with the corresponding results for periodontal disease by age group, sex, location, and year to adjust for the proportion of the population that was excluded from the denominator of periodontal disease models.

Severity distributions and disability weights

We considered all estimated prevalent cases of chronic periodontal disease to experience the disability described by “bad breath, a bad taste in the mouth, and gums that bleed a little from time to time, but this does not interfere with daily activities”. The GBD disability survey differentiated between those who experience pain and those who do not, but the calculated disability weight was the same for both forms of the condition, 0.007 (0.003–0.014).

Other oral disorders

Other oral disorders encompass a wide variety of dental, tongue, and jaw disorders and malformations, including all oral disorders that are not included in the case definitions of permanent or deciduous dental caries, periodontal disease, or edentulism and severe tooth loss. All data on the prevalence of other oral disorders were obtained from the USA MEPS, a nationally representative survey conducted yearly from 1996 to 2011 by the US Agency for Healthcare Research and Quality. These data were

modelled in DisMod-MR 2.1 using a prevalence-only model. Disability weights and severity distribution for these causes were also derived from MEPS.

References

1. C Mason, SR Porter, G Madland, J Parry. Early management of dental pain in children and adolescents. *J Dent.* Jan 1997; 25(1): 31-4.
2. RA Whyman, ET Treasure, KM Ayers. Dental disease levels and reasons for emergency clinic attendance in patients seeking relief of pain in Auckland. *NZ Dent J.* Dec 1996; 92(410): 114-7
3. RC Page and PI Eke. Case Definitions for Use in Population-Based Surveillance of Periodontitis. *J Periodont*. Jul 2007. 78(7 Suppl): 1387-99
4. Petersen PE, Baez RJ. Oral health surveys: basic methods. Geneva: World Health Organization; 2013.

Osteoarthritis

Flowchart

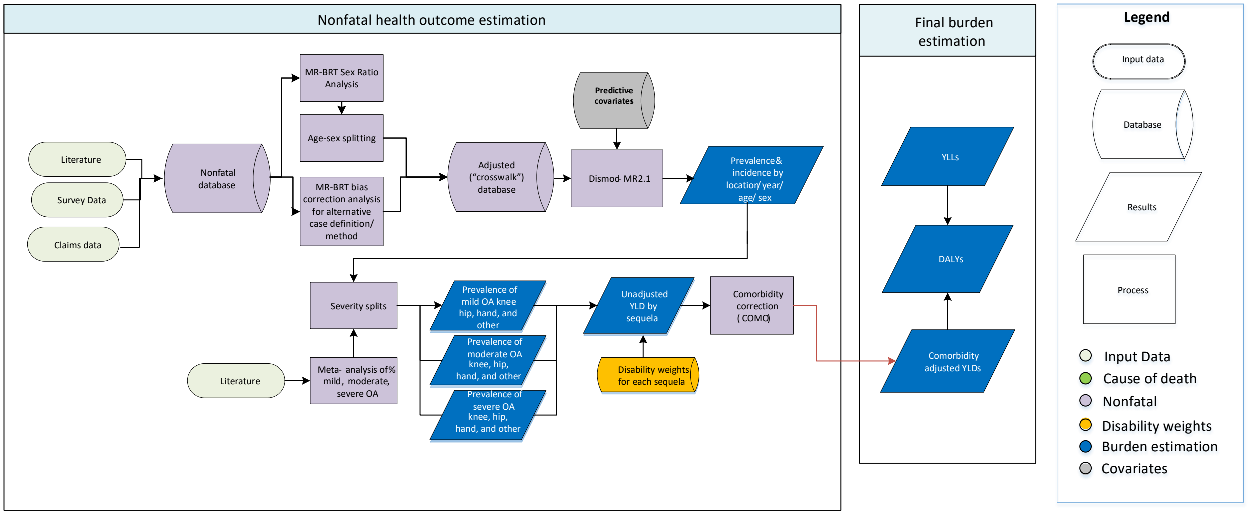

Input data and methodological summary for osteoarthritis

Case definition

Osteoarthritis (OA) is the most common form of arthritis, involving chronic inflammation, breakdown, and structural changes of whole joints. For the GBD study, four individual sites—hip, knee, hand, and other sites—were separately estimated. The hip, knee, and hand are the most common sites of OA. OA in the larger joints, such as the hip and knee, is considered to produce the greatest disability. Failure of these joints can lead to the need for joint replacement surgery, if available, and thus contributes to a significant proportion of the high direct health-care costs attributable to arthritis. OA of the spine is also common; however, it was considered that any symptoms and disability related to the cervical, thoracic, and/or lumbar spine would be captured in the estimates of low back pain and neck pain.

The osteoarthritis (OA) reference case definition is symptomatic osteoarthritis radiologically confirmed as Kellgren-Lawrence grade 2–4. Prior to GBD 2019, we only estimated OA of the hip and knee. For GBD 2019, two new sites of OA were added, OA of the hand, with the same reference criteria present in any single hand joint type, and OA other, with the same reference criteria present in any joint other than those of the hand, hip, knee, or spine. Grade 2 symptomatic requires one defined osteophyte in the affected joint and pain for at least one month out of the last 12. Grade 3–4 symptomatic requires osteophytes and joint space narrowing in the affected joint with deformity also present for grade 4, and pain for at least one month out of the last 12 months.

ICD-10 codes for OA of the hip, knee, hand, and other are M16, M17, M18, and M19, respectively. The ICD-9 code for OA is 715, without specific codes for various sites.

The case definitions accepted for osteoarthritis are shown in the table below.

Reference or alternative	Definition
Reference	Symptomatic osteoarthritis radiologically confirmed as Kellgren-Lawrence grade 2–4.
Alternative	Self-reported symptomatic OA physician diagnosis without the use of radiography
Alternative	Self-reported pain only without physician diagnosis
Alternative	Radiographically confirmed OA without pain or without mention of the presence of pain
Alternative	USA claims data
Alternative	Taiwan claims data

Input data

A systematic review for OA hip and OA knee was last conducted in 2017 for studies published between 2013 and 2017. A systematic review of the prevalence, incidence, and mortality was performed on MEDLINE, EMBASE, CINAHL, CAB Abstracts, WHO Library (WHOLIS), and OpenSIGLE. For prevalence and incidence, the following search terms were used: (osteoarth* OR gonarthr*) AND (prevalen* OR inciden* OR cross-sectional OR cross sectional OR epidemiol* OR survey OR population-based OR population based OR population study OR population sample OR cohort OR follow-up OR follow up OR longitudinal OR regist*) AND (list of names of all GBD countries).

Exclusion criteria were:

1. Sub-populations clearly not representative of the national population
2. Not a population-based study
3. Low sample size (less than 150)
4. Review rather than original studies

We identified 1864 articles and extracted data from 26. These studies were from 19 locations: Australia, Brazil, Canada, China, Ecuador, Egypt, India, Iran, the UK, France, Japan, the USA, Mongolia, Portugal, Spain, Mexico, Turkey, Venezuela, and Vietnam.

Figure 1: PRISMA diagram of osteoarthritis systematic review from 2013–2017

From: Moher D, Liberati A, Tetzlaff J, Altman DG, The PRISMA Group (2009). *P*referred *R*eporting *I*tems for *S*ystematic Reviews and *M*eta-*A*nalyses: The PRISMA Statement. PLoS Med 6(7): e1000097. doi:10.1371/journal.pmed1000097

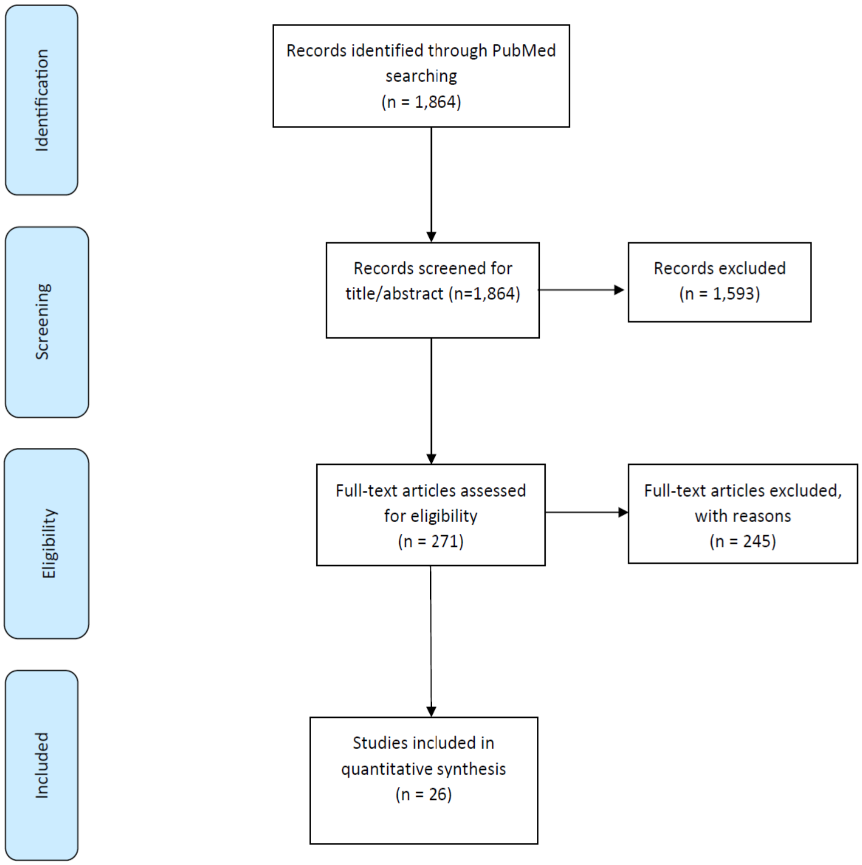

All existing sources used in the hip and knee models were re-reviewed for mention of prevalence and incidence of OA hand or OA other specifically. To gather more input data on prevalence for the new OA hand and OA other models, a broad systematic review was also conducted in 2019 specifically for data on these sites. A PubMed search was conducted for studies published between 1980 and 2019 using the following search terms: (("osteoarthritis" AND ("epidemiology" OR "prevalence")) AND "humans") AND ("population" OR "population groups" OR ("population" AND "groups")).

Figure 2: PRISMA diagram of osteoarthritis systematic review from 1980–2019

Figure 2: PRISMA diagram of osteoarthritis systematic review from 1980–2019

From: Moher D, Liberati A, Tetzlaff J, Altman DG, The PRISMA Group (2009). *P*referred *R*eporting *I*tems for *S*ystematic Reviews and *M*eta-*A*nalyses: The PRISMA Statement. PLoS Med 6(7): e1000097. doi:10.1371/journal.pmed1000097

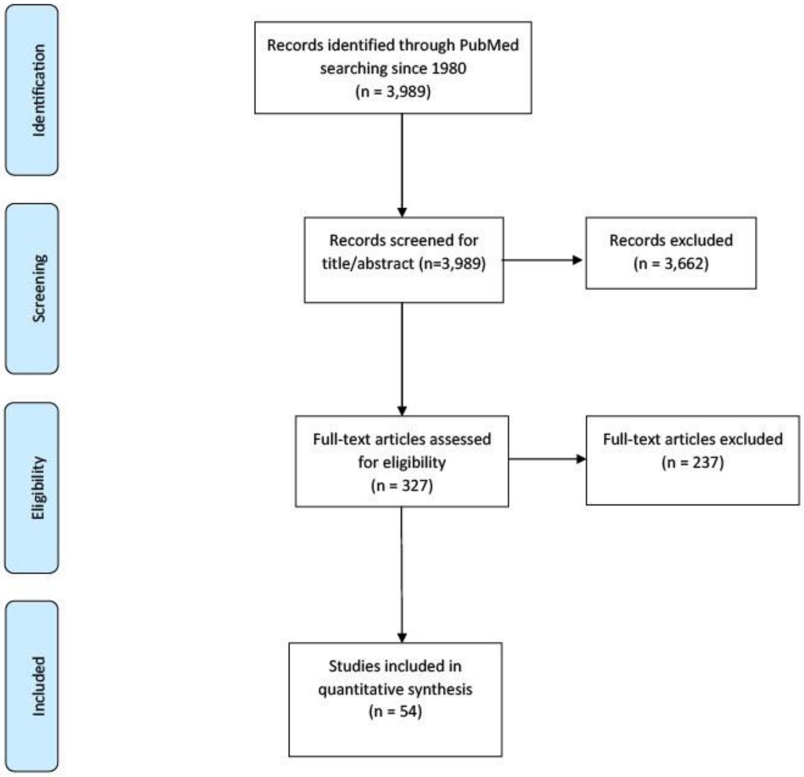

As in past rounds of the GBD, we decided not to use hospital inpatient data as we considered it would not be representative of true prevalence, and that variation between countries in the proportion of true prevalent cases captured in hospital inpatient data systems would likely vary more than can be captured by a single crosswalk in DisMod-MR 2.1. Data from USA claims data for 2000 and 2010–2016 by state and Taiwan claims data from 2016 were considered for inclusion. Very few sources were identified through data re-review and systematic review for OA other, with minimal overlap in reported site. As a result, USA claims data constituted the only data input source for this model.

Age and sex splitting

Reported estimates of prevalence were split by age and sex where possible. First, if studies reported prevalence for broad age groups by sex (eg, prevalence in 15- to 65-year-old males and females separately), and also by specific age groups but for both sexes combined (eg, prevalence in 15- to 30-year-olds, then in 31- to 65-year-olds, for males and females combined), age-specific estimates were split by sex using the reported sex ratio and bounds of uncertainty. Second, input data reporting prevalence of OA for both sexes that could not be split using a within-study ratio were split using a sex ratio derived from a meta-analysis of existing sex-specific data for each type of OA using MR-BRT (meta-regression—Bayesian, regularised, trimmed, see appendix 1 section 2). The female to male ratio was 1.10 (1.09–1.12) for the hip, 1.44 (1.43–1.45) for the knee, and 2.36 (2.33–2.38) for the hand. There were no both-sex input data for OA other. Finally, after the application of bias adjustments, where studies on OA hip and OA knee reported estimates across age groups spanning 25 years or more, these were split into five-year age groups using the prevalence age pattern estimated by DisMod-MR 2.1 (disease model—Bayesian meta-regression, appendix 1 section 2) for each type of OA. Wide age bin data for OA hand were split into five-year age groups using the prevalence age pattern of the USA claims input data. There were no wide age bin input data for OA other.

Data adjustment

For OA hip and OA knee, we marked studies that reported on X-rays only, self-reported OA with pain, or self-reported OA with no information on pain. Other studies identified cases of OA through a review of medical charts. We assumed that these cases were diagnosed by X-ray with pain present. We added three additional covariates for claims data in the USA from the year 2000 and from 2010 onward and for Taiwan claims data. For all these alternative case definitions, we derived adjustment factors using MR-BRT. Claims data from Taiwan were excluded from the model, as we did not have data on the reference case definition from Taiwan to inform a reliable adjustment. Betas and exponentiated values (which can be interpreted as an odds ratio) for these two covariates are shown in the tables 2 and 3:

Table 2: MR-BRT crosswalk adjustment factors for OA hip

Data input	Reference or alternative case definition	Gamma	Beta coefficient, log (95% UI) *	Adjustment factor**
Radiography with pain	Ref	0.26	---	---
Radiography only	Alt		1.09 (0.89 to 1.28)	2.96 (2.44 to 3.6)
Self-reported OA with pain	Alt		1.32 (1.15 to 1.48)	3.73 (3.16 to 4.39)
Self-reported OA, no mention of pain	Alt		1.60 (1.18 to 2.01)	4.94 (3.26 to 7.49)
USA claims data – 2000	Alt		–2.50 (–2.96 to –2.01)	0.082 (0.052 to 0.13)

USA claims data – 2010–2016	Alt		–2.03 (–2.08 to –1.97)	0.13 (0.12 to 0.14)

**MR-BRT crosswalk adjustments can be interpreted as the factor the alternative case definition is adjusted by to reflect what it would have been had it been measured using the reference case definition. If the log/logit beta coefficient is negative, then the alternative is adjusted up to the reference. If the log/logit beta coefficient is positive, then the alternative is adjusted down to the reference.*

***The adjustment factor column is the exponentiated beta coefficient. For log beta coefficients, this is the relative rate between the two case definitions. For logit beta coefficients, this is the relative odds between the two case definitions.*

Table 3: MR-BRT crosswalk adjustment factors for OA knee

Data input	Reference or alternative case definition	Gamma	Beta coefficient, log (95% UI)*	Adjustment factor**
Radiography with pain	Ref	0.38	---	---
Radiography only	Alt		0.21 (0.14 to 0.27)	1.23 91.15 to 1.32)
Self-reported OA with pain	Alt		0.063 (–0.027 to 0.15)	1.065 (0.97 to 1.17)
Self-reported OA, no mention of pain	Alt		–0.77 (–0.81 to –0.72)	0.46 (0.44 to 0.48)
USA claims data – 2000	Alt		–2.26 (–2.64 to –1.88)	0.10 (0.072 to 0.15)
USA claims data – 2010–2016	Alt		–1.60 (–2.43 to –0.77)	0.20 (0.088 to 0.46)

**MR-BRT crosswalk adjustments can be interpreted as the factor the alternative case definition is adjusted by to reflect what it would have been had it been measured using the reference case definition. If the log/logit beta coefficient is negative, then the alternative is adjusted up to the reference. If the log/logit beta coefficient is positive, then the alternative is adjusted down to the reference.*

***The adjustment factor column is the exponentiated beta coefficient. For log beta coefficients, this is the relative rate between the two case definitions. For logit beta coefficients, this is the relative odds between the two case definitions.*

For OA hand, we allowed for alternatives to two dimensions of case definition: affected joint and diagnostic criteria. These alternative case definitions concerned studies reporting on the presence of OA in any single joint type (eg, distal interphalangeal), present in the first carpometacarpal joint of the thumb specifically, present in multiple joint types, or diagnosed as generalised hand OA. Adjustments were also considered for studies that used X-rays, studies in which a physician diagnosed OA without X-rays, studies that used reported pain, and studies that used self-report. We added two additional covariates for claims data in the USA from the year 2000 and from 2010 onward. The mean and standard error for the coefficients were calculated using the MR-BRT crosswalk adjustment method. Data concerning the presence of OA in the thumb base and through self-report were not included in the final model, as we were unable to find matches to inform a reliable crosswalk. Claims data in the USA

were not included in the final model for the same reason. Betas and exponentiated values (which can be interpreted as an odds ratio) for these two covariates are shown in table 4:

Table 4: MR-BRT crosswalk adjustment factors for OA hand

Data input	Reference or alternative case definition	Gamma	Beta coefficient, log (95% UI)*	Adjustment factor**
Radiography with pain in a single joint type	Ref	0.36	---	---
OA in a single joint type	Alt		0.32 (0.29 to 0.34)	1.37 (1.34 to 1.40)
OA in multiple joint types	Alt		0.32 (0.30 to 0.34)	1.38 (1.35 to 1.41)
Generalised hand OA	Alt		–0.74 (–0.80 to –0.68)	0.48 (0.45 to 0.51)
Radiography only	Alt		1.09 (1.03 to 1.15)	2.97 (2.79 to 3.16)
Physician diagnosis only	Alt		0.58 (0.51 to 0.65)	1.78 (1.66 to 1.92)
Pain only	Alt		0.055 (0.0077 to 0.10)	1.06 (1.01 to 1.11)
Radiography with pain	Alt		0.31 (0.23 to 0.39)	1.36 (1.26 to 1.48)
Physician diagnosis with pain	Alt		0.28 (0.20 to 0.35)	1.32 (1.22 to 1.42)

**MR-BRT crosswalk adjustments can be interpreted as the factor the alternative case definition is adjusted by to reflect what it would have been had it been measured using the reference case definition. If the log/logit beta coefficient is negative, then the alternative is adjusted up to the reference. If the log/logit beta coefficient is positive, then the alternative is adjusted down to the reference.*

***The adjustment factor column is the exponentiated beta coefficient. For log beta coefficients, this is the relative rate between the two case definitions. For logit beta coefficients, this is the relative odds between the two case definitions.*

Modelling strategy

For OA hip and OA knee, prior settings in the DisMod-MR model included setting remission to zero, and it was assumed that there was no incidence or prevalence of OA before the age of 30 years. We assumed that excess mortality is zero. While there are some data on excess mortality risk, the values of hazard ratios or standardised mortality ratios are close to one, with some studies reporting mean estimates less than one. We included mean BMI and the SEV scalar for osteoarthritis as country covariates on prevalence. The OA SEV scalar combines the exposure measures for risk estimated to impinge on OA in GBD: increased BMI.

Table 5. Covariates. Summary of covariates used in the OA hip and OA knee DisMod-MR meta-regression models

Covariate	Beta, log (95% uncertainty interval), OA hip	Exponentiated beta (95% uncertainty interval), OA hip	Beta, log (95% uncertainty interval), OA knee	Exponentiated beta (95% uncertainty interval), OA knee
Mean BMI	0.98 (0.86–1.00	2.66 (2.37–2.72)	0.72 (0.54–0.91)	2.06 (1.72–2.48)
Log-transformed age-standardised SEV scalar: OA	1.89 (0.0017–2.00)	6.62 (1.00–7.38)	0.77 (0.75–0.81)	2.16 (2.12–2.24)

For the OA hand and OA other models, settings in DisMod-MR included setting remission to zero and assuming no incidence or prevalence of OA before the age of 30 years. In addition, we included the SEV scalar for OA as a country covariate on prevalence for OA other in order to provide a basis for some geographical variation in a model that only has input data in the USA. This covariate was not used in the OA hand model because we did not have reason to believe that there is a reliable relationship between increased BMI and OA in hand joints.

Table 6. Covariates. Summary of covariates used in the OA other DisMod-MR meta-regression model

Covariate	Beta, log (95% uncertainty interval)	Exponentiated beta (95% uncertainty interval)
Log-transformed age-standardised SEV scalar: OA	1.23 (1.20–1.25)	3.43 (3.32–3.49)

Severity and disability

The basis of the GBD disability weight survey assessments are lay descriptions of sequelae highlighting major functional consequences and symptoms. The lay descriptions and disability weights for OA severity levels are shown below.

Table 7. Severity distribution, details on the severity levels for OA in GBD 2019 and the associated disability weight (DW) with that severity

Severity level	Lay description	DW (95% CI)
Asymptomatic		0
Mild	This person has pain in the leg, which causes some difficulty running, walking long distances, and getting up and down.	0.023 (0.013–0.037)
Moderate	This person has moderate pain in the leg, which makes the person limp, and causes some difficulty walking, standing, lifting and carrying heavy things, getting up and down, and sleeping.	0.079 (0.054–0.110)
Severe	This person has severe pain in the leg, which makes the person limp and causes a lot of difficulty walking,	0.165 (0.112–0.232)

	standing, lifting and carrying heavy things, getting up and down, and sleeping.	

In past GBD rounds, to determine the proportion of people with OA within each of the severity levels, four studies representing the high-income, south Asia, and southeast Asia, dast Asia, and Oceania super-regions provided information on the severity of OA. In GBD 2017, data from the USA Osteoarthritis Initiative study were included as well. The OA Initiative is a large cohort study that follows individuals with OA of the knee recruited from four centres around the USA. In all five studies, severity was classified based on the Western Ontario and McMaster Universities Arthritis Index (WOMAC) with scores 0–5 taken as mild, 6–13 as moderate, and 14 and higher as severe. Estimates were pooled across studies using a random effects meta-analysis model. The pooled percentages were mild 47.0% (42.2–51.9), moderate 35.9% (31.3–40.7), and severe 17.1% (12.9–21.6) pooled between patient and physician ratings in a study from Bangladesh, which we apply to low- and middle-income countries. The pooled proportions from three high-income countries were mild 74.3% (64.8–82.7), moderate 24.3% (16.4–33.1), and severe 1.1% (0.6–1.7). After streaming out 1000 draws assuming a binomial distribution, percentages were scaled to sum to 1 at each draw. For the sake of consistency, the same severity distribution and disability weights were applied to OA hand and OA other, to be reconsidered in the subsequent modelling round.

Other cardiovascular diseases

Flowchart

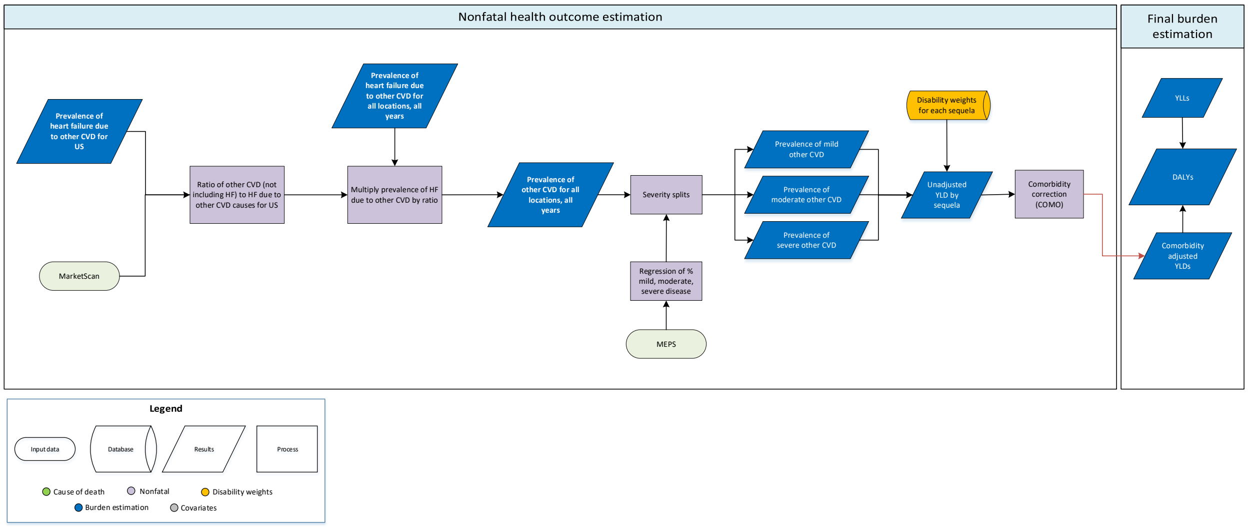

Case definition

Other cardiovascular disease is a residual category resulting from the GBD approach of estimating the total burden of all causes. Prevalence estimates are produced in order to provide YLDs consistent with the estimated YLLs from the death modelling process and to enable the calculation of DALYs.

Conditions included in this cause, based on ICD codes used for both fatal and non-fatal modelling, are other diseases of pulmonary vessels; acute pericarditis; other diseases of pericardium; pericarditis in diseases classified elsewhere; paroxysmal tachycardia; cardiac septal defect, acquired; rupture of chordae tendineae, not elsewhere classified; rupture of papillary muscle, not elsewhere classified; intracardiac thrombosis, not elsewhere classified; cerebral amyloid angiopathy; other aneurysm; other disorders of arteries and arterioles; diseases of capillaries; disorders of arteries, arterioles, and capillaries in diseases classified elsewhere; phlebitis and thrombophlebitis; portal vein thrombosis; other venous embolism and thrombosis; varicose veins of lower extremities; varicose veins of other sites; other disorders of veins; non-specific lymphadenitis; other non-infective disorders of lymphatic vessels and lymph nodes; other disorders of circulatory system in diseases classified elsewhere. As of GBD 2021, codes for pulmonary arterial hypertension (PAH) are no longer included, as PAH is modelled separately.

Input data

As this is a residual category, we used inpatient and outpatient claims data from the USA (MarketScan) and modelled estimates from heart failure due to other cardiovascular disease to estimate prevalence of other cardiovascular disease. MarketScan replaced data from the Medical Expenditure Panel Survey, used in GBD 2021 and before. Details on MarketScan and methods used to extract cause-specific prevalence estimates are detailed in the “Claims data” section of the appendix.

Severity split inputs

The proportions of asymptomatic, mild, moderate, and severe cases for other cardiovascular diseases were determined by the standard approach for severity splitting for GBD 2023 that utilised the Medical Expenditure Panel Survey (MEPS) to map other cardiovascular diseases ICD codes (see table 1) to quality-of-life metrics to quantify disability. More information on methodology on the proportion split using MEPS can be found in the appendix section 4.7: Severity distribution. The table below includes lay descriptions and disability weights for the severity levels of other cardiovascular disease for GBD 2023.

Severity level	Lay description	DW (95% CI)
Asymptomatic		N/A
Mild	Is short of breath and easily tires with moderate physical activity, such as walking uphill or more than a quarter-mile on level ground. The person feels comfortable at rest or during activities requiring less effort.	0.041 (0.026–0.062)
Moderate	Is short of breath and easily tires with minimal physical activity, such as walking only a short distance. The person feels comfortable at rest but avoids moderate activity.	0.072 (0.047–0.103)
Severe	Is short of breath and feels tired when at rest. The person avoids any physical activity, for fear of worsening the breathing problems.	0.179 (0.122–0.251)

Modelling strategy

To obtain prevalence estimates of other cardiovascular disease, we used MarketScan data combined with prevalence estimates of heart failure due to other CVD for the USA to estimate the ratio of the prevalence of heart failure due to other CVD causes in 2015 to the prevalence of other CVD causes in 2015. We then applied this ratio to the age-, sex-, and year-specific prevalence estimates for heart failure due to other CVD causes for all locations to generate prevalence estimates of other cardiovascular disease. Estimation of heart failure due to other CVD is detailed elsewhere in the appendix.

In GBD 2023, updates to heart failure methods made between GBD 2017 and GBD 2021 were applied to this cause. Estimates of heart failure due to other CVD causes are now informed by person-level multiple cause of death data from the USA, Mexico, Colombia, Brazil, and Taiwan (province of China), linked longitudinal data from Italy, and cause-specific mortality estimates from all locations. This substantial methodological improvement led to changes in estimates of heart failure due to other CVD, and therefore changes in estimates of other CVD.

Other chronic respiratory diseases

In addition to chronic obstructive pulmonary disease, asthma, interstitial lung disease and pulmonary sarcoidosis, and pneumoconiosis, there are other types of chronic respiratory diseases with a range of severities and associated sequelae. Because these chronic respiratory diseases are diverse in their underlying causes and risk factors, as well as in their associated health outcomes, modelling them together in a DisMod-MR model would not produce reliable estimates of prevalence. Instead, we calculated the YLDs caused by other chronic respiratory diseases directly using a YLD/YLL ratio.

We calculated the ratio of YLDs to YLLs across the specified chronic respiratory diseases for which non-fatal outcomes were modelled, using YLL estimates from the GBD 2023 cause of death analysis. We then multiplied this YLD/YLL ratio by the YLL estimates for other chronic respiratory diseases.

Other digestive diseases

In addition to specified digestive diseases including inguinal, femoral, and abdominal hernia, inflammatory bowel disease, gastro-oesophageal reflux disease, gastritis and duodenitis, peptic ulcer disease, gallbladder and biliary diseases, appendicitis, paralytic ileus and intestinal obstruction, vascular intestinal disorders, pancreatitis, and diverticular disease, there are a number of other types of digestive diseases with a range of severities and associated sequelae. Because these digestive diseases are diverse in their underlying causes and risk factors, as well as in their associated health outcomes, modelling them together in a DisMod-MR model would not produce reliable estimates of prevalence. Instead, we calculated the YLDs caused by other digestive diseases directly using an YLD/YLL ratio as a "placeholder".

We calculated the ratio of YLDs to YLLs across the specified digestive diseases for which non-fatal outcomes were modelled, using YLL estimates from the GBD 2023 cause of death analysis. We then multiplied this YLD/YLL ratio by the YLL estimates for other digestive diseases.

Other drug use disorders

Flowchart

Other drug use disorders

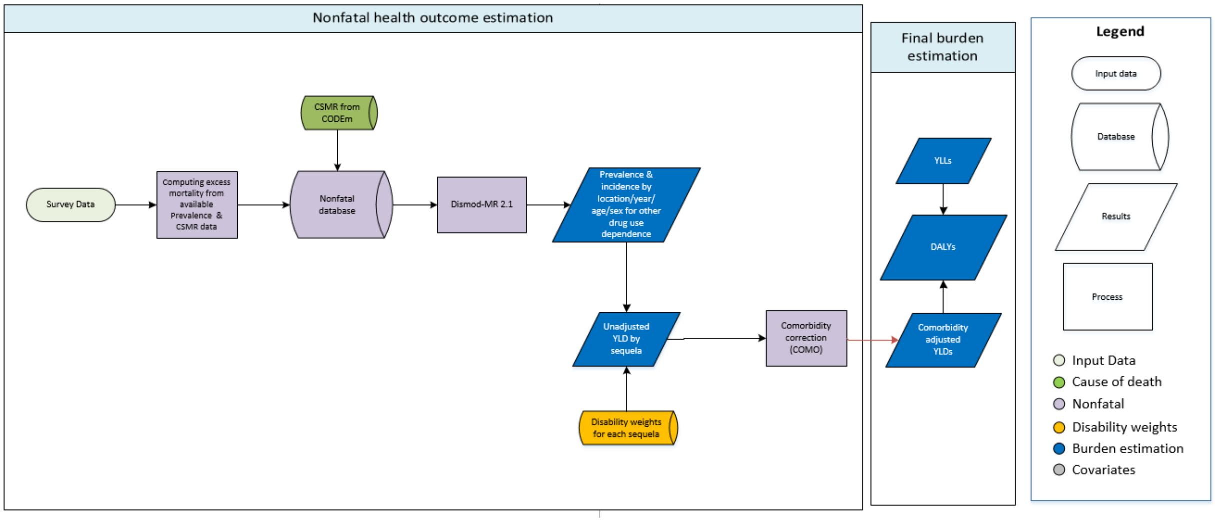

Input data and methodological summary for other drug use disorders

Case definition

In addition to the four drug use disorders for which we specifically estimate non-fatal burden (opioid, cocaine, amphetamine, and cannabis dependence), we also estimate the burden attributable to a residual cause of "other drug use disorders." This is made up of an aggregate group of other forms of drug dependence. Included in the Global Burden of Disease (GBD) modelling were cases meeting the Diagnostic and Statistical Manual of Mental Disorders (DSM-IV-TR)[1] or the International Classification of Diseases (ICD-10)[2,3]diagnostic criteria for:

- Hallucinogen dependence: ICD-9 304.50, corresponding to ICD-10 F16.2
- Inhalant or solvent dependence: ICD-9 304.60, corresponding to ICD-10 F18.2
- Sedative dependence: ICD-9 304.10, corresponding to ICD-10 F13.2
- Tranquilisers dependence: ICD-9 304.10, corresponding to ICD-10 F13.2
- Other medicines, drugs, substance dependence: ICD-9 304.80 or 304.90, corresponding to ICD-10 F19.2

Table 1. Case definitions accepted for other drug use disorders.

**Quantity of interest**	**Reference or alternative**	**Definition**
Other drug dependence prevalence	Reference	Dependence on other drugs based on ICD or DSM criteria. "Other drugs" include hallucinogens, inhalant and solvents, sedatives, tranquilisers, and any other drugs that are not classified as opioids, amphetamines, cannabis, or cocaine.

Other drug dependence prevalence	Alternative	A chronic condition in which a person craves a drug other than opioids, cocaine, amphetamines or cannabis and is unable to control taking the drug. The person needs greater amounts to get the same effect and has withdrawal symptoms after stopping

Input data

Prevalence estimates for drug dependence were derived from two major surveys: the Australian National Survey of Mental Health and Wellbeing (NSMHWB)[4] conducted in 1997, and the US National Epidemiological Survey on Alcohol and Related Conditions (NESARC),[5] which had two waves in 2001–2002 and 2004–2005. Considering the frequent co-occurrence of various forms of drug dependence—specifically opioid, cocaine, amphetamine, and cannabis dependence—it was crucial to adjust for co-morbidity to avoid overestimating the overall burden of drug dependence. In these surveys, individuals were only classified as having a prevalent case of drug dependence if they did not meet the criteria for dependence on opioids, cocaine, amphetamines, or cannabis simultaneously.

Modelling strategy

We have made no substantive changes in the modelling strategy from GBD 2021. The epidemiological modelling strategy made use of our disease model—Bayesian meta-regression tool[7] (DisMod-MR 2.1, see appendix 1 section 2).

A number of additional expert priors were used in order to run a full parameter model. We assumed no incidence before age 14, a maximum of 0.0004 on incidence from the age of 60 years onward, and a maximum remission of 0.2. These priors were corroborated with expert feedback and existing literature on drug use disorders including the European Monitoring Centre for Drugs and Drug Addiction.[6] Finally, cause-specific mortality rates (CSMR) from the GBD 2023 cause of death model for other drug use disorders were included as datapoints in the DisMod-MR model. Prior to GBD 2016, a YLL (years of life lost) to YLD (years lived with disability) ratio analysis was used to estimate prevalence. However, in GBD 2021, this methodology was discontinued to prevent the issue of double-counting. Consequently, there has been a global reduction in the reported YLDs.

Severity and disability

The basis of the GBD disability weight survey assessments are lay descriptions of sequelae highlighting major functional consequences and symptoms. The average disability weight estimated for cocaine and amphetamine dependence was applied to all cases in this residual group of other drug use disorders.[7] The cocaine and amphetamine lay descriptions and disability weights are shown below.

Table 2. Severity distribution, details on the severity levels for amphetamine use and cocaine use disorders in GBD 2021, and the associated disability weight (DW) with that severity

Severity level	Lay description	DW (95% CI)
**Amphetamine dependence**		

Mild	Uses stimulants (drugs) at least once a week and has some difficulty controlling the habit. When not using, the person functions normally.	0.079 (0.051–0.114)
Moderate to severe	Uses stimulants (drugs) and has difficulty controlling the habit. The person sometimes has depression, hallucinations, and mood swings, and has difficulty in daily activities.	0.486 (0.329–0.637)
**Cocaine dependence**		
Mild	Uses cocaine at least once a week and has some difficulty controlling the habit. When not using, the person functions normally.	0.116 (0.074–0.165)
Moderate to severe	Uses cocaine and has difficulty controlling the habit. The person sometimes has mood swings, anxiety, paranoia, hallucinations, and sleep problems, and has some difficulty in daily activities.	0.479 (0.324–0.634)

**Asymptomatic cases carried no disability weight.*

As in GBD 2021, lag-distributed income (LDI) was included as a country covariate on EMR with bounds set at –1 and –0.1.

Table 3. Covariates. Summary of covariates used in the other drug use disorders DisMod-MR meta-regression model

Covariate	Type	Parameter	Exponentiated beta (95% uncertainty interval)
LDI ($ per capita)	Country-level	Excess mortality rate	0.46 (0.38–0.58)

References

1. American Psychiatric Association. Diagnostic and Statistical Manual of Mental Disorders (DSM-IV-TR). 4th, Text Revision ed. Washington DC: 2000.
2. World Health Organization. The ICD-10 Classification of Mental and Behavioural Disorders. Clinical descriptions and diagnostic guidelines. Geneva: 1992.
3. ICD10Data.com. Convert ICD-9-CM codes to ICD-10-CM codes. Available from: https://www.icd10data.com/Convert. Accessed 1/27/2025].

4. Australia Bureau of Statistics. Australia National Survey of Mental Health and Wellbeing 1997. Canberra: 1997.

5. National Institute on Alcohol Abuse and Alcoholism (NIAAA), National Institutes of Health (NIH). United States National Epidemiologic Survey on Alcohol and Related Conditions 2001-2002. 2002.

6. European Monitoring Centre for Drugs and Drug Addiction. Lisbon, Portugal: 2014.

7. Vos T, Lim SS, Abbafati C, et al. Global burden of 369 diseases and injuries in 204 countries and territories, 1990–2019: a systematic analysis for the Global Burden of Disease Study 2019. *The Lancet* 2020; 396: 1204–22. doi: https://doi.org/10.1016/S0140-6736(20)30925-9

Other intestinal infectious diseases

There are many diverse types of intestinal infectious diseases. Because these intestinal infectious diseases are diverse in their underlying causes and risk factors as well as in their associated health outcomes, modelling them together in a DisMod-MR model would not produce reliable estimates of prevalence or excess mortality. Instead, we calculated the YLDs caused by intestinal infectious diseases directly using a YLD/YLL ratio.

We calculated the ratio of YLDs to YLLs across the specified intestinal infectious diseases for which non-fatal outcomes were modelled, using YLL estimates from the GBD 2023 cause of death (CoD) analysis. We then multiplied this YLD/YLL ratio by the YLL estimates for other intestinal infectious diseases from the GBD 2023 CoD analysis, providing us with an estimate of the YLDs associated with other intestinal infectious diseases.

Other mental disorders

Flowchart

Other mental disorders: Personality disorders

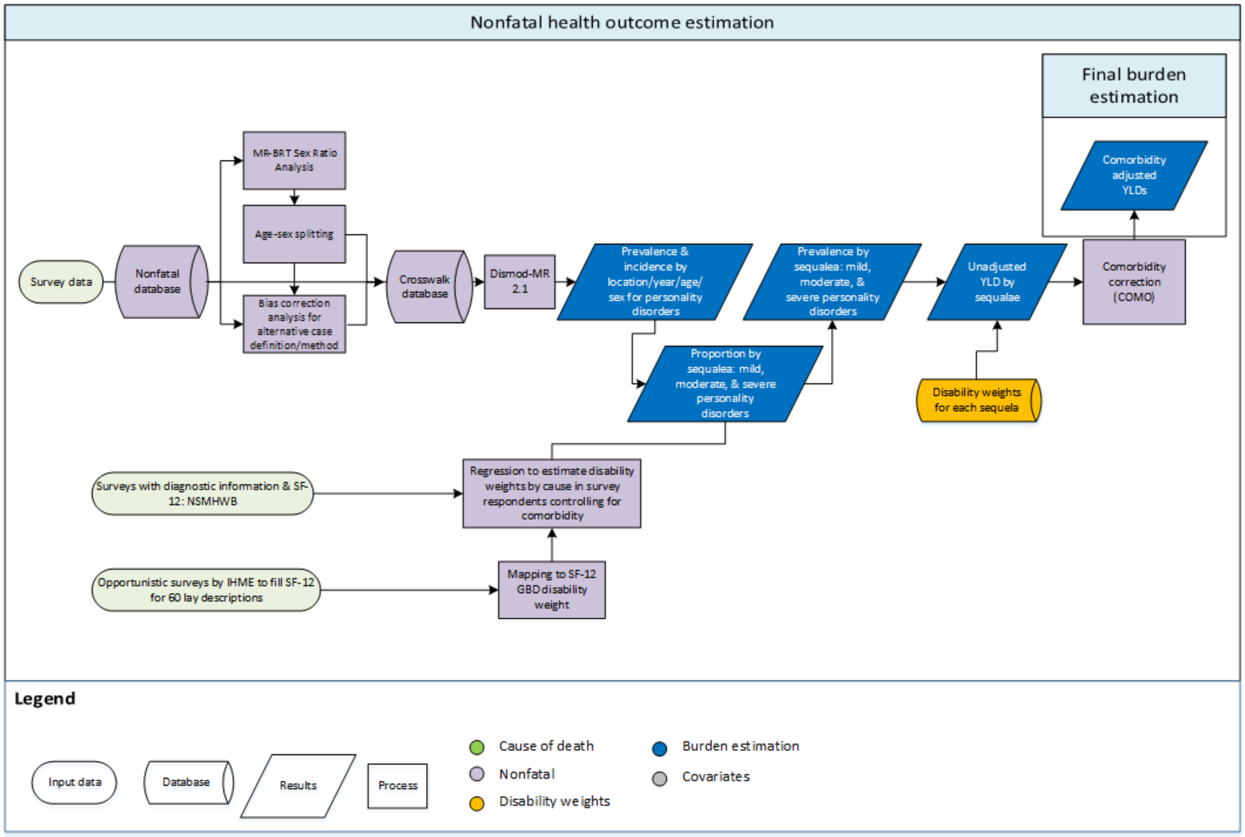

Input data and methodological summary for other mental disorders

Case definition

In addition to the individual mental disorders for which we estimate burden, we also estimate the non-fatal burden attributable to a residual cause of "other mental disorders." This is made up of an aggregate group of personality disorders. Personality disorders are characterised by pervasive, inflexible and maladaptive patterns of behaviour and inner experience which are markedly different from what is considered to be acceptable in the individual's culture. These disorders tend to be chronic and are associated with significant distress or disability. Included in GBD 2023 were cases meeting diagnostic criteria for personality disorders according to the Diagnostic and Statistical Manual of Mental Disorders (DSM-IV-TR), or the equivalent diagnosis in the International Classification of Diseases (ICD-10: F60)[1,2] that did not meet criteria for another mental disorder or substance use disorder. The aggregated group of DSM personality disorders used in GBD 2023 captured any of the following:

- Paranoid personality disorder
- Schizoid personality disorder

- Schizotypal personality disorder
- Antisocial personality disorder
- Borderline personality disorder
- Histrionic personality disorder
- Narcissistic personality disorder
- Avoidant personality disorder
- Dependent personality disorder
- Obsessive-compulsive personality disorder
- Personality disorder not otherwise specified

Input data

Prevalence estimates for the above personality disorders were obtained from the US National Epidemiological Survey on Alcohol and Related Conditions (NESARC, conducted in two waves from 2001–2002 and 2004–2005)[3] and the Australian National Survey of Mental Health and Wellbeing of Adults (NSMHWB, conducted in 1997).[4] Given that personality disorders often co-occur with other mental and substance use disorders, an adjustment for comorbidity is important so as not to overestimate the overall burden attributable to mental and substance use disorders. Participants meeting criteria for any type of personality disorder from the NESARC and NSMHWB surveys were counted as a prevalent case only if they did not simultaneously meet criteria for another mental and substance use disorders featured in GBD 2023.

Bias corrections/crosswalks

Estimates with known biases were adjusted/crosswalked accordingly prior to DisMod-MR 2.1. A NESARC:NSMHWB prevalence ratio of 2.04 (95% uncertainty interval [UI] 1.82–2.34) was used to adjust all datapoints derived from NESARC towards the level of datapoints from the NSMHWB. The latter survey was made up of a more representative list of personality disorders and produced estimates along the levels of what we would expect for personality disorders. As this ratio was informed by only two data sources it was estimated outside of the meta-regression—Bayesian, regularised, trimmed (MR-BRT) analysis typically used for bias correction in GBD 2023.

Modelling strategy

We have made no substantive changes in the modelling strategy from GBD 2021.

After the above data processes were applied, DisMod MR 2.1 was used to model the epidemiological data for personality disorders. Adjustments to model priors or the dataset were made where appropriate. Where outliers were identified in the data, we reassessed the study's methodology and quality before a decision was made to exclude or include the data.

As we only had prevalence data available, a number of expert priors were used in order to run a full-parameter model. We assumed no incidence and prevalence before age 14. This minimum age of onset was corroborated with expert feedback and DSM criteria highlighting the fact that personality disorders typically become recognisable during adolescence and early adulthood. Remission was set to a maximum of 0.01, given that these are understood to be chronic disorders with little or no complete remission. Excess mortality was set to zero in this model, in the absence of mortality data required for

DisMod-MR 2.1 modelling purposes. Given the sparsity of data, we applied a restriction on location random effects of –0.1 to 0.1 to further guide prevalence estimation.

Severity splits and disability weights

The GBD disability weight survey assessments include lay descriptions of sequelae highlighting major functional consequences and symptoms. The lay descriptions and disability weights applied to the personality disorders within this residual group are shown below and were those estimated for anxiety disorders (See Table 1). To determine the proportion of people with personality disorders within each of the severity levels, the NSMHWB survey was used to estimate the proportion of cases asymptomatic (30%, 28–32), mild (41%, 33–47), moderate (15%, 11–20), and severe (14%, 10–18).

Table 1. Severity distribution, details on the severity levels for other mental disorders and the associated disability weight with that severity

**Severity level**	**Lay description**	**Disability weight (95% UI)**
Mild	Feels mildly anxious and worried, which makes it slightly difficult to concentrate, remember things, and sleep. The person tires easily but is able to perform daily activities.	0.03 (0.018–0.046)
Moderate	Feels anxious and worried, which makes it difficult to concentrate, remember things, and sleep. The person tires easily and finds it difficult to perform daily activities.	0.133 (0.091–0.186)
Severe	Constantly feels very anxious and worried, which makes it difficult to concentrate, remember things, and sleep. The person has lost pleasure in life and thinks about suicide.	0.523 (0.362–0.677)

There were no significant changes in GBD 2023 results for other mental disorders compared to GBD 2021. In this model, global prevalence was exclusively estimated using prevalence estimates from two surveys from the USA and Australia where we had unit record data available to estimate the prevalence of personality disorders, excluding those not simultaneously meeting criteria for another mental or substance use disorder. The sparsity of data leads to modelled prevalence estimates with large uncertainty bounds, which are sensitive to model re-runs and small changes to model settings. We are currently undertaking a literature review of population-survey data on the epidemiology of personality disorders across low-, middle-, and high-income countries with the aim of providing more robust and globally representative burden estimates for personality disorders in future GBD studies.

References

1. American Psychiatric Association. Diagnostic and Statistical Manual of Mental Disorders (DSM). Washington: American Psychiatric Association, 1952.
2. World Health Organization. The ICD-10 Classification of Mental and Behavioural Disorders. Clinical descriptions and diagnostic guidelines. Geneva: World Health Organization; 1992.

3. Introduction to the National Epidemiologic Survey on Alcohol and Related Conditions [http://pubs.niaaa.nih.gov/publications/arh29-2/74-78.htm]. Access date 1 December 2014.
4. Australian Bureau of Statistics. National Survey of Mental Health and Wellbeing of Adults 1997. Canberra: Australian Bureau of Statistics.

Other musculoskeletal disorders (other MSK)

Flowchart

Other musculoskeletal disorders

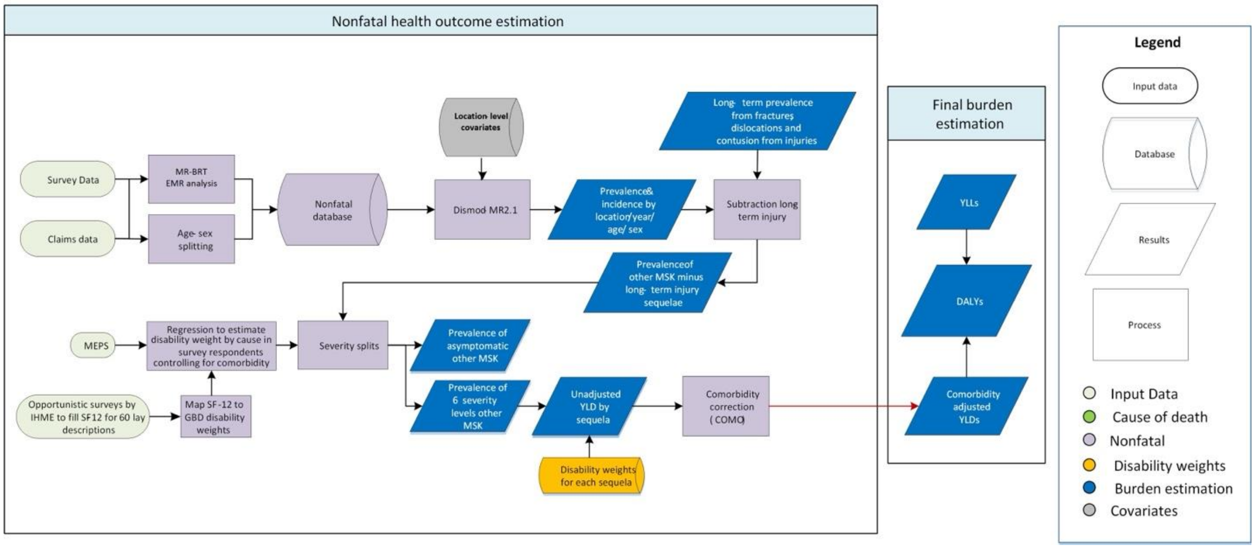

Input data and methodological summary for other musculoskeletal disorders

Case definition

Other musculoskeletal disorders is a heterogeneous rest category comprising a wide range of disorders of muscles, bones, and ligaments that are not included in the five GBD defined musculoskeletal diseases – rheumatoid arthritis, osteoarthritis, low back pain, neck pain, and gout – and are not captured as long-term sequelae of injuries.

The utilised case definitions for other musculoskeletal disorders are listed below.

**Reference or alternative**	**Definition**
Reference	Prevalence of any of the following conditions: lupus erythematosus, infectious arthropathies, inflammatory polyarthropathies, other joint disorders, systemic connective tissue disorders, deforming dorsopathies, spondylopathies, disorders of the muscles, disorders of synovium and tendon, other soft tissue disorders, disorders of bone density and structure, osteomyelitis, other osteopathies, chondropathies, other disorders of the musculoskeletal system and connective tissue not included under gout, rheumatoid arthritis, osteoarthritis, low back pain, or neck pain
Alternative	USA claims data 2010–2015
Alternative	USA claims data 2000

The table below provides detail of the ICD-10 and ICD-9 codes included in this category.

ICD-10 codes	ICD-9 codes
L93—Lupus erythematosus<br>M00-M02—Infectious arthropathies<br>M08, M11-M13—Inflammatory polyarthropathies<br>M20-M25—Other joint disorders<br>M30-M35—Systemic connective tissue disorders<br>M40-M43—Deforming dorsopathies<br>M45-M46—Spondylopathies<br>M60 -M63—Disorders of muscles<br>M65-M68—Disorders of synovium and tendon<br>M70- M73, M75-M79—Other soft tissue disorders<br>M80-M85—Disorders of bone density and structure<br>M86—Osteomyelitis<br>M87-M90—Other osteopathies<br>M91-M94—Chondropathies<br>M95-M99—Other disorders of the MSK system and connective tissue	710.0<br>711<br>712–713<br>716–719<br>710.1–710.9<br>737<br>720–721<br>725<br>726–728<br>729<br>733.0-2<br>730.1–730.3, 730.7-9<br>731, 733.3-9<br>732<br>734–736, 738–739

Input data

The above ICD codes were used to extract other MSK prevalence from USA claims data for 2000 and 2010–2016 by state. The systematic review concentrated on finding health surveys that measured an overall amount of musculoskeletal disorders and reported information to distinguish a rest category that was not OA, RA, gout, or low back or neck pain. These data sources are based on self-reported musculoskeletal conditions or symptoms and did not use the listed ICD codes.

Age and sex splitting

Reported estimates of prevalence were split by age and sex where possible. First, if studies reported prevalence for broad age groups by sex (eg, prevalence in 15- to 65-year-old males and females separately), and by specific age groups but for both sexes combined (eg, prevalence in 15- to 30-year-olds, then in 31- to 65-year-olds, for males and females combined), age-specific estimates were split by sex using the reported sex ratio and bounds of uncertainty. Second, prevalence data for both sexes that could not be split using a within-study ratio were split using a sex ratio derived from a meta-analysis of existing sex-specific data using MR-BRT (meta-regression—Bayesian, regularised, trimmed; described in See appendix 1 section 2). The female to male ratio was 1.37 (1.37–1.38). Finally, where studies reported estimates across age groups spanning 25 years or more, these were split into five-year age groups using the prevalence age pattern estimated by DisMod-MR 2.1 (see appendix 1 section 2).

Data adjustment

In previous rounds, we used two study covariates to adjust claims data from the USA by state from the year 2000 and from 2010 onward. For GBD 2019 onward, we did not carry out bias adjustments for claims data because claims sources are more likely to capture all of the ICD codes included in the other MSK category and reflect the assumed mutual exclusivity of component disorders than study and survey data. In future rounds of the GBD, we intend to begin the process of modelling certain component

disorders independently to more accurately reflect their prevalence and reduce variability of input data for the remaining disorders in the other MSK model.

Modelling strategy

There have been no significant changes to the modelling strategy in GBD 2023.

Prior settings in the DisMod-MR model included the assumption of no incidence or prevalence of other MSK before the age of 10 years. In the absence of any meaningful data on incidence and remission for such a heterogeneous category of disorders, we made a rather arbitrary decision of remission of 0.5–1, ie, an average duration of 1–2 years. We also included the Socio-demographic Index country covariate with bounds set at −1 and 1.

Despite its inconsistencies between CSMR and prevalence prior to the inclusion of the modelled EMR data, the final other MSK model both excludes predicted data for the EMR (excess mortality rate) parameter and has the GBD 2017 DisMod-MR EMR calculation disabled. This is because the input data for the EMR MR-BRT analysis represented a narrow range of relatively high Healthcare Access and Quality (HAQ) Index locations, which resulted in far greater predicted EMR in data-sparse, lower HAQ Index locations than in prior rounds, suppressing prevalence to implausibly low levels. Data for cause-specific mortality rate were also excluded from the model (arguing that the pattern of mortality comes from autoimmune diseases, which constitute only a small fraction of the non-fatal manifestations captured in this residual category), a 15-year time window was set, and bounds of 0 to 0 were set on EMR, while retaining the HAQ Index country covariate on the parameter.

Table 2. Covariates. Summary of covariates used in the other MSK DisMod-MR meta-regression model

**Covariate**	**Type**	**Parameter**	**Exponentiated beta (95% uncertainty interval)**
Healthcare Access and Quality Index	Country-level	Excess mortality rate	0.95 (0.94–0.96)
Socio-demographic Index	Country-level	Prevalence	2.71 (2.69–2.72)

Severity and disability

The basis of the GBD disability weight survey assessments are lay descriptions of health states highlighting major functional consequences and symptoms. The lay descriptions and disability weights for other MSK severity levels are shown below. They include the three levels of health states that are used for osteoarthritis and rheumatoid arthritis, each.

Table 3. Severity distribution, details on the severity levels for other MSK in GBD 2019 and the associated disability weight (DW) with that severity.

Severity level	Lay description	DW (95% CI)	Proportions
Asymptomatic			0.28 (0.27–0.29)
Musculoskeletal problems, lower limbs, mild	This person has pain in the leg, which causes some difficulty running,	0.023 (0.013–0.040)	0.22 (0.15–0.30)

	walking long distances, and getting up and down.		
Musculoskeletal problems, upper limbs, mild	This person has mild pain and stiffness in the arms and hands. The person has some difficulty lifting, carrying, and holding things.	0.028 (0.017–0.046)	0.20 (0.15–0.29)
Musculoskeletal problems, upper limbs, moderate	This person has moderate pain and stiffness in the arms and hands, which causes difficulty lifting, carrying, and holding things, and trouble sleeping because of the pain.	0.115 (0.079–0.163)	0.10 (0.06–0.15)
Musculoskeletal problems, lower limbs, severe	This person has severe pain in the leg, which makes the person limp and causes a lot of difficulty walking, standing, lifting and carrying heavy things, getting up and down, and sleeping.	0.163 (0.109–0.224)	0.06 (0.04–0.07)
Musculoskeletal problems, generalised, moderate	This person has pain and deformity in most joints, causing difficulty moving around, getting up and down, and using the hands for lifting and carrying. The person often feels fatigue.	0.312 (0.201–0.438)	0.07 (0.06–0.08)
Musculoskeletal problems, generalised, severe	This person has severe, constant pain and deformity in most joints, causing difficulty moving around, getting up and down, eating, dressing, lifting, carrying, and using the hands. The person often feels sadness, anxiety, and extreme fatigue.	0.572 (0.370–0.758)	0.07 (0.07–0.08)

The severity distributions were derived from an analysis of the Medical Expenditure Panel Surveys (MEPS) in the USA. MEPS is an overlapping continuous panel survey of the United States non-institutionalised population whose primary purpose is to collect information on the use and cost of health care. Panels are two years long and are conducted in five rounds, which are conducted every five to six months. A new panel begins annually, while the last panel is in its second year (http://www.meps.ahrq.gov/survey_comp/hc_data_collection.jsp). Each panel typically contains about 30,000 to 35,000 individual respondents.

MEPS was initiated in 1996 but only began collecting health status data in the form of 12-Item Short Form Survey (SF-12) responses in 2000. For GBD 2016, we used data from 2000–2014. Respondents self-administer the SF-12 twice per panel, at rounds two and four, typically about a year apart. Only adults 18 years and older completed the SF-12. MEPS also usually collects information on diagnoses based on self-report of reasons for encounters with health services. In addition, diagnoses are derived through additional questions on “problems that bother you” or conditions that led to “disability days,” ie, days

out of role due to illness. Professional coders translate the verbatim text into three-digit ICD-9 codes. The main reason for other MSK being measured in MEPS relates to health care contact.

To derive a crosswalk of SF-12 values into a scale comparable with that used by the GBD disability weights, small studies on convenience samples were conducted asking respondents to fill in SF-12 to reflect 62 lay descriptions of diverse severity that were used to derive the GBD disability weights. From these responses, a relationship between SF-12 summary score and the GBD DWs was derived. With regression methods, average disability weights were calculated for each of 156 conditions for which there were corresponding diagnoses in MEPS, while controlling for any comorbid other condition by adding dummy variables for each condition. We binned the amount of DW attributed to other MSK across the seven health states assuming thresholds at the midpoints between DW values.

Other neglected tropical diseases

Other neglected tropical diseases is a residual category in addition to the specific neglected tropical diseases that were modelled separately. There are many diverse types of neglected tropical diseases included in this category, which are encompassed by the following ICD-10 codes:

A68 Relapsing fevers

A68.0 Louse-borne relapsing fever

A68.1 Tick-borne relapsing fever

A68.9 Relapsing fever, unspecified

A69.5 (Code not listed in ICD-10 categorisations, but present in mortality data)

A69.8 Other specified spirochetal infections

A69.9 Spirochetal infection, unspecified

A75 Typhus fever

A75.0 Epidemic louse-borne typhus fever due to *Rickettsia prowazekii*

A75.1 Recrudescent typhus [Brill's disease]

A75.2 Typhus fever due to *Rickettsia typhi*

A75.3 Typhus fever due to *Rickettsia tsutsugamushi*

A75.9 Typhus fever, unspecified

A77 Spotted fever [tick-borne rickettsioses]

A77.0 Spotted fever due to *Rickettsia rickettsii*

A77.1 Spotted fever due to *Rickettsia conorii*

A77.2 Spotted fever due to *Rickettsia siberica*

A77.3 Spotted fever due to *Rickettsia australis*

A77.8 Other spotted fevers

A77.9 Spotted fever, unspecified

A78 Q fever

A79 Other rickettsioses

A79.0 Trench fever

A79.1 Rickettsialpox due to *Rickettsia akari*

A79.8 Other specified rickettsioses

A79.81 Rickettsiosis due to *Ehrlichia sennetsu*

A79.89 Other specified rickettsioses

A79.9 Rickettsiosis, unspecified

A92 Other mosquito-borne viral fevers

A92.0 Chikungunya virus disease

A92.1 O’nyong-nyong fever

A92.2 Venezuelan equine fever

A92.3 West Nile virus infection

A92.30 West Nile virus infection, unspecified

A92.31 West Nile virus infection with encephalitis

A92.32 West Nile virus infection with other neurological manifestation

A92.39 West Nile virus infection with other complications

A92.4 Rift Valley fever

A92.8 Other specified mosquito-borne viral fevers

A92.9 Mosquito-borne viral fever, unspecified

A93 Other arthropod-borne viral fevers, not elsewhere classified

A93.0 Oropouche virus disease

A93.1 Sandfly fever

A93.2 Colorado tick fever

A93.8 Other specified arthropod-borne viral fevers

A94 Unspecified arthropod-borne viral fever

A94.0 Unspecified arthropod-borne viral fever

A96 Arenaviral haemorrhagic fever

A96.0 Junin haemorrhagic fever

A96.1 Machupo haemorrhagic fever

A96.2 Lassa fever

A96.8 Other arenaviral haemorrhagic fevers

A96.9 Arenaviral haemorrhagic fever, unspecified

A98 Other viral haemorrhagic fevers, not elsewhere classified

A98.0 Crimean-Congo haemorrhagic fever

A98.1 Omsk haemorrhagic fever

A98.2 Kyasanur Forest disease

A98.3 Marburg virus disease

A98.5 Haemorrhagic fever with renal syndrome

A98.8 Other specified viral haemorrhagic fevers

B33.1 Ross River disease

B60 Other protozoal diseases, not elsewhere classified

B60.0 Babesiosis

B60.1 Acanthamebiasis

B60.10 Acanthamebiasis, unspecified

B60.11 Meningoencephalitis due to *Acanthamoeba* (culbertsoni)

B60.12 Conjunctivitis due to *Acanthamoeba*

B60.13 Keratoconjunctivitis due to *Acanthamoeba*

B60.19 Other acanthamebic disease

B60.2 Naegleriasis

B60.8 Other specified protozoal diseases

B67.5 Echinococcus multilocularis infection of liver

B67.6 Echinococcus multilocularis infection, other and multiple sites

B67.61 Echinococcus multilocularis infection, multiple sites

B67.69 Echinococcus multilocularis infection, other sites

B67.7 Echinococcus multilocularis infection, unspecified

B70 Diphyllobothriasis and sparganosis

B70.0 Diphyllobothriasis

B70.1 Sparganosis

B71 Other cestode infections

B71.0 Hymenolepiasis

B71.1 Dipylidiasis

B71.8 Other specified cestode infections

B71.9 Cestode infection, unspecified

B74.3 Loiasis

B74.4 Mansonelliasis

B74.8 Other filariases

B74.9 Filariasis, unspecified

B75 Trichinellosis

B78 Strongyloidiasis

B78.0 Intestinal strongyloidiasis

B78.1 Cutaneous strongyloidiasis

B78.7 Disseminated strongyloidiasis

B78.9 Strongyloidiasis, unspecified

B83 Other helminthiases

B83.0 Visceral larva migrans

B83.1 Gnathostomiasis

B83.2 Angiostrongyliasis due to Parastrongylus cantonensis

B83.3 Syngamiasis

B83.4 Internal hirudiniasis

B83.8 Other specified helminthiases

Because these neglected tropical diseases are diverse in their underlying causes and risk factors, as well as in their associated health outcomes, modelling them together in a DisMod-MR model would not produce reliable estimates of prevalence or excess mortality. Instead, we calculated the YLDs caused by neglected tropical diseases directly using a YLD/YLL ratio.

We calculated the ratio of YLLs for other neglected tropical diseases to the sum of YLLs across the specified neglected tropical disease, using YLL estimates from the GBD 2023 cause of death analysis. We then multiplied this ratio by the YLDs estimated for the specified neglected tropical diseases from the GBD 2023 non-fatal analysis, providing us with an estimate of the YLDs associated with other neglected tropical diseases. The YLDs of anaemia due to other neglected tropical diseases were estimated using a different approach (see anaemia documentation for details).

We did not apply any adjustments for the COVID-19 pandemic to other neglected tropical diseases due to a lack of available data quantifying the impacts of the pandemic on NTD epidemiology.

Changes from GBD 2021 to GBD 2023

There were no substantive changes to the modelling strategy for GBD 2023.

Other neurological disorders

In addition to neurological disorders modeled separately in the GBD, we include an "Other neurological disorders" category to encompass a diverse range of neurological disorders with varying severities and associated sequelae (eg, muscular dystrophy; Huntington's disease – see below for a complete list of ICD-9-CM and ICD-10-CM codes and condition types included in this category). Because these neurological disorders are diverse in their underlying causes and risk factors as well as in their associated health outcomes, modelling them together in a DisMod-MR model would not produce reliable estimates of prevalence or excess mortality. Instead, we calculated the YLDs caused by neurological disorders directly using a YLD/YLL ratio.

We calculated the ratio of YLDs to YLLs across the specified neurological disorders for which fatal and non-fatal outcomes were modelled, including motor neuron disease, multiple sclerosis, epilepsy, dementia, and Parkinson's disease. We used YLL estimates from the GBD 2023 cause of death (CoD) analysis. We then multiplied this YLD/YLL ratio by the YLL estimates for other neurological disorders from the GBD 2023 CoD analysis, providing us with an estimate of the YLDs associated with other neurological disorders.

Table 1. ICD-9-CM and ICD-10-CM codes included in other neurological disorders category

Condition type	ICD codes	Conditions covered
Neurodegenerative disorders	**ICD-9**: 330, 330.1, 330.2, 330.3, 330.4, 330.8, 330.9, 331.7 **ICD-10**: F02.2, G13, G13.1, G13.2, G13.8, G23, G23.0, G23.1, G23.2, G23.5, G23.8, G23.9	Cerebral degenerations usually manifest in childhood; Cerebral lipidoses; Cerebral degeneration in generalized lipidoses; Cerebral degeneration of childhood in other diseases classified elsewhere; Other specified cerebral degenerations in childhood; Unspecified cerebral degeneration in childhood; Cerebral degeneration in diseases classified elsewhere; Dementia in other diseases classified elsewhere; Systemic atrophies primarily affecting central nervous system in diseases classified elsewhere; Other systemic atrophy primarily affecting central nervous system in neoplastic disease; Systemic atrophy primarily affecting the central nervous system in myxedema; Systemic atrophy primarily affecting central nervous system in other diseases classified elsewhere; Other degenerative diseases of basal ganglia; Hallervorden-Spatz disease; Progressive supranuclear ophthalmoplegia [Steele-Richardson-Olszewski]; Striatonigral degeneration; Other specified degenerative diseases of basal ganglia; Degenerative disease of basal ganglia, unspecified
Movement disorders	**ICD-9**: 333, 333.1, 333.2, 333.3, 333.5,	Other extrapyramidal disease and abnormal movement disorders; Essential and other specified

	333.6, 333.7, 333.71, 333.72, 333.79, 333.8, 333.81, 333.83, 333.84, 333.85, 333.89, 333.9, 333.91, 333.92, 333.93, 333.94, 333.99<br>**ICD-10**: G24, G24.1, G24.2, G24.3, G24.4, G24.5, G24.8, G24.9, G25, G25.0, G25.2, G25.3, G25.5, G25.8, G25.81, G25.82, G25.83, G25.89, G25.9, G26, G26.0	forms of tremor; Myoclonus; Tics of organic origin; Other choreas; Genetic torsion dystonia; Acquired torsion dystonia; Athetoid cerebral palsy; Acute dystonia due to drugs;<br>Other acquired torsion dystonia; Fragments of torsion dystonia; Blepharospasm; Orofacial dyskinesia; Spasmodic torticollis; Organic writers' cramp; Subacute dyskinesia due to drugs; Other fragments of torsion dystonia; Unspecified extrapyramidal disease and abnormal movement disorder; Stiff-man syndrome; Neuroleptic malignant syndrome; Benign shuddering attacks; Restless legs syndrome (RLS); Idiopathic nonfamilial dystonia
Spinocerebellar disorders	**ICD-9**: 334, 334.1, 334.2, 334.3, 334.4, 334.8, 334.9<br>**ICD-10**: G11, G11.0, G11.1, G11.2, G11.3, G11.4, G11.8, G11.9	Spinocerebellar disease; Hereditary spastic paraplegia; Primary cerebellar degeneration; Other cerebellar ataxia; Cerebellar ataxia in diseases classified elsewhere; Other spinocerebellar diseases; Spinocerebellar disease unspecified; Hereditary ataxia; Congenital nonprogressive ataxia; Early-onset cerebellar ataxia; Late-onset cerebellar ataxia; Cerebellar ataxia with defective DNA repair; Hereditary spastic paraplegia; Other hereditary ataxias; Hereditary ataxia, unspecified
Neuromuscular disorders	**ICD-9**: 358, 358.01, 358.2, 358.3, 358.31, 358.39, 358.8, 358.9, 359, 359.3, 359.4, 359.5, 359.6, 359.7, 359.71, 359.79, 359.8, 359.81, 359.89, 359.9<br>**ICD-10:** G13.0, G70.8, G70.80, G70.81, G70.89, G70.9, G73, G73.0, G73.1, G73.2, G73.3, G73.4, G73.5, G73.6, G73.7	Myoneural disorders; Myoneural disorders without (acute) exacerbation; Myoneural disorders with (acute) exacerbation; Toxic myoneural disorders; Lambert-Eaton syndrome; Lambert-Eaton syndrome in neoplastic disease; Lambert-Eaton syndrome in other diseases classified elsewhere; Other specified myoneural disorders; Myoneural disorders unspecified; Muscular dystrophies and other myopathies; Periodic paralysis; Toxic myopathy; Myopathy in endocrine diseases classified elsewhere; Symptomatic inflammatory myopathy in diseases classified elsewhere; Inflammatory and immune myopathies, NEC; Inclusion body myositis; Other inflammatory and immune myopathies, NEC; Other myopathies; Critical illness myopathy; Other myopathies; Myopathy, unspecified; Paraneoplastic neuromyopathy and neuropathy; Disorders of myoneural junction and muscle in diseases classified elsewhere; Myasthenic syndromes in other diseases classified elsewhere; Myopathy in infectious and parasitic diseases classified elsewhere

Peripheral neuropathies	**ICD-9**: 356, 356.1, 356.2, 356.3, 356.4, 356.8, 356.9, 357, 357.1, 357.3, 357.4, 357.7<br>**ICD-10:** G61, G61.0, G61.1, G61.8, G61.81, G61.89, G61.9	Hereditary and idiopathic peripheral neuropathy; Peroneal muscular atrophy; Hereditary sensory neuropathy; Refsum's disease; Idiopathic progressive polyneuropathy; Other specified idiopathic peripheral neuropathy; Unspecified hereditary and idiopathic peripheral neuropathy; Acute infective polyneuritis; Polyneuropathy in collagen vascular disease; Polyneuropathy in malignant disease; Polyneuropathy in other diseases classified elsewhere; Polyneuropathy due to other toxic agents
Spinal cord disorders	**ICD-9**: 336, 336.1, 336.2, 336.3, 336.8, 336.9<br>**ICD-10:** G95, G95.0, G95.1, G95.11, G95.19, G95.2, G95.29, G95.3, G95.6, G95.8, G95.81, G95.89, G95.9	Other diseases of spinal cord; Syringomyelia and syringobulbia; Vascular myelopathies; Subacute combined degeneration of spinal cord in diseases classified elsewhere; Myelopathy in other diseases classified elsewhere; Other myelopathy; Unspecified disease of spinal cord; Acute infarction of spinal cord (embolic) (non-embolic); Other vascular myelopathies; Other and unspecified cord compression; Unspecified cord compression; Other cord compression; Conus medullaris syndrome
Demyelinating disorders	**ICD-9**: 341, 341.1, 341.2, 341.21, 341.22, 341.8, 341.9<br>**ICD-10:** G36, G36.0, G36.1, G36.8, G36.9, G37, G37.0, G37.1, G37.2, G37.3, G37.4, G37.5, G37.8, G37.9	Other demyelinating diseases of central nervous system; Neuromyelitis optica; Schilder's disease; Acute (transverse) myelitis; Other demyelinating diseases of central nervous system NOS; Other demyelinating diseases of central nervous system in conditions classified elsewhere; Idiopathic transverse myelitis; Other demyelinating diseases of central nervous system; Demyelinating disease of central nervous system, unspecified; Acute and subacute haemorrhagic leukoencephalitis [Hurst]; Other specified acute disseminated demyelination; Acute disseminated demyelination, unspecified; Diffuse sclerosis of central nervous system; Central demyelination of corpus callosum; Central pontine myelinolysis; Acute transverse myelitis in demyelinating disease of central nervous system; Subacute necrotizing myelitis of central nervous system; Concentric sclerosis [Balo] of central nervous system; Other specified demyelinating diseases of central nervous system; Demyelinating disease of central nervous system, unspecified
Autonomic nervous system disorders	**ICD-9**: 337, 337.01, 337.09, 337.1, 337.2, 337.21, 337.22, 337.29, 337.3, 337.9	Disorders of the autonomic nervous system; Carotid sinus syndrome; Other idiopathic peripheral autonomic neuropathy; Peripheral autonomic neuropathy in disorders classified elsewhere; Reflex sympathetic dystrophy;

	**ICD-10**: G90, G90.0, G90.01, G90.09, G90.1, G90.2, G90.3, G90.4, G90.5, G90.50, G90.51, G90.52, G90.59, G90.8, G90.9	Disorders of the autonomic nervous system unspecified; Disorders of the autonomic nervous system of the upper limb; Disorders of the autonomic nervous system of the lower limb; Disorders of the autonomic nervous system of other specified site; Autonomic dysreflexia; Unspecified disorder of autonomic nervous system; Idiopathic peripheral autonomic neuropathy; Familial dysautonomia [Riley-Day]; Horner's syndrome; Multi-system degeneration of the autonomic nervous system; Complex regional pain syndrome I (CRPS I); Complex regional pain syndrome I, unspecified; Complex regional pain syndrome I of upper limb; Complex regional pain syndrome I of right upper limb; Complex regional pain syndrome I of left upper limb; Complex regional pain syndrome I of upper limb, bilateral; Complex regional pain syndrome I of unspecified upper limb; Complex regional pain syndrome I of lower limb; Complex regional pain syndrome I of right lower limb; Complex regional pain syndrome I of left lower limb; Complex regional pain syndrome I of lower limb, bilateral; Complex regional pain syndrome I of unspecified lower limb; Complex regional pain syndrome I of other specified site
Other disorders of nervous system	**ICD-9**: 349, 349.2, 349.3, 349.39, 349.8, 349.9	Other and unspecified disorders of the nervous system; Disorders of meninges, not elsewhere classified; Dural tear; Other dural tear; Other specified disorders of nervous system; Other nerve root and plexus disorders; Unspecified nerve root and plexus disorder

Other sense organ diseases

Flowchart

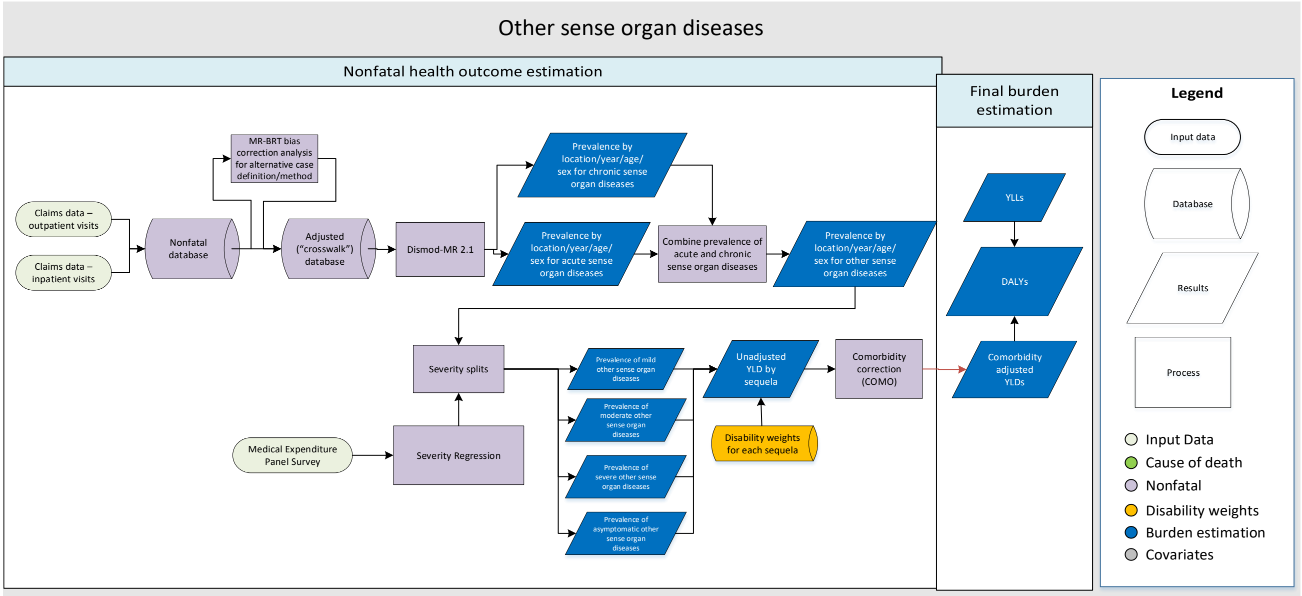

Input Data and Methodological Summary for other sense organ diseases

Case definition

Other sense organ diseases is a residual cause capturing both acute and chronic eye and ear conditions that do not map to other causes but lead to non-trivial morbidity. These include the following codes that encompass a plethora of eye and ear disorders and conditions. Because all data come from clinical ICD-coded claims data, we do not have data from alternative case definitions that require adjustment.

ICD-9: 077, 360, 370, 372-76, 379, 380, and 388

ICD-10: B30, H00-06, H10-11, H13, H16, H19, H30, H44, H57-58, H60-62, and H92

Table 1. ICD-9 codes included in other sense organs disease category

ICD Code	Description
077	Other diseases of conjunctiva due to viruses and chlamydiae
360	Disorders of the globe
370	Keratitis, or diseases of the cornea
372-76	Disorders of conjunctiva, inflammation of eyelids, other disorders of eyelids, disorders of the lacrimal system, disorders of the orbit
379	Other disorders of eye
380	Disorders of external ear
388	Other disorders of ear

Input data

Model inputs

For GBD 2021, we used claims data from the USA and Poland to model other sense organ diseases; in GBD 2023 we also added claims data from Mongolia and USA Medicare data. These conditions do not appear in inpatient hospital data. All USA insurance claims data were outliered over the age of 65 years and replaced with Medicare data, which are more representative of the general population for this age group. ICD codes were assigned up to the five-digit level to either acute or chronic conditions as listed elsewhere in the appendix table of all ICD codes.

Modelling strategy

Data were extracted separately for the chronic and acute conditions included in other sense organ diseases. Chronic data were extracted as prevalence, and acute data as incidence. We then ran two separate DisMod-MR 2.1 models (disease model—Bayesian meta-regression; for details on this method see appendix 1, section 2). The chronic model, with prevalence data, was run as a prevalence-only model. The acute model was run as a full compartmental model with incidence data, assuming zero excess mortality and duration of one week (remission = 52). We included Socio-demographic Index (SDI) as a country-level predictive covariate for incidence and prevalence, respectively, in the acute and chronic model. This covariate is used because many of the conditions included in other sense organ diseases can be addressed through health care.

Covariates. Summary of county-level covariates used in the other sense organ disease DisMod-MR models

Cause	Covariate	Type	Coefficient	Exponentiated beta (95% Uncertainty Interval)
Acute other sense organ diseases	Socio-demographic Index	Incidence	0.30 (0.29–0.30)	1.35 (1.34–1.35)
Chronic other sense organ diseases	Socio-demographic Index	Prevalence	0.30 (0.30–0.30)	1.35 (1.35–1.35)

We then aggregated chronic and acute prevalence outputs, resulting in the prevalence of other sense organ diseases by country, age, year, and sex.

Severity splits and disability weights

The basis of the GBD disability weight survey assessments are lay descriptions of sequelae highlighting major functional consequences and symptoms. Severity splits for other sense organ diseases were calculated via the Medical Expenditure Panel Survey (MEPS) regression borrowing from disability weights used for infectious disease for acute other sense organ diseases and from vertigo and physical disfigurement for chronic other sense organ disease.[1]

Severity distributions are listed in the table below and provide details on the severity levels for other sense organ diseases in GBD 2021 and the disability weight (DW) associated with that severity.

Table 5. Disability weights for chronic and acute other sense organ disease severity levels

Chronic:

Severity	Proportion	Health state	Disability weight
Moderate (vertigo)	0.21 (0.15–0.28)	Has short spells of dizziness and loss of balance; between spells the person is worried the spells will occur again	0.113 (0.078–0.159)
Mild (disfigurement)	0.37 (0.30–0.42)	This person has slight physical deformity which causes some worry and discomfort	0.011 (0.005–0.021)
Asymptomatic	0.42 (0.41–0.44)	Asymptomatic	N/A

Acute:

Severity	Proportion	Health state	Disability weight
Moderate (moderate infectious disease)	0.25 (0.18–0.32)	Has a fever and aches, and feels weak, which causes some difficulty with daily activities	0.05 (0.033–0.073)
Mild (mild infectious disease)	0.30 (0.23–0.37)	This person has low fever and mild discomfort but no difficulty with daily activities	0.006 (0.002–0.012)
Asymptomatic	0.45 (0.43–0.46)	Asymptomatic	N/A

References

[1] Salomon JA, Haagsma JA, Davis A, *et al.* Disability weights for the Global Burden of Disease 2013 study. *Lancet Global Health* 2015; **3**: e712-23. doi: https://doi.org/10.1016/S2214-109X(15)00069-8

Other skin and subcutaneous diseases

Flowchart

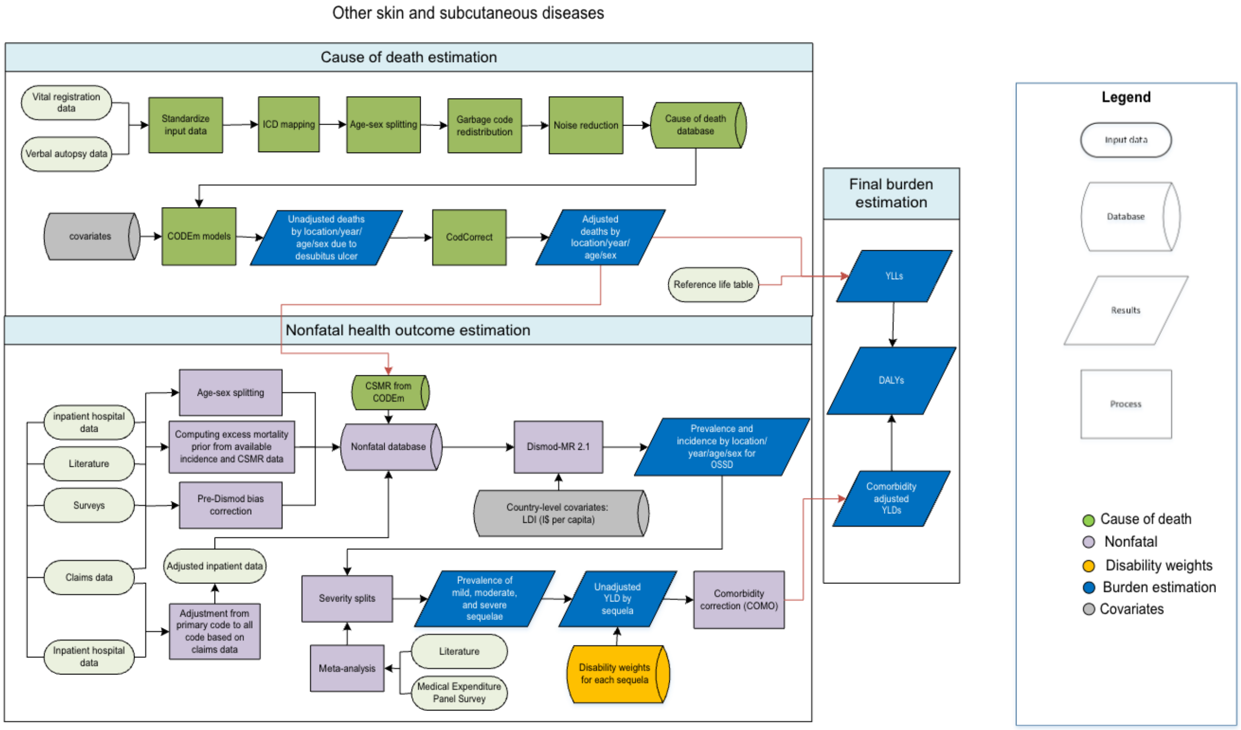

Input data and methodological summary for other skin and subcutaneous diseases

Case definition

The other skin and subcutaneous diseases category encompasses a large group of skin conditions not captured in other skin categories: other viral infections characterised by skin and mucous membrane lesions, not elsewhere classified (B08), unspecified viral infection characterised by skin and mucous membrane lesions (B09), pediculosis and phthiriasis (B85), myiasis (B87), other infestations (B88), sarcoidosis of skin (D86.3), porphyria cutanea tarda (E80.1), other and unspecified porphyria (E80.2), pemphigus (L10), other acantholytic disorders (L11), pemphigoid (L12), other bullous disorders (L13), bullous disorders in diseases classified elsewhere (L14), lichen simplex chronicus and prurigo (L28), pityriasis rosea (L42), lichen planus (L43), other papulosquamous disorders (L44), papulosquamous disorders in diseases classified elsewhere (L45), exfoliation due to erythematous conditions according to extent of body surface involved (L49), erythema multiforme (L51), erythema nodosum (L52), other erythematous conditions (L53), erythema in diseases classified elsewhere (L54), other acute skin changes due to ultraviolet radiation (L56), skin changes due to chronic exposure to nonionising radiation (L57), other disorders of skin and subcutaneous tissue related to radiation (L59), nail disorders (L60), nail disorders in diseases classified elsewhere (L62), androgenic alopecia (L64), other nonscarring hair loss (L65), cicatricial alopecia [scarring hair loss] (L66), hair colour and hair shaft abnormalities (L67), hypertrichosis (L68), rosacea (L71), follicular cysts of skin and subcutaneous tissue (L72), other follicular disorders (L73), eccrine sweat disorders (L74), apocrine sweat disorders (L75), vitiligo (L80), other

disorders of pigmentation (L81), seborrheic keratosis (L82), acanthosis nigricans (L83), corns and callosities (L84), other epidermal thickening (L85), keratoderma in diseases classified elsewhere (L86), transepidermal elimination disorders (L87), atrophic disorders of skin (L90), hypertrophic disorders of skin (L91), granulomatous disorders of skin and subcutaneous tissue (L92), other localised connective tissue disorders (L94), vasculitis limited to skin, not elsewhere classified (L95), and other disorders of skin and subcutaneous tissue in diseases classified elsewhere (L99).

Other skin and subcutaneous diseases

Quantity of interest	Reference or alternative	Definition
Other skin and subcutaneous diseases	Reference	Other skin and subcutaneous diseases as determined by Poland National Health Fund Patient Claims 2015–2019
Other skin and subcutaneous diseases	Alternative	All other data for other skin and subcutaneous diseases

Input data

In the GBD 2010 study, a systematic review of the literature was conducted using PubMed and Google Scholar to capture epidemiological data for other skin and subcutaneous diseases. The inclusion criteria stipulated that studies (1) must be published between 1980 and 2012; (2) must provide data on the incidence or prevalence of Other skin and subcutaneous diseases; (3) must use samples representative of the general population (ie, samples derived from the experimental arm of clinical trials or based in dermatology clinics were excluded); (4) must use a sample size larger than 100; and (5) must provide sufficient information on study method and sample characteristics to assess the quality of the study. For GBD 2016, the GBD 2010 search strategy was replicated in PubMed to capture epidemiological studies published between 2013 and 2016. Since then, USA claims data from 2010–2019 and Poland National Health Fund Patient Claims 2015–2019 have been added to our data sources. Data were further considered for exclusion when they led to underestimation of subnational pseudo-random effects and poor model fit, or if we found them unreasonable when compared to regional, super-regional, and global rates.

Data processing

For other skin and subcutaneous diseases, we crosswalked all data to the reference definition. We began by evaluating the number of observations of each alternate definition that matched with a corresponding observation from the reference definition. We considered “between” study matches, where the alternative was from the same GBD age group and sex, and the midpoint year of the study was within five years of the midpoint of the reference definition observation.

$$\log i\,t\left(y_i^{alt}\right) - \log i\,t\left(y_i^{ref}\right) = \beta_0 + \epsilon_i$$

Table 1: MR-BRT crosswalk adjustment factors for other skin and subcutaneous diseases

Data input	Reference or alternative case definition	Gamma	Beta coefficient, log/logit (95% UI)*	Adjustment factor**
Poland National Health Fund Patient Claims 2015–2019	Ref	0.0907	---	---
All other data	Alt		–0.1807 (–0.7709 to 0.4094)	0.8346 (0.4626, 1.5059)

**MR-BRT crosswalk adjustments can be interpreted as the factor the alternative case definition is adjusted by to reflect what it would have been had it been measured using the reference case definition. If the log/logit beta coefficient is negative, then the alternative is adjusted up to the reference. If the log/logit beta coefficient is positive, then the alternative is adjusted down to the reference.*
***The adjustment factor column is the exponentiated beta coefficient. For log beta coefficients, this is the relative rate between the two case definitions. For logit beta coefficients, this is the relative odds between the two case definitions.*

Modelling strategy

In GBD 2023, we have made no substantive changes in the modelling strategy from GBD 2021.

DisMod-MR 2.1, a Bayesian meta-regression tool, was used to estimate prevalence by age, sex, year, and geography (subnational, country, region, super-region) for other skin and subcutaneous diseases. The available data were mainly composed of prevalence estimates. For GBD 2023, we made both prevalence and incidence estimates. We used a time window set to 25 years. We assumed remission of one, implying a duration of 12 months. This was in line with the available epidemiological data, expert opinion, and previous GBD work. Since GBD 2019, we have replaced our within-DisMod crosswalks with crosswalks completed using the MR-BRT modelling tool. We adjusted all our other skin and subcutaneous diseases data toward the level of Poland National Health Fund Patient Claims 2015–2019, which were more representative of the general population. In addition, log-transformed lagged distributed income (LDI) was used as a country-level covariate to guide estimates for locations with few or no data.

$$prevalence = incidence * duration$$

$$Remission = \frac{Cured\ cases}{person - year\ of follow - up\ in\ prevalent\ cases}$$

Table 2. Severity distribution, details on the severity levels for other skin and subcutaneous diseases and the associated disability weight (DW) with that severity.

Sequela	Severity level	Lay description	DW (95% CI)
Asymptomatic other skin and subcutaneous diseases	Asymptomatic		0.598 (0.596, 0.6)

Symptomatic other skin and subcutaneous diseases	Disfigurement, level 1	The person has a slight, visible physical deformity that others notice, which causes some worry and discomfort.	0.402 (0.4, 0.404)

Table 3. Covariates. Summary of covariates used in the other skin and subcutaneous diseases DisMod-MR meta-regression model

Covariate	Type	Parameter	value	Exponentiated beta (95% uncertainty interval)
LDI (I$ per capita)	Country-level	Prevalence	0.16 (–0.11, 0.30)	1.17 (0.90, 1.35)

Other unspecified infectious diseases

Flowchart

Anemia Impairment Estimation

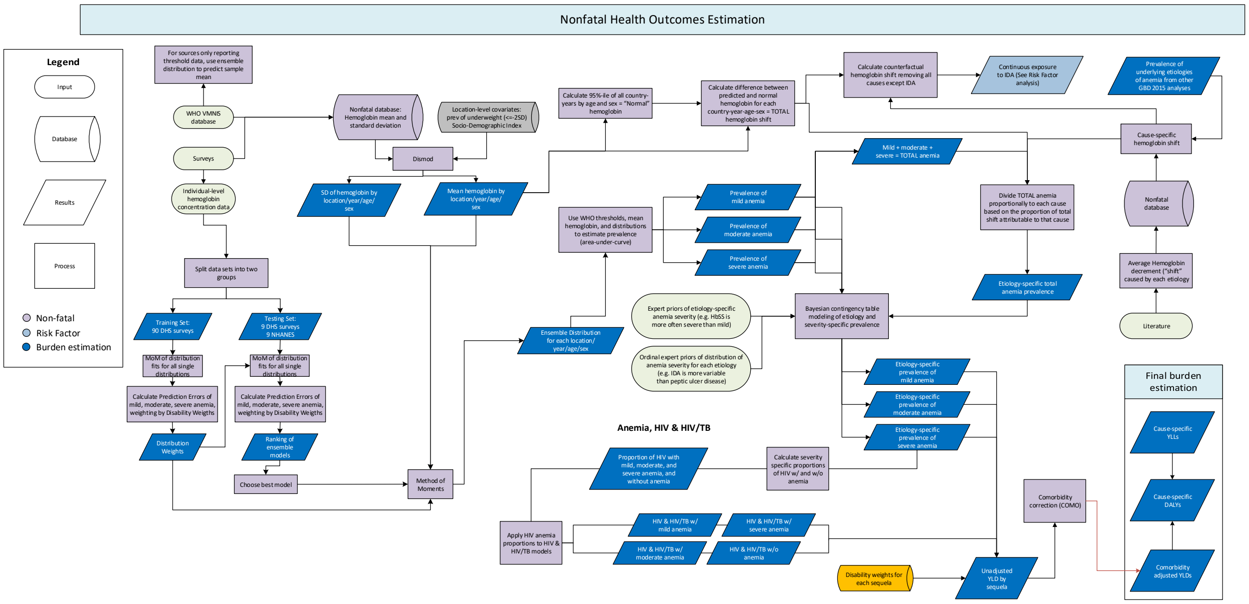

Input data and methodological summary for other unspecified infectious diseases

We estimate other unspecified infectious diseases using the residual anaemia impairment envelope based on a fixed proportion of redistribution. The resulting models of mild anaemia due to other infectious diseases, moderate anaemia due to other infectious diseases, and severe anaemia due to other infectious diseases go into our central computation to generate YLDs based on our prevalence values.

Causes for which allocation of residual anaemia envelope was based on fixed proportion redistribution methods*:

Iron-deficiency anaemia (IDA)
Other infectious diseases
Other neglected tropical diseases
Other endocrine, nutrition, blood, and immune disorders
Other haemoglobinopathies and haemolytic anemias

* A minimum of 10% of all anaemia was assigned to residual categories based on analysis of NHANES-III data from the USA.

References

1. Kassebaum NJ. The Global Burden of Anemia. *Hematology/Oncology Clinics* 2016; **30**: 247–308.
2. Kassebaum NJ, Jasrasaria R, Naghavi M, *et al.* A systematic analysis of global anemia burden from 1990 to 2010. *Blood* 2014; **123**: 615–24.

Otitis media

Flowchart

Otitis media (OM)

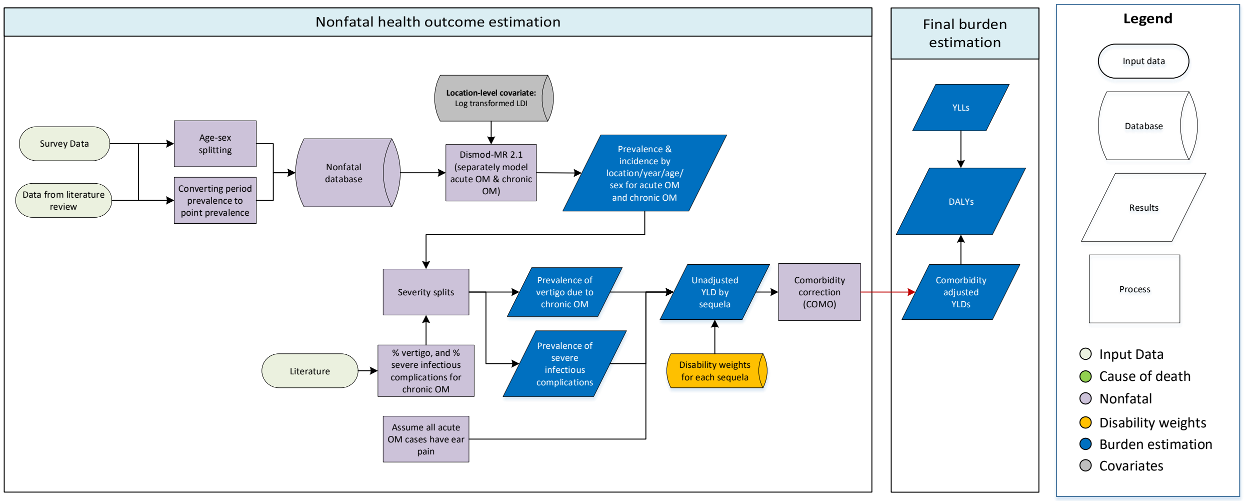

Input data and methodological summary for otitis media

Case definition

Otitis media is an infection of the middle ear space. We included acute otitis media, chronic otitis media, and hearing loss due to chronic otitis media in the GBD non-fatal outcome modelling. Hearing loss due to chronic otitis media estimation is included in the hearing loss report provided separately. The ICD-10 codes are H65-H75.83, and ICD-9 codes are 381-384.9.

**Quantity of interest**	**Reference or Alternative**	**Definition**
Incidence of acute otitis media	Reference	Cases of acute otitis media from clinical diagnosis, surveys, or literature.
Incidence of chronic otitis media	Reference	Cases of chronic otitis media from surveys or literature.
Prevalence of acute otitis media	Reference	Cases of acute otitis media from clinical diagnosis, surveys, or literature.
Prevalence of chronic otitis media	Reference	Cases of chronic otitis media from surveys or literature.
Remission of chronic otitis media	Reference	The rate at which chronic otitis media cases stop meeting the ICD diagnostic criteria.

Input data

We used the same input data for otitis media as in GBD 2021, including the extracted literature data from our previous systematic review published in GBD 2021.[1]

Table 1: Data Inputs for otitis media morbidity modelling by parameter.

	Countries with data	New sources	Total sources
Incidence	10	3	52
Prevalence	21	3	33
Remission	4	0	5
Other	0	0	0

Modelling strategy

We assume that all acute otitis media cases would experience ear pain. The severity distributions for chronic otitis media based on the study by Lin and colleagues (2009) were as follows: (i) vertigo (2.9%, 95% CI: 2.4–3.6), and (ii) severe infectious complications (0.05%, 0.01–0.2).[2] We assumed that all chronic otitis media cases experience either mild or moderate hearing loss. The lay descriptions and disability weights for severity levels derived from the GBD disability weights study are shown below.

Table 2. Severity distribution, details on the severity levels for otitis media and the associated disability weight (DW) with that severity.

Severity level	Lay description	DW (95% CI)
Acute otitis media	Has an earache that causes some difficulty with daily activities.	0.013 (0.007–0.024)
Severe infectious complications due to chronic otitis media	Has an earache that causes some difficulty with daily activities.	0.013 (0.009–0.019)
Mild hearing loss due to chronic otitis media	Has great difficulty hearing and understanding another person talking in a noisy place (for example, on an urban street).	0.01 (0.004–0.019)
Moderate hearing loss due to chronic otitis media	Is unable to hear and understand another person talking in a noisy place (for example, on an urban street), and has difficulty hearing another person talking even in a quiet place or on the phone.	0.027 (0.015–0.042)
Mild hearing loss with ringing due to chronic otitis media	Has great difficulty hearing and understanding another person talking in a noisy place (for example, on an urban street), and sometimes has annoying ringing in the ears.	0.021 (0.012–0.036)
Moderate hearing loss with ringing due to chronic otitis media	Is unable to hear and understand another person talking in a noisy	0.074 (0.049–0.107)

	place (for example, on an urban street), and has difficulty hearing another person talking even in a quiet place or on the phone, and has annoying ringing in the ears for more than 5 minutes at a time, almost every day.	
Vertigo with mild hearing loss due to chronic otitis media	*	0.122 (0.079–0.17)
Vertigo with mild hearing loss and ringing due to chronic otitis media	*	0.132 (0.086–0.184)
Vertigo with moderate hearing loss due to chronic otitis media	*	0.137 (0.089–0.189)
Vertigo with moderate hearing loss and ringing due to chronic otitis media	*	0.179 (0.12–0.247)

* See the hearing loss report for the lay descriptions and disability weights for different severity levels.

We modelled acute and chronic otitis media as separate non-fatal health outcomes using DisMod-MR 2.1. Log-transformed LDI covariate was used as a country-level covariate to model chronic otitis media.

Table 4a. Covariates. Summary of covariates used in the acute otitis media DisMod-MR meta-regression model

**Covariate**	**Type**	**Parameter**	**Exponentiated beta (95% CI)**
Sex	Study-level	Prevalence	0.99 (0.66–1.50)
Sex	Study-level	Incidence	0.79 (0.78–0.80)

Table 4b. Covariates. Summary of covariates used in the chronic otitis media DisMod-MR meta-regression model

**Covariate**	**Type**	**Parameter**	**Exponentiated beta (95% CI)**
Log LDI	Country-level	Prevalence	0.72 (0.63–0.82)
Sex	Study-level	Prevalence	1.14 (0.98–1.32)
Sex	Study-level	Incidence	1.26 (0.66–2.49)

References

1. Sirota SB, Doxey MC, Dominguez RMV, Bender RG, Vongpradith A, Albertson SB, et al. Global, regional, and national burden of upper respiratory infections and otitis media, 1990–2021: a systematic analysis from the Global Burden of Disease Study 2021. *The Lancet Infectious Diseases*. 2025 Jan 1;25(1):36–51.

2. Lin YS, Lin LC, Lee FP, Lee KJ. The prevalence of chronic otitis media and its complication rates in teenagers and adult patients. *Otolaryngol Head Neck Surg*. 2009 Feb;140(2):165–70.

Pancreatitis

Flowchart

Pancreatitis

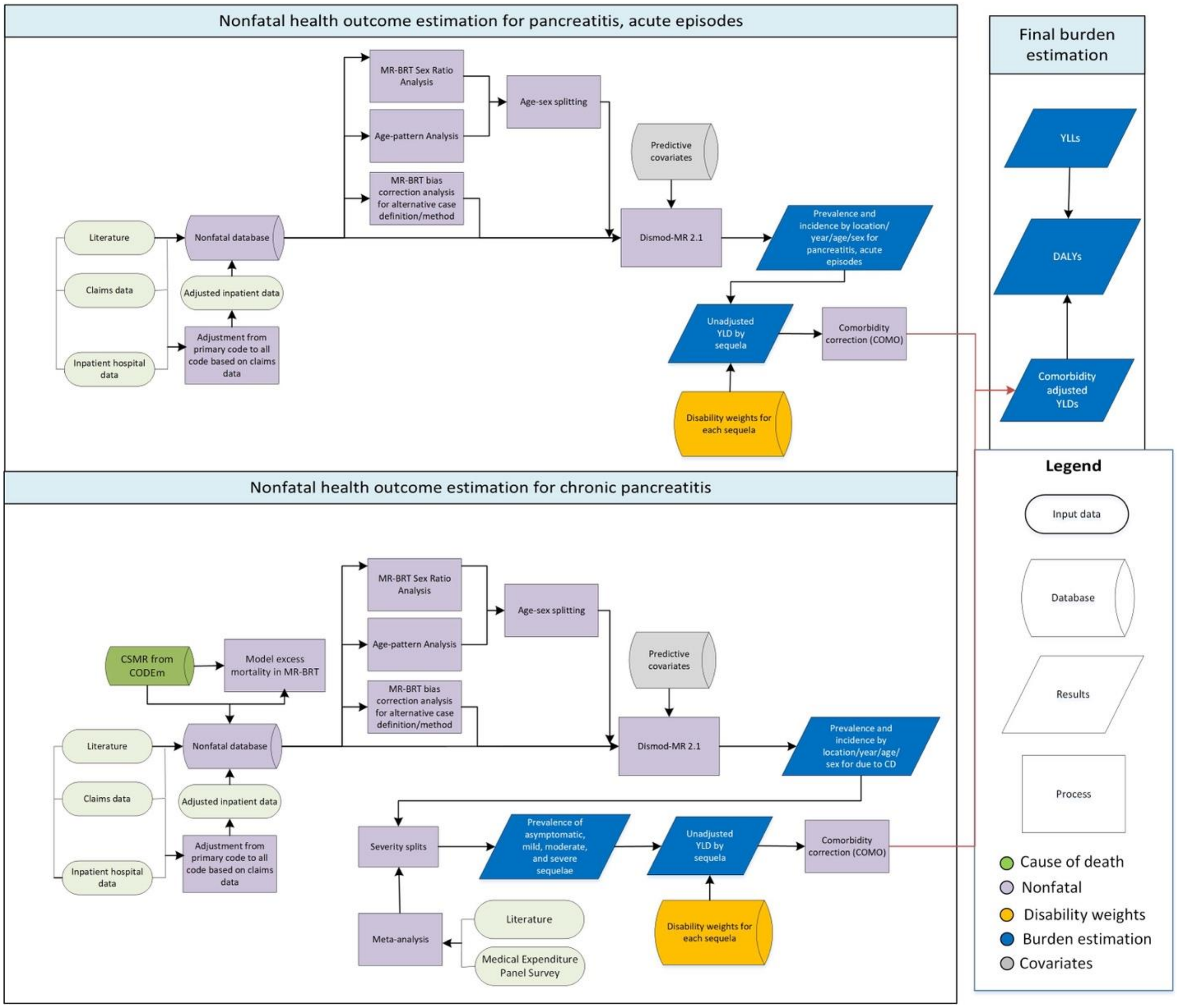

Input data and methodological summary for chronic pancreatitis and pancreatitis, acute episodes

Case definition

Pancreatitis is inflammation of the pancreas, acutely or chronically. Acute pancreatitis involves active inflammation and injury to the pancreas, generally presenting with severe upper abdominal pain and nausea, inappropriate release of pancreatic contents, and a systemic inflammatory response with fever, low blood pressure, and, in some cases, failure of one or more organs. Chronic pancreatitis involves permanent damage to the pancreas from longstanding or recurrent inflammation; this produces chronic

or episodic abdominal pain and nausea and ultimately failure of the pancreas to produce and release digestive enzymes and hormones, leading to chronic diarrhoea, poor absorption of nutrients from food, and diabetes.

Individuals with chronic pancreatitis can have superimposed episodes of acute pancreatitis, but acute episodes can also occur in individuals without chronic pancreatitis. In early rounds of GBD, we modelled acute and chronic pancreatitis together, but starting in GBD 2017, we developed separate models for these two diseases.

ICD-10 codes are K85 for acute and K86 for chronic pancreatitis. ICD-9 code 577.0 corresponds to acute pancreatitis, and 577 and the remainder of its four-digit and five-digit constituents refer to chronic or unspecified pancreatitis.

**Quantity of interest**	**Reference or alternative**	**Definition**
Incidence of acute pancreatitis	Alternative	Acute pancreatitis diagnosed by clinic or study personnel based on imaging (eg, CT scan) and/or lab studies (eg, serum amylase, lipase level) in a patient with appropriate clinical signs and symptoms from a representative sample of the general population, or cases that are identified via a patient database and confirmed through detailed chart review
Incidence of acute pancreatitis	Alternative	Cases identified from database of commercial claims from USA in 2010–2017 using ICD codes.
Incidence of acute pancreatitis	Alternative	Cases identified from database of commercial claims from USA in 2000 using ICD codes.
Incidence of acute pancreatitis	Reference	Cases of acute pancreatitis identified using ICD codes in administrative records that are considered population-representative. (Sources processed by IHME in this group define cases based on the ICD codes listed above, but this group can also include data extracted from studies reported in peer-reviewed journals that extracted data from administrative records using slightly different ICD code lists, if no appreciable bias is detected.)
Incidence of chronic pancreatitis	Alternative	Chronic pancreatitis diagnosed by clinic or study personnel based on laboratory tests, radiological tests, and/or histological examination in a patient with appropriate clinical signs and symptoms from a representative sample of the general population, or cases that are identified via a patient database and confirmed through detailed chart review.
Incidence of chronic pancreatitis	Reference	Cases of chronic pancreatitis identified using ICD codes in administrative records that are considered population-representative.
Prevalence of chronic pancreatitis	Alternative	Chronic pancreatitis diagnosed by clinic or study personnel based on laboratory tests, radiological tests, and/or histological examination in a patient with appropriate clinical signs and symptoms from a representative sample of the general population, or cases that are identified via a patient database and confirmed through detailed chart review.
Prevalence of chronic pancreatitis	Alternative	Cases identified from database of commercial claims from USA in 2010–2017 using ICD codes.
Prevalence of chronic pancreatitis	Alternative	Cases identified from database of commercial claims from USA in 2010–2017 using ICD codes.

Prevalence of chronic pancreatitis	Reference	Cases of chronic pancreatitis identified using ICD codes in administrative records that are considered population-representative. (Sources processed by IHME in this group define cases based on the ICD codes listed above, but this group can also include data extracted from studies reported in peer-reviewed journals that extracted data from administrative records using slightly different ICD code lists, if no appreciable bias is detected.)

Overall strategy

Two databases were used as inputs to two separate, complete compartmental DisMod models: pancreatitis, acute episodes, and chronic pancreatitis.

Input data

Input data

For GBD 2013, a systematic literature review was conducted to capture studies of prevalence and incidence of pancreatitis throughout the world. This search was updated for GBD 2015 and again for GBD 2016. A PubMed search was conducted using the following search terms: Pancreatitis[Title/Abstract] OR "Pancreatitis"[Mesh] OR "Pancreatitis, Acute Necrotizing"[Mesh] OR "Pancreatitis, Chronic"[Mesh]) AND (prevalence[Title/Abstract] OR incidence[Title/Abstract]) AND ("2010/01/01"[Date - Publication] : "2016/11/01"[Date - Publication]) NOT(animals[MeSH] NOT humans[MeSH])) NOT("comment"[Publication Type])

The exclusion criteria were:

1. Studies clearly not representative of the national population (ie, alcoholics or smokers).
2. Studies that did not provide primary data on epidemiological parameters (eg, a commentary piece).

Studies were added to the acute database if they measured the incidence of acute pancreatitis as defined by appropriate ICD codes, or by a combination of clinical, biochemical, and radiographic criteria. The acute database included studies that measured incidence of first episode of acute pancreatitis only, and studies that measured incidence of all acute pancreatitis, including recurrent episodes. Studies that included individuals with underlying chronic pancreatitis were excluded from the acute database. Studies were added to the chronic database if they employed appropriate ICD codes or appropriate clinical, biochemical, and radiographic criteria of chronic pancreatitis. Some studies reported incidence of acute and chronic disease separately and data were extracted to both databases, but those few studies that reported only a single combined estimate for both acute and chronic disorders were excluded.

In GBD 2017, the acute database included literature data extracted as prevalence from six countries, such as Ireland, Japan, and Poland. These data were excluded from analysis in subsequent rounds.

In addition to studies from systematic reviews, both databases included administrative data that were extracted and processed by IHME, as described elsewhere in this appendix. The input data and processing steps for modelling in GBD 2023 was the same as in GBD 2021.

Incidence and prevalence input processing

Hospital discharge data provide observations about encounters, generally with only the primary diagnostic code for the encounter. Claims data, on the other hand, link claims for all inpatient and outpatient encounters for a single individual and provide primary and secondary diagnoses for all encounters.

Similar to GBD 2019, in the acute database, an individual was extracted from claims data as an incident case if that individual had one or more inpatient encounters with an appropriate ICD code as any diagnosis; readmissions within 28 days were assumed to be for the same episodes of illness. Hospital discharges were included only if the primary discharge diagnosis was a code for acute pancreatitis, and incident cases were estimated from number of discharges using a correction factor (ie, correction factor 1) from claims data.

In the chronic database, individuals were extracted from claims data as prevalent cases if they had at least one inpatient or two outpatient encounters with a chronic pancreatitis ICD code as any diagnosis. Hospital discharge data were processed by extracting discharges with an appropriate ICD code as primary diagnosis and adjusting using a correction factor (ie, correction factor 3) derived from claims data. Specifically, we modelled from the ratio of inpatient claims with chronic pancreatitis as primary diagnosis to total prevalent cases of chronic pancreatitis seen in claims data.

After extraction of data from the systematic reviews and the administrative sources, the next step in data processing was to identify datapoints from published reports that refer to both sexes combined using the pooled sex-ratio estimated from studies that reported incidence or prevalence in males and females separately. The ratios of female to male cases derived from MR-BRT analysis were 0.81 (CI: 0.54, 1.20) and 0.66 (CI: 0.36, 1.22) for acute and chronic pancreatitis, respectively.

After sex-splitting, we adjusted incidence and prevalence measurements that used non-reference case definitions or data collection methods towards a reference standard. In GBD 2019, we improved the bias adjustment methods to allow a more direct comparison between different case definitions and/or study designs. In GBD 2017, we used data from published studies that employed rigourous case definitions as our reference standard for acute pancreatitis and adjusted clinical administrative data toward this reference standard by marking administrative data with binary covariates and estimating a fixed effect for this covariate in our DisMod meta-regression modelling process. This amounts to adjusting data using an ecological comparison and is vulnerable to compositional bias; if data from different location-years were collected using different methods or case definitions, true spatiotemporal differences in epidemiology can be erroneously adjusted, and differences truly due to differences in methods can be erroneously estimated as differences in underlying epidemiology. In GBD 2019, we avoided this risk by making pre-modelling bias adjustments and dropping data types that could not be rigorously adjusted. This was done by conducting a meta-regression of the relationship between datapoints matched on year, age, sex, and location, but differing with regard to one or more study design characteristic. This pre-modelling bias adjustment approach was used in in all rounds subsequent to GBD 2017, including GBD 2023.

As in GBD 2017, we desired to use data from published studies that identified cases through detailed chart review as the reference standard for the acute pancreatitis model. These studies used a combination of clinical presentation, and biochemical and radiographic findings to validate a case

definition, which we refer to as “stringent criteria” in shorthand. Using the stringent criteria, ideally, we would then adjust data collected by identifying cases using ICD codes in administrative data. However, the number of matched pairs between reference and alternative (based on year, age, sex, and location) was small and yielded highly uncertain adjustment factors for the alternative case definitions. As a result, a new case definition was adopted in GBD 2019: diagnosis of acute pancreatitis as indicated by ICD code in a clinical encounter. Other case definitions and study design characteristics were adjusted toward this new reference standard. This choice of reference and adjustment approach remained the same in GBD 2021 and GBD 2023.

The chronic pancreatitis model used ICD-code-based administrative data as the reference standard in GBD 2017 due to scant literature data that were available. In GBD 2019, we attempted to employ the new bias adjustment method for chronic pancreatitis using the more rigorous case definition based on clinical, biochemical, and radiographic findings, but, as in the acute pancreatitis model, we could not find an adequate number of comparison pairs to inform reliable adjustment factors. Therefore, we decided to use the same ICD-based administrative data as the reference standard in GBD 2019, GBD 2021 and GBD 2023, adjusting other case definitions and study design characteristics to this reference standard.

For both acute and chronic pancreatitis models, the USA claims data from the year 2000 and from the years 2010–2017 were each adjusted to the reference to adjust for selection bias due to commercial insurance. This was done by matching datapoints of this type to datapoints from more population-representative sources by year, age group, sex and location, calculating the log ratio of the incidence or prevalence measured by the two sources, and modelling that log-ratio using MR-BRT. This adjustment method, termed “crosswalking”, is described in greater detail elsewhere in this appendix.

The table below shows bias correction factors estimated using MR-BRT.

Table 1. MR-BRT crosswalk adjustment factors for pancreatitis

<u>Acute pancreatitis episode: incidence</u>

Data input	Reference or alternative case definition	Gamma	Beta coefficient, log (95% CI)	Adjustment factor*
ICD-code based administrative data	Ref	0.30		
USA claims from year 2000	Alt		–0.18 (–1.12, 0.75)	0.83 (0.33, 2.12)
USA claims from years 2010–2017	Alt		0.19 (–0.44, 0.82)	1.21 (0.65, 2.26)
Stringent criteria	Alt		–0.22 (–1.05, 0.60)	0.80 (0.35, 1.82)

**Adjustment factor is the transformed beta coefficient in normal space and can be interpreted as the factor by which the alternative case definition is adjusted to reflect what it would have been if measured as the reference.*

Chronic pancreatitis: incidence

Data input	Reference or alternative case definition	Gamma	Beta coefficient, log (95% CI)	Adjustment factor*
ICD-code based administrative data	Ref	0.61		
Stringent criteria	Alt		–0.66 (–2.14, 0.82)	0.52 (0.12, 2.28)

**Adjustment factor is the transformed beta coefficient in normal space and can be interpreted as the factor by which the alternative case definition is adjusted to reflect what it would have been if measured as the reference.*

Chronic pancreatitis: prevalence

Data input	Reference or alternative case definition	Gamma	Beta coefficient, log (95% CI)	Adjustment factor*
ICD-code based administrative data	Ref	0.18		
USA claims from year 2000	Alt		–0.89 (–1.83, 0.05)	0.41 (0.16, 1.05)
USA claims from years 2010–2016	Alt		0.10 (–0.35, 0.55)	1.11 (0.70, 1.73)
Stringent criteria	Alt		0.09 (–2.74, 2.93)	1.10 (0.06, 18.79)

**Adjustment factor is the transformed beta coefficient in normal space and can be interpreted as the factor by which the alternative case definition is adjusted to reflect what it would have been if measured as the reference.*

After sex-splitting and crosswalking, we split datapoints where the age range was greater than 20 years using the global age pattern informed by the datapoints with fine age groups (ie, ages 5–9, 10–14, and 15–20…).

Datapoints with an age-standardised prevalence greater than three median absolute deviations from the median of the age-standardised prevalence for all inpatient and non-USA claims data were marked as outliers and excluded from analysis. Data from Nepal, Turkey, and the Philippines were also marked as outliers in the chronic pancreatitis model because their estimates were unreasonably low or high when compared to regional, super-regional, and global rates.

EMR processing

In GBD 2017, EMR inputs were produced by matching prevalence datapoints with their corresponding CSMR values within the same age, sex, year, and location (by dividing CSMR by prevalence). For short-duration conditions (remission >1), the corresponding prevalence was derived by running an initial model and then applying the same CSMR/prevalence method. However, this method of producing EMR inputs demonstrated a rather unrealistic pattern of EMR compared to an expected pattern of decreasing EMR with greater access to quality health care. Such unexpected patterns often signal inconsistencies between CSMR estimates and the measures of prevalence and/or incidence. Thus, in an effort to provide greater guidance on the expected pattern of EMR in the chronic pancreatitis model, in GBD 2019, EMR data produced per above in GBD 2017 were modelled by age, sex, and Healthcare Access and

Quality (HAQ) Index using MR-BRT, with a prior on HAQ Index having a negative coefficient. In GBD 2021, we employed the same MR-BRT method to predict EMR for each location, year, sex, and for ages 0, 10, 20….100, and these predictions were used as inputs to our non-fatal model. These inputs were also used in GBD 2023.

Modelling strategy

Acute pancreatitis episodes

Similar to previous rounds of GBD, we ran a DisMod-MR 2.1 model to produce estimates by age, sex, year, and country, and no substantial model changes were made in GBD 2023. Inputs to DisMod for acute pancreatitis included incidence data processed as described above. The prior value of remission was bounded from 8 to 9 (a duration of about six weeks) for all ages. The minimum coefficient of variation at the regional, super-regional, and global level was set at 0.8. Predictive covariates were per capita alcohol consumption on incidence and HAQ Index on EMR.

Chronic pancreatitis

Similar to previous rounds of GBD, we ran a DisMod-MR 2.1 model to produce estimates by age, sex, year, and country. Inputs to DisMod for chronic pancreatitis included prevalence data processed as described above. We assumed chronic pancreatitis does not remit. The minimum coefficient of variation at the regional, super-regional, and global level was set at 0.8. Predictive covariates included a log-transformed age-standardised SEV scalar covariate for pancreatitis on prevalence, and HAQ Index on EMR.

Betas and exponentiated values of predictive covariates (which can be interpreted as an odds ratio) are shown in the table below.

Table 2. Covariates. Summary of covariates used in the pancreatitis DisMod-MR meta-regression model

Acute pancreatitis episodes

Covariate	Parameter	Exponentiated beta (95% uncertainty interval)
Alcohol (litres per capita)	Incidence	1.00<br>(1.00–1.00)
Healthcare Access and Quality Index	Excess mortality rate	0.98<br>(0.15–7.31)

Chronic pancreatitis

Covariate	Parameter	Exponentiated beta (95% uncertainty interval)
Log-transformed age-standardised scaled exposure variable for pancreatitis risk factors	Prevalence	2.51<br>(2.43–2.60)

Healthcare Access and Quality Index	Excess mortality rate	0.98 (0.98–0.98)

Severity split and disability weight

The basis of the GBD disability weight survey assessments are lay descriptions of sequelae highlighting major functional consequences and symptoms. The lay descriptions and disability weights for pancreatitis are shown below. All prevalent cases from the pancreatitis, acute episode model were assigned a single, combined disability weight for severe abdominal pain and severe infectious disease symptoms. Prevalent cases from the chronic pancreatitis disease model were divided into symptomatic and asymptomatic groups using proportions found in a review of published studies of the natural history of chronic pancreatitis. The symptomatic group was divided into mild, moderate, and severe groups using proportions from the Medical Expenditure Panel Survey (MEPS).

Table 3. Severity distribution, details on the severity levels for pancreatitis in GBD 2021 and the associated disability weight (DW) with that severity.

Severity split	Lay description	DW (95% CI)
Acute pancreatitis episodes	This person has severe pain in the belly and feels nauseated. The person has high fevers, pain and feels very weak. This causes great difficulty with daily activities.	*Combined DW: 0.324 (0.220–0.442) 0.133 (0.088–0.190)
Asymptomatic chronic pancreatitis	--	0
Mild chronic pancreatitis	This person has some pain in the belly that causes nausea but does not interfere with daily activities.	0.011 (0.005–0.021)
Moderate chronic pancreatitis	This person has pain in the belly and feels nauseous. The person has difficulties with daily activities.	0.114 (0.080–0.159)
Severe chronic pancreatitis	This person has severe pain in the belly and feels nauseated. The person is anxious and unable to carry out daily activities.	0.324 (0.219–0.442)

*Acute pancreatitis episodes have a custom disability weight combining abdominal pain and infectious disease. More information can be found in the appendix detailing disability weights.

Paralytic ileus and intestinal obstruction

Flowchart

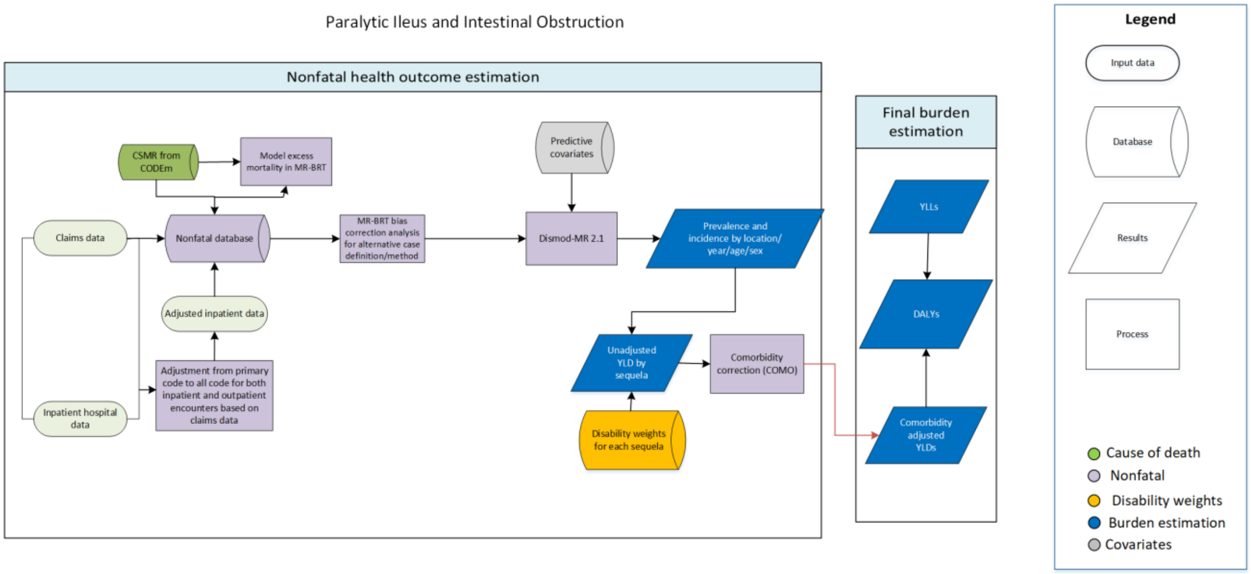

Input data and methodological summary for paralytic ileus and intestinal obstruction

Case definition

Paralytic ileus and intestinal obstruction is a lack of digestive propulsion caused by failed peristalsis, commonly presenting with abdominal bloating, abdominal distension, gas, constipation, nausea and vomiting, and dehydration.

ICD code for paralytic ileus and intestinal obstruction is K56.

Quantity of interest	Reference or alternative	Definition
Incidence of paralytic ileus and intestinal obstruction	Alternative	Cases identified from database of commercial claims from USA in 2010–2017 using ICD codes.
Incidence of paralytic ileus and intestinal obstruction	Alternative	Cases identified from database of commercial claims from USA in 2000 using ICD codes.
Incidence of paralytic ileus and intestinal obstruction	Reference	Cases of paralytic ileus and intestinal obstruction identified using ICD codes in administrative records that are considered population-representative.

Input data and data processing

Inputs

As in previous rounds of GBD, in GBD 2023 we modelled paralytic ileus and intestinal obstruction using incidence data extracted from the IHME clinical administrative data library aggregated and processed for GBD as described in "Clinical input data and methods summary" section of this appendix. Notably, in GBD 2023, this library of hospital discharges and claims expanded with additional years of data from USA claims (years 2018, 2019), Poland claims (year 2019), new sources in Mongolia claims (year 2019), and South Korea (years 2018, 2019). Additional years of hospital discharges were also included in GBD 2023 from USA (HCUP year 2016-2019), Austria, Brazil, Chile, Georgia, Italy, New Zealand, and the Philippines, as well as the new sources of hospital discharges from Germany and Mexico and data from the USA Medicare programme for age-groups 65–69 and older, (years 2000, 2010, 2014–16).

Prior to GBD 2023, given the heterogeneity within the clinical administrative data for this cause, we systematically excluded data series with an age-standardised incidence rate greater than two median absolute deviations from the median of the age-standardised incidence rate for all data. In GBD 2023, however, we explored this heterogeneity in greater detail and found that many extreme values were seen in inpatient data from sources either do not cover the entire population or do not include a representative population sample, thus requiring extraction as discharge diagnosis cause fractions and conversion to incidence using GBD estimates of hospital admission rate per capita. (See the section of this appendix on the hospital utilisation estimates for information on this input.) In GBD 2023, we decided to exclude all inpatient sources that require this adjustment and only include the sources with full coverage of the target population. Lastly, we excluded USA MarketScan data for individuals aged 65 and older, replacing them with CMS data, which are more representative of the US population in these age-groups.

Inputs to our non-fatal modelling also included cause-specific mortality rate (CSMR) estimates taken from our fatal modelling process (see CoD cause-specific modelling description for ileus in this appendix) and excess mortality rates (EMR) estimates modelled outside of DisMod (see the EMR data processing section below).

Incidence data processing

Hospital discharge data provide observations about encounters, generally with only the primary diagnostic code for the encounter. Claims data, on the other hand, link claims for all inpatient and outpatient encounters for a single individual and provide primary and secondary diagnoses for all encounters.

In GBD 2017, an individual was extracted from claims data as an incident case if that individual had one or more inpatient encounters with an appropriate ICD code of paralytic ileus and intestinal obstruction as any diagnosis. Hospital discharges with an appropriate ICD code as primary diagnosis were extracted and adjusted for readmission.

In both GBD 2019 and GBD 2021, however, we employed data processing methods to capture cases that were diagnosed and/or treated in both inpatient and outpatient settings. Specifically, an individual was extracted from claims data as an incident case if that individual had at least one inpatient or outpatient encounter with an appropriate ICD code as any diagnosis within 28 days. Hospital discharge data were processed by extracting discharges with an appropriate ICD code as primary diagnosis and adjusting using correction factors derived from claims data. Specifically, we modelled the ratio of inpatient claims with paralytic ileus and intestinal obstruction as primary diagnosis to total incident cases of paralytic ileus and intestinal obstruction seen in claims data. In GBD 2021, we updated the method of estimating

these correction factors by assigning three frequency-placed knots, instead of two, in the age-spline parameter of MR-BRT analysis. Other processing methods remained the same as in GBD 2019.

In GBD 2023, we refined our approach by updating the correction factor used for hospital discharge data adjustments. Instead of using correction factor 3, which was a single model of the ratio of inpatient claims with a primary diagnosis to total incident cases seen in claims data with complete information on all diagnoses in all encounter types, we now apply correction factors 1 and 5 (CF1 and CF5). CF1 is defined as the ratio of deduplicated primary diagnoses among inpatient admissions to adjusted primary diagnoses among inpatient admissions, while CF5 is defined as the ratio of deduplicated primary and secondary diagnoses among inpatient encounters and outpatient encounters to deduplicated primary diagnoses among inpatient encounters. Like the previous approach, the approach this year accounts for non-primary inpatient diagnoses and outpatient cases, does so serially, to allow data sources to contribute to estimation of de-duplication for readmission, multiple diagnosis in inpatient encounters and/or outpatient care depending on which of these relationships are captured in the dataset, rather than only employing sources that have complete information on all diagnoses and encounter types. This approach makes use of more diverse data inputs. This final scalar is applied to extracted primary diagnosis admission rate data as a product of CF1 and 1/CF5. See the section of this appendix on the processing of hospital data for more details.

As first done in GBD 2019, USA claims data (extracted and processed as described above) were adjusted to account for selection bias due to commercial insurance, using MR-BRT analysis. The USA MarketScan claims data from the year 2000 and from the years 2010–2019 were separately adjusted to account for selection bias due to commercial insurance, which was suspected to be differential over the years as coverage expanded. In contrast to GBD 2019, in GBD 2021 and GBD 2023, we used age as a covariate to estimate bias adjustment factors. (As mentioned, these data were excluded for individuals aged 65 years and older, due to the new availability of CMS data, but this adjustment was applied to data for younger age-groups, which were retained.)

The process of adjusting for non-reference data using MR-BRT with the logit-transformation method is described below:

1. Identify datapoints with overlapping year, age, sex, and location between reference and non-reference population data.
2. Logit transform overlapping datapoints of alternative and reference types.
3. Convert overlapping datapoints into a difference in logit space using the following equation: $logit(altnerative) - logit(reference)$
4. Use the delta method to compute standard errors of overlapping datapoints in logit space, then calculate standard error of logit difference using the following equation: $\sqrt{(variance\ of\ logit(alternative)) + (variance\ of\ logit(reference))}$
5. Using MR-BRT, conduct a random effects meta-regression to obtain the pooled logit difference of alternative to reference
6. Apply the pooled logit difference to all data points of non-reference types using the following equation:
$$new_{estimate} = inverse.logit((logit(alternative)) - (pooled\ logit\ difference))$$
7. Calculate new standard errors using the delta method, accounting for gamma (between-study heterogeneity)

The table below shows bias correction factors estimated using MR-BRT.

Table 1. MR-BRT crosswalk adjustment factors for paralytic ileus and intestinal obstruction

Data input	Reference or alternative data collection	Gamma	Covariate	Beta coefficient, logit (95% CI)	Adjustment factor*
Hospital + non-USA claims	Ref	---		---	---
USA claims from year 2000	Alt	0	Age (continuous from 0 to 95+)	–0.005 (–0.007 to –0.002)	0.995 (0.993 to 0.998)
			Sex (female to male)	0.08 (–0.004 to 0.17)	1.085 (0.996 to 1.181)
			Intercept	–0.08 (–0.27 to 0.11)	0.92 (0.76 to 1.11)
USA claims from years 2010–2019	Alt	0.004	Age (continuous from 0 to 95+)	–0.008 (–0.008 to –0.007)	0.992 (0.984 to 1.001)
			Sex (female to male)	0.11 (0.1 to 0.12)	1.117 (1.101to 1.133)
			Intercept	0.12 (0.08 to 0.16)	1.13 (1.083 to 1.179)

**MR-BRT crosswalk adjustments can be interpreted as the factor the alternative case definition is adjusted by to reflect what it would have been had it been measured using the reference case definition. If the log/logit beta coefficient is negative, then the alternative is adjusted up to the reference. If the log/logit beta coefficient is positive, then the alternative is adjusted down to the reference.*

***The adjustment factor column is the exponentiated beta coefficient. For log beta coefficients, this is the relative rate between the two case definitions. For logit beta coefficients, this is the relative odds between the two case definitions.*

Prior to GBD 2023, given the heterogeneity within the clinical administrative data, we systematically excluded data series with an age-standardised incidence rate greater than two median absolute deviations from the median of the age-standardised incidence rate for all data. In GBD 2023, we explored sources of heterogeneity in greater detail and identified that many data with extreme values were hospital discharge datasets that do not cover the entire population, which we extract as discharge diagnosis cause fractions and combine with GBD estimates of hospital admission rates in the relevant population, matched by year, age group, sex, and location. In GBD 2023, we decided to exclude all inpatient sources that require this adjustment and only include the sources that have full coverage of the target population. We also excluded USA MarketScan data for individuals aged 65 and older, retaining USA CMS data as the sole source for the USA in these age groups.

EMR processing

In GBD 2017, EMR inputs were produced by fitting a preliminary compartment model to estimate prevalence from incidence data, matching preliminary prevalence resulting from each incidence

datapoint with the corresponding CSMR value within the same age, sex, year, and location, and dividing CSMR by prevalence. This method of producing EMR inputs, however, demonstrated a rather unrealistic pattern of EMR compared to an expected pattern of decreasing EMR with greater access to quality health care. Such unexpected patterns often signal inconsistencies between CSMR estimates and data on prevalence and/or incidence. Thus, in an effort to provide greater guidance on the expected pattern of EMR, in GBD 2019, EMR estimates produced per above were used in an MR-BRT model with age, sex, and Healthcare Access and Quality (HAQ) Index as predictors, with a prior on HAQ Index to have a negative coefficient. In GBD 2021, we employed the same MR-BRT method to predict EMR for each location, year, sex, and for ages 0, 10, 20….100. Predictions from this model were used as inputs to our non-fatal model, described below.

Modelling strategy

DisMod model

Similar to previous rounds, we ran a DisMod-MR 2.1 model to produce estimates by age, sex, year, and location. Inputs to DisMod for intestinal obstruction and paralytic ileus include incidence, CSMR, and EMR inputs processed as described above. A prior value was set on remission so that all cases remit within two weeks. The minimum coefficient of variation at the regional, super-regional, and global level was set at 0.8. We included HAQ Index as a predictive covariate on EMR with a mean and standard deviation produced from the MR-BRT model described above. The beta and exponentiated values of this predictive covariate (which can be interpreted as an odds ratio) are shown in the table below.

Table 2. Covariates. Summary of covariates used in the paralytic ileus and intestinal obstruction DisMod-MR meta-regression model

Covariate	Parameter	Exponentiated beta (95% uncertainty interval)
Healthcare Access and Quality Index	Excess mortality rate	0.98<br>(0.98–0.98)

Severity split and disability weight

The basis of the GBD disability weight survey assessments are lay descriptions of sequelae highlighting major functional consequences and symptoms. The lay descriptions and disability weights for paralytic ileus and intestinal obstruction are shown below.

Table 3. Severity distribution, details on the severity levels for paralytic ileus and intestinal obstruction in GBD 2023 and the associated disability weight (DW) with that severity.

Severity level	Lay description	DW (95% CI)
Severe	This person has severe pain in the belly and feels nauseated. The person is anxious and unable to carry out daily activities.	0.324 (0.219–0.442)

Parkinson's disease

Flowchart

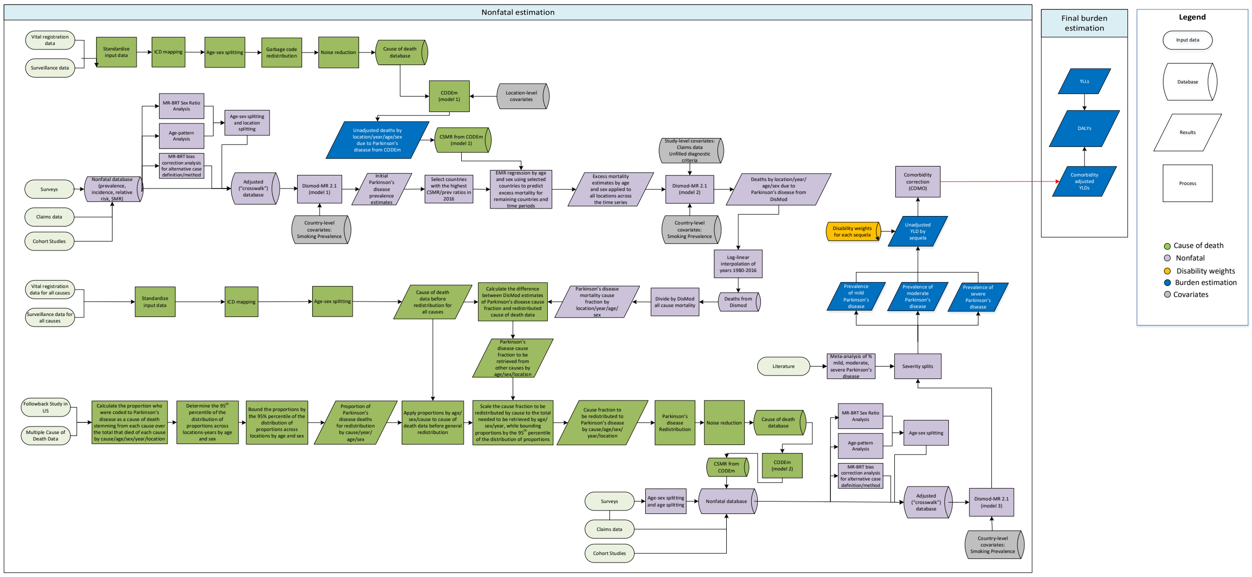

Input Data and Methodological Summary for Parkinson's disease

Case definition

Parkinson's disease (PD) is a chronic, degenerative, and progressive neurological condition typified by the loss of motor mobility and control – most notably tremors. Our case definition for GBD is the presence of at least two of the four primary symptoms: (1) tremors/trembling, (2) bradykinesia, (3) stiffness of limbs and torso, and (4) posture instability. The non-fatal ICD codes for Parkinson's disease are G20 for ICD-10 and 332 for ICD-9; these codes specifically exclude parkinsonism.

Unlike most causes in the Global Burden of Disease project, Parkinson's disease mortality and morbidity estimates are modelled jointly. This is because of marked discrepancies between prevalence data and cause of death data. Specifically, prevalence data suggest little to no variation over time (eg, 1990–2017) whereas age-standardised mortality rates in vital registrations in high-income countries have increased multiple times over this same period. Additionally, prevalence variation between countries is much smaller than the variation in death rates assigned to Parkinson's disease in vital registration. We attribute these discrepancies to changing coding practices rather than epidemiological change.

Because of this joint procedure, descriptions of the mortality estimation process are included where relevant, but see the Parkinson's disease appendix describing fatal modelling for more details.

Input data

Model inputs

To inform our estimates of burden due to Parkinson's disease, we use mortality data from vital registration systems, as well as prevalence data from surveys and administrative data such as claims sources.

The last systematic review was conducted from August 2017 to March 2023 in PubMed, and the search terms were set to capture studies of incidence or prevalence of Parkinson's disease: (Parkinson disease[TiAb] OR Parkinson's disease[TiAb] OR "Parkinson Disease"[MeSH Terms]) AND (epidemiology[TiAb] OR prevalence[TiAb] OR prevalent[TiAb]OR incidence[TiAb] OR incident[TiAb]) NOT (animals[MeSH] NOT humans[MeSH]) AND (2021/09/03[PDAT] : 3000[PDAT]) NOT (Review[Publication Type])

As depicted in the PRISMA diagram, the systematic review identified 3144 sources for review and resulted in 106 newly added sources to our model. We also performed a further opportunistic search in April 2024 for world regions with limited data coverage and found an additional seven articles to include.

Figure 1: PRISMA flow diagram

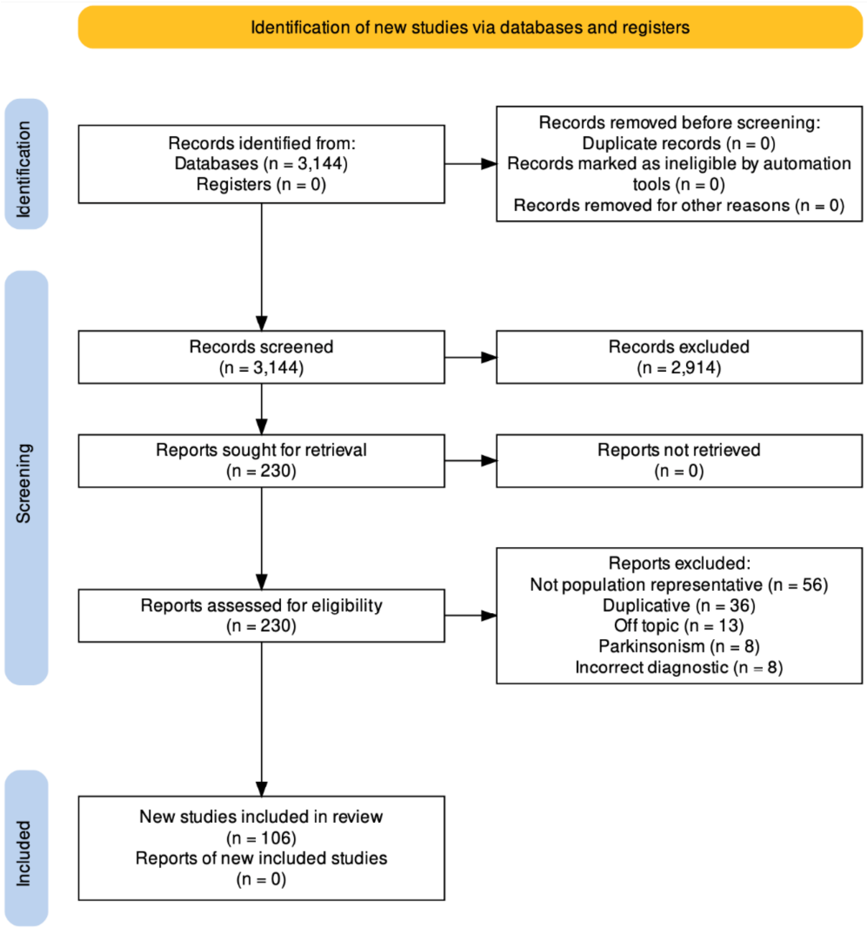

Prior to our most recent systematic review, a review was conducted from September 2015 to August 2017. This search term resulted in 660 initial hits with 20 sources marked for extraction. Studies with no clearly defined sample or that drew from specific clinic/patient organisations were excluded.

Studies using non-representative populations were excluded from modelling. Certain studies were outliered on a case-by-case basis due to subsequent review and exclusion due to inappropriateness of the study design, or case ascertainment that conflict with existing gold-standard data. We excluded US MarketScan claims data from the year 2000 because these data were systematically lower than other years. We also excluded MarketScan claims data for individuals over the age of 65 years, and instead used CMS Medicare data available for ages 65+ years, as we expect this to be more representative of the population. In claims data, a prevalent case was identified from claims data where an individual had one inpatient visit, two outpatient visits, or one outpatient and one inpatient visit (arguing that a single mention of a code for PD in an individual could be a provisional diagnosis prior to confirmation).

Incidence data were incorporated into modelling for the first time in GBD 2023, with data from 94 unique sources.

Modelling strategy

Studies with age and sex detail separately were split into age- and sex-specific datapoints. Standard GBD sex-splitting methods were used for studies with only "both"-sex datapoints: we modelled the ratio of female/male prevalence in MR-BRT[1] and then calculated male prevalence:

$$prev_{male} = prev_{both} * \frac{pop_{both}}{(pop_{male} + ratio * pop_{female})}$$

And then calculated female prevalence:

$$prev_{female} = ratio * prev_{male}$$

We also split datapoints where the age range was greater than 25 years. In GBD 2023, age splitting was based on the global age pattern from a DisMod-MR 2.1 model (disease model—Bayesian meta-regression, for details on this method see appendix 1, section 2) that only used input data with less than a 25-year age range. Data were location split if they are at country level and cover a number of subnationals (or are UK data) either by population if the study sampled from different units proportional to the population or evenly if the study sampled the same number of individuals from different units.

For GBD 2023, adjustments were not made for non-reference case definitions because a GBD 2021 analysis demonstrated no systematic difference to reference. Adjustment factors (crosswalks) for all studies that did not use reference methodology were determined using matched data (by year, age, sex, location) for reference and alternative case definitions in a logit difference network meta-regression using the MR-BRT tool[1] (meta-regression—Bayesian, regularised, trimmed; additional information can be found in appendix 1, section 4.4.1 of the cited paper). These covariates included studies that were not population representative (if records of Parkinson's only came from a particular hospital/department), excluded nursing homes from their estimates, followed UKPD Brain Bank diagnosis criteria, followed Movement Disorder Society (MDS) diagnosis criteria, or did not explicitly define diagnosis criteria. The following table provides an overview of the study-level covariates tested.

MR-BRT crosswalk adjustment factors for Parkinson's disease

Data input	Reference or alternative case definition	Gamma	Beta coefficient, logit* (95% CI)	Adjustment factor**
Two of four diagnostic criteria	Ref	0.48	---	---
Not population representative	Alt		0.03 (–0.95 to 1.04)	01.03
Excluded nursing homes	Alt		0.01 (–0.95 to 0.95)	1.01
UKPD Brain Bank criteria	Alt		0.01 (–1.46 to 0.47)	1.01
MDS criteria	Alt		0.14 (–0.83 to 1.54)	1.15
No explicit criteria	Alt		0.01 (–0.56 to 1.37)	1.01

**MR-BRT crosswalk adjustments can be interpreted as the factor the alternative case definition is adjusted by to reflect what it would have been had it been measured using the reference case definition. If the log/logit beta coefficient is negative, then the alternative is adjusted up to the reference. If the log/logit beta coefficient is positive, then the alternative is adjusted down to the reference.*

***The adjustment factor column is the exponentiated beta coefficient. For logit beta coefficients, this is the relative odds between the two case definitions.*

Country covariates are used to inform global patterns. A DisMod-MR model was run including two country-level covariates, smoking prevalence and Healthcare Access and Quality Index. Excess mortality rate input data from fatal modelling process were included in the model, and cause-specific mortality results from the final fatal Parkinson's disease model were pulled into the final non-fatal DisMod-MR model. The following table provides an overview of country covariates used in the Parkinson's disease DisMod-MR model.

Covariates. Summary of county-level covariates used in the Parkinson's disease DisMod-MR model

**Covariate**	**Type**	**Coefficient**	**Exponentiated beta (95% uncertainty interval)**
Smoking prevalence (age-standardised)	Prevalence	–1.99 (–2.00 to –1.95)	0.14 (0.14–0.14)
Healthcare Access and Quality Index	Excess mortality rate	–0.017 (–0.025 to –0.013)	0.98 (0.98–0.99)

Severity splits

We used the Hoehn and Yahr stages[2] to determine severity, as shown in the table below.

Hoehn and Yahr stages mapped to Parkinson's disease severity in the GBD.

Severity	Stage
Mild	≤2.0
Moderate	2.5–3.5

Severe	≥4

The following figures show the results of the meta-analysis on Hoehn and Yahr stages to split Parkinson's prevalence by severity.

Figure 1. Percentage of mild cases of Parkinson's disease in population-based studies

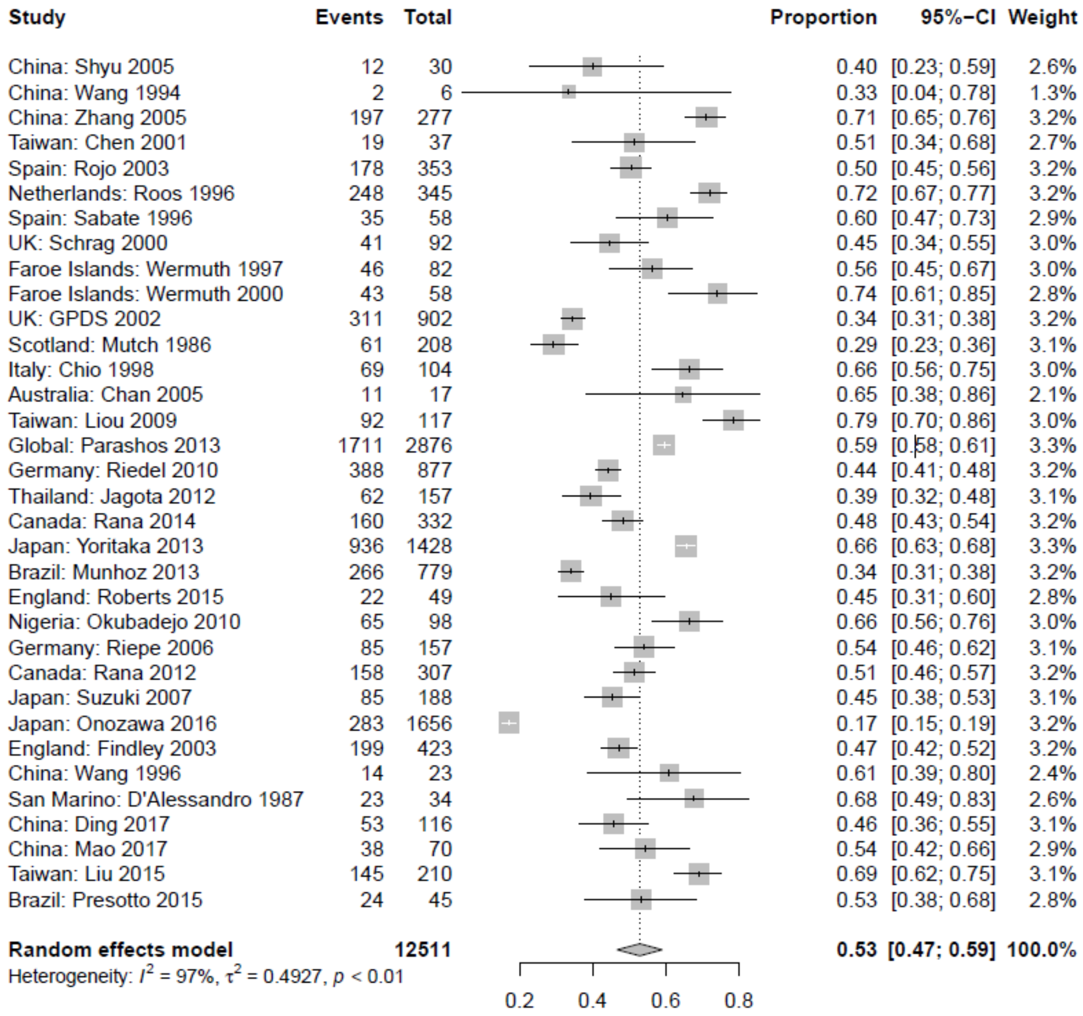

Figure 2. Percentage of moderate cases of Parkinson’s disease in population-based studies

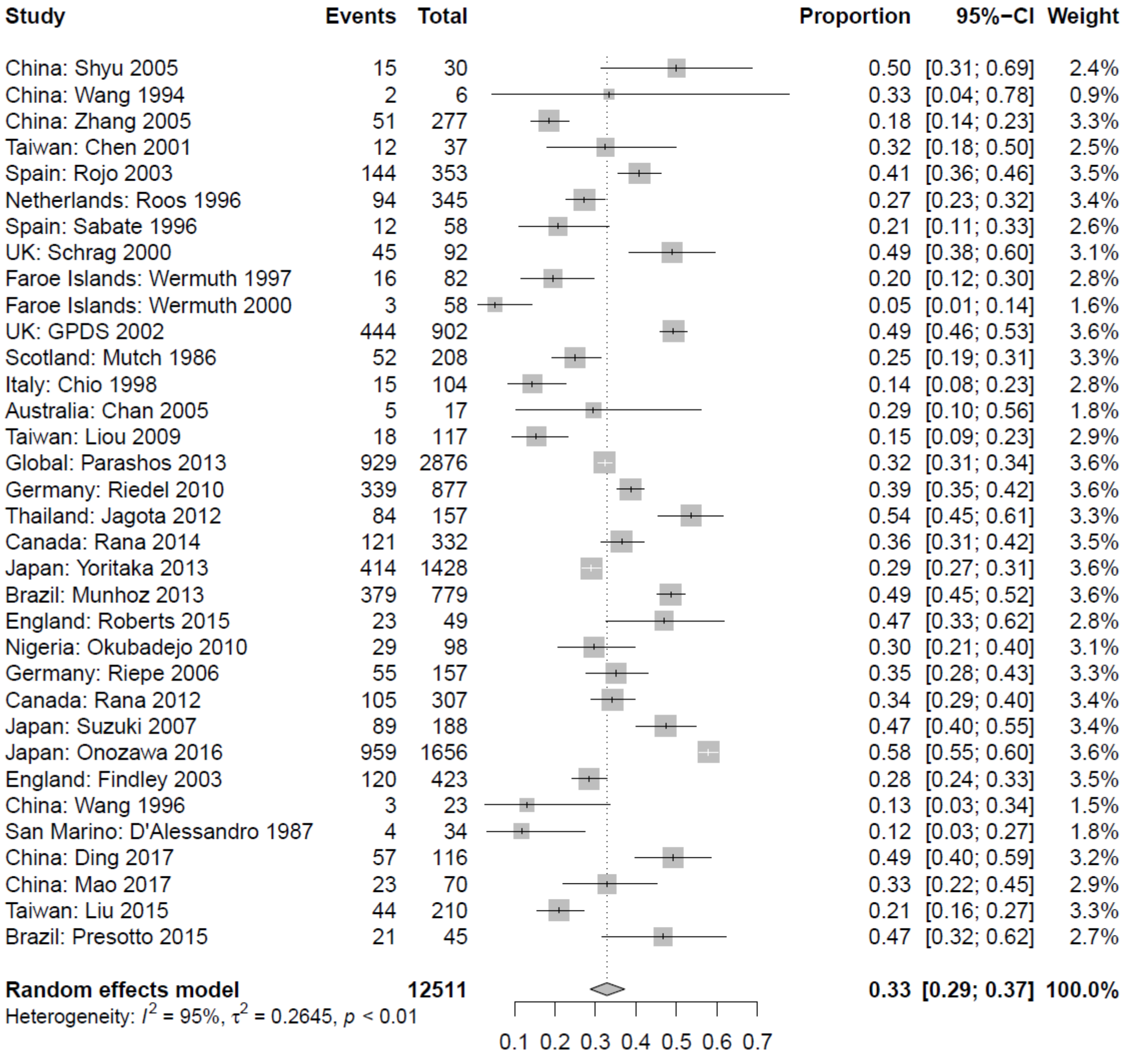

Figure 3. Percentage of severe cases of Parkinson's disease in population-based studies

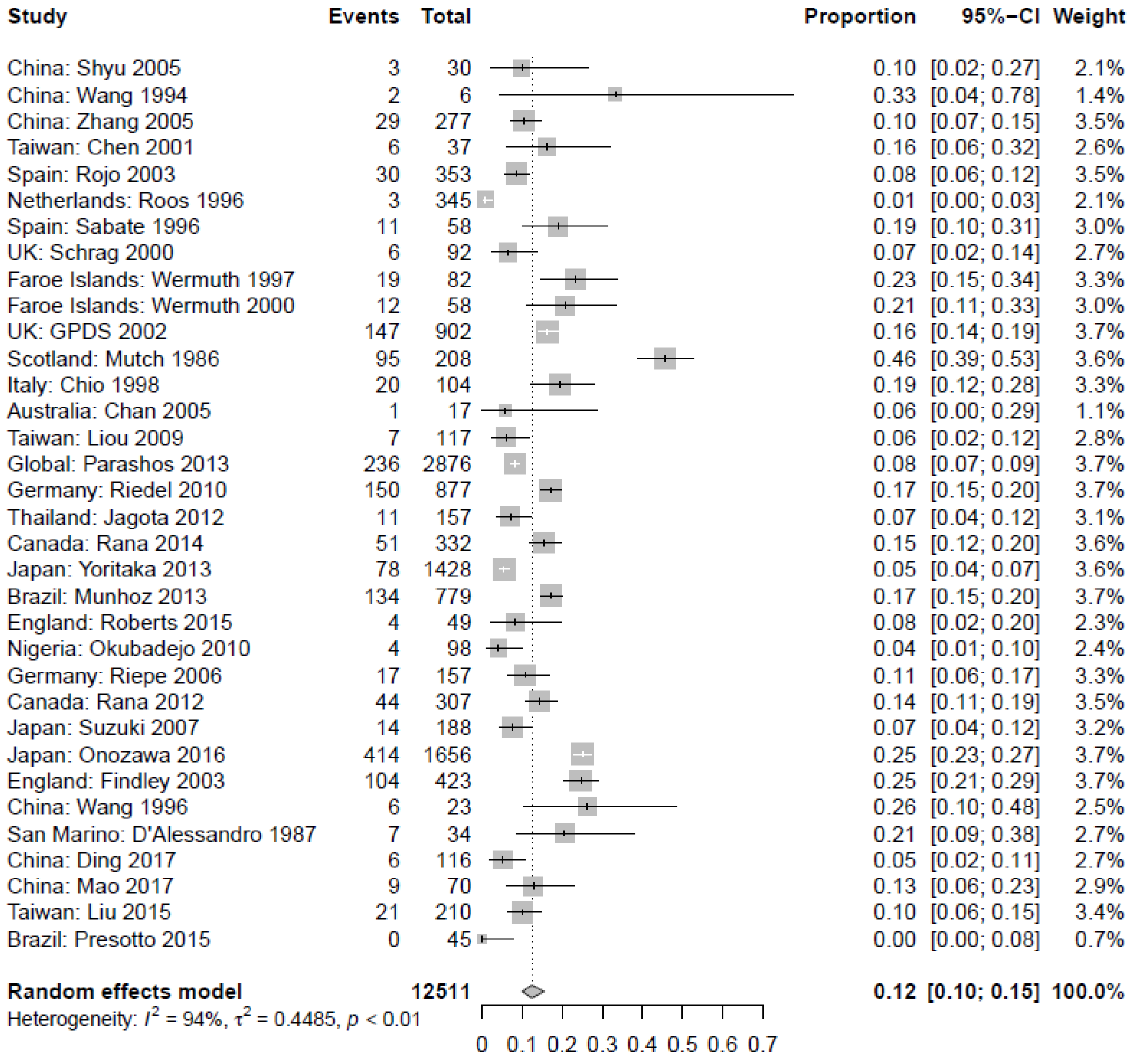

Severity estimates were generated by multiplying estimates of prevalence (country-year-sex-age-specific) by the fractions of mild, moderate, and severe PD, and 95% confidence intervals were estimated by taking 1000 draws.

The following table provides the lay description and disability weights associated with Parkinson's disease.[3]

Severity level	Lay description	DW (95% CI)
Mild	Has mild tremors and moves a little slowly, but is able to walk and do daily activities without assistance.	0.01 (0.005–0.019)
Moderate	Has moderate tremors and moves slowly, which causes some difficulty in walking and daily activities. The person has some trouble swallowing, talking, sleeping, and remembering things.	0.267 (0.181–0.372)
Severe	Has severe tremors and moves very slowly, which causes great difficulty in walking and daily activities. The person falls easily and has a lot of difficulty talking, swallowing, sleeping, and remembering things.	0.575 (0.396–0.73)

References

[1] Vos T, Lim SS, Abbafati C, *et al.* Global burden of 369 diseases and injuries in 204 countries and territories, 1990–2019: a systematic analysis for the Global Burden of Disease Study 2019. *The Lancet* 2020; **396**: 1204–22. doi: https://doi.org/10.1016/S0140-6736(20)30925-9

[2] Hoehn MM, Yahr MD. Parkinsonism: onset, progression, and mortality. *Neurology* 1967; **17(5)**: 427-42. doi: https://doi.org/10.1212/WNL.17.5.427

[3] Salomon JA, Haagsma JA, Davis A, *et al.* Disability weights for the Global Burden of Disease 2013 study. *Lancet Global Health* 2015; **3**: e712-23. doi: https://doi.org/10.1016/S2214-109X(15)00069-8

Pelvic inflammatory disease (impairment)

Flowchart

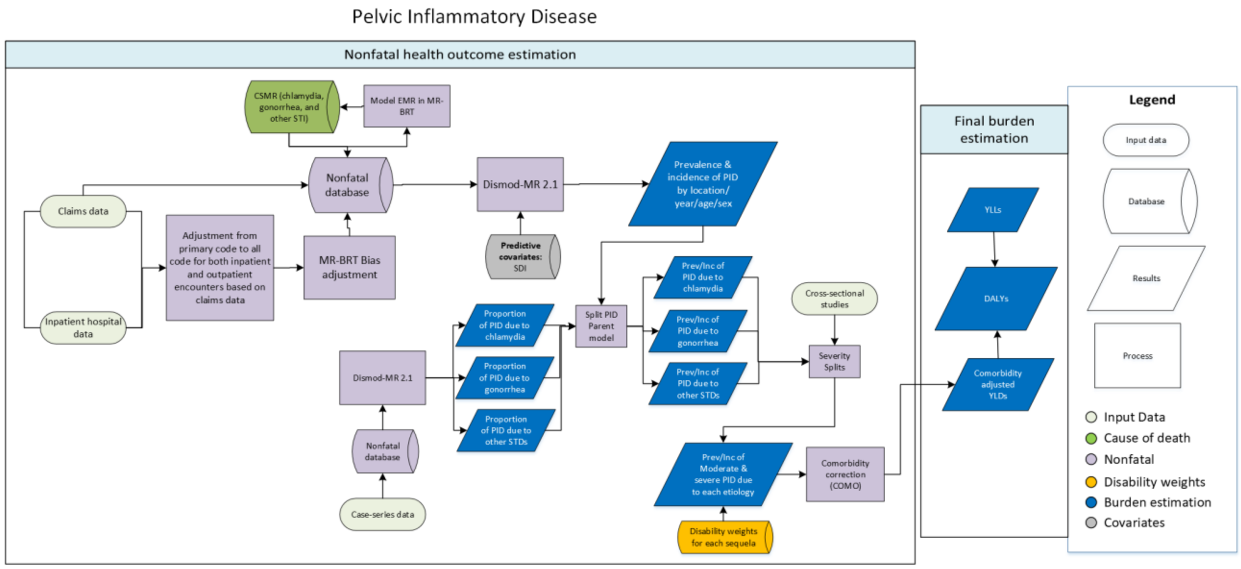

Input data and methodological summary for pelvic inflammatory disease

Case definition

Pelvic inflammatory disease (PID) is an infection of the female reproductive organs that affects the upper portion of the female reproductive tract and can be caused by multiple sexually transmitted infections (such as chlamydia, gonorrhoea, and other sexually transmitted diseases), as well as non-sexually transmitted infections; this generally presents with abdominal or pelvic pain, which can be severe.

The following International Classification of Disease (ICD) codes are relevant for PID.

Table 1: ICD codes for PID

ICD set	Code
ICD-9	98.1, 98.17, 98.19, 98.2, 98.3, 98.36, 98.37, 98.39, 99.54, 99.56,<br>613, 614.0–614.9, 615.0–615.1, 615.9
ICD-10	A54.24, A56.1–56.11,<br>K67.0, K67.1,<br>N74.3, N70.00–70.03, N70.1, N70.11, N70.12, N70.13, N70.90-70.93, N71.0–71.1,<br>N71.9, N73, N73.0–N73.9, N74, N74.2, N74.8, N74.4

Quantity of interest	Reference or alternative	Definition

Pelvic inflammatory disease due to any sexually transmitted infection	Reference	Prevalent cases of pelvic inflammatory disease identified using ICD codes in administrative records that are considered population-representative.
Pelvic inflammatory disease due to any sexually transmitted infection	Alternate	Cases identified from database of commercial claims from USA using ICD codes.

Pelvic inflammatory disease is also defined according to its aetiology:

- Pelvic inflammatory disease due to chlamydial infection: The proportion of cases of PID diagnosed by physical examination, laparoscopy, or ultrasound who are diagnosed with *Chlamydia trachomatis* as the causal pathogen diagnosed via endocervical swab, antigen and/or antibody testing in a representative series of PID cases in cross-sectional, retrospective cohort studies in clinical settings (hospital, STD clinic, OBGYN office).
- Pelvic inflammatory disease due to gonococcal infection: The proportion of cases of PID diagnosed by physical examination, laparoscopy, or ultrasound who are diagnosed with *Neisseria gonorrhoeae* as the causal pathogen diagnosed via endocervical swab, antigen and/or antibody testing in a representative series of PID cases in cross-sectional, retrospective cohort studies in clinical settings (hospital, STD clinic, OBGYN office).
- Pelvic inflammatory disease due to other sexually transmitted infections: The proportion of cases of PID diagnosed by physical examination, laparoscopy, or ultrasound who are diagnosed with other sexually transmitted infections as the causal pathogen diagnosed via endocervical swab, antigen and/or antibody testing in a representative series of PID cases in cross-sectional, retrospective cohort studies in clinical settings (hospital, STD clinic, OBGYN office).

Input data

A systematic review was completed for GBD 2013 on October 28, 2013, using the following search terms: (("pelvic inflammatory disease"[Title/Abstract] OR "salpingitis"[Title/Abstract]) AND ((chlamydia[Title/Abstract] OR gonorrhoea[Title/Abstract]) OR aetiology[Title/Abstract] OR aetiology[Title/Abstract] OR pathogen[Title/Abstract])) AND (“1994”[Date – Publication] : “2013”[Date – Publication]))

In GBD 2013, data extracted from published studies identified in our systematic review included measurements of incidence, prevalence, and aetiological proportions. That is to say, a subset of the studies from the systematic review reported the underlying aetiology of PID, allowing us to estimate the proportion of PID due to chlamydia, gonorrhoea, or other sexually transmitted diseases. Starting in GBD 2015 and continuing through GBD 2023, only data from hospital discharges and claims were used to model the incidence and prevalence of PID, but published studies from the original systematic review were retained for the estimation of aetiological proportions.

There are also fatal data inputs to the model used to estimate the incidence and prevalence of PID. These include cause-specific mortality rate (CSMR) and excess mortality rate (EMR), which are explained in further detail below.

Data processing

PID envelope

In GBD 2017, an individual was extracted from claims data as an incident case if that individual had one or more inpatient encounters with an appropriate ICD code as any diagnosis. Hospital discharges with an appropriate ICD code as primary diagnosis were extracted and adjusted for readmissions.

Beginning in GBD 2019, however, data processing methods were employed to capture cases that were diagnosed and/or treated in both inpatient and outpatient settings. Specifically, an individual was extracted from claims data as an incident case if that individual had at least one inpatient or outpatient encounter with an appropriate ICD code as any diagnosis within one year. Hospital discharge data were processed by extracting discharges with an appropriate ICD code as primary diagnosis and adjusting using correction factors derived from claims data. Please see the non-fatal modelling methods "Inpatient hospital admissions" section of the appendix for further details.

A priori, we believed that claims data from the USA MarketScan database reflect a certain level of selection bias due to commercial insurance, while other sources of claims and hospital data are more reflective of the general population. We therefore adjusted USA MarketScan claims data to USA inpatient hospital data using meta-regression—Bayesian, regularised, trimmed (MR-BRT), prior to analysis in DisMod. We included a covariate on age to account for the age pattern seen in the relationship between the incidence of PID reported through MarketScan and incidence reported from hospital discharges. The adjustment factors were modelled in a random effects meta-regression in MR-BRT, with the log-transformed ratios between claims data sources and inpatient data sources as data inputs. Ratios were formed by matching sources by year, age, and location.

Table 2: MR-BRT crosswalk adjustment factors for pelvic inflammatory disease

Data input	Reference or alternative case definition	Gamma	Beta coefficient, log (95% UI)*	Adjustment factor**
Inpatient hospital	Ref	0.08	---	---
Claims (10–14 yrs)	Alt		–0.43 (–0.65 to –0.21)	0.65 (0.52–0.81)
Claims (15–19 yrs)	Alt		–0.41 (–0.62 to –0.22)	0.66 (0.53–0.80)
Claims (20–24 yrs)	Alt		–0.39 (–0.58 to –0.21)	0.67 (0.55–0.81)
Claims (25–29 yrs)	Alt		–0.39 (–0.57 to –0.20)	0.67 (0.56–0.81)
Claims (30–34 yrs)	Alt		–0.38 (–0.57 to –0.20)	0.68 (0.56–0.81)
Claims (35–39 yrs)	Alt		–0.34 (–0.52 to –0.16)	0.71 (0.59–0.85)
Claims (40–44 yrs)	Alt		–0.23 (–0.41 to –0.04)	0.79 (0.66–0.96)
Claims (45–49 yrs)	Alt		–0.01 (–0.19 to 0.17)	0.99 (0.82–1.18)

Claims (50–54 yrs)	Alt		0.29 (0.10 to 0.47)	1.33 (1.10–1.59)
Claims (55–59 yrs)	Alt		0.65 (0.45 to 0.84)	1.91 (1.56–2.31)

**MR-BRT crosswalk adjustments can be interpreted as the factor the alternative case definition is adjusted by to reflect what it would have been had it been measured using the reference case definition. If the log/logit beta coefficient is negative, then the alternative is adjusted up to the reference. If the log/logit beta coefficient is positive, then the alternative is adjusted down to the reference.*
***The adjustment factor column is the exponentiated beta coefficient. For log beta coefficients, this is the relative rate between the two case definitions. For logit beta coefficients, this is the relative odds between the two case definitions.*

After the relevant datapoints were adjusted to account for selection bias, datapoints with an age-standardised prevalence greater than one median absolute deviation from the median of the age-standardised prevalence for all inpatient and non-USA claims data were marked as outliers and excluded from analysis.

Hospital inpatient data and claims data provided by the GBD Clinical Informatics team are processed into small age bins (eg, 10–14 years, 15–19 years, 20–24 years) that are ideal for input to a DisMod model. Thus, no further age splitting was performed on the data prior to modelling.

Fatal inputs to the PID model are also processed. For each non-fatal cause, the CSMR data inputs come from the cause's corresponding fatal estimates. PID, however, is a GBD impairment that can be the result of multiple GBD causes, so CSMR must be aggregated from multiple causes of death. We assume that for women, most deaths due chlamydia, gonorrhoea, and other STIs (excluding HIV, HSV and syphilis) are mediated via extension of lower genital tract infections to the upper genital tract (ie, PID), followed by systemic infection and sepsis. Thus, the CSMR from the fatal estimates of each of the aetiologies of PID (chlamydia, gonorrhoea, other STI) were aggregated to create CSMR representative of all aetiologies for input to the PID model.

Prior to GBD 2019, EMR inputs were produced by fitting a preliminary compartment model to estimate prevalence from incidence data, matching preliminary prevalence resulting from each incidence datapoint with the corresponding CSMR value within the same age, sex, year, and location, and dividing CSMR by prevalence. This method of producing EMR inputs, however, demonstrated a rather unrealistic pattern of EMR compared to an expected pattern of decreasing EMR with greater access to quality health care. Such unexpected patterns often signal inconsistencies between CSMR estimates and data on prevalence and/or incidence. Thus, in an effort to provide greater guidance on the expected pattern of EMR, in GBD 2019, EMR data produced per above were modelled by age, sex, and Healthcare Access and Quality (HAQ) Index using MR-BRT, with a prior on HAQ Index having a negative coefficient. In GBD 2021, we employed the same MR-BRT method to predict EMR for each location, year, sex, and for ages 0, 10, 20….100; these predictions were used as inputs to our non-fatal models in GBD 2021 and GBD 2023, with the latter described below.

PID aetiological proportions

Data on the aetiological proportions with age ranges greater than five years were split into distinct age bins prior to input into DisMod. No other pre-modelling adjustments were made.

Modeling strategy

DisMod models

First, we estimated the total incidence and prevalence of PID using DisMod-MR 2.1. We used a Bayesian prior on remission (13–17) for all ages and set the incidence of PID to 0 for ages 0–10 years. The Socio-demographic Index was used as a predictive covariate on incidence.

Table 3. Covariates. Summary of covariates used in the pelvic inflammatory disease envelope DisMod-MR meta-regression model

Covariate	Parameter	Exponentiated beta (95% uncertainty interval)
Socio-demographic index	Incidence	0.99<br>(0.96–1.00)

Second, we ran three separate DisMod models for the *proportion* of PID due to the following three causes: chlamydia, gonococcal infection, and other sexually transmitted infections. Each of these models incorporated the cause-specific age-standardised death rate (ASDR) as a covariate.

Table 4. Covariates. Summary of covariates used in the pelvic inflammatory disease due to chlamydial infection DisMod-MR proportion model

Covariate	Parameter	Exponentiated beta (95% uncertainty interval)
Age-standardised death rate (lnASDR) for chlamydial infection	Proportion	1.15<br>(1.02–1.32)

Table 5. Covariates. Summary of covariates used in the pelvic inflammatory disease due to gonococcal infection DisMod-MR proportion model

Covariate	Parameter	Exponentiated beta (95% uncertainty interval)
Age-standardised death rate (lnASDR) for gonococcal infection	Proportion	1.22<br>(1.06–1.34)

Table 6. Covariates. Summary of covariates used in the pelvic inflammatory disease due to other sexually transmitted infections DisMod-MR proportion model

Covariate	Parameter	Exponentiated beta (95% uncertainty interval)
Age-standardised death rate (lnASDR) for other sexually transmitted infections	Proportion	1.09<br>(1.01–1.20)

Assigning PID envelope to aetiological proportions

Once the PID envelope model is completed, the estimates are restricted to ages 10–59 years, as experts advise that PID hospital data in older ages are likely not due to STIs. Estimates outside of this age range are dropped to zero. This creates an age-restricted PID envelope model, which is then split according to the estimates generated in each aetiological proportion model for a given year, age, and location.

Table 7: PID aetiological assignment

Input models	Output model
1. Age-restricted PID envelope<br>2. PID due to chlamydial infection proportion	PID due to chlamydial infection
1. Age-restricted PID envelope<br>2. Pelvic inflammatory disease due to gonococcal infection proportion	PID due to gonococcal infection
1. Age-restricted PID envelope<br>2. Pelvic inflammatory disease due to other sexually transmitted infections proportion	PID due to other sexually transmitted infections

Severity splits and disability weights

Sequelae highlight major functional consequences and symptoms of disease, represented in the GBD by health states and disability weights.[1] The lay descriptions and disability weights for PID are shown below. Because PID has underlying aetiologies, the YLDs and DALYs measures are ultimately assigned to the aetiologies rather than to PID itself. Thus, the PID-related sequela are moderate pelvic inflammatory disease due to chlamydial infection, severe pelvic inflammatory disease due to chlamydial infection, moderate pelvic inflammatory disease due to gonococcal infection, severe pelvic inflammatory disease due to gonococcal infection, moderate pelvic inflammatory disease due to other STIs, and severe pelvic inflammatory disease due to other STIs.

Table 8. Severity distribution, details on the severity levels for pelvic inflammatory disease and the associated disability weight (DW) with that severity.

Severity level	Lay description	DW (95% CI)	Distribution (95% CI)
Abdominopelvic problem, moderate	This person has pain in the belly and feels nauseated. The person has difficulties with daily activities.	0.114<br>(0.078, 0.159)	0.767<br>(0.7666, 0.7675)
Abdominopelvic problem, severe	This person has severe pain in the belly and feels nauseated. The person is anxious and unable to carry out daily activities.	0.324<br>(0.219, 0.442)	0.233<br>(0.2326, 0.2335)

The prevalence proportions of moderate and severe PID listed in the table below are the same for each aetiology. These proportions came from data found in the PID systematic review.[2] In GBD 2023, we detected an extraction error, reextracted these values, and updated the proportions accordingly.

References

1. Salomon JA, Vos T, Hogan DR, et al. Common values in assessing health outcomes from disease and injury: disability weights measurement study for the Global Burden of Disease Study 2010. *Lancet* 2012; 380: 2129–43.
2. Weström L, Joesoef R, Reynolds G, Hagdu A, Thompson SE. Pelvic inflammatory disease and fertility. A cohort study of 1,844 women with laparoscopically verified disease and 657 control women with normal laparoscopic results. *Sex Transm Dis* 1992; 19: 185–92.

Peptic ulcer disease

Flowchart

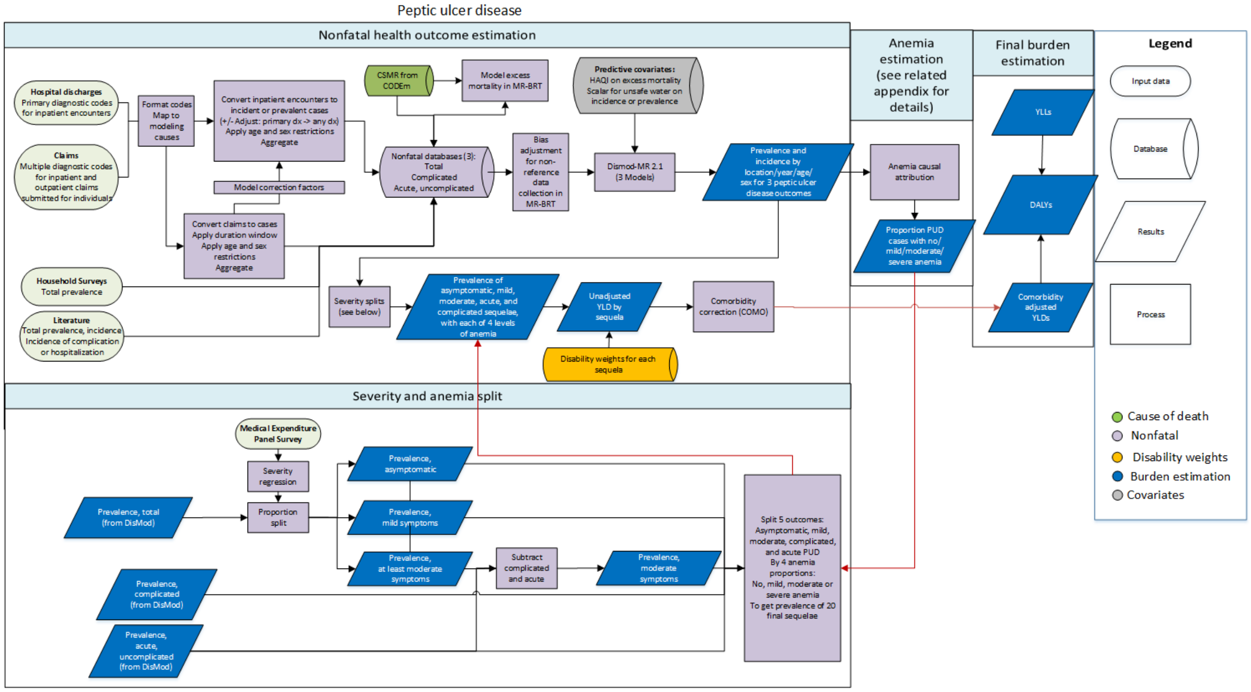

Input data and methodological summary for peptic ulcer disease

Case definition

Peptic ulcer disease is a digestive disorder defined by defects in the lining of the stomach (gastric ulcers) or the duodenum (duodenal ulcers) that extend through the muscularis mucosa. Peptic ulcers can develop marked abdominal pain acutely or have a more insidious onset and develop into a chronic disease with asymptomatic and symptomatic periods. Symptomatic periods are characterised by abdominal pain, bloating, nausea, and early satiety. Regardless of the duration of the disease, acute, life-threatening complications of bleeding, perforation, or gastric outlet obstruction can develop.

For GBD, cases were defined by diagnostic codes in administrative data. ICD-10 codes used to identify cases of peptic ulcer disease are K25, K26, K27, K28, and K31. ICD-10 codes for complicated peptic ulcer disease are K25.0-2, K25.4-6, K26.0-2, K26.4-6, K27.0-2, K27.4-6, K28.0-2, and K28.4-6. ICD-10 codes for acute peptic ulcer disease without complication are K25.3, K26.3, K27.3 and K28.3. Equivalent ICD-9 codes were used where appropriate.

Quantity of interest	Reference or alternative	Definition
Incidence of complicated PUD	Alternative	Cases identified from database of commercial claims from USA in 2000 using ICD codes.
Incidence of complicated PUD	Alternative	Cases identified from database of commercial claims from USA in 2010-2016 using ICD codes.

Incidence of complicated PUD	Reference	Cases of complicated peptic ulcer disease identified using ICD codes in administrative records that are considered population-representative.
Prevalence of total peptic ulcer disease	Alternative	Cases identified from database of commercial claims from USA in 2000 using ICD codes.
Prevalence of total peptic ulcer disease	Alternative	Cases identified from database of commercial claims from USA in 2010-2016 using ICD codes.
Prevalence of total peptic ulcer disease	Alternative	Cases of peptic ulcer disease identified by self-report in general, population-based surveys.
Prevalence of total peptic ulcer disease	Reference	Cases of peptic ulcer disease identified using ICD codes in administrative records that are considered population-representative.
Incidence of uncomplicated, acute PUD	Alternative	Cases identified from database of commercial claims from USA in 2000 using ICD codes.
Incidence of uncomplicated, acute PUD	Alternative	Cases identified from database of commercial claims from USA in 2010-2016 using ICD codes.
Incidence of uncomplicated, acute PUD	Reference	Cases of uncomplicated, acute peptic ulcer disease identified using ICD codes in administrative records that are considered population-representative.

Overall strategy

As in GBD 2017, GBD 2019, and GBD 2021, the GBD 2023 non-fatal estimation strategy for peptic ulcer disease consisted of:

- Estimating the prevalence of total peptic ulcer disease.
- Dividing the total prevalent cases into asymptomatic, mild, and at least moderate severity levels.
- Separately estimating the prevalence of peptic ulcer disease with complication.
- Separately estimating the prevalence of peptic ulcer disease, acute, without complication (but with sufficient severity to require hospitalisation).
- Subtracting prevalent cases of peptic ulcer disease with complication and peptic ulcer disease, acute, without complication (but with sufficient severity to require hospitalisation) from prevalent cases of peptic ulcer disease of at least moderate severity.

Input data

Data sources

As in previous rounds, our GBD 2023 peptic ulcer disease models relied primarily on data from hospital discharges and claims, as processed by IHME and described elsewhere in this appendix. Additional sources of data for peptic ulcer disease included peer-reviewed publications identified via systematic reviews of the literature conducted using recognised search engines (PubMed, Embase) for previous rounds of GBD, most recently GBD 2016. They also included studies contributed to the Global Health Data Exchange by GBD Collaborators and identified by a keyword search. In brief, to be included, studies from all sources needed to:

1) Report a standard epidemiological measure (incidence, prevalence, case-fatality ratio, standardised mortality rate, etc.) of peptic ulcer disease or its complications (bleeding, perforation, hospital admission).
2) Provide sufficient information on study methods and sample characteristics to assess its quality and make appropriate adjustments.
3) Use a gold-standard endoscopic case definition or use a well-defined alternative case definition that could be adjusted toward a reference standard.
4) Be conducted in a representative sample of a general population defined only by year, age, sex, and location, or be conducted in a representative sample of a well-defined sub-population for which valid adjustments could be made or ascertain all cases for a defined catchment area for which GBD population estimates are available.

The peptic ulcer disease modelling strategy uses three separate databases: total peptic ulcer disease, peptic ulcer disease with complication (such as haemorrhage or perforation), and peptic ulcer disease, acute, without complication (but with sufficient severity to require hospitalisation). The total peptic ulcer disease model included data from hospital discharges and claims coded with any peptic ulcer disease ICD code, as well as data from peer-reviewed publications and household surveys. The peptic ulcer disease with complication dataset included hospital discharges and inpatient claims with ICD codes specifying the occurrence of complications, as well as data from peer-reviewed publications. The peptic ulcer disease, uncomplicated, acute dataset included only hospital discharges and inpatient claims with ICD codes specifying that a complication did not occur.

No new data were added in GBD 2023. The input data and processing steps for modelling in GBD 2023 were the same as in GBD 2021.

Inputs to our non-fatal modelling also included cause-specific mortality rate (CSMR) estimates taken from our fatal modelling process (see CoD cause-specific modelling description for appendicitis in this appendix) and excess mortality rate (EMR) estimates modelled outside of DisMod (see the EMR data processing section below).

Prevalence and incidence data processing

The extraction and processing of prevalence and incidence data for peptic ulcer disease were identical in GBD 2021 and GBD 2023. The preponderance of these data came from claims and hospital discharges. Hospital discharge data provide observations about encounters, generally with only the primary diagnostic code for the encounter. Claims data, on the other hand, link claims for all inpatient and outpatient encounters for a single individual and provide primary and secondary diagnoses for all encounters.

For the total peptic ulcer disease database, an individual was extracted from claims data as a prevalent case if they had any peptic ulcer disease ICD code as any diagnosis in one or more inpatient encounters or two or more outpatient encounters. Hospital discharges were extracted if an appropriate ICD code appeared as the primary discharge diagnosis, and the discharges were then adjusted using a correction factor estimated from claims data. Specifically, the correction factor (known as CF3) was modelled as the ratio of inpatient claims with an appropriate primary diagnostic code to all prevalent cases (inpatient and outpatient) in claims data, using MR-BRT.

For the peptic ulcer disease with complication dataset and the peptic ulcer disease, uncomplicated, acute dataset, individuals were extracted from claims as incident cases if they had an inpatient claim with an appropriate ICD code as any diagnosis. These incident cases were extracted linking multiple encounters for an individual and assuming multiple claims within a 60-day window represented a single incident case, and multiple claims separated by more than 60 days represented separate episodes of illness and, thus, additional incident cases. Hospital discharges were extracted if an appropriate ICD code appeared as the primary diagnosis, and the discharges were then adjusted using a correction factor estimated from claims data. Specifically, the correction factor (known as CF2), was modelled as the ratio of inpatient claims with an appropriate primary diagnostic code to all incident (inpatient) cases in claims data, using MR-BRT.

Details of the extraction, utilisation envelope, and correction factor models used to process hospital discharge and claims data for peptic ulcer disease are found elsewhere in this appendix.

Epidemiological measurements from peer-reviewed publications were manually extracted for the most granular age-sex groups reported, with a measure of uncertainty and information on the study design. Prevalence measurements were extracted from individual-level data from household surveys using questionnaire text, skip-pattern, and weights for complex sampling strategies provided in the documentation from original study investigators. Extracted measurements were marked with dichotomous variables to indicate alternative (non-reference) case definitions, study populations, or other study design features. Where a single study measured using more than one case definition, multiple measurements were extracted to create paired data for modelling adjustment factors.

For total peptic ulcer disease, we sought to use a gold-standard case definition of endoscopy without clinical indication and to develop adjustments for alternative case definitions of endoscopy with clinical indication, diagnostic code in administrative data, and self-reported diagnosis (current or with 12-month recall). Unfortunately, the few (four) endoscopy-based studies in our database were not performed in samples from locations for which we had data with alternative case definitions available. Thus, we dropped the endoscopy-based data and adopted diagnostic code in administrative data as our reference case definition.

Two pre-modelling adjustments were made to non-reference data sources: data using self-reported diagnosis and data from a claims database that only covers a commercially insured sub-population. 26 sources used self-reported diagnosis, and 18 of these were matched to hospital discharge data, claims data, or both. Commercial claims data were available for all 51 USA subnational locations, and matched hospital discharge data covering the general population were available for one or more years for 24 USA subnational locations. These sets of paired data were used as inputs to a model of the difference in logit prevalence between alternative and reference data types using a network model in MR-BRT. The estimated mean logit differences were applied to non-reference data types as bias correction prior to modelling in DisMod-MR 2.1 (below).

The process of adjusting for non-reference (alternative) data types using MR-BRT with the logit-transformation method is described below:

1. Identify datapoints with overlapping year, age, sex, and location between reference and non-reference population data.
2. Logit transform overlapping datapoints of alternative and reference types.
3. Convert overlapping datapoints into a difference in logit space using the following equation: $logit(altnerative) - logit(reference)$
4. Use the delta method to compute standard errors of overlapping datapoints in logit space, then calculate standard error of logit difference using the following equation: $\sqrt{(variance\ of\ logit(alternative)) + (variance\ of\ logit(reference))}$
5. Using MR-BRT, conduct a random effects meta-regression to obtain the pooled logit difference of alternative to reference
6. Apply the pooled logit difference to all data points of non-reference types using the following equation:
$$new_{estimate} = inverse.logit((logit(alternative)) - (pooled\ logit\ difference))$$
7. Calculate new standard errors using the delta method, accounting for gamma (between-study heterogeneity)

The table below shows bias correction factors estimated using MR-BRT.

Table 1.1: MR-BRT crosswalk adjustment factors for total peptic ulcer disease

Data input	Reference or alternative data collection	Gamma	Beta coefficient, logit difference (95% CI)	Adjustment factor*
Hospital + non-USA claims	Reference	0.163	---	---
USA claims from year 2000	Alternative		0.00936 (–0.319 to 0.340)	0.50 (0.42 to 0.58)
USA claims from years 2010–2016	Alternative		–0.138 (–0.463 to 0.193)	0.47 (0.39 to 0.55)
Self-reported diagnosis	Alternative		2.37 (2.05 to 2.70)	0.91 (0.89 to 0.94)

**Adjustment factor is the inverse-logit transformed beta coefficient; <0.5 represents that alternative is adjusted upward; >0.5 represents that alternative is adjusted downward.*

For peptic ulcer disease with complication, similar to the total peptic ulcer disease model, we sought to use a gold-standard endoscopic case definition and to develop adjustments for the alternative case definitions by diagnostic code in administrative data. Unfortunately, there were only five studies that used endoscopy to define peptic ulcer disease with complications in our database, and they were not conducted in the same year, age, sex, and location as studies with other designs, so these data were dropped, and diagnosis in administrative data was adopted as the reference case definition. Pre-modelling adjustments were made to data from commercial claims, using an approach similar to that described above for total peptic ulcer disease data.

Table 1.2: MR-BRT crosswalk adjustment factors for peptic ulcer disease with complication

Data input	Reference or alternative data collection	Gamma	Beta coefficient, logit difference (95% CI)	Adjustment factor*

Hospital + non-USA claims	Reference	0.118	---	---
USA claims from year 2000	Alternative		0.861 (0.214 to 1.50)	0.70 (0.55 to 0.82)
USA claims from years 2010–2016	Alternative		0.778 (0.511 to 1.03)	0.69 (0.62 to 0.74)

**Adjustment factor is the inverse-logit transformed beta coefficient; <0.5 represents that alternative is adjusted upward; >0.5 represents that alternative is adjusted downward.*

For peptic ulcer disease, uncomplicated, acute, all data were based on diagnostic codes in administrative data. Pre-modelling adjustments were made to data from commercial claims, as described above.

Table 1.3: MR-BRT crosswalk adjustment factors for peptic ulcer disease, uncomplicated, acute

Data input	Reference or alternative data collection	Gamma	Beta coefficient, logit difference (95% CI)	Adjustment factor*
Hospital + non-USA claims	Reference	0.550	---	---
USA claims from year 2000	Alternative		0.291 (–1.22 to 1.72)	0.57 (0.23 to 0.85)
USA claims from years 2010–2016	Alternative		0.220 (–0.903 to 1.39)	0.55 (0.29 to 0.80)

**Adjustment factor is the inverse-logit transformed beta coefficient; <0.5 represents that alternative is adjusted upward; >0.5 represents that alternative is adjusted downward.*

After adjustment, for each source-location-year-sex combination, age-standardised mean was calculated, and the data series was excluded if this was zero or was greater than two times the median absolute deviation above or below the median for the database.

EMR processing

EMR inputs have evolved in recent rounds of GBD. In GBD 2017, EMR inputs were produced by matching total peptic ulcer disease prevalence datapoints with their corresponding CSMR values within the same age, sex, year, and location (by dividing CSMR by prevalence). However, this method of producing EMR inputs demonstrated a rather unrealistic pattern of EMR compared to an expected pattern of decreasing EMR with greater access to quality health care. (Such unexpected patterns often signal inconsistencies between CSMR estimates and the measures of prevalence and/or incidence.) Thus, in an effort to provide greater guidance on the expected pattern of EMR, in GBD 2019, EMR data produced per above in GBD 2017 were modelled by age, sex, and Healthcare Access and Quality (HAQ) Index using MR-BRT, with a prior on HAQ Index having a negative coefficient. We then predicted EMR for each country, year, sex, and for ages 0, 10, 20….100. These predictions were used as inputs to our total peptic ulcer disease DisMod model in GBD 2019, GBD 2021, and GBD 2023.

Modelling strategy

Total peptic ulcer disease, symptomatic and asymptomatic

Similar to previous rounds, we ran a DisMod-MR 2.1 model to produce estimates by age, sex, year, and location. Inputs to DisMod for total peptic ulcer disease included prevalence, CSMR, and EMR inputs processed as described above, and expert priors for other epidemiological measures.

The prior value of remission was bounded from 0.1 to 0.5 (a duration of two to ten years), and the prior value of incidence was that no incidence occurs before age 5. The minimum coefficient of variation at the regional, super-regional, and global level was set at 0.8, and the time window of data to include for fitting was five years. We included HAQ Index as a predictive covariate on EMR with a mean and standard deviation produced from the MR-BRT model described above. The summary exposure variable (SEV) for access to safe water was applied as a covariate to predict prevalence. Betas and exponentiated values (which can be interpreted as an odds ratio) are shown in the tables below for all predictive covariates in the DisMod model.

Table 2.1. Covariates. DisMod-MR 2.1 predictive covariates for total peptic ulcer disease

**Covariate**	**Parameter**	**Exponentiated beta**
Summary exposure variable for unsafe water	Prevalence	2.12 (2.12 to 2.13)
Healthcare Access and Quality Index	Excess mortality	0.98 (0.98 to 0.98)

Complicated peptic ulcer disease

The DisMod model for complicated peptic ulcer disease included incidence data as described above. The prior value of incidence was set to zero before age 5, the prior value of excess mortality rate was bounded to 0.1 to 10, and the prior value of remission was bounded to 6 to 13 cases of remission per person-year (disease duration 4 to 8.7 weeks). A covariate for HAQ Index was applied to EMR, and a covariate for the log-transformed age-standardised death rate due to peptic ulcer disease was applied to incidence, but neither of these were found to be predictive.

Table 2.2. Covariates. DisMod-MR 2.1 predictive covariates for peptic ulcer disease with complication

**Covariate**	**Parameter**	**Exponentiated beta**
Natural log of age-standardised death rate	Incidence	1.00 (1.00 to 1.00)
Healthcare Access and Quality Index	Excess mortality	1.06 (0.15 to 6.87)

Acute peptic ulcer disease, without complication

The DisMod model for acute, uncomplicated peptic ulcer disease included incidence data as described above. The prior value on incidence was set to zero through age 5 years, the range of prior values on EMR was bounded to 0 to 0.1, and the range of prior values on remission was bounded to 16.5 to 17.5 cases per person-year (duration of approximately three weeks). Covariates were applied for HAQ Index (on EMR), log-transformed age-standardised death rate due to peptic ulcer disease (on incidence), and unsafe water (on incidence).

Table 2.3. Covariates. DisMod-MR 2.1 Predictive covariates for peptic ulcer disease, uncomplicated, acute

Covariate	Parameter	Exponentiated beta
Natural log of age-standardised death rate	Incidence	1.00 (1.00 to 1.00)
Healthcare Access and Quality Index	Excess mortality	0.60 (0.37 to 0.97)

Severity split & disability weight

The basis of the GBD disability weight survey assessments are lay descriptions of health states highlighting major functional consequences and symptoms.

Table 3.1. Severity distribution, details on the severity levels for peptic ulcer disease, with complication, and peptic ulcer disease, uncomplicated, acute, and the associated disability weight (DW) with that severity.

Severity level	Lay description	DW (95% CI)
Peptic ulcer disease, with complication	This person vomits blood and feels nauseous.	0.325 (0.209–0.462)
Peptic ulcer disease, uncomplicated, acute	This person has severe pain in the belly and feels nauseous. The person is anxious and unable to carry out daily activities.	0.324 (0.220–0.442)

Prevalence draws from the total peptic ulcer disease model were divided into asymptomatic, mild, and at least moderate severity levels using proportions derived from the Medical Expenditure Panel Survey (MEPS). It must be noted that the MEPS analysis uses quality-of-life data from individuals who had a health-care encounter for peptic ulcer disease within the preceding 12 months and were interviewed about their quality of life in the preceding four weeks, so the asymptomatic proportion represents those with diagnosed disease who were asymptomatic in a given period of time, not those always asymptomatic who may have peptic ulcer disease on endoscopy if examined for study or screening purposes. After dividing the total prevalence draws by these three proportions, the complicated and uncomplicated, acute prevalence draws were subtracted from the at least moderate draws.

Table 3.2. Severity distribution, details on the asymptomatic, mild, and remaining moderate prevalent cases and the associated disability weight (DW) with that severity.

Severity level	Lay description	DW (95% CI)
Diagnosed peptic ulcer disease, not in a symptomatic episode	--	0
Mild peptic ulcer disease episode	This person has some pain in the belly that causes nausea but does not interfere with daily activities.	0.011 (0.005–0.021)
Moderate peptic ulcer disease episode	This person has pain in the belly and feels nauseous. The person has difficulties with daily activities.	0.114 (0.080–0.159)

*The numerous sequelae generated from exclusive combinations of anaemia and peptic ulcer disease each contain custom disability weights. More information can be found in the appendix detailing disability weights.

These five final health states were then combined with health states for anaemia. Methods for causal attribution of anaemia due to peptic ulcer disease can be found in the section of this appendix on the anaemia impairment.

Pertussis (whooping cough)

Flowchart

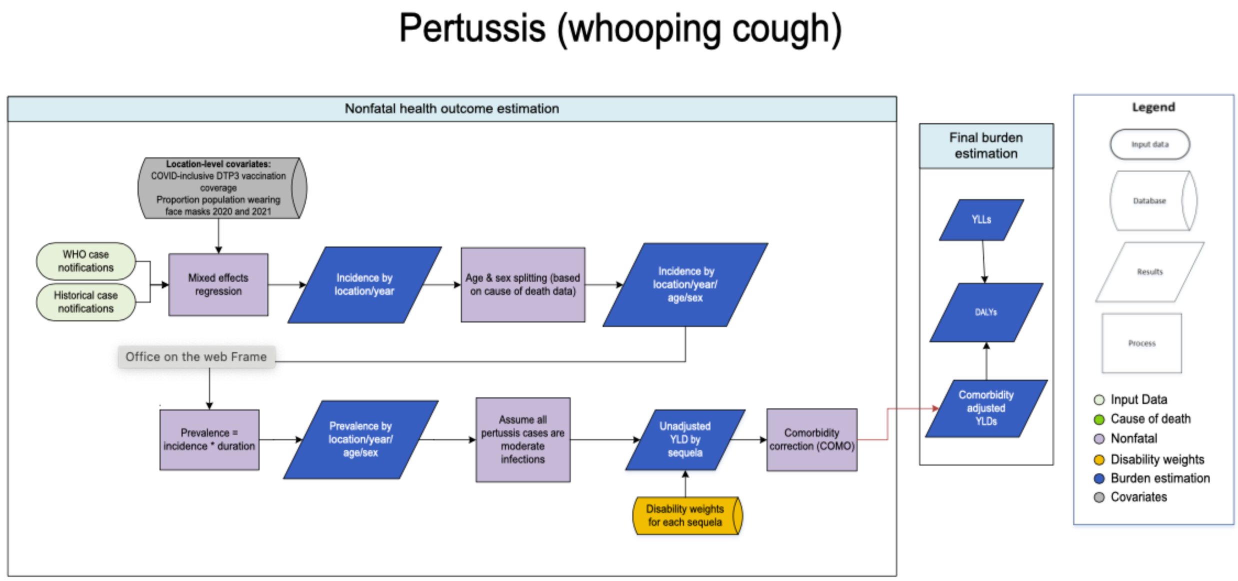

Input data and methodological summary for pertussis

Case definition

Pertussis (whooping cough) is a subacute respiratory illness caused by infection with the bacterium *Bordetella pertussis*. Early symptoms include fever, rhinitis, and conjunctivitis. Progression to lower respiratory tract infection results in classic paroxysmal coughing which can be complicated by pneumonia, apnea, and death in severe cases. For pertussis, ICD-10 codes are A37-A37.91, Z23.7, and ICD-9 codes are 033-033.9, 484.3, V03.6.

Quantity of interest	Reference or alternative	Definition
Pertussis incidence	Reference	Cases reported by national pertussis surveillance systems to WHO. Cases may be laboratory-confirmed, clinically diagnosed, or epidemiologically linked
Pertussis case fatality rate	Reference	Ratio of fatal cases of pertussis to total confirmed cases of pertussis in the sample

Input data

Model inputs

To estimate pertussis incidence and prevalence rates, our primary input data are the pertussis case notifications released annually by the World Health Organization (WHO) through the Joint Reporting Form (JRF). Historical case notifications and vaccination coverage for the UK since 1942 were also included in the model to better inform the relationship between vaccination and incidence.

Modelling strategy

As in GBD 2021, we used a mixed-effects linear regression model to predict pertussis cases for each GBD location. In GBD 2023, we used COVID-inclusive diphtheria-tetanus-pertussis third dose (DTP3) vaccine coverage as a predictor of pertussis incidence. We use a mean of DTP3 coverage calculated over a rolling, five-year interval to capture population-level vaccine-derived immunity among under 5-year-olds, including coverage both in the current year and in recent years. New in GBD 2023 was the inclusion of a masking covariate to account for the impact of COVID-19 mitigation strategies on the incidence of pertussis. This approach differs from the COVID-19 adjustment used in GBD 2021, wherein COVID-free counterfactual estimates of pertussis incidence were modelled, followed by a post-hoc adjustment for the impact of the pandemic.

The model includes location-specific random effects to capture variation in reported pertussis incidence not explained by DTP3 coverage:

$$Y_{ij} = \beta_0 + \beta_1 (1\text{-}DTP3_{ij}) + \beta_2 \ln(1\text{-}masking) + u_j + e_{ij} ,$$

where Y_{ij} is the log-transformed incidence rate (in cases per 100,000 persons using WHO case notifications and GBD populations); β_0 is the fixed effect intercept; β_1 is the fixed effects slope on the log-transformed proportion of unvaccinated individuals (using the rolling mean of COVID-inclusive DTP3 coverage over the past five years); β_2 is the natural log of the proportion of population not wearing masks outside the home; u_j is the country random effect; e_{ij} is the residual; i is the year; and j is the location.

As in GBD 2021, we corrected for pertussis under-reporting by substituting the location-specific random effect with the random effect obtained for Switzerland. In the last few GBD cycles, Switzerland has consistently had the largest random effect in the model and is known to have a robust pertussis monitoring system. We account for under-reporting by substituting Switzerland's random effect for the location-specific random effect when making predictions from the model. This approach assumes no under-reporting in Switzerland and a stable attack rate across unvaccinated populations. With the addition of updated case notification data in this GBD cycle, the random effect of Switzerland changed little compared to GBD 2021.

From this model, 1000 incidence predictions were generated using the estimated variance-covariance matrix to capture uncertainty. The results of this model were used to predict incidence and prevalence rates. For all countries, we produced estimates for all age groups between post-neonatal and 59 years. New in GBD 2023, we replaced our modelled estimates with case notifications when our modelled results were lower than notified cases. Incidence rate was the result of predicted cases divided by GBD-estimated populations. Prevalence rate was calculated as the product of incidence and case duration divided by GBD-estimated populations. We assumed an average case duration of 50 days. We obtained 1000 samples (draws) from the posterior distribution and report the means of all samples and 95% uncertainty intervals (the 2.5th and 97.5th percentiles of all draws).

Severity splits

Each pertussis case was assumed to be a moderate episode of acute infectious disease. The lay description and disability weight (DW) derived from the GBD disability weights study are shown in Table 1.

Table 1. Severity splits, lay descriptions, and disability weights

**Severity level**	**Lay description**	**DW (95% CI)**
Moderate	Has a fever and aches and feels weak, which causes some difficulty with daily activities.	0.051<br>(0.032–0.074)

Changes from GBD 2021 to GBD 2023

The major substantive change made to the modelling strategy for GBD 2023 was in the handling of COVID-19 impacts on pertussis incidence. In GBD 2023, we used a five-year lagged COVID-inclusive DTP3 mean vaccine coverage covariate and a masking covariate to account for COVID effects directly within the model.

Pneumoconiosis

Silicosis, asbestosis, coal workers pneumoconiosis, and other pneumoconiosis

Flowchart

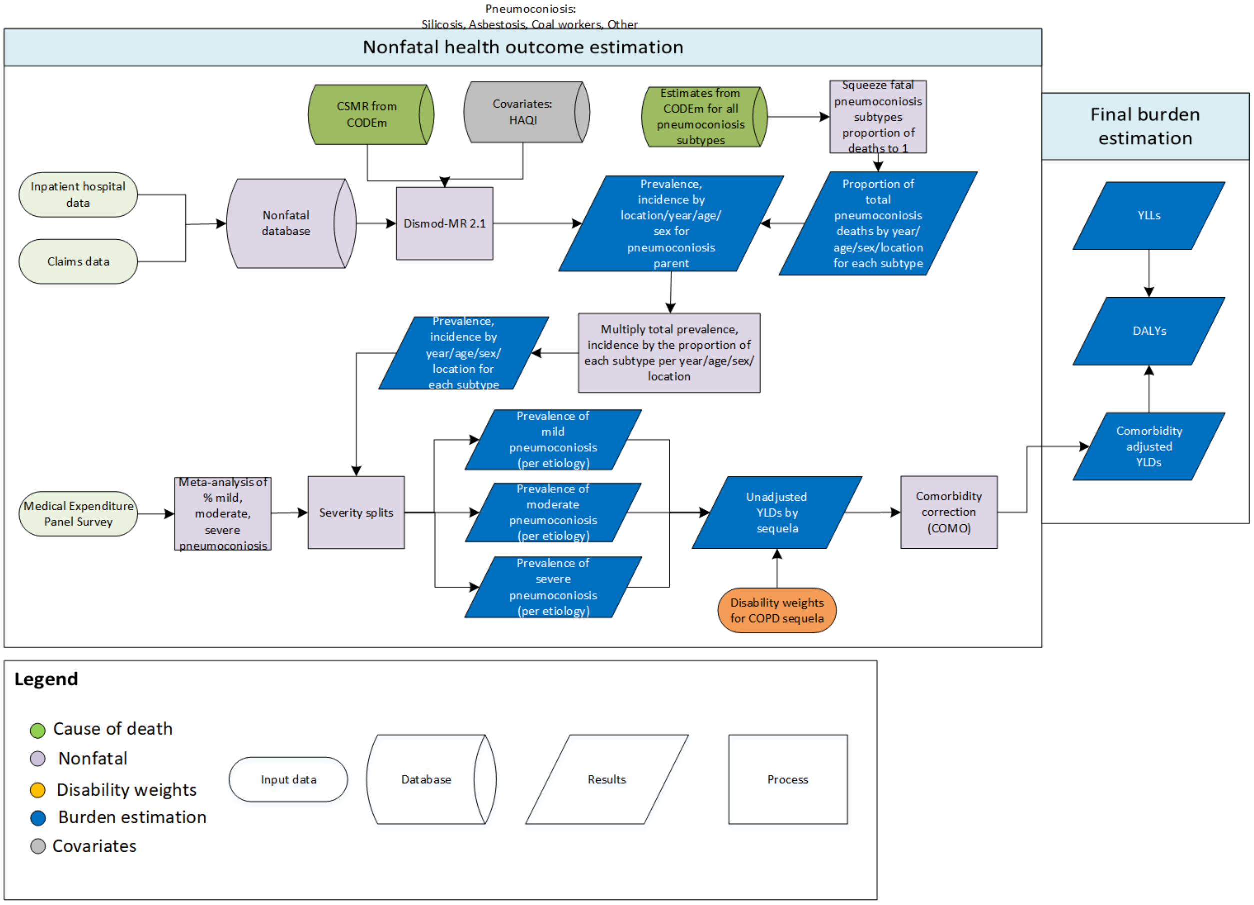

Input data and methodological appendix

Case definition

Pneumoconiosis is a chronic lung disease characterised by lung scarring and other interstitial damage caused by exposure to dust and other containments – usually through occupational exposure, typified by lung fibrosis and other interstitial damage, and symptoms of coughing, shortness of breath, and phlegm production. For GBD 2023, we produce estimates of pneumoconiosis by exposure type: silica, asbestos, coal, and other. Corresponding ICD-9 codes for pneumoconiosis include 500-504.9, and the ICD-10 codes are J60-J63.8, J92.

Input data

Two types of data are used to make estimates for pneumoconiosis: inpatient hospital reports and health insurance claims data. The inpatient hospital data are processed by the clinical informatics team and receive a correction that adjusts for non-primary diagnoses, patient readmission, and outpatient visits. There are non-fatal data for 42 different locations. We currently do not use any scientific literature data in our pneumoconioses models because pneumoconiosis is a rare condition and studies generally pool from non-representative sources (ie, occupational workers).

Data processing

Bias adjustments

In GBD 2023, we model bias adjustment methods by utilising a MR-BRT (meta-regression—Bayesian, regularised, trimmed, described in appendix 1, section 2) model outside of DisMod-MR 2.1 (disease model—Bayesian meta-regression, described in appendix 1, section 2) to allow a more direct comparison between different case definitions and/or study designs.

For pneumoconiosis, we adjusted USA MarketScan claims data collected in the year 2000 to all other USA MarketScan data. To do so, we used the logit difference for datapoints from reference (non-2000 claims data) and alternative (2000 claims data) matched on age, sex, and location as input into MR-BRT.

The adjustment is a logit-transformation method in MR-BRT. The general process is described below:

1. Identify datapoints with overlapping age, sex, and location between reference and alternative definitions.
2. Logit transform overlapping datapoints of alternative and reference case definitions.
3. Convert overlapping datapoints into a difference in logit space using the following equation: $logit(altnerative) - logit(reference)$
4. Use the delta method to compute standard errors of overlapping datapoints in logit space, then calculate standard error of logit difference using the following equation: $\sqrt{(variance\ of\ alternative) + (variance\ of\ reference)}$
5. Using MR-BRT, conduct a random effects meta-regression to obtain the pooled logit difference of alternative to reference.
6. Apply the pooled logit difference to all datapoints of alternative case definitions using the following equation:
$$new_{estimate} = inverse.logit((logit(alternative)) - (pooled\ logit\ difference))$$
7. Calculate new standard errors using the delta method, accounting for gamma (between-study heterogeneity).

The coefficients for bias adjustments are shown below:

Table 1. MR-BRT crosswalk adjustment factor: pneumoconiosis

Data input	Status	Gamma	Beta coefficient, logit (95% UI)*	Adjustment factor**
MarketScan (not 2000)	Ref		---	---
MarketScan 2000	Alt	0.0	–0.23 (–0.34 to –0.12)	0.44

**MR-BRT crosswalk adjustments can be interpreted as the factor the alternative case definition is adjusted by to reflect what it would have been had it been measured using the reference case definition. If the log/logit beta coefficient is negative, then the alternative is adjusted up to the reference. If the log/logit beta coefficient is positive, then the alternative is adjusted down to the reference.*
***The adjustment factor column is the exponentiated beta coefficient. For log beta coefficients, this is the relative rate between the two case definitions. For logit beta coefficients, this is the relative odds between the two case definitions.*

Modelling strategy

Estimates for the pneumoconioses are produced using a modified DisMod-MR 2.1 approach.

We first ran a single pneumoconiosis model, grouping together all the pneumoconiosis data (asbestosis, coal workers, silicosis, and other pneumoconiosis) and ran a single DisMod model. We set remission to zero and assumed no prevalence or incidence before the age of 15. We include Healthcare Access and Quality Index as a predictive covariate on excess mortality rate in our parent pneumoconiosis model. Location random effects are set at –1 to 1 for prevalence.

This single pneumoconiosis model estimated all-pneumoconiosis prevalence by year, age, sex, and location. We then split these estimates by taking the proportion of estimated pneumoconiosis deaths produced by our CODEm (Cause of Death Ensemble modeling, details in appendix 1, section 4 of reference) model assigned to each pneumoconiosis subtype. Thus, each non-fatal pneumoconiosis subtype estimate is the non-fatal pneumoconiosis model estimate multiplied by the proportion of deaths assigned to each subtype in each year, age, sex, and location.

Severity split inputs

Data to inform estimates of the severity gradient due to pneumoconiosis aetiologies are derived from previous analyses of the Medical Expenditure Panel Survey (MEPS). The disability weights are taken from chronic obstructive pulmonary disease (COPD) and shared by all pneumoconiosis aetiologies. Severity distribution is also shared by all pneumoconiosis aetiologies.

Table 2. Severity distribution, details on the severity levels for pneumoconiosis and the associated disability weight (DW) with that severity.

Severity level	Lay description	DW (95% CI)	Severity distributions
Asymptomatic			23.0% (20.8–25.0)
Mild	Has cough and shortness of breath after heavy physical activity, but is able to walk long distances and climb stairs.	0.019 (0.011–0.033)	34.2% (26.4–37.5)
Moderate	Has cough, wheezing, and shortness of breath, even after light physical activity. The person feels tired and can walk only short distances or climb only a few stairs.	0.225 (0.153–0.312)	13.3% (9.7–19.4)

Severe	Has cough, wheezing, and shortness of breath all the time. The person has great difficulty walking even short distances or climbing any stairs, feels tired when at rest, and is anxious.	0.408 (0.273–0.556)	29.5 (20.8–36.1)

Table 3. Covariates. Summary of covariates used in the pneumoconiosis DisMod-MR meta-regression model

Covariate	Type	Parameter	Exponentiated beta (95% uncertainty interval)
Healthcare Access and Quality (HAQ) Index	Country-level	Excess mortality rate	0.99 (0.38–2.49)

Pruritus

Flowchart

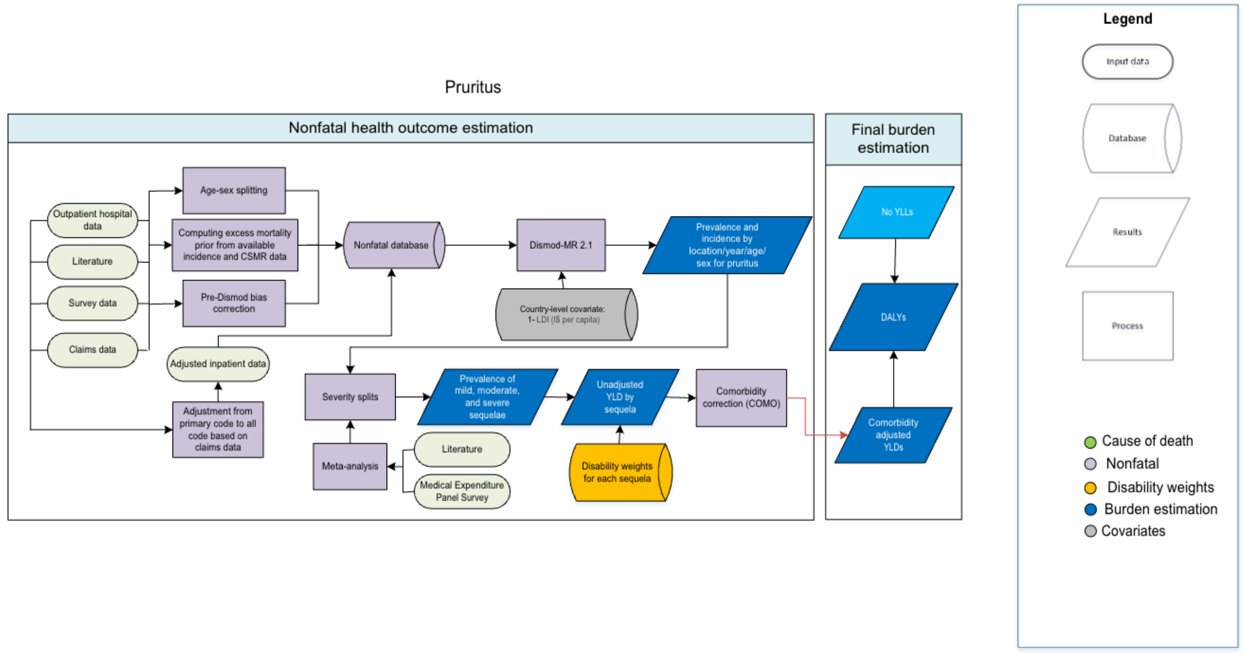

Input data and methodological summary for pruritus

Case definition

Pruritus is defined as an unpleasant sensation on the skin that provokes the desire to scratch (ICD-10: L29). Pruritus was included in the GBD 2021 cause group of skin and subcutaneous conditions.

Pruritus

Quantity of interest	Reference or alternative	Definition
Pruritus	Reference	Pruritus as determined by Poland National Health Fund Patient Claims 2015–2019
Pruritus	Alternative	All other data for pruritus

Input data

In the GBD 2010 study, a systematic review of the literature was conducted using PubMed and Google Scholar to capture epidemiological data for pruritus. The inclusion criteria stipulated that studies (1) must be published between 1980 and 2012; (2) must provide data on the incidence or prevalence of pruritus; (3) must use samples representative of the general population (ie, samples derived from the experimental arm of clinical trials or based in dermatology clinics were excluded); (4) must use a sample size larger than 100; and (5) must provide sufficient information on study method and sample characteristics to assess the quality of the study. For GBD 2016, the GBD 2010 search strategy was replicated in PubMed to capture epidemiological studies published between 2013 and 2016. Since then,

USA claims data from 2010–2019 were included along with Poland National Health Fund Patient Claims 2015–2019. Data were further considered for exclusion when they led to underestimation of subnational pseudo-random effects and poor model fit, or if we found them unreasonable when compared to regional, super-regional, and global rates.

Data processing

For pruritus, we crosswalked all data to the reference definition. We began by evaluating the number of observations of each alternate definition that matched with a corresponding observation from the reference definition. We considered "between" study matches, where the alternative was from the same GBD age group and sex, and the midpoint year of the study was within five years of the midpoint of the reference definition observation.

$$\log i\, t\left(y_i^{alt}\right) - \log i\, t\left(y_i^{ref}\right) \;=\; \beta_0 + \epsilon_i$$

Table 1: MR-BRT crosswalk adjustment factors for pruritus

Data input	Reference or alternative case definition	Gamma	Beta coefficient, log/logit (95% UI)*	Adjustment factor**
Poland National Health Fund Patient Claims 2015–2019	Ref	0.1012	---	---
All other data	Alt		2.0102 (1.3866–2.6337)	7.4647 (4.0014–13.9258)

**MR-BRT crosswalk adjustments can be interpreted as the factor the alternative case definition is adjusted by to reflect what it would have been had it been measured using the reference case definition. If the log/logit beta coefficient is negative, then the alternative is adjusted up to the reference. If the log/logit beta coefficient is positive, then the alternative is adjusted down to the reference.*
***The adjustment factor column is the exponentiated beta coefficient. For log beta coefficients, this is the relative rate between the two case definitions. For logit beta coefficients, this is the relative odds between the two case definitions.*

Modelling strategy

In GBD 2023, we have made no substantive changes in the modelling strategy from GBD 2021. DisMod-MR 2.1, a Bayesian meta-regression tool, was used to estimate prevalence by age, sex, year, and geography (subnational, country, region, super-region) for pruritus. Pruritus was modelled with remission set between 0.2 and 1, implying a duration between three months and one year. This was in line with the available epidemiological data, expert opinion, and previous GBD work. Excess mortality was assumed to be zero. We used a time window of 25 years to determine which datapoints were used for a particular year of fit. LDI was used as a location-level covariate to guide estimates for countries with few or no data. Since GBD 2019, we have replaced our within-DisMod crosswalks with crosswalks completed using the MR-BRT modelling tool. We adjusted all our pruritus data toward the level of

Poland National Health Fund Patient Claims 2015–2019, which were more representative of the general population.

$$prevalence = incidence * duration$$

$$Remission = \frac{Cured\ cases}{person - year\ of follow - up\ in\ prevalent\ cases}$$

Table 2. Severity distribution, details on the severity levels for pruritus and the associated disability weight (DW) with that severity

Severity level	Lay description	DW (95% CI)
Mild	The individual has a slight, visible physical deformity that is sometimes sore or itchy. Others notice deformity, which causes some worry and discomfort.	0.011 (0.005, 0.021)

Table 3. Covariates. Summary of covariates used in the pruritus DisMod-MR meta-regression model

Covariate	Type	Parameter	Value	Exponentiated beta (95% uncertainty interval)
LDI (I$ per capita)	Country-level	Prevalence	0.19 (–0.069, 0.30)	1.21 (0.93–1.35)

Psoriasis

Flowchart

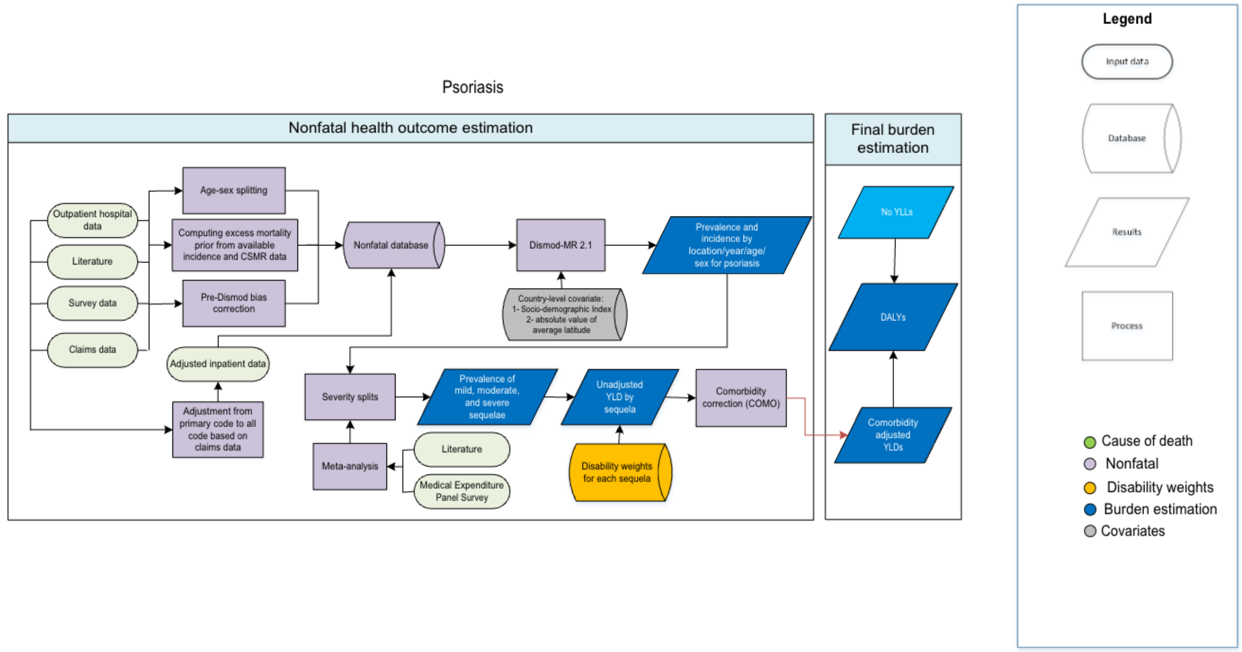

Input data and methodological summary for psoriasis

Case definition

Psoriasis is an immune-mediated disease that involves inflammation and excess growth and abnormal behavior of certain skin cells. This disease is characterised by areas of raised, red skin with silvery scales (ICD-10: L40, L41).

Psoriasis

Quantity of interest	Reference or alternative	Definition
Psoriasis	Reference	Psoriasis as determined by Poland National Health Fund Patient Claims 2015–2019
Psoriasis	Alternative	All other data for psoriasis

Input data

The data for the psoriasis model come from claims data from the USA and Poland. In the GBD 2010 study, a systematic review of the literature was conducted using PubMed and Google Scholar to capture epidemiological data for psoriasis. In GBD 2013, the 2010 search strategy was replicated to capture studies from 2012 to 2014. This process was repeated in GBD 2016 to capture studies through October 1, 2016. The inclusion criteria stipulated that studies (1) must provide data on the incidence or prevalence of psoriasis; (2) must use samples representative of the general population (ie, samples derived from the experimental arm of clinical trials or based in dermatology clinics were excluded); (3)

must use a sample size larger than 100; and (4) must provide sufficient information on study method and sample characteristics to assess the quality of the study. Surveys used include the Medical Expenditure Panel Survey (MEPS) in the USA for 2000–2009, the Australian National Health Survey 1995–1996, 2001, 2004–2005, 2007–2008, and the USA National Health and Nutrition Examination Survey (NHANES) in 2002 and 2005. Claims data from the USA and Poland link claims for multiple inpatient and outpatient encounters to a single individual. An individual was extracted as a prevalent case if they had one or more inpatient or outpatient encounter with a psoriasis ICD code as any encounter diagnosis. Data were further considered for exclusion when they led to underestimation of subnational pseudo-random effects and poor model fit, or if we found them unreasonable when compared to regional, super-regional, and global rates.

Data processing

For psoriasis, we crosswalked all data to the reference definition. We began by evaluating the number of observations of each alternate definition that matched with a corresponding observation from the reference definition. We considered "between" study matches, where the alternative was from the same GBD age group and sex, and the midpoint year of the study was within five years of the midpoint of the reference definition observation.

$$\log i\,t\left(y_i^{alt}\right) - \log i\,t\left(y_i^{ref}\right) \;=\; \beta_0 \;+ \epsilon_i$$

Table 1: MR-BRT crosswalk adjustment factors for psoriasis

Data input	Reference or alternative case definition	Gamma	Beta coefficient, log/logit (95% UI)*	Adjustment factor**
Poland National Health Fund Patient Claims 2015–2019	Ref	0.0651	---	---
All other data	Alt		0.3585 (–0.1414 to 0.8584)	1.4311 (0.8681 ,2.3594)

**MR-BRT crosswalk adjustments can be interpreted as the factor the alternative case definition is adjusted by to reflect what it would have been had it been measured using the reference case definition. If the log/logit beta coefficient is negative, then the alternative is adjusted up to the reference. If the log/logit beta coefficient is positive, then the alternative is adjusted down to the reference.*

***The adjustment factor column is the exponentiated beta coefficient. For log beta coefficients, this is the relative rate between the two case definitions. For logit beta coefficients, this is the relative odds between the two case definitions.*

Modelling strategy

In GBD 2023, we have made no substantive changes in the modelling strategy from GBD 2021. DisMod-MR 2.1, a Bayesian meta-regression tool, was used to estimate prevalence by age, sex, year, and geography (subnational [select countries], country, region, super-region) for psoriasis. Psoriasis was modelled with remission set between 0.05 and 0.15, implying a duration between 6.6 and 20 years. This was in line with the available epidemiological data, expert opinion, and previous GBD work. Excess mortality was assumed to be zero. The datasets for psoriasis were sufficiently large to make use of a

relatively short time window of ten years to determine which datapoints were used for a particular year of fit. Socio-demographic Index and absolute value of average latitude were used as location-level covariates to guide estimates for countries with few or no data. In GBD 2019, we replaced our within-DisMod crosswalks with crosswalks completed using the MR-BRT modelling tool. We adjusted USA MarketScan data toward the level of other prevalence datapoints, which were more representative of the general population. In addition, Socio-demographic Index and absolute value of average latitude were used as country-level covariates to guide estimates for countries with few or no data.

$$prevalence = incidence * duration$$

$$Remission = \frac{Cured\ cases}{person - year\ of follow - up\ in\ prevalent\ cases}$$

Table 2. Severity distribution, details on the severity levels for psoriasis and the associated disability weight (DW) with that severity

Severity level	Lay description	DW (95% CI)
Mild	The individual has a slight, visible physical deformity that is sometimes sore or itchy. Others notice deformity, which causes some worry and discomfort.	0.649 (0.538–0.749)
Moderate	The individual has a visible physical deformity that is sore and itchy. Other people stare and comment, which causes the person to worry. The person has trouble sleeping and concentrating.	0.229 (0.157–0.311)
Severe	The individual has an obvious physical deformity that is very painful and itchy. Physical deformity makes others uncomfortable, which causes the person to avoid social contact, feel worried, sleep poorly, and think about suicide.	0.122 (0.083–0.167)

Table 3. Covariates. Summary of covariates used in the psoriasis DisMod-MR meta-regression model

Covariate	Type	Parameter	Value	Exponentiated beta (95% uncertainty interval)
Socio-demographic Index	Country-level	Prevalence	0.0011 (0.000016, 0.0021)	1.00 (1.00–1.00)
Absolute value of average latitude	Country-level	Prevalence	1.99 (1.97, 2.00)	7.33 (7.14–7.39)

Pulmonary arterial hypertension

Flowchart

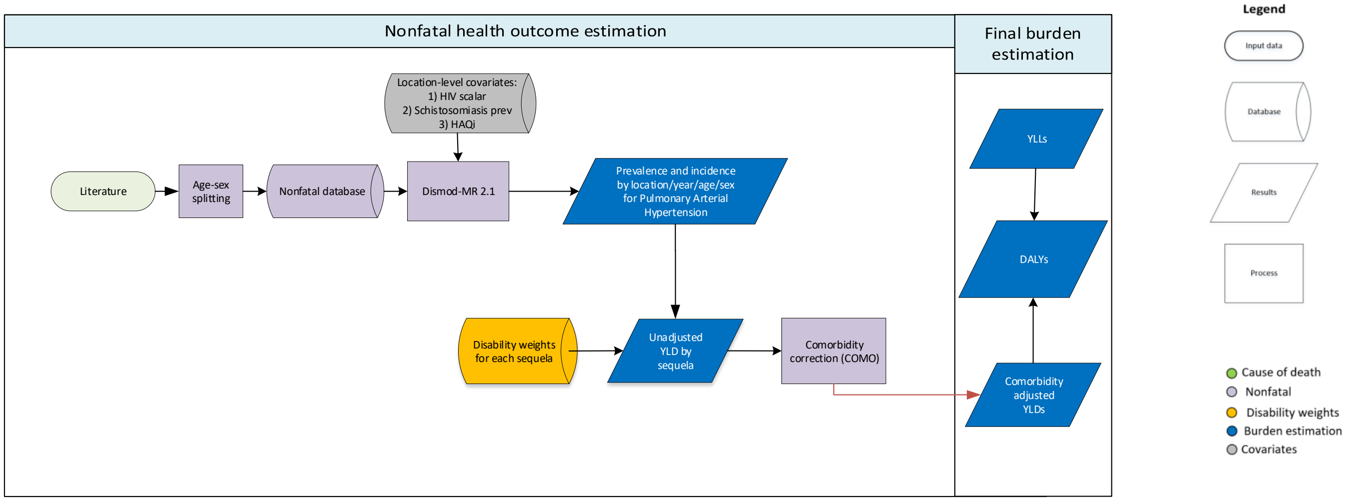

Input data and methodological summary for pulmonary arterial hypertension

Case definition

Pulmonary arterial hypertension (PAH) is a vascular disease in which remodelling of the pulmonary arteries leads to high pulmonary pressures, increased vascular resistance, and eventual right heart dysfunction. It is a form of pulmonary hypertension (PH) characterised by high pressures in the pulmonary system; PAH is consistent with WHO Group 1 pulmonary hypertension (Figure 1).[1] We restrict our case definition to PAH or Group 1 PH, as other forms of PH are captured in other GBD causes.

The GBD case definition of PAH is clinically diagnosed pulmonary arterial hypertension, with supporting diagnostic evidence either via right heart catheterisation or echocardiogram. We include PAH identified through ICD codes if the study authors have confirmed the diagnosis by reviewing medical records for results from catheterisation or echocardiography. All other forms of pulmonary hypertension are excluded from this cause.

Figure 1: WHO classification of pulmonary hypertension groups 1–5

WHO Classification	Description
Group 1	Pulmonary Arterial Hypertension (PAH)
Group 2	Pulmonary hypertension due to left heart disease
Group 3	Pulmonary hypertension due to lung disease
Group 4	Pulmonary hypertension due to thromboembolic disease
Group 5	Pulmonary hypertension with unclear mechanism

Input data

As systematic review for PAH was not done in GBD 2023. A systematic review for incidence, prevalence, mortality, and aetiological breakdown for pulmonary arterial hypertension was conducted for GBD 2019 and updated for GBD 2021. The search strings can be found below.

We searched the Global Index Medicus, which indexes PubMed as well as several international journals, on 11/13/2018 with the following string: tw:(“pulmonary arterial hypertension”) OR tw:(“pulmonary artery hypertension”) OR tw:(“primary pulmonary hypertension”) OR tw:(“group 1 pulmonary hypertension”) OR tw:(“group one pulmonary hypertension”)) AND (tw:(epidemiology) OR tw:("prevalent cases") OR tw:(prevalence) OR tw:("incident cases") OR tw:(incidence) OR tw:(“standardized mortality ratio”) OR tw:(“case fatality”) OR tw:(“relative risk of death”) OR tw:(“excess mortality”) OR tw:(survival)) AND NOT (tw:(rats) OR tw:(mice) OR tw:(dogs) OR tw:(apes) OR tw:(monkeys) OR tw:(chickens) OR tw:(pigs) OR tw:(sheep)).

Since the original search, GIM has removed PubMed from its indexing; to account for this, we searched PubMed independently for results from 2018–2020 and de-duplicated the results in the final count. We searched PubMed with the following string: (“pulmonary arterial hypertension”[Title] OR “pulmonary arterial hypertension”[Abstract] OR “pulmonary artery hypertension”[Title] OR “pulmonary artery hypertension”[Abstract] OR “primary pulmonary hypertension”[Title] OR “primary pulmonary hypertension”[Abstract] OR “group 1 pulmonary hypertension”[Title] OR “group 1 pulmonary hypertension”[Abstract] OR “group one pulmonary hypertension”[Title] OR “group one pulmonary hypertension”[Abstract]) AND (“epidemiology”[Abstract] OR “prevalent cases”[Abstract] OR “prevalence”[Abstract] OR "incident cases"[Abstract] OR “incidence”[Abstract] OR “standardized mortality ratio”[Abstract] OR “case fatality”[Abstract] OR “relative risk of death”[Abstract] OR “excess mortality”[Abstract] OR “survival”[Abstract]) NOT (animals[MeSH] NOT humans[MeSH])

The dates of the search were 01/01/1980–2/5/2021. 7106 hits were returned, of which 65 were extracted (see PRISMA diagram below). We excluded literature that was not representative of the general population or included pulmonary hypertension Groups 2–5.

Figure 2: PRISMA 2020 flow diagram

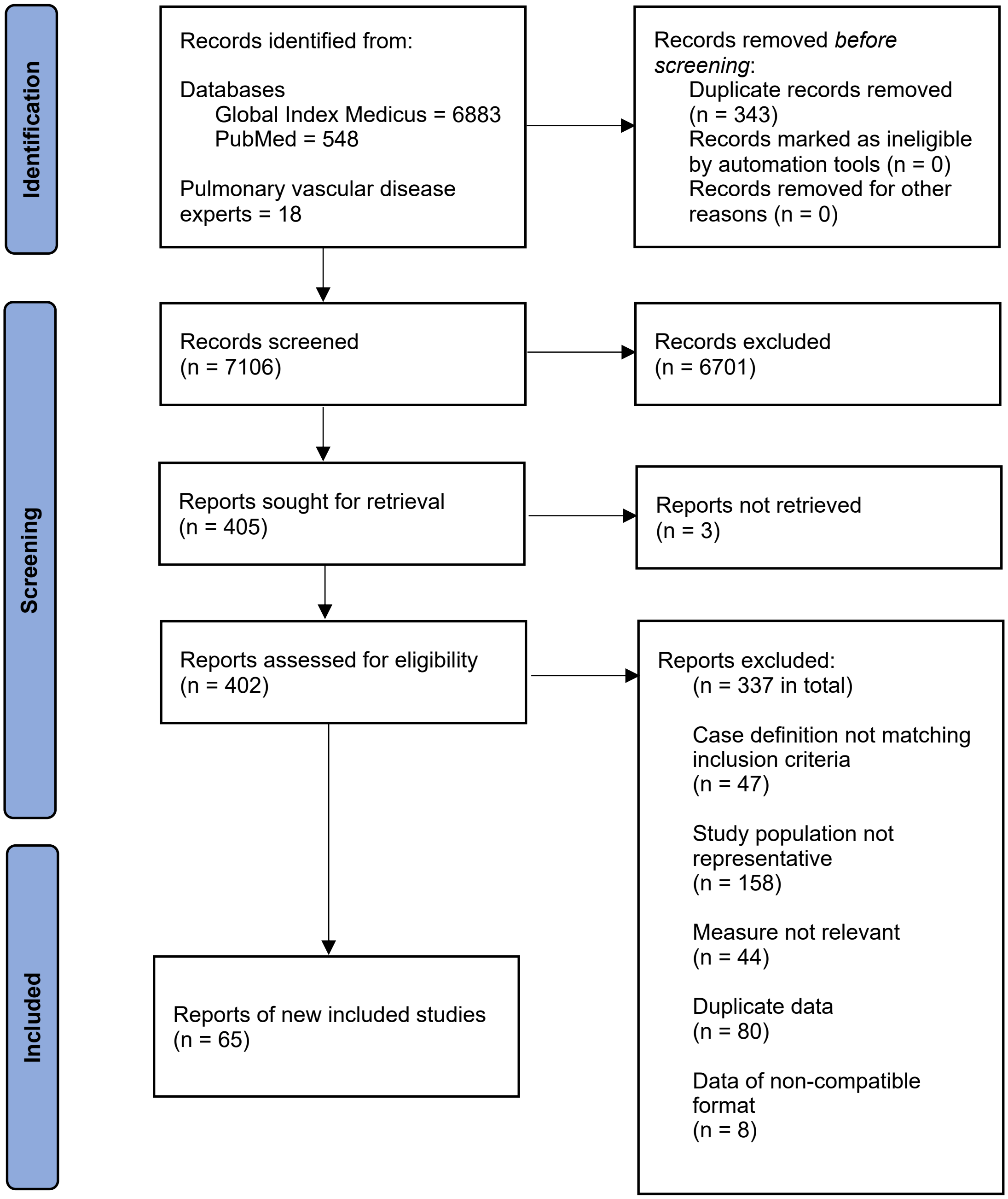

Data processing

We used the modelling software meta-regression—Bayesian, regularised, trimmed (MR-BRT) to split both-sex datapoints for incidence, prevalence, and with-condition mortality into sex-specific estimates. This methodology is detailed elsewhere in the appendix. We also split datapoints where the age range was greater than 25 years. Age splitting was based on the global sex-specific age pattern from a DisMod-MR 2.1 model that only used input data from scientific literature with less than a 25-year age range.

We relied on published estimates of PAH survival or case fatality, transformed from case fatality into with-condition mortality rate using the following formula:

$$mtwith = -\ln{(1-cfr)}/time(years)$$

We did not incorporate cause-specific mortality estimates from death certificates as estimates of survival or case fatality were commonly found in the literature and were measured with a higher degree of precision and alignment with the GBD case definitions than could be determined for death certificates. Due to evolving ICD codes and PAH coding practices on death certificates, we decided published estimates of survival from cohort and other population-based studies of patients with PAH would more closely approximate non-fatal patterns than CSMR from death certificates.

Severity distributions, details on the health states for pulmonary arterial hypertension in GBD 2023, and the associated disability weight (DW) are shown in Table 1. We selected heart failure disability weights as most closely representing the disability due to PAH, based on lay descriptions of the health states.

Table 1. Severity distributions and associated disability weights (DW)

Severity level	Lay description	DW (95% CI)
Controlled, medically managed	Has been diagnosed with clinical heart failure, a chronic disease that requires medication every day and causes some worry but minimal interference with daily activities.	0.049 (0.031–0.072)
Mild	Is short of breath and easily tires with moderate physical activity, such as walking uphill or more than a quarter-mile on level ground. The person feels comfortable at rest or during activities requiring less effort.	0.041 (0.026–0.062)
Moderate	Is short of breath and easily tires with minimal physical activity, such as walking only a short distance. The person feels comfortable at rest but avoids moderate activity.	0.072 (0.047–0.103)
Severe	Is short of breath and feels tired when at rest. The person avoids any physical activity, for fear of worsening the breathing problems.	0.179 (0.122–0.251)

Modelling strategy

We used DisMod to model the incidence and prevalence of PAH, informed by the input data described above. We set a prior of no remission and used the Healthcare Access and Quality (HAQ) Index, the natural log of age-standardised schistosomiasis prevalence, and an age-standardised summary exposure value (SEV) scalar for HIV prevalence as covariates. HIV and schistosomiasis were chosen as covariates because these diseases can cause PAH and are drivers of PAH prevalence in locations where those diseases are common. Information on covariates, including parameters and coefficients, can be found in Table 2. All data adjustments were done outside of DisMod, described above.

The prevalence of heart failure due to pulmonary arterial hypertension was modelled separately and is detailed elsewhere in the appendix.

Table 2. Summary of covariates used in the PAH DisMod meta-regression model

Covariate	Type	Parameter	Beta (95% uncertainty interval)	Exponentiated beta (95% uncertainty interval)
Log-transformed age-standardised SEV scalar: HIV	Country-level	Prevalence	0.38 (0.023 to 0.73)	1.46 (1.02 to 2.07)
Log-transformed age-standardised prevalence of schistosomiasis	Country-level	Prevalence	4.21 (–2.65 to 9.42)	67.37 (0.071 to 12369.64)
Healthcare Access and Quality Index	Country-level	Excess mortality rate	–1.00 (–1.95 to –0.049)	0.37 (0.14 to 0.95)

There have been no substantial updates in modelling strategy for PAH since GBD 2021.

Pyoderma

Flowchart

Abscess and Other Bacterial Skin Diseases

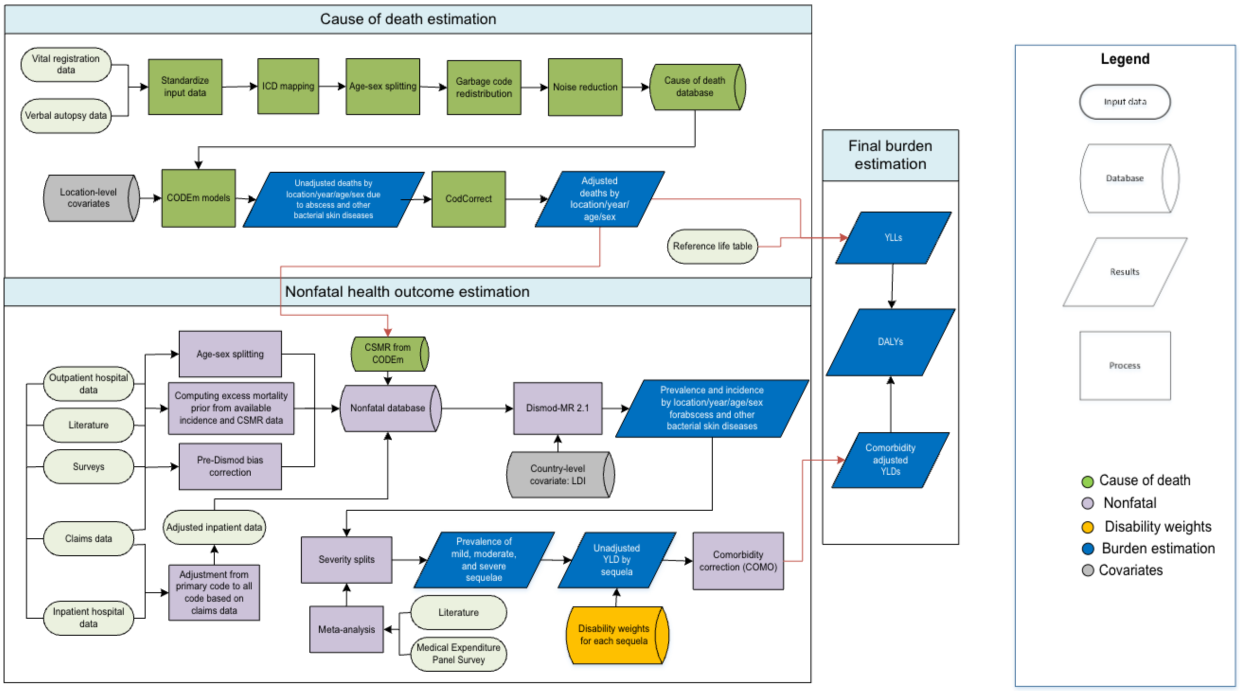

Impetigo

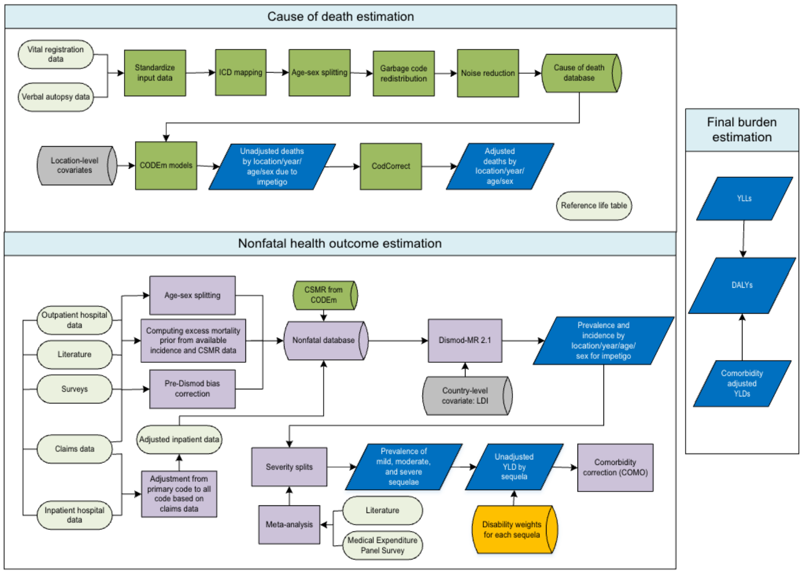

Input data and methodological summary for pyoderma

Case definition

Pyoderma refers to any skin disease that is pyogenic, ie, involves the development of pus. These include superficial bacterial conditions such as impetigo, furuncles, ulcers, and abscesses. In line with previous

GBD rounds, for GBD 2023, pyoderma was modelled as two separate groups: impetigo, and abscess and other bacterial skin diseases. Impetigo is a highly contagious bacterial skin infection often characterised by red sores, which eventually leak pus or fluid (ICD-10: L01). An abscess is a collection of pus that builds up within the tissue of the body, with carbuncles and furuncles being examples of specific types of abscesses.

The abscess and other bacterial skin diseases group included all bacterial skin diseases except impetigo (ICD-10: L00, L02, L04, L05, L08).

Pyoderma:

Quantity of interest	Reference or alternative	Definition
Impetigo	Reference	Impetigo as determined by Poland National Health Fund Patient Claims 2015–2019
	Alternative	Other data inputs for impetigo
Abscess and other bacterial skin diseases	Reference	Abscess and other bacterial skin diseases as determined by Poland National Health Fund Patient Claims 2015–2019
	Alternative	Other data inputs for impetigo

Input data

For GBD 2010, a systematic review of the literature using PubMed and Google Scholar was conducted to capture epidemiological data for pyoderma. The literature search also included any relevant data from the Medical Expenditure Panel Survey (MEPS) in the USA in 2000–2009. The inclusion criteria stipulated that studies (1) must be published between 1980 and 2012; (2) must provide data on the incidence or prevalence of pyoderma; (3) must use samples representative of the general population (ie, samples derived from the experimental arm of clinical trials or based in dermatology clinics were excluded); (4) must use a sample size larger than 100; and (5) must provide sufficient information on study method and sample characteristics to assess the quality of the study. For GBD 2013, the GBD 2010 search strategy was replicated to capture epidemiological studies published between 2012 and 2013. For GBD 2017, the GBD 2010 search strategy was replicated in PubMed to capture epidemiological studies published between 2013 and 2017. For GBD 2023, we have added year-locations of outpatient clinical data. The new data sources for impetigo include the USA, Mongolia, Poland, South Korea, and Sweden. For abscess and other bacterial skin diseases, the new sources for clinical data are from the USA, Poland, and South Korea. Data were outliered or excluded if we found them unreasonable when compared to regional, super-regional, and global rates.

Data processing

For pyoderma, we crosswalked all data to the reference definition. We began by evaluating the number of observations of each alternate definition that matched with a corresponding observation from the reference definition. We considered "between" study matches, where the alternative was from the same GBD age group and sex, and the midpoint year of the study was within five years of the midpoint of the reference definition observation.

$$\log i\, t\left(y_i^{alt}\right) - \log i\, t\left(y_i^{ref}\right) = \beta_0 + \epsilon_i$$

Table 1: MR-BRT crosswalk adjustment factors for pyoderma

Data input	Reference or alternative case definition	Gamma	Beta coefficient, log/logit (95% UI)*	Adjustment factor**
Impetigo	Ref	0	---	---
	Alt		1.4169 (1.4147 to 1.4192)	4.1245 (4.1151, 4.1340)
Abscess and other bacterial skin diseases	Ref	0.1014	---	---
	Alt		–0.1611 (–0.7851 to 0.4629)	0.8512 (0.4560, 1.5886)

**MR-BRT crosswalk adjustments can be interpreted as the factor the alternative case definition is adjusted by to reflect what it would have been had it been measured using the reference case definition. If the log/logit beta coefficient is negative, then the alternative is adjusted up to the reference. If the log/logit beta coefficient is positive, then the alternative is adjusted down to the reference.*

***The adjustment factor column is the exponentiated beta coefficient. For log beta coefficients, this is the relative rate between the two case definitions. For logit beta coefficients, this is the relative odds between the two case definitions.*

Modelling strategy

DisMod-MR 2.1, a Bayesian meta-regression tool, was used to estimate impetigo and abscess and other bacterial skin diseases prevalence by age, sex, year, and geography (subnational, country, region, super-region). Separate models were run for impetigo and abscess and other bacterial skin diseases.

Impetigo

To help inform the distribution of impetigo across the lifespan, excess mortality was set at 0 to 2, remission was set at 17 to 20, and incidence was set at 0 to 1. This was in agreement with the available prevalence data and expert advice. We made use of a relatively long time window of 20 years to determine which datapoints were used for a particular year of fit. This means that for the year 2000, for instance, DisMod-MR 2.1 incorporated all datapoints ranging from 1980 to present to estimate prevalence. Since GBD 2019, we replaced our within-DisMod crosswalks with crosswalks completed using the MR-BRT modelling tool. We adjusted all our datapoints toward the Poland National Health Fund Patient Claims 2015–2019, which were more representative of the general population. In addition, LDI was used as country-level covariates to guide estimates for countries with few or no data.

Abscess and other bacterial skin diseases

For abscess and other bacterial skin diseases, remission is set between 17 and 30. In GBD 2023, we have crosswalks completed using the MR-BRT modelling tool. We adjusted all clinical and literature data sources toward the level of Poland claims datapoints which were more representative of the general population. In addition, LDI was used as a country-level covariate to guide estimates for countries with few or no data.

We have made no substantive changes in the modelling strategy from GBD 2021.

$$prevalence = incidence * duration$$

$$Remission = \frac{Cured\ cases}{person - year of follow - up\ in\ prevalent\ cases}$$

Table 2. Severity distribution, details on the severity levels for pyoderma and the associated disability weight (DW) with that severity.

Sequela	Severity level	Lay description	DW (95% CI)
Impetigo	Infectious disease, acute episode, mild	The person has a low fever and mild discomfort but no difficulty with daily activities.	0.006 (0.002,0.012)
Abscess and other bacterial skin diseases	Infectious disease, acute episode, mild	The person has a low fever and mild discomfort, but no difficulty with daily activities.	0.006 (0.002, 0.012)

Table 3. Covariates. Summary of covariates used in pyoderma DisMod-MR meta-regression model

Sequela	Covariate	Type	Parameter	Value	Exponentiated beta (95% uncertainty interval)
Impetigo	LDI (I$ per capita)	Country-level	Prevalence	0.14 (0.0088, 0.31)	1.15 (1.01–1.36)
Abscess and other bacterial skin diseases	LDI (I$ per capita)	Country-level	Excess mortality rate	–0.5 (–0.99, –0.026)	0.61 (0.37–0.97)

Rabies

Flowchart

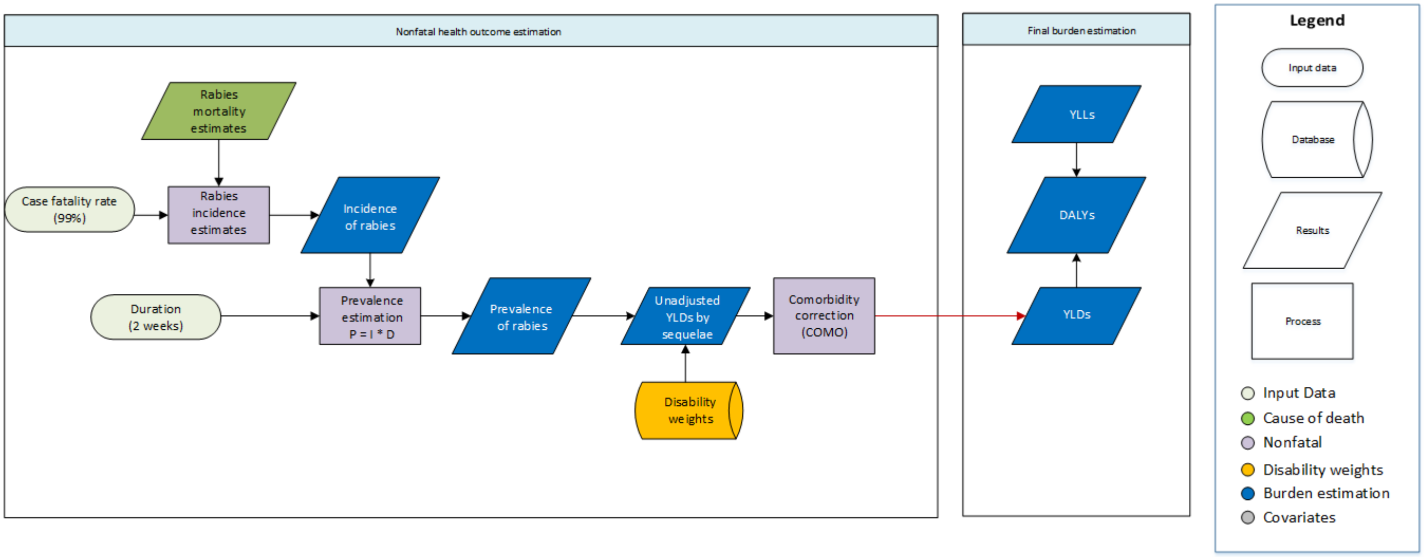

Input data and methodological summary for rabies

Case definition

Rabies is a fatal viral infection transmitted by animal bites. Without prophylactic vaccination, the disease is almost universally fatal. The disease has a long incubation period (1–3 months), and early intervention with prophylactic vaccination is nearly 100% effective in preventing symptomatic disease. It is considered a neglected tropical disease (NTD). We model symptomatic infections, not including those infections in which intervention prevented the onset of symptomatic disease, corresponding to the ICD-10 code A82. We used the following case definition for GBD 2023:

Quantity of interest	Reference or alternative	Definition
Rabies	Reference	Clinical diagnosis of rabies (not including cases where intervention prevented disease after an animal bite), corresponding to the ICD-10 code A82.

Input data

As we derived our estimate of cases from our estimate of deaths, no incidence data were used in the model. For GBD 2023, we modelled rabies mortality using all available data in the cause of death (CoD) database. Datapoints were outliered if they reported an improbable number of rabies deaths (eg, zero rabies deaths in a hyperendemic country), or if their inclusion in the model yielded distorted trends. In some cases, multiple data sources for the same location differed dramatically both in their quality and reported rabies mortality (eg, a verbal autopsy and vital registration source). In these cases, the lower-quality data source was outliered.

Modelling strategy

We derive estimates of the number of symptomatic rabies infections (ie, those not averted through prophylactic vaccination) based on rabies mortality estimates, assuming 99% case fatality. All cases are assumed to be severe. Prevalence estimates are calculated from incident cases assuming a two-week duration.

We modelled rabies mortality using a two-model hybrid approach 1) a global Cause of Death Ensemble model (CODEm) of all locations, using all data in the CoD database; and 2) a CODEm model restricted to data-rich countries.

Sequela description and disability weight

There is only one sequela and associated disability weight for rabies, which is severe. The lay description is included in the table below.

Table 1. Severity distribution, details on the severity levels for rabies and the associated disability weight (DW) with that severity.

Sequela	Lay description	DW (95% CI)
Severe	Has a high fever and pain, and feels very weak, which causes great difficulty with daily activities	0.133 (0.088–0.190)

We did not apply any adjustments for the COVID-19 pandemic to rabies due to a lack of available data quantifying the impacts of the pandemic on NTD epidemiology.

Changes from GBD 2021 to GBD 2023

There have been no substantive changes to the modelling strategy for GBD 2023.

Rheumatic heart disease

Flowchart

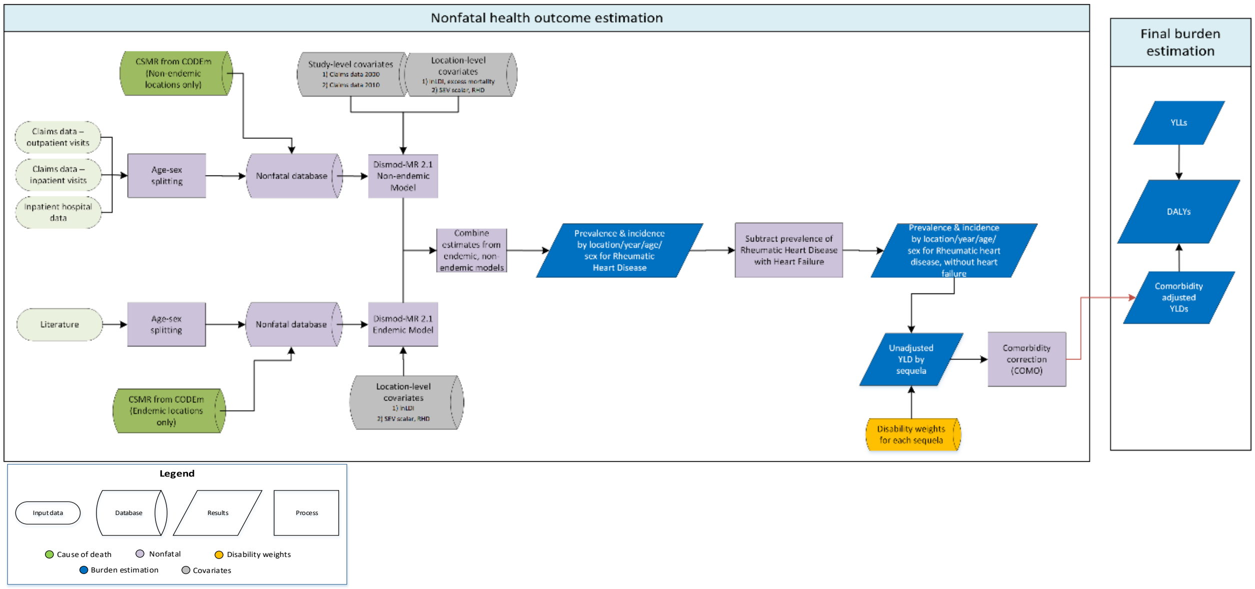

Input data and methodological summary for rheumatic heart disease

Case definition

Rheumatic heart disease (RHD) was defined as condition where the valves of the heart become damaged due to acute rheumatic fever, an autoimmune response to infection with group A streptococcal bacteria. The GBD case definition (Table 1) requires echocardiographic confirmation of RHD and follows the World Heart Federation criteria for echocardiographic diagnosis of RHD.[1] International Classification of Disease (ICD) codes mapped to RHD are shown in Table 2. We do not include cases of acute rheumatic fever in modelling.

Table 1: Criteria used to define rheumatic heart disease

**Criterion**	**Reference or alternative**	**Definition**
Echocardiography	Reference	Prevalent rheumatic heart disease based on echocardiographic assessment and clinical confirmation
Clinical diagnosis	Alternative	Prevalent rheumatic heart disease based on physician diagnosis

Table 2: ICD codes mapped to rheumatic heart disease

ICD code	ICD cause name
391<br>392.0	Rheumatic fever with heart involvement<br>Rheumatic chorea with heart involvement
I01<br>I02.0<br>I05	Rheumatic fever with heart involvement<br>Rheumatic chorea with heart involvement<br>Rheumatic mitral valve diseases

I06	Rheumatic aortic valve diseases
I07	Rheumatic tricuspid valve diseases
I08	Multiple valve diseases
I09	Other rheumatic heart diseases

Input data

We performed a systematic review for GBD 2023; previous reviews were completed in the GBD 2013 and GBD 2015 cycles.

The search terms for GBD 2023 were:
(’rheumatic heart disease’ AND epidemiology[MeSH Subheading]) OR (’acute rheumatic fever’ AND epidemiology[MeSH Subheading]) OR (’rheumatic fever’ AND epidemiology[MeSH Subheading]) OR (RHD AND epidemiology[MeSH Subheading]) OR (((streptococcus OR streptococci) AND heart) AND epidemiology[MeSH Subheading]) OR (heart AND valve AND disease AND epidemiology[MeSH Subheading] AND ”rheumatic”[TIAB]) OR (’mitral valve stenosis’ AND rheumatic [TIAB] AND epidemiology[MeSH Subheading]) OR ((’rheumatic heart disease’ OR ’rheumatic fever’) AND prevalence) OR ((’rheumatic heart disease’ OR ’rheumatic fever’) AND incidence) OR ((’rheumatic heart disease’ OR ’rheumatic fever’) AND (’standardized mortality ratio’ OR SMR)) OR (’rheumatic heart disease’ OR ’rheumatic fever’ AND ’case fatality’) NOT ”animal model” NOT rat NOT mice NOT ”tissue culture”[TIAB] AND 2015/02/16:2023/01/31[dp]

The results of the systematic review were reported according to the PRISMA (Preferred Reporting Items for Systematic reviews and Meta-Analyses) statement and registered with PROSPERO under record ID CRD42022355770. Figure 1 shows the PRISMA diagram for the systematic review. In the diagram, screening refers to reviewing of the title and abstract of an article for relevant information, not screening of the entire article. Reasons for exclusion include non-representativeness, use of different case definitions, studies reporting age-standardised data, and data reported in a non-compatible format. We initiated a second round of screening at the beginning of 2023 to ensure capture of all publications through 2022. In total, 4857 studies were returned after deduplication, (1803 from EMBASE, 2056 from PubMed, and 998 from VHL). Following full text review, 70 articles were extracted.

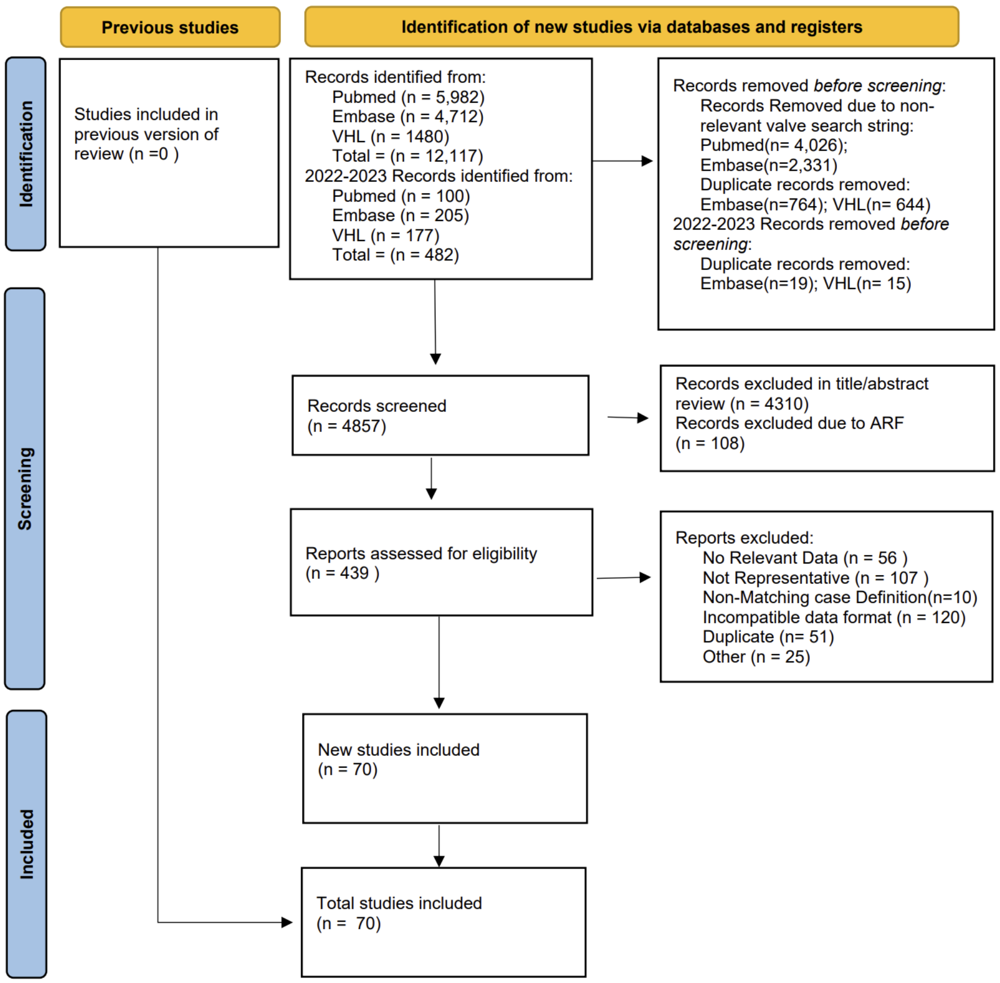

Figure 1. PRISMA diagram of RHD 2022 literature review

We also included administrative health facility data. Descriptions of search strategies and the methodology used to process these data are described in Appendix 1, Section 2. Inpatient data were adjusted to reflect multiple visits in the same year, non-primary diagnoses, and inpatient to outpatient utilisation ratios. Prevalence from hospital and claims data sources were included only for the non-endemic country model, described below. We did not include hospital or claims data in the endemic country model due to these data being from non-population-representative tertiary referral hospital facilities.

No adjustments were performed to account for different methods of case ascertainment. The estimation process for these adjustments requires overlap in location, year, and age between data sources which use the reference definition to identify cases and any other methodologies to produce stable adjustment factors. Due to the sparsity of the current RHD data in the endemic country model, this is currently not possible. Age-splitting was based on the global sex-specific age patterns obtained from a disease model—Bayesian meta-regression (DisMod-MR 2.1) models computed using data with age ranges of less than 25 years.

Modelling strategy

Rheumatic Heart Disease Endemicity Index

For GBD 2023 non-fatal rheumatic heart disease estimation, we ran separate models for non-endemic locations and one for endemic locations. To define which locations were included in each model, we instituted a new method of endemicity determination called the Rheumatic Heart Disease Endemicity Index (RHDEI).

The RHDEI first uses population-level covariates produced in GBD identified through themes in the literature as leading to higher rates of RHD in young populations. It then utilises principal component analysis (PCA) and Gaussian mixed modelling (GMM) to create a single index categorised into two clusters for modelling. This was applied to each year of GBD to produce a spatiotemporal definition of endemicity from 1990 to 2023.

The first step of the RHDEI was a small, targeted review of literature to identify covariates that are associated with higher numbers of RHD cases in young populations.[2–13] These were matched to covariates that the GBD produces estimates for as there would then be consistent variables across space and time for the index calculation. The review resulted in the following variables being chosen for inclusion in the index: Socio-demographic Index (SDI), Healthcare Access and Quality (HAQ) Index, sanitation access, water access, proportion of children underweight, and all-cause mortality for ages 10-14 (used to capture areas that have poor child health outcomes). Definitions for these variables can be seen in Table 3.

Table 3: RHDEI variables

Variable	Definition
**Socio-demographic Index (SDI)**	A measure of development using the geometric mean of 1) log-transformed lag-distributed income; 2) total fertility rate (ages 25+); and 3) education years per capita over age 15
**Healthcare Access and Quality (HAQ) Index**	Uses 32 causes from which death should not occur in the presence of effective care to approximate personal health-care access and quality by location and time
**Sanitation access**	Percentage of population with access to improved toilet types as defined by the Joint Monitoring Program
**Water access**	Percentage of population with access to improved water sources as defined by the Joint Monitoring Program
**Underweight**	Age-standardised excess risk for the proportion of children underweight
**All-cause death**	All-cause mortality rate for ages 10–14

Next, we applied principal component analysis (PCA) methodologies to integrate the covariates into a single value index ranging from zero to one. PCA is a dimensionality reduction method that takes a dataset of variables for *n* entities (subjects, locations, etc.) and identifies the linear combinations that result in the highest variance via orthogonal lines of best fit.[14,15] This method was selected for the creation of the RHDEI because it provides a single measure (the first principal component) based on correlated variables which is much simpler to interpret and apply to subsequent methodological steps. The basic equation for the PCA computation is as follows:

$$c_1 = \beta_{1p}x_1 + \beta_{12}x_2 + \ldots + \beta_{1p}x_p$$

Where, $c_1$is the entity's principal component score for the first component, β_{1p} is the weight for observed variable p, and $x_p$is the value of the observed variable p for that entity. This equation is calculated the same number of times as there are variables in the dataset, resulting in multiple c's. The PCA was completed in R, utilising the prcomp() command.[16] We used the scaling and centering options provided by the function as PCA is affected by scale. In addition, variables for all-cause death and underweight were inverted so that the direction of all variables would uniformly indicate better health outcomes with increasing values (ie, locations with higher values of each variable would have lower rates of the drivers associated with RHD). The results of the PCA indicated that around 80% of the variance in the data was explained by the first principal component. Figure 2 shows the variance captured across all dimensions of the PCA via a scree plot. All values also loaded in the same direction, indicating that the first principal component captured a theme associated with the increase of each of these variables (such as lower RHD risk).

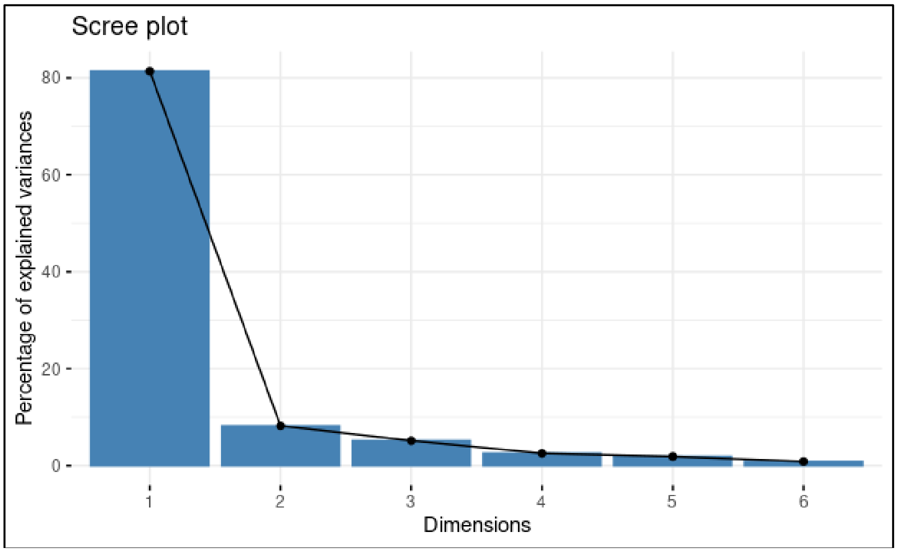

Figure 2. Scree plot of RHDEI PCA

Once the first principal component was extracted for each location included in the GBD study, the final step was running a Gaussian mixed model (GMM) clustering function. We utilised the Mclust package in R, which assumes a Gaussian mixture model via the function:[17]

$$\prod_{i=1}^{n} \sum_{k=1}^{G} \tau_k \, \phi_k(x_i|\mu_k, \Sigma_k),$$

$$\phi_k(x|\mu_k, \Sigma_k) = (2\pi^{-\frac{P}{2}}|\Sigma_k|^{-\frac{1}{2}} \, exp\left\{-\frac{1}{2}(x_i - \mu_k)^{\tau}\Sigma_k^{-1}(x_i - \mu_k)\right\}.$$

Where x is the data we are clustering, G is the number of components, τ_k is the probability that a datapoint belongs to component k. Each cluster is ellipsoidal, with the mean centered at μ_k and covariances Σ_k. The GMM clustering function assumes that a total distribution, in this case the values of PC1, is the result of multiple Gaussian distributions. Through BIC optimisation, the GMM clustering function identifies the number of Gaussian distributions that results in the best fit, and then identifies

which cluster each datapoint falls into based on probability. For our dataset, the model identified a four-cluster solution as optimal, seen in figure 3, which was then split into two clusters that resulted in the final endemic (clusters 1 and 2) and non-endemic (clusters 3 and 4) classification.

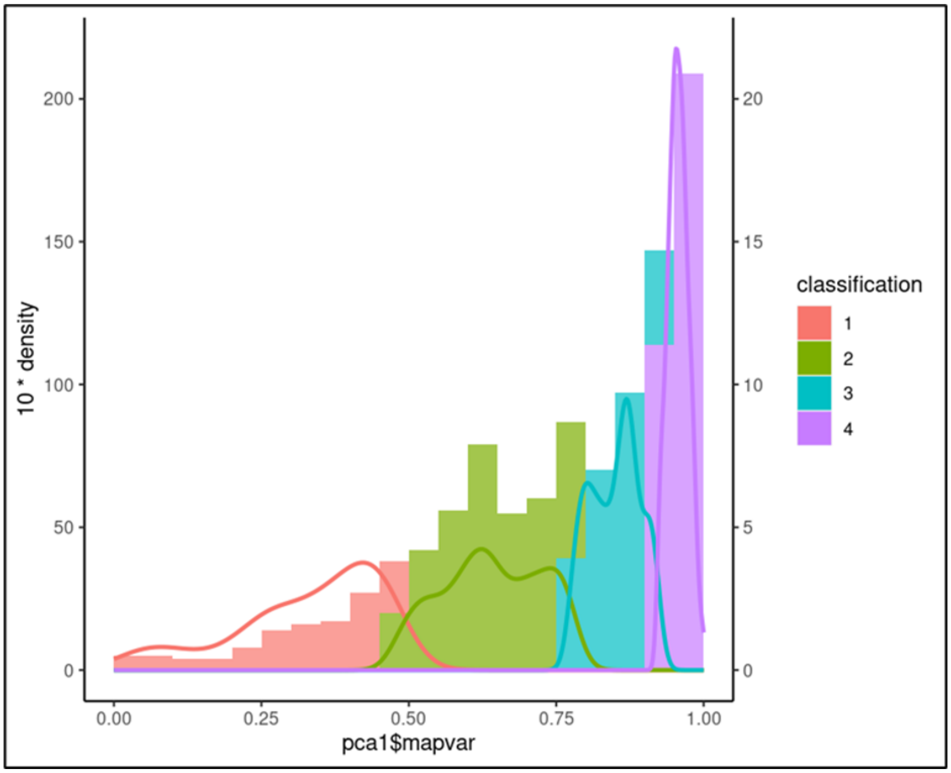

Figure 3. GMM cluster classification. Count of locations on y-axis and principal component 1 on x-axis.

Incidence and prevalence estimation

After categorising locations as either endemic or non-endemic based on the RHEI, we then ran two models in DisMod-MR 2.1, restricting the input data to endemic or non-endemic locations, as appropriate. Details of the DisMod-MR software can be found in Appendix 1, Section 2 of this manuscript. Specifics of the RHD modelling process can be found below.

Disability weights associated with rheumatic heart disease without heart failure are shown in Table 4.

Table 4. Severity distribution, details on the severity levels for rheumatic heart disease, and the associated disability weight (DW) with that severity

Severity level	Lay description	DW (95% CI)
Rheumatic heart disease, not including heart failure	Has a chronic disease that requires medication every day and causes some worry but minimal interference with daily activities.	0.049 (0.031–0.072)

Non-endemic model: We included data on prevalence from the published literature and administrative health care data sources and cause-specific mortality rates from our mortality estimates of RHD. A prior of no remission was set for all ages. Coefficients for selected covariates are listed in Table 5.

Endemic model: We included data on prevalence, remission, and excess mortality published in the literature. For remission, a prior of zero was set on remission for ages 20 years and above. Excess mortality was capped at 0.07, the highest observed mean excess mortality rate datapoint observed in this model. We also set priors of zero on incidence for ages 0–1 and 50–100 to account for patterns of incidence in endemic countries. Coefficients for selected covariates are listed in Table 5.

Table 5: Coefficients for country covariates included in the DisMod-MR models

Covariate	Parameter	Beta	Exponentiated beta
Endemic model			
LDI (I$ per capita)	Excess mortality rate	–0.3 (–0.5 to –0.12)	0.74 (0.61 to 0.89)
Non-endemic model			
LDI (I$ per capita)	Excess mortality rate	–0.4 (–0.43 to –0.36)	0.67 (0.65 to 0.70)

Final estimation processing

We combined estimates of prevalence and incidence from the endemic and non-endemic models, selecting estimates for the locations identified as non-endemic from the non-endemic model and estimates for the locations identified as endemic from the endemic model. Estimates of heart failure due to RHD were then subtracted from the estimates for RHD, giving the overall prevalence of RHD without heart failure. A description of the modelling strategy for heart failure due to RHD can be found in the heart failure appendix.

We evaluated models based on comparing estimates with input data as well as estimates from previous rounds of GBD.

Endemic locations: Abra, Aceh, Acre, Addis Ababa, Afar, Afghanistan, Agusan Del Norte, Agusan Del Sur, Aklan, Alagoas, Albay, Algeria, Amapá, Amazonas, American Samoa, Amhara, Angola, Antique, Apayao, Ardebil, Aurora, Azad Jammu & Kashmir, Azerbaijan, Bahia, Bali, Balochistan, Bangka-Belitung Islands, Bangladesh, Banten, Baringo, Basilan, Bataan, Batanes, Batangas, Belize, Bengkulu, Benguet, Benin, Benishangul-Gumuz, Bhutan, Biliran, Bohol, Bolivia, Bomet, Botswana, Brazil, Bukidnon, Bulacan, Bungoma, Burkina Faso, Burundi, Busia, Cabo Verde, Cagayan, Camarines Norte, Camarines Sur, Cambodia, Cameroon, Camiguin, Campeche, Capiz, Catanduanes, Cavite, Ceará, Cebu, Central African Republic, Central Java, Central Kalimantan, Central Sulawesi, Chad, Chiapas, Chihuahua, China, Colombia, Comoros, Congo, Cotabato (North Cotabato), Côte d'Ivoire, Davao de Oro, Davao Del Norte, Davao Del Sur, Davao Occidental, Davao Oriental, Democratic Republic of the Congo, Dinagat Islands, Dire Dawa, Djibouti, Dominica, Dominican Republic, Durango, East Azarbayejan, East Java, East Kalimantan, East Nusa Tenggara, Eastern Cape, Eastern Samar, Ecuador, Egypt, El Salvador, Elgeyo-Marakwet, Embu, Equatorial Guinea, Eritrea, Espírito Santo, Eswatini, Ethiopia, Fars, Federated States of Micronesia, Fiji, Free State, Gabon, Gambella, Gambia, Garissa, Gauteng, Ghana, Gilgit-Baltistan, Goiás, Golestan, Gorontalo, Grenada, Guanajuato, Guatemala, Guerrero, Guimaras, Guinea, Guinea-Bissau, Guyana, Haiti, Hamadan, Harari, Hidalgo, Homa Bay, Honduras, Hormozgan, Ifugao, Ilocos Norte, Ilocos Sur, Iloilo, India, Indonesia, Iran, Iraq, Isabela, Isiolo, Islamabad Capital Territory, Jakarta, Jalisco, Jamaica, Jambi, Kajiado, Kakamega, Kalinga, Kenya, Kericho, Kerman, Kermanshah, Khorasan-e-Razavi, Khuzestan, Khyber Pakhtunkhwa, Kiambu, Kilifi, Kiribati, Kirinyaga, Kisii, Kisumu, Kitui, Kohgiluyeh and Boyer-Ahmad, Kurdistan, Kwale, KwaZulu-Natal, Kyrgyzstan, La Union, Laguna, Laikipia, Lampung, Lamu, Lanao Del

Norte, Lanao Del Sur, Laos, Lesotho, Leyte, Liberia, Libya, Limpopo, Machakos, Madagascar, Maguindanao, Makueni, Malawi, Malaysia, Maldives, Mali, Maluku, Mandera, Maranhão, Marinduque, Markazi, Marsabit, Marshall Islands, Masbate, Mato Grosso, Mato Grosso do Sul, Mauritania, Mauritius, Meru, Mexico, México, Michoacán de Ocampo, Migori, Minas Gerais, Misamis Occidental, Misamis Oriental, Mombasa, Mongolia, Morelos, Morocco, Mountain Province, Mozambique, Mpumalanga, Murang'a, Myanmar, Nairobi, Nakuru, Namibia, Nandi, Narok, National Capital Region, Nauru, Nayarit, Negros Occidental, Negros Oriental, Nepal, Nicaragua, Niger, Nigeria, Niue, North Kalimantan, North Khorasan, North Korea, North Maluku, North Sulawesi, North Sumatra, North-West, Northern Cape, Northern Samar, Nueva Ecija, Nueva Vizcaya, Nyamira, Nyandarua, Nyeri, Oaxaca, Occidental Mindoro, Oriental Mindoro, Oromia, Pakistan, Palau, Palawan, Palestine, Pampanga, Panama, Pangasinan, Papua, Papua New Guinea, Pará, Paraguay, Paraíba, Pernambuco, Peru, Philippines, Piaui, Puebla, Punjab, Querétaro, Quezon, Quintana Roo, Quirino, Riau, Riau Islands, Rio Grande do Norte, Rizal, Romblon, Rondônia, Roraima, Rwanda, Saint Vincent and the Grenadines, Samar (Western Samar), Samburu, Samoa, San Luis Potosí, São Tomé and Príncipe, Sarangani, Senegal, Sergipe, Seychelles, Siaya, Sierra Leone, Sindh, Siquijor, Sistan and Baluchistan, Solomon Islands, Somali, Somalia, Sorsogon, South Africa, South Cotabato, South Kalimantan, South Khorasan, South Sudan, South Sulawesi, South Sumatra, Southeast Sulawesi, Southern Leyte, Southern Nations, Nationalities, and Peoples, Sri Lanka, Sudan, Sultan Kudarat, Sulu, Surigao Del Norte, Surigao Del Sur, Suriname, Syria, Tabasco, Taita Taveta, Tajikistan, Tana River, Tanzania, Tarlac, Tawi-Tawi, Thailand, Tharaka Nithi, Tigray, Timor-Leste, Tlaxcala, Tocantins, Togo, Tokelau, Tonga, Trans Nzoia, Turkana, Turkmenistan, Tuvalu, Uasin Gishu, Uganda, Uzbekistan, Vanuatu, Venezuela, Veracruz de Ignacio de la Llave, Viet Nam, Vihiga, Wajir, West Azarbayejan, West Java, West Kalimantan, West Nusa Tenggara, West Papua, West Pokot, West Sulawesi, West Sumatra, Western Cape, Yemen, Yogyakarta, Yucatán, Zacatecas, Zambales, Zambia, Zamboanga Del Norte, Zamboanga Del Sur, Zamboanga Sibugay, Zanjan, Zimbabwe

Non-endemic locations: Abruzzo, Agder, Aguascalientes, Aichi, Akita, Alabama, Alaska, Albania, Alborz, Andorra, Antigua and Barbuda, Aomori, Argentina, Arizona, Arkansas, Armenia, Australia, Austria, Bahamas, Bahrain, Baja California, Baja California Sur, Barbados, Barking and Dagenham, Barnet, Barnsley, Basilicata, Bath and North East Somerset, Bedford, Belarus, Belgium, Bermuda, Bexley, Birmingham, Blackburn with Darwen, Blackpool, Bolton, Bosnia and Herzegovina, Bournemouth, Bracknell Forest, Bradford, Brent, Brighton and Hove, Bristol, City of, Bromley, Brunei, Buckinghamshire, Bulgaria, Bury, Bushehr, Calabria, Calderdale, California, Cambridgeshire, Camden, Campania, Canada, Central Bedfordshire, Chahar Mahaal and Bakhtiari, Cheshire East, Cheshire West and Chester, Chiba, Chile, Coahuila, Colima, Colorado, Connecticut, Cook Islands, Cornwall, Costa Rica, County Durham, Coventry, Croatia, Croydon, Cuba, Cumbria, Cyprus, Czechia, Darlington, Delaware, Denmark, Derby, Derbyshire, Devon, District of Columbia, Distrito Federal, Doncaster, Dorset, Dudley, Ealing, East Midlands, East of England, East Riding of Yorkshire, East Sussex, Ehime, Emilia-Romagna, Enfield, England, Essex, Estonia, Finland, Florida, France, Friuli-Venezia Giulia, Fukui, Fukuoka, Fukushima, Gateshead, Georgia, Georgia, Germany, Gifu, Gilan, Gloucestershire, Greater London, Greece, Greenland, Greenwich, Guam, Gunma, Hackney, Halton, Hammersmith and Fulham, Hampshire, Haringey, Harrow, Hartlepool, Havering, Hawaii, County of Herefordshire, Hertfordshire, Hillingdon, Hiroshima, Hokkaidō, Hounslow, Hungary, Hyōgo, Ibaraki, Iceland, Idaho, Ilam, Illinois, Indiana, Innlandet, Iowa, Ireland, Isfahan, Ishikawa, Isle of Wight, Islington, Israel, Italy, Iwate, Japan, Jordan, Kagawa, Kagoshima, Kanagawa, Kansas, Kazakhstan, Kensington and Chelsea, Kent, Kentucky, City of Kingston upon Hull, Kingston upon Thames, Kirklees, Knowsley, Kōchi, Kumamoto, Kuwait, Kyōto, Lambeth, Lancashire, Latvia, Lazio, Lebanon, Leeds, Leicester, Leicestershire, Lewisham, Liguria, Lincolnshire, Lithuania, Liverpool, Lombardia, Lorestan, Louisiana, Luton, Luxembourg, Maine, Malta, Manchester, Marche, Maryland, Massachusetts, Mazandaran, Medway, Merton, Mexico City, Michigan, Middlesbrough, Mie,

Milton Keynes, Minnesota, Mississippi, Missouri, Miyagi, Miyazaki, Molise, Monaco, Montana, Montenegro, Møre og Romsdal, Nagano, Nagasaki, Nara, Nebraska, Netherlands, Nevada, New Hampshire, New Jersey, New Mexico, New York, New Zealand, Newcastle upon Tyne, Newham, Niigata, Nordland, Norfolk, North Carolina, North Dakota, North East England, North East Lincolnshire, North Lincolnshire, North Macedonia, North Somerset, North Tyneside, North West England, North Yorkshire, Northamptonshire, Northern Ireland, Northern Mariana Islands, Northumberland, Norway, Nottingham, Nottinghamshire, Nuevo León, Ohio, Ōita, Okayama, Okinawa, Oklahoma, Oldham, Oman, Oregon, Ōsaka, Oslo, Oxfordshire, Paraná, Pennsylvania, Peterborough, Piemonte, Plymouth, Poland, Poole, Portsmouth, Portugal, Provincia autonoma di Bolzano, Provincia autonoma di Trento, Puerto Rico, Puglia, Qatar, Qazvin, Qom, Reading, Redbridge, Redcar and Cleveland, Republic of Moldova, Rhode Island, Richmond upon Thames, Rio de Janeiro, Rio Grande do Sul, Rochdale, Rogaland, Romania, Rotherham, Russia, Rutland, Saga, Saint Kitts and Nevis, Saint Lucia, Saitama, Salford, San Marino, Sandwell, Santa Catarina, São Paulo, Sardegna, Saudi Arabia, Scotland, Sefton, Semnan, Serbia, Sheffield, Shiga, Shimane, Shizuoka, Shropshire, Sicilia, Sinaloa, Singapore, Slough, Slovakia, Slovenia, Solihull, Somerset, Sonora, South Carolina, South Dakota, South East England, South Gloucestershire, South Korea, South Tyneside, South West England, Southampton, Southend-on-Sea, Southwark, Spain, St Helens, Staffordshire, Stockholm, Stockport, Stockton-on-Tees, Stoke-on-Trent, Suffolk, Sunderland, Surrey, Sutton, Sweden, Sweden except Stockholm, Swindon, Switzerland, Taiwan (Province of China), Tamaulipas, Tameside, Tehran, Telford and Wrekin, Tennessee, Texas, Thurrock, Tochigi, Tokushima, Tōkyō, Torbay, Toscana, Tottori, Tower Hamlets, Toyama, Trafford, Trinidad and Tobago, Troms og Finnmark, Trøndelag, Tunisia, Türkiye, Ukraine, Umbria, United Arab Emirates, UK, USA, Uruguay, Utah, Valle d'Aosta, Veneto, Vermont, Vestfold og Telemark, Vestland, Viken, Virgin Islands, Virginia, Wakayama, Wakefield, Wales, Walsall, Waltham Forest, Wandsworth, Warrington, Warwickshire, Washington, West Berkshire, West Midlands, West Sussex, West Virginia, Westminster, Wigan, Wiltshire, Windsor and Maidenhead, Wirral, Wisconsin, Wokingham, Wolverhampton, Worcestershire, Wyoming, Yamagata, Yamaguchi, Yamanashi, Yazd, York, Yorkshire and the Humber.

References

1 Reményi B, Wilson N, Steer A, *et al.* World Heart Federation criteria for echocardiographic diagnosis of rheumatic heart disease--an evidence-based guideline. *Nat Rev Cardiol* 2012; **9**: 297–309.

2 Beaton A, Okello E, Rwebembera J, *et al.* Secondary Antibiotic Prophylaxis for Latent Rheumatic Heart Disease. *N Engl J Med* 2022; **386**: 230–40.

3 Carapetis JR, Beaton A, Cunningham MW, *et al.* Acute rheumatic fever and rheumatic heart disease. *Nat Rev Dis Primers* 2016; **2**: 15084.

4 Jaine R, Baker M, Venugopal K. Acute rheumatic fever associated with household crowding in a developed country. *Pediatr Infect Dis J* 2011; **30**: 315–9.

5 Land MA, Bisno AL. Acute rheumatic fever. A vanishing disease in suburbia. *JAMA* 1983; **249**: 895–8.

6 Gordis L. The virtual disappearance of rheumatic fever in the United States: lessons in the rise and fall of disease. T. Duckett Jones memorial lecture. *Circulation* 1985; **72**: 1155–62.

7 Zühlke LJ, Steer AC. Estimates of the global burden of rheumatic heart disease. *Glob Heart* 2013; **8**: 189–95.

8 Okello E, Kakande B, Sebatta E, *et al.* Socioeconomic and environmental risk factors among rheumatic heart disease patients in Uganda. *PLoS One* 2012; **7**: e43917.

9 Vlajinac H, Adanja B, Marinković J, Jarebinski M. Influence of socio-economic and other factors on rheumatic fever occurrence. *Eur J Epidemiol* 1991; **7**: 702–4.

10 Beaton A, Aliku T, Dewyer A, *et al.* Latent Rheumatic Heart Disease: Identifying the Children at Highest Risk of Unfavorable Outcome. *Circulation* 2017; **136**: 2233–44.

11 Nascimento BR, Beaton AZ. Rheumatic heart disease and socioeconomic development. *Lancet Glob Health* 2019; **7**: e1297–9.

12 Dougherty S, Beaton A, Nascimento BR, Zühlke LJ, Khorsandi M, Wilson N. Prevention and control of rheumatic heart disease: Overcoming core challenges in resource-poor environments. *Ann Pediatr Cardiol* 2018; **11**: 68–78.

13 Kang K, Chau KWT, Howell E, *et al.* The temporospatial epidemiology of rheumatic heart disease in Far North Queensland, tropical Australia 1997-2017; impact of socioeconomic status on disease burden, severity and access to care. *PLoS Negl Trop Dis* 2021; **15**: e0008990.

14 Pearson K. LIII. *On lines and planes of closest fit to systems of points in space*. *The London, Edinburgh, and Dublin Philosophical Magazine and Journal of Science* 1901; **2**: 559–72.

15 Hotelling H. Analysis of a complex of statistical variables into principal components. *Journal of Educational Psychology* 1933; **24**: 417–41.

16 R a language and environment for statistical computing. 2022.

17 Scrucca L, Fop M, Murphy T Brendan, Raftery A E. mclust 5: Clustering, Classification and Density Estimation Using Gaussian Finite Mixture Models. *The R Journal* 2016; **8**: 289.

Rheumatoid arthritis

Flowchart

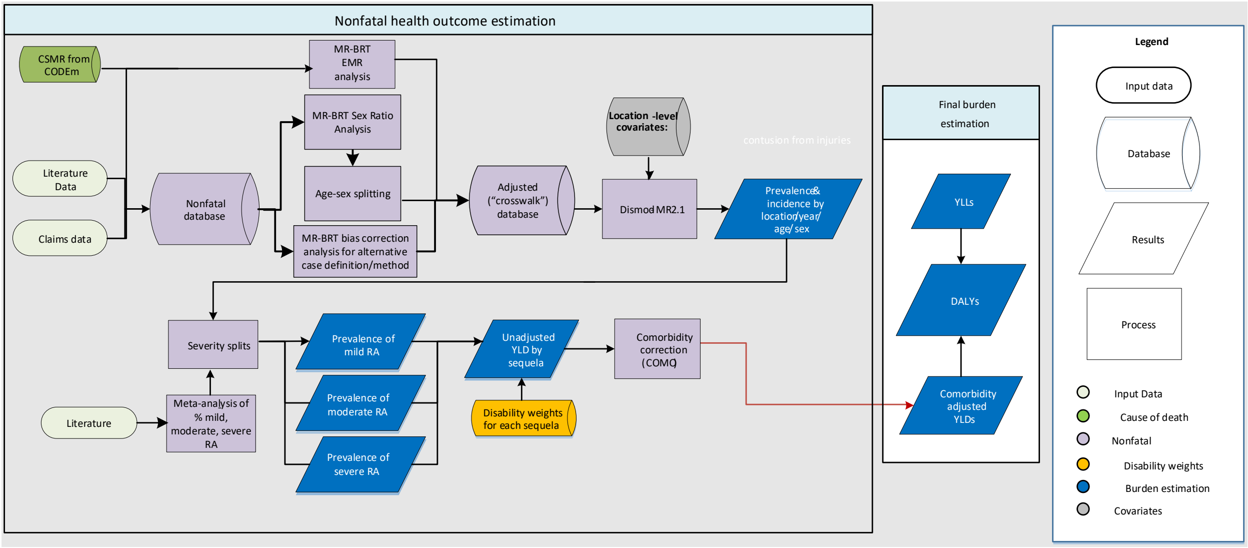

Input data and methodological summary for rheumatoid arthritis

Case definition

Rheumatoid arthritis (RA) is a systemic autoimmune disorder that causes pain, swelling, and deformation of the joints and may be accompanied by systemic symptoms. While RA is known to affect internal organs in addition to the joints, these extra-articular effects are currently not quantified in GBD. The reference case definition for RA is based on the 1987 criteria by the American College of Rheumatology (ACR 1987) which stipulates seven diagnostic criteria, of which four need to be satisfied for a diagnosis and the first four of which need to have been present for at least six weeks:

1. Morning stiffness
2. Arthritis of three or more joint areas
3. Arthritis of hand joints
4. Symmetric arthritis
5. Rheumatoid nodules
6. Serum rheumatoid factor
7. Radiographic changes

For RA, ICD-10 codes are M05, M06, and M08, and ICD-9 codes are 714.0–714.9.

The case definitions accepted for rheumatoid arthritis are shown in the table below.

**Reference or alternative**	**Definition**

Reference	Four of the 1987 criteria by the American College of Rheumatology (ACR 1987), which stipulate seven diagnostic criteria, need to be satisfied for a diagnosis.
Alternative	Physician diagnosis of rheumatoid arthritis using diagnostic criteria other than ACR 1987
Alternative	Diagnosis of rheumatoid arthritis from administrative health system records
Alternative	USA claims data 2000

Input data

Input data for RA were obtained from a previous systematic review of RA prevalence conducted for GBD 2010. Opportunistically, additional studies encountered during data review were added for GBD 2019. In addition, data from USA claims data for 2000, 2010–2012, and 2014–2016 by state and Taiwan claims for 2016 were included. We decided not to use hospital inpatient data as we considered they would not be representative of true prevalence and that variation between countries in the proportion of true prevalent cases captured in hospital inpatient data systems would likely vary more than can be captured by a single crosswalk. We compared the rates of RA in the outpatient data from Norway, Sweden, Canada, and the USA and found implausibly large differences with the rates from the claims data. The USA outpatient rates were half the value of the claims data and those for the other countries much lower still. For those reasons we decided not to use the outpatient data.

For GBD 2021, we conducted a systematic review of rheumatoid arthritis prevalence using the following search string:

> ("Arthritis, Rheumatoid"[Mesh] OR arthrit*) AND (prevalen*[Mesh] OR inciden*[Mesh]) AND ("2017/12/18"[PDAT] : "2019/10/18"[PDAT])

The exclusion criteria were:

1. Studies clearly not representative of the national population
2. Studies that were not population-based, eg, hospital or clinic-based studies
3. Studies that did not provide primary data on epidemiological parameters, eg, a commentary piece
4. Studies of a specific type of RA, eg, seropositive RA
5. Studies with a sample size of less than 150
6. Reviews

This search returned 641 sources, of which five were extracted.

Figure 1: PRISMA 2021 flow diagram, rheumatoid arthritis

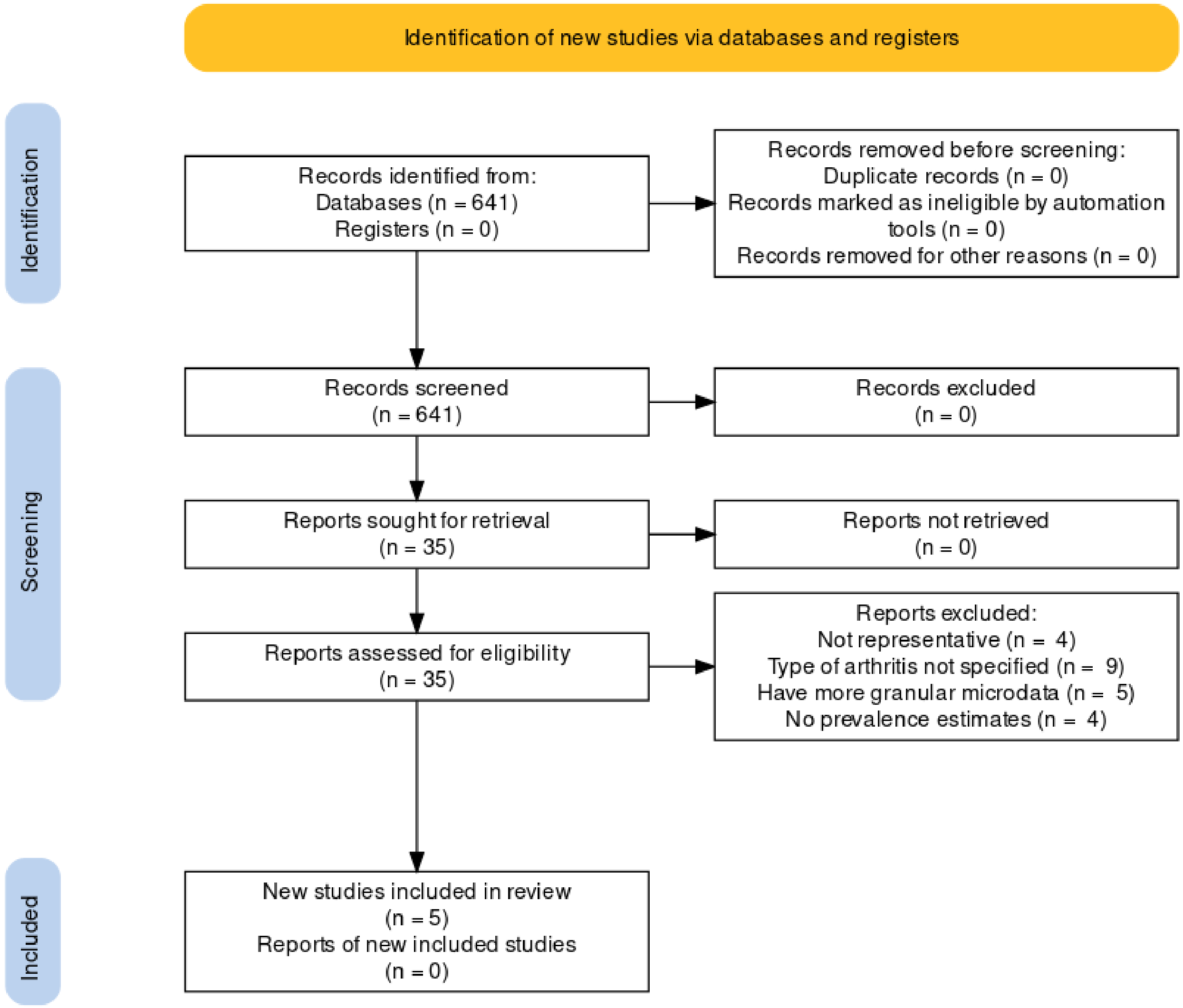

Age and sex splitting

Reported estimates of prevalence were split by age and sex where possible. First, if studies reported prevalence for broad age groups by sex (eg, prevalence in 15- to 65-year-old males and females separately), and also by specific age groups for both sexes combined (eg, prevalence in 15- to 30-year-olds, then in 31- to 65-year-olds, for males and females combined), age-specific estimates were split by sex using the reported sex ratio and bounds of uncertainty. Second, prevalence data for both sexes that could not be split using a within-study ratio were split using a sex ratio derived from a meta-analysis of existing sex-specific data using MR-BRT (meta-regression—Bayesian, regularised, trimmed; see appendix 1 section 2). The female to male ratio was 2.60 (2.58–2.62). Finally, after the application of bias adjustments, where studies reported estimates across age groups spanning 25 years or more, these were split into five-year age groups using the prevalence age pattern estimated by DisMod-MR 2.1 (disease model—Bayesian meta-regression, appendix 1 section 2).

Data adjustment

We used a single study covariate for studies using diagnostic criteria that did not match our reference case definition based on ACR 1987 criteria. We added an additional covariate for claims data in the USA from the year 2000. We treat claims data from the USA from 2010 onward and Taiwan as reference case definition data; rarely would cases of RA not intersect with the health system in the USA and Taiwan. Betas and exponentiated values (which can be interpreted as an odds ratio) for these two covariates are shown in the table below:

Table 2: MR-BRT crosswalk adjustment factors for rheumatoid arthritis

Data input	Reference or alternative case definition	Gamma	Beta coefficient, log (95% UI)*	Adjustment factor**
ACR 1987	Ref	0.09	---	---
RA criteria other than RA 1987	Alt		0.23 (–0.47 to 0.94)	1.26 (0.63 to 1.56)
USA claims data – 2000	Alt		0.69 (–0.46 to 1.84)	1.72 (0.63 to 6.30)

**MR-BRT crosswalk adjustments can be interpreted as the factor the alternative case definition is adjusted by to reflect what it would have been had it been measured using the reference case definition. If the log/logit beta coefficient is negative, then the alternative is adjusted up to the reference. If the log/logit beta coefficient is positive, then the alternative is adjusted down to the reference.*

***The adjustment factor column is the exponentiated beta coefficient. For log beta coefficients, this is the relative rate between the two case definitions. For logit beta coefficients, this is the relative odds between the two case definitions.*

After adjusting data for case definition, we outliered data with a median age-standardised absolute deviation of 2 or more above the mean to cull data that were implausibly high.

Modelling strategy

There have been no significant changes to the modelling strategy in GBD 2023.

Prior settings in the DisMod-MR model included the assumption of no incidence or prevalence of RA before the age of 5 years. Remission was set to 0–0.02 for ages up to 65 and 0–0.05 for ages 65+, reflecting the chronic nature of the condition.

For many causes, DisMod-MR estimated an unrealistic pattern of EMR, deviating from the expected trend of decreasing EMR with greater access to quality health care. Such discrepancies often indicate inconsistencies between CSMR estimates and measures of prevalence and/or incidence. To address this, EMR data from the previous round were modelled using the MR-BRT approach by age and sex, incorporating a prior with the Healthcare Access and Quality (HAQ) Index set to have a negative coefficient. The MR-BRT results were then predicted for each location, year, sex, and age from 0 to 100 in ten-year increments. We included HAQ Index as a country-level covariate to inform EMR with a mean and standard deviation produced from MR-BRT. However, even without this setting, DisMod-MR would tend to estimate a coefficient that was consistent with the MR-BRT analysis.

Table 3. Covariates. Summary of covariates used in the rheumatoid arthritis DisMod-MR meta-regression model

Covariate	Type	Parameter	Beta (95% uncertainty interval)	Exponentiated beta (95% uncertainty interval)
Healthcare Access and Quality Index	Country-level	Excess mortality rate	–0.025 (–0.025 to –0.025)	0.98 (0.98 to 0.98)
Mean BMI	Country-level	Prevalence	0.16 (0.13 to 0.18)	1.16 (1.12 to 1.18)

Severity and disability

The basis of the GBD disability weight survey assessments are lay descriptions of health states highlighting major functional consequences and symptoms. The lay descriptions and disability weights for RA severity levels are shown below.

Table 4. Severity distribution, details on the severity levels for rheumatoid arthritis in GBD 2023 and the associated disability weight (DW) with that severity.

Severity level	Lay description	DW (95% CI)
Mild	This person has moderate pain and stiffness in the arms and hands which causes difficulty lifting, carrying, and holding things, and trouble sleeping because of the pain.	0.117 (0.080–0.163)
Moderate	This person has pain and deformity in most joints, causing difficulty moving around, getting up and down, and using the hands for lifting and carrying. The person often feels fatigue.	0.317 (0.216–0.440)
Severe	This person has severe, constant pain, and deformity in most joints, causing difficulty moving around, getting up and down, eating, dressing, lifting, carrying, and using the hands. The person often feels sadness, anxiety, and extreme fatigue.	0.581 (0.403–0.739)

To determine the proportion of people with rheumatoid arthritis (RA) across severity levels, seven studies from three regions provided information on RA severity. Severity was classified based on Health Assessment Questionnaire (HAQ) scores, with cutoff values for each severity level defined as follows: <1 for mild, 1–2 for moderate, and ≥2 for severe. Estimates were drawn from multiple studies, and a random-effects meta-analysis model was used to pool the data. These estimates were generated using 1000 draws assuming a binomial distribution, and percentages were scaled to sum to 1 for each draw.

Figure 1. Severity distribution meta-analysis, details on the studies included in the meta-analysis calculating the proportion of mild RA.

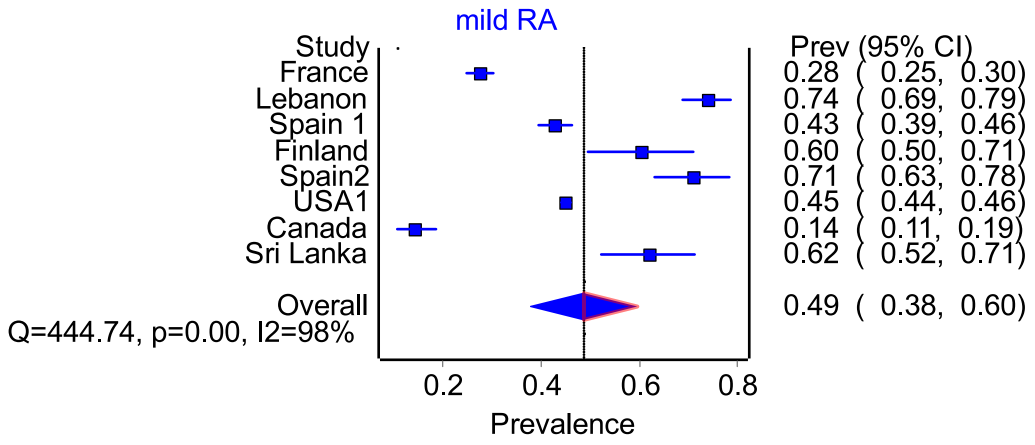

Figure 2. Severity distribution meta-analysis, details on the studies included in the meta-analysis calculating the proportion of moderate RA.

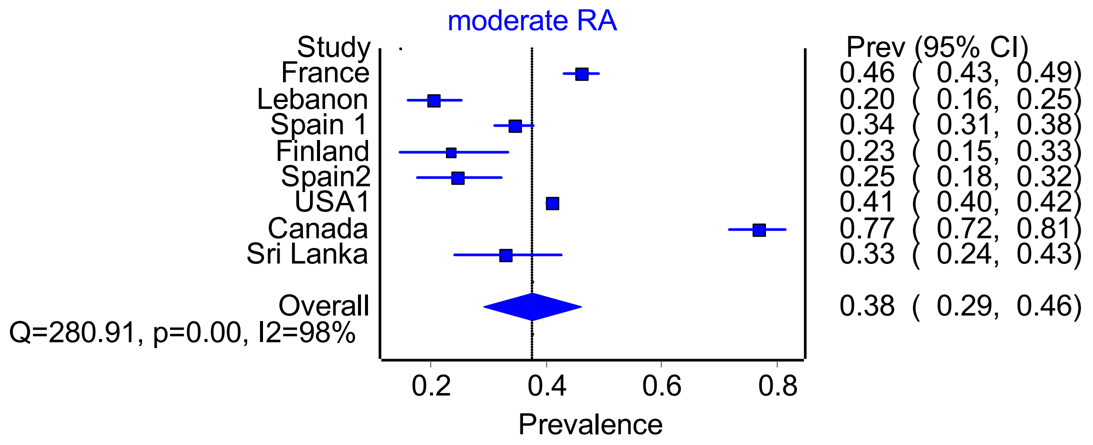

Figure 3. Severity distribution meta-analysis, details on the studies included in the meta-analysis calculating the proportion of severe RA.

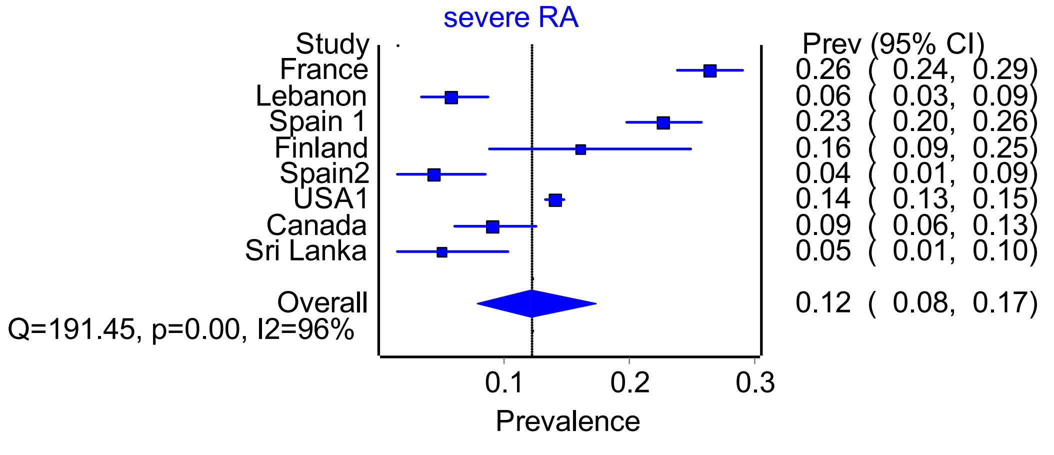

Scabies

Flowchart

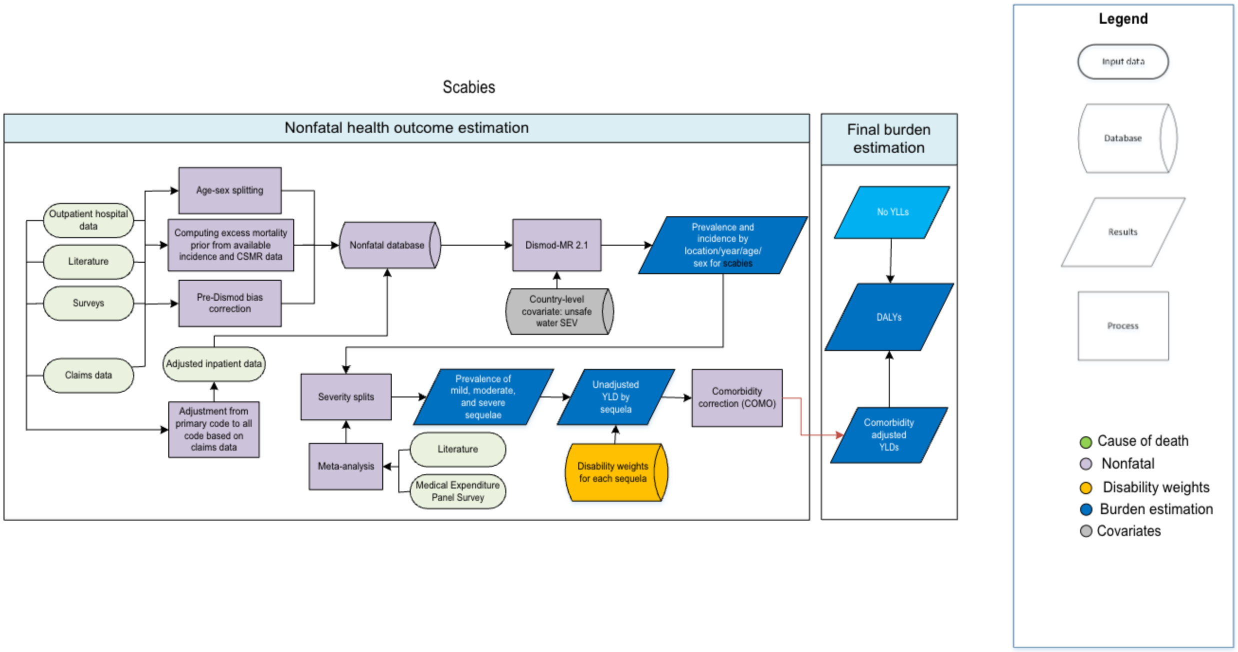

Input data and methodological summary for scabies

Case definition

According to the International Classification of Diseases (ICD-10), scabies is a skin disease caused by an infestation of the skin by the microscopic mite *Sarcoptes scabiei*, causing intense itching and a pimple-like skin rash (ICD-10: B86).

Quantity of interest	Reference or alternative	Definition
Scabies	Reference	Scabies as determined by Poland National Health Fund Patient Claims 2015–2019
Scabies	Alternative	All other scabies data

Input data

In the GBD 2010 study, a systematic review of the literature was conducted using PubMed and Google Scholar to capture epidemiological data for scabies. The inclusion criteria stipulated that studies (1) must be published between 1980 and 2012; (2) must provide data on the incidence or prevalence of scabies; (3) must use samples representative of the general population (ie, samples derived from the experimental arm of clinical trials or based in dermatology clinics were excluded); (4) must use a sample size larger than 100; and (5) must provide sufficient information on study method and sample characteristics to assess the quality of the study. For GBD 2013, the GBD 2010 search strategy was replicated to capture epidemiological studies published between 2011 and 2013. Therefore, we updated the systematic review through October 6, 2016, for GBD 2016. Additionally, clinical data from Poland

National Health Fund Patient Claims 2015–2019, United States Census Bureau (USCB) data 1993–2006, USA MarketScan data 2000–2019, and clinical data from Mongolia, Fiji, South Korea, Australia, Sweden, and Nigeria were added for GBD 2023. Data were outliered or excluded if we found them unreasonable when compared to regional, super-regional, and global rates.

Data processing

For scabies, we crosswalked all data to the reference definition. We began by evaluating the number of observations of each alternate definition that matched with a corresponding observation from the reference definition. We considered "between" study matches, where the alternative was from the same GBD age group and sex, and the midpoint year of the study was within five years of the midpoint of the reference definition observation.

$$\log i\, t\left(y_i^{alt}\right) - \log i\, t\left(y_i^{ref}\right) \;=\; \beta_0 + \epsilon_i$$

Table 1: MR-BRT crosswalk adjustment factors for scabies

Data input	Reference or alternative case definition	Gamma	Beta coefficient, log/logit (95% UI)*	Adjustment factor**
Scabies as determined by Poland National Health Fund Patient Claims 2015–2019	Ref	0	---	---
All other data	Alt		–5.3462 (–5.3532 to –5.3392)	0.0048 (0.0047, 0.9948)

**MR-BRT crosswalk adjustments can be interpreted as the factor the alternative case definition is adjusted by to reflect what it would have been had it been measured using the reference case definition. If the log/logit beta coefficient is negative, then the alternative is adjusted up to the reference. If the log/logit beta coefficient is positive, then the alternative is adjusted down to the reference.*
***The adjustment factor column is the exponentiated beta coefficient. For log beta coefficients, this is the relative rate between the two case definitions. For logit beta coefficients, this is the relative odds between the two case definitions.*

Modelling strategy

In GBD 2023, we have made no substantive changes in the modelling strategy from GBD 2021. DisMod-MR 2.1, a Bayesian meta-regression tool, was used to estimate scabies prevalence by age, sex, year, and geography (subnational [select countries], country, region, super-region). The cause scabies was modelled with remission set between 2.5 and 3.5, implying four to five months of duration, and excess mortality was assumed to be zero. This was in line with the available epidemiological data, expert opinion, and previous GBD work. The datasets for scabies were sufficiently large to make use of a relatively short time window of five years to determine which datapoints were used for a particular year of fit. Additionally, to improve estimation across all regions, we restricted location random effects to (–0.25, 0.25) in Cambodia, Mali, Nepal, Fiji, Timor-Leste, Vanuatu; the Oceania, southeast Asia, and east Asia GBD regions; and the corresponding super-region. We also restricted the random effect in Kenya (0, 0.5). In GBD 2023, we complete crosswalks using the MR-BRT modelling tool. We adjusted our input

data to reflect the reference data, Poland clinical claims 2015–2019 datapoints, which were more representative of the general population. We used the unsafe water SEV (summary exposure value) as a location-level covariate.

$$prevalence = incidence * duration$$

$$Remission = \frac{Cured\ cases}{person - year of f ollow - up\ in\ prevalent\ cases}$$

Table 2. Severity distribution, details on the severity levels for scabies and the associated disability weight (DW) with that severity

Severity level	Lay description	DW (95% CI)
Disfigurement, level 1 with itch/pain	The individual has a slight, visible physical deformity that is sometimes sore or itchy. Others notice deformity, which causes some worry and discomfort.	0.027 (0.015–0.042)

Table 3. Covariates. Summary of covariates used in the scabies DisMod-MR meta-regression model

Covariate	Type	Parameter	Log beta (95% uncertainty interval)	Exponentiated beta (95% uncertainty interval)
Log-transformed age-standardised SEV scalar: Scabies	Country-level	Prevalence	0.0015 (0.0001–0.0038)	1.00 (1.00–1.00)

Schistosomiasis

Flowchart

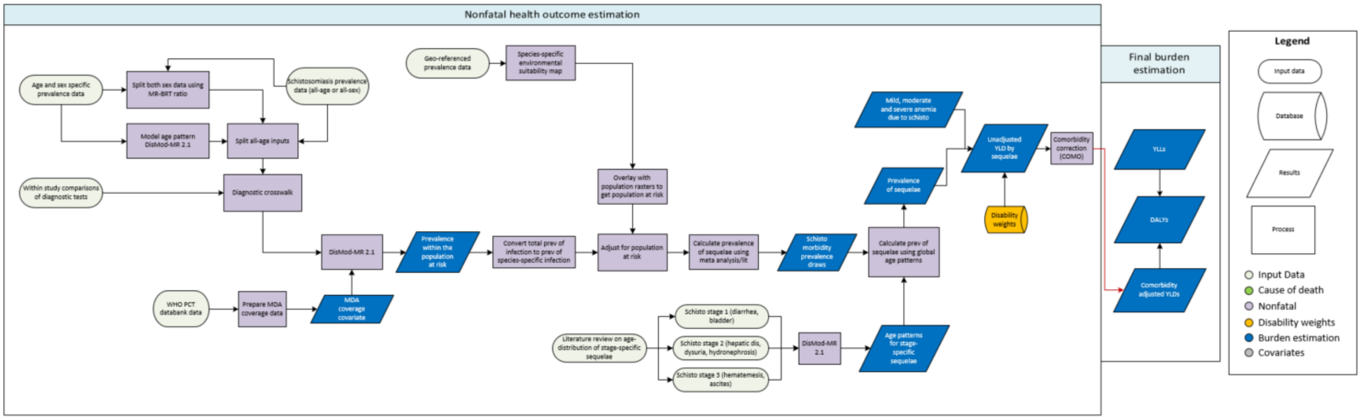

Input data and methodological summary for schistosomiasis

Case definition

Schistosomiasis, also known as bilharzia or “snail fever,” is a helminth disease caused by infection with five species of the parasite *Schistosoma,* namely, *S mansoni, S japonicum, S haematobium, S mekongi, and S intercalatum*. It is considered a neglected tropical disease (NTD). The first three species cause the most infection and the last two rarely cause disease. Diagnosis is made by microscopic exam of stool or urine for parasite eggs. For less advanced infections, serological techniques are used. The ICD-10 codes for schistosomiasis are B65-B65.9. We used the following case definitions for GBD 2023:

**Quantity of interest**	**Reference or Alternative**	**Definition**
Schistosomiasis - *S mansoni*	Reference	Diagnosis is made by microscopic examination of stool (Kato-Katz, 3 samples)
Schistosomiasis - *S mansoni*	Alternative	Diagnosis is made by microscopic examination of stool (Kato-Katz, 2 samples)
Schistosomiasis - *S mansoni*	Alternative	Diagnosis is made by microscopic examination of stool (Kato-Katz, 1 sample)
Schistosomiasis - *S mansoni*	Alternative	Diagnosis is made by detection of circulating cathodic antigen (CCA)
Schistosomiasis - *S mansoni*	Alternative	Diagnosis is made by microscopic examination of stool using sedimentation
Schistosomiasis - *S mansoni*	Alternative	Diagnosis is made by microscopic examination of stool using formol-ether
Schistosomiasis - *S mansoni*	Alternative	Diagnosis is made by PCR of stool or serum
Schistosomiasis - *S mansoni*	Alternative	Diagnosis is made by analysis of serum using ELISA

Schistosomiasis - *S haematobium*	Reference	Diagnosis is made by microscopic examination of urine using filtration
Schistosomiasis - *S haematobium*	Alternative	Diagnosis is made by analysis of urine using dipstick to identify haematuria
Schistosomiasis - *S haematobium*	Alternative	Diagnosis is made by PCR of urine or serum
Schistosomiasis - *S haematobium*	Alternative	Diagnosis is made by microscopic examination of urine using centrifugation
Schistosomiasis - *S haematobium*	Alternative	Diagnosis is made by microscopic examination of urine using sedimentation
Schistosomiasis - *S japonicum*	Reference	Diagnosis is made by analysis of serum using indirect hemagglutination (IHA)
Schistosomiasis - *S japonicum*	Alternative	Diagnosis is made by microscopic exam of stool, (Kato-Katz, 3 samples)
Schistosomiasis - *S japonicum*	Alternative	Diagnosis is made by microscopic exam of stool, (Kato-Katz, 2 samples)
Schistosomiasis - *S japonicum*	Alternative	Diagnosis is made by microscopic exam of stool, (Kato-Katz, 1 sample)
Schistosomiasis - *S mekongi* and *iterkalatum*	Reference	Diagnosis is made by microscopic exam of stool, (Kato-Katz, with either 3, 2, or 1 samples)

Input data

Prevalence

To model non-fatal outcomes due to schistosomiasis, we conducted a systematic literature review, extracting prevalence data from 1980 to 2016 for the five species of schistosomiasis listed above. The search string used in the systematic review is (schistosom*[Title/Abstract] OR bilharzia*[Title/Abstract] OR "snail fever"[Title/Abstract]) AND ("1990"[Date - Publication] : "3000"[Date - Publication]) AND (epidemiolog* OR inciden* OR prevalen* OR seroprevalen*) NOT (animals[mesh] NOT humans[mesh]). Additionally, we used data obtained through the Expanded Special Project for the Elimination of Neglected Tropical Diseases (ESPEN) data portal (maintained by WHO African Region) and data compiled by the Global Atlas of Helminth Infections (GAHI), which includes grey literature and unpublished data. Site-specific prevalence data are aggregated by GBD location and year.

Mass drug administration

Mass drug administration data were extracted from the WHO Preventive Chemotherapy and Transmission Control (PCT) Databank.[1]

Modelling strategy

Data processing

Schistosomiasis prevalence data reported for both sexes was first split into sex-specific inputs. To sex-split our both-sex datapoints, we used sex-specific inputs in a Bayesian meta-regression (MR-BRT) model to derive a ratio of female schistosomiasis prevalence to both-sex prevalence (scientific literature data). The resultant logit ratio was applied to both-sex datapoints to calculate out females, and males were calculated via subtraction. The beta coefficients of the adjustment are presented in Table 1.

Table 1: MR-BRT sex split adjustment factors for schistosomiasis

Data input Species	Reference or alternative case definition	Gamma	Beta coefficient, logit* (95% CI)	Adjustment factor**
S mansoni	Female (reference)	0.15	---	
	Both sexes		0.16 (–0.61; 0.94)	1.18
S haematobium	Female (reference)	0.03	---	
	Both sexes		0.27 (–0.09; 0.63)	1.32
S japonicum	Female (reference)	0.01	---	
	Both sexes		0.30 (0.07; 0.52)	1.34

**MR-BRT crosswalk adjustments can be interpreted as the factor the alternative case definition is adjusted by to reflect what it would have been had it been measured using the reference case definition. If the log/logit beta coefficient is negative, then the alternative is adjusted up to the reference. If the log/logit beta coefficient is positive, then the alternative is adjusted down to the reference.*
***The adjustment factor column is the exponentiated beta coefficient. For log beta coefficients, this is the relative rate between the two case definitions. For logit beta coefficients, this is the relative odds between the two case definitions.*

Diagnostic crosswalks

Next, we used a method for diagnostic adjustment to account for species-specific diagnostic tests, generating an adjustment for *S haematobium*, *S mansoni,* and *S japonicum,* separately. For *S mansoni*, we identified 81 within-study comparisons including at least two of the following diagnostic methods: Kato-Katz (1, 2, or 3 stool smears); ELISA; CCA; formol-ether concentration; sedimentation, and PCR. A total of 55 diagnostic comparisons were identified for *S haematobium*: CCA; urine filtration, dipstick tests, centrifugation, and sedimentation. 47 comparisons were identified for *S japonicum*, including Kato-Katz, IHA, hatch test, and ELISA. The reference categories by species were defined as Kato-Katz for *S mansoni*, urine filtration for *S haematobium* and PCR for *S japonicum* (adjustment factors presented in Tables 2-4).

Table 2: MR-BRT crosswalk adjustment factors for *S mansoni*

Data input	Reference or alternative case definition	Gamma	Beta coefficient, logit* (95% CI)	Adjustment factor**
Kato-Katz 3 sample	Ref	0.62	---	
Kato-Katz 2 sample	Alt		–0.17 (–1.74; 1.39)	0.84
Kato-Katz 1 sample	Alt		–0.62 (–2.17; 0.94)	0.53
CCA	Alt		1.04 (–0.51; 2.58)	2.83
Sedimentation	Alt		–0.16 (–1.77; 1.47)	0.85
Formol-ether	Alt		–0.83 (–2.63; 0.96)	0.43
PCR	Alt		1.90 (0.13; 3.66)	6.69
ELISA	Alt		0.75 (–0.83; 2.33)	2.12

**MR-BRT crosswalk adjustments can be interpreted as the factor the alternative case definition is adjusted by to reflect what it would have been had it been measured using the reference case definition. If the log/logit beta*

coefficient is negative, then the alternative is adjusted up to the reference. If the log/logit beta coefficient is positive, then the alternative is adjusted down to the reference.
***The adjustment factor column is the exponentiated beta coefficient. For log beta coefficients, this is the relative rate between the two case definitions. For logit beta coefficients, this is the relative odds between the two case definitions.*

Table 3: MR-BRT crosswalk adjustment factors for *S haematobium*

**Data input**	**Reference or alternative case definition**	**Gamma**	**Beta coefficient, logit* (95% CI)**	**Adjustment factor****
Urine filtration	Ref	0.54	---	
CCA	Alt		2.38 (0.85; 3.91)	10.80
Dipstick	Alt		–0.23 (–1.70; 1.23)	0.79
PCR	Alt		–0.07 (–2.22; 2.09)	0.93
Centrifugation	Alt		–0.20 (–177; 1.37)	0.82
Sedimentation	Alt		–0.58 (–2.37; 1.21)	0.56

**MR-BRT crosswalk adjustments can be interpreted as the factor the alternative case definition is adjusted by to reflect what it would have been had it been measured using the reference case definition. If the log/logit beta coefficient is negative, then the alternative is adjusted up to the reference. If the log/logit beta coefficient is positive, then the alternative is adjusted down to the reference.*
***The adjustment factor column is the exponentiated beta coefficient. For log beta coefficients, this is the relative rate between the two case definitions. For logit beta coefficients, this is the relative odds between the two case definitions.*

Table 4: MR-BRT crosswalk adjustment factors for *S japonicum*

**Data input**	**Reference or alternative case definition**	**Gamma**	**Beta coefficient, logit* (95% CI)**	**Adjustment factor****
IHA	Ref	0.11	---	
ELISA	Alt		0.99 (0.20; 1.77)	2.69
Hatch test	Alt		–1.76 (–2.49; –1.02)	0.17
Kato-Katz 1 sample	Alt		–1.71 (–2.40; –1.02)	0.18
Kato-Katz 2 sample	Alt		–1.59 (–2.29; –0.88)	0.20
Kato-Katz 3 sample	Alt		–2.00 (–3.00; –1.00)	0.14

**MR-BRT crosswalk adjustments can be interpreted as the factor the alternative case definition is adjusted by to reflect what it would have been had it been measured using the reference case definition. If the log/logit beta coefficient is negative, then the alternative is adjusted up to the reference. If the log/logit beta coefficient is positive, then the alternative is adjusted down to the reference.*

***The adjustment factor column is the exponentiated beta coefficient. For log beta coefficients, this is the relative rate between the two case definitions. For logit beta coefficients, this is the relative odds between the two case definitions.*

Age splitting

Finally, the all-age data were split into five-year age groups by using a global age pattern obtained via DisMod Bayesian meta-regression (DisMod-MR) model, illustrated in Figure 1. Uncertainty was propagated throughout the sex, diagnostic, and age-splitting processes, such that final sex- and age-specific prevalence estimates reflect uncertainty of the original data.

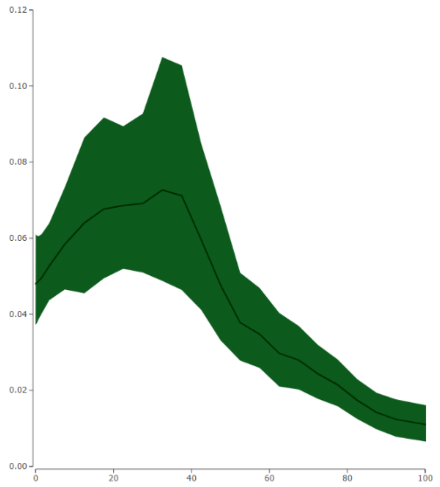

Figure 1. Global age pattern of schistosomiasis prevalence produced by DisMod-MR

Health states/sequelae

The morbidity model for schistosomiasis involved a multi-step process. First, we ran a single-parameter prevalence model in DisMod-MR 2.1 using the prevalence data after adjusting for age, sex, and diagnostic. We assume that all of our data are measured within a population at risk – therefore, the estimates from the DisMod model represent prevalence estimates among the population at risk for schistosomiasis. Additionally, we included the MDA treatment data from WHO, sanitation (proportion with access), and 90th percentile climatic temperature in the given country-year as country-level covariates in the DisMod model (Table 5).

Table 5. Covariates. Summary of covariates used in the schistosomiasis DisMod-MR model

**Covariate**	**Type**	**Parameter**	**Exponentiated beta (95% UI)**
Socio-demographic Index	Country-level	Prevalence	7.00 (6.13–8.04)
MDA treatments	Country-level	Prevalence	0.79 (0.78–0.80)
Sanitation (proportion with access)	Country-level	Prevalence	0.13 (0.12–0.14)
90th percentile climatic temperature	Country-level	Prevalence	0.54 (0.51–0.57)

Second, we ran three separate ecological niche maps for the three major species of schistosomiasis (*S mansoni, S haematobium,* and *S japonicum*) using a boosted regression tree and all geolocated data that were extracted from both the literature review and the GAHI database. The output was 1000 maps (representing 1000 draws) for each of the three species representing the suitability for schistosomiasis to exist in each 5x5 km square. Then, we extracted population at risk by optimising the area under the curve for each of the 1000 maps for each of the three species, overlaid the three species maps over one another, and extracted 1000 draws of proportion of the population at risk for schistosomiasis at the GBD location level. To avoid over-estimation of prevalence using the population at risk raster in urban areas in Brazil and China, we masked out urban areas. In China, we used year-specific masks based on published literature on county-specific elimination of schistosomiasis, allowing the geographical restrictions to be implemented at a more detailed level where information is available.[5]

We then scaled the prevalence estimates to the population at risk estimates from the ecological niche map to get age/sex/location/year all-schistosomiasis prevalence envelopes. For co-endemic locations of both *S haematobium* and *S mansoni*, we ran a generalised linear model to obtain regional species-specific proportional prevalence using data from 58 studies that reported both *S haematobium* and *S mansoni* infection. These regional proportions were used to distribute prevalent cases of schistosomiasis between *S haematobium* and *S mansoni* for each location. For the other, non-co-endemic locations, we assumed that all schistosomiasis cases were attributable to the sole endemic species. Then, we used the species-specific all-age prevalence to estimate morbidity. We used literature-informed parameters (a, b, c) for translating infection (x) to morbidity (y):

$$y = (a + bx\hat{}c)/\ (1 + bx\hat{}c) - ay = (a + bx\hat{}c)/\ (1 + bx\hat{}c) - a$$

where a = baseline morbidity, which we set to be 0, and the parameters b and c were estimated in previous studies.[2-4] The sequelae were then age-split based on DisMod-MR stage age patterns (see Figure 1). The burden of anaemia due to schistosomiasis was estimated (see anaemia documentation for details).

Model evaluation was done by separately assessing the fit of the single-parameter DisMod models and checking the final estimates produced after age-sex splits. Plots of time trends of prevalence across locations and age were used to evaluate the results. In addition, maps of the global distribution of total schistosomiasis prevalence and prevalence of sequelae due to schistosomiasis were also assessed across time.

Disability weights

Table 6 shows the list of clinical sequelae (including mild, moderate, and severe anaemia) due to schistosomiasis, their lay descriptions, and the associated disease stages and disability weights. Using literature,[1] a list of eight possible clinical sequelae and anaemia sequelae were defined: mild infection, mild diarrhoea, haematemesis (vomiting blood), hepatomegaly, ascites (buildup of fluid in the peritoneal cavity), dysuria (painful urination), bladder pathology, hydronephrosis (swelling of kidney due to buildup of urine in the kidney), mild anaemia, moderate anaemia, and severe anaemia.

Table 6. Clinical sequelae, lay descriptions, disease stages, and disability weights (DWs)

Clinical sequela	Lay description	Disease stage	DW (95% CI)
Mild infection	has a low fever and mild discomfort, but no difficulty with daily activities	1	0.006 (0.002–0.012)
Mild diarrhoea	has diarrhea three or more times a day with occasional discomfort in the belly	1	0.074 (0.049-0.104)
Hepatomegaly	has some pain in the belly that causes nausea but does not interfere with daily activities	2	0.011 (0.005–0.021)
Dysuria	has some pain in the belly that causes nausea but does not interfere with daily activities	2	0.011 (0.005–0.021)
Hydronephrosis	has some pain in the belly that causes nausea but does not interfere with daily activities	2	0.011 (0.005–0.021)
Haematemesis	vomits blood and feels nauseated	3	0.325 (0.209–0.463)
Ascites	has pain in the belly and feels nauseated. The person has difficulties with daily activities	3	0.114 (0.078–0.159)
Bladder pathology	has some pain in the belly that causes nausea but does not interfere with daily activities	3	0.011 (0.005–0.021)
Mild anaemia	feels slightly tired and weak at times, but this does not interfere with normal daily activities	NA	0.004 (0.001–0.008)
Moderate anaemia	feels moderate fatigue, weakness, and shortness of breath after exercise, making daily activities more difficult	NA	0.052 (0.034–0.076)
Severe anaemia	feels very weak, tired, and short of breath, and has problems with activities that require physical effort or deep concentration	NA	0.149 (0.101–0.210)

We did not apply any adjustments for the COVID-19 pandemic to schistosomiasis due to a lack of available data quantifying the impacts of the pandemic on NTD epidemiology.

Changes from GBD 2021 to GBD 2023

The major change that we implemented in this cycle was the addition of MDA data to the covariate used in the DisMod prevalence model. The additional years of data better account for proportion of the population receiving MDA within a location.

References

1. World Health Organization (WHO). WHO PCT Databank - Schistosomiasis. Geneva, Switzerland: World Health Organization (WHO).
2. van der Werf MJ, de Vlas SJ, Brooker S, et al. Quantification of clinical morbidity associated with schistosome infection in sub-Saharan Africa. Acta Trop. 2003;86(2-3):125-39
3. van der Werf MJ, de Vlas SJ, Looman CW, Nagelkerke NJ, Habbema JD, Engels D. Associating community prevalence of Schistosoma mansoni infection with prevalence of signs and symptoms. Acta Trop. 2002;82(2):127-37

4. van der Werf MJ, de Vlas SJ. Diagnosis of urinary schistosomiasis: A novel approach to compare bladder pathology measured by ultrasound and three methods for hematuria detection. Am. J. Trop. Med. Hyg. 2004;82:98-106
5. Zhou, Xiao-Nong & Bergquist, Robert & Leonardo, Lydia & Olveda, Remigio. (2018). Schistosomiasis: The Disease and its Control.

Schizophrenia

Flowchart

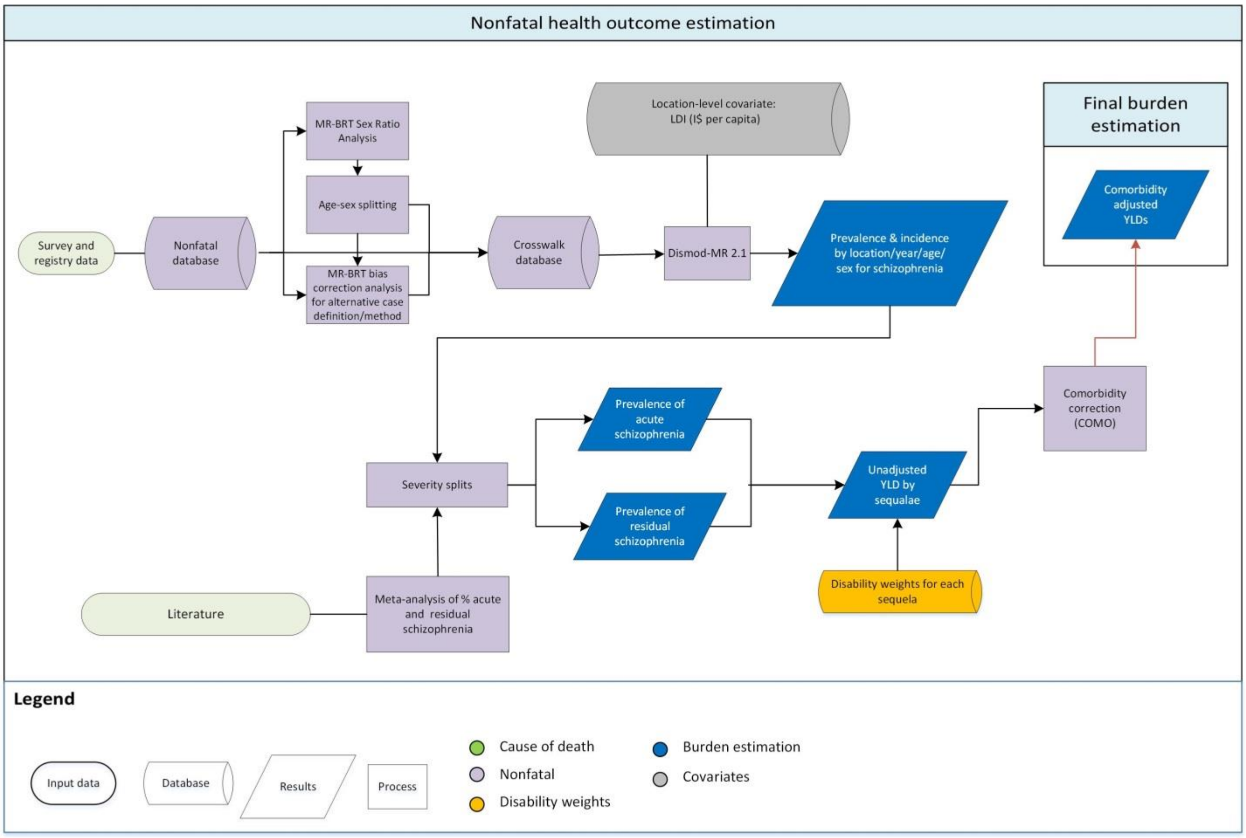

Input data and methodological summary for schizophrenia

Case definition

Schizophrenia is a chronic psychotic disorder which involves the experience of positive symptoms (eg, delusions, hallucinations, thought disorder) and negative symptoms (eg, flat affect, loss of interest, and emotional withdrawal). Included in the GBD disease modelling were cases meeting the Diagnostic and Statistical Manual of Mental Disorders (DSM) or the International Classification of Diseases (ICD) diagnostic criteria for schizophrenia.[1,2] These were identified by the following ICD-10 code: F20. Different versions of DSM (DSM-III, DSM-III-R, DSM-IV, DSM-IV-TR, DSM-5, and DSM-5-TR) and ICD (ICD-9, ICD-10, and ICD-11) were accepted. Diagnostic criteria are:

A. Two (or more) of the following, each present for a significant portion of time during a one-month period (or less if successfully treated): i) delusions, ii) hallucinations, iii) disorganised speech, iv) grossly disorganised or catatonic behaviour, v) negative symptoms

B. Social/occupational dysfunction

C. Continuous signs of the disturbance persist for at least 6 months

D. Exclusions must be met for schizoaffective disorder and mood disorders

E. The disturbance is not due to the direct physiological effects of a substance or a general medical condition

F. If there is a history of a pervasive development disorder, the diagnosis of schizophrenia is made if prominent delusions of hallucinations are also present for at least one month (or less if successfully treated)

Input data

The epidemiological systematic literature review for schizophrenia was conducted in three stages involving electronic searches of the peer-reviewed literature (ie, via PsycInfo, Embase, and PubMed), the grey literature, and expert consultation. For mental disorders, we update our GBD electronic database searches on a rolling basis. A systematic review update for schizophrenia was completed for GBD 2023. Databases were searched on October 30, 2023, for publications after January 1, 2017. Below are the search terms used for each database:

PubMed: ("Schizophrenia"[All Fields] OR "Schizophrenia"[MeSH Terms] OR "schizophre*"[Title/Abstract]) AND ("prevalen*"[Title/Abstract] OR "prevalence"[MeSH Terms] OR "inciden*"[Title/Abstract] OR "incidence"[MeSH Terms] OR "remit*"[Title/Abstract] OR "remission"[Title/Abstract] OR "recovery"[Title/Abstract] OR "recurrence"[MeSH Terms:noexp] OR "recurren*"[Title/Abstract] OR "duration"[Title/Abstract] OR "mortality"[Title/Abstract] OR "death*"[Title/Abstract] OR "mortality"[MeSH Terms] OR "epidemiolog*"[Title/Abstract] OR "Epidemiology"[MeSH Major Topic] OR "Epidemiology"[MeSH Terms:noexp] OR "Morbidity"[MeSH Terms:noexp]).

Embase: (schizophre*:ab,ti OR 'schizophrenia'/exp/mj OR schizophrenia) AND (prevalen*:ab,ti OR mortalit*:ab,ti OR death*:ab,ti OR inciden*:ab,ti OR remission:ab,ti OR recurren*:ab,ti OR duration:ab,ti OR remit*:ab,ti OR epidemiolog*:ab,ti OR 'incidence'/exp OR 'mortality'/exp OR 'morbidity'/mj OR 'remission'/exp OR 'prevalence'/exp OR 'epidemiology'/exp)

PsycInfo: (TX Schizophrenia OR TI Schizophre* OR AB Schizophre* OR (DE "Schizophrenia") OR (MM "Schizophrenia")) AND ((TI prevalen* OR AB prevalen*) OR (TI mortalit* OR AB mortalit*) OR (TI death* OR AB death*) OR (TI inciden* OR AB inciden*) OR (TI recurren* OR AB recurren*) OR (TI remission OR AB remission) OR (TI duration OR AB duration) OR (TI remit* OR AB remit*) OR (TI epidemiolog* OR AB epidemiolog*) OR (DE "Epidemiology") OR (DE "Morbidity") OR (DE "Mortality Rate") OR (DE "Remission (Disorders)")

In addition to the database search, a grey literature search and expert consultation were conducted. The systematic review update was conducted according to Preferred Reporting Items for Systematic Reviews and Meta-Analyses (PRISMA; see Figure 1).

Figure 1: PRISMA 2009 flow diagram

From: Moher D, Liberati A, Tetzlaff J, Altman DG, The PRISMA Group (2009). *P*referred *R*eporting *I*tems for *S*ystematic Reviews and *M*eta-*A*nalyses: The PRISMA Statement. PLoS Med 6(7): e1000097. doi:10.1371/journal.pmed1000097

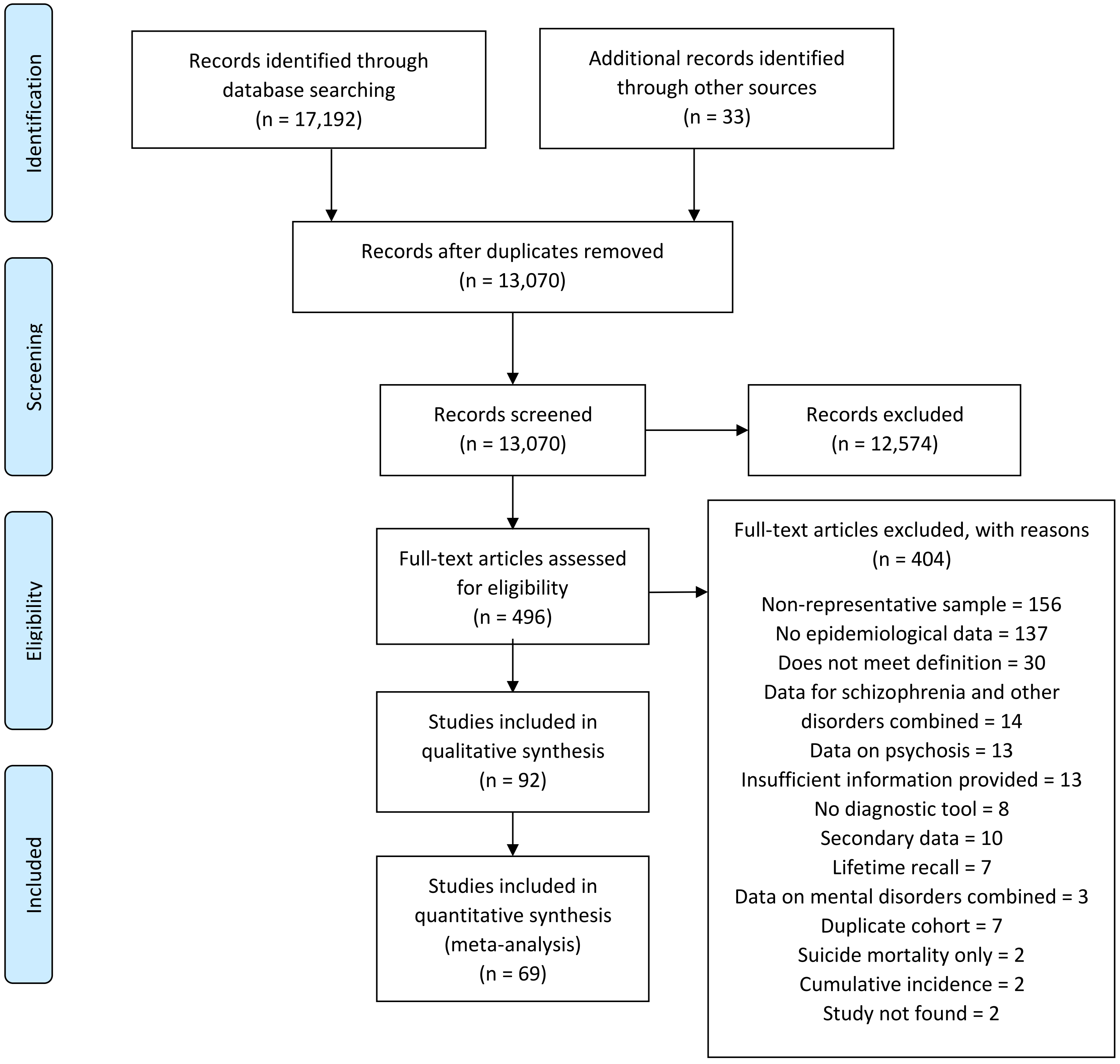

**The qualitative analysis led to the exclusion of additional studies with duplicative cohorts or methodological limitations impacting their eligibility and increasing measurement error within the data.*

The GBD inclusion criteria stipulated that: 1) the publication year must be from 1980 onward; 2) “caseness” must be based on clinical threshold as established by the DSM or ICD; 3) sufficient information must be provided on study method and sample characteristics to assess the quality of the study; and 4) study samples must be representative of the general population (ie, inpatient or pharmacological treatment samples, case studies, veterans, or refugee samples were excluded). No limitation was set on the language of publication. Methods used for this systematic review have been reported in greater detail elsewhere.[3]

Age-sex splitting

The extracted data, where possible, underwent three types of age-sex splitting processes:

1. Estimates were further split by sex and age based on the available data. For instance, if studies reported prevalence for broad age groups by sex (eg, prevalence in 15–65-year-old males and females separately), and also by specific age groups but for both sexes combined (eg, prevalence in 15–30-year-olds, then in 31–65-year-olds, for males and females combined); age-specific estimates were split by sex using the reported sex ratio and bounds of uncertainty.

2. A meta-regression—Bayesian, regularised, trimmed (MR-BRT) analysis was used to split the remaining both-sex estimates in the dataset. For each parameter, sex-specific estimates were matched by location, age, and year. A MR-BRT regression analysis was then used to estimate pooled sex ratios and bounds of uncertainty. These were then used to split the both-sex estimates in the dataset. The male-to-female ratio for prevalence, remission, and incidence was estimated to be 1.24 (95% uncertainty interval [UI] 1.22–1.26), 0.91 (0.36–2.30), and 1.56 (1.38–1.77), respectively. The male-to-female ratio for standardised mortality ratio, relative risk of mortality, with-condition mortality rate, and excess mortality rate were found to be 0.84 (0.83–0.84), 1.16 (1.06–1.27), 1.31 (1.26–1.36), and 1.22 (1.16–1.28), respectively.

Bias corrections/crosswalks

Estimates with known biases were adjusted/crosswalked accordingly prior to DisMod-MR 2.1. For each crosswalk of interest, parts of the reference and the alternative estimates were matched by age, sex, location, and year. This was done for both within-study (where possible) and between-study pairs. These pairs were then used as inputs in a MR-BRT network meta-analysis. The MR-BRT analysis produced a pooled adjustment factor between the reference estimates and alternative estimates, which was used to adjust all alternative estimates in the dataset. Reference data informing the prevalence of schizophrenia consisted of estimates reporting past-month/point or past-year prevalence of schizophrenia only. Four adjustment ratios were used for alternative data:

1. A lifetime correction adjusted all schizophrenia datapoints derived from lifetime prevalence towards the level they would have been if the study had captured past year prevalence. Lifetime estimates were included as recall would not be a significant source of bias in a chronic and severe disorder such as schizophrenia.
2. A point recall schizophrenia broad definition correction adjusted prevalence estimates derived for schizophrenia spectrum disorders, which includes disorders such as schizoaffective, schizophreniform, and delusional disorder, using a point recall, towards the level they would have been if the study had captured past-year prevalence of schizophrenia only.
3. A one-year recall schizophrenia broad definition correction adjusted prevalence estimates derived for schizophrenia spectrum disorders, which includes disorders such as schizoaffective, schizophreniform, and delusional disorder, using a one-year recall, towards the level they would have been if the study had captured past year prevalence of schizophrenia only.
4. A lifetime schizophrenia broad definition correction adjusted prevalence estimates derived for schizophrenia spectrum disorders, which includes disorders such as schizoaffective,

schizophreniform, and delusional disorder, using a lifetime recall, towards the level they would have been if the study had captured past-year prevalence of schizophrenia only.

See Table 1 for adjustment factors using for schizophrenia. The estimated uncertainty intervals (UIs) around the adjustment factor incorporate gamma, which represents the between-study variance across all input data in the model. This added uncertainty widens the UIs for crosswalks with significant fixed effects.

Table 1: MR-BRT crosswalk adjustment factors for dysthymia

**Data input**	**Reference or alternative case definition**	**Gamma**	**Beta coefficient, log (95% UI)***	**Adjustment factor****
Population survey	Reference: past-year schizophrenia prevalence	0.035		
Population survey	Alternative: lifetime schizophrenia prevalence		0.28 (0.18 to 0.39)	1.33 (1.20–1.47)
Population survey	Alternative: point schizophrenia spectrum prevalence		0.11 (–0.02 to 0.25)	1.12 (0.98–1.29)
Population survey	Alternative: past-year schizophrenia spectrum prevalence		0.35 (0.23 to 0.47)	1.42 (1.26–1.59)
Population survey	Alternative: lifetime schizophrenia spectrum prevalence		0.60 (0.46 to 0.74)	1.82 (1.59–2.10)

**MR-BRT crosswalk adjustments can be interpreted as the factor the alternative case definition is adjusted by to reflect what it would have been had it been measured using the reference case definition. If the log/logit beta coefficient is negative, then the alternative is adjusted up to the reference. If the log/logit beta coefficient is positive, then the alternative is adjusted down to the reference.*

***The adjustment factor column is the exponentiated beta coefficient. For log beta coefficients, this is the relative rate between the two case definitions. For logit beta coefficients, this is the relative odds between the two case definitions.*

Modelling strategy

After the above data processes were applied, DisMod-MR 2.1 was used to model the epidemiological data for schizophrenia. Adjustments to model priors or the dataset were made where appropriate. Where outliers were identified in the data, we reassessed the study's methodology and quality before a decision was made to exclude or include the data.

Data across all epidemiological parameters were included in the modelling process. We assumed no incidence and prevalence before age 10 and after age 80 and similarly, no excess-mortality before age 10. This minimum age of onset was corroborated with expert feedback and existing literature on schizophrenia. Remission was also restricted to a maximum of 0.04 as guided by data available in the dataset.

Severity splits and disability weights

The GBD disability weight survey assessments include lay descriptions of sequelae highlighting major functional consequences and symptoms. The lay descriptions and disability weights for schizophrenia severity levels are shown in Table 2. Severity splits used in GBD 2023 were consistent with those used in GBD 2021 for schizophrenia. Information on the distribution of acute and residual states of schizophrenia was obtained from a separate systematic review of the literature.[4] Meta-XL (a Microsoft Excel add-in for meta-analysis) was used to pool estimates across all studies to calculate the overall proportion of schizophrenia cases in each health state. The proportion of schizophrenia cases in each health state were as follows: acute 63% (29–91), and residual 37% (9–71).

Table 2. Severity distribution, details on the severity levels for schizophrenia and the associated disability weight with that severity

Severity level	Lay description	Disability weight (95% UI)
Acute state	Hears and sees things that are not real and is afraid, confused, and sometimes violent. The person has great difficulty with communication and daily activities, and sometimes wants to harm or kill himself (or herself).	0.778 (0.606–0.9)
Residual state	Hears and sees things that are not real and has trouble communicating. The person can be forgetful, has difficulty with daily activities, and thinks about hurting himself (or herself).	0.588 (0.411–0.754)

Location-level covariates were used to inform the estimation of prevalence in locations with no available data. For schizophrenia, one location-level covariate, lag distributed income (LDI), was used. This covariate represents a moving average of gross domestic product (GDP) over time. LDI was applied to excess mortality data with a negative relationship assumed. Table 3 below illustrates the covariate, parameter, beta, and exponentiated beta values for the model.

Table 3. Summary of covariates used in the schizophrenia DisMod-MR meta-regression model

Covariate	Type	Parameter	Exponentiated beta (95% UI)
LDI	Location-level	Excess mortality rate	0.89 (0.87–0.90)

There were no substantial changes in GBD 2023 results for schizophrenia compared to GBD 2021. While we continue to improve on the data and methods used to estimate the burden of mental disorders, some challenges need to be acknowledged. Firstly, we still have a large number of locations with no high-quality raw data available. Secondly, it is difficult to quantify and remove all variation due to measurement error in our prevalence estimates. While we have improved the methodology used to account for known sources of bias (eg, survey methods or case definitions), we still have very few datapoints to inform such adjustments. Thirdly, there is a paucity of research on the risk factors of mental disorders which can be used as predictive covariates in our epidemiological models.

References

1. American Psychiatric Association. Diagnostic and Statistical Manual of Mental Disorders (DSM-IV-TR). Fourth Edition, Text Revision ed. Washington DC: American Psychiatric Association; 2000.
2. World Health Organization. The ICD-10 Classification of Mental and Behavioural Disorders. Clinical descriptions and diagnostic guidelines. Geneva: World Health Organization; 1992.
3. Charlson FJ, Ferrari AJ, Santomauro DF, et al. Global Epidemiology and Burden of Schizophrenia: Findings From the Global Burden of Disease Study 2016. *Schizophr Bull* 2018; **44**(6): 1195-203.
4. Ferrari AJ, Saha S, McGrath JJ, et al. Health states for schizophrenia and bipolar disorder within the Global Burden of Disease 2010 Study. *Population health metrics* 2012; **10**(1): 16.

Sexual violence

Flowchart

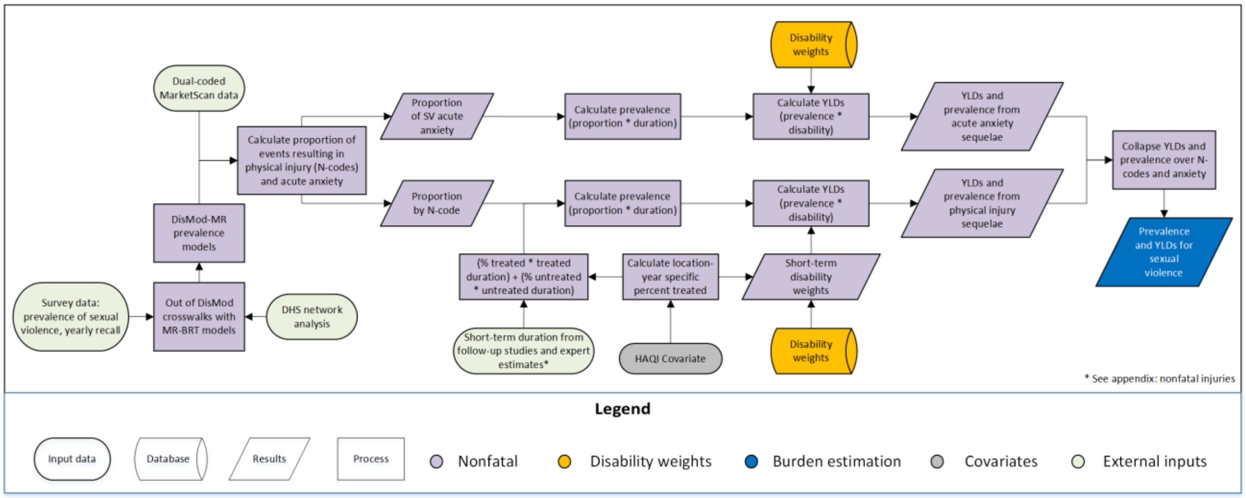

Input data and methodological summary

Definition

For the sexual violence cause, we estimate the yearly prevalence of sexual violence (ie, the proportion of the population that experienced at least one event of sexual violence in the last year). We define sexual violence as any sexual assault, including both penetrative sexual violence (rape) and non-penetrative sexual violence (other forms of unwanted sexual touching).

Input data

Exposure

The majority of the data for sexual violence come from various health and demographic surveys. We include many Demographic and Health Surveys (DHS) and Reproductive Health Surveys (RHS). Other survey series include the USA Behavioral Risk Factor Surveillance Survey (BRFSS) and the British Crime Surveys. **Table 1** contains information about our input data for the sexual violence modelling process. **Table 2** provides more information about data coverage in the seven Global Burden of Disease super-regions.

Table 1: Data inputs for sexual violence morbidity modelling by parameter

Measure	Total sources	Countries with data
Prevalence	121	96

Many other non-survey data sources exist for sexual violence. For example, we explored the use of the United Nations Office on Drugs and Crime (UNODC) Statistics [1]. However, these estimates are based only on police reports, and their incidence is about 20 times lower than the incidence seen in the same location-years from survey data. Although we could include a covariate in our models to adjust for this underreporting, we deemed the source unusable because of the magnitude of the difference between

the police reports and survey data. Survey data typically range between 1% and 10% of individuals experiencing sexual violence in the last year. Figure 1 shows the incidence estimates from the UNODC data, where most of the estimates are below about 0.05%. The geographical pattern is the opposite of what we see in survey data, with higher-income countries having higher estimates in the UNODC data. Additionally, the reports were not age-sex-specific, and the definition for what constitutes sexual violence varies across countries.

Figure 1: United Nations Office of Drugs and Crime Statistics: estimates of sexual violence (incidence per person), coloured by Global Burden of Disease super-regions

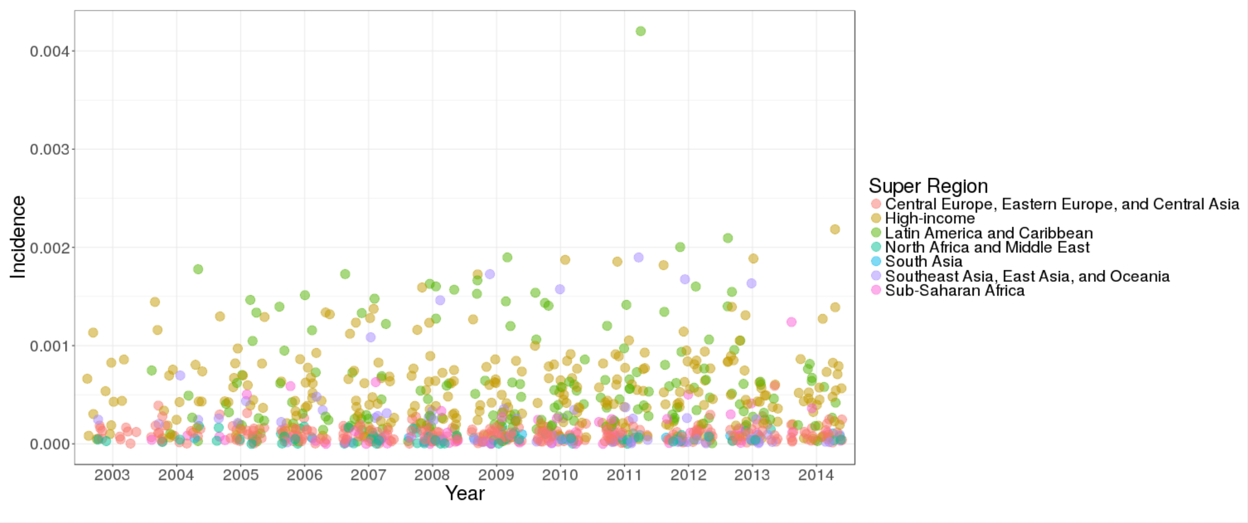

We also chose not to include the Centers for Disease Control non-fatal injury reports of sexual violence. Although this data source includes age- and sex-specific estimates for sexual violence in the USA, only sexual violence cases which resulted in physical injury are reported. These estimates are also systematically lower than the survey data, to the degree at which any adjustment with covariates would be unreliable. Lastly, we excluded a source from the USA Federal Bureau of Investigation: the Uniform Crime Reporting (UCR) program. The FBI estimates are produced at the state level for the United States and are meant to be comparable across states. However, police report data for sexual violence are systematically lower, similar in magnitude to the UNODC data, so we chose to exclude them.

Data searches

No new data searches were run for GBD 2023.

Modelling strategy

Prevalence of sexual violence

To produce estimates of the yearly prevalence of sexual violence, we used the Bayesian meta-regression method DisMod-MR 2.1 *(DisMod-MR 2.1 estimation is described in detail in a separate section of this appendix)*. To preserve variation between male- and female-specific estimates, we have separate models for men and women. We make various assumptions within DisMod-MR 2.1, including no excess mortality due to sexual violence, given that sexual violence is not a cause of death in the GBD, and we age restrict the model between 0–2 and 98–100.

Adjusting data

Because of the different ways that questions about sexual violence in the last year can be asked, we include multiple study-level covariates (for coefficient estimates, see Table 3). We bounded the covariates at logical values to minimise the effect of collinearity between the covariates, ie, we expect studies that ask about penetrative sexual violence only to have lower estimates of sexual violence overall, so that covariate has an upper bound of 1. Using these study-level covariates, we can extract data that do not meet our case definition and adjust the data accordingly. We performed a network analysis on Demographic Health Survey data to obtain within-study covariate comparisons and used coefficients output by the modelling tool meta-regression—Bayesian, regularised, trimmed (MR-BRT) to make necessary adjustments *(MR-BRT is described in detail in a separate section of this appendix)*.

Table 2: Covariates. Study-level covariates for DisMod-MR 2.1 yearly recall prevalence models for sexual violence

Covariate	Covariate bounds	Exponentiated beta (95% uncertainty interval)
Physically forced sexual violence only	Upper: 1	1.03 (1.00–1.05)
Ever-partnered people only	None	1.04 (1.02–1.05)
Ever-married people only	None	0.95 (0.92–0.97)
Ever had sex	None	0.96 (0.95–0.96)
Penetrative sexual violence only	Upper: 1	0.71 (0.69–0.73)
Only includes partner sexual violence	Upper: 1	0.93 (0.92–0.93)

Years lived with disability (YLDs) due to sexual violence

To calculate the years lived with disability (YLDs) due to having experienced sexual violence in the past year, we utilised claims data from the USA from the years 2000 and 2010–2017 to assess sexual violence sequelae. We searched through the claims database for the following ICD-9 diagnosis codes: 995.53 (child sexual abuse), 995.83 (adult sexual abuse), and E960.1 (rape) for claims before October 1, 2015. After October 2015, the following ICD-10 codes were queried: T74.2, T74.5, T76.2, T76.5. We considered sequelae relating to both physical injuries and mental health consequences in the short term.

In this process of calculating of YLDs due to sexual violence, we currently measure only the short-term physical and psychological effects of sexual violence. The long-term consequences of sexual violence as a risk factor are shown for two risk factors: sexual violence against children and intimate partner violence. The long-term consequences of non-partner sexual violence during adulthood are not currently captured in the risk factor section of the GBD study.

Physical injury

For the physical injury sequelae, we looked for any nature-of-injury ICD-9 or ICD-10 code on the same date of contact with medical service providers for a sexual violence code (above) and categorised the nature-of-injury codes as we do for the general injuries non-fatal modelling process (see appendix: non-fatal injuries). We calculate the proportion of individuals with any sexual violence code that results in each of the physical injuries categories. This strategy is similar to the strategy that we use for the cause-nature of injury matrices in the general injuries modelling process, but we have an additional category for no physical injury result as the majority of sexual violence incidents do not result in physical injury in the claims database. Additionally, because we only have one data source, we do not model these proportions with Dirichlet regression like we do for the injuries cause-nature of injury matrices but just compute them directly from the claims data. To estimate the physical injuries component of YLDs, we multiply the DisMod-MR 2.1 estimates of yearly prevalence of sexual violence by these proportions and then multiply by each physical injury's respective short-term duration and disability weight that we use in the general injuries process (see appendix: non-fatal injuries).

Acute anxiety and/or reaction to stress

For the mental and psychological sequelae of sexual violence, we searched an individual being coded to any of the following diagnosis codes at any point *after* a sexual violence incident was noted. The codes are meant to reflect conditions relating to an "acute anxiety and/or reaction to stress" condition following a traumatic incident, displayed in Table 4.

Table 3: ICD diagnosis codes included in the "acute anxiety and/or reaction to stress" condition as a sequela for sexual violence

ICD-9 code	Condition description
308	Acute reaction to stress
308	Predominant disturbance of emotions
308.1	Predominant disturbance of consciousness
308.2	Predominant psychomotor disturbance
308.3	Other acute reactions to stress
308.4	Mixed disorders as reaction to stress
308.9	Unspecified acute reaction to stress
309	Adjustment reaction
309	Adjustment disorder with depressed mood
309.1	Prolonged depressive reaction
309.2	Adjustment reaction with predominant disturbance of other emotions
309.21	Separation anxiety disorder
309.22	Emancipation disorder of adolescence and early adult life
309.23	Specific academic or work inhibition
309.24	Adjustment disorder with anxiety
309.28	Adjustment disorder with mixed anxiety and depressed mood
309.29	Other adjustment reactions with predominant disturbance of other emotions
309.3	Adjustment disorder with disturbance of conduct
309.4	Adjustment disorder with mixed disturbance of emotions and conduct
309.8	Other specified adjustment reactions

309.81	Posttraumatic stress disorder
309.82	Adjustment reaction with physical symptoms
309.83	Adjustment reaction with withdrawal
309.89	Other specified adjustment reactions
309.9	Unspecified adjustment reaction
F41	Other anxiety disorders
F41.0	Panic disorder [episodic paroxysmal anxiety] without agoraphobia
F41.1	Generalised anxiety disorder
F41.2	Mixed anxiety and depressive disorder
F41.3	Other mixed anxiety disorders
F41.8	Other specified anxiety disorders
F41.9	Anxiety disorder unspecified

It is possible that the appearance of one of these ICD codes is entirely unrelated to the sexual violence incident. Additionally, the appearance of one of these codes could be related instead to underlying depression and anxiety. To control for these confounding factors, we also searched for these ICD codes among individuals that were not victims of sexual violence in the past year. We used Poisson regression with robust standard errors to model the relative risk of the "acute anxiety and/or reaction to stress" comparing individuals with and without sexual violence within the year, controlling for underlying diagnoses of depression and anxiety:

$$\log(\lambda) = \beta_0 + \beta_1(sexual\ violence) + \beta_2(depression) + \beta_3(anxiety) + \beta_4(female) + \beta_5(age)$$

where λ is the risk of "acute anxiety and/or reaction to stress," and e^{β_1} is the relative risk of "acute anxiety and/or reaction to stress" comparing those experiencing at least one sexual violence incident to those with no sexual violence incidence, holding underlying depression, anxiety, sex, and age constant. We can approximate the risk of "acute anxiety and/or reaction to stress" for each age and sex experiencing sexual violence by:

$$\lambda_{age,sex} = e^{\beta_1} * (e^{\beta_0} * e^{sex*\beta_4+age*\beta_5}) - (e^{\beta_0} * e^{sex*\beta_4+age*\beta_5})$$

Using the equation above, the transformed coefficients and transformed robust standard errors (transformations were performed with the Delta method) are shown in **Table 4**.

Table 4: Estimates of the risk of "acute anxiety and/or reaction to stress" ($\boldsymbol{\lambda_{age,sex}}$) among people experiencing sexual violence over a year time period, specific to age and sex

Age	Male		Female	
	Estimate	*Standard error*	*Estimate*	*Standard error*
0-4	0.0967	0.0023	0.1205	0.0028
5-9	0.0933	0.0021	0.1162	0.0027
10-14	0.0899	0.0021	0.1120	0.0026
15-19	0.0867	0.0020	0.1080	0.0025
20-24	0.0836	0.0020	0.1042	0.0024

25-29	0.0806	0.0019	0.1004	0.0024
30-34	0.0777	0.0018	0.0968	0.0023
35-39	0.0749	0.0018	0.0934	0.0022
40-44	0.0722	0.0017	0.0900	0.0021
45-49	0.0697	0.0016	0.0868	0.0020
50-54	0.0672	0.0016	0.0837	0.0020
55-59	0.0648	0.0015	0.0807	0.0019
60-64	0.0624	0.0015	0.0778	0.0018
65-69	0.0602	0.0014	0.0750	0.0018
70-74	0.0581	0.0014	0.0723	0.0017
75-79	0.0560	0.0013	0.0697	0.0016
80-84	0.0540	0.0013	0.0672	0.0016
85-89	0.0520	0.0012	0.0648	0.0015
90-94	0.0502	0.0012	0.0625	0.0015
95-99	0.0484	0.0011	0.0603	0.0014

We multiplied the prevalence of yearly sexual violence by $\lambda_{age,sex}$ to get the prevalence of "acute anxiety and/or reaction to stress" due exclusively to sexual violence. To estimate YLDs for this sexual violence sequela, we used the average of the disability weights for mild depression and anxiety. For simplicity, we assume a duration of one year; thus, the YLDs for the mental and psychological stress component of sexual violence is the product of the residual probability of "acute anxiety and/or reaction to stress" and the disability weight.

The long-term disability from sexual violence is captured in the risk factors component of the GBD study, under sexual violence against children and intimate partner violence.

References

[1] United Nations Office on Drugs and Crime (UNODC). United Nations Office on Drugs and Crime Global Study on Homicide. Vienna, Austria: United Nations Office on Drugs and Crime (UNODC).

Sexually transmitted infections excluding HIV

Syphilis: adult syphilis seroprevalence, adult tertiary syphilis, congenital syphilis

Flowcharts

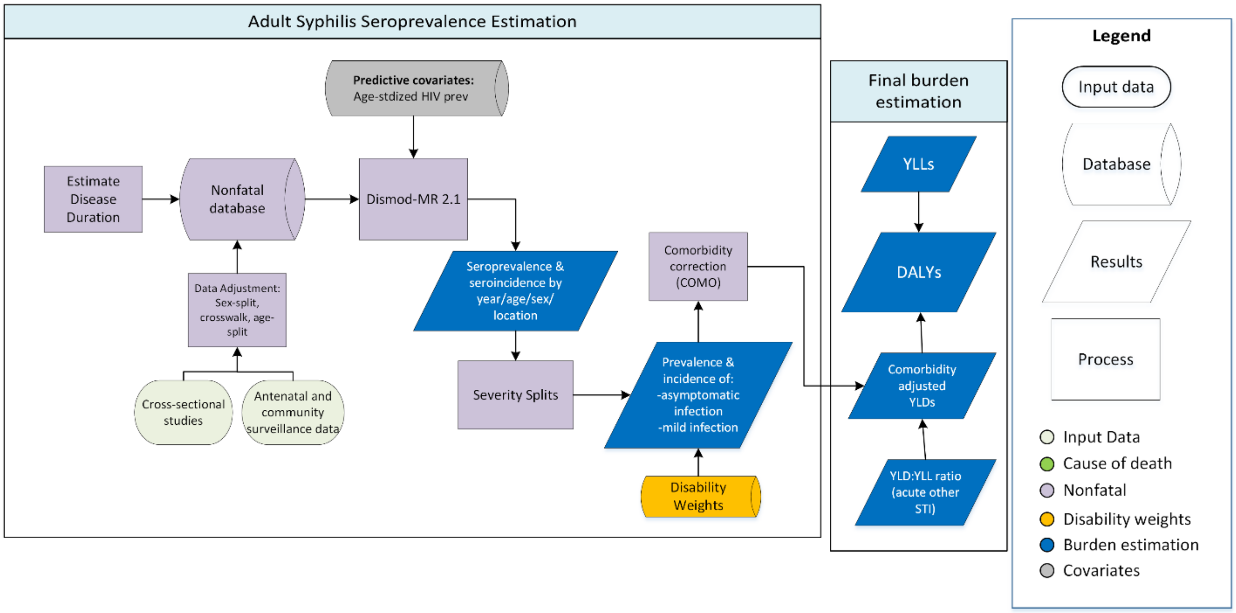

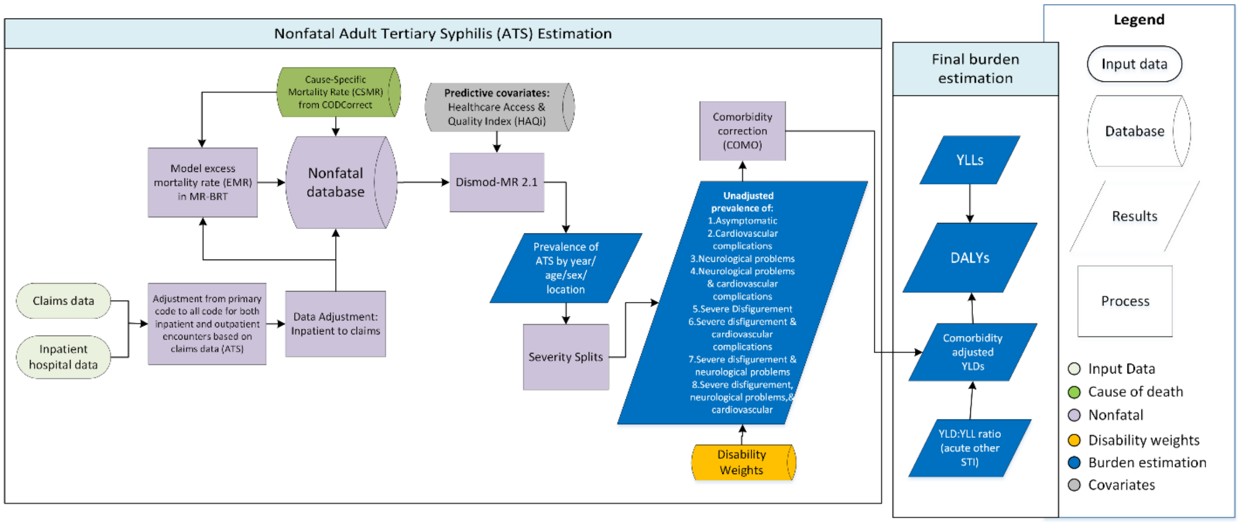

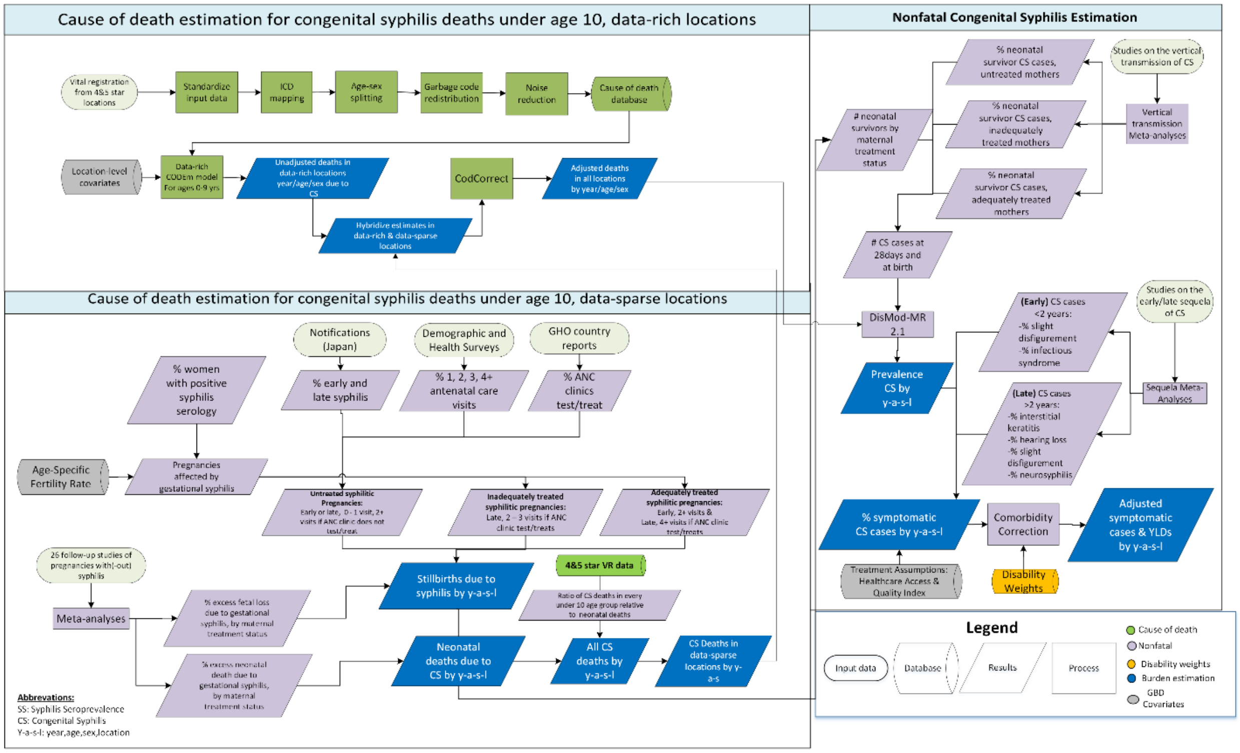

Input data and methodological summary for syphilis

Case definition

Syphilis is an infection with the *Treponema pallidum* bacterium usually spread by sexual contact or from a pregnant person to offspring; we account here for acute and chronic infection, with or without symptoms, and sequelae of congenital cases that persist after treatment.

Adult syphilis was estimated in two separate models, an adult seroprevalence model, from which we estimated the occurrence of early (primary, secondary, and early latent) sexually acquired syphilis, and a separate model of adult tertiary syphilis. The adult seroprevalence model also served as a covariate in other estimation processes in GBD; see separate appendix sections on estimation of fatal burden of STI for details. In GBD 2021, we estimated the non-fatal burden of congenital syphilis for the first time. Case definitions for early syphilis and congenital syphilis were based on laboratory findings (see below for details), while tertiary syphilis is ascertained from administrative data using ICD-9 (093–095) and ICD-10 (A52 and I98.0).

Quantity of interest	Reference or alternative	Definition
Early syphilis	Reference	Infection due to *Treponema pallidum* at the primary, secondary, or latent stage, diagnosed by the combination of a treponemal serologic test, such as *T pallidum* particle (haem)aglutination assay (TPHA), and a nontreponemal serologic test, such as rapid plasma reagin (RPR) test and/or Venereal

		Disease Research Laboratory (VDRL), in a sample of representative of the population in a cross-sectional study design.
Early syphilis	Alternative	Infection with *Treponema pallidum* at the primary, secondary, or latent stage confirmed only by a treponemal serologic test, such as *T pallidum* particle (haem)aglutination assay, in a sample representative of the population in a cross-sectional study design.
Early syphilis	Alternative	Infection with *Treponema pallidum* at the primary, secondary, or latent stage confirmed only by a nontreponemal serologic test, such as rapid plasma reagin (RPR) test and/or Venereal Disease Research Laboratory (VDRL), in a sample representative of the population in a cross-sectional study design.
Adult tertiary syphilis	Reference	Cases of *Treponema pallidum* infection at the tertiary stage identified using ICD codes in administrative records that are considered population-representative.
Adult tertiary syphilis	Alternate	Cases identified from database of commercial claims from USA using ICD codes.
Congenital syphilis	Reference	Cases of *Treponema pallidum* infection transmitted by a mother to a fetus, either during pregnancy or childbirth. This includes both liveborn infants who die in the first 28 days of life due to exposure to gestational syphilis, as estimated via CODEm or natural history methods, as well as exposed 28-day survivors who meet the case definition of follow-up of an IgG finding at birth, subsequently confirmed by IgM positivity, direct detection of treponemes, quantitative titers 4x higher than mother's, persistent antibody response at 18 months, or specific radiographic or physical exam findings. Cases of CS that were based on maternal treatment status only were excluded.

For early syphilis, these case definitions were variably employed for different subpopulations as described below.

Input data

A systematic literature review for adult syphilis seroprevalence was completed on April 17, 2015, during GBD 2015. From the review, we identified data on the seroprevalence of syphilis in populations aged 10 years and older for extraction. Our inclusion criteria were syphilis seroprevalence diagnosed with a treponemal and/or non-treponemal diagnostic test among the general population, or among sub-populations for which bias adjustments to the general population could be made. We excluded self-reported data. We also excluded data in high-risk populations for which there are not enough data currently to make a bias adjustment.

> **1265 initial hits; 178 sources selected from full text review for data extraction:**
> ("syphilis"[MeSH] OR "Treponema pallidum"[MeSH]) NOT "Yaws"[MeSH] AND "prevalence"[MeSH] AND "1990"[PDAT] : "2015"[PDAT] AND "humans"[MeSH] **///** ("syphilis"[MeSH] OR "Treponema pallidum"[Mesh]) NOT "Yaws"[MeSH] AND ("incidence"[MeSH]) AND ("1990"[PDAT] : "2015"[PDAT]) AND "humans"[MeSH])

For the adult seroprevalence model, we supplemented our datasets with antenatal clinic surveillance reports, data from the GBD Collaborator Network. Case-notification data were identified and extracted as incidence datapoints from locations where centralised reporting is mandatory, but were not employed in modelling. Additionally, in the congenital syphilis systematic review, described below,

studies including data on syphilis seroprevalence among pregnant women were added to the adult seroprevalence model if identified.

Adult tertiary syphilis is defined by clinical syndrome rather than acquisition of an infectious agent and is modelled using prevalence data from claims and hospital discharges as prepared by the GBD Clinical Informatics team. We employed data processing methods to capture cases that were diagnosed or treated in both inpatient and outpatient settings. Specifically, an individual was extracted from claims data as a prevalent case if that individual had at least one inpatient or outpatient encounter with an appropriate ICD code as any diagnosis within one year. Hospital discharge data were processed by extracting discharges with an appropriate ICD code as primary diagnosis and adjusted using correction factors derived from claims data. Specifically, the IHME Clinical Informatics team modelled the ratio of inpatient claims as primary diagnosis to total incident cases seen in claims data. The method of estimating each correction factor was meta-regression—Bayesian, regularised, trimmed (MR-BRT) analysis, using three frequency-placed knots in the age-spline parameter.

Data for the adult tertiary syphilis model also included estimates of syphilis cause-specific mortality rate (CSMR) in ages 10 years and older, as well as estimates of excess mortality rate (EMR) due to syphilis modelled in MR-BRT. Please see the Cause of Death modelling methods “Adult sexually transmitted infections” section of the appendix for more information about the estimation of syphilis CSMR. Please see the adult tertiary syphilis (ATS) data processing section below for more information about the estimation of EMR.

A systematic literature review for congenital syphilis was completed on April 4, 2019, for GBD 2021. From the review, we identified data on the birth outcomes of pregnancies that are positive for syphilis for extraction: stillbirth, spontaneous abortion, preterm birth, low birthweight, neonatal death, vertical transmission of congenital syphilis, and infants not infected with syphilis. The review additionally identified data on some of the symptoms that infants with congenital syphilis exhibited in the short and long term.

> **1675 initial hits; 191 sources selected from full text review for data extraction:** (syphilis[tiab] OR "treponema pallidum"[tiab]) AND ((pregnan*[tiab] OR fetal[tiab] OR foetal[tiab] or fetus*[tiab] OR foetus*[tiab] OR neonat*[tiab] OR infan*[tiab] OR newborn*[tiab] OR congenital[tiab]) OR ((vertical*[tiab] OR maternal[tiab] OR mother[tiab] OR fetomaternal[tiab]) AND transmi*[tiab])) AND (outcomes[tiab] OR sequela*[tiab] OR manifestation*[tiab] OR morbidity*[tiab] OR diagnos*[tiab] OR hutchinson*[tiab])

For congenital syphilis, we supplemented our datasets with modelled estimates of CSMR, EMR, neonatal death counts, and the number of stillbirths from the fatal estimation of congenital syphilis. The methodology for utilising fatal estimates in non-fatal modelling is described later in this write-up. For information on data inputs and methodology for creating the fatal estimates, please see the causes of death modelling methods “Congenital syphilis” section of the appendix.

Adult early syphilis data processing

To sex-split data sources reported for both sexes combined, sources reporting for each sex separately were matched by age and location. Log ratios between seroprevalence in females and seroprevalence in males were put into meta-regression—Bayesian, regularised, trimmed (MR-BRT), a meta-analytic tool

developed for the Global Burden of Disease study. MR-BRT was used to estimate an adjustment factor to split both-sex datapoints into sex-specific datapoints. The values are specific to age and pooled across all geographies. The model utilised a spline on age with knots at ages 12, 30, 60, and 80 years.

Figure 1: Female to male ratios of syphilis prevalence

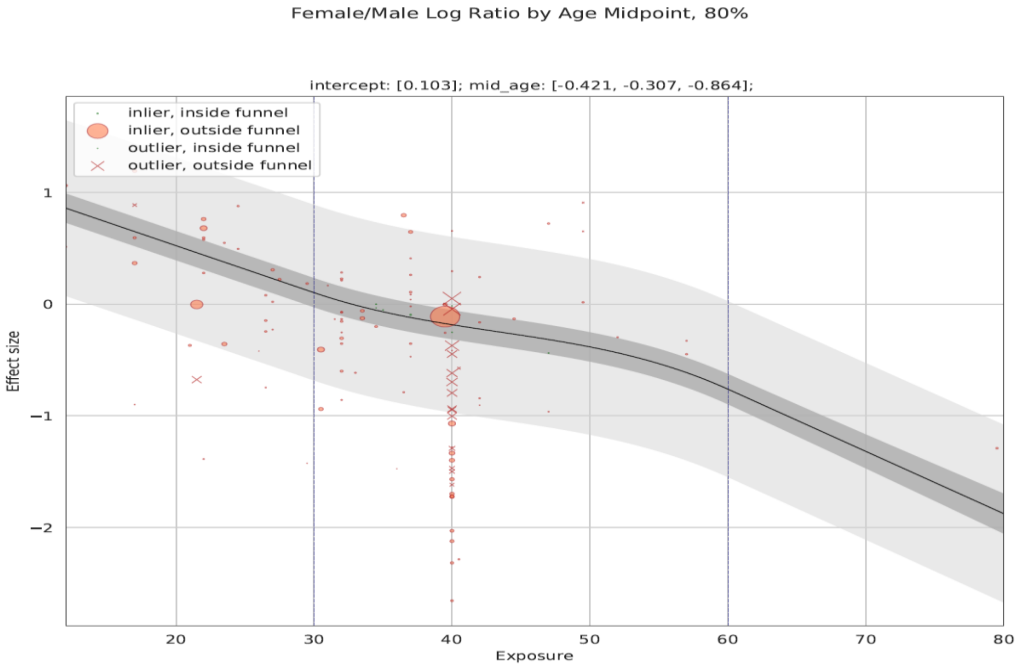

Figure 2: Pre and post comparison of prevalence sex-splitting by age group

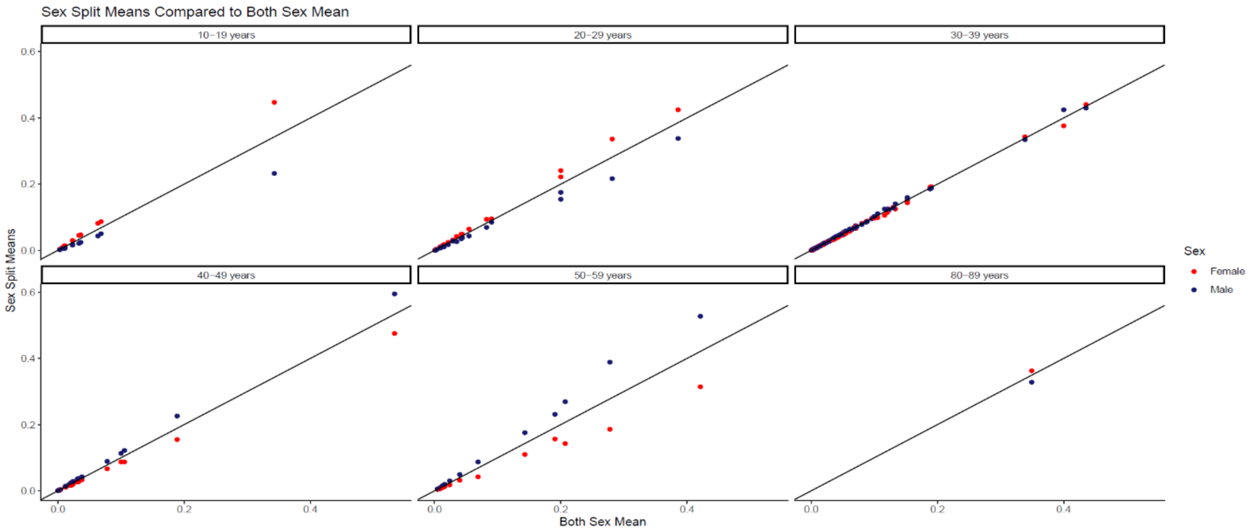

For syphilis seroprevalence, the reference case definition was initial and confirmatory diagnosis with both treponemal and non-treponemal serological tests. The alternative case definitions were diagnosis with only a treponemal test or diagnosis with only a non-treponemal test. To adjust data collected with alternative methods to the level of the reference case definition, we ran a meta-regression in MR-BRT. Data inputs for this model were log ratios between data collected with alternative case definitions and

data collected with the reference case definition estimated by matching sources by age, sex, and location to find comparisons. We also adjusted data collected from samples of blood donors to the seroprevalence expected in the general population by using similarly matched sources as inputs to MR-BRT.

Table 1: MR-BRT crosswalk adjustment factors for early syphilis seroprevalence

Data input	Reference or alternative case definition	Gamma	Beta coefficient, logit (95% UI)*	Adjustment factor**
Both treponemal and non-treponemal diagnostic tests	Ref	0.028	---	---
Treponemal diagnostic only	Alt		0.14 (–0.007, 0.29)	1.15 (0.99, 1.34)
Non-treponemal diagnostic only	Alt		0.30 (0.16, 0.46)	1.36 (1.17, 1.58)
General population	Ref		---	---
Blood donors	Alt		–0.31 (–0.92, 0.29)	0.73 (0.40, 1.34)

**MR-BRT crosswalk adjustments can be interpreted as the factor the alternative case definition is adjusted by to reflect what it would have been had it been measured using the reference case definition. If the log/logit beta coefficient is negative, then the alternative is adjusted up to the reference. If the log/logit beta coefficient is positive, then the alternative is adjusted down to the reference.*
***The adjustment factor column is the exponentiated beta coefficient. For log beta coefficients, this is the relative rate between the two case definitions. For logit beta coefficients, this is the relative odds between the two case definitions.*

Data on syphilis seroprevalence were excluded if the sample population was drawn exclusively from a high-risk group (eg, HIV-positive, men who have sex with men [MSM], or sex workers). For sources reported for age groups spanning more than 15 years, these datapoints were disaggregated by imposing an age pattern from the best GBD 2019 model.

Due to difficulty in reconciling differences between prevalence and incidence sources, likely due to under-reporting in surveillance data, incidence data were ignored for all adult STIs.

Remission inputs for syphilis seroprevalence were estimated from disease duration ranges calculated as follows: Duration ranges were calculated using a sum of the duration of untreated and treated disease, weighted by the stage of syphilis and the percentage of individuals in each stage that are symptomatic and the probability of receiving treatment if symptomatic with the formula below.

$$\begin{aligned}\textbf{Duration} \quad &= (\%\,Symptomatic)(Prob_{Rx})(Duration_{Rx}) \\ &+ (1 - \%\,Symptomatic)(Duration_{\,not\,Rx}) \\ &+ (\%\,Symptomatic)(1 - Prob_{Rx})\,(Duration_{\,not\,Rx})\end{aligned}$$

The durations and probabilities of symptoms used in this formula were taken from GBD 2000 and WHO 2005 and were largely expert-driven. The probability of treatment if symptomatic was modelled using the Healthcare Access and Quality (HAQ) Index to compute this probability for each location and year.

For syphilis seroprevalence, durations per disease stage (primary, secondary, latent, and tertiary) were calculated individually and summed along with the average seroreversion by stage, weighting by the proportion of cases remaining at each stage and including the time it would take to serorevert after adequate treatment.

Adult tertiary syphilis data processing – prevalence

For adult tertiary syphilis, claims data from the USA (MarketScan) were adjusted to inpatient hospital data prior to analysis in DisMod. A priori, we believed that MarketScan data reflected a certain level of selection bias due to commercial insurance, while hospital data and claims databases from other countries were more reflective of the general population. The adjustment factor was modelled in MR-BRT as a meta-regression of log-transformed ratios between USA claims data sources and USA inpatient data sources. The model utilised a spline with knots at ages 15, 42, 72, and 104 years. Ratios were formed between sources matched by age and location.

Table 2: MR-BRT crosswalk adjustment factors for adult tertiary syphilis

**Data input**	**Reference or alternative case definition**	**Spline knot (age)**	**Gamma**	**Beta coefficient, logit (95% CI)***	**Adjustment factor****
Inpatient data	Reference	--	0	---	---
USA claims (MarketScan)	Alternative	15 years		1.48 (1.36–1.60)	4.36 (3.89–4.95)
		42 years		0.54 (0.36–0.72)	1.72 (1.43–2.05)
		72 years		0.45 (0.19–0.70)	1.57 (1.21–2.01)
		104 years		0.23 (0.05–0.41)	1.26 (1.05–1.51)

**MR-BRT crosswalk adjustments can be interpreted as the factor the alternative case definition is adjusted by to reflect what it would have been had it been measured using the reference case definition. If the log/logit beta coefficient is negative, then the alternative is adjusted up to the reference. If the log/logit beta coefficient is positive, then the alternative is adjusted down to the reference.*

***The adjustment factor column is the exponentiated beta coefficient. For log beta coefficients, this is the relative rate between the two case definitions. For logit beta coefficients, this is the relative odds between the two case definitions.*

Figure 3: Prevalence ratios between claims and inpatient discharge data

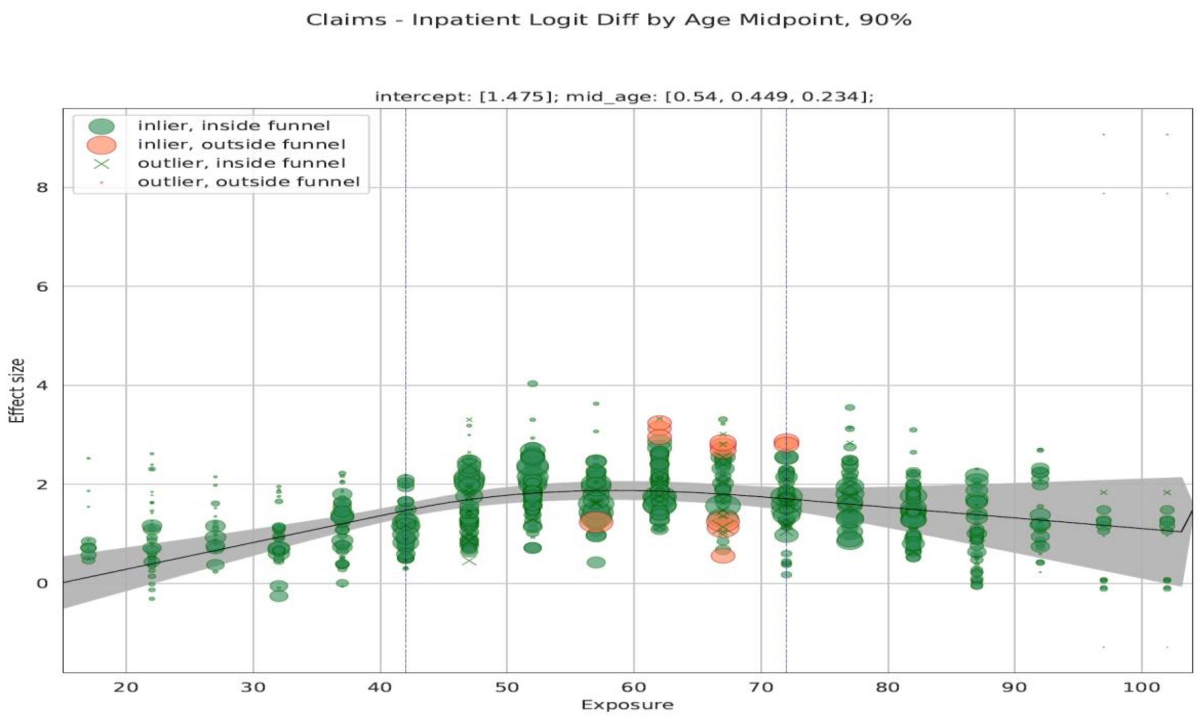

Figure 4: Pre and post comparison of prevalence data in adult tertiary syphilis in Arizona

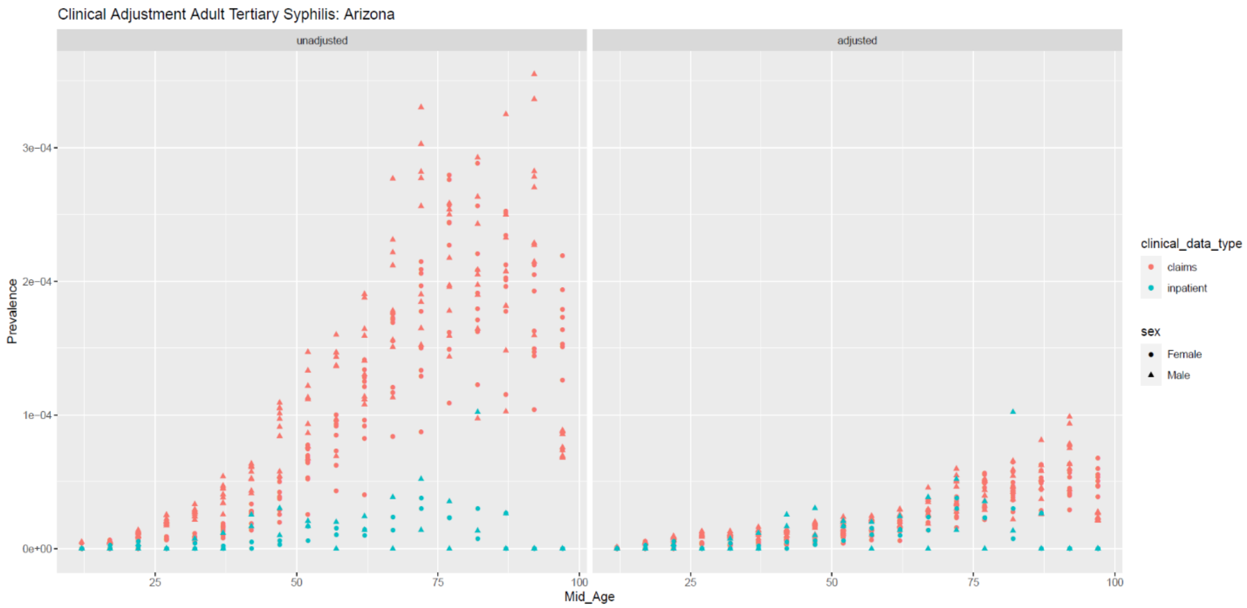

After adjustments were made, all datapoints with an age-standardised prevalence greater than one median absolute deviation from the median of the age-standardised prevalence were marked as outliers and excluded from analysis.

Adult tertiary syphilis data processing – EMR data processing

Prior to GBD 2019, EMR datapoints were created in DisMod-MR when the model matched prevalence datapoints to CSMR datapoints in the same year, age, sex, and location, and divided the CSMR value by the prevalence value. For many causes, including adult tertiary syphilis, this method of producing EMR inputs created an implausible geographical pattern, compared to the expected pattern of decreasing EMR with greater access to quality health care. Such unexpected patterns often signal inconsistencies between CSMR estimates and the measures of prevalence and/or incidence. To rectify this, the following method was applied beginning in GBD 2021. In an effort to provide greater guidance on the expected pattern of EMR, we generated EMR datapoints in the manner described above (by matching prevalence and CSMR datapoints). Then, those EMR datapoints were modelled by age, sex, and the HAQ Index in MR-BRT. The MR-BRT model included a prior on HAQ Index with a negative coefficient. This model was utilised to predict EMR for each year, sex, location, and ages 0, 10, 20….100. The predictions were then used as inputs to the non-fatal DisMod model.

Congenital syphilis data processing

We modelled non-fatal congenital syphilis (CS) in a natural history model. The natural history model was first utilised in the fatal estimation pipeline of CS and was leveraged for use in the non-fatal estimation pipeline of CS. It is explained briefly below, and a more detailed description is present in the fatal estimation appendix.

Briefly, this natural history model starts by estimating the number of pregnancies at risk of vertical transmission. Next, it incorporates data on access to comprehensive antenatal care and the disease stage of syphilis during pregnancy to estimate the number of pregnancies that are untreated, inadequately treated, or adequately treated. The model then incorporates estimates of excess stillbirth rate for pregnancies exposed to gestational syphilis to adjust the number of at-risk pregnancies to at-risk livebirths. Next, the model incorporates estimates of excess neonatal death rate among pregnancies exposed to gestational syphilis in order to adjust the number of at-risk livebirths to the number of at-risk 28-day survivors. The number of exposed 28-day survivors is distinct to each maternal treatment status.

Beginning in GBD 2021, we incorporated new estimates of the proportions of at-risk 28-day survivors who acquire congenital syphilis for infants born to mothers of each treatment status. These vertical transmission proportions – described in the paragraphs below – are applied to the number of exposed 28-day survivors to get the number of cases of congenital syphilis at 28 days of life. The CS cases at 28 days act as the numerator for estimating the 28-day prevalence of CS. The denominator is the number of 28-day infants in a given year, sex, and location. To estimate the number of 28-day infants, we started with the number of livebirths, converted the early and late all-cause neonatal death rates to counts, then subtracted the total number of all-cause neonatal deaths from the number of livebirths to get the number of infants at 28 days. We also estimated the birth prevalence of CS. The numerator is the number of 28-day CS cases increased by the number of neonatal deaths due to CS. The denominator is the number of livebirths in a given year, sex, location.

Estimation of the vertical transmission proportions of CS will now be described in further detail. The case definition of the vertical transmission of congenital syphilis is diagnosis with both positive immunoglobulin G (IgG) at birth and a specific confirmatory finding, which can include immunoglobulin M (IgM) positivity, direct detection of treponemes, quantitative titres 4x higher than mother, positive

result with *Treponema pallidum* particle agglutination (TPPA) at 18 months, or specific radiographic or physical exam findings. We excluded cases of CS based on maternal status only or cases of CS diagnosed without a confirmatory test.

From the included sources, we modelled the vertical transmission proportions in MR-BRT as a meta-regression of the logit-transformed proportions, with covariates on maternal treatment status. Our analysis found that of infants alive at 28 days and exposed to congenital syphilis, 17.5% of infants born to untreated mothers acquired the disease, 14.6% of infants born to inadequately treated mothers acquired the disease, and 3.7% of infants born to adequately treated mothers acquired the disease.

Figure 5: Forest plots of vertical transmission of congenital syphilis based on maternal treatment status

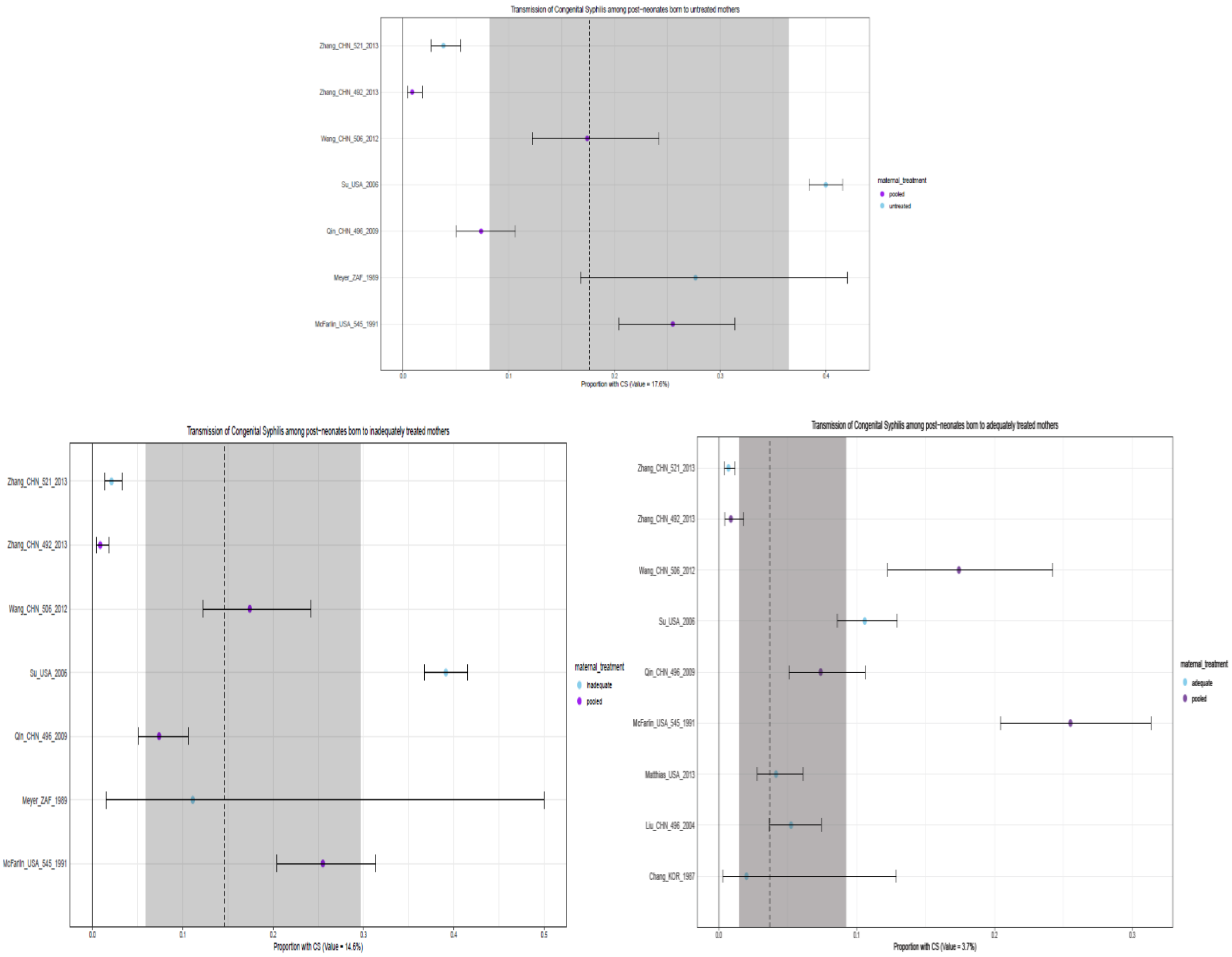

Table 3: MR-BRT vertical transmission proportions for congenital syphilis

Maternal treatment status	Reference or alternative case definition	Beta coefficient, logit (95% CI)*	Adjustment factor**
Untreated	Ref	–1.54 (–2.42 to –0.56)	0.175 (0.08–0.36)
Inadequately	Alt	–1.77 (–2.77 to –0.86)	0.146 (0.06–0.29)
Adequately	Alt	–3.26 (–4.24 to –2.33)	0.037 (0.01–0.09)

**MR-BRT crosswalk adjustments can be interpreted as the factor the alternative case definition is adjusted by to reflect what it would have been had it been measured using the reference case definition. If the log/logit beta*

coefficient is negative, then the alternative is adjusted up to the reference. If the log/logit beta coefficient is positive, then the alternative is adjusted down to the reference.
***The adjustment factor column is the exponentiated beta coefficient. For log beta coefficients, this is the relative rate between the two case definitions. For logit beta coefficients, this is the relative odds between the two case definitions.*

Pre-existing estimates of CSMR, EMR, and neonatal death counts of CS were utilised in conjunction with new estimates of the 28-day prevalence of CS and birth prevalence of CS for input to DisMod. Estimates were available for every year, sex, location, and for the specified age groups. Please see the causes of death modelling methods "Congenital syphilis" section of the appendix for more information on the CS mortality estimates.

Modelling strategy

Adult syphilis seroprevalence

There were no changes to the modeling strategy and settings between GBD 2021 and GBD 2023. The primary inputs to the adult seroprevalence model were seroprevalence data from cross-sectional studies and antenatal care (ANC) clinic reports, and modelled remission rates. Data using alternative case definitions were adjusted to the reference case definition as described above.

Incidence was restricted to occur only between ages 10 and 69. Remission was bounded 0-4 for all age groups. HIV age-standardised prevalence was included as a predictive covariate on prevalence.

Table 4. Covariates. Summary of covariates used in the adult syphilis seroprevalence DisMod-MR meta-regression model

Covariate	Parameter	Exponentiated beta (95% uncertainty interval)
HIV age-standardised prevalence	Prevalence	1.04 (1.00, 1.14)

Adult tertiary syphilis

There were no changes to the modelling strategy and settings between GBD 2021 and GBD 2023. Inputs for this model included prevalence data from hospital discharge and claims data, as described above, and CSMR estimates for syphilis from the GBD causes of death analysis. It also includes modelled EMR data, as described above. Incidence was restricted to not occur until age 15, and the maximum value of incidence for all other ages was set to 0.00001. Remission was set to zero. HAQ Index was included as a covariate on EMR with a mean and standard deviation produced from MR-BRT.

Table 5. Covariates. Summary of covariates used in the adult tertiary syphilis DisMod-MR meta-regression model

Covariate	Parameter	Exponentiated beta (95% uncertainty interval)
HAQ Index	Excess mortality rate	0.99 (0.99, 0.99)

Congenital syphilis

There were no changes to the modelling strategy and settings between GBD 2021 and GBD 2023. Inputs for this model included modelled estimates of the prevalence of congenital syphilis at birth and at 28 days of life. It also included cause-specific mortality estimates for ages 0–9 years and EMR estimates for the neonatal age group. This model assumed no incidence or remission. The modelled estimates were informed by covariates during data processing, and thus no covariates were included in the model.

Additionally, by default, DisMod uses a cascade of geographical priors to inform estimates at each level of the location hierarchy. Data and estimates at the global level act as priors for estimates at the super-region level, which act as priors for estimates at the region level, which act as priors for estimates at the country level, which act as priors for estimates at the subnational level. This is useful in the case of data scarcity, because it allows data from higher levels of the location hierarchy to be leveraged to produce more informed estimates at lower levels of the location hierarchy. However, because data inputs for the CS DisMod model are modelled for prevalence, CSMR, and EMR at every national and subnational location, the cascading behaviour of DisMod and the estimation of priors became unnecessary. Thus, the CS DisMod model creates estimates for each parameter at the finest levels of geography without priors, then aggregates back up. Please see the non-fatal outcome estimation "DisMod-MR 2.1 estimation" section of the appendix for further details on estimation and utility of priors in DisMod-MR.

Sequelae and disability weights

Adult early syphilis

We assumed that 0.043 (0.014–0.073) of adults seropositive for syphilis (encompassing primary, secondary, and early latent syphilis infections and treated persons who have not yet seroreverted) are symptomatic; these were assigned a health state of mild, acute, infectious disease. The remainder were considered asymptomatic.

Table 6. Severity distribution, details on the severity levels for adult early syphilis and the associated disability weight (DW) with that severity.

Severity level	Lay description	DW (95% CI)	Distribution (95% CI)
Asymptomatic		---	0.957<br><br>(0.927–0.986)
Mild early syphilis infection	Has a low fever and mild discomfort, but no difficulty with daily activities.	0.006<br>(0.002–0.012)	0.043<br><br>(0.014–0.073)

Adult tertiary syphilis

For adult tertiary syphilis, there are eight sequelae, including asymptomatic.

Table 7. Severity distribution, details on the severity levels for adult tertiary syphilis and the associated disability weight (DW) with that severity.

Sequela name	DW (95% UI)	Distribution (95% UI) – males	Distribution (95% UI) – females
Asymptomatic	--	0.3932 (0.338–0.448)	0.689 (0.652–0.727)
Cardiovascular complications	0.0505 (0.0323–0.074)	0.0999 (0.0662–0.1337)	0.058 (0.0391–0.0769)
Neurological problems	0.2029 (0.1339–0.2895)	0.0193(0.0038–0.0348)	0.034 (0.0196–0.0492)
Neurological problems and cardiovascular complications	0.2430 (0.168–0.3331)	0.0845 (0.0532–0.1158)	0.004 (0.0–0.0091)
Severe disfigurement	0.4047 (0.2745–0.5455)	0.1283 (0.0906–0.1659)	0.1853 (0.1538–0.2168)
Severe disfigurement and cardiovascular complications	0.4346 (0.3056–0.5713)	0.1475 (0.1076–0.1874)	0.0171 (0.0066–0.0276)
Severe disfigurement and neurological problems	0.5232 (0.3784–0.6693)	0.0931 (0.0604–0.1258)	0.0107 (0.0024–0.019)
Severe disfigurement, neurological problems, and cardiovascular	0.5469 (0.4020–0.6907)	0.0341 (0.0136–0.0545)	0.000856 (0.0–0.0032)

Congenital syphilis

There are seven sequelae for congenital syphilis, including asymptomatic. Symptoms arising before infants reach 2 years of age are called early symptomatic CS. Symptoms arising after infants reach 2 years of age are called late symptomatic CS.

Data on the frequency of occurrence of early symptomatic CS in a prospectively identified cohort of infected, untreated infants were scant. We employed the case series of Wile and Mundt[1] from the pre-penicillin era, which reported that 52% of all congenital syphilis cases become symptomatic in the early stage. Data on the frequency of occurrence of late symptomatic CS in a prospectively identified cohort of infected, untreated infants were not found, so we assumed the same 52% with early symptomatic CS were also at risk for late symptomatic CS.

We did, however, identify studies that report the distribution of symptoms among identified CS cases. Symptoms were then matched to a GBD health state that best represented the severity of symptoms that most CS cases experience. We conducted meta-analyses using the Metafor package in RStudio to get the proportion of symptomatic CS cases that showed symptoms of each health state.

Figure 6: Distribution of symptoms among identified cases of congenital syphilis

Moderate Infection in Infants with Early Congenital Syphilis

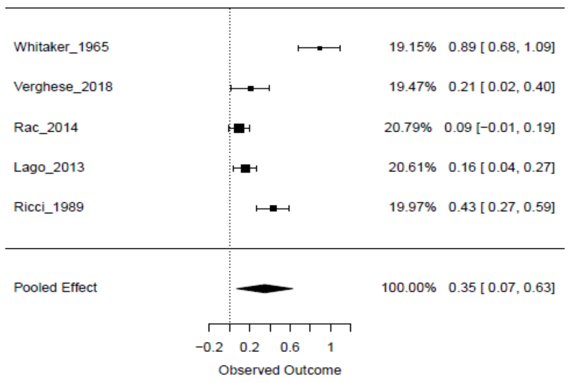

Slight Disfigurement in Infants with Early Congenital Syphilis

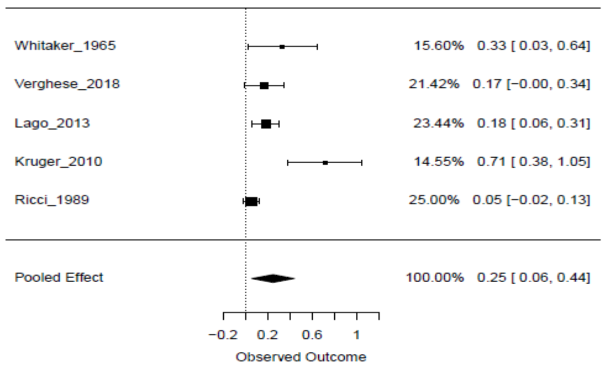

Motor & Cognitive Impairment in Persons with Late Congenital Syphilis

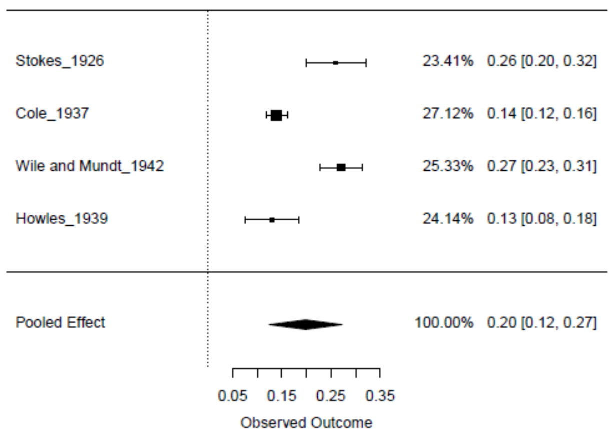

Slight Disfigurement in Persons with Late Congenital Syphilis

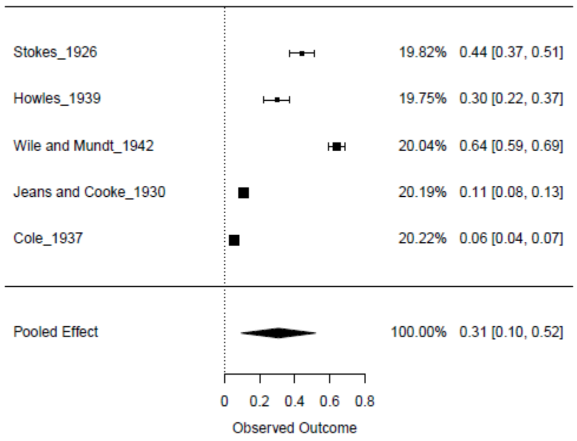

Unilateral Hearing Loss in Persons with Late Congenital Syphilis

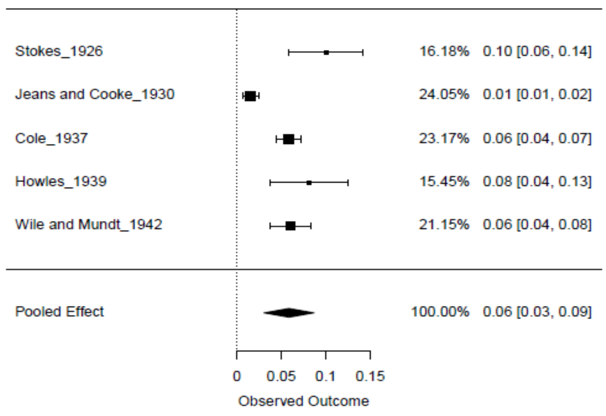

Interstitial Keratitis in Persons with Late Congenital Syphilis

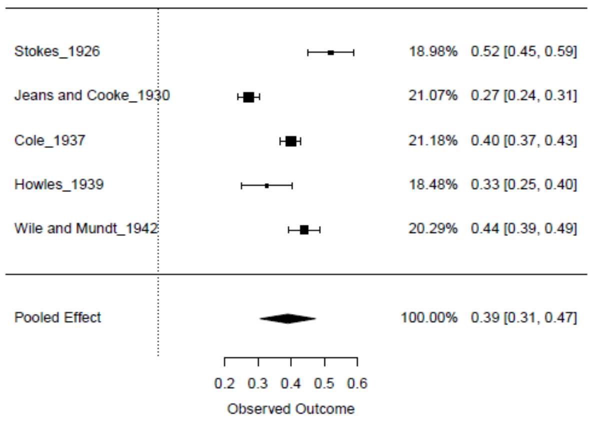

We then rescaled the health state proportions into the number of cases that are symptomatic for each given stage of CS and age group that symptoms arise. The health states and proportions are the same for both sexes.

Table 8. Severity distribution, details on the severity levels for congenital syphilis and the associated disability weight (DW) with that severity.

Sequela name	DW (95% UI)	Proportion of symptomatic cases experiencing health state, pre-squeeze
Asymptomatic CS	--	NA
Early symptomatic CS, slight disfigurement	0.011 (0.005–0.021)	0.35 (0.07–0.63)
Early symptomatic CS, infectious syndrome	0.051 (0.032–0.074)	0.25 (0.06–0.44)
Late symptomatic CS, interstitial keratitis	0.003 (0.001–0.007)	0.39 (0.31–0.47)
Late symptomatic CS, unilateral hearing loss	0.008 (0.003–0.020)	0.06 (0.03–0.09)
Late symptomatic CS, slight disfigurement	0.011 (0.005–0.021)	0.31 (0.10–0.52)
Late symptomatic CS, neurosyphilis	0.203 (0.134–0.29)	0.20 (0.12–0.27)

The above steps to produce initial sequela estimates have not accounted for treatment with penicillin. This is important to consider because penicillin has the ability to decrease the amount of time that a case spends symptomatic or to prevent certain health states from ever occurring. Using information from experts in the field of CS, we incorporated three different circumstances in which CS might be treated.

Presumptive treatment: Infants are treated based on maternal syphilis status alone. Rather than waiting for a radiographic, clinical, or laboratory-confirmed diagnosis of CS, all infants born to mothers with syphilis are automatically given treatment in the first week of life. Infants treated presumptively only experience early symptomatic CS health states during the early neonatal stage of life and will not experience late symptomatic CS. Infants not treated presumptively experience early symptomatic CS health states from the late neonatal stage until age 2 years and are at risk of experiencing late symptomatic CS.

Treatment of early syndrome: Infants are treated when symptoms of early symptomatic CS arise, which we assume prevents the development of late symptomatic CS, with the exception of hearing loss, which is not preventable with treatment. Infants that are not treated due to early syndrome of CS are at risk of all late-stage sequelae – hearing loss, interstitial keratitis, disfigurement, and neurosyphilis. The treatment of early syndrome does not decrease estimates of early symptomatic CS burden; rather, it decreases estimates of late symptomatic CS burden.

Treatment of late congenital CS: CS cases that experience interstitial keratitis are treated when blurred vision becomes concerning enough. Cases treated for interstitial keratitis experience it for six months in the year it arises. Cases that do not receive treatment for interstitial keratitis experience it for the

remainder of their lives. We assume that children seeking care for other sequelae of late congenital CS halt the progression of their disfigurement or neurological sequelae but do not reverse the sequelae they already have; we do not currently account for the difference in severity of these sequelae that late treatment could provide. CS cases treated late are assumed to experience hearing loss at the same frequency as untreated CS cases.

In each of the treatment circumstances outlined above, it is vital that CS cases are able to access care, receive the appropriate diagnosis, and receive comprehensive treatment. To determine what proportion of symptomatic cases in each country receive treatment, we leveraged the HAQ Index to create a treatment gradient of the likelihood of CS cases to receive penicillin. This covariate is a measure of health system performance[2] estimated by the GBD Health Systems team and is available for every national and subnational location.

For the presumptive treatment scenario, we assumed that all countries at the 75th percentile HAQ Index and above treat 100% of infants presumptively. In countries lower than the 75th percentile, we assume that the proportion of infants treated is equal to the HAQ Index value multiplied by the slope of treatment over a range of HAQ Index values spanning the 0–75th percentile. This strategy is reflected by the equations below. For the other two treatment scenarios, we use the same strategy described to account for treatment. However, the cutoff for 100% treatment during early syndrome or when experiencing interstitial keratitis is the 50th percentile of HAQ Index values.

$$Proportion\ Treated_{location,\ year, HAQi_{\geq P75\%}} = 1.0$$

$$Proportion\ Treated_{location,\ year, HAQi_{<P75\%}} = HAQi\ value_{location, year} * \frac{1}{HAQi\ value_{P75\%_{year}}}$$

$$Proportion\ Untreated_{location,\ year} = 1 - Proportion\ Treated_{location,\ year}$$

References

1. Stokes, John Hinchman, Herman Beerman, and Norman Reeh Ingraham. *Modern clinical syphilology: diagnosis, treatment, case study*. WB Saunders, 1944.
2. Fullman, Nancy, et al. Measuring performance on the Healthcare Access and Quality Index for 195 countries and territories and selected subnational locations: a systematic analysis from the Global Burden of Disease Study 2016. *The Lancet* 391.10136 (2018): 2236-2271.

Chlamydial infection

Flowchart

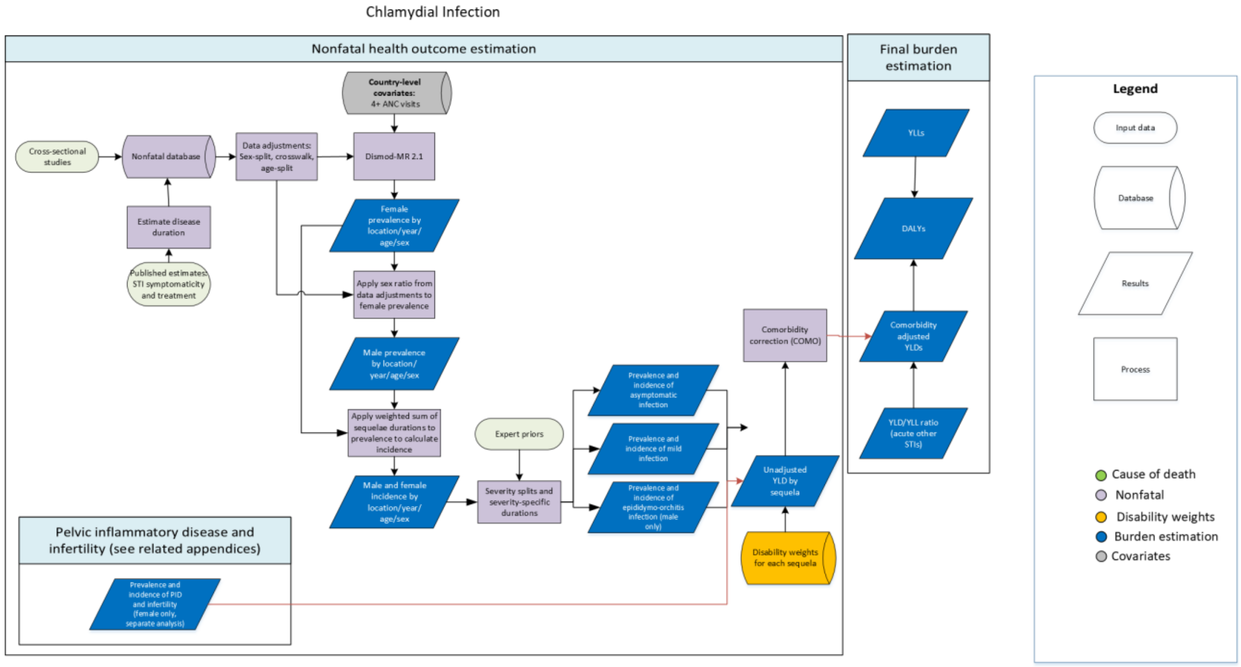

Input data and methodological summary for chlamydial infection

Case definition

Chlamydial infection is a genital infection with *Chlamydia trachomatis* bacteria. Transmission occurs through sexual contact. It can infect both males and females. If left untreated, chlamydia may manifest as epididymo-orchitis in males or as pelvic inflammatory disease in females. Case definitions were based on laboratory findings, including diagnosis with culture or nucleic acid amplification tests for chlamydial infections.

Quantity of interest	Reference or alternative	Definition
Prevalence of chlamydial infection	Reference	Genital infection due to the *Chlamydia trachomatis* bacteria, diagnosed by a nucleic acid amplification test, in a sample of individuals assumed to be representative of the general population, regardless of symptoms.
Prevalence of chlamydial infection	Alternative	Genital infection with *Chlamydia trachomatis* bacteria, diagnosed by culture, in a sample of individuals representative of the general population, regardless of symptoms.

Input data

Systematic literature reviews for chlamydial infection, gonococcal infection, and trichomoniasis were conducted for GBD 2013 and GBD 2015. A related search string was used for all three infections, as many studies report on multiple infections.

462 initial hits; 54 sources selected from full text review for data extraction:
(((chlamydia[Title/Abstract] OR chlamydia tracomatis[Title/Abstract] OR trachoma[Title/Abstract]) AND prevalence[Title/Abstract]) AND ('2013'[Date - Publication] : '2015'[Date - Publication])) /// ((gonorrhoea[Title/Abstract] OR Neisseria[Title/Abstract] OR gonococcal[Title/Abstract]) AND prevalence[Title/Abstract]) AND ("2013"[PDAT] : "2015"[PDAT]) /// ((trichomonal[Title/Abstract] OR trichomonas[Title/Abstract]) AND prevalence[Title/Abstract]) AND ('2013'[PDAT] : '2015'[PDAT])

We supplemented the peer-reviewed reports identified by this search string with a manual search of national ministry of health websites. In rounds of GBD subsequent to 2015, we added data contributed by the GBD Collaborator Network. For GBD 2023, 56 new sources were added based on mutual review by IHME of sources identified by Rowley and colleagues as part of the WHO Global Progress Report for 2021.[1]

Regardless of the manner of identification, sources were included if the sample was representative of the general population, or representative of a specific population for which an adjustment could be made, and if the source reported prevalence of infection diagnosed by laboratory methods. Sources were excluded if there was non-representative sampling or sampling of a specific population for which no valid adjustment could be made (eg, populations that include men who have sex with men [MSM], sex workers, HIV+, prisoners, other sub-populations, etc.).

Validation studies on the prevalence of chlamydial infection measured using different diagnostic techniques were found with a non-systematic literature search in Google Scholar, and then searching the citations of chosen studies to look further into and find more validation studies. These studies were used in data processing, as described below.

Prior to GBD 2017, case-notification data from locations with mandatory centralised reporting were included as incidence data in modelling, but the implied duration of infection that resulted from combining these incidence data with prevalence data described above was very long, suggesting potential under-reporting in the incidence data. From GBD 2017, these data have been retained in the database for vetting purposes but not used to inform model fits.

Data processing

Prevalence data reported for both sexes combined were split into estimated male-only and female-only data prior to modelling. To do this, sources reporting prevalence for each sex separately were matched by age, year, and location. Logit differences between the prevalence of each STI in females and the prevalence of each STI in males were input into meta-regression—Bayesian, regularised, trimmed (MR-BRT) to estimate an adjustment factor. Prior to GBD 2023, we used log ratios to determine the adjustment factor, but in GBD 2023 we switched to logit difference to ensure prevalence was bounded between 0 and 1.

The logit adjustment factor for both-sex datapoints was 0.14 (–0.43, 0.71) for chlamydial infection in GBD 2023. The MR-BRT model was fit with 10% trimming and no covariates. To be included, a study had to report on laboratory-confirmed diagnosis of an STI. For chlamydial infection, the reference case definition was diagnosis with a nucleic acid amplification test (NAAT). Data from high-quality sources using any other diagnostic test were considered for inclusion. For these data collected with alternative

methods, we estimated an adjustment factor in MR-BRT by running a meta-regression on the logit difference of the prevalence of infection diagnosed with an alternative test to the prevalence of infection diagnosed with a NAAT. Please see the non-fatal outcome estimation, "Bias adjustment for alternative case definitions and study methods" section of the appendix for further information. To estimate these logit differences, we searched for validation studies that compared the sensitivity of alternative tests to the reference, DNA-based tests for each respective STI. Thus, we could quantitatively adjust data collected with alternative tests to the level expected had the reference test been used. For reference, the process of adjusting for biases in non-reference data using MR-BRT with the logit-transformation method is described below:

1. Identify datapoints with overlapping year, age, sex, and location between reference and non-reference population data.
2. Logit transform overlapping datapoints of alternative and reference types.
3. Convert overlapping datapoints into a difference in logit space using the following equation: $logit(altnerative) - logit(reference)$
4. Use the delta method to compute standard errors of overlapping datapoints in logit space, then calculate standard error of logit difference using the following equation: $\sqrt{(variance\ of\ logit(alternative)) + (variance\ of\ logit(reference))}$
5. Using MR-BRT, conduct a random effects meta-regression to obtain the pooled logit difference of alternative to reference
6. Apply the pooled logit difference to all data points of non-reference types using the following equation:
$$new_{estimate} = inverse.logit((logit(alternative)) - (pooled\ logit\ difference))$$
7. Calculate new standard errors using the delta method, accounting for gamma (between-study heterogeneity)

In GBD 2021, we used data that employed any of three categories of tests in chlamydial infection: NAATs, culture, and "other" tests, crosswalking the latter two to NAAT as reference by means of estimating the log-ratio of prevalence measured by each test type as observed in validation studies. In GBD 2023, in contrast, we modelled crosswalks as logit-differences. Additionally, in GBD 2023, we determined that the "other" category of tests was sufficiently heterogeneous as to be difficult to capture a consistent shared bias of "other" tests on prevalence. Thus, we decided to drop data that employed tests from the "other" category, including only data measured using NAAT or culture diagnostics. The crosswalk adjustment factor for the latter (culture) is shown in the table below.

Table 1: MR-BRT crosswalk adjustment factors for chlamydial infection

Data input	Reference or alternative case definition	Gamma	Beta coefficient, logit (95% UI)*	Adjustment factor**
Nucleic acid amplification test	Ref	0.003	---	---
Culture diagnostic	Alt		–0.56 (–0.79, –0.34)	0.36 (0.31, 0.42)

**MR-BRT crosswalk adjustments can be interpreted as the factor the alternative case definition is adjusted by to reflect what it would have been had it been measured using the reference case definition. If the log/logit beta coefficient is negative, then the alternative is adjusted up to the reference. If the log/logit beta coefficient is positive, then the alternative is adjusted down to the reference.*

***The adjustment factor column is the exponentiated beta coefficient. For log beta coefficients, this is the relative*

rate between the two case definitions. For logit beta coefficients, this is the relative odds between the two case definitions.

Additionally, for sources reported for age groups spanning more than 15 years, these datapoints were disaggregated by imposing an age pattern from the best model of the prior GBD round.

As mentioned above, due to difficulty in reconciling differences between prevalence and incidence sources, likely due to under-reporting in surveillance data, incidence data were ignored for all STIs.

Remission inputs

Remission inputs for chlamydial infection were estimated from disease duration ranges beginning in GBD 2019. Duration ranges were calculated using a sum of the duration of untreated and treated disease, weighted by the percentage of individuals that are symptomatic and the probability of receiving treatment if symptomatic with the formula below.

$$\textbf{Duration} = (\%\,Symptomatic)(Prob_{Rx})(Duration_{Rx}) + (1 - \%\,Symptomatic)(Duration_{not\,Rx}) + (\%\,Symptomatic)(1 - Prob_{Rx})\,(Duration_{not\,Rx})$$

Early GBD rounds used static values from a WHO 2005 report[2] to determine remission by WHO region. Beginning in GBD 2019, uncertainty was incorporated into the remission inputs by using a binomial or normal distribution of the values as shown in the below table. Additionally, the probability of treatment if symptomatic was calculated using the Healthcare Access and Quality Index (a covariate in GBD produced by location and year) to scale each location's probability of treatment between the WHO Region A (maximum) probability and WHO Region C (minimum) probability, therefore modelling a location-year-sex probability of treatment, rather than a region-sex probability applied to all years. All inputs to the above formula are summarised in the table below.

Table 2. Remission inputs for chlamydial infection

	Sex	WHO and early GBD	GBD 2019 and since
% Symptomatic	Male	0.54	Binomial distribution with previous mean and standard error = mean*0.1
	Female	0.17	
Duration treated (yrs)	Male	0.08	Normal distribution with previous mean and standard error = mean*0.1
	Female	0.15	
Duration untreated (yrs)	Male	1.25	
	Female	1.25	
Probability of treatment if symptomatic			
WHO Region A*	Male	0.80	
	Female	0.75	
WHO Region B*	Male	0.65	
	Female	0.50	
WHO Region C*	Male	0.35	
	Female	0.225	
Maximum probability	Male		

	Female		Binomial distribution with mean WHO Region A and standard error = mean*0.1
Minimum probability	Male		Binomial distribution with mean WHO Region C and standard error = mean*0.1
	Female		
Healthcare access and quality index	Male		Location-specific GBD 2017 covariate, scaled 0 to 1
	Female		
Final location-year-sex probability	Male		ProbRx = HAQ * (max probability - min probability) + (min probability)
	Female		

**Location A - WHO European Region, North America (Canada and USA)*
Location B - WHO Eastern Mediterranean Region, WHO Region of the Americas (excluding North America)
Location C - WHO African Region, WHO South-East Asia Region

Modelling strategy

First, we estimated the prevalence of chlamydial infection in DisMod-MR 2.1. The incidence of chlamydial infection was estimated in a custom process outside of DisMod, as described in the post-processing section below. Specific modelling considerations in DisMod for each of these entities are also described below, except PID, which is described in detail in a separate section of this appendix.

Second, we split cases into asymptomatic and symptomatic health states, based on assumptions about probability and duration of symptoms. This included estimating the proportion of chlamydia cases that experienced epididymo-orchitis. The subset of chlamydia cases that experienced PID was determined by separately estimating the incidence and prevalence of PID and the proportion of those cases due to each aetiology: chlamydial infection, gonococcal infection, and other STIs.

The inputs to the chlamydial infection model were prevalence data from cross-sectional studies and modelled remission rates as described above. Incidence was restricted to occur only between ages 10 and 69. EMR was set to have a maximum value of 0.0001. The proportion of pregnant women estimated to experience four visits to antenatal care clinics (ANC4) was used as a covariate to help predict prevalence.

Table 3. Covariates. Summary of covariates used in the chlamydial infection DisMod-MR meta-regression model

Covariate	Parameter	Exponentiated beta (95% uncertainty interval)
Antenatal care (4 visits) coverage (proportion)	Prevalence	0.95<br>(0.91, 1.00)

Post-processing: sex ratio method

In GBD 2019 and GBD 2021, estimates of the prevalence of chlamydial infection were flagged as having highly variable and often implausible sex ratios. As described in the section of this appendix about DisMod-MR 2.1, this tool uses a geographical cascade, in which an initial model is fit with all data, which is used to generate Bayesian priors for models fit with only the data for a specific super-region, which in turn produce Bayesian priors for models fit with only the data-specific regions, and so on to the most local level. This approach leverages geographical proximity to produce estimates for data-scarce locations. These models are fit with data for both sexes at the global level but fit separately by sex at

lower levels of the geographical cascade. Because chlamydial infection – and all STIs excluding HIV – are studied more often in antenatal care populations than in general populations, the available data for estimating its prevalence is predominantly only available in females. Thus, the DisMod-MR 2.1 geographical cascade is updated with additional data at many stages and locations in fitting female models, but tends toward the global prior for males. Given the sexual transmission of these infections, we sought a method to better leverage the relatively rich data for females for males. Thus, we used prevalence estimates output from DisMod for females, but for males ignored DisMod results and generated prevalence estimates by applying modelled sex ratios (described above in "Data processing") to female estimates from DisMod.

PID and infertility due to chlamydial infection

The incidence and prevalence of pelvic inflammatory disease due to any cause was estimated using DisMod-MR 2.1, and proportion of incident and prevalent cases were assigned to chlamydia based on proportions also estimated in a separate DisMod model; the data inputs, settings, and synthesis of these models are described separately in the section of the this appendix on the PID impairment. The incidence of PID due to chlamydia was used, along with a single proportion identified from a cohort study,[3] to produce preliminary estimates of the incidence of infertility due to PID due to chlamydia, which were entered into another DisMod model to produce estimates of prevalence; modelling infertility due to PID due to chlamydia is described in the section of this appendix on the infertility impairment.

Incidence estimation

Prevalence estimates obtained for females from DisMod and for males from the application of sex ratio to female prevalence estimates were divided by a weighted duration to estimate incidence.

Estimation of female incidence:

$$1)\ prevalence_{female} = prevalence_{asymptomatic} + prevalence_{mild}$$

$$2)\ prevalence_{female} = (proportion_{asymptomatic} * duration_{asymptomatic} * incidence_{female}) + (proportion_{mild} * duration_{mild} * incidence_{female})$$

$$3)\ incidence_{female} = \frac{prevalence_{female}}{(proportion_{asymptomatic} * duration_{asymptomatic}) + (proportion_{mild} * duration_{mild})}$$

Estimation of male incidence:

$$1)\ prevalence_{male} = prevalence_{asymptomatic} + prevalence_{mild} + prevalence_{EO}$$

$$2)\ prevalence_{male} = (proportion_{asymptomatic} * duration_{asymptomatic} * incidence_{male}) + (proportion_{mild} * duration_{mild} * incidence_{male}) + (proportion_{EO} * duration_{EO} * incidence_{male})$$

$$3)\ incidence_{male} = \frac{prevalence_{male}}{(proportion_{asymptomatic} * duration_{asymptomatic}) + (proportion_{mild} * duration_{mild}) + (proportion_{EO} * duration_{EO})}$$

In these formulae, the following values were used for proportion symptomatic, duration symptomatic, proportion asymptomatic, and duration asymptomatic.

Sequelae and disability weights

The burden of chlamydial infection in females is the sum of the burden due to asymptomatic cases, symptomatic cases with mild infection (urethritis or cervicitis without upper tract involvement), PID cases, and individuals in the population with infertility due to past chlamydial infection. In males, chlamydial infection burden is the sum of the burden due to asymptomatic cases, symptomatic cases with mild infection (urethritis), and cases of epididymo-orchitis (EO).

For females, 0.17 (0.153–0.187) of chlamydia incidence was estimated to be symptomatic mild infection and the remainder was considered asymptomatic;[2] incident mild and asymptomatic cases were multiplied by assumed durations of 1 week and 1 year, respectively, to obtain estimates of the prevalence of these severity levels. The prevalence of PID due to chlamydia was estimated and split into moderate and severe levels in a separate process described elsewhere in this appendix. Likewise, the prevalence of infertility due to PID due to chlamydial infection was estimated in a separate process described elsewhere in this appendix.

For males, 0.505 (0.4545–0.5555) of chlamydia incidence was estimated to be symptomatic mild infection, a variable proportion was estimated to be EO, and the remainder was considered asymptomatic. The proportion of incident cases that developed EO was assumed to differ with better health-care access, and health-care access was assumed to correspond to high-quality vital registration systems. Thus, GBD locations with long time-series of high-quality vital registration data were labelled as "developed," while all others were marked as "developing." The proportion of incident cases thought to experience EO in locations considered "developed" was 0.02 (0.01–0.03) for chlamydia. The proportion of incident cases thought to experience EO in "developing" locations was 0.0625 (0.0325–0.0975) for chlamydia. We assumed a duration of 3 weeks for EO, a duration of 1 week for mild, symptomatic infection, and a duration of 1 year for asymptomatic infection. A weighted sum of these durations was applied to calculate the incidence of chlamydial infection in males from the prevalence produced by the application of sex-ratios to female prevalence estimates.

Table 4. Severity distribution, details on the severity levels for chlamydial infection and the associated disability weight (DW) with that severity.

Severity level	Lay description	DW (95% CI)
Asymptomatic		---
Mild chlamydial infection	Has a low fever and mild discomfort, but no difficulty with daily activities.	0.006 (0.002, 0.012)
Epididymo-orchitis due to chlamydial infection	Has swelling and tenderness in the testicles and pain during urination.	0.128 (0.086, 0.180)
Primary infertility due to chlamydial infection	Wants to have a child and has a fertile partner, but the couple cannot conceive.	0.008 (0.003, 0.015)
Secondary infertility due to chlamydial infection	Has at least one child, and wants to have more children. The person has a fertile partner, but the couple cannot conceive.	0.005 (0.002, 0.011)

Moderate pelvic inflammatory diseases due to chlamydial infection	Has pain in the belly and feels nauseous. The person has difficulties with daily activities.	0.114<br>(0.078, 0.159)
Severe pelvic inflammatory diseases due to chlamydial infection	Has severe pain in the belly and feels nauseous. The person is anxious and unable to carry out daily activities.	0.324<br>(0.220, 0.442)

References

1. World Health Organization. Global progress report on HIV, viral hepatitis and sexually transmitted infections, 2021. 2021; published online July 15. https://www.who.int/publications/i/item/9789240027077 (accessed March 3, 2025).
2. World Health Organization (WHO). Prevalence and incidence of selected sexually transmitted infections, *Chlamydia trachomatis*, *Neisseria gonorrhoeae*, syphilis and *Trichomonas vaginalis*: methods and results used by WHO to generate 2005 estimates. 2011.
3. Weström L, Joesoef R, Reynolds G, Hagdu A, Thompson SE. Pelvic Inflammatory Disease and Fertility. a Cohort Study of 1,844 Women with Laparoscopically Verified Disease and 657 Control Women with Normal Laparoscopic Results. *Sex Transm Dis*. 1992; 19(4): 185-92.

Gonococcal infection

Flowchart

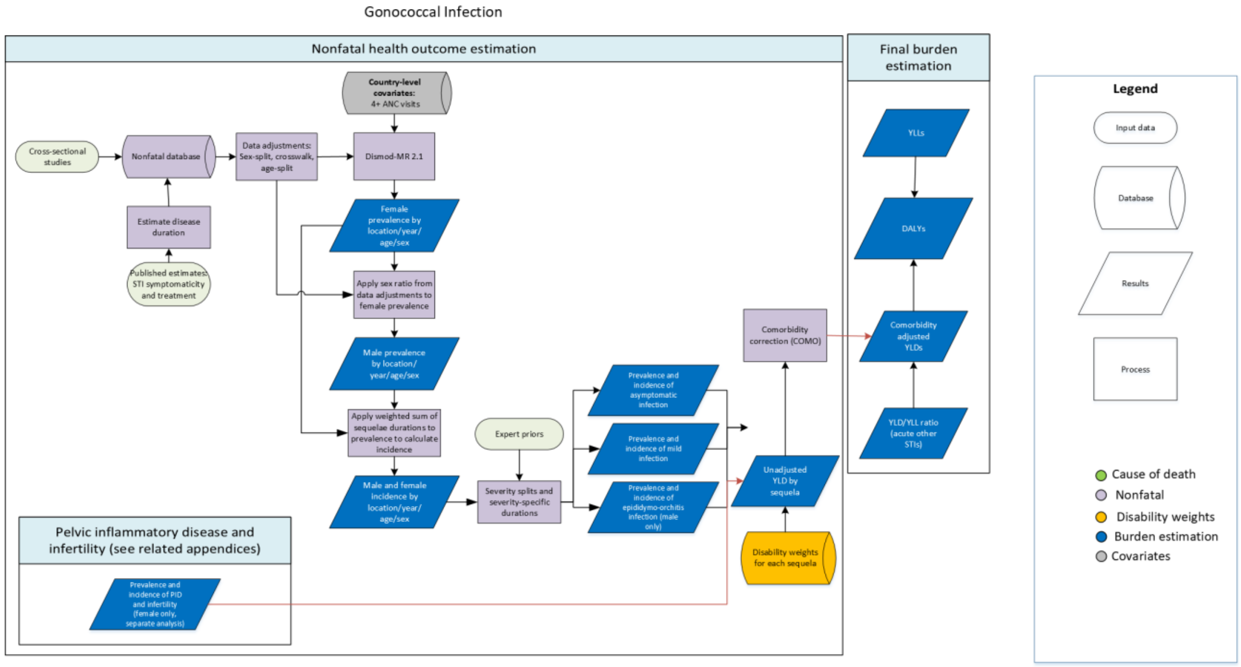

Input data and methodological summary for gonococcal infection

Case definition

Gonorrhea is a sexually transmitted infection caused by the *Neisseria gonorrhea* bacteria. If untreated, gonorrhea may manifest as pelvic inflammatory disease in females or as epididymo-orchitis in males. Case definition: genital infection with *Neisseria gonorrhea* bacteria; we account here both for acute or ongoing infections, with or without symptoms, and cases of infertility that are the result of an infection in the past. Case definitions for were based on laboratory findings, including diagnosis with culture or nucleic acid amplification tests.

**Quantity of interest**	**Reference or alternative**	**Definition**
Prevalence of gonococcal infection	Reference	Genital infection due to the *Neisseria gonorrhea* bacteria, diagnosed by a nucleic acid amplification test, in a sample of individuals representative of the general population, regardless of symptoms.
Prevalence of gonococcal infection	Alternative	Genital infection with *Neisseria gonorrhea* bacteria, diagnosed by culture, in a sample of individuals representative of the general population, regardless of symptoms.

Input data

Systematic literature reviews for chlamydial infection, gonococcal infection, and trichomoniasis were conducted for GBD 2013 and GBD 2015. A related search string was used for all three infections, as many studies report on multiple infections.

462 initial hits; 54 sources selected from full text review for data extraction: (((chlamydia[Title/Abstract] OR chlamydia tracomatis[Title/Abstract] OR trachoma[Title/Abstract]) AND prevalence[Title/Abstract]) AND ('2013'[Date - Publication] : '2015'[Date - Publication])) /// ((gonorrhoea[Title/Abstract] OR Neisseria[Title/Abstract] OR gonococcal[Title/Abstract]) AND prevalence[Title/Abstract]) AND ("2013"[PDAT] : "2015"[PDAT]) /// ((trichomonal[Title/Abstract] OR trichomonas[Title/Abstract]) AND prevalence[Title/Abstract]) AND ('2013'[PDAT] : '2015'[PDAT])

We supplemented the peer-reviewed reports identified by this search string with a manual search of national ministry of health websites. In rounds of GBD subsequent to 2015, we added data contributed by the GBD Collaborator Network. For GBD 2023, 63 new sources were added based on mutual review by IHME of sources identified by Rowley and colleagues as part of the WHO Global Progress Report for 2021.[1]

Regardless of the manner of identification, sources were included if the sample was representative of the general population, or representative of a specific population for which an adjustment could be made, and if the source reported prevalence of infection diagnosed by laboratory methods. Sources were excluded if there was non-representative sampling or sampling of a specific population for which no valid adjustment could be made (eg, populations that include men who have sex with men [MSM], sex workers, HIV+, prisoners, other sub-populations, etc.).

Validation studies on the prevalence of gonococcal infection measured using different diagnostic techniques were found with a non-systematic literature search in Google Scholar and then searching the citations of chosen studies to look further into and find more validation studies. These studies were used in data processing, as described below.

Data processing

Data processing was completed as described in the chlamydia data processing section.

The logit adjustment factor for both-sex datapoints was 0.48 (–0.27, 1.22) for gonococcal infection in GBD 2023. The MR-BRT model was fit with 10% trimming and no covariates.

For gonococcal infection, we crosswalked only data that had one of two categories of diagnostic tests: nucleic acid amplification tests (NAATs) and culture. The crosswalk adjustment factor for the latter is shown in the table below.

Table 1: MR-BRT crosswalk adjustment factors for gonococcal infection

Data input	Reference or alternative case definition	Gamma	Beta coefficient, logit (95% UI)*	Adjustment factor**
Nucleic acid amplification test	Ref	0.50	---	---
Culture diagnostic	Alt		–0.94 (–2.56, 0.67)	0.28 (0.07, 0.66)

**MR-BRT crosswalk adjustments can be interpreted as the factor the alternative case definition is adjusted by to reflect what it would have been had it been measured using the reference case definition. If the log/logit beta*

coefficient is negative, then the alternative is adjusted up to the reference. If the log/logit beta coefficient is positive, then the alternative is adjusted down to the reference.
***The adjustment factor column is the exponentiated beta coefficient. For log beta coefficients, this is the relative rate between the two case definitions. For logit beta coefficients, this is the relative odds between the two case definitions.*

Additionally, for sources reported for age groups spanning more than 15 years, these datapoints were disaggregated by imposing an age pattern from the best model of the prior GBD round.

As mentioned above, due to difficulty in reconciling differences between prevalence and incidence sources, likely due to under-reporting in surveillance data, incidence data were ignored for all STIs.

Remission inputs

Remission inputs for gonococcal infection were estimated from disease duration ranges beginning in GBD 2019. Duration ranges were calculated using a sum of the duration of untreated and treated disease, weighted by the percentage of individuals that are symptomatic and the probability of receiving treatment if symptomatic with the formula below.

$$\textbf{Duration} = (\%\,Symptomatic)(Prob_{Rx})(Duration_{Rx}) + (1 - \%\,Symptomatic)(Duration_{\,not\,Rx}) + (\%\,Symptomatic)(1 - Prob_{Rx})\,(Duration_{\,not\,Rx})$$

Early GBD rounds used static values from a WHO 2005 report[2] to determine remission by WHO region. Beginning in GBD 2019, uncertainty was incorporated into the remission inputs by using a binomial or normal distribution of the values as shown in the below table. Additionally, the probability of treatment if symptomatic was calculated using the Healthcare Access and Quality Index (a covariate in GBD produced by location and year) to scale each location's probability of treatment between the WHO Region A (maximum) probability and WHO Region C (minimum) probability, therefore modelling a location-year-sex probability of treatment, rather than a region-sex probability applied to all years. All inputs to the above formula are summarised in the table below.

Table 2. Remission inputs for gonococcal infection

	Sex	WHO and early GBD	GBD 2019 and since
% Symptomatic	Male	0.64	Binomial distribution with previous mean and standard error = mean*0.1
	Female	0.34	
Duration treated (yrs)	Male	0.04	Normal distribution with previous mean and standard error = mean*0.1
	Female	0.08	
Duration untreated (yrs)	Male	0.4167	
	Female	0.5	
Probability of treatment if symptomatic			
WHO Region A*	Male	0.80	
	Female	0.75	

WHO Region B*	Male<br>Female	0.65<br>0.50	
WHO Region C*	Male<br>Female	0.35<br>0.225	
Maximum probability	Male<br>Female		Binomial distribution with mean WHO Region A and standard error = mean*0.1
Minimum probability	Male<br>Female		Binomial distribution with mean WHO Region C and standard error = mean*0.1
Healthcare Access and Quality Index	Male<br>Female		Location-specific GBD 2017 covariate, scaled 0 to 1
Final location-year-sex probability	Male<br>Female		ProbRx = HAQ * (max probability - min probability) + (min probability)

**Location A - WHO European Region, North America (Canada and USA)*
Location B - WHO Eastern Mediterranean Region, WHO Region of the Americas (excluding North America)
Location C - WHO African Region, WHO South-East Asia Region

Modelling strategy

First, we estimated the prevalence of gonococcal infection in DisMod-MR 2.1. The incidence of gonococcal infection was estimated in a custom process outside of DisMod, as described in the post-processing section below. Specific modelling considerations in DisMod for each of these entities are also described below, except PID, which is described in detail in a separate section of this appendix.

Second, we split cases into asymptomatic and symptomatic health states, based on assumptions about probability and duration of symptoms. This included estimating the proportion of gonococcal cases that experienced epididymo-orchitis. The subset of gonococcal cases that experienced PID was determined by separately estimating the incidence and prevalence of PID and the proportion of those cases due to each aetiology: chlamydial infection, gonococcal infection, and other STIs.

The inputs to the gonococcal infection model were prevalence data from cross-sectional studies and modelled remission rates as described above. Incidence was restricted to occur only between ages 10 and 69. EMR was set to have a maximum value of 0.0001. The proportion of pregnant women estimated to experience four visits to antenatal care clinics (ANC4) was used as a covariate to help predict prevalence.

Table 3. Covariates. Summary of covariates used in the gonococcal infection DisMod-MR meta-regression model

Covariate	Parameter	Exponentiated beta (95% uncertainty interval)
Antenatal care (4 visits) coverage (proportion)	Prevalence	0.96<br>(0.92–1.00)
Age-standardised death rate (lnASDR) for gonococcal infection	Prevalence	1.04<br>(1.01–1.07)

Post-processing: sex ratio method

The sex ratio method was applied in gonococcal infection as described for chlamydial infection above.

PID and infertility due to gonococcal infection

The incidence and prevalence of pelvic inflammatory disease and infertility due to gonococcal infection were estimated as described above in the PID and infertility due to chlamydial infection section. Further information on these processes is described in the sections of this appendix on the PID and infertility impairments.

Incidence estimation

Prevalence estimates obtained for females from DisMod and for males from the application of sex-ratio to female prevalence estimates were divided by a weighted duration to estimate incidence of gonococcal infection. Formulae were applied as described above for chlamydial infection.

Sequelae and disability weights

As with chlamydial infection, the burden of gonococcal infection in females is the sum of the burden due to asymptomatic cases, symptomatic cases with mild infection (urethritis or cervicitis without upper tract involvement), PID cases, and individuals in the population with infertility due to past gonococcal infection. In males, gonococcal infection burden is the sum of the burden due to asymptomatic cases, symptomatic cases with mild infection (urethritis), and cases of epididymo-orchitis (EO).

For females, 0.34 (0.306–0.374) of gonococcal incidence was estimated to be symptomatic mild infection and the remainder was considered asymptomatic;[2] incident mild and asymptomatic cases were multiplied by assumed durations of 1 week and 1 year, respectively, to obtain estimates of the prevalence of these severity levels. The prevalence of PID due to gonorrhea was estimated and split into moderate and severe levels in a separate process described elsewhere in this appendix. Likewise, the prevalence of infertility due to PID due to chlamydial infection was estimated in a separate process described elsewhere in this appendix.

For males, 0.5875 (0.5288–0.6463) of gonococcal incidence was estimated to be symptomatic mild infection, a variable proportion was estimated to be EO, and the remainder was considered asymptomatic. The proportion of incident cases that developed EO was assumed to differ with better health-care access, and health-care access was assumed to correspond to high-quality vital registration systems. Thus, GBD locations with long time-series of high-quality vital registration data were labelled as “developed,” while all others were marked as “developing.” The proportion of incident cases thought to experience EO in locations considered “developed” was 0.03 (0.015–0.045) for gonococcal infection. The proportion of incident cases thought to experience EO in “developing” locations was 0.0975 (0.0483–0.143) for gonococcal infection. We assumed a duration of 3 weeks for EO, a duration of 1 week for mild, symptomatic infection, and a duration of 1 year for asymptomatic infection. A weighted sum of these durations was applied to calculate the incidence of chlamydial infection in males from the prevalence produced by the application of sex ratios to female prevalence estimates.

Table 4. Severity distribution, details on the severity levels for gonococcal infection and the associated disability weight (DW) with that severity.

Severity level	Lay description	DW (95% CI)
Asymptomatic		---
Mild gonococcal infection	Has a low fever and mild discomfort, but no difficulty with daily activities.	0.006 (0.002, 0.012)

Epididymo-orchitis due to gonococcal infection	Has swelling and tenderness in the testicles and pain during urination.	0.128 (0.086, 0.180)
Primary infertility due to gonococcal infection	Wants to have a child and has a fertile partner, but the couple cannot conceive.	0.008 (0.003, 0.015)
Secondary infertility due to gonococcal infection	Has at least one child, and wants to have more children. The person has a fertile partner, but the couple cannot conceive.	0.005 (0.002, 0.011)
Moderate pelvic inflammatory disease due to gonococcal infection	Has pain in the belly and feels nauseous. The person has difficulties with daily activities.	0.114 (0.078, 0.159)
Severe pelvic inflammatory disease due to gonococcal infection	Has severe pain in the belly and feels nauseous. The person is anxious and unable to carry out daily activities.	0.324 (0.220, 0.442)

References

1. World Health Organization. Global progress report on HIV, viral hepatitis and sexually transmitted infections, 2021. 2021; published online July 15. https://www.who.int/publications/i/item/9789240027077 (accessed March 3, 2025).
2. World Health Organization (WHO). Prevalence and incidence of selected sexually transmitted infections, *Chlamydia trachomatis*, *Neisseria gonorrhoeae*, syphilis and *Trichomonas vaginalis*: methods and results used by WHO to generate 2005 estimates. 2011.

Trichomoniasis

Flowchart

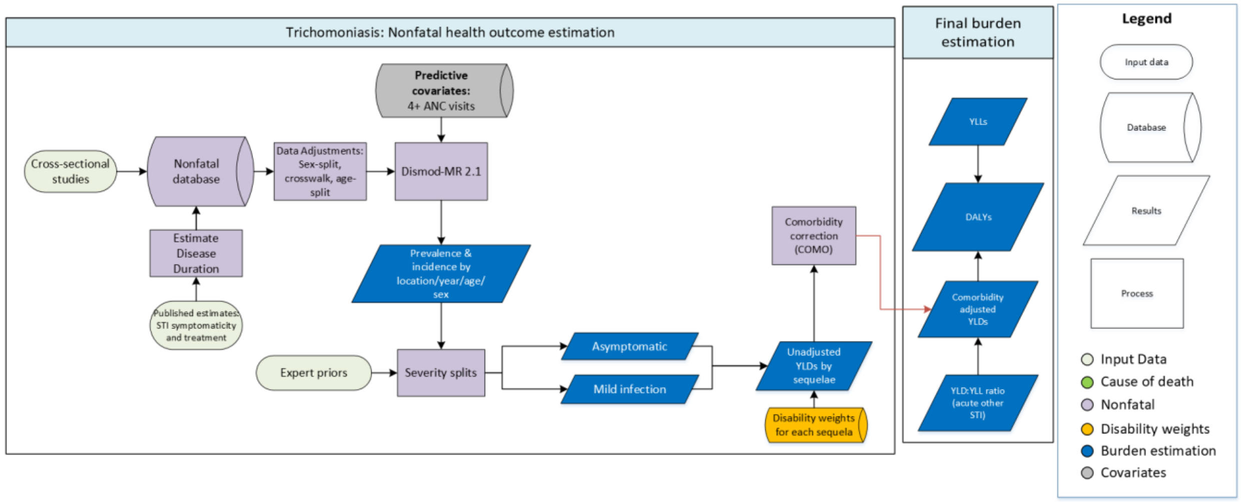

Input data and methodological summary for trichomoniasis

Case definition

Trichomoniasis is a sexually transmitted infection caused by infection with a protozoan parasite called *Trichomonas vaginalis*. We account here both for acute and chronic infections, with or without symptoms. Case definitions for were based on laboratory findings, including diagnosis with culture, wet mount, or nucleic acid amplification tests.

**Quantity of interest**	**Reference or alternative**	**Definition**
Prevalence of trichomoniasis	Reference	Infection with *Trichomonas vaginalis*, diagnosed via a nucleic acid amplification test collected through vaginal and endocervical swabs in a sample of individuals representative of a given population in cross-sectional surveys.
Prevalence of trichomoniasis	Alternative	Infection with *Trichomonas vaginalis*, diagnosed via culture, identified through vaginal and endocervical swabs in a sample of individuals representative of a given population in cross-sectional surveys.
Prevalence of trichomoniasis	Alternative	Infection with *Trichomonas vaginalis*, diagnosed via wet-mount test, identified through vaginal and endocervical swabs in a sample of individuals representative of a given population in cross-sectional surveys.

Input data

Systematic literature reviews for chlamydial infection, gonococcal infection, and trichomoniasis were conducted for GBD 2013 and GBD 2015. A related search string was used for all three infections, as many studies report on multiple infections.

462 initial hits; 54 sources selected from full text review for data extraction: (((chlamydia[Title/Abstract] OR chlamydia tracomatis[Title/Abstract] OR trachoma[Title/Abstract]) AND prevalence[Title/Abstract]) AND ('2013'[Date - Publication] : '2015'[Date - Publication])) /// ((gonorrhoea[Title/Abstract] OR Neisseria[Title/Abstract] OR gonococcal[Title/Abstract]) AND prevalence[Title/Abstract]) AND ("2013"[PDAT] : "2015"[PDAT]) /// ((trichomonal[Title/Abstract] OR trichomonas[Title/Abstract]) AND prevalence[Title/Abstract]) AND ('2013'[PDAT] : '2015'[PDAT])

We supplemented the peer-reviewed reports identified by this search string with a manual search of national ministry of health websites. In the rounds of GBD subsequent to 2015, we added data contributed by the GBD Collaborator Network. For GBD 2023, 74 new sources were added based on mutual review by IHME of sources identified by Rowley and colleagues as part of the WHO Global Progress Report for 2021.[1]

Regardless of the manner of identification, sources were included if the sample was representative of the general population, or representative of a specific population for which an adjustment could be made, and if the source reported prevalence of infection diagnosed by laboratory methods. Sources were excluded if there was non-representative sampling or sampling of a specific population for which no valid adjustment could be made (eg, populations that include men who have sex with men [MSM], sex workers, HIV+, prisoners, other sub-populations, etc.).

Validation studies on the prevalence of gonococcal infection measured using different diagnostic techniques were found with a non-systematic literature search in Google Scholar, and then searching the citations of chosen studies to look further into and find more validation studies. These studies were used in data processing, as described below.

Data processing

Data processing was completed as described in the chlamydia data processing section.

The logit adjustment factor for both-sex datapoints was 1.16 (0.51, 1.80) for trichomoniasis in GBD 2023. The MR-BRT model was fit with 10% trimming and no covariates.

For trichomoniasis, we crosswalked only data that had one of three categories of diagnostic tests: nucleic acid amplification tests (NAATs), culture, or wet mount. The crosswalk adjustment factors are shown in the table below.

Table 1: MR-BRT crosswalk adjustment factors for trichomoniasis

Data input	Reference or alternative case definition	Gamma	Beta coefficient, logit (95% UI)*	Adjustment factor**
Nucleic acid amplification test	Ref	0.29	---	---
Culture diagnostic	Alt		–0.23 (–1.33, 0.86)	0.44 (0.21, 0.70)
Wet mount diagnostic	Alt		–0.62 (–1.75, 0.51)	0.35 (0.15, 0.62)

**MR-BRT crosswalk adjustments can be interpreted as the factor the alternative case definition is adjusted by to reflect what it would have been had it been measured using the reference case definition. If the log/logit beta coefficient is negative, then the alternative is adjusted up to the reference. If the log/logit beta coefficient is positive, then the alternative is adjusted down to the reference.*
***The adjustment factor column is the exponentiated beta coefficient. For log beta coefficients, this is the relative rate between the two case definitions. For logit beta coefficients, this is the relative odds between the two case definitions.*

Additionally, for sources reported for age groups spanning more than 15 years, these datapoints were disaggregated by imposing an age pattern from the best model of the prior GBD round.

As mentioned above, due to difficulty in reconciling differences between prevalence and incidence sources, likely due to under-reporting in surveillance data, incidence data were ignored for all STIs.

Remission inputs

Remission inputs for trichomoniasis were estimated from disease duration ranges beginning in GBD 2019. Duration ranges were calculated using a sum of the duration of untreated and treated disease, weighted by the percentage of individuals who are symptomatic and the probability of receiving treatment if symptomatic with the formula below.

$$\begin{aligned}\textbf{Duration} \quad &= (\%\,Symptomatic)(Prob_{Rx})(Duration_{Rx}) \\ &+(1 - \%\,Symptomatic)(Duration_{\,not\,Rx}) \\ &+(\%\,Symptomatic)(1 - Prob_{Rx})\,(Duration_{\,not\,Rx})\end{aligned}$$

Early GBD rounds used static values from a WHO 2005 report[2] to determine remission by WHO region. Beginning in GBD 2019, uncertainty was incorporated into the remission inputs by using a binomial or normal distribution of the values as shown in the below table. Additionally, the probability of treatment if symptomatic was calculated using the Healthcare Access and Quality Index (a covariate in GBD produced by location and year) to scale each location's probability of treatment between the WHO Region A (maximum) probability and WHO Region C (minimum) probability, therefore modelling a location-year-sex probability of treatment, rather than a region-sex probability applied to all years. All inputs to the above formula are summarised in the table below.

Table 2. Remission inputs for trichomoniasis

	Sex	WHO and early GBD	GBD 2019 and since
% Symptomatic	Male	0.067	Binomial distribution with previous mean and standard error = mean*0.1
	Female	0.34	
Duration treated (yrs)	Male	0.08	Normal distribution with previous mean and standard error = mean*0.1
	Female	0.25	
Duration untreated (yrs)	Male	0.125	
	Female	1.5	
Probability of treatment if symptomatic			
WHO Region A*	Male	0.80	
	Female	0.75	

WHO Region B*	Male<br>Female	0.65<br>0.50	
WHO Region C*	Male<br>Female	0.35<br>0.225	
Maximum probability	Male<br>Female		Binomial distribution with mean WHO Region A and standard error = mean*0.1
Minimum probability	Male<br>Female		Binomial distribution with mean WHO Region C and standard error = mean*0.1
Healthcare access and quality index	Male<br>Female		Location-specific GBD 2017 covariate, scaled 0 to 1
Final location-year-sex probability	Male<br>Female		ProbRx = HAQ * (max probability - min probability) + (min probability)

**Location A - WHO European Region, North America (Canada and USA)*
Location B - WHO Eastern Mediterranean Region, WHO Region of the Americas (excluding North America)
Location C - WHO African Region, WHO South-East Asia Region

Modelling strategy

First, we estimated the prevalence and incidence of trichomoniasis in DisMod-MR 2.1. Second, we split cases into asymptomatic and symptomatic health states, based on assumptions about the proportion of prevalent cases experiencing symptoms at any given time.

The inputs to the trichomoniasis model were prevalence data from cross-sectional studies and modelled remission rates as described above. Incidence was restricted to occur only between ages 10 and 64 years of age. EMR was set to have a maximum value of 0. The proportion of pregnant women estimated to experience four visits to antenatal care clinics (ANC4) was used as a covariate to help predict prevalence.

Table 3. Covariates. Summary of covariates used in the trichomoniasis DisMod-MR meta-regression model

Covariate	Parameter	Exponentiated beta (95% uncertainty interval)
Antenatal care (4 visits) coverage (proportion)	Prevalence	0.93<br>(0.91, 0.97)

Sequelae and disability weights

For trichomoniasis, 0.067 (0.063–0.073) of prevalent male cases were assumed to be symptomatic, and assigned a health state of mild, acute infectious disease. For females, 0.34 (0.306–0.374) were assumed symptomatic and assigned a health state of mild, acute infectious disease.[2] For each sex, the remaining proportion was assumed to be asymptomatic.

Table 4. Severity distribution, details on the severity levels for trichomoniasis and the associated disability weight (DW) with that severity.

Severity level	Lay description	DW (95% CI)
Asymptomatic		---

Acute trichomoniasis infection	Has a low fever and mild discomfort, but no difficulty with daily activities.	0.006 (0.002, 0.012)

References

1. World Health Organization. Global progress report on HIV, viral hepatitis and sexually transmitted infections, 2021. 2021; published online July 15. https://www.who.int/publications/i/item/9789240027077 (accessed March 3, 2025).
2. World Health Organization (WHO). Prevalence and incidence of selected sexually transmitted infections, *Chlamydia trachomatis*, *Neisseria gonorrhoeae*, syphilis and *Trichomonas vaginalis*: methods and results used by WHO to generate 2005 estimates. 2011.

Genital herpes

Flowchart

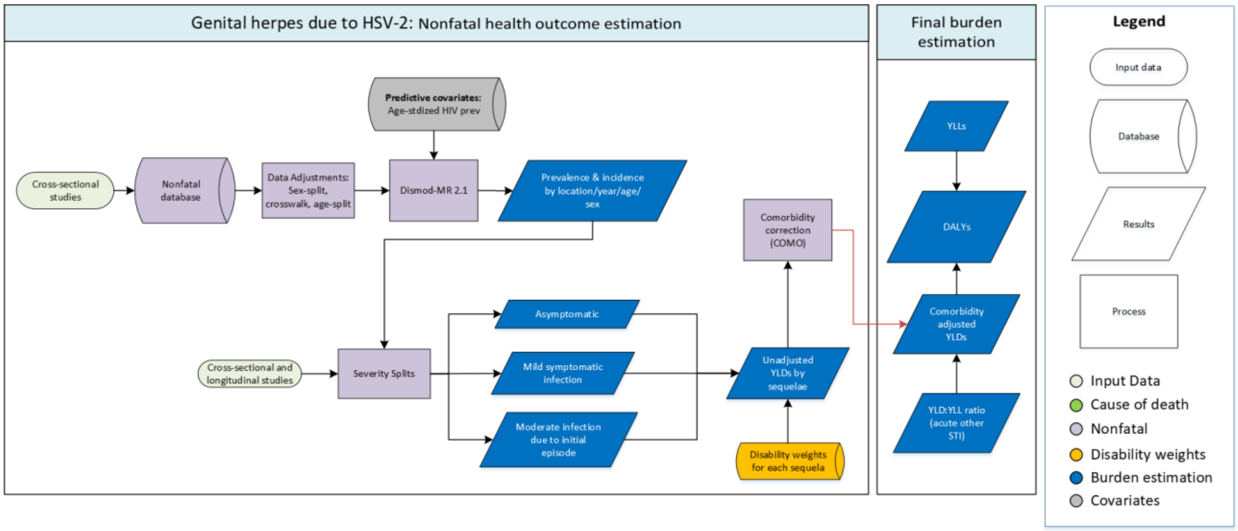

Input data and methodological summary for genital herpes

Case definition

Genital herpes is a sexually transmitted infection, defined as a genital infection with herpes simplex 2 virus, regardless of symptoms. Case definitions were based on laboratory findings, including diagnosis with a type-specific blood test for antibodies against HSV-2, such as the enzyme-linked immunoassay (ELISA), enzyme immunoassay (EIA), and others

**Quantity of interest**	**Reference or alternative**	**Definition**
Prevalence of genital herpes	Reference	Seroprevalence of HSV2 G 2 antigen or IgG antibody measured in a sample of the general population.
Prevalence of genital herpes	Alternative	Seroprevalence of HSV2 G 2 antigen or IgG antibody measured in a sample of blood donors.
Prevalence of genital herpes	Alternative	Seroprevalence of HSV2 G 2 antigen or IgG antibody measured in a sample of pregnant women.

Input data

A systematic literature review for genital herpes was conducted for GBD 2013 and GBD 2015.

> **13 initial hits; 1 selected from full text review for data extraction:** herpes"[Title/Abstract] OR "Herpesvirus 2, Human"[Mesh]) AND ("Prevalence"[Title/Abstract] OR "Incidence"[Title/Abstract] AND ("2015"[PDAT] : "2015"[PDAT])

We supplemented the peer-reviewed reports identified by this search string with a manual search of national ministry of health websites and data contributed by the GBD Collaborator Network. No new data were added for GBD 2023.

Regardless of the manner of identification, sources were included if the sample was representative of the general population, or representative of a specific population for which an adjustment could be made, and if the source reported prevalence of infection diagnosed by laboratory methods. Sources were excluded if there was non-representative sampling or sampling of a specific population for which no valid adjustment could be made (eg, populations that include men who have sex with men [MSM], sex workers, HIV+, prisoners, other sub-populations, etc.).

Data processing

Prevalence data reported for both sexes combined were split into estimated male-only and female-only data prior to modelling. To do this, sources reporting prevalence for each sex separately were matched by age, year, and location. Log ratios between the prevalence of genital herpes in females and the prevalence of genital herpes in males were input into meta-regression—Bayesian, regularised, trimmed (MR-BRT) to estimate an adjustment factor.

The log adjustment factor for both-sex datapoints was 0.46 (–0.09 to 1.05) for genital herpes due to HSV-2. We then used the modelled sex ratio to adjust "both"-sex data values to expected "male" and "female" values. We calculated the male values as $val_{male} = val_{both} * \frac{pop_{both}}{(pop_{male}+ratio*pop_{female})}$. We calculated the female values as $val_{female} = ratio * val_{male}$.

To be included, a study had to report on laboratory-confirmed diagnosis of an STI. For genital herpes, neither validation studies nor matched studies could be found to estimate adjustment factors, so any sources that did not use blood tests for HSV-2 were excluded. However, adjustments were made for non-representative populations. Adjustment factors were calculated in MR-BRT for populations of blood donors and pregnant women. The log ratios that were inputs to MR-BRT were estimated from matched comparisons by age, sex, and location using all data in the genital herpes database. Please see the non-fatal outcome estimation "Bias adjustment for alternative case definitions and study methods" section of the appendix for further information.

Table 1: MR-BRT crosswalk adjustment factors for genital herpes

Data input	Reference or alternative case definition	Gamma	Beta coefficient, log (95% UI)*	Adjustment factor**
General population	Ref	0.35	---	---
Population of pregnant women	Alt		–0.24 (–0.97, 0.46)	0.78 (0.37, 1.58)
Population of blood donors	Alt		0.64 (–0.13, 1.39)	1.89 (0.88, 4.01)

**MR-BRT crosswalk adjustments can be interpreted as the factor the alternative case definition is adjusted by to reflect what it would have been had it been measured using the reference case definition. If the log/logit beta coefficient is negative, then the alternative is adjusted up to the reference. If the log/logit beta coefficient is positive, then the alternative is adjusted down to the reference.*

***The adjustment factor column is the exponentiated beta coefficient. For log beta coefficients, this is the relative rate between the two case definitions. For logit beta coefficients, this is the relative odds between the two case definitions.*

Additionally, for sources reported for age groups spanning more than 15 years, these datapoints were disaggregated by imposing an age pattern from the best model of the prior GBD round.

As mentioned above, due to difficulty in reconciling differences between prevalence and incidence sources, likely due to under-reporting in surveillance data, incidence data were ignored for all STIs.

Modelling strategy

There were no changes to the modelling strategy and settings between GBD 2021 and GBD 2023. First, we estimated the prevalence and incidence of genital herpes in DisMod-MR 2.1. Second, we split cases into asymptomatic and symptomatic health states, based on assumptions about probability and duration of symptoms.

Prevalence data from cross-sectional studies were the input to the HSV-2 infection model. Genital herpes estimation assumed mortality is zero and remission is a small value (0–0.02) to account for a subset of herpes-infected patients who experience seroreversion. Incidence was restricted to occur between ages 10 and 79. A predictive covariate for age-standardised HIV prevalence was used to guide estimates in geographies with sparse data in recognition of the strong relationship between HSV-2 and HIV transmission.

Table 2. **Covariates.** Summary of covariates used in the genital herpes DisMod-MR meta-regression model

Covariate	Parameter	Exponentiated beta (95% uncertainty interval)
HIV, age-standardised prevalence	Prevalence	2.60 (2.37, 2.71)

Sequelae and disability weights

A systematic literature review revealed a few studies that informed our estimation that 0.175 (0.10–0.25) of herpes cases experience initial episodes that have symptoms of moderate, acute infectious disease and that last 3 (2–4) weeks. Additionally, 0.189 of prevalent cases experience an average of 6 (5–7) recurrent episodes per year, each lasting for a duration of 2 (1–3) weeks.

Table 3. **Severity distribution,** details on the severity levels for genital herpes and the associated disability weight (DW) with that severity.

Severity level	Lay description	DW (95% CI)
Asymptomatic		---
Initial symptomatic episode of genital herpes	Has a fever and aches, and feels weak, which causes some difficulty with daily activities.	0.051 (0.032, 0.074)
Recurrent symptomatic episode of genital herpes	Has a low fever and mild discomfort, but no difficulty with daily activities.	0.006 (0.002, 0.012)

Other sexually transmitted infections

Input data and methodological summary for other sexually transmitted infections

Case definition

Other sexually transmitted infections (STIs) encompasses all sexually transmitted infections that are not syphilis, chlamydial infection, gonococcal infection, trichomoniasis, genital herpes simplex virus 2, or HIV.

Modelling strategy

To calculate YLDs due to acute infection with other STI, we implemented a strategy of indirect YLD estimation. We calculated the YLD to YLL ratio for all STI (excluding other STI) and then applied that same ratio to other STI YLLs.

Sequelae and disability weights

YLDs were also estimated to other STIs as a result of the proportion of PID and PID-induced infertility that was not due to chlamydial or gonococcal infection. Please see the PID and infertility sections of this appendix for more details.

Table 1. Severity distribution, details on the severity levels for other sexually transmitted infections and the associated disability weight (DW) with that severity.

Severity level	Lay description	DW (95% CI)
Primary infertility due to other sexually transmitted infections	Wants to have a child and has a fertile partner, but the couple cannot conceive.	0.008 (0.003, 0.015)
Secondary infertility due to other sexually transmitted infections	Has at least one child, and wants to have more children. The person has a fertile partner, but the couple cannot conceive.	0.005 (0.002, 0.011)
Moderate pelvic inflammatory diseases due to other sexually transmitted infections	Has pain in the belly and feels nauseous. The person has difficulties with daily activities.	0.114 (0.078, 0.159)
Severe pelvic inflammatory diseases due to other sexually transmitted infections	Has severe pain in the belly and feels nauseous. The person is anxious and unable to carry out daily activities.	0.324 (0.220, 0.442)

Tetanus

Flowchart

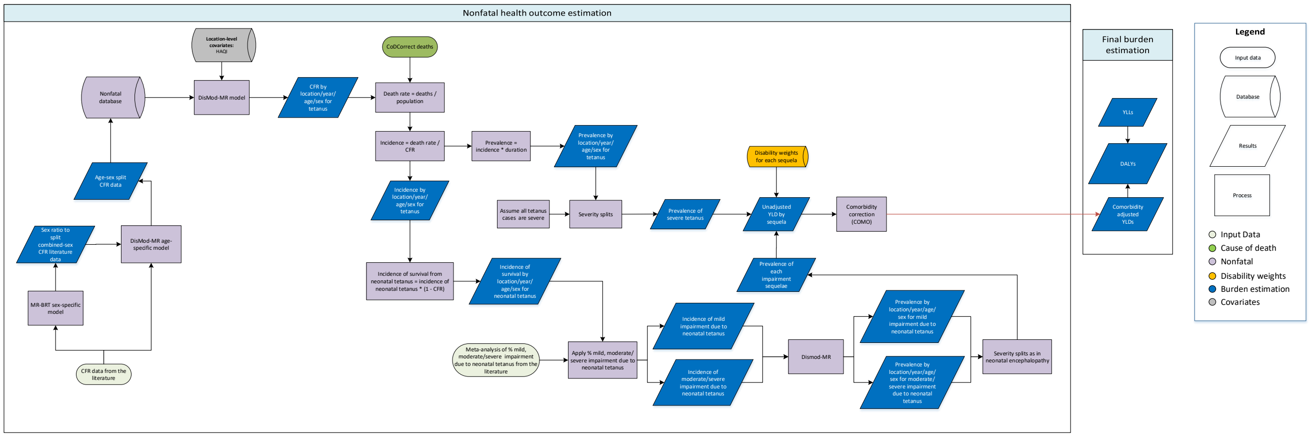

Input data and methodological summary for tetanus

Case definition

Tetanus is a life-threatening disease caused by infection with the toxin-producing bacterium *Clostridium tetani.* Tetanus is usually acquired via contamination of wounds. Neonatal tetanus is often caused by contamination of the umbilical stump. Initial symptoms include failure to feed and excessive crying, progressing to the typical clinical presentation of tetanus. Severe tetanus is characterised by generalised, painful muscular spasms, with complications including respiratory failure, autonomic dysfunction, and death. For tetanus, the ICD-10 codes are A33-A35.0, Z23.5, and ICD-9 codes are 037-037.9,771.3, V03.7.

**Quantity of interest**	**Reference or alternative**	**Definition**
Tetanus case fatality rate	Reference	Ratio of fatal cases of tetanus over total confirmed cases of tetanus in the sample

Input data

Model inputs

The non-fatal tetanus model has two primary inputs. The first is data obtained from systematic reviews of the tetanus case-fatality ratio (CFR) literature. The second is GBD mortality estimates for tetanus, calculated per country using Cause of Death Ensemble modelling (CODEm).

A new systematic review of the tetanus CFR literature was completed for GBD 2023. We used the following search string in PubMed: *(tetanus [TiAb] OR "tetanus"[MeSH Terms]) AND ("case fatality" [TiAb] OR death*[TiAb] OR died[TiAb] OR mortality[TiAb]) AND (1980[PDAT]: 3000[PDAT])*. The GBD 2023 systematic review spanned publication years 1980–2020. We excluded studies with fewer than ten cases.

Input data processing

Tetanus CFR data that were not sex-specific or were reported in age range widths of greater than ten years were split into sex- and age-specific groups prior to modelling. Because scant sex- and age-specific data are available, location- or year-specific sex and age patterns could not be estimated. Instead, global sex ratios and age patterns were generated using all available sex- and age-specific tetanus CFR data. These ratios were then used to split all non-age- or sex-specific data prior to inclusion in the model. Uncertainty was propagated throughout the splitting process.

The sex ratio used to make the sex splits was calculated using MR-BRT, a Bayesian meta-regression tool. The female-to-male sex adjustment factor calculated for use in GBD 2023 modelling was 1.20 (1.16–1.24) compared to 1.15 (1.01–1.29) in GBD 2021 (Table 1).

Table 1: MR-BRT sex-splitting adjustment factor for tetanus CFR

**Data input**	**Reference or alternative case definition**	**Beta coefficient, log (95% UI)**	**Adjustment factor***
Sex	N/A	0.183 (0.149, 0.216)	1.201 (1.161, 1.241)

**MR-BRT crosswalk adjustments can be interpreted as the factor the alternative case definition is adjusted by to reflect what it would have been had it been measured using the reference case definition. If the log/logit beta coefficient is negative, then the alternative is adjusted up to the reference. If the log/logit beta coefficient is positive, then the alternative is adjusted down to the reference.*

***The adjustment factor column is the exponentiated beta coefficient. For log beta coefficients, this is the relative rate between the two case definitions. For logit beta coefficients, this is the relative odds between the two case definitions.*

To generate a global age pattern, tetanus CFR data representing age ranges of ten years in width or less were used to fit a DisMod-MR model. The Healthcare Access and Quality (HAQ) Index was included as a location-level covariate. The final global age pattern – produced by DisMod for each sex and age group from early neonatal to 95+ years – was used to split the remaining data.

Figure 1. Global age pattern for tetanus CFR (L: female, R: male)

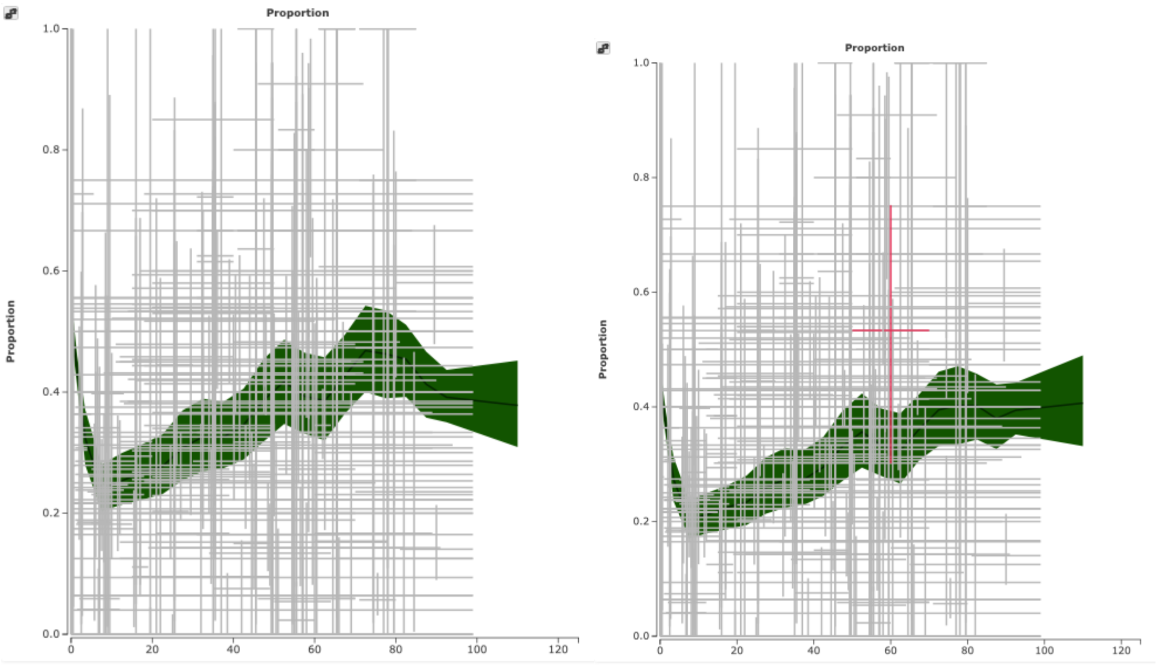

Modelling strategy

We utilised DisMod-MR to produce location-, year-, age-, and sex-specific tetanus CFR estimates from sex- and age-specific input data, following the age- and sex-splitting process described above. In the model, we used the HAQ Index as a location-level covariate, enforcing a directional prior such that locations with higher HAQ Index are predicted to have lower tetanus CFR. Table 3 displays the raw and exponentiated magnitude of covariate influence, which can be interpreted as odds ratios. With the addition of new CFR sources, CFR estimates changed globally. Estimates in some super-regions (including south Asia and north Africa and the Middle East) increased substantially.

Incidence rates were calculated by dividing GBD 2023 mortality rate estimates by the predicted CFR ratio. As in GBD 2021, GBD 2023 tetanus mortality rates were produced using separate CODEm models for all combinations of children under 1 year of age and children and adults over 1, data-rich and non-data-rich countries, and for females and males. Prevalence was calculated by multiplying the incidence rate by tetanus case duration, estimated to last an average of 20 days. These calculations were completed for each of 1000 samples (draws) from the posterior distribution, then summarised as a mean of draws and a 95% uncertainty interval (the 2.5th and 97.5th percentile of all draws).

Severity splits and disability weights

All tetanus cases are assumed to be severe, acute infections. Table 2 presents our lay description of severe tetanus in addition to the disability weight applied. For neonatal tetanus impairments, our distribution matches the distribution of neonatal encephalopathy.

Table 2. Severity distribution, details on the severity levels for tetanus in GBD 2023 and the associated disability weight (DW) with that severity

**Severity level**	**Lay description**	**DW (95% CI)**
Severe	Has a high fever and pain, and feels very weak, which causes great difficulty with daily activities.	0.133<br>(0.088–0.19)

Table 3. Covariates. Summary of covariates used in the tetanus CFR DisMod-MR meta-regression model

**Covariate**	**Type**	**Parameter**	**Exponentiated beta (95% CI)**
Healthcare Access and Quality (HAQ) Index	Country-level	Case fatality ratio	0.78 (0.73–0.83)

To estimate mild and moderate impairment due to neonatal tetanus, we first computed the incidence of survival from neonatal tetanus as:

$$incidence\ of\ survival = incidence * (1 - CFR)\ .$$

To appropriately proportion impairments as either mild or moderate-to-severe, we used estimates from the literature. We applied a split of 0.11 for mild impairments and 0.07 for moderate-to-severe impairments to the incidence of survival and calculated the incidence of survival from neonatal tetanus with mild impairment and with moderate-to-severe impairment. These estimates were each then used as input data for separate DisMod-MR models, which in turn produced estimates of the prevalence of mild or moderate-to-severe impairment due to neonatal tetanus for all ages, sexes, years, and locations. As in GBD 2021, we included neonatal encephalopathy excess mortality rate as a prior on excess mortality in the moderate-to-severe impairments model.

Changes from GBD 2021 to GBD 2023

There were no substantive changes to the modelling strategy for GBD 2023. Changes in the non-fatal tetanus estimates between GBD 2021 and GBD 2023 were primarily driven by the addition of new cause of death data and updates to data sources used in the tetanus CFR model, as described above.

Figure 2: PRISMA 2020 flow diagram for tetanus CFR

From: Page MJ, McKenzie JE, Bossuyt PM, et al. The PRISMA 2020 statement: an updated guideline for reporting systematic reviews. *BMJ*. 2021;372:n71. Published 2021 Mar 29. doi:10.1136/bmj.n71

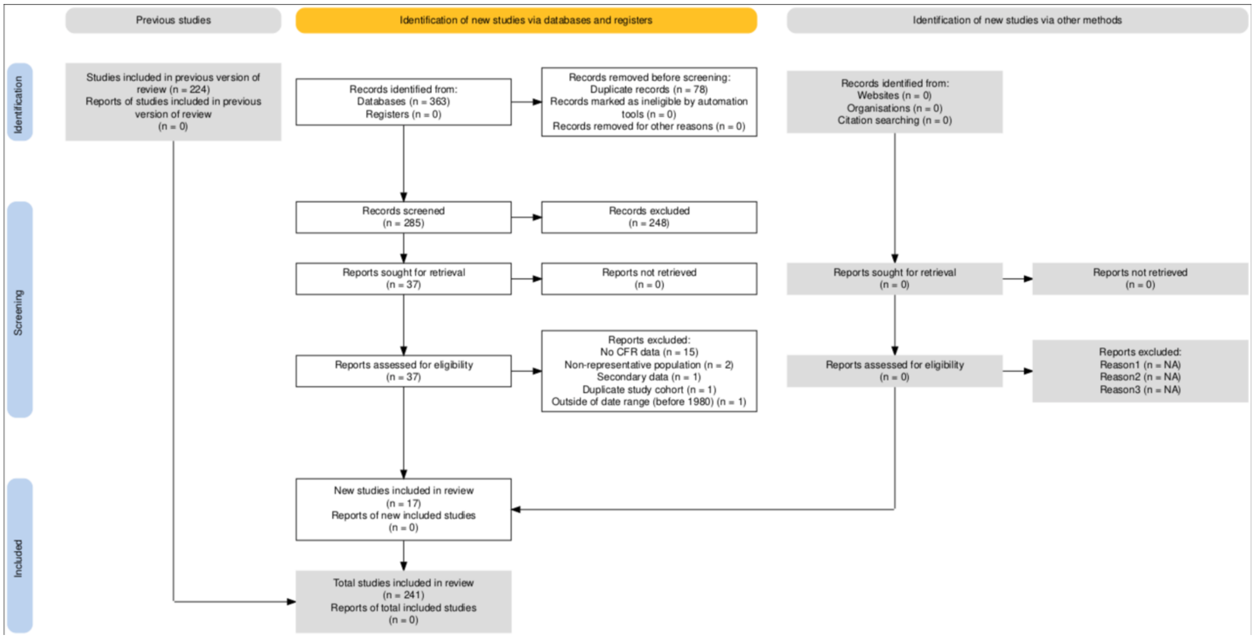

Trichuriasis

Flowchart

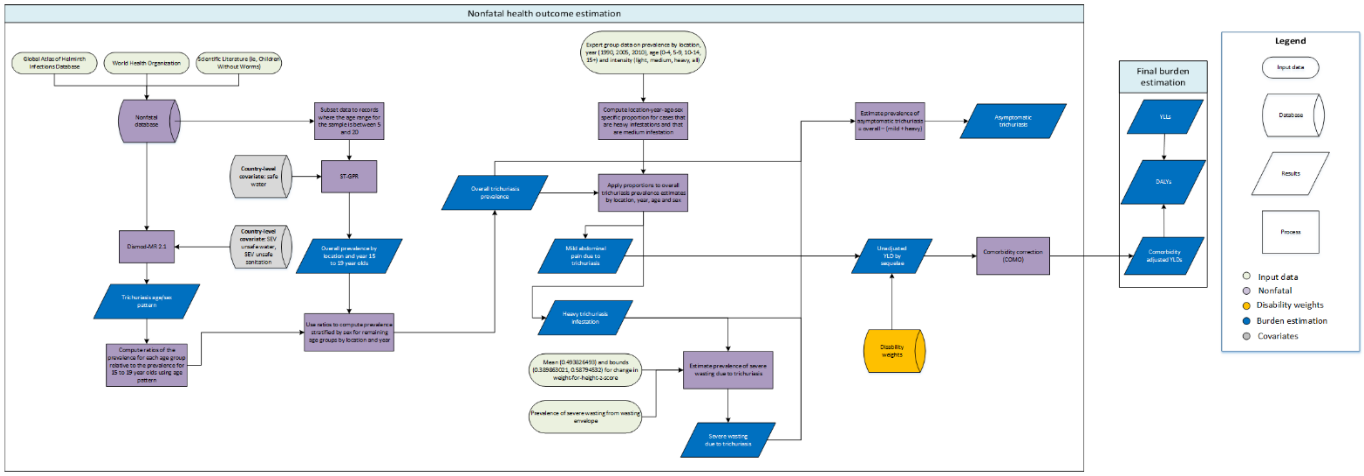

Input data and methodological summary for trichuriasis

Case definition

Trichuriasis is a helminth disease caused by the parasitic whipworm *Trichuris trichiura*. It is one of the three intestinal nematode infections (INI), or soil-transmitted helminthiasis (STH), that we model in GBD. Diagnosis is made by examination of stool by microscope or PCR, with or without concentration procedures. The ICD-10 code for trichuriasis is B79. We used the following case definition for GBD 2023:

Quantity of interest	Reference or alternative	Definition
Trichuriasis	Reference	Diagnosis made by examination of stool using Kato-Katz technique, resulting in positive for intestinal helminth eggs of type *T trichiura*.

Input data

The primary input data for this model was from the Global Atlas of Helminth Infections (GAHI) database and the Expanded Special Project for the Elimination of Neglected Tropical Diseases (ESPEN). The GAHI and ESPEN databases include surveys and studies conducted to measure the prevalence of STH.[1] Each record in the database contained metadata (ie, location, year, age range, sex) of each study sample and the prevalence of trichuriasis in that sample.

We supplemented the GAHI data with survey-data collected in a literature review performed by Children Without Worms (CWW), which included countries outside of sub-Saharan Africa; a 2001–2004 China subnational survey to better inform our estimates in China; and additional data provided by the World Health Organization (WHO). For GBD 2023, we added data from systematic reviews (Figure 2a) and additional extracted data from the GAHI, CWW, and WHO datasets (Figure 2b). For all input data, we

excluded datapoints where the age range of the sample was unknown and retained only those surveys utilising the Kato-Katz diagnostic method.

Geographical restrictions

We conducted a literature review (last updated for GBD 2017) to determine the geographical extent of the disease and classify locations based on whether the disease is absent or present in each year. Locations that were geographically restricted in any given year did not have estimates made for them. Of note, we did not attempt a complete systematic review, since a single high-quality source could offer sufficient evidence of presence. Evidence of absence or presence was not available for every location for each year. Assumptions made for missing years took into consideration the epidemiological characteristics of the disease.

If evidence indicated disease presence for two non-consecutive years, we assumed presence for all years between the two. If evidence indicated disease absence for two non-consecutive years, we assumed absence for all years between the two. If evidence indicated a change in status (ie, from absent to present, or present to absent) between two non-consecutive years, then we conducted targeted searches to ascertain the relevant year of introduction or elimination for that location. In the cases where presence or absence information was missing for the start or end years of our study interval without evidence of any introduction or elimination events within the interval, we applied the status of the first and last presence/absence observations, respectively, to all years between the interval bound and the observation year. Table 1 shows the search strings and associated yield for each of the databases queried.

Table 1. Geographical restriction search strings

**Database**	**Search String**	**Yield**
PubMed	(Ascariasis[Title/Abstract] OR Ascaris[Title/Abstract] OR "A. lumbricoides"[Title/Abstract] OR Ascaris[MeSH] OR Trichuris[Title/Abstract] OR Trichuriasis[Title/Abstract] OR "Whip Worm"[Title/Abstract] OR "T. trichura"[Title/Abstract] OR Trichuris[MeSH] OR Hookworm[Title/Abstract] OR "A. duodenale"[Title/Abstract] OR "Ancylostoma duodenale"[Title/Abstract] OR ancylostomiasis[Title/Abstract] OR "N. americanus"[Title/Abstract] OR "Necator americanus"[Title/Abstract] OR necatoriasis[Title/Abstract] OR Ancylostoma [MeSH] OR Necator[MeSH]) AND (prevalence[Title/Abstract] OR incidence[Title/Abstract] OR epidemiology[Title/Abstract] OR surveillance[Title/Abstract]) NOT(Animals[MeSH] NOT Humans[MeSH])	2376
Web of Science	(Ascariasis OR Ascaris OR A. lumbricoides OR Trichuris OR Trichuriasis OR Whip Worm OR T. trichura OR Hookworm OR A. duodenale OR Ancylostoma duodenale OR anclyostomiasis OR N. americanus OR Necator americanus OR necatoriasis) AND TOPIC:(prevalence OR incidence OR epidemiology OR surveillance) NOTTOPIC: ((Animals NOT Humans)) Timespan: 1980-2016. Indexes: SCI-EXPANDED, SSCI, A&HCI, ESCI.	2266
SCOPUS	TITLE-ABS_KEY (ascariasis OR ascaris OR a. lumbricoides OR trichuris OR trichuriasis OR whip worm OR t. trichura OR hookworm OR a. duodenale	29

	OR ancylostoma duodenale OR anclyostomiasis OR n. americanus OR necator americanus OR necatoriasis) AND PUBYEAR>1979	

These papers were used to classify location-years for all locations and years present in the literature. We only utilised papers that are explicitly concerned with trichuriasis. Additionally, systematic literature reviews, meta-analyses, national health statistics publications, and collaborator input supported classification of location-years not present in the literature review wherever possible.

Modelling strategy

Prevalence model

In the estimation of overall morbidity due to hookworm disease, we implemented a three-stage modelling framework. We first utilised a spatiotemporal Gaussian process regression (ST-GPR) to generate a complete time series of estimates for each location where there are no geographical restrictions. ST-GPR attempts to model non-linear trends utilising a Gaussian process to fit a trend. We ran an age-restricted ST-GPR model, using all data with age bins between 5 and 20 because these data fall within the peak in prevalence across all age groups, the majority of data fall within these age ranges, and these data provide sufficient statistical power for our model. The following were the model specifications:

$$prevalence = improved\ water\ source + (1|level\ 1/level\ 2/level\ 3)$$

Levels 1, 2, and 3 refer to GBD location hierarchies, or nested random effects for super-region, region, and location. Covariate selection was based on directionality of the resulting beta values from the linear model, significant effect size, and subsequently out-of-sample testing for the resulting "best" combinations. The covariate used for the GBD 2023 model was proportion of population with access to improved water sources. Safe and improved water sources are defined by the Joint Monitoring Programme.[2] The following hyperparameters were used: st-lambda = 0.25, st-omega = 2, st-zeta = 0.005, gpr-scale = 15. We selected these hyperparameters as they provided more weight to country-level data rather than region-level data when estimating the prevalence for a given location-year, ensuring that the Gaussian process regressions follow country-specific data rather than region-specific data when estimating a time series for a location.

Table 2b. Covariates. Summary of covariates used in the trichuriasis ST-GPR model

Covariate	Beta coefficient, log (95% UI)	Standard error	Exponentiated beta (95% UI)
Improved water	–6.024 (–6.606 to –5.443)	0.297	0.002 (0.001–0.004)

Age pattern model

The next stage of the modelling process used a DisMod Bayesian meta-regression (DisMod-MR) model, to generate a global age-sex curve to disaggregate all-age, both-sex prevalence data. DisMod-MR is an integrated meta-regression framework that allows multiple datasets to be used within a singular analysis regardless of age-binning, sources, and geographies. As a result, a variety of differently

aggregated information combine to generate a consensus output. Our final model contained all processed GAHI data as input informed by two country-level covariates (ie, all risk factors summary exposure values [SEVs] for unsafe water and unsafe sanitation).

Table 2a. Covariates. Summary of covariates used in the trichuriasis DisMod-MR model.

**Covariate**	**Type**	**Parameter**	**Exponentiated beta (95% UI)**
SEV unsafe water	Country-level	Proportion	4.43 (4.34–4.48)
SEV unsafe sanitation	Country-level	Proportion	4.43 (4.33–4.48)

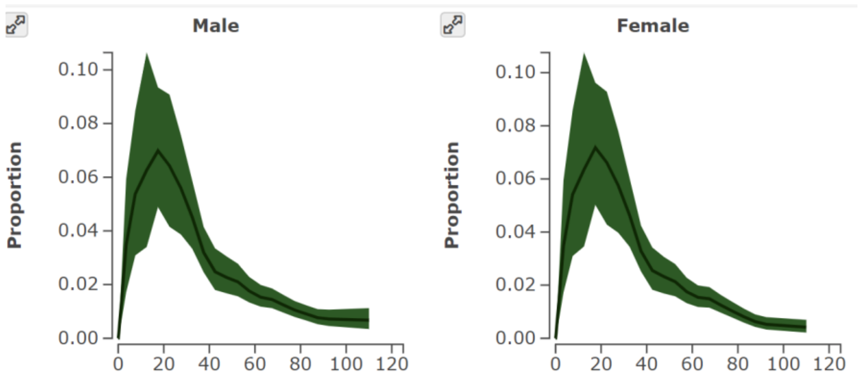

Figure 1: Global age-specific prevalence estimates for males (left) and females (right) for the year 2010. Proportion (prevalence) is on the Y-axis, and age in years on the X-axis. Screenshot from EpiViz tool.

Figure 1 shows the age-specific variation in prevalence rates, differentiated by sex. When considered as a global aggregate, we see that reported male and female prevalence are very similar. This is mostly a function of data used for modelling mainly being reported for both sexes. The highest prevalence rates are among young adults and then decline among adults.

Imputations

The final stage of the overall prevalence modelling process is to impute the remaining age groups by borrowing information from the DisMod-MR global age-sex pattern and ST-GPR time series, by first assuming the estimates from ST-GPR are representative of the 15–19-year-old age group. Each additional age group is assigned a ratio representing how much larger or smaller the prevalence is compared to the prevalence of the reference group (15–19-year-olds) using the DisMod-MR global age-sex pattern. The following is the computation for each age group:

$$Ratio = \frac{prevalence_{[age\ start]to\ [age\ end]}}{prevalence_{15\ to\ 19}}$$

With a ratio for every age group by sex, we multiplied the ratio by the ST-GPR location-year estimates to impute estimates for the remaining age groups.

Health states/sequelae

The table below shows the list of sequelae due to trichuriasis and the associated disability weights (DWs). Prevalence of medium infection and heavy infection were mapped to *mild abdominopelvic problems* and *heavy infestation of trichuriasis,* respectively. Light infection or asymptomatic were not attributed any disability.

Table 3. Severity distribution, details on the severity levels for trichuriasis and the associated disability weight (DW) with that severity

**Sequela**	**Lay description**	**DW (95% CI)**
Mild abdominopelvic problems	Has some pain in the belly that causes nausea but does not interfere with daily activities	0.011 (0.005–0.021)
Heavy infestation	Has cramping pain and a bloated feeling in the belly	0.027 (0.015–0.044)
Severe wasting	Is extremely skinny and has no energy	0.128 (0.082–0.183)
Asymptomatic trichuriasis	N/A	N/A

Following computations of location-year-age-sex-specific prevalence of trichuriasis, we leverage information from the 2010 Expert Group (EG) data to conduct sequelae splits. The 2010 EG data provided estimates for heavy infestation, mild abdominopelvic problems, and asymptomatic trichuriasis by location and for 1990, 2005, and 2010. These three values add up to *all cases* of trichuriasis. Thus, for heavy infestation and mild abdominopelvic problems, we computed the proportion of cases that belong to our sequelae of interest over *all cases* of trichuriasis. More specifically, the following is the computation by heavy infestation and mild abdominopelvic problems:

$$Proportion_{sequelae} = \frac{prevalence_{sequelae}}{prevalence_{all\ cases}}$$

This calculates proportions for every location, year, and age group available. The EG data only had four age groups (0–4, 5–9, 10–14, 15+ years), so we applied the 15+ age group proportion for all remaining age groups. In addition, for the years 1995 and 2000, we applied the 1990 proportions, and for years 2015, 2019, and 2020–2021, we applied the 2010 proportions. Using these location-year-age-specific proportions, we multiplied the total trichuriasis estimates to compute heavy infestation and mild abdominopelvic prevalence. To estimate the prevalence of asymptomatic trichuriasis, prevalence of mild and heavy infestation were each subtracted from the overall trichuriasis prevalence.

The final step in the modelling process was to estimate the prevalence of severe wasting due to trichuriasis in age groups 1–5 months, 6–11 months, 12–23 months and 2–4 years. This was done separately using 1000 draws of prevalence of heavy infestation due to trichuriasis and the wasting envelope prevalence. The initial step in determining prevalence of severe wasting due to trichuriasis was generating 1000 draws of change in weight-for-height z-score per heavy prevalent case from a random normal distribution with mean = 0.493826493 and standard deviation = 0.04972834 (calculated from upper and lower bounds of the mean estimate). The mean, upper, and lower bounds were based on a published article.[3] The prevalence of severe wasting due to trichuriasis was then obtained as a function of change in weight-for-height z-score. The following are the computations:

$$Prevalence_{wasting\ due\ to\ trichuriasis} = wasting - \Phi(\Phi^{-1}(\text{wasting}) - \text{z score} * \text{heavy infestation})$$

Where Φ is the standard normal cumulative distribution function and Φ^{-1} is the inverse standard normal cumulative distribution function.

Changes from GBD 2021 to GBD 2023

The major change from GBD 2021 was the addition of new data between the rounds. New data inputs from scientific literature and ESPEN and expansion of age-specific data from the CWW and GAHI datasets were added to the model.

Limitations

As we attempt to improve the modelling processes for trichuriasis, we recognise several limitations. We only include studies where Kato-Katz identifies infected individuals. Future updates to the model will include a systematic review for within-study comparisons of diagnostic performance to facilitate a diagnostic crosswalk model.

A secondary limitation to our data is that several included studies are not nationally representative, and therefore at a location level, the data are highly heterogeneous. Numerous studies within the database come from districts or villages, and in most cases, the studies were done in areas where prevalence is known to be high.

In addition, our current model does not include the impact of mass drug administration (MDA). In future rounds, we plan to integrate MDA coverage into the estimate of prevalence.

Furthermore, we made a large assumption that the global age-sex distributions were applicable to all locations. While we believe that prevalence should peak among adolescents and slowly decline afterward, there is likely variation across regions and locations. Given that our data are among children or all-age, it is very difficult to build an age trend at granular location levels. Thus, we allowed DisMod-MR to disaggregate our heterogeneous data in an effort to provide sensible age-sex curves.

We did not apply any adjustments for the COVID pandemic to trichuriasis due to a lack of available data quantifying the impacts of the pandemic on NTD epidemiology.

We believe that more work will improve our sequelae split methods. Since the EG data do not provide all estimation years and age groups, several assumptions had to be made to estimate sequelae for all years, locations, and age groups in GBD. Further work will be required to gather additional data and improve these sequelae estimates.

References

1. London School of Hygiene and Tropical Medicine. Global Atlas of Helminth Infections – Soil Transmitted Helminths. London, United Kingdom: London School of Hygiene and Tropical Medicine.

2. “Improved and Unimproved Water Sources and Sanitation Facilities.” *WHO / UNICEF Joint Monitoring Programme: Wat/san Categories*. The WHO/UNICEF, n.d. Web. 08 June 2016
3. Hall A, Hewitt G, Tuffrey V, de Silva N. A review and meta-analysis of the impact of intestinal worms on child growth and nutrition. *Maternal and Child Nutrition*. 2008. 4. 118-236.

Figure 2a. PRISMA 2023 flow diagram – systematic review of trichuriasis prevalence literature (updates for GBD 2023)

Source: Page MJ, et al. BMJ 2021;372:n71. doi: 10.1136/bmj.n71. This work is licensed under CC BY 4.0. To view a copy of this license, visit https://creativecommons.org/licenses/by/4.0/

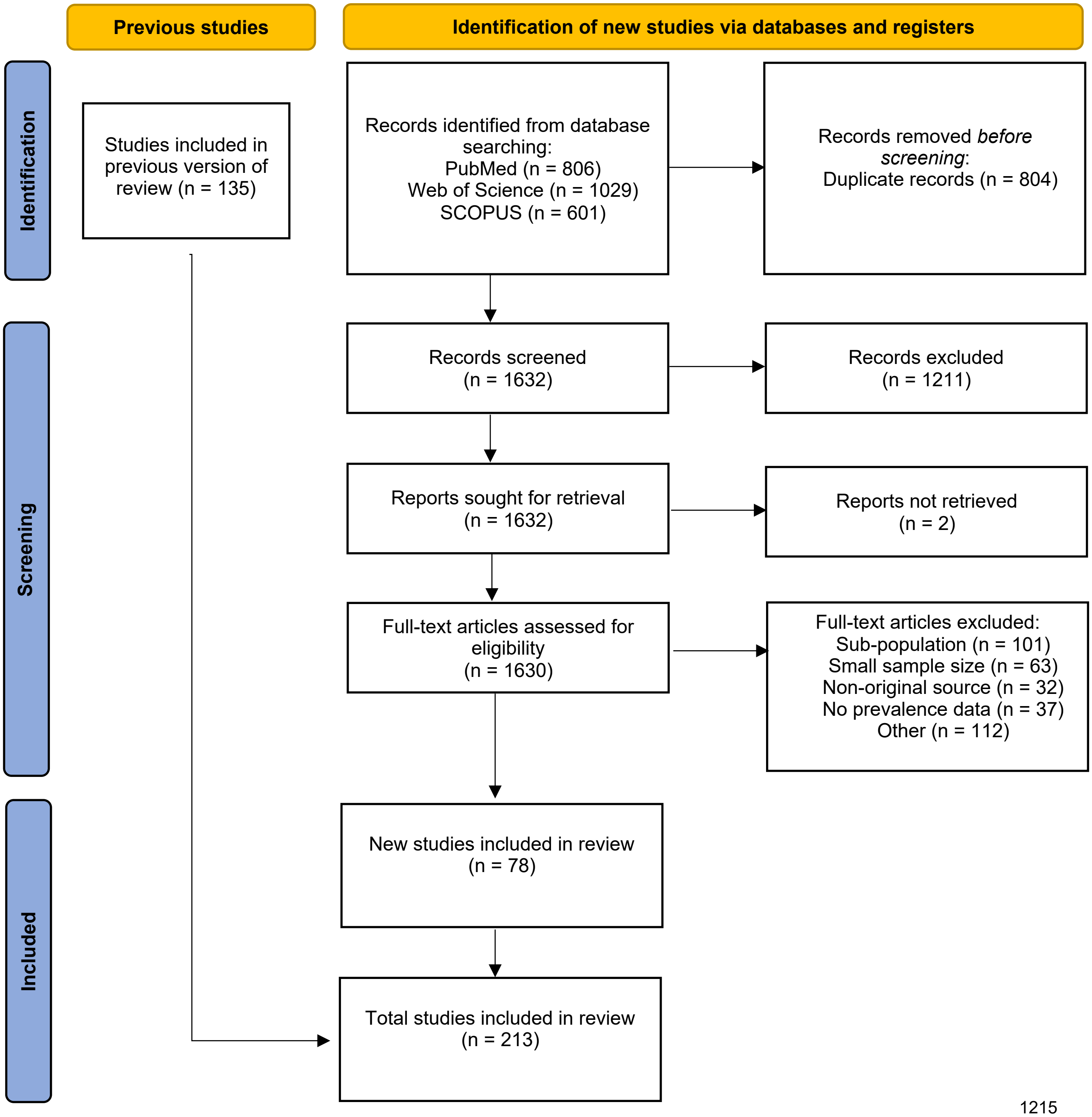

Figure 2b. PRISMA 2023 flow diagram – expansion of prevalence data from GAHI, CWW, and WHO databases (updates for GBD 2023)

Source: Page MJ, et al. BMJ 2021;372:n71. doi: 10.1136/bmj.n71. This work is licensed under CC BY 4.0. To view a copy of this license, visit https://creativecommons.org/licenses/by/4.0/

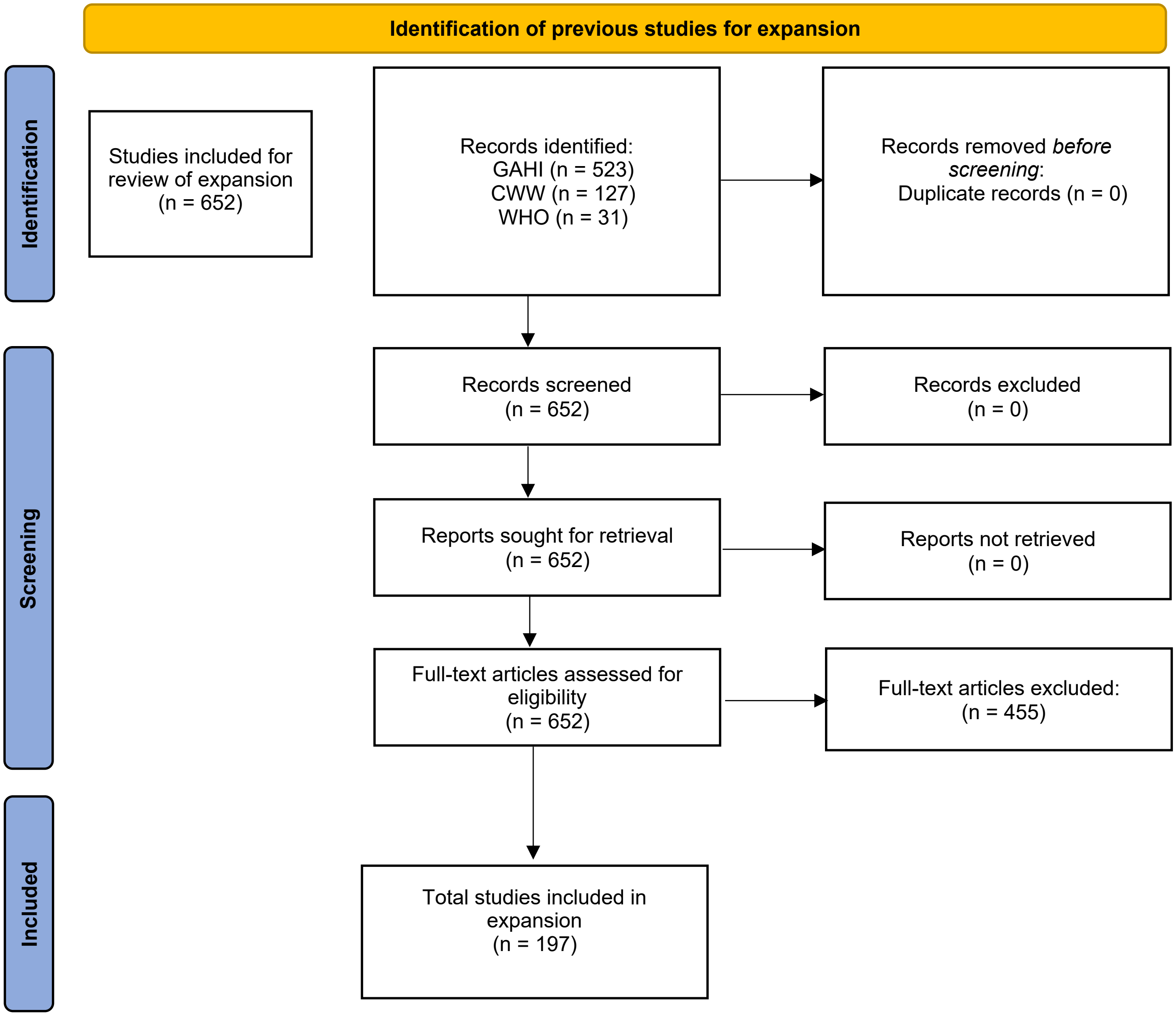

Tuberculosis

Flowchart

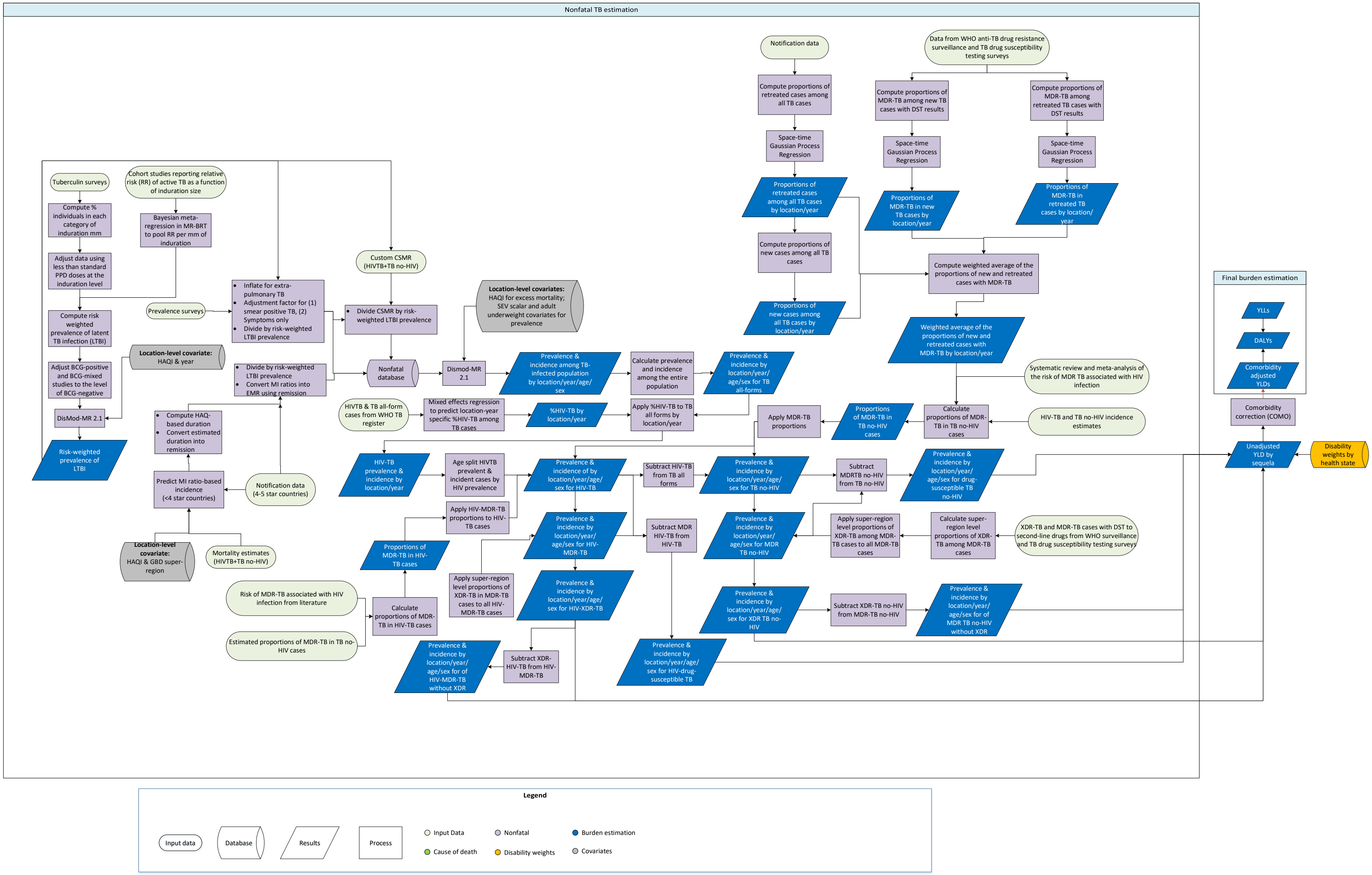

Input data and methodological summary for tuberculosis

Case definition

Tuberculosis (TB) is an infectious disease caused by *Mycobacterium tuberculosis*. The case definition includes all forms of TB, including pulmonary TB and extrapulmonary TB, which are bacteriologically confirmed or clinically diagnosed. For TB, the ICD-10 codes are A10-A19.9, B90-B90.9, K67.3, K93.0, M49.0, P37.0, and ICD-9 codes are 010-019.9, 137-137.9, 138.0, 138.9, 139.9, 320.4, 730.4-730.6. For HIV-TB, the ICD-10 code is B20.0.

Latent TB infection is defined as an infection with *Mycobacterium tuberculosis* without any symptoms or signs of active TB disease.

**Quantity of interest**	**Reference or alternative**	**Case definition**
Prevalence of LTBI	Clinical case definition	Individuals infected with *Mycobacterium tuberculosis*, but do not have TB disease. The only sign of TB infection is a positive reaction to the tuberculin skin test or TB blood test.
Prevalence of LTBI	Reference	Infected with *Mycobacterium tuberculosis* without any symptoms or signs of active TB disease among those without BCG vaccination (among those with TST).
Prevalence of LTBI	Alternative	Infected with *Mycobacterium tuberculosis* without any symptoms or signs of active TB disease among those with BCG vaccination.
Prevalence of LTBI	Alternative	Infected with *Mycobacterium tuberculosis* without any symptoms or signs of active TB disease among those with and without BCG vaccination.
Prevalence of LTBI	Reference	Infected with *Mycobacterium tuberculosis*, measured with a tuberculin skin test with 5 TU, without any symptoms or signs of active TB disease.
Prevalence of LTBI	Alternative	Infected with *Mycobacterium tuberculosis*, measured with a tuberculin skin test with TU between 1 and 3, without any symptoms or signs of active TB disease.
Prevalence of LTBI	Alternative	Infected with *Mycobacterium tuberculosis*, measured with a tuberculin skin test with 10 TU, without any symptoms or signs of active TB disease.
Risk of progression to active TB	Reference	The relative risk of developing active TB as a function of tuberculin skin test induration size.

We separately estimated the incidence and prevalence of multidrug-resistant tuberculosis and extensively drug-resistant tuberculosis by HIV status. The case definitions are shown below.

Multidrug-resistant TB without extensive drug resistance: a form of TB (among HIV-negative individuals) that is resistant to the two most effective first-line anti-tuberculosis drugs (isoniazid and rifampicin) but is not resistant to any fluoroquinolone and any second-line injectable drugs (amikacin, kanamycin, or capreomycin).

Quantity of interest	Reference or alternative	Case definition
Proportion of all-form TB cases that are multidrug-resistant	Clinical case definition	Multidrug-resistant TB (MDR TB) is caused by *Mycobacterium tuberculosis* that is resistant to at least isoniazid and rifampin.
Proportion of all-form TB cases that are multidrug-resistant	Reference	A form of tuberculosis that is resistant to the two most effective first-line anti-tuberculosis drugs (isoniazid and rifampicin) but is not resistant to any fluoroquinolone and any second-line injectable drugs (amikacin, kanamycin, or capreomycin).

Extensively drug-resistant TB: a form of TB (among HIV-negative individuals) that is resistant to isoniazid and rifampicin, plus any fluoroquinolone and any second-line injectable drugs.

Quantity of interest	Reference or alternative	Case definition
Proportion of multi-drug-resistant TB cases that are extensively drug-resistant tuberculosis	Clinical case definition	A type of TB that is resistant to isoniazid and rifampin, plus any fluoroquinolone and at least one of three injectable second-line drugs (ie, amikacin, kanamycin, or capreomycin).
Proportion of multi-drug-resistant TB cases that are extensively drug-resistant tuberculosis	Reference	A form of tuberculosis that is resistant to isoniazid and rifampicin, plus any fluoroquinolone and any second-line injectable drugs.

Drug-susceptible TB: TB (among HIV-negative individuals) that is susceptible to isoniazid and rifampicin.

Quantity of interest	Reference or alternative	Case definition
Proportion of all-form TB cases that are sensitive to first-line drugs	Clinical case definition	A form of tuberculosis that is susceptible to at least isoniazid and rifampicin.
Proportion of all-form TB cases that are sensitive to first-line drugs	Reference	A form of tuberculosis that is susceptible to isoniazid and rifampicin

Tuberculosis is estimated by HIV status using the case definitions for the drug resistance splits listed above and HIV/AIDS-TB listed below. The HIV-TB child causes are:

- HIV/AIDS – multidrug-resistant TB without extensive drug resistance: a form of TB (among HIV-positive individuals) that is resistant to the two most effective first-line anti-tuberculosis drugs (isoniazid and rifampicin) but is not resistant to any fluoroquinolone and any second-line injectable drugs (amikacin, kanamycin, or capreomycin).
- HIV/AIDS – extensively drug-resistant TB: a form of TB (among HIV-positive individuals) that is resistant to isoniazid and rifampicin, plus any fluoroquinolone and any second-line injectable drugs.
- HIV/AIDS – drug-susceptible TB: TB (among HIV-positive individuals) that is susceptible to isoniazid and rifampicin.

HIV/AIDS – tuberculosis

**Quantity of interest**	**Reference or alternative**	**Case definition**
Proportion of all-form TB cases are also infected with HIV	Clinical case definition	Co-infection with both HIV and TB.
Proportion of all-form TB cases are also infected with HIV	Reference	All-form tuberculosis cases that are recorded as positive in an HIV test

Input data

Input data for TB include annual case notifications, data from prevalence surveys, and estimated cause-specific mortality rates (CSMR) of TB among HIV-positive and HIV-negative individuals. For latent TB infection (LTBI), input data include (1) population-based tuberculin surveys, and (2) cohort studies examining the risk of developing active TB disease as a function of induration size. An updated systematic review looking for tuberculosis prevalence data was done for GBD 2023. The search terms, number of studies identified, and number of studies included are shown in the table below.

Outcome	Search terms	Total number of studies identified	Number of studies included
Tuberculosis	Pubmed: *("tuberculosis"[MeSH] OR tuberculosis[Title/Abstract] OR TB[Title/Abstract] OR Mycobacterium tuberculosis[Title/Abstract] AND prevalence[Title/Abstract] AND ("2020/03/30"[PDAT] : "2024/04/02"[PDAT]) NOT (animals\{SESH] NOT humans[MESH])*	479	3

Input data for multidrug-resistant TB (MDR-TB) and extensively drug-resistant TB (XDR-TB) include (i) the number of MDR-TB cases, XDR-TB cases, new and retreated TB cases with a drug sensitivity testing (DST) result for isoniazid and rifampicin, and MDR-TB cases with DST for second-line drugs from routine surveillance and surveys reported to the World Health Organization, and (ii) the risk of MDR-TB associated with HIV infection from the literature.[1]

Figure 1. PRISMA diagram of TB all forms prevalence in GBD 2023

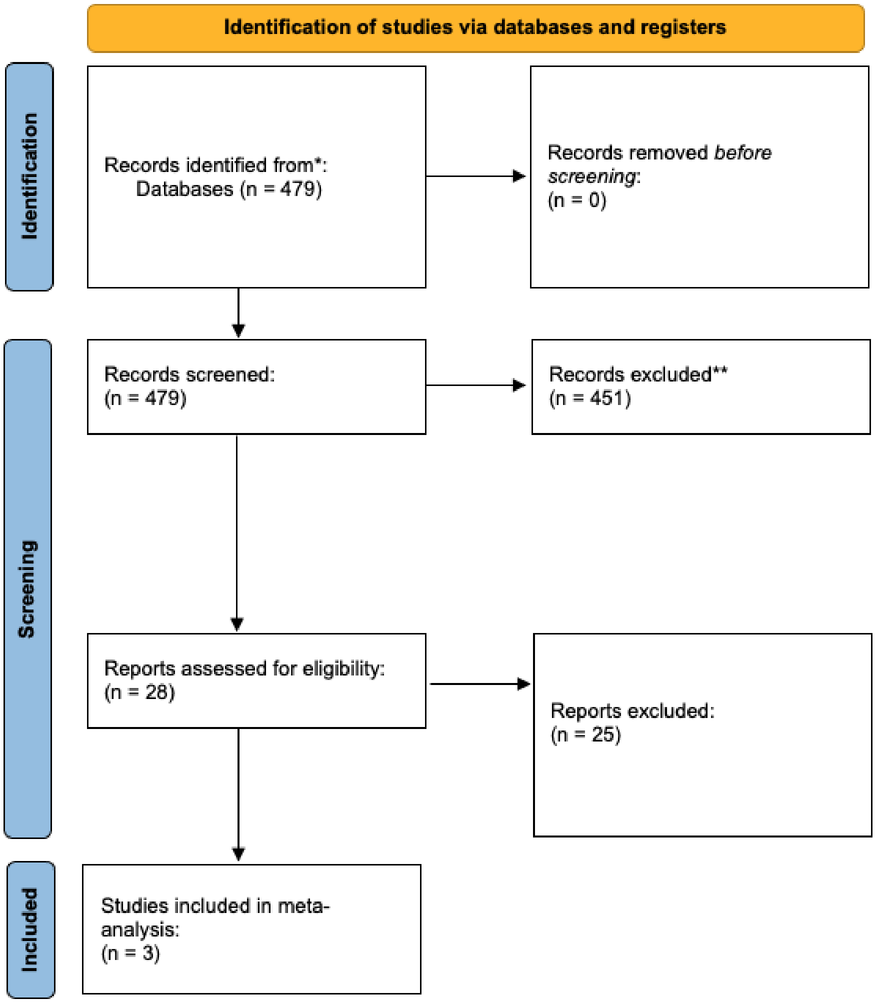

From: Page MJ, McKenzie JE, Bossuyt PM, Boutron I, Hoffmann TC, Mulrow CD, et al. The PRISMA 2020 statement: an updated guideline for reporting systematic reviews. BMJ 2021;372:n71. doi: 10.1136/bmj.n71

For more information, visit: http://www.prisma-statement.org/

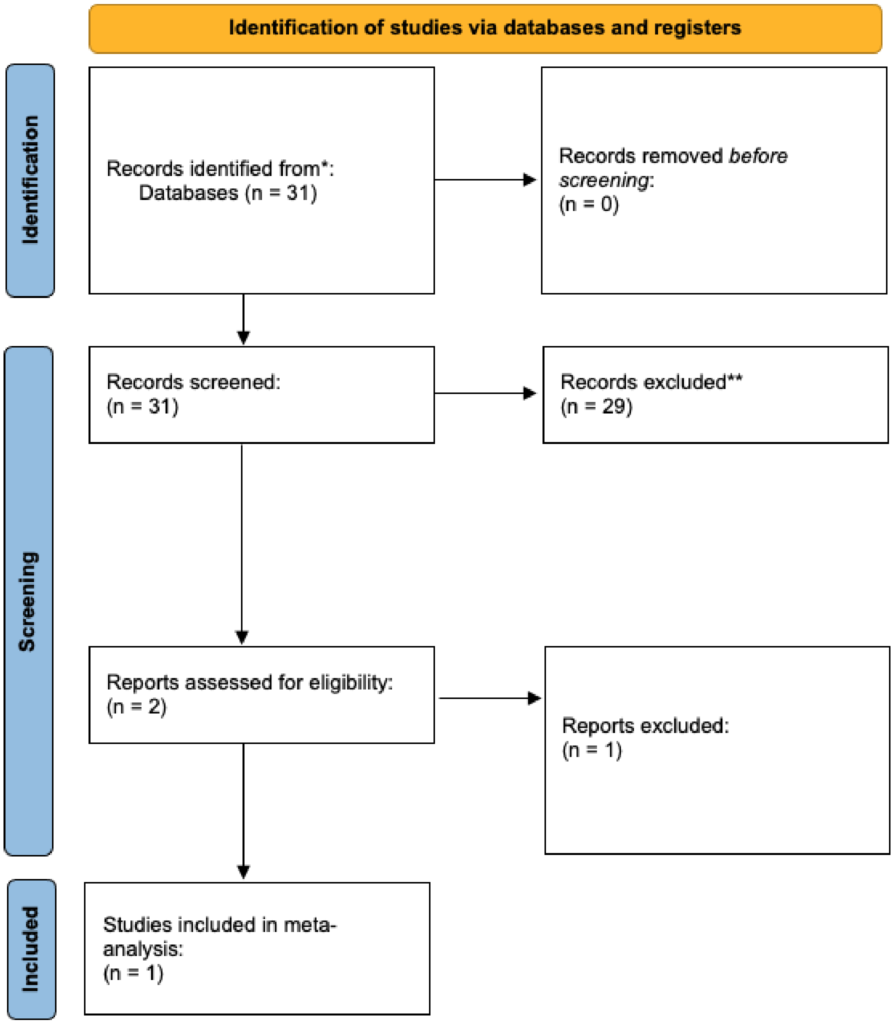

From: Page MJ, McKenzie JE, Bossuyt PM, Boutron I, Hoffmann TC, Mulrow CD, et al. The PRISMA 2020 statement: an updated guideline for reporting systematic reviews. BMJ 2021;372:n71. doi: 10.1136/bmj.n71

For more information, visit: http://www.prisma-statement.org/

Modelling strategy

Overview

We used the meta-regression with Bayesian priors, regularisation, and trimming (MR-BRT) model as the primary analytical engine to predict MI ratios, and we used modelled excess mortality rate (EMR) as input in DisMod. First, we estimated risk-weighted prevalence of LTBI by location, year, age, and sex using data from population-based tuberculin surveys and cohort studies reporting the risk of developing active TB disease as a function of induration size. Next, we divided the inputs on prevalence (from surveys in low- and middle-income countries), incidence (notification data from countries with a four- or five-star rating, and estimated incidence for countries with a less than four-star rating), and cause-

specific mortality rate (CSMR) by the risk-weighted LTBI prevalence to model TB among those at risk in each country. Next we ran MR-BRT (with GBD super-region fixed effects) using MI ratios (logit transformed) from locations with a 4- or 5-star rating on causes of death with HAQ Index as a covariate anchoring the lower end of the HAQ Index scale with a datapoint from the Bangalore study[2] reporting that 49.2% of 126 untreated new pulmonary TB cases were dead at the end of the five-year follow-up period, to predict age-sex-specific MI ratios for all locations and years. We then estimated age-sex-specific incidence using the predicted MI ratios and CSMR estimates. Finally, we modelled remission as a function of the HAQ Index and used estimated remission to convert MI ratios into excess mortality rates (EMR).

We used DisMod-MR 2.1, the GBD Bayesian meta-regression tool, to generate consistent trends in all parameters. We then multiplied the DisMod-MR 2.1 outputs by the risk-weighted prevalence of LTBI to get population-level estimates of incidence and prevalence. Because the outputs from DisMod-MR 2.1 are for all forms of TB, we split them into MDR-TB and XDR-TB by HIV status. To do so, we estimated the proportions of TB cases with MDR-TB for all locations and years, using data from notifications and survey data. We then estimated the proportions of MDR-TB among HIV-negative individuals and MDR-TB among HIV-positive individuals based on the risk of MDR-TB associated with HIV infection from a meta-analysis.[1] To split MDR-TB into MDR-TB with and without extensive drug resistance, we pooled the limited notification and survey data on the proportion of MDR-TB cases with extensive drug resistance by super-region, and applied these proportions to MDR-TB cases among HIV-negative and HIV-positive individuals, respectively.

Modelling risk-weighted latent TB infection prevalence

Input data for modelling risk-weighted LTBI prevalence were from two sources: (i) population-based tuberculin skin test (TST) surveys, and (ii) cohort studies examining the risk of developing active TB disease as a function of induration size. First, we extracted the prevalence of tuberculin skin testing results by induration size using the most detailed induration categories reported by studies. Second, we extracted relative risk data from cohort studies reporting on the risk of developing active TB disease as a function of induration size. We then pooled the risk of developing active TB by induration size in millimeters using MR-BRT to allow for integration over binned data. Third, we multiplied the LTBI prevalence by induration in millimeters ranging from 0-20+ with the relative risk of developing active TB at each induration size and summed them up to derive risk-weighted LTBI prevalence for each age group.

Available evidence[3] suggests that people with very advanced HIV infection (CD4 counts <200 cells/mm^3) may have a false-negative TST (0 mm induration) due to profound immune suppression, but still have very high risk for TB. For those who are HIV-positive, but with higher CD4 counts, the risk for active TB increases with greater induration size as in HIV-negative individuals (ie, the shape of the tuberculin response curve is similar to that for the general population). To take into account the false-negative TST response in HIV cases with profound immune suppression, we first computed the proportion of HIV-positive individuals with CD4 counts <200 cells/mm^3 for the 0 mm induration group using our HIV prevalence estimates for that particular category. We then multiplied that proportion by the relative risk of developing active TB disease in the 0 mm induration group compared with the 20+ mm induration

group among HIV-positive individuals. The relative risk was computed using data from a prospective, multicenter cohort study of HIV-positive people in the USA.[3]

Additional evidence[4] indicates that lower doses of PPD (eg, 1 TU RT23) in a tuberculin skin test yields smaller reactions compared to the standard dose (2 TU RT23; 5 TU PPD-S). In GBD 2023, we adjusted for this bias by collating data from studies that report the difference in reactivity between the standard dose and smaller doses in the same population. We used the reported mean difference from two studies[4,5] in the MR-BRT model to derive a pooled difference. We then added this pooled difference to every reported induration category from studies using lower doses of PPD to adjust the data to the level of the standard dose. In GBD 2023 we also utilised the MR-BRT model to derive adjustment factors for studies where the entire sample is BCG-positive and for studies where BCG status is mixed. The table below contains adjustment factors for BCG status in GBD 2023:

Table 1: MR-BRT crosswalk odds ratio for latent tuberculosis infection

Reference or alternative case definition	Gamma	Beta coefficient, logit (95% CI)	Relative odds ratio*
BCG negative	0.36	---	---
BCG mixed		0.09 (–0.04 to 0.22)	1.09 (0.97 to 1.25)
BCG positive		0.42 (0.39 to 0.45)	1.52 (1.48 to 1.57)

**MR-BRT crosswalk adjustments can be interpreted as the factor the alternative case definition is adjusted by to reflect what it would have been had it been measured using the reference case definition. If the log/logit beta coefficient is negative, then the alternative is adjusted up to the reference. If the log/logit beta coefficient is positive, then the alternative is adjusted down to the reference.*

***The adjustment factor column is the exponentiated beta coefficient. For log beta coefficients, this is the relative rate between the two case definitions. For logit beta coefficients, this is the relative odds between the two case definitions.*

Using the risk-weighted LTBI prevalence (adjusted for a false-negative TST among people with advanced HIV infection, for non-standard PPD doses, and for BCG status) as input data, we ran a DisMod-MR 2.1 model with the HAQ Index covariate to help inform variation over year and geography, with priors that at higher HAQ Index values, LTBI prevalence decreases. To stabilise temporal trends, we included a covariate for year with priors such that LTBI prevalence decreases over time.

Modelling TB incidence

Incidence inputs were from two different sources: (1) incidence from notification data for countries with a 4- or 5-star rating on their cause of death data[6] as a proxy for the quality of health-related administrative data systems, and (2) estimated incidence for countries with a less than four-star rating. We used the age- and sex-specific notifications (all new and relapse cases combined) in our analysis. Prior to 2013, notification data were available by case type (new pulmonary smear-positive, new pulmonary smear-negative, and new extrapulmonary) and there were missing age data, especially for younger age groups in some countries. We imputed the missing age groups for the three forms of TB

notifications. Smear-positive age-specific notifications were inflated with the proportion smear-unknown and relapsed cases only reported at the country-year level. Some countries reported only pulmonary smear-positive cases for selected years. Missing smear-negative and extrapulmonary cases were predicted from the adjusted smear-positive cases using a seemingly unrelated regression. All three types of notifications were added together to represent TB-all-form incidence for countries with a four- or five-star rating.

To generate incidence estimates for locations with a less than four-star rating, we implemented the MR-BRT model with age and sex dummies and super-region fixed effects, using MI ratios (logit transformed) from locations with a 4- or 5-star rating on causes of death as input data with HAQ Index as a covariate anchoring the lower end of the HAQ Index scale with a datapoint from a cohort study in the 1960s[2] reporting that 49.2% of 126 untreated new pulmonary TB cases were dead at the end of the five-year follow-up period, in order to predict age-sex-specific MI ratios for all locations and years. We then used the MI ratios and cause-specific mortality estimates to compute the incidence input for DisMod-MR 2.1 for locations with a less than four-star rating. Finally, we computed the age-sex-specific incidence of TB among the latent TB-infected population, using TB incidence as the numerator and our estimated risk-weighted latent TB infection prevalence as the denominator.

Since this method may result in incidence estimates that diverge from case notifications, we made an update to our approach in GBD 2023 to better align with case notification. We first determine the upper limit (99th percentile) of the fraction of all TB case notifications that are likely true TB cases (i.e., those that are bacteriologically confirmed) from countries with high quality information systems (countries with 4-5-star ratings as determined by our cause of death star rating system). We took the 99th percentile value and created a ratio with TB incidence estimates from DisMod MR 2.1. This resulting ratio was then applied to countries with lower-quality data to determine the likely true TB case notification rate. The end goal was for our methods to account for the fact that not all notified cases are bacteriologically confirmed and might lead to overestimation for TB incidence.

Modelling TB prevalence

Data from prevalence surveys reporting on pulmonary smear-positive TB and bacteriologically positive TB were included. Because incidence data are for all forms of TB, we adjusted prevalence surveys to account for extrapulmonary cases. We ran a spatiotemporal Gaussian process regression to predict location-year-age-sex-specific proportions of extrapulmonary TB among all TB cases using data on the three forms of TB from the incidence data above. We then computed the extrapulmonary inflation factor as *1+(proportion of extrapulmonary TB /(1- proportion of extrapulmonary TB))*, and applied it to data from prevalence surveys.

In GBD 2023, we used the MR-BRT model to derive adjustment factors for studies where the case definition was smear-positive TB rather than bacteriologically positive TB (reference). For the adjustment, we identified all prevalence surveys that provided comparisons of smear-positive TB and bacteriologically positive TB from the same sample. Overall, 16 prevalence surveys from Cambodia, China, Ethiopia, the Gambia, India, Myanmar, South Korea, the Philippines, Rwanda, and Viet Nam were included as inputs in the MR-BRT model. The model also contained covariates for sex and age to reflect gradients across demographics. In GBD 2023, we also computed an adjustment factor to adjust studies that used symptoms only as a screening method compared to studies using both symptoms and chest X-

ray during screening (reference). To derive the adjustment factor, we ran a MR-BRT model with data from six studies[7,8,9,10,11,12] comparing prevalence between using symptoms only as opposed to symptoms and chest X-ray in the same population as input. The adjustment factors are in the table below.

Finally, we computed the prevalence of TB among the TB-infected population, using TB prevalence as the numerator and our estimated risk-weighted LTBI prevalence as the denominator. We included two location-level covariates, namely, age-standardised adult underweight prevalence and log-transformed age-standardised summary exposure value (SEV) scalar for TB (a summary variable of the exposure levels of TB risk factors weighted by relative risk) to help inform variation of TB prevalence over year and geography.

Table 2: MR-BRT crosswalk relative odds ratio for tuberculosis prevalence

Reference or alternative case definition	Gamma	Beta coefficient, log (95% CI)	Relative odds ratio*
Bacteriologically positive	0.23	---	---
Smear positive		–0.46 (–0.70 to –0.22)	0.63 (0.50 to 0.80)
Symptoms and chest X-ray	0	---	---
Symptoms only		–0.37 (–0.50 to –0.25)	0.69 (0.61 to 0.78)

**MR-BRT crosswalk adjustments can be interpreted as the factor the alternative case definition is adjusted by to reflect what it would have been had it been measured using the reference case definition. If the log/logit beta coefficient is negative, then the alternative is adjusted up to the reference. If the log/logit beta coefficient is positive, then the alternative is adjusted down to the reference.*

***The adjustment factor column is the exponentiated beta coefficient. For log beta coefficients, this is the relative rate between the two case definitions. For logit beta coefficients, this is the relative odds between the two case definitions.*

Modelling TB remission and excess mortality

In GBD 2023, we computed TB duration based on a systematic review of studies during the pre-chemotherapy era finding that duration from onset to cure or death is 3 years.[13] To anchor the lowest end of TB duration we assumed a duration of 6 months based on treatment regimens. We then linearly interpolated between 6 months and 3 years across the HAQ Index to compute TB duration for every country-year. We converted duration into remission by taking the inverse (ie, remission = 1/duration). Using HAQ Index-based remission and estimated MI ratios, we computed excess mortality rate (EMR) with the following computation: EMR = MI*Remission (formula derived from Prevalence=Incidence*Duration).

DisMod-MR 2.1

For each location, we included the following as input in the DisMod model: case notifications for locations with a 4- or 5-star rating, predicted MI-ratio-based incidence for locations with a less than 4-star rating, prevalence survey data where available, predicted excess mortality estimates, HAQ Index-based remission, and CSMR (TB and HIV-TB combined) by age and sex.

The output from the DisMod model was for all forms of TB in TB-infected populations, including both HIV-negative and HIV-positive individuals. We computed the incidence and prevalence of TB among the

entire population by multiplying the prevalence of LTBI with the DisMod model estimates. Betas and exponentiated values from the DisMod model are shown in the table below.

Covariate	Parameter	Beta (95% CI)	Exponentiated beta (95% CI)
Sex (male)	Prevalence	0.45 (0.48–0.50)	1.61 (1.56–1.66)
Sex (male)	Incidence	0.33 (0.33–0.34)	1.40 (1.39–1.40)
Age-standardised proportion adult underweight	Prevalence	1.85 (1.68–2.00)	6.38 (5.35–7.43)
Age-standardised SEV scalar (log-transformed)	Prevalence	0.76 (0.75–0.76)	2.13 (2.12–2.15)

**MR-BRT crosswalk adjustments can be interpreted as the factor the alternative case definition is adjusted by to reflect what it would have been had it been measured using the reference case definition. If the log/logit beta coefficient is negative, then the alternative is adjusted up to the reference. If the log/logit beta coefficient is positive, then the alternative is adjusted down to the reference.*

***The adjustment factor column is the exponentiated beta coefficient. For log beta coefficients, this is the relative rate between the two case definitions. For logit beta coefficients, this is the relative odds between the two case definitions.*

HIV-TB incidence and prevalence

To distinguish HIV-TB from all forms of TB, we first estimated the proportions of HIV-TB cases among all TB cases using data on the number of TB cases recorded as HIV-positive and the number of TB cases with an HIV test result recorded in the WHO TB notifications register. We ran a mixed effects regression using the adult HIV death rate as a covariate to predict location-year-specific HIV-TB proportions, which were then applied to TB incident and prevalent cases from DisMod, to generate HIV-TB incident and prevalent cases by location and year. These cases were then age-sex split based on the age-sex pattern of estimated HIV prevalence by location-year to generate location-year-age-sex-specific HIV-TB incident and prevalent cases.

Multidrug-resistant TB, extensively drug-resistant TB, and drug-susceptible TB

We ran spatiotemporal Gaussian process regressions to predict the proportions of new TB cases with MDR-TB, proportions of retreated TB cases with MDR-TB, and proportions of retreated cases among all TB cases for all locations and years. We calculated the proportions of new TB cases among all TB cases as *1 – estimated proportions of retreated cases*. Next, we computed the weighted average of the proportions of new and retreated cases with MDR-TB at the 1000 draw level. We then used the weighted average proportions of MDR-TB, along with the HIV-TB and TB no-HIV incidence estimates, and the relative risk of MDR-TB associated with HIV infection from the literature[1] to compute the proportions of MDR-TB cases among HIV-negative TB cases $(PnoHIV_{c,y,a,s})$ by location, year, age, and sex using the following formula:

$$PnoHIV_{c,y,a,s} = \frac{MDR_{c,y}}{\left(1 + \left(RR\frac{HIVTB_{c,y,a,s}}{TBnoHIV_{c,y,a,s}}\right)\right) TBnoHIV_{c,y,a,s}}$$

where $MDR_{c,y}$ is the number of all MDR-TB cases among HIV-positive and HIV-negative individuals by location and year, RR is the relative risk of MDR-TB associated with HIV infection, $HIVTB_{c,y,a,s}$ is the number of HIV-TB incident cases by location, year, age, and sex, and $TBnoHIV_{c,y,a,s}$ is the number of TB no-HIV incident cases by location, year, age, and sex.

We then applied the predicted proportions of MDR-TB cases among HIV-negative TB cases to our predicted HIV-negative TB incident and prevalent cases to generate MDR-TB incident and prevalent cases by location, year, age, and sex. Next, we subtracted MDR-TB cases from all HIV-negative TB cases to generate drug-susceptible TB cases by location, year, age, and sex. To distinguish XDR-TB from MDR-TB, we aggregated the XDR-TB cases and MDR-TB cases (with drug sensitivity testing for second-line drugs) up to the super-region level and calculated the super-region-level proportions of XDR-TB among MDR-TB cases, which were then applied to MDR-TB cases in corresponding countries within the super-regions to produce XDR-TB cases by location, year, age, and sex. We linearly extrapolated XDR-TB prevalence and incidence back assuming the rates were zero in 1992, one year before 1993 when XDR-TB was first recorded in USA surveillance data.[14] Finally, we subtracted XDR-TB cases from MDR-TB cases to generate MDR-TB (without XDR) cases by location, year, age, and sex.
`

HIV/AIDS – multidrug-resistant TB, HIV/AIDS – extensively drug-resistant TB, and HIV/AIDS – drug-susceptible TB

To split HIV-TB into HIV-MDR-TB and HIV-drug-susceptible-TB, we first calculated the proportions of HIV-MDR-TB among all HIV-TB cases ($PHIV_{c,y,a,s}$) for each location, year, age, and sex using the following formula:

$$PHIV_{c,y,a,s} = PnoHIV_{c,y,a,s}RR$$

where $PnoHIV_{c,y,a,s}$ is the proportions of MDR-TB among all HIV-negative TB cases for each location, year, age, and sex, and RR is the relative risk of MDR-TB associated with HIV infection. We then applied the predicted proportions of MDR-TB cases among HIV-TB cases to our estimated HIV-TB incident and prevalent cases to generate HIV-MDR-TB incident and prevalent cases by location, year, age, and sex. Next, we subtracted HIV-MDR-TB cases from all HIV-TB cases to generate HIV-drug-susceptible-TB cases by location, year, age, and sex. To separate out HIV-XDR-TB from HIV-MDR-TB, we applied the super-region-level proportions of XDR-TB among MDR-TB cases, to HIV-MDR-TB cases in corresponding countries within the super-regions to produce HIV-XDR-TB cases by location, year, age, and sex. We linearly extrapolated HIV-XDR-TB prevalence and incidence back assuming the rates were zero in 1992, one year before 1993 when XDR-TB was first recorded in USA surveillance data.[14] Finally, we subtracted HIV-XDR-TB cases from HIV-MDR-TB cases to generate HIV-MDR-TB (without extensive drug resistance) cases by location, year, age, and sex.

New MDR-TB and XDR-TB cases among retreated cases by HIV status

Because we split TB incidence (new and relapse cases combined) by drug-resistance type, the above estimation did not capture new MDR-TB and XDR-TB cases arising from retreated TB cases other than relapse cases. We therefore separately estimated new MDR-TB and XDR-TB cases arising from retreated TB cases and added them to the incident cases estimated above. To do so, we first ran a spatiotemporal Gaussian process regression using notification data and HAQ Index as a covariate to predict the proportion of retreated cases (excluding relapse cases) among all TB patients for all locations and years. Next, we computed retreated cases as *(retreated proportion*estimated incident cases)/(1 – retreated proportion)*. We then computed the total number of TB cases by summing estimated incident cases and retreated cases. Similar to our estimation for MDR-TB and XDR-TB among TB incident cases by HIV status, we estimated MDR-TB and XDR-TB cases among all TB cases (incident cases and retreated cases combined) by HIV status. Finally, the number of retreated cases with MDR-TB was computed by subtracting MDR-TB among TB incident cases from MDR-TB among all TB cases (incident cases and retreated cases combined), separately for HIV-positive and HIV-negative individuals. Similarly, the number of retreated cases with XDR-TB was computed by subtracting XDR-TB among TB incident cases from XDR-TB among all TB cases, separately for HIV-positive and HIV-negative individuals. All computations were done at the 1000-draw level.

Disability weights

The lay descriptions and disability weights for severity levels derived from the GBD disability weights study are shown below.

Health state name	Lay description	Disability weights (95% CI)
Tuberculosis, not HIV-infected	has a persistent cough and fever, is short of breath, feels weak, and has lost a lot of weight	0.333 (0.224–0.454)
Tuberculosis, HIV-infected	has a persistent cough and fever, shortness of breath, night sweats, weakness and fatigue and severe weight loss	0.408 (0.274–0.549)

For drug-susceptible TB, MDR-TB without extensive drug resistance, and XDR-TB, we used the same disability weight [0.333 (0.224–0.454)] as in non-HIV-infected TB. For HIV-drug-susceptible-TB, HIV-MDR-TB without extensive drug resistance, and HIV-XDR-TB, we used the same disability weight [0.408 (0.274–0.549)] as in HIV-infected TB.

References

1. Mesfin YM, Hailemariam D, Biadglign S, Kibret KT. Association between HIV/AIDS and multi-drug resistance tuberculosis: a systematic review and meta-analysis. *PLoS One*. 2014 Jan 8;9(1):e82235.
2. Institute NT. Tuberculosis in a rural population of South India: a five-year epidemiological study. *Bulletin of the World Health Organization*. 1974;51(5):473.

3. Markowitz N, Hansen NI, Hopewell PC, Glassroth J, Kvale PA, Mangura BT, Wilcosky TC, Wallace JM, Rosen MJ, Reichman LB. Incidence of tuberculosis in the United States among HIV-infected persons. *Annals of internal medicine*. 1997 Jan 15;126(2):123-32.
4. Chadha VK, Jagannath PS, Nagaraj AV, Prasad DN, Anantha A. A comparative study of tuberculin reactions to 1 TU and 2 TU of PPD-RT23. *Indian Journal of Tuberculosis*. 2000;47(15):15-20.
5. Chadha VK, Jagannath PS, Vaidyanathan PS, Jagota P. PPD RT23 for tuberculin surveys in India. *International Journal of Tuberculosis and Lung Disease*. 2003;7(2):172-179.
6. GBD 2017 Mortality and Causes of Death Collaborators. Global, regional, and national age-sex specific mortality and life expectancy, 1950–2017: a systematic analysis for the Global Burden of Disease Study 2017. *The Lancet* (under review)
7. Gothi GD, Narayan R, Nair S, Chakraborty A, Srikantaramu N. Estimation of prevalence of bacillary on the bases if of chest X-ray and/or symptomatic screening. *Indian Journal of Tuberculosis*. 1976;64(8):1150-1159.
8. Chadha VK, Kumar P, Anjinappa SM, Singh S, Narasimhaiah S, et al. Prevalence of Pulmonary Tuberculosis among Adults in a Rural Sub-District of South India. *PLoS ONE* 2012;7(8): e42625.
9. Datta M, Radhamani MP, Sadacharam K, Selvaraj R, Satyanarayana Rao DL Nagabushana Rao RS, Gopalan BN, Prabhakar R. Survey for tuberculosis in a tribal population in North Arcot District. *International Journal of Tuberculosis and Lung Disease*. 2001;5(2):240-249.
10. Datta M, Gopi PG, Appeqgowda BN, Bhima Rao KR, Gopalan BN. *Indian Journal of Tuberculosis*. 2000;47:147-154.
11. Gopi PG, Subramani R, Sadacharam K, Narayanan R. Yield of pulmonary tuberculosis cases by employing two screening methods in a community survey. *International Journal of Tuberculosis and Lung Disease*. 2006;10(3):343-345.
12. Revised National Tuberculosis Control Program (India). Tuberculosis Survey in Gujarat, Gujarat, 2011-2012. [Unpublished].
13. Tiemersma EW, Van der Werf MJ, Borgdorff MW, Williams BG, Nagelkerke N. Natural History of Tuberculosis: Duration and Fatality of Untreated Pulmonary Tuberculosis in HIV Negative Patients: A Systematic Review. *PLoS ONE*. 2011;6(4): e17601.
14. Centers for Disease Control and Prevention (CDC). Extensively Drug-Resistant Tuberculosis --- United States, 1993—2006. *MMWR*. 2007; 56(11);250-253.

Typhoid and paratyphoid fevers

Flowchart

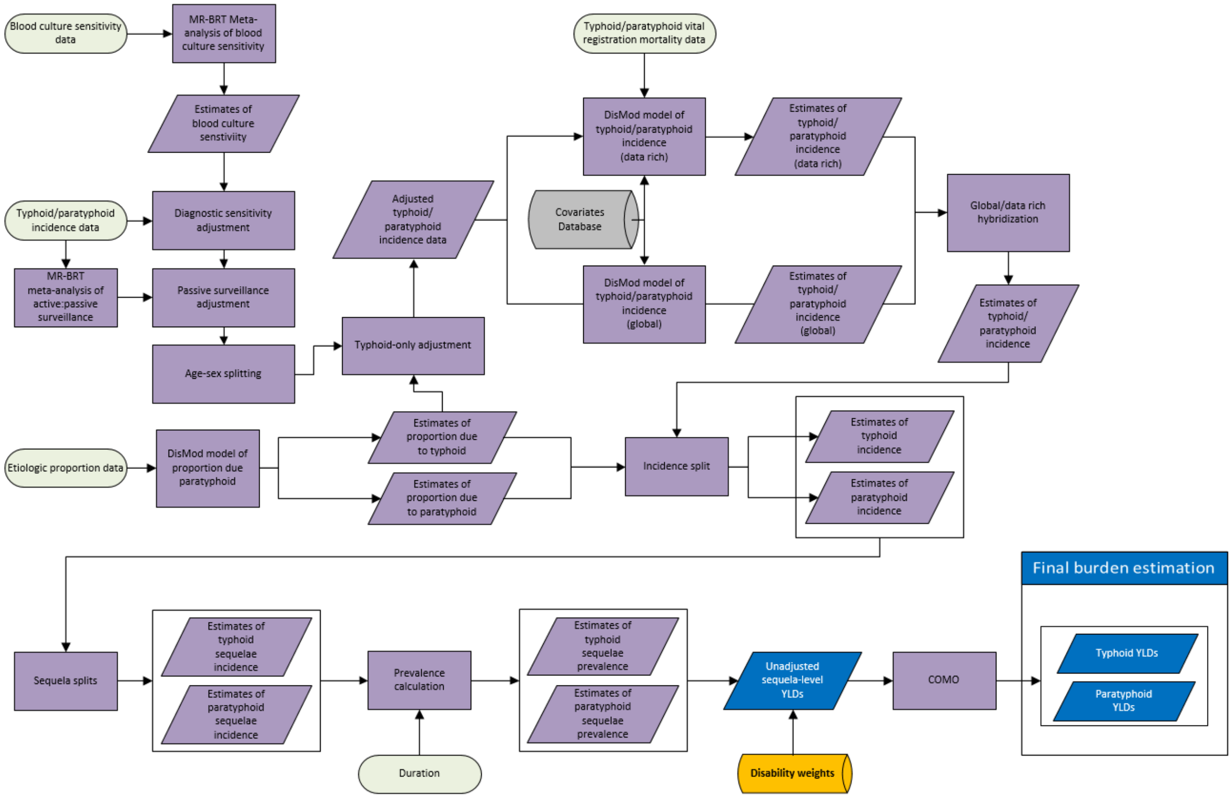

Case definition

Typhoid and paratyphoid are acute bacterial infections that most commonly cause febrile illness and gastrointestinal symptoms. Severe cases are associated with intestinal bleeding and perforation, altered mental state and, in some cases, death. We define a confirmed case as one for which there has been a positive blood culture test for either *Salmonella enterica* typhi or paratyphi. Diagnostic criteria do not typically accompany national surveillance reports; however, with blood culture being the standard diagnostic, we treat reported cases as confirmed. Given the poor sensitivity of blood culture, however, we estimated case definition as simply febrile illness resulting from an infection with *Salmonella enterica* typhi or paratyphi. This is effectively a counterfactual definition in which we attempt to estimate the number of true infections regardless of test result. These causes include all ICD-10 codes under the heading A01 (typhoid and paratyphoid fevers).

Input data

Model inputs

Our incidence dataset included a combination of data from prospective cohort studies and national surveillance systems. Similarly, data on proportions due to typhoid and paratyphoid included a combination of prospective cohort studies, national surveillance systems, and facility- or lab-based studies. We updated our systematic reviews of typhoid and paratyphoid data for GBD 2023 (Figure 1).

Severity splits

For GBD 2019, we derived severity splits based on a published review of enteric fever outcomes from (Azmatullah A, Qamar FN, Thaver D, et al. 2005).

Paratyphoid is split into four sequelae: mild (28.5% [15.6–44.2]), moderate (52.25% [27.2–77.7]), severe (14.25% [8.2–21.8]), and abdominal pain and distention (5.0% [2.8–7.6]):

Table 1: Severity distribution for paratyphoid fever

Sequela	Description	Disability weight
Mild	Has a low fever and mild discomfort, but no difficulty with daily activities.	0.006 (0.002–0.012)
Moderate	Has a fever and aches, and feels weak, which causes some difficulty with daily activities.	0.051 (0.032–0.074)
Severe	Has a high fever and pain, and feels very weak, which causes great difficulty with daily activities.	0.133 (0.088–0.19)
Abdominal pain & distention due to paratyphoid	Has pain in the belly and feels nauseated. The person has difficulties with daily activities.	0.114 (0.078–0.159)

Similarly, typhoid is split into four sequelae: moderate (35.0% [26.0–44.3]), severe (47.75% [38.0–57.4]), severe abdominal pain and distention (17.0% [10.0–25.7]), and intestinal bleeding (0.25% [0–2.0]):

Table 2: Severity distribution for typhoid fever

Sequela	Description	Disability weight
Moderate	Has a fever and aches, and feels weak, which causes some difficulty with daily activities.	0.051 (0.032–0.074)
Severe	Has a high fever and pain, and feels very weak, which causes great difficulty with daily activities.	0.133 (0.088–0.19)
Gastrointestinal bleeding	Vomits blood and feels nauseated.	0.325 (0.209–0.462)
Abdominal pain and distention (includes intestinal perforation)	Has severe pain in the belly and feels nauseated. The person is anxious and unable to carry out daily activities.	0.324 (0.22–0.442)

Modelling strategy

We first model total incidence of typhoid and paratyphoid combined. Second, we model the proportion of this total due to typhoid and the proportion due to paratyphoid. Finally, we split the case estimates into sequelae representing different major symptoms and levels of severity.

Before modelling, we applied four adjustments to the incidence data: 1) diagnostic sensitivity adjustment, 2) passive surveillance adjustment, 3) typhoid-only adjustment, and 4) age/sex splits. Incidence data were inflated to account for poor diagnostic sensitivity, based on an internal meta-analysis of the sensitivity of blood culture, the most common diagnostic used for typhoid: we estimate a sensitivity of 60.3% (50.3–68.8). We performed a crosswalk to adjust for incomplete case capture data from passive versus active surveillance, with active surveillance as the reference using a MR-BRT model and adjusted the data before modelling. Where incidence data were from studies that only tested for typhoid fever, we used estimates from our aetiological proportion models to adjust these typhoid-only sources and calculated an adjusted joint incidence (ie, including both typhoid and paratyphoid cases) by dividing the typhoid-only incidence by the estimated proportion due to typhoid. We performed this calculation using posterior simulation with 1000 draws to propagate uncertainty from both the incidence data and the proportion estimate. Finally, where incidence data were reported for both sexes combined or for age categories spanning more than 25 years, we produced datapoints that were age- and sex-specific based on a MR-BRT model of sex ratios, and a DisMod model of age patterns.

Total incidence was modelled using DisMod-MR, using the summary exposure values (SEV) for unsafe sanitation and the proportion of the population living in the Indian Ocean monsoon belt as covariates. Similarly, we used a DisMod model to estimate aetiological proportions, using a single model of the proportion of enteric fever due to *Salmonella* paratyphi. We model the proportion due to paratyphoid instead of the proportion due to typhoid as we've found that this approach results in better a better model fit for sub-Saharan Africa, where the proportion of enteric fever due to *Salmonella* typhi approaches 1.0, as DisMod performs better with proportions that are near zero than with proportions that are near one.

Typhoid cases are split between four sequelae: moderate typhoid fever, severe typhoid fever, severe typhoid fever with intestinal bleeding, and typhoid fever with abdominal complications. Paratyphoid cases are split between four sequelae: mild paratyphoid fever, moderate paratyphoid fever, severe paratyphoid fever, and paratyphoid fever with abdominal complications.

Changes from GBD 2021 to GBD 2023

We updated our systematic review for GBD 2023 (Figure 1) and updated the code pipeline to improve robustness and efficiency, but made no substantive changes to our modelling strategy for typhoid and paratyphoid between GBD 2021 and GBD 2023.

Figure 1: PRISMA flow diagram

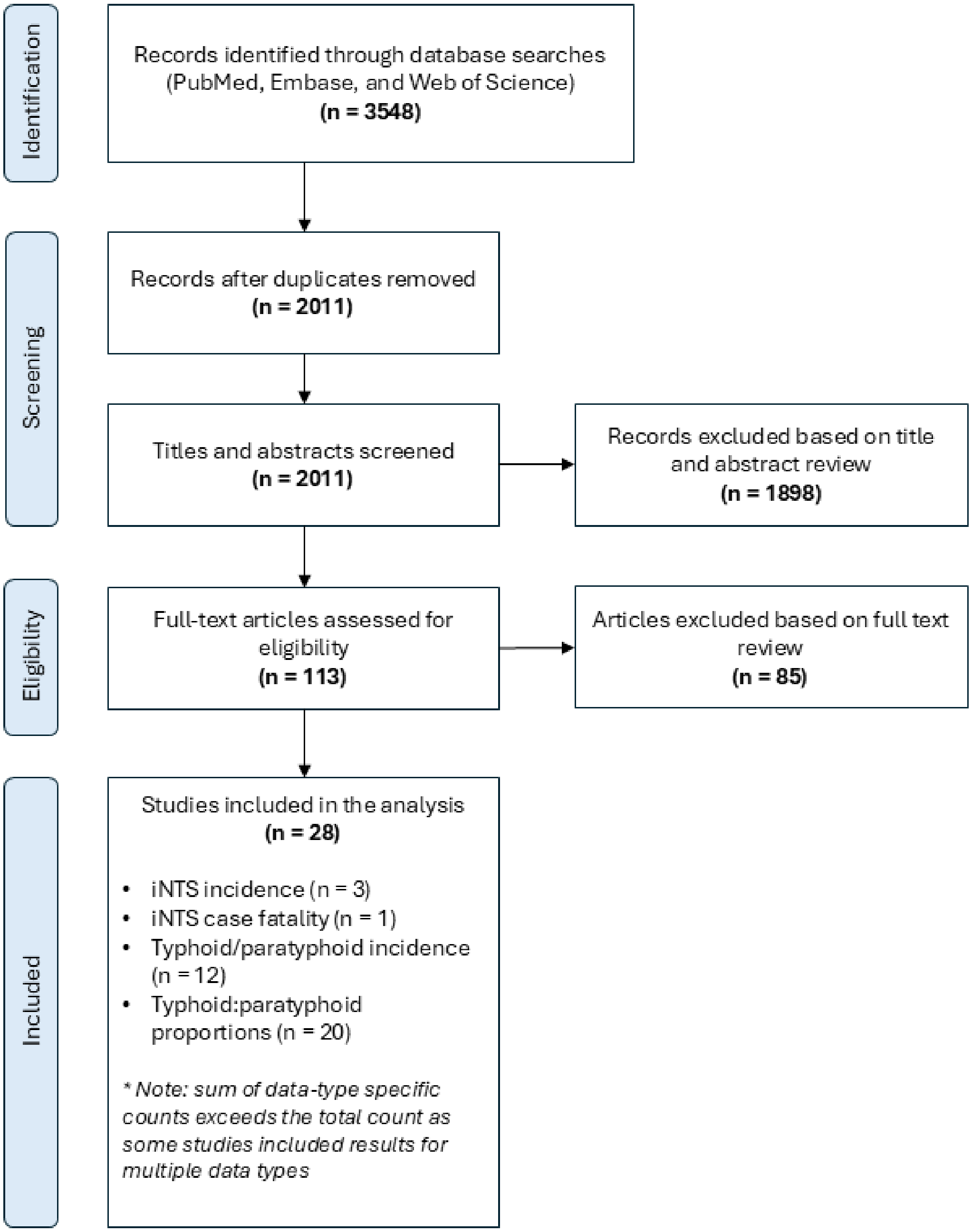

Upper respiratory infections

Flowchart

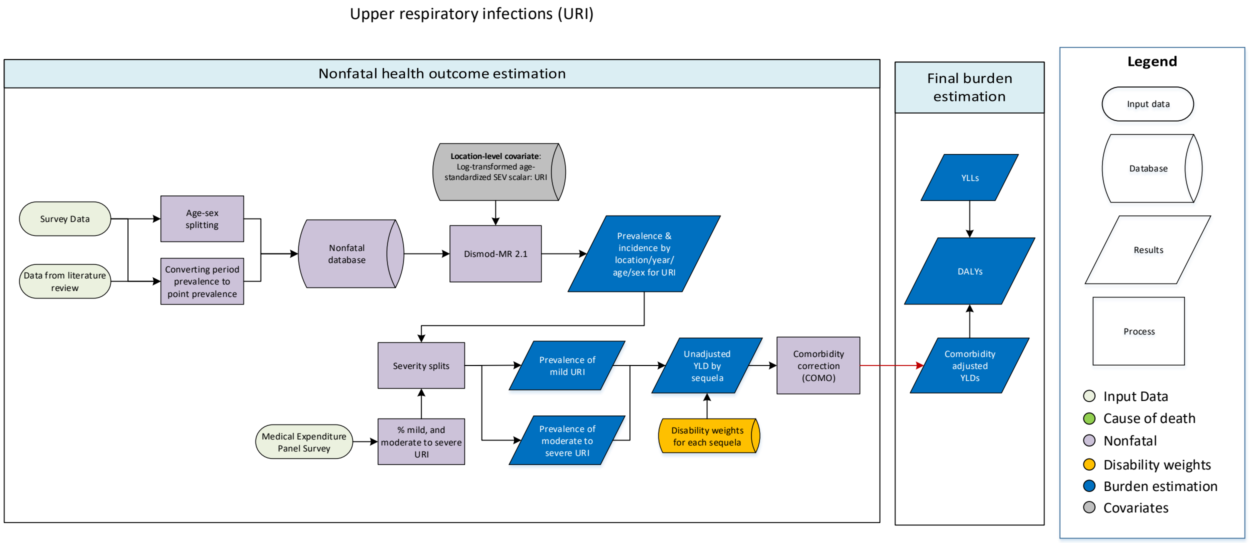

Input data and methodological summary for upper respiratory infections

Case definition

Upper respiratory infections (URI) are characterised by sore throat, low-grade fever, and an adherent membrane of the tonsil(s), pharynx, and/or nose without other apparent cause. URIs include cough, acute nasopharyngitis, sinusitis, pharyngitis, tonsillitis, laryngitis/tracheitis, epiglottitis, rhinitis, rhinosinusitis, rhinopharyngitis, supraglottitis, and the common cold. For URI, ICD-10 codes are J00-J02, J02.8-J03, J03.8-J06.9, J36, J36.0, and ICD-9 codes are 460-465.9, 475-475.9, 476.9.

Input data

Model inputs

We used the same literature data and nationally representative survey data, such as Demographic and Health Surveys, as in GBD 2021.[1]

The definition of upper respiratory infections from these surveys was the two-week period prevalence of cough. We assume that cough without difficulty breathing, along with or without a fever, is the definition of upper respiratory infection. We converted these data from two-week period prevalence to point prevalence assuming a duration of five days. The equation for this adjustment is:

$$Point\ Prevalence = \frac{Period\ Prevalence\ *\ Duration}{(Recall\ Period + Duration - 1)}$$

Severity splits

The table below shows the severity distributions based on the data from Medical Expenditure Panel Surveys where we categorised “acute nasopharyngitis or acute URI multi sites/nos” as mild URI and

"acute sinusitis, acute pharyngitis, acute tonsillitis, and acute laryngitis/tracheitis and epiglottitis" as moderate URI.

Table 2. URI severity split proportions

**Mild URI proportion**	**Moderate URI proportion**
0.56 (0.43–0.68)	0.44 (0.32–0.57)

The lay descriptions and disability weights for severity levels derived from the GBD disability weights study are shown below.

Table 3. Severity split disability weights

Severity level	Lay description		DW (95% CI)
Mild upper respiratory infections	Has a low fever and mild discomfort, but no difficulty with daily activities		0.006 (0.002–0.012)
Moderate/severe upper respiratory infections	Has a fever and aches, and feels weak, which causes some difficulty with daily activities		0.051 (0.032–0.074)

Modelling strategy

URI was modelled using a standard DisMod-MR 2.1 model using secondhand smoke as the location-level covariate.

Betas and exponentiated values are shown in the table below:

Table 3. URI DisMod covariates

Covariate	Parameter	Beta	Exponentiated beta
Secondhand smoke	Prevalence	0.11	1.15 (1.01–1.31)
Sex	Prevalence	–0.027	1.00 (0.99–1.02)

References

1. Sirota SB, Doxey MC, Dominguez RMV, Bender RG, Vongpradith A, Albertson SB, et al. Global, regional, and national burden of upper respiratory infections and otitis media, 1990–2021: a systematic analysis from the Global Burden of Disease Study 2021. The Lancet Infectious Diseases. 2025 Jan 1;25(1):36–51.

Urinary diseases and male infertility

Urinary tract infections and interstitial nephritis

Flowchart

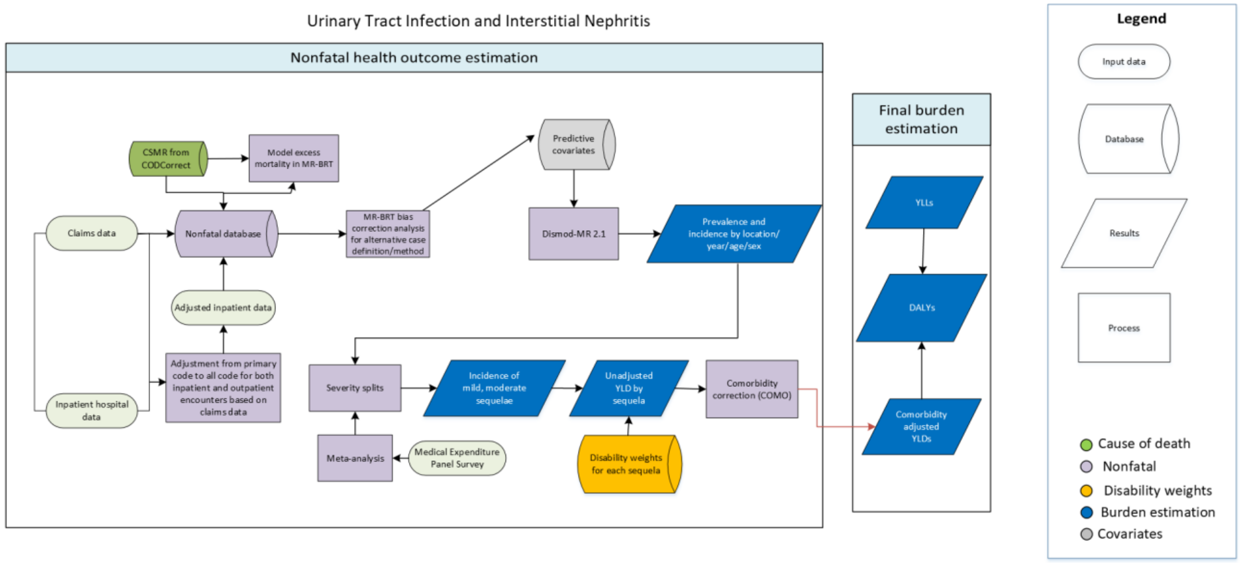

Input data and methodological summary for urinary tract infections and interstitial nephritis

Case definition

Urinary tract infection (UTI) encompasses symptomatic pyelonephritis, cystitis, urethritis, and other unspecified infections along the urinary tract caused by bacteria. Asymptomatic bacteriuria is excluded from this cause. Interstitial nephritis is grouped with UTI for GBD modelling due to at least some cases of interstitial inflammation being attributable to infection and limited information on aetiology being available in most ICD-coded datasets. ICD-10 codes include N10, N10.0, N10.9, N11, N11.0, N11.1, N11.8, N11.9, N12, N12.0, N12.9, N13.6, N15, N15.1, N15.8, N15.9, N16, N16.0-N16.5, N16.8, N30, N30.0-N30.3, N30.8-N30.9, N34, N34.0-N34-3, and N39.0.

**Quantity of interest**	**Reference or alternative**	**Definition**
Incidence of UTI	Reference	Cases of urinary tract infections identified using ICD codes in administrative records that are considered population-representative.
Incidence of UTI	Alternative	Cases identified from database of commercial claims from USA in 2010-2017 using ICD codes.
Incidence of UTI	Alternative	Cases identified from database of commercial claims from USA in 2000 using ICD codes.

Input data

Input data

As in GBD 2021, the GBD 2023 non-fatal UTI model included incidence data extracted from the GBD library of hospital discharges and claims aggregated and processed by IHME. No new incidence data were added in GBD 2023. Inputs to the non-fatal model also included cause-specific mortality rate (CSMR) estimates taken from our fatal modelling process (see CoD cause-specific modelling description for UTI in this appendix) and excess mortality rate (EMR) estimates modelled using MR-BRT (see the EMR data processing section below).

Incidence data processing

Hospital discharge data provide observations about encounters, generally with only the primary diagnostic code for the encounter. Claims data, on the other hand, link claims for all inpatient and outpatient encounters for a single individual and provide primary and secondary diagnoses for all encounters.

In GBD 2017, an individual was extracted from claims data as an incident case if that individual had one or more inpatient encounters with an appropriate ICD code as any diagnosis. Hospital discharges with an appropriate ICD code as primary diagnosis were extracted and adjusted to account for typical patterns of readmission.

Beginning in GBD 2019, however, we employed data processed with methods to capture cases that were diagnosed and/or treated in both inpatient and outpatient settings. Specifically, an individual was extracted from claims data as an incident case if that individual had at least one inpatient or outpatient encounter with an appropriate ICD code as any diagnosis within 28 days. Hospital discharge data were processed by extracting discharges with an appropriate ICD code as primary diagnosis and adjusting using correction factors derived from claims data. Specifically, we calculated the ratio of inpatient claims with UTI as primary diagnosis to total incident cases of UTI seen in claims data, and modelled these ratios as functions of age, sex, and Healthcare Access and Quality Index using MR-BRT (meta-regression—Bayesian, regularised, trimmed). Details of modelling and applying these ratios remained the same as in GBD 2019.

Furthermore, in GBD 2019, GBD 2021, and GBD 2023, USA claims data (extracted as described above) were adjusted to account for selection bias due to commercial insurance, using MR-BRT analysis. The process of adjusting for biases in non-reference data using MR-BRT with the logit-transformation method is described below:

1. Identify datapoints with overlapping year, age, sex, and location between reference and non-reference population data.
2. Logit transform overlapping datapoints of alternative and reference types.
3. Convert overlapping datapoints into a difference in logit space using the following equation: $logit(altnerative) - logit(reference)$
4. Use the delta method to compute standard errors of overlapping datapoints in logit space, then calculate standard error of logit difference using the following equation: $\sqrt{(variance\ of\ logit(alternative)) + (variance\ of\ logit(reference))}$
5. Using MR-BRT, conduct a random effects meta-regression to obtain the pooled logit difference of alternative to reference
6. Apply the pooled logit difference to all data points of non-reference types using the following equation:
$$new_{estimate} = inverse.logit((logit(alternative)) - (pooled\ logit\ difference))$$
7. Calculate new standard errors using the delta method, accounting for gamma (between-study heterogeneity)

The table below shows bias correction factors estimated using MR-BRT.

Table 1: MR-BRT crosswalk adjustment factors for urinary tract infection

Data input	Reference or alternative case definition	Gamma	Beta coefficient, logit (95% UI)*	Adjustment factor**
Hospital + non-USA claims	Ref	0.36	---	---
USA claims from year 2000	Alt		–0.40 (–1.40, 0.59)	0.40 (0.20, 0.64)
USA claims from year 2010–2017	Alt		–0.18 (–1.03, 0.68)	0.46 (0.26, 0.66)

**MR-BRT crosswalk adjustments can be interpreted as the factor the alternative case definition is adjusted by to reflect what it would have been had it been measured using the reference case definition. If the log/logit beta coefficient is negative, then the alternative is adjusted up to the reference. If the log/logit beta coefficient is positive, then the alternative is adjusted down to the reference.*
***The adjustment factor column is the exponentiated beta coefficient. For log beta coefficients, this is the relative rate between the two case definitions. For logit beta coefficients, this is the relative odds between the two case definitions.*

Datapoints with an age-standardised incidence rate greater than two median absolute deviations from the median of the age-standardised incidence rate for all inpatient and non-USA claims data were marked as outliers and excluded from analysis. Datapoints in Taiwan (province of China) and Indonesia, particularly in older age groups, were also marked as outliers because they were implausibly high when compared to the regional, super-regional, and global rates.

EMR processing

In GBD 2017, EMR inputs were produced by fitting a preliminary compartment model to estimate prevalence from incidence data, matching preliminary prevalence resulting from each incidence datapoint with the corresponding CSMR value within the same age, sex, year, and location, and dividing CSMR by prevalence. This method of producing EMR inputs, however, demonstrated a rather unrealistic

pattern of EMR compared to an expected pattern of decreasing EMR with greater access to quality health care. Such unexpected patterns often signal inconsistencies between CSMR estimates and data on prevalence and/or incidence. Thus, in an effort to provide greater guidance on the expected pattern of EMR, in GBD 2019, EMR data produced per above were modelled by age, sex, and Healthcare Access and Quality (HAQ) Index using MR-BRT, with a prior on HAQ Index having a negative coefficient. In GBD 2021, we employed the same MR-BRT method to predict EMR for each location, year, sex, and for ages 0, 10, 20….100; these predictions were used as inputs to our non-fatal models in GBD 2021 and GBD 2023, with the latter described below.

Modelling strategy

We have made no changes in the modelling strategy from GBD 2021 for GBD 2023. Similar to previous rounds, we ran a DisMod-MR 2.1 model to produce estimates by age, sex, year, and location. Inputs to DisMod-MR for UTI included incidence, CSMR, and EMR inputs processed as described above. A prior value was set on remission so that all cases remit within one week. The minimum coefficient of variation at the regional, super-regional, and global level was set at 0.8. The HAQ Index covariate was included as a predictive covariate on EMR. Beta and exponentiated value (which can be interpreted as odds ratios) of this predictive covariate are shown in the table below.

Table 2. Covariates. Summary of covariates used in the urinary tract infection DisMod-MR meta-regression model

Covariate	Type	Parameter	Exponentiated beta (95% uncertainty interval)
Healthcare Access and Quality Index	Country-level	Excess mortality rate	1.00 (1.00–1.00)

Severity split & disability weight

The basis of the GBD disability weight survey assessments is lay descriptions of sequelae highlighting major functional consequences and symptoms.[1] UTI is split into mild and moderate severity. Mild severity is associated with a disability weight that correlates with low fever and mild discomfort but no difficulty with daily activities. Moderate severity is associated with a disability weight that correlates with systemic symptoms of fever, aches, weakness, and some difficulty with daily activities. The lay descriptions and disability weights for UTI are shown below.

Table 3. Severity distribution, details on the severity levels for Urinary tract infection and the associated disability weight (DW) with that severity.

Severity level	Lay description	DW (95% CI)	Distribution (95% CI)
Mild	Has a low fever and mild discomfort, but no difficulty with daily activities.	0.006 (0.002, 0.012)	0.362 (0.258, 0.478)
Moderate	Has a fever and aches, and feels weak, which causes	0.051 (0.032, 0.074)	0.638 (0.522, 0.742)

	some difficulty with daily activities.		

The distribution of incident cases of UTI between the two severity levels was derived from analysis of the Medical Expenditure Panel Surveys (MEPS).[2] MEPS is an overlapping panel survey of the non-institutionalised USA population that collects data on respondents' health service interactions. Panels are initiated every year. Each panel is two years long and consists of five rounds. In 2000, MEPS began using 12-Item Short Form Surveys (SF-12) to collect data on functional health status. The SF-12 survey is administered twice per panel (about once per year). This survey has been employed to estimate distributions of severity levels for diverse GBD diseases as previously described and summarised below.[2]

In order to translate SF-12 scores into GBD disability weights, 62 lay descriptions for conditions representing the full range of disability weight values (from most mild to most severe) were selected. A convenience sample of respondents was then asked to complete an SF-12 form for an individual with the health state described in the lay descriptions of these conditions. Composite mental and physical SF-12 score was regressed on GBD disability weight to derive the relationship between disability weight and SF-12 score. Individual respondent scores were then regressed on reported conditions to predict cumulative disability from the individuals' diagnoses, and a counterfactual cumulative disability excluding a single condition was predicted to obtain a comorbidity-corrected condition-specific disability weights for all individual with that single condition. The distribution of these condition-specific disability weights was used to derive the proportion of individuals with the conditions that fall within each GBD severity category.

References

1. Salomon JA, Vos T, Hogan DR, *et al.* Common values in assessing health outcomes from disease and injury: disability weights measurement study for the Global Burden of Disease Study 2010. *Lancet* 2012; **380**: 2129–43.
2. Burstein R, Fleming T, Haagsma J, Salomon JA, Vos T, Murray CJL. Estimating distributions of health state severity for the global burden of disease study. *Popul Health Metr* 2015; 13: 31.

Urolithiasis

Flowchart

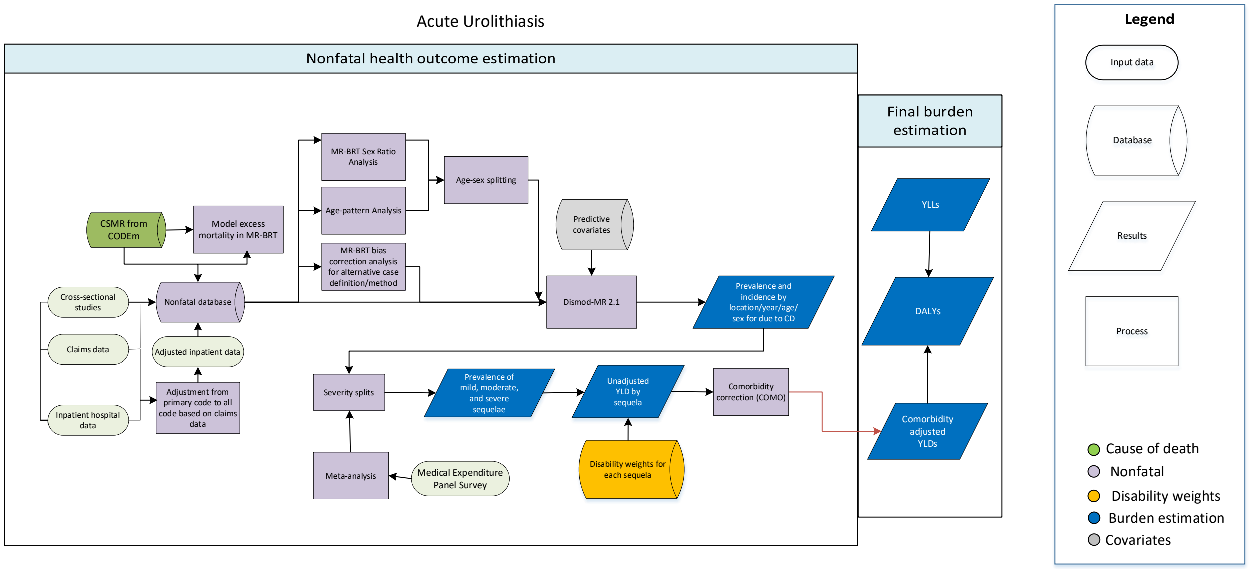

Input data and methodological summary for urolithiasis

Case definition

Acute urolithiasis (AU) is defined as abnormal formation of crystalline masses along the urinary tract, commonly from calcium compounds, uric acid, struvite, or cystine, generally presenting with waves of severe abdominal or flank pain, haematuria, nausea, or painful or difficult urination. Associated ICD codes include N20, N20.0, N20.1, N20.2, N20.9, N21, N21.1, N21.8, N21.9, N22, N22.0, N22.8, N23, and N23.0.

Quantity of interest	Reference or alternative	Definition
Incidence of urolithiasis	Reference	Cases of urolithiasis infections identified using ICD codes in administrative records that are considered population-representative.
Incidence of urolithiasis	Alternative	Cases identified from database of commercial claims from USA in 2010-2017 using ICD codes.
Incidence of urolithiasis	Alternative	Cases identified from database of commercial claims from USA in 2000 using ICD codes.

Input data

Input data

A systematic literature review was first conducted in 2010 and updated in 2013 and 2016. Data from the systematic reviews were combined with clinical administrative data assembled, extracted, and processed by IHME, most recently in GBD 2021. No new data were added in GBD 2023.

Inputs to the non-fatal model also included cause-specific mortality rate (CSMR) estimates taken from our fatal modelling process (see CoD cause-specific modelling description for AU in this appendix) and excess mortality rates (EMR) estimates modelled using MR-BRT (see the EMR data processing section below).

Data processing

Hospital discharge data provide observations about encounters, generally with only the primary diagnostic code for the encounter. Claims data, on the other hand, link claims for all inpatient and outpatient encounters for a single individual and provide primary and secondary diagnoses for all encounters.

In GBD 2017, an individual was extracted from claims data as an incident case if that individual had one or more inpatient encounters with an appropriate ICD code as any diagnosis. Hospital discharges with an appropriate ICD code as primary diagnosis were extracted and adjusted to account for typical patterns of readmission.

Beginning in GBD 2019, however, we employed data processed with methods to capture cases that were diagnosed and/or treated in both inpatient and outpatient settings. Specifically, incident cases were extracted from claims data if an individual had at least one inpatient or outpatient encounter with an appropriate ICD code as any diagnosis; repeat encounters within 28 days, regardless of setting, were assumed to represent care for the same episode. Hospital discharge data were processed by extracting discharges with an appropriate ICD code as primary diagnosis and adjusting using correction factors (ie, correction factor 3) derived from claims data. Specifically, we calculated the ratio of inpatient claims with AU as primary diagnosis to total incident cases of AU seen in claims data, and modelled these ratios as functions of age, sex, and Healthcare Access and Quality Index using MR-BRT (meta-regression—Bayesian, regularised, trimmed).

In addition to broadening the inclusion criteria for cases of AU in claims data and employing more inclusive ratios for adjusting hospital discharge data, the methods for subsequently adjusting for systematic biases between source types were also updated in GBD 2019. The method used in GBD 2019, 2021, and 2023 allow a more direct comparison between different case definitions and/or study designs than methods used in earlier GBD cycles. Prior to GBD 2019, we used data from published studies that employed rigorous case definitions for AU as our reference standard and adjusted clinical administrative data toward this reference standard by marking administrative data with binary covariates and estimating a fixed effect for this covariate in our DisMod-MR meta-regression modelling process. This amounts to adjusting data using an ecological comparison and is vulnerable to compositional bias; if data from different location-years were collected using different methods or case definitions, true spatiotemporal differences in epidemiology can be erroneously adjusted, and differences truly due to differences in methods can be erroneously estimated as differences in underlying epidemiology. In GBD 2019, we avoided this risk by making pre-modelling bias adjustments and dropping data types that could not be rigorously adjusted. This was done by conducting a meta-regression of the relationship between datapoints matched with regard to year, age, sex, and location, but differing with regard to one or more study design characteristic. This type of adjustment is referred to as “crosswalking”. Data from studies that identified cases of AU based on urinalysis and/or imaging findings were scarce, and we were not able to find overlapping datapoints from administrative data sources to estimate adjustment factors. As a result, these data were excluded and a new case definition was adopted: diagnosis of AU of any

aetiology as indicated by ICD code in a clinical encounter. Data excluded because they were collected with non-reference methods that could not be crosswalked were retained in the database for visualisation and vetting, and will be considered for inclusion in future modelling should sufficient data for estimating adjustment factors become available.

Sufficient matched data were available, however, to estimate adjustment factors to crosswalk USA claims data (extracted as described above) to account for selection bias due to commercial insurance status starting in GBD 2019 and continuing in subsequent rounds. The process of adjusting for biases in non-reference data using MR-BRT with the logit-transformation method is described below:

1. Identify datapoints with overlapping year, age, sex, and location between reference and non-reference population data.
2. Logit transform overlapping datapoints of alternative and reference types.
3. Convert overlapping datapoints into a difference in logit space using the following equation: $logit(altnerative) - logit(reference)$
4. Use the delta method to compute standard errors of overlapping datapoints in logit space, then calculate standard error of logit difference using the following equation: $\sqrt{(variance\ of\ logit(alternative)) + (variance\ of\ logit(reference))}$
5. Using MR-BRT, conduct a random effects meta-regression to obtain the pooled logit difference of alternative to reference
6. Apply the pooled logit difference to all data points of non-reference types using the following equation:
$$new_{estimate} = inverse.logit((logit(alternative)) - (pooled\ logit\ difference))$$
7. Calculate new standard errors using the delta method, accounting for gamma (between-study heterogeneity)

The table below shows these bias correction factors. Beta coefficients and adjustment factors incorporate study heterogeneity (gamma).

Table 1: MR-BRT crosswalk adjustment factors for urolithiasis

Data input	Reference or alternative case definition	Gamma	Beta coefficient, logit (95% UI)*	Adjustment factor**
Hospital + non-USA claims	Ref	0.36	---	---
USA claims from year 2000	Alt		–0.73 (–0.86, –0.60)	0.33 (0.30, 0.35)
USA claims from year 2010–2017	Alt		0.12 (0.09, 0.15)	0.53 (0.52, 0.54)

**MR-BRT crosswalk adjustments can be interpreted as the factor the alternative case definition is adjusted by to reflect what it would have been had it been measured using the reference case definition. If the log/logit beta coefficient is negative, then the alternative is adjusted up to the reference. If the log/logit beta coefficient is positive, then the alternative is adjusted down to the reference.*

***The adjustment factor column is the exponentiated beta coefficient. For log beta coefficients, this is the relative rate between the two case definitions. For logit beta coefficients, this is the relative odds between the two case definitions.*

Datapoints with an age-standardised incidence rate greater than two median absolute deviations from the median of the age-standardised incidence rate for all inpatient and non-USA claims data were marked as outliers and excluded from analysis. Data from Nepal, Iran, Qatar, Turkey, and Russia were also marked as outliers because they were implausibly low when compared to regional, super-regional, and global rates.

EMR input processing

In GBD 2017, EMR inputs were produced by fitting a preliminary compartment model to estimate prevalence from incidence data, matching preliminary prevalence resulting from each incidence datapoint with the corresponding CSMR values within the same age, sex, year, and location, and dividing CSMR by prevalence. This method of producing EMR inputs, however, demonstrated a rather unrealistic pattern of EMR compared to an expected pattern of decreasing EMR with greater access to quality health care. Such unexpected patterns often signal inconsistencies between CSMR estimates and data on prevalence and/or incidence. Thus, in an effort to provide greater guidance on the expected pattern of EMR, in GBD 2019, EMR data produced per above in GBD 2017 were modelled by age, sex, and Healthcare Access and Quality (HAQ) Index using MR-BRT, with a prior on HAQ Index having a negative coefficient. In GBD 2021, we employed the same MR-BRT method to predict EMR for each location, year, sex, and for ages 0, 10, 20….100; these predictions were used as inputs to our non-fatal models in GBD 2021 and GBD 2023, with the latter described below.

Modelling strategy

There were no changes to the modelling strategy and settings between GBD 2021 and GBD 2023. We modelled prevalence, incidence, and remission of urolithiasis using DisMod-MR 2.1 to produce estimates by age, sex, year, and location. Inputs to DisMod-MR for acute urolithiasis included incidence, CSMR, and EMR inputs processed as described above. Prior settings in the DisMod-MR model included setting remission of two weeks. The minimum coefficient of variation at the regional, super-regional, and global level was set at 0.8. We included HAQ Index as a predictive covariate on EMR with a mean and standard deviation produced from the MR-BRT model described above. The beta and exponentiated values of this predictive covariate (which can be interpreted as an odds ratio) are shown in the table below.

Table 2. Covariates. Summary of covariates used in the urolithiasis DisMod-MR meta-regression model

Covariate	Type	Parameter	Exponentiated beta (95% uncertainty interval)
Healthcare Access and Quality Index	Country-level	Excess mortality rate	0.98 (0.98–0.98)

Severity split & disability weight

The basis of the GBD disability weight survey assessments is lay descriptions of sequelae highlighting major functional consequences and symptoms.[1] Urolithiasis is split into mild, moderate, and severe categories. The lay descriptions and disability weights for urolithiasis are shown below.

Table 3. Severity distribution, details on the severity levels for urolithiasis and the associated disability weight (DW) with that severity.

Severity level	Lay description	DW (95% CI)	Distribution (95% CI)
Mild	Has some pain in the belly that causes nausea but does not interfere with daily activities.	0.011 (0.005–0.021)	0.642 (0.536, 0.734)
Moderate	Has pain in the belly and feels nauseous. The person has difficulties with daily activities.	0.114 (0.078–0.159)	0.217 (0.149, 0.296)
Severe	Has severe pain in the belly and feels nauseous. The person is anxious and unable to carry out daily activities.	0.324 (0.220–0.442)	0.141 (0.108, 0.178)

The distribution of incident cases of urolithiasis between the three severity levels was derived from analysis of the Medical Expenditure Panel Surveys (MEPS).[2] MEPS is an overlapping panel survey of the non-institutionalised USA population that collects data on respondents' health service interactions. Panels are initiated every year. Each panel is two years long and consists of five rounds. In 2000, MEPS began using 12-Item Short Form Surveys (SF-12) to collect data on functional health status. The SF-12 survey is administered twice per panel (about once per year).

In order to translate SF-12 scores into GBD disability weights, 62 lay descriptions for conditions representing the full range of disability weight values (from most mild to most severe) were selected. A convenience sample of respondents was then asked to complete an SF-12 form for an individual with the health state described in the lay descriptions of these conditions. Composite mental and physical SF-12 score was regressed on GBD disability weight to derive the relationship between disability weight and SF-12 score. Individual respondent scores were then regressed on reported conditions to obtain a comorbidity-corrected condition-specific disability weight. The distribution of these condition-specific weights was used to derive the proportion of individuals with the conditions that fall within each GBD severity category.

References

1. Salomon JA, Vos T, Hogan DR, *et al.* Common values in assessing health outcomes from disease and injury: disability weights measurement study for the Global Burden of Disease Study 2010. *Lancet* 2012; **380**: 2129–43.
2. Burstein R, Fleming T, Haagsma J, Salomon JA, Vos T, Murray CJL. Estimating distributions of health state severity for the global burden of disease study. *Popul Health Metr* 2015; 13: 31.

Benign prostatic hyperplasia

Flowchart

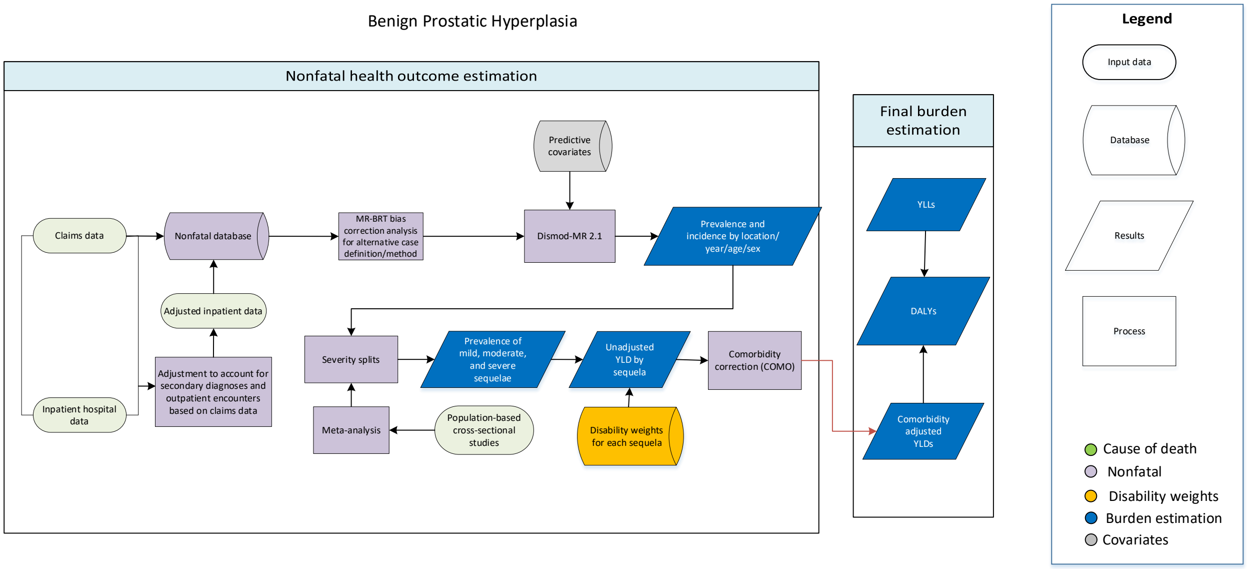

Input data and methodological summary for benign prostatic hyperplasia

Case definition

Benign prostatic hyperplasia (BPH) is defined as a chronic, non-cancerous proliferation of prostatic tissue regardless of symptoms. The ICD codes for BPH include N40, N40.0, N40.1, N40.2, N40.3, and N40.9.

Quantity of interest	Reference or alternative	Definition
Prevalence of benign prostatic hyperplasia	Reference	Cases of benign prostatic hyperplasia identified using ICD codes in administrative records that are considered population-representative.
Prevalence of benign prostatic hyperplasia	Alternative	Cases identified from database of commercial claims from USA in 2010-2017 using ICD codes.
Prevalence of benign prostatic hyperplasia	Alternative	Cases identified from database of commercial claims from USA in 2000 using ICD codes.

Input data

Input data

As in GBD 2021, the model included prevalence data from hospital discharges and claims. No new data were added in GBD 2023.

Data processing

Hospital discharge data provide observations about encounters, generally with only the primary diagnostic code for the encounter. Claims data, on the other hand, link claims for all inpatient and outpatient encounters for a single individual and provide primary and secondary diagnoses for all encounters.

An individual was extracted from claims data as a prevalent case if that individual had at least one inpatient or two outpatient encounters with an appropriate ICD code as any diagnosis within one year. Hospital discharge data were processed by extracting discharges with an appropriate ICD code as primary diagnosis and adjusting using correction factors (ie, correction factor 3) derived from claims data. Specifically, we calculated the ratio of inpatient claims with BPH as primary diagnosis to total prevalent cases of BPH seen in claims data and modelled these ratios using MR-BRT (meta-regression—Bayesian, regularised, trimmed).

As first done in GBD 2019, USA claims data (extracted and processed as described above) were adjusted to account for selection bias due to commercial insurance using MR-BRT analysis. The process of adjusting for biases in non-reference data using MR-BRT with the logit-transformation method is described below:

1. Identify datapoints with overlapping year, age, sex, and location between reference and non-reference population data.
2. Logit transform overlapping datapoints of alternative and reference types.
3. Convert overlapping datapoints into a difference in logit space using the following equation: $logit(altnerative) - logit(reference)$
4. Use the delta method to compute standard errors of overlapping datapoints in logit space, then calculate standard error of logit difference using the following equation: $\sqrt{(variance\ of\ logit(alternative)) + (variance\ of\ logit(reference))}$
5. Using MR-BRT, conduct a random effects meta-regression to obtain the pooled logit difference of alternative to reference
6. Apply the pooled logit difference to all data points of non-reference types using the following equation:
$$new_{estimate} = inverse.logit((logit(alternative)) - (pooled\ logit\ difference))$$
7. Calculate new standard errors using the delta method, accounting for gamma (between-study heterogeneity)

The table below shows bias correction factors estimated using MR-BRT.

Table 1: MR-BRT crosswalk adjustment factors for benign prostatic hyperplasia

Data input	Reference or alternative case definition	Gamma	Beta coefficient, logit (95% UI)*	Adjustment factor**
Hospital + non-USA claims	Ref	0.000025	---	---
USA claims from year 2000	Alt		–0.87 (–0.94, –0.79)	0.29 (0.28, 0.31)
USA claims from year 2010–2017	Alt		–0.28 (–0.36, –0.21)	0.43 (0.41, 0.45)

**MR-BRT crosswalk adjustments can be interpreted as the factor the alternative case definition is adjusted by to reflect what it would have been had it been measured using the reference case definition. If the log/logit beta*

coefficient is negative, then the alternative is adjusted up to the reference. If the log/logit beta coefficient is positive, then the alternative is adjusted down to the reference.
***The adjustment factor column is the exponentiated beta coefficient. For log beta coefficients, this is the relative rate between the two case definitions. For logit beta coefficients, this is the relative odds between the two case definitions.*

Datapoints with an age-standardised prevalence rate greater than two median absolute deviations from the median of the age-standardised prevalence rate for all inpatient and non-USA claims data were marked as outliers and excluded from analysis.

Modelling strategy

There were no changes to the modelling strategy and settings between GBD 2021 and GBD 2023. We modelled prevalence, incidence, and remission of BPH using DisMod-MR 2.1 model to produce estimates by age, sex, year, and country. Settings in the DisMod-MR model included a prior value of zero incidence and remission for ages less than 40 years. We set an upper bound on remission for ages 40 years and older to 0.1, corresponding to a maximum disease duration of ten years. We also assumed that there was no excess mortality related to BPH. The minimum coefficient of variation at the regional, super-regional, and global level was changed from 0.4 to 0.8 in GBD 2019 to improve model fit against input data, and this setting has been carried forward through GBD 2023.

We included the age-standardised prevalence of diabetes as a predictive covariate to inform prevalence, which was a better predictor than the mean BMI that was used in GBD 2017. The beta and exponentiated values of this covariate (which can be interpreted as an odds ratio) are shown in the table below.

Table 2. Covariates. Summary of covariates used in the benign prostatic hyperplasia DisMod-MR meta-regression model

Covariate	Type	Parameter	Exponentiated beta (95% uncertainty interval)
Age-standardised prevalence of diabetes	Country-level	Prevalence	11.30 (7.89–15.38)

Severity split & disability weight

The basis of the GBD disability weight survey assessments are lay descriptions of sequelae highlighting major functional consequences and symptoms of a given cause.[1] Prevalent cases of BPH are split into symptomatic and asymptomatic groups. There is no disability weight (DW) assigned to asymptomatic cases of BPH. The DW associated with symptomatic BPH, such as urinary frequency, that is sometimes associated with pain – as seen in the table below, along with the proportion of BPH cases estimated to be symptomatic, estimated as described below.

Table 3. Severity distribution, details on the severity levels for benign prostatic hyperplasia and the associated disability weight (DW) with that severity.

Severity level	Lay description	DW (95% CI)	Distribution (95% CI)

Asymptomatic	N/A	0	0.673 (0.655–0.692)
Symptomatic	Feels the urge to urinate frequently, but when passing urine, it comes out slowly and sometimes is painful.	0.067 (0.043–0.097)	0.327 (0.245–0.436)

Beginning in GBD 2019, we started estimating the proportion of BPH cases that are symptomatic using a method that employs the International Prostate Symptom Score (I-PSS) reported in four population-based studies in Japan, USA, France, and Scotland.[2] I-PSS is a widely used validated questionnaire that is developed to assess severity of lower urinary tract symptoms (LUTS) related to BPH. The questionnaire consists of seven questions on incomplete emptying, frequency, intermittency, urgency, weak stream, straining, and nocturia, and one question on quality of life. Four studies recruited a representative sample of men between ages 40 and 79 in Japan, USA, and Scotland, and ages 50–84 in France. I-PSS was either self-administered in the presence of a research assistant or through face-to-face interviews. We modelled cumulative distribution of the I-PSS scores in the survey participants using MR-BRT to estimate the mean proportion of individuals with symptomatic LUTS.

References

1. Salomon JA, Vos T, Hogan DR, *et al.* Common values in assessing health outcomes from disease and injury: disability weights measurement study for the Global Burden of Disease Study 2010. *Lancet* 2012; **380**: 2129–43.
2. Sagnier P-P, Girman CJ, Garraway M, Kumamoto Y, Lieber MM, Richard F, et al. International Comparison of the Community Prevalence of Symptoms of Prostatism in Four Countries. *EUR*. 1996;29:15–20.

Other urinary diseases

In addition to the above-specified urinary diseases of urinary tract infections and interstitial nephritis, acute urolithiasis, and benign prostatic hyperplasia, there are other types of urinary diseases with a range of severities and associated sequelae. Because these urinary diseases are diverse in their underlying causes and risk factors as well as in their associated health outcomes, modelling them together in a DisMod-MR model would not produce reliable estimates of prevalence. Instead, we calculated the YLDs caused by other urinary disorders directly using an YLD/YLL ratio.

We calculated the ratio of YLDs to YLLs across the specified urinary diseases for which non-fatal outcomes were modelled, using YLL estimates from the GBD 2023 cause of death analysis. We then multiplied this YLD/YLL ratio by the YLL estimates for other urinary diseases.

Urticaria

Flowchart

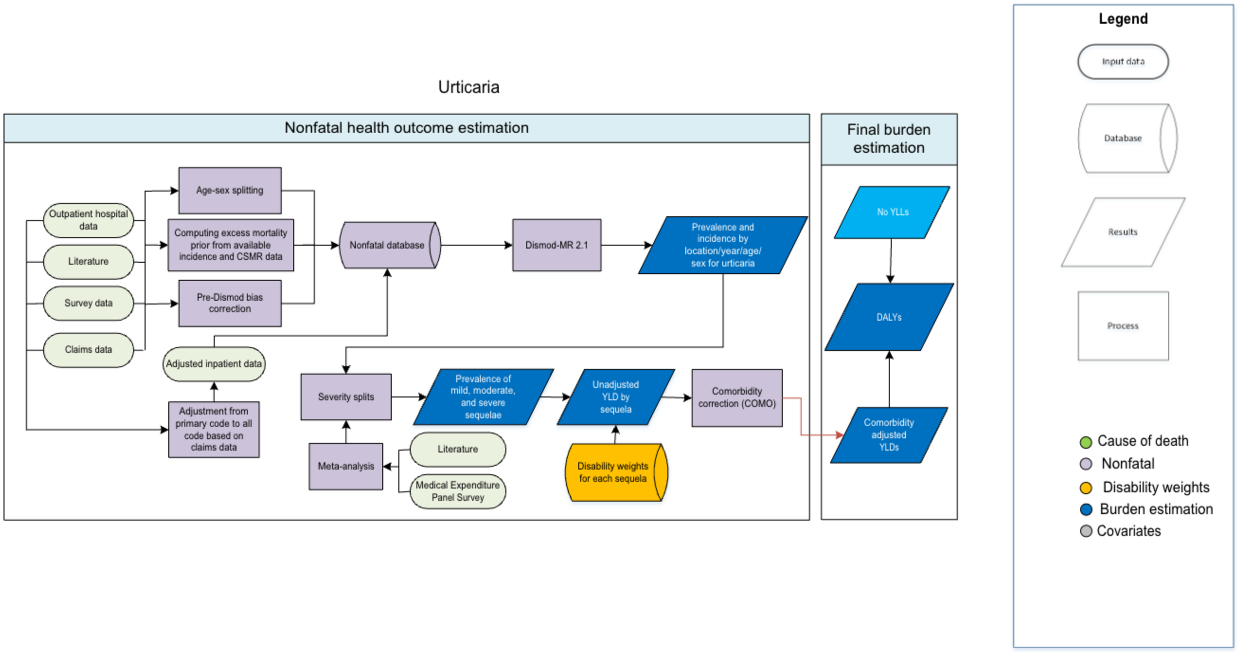

Input data and methodological summary for urticaria

Case definition

Urticaria is defined as a skin rash triggered by a reaction to food, medicine, or other irritants (ICD-10: L50). Urticaria was included in the GBD 2021 cause group of skin and subcutaneous conditions.

Urticaria

Quantity of interest	Reference or alternative	Definition
Urticaria	Reference	Urticaria as determined by Poland National Health Fund Patient Claims 2015–2019
Urticaria	Alternative	All other data for urticaria

Input data

In the GBD 2010 study, a systematic review of the literature was conducted using PubMed and Google Scholar to capture epidemiological data for urticaria. The inclusion criteria stipulated that studies (1) must be published between 1980 and 2012; (2) must provide data on the incidence or prevalence of urticaria; (3) must use samples representative of the general population (ie, samples derived from the experimental arm of clinical trials or based in dermatology clinics were excluded); (4) must use a sample size larger than 100; and (5) must provide sufficient information on study method and sample characteristics to assess the quality of the study. For GBD 2016, the GBD 2010 search strategy was replicated in PubMed to capture epidemiological studies published between 2013 and 2016. Since then, USA claims data from 2010–2019 and Poland National Health Fund Patient Claims 2015–2019 as clinical

sources, along with new literature data from Hong Kong, Nepal, Romania, Egypt, and Israel have been added to our data sources. Data were further considered for exclusion when they led to underestimation of subnational pseudo-random effects and poor model fit, or if we found them unreasonable when compared to regional, super-regional, and global rates.

Data processing

For urticaria, we crosswalked all data to the reference definition. We began by evaluating the number of observations of each alternate definition that matched with a corresponding observation from the reference definition. We considered "between" study matches, where the alternative was from the same GBD age group and sex, and the midpoint year of the study was within five years of the midpoint of the reference definition observation.

$$\log i\, t\left(y_i^{alt}\right) - \log i\, t\left(y_i^{ref}\right) \;=\; \beta_0 + \epsilon_i$$

Table 1: MR-BRT crosswalk adjustment factors for urticaria

Data input	Reference or alternative case definition	Gamma	Beta coefficient, log/logit (95% UI)*	Adjustment factor**
Poland National Health Fund Patient Claims 2015–2019	Ref	0.0907	---	---
All other data	Alt		–0.1807 (–0.7709 to 0.4094)	0.8346 (0.4626, 1.5059)

**MR-BRT crosswalk adjustments can be interpreted as the factor the alternative case definition is adjusted by to reflect what it would have been had it been measured using the reference case definition. If the log/logit beta coefficient is negative, then the alternative is adjusted up to the reference. If the log/logit beta coefficient is positive, then the alternative is adjusted down to the reference.*
***The adjustment factor column is the exponentiated beta coefficient. For log beta coefficients, this is the relative rate between the two case definitions. For logit beta coefficients, this is the relative odds between the two case definitions.*

Modelling strategy

In GBD 2023, we have made no substantive changes in the modelling strategy from GBD 2021.

DisMod-MR 2.1, a Bayesian meta-regression tool, was used to estimate prevalence by age, sex, year, and geography (subnational, country, region, super-region) for urticaria. The available data were mainly composed of prevalence estimates with a few incidence datapoints. For GBD 2023, we made both prevalence and incidence estimates. We used a time window set to 25 years. We set excess mortality to zero and remission between 1.5 to 2, implying a duration between eighteen months and two years. This was in line with the available epidemiological data, expert opinion, and previous GBD work. Excess mortality was assumed to be zero. Since GBD 2019, we have replaced our within-DisMod crosswalks with crosswalks completed using the MR-BRT modelling tool. We adjusted all our urticaria data toward

the level of Poland National Health Fund Patient Claims 2015–2019, which were more representative of the general population.

$$prevalence = incidence * duration$$

$$Remission = \frac{Cured\ cases}{person - year\ of follow - up\ in\ prevalent\ cases}$$

Table 2. Severity distribution, details on the severity levels for urticaria and the associated disability weight (DW) with that severity.

Sequela	Severity level	Lay description	DW (95% CI)
Mild urticaria	Disfigurement, level 1 with itch/pain	The person has a slight, visible physical deformity that is sometimes sore or itchy. Others notice deformity, which causes some worry and discomfort.	0.776 (0.692, 0.819)
Severe urticaria	Disfigurement, level 2, with itch/pain	The person has a visible physical deformity that is sore and itchy. Other people stare and comment, which causes the person to worry. The person has trouble sleeping and concentrating.	0.224 (0.181, 0.308)

Varicella (chickenpox) and herpes zoster

Flowchart

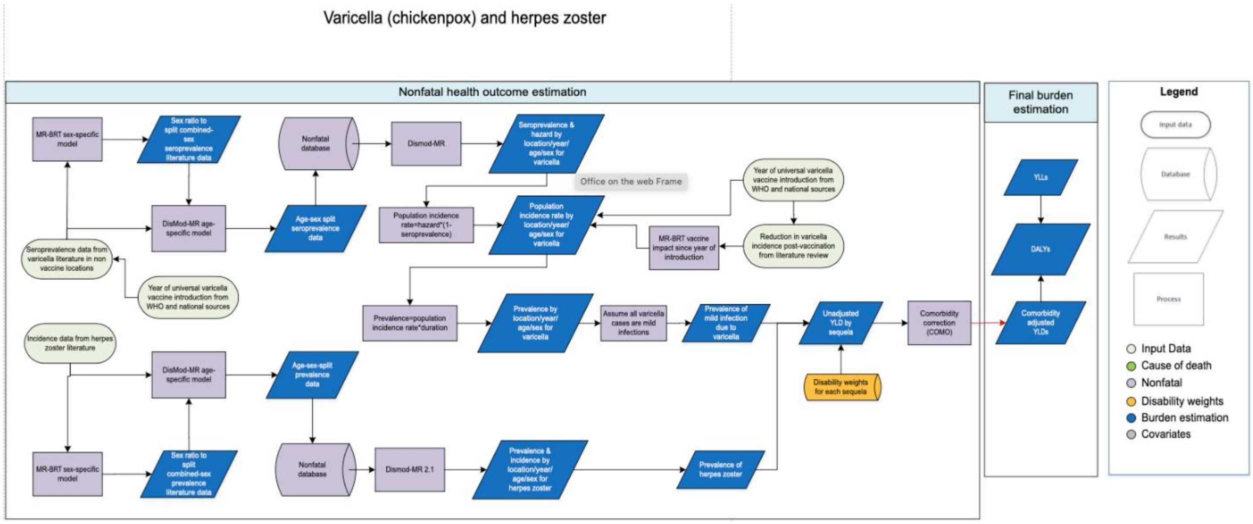

Input data and methodological summary for varicella and herpes zoster

Case definition

Varicella (chickenpox) is caused by primary infection with the varicella-zoster virus and is characterised by a diffuse vesicular rash, malaise, and fever. In healthy children, varicella is typically a mild, self-limiting illness. Complications include pneumonia, encephalitis, and secondary bacterial infection of skin lesions. Herpes zoster (shingles) is caused by reactivation of the varicella-zoster virus, primarily in older and immune-compromised adults. Herpes zoster is characterised by a painful, vesicular rash with a dermatomal distribution.

For varicella and herpes zoster, the ICD-10 codes are B01-B02.9, P35.8, Z20.820, and ICD-9 codes are 052-053.9, V01.71, V01.79, V05.4.

Quantity of interest	Reference or alternative	Definition
Seroprevalence of varicella-zoster virus	Reference	Proportion of sample seropositive for varicella-zoster antibody
Incidence of herpes zoster	Reference	Incidence of herpes zoster virus, as reported in the literature, including cases diagnosed clinically or by lab testing

Input data

Model inputs

The varicella and herpes zoster non-fatal models rely on two distinct data inputs. For varicella, we use seroprevalence estimates from a systematic review of the varicella seroprevalence scientific literature.

For herpes zoster, we use herpes zoster incidence data extracted from scientific literature. For GBD 2023, we updated both the varicella seroprevalence and herpes zoster incidence systematic reviews.

For the varicella seroprevalence systematic review, the following search string was used:

(("varicella") OR (varicella[Title/Abstract]) OR (chickenpox [Title/Abstract]) OR (chickenpox [MeSH Terms])) AND ((seroprevalence[Title/Abstract]) OR (seroprevalence[MeSH Terms]) OR (serology [MeSH Terms]) OR (serology [Title/Abstract])) AND (incidence[Title/Abstract] OR prevalence[Title/Abstract]) NOT (herpes zoster[Title/Abstract] OR shingles[Title/Abstract]) AND ("1980"[Date - Publication] : "2024"[Date - Publication]).

In our varicella seroprevalence systematic review we excluded data from children under 6 months of age, in order to avoid confounding by maternal antibody status. New in GBD 2023, we also excluded seroprevalence data from location-years following introduction of universal varicella vaccination (UVV) to avoid confounding by vaccination status.

New in GBD 2023, we accounted for the impact of UVV on the incidence of chickenpox. Data informing our vaccine adjustments to the model included a) the World Health Organization (WHO) Joint Reporting Form (JRF) on year of varicella vaccine introduction, and b) a targeted literature review on the impact of varicella vaccination on population-level chickenpox incidence.

For the UVV impact literature review, the following search string was used:

("varicella zoster"[Title/Abstract]) OR (varicella[Title/Abstract]) OR (chickenpox [Title/Abstract]) OR (VZV[Title/Abstract])) AND ((incidence[Title/Abstract]) OR (incidence[MeSH Terms])) AND ((vaccination[Title/Abstract]) OR (vaccine[Title/Abstract]) OR (immunization [Title/Abstract])) NOT "herpes zoster"

For the herpes zoster incidence systematic review, the following search string was used:

(("herpes zoster"[Title/Abstract] OR "shingles"[Title/Abstract] OR "herpes zoster"[MeSH Terms]) AND incidence[Title/Abstract]) NOT ("varicella"[Title/Abstract] OR "chicken pox"[Title/Abstract]) AND ("2016"[PDAT] : "2030"[PDAT]).

Input data processing

All extracted varicella seroprevalence and herpes zoster incidence data that were not sex-specific or reported age range widths of greater than ten years were split into sex- and age-specific groups prior to modelling. Because scant age- and sex-specific data on varicella seroprevalence and herpes zoster incidence are available, global sex ratios and age patterns were generated as described below and used to split non-sex- or age-specific data. The ratios used to make the sex splits were calculated using meta-regression—Bayesian, regularised, trimmed (MR-BRT), a Bayesian meta-regression tool. The sex adjustment factors (female/male ratios) calculated for GBD 2023 were 1.01 for varicella seroprevalence, and 1.17 for herpes zoster incidence (Tables 1a, 1b).

Table 1a: MR-BRT sex-splitting adjustment factor for varicella seroprevalence

Data input	Reference or alternative case definition	Beta coefficient, log (95% CI)	Adjustment factor*
Sex (female/male)	N/A	0.007 (-0.004, 0.017)	1.01 (0.97 - 1.05)

Table 1b: MR-BRT sex-splitting adjustment factor for herpes zoster incidence

Data input	Reference or alternative case definition	Beta coefficient, log (95% CI)	Adjustment factor*
Sex (female/male)	N/A	0.155<br>(0.0502 - 0.250)	1.17

**MR-BRT crosswalk adjustments can be interpreted as the factor the alternative case definition is adjusted by to reflect what it would have been had it been measured using the reference case definition. If the log/logit beta coefficient is negative, then the alternative is adjusted up to the reference. If the log/logit beta coefficient is positive, then the alternative is adjusted down to the reference.*
***The adjustment factor column is the exponentiated beta coefficient. For log beta coefficients, this is the relative rate between the two case definitions. For logit beta coefficients, this is the relative odds between the two case definitions.*

To estimate global age patterns, we fit two separate DisMod-MR models, one for herpes zoster incidence and one for varicella seroprevalence. For each model, we used only age-specific data (defined as a data representing an age band of <10 years). The global age pattern output – produced by DisMod in five-year age-bins – spanned early neonatal to 95+ age groups. For varicella seroprevalence, scant data were available for older adults. We therefore assumed that seroprevalence for individuals age 65+ was the same as that estimated for individuals aged 60–64 years. These DisMod-MR age patterns were used to split data from the remaining non-age-specific data sources.

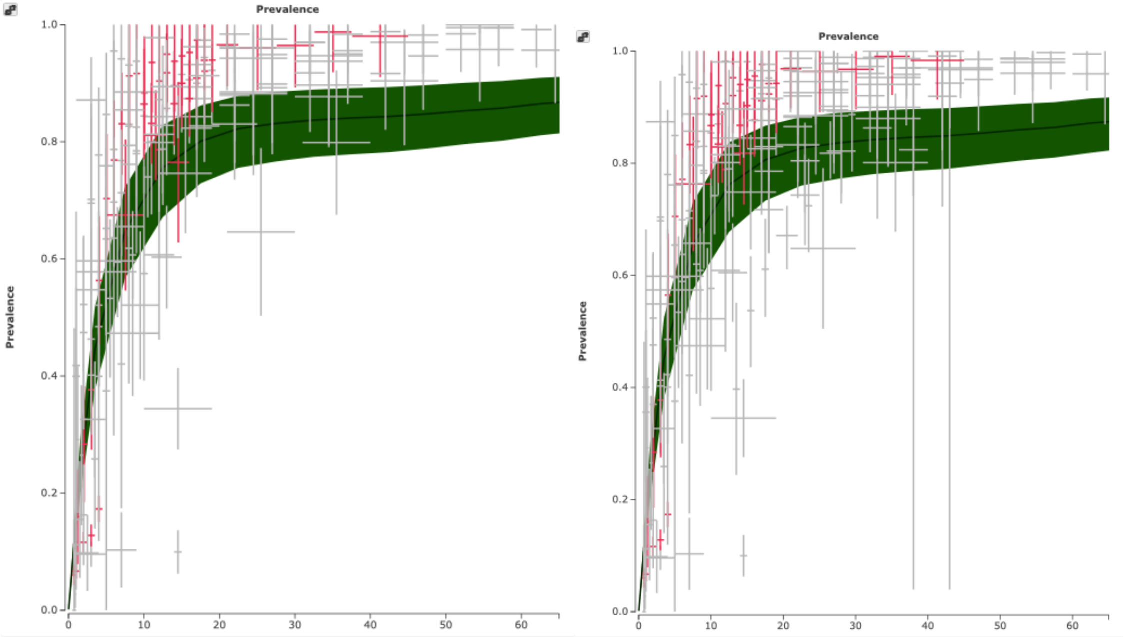

Figure 1. Global age pattern for varicella seroprevalence (L: male, R: female)

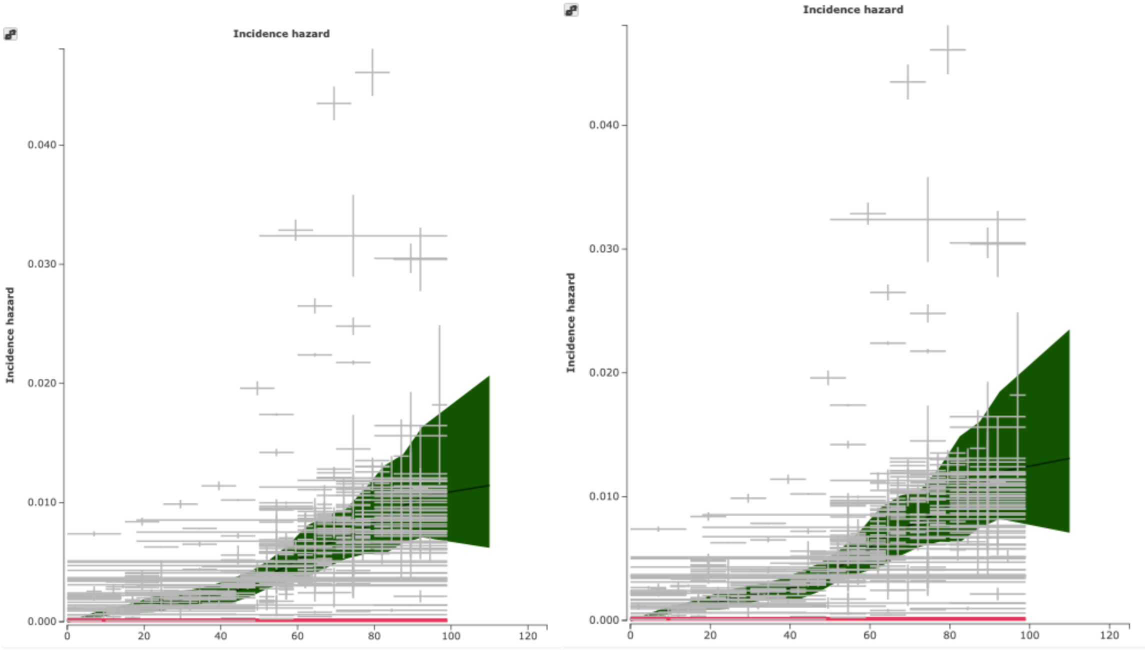

Figure 2. Global age pattern for herpes zoster incidence (L: male, R: female)

Modelling strategy

We used our sex- and age-split varicella seroprevalence data to model seroprevalence for all locations and years in DisMod-MR. Because the model is fit to seroprevalence data from non-UVV locations, the DisMod results represent expected seroprevalence in the absence of vaccination. We used the Healthcare Access and Quality (HAQ) Index as the sole covariate (Table 3). Model parameters were

constrained such that there is zero remission and no excess mortality. Using the incidence hazard and prevalence outputs of the seroprevalence model, varicella incidence rate was calculated as follows:

$$incidence\ rate\ =\ hazard\ *(1-prevalence)$$

In GBD 2023, we adjusted our estimates to reflect the impact of UVV on varicella incidence. We used the WHO JRF to identify locations and years where UVV was introduced. Where national data on the year of UVV introduction differed from the JRF, the national source was used. We then identified high-quality epidemiological studies estimating the impact of UVV on varicella incidence. We fit a simple regression model in MR-BRT where the relative change in pre- to post-vaccination incidence was a function of years since introduction of UVV.

$$\frac{(postvaccine\ incidence-prevaccine\ incidence)}{prevaccine\ incidence}\sim years\ post\ UVV\ introduction$$

We then predicted the expected change in varicella incidence for each year following UVV introduction, assuming a constant vaccine impact after ten years. We used the predicted reductions in varicella incidence to apply a post-hoc adjustment to our modelled incidence estimates. For each location where UVV has been implemented, we multiplied the without-vaccine estimate from the year of UVV introduction by the predicted change in incidence to produce estimates for each year following UVV introduction.

Once incidence estimates had been adjusted, we calculated varicella prevalence as the product of the incidence rate * duration. We assumed a mean case duration of seven days:

$$prevalence=incidence\ rate*duration$$

To produce our non-fatal herpes zoster estimates, we used the age- and sex-split herpes zoster incidence data directly in a DisMod model. No covariates were included.

For both varicella and herpes zoster, we assumed that excess mortality would be negligible relative to the total number of cases and therefore set this parameter to zero in the DisMod models.

As expected, in locations where UVV has been implemented, significant reductions in varicella incidence and prevalence were observed in the years following UVV introduction. Inclusion of new data in our herpes zoster model led to lower estimated incidence in southeast Asia, east Asia and Oceania and higher estimates in high-income regions.

Severity splits and disability weights

We assume all varicella cases are mild episodes of acute infectious disease and treat herpes zoster as a sequela. The lay descriptions and corresponding disability weights (DW) are presented in Table 2.

Table 2. Severity distribution, details on the severity levels for varicella-related non-fatal burden in GBD 2019 and the associated disability weight (DW) with that severity.

Severity level	Lay description	DW (95% CI)
Mild acute infectious disease	Has a low fever and mild discomfort but no difficulty with daily activities.	0.006<br>(0.002–0.012)
Herpes zoster	Has a blistering skin rash that causes pain, with some burning and itching.	0.058<br>(0.035–0.09)

Table 3. Covariates. Summary of covariates used in the varicella seroprevalence DisMod-MR meta-regression model

Covariate	Type	Parameter	Exponentiated beta (95% CI)
Healthcare Access and Quality (HAQ) Index	Country-level	Seroprevalence	0.61 (0.37–0.98)

Changes from GBD 2021 to GBD 2023

In GBD 2023, we included varicella vaccination in the model and modified our estimates to reflect the impact of vaccination on varicella incidence. There were no significant methodological changes to the herpes zoster modelling.

Figure 3: PRISMA 2020 flow diagram for varicella seroprevalence

From: Page MJ, McKenzie JE, Bossuyt PM, et al. The PRISMA 2020 statement: an updated guideline for reporting systematic reviews. *BMJ*. 2021;372:n71. Published 2021 Mar 29. doi:10.1136/bmj.n71

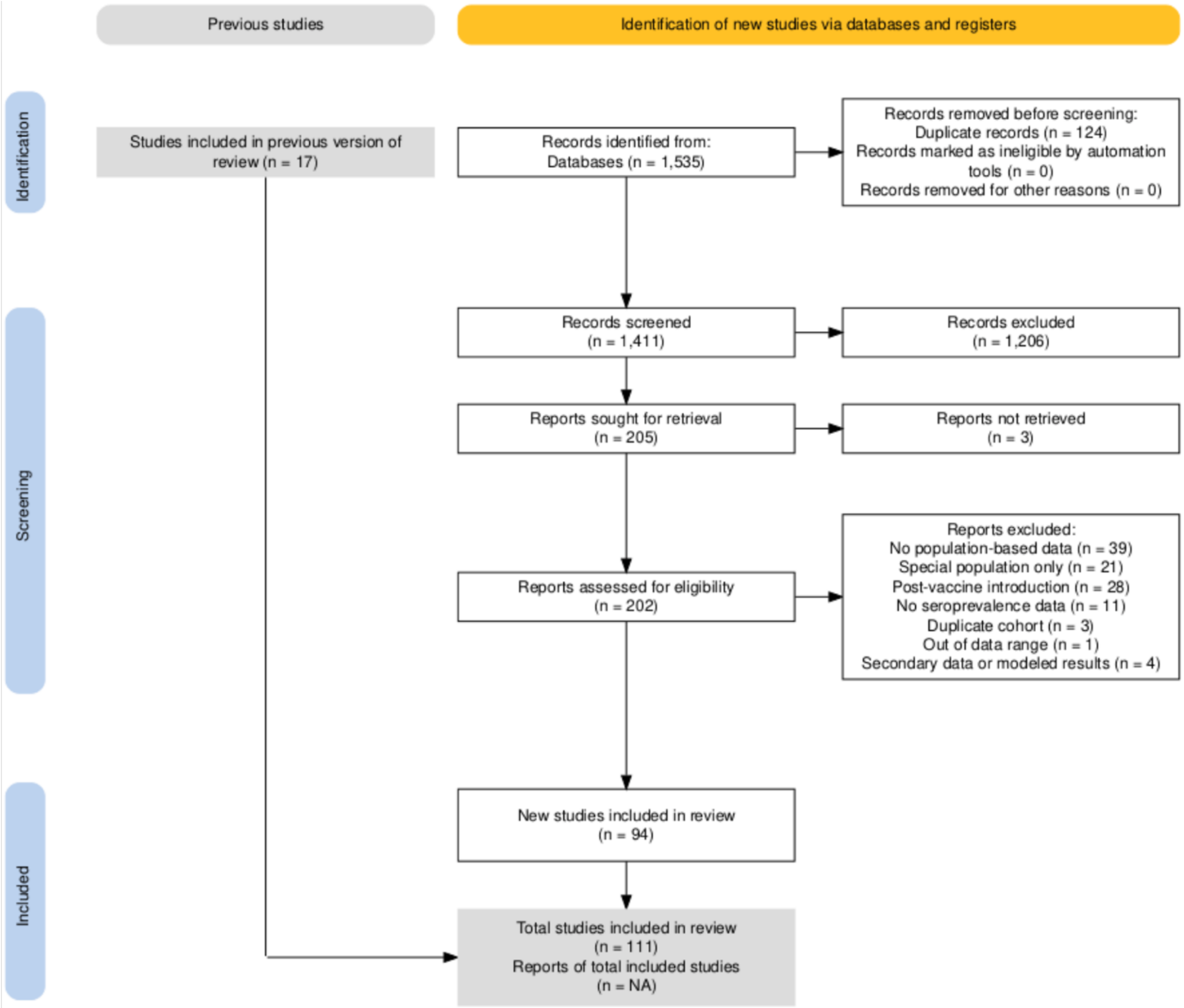

Previous studies
Identification of new studies via databases and registers
Identification
Studies included in previous version of review (n = 17)
Records identified from:
Databases (n = 1,535)
Records removed before screening:
Duplicate records (n = 124)
Records marked as ineligible by automation tools (n = 0)
Records removed for other reasons (n = 0)
Screening
Records screened
(n = 1,411)
Records excluded
(n = 1,206)
Reports sought for retrieval
(n = 205)
Reports not retrieved
(n = 3)
Reports assessed for eligibility
(n = 202)
Reports excluded:
No population-based data (n = 39)
Special population only (n = 21)
Post-vaccine introduction (n = 28)
No seroprevalence data (n = 11)
Duplicate cohort (n = 3)
Out of data range (n = 1)
Secondary data or modeled results (n = 4)
Included
New studies included in review
(n = 94)
Total studies included in review
(n = 111)
Reports of total included studies
(n = NA)

Figure 4: PRISMA 2020 flow diagram for herpes zoster incidence

From: Page MJ, McKenzie JE, Bossuyt PM, et al. The PRISMA 2020 statement: an updated guideline for reporting systematic reviews. *BMJ*. 2021;372:n71. Published 2021 Mar 29. doi:10.1136/bmj.n71

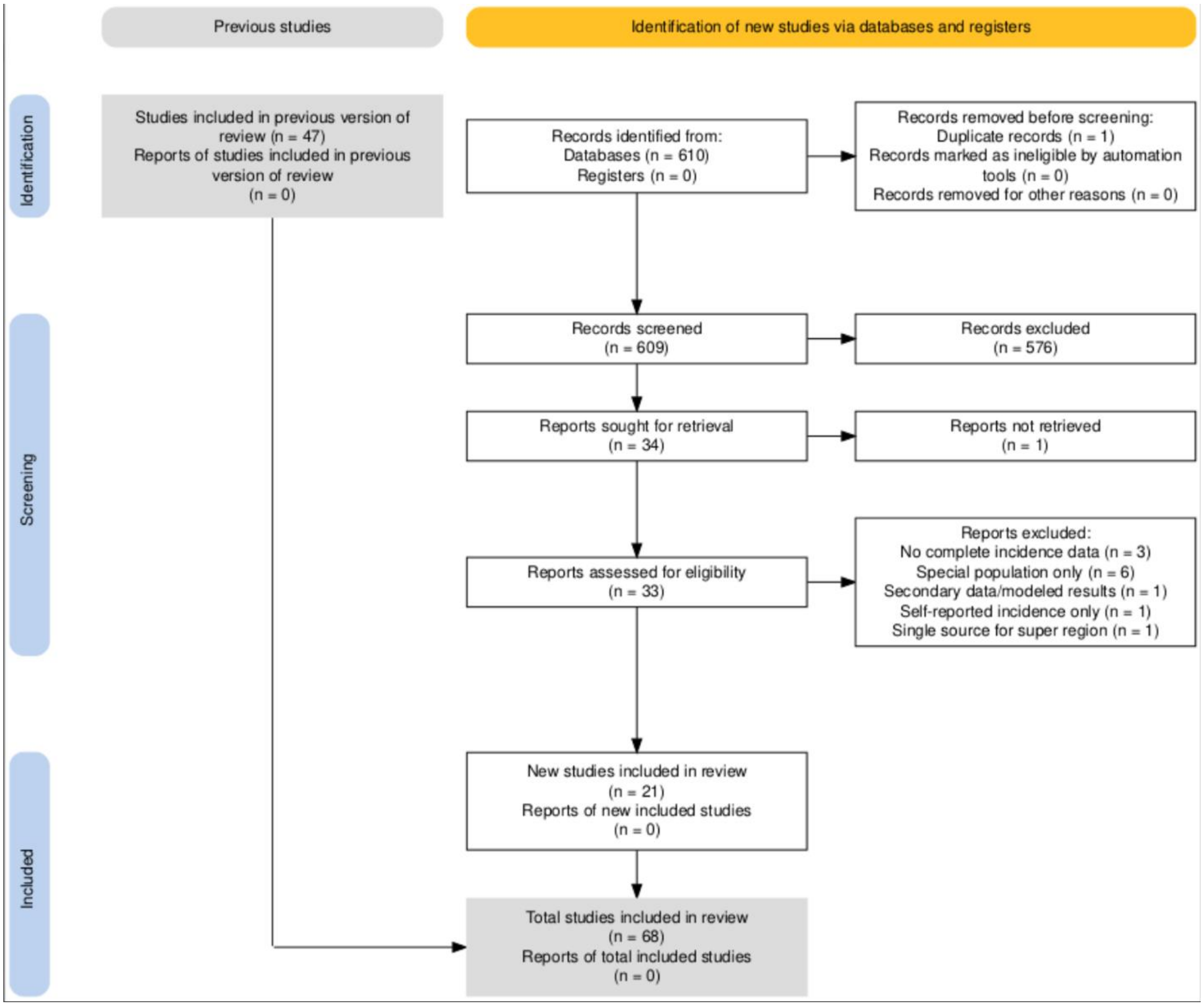

Vascular intestinal disorders

Flowchart

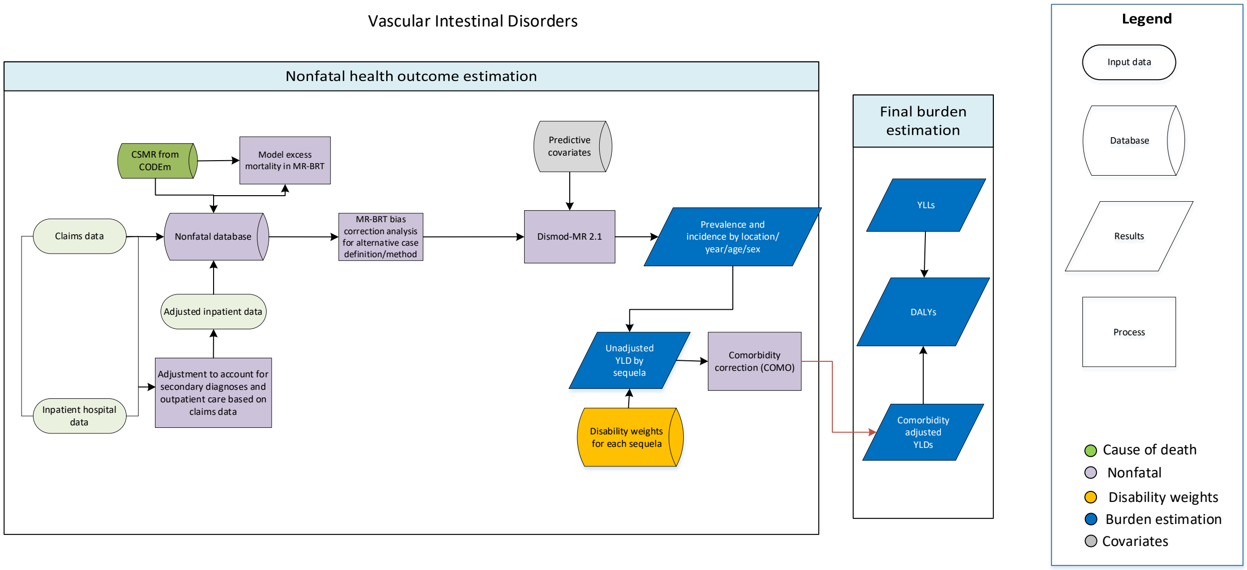

Input data and methodological summary for vascular intestinal disorders

Case definition

Vascular intestinal disorders encompass ischaemic disorders and vascular malformations (eg, angiodysplasias). Ischaemia refers to a condition in which blood flow to the gastrointestinal tract is restricted, causing injury to the bowel and severe pain and complications. Vascular malformations refers to inappropriate growth of blood vessels in the bowel, predisposing to bleeding.

Vascular intestinal disorders typically require surgical or endoscopic treatment. The ICD-10 code for vascular intestinal disorders is K55; ischaemia and angiodysplasia are only distinguished at the level of 4-digit and 5-digit codes. Equivalent codes for ICD-9 are 569.84, 569.85 and 569.86 (for angiodysplasia), and 557 and its 4- and 5-digit constituents (for ischaemia).

Quantity of interest	Reference or alternative	Definition
Incidence of vascular intestinal disorders	Alternative	Cases identified from database of commercial claims from USA in 2010-2017 using ICD codes.
Incidence of vascular intestinal disorders	Alternative	Cases identified from database of commercial claims from USA in 2000 using ICD codes.
Incidence of vascular intestinal disorders	Reference	Cases of vascular intestinal disorders identified using ICD codes in administrative records that are considered population-representative.

Input data

Inputs

Like GBD 2021, the model included incidence data from hospital discharges and claims. No new data were added in GBD 2023. The input data and processing steps for modelling in GBD 2023 were the same as in GBD 2021.

Inputs to our non-fatal modelling also included cause-specific mortality rate (CSMR) estimates taken from our fatal modelling process (see CoD cause-specific modelling description for vascular intestinal disorders in this appendix) and excess mortality rates (EMR) estimates modelled outside of DisMod (see the EMR data processing section below).

Incidence data processing

Hospital discharge data provide observations about encounters, generally with only the primary diagnostic code for the encounter. Claims data, on the other hand, link claims for all inpatient and outpatient encounters for a single individual and provide primary and secondary diagnoses for all encounters.

In GBD 2017, an individual was extracted from claims data as an incident case if that individual had one or more inpatient encounters with an appropriate ICD code as any diagnosis. Hospital discharges with an appropriate ICD code as primary diagnosis were extracted and adjusted for readmissions.

In GBD 2019, GBD 2021, and GBD 2023, however, we employed data processing methods to capture cases that were diagnosed and/or treated in both inpatient and outpatient settings. Specifically, an individual was extracted from claims data as an incident case if that individual had at least one inpatient or outpatient encounter with an appropriate ICD code as any diagnosis within one year. Hospital discharge data were processed by extracting discharges with an appropriate ICD code as primary diagnosis and adjusting using correction factors (ie, correction factor 3) derived from claims data. Specifically, we modelled from the ratio of inpatient claims with vascular intestinal disorders as primary diagnosis to total incident cases of vascular intestinal disorders seen in claims data.

As first done in GBD 2019, USA claims data (extracted and processed as described above) were then adjusted to account for selection bias due to commercial insurance using MR-BRT analysis.

The process of adjusting for biases in non-reference data using MR-BRT with the logit-transformation method is described below:

1. Identify datapoints with overlapping year, age, sex, and location between reference and non-reference population data.
2. Logit transform overlapping datapoints of alternative and reference types.
3. Convert overlapping datapoints into a difference in logit space using the following equation: $logit(altnerative) - logit(reference)$
4. Use the delta method to compute standard errors of overlapping datapoints in logit space, then calculate standard error of logit difference using the following equation: $\sqrt{(variance\ of\ logit(alternative)) + (variance\ of\ logit(reference))}$
5. Using MR-BRT, conduct a random effects meta-regression to obtain the pooled logit difference of alternative to reference
6. Apply the pooled logit difference to all data points of non-reference types using the following equation:
$$new_{estimate} = inverse.logit((logit(alternative)) - (pooled\ logit\ difference))$$
7. Calculate new standard errors using the delta method, accounting for gamma (between-study heterogeneity)

The table below shows bias correction factors estimated using MR-BRT.

Table 1. MR-BRT crosswalk adjustment factors for vascular intestinal disorders

Data input	Reference or alternative data collection	Gamma	Beta coefficient, logit (95% CI)	Adjustment factor*
Hospital + non-USA claims	Ref	0.05	---	---
USA claims from year 2000	Alt		–0.24 (–0.71, 0.22)	0.44 (0.33, 0.55)
USA claims from years 2010–2017	Alt		0.12 (–0.02, 0.26)	0.53 (0.50, 0.56)

**MR-BRT crosswalk adjustments can be interpreted as the factor the alternative case definition is adjusted by to reflect what it would have been had it been measured using the reference case definition. If the log/logit beta coefficient is negative, then the alternative is adjusted up to the reference. If the log/logit beta coefficient is positive, then the alternative is adjusted down to the reference.*
***The adjustment factor column is the exponentiated beta coefficient. For log beta coefficients, this is the relative rate between the two case definitions. For logit beta coefficients, this is the relative odds between the two case definitions.*

Datapoints with an age-standardised incidence rate greater than two median absolute deviations from the median of the age-standardised incidence rate for all data were marked as outliers and excluded from analysis.

EMR processing

In GBD 2017, EMR inputs were produced by matching prevalence datapoints with their corresponding CSMR values within the same age, sex, year, and location (by dividing CSMR by prevalence). For short-duration conditions (remission >1), the corresponding prevalence was derived by running an initial model and then applying the same CSMR/prevalence method. However, this method of producing EMR inputs demonstrated a rather unrealistic pattern of EMR compared to an expected pattern of decreasing

EMR with greater access to quality health care. Such unexpected patterns often signal inconsistencies between CSMR estimates and the measures of prevalence and/or incidence. Thus, in an effort to provide greater guidance on the expected pattern of EMR, in GBD 2019, EMR data produced per above in GBD 2017 were modelled by age, sex, and Healthcare Access and Quality (HAQ) Index using MR-BRT, with a prior on HAQ Index having a negative coefficient. In GBD 2021, we employed the same MR-BRT method to predict EMR for each location, year, sex, and for ages 0, 10, 20….100, and these predictions were used as inputs to our non-fatal model in GBD 2019 and subsequent rounds, described below.

Modelling strategy

DisMod model

Similar to previous rounds, we ran a DisMod-MR 2.1 model to produce estimates by age, sex, year, and location. Inputs to DisMod for vascular intestinal disorders include incidence, CSMR, and EMR inputs processed as described above. Prior settings included bounding remission between 2 and 12 (a disease duration of four weeks to half a year) for all age groups. The minimum coefficient of variation at the regional, super-regional, and global level was set at 0.8. A lag-distributed income covariate (log transformed) and a mean total cholesterol covariate were applied to incidence as predictive covariates. Betas and exponentiated values (which can be interpreted as an odds ratio) are shown in the table below.

Table 2. Covariates. Summary of covariates used in the vascular intestinal disorders DisMod-MR meta-regression model.

Covariate	Parameter	Exponentiated beta (95% uncertainty interval)
Low-density lipoprotein (mmol/L)	Incidence	0.79 (0.75–0.84)
LDI (I$ per capita)	Incidence	1.32 (1.29–1.35)
Healthcare Access and Quality Index	Excess mortality rate	0.96 (0.96–0.97)

Severity split and disability weight

The basis of the GBD disability weight survey assessments are lay descriptions of sequelae highlighting major functional consequences and symptoms. The lay description and disability weights for vascular intestinal disorders are shown below.

Table 3. Severity distribution, details on the severity levels for vascular intestinal disorders in GBD 2023 and the associated disability weight (DW) with that severity.

Severity level	Lay description	DW (95% CI)
Severe	This person has severe pain in the belly and feels nauseated. The person is anxious and	0.324 (0.219–0.442)

	unable to carry out daily activities.	

Viral skin diseases

Flowchart

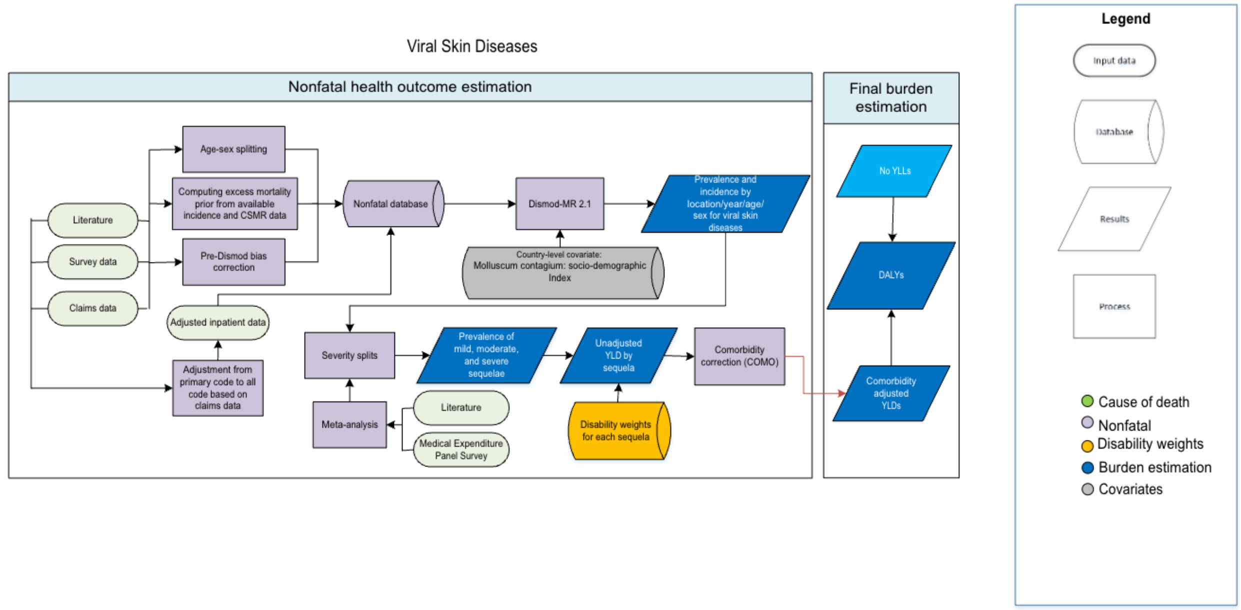

Input data and methodological summary for viral skin diseases

Case definition

Viral skin diseases consist of viral warts and molluscum contagiosum and consist of raised growths on the surface of the skin caused by an infection with the human papillomavirus (viral warts) (ICD-10: B07) or a viral infection of the skin or occasionally mucous membranes characterised by the appearance of waxy, dome-shaped nodules (molluscum contagiosum) (ICD-10: B08.1). In GBD 2021, we modelled viral warts and molluscum contagiosum separately in order to better accommodate differences in burden between the subtypes of viral skin diseases.

Viral skin diseases

Quantity of interest	Reference or alternative	Definition
Viral skin diseases	Reference	Viral skin diseases as determined by Poland National Health Fund Patient Claims 2015–2019
	Alternative	Other data inputs for viral skin diseases

Input data

In the GBD 2010 study, a systematic review of the literature was conducted using PubMed and Google Scholar to capture epidemiological data for viral skin diseases. Due to lack of published data on the epidemiology of viral skin diseases, the literature search also included relevant incidence data from national inpatient or outpatient records in the USA. The inclusion criteria stipulated that studies (1) must be published between 1980 and 2012; (2) must provide data on the incidence or prevalence of viral

warts or molluscum contagiosum; (3) must use samples representative of the general population (ie, samples derived from the experimental arm of clinical trials or based in dermatology clinics were excluded); (4) must use a sample size larger than 100; and (5) must provide sufficient information on study method and sample characteristics to assess the quality of the study. For GBD 2013, the GBD 2010 search strategy was replicated to capture epidemiological studies published between 2012 and 2013. For GBD 2017, the GBD 2010 search strategy was replicated to capture epidemiological studies published between 2013 and 2017.

For GBD 2023, we have added year-locations of outpatient clinical data. The new data sources for molluscum contagium include the USA, Poland, and Ethiopia. For viral warts, the new sources for clinical data are from the USA, Poland, Sri Lanka, Nepal, Romania, Norway, Ethiopia, Nigeria, Taiwan, Tanzania, India, the Netherlands, Portugal, England, Gabon, and South Korea. Data were outliered or excluded if we found them unreasonable when compared to regional, super-regional, and global rates.

Data processing

For viral skin diseases, we crosswalked all data to the reference definition. We began by evaluating the number of observations of each alternate definition that matched with a corresponding observation from the reference definition. We considered "between" study matches, where the alternative was from the same GBD age group and sex, and the midpoint year of the study was within five years of the midpoint of the reference definition observation.

$$\log i\, t\left(y_i^{alt}\right) - \log i\, t\left(y_i^{ref}\right) \; = \; \beta_0 \; + \epsilon_i$$

Table 1: MR-BRT crosswalk adjustment factors for viral skin diseases

Data input	Reference or alternative case definition	Gamma	Beta coefficient, log/logit (95% UI)*	Adjustment factor**
Molluscum contagium	Ref	0.0987	---	---
	Alt		2.4032 (1.7876–3.0189)	11.0587 (5.9749, 20.4682)
Viral warts	Ref	0.1014	---	---
	Alt		0.8386 (0.2146–1.4626)	2.3131 (1.2394, 4.3172)

**MR-BRT crosswalk adjustments can be interpreted as the factor the alternative case definition is adjusted by to reflect what it would have been had it been measured using the reference case definition. If the log/logit beta coefficient is negative, then the alternative is adjusted up to the reference. If the log/logit beta coefficient is positive, then the alternative is adjusted down to the reference.*

***The adjustment factor column is the exponentiated beta coefficient. For log beta coefficients, this is the relative rate between the two case definitions. For logit beta coefficients, this is the relative odds between the two case definitions.*

Modelling strategy

DisMod-MR 2.1, a Bayesian meta-regression tool, was used to estimate viral skin diseases prevalence by age, sex, year, and geography (subnational, country, region, super-region). Separate models were run for molluscum contagium and viral warts.

Molluscum contagium

To help inform the distribution of molluscum contagium across the lifespan, excess mortality was set at zero, and remission was set at 0.5 to 2. This was in agreement with the available prevalence data and expert advice. We made use of a relatively long time window of 20 years to determine which datapoints were used for a particular year of fit. This means that for the year 2000, for instance, DisMod-MR 2.1 incorporated all datapoints ranging from 1980 to present to estimate prevalence.

Since GBD 2019, we replaced our within-DisMod crosswalks with crosswalks completed using the MR-BRT modelling tool. We adjusted all our datapoints toward the Poland National Health Fund Patient Claims 2015–2019, which were more representative of the general population. In addition, Socio-demographic Index was used as a country-level covariate to guide estimates for countries with few or no data.

Viral warts

For viral warts, excess mortality rate was set at zero, and remission set between 0.25 and 2, and incidence hazard set at 0 to 0.1. In GBD 2023, we have crosswalks completed using the MR-BRT modelling tool. We adjusted all clinical and literature data sources toward the level of Poland claims datapoints which were more representative of the general population.

We have made no substantive changes in the modelling strategy from GBD 2021.

$$prevalence = incidence * duration$$

$$Remission = \frac{Cured\ cases}{person - year of follow - up\ in\ prevalent\ cases}$$

Table 2. Severity distribution, details on the severity levels for viral skin diseases and the associated disability weight (DW) with that severity

sequela	Severity level	Lay description	DW (95% CI)
Mild molluscum contagium	Infectious disease, acute episode, mild	The person has a low fever and mild discomfort but no difficulty with daily activities.	0.006 (0.002, 0.012)
Severe molluscum contagium	Disfigurement, level 2	The person has a visible physical deformity that causes others to stare and comment. As a result, the person is worried and has trouble sleeping and concentrating.	0.067 (0.044, 0.096)

Mild viral warts	Infectious disease, acute episode, mild	The person has a low fever and mild discomfort, but no difficulty with daily activities.	0.006 (0.002, 0.012)
Severe viral warts	Disfigurement, level 2	The person has a visible physical deformity that causes others to stare and comment. As a result, the person is worried and has trouble sleeping and concentrating.	0.067 (0.044, 0.096)

Table 3. Covariates. Summary of covariates used in viral skin diseases DisMod-MR meta-regression model

Sequela	Covariate	Type	Parameter	Value	Exponentiated beta (95% uncertainty interval)
Molluscum contagium	Socio-demographic Index	Country-level	Prevalence	1.48 (–0.12, 2.00)	4.38 (0.89–7.38)

Visceral leishmaniasis

Flowchart

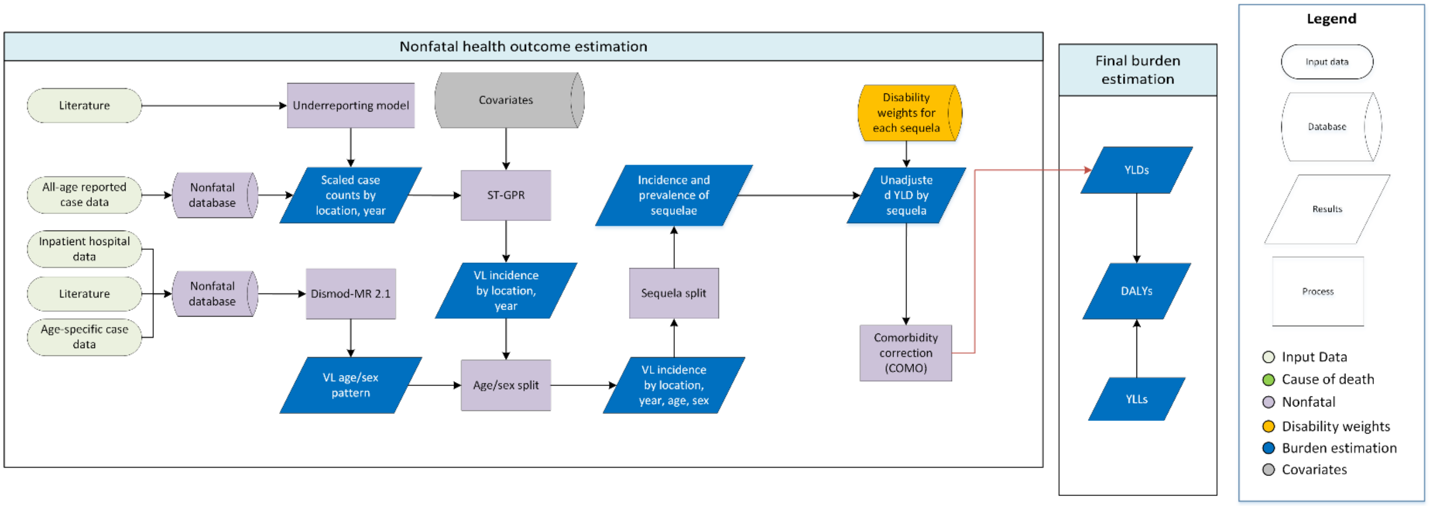

Input data and methodological summary for visceral leishmaniasis

Case definition

Visceral leishmaniasis (VL) is the most serious manifestation of disease caused by the *Leishmania* parasite, transmitted through the bite of phlebotomine sandflies. Those infected typically present with fever, weight loss, anaemia, leukopenia, thrombocytopenia, and enlargement of the spleen and liver. If left untreated, it can be fatal. Transmission varies by geographical region, with a variety of reservoir hosts implicated, and different vector species associated, maintaining both zoonotic and anthroponotic transmission cycles. The ICD-9 code related to visceral leishmaniasis is 085.0, and the ICD-10 code is B55.0. We used the following case definition for GBD 2023:

Quantity of interest	Reference or Alternative	Definition
Visceral leishmaniasis	Reference	Cases reported to public health surveillance systems and published by WHO or other national health organisations. Diagnosis can be made via clinical manifestations such as prolonged fever, splenomegaly, pallor, and weight loss, and then confirmed through parasitological testing.

Input data

Incidence

Current estimation for the all-age, both-sex incidence envelope is based upon location-representative information rather than site-specific epidemiological measures due to the absence of global foci maps allowing for upscaling of geographically precise information. The primary input data are case notification time-series reported by national control programmes, ministries of health, and the World Health Organization (WHO). This is supplemented by systematic literature review (last updated for GBD 2015) to identify alternate sources of data for years missing information. For countries with subnational

estimates, in-country collaborators have compiled information for respective programmes or identified key resources.

Under-reporting

It is recognised that case notification series record only a subset of the true cases present. A review was undertaken (from GBD 2017) to identify articles that compared reported cases with alternate measures to estimate the degree of under-reporting. The following search strings were used: 'leish* AND under*'; 'active passive leish*'. Inclusion criteria were broad to maximise spatiotemporal coverage in potential estimates – any report that compared reported statistics with some notion of "truth" (whether capture-recapture, active surveillance, etc.) were extracted. Values for both cutaneous and visceral leishmaniasis were included. Nine articles were included, summarised in Table 1. Studies[13,14] with case detection less than 15% were outliered due to concerns of their representativeness to other locations.

Table 1. Metadata for underreporting scalars. Each record lists a citation, GBD location of relevance, year, pathogen, brief summary of methods, and output values used in modelling.

**Citation**	**GBD location**	**Time period**	**Pathogen**	**Method synopsis**	**Proportion of "true" cases reported**
Yadon *et al.* 2001[2] "Assessment of Leishmaniasis notification system in Santiago del Estero, Argentina, 1990-1993"	Argentina	1990–1993	CL	Capture-recapture methods were used to evaluate four reporting sources.	94/210
Sesma *et al.* 1997[3] "Leishmaniasis in Navarra: a review of activities"	Spain	1990–1997	CL, VL	Comparison of active searching within the region with reporting via Epidemiological Surveillance System	8/21
Maia-Elkhoury *et al.* 2007[4] "Analysis of visceral leishmaniasis reports by the capture-recapture method"	Brazil	2002–2003	VL	Comparison of three notification systems for completeness	5896/10,691
Gkolfinopoulou *et al.* 2013[5] "Epidemiology of human leishmaniasis in Greece, 1981-2011"	Greece	2004–2009	VL	Comparing number of cases identified at national reference laboratory with mandatory notification system.	260/361
Singh *et al.* 2010[6] "Estimation of under-reporting of Visceral Leishmaniasis cases in Bihar India"	Bihar, India	2006	VL	Comparison of actual reported number of cases with estimated age-sex-stratified incidence proportions for a cohort of 31,324 persons	34/177
Hirve *et al.* 2010[7] "Effectiveness and feasibility of active and	Bihar, India<br>Nepal<br>Bangladesh	2008	VL	Comparing active case detection evaluations (conducting via house-to-	111/130<br>119/127<br>18/25

passive case detection in the Visceral Leishmaniasis Elimination Initiative in India, Bangladesh, and Nepal"				house screening) with passive case detection systems	20/32
Faraj *et al.* 2016[8] "Effectiveness and cost of insecticide-treated bed nets and indoor residual spraying for the control of cutaneous leishmaniasis: A cluster-randomized control trial in Morocco"	Morocco	2008–2013	CL	Comparison of incidence of new CL cases by both active and passive case detection	409/670
Das *et al.* 2014[9] "Active and passive case detection strategies for the control of leishmaniasis in Bangladesh"	Bangladesh	2010–2011	VL	Comparing two districts' estimates [identified in the paper as being directly comparable] of cases, one via active case detection, the other via passive case detection. Active case detection was via community education and outreach workers targeting households	756/1087
Rahman *et al.* 2015[10] "Performance of Kala-azar surveillance in Gaffargaon subdistrict of Mymensingh, Bangladesh"	Bangladesh	2010–2011	VL	Comparison of cases reported to the local health complex versus active search for kala-azar cases	29/58
Eid *et al.* 2017[11] "Assessment of a Leishmaniasis reporting system in tropical Bolivia using the capture-recapture method"	Bolivia	2013–2014	CL	Active surveillance during medical campaigns were compared to registered cases reported by the National Program of Leishmaniasis Control	23/86

Age-sex splitting

Where possible, information disaggregating location-level statistics by age and sex were extracted.

Geographical restrictions

There are strong climatic and biogeographical constraints on the geographical distribution of VL resulting in a focal rather than global distribution. As a result, it is necessary to identify locations burdened by the disease through space and time as distinct from countries where VL is absent. Tags were assigned to each location-year based upon the outcome of a search of IHME databases, as well as location-specific searches of PubMed. Each location-year was tagged as follows:

- Present – where a specific citation of either an autochthonous laboratory-confirmed case (ie, a case with PCR, serological, or parasitological diagnosis), reported case (ie, a case noted as VL, but with no supporting diagnostic), or supporting evidence (ie, confirmed infection in animal reservoirs or sandfly vectors)
- Protocol Present – for a given location-year, where no specific citation is used, but is present for another year in the same location, it is assumed that VL is present given that eradication of the pathogen has not been achieved
- Absent – where PubMed location-specific searches returned zero relevant results, in locations scoring –25 or lower as evaluated by study[12] [the threshold for "absence"]
- Protocol Absent – as with Absent, locations with zero relevant PubMed results, but with a score greater than –25 as evaluated by study,[12] were tagged as Protocol Absent

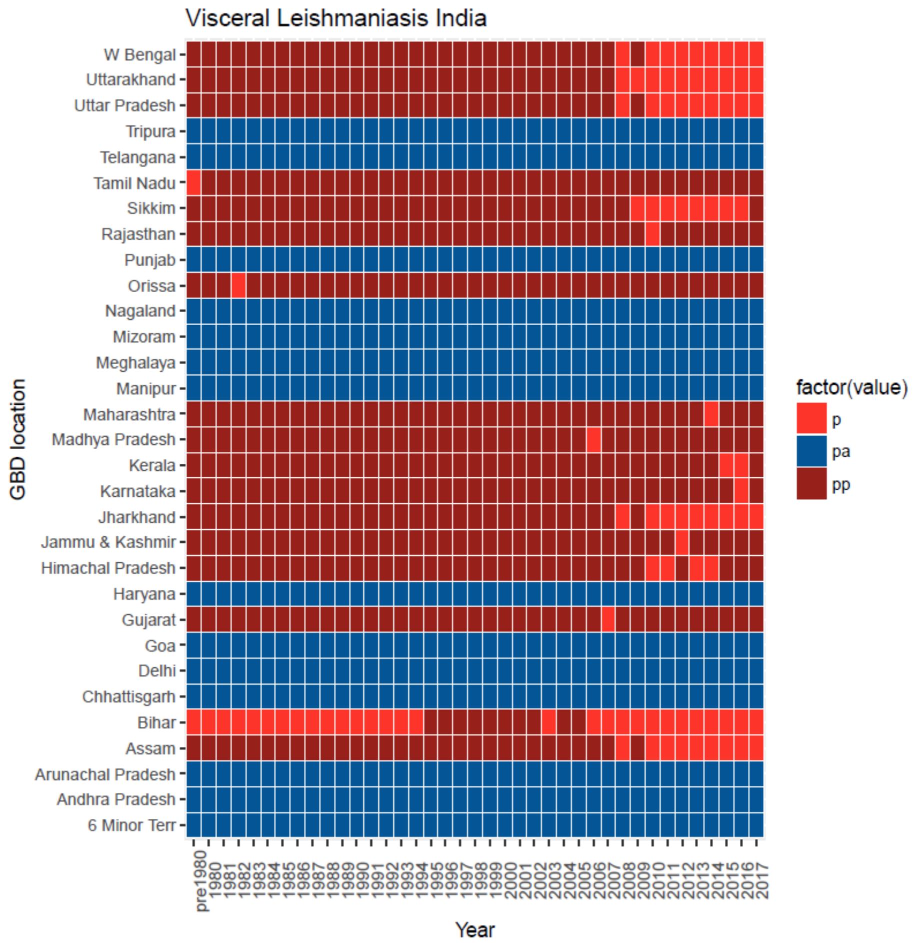

Figure 1: Visceral Leishmaniasis geographical restrictions for Indian subnationals. Locations tagged as present are coloured in light red, dark red represents protocol presence, and dark blue represents protocol absence.

Modelling strategy

Under-reporting

Under-reporting scalars were modelled as a generalised linear model estimating the proportion of true cases captured by reporting systems. A value of 1 represents all actual cases of leishmaniasis being reported through notification systems. The model is as follows:

$$\frac{reported\ cases}{"true"\ cases} = Pathogen + Year + Sociodemographic\ Index$$

To account for potential biases inherently present based upon differing survey methods or location-specific confounders, 1000 models were run, with each model randomly dropping all data from a specific location and dropping 10% of the additional data from the remaining dataset. Similarly, for estimates that spanned multiple years, each model was randomly assigned a year within the range of possible years.

From each of these 1000 models, a prediction was made for each location. To predict the under-reporting rate and propagate uncertainty, we sampled from a logit normal distribution centered around the mean value estimated by that model. Standard deviation was calculated as 1.96*standard error of the predicted under-reporting rate.

Incidence

The summarised values were modelled using spatiotemporal Gaussian process regression (ST-GPR) to produce a complete time series of estimates for each location-year tagged “Present” or “Protocol Present”. In short, ST-GPR attempts to model non-linear trends utilising a Gaussian process to fit a trend, rather than a definitive functional form. The following were the model specifications:

Incidence = haqi + sdi + (1|level 1) + (1|level 2) + (1|level 3)

Levels 1, 2, and 3 refer to GBD location hierarchies, treated as random intercepts by super-region, region and country, respectively. The following hyperparameters were used: st-lambda = 0.4, st-omega = 1, st-zeta = 0.01, gpr-scale = 10. The table below lists coefficients of the covariates.

Table 2. Covariates. Summary of covariates used in the VL ST-GPR model

**Covariate**	**Beta coefficient, logit (95% UI)**	**Standard error**	**Exponentiated beta (95% UI)**
Socio-demographic Index	–6.66 (–8.67 to –4.66)	1.023	$1.28 * 10^{-3}$ ($1.72 * 10^{-4}$ to $9.47 * 10^{-3}$)
Healthcare Access and Quality Index	–0.037 (–0.057 to –0.017)	0.010	0.964 (0.945 to 0.983)

Age-sex pattern

A DisMod Bayesian meta-regression (DisMod-MR) model was used to generate an age-sex curve to disaggregate all-age, both-sex incidence data. DisMod-MR is an integrated meta-regression framework allowing multiple datasets to be integrated into a singular analysis regardless of age-binning, sources,

and geographies. This allows a variety of differently aggregated information to be evaluated and generate a consensus output. From this model, the global fit was used.

Sequelae

Following standard GBD estimation protocols, incidence estimates were used to calculate disease prevalence (by multiplication with duration), disaggregated by disease sequelae. In total, two health states are assigned to visceral leishmaniasis, "moderate visceral leishmaniasis" and "severe visceral leishmaniasis" (Table 3). Duration values derive from a published study.[15]

Table 3. Severity distribution, details on the severity levels for VL and the associated disability weight (DW) with that severity

**Sequela**	**Health state lay description**	**DW (95% CI)**	**Duration**
Moderate visceral leishmaniasis	Infectious disease, acute episode, moderate<br>"has a fever and aches, and feels weak, which causes some difficulty in daily activities"	0.051 (0.032–0.074)	2.5 months
Severe visceral leishmaniasis	Infectious disease, acute episode, severe<br>"has a high fever and pain, and feels very weak, which causes great difficulty with daily activities"	0.133 (0.088–0.19)	15 days

Central processing generated the final estimates, including comorbidity simulations.

We did not apply any adjustments for the COVID pandemic to VL due to a lack of available data quantifying the impacts of the pandemic on NTD epidemiology.

Changes from GBD 2021 to GBD 2023

There were no substantive changes to the modelling strategy for GBD 2023.

References

1. Alvar, Jorge, Iván D Vélez, Caryn Bern, Mercé Herrero, Philippe Desjeux, Jorge Cano, Jean Jannin, and Margriet den Boer. 2012. "Leishmaniasis Worldwide and Global Estimates of Its Incidence." *PLoS One* 7 (5): e35671.
2. Yadón, Z E, M A Quigley, C R Davies, L C Rodrigues, and E L Segura. 2001. "Assessment of Leishmaniasis Notification System in Santiago Del Estero, Argentina, 1990-1993." *Am. J. Trop. Med. Hyg.* 65 (1): 27–30.
3. Sesma, B, and A Barricarte. 1997. "[Leishmaniasis in Navarra: review of activities]." *An. Sist. Sanit. Navar.* 20 (2): 209–216.

4. Maia-Elkhoury, Ana Nilce Silveira, Eduardo Hage Carmo, Marcia Leite Sousa-Gomes, and Eduardo Mota. 2007. "[Analysis of visceral leishmaniasis reports by the capture-recapture method]." *Rev. Saude Publica* 41 (6): 931–937.
5. Gkolfinopoulou, K, N Bitsolas, S Patrinos, L Veneti, A Marka, G Dougas, D Pervanidou, et al. 2013. "Epidemiology of Human Leishmaniasis in Greece, 1981-2011." *Euro Surveill.* 18 (29): 20532.
6. Singh, V P, A Ranjan, R K Topno, R B Verma, N A Siddique, V N Ravidas, N Kumar, K Pandey, and P Das. 2010. "Estimation of Under-Reporting of Visceral Leishmaniasis Cases in Bihar, India." *Am. J. Trop. Med. Hyg.* 82 (1): 9–11.
7. Hirve, S, S P Singh, N Kumar, M R Banjara, P Das, S Sundar, S Rijal, et al. 2010. "Effectiveness and Feasibility of Active and Passive Case Detection in the Visceral Leishmaniasis Elimination Initiative in India, Bangladesh, and Nepal." *Am. J. Trop. Med. Hyg.* 83 (3): 507–511.
8. Faraj, Chafika, Joshua Yukich, El Bachir Adlaoui, Rachid Wahabi, Abraham Peter Mnzava, Mustapha Kaddaf, Abderrahmane Laamrani El Idrissi, Btissam Ameur, and Immo Kleinschmidt. 2016. "Effectiveness and Cost of Insecticide-Treated Bed Nets and Indoor Residual Spraying for the Control of Cutaneous Leishmaniasis: A Cluster-Randomized Control Trial in Morocco." *Am. J. Trop. Med. Hyg.* 94 (3): 679–685.
9. Das, A K, A D Harries, S G Hinderaker, R Zachariah, B Ahmed, G N Shah, M A Khogali, G I Das, E M Ahmed, and K Ritmeijer. 2014. "Active and Passive Case Detection Strategies for the Control of Leishmaniasis in Bangladesh." *Public Health Action* 4 (1): 15–21.
10. Rahman, Kazi Mizanur, Indira V M Samarawickrema, David Harley, Anna Olsen, Colin D Butler, Shariful Amin Sumon, Subrata Kumar Biswas, Stephen P Luby, and Adrian C Sleigh. 2015. "Performance of Kala-Azar Surveillance in Gaffargaon Subdistrict of Mymensingh, Bangladesh." Edited by Carlos Franco-Paredes. *PLoS Negl. Trop. Dis.* 9 (4): e0003531.
11. Eid, Daniel, Miguel Guzman-Rivero, Ernesto Rojas, Isabel Goicolea, Anna-Karin Hurtig, Daniel Illanes, and Miguel San Sebastian. 2017. "Assessment of a Leishmaniasis Reporting System in Tropical Bolivia Using the Capture-Recapture Method," October, tpmd170308.
12. Pigott, David M, Samir Bhatt, Nick Golding, Kirsten A Duda, Katherine E Battle, Oliver J Brady, Jane P Messina, et al. 2014. "Global Distribution Maps of the Leishmaniases." *Elife* 3 (January): e02851.
13. Copeland, H W, B A Arana, and T R Navin. 1990. "Comparison of Active and Passive Case Detection of Cutaneous Leishmaniasis in Guatemala." *Am. J. Trop. Med. Hyg.* 43 (3): 257–259.
14. Singh, S P, D C S Reddy, M Rai, and S Sundar. 2006. "Serious Underreporting of Visceral Leishmaniasis through Passive Case Reporting in Bihar, India." *Trop. Med. Int. Health* 11 (6): 899–905.
15. Murray, H. W., Berman, J. D., Davies, C. R., & Saravia, N. G. 2005. "Advances in leishmaniasis." *Lancet*, *366*(9496), 1561–1577.

Yellow fever

Flowchart

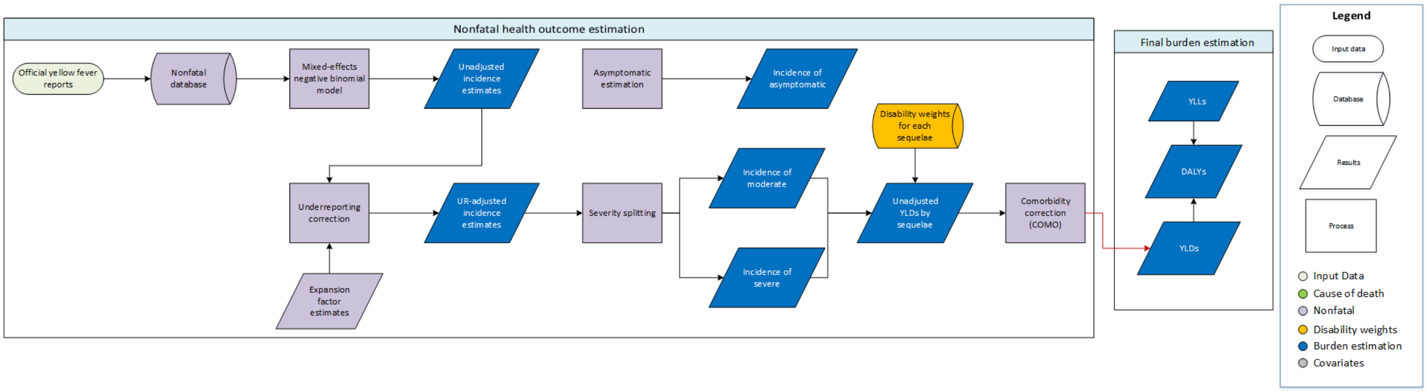

Input data and methodological summary for yellow fever

Case definition

Yellow fever is mosquito-borne viral infection that causes febrile illness and, in severe cases, jaundice, haemorrhage, and death. It includes all ICD-10 codes under the heading A95 (yellow fever).
We used the following case definition for GBD 2023:

Quantity of interest	Reference or Alternative	Definition
Yellow fever	Reference	One of the following: (i) presence of yellow fever IgM antibody in the absence of yellow fever immunisation within 30 days before onset of illness; or (ii) positive postmortem liver histopathology; or (iii) epidemiological link to a confirmed case or an outbreak (based on WHO definition);<br>and either:<br>(a) Absence of yellow fever immunisation within 30 days before onset of illness; and one of the following: (i) detection of yellow fever-specific* IgM; or (ii) detection of fourfold increase in yellow fever IgM, or IgG antibody titres between acute and convalescent serum samples, or both; or (iii) detection of yellow fever-specific* neutralising antibodies<br>(b) Absence of yellow fever immunisation within 14 days before onset of illness; and one of the following: (i) detection of yellow fever virus genome in blood or other organs by PCR; or (ii) detection of yellow fever antigen in blood, liver or other organs by immunoassay; or (iii) isolation of yellow fever virus.<br>*Yellow fever-specific means that the results of antibody tests (such as IgM or neutralising antibody) for other prevalent flaviviruses are negative or not significant. Testing should include at least IgM for dengue fever and West Nile virus but may include other flaviviruses according to local epidemiology (for example, Zika virus; based on WHO definition).
Yellow fever	Reference	Cases of yellow fever notified to public health agencies.

Yellow fever	Alternative	Acute onset of fever, with jaundice appearing within 14 days of onset of the first symptoms. (Based on WHO definition.)
Yellow fever	Alternative	A probable case as determined by one of the following: (i) presence of yellow fever IgM antibody in the absence of yellow fever immunisation within 30 days before onset of illness; or (ii) positive postmortem liver histopathology; or (iii) epidemiological link to a confirmed case or an outbreak (based on WHO definition).

Input data

Case data for the yellow fever estimate process come from official case reports filed with the World Health Organization (WHO).

Modelling strategy

We modelled reported cases of yellow fever using a mixed-effects negative binomial model, with fixed effects for year and Socio-demographic Index and random effects for super-region, region, and country. We use GBD population estimates for the location level as the offset. We assume that yellow fever cases are under-reported, and that this under-reporting mirrors that for dengue (a disease for which we have better data on under-reporting). With that, we estimate symptomatic cases as the product of our base case estimates and dengue expansion factors (ie, the factor by which you must multiply reported cases to derive true cases). Expansion factors are applied to the all-age modelled incidence prior to splitting incidence by age and sex. Data that are age- and sex-specific are used to generate an age- and sex-specific incidence pattern via a negative binomial regression with fixed effects for sex and age group (with cubic splines). Based on published estimates,[1] we split yellow fever into the following proportions: moderate (33% [13–52]), severe (12% [5–26]), and asymptomatic (55% [37–74]).

Sequelae

The table below shows the list of sequelae due to yellow fever and the associated disability weights. Asymptomatic infection was not attributed to any disability. Table 1 below illustrates this breakdown.

Table 1. Severity distribution, details on the severity levels for yellow fever and the associated disability weight (DW) with that severity

**Sequela**	**Description**	**DW (95% CI)**
Moderate	Has a fever and aches, and feels weak, which causes some difficulty with daily activities.	0.051 (0.032–0.074)
Severe	Has a high fever and pain, and feels very weak, which causes great difficulty with daily activities.	0.133 (0.088–0.19)
Asymptomatic	Infection with no apparent illness.	N/A

We did not apply any adjustments for the COVID pandemic to yellow fever due to a lack of available data quantifying the impacts of the pandemic on NTD epidemiology.

Changes from GBD 2021 to GBD 2023

There have been no substantive changes to the modelling strategy for endemic countries for GBD 2023.

References

1. Johansson MA, Vasconcelos PFC, Staples JE. The whole iceberg: estimating the incidence of yellow fever virus infection from the number of severe cases. *Trans R Soc Trop Med Hyg* 2014; **108**: 482–7.

Zika virus disease

Flowchart

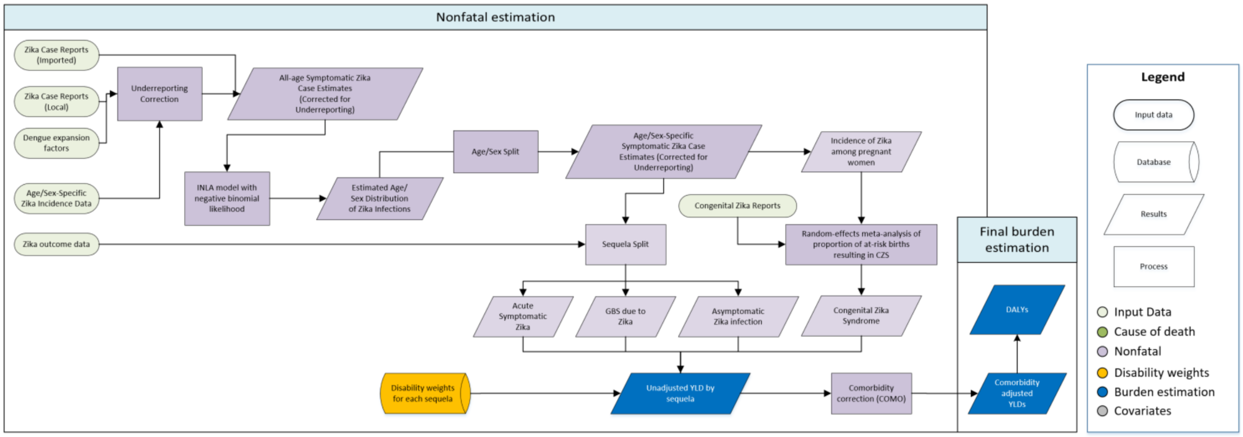

Input data and methodological summary for Zika virus disease

Case definition

Zika virus is transmitted via mosquito bites; symptoms include rash, fever, headache, arthralgia, conjunctivitis, and myalgias. Maternal Zika infection during pregnancy can lead to congenital Zika syndrome; symptoms include severe microcephaly, contractures, hypotonia, and other central nervous system and ocular abnormalities. We used the following case definitions for estimation of non-fatal burden of Zika virus disease in GBD 2023:

Quantity of interest	Quantity of interest	Reference or alternative	Definition
Zika virus	Suspected case	Reference	Patient with rash with two or more of the following signs or symptoms: fever, usually <38.5°C; conjunctivitis (non-purulent/hyperaemic); arthralgia; myalgia; peri-articular oedema (PAHO 2016)
Zika virus	Suspected allochthonous case	Reference	Patient who meets the criteria for a suspected case (above) AND who EITHER (i) in the 2 weeks prior to onset, traveled to, or resided in, a geographical area where there is known local transmission of the Zika virus or there is known vector presence; OR (ii) had un-protected sex, in the 2 weeks prior to onset, with a person who traveled, in the previous 8 weeks, to a geographical area with (a) known local transmission of the Zika virus or (b) and area with known vector presence (PAHO 2016)
Zika virus	Probable case	Reference	Patient who meets the criteria of a suspected case AND has Zika IgM antibodies, with no evidence of infection with other flaviviruses (PAHO 2016)
Zika virus	Confirmed case	Reference	Patient who meets the criteria for a suspected case AND has laboratory confirmation of a recent Zika virus infection via EITHER (i)

			RNA or Zika virus antigen in any specimen (serum, urine, saliva, tissue, or whole blood); OR (ii) Positive Zika IgM antibodies AND plaque reduction neutralisation (PRNT90) for Zika virus titers = 20 AND four or more times greater than the titers for other flaviviruses; AND exclusion of other flavivirus; OR (iii) In autopsy specimens, detection of the viral genome (in fresh or paraffin tissue) by molecular techniques or immuno-histochemistry (PAHO 2016)

Input data

Data on cases of acute Zika virus disease and congenital Zika syndrome (CZS) come from official reports, primarily from the Pan American Health Organization (PAHO) from 2014 to 2019.

Modelling strategy

We estimate the all-age incidence of symptomatic Zika virus disease as the product of reported Zika cases and country-specific expansion factors that adjust for under-reporting. Those expansion factors are derived from our dengue model, and the methods used for their estimation are detailed in the dengue model documentation and by Stanaway and colleagues.[1] First, we used the expansion factor to inflate the raw data. Then, we used the integrated nested Laplace approximation (INLA) method, as implemented in R-INLA,[2] with a negative binomial likelihood and using location as a random effect to predict incidence. These random effects consisted of independent and identically distributed (i.i.d.) effects by most-detailed locations (including subnationals, where appropriate), country, and GBD region.

We used the Healthcare Access and Quality (HAQ) Index, the proportion of the population living above 1500 meters of elevation, the Enhanced Vegetation Index's long-term average for 2000–2012, and the population-weighted mean temperature as random effect covariates, using second-order random walk (RW2) models to accommodate non-linearity. As fixed effect covariates, we used rainfall, sanitation, and solar radiation. The model also included a single-order random walk (RW1) model on years since the peak of the initial outbreak in each location. We used age-specific data to estimate age- and sex-specific incidence curves using the INLA method, with a RW1 model on the midpoint of each age bin, replicated by sex. We obtained 1000 draws of the posterior distribution of incidence, and subsequent estimation steps took place at the draw level to preserve and propagate uncertainty. We then split the estimated incidence of acute symptomatic Zika virus disease by location, year, age, and sex using the age and sex-distribution model. To estimate acute symptomatic Zika virus disease prevalence, we assumed a duration of six days.

We conducted a meta-analysis of three studies[3–5] to estimate the proportion of all Zika infections that are symptomatic. We estimated that 41% of Zika infections are symptomatic (14–68%), with 59% being asymptomatic. We then estimated incidence of asymptomatic infections as

$$I_{asymp} = \frac{I_{symp}}{Pr_{symp}} - I_{symp}$$

Where I_{asymp} is the incidence of asymptomatic infections, I_{symp} is the incidence of symptomatic Zika virus disease, and Pr_{symp} is the proportion of infections that are symptomatic (ie, 41%).

We assume that the incidence of Zika virus disease among pregnant women equals the incidence of Zika virus disease among all women within a given location, year, and age group. We then estimated the number of pregnant women infected with Zika virus as the product of the incidence of Zika virus infection and the number of pregnant women in every location, year, and age group. We then accounted for the lag between first trimester infection and date of birth. Finally, we fit an INLA model to reported congenital Zika syndrome cases from country reports, with random effects by location, an offset of the number of births to Zika-infected mothers, and RW1 on year.

To estimate the incidence of Guillain-Barré Syndrome (GBS) due to Zika virus infection, we used data from Daudens-Vaysse 2016[6] to fit a beta distribution of the probability of GBS. We estimated the incidence of GBS due to Zika virus infection by multiplying the 1000 draws obtained from this distribution by the 1000 draws of symptomatic Zika disease incidence estimates, described previously. To estimate the prevalence, we divided the incidence draws by the remission rate from the GBS impairment envelope model.

Table 1. Zika virus sequelae disability weights (DWs)

**Sequela**	**Health state**	**Lay description**	**DW**
Symptomatic acute Zika cases	Infectious disease, acute episode, moderate	Has a fever and aches, and feels weak, which causes some difficulty with daily activities.	0.051 (0.032–0.074)
Asymptomatic Zika infections	Asymptomatic	-	0
Guillain–Barré syndrome due to Zika infection	Spinal cord lesion below neck level (treated)	Is paralyzed from the waist down, cannot feel or move the legs and has difficulties with urine and bowel control. The person uses a wheelchair to move around.	0.296 (0.198–0.414)
Congenital Zika syndrome	Severe motor plus cognitive impairment with epilepsy	(combined DW)	-

We did not apply any adjustments for the COVID-19 pandemic to Zika virus disease due to a lack of available data quantifying the impacts of the pandemic on NTD epidemiology.

Changes from GBD 2021 to GBD 2023

There were no substantial changes to the modelling strategy for GBD 2023.

References

1. Stanaway JD, Shepard DS, Undurraga EA, Halasa YA, Coffeng LE, Brady OJ, et al. The global burden of dengue: an analysis from the Global Burden of Disease Study 2013. *Lancet Infectious Disease* [Internet]. 2016 Feb [cited 2016 May 23]; Available from: http://linkinghub.elsevier.com/retrieve/pii/S1473309916000268
2. Lindgren F, Rue H. Bayesian spatial modelling with R-INLA. *J Stat Softw*. 2015;63(19):1–25.
3. Gallian P, Cabié A, Richard P, Paturel L, Charrel RN, Pastorino B, et al. Zika virus in asymptomatic blood donors in Martinique. *Blood*. 2017 Jan 12;129(2):263–6.
4. Duffy MR, Chen T-H, Hancock WT, Powers AM, Kool JL, Lanciotti RS, et al. Zika virus outbreak on Yap Island, Federated States of Micronesia. *N Engl J Med*. 2009 Jun 11;360(24):2536–43.
5. Aubry M, Teissier A, Huart M, Merceron S, Vanhomwegen J, Roche C, et al. Zika Virus Seroprevalence, French Polynesia, 2014–2015. *Emerg Infect Dis*. 2017 Apr;23(4):669–72.
6. Daudens-Vaysse E, Zika Surveillance Working Group. Zika emergence in the French Territories of America and description of first confirmed cases of Zika virus infection on Martinique, November 2015 to February 2016. *Euro Surveill.* 2016; 21(28): 30285.
