# Supplementary material for: Burden of 375 diseases and injuries, risk-attributable burden of 88 risk factors, and healthy life expectancy in 204 countries and territories, including 660 subnational locations, 1990–2023: a systematic analysis for the Global Burden of Disease Study 2023
Source: Lancet. 2025 Oct 18;406(10513):1873–922. doi: 10.1016/S0140-6736(25)01637-X (PMC12535840; doi:10.1016/S0140-6736(25)01637-X)
Supplement: Supplementary appendix 2 [file mmc2.pdf]

THE LANCET

Supplementary appendix 2

This appendix formed part of the original submission and has been peer reviewed. We post it as supplied by the authors.

Supplement to: GBD 2023 Disease and Injury and Risk Factor Collaborators. Burden of 375 diseases and injuries, risk-attributable burden of 88 risk factors, and healthy life expectancy in 204 countries and territories, including 660 subnational locations, 1990–2023: a systematic analysis for the Global Burden of Disease Study 2023. *Lancet* 2025; published online Oct 12. https://doi.org/10.1016/S0140-6736(25)01637-X.

Appendix 2: Risk factor methods appendix to "Burden of 375 diseases and injuries, risk-attributable burden of 88 risk factors, and healthy life expectancy in 204 countries and territories, including 660 subnational locations, 1990–2023: a systematic analysis for the Global Burden of Disease Study 2023"

Preamble

This appendix provides further methodological detail for "Burden of 375 diseases and injuries, risk-attributable burden of 88 risk factors, and healthy life expectancy in 204 countries and territories, including 660 subnational locations, 1990–2023: a systematic analysis for the Global Burden of Disease Study 2023." This study complies with the Guidelines for Accurate and Transparent Health Estimates Reporting (GATHER) recommendations.[1] It includes detailed tables and information on data in an effort to maximise transparency in our estimation processes and provide a comprehensive description of analytical steps. We intend this appendix to be a living document, to be updated with each iteration of the Global Burden of Disease Study.

Portions of this appendix have been reproduced or adapted from the appendices of Lim et al 2012,[2] GBD 2015 Risk Factors Collaborators,[3] GBD 2016 Risk Factors Collaborators,[4] GBD 2017 Risk Factor Collaborators,[5] GBD 2019 Risk Factors Collaborators,[6] and GBD 2021 Risk Factor Collaborators.[7] References are provided for reproduced or adapted sections.

Contents

Preamble 2

List of methods appendix tables 5

Section 1: GBD overview 6

Section 1.1: Global Burden of Diseases, Injuries, and Risk Factors Study 2023 6

Section 1.2: Geographical locations of the analysis 6

Section 1.3: Time period of the analysis 6

Section 1.5: GBD risk factor hierarchy 6

Section 1.6: List of abbreviations 6

Section 1.7: Data input sources overview 9

Section 1.8: Funding sources 9

Section 2: Risk factor estimation 9

Overview 9

Step 1. Effect size estimation 10

Section 2.1.1: Criteria for inclusion of risk–outcome pairs[7] 10

Section 2.1.2: Overview of the effect size estimation pathway 11

Section 2.1.3: Collate relative risk data 11

Section 2.1.4: Estimating the shape of the risk–outcome relationship 16

Section 2.1.6: Estimating the burden of proof risk function 22

Section 2.1.7: Evaluating potential for publication or reporting bias 23

Section 2.1.8: MR-BRT and temperature 23

Step 2. Exposure estimation[5] 24

Section 2.2.1: Collate exposure data 24

Section 2.2.2: Adjust exposure data 25

Section 2.2.3: Estimate exposure 27

Step 3. TMREL[5] 38

Step 4. Estimate population attributable fractions[5] 38

Step 5. Estimate summary exposure values[5] 39

Step 6. Mediation[5] 40

Section 2.6.1: Summary 40

Section 2.6.2: Calculating the burden of multiple risk factors 41

Section 2.6.3: Computing mediation factors using linear relationships 42

Section 2.6.4: Adjusting for mediation 42

Section 2.6.5: Calculating mediation factor 43
Section 2.6.6: Piecewise aggregation (Pattern 3) 44
Section 2.6.7: Uncertainty of aggregated and mediated PAFs 46
Section 2.6.8: Important assumptions in aggregating risk factors and including mediation 46
Step 7. Estimate attributable burden[5] 46
Section 3: References 47
Section 4: GBD 2023 risk factor-specific modelling descriptions 51

List of methods appendix tables

All supplementary tables can be accessed here:
https://ghdx.healthdata.org/record/ihme-data/gbd-2023-yld-daly-hale-risk-1990-2023

Table S1. GBD risk hierarchy with levels

Table S2. Types of comparative risk assessments (CRA) based on the time perspective and the nature of the counterfactual level or distribution of exposure

Table S3. Status of risk–outcome pairs considered for inclusion in GBD 2023: included in both GBD 2021 and GBD 2023, added in GBD 2023, or removed in GBD 2023

Table S4. Theoretical minimum risk exposure levels (TMREL) changes compared to GBD 2021

Table S5. Mediation factor matrix

Table S6. Status of mediated risk–outcome pairs considered for inclusion in GBD 2023: included in both GBD 2021 and GBD 2023, added in GBD 2023, or removed in GBD 2023

Table S7. Source counts by risk factor for GBD 2023

Table S8. BPRF Risk-Outcome Score ranges associated with each star rating, and number of risk-outcome pairs assigned to each star rating.

Section 1: GBD overview

Section 1.1: Global Burden of Diseases, Injuries, and Risk Factors Study 2023

The Global Burden of Diseases, Injuries, and Risk Factors Study (GBD) is a collaborative research effort aimed at estimating morbidity and mortality from a comprehensive set of diseases, injuries, and risk factors. The GBD Collaborator Network draws on the expertise of over 14,000 contributors from around the world. For this paper, we estimated risk factor exposure levels, relative health risk by exposure, and risk-attributable burden by age, sex, and location from 1990 to 2023.

Section 1.2: Geographical locations of the analysis

We produced estimates for 204 countries and territories that were grouped into 21 regions and seven super-regions (appendix 1, table S1). The seven super-regions are central Europe, eastern Europe, and central Asia; high income; Latin America and the Caribbean; north Africa and the Middle East; south Asia; southeast Asia, east Asia, and Oceania; and sub-Saharan Africa. In GBD 2023, we continue to analyse at subnational levels countries that were added in previous cycles, including Brazil, China, Ethiopia, India, Indonesia, Iran, Italy, Japan, Kenya, Mexico, New Zealand, Nigeria, Norway, Pakistan, the Philippines, Poland, Russia, South Africa , the United Kingdom, and the United States of America. All analyses are at the first level of administrative organisation within each country except for New Zealand (by Māori ethnicity)), the Philippines (by provinces), and the UK. To meet data use requirements, in this publication we present subnational estimates for Brazil, India, Indonesia, Japan, Kenya, Mexico, and the USA; given space constraints, these results are presented in Appendix 3 instead of the main text. Subnational estimates for China are included in maps but are not reported in appendix tables. Subnational estimates for other countries will be released in separate publications, although please note that we only release estimates for a subset of these countries, per agreements with country partners.

At the most detailed spatial resolution, we generated estimates for 843 unique locations. As was done in GBD 2023, in GBD 2023 we continue to use the set of locations defined as standard locations and non-standard locations. Standard GBD locations are defined as the set of all subnationals belonging to countries where data quality is high and with populations over 200 million, in addition to all other countries. Standard locations include the subnationals for China, India, the USA, and Brazil, but not Indonesia; data for China, India, the USA, and Brazil are also included at the country level. All other countries with subnational estimates are defined as non-standard locations.

Section 1.3: Time period of the analysis

A complete set of risk-specific exposures, relative risks (RRs), theoretical minimum risk exposure levels (TMRELs), and population attributable fractions (PAFs) were computed for the years 1990 to 2023.

Section 1.5: GBD risk factor hierarchy

The GBD 2023 risk factors hierarchy and levels are summarised in table S1. The risk hierarchy is based on common features of individual risks; for example, risk factors that represent behavioural factors are grouped together.

The GBD risk factor list continues to evolve to reflect the policy relevance, public health, and medical care importance of major risk factors. 2023

Section 1.6: List of abbreviations

APCSC	Asia-Pacific Cohort Studies Collaboration

ARC	annualised rate of change
BMI	body-mass index
BoP	Burden of Proof
BPRF	Burden of Proof risk functions
BMD	bone mineral density
CDC	Centers for Disease Control and Prevention
CF	correction factor
CKD	chronic kidney disease
COD	causes of death
CODEm	Cause of Death Ensemble modelling
COPD	chronic obstructive pulmonary disease
COVID-19	coronavirus disease 2019
CRA	comparative risk assessment
CSV	comma-separated values
CRA	comparative risk assessment
CSMR	cause-specific mortality rate
CVD	cardiovascular disease
DALY	disability-adjusted life-year
DHS	Demographic and Health Survey
DRI	data representativeness index
EDU15+	mean education for those aged 15 years or older
EMR	excess mortality rate
FAO	Food and Agriculture Organization
FPG	fasting plasma glucose
GAM	generalised additive model
GATHER	Guidelines for Accurate and Transparent Health Estimates Reporting
GBD	Global Burden of Diseases, Injuries, and Risk Factors Study
GHDx	Global Health Data Exchange
GoF	goodness of fit
HAP	household air pollution
ID	iron deficiency
IDA	Iron-deficiency anaemia
IER	integrated exposure response
IHD	ischaemic heart disease
ILO	International Labour Organization
IPV	intimate partner violence
IQ	intelligence quotient
JMP	Joint Monitoring Project
KS	Kolmogorov-Smirnov
LDI	lag-distributed income
LDL	low-density lipoprotein
LMICs	low- and middle-income countries
LOESS	locally estimated scatterplot smoothing
LRI	lower respiratory infection

MCMC	Markov Chain Monte Carlo simulations
MDG	Millennium Development Goal
MF	mediation factor
MICS	Multiple Indicator Cluster Surveys
MoM	method of moments
MR-BRT	meta-regression—Bayesian, regularised, trimmed
NCD	non-communicable disease
NCD-RisC	Non-communicable Disease Risk Factor Collaboration
OER	observed-to-expected ratio
PAF	population attributable fraction
PDF	probability distribution factor
$PM_{2.5}$	particulate matter <2.5 μm in aerodynamic diameter
PRISMA	Preferred Reporting Items for Systematic Reviews and Meta-Analyses
PCS	prospective cohort study
RCT	randomised controlled trial
PURE	Prospective Urban and Rural Epidemiological Study
REDCap	Research Electronic Data Capture
RMSE	root mean square error
ROS	risk–outcome score
RR	relative risk
SARS-CoV 2	Severe acute respiratory syndrome coronavirus 2
SBP	systolic blood pressure
SD	standard deviation
SDG	Sustainable Development Goal
SDI	Socio-demographic Index
SEER	Surveillance, Epidemiology, and End Results Program
SEV	summary exposure value
SHS	secondhand smoke
SIR	smoking impact ratio
SSB	sugar-sweetened beverages
ST-GPR	spatiotemporal Gaussian process regression
SVAC	sexual violence against children
TB	tuberculosis
TFU25	total fertility rate in those under 25 years old
TMREL	theoretical minimum risk exposure level
TSNA	tobacco-specific nitrosamines
UI	uncertainty interval
USD	United States dollars
WaSH	Water, sanitation, and handwashing
WCRF	World Cancer Research Fund
WHO	World Health Organization
YLDs	years lived with disability
YLLs	years of life lost

Section 1.7: Data input sources overview

GBD 2023 incorporated a large number and wide variety of input sources to estimate mortality, causes of death and illness, and risk factors for 204 countries and territories from 1990 to 2023. These input sources are accessible through an interactive citation tool available in the GHDx [https://ghdx.healthdata.org/].

Users can retrieve citations for a specific GBD component, cause or risk, and location by choosing from the available selection boxes. They can then view and access GHDx records for input sources and export a comma-separated value (CSV) file that includes the GHDx metadata, citations, and information about where the data were used in GBD. Additional metadata for each input source are available through the citation tool as required by the GATHER statement.

The citation tool is available online via the GBD 2023 Sources Tool in the GHDx [https://ghdx.healthdata.org/gbd-2023/sources].

Section 1.8: Funding sources

This publication and the research it presents were funded by the Gates Foundation (OPP1152504); Bloomberg Philanthropies; Queensland Department of Health, Australia;UK Department of Health and Social Care; the Norwegian Institute of Public Health; the New Zealand Ministry of Health; St. Jude Children's Research Hospital. The funders of the study had no role in study design, data collection, data analysis, data interpretation, or writing of the report. All authors had full access to all data in the study and had final responsibility for the decision to submit for publication.

Section 2: Risk factor estimation

Overview

The comparative risk assessment (CRA) conceptual framework was developed by Murray and Lopez,[8] who established a causal web of hierarchically organised risks or causes that contribute to health outcomes, which allows for quantification of risks or causes at any level in the framework. In GBD 2023, as in previous iterations of the GBD study, we evaluated a set of behavioural, environmental and occupational, and metabolic risks, in which risk–outcome pairs were included based on evidence rules. These risks were organised in four hierarchical levels, where Level 1 represents the overarching categories (behavioural, environmental and occupational, and metabolic) nested within Level 1 risks; Level 2 contains both single risks and risk clusters (such as child and maternal malnutrition); Level 3 contains the disaggregated single risks from within Level 2 risk clusters (such as low birthweight and short gestation); and Level 4 details risks with the most granular disaggregation, such as for specific occupational carcinogens, the subcomponents of child growth failure (stunting, wasting, underweight), and suboptimal breastfeeding (discontinued and non-exclusive breastfeeding). At each level of risk, we evaluated whether risk combinations were additive, multiplicative, or shared common pathways for intervention. This approach allows the quantification of the proportion of risk-attributable burden shared with another risk or combination of risks and the measurement of potential overlaps between behavioural, environmental and occupational, and metabolic risks. To date in GBD, we have not quantified the contribution of other classes of risk factors illustrated in table S2. We do provide some

insights into the potential magnitude of distal social, cultural, and economic factors through an analysis of the relationship between risk exposures and development measured by using the Socio-demographic Index (SDI) (more details in appendix 1, section 3).

Two types of risk assessments are possible within the CRA framework: attributable burden and avoidable burden. Attributable burden is the reduction in current disease burden that would have been possible if past population exposure had shifted to an alternative or counterfactual distribution of risk exposure. Avoidable burden is the potential reduction in future disease burden that could be achieved by changing the current distribution of exposure to a counterfactual distribution of exposure. Murray and Lopez identified four types of counterfactual exposure distributions: (1) theoretical minimum risk; (2) plausible minimum risk; (3) feasible minimum risk; and (4) cost-effective minimum risk.[9] The theoretical minimum risk exposure level (TMREL) is the level of risk exposure that minimises risk at the population level. Other possible forms of risk quantification include plausible minimum risk and feasible minimum risk. Plausible minimum risk reflects the distribution of risk that is conceivably possible and would minimise population-level risk if achieved. Feasible minimum risk describes the lowest risk distribution that has been attained within a population, and cost-effective minimum risk is the lowest risk distribution for a population that can be attained in a cost-effective manner. Because no robust set of forecasts for all components of GBD is available, in this study we focus on quantifying attributable burden by using the theoretical minimum risk counterfactual distribution. Table S2 shows the eight possible types of risk quantification within the CRA framework; the grey box represents the type of CRA currently undertaken by the GBD study. According to the definition of avoidable burden, risk reversibility would be incorporated into this type of assessment because it would involve reducing risk to the counterfactual for the index year, given a history of past risk exposure. Given the focus in this study on attributable burden, risk reversibility is not a criterion used in estimation here.

In general, this analysis follows the CRA methods used since GBD 2015.[3] The methods described here provide a high-level overview of the analytical logic and focus on areas of notable change from the methods employed in GBD 2015 and since GBD 2021. Here we aim to provide sufficient detail on the methods and overall structure of the estimation process. This study complies with the GATHER recommendations proposed by the World Health Organization (WHO) and others, which include recommendations on documentation of data sources, estimation methods, and statistical analysis (table S3).[1]

Step 1. Effect size estimation

Section 2.1.1: Criteria for inclusion of risk–outcome pairs[7]

From GBD 2010 until GBD 2019, we included risk–outcome pairs that met the World Cancer Research Fund (WCRF) grades of convincing or probable evidence.[10] In this framework, convincing evidence consists of biologically plausible associations between exposure and disease established from multiple epidemiological studies in different populations. Evidentiary studies must be substantial, include prospective observational studies, and, where relevant, randomised controlled trials (RCTs) of sufficient size, duration, and quality that show consistent effects. Probable evidence is similarly based on epidemiological studies with consistent associations between exposure and disease, but for which shortcomings in the evidence exist, such as insufficient available trials (or prospective observational studies). For GBD 2023, we retained risk–outcome pairs included in GBD 2021; the majority of these (table S3) were evaluated using the Burden of Proof (BoP) methodology introduced in GBD 2021,

described in more detail in section 2.1.6 below. Risk–outcome pairs previously included in GBD 2021 were retained in GBD 2023 unless subsequent BoP analysis indicated exclusion. Entirely new risk factors were added based upon a minimal one-star Burden of Proof risk function (BPRF) rating (methods on BPRF detailed in section 2.1.6) and review and majority vote for inclusion by the GBD Scientific Council. More specifically, our inclusion criteria were that the RR estimate's 95% uncertainty interval (UI), conventionally calculated, without accounting for unexplained between-study heterogeneity, must not cross the null RR value of 1 (ie, the mean RR estimate must be significantly higher [for harmful risks] or lower [for protective risks] than 1) for a risk–outcome pair to be included in GBD. To maintain stability in included risk factors and risk–outcome pairs between GBD cycles, exclusion criteria for those pairs already included in GBD 2019 were less stringent; previously included pairs were excluded only if the conventionally calculated 90% UI crossed the null.

Section 2.1.2: Overview of the effect size estimation pathway

For most relative risks, our meta-analytic approach followed six main steps: 1) search and extract data from published studies using a standardised approach; 2) estimate the shape of the exposure versus relative risk relationship, integrating over exposure ranges in different comparison groups and avoiding the distorting effect of outliers; 3) test and adjust for systematic biases as a function of study attributes; 4) quantify remaining between-study heterogeneity while adjusting for within-study correlation induced by computing relative risks for several alternatives with the same reference, as well as the number of studies; 5) evaluate evidence for small-study effects to evaluate a potential risk of publication or reporting bias; and 6) estimate the mean risk function as well as the BPRF. The BPRF quantifies a conservative interpretation of the average risk increase across the range of exposure supported by the evidence. The BPRF is summarized across exposure to compute the risk–outcome scores (ROS), which are then mapped into five categories of risk as star ratings. Zheng and colleagues[11] published the technical developments required to implement this approach, which are also disseminated using open-source Python libraries.[12,13] Implementation details for each step of the approach used to find the ROS are described below. Custom models were used for some risk factors such as temperature (see GBD 2023 risk factor–specific modelling descriptions below for more details).

Section 2.1.3: Collate relative risk data

The relative risk (RR) by level of exposure or by cause for mortality or morbidity can be found in published and unpublished primary studies or in secondary studies that summarise RRs. We collated information from primarily RCTs, cohort, and pooled cohort studies; and in some instances, case-control studies. We used these data to determine the RR for the risk–outcome pairs included in GBD 2023 (table S3). For most risks, data from pooled cohorts or meta-analyses of cohorts were used; in the case of the risk of cataracts from household air pollution (HAP), cohort data were not available, and instead we used case-control data. We estimated RRs of mortality and morbidity for 88 risk factors for which we determined attributable burden by using RR and exposure. To the extent possible, we incorporated RRs from studies that controlled for confounding but not for factors along the causal pathway between exposure and outcome. For risk–outcome pairs with evidence available for only one element of mortality or morbidity, we generally assumed that the estimated RRs applied equally to both. Given evidence of statistically different RRs for mortality and morbidity, we incorporated different RRs for each. Details and citation information for the data sources used for RRs are provided in searchable form

through a web tool (http://ghdx.healthdata.org/). Available data sources for determining RRs varied across risks. Details on how RRs were calculated for each risk can be found in section 4.

Systematic review protocol for relative risks

Task	Protocol
Develop inclusion criteria and search string	• Develop inclusion/exclusion criteria for systematic review based on GBD definition and expert knowledge.<br>• Develop search string in collaboration with GBD risk factor and cause teams and UW librarian (or host institution librarian as needed).
Identify existing meta-analysis / systematic review	• Use PubMed to identify existing meta-analysis/systematic review for risk-outcome pair. Criteria to identify meta-analysis:<br>1. PRISMA compliant – meta-analysis follows PRISMA reporting guidelines<br>2. Published in quality journal – journal ranks in the top two quartiles based on: https://www.scimagojr.com/journalrank.php?area=270 [scimagojr.com] (using the most appropriate subjects categories for the R-O pair)<br>3. Incorporates inclusion criteria that are the same as or more inclusive of final inclusion criteria for risk-outcome pair (e.g. include 'diarrhea' as outcome; whereas final inclusion criteria specifies 'WHO diarrhea definition')<br>4. Most recent
Pre-registration	• Pre-register systematic review on PROSPERO; example linked here.
Screening studies in meta-analysis	• One reviewer will be required to include an article in the data extraction phase, and two reviewers will be required to exclude an article. Discussion and consultation with senior personnel will occur as needed.<br>• Other considerations for data sparse topics – if meta-analysis yields 5 or fewer studies for inclusion:<br>1. Screen at least 2 additional published meta-analyses.<br>2. If still less than 5 included studies, conduct full literature review.
Updated search	• Conduct updated literature search from date published meta-analysis completed its search to present day (if applicable) or conduct literature review for studies published from at least 1985 to present if completing a full literature review.<br>• Use at least three databases in search that include PubMed (https://www.pubmed.gov), Embase + one topic-specific database<br>• Additional databases to consider:<br>○ Web of Science<br>○ Scopus<br>○ CINAHL: Cumulative Index to Nursing & Allied Health Literature<br>○ PsychINFO<br>○ Cochrane Library<br>○ LILACS: Latin American and Caribbean Health Sciences Literature

	○ CNKI: Chinese National Knowledge Infrastructure<br>○ Global Index Medicus<br>○ SciELO: Scientific Electronic Library Online
Title/abstract screening	• De-duplicate articles from multiple databases using DistillerSR (or similar).<br>• For reviews utilizing a single screener, a second reviewer will duplicate screen 100 studies or 10%, whichever is higher, of *excluded* articles as a quality check.<br>• Resolve conflicts with full-text screening and third reviewer (content expert) as needed. If the second reviewer identifies 2 or more studies that were incorrectly excluded after resolving conflicts with full-text screening, then retrain first reviewer on inclusion/exclusion criteria and redo full title/abstract screening from the beginning.<br>• If dual screening is employed, then this is considered to be above the minimum standard outlined in this document, so different criteria for % agreement and kappa can be used.
Full-text screening	• One reviewer will be required to include an article in the data extraction phase, and two reviewers will be required to exclude an article. Discussion and consultation with senior personnel will occur as needed.<br>• Other considerations: use team-level input to resolve edge cases; include specific information on how to handle data related to bias covariates and outcomes during team-specific training process.
The following steps apply to all included studies from meta-analyses and updated literature reviews:	
Duplicate cohorts or case-control (if applicable)	• Check for duplicate cohorts or case-control.<br>• If duplicates exist, select study to include based on exposure time, follow-up period, and covariates included in RR estimation (or other pre-determined criteria).
Data extraction	• Use one reviewer to extract data using the relative risk extraction template and validations.<br>• Use a second reviewer (content expert) to check for correctness and completeness of extracted data from at least 10% or 25 articles, whichever is higher.
Documentation and training	• Complete PRISMA flowchart (2020 version)<br>• Complete REDCap documentation. REDCap is a centralized web-based database used to document all GBD systematic reviews.<br>• Complete RR systematic review extended documentation.<br>○ Team-specific and GBD-wide trainings related to risk factors training, bundles, study design and measures of association, Distiller SR, evidence score/burden of proof.

Bias covariates: categories, conventions, and cases precluding inclusion of bias covariates

In the BoP analysis, each source of bias is represented as a binary bias covariate, taking on the value ‘0’ if the study has the gold standard in the bias covariate, and otherwise taking on the value ‘1.’ The gold standard should be defined prior to data extraction. A value must be assigned for each bias covariate for each study, and each bias covariate must have at least two studies per group (‘0’ and ‘1’).

The bias covariate selection methodology is entirely data driven. That means that any bias covariate included in the data must have sufficient studies with labels of (‘0’) and (‘1’); in particular, a user cannot evaluate a bias covariate with no gold standard representatives, or only gold standard representatives.

Data sparsity issues that preclude bias covariate inclusion based on available data are:

- A bias covariate that has only zeros or ones, or only one study with the value ‘0’ or ‘1,’ cannot be included in the analysis. For inclusion, every bias covariate must have some studies that are the gold standard (have a value of ‘0’), and there must be at least two studies in both groups (‘0’ and ‘1’). Even with the use of priors, the model cannot include variables for which there is no variation in the covariate across observations.
- Redundant bias covariates cannot be included. If two or more bias covariates all have the same labels across studies, or nearly all the same labels across studies, all but one of the redundant bias covariates must be removed.

There are six categories of bias covariates, based on the GRADE criteria, that must be evaluated in a BPRF analysis (assuming sufficient data as discussed above). It is possible to include more than one potential bias covariate within each category, again assuming there is sufficient data to do this. These six study-level bias sources are listed below, with multiple examples of potential bias covariates:

1. **Representativeness of the study population**: assesses whether the study participants are representative of the target population, in terms of demographics and other relevant factors.
    - Typically named cov_representativeness
    - ‘0’ for studies whose results are likely generalizable to the total population: sample was based on the general population with reasonable exclusions for pre-existing disease states.
    - ‘1’ for studies performed on non-representative subpopulations, e.g., a high-risk group.

2. **Exposure**: assesses whether the exposure (i.e., risk factor) of interest is well-defined and measured accurately in the study.
    - Typically named cov_exposure_quality
    - ‘0’ for studies whose exposure measurement characteristics are considered the gold standard for that risk-outcome pair and ‘1’ otherwise.
    - May be broken into subgroups if sufficient information exists for each subgroup:
        - cov_exposure_population: ‘0’ for individual-level exposure and ‘1’ for population-level exposure.
        - cov_exposure_selfreport: ‘0’ for measurements based on assays, tests, or physician observations and ‘1’ for self-report.
        - cov_exposure_study: ‘0’ if exposure was measured multiple times and ‘1’ for only a baseline measurement. Case-control studies should be scored as ‘1’ unless a detailed exposure history was solicited that allows for quantification of variation in exposure.

3. **Outcome**: assesses whether the outcome of interest is clearly defined, clinically relevant, and accurately measured in the study.
    - Typically named cov_outcome_quality

- ‘0’ for studies whose outcome measurement characteristics are considered the gold standard for that risk-outcome pair and ‘1’ otherwise.
- May be broken into subgroups if sufficient information exists for each subgroup:
    - cov_outcome_selfreport: ‘0’ if outcome measurement was based on death certificates, physician diagnosis or medical records and ‘1’ if based on self-report.
    - cov_outcome_unblinded: ‘0’ if outcome assessment is blind to the individual level of exposure and ‘1’ if unblinded.

4. **Reverse causation**: assesses the possibility that the observed relationship between the exposure and outcome could be due to the outcome causing the exposure, rather than the other way around.

- Typically named cov_reverse_causation
- ‘0’ for studies where reverse causation was accounted for (there is minimal or no risk of reverse causation).
- ‘1’ for studies where reverse causation was not accounted for (there is a risk of reverse causation).

5. **Control for confounding**: assesses whether the study design and analysis adequately account for potential confounding factors that could affect the relationship between the exposure and outcome.

- Typically named cov_confounder_quality
- ‘0’ for studies where confounding factors were accounted for.
- ‘1’ for studies where confounding factors were not accounted for.
- May be broken into subgroups if sufficient information exists for each subgroup:
    - cov_confounder_nonrandom: ‘0’ if the study was randomized and ‘1’ if the study was nonrandomized.
    - cov_confounder_uncontrolled: ‘0’ for randomization or for a non-randomized study where the outcome is controlled for all major known confounders including age, sex, education, income, and other critical determinants of the outcome. ‘1’ for non-randomized studies with control for only some determinants.

6. **Selection bias**: assesses whether the study sample was selected in a way that could introduce bias and whether efforts were made to minimize bias in the study design and analysis.

- Typically named cov_selection_bias
- ‘0’ for studies with greater than 95% follow-up and ‘1’ for studies with less than 95% follow-up.
- Case-control studies should be scored based on the percentage of cases and controls for which exposure data could be ascertained.

Additional bias covariates, following the same general criteria for collecting and assigning values, may be considered for certain risk factors. The decision to include any additional bias covariates is up to the modeler and their team leads. The names of the additional bias covariates may be defined arbitrarily but should reflect the content of the bias.

Because our methods to control for bias (see **Testing for bias across different study designs and characteristics** in Section 2.1.4 for further details) are data-driven and rely on high-level information available about input studies to meta-analyses (eg, which studies were gold standard vs. not), they are able to test and adjust for bias, but are unable to capture this bias in a more nuanced manner. Moreover, if the data available are insufficient to generate bias covariates with adequate spread, bias adjustment cannot be performed. We are presently exploring enhanced diagnostic tools to assess covariate coverage and leverage across models to better assess bias adjustment feasibility and to identify where additional data collection or alternative methods are necessary. More efforts will be made in future GBD iterations to systematize bias coding across modelers and research teams.

Section 2.1.4: Estimating the shape of the risk–outcome relationship

Most classic epidemiological analyses of exposure or dose–response risk relationships have either assumed the relationship between risk and outcome to be log-linear or have converted continuous exposure variables into dichotomous exposure categories. This assumption simplifies the analysis considerably. Unfortunately, while assuming a log-linear relationship is analytically convenient and allows for the use of simple open-source tools,[14] it is not necessarily biologically or clinically plausible (see model validation section for more details). For some risks, such as smoking, log-relative risk of outcome flattens at higher exposures. For others, such as BMI, the log-relative risk curves are J-shaped. We therefore chose to estimate the shape of the relationship directly from the data using a regularised spline.

In GBD 2021, for continuous and dichotomous risk factors, we modelled RRs using meta-regression—Bayesian, regularised, trimmed (MR-BRT) (see details below), relaxing the log-linear assumption to allow for monotonically increasing or decreasing non-linear functions using splines, typically with degrees 2 or 3 (quadratic and cubic splines, respectively). Monotonicity assumptions, which are less stringent than the log-linearity assumed in classic analyses, serve primarily to stabilize model fits in areas of sparse data. Any artifacts created by deviations from monotonicity are identified by examination of the funnel plot residuals. Risk factors for which we undertook this reanalysis include all dietary risk factors, low physical activity, kidney dysfunction, unsafe water and sanitation, no access to handwashing facility, particulate matter air pollution, lead exposure, vitamin A deficiency, secondhand smoke, bullying victimisation, high body-mass index, high fasting plasma glucose, and high alcohol use. In GBD 2023, we completed the reanalysis for sexual violence against children, intimate partner violence, and chewing tobacco. We did not conduct this reanalysis for risk factors with direct PAFs or PAFs=1 as well as select risk factors such as occupational risk factors, low bone mineral density, iron deficiency, suboptimal breastfeeding, and low birthweight and short gestation.

Because knot placement can affect the shape of the risk function when modelling with a cubic spline, we generated a wide range of knot placements and created an ensemble across these different knot placements. We also included in the final estimation 10% trimming of the data to avoid the results being sensitive to outliers.

Many meta-analyses convert RRs to per unit increase for convenience, particularly when studies choose different categories that could not otherwise be compared. If samples in the primary studies at high levels of exposure were sufficient to inform the shape of the tail of the distribution, we applied a cap to the maximum RR by using the midpoint of the last category for which a RR was reported.

Basis splines, measurement mechanism, and shape constraints

First, we used a Bayesian regularised spline to obtain the general shape of the non-linear relationship. Basis splines represent nonlinear curves as linear combination of recursively generated basis elements.[15] The basis elements were recursively generated using piecewise smooth polynomials and were roughly localised to certain regions of the exposure variable in the data. In most cases, quadratic or cubic polynomials were used, often with linear tails in the presence of sparse data. This approach allowed the use of the common restricted cubic spline, as well as constraints on the shape of the relationship (including non-decreasing and non-increasing).

Given basis functions $f_1, \ldots, f_k$, the final curve is obtained as a β-linear combination

$$signal = \beta_1 f_1 + \cdots + \beta_k f_k.$$

Specifically, for any given exposure x, the prediction using the spline model is given by

$$signal(x) = \beta_1 f_1(x) + \cdots + \cdots + \beta_k f_k\ (x) = \langle X, \beta \rangle \tag{1}$$

where X is a vector containing $(f_1(x), \ldots, f_k(x))$. Derivatives and integrals of splines can likewise be expressed as linear combinations of spline coefficient sβ. For additional details about B-splines see Zheng et al.[11]

Many studies of exposure–response relationships report relative risks between categories defined by intervals of exposure. The relative risk between two exposure groups is a ratio of integrals of the spline across two specified intervals, so we used this exact non-linear mechanism to inform the fit.[11] Data from studies usually compare outcome rates in one exposure alternative group to those in a separate reference group. In mathematical notation, such observations are given by

$$y_{ij} = \frac{\frac{1}{d_{ij} - c_{ij}} \int_{c_{ij}}^{d_{ij}} f(x)dx}{\frac{1}{b_{ij} - a_{ij}} \int_{a_{ij}}^{b_{ij}} f(x)dx}, \tag{2}$$

where y_{ij} is the reported relative risk corresponding to measurement j in study i, $[a_{ij},\ b_{ij}]$ delineates the reference group exposure interval, and $[c_{ij},\ d_{ij}]$ delineates the alternative group exposure interval. When $f(x)$ is represented using a spline, each integral is a linear function of β similar to (1). The observation model (2) is then a non-linear function given as the ratio of linear functions,

$$y_{ij} = f_{ij}(\beta) := \frac{\langle X_{ij}^1, \beta \rangle}{X_{ij}^2, \beta}. \tag{3}$$

with the associated log-relative risk given by

$$\ln(y_{ij}) = \ln(\langle X_{ij}^1, \beta \rangle) - ln\left(\langle X_{ij}^2, \beta \rangle\right). \tag{4}$$

Equation (4) is the main model used to infer the spline, and it is a simple but non-linear function of the spline coefficient β.

When studying exposure–response relationships, we allow for shape constraints of the inferred mean response. For example, for some harmful risks, such as smoking and air pollution, we allow the relative risk to be specified as monotonically increasing with exposure. In order to introduce each of these constraints, we used the fact that all derivatives of splines are linear functions of spline coefficients, similar to (1).

Monotonicity. Monotonicity constraints can be imposed using linear inequality constraints based on exemplar exposures. Given an exemplar exposure x_i, the requirement that the slope of the spline at exposure x_i be non-negative can be formulated as

$$\langle X_{ij}, \beta \rangle \geq 0$$

for a particular vector X_i. Linear inequality constraints are strictly enforced by the optimisation solver used to fit the model; see Zheng et al.[11]

Robust trimming strategy

To make the estimation of the overall relationship less sensitive to potential outlying studies or observations within studies, we applied a robust, likelihood-based statistical approach—least trimmed squares (LTS)[16]—to our mixed effects models.[11] The goal of robust statistical methods is to ensure that estimates are robust to such outlying observations. Trimming approaches form a subclass of robust statistical methods, and LTS was originally developed in the context of linear regression.[17] LTS works by classifying observations into a majority of inliers and minority of outliers, while simultaneously fitting the model with respect to which the inlier/outlier classification is made. Compared with other robust approaches, such as M-estimators,[18] trimming methods are more effective in limiting influence than outliers, and have a high breakdown point[19]; ie, the proportion of the data than can be arbitrarily corrupted before the estimator becomes invalid.

Trimming estimators have been applied to a broad range of problems, from linear regression[16] to high-dimensional sparse regression and general machine learning problems.[20] In the context of mixed effects models, trimming methods are the most effective robust tools currently available for meta-analysis.[11] In practice, the approach requires only a specified inlier proportion, which was set to 90% across all examples, ie, we fit the 90% most self-coherent datapoints.

Using this approach, we trimmed 10% of the observations as part of the model fitting process, simultaneously discovering and fitting the most self-coherent 90% of the observations.[11] Numerical studies in data-rich cases have shown that quality of estimation is unaffected by trimming, even when there are no outliers in the data.[20] In the meta-analytic regime, the 90% level is a heuristic that balances the sparsity of available data with the need to improve estimates in the presence of outliers. As noted below, this step also substantially decreased the number of risk–outcome pairs with evidence of residual publication or reporting bias.

Spline ensemble

Third, to make non-linear risk function estimates robust to knot placement, we created 50 models based on random knot placement samples. Spline estimates depend on the choice of spline parameters, including spline degree, number of knots, and knot placement. To mitigate the effect of spline parameter selection on results, we developed an ensemble approach over knot placement, so that the modeller only had to specify the spline degree and number of knots.

Given the degree and number of knots, we automatically sampled a set of knot placements for a feasible knot distribution (described below). For each knot placement, we fit a spline (including non-linear measurements, shape constraints, and trimming as discussed above), evaluated each resulting model by computing its fit and curvature, and aggregated the final model as a weighted combination of the ensemble.

Sampling knots from simplex. We used a minimal set of rules that describe a feasible set from which to sample knots, and sample from this set uniformly. Given a number of knots, the rules specify feasible ranges for each knot and feasible gaps between knots. The set of knot placements that satisfy these four rules form a closed polyhedron (a volume in high-dimensional space delineated by hyperplanes). We calculated the vertices of the polyhedron using the double description method[21] and uniformly sampled knot placements from within the polyhedron. Each knot placement yielded a model, fit using the trimmed constrained spline approach described above.

Ensemble performance evaluation. Once the ensemble was created, we scored the resulting risk curves using two criteria: model fit (measured using the log-likelihood) and total variation (measured using the highest-order derivative). These scores balanced competing objectives of fit and generalisability. Once we had these scores, we normalised them to the range $[0,1]$ and applied a logistic transformation. The transformation was used to make the scoring meaningful even in the presence of spurious curves in a large ensemble. We then multiplied the scores to down-weight models that were low under either criterion (fit or total variation). The final weights were normalised to sum to 1. Using a weighted combination of these metrics, we weighted the 50 models to create the ensemble model.

New non-linear covariates

Fourth, for risk–outcome pairs with non-linear relationships, we evaluated exposure levels since this information matters for non-log-linear pairs. To do this, we took advantage of the spline model and directly captured the typical data-generating mechanism. Specifically, we used the final model that we had estimated using the robust spline ensemble to generate a non-linear dose–response curve, which we encoded into new non-linear "signal" covariates that were later used to enable linear mixed effects analyses. Once the non-linear estimation was complete, the log-relative risk for each datapoint was a function of four parameters:

$$F(a_{ij}, b_{ij}, c_{ij}, d_{ij}) = \frac{\frac{1}{d_{ij} - c_{ij}} \int_{c_{ij}}^{d_{ij}} \hat{f}(x)dx}{\frac{1}{b_{ij} - a_{ij}} \int_{a_{ij}}^{b_{ij}} \hat{f}(x)dx}$$

where $\hat{f}$ is the non-linear function obtained by estimating spline coefficients $\hat{\beta}$, see (4), $[a_{ij}, b_{ij}]$ delineates the reference group exposure interval, and $[c_{ij}, d_{ij}]$ delineates the alternate group exposure interval.

We produced non-linear covariates for fixed and random effects. The non-linear fixed effects covariate, denoted $signal^f$, is given by

$$signal_{ij}^f = F(a_{ij}, b_{ij}, c_{ij}, d_{ij}). \tag{5}$$

The new non-linear random effect covariate, denoted by $signal^r$, is given by

$$signal_{ij}^{r} = F(t, t, c_{ij}, d_{ij}), \tag{6}$$

where t denotes a fixed reference, eg, the theoretical minimum risk exposure level (TMREL). We used these new covariates in linear mixed effects models in further stages of analysis:

$$y_{ij} = signal_{ij}^{f}\beta_s + signal_{ij}^{r}u_i + \epsilon_{ij} \tag{7}$$

where $\epsilon_{ij} \sim N(0, \sigma_{ij}^2)$ are known by each observation, β_s is a scalar linear covariate multiplier on the $signal^f$ covariate, and u_i is a random study-specific slope on the $signal^r$ covariate with unknown variance γ. The posterior for β_s in (7) was used as a reference for the prior in bias covariate selection, described in step 3.

For our visualisations (figures 1A–6A), we plotted each datapoint with x-value at the midpoint exposure of the alternative group, and y-value corresponding to the sum of the log relative risk and estimated curve evaluated at the midpoint of the reference group. These visualisations allow the standard assessment of fit quality, with a perfect fit corresponding to the estimated non-linear relationship passing through the data.

Testing for bias across different study designs and characteristics

Following the approach of the GRADE criteria,[22] we quantified common sources of bias across six general domains: representativeness of the study population, exposure assessment, outcome ascertainment, reverse causation, control for confounding, and selection bias. In the illustrative cases presented here, these variables were quantified for each study during the study extraction phase. For the set of studies on a risk–outcome association, we tested systematic variation as a function of these risk of bias variables through meta-regression. We converted the dose–response relationship identified in step 1 into a new "signal" covariate, effectively linearising the non-log-linear relationship. For each bias covariate x (coded as an indicator variable), we defined a corresponding interaction covariate (ie, an effect modifier):

$$y_{ij} = signal_{ij}^{f} \times \left(\beta_s + x_{ij}^{1}\beta_1 + \cdots + x_{ij}^{k}\beta_k\right) + \epsilon_{ij}$$

that modified the slope of the "signal" covariate. We then tested risks of bias of the effect modifiers through linear meta-regression. To be included, every bias covariate must have some studies that are the "gold standard" (ie, at the standard of the best studies that have been conducted) for that covariate; otherwise, it is not possible to incorporate it into the regression framework. Further, in considering potential covariates, we enforced that every categorical covariate had at least two studies in each category. Since bias covariates were already study-specific, we only considered the fixed-effects model in bias covariate selection.

We used a robust approach to test for bias that limited the risk of over-interpreting differences with limited numbers of studies. We used the Lasso[23,24] approach—which augments the least squares loss typically solved in a linear regression by penalising the sum of absolute values of the bias covariate multipliers—to obtain a ranked list of bias covariates using the following equation:

$$\min_{\beta} \sum_{i,j} \frac{1}{2\sigma_{ij}^2} (y_{ij} - signal_{ij}^f \times (\beta_s + x_{ij}^1\beta_1 + \cdots + x_{ij}^k\beta_k))^2 + \frac{1}{2}\beta^T\Sigma^{\{-1\}}\beta + \lambda \parallel \beta \parallel_1 \quad (8)$$

where β contains specifically bias covariate multipliers, $\sum$ is a diagonal matrix linked to the posterior on β_s from the basic linear model (7), and the term $\lambda \parallel \beta \parallel_1$ penalises the sum of the absolute values, pushing the bias covariate multipliers β to 0, with a strength determined by λ.[24]

We then selected bias covariates based on their Lasso ranking, obtained by sweeping through from high to low values of λ in (8). We then added the selected covariates to the linear meta-regression model one at a time, following this ranking. To stabilise the selection process and follow through on the "burden of proof" philosophy, we tested for significance of covariates using a Gaussian prior that biased all bias coefficients to 0 with a strength proportional to the posterior of the main dose–response relationship. If the coefficients were significant, they stayed in the model as the process continued; we terminated the process when the last added bias covariate was no longer significant after accounting for "signal" and higher-ranked covariates in the model. We predicted the risk function using the values of the included bias covariates that reflected the preferred level of the covariate, such as the highest level of control for confounding.

Section 2.1.5: Fitting the mean and quantifying between-study heterogeneity. Estimation of between-study heterogeneity is an important aspect of meta-analysis. It reflects the variation between studies and consistency across literature. In the following, we describe how we used the signal and bias covariates obtained in steps 2.4 and 3 to build a simple linear mixed effects model to capture the between-study heterogeneity.

After the selection procedure, we fit a final linear mixed-effects model that included the "signal" as well as selected bias covariates. Division by a common referent in the typical measurement mechanism induces correlation, (via an intercept shift in log-relative risk space); we therefore used a random intercept in the mixed-effects model to account for this induced within-study correlation. To capture the between-study heterogeneity, we used a study-specific random slope with respect to the "signal" model so that the random effect for each study effectively scaled the non-linear relative risk curve. Formally, we fit a linear mixed effects model of the form

$$y_{ij} = signal_{ij}^f \times (\beta_s + x_1\beta_1 + \cdots + x_k\beta_k) + signal_{ij}^f u_i + \epsilon_{ij}$$

where $\epsilon_{ij} \sim N(0, \sigma_{ij}^2)$ are the reported observation standard errors, and u_i are random effects with a common unknown variance,

$$u_i \sim N(0, \gamma).$$

Parameters β and γ were estimated simultaneously using maximum likelihood; see Zheng et al[11] for more details. The fixed effects portion of the formula estimates the mean log-relative risk. We used the same prior on bias covariates in this analysis as we used in equation (8), ie, $\beta \sim N(0, \sum)$. For log-linear relative risks, this modelling choice reduced to the classic analysis, where the random slope with respect to exposure was equivalent to the random intercept for log-linear relative risk.

To account for the small studies problem—where in the setting of small numbers of studies, between-study heterogeneity (γ) can easily be under-estimated,[25] and in particular the estimate may be zero when too few studies are available—we quantified the uncertainty in heterogeneity estimation.[26] This estimate allowed a quantile of the heterogeneity parameter to be used, increasing the robustness of the estimate against the small study problem. Among several alternatives in the literature,[27,28] we used the Fisher information matrix (FIM)[27] to estimate the uncertainty of the between-study heterogeneity. The FIM is weakly dependent on observed data but is sensitive to the non-linear relationship, selected bias covariates, reported standard errors, and the number of studies. The final uncertainty intervals we report are composed of two components: (1) posterior uncertainty corresponding to fixed effect β_S, and (2) 95% quantile of γ, which depends on the estimate of γ and the estimate of the variance of γ using the inverse of Fisher information.

Section 2.1.6: Estimating the burden of proof risk function

The combined uncertainty for the mean, estimated between-study heterogeneity, and 95th quantile of the between-study heterogeneity obtained from the FIM estimate were used to generate a BPRF. The BPRF is defined as either the 5th (for harmful risks) or 95th (for protective risks) quantile curve closest to the line of relative risk equal to 1 (the null) and can be interpreted as the smallest harmful or protective effect at each level of exposure consistent with the available evidence.

In the range of exposures defined by the 15th and 85th percentiles of exposure levels observed for each risk across available studies, the ROS is defined as the signed value of the average log BPRF. For example, a log BPRF of 0.4 for a harmful risk (where null = 0) and a log BPRF of –0.4 for a protective risk would both have an ROS of 0.4 because the magnitude of the log relative risk is the same. In contrast, for risk–outcome pairs with a BPRF opposite the null from the mean risk (ie, the BPRF suggests that the relationship is opposite of the expected relationship—a BPRF below 1 for a harmful risk and a BPRF above 1 for a protective risk), ROS would be calculated as negative.

Network analysis

Some relative risks were modelled using network analysis. Network analysis is a special case of the mixed effects linear model that is used to compare multiple treatment effects, for example, in the case of drinking water. To explain the coding, we use an example with four treatments A, B, C, D.

For simplicity, assume *A* is this reference treatment. We then have the following coding.

$$AB \to B - A: \quad [1 \quad 0 \quad 0]$$

$$AC \to C - A: \quad [0 \quad 1 \quad 0]$$

$$AD \to D - A: \quad [0 \quad 0 \quad 1].$$

We see from this example that the design matrix under the basic network assumption is always full rank, since a subset of rows forms the identity matrix.

Comparisons that do not include the reference can be computed. For example,

$$BC \to C - B = (C - A) - (B - A)$$

$$= [0 \quad 1 \quad 0] - [1 \quad 0 \quad 0]$$

$$= [-1 \quad 1 \quad 0]$$

Using this simple algebra, we obtain the remaining codings.

$$BC \rightarrow C - B: \quad [-1 \quad 1 \quad 0]$$

$$BD \rightarrow D - B: \quad [-1 \quad 0 \quad 1]$$

$$CD \rightarrow D - C: \quad [0 \quad -1 \quad 1]$$

Each row of the design matrix $\boldsymbol{X}$ is coded according to the comparison.

When doing network analysis, the design matrix $\boldsymbol{X}$ does not include the intercept term (**1** column).

Section 2.1.7: Evaluating potential for publication or reporting bias

A significant association between mean effect and standard deviation may indicate potential for publication or reporting bias, or methodological differences between large and small studies, which likewise lead to biased results. Publication bias is an important issue in meta-analysis,[29] and a formal test is typically done in addition to visual inspection of the funnel plot to decrease the chances of flagging apparent bias due to chance alone. In the proposed approach, we checked whether the standard deviations were significant predictors of the observations in the presence of the "signal" and bias covariates. To detect publication bias, we used a data-driven approach known as Egger's regression.[30] The approach detects if there is a significant correlation between the residuals and their standard deviations. When Egger's regression failed to detect significant evidence of publication bias, we terminated the process. While we identified these pairs as having potential for publication or reporting bias, we followed the general literature and did not incorporate any correction to the risk function based on this finding.

Section 2.1.8: MR-BRT and temperature

While meta-regression of literature studies was applied to estimate relationships for risk–outcome pairs, for temperature, we conducted primary analysis of relationships with cause-specific mortality as described previously.[31] The relative risk, RR, of mortality was calculated for each daily and mean annual temperature category in each administrative unit. For this purpose, we calculated the daily mean temperature and aggregated the daily cause-specific death counts for each administration. We then calculated mortality rates for each cause, c, location, l, and daily mean temperature, ie, temperature category, t:t

$$MR_{clt} = \frac{deaths_{clt}}{person - days_{lt}}$$

With *MR* representing the mortality rate, *deaths* being the absolute number of cause-specific deaths, and *person-days* depicting the sum of the population in location, *l*, across all days with a daily temperature of *t*.

Following, we calculated the mean MR, $\overline{MR}$, for each cause, *c*, and location, *l*:

$$\overline{MR_{cl}} = \frac{deaths_{cl}}{person - days_{l}}$$

The daily temperature-specific mortality rate ratio, *MRR*, was then calculated as the ratio of the MR for each temperature category, location, and cause, and the average $\overline{MR}$:

$$MRR_{clt} = \frac{MR_{clt}}{\overline{MR_{cl}}}$$

In order to aggregate the *MMRs* to the first-level administrative unit, we calculate the population-weighted mean temperature (PWMT) for each location and across all days and then pooled all *MRRs* for each combination of daily temperature and PWMT.

Step 2. Exposure estimation[5]

Section 2.2.1: Collate exposure data

Systematic reviews

For GBD 2023, we conducted updated systematic literature reviews of risk factor exposure for three risks (lead exposure, high fasting plasma glucose, and high systolic blood pressure). For other risk factors, only a fraction of the existing data appears in the published literature, and other sources predominate, such as survey, measurement, or satellite data. Data were systematically screened from household surveys archived in the GHDx (http://ghdx.healthdata.org), including Demographic and Health Surveys, Multiple Indicator Cluster Surveys, Living Standards Measurement Surveys, and Reproductive Health Surveys. Other national health surveys were identified based on survey series that had yielded usable data for past rounds of GBD, sources suggested to us by in-country collaborators, and surveys identified in major multinational survey data catalogs, such as the International Household Survey Network and the WHO Central Data Catalog, as well as through country ministry of health and central statistical office websites. Certain risks, such as poor diet and excessive alcohol consumption, also incorporated administrative record systems. Citations for all data sources used for risk factor estimation in GBD 2023 are provided in searchable form through a web tool (http://ghdx.healthdata.org). A description of the search terms employed for risk-specific systematic reviews are detailed by cause in appendix section 4.

Information on systematic reviews were managed by using Research Electronic Data Capture (REDCap) electronic data capture tools hosted at the University of Washington.[32] REDCap is a secure, web-based application designed to support data capture for research studies that provides 1) an intuitive interface for validated data entry; 2) audit trails for tracking data manipulation and export procedures; 3) automated export procedures for seamless data downloads to common statistical packages; and 4) procedures for importing data from external sources.

Search terms

Search terms for updates of systematic reviews for GBD 2023 are shown by risk factor in section 4.

Survey data preparation

Survey data constitute a substantial part of the underlying data used in the estimation process. During extraction, we concentrated on demographic variables (such as location, gender, age), survey design variables (such as sampling strategy and sampling weights), and the variables used to define the

population estimate (such a prevalence or a proportion) and a measure of uncertainty (standard error, confidence interval, or sample size and number of cases).

Section 2.2.2: Adjust exposure data

Compiled several adjustments were applied to extracted exposure sources to make the data more consistent and suitable for modelling. In GBD 2023, we implemented adjustments of risk exposure data to deal with alternative case definitions or study methods prior to entering data into our main analytical tools of DisMod-MR 2.1, ST-GPR, and MR-BRT. This decision also included the adjustment of data presented for both sexes to a male and female equivalent. The starting point was to explicitly state the reference case definition and study method and identify alternative definitions and study characteristics that fall without our inclusion criteria.

We compiled data from both within-study comparisons (ie, data that used alternative and reference definitions in the same population) and between-study comparisons (ie, data that used an alternative definition in one population and a reference definition in another population that overlap in location, time, age, and sex) of different case definitions. For between-study comparisons, we allowed a maximum calendar year difference between studies of five years. Where validation studies (ie, those carried out at the introduction of a new set of diagnostic criteria comparing to previous criteria) were available, we extracted data on the comparison of alternative to reference. For quantities of interest with multiple alternative definitions/methods, we also look for pairs comparing two alternatives. In a network analysis, if A is the reference and B and C are two alternatives, a comparison of A versus B and B versus C provides an indirect comparison of the alternative C against the reference A.

We pooled either the logit difference between alternative and reference or the natural log of the ratio of alternative to reference. From simulations, we found that the two methods provide almost identical results for quantities that after adjustment do not exceed a value of 0.5 (eg, prevalence or proportion). The logit difference method much better dealt with higher values and avoided prevalence or proportions to exceed 1. If the values of either the reference or alternative were zero, we aggregated values across age groups until both values had non-zero observations. We used the delta method to compute the standard error of the reference and alternative measures in logit space. The standard error of the logit difference was computed as the square root of the sum of the variances of each datapoint in a pair.

Data analysis

We used a network random effects meta-regression in MR-BRT (see section 2.1.4). In a network analysis, if A is the reference and B and C are two alternatives, a comparison of A versus B and B versus C provides an indirect comparison of the alternative C against the reference A. To implement the network, we included dummy variables with a particular structure. This was implemented as follows, where A is the reference definition/method:

- Create *k* dummy variables where *k* are all definitions/methods other than A (eg, *k* = B, C)
- Code dummy *k* as
  - 1 if the first term of the logit difference is *k*;
  - –1 if k is second term of the logit difference;
  - 0 otherwise

For example:

Study	Comparison	DummyB	DummyC
1	logit(B)-logit(A)	1	0
2	logit(B)-logit(A)	1	0
3	logit(C)-logit(A)	0	1
4	logit(C)-logit(A)	0	1
5	logit(C)-logit(B)	-1	1
6	logit(C)-logit(B)	-1	1

The coding structure outlined above in step 1 assumes that all case definitions are mutually exclusive. In some cases, however, individual case definitions are a function of different components or dimensions. For example, case definitions may vary by the type of symptoms that a respondent experiences as well as the recall period over which those symptoms are experienced. In the presence of sparse data, it may be difficult to find both direct and indirect comparisons of all individual case definitions. In these cases, an alternative approach is to assume different dimensions of case definitions have a multiplicative effect. In other words, the effect of recall period has the same relative effect across different categories of symptoms reported by respondents. To implement this coding scheme:

- Create *k* dummy variable columns for each case definition dimension
- For each dummy variable k:
  - Add 1 if *k* is a component of the first term in the logit difference
  - Subtract 1 if *k* is a component of the second term in the logit difference

In MR-BRT, we ran random effects meta-regression of the logit difference (or log ratio) with all the *k* dummy variables as covariates, omitting the intercept in the meta-regression. We used a study_id variable for be the unique combination of the NIDs of the reference and alternative studies (or alternative1 to alternative2). The coefficients on the *k* dummy variables represent the pooled logit difference of the *k* alternative definition to the reference taking into account evidence from both direct and indirect comparisons. In the example above, the coefficient on DummyA is the pooled logit difference of B minus A; the coefficient on DummyB is the pooled logit difference of C minus A. The standard error of the pooled logit difference incorporating the between-study variance was calculated as:

$$se(\text{logit}(difference_k)) = \sqrt{var_k + \gamma^2}$$

Where:

$se(\text{logit}(difference_k))$ = standard error of the pooled logit difference of alternative k to the reference
var_k= variance of the coefficient on dummy variable k
γ^2 = between-study variance

If both between- and within-study pairs were available, we examined whether there was a systematic difference between these. If there was a significant difference, we made judgement call as to whether within-study or between-study data comparisons were most appropriate. In general, this was the within-study data; however, there were important measurement or conceptual reasons for choosing between-study data. For example, for crosswalks between self-reported height and weight compared to measured height and weight, between-study comparisons may be preferable if respondents knew they would be measured and, therefore, were less likely to misreport their height and weight.

We also examined whether there were systematic differences in the adjustments by key demographics (age, sex, geographical location, year) and other potential factors that may lead to variation in crosswalks. This could only be done at present in a direct comparison model and not in a network. We did this when there was a strong rationale, eg, biological plausibility, for variation by such characteristics.

After obtaining the pooled logit difference or log ratio estimates, we predicted adjustments based on the statistical model, including uncertainty in the adjustment and sampling error of each datapoint. For non-significant logit differences or log ratios, we still applied the adjustments if there was a conceptual reason to believe that the alternative definition is biased. This expands the variance of these alternative definition datapoints.

Section 2.2.3: Estimate exposure

Mean exposure estimation

Once data were collected and compiled, the next step of the analytical flowchart was to apply adjustments, where necessary, to correct for bias. Examples of these adjustments include use of urban studies for lead; crosswalks between different measurements, methods, and definitions, such as for self-

report of obesity and glycated haemoglobin (HbA_{1C}) for diabetes; and age-sex splitting of data, such as for fasting plasma glucose (FPG) level, cholesterol level, and systolic blood pressure that may be reported from broad age groups.

For the GBD, we developed two modelling approaches, a Bayesian meta-regression model (DisMod-MR 2.1) and a spatiotemporal Gaussian process regression model (ST-GPR), to pool data from different sources, control and adjust for bias in data, and incorporate other types of information such as country-level covariates. DisMod-MR 2.1 and ST-GPR are mixed effect models that borrow information across age, time, and locations to synthesise multiple data sources into unified estimates of levels and trends. A detailed description of the likelihood used for estimation and a full description of improvements made for DisMod-MR 2.1 were detailed by Vos and colleagues,[33] who provided additional detail in the appendix to that paper.[20] The ST-GPR model has three main hyper-parameters that control for smoothing across time, age, and location. Values for these hyper-parameters were selected on the basis of cross-validation. Cross-validation tests were conducted for different combinations of the hyper-parameters for three types of models: one data-sparse model, one data-moderate model, and one data-dense model. In each test, 20% of the data were held out, and the performance of each combination of hyper-parameters was evaluated on the held-out data. For each hyper-parameter combination, ten cross-validation tests were conducted. The performance of each model in predicting the withheld 20% of the data was evaluated by using a combined measure based on root mean square error (RMSE) and uncertainty interval (UI) coverage. A detailed description of the ST-GPR process regression can be found below.

The main difference between these methods is their power to include unstructured types of data by sex and age group and their degree of flexibility. DisMod-MR 2.1 is the preferred tool in these cases because of its ability to integrate over age and adjust for different exposure definitions in the data; however, the use of Bayesian Markov Chain Monte Carlo (MCMC) simulations with large volumes of data renders the analysis computationally intensive and reduces the number of iterations that are possible. If standard age-group data are available – as is generally the case for metabolic risks – using ST-GPR becomes the preferred approach.

In some cases, we adapted our methods of modelling exposure to risks where necessary to account for complexities in the risk–outcome relationship or the need for particular handling of data, for example, dietary risks and ambient air pollution (see appendix section 4 for more detail). A complete list of risks is reported in table S1. Additional details for adjustments or adaptations to particular risk models are provided in appendix section 4.

DisMod-MR 2.1 description

Until GBD 2010, non-fatal estimates in burden of disease assessments were based on a single data source on prevalence, incidence, remission, or a mortality risk selected by the researcher as most relevant to a particular location and time. For GBD 2010, we set a more ambitious goal: to evaluate all available information on a disease that passes a minimum quality standard. That required a different analytical tool that would be able to pool disparate information presented for varying age groupings and from data sources by using different methods. The DisMod-MR 1.0 tool used in GBD 2010 evaluated and pooled all available data, adjusted data for systematic bias associated with methods that varied from the reference, and produced estimates by world regions with UIs by using Bayesian statistical methods. For GBD 2013, the improved DisMod-MR 2.0 increased computational speed, which allowed computations

to be consistent between all disease parameters at the country rather than the region level. The hundred-fold increase in speed of DisMod-MR 2.0 was partly due to a more efficient rewrite of the code in C++ but also to changing to a model specification by using log rates rather than a negative binomial model used in DisMod-MR 1.0. In cross-validation tests, the log rates specification worked as well as or better than the negative binomial specification.[34]

In GBD 2015, we also improved how country covariates differentiate non-fatal estimates for diseases with sparse data. The coefficients for country covariates are re-estimated at each level of the cascade. For a given location, country coefficients are calculated by using both data and prior information available for that location. In the absence of data, the coefficient of its parent location is used to utilise the predictive power of our covariates in data-sparse situations.

For GBD 2016, the computational engine (DisMod-MR 2.1) remained substantively unchanged from GBD 2015. We changed the prediction year set to generate fits for the years 1990, 1995, 2000, 2005, 2010, and 2016. We updated the age prediction sets to include age groups 80–84 years, 85–89 years, 90–94 years, and 95+ years to comply with changes across all functional areas of the GBD. We also expanded the set of locations where subnational units are modelled; the set now includes Brazil, China, England, India, Indonesia, Japan, Kenya, Mexico, South Africa, Sweden, and the USA.

In GBD 2017, we continued to use DisMod-MR 2.1 because no substantial changes were made. Updates to computation include extending the terminal prediction year to 2017 and additional subnational units in Ethiopia, Iran, New Zealand, Norway, and Russia. Saudi Arabia was also modelled only at the national level in 2017.

In GBD 2019, 2021 and 2023, no substantial changes were made to DisMod-MR 2.1, but we made more substantial changes to how we use the tool. First, we added the years 2019, 2020, and 2021, 2022 and 2023 as additional years of estimation. Second, we also included the option again to have random effects on cause-specific mortality rates (CSMR) and EMR. This functionality had been dropped a couple of GBD rounds earlier. Third, as we did all our adjustments for alternative case definitions and study methods as well as adjustments to combined-sex data points prior to entering data into DisMod-MR 2.1, we no longer used the functionality in DisMod-MR 2.1 to estimate coefficients for study and sex covariates and instead, processed data prior to entry in DisMod-MR 2.1 as described in the section above on data adjustments. Fourth, based on simulation testing conducted in GBD 2019 we found that coverage improved, and errors reduced when passing down priors with a wider setting of minimum coefficient of variation (which determines the uncertainty around priors and hence how 'informative' the priors are) than had generally been used in past GBD iterations. We settled on a default value of 0.8 where in the past values of 0.4 or less had been more commonly used. We made some exceptions for highly prevalent conditions where a lower minimum coefficient of variation (CV) setting achieved the task of making priors less informative, but not completely uninformative.

DisMod-MR 2.1 simultaneously fits several epidemiologic measures using a Bayesian, nonlinear, mixed-effects regression model, which produces estimates of a consistent set of epidemiologic measures in a compartmental disease model, meaning that they are the solution to the set of differential equations that specify the model.

A three-compartment model of a disease in a population consisting of susceptibles, i.e. those in the population without disease, cases of the disease and deaths. Individuals move from susceptible to case by the incidence rate *i*, and can return to susceptible by remission rate *r*. Cases are susceptible to a mortality rate, m_i, that reflects the excess mortality in cases of the disease. Cases and susceptibles are subject to a mortality rate from other causes, m_o. Each quantity in figure 15 is a function of age and time, governed by the following differential equations:

$$\frac{d}{d\tau}S(a+\tau, t+\tau) = -(i+m_o)S + rC$$

$$\frac{d}{d\tau}C(a+\tau, t+\tau) = iS - (r+m_o+m_c)C$$

where:

$S = S(a,t)$ = stock of susceptibles

$C = C(a,t)$ = stock of cases, i.e. those in population with disease

$i = i(a,t)$ = incidence hazard for susceptibles S

$r = r(a,t)$ = remission ('cure') hazard for individuals with the disease C

$m_o = m_o(a,t)$ = without condition mortality hazard for susceptibles S

$m_c = m_c(a,t)$ = excess mortality hazard for individuals with the disease C

a and *t* denote age and time.

A Monte Carlo Markov Chain simulation starts with the prevalence at birth, and samples the posterior distribution integrating the observed values for all observations of all epidemiologic quantities, ensuring that susceptibles and cases sum to population size, and that the partial derivatives of each differential equation in the system with respect to time zero. For most diseases the birth prevalence is set to zero. For disease that can be present at birth, a non-zero birth prevalence is specified. Population sizes for all combinations of year, age, sex, and location are input to DisMod-MR 2.1 from the latest GBD estimates, which are published iteratively and available by download from the GBD results tool.

The sequence of estimation occurs at five levels: global, super-region, region, country and, where applicable, subnational location. The super-region priors are generated at the global level with mixed-effects, non-linear regression by using all available data; the super-region fit, in turn, informs the region fit, and so on down the cascade. For some countries, additional models are fit for subnational locations. At each step in this cascade, only the data appropriate to that year, sex and geography are used to update the prior and yield the posterior.

Once fitting is complete for the finest granularity, results are summed, at the draw level, weighting by population size, to produce final results for larger geographic units from the finest results.

Analysts can choose to branch the cascade in terms of time and sex at different levels depending on data density. The default used in most models is to branch by sex after the global fit but to retain all years of data until the lowest level in the cascade is reached.

The computational engine is limited to three levels of random effects; we differentiate estimates at the super-region, region, and country level. In GBD 2013, the subnational units of China, the United Kingdom and Mexico were treated as "countries" to enable a random effect to be estimated for every location with contributing data. However, the lack of a hierarchy between country and subnational units meant that the fit to country data contributed as much to the estimation of a subnational unit as the fits for all other countries in the region. We found inconsistency between the country fit and the aggregation of subnational estimates when the country's epidemiology varied from the average of the region. Adding an additional level of random effects required a prohibitively comprehensive rewrite of the underlying DisMod-MR engine. Instead, we added a fifth layer to the cascade, with subnational estimation informed by the country fit and country covariates, plus an adjustment based on the average of the residuals between the subnational location's available data and its prior. This technique mimicked the impact of a random effect on estimates among subnationals.

In GBD 2017 and 2019 GBD rounds we calculated priors on excess mortality and entered these as data points by matching sex-specific prevalence data with an age width of 20 or less with the corresponding CSMR for the same location and year. For stability, we excluded calculation of EMR for prevalence data points of less than 1 in a million. EMR is simply calculated as CSMR divided by prevalence. As with previous GBD years, for diseases with an average duration of less than a year (as indicated by a setting of remission greater than one), we ran an initial global model to get an equivalent prevalence and used the following formula to calculate EMR:

$$EMR = {CSMR * (remission + (ACMR - CSMR) + EMR\_pred)}/{incidence}$$

where,

ACMR is the all-cause mortality rate

EMR_pred is the EMR fit from an initial global DisMod model

Despite using the log of LDI or the HAQ Index as a covariate with a prior that the coefficient had to be negative, we found many disease models with an implausible distribution of mortality to prevalence (or incidence) ratios implying lower case fatality in locations with lower HAQ Index than in countries with higher HAQ Index. This likely signals an inconsistency between fatal and non-fatal data inputs. For GBD 2019, we decided to run regressions on EMR data (calculated as described above) first using MR-BRT with HAQ Index as a predictor. In general, we tend to think that CSMR estimates are more robust than non-fatal data because of much greater data availability and a lesser task in adjusting cause death data for garbage coding than the complex task of adjusting non-fatal data sources for alternative case definitions and study methods. To indicate that we would reduce the random effects on EMR and the minimum coefficient of variation for priors on EMR being created at each next level down the cascade.

DisMod-MR 2.1 likelihood estimation

Analysts have the choice of using a Gaussian, log-Gaussian, Laplace, or Log-Laplace likelihood function in DisMod-MR 2.1. The default log-Gaussian equation for the data likelihood is

$$-log\left[p\left(y_j|\Phi\right)\right] = \log\left(\sqrt{2\pi}\right) + \log\left(\delta_j + s_j\right) + \frac{1}{2}\left(\frac{\log\left(a_j + \eta_j\right) - \log\left(m_j + \eta_j\right)}{\delta_j + s_j}\right)^2$$

where

y_j is a "measurement value" (ie, datapoint)

Φ denotes all model random variables

η_j is the offset value, *eta*, for a particular "integrand" (prevalence, incidence, remission, excess mortality rate, with-condition mortality rate, cause-specific mortality rate, relative risk, or standardised mortality ratio)

a_j is the adjusted measurement for datapoint j, defined by

$$a_j = e^{(-u_j - c_j)} y_j$$

Where:

u_j is the total "area effect" (ie, the sum of the random effects at three levels of the cascade: super-region, region, and country) and

c_j is the total covariate effect (ie, the mean combined fixed effects for sex, study-level, and country-level covariates), defined by

$$c_j = \sum_{k=0}^{K[I(j)]-1} \beta_{I(j),k} \hat{X}_{k,j}$$

with standard deviation

$$s_j = \sum_{l=0}^{L[I(j)]-1} \zeta_{I(j),l} \hat{Z}_{k,j}$$

Where:

k denotes the mean value of each datapoint in relation to a covariate (also called x-covariate)

I(j) denotes a datapoint for a particular integrand, *j*

$\beta_{I(j),k}$ is the multiplier of the kth x-covariate for the ith integrand

$\hat{X}_{k,j}$ is the covariate value corresponding to the datapoint *j* for covariate *k*;

l denotes the standard deviation of each datapoint in relation to a covariate (also called z-covariate)

$\zeta_{I(j),k}$ is the multiplier of the lth z-covariate for the ith integrand

δ_j is the standard deviation for adjusted measurement *j*, defined by:

$$\delta_j = log\left[y_j + e^{(-u_j - c_j)}\eta_j + c_j\right] - log\left[y_j + e^{(-u_j - c_j)}\eta_j\right]$$

Where:

m_j denotes the model for the jth measurement, not counting effects or measurement noise, and defined by:

$$m_j = \frac{1}{B(j) - A(j)} \int_{A(j)}^{B(j)} I_j(\text{a})\ \text{da}$$

Where:

A(j) is the lower bound of the age range for a datapoint

B(j) is the upper bound of the age range for a datapoint

I_j denotes the function of age corresponding to the integrand for datapoint *j*

Spatiotemporal Gaussian process regression

This type of regression has been used for risk factors for which the data density is sufficient to estimate a very flexible time trend. The approach is a stochastic modelling technique that is designed to detect signals amidst noisy data. It also serves as a powerful tool for interpolating non-linear trends.[35,36] Unlike classical linear models that assume that the trend underlying data follows a definitive functional form, GPR assumes that the specific trend of interest follows a Gaussian process, which is defined by a mean function $m(\cdot)$ and a covariance function $Cov(\cdot)$. For example, let $p_{c,a,s,t}$ be the exposure, in normal, log, or logit space, observed in country *c*, for age group *a*, and sex *s* at time t:

$$(p_{c,a,s,t}) = g_{c,a,s}(t) + \epsilon_{c,a,s,t}$$

where

$$\epsilon_{c,a,s,t} \sim Normal(0, \sigma_p^2),$$

$$g_{c,a,s}(t) \sim GP\left(m_{c,a,s}(t), Cov\left(g_{c,a,s}(t)\right)\right).$$

The derivation of the mean and covariance functions, $m_{c,a,s}(t)$ and $Cov\left(g_{c,a,s}(t)\right)$, along with a more detailed description of the error variance (σ_p^2), is described below.

Estimating mean functions

We estimated mean functions by using a two-step approach. To be more specific, $m_{c,a,s}(t)$ can be expressed, depending on the exposure transformation, as:

$$log\left(p_{c,a,s}(t)\right) = X_{c,a,s}\beta + h(r_{c,a,s,t})$$

$$logit\left(p_{c,a,s}(t)\right) = X_{c,a,s}\beta + h(r_{c,a,s,t})$$

$$p_{c,a,s}(t) = X_{c,a,s}\beta + h(r_{c,a,s,t})$$

where $X\beta$ is the summation of the components of a hierarchical mixed-effects linear regression, including the intercept and the product of covariates with their corresponding fixed-effect coefficients. Some models were run as hierarchical mixed-effects linear regressions with random effects on the levels of the geographical hierarchy. For most mixed-effects models, random effects were only used in the fit, not in the prediction. The second part of the equation, $h(r_{c,a,s,t})$, is a smoothing function for the residuals, $r_{c,a,s,t}$, derived from the linear model.[8] Descriptions of exposure transformations and which covariates were used in linear models can be found in appendix section 4, which described the risk-specific estimation approaches. Some models used a custom stage-1 estimate. Detailed information on the mixed-effect estimation process for these risks may be found in the risk-specific appendix section 4.

Although the linear component captures the general trend in exposures over time, much of the data variability may still not be adequately accounted for. To address this, we fit a locally weighted polynomial regression (locally estimated scatterplot smoothing, or LOESS) function $h(r_{c,a,s,t})$ to systematically estimate this residual variability by borrowing strength across time, age, and space patterns (the spatiotemporal component of ST-GPR).[37,38] The time adjustment parameter, defined by λ , aims to borrow strength from neighbouring time points (ie, the exposure in this year is highly correlated with exposure in the previous year but less so further back in time). The age-adjustment parameter, defined by ω, borrows strength from data in neighbouring age groups. The space-adjustment parameter, defined by ξ, aims to borrow strength across the hierarchy of geographical locations. The spatial and temporal weights are combined into a single space-time weight to allow the amount of spatial weight given to a particular point $r_{c,a,s,t}$ to fluctuate given the data availability at each time *t* and location-level *l* in the location hierarchy.

Let $w_{c,a,s,t}$ be the final weight assigned to observation $r_{c,a,s,t}$ with reference to a focal observation r_{c_0,a_0,s_0,t_0}. We first generated a temporal weight $t.w_{c,a,s,t}$ for smoothing over time, which was based on the scaled distance along the time dimension of the two observations:[38]

$$t.w_{c,a,s,t} = \frac{1}{e^{\lambda|t-t_0|}}$$

Next, we generated a spatial weight to smooth over geography. Specifically, we defined a geospatial relationship by categorising data based on the GBD location hierarchy (table S4). *Zeta* acts as a scalar on a given datapoint given its proximity to the target location:

$$t.w_{c,a,s,t} = \zeta^{|c-c_0|}$$

For example, estimating a country would use the following weighting scheme:

- Country data: ζ^0 = 1
- Regional data not from the country being estimated: ζ^1
- Data from other regions in the same super-region: ξ^2
- Global data from other super-regions: ζ^3

Under the spatial weighting specification, typical values of ζ range from [0.001, 0.2], where ζ can be interpreted as the amount to down-weight regional datapoints compared to country datapoints for a given estimating country. For example, for a given datapoint $r_{c,a,s,t}$ and ζ = 0.01, a datapoint not within

country *c* but within the same region *r* as $r_{c,a,s,t}$ would be assigned $\frac{1}{100}$ the weight of a datapoint within the country.

The spatial and temporal weights were then multiplied and summed across each level of the location hierarchy and normalised for each time period t . This procedure allowed the space-time weight to implicitly take into account the amount of data available at the country versus region versus super-region level and attribute spatial weight accordingly.

Given a normalisation constant,

$$K_i = \sum_{c \epsilon C} s.w_{c,t} * t.w_{c.t} + \sum_{c \epsilon R} s.w_{c,t} * t.w_{c.t} + \sum_{c \epsilon SR} s.w_{c,t} * t.w_{c.t}$$

the final space-time weight would then equal

$$w'_{c,a,s,t} = \frac{s.w_{c,t} * t.w_{c,t}}{K_i}$$

Finally, we calculated the weight $w''_{c,a,s,t}$ to smooth over age, which is based on a distance along the age dimension of two observations. For a point between the age a of the observation $r_{c,a,s,t}$ and a focal observation r_{c_0,a_0,s_0,t_0}, the weight is defined as follows:

$$w''_{c,a,s,t} = \frac{1}{e^{\omega|a-a_0|}}$$

The final weights would then be computed by simply multiplying the space-time weights and age weights and normalising so all weights for a given time period *t* sum to 1. A full derivation of weights for each category, assuming the location being estimated was a country, follows:

1) If the observation $r_{c,t}$ belongs to the same country c_0 of the focal observation r_{c_0,t_0}:

$$w_{c,a,s,t} = \frac{(w'_{c,a,s,t} w''_{c,a,s,t})}{\sum_{c=c_0}(w'_{c,a,s,t} w''_{c,a,s,t})} \qquad \forall c = c_0$$

2) If the observation $r_{c,t}$ belongs to a different country than the focal observation r_{c_0,t_0}, but both belong to the same region *R*:

$$w_{c,a,s,t} = \frac{(w'_{c,a,s,t} w''_{c,a,s,t})}{\sum_{c \neq c_0}(w'_{c,a,s,t} w''_{c,a,s,t})} \qquad \forall c \neq c_0 \cap \ R[c] = R[c_0]$$

3) If the observation $r_{c,t}$ belongs to the same super-region *SR* but to both a different country c_0 and a different region $R[c_0]$ than the focal observation r_{c_0,t_0}:

$$w_{c,a,s,t} = \frac{(w'_{c,a,s,t} w''_{c,a,s,t})}{\sum_{c \neq c_0}(w'_{c,a,s,t} w''_{c,a,s,t})} \qquad \forall c \neq c_0 \cap \ R[c] \neq R[c_0] \cap \ SR[c] = SR[c_0]$$

4) If the observation $r_{c,t}$ is from a different super-region than the focal observation r_{c_0,t_0}(Ie, all other data currently not receiving a weight):

$$w_{c,a,s,t} = \frac{(w'_{c,a,s,t} w''_{c,a,s,t})}{\sum_{c \neq c_0} (w'_{c,a,s,t} w''_{c,a,s,t})} \qquad \forall c \neq c_0 \cap\ R[c] \neq R[c_0] \cap\ SR[c] \neq SR[c_0]$$

Observations could be down-weighted by a factor of 0.1, usually because they were not geographically representative at the unit of estimation. Details of reasons for down-weighting can be found in risk-specific modeling summaries. The final weights were then normalised such that the sum of weights across age, time, and geographical hierarchy for a reference group was 1.

Estimating error variance

σ_p^2 represents the error variance in normal or transformed space including the sampling variance of the estimates and prediction error from any crosswalks performed. First, variance was systematically imputed if the data extraction did not include any measure of uncertainty. When some sample sizes for data were available, missing sample sizes were imputed as the fifth percentile of available sample sizes. Missing variances were then calculated as $\sigma_p^2 = \frac{p*(1-p)}{n}$ for proportions or were predicted from the mean by using a regression for continuous values. When sample sizes were entirely missing and could not be imputed, the 95th percentile of available variances at the most granular geographical level (ie, first country, then region, etc.) were used to impute missing variances. For proportions where *p*n* or *(1-p)*n* is <20, variance was replaced by using the Wilson Interval Score method.

Next, if the exposure was modelled as a log transformation, the error variance was transformed into log-space by using the delta method approximation as follows:

$$\sigma_p^2 \cong \frac{\sigma_{p'}^2}{p_{c,a,s,t}^2}$$

where $\sigma_{p'}^2$ represents the error variance in normal space. If the exposure was modelled as a logit transformation, the error variance was transformed into logit-space by using the delta method approximation as follows:

$$\sigma_p^2 \cong \frac{\sigma_{p'}^2}{(p_{c,a,s,t} * (1 - p_{c,a,s,t}))^2}$$

Finally, prior to GPR, an approximation of non-sampling variance was added to the error variance. Calculations of non-sampling variance were done on normal-space variances. Non-sampling variance was calculated as the variance of inverse-variance-weighted residuals from the space-time estimate at a given location-level hierarchy. If there were <10 datapoints at a given level of the location hierarchy, the non-sampling variance was replaced with that of the next highest geography level with >10 datapoints.

Estimating the covariance function

The final input into GPR is the covariance function, which defines the shape and distribution of the trends. Here, we have chosen the Matern-Euclidian covariance function, which offers the flexibility to

model a wide spectrum of trends with varying degrees of smoothness. The function is defined as follows:

$$M(t,t') = \sigma^2 \frac{2^{1-\nu}}{\Gamma(\nu)} \left(\frac{d(t,t')\sqrt{2\nu}}{l}\right)^{\nu} K_{\nu}\left(\frac{d(t,t')\sqrt{2\nu}}{l}\right)$$

where $d(\cdot)$is a distance function; σ^2, ν, l, and K_ν are hyperparameters of the covariance function—specifically σ^2 is the marginal variance, ν is the smoothness parameter that defines the differentiability of the function, l is the length scale, which roughly defines the distance between which two points become uncorrelated, and K_ν is the Bessel function. We approximated σ^2 by taking the normalised median absolute deviation $MADN(r_c')$ of the difference, which is the normalised absolute deviation of the difference of the first-stage linear regression estimate from the second-stage spatiotemporal smoothing step for each country. We then took the mean of these country-level MADN estimates for all countries with 10+ country-years of data to ensure that differences between first- and second-stage estimates had sufficient data to truly convey meaningful information on model uncertainty. We used the parameter specification $\nu = 2$ for all models. The scale parameter l used for each risk is reported in appendix section 4.

Prediction using GPR

We integrated over $g_{c,t}(t_*)$ to predict a full time series for country c, age a, sex s, and prediction time t_*as follows:

$$p_{c,a,s}(t_*) \sim N\left(m_{c,a,s,t}(t_*), \sigma_p^2 I + Cov\left(g_{c,a,s,t}(t_*)\right)\right)$$

Random draws of 500 samples were obtained from the distributions above for every country for a given indicator. The final estimated mean for each country was the mean of the draws. In addition, 95% UIs were calculated by taking the 2.5 and 97.5 percentile of the sample distribution. The linear modelling process was implemented by using the lmer4 package in R, and the ST-GPR analysis was implemented through the PyMC2 package in Python.

Subnational scaling and aggregation

To ensure internal consistency of the estimates between countries and their respective subnational locations, national estimates were either created by population-weighted aggregation or subnational estimates were adjusted by population-weighted scaling to the national estimates, depending on the data coverage of a given country compared to that of its subnational locations. For example, if data coverage was better at the national level than at its corresponding subnational locations for a given country and risk across age, sex, and time, estimates were rescaled to be consistent with the national level. Conversely, if data coverage was better at the subnational level, estimates for its parent country were generated through population-weighted aggregation of subnational estimates.

Estimates can also be scaled within logit space. Scaling in logit space ensures that subnational estimates of proportion models do not exceed 1 after being rescaled to the national estimate.

Fitting a distribution to exposure data

The most informative data describing the distribution of risk factors within a population come from individual-level data; additional sources of data include reported means and variances. In cases in which a risk factor also defines a disease or disease severity cut-off, such as haemoglobin level and mild,

moderate, or severe anaemia, or diabetes and FPG level, the prevalence of disease is also frequently reported. To model the distribution of any particular risk factor, we seek a family of probability density functions (PDF), a fitting method, and a model selection criterion. To make use of the most commonly available data describing most populations, we used the method of moments (MoM); the first two empirical moments from a population, the mean and the variance, were used to determine the parameters of two-parameter PDF describing the distribution of risk within any population. Exceptions to this rule are justified by context. We used the Kolmogorov-Smirnov[39] (KS) test to measure the goodness of fit (GoF) and compared the distance between the empirical and ensemble distributions, but in some cases, the GoF was based on the prediction error for the prevalence of disease.

We used an ensemble technique in which a model selection algorithm is used to choose the best model for each continuous risk factor.[39] We drew the initial set of candidate models from commonly used PDF families, including both right-skewed and left-skewed distributions. These included beta, exponential, gamma, gumbel, inverse gamma, inverse Weibull, log-logistic, lognormal, mirrored gamma, mirrored gumbel, normal, and Weibull. We fitted each PDF family candidate to each dataset by using the MoM and used the KS test as the measure of GoF. Preliminary analysis showed that the GoF ranking of PDF families varied across datasets for any particular risk factor and that combining the predictions of differently fitted PDF families could dramatically improve the GoF for each dataset. Therefore, we developed a new model for prediction by using the ensemble of candidate models, which is a weighted linear combination of all candidate models, $\{f\}$, where a set of weights $\{w\}$ is chosen such that $\sum_i w_i = 1$, and the values of the weights were determined by a second GoF criterion with its own validation process. For each risk, we pooled all available microdata and performed Nelder-Mead numeric optimisation across demographics subsets of data to derive a set of distribution-specific weights such that the average KS statistic across datasets would be minimised. The details can be summarised by 1) the summary statistics for each dataset; 2) a table showing the KS statistic for each candidate model; and 3) the weights defining the final ensemble model for each dataset. We then averaged across demographic subsets and datasets to determine the final weights for modelling the distribution of any particular risk factor.

Step 3. TMREL[5]

In this and all previous GBD studies, the counterfactual level of risk exposure used is the risk exposure that is both theoretically possible and minimises risk in the exposed population that consequently captures the maximum population attributable burden.[9] For each risk evaluated in GBD 2023, Step 3 in the analytical flowchart involves taking the best available epidemiological evidence used to estimate relative risk by level of exposure and the lowest observed level of exposure from cohorts. This is used to identify a single level of risk exposure that minimises risk from all causes of death combined to the establish the TMREL. In principle, the TMREL for a given risk may vary by age, sex, and location if supported by clear evidence. Based on the available evidence, the TMREL itself can be uncertain, which is reflected in the 95% UIs in table S4.

Step 4. Estimate population attributable fractions[5]

Risks are categorised on the basis of how exposure was measured: dichotomous, polytomous, and continuous. High low-density lipoprotein (LDL) cholesterol level is an example of a risk measured on a continuous scale. The PAF, which represents the proportion of risk that would be reduced in a given year

if the exposure to a risk factor in the past were reduced to an ideal exposure scenario, is defined for a continuous risk factor as:[40]

$$PAF_{joasgt} = \frac{\int_{x=l}^{u} RR_{joasg}(x)P_{jasgt}(x)dx - RR_{joasg}(TMREL_{jas})}{\int_{x=l}^{u} RR_{joasg}(x)P_{jasgt}(x)dx}$$

Where PAF_{joasgt} is the PAF for cause o due to risk factor j for age group a, sex s, location g, and year t. $RR_{joasg}(x)$ is the RR as a function of exposure level x for risk factor j for cause o, age group a, sex s, and location g with the lowest level of observed exposure as l and the highest as u; $P_{jasgt}(x)$ is the distribution of exposure at x for age group a, sex s, location g, and year t; and $TMREL_{jas}$ is the TMREL for risk factor *j*, age group *a*, and sex *s*.

The PAF_{joasgt} for dichotomous and polytomous risk factors for every country is defined as:

$$PAF_{joasgt} = \frac{\sum_{x=l}^{u} RR_{joasg}(x)P_{jasgt}(x) - RR_{joasg}(TMREL_{jas})}{\sum_{x=l}^{u} RR_{joasg}(x)P_{jasgt}(x)}$$

where PAF_{joasgt} is the PAF for cause o due to risk factor j for age group a, sex s, location g, and year t. $RR_{joasg}(x)$ is the RR as a function of exposure level x for risk factor j for cause o, age group a, sex s, and location g on a plausible range of exposure levels from *l* to *u*; $P_{jasgt}(x)$ is the proportion of the population in risk group (prevalence) for age group a, sex s, location g, and year t; and $TMREL_{jas}$ is the TMREL for risk factor *j*, age group *a*, and sex *s*.

Step 5. Estimate summary exposure values[5]

Summary exposure value (SEV) is the RR-weighted prevalence of exposure, a univariate measure of risk-weighted exposure, taking the value zero when no excess risk for a population exists and the value 1 when the population is at the highest level of risk. We report SEVs on a scale from 0% to 100% on which a decline in SEV indicates reduced exposure to a given risk factor and an increase in SEV indicates increased exposure.

In words, the formula is:

$$\text{SEV} = \frac{\text{average excess relative risk in a population}}{\text{maximum global, all-time excess relative risk}}$$

We first calculate SEVS for risk-cause pairs, rc, using the following equation,

$$SEV_{rc} = \frac{\frac{PAF_{rc}}{1 - PAF_{rc}}}{RR_{max,rc} - 1}$$

for each most-detailed age, sex, location, year, and outcome. PAF is the YLL (except for any outcomes which are YLD only and thus use the YLD) PAF. RR_{max} for categorical risks is the RR of the exposure category with the highest RR. For continuous risks, the RRmax is the RR at the 5th exposure percentile if the risk is protective, and at the 95th exposure percentile if the risk is harmful. For custom modelled

risks like ambient particulate matter pollution, HAP from solid fuels, ozone pollution, alcohol, smoking, and bullying, the modeller provides draws of RR_{max}.

Generally, RRs do not vary across time and space. However, exceptions exist, such as risks from secondhand smoke (SHS) for which the RR is based on the integrated exposure response (IER) curve. In these cases, the RR is averaged across location and year to ensure no time or space variation. If the PAF is negative, which signifies a protective effect for that outcome, the PAF is set to 0 and the SEV is then also 0 because the SEV is univariate and constrained to be a value between 0 and 1.

In most cases, risk – cause PAFs of 1 were not included in SEV calculations as the SEV function is undefined when the PAF value is 1. However, an alternate definition of SEV was used for a select set of risks for PAFs of 1: fasting plasma glucose, systolic blood pressure, and iron deficiency. For fasting plasma glucose, SEVs were set to risk prevalence above 7 mmol/L. For systolic blood pressure, SEVs were set to risk prevalence above 140 mmHg. Lastly, for iron deficiency, SEVs were set to the prevalence of moderate or severe anaemia.

Once we obtained a set of risk-cause-specific SEVs at the most-detailed risk, cause, age, sex, and location for all years, we averaged across causes to produce the final risk-specific SEV_r,

$$SEV_r = \frac{1}{N(c)} \sum_{c} SEV_{rc}$$

where $N(c)$is the total number of outcomes for a risk.

Step 6. Mediation[5]

Section 2.6.1: Summary

The portion of the burden of disease that is attributable to various combinations of risk factors or to all risk factors combined has been a topic of broad interest.[41] In GBD 2010, we only aggregated the burden of risk factors for some clusters of risks, including access to improved water and sanitation, child and maternal malnutrition, tobacco smoking, alcohol use, dietary risk factors, occupational risk factors, and sexual abuse and violence. We did not aggregate air pollution and metabolic risk factors. For GBD 2013 onward, we aggregated all risk factors into three large categories—behavioural, environmental and occupational, and metabolic risks—and aggregated all GBD risk factors into a single attributable fraction for each disease and eventually for all causes of burden. Please note that mediation is conducted as a separate process and is not part of the BoP methodology. In our relative risk estimation, we include RRs that do not adjust for mediation as our goal is to capture the direct effect of a risk factor on an outcome.

Aggregating risk factors at different levels shares three essential challenges:

1. Risk factor coexistence or aggregation: for example, metabolic risk factors often occur together, or high-risk behaviours such as drug abuse and unsafe sex are related.
2. Mediation: a risk factor may affect another risk factor that lies in the physiological pathway to a disease outcome. It can be inside a cluster of risk factors, such as the effect of obesity through an increase in FPG level and later cardiovascular disease (CVD) outcomes, or between clusters of risk factors, such as the effect of fibre on cholesterol.

3. The formula used to calculate the aggregated PAF.

The aggregation method is conceptually applicable to other aggregations such as socioeconomic factors, education, homelessness, and refugee status that are being considered for inclusion in future GBD iterations. In the next section, we explain our approach to dealing with these challenges.

There are three patterns of associations between risk factors to consider (Figure C). The first concerns confounding; risk B affects risk A and outcome C (Pattern 1 in *Patterns of associations between risk factors*). In these cases, the RR for A should be adjusted for B; for example, the fruit RR is adjusted for smoking. If part of the effect of A is through B, a mediator, we do not adjust the effect of A for B. For example, we do not adjust the RR of body-mass index (BMI) for cholesterol because cholesterol lies in the biological pathway between BMI and cardiovascular outcomes (Pattern 2 in *Patterns of associations between risk factors*). The third pattern occurs when risks A and B are proxies of a third variable Z and aggregation aims to estimate the total effect of a latent variable Z on C. An example is child growth failure, which is measured by stunting, wasting, and underweight as proxies.

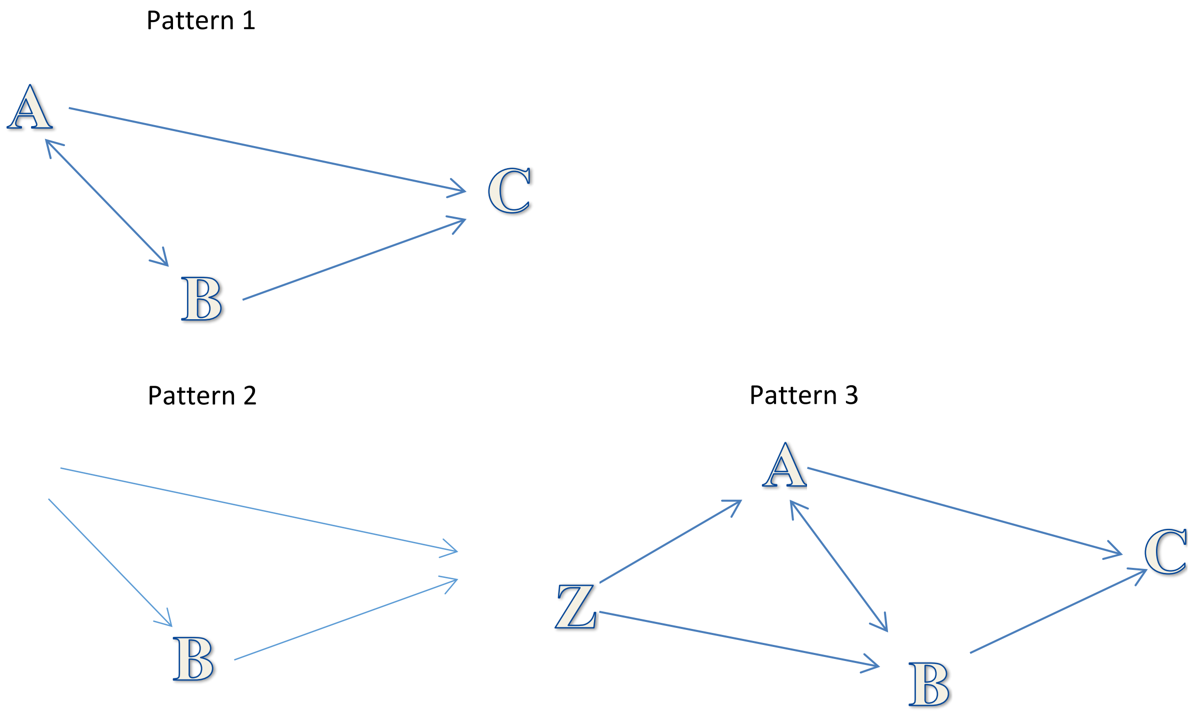

Figure C. Patterns of associations between risk factors

Section 2.6.2: Calculating the burden of multiple risk factors

Validation studies have reported congruency between the true risk associated with multiple risk factors affecting the same outcome and a multiplicative aggregation of the PAFs of the individual risk factors (formula below)[42]

$$PAF_{1..i} = 1 - \prod_{i=1}^{n}(1 - PAF_i)$$

where *PAF* is the population attributable fraction and *i* is each individual risk factor.

The same validation studies also found that the overestimation from ignoring the covariance between risk factors is small. This small overestimation was important to note because few data sources exist from which we can draw information on covariance.

We endeavoured to evaluate RRs that were controlled for confounders. However, because we had to rely on the literature for many RRs, we did not always have full control over the choice of confounders controlled for in each study.

Section 2.6.3: Computing mediation factors using linear relationships

If the relationship between the distal risk and the mediating risk factor is linear, eg, an increase in BMI of 1 kg/m^2 leads to an increase in FPG of $\delta_{B,A}$ mmol/L, we can use the linear relationship to estimate the mediated risk and hence the mediation factor. Specifically, the relative risk of C due to A mediated by B is computed as follows:

$$RR_{C,A}^{B} = \int_{b_{start}}^{b_{end}} \frac{1}{RR_{C,B}(b)} R_{C,B}\left(\delta_{B,A}(a - a_0)_{(+)}\right)p(b)db$$

The linear factor $\delta_{B,A}$ may be available from the literature or may be found using root finding in certain cases, as for example when the cause is defined purely by mediator exposure, such as for diabetes.

Section 2.6.4: Adjusting for mediation

When aggregating the effects of multiple risk factors, we included a mediation factor (MF) if a part of the effect of one risk factor was included in the effect estimated for in the mediator. First, we prepared a list of possible mediations, and especially between behavioural risks and metabolic risk factors with cardiometabolic outcomes. We did not assume any mediation effect between risk factors for cancers.

Danaei and colleagues assumed that part of the effect of BMI on ischaemic heart disease (IHD) is through high systolic blood pressure (SBP), cholesterol level, and FPG.[43] The proportion of the BMI effect that can be explained by other metabolic risk factors is the amount of mediation. The difference between the crude RR of BMI on IHD with the RR adjusted for SBP, FPG, and cholesterol level reflects the amount of BMI effect on IHD that is mediated and already included in SBP, FPG, and cholesterol level:

$$MF = \frac{RR_{crude} - RR_{adjusted}}{RR_{crude} - 1}$$

So, to aggregate the PAF of multiple risk factors, we first calculated the part of the excess risk (RR – 1) of every risk factor that is not mediated, re-compute the PAF so that it only includes the non-mediated risk then aggregated PAFs by assuming they are independent.

Therefore, if MF is the mediation factor of R2 through R1, the adjusted RR for R2 including only the non-mediated component of risk is:

$$RR_{1,2} = MF_{2/1}(RR_2 - 1) + 1$$

The PAF accounting for mediation is then computed using the adjusted RR and the joint PAF computed as detailed in Section 2.7.2. For every paired risk factor and outcome, the matrix of possible mediations was calculated and used.

Section 2.6.5: Calculating mediation factor

1 – Comparing crude RR versus mediator-adjusted RR

The best example is the mediation of BMI through SBP, FPG, and cholesterol level reported by Danaei et al.[43] In their meta-analysis, they report the adjusted and unadjusted RR of BMI on IHD and stroke based on combined data from individual cohorts. They calculated the MF by using the following equation, and we used it directly as the MF in risk factor aggregation. Using individual-level data from cohort studies, we estimated the MF for other metabolic risk factors and some dietary risks.

$$MF = \frac{RR_{crude} - RR_{adjusted}}{RR_{crude} - 1}$$

2 – Estimating the mediation factor by pathway of the effect

For many other risk factors, no data are available to enable the use of the first method. Instead, we searched studies to estimate the effect of the risk factor on the mediator and, finally, the expected increase in IHD risk. We pooled available studies to calculate the unit increase in the mediator per unit increase in the risk factor to calculate the size of the IHD RR (Figure D).

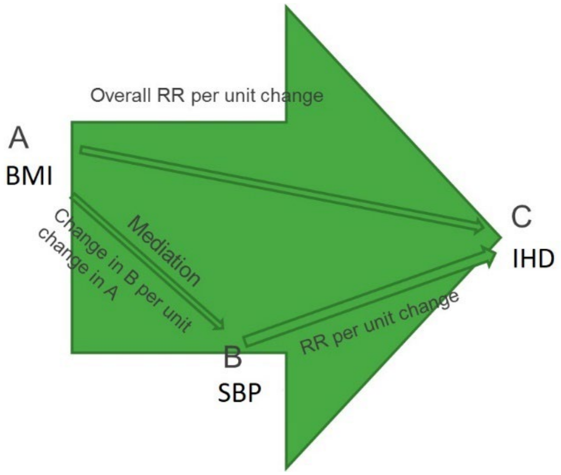

Figure D. Example of pathway between BMI, high systolic blood pressure, and ischemic heart disease

We have RRs for the effect of A on C and B on C in GBD from a meta-analysis of studies in the literature. The effect of A on B was estimated by analysis of trials.

$$RR_{ABC} = {RR_{BC}}^{\Delta_{AB}}$$

RR_{ABC} is the expected effect of A through B on C

RR_{BC} is the RR of each unit increase in mediator on outcome C

Δ_{AB} is the change in mediator level B per each unit change in A

If RR_{AB} is the overall effect of A on B, then:

$$MF = \frac{RR_{ABC} - 1}{RR_{AB} - 1}$$

We kept the uncertainty of each parameter to a minimum by generating and following 1000 draws of the estimates to calculate 1000 draws of the posterior distribution of the MF. We did not include risk-mediator pairs if the MF was not significant at the 5% level (more than 50 of 1000 draws were negative). We truncated the MF distribution at 1 when the whole effect of the risk factor on the outcome would be assumed to be exerted through the mediator pathway.

Some MFs equalled 1 when the whole effect was calculated through another risk factor (eg, the effect of salt through SBP) or when we assumed other risk factors were sources of the exposure (eg, fibre is provided by consuming fruit, vegetables, and whole grains, and all the beneficial effect of milk on colorectal cancer is mediated through calcium).

Air pollution
In GBD 2019, we considered mediation for particulate matter air pollution and SBP, FPG level, and cholesterol level, but in no case was the evidence strongly supportive. Review of the epidemiological evidence identified several cohort studies that reported increased prevalence and/or risk of hypertension due to long-term exposure to ambient $PM_{2.5}$, and several studies have found elevated SBP due to household solid fuel use as well. Studies of short-term exposure also reported acute elevations in blood pressure. However, there is not consensus as to whether the existing evidence with regard to the effects of long-term exposure is consistent with the current mechanistic understanding of the effect of air pollution exposure on blood pressure, and whether existing cohort studies have properly modelled that exposure.

Assumed mediations
For the risk factors with PAFs of 100%, such as FPG and diabetes, impaired kidney function and chronic kidney disease (CKD), SBP and hypertensive heart disease, alcohol and alcohol use disorders, child underweight and protein-energy malnutrition, child wasting and protein-energy malnutrition, and drug use and drug use disorders, no mediation is needed.

Section 2.6.6: Piecewise aggregation (Pattern 3)
There are three anthropometric indicators that are highly correlated: child underweight, stunting, and wasting, as shown in Figure E in this section. Available RRs for each indicator are not adjusted for the other two because these indicators are highly correlated and most of the burden occurs in an interaction. Estimating the total burden due to child growth failure, a latent variable, is difficult. The three anthropometric indicators are not independent, so the covariance between them should be considered. This consideration was the main reason that GBD 2010 only included child underweight. If covariance between these indicators is significant (as is shown in Figure E), aggregating these indicators by assuming they are independent would overestimate the total burden significantly.

To account for the high degree of correlation between CGF indicators, GBD uses a constrained optimisation method to adjust the observed univariate RRs that come out of the Burden of Proof analysis. First, we created a joint distribution of stunting, underweight, and wasting from a population of children. Second, we generated 500 RR draws for each univariate indicator and severity based on the

Burden of Proof analysis. Third, we altered these univariate RRs for the four causes (diarrhoea, LRI, malaria, and measles) and the two outcomes (mortality and morbidity) based upon interactions among the CGF indicators. An interaction occurs when the effect of one CGF indicator variable (eg, stunting) has a different effect on the outcome depending on the value of another CGF indicator variable (eg, underweight). Interaction terms alter the risk of the outcome among children with more than one indicator of CGF. These interaction terms were extracted from a pooled cohort analysis of all-cause mortality published by McDonald et al.[44] Lastly, we optimised the adjusted relative risks by minimising the error between the observed RRs (generated from the Burden of Proof analysis) and the altered RRs derived from the joint distribution and accounting for the interaction terms while ensuring that no alteration resulted in a previously identified increase in relative risk becoming protective.

For GBD 2021, we made several changes to improve the four main steps of RR adjustment. From GBD 2013 to GBD 2019, a simulated joint distribution of stunting, underweight, and wasting measures was created from the Olofin et al. meta-analysis.[45] Sources in this meta-analysis were cross-sectional Demographic and Health Surveys (DHS).[45] In GBD 2021, we created age-specific joint distributions of stunting, underweight, and wasting measures from 15 longitudinal studies (from 26 locations) in the Ki database.[46] The RR adjustment method was strengthened in GBD 2021 by constraining optimisation in two ways. Optimisation was only permitted to alter the RR for an indicator/severity in draws where the observed RR was greater than 1, and constraints were placed on the error that penalise larger alterations to the RR. These changes enabled the estimation and utilisation of age-specific adjusted RRs for GBD 2021 burden estimation. The largest change for GBD 2021 was conducting Burden of Proof analyses for each cause/outcome/risk triplet using both data from Olofin et al as well as KI data. These changes result in identifying large differences in the relationship between CGF and mortality versus morbidity as well as identifying some impact of CGF on malaria.

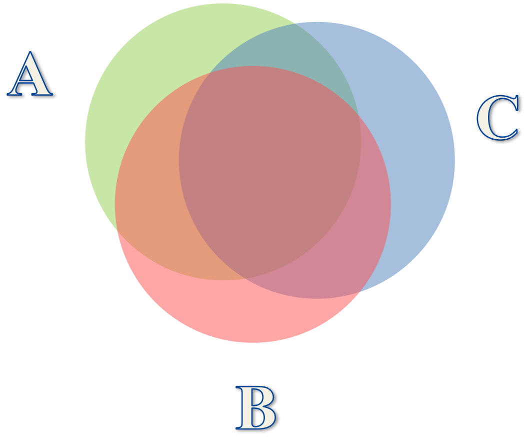

Figure E. Venn diagram demonstrating the correlation between child underweight, stunting, and wasting

After adjusting for the three risk factors, we calculated the PAFs and aggregated the underweight, stunting, and wasting burden.

Section 2.6.7: Uncertainty of aggregated and mediated PAFs

We generated 1000 draws of the posterior distribution of the MF calculated by different methods to use beside draws of other inputs to the PAF aggregation.

Section 2.6.8: Important assumptions in aggregating risk factors and including mediation

1 – The MFs or PAF adjustments are similar across countries, age, sex, and years. Although the size of mediation is probably different in different populations, few data are available to inform the covariance between different risk factors or the MF amount by age and country. For example, in some countries, the size of the mediated BMI-IHD PAF exerted through cholesterol level, as calculated by the MF, was even bigger than the total burden of cholesterol level. This finding indicated that less of the effect of BMI is mediated through cholesterol level and MFs are not similar across countries.

2 – For many risk-mediator-outcome pairs, no data are available, so we assumed the mediation is zero or one.

3 – Because the covariance between undernutrition indicators differs by location (and across time, but results were not reported), and an interaction exists between these indicators, the total burden might be underestimated.

4 – We assumed no significant covariance between PAFs, which might not be true between some risk factors, such as metabolic risk factors. Although this overestimation can be controlled by using adjusted RRs, using crude RRs for BMI and other metabolic risk factors may cause significant overestimation of the aggregated metabolic risks burden.

Step 7. Estimate attributable burden[5]

Four key components are included in the estimation of the burden attributable to a given risk factor: the metric of burden being assessed (the number of deaths, YLLs, YLDs, or DALYs [the sum of YLLs and YLDs]); the exposure levels for a risk factor; the RR of a given outcome due to exposure; and the counterfactual level of risk factor exposure. Uncertainty intervals for attributable burden were calculated using 250 draws for GBD 2023. Estimates of attributable burden as DALYs for risk–outcome pairs were generated by using the following model:

$$AB_{jasgt} = \sum_{o=1}^{w} DALY_{joasgt}\, PAF_{joasgt}$$

where AB_{jasgt} is the attributable burden for risk factor j for age group a, sex s, location g, and year t; $DALY_{joasgt}$ is total DALYs for cause o (of w relevant outcomes for risk factor j) for age group a, sex s, location g, and year t; and PAF_{joasgt} is the PAF for cause o due to risk factor j for age group a, sex s, location g, and year t. The proportions of deaths, YLLs, or YLDs attributable to a given risk factor or risk factor cluster were analogously computed by sequentially substituting each metric in place of DALYs in the equation provided.

Section 3: References

1 Stevens GA, Alkema L, Black RE. Guidelines for Accurate and Transparent Health Estimates Reporting: the GATHER statement. *Lancet* 2016.

2 Lim SS, Vos T, Flaxman AD. A comparative risk assessment of burden of disease and injury attributable to 67 risk factors and risk factor clusters in 21 regions, 1990–2010: a systematic analysis for the Global Burden of Disease Study 2010. *The Lancet* 2012; **380**: 2224–60.

3 Forouzanfar M, Afshin A, Alexander LT, Anderson H, Bhutta Z, Murray CJL. Global, regional, and national comparative risk assessment of 79 behavioural, environmental and occupational, and metabolic risks or clusters of risks, 1990-2015: a systematic analysis for the Global Burden of Disease Study 2015. *Lancet* 2016; **388**: 1659–724.

4 Gakidou E, Afshin A, Abajobir AA, *et al.* Global, regional, and national comparative risk assessment of 84 behavioural, environmental and occupational, and metabolic risks or clusters of risks, 1990–2016: a systematic analysis for the Global Burden of Disease Study 2016. *The Lancet* 2017; **390**: 1345–422.

5 Stanaway JD, Afshin A, Gakidou E, *et al.* Global, regional, and national comparative risk assessment of 84 behavioural, environmental and occupational, and metabolic risks or clusters of risks for 195 countries and territories, 1990–2017: a systematic analysis for the Global Burden of Disease Study 2017. *The Lancet* 2018; **392**: 1923–94.

6 Murray CJL, Aravkin AY, Zheng P. Global burden of 87 risk factors in 204 countries and territories, 1990–2019: a systematic analysis for the Global Burden of Disease Study 2019. *The Lancet* 2020; **396**: 1223–49.

7 GBD 2021 Risk Factors Collaborators. Global burden and strength of evidence for 88 risk factors in 204 countries and 811 subnational locations, 1990-2021: a systematic analysis for the Global Burden of Disease Study 2021. *Lancet* 2024; **403**: 2162–203.

8 Murray CJ, Lopez AD. Global mortality, disability, and the contribution of risk factors: Global Burden of Disease Study. *Lancet* 1997; **349**: 1436–42.

9 Murray CJ, Lopez AD. On the comparable quantification of health risks: lessons from the Global Burden of Disease Study. *Epidemiology* 1999; **10**: 594–605.

10 Food, nutrition, physical activity and the prevention of cancer: a global perspective. Washington, D.C: World Cancer Research Fund & American Institute for Cancer Research, 2007.

11 Zheng P, Barber R, Sorensen RJ, Murray CJ, Aravkin AY. Trimmed constrained mixed effects models: formulations and algorithms. *Journal of Computational and Graphical Statistics* 2021; : 1–13.

12 Zheng P. limetr: limetr: linear mixed effects model with trimming. https://github.com/zhengp0/limetr (accessed July 28, 2021).

13 Zheng P. xspline: xspline: Advanced spline tools. https://github.com/zhengp0/xspline (accessed July 28, 2021).

14 Viechtbauer W. Conducting meta-analyses in R with the metafor package. *Journal of statistical software* 2010; **36**: 1–48.

15 de Boor C. A practical guide to splines (applied mathematical sciences, 27). New York: Springer, 2001 https://link.springer.com/book/9780387953663 (accessed April 4, 2022).

16 Rousseeuw PJ, Leroy AM. Robust regression and outlier detection. John wiley & sons, 2005.

17 Rousseeuw PJ. Least median of squares regression. *Journal of the American Statistical Association* 1984; **79**: 871–80.

18 Huber PJ. Robust Statistics. John Wiley & Sons, 2004.

19 Rousseeuw P. Multivariate estimation with high breakdown point. *Mathematical Statistics and Applications Vol B* 1985; : 283–97.

20 Aravkin A, Davis D. Trimmed statistical estimation via variance reduction. *Mathematics of Operations Research* 2020; **45**: 292–322.

21 Motzkin TS, Raiffa H, Thompson GL, Thrall RM. 3. The Double Description Method. In: 3. The Double Description Method. Princeton University Press, 2016: 51–74.

22 Guyatt GH, Oxman AD, Vist G, *et al.* GRADE guidelines: 4. Rating the quality of evidence—study limitations (risk of bias). *Journal of clinical epidemiology* 2011; **64**: 407–15.

23 Efron B, Hastie T, Johnstone I, Tibshirani R. Least angle regression. *The Annals of statistics* 2004; **32**: 407–99.

24 Tibshirani R. Regression shrinkage and selection via the lasso. *Journal of the Royal Statistical Society: Series B (Methodological)* 1996; **58**: 267–88.

25 Kontopantelis E, Springate DA, Reeves D. A re-analysis of the Cochrane Library data: the dangers of unobserved heterogeneity in meta-analyses. *PloS one* 2013; **8**: e69930.

26 Ioannidis JP, Patsopoulos NA, Evangelou E. Uncertainty in heterogeneity estimates in meta-analyses. *Bmj* 2007; **335**: 914–6.

27 Biggerstaff BJ, Tweedie RL. Incorporating variability in estimates of heterogeneity in the random effects model in meta-analysis. *Statistics in medicine* 1997; **16**: 753–68.

28 Higgins JP, Thompson SG. Quantifying heterogeneity in a meta-analysis. *Statistics in medicine* 2002; **21**: 1539–58.

29 Sterne JA, Egger M. Chapter 6: Regression methods to detect publication and other bias in meta-analysis. In: Rothstein H, Sutton A, Borenstein M, eds. Publication bias in meta-analysis: Prevention, assessment and adjustments. John Wiley & Sons, Ltd, 2005.

30 Egger M, Smith GD, Schneider M, Minder C. Bias in meta-analysis detected by a simple, graphical test. *BMJ* 1997; **315**: 629–34.

31 Burkart KG, Brauer M, Aravkin AY. Estimating the cause-specific relative risks of non-optimal temperature on daily mortality: a two-part modelling approach applied to the Global Burden of Disease Study. *The Lancet* 2021; **398**: 685–97.

32 Harris PA, Taylor R, Thielke R, Payne J, Gonzalez N, Conde JG. Research Electronic Data Capture (REDCap) - A metadata-driven methodology and workflow process for providing translational research informatics support. *J Biomed Inform* 2009; **42**: 377–81.

33 GBD 2015 Diseases and Injury Incidence and prevalence Collaborators. Global, regional, and national incidence, prevalence, and years lived with disability (YLDs) for 310 acute and chronic diseases and injuries, 1990-2015: a systematic analysis for the Global Burden of Disease Study 2015. *The Lancet Under review*.

34 Flaxman AD, Vos T, Murray CJL, Kiyono P, editors. An integrative metaregression framework for descriptive epidemiology, 1 edition. Seattle: University of Washington Press, 2015.

35 Vasudevan S, Ramos F, Nettleton E, Durrant-Whyte H, Blair A. Gaussian Process modeling of large scale terrain. In: 2009 IEEE International Conference on Robotics and Automation. 2009: 1047–53.

36 Rasmussen CE, Williams CKI. Gaussian Processes for Machine Learning. Cambridge, Mass: The MIT Press, 2005.

37 Ng M, Fleming T, Robinson M, *et al.* Global, regional, and national prevalence of overweight and obesity in children and adults during 1980–2013: a systematic analysis for the Global Burden of Disease Study 2013. *Lancet* 2014; **384**: 766–81.

38 Ng M, Freeman MK, Fleming TD, *et al.* Smoking Prevalence and Cigarette Consumption in 187 Countries, 1980-2012. *JAMA* 2014; **311**: 183–92.

39 Massey Jr FJ. The Kolmogorov-Smirnov test for goodness of fit. *Journal of the American statistical Association* 1951; **46**: 68–78.

40 Vander Hoorn S, Ezzati M, Rodgers A, Lopez AD, Murray CJL. Estimating attributable burden of disease from exposure and hazard data. In: Comparative Quanitifcation of Health Risks: Global and regional burden of disease attribution to selected major risk factors. World Health Organisation, 2004: 2129–40.

41 Preston SH. Causes and Consequences of Mortality Declines in Less Developed Countries during the Twentieth Century. In: Population and economic change in developing countries. Chicago: Univ. of Chicago Pr, 1980: 289–360.

42 Carnahan E, Lim SS, Nelson EC, *et al.* Validation of a new predictive risk model: measuring the impact of major modifiable risks of death for patients and populations. *The Lancet* 2013; **381**: S26.

43 Danaei G, Singh GM, Paciorek CJ, *et al.* The global cardiovascular risk transition: associations of four metabolic risk factors with national income, urbanization, and Western diet in 1980 and 2008. *Circulation* 2013; **127**: 1493–502, 1502e1-8.

44 McDonald CM, Olofin I, Flaxman S. The effect of multiple anthropometric deficits on child mortality: meta-analysis of individual data in 10 prospective studies from developing countries. *Am J Clin Nutr* 2013; **97**: 896–901.

45 Olofin I, McDonald CM, Ezzati M, et al. Associations of suboptimal growth with all-cause and cause specific mortality in children under five years: a pooled analysis of ten prospective studies. *PloS ONE*; **8**: e64636.

46 Ki Global Health. (n.d.). https://www.kiglobalhealth.org/.

Section 4: GBD 2023 risk factor-specific modelling descriptions

1. Ambient nitrogen dioxide pollution
2. Ambient ozone pollution
3. Ambient particulate matter pollution
4. Bullying victimisation
5. Chewing tobacco
6. Child growth failure
7. Dietary risks
8. Drug use
9. High alcohol use
10. High body-mass index
11. High fasting plasma glucose
12. High LDL cholesterol
13. High systolic blood pressure
14. Household air pollution
15. Intimate partner violence
16. Iron deficiency
17. Kidney dysfunction
18. Lead exposure
19. Low birthweight and short gestation
20. Low bone mineral density
21. Low physical activity
22. No access to handwashing facility
23. Non-optimal temperature
24. Occupational risk factors
25. Residential radon pollution
26. Secondhand smoke
27. Sexual violence against children
28. Smoking
29. Suboptimal breastfeeding
30. Unsafe sanitation
31. Unsafe sex
32. Unsafe water
33. Vitamin A deficiency
34. Zinc deficiency

Ambient nitrogen dioxide pollution

Flowchart

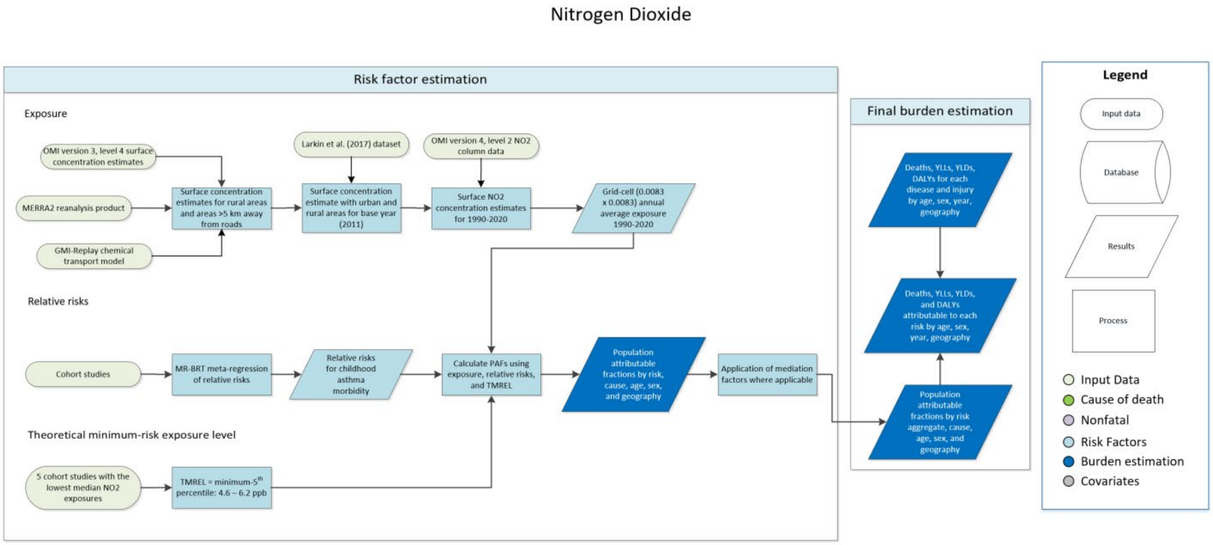

Input data and methodological summary

Exposure

Definition

Exposure to nitrogen dioxide (NO_2) pollution is defined as the population-weighted annual average ambient concentration of NO_2 gas measured in parts per billion (ppb).

Input data

The NO_2 exposure modelling process for the Global Burden of Disease (GBD) Study 2023 combines multiple and varied input data sources. These include ground measurements, satellite column measurements and satellite-based surface concentration estimates, land-use regression-based surface concentration estimates, urbanicity data, and population estimates.

Land-use regression estimates

The first NO_2 exposure model input was a global NO_2 pollution surface concentration dataset published in 2017 by Larkin and colleagues, hereafter referred to as the Larkin et al. (2017) NO_2 dataset.[1] This dataset estimates a three-year average annual NO_2 concentration from the years 2010, 2011, and 2012. Land-use regression modelling was used to produce this dataset, with data inputs from road networks, other land use variables, ground measurements of NO_2, and satellite NO_2 column observations from the SCIAMACHY and GOME-2 satellite products (Larkin et al., 2017; Geddes et al., 2016).

Satellite-based and chemical transport model products

Satellite-based and chemical transport model inputs include the OMI NO_2 version 3, level 4 surface concentration dataset, MERRA2 reanalysis product (satellite-derived surface concentration estimates), GMI-Replay chemical transport model, and OMI version 4.0 level 2 annual average NO_2 column product. The OMI version 3, level 4 surface concentration estimates were derived from OMI satellite instrument NO_2 column observations following methods described by Lamsal et al., 2008 and were obtained from

the NASA Goddard Space Flight Center (GSFC).[2] Though the newer version 4 OMI retrieval uses enhanced surface reflectivities in the calculation of the tropospheric column amounts, surface concentrations prepared by NASA GSFC are not currently available from the version 4 product.

Population and urbanicity data

The Global Human Settlement Model grid (Pesaresi et al. 2019) was used to classify grid cells as "urban" or "rural." Global population data on a high-resolution grid for the years 2000–2020 was obtained from the WorldPop database.[3] These data were provided at a resolution of 0.0083° × 0.0083°, which corresponds to the resolution of the GBD 2023 global NO_2 surface concentration estimates. Population estimates for 1990 and 1995 were estimated through an extrapolation process. For each 0.0083° × 0.0083° grid cell, where the population growth rate for 2000–2005 was positive, the growth rate was applied backward in time to extrapolate prior population concentrations. Where the growth rate was negative, the population value for the year 2000 was used directly for prior years.

Modelling strategy

Surface concentration estimates

The following is a summary of the GBD 2021 global NO_2 exposure estimation approach, developed and implemented by Arash Mohegh, Dan Goldberg, and Susan Anenberg at George Washington University. This approach was retained as part of the GBDF 2023 estimation process with subsequent application of scaling factors for years prior to 2020 as described in more detail below. For GBD 2021, surface annual average NO_2 concentrations were estimated at 1 km x 1 km resolution in five-year increments from 1990 to 2010 and annually from 2010 to 2019. Estimation was first conducted for 2011, the base year available from the Larkin et al. (2017) dataset.

To begin, the Larkin et al. (2017) NO_2 concentration dataset was aggregated from its native 100 m x 100 m resolution globally to 1 km x 1 km grid cells. In a previous study, Mohegh and colleagues determined that 1 km x 1 km is the optimum resolution for NO_2-attributable burden estimation to minimise computational resources while retaining a high enough resolution for accurate estimates.[4] Due to the lack of ground measurements in rural areas, the Larkin et al. (2017) dataset is finely tuned toward urban areas and overestimates NO_2 concentrations in rural areas, likely due to a high sensitivity to the normalised difference vegetation index (NDVI), one of the model's input land-use variables.

To better estimate NO_2 surface concentrations across urban and rural locations, multiple global NO_2 surface concentration estimates were combined. Given its good performance in urban areas, the Larkin et al. (2017) NO_2 dataset was applied in all 1 km x 1 km grid cells globally that are categorised as "urban" according to the Global Human Settlement Model grid.[5] The Larkin et al. (2017) dataset was also used for grid cells situated near major roadways. For grid cells >5 km away from roadways and in urban areas, new NO_2 surface concentration estimates were developed using a combination of satellite-based and chemical transport model products as described below.

For rural areas, NO_2 estimates derived from the OMI satellite instrument were used, with some adjustments to fill spatial and temporal gaps in the OMI satellite record and to estimate 24-hour averages from the early afternoon OMI overpass time. The OMI NO_2 version 3, level 4 surface concentration dataset (0.1° x 0.1° resolution) was the basis for estimating NO_2 annual 2011 concentrations. Due to the lack of satellite dataset coverage over snow- and ice-covered areas, some grid cells, primarily in higher latitudes, were missing OMI observations for some months. The MERRA 2

surface-level reanalysis product (0.625° x 0.625° resolution) was used to generate a set of grid-cell-specific correction factors to ensure availability of NO_2 concentrations in all locations and months. Correction factors were calculated and applied to each grid cell missing complete OMI coverage in 2011 using the following approach:

$$Correction\ factor\ 1 = \frac{MERRA2_{annual\ average}}{MERRA2_{average\ for\ months\ with\ OMI\ level\ 4\ availability}}$$

A second correction factor was applied to convert surface NO_2 concentrations from the early afternoon OMI overpass time (13:00 local time) to 24-hour averages. NO_2 surface concentration estimates from the GMI-Replay chemical transport model simulations (2° x 2.5° resolution) were used to generate a correction factor for each grid cell, following the protocol described by Anenberg and colleagues (2018). These correction factors were calculated as follows:

$$Correction\ factor\ 2 = \frac{GMI_{24-hour\ average}}{GMI_{13:00}}$$

The NO_2 surface concentration estimates used for grid cells >5 km away from roads and in rural areas were then generated using the following formula:

$$Adjusted\ rural\ concentrations = OMI\ level\ 4\ \times Correction\ factor\ 1\ \times Correction\ factor\ 2$$

For rural grid cells within 5 km of major roadways, NO_2 surface concentration estimates were linearly scaled between the Larkin et al. (2017) values and the new adjusted rural concentrations for the span of the 5 km distance.

The methods described above produced a 1 km x 1 km annual average surface NO_2 concentration dataset for 2011 that uses Larkin et al. (2017) values in grid cells categorised as urban or near roadways, and a new surface concentration dataset derived from OMI satellite observations in rural areas and those >5 km away from roadways.

Figure 1: NO_2 surface concentration estimates, 2011

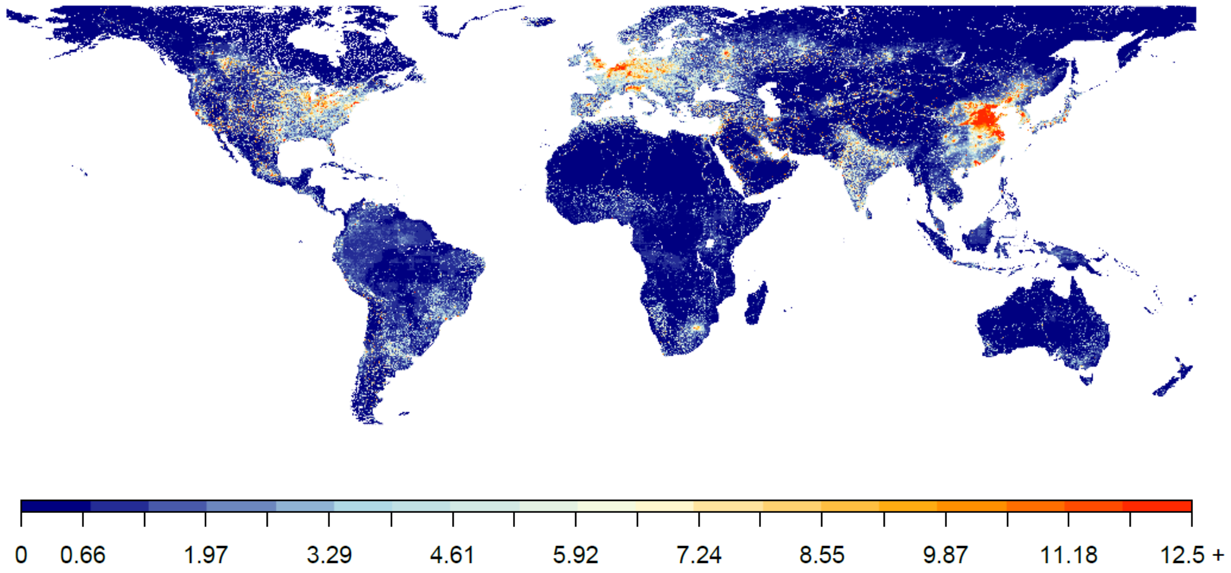

Scaling to GBD estimation years

This 2011 base-year surface NO_2 concentration dataset was then scaled to each year required for GBD burden analysis: 1990–2020 in five-year increments from 1990 to 2005 and annually from 2010 to 2020. For 2010–2018, surface NO_2 concentrations for 2011 were scaled to each year using three-year rolling averages of annual average NO_2 columns from the OMI version 4.0 level 2 product (13 km x 25 km resolution at nadir). NO_2 columns were used because surface concentrations derived from the version 4 OMI retrieval are not yet available. The NO_2 column dataset was oversampled to a 0.1° x 0.1° resolution and regridded to a resolution of 0.0083° x 0.0083° (approximately 1 km x 1 km). Three-year rolling averages were used to remove noise from the satellite data.

Estimates for 2005, 2019, and 2020 were scaled with satellite data from their respective years as described above; however, three-year rolling averages were not used. Instead, each year's NO_2 columns were used directly. 2005 and 2020 lacked sufficient data to create three-year averages, and due to the dramatic changes from the COVID-19 pandemic, 2020 estimates were not used to inform estimates for 2019.

The years 1990, 1995, and 2000 predate the OMI observational record. For these years, the NO_2 concentrations from the MERRA2 reanalysis product were used to scale the 2011 NO_2 surface concentrations to those years.[6] To remove model noise, these scaling factors were created across world regions (Giorgi Regions) as opposed to applying grid-cell-specific scaling factors.[7] These steps resulted in a global 0.0083° x 0.0083° (approximately 1 km x 1 km) resolution dataset of annual average surface NO_2 concentrations from 1990 to 2020.

Updates for GBD 2023

Since GBD 2023, we have integrated updated global land-use regression (LUR) concentration raster, developed by Larkin and colleagues,[11] for the years 2020–2023. To ensure consistency with previous data, we calculated a scaling factor based on the overlap in data availability for 2020 between the new LUR dataset and the dataset used for GBD 2021. This scaling factor was applied to the GBD 2021 inputs for the years 1990, 1995, 2000, and 2005–2019. The rescaled inputs, combined with the new data for 2020–2023, form the basis of our updated exposure modelling.

Figure 2: Updated NO_2 surface concentration estimates for GBD 2023

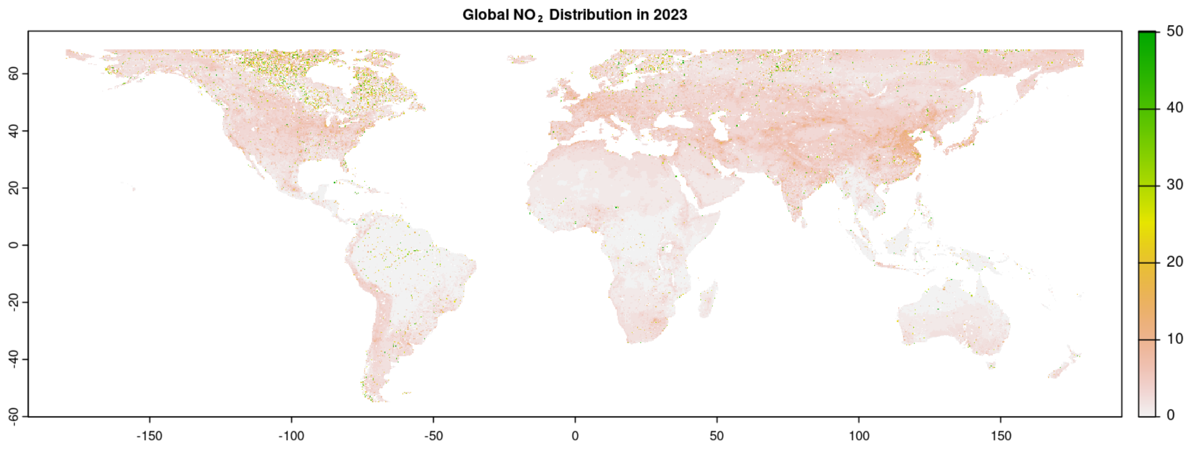

Uncertainty estimation

To incorporate estimates of model uncertainty, evaluation results for the Larkin et al. (2017) NO_2 concentration dataset were used. The mean absolute error (MAE) results in the table below were used to generate distributions of each grid cell's exposure for use in burden assessment. The Larkin et al. (2017) evaluation regions are not equivalent to GBD regions; for the north Africa and the Middle East region, the mean of the Larkin et al. (2017) MAE estimates for Asia and Africa was used. For the central Latin America region, the MAE estimate for South America was used. Due to lack of information on posterior correlation relationships, we chose a conservative approach and assumed complete correlation between deviation estimates when generating exposure distributions for each grid cell.

Table 2: Mean absolute error (ppb) of NO_2 surface concentration estimate for the Larkin et al. (2017) and Larkin et al (2023) dataset by region

Region	Mean absolute error (ppb) Larkin et al. (2017)	Mean absolute error (ppb) Larkin et al. (2017)
North America	3.8	3.1
South America	2.6	3.3
Europe	3.3	2.9
Africa	2.4	2.6
Asia	3.5	3.2
Oceania	2.3	4.0

Theoretical minimum risk exposure level

To calculate population attributable fractions (PAFs), we used a theoretical minimum risk exposure level (TMREL) of a uniform distribution between 4.6 and 6.2 ppb. This TMREL was calculated by imputing the 0.1 and 5th percentiles of study-specific exposure distributions (when the minimum and/or 5th percentile was unreported) for all NO_2 relative risk cohort studies that reported a median and standard deviation. The TMREL bounds are the means of the minimum/0.1 and 5th percentiles for the five studies with the lowest reported median values. A uniform distribution was used instead of a single fixed value to convey uncertainty in scientific literature regarding the level at which no harmful effects of NO_2 pollution exist. This TMREL calculation method is consistent with that used for the other GBD ambient air pollution risk factors, ambient particulate matter pollution and ozone.

Relative risks and population attributable fractions

Input data and systematic review

To assess the dose–response relationship between NO_2 exposure and paediatric asthma, we first extracted the 31 NO_2-related component studies from a recent (2017) and comprehensive meta-analysis of traffic-related air pollution's effects on childhood asthma development conducted by Khreis and colleagues.[8] We then searched PubMed and Embase for updates to relevant literature published from September 9, 2016, to December 19, 2019 (date of search), using the search string below.

Search string: ((((("child*") AND "air pollution") AND "asthma")) OR ((("child*") AND "air quality") AND "asthma")) OR ((("child*") AND "vehicle emissions") AND "asthma")) OR ((("child*") AND "ultra-fine particles") AND "asthma")

We excluded all cross-sectional studies, as this type of study design is less robust. Apart from this exclusion, we employed inclusion and exclusion criteria identical to those used in the Khreis et al., 2017 meta-analysis.

Figure 3: PRISMA diagram for systematic review of nitrogen dioxide pollution and childhood asthma

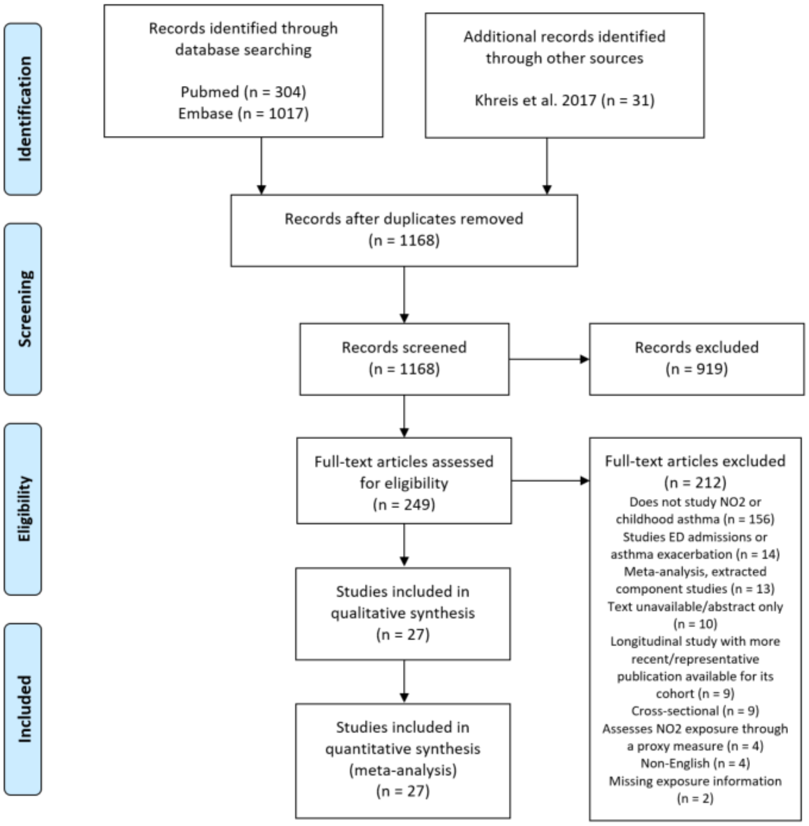

Table 4: Studies included in NO_2-paediatric asthma meta-analysis

Source	Citation
1	Brauer M, Hoek G, Smit HA, de Jongste JC, Gerritsen J, Postma DS, Kerkhof M, Brunekreef B. Air pollution and development of asthma, allergy and infections in a birth cohort. Eur Respir J. 2007; 29(5): 879-88.
2	Carlsten C, Dybuncio A, Becker A, Chan-Yeung M, Brauer M. Traffic-related air pollution and incident asthma in a high-risk birth cohort. Occup Environ Med. 2011; 68(4): 291-5.
3	Clark NA, Demers PA, Karr CJ, Koehoorn M, Lencar C, Tamburic L, Brauer M. Effect of Early Life Exposure to Air Pollution on Development of Childhood Asthma. Environ Health Perspect. 2010; 118(2): 284-90.
4	Clougherty JE, Levy JI, Kubzansky LD, Ryan PB, Suglia SF, Canner MJ, Wright RJ. Synergistic effects of traffic-related air pollution and exposure to violence on urban asthma etiology. Environ Health Perspect. 2007; 115: 1140-6.

5	Dell SD, Jerrett M, Beckerman B, Brook JR, Foty RG, Gilbert NL, Marshall L, Miller JD, To T, Walter SD, Stieb DM. Presence of other allergic disease modifies the effect of early childhood traffic-related air pollution exposure on asthma prevalence. Environ Int. 2014; 65: 83-92.
6	Deng Q, Lu C, Norbäck D, Bornehag CG, Zhang Y, Liu W, Yuan H, Sundell J. Early life exposure to ambient air pollution and childhood asthma in China. Environ Res. 2015; 143: 83-92.
7	Deng Q, Lu C, Ou C, Chen L, Yuan H. Preconceptional, prenatal and postnatal exposure to outdoor and indoor environmental factors on allergic diseases/symptoms in preschool children. Chemosphere. 2016; 152: 459-67.
8	Fuertes E, Standl M, Cyrys J, Berdel D, von Berg A, Bauer CP, Krämer U, Sugiri D, Lehmann I, Koletzko S, Carlsten C, Brauer M, Heinrich J. A longitudinal analysis of associations between traffic-related air pollution with asthma, allergies and sensitization in the GINIplus and LISAplus birth cohorts. PeerJ. 2003; 1: e193.
9	Gehring U, Wijga AH, Hoek G, Bellander T, Berdel D, Brüske I, Fuertes E, Gruzieva O, Heinrich J, Hoffmann B, de Jongste JC, Klümper C, Koppelman GH, Korek M, Krämer U, Maier D, Melén E, Pershagen G, Postma DS, Standl M, von Berg A, Anto JM, Bousquet J, Keil T, Smit HA, Brunekreef B. Exposure to air pollution and development of asthma and rhinoconjunctivitis throughout childhood and adolescence: a population-based birth cohort study. Lancet Respir Med. 2015; 3(12): 933-42.
10	Jerrett M, Shankardass K, Berhane K, Gauderman WJ, Kunzli N, Avol E, Gilliland F, Lurmann F, Molitor JN, Molitor JT, Thomas DC, Peters J, McConnell R. Traffic-Related Air Pollution and Asthma Onset in Children: A Prospective Cohort Study with Individual Exposure Measurement. Environ Health Perspect. 2008; 116(10): 1433-8.
11	Kravitz-Wirtz N, Teixeira S, Hajat A, Woo B, Crowder K, Takeuchi D. Early-Life Air Pollution Exposure, Neighborhood Poverty, and Childhood Asthma in the United States, 19902014. Int J Environ Res Public Health. 2018; 15(6).
12	Krämer U, Sugiri D, Ranft U, Krutmann J, von Berg A, Berdel D, Behrendt H, Kuhlbusch T, Hochadel M, Wichmann HE, Heinrich J, GINIplus and LISAplus study groups. Eczema, respiratory allergies, and traffic-related air pollution in birth cohorts from small-town areas. J Dermatol Sci. 2009; 56(2): 99-105.
13	Lavigne É, Bélair MA, Rodriguez Duque D, Do MT, Stieb DM, Hystad P, van Donkelaar A, Martin RV, Crouse DL, Crighton E, Chen H, Burnett RT, Weichenthal S, Villeneuve PJ, To T, Brook JR, Johnson M, Cakmak S, Yasseen AS 3rd, Walker M. Effect modification of perinatal exposure to air pollution and childhood asthma incidence. Eur Respir J. 2018.
14	Liu W, Huang C, Hu Y, Fu Q, Zou Z, Sun C, Shen L, Wang X, Cai J, Pan J, Huang Y, Chang J, Sun Y, Sundell J. Associations of gestational and early life exposures to ambient air pollution with childhood respiratory diseases in Shanghai, China: A retrospective cohort study. Environ Int. 2016; 92-93: 284-93.
15	McConnell R, Islam T, Shankardass K, Jerrett M, Lurmann F, Gilliland F, Gauderman J, Avol E, Künzli N, Yao L, Peters J, Berhane K. Childhood incident asthma and traffic-related air pollution at home and school. Environ Health Perspect. 2010; 118(7): 1021-6.
16	Morgenstern V, Zutavern A, Cyrys J, Brockow I, Koletzko S, Krämer U, Behrendt H, Herbarth O, von Berg A, Bauer CP, Wichmann HE, Heinrich J, GINI Study Group, LISA Study Group. Atopic diseases, allergic sensitization, and exposure to traffic-related air pollution in children. Am J Respir Crit Care Med. 2008; 177(12): 1331-7.
17	Mölter A, Agius R, de Vocht F, Lindley S, Gerrard W, Custovic A, Simpson A. Effects of long-term exposure to PM10 and NO2 on asthma and wheeze in a prospective birth cohort. J Epidemiol Community Health. 2014; 68(1): 21-8.
18	Nishimura KK, Galanter JM, Roth LA, Oh SS, Thakur N, Nguyen EA, Thyne S, Farber HJ, Serebrisky D, Kumar R, Brigino-Buenaventura E, Davis A, LeNoir MA, Meade K, Rodriguez-Cintron W, Avila PC, Borrell LN, Bibbins-Domingo K, Rodriguez-Santana JR, Sen Ś, Lurmann F, Balmes JR, Burchard EG. Early-life air pollution and asthma risk in minority children. The GALA II and SAGE II studies. Am J Respir Crit Care Med. 2013; 188(3); 309-18.
19	Norbäck D, Lu C, Wang J, Zhang Y, Li B, Zhao Z, Huang C, Zhang X, Qian H, Sun Y, Sundell J, Deng Q. Asthma and rhinitis among Chinese children - Indoor and outdoor air pollution and indicators of socioeconomic status (SES). Environ Int. 2018; 115: 1-8.
20	Oftedal B, Nystad W, Brunekreef B, Nafstad P. Long-term traffic-related exposures and asthma onset in schoolchildren in Oslo, Norway. Environ Health Perspect. 2009; 117(5): 839-44.
21	Ranzi A, Porta D, Badaloni C, Cesaroni G, Lauriola P, Davoli M, Forastiere F. Exposure to air pollution and respiratory symptoms during the first 7 years of life in an Italian birth cohort. Occup Environ Med. 2014; 71(6): 430-6.
22	Sbihi H, Koehoorn M, Tamburic L, Brauer M. Asthma Trajectories in a Population-based Birth Cohort. Impacts of Air Pollution and Greenness. Am J Respir Crit Care Med. 2017; 195(5): 607-613.
23	Shima M, Adachi M. Effect of outdoor and indoor nitrogen dioxide on respiratory symptoms in schoolchildren. Int J Epidemiol. 2000; 29(5): 862-70.

24	Shima M, Nitta Y, Ando M, Adachi M. Effects of air pollution on the prevalence and incidence of asthma in children. Arch Environ Health. 2002; 57(6): 529-35.
25	To T, Zhu J, Stieb D, Gray N, Fong I, Pinault L, Jerrett M, Robichaud A, Ménard R, van Donkelaar A, Martin RV, Hystad P, Brook JR, Dell S. Early Life Exposure to Air Pollution and Incidence of Childhood Asthma, Allergic Rhinitis and Eczema. Eur Respir J. 2019.
26	Tétreault LF, Doucet M, Gamache P, Fournier M, Brand A, Kosatsky T, Smargiassi A. Childhood Exposure to Ambient Air Pollutants and the Onset of Asthma: An Administrative Cohort Study in Québec. Environ Health Perspect. 2016; 124(8): 1276-82.
27	Voros K, Koi T, Magyar D, Rudnai P, Paldy A. The influence of air pollution on respiratory allergies, asthma and wheeze in childhood in Hungary. Minerva Pediatr. 2019.

When extracting data for analysis, we also extracted a set of study-specific covariates for use in quantifying unexplained between-study heterogeneity. These covariates include subpopulation, individual- or population-level exposure, self-reported exposure, exposure measured multiple times throughout the study or only at baseline, unblinded assessment of outcome or exposure, a randomised study design, uncontrolled confounders (age, sex, secondhand smoke, parental asthma/allergy), selection bias, follow-up duration (gold standard data extracted as greater than or equal to 85% follow-up), and controlled for PM (2.5 or 10).

Modelling strategy

Meta-analysis

To assess the summary effect size of NO_2 exposure on childhood asthma, we conducted a meta-analysis using the meta-regression—Bayesian, regularised, trimmed (MR-BRT) tool, a meta-regression tool used across the GBD in the Burden of Proof (BoP) framework.[9] We first tested fitting a third-order spline with three interior knots on the dataset; however, this analysis determined that there is currently insufficient evidence across the global NO_2 exposure range to support a non-linear relationship between NO_2 and childhood asthma. The final model we used was a log-linear meta-analysis weighting each estimated effect size by its inverse standard error and allowing each estimate to inform the meta-analysis between the 5th and 95th percentiles of its study-specific exposure distribution. 10% of all observations were trimmed during model fitting, in accordance with GBD protocol across risk factor teams.

We made two adjustments to prevent a single cohort or study from unduly weighting the final estimate. When multiple individual publications were available for the same cohort, we extracted only the peak RR estimate across follow-up time to account for patterns of asthma incidence and remission across age. Additionally, the standard error of observations from studies with multiple observations for a single cohort reporting an unstratified sample size were multiplied by the square root of n, where n is the total number of observations for a given cohort.

We performed covariate selection to empirically select significant covariates from those extracted to quantify between-study heterogeneity. The MR-BRT automated covariate selection tool implements a two-step process. First, a series of loosening Lasso penalty parameters are applied to a log-linear meta-regression on all input effect size observations. Then, covariates with a non-zero coefficient are tested for significance using a Gaussian prior (significance threshold = 0.05). Significant covariates selected were confounding uncontrolled, selection bias, and self-reported outcome. A Gaussian prior was used on each covariate's beta during curve fitting with a mean 0 and variance of 0.1 multiplied by the standard deviation of the beta from the initial log-linear meta-regression.

1000 predictions of the effect size were generated across the exposure distribution for use in calculating burden estimates. These predictions were created incorporating predictions of between-study heterogeneity and Fisher information. The Fisher scoring correction corrects for data-sparse situations when applied to the heterogeneity parameter. In such cases, the between-study heterogeneity parameter estimate may be 0, simply from lack of data. The Fisher scoring correction uses a quantile of gamma, which is sensitive to the number of studies, study design, and reported uncertainty. The summary relative risk for childhood asthma at 5 ppb NO_2 exposure is 1.082 (95% UI 1.010–1.160) relative to 0 ppb.

Table 5: MR-BRT relative risk model parameters for nitrogen dioxide pollution

Covariate	Gamma (95% UI)	Beta coefficient, log (95% UI)
Exposure (per 1 ppb)	3.536 x 10^{-5} (1.658 x 10^{-5} – 6.455 x 10^{-5})	0.0155 (0.0085 – 0.0224)
Confounding uncontrolled		–1.344 x 10^{-6} (–6.955 x 10^{-4} to 7.007 x 10^{-4})
Selection bias (<85%)		1.201 x 10^{-6} (–6.729 x 10^{-4} to 6.607 x 10^{-4})
Self-reported outcome		7.709 x 10^{-7} (–6.359 x 10^{-4} to 7.156 x 10^{-4})

Figure 4: Nitrogen dioxide pollution and childhood asthma risk literature funnel plot

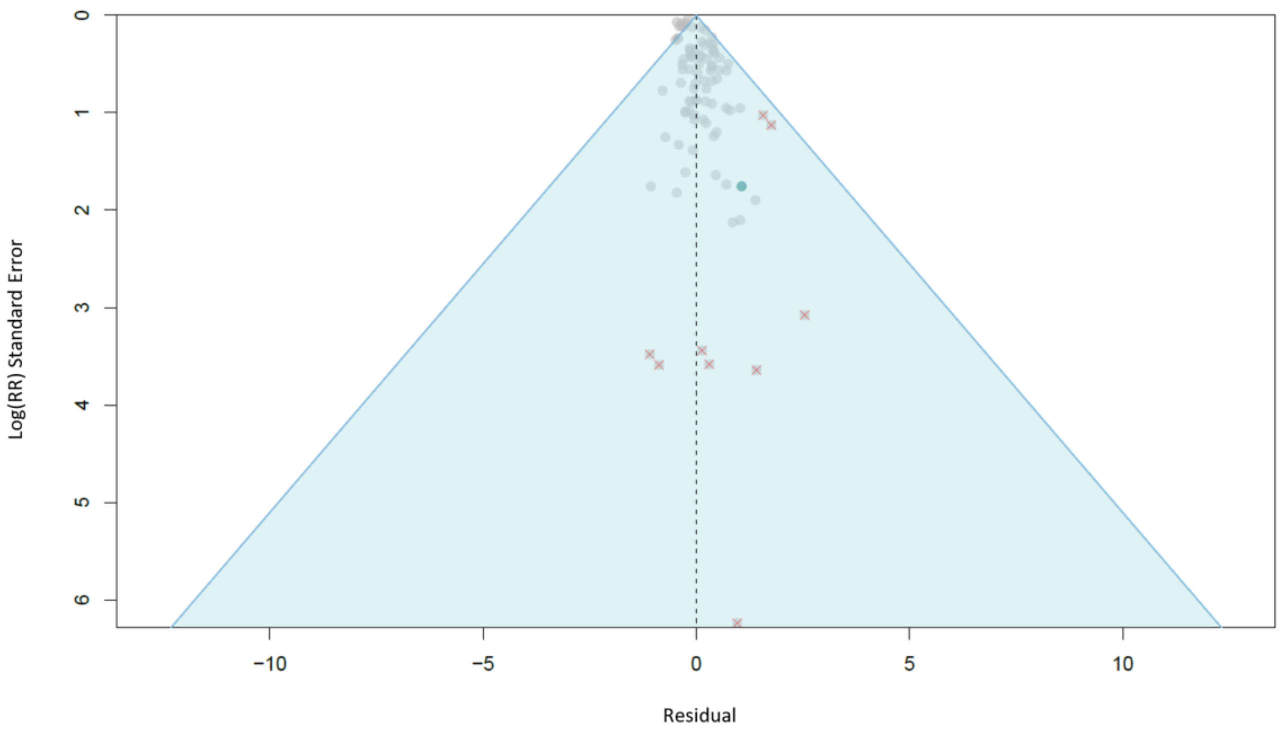

Figure 5: Nitrogen dioxide pollution and childhood asthma log-linear relative risk curve

Each number represents one study effect size. Each study's effect size is plotted at the 95th percentile of its study-specific NO_2 exposure distribution. The relative risk is plotted relative to the predicted relative risk at the 5th percentile of the study-specific exposure distribution.

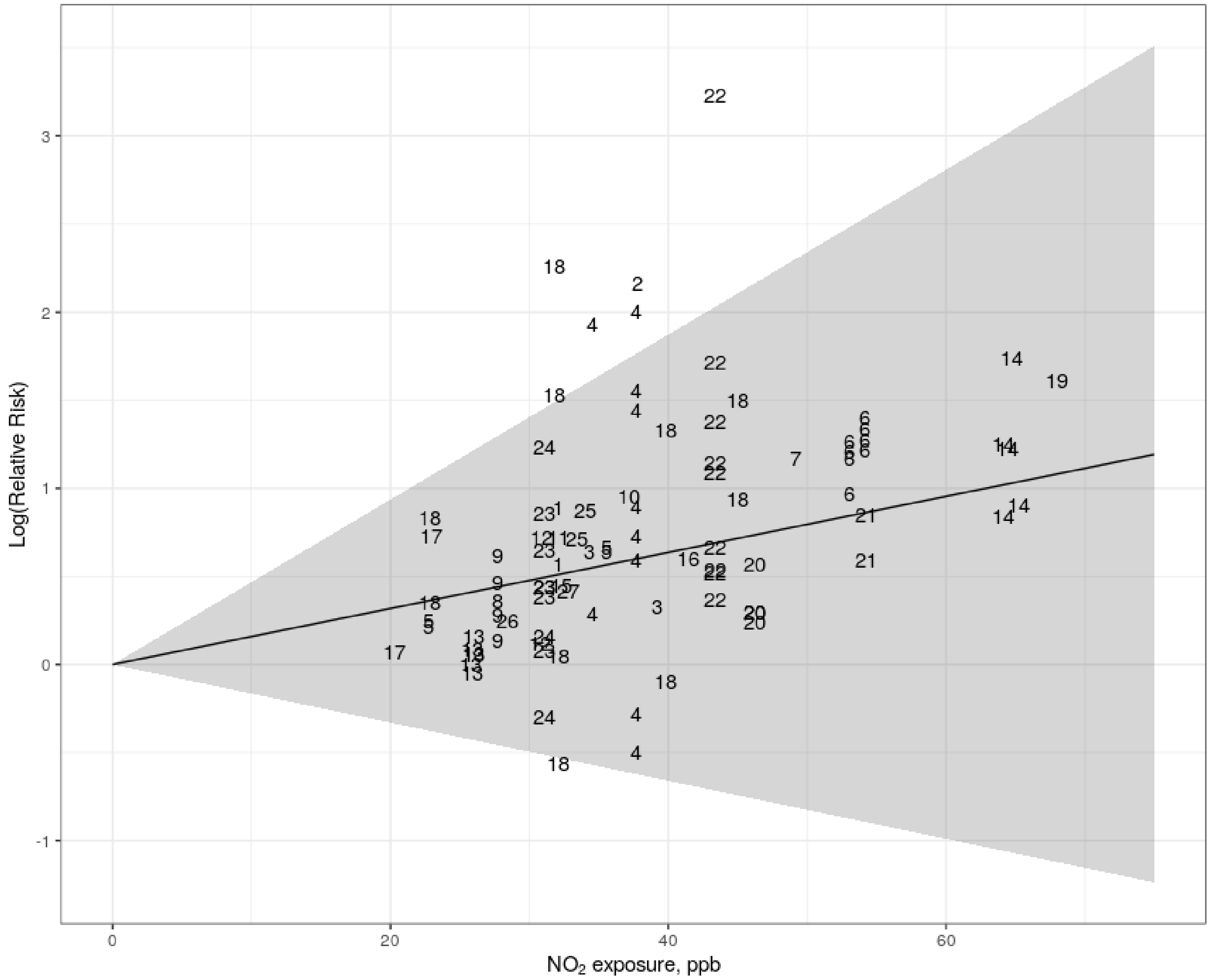

Risk–outcome scoring

Risk–outcome scores provide an empirical measure of the strength of evidence for risk–outcome pairs across risk factors in the GBD and are therefore useful for standardised comparison. Risk–outcome scores evaluate the area between the lower bound of the 95% uncertainty interval and the x-axis for harmful risk factors, including NO_2 pollution.

Prior to generating a risk–outcome score, we conducted an additional post-analysis step to detect and flag publication bias in the input data. This approach is based on the classic Egger's regression strategy, which is applied to the residuals in our model. In the current implementation, we do not correct for publication bias but flag the risk–outcome pairs where the risk for publication bias is significant. We flagged NO_2-childhood asthma for publication bias after detecting significant association between observation residuals and their standard errors (p-value = 0.014, Egger mean = –0.244, Egger SD =

0.111). To calculate the risk–outcome score, we generated an uncertainty interval from 1000 draws of the adjusted summary effect size (retaining uncertainty information from between-study heterogeneity predictions and the Fisher information boost). We then evaluated the risk–outcome score between the 15th and 85th percentiles of the input data exposure distribution (9.99–53.00 ppb). The final risk-outcome score is –0.51, which corresponds to a star rating of 1.

Population attributable fractions

PAFs were calculated for individuals between 0 and 19 years of age and contributed to morbidity estimates only. This is because available literature provides sufficient evidence for NO_2's association with childhood asthma incidence (not adult asthma or asthma-related mortality).[10] PAFs were calculated on the 0.0083° x 0.0083° (~1 x 1 km) grid-cell level. We aggregated PAFs to most-detailed GBD modeling locations using population rasters from the WorldPop Database (described above).

References

1. Larkin, A., Geddes, J. A., Martin, R. V, Xiao, Q., Liu, Y., Marshall, J. D., … Hystad, P. (2017). Global Land Use Regression Model for Nitrogen Dioxide Air Pollution. Environmental Science & Technology, 51(12), 6957–6964. https://doi.org/10.1021/acs.est.7b01148.
2. Lamsal LN, Martin R V., van Donkelaar A, Steinbacher M, Celarier EA, Bucsela E, et al. 2008. Ground-level nitrogen dioxide concentrations inferred from the satellite-borne Ozone Monitoring Instrument. J Geophys Res Atmos 113:1–15; doi:10.1029/2007JD009235.
3. Geography and Environmental Science, University of Southampton, WorldPop. Age and Sex Structures, Global Per Country 2000-2020. Southampton, United Kingdom: WorldPop, 2018.
4. Mohegh A., Goldberg D., Achakulwisut P., Anenberg S. Sensitivity of estimated NO2-attributable pediatric asthma incidence to grid resolution and urbanicity. Environ. Res. Lett. 2021; 16: 14019. https://doi.org/10.1088/1748-9326/abce25
5. Pesaresi, Martino; Florczyk, Aneta; Schiavina, Marcello; Melchiorri, Michele; Maffenini, Luca (2019): GHS settlement grid, updated and refined REGIO model 2014 in application to GHS-BUILT R2018A and GHS-POP R2019A, multitemporal (1975-1990-2000-2015), R2019A. European Commission, Joint Research Centre (JRC) DOI: 10.2905/42E8BE89-54FF-464E-BE7B-BF9E64DA5218
6. Gelaro, R., McCarty, W., Suárez, M. J., Todling, R., Molod, A., Takacs, L., Randles, C. A., Darmenov, A., Bosilovich, M. G., Reichle, R., Wargan, K., Coy, L., Cullather, R., Draper, C., Akella, S., Buchard, V., Conaty, A., da Silva, A. M., Gu, W., … Zhao, B. (2017): The Modern-Era Retrospective Analysis for Research and Applications, Version 2 (MERRA-2). J. Climate, 30, 5419–5454, https://doi.org/10.1175/JCLI-D-16-0758.1.
7. Giorgi, F., Francisco, R. Uncertainties in regional climate change prediction: a regional analysis of ensemble simulations with the HADCM2 coupled AOGCM. Climate Dynamics **16,** 169–182 (2000). https://doi-org.offcampus.lib.washington.edu/10.1007/PL00013733
8. Khreis H, Kelly C, Tate J, Parslow R, Lucas K, Nieuwenhuijsen M. Exposure to traffic-related air pollution and risk of development of childhood asthma: A systematic review and meta-analysis. Environ Int. 2017 Mar; 100:1-31. doi: 10.1016/j.envint.2016.11.012. Epub 2016 Nov 21. PMID: 27881237.

9. Zheng P, Barber R, Sorensen RJD, Murray CJL, Aravkin AY. Trimmed Constrained Mixed Effects Models: Formulations and Algorithms. J Comput Graph Stat. Published online February 12, 2021:1-13. doi:10.1080/10618600.2020.1868303
10. Achakulwisut P, Brauer M, Hystad P, Anenberg SC. Global, national, and urban burdens of paediatric asthma incidence attributable to ambient NO_2 pollution: estimates from global datasets. Lancet Planet Health. 2019; 3 (4): E166-E178. DOI:https://doi.org/10.1016/S2542-5196(19)30046-4.
11. Larkin, A. et al. A global spatial-temporal land use regression model for nitrogen dioxide air pollution. Front. Environ. Sci. 11, (2023).

Ambient ozone pollution

Flowchart

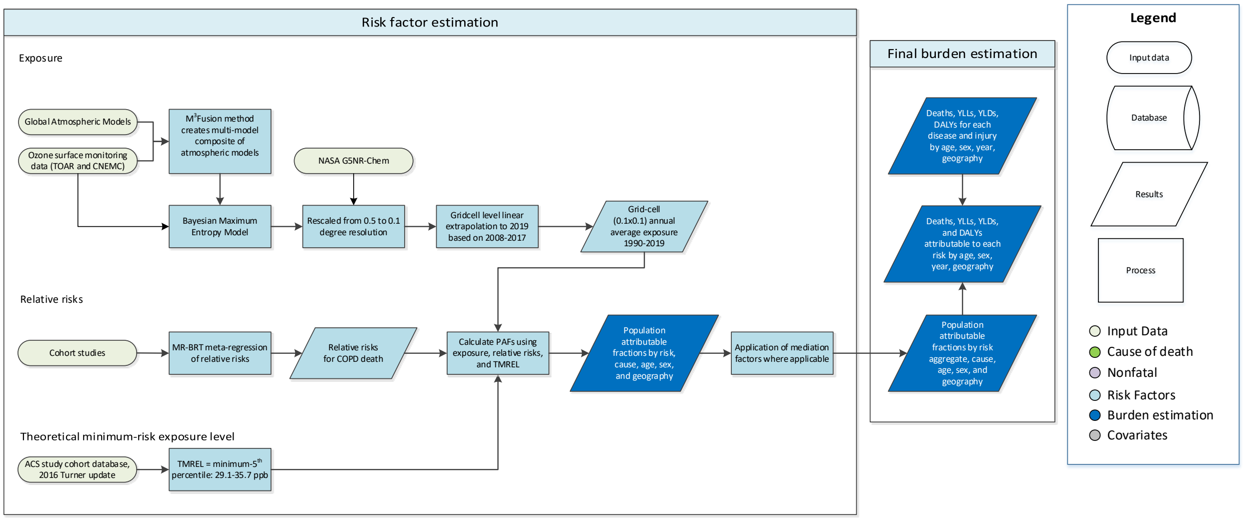

Input data and methodological summary

Exposure

Definition

Exposure to ambient ozone pollution is defined as the population-weighted highest seasonal (six-month) average of eight-hour daily maximum ozone concentrations. This measurement is reported in parts per billion (ppb).

Input data

To estimate the global distribution of exposure to ambient ozone pollution for the years 1990 to 2017, ozone ground measurement data were combined with chemical transport model estimates using Bayesian maximum entropy. Exposure estimates for 2018–2023 were extrapolated from these results, as described below.

1) Measurements and model combination

1.1) Measurement data

Ozone monitoring data were obtained from the Tropospheric Ozone Assessment Report (TOAR), which contains the world's largest collection of surface ozone metrics (DeLang et al., 2021; Schultz et al., 2017). Since TOAR has released data until 2015 to the public, an update was made to include readily available datasets until 2017. In addition to TOAR, our analysis included ozone data from the China National Environmental Monitoring Center (CNEMC) Network, which contains surface ozone measurements for 2013–2017 in China (Lu et al., 2018). All observations were processed to provide the six-month ozone season average of eight-hour daily maximum ozone concentrations.

1.2) Model combination

We used a combination of global atmospheric chemical transport models in our analysis, many of which simulated specified dynamics for the Chemistry-Climate Model Initiative (CCMI). Note that some of these modelling teams completed extra years of simulations beyond 2010 specifically for this project. The eight models and years available include the following: CHASER (1990–2010), MOCAGE (1988–2016), MRI-ESM (1988–2017), NASA MERRA2-GMI (1988–2017), NCAR CESM-Chem (1988–2010), NCAR WACCM (1988–2010), GFDL AM3 (1988–2014), and GFDL AM4 (2010–2016).

We obtained hourly ozone data for each of these models and then calculated the six-month maximum daily eight-hour maximum ozone mixing ratio (ppb). The M^3Fusion method (Chang et al., 2019) was used to create a multi-model composite of the specified-dynamics models in each year from 1990 to 2017. This multi-model composite finds the linear combination of models available for each year that minimises the mean square error as compared to the observations in each world region, and in the process, it corrects to minimise the mean model bias in each region. The world was divided geographically into eight regions: North America, South America, Europe, Africa, south central Asia, east Asia, Russia, and Oceania. In every region, each model was weighted to minimise the difference between the multi-model average and observations as described by the following:

Let s_g be the grid cell at resolution 0.5° × 0.5°, $\hat{y}(sg)$ be the interpolated observations, $\{\eta_k(sg); k = 1, . . . , n\}$ be the model output registered onto the same grid from the n models available in a given year. α_r is a constant that allows adjustment to the overall (regional) underestimation or overestimation and β_{rk} is an optimal weight for the k-th model in region r.

$$\underset{\{\alpha_r, \beta_{rk}; k=1,..,n\}}{minimize} \sum_{s_g \in Region\ r} \left(\hat{y}(s_g) - \alpha_r - \sum_{k=1}^{n} \beta_{rk}\eta_k(s_g) \right)^2,$$

$$subject\ to \sum_{k=1}^{n} \beta_{rk} = 1\ and\ \beta_{rk} \geq 0$$

In the M^3Fusion method, weights are constrained to be positive and sum to 1. A constant offset, α_r, was included to guarantee that the residuals from this optimisation have a zero mean, through which the mean model bias is corrected (Chang et al., 2019). In most regions and years, the multi-model mean ozone was biased high, so this method tends to decrease the average ozone.

Since the M^3Fusion method relies on surface measurements to change the weights, regions with sparse data had to be taken into account. North America and Europe use weights-based model and observation values for each individual year. The rest of the world regions (South America, Africa, south central Asia, east Asia, Russia, and Oceania) use individual year weights for 2000–2010, and apply weights calculated from the aggregated 2000 to 2010 period for 1990–1999. For 2011–2017, east Asia uses individual year weights, while South America, Africa, south central Asia, Russia, Oceania, and Antarctica use weights from the aggregated 2011–2014 period.

An example of the weighted values used to create the M^3Fusion model in North America and Europe are shown below, accompanied by a map of the M^3Fusion model in 2005.

	North America		Europe	
Year	*1995*	*2005*	*1995*	*2005*
CESM-Chem	0.38	0.29		
CHASER	0.04			
GFDL-AM3			0.18	0.62
MERRA		0.32	0.82	0.38
MOCAGE	0.36	0.01		
MRI-ESM	0.22	0.27		
WACCM		0.11		

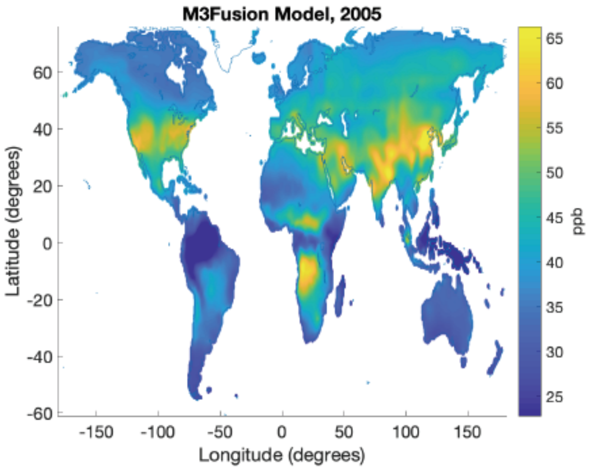

2) Bayesian maximum entropy (BME)

BME is a geostatistical modelling tool that can be used to combine various knowledge bases for an air pollutant and combine them to create a single product. In this case, we use BME to combine site-specific measurements and modelled concentrations, making use of the correlations between measurement locations. BME uses the measurement values to correct the M^3Fusion Model locally around each station spatially and temporally, allowing future and past observations to provide input. Since more measurement locations became available through time, this method allows later measurements to influence ozone surfaces earlier in the period, which is particularly important in China and data-sparse regions. The range over which each measurement can correct the M^3Fusion Model and how each measurement's impact decreases over distance in time and space are calculated as part of BME.

Beyond combining these knowledge bases to provide an estimate of ozone pollution, BME also estimates a variance, which can be used to assess estimation confidence at different locations.

In short, the steps are:

1. Let **Z(p)** be a field of ozone concentration estimations in space and time and let **mo(p)** be the M^3Fusion model output values in space and time.
2. Subtract the M^3Fusion model output values at each measure point from the observed values, **z(p)**, to obtain residuals **x(p)**=**z(p)**-**mo(p)**. Examples for 2005 of **z** and **x** are shown below on the left and right, respectively:

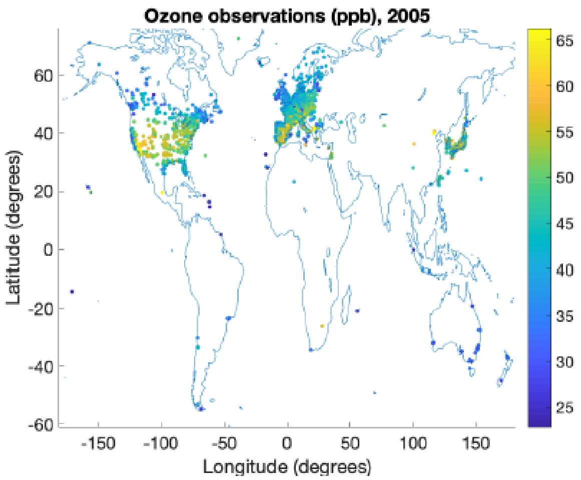

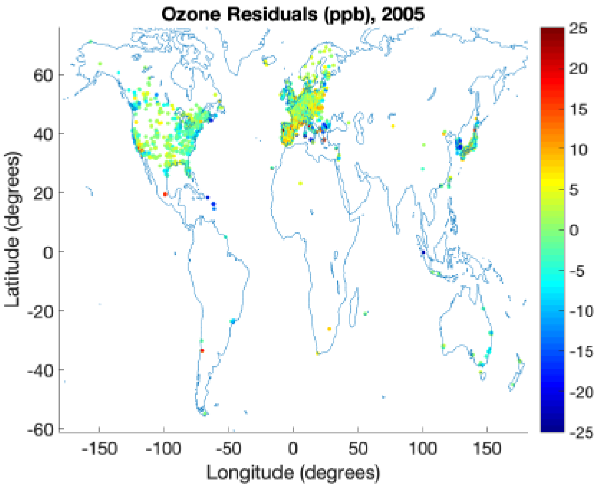

3. Model the covariance (the correlation between locations in space and time) $\mathbf{c_x}$ based on the residuals $\mathbf{x}$. The covariance of the residuals is the range of influence of a measurement to predict other concentrations in space and time. A shallower curve indicates that ozone values are correlated over a greater distance, while a steep drop-off indicates the reverse. The modelled spatial and temporal covariance are displayed below with the corresponding equation:

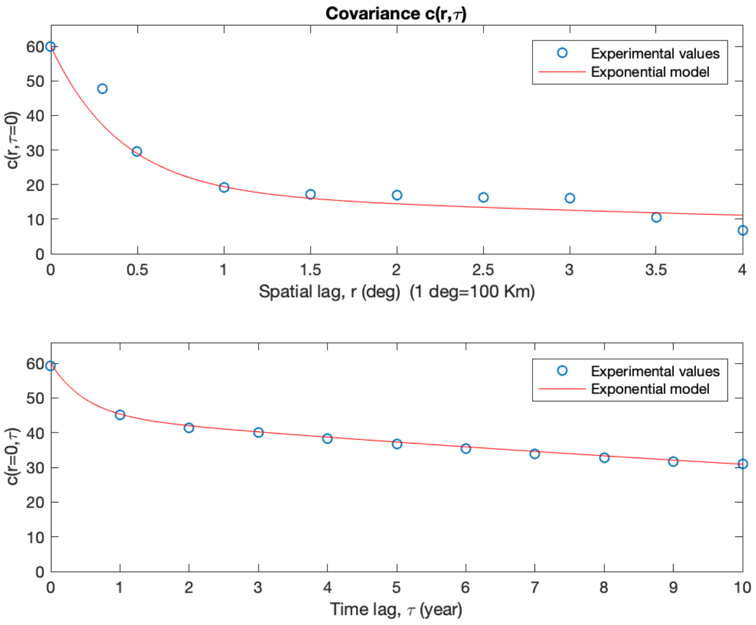

$$C_x(r,\tau) = 59.9938\ ppb^2(0.7\exp\left(-\frac{3r}{1.2\ degrees}\right)\exp\left(-\frac{3\tau}{80\ years}\right) + 0.05\exp\left(-\frac{3r}{25\ degrees}\right)\exp\left(-\frac{3\tau}{80\ years}\right) + 0.25\exp\left(-\frac{3r}{25\ degrees}\right)\exp\left(-\frac{3\tau}{1.5\ years}\right)$$

Where $\boldsymbol{\tau}$ *is temporal distance and r is spatial distance*

Note that the spatial covariance drops off steeply spatially such that the influence of a measurement location becomes very small beyond 1 degree of distance. However, the temporal covariance remains high, meaning that observations can influence ozone estimates through time over several years.

4. Combine the observation data residuals **(x)**, covariance **(cx),** and estimation parameters to get the BME estimation **(xk)** and variance **(vk)** on a 0.5º by 0.5º grid, shown for 2005.

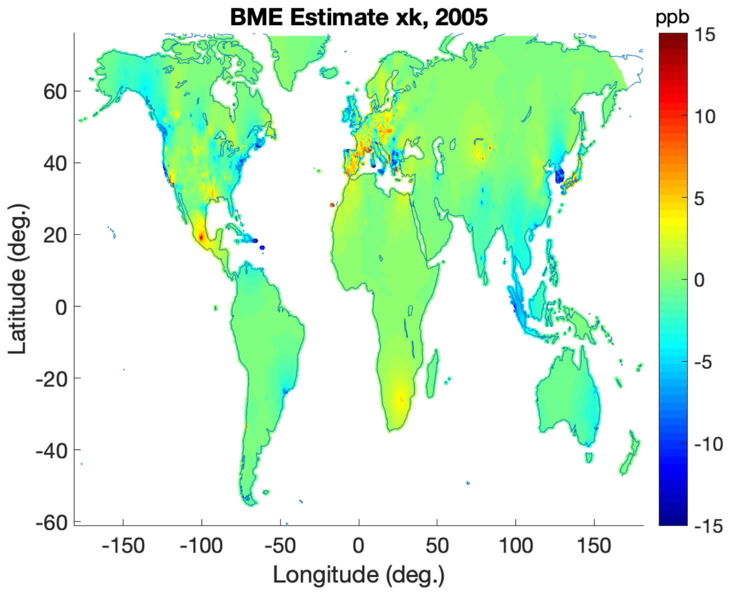

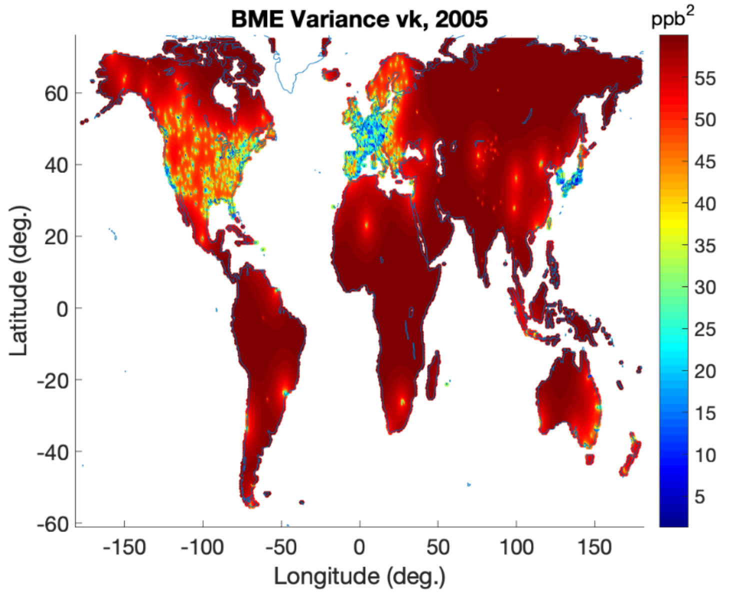

The variance is zero where the location of the estimation point matches an observation point in that year. The variance increases as the space time distance from an observation increases, until the variance reaches a maximum value equal to the sill of the covariance equation (59.9938 ppb^2).

5. Obtain final BME estimation values **(z_k)**, shown for 2005 in the figure below, by adding back the previously subtracted model values **mo(p_k)** to the BME estimation **(x_k)**.

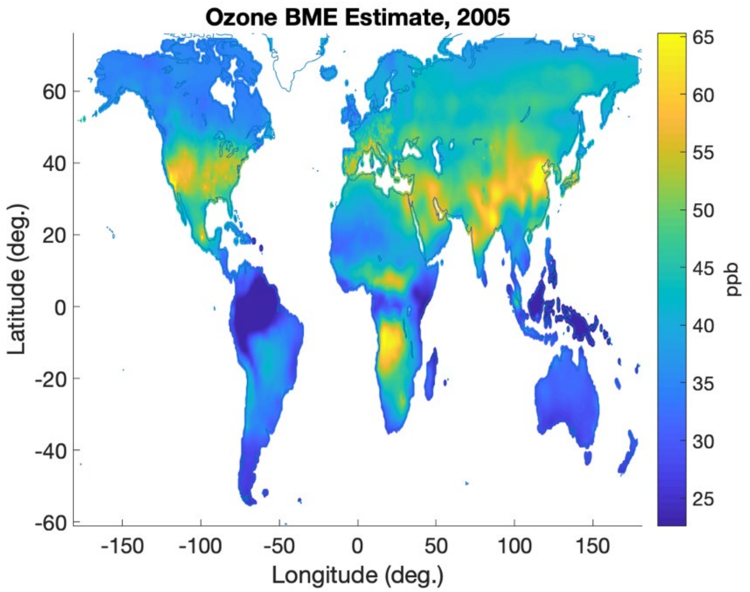

3) Adding fine resolution

Our results were calculated at 0.5° resolution, so to downscale estimates at finer resolution, we used the NASA G5NR-Chem model. The NASA G5NR-Chem model simulates surface ozone concentrations at 0.125° by 0.125° resolution for July 2013 to June 2014 (Hu et al., 2018). We regridded the G5NR-Chem model from 0.125° resolution to 0.1° resolution. While we do not expect that the raw values for 2013–2014 hold true for every year, we believe that the spatial distribution of this model can be used to inform the fine-scale spatial pattern for each year. To add fine resolution, we performed the following steps:

1. Regrid NASA G5NR-Chem from 0.125° resolution to 0.1° resolution
2. Average each 0.5° NASA G5NR-Chem grid cell
3. Calculate the difference between our BME estimation results at 0.5° and the average NASA G5NR-Chem at 0.5°
4. Add the calculated difference to NASA G5NR-Chem at 0.1° to obtain our BME estimation at 0.1°

Adding fine resolution to our results keeps the average of each 0.5° grid cell the same as the original estimation at 0.5°, as well as the global average.

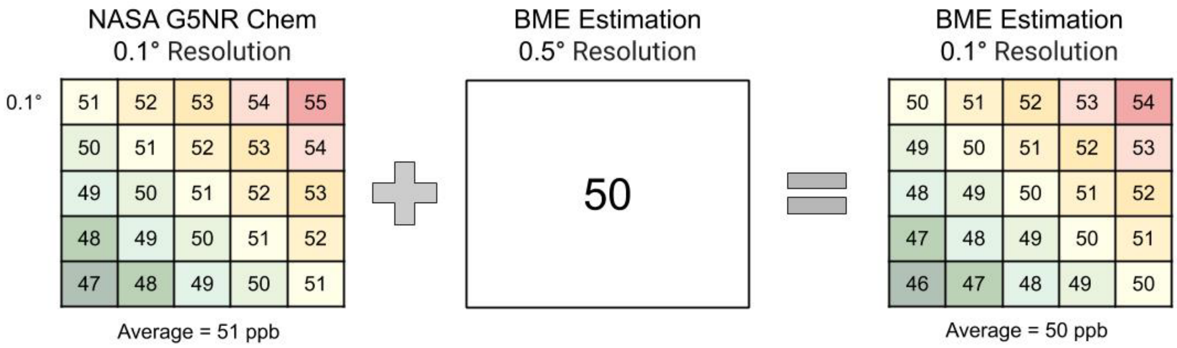

4) Final output

Three years are shown as an example below: 1995, 2005, and 2015. For each year, there are five maps displayed: the observations, M^{3}Fusion Model, BME Estimate, the difference between the BME Estimate and the M^{3}Fusion Model, and the variance. The difference map shows that the BME method corrects the M^{3}Fusion Model near monitoring stations, including stations in other years.

1995

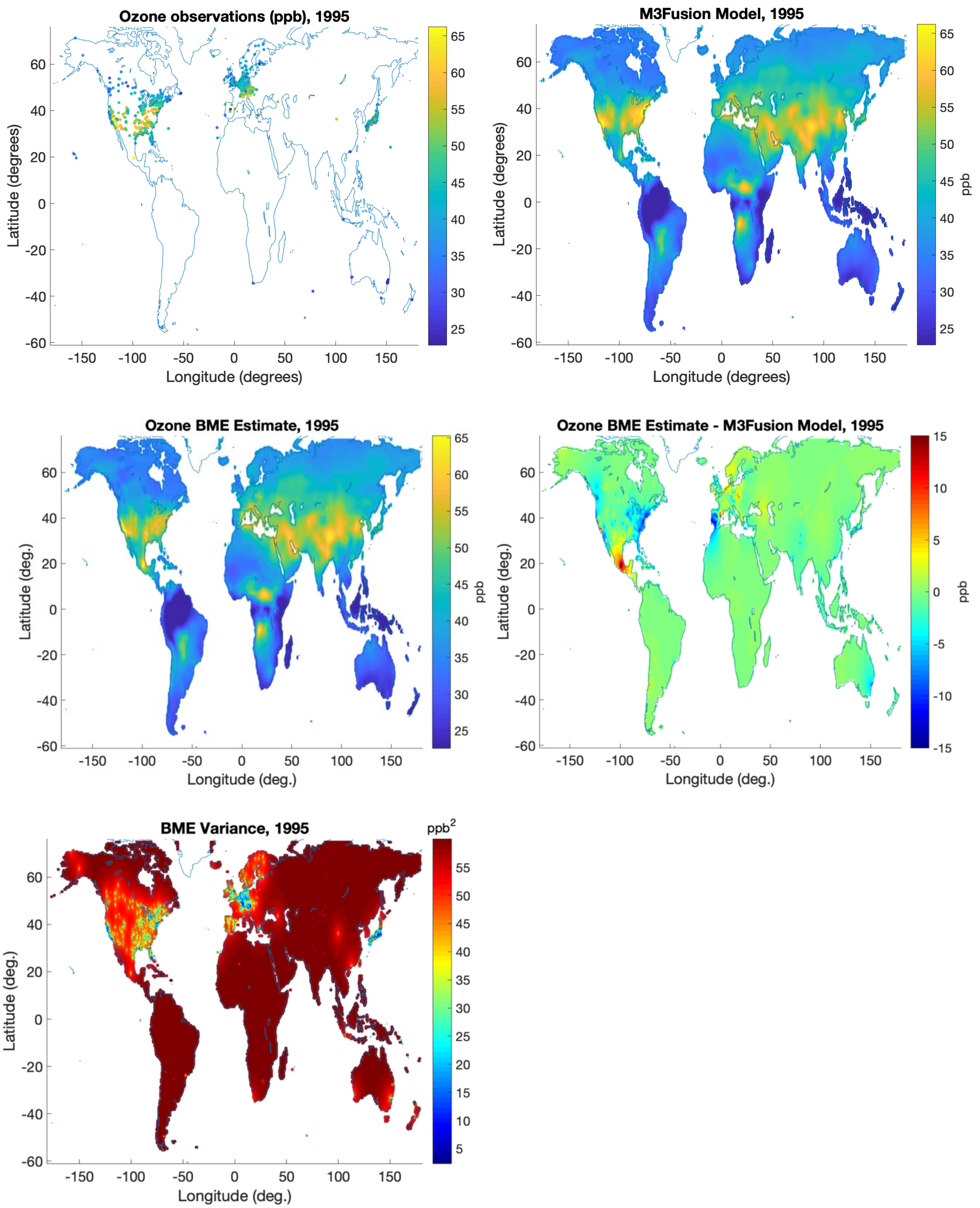

Ozone observations (ppb), 1995
Latitude (degrees)
Longitude (degrees)
M3Fusion Model, 1995
Latitude (degrees)
Longitude (degrees)
ppb
Ozone BME Estimate, 1995
Latitude (deg.)
Longitude (deg.)
ppb
Ozone BME Estimate - M3Fusion Model, 1995
Latitude (deg.)
Longitude (deg.)
ppb
BME Variance, 1995
Latitude (deg.)
Longitude (deg.)
ppb^2

2005

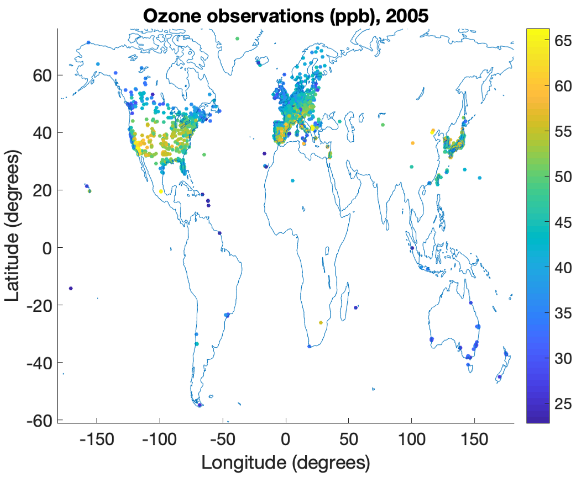
Ozone observations (ppb), 2005
Latitude (degrees)
60
40
20
0
-20
-40
-60
-150 -100 -50 0 50 100 150
Longitude (degrees)
65
60
55
50
45
40
35
30
25

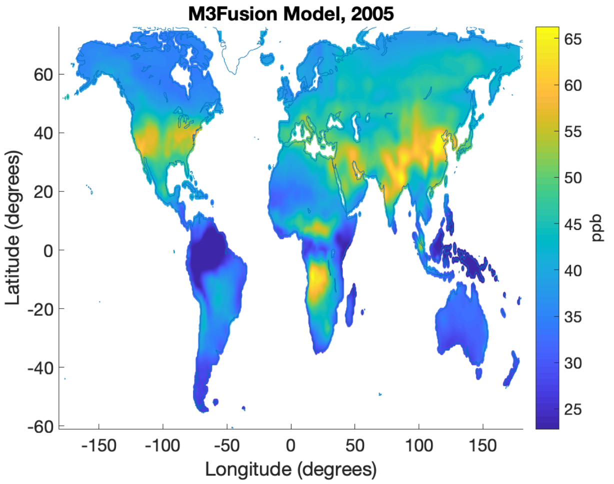
M3Fusion Model, 2005
Latitude (degrees)
60
40
20
0
-20
-40
-60
-150 -100 -50 0 50 100 150
Longitude (degrees)
65
60
55
50
45
40
35
30
25
ppb

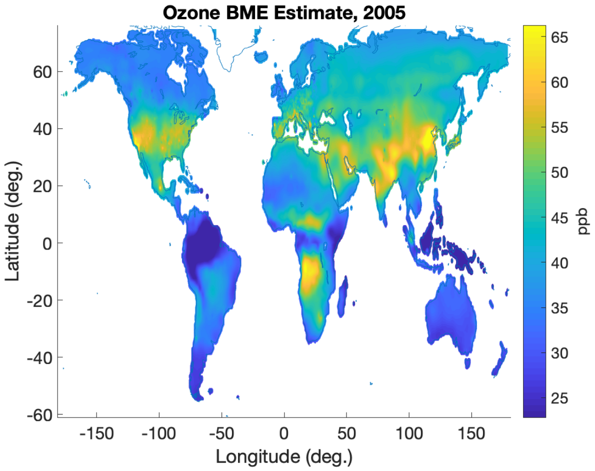
Ozone BME Estimate, 2005
Latitude (deg.)
60
40
20
0
-20
-40
-60
-150 -100 -50 0 50 100 150
Longitude (deg.)
65
60
55
50
45
40
35
30
25
ppb

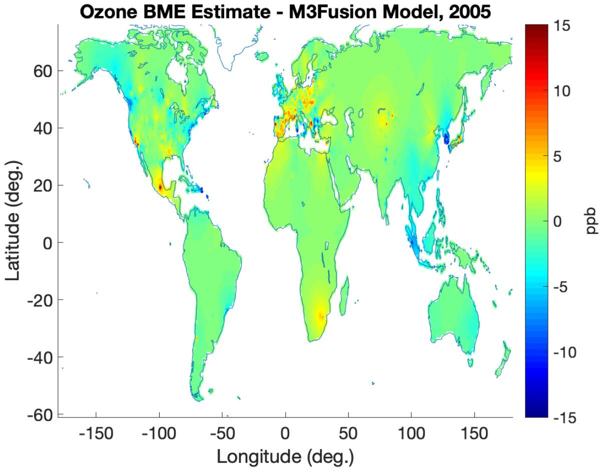
Ozone BME Estimate - M3Fusion Model, 2005
Latitude (deg.)
60
40
20
0
-20
-40
-60
-150 -100 -50 0 50 100 150
Longitude (deg.)
15
10
5
0
-5
-10
-15
ppb

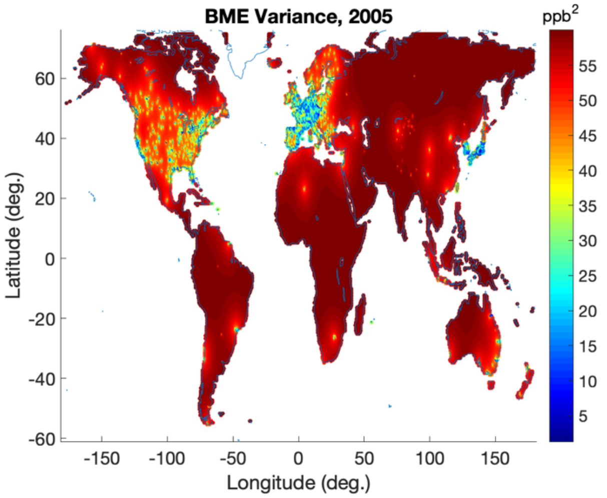
BME Variance, 2005
ppb^2
Latitude (deg.)
60
40
20
0
-20
-40
-60
-150 -100 -50 0 50 100 150
Longitude (deg.)
55
50
45
40
35
30
25
20
15
10
5

2015

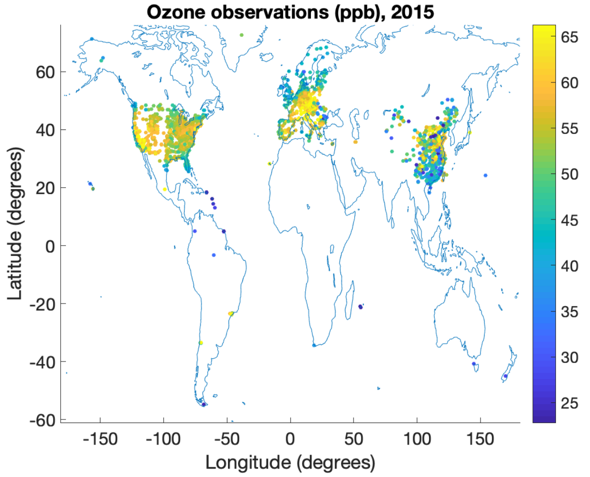
Ozone observations (ppb), 2015
Latitude (degrees)
Longitude (degrees)
60
40
20
0
-20
-40
-60
-150
-100
-50
0
50
100
150
65
60
55
50
45
40
35
30
25

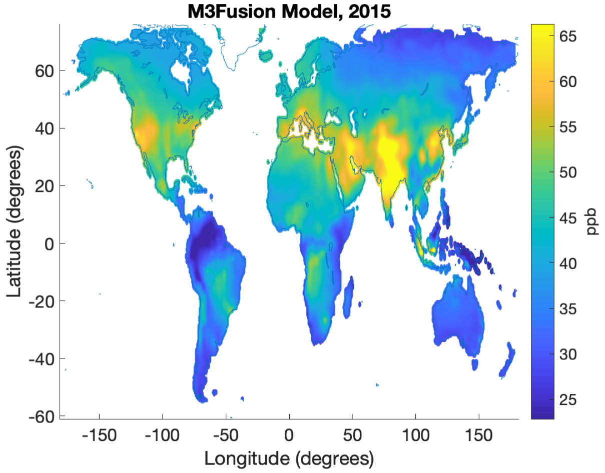
M3Fusion Model, 2015
Latitude (degrees)
Longitude (degrees)
ppb
60
40
20
0
-20
-40
-60
-150
-100
-50
0
50
100
150
65
60
55
50
45
40
35
30
25

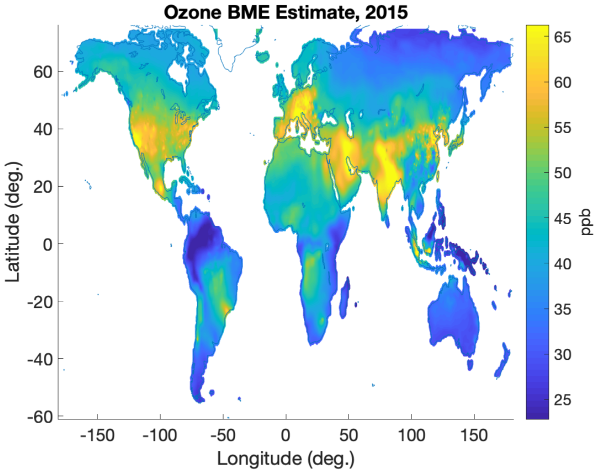
Ozone BME Estimate, 2015
Latitude (deg.)
Longitude (deg.)
ppb
60
40
20
0
-20
-40
-60
-150
-100
-50
0
50
100
150
65
60
55
50
45
40
35
30
25

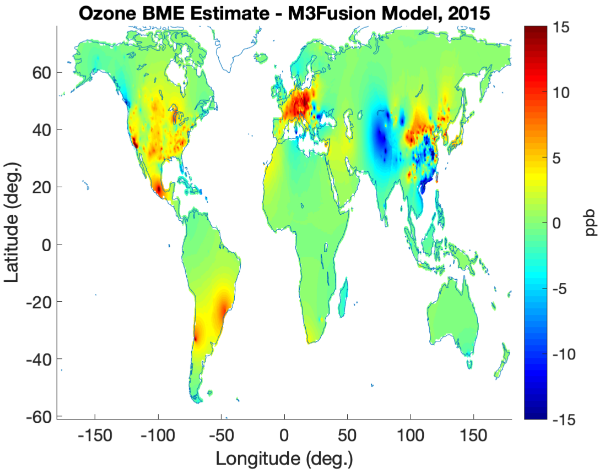
Ozone BME Estimate - M3Fusion Model, 2015
Latitude (deg.)
Longitude (deg.)
ppb
60
40
20
0
-20
-40
-60
-150
-100
-50
0
50
100
150
15
10
5
0
-5
-10
-15

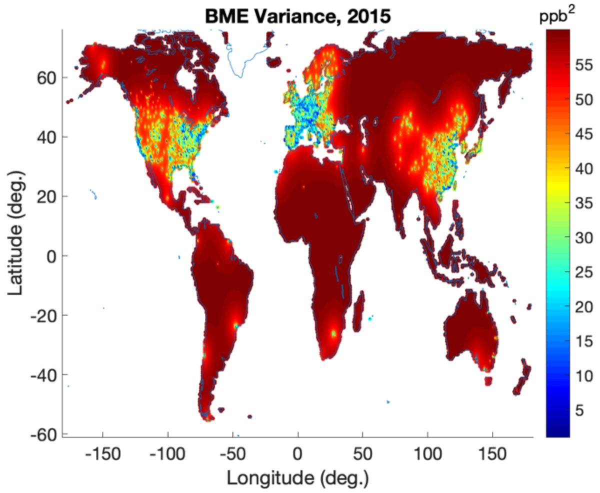
BME Variance, 2015
ppb^2
Latitude (deg.)
Longitude (deg.)
60
40
20
0
-20
-40
-60
-150
-100
-50
0
50
100
150
55
50
45
40
35
30
25
20
15
10
5

5) Extrapolation and annual means

To estimate global ozone exposures in 2018–2023 for each 0.1° grid cell, we ran a log-linear model of the ozone estimates on year for the most recent ten years (2008–2017) of the following form:

$$\log(\text{ozone}) \sim \text{year}+1.$$

We considered using splines, but due to annual variation of ozone, we found this to be the most reasonable prediction.

For burden estimation, we are more interested in long-term trends and effects than annual variation; therefore, for the years 1991–2016, we used a three-year mean of exposure centered on the year of interest. This strategy aligns with the methodology used in ambient air pollution. For 1990 and 2017, we used two-year means (1990/1991 and 2016/2017, respectively) because 1989 and 2018 were not available in the estimates.

To estimate the variance for the three-year mean to generate confidence intervals, we did not have information on the covariance between years, so a conservative estimate of the variance was made:

Let *X*, *Y*, and *Z*, be random variables describing ozone exposure in a given 0.1-degree grid cell for years *i-1*, *i,* and *i+1*, respectively. By the laws of variance,

$$Var\left(\frac{1}{3}(X+Y+Z)\right)=\frac{1}{9}\left(Var(X)+Var(Y)+Var(Z)+2Cov(X,Y)+2Cov(X,Z)+2Cov(Y,Z)\right).$$

We do not know the covariance, but by the Cauchy-Schwartz inequality,

$$Cov(A,B)\le\sqrt{Var(A)*Var(B)}.$$

Therefore,

$$Var\left(\frac{1}{3}(X+Y+Z)\right)\le\frac{1}{9}\Big(Var(X)+Var(Y)+Var(Z)+2\sqrt{Var(X)*Var(Y)}+ 2\sqrt{Var(X)*Var(Z)}+2\sqrt{Var(Y)*Var(Z)}\Big).$$

This is a conservative estimate of the variance used when taking a three-year mean.

6) Difference from previous estimations

This method, implemented in the Global Burden of Disease (GBD) Study 2019 and unchanged for GBD 2023, improves upon the GBD 2017 ozone exposure estimates (Chang et al, 2019) in the following ways:

1. The previous estimates used observations in a certain year to correct the model within 2° of a monitoring station. In the current method, the radius of influence of each observation is defined by the spatial covariance. The spatial covariance shows that much of the influence of an observation is lost after 1°.
2. Measurements not only bias-correct the model in the year in which they were observed, but also influence other years according to the temporal covariance. This is important for regions that were not monitored over the entire 1990–2017 period.
3. The fine spatial structure of the final product represents the spatial distribution of the 0.125° NASA G5NR-Chem model.

Theoretical minimum risk exposure level

As in GBD 2019, the TMREL is based on the exposure distribution from the ACS CPS-II study (Turner et al., 2016). It is a uniform distribution around the minimum and 5th percentile values observed in the cohort, ~U(29.1, 35.7), in ppb.

Relative risks

COPD is the only included outcome for ambient ozone pollution.

In GBD 2017, we performed a literature review of studies examining long-term ozone exposure and COPD. We included five cohorts from Canada, the UK, and the USA, all of which reported ozone effects on COPD mortality (Turner et al., 2016; Carey et al., 2013, and Burnett RT. "Cox…").

As in GBD 2019, we used the meta-regression—Bayesian, regularised, trimmed (MR-BRT) meta-regression tool to conduct a meta-analysis on these five observations. For GBD 2021, there were several key updates to the meta-regression process to standardise in as part of the Burden of Proof framework. First, we implemented automated covariate selection to detect significant covariates from those extracted (see table below) to quantify between-study heterogeneity. The MR-BRT automated covariate selection tool implements a two-step process. First, a series of loosening Lasso penalty parameters are applied to a log-linear meta-regression on all input effect size observations. Then, covariates with a non-zero coefficient are tested for significance using a Gaussian prior (significance threshold = 0.05). No significant covariates were detected for ozone.

Table 1: Extracted covariates for ambient ozone pollution and COPD risk literature

Covariate name	Covariate description
cv_subpopulation	Study represents the general population; study represents a subgroup (eg, high-risk group)
cv_exposure_population	Study measures individual-level exposure (≤1 km radius); study measures population-level exposure
cv_exposure_selfreport	Exposure is self-reported; exposure is measured externally
cv_exposure_study	Exposure is measured multiple times; exposure is measured only at baseline
cv_outcome_selfreport	Outcome is self-reported; outcomes is based on death certificate or medical record
cv_outcome_unblinded	Study implements unblinded assessment; assessment of outcome is blind to exposure (and vice versa)
cv_reverse_causation	Study presents no risk of reverse causation; risk of reverse causation
cv_confounding_nonrandom	Non-randomised study; randomised study
cv_confounding_uncontrolled	Study is randomised/outcome controlled for age, sex, education, income, and all critical determinants of outcome; study is controlled for age, sex, and other critical determinants of outcome; study is controlled for only age and sex
cv_selection_bias	Study reports >95% follow-up; study reports 85–95% follow-up; study reports <85% follow-up

The standard error of observations based on a single cohort that reported an unstratified sample size were multiplied by the square root of n, where n is the total number of observations for a given cohort. This adjustment was made to prevent a single cohort or study from having an outsize weight on the summary effect size. Additionally, we trimmed one of the input observations (Carey et al., 2013) during model fitting in accordance with GBD protocol across risk factor teams to trim 10% of input data.

We generated 1000 predictions of the effect size for use in calculating burden estimates. These predictions were created using predictions of between-study heterogeneity. We implemented the Fisher scoring correction to the heterogeneity parameter, which corrects for data-sparse situations. In such cases, the between-study heterogeneity parameter estimate may be zero, simply from lack of data. The Fisher scoring correction uses a quantile of gamma, which is sensitive to the number of studies, study design, and reported uncertainty.

The inverse-standard error weighted meta-analysis provided an estimated relative risk of 1.074 (95% CI 1.014–1.137) per 10 ppb with an estimated gamma (including between-study heterogeneity) of zero.

Table 4: MR-BRT relative risk model parameters for ambient ozone pollution

Covariate	Gamma (95% CI)	Beta coefficient, log (95% UI)	Exponentiated coefficient (95% UI)
Exposure (per 1 ppb)	0 (0–0)	7.133e-3 (4.488e-3 to 9.925e-3)	1.007 (1.004–1.010)

Ambient ozone pollution and COPD risk literature funnel plot

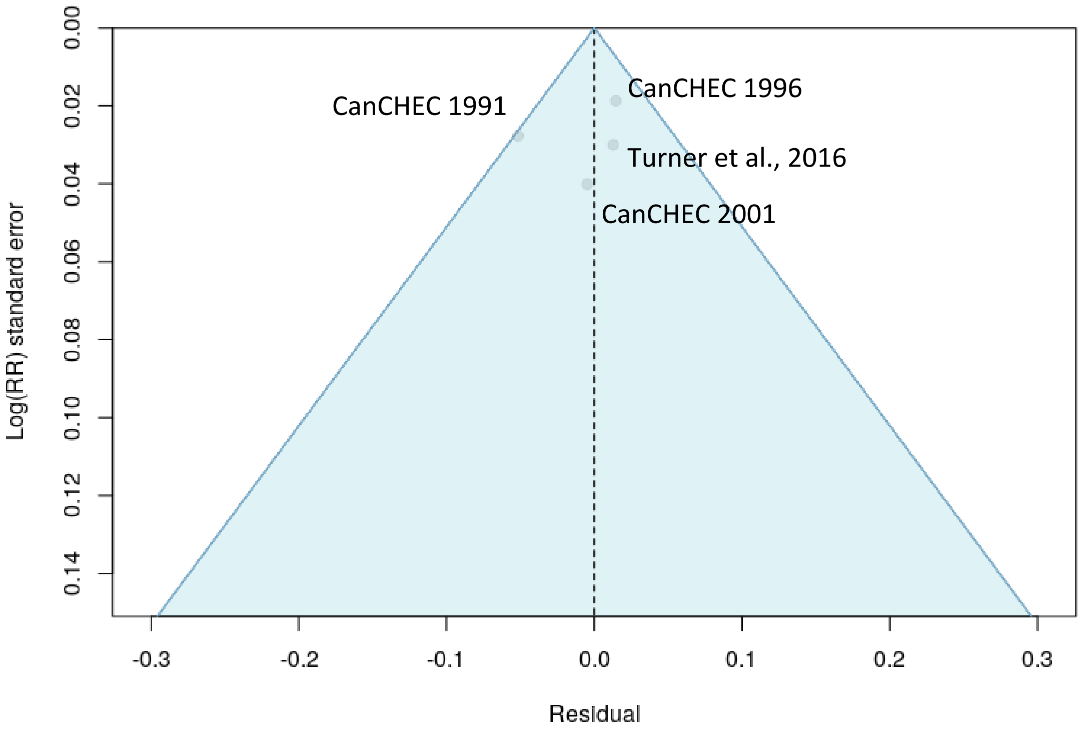

Risk–outcome scoring

Since GBD 2021, we also implemented risk–outcome scoring. Risk–outcome scores provide an empirical measure of the strength of evidence for risk–outcome pairs across risk factors in the GBD and are therefore useful for standardised comparison. Risk–outcome scores evaluate the area between the lower bound of the 95% uncertainty interval and the x-axis for harmful risk factors, including ambient ozone pollution. Prior to generating a risk–outcome score, we conducted an additional post-analysis step to detect and flag publication bias in the input data. This approach is based on the classic Egger's regression strategy, which is applied to the residuals in our model. In the current implementation, we do not correct for publication bias but flag the risk–outcome pairs where the risk for publication bias is significant. Publication bias was not detected for ambient ozone pollution and COPD risk literature.

To calculate the risk–outcome score, we generated an uncertainty interval from 1000 draws of the adjusted summary effect size (retaining uncertainty information from between-study heterogeneity predictions and the Fisher information boost). We then evaluated the risk–outcome score between the 15th and 85th percentiles of the input data exposure distribution (0–10 ppb). The final risk–outcome score is 0.011, which corresponds to a star rating of 2.

We calculated PAFs at the grid-cell level and aggregated up to GBD locations using population data from the Gridded Population of the World database. Estimates came from version 4 except for estimates for 1990 and 1995 from version 3. More details on these estimates are available in the Ambient Particulate Matter Pollution Methods Appendix.

Citations

1. DeLang MN, Becker JS, Chang KL, Serre ML, Cooper OR, Schultz MG, Schröder S, Lu X, Zhang L, Deushi M, Josse B, Keller CA, Lamarque JF, Lin M, Liu J, Marécal V, Strode AS, Sudo K, Tilmes S, Zhang L, Cleland SE, Collins EL, Brauer M, West JJ. Mapping Yearly Fine Resolution Global Surface Ozone through the Bayesian Maximum Entropy Data Fusion of Observations and Model Output for 1990–2017. Environ Sci Technol. 2021; 55 (8): 4389-4398. DOI: 10.1021/acs.est.0c07742.
2. Schultz MG, Schröder S, Lyapina O, Cooper O, Galbally I, Petropavlovskikh I, et al. Tropospheric Ozone Assessment Report: Database and Metrics Data of Global Surface Ozone Observations. *Elem Sci Anth*. 2017;5:58. DOI: http://doi.org/10.1525/elementa.244.
3. Lu, Xiao, Jiayun Hong, Lin Zhang, Owen R. Cooper, Martin G. Schultz, Xiaobin Xu, Tao Wang, Meng Gao, Yuanhong Zhao, and Yuanhang Zhang. Severe surface ozone pollution in China: a global perspective. *Environmental Science & Technology Letters* 2018 *5* (8), 487-49 DOI: 10.1021/acs.estlett.8b00366.
4. Chang, Kai-Lan, R. Cooper, Owen, West, Jason, L. Serre, Marc, G. Schultz, Martin, Lin, Meiyun, Marecal, Virginie, Josse, B, Deushi, Makoto, Sudo, Kengo, Liu, Junhua & A. Keller, Christoph. (2019). A new method (M^{3}Fusion v1) for combining observations and multiple model output for an improved estimate of the global surface ozone distribution. *Geoscientific Model Development*. 12. 955-978. 10.5194/gmd-12-955-2019.
5. Hu, L., C. A. Keller, M. S. Long, T. Sherwen, B. Auer, A. Da Silva, J. E. Nielsen, S. Pawson, M. A. Thompson, A. L. Trayanov, K. R. Travis, S. K. Grange, M. J. Evans, D. J. Jacob (2018) Global simulation of tropospheric chemistry at 12.5 km resolution: performance and evaluation of the GEOS-Chem chemical module (v10-1) within the NASA GEOS Earth system model (GEOS-5 ESM), *Geoscientific Model Development*, 11, 4603-4620. 10.5194/gmd-11-4603-2018.

6. Turner MC,Jerrett M, Pope CA 3rd, Krewski D, Gapstur SM, Diver WR, Beckerman BS, Marshall JD, Su J, Crouse DL, Burnett RT. Long-term ozone exposure and mortality in a large prospective study. *Am J Respir Crit Care Med*. 2016; 193(10): 1134-42.
7. Carey IM, Atkinson RW, Kent AJ, van Staa T, Cook DG, Anderson HR. Mortality associations with long-term exposure to outdoor air pollution in a national English cohort. *Am J Respir Crit Care Med.* 2013; 187(11): 1226-33.
8. Burnett RT. Cox Proportional Survival Model Hazard Ratios from Census Years (1991, 1996, 2001) to 2011 for Adults Aged 25 to 89 in CanCHEC Cohort. Custom Analysis for GBD 2017.

Citations for atmospheric chemical transport models

Ozone exposure information was provided by the following collaborators:

- Marissa DeLang, Jacob S. Becker, Stephanie Cleland, Elyssa Collins, Marc L. Serre, J. Jason West, University of North Carolina at Chapel Hill
- Owen R. Cooper and Kai-Lan Chang, CIRES, University of Colorado, Boulder/NOAA Earth System Research Laboratory, Boulder, USA
- Martin G. Schultz and Sabine Schröder, Jülich Supercomputing Centre (JSC), Forschungszentrum Jülich, Jülich, DE
- Xiao Lu and Lin Zhang, Laboratory for Climate and Ocean-Atmosphere Studies, Dept. of Atmospheric and Oceanic Sciences, School of Physics, Peking University, Beijing, China
- CCMI and NASA modellers

- CESM:
    - Tilmes, S., Lamarque, J.-F., Emmons, L. K., Kinnison, D. E., Ma, P.-L., Liu, X., Ghan, S., Bardeen, C., Arnold, S., Deeter, M., Vitt, F., Ryerson, T., Elkins, J. W., Moore, F., Spackman, J. R., and Val Martin, M.: Description and evaluation of tropospheric chemistry and aerosols in the Community Earth System Model (CESM1.2), *Geosci. Model Dev*., 8, 1395–1426, doi:10.5194/gmd-8-1395-2015, 2015
- CESM WACCM:
    - Garcia, R. R., Smith, A. K., Kinnison, D. E., de la Cámara, Á., and Murphy, D.: Modifications of the gravity wave parameterisation in the Whole Atmosphere Community Climate Model: Motivation and results, *J. Geophys. Res.-Atmos*., doi:10.1175/JAS-D16-0104.1, 2016.
    - Marsh, D., Mills, M. J., Kinnison, D. E., Garcia, R. R., Lamarque, J.-F., and Calvo, N.: Climate change from 1850–2005 simulated in CESM1 (WACCM), *J. Climate*, 26, 7372–7391, doi:10.1175/JCLI-D-12-00558.1, 2013
- CHASER:
    - Sudo, K., Takahashi, M., and Akimoto, H.: CHASER: A global chemical model of the troposphere, 2. Model results and evaluation, *J. Geophys. Res*., 107, 4586, https://doi.org/10.1029/2001JD001114, 2002a.

  - Sudo, K., Takahashi, M., Kurokawa, J., and Akimoto, H.: CHASER: A global chemical model of the troposphere, 1. Model description, *J. Geophys. Res*., 107, 4339, https://doi.org/10.1029/2001JD001113, 2002b.

  - Watanabe, S., Hajima, T., Sudo, K., Nagashima, T., Takemura, T., Okajima, H., Nozawa, T., Kawase, H., Abe, M., Yokohata, T., Ise, T., Sato, H., Kato, E., Takata, K., Emori, S., and Kawamiya, M.: MIROC-ESM 2010: model description and basic results of CMIP5-20c3m experiments, *Geosci. Model Dev*., 4, 845–872, https://doi.org/10.5194/gmd-4-845-2011, 2011.
- GEOSCCM:
  - Oman, L. D., Ziemke, J. R., Douglass, A. R., Waugh, D. W., Lang, C., Rodriguez, J. M., and Nielsen, J. E.: The response of tropical tropospheric ozone to ENSO, *Geophys. Res. Lett*., 38, L13706, https://doi.org/10.1029/2011GL047865, 2011.
- GFDL AM3 & AM4:
  - Lin, M., Fiore, A. M., Horowitz, L. W., Cooper, O. R., Naik, V., Holloway, J., Johnson, B. J., Middlebrook, A. M., Oltmans, S. J., Pollack, I. B., Ryerson, T. B., Warner, J. X., Wiedinmyer, C., Wilson, J., and Wyman, B.: Transport of Asian ozone pollution into surface air over the western United States in spring, *J. Geophys. Res*., 117, D00V07, https://doi.org/10.1029/2011JD016961, 2012.
  - Lin, M., Horowitz, L. W., Oltmans, S. J., Fiore, A. M., and Fan, S.: Tropospheric ozone trends at Mauna Loa Observatory tied to decadal climate variability, *Nat. Geosci*., 7, 136–143, https://doi.org/10.1038/NGEO2066, 2014.
  - Lin, M., Horowitz, L. W., Payton, R., Fiore, A. M., and Tonnesen, G.: US surface ozone trends and extremes from 1980 to 2014: quantifying the roles of rising Asian emissions, domestic controls, wildfires, and climate, *Atmos. Chem. Phys*., 17, 2943–2970, https://doi.org/10.5194/acp-17-2943-2017, 2017.
- MERRA GMI:
  - Ziemke, J. R., Oman, L. D., Strode, S. A., Douglass, A. R., Olsen, M. A., McPeters, R. D., Bhartia, P. K., Froidevaux, L., Labow, G. J., Witte, J. C., Thompson, A. M., Haffner, D. P., Kramarova, N. A., Frith, S. M., Huang, L.-K., Jaross, G. R., Seftor, C. J., Deland, M. T., and Taylor, S. L.: Trends in global tropospheric ozone inferred from a composite record of TOMS/OMI/MLS/OMPS satellite measurements and the MERRA-2 GMI simulation , *Atmos. Chem. Phys*., 19, 3257–3269, https://doi.org/10.5194/acp-19-3257-2019, 2019.
- MOCAGE:
  - Josse, B., Simon, P., and Peuch, V.-H.: Radon global simulations with the multiscale chemistry and transport model MOCAGE, Tellus B, 56, 339–356, https://doi.org/10.1111/j.1600-0889.2004.00112.x, 2004.
  - Teyssèdre, H., Michou, M., Clark, H. L., Josse, B., Karcher, F., Olivié, D., Peuch, V.-H., Saint-Martin, D., Cariolle, D., Attié, J.-L., Nédélec, P., Ricaud, P., Thouret, V., van der A, R. J., Volz-Thomas, A., and Chéroux, F.: A new tropospheric and stratospheric Chemistry and Transport Model MOCAGE-Climat for multi-year studies: evaluation of the present-day climatology and sensitivity to surface processes, *Atmos. Chem. Phys*., 7, 5815–5860, https://doi.org/10.5194/acp-7-5815-2007, 2007.
- MRI-ESM1r1:

- Adachi, Y., Yukimoto, S., Deushi, M., Obata, A., Taichu. Y. Tanaka, H. N., Hosaka, M., Sakami, T., Yoshimura, H., Hirabara, M., Shindo, E., Tsujino, H., Mizuta, R., Yabu, S., Koshiro, T., Ose, T., and Kitoh, A.: Basic performance of a new earth system model of the Meteorological Research Institute (MRI-ESM1), *Pap. Meteorol. Geophys*, 64, 1–18, https://doi.org/10.2467/mripapers.64.1, 2013.

Ambient particulate matter pollution

Flowchart

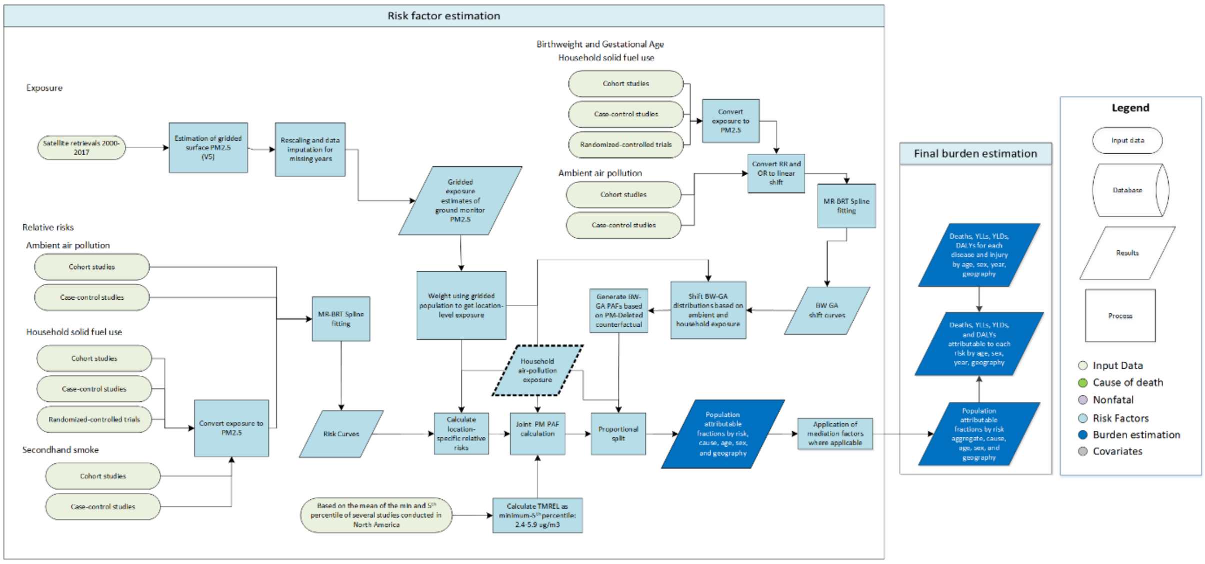

Input data and methodological summary

Exposure

Definition

Exposure to ambient particulate matter pollution is defined as the population-weighted annual average mass concentration of particles with an aerodynamic diameter less than 2.5 micrometers ($PM_{2.5}$) in a cubic meter of air. This measurement is reported in µg/m^3.

Input data

Ambient air pollution exposure estimates use input data from multiple sources. These include satellite observations of aerosols in the atmosphere, ground monitor measurements, chemical transport model simulations, population estimates, and land-use data.

Details for updates in exposure methodology and input data for the Global Burden of Disease (GBD) Study 2023 are as follows.

Ground-calibrated satellite-based $PM_{2.5}$ measurements

For GBD 2023, global satellite-derived estimates (V5.GL.04) were utilised as inputs for the period 2006–2022, at a resolution of 0.1° x 0.1° (~11 x 11 km at the equator). Aerosol optical depth (AOD) data from multiple satellites (MODIS, MISR, SeaWIFS, VIIRS) were integrated with the GEOS-Chem chemical transport model to establish geophysical relationships between surface $PM_{2.5}$ and AOD. These satellite products were calibrated against global ground-based observations using a Geographically Weighted Regression methodology, as detailed in van Donkelaar et al.[1] Data source: https://sites.wustl.edu/acag/datasets/surface-pm2-5/#V5.GL.04. This was a change from GBD 2021 and prior cycles when the Data Integration Model for Air Quality (DIMAQ) model was used for exposure estimation.

GBD 2021 exposures for years 1990, 1995, and 2000–2005 were rescaled for GBD 2023 by location using overlapping years available between the new exposures generated with V5 product and the

previous exposures. Then, a three-year rolling average is applied for years 2006–2008 to smooth the transition between both outputs.

Theoretical minimum risk exposure level

The theoretical minimum risk exposure level (TMREL) was assigned a uniform distribution with lower/upper bounds given by the average of the minimum and 5th percentiles of outdoor air pollution cohort studies exposure distributions conducted in North America, with the assumption that current evidence was insufficient to precisely characterise the shape of the concentration–response function below the 5th percentile of the exposure distributions. The TMREL was defined as a uniform distribution rather than a fixed value in order to represent the uncertainty regarding the level at which the scientific evidence was consistent with adverse effects of exposure. The specific outdoor air pollution cohort studies selected for this averaging were based on the criteria that their 5th percentiles were less than that of the American Cancer Society Cancer Prevention II (CPSII) cohort's 5th percentile of 8.2 based on Turner et al. (2016).[2] This criterion was selected because GBD 2010 used the minimum, 5.8, and 5th percentile solely from the CPS II cohort. The resulting lower/upper bounds of the distribution for GBD 2023 were 2.4 and 5.9. This has not changed since GBD 2015.

Relative risks and population attributable fractions

Input data

For GBD 2023, as in previous GBD cycles, we created one set of cause-specific risk curves for both household air pollution and ambient particulate matter pollution as two different sources of $PM_{2.5}$. In GBD 2017, we estimated the particulate-matter-attributable burden of disease based on the relation of long-term exposure to $PM_{2.5}$ with ischaemic heart disease, stroke (ischaemic and haemorrhagic), COPD, lung cancer, acute lower respiratory infection, and type 2 diabetes. In GBD 2019, we added adverse birth outcomes including low birthweight and short gestation as contributors to $PM_{2.5}$-attributable burden. Because these are risk factors (not outcomes) included in the GBD study, we performed a mediation analysis, in which a proportion of the burden attributable to low birthweight and short gestation is attributed to $PM_{2.5}$ pollution. For GBD 2023, as in previous cycles, we used risk curves to calculate burden for ages 25+ for ischaemic heart disease, stroke (ischaemic and haemorrhagic), COPD, lung cancer, and type 2 diabetes and for all ages for acute lower respiratory infection. For GBD 2023, we calculate burden for the same list of outcomes in addition to dementia. Burden calculation for mediated outcomes is described below.

For the six non-mediated outcomes, we used results from cohort and case-control studies of ambient $PM_{2.5}$ pollution and cohort studies, case-control studies, and randomised-controlled trials of household use of solid fuel for cooking. Since GBD 2021, we excluded secondhand smoke cohort and case-control studies from risk curve input data.

We conducted a literature review for studies of $PM_{2.5}$ (ambient and household air pollution) and risk of lower respiratory infection using the search string below. We searched the PubMed database for studies published between January 1, 2017, and July 22, 2020 (date of search). 32 initial results were obtained from the database, 31 of which were excluded during title-abstract and full-text screening. The remaining study was later excluded due to insufficient information reported on the study-specific exposure distribution.

Search string: ((("Air Pollution"[Mesh] OR "Particulate Matter"[Mesh] OR "air pollution"[Title/Abstract] OR "urban air pollution"[Title/Abstract] OR "ambient air pollution"[Title/Abstract] OR "airborne particulate matter"[Title/Abstract]) OR ("Air Pollution,

Indoor"[Mesh] OR "Household air"[Title/Abstract] OR "Indoor air pollution"[Title/Abstract] OR "Indoor fine particulate matter"[Title/Abstract] OR "Indoor particulate matter"[Title/Abstract] OR "Indoor air quality"[Title/Abstract])) AND ("lower respiratory infection"[Title/Abstract] OR "LRI"[Title/Abstract]))

For GBD 2023, as in GBD 2021 and 2019, the meta-regression—Bayesian, regularised, trimmed (MR-BRT) meta-regression tool was used to create relative risk estimates, with three key updates to input data. In GBD 2017, we used relative estimates for active smoking and secondhand smoke (converting cigarettes per day to $PM_{2.5}$ exposure) to estimate relative risk predictions for $PM_{2.5}$ exposure at the highest end of the exposure–response curve. These data were included because the majority of the air pollution epidemiological studies have been performed in high-income countries which have lower levels of ambient $PM_{2.5}$ pollution. This posed a barrier to extrapolating relative risk estimates from the steep relationship at the beginning of the exposure range to locations with high exposures but no relative risk estimates, such as India and China. In GBD 2019, we incorporated estimates at high $PM_{2.5}$ levels by adding recently published ambient $PM_{2.5}$ studies conducted in China and other higher-exposure settings and additional HAP studies.[3,4,5,6,7] Additionally, the switch to MR-BRT splines in GBD 2019 (instead of the integrated exposure–response function employed in GBD 2017) presented a more flexible approach that allowed the curve to fit ambient and household data and removed the need for active smoking data to anchor the curve at higher exposures. The inclusion of active smoking and secondhand smoking data in previous GBD cycles required conversion from cigarettes per day to $PM_{2.5}$ exposure and introduced other differences, including differences in dose rates and those between voluntary (active smoking) and involuntary (ambient $PM_{2.5}$, household air pollution, secondhand smoke) exposures. Due to these factors, in GBD 2019, we removed active smoking data from the relative risk model's input data. Since GBD 2021, we have removed secondhand smoking data, completing the transition to only using $PM_{2.5}$ and HAP relative risk input data. This removes important sources of uncertainty in our earlier estimates.[8,9]

New risk curves were developed for GBD 2023 for ischaemic heart disease, stroke, and lower respiratory infections with the inclusion of new input data. A new risk curve was also developed for dementia which was included as a new $PM_{2.5}$ risk–outcome pair in GBD 2023. For all other causes, there were no updates to the risk curves in GBD 2023. The following plot displays $PM_{2.5}$ risk curves from GBD 2021 and those updated for GBD 2023:

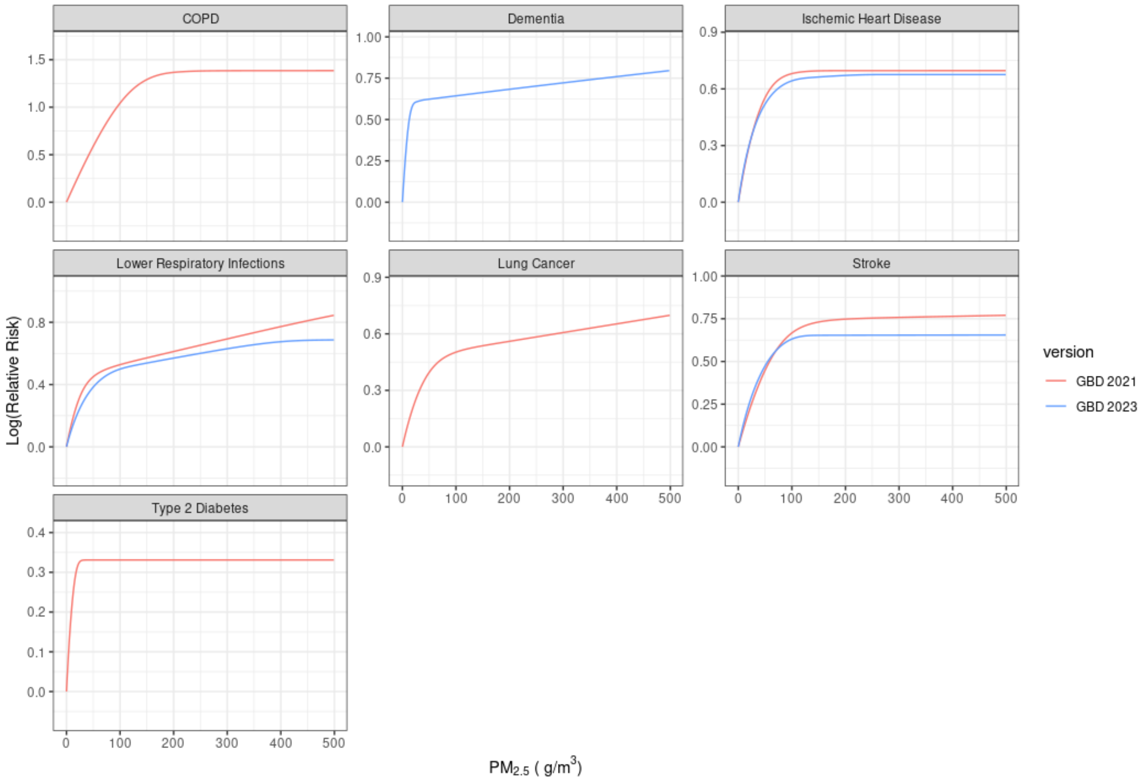

Since GBD 2021, we dropped the use of age-specific risk curves for cardiovascular disease outcomes. Linear regressions on cardiovascular disease input data predicting log(RR) by mean cohort age, with and without random effects on study ID, were fit to ischaemic heart disease and stroke input data separately. None of these regressions showed evidence for a significant association between the two variables. Additionally, we used the MR-BRT automated covariate selection tool (detailed below) to test mean cohort age for significance as a bias covariate and found no significant results. As in GBD 2021, single risk curves for each of the cardiovascular outcomes were applied across all age groups.

For all $PM_{2.5}$ outcomes, the standard error of observations from studies with multiple observations for a single cohort that reported an unstratified sample size were multiplied by the square root of *n*, where *n* is the total number of observations for a given cohort. This adjustment was made to prevent a single cohort or study from unduly weighting the final risk curve.

As in previous GBD cycles, we considered the published relative risk over a range of exposure data when fitting the risk curves. For OAP studies, the relative risk informs the curve from the 5th to the 95th percentile of observed exposure. When this is not available in the published study, we estimate the distribution from the provided information (mean and standard deviation, mean and IQR, etc.). We scale the RR to this range. For HAP studies, we allow each study to inform the curve from the Exp_{OAP} to the Exp_{OAP}+Exp_{HAP}, where Exp_{OAP} is the GBD 2023 estimate of the ambient exposure level in the study location and year, and Exp_{HAP} is the GBD 2023 estimate of the excess exposure for those who use solid fuel for cooking in the study location and year.

MR-BRT risk splines

To estimate relative risk curves for each of the $PM_{2.5}$ outcomes, we used the MR-BRT meta-regression tool to fit splines on the input datasets of OAP and HAP studies. We used the following functional form, where X and X_{CF} represent the range of exposure characterised by the effect size:

$$log\left(\frac{MRBRT(X)}{MRBRT(X_{CF})}\right) \sim \log\,(Published\ Effect\ Size)$$

Several key updates were made to the model fitting methods. For each risk–outcome pair, model settings and priors were tested when fitting the MR-BRT splines. The final models used third-order splines with three interior knots and a constraint on the right-most segment forcing the fit to be linear rather than cubic. Splines were also constrained to be concave and monotonically increasing, the most biologically plausible shape for the $PM_{2.5}$ risk curve. We used an ensemble approach to generate final spline predictions, in which 50 different models were run with randomly placed knots, then weighted and combined based on a measure of fit that penalises excessive changes in the maximum derivative of the curve. Knots were free to be placed across the entire domain of the input exposure data. To prevent over-fitting, on the non-linear segments, we implemented a Gaussian prior on the third derivative of mean 0 and variance 1e-4. On the linear segment, a stronger prior of mean 0 and variance 1e-6 was used to ensure that the risk curves do not continue to increase beyond the range of the data. 10% of all observations were trimmed during model fitting, in accordance with GBD protocol across risk factor teams.

To select significant covariates from those extracted (see table below) to quantify between-study heterogeneity, we performed covariate selection. The MR-BRT automated covariate selection tool implements a two-step process. First, a series of loosening Lasso penalty parameters are applied to a log-linear meta-regression on all input effect size observations. Then, covariates with a non-zero coefficient are tested for significance using a Gaussian prior (significance threshold = 0.05). A Gaussian prior was used on each covariate's beta during spline fitting with a mean 0 and variance of 0.1 multiplied by the standard deviation of the beta from the log-linear meta-regression. Type 2 diabetes was the only outcome for which a significant covariate was identified. Its selected covariate was cv_hap, a binary indicator for whether or not an observation was from a household air pollution study.

Covariate name	Covariate description
cv_subpopulation	Study represents the general population; study represents a subgroup (eg, high-risk group)
cv_exposure_population	Study measures individual-level exposure (≤1 km radius); study measures population-level exposure
cv_exposure_self_report	Exposure is self-reported; exposure is measured externally
cv_exposure_study	Exposure is measured multiple times; exposure is measured only at baseline
cv_outcome_self_report	Outcome is self-reported; outcomes is based on death certificate or medical record
cv_outcome_unblinded	Study implements unblinded assessment; assessment of outcome is blind to exposure (and vice versa)
cv_reverse_causation	Study presents no risk of reverse causation; risk of reverse causation
cv_confounding_nonrandom	Non-randomised study; randomised study
cv_confounding_uncontrolled	Study is randomised/outcome controlled for age, sex, education, income, and all critical determinants of outcome; study is controlled for age, sex, and other critical determinants of outcome; study is controlled for only age and sex
cv_selection_bias	Study reports >95% follow-up; study reports 85–95% follow-up; study reports <85% follow-up
cv_hap	Studies household air pollution; studies ambient air pollution

1000 predictions of the effect size were generated across the exposure distribution for use in calculating burden estimates. These predictions were created by incorporating predictions of between-study heterogeneity to characterise the model's uncertainty. We implemented the Fisher scoring correction to the heterogeneity parameter, which corrects for data-sparse situations. In such cases, the between-study heterogeneity parameter estimate may be 0, simply from lack of data. The Fisher scoring correction uses a quantile of gamma, which is sensitive to the number of studies, study design, and reported uncertainty.

Risk–outcome scoring

Risk–outcome scores provide an empirical measure of the strength of evidence for risk–outcome pairs across risk factors in the GBD and are therefore useful for standardised comparison. Risk-outcome scores evaluate the area between the lower bound of the 95% uncertainty interval and the x-axis for harmful risk factors, including $PM_{2.5}$ pollution.

Prior to generating a risk–outcome score, we conducted an additional post-analysis step to detect and flag publication bias in the input data. This approach is based on the classic Egger's regression strategy, which is applied to the residuals in our model. In the current implementation, we do not correct for publication bias but flag the risk–outcome pairs where the risk for publication bias is significant. Of the $PM_{2.5}$ outcomes, three were flagged for publication bias: birthweight, ischaemic heart disease, and type 2 diabetes. Highlighted rows are outcomes updated in GBD 2023.

Outcome	Egger p-value	Publication bias
Birthweight	0.0208	X
Gestational age	0.249	
Ischaemic heart disease	0.123	
Stroke	0.117	
LRI	0.1738	
Lung cancer	0.191	
COPD	0.423	
Type 2 diabetes	0.0419	X
Dementia	0.0253	X

To calculate the risk–outcome score, we generated an uncertainty interval from 1000 draws of the adjusted summary effect size (retaining uncertainty information from between-study heterogeneity predictions and the Fisher information correction). We then evaluated the risk–outcome score between the 15th and 85th percentiles of the input data exposure distribution. Risk–outcome scores and star ratings are below. Risk–outcome scores are not reported for birthweight and gestational age because these are mediated outcomes. Highlighted rows are outcomes updated in GBD 2023.

Outcome	Risk–outcome score	Star rating
Ischaemic heart disease	0.280	3

Stroke	0.185	3
LRI	0.268	3
Lung cancer	0.342	3
COPD	0.441	4
Type 2 diabetes	0.188	3
Dementia	0.130	2

The following table includes all ambient and household sources used to generate GBD 2023 risk curves. New studies added for GBD 2023 are indicated in **bold** text.

Source	nid	Reference
1	389741	Tielsch JM, Katz J, Thulasiraj RD, Coles CL, Sheeladevi S, Yanik EL, Rahmathullah L. Exposure to indoor biomass fuel and tobacco smoke and risk of adverse reproductive outcomes, mortality, respiratory morbidity and growth among newborn infants in south India. Int J Epidemiol. 2009; 38(5): 1351-63.
2	193971	Victora CG, Fuchs SC, Flores JA, Fonseca W, Kirkwood B. Risk factors for pneumonia among children in a Brazilian metropolitan area. Pediatrics. 1994; 977-85.
3	332647	Azizi BH, Zulkifli HI, Kasim MS. Protective and risk factors for acute respiratory infections in hospitalized urban Malaysian children: a case control study. Southeast Asian J Trop Med Public Health. 1995; 26(2): 280-5.
4	333170	Fonseca W, Kirkwood BR, Victora CG, Fuchs SR, Flores JA, Misago C. Risk factors for childhood pneumonia among the urban poor in Fortaleza, Brazil: a case-control study. Bull World Health Organ. 1996; 74(2): 199-208.
5	193985	Broor S, Pandey RM, Ghosh M, Maitreyi RS, Lodha R, Singhal T, Kabra SK. Risk factors for severe acute lower respiratory tract infection in under-five children. Indian Pediatr. 2001; 1361-9.
6	333174	Mahalanabis D, Gupta S, Paul D, Gupta A, Lahiri M, Khaled MA. Risk factors for pneumonia in infants and young children and the role of solid fuel for cooking: a case-control study. Epidemiol Infect. 2002; 129(1): 65-71.
7	333176	Kumar S, Awasthi S, Jain A, Srivastava RC. Blood zinc levels in children hospitalized with severe pneumonia: a case control study. Indian Pediatr. 2004; 41(5): 486-91.
8	333178	Wayse V, Yousafzai A, Mogale K, Filteau S. Association of subclinical vitamin D deficiency with severe acute lower respiratory infection in Indian children under 5 y. Eur J Clin Nutr. 2004; 58(4): 563-7.
9	333182	Robin LF, Less PS, Winget M, Steinhoff M, Moulton LH, Santosham M, Correa A. Wood-burning stoves and lower respiratory illnesses in Navajo children. Pediatr Infect Dis J. 1996; 15(10): 859-65.
10	173862	Smith KR, McCracken JP, Weber MW, Hubbard A, Jenny A, Thompson LM, Balmes J, Diaz A, Arana B, Bruce N. Effect of reduction in household air pollution on childhood pneumonia in Guatemala (RESPIRE): a randomised controlled trial. Lancet. 2011; 378(9804): 1717-26.
11	335978	Wesley AG, Loening WE. Assessment and 2-year follow-up of some factors associated with severity of respiratory infections in early childhood. *S Afr Med J*. 1996; 86(4): 365–8.
12	333188	Jin C, Rossignol AM. Effects of passive smoking on respiratory illness from birth to age eighteen months, in Shanghai, People's Republic of China. J Pediatr. 1993; 123(4): 553-8.
13	357895	Jeena PM, Ayannusi OE, Annamalai K, Naidoo P, Coovadia HM, Guldner P. Risk factors for admission and the role of respiratory syncytial virus-specific cytotoxic T-lymphocyte responses in children with acute bronchiolitis. S Afr Med J. 2003; 93(4): 291-4.

14	335980	Al-Sonboli N, Hart CA, Al-Aghbari N, Al-Ansi A, Ashoor O, Cuevas LE. Human metapneumovirus and respiratory syncytial virus disease in children, Yemen. *Emerg Infect Dis*. 2006; 12(9): 1437–9.
15	333200	Shen M, Chapman RS, Vermeulen R, Tian L, Zheng T, Chen BE, Engels EA, He X, Blair A, Lan Q. Coal use, stove improvement, and adult pneumonia mortality in Xuanwei, China: a retrospective cohort study. Environ Health Perspect. 2009; 117(2): 261-6.
16	335982	Sasco AJ, Merrill RM, Dari I, Benhaïm-Luzon V, Carriot F, Cann CI, Bartal M. A case-control study of lung cancer in Casablanca, Morocco. *Cancer Causes Control*. 2002; 13(7): 609–16
17	335984	Sapkota A, Gajalakshmi V, Jetly DH, Roychowdhury S, Dikshit RP, Brennan P, Hashibe M, Boffetta P. Indoor air pollution from solid fuels and risk of hypopharyngeal/laryngeal and lung cancers: a multicentric case-control study from India. *Int J Epidemiol*. 2008; 37(2): 321–8.
18	335986	Johnson AW, Aderele WI. The association of household pollutants and socio-economic risk factors with the short-term outcome of acute lower respiratory infections in hospitalized pre-school Nigerian children. *Ann Trop Paediatr*. 1992; 12(4): 421–32.
19	335988	Shah N, Ramankutty V, Premila PG, Sathy N. Risk factors for severe pneumonia in children in south Kerala: a hospital-based case-control study. *J Trop Pediatr*. 1994; 40(4): 201–6
20	336027	Lissowska J, Bardin-Mikolajczak A, Fletcher T, Zaridze D, Szeszenia-Dabrowska N, Rudnai P, Fabianova E, Cassidy A, Mates D, Holcatova I, Vitova V, Janout V, Mannetje A, Brennan P, Boffetta P. Lung cancer and indoor pollution from heating and cooking with solid fuels: the IARC international multicentre case-control study in Eastern/Central Europe and the United Kingdom. *Am J Epidemiol*. 2005; 162(4): 326–33.
21	335990	Gupta D, Boffetta P, Gaborieau V, Jindal SK. Risk factors of lung cancer in Chandigarh, India. *Indian J Med Res*. 2001; 113: 142–50
22	193976	Wu AH, Henderson BE, Pike MC, Yu MC. Smoking and other risk factors for lung cancer in women. J Natl Cancer Inst. 1985; 74(4): 747-51.
23	335992	Luo RX, Wu B, Yi YN, Huang ZW, Lin RT. Indoor burning coal air pollution and lung cancer--a case-control study in Fuzhou, China. *Lung Cancer*. 1996; 14 Suppl 1: S113-119.
24	193940	Ko YC, Lee CH, Chen MJ, Huang CC, Chang WY, Lin HJ, Wang HZ, Chang PY. Risk factors for primary lung cancer among non-smoking women in Taiwan. Int J Epidemiol. 1997; 26(1): 24-31.
25	335997	Le CH, Ko YC, Cheng LS, Lin YC, Lin HJ, Huang MS, Huang JJ, Kao EL, Wang HZ. The heterogeneity in risk factors of lung cancer and the difference of histologic distribution between genders in Taiwan. *Cancer Causes Control*. 2001; 12(4): 289–300.
26	335999	Kleinerman RA, Wang Z, Wang L, Metayer C, Zhang S, Brenner AV, Zhang S, Xia Y, Shang B, Lubin JH. Lung cancer and indoor exposure to coal and biomass in rural China. *J Occup Environ Med*. 2002; 44(4): 338–44.
27	336001	Collings DA, Sithole SD, Martin KS. Indoor woodsmoke pollution causing lower respiratory disease in children. *Trop Doct*. 1990; 20(4): 151–5.
28	336003	Galeone C, Pelucchi C, La Vecchia C, Negri E, Bosetti C, Hu J. Indoor air pollution from solid fuel use, chronic lung diseases and lung cancer in Harbin, Northeast China. *Eur J Cancer Prev*. 2008; 17(5): 473–8
29	336221	Lan Q, He X, Shen M, Tian L, Liu LZ, Lai H, Chen W, Berndt SI, Hosgood HD, Lee K-M, Zheng T, Blair A, Chapman RS. Variation in lung cancer risk by smoky coal subtype in Xuanwei, China. *Int J Cancer*. 2008; 123(9): 2164–9.
30	336005	Akhtar T, Ullah Z, Khan MH, Nazli R. Chronic bronchitis in women using solid biomass fuel in rural Peshawar, Pakistan. *Chest*. 2007; 132(5): 1472–5.
31	336007	Dennis RJ, Maldonado D, Norman S, Baena E, Martinez G. Woodsmoke exposure and risk for obstructive airways disease among women. *Chest*. 1996; 109(1): 115–9.

32	336009	Savitha MR, Nandeeshwara SB, Pradeep Kumar MJ, ul-Haque F, Raju CK. Modifiable risk factors for acute lower respiratory tract infections. *Indian J Pediatr*. 2007; 74(5): 477–82.
33	336011	Sezer H, Akkurt I, Guler N, Marakoğlu K, Berk S. A case-control study on the effect of exposure to different substances on the development of COPD. *Ann Epidemiol*. 2006; 16(1): 59–62.
34	336022	Huang C, Zhang X, Qiao Z, Guan L, Peng S, Liu J, Xie R, Zheng L. A case-control study of dietary factors in patients with lung cancer. *Biomed Environ Sci*. 1992; 5(3): 257–65.
35	336024	Ger LP, Hsu WL, Chen KT, Chen CJ. Risk Factors of Lung Cancer by Histological Category in Taiwan. Anticancer Res. 1993; 13(5A): 1491–500.
36	352222	Kim C, Seow WJ, Shu X-O, Bassig BA, Rothman N, Chen BE, Xiang Y-B, Hosgood HD, Ji B-T, Hu W, Wen C, Chow W-H, Cai Q, Yang G, Gao Y-T, Zheng W, Lan Q. Cooking Coal Use and All-Cause and Cause-Specific Mortality in a Prospective Cohort Study of Women in Shanghai, China. Environ Health Perspect. 2016; 124(9): 1384-9.
37	352226	Alam DS, Chowdhury MAH, Siddiquee AT, Ahmed S, Hossain MD, Pervin S, Streatfield K, Cravioto A, Niessen LW. Adult Cardiopulmonary Mortality and Indoor Air Pollution: A 10-Year Retrospective Cohort Study in a Low-Income Rural Setting. Glob Heart. 2012; 7(3): 215-21.
38	352228	Yu K, Qiu G, Chan K-H, Lam K-BH, Kurmi OP, Bennett DA, Yu C, Pan A, Lv J, Guo Y, Bian Z, Yang L, Chen Y, Hu FB, Chen Z, Li L, Wu T. Association of Solid Fuel Use With Risk of Cardiovascular and All-Cause Mortality in Rural China. JAMA. 2018; 319(13): 1351-61.
39	418511	Hystad P, Duong M, Brauer M, Larkin A, Arku R, Kurmi OP, Fan WQ, Avezum A, Azam I, Chifamba J, Dans A, du Plessis JL, Gupta R, Kumar R, Lanas F, Liu Z, Lu Y, Lopez-Jaramillo P, Mony P, Mohan V, Mohan D, Nair S, Puoane T, Rahman O, Lap AT, Wang Y, Wei L, Yeates K, Rangarajan S, Teo K, Yusuf S, on behalf of Prospective Urban and Rural Epidemiological (PURE) Study investigators. Health Effects of Household Solid Fuel Use: Findings from 11 Countries within the Prospective Urban and Rural Epidemiology Study [Unpublished]. Environ Health Perspect. 2019; 127(5): 57003.
40	415878	Hystad P, Duong M, Brauer M, Larkin A, Arku R, Kurmi OP, Fan WQ, Avezum A, Azam I, Chifamba J, Dans A, du Plessis JL, Gupta R, Kumar R, Lanas F, Liu Z, Lu Y, Lopez-Jaramillo P, Mony P, Mohan V, Mohan D, Nair S, Puoane T, Rahman O, Lap AT, Wang Y, Wei L, Yeates K, Rangarajan S, Teo K, Yusuf S, on behalf of Prospective Urban and Rural Epidemiological (PURE) Study investigators. Health Effects of Household Solid Fuel Use: Findings from 11 Countries within the Prospective Urban and Rural Epidemiology Study. Environ Health Perspect. 2019; 127(5): 57003.
**41**	**561027**	**McCollum ED, McCracken JP, Kirby MA, Grajeda LM, Hossen S, Moulton LH, Simkovich SM, Goodman-Palmer D, Rosa G, Mukeshimana A, Balakrishnan K, Thangavel G, Garg SS, Castanaza A, Thompson LM, Diaz-Artiga A, Papageorghiou AT, Davila-Roman VG, Underhill LJ, Hartinger SM, Williams KN, Nicolaou L, Chang HH, Lovvorn AE, Rosenthal JP, Pillarisetti A, Ye W, Naeher LP, Johnson MA, Waller LA, Jabbarzadeh S, Wang J, Chen Y, Steenland K, Clasen TF, Peel JL, Checkley W, HAPIN Investigators. Liquefied Petroleum Gas or Biomass Cooking and Severe Infant Pneumonia. N Engl J Med. 2024; 390(1): 32-43.**
**42**	**561033**	**Liang R, Chen R, Yin P, van Donkelaar A, Martin RV, Burnett R, Cohen AJ, Brauer M, Liu C, Wang W, Lei J, Wang L, Wang L, Zhang M, Kan H, Zhou M. Associations of long-term exposure to fine particulate matter and its constituents with cardiovascular mortality: A prospective cohort study in China. Environ Int. 2022; 162(107156): 1-9.**
43	174243	Miller KA, Siscovick DS, Sheppard L, Shepherd K, Sullivan JH, Anderson GL, Kaufman JD. Long-term exposure to air pollution and incidence of cardiovascular events in women. N Engl J Med. 2007; 356(5): 447-58.

44	356387	Bowe B, Xie Y, Li T, Yan Y, Xian H, Al-Aly Z. The 2016 global and national burden of diabetes mellitus attributable to PM2¬∑5 air pollution. The Lancet Planetary Health. 2018; 2(7): e301-12.
45	329943	Hart J, Garshick E, Dockery D, Smith T, Ryan L, Laden F. Long-Term Ambient Multipollutant Exposures and Mortality. Am J Respir Crit Care Med. 2011; 183: 75-8.
46	418513	Puett RC, Hart JE, Yanosky JD, Paciorek C, Schwartz J, Suh H, Speizer FE, Laden F. Chronic fine and coarse particulate exposure, mortality, and coronary heart disease in the Nurses' Health Study. Environ Health Perspect. 2009; 117(11): 1697-701.
47	236193	Hart JE, Puett RC, Rexrode KM, Albert CM, Laden F. Effect Modification of Long-Term Air Pollution Exposures and the Risk of Incident Cardiovascular Disease in US Women. J Am Heart Assoc. 2015; 4(12).
48	356402	Honda T, Pun VC, Manjourides J, et al. Associations between long-term exposure to air pollution, glycosylated hemoglobin and diabetes. Int J Hyg Environ Health. 2017, 220 (7): 1124-1132.
49	454724	Puett RC, Hart JE, Schwartz J, et al. Are particulate matter exposures associated with risk of type 2 diabetes? Environ Health Perspect. 2011,119 (3): 384-9.
50	418510	Beelen R, Hoek G, van den Brandt PA, Goldbohm RA, Fischer P, Schouten LJ, Jerrett M, Hughes E, Armstrong B, Brunekreef B. Long-Term Effects of Traffic-Related Air Pollution on Mortality in a Dutch Cohort (NLCS-AIR Study). Environ Health Perspect. 2008; 116(2): 196-202.
51	259386	Beelen R, Hoek G, van den Brandt PA, Goldbohm RA, Fischer P, Schouten LJ, Jerrett M, Hughes E, Armstrong B, Brunekreef B. Long-Term Effects of Traffic-Related Air Pollution on Mortality in a Dutch Cohort (NLCS-AIR Study). Environ Health Perspect. 2008; 116(2): 196-202.
52	174242	Hertz-Picciotto I, Baker RJ, Yap P-S, Dostál M, Joad JP, Lipsett M, Greenfield T, Herr CEW, Benes I, Shumway RH, Pinkerton KE, Srám R. Early childhood lower respiratory illness and air pollution. Environ Health Perspect. 2007; 115(10): 1510-8.
53	454729	Pope CA, Lefler JS, Ezzati M, et al. 2019. Mortality Risk and Fine Particulate Air Pollution in a Large, Representative Cohort of US Adults. Environmental Health Perspectives, 127(7), 077007.
54	454735	Parker JD, Kravets N, Vaidyanathan A. 2018. Particulate matter air pollution exposure and heart disease mortality risks by race and ethnicity in the United States: 1997 to 2009 National Health Interview Survey with mortality follow-up through 2011. Circulation, 137(16), 1688-1697.
55	174250	Carey IM, Atkinson RW, Kent AJ, van Staa T, Cook DG, Anderson HR. Mortality associations with long-term exposure to outdoor air pollution in a national English cohort. Am J Respir Crit Care Med. 2013; 187(11): 1226-33.
56	174256	Hystad P, Demers PA, Johnson KC, Carpiano RM, Brauer M. Long-term residential exposure to air pollution and lung cancer risk. Epidemiology. 2013; 24(5): 762-72.
57	174257	Katanoda K, Sobue T, Satoh H, Tajima K, Suzuki T, Nakatsuka H, Takezaki T, Nakayama T, Nitta H, Tanabe K, Tominaga S. An association between long-term exposure to ambient air pollution and mortality from lung cancer and respiratory diseases in Japan. J Epidemiol. 2011; 21(2): 132-43.
58	236192	Gan WQ, FitzGerald JM, Carlsten C, Sadatsafavi M, Brauer M. Associations of ambient air pollution with chronic obstructive pulmonary disease hospitalization and mortality. Am J Respir Crit Care Med. 2013; 187(7): 721-7.
59	236200	Cesaroni G, Badaloni C, Gariazzo C, Stafoggia M, Sozzi R, Davoli M, Forastiere F. Long-term exposure to urban air pollution and mortality in a cohort of more than a million adults in Rome. Environ Health Perspect. 2013; 121(3): 324-31.
60	251023	Pinault L, Tjepkema M, Crouse DL, Weichenthal S, van Donkelaar A, Martin RV, Brauer M, Chen H, Burnett RT. Risk estimates of mortality attributed to low concentrations of ambient fine particulate matter in the Canadian community health survey cohort. Environ Health. 2016; 15(1): 18.
61	418468	Pinault L, Tjepkema M, Crouse DL, Weichenthal S, van Donkelaar A, Martin RV, Brauer M, Chen H, Burnett RT. Risk estimates of mortality attributed to low

		concentrations of ambient fine particulate matter in the Canadian community health survey cohort. Environ Health. 2016; 15(1): 18.
62	251335	Naess Ø, Nafstad P, Aamodt G, Claussen B, Rosland P. Relation between concentration of air pollution and cause-specific mortality: four-year exposures to nitrogen dioxide and particulate matter pollutants in 470 neighborhoods in Oslo, Norway. Am J Epidemiol. 2007; 165(4): 435-43.
63	259376	Villeneuve PJ, Weichenthal SA, Crouse D, Miller AB, To T, Martin RV, van Donkelaar A, Wall C, Burnett RT. Long-term exposure to fine particulate matter air pollution and mortality among Canadian women. Epidemiology. 2015; 26(4): 536-45.
64	234298	Atkinson RW, Carey IM, Kent AJ, van Staa TP, Anderson HR, Cook DG. Long-term exposure to outdoor air pollution and the incidence of chronic obstructive pulmonary disease in a national English cohort. Occup Environ Med. 2015; 72(1): 42-8.
65	367916	Beelen R, Stafoggia M, Raaschou-Nielsen O, Andersen ZJ, et al. Long-term exposure to air pollution and cardiovascular mortality: an analysis of 22 European cohorts. Epidemiology. 2014; 25(3): 368-378.
66	236188	Stafoggia M, Cesaroni G, Peters A, Andersen ZJ, Badaloni C, Beelen R, Caracciolo B, Cyrys J, de Faire U, de Hoogh K, Eriksen KT, Fratiglioni L, Galassi C, Gigante B, Havulinna AS, Hennig F, Hilding A, Hoek G, Hoffmann B, Houthuijs D, Korek M, Lanki T, Leander K, Magnusson PK, Meisinger C, Migliore E, Overvad K, Ostenson C-G, Pedersen NL, Pekkanen J, Penell J, Pershagen G, Pundt N, Pyko A, Raaschou-Nielsen O, Ranzi A, Ricceri F, Sacerdote C, Swart WJR, Turunen AW, Vineis P, Weimar C, Weinmayr G, Wolf K, Brunekreef B, Forastiere F. Long-term exposure to ambient air pollution and incidence of cerebrovascular events: results from 11 European cohorts within the ESCAPE project. Environ Health Perspect. 2014; 122(9): 919-25.
67	367753	MacIntyre EA, Gehring U, Mölter A, et al. Air Pollution and Respiratory Infections during Early Childhood: An Analysis of 10 European Birth Cohorts within the ESCAPE Project. Environ Health Perspect. 2014; 122(1): 107-13.
68	235380	Raaschou-Nielsen O, Andersen ZJ, Beelen R, et al. Air pollution and lung cancer incidence in 17 European cohorts: prospective analyses from the European Study of Cohorts for Air Pollution Effects (ESCAPE). Lancet Oncol. 2013; 14(9): 813-22.
69	329945	Gan WQ, Koehoorn M, Davies H, Demers P, Tamburic L, Brauer M. Long-Term Exposure to Traffic-Related Air Pollution and the Risk of Coronary Heart Disease Hospitalization and Mortality. Environ Health Perspect. 2011; 119(4): 501-7.
70	335965	Park, Sung Kyun, Sara D. Adar, Marie S. O'Neill, Amy H. Auchincloss, Adam Szpiro, Alain G. Bertoni, Ana Navas-Acien, Joel D. Kaufman, and Ana V. Diez-Roux. Long-Term Exposure to Air Pollution and Type 2 Diabetes Mellitus in a Multiethnic Cohort. American Journal of Epidemiology 181, no. 5 (March 1, 2015): 327-36. https://doi.org/10.1093/aje/kwu280.
71	174240	Karr CJ, Rudra CB, Miller KA, Gould TR, Larson T, Sathyanarayana S, Koenig JQ. Infant exposure to fine particulate matter and traffic and risk of hospitalization for RSV bronchiolitis in a region with lower ambient air pollution. Environ Res. 2009; 109(3): 321-7.
72	335959	Chen, Hong, Richard T. Burnett, Jeffrey C. Kwong, Paul J. Villeneuve, Mark S. Goldberg, Robert D. Brook, Aaron van Donkelaar, et al. ‚ÄúRisk of Incident Diabetes in Relation to Long-Term Exposure to Fine Particulate Matter in Ontario, Canada.‚Äù Environmental Health Perspectives 121, no. 7 (July 2013): 804-10. https://doi.org/10.1289/ehp.1205958.
73	174247	Karr C, Lumley T, Schreuder A, Davis R, Larson T, Ritz B, Kaufman J. Effects of subchronic and chronic exposure to ambient air pollutants on infant bronchiolitis. Am J Epidemiol. 2007; 165(5): 553-60.
74	335963	Weinmayr, Gudrun, Frauke Hennig, Kateryna Fuks, et al. Long-Term Exposure to Fine Particulate Matter and Incidence of Type 2 Diabetes Mellitus in a Cohort Study: Effects of Total and Traffic-Specific Air Pollution. Environmental Health 14 (June 19, 2015): 53. https://doi.org/10.1186/s12940-015-0031-x.

75	335967	To T, Zhu J, Villeneuve PJ, Simatovic J, Feldman L, Gao C, Williams D, Chen H, Weichenthal S, Wall C, Miller AB. Chronic disease prevalence in women and air pollution--A 30-year longitudinal cohort study. Environ Int. 2015; 80: 26-32.
76	335971	Clark C, Sbihi H, Tamburic L, Brauer M, Frank LD, Davies HW. Association of Long-Term Exposure to Transportation Noise and Traffic-Related Air Pollution with the Incidence of Diabetes: A Prospective Cohort Study. Environ Health Perspect. 2017; 125(8): 087025.
77	174254	Puett RC, Hart JE, Suh H, Mittleman M, Laden F. Particulate matter exposures, mortality, and cardiovascular disease in the health professionals follow-up study. Environ Health Perspect. 2011; 119(8): 1130-5.
78	336031	Tseng E, Ho W-C, Lin M-H, Cheng T-J, Chen P-C, Lin H-H. Chronic exposure to particulate matter and risk of cardiovascular mortality: cohort study from Taiwan. BMC Public Health. 2015; 15: 936.
79	336033	Yin P, Brauer M, Cohen A, Burnett RT, Liu J, Liu Y, Liang R, Wang W, Qi J, Wang L, Zhou M. Long-term Fine Particulate Matter Exposure and Nonaccidental and Cause-specific Mortality in a Large National Cohort of Chinese Men. Environ Health Perspect. 2017; 125(11): 117002.
80	418507	Yin P, Brauer M, Cohen A, Burnett RT, Liu J, Liu Y, Liang R, Wang W, Qi J, Wang L, Zhou M. Long-term Fine Particulate Matter Exposure and Nonaccidental and Cause-specific Mortality in a Large National Cohort of Chinese Men. Environ Health Perspect. 2017; 125(11): 117002.
81	352234	Wong CM, Lai HK, Tsang H, Thach TQ, Thomas GN, Lam KBH, Chan KP, Yang L, Lau AKH, Ayres JG, Lee SY, Man Chan W, Hedley AJ, Lam TH. Satellite-Based Estimates of Long-Term Exposure to Fine Particles and Association with Mortality in Elderly Hong Kong Residents. Environ Health Perspect. 2015; 123(11): 1167-72.
82	259390	Lepeule J, Laden F, Dockery D, Schwartz J. Chronic exposure to fine particles and mortality: an extended follow-up of the Harvard Six Cities study from 1974 to 2009. Environ Health Perspect. 2012; 120(7): 965-70.
83	418531	Lepeule J, Laden F, Dockery D, Schwartz J. Chronic exposure to fine particles and mortality: an extended follow-up of the Harvard Six Cities study from 1974 to 2009. Environ Health Perspect. 2012; 120(7): 965-70.
84	356414	Hansen AB, Ravnskjær L, Loft S, Andersen KK, Bräuner EV, Baastrup R, Yao C, Ketzel M, Becker T, Brandt J, Hertel O, Andersen ZJ. Long-term exposure to fine particulate matter and incidence of diabetes in the Danish Nurse Cohort. Environ Int. 2016; 91: 243–50.
85	415478	Qiu H, Schooling CM, Sun S, Tsang H, Yang Y, Lee RS, Wong CM, Tian L. Long-term exposure to fine particulate matter air pollution and type 2 diabetes mellitus in elderly: A cohort study in Hong Kong. *Environ Int*. 2018; 113: 350-356.
86	329948	Pope CA, Burnett R, Thun M, Calle E, Krewski D, Ito K, Thurston G. Lung Cancer, Cardiopulmonary Mortality, and Long-term Exposure to Fine Particulate Air Pollution. JAMA. 2002; 287(9): 1132-41.
87	345241	Burnett RT. Cox Proportional Survival Model Hazard Ratios from Census Year to 2011 for Adults Aged 25 to 89 in CanCHEC Cohort.
88	421200	Hystad P et al. Outdoor fine particulate matter air pollution and cardiovascular disease: Results from 747 communities across 21 countries in the PURE study
89	438582	Huang K, Liang F, Yang X, Liu F, Li J, Xiao Q, Chen J, Liu X, Cao J, Shen C, Yu L, Lu F, Wu X, Zhao L, Wu X, Li Y, Hu D, Huang J, Liu Y, Lu X, Gu D. Long term exposure to ambient fine particulate matter and incidence of stroke: prospective cohort study from the China-PAR project. BMJ. 2019; 367: l6720.
90	438580	Qiu H, Sun S, Tsang H, Wong CM, Lee RS, Schooling CM, Tian L. Fine particulate matter exposure and incidence of stroke: A cohort study in Hong Kong. Neurology. 2017; 88(18): 1709-1717.
91	174244	Lipsett MJ, Ostro BD, Reynolds P, Goldberg D, Hertz A, Jerrett M, Smith DF, Garcia C, Chang ET, Bernstein L. Long-term exposure to air pollution and cardiorespiratory disease in the California teachers study cohort. Am J Respir Crit Care Med. 2011; 184(7): 828-35.

92	259372	Lipsett MJ, Ostro BD, Reynolds P, Goldberg D, Hertz A, Jerrett M, Smith DF, Garcia C, Chang ET, Bernstein L. Long-term exposure to air pollution and cardiorespiratory disease in the California teachers study cohort. Am J Respir Crit Care Med. 2011; 184(7): 828-35.
93	335969	Coogan PF, White LF, Yu J, Burnett RT, Seto E, Brook RD, Palmer JR, Rosenberg L, Jerrett M. PM2.5 and Diabetes and Hypertension Incidence in the Black Women's Health Study. Epidemiology. 2016; 27(2): 202-10.
94	452892	Cakmak S, Hebbern C, Pinault L, Lavigne E, Vanos J, Crouse DL, Tjepkema M. Associations between long-term PM_{2.5} and ozone exposure and mortality in the Canadian Census Health and Environment Cohort (CANCHEC), by spatial synoptic classification zone. Environ Int. 2018; 111: 200-211.
95	174252	Chen LH, Knutsen SF, Shavlik D, Beeson WL, Petersen F, Ghamsary M, Abbey D. The association between fatal coronary heart disease and ambient particulate air pollution: Are females at greater risk? Environ Health Perspect. 2005; 113(12): 1723-9.
96	454716	Garcia CA, Yap PS, Park HY, Weller BL. 2016. Association of long-term PM2.5 exposure with mortality using different air pollution exposure models: impacts in rural and urban California. International Journal of Environmental Health Research, 26(2), 145-157
97	454718	Jerrett M, Burnett RT, Beckerman BS, et al. 2013. Spatial analysis of air pollution and mortality in California. American Journal of Respiratory and Critical Care Medicine, 188(5), 593-599.
98	369122	Weichenthal S, Villeneuve PJ, Burnett RT, van Donkelaar A, Martin RV, Jones RR, DellaValle CT, Sandler DP, Ward MH, Hoppin JA. Long-term exposure to fine particulate matter: association with nonaccidental and cardiovascular mortality in the agricultural health study cohort. Environ Health Perspect. 2014; 122(6): 609-15.
99	174252	Chen LH, Knutsen SF, Shavlik D, Beeson WL, Petersen F, Ghamsary M, Abbey D. The association between fatal coronary heart disease and ambient particulate air pollution: Are females at greater risk? Environ Health Perspect. 2005; 113(12): 1723-9.
100	454746	Turner MC, Krewski D, Pope CA, et al. 2011. Long-term ambient fine particulate matter air pollution and lung cancer in a large cohort of never-smokers. American Journal of Respiratory and Critical Care Medicine, 184(12), 1374-1381.
101	259393	Turner MC,Jerrett M, Pope CA 3rd, Krewski D, Gapstur SM, Diver WR, Beckerman BS, Marshall JD, Su J, Crouse DL, Burnett RT. Long-term ozone exposure and mortality in a large prospective study . Am J Respir Crit Care Med. 2016; 193(10): 1134-42.
102	454732	Pinault L, Brauer M, Crouse DL, et al. 2018. Diabetes status and susceptibility to the effects of PM2.5 exposure on cardiovascular mortality in a National Canadian Cohort. Epidemiology, 29(6), 784-794.
103	454739	Ostro B, Hu J, Goldberg D, et al. 2015. Associations of mortality with long-term exposures to fine and ultrafine particles, species and sources: results from the California Teachers Study Cohort. Environmental Health Perspectives, 123(6), 549-556.
104	418506	Thurston GD, Ahn J, Cromar KR, Shao Y, Reynolds HR, Jerrett M, Lim CC, Shanley R, Park Y, Hayes RB. Ambient particulate matter air pollution exposure and mortality in the NIH-AARP diet and health cohort. Environ Health Perspect. 2016; 124(4): 484-90.
105	454748	Renzi M, Cerza F, Gariazzo C, et al. Air pollution and occurrence of type 2 diabetes in a large cohort study. Environ Int. 2018, 112: 68-76.
106	415814	lim et al 2018
107	415478	Qiu H, Schooling CM, Sun S, et al. Long-term exposure to fine particulate matter air pollution and type 2 diabetes mellitus in elderly: A cohort study in Hong Kong. Environ Int. 2018, 113: 350-356.
108	454753	Cramer J, Jørgensen JT, Hoffmann B, Loft S, Bräuner EV, Prescott E, Ketzel M, Hertel O, Brandt J, Jensen SS, Backalarz C, Simonsen MK, Andersen ZJ. Long-Term Exposure

		to Air Pollution and Incidence of Myocardial Infarction: A Danish Nurse Cohort Study. *Environ Health Perspect*. 2020; 128(5): 57003.
**109**	**464987**	**Chen H, Kwong JC, Copes R, Hystad P, van Donkelaar A, Tu K, Brook JR, Goldberg MS, Martin RV, Murray BJ, Wilton AS, Kopp A, Burnett RT. Exposure to ambient air pollution and the incidence of dementia: A population-based cohort study. Environ Int. 2017 Nov;108:271-277. doi: 10.1016/j.envint.2017.08.020. Epub 2017 Sep 13. PMID: 28917207.**
**110**	**464992**	**Carey IM, Anderson HR, Atkinson RW, Beevers SD, Cook DG, Strachan DP, Dajnak D, Gulliver J, Kelly FJ. Are noise and air pollution related to the incidence of dementia? A cohort study in London, England. BMJ Open. 2018 Sep 11;8(9):e022404. doi: 10.1136/bmjopen-2018-022404. PMID: 30206085; PMCID: PMC6144407.**
**111**	**465331**	**Jung CR, Lin YT, Hwang BF. Ozone, particulate matter, and newly diagnosed Alzheimer's disease: a population-based cohort study in Taiwan. J Alzheimers Dis. 2015;44(2):573-84. doi: 10.3233/JAD-140855. PMID: 25310992.**
**112**	**465328**	**Oudin A, Segersson D, Adolfsson R, Forsberg B. Association between air pollution from residential wood burning and dementia incidence in a longitudinal study in Northern Sweden. PLoS One. 2018 Jun 13;13(6):e0198283. doi: 10.1371/journal.pone.0198283. PMID: 29897947; PMCID: PMC5999109.**
**113**	**472344**	**Smargiassi A, Sidi EAL, Robert LE, Plante C, Haddad M, Gamache P, Burnett R, Goudreau S, Liu L, Fournier M, Pelletier E, Yankoty I. Exposure to ambient air pollutants and the onset of dementia in Québec, Canada. Environ Res. 2020 Nov;190:109870. doi: 10.1016/j.envres.2020.109870. Epub 2020 Jul 23. PMID: 32739624.**
**114**	**472333**	**Younan D, Wang X, Casanova R, Barnard R, Gaussoin SA, Saldana S, Petkus AJ, Beavers DP, Resnick SM, Manson JE, Serre ML, Vizuete W, Henderson VW, Sachs BC, Salinas JA, Gatz M, Espeland MA, Chui HC, Shumaker SA, Rapp SR, Chen JC; Women's Health Initiative. PM2.5 associated with gray matter atrophy reflecting increased Alzheimer's risk in older women. Neurology. 2020 Nov 18:10.1212/WNL.0000000000011149. doi: 10.1212/WNL.0000000000011149. Epub ahead of print. PMID: 33208540.**
**115**	**472335**	**Ran J, Schooling CM, Han L, Sun S, Zhao S, Zhang X, Chan KP, Guo F, Lee RS, Qiu Y, Tian L. Long-term exposure to fine particulate matter and dementia incidence: A cohort study in Hong Kong. Environ Pollut. 2021 Feb 15;271:116303. doi: 10.1016/j.envpol.2020.116303. Epub 2020 Dec 25. PMID: 33370610.**
**116**	**472337**	**Shi L, Wu X, Danesh Yazdi M, Braun D, Abu Awad Y, Wei Y, Liu P, Di Q, Wang Y, Schwartz J, Dominici F, Kioumourtzoglou MA, Zanobetti A. Long-term effects of PM2¬∑5 on neurological disorders in the American Medicare population: a longitudinal cohort study. Lancet Planet Health. 2020 Dec;4(12):e557-e565. doi: 10.1016/S2542-5196(20)30227-8. Epub 2020 Oct 19. PMID: 33091388; PMCID: PMC7720425.**
**117**	**472579**	**Grande G, Ljungman PLS, Eneroth K, Bellander T, Rizzuto D. Association Between Cardiovascular Disease and Long-term Exposure to Air Pollution With the Risk of Dementia. JAMA Neurol. 2020 Jul 1;77(7):801-809. doi: 10.1001/jamaneurol.2019.4914. PMID: 32227140; PMCID: PMC7105952.**
**118**	**472576**	**Mortamais M, Gutierrez LA, de Hoogh K, Chen J, Vienneau D, Carrière I, Letellier N, Helmer C, Gabelle A, Mura T, Sunyer J, Benmarhnia T, Jacquemin B, Berr C. Long-term exposure to ambient air pollution and risk of dementia: Results of the prospective Three-City Study. Environ Int. 2021 Mar;148:106376. doi: 10.1016/j.envint.2020.106376. Epub 2021 Jan 20. PMID: 33484961.**
**119**	**486780**	**Shaffer RM, Blanco MN, Li G, Adar SD, Carone M, Szpiro AA, Kaufman JD, Larson TV, Larson EB, Crane PK, Sheppard L. Fine Particulate Matter and Dementia Incidence in the Adult Changes in Thought Study. Environ Health Perspect. 2021; 129(8): 87001.**
**120**	**490458**	**Younan D, Wang X, Gruenewald T, Gatz M, Serre ML, Vizuete W, Braskie MN, Woods NF, Kahe K, Garcia L, Lurmann F, Manson JE, Chui HC, Wallace RB,**

		**Espeland MA, Chen JC. Racial/Ethnic Disparities in Alzheimer's Disease Risk: Role of Exposure to Ambient Fine Particles. J Gerontol A Biol Sci Med Sci. 2021.**
**121**	**490462**	**Sullivan KJ, Ran X, Wu F, Chang CH, Sharma R, Jacobsen E, Berman S, Snitz BE, Sekikawa A, Talbott EO, Ganguli M. Ambient fine particulate matter exposure and incident mild cognitive impairment and dementia. J Am Geriatr Soc. 2021; 69(8): 2185-2194.**
**122**	**490464**	**Cerza F, Renzi M, Gariazzo C, Davoli M, Michelozzi P, Forastiere F, Cesaroni G. Long-term exposure to air pollution and hospitalization for dementia in the Rome longitudinal study. Environ Health. 2019; 18(1): 72.**
**123**	**490466**	**Ilango SD, Chen H, Hystad P, van Donkelaar A, Kwong JC, Tu K, Martin RV, Benmarhnia T. The role of cardiovascular disease in the relationship between air pollution and incident dementia: a population-based cohort study. Int J Epidemiol. 2020; 49(1): 36-44.**
**124**	**490468**	**Yuchi W, Sbihi H, Davies H, Tamburic L, Brauer M. Road proximity, air pollution, noise, green space and neurologic disease incidence: a population-based cohort study. Environ Health. 2020; 19(1): 8.**
**125**	**490761**	**Bowe B, Xie Y, Yan Y, Al-Aly Z. Burden of Cause-Specific Mortality Associated With PM2.5 Air Pollution in the United States. *JAMA Netw Open*. 2019; 2(11): e1915834.**
**126**	**492954**	**Shi L, Steenland K, Li H, Liu P, Zhang Y, Lyles RH, Requia WJ, Ilango SD, Chang HH, Wingo T, Weber RJ, Schwartz J. A national cohort study (2000-2018) of long-term air pollution exposure and incident dementia in older adults in the United States. Nat Commun. 2021; 12(1): 6754.**

The following figures display new risk curves developed for GBD 2023. We included new data for lower respiratory infections, ischaemic heart disease and stroke; and added dementia as a new risk factor. Risk curves for chronic obstructive pulmonary disease, type 2 diabetes, and lung cancer are available in the burden of proof visualization tool and from the GHDx. In each figure, the first plot depicts the typical range of outdoor exposures, whereas the second plot includes higher levels typical of household air pollution exposure. The grey shaded areas represent 95% UI.

Each point or number represents one study effect size. Each is plotted at the 95th percentile of the exposure distribution (OAP) or the expected level of exposure for individual using solid fuel (HAP). The relative risk is plotted relative to the predicted relative risk at the 5th percentile of exposure distribution (OAP) or the expected (ambient only) level of exposure for individuals not using solid fuel (HAP). For example, a study predicting a relative risk of 1.5 for an exposure range of 10 to 20 would be plotted at (20, MRBRT(10)*1.5). Arrows represent studies that would have been outside the range of the plot but have been shifted to be included in the figure.

Ischemic Heart Disease, Low Exposure Range

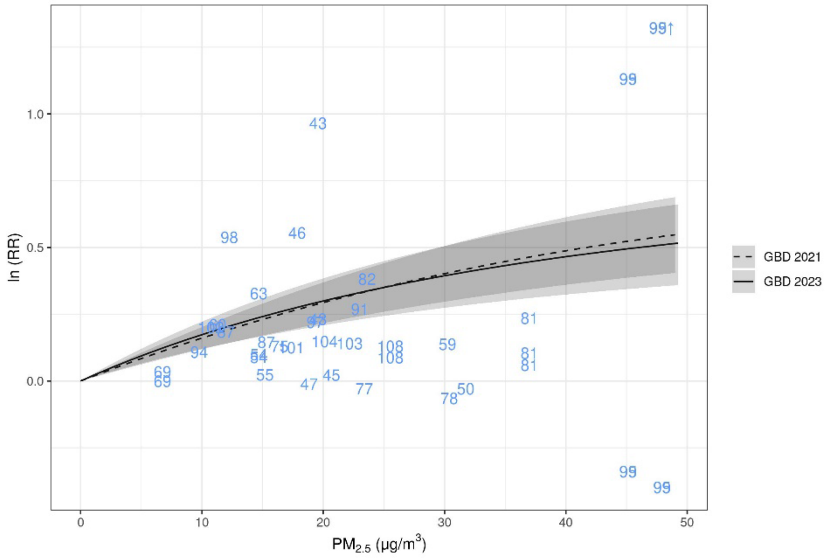

Ischemic Heart Disease, Full Exposure Range

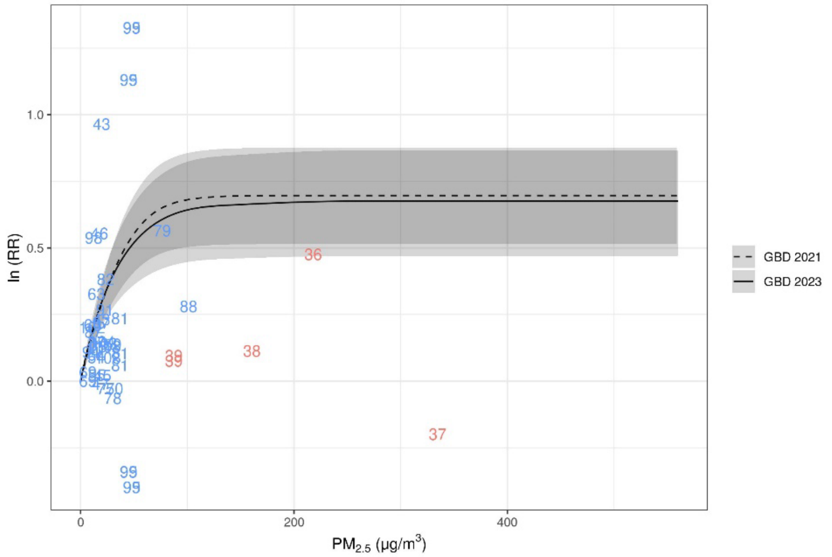

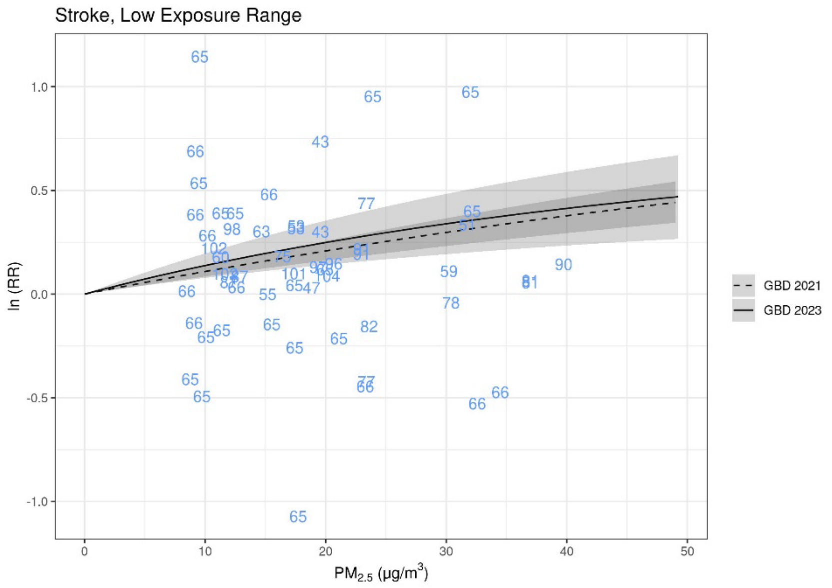
Stroke, Low Exposure Range
ln (RR)
PM$_{2.5}$ (μg/m^3)
GBD 2021
GBD 2023

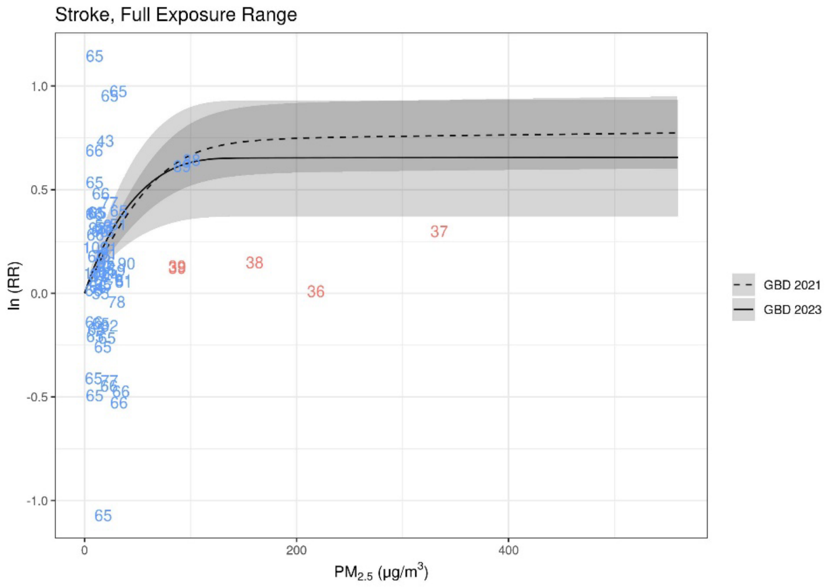
Stroke, Full Exposure Range
ln (RR)
PM$_{2.5}$ (μg/m^3)
GBD 2021
GBD 2023

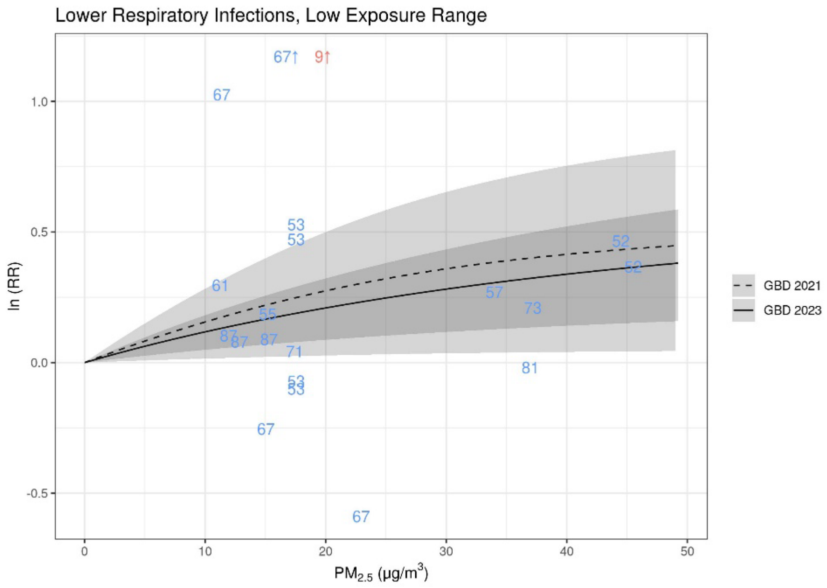
Lower Respiratory Infections, Low Exposure Range
ln (RR)
PM2.5 (μg/m3)
GBD 2021
GBD 2023

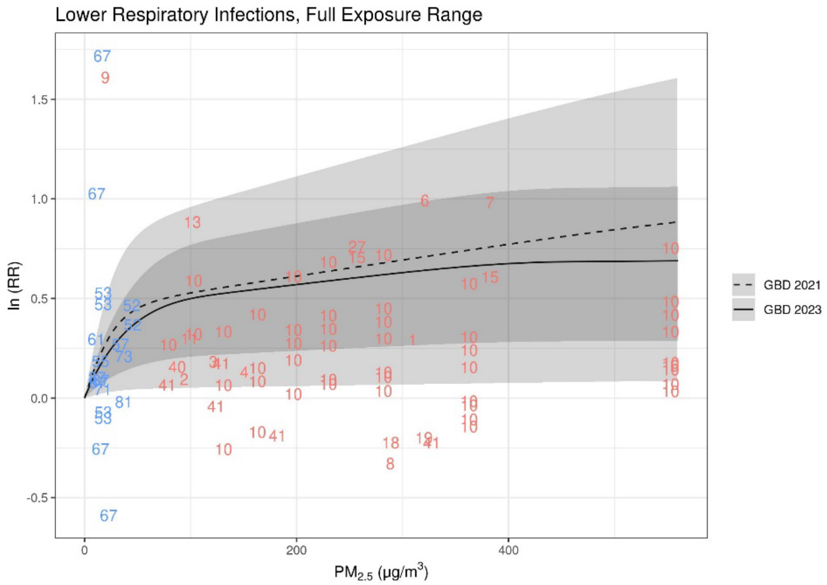
Lower Respiratory Infections, Full Exposure Range
ln (RR)
PM2.5 (μg/m3)
GBD 2021
GBD 2023

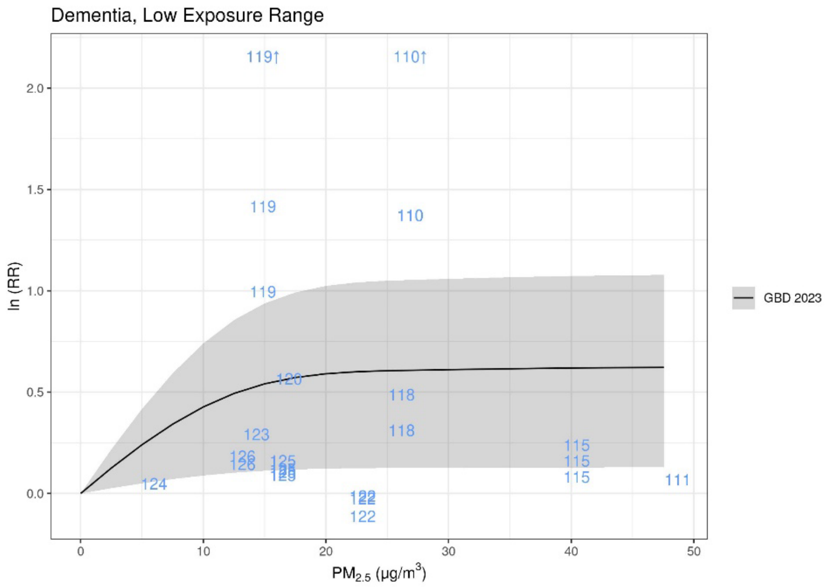

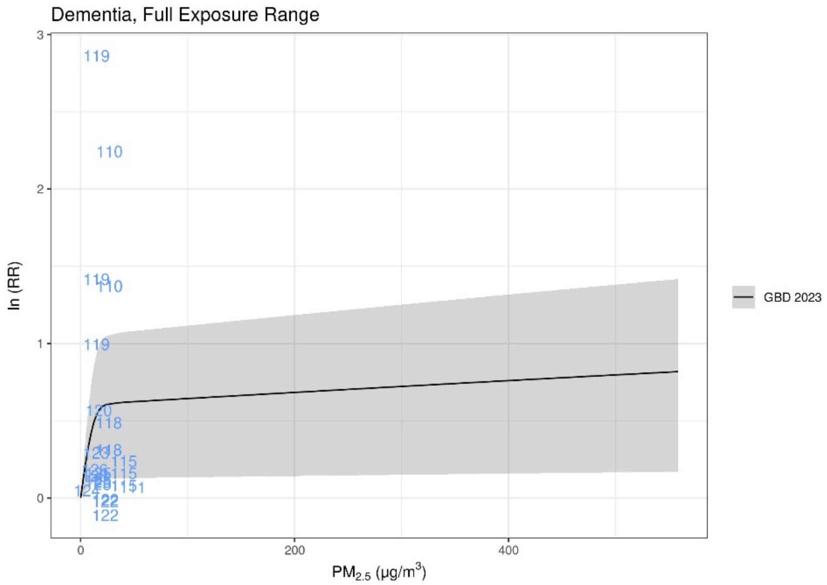

Low birthweight and short gestation mediation analysis

Since GBD 2019, low birthweight and short gestation were included as $PM_{2.5}$ outcomes via a mediation analysis. No changes were made to the modelling for GBD 2023. Low birthweight and short gestation includes mortality due to diarrhoeal diseases, lower respiratory infections, upper respiratory infections, otitis media, meningitis, encephalitis, neonatal preterm birth, neonatal encephalopathy due to birth asphyxia and trauma, neonatal sepsis and other neonatal infections, haemolytic disease and other neonatal jaundice, and other neonatal disorders. Morbidity estimates were also calculated for neonatal preterm birth. These outcomes are specific to the neonatal ages: 0–6 days and 7–27 days.

The following is a summary of methods used to conduct the mediation analysis. For GBD 2019, we conducted a systematic review of all cohort, case-control, or randomised-controlled trial studies of ambient $PM_{2.5}$ pollution or household air pollution and birthweight or gestational age outcomes for GBD 2019.[10] Outcomes measured included continuous birthweight (bw), continuous gestational age

(ga), low birthweight (LBW) (<2500 g), preterm birth (PTB) (<37 weeks), and very preterm birth (VPTB) (<32 weeks). We included any papers published until April 4, 2021.

Birthweight and gestational age are modelled using a continuous joint distribution for the GBD. To determine how these distributions are influenced by $PM_{2.5}$ pollution, we used available literature to model the continuous shift in birthweight (bw, grams) and gestational age (ga, weeks) at a given $PM_{2.5}$ exposure level. When available, we used estimates of continuous shifts in bw or ga directly from each study. When shifts were not available, we converted the published OR/RR/HR for LBW, PTB, or VPTB using the following strategy:

1. Extract the OR/RR/HR from the study.
2. Select the GBD 2017 estimated bw-ga joint distribution for the study location and year.
3. Calculate the number of grams or weeks required to shift the distribution such that the proportion of births under the specified threshold (P) is reduced by the study effect size to a counterfactual level (P_{cf}).
4. Save the resulting shift and 95% CI as the continuous effect.

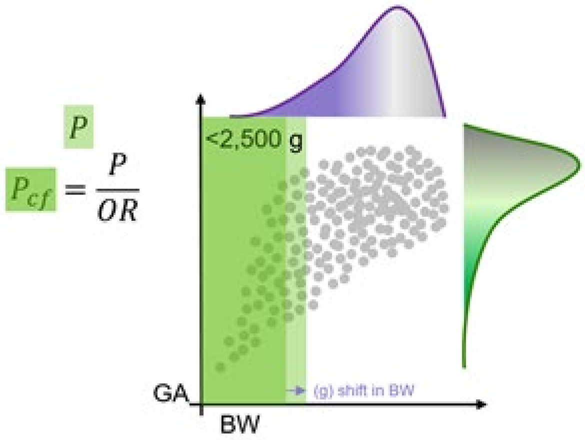

When preparing HAP data to fit splines, we used the same strategy described above for other outcomes to map HAP input data to $PM_{2.5}$ exposure values. We then fit MR-BRT splines to the input studies, where the difference in the value of the model at the upper concentration (X) and the value of the model at the counterfactual concentration (X_{CF}) is equal to the published or calculated shift in bw or ga:

$$MRBRT(X) - MRBRT(X_{CF}) \sim Shift$$

We used the same model fitting process, settings, and covariate selection process as described above for the other outcomes. The only exception is that, because the change in birthweight and gestational age was expected to be negative, the splines were constrained to be monotonically decreasing.

No changes were made to risk curves for GBD 2023.

We used the curves of estimated shifts across the exposure range to predict the shift in both birthweight and gestational age for total female particulate matter pollution exposure in each location and year. Because the epidemiological studies mutually controlled for birthweight and gestational age, we assumed these shifts are independent. We then shifted the observed distributions to reflect the expected bwga distribution in the absence of particulate matter pollution. These shifted distributions were used as the counterfactual in the PAF calculation equation to calculate the burden attributable to $PM_{2.5}$ pollution.

To calculate PAFs, the distribution is divided into 56 bw-ga categories, each with a unique RR. Let p_i be the observed proportion of babies in category, *i* and p_i' be the counterfactual proportion of babies in category, *i* if there were no particulate matter pollution.

$$PAF_{PM} = \frac{\sum_{i\, \in bwga\ category} RR_i p_i - \sum_{i \in bwga\ category} RR_i p_i'}{\sum_{i\, \in bwga} RR_i p_i}$$

We proportionately split this PAF to ambient and HAP based on exposure as described below. One important assumption to note is that we assume the shift in bw and ga is linear across the bwga distribution.

For lower respiratory infections, $PM_{2.5}$-attributable PAFs are directly estimated in addition to estimated through bwga mediation. We expect that some of the directly estimated PAFs are mediated through bw and ga. Additionally, the directly estimated PAF is based on a summary of relative risks for all children under 5 years, so there is a possibility that the mediated PAF, which is more finely resolved, could be greater. To avoid double counting, for the two neonatal age groups (0–6 days and 0–27 days), we take the maximum of the two PAF estimates. If the directly estimated PAF is greater than the bwga-mediated PAF, we take the direct estimate, and if the mediated PAF is greater, we take the mediated estimate.

PTB incidence and mortality are both outcomes measured in the GBD. 100% of the burden for this cause is attributable to short gestation. To calculate the percentage attributable to particulate matter pollution, we estimated the percentage of babies born at less than 37 weeks (p_{ptb}) and the percentage of babies that would have been born at less than 37 weeks in the counterfactual scenario of no particulate matter pollution (p_{ptb}').

$$PAF_{ptb,pm} = 1 - \frac{p_{ptb}'}{p_{ptb}}$$

Limitations

Since GBD 2021 we have not used active smoking or secondhand smoking data to estimate $PM_{2.5}$ risk curves; however, we still use an integrated exposure–response approach because we integrate relative risk estimates across ambient and HAP sources. The use of both source types to construct a risk curve with $PM_{2.5}$ as the exposure indicator assumes equitoxicity of particles regardless of source, despite evidence suggesting differences in health impacts by specific PM source (eg, motor vehicles, coal-fired power plant), size, and/or chemical composition. However, in the absence of sufficient estimates of source- or composition-specific exposure–response relationships and consistent and robust evidence of differential toxicity by source, integrating across all OAP and HAP studies is the approach most consistent with the current evidence, as reviewed by USA EPA and WHO.[11,12]

Proportional PAF approach

Prior to GBD 2017, relative risks for both ambient and HAP exposures were obtained from the risk curve as a function of exposure, relative to the same TMREL. In reality, were a country to reduce only one of these risk factors, the other would remain. We did not consider the joint effects of particulate matter from outdoor exposure and burning solid fuels for cooking. For GBD 2017, we developed a new approach to use the risk curve for obtaining PAFs for both OAP and HAP, which was also implemented in GBD 2019, 2021, and 2023.

Let Exp_{OAP} be the ambient $PM_{2.5}$ exposure level and Exp_{HAP} be the excess exposure for those who use solid fuel for cooking. Let P_{HAP} be the proportion of the population using solid fuel for cooking. We calculated PAFs at each $0.1^{\circ} \times 0.1^{\circ}$ grid cell. We assumed that the distribution of those using solid fuel for cooking (HAP) was equivalent across all grid cells of the GBD location.

For the proportion of the population not exposed to HAP the relative risk was:

$$RR_{OAP} = MRBRT(z = Exp_{OAP})/MRBRT(z = TMREL),$$

And for those exposed to HAP, the relative risk was

$$RR_{HAP} = MRBRT(z = Exp_{OAP} + Exp_{HAP})/MRBRT(z = TMREL).$$

We then calculate a population-level RR and PAF for all particulate matter exposure:

$$RR_{PM} = RR_{OAP}(1 - P_{HAP}) + RR_{HAP}P_{HAP}$$

$$PAF_{PM} = \frac{RR_{PM} - 1}{RR_{PM}}$$

We population weight the grid-cell level particulate matter PAFs to get a country-level PAF, and finally, we split this PAF based on the average exposure to each OAP and HAP:

$$PAF_{OAP} = \frac{Exp_{OAP}}{Exp_{OAP}+P_{HAP}*Exp_{HAP}} PAF_{PM}, \text{ and } PAF_{HAP} = \frac{P_{HAP}*Exp_{HAP}}{Exp_{OAP}+P_{HAP}*Exp_{HAP}} PAF_{PM}.$$

With this strategy, $PAF_{PM} = PAF_{HAP} + PAF_{OAP}$, and no burden is counted twice.

References

1. Aaron van Donkelaar, Melanie S. Hammer, Liam Bindle, Michael Brauer, Jeffery R. Brook, Michael J. Garay, N. Christina Hsu, Olga V. Kalashnikova, Ralph A. Kahn, Colin Lee, Robert C. Levy, Alexei Lyapustin, Andrew M. Sayer and Randall V. Martin (2021). Monthly Global Estimates of Fine Particulate Matter and Their Uncertainty Environmental Science & Technology, 2021
2. Turner MC, Jerrett M, Pope CA 3rd, Krewski D, Gapstur SM, Diver WR, Beckerman BS, Marshall JD, Su J, Crouse DL, Burnett RT. Long-term ozone exposure and mortality in a large prospective study. Am J Respir Crit Care Med. 2016; 193(10): 1134-42.
3. Thomas, M. L., Shaddick, G., Simpson, D., de Hoogh, K. and Zidek, J. V. Spatio-temporal downscaling for continental–scale estimation of air pollution concentrations. arXiv preprint arXiv:1907.00093 (also been Submitted to the Journal of the Royal Statistical Society: Series C (Applied Statistics)).
4. Yin P, Brauer M, Cohen A, et al. Long-term Fine Particulate Matter Exposure and Nonaccidental and Cause-specific Mortality in a Large National Cohort of Chinese Men. Environ Health Perspect 2017; 125: 117002.
5. Li T, Zhang Y, Wang J, et al. All-cause mortality risk associated with long-term exposure to ambient PM2·5 in China: a cohort study. Lancet Public Health 2018; 3: e470–7.
6. Yang Y, Tang R, Qiu H, et al. Long term exposure to air pollution and mortality in an elderly cohort in Hong Kong. Environ Int 2018; 117: 99–106.
7. Hystad P, Larkin A, Rangarajan S, AlHabib KF, Avezum A, Tumerdem Calik KB; Chifamba J, Dans A, Diaz R, du Plessis JL, Gupta R, Iqbal R, Khatib R, Kelishadi R, Lanas F, Liu Z, Lopez-Jaramillo P, Nair S, Poirier P, Rahman O, Rosengren A, Swidan H, Tse L-A, Wei L, Wielgosz A, Yeates K, Yusoff K, Zatoński T, Yusuf S, Brauer M. Outdoor fine particulate matter air pollution and cardiovascular disease: Results from 747 communities across 21 countries in the PURE Study. (Submitted to Lancet Global Health)
8. Joseph P, Rangarajan S, Islam S, Mente A, Hystad P, Brauer M, Raman Kutty V, Gupta R, Wielgosz A, AlHabib KF, Dans A, Lopez-Jaramillo P, Avezum A, Lanas F, Oguz A, Kruger IM,

Diaz R, Yusoff K, Mony P, Chifamba J, Yeates K, Kelishadi R, Yusufali A, Khatib R, Rahman O, Zatonska K, Iqbal R, Wei L, Bo H, Rosengren A, Kaur M, Mohan V, Lear SA, Teo KK, O'Donnell M, McKee M, Dagenais G, Yusuf S. Modifiable risk factors, cardiovascular disease and mortality in 155,722 individuals from 21 high-, middle-, and low-income countries (PURE): a prospective cohort study. The Lancet. 2019. doi:10.1016/S0140-6736(19)32008-2

9. Burnett RT, Pope CA 3rd, Ezzati M, Olives C, Lim SS, Mehta S, Shin HH, Singh G, Hubbell B, Brauer M, Anderson HR, Smith KR, Balmes JR, Bruce NG, Kan H, Laden F, Prüss-Ustün A, Turner MC, Gapstur SM, Diver WR, Cohen A. An integrated risk function for estimating the global burden of disease attributable to ambient fine particulate matter exposure. *Environ Health Perspect*. 2014; 122(4): 397-403.
10. Lind L, Sundström J, Ärnlöv J, Lampa E. Impact of Aging on the Strength of Cardiovascular Risk Factors: A Longitudinal Study Over 40 Years. *J Am Heart Assoc.* 2018;7(1):e007061. Published 2018 Jan 6. doi:10.1161/JAHA.117.007061
11. Ghosh R, Causey K, Burkart K, Wozniak S, Cohen A, Brauer M. Ambient and household $PM_{2.5}$ pollution and adverse perinatal outcomes: A meta-regression and analysis of attributable global burden for 204 countries and territories. *PLoS Med* 2021. Accepted; in press.
12. US Environmental Protection Agency. Integrated science assessment (ISA) for particulate matter (Final Report, Dec 2009). EPA/600/R-08/139F, 2009. Washington, DC: US Environmental Protection Agency; 2009. Available at: http://cfpub.epa.gov/ncea/risk/recordisplay.cfm?deid=216546

Bullying victimisation

Flowchart

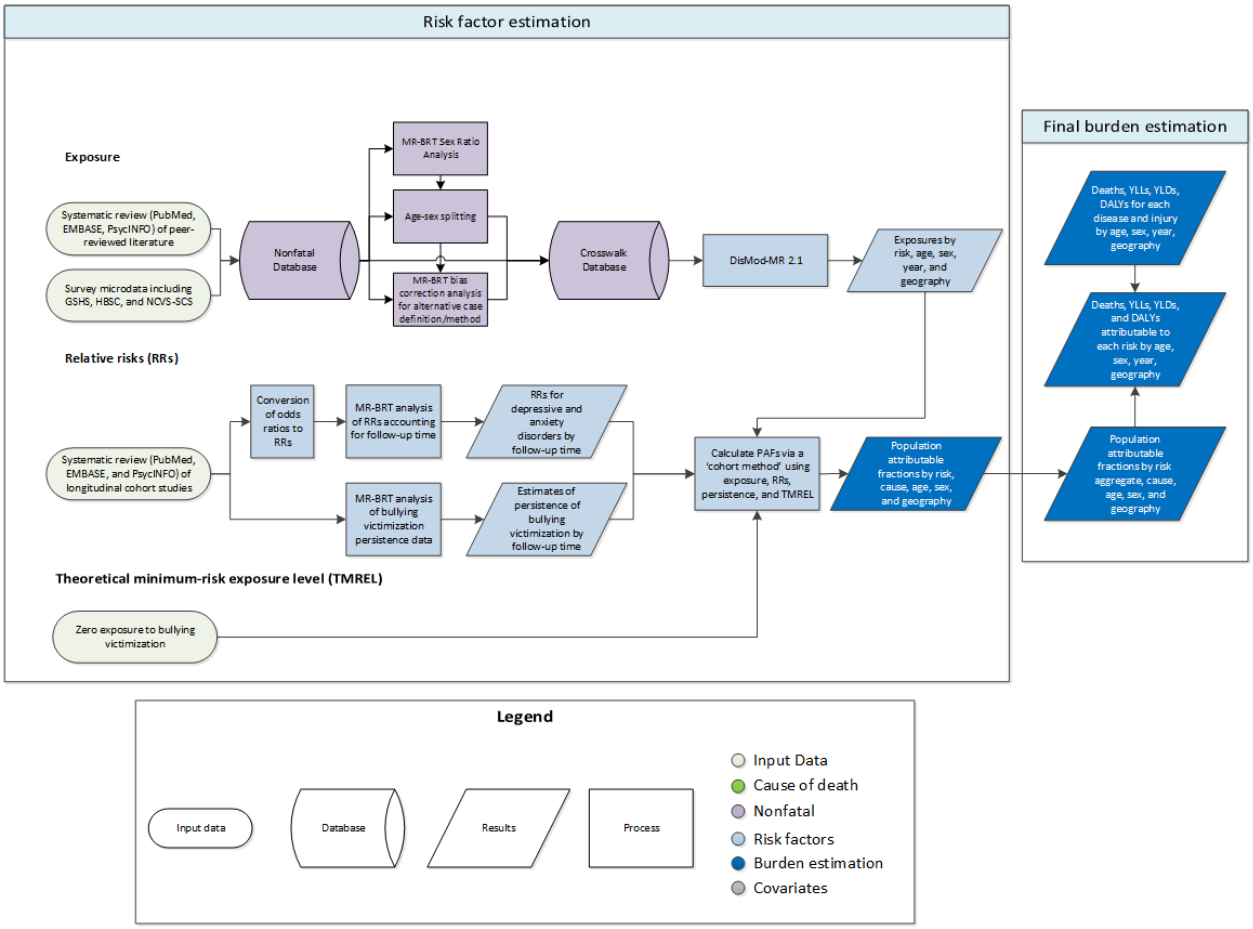

Input data and methodological summary

Exposure

Case definition

Bullying victimisation is commonly conceptualised as the intentional and repeated harm of a less powerful individual by peers.[1] This differentiates bullying victimisation from disagreements, conflicts, or playful teasing. The case definition of bullying victimisation in the GBD context is "bullying victimisation of children and adolescents attending school by peers". This definition includes the global concept of bullying victimisation, which incorporates combined estimates of subtypes such as physical, verbal, relational, and cyberbullying victimisation. It excludes abuse/harassment by siblings, intimate partners, and adults (eg, teachers). While bullying can be experienced as either a victim or perpetrator, perpetration (ie, those who bully others) is not included in this definition although some victims will also be perpetrators.

Input data

In order for a study to be included, it must report the prevalence of bullying victimisation and 1) have been published since 1980, 2) ask participants about bullying victimisation in the previous year or more recently, 3) use an appropriate frequency threshold to define bullying victimisation (approximating at least once a week or greater than “occasionally”), 4) be representative of the general population rather than a special population (eg, ethnic minorities), and 5) report prevalence for bullying victimisation overall rather than a subtype (eg, physical bullying victimisation).

The systematic literature review for bullying exposure was conducted in three stages, involving electronic searches of the peer-reviewed literature (ie, via PsycInfo, Embase, and PubMed), the grey literature, and expert consultation. An update to the systematic literature review was conducted and informed GBD 2023. Databases were searched on October 24, 2021, for publications after 25 October 2017. Below are the search terms used for each database:

Pubmed: (((((((bully*[Title/Abstract]) OR bulli*[Title/Abstract]) OR "peer victimisation"[Title/Abstract]) OR "peer victimization"[Title/Abstract]) OR "peer aggression"[Title/Abstract]) OR bullying[MeSH Terms] OR cyberbull*[Title/Abstract] OR cyber-bull*[Title/Abstract]))) AND ((prevalen*[Title/Abstract]) OR prevalence[MeSH Terms])

EMBASE: ('bulli*':ab,ti OR 'bully*':ab,ti OR 'peer victimisation':ab,ti OR 'peer victimization':ab,ti OR 'peer aggression':ab,ti OR 'cyber-bull*':ab,ti OR 'cyberbull*':ab,ti OR 'bullying'/exp/mj) AND ('prevalen*':ab,ti OR 'prevalence'/exp/mj)

PsycInfo: (TI bulli* OR AB bulli* OR TI bully* OR AB bully* OR TI "peer victimization" OR AB "peer victimization" OR TI "peer victimisation" OR AB "peer victimisation" OR TI "peer aggression" OR AB "peer aggression" OR MA bullying OR TI cyberbull* OR AB cyberbull* OR TI cyber-bull* OR AB cyber-bull*) AND (TI prevalen* OR AB prevalen* OR MA prevalence)

The Global Health Data Exchange (GHDx) was also used to source microdata from survey series meeting the above inclusion criteria. Estimates from the Global School-based Student Health Survey (GSHS), the Health Behavior in School-aged Children (HBSC), and the National Crime Victimization Survey – School Crime Supplement (NCVS-SCS) were extracted and included in the dataset. The systematic literature review was conducted according to Preferred Reporting Items for Systematic Reviews and Meta-Analyses (PRISMA; see Figure 1).

Figure 1: PRISMA 2009 flow diagram for bullying exposure data

From: Moher D, Liberati A, Tetzlaff J, Altman DG, The PRISMA Group (2009). *P*referred *R*eporting *I*tems for *S*ystematic Reviews and *M*eta-*A*nalyses: The PRISMA Statement. PLoS Med 6(7): e1000097. doi:10.1371/journal.pmed1000097

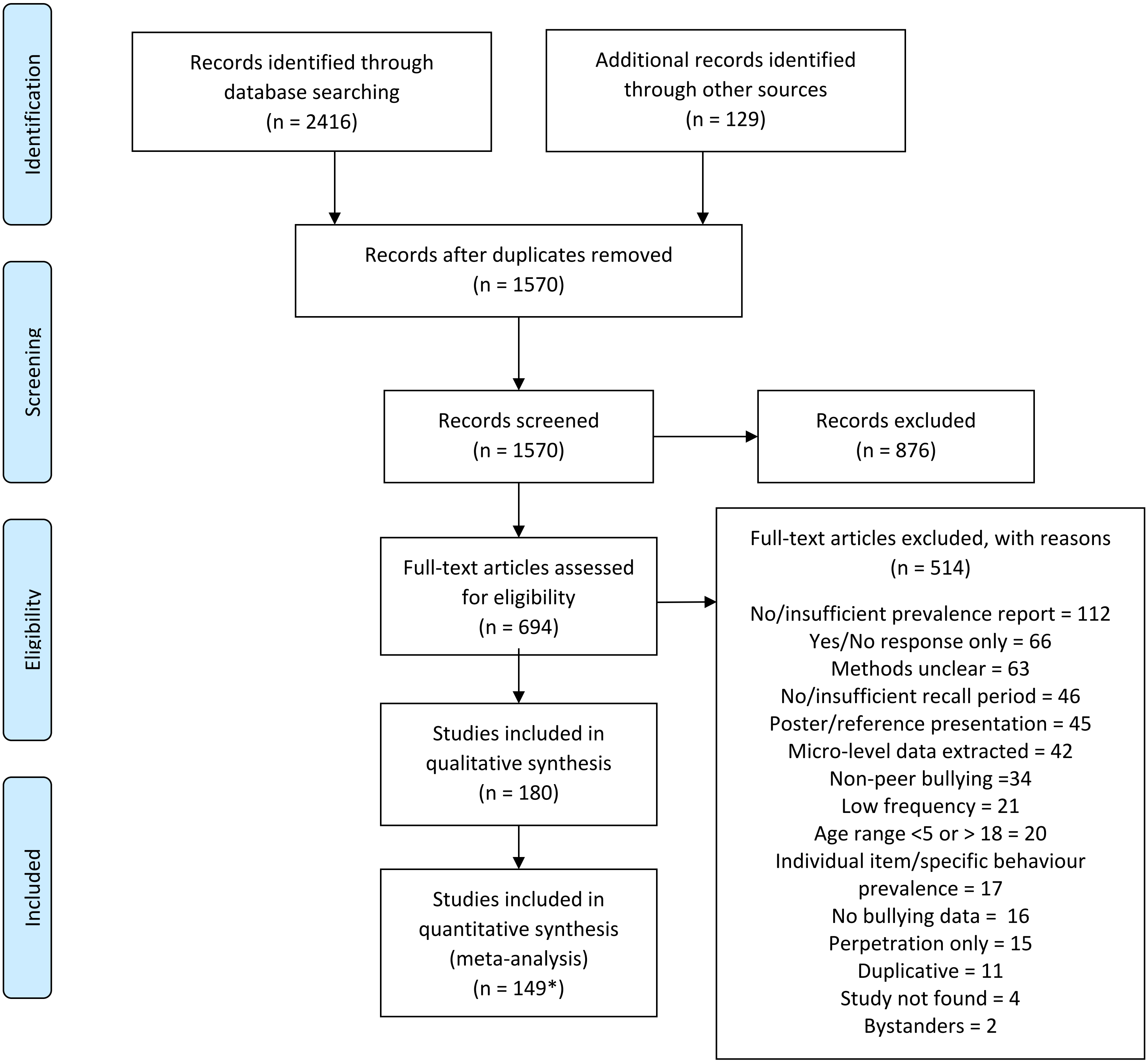

**The qualitative analysis led to the exclusion of additional studies with duplicative cohorts or methodological limitations impacting their eligibility and increasing measurement error within the data.*

Age and sex splitting

The extracted data underwent two types of age and sex splitting processes:

1. Where possible, estimates were further split by sex and age based on the data that were available. For instance, if studies reported prevalence for broad age groups by sex (eg, prevalence in 5–17-year-old males and females separately), and also by specific age groups, but for both sexes combined (eg, prevalence in 5–12-year-olds, then in 13–17-year-olds, for males and females combined), age-specific estimates were split by sex using the reported sex ratio and bounds of uncertainty.
2. A meta-regression—Bayesian, regularised, trimmed (MR-BRT) analysis was used to split the remaining both-sex estimates in the dataset. For each parameter, sex-specific estimates were matched by location, age, and year, and a MR-BRT network meta-analysis was used to estimate pooled sex ratios and bounds of uncertainty. These were then used to split the both-sex estimates in the dataset. The male:female proportion ratio was 1.20 (95% CI: 1.18–1.21).

Bias corrections/crosswalks

Estimates with known biases were adjusted/crosswalked accordingly prior to DisMod-MR 2.1. Within the bullying victimisation epidemiological dataset, within- and between-study estimates were paired by age, sex, location, and year, between the reference and alternative estimates. Pairs were also made between the different alternative estimates. These pairs were then used as inputs in a MR-BRT network meta-analysis. The MR-BRT analysis produced pooled adjustment factors between the reference estimates and alternative estimates. These adjustment factors (see Table 1) were used to adjust all alternative estimates in the dataset. Bullying victimisation had four alternative definitions to crosswalk:

1. Qualitative suboptimal frequency threshold used, eg, “sometimes + frequently”
2. Quantitative suboptimal frequency of once a month used
3. Quantitative suboptimal frequency of twice a month used
4. No definition of bullying victimisation presented to participants or not specified
5. Asked about bullying victimisation in the past year

Table 2: MR-BRT crosswalk adjustment factors for bullying victimisation

Data input	Reference or alternative case definition	Gamma	Beta coefficient, log (95% CI)*	Adjustment factor (95% CI)**
School survey	Reference: Point proportion of children and adolescents attending school who have been exposed to bullying victimisation by peers at least once a week or greater than “occasionally”. Participants are given a definition of bullying victimisation prior to being asked about exposure.	0.084	---	---
School survey	Alternative: Qualitative suboptimal frequency threshold used, eg, “sometimes + frequently”		1.10 (0.99–1.21)	3.00 (2.69–3.35)

School survey	Alternative: Quantitative suboptimal frequency of once a month used		0.61 (0.51–0.71)	1.85 (1.67–2.04)
School survey	Alternative: Quantitative suboptimal frequency of twice a month used		0.54 (0.49–0.59)	1.72 (1.62–1.81)
School survey	Alternative: No definition of bullying victimisation presented to participants or not specified		0.25 (0.11 – 0.40)	1.29 (1.12–1.49)
School survey	Alternative: Past-year proportion		0.29 (0.12–0.47)	1.34 (1.12–1.60)

**MR-BRT crosswalk adjustments can be interpreted as the factor the alternative case definition is adjusted by to reflect what it would have been had it been measured using the reference case definition. If the log/logit beta coefficient is negative, then the alternative is adjusted up to the reference. If the log/logit beta coefficient is positive, then the alternative is adjusted down to the reference.*

***The adjustment factor column is the exponentiated beta coefficient. For log beta coefficients, this is the relative rate between the two case definitions. For logit beta coefficients, this is the relative odds between the two case definitions.*

Confidence intervals incorporate gamma, which represents the between-study variance across all input data in the model. This added uncertainty widens the confidence intervals for crosswalks with significant fixed effects.

Relative risk

Case definition

We estimate burden attributable to bullying victimisation for major depressive disorder (MDD) and anxiety disorders. Data on the association between bullying victimisation and self-harm was also reviewed but not included due to variation in the definition of "self-harm" and only one study looking at suicide.

Input data

Studies had to report relative risks (RRs), odds ratios, or sufficient data to calculate RRs (ie, exposed/non-exposed cases/non-cases). For GBD 2023, an updated systematic review search was conducted for studies reporting the prospective longitudinal association between the outcomes listed above and bullying victimisation. The updated search was conducted via three databases (PubMed, EMBASE, and PsycINFO). No restriction was set on the language of publication. Databases were searched on March 23, 2021, for publications after April 1, 2018. Below are the search terms used for each database:

PubMed: (bulli*[Title/Abstract] OR bully*[Title/Abstract] OR harass*[Title/Abstract] OR 'peer victimisation'[Title/Abstract] OR 'peer aggression'[Title/Abstract] OR 'peer victimization'[Title/Abstract] OR intimidat*[Title/Abstract] OR bullying[MeSH Terms]) AND (mood*[Title/Abstract] OR "mood disorders"[Title/Abstract] OR "mood disorder"[Title/Abstract] OR "Mood Disorders"[Mesh] OR depress*[Title/Abstract] OR dysthymi*[Title/Abstract] OR "Dysthymic Disorder"[Mesh] OR anxiety[Title/Abstract] OR "Anxiety Disorders"[Mesh] OR "obsessive compulsive disorder"[Title/Abstract] OR "obsessive compulsive disorders"[Title/Abstract] OR "OCD"[Title/Abstract] OR "post traumatic stress disorder"[Title/Abstract] OR "post traumatic stress disorders"[Title/Abstract] OR "PTSD"[Title/Abstract] OR "Stress Disorders, Post-Traumatic"[Mesh] OR internali*[Title/Abstract] OR suici*[Title/Abstract] OR 'self injury'[Title/Abstract] OR 'self injure'[Title/Abstract] OR 'self

harm'[Title/Abstract] OR 'self harmed'[Title/Abstract] OR mutilat*[Title/Abstract] OR poiso*[Title/Abstract] OR 'self inflict'[Title/Abstract] OR 'self inflicted'[Title/Abstract] OR DSH[Title/Abstract] OR "Self-Injurious Behavior"[Mesh]) AND (outcome*[Title/Abstract] OR consequence*[Title/Abstract] OR associat*[Title/Abstract] OR effect[Title/Abstract] OR risk*[Title/Abstract] OR longitudinal [Title/Abstract] OR follow*[Title/Abstract] OR odds[Title/Abstract])

EMBASE: (“bulli*”:ab,ti OR “bully*”:ab,ti OR “harass*”:ab,ti OR “peer victimization”:ab,ti OR “peer victimisation”:ab,ti OR “peer aggression”:ab,ti OR “intimidat*”:ab,ti OR “bullying”/exp) AND (“mood*”:ab,ti OR “mood disorder”:ab,ti OR “mood disorders”:ab,ti OR 'mood disorder'/exp OR “depress*”:ab,ti OR “dysthymi*”:ab,ti OR “depression”/exp OR “anxiety”:ab,ti OR “anxiety disorder”/exp "obsessive compulsive disorder":ab,ti OR "obsessive compulsive disorders":ab,ti OR “OCD”:ab,ti OR "post traumatic stress disorder":ab,ti OR "post traumatic stress disorders":ab,ti OR “PTSD”:ab,ti OR “internali*”:ab,ti OR “suici*”:ab,ti OR “self injury”:ab,ti OR “self injure”:ab,ti OR “self harm”:ab,ti OR “self harmed”:ab,ti OR “mutilat*”:ab,ti OR “poiso*”:ab,ti OR “self inflict”:ab,ti OR “self inflicted”:ab,ti OR “dsh”:ab,ti OR “suicidal behavior”/exp OR “automutilation”/exp) AND (“outcome*”:ab,ti OR “consequence*”:ab,ti OR “associat*”:ab,ti OR “effect”:ab,ti OR “risk*”:ab,ti OR “longitudinal”:ab,ti OR “follow*”:ab,ti OR “odds”:ab,ti)

PsycInfo: (TI bulli* OR AB bulli* OR TI bully* OR AB bully* OR TI harass* OR AB harass* OR TI “peer victimisation” OR AB “peer victimisation” OR TI “peer victimization” OR AB “peer victimization” OR TI “peer aggression” OR AB “peer aggression” OR TI intimidat* OR AB intimidat* OR DE "Bullying" OR DE "Cyberbullying") AND (TI mood* OR AB mood* OR TI "mood disorders" OR AB "mood disorders" OR TI "mood disorder" OR AB "mood disorder" OR DE "Affective Disorders" OR TI depress* OR AB depress* OR DE "Major Depression" OR TI dysthymi* OR AB dysthymi* OR DE "Persistent Depressive Disorder" OR DE "Dysthymic Disorder" OR TI anxiety OR AB anxiety OR DE "Anxiety Disorders" OR DE "Generalized Anxiety Disorder" OR DE "Panic Attack" OR DE "Panic Disorder" OR DE "Phobias" OR DE "Separation Anxiety Disorder" OR TI "obsessive compulsive disorder" OR AB "obsessive compulsive disorder" OR TI "obsessive compulsive disorders" OR AB "obsessive compulsive disorders" OR TI OCD OR AB OCD DE "Obsessive Compulsive Disorder" OR TI "post traumatic stress disorder" OR AB "post traumatic stress disorder" OR TI "post traumatic stress disorders" OR AB "post traumatic stress disorders" OR TI PTSD OR AB PTSD OR DE "Posttraumatic Stress Disorder" OR DE "Complex PTSD" OR internali* OR AB internali* OR TI suici* OR AB suici* OR TI self injury OR AB self injury OR TI self injure OR AB self injure OR TI self harm OR AB self harm OR TI self harmed OR AB self harmed OR TI mutilate* OR AB mutilate* OR TI poiso* OR AB poiso* OR TI self inflict OR AB self inflict OR TI self inflicted OR AB self inflicted OR TI DSH OR AB DSH OR DE "Suicidal Behavior" OR DE "Attempted Suicide" OR DE "Suicidal Ideation" OR DE "Suicide") AND (TI outcome* OR AB outcome* OR TI consequence* OR AB consequence* OR TI associat* OR AB associat* OR TI effect OR AB effect OR TI risk* OR AB risk* OR TI longitudinal OR AB longi* OR TI follow* OR AB follow* OR TI odds OR AB odds)

The systematic literature review was conducted according to Preferred Reporting Items for Systematic Reviews and Meta-Analyses (PRISMA; see Figure 2). No studies from the updated systematic review met eligibility criteria.

Figure 2: PRISMA 2009 flow diagram for bullying relative risk data

From: Moher D, Liberati A, Tetzlaff J, Altman DG, The PRISMA Group (2009). *P*referred *R*eporting *I*tems for *S*ystematic Reviews and *M*eta-*A*nalyses: The PRISMA Statement. PLoS Med 6(7): e1000097. doi:10.1371/journal.pmed1000097

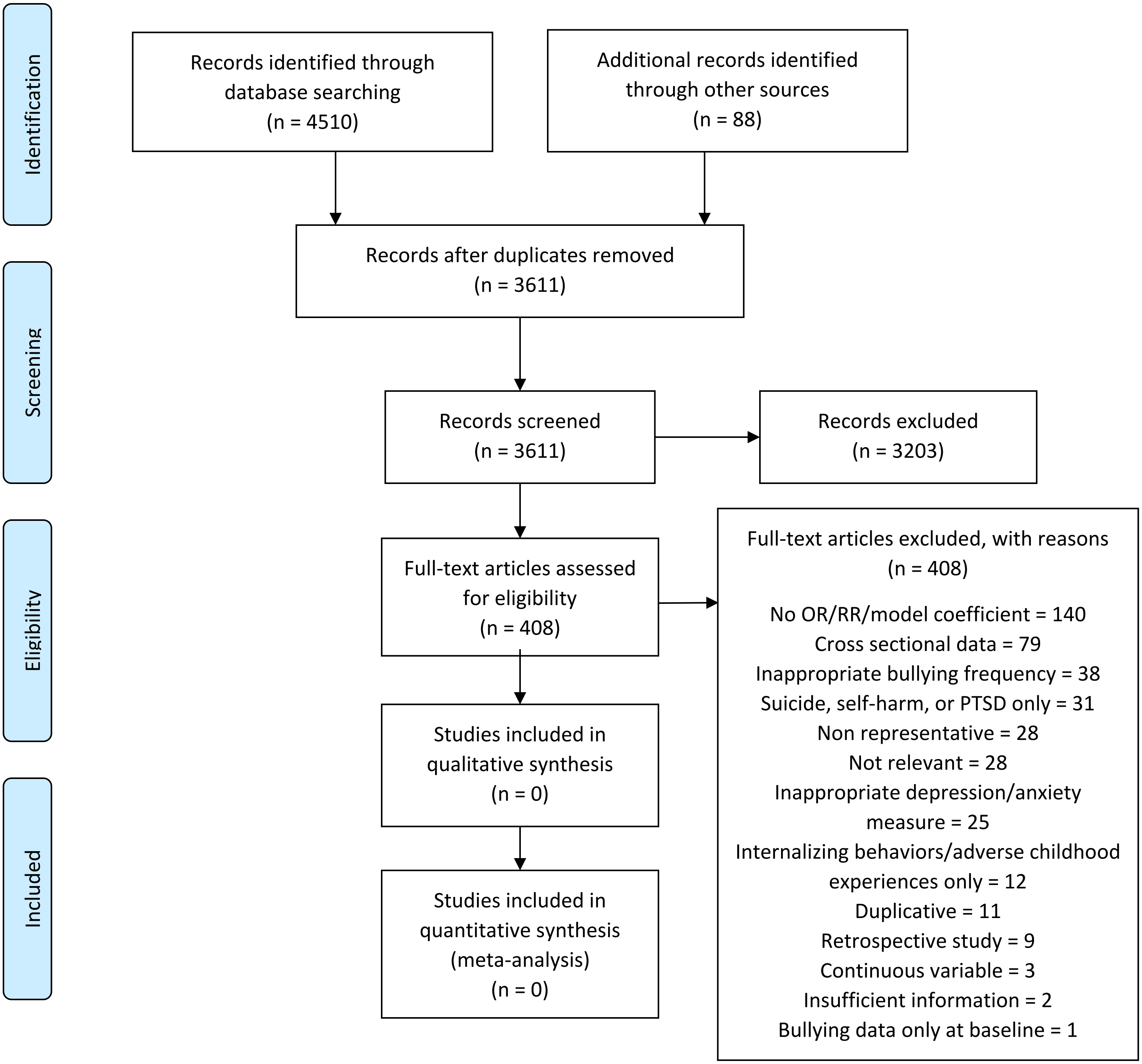

Modelling strategy

Exposure

After the above data processes were applied, DisMod-MR 2.1 was used to model the prevalence of bullying victimisation. Bullying victimisation prevalence was modelled as a single parameter prevalence model. Adjustments to model priors or the dataset were made where appropriate. Where outliers were identified in the data, we reassessed the study's methodology and quality before a decision was made to exclude or include the data. We assumed no prevalence prior to 5 years or after 20 years of age.

Adjustment for years of schooling

In order to better represent the prevalence of bullying victimisation, prevalence estimates were adjusted for the proportion of children and adolescents attending school by ages 5–9, 10–14, and 15–19 years by sex, location, and year. Data on the proportion of children and adolescents attending school were sourced from the online database (http://data.uis.unesco.org/) published by the United Nations Educational, Scientific, and Culture Organization (UNESCO). The data covered 26,592 country-years for age groups 6–11, 12–14, and 15–17 years by sex. These data were modelled in ST-GPR, with average years of education as a country-level covariate, to predict the proportion of children and adolescents attending school by these age groups. This gave estimates of the proportion of children and adolescents attending school by age, sex, year, and location.

Theoretical minimum risk exposure level

The theoretical minimum risk exposure level was assumed to be zero exposure to bullying victimisation.

Relative risk

Estimation of pooled relative risks

MR-BRT meta-regressions were conducted to determine the impact of follow-up time on the risk of MDD and anxiety disorders following exposure to bullying victimisation. These analyses controlled for percentage of the sample female and four bias covariates: 1) Exposure reported by parent only, 2) Estimate derived from multiple logistic regression, 3) Suboptimal exposure frequency threshold, 4) 15%+ attrition at follow-up. All available estimates by cohort that varied across the covariates were extracted to better inform the covariates. An initial log-linear model containing both MDD and anxiety disorders was run to obtain priors for sex and the bias covariates for disorder-specific ensemble random splines models with decreasing monotonicity priors.

Population attributable fractions (PAFs)

A cohort method was developed to accommodate the waning risk over time observed in the MR-BRT meta-regression of the RRs. The following steps are conducted for each point of estimation (ie, by age, sex, location, and year), hereafter referred to as a "cohort":

1. Pull current and past bullying victimisation prevalence for the cohort from the DisMod-MR 2.1 exposure model.
2. Adjust each bullying victimisation prevalence estimate for the proportion of the cohort attending school in that year.
3. Estimate incidence of bullying victimisation within the cohort for each year using the following formula:

$$I_k = P_k - \sum_{n=0}^{k-1}(I_n \times r_{k-n})$$

Where *I* represents incidence, *P* represents prevalence, *r* represents the estimate of persistence, and *k* represents the time between the incidence estimate and the earliest possible time of exposure in the cohort. I_k requires I_0 through to I_{k-1} to first be calculated and so we complete this process by first estimating I_0, then I_1, and so on until we have estimated incidence for the latest possible year of exposure for this cohort. The persistence estimate is based on a separate MR-BRT meta-regression of seven studies.[2-8]

4. Use the incidence estimates to divide the cohort into proportions based on time since first exposed to bullying victimisation:

$$p_t = I_{\max(k)-k}$$

Where t is the time since first exposed to bullying victimisation, and p is the proportion of the cohort first exposed to bullying victimisation at time t.

5. Estimate PAFs via the following formula:

$$PAF = \frac{\sum(p_t \times RR_t) + p_{no\ exposure} - 1}{\sum(p_t \times RR_t) + p_{no\ exposure}}$$

Where *t* is the time since first exposed to bullying victimisation, *p* is the proportion of the cohort first exposed to bullying victimisation at time *t* or the proportion not exposed to bullying victimisation, and *RR* is the relative risk for depressive and anxiety disorders given *t*.

GBD 2023 prevalence and burden estimates for MDD and anxiety disorders incorporated the impact of the COVID-19 pandemic, leading to an increase in estimated prevalent cases and burden from 2020 onward for these two disorders. After consultation with our GBD collaborators regarding this, we removed the impact of COVID-19 from the PAF estimation for bullying victimisation. This was achieved by removing the increase in prevalence following the start of the COVID-19 pandemic from the final PAFs for years 2020 to 2023. To date, there was insufficient evidence to suggest that bullying victimisation would explain part of this additional prevalence or burden. This will be reviewed as more epidemiological data relating to the impacts of the pandemic on bullying victimisation emerge.

Changes between GBD 2021 and GBD 2023

There was one main changes in the GBD 2023 modelling strategy compared to GBD 2021. For GBD 2023, the suboptimal frequency covariate was split based on whether the suboptimal frequency was qualitative or quantitative in nature. In addition, the qualitative suboptimal frequency was further split for a frequency of once a month and twice a month.

References

1. Olweus D. Bullying at school: What we know and what we can do. MA, USA; Oxford, UK; Vic, Australia: Blackwell; 1993.
2. Baly MW, Cornell DG, Lovegrove P. A Longitudinal Investigation of Self- and Peer Reports of Bullying Victimization Across Middle School. *Psychology in the Schools* 2014; **51**(3): 217-40.
3. Bowes L, Maughan B, Ball H, et al. Chronic bullying victimization across school transitions: the role of genetic and environmental influences. *Development and psychopathology* 2013; **25**(2): 333-46.
4. Kumpulainen K, Rasanen E, Henttonen I. Children involved in bullying: psychological disturbance and the persistence of the involvement. *Child Abuse Negl* 1999; **23**(12): 1253-62.
5. Lereya ST, Copeland WE, Zammit S, Wolke D. Bully/victims: A longitudinal, population-based cohort study of their mental health. *European Child & Adolescent Psychiatry* 2015; **24**(12): 1461-71.

6. Lien L, Welander-Vatn A. Factors Associated with the Persistence of Bullying Victimization From 10th grade to 13th Grade: A Longitudinal Study. *Clinical practice and epidemiology in mental health : CP & EMH* 2013; **9**: 243-50.
7. Sourander A, Helstela L, Helenius H, Piha J. Persistence of bullying from childhood to adolescence--a longitudinal 8-year follow-up study. *Child Abuse Negl* 2000; **24**(7): 873-81.
8. Winsper C, Lereya T, Zanarini M, Wolke D. Involvement in bullying and suicide-related behavior at 11 years: a prospective birth cohort study. *Journal of the American Academy of Child and Adolescent Psychiatry* 2012; **51**(3): 271-82.e3.

Chewing tobacco

Flowchart

Chewing Tobacco

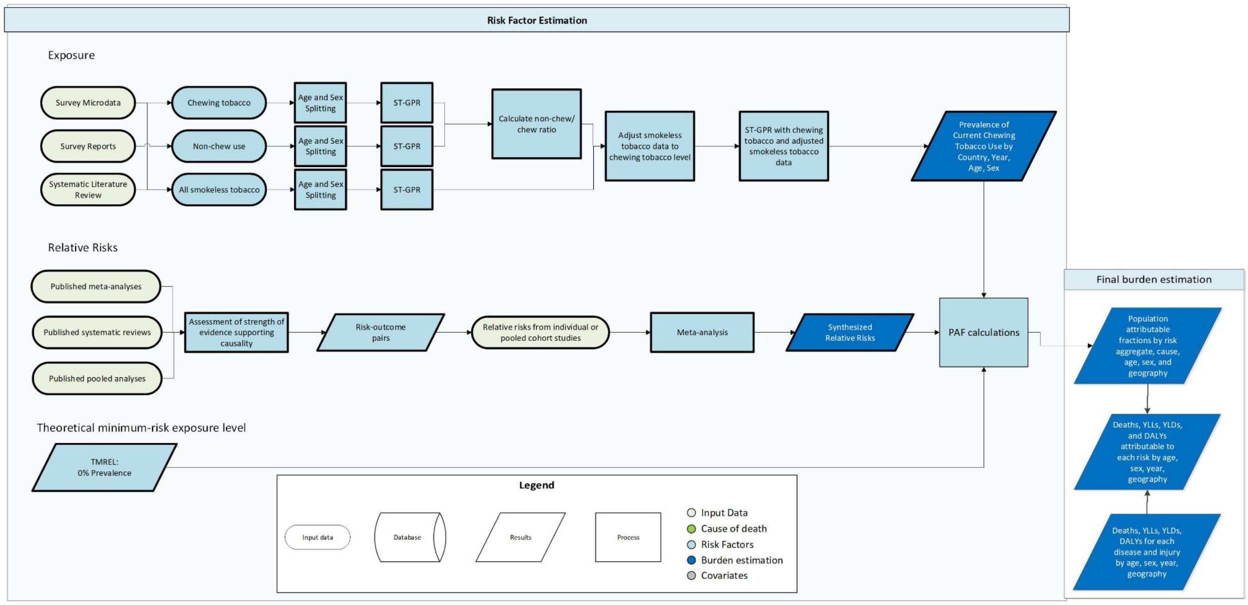

Input data and methodological summary

Definition

Exposure

Current chewing tobacco use is defined as current use (use within the last 30 days where possible, or according to the closest definition available from the survey) of any frequency (any, daily, or less than daily). Chewing tobacco includes local products, such as betel quid.

Input data

Exposure

As in GBD 2021, we included sources that reported primary chewing tobacco, non-chew smokeless tobacco, and all smokeless tobacco use among respondents over age 10. To be eligible for inclusion, sources had to be representative for their level of estimation (ie, national sources needed to be nationally representative, and subnational sources had to be subnationally representative). We included only data on self-reported use and excluded data from questions asking about others' tobacco use behaviours. Surveys from large, standardised series (such as NHANES, STEPS NCD, GATS, etc.) as well as those covering data-sparse locations or time periods were prioritised for extraction.

We extracted primary data from individual-level microdata and survey report tabulations on chewing tobacco, non-chewing smokeless tobacco, and any smokeless tobacco use. We extracted data on current, former, and/or ever use as well as frequency of use (daily, occasional, and unspecified, which includes both daily and occasional users). Products that do not include tobacco, such as betel quid without tobacco, were excluded or estimated separately as part of the drug use risk factor, if applicable.

For microdata, we extracted relevant demographic information, including age, sex, location, and year, as well as survey metadata, including survey weights, primary sampling units, and strata. This information allowed us to tabulate individual-level data in the standard GBD five-year age-sex groups and produce accurate estimates of uncertainty. Due to data sparsity among individuals aged 80 years and older, we were unable to directly model older GBD age sets. Instead, data for those 80 years and older was collapsed into a single age set, and later results were duplicated to fulfill the GBD age bins under the assumption that patterns among these groups would prove stable. For survey report tabulations, we extracted data at the most granular age-sex group provided. Compared to GBD 2021, we identified 239 new input sources to inform our chewing tobacco exposure estimates.

Relative risk

For GBD 2023, new relative risks (RRs) for chewing tobacco were generated using the Burden of Proof approach. Seven risk–outcome pairs were evaluated: two from prior rounds (oesophageal cancer and lip and oral cavity cancer), as well as five new outcomes (stroke, larynx cancer, nasopharynx cancer, other pharynx cancers, and ischaemic heart disease). Ultimately, the strength of the current evidence was sufficient to determine each to be causally associated with chewing tobacco exposure, except ischaemic heart disease. Therefore, RRs for six outcomes were included for chewing tobacco in GBD 2023.

Table 1: Changes in relative risks used in GBD rounds

	Relative Risk		
Outcome	GBD 2021	GBD 2023 (with gamma)	GBD 2023 (without gamma)
Esophageal cancer	2.71 (1.85-3.42)	2.14 (0.89, 5.15)	2.14 (1.77, 2.57)
Lip and oral cavity cancer	Men: 3.02 (2.01-4.47)<br>Women: 8.09 (5.46-11.77)	3.64 (0.66, 19.95)	3.64 (3.00, 4.41)
Larynx cancer	n/a	2.66 (0.52, 13.63)	2.66 (1.98, 3.57)
Nasopharynx cancer	n/a	2.50 (0.49, 12.66)	2.50 (1.79, 3.49)
Other pharynx cancer	n/a	2.33 (0.45, 12.04)	2.33 (1.80, 3.01)
Stroke	n/a	1.46 (1.11, 1.93)	1.46 (1.28, 1.68)

Data processing

Age- and sex-splitting

We split data reported in broader age groups than the GBD five-year age groups or as both sexes combined by adapting the method reported in Ng and colleagues[1] to split using a sex-geography-time-specific reference age pattern. Mirroring the data preparation step, tabulated groups including those over the age of 80 were split/collapsed into a single 80+ age group by source-sex-geography-time. We separated the data into two sets: a training dataset, with data already falling into GBD sex-specific five-year age groups, and a split dataset, which reported data in aggregated age or sex groups. We then used spatiotemporal Gaussian process regression (ST-GPR) to estimate sex-geography-time-specific age patterns using data in the training dataset. The estimated age patterns were then used to split each source in the split dataset.

The ST-GPR model used to estimate the age patterns for age-sex splitting used an age weight parameter value that minimises the effect of any age smoothing. This parameter choice allows the estimated age pattern to be driven by data, rather than being enforced by any smoothing parameters of the model. Because these age-sex-split datapoints will be incorporated in the final ST-GPR exposure model, we do not want to doubly enforce a modelled age pattern for a given sex-location-year on a given aggregate datapoint. We run three separate age-sex splitting ST-GPR models: one for each smokeless tobacco category (chew, non-chew, and all smokeless).

Table 2: Data inputs for exposure for chewing tobacco

	Countries with data	New sources	Total sources
Exposure	180	239	944

Table 3: Data inputs for relative risks for chewing tobacco

	Countries with data	New sources	Total sources
Relative risks	8	98	103

Modelling strategy

Exposure

We used ST-GPR to model chewing tobacco prevalence. Full details on the ST-GPR method are reported elsewhere in the appendix. Briefly, the mean function input to GPR is a complete time series of estimates generated from a mixed effects hierarchical linear model plus weighted residuals smoothed

across time, space, and age. The linear model formula for chewing tobacco, fit separately by sex using restricted maximum likelihood in R, is:

$$logit(p_{g,a,t}) = \beta_0 + \sum_{k=1}^{18} \beta_k I_{A[a]} + \alpha_s + \alpha_r + \alpha_g + \epsilon_{g,a,t}$$

Where $I_{A[a]}$ is a dummy variable indicating a specific age group A that the prevalence point $p_{g,a,t}$captures, and α_s, α_r, and α_g are super-region, region, and geography random intercepts, respectively. The hyperparameters are the same as in GBD 2021. We ran three ST-GPR models for each prevalence category – one for each smokeless tobacco category (chew, non-chew, and all smokeless).

All smokeless tobacco prevalence adjustment

Using the 1000 draws from each of the prevalence ST-GPR models, we calculated 1000 draws of chewing tobacco prevalence divided by the sum of chewing tobacco and non-chewing tobacco prevalence for each location, age group, sex, and year. The draws were unordered, as we did not want to enforce an assumption about the relationship between the levels of chewing tobacco and non-chewing tobacco prevalence.

The draws of the ratio of chewing to non-chewing tobacco were then multiplied by the draws from the smokeless tobacco prevalence model to adjust the estimates to chewing tobacco prevalence. These were then averaged to get the mean estimate. The variance across the ratios was calculated for each location, year, age, and sex, and was added to the variance from the original all smokeless tobacco draws.

Final chewing tobacco prevalence model

To calculate the final chewing tobacco prevalence, we ran an additional ST-GPR model with both the original chewing tobacco data (post-age-sex splitting), as well as the adjusted data. These adjusted data add more information to the model – as surveys will often only ask about all smokeless tobacco consumption – while taking into consideration the uncertainty from the ratio calculation. Upon completion of the model, detailed GBD age sets for older age groups (80–84, 85–89, 90–94, and 95–124) were imputed as duplicates of the modelled 80+ age set under the assumption that patterns among these groups would be reasonably stable. This strategy will be interrogated in future rounds. As we add more data, we will be able to shift to directly modelling detailed older age groups.

Relative risk

In detail, the Burden of Proof approach included a systematic review, synthesising data from case-control and cohort study designs, while accounting for between-study heterogeneity and biases. Our models included study design, population characteristics, exposure definitions, and other confounders to account for bias in the estimation of RR. For models with sufficient data (≥10 studies), likelihood-based trimming ensured that outlying estimates were excluded. After screening, the systematic review included 103 studies comprising over 100,000 participants. In addition to RR, other Burden of Proof-

specific metrics were calculated, including the Burden of Proof Risk Function and Risk–Outcome Score, both of which quantify the strength of evidence for each association. As seen below, this was translated into a star rating system, reflecting moderate but consistent evidence linking chewing tobacco exposure to these outcomes. The addition of stroke and cancers of the nasopharynx, pharynx (other), and larynx represent a significant enhancement of the comprehensiveness of the GBD framework as it relates to chewing tobacco. For additional information on the systematic reviews, including PRISMA diagrams for each RR pair, please see our publication titled “Health effects associated with chewing tobacco: a Burden of Proof study” by Gil and colleagues in *Nature Communications.*[2]

Table 4: Relative risk results and star ratings of chewing tobacco health outcomes

Health outcome	RR (95% UI without between-study heterogeneity)	RR (95% UI with between-study heterogeneity)	BPRF	ROS	Star rating	Pub. bias	No. of studies	Selected bias covariates	Risk–outcome pair in GBD 2021
Stroke	1.46 (1.28, 1.68)	1.46 (1.11, 1.93)	1.16	0.07	★★	No	3	None	N
Oesophageal Cancer	2.14 (1.77, 2.57)	2.14 (0.89, 5.15)	1.02	0.01	★★	No	22	Maximally adjusted; adjusted for smoking, age, and sex	Y
Lip and oral cavity cancer	3.64 (3.00, 4.41)	3.64 (0.66, 19.95)	0.87	-0.07	★	No	70	Chewing tobacco product; study subpopulation	Y
Larynx cancer	2.66 (1.98, 3.57)	2.66 (0.52, 13.63)	0.68	-0.20	★	No	24	Aggregate outcome definition; adjusted for age and sex	N
Nasopharynx cancer	2.50 (1.79, 3.49)	2.50 (0.49, 12.66)	0.64	-0.22	★	No	17	Maximally adjusted; adjusted for age and sex	N
Other pharynx cancer	2.33 (1.80, 3.01)	2.33 (0.45, 12.04)	0.59	-0.27	★	No	31	Aggregate outcome definition; adjusted for age and sex	N
Ischaemic heart disease	1.30 (0.88, 1.92)	1.30 (0.29, 5.83)	N/A	N/A		No	8	None	N

Theoretical minimum risk exposure level

The theoretical minimum risk exposure level is that everyone in the population has been a lifelong non-user of chewing tobacco.

Population attributable fraction

In order to estimate population attributable fraction (PAF) for chewing tobacco, we utilised the standard GBD PAF equation for dichotomous risks to estimate burden based on exposure, relative risks, and theoretical minimum risk exposure level.

Citations

[1] Ng, M., Freeman, M. K., Fleming, T. D., Robinson, M., Dwyer-Lindgren, L., Thomson, B., Wollum, A., Sanman, E., Wulf, S., Lopez, A. D., Murray, C. J. L., & Gakidou, E. (2014). Smoking Prevalence and Cigarette Consumption in 187 Countries, 1980-2012. *JAMA*, 311(2), 183.https://doi.org/10.1001/jama.2013.284692

[2] Gil GF, Anderson JA, Aravkin A, Bhangdia K, Carr S, Dai X, et al. Health effects associated with chewing tobacco: a Burden of Proof study. *Nat Commun*. 2024 Feb 5;15(1):1082.

Child growth failure

Combined flowchart for child growth failure and protein-energy malnutrition

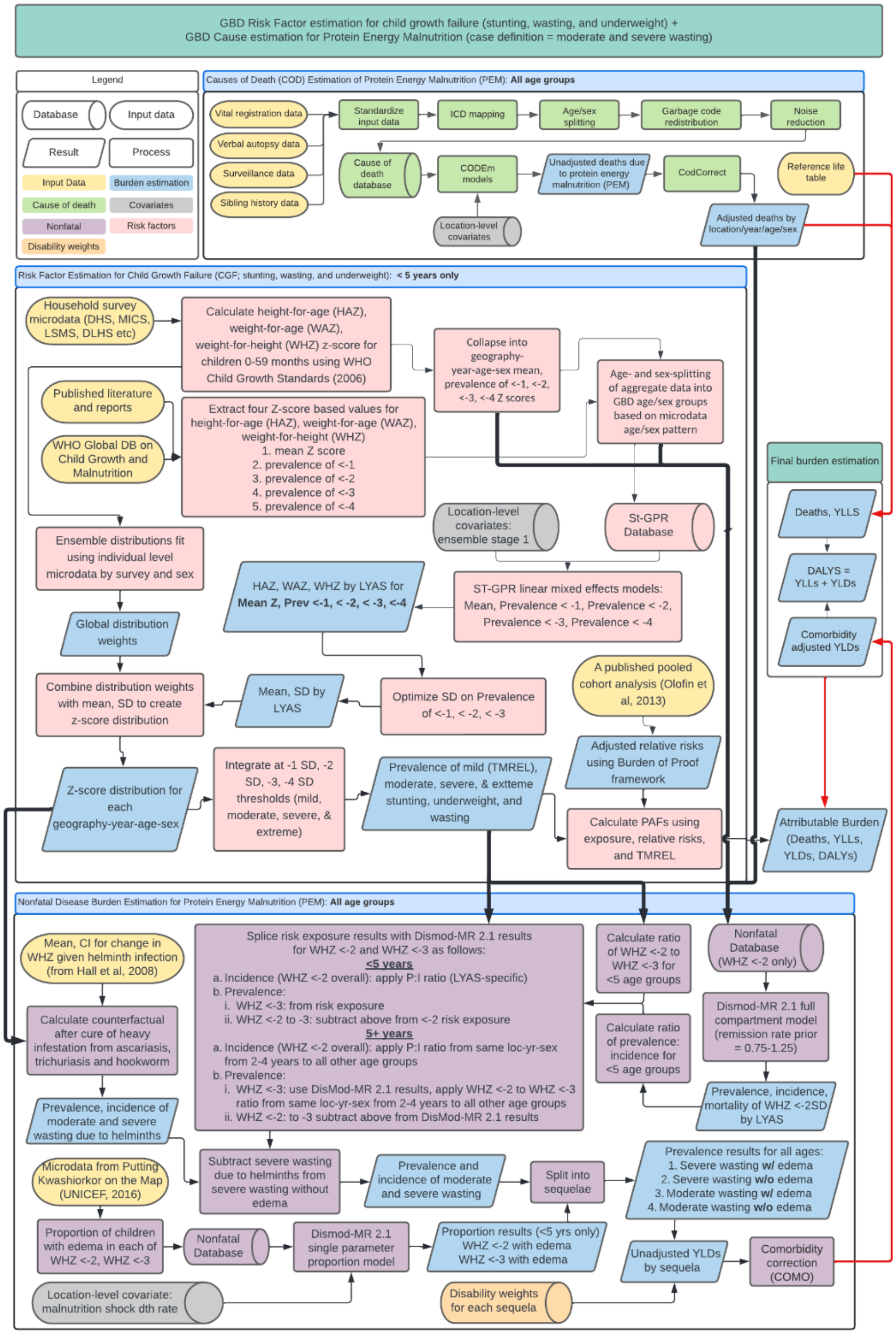

Input data and methodological summary

Exposure

Case definition

Child growth failure (CGF) is estimated using three indicators (stunting, wasting, and underweight), all of which are based on categorical definitions using the WHO 2006 growth standards for children 0–59 months. Definitions are based on Z scores from the growth standards, which were derived from an international reference population. Mild (< –1 to –2 Z score), moderate (< –2 to –3 Z score), and severe (<3 Z score) categorical prevalences were estimated for each of the three indicators.

Input data

There are three main inputs for the GBD child growth failure models: microdata from population surveys, tabulated data from reports and published literature, and the WHO Global Database on Child Growth and Malnutrition.[1] Population surveys include a variety of multi-country and country-specific survey series such as Multiple Indicator Cluster Surveys (MICS), Demographic and Health Surveys (DHS), Living Standards Measurement Surveys (LSMS), and the China Health and Nutrition Survey (CHNS), as well as other one-time country-specific surveys such as the Indonesia Family Life Survey and the Brazil National Demographic and Health Survey of Children and Women. These microdata contain information about each individual child's age (from which age in weeks and age in months are calculated), as well as height and/or weight. From that information, a height-for-age Z-score (HAZ), weight-for-age Z-score (WAZ), and weight-for-height Z-score (WHZ) are calculated using the WHO 2006 Child Growth Standards and the LMS method.[2] Data that did not meet the following three criteria were dropped: 1) non-sex-specific data, 2) data with invalid Z-scores (HAZ, WAZ, WHZ, or BMI above 6 SD or below -6 SD), and 3) data with impossible values (eg, negative height, weight, or age).

All available data from the WHO Global Database on Child Growth and Malnutrition were extracted in GBD 2016 – much of which are from published studies. Exclusions included examination date prior to 1985, non-population-representative studies, and those based on self-report. A systematic literature review was last completed in GBD 2010. We looked for four metrics from all sources with tabulated data: mean Z score, prevalence < –1 Z score, prevalence < –2 Z score, and prevalence < –3 Z score, and prevalence < –4 Z score. All data for each metric were extracted for each of stunting (height-for-age Z score; HAZ), wasting (weight-for-height Z score; WHZ), and underweight (weight-for-age Z score; WAZ).

Data processing

Any data that were presented as both sexes combined or for 0–59 months combined were split into standard GBD age and sex categories according to the age and sex pattern from all data sources that report estimates by GBD age and sex categories. The primary focus of GBD 2023 was streamlining our data processing and modelling approach to reduce redundancy and eliminate modelling pipeline bottlenecks.

Modelling strategy

Exposure estimation

The following four-step modelling process was applied in parallel to each of stunting, wasting, and underweight.

First, all microdata were fit using ensemble modelling. A series of ten individual distributions (normal, log-normal, log-logistic, exponential, gamma, mirror gamma, inverse gamma, gumbel, mirror gumbel, and Weibull) were fit simultaneously to each microdata source in the dataset. All component distributions that were used to derive weights were parameterised using “method of moments,” meaning that each corresponding probability density function (PDF) could be described as a function of the mean and variance of the quantity of interest. From these distribution families, an ensemble distribution was parameterised using an updated methodology first introduced in GBD 2021, which had the advantages of (1) specifically fitting on the portions of the distributions that constitute mild, moderate, and severe CGF to minimise absolute prediction error in highly relevant areas of the curve, and (2) considering the fit across all microdata sources simultaneously in the optimisation process to target the set of ensemble weights that minimises the predictive error across all microdata sources collectively, as opposed to finding one set of weights for each individual microdata source and averaging those sets of weights together.

Second, after ensemble distributions have been parameterised, models were developed for mean Z scores and prevalence of moderate and severe growth failure. Individual-level microdata were collapsed to calculate five metrics: mean z-score, mild prevalence, moderate prevalence, severe prevalence, and extreme prevalence. These data were combined with those derived from literature, GHDx review, and the WHO Global Database on Child Growth and Malnutrition. Each of the three metrics was then modelled using spatiotemporal Gaussian process regression (ST-GPR), a common modelling framework used across GBD, generating estimates for each age group, sex, year, and location. Location-level covariates used in all models included Socio-demographic Index (SDI) and logit-transformed proportion of households with improved sanitation. An ensemble linear prior incorporating total available calories per person per day, age-standardised prevalence of severe anaemia, and Healthcare Access and Quality (HAQ) Index, maternal care and immunisation index (MCI), Socio-demographic Index, age- and sex-specific summary exposure value (SEV) for unsafe sanitation, and proportion with access to sanitation covariates were generated for each severity level-CGF type-location-year-age-sex combination.

Third, we combined estimates of mean, prevalence (moderate and severe) with ensemble weights in an optimisation framework to derive the variance that would best correspond to the predicted mean and prevalence. This variance was then paired with the mean and, using the method of moments equation for each of the component distributions of the ensemble, a PDF of the distribution of Z-scores were calculated for each location, year, age group, and sex.

Fourth, PDFs were integrated to determine the prevalence between –1 and –2 Z scores (mild), between –2 and –3 Z scores (moderate), and below –3 Z scores (severe). These were categorical exposures used for subsequent attributable risk analyses.

Theoretical minimum risk exposure level

Theoretical minimum risk exposure level (TMREL) for underweight, stunting, and wasting was assigned to be greater than or equal to –1 SD of the WHO 2006 standard weight-for-age, height-for-age, and weight-for-height curves, respectively. This has not changed since GBD 2010.

Relative risks

The final list of outcomes paired with child growth failure risks included mortality and morbidity for lower respiratory infections (LRI), diarrhoea, malaria, measles, and protein-energy malnutrition (PEM), as shown in Table 1. These were derived from a Burden of Proof analysis that incorporated both a pooled analysis of ten prospective cohort studies by Olofin and colleagues[4] as well as relative risk estimates from Knowledge Integration (KI) studies (Table 2). For the KI studies, aggregated relative risks of disease or cause-specific mortality were calculated for 1-unit z-score bins for stunting, wasting, and underweight (eg, relative risk of diarrhoea-attributable death in children 1 to 2 years of age and with a HAZ score between –4 and –3). The burden of proof analysis uses all available relative risks with corresponding uncertainty to create continuous relative risk curves for each risk–outcome pair. These continuous risk curves are then combined with the global exposure curves for HAZ, WAZ, and WHZ to calculate exposure-weighted relative risks for severe, moderate, and mild stunting, wasting, and underweight with uncertainty. Of historical note, upper respiratory infections and otitis media were included as outcomes in the GBD 2013 risk analysis, based on the “analogy” causal criterion, assuming there is similar pathway as LRI outcome. However, closer review for GBD 2015 did not find sufficient evidence to support their inclusion and they were excluded, a decision that was carried forward to the current round. We also attributed 100% of PEM to childhood wasting and underweight but not stunting. To build on the existing literature base for GBD on risk–outcome pairs, a literature search was conducted for GBD 2017 searching for case-control studies published after January 1, 1985, and did not return any sources that were usable.

There is a high degree of correlation between stunting, wasting, and underweight. Failing to account for their covariance and assuming independence would overestimate the total burden significantly and misrepresent the attributable burden of individual CGF indicators. Inability to address these correlations is the main reason that GBD 2010 only included childhood underweight. To account for the high degree of correlation between CGF indicators, GBD uses a constrained optimisation method to adjust the observed univariate RRs that come out of the Burden of Proof analysis. First, we created a joint distribution of stunting, underweight, and wasting from a population of children. Second, we generated 1000 RR draws for each univariate indicator and severity based from the Burden of Proof analysis. Third, we altered these univariate RRs for the four causes (diarrhoea, LRI, malaria, and measles) and the two outcomes (mortality and morbidity) based upon interactions among the CGF indicators. An interaction occurs when the effect of one CGF indicator variable (eg, stunting) has a different effect on the outcome depending on the value of another CGF indicator variable (eg, underweight). Interaction terms alter the risk of the outcome among children with more than one indicator of CGF. These interaction terms were extracted from a pooled cohort analysis of all-cause mortality published by McDonald and colleagues.[5] Lastly, we optimised the adjusted relative risks by minimising the error between the observed RRs (generated from Olofin et al.[4]) and the altered RRs derived from the joint distribution and accounting for the interaction terms while ensuring that no alteration resulted in a previously identified increase in relative risk becoming protective.

Several changes to improve the four main steps of RR adjustment were first introduced for GBD 2021. From GBD 2013 to GBD 2019, a simulated joint distribution of stunting, underweight, and wasting measures was created from the Olofin et al.[4] meta-analysis. Sources in this meta-analysis were cross-sectional Demographic and Health Surveys (DHS). Starting with GBD 2021, we created age-specific joint distributions of stunting, underweight, and wasting measures from 15 longitudinal studies (from 26 locations) in the Bill and Melinda Gates Foundation's Knowledge Integration (Ki) database.[6] (Study details are provided in Table 1.) The RR adjustment method was strengthened for GBD 2021 by constraining optimisation in two ways: optimisation was only permitted to alter the RR for an indicator/severity in draws where the observed RR was greater than 1, and constraints were placed on the error that penalise larger alterations to the RR. Starting with GBD 2021, these changes enabled the estimation and utilisation of age-specific adjusted RRs for burden estimation.

Table 1: Bill and Melinda Gates Foundation Knowledge Integration (KI) database study details

Study name	Country	Sample Size	Years conducted
Zimbabwe Vitamin A for Mothers and Babies Trial	ZWE	14,110	1997-2001
CMC Vellore Birth Cohort Study	IND	373	2002-2006
International Lipid-Based Nutrient Supplements Project	MWI	1206	2011-2014
Malnutrition and Enteric Disease Study	BGD	265	2009-2017
Malnutrition and Enteric Disease Study	IND	251	2009-2017
Malnutrition and Enteric Disease Study	NEP	240	2009-2017
Malnutrition and Enteric Disease Study	PER	303	2009-2017
Malnutrition and Enteric Disease Study	BRA	233	2009-2017
Malnutrition and Enteric Disease Study	ZAF	314	2009-2017
Malnutrition and Enteric Disease Study	TZA	262	2009-2017
Medical Research Council Keneba	GMB	2867	-
Performance of Rotavirus and Oral Polio Vaccines in Developing Countries	BGD	700	2011-2014
Community-based Intervention Trial to Compare the Impact of Preventive and Therapeutic Zinc Supplementation Programs Among Young Children in Burkina Faso	BFA	7634	2010-2012
WASH Benefits Bangladesh	BGD	4423	2011-2014
WASH Benefits Kenya	KEN	5649	2012-2016
Promotion of Breastfeeding Intervention Trial	BLR	16,897	1996-1998
Childhood Malnutrition and Infection Network	BGD	477	1993-1996
Childhood Malnutrition and Infection Network	BRA	119	1989-1998
Childhood Malnutrition and Infection Network	GNB	350	1987-1990
Childhood Malnutrition and Infection Network	GNB	885	1996-1997
Childhood Malnutrition and Infection Network	PER	210	1989-1991
Childhood Malnutrition and Infection Network	PER	224	1995-1998
Delhi Infant Vitamin D Study	IND	2100	2007-2010
Characterization of Respiratory pathogens endemic to Pakistan in pregnant women and newborns in urban settings	PAK	380	2012-2013
Impact of Zinc Supplementation in Low Birth Weight Infants on Severe Morbidity, Mortality and Zinc Status: A Randomized Controlled Trial	IND	2052	2005-2007
A Trial of Zinc and Micronutrients in Tanzanian Children	TZA	2400	2007-2012

Table 2: Age-specific adjusted RRs for each risk–outcome pair for child growth failure

1 to 5 months		Incidence			Mortality		
**Cause**		**< –3**	**–3, –2**	**–2, –1**	**< –3**	**–3, –2**	**–2, –1**
**Diarrhoea**	HAZ	1.2 (0.8, 1.7)	1.2 (0.8, 1.6)	1.1 (0.9, 1.5)	3.6 (2.1, 4.4)	2.1 (1.6, 2.6)	1.4 (1.2, 1.6)
	WAZ	1.6 (0.9, 2.7)	1.6 (0.9, 2.6)	1.5 (0.9, 2.4)	6.7 (4.4, 9.3)	3.4 (2.2, 4.7)	1.8 (1.3, 2.2)
	WHZ	1.3 (0.8, 1.8)	1.2 (0.9, 1.7)	1.2 (0.9, 1.6)	40.8 (0.8, 224.7)	12.8 (0.8, 51.5)	4.1 (0.9, 10.6)
**LRI**	HAZ	1.3 (0.6, 2.5)	1.1 (0.7, 1.8)	1.1 (0.8, 1.5)	5.1 (2.7, 7.6)	2.9 (1.7, 4.2)	1.8 (1.2, 2.4)
	WAZ	1.6 (0.6, 4.1)	1.3 (0.7, 2.2)	1.2 (0.8, 1.7)	31.1 (1.0, 191.4)	13.9 (1.0, 69.2)	4.9 (1.0, 16.8)
	WHZ	1.3 (0.9, 1.8)	1.1 (1.0, 1.2)	1.0 (1.0, 1.1)	6.5 (4.5, 8.8)	3.6 (2.6, 4.6)	1.8 (1.5, 2.1)
**Malaria**	HAZ	1.0 (1.0, 1.0)	1.0 (1.0, 1.0)	1.0 (1.0, 1.0)	3.0 (0.6, 12.4)	1.1 (0.9, 1.2)	1.0 (1.0, 1.0)
	WAZ	2.3 (0.6, 6.6)	2.3 (0.6, 6.6)	2.3 (0.6, 6.6)	2.7 (0.6, 8.4)	1.8 (0.7, 3.9)	1.5 (0.8, 2.5)
	WHZ	1.0 (1.0, 1.0)	1.0 (1.0, 1.0)	1.0 (1.0, 1.0)	1.0 (1.0, 1.0)	1.0 (1.0, 1.0)	1.0 (1.0, 1.0)
**Measles**	HAZ	1.3 (0.9, 2.3)	1.0 (1.0, 1.0)	1.0 (1.0, 1.0)	4.1 (1.8, 5.7)	2.2 (1.4, 2.9)	1.4 (1.1, 1.5)
	WAZ	1.0 (1.0, 1.0)	1.0 (1.0, 1.0)	1.0 (1.0, 1.0)	5.1 (2.2, 7.8)	2.7 (1.6, 3.5)	1.4 (1.2, 1.6)
	WHZ	1.7 (0.8, 3.3)	1.3 (0.9, 1.8)	1.3 (0.9, 1.8)	6.7 (2.5, 15.4)	2.6 (1.6, 4.2)	1.2 (1.1, 1.4)
**PEM**	HAZ		0% PAF			0% PAF	
	WAZ		100% PAF			100% PAF	
	WHZ		100% PAF			100% PAF	
**6 to 11 months**		**Incidence**			**Mortality**		
**Cause**		**< –3**	**–3, –2**	**–2, –1**	**< –3**	**–3, –2**	**–2, –1**
**Diarrhoea**	HAZ	1.2 (0.8, 1.7)	1.2 (0.8, 1.6)	1.1 (0.9, 1.5)	3.1 (2.2, 3.9)	1.9 (1.5, 2.4)	1.3 (1.2, 1.6)
	WAZ	1.6 (0.9, 2.7)	1.6 (0.9, 2.6)	1.5 (0.9, 2.4)	5.9 (3.7, 8.9)	3.1 (2.0, 4.5)	1.7 (1.3, 2.2)
	WHZ	1.3 (0.8, 1.8)	1.2 (0.9, 1.7)	1.2 (0.9, 1.6)	40.7 (0.8, 224.7)	12.7 (0.8, 51.5)	4.1 (0.9, 10.6)
**LRI**	HAZ	1.3 (0.6, 2.5)	1.1 (0.7, 1.8)	1.1 (0.8, 1.5)	4.3 (2.2, 6.8)	2.6 (1.5, 3.9)	1.7 (1.2, 2.3)
	WAZ	1.6 (0.6, 4.1)	1.3 (0.7, 2.2)	1.2 (0.8, 1.7)	31.0 (1.0, 191.4)	13.8 (1.0, 69.2)	4.9 (1.0, 16.8)
	WHZ	1.3 (0.9, 1.8)	1.1 (1.0, 1.2)	1.0 (1.0, 1.1)	6.2 (4.3, 8.4)	3.5 (2.5, 4.5)	1.8 (1.5, 2.1)
**Malaria**	HAZ	1.0 (1.0, 1.0)	1.0 (1.0, 1.0)	1.0 (1.0, 1.0)	3.0 (0.6, 12.4)	1.1 (0.9, 1.2)	1.0 (1.0, 1.0)
	WAZ	2.3 (0.6, 6.6)	2.3 (0.6, 6.6)	2.3 (0.6, 6.6)	2.7 (0.6, 8.4)	1.8 (0.7, 3.9)	1.5 (0.8, 2.5)
	WHZ	1.0 (1.0, 1.0)	1.0 (1.0, 1.0)	1.0 (1.0, 1.0)	1.0 (1.0, 1.0)	1.0 (1.0, 1.0)	1.0 (1.0, 1.0)
**Measles**	HAZ	1.3 (0.9, 2.3)	1.0 (1.0, 1.0)	1.0 (1.0, 1.0)	3.6 (1.8, 4.9)	2.1 (1.4, 2.6)	1.3 (1.1, 1.5)

	WAZ	1.0 (1.0, 1.0)	1.0 (1.0, 1.0)	1.0 (1.0, 1.0)	5.2 (2.2, 8.1)	2.7 (1.6, 3.6)	1.4 (1.2, 1.6)
	WHZ	1.7 (0.8, 3.3)	1.3 (0.9, 1.8)	1.3 (0.9, 1.8)	6.7 (2.5, 15.4)	2.6 (1.6, 4.2)	1.2 (1.1, 1.4)
**PEM**	HAZ		0% PAF			0% PAF	
	WAZ		100% PAF			100% PAF	
	WHZ		100% PAF			100% PAF	

**12 to 23 months**		**Incidence**			**Mortality**		
**Cause**		**< –3**	**–3, –2**	**–2, –1**	**< –3**	**–3, –2**	**–2, –1**
**Diarrhoea**	HAZ	1.2 (0.8, 1.7)	1.2 (0.8, 1.6)	1.1 (0.9, 1.5)	2.7 (2.2, 3.4)	1.7 (1.4, 2.2)	1.3 (1.1, 1.5)
	WAZ	1.6 (0.9, 2.7)	1.6 (0.9, 2.6)	1.5 (0.9, 2.4)	5.4 (3.7, 8.4)	2.9 (2.0, 4.4)	1.6 (1.3, 2.1)
	WHZ	1.3 (0.8, 1.8)	1.2 (0.9, 1.7)	1.2 (0.9, 1.6)	40.7 (0.8, 225.5)	12.7 (0.8, 51.7)	4.0 (0.9, 10.6)
**LRI**	HAZ	1.3 (0.6, 2.5)	1.1 (0.7, 1.8)	1.1 (0.8, 1.5)	4.1 (2.3, 5.9)	2.5 (1.5, 3.5)	1.6 (1.2, 2.2)
	WAZ	1.6 (0.6, 4.1)	1.3 (0.7, 2.2)	1.2 (0.8, 1.7)	29.3 (1.0, 186.4)	13.0 (1.0, 67.8)	4.6 (1.0, 16.5)
	WHZ	1.3 (0.9, 1.8)	1.1 (1.0, 1.2)	1.0 (1.0, 1.1)	5.4 (3.5, 7.6)	3.1 (2.1, 4.2)	1.7 (1.3, 2.0)
**Malaria**	HAZ	1.0 (1.0, 1.0)	1.0 (1.0, 1.0)	1.0 (1.0, 1.0)	3.0 (0.6, 12.4)	1.1 (0.9, 1.2)	1.0 (1.0, 1.0)
	WAZ	2.3 (0.6, 6.6)	2.3 (0.6, 6.6)	2.3 (0.6, 6.6)	2.7 (0.6, 8.4)	1.8 (0.7, 3.9)	1.5 (0.8, 2.5)
	WHZ	1.0 (1.0, 1.0)	1.0 (1.0, 1.0)	1.0 (1.0, 1.0)	1.0 (1.0, 1.0)	1.0 (1.0, 1.0)	1.0 (1.0, 1.0)
**Measles**	HAZ	1.3 (0.9, 2.3)	1.0 (1.0, 1.0)	1.0 (1.0, 1.0)	3.5 (1.8, 4.4)	2.0 (1.4, 2.5)	1.3 (1.1, 1.4)
	WAZ	1.0 (1.0, 1.0)	1.0 (1.0, 1.0)	1.0 (1.0, 1.0)	4.6 (2.2, 6.1)	2.5 (1.6, 3.0)	1.4 (1.2, 1.5)
	WHZ	1.7 (0.8, 3.3)	1.3 (0.9, 1.8)	1.3 (0.9, 1.8)	6.7 (2.5, 15.4)	2.6 (1.6, 4.2)	1.2 (1.1, 1.4)
**PEM**	HAZ		0% PAF			0% PAF	
	WAZ		100% PAF			100% PAF	
	WHZ		100% PAF			100% PAF	

**2 to 4 years**		**Incidence**			**Mortality**		
**Cause**		**< –3**	**–3, –2**	**–2, –1**	**< –3**	**–3, –2**	**–2, –1**
**Diarrhoea**	HAZ	1.2 (0.8, 1.7)	1.2 (0.8, 1.6)	1.1 (0.9, 1.5)	2.8 (2.1, 3.4)	1.8 (1.5, 2.2)	1.3 (1.2, 1.5)
	WAZ	1.6 (0.9, 2.7)	1.6 (0.9, 2.6)	1.5 (0.9, 2.4)	5.6 (4.0, 8.3)	2.9 (2.1, 4.3)	1.6 (1.3, 2.1)
	WHZ	1.3 (0.8, 1.8)	1.2 (0.9, 1.7)	1.2 (0.9, 1.6)	41.3 (0.8, 229.9)	12.8 (0.8, 52.4)	4.0 (0.9, 10.7)
**LRI**	HAZ	1.3 (0.6, 2.5)	1.1 (0.7, 1.8)	1.1 (0.8, 1.5)	9.5 (4.7, 21.2)	4.7 (3.0, 8.6)	2.5 (2.0, 3.8)
	WAZ	1.6 (0.6, 4.1)	1.3 (0.7, 2.2)	1.2 (0.8, 1.7)	14.5 (1.0, 60.4)	7.1 (1.0, 21.7)	3.1 (1.0, 5.6)

	WHZ	1.3 (0.9, 1.8)	1.1 (1.0, 1.2)	1.0 (1.0, 1.1)	5.1 (2.3, 7.9)	3.0 (1.5, 4.3)	1.6 (1.2, 2.1)
**Malaria**	HAZ	1.0 (1.0, 1.0)	1.0 (1.0, 1.0)	1.0 (1.0, 1.0)	3.0 (0.6, 12.4)	1.1 (0.9, 1.2)	1.0 (1.0, 1.0)
	WAZ	2.3 (0.6, 6.6)	2.3 (0.6, 6.6)	2.3 (0.6, 6.6)	2.7 (0.6, 8.4)	1.8 (0.7, 3.9)	1.5 (0.8, 2.5)
	WHZ	1.0 (1.0, 1.0)	1.0 (1.0, 1.0)	1.0 (1.0, 1.0)	1.0 (1.0, 1.0)	1.0 (1.0, 1.0)	1.0 (1.0, 1.0)
**Measles**	HAZ	1.3 (0.9, 2.3)	1.0 (1.0, 1.0)	1.0 (1.0, 1.0)	3.5 (1.8, 4.4)	2.0 (1.4, 2.5)	1.3 (1.1, 1.4)
	WAZ	1.0 (1.0, 1.0)	1.0 (1.0, 1.0)	1.0 (1.0, 1.0)	4.6 (2.2, 6.1)	2.5 (1.6, 3.0)	1.4 (1.2, 1.5)
	WHZ	1.7 (0.8, 3.3)	1.3 (0.9, 1.8)	1.3 (0.9, 1.8)	6.7 (2.5, 15.4)	2.6 (1.6, 4.2)	1.2 (1.1, 1.4)
**PEM**	HAZ		0% PAF			0% PAF	
	WAZ		100% PAF			100% PAF	
	WHZ		100% PAF			100% PAF	

References

1 WHO | WHO Global Database on Child Growth and Malnutrition. WHO. http://www.who.int/nutgrowthdb/en/ (accessed July 30, 2018).

2 Wang Y, Chen H-J. Use of Percentiles and Z-Scores in Anthropometry. In: Preedy VR, ed. Handbook of Anthropometry. New York, NY: Springer New York, 2012: 29–48.

3 Uribe Á, Cecilia M, López Gaviria A, Estrada Restrepo A. Concordance between Z scores from WHO 2006 and the NCHS 1978 growth standards of children younger than five. Antioquia-Colombia. *Perspectivas en Nutrición Humana* 2008; 10: 177–87.

4 Olofin I, McDonald CM, Ezzati M, *et al.* Associations of Suboptimal Growth with All-Cause and Cause-Specific Mortality in Children under Five Years: A Pooled Analysis of Ten Prospective Studies. *PLOS ONE* 2013; 8: e64636.

5 McDonald CM, Olofin I, Flaxman S, *et al.* The effect of multiple anthropometric deficits on child mortality: meta-analysis of individual data in 10 prospective studies from developing countries. *Am J Clin Nutr* 2013; 97: 896–901.

6 Jumbe NL, Murray JC, Kern S. Data Sharing and Inductive Learning – Toward Healthy Birth, Growth, and Development. *N Engl J Med*. 2016 Jun 23;374(25):2415-7. doj: 10.1056/NEJMp1605441. Epub 2016 May 11. PMID: 27168111.

Dietary risks

Flowchart

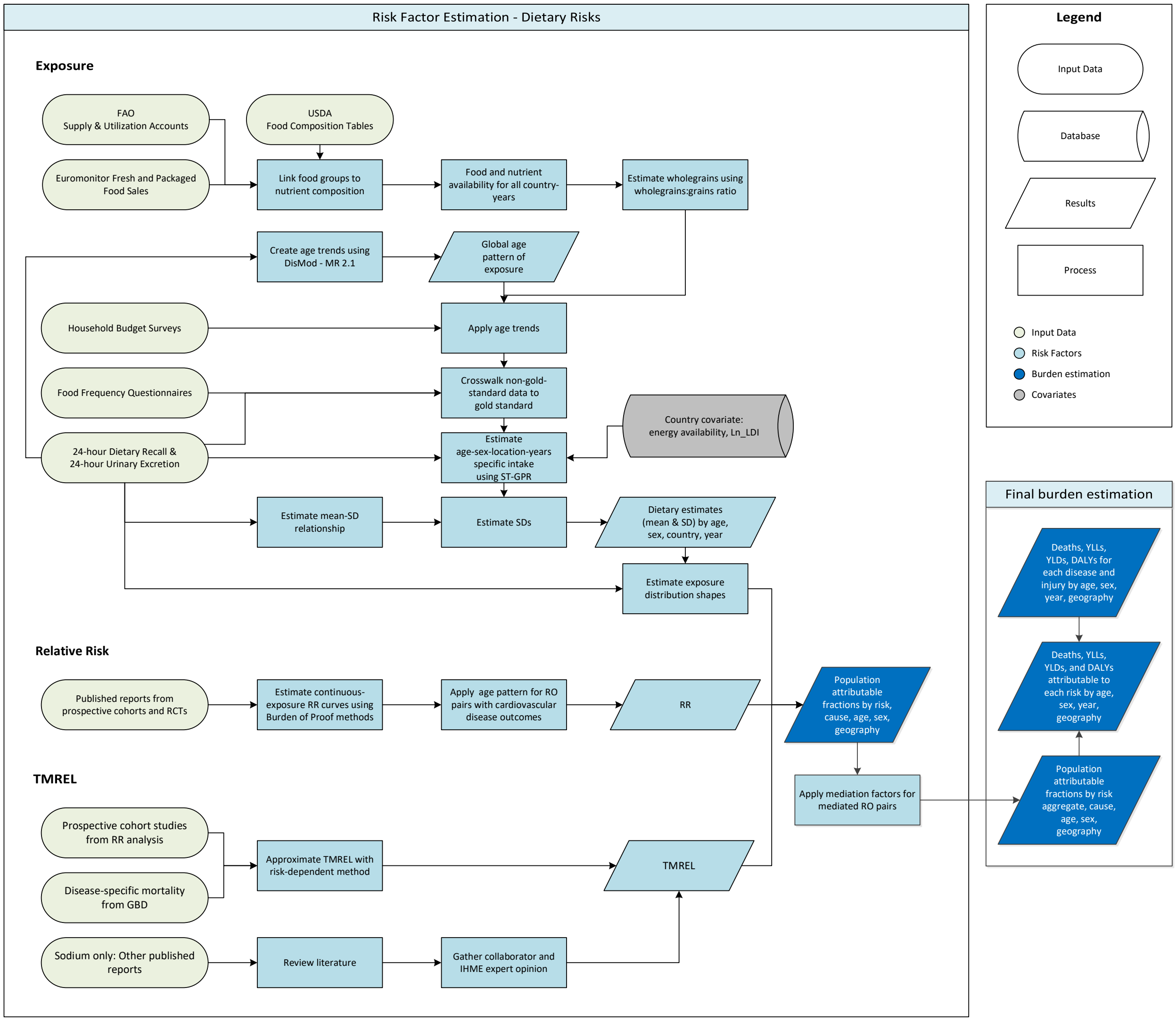

"Ln-LDI" stands for log lag-distributed income per capita; "RO pair" stands for risk–outcome pair; "SD" stands for standard deviation; "RR" stands for relative risk. Other abbreviations defined in list of abbreviations for this section.

Input data and methodological summary

Note that GBD dietary risk factor modelling is for adult populations only (ages 25+).

Definition

Exposure

Table 1: Dietary risk factor exposure definitions and optimal level of exposure as defined by GBD 2023

Dietary risk factor	Definition of exposure	Optimal level or range of intake*

**Diet low in fruit**	Average daily consumption (in grams per day) of fruit including fresh, frozen, cooked, canned, or dried fruit, excluding fruit juices and salted or pickled fruits	340–350 g/day
**Diet low in vegetables**	Average daily consumption (in grams per day) of vegetables, including fresh, frozen, cooked, canned, or dried vegetables and excluding legumes and salted or pickled vegetables, juices, nuts and seeds, and starchy vegetables such as potatoes or corn	306–372 g/day
**Diet low in whole grains**	Average daily consumption (in grams per day) of whole grains (bran, germ, and endosperm in their natural proportion) from breakfast cereals, bread, rice, pasta, biscuits, muffins, tortillas, pancakes, and other sources	160–210 g/day
**Diet low in nuts and seeds**	Average daily consumption (in grams per day) of nuts and seeds, including tree nuts and seeds and peanuts	19–24 g/day
**Diet low in fibre**	Average daily consumption (in grams per day) of fibre from all sources including fruits, vegetables, grains, legumes, and pulses	22–25 g/day
**Diet low in seafood omega-3 fatty acids**	Average daily consumption (in milligrams per day) of eicosapentaenoic acid (EPA) and docosahexaenoic acid (DHA)	470–660 mg/day
**Diet low in omega-6 polyunsaturated fatty acids**	Average daily consumption (in % daily energy) from omega-6 polyunsaturated fatty acids (PUFA) (specifically linoleic acid, γ-linolenic acid, eicosadienoic acid, dihomo-γ-linolenic acid, arachidonic acid)	9–10% of total daily energy
**Diet low in calcium**	Average daily consumption (in grams per day) of calcium from all sources, including milk, yoghurt, and cheese	0.72–0.86 g/day (males)<br>1.1–1.2 g/day (females)
**Diet low in milk**	Average daily consumption (in grams per day) of dairy milk including non-fat, low-fat, and full-fat milk, but excluding plant-based milks, fermented milk products such as buttermilk, and other dairy products such as cheese	280–340 g/day (males)<br>500–610 g/day (females)
**Diet low in legumes**	Average daily consumption (in grams per day) of legumes and pulses, including fresh, frozen, cooked, canned, or dried legumes	100–110 g/day
**Diet high in red meat**	Average daily consumption (in grams per day) of unprocessed red meat including pork and bovine meats such as beef, pork, lamb, and goat, but excluding all processed meats, poultry, fish, and eggs	0–200 g/day

**Diet high in processed meat**	Average daily consumption (in grams per day) of meat preserved by smoking, curing, salting, or addition of chemical preservatives	0 g/day
**Diet high in sugar-sweetened beverages (SSBs)**	Average daily consumption (in grams per day) of beverages with ≥50 kcal per 226.8 gram serving, including carbonated beverages, sodas, energy drinks, and fruit drinks, but excluding 100% fruit and vegetable juices	0 g/day
**Diet high in trans fatty acids**	Average daily consumption (in percent daily energy) of trans fat from industrially produced trans-fat mainly from partially hydrogenated vegetable oils	0% of total daily energy
**Diet high in sodium**	Average 24-hour urinary sodium excretion (in grams per day)	1–5 g/day

** "Optimal level" is equivalent to "TMREL" throughout this document.*

List of abbreviations used in this section

DR	dietary recall surveys
FAO	Food and Agriculture Organization of the United Nations
FBS	FAO Food Balance Sheets
FFQ	food frequency questionnaires
FPG	fasting plasma glucose
GHDx	IHME Global Health Data Exchange
HBS	household budget surveys
IHD	ischaemic heart disease
IHME	Institute for Health Metrics and Evaluation
MR-BRT	meta-regression—Bayesian, regularised, trimmed
PRISMA	Preferred Reporting Items for Systematic Reviews and Meta-Analyses
PUFA	polyunsaturated fatty acids
Sales	Euromonitor Fresh and Packaged Food Sales data
SBP	systolic blood pressure
SSBs	sugar-sweetened beverages
ST-GPR	spatiotemporal Gaussian process regression
SUA	FAO Supply Utilization Accounts
TMREL	theoretical minimum risk exposure level
USDA	United States Department of Agriculture

Input data

Exposure

The dietary data that we used in the exposure models come from multiple sources, including nationally and subnationally representative nutrition surveys using 24-hour dietary recall methodology (DR), food frequency questionnaires (FFQ), household budget surveys (HBS), accounts of national sales from Euromonitor ("sales"), and food availability data from the Food and Agriculture Organization of the

United Nations (FAO). For sodium, data from 24-hour urinary sodium excretion and 24-hour dietary recall surveys were used. In GBD 2023, we have added seven new years of FAO data (2014–2021), as well as updated our database on trans fatty acid bans that have been implemented. Specifically, while in the year 2021, 16 countries had a TFA ban in effect, by 2024, that number had increased nearly four-fold to 59 countries.

Through GBD 2017, the availability data for food groups in GBD was based on the FAO Food Balance Sheets (FBS), which provide tabulated and processed data of national food supply. In GBD 2019, to characterise the national availability of various food groups more accurately, we used more disaggregated data on food commodities that were included in FAO Supply Utilization Accounts (SUA) and recreated the national availability of each food group based on the GBD definition of the food group. In some cases where it serves as an appropriate supplement to FAO SUA data, we continued to use FAO FBS data. This approach has been used since GBD 2019, including in GBD 2023.
To estimate nutrient availability, we used USDA Food Composition Tables and constructed a global nutrient database, as indicated in Schmidhuber et al., 2018.[1,2]

Our primary, gold-standard data sources are 24-hour dietary recall surveys where food and nutrient intake are reported or convertible to grams/milligrams per person per day for all dietary risk factors; for sodium, the gold standard is 24-hour urinary sodium excretion. For other data types, not every type was used for every dietary risk factor. Table 2 summarises which sources were used in modelling each dietary risk factor.

Table 2. Types of dietary data sources (other than 24-hour dietary recall) and covariates used in modelling of each dietary risk factor

**Dietary risk factor**	**Data sources**				**Country-level covariate**
	**Sales**	**FFQ[1]**	**HBS[2]**	**FAO [3]**	
Diet low in fruits	•	•	•	•	Lag-distributed income
Diet low in vegetables	•	•	•	•	Energy availability (kcal)
Diet low in whole grains	-	•	-	•	Energy availability (kcal)
Diet low in nuts and seeds	-	-	•	•	Energy availability (kcal)
Diet low in milk	•	•	•	•	Energy availability (kcal)
Diet high in red meat	•	•	•	•	Energy availability (kcal)
Diet high in processed meat	•	•	•	-	Energy availability (kcal), red meat availability (g), lag-distributed income
Diet low in legumes	•	•	-	•	Energy availability (kcal)
Diet high in sugar-sweetened beverages	•	•	•	-	Energy availability (kcal)
Diet low in fibre	-	•	-	•	Energy availability (kcal)
Diet low in calcium	-	•	-	•	Energy availability (kcal)
Diet low in seafood omega-3 fatty acids	-	-	-	•	Lag-distributed income, proportion landlocked area
Diet low in omega-6 polyunsaturated fatty acids	-	•	-	•	Lag-distributed income
Diet high in trans fatty acids	•	•	-	-	

Diet high in sodium[4]	-	-	-	-	

[1] Food Frequency Questionnaire
[2] Household Budget Survey
[3] Food and Agriculture Organization
[4] For sodium, we used data from the 24-hour urinary sodium excretion and 24-hour dietary recall.

Relative risk

In GBD 2023, we conducted three additional systematic reviews that examined the health effects of processed meat, sugar-sweetened beverage and trans fatty acid consumption.

In the processed meat systematic review, we evaluated the association between processed meat consumption and type 2 diabetes, IHD, and colorectal cancer. The workflow is detailed for each health outcome in the PRISMA flow diagrams (**Figure 1-3**). We identified 44 eligible studies presenting a total of 208 estimates of relative risk (RR) for processed meat consumption in relation to the three health outcomes included. All included studies except one were prospective cohort study designs. A total of 41 studies used food frequency questionnaires; two studies used dietary recall, and only one study used food diary methods to assess processed meat consumption. The detailed methods and results of the study were published elsewhere.[3]

In the SSB systematic review, we evaluated the association between SSB consumption and type 2 diabetes and IHD. The workflow is detailed for each of the health outcomes in the PRISMA flow diagrams (**Figure 4-5**). We synthesised data from a total of 27 eligible studies presenting a total of 122 estimates of RRs for SSB consumption and the two health outcomes included. All included studies except one were prospective cohort study designs. A total of 26 studies used food frequency questionnaires to assess SSB consumption; one study used dietary recall. The detailed methods and full findings of the systematic review are available elsewhere.[3]

In the TFA systematic review and meta-analysis, we evaluated the association between TFA consumption and IHD. The workflow is detailed in the PRISMA flow diagram (**Figure 6**). For those studies examining total TFA consumption, we assumed the major contributor to be industrially produced TFAs. We excluded studies that examined the effect of TFAs from ruminant-only sources on IHD because our focus in this systematic review is industrially produced trans fats. A total of six eligible studies presenting a total of 31 estimates of RRs for TFA consumption and IHD were included. All of the studies were prospective cohort study designs. A total of five studies used food frequency questionnaires to assess TFA consumption, and one study used dietary recall. The detailed methods and full findings of the systematic review are available elsewhere.[3]

Figure 1: PRISMA flow diagram of processed meat consumption and type 2 diabetes

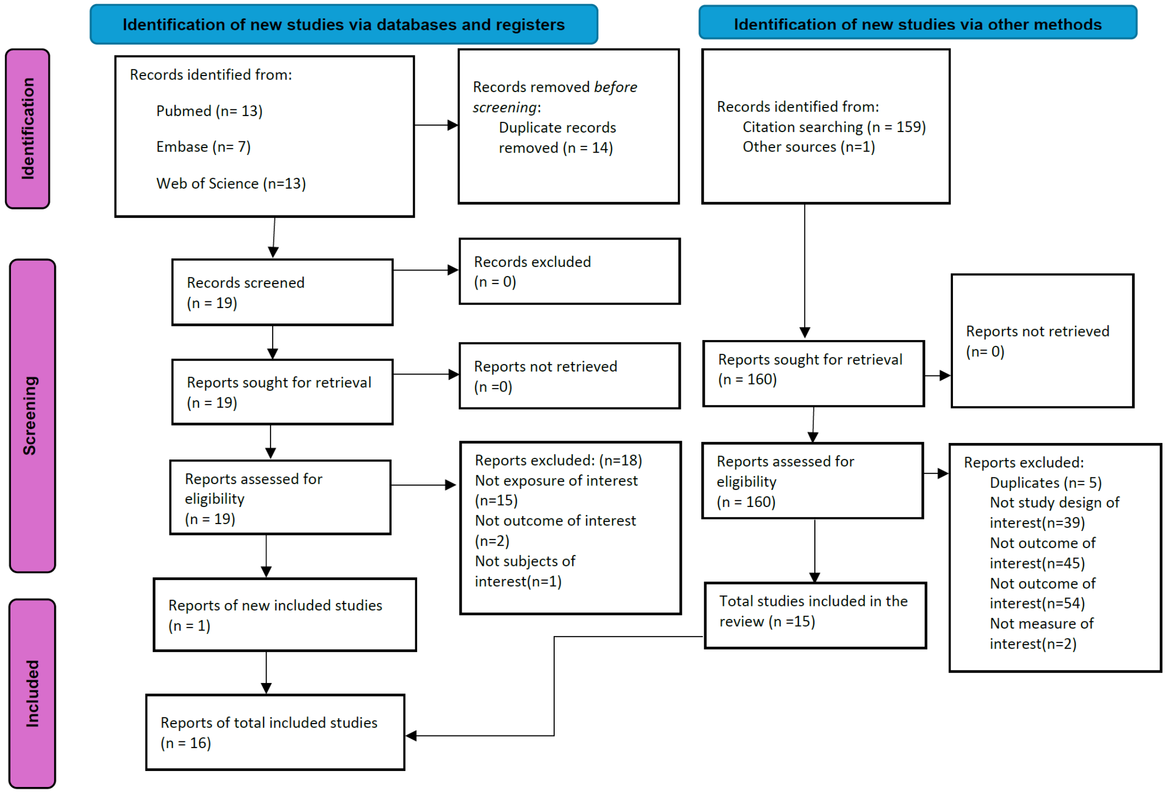

Figure 2: PRISMA flow diagram of processed meat consumption and ischaemic heart disease

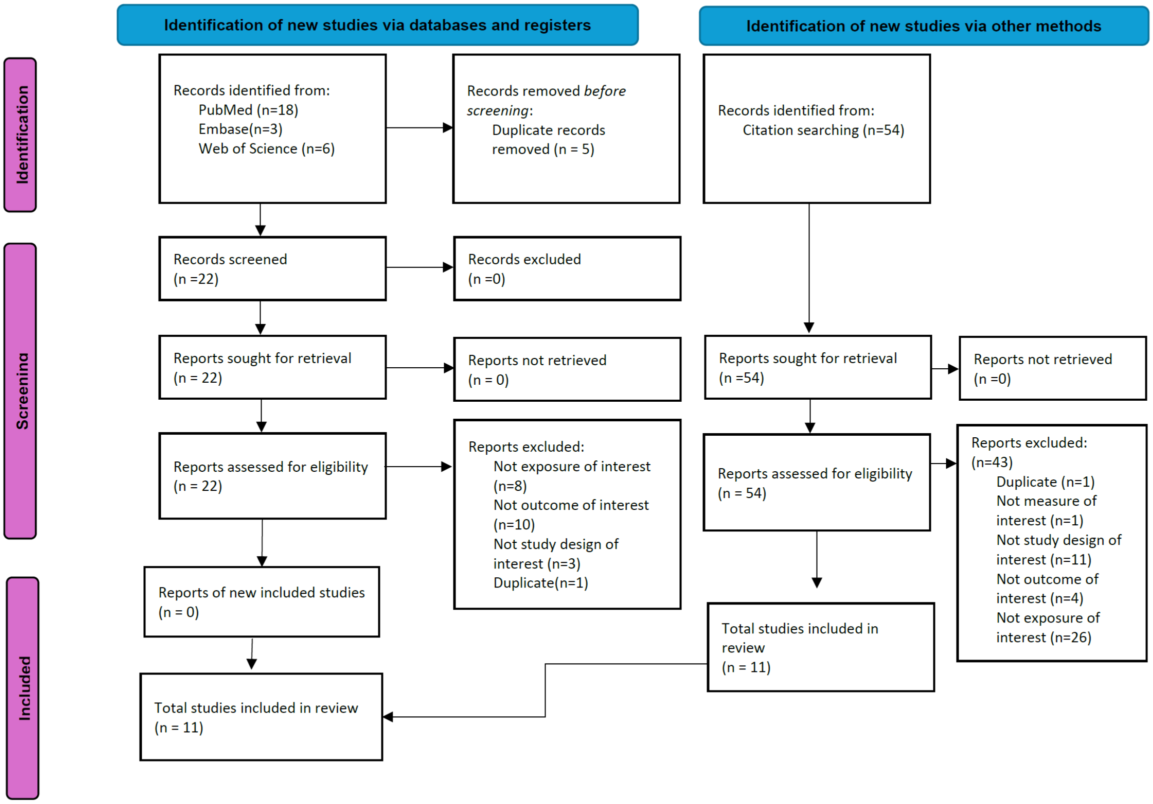

Figure 3: PRISMA flow diagram of processed meat consumption and colorectal cancer

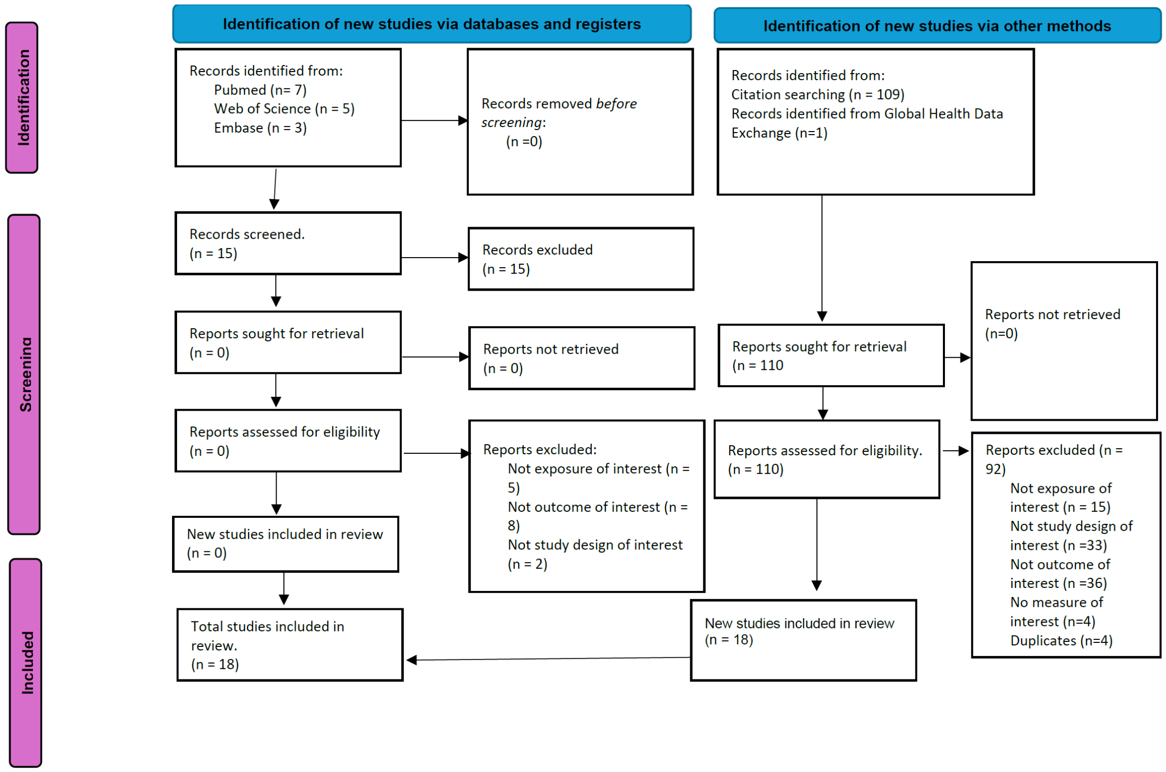

Figure 4: PRISMA flow diagram of sugar-sweetened beverages and type 2 diabetes

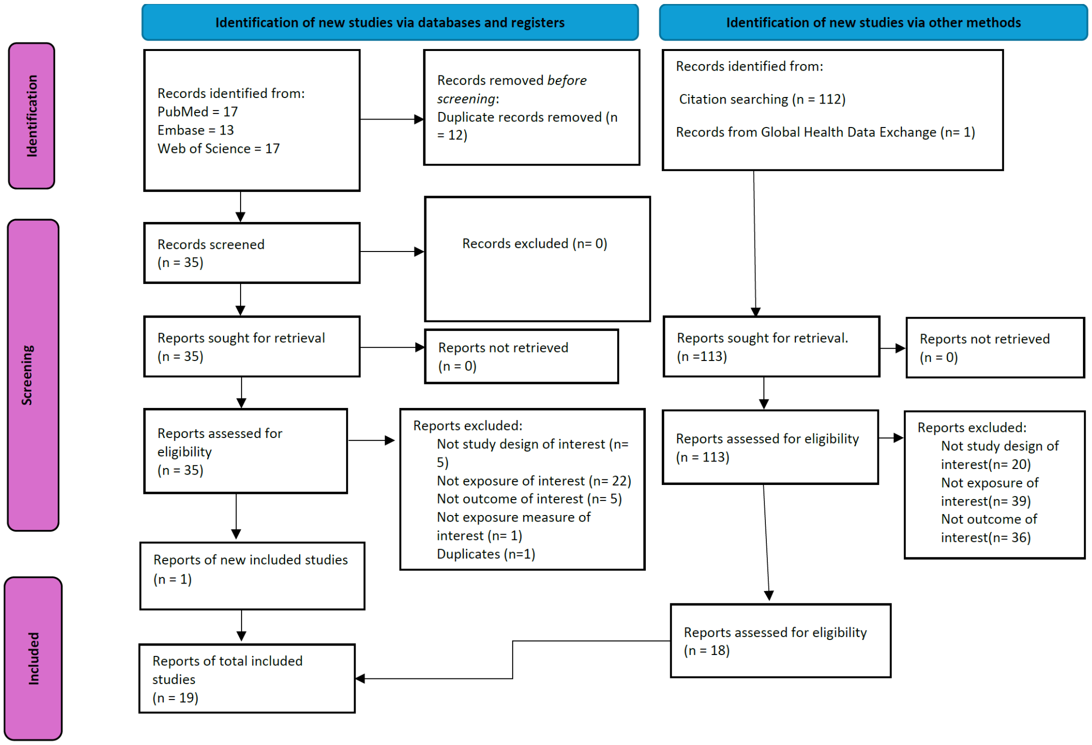

Figure 5: PRISMA flow diagram of sugar-sweetened beverages and ischaemic heart disease

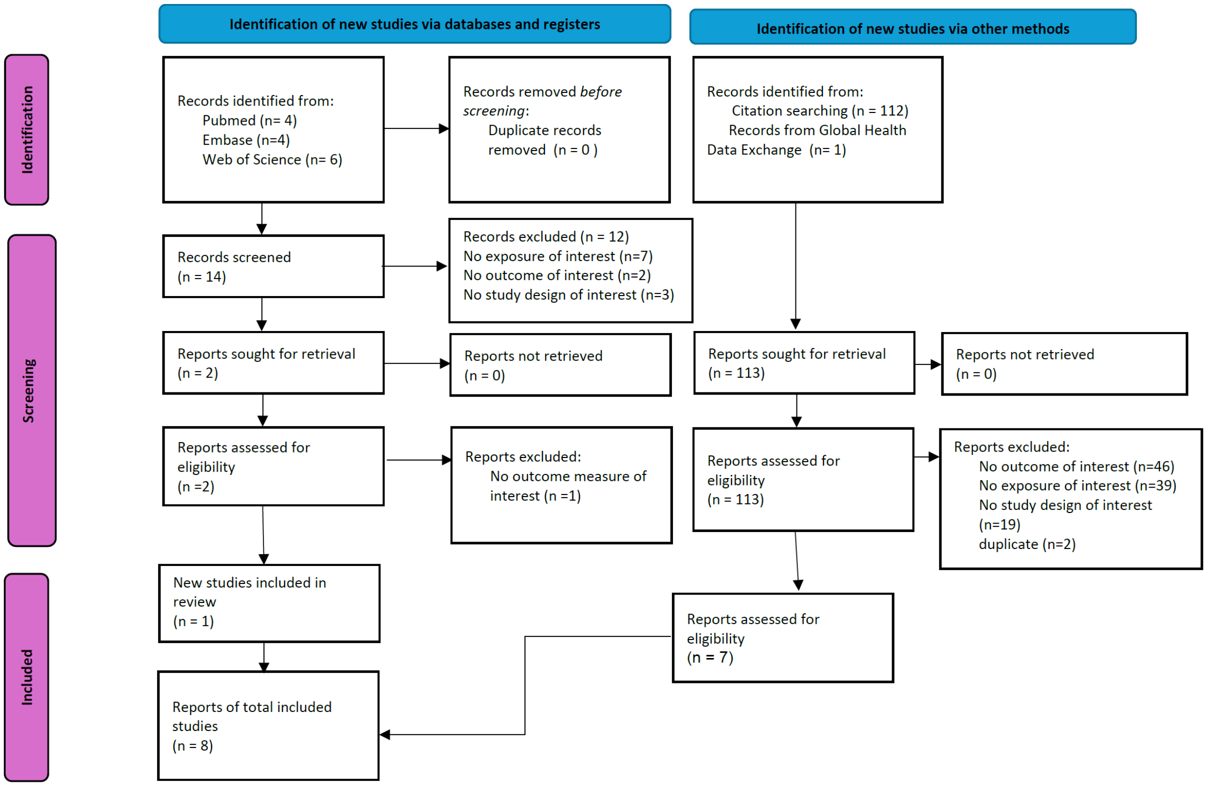

Figure 6: PRISMA flow diagram of trans-fat consumption and ischaemic heart disease

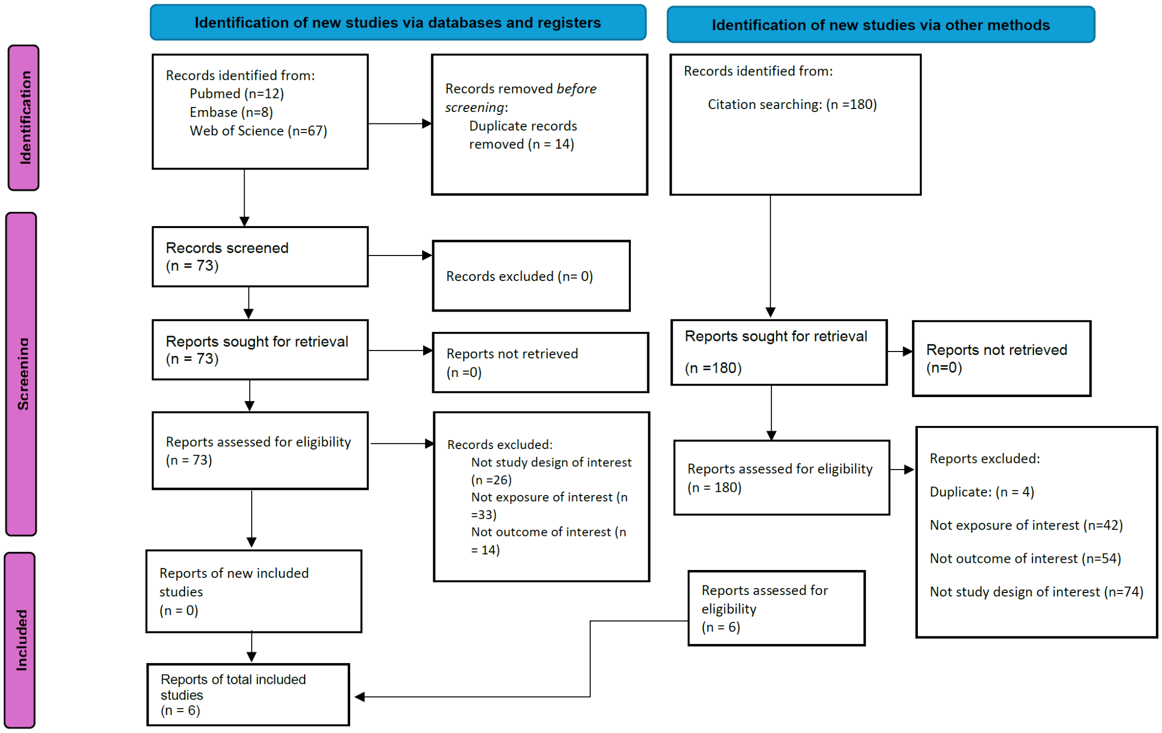

Data processing

Data processing for exposure data largely followed the methodology of previous GBD rounds, apart from a change for whole grains. For whole grains, we revised the approach to estimating whole grains availability from FAO data. Previously, this was based on a relationship between SUA and FBS grains data, where a value for total grains was estimated from SUA data and a value of refined grains estimated from FBS data. The difference of these values yielded whole grains. In GBD 2023, we estimate whole grains from SUA data based on "high-fibre" grains.[4] High-fibre grains include barley, buckwheat, bulgur, maize, malt, millet, oats, quinoa, rye, sorghum, and other cereals. Additionally, 18.5% of wheat was included in this estimation, an estimated proportion based on literature.[5] Rice, another major grain, was not included in this estimation of whole grain, as we were unable to find a reasonable estimation of the proportion of rice consumed as whole grain. Furthermore, in countries where rice is the major staple, rice is primarily consumed as refined (white) rice. While the definition of our whole grain risk factor has not changed nor have the gold-standard data used in our model changed, the revision to our estimation process through FAO data does have implications. Namely, maize was under-represented in the previous approach, which means that countries with maize as a major staple are likely to see increases in whole grain exposure estimates. Countries with rice as a major staple are likely to see decreases in whole grain exposure estimates.

The general data processing process for all dietary risk factors is described in more detail in the following paragraphs.

First, we prepared the FAO data. We mapped specific food items in the FAO data to our risk factors (eg, a type of vegetable is included in the risk factor category “vegetables”). For omega-3, omega-6, calcium, and fibre, availability was estimated by mapping the FAO food item to a corresponding entry in the USDA Food and Nutrient Database. In GBD 2021, missing country-year data for each risk was estimated by using a spatiotemporal Gaussian process regression with log lag-distributed income per capita as the covariate. In GBD 2023, this modelling step was removed after analysis indicated it was unnecessary. FAO changed their methodology with SUA and FBS data, which is reflected in data from 2010 and onward (FAO 2024), and was evident for GBD with the addition of newer FAO data. To account for the discontinuity across time at the point of changed methodology, a ratio shift in the data prior to 2010 was applied to move those data in alignment with the trend of the 2010 and onward data. For many locations this had little effect on the values, while for others we saw a substantial shift up or down.

Second, we applied information about consumption by age group to data sources that are not age specific. For each dietary risk factor, we estimated the global age pattern of consumption based on 24-hour dietary recall surveys and applied that age pattern to the all-age data (FAO, sales, and HBS). The age-patterns previously established for each dietary risk factor did not change for GBD 2023, though we did update the age-splitting methodology. We used a new age-splitting package called “PyDisagg” to conduct the age splitting. PyDisagg[6] disaggregates proportion, rate, and count observations based on a proportionality assumption. We seek to disaggregate a user-provided datum D into subcomponents D_i, guaranteeing that:

$$sum(D_i) = D$$

$$D_i = g_i p_i$$

where p_i is population weight and g_i a transform of the global rate (f_i).

$$g_i = T^{-1}\big(\beta + T(f_i)\big)$$

The basic use case has T = ln, guaranteeing that the recovered rates are proportional to the global. In addition, PyDisagg allows splitting of bounded quantities using the appropriate logit transform for T. PyDisagg version 0.6.0 disaggregates observations of both continuous quantities (eg, age-splitting of age-aggregated observations) as well as discrete quantities (including sex and multiple categories). Uncertainty for split datapoints is obtained using asymptotic statistics. Specifically, we use uncertainty of the inputs (ie, uncertainty of the aggregate and uncertainty of the age pattern) together with a generalised delta method that effectively computes a linearised map from the inputs to the outputs and uses that to obtain posterior uncertainty intervals for the split datapoints.

Third, we used gold-standard data to adjust non-gold-standard data for bias. Our gold-standard data source for all dietary risks (except sodium) is 24-hour dietary recall surveys where food and nutrient intake are reported or convertible to grams per person per day or, in the case of trans fatty acids and omega-6 polyunsaturated fatty acids, as percentage of total energy per person per day; the gold-standard data source for sodium is 24-hour urinary sodium excretion. The other data sources we used—household budget surveys, food frequency questionnaires, sales, and availability—were treated as

alternate definitions for dietary intake and crosswalked to the gold-standard definition. Non-sex-specific sources (FAO, sales, and HBS) were accounted for in the crosswalk, where we applied a sex split of these data, and conducted the crosswalk as sex-specific, using gold-standard intake data to generate sex-specific coefficients. In GBD 2016 and GBD 2017, we determined the bias adjustment factors from a mixed-effects linear regression. In GBD 2019 and GBD 2021, we used MR-BRT (a tool developed by researchers at IHME, standing for meta-regression—Bayesian, regularised, trimmed) to determine the adjustment factors for non-gold-standard datapoints, and a network meta-regression was implemented with MR-BRT for dietary risk factors with more than one non-gold-standard data input type. In GBD 2023, a MR-BRT package version 0.0.1 specifically designed for the crosswalk process by researchers at IHME replaced the previous models. In addition, fixes were made to how the models were being applied that has improved the sex-specific estimates, as well as the measurement of uncertainty. Notably, where sufficient data existed, we allowed super-region-specific data to weight more heavily for corresponding super-region crosswalks while still grounding estimates with a global model prior (theta=2).

The coefficients for these models can be found in Tables 3 through 17.

Table 3: MR-BRT crosswalk adjustment factors for fruits exposure

Level	Sex	Data input	Reference or alternative case definition	Gamma	Beta coefficient, log (95% UI)*	Adjustment factor**
Global	---	DR	Reference	0.6687	---	---
Global	Female	FAO	Alternative		0.2 (0.04 to 0.37)	0.82 (–0.99 to 2.62)
Global	Female	FFQ	Alternative		0 (–0.2 to 0.21)	1 (–0.77 to 2.76)
Global	Female	HHBS	Alternative		0.12 (–0.16 to 0.4)	0.88 (–0.81 to 2.58)
Global	Female	Sales	Alternative		0.61 (0.41 to 0.81)	0.54 (–1.22 to 2.31)
Global	Male	FAO	Alternative		0.43 (0.26 to 0.59)	0.65 (–1.15 to 2.45)
Global	Male	FFQ	Alternative		0.11 (–0.1 to 0.32)	0.9 (–0.87 to 2.66)
Global	Male	HHBS	Alternative		0.35 (0.07 to 0.63)	0.71 (–0.99 to 2.4)
Global	Male	Sales	Alternative		0.82 (0.62 to 1.02)	0.44 (–1.33 to 2.21)
Central Europe, eastern Europe, and central Asia	---	DR	Reference	0.4388	---	---
Central Europe, eastern Europe, and central Asia	Female	FAO	Alternative		0.28 (0.01 to 0.55)	0.75 (–0.95 to 2.46)
Central Europe, eastern Europe, and central Asia	Female	FFQ	Alternative		0.14 (–0.23 to 0.51)	0.87 (–0.76 to 2.5)
Central Europe, eastern Europe, and central Asia	Female	HHBS	Alternative		0.59 (0.24 to 0.94)	0.55 (–1.08 to 2.19)

Central Europe, eastern Europe, and central Asia	Female	Sales	Alternative		0.59 (0.27 to 0.92)	0.55 (–1.11 to 2.21)
Central Europe, eastern Europe, and central Asia	Male	FAO	Alternative		0.52 (0.19 to 0.85)	0.59 (–1.06 to 2.25)
Central Europe, eastern Europe, and central Asia	Male	FFQ	Alternative		–0.04 (–0.46 to 0.38)	1.04 (–0.54 to 2.62)
Central Europe, eastern Europe, and central Asia	Male	HHBS	Alternative		0.83 (0.43 to 1.23)	0.44 (–1.16 to 2.03)
Central Europe, eastern Europe, and central Asia	Male	Sales	Alternative		0.83 (0.46 to 1.21)	0.44 (–1.18 to 2.05)
High-income	---	DR	Reference	0.214	---	---
High-income	Female	FAO	Alternative		0.01 (–0.09 to 0.11)	0.99 (–0.88 to 2.85)
High-income	Female	FFQ	Alternative		0.37 (0.25 to 0.5)	0.69 (–1.15 to 2.53)
High-income	Female	HHBS	Alternative		0.14 (–0.12 to 0.39)	0.87 (–0.85 to 2.59)
High-income	Female	Sales	Alternative		0.64 (0.52 to 0.77)	0.53 (–1.32 to 2.37)
High-income	Male	FAO	Alternative		0.24 (0.14 to 0.34)	0.78 (–1.08 to 2.65)
High-income	Male	FFQ	Alternative		0.48 (0.35 to 0.6)	0.62 (–1.22 to 2.46)
High-income	Male	HHBS	Alternative		0.36 (0.11 to 0.62)	0.7 (–1.02 to 2.41)
High-income	Male	Sales	Alternative		0.86 (0.74 to 0.98)	0.42 (–1.42 to 2.26)
Latin America and Caribbean	---	DR	Reference	0.1535	---	---
Latin America and Caribbean	Female	FAO	Alternative		0.46 (0.21 to 0.7)	0.63 (–1.1 to 2.36)
Latin America and Caribbean	Female	FFQ	Alternative		–0.21 (–0.52 to 0.09)	1.23 (–0.44 to 2.91)
Latin America and Caribbean	Female	HHBS	Alternative		–0.44 (–0.88 to 0.01)	1.55 (–0.01 to 3.11)
Latin America and Caribbean	Female	Sales	Alternative		0.74 (0.44 to 1.04)	0.48 (–1.2 to 2.16)
Latin America and Caribbean	Male	FAO	Alternative		0.42 (0.15 to 0.69)	0.66 (–1.05 to 2.37)
Latin America and Caribbean	Male	FFQ	Alternative		–0.26 (–0.58 to 0.06)	1.29 (–0.37 to 2.96)
Latin America and Caribbean	Male	HHBS	Alternative		–0.45 (–0.9 to 0.01)	1.56 (0.01 to 3.11)
Latin America and Caribbean	Male	Sales	Alternative		0.7 (0.38 to 1.01)	0.5 (–1.17 to 2.17)
Southeast Asia, east Asia, and Oceania	---	DR	Reference	0.374	---	---
Southeast Asia, east Asia, and Oceania	Female	FAO	Alternative		0.4 (0.12 to 0.68)	0.67 (–1.03 to 2.37)
Southeast Asia, east Asia, and Oceania	Female	FFQ	Alternative		–0.06 (–0.41 to 0.28)	1.07 (–0.58 to 2.71)
Southeast Asia, east Asia, and Oceania	Female	Sales	Alternative		0.87 (0.57 to 1.17)	0.42 (–1.26 to 2.1)

Southeast Asia, east Asia, and Oceania	Male	FAO	Alternative		0.52 (0.24 to 0.81)	0.59 (–1.1 to 2.29)
Southeast Asia, east Asia, and Oceania	Male	FFQ	Alternative		0.84 (–0.15 to 1.83)	0.43 (–0.75 to 1.61)
Southeast Asia, east Asia, and Oceania	Male	Sales	Alternative		0.99 (0.69 to 1.3)	0.37 (–1.31 to 2.05)
Sub-Saharan Africa	---	DR	Reference	2.1937	---	---
Sub-Saharan Africa	Female	FAO	Alternative		0.48 (0.18 to 0.79)	0.62 (–1.06 to 2.29)
Sub-Saharan Africa	Female	FFQ	Alternative		–0.24 (–0.61 to 0.14)	1.27 (–0.35 to 2.88)
Sub-Saharan Africa	Female	HHBS	Alternative		–0.98 (–2.69 to 0.72)	2.67 (1.85 to 3.5)
Sub-Saharan Africa	Female	Sales	Alternative		0.72 (0.32 to 1.11)	0.49 (–1.12 to 2.09)
Sub-Saharan Africa	Male	FAO	Alternative		0.46 (0.11 to 0.8)	0.63 (–1.01 to 2.28)
Sub-Saharan Africa	Male	FFQ	Alternative		–0.48 (–0.93 to –0.04)	1.62 (0.06 to 3.18)
Sub-Saharan Africa	Male	HHBS	Alternative		–1.01 (–2.72 to 0.7)	2.75 (1.93 to 3.56)
Sub-Saharan Africa	Male	Sales	Alternative		0.69 (0.26 to 1.12)	0.5 (–1.07 to 2.08)

Table 4: MR-BRT crosswalk adjustment factors for vegetables exposure

Level	Sex	Data input	Reference or alternative case definition	Gamma	Beta coefficient, log (95% UI)*	Adjustment factor**
Global	Female	---	Reference	0.4659	---	---
Global	Female	FAO	Alternative		0.38 (0.24 to 0.53)	0.68 (–1.14 to 2.5)
Global	Female	FFQ	Alternative		0.33 (0.16 to 0.49)	0.72 (–1.08 to 2.52)
Global	Female	HHBS	Alternative		0.1 (–0.23 to 0.43)	0.9 (–0.75 to 2.56)
Global	Female	Sales	Alternative		0.8 (0.63 to 0.97)	0.45 (–1.35 to 2.25)
Global	Male	FAO	Alternative		0.36 (0.22 to 0.5)	0.7 (–1.13 to 2.52)
Global	Male	FFQ	Alternative		0.2 (0.03 to 0.36)	0.82 (–0.98 to 2.62)
Global	Male	HHBS	Alternative		0.07 (–0.26 to 0.4)	0.93 (–0.72 to 2.59)
Global	Male	Sales	Alternative		0.76 (0.59 to 0.93)	0.47 (–1.33 to 2.27)
High-income	---	DR	Reference	0.3083	---	---
High-income	Female	FAO	Alternative		0.39 (0.28 to 0.5)	0.68 (–1.17 to 2.53)
High-income	Female	FFQ	Alternative		0.48 (0.35 to 0.62)	0.62 (–1.21 to 2.45)
High-income	Female	HHBS	Alternative		0.23 (–0.09 to 0.55)	0.8 (–0.87 to 2.46)
High-income	Female	Sales	Alternative		0.76 (0.63 to 0.89)	0.47 (–1.36 to 2.3)
High-income	Male	FAO	Alternative		0.37 (0.26 to 0.48)	0.69 (–1.16 to 2.54)
High-income	Male	FFQ	Alternative		0.35 (0.21 to 0.48)	0.71 (–1.12 to 2.54)

High-income	Male	HHBS	Alternative		0.2 (–0.12 to 0.52)	0.82 (–0.85 to 2.48)
High-income	Male	Sales	Alternative		0.73 (0.59 to 0.86)	0.48 (–1.35 to 2.31)
Latin America and Caribbean	---	DR	Reference	0.42448	---	---
Latin America and Caribbean	Female	FAO	Alternative		0.31 (0.05 to 0.56)	0.74 (–0.99 to 2.46)
Latin America and Caribbean	Female	FFQ	Alternative		0.19 (–0.11 to 0.5)	0.82 (–0.86 to 2.5)
Latin America and Caribbean	Female	HHBS	Alternative		–0.2 (–1.13 to 0.72)	1.23 (0.01 to 2.45)
Latin America and Caribbean	Female	Sales	Alternative		0.85 (0.54 to 1.15)	0.43 (–1.25 to 2.11)
Latin America and Caribbean	Male	FAO	Alternative		0.2 (–0.06 to 0.46)	0.82 (–0.89 to 2.53)
Latin America and Caribbean	Male	FFQ	Alternative		–0.01 (–0.33 to 0.31)	1.01 (–0.66 to 2.68)
Latin America and Caribbean	Male	HHBS	Alternative		–0.27 (–1.2 to 0.66)	1.31 (0.09 to 2.52)
Latin America and Caribbean	Male	Sales	Alternative		0.74 (0.43 to 1.05)	0.48 (–1.19 to 2.15)
Southeast Asia, east Asia, and Oceania	---	DR	Reference	0.2953	---	---
Southeast Asia, east Asia, and Oceania	Female	FAO	Alternative		0.28 (0.04 to 0.52)	0.76 (–0.98 to 2.49)
Southeast Asia, east Asia, and Oceania	Female	FFQ	Alternative		0.08 (–0.19 to 0.34)	0.93 (–0.79 to 2.64)
Southeast Asia, east Asia, and Oceania	Female	Sales	Alternative		0.91 (0.67 to 1.15)	0.4 (–1.33 to 2.13)

Southeast Asia, east Asia, and Oceania	Male	FAO	Alternative		0.22 (–0.03 to 0.46)	0.81 (–0.93 to 2.54)
Southeast Asia, east Asia, and Oceania	Male	FFQ	Alternative		0.04 (–0.23 to 0.31)	0.96 (–0.75 to 2.67)
Southeast Asia, east Asia, and Oceania	Male	Sales	Alternative		0.85 (0.6 to 1.09)	0.43 (–1.3 to 2.16)
Sub-Saharan Africa	---	DR	Reference	1.9464	---	---
Sub-Saharan Africa	Female	FAO	Alternative		0.38 (0.12 to 0.65)	0.68 (–1.03 to 2.4)
Sub-Saharan Africa	Female	FFQ	Alternative		0.21 (–0.1 to 0.52)	0.81 (–0.86 to 2.48)
Sub-Saharan Africa	Female	HHBS	Alternative		–0.05 (–2 to 1.91)	1.05 (0.32 to 1.77)
Sub-Saharan Africa	Female	Sales	Alternative		0.79 (0.47 to 1.12)	0.45 (–1.21 to 2.11)
Sub-Saharan Africa	Male	FAO	Alternative		0.38 (0.06 to 0.7)	0.68 (–0.98 to 2.35)
Sub-Saharan Africa	Male	FFQ	Alternative		0.19 (–0.19 to 0.57)	0.83 (–0.79 to 2.44)
Sub-Saharan Africa	Male	HHBS	Alternative		–0.04 (–2 to 1.92)	1.05 (0.32 to 1.77)
Sub-Saharan Africa	Male	Sales	Alternative		0.8 (0.42 to 1.17)	0.45 (–1.17 to 2.07)

Table 5: MR-BRT crosswalk adjustment factors for legumes exposure

**Level**	**Sex**	**Data input**	**Reference or alternative case definition**	**Gamma**	**Beta coefficient, log (95% UI)***	**Adjustment factor****
Global	---	DR	Reference	0.658	---	---
Global	Female	FAO	Alternative		0.71 (0.42 to 1.01)	0.49 (–1.2 to 2.18)
Global	Female	FFQ	Alternative		0.32 (–0.06 to 0.69)	0.73 (–0.89 to 2.35)
Global	Female	Sales	Alternative		–0.29 (–0.6 to 0.03)	1.33 (–0.34 to 3)
Global	Male	FAO	Alternative		0.58 (0.29 to 0.88)	0.56 (–1.13 to 2.24)
Global	Male	FFQ	Alternative		0.34 (–0.04 to 0.71)	0.72 (–0.9 to 2.33)

Global	Male	Sales	Alternative		–0.52 (–0.84 to –0.21)	1.69 (0.02 to 3.36)

Table 6: MR-BRT crosswalk adjustment factors for whole grains exposure

Level	Sex	Data input	Reference or alternative case definition	Gamma	Beta coefficient, log (95% UI)*	Adjustment factor**
Global	Female	---	Reference	0.5884	---	---
Global	Female	FAO	Alternative		1.03 (0.79 to 1.27)	0.36 (–1.37 to 2.09)
Global	Female	FFQ	Alternative		0.17 (–0.52 to 0.86)	0.85 (–0.53 to 2.22)
Global	Male	FAO	Alternative		0.91 (0.67 to 1.15)	0.4 (–1.33 to 2.13)
Global	Male	FFQ	Alternative		0.13 (–0.57 to 0.82)	0.88 (–0.5 to 2.26)

Table 7: MR-BRT crosswalk adjustment factors for nuts and seeds exposure

Level	Sex	Data input	Reference or alternative case definition	Gamma	Beta coefficient, log (95% UI)*	Adjustment factor**
Global	---	DR	Reference	0.5583	---	---
Global	Female	FAO	Alternative		0.92 (0.67 to 1.16)	0.4 (–1.33 to 2.13)
Global	Female	FFQ	Alternative		0 (–0.88 to 0.87)	1 (–0.25 to 2.26)
Global	Male	DR	Reference		---	---
Global	Male	FAO	Alternative		0.91 (0.66 to 1.16)	0.4 (–1.32 to 2.13)
Global	Male	FFQ	Alternative		–0.01 (–0.92 to 0.89)	1.01 (–0.22 to 2.25)
High-income	---	DR	Reference	0.5714	---	---
High-income	Female	FAO	Alternative		0.92 (0.7 to 1.15)	0.4 (–1.35 to 2.14)
High-income	Female	FFQ	Alternative		0 (–0.79 to 0.78)	1 (–0.31 to 2.32)
High-income	Male	FAO	Alternative		0.92 (0.69 to 1.14)	0.4 (–1.34 to 2.14)
High-income	Male	FFQ	Alternative		–0.01 (–0.83 to 0.81)	1.01 (–0.28 to 2.3)

Table 8: MR-BRT crosswalk adjustment factors for milk exposure

Level	Sex	Data input	Reference or alternative case definition	Gamma	Beta coefficient, log (95% UI)*	Adjustment factor**
Global	---	DR	Reference	1.0771	---	---

Global	Female	FAO	Alternative		–0.06 (–0.31 to 0.2)	1.06 (–0.66 to 2.78)
Global	Female	FFQ	Alternative		0.59 (0.24 to 0.94)	0.55 (–1.09 to 2.19)
Global	Female	HHBS	Alternative		–0.23 (–0.64 to 0.18)	1.25 (–0.34 to 2.84)
Global	Female	Sales	Alternative		0.08 (–0.21 to 0.38)	0.92 (–0.77 to 2.61)
Global	Male	FAO	Alternative		–0.12 (–0.37 to 0.14)	1.13 (–0.59 to 2.85)
Global	Male	FFQ	Alternative		0.68 (0.33 to 1.04)	0.5 (–1.13 to 2.14)
Global	Male	HHBS	Alternative		–0.28 (–0.69 to 0.13)	1.32 (–0.27 to 2.91)
Global	Male	Sales	Alternative		0.04 (–0.26 to 0.33)	0.96 (–0.72 to 2.65)
Central Europe, eastern Europe, and central Asia	---	DR	Reference	0.4568	---	---
Central Europe, eastern Europe, and central Asia	Female	FAO	Alternative		0.13 (–0.23 to 0.49)	0.88 (–0.75 to 2.51)
Central Europe, eastern Europe, and central Asia	Female	FFQ	Alternative		0.86 (0.38 to 1.34)	0.42 (–1.11 to 1.96)
Central Europe, eastern Europe, and central Asia	Female	HHBS	Alternative		0.11 (–0.32 to 0.54)	0.9 (–0.68 to 2.47)
Central Europe, eastern Europe, and central Asia	Female	Sales	Alternative		–0.43 (–0.84 to –0.02)	1.54 (–0.05 to 3.13)
Central Europe, eastern Europe, and central Asia	Male	FAO	Alternative		0.05 (–0.33 to 0.43)	0.95 (–0.66 to 2.57)
Central Europe, eastern Europe, and central Asia	Male	FFQ	Alternative		0.79 (0.29 to 1.28)	0.45 (–1.07 to 1.98)
Central Europe, eastern Europe, and central Asia	Male	HHBS	Alternative		0.03 (–0.41 to 0.48)	0.97 (–0.59 to 2.53)
Central Europe, eastern Europe, and central Asia	Male	Sales	Alternative		–0.51 (–0.94 to –0.08)	1.67 (0.09 to 3.24)
High-income	---	DR	Reference	0.74147	---	---
High-income	Female	FAO	Alternative		–0.06 (–0.27 to 0.15)	1.06 (–0.7 to 2.82)
High-income	Female	FFQ	Alternative		0.57 (0.28 to 0.85)	0.57 (–1.13 to 2.26)
High-income	Female	HHBS	Alternative		–1.3 (–1.95 to –0.64)	3.66 (2.25 to 5.06)
High-income	Female	Sales	Alternative		0.49 (0.23 to 0.74)	0.62 (–1.11 to 2.34)
High-income	Male	FAO	Alternative		–0.14 (–0.35 to 0.07)	1.15 (–0.61 to 2.91)
High-income	Male	FFQ	Alternative		0.66 (0.37 to 0.94)	0.52 (–1.17 to 2.21)
High-income	Male	HHBS	Alternative		–1.36 (–2.02 to –0.71)	3.92 (2.51 to 5.32)
High-income	Male	Sales	Alternative		0.43 (0.18 to 0.69)	0.65 (–1.07 to 2.37)
Latin America and Caribbean	---	DR	Reference	0.00839	---	---
Latin America and Caribbean	Female	FAO	Alternative		0.5 (0.37 to 0.63)	0.61 (–1.23 to 2.44)
Latin America and Caribbean	Female	HHBS	Alternative		–0.67 (–0.85 to –0.49)	1.96 (0.17 to 3.75)
Latin America and Caribbean	Female	Sales	Alternative		–0.09 (–0.22 to 0.05)	1.09 (–0.74 to 2.92)
Latin America and Caribbean	Male	FAO	Alternative		0.57 (0.43 to 0.71)	0.56 (–1.26 to 2.39)

Latin America and Caribbean	Male	HHBS	Alternative		–0.57 (–0.76 to –0.38)	1.77 (–0.01 to 3.55)
Latin America and Caribbean	Male	Sales	Alternative		–0.02 (–0.15 to 0.12)	1.02 (–0.81 to 2.84)

Table 9: MR-BRT crosswalk adjustment factors for calcium exposure

Level	Sex	Data input type	Reference or alternative case definition	Gamma	Beta coefficient, log (95% UI)*	Adjustment factor**
Global	---	DR	Reference	0.1126	---	---
Global	Female	FAO	Alternative		0.22 (0.16 to 0.29)	0.8 (–1.1 to 2.7)
Global	Female	FFQ	Alternative		0.32 (0.23 to 0.42)	0.72 (–1.14 to 2.59)
Global	Male	FAO	Alternative		0.08 (0.01 to 0.14)	0.92 (–0.97 to 2.82)
Global	Male	FFQ	Alternative		0.15 (0.06 to 0.24)	0.86 (–1.01 to 2.73)
Central Europe, eastern Europe, and central Asia	---	DR	Reference	0.0671	---	---
Central Europe, eastern Europe, and central Asia	Female	FAO	Alternative		0.19 (0.07 to 0.3)	0.83 (–1.02 to 2.68)
Central Europe, eastern Europe, and central Asia	Female	FFQ	Alternative		0.22 (0.05 to 0.4)	0.8 (–0.99 to 2.59)
Central Europe, eastern Europe, and central Asia	Male	FAO	Alternative		0.09 (–0.04 to 0.22)	0.91 (–0.92 to 2.75)
Central Europe, eastern Europe, and central Asia	Male	FFQ	Alternative		0.11 (–0.08 to 0.3)	0.9 (–0.88 to 2.68)
High-income	---	DR	Reference	0.0727	---	---
High-income	Female	FAO	Alternative		0.23 (0.18 to 0.28)	0.8 (–1.11 to 2.7)
High-income	Female	FFQ	Alternative		0.26 (0.18 to 0.33)	0.77 (–1.11 to 2.66)
High-income	Male	FAO	Alternative		0.09 (0.03 to 0.14)	0.92 (–0.99 to 2.82)
High-income	Male	FFQ	Alternative		0.08 (0 to 0.16)	0.92 (–0.96 to 2.81)
Latin America and Caribbean	---	DR	Reference	0.3445	---	---
Latin America and Caribbean	Female	FAO	Alternative		0.15 (0.02 to 0.27)	0.86 (–0.97 to 2.7)
Latin America and Caribbean	Female	FFQ	Alternative		0.25 (0.07 to 0.43)	0.78 (–1.01 to 2.57)
Latin America and Caribbean	Male	FAO	Alternative		0.09 (-0.04 to 0.23)	0.91 (–0.92 to 2.74)
Latin America and Caribbean	Male	FFQ	Alternative		0.35 (0.16 to 0.53)	0.71 (–1.08 to 2.49)

North Africa and Middle East	---	DR	Reference	0.051	---	---
North Africa and Middle East	Female	FAO	Alternative		0.16 (0.06 to 0.27)	0.85 (–1.01 to 2.71)
North Africa and Middle East	Female	FFQ	Alternative		0.26 (0.11 to 0.41)	0.77 (–1.04 to 2.59)
North Africa and Middle East	Male	FAO	Alternative		0 (–0.12 to 0.12)	1 (–0.85 to 2.85)
North Africa and Middle East	Male	FFQ	Alternative		0.26 (0.09 to 0.43)	0.77 (–1.03 to 2.57)
Southeast Asia, east Asia, and Oceania	---	DR	Reference	0.2306	---	---
Southeast Asia, east Asia, and Oceania	Female	FAO	Alternative		0.11 (–0.01 to 0.23)	0.9 (–0.94 to 2.74)
Southeast Asia, east Asia, and Oceania	Female	FFQ	Alternative		0.27 (0.09 to 0.46)	0.76 (–1.03 to 2.55)
Southeast Asia, east Asia, and Oceania	Male	FAO	Alternative		–0.09 (–0.23 to 0.04)	1.1 (–0.73 to 2.93)
Southeast Asia, east Asia, and Oceania	Male	FFQ	Alternative		0.1 (–0.1 to 0.29)	0.91 (–0.86 to 2.68)
Sub-Saharan Africa	---	DR	Reference	0.58146	---	---
Sub-Saharan Africa	Female	FAO	Alternative		0.15 (0.02 to 0.28)	0.86 (–0.98 to 2.69)
Sub-Saharan Africa	Female	FFQ	Alternative		0.24 (0.06 to 0.43)	0.78 (–1 to 2.56)
Sub-Saharan Africa	Male	FAO	Alternative		0.17 (–0.13 to 0.47)	0.84 (–0.84 to 2.52)
Sub-Saharan Africa	Male	FFQ	Alternative		0.55 (0.21 to 0.89)	0.58 (–1.07 to 2.22)

Table 10: MR-BRT crosswalk adjustment factors for red meat exposure

Level	Sex	Data input	Reference or alternative case definition	Gamma	Beta coefficient, log (95% UI)*	Adjustment factor**
Global	---	DR	Reference	0.3043	---	---
Global	Female	FAO	Alternative		1.02 (0.85 to 1.2)	0.36 (–1.43 to 2.15)
Global	Female	FFQ	Alternative		0.35 (–0.05 to 0.75)	0.71 (–0.89 to 2.3)
Global	Female	HHBS	Alternative		1.33 (1.07 to 1.59)	0.26 (–1.45 to 1.98)
Global	Female	Sales	Alternative		1.32 (1.12 to 1.52)	0.27 (–1.5 to 2.04)
Global	Male	FAO	Alternative		0.58 (0.4 to 0.76)	0.56 (–1.23 to 2.35)
Global	Male	FFQ	Alternative		0.14 (–0.26 to 0.54)	0.87 (–0.73 to 2.47)
Global	Male	HHBS	Alternative		0.9 (0.64 to 1.16)	0.41 (–1.31 to 2.12)
Global	Male	Sales	Alternative		0.88 (0.68 to 1.08)	0.42 (–1.35 to 2.18)
High-income	---	DR	Reference	0.1302	---	---
High-income	Female	FAO	Alternative		1 (0.88 to 1.12)	0.37 (–1.47 to 2.21)
High-income	Female	FFQ	Alternative		0.32 (–0.09 to 0.74)	0.72 (–0.86 to 2.31)
High-income	Female	HHBS	Alternative		0.28 (–0.15 to 0.71)	0.76 (–0.82 to 2.33)
High-income	Female	Sales	Alternative		1.4 (1.26 to 1.54)	0.25 (–1.58 to 2.08)
High-income	Male	FAO	Alternative		0.55 (0.43 to 0.67)	0.58 (–1.27 to 2.42)

High-income	Male	FFQ	Alternative		0.12 (–0.3 to 0.53)	0.89 (–0.7 to 2.48)
High-income	Male	HHBS	Alternative		–0.17 (–0.59 to 0.26)	1.18 (–0.4 to 2.76)
High-income	Male	Sales	Alternative		0.96 (0.82 to 1.09)	0.38 (–1.44 to 2.21)

Table 11: MR-BRT crosswalk adjustment factors for processed meat exposure

Level	Sex	Data input	Reference or alternative case definition	Gamma	Beta coefficient, log (95% UI)*	Adjustment factor**
Global	---	DR	Reference	0.2757	---	---
Global	Female	FFQ	Alternative		–0.27 (–0.88 to 0.34)	1.31 (–0.12 to 2.75)
Global	Female	HHBS	Alternative		0.13 (–0.69 to 0.96)	0.87 (–0.41 to 2.16)
Global	Female	Sales	Alternative		1.52 (1.25 to 1.79)	0.22 (–1.49 to 1.93)
Global	Male	FFQ	Alternative		–0.13 (–0.74 to 0.48)	1.14 (–0.3 to 2.57)
Global	Male	HHBS	Alternative		–0.02 (–0.85 to 0.8)	1.02 (–0.26 to 2.31)
Global	Male	Sales	Alternative		1.36 (1.1 to 1.63)	0.26 (–1.46 to 1.97)

Table 12: MR-BRT crosswalk adjustment factors for SSB exposure

Level	Sex	Data input	Reference or alternative case definition	Gamma	Beta coefficient, log (95% UI)*	Adjustment factor**
Global	---	DR	Reference	0.3266	---	---
Global	Female	FFQ	Alternative		0.39 (–0.49 to 1.27)	0.68 (–0.58 to 1.93)
Global	Female	HHBS	Alternative		0.19 (–0.44 to 0.82)	0.83 (–0.59 to 2.25)
Global	Female	Sales	Alternative		0.44 (0.17 to 0.71)	0.64 (–1.07 to 2.35)
Global	Male	FFQ	Alternative		0.06 (–0.82 to 0.94)	0.94 (–0.31 to 2.19)
Global	Male	HHBS	Alternative		–0.14 (–0.77 to 0.49)	1.15 (–0.27 to 2.57)
Global	Male	Sales	Alternative		0.11 (–0.16 to 0.38)	0.9 (–0.81 to 2.61)

Table 13: MR-BRT crosswalk adjustment factors for fibre exposure

Level	Sex	Data input	Reference or alternative case definition	Gamma	Beta coefficient, log (95% UI) *	Adjustment factor**
Global	---	DR	Reference	0.189	---	---
Global	Female	FAO	Alternative		0.47 (0.38 to 0.56)	0.62 (–1.25 to 2.49)

Global	Male	FAO	Alternative		0.41 (0.32 to 0.5)	0.66 (–1.21 to 2.54)
Central Europe, eastern Europe, and central Asia	---	DR	Reference	0.0872	---	---
Central Europe, eastern Europe, and central Asia	Female	FAO	Alternative		0.4 (0.24 to 0.56)	0.67 (–1.14 to 2.48)
Central Europe, eastern Europe, and central Asia	Male	FAO	Alternative		0.32 (0.15 to 0.48)	0.73 (–1.07 to 2.53)
High-income	---	DR	Reference	0.1497	---	---
High-income	Female	FAO	Alternative		0.44 (0.36 to 0.52)	0.64 (–1.24 to 2.53)
High-income	Male	FAO	Alternative		0.38 (0.3 to 0.46)	0.69 (–1.2 to 2.57)
Latin America and Caribbean	---	DR	Reference	0.4733	---	---
Latin America and Caribbean	Female	FAO	Alternative		0.44 (0.26 to 0.62)	0.64 (–1.15 to 2.44)
Latin America and Caribbean	Male	FAO	Alternative		0.34 (0.15 to 0.53)	0.71 (–1.07 to 2.49)
North Africa and Middle East	---	DR	Reference	0.2967	---	---
North Africa and Middle East	Female	FAO	Alternative		0.49 (0.32 to 0.65)	0.62 (–1.18 to 2.41)
North Africa and Middle East	Male	FAO	Alternative		0.34 (0.17 to 0.52)	0.71 (–1.08 to 2.5)
South Asia	---	DR	Reference	0.1307	---	---
South Asia	Female	FAO	Alternative		0.47 (0.38 to 0.56)	0.62 (–1.25 to 2.49)
South Asia	Male	FAO	Alternative		0.41 (0.32 to 0.5)	0.66 (–1.21 to 2.54)
Southeast Asia, east Asia, and Oceania	---	DR	Reference	0.7261	---	---
Southeast Asia, east Asia, and Oceania	Female	FAO	Alternative		0.46 (0.28 to 0.64)	0.63 (–1.16 to 2.42)
Southeast Asia, east Asia, and Oceania	Male	FAO	Alternative		0.47 (0.25 to 0.68)	0.63 (–1.13 to 2.39)

Table 14: MR-BRT crosswalk adjustment factors for seafoods omega-3 fatty acid exposure

Level	Sex	Data input	Reference or alternative case definition	Gamma	Beta coefficient, log (95% UI)*	Adjustment factor**
Global	---	DR	Reference	0.8953	---	---
Global	Female	FAO	Alternative		–0.69 (–1.37 to –0.02)	2 (0.61 to 3.39)
Global	Male	DR	Reference		---	---
Global	Male	FAO	Alternative		–0.9 (–1.57 to –0.23)	2.46 (1.07 to 3.85)

Table 15: MR-BRT crosswalk adjustment factors for omega-6 polyunsaturated fatty acids exposure

Level	Sex	Data input	Reference or alternative case definition	Gamma	Beta coefficient, log (95% UI)*	Adjustment factor**
Global	---	DR	Reference	0.1378	---	---
Global	Female	FAO	Alternative		–0.57 (–0.74 to –0.4)	1.78 (–0.02 to 3.57)
Global	Female	FFQ	Alternative		0.42 (0.03 to 0.81)	0.66 (–0.95 to 2.26)
Global	Male	FAO	Alternative		–0.52 (–0.69 to –0.35)	1.68 (–0.12 to 3.47)
Global	Male	FFQ	Alternative		0.48 (-0.09 to 1.05)	0.62 (–0.84 to 2.08)
High-income	---	DR	Reference	0.1197	---	---
High-income	Female	FAO	Alternative		–0.54 (–0.68 to –0.39)	1.71 (–0.11 to 3.53)
High-income	Female	FFQ	Alternative		0.31 (–0.06 to 0.68)	0.73 (–0.89 to 2.36)
High-income	Male	FAO	Alternative		–0.48 (–0.62 to –0.33)	1.61 (–0.21 to 3.43)
High-income	Male	FFQ	Alternative		0.34 (–0.27 to 0.95)	0.71 (–0.72 to 2.15)

Table16: MR-BRT crosswalk adjustment factors for sodium exposure

Level	Sex	Data input	Reference or alternative case definition	Gamma	Beta coefficient, log (95% UI)*	Adjustment factor**
Global	---	Urinary sodium excretion	Reference	0.0493	---	---
Global	Female	DR	Alternative		0 (–0.12 to 0.13)	1 (–0.84 to 2.83)
Global	Female	FFQ	Alternative		0.1 (–0.08 to 0.27)	0.91 (–0.89 to 2.7)
Global	Male	DR	Alternative		–0.09 (–0.21 to 0.04)	1.09 (–0.75 to 2.93)
Global	Male	FFQ	Alternative		0.02 (–0.16 to 0.2)	0.98 (–0.81 to 2.77)
High-income	---	Urinary sodium excretion	Reference	0.0498	---	---
High-income	Female	DR	Alternative		0 (–0.12 to 0.13)	1 (–0.84 to 2.83)
High-income	Female	FFQ	Alternative		0.11 (–0.05 to 0.28)	0.89 (–0.91 to 2.7)
High-income	Male	DR	Alternative		–0.09 (–0.21 to 0.04)	1.09 (–0.75 to 2.93)
High-income	Male	FFQ	Alternative		0.04 (–0.13 to 0.21)	0.96 (–0.84 to 2.76)
Southeast Asia, east Asia, and Oceania	---	Urinary sodium excretion	Reference	0	---	---
Southeast Asia, east Asia, and Oceania	Female	DR	Alternative		–0.15 (–0.21 to –0.09)	1.16 (–0.74 to 3.06)
Southeast Asia, east Asia, and Oceania	Male	DR	Alternative		–0.18 (–0.29 to –0.07)	1.2 (–0.65 to 3.06)

Table 17: MR-BRT crosswalk adjustment factors for trans-fat exposure

Level	Sex	Data input	Reference or alternative case definition	Gamma	Beta coefficient, log (95% UI)*	Adjustment factor**
Global	---	DR	Reference	0	---	---
Global	Female	FFQ	Alternative		0.72 (–1.49 to 2.93)	0.49 (–0.15 to 1.12)
Global	Female	Sales	Alternative		–0.37 (–2.52 to 1.78)	1.45 (0.79 to 2.1)
Global	Male	FFQ	Alternative		0.64 (–3.17 to 4.46)	0.53 (0.25 to 0.81)
Global	Male	Sales	Alternative		–0.45 (–4.17 to 3.26)	1.57 (1.28 to 1.87)

** MR-BRT crosswalk adjustments can be interpreted as the factor the alternative case definition is adjusted by to reflect what it would have been had it been measured using the reference case definition. If the log/logit beta coefficient is negative, then the alternative is adjusted up to the reference. If the log/logit beta coefficient is positive, then the alternative is adjusted down to the reference.*

*** The adjustment factor column is the exponentiated beta coefficient. For log beta coefficients, this is the relative rate between the two case definitions. For logit beta coefficients, this is the relative odds between the two case definitions.*

Modelling strategy

Exposure

We made no changes to the exposure modelling strategy in GBD 2023, though minor changes in covariates to the models were made. These changes are reflected in Table 2.

We used a spatiotemporal Gaussian process regression (ST-GPR) framework to estimate the mean intake of each dietary risk factor by age, sex, location, and year. The covariates used in the model can be seen in Table 2.

We determined the standard deviation of each population's consumption through a linear regression that captured the relationship between the standard deviation and mean intake. We estimated this model using a subset of our gold-standard data (ie, nationally representative surveys capturing 24-hour urinary sodium excretion for the sodium risk factor and 24-hour dietary recall data for other dietary risk factors):

$$ln\ (Standard\ deviation) = \beta_0 + \beta_1 \times ln\ (Mean_i)$$

Then we applied the coefficients of this regression to the outputs of our ST-GPR model to calculate the standard deviation of intake by age, sex, year, and location. The standard deviations were adjusted for within-person variation. As no new gold-standard data were added to our estimates for this cycle, neither the regression coefficients nor the adjustment factors changed from GBD 2021 to GBD 2023.

We characterised the shape of the distribution of exposure for each dietary risk factor with an approach that uses an ensemble of distribution types. First, we selected microdata suitable for estimating the shape of the distribution of dietary risk factor exposure within a population. The selected microdata we

used to create the fit include a subset of 24-hour urinary sodium excretion for sodium and a subset of 24-hour dietary recall data for other dietary risk factors. Second, we separately fit 12 types of distributions (including, for example, normal, log normal, gamma, etc.) to this microdata. Then, the respective goodness of fit of each distribution type was assessed to determine the weight for each distribution type. Finally, we created the global weighted ensemble distribution shape, which is applied during PAF calculation.

Theoretical minimum risk exposure level

We used several different approaches to set the dietary theoretical minimum risk exposure levels (TMRELs).

Sodium: For sodium, we use a TMREL range of 1–5 grams per day of urinary sodium excretion, based on a literature review, collaborator discussion, and a final decision taken by the GBD Scientific Council. This TMREL has remained the same since GBD 2013.[7]

Harmful risks: For strictly harmful dietary risks, the TMREL was set to zero, following the same approach as GBD 2019. Based on updated relative risk curves, trans-fatty acid was updated from a j-shaped risk in GBD 2021 to a harmful risk in GBD 2023.[3] We also updated the case definition of trans-fatty acid exposure to focus on industrially produced trans-fatty acids, primarily from partially hydrogenated oils.

Protective risks: For strictly protective dietary risks except vegetables, we used data from the cohort studies used in the relative risk analysis to create a TMREL. The aim of the approach was to select an exposure level that reflects high real-world consumption and for which we have solid evidence about the associated risks. First, for each risk–outcome pair, we calculated the 85th percentile of each the lower bound and the midpoints of all alternative (non-lowest) exposure ranges reported by the underlying study. Second, for each the lower bound and the midpoint statistic, we took a weighted average across outcomes, weighting by the global number of deaths due to each outcome as reported by GBD 2019. Finally, we constructed a uniform distribution between these two values to serve as the TMREL. For vegetables, a similar but slightly different approach was followed, as described in Stanaway et al., 2022.[8] This calculation method differs only slightly from GBD 2019.

Mixed protective and harmful risks: For risks that have both protective and harmful associations with outcomes (calcium and milk), the approach was similar to protective risk TMREL calculation. However, in the first step, the 15th percentile of the midpoints of the reference exposure ranges (ie, the lowest exposure range reported by the study) and the 15th percentile of the upper bound of the reference exposure ranges were used for harmful risk–outcome pairs. In the second step, sex-specific death weights were used. The final step of constructing the uniform distribution remained the same. In GBD 2019, calcium and milk were both protective risks, so the TMREL calculation followed the protective risk methodology. Furthermore, these TMRELs were not sex specific in GBD 2019. In GBD 2021, new, harmful associations with prostate cancer were added for both calcium and milk, so these risks became "mixed" and the TMREL became sex-specific due to the sex-specificity of prostate cancer. This updated approach that was applied in GBD 2021 has remained for GBD 2023.

J-shaped risks: For J-shaped risks, we used an approach that minimises the mean relative risk curve. The only J-shaped dietary risk in GBD 2023 is red meat. For this risk factor we first estimated 1000 draws of the relative risk curve for each risk–outcome pair. Second, relative risk draws from each risk–outcome pair were weighted by global number of deaths from the outcome, as estimated by GBD 2019, and

combined, resulting in 1000 draws of an across-outcome, death-weighted relative risk curve for red meat. Finally, each draw of the death-weighted relative risk curve was minimised, yielding 1000 draws of the TMREL. The non-normal distribution can be seen in Figure 7.

Because the distribution of the TMREL is not normal for the J-shaped risks, 1000 draws of the TMREL were used in downstream calculations. The 95% uncertainty interval of these TMRELs are reported in Table 18. In GBD 2019, all dietary risk factors were modelled with a monotonic constraint, so red meat and trans-fat both followed the harmful risk of TMREL calculation in that round.

An overarching limitation in the calculation of dietary TMRELs was that only direct risk–outcome relationships are considered in calculation; mediation is not accounted for. Furthermore, while GBD accounts for key risk–outcome relationships, risk–outcome relationships that were not accounted for in GBD were not accounted for in TMREL calculation. A summary of GBD 2021 and GBD 2023 TMRELs for dietary risks can be found in Table 18. Notably, the only TMREL that has changed from GBD 2021 is trans fatty acids, moving from a J-shaped risk with a TMREL range, to a harmful risk with a TMREL of 0% of total daily energy.

Table 18. Theoretical minimum risk exposure level for dietary risk factors, GBD 2021 and GBD 2023

**Risk type**	**Dietary risk factor**	**GBD 2021**	**GBD 2023**
Protective	**Fruits**	340–350 g/day	340–350 g/day
	**Vegetables**	306–372 g/day	306–372 g/day
	**Whole grains**	160–210 g/day	160–210 g/day
	**Nuts and seeds**	19–24 g/day	19–24 g/day
	**Dietary fibre**	22–25 g/day	22–25 g/day
	**Legumes**	100–110 g/day	100–110 g/day
	**Omega-6 polyunsaturated fatty acids**	9–10% of total daily energy	9–10% of total daily energy
	**Seafood omega-3 fatty acids**	470–660 mg/day	470–660 mg/day
Harmful	**Processed meats**	0 g/day	0 g/day
	**Dietary sodium**	1–5 g/day	1–5 g/day
	**Sugar-sweetened beverages**	0 g/day	0 g/day
	**Trans fatty acids**	0–1.1% of total daily energy	0% of total daily energy
J-shaped	**Red meats**	0–200 g/day	0–200 g/day
Mixed protective and harmful	**Dietary calcium**	0.72–0.86 g/day (males)<br>1.1–1.2 g/day (females)	0.72–0.86 g/day (males)<br>1.1–1.2 g/day (females)
	**Milk**	280–340 g/day (males)<br>500–610 g/day (females)	280–340 g/day (males)<br>500–610 g/day (females)

Figure 7. The TMREL draws for red meat, as based on the cause-weighted mortality curve. Note that about 50 draws of the relative risk curve are monotonically decreasing; therefore, relative risk is minimised at the maximum exposure value (in this case, 200 g/day).

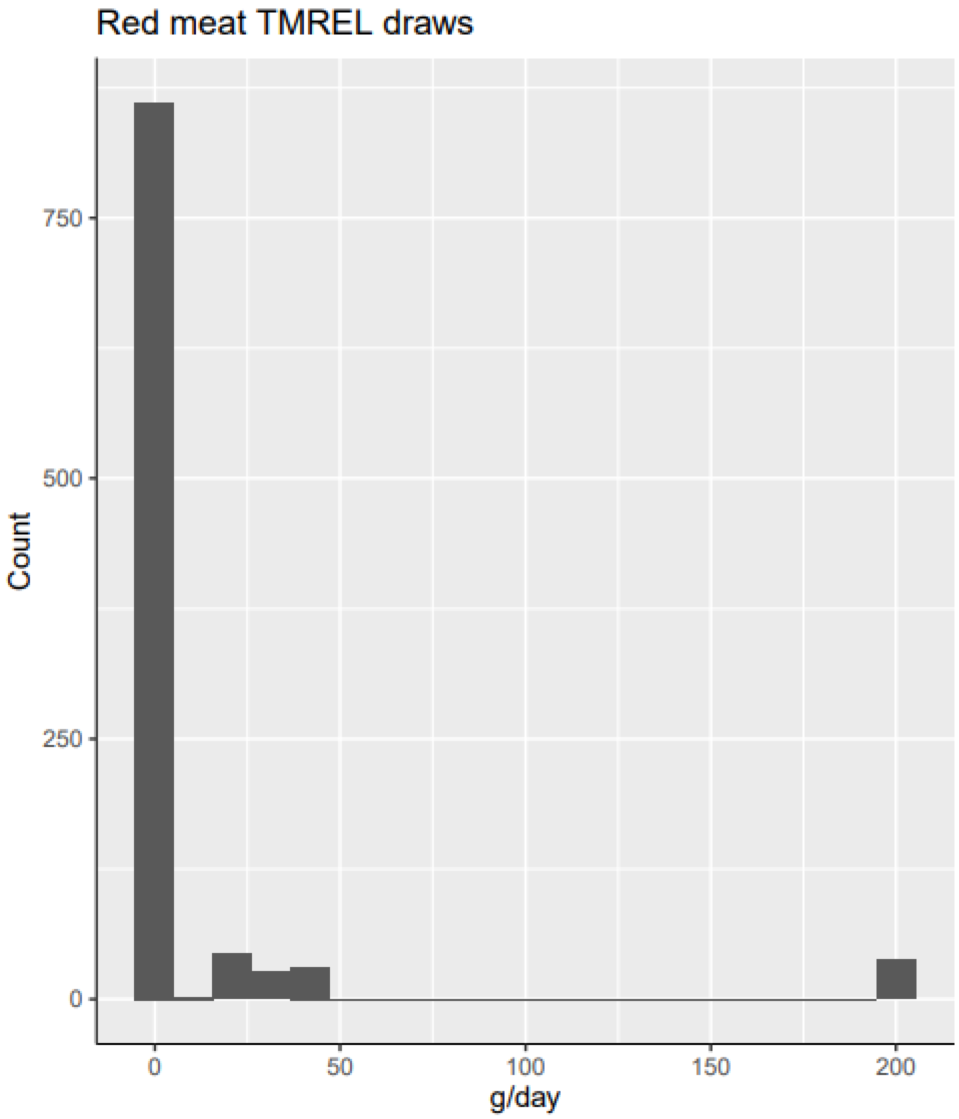

Relative risk

In GBD 2021, we updated the previous relative risk curves using the burden of proof method[9] and new model specifications, which has remained the approach for GBD 2023. To determine the shape of the risk curve for each risk–outcome pair, we first modelled it without any constraints. If a risk curve was generally increasing or decreasing across the full exposure domain, it was considered a monotonic (ie, strictly protective or strictly harmful) risk. Otherwise, if the risk curve had J-shape behaviour (ie, it decreases before increasing, or the inverse, or does not demonstrate a change in relative risk until some threshold), it was considered a J-shaped risk. Then, each risk–outcome pair was modelled according to the priors and constraints outlined in Table 19.

For each risk–outcome pair meta-regression, we considered study-level covariates that could potentially bias the study's reported effect size estimates. These study-level covariates included indication of whether the study had a follow-up period >10 years, whether the study used a wider definition of the exposure or outcome, whether the study was a randomised controlled trial or a cohort study, whether the effect sizes were relative risks or odds ratios, whether the effect sizes were for incidence or mortality, whether the study measured intake with a single or repeat measurements, whether the study determined outcomes based on administrative records or self-reports, and the level of adjustment for relevant confounders like age, sex, smoking, education, physical activity, and income. Additionally for our vegetable risk–outcome pairs, we considered whether the studies adjusted for energy intake as a covariate.[4] We adjusted for these covariates in our meta-regression if they significantly biased our estimated relative risk function. For processed meat, SSB, and trans-fat, we considered whether the studies adjusted for energy intake, fruit and vegetable intake, saturated fat intake, and unprocessed meat intake. All the studies included in SSB risk curves adjusted their effect size for BMI. The details are published elsewhere.[3]For the risk-outcome pairs updated for GBD 2023, we presented a comparison of relative risk curves between GBD 2021 and GBD 2023 in Figures 8 through 13. For causes with age-specific relative risks, we included a comparison figure for one age group as a representative sample to illustrate the changes in relative risk between GBD 2021 and GBD 2023. The same age trend is applied in both GBD 2021 and GBD 2023 for causes with age-specific relative risks.

After using the aforementioned study-level covariates to account for known differences in study design characteristics, we used a linear mixed-effects model to quantify the remaining unexplained between-study heterogeneity, as captured by gamma (γ). The remaining between-study heterogeneity captured by γ contributes to the overall assessment of effect size and evidence strength as reflected in the Burden of Proof risk function (BPRF). The BPRF represents a conservative estimate, based on the available evidence, of the magnitude of the association between disease outcome and risk factor exposure, in addition to the strength of the evidence supporting the association. We used the Fisher information matrix to estimate the uncertainty of the heterogeneity. The Fisher information matrix is weakly dependent on observed data, but is sensitive to the non-linear relationship, selected bias covariates, reported standard errors, and the number of studies. The details about the Burden of Proof methods are described elsewhere.[9]

We examined the presence of publication and reporting bias using Egger's regression and by visually inspecting funnel plots. In the current implementation, we do not adjust the risk curves for publication bias but flag the risk–outcome pairs where the risk of publication bias is significant.

In the tables 19 and 20, we list each dietary risk–outcome pair used in GBD 2023 along with several of the key modelling parameters and results.

Table 19: The burden of proof models specifications

Risk–outcome	Spline degree, # interior knots	Priors & constraints
Calcium & colorectal cancer	Quadratic, 2 Interior knots	Monotonic decreasing, right linear tail, Gaussian max derivative prior on the right tail (0, 0.001)
Calcium & prostate cancer	Quadratic, 2 Interior knots	Monotonic increasing, right linear tail, Gaussian max derivative prior on the right tail (0, 0.001)

Fibre & colorectal cancer	Quadratic, 2 Interior knots	Monotonic decreasing, right linear tail, Gaussian max derivative prior on the right tail (0, 0.001)
Fibre & type 2 diabetes	Quadratic, 2 Interior knots	Monotonic decreasing, right linear tail, Gaussian max derivative prior on the right tail (0, 0.001)
Fibre & haemorrhagic stroke	Quadratic, 2 Interior knots	Monotonic decreasing, right linear tail, Gaussian max derivative prior on the right tail (0, 0.001)
Fibre & IHD	Quadratic, 2 Interior knots	Monotonic decreasing, right linear tail, Gaussian max derivative prior on the right tail (0, 0.001)
Fibre & ischaemic stroke	Quadratic, 2 Interior knots	Monotonic decreasing, right linear tail, Gaussian max derivative prior on the right tail (0, 0.001)
Fruit & type 2 diabetes	Quadratic, 2 Interior knots	Monotonic decreasing, right linear tail, Gaussian max derivative prior on the right tail (0, 0.001)
Fruit & haemorrhagic stroke	Quadratic, 2 Interior knots	Monotonic decreasing, right linear tail, Gaussian max derivative prior on the right tail (0, 0.001)
Fruit & IHD	Quadratic, 2 Interior knots	Monotonic decreasing, right linear tail, Gaussian max derivative prior on the right tail (0, 0.001)
Fruit & ischaemic stroke	Quadratic, 2 Interior knots	Monotonic decreasing, right linear tail, Gaussian max derivative prior on the right tail (0, 0.001)
Fruit & lung cancer	Quadratic, 2 Interior knots	Monotonic decreasing, right linear tail, Gaussian max derivative prior on the right tail (0, 0.001)
Legumes & IHD	Quadratic, 2 Interior knots	Monotonic decreasing, right linear tail, Gaussian max derivative prior on the right tail (0, 0.001)
Milk & colorectal cancer	Quadratic, 2 Interior knots	Monotonic decreasing, right linear tail, Gaussian max derivative prior on the right tail (0, 0.001)
Milk & prostate cancer	Quadratic, 2 Interior knots	Monotonic decreasing, right linear tail, Gaussian max derivative prior on the right tail (0, 0.001)
Nuts and seeds & IHD	Quadratic, 2 Interior knots	Monotonic decreasing, right linear tail, Gaussian max derivative prior on the right tail (0, 0.001)
Omega-3 & IHD	Quadratic, 2 Interior knots	Monotonic decreasing, right linear tail, Gaussian max derivative prior on the right tail (0, 0.001)
Processed meat & colorectal cancer	Quadratic, 3 interior knots	Monotonic increasing, right linear tail, Gaussian max derivative prior on the right tail (0, 0.001)

Processed meat & type 2 diabetes	Quadratic, 3 interior knots	Monotonic increasing, right linear tail, Gaussian max derivative prior on the right tail (0, 0.001)
Processed meat & IHD	Quadratic, 3 interior knots	Monotonic increasing, right linear tail, Gaussian max derivative prior on the right tail (0, 0.001)
Omega-6 & IHD	Quadratic, 2 Interior knots	Monotonic decreasing, right linear tail, Gaussian max derivative prior on the right tail (0, 0.001)
Red meat & breast cancer	Quadratic, 2 Interior knots	Monotonic increasing, right linear tail, Gaussian max derivative prior on the right tail (0, 0.001)
Red meat & colorectal cancer	Quadratic, 2 Interior knots	Monotonic increasing, right linear tail, Gaussian max derivative prior on the right tail (0, 0.001)
Red meat & type 2 diabetes	Quadratic, 3 Interior knots	No monotonicity constraint, right and left linear tails, Gaussian max derivative prior on the right and left tail (0, 0.001)
Red meat & haemorrhagic stroke	Quadratic, 2 Interior knots	Monotonic decreasing, right linear tail, Gaussian max derivative prior on the right tail (0, 0.001)
Red meat & IHD	Quadratic, 3 Interior knots	No monotonicity constraint, right and left linear tails, Gaussian max derivative prior on the right and left tail (0, 0.001)
Red meat & ischaemic stroke	Quadratic, 3 Interior knots	No monotonicity constraint, right and left linear tails, Gaussian max derivative prior on the right and left tail (0, 0.001)
Sodium & stomach cancer	Quadratic, 2 Interior knots	Monotonic increasing, right linear tail, Gaussian max derivative prior on the right tail (0, 0.001)
SSB & type 2 diabetes	Quadratic, 3 interior knots	Monotonic increasing, right linear tail, Gaussian max derivative prior on the right tail (0, 0.001)
SSB & IHD	Quadratic, 3 interior knots	Monotonic increasing, right linear tail, Gaussian max derivative prior on the right tail (0, 0.001)
Trans fatty acids & IHD	Cubic, 3 interior knots	Monotonic increasing, right linear tail, Gaussian max derivative prior on the right tail (0, 0.001)
Veg & type 2 diabetes	Quadratic, 2 Interior knots	Monotonic decreasing, right linear tail, Gaussian max derivative prior on the right tail (0, 0.001)
Veg & haemorrhagic stroke	Quadratic, 2 Interior knots	Monotonic decreasing, right linear tail, Gaussian max derivative prior on the right tail (0, 0.001)
Veg & IHD	Quadratic, 2 Interior knots	Monotonic decreasing, right linear tail, Gaussian max derivative prior on the right tail (0, 0.001)

Veg & ischaemic stroke	Quadratic, 2 Interior knots	Monotonic decreasing, right linear tail, Gaussian max derivative prior on the right tail (0, 0.001)
Veg & oesophageal cancer	Quadratic, 2 Interior knots	Monotonic decreasing, right linear tail, Gaussian max derivative prior on the right tail (0, 0.001)
Whole grain & colorectal cancer	Quadratic, 2 Interior knots	Monotonic decreasing, right linear tail, Gaussian max derivative prior on the right tail (0, 0.001)
Whole grain & type 2 diabetes	Quadratic, 2 Interior knots	Monotonic decreasing, right linear tail, Gaussian max derivative prior on the right tail (0, 0.001)
Whole grain & IHD	Quadratic, 2 Interior knots	Monotonic decreasing, right linear tail, Gaussian max derivative prior on the right tail (0, 0.001)
Whole grain & ischaemic stroke	Quadratic, 2 Interior knots	Monotonic decreasing, right linear tail, Gaussian max derivative prior on the right tail (0, 0.001)

Table 20: Risk–outcome pair, shape of risk curve, selected bias covariates, gamma, and publication bias

**Risk–outcome**	**Shape of risk**	**Selected covariates**	**Mean gamma solution**	**Publication bias**
Calcium & colorectal cancer	Protective	--	0	No publication bias
Calcium & prostate cancer	Harmful	--	0	No publication bias
Fibre & colorectal cancer	Protective	--	0.012	No publication bias
Fibre & type 2 diabetes	Protective	--	0	Publication bias observed
Fibre & haemorrhagic stroke	Protective	--	0	No publication bias
Fibre & IHD	Protective	--	0	No publication bias
Fibre & ischaemic stroke	Protective	Total stroke	0	No publication bias
Fruit & type 2 diabetes	Protective	--	0	No publication bias
Fruit & haemorrhagic stroke	Protective	Total stroke	0.079	Publication bias observed
Fruit & IHD	Protective	--	0.006	No publication bias
Fruit & ischaemic stroke	Protective	Total stroke	0	Publication bias observed
Fruit & lung cancer	Protective	--	0	No publication bias
Legumes & IHD	Protective	--	0.250	No publication bias

Risk–outcome	Shape of risk	Selected covariates	Mean gamma solution	Publication bias
Milk & colorectal cancer	Protective	--	0	No publication bias
Milk & prostate cancer	Harmful	--	0.434	No publication bias
Nuts and seeds & IHD	Protective	--	0.018	No publication bias
Omega-3 & IHD	Protective	Study type	0.028	No publication bias
Processed meat & colorectal cancer	Harmful	--	0	No publication bias
Processed meat & type 2 diabetes	Harmful	Adjusting for hypertension, Having multiple exposure assessment.	0	Publication bias observed
Processed meat & IHD	Harmful	Having multiple exposure assessment	0	No publication bias
Omega-6 & IHD	Protective	Study type	0.809	No publication bias
Red meat & breast cancer	Harmful		0	No publication bias
Red meat & colorectal cancer	Harmful		0.099	No publication bias
Red meat & type 2 diabetes	J-shaped	--	0.096	No publication bias
Red meat & haemorrhagic stroke	Protective	--	0.930	No publication bias
Red meat & IHD	J-shaped	--	0	No publication bias
Red meat & ischaemic stroke	J-shaped		0.016	Publication bias observed
Sodium & stomach cancer	Harmful	--	0.006	No publication bias
SSB & type 2 diabetes	Harmful	Adujsting effect size for hypertension or/and Hypercholesterolemia, Having clear definition of SSB (ie, sugar was explicitly mentioned as a sweetener)	0.01	No publication bias
SSB & IHD	Harmful	--	0	No publication bias
Trans fatty acids & IHD	Harmful	--	0.04	No publication bias
Veg & type 2 diabetes	Protective	Outcome assessment, confounder	0.236	No publication bias

Risk–outcome	Shape of risk	Selected covariates	Mean gamma solution	Publication bias
		adjustment, exposure measurement, length of follow-up		
Veg & haemorrhagic stroke	Protective		0	No publication bias
Veg & IHD	Protective	Confounder measurement, incidence	0.020	Publication bias observed
Veg & ischaemic stroke	Protective		0	No publication bias
Veg & oesophageal cancer	Protective	--	0.006	Publication bias observed
Whole grain & colorectal cancer	Protective	--	0	No publication bias
Whole grain & type 2 diabetes	Protective	Follow-up period, odds ratio	0	No publication bias
Whole grain & IHD	Protective	--	0	No publication bias
Whole grain & ischaemic stroke	Protective	Total stroke	0.211	No publication bias

There is a well-documented attenuation of the risk for cardiovascular disease due to metabolic risks factors throughout one's life.[10] To incorporate this age trend in the relative risks, we first identified the median age-at-event across all cohorts and considered that as the reference age group. We then assigned our risk curves to this reference age group. Then, we derived attenuation factors by taking the ratio of excess risk between each age group and the reference. Finally, we applied 1000 draws of the age-specific attenuation factors to 1000 draws of the reference age group's risk curve to determine age-specific risk curves that propagated the uncertainty of both the risk function and age pattern. The three cardiovascular disease outcomes for dietary risks are haemorrhagic stroke (including intracerebral haemorrhage and subarachnoid haemorrhage), ischaemic stroke, and IHD; the effects of dietary risks on them are mediated through high systolic blood pressure and cholesterol (not included for haemorrhagic stroke). There was no evidence found for an age pattern in fasting plasma glucose, and since the effect of diet is estimated independently of body-mass index in the GBD study, body-mass index was not included as a mediator in the relative risk age trend analysis.

Figure 8. Comparison of relative risk between GBD 2021 and GBD 2023 for processed meat consumption and type 2 diabetes.

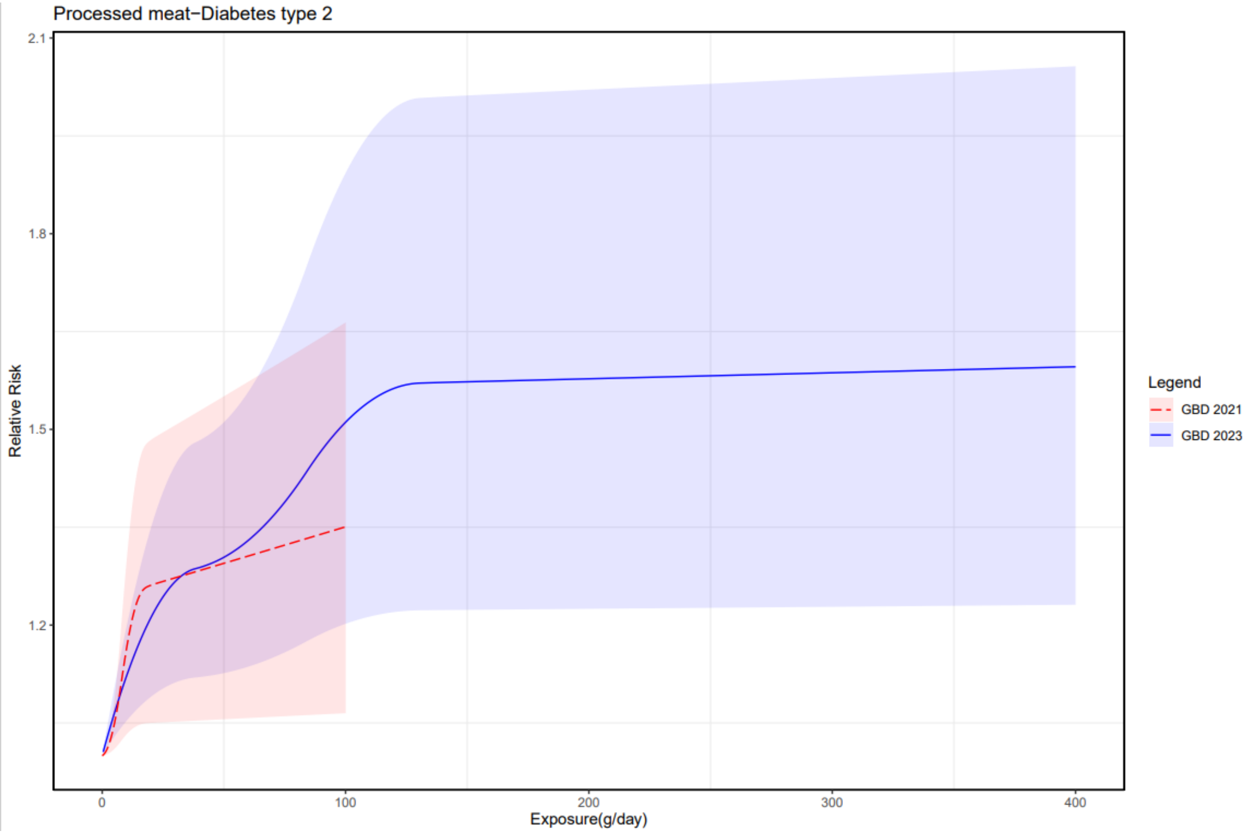

Figure 9. Comparison of relative risk between GBD 2021 and GBD 2023 for processed meat consumption and ischemic heart diseases.

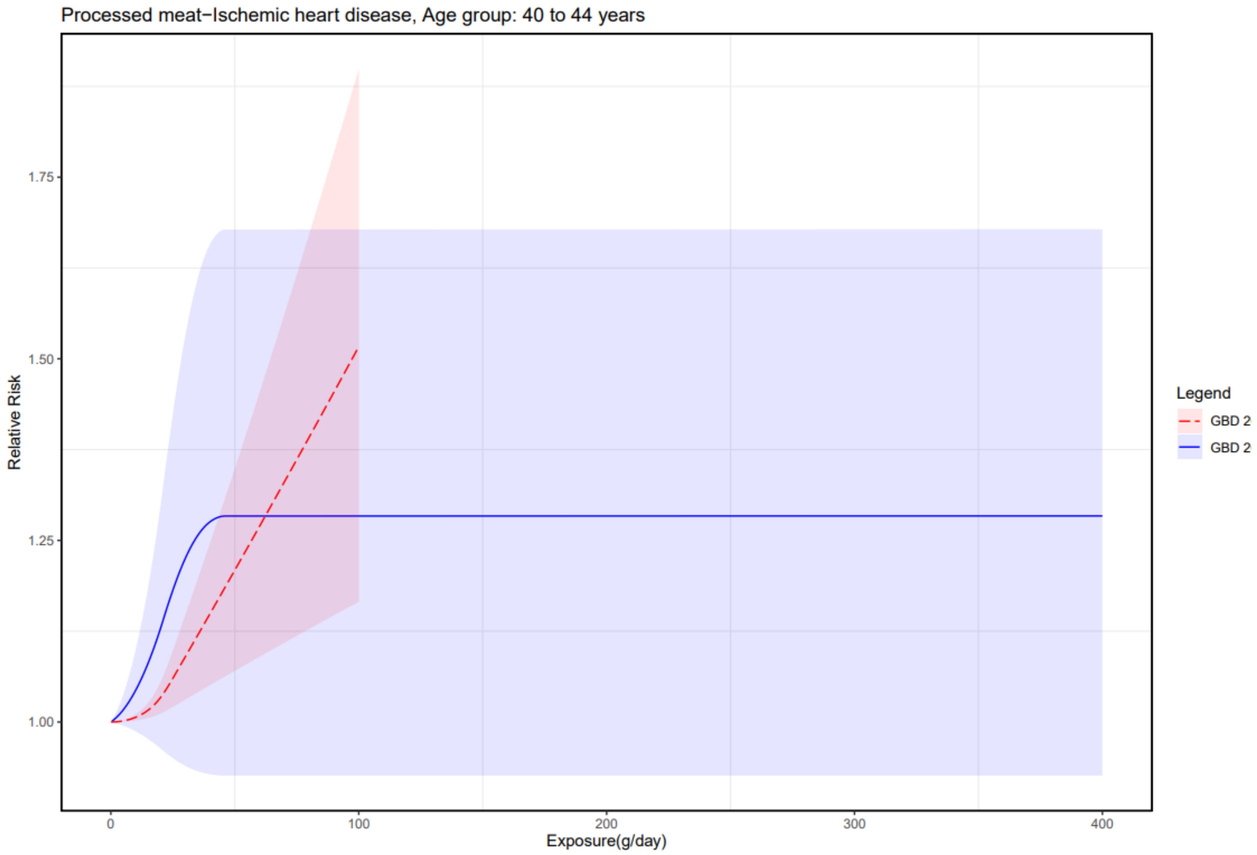

Figure 10. Comparison of relative risk between GBD 2021 and GBD 2023 for processed meat consumption and colorectal cancer.

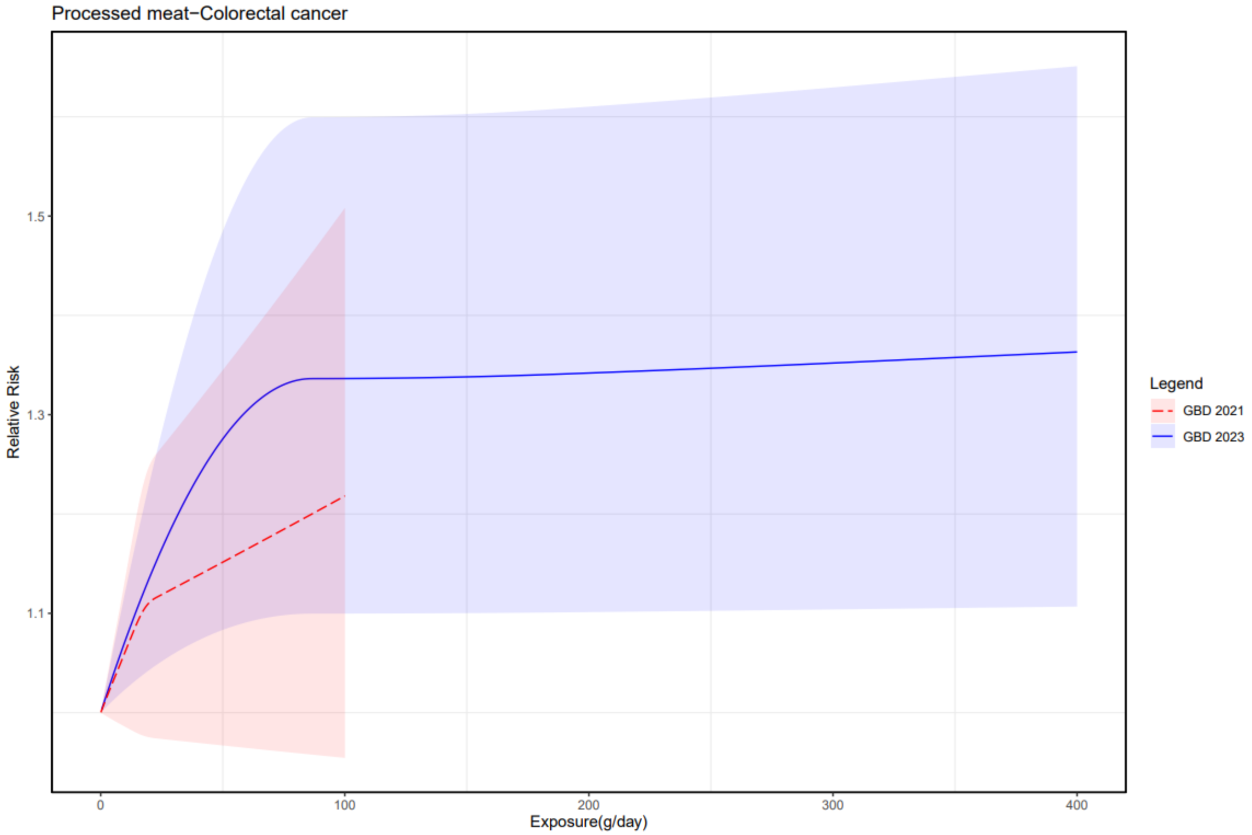

Figure 11. Comparison of relative risk between GBD 2021 and GBD 2023 for SSB consumption and type 2 diabetes

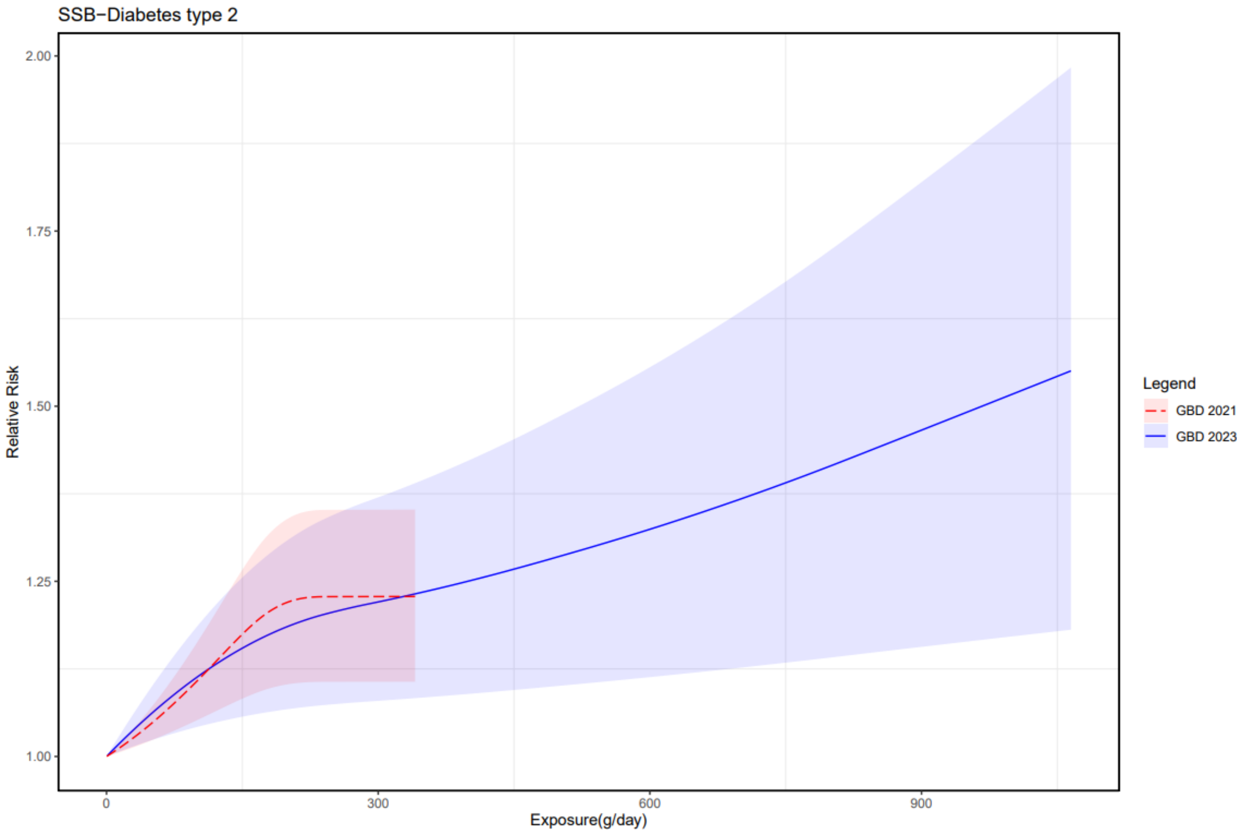

Figure 12. Comparison of relative risk between GBD 2021 and GBD 2023 for SSB consumption and ischemic heart diseases.

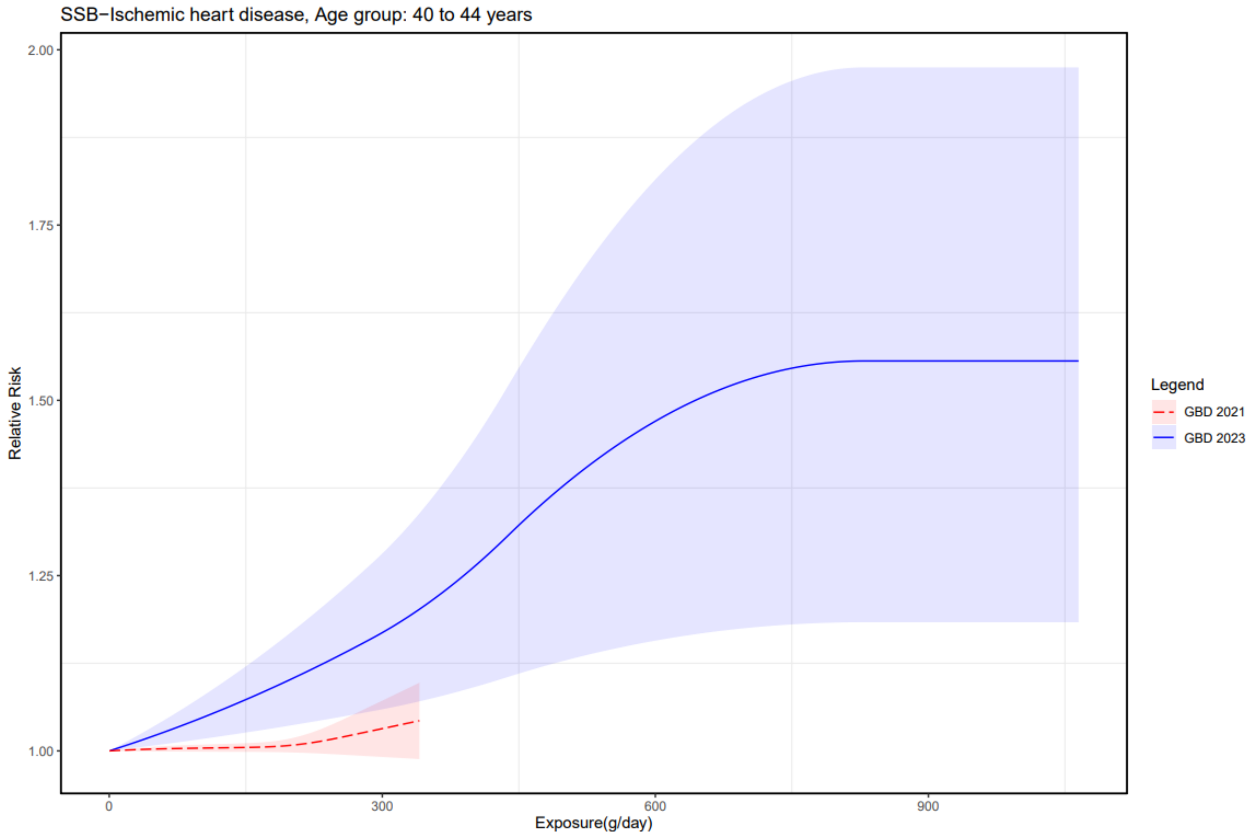

Figure 13. Comparison of relative risk between GBD 2021 and GBD 2023 for trans-fat consumption (Industrial sources) and ischemic heart diseases.

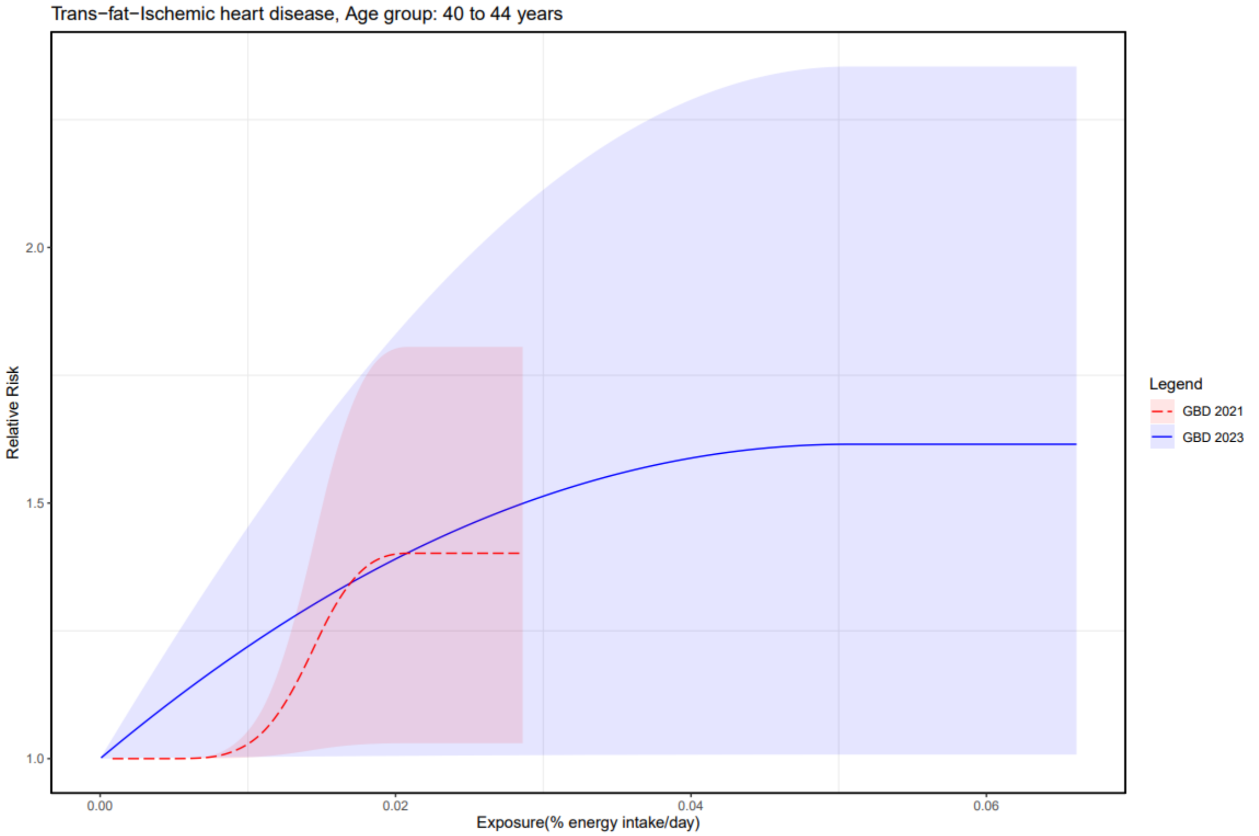

Risk–outcome pairs modelled through mediation

This dietary-risk-specific modelling section has dealt almost exclusively with dietary risks associated *directly* with outcomes in the GBD 2023 framework. Dietary risks were also associated with outcomes *indirectly* through mediation.

Below is a list of the dietary risk–outcome pairs modelled through mediation in GBD 2023, where dietary risk factors (first column) affect mediators (middle column), which in turn affect outcomes (last column). Mediators themselves are risk factors. The last column contains the value of the mediation factor.

Table 21. Mediation factor matrix for dietary risk factors

Risk Factor	Mediator	Cause	Mediation factor (95% UI)
Diet low in fruits	High fasting plasma glucose	Ischaemic stroke	0.050 (0.040–0.062)
Diet low in fruits	High fasting plasma glucose	Lower extremity peripheral arterial disease	1 (1–1)
Diet low in fruits	High fasting plasma glucose	Chronic kidney disease	0.151 (0.016–0.303)
Diet low in fruits	High fasting plasma glucose	Drug-susceptible tuberculosis	1 (1–1)

Diet low in fruits	High fasting plasma glucose	Multidrug-resistant tuberculosis without extensive drug resistance	1 (1–1)
Diet low in fruits	High fasting plasma glucose	Extensively drug-resistant tuberculosis	1 (1–1)
Diet low in fruits	High fasting plasma glucose	Diabetes mellitus type 2	1 (1–1)
Diet low in fruits	High systolic blood pressure	Ischaemic heart disease	0.065 (0.051–0.079)
Diet low in fruits	High systolic blood pressure	Ischaemic stroke	0.050 (0.040–0.062)
Diet low in fruits	High systolic blood pressure	Intracerebral haemorrhage	0.024 (0.019–0.028)
Diet low in fruits	High systolic blood pressure	Subarachnoid haemorrhage	0.024 (0.019–0.028)
Diet low in fruits	High systolic blood pressure	Hypertensive heart disease	1 (1–1)
Diet low in fruits	High systolic blood pressure	Aortic aneurysm	1 (1–1)
Diet low in fruits	High systolic blood pressure	Chronic kidney disease	0.849 (0.697–0.984)
Diet low in fruits	High LDL cholesterol	Ischaemic heart disease	0.065 (0.051–0.079)
Diet low in fruits	High LDL cholesterol	Ischaemic stroke	0.050 (0.040–0.062)
Diet low in vegetables	High fasting plasma glucose	Ischaemic heart disease	0.064 (0.014–0.179)
Diet low in vegetables	High fasting plasma glucose	Ischaemic stroke	0.084 (0.037–0.163)
Diet low in vegetables	High fasting plasma glucose	Intracerebral haemorrhage	0.084 (0.037–0.163)
Diet low in vegetables	High fasting plasma glucose	Lower extremity peripheral arterial disease	1 (1–1)
Diet low in vegetables	High fasting plasma glucose	Chronic kidney disease	0.055 (–0.016 to 0.135)
Diet low in vegetables	High fasting plasma glucose	Drug-susceptible tuberculosis	1 (1–1)
Diet low in vegetables	High fasting plasma glucose	Multidrug-resistant tuberculosis without extensive drug resistance	1 (1–1)
Diet low in vegetables	High fasting plasma glucose	Extensively drug-resistant tuberculosis	1 (1–1)
Diet low in vegetables	High fasting plasma glucose	Diabetes mellitus type 2	1 (1–1)
Diet low in vegetables	High systolic blood pressure	Ischaemic heart disease	0.041 (0.025–0.054)
Diet low in vegetables	High systolic blood pressure	Ischaemic stroke	0.027 (0.016–0.038)
Diet low in vegetables	High systolic blood pressure	Intracerebral haemorrhage	0.036 (0.022–0.051)

Diet low in vegetables	High systolic blood pressure	Subarachnoid haemorrhage	0.036 (0.022–0.051)
Diet low in vegetables	High systolic blood pressure	Hypertensive heart disease	1 (1–1)
Diet low in vegetables	High systolic blood pressure	Aortic aneurysm	1 (1–1)
Diet low in vegetables	High systolic blood pressure	Chronic kidney disease	0.945 (0.865–1.016)
Diet low in vegetables	High LDL cholesterol	Ischaemic heart disease	0.041 (0.025–0.054)
Diet low in vegetables	High LDL cholesterol	Ischaemic stroke	0.086 (0.041–0.156)
Diet low in whole grains	High fasting plasma glucose	Lower extremity peripheral arterial disease	1 (1–1)
Diet low in whole grains	High fasting plasma glucose	Chronic kidney disease	1 (1–1)
Diet low in whole grains	High fasting plasma glucose	Drug-susceptible tuberculosis	1 (1–1)
Diet low in whole grains	High fasting plasma glucose	Multidrug-resistant tuberculosis without extensive drug resistance	1 (1–1)
Diet low in whole grains	High fasting plasma glucose	Extensively drug-resistant tuberculosis	1 (1–1)
Diet low in whole grains	High fasting plasma glucose	Diabetes mellitus type 2	1 (1–1)
Diet low in whole grains	High LDL cholesterol	Ischaemic heart disease	0.385 (0.170–0.544)
Diet low in whole grains	High LDL cholesterol	Ischaemic stroke	0.159 (0.054–0.353)
Diet low in nuts and seeds	High LDL cholesterol	Ischaemic heart disease	0.197 (0.011–0.791)
Diet low in milk	Diet low in calcium	Colon and rectum cancer	1 (1–1)
Diet high in red meat	High fasting plasma glucose	Lower extremity peripheral arterial disease	1 (1–1)
Diet high in red meat	High fasting plasma glucose	Chronic kidney disease	1 (1–1)
Diet high in red meat	High fasting plasma glucose	Drug-susceptible tuberculosis	1 (1–1)
Diet high in red meat	High fasting plasma glucose	Multidrug-resistant tuberculosis without extensive drug resistance	1 (1–1)
Diet high in red meat	High fasting plasma glucose	Extensively drug-resistant tuberculosis	1 (1–1)
Diet high in red meat	High fasting plasma glucose	Diabetes mellitus type 2	1 (1–1)
Diet high in processed meat	High fasting plasma glucose	Ischaemic heart disease	0.010 (0.006–0.016)

Diet high in processed meat	High fasting plasma glucose	Ischaemic stroke	1 (1–1)
Diet high in processed meat	High fasting plasma glucose	Lower extremity peripheral arterial disease	1 (1–1)
Diet high in processed meat	High fasting plasma glucose	Chronic kidney disease	1 (1–1)
Diet high in processed meat	High fasting plasma glucose	Drug-susceptible tuberculosis	1 (1–1)
Diet high in processed meat	High fasting plasma glucose	Multidrug-resistant tuberculosis without extensive drug resistance	1 (1–1)
Diet high in processed meat	High fasting plasma glucose	Extensively drug-resistant tuberculosis	1 (1–1)
Diet high in processed meat	High fasting plasma glucose	Diabetes mellitus type 2	1 (1–1)
Diet high in sugar-sweetened beverages	High fasting plasma glucose	Ischaemic stroke	1 (1–1)
Diet high in sugar-sweetened beverages	High fasting plasma glucose	Lower extremity peripheral arterial disease	1 (1–1)
Diet high in sugar-sweetened beverages	High fasting plasma glucose	Chronic kidney disease	1 (1–1)
Diet high in sugar-sweetened beverages	High fasting plasma glucose	Drug-susceptible tuberculosis	1 (1–1)
Diet high in sugar-sweetened beverages	High fasting plasma glucose	Multidrug-resistant tuberculosis without extensive drug resistance	1 (1–1)
Diet high in sugar-sweetened beverages	High fasting plasma glucose	Extensively drug-resistant tuberculosis	1 (1–1)
Diet high in sugar-sweetened beverages	High fasting plasma glucose	Diabetes mellitus type 2	1 (1–1)
Diet low in polyunsaturated fatty acids	High LDL cholesterol	Ischaemic heart disease	–0.005 (–0.03 to 0.034)
Diet low in polyunsaturated fatty acids	High LDL cholesterol	Ischaemic stroke	1 (1–1)
Diet high in trans fatty acids	High LDL cholesterol	Ischaemic heart disease	0.150 (0.024–0.238)

Diet high in sodium	High systolic blood pressure	Ischaemic heart disease	1 (1–1)
Diet high in sodium	High systolic blood pressure	Ischaemic stroke	1 (1–1)
Diet high in sodium	High systolic blood pressure	Intracerebral haemorrhage	1 (1–1)
Diet high in sodium	High systolic blood pressure	Subarachnoid haemorrhage	1 (1–1)
Diet high in sodium	High systolic blood pressure	Hypertensive heart disease	1 (1–1)
Diet high in sodium	High systolic blood pressure	Aortic aneurysm	1 (1–1)
Diet high in sodium	High systolic blood pressure	Aortic aneurysm	1 (1–1)
Diet high in sodium	High systolic blood pressure	Lower extremity peripheral arterial disease	1 (1–1)
Diet high in sodium	High systolic blood pressure	Chronic kidney disease	1 (1–1)
For IHD, stroke, and diabetes we pooled all available cohorts and estimated relative risks with and without adjustment across all combinations of metabolic risk factors. We then computed the excess attenuated risk for each mediation-risk-cause set.			

Citations

1 Schmidhuber J, Sur P, Fay K, *et al.* The Global Nutrient Database: availability of macronutrients and micronutrients in 195 countries from 1980 to 2013. *The Lancet Planetary Health* 2018; **2**: e353–68.

2 USDA. United States Department of Agriculture, Agricultural Research Service, Nutrient Data Laboratory. USDA National Nutrient Database for Standard Reference, Release 28 (Slightly revised). Version Current: May 2016. 2016. http://www.ars.usda.gov/ba/bhnrc/ndl.

3 Haile D, Harding KL, McLaughlin SA, *et al.* Health effects associated with consumption of processed meat, sugar-sweetened beverages, and trans fatty acids: a Burden of Proof study. *Nature Medicine*, Under review. 2025.

4 Del Gobbo LC, Khatibzadeh S, Imamura F, *et al.* Assessing global dietary habits: a comparison of national estimatesfrom the FAO and the Global Dietary Database. *The American Journal of Clinical Nutrition* 2015; **101**: 1038–46.

5 Dunford EK, Miles DR, Popkin B, Ng SW. Whole Grain and Refined Grains: An Examination of US Household Grocery Store Purchases. *The Journal of Nutrition* 2022; **152**: 550–8.

6 Alexander Hsu, Peng Zheng, Kelsey Maass, Aleksandr Aravkin, Sameer Ali. pyDisagg: Dissaggregation under Generalized Proportionality Assumptions. 2025; published online Jan 13. DOI:10.5281/ZENODO.14641582.

7 GBD 2013 Risk Factors Collaborators, Forouzanfar MH, Alexander L, *et al.* Global, regional, and national comparative risk assessment of 79 behavioural, environmental and occupational, and metabolic risks or clusters of risks in 188 countries, 1990-2013: a systematic analysis for the Global Burden of Disease Study 2013. *Lancet* 2015; **386**: 2287–323.

8 Stanaway JD, Afshin A, Ashbaugh C, *et al.* Health effects associated with vegetable consumption: a Burden of Proof study. *Nat Med* 2022; **28**: 2066–74.

9 Zheng P, Afshin A, Biryukov S, *et al.* The Burden of Proof studies: assessing the evidence of risk. *Nat Med* 2022; **28**: 2038–44.

10 Singh GM, Danaei G, Farzadfar F, *et al.* The age-specific quantitative effects of metabolic risk factors on cardiovascular diseases and diabetes: a pooled analysis. *PLoS One* 2013; **8**: e65174.

Drug use

Flowchart

Drug Use

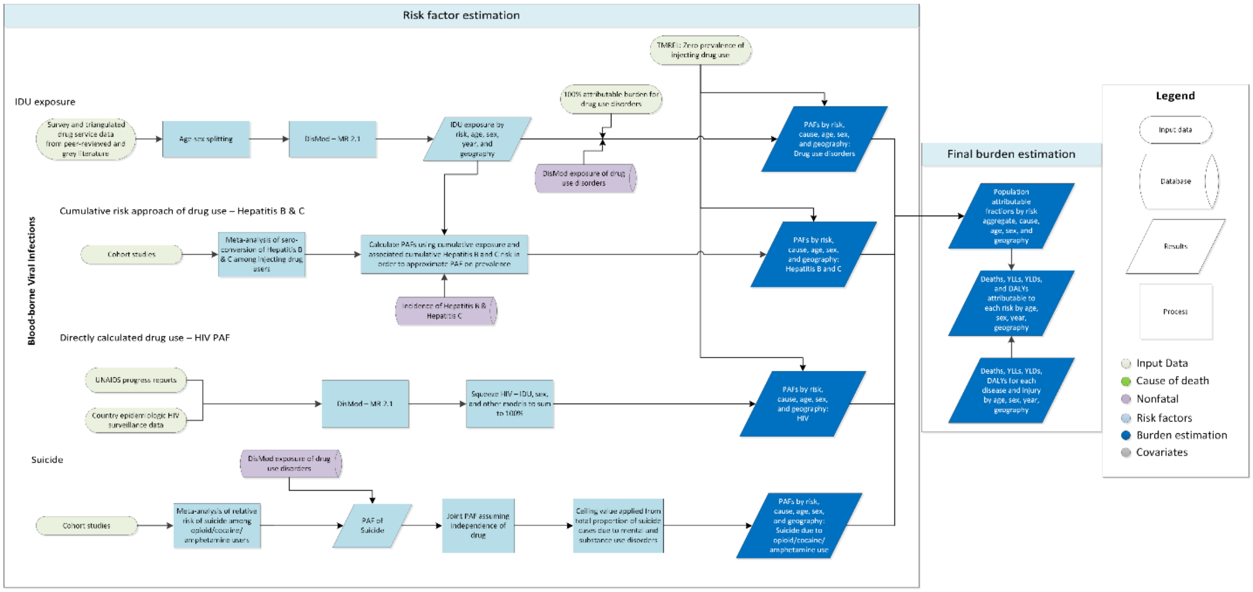

Input data and methodological summary

Exposure definition

The drug use risk factor includes four dimensions of exposure. First, we include 100% attribution of drug use disorder estimates. Second, estimates of prevalence of opioid, amphetamine, and cocaine use disorder are used as exposures for risk of suicide. These drug use disorders are defined based on DSM or ICD diagnostic criteria. Third, instead of starting with an exposure model to estimate the proportion of HIV cases due to injection drug use (IDU), we model the PAF (population attributable fractions, described in appendix 2 section 2) directly, alongside proportion of HIV cases due to sexual transmission and other routes of transmission, which mainly includes blood transfusions. Finally, prevalence of IDU is used to model risk of Hepatitis B and C viruses (HBV and HCV, respectively). Injecting drug users are at high risk of bloodborne infections due to the use of shared needles and injection equipment. Injecting drug use is defined as current IDU among individuals aged 15–64. The theoretical minimum risk exposure level (TMREL, described in appendix 2 section 2) for drug use is defined as zero exposure to drug use.

Input data

We made no substantial changes to the data input for this round.

To estimate the burden of HIV cases attributable to IDU, we extracted data on the proportion of notified HIV cases by transmission route – sexual intercourse, injecting drug use, and other – from a number of agencies that conduct surveillance of HIV across the globe.[1-8]

The prevalence of current injecting drug use was estimated using data from a multistage process of systematic review. It involved multiple stages of peer and expert review, including by the Reference Group to the UN on HIV and injecting drug use,[9] with searches of the peer-reviewed literature in

addition to an extensive review of online grey literature databases in the drug and alcohol and HIV fields.

To generate a pooled incidence rate/absolute relative risk for viral hepatitis among people who inject drugs, we conducted a meta-analysis of longitudinal epidemiological studies that reported a hepatitis B or hepatitis C incidence rate among persons who inject drugs.[10-25] We calculated confidence intervals for the incidence rate (where no confidence interval was reported) from a Poisson distribution around the number of cases.

We excluded studies that focused on non-representative subgroups, such as recent injectors or adolescents, because hepatitis incidence is far higher in those groups than for all people who inject drugs (eg, Larney and colleagues[26]). We did not vary incidence among active injectors according to the availability of bloodborne virus-prevention strategies (eg, needle and syringe programs [NSPs], opioid substitution therapy) because too few studies have examined different levels of incidence according to variable coverage, and we were not able to estimate coverage by country over time. In any case, in most countries, effective coverage of virus-prevention strategies remains low among people who inject drugs.[27]

Inputs to the model also include estimates of the incidence of hepatitis B and hepatitis C, coming from estimation of non-fatal health outcomes in GBD. Full details on the inputs and modelling process to produce these estimates are available in the disease-specific appendices in the GBD 2023 diseases and injuries manuscript.

Modelling strategy

We made no substantial changes to the modelling strategy for GBD 2023.

Burden of HIV attributable to injecting drug use

We estimated the proportion of HIV cases attributable to three transmission categories (sex, IDU, and other) for all country-time periods using DisMod-MR 2.1 (disease model—Bayesian meta-regression, described in appendix 1, section 2). Prior to GBD 2019, data for estimating the proportion of HIV cases attributable to IDU were age-split using the age pattern of the IDU exposure model and sex-split in DisMod. In GBD 2019, these data were age- and sex-split using the estimated IDU exposure age-sex pattern, resulting in increases in the proportion of HIV due to IDU among men and decreases among women. We scaled the proportions from each of the three transmission models (sex, IDU, and other) to ensure that they fit the total HIV transmission envelope by country, year, age, and sex. Scaled estimates are used as direct PAFs, meaning that the proportion coming from the model is the proportion of HIV deaths or DALYs attributable to IDU.

Burden of hepatitis B and hepatitis C attributable to injecting drug use

To estimate the relative contribution of IDU to hepatitis B and C disease burden at the country, regional, and global level, we used a cohort method. We recalibrated individuals according to history of injecting drug use and their accumulated risk of incident hepatitis B and C due to IDU. We made use of data on prevalence of current injecting drug use, pooled in DisMod-MR 2.1; a meta-analysis of incidence rates of hepatitis B and hepatitis C among people who inject drugs; and estimates of population-level incidence

of hepatitis B and C between 1990 and 2023. We used back-extrapolations to estimate incidence before 1990. These steps are detailed below.

To estimate the lifetime risk of being infected with hepatitis B or C, we undertook a cohort analysis for each country, year, age, and sex category and estimated the probability of an individual having been infected in each preceding year. One of the main inputs to this cohort method was the probability of having injected drugs in a specific age cohort in a given calendar year. For example, for a cohort of 40-year-olds in 2015, the relevant probability in 2005 is the estimated prevalence of injecting drug use among 30-year-olds.

DisMod-MR 2.1 was used to estimate the prevalence of injecting drug use with year as a covariate to estimate the trends over time. DisMod makes an average estimate of the change in drug use over the time period 1990–2023, and we took draws from a normal distribution of the coefficient to project IDU prevalence backward in time to 1960 from baseline level in 1990 (assuming there was little injecting drug use before the 1960s). Prevalence of IDU was estimated as a single parameter prevalence model in DisMod, as opposed to a full compartmental model, because factoring in cause-specific mortality resulted in underestimating prevalence in certain locations, particularly in the north Africa and the Middle East and south Asia super-regions. In future rounds, we plan to test bias covariates for potential use in these models.

Theoretical minimum risk exposure level

TMREL is defined as zero exposure to drug use.

Relative risk

We used a pooled absolute risk of hepatitis C and hepatitis B among those who have ever used injecting drugs. Input data for this pooled absolute risk are described above, and there were no methodological or data changes to this parameter in GBD 2023. Data used to pool the absolute risk of hepatitis C and hepatitis B are shown in Figures 1 and 2.

Figure 1: Forest plot of absolute risk of HBV incidence among cohorts of people who inject drugs

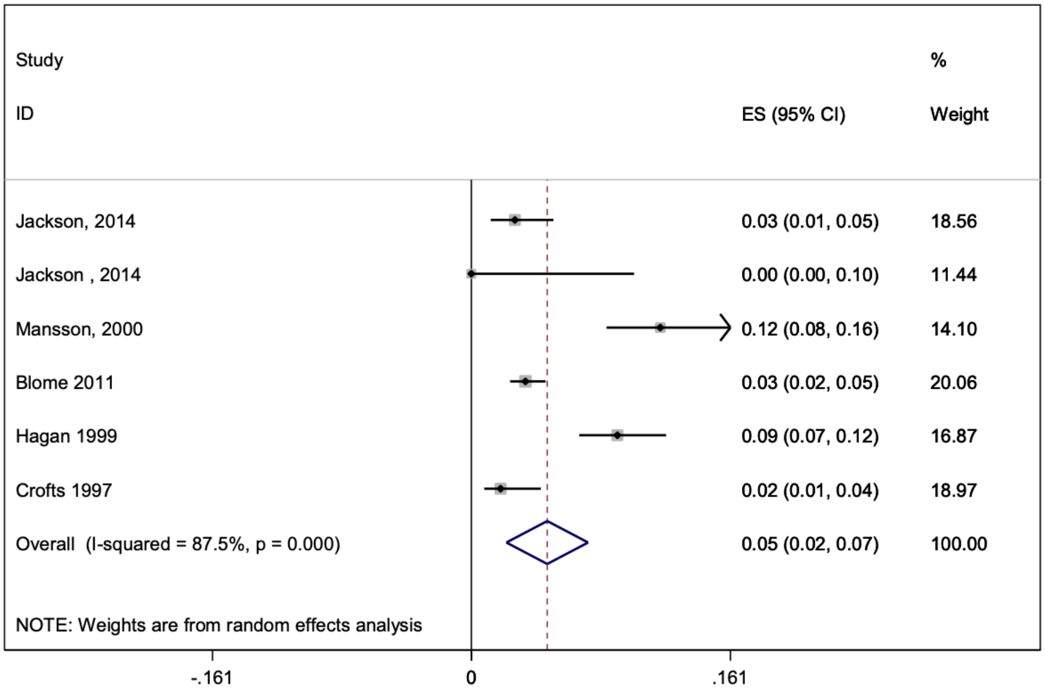

Figure 2: Forest plot of absolute risk of HCV incidence among cohorts of people who inject drugs

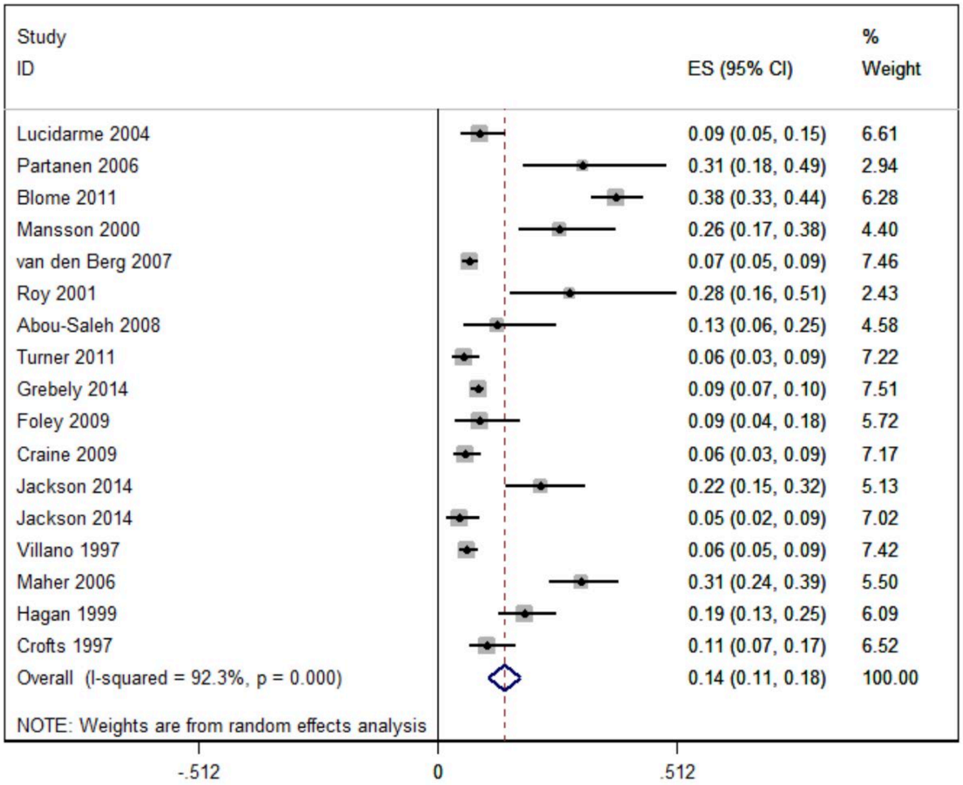

We used previously published data[28-54] and estimated the relative risk – as well as evaluated the strength of evidence of the association between drug use disorders and outcomes – for relationships that are not a PAF of 1 or use a direct PAF approach. We used a standard BoP method; a description of the methods and approach can be found in the evidence score documentation (binary risk–outcome pairs, appendix 2 section 2). Funnel plots of the results are shown in Figures 3-6.

Figure 3: Amphetamine use and cocaine use (risk) and suicide (outcome): The data sources used are the same.

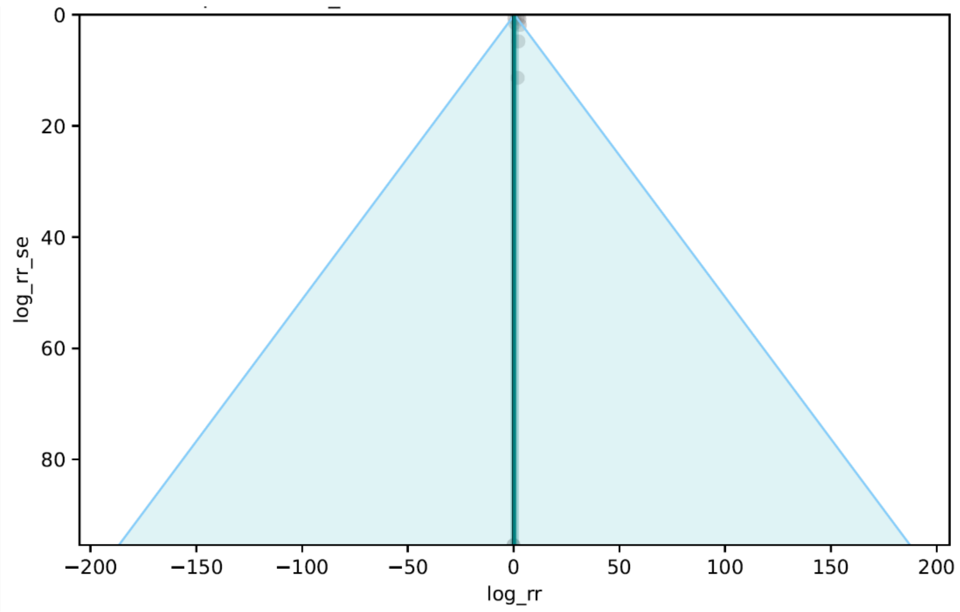

Figure 4: Opioid use (risk) and suicide (outcome)

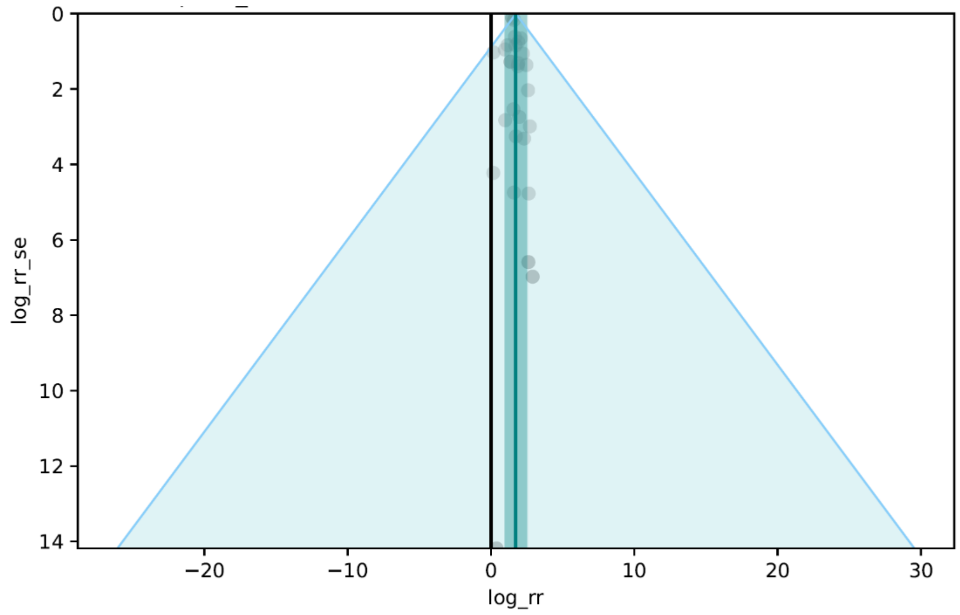

Figure 5: Injection drug use (risk) and hepatitis B (outcome)

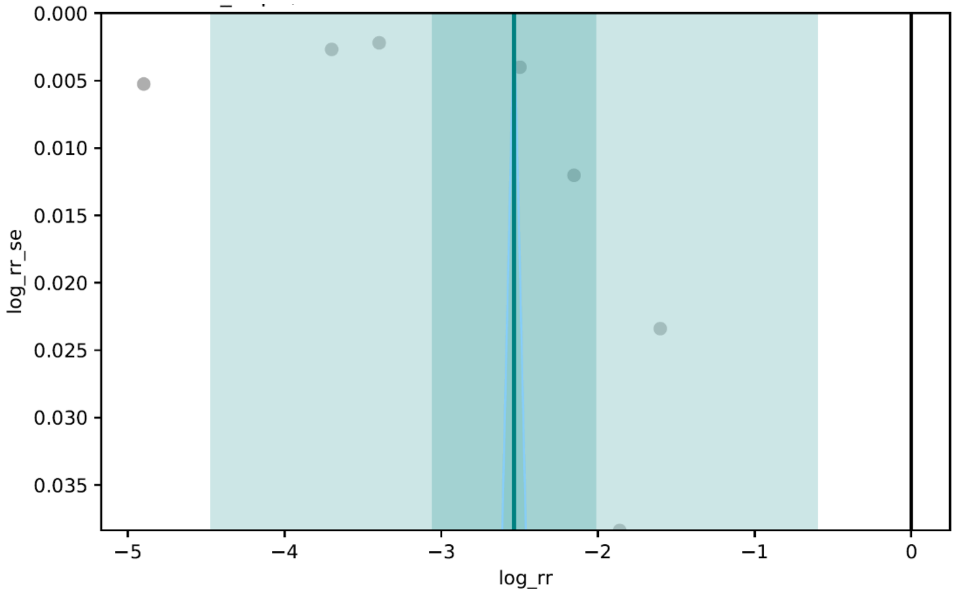

Figure 6: Injection drug use (risk) and hepatitis C (outcome)

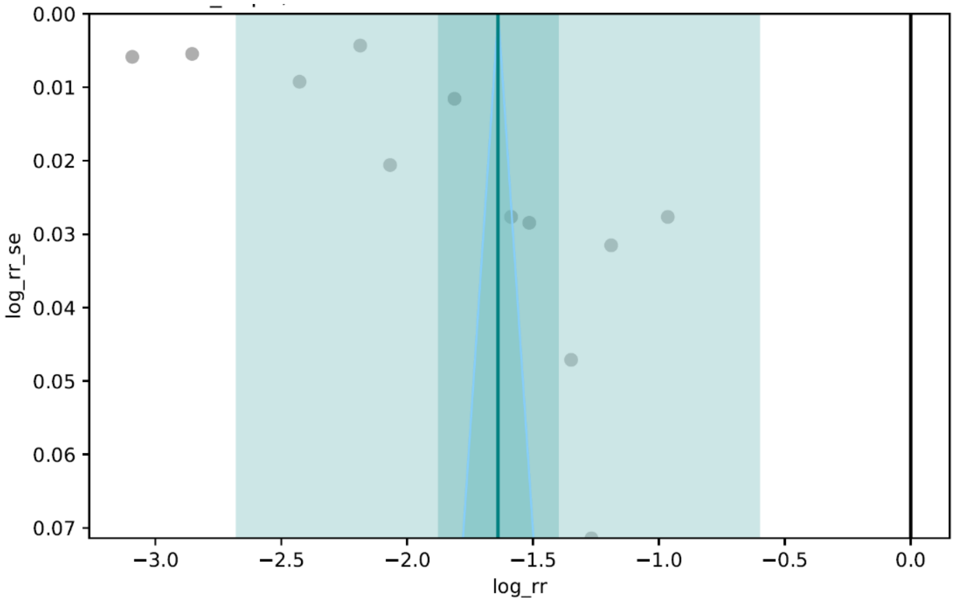

References

1. European Centre for Disease Prevention. HIV/AIDS surveillance in Europe 2014 Solna, Sweden. http://ecdc.europa.eu/en/publications/surveillance_reports/HIV_STI_and_blood_borne_viruses/Pages/HIV_STI_and_blood_borne_viruses.aspx: ECDC, 2014.
2. Family Health International, Bureau of AIDS TB and STIs Department of Disease Control. The Asian Epidemic Model (AEM) Projections for HIV/AIDS in Thailand:2005-2025. Bangkok: Family Health International (FHI) and Bureau of AIDS, TB and STIs, Department of Disease Control, Ministry of Public Health, Thailand, 2008.
3. Kirby Institute. 2015 Annual Surveillance Report of HIV, viral hepatitis, STIs. Sydney, New South Wales. https://kirby.unsw.edu.au/surveillance/2015-annual-surveillance-report-hiv-viral-hepatitis-stis: Kirby Institute, UNSW Australia, 2015.
4. Kirby Institute. Australian NSP survey national data report 2015. Sydney, New South Wales: Kirby Institute, University of New South Wales, 2015.
5. Country reports for Global AIDS Response Progress Reporting [Internet]. UNAIDS. 2014.
6. UNAIDS. UNAIDS Country reports. Geneva: Joint United Nations Programme on HIV/AIDS. http://www.unaids.org/en/regionscountries/countries, 2015.
7. United States Center for Disease Control and Prevention. HIV/AIDS Statistics. Atlanta, Georgia: US CDC. http://www.cdc.gov/hiv/statistics/index.html, 2015.
8. Gouws E, White PJ, Stover J, Brown T. Short term estimates of adult HIV incidence by mode of transmission: Kenya and Thailand as examples. Sex Transm Infect. 2006;82 Suppl 3:iii51-5.
9. Mathers BM, Degenhardt L, Phillips B, Wiessing L, Hickman M, Strathdee SA, et al. Global epidemiology of injecting drug use and HIV among people who inject drugs: a systematic review. Lancet. 2008;372(9651):1733-45.
10. Jackson JB, Wei L, Liping F, Aramrattana A, Celentano DD, Walshe L, et al. Prevalence and Seroincidence of Hepatitis B and Hepatitis C Infection in High Risk People Who Inject Drugs in China and Thailand. Hepatitis research and treatment. 2014;2014.
11. Månsson A-S, Moestrup T, Nordenfelt E, Widell A. Continued transmission of hepatitis B and C viruses, but no transmission of human immunodeficiency virus among intravenous drug users participating in a syringe/needle exchange program. Scandinavian Journal of Infectious Diseases. 2000;32(3):253-8.
12. Blomé MA, Björkman P, Flamholc L, Jacobsson H, Molnegren V, Widell A. Minimal transmission of HIV despite persistently high transmission of hepatitis C virus in a Swedish needle exchange program. Journal of viral hepatitis. 2011;18(12):831-9.
13. Hagan H, McGough JP, Thiede H, Weiss NS, Hopkins S, Alexander ER. Syringe exchange and risk of infection with hepatitis B and C viruses. American journal of epidemiology. 1999;149(3):203-13.
14. Crofts N, Aitken CK. Incidence of bloodborne virus infection and risk behaviours in a cohort of injecting drug users in Victoria in 1990-1995. Medical Journal of Australia. 1997;167(1):17-20.
15. Roy K, Goldberg D, Taylor A, Hutchinson S, MacDonald L, Wilson K, et al. A method to detect the incidence of hepatitis C infection among injecting drug users in Glasgow 1993–98. Journal of Infection. 2001;43(3):200-5.

16. Abou-Saleh M, Davis P, Rice P, Checinski K, Drummond C, Maxwell D, et al. The effectiveness of behavioural interventions in the primary prevention of hepatitis C amongst injecting drug users: a randomised controlled trial and lessons learned. Harm reduction journal. 2008;5(1):1.
17. Turner KM, Hutchinson S, Vickerman P, Hope V, Craine N, Palmateer N, et al. The impact of needle and syringe provision and opiate substitution therapy on the incidence of hepatitis C virus in injecting drug users: pooling of UK evidence. Addiction. 2011;106(11):1978-88.
18. Grebely J, Lima VD, Marshall BD, Milloy M, DeBeck K, Montaner J, et al. Declining incidence of hepatitis C virus infection among people who inject drugs in a Canadian setting, 1996-2012. PloS one. 2014;9(6):e97726.
19. Foley S, Abou-Saleh MT. Risk behaviors and transmission of hepatitis C in injecting drug users. Addictive Disorders & Their Treatment. 2009;8(1):13-21.
20. Craine N, Hickman M, Parry J, Smith J, Walker A, Russell D, et al. Incidence of hepatitis C in drug injectors: the role of homelessness, opiate substitution treatment, equipment sharing, and community size. Epidemiology and Infection. 2009;137(09):1255-65.
21. Villano SA, Vlahov D, Nelson KE, Lyles CM, Cohn S, Thomas DL. Incidence and risk factors for hepatitis C among injection drug users in Baltimore, Maryland. Journal of clinical microbiology. 1997;35(12):3274-7.
22. Maher L, Jalaludin B, Chant KG, Jayasuriya R, Sladden T, Kaldor JM, et al. Incidence and risk factors for hepatitis C seroconversion in injecting drug users in Australia. Addiction. 2006;101(10):1499-508.
23. Lucidarme D, Bruandet A, Ilef D, Harbonnier J, Jacob C, Decoster A, et al. Incidence and risk factors of HCV and HIV infections in a cohort of intravenous drug users in the North and East of France. Epidemiology and infection. 2004;132(04):699-708.
24. Partanen A, Malin K, Perälä R, Harju O, Holopainen A, Holmström P, et al. Riski-tutkimus 2000-2003. Pistämällä huumeita käyttävien seurantatutkimus. A-Klinikkasäätiön Raporttisarja nro 52. Helsinki: A-Klinikkasäätiön, 2006.
25. Van Den Berg C, Smit C, Van Brussel G, Coutinho R, Prins M. Full participation in harm reduction programmes is associated with decreased risk for human immunodeficiency virus and hepatitis C virus: evidence from the Amsterdam Cohort Studies among drug users. Addiction. 2007;102(9):1454-62.
26. Larney S, Kopinski H, Beckwith CG, Zaller ND, Jarlais DD, Hagan H, et al. Incidence and prevalence of hepatitis C in prisons and other closed settings: results of a systematic review and meta-analysis. Hepatology. 2013;58(4):1215-24.
27. Degenhardt L, Mathers B, Vickerman P, Rhodes T, Latkin C, Hickman M. Prevention of HIV infection for people who inject drugs: Why individual, structural, and combination approaches are needed. The Lancet. 2010;376:285-301.
28. Pavarin RM. Cocaine consumption and death risk: a follow-up study on 347 cocaine addicts in the metropolitan area of Bologna. Ann Ist Super Sanita. 2008; 44(1): 91-8.
29. Tyndall MW, Craib KJ, Currie S, Li K, O'Shaughnessy MV, Schechter MT. Impact of HIV infection on mortality in a cohort of injection drug users. J Acquir Immune Defic Syndr. 2001; 28(4): 351-7.
30. Miller CL, Kerr T, Strathdee SA, Li K, Wood E. Factors associated with premature mortality among young injection drug users in Vancouver. Harm Reduct J. 2007; 4: 1.

31. Galli M, Musicco M. Mortality of intravenous drug users living in Milan, Italy: role of HIV-1 infection. COMCAT Study Group. AIDS. 1994; 8(10): 1457-63.
32. Manfredi R, Sabbatani S, Agostini D. Trend of mortality observed in a cohort of drug addicts of the metropolitan area of Bologna, North-Eastern Italy, during a 25-year-period. Coll Antropol. 2006; 30(3): 479-88.
33. Eskild A, Magnus P, Samuelsen SO, Sohlberg C, Kittelsen P. Differences in mortality rates and causes of death between HIV positive and HIV negative intravenous drug users. Int J Epidemiol. 1993; 22(2): 315-20.
34. Ødegård E, Amundsen EJ, Kielland KB. Fatal overdoses and deaths by other causes in a cohort of Norwegian drug abusers – a competing risk approach. Drug Alcohol Depend. 2007; 89(2-3): 176-82.
35. Rossow I. Suicide among drug addicts in Norway. Addiction. 1994; 89(12): 1667-73.
36. Risser D, Hönigschnabl S, Stichenwirth M, Pfudl S, Sebald D, Kaff A, Bauer G. Mortality of opiate users in Vienna, Austria. Drug Alcohol Depend. 2001; 64(3): 251-6.
37. Bartu A, Freeman NC, Gawthorne GS, Codde JP, Holman CDJ. Mortality in a cohort of opiate and amphetamine users in Perth, Western Australia. Addiction. 2004; 99(1): 53-60.
38. Degenhardt L, Randall D, Hall W, Law M, Butler T, Burns L. Mortality among clients of a state-wide opioid pharmacotherapy program over 20 years: risk factors and lives saved. Drug Alcohol Depend. 2009; 105(1): 9–15.
39. Tait RJ, Ngo HTT, Hulse GK. Mortality in heroin users 3 years after naltrexone implant or methadone maintenance treatment. J Subst Abuse Treat. 2008; 35(2): 116-24.
40. Vlahov D, Galai N, Safaeian M, Galea S, Kirk GD, Lucas GM, Sterling TR. Effectiveness of highly active antiretroviral therapy among injection drug users with late-stage human immunodeficiency virus infection. Am J Epidemiol. 2005; 161(11): 999-1012.
41. Vlahov D, Wang C, Ompad D, Fuller CM, Caceres W, Ouellet L, Kerndt P, Jarlais DCD, Garfein RS, Collaborative Injection Drug User Study. Mortality risk among recent-onset injection drug users in five U.S. cities. Subst Use Misuse. 2008; 43(3-4): 413-28.
42. Oppenheimer E, Tobutt C, Taylor C, Andrew T. Death and survival in a cohort of heroin addicts from London clinics: a 22-year follow-up study. Addiction. 1994; 89(10): 1299-308.
43. Goldstein A, Herrera J. Heroin addicts and methadone treatment in Albuquerque: a 22-year follow-up. Drug Alcohol Depend. 1995; 40(2): 139-50.
44. Soyka M, Apelt SM, Lieb M, Wittchen H-U. One-year mortality rates of patients receiving methadone and buprenorphine maintenance therapy: a nationally representative cohort study in 2694 patients. J Clin Psychopharmacol. 2006; 26(6): 657-60.
45. Fugelstad A, Agren G, Romelsjö A. Changes in mortality, arrests, and hospitalizations in nonvoluntarily treated heroin addicts in relation to methadone treatment. Subst Use Misuse. 1998; 33(14): 2803-17.
46. Stenbacka M, Leifman A, Romelsjo A. Mortality Among Opiate Abusers in Stockholm: A Longitudinal Study. Heroin Addict Relate Clin Probl. 2007; 9(3): 41-50.
47. Fugelstad A, Annell A, Rajs J, Agren G. Mortality and causes and manner of death among drug addicts in Stockholm during the period 1981-1992. Acta Psychiatr Scand. 1997; 96(3): 169-75.

48. Antolini G, Pirani M, Morandi G, Sorio C. [Gender difference and mortality in a cohort of heroin users in the Provinces of Modena and Ferrara, 1975-1999]. Epidemiol Prev. 2006; 30(2): 91-9.
49. Digiusto E, Shakeshaft A, Ritter A, O'Brien S, Mattick RP, NEPOD Research Group. Serious adverse events in the Australian National Evaluation of Pharmacotherapies for Opioid Dependence (NEPOD). Addiction. 2004; 99(4): 450-60.
50. Brancato V, Delvecchio G, Simone P. [Survival and mortality in a cohort of heroin addicts in 1985-1994]. Minerva Med. 1995; 86(3): 97-9.
51. Wang C, Vlahov D, Galai N, Cole SR, Bareta J, Pollini R, Mehta SH, Nelson KE, Galea S. The effect of HIV infection on overdose mortality. AIDS. 2005; 19(9): 935-42.
52. Auckloo MBKM, Davies BB. Post-mortem toxicology in violent fatalities in Capte Town, South Africa: A preliminary investigation. J Foresnsic Leg Med. 2019; 63:18-25.
53. Brådvik L. Suicide risk and mental disorders. Int J Environ Res Publ Health. 2019; 15(9):2028.
54 Merrall E, Bird S, Hutchinson SJ. A record-linkage study of drug-related death and suicide after hospital discharge among drug-treatment clients in Scotland, 1996-2006. Addiction. 2012; 102(2).

High alcohol use

Flowchart

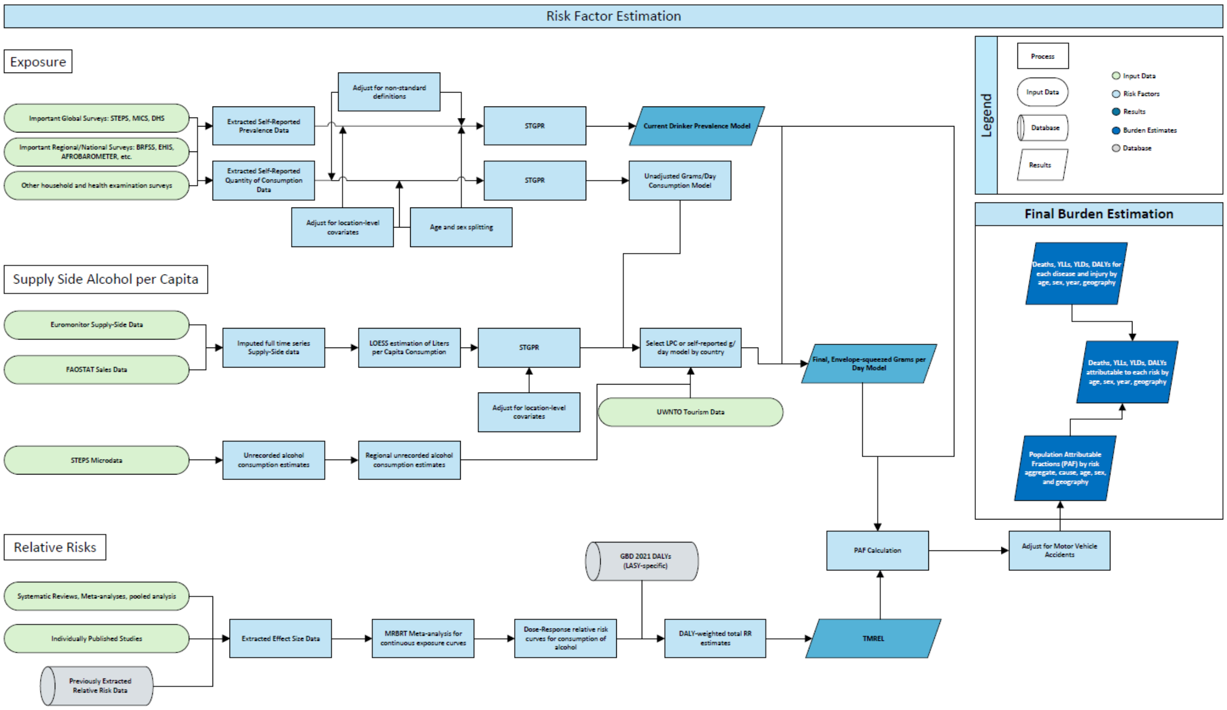

Input data and methodological summary

Definition

Exposure

As considered within the GBD, high alcohol use refers to alcohol consumption greater than the theoretical minimum risk exposure level (TMREL), which is the calculated grams per day exposure level of alcohol consumption at which the relevant weighted all-cause risk curve is minimised. Because the TMREL is estimated specific to age, sex, location, and year, the number of persons exposed to high alcohol use as a risk factor is variable. The definitions and modelling strategies for high alcohol use as a risk factor are largely consistent with methods used in GBD 2021.

We define high alcohol use exposure as typical consumption in grams per day of pure alcohol consumed among current drinkers (ie, not former or never drinkers). This exposure is calculated by coalescing three major estimates:

1. **The percentage of current drinkers within a population**, the proportion of individuals who have consumed at least one alcoholic beverage (~10 g ethanol) within the past year.
2. **The typical amount of pure alcohol consumed**, in grams per day, by current drinkers in the past year.
3. **The amount of alcohol stock**, in liters per capita (LPC) of pure alcohol, purchased over a 12-month period for a given location.

a. **Adjusted for number of tourists –** the number of total visitors to a location within a 12-month period.
b. **Adjusted for length of tourist stay –** the average number of days tourists reside in the given location.
c. **Adjusted for unrecorded alcohol stock –** the percentage of total alcohol stock estimated to be available that was produced outside of traditional, trackable markets.

Input data

Exposure

The GHDx, IHME's internal data catalog, was searched for any applicable sources containing information regarding individual alcohol consumption. Data sources were considered for inclusion if they 1) described a representative sample of the population at the relevant geographical location and 2) contained pertinent alcohol-consumption data. To ensure completeness, key survey series for major countries were identified and searched individually for additional sources: DHS, MICS, and STEPS. Data were extracted using R and Winnower.

At the supply-side level, four major data sources are used to generate estimates: FAOSTAT,[1] Euromonitor,[2] UNWTO,[4] and STEPS.[42]

Relative risk

This systematic review was performed searching four databases: PubMed, CINAHL, EMBASE, and Web of Science. For each outcome, a best, most-recent systematic review was determined. These reviews were citation searched, and the end date of the relevant search was used as the beginning date for our updated search. This constituted a "pragmatic systematic review", which greatly improved the efficiency of our search.

Below is a table with each modelled outcome and the relevant systematic review considered as a basis for our review.

Table 1: Best previously published systematic review for each outcome

outcome	Best MASR	Dates of review	#underlying sources
**A-fib**	Gallagher, Celine et al.<br>Alcohol and incident atrial fibrillation – A systematic review and meta-analysis<br>*International Journal of Cardiology*, Volume 246, 46 – 52	2/1/2016	30
**Breast cancer**	Poorolajal J, Heidarimoghis F, Karami M, Cheraghi Z, Gohari-Ensaf F, Shahbazi F, Zareie B, Ameri P, Sahraee F. Factors for the Primary Prevention of Breast Cancer: A Meta-Analysis of Prospective Cohort Studies. *J Res Health Sci*. 2021 Jul 20;21(3):e00520. doi: 10.34172/jrhs.2021.57. PMID: 34698654; PMCID: PMC8957681.	1/1/2021	53

**Cirrhosis**	Glyn-Owen K, Böhning D, Parkes J, Roderick P, Buchanan R, 2021. The combined effect of alcohol and body mass index on risk of chronic liver disease: A systematic review and meta-analysis of cohort studies. *Liver International* 41, 1216–1226. https://doi.org/10.1111/liv.14754	6/17/2020	9
**colorectal cancer**	Ubago-Guisado E; Rodríguez-Barranco M; ChingLópez A; Petrova D; MolinaMontes E; Amiano P; BarricarteGurrea A; Chirlaque M-D; Agudo A; Sánchez M-J. Evidence Update on the Relationship between Diet and the Most Common Cancers from the European Prospective Investigation into Cancer and Nutrition (EPIC) Study: A Systematic Review. *Nutrients* 2021, 13, 3582. https:// doi.org/10.3390/nu13103582	3/1/2021	110
**Dementia**	Dementia prevention, intervention, and care: 2020 report of the *Lancet* Commission Livingston, Gill et al. *The Lancet*, Volume 396, Issue 10248, 413 - 446	9/8/2018	7
**Diabetes**	Schlesinger S, Neuenschwander M, Ballon A, et al. *J Epidemiol Community Health* 2020;74:481–487.	9/1/2019	19
**Epilepsy**	Walsh S, Donnan J, Fortin Y, Sikora L, Morrissey A, Collins K, MacDonald D. A systematic review of the risk factors associated with the onset and natural progression of epilepsy. *NeuroToxicology*. 2017; 61:64-77. ISSN 0161-813X. https://doi.org/10.1016/j.neuro.2016.03.011.	2/1/2013	8
**Oesophageal cancer**	Sun LP, Yan LB, Liu ZZ, Zhao WJ, Zhang CX, Chen YM, Lao XQ, Liu X. Dietary factors and risk of mortality among patients with esophageal cancer: a systematic review. *BMC Cancer*. 2020 Apr 6;20(1):287. doi: 10.1186/s12885-020-06767-8. PMID: 32252671; PMCID: PMC7137267.	1/1/2020	15
**Larynx cancer**	Islami F, Tramacere I, Rota M, Bagnardi V, Fedirko V, Scotti L, Garavello W, Jenab M, Corrao G, Straif K, Negri E, Boffetta P, La Vecchia C. Alcohol drinking and laryngeal cancer: Overall and dose–risk relation – A systematic review and meta-analysis. Oral Oncology. 2010; 46(11):802-810. ISSN 1368-8375. https://doi.org/10.1016/j.oraloncology.2010.07.015. (https://www.sciencedirect.com/science/article/pii/S1368837510002319)	5/13/2010	40

**Mouth cancer**	Bagnardi V, Rota M, Botteri E, et al. Alcohol consumption and site-specific cancer risk: a comprehensive dose–response meta-analysis. *Br J Cancer* 112, 580–593 (2015). https://doi.org/10.1038/bjc.2014.579	7/9/2021	572
**Other pharynx cancer**	Batistella EÂ, Gondak R, Rivero ERC, et al. Comparison of tobacco and alcohol consumption in young and older patients with oral squamous cell carcinoma: a systematic review and meta-analysis. *Clin Oral Invest* 26, 6855–6869 (2022). https://doi-org.offcampus.lib.washington.edu/10.1007/s00784-022-04719-z	1/1/2013	38
**Hypertensive heart disease**	Benenson I, Waldron FA, Jadotte YT, Dreker M, Holly C. Risk factors for hypertensive crisis in adult patients: a systematic review. *JBI Evidence* Synthesis 19(6):p 1292-1327, June 2021. | DOI: 10.11124/JBIES-20-00243	12/1/2019	19
**Ischaemic heart disease**	Ding C, O'Neill D, Bell S, et al. Association of alcohol consumption with morbidity and mortality in patients with cardiovascular disease: original data and meta-analysis of 48,423 men and women. *BMC Med* 19, 167 (2021). https://doi.org/10.1186/s12916-021-02040-2	7/30/2021	14
**Injuries**	Valentin G, Ravn M, Jensen E, et al. Socio-economic inequalities in fragility fracture incidence: a systematic review and meta-analysis of 61 observational studies. *Osteoporosis Int* 32, 2433–2448 (2021). https://doi.org/10.1007/s00198-021-06038-7	4/28/2021	61
**Interpersonal violence**	Wright MMM, Kankkunen PM, Jokiniemi KS, 2023. Prevention interventions for interpersonal violence occurring under the influence of alcohol: A mixed method systematic review. *Journal of Advanced Nursing* 79, 1247–1266. https://doi.org/10.1111/jan.15335	10/31/2021	16
**Liver cancer**	Park H, Shin SK, Joo I, Song DS, Jang JW, Park J-W, 2020. Systematic Review with Meta-Analysis: Low-Level Alcohol Consumption and the Risk of Liver Cancer. *Gut and Liver* 14, 792–807. https://doi.org/10.5009/gnl19163	7/1/2019	28
**Lower respiratory infection**	Wood S, Harrison SE, Judd N, Bellis MA, Hughes K, Jones A. 2021. The impact of behavioural risk factors on communicable diseases: a systematic review of reviews. *BMC Public Health* 21. https://doi.org/10.1186/s12889-021-12148-y	10/28/2020	14

**Pancreatitis**	Samokhvalov AV, Rehm J, Roerecke M. Alcohol Consumption as a Risk Factor for Acute and Chronic Pancreatitis: A Systematic Review and a Series of Meta-analyses. *EBioMedicine*. 2015 Nov 14;2(12):1996-2002. doi: 10.1016/j.ebiom.2015.11.023. PMID: 26844279; PMCID: PMC4703772.	5/1/2015	3
**Pancreatic cancer**	Lu P-Y, Shu L, Shen S-S, Chen X-J, Zhang X-Y, 2017. Dietary Patterns and Pancreatic Cancer Risk: A Meta-Analysis. *Nutrients* 9, 38. https://doi.org/10.3390/nu9010038	5/1/2016	32
**Prostate cancer**	Hong S, Khil H, Lee DH, Keum N, Giovannucci EL, 2020. Alcohol Consumption and the Risk of Prostate Cancer: A Dose-Response Meta-Analysis. *Nutrients* 12, 2188. https://doi.org/10.3390/nu12082188	4/1/2020	11
**Self-harm**	Isaacs JY, Smith MM, Sherry SB, Seno M, Moore ML, Stewart SH, 2022. Alcohol use and death by suicide: A meta-analysis of 33 studies. *Suicide and Life-Threatening Behavior* 52, 600–614. https://doi.org/10.1111/sltb.12846	12/1/2020	33
**Stomach cancer**	Deng W, Jin L, Zhuo H, Vasiliou V, Zhang Y. Alcohol consumption and risk of stomach cancer: A meta-analysis. *Chemico-Biological Interactions*. 2021; 336: 109365. ISSN 0009-2797. https://doi.org/10.1016/j.cbi.2021.109365. (https://www.sciencedirect.com/science/article/pii/S0009279721000016)	6/1/2019	81
**Ischaemic stroke**	Wang S, Zou XL, Wu LX, Zhou HF, Xiao L, Yao T, Zhang Y, Ma J, Zeng Y, Zhang L. Epidemiology of intracerebral hemorrhage: A systematic review and meta-analysis. *Front Neurol*. 2022 Sep 16;13:915813. doi: 10.3389/fneur.2022.915813. PMID: 36188383; PMCID: PMC9523083.	1/1/2020	52
**Haemorrhagic stroke**	Wang S, Zou XL, Wu LX, Zhou HF, Xiao L, Yao T, Zhang Y, Ma J, Zeng Y, Zhang L. Epidemiology of intracerebral hemorrhage: A systematic review and meta-analysis. *Front Neurol*. 2022 Sep 16;13:915813. doi: 10.3389/fneur.2022.915813. PMID: 36188383; PMCID: PMC9523083.	1/1/2020	52
**Tuberculosis**	Wood S, Harrison SE, Judd N, Bellis MA, Hughes K, Jones A. 2021. The impact of behavioural risk factors on communicable diseases: a systematic review of reviews. *BMC Public Health* 21. https://doi.org/10.1186/s12889-021-12148-y	10/28/2020	44

Sources from all four databases were combined and deduplicated using DistillerSR's[17] matching programme with a confidence level of 63%. Any studies that flagged as matching below a 63% confidence level were individually reviewed for duplication.

Once deduplicated, sources were screened by two independent screeners using DistillerSR's[17] prioritisation algorithm. When 50% of studies were screened, we enabled the programme's AI screening tool to screen the remaining sources, using a 0.5 likelihood of inclusion threshold.

Data processing

Any current drinking estimates that were not already specific to age and sex were split using the following technique. First, sources reporting data for both sexes were split using a region-specific sex ratio estimated by calculating the mean ratio of males to females for each location and then applying this ratio to the sample size. Sources which did not report data in GBD-relevant age groups were split into standard five-year age groups using the global age pattern from ST-GPR.

To include additional data that did not meet our reference definition for current drinking, but still contained valuable alcohol consumption data, two crosswalks were performed using MR-BRT. The first crosswalk converted estimates of one-month drinking prevalence to what they would be if data represented estimates of 12-month drinking prevalence. This crosswalk incorporated two binary covariates: male sex and age ≥50. The second crosswalk converted estimates of one-week drinking prevalence to 12-month drinking prevalence. This crosswalk incorporated age <20 and male sex as covariates. The covariates used in both crosswalks were included as both x and z covariates. A uniform prior of zero was set as the upper bound for the beta coefficients to enforce the logical constraint that one-month and one-week prevalence could not be greater than 12-month prevalence.

Table 2: MR-BRT crosswalk adjustment factors for alcohol use current drinking model

**Data input**	**Reference or alternative case definition**	**Gamma**	**Beta coefficient, logit (95% UI)***	**Adjustment factor****
12-month prevalence	Ref	---	---	---
1-month prevalence	Alt	0.22	–0.60 (–1.05, –0.16)	0.55 (0.35, 0.85)
Age ≥50		0.13	0.16 (–0.10, 0.43)	1.17 (0.9, 1.54)
Male		0.29	0.01 (–0.57, 0.59)	1.01 (0.57, 1.8)
1-week prevalence	Alt	0.46	–1.51 (–2.42, –0.59)	0.22 (0.09, 0.55)

Age <20		0.47	–0.29 (–1.34, 0.76)	0.75 (0.26, 2.14)
Male		0.00	0.38 (0.15, 0.60)	1.46 (1.16, 1.82)

***MR-BRT crosswalk adjustments can be interpreted as the factor the alternative case definition is adjusted by to reflect what it would have been had it been measured using the reference case definition. If the log/logit beta coefficient is negative, then the alternative is adjusted up to the reference. If the log/logit beta coefficient is positive, then the alternative is adjusted down to the reference.**
****The adjustment factor column is the exponentiated beta coefficient. For log beta coefficients, this is the relative rate between the two case definitions. For logit beta coefficients, this is the relative odds between the two case definitions.***

The raw data included in the supply-side model are domestic supply (FAO[1]) and retail supply (Euromonitor[2]) of litres of pure ethanol consumed. Domestic supply is calculated as the sum of production and imports, subtracting exports. In GBD 2021, WHO GISAH[3] estimates were included in the domestic supply estimate; however, after thorough examination, we did not include WHO GISAH data in GBD 2023 as they do not include any additional primary data inputs apart from FAO and Euromonitor, which we are already considering. Where necessary, the following adjustments were made when alcohol consumption was presented more granularly than in pure ethanol.

$$LPC\ Pure\ Ethanol = 0.13 * \left(\frac{Wine}{0.973}\right)$$

$$LPC\ Pure\ Ethanol = 0.05 * \left(\frac{Beer}{0.989}\right)$$

$$LPC\ Pure\ Ethanol = 0.4 * \left(\frac{Spirits}{0.91}\right)$$

We then outliered the data systematically using the following techniques: First, we calculated the grams of alcohol consumed per drinker per day using the current drinker model. A point was outliered if the grams of pure ethanol per drinker per day estimate for a given source-location-year was greater than 100 (approximately ten standard drinks). These thresholds were selected by consulting expert knowledge about reasonable consumption levels. In the second round of outliering, the mean LPC value over a ten-year window was calculated. If an individual datapoint differed from the calculated mean value by greater than 70% of the calculated mean value, it was considered an outlier. This is consistent with our methodology in previous GBD rounds. Manual outliering was performed and reviewed by multiple team members to account for edge cases. In previous years, data smoothing was performed by taking a three-year rolling mean over each location-year; however, for GBD 2023, this process resulted in masking fluctuations during the global COVID-19 pandemic and was thus abandoned to preserve these fluctuations.

An imputation to fill in missing years was performed for all series to compensate for compositional bias from our final estimates. Because our main sources report data from different time periods, imputing a complete time series for each data series reduces the probability that compositional bias of the sources

results in biased final estimates. To impute missing years for each series, we modelled the log ratio of each pair of sources as a function of an intercept and nested random effects on super-region, region, and location. The corresponding predicted ratio was applied to the relevant existing source data, generating an estimated value for the missing source. For locations where there was limited overlap between series, the predicted ratio appeared nonsensical, and thus a regional ratio was used.

Finally, variance was calculated both across series (within a location-year) as well as across years (within a location-source). The average estimates in each location-year served as the input to an ST-GPR model. This used a mixed-effects model modelled in log space with nested location random effects.

We obtained data on the number of tourists and their duration of stay from the UN World Tourism Organization.[4] We applied a crosswalk across different tourist categories, mimicking the technique applied to the LPC data, to arrive at a consistent definition (ie, visitors to a country).

In GBD 2021, we used unrecorded alcohol stock estimates from the WHO GISAH database[2] to generate an overall estimate of alcohol stock available in the country. However, after significant review, we were not able to assess what the primary data input for the WHO GISAH estimates is. So, in GBD 2023, we explored an alternative source of data for unrecorded consumption, based on all of the STEPS[5] surveys where respondents were asked questions about unrecorded alcohol consumption. These individual surveys were combined to create regional models of unrecorded alcohol consumption, which were subsequently used to calculate overall alcohol stock.

Modelling strategy

Exposure

Alcohol exposure was constructed by combining supply-side alcohol purchase data and self-reported consumption data to provide comprehensive estimates of alcohol consumption at the individual level. Because of the risk of underreporting bias, we considered LPC as a better estimate of overall volume of consumption when compared to self-reported consumption.

Self-reported grams per day alcohol consumption data was adjusted (as detailed above) for age, sex, and definition, and then modelled in ST-GPR to produce complete age/sex/location/year-specific estimates. Similarly, a binary (% current drinker) model was generated using self-reported consumption data and ST-GPR.

The litres per capita estimates (LPC) were modelled in STGPR to generate age/sex/location/year-specific estimates. Then, the tourism and unrecorded adjustments were applied as ratios.

We adjusted the alcohol LPC for unrecorded consumption using the following equation:

$$Alcohol\ LPC = \frac{Alcohol\ LPC}{(1 - \%\ Unrecorded)}$$

We then adjusted the estimates for alcohol LPC for tourist consumption by adding in the per capita rate of consumption abroad and subtracting the per capita rate of tourist consumption domestically.

$$Alcohol\ LPC_d = Unadjusted\ Alcohol\ LPC_d + Alcohol\ LPC_{Domestic\ consumption\ abroad} - Alcohol\ LPC_{Tourist\ consumption\ domestically}$$

$$Alcohol\ LPC_i = \frac{\sum_l Tourist\ Population_l * Proportion\ of\ tourists_{i,l} * Unadjusted\ Alcohol\ LPC_l * \frac{Average\ length\ of\ stay_{i,l}}{365} *}{Population_d}$$

where:

l *is the set of all locations*, i *is either* domestic consumption abroad *or* tourist consumption domestically, *and* d *is a domestic location.*

After adjusting alcohol LPC by tourist consumption and unrecorded consumption for all location/years reported, sex-specific and age-specific estimates were generated by incorporating estimates modelled in ST-GPR to determine the overall percentage of current drinkers within a location/year/sex/age. Consumption trends were also modelled in ST-GPR using the grams per day model by calculating the proportion of total consumption for a given location/year by age and sex, using the estimates of alcohol consumed per day, the population size, and the percentage of current drinkers. We then multiplied this proportion of total stock for a given location/year/sex/age by the total stock for a given location/year to calculate the consumption in terms of LPC for a given location/year/sex/age. We then convert these estimates to be in terms of grams/per day. The following equations describe these calculations:

$$Proportion\ of\ total\ consumption_{l,y,s,a} = \frac{Alcohol\ \ g/day_{l,y,s,a} * Population_{l,y,s,a} * \%\ Current\ drinkers_{l,y,s,a}}{\sum_{s,a} Alcohol\ \ g/day_{l,y,s,a} * Population_{l,y,s,a} * \%\ Current\ drinkers_{l,y,s,a}}$$

$$Alcohol\ LPC_{l,y,s,a} = \frac{Alcohol\ LPC_{l,y} * Population_{l,y} * Proportion\ of\ total\ consumption_{l,y,s,a}}{\%\ Current\ drinkers_{l,y,s,a} * Population_{l,y,s,a}}$$

$$Alcohol\ g/day_{l,y,s,a} = Alcohol\ LPC_{l,y,s,a} * \frac{789\ g/L}{365}$$

where:

$$l \text{ is a location}, y \text{ is a year}, s \text{ is a sex}, \text{and } a \text{ is an age group}.$$

Finally, we used the gamma distribution to estimate individual-level variation within location, year, sex, age drinking populations, following the recommendations of other published alcohol studies.[7,8] We chose parameters of the gamma distribution based on the mean and standard deviation of the 1000 draws of alcohol g/day exposure for a given population. Standard deviation was calculated using the following formula:

$$standard\ deviation = mean * (0.087 * female + 1.171)$$

Theoretical minimum risk exposure level

TMREL is an acronym for the "theoretical minimum risk exposure level", or, in the case of alcohol, the amount of alcohol a person can/should drink (based on their age, sex, location) to accrue the minimal risk possible. This occurs at the point at which the overall weighted relative risk curve (which is specific to age-sex-location-decade) is minimised. For populations which have a larger weighted burden from outcomes for which alcohol is protective, for example, older people who have a heightened burden from IHD, the overall weighted RR curve is j-shaped, and thus the TMREL >0. This is due to the complicated nature of alcohol consumption on relative risk. In the case of tobacco, which is always harmful, the TMREL would be 0. The same cannot be said for alcohol because there is a body of evidence (see "relative risk") suggesting that alcohol may be somewhat protective against vascular outcomes.

Calculating the TMREL is how we get from abstracted relative risk curves to concrete estimates of how alcohol affects populations. We weight each respective RR curve by its relative burden and then combine these to get an overall, all-cause RR curve. The x-value (exposure) at which this weighted curve is minimized is the TMREL (definition). See equation below.

$$TMREL = argmin\ average\ overall\ risk_{\omega}(g/day)$$

$$Average\ overall\ risk_{\omega,l,y,a,s}(g/day) = \sum_{i}^{\omega} \log\left(RR_i(g/day)\right) * \frac{DALY_{i,l,y,a,s}}{\sum_i^{\omega} DALY_{i,l,y,a,s}}$$

Where:

$$\omega \text{ is the set of causes associated with alcohol}, i \text{ is a given cause from that set},$$

$$l \text{ is a location}, y \text{ is a year}, s \text{ is a sex}, a \text{ is an age group}, DALY \text{ is the DALY rate},$$

$$\text{and } RR \text{ is the dose response curve for a given cause and exposure level in grams per day}.$$

For the GBD, we calculate the burden due to alcohol consumption among those consuming GREATER than the TMREL, or "high alcohol users". Thus, when we calculate PAFs, which are used further

downstream to calculate the attributable burden of high alcohol use, everything is defined by the relationship to the TMREL.

In GBD 2021, we started calculating TMRELs that are specific to age, sex, super-region and year. Since the RR of 20 outcomes were updated for GBD 2023, the resulting TMRELs were also updated. **Table 3** details the TMRELs calculated for GBD 2021, and the new TMRELs for GBD 2023 for the year 2020.

Table 3: Theoretical minimum risk exposure level

Region	Age group	sex_id	GBD 2021	GBD 2023	change_since_2021
Andean Latin America	15 to 19	2	0.616	0	-0.616
Andean Latin America	15 to 19	1	0.139	0	-0.139
Andean Latin America	20 to 24	1	0.133	0	-0.133
Andean Latin America	20 to 24	2	0.897	0	-0.897
Andean Latin America	25 to 29	1	0.229	0	-0.229
Andean Latin America	25 to 29	2	1.703	0	-1.703
Andean Latin America	30 to 34	1	0.721	0	-0.721
Andean Latin America	30 to 34	2	2.346	0.004	-2.342
Andean Latin America	35 to 39	1	1.463	0.004	-1.459
Andean Latin America	35 to 39	2	3.008	0.012	-2.996
Andean Latin America	40 to 44	1	2.593	0.02	-2.573
Andean Latin America	40 to 44	2	3.8	0.128	-3.672
Andean Latin America	45 to 49	1	3.848	0.308	-3.54
Andean Latin America	45 to 49	2	4.873	1.016	-3.857
Andean Latin America	50 to 54	2	5.76	3.16	-2.6
Andean Latin America	50 to 54	1	4.897	1.512	-3.385
Andean Latin America	55 to 59	1	5.816	3.624	-2.192
Andean Latin America	55 to 59	2	6.444	6.096	-0.348
Andean Latin America	60 to 64	1	6.333	6.048	-0.285
Andean Latin America	60 to 64	2	6.911	8.648	1.737
Andean Latin America	65 to 69	1	6.739	7.608	0.869
Andean Latin America	65 to 69	2	7.12	10.152	3.032
Andean Latin America	70 to 74	1	6.919	9.024	2.105
Andean Latin America	70 to 74	2	7.165	11.72	4.555
Andean Latin America	75 to 79	1	6.961	10.8	3.839
Andean Latin America	75 to 79	2	7.153	13.232	6.079
Andean Latin America	80 to 84	2	7.215	16.408	9.193
Andean Latin America	80 to 84	1	7.072	13.272	6.2
Andean Latin America	85 to 89	2	7.388	20.664	13.276
Andean Latin America	85 to 89	1	7.349	16.772	9.423
Andean Latin America	90 to 94	1	7.881	21.156	13.275
Andean Latin America	90 to 94	2	7.866	24.672	16.806

Andean Latin America	95 plus	1	8.452	26.536	18.084
Andean Latin America	95 plus	2	8.834	28.66	19.826
Australasia	15 to 19	1	0.06	0.12	0.06
Australasia	15 to 19	2	0.029	0.08	0.051
Australasia	20 to 24	1	0.143	0.16	0.017
Australasia	20 to 24	2	0.049	0.08	0.031
Australasia	25 to 29	1	0.203	0.12	-0.083
Australasia	25 to 29	2	0.066	0.04	-0.026
Australasia	30 to 34	1	0.492	0.12	-0.372
Australasia	30 to 34	2	0.188	0	-0.188
Australasia	35 to 39	1	1.482	0.04	-1.442
Australasia	35 to 39	2	0.509	0	-0.509
Australasia	40 to 44	2	1.135	0	-1.135
Australasia	40 to 44	1	2.941	0.268	-2.673
Australasia	45 to 49	2	1.964	0	-1.964
Australasia	45 to 49	1	4.14	0.628	-3.512
Australasia	50 to 54	1	4.993	1.468	-3.525
Australasia	50 to 54	2	2.789	0.016	-2.773
Australasia	55 to 59	2	3.807	0.208	-3.599
Australasia	55 to 59	1	5.495	2.292	-3.203
Australasia	60 to 64	1	6.101	3.84	-2.261
Australasia	60 to 64	2	4.699	1.764	-2.935
Australasia	65 to 69	1	6.708	5.656	-1.052
Australasia	65 to 69	2	5.335	6.464	1.129
Australasia	70 to 74	2	5.79	12.332	6.542
Australasia	70 to 74	1	7.025	8.66	1.635
Australasia	75 to 79	1	7.431	12.64	5.209
Australasia	75 to 79	2	6.088	17.524	11.436
Australasia	80 to 84	1	8.074	19.372	11.298
Australasia	80 to 84	2	6.298	24.68	18.382
Australasia	85 to 89	2	6.686	27.72	21.034
Australasia	85 to 89	1	9.389	24.16	14.771
Australasia	90 to 94	2	7.533	29.496	21.963
Australasia	90 to 94	1	10.738	27.232	16.494
Australasia	95 plus	1	12.118	30.432	18.314
Australasia	95 plus	2	9.162	31.264	22.102
Caribbean	15 to 19	2	0.764	0.004	-0.76
Caribbean	15 to 19	1	0.1	0.12	0.02
Caribbean	20 to 24	1	0.164	0.12	-0.044
Caribbean	20 to 24	2	1.753	0.084	-1.669
Caribbean	25 to 29	1	0.321	0.188	-0.133

Caribbean	25 to 29	2	2.797	0.136	-2.661
Caribbean	30 to 34	1	1.05	0.16	-0.89
Caribbean	30 to 34	2	3.894	0.664	-3.23
Caribbean	35 to 39	1	2.628	0.236	-2.392
Caribbean	35 to 39	2	4.806	1.832	-2.974
Caribbean	40 to 44	2	5.703	4.272	-1.431
Caribbean	40 to 44	1	4.216	0.652	-3.564
Caribbean	45 to 49	1	5.406	2.92	-2.486
Caribbean	45 to 49	2	6.433	7.5	1.067
Caribbean	50 to 54	2	7.043	9.744	2.701
Caribbean	50 to 54	1	6.374	5.904	-0.47
Caribbean	55 to 59	1	6.974	7.828	0.854
Caribbean	55 to 59	2	7.497	11.156	3.659
Caribbean	60 to 64	1	7.332	9.84	2.508
Caribbean	60 to 64	2	7.858	12.572	4.714
Caribbean	65 to 69	2	7.982	13.384	5.402
Caribbean	65 to 69	1	7.697	11.364	3.667
Caribbean	70 to 74	1	7.743	12.132	4.389
Caribbean	70 to 74	2	7.742	13.876	6.134
Caribbean	75 to 79	2	7.639	14.604	6.965
Caribbean	75 to 79	1	7.779	13.14	5.361
Caribbean	80 to 84	2	7.519	16.824	9.305
Caribbean	80 to 84	1	7.88	14.712	6.832
Caribbean	85 to 89	1	8.136	17.488	9.352
Caribbean	85 to 89	2	7.796	20.52	12.724
Caribbean	90 to 94	1	8.47	21.792	13.322
Caribbean	90 to 94	2	8.033	23.936	15.903
Caribbean	95 plus	1	9.16	26.072	16.912
Caribbean	95 plus	2	8.584	28.04	19.456
Central Asia	15 to 19	1	0.212	0	-0.212
Central Asia	15 to 19	2	1.121	0.06	-1.061
Central Asia	20 to 24	1	0.416	0	-0.416
Central Asia	20 to 24	2	2.207	0.04	-2.167
Central Asia	25 to 29	1	0.921	0	-0.921
Central Asia	25 to 29	2	2.931	0	-2.931
Central Asia	30 to 34	1	2.242	0	-2.242
Central Asia	30 to 34	2	3.439	0.036	-3.403
Central Asia	35 to 39	1	3.513	0.024	-3.489
Central Asia	35 to 39	2	4.02	0.22	-3.8
Central Asia	40 to 44	1	4.663	0.544	-4.119
Central Asia	40 to 44	2	4.707	0.892	-3.815

Central Asia	45 to 49	1	5.683	3.156	-2.527
Central Asia	45 to 49	2	5.351	3.616	-1.735
Central Asia	50 to 54	1	6.716	7.52	0.804
Central Asia	50 to 54	2	5.948	6.576	0.628
Central Asia	55 to 59	1	7.529	10.472	2.943
Central Asia	55 to 59	2	6.683	9.372	2.689
Central Asia	60 to 64	1	8.227	13.38	5.153
Central Asia	60 to 64	2	7.512	12.612	5.1
Central Asia	65 to 69	1	9.369	15.772	6.403
Central Asia	65 to 69	2	8.519	15.06	6.541
Central Asia	70 to 74	1	10.186	17.816	7.63
Central Asia	70 to 74	2	9.214	17.856	8.642
Central Asia	75 to 79	1	13.794	22.396	8.602
Central Asia	75 to 79	2	12.349	22.592	10.243
Central Asia	80 to 84	1	13.897	26.812	12.915
Central Asia	80 to 84	2	13.571	27.544	13.973
Central Asia	85 to 89	1	14.935	31.752	16.817
Central Asia	85 to 89	2	13.594	31.788	18.194
Central Asia	90 to 94	1	16.62	34.504	17.884
Central Asia	90 to 94	2	14.341	35.388	21.047
Central Asia	95 plus	1	17.313	36.736	19.423
Central Asia	95 plus	2	15.186	37.188	22.002
Central Europe	15 to 19	2	0.072	0	-0.072
Central Europe	15 to 19	1	0.015	0	-0.015
Central Europe	20 to 24	1	0.083	0.04	-0.043
Central Europe	20 to 24	2	0.411	0	-0.411
Central Europe	25 to 29	1	0.237	0	-0.237
Central Europe	25 to 29	2	1.176	0	-1.176
Central Europe	30 to 34	2	1.943	0	-1.943
Central Europe	30 to 34	1	1.094	0.04	-1.054
Central Europe	35 to 39	1	2.6	0	-2.6
Central Europe	35 to 39	2	2.672	0	-2.672
Central Europe	40 to 44	1	3.789	0.144	-3.645
Central Europe	40 to 44	2	3.55	0.028	-3.522
Central Europe	45 to 49	2	4.33	0.348	-3.982
Central Europe	45 to 49	1	4.619	0.868	-3.751
Central Europe	50 to 54	1	5.097	2.12	-2.977
Central Europe	50 to 54	2	4.957	1.344	-3.613
Central Europe	55 to 59	1	5.761	3.772	-1.989
Central Europe	55 to 59	2	5.67	3.816	-1.854
Central Europe	60 to 64	1	6.261	6.184	-0.077

Central Europe	60 to 64	2	6.244	7.304	1.06
Central Europe	65 to 69	1	6.649	8.46	1.811
Central Europe	65 to 69	2	6.608	10.16	3.552
Central Europe	70 to 74	1	6.942	10.684	3.742
Central Europe	70 to 74	2	6.773	12.36	5.587
Central Europe	75 to 79	1	7.284	12.52	5.236
Central Europe	75 to 79	2	7.043	14.02	6.977
Central Europe	80 to 84	1	8.178	15.532	7.354
Central Europe	80 to 84	2	7.758	17.292	9.534
Central Europe	85 to 89	1	9.848	20.332	10.484
Central Europe	85 to 89	2	9.105	22.436	13.331
Central Europe	90 to 94	1	12.037	27.176	15.139
Central Europe	90 to 94	2	10.514	27.768	17.254
Central Europe	95 plus	2	12.741	31.78	19.039
Central Europe	95 plus	1	14.241	31.276	17.035
Central Latin America	15 to 19	1	0.195	0.36	0.165
Central Latin America	15 to 19	2	0.208	0.04	-0.168
Central Latin America	20 to 24	1	0.267	0.4	0.133
Central Latin America	20 to 24	2	0.664	0.124	-0.54
Central Latin America	25 to 29	1	0.352	0.44	0.088
Central Latin America	25 to 29	2	1.795	0.112	-1.683
Central Latin America	30 to 34	1	0.638	0.508	-0.13
Central Latin America	30 to 34	2	3.302	0.4	-2.902
Central Latin America	35 to 39	2	4.698	1.252	-3.446
Central Latin America	35 to 39	1	1.596	0.488	-1.108
Central Latin America	40 to 44	2	6.103	3.744	-2.359
Central Latin America	40 to 44	1	3.407	0.776	-2.631
Central Latin America	45 to 49	1	5.314	2.232	-3.082
Central Latin America	45 to 49	2	7.05	7.392	0.342
Central Latin America	50 to 54	2	7.698	9.896	2.198
Central Latin America	50 to 54	1	6.735	5.388	-1.347
Central Latin America	55 to 59	1	7.677	8.172	0.495
Central Latin America	55 to 59	2	8.136	11.624	3.488
Central Latin America	60 to 64	2	8.411	13.128	4.717
Central Latin America	60 to 64	1	8.246	10.452	2.206
Central Latin America	65 to 69	1	8.638	12.364	3.726
Central Latin America	65 to 69	2	8.626	14.224	5.598
Central Latin America	70 to 74	2	8.572	15.536	6.964
Central Latin America	70 to 74	1	8.697	14.008	5.311
Central Latin America	75 to 79	2	8.616	17.32	8.704
Central Latin America	75 to 79	1	8.876	15.74	6.864

Central Latin America	80 to 84	1	9.145	19.54	10.395
Central Latin America	80 to 84	2	8.753	21.928	13.175
Central Latin America	85 to 89	1	10.013	25.52	15.507
Central Latin America	85 to 89	2	9.584	27.42	17.836
Central Latin America	90 to 94	1	12.145	30.596	18.451
Central Latin America	90 to 94	2	11.625	32.528	20.903
Central Latin America	95 plus	1	14.575	35.56	20.985
Central Latin America	95 plus	2	14.027	36.376	22.349
Central sub-Saharan Africa	15 to 19	1	0.137	0	-0.137
Central sub-Saharan Africa	15 to 19	2	2.121	0	-2.121
Central sub-Saharan Africa	20 to 24	2	3.548	0	-3.548
Central sub-Saharan Africa	20 to 24	1	0.195	0	-0.195
Central sub-Saharan Africa	25 to 29	2	4.586	0	-4.586
Central sub-Saharan Africa	25 to 29	1	0.63	0	-0.63
Central sub-Saharan Africa	30 to 34	1	1.273	0	-1.273
Central sub-Saharan Africa	30 to 34	2	4.757	0	-4.757
Central sub-Saharan Africa	35 to 39	1	2.112	0	-2.112
Central sub-Saharan Africa	35 to 39	2	5.183	0	-5.183
Central sub-Saharan Africa	40 to 44	2	5.027	0	-5.027
Central sub-Saharan Africa	40 to 44	1	3.528	0	-3.528
Central sub-Saharan Africa	45 to 49	1	4.635	0.008	-4.627
Central sub-Saharan Africa	45 to 49	2	5.337	0.148	-5.189
Central sub-Saharan Africa	50 to 54	1	5.557	0.184	-5.373
Central sub-Saharan Africa	50 to 54	2	5.71	0.656	-5.054
Central sub-Saharan Africa	55 to 59	2	6.109	2.948	-3.161
Central sub-Saharan Africa	55 to 59	1	5.987	1.148	-4.839
Central sub-Saharan Africa	60 to 64	1	6.247	3.932	-2.315
Central sub-Saharan Africa	60 to 64	2	6.243	5.848	-0.395
Central sub-Saharan Africa	65 to 69	2	6.246	7.856	1.61
Central sub-Saharan Africa	65 to 69	1	6.21	5.772	-0.438
Central sub-Saharan Africa	70 to 74	2	6.256	9.592	3.336
Central sub-Saharan Africa	70 to 74	1	6.487	7.732	1.245
Central sub-Saharan Africa	75 to 79	1	6.499	8.744	2.245
Central sub-Saharan Africa	75 to 79	2	6.251	10.556	4.305
Central sub-Saharan Africa	80 to 84	1	6.756	10.82	4.064
Central sub-Saharan Africa	80 to 84	2	6.319	12.084	5.765
Central sub-Saharan Africa	85 to 89	1	6.709	12.372	5.663
Central sub-Saharan Africa	85 to 89	2	6.36	14.672	8.312
Central sub-Saharan Africa	90 to 94	1	6.886	14.924	8.038
Central sub-Saharan Africa	90 to 94	2	6.509	17.848	11.339
Central sub-Saharan Africa	95 plus	1	7.045	18.44	11.395

Central sub-Saharan Africa	95 plus	2	6.421	21.96	15.539
East Asia	15 to 19	1	0.362	0	-0.362
East Asia	15 to 19	2	1.119	0	-1.119
East Asia	20 to 24	1	0.868	0	-0.868
East Asia	20 to 24	2	2.116	0.1	-2.016
East Asia	25 to 29	2	3.007	0.388	-2.619
East Asia	25 to 29	1	1.715	0.016	-1.699
East Asia	30 to 34	2	3.437	0.476	-2.961
East Asia	30 to 34	1	2.818	0.088	-2.73
East Asia	35 to 39	1	3.522	0.28	-3.242
East Asia	35 to 39	2	3.788	0.568	-3.22
East Asia	40 to 44	2	4.196	1.072	-3.124
East Asia	40 to 44	1	4.025	0.692	-3.333
East Asia	45 to 49	1	4.3	1.388	-2.912
East Asia	45 to 49	2	4.678	2.756	-1.922
East Asia	50 to 54	1	4.587	2.696	-1.891
East Asia	50 to 54	2	5.048	5.14	0.092
East Asia	55 to 59	2	5.299	7.76	2.461
East Asia	55 to 59	1	4.81	4.32	-0.49
East Asia	60 to 64	1	5.017	6.26	1.243
East Asia	60 to 64	2	5.554	9.928	4.374
East Asia	65 to 69	1	5.13	7.972	2.842
East Asia	65 to 69	2	5.692	11.456	5.764
East Asia	70 to 74	1	5.248	9.404	4.156
East Asia	70 to 74	2	5.788	12.56	6.772
East Asia	75 to 79	1	5.4	11.136	5.736
East Asia	75 to 79	2	5.935	14.244	8.309
East Asia	80 to 84	2	6.351	18.276	11.925
East Asia	80 to 84	1	5.861	13.74	7.879
East Asia	85 to 89	2	6.729	22.492	15.763
East Asia	85 to 89	1	6.398	15.136	8.738
East Asia	90 to 94	1	7.228	19.044	11.816
East Asia	90 to 94	2	7.789	27.924	20.135
East Asia	95 plus	1	7.158	26.484	19.326
East Asia	95 plus	2	8.876	31.928	23.052
Eastern Europe	15 to 19	1	0.1	0.16	0.06
Eastern Europe	15 to 19	2	0.134	0	-0.134
Eastern Europe	20 to 24	1	0.136	0.16	0.024
Eastern Europe	20 to 24	2	0.315	0	-0.315
Eastern Europe	25 to 29	1	0.315	0.1	-0.215
Eastern Europe	25 to 29	2	0.8	0	-0.8

Eastern Europe	30 to 34	1	1.109	0.052	-1.057
Eastern Europe	30 to 34	2	1.559	0	-1.559
Eastern Europe	35 to 39	1	2.432	0.044	-2.388
Eastern Europe	35 to 39	2	2.549	0.004	-2.545
Eastern Europe	40 to 44	1	3.621	0.388	-3.233
Eastern Europe	40 to 44	2	3.419	0.052	-3.367
Eastern Europe	45 to 49	1	4.478	1.536	-2.942
Eastern Europe	45 to 49	2	4.18	0.524	-3.656
Eastern Europe	50 to 54	1	5.342	4.532	-0.81
Eastern Europe	50 to 54	2	4.832	2.132	-2.7
Eastern Europe	55 to 59	1	6.241	8.868	2.627
Eastern Europe	55 to 59	2	5.715	5.852	0.137
Eastern Europe	60 to 64	1	7.788	13.384	5.596
Eastern Europe	60 to 64	2	6.695	11.072	4.377
Eastern Europe	65 to 69	1	9.606	16.496	6.89
Eastern Europe	65 to 69	2	8.169	15.564	7.395
Eastern Europe	70 to 74	1	12.072	19.556	7.484
Eastern Europe	70 to 74	2	10.777	20.456	9.679
Eastern Europe	75 to 79	1	15.222	24.04	8.818
Eastern Europe	75 to 79	2	13.726	25.78	12.054
Eastern Europe	80 to 84	1	17.694	29.964	12.27
Eastern Europe	80 to 84	2	15.801	31.356	15.555
Eastern Europe	85 to 89	1	19.613	33.82	14.207
Eastern Europe	85 to 89	2	17.282	35.216	17.934
Eastern Europe	90 to 94	2	19.234	37.12	17.886
Eastern Europe	90 to 94	1	21.062	36.3	15.238
Eastern Europe	95 plus	1	21.873	37.352	15.479
Eastern Europe	95 plus	2	20.883	38.256	17.373
Eastern sub-Saharan Africa	15 to 19	1	0.239	0	-0.239
Eastern sub-Saharan Africa	15 to 19	2	1.537	0	-1.537
Eastern sub-Saharan Africa	20 to 24	2	2.687	0	-2.687
Eastern sub-Saharan Africa	20 to 24	1	0.452	0	-0.452
Eastern sub-Saharan Africa	25 to 29	1	0.93	0	-0.93
Eastern sub-Saharan Africa	25 to 29	2	3.039	0	-3.039
Eastern sub-Saharan Africa	30 to 34	1	2.739	0	-2.739
Eastern sub-Saharan Africa	30 to 34	2	3.691	0	-3.691
Eastern sub-Saharan Africa	35 to 39	1	3.129	0	-3.129
Eastern sub-Saharan Africa	35 to 39	2	4.076	0	-4.076
Eastern sub-Saharan Africa	40 to 44	1	3.58	0	-3.58
Eastern sub-Saharan Africa	40 to 44	2	4.026	0.008	-4.018
Eastern sub-Saharan Africa	45 to 49	1	4.266	0.012	-4.254

Eastern sub-Saharan Africa	45 to 49	2	4.37	0.14	-4.23
Eastern sub-Saharan Africa	50 to 54	1	4.571	0.048	-4.523
Eastern sub-Saharan Africa	50 to 54	2	4.611	0.212	-4.399
Eastern sub-Saharan Africa	55 to 59	1	4.916	0.564	-4.352
Eastern sub-Saharan Africa	55 to 59	2	5.002	1.6	-3.402
Eastern sub-Saharan Africa	60 to 64	1	5.06	3.224	-1.836
Eastern sub-Saharan Africa	60 to 64	2	5.189	4.016	-1.173
Eastern sub-Saharan Africa	65 to 69	1	5.042	4.416	-0.626
Eastern sub-Saharan Africa	65 to 69	2	5.236	5.872	0.636
Eastern sub-Saharan Africa	70 to 74	1	5.434	5.532	0.098
Eastern sub-Saharan Africa	70 to 74	2	5.45	7.432	1.982
Eastern sub-Saharan Africa	75 to 79	1	5.469	6.452	0.983
Eastern sub-Saharan Africa	75 to 79	2	5.495	9.02	3.525
Eastern sub-Saharan Africa	80 to 84	1	5.777	8.036	2.259
Eastern sub-Saharan Africa	80 to 84	2	5.626	10.16	4.534
Eastern sub-Saharan Africa	85 to 89	1	5.819	10.108	4.289
Eastern sub-Saharan Africa	85 to 89	2	5.776	12.408	6.632
Eastern sub-Saharan Africa	90 to 94	1	5.944	11.384	5.44
Eastern sub-Saharan Africa	90 to 94	2	5.722	15.596	9.874
Eastern sub-Saharan Africa	95 plus	1	5.998	15.72	9.722
Eastern sub-Saharan Africa	95 plus	2	5.617	16.784	11.167
High-income Asia Pacific	15 to 19	2	0.433	0.52	0.087
High-income Asia Pacific	15 to 19	1	0.179	0.36	0.181
High-income Asia Pacific	20 to 24	1	0.743	1.08	0.337
High-income Asia Pacific	20 to 24	2	0.888	0.868	-0.02
High-income Asia Pacific	25 to 29	1	1.149	1.12	-0.029
High-income Asia Pacific	25 to 29	2	1.225	0.964	-0.261
High-income Asia Pacific	30 to 34	1	1.955	1.256	-0.699
High-income Asia Pacific	30 to 34	2	1.533	0.724	-0.809
High-income Asia Pacific	35 to 39	1	2.908	1.304	-1.604
High-income Asia Pacific	35 to 39	2	1.945	0.472	-1.473
High-income Asia Pacific	40 to 44	2	2.557	0.412	-2.145
High-income Asia Pacific	40 to 44	1	3.803	1.552	-2.251
High-income Asia Pacific	45 to 49	1	4.362	2.056	-2.306
High-income Asia Pacific	45 to 49	2	3.219	0.496	-2.723
High-income Asia Pacific	50 to 54	2	3.75	0.984	-2.766
High-income Asia Pacific	50 to 54	1	4.622	2.512	-2.11
High-income Asia Pacific	55 to 59	1	4.796	2.96	-1.836
High-income Asia Pacific	55 to 59	2	4.378	2.752	-1.626
High-income Asia Pacific	60 to 64	1	5.003	4.02	-0.983
High-income Asia Pacific	60 to 64	2	5.046	7.044	1.998

High-income Asia Pacific	65 to 69	1	5.107	4.64	-0.467
High-income Asia Pacific	65 to 69	2	5.31	10.284	4.974
High-income Asia Pacific	70 to 74	1	5.215	6.676	1.461
High-income Asia Pacific	70 to 74	2	5.479	14.86	9.381
High-income Asia Pacific	75 to 79	1	5.3	9.028	3.728
High-income Asia Pacific	75 to 79	2	5.571	18.964	13.393
High-income Asia Pacific	80 to 84	1	5.415	14.18	8.765
High-income Asia Pacific	80 to 84	2	5.637	25.652	20.015
High-income Asia Pacific	85 to 89	1	5.556	17.648	12.092
High-income Asia Pacific	85 to 89	2	5.651	28.156	22.505
High-income Asia Pacific	90 to 94	1	5.785	20.804	15.019
High-income Asia Pacific	90 to 94	2	5.798	28.936	23.138
High-income Asia Pacific	95 plus	2	6.014	30.212	24.198
High-income Asia Pacific	95 plus	1	6.031	26.404	20.373
High-income North America	15 to 19	1	0.132	0.24	0.108
High-income North America	15 to 19	2	0.207	0.08	-0.127
High-income North America	20 to 24	1	0.168	0.24	0.072
High-income North America	20 to 24	2	0.359	0.12	-0.239
High-income North America	25 to 29	2	0.951	0	-0.951
High-income North America	25 to 29	1	0.319	0.24	-0.079
High-income North America	30 to 34	2	2.106	0	-2.106
High-income North America	30 to 34	1	1.19	0.16	-1.03
High-income North America	35 to 39	2	3.176	0.028	-3.148
High-income North America	35 to 39	1	3.049	0.204	-2.845
High-income North America	40 to 44	1	4.746	0.724	-4.022
High-income North America	40 to 44	2	4.187	0.22	-3.967
High-income North America	45 to 49	1	5.891	2.472	-3.419
High-income North America	45 to 49	2	4.976	0.956	-4.02
High-income North America	50 to 54	1	6.777	4.388	-2.389
High-income North America	50 to 54	2	5.674	2.088	-3.586
High-income North America	55 to 59	1	7.447	5.96	-1.487
High-income North America	55 to 59	2	6.364	4.14	-2.224
High-income North America	60 to 64	1	8.005	8.34	0.335
High-income North America	60 to 64	2	6.887	7.556	0.669
High-income North America	65 to 69	2	7.086	10.736	3.65
High-income North America	65 to 69	1	8.465	10.464	1.999
High-income North America	70 to 74	2	7.054	14.064	7.01
High-income North America	70 to 74	1	8.594	12.78	4.186
High-income North America	75 to 79	1	9.014	15.992	6.978
High-income North America	75 to 79	2	6.978	18.28	11.302
High-income North America	80 to 84	1	9.953	22.712	12.759

High-income North America	80 to 84	2	7.035	25.532	18.497
High-income North America	85 to 89	2	7.682	29.452	21.77
High-income North America	85 to 89	1	11.942	28.22	16.278
High-income North America	90 to 94	1	13.39	32.516	19.126
High-income North America	90 to 94	2	9.038	31.496	22.458
High-income North America	95 plus	2	10.802	32.964	22.162
High-income North America	95 plus	1	14.47	34.984	20.514
North Africa and Middle East	15 to 19	1	0.749	0	-0.749
North Africa and Middle East	15 to 19	2	2.664	0.008	-2.656
North Africa and Middle East	20 to 24	1	0.741	0	-0.741
North Africa and Middle East	20 to 24	2	3.025	0.048	-2.977
North Africa and Middle East	25 to 29	2	3.488	0.24	-3.248
North Africa and Middle East	25 to 29	1	1.677	0.008	-1.669
North Africa and Middle East	30 to 34	2	4.164	0.996	-3.168
North Africa and Middle East	30 to 34	1	2.66	0.164	-2.496
North Africa and Middle East	35 to 39	1	3.725	1.164	-2.561
North Africa and Middle East	35 to 39	2	4.652	2.412	-2.24
North Africa and Middle East	40 to 44	1	5.079	5.524	0.445
North Africa and Middle East	40 to 44	2	5.403	5.4	-0.003
North Africa and Middle East	45 to 49	1	6.954	10.772	3.818
North Africa and Middle East	45 to 49	2	6.39	8.36	1.97
North Africa and Middle East	50 to 54	1	8.516	13.528	5.012
North Africa and Middle East	50 to 54	2	7.226	10.6	3.374
North Africa and Middle East	55 to 59	1	9.401	14.388	4.987
North Africa and Middle East	55 to 59	2	7.984	12.288	4.304
North Africa and Middle East	60 to 64	2	8.598	13.536	4.938
North Africa and Middle East	60 to 64	1	9.495	14.612	5.117
North Africa and Middle East	65 to 69	1	9.575	15.088	5.513
North Africa and Middle East	65 to 69	2	8.921	14.196	5.275
North Africa and Middle East	70 to 74	1	8.897	14.604	5.707
North Africa and Middle East	70 to 74	2	8.734	14.512	5.778
North Africa and Middle East	75 to 79	1	8.815	15.528	6.713
North Africa and Middle East	75 to 79	2	8.844	15.976	7.132
North Africa and Middle East	80 to 84	1	8.889	17.98	9.091
North Africa and Middle East	80 to 84	2	8.632	18.084	9.452
North Africa and Middle East	85 to 89	1	9.09	21.5	12.41
North Africa and Middle East	85 to 89	2	9.218	21.472	12.254
North Africa and Middle East	90 to 94	1	9.456	26.46	17.004
North Africa and Middle East	90 to 94	2	9.503	25.184	15.681
North Africa and Middle East	95 plus	2	10.648	30.564	19.916
North Africa and Middle East	95 plus	1	10.149	30.48	20.331

Oceania	15 to 19	2	2.245	0.004	-2.241
Oceania	15 to 19	1	0.216	0	-0.216
Oceania	20 to 24	1	0.674	0	-0.674
Oceania	20 to 24	2	2.982	0.004	-2.978
Oceania	25 to 29	2	3.963	0.324	-3.639
Oceania	25 to 29	1	2.49	0	-2.49
Oceania	30 to 34	1	4.823	0.236	-4.587
Oceania	30 to 34	2	5.239	1.576	-3.663
Oceania	35 to 39	1	5.827	2.276	-3.551
Oceania	35 to 39	2	6.025	4.196	-1.829
Oceania	40 to 44	2	6.85	8.564	1.714
Oceania	40 to 44	1	7.498	7.944	0.446
Oceania	45 to 49	2	6.957	10.736	3.779
Oceania	45 to 49	1	8.204	10.72	2.516
Oceania	50 to 54	1	8.686	13.02	4.334
Oceania	50 to 54	2	7.367	12.184	4.817
Oceania	55 to 59	1	8.984	14.252	5.268
Oceania	55 to 59	2	7.609	12.836	5.227
Oceania	60 to 64	1	8.815	14.588	5.773
Oceania	60 to 64	2	7.741	14.232	6.491
Oceania	65 to 69	1	8.677	14.456	5.779
Oceania	65 to 69	2	7.704	14.6	6.896
Oceania	70 to 74	1	8.166	14.344	6.178
Oceania	70 to 74	2	7.534	14.648	7.114
Oceania	75 to 79	2	7.145	14.724	7.579
Oceania	75 to 79	1	8.136	14.892	6.756
Oceania	80 to 84	2	6.926	16.6	9.674
Oceania	80 to 84	1	7.884	15.78	7.896
Oceania	85 to 89	2	7.241	19.496	12.255
Oceania	85 to 89	1	8.059	16.9	8.841
Oceania	90 to 94	1	8.718	20.356	11.638
Oceania	90 to 94	2	7.138	22.148	15.01
Oceania	95 plus	1	9.085	25.74	16.655
Oceania	95 plus	2	7.564	28.58	21.016
South Asia	15 to 19	1	0.064	0	-0.064
South Asia	15 to 19	2	1.033	0.06	-0.973
South Asia	20 to 24	2	1.475	0.04	-1.435
South Asia	20 to 24	1	0.427	0	-0.427
South Asia	25 to 29	1	0.943	0	-0.943
South Asia	25 to 29	2	2.414	0.04	-2.374
South Asia	30 to 34	1	2.774	0	-2.774

South Asia	30 to 34	2	3.618	0	-3.618
South Asia	35 to 39	1	3.669	0.016	-3.653
South Asia	35 to 39	2	4.159	0.044	-4.115
South Asia	40 to 44	1	4.977	0.416	-4.561
South Asia	40 to 44	2	5.252	0.676	-4.576
South Asia	45 to 49	2	5.686	3.424	-2.262
South Asia	45 to 49	1	5.838	2.176	-3.662
South Asia	50 to 54	2	6.227	4.936	-1.291
South Asia	50 to 54	1	6.469	5.356	-1.113
South Asia	55 to 59	1	6.951	7.708	0.757
South Asia	55 to 59	2	6.787	8.324	1.537
South Asia	60 to 64	1	7.114	9.748	2.634
South Asia	60 to 64	2	6.775	10.652	3.877
South Asia	65 to 69	1	7.177	11.092	3.915
South Asia	65 to 69	2	6.847	11.612	4.765
South Asia	70 to 74	1	7.406	12.028	4.622
South Asia	70 to 74	2	6.807	11.68	4.873
South Asia	75 to 79	1	7.65	13.392	5.742
South Asia	75 to 79	2	7.185	12.852	5.667
South Asia	80 to 84	1	8.231	15.316	7.085
South Asia	80 to 84	2	7.543	15.304	7.761
South Asia	85 to 89	2	6.941	16.728	9.787
South Asia	85 to 89	1	7.973	17.4	9.427
South Asia	90 to 94	2	6.983	20.472	13.489
South Asia	90 to 94	1	8.266	20.172	11.906
South Asia	95 plus	2	7.058	25.104	18.046
South Asia	95 plus	1	8.295	22.568	14.273
Southeast Asia	15 to 19	2	2.201	0	-2.201
Southeast Asia	15 to 19	1	0.244	0	-0.244
Southeast Asia	20 to 24	1	0.575	0	-0.575
Southeast Asia	20 to 24	2	3.676	0	-3.676
Southeast Asia	25 to 29	1	1.664	0	-1.664
Southeast Asia	25 to 29	2	4.221	0.008	-4.213
Southeast Asia	30 to 34	1	2.987	0.008	-2.979
Southeast Asia	30 to 34	2	4.136	0.156	-3.98
Southeast Asia	35 to 39	1	3.785	0.172	-3.613
Southeast Asia	35 to 39	2	4.539	0.84	-3.699
Southeast Asia	40 to 44	1	4.444	1.32	-3.124
Southeast Asia	40 to 44	2	4.846	2.384	-2.462
Southeast Asia	45 to 49	1	4.813	3.392	-1.421
Southeast Asia	45 to 49	2	5.11	4.932	-0.178

Southeast Asia	50 to 54	1	5.178	5.88	0.702
Southeast Asia	50 to 54	2	5.43	7.22	1.79
Southeast Asia	55 to 59	1	5.39	7.492	2.102
Southeast Asia	55 to 59	2	5.733	8.712	2.979
Southeast Asia	60 to 64	2	5.942	10.168	4.226
Southeast Asia	60 to 64	1	5.599	8.652	3.053
Southeast Asia	65 to 69	1	5.718	9.532	3.814
Southeast Asia	65 to 69	2	6.081	11.44	5.359
Southeast Asia	70 to 74	2	6.076	11.872	5.796
Southeast Asia	70 to 74	1	5.865	10.36	4.495
Southeast Asia	75 to 79	1	5.881	11.06	5.179
Southeast Asia	75 to 79	2	6	12.472	6.472
Southeast Asia	80 to 84	1	6.137	12.212	6.075
Southeast Asia	80 to 84	2	6.122	14.08	7.958
Southeast Asia	85 to 89	1	6.232	13.652	7.42
Southeast Asia	85 to 89	2	6.163	15.932	9.769
Southeast Asia	90 to 94	1	6.479	16.776	10.297
Southeast Asia	90 to 94	2	6.406	19.724	13.318
Southeast Asia	95 plus	2	6.557	23.336	16.779
Southeast Asia	95 plus	1	6.774	21.784	15.01
Southern Latin America	15 to 19	1	0.063	0.12	0.057
Southern Latin America	15 to 19	2	0.116	0	-0.116
Southern Latin America	20 to 24	1	0.089	0.08	-0.009
Southern Latin America	20 to 24	2	0.202	0	-0.202
Southern Latin America	25 to 29	2	0.446	0	-0.446
Southern Latin America	25 to 29	1	0.115	0.04	-0.075
Southern Latin America	30 to 34	2	0.972	0	-0.972
Southern Latin America	30 to 34	1	0.331	0	-0.331
Southern Latin America	35 to 39	1	1.295	0	-1.295
Southern Latin America	35 to 39	2	1.745	0	-1.745
Southern Latin America	40 to 44	1	2.84	0.008	-2.832
Southern Latin America	40 to 44	2	2.832	0	-2.832
Southern Latin America	45 to 49	1	4.104	0.42	-3.684
Southern Latin America	45 to 49	2	3.764	0.116	-3.648
Southern Latin America	50 to 54	1	5.067	1.556	-3.511
Southern Latin America	50 to 54	2	4.673	0.808	-3.865
Southern Latin America	55 to 59	2	5.497	2.212	-3.285
Southern Latin America	55 to 59	1	5.72	3.136	-2.584
Southern Latin America	60 to 64	1	6.213	5.032	-1.181
Southern Latin America	60 to 64	2	6.176	5.504	-0.672
Southern Latin America	65 to 69	1	6.555	6.612	0.057

Southern Latin America	65 to 69	2	6.413	7.676	1.263
Southern Latin America	70 to 74	2	6.456	10.252	3.796
Southern Latin America	70 to 74	1	6.63	8.332	1.702
Southern Latin America	75 to 79	1	6.676	10.192	3.516
Southern Latin America	75 to 79	2	6.408	12.852	6.444
Southern Latin America	80 to 84	2	6.453	18.388	11.935
Southern Latin America	80 to 84	1	6.72	12.76	6.04
Southern Latin America	85 to 89	2	6.569	21.544	14.975
Southern Latin America	85 to 89	1	6.906	15.92	9.014
Southern Latin America	90 to 94	1	7.205	18.904	11.699
Southern Latin America	90 to 94	2	6.879	23.536	16.657
Southern Latin America	95 plus	1	7.569	25.14	17.571
Southern Latin America	95 plus	2	7.455	25.188	17.733
Southern sub-Saharan Africa	15 to 19	2	3.32	0	-3.32
Southern sub-Saharan Africa	15 to 19	1	0.182	0.096	-0.086
Southern sub-Saharan Africa	20 to 24	1	0.194	0.12	-0.074
Southern sub-Saharan Africa	20 to 24	2	4.314	0	-4.314
Southern sub-Saharan Africa	25 to 29	1	0.215	0.08	-0.135
Southern sub-Saharan Africa	25 to 29	2	3.537	0	-3.537
Southern sub-Saharan Africa	30 to 34	1	0.351	0.08	-0.271
Southern sub-Saharan Africa	30 to 34	2	3.063	0	-3.063
Southern sub-Saharan Africa	35 to 39	1	1.117	0.04	-1.077
Southern sub-Saharan Africa	35 to 39	2	3.211	0	-3.211
Southern sub-Saharan Africa	40 to 44	1	2.705	0	-2.705
Southern sub-Saharan Africa	40 to 44	2	3.815	0	-3.815
Southern sub-Saharan Africa	45 to 49	1	4.224	0	-4.224
Southern sub-Saharan Africa	45 to 49	2	4.934	0.532	-4.402
Southern sub-Saharan Africa	50 to 54	1	4.891	0.124	-4.767
Southern sub-Saharan Africa	50 to 54	2	5.685	3.42	-2.265
Southern sub-Saharan Africa	55 to 59	1	5.647	1.248	-4.399
Southern sub-Saharan Africa	55 to 59	2	6.298	6.436	0.138
Southern sub-Saharan Africa	60 to 64	1	6.404	4.984	-1.42
Southern sub-Saharan Africa	60 to 64	2	6.83	9.548	2.718
Southern sub-Saharan Africa	65 to 69	2	6.854	10.6	3.746
Southern sub-Saharan Africa	65 to 69	1	6.426	6.908	0.482
Southern sub-Saharan Africa	70 to 74	1	6.557	8.22	1.663
Southern sub-Saharan Africa	70 to 74	2	6.757	11.444	4.687
Southern sub-Saharan Africa	75 to 79	1	6.581	9.42	2.839
Southern sub-Saharan Africa	75 to 79	2	6.662	11.964	5.302
Southern sub-Saharan Africa	80 to 84	2	6.621	12.828	6.207
Southern sub-Saharan Africa	80 to 84	1	6.699	10.94	4.241

Southern sub-Saharan Africa	85 to 89	1	6.694	11.996	5.302
Southern sub-Saharan Africa	85 to 89	2	6.582	13.856	7.274
Southern sub-Saharan Africa	90 to 94	1	6.722	13.328	6.606
Southern sub-Saharan Africa	90 to 94	2	6.592	15.144	8.552
Southern sub-Saharan Africa	95 plus	2	6.508	17.204	10.696
Southern sub-Saharan Africa	95 plus	1	6.465	14.228	7.763
Tropical Latin America	15 to 19	1	0.352	0.52	0.168
Tropical Latin America	15 to 19	2	0.168	0.08	-0.088
Tropical Latin America	20 to 24	1	0.306	0.44	0.134
Tropical Latin America	20 to 24	2	0.371	0	-0.371
Tropical Latin America	25 to 29	1	0.266	0.36	0.094
Tropical Latin America	25 to 29	2	0.96	0	-0.96
Tropical Latin America	30 to 34	2	2.272	0	-2.272
Tropical Latin America	30 to 34	1	0.38	0.28	-0.1
Tropical Latin America	35 to 39	2	3.521	0.056	-3.465
Tropical Latin America	35 to 39	1	1.031	0.176	-0.855
Tropical Latin America	40 to 44	1	2.505	0.08	-2.425
Tropical Latin America	40 to 44	2	4.694	0.56	-4.134
Tropical Latin America	45 to 49	2	5.682	2.868	-2.814
Tropical Latin America	45 to 49	1	3.823	0.564	-3.259
Tropical Latin America	50 to 54	1	4.829	1.792	-3.037
Tropical Latin America	50 to 54	2	6.423	5.656	-0.767
Tropical Latin America	55 to 59	1	5.634	3.944	-1.69
Tropical Latin America	55 to 59	2	7.091	8.504	1.413
Tropical Latin America	60 to 64	2	7.374	11.748	4.374
Tropical Latin America	60 to 64	1	6.282	6.912	0.63
Tropical Latin America	65 to 69	1	6.693	9.2	2.507
Tropical Latin America	65 to 69	2	7.554	13.576	6.022
Tropical Latin America	70 to 74	2	7.288	15.148	7.86
Tropical Latin America	70 to 74	1	6.809	11.224	4.415
Tropical Latin America	75 to 79	1	6.828	12.788	5.96
Tropical Latin America	75 to 79	2	7.14	16.876	9.736
Tropical Latin America	80 to 84	1	6.832	15.724	8.892
Tropical Latin America	80 to 84	2	6.945	21.032	14.087
Tropical Latin America	85 to 89	1	6.811	18.796	11.985
Tropical Latin America	85 to 89	2	6.822	23.832	17.01
Tropical Latin America	90 to 94	1	6.813	22.116	15.303
Tropical Latin America	90 to 94	2	6.761	26.296	19.535
Tropical Latin America	95 plus	2	6.826	28.328	21.502
Tropical Latin America	95 plus	1	6.895	27.476	20.581
Western Europe	15 to 19	2	0.024	0	-0.024

Western Europe	15 to 19	1	0.013	0	-0.013
Western Europe	20 to 24	2	0.305	0	-0.305
Western Europe	20 to 24	1	0.106	0.12	0.014
Western Europe	25 to 29	2	1.14	0.02	-1.12
Western Europe	25 to 29	1	0.381	0.12	-0.261
Western Europe	30 to 34	1	1.237	0.056	-1.181
Western Europe	30 to 34	2	1.568	0.02	-1.548
Western Europe	35 to 39	1	2.537	0.072	-2.465
Western Europe	35 to 39	2	1.746	0.012	-1.734
Western Europe	40 to 44	1	3.581	0.288	-3.293
Western Europe	40 to 44	2	2.066	0.016	-2.05
Western Europe	45 to 49	1	4.355	0.732	-3.623
Western Europe	45 to 49	2	2.639	0.02	-2.619
Western Europe	50 to 54	1	5.02	1.204	-3.816
Western Europe	50 to 54	2	3.391	0.04	-3.351
Western Europe	55 to 59	1	5.534	1.856	-3.678
Western Europe	55 to 59	2	4.286	0.16	-4.126
Western Europe	60 to 64	2	5.237	1.308	-3.929
Western Europe	60 to 64	1	6.113	3.128	-2.985
Western Europe	65 to 69	2	5.759	4.196	-1.563
Western Europe	65 to 69	1	6.585	4.928	-1.657
Western Europe	70 to 74	1	6.801	8.112	1.311
Western Europe	70 to 74	2	6.018	9.856	3.838
Western Europe	75 to 79	1	7.022	12.176	5.154
Western Europe	75 to 79	2	6.164	14.88	8.716
Western Europe	80 to 84	1	7.218	18.572	11.354
Western Europe	80 to 84	2	6.305	22.832	16.527
Western Europe	85 to 89	1	7.482	21.928	14.446
Western Europe	85 to 89	2	6.414	25.576	19.162
Western Europe	90 to 94	2	6.694	26.392	19.698
Western Europe	90 to 94	1	7.931	24.348	16.417
Western Europe	95 plus	1	8.444	26.76	18.316
Western Europe	95 plus	2	7.081	27.092	20.011
Western sub-Saharan Africa	15 to 19	1	0.169	0	-0.169
Western sub-Saharan Africa	15 to 19	2	1.74	0	-1.74
Western sub-Saharan Africa	20 to 24	1	0.267	0	-0.267
Western sub-Saharan Africa	20 to 24	2	2.21	0	-2.21
Western sub-Saharan Africa	25 to 29	1	0.484	0	-0.484
Western sub-Saharan Africa	25 to 29	2	2.41	0	-2.41
Western sub-Saharan Africa	30 to 34	1	1.488	0	-1.488
Western sub-Saharan Africa	30 to 34	2	3.386	0.02	-3.366

Western sub-Saharan Africa	35 to 39	1	2.163	0	-2.163
Western sub-Saharan Africa	35 to 39	2	3.547	0.036	-3.511
Western sub-Saharan Africa	40 to 44	1	3.597	0.092	-3.505
Western sub-Saharan Africa	40 to 44	2	3.973	0.288	-3.685
Western sub-Saharan Africa	45 to 49	1	4.256	0.74	-3.516
Western sub-Saharan Africa	45 to 49	2	4.671	1.372	-3.299
Western sub-Saharan Africa	50 to 54	1	4.849	2.268	-2.581
Western sub-Saharan Africa	50 to 54	2	4.976	3.544	-1.432
Western sub-Saharan Africa	55 to 59	1	5.121	4.14	-0.981
Western sub-Saharan Africa	55 to 59	2	5.187	5.232	0.045
Western sub-Saharan Africa	60 to 64	1	5.286	6.204	0.918
Western sub-Saharan Africa	60 to 64	2	5.912	7.776	1.864
Western sub-Saharan Africa	65 to 69	1	5.74	7.456	1.716
Western sub-Saharan Africa	65 to 69	2	6.03	9.232	3.202
Western sub-Saharan Africa	70 to 74	1	5.831	8.856	3.025
Western sub-Saharan Africa	70 to 74	2	6.08	9.632	3.552
Western sub-Saharan Africa	75 to 79	1	6.143	9.752	3.609
Western sub-Saharan Africa	75 to 79	2	6.04	10.648	4.608
Western sub-Saharan Africa	80 to 84	1	6.397	11.244	4.847
Western sub-Saharan Africa	80 to 84	2	6.338	12.148	5.81
Western sub-Saharan Africa	85 to 89	1	6.529	12.244	5.715
Western sub-Saharan Africa	85 to 89	2	6.29	13.46	7.17
Western sub-Saharan Africa	90 to 94	1	6.519	13.656	7.137
Western sub-Saharan Africa	90 to 94	2	6.382	16.772	10.39
Western sub-Saharan Africa	95 plus	1	6.583	17.472	10.889
Western sub-Saharan Africa	95 plus	2	6.258	19.448	13.19

Relative risk

Relative risk due to alcohol consumption in grams per day was updated for 20 outcomes in GBD 2023. The following table lists each outcome, along with the MR-BRT model settings used and result of the MR-BRT estimation. Additional details of the methodology are described below the table.

Table 4: MR-BRT RR model settings, scores, and star ratings

Outcome	Model settings	Model score	Star rating	Number of studies

Tuberculosis	right linear tail, monotonically increasing, 2 knots, 1 round outliered	-0.0156	1	9
Oesophageal cancer	right linear tail, monotonically increasing, 2 knots, 2 rounds outliered	0.1373	2	66
Liver cancer	right linear tail, monotonically increasing, 2 knots, 3 rounds outliered	0.0344	2	59
Larynx cancer	right linear tail, monotonically increasing, 2 knots, 0 rounds outliered	0.3591	3	30
Breast cancer	right linear tail, monotonically increasing, 2 knots, 1 round outliered	0.1082	2	68
Colon and rectum cancer	right linear tail, monotonically increasing, 2 knots, 2 rounds outliered	0.1665	3	38

Lip and oral cavity cancer	no linear tail, monotonically increasing, 2 knots, 1 round outliered	0.1759	3	19
Other pharynx cancer	right linear tail, monotonically increasing, 2 knots, 0 rounds outliered	0.7171	5	23
Ischaemic heart disease	left linear tail, no monotonicity constraint, 2 knots, 3 rounds outliered	-0.0687	1	164
Ischaemic stroke	right linear tail, no monotonicity constraint, 2 knots, 3 rounds outliered	-0.0082	1	67
Intracerebral haemorrhage	right linear tail, no monotonicity constraint, 2 knots, 3 rounds outliered	-0.0046	1	57

Atrial fibrillation and flutter	right linear tail, monotonically increasing, 2 knots, 3 rounds outliered	0.0581	2	32
Cirrhosis and other chronic liver diseases	right linear tail, monotonically increasing, 2 knots, 3 rounds outliered	0.3367	3	14
Pancreatitis	no linear tail, monotonically increasing, 2 knots, 1 round outliered	0.1571	3	7
Diabetes mellitus type 2	right linear tail, no monotonicity constraint, 2 knots, 2 rounds outliered	0.0456	2	83
Lower respiratory Infections	no linear tail, monotonically increasing, 2 knots, 1 round outliered	0.0171	2	7

Stomach cancer	right linear tail, monotonically increasing, 2 knots, 3 rounds outliered	-0.0006	1	35
Prostate cancer	right linear tail, monotonically increasing, 2 knots, 1 round outliered	0.0125	2	25
Pancreatic cancer	right linear tail, monotonically increasing, 2 knots, 3 rounds outliered	0.0328	2	16
Alzheimer's disease and other dementias	left linear tail, no monotonicity constraint, 2 knots, 1 round ouliered	0.0037	2	17

Table 5: MR-BRT bias covariates and gamma solutions for alcohol relative risk models by outcome

**Selected bias covariates and gamma solution**		
Health outcome	Selected bias covariates	Gamma solution (mean and sd)
Cirrhosis and other chronic liver diseases	em_age, em_incidence	0.02 (0.03)
Atrial fibrillation and flutter	em_incidence, em_age	0.02 (0.03)

Selected bias covariates and gamma solution		
Health outcome	Selected bias covariates	Gamma solution (mean and sd)
Ischaemic heart disease	None	0.25 (0.06)
Intracerebral haemorrhage	em_sex	0.38 (0.22)
Ischaemic stroke	None	0.4 (0.2)
Diabetes mellitus type 2	None	0.08 (0.08)
Pancreatitis	None	0 (0.02)
Lower respiratory infections	None	0 (0.09)
Breast cancer	None	0 (0.02)
Colon and rectum cancer	em_incidence	0 (0.02)
Oesophageal cancer	em_age	0.24 (0.07)
Larynx cancer	em_age, em_incidence	0.03 (0.04)
Liver cancer	em_incidence, em_age	0.35 (0.14)
Lip and oral cavity cancer	None	0.03 (0.04)
Other pharynx cancer	None	0 (0)
Pancreatic cancer	None	0 (0.07)
Prostate cancer	None	0 (0.09)

Selected bias covariates and gamma solution		
Health outcome	Selected bias covariates	Gamma solution (mean and sd)
Stomach cancer	None	0 (0.24)
Alzheimer's disease and other dementias	em_followup_excl_comment	0.24 (0.17)
Tuberculosis	em_age, em_representativeness	0.12 (0.15)

Table 5. em_age: study does not control for age; em_incidence: extracted OR/RR/HR is for mortality (as opposed to incidence); em_sex: study does not control for sex; em_followup_excl_commen: study excluded participants based on follow-up; em_representativeness: study population is a non-representative sample.

For each outcome, we converted all data into comparable g/day exposure. Relative risk was abstracted from each source and collated for analysis with MR-BRT. The natural log of relative risk for alcohol consumption across all ages and both sexes was calculated using MR-BRT.

Estimating the relative risk (RR) of each outcome listed above occurring as a function of exposure to alcohol consumption in g/day followed the burden of proof approach established by Zheng and colleagues[1,2] and instantiated in the meta-regression—Bayesian, regularised, trimmed (MR-BRT) tool. MR-BRT synthesises input data to generate a RR curve by relying on an ensemble spline method to capture the potentially non-linear shape of the risk–outcome relationship; integrating over varying exposure ranges in different comparison groups; trimming potentially distorting outliers; testing, selecting, and adjusting for bias covariates to account for known heterogeneity in input study-design characteristics (eg, confounding, selection bias, exposure measurement); and quantifying remaining between-study heterogeneity (gamma) through random effects modelling and incorporating this value into uncertainty around the mean RR curve.

MR-BRT further evaluates evidence for small-study effects and generates funnel plots that represent potential risk of publication or reporting bias and generates the burden of proof risk function (BPRF), defined for harmful risks as the 5th and for protective risks as the 95th quantile risk curve – inclusive of between-study heterogeneity/gamma – that is closest to null. The BPRF is transformed into a risk–outcome score (ROS: the signed value of the average log BPRF between the 15th and 85th percentiles of risk exposure levels observed across included studies) and mapped onto a star-rating system from one to five stars. These metrics complement RR estimates by providing an alternative, conservative measure of effect size and evidence strength that formally and systematically incorporates divergence/convergence across input findings, with higher positive ROS values and more stars corresponding to incrementally larger effects and stronger evidence for the risk–outcome relationship.

Relative risk curves

Atrial fibrillation

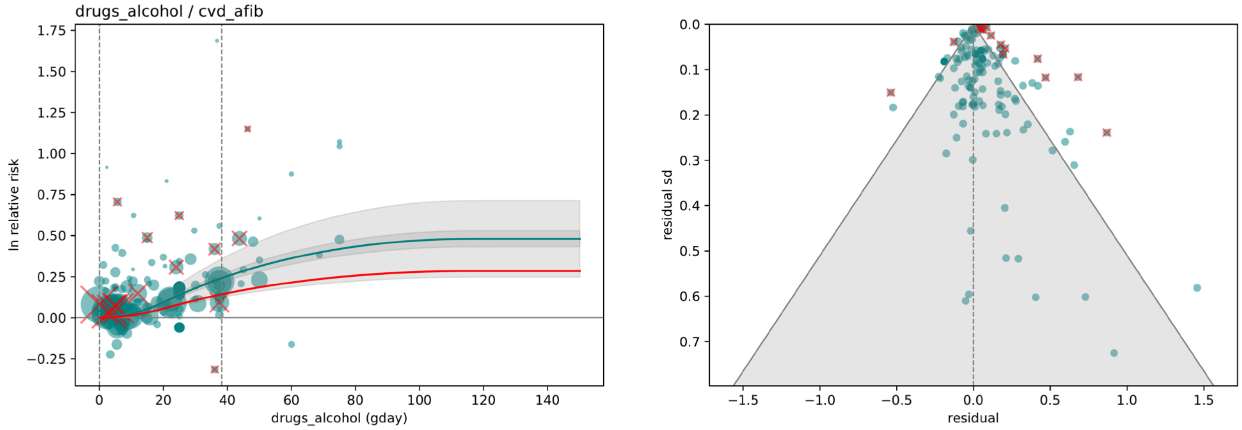

Breast cancer

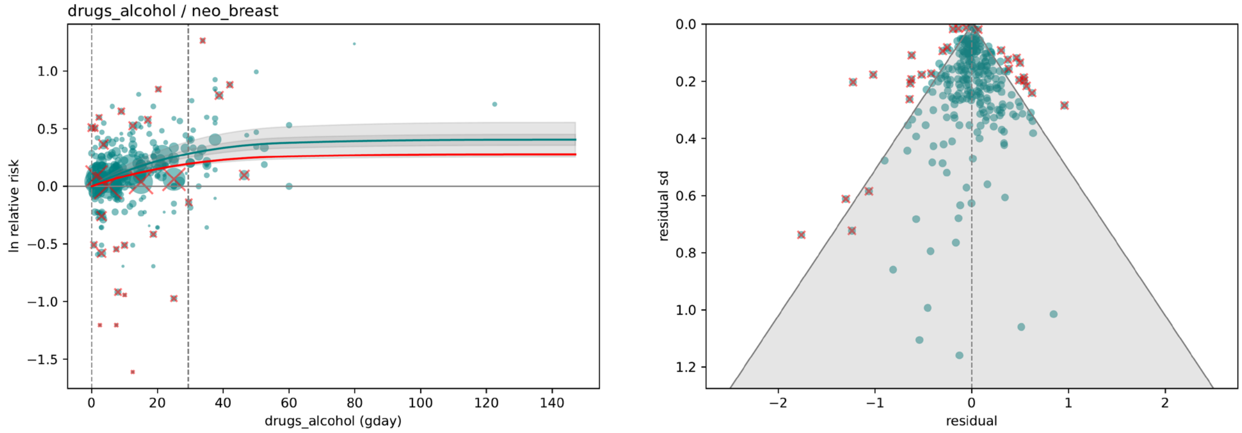

Cirrhosis

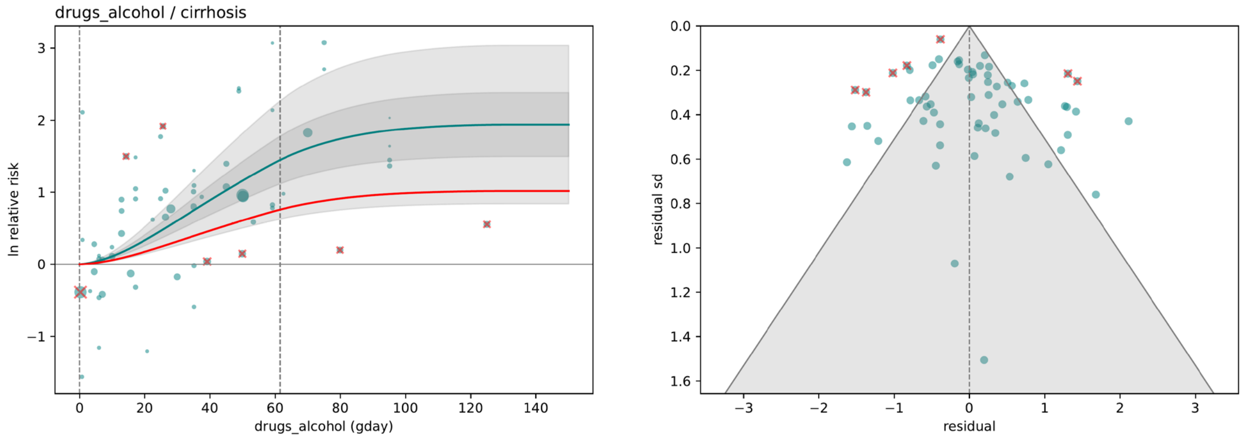

Colorectal cancer

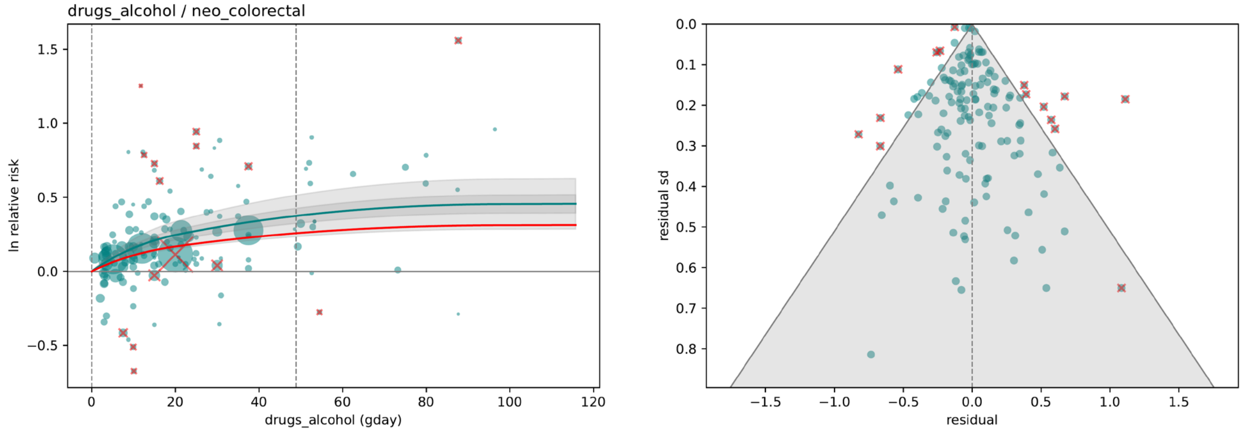

Dementia

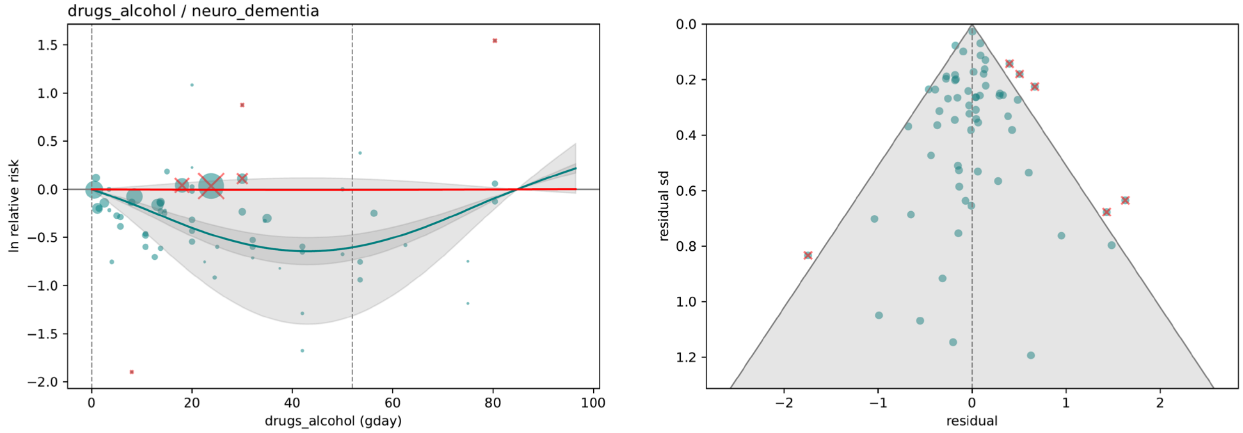

Diabetes

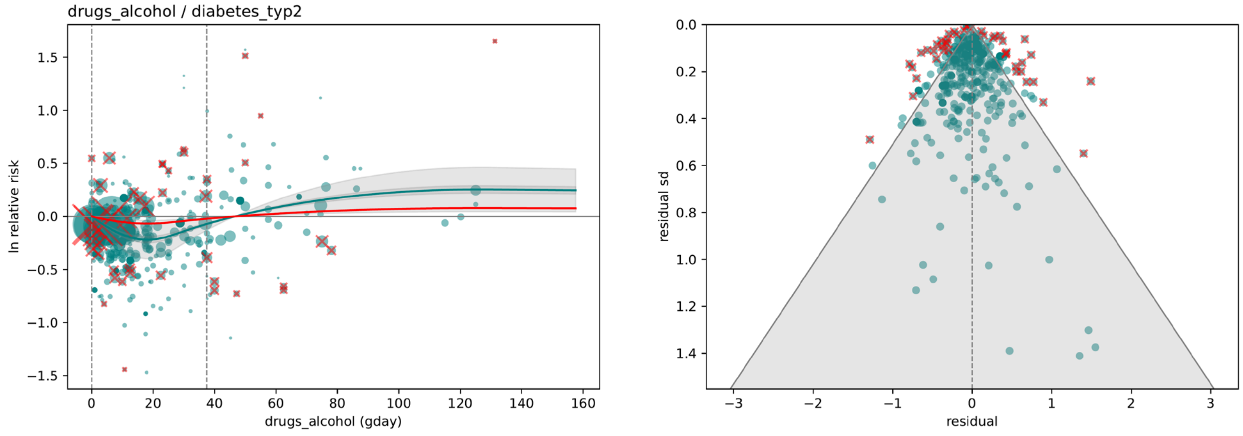

Oesophageal cancer

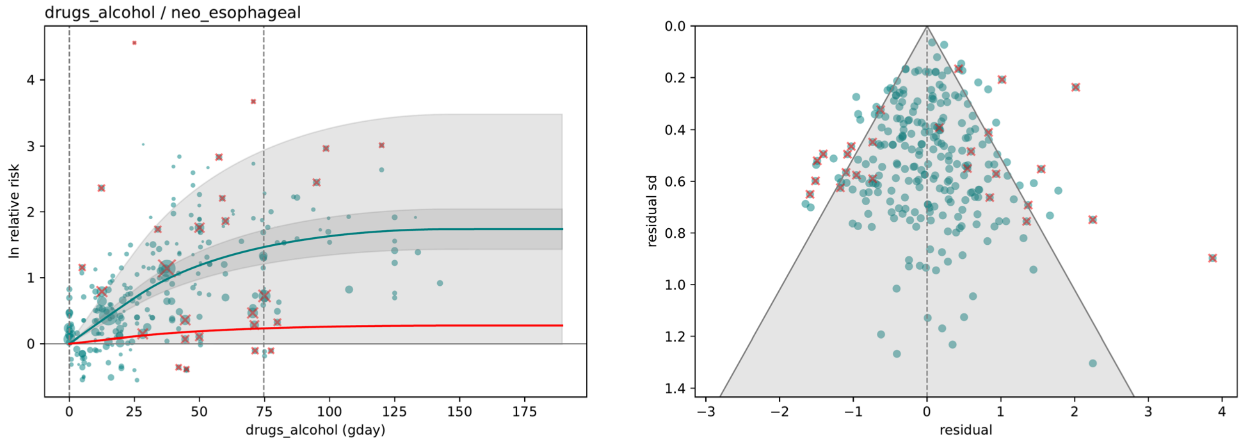

Larynx cancer

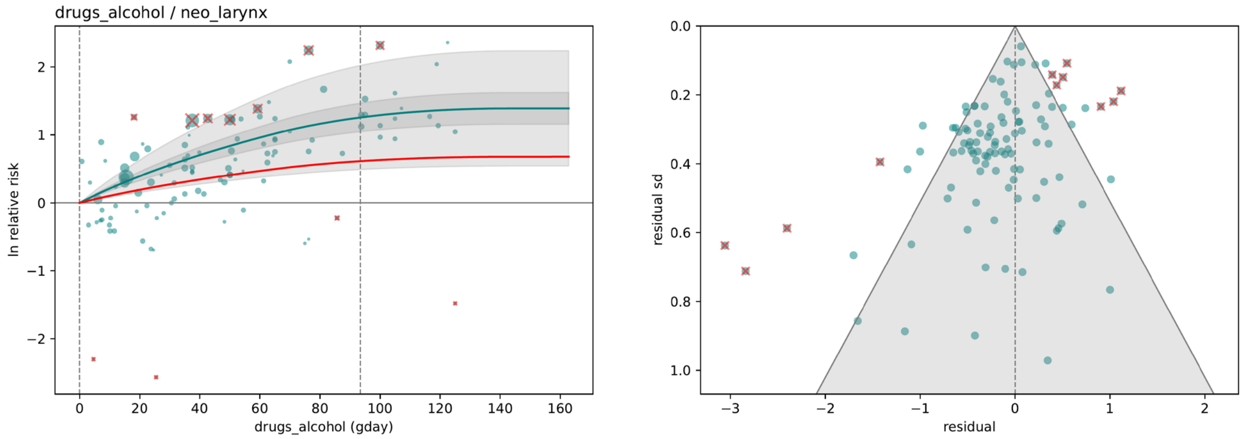

Mouth cancer

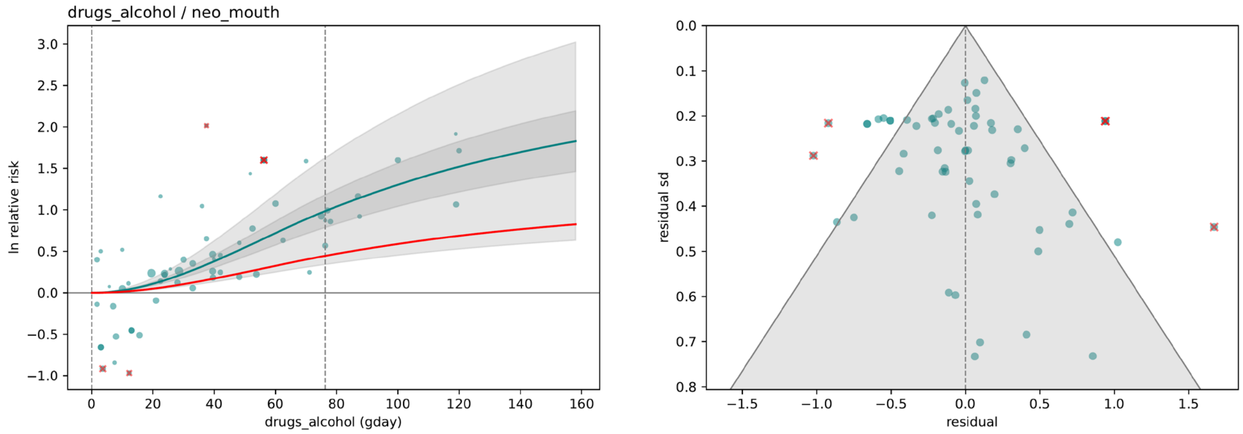

Other pharynx cancer

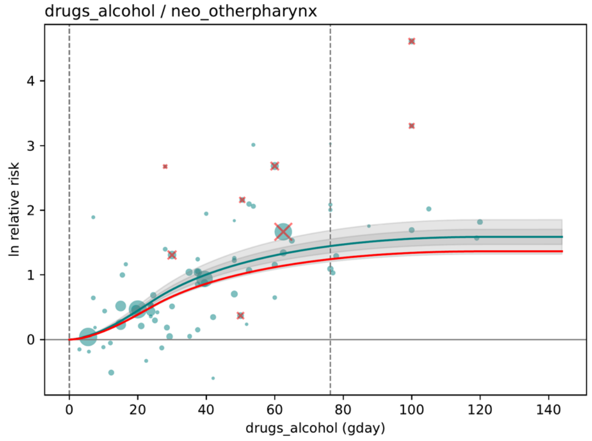

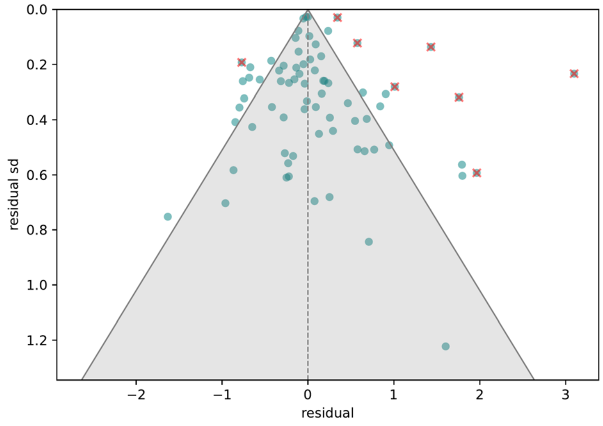

Ischaemic heart disease

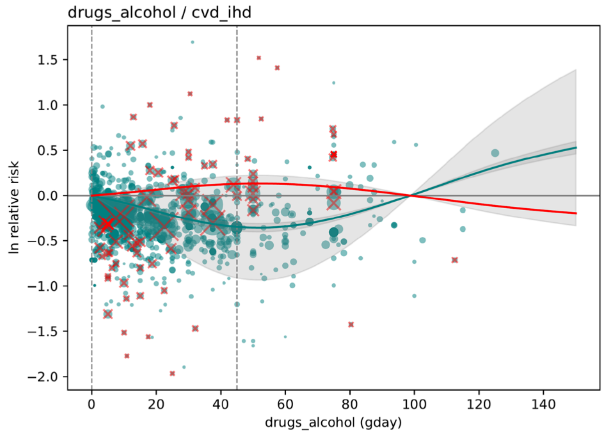

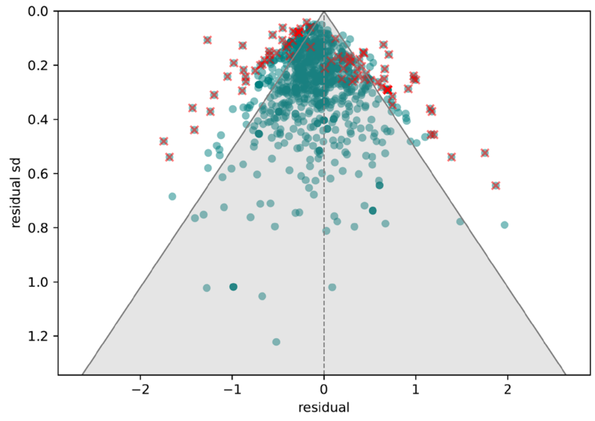

Liver cancer

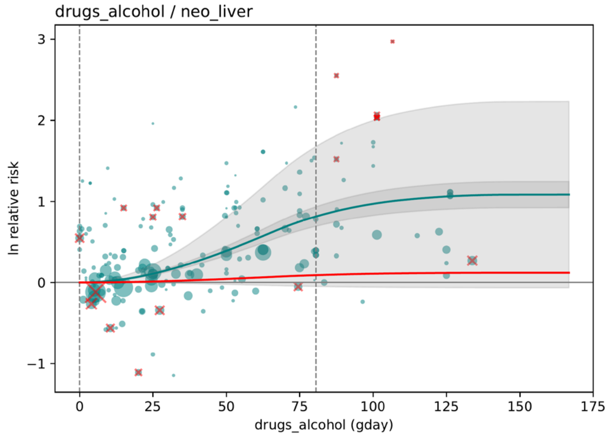

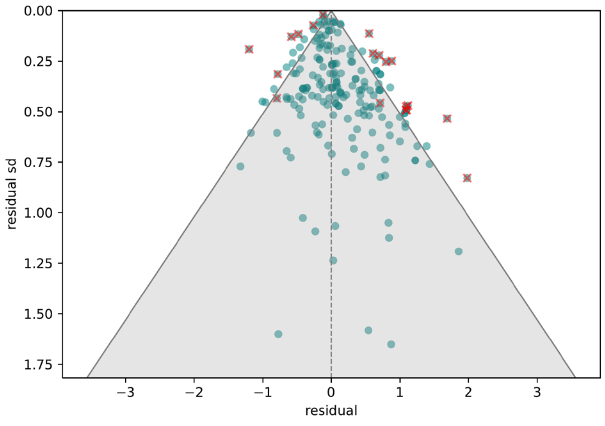

Lower respiratory infection

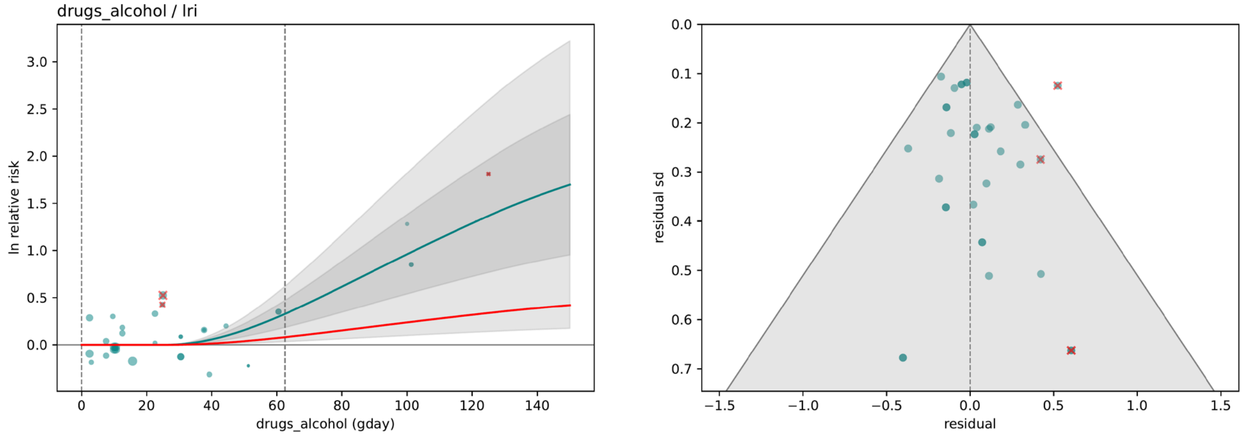

Pancreatitis

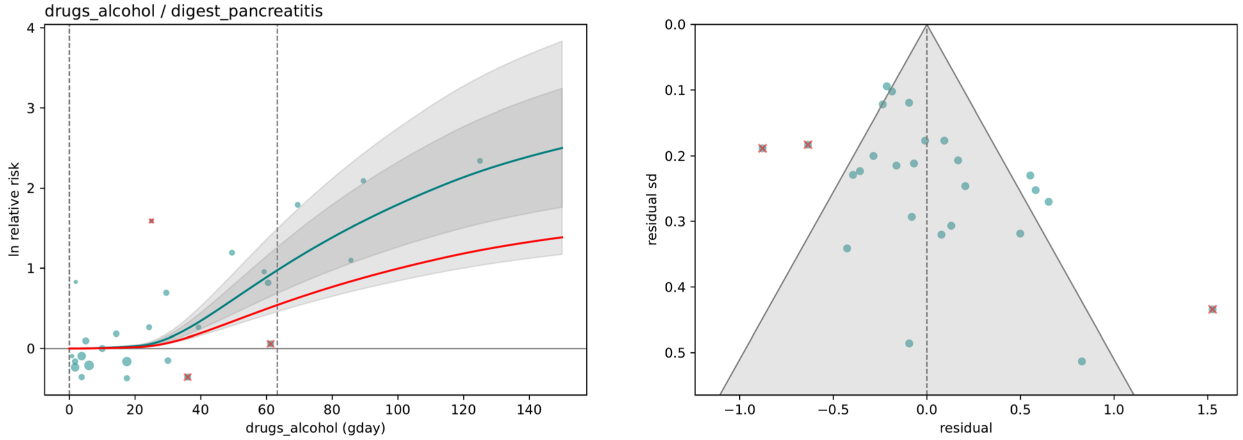

Pancreatic cancer

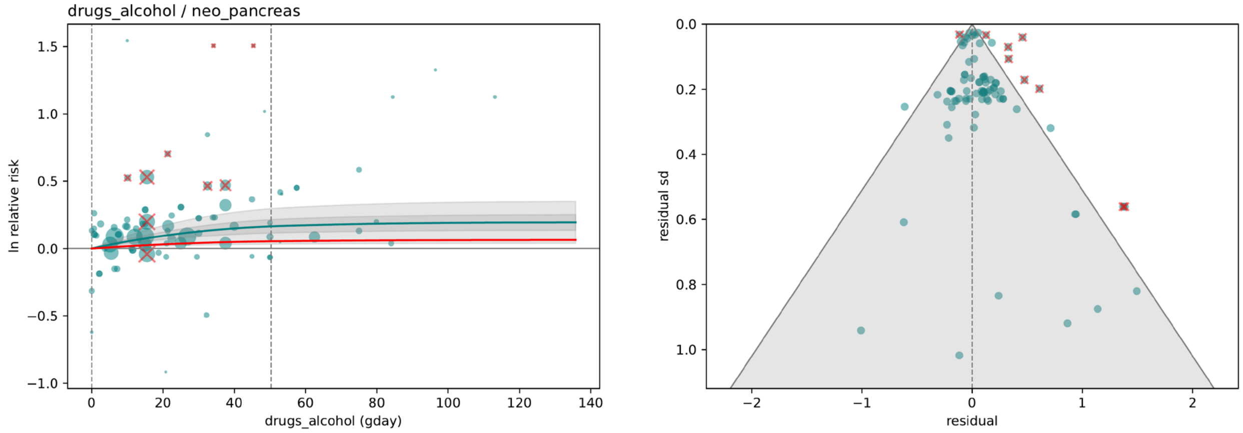

Prostate cancer

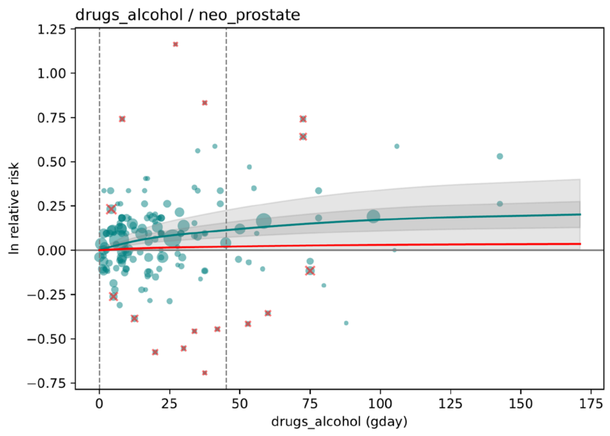

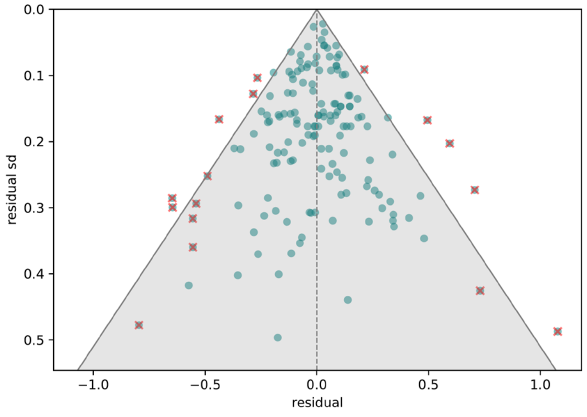

Stomach cancer

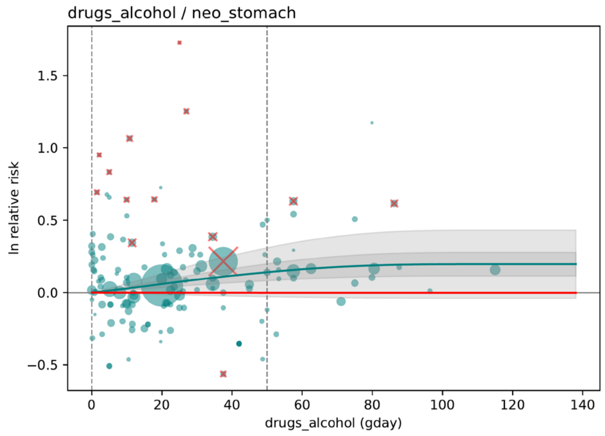

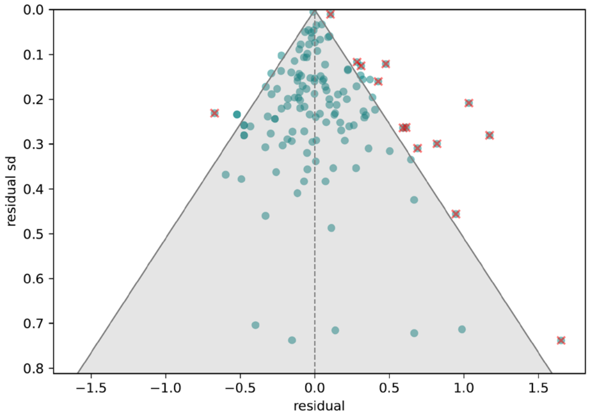

Ischaemic stroke

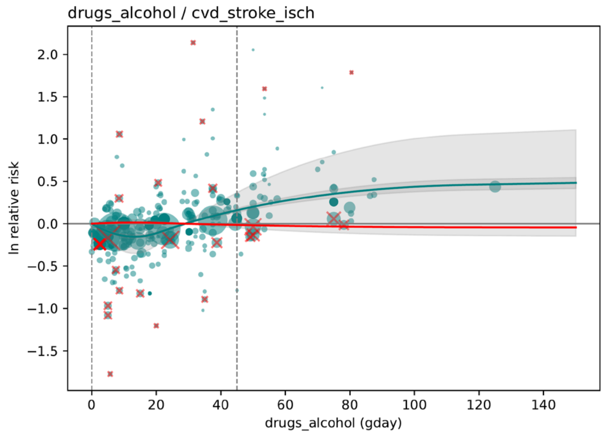

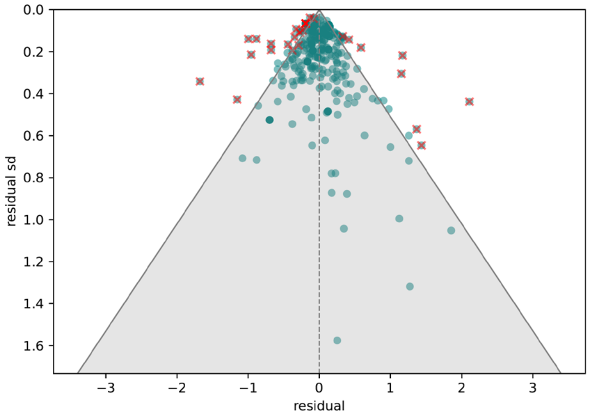

Haemorrhagic stroke

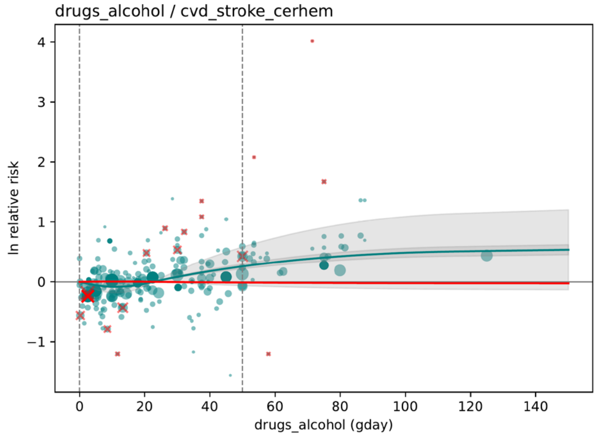

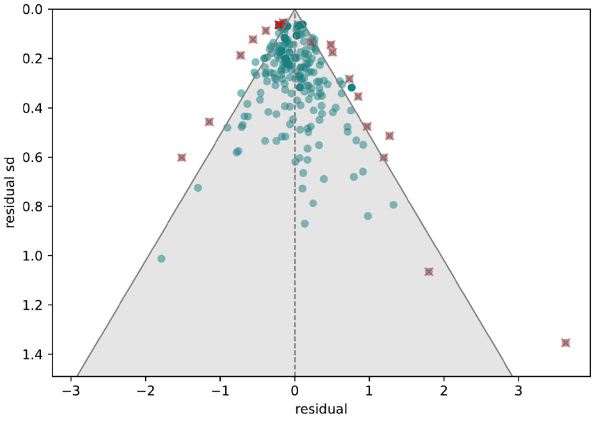

Tuberculosis

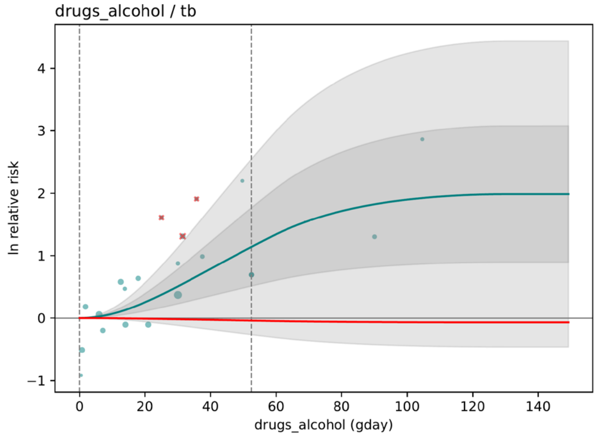

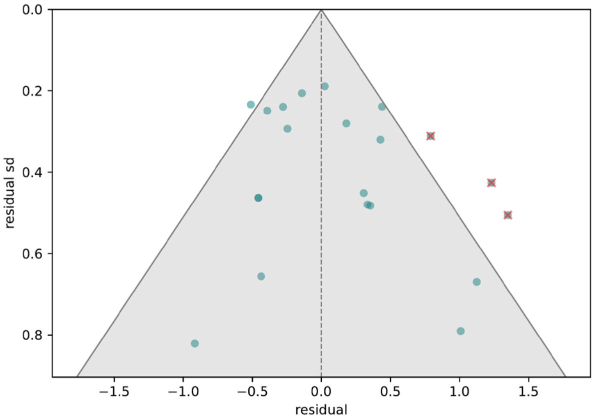

GBD 2021 vs GBD 2023 Relative Risk Curves

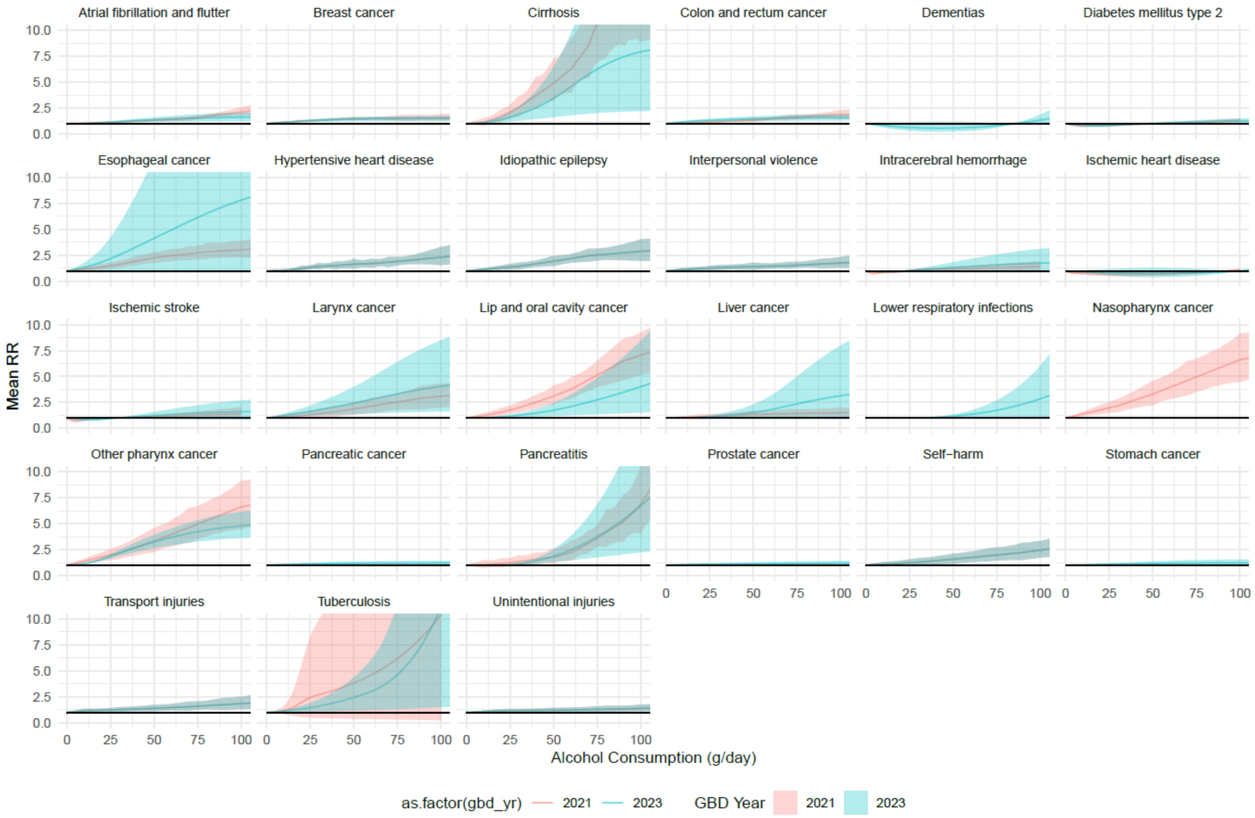

Dementias, Pancreatic Cancer, Stomach Cancer, and Prostate Cancer are new for GBD 2023. Hypertensive heart disease, Idiopathic Epilepsy, Interpersonal Violence, Self Harm, Transport Injuries, and Unintentional Injuries were not updated in GBD 2023, thus the GBD 2021 curve was used.

Prisma diagrams

Below are PRISMA diagrams for each outcome updated during the scope of this review.

Atrial fibrillation

Alcohol-Atrial Fibrillation

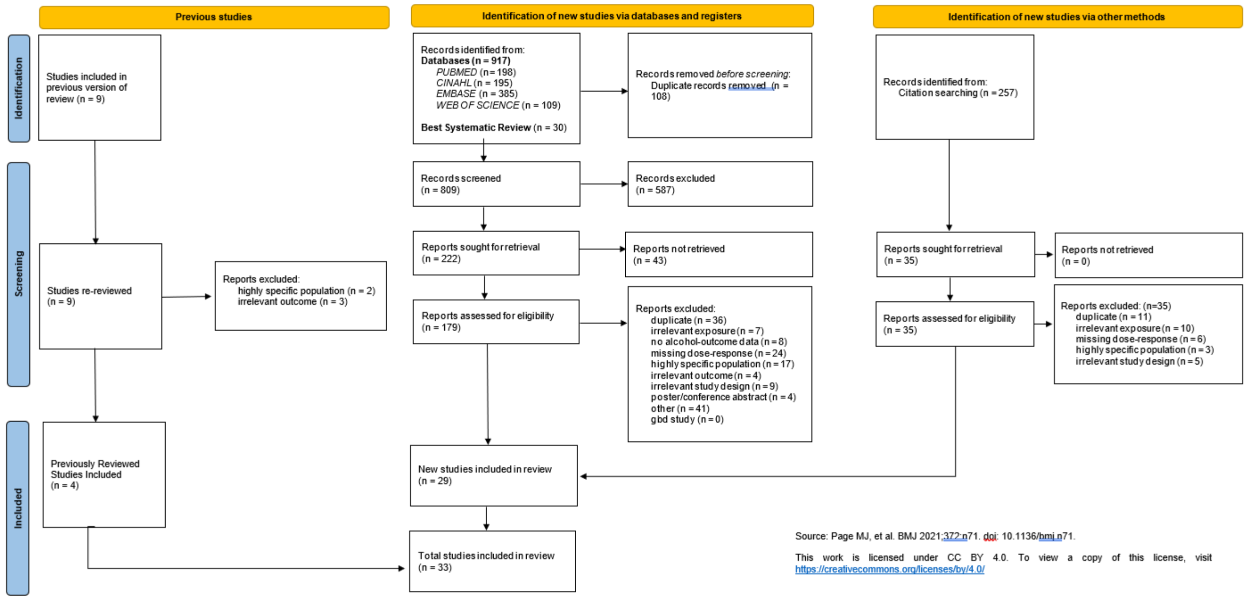

Breast cancer

Alcohol-Breast Cancer

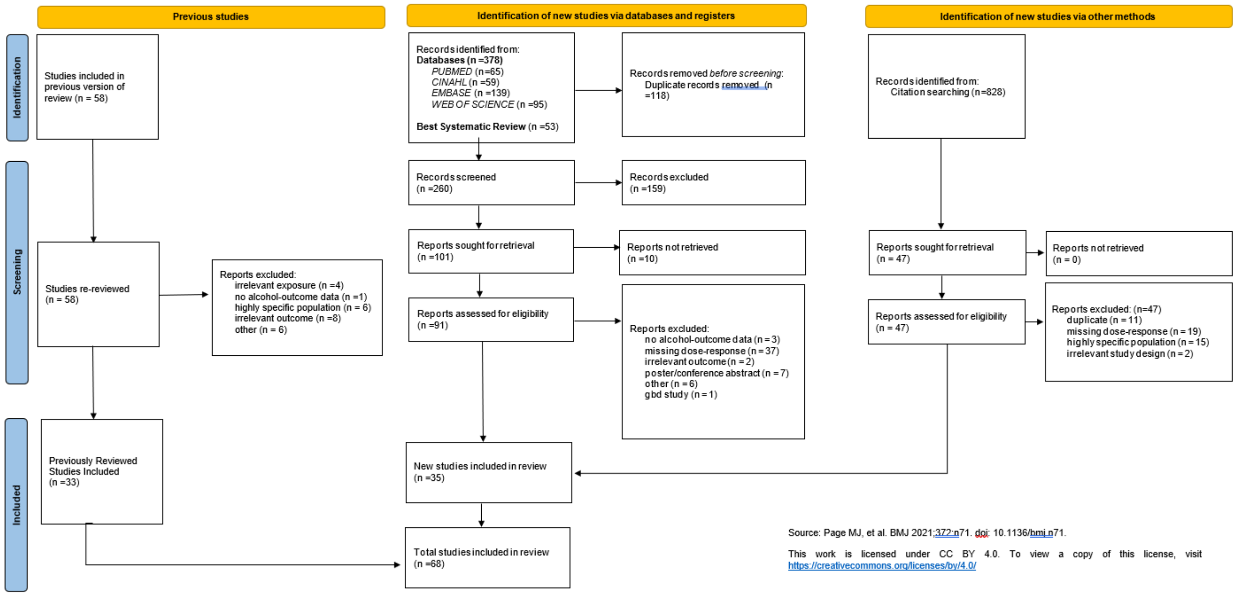

Cirrhosis

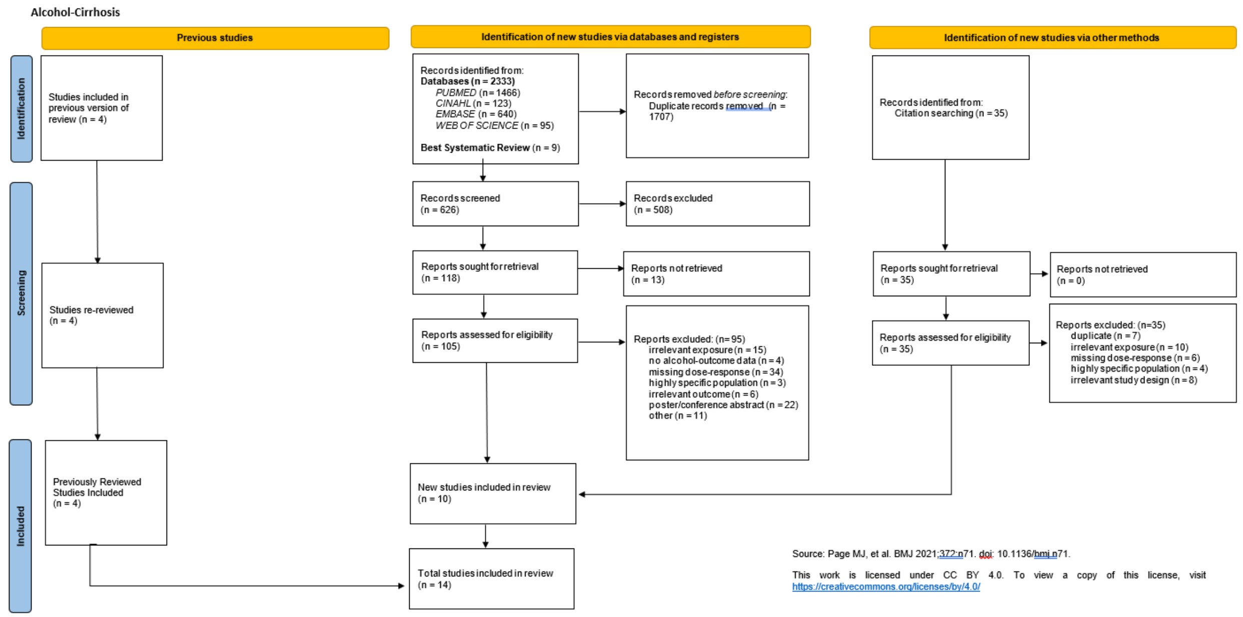

Colorectal cancer

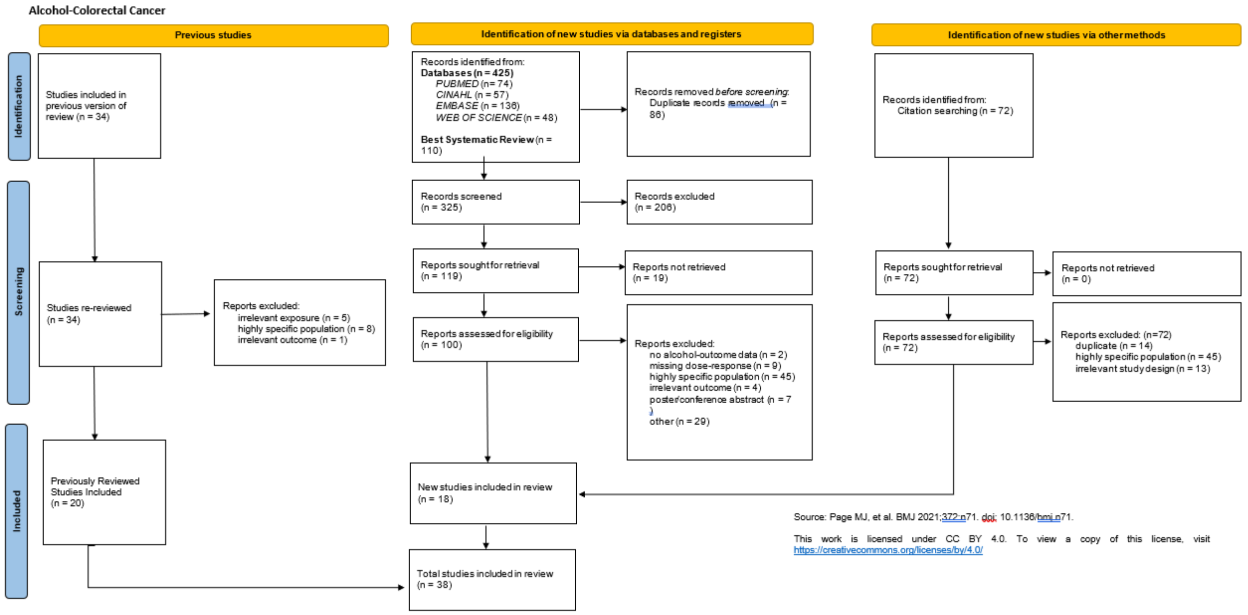

Dementia

Alcohol-Dementia

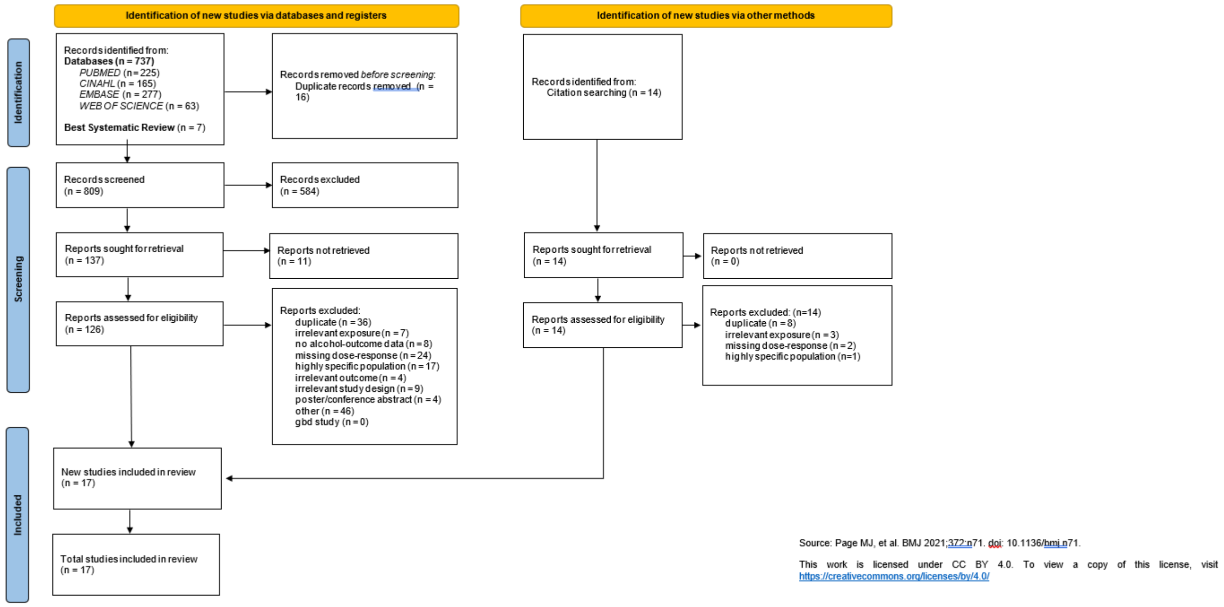

Type 2 diabetes

Alcohol-Diabetes

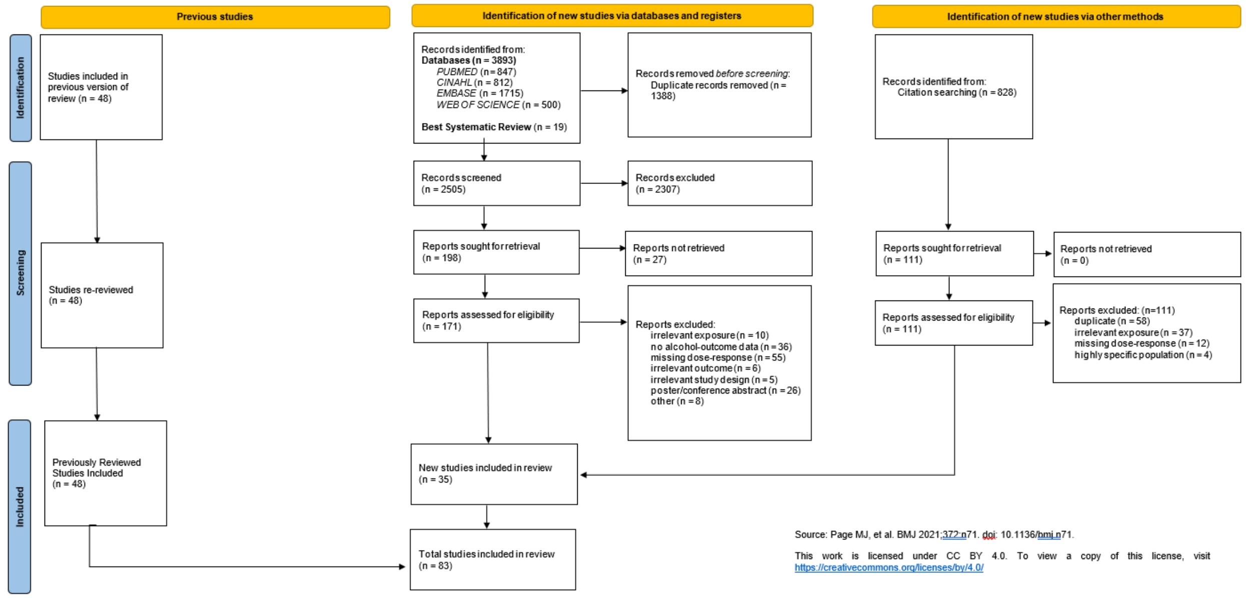

Head and neck cancers

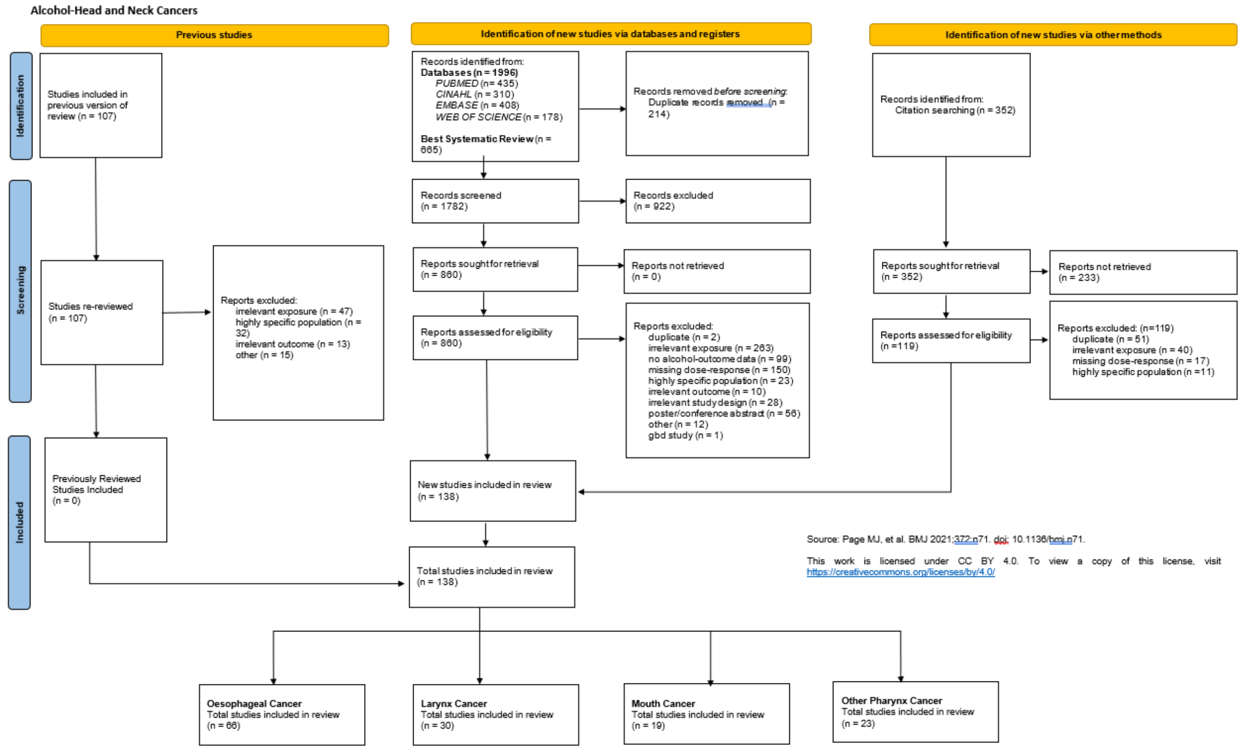

Ischaemic heart disease

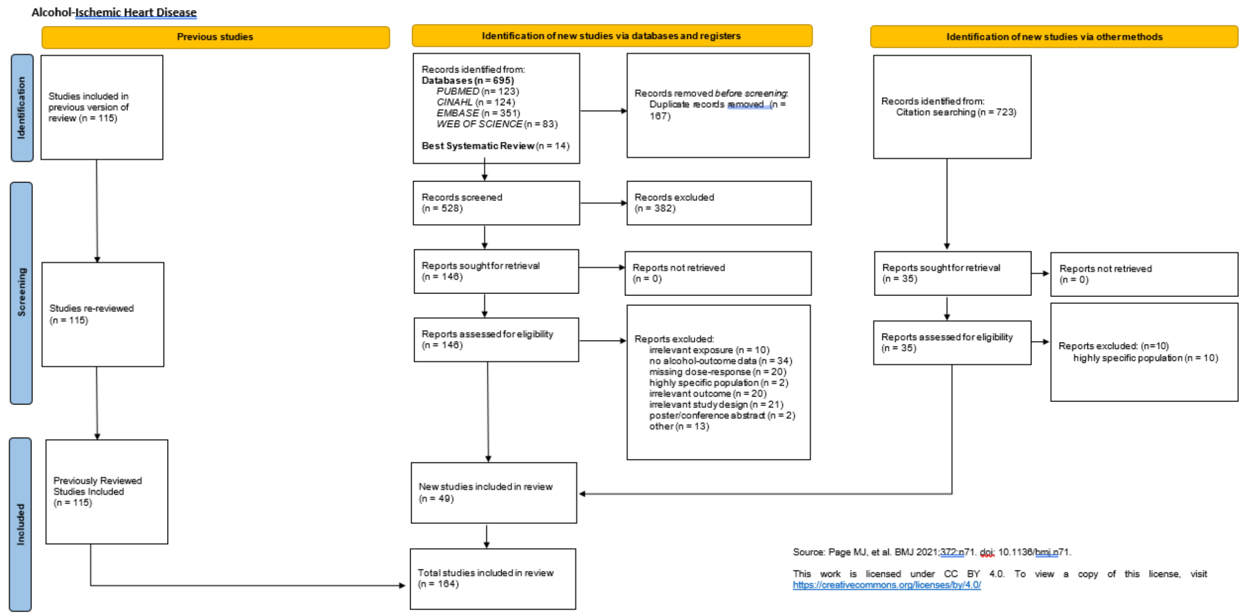

Liver cancer

Alcohol-Liver Cancer

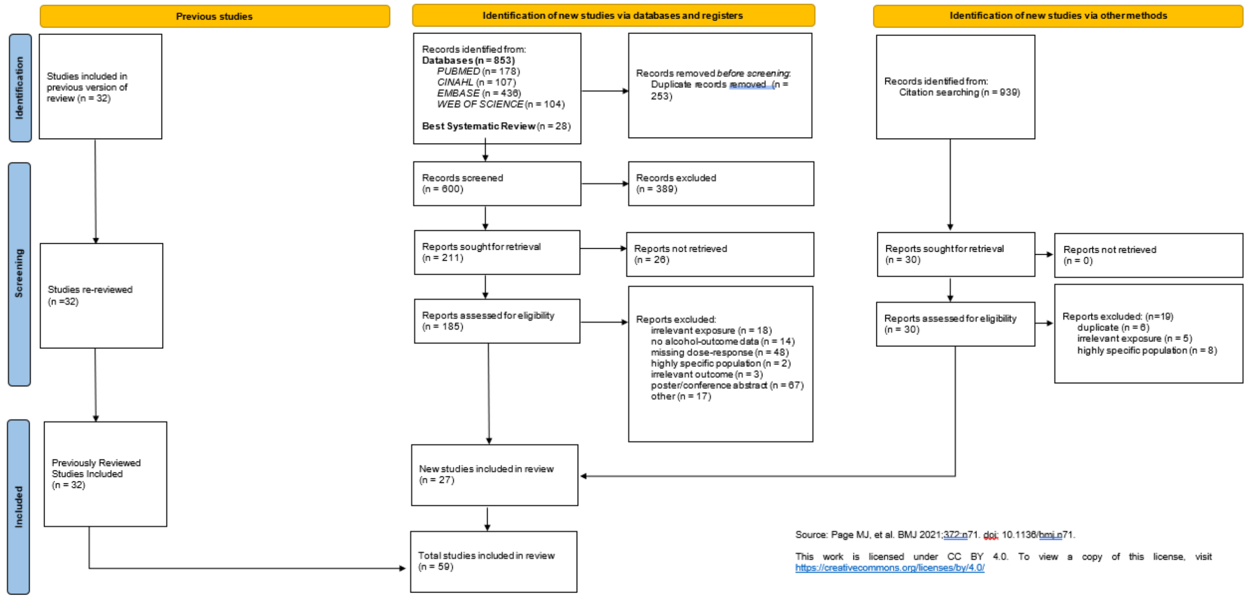

Source: Page MJ, et al. BMJ 2021;372:n71. doi: 10.1136/bmj.n71.

This work is licensed under CC BY 4.0. To view a copy of this license, visit https://creativecommons.org/licenses/by/4.0/

Lower respiratory infections

Alcohol-Lower Respiratory Infections

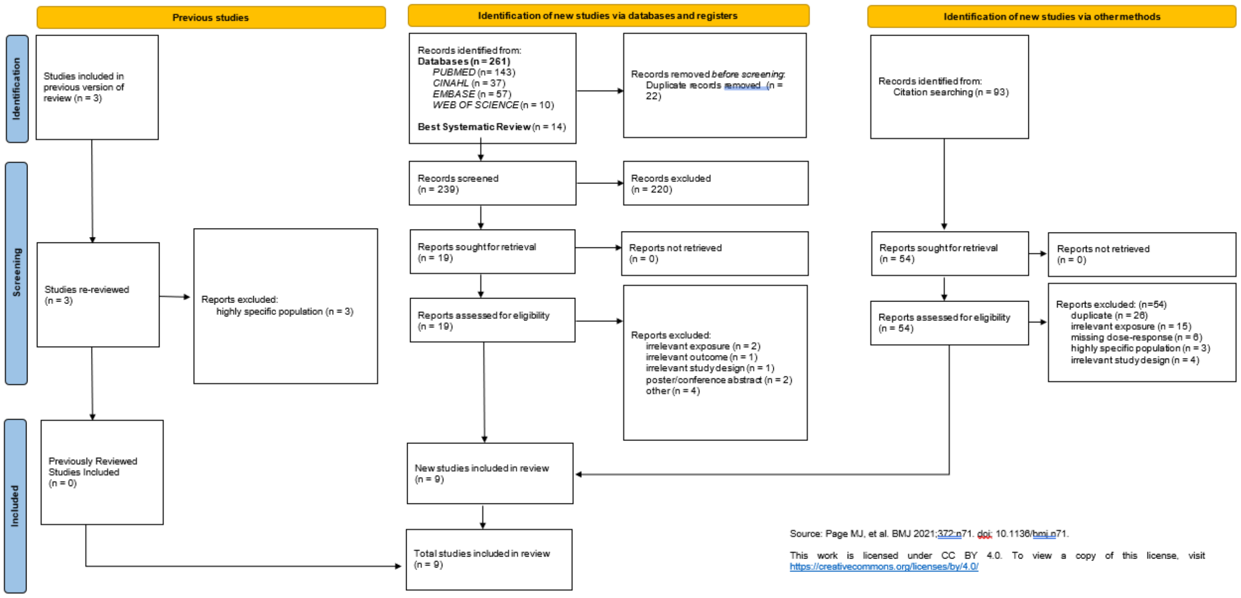

Source: Page MJ, et al. BMJ 2021;372:n71. doi: 10.1136/bmj.n71.

This work is licensed under CC BY 4.0. To view a copy of this license, visit https://creativecommons.org/licenses/by/4.0/

Pancreatitis

Alcohol-Pancreatitis

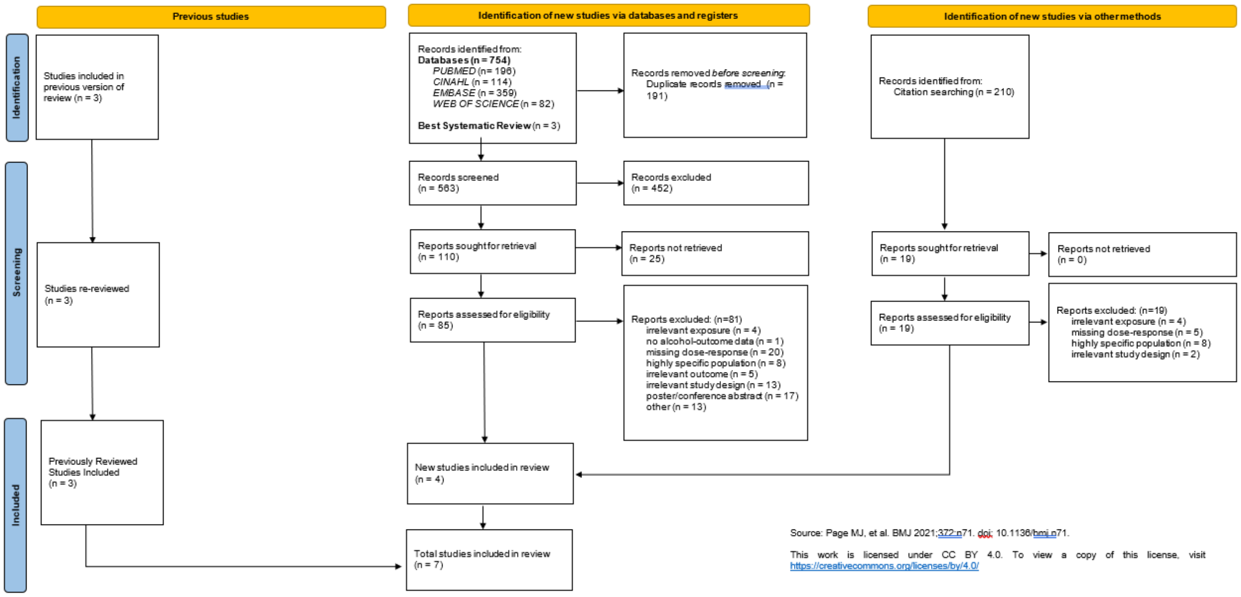

Pancreatic cancer

Alcohol-Pancreatic Cancer

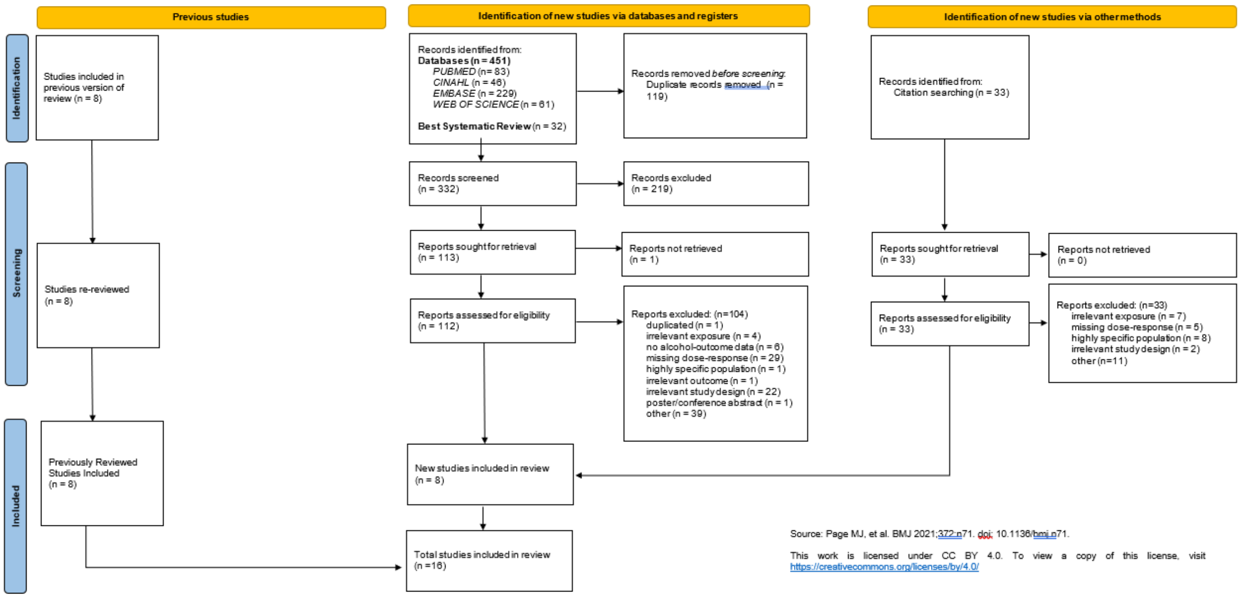

Prostate cancer

Alcohol-Prostate Cancer

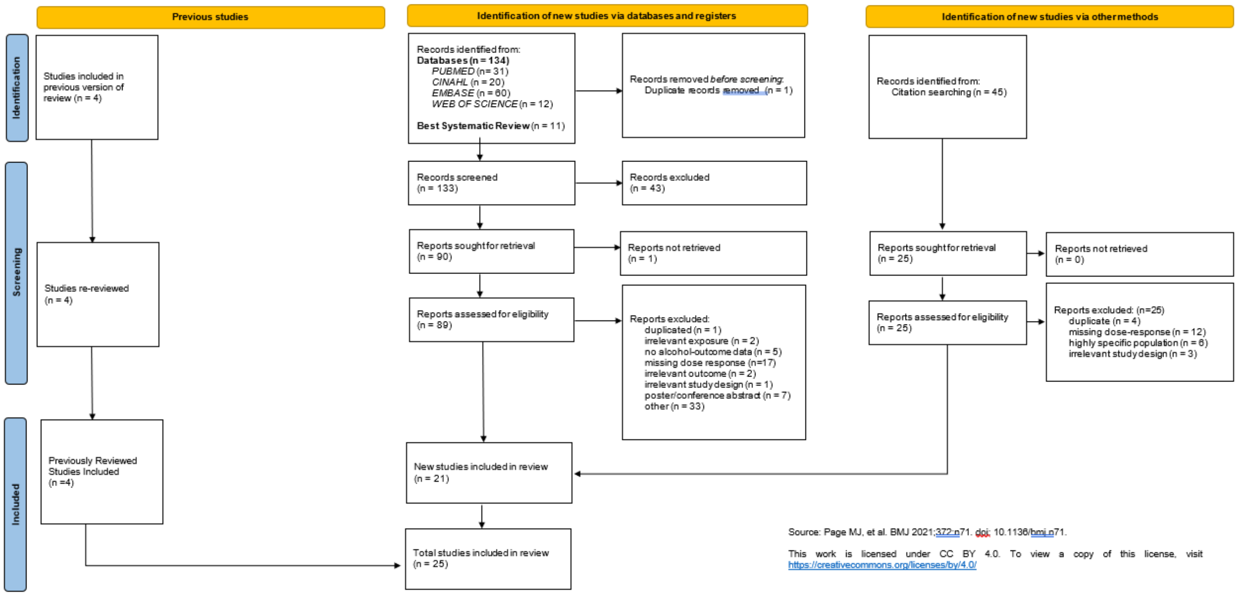

Stomach cancer

Alcohol-Stomach Cancer

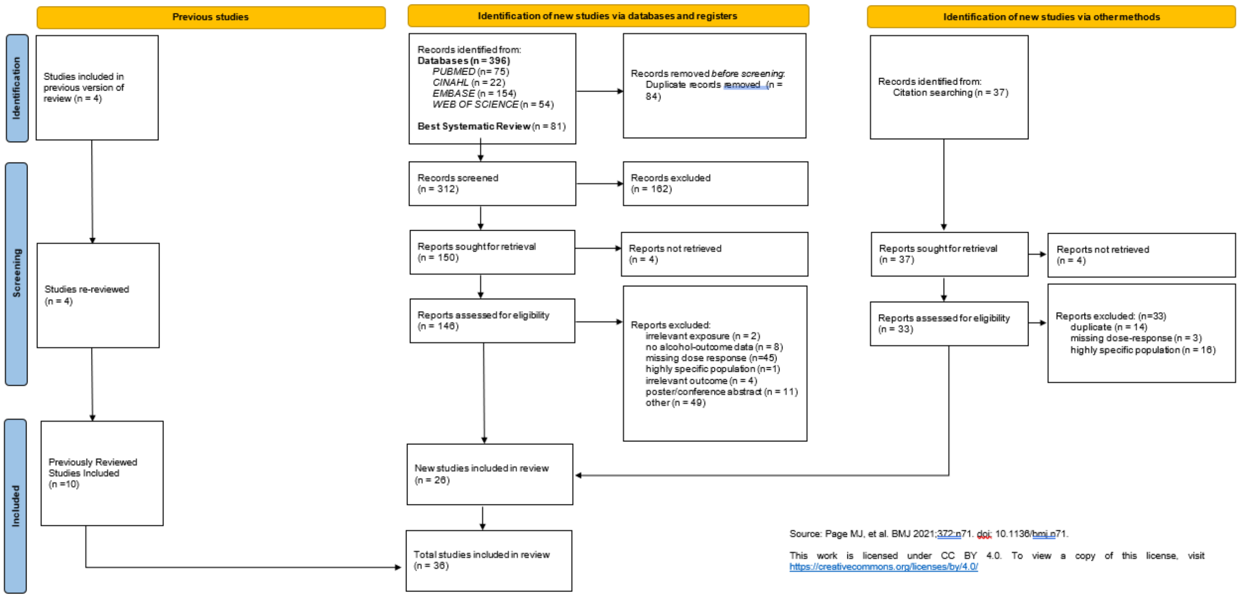

Ischaemic and haemorrhagic stroke

Alcohol-Stroke

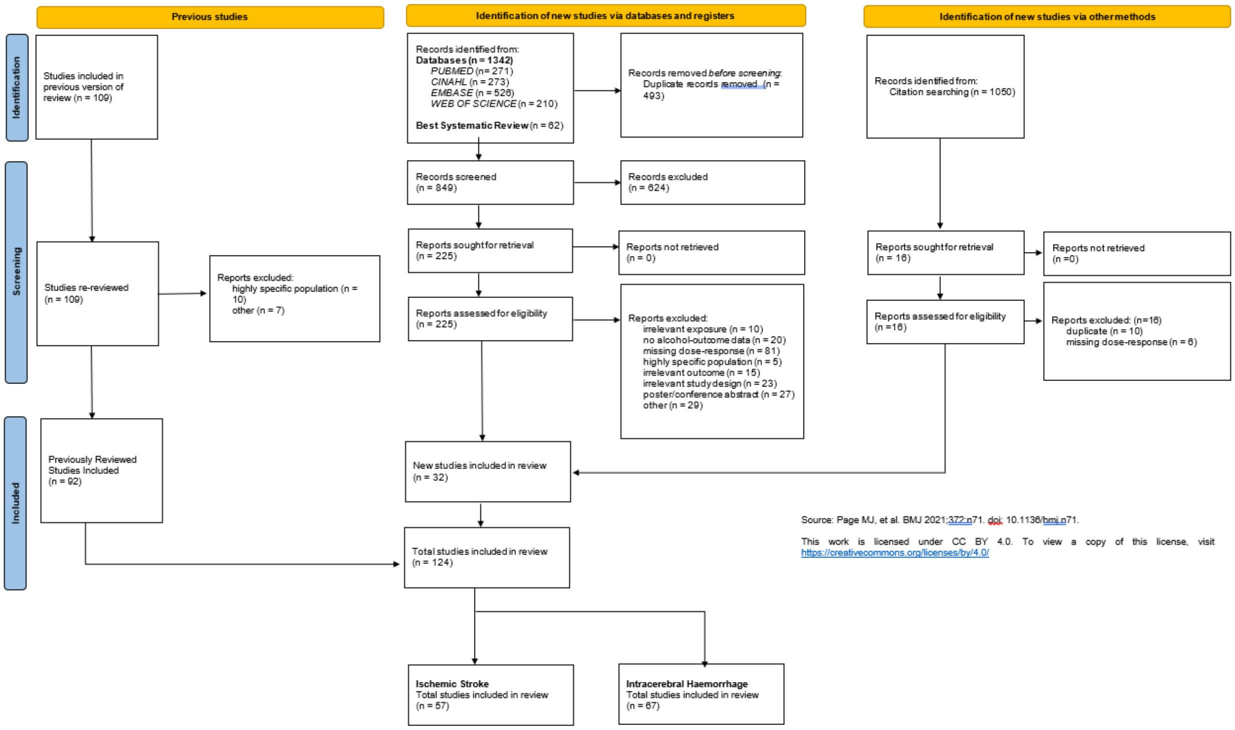

Source: Page MJ, et al. BMJ 2021;372:n71. doi: 10.1136/bmj.n71.

This work is licensed under CC BY 4.0. To view a copy of this license, visit https://creativecommons.org/licenses/by/4.0/

Tuberculosis

Alcohol-Tuberculosis

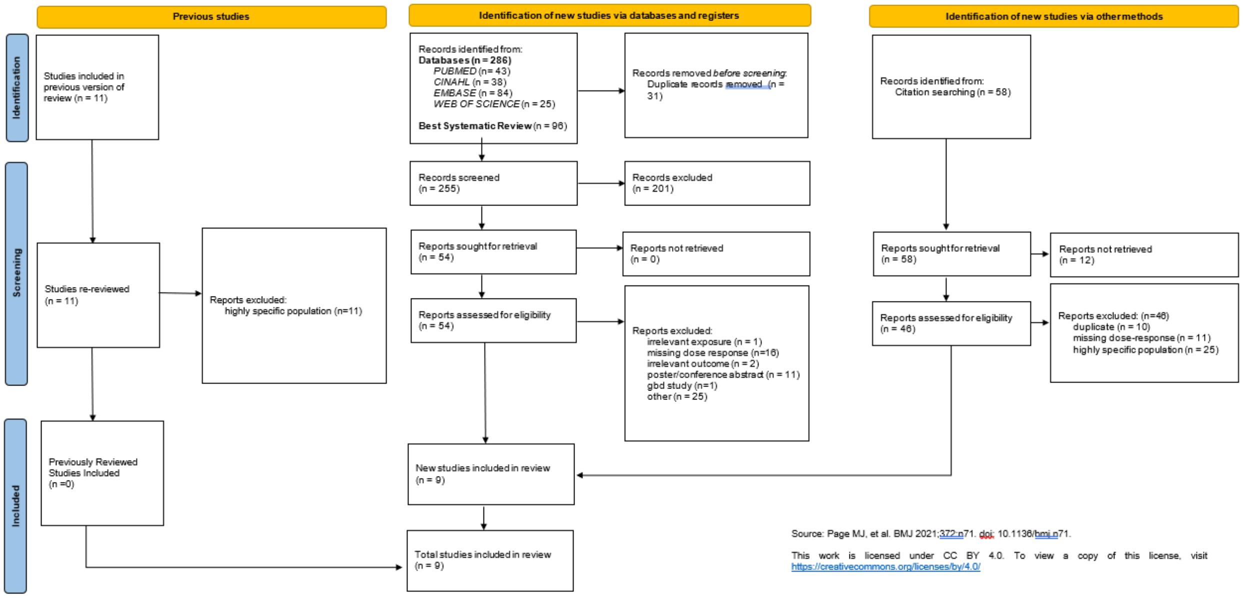

Source: Page MJ, et al. BMJ 2021;372:n71. doi: 10.1136/bmj.n71.

This work is licensed under CC BY 4.0. To view a copy of this license, visit https://creativecommons.org/licenses/by/4.0/

Not updated for GBD 2023

The following outcomes were not updated for GBD 2023. The following PRISMA diagrams are from the previous updated systematic review for these outcomes, GBD 2016 (44).

Unintentional injury

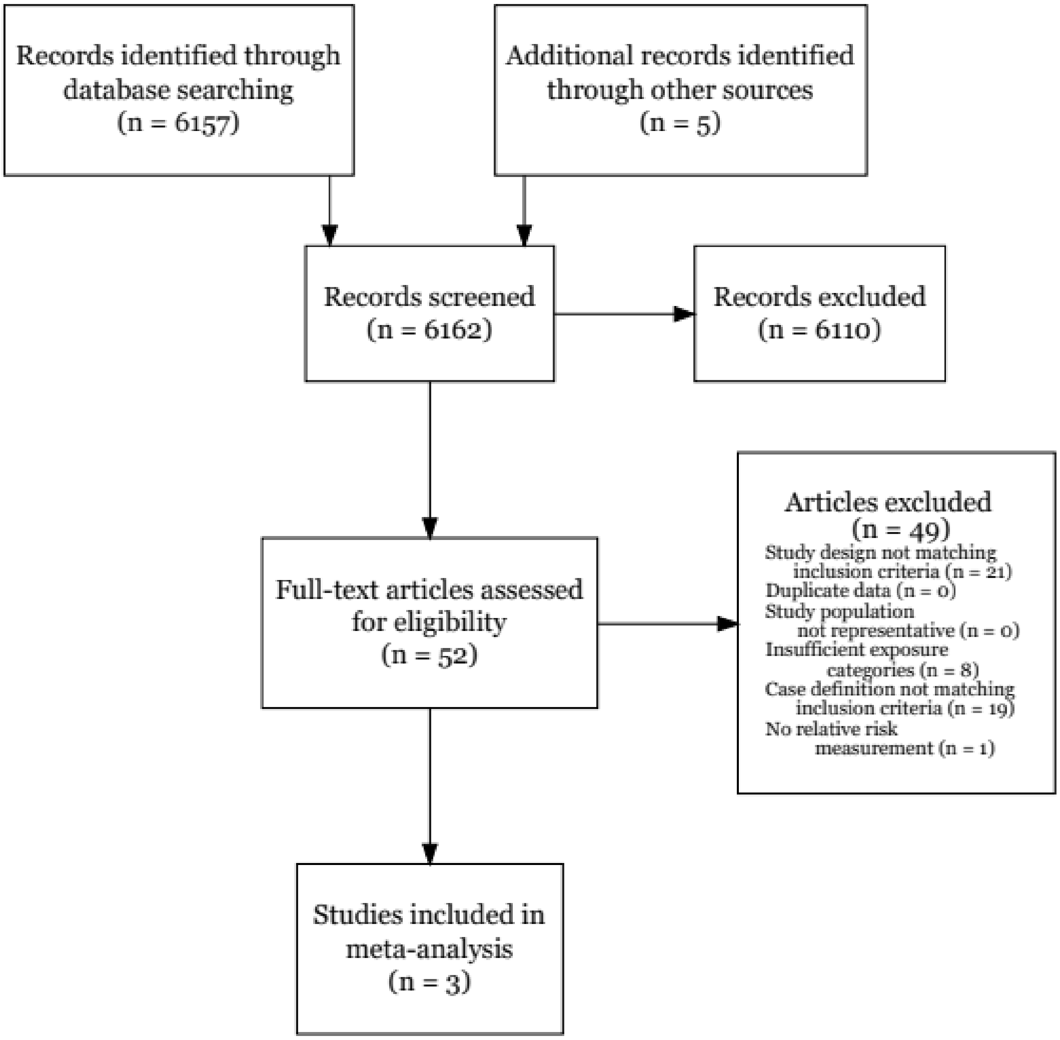

Self-harm/suicide

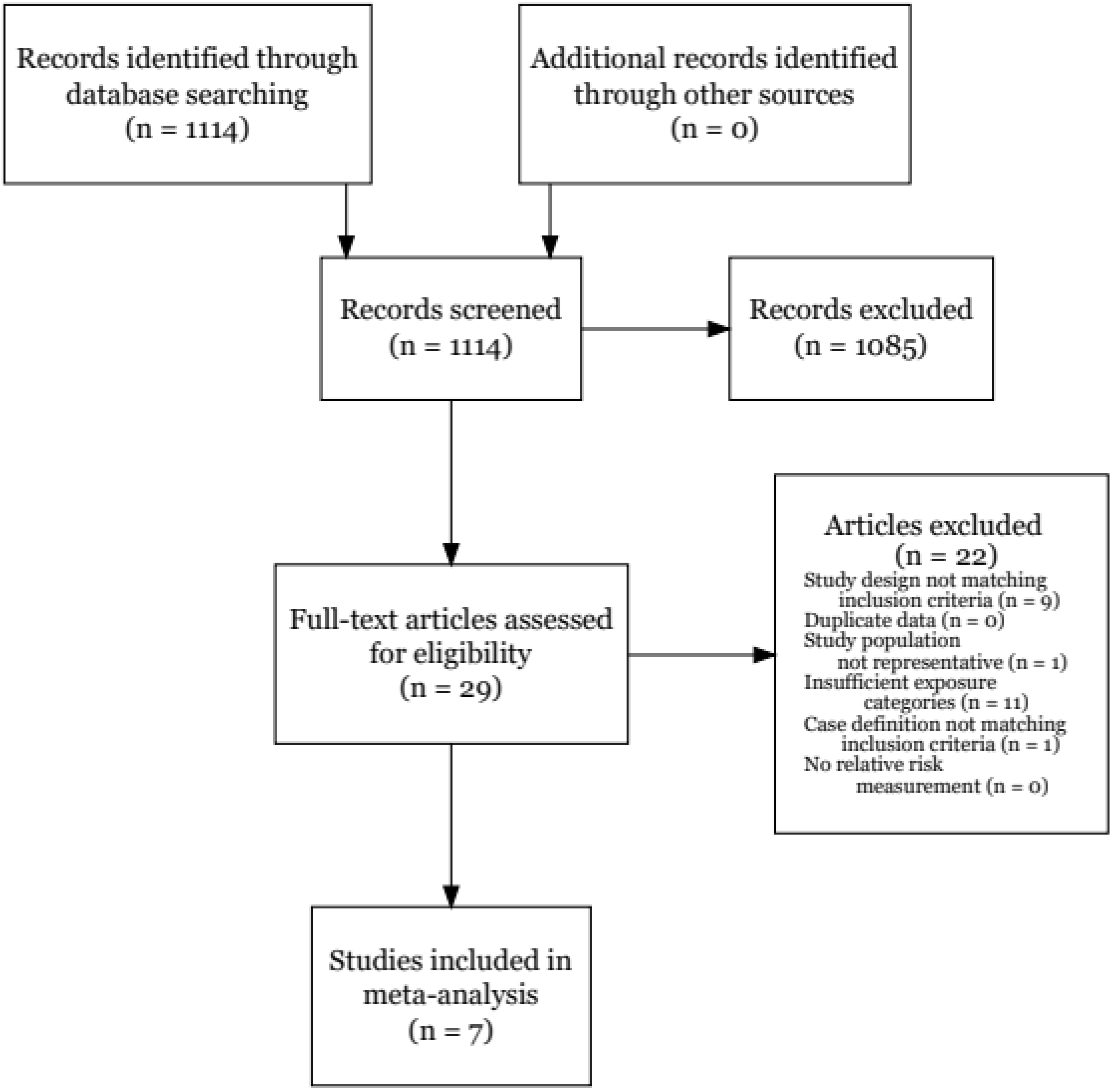

Transport injury

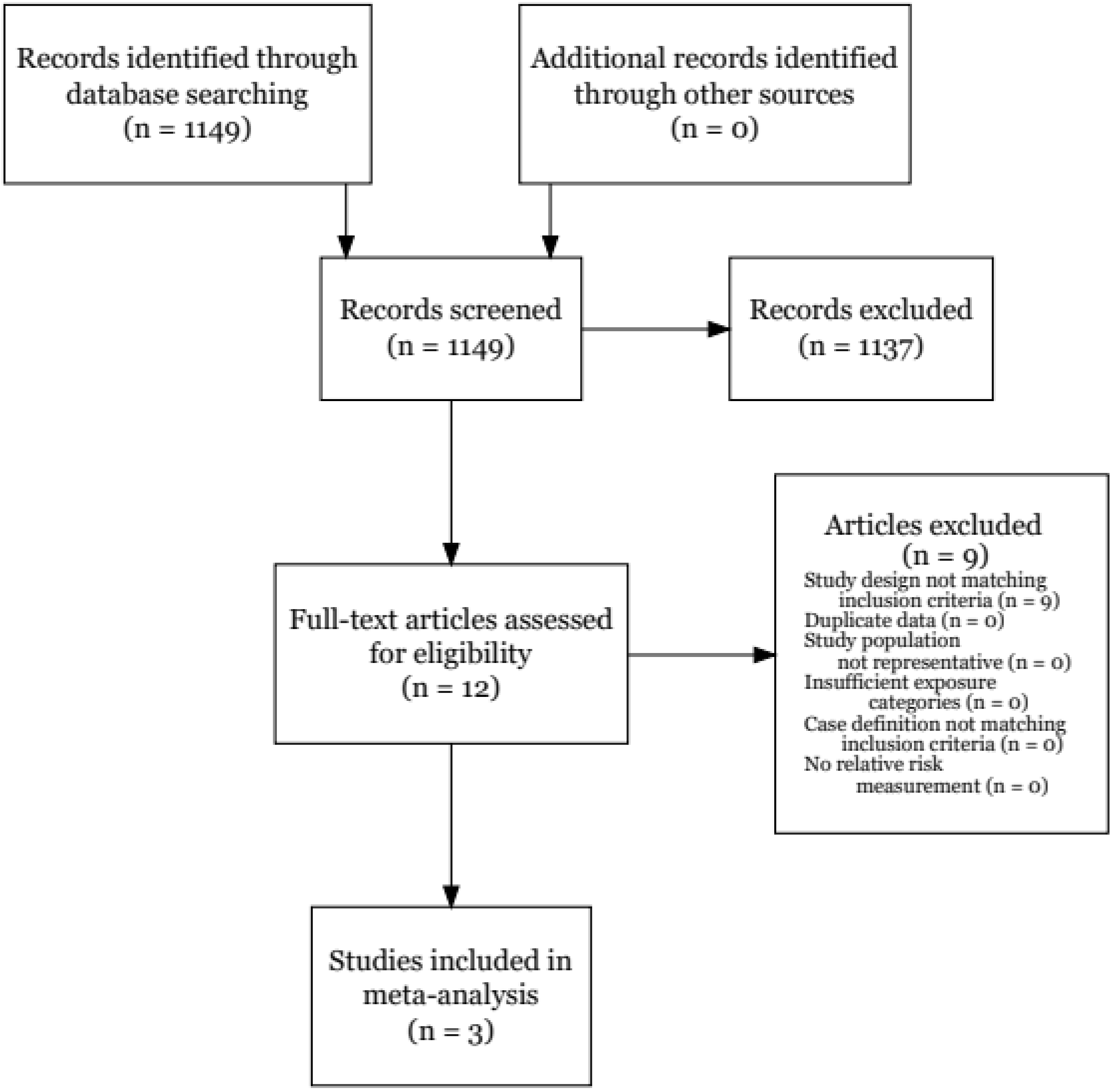

Interpersonal violence

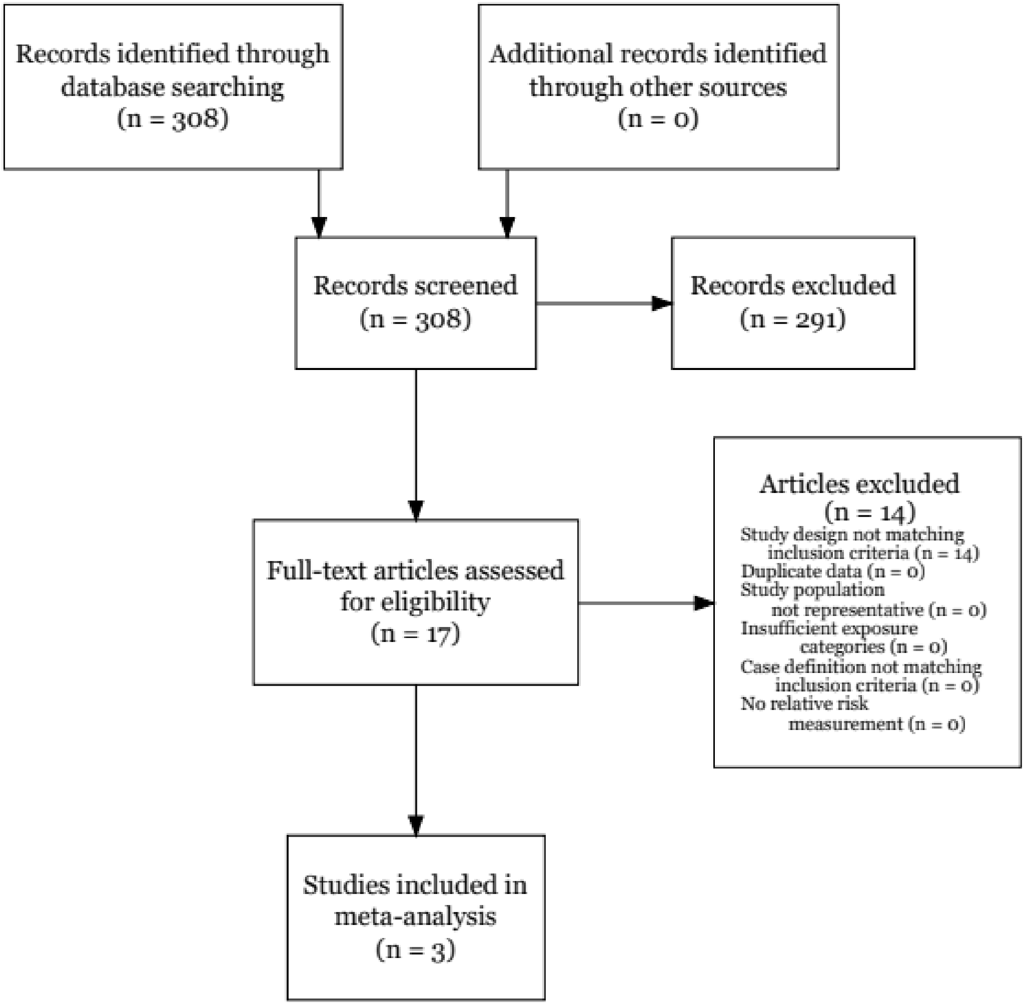

Epilepsy

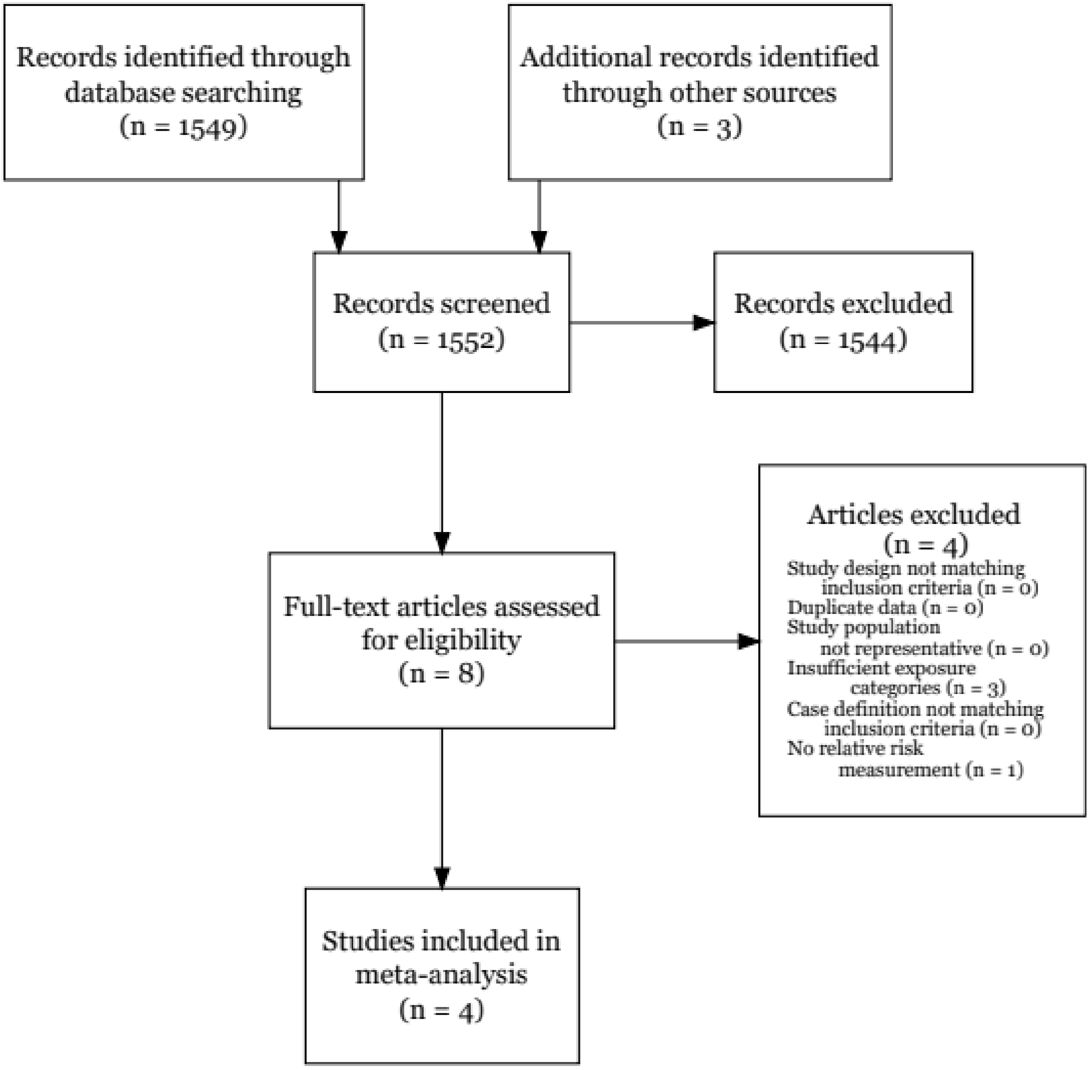

Population attributable fraction (PAF)

Population attributable fractions (PAFs) were estimated by setting the relative risk of alcohol consumption among abstainers and drinkers consuming alcohol below the TMREL to be 1, and calculating PAFs for drinkers consuming alcohol in excess of the TMREL as in previous rounds. For each location, age, sex, year, and cause, we defined PAF as:

$$PAF(x) = \frac{P_A + \int_0^{TMREL} P(x)\ dx + \int_{TMREL}^{100} P(x) * RR_C(x)\ dx - RR_C(TMREL)}{P_A + \int_0^{TMREL} P(x)\ dx + \int_{TMREL}^{100} P(x) * RR_C(x)\ dx} \qquad P(x) = P_C * \Gamma(\boldsymbol{p})$$

where:

$$P_C \text{ is the prevalence of current drinkers}, P_A \text{ is the prevalence of abstainers, and}$$

$$\boldsymbol{p} \text{ are parameters determined by the mean and sd of exposure for that location, age, sex, and year};$$

$$RR_c(x) \text{ is the global relative risk function for current drinkers for a given cause, and}$$

$$TMREL \text{ is the theoretical minimum risk exposure level for that location's region, age, sex, and year}$$

We performed the above equation for 1000 draws of the exposure and relative risk models. We then used the estimated PAF draws to calculate YLL, YLDs, and DALYs, as per the other risk factors.

For outcomes that are caused by alcohol by definition, such as liver cancer or cirrhosis due to alcohol use, PAFs were set to 1. PAFs for cirrhosis due to all causes other than non-alcohol-related cirrhosis (PAF=0) and alcohol-related cirrhosis (PAF=1) were proportionally redistributed over cirrhosis due to hepatitis B, hepatitis C, and other causes. PAFs for liver cancer due to all causes other than NAFLD (PAF=0) and alcohol-related liver cancer (PAF=1) were similarly redistributed to liver cancer due to hepatitis B, hepatitis C, and other causes. The proportions for these redistributions were obtained from the relative proportions of estimated deaths produced through cause-of-death estimation pipeline.

In the case of motor vehicle accidents, we adjusted the PAF for accidental injuries to account for victims of drunk drivers. This methodology remained consistent with GBD 2021 estimation. To adjust PAFs, we multiplied attributable deaths by the average number of fatalities from FARS[16] and redistributed the PAF among each population, based on the probability of being a victim to a certain drunk driver by age and sex, based on the FARS data. The following equation describes this process:

$$Adjusted\ PAF_i = \frac{\sum_d PAF_d * DALY_d * Avg\ Fatalities_d * \ P(i \text{ is a victim})_d}{DALY_i}$$

where:

$$i \text{ is a population by location, year, age, sex and}$$

$$d \text{ is the set of all age and sex exposed groups within that location and year.}$$

Citations

[1] Food and Agriculture Organization of the United Nations (FAO). FAOSTAT Food Balance Sheets, October 2014. Rome, Italy: Food and Agriculture Organization of the United Nations (FAO).

[2] Euromonitor International. Euromonitor Passport - Alcoholic Drinks Statistics. London, United Kingdom: Euromonitor International.

[3] World Health Organization (WHO). WHO Global Health Observatory - Recorded adult per capita alcohol consumption, Total per country. Geneva, Switzerland: World Health Organization (WHO).

[4] UN World Tourism Organization (UNWTO). UN World Tourism Organization Compendium of Tourism Statistics 2015 [Electronic]. Madrid, Spain: UN World Tourism Organization (UNWTO), 2016.

[5] Ramstedt M. "How much alcohol do you buy? A comparison of self-reported alcohol purchases with actual sales." *Addiction* 105.4 (2010): 649-654.

[6] Stockwell T, et al. "Under-reporting of alcohol consumption in household surveys: a comparison of quantity–frequency, graduated–frequency and recent recall." *Addiction* 99.8 (2004): 1024-1033.

[7] Kerr WC, Greenfield TK. "Distribution of alcohol consumption and expenditures and the impact of improved measurement on coverage of alcohol sales in the 2000 National Alcohol Survey." *Alcoholism: Clinical and Experimental Research* 31.10 (2007): 1714-1722.

[8] Kehoe T, et al. "Determining the best population-level alcohol consumption model and its impact on estimates of alcohol-attributable harms." *Population Health Metrics* 10 6. (2012)

[9] Taylor B, et al. "The more you drink, the harder you fall: a systematic review and meta-analysis of how acute alcohol consumption and injury or collision risk increase together." *Drug and Alcohol Dependence* 110.1 (2010): 108-116.

[10] Vinson DC, Borges G, Cherpitel CJ. "The risk of intentional injury with acute and chronic alcohol exposures: a case-control and case-crossover study." *Journal of Studies on Alcohol* 64.3 (2003): 350-357.

[11] Vinson DC, et al. "A population-based case-crossover and case-control study of alcohol and the risk of injury." *Journal of Studies on Alcohol* 64.3 (2003): 358-366.

[12] Chen, Li-Hui, Susan P. Baker, and Guohua Li. "Drinking history and risk of fatal injury: comparison among specific injury causes." Accident Analysis & Prevention 37.2 (2005): 245-251.

[13] Bell NS, et al. "Self-reported risk-taking behaviors and hospitalization for motor vehicle injury among active-duty army personnel." *American Journal of Preventive Medicine* 18.3 (2000): 85-95.

[14] Margolis KL, et al. "Risk factors for motor vehicle crashes in older women." *The Journals of Gerontology Series A: Biological Sciences and Medical Sciences* 57.3 (2002): M186-M191.

[15] Sorock GS, et al. "Alcohol-drinking history and fatal injury in older adults." *Alcohol* 40.3 (2006): 193-199.

[16] Fatal Accident Reporting System (FARS). National Highway Traffic Safety Administration, National Center for Statistics and Analysis Data Reporting and Information Division (NVS-424); 1985, 1990, 1995, 2000, 2005, 2010, 2015

[17] DistillerSR. Version 2.35. DistillerSR Inc.; 2023. Accessed June 2022-Dec 2024. https://www.distillersr.com/.Alcohol and incident atrial fibrillation – A systematic review and meta-analysis.

[18] Gallagher C, Hendriks JML, Elliott AD, Wong CX, Rangnekar G, Middeldorp ME, Mahajan R, Lau DH, Sanders P. Alcohol and incident atrial fibrillation - A systematic review and meta-analysis. *Int J Cardiol* 2017;Nov 1;246:46-52.

[19] Poorolajal J, Heidarimoghis F, Karami M, Cheraghi Z, Gohari-Ensaf F, Shahbazi F, Zareie B, Ameri P, Sahraee F. Factors for the Primary Prevention of Breast Cancer: A Meta-Analysis of Prospective Cohort Studies. *J Res Health Sci*. 2021 Jul 20;21(3):e00520. doi: 10.34172/jrhs.2021.57. PMID: 34698654; PMCID: PMC8957681.

[201] Glyn-Owen K, Böhning D, Parkes J, Roderick P, Buchanan R, 2021. The combined effect of alcohol and body mass index on risk of chronic liver disease: A systematic review and meta-analysis of cohort studies. *Liver International* 41, 1216–1226. https://doi.org/10.1111/liv.14754

[21] Ubago-Guisado, E.; Rodríguez-Barranco, M.; ChingLópez, A.; Petrova, D.; MolinaMontes, E.; Amiano, P.; BarricarteGurrea, A.; Chirlaque, M.-D.; Agudo, A.; Sánchez, M.-J. Evidence Update on the Relationship between Diet and the Most Common Cancers from the European Prospective Investigation into Cancer and Nutrition (EPIC) Study: A Systematic Review. *Nutrients* 2021, 13, 3582. https:// doi.org/10.3390/nu13103582

[22] Dementia prevention, intervention, and care: 2020 report of the *Lancet* Commission. Livingston G, et al. *The Lancet*, Volume 396, Issue 10248, 413-446

[23] Schlesinger, S., Neuenschwander, M., Ballon, et al. Adherence to healthy lifestyles and incidence of diabetes and mortality among individuals with diabetes: a systematic review and meta-analysis of prospective studies. *Journal of Epidemiology and Community Health* 2020;74(5):481–487.

[24] Walsh S, Donnan J, Fortin Y, Sikora L, Morrissey A, Collins K, MacDonald D, A systematic review of the risks factors associated with the onset and natural progression of epilepsy, *NeuroToxicology*, Volume 61, 2017, Pages 64-77, ISSN 0161-813X, https://doi.org/10.1016/j.neuro.2016.03.011.

[25] Sun LP, Yan LB, Liu ZZ, Zhao WJ, Zhang CX, Chen YM, Lao XQ, Liu X. Dietary factors and risk of mortality among patients with esophageal cancer: a systematic review. *BMC Cancer*. 2020 Apr 6;20(1):287. doi: 10.1186/s12885-020-06767-8. PMID: 32252671; PMCID: PMC7137267.

[26] Islami F, Tramacere I, Rota M, Bagnardi V, Fedirko V, Scotti L, Garavello W, Jenab M, Corrao G, Straif K, Negri E, Boffetta P, La Vecchia C, Alcohol drinking and laryngeal cancer: Overall and dose–risk relation – A systematic review and meta-analysis, *Oral Oncology*, Volume 46, Issue 11, 2010, Pages 802-810, ISSN 1368-8375, https://doi.org/10.1016/j.oraloncology.2010.07.015. (https://www.sciencedirect.com/science/article/pii/S1368837510002319)

[27] Bagnardi, V., Rota, M., Botteri, E. et al. Alcohol consumption and site-specific cancer risk: a comprehensive dose–response meta-analysis. *Br J Cancer* 112, 580–593 (2015). https://doi.org/10.1038/bjc.2014.579

[28] Batistella, E.Â., Gondak, R., Rivero, E.R.C. et al. Comparison of tobacco and alcohol consumption in young and older patients with oral squamous cell carcinoma: a systematic review and meta-analysis. *Clin Oral Invest* 26, 6855–6869 (2022). https://doi-org.offcampus.lib.washington.edu/10.1007/s00784-022-04719-z

[29] Benenson I, Waldron FA, Jadotte YT, Dreker M, Holly C, Risk factors for hypertensive crisis in adult patients: a systematic review. *JBI Evidence Synthesis* 19(6):p 1292-1327, June 2021. | DOI: 10.11124/JBIES-20-00243

[30] Ding C, O'Neill D, Bell S, et al. Association of alcohol consumption with morbidity and mortality in patients with cardiovascular disease: original data and meta-analysis of 48,423 men and women. *BMC Med* 19, 167 (2021). https://doi.org/10.1186/s12916-021-02040-2

[31] Valentin G, Ravn M, Jensen E, et al. Socio-economic inequalities in fragility fracture incidence: a systematic review and meta-analysis of 61 observational studies. *Osteoporosis Int* 32, 2433–2448 (2021). https://doi.org/10.1007/s00198-021-06038-7

[32] Wright MMM, Kankkunen PM, Jokiniemi KS, 2023. Prevention interventions for interpersonal violence occurring under the influence of alcohol: A mixed method systematic review. *Journal of Advanced Nursing* 79, 1247–1266. https://doi.org/10.1111/jan.15335

[33] Park, H., Shin, S.K., Joo, I., Song, D.S., Jang, J.W., Park, J.-W., 2020. Systematic Review with Meta-Analysis: Low-Level Alcohol Consumption and the Risk of Liver Cancer. *Gut and Liver* 14, 792–807. https://doi.org/10.5009/gnl19163

[34] Wood, S., Harrison, S.E., Judd, N., Bellis, M.A., Hughes, K., Jones, A., 2021. The impact of behavioral risk factors on communicable diseases: a systematic review of reviews. *BMC Public Health* 21. https://doi.org/10.1186/s12889-021-12148-y

[35] Samokhvalov AV, Rehm J, Roerecke M. Alcohol Consumption as a Risk Factor for Acute and Chronic Pancreatitis: A Systematic Review and a Series of Meta-analyses. *EBioMedicine*. 2015 Nov 14;2(12):1996-2002. doi: 10.1016/j.ebiom.2015.11.023. PMID: 26844279; PMCID: PMC4703772.

[36] Lu P-Y, Shu L, Shen S-S, Chen X-J, Zhang X-Y, 2017. Dietary Patterns and Pancreatic Cancer Risk: A Meta-Analysis. *Nutrients* 9, 38. https://doi.org/10.3390/nu9010038

[37] Hong, S., Khil, H., Lee, D.H., Keum, N., Giovannucci, E.L., 2020. Alcohol Consumption and the Risk of Prostate Cancer: A Dose-Response Meta-Analysis. Nutrients 12, 2188. https://doi.org/10.3390/nu12082188

[38] Isaacs, J.Y., Smith, M.M., Sherry, S.B., Seno, M., Moore, M.L., Stewart, S.H., 2022. Alcohol use and death by suicide: A meta-analysis of 33 studies. *Suicide and Life-Threatening Behavior* 52, 600–614. https://doi.org/10.1111/sltb.12846

[39] Deng W, Jin L, Zhuo H, Vasiliou V, Zhang Y, Alcohol consumption and risk of stomach cancer: A meta-analysis, *Chemico-Biological Interactions*, Volume 336, 2021, 109365, ISSN 0009-2797, https://doi.org/10.1016/j.cbi.2021.109365. (https://www.sciencedirect.com/science/article/pii/S0009279721000016)

[40] Wang S, Zou XL, Wu LX, Zhou HF, Xiao L, Yao T, Zhang Y, Ma J, Zeng Y, Zhang L. Epidemiology of intracerebral hemorrhage: A systematic review and meta-analysis. *Front Neurol*. 2022 Sep 16;13:915813. doi: 10.3389/fneur.2022.915813. PMID: 36188383; PMCID: PMC9523083.

[41] Wood, S., Harrison, S.E., Judd, N., Bellis, M.A., Hughes, K., Jones, A., 2021. The impact of behavioral risk factors on communicable diseases: a systematic review of reviews. *BMC Public Health* 21. https://doi.org/10.1186/s12889-021-12148-y

[42] World Health Organization (WHO). STEPS Noncommunicable Disease Risk Factors Survey 2023.

[43] Demographic and Health Survey (DHS) 2023. Fairfax, United States of America: ICF International.

[44] Multiple Indicator Cluster Survey 2021-2022, Wave 5. New York, United States of America: United Nations Children's Fund (UNICEF).

[45] Griswold, Max G et al. Alcohol use and burden for 195 countries and territories, 1990–2016: a systematic analysis for the Global Burden of Disease Study 2016. *The Lancet*, Volume 392, Issue 10152, 1015-1035

High body-mass index

Flowchart

Childhood (Ages 2-19) High Body-Mass Index: Data and Model Flow Chart

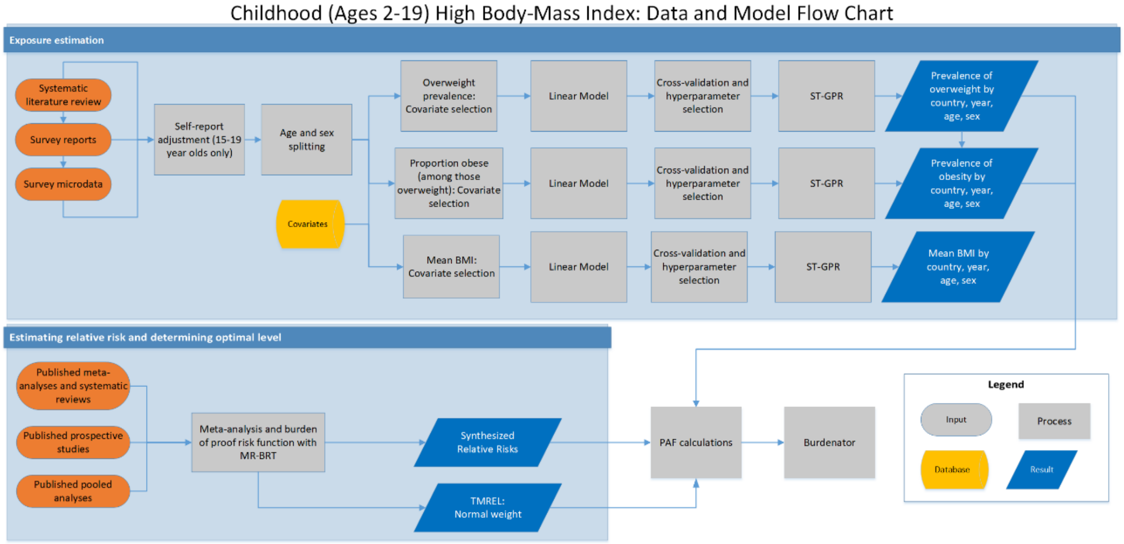

Adult (Ages 20+) High Body-Mass Index: Data and Model Flow Chart

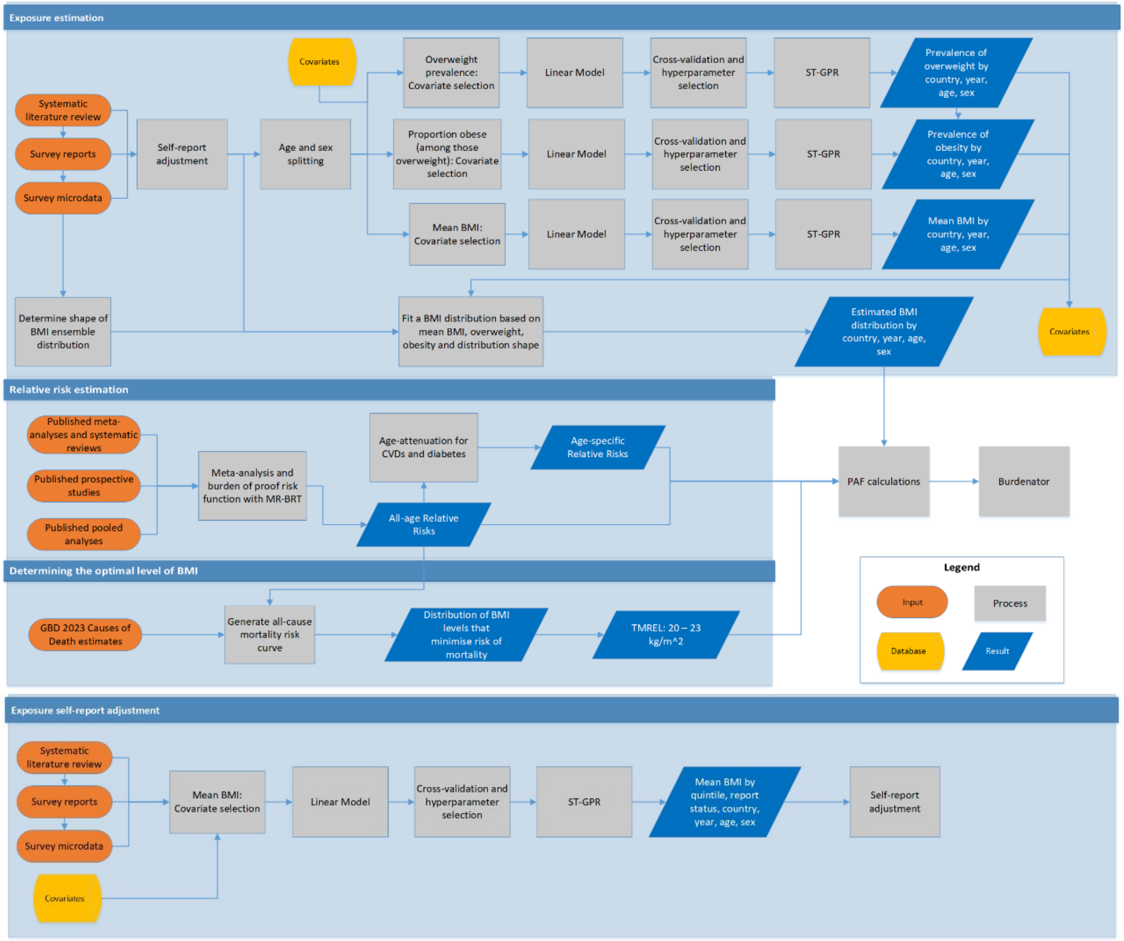

Input data and methodological summary

Definition

Exposure

High body-mass index (BMI) for adults (ages 20+) is defined as BMI greater than 20 to 21 kg/m^2. High BMI for children and adolescents (ages 2–19) is defined as being overweight or obese based on International Obesity Task Force standards.[1]

Input data

Exposure

In GBD 2023, new data were added from sources included in the annual GHDx update of known survey series. We conducted a systematic review in GBD 2017 to identify studies providing nationally or subnationally representative estimates of overweight prevalence, obesity prevalence, or mean body-mass index (BMI). We limited the search to literature published between January 1, 2016, and December 31, 2016, to update the systematic literature search previously performed as part of GBD 2015.

The search for adults was conducted on 4 January 2017, using the following terms:

((("Body Mass Index"[Mesh] OR "Overweight"[Mesh] OR "Obesity"[Mesh]) AND ("Geographic Locations"[Mesh] NOT “United States”[Mesh]) AND ("humans"[Mesh] AND "adult"[MeSH]) AND ("Data Collection"[Mesh] OR "Health Services Research"[Mesh] OR "Population Surveillance"[Mesh] OR "Vital statistics"[Mesh] OR "Population"[Mesh] OR "Epidemiology"[Mesh] OR "surve*"[TiAb]) NOT (Comment[ptyp] OR Case Reports[ptyp] OR "hospital"[TiAb])) AND ("2016/01/01"[Date - Publication] : "2016/12/31"[Date - Publication]))

The search for children was conducted on 4 August 2016, using the following terms:
((("Body Mass Index"[Mesh] OR "Overweight"[Mesh] OR "Obesity"[Mesh]) AND ("Geographic Locations"[Mesh] NOT “United States”[Mesh]) AND ("humans"[Mesh] AND "child"[MeSH]) AND ("Data Collection"[Mesh] OR "Health Services Research"[Mesh] OR "Population Surveillance"[Mesh] OR "Vital statistics"[Mesh] OR "Population"[Mesh] OR "Epidemiology"[Mesh] OR "surve*"[TiAb]) NOT (Comment[ptyp] OR Case Reports[ptyp] OR "hospital"[TiAb])) AND ("2016/01/01"[Date - Publication] : "2016/12/31"[Date - Publication]))

Figure 1: PRISMA diagram for exposure

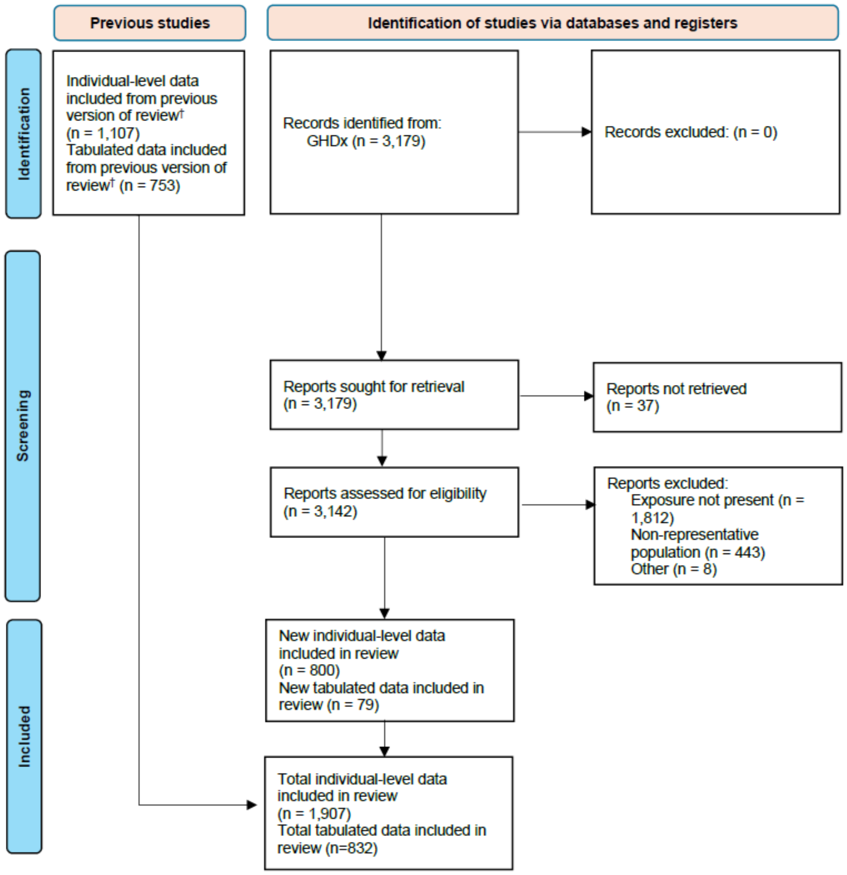

†The number of studies included in previous version of review that met inclusion criteria after data audit.

Eligibility criteria

We included representative studies providing data on mean BMI or prevalence of overweight or obesity among adults or children. For adults, studies were included if they defined overweight as BMI ≥25 kg/m^2 and obesity as BMI ≥30 kg/m^2, or if estimates using those cutoffs could be back-calculated from reported categories. For children (children ages 2–19), studies were included if they used International Obesity Task Force (IOTF) standards to define overweight and obesity thresholds.[1] We only included studies reporting data collected after January 1, 1980. Studies were excluded if they used non-random samples (eg, case-control studies or convenience samples), conducted among specific subpopulations (eg, pregnant women, racial or ethnic minorities, immigrants, or individuals with specific diseases), used alternative methods to assess adiposity (eg, waist circumference, skin-fold thickness, or hydrodensitometry), had sample sizes of less than 20 per age-sex group, or provided inadequate information on any of the inclusion criteria. We also excluded review articles and non-English-language articles.

Data collection process

Where individual-level survey data were available, we computed mean BMI using weight and height. We then used BMI to determine the prevalence of overweight and obesity. For individuals aged over 19 years, we considered them to be overweight if their BMI was greater than or equal to 25 kg/m^2, and

obese if their BMI was greater than or equal to 30 kg/m^2. For individuals aged 2 to 19 years, we used monthly IOTF cutoffs[2] to determine overweight and obese status when age in months was available. When only age in years was available, we used the cutoff for the midpoint of that year. Obese individuals were also considered to be overweight. We excluded studies using the World Health Organization (WHO) standards or country-specific cutoffs to define childhood overweight and obesity. At the individual level, we considered BMI <5 kg/m^2 and BMI >90 kg/m^2 to be biologically implausible and excluded those observations.

The rationale for choosing to use the IOTF cutoffs over the WHO standards has been described elsewhere.[1] Briefly, the IOTF cutoffs provide consistent child-specific standards for ages 2–18 derived from surveys covering multiple countries. By contrast, the WHO growth standards apply to children under age 5, and the WHO growth reference applies to children ages 5–19. The WHO growth reference for children ages 5–19 was derived from USA data, which are less representative than the multinational data used by IOTF. Additionally, the switch between references at age 5 can produce artificial discontinuities. Given that we estimate global childhood overweight and obesity for ages 2–19 (with age 19 using standard adult cutoffs), the IOTF cutoffs were preferable. Additionally, we found that IOTF cutoffs were more commonly used in scientific literature covering childhood obesity.

From report and literature data, we extracted data on mean BMI, prevalence of overweight, and prevalence of obesity, measures of uncertainty for each, and sample size, by the most granular age and sex groups available. Additionally, we extracted the same study-level covariates as were extracted from microdata (measurement, urbanicity, and representativeness), as well as location and year.

In addition to the primary indicators described above, we extracted relevant survey-design variables, including primary sampling unit, strata, and survey weights, which were used to tabulate individual-level microdata and produce accurate measures of uncertainty. We extracted three study-level covariates: 1) whether height and weight data were measured or self-reported; 2) whether the study was predominantly conducted in an urban area, rural area, or both; and 3) the level of representativeness of the study (national or subnational).

Finally, we extracted relevant demographic indicators, including location, year, age, and sex. We estimated the standard error of the mean from individual-level data, where available, and used the reported standard error of the mean for published data. When multiple data sources were available for the same country, we included all of them in our analysis. If data from the same data source were available in multiple formats such as individual-level data and tabulated data, we used individual-level data.

Relative risk

In GBD 2023, we did not conduct an updated systematic review to identify new relative risk data sources. The last date of search in PubMed for evidence studying the health effects of high BMI on cardiovascular diseases and diabetes was 6 June 2019, using the following terms: ("Diabetes Mellitus"[Mesh] OR “diabetes"[title] OR "Stroke"[Mesh] OR “stroke[title]” OR "Heart Diseases"[Mesh] OR “Heart Diseases"[title] OR "Cardiovascular Diseases"[Mesh] OR "Cardiovascular Diseases"[title]) AND ("Obesity"[Mesh] OR "Obesity"[title] OR "Overweight"[Mesh] OR "Overweight"[title] OR "Body Mass Index"[Mesh] OR "Body Mass Index"[title]) AND (“cohort”[tiab]). For other risk–outcome pairs, we used

existing meta-analyses and systematic reviews to identify and extract pooled cohorts and prospective studies for analysis.

Data processing

Age and sex splitting

Any report or literature data provided in age groups wider than the standard five-year age groups or as both sexes combined were split using the approach used by Ng and colleagues.[2] We first modelled age-sex patterns with spatiotemporal Gaussian process regression (ST-GPR) using data sources reporting in sex-specific, standard five-year age units. To account for the large heterogeneity in overweight and obesity prevalence across geographical regions, we categorised each location into three categories of overweight and obesity prevalence. We then aggregated the modelled age and sex patterns into tertiles of overweight and obesity prevalence. Finally, the aggregated patterns were applied to split report and literature data based on the data source's location and its respective tertile of overweight or obesity prevalence. We did not propagate the uncertainty in the age pattern and sex pattern used to split the data as they seemed to have small effect.

Self-report bias adjustment

We included both measured and self-reported data. We tested for bias in self-report data compared to measured data, which is considered to be the gold standard. There was no clear direction of bias for children ages 2–14, so for these age groups we only included measured data. For individuals ages 15 and older, we adjusted self-reported data for BMI at the individual level. We used ST-GPR to determine the level of self-report bias adjustment. Within quintiles of mean BMI, we fit data-type-specific (measured or self-report) ST-GPR models at three different location levels: country, region, and super-region. Multiple location-level models were run to take data availability into consideration. No covariates were selected for these models.

Within each country, sex, and age group combination, model location level was determined by data availability. Groups with ≥5 measured and ≥5 self-report sources used a country-specific model; groups that did not meet the country-specific conditions and were within a region with ≥5 measured and ≥5 self-report sources used a region-specific model; and all other groups used a super-region-specific model. Self-report adjustment factors were calculated from the ratio between measured and self-report ST-GPR model results for each country, year, sex, and five-year age group of the appropriate location-level model.

A separate self-report bias adjustment was completed at the state and national level for the USA. Self-report data from Gallup and BRFSS were aligned with measured data from the NHANES survey series, which was selected as the gold standard for the USA. Microdata were collapsed to mean BMI by age group (ten-year intervals), sex, race, and BMI quantile bin (15 bins), and each self-report source was matched to measured NHANES data within a six-year range and equivalent population. Log-ratio of matched measured to self-reported BMI was calculated as the response variable in an INLA model using random effects on age (ten-year intervals), race, data source, quantile bin, and two-way interactions between age group and race, source and race, and quantile bin and age group, with an assumed Gaussian distribution. Models were fit separately by sex, and a correction ratio was predicted for all

combinations of sex, age, race, source, and quantile bin. All-race adjustment factors were created by taking the population-weighted average of race-specific adjustment factors. All-source adjustment factors were created based on the linear predictor of the INLA model, excluding source-specific effects. For individuals ages 15 and older, the correction ratio was used to adjust all USA self-reported data for BMI at the individual level.

The adjustment factors derived from these two methods are available on the GHDx. GHDX files currently saved: "/snfs1/DATA/Incoming Data/IHME/GBD_2023/BMI"

Modelling strategy

Exposure

Prevalence estimation for mean BMI, overweight, and obesity

After adjusting for self-report bias and splitting aggregated data into five-year age-sex groups, we used ST-GPR to estimate the mean BMI, as well as the prevalence of overweight and obesity. This modelling approach has been described in detail elsewhere.

The linear model, which when added to the smoothed residuals forms the mean prior for GPR, is as follows:

$$\mathrm{logit}(\mathrm{overweight})_{c,a,t} = \beta_0 + \beta_1 \mathrm{educ}_{c,t} + \sum_{k=1}^{16} \beta_k I_{A[a]} + \alpha_s + \alpha_r + \alpha_c$$

$$\mathrm{logit}(\mathrm{obesity/overweight})_{c,a,t} = \beta_0 + \beta_1 \mathrm{educ}_{c,t} + \sum_{k=1}^{16} \beta_k I_{A[a]} + \alpha_s + \alpha_r + \alpha_c$$

$$\log(\mathrm{meanBMI})_{c,a,t} = \beta_0 + \beta_1 \mathrm{educ}_{c,t} + \sum_{k=1}^{16} \beta_k I_{A[a]} + \alpha_s + \alpha_r + \alpha_c$$

where educ is the age-standardised level of educational attainment. $I_{A[a]}$ is a dummy variable indicating specific age group A that the prevalence point captures, and α_s, α_r, and α_c are super-region, region, and country nested random intercepts, respectively. Random effects were used in model fitting but were not used in prediction.

We tested all combinations of the following covariates to see which performed best in terms of in-sample AIC for the overweight linear model and the obesity as a proportion of overweight linear model: ten-year lag-distributed energy per capita, proportion of the population living in urban areas, SDI, lag-distributed income per capita, educational attainment (years) per capita, proportion of the population working in agriculture, grams of sugar adjusted for energy per capita, grams of sugar not adjusted for energy per capita, and the number of two- or four-wheeled vehicles per capita. We selected these candidate covariates based on theory as well as reviewing covariates used in other publications. The final linear model was selected based on 1) if the direction of covariates matched what is expected from theory, 2) all the included covariates were significant, and 3) minimising in-sample AIC. The covariate selection process was performed using the dredge package in R.

Estimating BMI distribution

We used the ensemble distribution approach described in the manuscript. We fit ensemble weights by source, sex, and age groups, with source-, sex-, and age-group-specific weights averaged across all sources included to produce the final global weights. The ensemble weights were fit on measured microdata. The final ensemble weights were exponential = 0.003, gamma = 0.086, inverse gamma = 0.050, log-logistic = 0.352, Gumbel = 0.253, Weibull = 0.041, log-normal = 0.058, normal = 0.020, mirror gamma = 0.015, and mirror Gumbel = 0.122.

250 draws of BMI distributions for each location, year, age group, and sex estimated were produced by fitting an ensemble distribution using 250 draws of estimated mean BMI, 250 draws of estimated standard deviation, and the ensemble weights. Estimated standard deviation was produced by optimising a standard deviation to fit estimated overweight prevalence draws and estimated obesity prevalence draws.

Theoretical minimum risk exposure level

For adults (ages 20+), the theoretical minimum risk exposure level (TMREL) of BMI (20–21 kg/m^2) was determined based on the BMI level that was associated with the lowest risk of all-cause mortality. Briefly, after estimating all-age, cause-specific dose–response risk curves, we generated 250 draws of an all-cause mortality risk curve by taking weighted averages of 250 draws of cause-specific risk curves. The weights were determined from the number of cause-specific global deaths from the GBD 2021 Causes of Death analysis. By generating the all-cause risk curve at the draw level, we were able to determine a distribution of the BMI levels that minimised all-cause mortality by assessing the level of BMI that minimised the risk for each of the 250 draws.

For children and adolescents (ages 2–19), the TMREL is “normal weight,” that is, not overweight or obese, based on IOTF cutoffs.[1]

Table 1: Theoretical minimum-risk exposure level

TMREL definition:	
GBD 2021*	GBD 2023
20–21 kg/m^2	20–21 kg/m^2

*Methods appendix from GBD 2021 mistakenly reported TMREL as 20–23 kg/m^2

Relative risk

Estimating the relative risk (RR) of 26 high-BMI related outcomes occurring as a function of exposure to BMI followed the burden of proof approach established by Zheng and colleagues[3,4] and instantiated in the meta-regression—Bayesian, regularised, trimmed (MR-BRT) tool. MR-BRT synthesises input data to generate a RR curve by relying on an ensemble spline method to capture the potentially non-linear shape of the risk–outcome relationship; integrating over varying exposure ranges in different comparison groups; trimming potentially distorting outliers; testing, selecting, and adjusting for bias covariates to account for known heterogeneity in input study-design characteristics (eg, confounding, selection bias,

exposure measurement); and quantifying remaining between-study heterogeneity (gamma) through random effects modelling and incorporating this value into uncertainty around the mean RR curve.
In GBD 2023, we used MR-BRT to estimate the non-linear dose–response relationships between high BMI and risk for 26 disease endpoints. Specifically, we used the evidence score framework to systematically determine the risk function and evaluate the strength of evidence for each risk–outcome pair. Further details on the evidence score framework are available in the general methods of the Appendix.

The shape of dose–response relationships between BMI and risk for diseases has been well defined.[5,6] To best account for the various shapes (eg, J-shaped, increasing, and decreasing) of these relationships, we used the MR-BRT tool to estimate the log relative risk associated with each level of BMI on a continuous scale. Outcome-specific model characteristics are described in Table 2.

For each risk–outcome pair meta-regression, we considered study-level covariates that could potentially bias the study's reported effect size estimates. These study-level covariates included indication of whether the study used a washout period, whether the study population was randomly sampled from the general population, whether the study measured or asked participants to self-report baseline BMI levels, whether the study determined outcomes based on administrative records or self-reports, and the level of adjustment for relevant confounders like age, sex, smoking, education, and income. We adjusted for these covariates in our meta-regression if they significantly biased our estimated relative risk function.

We implemented the Fisher scoring correction to the heterogeneity parameter, which corrects for data-sparse situations. In such cases, the between-study heterogeneity parameter estimate may be zero, simply from lack of data. The Fisher scoring correction uses a quantile of gamma, which is sensitive to the number of studies, study design, and reported uncertainty.

We also added methodology to detect and flag publication bias. The approach is based on the classic Egger's regression strategy, which is applied to the residuals in our model. In the current implementation, we do not correct for publication bias but flag the risk–outcome pairs where the risk for publication bias is significant. We found no evidence of publication bias for the outcomes associated with high body-mass index.

There is a well-documented attenuation of the risk for cardiovascular disease and diabetes due to metabolic risks factors throughout one's life.[7] To incorporate this age trend in the relative risks, we first identified the median age-at-event across all cohorts and considered that as the reference age group. We then assigned our risk curves to this reference age group. Then, we derived attenuation factors by taking the ratio of excess risk between each age group and the reference. Finally, we applied 1000 draws of the age-specific attenuation factors to 1000 draws of the reference age group's risk curve to determine age-specific risk curves that propagated the uncertainty of both the risk function and age pattern.
For children and adolescent outcomes (ages 2–19), we computed dichotomous relative risks for overweight and obesity by modelling the log difference in relative risk between alternative groups (ie, overweight or obese) and reference groups (ie, normal weight) from prospective cohort studies.

Table 2: Model characteristics for outcomes related to high body-mass index in adults

Outcome	Non-linear specifications and constraints	Selected covariates	Mean gamma solution	Publication bias
Alzheimer’s disease and other dementias	*	Reverse causality; representative population	0.332	No
Asthma	*		0.020	No
Atrial fibrillation and flutter	*		0.016	No
Breast cancer (in premenopausal women)	*		0.000	No
Breast cancer (in postmenopausal women)	*	Representative population	0.110	No
Cataract	**		0.157	No
Colon and rectum cancer	*		0.000	No
Diabetes mellitus type 2	*	Objective exposure measurement; objective outcome ascertainment	0.087	No
Gallbladder and biliary diseases	*	Objective outcome ascertainment; level of adjusted confounders	0.049	No
Gallbladder and biliary tract cancer	*		0.000	No
Gout	*		0.000	No
Intracerebral haemorrhage and subarachnoid haemorrhage	*	Objective exposure measurement	0.118	No
Ischaemic heart disease	*	Objective exposure measurement	0.106	No
Ischaemic stroke	*		0.458	No

Kidney cancer	*		0.036	No
Leukaemia	*		0.000	No
Liver cancer	*		0.032	No
Low back pain	*		0.000	No
Multiple myeloma	**		0.000	No
Non-Hodgkin lymphoma	*		0.058	No
Osteoarthritis	*		0.045	No
Ovarian cancer	*		0.000	No
Pancreatic cancer	*		0.019	No
Thyroid cancer	*		0.000	No
Uterine cancer	*		0.008	No

** Cubic splines with 5 knots; left and right linear tails; Gaussian prior (0, 0.01) on max derivative of non-linear intervals.*

*** Cubic splines with 5 knots; left and right linear tails; Gaussian prior (0, 0.01) on max derivative of non-linear intervals.*

References

[1] Cole TJ, Lobstein T. Extended international (IOTF) body mass index cut-offs for thinness, overweight and obesity. *Pediatr Obes* 2012;7(4):284—94.

[2] Ng M, Fleming T, Robinson M, et al. Global, regional, and national prevalence of overweight and obesity in children and adults during 1980–2013: a systematic analysis for the Global Burden of Disease Study 2013. *Lancet* 2014; 384: 766–81.

[3] Zheng P, Barber R, Sorensen RJD, Murray CJL, Aravkin AY. Trimmed constrained mixed effects models: formulations and algorithms. *J Comput Graph Stat* 2021; 30: 544–56.

[4] Zheng P, Afshin A, Biryukov S, et al. The Burden of Proof studies: assessing the evidence of risk. *Nat Med* 2022; 28: 2038–44.

[5] Angelantonio ED, Bhupathiraju SN, Wormser D, et al. Body-mass index and all-cause mortality: individual-participant-data meta-analysis of 239 prospective studies in four continents. *Lancet* 2016; 388: 776–86. doi: 10.1016/S0140-6736(16)30175-1.

[6] Bhaskaran K, Dos-Santos-Silva I, Leon DA, Douglas IJ, Smeeth L. Association of BMI with overall and cause-specific mortality: a population-based cohort study of 3·6 million adults in the UK. *Lancet Diabetes Endocrinol* 2018; 6(12): 944–53.

[7] Singh GM, Danaei G, Farzadfar F, et al. The age-specific quantitative effects of metabolic risk factors on cardiovascular diseases and diabetes: a pooled analysis. *PLoS One* 2013; 8(7): e65174.

High fasting plasma glucose

Flowchart

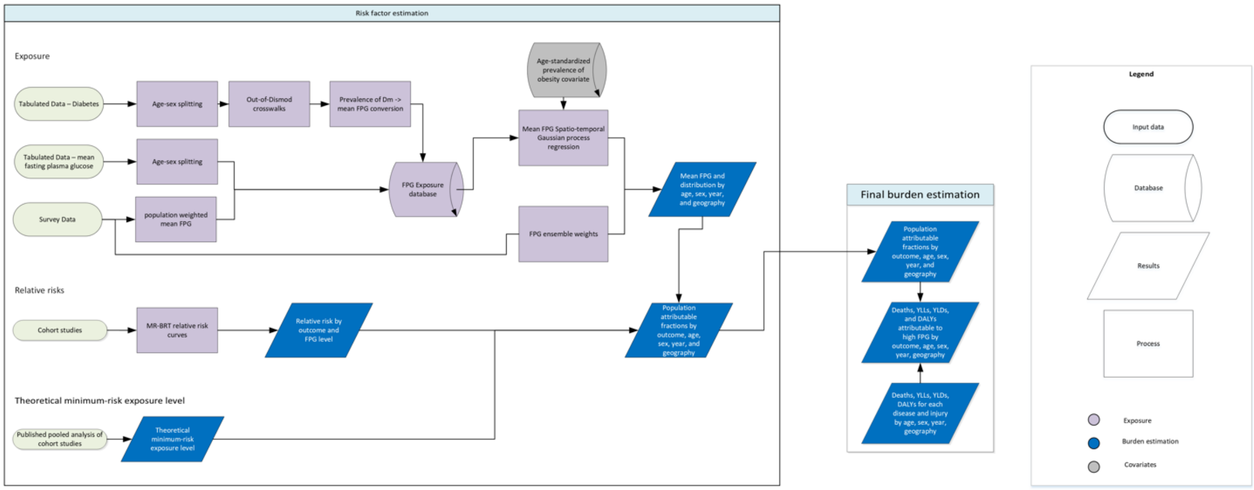

Input data and methodological summary

Definition

Exposure

High fasting plasma glucose (FPG) is measured as the mean FPG in a population, where FPG is a continuous exposure in units of mmol/L. Since FPG is along a continuum, we define high FPG as any level above the theoretical minimum risk exposure level (TMREL), which is 4.9–5.3 mmol/L.

Input data

Exposure

We used all available sources on FPG and prevalence of diabetes in the FPG model. Data inputs came from three sources:

- Estimates of mean FPG in a representative population
- Individual-level data of FPG measured from surveys
- Estimates of diabetes prevalence in a representative population

Data sources that did not report mean FPG or prevalence of diabetes were excluded from analysis. When a study reported both mean FPG and prevalence of diabetes, we used the mean FPG for exposure estimates. Where possible, individual-level data supersede any data presented in a published study or report. Individual-level data were aggregated to produce estimates for each five-year age group, sex, location, and year of a survey.

In GBD 2023, a total of 236 new sources were added to the FPG exposure model compared to GBD 2021.

An updated systematic review was conducted for the high FPG exposure model alongside non-fatal diabetes model for GBD 2023; details of the systematic review have been described in the non-fatal write-up for diabetes. Briefly, the systematic review search dates were Oct 17, 2018 – Feb 11, 2022. A total of 75 new sources were identified for diabetes and high FPG. Common exclusion reasons include duplicative studies and not population representative.

Figure 2: *Prisma flow diagram for high FPG exposure model GBD 2023 systematic review*

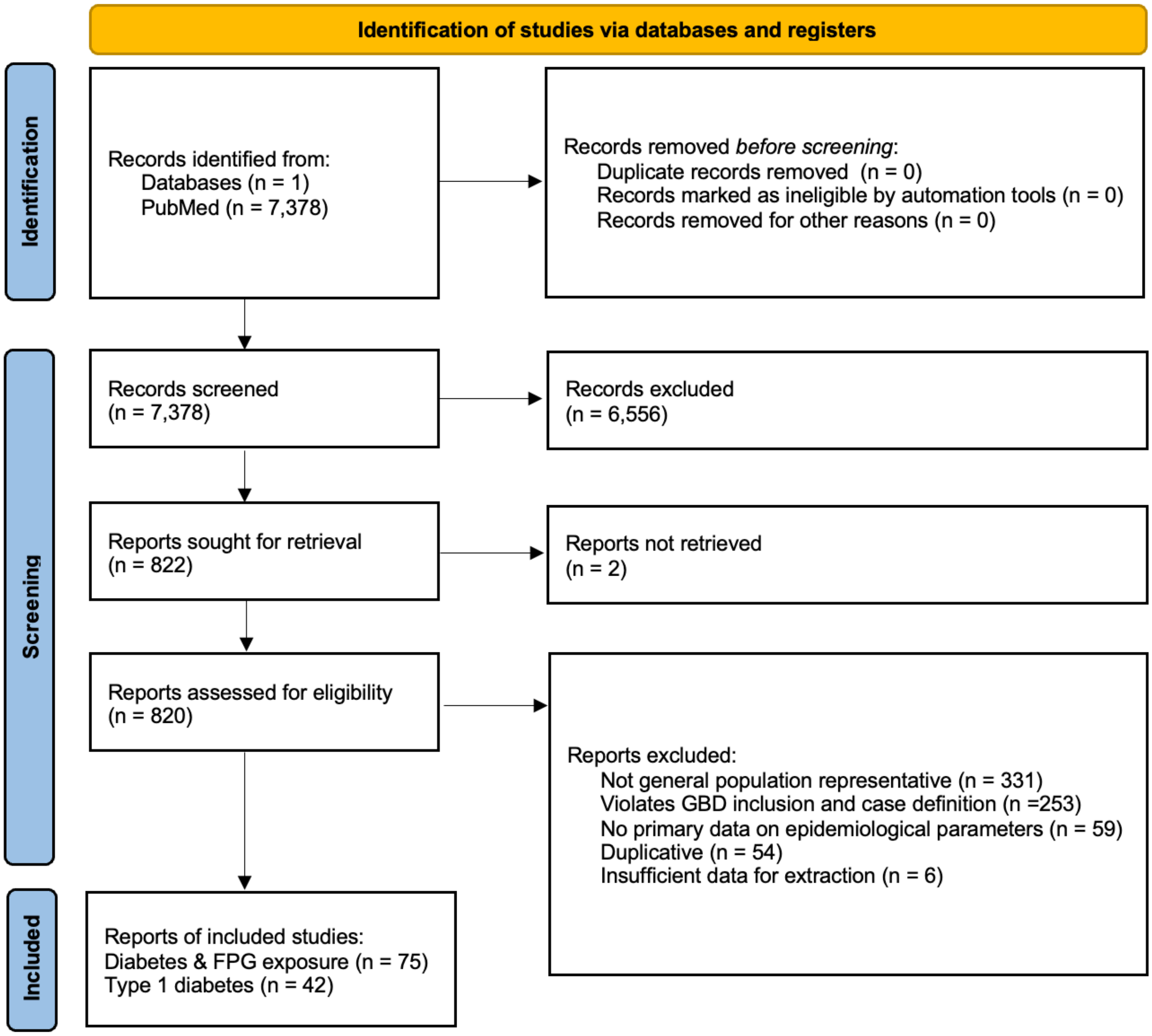

Additionally, collaborator-provided sources that were either shared directly with us or were identified through searching the Global Health Data Exchange (GHDx) were reviewed for inclusion.

Relative risk

For each outcome, we extract the relative risk estimates from original cohort studies. In GBD 2023, all cancer-related and dementia outcomes had updated systematic reviews. Other outcomes continue to

use the same input data described in detail previously in GBD 2021. Briefly, prior studies used in earlier GBD rounds were reviewed to replace meta-analysis sources with primary data from original cohorts, and additional opportunistic reviews were done based on the search string.

Outcomes: bladder cancer, breast cancer, colon and rectum cancer, liver cancer, tracheal, bronchus and lung cancer, pancreatic cancer

In GBD 2023, an updated systematic review was conducted for cancer-related outcomes. PubMed was the only database searched due to time constraints. However, we reviewed references in meta-analysis and systematic reviews that were identified in the search, in which most included searches in multiple databases. Additionally, input data used in previous GBD rounds were reviewed for extraction errors and cohort duplications.

- Search string (Dates: **2020/01/01 - 2023/09/07)**
  (((((diabetes[MeSH Terms] OR diabetes[Title/Abstract] OR hyperglycemia[MeSH Terms] OR hyperglycemia[Title/Abstract] OR blood glucose[MeSH Terms] OR blood glucose[Title])) AND (Case-Control Studies[MeSH Terms] OR Cross-Over Studies[MeSH Terms] OR Cohort Studies[MeSH Terms] OR Systematic Review[Publication Type] OR Meta-Analysis[Publication Type] OR systematic review[Title/Abstract] OR Meta-analysis[Title/Abstract] OR cohort[Title/Abstract] OR cross-over[Title/Abstract] OR crossover[Title/Abstract] OR case-control[Title/Abstract] OR prospective[Title/Abstract] OR retrospective[Title/Abstract] OR longitudinal[Title/Abstract] OR follow-up[Title/Abstract] OR Dose-Response Relationship, Drug[MeSH Terms] OR dose-response[Title/Abstract]) AND (Risk[MeSH Terms] OR Odds Ratio[MeSH Terms] OR risk[Title/Abstract] OR odds ratio[Title/Abstract] OR cross-product ratio[Title/Abstract] OR hazards ratio[Title/Abstract] OR hazard ratio[Title/Abstract])) NOT (animals[MeSH Terms] NOT Humans[MeSH Terms])))) AND (cancer[MeSH Terms] OR neoplasm[MeSH Terms] OR cancer[Title/Abstract] OR neoplasm[Title/Abstract])

Figure 3: Prisma diagram for updated cancer-related outcomes systematic review for GBD 2023

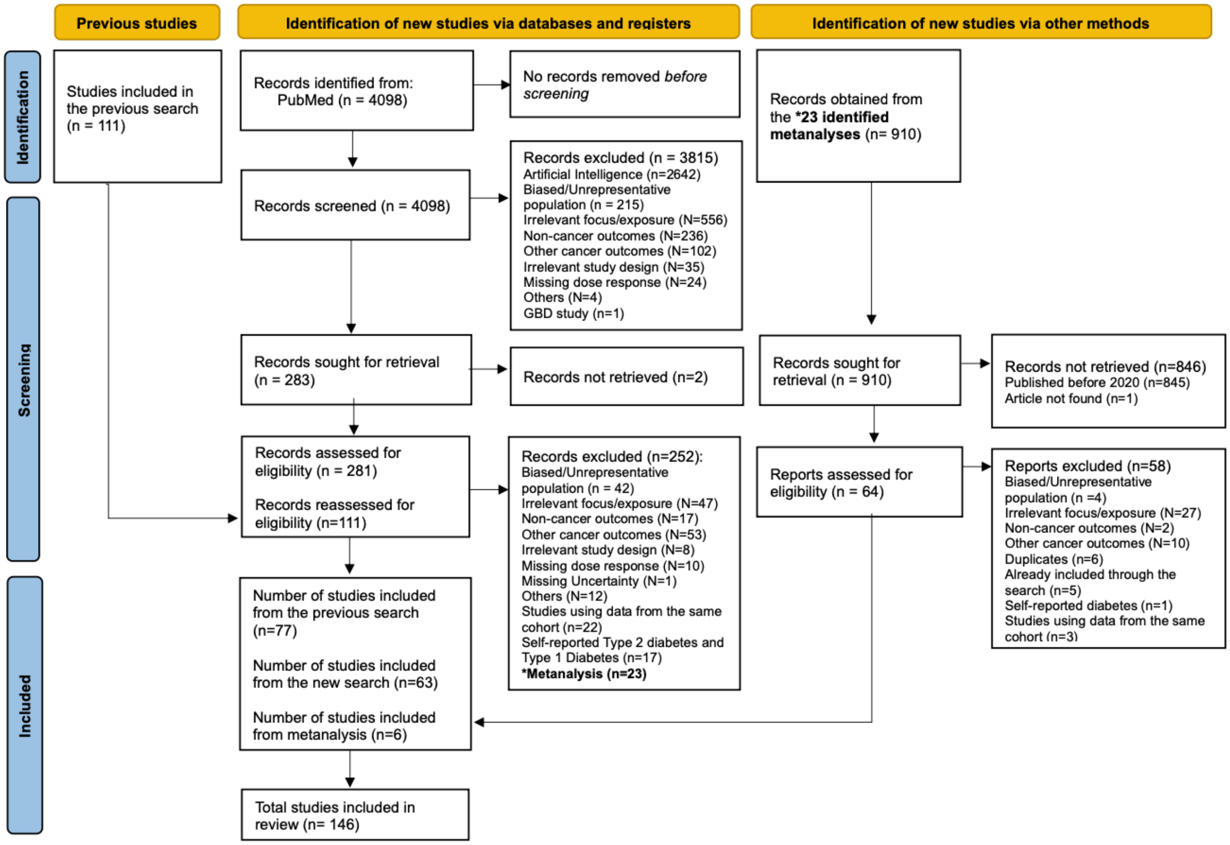

Below are the final sources count by outcome:

- Bladder cancer: 34
- Breast cancer: 49
- Colon and rectum cancer: 58
- Pancreatic cancer: 55
- Liver cancer: 50
- Tracheal, bronchus, and lung cancer: 32

Outcome: Alzheimer's disease and other dementia

In GBD 2023, an updated systematic review was conducted for Alzheimer's disease and other dementia outcome in three different databases. Additionally, input data used in previous GBD rounds were reviewed for extraction errors and cohort duplications.

- Search string by database (Dates: 2020/01/01 - 2024/01/04)

PubMed	Embase	Web of Science
(((((diabetes[MeSH Terms] OR diabetes[Title/Abstract] OR hyperglycemia[MeSH Terms] OR hyperglycemia[Title/Abstract] OR blood glucose[MeSH Terms] OR blood glucose[Title])) AND (Case-Control Studies[MeSH	('diabetes mellitus'/exp OR 'diabetes mellitus' OR 'hyperglycemia'/exp OR 'hyperglycemia' OR 'blood glucose level'/exp OR 'blood glucose level' OR 'diabetes':ab,ti,kw OR	((TI=(("diabetes" OR "hyperglycemia" OR "blood glucose") AND ("alzheimer disease" OR "dementia" OR "alzheimer") AND (("Case-Control

Terms] OR Cross-Over Studies[MeSH Terms] OR Cohort Studies[MeSH Terms] OR Systematic Review[Publication Type] OR Meta-Analysis[Publication Type] OR systematic review[Title/Abstract] OR meta-analysis[Title/Abstract] OR cohort[Title/Abstract] OR cross-over[Title/Abstract] OR crossover[Title/Abstract] OR case-control[Title/Abstract] OR prospective[Title/Abstract] OR retrospective[Title/Abstract] OR longitudinal[Title/Abstract] OR follow-up[Title/Abstract] OR Dose-Response Relationship, Drug[MeSH Terms] OR dose-response[Title/Abstract]) AND (Risk[MeSH Terms] OR Odds Ratio[MeSH Terms] OR risk[Title/Abstract] OR odds ratio[Title/Abstract] OR cross-product ratio[Title/Abstract] OR hazards ratio[Title/Abstract] OR hazard ratio[Title/Abstract])) AND ((2020/01/01[PDat] : 3000[PDat]) NOT (animals[MeSH Terms] NOT Humans[MeSH Terms])))) AND (alzheimer disease[MeSH Terms] OR dementia[MeSH Terms] OR alzheimer[Title/Abstract] OR dementia[Title/Abstract])	'hyperglycemia':ab,ti,kw OR 'blood glucose':ab,ti,kw) AND ('case control study'/exp OR 'case control study' OR 'cohort analysis'/exp OR 'cohort analysis' OR 'systematic review'/exp OR 'systematic review' OR 'meta analysis'/exp OR 'meta analysis' OR 'dose response'/exp OR 'dose response' OR 'systematic review':ab,ti,kw OR 'meta-analysis':ab,ti,kw OR 'cohort':ab,ti,kw OR 'cross-over':ab,ti,kw OR 'crossover':ab,ti,kw OR 'case-control':ab,ti,kw OR 'prospective':ab,ti,kw OR 'retrospective':ab,ti,kw OR 'longitudinal':ab,ti,kw OR 'follow-up':ab,ti,kw OR 'dose-response':ab,ti,kw) AND ('risk'/exp OR 'risk' OR 'odds ratio'/exp OR 'odds ratio' OR 'hazard ratio'/exp OR 'hazard ratio' OR 'attributable risk'/exp OR 'attributable risk' OR 'risk':ab,ti,kw OR 'odds ratio':ab,ti,kw OR 'cross-product ratio':ab,ti,kw OR 'hazards ratio':ab,ti,kw OR 'hazard ratio':ab,ti,kw) AND [2020-2023]/py NOT (('animal'/exp OR 'animal') NOT ('human'/exp OR 'human')) AND ('dementia'/exp OR 'dementia' OR 'alzheimer disease'/exp OR 'alzheimer disease' OR 'dementia':ab,ti,kw OR 'cognition':ab,ti,kw OR 'cognitive decline':ab,ti,kw OR 'alzheimer':ab,ti,kw) AND [embase]/lim NOT [medline]/lim	Studies" OR "Cross-Over Studies" OR "Cohort Studies" OR "Systematic Review" OR "Meta-Analysis" OR "systematic review" OR "meta-analysis" OR "cohort" OR "cross-over" OR "crossover" OR "case-control" OR "prospective" OR "retrospective" OR "longitudinal" OR "follow-up" OR "Dose-Response") AND ("Risk" OR "Odds Ratio" OR "cross-product ratio" OR "hazards ratio" OR "hazard ratio")))) OR AB=(("diabetes" OR "hyperglycemia" OR "blood glucose") AND ("alzheimer disease" OR "dementia" OR "alzheimer") AND (("Case-Control Studies" OR "Cross-Over Studies" OR "Cohort Studies" OR "Systematic Review" OR "Meta-Analysis" OR "systematic review" OR "meta-analysis" OR "cohort" OR "cross-over" OR "crossover" OR "case-control" OR "prospective" OR "retrospective" OR "longitudinal" OR "follow-up" OR "Dose-Response") AND ("Risk" OR "Odds Ratio" OR "cross-product ratio" OR "hazards ratio" OR "hazard ratio")))) NOT ALL=(animals)

Figure 4: Prisma diagram for updated Alzheimer's disease and other dementia systematic review for GBD 2023

Final source count for dementia is 48.

Outcomes: ischaemic stroke, ischaemic heart disease, intracerebral haemorrhage

No updates in GBD 2023. Described below are updates in GBD 2021 and data that were used for GBD 2023.

- Search string

(((((diabetes[MeSH Terms] OR diabetes[Title/Abstract] OR hyperglycemia[MeSH Terms] OR hyperglycemia[Title/Abstract] OR blood glucose[MeSH Terms] OR blood glucose[Title])) AND (Case-Control Studies[MeSH Terms] OR Cross-Over Studies[MeSH Terms] OR Cohort Studies[MeSH Terms] OR Systematic Review[Publication Type] OR Meta-Analysis[Publication Type] OR systematic review[Title/Abstract] OR meta-analysis[Title/Abstract] OR cohort[Title/Abstract] OR cross-over[Title/Abstract] OR crossover[Title/Abstract] OR case-control[Title/Abstract] OR prospective[Title/Abstract] OR retrospective[Title/Abstract] OR longitudinal[Title/Abstract] OR follow-up[Title/Abstract] OR Dose-Response Relationship, Drug[MeSH Terms] OR dose-response[Title/Abstract]) AND (Risk[MeSH Terms] OR Odds Ratio[MeSH Terms] OR risk[Title/Abstract] OR odds ratio[Title/Abstract] OR cross-product ratio[Title/Abstract] OR hazards ratio[Title/Abstract] OR hazard ratio[Title/Abstract])) AND ((1970/01/01[PDat] : 2019/12/31[PDat]) NOT (animals[MeSH Terms] NOT Humans[MeSH Terms])))) AND (intracranial hemorrhage[MeSH Terms] OR stroke[MeSH Terms] OR brain infarction[MeSH Terms] OR cerebral infarction[MeSH Terms] OR intracerebral hemorrhage[MeSH Terms] OR subarachnoid hemorrhage[MeSH Terms] OR cerebrovascular stroke[MeSH Terms] OR heart disease[MeSH Terms] OR peripheral artery disease[MeSH Terms] OR intracranial hemorrhage[Title/Abstract] OR stroke[Title/Abstract] OR brain infarction[Title/Abstract] OR cerebral infarction[Title/Abstract] OR intracerebral hemorrhage[Title/Abstract] OR subarachnoid

hemorrhage[Title/Abstract] OR cerebrovascular stroke[Title/Abstract] OR heart disease[Title/Abstract] OR peripheral artery disease[Title/Abstract])

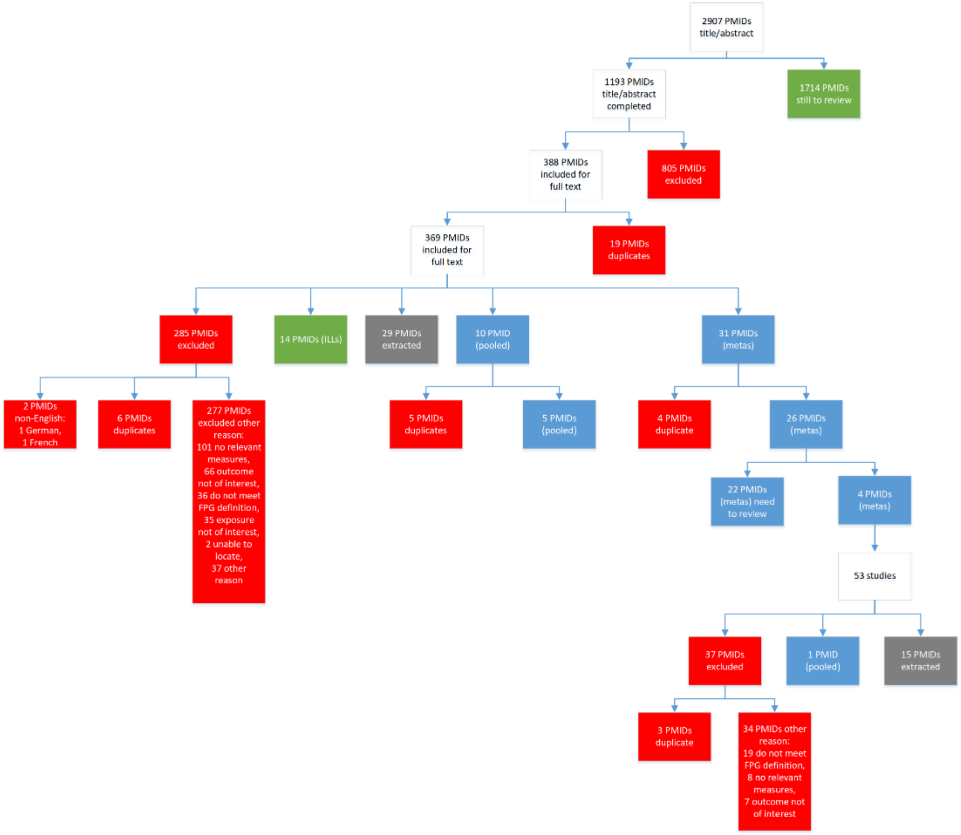

- Previously accepted studies

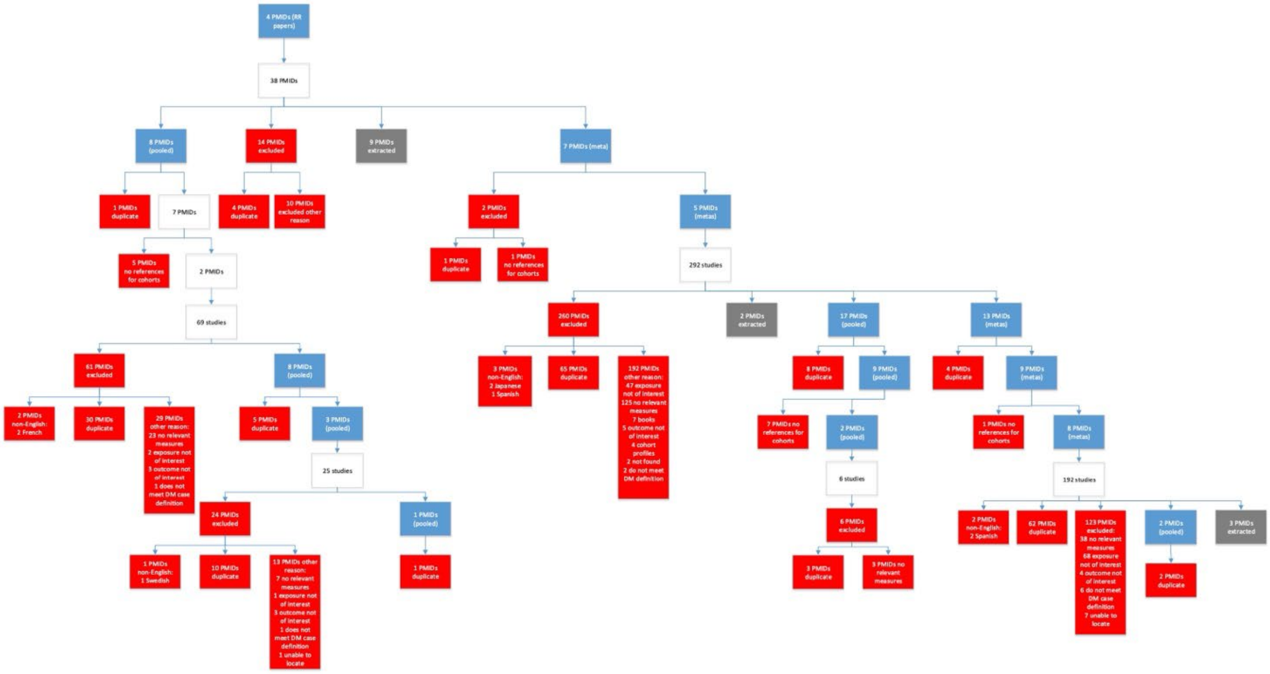

- Prospectively found additional meta-analyses

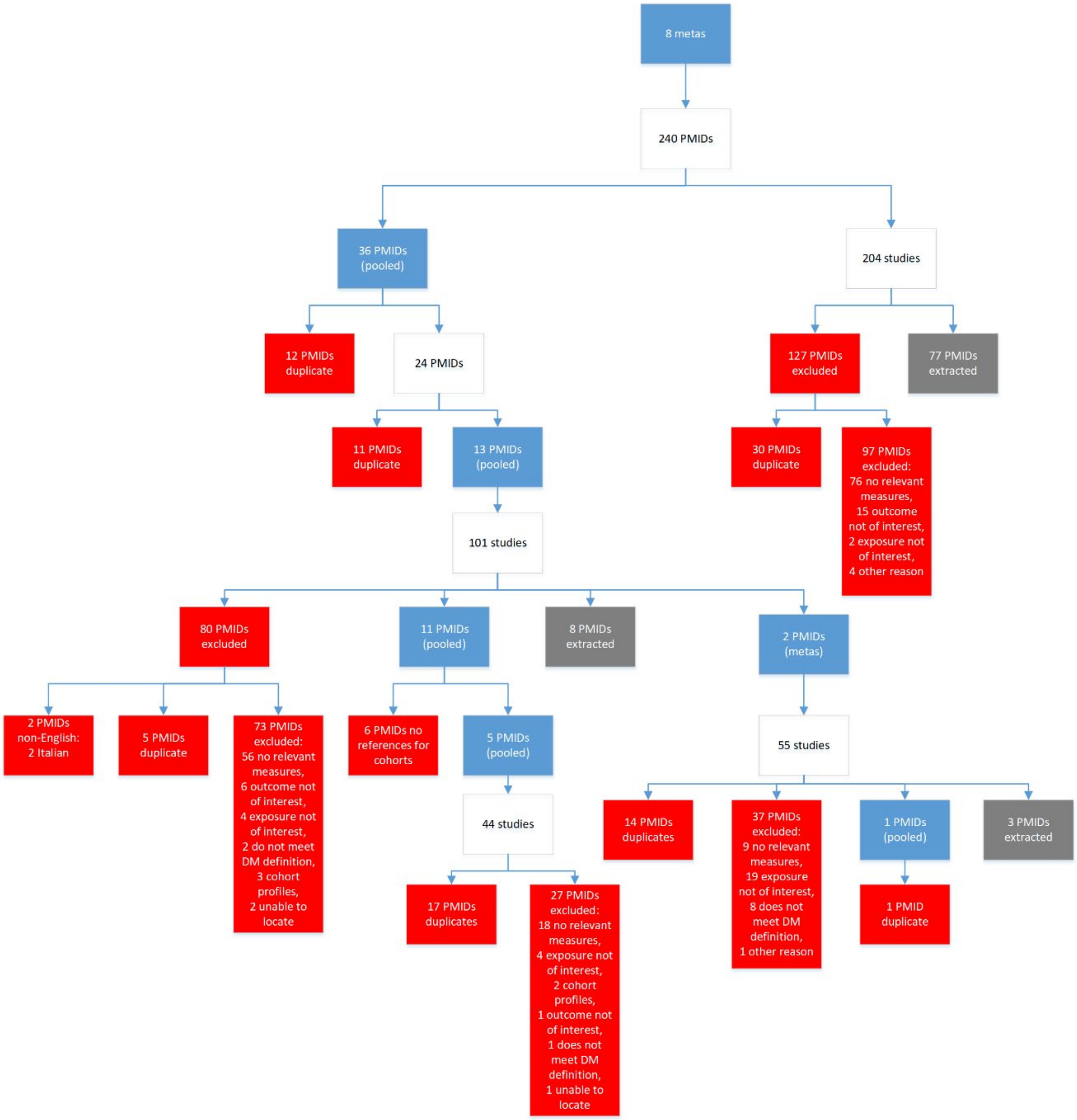

An additional effort for the ischaemic stroke, ischaemic heart disease, and intracerebral haemorrhage outcomes was undertaken in 2022 to re-review the relative risk data. Major updates included excluding data for composite cardiovascular outcomes that were previously included in the ischaemic heart disease model and excluding data for unspecified stroke that were previously included in the ischaemic stroke model. An opportunistic search of PubMed was also undertaken to add recently published cohort studies (post-2010) and data reporting exposure using glycated hemoglobin (HbA1c) or oral glucose tolerance tests (OGTT). Below are the final sources count:

- Ischaemic stroke: 26 sources
- Ischaemic heart disease: 49 sources
- Intracerebral haemorrhage: 9 sources

As a result of this additional effort, subarachnoid haemorrhage was dropped as an outcome of high FPG during GBD 2021, due to an implausible biological mechanism for this risk–outcome pair and a lack of data informing this relationship (only one datapoint specific to subarachnoid haemorrhage was found acceptable for inclusion).

Outcome: chronic kidney disease

No updates in GBD 2023. Described below are updates in GBD 2021 and data that were used for GBD 2023.

- Previously accepted studies

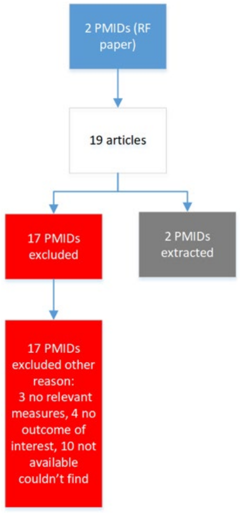

- Search string

((((((chronic kidney failure[MeSH Terms]) OR chronic kidney disease[Title/Abstract]) OR glomerulonephritis[MeSH Terms]) OR glomerulonephritis[Title/Abstract]) OR chronic kidney failure[Title/Abstract])) AND (((((diabetes[MeSH Terms]) OR diabetes[Title/Abstract]) OR hyperglycemia[MeSH Terms]) OR hyperglycemia[Title/Abstract]) OR blood glucose[MeSH Terms]) OR blood glucose[Title])) AND (((((((((diabetes[MeSH Terms]) OR diabetes[Title/Abstract]) OR hyperglycemia[MeSH Terms]) OR hyperglycemia[Title/Abstract]) OR blood glucose[MeSH Terms]) OR blood glucose[Title])) AND ((Case-Control Studies[MeSH Terms] OR Cross-Over Studies[MeSH Terms] OR Cohort Studies[MeSH Terms] OR Systematic Review[Publication Type] OR Meta-Analysis[Publication Type] OR systematic review[Title/Abstract] OR meta-analysis[Title/Abstract] OR cohort[Title/Abstract] OR cross-over[Title/Abstract] OR crossover[Title/Abstract] OR case-control[Title/Abstract] OR prospective[Title/Abstract] OR retrospective[Title/Abstract] OR longitudinal[Title/Abstract] OR follow-up[Title/Abstract] OR Dose-Response Relationship, Drug[MeSH Terms] OR dose-response[Title/Abstract]) AND (Risk[MeSH Terms] OR Odds Ratio[MeSH Terms] OR risk[Title/Abstract] OR odds ratio[Title/Abstract] OR cross-product ratio[Title/Abstract] OR hazards ratio[Title/Abstract] OR

hazard ratio[Title/Abstract]) AND (1970/01/01[PDat] : 2019/12/31[PDat]) NOT (animals[MeSH Terms] NOT Humans[MeSH Terms])))

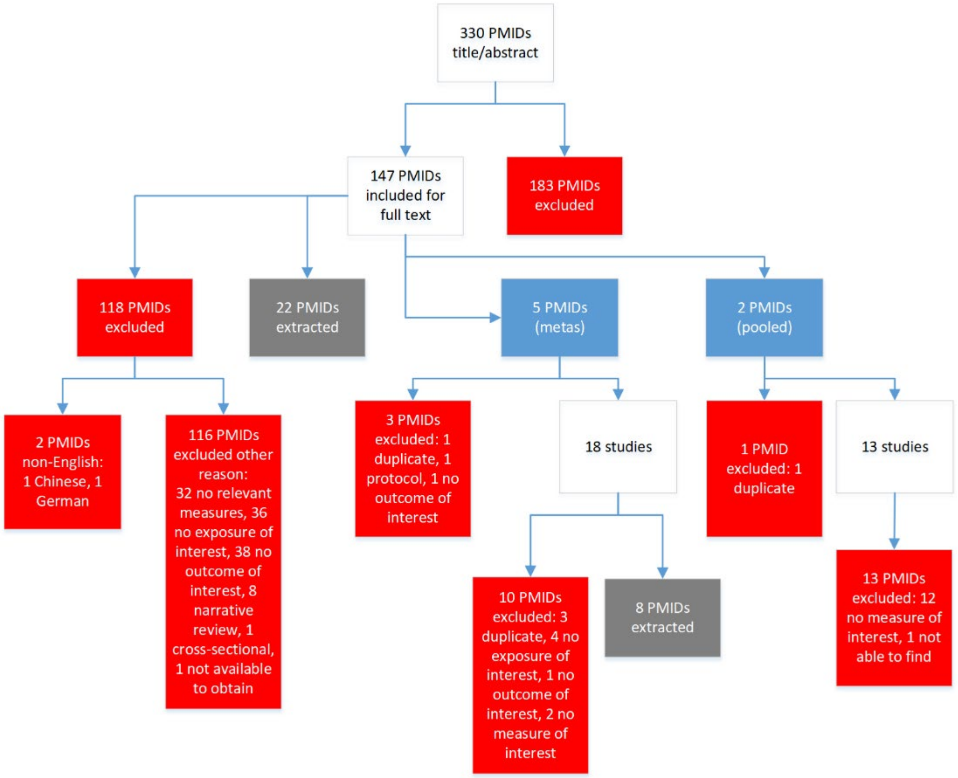

Final number of sources: 17

Outcome: tuberculosis

No updates in GBD 2023. Described below are updates in GBD 2021 and data that were used for GBD 2023.

- Previously accepted studies

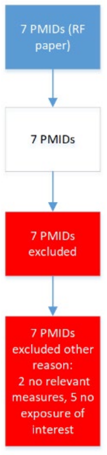

- Search string

((((tuberculosis[MeSH Terms]) OR tuberculosis[Title/Abstract])) AND (((((((Case-Control Studies[MeSH Terms] OR Cross-Over Studies[MeSH Terms] OR Cohort Studies[MeSH Terms] OR Systematic Review[Publication Type] OR Meta-Analysis[Publication Type] OR systematic review[Title/Abstract] OR meta-analysis[Title/Abstract] OR cohort[Title/Abstract] OR cross-over[Title/Abstract] OR crossover[Title/Abstract] OR case-control[Title/Abstract] OR prospective[Title/Abstract] OR retrospective[Title/Abstract] OR longitudinal[Title/Abstract] OR follow-up[Title/Abstract] OR Dose-Response Relationship, Drug[MeSH Terms] OR dose-response[Title/Abstract]) AND (Risk[MeSH Terms] OR Odds Ratio[MeSH Terms] OR risk[Title/Abstract] OR odds ratio[Title/Abstract] OR cross-product ratio[Title/Abstract] OR hazards ratio[Title/Abstract] OR hazard ratio[Title/Abstract]) AND (1970/01/01[PDat] : 2019/12/31[PDat]) NOT (animals[MeSH Terms] NOT Humans[MeSH Terms]))))))) AND ((((((diabetes[MeSH Terms]) OR diabetes[Title/Abstract]) OR hyperglycemia[MeSH Terms]) OR hyperglycemia[Title/Abstract]) OR blood glucose[MeSH Terms]) OR blood glucose[Title])

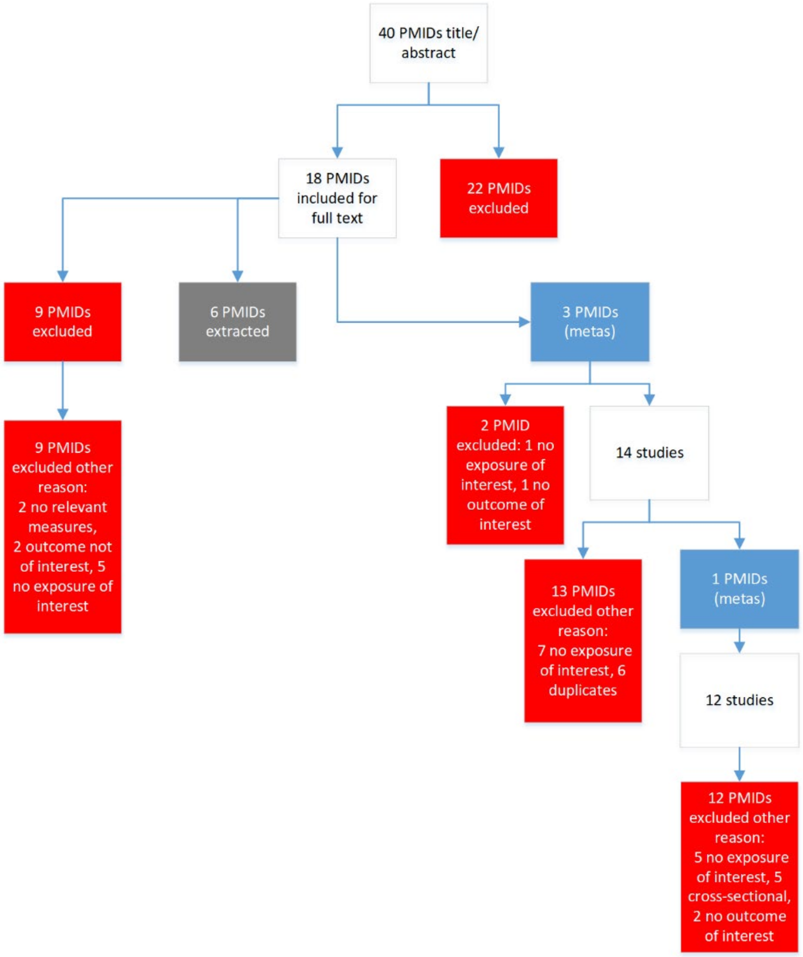

Final number of sources: 12

Outcomes: glaucoma, cataracts

No updates in GBD 2023. Described below are updates in GBD 2021 and data that were used for GBD 2023.

- Previously accepted studies

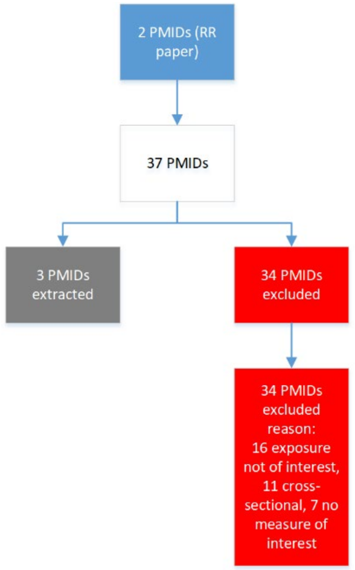

- Search string

(((((diabetes[MeSH Terms] OR diabetes[Title/Abstract] OR hyperglycemia[MeSH Terms] OR hyperglycemia[Title/Abstract] OR blood glucose[MeSH Terms] OR blood glucose[Title])) AND (Case-Control Studies[MeSH Terms] OR Cross-Over Studies[MeSH Terms] OR Cohort Studies[MeSH Terms] OR Systematic Review[Publication Type] OR Meta-Analysis[Publication Type] OR systematic review[Title/Abstract] OR meta-analysis[Title/Abstract] OR cohort[Title/Abstract] OR cross-over[Title/Abstract] OR crossover[Title/Abstract] OR case-control[Title/Abstract] OR prospective[Title/Abstract] OR retrospective[Title/Abstract] OR longitudinal[Title/Abstract] OR follow-up[Title/Abstract] OR Dose-Response Relationship, Drug[MeSH Terms] OR dose-response[Title/Abstract]) AND (Risk[MeSH Terms] OR Odds Ratio[MeSH Terms] OR risk[Title/Abstract] OR odds ratio[Title/Abstract] OR cross-product ratio[Title/Abstract] OR hazards ratio[Title/Abstract] OR hazard ratio[Title/Abstract])) AND ((1970/01/01[PDat] : 2019/12/31[PDat]) NOT (animals[MeSH Terms] NOT Humans[MeSH Terms])))) AND (glaucoma[MeSH Terms] OR cataract[MeSH Terms] OR glaucoma[Title/Abstract] OR cataract[Title/Abstract])

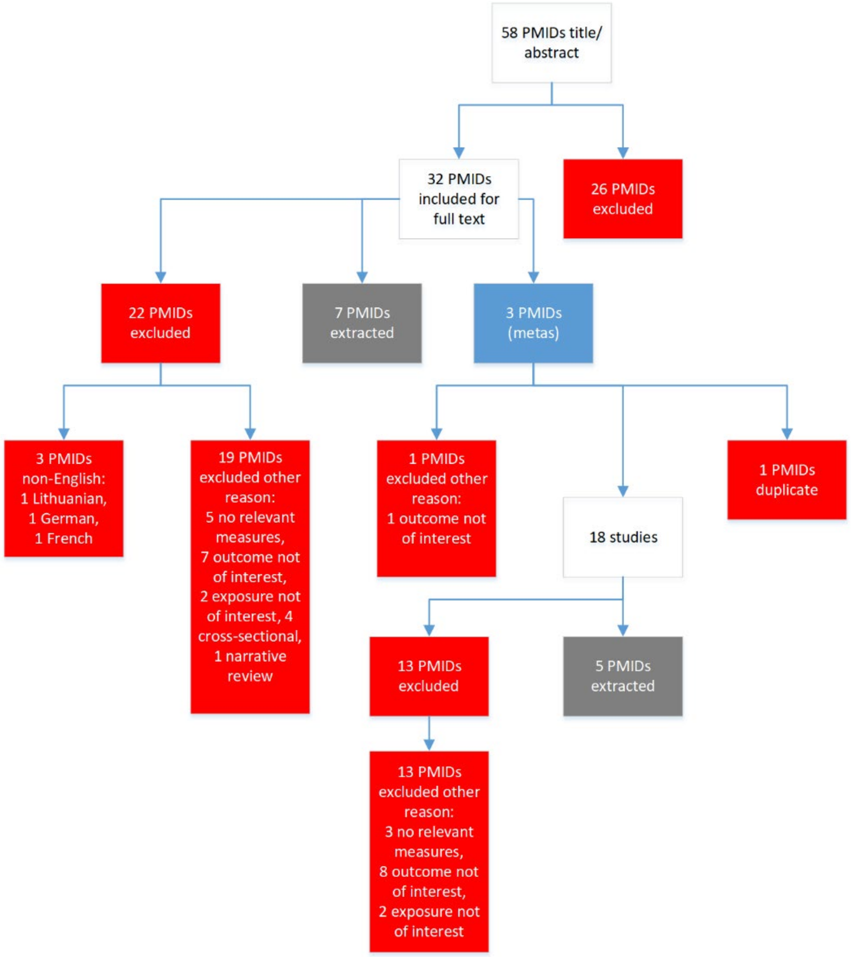

Final number of sources:

- Glaucoma: 25
- Cataract: 5

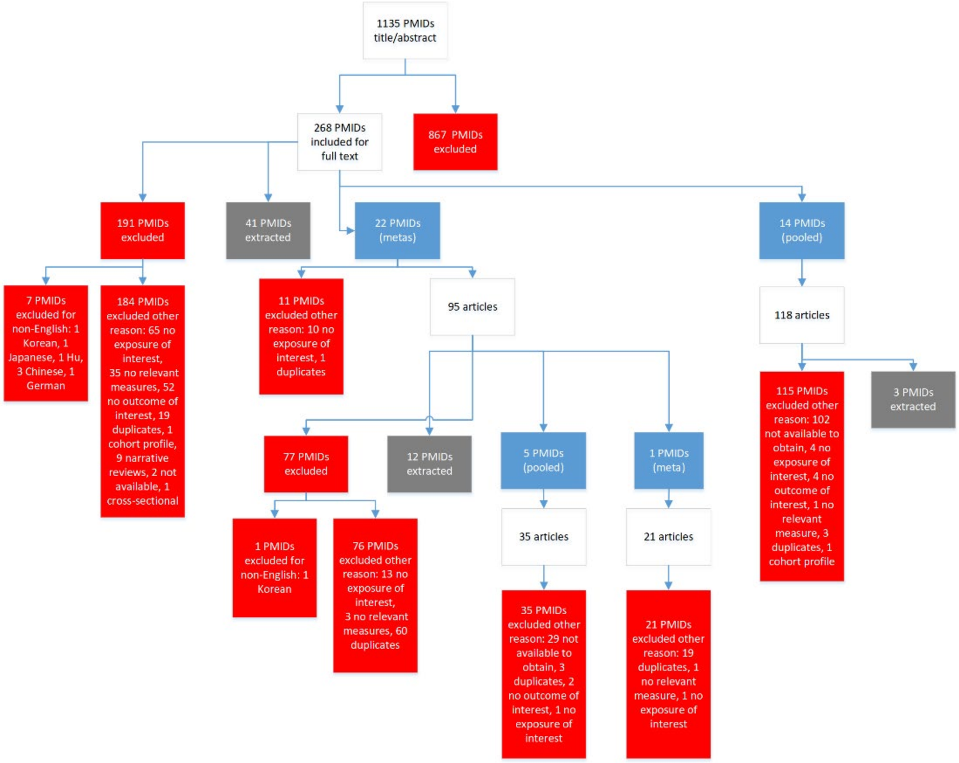

Outcome: peripheral arterial disease

No updates in GBD 2023. Described below are updates in GBD 2021 and data that were used for GBD 2023.

An opportunistic search was undertaken for peripheral arterial disease in 2022. A review of studies included in previous GBD rounds and original studies described in meta-analyses for the relationship between peripheral arterial disease and high FPG was prioritised due to resource constraints. As a result of this opportunistic search, ten sources and 15 datapoints were included for peripheral arterial disease in GBD 2021.

Final number of sources: 10

Data processing

Exposure

We performed several processing steps to the data to address sampling and measurement inconsistencies and to ensure the data are comparable across data sources and between diabetes mellitus prevalence modelling efforts.

1. *Small sample size*
   Data with a sample size of ten or less were outliered prior to modelling.
2. Imputing missing standard deviation

We imputed standard deviation for sources which this information was missing and only reported mean FPG value. This was imputed using mean FPG ensemble distributions described in more detail below in the modelling section.

3. *Diabetes prevalence processing*
   We used an ensemble distribution to estimate mean FPG based on prevalence of diabetes for sources where data on mean FPG were not available, but there were data on diabetes prevalence. Essentially, we constructed a distribution based on unit-level data available in 31 countries. Before predicting mean FPG from prevalence of diabetes, we ensured that the prevalence of diabetes was based on the reference case definition: FPG greater than or equal to 126 mg/dL (7 mmol/L) or on treatment. For more details on how the case definition crosswalk is conducted, please see the diabetes mellitus appendix section. Then, we predicted out the mean FPG by age and sex.
4. *Age and sex splitting*: Reported estimates of mean FPG were split by age and sex where possible.
   a. First, if studies reported mean FPG for broad age groups by sex, and by specific age groups but for both sexes combined, age-specific estimates were split by sex using the sex ratio from within the study.
   b. Second, input data reporting mean FPG for both sexes that could not be split using a within-study ratio were split using a sex ratio derived from a meta-analysis of existing sex-specific data using meta-regression—Bayesian, regularised, trimmed (MR-BRT).[1]

Figure 5: Mean FPG MR-BRT input and output sex ratio

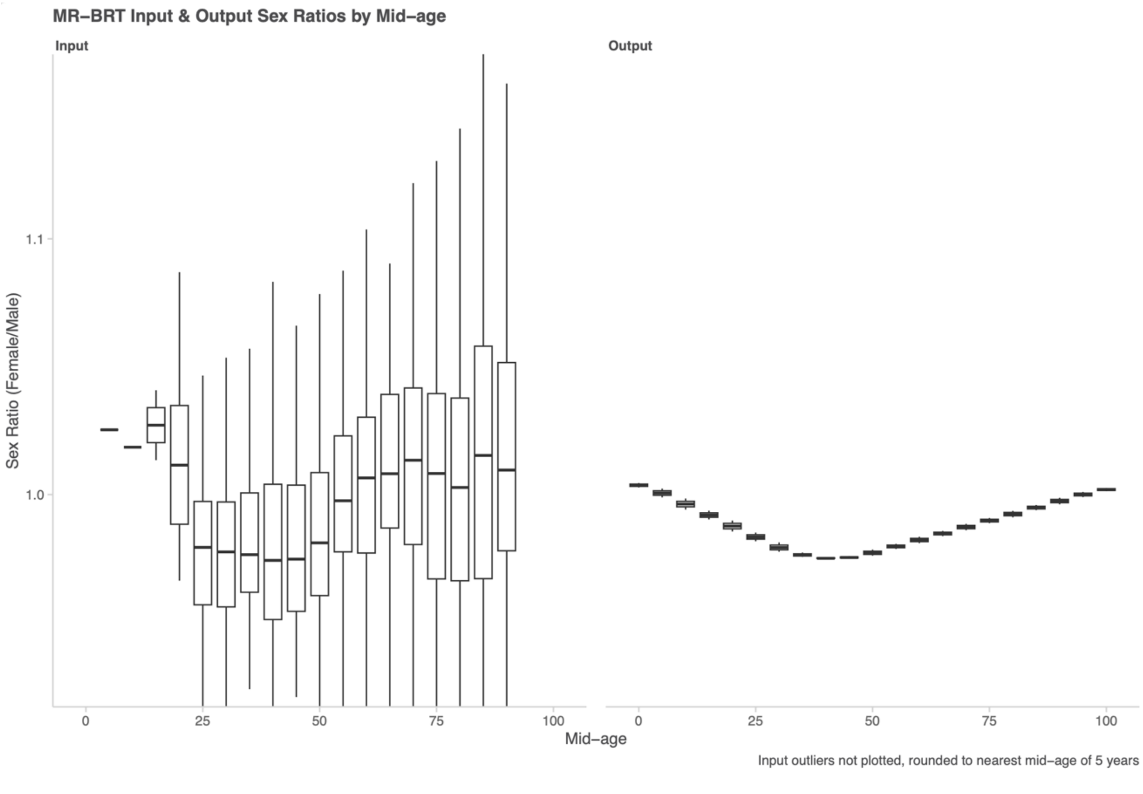

   c. Finally, where studies reported estimates across age groups spanning more than five years, these were split into five-year age groups using the age midpoint of the estimate.

Figure 6: Mean FPG male and female age pattern

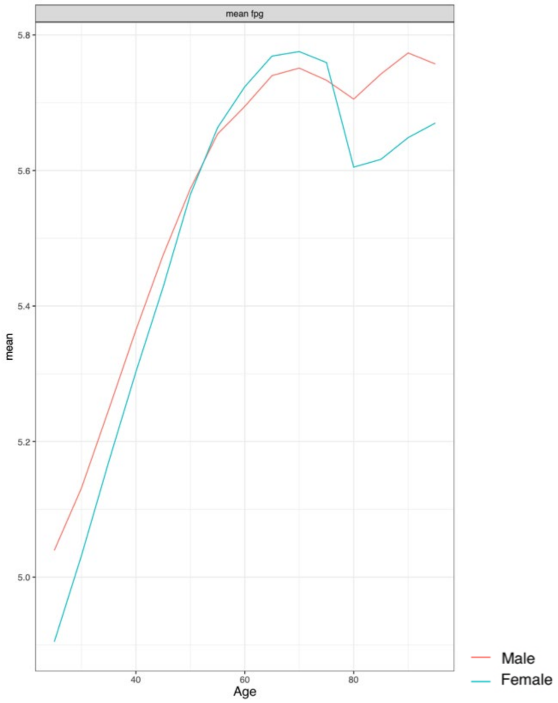

Relative risk

We performed some data processing steps to the relative risk data prior to modelling the risk function. These include:

1. Where exposure is defined using HbA1c (%) and OGTT (mmol/L), they were converted to FPG (mmol/L) equivalent exposure levels using the conversion values of 7/6.5 and 7/11.1, respectively, in order to standardise exposure ranges to FPG equivalent values.
2. Where exposure is defined dichotomously by diabetes status (ie, having diabetes versus not having diabetes), we assume that the lower bound of the alternative exposure range to be 7 mmol/L of FPG, and the upper bound of the reference to be 6.99 mmol/L.
3. Where bound of the exposure ranges are missing, including sources where exposure is defined based on diabetes status, we use super-region specific FPG ensemble distributions to impute any missing lower or upper bound of the exposure range based on the given cutoff provided in the source. For example, if the lower bound of the alternative exposure range is 7 mmol/L and the source did not define the upper exposure range, we take the 85th percentile value above 7 mmol/L for the given FPG distribution for this specific super-region. The lower bound is in turn the 15th percentile below the cut point of each corresponding FPG distribution.

Modelling strategy

Exposure

There are no substantial changes to the modelling strategy in GBD 2023.

Exposure estimates were produced for every year between 1980 and 2023 for each national and subnational location, sex, and for each five-year age group starting from age 25 onward. As in previous rounds of GBD, we used a spatiotemporal Gaussian process regression (ST-GPR) framework to model

the mean FPG at the location, year, age, and sex level. Additional information on ST-GPR can be found in appendix 1, section 2.

To inform our estimates in data-sparse countries, we systematically tested a range of covariates and selected age-specific prevalence of obesity as a covariate based on direction of the coefficient and significance level.

Mean FPG was estimated using a mixed-effects linear regression, run separately by sex:

$$\log(\mathrm{FPG}_{c,a,t}) = \beta_0 + \beta_1 p_{\mathrm{overweight}_{c,a,t}} + \sum_{k=2}^{16} \beta_k I_{A[a]} + \alpha_s + \alpha_r + \alpha_c + \epsilon_{c,a,t}$$

where $p_{\mathrm{overweight}_{c,a,t}}$ is the prevalence of overweight, $I_{A[a]}$ is an indicator variable for a fixed effect on a given five-year age group, and α_s α_r α_c are random effects at the super-region, region, and country level, respectively. The estimates were then propagated through the ST-GPR framework to obtain 1000 draws for each location, year, age, and sex.

Ensemble distributions

We used a set of 12 different distribution types, specifically (exp, gamma, inverse gamma, llogis, invweigbul, gumbel, Weibull, lnorm, norm, mgamma, m gumbel, beta sr) that make up the ensemble distributions. They are fitted to 24 individual-level data. Once all available sets of data are fitted, ensemble weights generated from each dataset are averaged to generate global ensemble weights.

FPG exposure standard deviation

FPG exposure standard deviations is then back-calculated using the ensemble weights derived and the modelled mean FPG exposure, along with estimates of prevalence of diabetes from the non-fatal total diabetes model, to ensure the right tail end of the distribution will capture the same prevalence informed by the diabetes model.

FPG ensemble distribution

FPG ensemble distributions are then generated using the mean FPG exposure, FPG exposure standard deviation, and the global ensemble weights for each year, age, sex, and location.

Theoretical minimum risk exposure level

The TMREL for FPG is 4.9–5.3 mmol/L. This was calculated by taking the person-year weighted average of the levels of FPG that were associated with the lowest risk of mortality in the pooled analyses of prospective cohort studies.[1]

Relative risk

In GBD 2023, we attributed burden of 25 Level 4 diseases to high FPG. We made updates in relative risk estimation for cancer-related outcomes and dementia outcomes. The remainder outcomes have maintained their previous relative risk values from GBD 2021. All FPG and its associated outcomes have continuous risk curves.

Estimating the relative risk (RR) of *all associated outcomes* occurring as a function of exposure to high *FPG* followed the burden of proof approach established by Zheng and colleagues (see appendix 2 section

2) and instantiated in the meta-regression—Bayesian, regularised, trimmed (MR-BRT) tool. MR-BRT synthesises input data to generate a RR curve by relying on an ensemble spline method to capture the potentially non-linear shape of the risk–outcome relationship; integrating over varying exposure ranges in different comparison groups; trimming potentially distorting outliers; testing, selecting, and adjusting for bias covariates to account for known heterogeneity in input study-design characteristics (eg, confounding, selection bias, exposure measurement); and quantifying remaining between-study heterogeneity (gamma) through random effects modelling and incorporating this value into uncertainty around the mean RR curve.

MR-BRT further evaluates evidence for small-study effects and generates funnel plots that represent potential risk of publication or reporting bias, and also generates the burden of proof risk function (BPRF), defined for harmful risks as the 5th and for protective risks as the 95th quantile risk curve – inclusive of between-study heterogeneity/gamma – that is closest to null. The BPRF is transformed into a risk–outcome score (ROS: the signed value of the average log BPRF between the 15th and 85th percentiles of risk exposure levels observed across included studies) and mapped onto a star-rating system from one to five stars. These metrics complement RR estimates by providing an alternative, conservative measure of effect size and evidence strength that formally and systematically incorporates divergence/convergence across input findings, with higher positive ROS values and more stars corresponding to incrementally larger effects and stronger evidence for the risk–outcome relationship.

Study-level bias covariates that were adjusted for in the analysis include study representativeness, quality of confounder adjustment, imputed FPG bounds, exposure range reported in HbA1c values, exposure range reported in OGTT values, exposure defined based on diabetes diagnosis based on anti-diabetic medication use as part of the criteria, studies that exclude people with known/previously diagnosed diabetes from analysis. Additionally, for CVD outcomes, the relative risk was also adjusted for studies where total haemorrhage was reported instead of intracerebral haemorrhage, and the quality of PAD outcome assessment/definition; for CKD outcome, stage of kidney disease was adjusted for.

For relationships that are PAF of 1, which include type 1 and type 2 diabetes, 100% of type 1 and type 2 diabetes burden is attributable to high FPG. For all other outcomes, a description of the methods and approach can be found in the evidence score documentation, appendix 2 section 2 (continuous risk–outcome pairs). Risk curves for each outcome can be found in the Burden of Proof tool.

Finally, we made updates to the relative risk modelling for chronic kidney disease (CKD). We used the risk curve for CKD for total CKD. We do not have separate risk curves for each CKD aetiology due to the paucity of data. Instead, we assumed that estimates from end-stage renal disease registries of the proportion of CKD due to diabetes is the PAF and matched that information by age, sex, year, and location to estimates of diabetes prevalence. Then we back-calculated the relative risk. That modelled data, in addition to data from studies where end-stage renal disease was the outcome, were used to calculate the PAF for CKD and high FPG. Then, we subtracted out 100% of the burden of CKD due to type 1 and type 2 diabetes (where the PAF is 1) and proportionally allocated the remaining burden among the remaining three aetiologies and back-calculated the PAF for each aetiology. The GBD 2023 risk curves can be found in the Burden of Proof tool.

Outcome	Score	Star rating	Relative risk curve (log normal) and funnel plot
CKD	1.70	5	
Alzheimer's disease and other dementia	0.34	3	
Tuberculosis	0.2	3	
Colorectal cancer	0.20	3	
Peripheral artery disease	0.18	3	

Ischaemic stroke	0.15	3	
Ischaemic heart disease	0.15	3	
Pancreatic cancer	0.14	3	
Breast cancer	0.14	3	
Liver cancer	0.05	2	

Intracerebral haemorrhage	0.048	2	
Lung cancer	0.04	2	
Bladder cancer	0.02	2	
Cataract	0.013	2	
Glaucoma	-0.092	1	

Model settings and covariates

Outcome	Model priors	Bias covariates tested	Bias covariates selected
Chronic kidney disease (CKD)	Spline degree=2<br>Right linear tail = T<br>Inlier_pct=0.9<br>Monotonicity= increasing	CKD stage	CKD stage
Alzheimer's disease and other dementia	Spline degree=3<br>Right linear tail=T<br>Inlier_pct=0.9	Representativeness, outcome quality, confounder quality, imputed FPG, diabetes diagnosis based on HbA1c or OGTT or treatment or medical records or doctor's diagnosis or multiple tests, exclusion of known diabetes, dementia subtype, APOE, dementia ICD codes, dementia clinical records, dementia clinical sample, dementia screening phase, dementia claims data.	Diabetes diagnosis based on doctor's diagnosis, dementia subtype
Tuberculosis	Spline degree =3<br>Left linear tail =T	-	-
Peripheral artery disease (PAD)	Spline degree =3<br>Right linear tail =T<br>Left linear tail =T<br>Monotonicity= increasing	Confounder quality, diabetes diagnosis based on HbA1c or OGTT or treatment or medical records, exclude known DM<br>For PAD: Pad measurement quality<br>For intracerebral haemorrhage: total haemorrhage	none
Ischaemic stroke	Spline degree=3<br>Right linear tail =T<br>Left linear tail =T<br>Inlier_pct=0.9		none
Ischaemic heart disease	Spline degree=2		Diagnosis based on treatment

	Right linear tail =T<br>Inlier_pct=0.9<br>Monotonicity= increasing		
Intracerebral haemorrhage	Spline degree=3<br>Right linear tail =T<br>Left linear tail =T<br>Inlier_pct=0.9		none
Liver cancer	Spline degree=2<br>Right linear tail =T<br>Left linear tail =T<br>Inlier_pct=0.9	Representativeness, outcome quality, confounder quality, imputed FPG, diagnosis based on HbA1c or OGTT or treatment or medical records or doctor's diagnosis or multiple tests, exclusion of known diabetes, mortality, hazard ratio, sex specific, geography representativeness<br>Outcome ICD code alignment	Confounder quality, exclude known diabetes, diagnosis based on treatment, doctor's diagnosis or multiple tests, mortality, sex specific, geography representation, outcome alignment
Pancreatic cancer	Spline degree=3<br>Right linear tail=T<br>Inlier_pct=0.9		Diagnosis based on OGTT, and sex specific
Colorectal cancer			none
Bladder cancer			none
Lung cancer			Exclude known diabetes, diagnosis based on treatment
Breast cancer			none
Glaucoma	Spline degree =3<br>Left linear tail =T	-	-
Cataract	Spline degree =3<br>Left linear tail =T	-	-

Figure 7. Comparing RR curves* for outcomes that were updated in GBD 2023 to previous version in GBD 2021

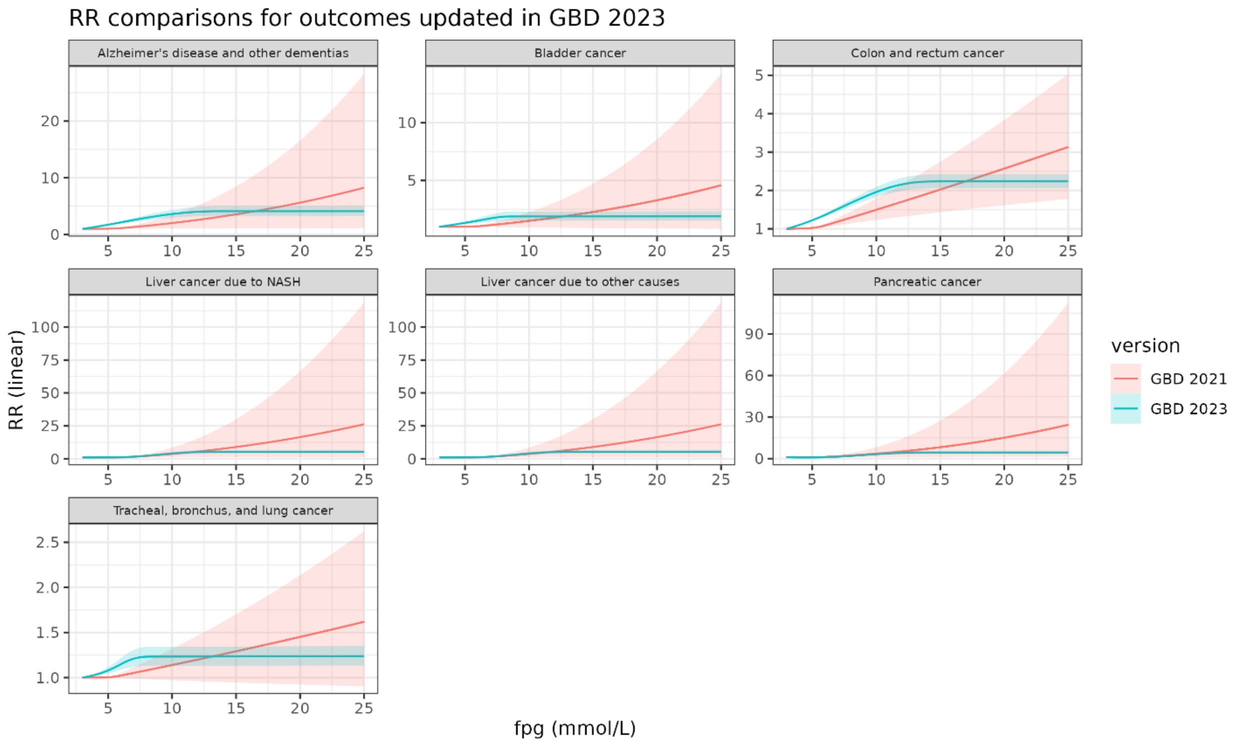

*Note: the risk function model produced by the BOP pipeline was used to predict RR values for a broader FPG exposure range of 3-25mmol/L for computational purposes, which is presented here.

References

1. Singh GM, Danaei G, Farzadfar F, et al. The age-specific quantitative effects of metabolic risk factors on cardiovascular diseases and diabetes: a pooled analysis. *PloS One* 2013; 8: e65174.

High LDL cholesterol

Flowchart

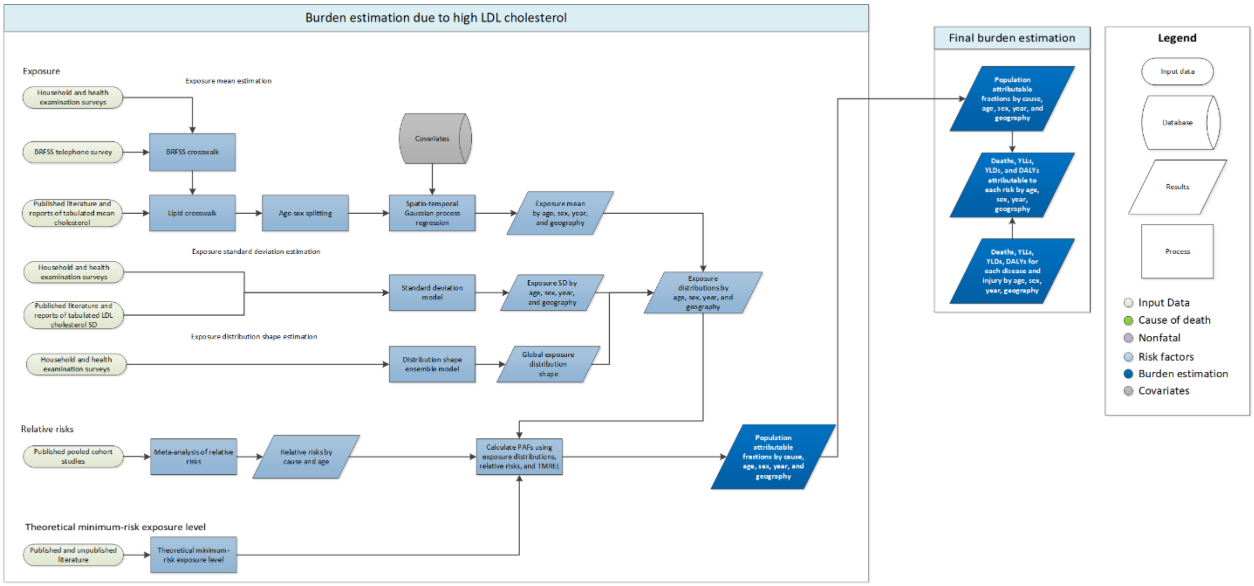

Input data and methodological summary

Definition

Exposure

Exposure is defined as blood concentration of low-density lipoprotein (LDL) cholesterol in units of mmol/L. We adjust data for total cholesterol (TC), triglycerides (TGL), and high-density lipoprotein (HDL) cholesterol using the correction approach described in the "lipid crosswalk" section below.

Input data

Exposure

We used data on levels of LDL, TC, TGL, and HDL from literature and from household survey microdata and reports (eg, WHO STEPwise approach to NCD risk factor surveillance [STEPS] and the National Health and Nutrition Examination Surveys [NHANES]). Sources reporting only prevalence of hyperlipidaemia but not the mean level of cholesterol in the population studied were not used to model LDL cholesterol, except for data from the Behavioral Risk Factors Surveillance System (BRFSS) where access to detailed person-level data from both BRFSS and NHANES for the same population allowed us to predict the mean LDL cholesterol for individuals based on reported prevalent hyperlipidaemia (see below regarding USA prevalence data under "data processing" section). For GBD 2023, we did not carry out a systematic review of the literature for new exposure data. However, we updated our list of sources utilised in previous GBD cycles by searching for relevant studies entered into the Global Health Data Exchange (GHDx) repository, based on metadata applied by trained data librarians indicating relevant content[1] and literature data that were found to include cholesterol measurements when seeking systolic blood pressure data. New data sources included STEPS surveys with microdata from

Cabo Verde, Saint Lucia, Sri Lanka, and Ukraine and literature data from Latvia. Details of inclusion and exclusion criteria and data processing steps follow.

Inclusion criteria

Studies were included if they were population-based and reported measurements of total LDL, TC, HDL, and/or TG from blood tests or if LDL was calculated using the Friedewald equation.[2] We assumed the data were representative of the location if the geography or population chosen was not related to hyperlipidaemia or associated outcomes.

Outliers

All data were used in the modelling process unless an assessment of data strongly suggested that the data were biased. A candidate source was excluded if the quality of study did not warrant a valid estimate because of selection (non-representative populations) or if the study did not provide methodological details for evaluation. In a small number of cases, a datapoint was an outlier candidate if it deviated significantly from other datapoints within the respective country or region or the level was implausibly low or high based on expert judgement.

Data extraction

Where possible, individual-level cholesterol data were extracted from survey microdata and tabulated by demographic groupings to produce mean estimates in the standard GBD five-year age-sex groups. If microdata were unavailable, information from survey reports or from literature were extracted along with any available measure of uncertainty, including standard error, uncertainty intervals, and sample size. Standard deviations associated with mean estimates were also extracted from tabulated data as well as calculated from survey microdata. Where mean cholesterol was reported split by groups other than age, sex, location, and year (eg, by diabetes status), a weighted mean was calculated.

Relative risk

For GBD 2023, a systematic review for the relative risk (RR) of exposure and selected outcomes was not performed. The most recent review was conducted for GBD 2021, during which we revisited the underlying 52 randomised trials used in a previously published systematic review and meta-analysis of intensive LDL-lowering treatment for the prevention of major vascular events conducted by Wang and colleagues[3] and re-analysed the evidence on LDL as a risk factor of ischaemic heart disease and ischaemic stroke. Additional details on the systematic review, including search strings and strategy utilized for each outcome, can be found in the GBD 2021 appendix.[4]

Data processing

Lipid crosswalk

Total cholesterol consists of three major components: LDL, HDL, and TGL. LDL is often calculated for an individual using the Friedewald equation,[2] shown below:

$$LDL = TC - \left(HDL + \frac{TGL}{2.2}\right)$$

We used this relationship at the individual level to impute the mean LDL for a study population when only data on TC, HDL, and/or TGL were available. Because studies report different combinations of TC, HDL, and TGL, we constructed a single regression to utilise all available data to evaluate the relationship between each lipid and LDL at the population level. We used the following regression:

$$LDL = ind_{tc}\beta_1 TC - \left(ind_{hdl}\beta_2 HDL + ind_{tgl}\beta_3 TGL\right) + \sum \alpha_l I_l$$

Where ind_{tc}, ind_{hdl}, and ind_{tgl} are indicator variables for whether data are available for a given lipid, I_l is an indicator variable for a given set of available lipids l, and α_l is a unique intercept for each set of available lipid combinations. For example, for sources that only reported TC and HDL, $\alpha_{l=TC,HDL}$ should account for the missing lipid data, ie, TGL. The form of this regression allows us to estimate the betas for each lipid using all available data. The coefficients for the model are reported in Table 1. As a sensitivity analysis, we also ran separate regressions for each set of available lipids and found that the single regression method had much lower root-mean-squared error. We found almost no relationship between LDL and HDL or TGL when TC was not available, so only studies that reported TC were adjusted to LDL.

Table 1: Coefficients in the lipid regression

TC (β_1)	HDL (β_2)	TGL (β_3)	α_{TC}	$\alpha_{TC,HDL}$	$\alpha_{TC,HDL,TGL}$	α_{HDL}	$\alpha_{HDL,TGL}$
0.7946	0.4471	0.1849	-0.8723	-0.3936	-0.2275	3.3274	3.7482

Incorporating USA prevalence data

Survey reports and literature often report information only on the prevalence of hypercholesterolemia, not the mean cholesterol levels, in the population studied. These sources were not used to model LDL, except for data from the Behavioral Risk Factors Surveillance System (BRFSS) because of the availability of a similarly structured exam survey that is representative of the same population: the National Health and Nutrition Examination Survey (NHANES), a nationally representative health examination survey of the USA adult population. This process was updated for GBD 2023 to include more recent years of BRFSS data as well as to update the methods of adjusting this data for use in the GBD.

BRFSS is a telephone survey conducted in the USA for all counties that collects self-reported diagnosis of hypercholesterolemia. These data were first adjusted for self-report bias using NHANES to estimate the probability of measured elevated TC for each individual. To do so, a logistic regression weighted by survey weight was run separately by sex and by whether the respondent reported a diagnosis of hypercholesterolemia. It was specified as:

$$\log\left(\frac{\text{Elevated TC}}{1 - \text{Elevated TC}}\right) = \beta_0 + \sum_{k=1}^{3}\beta_k(edu) + \beta_4\text{bmi} + \beta_5\text{dm} + \beta_6\text{insur} + \beta_7\text{smoke} + \beta_8\text{year} + \sum_{k=9}^{11}\beta_k s_k(age)$$

w*here* Elevated TC represents whether a respondent's measured total cholesterol was ≥6.2 mmol/L, edu represents the reported education level of the respondent (high school graduate, some college, or college graduate compared to less than high school), bmi is the reported BMI of the respondent corrected for self-report bias using a linear regression run separately by sex to estimate mean measured BMI in NHANES from mean self-reported BMI in BRFSS by survey year and five-year age group, dm is a binary indicator representing self-reported diagnosis of diabetes, insur is a binary indicator representing whether the respondent reports having health insurance, smoke is a binary indicator representing whether the respondent currently smokes, year is the survey year, and $s_k(age)$ corresponds to the

basis functions of a cubic spline of the respondent's age. The coefficients for the models are reported in Table 2. The models were applied to individual-level data from BRFSS by running ten simulations which were averaged to obtain an estimate of the probability of measured elevated TC for each individual. The data were then tabulated by reported diagnosis of hypercholesterolaemia, age group, sex, USA state, and year to obtain prevalence of elevated TC for each strata.

Table 2: Logit coefficients in the USA self-reported hypercholesterolaemia logistic regression

Term	Males		Females	
	Not diagnosed	Diagnosed	Not diagnosed	Diagnosed
Intercept (β_0)	29.0237	99.0016	40.1911	78.3417
Education, high school graduate (β_1)	-0.0132	-0.0880	-0.0463	0.0749
Education, some college (β_2)	-0.1019	0.1247	-0.0044	0.2335
Education, college graduate (β_3)	-0.2218	-0.1806	-0.0577	0.0378
BMI (β_4)	0.0213	-0.0234	0.0005	-0.0148
Diabetes (β_5)	-0.7304	-0.6161	-0.6921	-0.5447
Health insurance (β_6)	-0.3313	-0.1777	-0.0583	-0.4838
Smoker (β_7)	0.2539	0.0289	0.1355	0.0416
Survey year (β_8)	-0.0164	-0.0504	-0.0215	-0.0396
Age spline, basis function 1 (β_9)	2.6779	4.9413	-0.2239	1.5525
Age spline, basis function 2 (β_{10})	1.8041	0.5517	2.6005	1.5443
Age spline, basis function 3 (β_{11})	0.5864	0.7525	1.2834	0.7683

Prevalence estimates from BRFSS data were used to predict a mean TC for the same strata with a linear regression using data from NHANES. The regression was weighted by strata sample size and run separately by sex and by reported diagnosis of hypercholesterolemia. It was specified as:

$$\mathrm{TC}_{l,a,t,s,g} = \beta_0 + \beta_1 \mathrm{prev}_{l,a,t,s,g} + \beta_2 \mathrm{age} + \beta_3 (\mathrm{prev}_{l,a,t,s,g} * \mathrm{age})$$

where $TC_{l,a,t,s,g}$ is the location-, age-, time-, sex-, and diagnosis group-specific mean TC, $prev_{l,a,t,s,g}$ is the location-, age-, time-, sex-, and diagnosis group-specific prevalence of raised TC, and age is the starting age of the five-year age group. The coefficients for the models are reported in Table 3.

Table 3: Coefficients in the USA total cholesterol prediction models

Term	Males		Females	
	Not diagnosed	Diagnosed	Not diagnosed	Diagnosed
Intercept (β_0)	4.273	5.338	3.923	5.201
Prevalence (β_1)	6.077	1.532	7.874	1.803
Age (β_2)	0.003	-0.015	0.016	-0.008
Prevalence and age interaction (β_3)	-0.018	0.037	-0.091	0.018

Out-of-sample root-mean-square error (RMSE) was used to quantify the predictive validity of the model. The regression was repeated ten times for each sex and hypercholesterolaemia diagnosis group, each time randomly holding out 20% of the data. The RMSEs from each holdout analysis were averaged to get the average out-of-sample RMSE. The results of this holdout analysis are reported in Table 4.

Table 4: Out-of-sample RMSEs of the USA total cholesterol prediction models

	Males	Females
**Not diagnosed**	0.18 mmol/L	0.17 mmol/L
**Diagnosed**	0.26 mmol/L	0.17 mmol/L

After applying the models to BRFSS data, an average weighted by sample size was calculated across hypercholesterolaemia diagnosis group to obtain location-, age-, time-, and sex-specific mean total cholesterol. These total cholesterol estimates were used to impute mean LDL using the lipid crosswalk reported above.

Age and sex splitting

Prior to modelling, data provided in age groups wider than the GBD five-year age groups or for both sexes rather than males and females separately were processed using PyDisagg and the modelled global age-sex pattern.[5] Use of this tool was new for GBD 2023, reflecting a transition from the approach for age and sex splitting outlined by Ng and colleagues[6] used for LDL in previous GBD cycles.[4] Briefly, PyDisagg disaggregates rate and count observations based on a proportionality assumption. A user-provided datum D is disaggregated into subcomponents D_i, guaranteeing that
(1) $\sum D_i = D$
(2) $D_i = g_i \cdot p_i$
with p_i the user-provided population weight, and g_i a transform of the user-provided global rate f_i:
$g_i = T^{-1}(\beta + T(f_i))$.
The basic use case has $T = ln$, guaranteeing that the recovered rates are proportional to the global. In addition, PyDisagg allows splitting of bounded quantities using the appropriate logit transform for T. PyDisagg version 0.6.0 disaggregates observations of both continuous quantities (eg, age-splitting of age-aggregated observations) as well as discrete quantities (including sex and multiple categories). Uncertainty for split datapoints is obtained using asymptotic statistics. Specifically, uncertainty of the inputs (ie, uncertainty of the aggregate and uncertainty of the age pattern) is used together with a generalised delta method that effectively computes a linearised map from the inputs to the outputs and uses that to obtain posterior uncertainty intervals for the split datapoints.

Modelling strategy

Exposure

Exposure estimates were produced from 1980 to 2023 for each national and subnational location, sex, and five-year age group starting from ages 25–29.

Estimate of mean

As in GBD 2021, we used a spatiotemporal Gaussian process regression (ST-GPR) framework to model mean LDL at the location, year, age, and sex level. Details of the ST-GPR method used in GBD 2023 can be found elsewhere in the appendix.

The first step of the ST-GPR framework requires the creation of a linear model for predicting LDL at the location, year, age, sex level. Variables with an expected causal relationship with LDL were selected as covariates for this model based on significant association found within high-quality prospective cohort studies reported in the published scientific literature. These variables included: mean body-mass index, Socio-demographic Index, Healthcare Access and Quality Index, age- and sex-specific summary exposure value (SEV) for smoking, age- and sex-specific SEV for low fruit, age- and sex-specific SEV for low vegetables, age- and sex-specific SEV for low omega-3, and age- and sex-specific SEV for low nuts and seeds.

Smoothing parameters for the second and third steps of the ST-GPR framework were selected through cross-validation with out-of-sample root-mean-squared error used to assess model performance. The results of the ensemble linear model were used for the first stage in an ST-GPR model. The result of the ST-GPR model are estimates of the mean LDL for each age, sex, location, and year.

Estimate of standard deviation

The standard deviation of LDL within a population was estimated for each location, sex, year, and age group using the standard deviation from person-level and some tabulated data sources, which were updated for GBD 2023. Tabulated data were only used to model standard deviation if they were sex-specific and five-year-age-group-specific and reported a population standard deviation of LDL. LDL standard deviation was estimated using a linear regression:

$$\log\left(\mathrm{SD}_{\mathrm{l,a,t,s}}\right) = \beta_0 + \beta_1 \log\left(\mathrm{mean\_LDL}_{\mathrm{l,a,t,s}}\right) + \sum_{\mathrm{k}=2}^{7} \beta_{\mathrm{k}} \mathrm{s}_{\mathrm{k}}(\mathrm{age}) + \beta_8 \mathrm{sex} + \beta_9 \mathrm{year}$$

where $mean\_LDL_{l,a,t,s}$ is the location-, age-, time-, and sex-specific mean LDL estimate from ST-GPR and $s_k(age)$ corresponds to the basis functions of a cubic spline on the starting age of a given five-year age group with knots placed at ages 35, 50, and 65. The addition of year as a covariate and the switch from modelling age with dummy variables to a cubic spline was implemented for GBD 2023. The coefficients for the model are reported in Table 5.

Table 5: Log coefficients in the standard deviation model

Term	Coefficient
Intercept (β_0)	-7.446
Log mean SBP (β_1)	0.471
Age spline, basis function 1 (β_2)	-0.002
Age spline, basis function 2 (β_3)	0.009
Age spline, basis function 3 (β_4)	0.062
Age spline, basis function 4 (β_5)	0.063
Age spline, basis function 5 (β_6)	0.145
Age spline, basis function 6 (β_7)	0.024
Sex (β_8)	-0.002
Year (β_9)	0.003

Distribution shape modelling

The shape of the distribution of LDL was estimated using all available person-level microdata sources,

which was a subset of the input data into the modelling process. The distribution shape modelling framework is detailed elsewhere in the appendix. Briefly, an ensemble distribution created from a weighted average of distribution families was fit for each individual microdata source, separately by age and sex. The weights for the distribution families for each individual source were then averaged and weighted to create a global ensemble distribution for each sex. Figure 1 shows the final ensemble distribution for both sexes combined. Weights were not updated for GBD 2023.

Figure 1. Global ensemble distribution fit and distribution-specific weights for LDL

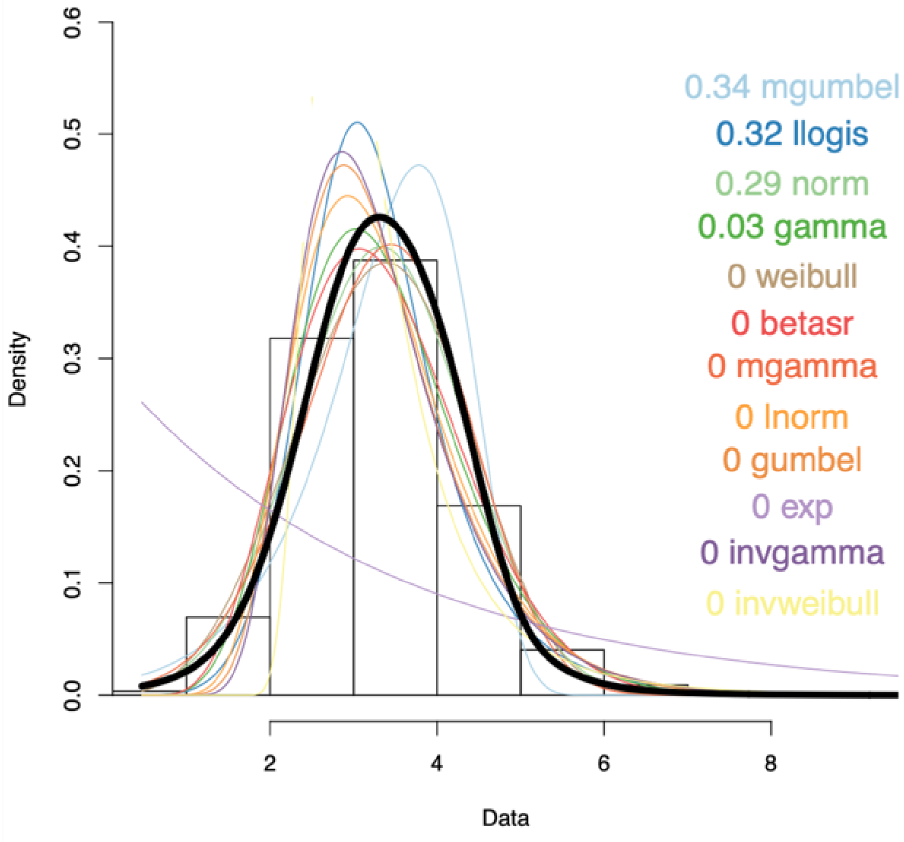

Theoretical minimum risk exposure level

The TMREL was updated for GBD 2021 and did not change for GBD 2023 (Table 6). For GBD 2021, we used the LDL values reported in the randomised trials used to estimate the LDL relative risks to update the TMREL as well. We first identified the LDL levels from the reference exposure (or control) and alternate (or comparison) groups of the studies for all outcomes associated with LDL. Then, we computed the midpoint of the lower and upper bounds of the reference exposure groups and defined the TMREL as a uniform distribution with lower/upper bounds of 0.9 and 1.4. The lower bound was given by the 15th percentile of the midpoints calculated above, and the upper bound was given by the 15th percentile of only the upper bounds of the reference exposure groups. We calculated these ranges for each RO pair and then took the weighted average of the ranges using global cause-specific deaths as the weights. The TMREL was defined as a uniform distribution rather than a fixed value to represent the uncertainty regarding the level at which the scientific evidence was consistent with adverse effects of exposure.

Table 6: Theoretical minimum risk exposure level

TMREL definition	
GBD 2021	GBD 2023
0.9–1.4 mmol/L	0.9–1.4 mmol/L

Relative risk

Estimating the relative risk (RR) of ischaemic heart disease and ischaemic stroke occurring as a function of exposure to LDL followed the burden of proof approach established by Zheng and colleagues[7,8] and instantiated in the meta-regression—Bayesian, regularised, trimmed (MR-BRT) tool. MR-BRT synthesises input data to generate an RR curve by relying on an ensemble spline method to capture the potentially non-linear shape of the risk–outcome relationship; integrating over varying exposure ranges in different comparison groups; trimming potentially distorting outliers; testing, selecting, and adjusting for bias covariates to account for known heterogeneity in input study-design characteristics (eg, confounding, selection bias, exposure measurement); and quantifying remaining between-study heterogeneity (gamma) through random effects modelling and incorporating this value into uncertainty around the mean RR curve.

MR-BRT further evaluates evidence for small-study effects and generates funnel plots that represent potential risk of publication or reporting bias and also generates the burden of proof risk function (BPRF), defined for harmful risks as the 5th and for protective risks as the 95th quantile risk curve – inclusive of between-study heterogeneity/gamma – that is closest to null. The BPRF is transformed into a risk-outcome score (ROS: the signed value of the average log BPRF between the 15th and 85th percentiles of risk exposure levels observed across included studies) and mapped onto a star-rating system from one to five stars. These metrics complement RR estimates by providing an alternative, conservative measure of effect size and evidence strength that formally and systematically incorporates divergence/convergence across input findings, with higher positive ROS values and more stars corresponding to incrementally larger effects and stronger evidence for the risk–outcome relationship.

LDL RRs were updated for GBD 2021 and did not change for GBD 2023. Relative risks for ischaemic heart disease and ischaemic stroke were modelled with log (RR) as the dependent variable and LDL exposure values as the independent variable. Due to data sparsity and given that most of the studies included in the meta-regression do not report information disaggregated by stroke subtypes (ie, ischaemic versus haemorrhagic stroke), we decided to combine data sources that reported “stroke” and “ischaemic stroke” as outcomes in a single model, assuming that physiologically LDL can be associated with ischaemic stroke only. We implemented the Fisher scoring correction to the heterogeneity parameter, which corrects for data-sparse situations. In such cases, the between-study heterogeneity parameter estimate may be zero, simply from lack of data. The Fisher scoring correction uses a quantile of gamma, which is sensitive to the number of studies, study design, and reported uncertainty. In addition, we have added methodology that can detect and flag publication bias. The approach is based on the classic Egger’s regression strategy,[9] which is applied to the residuals of the model. In the current implementation, we do not correct for publication bias but flag the risk–outcome pairs where the risk for publication bias is significant. For this analysis, no risk of publication bias was detected for LDL and related outcomes, as shown in the funnel plots in Figures 2 and 3. Risk–outcome scores and star ratings are reported in Table 7.

Figure 2. Low-density lipoprotein cholesterol and ischaemic heart disease log relative risk (a) and residuals by estimated standard deviation (b)

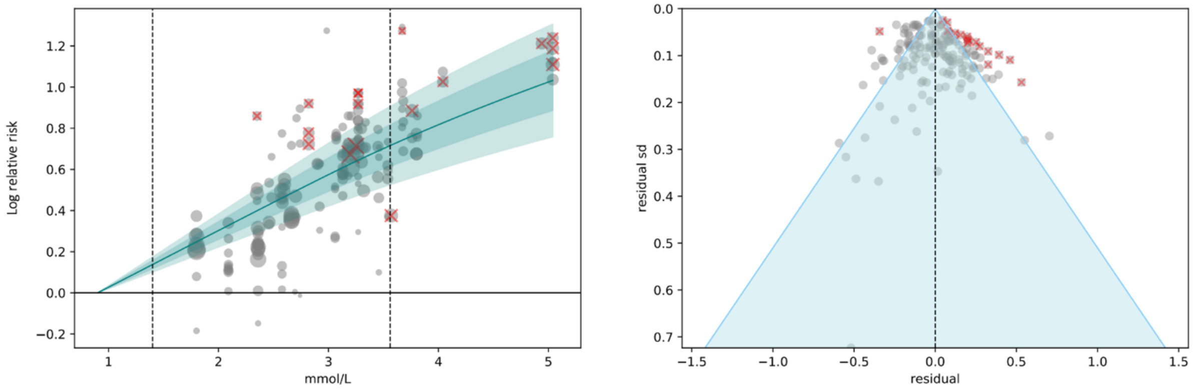

Figure 3: Low-density lipoprotein cholesterol and ischaemic stroke log relative risk (a) and residuals by estimated standard deviation (b)

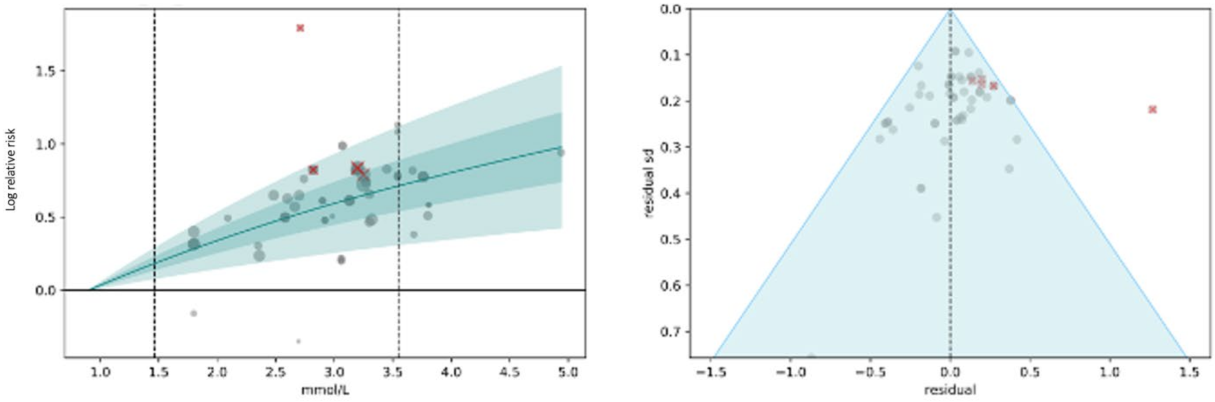

Figures 2 and 3. The risk curves are computed relative to an LDL cholesterol value of 0.9 mmol/L. In panel (a) the dark line indicates mean relative risk across LDL cholesterol exposure levels; the light and dark shading show 95% uncertainty intervals with and without between-study heterogeneity, respectively; the size of the datapoints corresponds to the inverse of the standard error, with those trimmed during the model fitting process marked by a red x; and the dashed lines represent the 15th percentile of the reference exposure and the 85th percentile of the alternative exposure. To visualise log-relative-risk points in panel (a), we plotted each datapoint with the x-value at the midpoint of the alternative group and the y-value offset by the difference between the reported and predicted log risk. Panel (b) depicts a customised funnel plot, with the x-axis representing residuals between predicted and observed relative risks, and the y-axis representing uncertainty from both measurement error and between-study heterogeneity.

Table 7: Risk–outcome scores and star ratings

Outcome	Risk–outcome score	Star rating
Ischaemic heart disease	0.32	3
Ischaemic stroke	0.21	3

We assumed that the estimated RRs were universal for all countries and sex categories and were the same for incidence and mortality. To account for the heterogeneity of the effect size by age and given the limitations of both the available data and MR-BRT in terms of lack of age-specific data and estimates, we estimated cause-specific age attenuation factors using a second MR-BRT model with log

(RR) as the dependent variable and age as an independent variable, including data for TC from the APCSC and the PSC cohorts only reported by Singh and colleagues.[10] We then applied these cause-specific attenuation factors to the corresponding RR curve using the mid age at event observed in these two cohort studies (60–64 years) as the reference group to finally generate RR for standard five-year GBD age categories starting at age 25. With this new methodology, we removed the previous assumption that there is not a protective effect of LDL and stroke after age 70. In future iterations of GBD, we plan to update the MR-BRT tool to be able to incorporate a second spline on age and generate more accurate age-specific RR curves.

Citations

1 Global Health Data Exchange | GHDx. https://ghdx.healthdata.org/ (accessed Jan 16, 2025).

2 Friedewald WT, Levy RI, Fredrickson DS. Estimation of the Concentration of Low-Density Lipoprotein Cholesterol in Plasma, Without Use of the Preparative Ultracentrifuge. *Clin Chem* 1972; **18**: 499–502.

3 Wang N, Fulcher J, Abeysuriya N, *et al.* Intensive LDL cholesterol-lowering treatment beyond current recommendations for the prevention of major vascular events: a systematic review and meta-analysis of randomised trials including 327 037 participants. *Lancet Diabetes Endocrinol* 2020; **8**: 36–49.

4 Brauer M, Roth GA, Aravkin AY, *et al.* Global burden and strength of evidence for 88 risk factors in 204 countries and 811 subnational locations, 1990–2021: a systematic analysis for the Global Burden of Disease Study 2021. *The Lancet* 2024; **403**: 2162–203.

5 Hsu A, Zheng P, Maass K, Aravkin A, Ali S. pyDisagg: Dissaggregation under Generalized Proportionality Assumptions. 2025; published online Jan 13. DOI:10.5281/zenodo.14641582.

6 Ng M, Fleming T, Robinson M, *et al.* Global, regional, and national prevalence of overweight and obesity in children and adults during 1980–2013: a systematic analysis for the Global Burden of Disease Study 2013. *The Lancet* 2014; **384**: 766–81.

7 Zheng P, Barber R, Sorensen RJD, Murray CJL, Aravkin AY. Trimmed Constrained Mixed Effects Models: Formulations and Algorithms. *J Comput Graph Stat* 2021; **30**: 544–56.

8 Zheng P, Afshin A, Biryukov S, *et al.* The Burden of Proof studies: assessing the evidence of risk. *Nat Med* 2022; **28**: 2038–44.

9 Egger M, Smith GD, Schneider M, Minder C. Bias in meta-analysis detected by a simple, graphical test. *BMJ* 1997; **315**: 629–34.

10 Singh GM, Danaei G, Farzadfar F, *et al.* The Age-Specific Quantitative Effects of Metabolic Risk Factors on Cardiovascular Diseases and Diabetes: A Pooled Analysis. *PLOS ONE* 2013; **8**: e65174.

High systolic blood pressure

Flowchart

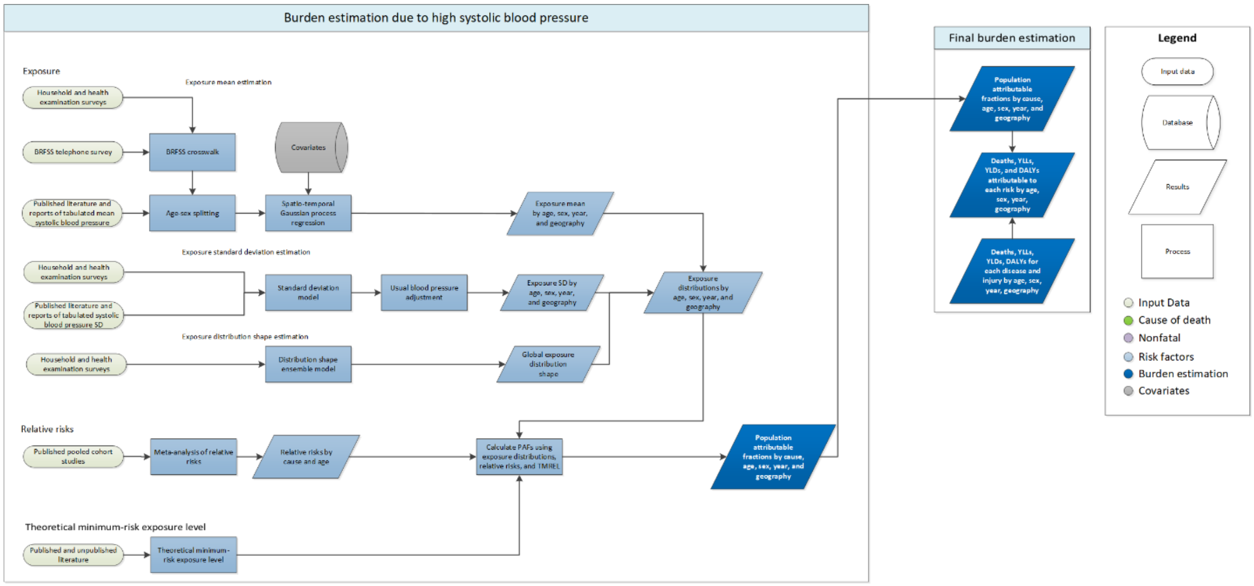

Input data and methodological summary

Definition

Exposure

Exposure is defined as brachial systolic blood pressure (SBP) in mmHg.

Input data

Exposure

We used data on mean systolic blood pressure from literature and from household surveys with microdata and reports (eg, WHO STEPwise approach to NCD risk factor surveillance [STEPS] and the National Health and Nutrition Examination Surveys [NHANES]). Sources reporting only prevalence of hypertension but not the mean level of systolic blood pressure in the population studied were not used to model systolic blood pressure, except for data from the Behavioral Risk Factors Surveillance System (BRFSS) where access to detailed person-level data from both BRFSS and NHANES for the same population allowed us to predict the mean systolic blood pressure for individuals based on reported prevalent hypertension (see below regarding USA prevalence data under "Data processing" section). For GBD 2023, a systematic review of the literature was conducted for new exposure data. The PubMed database was searched using the following search string:
("hypertension"[Mesh:noexp] OR "blood pressure"[Mesh] OR "blood pressure determination"[Mesh] OR "hypertension"[tiab] OR "blood pressure"[tiab])
AND ("Geographic Locations"[Mesh] OR "Population"[Mesh] OR "population*"[TiAb] OR "nation*"[TiAb] OR "communit*"[TiAb] OR "reside*"[TiAb] OR "residing"[TiAb] OR "inhabitants"[TiAb] OR "living in"[TiAb])
AND ("Health Surveys"[Mesh] OR "Health Services Research"[Mesh] OR "Epidemiologic Studies"[Mesh]

OR "surve*"[TiAb] OR “examination*”[TiAb] OR “screened”[TiAb] OR “sampl*”[TiAb] OR “cross-sectional”[TiAb] OR “cohort”[TiAb] OR “longitudinal”[TiAb] OR “observational”[TiAb])
NOT ('NHANES'[tiab] OR 'National Health and Nutrition Examination'[tiab])
NOT (Comment[pt] OR Case Reports[pt] OR Review[pt] OR Editorial[pt] OR Clinical Trial[pt] OR News[pt])
NOT ("hospital*"[tiab] OR “hospitalization”[Mesh] OR “patient*”[ti])
NOT (“Pregnancy”[Mesh] OR "Pregnancy Complications"[Mesh] OR "pregnant"[ti] OR "pregnanc*"[ti] OR "preeclampsia"[ti] OR "pre-eclampsia"[ti] OR "eclampsia"[ti] OR "gestational"[ti] OR "preterm"[ti])
NOT ("orthostatic"[ti] OR "hypotension"[ti])
NOT (“Animal Experimentation”[Mesh] OR “Models, Animal"[Mesh] OR “mouse”[tiab] OR “mice”[tiab] OR “rat”[tiab] OR “rats”[tiab] OR “dog”[tiab] OR “dogs”[tiab] OR “cat”[tiab] OR “cats”[tiab])
AND ("2022/01/01"[pdat] : "2022/12/31"[pdat])

The findings were reported according to the Preferred Reporting Items for Systematic reviews and Meta-Analyses (PRISMA) statement[1] and registered with PROSPERO (PROSPERO record CRD42024510285). Figure 1 shows the PRISMA 2020 flow diagram for the systematic review. Due to time constraints, a subset of articles was prioritised for screening. This subset was defined as articles reporting on data in locations for which there were not systolic blood pressure data in previous GBD cycles. Such articles were identified using GPT4 to extract the location where data were collected from each article title and abstract, if provided. GPT4 was similarly utilised to extract the name of the survey or study from which systolic blood pressure data were reported, if available in the title or abstract, to exclude articles reporting data from large-scale population health surveys for which microdata are already extracted and utilised in the GBD, such as STEPS and select national health surveys. In the PRISMA diagram, screening refers to reviewing the title and abstract of an article for relevant information. Review of the entire article occurred after title and abstract screening to assess articles for eligibility. Reasons for exclusion include a study sample not being representative of the general population, use of different exposure definitions and/or measurements, and studies that did not report any relevant data. In total, two articles reporting mean systolic blood pressure data collected in Guinea-Bissau and Latvia were included.

Figure 1: PRISMA diagram for systolic blood pressure exposure

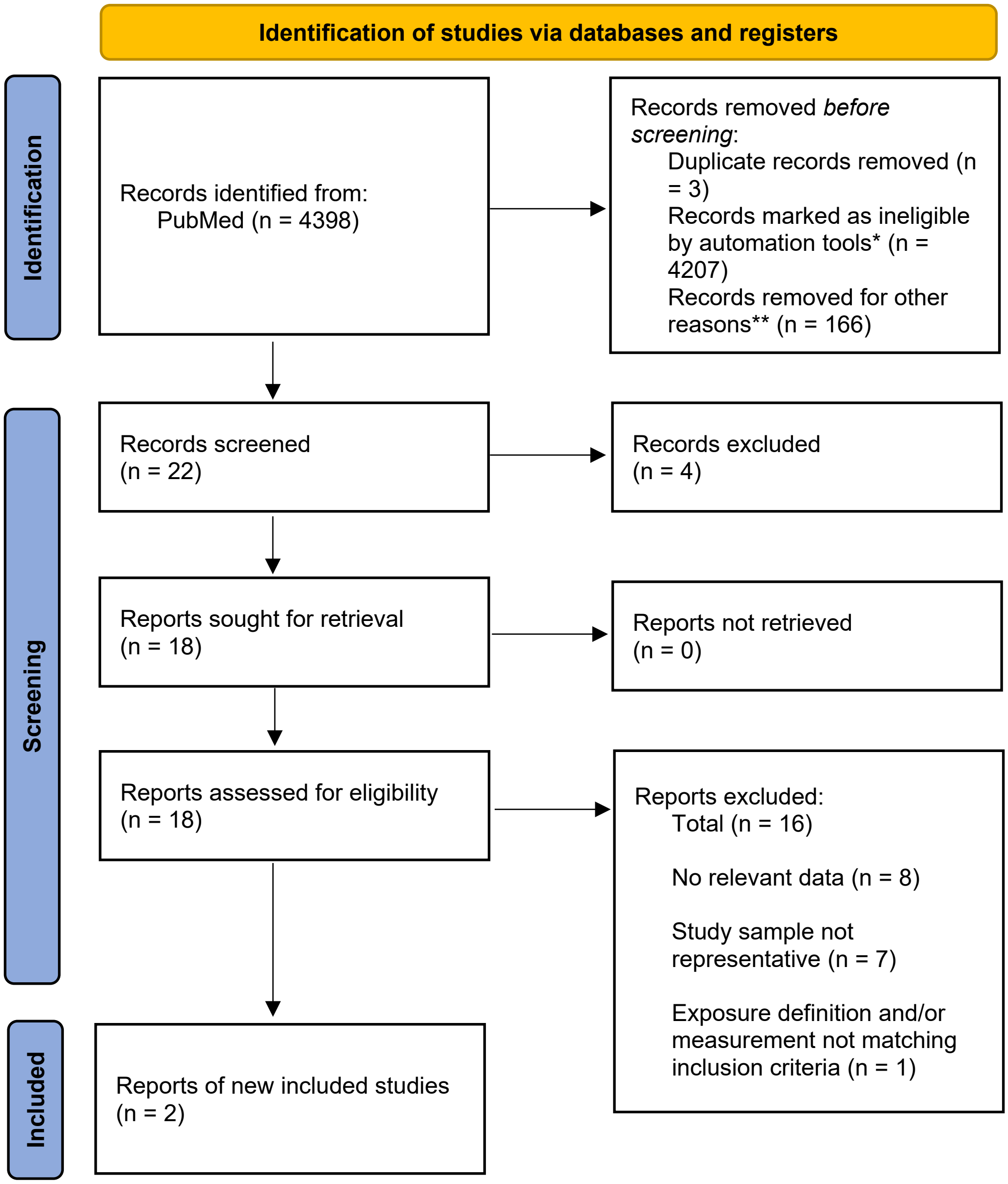

**Articles that were not prioritized for screening due to GPT4 not identifying them as reporting data from locations for which systolic blood pressure data is not currently available in the GBD*

***Articles that were removed due to GPT4 identifying them as reporting data from large-scale population health surveys for which microdata is already extracted and utilized in the GBD*

In addition to a systematic literature review, we updated the list of sources utilised in previous GBD cycles by searching for relevant studies entered into the Global Health Data Exchange (GHDx) repository, based on metadata applied by trained data librarians indicating relevant content.[2] We added new data sources with microdata that met our inclusion criteria, including STEPS surveys from Cabo Verde, Saint Lucia, Sri Lanka, and Ukraine as well as surveys from India, Guatemala, South Korea, Mexico, Gabon, South Africa, Uganda, Tanzania, Panama, Nepal, Brazil, and New Zealand. Details of inclusion and exclusion criteria and data processing steps follow.

Inclusion criteria

Studies were included if they were population-based and directly measured systolic blood pressure using a sphygmomanometer. We assumed the data were representative if the geography or the population was not selected because it was related to hypertension or hypertensive outcomes.

Outliers

Data were used in the modelling process unless an assessment strongly suggested that the source was biased. A candidate source was excluded if the quality of study did not warrant a valid estimate because of selection (non-representative populations) or if the study did not provide methodological details for evaluation. In a small number of cases, a datapoint was an outlier candidate if it deviated significantly from other datapoints within the respective country or region or the level was implausibly low or high based on expert judgement.

Data extraction

Where possible, individual-level systolic blood pressure data were extracted from survey microdata and tabulated by demographic groupings to produce mean estimates in the standard GBD five-year age-sex groups. If microdata were unavailable, information from survey reports or from literature were extracted along with any available measure of uncertainty, including standard error, uncertainty intervals, and sample size. Standard deviations associated with mean estimates were also extracted from tabulated data as well as calculated from survey microdata. Where mean systolic blood pressure was reported split out by groups other than age, sex, location, and year (eg, by hypertensive status), a weighted mean was calculated.

Relative risk

For GBD 2023, a systematic review for the relative risk (RR) of exposure and selected outcomes was not performed. The most recent review was conducted for GBD 2021, during which we built upon published systematic reviews and used a standardised approach to conduct literature reviews of randomised controlled trials (RCTs) and cohort studies for each disease outcome. Additional details on the systematic review, including search strings and strategy utilised for each outcome, can be found in the GBD 2021 appendix.[3]

Data processing

Incorporating USA prevalence data

Survey reports and literature often report information only on the prevalence of hypertension, not the mean systolic blood pressure, in the population studied. These sources were not used to model systolic blood pressure, except for data from the Behavioral Risk Factors Surveillance System (BRFSS) because of the availability of a similarly structured exam survey that is representative of the same population: the National Health and Nutrition Examination Survey (NHANES), a nationally representative health examination survey of the USA adult population. This process was updated for GBD 2023 to include

more recent years of BRFSS data as well as to update the methods of adjusting this data for use in the GBD.

BRFSS is a telephone survey conducted in the USA for all counties that collects self-reported diagnosis of hypertension. These data were first adjusted for self-report bias using NHANES to estimate the probability of measured elevated blood pressure for each individual. To do so, a logistic regression weighted by survey weight was run separately by sex and by whether the respondent reported a diagnosis of hypertension. It was specified as:

$$\log\left(\frac{\text{Elevated BP}}{1 - \text{Elevated BP}}\right) = \beta_0 + \sum_{k=1}^{3} \beta_k(edu) + \beta_4\text{bmi} + \beta_5\text{dm} + \beta_6\text{insur} + \beta_7\text{smoke} + \beta_8\text{year} + \sum_{k=9}^{11} \beta_k s_k(age)$$

where Elevated BP represents whether a respondent's averaged three systolic blood pressure measurements was ≥140 mmHg or averaged three diastolic blood pressure measurements was ≥90 mmHg, edu represents the reported education level of the respondent (high school graduate, some college, or college graduate compared to less than high school), bmi is the reported BMI of the respondent corrected for self-report bias using a linear regression run separately by sex to estimate mean measured BMI in NHANES from mean self-reported BMI in BRFSS by survey year and five-year age group, dm is a binary indicator representing self-reported diagnosis of diabetes, insur is a binary indicator representing whether the respondent reports having health insurance, smoke is a binary indicator representing whether the respondent currently smokes, year is the survey year, and $s_k(age)$ corresponds to the basis functions of a cubic spline of the respondent's age. The coefficients for the models are reported in Table 1. The models were applied to individual-level data from BRFSS by running ten simulations which were averaged to obtain an estimate of the probability of measured elevated blood pressure for each individual. These data were then tabulated by reported diagnosis of hypertension, age group, sex, USA state, and year to obtain prevalence of elevated blood pressure for each strata.

Table 1: Logit coefficients in the USA self-reported hypertension logistic regression

Term	Males		Females	
	Not diagnosed	Diagnosed	Not diagnosed	Diagnosed
Intercept (β_0)	20.5055	40.2127	61.1922	69.9189
Education, high school graduate (β_1)	-0.0187	-0.1069	-0.0243	-0.1451
Education, some college (β_2)	-0.0900	-0.1705	-0.2170	-0.0893
Education, college graduate (β_3)	-0.3219	-0.3066	-0.4732	-0.3856
BMI (β_4)	0.0512	0.0140	0.0283	0.0005
Diabetes (β_5)	0.0208	-0.1096	0.0772	-0.0364
Health insurance (β_6)	-0.0390	-0.5085	-0.1369	-0.4284
Smoker (β_7)	0.0627	0.0452	0.1197	-0.0736
Survey year (β_8)	-0.0125	-0.0210	-0.0335	-0.0362
Age spline, basis function 1 (β_9)	1.0160	2.1235	2.8480	3.1931

Age spline, basis function 2 (β_{10})	1.8100	0.9005	3.7554	1.7262
Age spline, basis function 3 (β_{11})	2.7547	2.0092	5.4570	3.5816

Prevalence estimates from BRFSS data were used to predict a mean systolic blood pressure for the same strata with a linear regression using data from NHANES. The regression was weighted by strata sample size and run separately by sex and by reported diagnosis of hypertension. It was specified as:

$$\mathrm{SBP}_{l,a,t,s,g} = \beta_0 + \beta_1 \mathrm{prev}_{l,a,t,s,g} + \beta_2 \mathrm{age} + \beta_3 (\mathrm{prev}_{l,a,t,s,g} * \mathrm{age})$$

where $SBP_{l,a,t,s,g}$ is the location-, age-, time-, sex-, and diagnosis group-specific mean systolic blood pressure, $prev_{l,a,t,s,g}$ is the location-, age-, time-, sex-, and diagnosis group-specific prevalence of raised blood pressure, and age is the starting age of a given five-year age group. The coefficients for the models are reported in Table 2.

Table 2: Coefficients in the USA blood pressure prediction models

**Term**	**Males**		**Females**	
	**Not diagnosed**	**Diagnosed**	**Not diagnosed**	**Diagnosed**
Intercept (β_0)	115.17	124.80	102.14	111.60
Prevalence (β_1)	21.07	4.25	43.32	31.28
Age (β_2)	0.04	-0.06	0.24	0.13
Prevalence and age interaction (β_3)	0.31	0.46	0.01	0.15

Out-of-sample root-mean-square error (RMSE) was used to quantify the predictive validity of the model. The regression was repeated ten times for each sex and hypertension diagnosis group, each time randomly holding out 20% of the data. The RMSEs from each holdout analysis were averaged to get the average out-of-sample RMSE. The results of this holdout analysis are reported in Table 3.

Table 3: Out-of-sample RMSEs of the USA blood pressure prediction models

	**Males**	**Females**
**Not diagnosed**	1.68 mmHg	2.35 mmHg
**Diagnosed**	2.73 mmHg	3.69 mmHg

After applying the models to BRFSS data, an average weighted by sample size was calculated across hypertension diagnosis group to obtain location-, age-, time-, and sex-specific mean systolic blood pressure.

Age and sex splitting

Prior to modelling, data provided in age groups wider than the GBD five-year age groups or for both sexes rather than males and females separately were processed using PyDisagg and the modelled global age-sex pattern.[4] Use of this tool was new for GBD 2023, reflecting a transition from the approach for age and sex splitting outlined by Ng and colleagues[5] used for SBP in previous GBD cycles.[3] Briefly, PyDisagg disaggregates rate and count observations based on a proportionality assumption. A user-provided datum D is disaggregated into subcomponents D_i, guaranteeing that

(1) $\sum D_i = D$

(2) $D_i = g_i \cdot p_i$

with p_i the user-provided population weight, and g_i a transform of the user-provided global rate f_i:

$g_i = T^{-1}\big(\beta + T(f_i)\big).$
The basic use case has $T = ln$, guaranteeing that the recovered rates are proportional to the global. In addition, PyDisagg allows splitting of bounded quantities using the appropriate logit transform for T. PyDisagg version 0.6.0 disaggregates observations of both continuous quantities (eg, age-splitting of age-aggregated observations) as well as discrete quantities (including sex and multiple categories). Uncertainty for split datapoints is obtained using asymptotic statistics. Specifically, uncertainty of the inputs (ie, uncertainty of the aggregate and uncertainty of the age pattern) is used together with a generalised delta method that effectively computes a linearised map from the inputs to the outputs and uses that to obtain posterior uncertainty intervals for the split datapoints.

Modelling strategy

Exposure

Exposure estimates were produced from 1980 to 2023 for each national and subnational location, sex, and five-year age group starting from ages 25–29.

Estimate of mean

As in GBD 2021, we used a spatiotemporal Gaussian process regression (ST-GPR) framework to model mean systolic blood pressure at the location, year, age, sex level. Details of the ST-GPR method used in GBD 2023 can be found elsewhere in the appendix.

The first step of the ST-GPR framework requires the creation of a linear model for predicting SBP at the location, year, age, sex level. Variables with an expected causal relationship with SBP were selected as covariates for this model based on significant association found within high-quality prospective cohort studies reported in the published scientific literature. These variables included: mean body-mass index, Socio-demographic Index, Healthcare Access and Quality Index, age- and sex-specific summary exposure value (SEV) for smoking, age- and sex-specific SEV for low fruit, age- and sex-specific SEV for low vegetables, age- and sex-specific SEV for low omega-3, age- and sex-specific SEV for high sodium, and litres of alcohol consumed per capita.

Smoothing parameters for the second and third steps of the ST-GPR framework were selected through cross validation with out-of-sample root-mean-squared error used to assess model performance. The result of the ST-GPR model are estimates of the mean SBP for each age, sex, location, and year.

Estimate of standard deviation

The standard deviation of systolic blood pressure within a population was estimated for each location, sex, year, and age group using the standard deviation from person-level and some tabulated data sources, which were updated for GBD 2023. Tabulated data were only used to model standard deviation if they were sex-specific and five-year-age-group-specific and reported a population standard deviation of systolic blood pressure. Systolic blood pressure standard deviation was estimated using a linear regression:

$$\log\big(\mathrm{SD}_{l,a,t,s}\big) = \beta_0 + \beta_1 \log\left(\mathrm{mean\_SBP}_{l,a,t,s}\right) + \sum_{k=2}^{7} \beta_k s_k(\mathrm{age}) + \beta_8 \mathrm{sex} + \beta_9 \mathrm{year}$$

where $mean\_SBP_{l,a,t,s}$ is the location-, age-, time-, and sex-specific mean SBP estimate from ST-GPR and $s_k(age)$ corresponds to the basis functions of a cubic spline on the starting age of a given five-year age group with knots placed at ages 35, 50, and 65. The addition of year as a covariate and the switch from

modelling age with dummy variables to a cubic spline was implemented for GBD 2023. The coefficients for the model are reported in Table 4.

Table 4: Log coefficients in the standard deviation model

Term	Coefficient
Intercept (β_0)	–9.845
Log mean SBP (β_1)	1.703
Age spline, basis function 1 (β_2)	0.015
Age spline, basis function 2 (β_3)	0.121
Age spline, basis function 3 (β_4)	0.326
Age spline, basis function 4 (β_5)	0.32
Age spline, basis function 5 (β_6)	0.359
Age spline, basis function 6 (β_7)	0.305
Sex (β_8)	0.099
Year (β_9)	0.002

Adjustment for usual levels of blood pressure

To account for in-person variation in systolic blood pressure, a "usual blood pressure" adjustment was done. The need for this adjustment has been described elsewhere.[6,7] Briefly, measurements of a risk factor taken at a single time point may not accurately capture an individual's true long-term exposure to that risk. Blood pressure readings are highly variable over time due to measurement error as well as diurnal, seasonal, or biological variation. These sources of variation result in an overestimation of the variation in cross-sectional studies of the distribution of SBP.

To adjust for this overestimation, we applied a correction factor to each location-, age-, time-, and sex-specific standard deviation. These correction factors were age-specific and represented the proportion of the variation in blood pressure within a population that would be observed if there were no within-person variation across time. Four longitudinal surveys were used to estimate these factors: the China Health and Retirement Longitudinal Survey (CHRLS), the Indonesia Family Life Survey (IFLS), the United States National Health and Nutrition Examination Survey I Epidemiological Follow-up Study (NHANES I/EFS), and the South Africa National Income Dynamics Survey (NIDS). The sample size and number of blood pressure measurements at each measurement period for each survey is reported in Table 5. For GBD 2023, more recent years of these surveys were added and the regression used to generate the correction factors was updated.

Table 5. Characteristics of longitudinal surveys used for the usual blood pressure adjustment

Source	Measurement periods	Number of measurements	Total sample size	Sample size from baseline cohort
CHRLS (pilot)	2008–2008	3	1967	--
	2012–2012	3	1849	1420
CHRLS	2011–2012	3	13,774	--
	2013–2013	3	12,990	9283
	2015–2015	3	16,249	9871

IFLS	1997–1997	1	19,418	--
	2000–2000	1	25,304	16,626
	2007–2007	3	29,283	14,136
	2014–2015	3	32,681	11,746
NIDS	2008–2008	2	14,084	--
	2010–2011	2	15,234	9098
	2012–2012	2	18,347	9612
	2014–2015	2	22,474	9809
	2017–2017	2	23,531	9007
NHANES I/EFS	1971–1975	2	20,716	--
	1982–1984	3	9972	9932

The following regression was run separately for each survey:

$$\mathrm{SBP_i} = \beta_0 + \beta_1 \mathrm{sex} + \sum_{k=2}^{4} \beta_k s_k(age) + \upsilon_\mathrm{i}$$

where SBP_i is the systolic blood pressure of an individual *i*, sex is a dummy variable for the sex of the individual, $s_k(age)$ corresponds to the basis functions of a cubic spline of the age of the individual, and υ_i is a random intercept for each individual. Then, a blood pressure value $\widehat{\mathrm{SBP}}_{\mathrm{i,b}}$ was predicted for each individual at baseline *b* given their sex and age at baseline. The correction factor $\mathrm{cf_a}$ for an age group *a* was calculated as the square root of the variation in these predicted blood pressures for a given age group divided by the variation in the observed blood pressures at baseline for a given age group:

$$\mathrm{cf_a} = \sqrt{\frac{\mathrm{var}\left(\widehat{\mathrm{SBP}}_{\mathrm{i,b,a}}\right)}{\mathrm{var}\left(\mathrm{SBP}_{\mathrm{i,b,a}}\right)}}$$

The average of the correction factors was taken over the four surveys to get one set of age-specific correction factors, which were then multiplied by the modelled standard deviations to estimate standard deviation of the “usual blood pressure” of each age, sex, location, and year. Because of low sample sizes, the correction factors for the 80–84 age group were used for all terminal age groups. The final correction factors for each age group are reported in Table 6. Figure 2 shows the correction factors by survey and age group.

Table 6. Age-specific usual blood pressure correction factors

Age group	Correction factor
25–29	0.763
30–34	0.812
35–39	0.825
40–44	0.801
45–49	0.793
50–54	0.779

55–59	0.761
60–64	0.757
65–69	0.738
70–74	0.719
75–79	0.745
80+	0.7

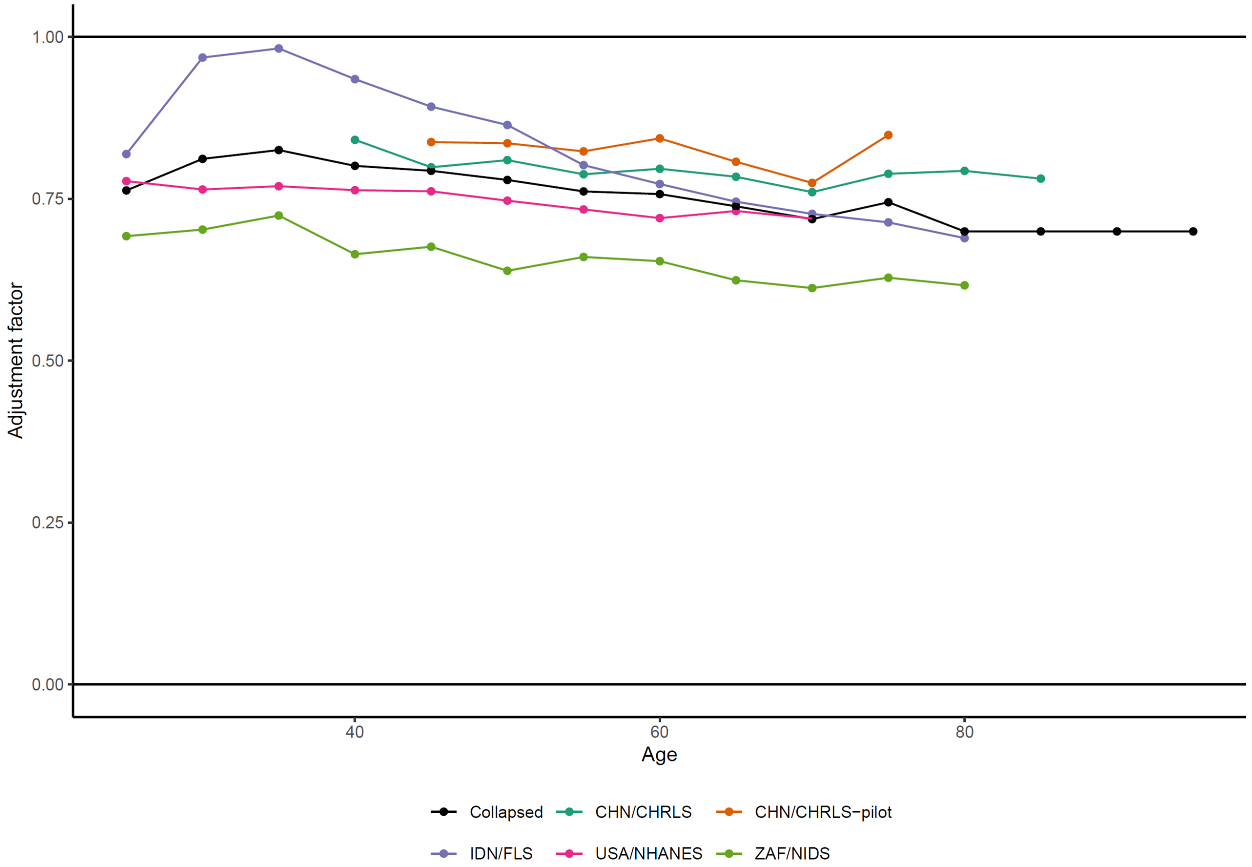

Figure 2: Correction factor by survey and age group. In black is the average correction factor for each age group, summarised in Table 6.

A visualisation of how the uncorrected blood pressure measurements overestimate the "usual" blood pressure variation is shown in Figure 3. This image shows the distribution of the observed blood pressure values $\mathrm{SBP}_{i,b}$ in participants at baseline in the South Africa National Income Dynamics Survey in black, and the distribution of the predicted blood pressure values $\widehat{\mathrm{SBP}}_{i,b}$ for participants at baseline in blue. The square root of the ratio of the variance of the blue distribution to the variance of the black distribution is an example of the scalar adjustment factor being applied to the modelled standard deviations.

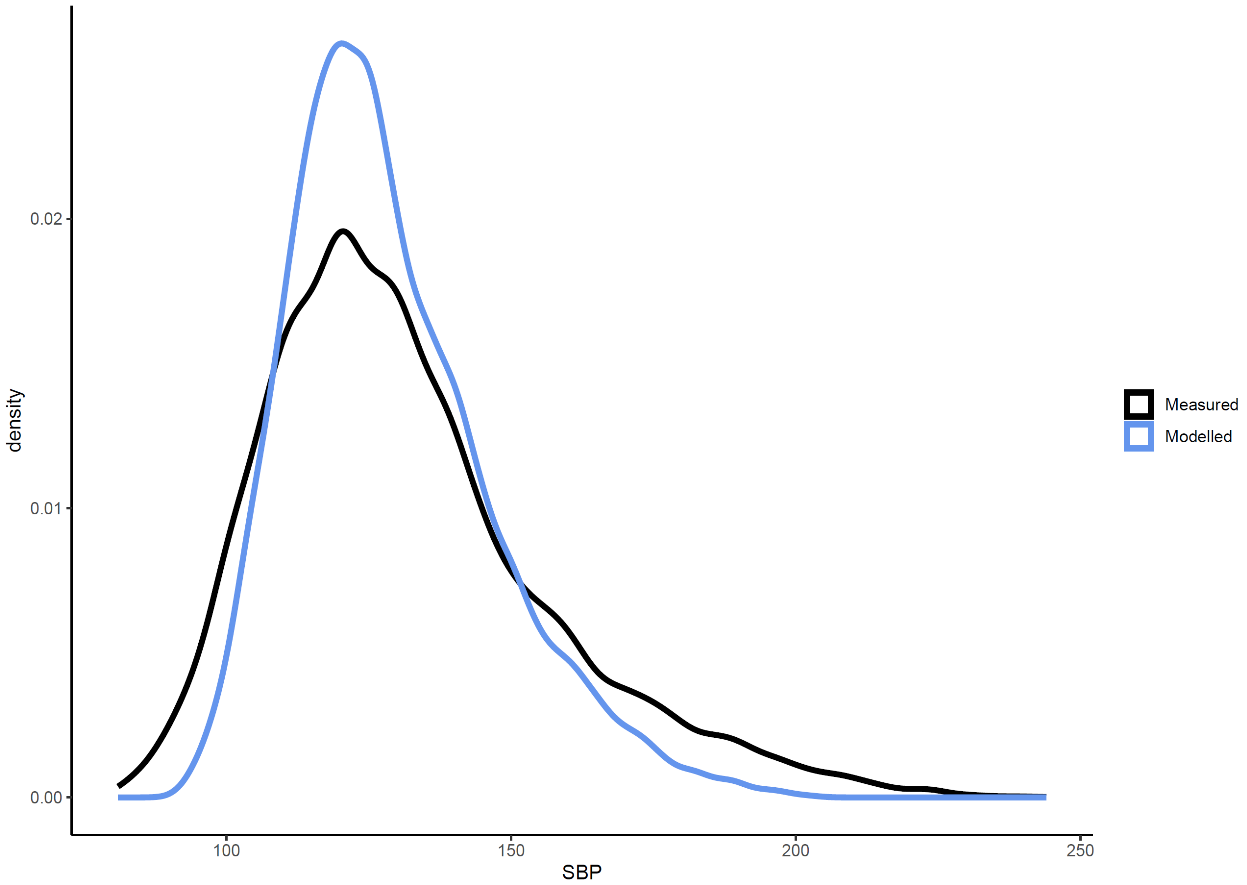

Figure 3: Raw and predicted distributions of blood pressure in the South Africa National Income Dynamics Survey at baseline

Estimating the exposure distribution shape

The shape of the distribution of systolic blood pressure was estimated using all available person-level microdata sources, which was a subset of the input data in the modelling process. The distribution shape modelling framework is detailed elsewhere in the appendix. Briefly, an ensemble distribution created from a weighted average of distribution families was fit for each individual microdata source, separately by sex. The weights for the distribution families for each individual source were then averaged and weighted to create a global ensemble distribution for each sex (Figure 4). Weights were not updated for GBD 2023.

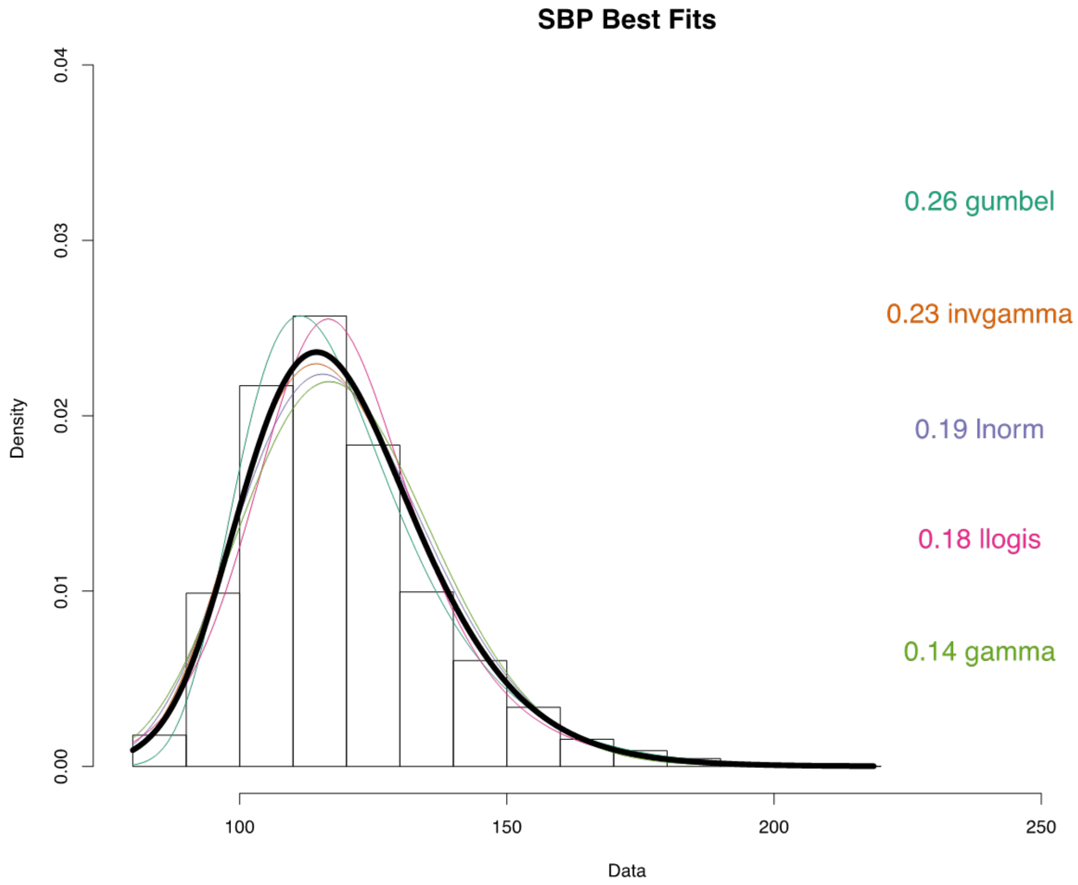

Figure 4: Global ensemble distribution fit and distribution-specific weights for systolic blood pressure

Theoretical minimum risk exposure level

The theoretical minimum risk exposure level (TMREL) is the level of exposure to a risk, in this case, systolic blood pressure, that, within the theoretically possible range of values at the population level, will minimise the risk of all outcomes associated with that risk. For harmful exposures, the TMREL is typically set to the exposure level that corresponds to the minimum of the risk curve or to zero. Defining the TMREL for metabolic risks factors such as systolic blood pressure levels is conceptually difficult and requires us to consider two factors: 1) setting the TMREL at zero is not physiologically plausible; 2) the TMREL can only be defined within the range of the exposure levels that are observed in the data sources (that is, there may plausibly be an exposure level with a true minimum risk level which occurs outside the range of the observed data).

The TMREL was updated for GBD 2021 and did not change for GBD 2023 (Table 7). For GBD 2021, we used the systolic blood pressure values reported in the cohort and RCT studies used to estimate the systolic blood pressure relative risks to update the TMREL as well. For each study, regardless the outcome, we took the range of systolic blood pressure levels for the reference group, defined as the group with the lowest systolic blood pressure levels. We defined the lower bound of the TMREL as the 15th percentile of the lower limit of that range across all studies, and we defined the upper bound of the TMREL as the 15th percentile of the midpoint between the lower and upper bounds of the exposure range across all studies. This approach yields a TMREL that corresponds to the lowest levels of systolic

blood pressure for which we have adequate data to draw robust conclusions about the outcomes included in this analysis. The TMREL was defined as a uniform distribution between 105 and 115 mmHg rather than a fixed value to represent the uncertainty regarding the level at which the scientific evidence was consistent with adverse effects of exposure. To include the uncertainty in the TMREL, we took a random draw from the uniform distribution of the interval between 105 mmHg and 115 mmHg each time the population attributable burden was calculated.

Table 7: Theoretical minimum risk exposure level

TMREL definition	
GBD 2021	GBD 2023
105–115 mmHg	105–115 mmHg

Relative risk

Estimating the relative risk (RR) of ischaemic heart disease, stroke, atrial fibrillation, aortic aneurysm, chronic kidney disease, and peripheral arterial disease occurring as a function of exposure to systolic blood pressure followed the burden of proof approach established by Zheng and colleagues[8,9] and instantiated in the meta-regression—Bayesian, regularised, trimmed (MR-BRT) tool. Details of the RR estimated for systolic blood pressure have been reported previously.[10] MR-BRT synthesises input data to generate an RR curve by relying on an ensemble spline method to capture the potentially non-linear shape of the risk–outcome relationship; integrating over varying exposure ranges in different comparison groups; trimming potentially distorting outliers; testing, selecting, and adjusting for bias covariates to account for known heterogeneity in input study-design characteristics (eg, confounding, selection bias, exposure measurement); and quantifying remaining between-study heterogeneity (gamma) through random effects modelling and incorporating this value into uncertainty around the mean RR curve.

MR-BRT further evaluates evidence for small-study effects and generates funnel plots that represent potential risk of publication or reporting bias and also generates the burden of proof risk function (BPRF), defined for harmful risks as the 5th and for protective risks as the 95th quantile risk curve – inclusive of between-study heterogeneity/gamma – that is closest to null. The BPRF is transformed into a risk–outcome score (ROS: the signed value of the average log BPRF between the 15th and 85th percentiles of risk exposure levels observed across included studies) and mapped onto a star-rating system from one to five stars. These metrics complement RR estimates by providing an alternative, conservative measure of effect size and evidence strength that formally and systematically incorporates divergence/convergence across input findings, with higher positive ROS values and more stars corresponding to incrementally larger effects and stronger evidence for the risk–outcome relationship.

Systolic blood pressure RRs were updated for GBD 2021 and did not change for GBD 2023. Relative risks for each outcome were modelled with log (RR) as the dependent variable and systolic blood pressure exposure values as the independent variable. Due to data sparsity and given that most of the studies included in the meta-regression do not report information disaggregated by stroke subtypes (ie, ischaemic versus haemorrhagic stroke), we decided to combine sources that reported any stroke subtype as outcomes in a single model data, assuming that physiologically, systolic blood pressure can be associated with any stroke. We implemented the Fisher scoring correction to the heterogeneity parameter, which corrects for data-sparse situations. In such cases, the between-study heterogeneity parameter estimate may be zero, simply from lack of data. The Fisher scoring correction uses a quantile

of gamma, which is sensitive to the number of studies, study design, and reported uncertainty. In addition, we have added methodology that can detect and flag publication bias. The approach is based on the classic Egger's regression strategy,[11] which is applied to the residuals in our model. In the current implementation, we do not correct for publication bias but flag the risk–outcome pairs where the risk for publication bias was significant. For this analysis, no risk of publication bias was detected for systolic blood pressure and related outcomes, as shown in the funnel plots below (Figures 5-10). Risk–outcome scores and star ratings are reported in Table 8.

We assumed that the RRs are universal for all countries and sex categories. To account for the heterogeneity of the effect size by age and given the limitations of both the available data and MR-BRT in terms of lack of age-specific data and estimates, we estimated cause-specific age attenuation factors using a second MR-BRT model with log (RR) as the dependent variable and age as an independent variable including data from the APCSC and the PSC cohorts only reported by Singh and colleagues.[12] We then applied these cause-specific attenuation factors to the corresponding RR curve using the mid age at event observed in these two cohort studies (60–64 years) as the reference group to finally generate RR for standard five-year GBD age categories starting at age 25. In future iterations of GBD, we plan to update the MR-BRT tool to be able to incorporate a second spline on age and generate more accurate age-specific RR curves.

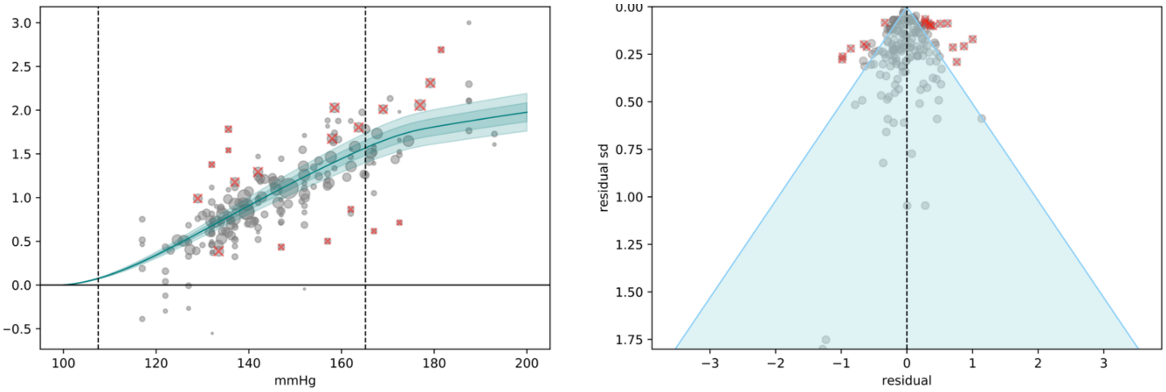

Figure 5: Systolic blood pressure and ischaemic heart disease log relative risk (a) and residuals by estimated standard deviation (b)

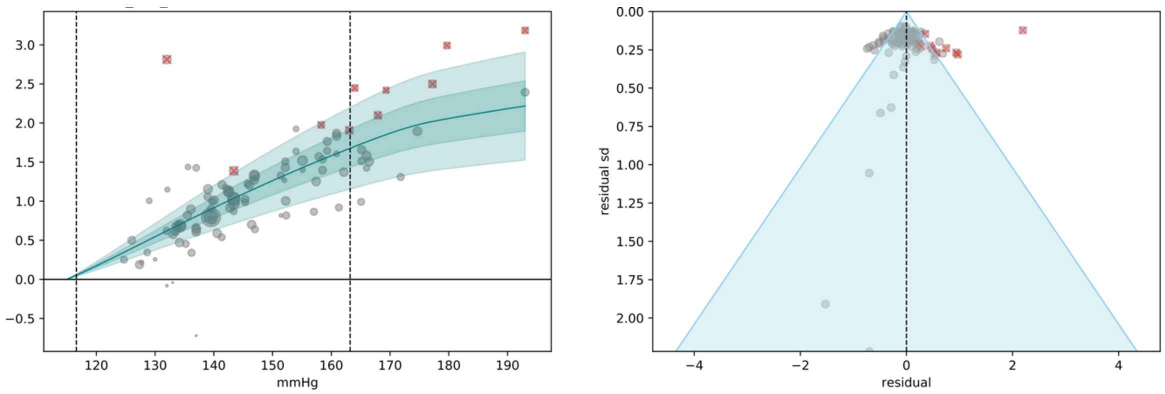

Figure 6. Systolic blood pressure and stroke log relative risk (a) and residuals by estimated standard deviation (b)

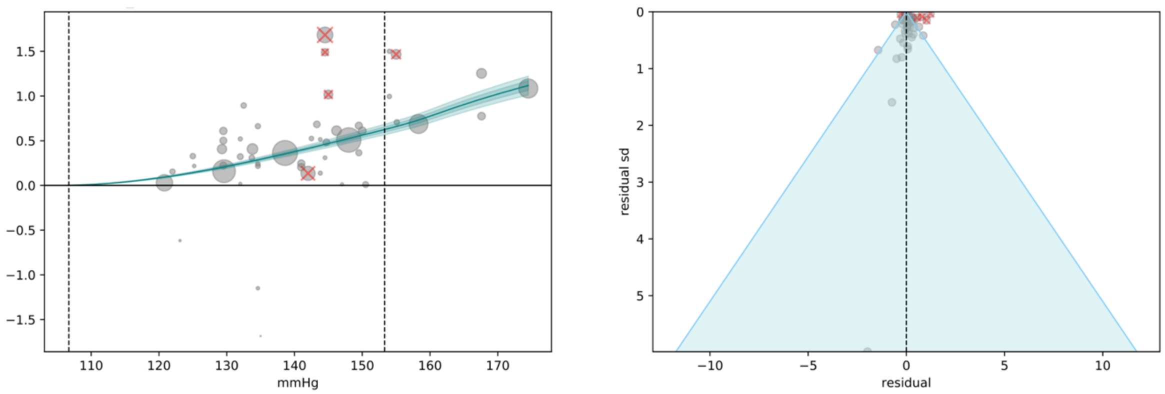

Figure 7. Systolic blood pressure and chronic kidney disease log relative risk (a) and residuals by estimated standard deviation (b)

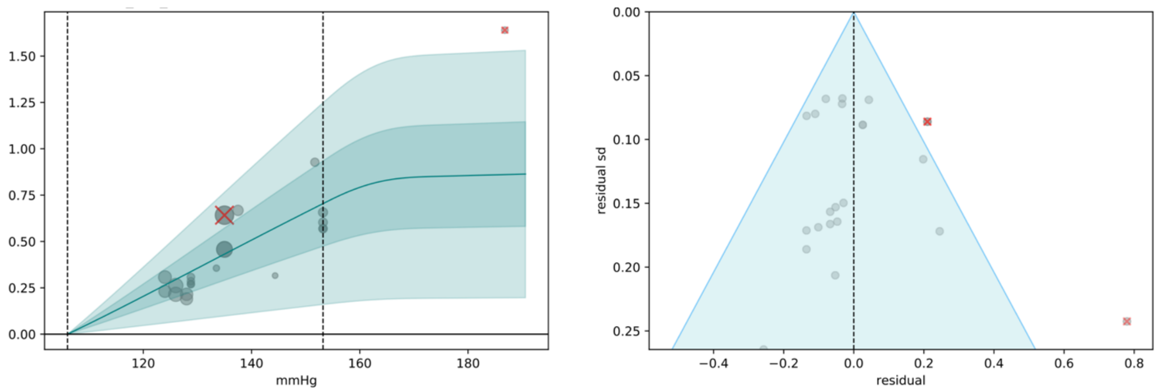

Figure 8. Systolic blood pressure and atrial fibrillation log relative risk (a) and residuals by estimated standard deviation (b)

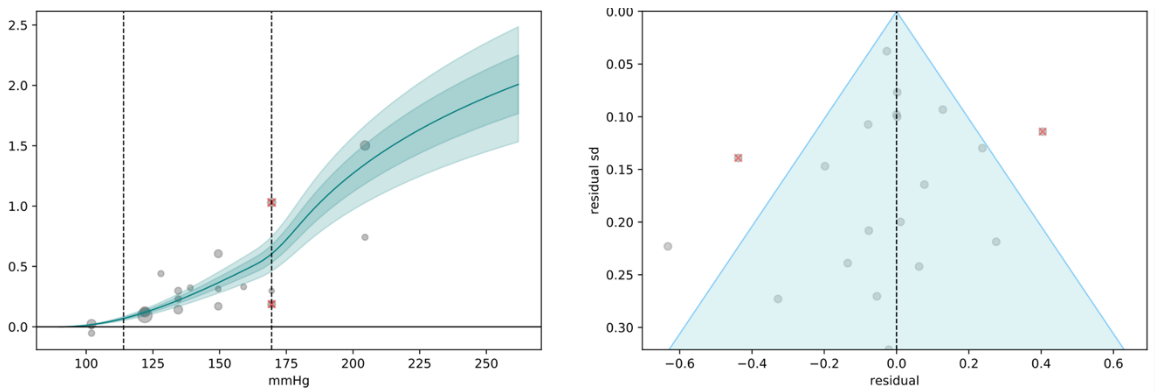

Figure 9. Systolic blood pressure and aortic aneurysm log relative risk (a) and residuals by estimated standard deviation (b)

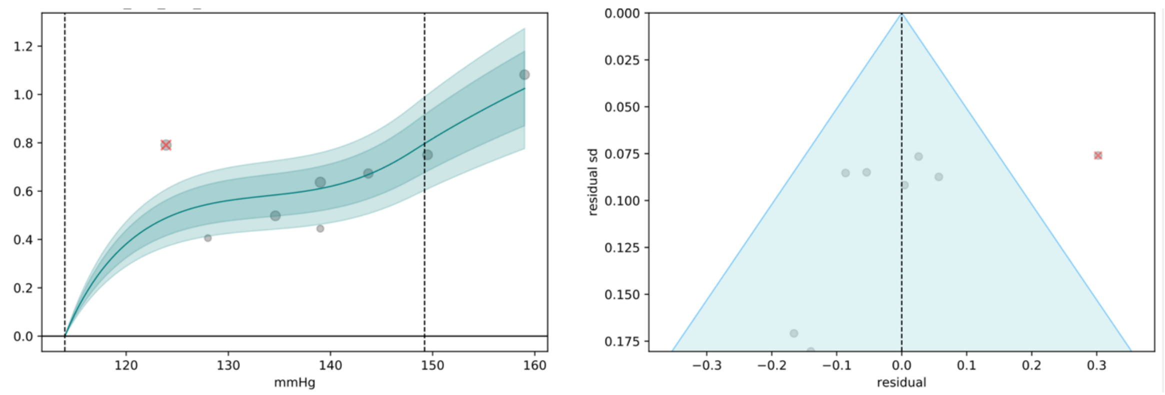

Figure 10. Systolic blood pressure and peripheral arterial disease log relative risk (a) and residuals by estimated standard deviation (b)

Figures 5 to 10. In panel (a) the dark line indicates mean relative risk across systolic blood pressure exposure levels; the light and dark shading show 95% uncertainty intervals with and without between-study heterogeneity, respectively; the size of the datapoints corresponds to the inverse of the standard error, with those trimmed during the model fitting process marked by a red x; and the dashed lines represent the 15th percentile of the reference exposure and the 85th percentile of the alternative exposure. To visualise log-relative-risk points in panel (a), we plotted each datapoint with the x-value at the midpoint of the alternative group and the y-value offset by the difference between the reported and predicted log risk. Panel (b) depicts a customised funnel plot, with the x-axis representing residuals between predicted and observed relative risks, and the y-axis representing uncertainty from both measurement error and between-study heterogeneity

Table 8: Risk–outcome scores and star ratings

Outcome	Risk–outcome score	Star rating
Ischaemic heart disease	0.70	5
Stroke	0.67	5
Chronic kidney disease	0.23	3
Atrial fibrillation	0.12	2
Aortic aneurysm	0.24	3
Peripheral arterial disease	0.42	4

Citations

1 Page MJ, McKenzie JE, Bossuyt PM, *et al.* The PRISMA 2020 statement: an updated guideline for reporting systematic reviews. *BMJ* 2021; **372**: n71.

2 Global Health Data Exchange | GHDx. https://ghdx.healthdata.org/ (accessed Jan 16, 2025).

3 Brauer M, Roth GA, Aravkin AY, *et al.* Global burden and strength of evidence for 88 risk factors in 204 countries and 811 subnational locations, 1990–2021: a systematic analysis for the Global Burden of Disease Study 2021. *The Lancet* 2024; **403**: 2162–203.

4 Hsu A, Zheng P, Maass K, Aravkin A, Ali S. pyDisagg: Dissaggregation under Generalized Proportionality Assumptions. 2025; published online Jan 13. DOI:10.5281/zenodo.14641582.

5 Ng M, Fleming T, Robinson M, *et al.* Global, regional, and national prevalence of overweight and obesity in children and adults during 1980–2013: a systematic analysis for the Global Burden of Disease Study 2013. *The Lancet* 2014; **384**: 766–81.

6 Prospective Studies Collaboration. Age-specific relevance of usual blood pressure to vascular mortality: a meta-analysis of individual data for one million adults in 61 prospective studies. *The Lancet* 2002; **360**: 1903–13.

7 Clarke R, Shipley M, Lewington S, *et al.* Underestimation of Risk Associations Due to Regression Dilution in Long-term Follow-up of Prospective Studies. *American Journal of Epidemiology* 1999; **150**: 341–53.

8 Zheng P, Barber R, Sorensen RJD, Murray CJL, Aravkin AY. Trimmed Constrained Mixed Effects Models: Formulations and Algorithms. *Journal of Computational and Graphical Statistics* 2021; **30**: 544–56.

9 Zheng P, Afshin A, Biryukov S, *et al.* The Burden of Proof studies: assessing the evidence of risk. *Nat Med* 2022; **28**: 2038–44.

10 Razo C, Welgan CA, Johnson CO, *et al.* Effects of elevated systolic blood pressure on ischemic heart disease: a Burden of Proof study. *Nat Med* 2022; **28**: 2056–65.

11 Egger M, Smith GD, Schneider M, Minder C. Bias in meta-analysis detected by a simple, graphical test. *BMJ* 1997; **315**: 629–34.

12 Singh GM, Danaei G, Farzadfar F, *et al.* The Age-Specific Quantitative Effects of Metabolic Risk Factors on Cardiovascular Diseases and Diabetes: A Pooled Analysis. *PLOS ONE* 2013; **8**: e65174.

Household air pollution

Flowchart

Household Air Pollution from Solid Fuels

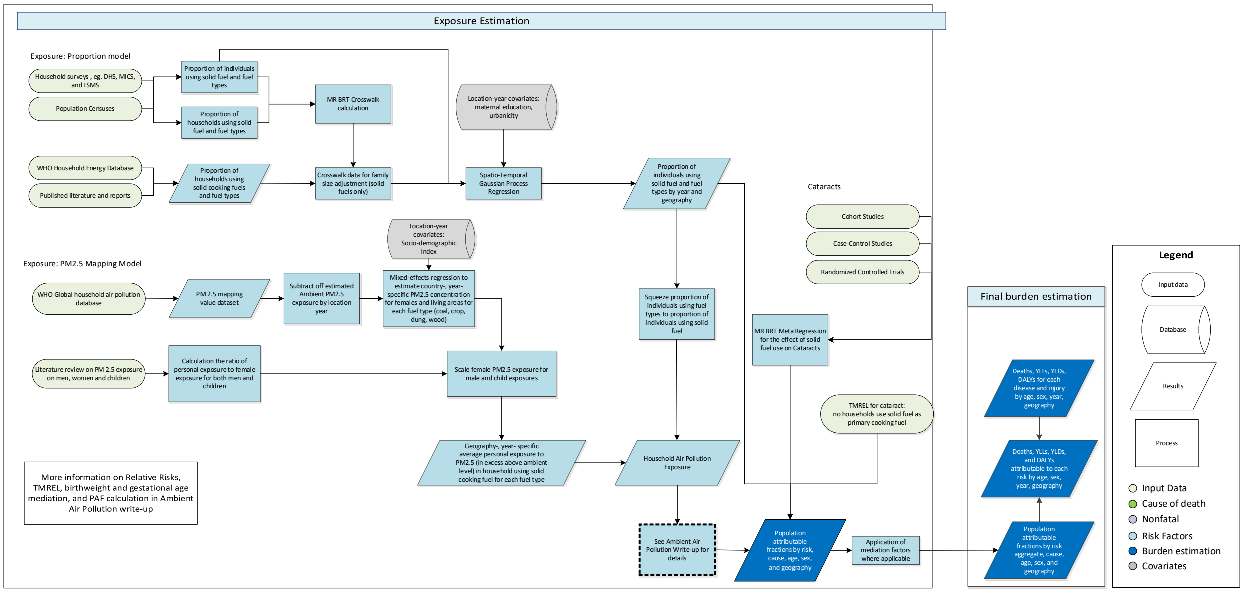

Input data and methodological summary

Definition

Exposure

Exposure to household air pollution from solid fuels (HAP) is estimated from both the proportion of individuals using solid cooking fuels and the level of exposure to particulate matter less than 2.5 micrometers in aerodynamic diameter ($PM_{2.5}$) air pollution for these individuals. Solid fuels in our analysis include wood, coal/charcoal, dung, and agricultural residues.

Input data

Exposure

We extracted information on the use of solid fuels for cooking from standard multi-country survey series, including the Demographic and Health Surveys (DHS), Living Standards Measurement Surveys (LSMS), Multiple Indicator Cluster Surveys (MICS), and World Health Surveys (WHS). We also used data from censuses and country-specific survey series, such as the Kenya Welfare Monitoring Survey and South Africa General Household Survey. To fill remaining gaps in survey and census data, we downloaded the WHO Household Energy Database and updated estimates using extracted information from literature through a systematic review.[1] From this combined body of input data, each nationally or subnationally representative datapoint provided an estimate of the percentage of households or individuals using solid cooking fuels.

We excluded sources that did not distinguish specific primary fuel types, estimated fuel used for purposes other than cooking (eg, lighting or heating), failed to report standard error or sample size,

reported over 15% missingness for households surveyed, reported fuel use in physical units, or were secondary sources referencing primary analyses.

Data processing

Many estimates in the WHO Energy Database and other reports quantify the proportion of households using solid fuel for cooking; however, we are interested in the proportion of individuals using solid fuel for cooking for exposure and burden assessment. To crosswalk these estimates, where available, we extracted fuel use at both the individual and household levels. We used studies that reported values for both household and individual solid fuel use. For studies that reported zero, we replaced the zero value with half of the lowest non-zero value for the region the datapoint is in. For studies that reported one, we replaced one with the value of the globally highest non-one value plus half of the difference between one and the globally highest non-one value. For the crosswalk model, we used the meta-regression—Bayesian, regularised, trimmed (MR-BRT) meta-regression tool.[2] The results of the model can be found in Table 1. We applied this crosswalk only to proportion estimates for the parent solid fuel category. We did not adjust fuel-specific (coal/charcoal, crop, dung, or wood) proportion estimates due to lack of sufficient data for each individual fuel type.

Table 1: MR-BRT crosswalk adjustment factors for household air pollution exposure

Data input	Reference or alternative case definition	Gamma	Beta coefficient, logit (95% UI)*	Adjustment factor**
Proportion of individuals	Ref	4.93	---	---
Proportion of households	Alt		–0.53 (–0.580 to –0.480)	1.699 (1.617–1.786)

**MR-BRT crosswalk adjustments can be interpreted as the factor the alternative case definition is adjusted by to reflect what it would have been had it been measured using the reference case definition. If the log/logit beta coefficient is negative, then the alternative is adjusted up to the reference. If the log/logit beta coefficient is positive, then the alternative is adjusted down to the reference.*

***The adjustment factor column is the exponentiated negative beta coefficient. For log beta coefficients, this is the relative rate between the two case definitions. For logit beta coefficients, this is the relative odds between the two case definitions.*

We applied this coefficient to household-only solid fuel reports with the following formula:

$prop_{individ}$ = the proportion of individuals using solid fuel for cooking, and

$prop_{hh}$ = the proportion of households using solid fuel for cooking.

$$\log\left(\frac{prop_{individ}}{1 - prop_{individ}}\right) = \log\left(\frac{prop_{hh}}{1 - prop_{hh}}\right) - \beta$$

or

$$prop_{individ} = \frac{prop_{hh} * e^{-\beta}}{1 - prop_{hh} + prop_{hh} * e^{-\beta}}$$

As a result, household studies were inflated to account for bias in size between households that use solid cooking fuels and those that do not. Larger households are more likely to use solid fuels for cooking.

Modelling strategy

Exposure

Household air pollution was modelled at the individual level using a three-step modelling strategy implementing linear regression, spatiotemporal regression, and Gaussian process regression (ST-GPR).[3]

In GBD 2021, we updated the HAP proportion model to disaggregate estimates of solid fuel use to estimate the proportion of individuals using each of the following component fuel type categories: 1) coal or charcoal, 2) crop residue, 3) dung, and 4) wood. With this strategy, we can more finely characterise individual exposure to $PM_{2.5}$ due to solid fuel use by applying fuel-specific mapping values to fuel-specific proportion estimates. This change addresses an important limitation in our model, in that it previously assumed equal $PM_{2.5}$ exposure for all solid fuel categories.

ST-GPR was used to create estimates for the parent solid fuel category, and each fuel type (coal, crop, dung, and wood) for a total of five models. Once an ST-GPR model is established, we use the covariates, but not the random effects, to predict for locations and years with insufficient data. Each model's linear regression equation is the same, with the following equation:

$$logit(data) \sim maternal\ education + prop_{urban} + (1|level_1) + (1|level_2)$$

Maternal education = mean years of maternal education per capita
$Prop_{urban}$ = proportion of population living in urban areas
$(1|level_1)$ = super-region-level random effects
$(1|level_2)$ = region-level random effects

$Prop_{urban}$'s methodologies can be found in the lead risk factor appendix.

The four fuel-type-specific proportion estimates were then squeezed to the estimates for the overall proportion of individuals using solid fuel for cooking. For each location and year, we used the following formula, where $prop_{coal}$, $prop_{crop}$, $prop_{dung}$, $prop_{wood}$, and $prop_{solid}$ indicate the proportion of individuals using coal, crop, dung, wood, or any type of solid fuel, respectively.

$$prop_{total} = prop_{coal} + prop_{wood} + prop_{crop} + prop_{dung}$$

$$S = prop_{total} \,/\, prop_{solid}$$

where *S* is the squeezed total value. For each fuel category, with coal shown below as an example, the adjusted (squeezed) proportion is calculated as

$$prop_{coal}' = prop_{coal} \,/\, S$$

In GBD 2021, during preliminary model iterations, we mapped mixed fuel strings (eg, "wood and agricultural residues") to the category associated with highest $PM_{2.5}$ exposure to avoid underestimating HAP exposure. However, fuel-specific ST-GPR models were unstable with this approach. We therefore excluded mixed-fuel string studies from final estimates for fuel-specific proportions, though we retained these studies when modelling the proportion of overall solid fuel use. No further changes were made for GBD 2023.

Theoretical minimum risk exposure level

For all HAP outcomes except cataract, burden is related to both ambient and household air pollution. These PAFs are estimated jointly, and the theoretical minimum risk exposure level (TMREL) is defined as a uniform distribution between 2.4 and 5.9 $\mu g/m^3$ $PM_{2.5}$. For cataract, the TMREL is defined as no individuals using solid cooking fuel.

Relative risk

The outcomes associated with household air pollution are lower respiratory infections (LRI), stroke, ischaemic heart disease (IHD), chronic obstructive pulmonary disease (COPD), lung cancer, type 2 diabetes, and cataract. Low birthweight and short gestation are also outcomes attributable to household air pollution through a mediation analysis. With the exception of cataract, all causes share risk curves and are calculated jointly with ambient particulate matter pollution.

Cataract relative risk meta-analysis

Prior to GBD 2019, we used the results of an external meta-analysis with a summary relative of 2.47 (95% UI 1.63–3.73) for cataract risk estimates.[4] While this effect estimate was for both sexes, in the past we estimated burden for women only because women are known to have higher HAP exposure than men. In GBD 2019, we performed our own meta-regression analysis of household air pollution and cataracts. We updated this meta-regression for GBD 2021. No further changes were made for GBD 2023.

We extracted all the component studies of the above meta-analysis paper but excluded one cross-sectional study. GBD risk factor analyses typically do not include cross-sectional analyses due to their weaker evidence base. In literature search conducted in GBD 2019, we found one additional paper describing different fuel types and cataracts.[5] We excluded this study because there was no comparison group without solid fuel use. We conducted an additional literature search in GBD 2021 but found no new studies to include. The following search string was used to identify studies in the PubMed database published between January 1, 2017, and July 22, 2020 (date of search).

Search string: (("Air Pollution, Indoor"[Mesh] OR "Household air"[Title/Abstract] OR "Indoor air pollution"[Title/Abstract] OR "Indoor fine particulate matter"[Title/Abstract] OR "Indoor particulate matter"[Title/Abstract] OR "Indoor air quality"[Title/Abstract] OR "Airborne particulate matter"[Title/Abstract]) AND ("Cataract"[Title/Abstract] OR "Cataracts"[Title/Abstract] OR "Cataracts"[Mesh] OR "Lens Opacities"[Mesh] OR "Lens Opacity"[Mesh] OR "Opacities, Lens"[Mesh] OR "Opacity, Lens"[Mesh] OR "Cataract, Membranous"[Mesh] OR "Cataracts, Membranous"[Mesh] OR "Membranous Cataract"[Mesh] OR "Membranous Cataracts"[Mesh] OR "Pseudoaphakia"[Mesh] OR "Pseudoaphakias"[Mesh]))

Our resulting dataset contained eight estimates from six sources in India and Nepal. We ran a MR-BRT meta-regression on these eight estimates to generate a summary effect size of 2.52 (95% UI 1.42–4.57). We did not trim any of the observations due to the relatively few input studies available compared to

other GBD risk factors. We used the MR-BRT automated covariate selection process to identify significant covariates from those extracted to quantify between-study heterogeneity. Briefly, a series of loosening Lasso penalty parameters were applied to a log-linear meta-regression on all input effect size observations. Then, covariates with a non-zero coefficient were tested for significance using a Gaussian prior (significance threshold = 0.05). No significant covariates were identified. Figure 1 provides a visual representation.

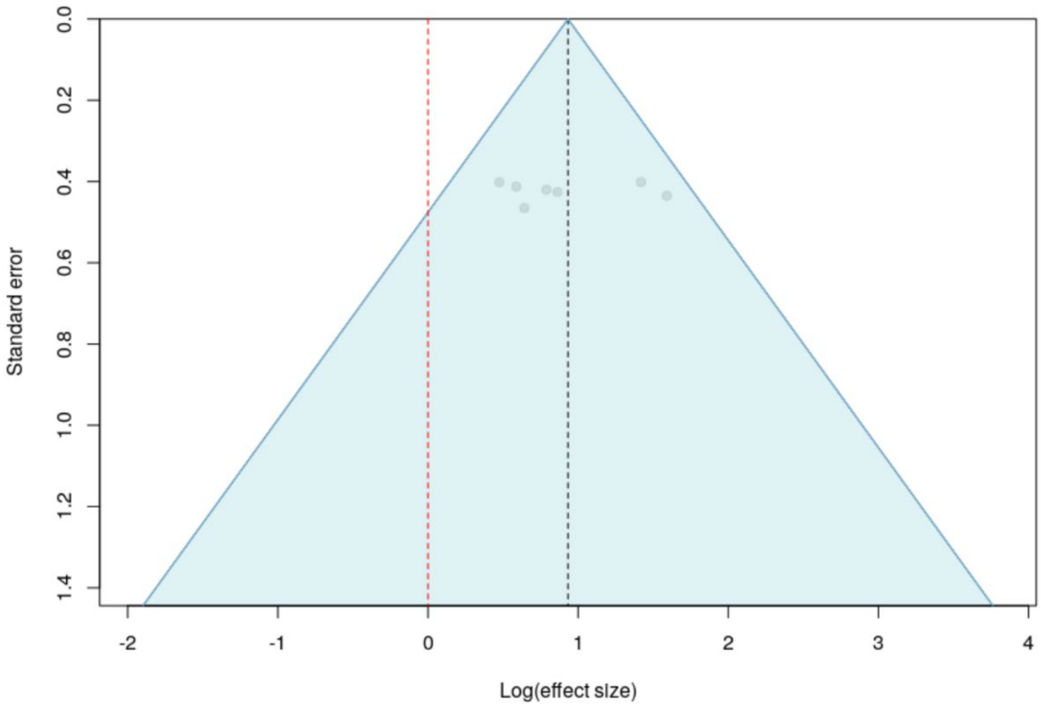

Figure 1: Household air pollution and cataract risk literature funnel plot

Studies reported effect sizes for males, females, and/or both sexes. In a sensitivity analysis conducted in GBD 2019 and repeated in GBD 2021, we included a covariate for sex and found no significant difference in effect size by sex. We therefore estimate cataract as an outcome of household air pollution in both males and females.

In GBD 2021, we also implemented risk–outcome scoring in the Burden of Proof (BoP) framework to provide an empirical measure of strength of evidence for risk–outcome pairs across risk factors in the GBD study (described in more detail elsewhere).[2,6] Prior to generating a risk–outcome score, we conducted an additional post-analysis step to detect and flag publication bias in the input data. This approach is based on the classic Egger's regression strategy, which is applied to the residuals in our model. In the current implementation, we do not correct for publication bias but flag the risk–outcome pairs where the risk for publication bias is significant. Publication bias was not detected for HAP–cataract risk literature. The resulting risk–outcome score for HAP and cataract was –0.009, which corresponds to a star rating of one.

$PM_{2.5}$ mapping value estimation

To calculate relative risks from particulate matter risk curves for individuals using solid fuels for cooking, we first estimated the $PM_{2.5}$ exposure level resulting from usage of each fuel type. Input data for the

HAP mapping model included indoor and personal measurement data from the WHO Global Household Air Pollution Measurements database.[7] In GBD 2021, we added data from the PURE-AIR study published in 2020, which included additional measurements from 120 rural locations in Bangladesh, Chile, China, Colombia, India, Pakistan, Tanzania, and Zimbabwe.[8] We included measurements for indoor exposure and personal monitors.

The following models were used to predict log-transformed estimates of excess $PM_{2.5}$ for each individual fuel type (coal, crop, dung, wood) and for the parent solid category. Predictions for the parent solid category were used only to prepare relative risk input data for analysis, not for predicting individual exposure to $PM_{2.5}$ from solid fuel use.

Fuel types:

$$\log(excess\ PM) \sim crop + coal + dung + wood + measure\ group + 24\ hr\ measurement + LDI + (1|study)$$

Solid:

$$\log(excess\ PM) \sim solid + measure\ group + 24\ hr\ measurement + LDI + (1|study)$$

Where

- *24-hour measurement*: binary variable equal to one if the measurement occurred over at least a 24-hour period and not only during mealtimes
- *Measure group*: categorical variable indicating indoor, female, male, or children
- *Solid*: indicator variable equal to one if the measurements were among households using solid fuel only, 0.5 if the measurements represented a mix of clean and solid fuels, and zero if the households only used clean fuels.

Prior to GBD 2021, we also included the Socio-demographic Index (SDI) as a variable to predict a unique value of HAP for each location and year based on development. In GBD 2021, we updated the HAP mapping model to predict unique values from the lag-distributed income per capita (LDI). Evaluations of model fit using root mean square error (RMSE) indicated that LDI is a more suitable predictor of excess $PM_{2.5}$. We also included a random effect on study and weighted each study by the square root of its sample size.

Before modelling, we subtracted off the prediction of ambient $PM_{2.5}$ in the study location and year to calculate the excess particulate matter for individuals using solid fuel. The final model coefficients are included below, in Table 2.

Table 2: HAP mapping model and coefficients

**Variable**	**Beta, log (95% UI)**	**Beta, exponentiated (95% UI)**
Intercept	3.22 (2.85–3.58)	24.97 (17.21–36.00)
Fuel type		
• Clean (ref)		
• Crop	3.25 (3.15–3.35)	25.80 (23.26–28.42)
• Coal	1.61 (1.53–1.71)	5.00 (4.61–5.53)
• Dung	2.49 (2.38–2.59)	12.05 (108.81–13.31)
• Wood	1.96 (1.91–2.02)	7.10 (6.76–7.50)

Measure group<br>• Indoor (ref)<br>• Female<br>• Male<br>• Child	 <br>–0.32 (–0.39 to –0.28)<br>–0.24 (–0.32 to –0.15)<br>–1.04 (–1.14 to –0.95)	 <br>0.72 (0.68–0.76)<br>0.79 (0.73–0.86)<br>0.35 (0.32–0.39)
24-hour measurement	0.19 (0.07–0.31)	1.20 (1.08–1.36)
LDI	1.01e-07 (7.09e-08 to 1.36e-07)	1.00 (1.00–1.00)

To derive final predicted $PM_{2.5}$ exposure values due to solid fuel usage, instead of using direct model outputs for males and children, we scaled $PM_{2.5}$ exposure values for females to the other two groups. There are few studies of personal monitoring in men and children, so we derived ratios of female-male and female-child exposures using studies that reported PM exposure values for females and one or both of the other groups. To calculate these ratios, we first subtracted off the outdoor value from each PM measurement (using ambient $PM_{2.5}$ predictions as above for $PM_{2.5}$ studies and the studies' published values for PM_4 and PM_{10} studies) and then calculated ratios weighted by sample size.

The final ratios, updated with information from the 2020 PURE-AIR study, were 0.85 (95% UI 0.67–1.09) for children and 0.64 (0.52–0.79) for males compared to 0.85 (0.56–1.31) for children and 0.64 (0.45–0.91) for males in GBD 2019. These results were used to scale the $PM_{2.5}$ mapping model fuel-type-specific predictions for these age and sex groups to calculate relative risks from the $PM_{2.5}$ risk curves.

Population attributable fraction

HAP population attributable fractions (PAFs) are calculated jointly with those for ambient particulate matter pollution. Details of PAF calculation, relative risks, and risk–outcome scores for all outcomes besides cataract are provided in the ambient particulate matter pollution appendix.

References

1. World Health Organization (WHO). WHO Household Energy Database 1960-2017. Geneva, Switzerland: World Health Organization (WHO), 2019.
2. Zheng P, Barber R, Sorensen RJD, Murray CJL, Aravkin AY. Trimmed constrained mixed effects models: formulations and algorithms. *J Comput Graph Stat* 2021; **30**: 544–56.
3. GBD 2021 Risk Factors Collaborators. Global burden and strength of evidence for 88 risk factors in 204 countries and 811 subnational locations, 1990–2021: a systematic analysis for the Global Burden of Disease Study 2021. *Lancet* 2024; 403: 2162–203.
4. Smith KR, Bruce N, Balakrishnan K, Adair-Rohani H, Balmes J, Chafe Z, *et al*. Millions Dead: How Do We Know and What Does It Mean? Methods Used in the Comparative Risk Assessment of Household Air Pollution. *Annu Rev Public Health*. 2014; **35**(1):185–206.
5. Tanchangya J, Geater AF. Use of traditional cooking fuels and the risk of young adult cataract in rural Bangladesh: a hospital-based case-control study. *BMC Ophthalmology*. 2011; **11**.
6. Zheng P, Afshin A, Biryukov S, *et al.* The Burden of Proof studies: assessing the evidence of risk. *Nat Med* 2022; **28**: 2038–44.
7. Shupler M, Balakrishnan K, Ghosh S, *et al*. Global household air pollution database: Kitchen concentrations and personal exposures of particulate matter and carbon monoxide. *Data in Brief* 2018; **21**: 1292–5.

8. Shupler M, Hystad P, Birch A, Miller-Lionberg D, Jeronimo M, Arku R, *et al*. Household and personal air pollution exposure measurements from 120 communities in eight countries: results from the PURE-AIR study. *Lancet Planetary Health*. 2020; **4**(10): E451-E462.

Intimate partner violence

Flowchart

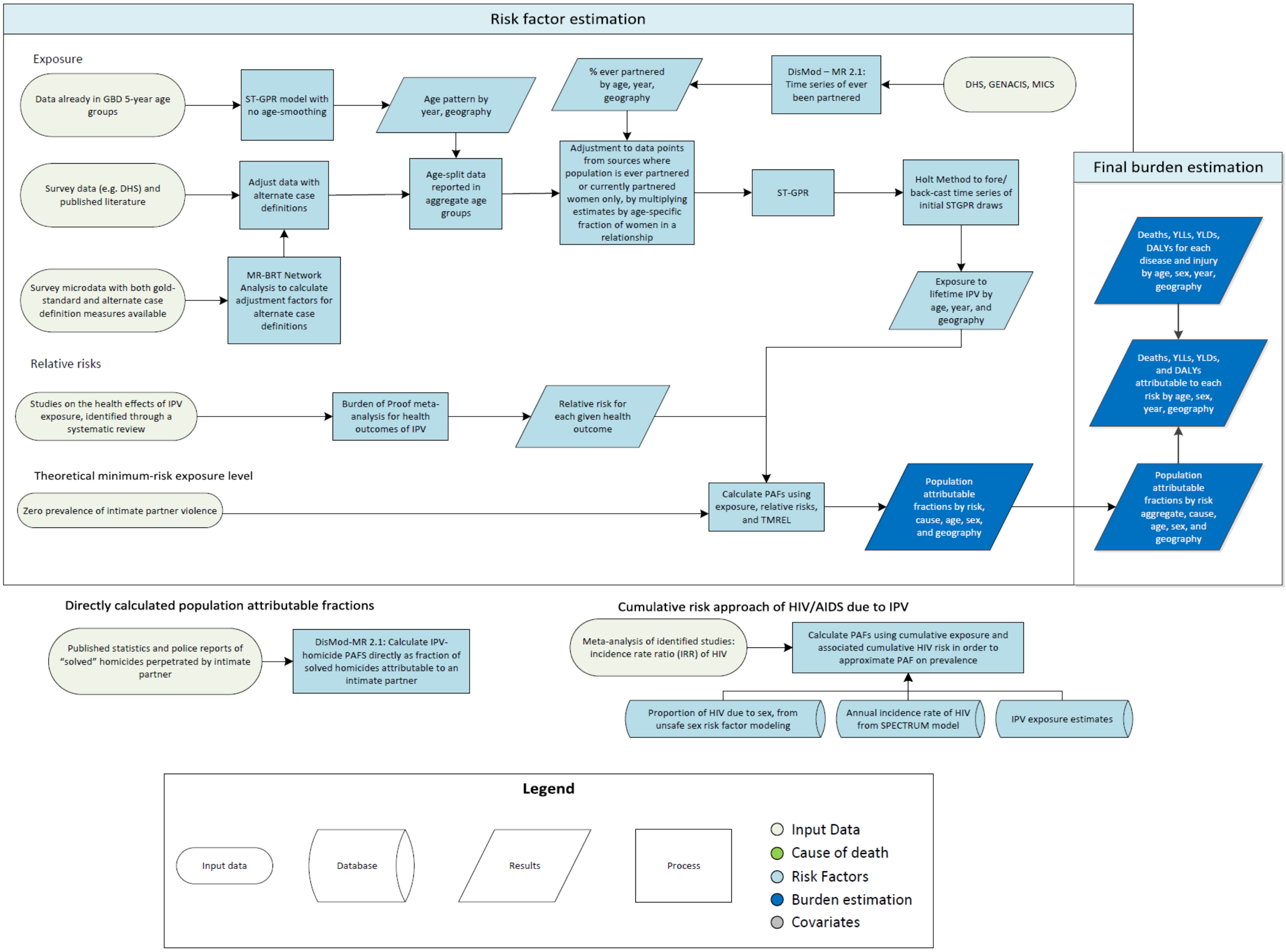

Input data and methodological summary

Definition

Exposure

The case definition for intimate partner violence (IPV) is having ever experienced one or more acts of physical and/or sexual violence by a current or former intimate partner since the age of 15 years. IPV is estimated in females only because the existing evidence of risk–outcomes for males does not meet our inclusion criteria.

- Physical violence is defined as "being slapped or having something thrown at you that could hurt you, being pushed or shoved, being hit with a fist or something else that could hurt, being kicked, dragged, or beaten up, being choked or burnt on purpose, and/or being threatened with or actually having a gun, knife, or other weapon used on you."

- Sexual violence is defined as "being physically forced to have intercourse when you did not want to, having sexual intercourse because you were afraid of what your partner might do, and/or being forced to do something that you found humiliating or degrading." The definition of humiliating and degrading may vary across studies depending on the regional and cultural setting.
- Intimate partner is defined as "a partner to whom you are married or with whom you cohabit." In countries where people date, dating partners will also be considered (a partner with whom you have an intimate [sexual] relationship with but are not married to or cohabiting).

Input data

Exposure

For GBD 2023, we incorporated new exposure data sources identified through the Global Health Data Exchange (GHDx), the World Health Organization (WHO) Global Database on the Prevalence of Violence against Women, the United Nations Entity for Gender Equality and the Empowerment of Women (UN Women) Global Database on Violence against Women, and the GBD Collaborator Network. In this review, we also included additional information for the fraction of homicides against women perpetrated by an intimate partner.

We included all sources that provided population-representative data on the proportion of women who have ever experienced physical or sexual violence by a current or former intimate partner. We also accepted sources reporting on the following alternate case definitions and non-reference populations:

1. Women who have ever experienced any physical IPV
2. Women who have ever experienced any sexual IPV
3. Women who have ever experienced severe physical IPV
4. Women who have experienced IPV in the past year
5. Women who have ever had an intimate partner who have experienced IPV
6. Women who currently have an intimate partner who have experienced IPV
7. Women who have experienced intimate partner violence by a spouse
8. Women who have experienced intimate partner violence by a current spouse

Relative risk

For GBD 2023, we conducted a new systematic review for IPV relative risks.[1] Briefly, we assessed all available literature on the health impacts of intimate partner violence by systematically searching PubMed, Embase/Elsevier, Cumulative Index to Nursing and Allied Health Literature (CINAHL), PsychInfo, Global Index Medicus, Cochrane, and Web of Science. Through this review, we identified peer-reviewed studies published from 1 January 1970 to 31 January 2024 reporting on any health effects associated with any form of violence against children or gender-based violence, including intimate partner violence. The search strings have been published elsewhere[1,2] and do not limit identified studies to any specific health outcome of interest. Instead, the search aims to identify all health outcomes that have been researched in association with violence against children or gender-based violence, allowing for meta-analyses of those that have been reported on by three or more studies.

The search was first conducted on 30 September 2021, and results up to that moment were published in an October 2024 study in Nature Medicine.[2] The search was updated on 26 February 2024 to include the

more recent breadth of literature for the current GBD cycle, which allowed us to analyse one new health outcome in association with IPV and incorporate newer studies across the previously analysed associations. During both phases of the systematic review, studies were included if they used study designs where temporality between exposure and outcome could be determined (eg, case-control or cohort studies), were conducted among a generalisable study population, and reported an estimate of association between an exposure and outcome of interest or enough data to construct a crude effect size. Studies were only further eligible for inclusion in the analysis of IPV's health effects if they had data on the association between a health outcome, defined in alignment with GBD standards, and physical and/or sexual violence perpetrated by a partner/former partner, specifically. Details on the included outcome and exposure definitions can be found elsewhere, as well as a thorough breakdown of the screening process.[1,2]

Figure 1: PRISMA diagram 2020 flow diagram for the health effects of physical and/or sexual intimate partner violence

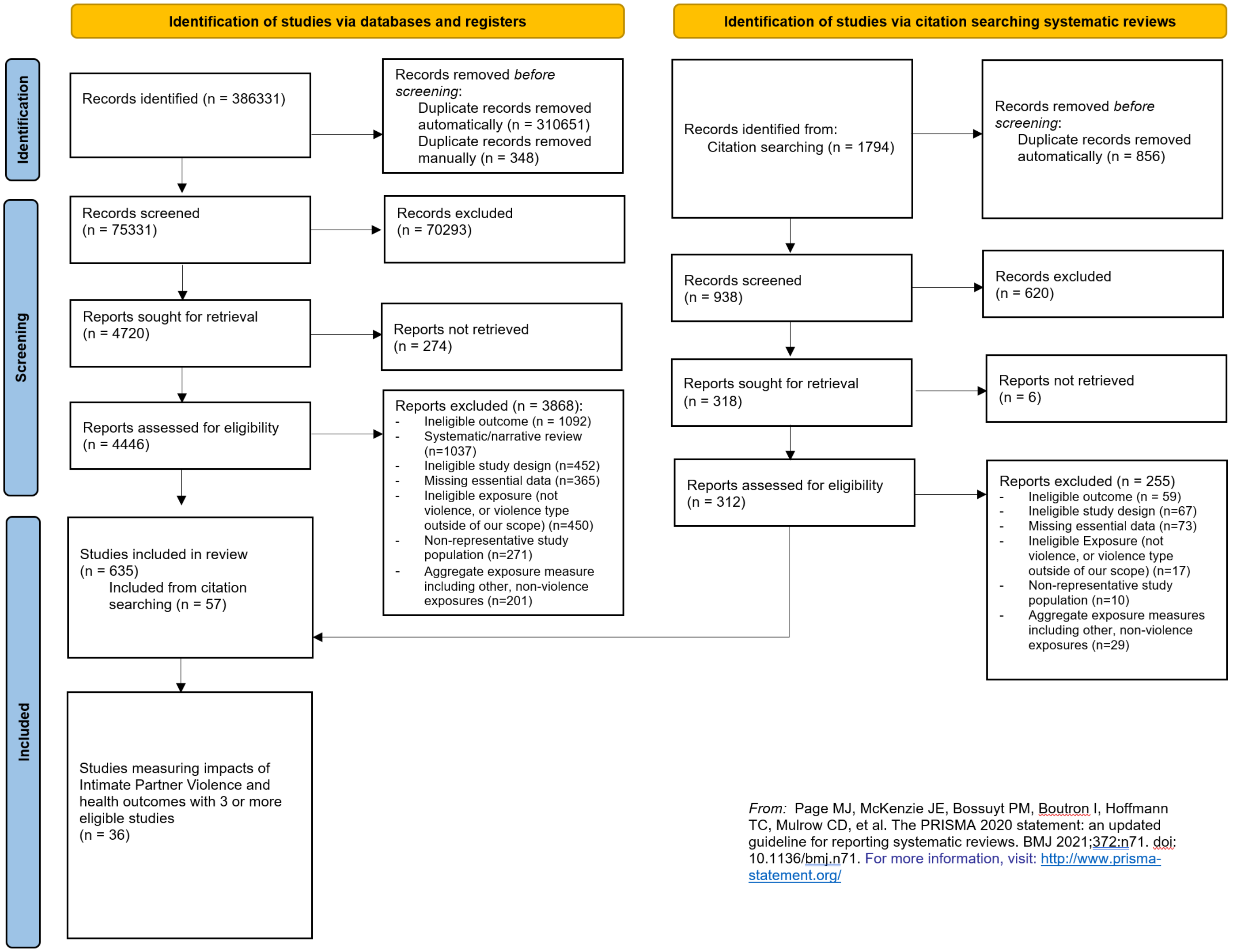

Data processing

Crosswalking

For data that reported IPV using an alternate case definition (ie, IPV experienced in the past year), we ran a logit-difference meta-regression with the MR-BRT tool to estimate correction factors. MR-BRT is described in detail in a separate section of this appendix. Only within-study comparisons were used to inform the meta-regression, and data from subnational locations were not used unless they were the only data available from that location. This helps avoid biasing crosswalk calculations towards locations with estimates available from multiple subnational units. In comparing alternate definitions against gold-standard, it was observed that the difference between 12-month recall and lifetime recall definitions differed by abuse type (ie, the difference in 12-month and lifetime recall was larger for physical IPV definitions when compared to sexual IPV definitions). In addition, the difference between definitions varied by age of respondent (ie, the difference between 12-month and lifetime recall widens as participants get older). For these reasons, a network meta-analysis was created with a spline on age and mutually exclusive definitions (eg, recall and abuse type alternate definition adjustment factors are interactive, not additive). The model was fit using 10% trimming and priors assuming that past 12-month prevalence would be less than lifetime prevalence of the same definition type and component definition prevalence would be less than aggregate within the same recall period (ie, severe physical IPV over the lifetime would be less than any physical and/or sexual IPV over the lifetime).

Age splitting

We split data reported in broader age groups than the GBD five-year age groups by adapting the method reported in Ng et al.[3] to split aggregate data using a reference age pattern. We divided the data into two sets: (1) a training dataset, containing data that already fell into GBD five-year age groups, and (2) a split dataset, which contained data reported in aggregate age groups broader than GBD five-year bins. We then used spatiotemporal Gaussian process regression (ST-GPR) to estimate geography-time-specific age patterns using the training dataset. The ST-GPR model used an age-weight parameter value that minimised the effect of any age smoothing within the model. This parameter choice allowed the estimated age pattern to be driven by data rather than enforced by smoothing parameters of the model. Due to data sparsity within the training dataset, estimated geography-age patterns were aggregated to the GBD region level. The age pattern from the GBD region with the most training datapoints (south Asia) was used to adjust data reported in aggregated age groups.

Ever-partnered sample adjustment

To correct for studies reporting IPV prevalence out of only ever- or currently partnered women, we multiplied estimates from these studies by the age-specific fraction of women who had ever been partnered. We generated ever-partnered estimates using MICS and DHS data in a single parameter DisMod-MR 2.1 model to reflect the most recent data on proportion of women who have ever been partnered.

For studies restricting the perpetrator to spouses or current spouses, due to insufficient data comparing our reference and alternate populations in specific age-location-years, we refrained from calculating under-informed correction factors.

Modelling strategy

We use three distinct approaches to estimate burden attributable to IPV, including (1) the direct PAF approach for estimating the proportion of homicides that are perpetrated by an intimate partner; (2) a

cumulative risk approach for estimating the burden of HIV/AIDS attributable to IPV; and (3) the traditional exposure and relative risk (RR) to population attributable fraction (PAF) method for all other health outcomes.

Exposure

We used ST-GPR to model lifetime IPV prevalence. Input data were prepared by first adjusting data with alternate case definitions and then splitting data in aggregate age groups by applying modelled reference age patterns, as described above. Full details on the ST-GPR method are reported elsewhere in the appendix. Briefly, the mean function input to GPR is a complete time series of estimates generated from a mixed effects hierarchical linear model plus weighted residuals smoothed across time, space, and age. The linear model formula for IPV is:

$$logit(p_{g,a,t}) = \beta_0 + \sum_{k=1}^{18} \beta_k I_{A[a]} + \alpha_s + \alpha_r + \alpha_g + \epsilon_{g,a,t}$$

Where, $I_{A[a]}$ is a dummy variable indicating specific age group A that the prevalence point $p_{g,a,t}$ captures, and α_s, α_r, and α_g are super-region, region, and geography random intercepts, respectively. Random effects were used in model fitting but not in prediction.

Data sparsity within the IPV model caused poor model fits over time. Thus, we introduced Holt's linear trend method (extended simple exponential smoothing) to forecast and back-cast draws from the initial ST-GPR model. Holt's linear trend method allows forecasting of data with a linear trend using a weighted average of past observations, with weights decaying exponentially as observations get older.[4] We applied this method to location-age-specific draws from our initial ST-GPR model, with the year range of the ST-GPR draws to be used as the initial time series defined based upon location-age data availability. For location-age combinations with available data spanning more than three years, draws were bounded from the minimum year to the maximum year of location-age-specific data. Otherwise, draws were bounded from the minimum year to the maximum year of super-region-age-specific data. To avoid over-forecasting for longer time periods (ie, in locations where only very old data were available), we used a damping parameter (phi=0.9) to enforce a zero-slope linear trend over time. Finally, due to our adjustment to ST-GPR draws we needed to re-enforce consistency between subnational and national means, so we logit-raked subnational draws to fit national means for countries with subnational estimation.

Theoretical minimum risk exposure level

The theoretical minimum risk exposure level for IPV is zero.

Direct PAF for female homicides

The burden of homicides attributable to intimate partner violence was modelled as a direct PAF. Input data sources provided the direct measurement of proportion of homicide cases where an intimate partner was the perpetrator. A single-parameter proportion DisMod-MR 2.1 model was run on input data to estimate geography-age-specific estimates of the fraction of homicides perpetrated by an intimate partner, which were then used as PAFs for homicide outcomes.

Cumulative risk approach for PAF of HIV/AIDS due to IPV

We used a cumulative risk approach to measure the burden of HIV/AIDS attributable to IPV. As we measure burden based on deaths and prevalence, we needed to quantify attributable fractions for

prevalence and death rather than incidence. To get a PAF for prevalence, we needed to consider the history of exposure to IPV and the accumulated associated risk of incident HIV due to IPV, relative to the overall risk of HIV at the population level. The ratio of cumulative IPV-attributable HIV incidence to total HIV incidence was an approximation of the relevant PAF for HIV prevalence, and we assumed this PAF can also be applied to mortality.

$$\frac{Cumulative\ HIV\ incidence\ due\ to\ IPV}{Cumulative\ HIV\ incidence\ overall} = \frac{1-\prod_{a=o}^{a=n}\left(1-PAF_{ay}*I_{ay}\right)}{1-\prod_{a=o}^{a=n}\left(1-I_{ay}\right)}$$

where:

I = annual incidence rate of HIV

a = age (15-95)

y = year (1990-2023)

$$PAF_{HIV\ incidence} = \frac{[Prevalence\ of\ IPV]_{ay}*\ (IRR-1)}{[Prevalence\ of\ IPV]_{ay}*\ (IRR-1)+1}$$

Relative risk

We re-examined existing risk–outcome pairs included in the GBD for IPV and evaluated the strength of evidence and association for new pairs drawing on the updated systematic review described above and the burden of proof risk function (BPRF) methodology developed by Zheng and colleagues.[5,6] Through the systematic review, we identified seven health outcomes with three or more eligible studies reporting IPV-related effect sizes (or sufficient data to derive crude effect sizes). The association between IPV and these health outcomes were consequently assessed through the BPRF methodology, using the meta-regression—Bayesian, regularised, trimmed (MR-BRT) tool to derive a pooled relative risk (RR) and associated measures. With MR-BRT, we synthesised input data to estimate the risk of developing each outcome for individuals who have experienced IPV compared to those not exposed by trimming potentially distorting outliers; testing, selecting, and adjusting for bias covariates to account for known heterogeneity in input study-design characteristics (eg, confounding, selection bias, sample characteristics, level of adjustment, deviations in outcome definitions, and aspects related to IPV exposure definitions, such as source recall period and exposure type); and quantifying any remaining between-study heterogeneity (gamma) through random effects modelling. Finally, gamma, and the uncertainty surrounding it, are incorporated into the draws used to derive the 95% uncertainty interval for the mean RR value. More details on the data processing and bias covariates tested can be found in a previously published iteration of these analyses, which reports on findings prior to the most recent systematic review update.[2]

As has been described elsewhere,[5,6] MR-BRT further evaluates evidence for small-study effects and generates funnel plots that represent potential risk of publication or reporting bias. It also produces the burden of proof risk function (BPRF), defined for IPV and other harmful dichotomous risk factors as the fifth quantile of draws closest to the null when incorporating between-study heterogeneity. The BPRF is transformed into a risk–outcome score (ROS: the signed natural log(BPRF) divided by two for dichotomous risks) and mapped onto a star-rating system from one to five stars. These metrics complement RR estimates by providing an alternative, conservative measure synthesising the effect size

and evidence strength by formally and systematically accounting for divergence/convergence across input findings. To this end, positive ROS values and more stars corresponding to incrementally larger effects and stronger evidence for the risk–outcome relationship, measures which can be compared across risk-outcome pairs.

In total, all seven health outcomes met GBD inclusion criteria of a statistically significant association based on conventional RR uncertainty estimates: major depressive disorder, maternal abortion and miscarriage, HIV/AIDS, anxiety disorders, maternal haemorrhage, drug use disorders, and self-harm. Of these, only HIV/AIDS and major depressive disorder had been included as outcomes associated with IPV in previous GBD cycles. Table 1 shows the main outputs from the burden of proof analysis for all seven health outcomes now attributable to IPV within the GBD.

Table 1: Summary of the Burden of Proof analysis results for the relationship between exposure to physical and/or sexual intimate partner violence and associated health outcomes

Health outcome	Pooled mean RR	95% RR UI without between-study heterogeneity	95% RR UI with between-study heterogeneity	BPRF	ROS	Star rating	Publication bias	No. of studies	Selected bias covariates
Major depressive disorder	1.91	1.69–2.17	1.35–2.72	1.42	0.18	★★★	No	12	Representativeness
Maternal abortion and miscarriage	1.93	1.55–2.41	1.15–3.26	1.25	0.11	★★	No	9	Non-lifetime recall period; risk of reverse causation
HIV/AIDS	1.58	1.36–1.84	1.06–2.34	1.13	0.063	★★	No	6	None
Drug use disorders	1.94	1.44–2.62	0.91–4.12	1.03	0.02	★★	No	5	Non-lifetime recall period; female-only study
Anxiety disorders	2.49	1.72–3.61	0.80–7.82	0.96	–0.023	★	No	5	Non-lifetime recall period; female-only study; risk of selection bias
Maternal haemorrhage	1.24	1.06–1.46	0.86–1.81	0.91	–0.05	★	No	3	None
Self-harm	2.99	1.36–6.57	0.29–30.25	0.43	–0.42	★	No	4	None

Table 2: Changes in relative risks used in GBD 2021 vs. GBD 2023

Outcome	Relative Risk		
	GBD 2021	GBD 2023 (with gamma)	GBD 2023 (without gamma)
HIV/AIDS	1.60 (1.31, 1.93)	1.58 (1.06, 2.34)	1.58 (1.36, 1.84)
Major depressive disorder	1.54 (1.00, 2.36)	1.91 (1.35, 2.72)	1.91 (1.69, 2.17)

Citations

[1] Spencer CN, Baeza MJ, Chandan JK, Debure A, Herbert M, Jewell T, et al. Estimating the global health impact of gender-based violence and violence against children: a systematic review and meta-analysis protocol. *BMJ Open*. 2022 Jun;12(6):e061248.

[2] Spencer CN, Khalil M, Herbert M, Aravkin AY, Arrieta A, Baeza MJ, et al. Health effects associated with exposure to intimate partner violence against women and childhood sexual abuse: a Burden of Proof study. *Nat Med*. 2023 Dec;29(12):3243–58.

[3] Ng M, Freeman MK, Fleming TD, Robinson M, Dwyer-Lindgren L, Thomson B, et al. Smoking prevalence and cigarette consumption in 187 countries, 1980-2012. *JAMA*. 2014 Jan 8;311(2):183–92.

[4] Hyndman RJ, Athanasopoulos G. Forecasting: principles and practice. 2nd edition. Lexington, Ky.: Otexts, online, open-access textbook; 2018. 382 p.

[5] Zheng P, Afshin A, Biryukov S, Bisignano C, Brauer M, Bryazka D, et al. The Burden of Proof studies: assessing the evidence of risk. *Nat Med*. 2022 Oct;28(10):2038–44.

[6] Zheng P, Barber R, Sorensen RJD, Murray CJL, Aravkin AY. Trimmed constrained mixed effects models: formulations and algorithms. *J Comput Graph Stat* 2021; 30: 544–56.

Iron deficiency

Flowchart

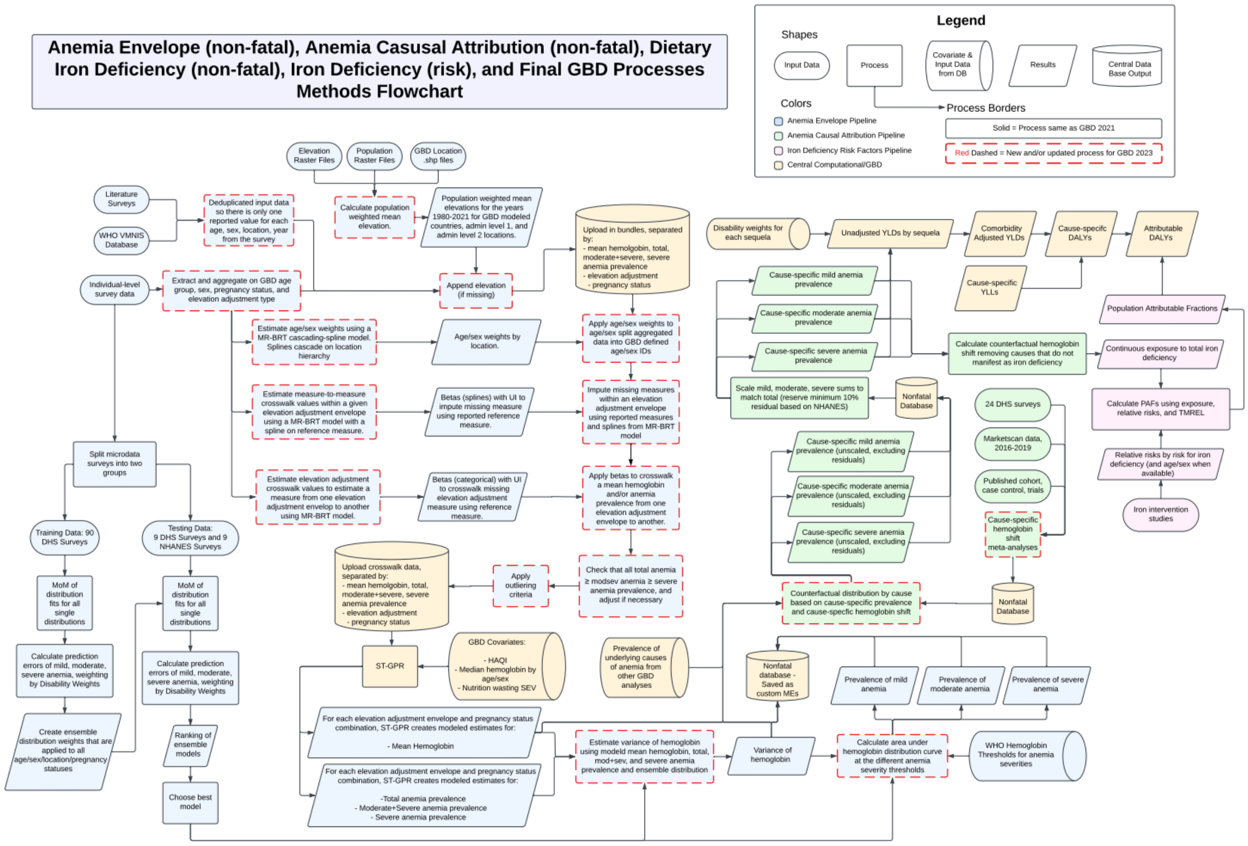

Input data and methodological summary

Definition

Exposure

Iron deficiency in the GBD risk factors analysis is defined as inadequate iron to meet the body's needs. Exposure is quantified in terms of mean haemoglobin concentration at the population level from the cumulative effect of all causes that lead to iron deficiency. This is distinct from the GBD cause of "dietary iron deficiency" that only includes the subset of anaemia that is due to inadequate intake of elemental iron and excludes other diseases that manifest as iron deficiency (eg, maternal haemorrhage, uterine fibroids, menstrual disorders, hookworm, schistosomiasis, gastritis and duodenitis, inflammatory bowel disease, etc.).

Input data

Exposure

Data informing estimates of population mean haemoglobin concentration were extracted from a variety of sources, primarily population-based household surveys such as the Demographic and Health Surveys, Multiple Indicator Cluster Surveys, and country-specific micronutrient and nutrition surveys. We supplemented these data with additional sources reporting mean haemoglobin from the WHO Vitamin and Mineral Nutrition Information System (available at http://www.who.int/vmnis/database/anaemia/countries/en/) and other literature sources where available. Additional detail on data sources and processing of haemoglobin data can be found in the "Anaemia (impairment)" documentation.

Relative risk

Data directly linking iron deficiency to other outcomes are sparse. The CHERG iron report[1] presents data supporting low haemoglobin as a risk factor for maternal outcomes, which has traditionally been used as a proxy for assigning the corresponding relative risks from that report to all maternal outcomes in the GBD study. No other outcomes have been identified as having sufficient evidence of causal relationship with iron deficiency.

In GBD 2021, we undertook a systematic review of iron supplementation trials. We identified 17 trials that reported the effect of oral or IV iron supplementation on haemoglobin levels. Fitting a network meta-regression—Bayesian, regularised, trimmed (MR-BRT) analysis of these studies, similar to the approach used for GBD crosswalking, yielded a mean haemoglobin shift of 14.3 g/L (95% UL: 3.58–25.59) resulting from IV iron supplementation. This shift best approximates the haemoglobin shift that would occur in the absence of all iron-responsive deficiencies. No updates were made to the GBD 2021 relative risk and hemoglobin shift for GBD 2023.

Data processing

For GBD 2023, we only reported final mean haemoglobin concentrations and anaemia prevalence values that were adjusted using the new WHO elevation-adjustment method. However, when processing all input data in the anaemia pipeline, we created three "anaemia envelopes" in parallel to be used to help inform the final new WHO elevation-adjusted dataset. The three envelopes consist of mean haemoglobin concentrations and anaemia prevalence values that were: 1) raw/unadjusted for elevation, 2) elevation-adjusted using the old WHO method, and 3) elevation-adjusted using the new WHO elevation adjustment method. The detailed methods are described in the "Anaemia (impairment)" documentation.

Modelling strategy

Exposure

Iron deficiency was quantified as an output of the GBD Anaemia Causal Attribution framework. The GBD anaemia model has two main steps – estimation of the anaemia envelope and causal attribution – both of which inherently impact estimates of iron deficiency. See the methodological description of "Anaemia (impairment)" for detailed description of the analytic approach and inputs.

Briefly, the first step is estimating the anaemia envelope – the prevalence of mild, moderate, and severe anaemia prevalence for each GBD location, age group, sex, and year. The inputs to the envelope model are mean and standard deviation (SD) of haemoglobin [Hb] concentration. Mean haemoglobin is modelled directly in spatiotemporal Gaussian process regression (ST-GPR), and SD is estimated using a variance optimisation algorithm that takes as inputs the modelled mean haemoglobin estimates and estimates of the prevalence of severe, moderate+severe, and total anaemia (also modelled in ST-GPR). For every location, year, age, and sex, we anchor the distributions at the estimated mean [Hb] value and find the variance value that minimises the error between our ST-GPR estimates of severe, moderate+severe, and total anaemia and the corresponding values implied by a given mean and variance [Hb] combination. A detailed description of ST-GPR and the covariates included is presented in the "Anaemia (impairment)" documentation.

Individual-level data sources are then used to develop a set of ensemble distribution weights using method of moments, which are then paired with mean and SD results to produce estimates of the entire distribution of haemoglobin for each population group. A population group is a specific geography, sex, age group, and year combination. A detailed description of the ensemble distribution modelling is provided in the anaemia (impairment) documentation. The second step is anaemia causal attribution, which generates counterfactual haemoglobin distributions for each cause of anaemia based on the cause-level prevalence (or incidence, in the case of maternal haemorrhage) estimates from the respective GBD analyses and cause-specific haemoglobin shifts that were determined via meta-analysis of data from the literature, population-based surveys and insurance claims databases for each cause. The counterfactual distribution methods used the same ensemble distribution weights as the overall anaemia envelope because there are inadequate data to guide alternate distributions for each sub-cause. Mild, moderate, and severe anaemia were assigned to each cause based on the difference between the counterfactual and observed haemoglobin distributions in each population group. The sum of severity-specific prevalence was then summed to match the total, with a minimum residual of 10%,[2] and then the remainder was distributed among five GBD causes using fixed proportion redistribution methods: 1) dietary iron deficiency (GBD cause), 2) other haemoglobinopathies and haemolytic anaemias, 3) other infectious diseases, 4) other neglected tropical diseases, and 5) endocrine, metabolic, blood, and immune disorders.

Iron deficiency exposure for GBD risk factors analysis is the haemoglobin concentration for each population group for all diseases and injuries that manifest with iron deficiency. This was operationalised by using the observed mean haemoglobin concentration in each population as the actual exposure and then calculating a separate theoretical minimum risk exposure level (TMREL) for each population group as described below.

The following substantive changes were made for GBD 2023:

- Applied elevation-adjustments to haemoglobin using the new WHO elevation adjustment method published in 2023,[3,4] which caused anaemia prevalence values to be higher for all modelled locations below 3000 meters for all estimated ages, sexes, and years.
- Calculated and assigned population-weighted mean elevations to surveys if elevation was not reported using the assigned GBD location for the given survey.
- Implemented new crosswalk steps to age-sex split means reported in tabulated sources that span multiple GBD age and/or sex categories and to impute missing measures among the various elevation-adjustment envelopes. The detail of the crosswalks is described in the

anaemia (impairment) documentation. An example of these elevation crosswalks and modelling steps is described here:

1. Load in the MR-BRT estimated betas for crosswalking total anaemia prevalence using the old WHO elevation-adjustment method to the new WHO elevation-adjustment method, shown in the table.
2. Identify the categories the data point to be crosswalked correlates to (highlighted in red).
3. Convert the reference total anaemia prevalence to logit space:

$$logit(0.250) = -1.099$$

4. Add the estimated betas to the value calculated in step 3.

$$logit(prev) + \beta_0 + \beta_{elevation} + \beta_{anemia} =$$

$$-1.099 + 0.156 + 0.337 - 0.108 = \mathbf{-0.713}$$

5. Convert back to linear space

$$inv\_logit(-0.713) = \mathbf{0.329}$$

Variable Type	Category	Beta
Intercept	---	0.156
Elevation Category	0 m - 400 m	REFERENCE
	400 m - 800 m	0.132
	800 m - 1200 m	0.337
	1200 m - 1600 m	0.516
	1600 m - 2000 m	0.510
	2000 m - 2400 m	0.461
	2400 m - 2800 m	0.413
	2800 m - 3200 m	0.209
	3200 m - 3600 m	-0.270
	3600 m - 4000 m	-0.259
	4000 m - 4400 m	-0.678
	4400 m - 4800 m	-0.877
	4800 m - 5200 m	-1.959
	5200 m - 5600 m	-2.624
Reference Anaemia Prevalence Category	0% to 20%	REFERENCE
	20% to 40%	-0.108
	40% to 60%	-0.164
	60% to 80%	-0.197
	80% to 100%	-0.128

- Utilised the stage-1 linear prior functionality within ST-GPR and passed in only three of the 13 covariates used in GBD 2021 to model mean haemoglobin and the various severities of anaemia prevalence, which were 1) median haemoglobin by age and sex, 2) Healthcare Access and Quality Index by GBD location and year, and 3) summary exposure values for nutrition wasting in children under 5 years old.
- Updated haemoglobin shift values for a subset of the input causes that are used in the anaemia causal attribution pipeline. The updated haemoglobin shift values for each cause is presented in the anaemia (impairment) documentation.

Theoretical minimum risk exposure level

The implied mean haemoglobin in the absence of iron deficiency is the TMREL. To calculate the TMREL, we took an estimate of "normal" haemoglobin concentration and subtracted the prevalence-weighted haemoglobin shifts corresponding to causes that are not "iron responsive" (including all haemoglobinopathies and malaria) for each location, year, age, and sex. Normal haemoglobin was operationalised as the 95th percentile of mean haemoglobin concentration by age and sex across all locations. From this calculation, we estimate a location-, year-, age-, and sex-specific TMREL that corresponds to the expected normal haemoglobin concentration after subtracting out causes that would not be responsive to supplemental iron.

Relative risk

For GBD 2023, we followed the same approach as GBD 2021. For GBD 2021, we updated the relative risk analysis to account for the mediation of low haemoglobin on the relationship between iron deficiency and maternal outcomes. As mentioned above, we still used the relative risk values from the CHERG iron report[1] but adjusted the exposure values based on the haemoglobin shift that would occur under iron supplementation.

We calculated the population attributable fraction (PAF) of maternal outcomes due to low haemoglobin under two scenarios: firstly, with the observed haemoglobin exposure, and secondly, with the haemoglobin level that would exist if everyone had received iron supplementation (the current haemoglobin exposure + iron supplementation haemoglobin shift). Then, we calculated the iron deficiency burden as the difference between these two PAFs such that the proportion of the risk that is from low haemoglobin and not amenable to iron supplementation is removed. In other words, the final PAF is the PAF of iron deficiency without low haemoglobin.

Notably, this approach to mediation only allows us to assign risk for iron-responsive iron deficiency; in future years of the GBD study, additional trials would be needed to assess functional iron deficiency (eg, EPO, inflammation, chronic disease).

Citations

1 Murray-Kolb L, Chen L, Chen P, Shapiro M, Caulfield L, CHERG Iron Report. Maternal Mortality, Child Mortality, Perinatal Mortality, Child Cognition, and Estimates of Prevalence of Anemia due to Iron Deficiency. Baltimore, USA: CHERG, 2012.

2 Looker AC, Dallman PR, Carroll MD, Gunter EW, Johnson CL. Prevalence of iron deficiency in the United States. *JAMA* 1997; **277**: 973–6.

3 Sharma AJ, Addo OY, Mei Z, Suchdev PS. Reexamination of hemoglobin adjustments to define anemia: altitude and smoking. *Ann N Y Acad Sci* 2019; **1450**: 190–203.

4 World Health Organization, editor. Guideline on haemoglobin cutoffs to define anaemia in individuals and populations. Geneva: World Health Organization, 2024.

Kidney dysfunction

Flowchart

Kidney Dysfunction

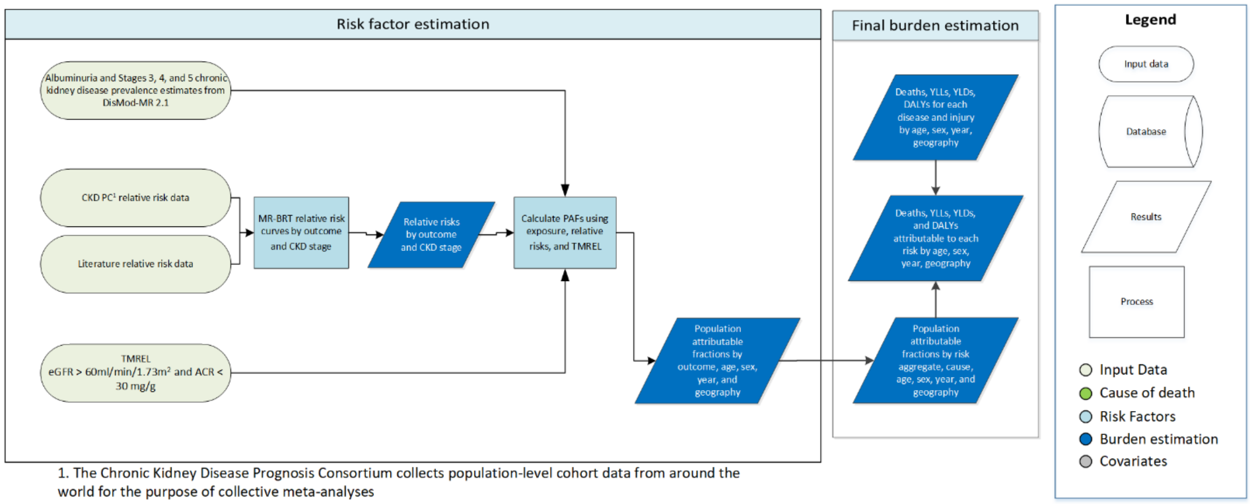

1. The Chronic Kidney Disease Prognosis Consortium collects population-level cohort data from around the world for the purpose of collective meta-analyses

Input data and methodological summary

Definition

Exposure

The kidney dysfunction (KD) risk factor exposure is divided into four categories of renal function defined by urinary albumin to creatinine ratio (ACR) and estimated glomerular filtration rate (eGFR). The definitions of KD exposures can be found in Table 1.

Table 1. GBD case definitions of KD exposure

Quantity of interest	Reference or alternative	Definition
Stages 1&2 chronic kidney disease	Reference	Albumin to creatinine ratio (ACR) of ≥30 mg/g and estimated glomerular filtration rate (eGFR) >60 mL/min/1.73 m^2 as estimated using the CKD-EPI equation for individuals age >18 and the Schwartz equation for those <18.
Stages 1&2 chronic kidney disease	Alternative	Albumin to creatinine ratio (ACR) of ≥ a threshold other than 30 mg/g and estimated glomerular filtration rate (eGFR) >60 mL/min/1.73 m^2 as estimated using the CKD-EPI equation for individuals age >18 and the Schwartz equation for those <18.
Stages 1&2 chronic kidney disease	Alternative	Albumin to creatinine ratio (ACR) of ≥30 mg/g and estimated glomerular filtration rate (eGFR) >60 mL/min/1.73 m^2 as estimated using the MDRD equation (or modifications thereof) for individuals age >18.

Stages 1&2 chronic kidney disease	Alternative	Albumin to creatinine ratio (ACR) of ≥30 mg/g and estimated glomerular filtration rate (eGFR) >60 mL/min/1.73 m^2 as estimated using the Cockcroft-Gault equation (standardised for body surface area) for individuals age >18.
Stages 1&2 chronic kidney disease	Alternative	Albumin to creatinine ratio (ACR) of ≥ a threshold other than 30 mg/g and estimated glomerular filtration rate (eGFR) >60 mL/min/1.73 m^2 as estimated using the MDRD equation (or modifications thereof) for individuals age >18.
Stages 1&2 chronic kidney disease	Alternative	Albumin to creatinine ratio (ACR) of ≥ a threshold other than 30 mg/g and estimated glomerular filtration rate (eGFR) >60 mL/min/1.73 m^2 as estimated using the Cockcroft-Gault equation (standardised for body surface area) for individuals age >18.
Stage 3 chronic kidney disease	Reference	Estimated glomerular filtration rate (eGFR) 30-60 mL/min/1.73 m^2 as estimated using the CKD-EPI equation for individuals age >18 and the Schwartz equation for those <18 not on renal replacement therapy.
Stage 3 chronic kidney disease	Alternative	Estimated glomerular filtration rate (eGFR) 30-60 mL/min/1.73 m^2 as estimated using the MDRD equation (or modifications thereof) for individuals age >18 not on renal replacement therapy.
Stage 3 chronic kidney disease	Alternative	Estimated glomerular filtration rate (eGFR) 30-60 mL/min/1.73 m^2 as estimated using the Cockcroft-Gault equation (standardised for body surface area) for individuals age >18 not on renal replacement therapy.
Stage 4 chronic kidney disease	Reference	Estimated glomerular filtration rate (eGFR) 15-30 mL/min/1.73 m^2 as estimated using the CKD-EPI equation for individuals age >18 and the Schwartz equation for those <18 not on renal replacement therapy.
Stage 4 chronic kidney disease	Alternative	Estimated glomerular filtration rate (eGFR) 15-30 mL/min/1.73 m^2 as estimated using the MDRD equation (or modifications thereof) for individuals age >18 not on renal replacement therapy.
Stage 4 chronic kidney disease	Alternative	Estimated glomerular filtration rate (eGFR) 15-30 mL/min/1.73 m^2 as estimated using the Cockcroft-Gault equation (standardised for body surface area) for individuals age >18 not on renal replacement therapy.
Stage 5 chronic kidney disease	Reference	Estimated glomerular filtration rate (eGFR) <15 mL/min/1.73 m^2 as estimated using the CKD-EPI equation for individuals age >18 and the Schwartz equation for those <18 not on renal replacement therapy.
Stage 5 chronic kidney disease	Alternative	Estimated glomerular filtration rate (eGFR) <15 mL/min/1.73 m^2 as estimated using the MDRD equation (or modifications thereof) for individuals age >18 not on renal replacement therapy.
Stage 5 chronic kidney disease	Alternative	Estimated glomerular filtration rate (eGFR) <15 mL/min/1.73 m^2 as estimated using the Cockcroft-Gault equation

		(standardised for body surface area) for individuals age >18 not on renal replacement therapy.

Input data

Exposure

A systematic review across three databases for CKD Stages 1&2, 3, 4, and 5 exposure estimates was conducted for GBD 2023. Databases were searched on September 13, 2022, for publications after January 1, 2017. Below are the search terms for each database:

PubMed: (("chronic kidney disease"[Title/Abstract] OR "CKD"[Title/Abstract] OR "eGFR"[Title/Abstract]) AND ("prevalence"[Title/Abstract] OR "incidence"[Title/Abstract])) NOT ("animals"[MeSH Terms:noexp] OR "animal"[All Fields] OR "humans"[MeSH Terms])

Web of Science: ((AB=("chronic kidney disease" OR "CKD" or "eGFR")) AND TI=("prevalence" OR "incidence")) AND PY=(2017-2022)

ScienceDirect: Title contains (chronic kidney disease OR ckd OR egfr) AND Title contains (prevalence OR incidence) SCOPE: Library Resources / Articles, Books and More Material Type: Articles; Collection: ScienceDirect Journals (5 years ago present)

Below is the PRISMA diagram for the systematic review.

Figure 1: PRISMA diagram for exposure

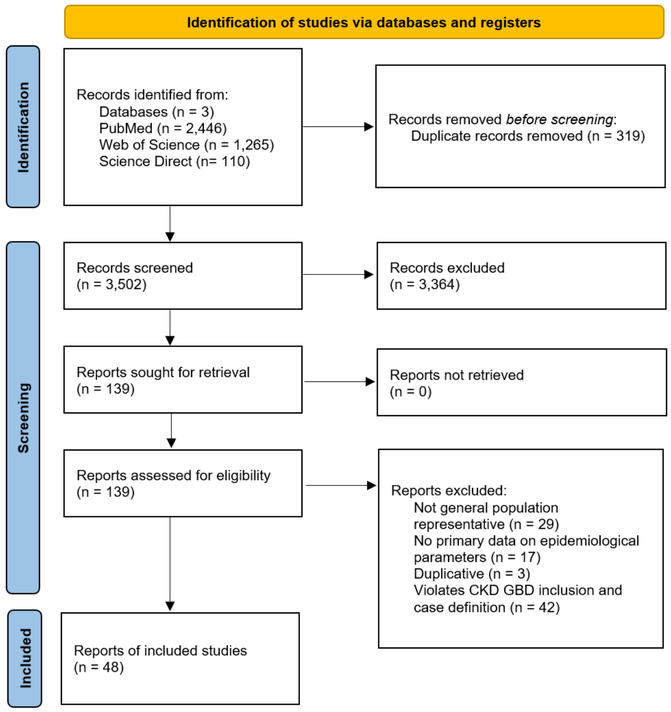

Relative risk

Gout

A systematic review across three databases for kidney dysfunction-gout relative risk estimates was conducted for GBD 2023. Databases were searched on October 4, 2022, for publications after January 1, 1990. Below are the search terms for each database:

PubMed: ("gout"[Title/Abstract] OR "gouty"[Title/Abstract] OR "hyperuricemia"[Title/Abstract]) AND ("chronic kidney disease"[Title/Abstract] OR "CKD"[Title/Abstract] OR "GFR"[Title/Abstract]) AND ("relative risk"[Text Word] OR "hazard ratio"[Text Word] OR "cohort"[Text Word])

Web of Science: ((AB=(chronic kidney disease OR CKD or eGFR)) AND AB=(gout OR gouty OR hyperuricemia)) AND ALL=(relative risk OR hazard ratio OR cohort)

Scopus: (TITLE-ABS(gout) OR TITLE-ABS(gouty) OR TITLE-ABS(hyperuricemia)) AND (TITLE-ABS("chronic kidney disease") OR TITLE-ABS(CKD) OR TITLE-ABS(GFR)) AND (TITLE-ABS-KEY("relative risk") OR TITLE-ABS-KEY("hazard ratio") OR TITLE-ABS-KEY(cohort))

Below is the PRISMA diagram for the systematic review.

Figure 2: PRISMA diagram for kidney dysfunction-gout relative risk

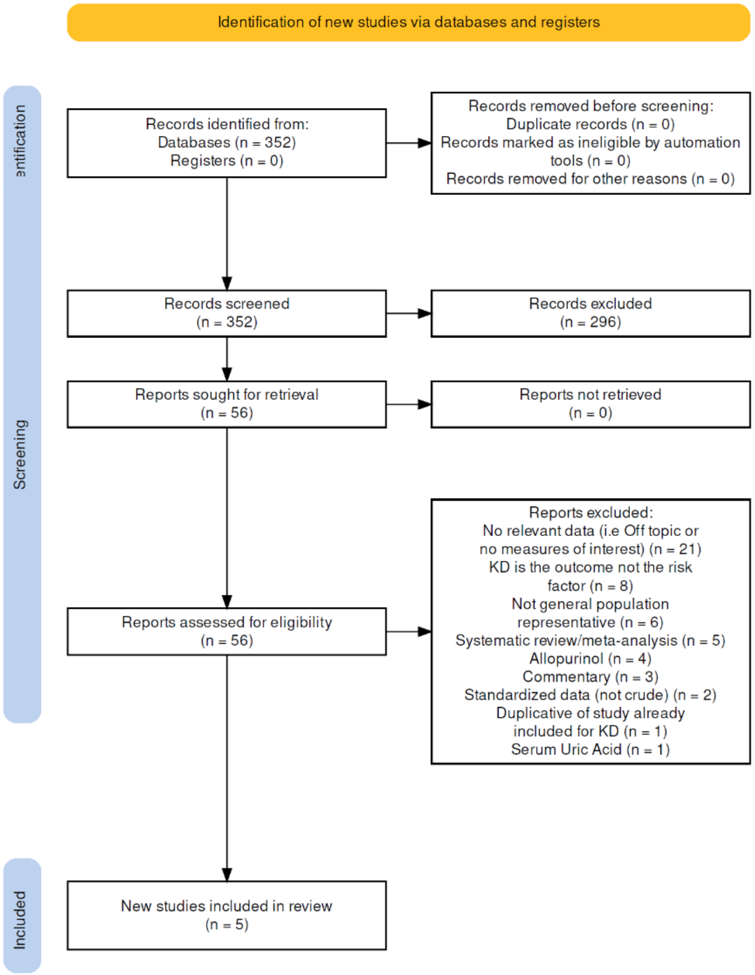

Ischaemic heart disease, peripheral artery disease, and stroke

A systematic review of PubMed for kidney dysfunction-cardiovascular outcomes (specifically, ischaemic heart disease, peripheral artery disease, and stroke) relative risk estimates was conducted for GBD 2023. PubMed was searched on April 25, 2023, for publications after January 1, 1990. Below is the search term used:

PubMed: ((((("chronic kidney disease"[Title/Abstract] OR "CKD"[Title/Abstract] OR "estimated glomerular filtration rate"[Title/Abstract] OR "Dialysis"[Title/Abstract] OR "Renal Transplant"[Title/Abstract] OR "Kidney Transplant"[Title/Abstract]) AND ("coronary heart disease"[Title/Abstract] OR "CAD"[Title/Abstract] OR "IHD"[Title/Abstract] OR "ischemic heart disease"[Title/Abstract] OR "coronary artery disease"[Title/Abstract] OR "angina"[Title/Abstract] OR "myocardial infarction"[Title/Abstract] OR "acute coronary syndrome"[Title/Abstract] OR "peripheral vascular disease"[Title/Abstract] OR "peripheral arterial disease"[Title/Abstract] OR "stroke"[Title/Abstract] OR "ischemic stroke"[Title/Abstract] OR "ischemic stroke"[Title/Abstract] OR "cerebral infarction"[Title/Abstract] OR "intracerebral hemorrhage"[Title/Abstract] OR "intracerebral hemorrhage"[Title/Abstract] OR "subarachnoid hemorrhage"[Title/Abstract] OR "subarachnoid hemorrhage"[Title/Abstract]) AND ("relative risk"[Title/Abstract] OR "hazard ratio"[Title/Abstract] OR "cohort"[Title/Abstract] OR "hazard"[Title/Abstract] OR "odds ratio"[Title/Abstract])) NOT "rat"[Title/Abstract]) NOT "mice"[Title/Abstract]) NOT "monkey"[Title/Abstract]) NOT "pig"[Title/Abstract]) NOT "animals"[Title/Abstract]

Below is the PRISMA diagram for the systematic review.

Figure 3: PRISMA diagram for kidney dysfunction-cardiovascular outcomes relative risk

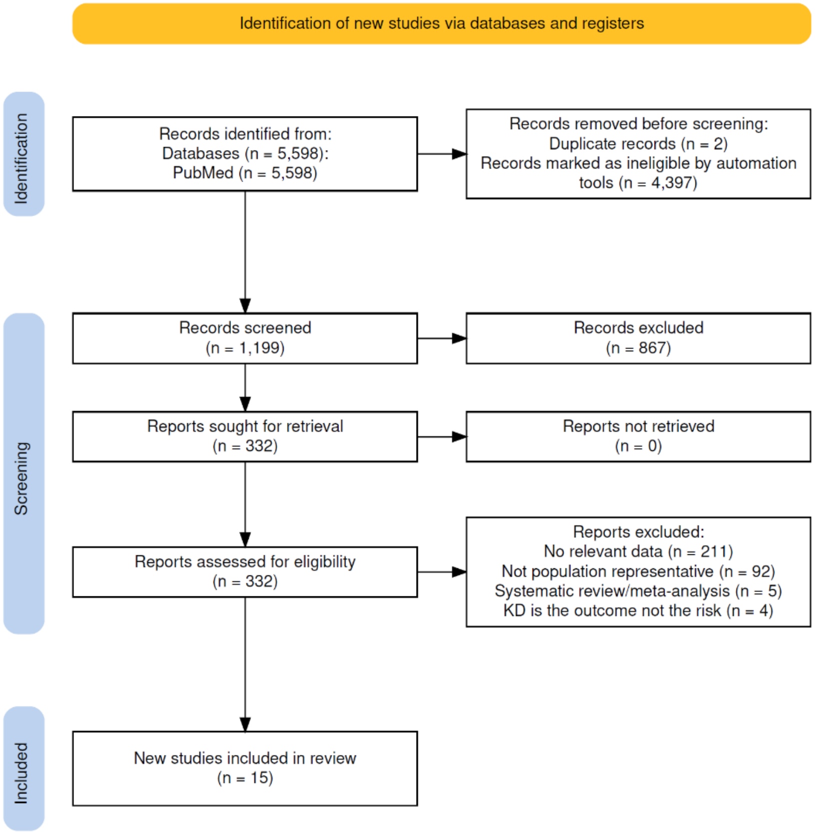

The included studies from the systematic review supplemented relative risk data for ischaemic heart disease, peripheral artery disease, and stroke that we obtained from the Chronic Kidney Disease Prognosis Consortium (CKD-PC) for GBD 2021. The CKD-PC is a research group composed of investigators representing cohorts from around the world. Investigators share data for the purpose of collaborative meta-analyses to study prognosis in CKD.

Data processing

For GBD 2023, we updated the adjustment factors applied to the alternative case definitions for CKD Stages 1&2, 3, 4, and 5. We adjusted data using alternative case definitions with adjustment factors from a meta-regression—Bayesian, regularised, trimmed[1] (MR-BRT) model to account for different estimates that result from these different equations (for methods details see appendix 1, section 2). The adjustment is a logit-transformation method in MR-BRT.

Additional information on data processing, including age splitting, sex splitting, and bias adjustment factors, for the CKD stage exposure models can be found in the non-fatal CKD appendix section.

Modelling strategy

Exposure

The prevalence of exposure to Stage 1-2, Stage 3, Stage 4, and Stage 5 CKD were obtained from the GBD 2023 non-fatal burden of disease analysis. More detailed information on the exposure modelling strategy can be found in the non-fatal CKD appendix section.

Briefly, we ran separate disease model—Bayesian meta-regression (DisMod-MR 2.1, for methods description see appendix 1, section 2) models for each stage of CKD and an aggregate CKD stage 3-5 model to produce estimates by age, sex, year, and country. Estimates from the CKD stage 3, 4, and 5 models were scaled by age, sex, year, and location to sum to the aggregate CKD stage 3-5 estimates.

Theoretical minimum risk exposure level

The theoretical minimum risk exposure level is ACR 30 mg/g or less and eGFR greater than 60 mL/min/1.73 m^2. An ACR above 30 mg/g and eGFR below 60 mL/min/1.73 m^2 have been demonstrated in the literature to be the thresholds at which increased cardiovascular and gout events occur secondary to KD.[2-11]

Relative risk

Estimating the relative risk (RR) of gout, ischaemic heart disease, peripheral artery disease, and stroke occurring as a function of exposure to kidney dysfunction followed the burden of proof approach established by Zheng and colleagues[1,12] and instantiated in the meta-regression—Bayesian, regularised, trimmed (MR-BRT) tool. MR-BRT synthesises input data to generate an RR curve by relying on an ensemble spline method to capture the potentially non-linear shape of the risk–outcome relationship; integrating over varying exposure ranges in different comparison groups; trimming potentially distorting outliers; testing, selecting, and adjusting for bias covariates to account for known heterogeneity in input study-design characteristics (eg, confounding, selection bias, exposure measurement); and quantifying remaining between-study heterogeneity (gamma) through random effects modelling and incorporating this value into uncertainty around the mean RR curve.

Due to data sparsity, the RR for gout was modelled using MR-BRT as a function of exposure to dichotomous kidney dysfunction not disaggregated by CKD stage, defined according to the TMREL above. No bias covariates or model priors were applied. Below is the funnel plot from the gout MR-BRT model:

Figure 8: Kidney dysfunction-gout MR-BRT funnel plot

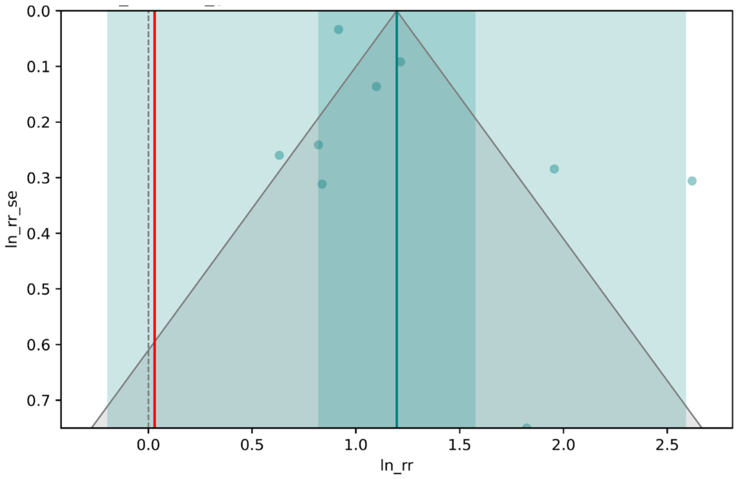

We estimated the RR of ischaemic heart disease, peripheral artery disease, and stroke separately by stage using MR-BRT with a cubic spline on age, and right and left linear tails. No bias covariates were applied. All relative risk estimates for these cardiovascular outcomes above age 85 were set equal to the risk at age 85 to control for lack of data in older age groups. The following plot shows the mean relative risks by each stage of CKD. Stage 5 and stage 4 CKD have higher risks overall. Risks are also higher at younger ages and lower at the oldest age, likely reflecting competing risk factors.

Figure 9: Kidney dysfunction-cardiovascular outcomes MR-BRT mean relative risks

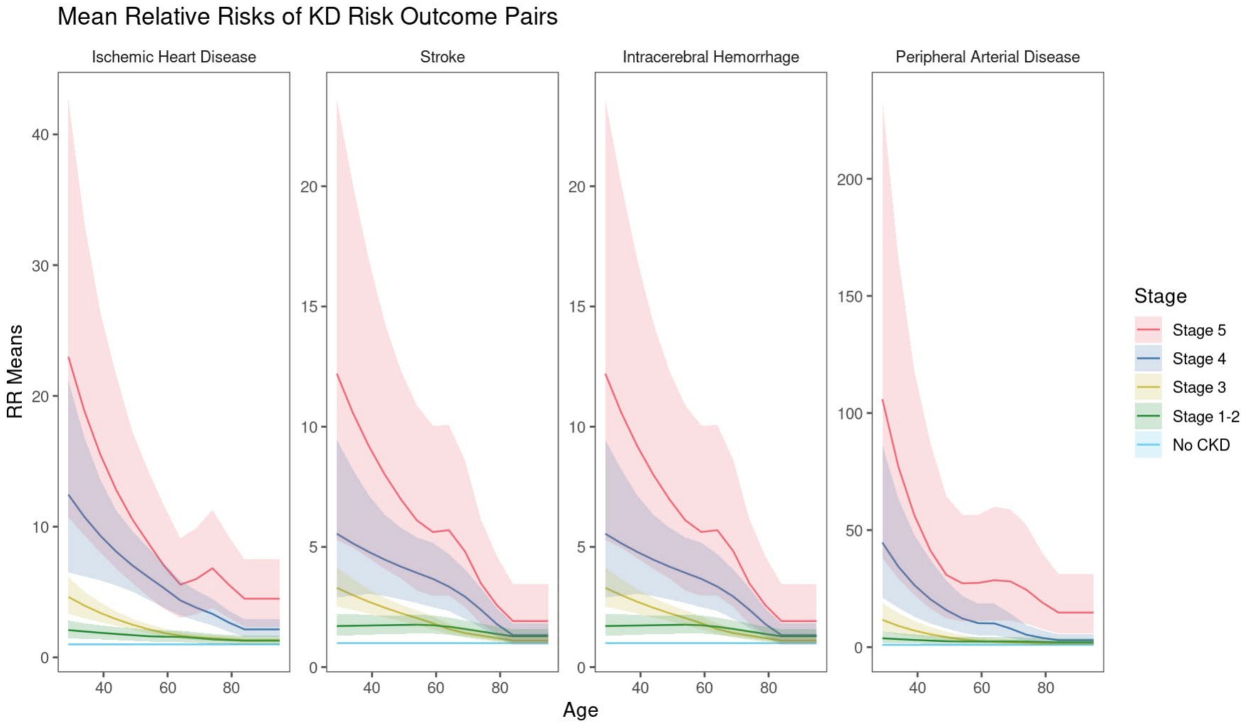

Figure 10: Kidney Dysfunction and Gout Outcomes: Mean Relative Risks Comparison Between GBD 2021 and GBD 2023

KD stage	GBD 2021	GBD 2023
Stage 3−5	2.52 (2.23 − 2.86)	4.24 (0.92 − 12.93)

Figure 11: Kidney Dysfunction and ischaemic heart disease Outcomes: Mean Relative Risks Comparison Between GBD 2021 and GBD 2023

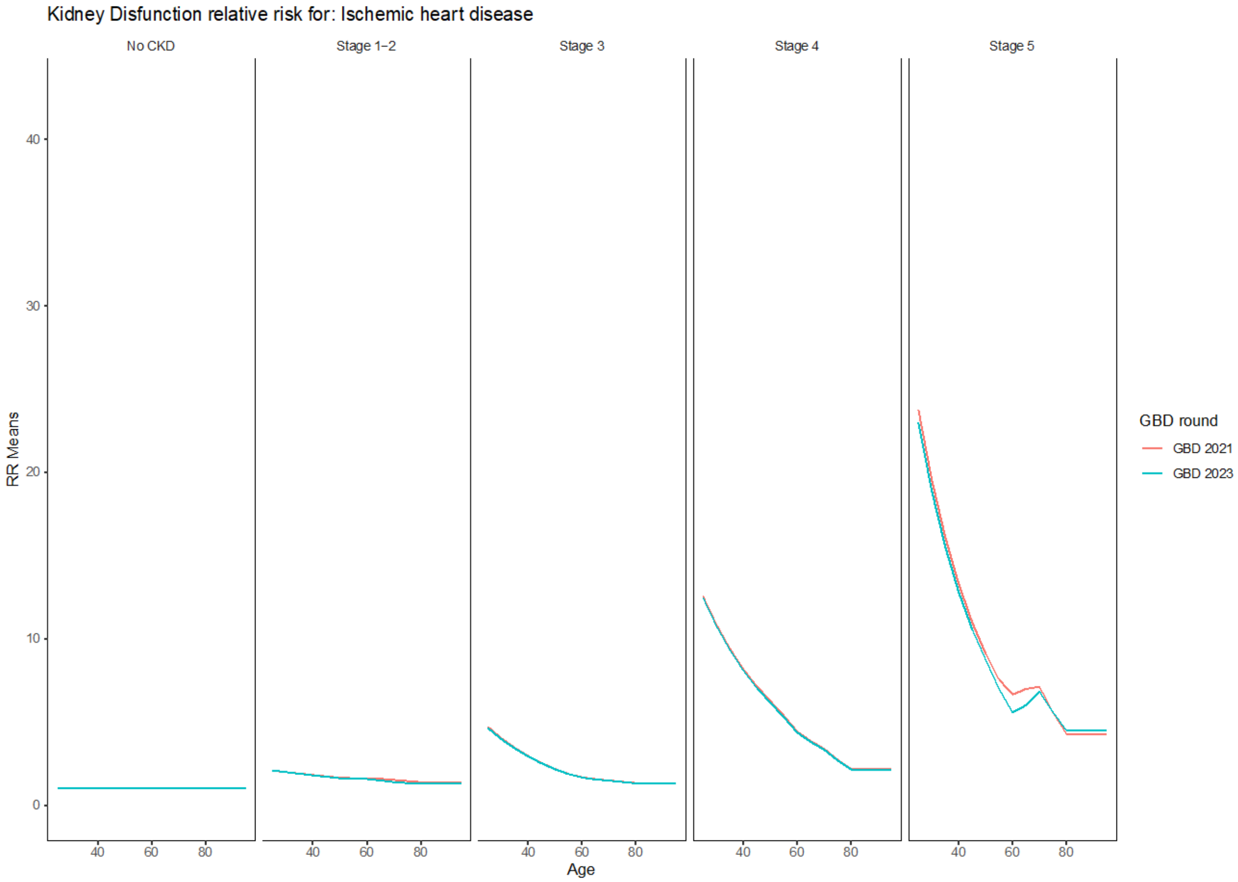

Figure 12: Kidney Dysfunction and peripheral artery disease Outcomes: Mean Relative Risks Comparison Between GBD 2021 and GBD 2023

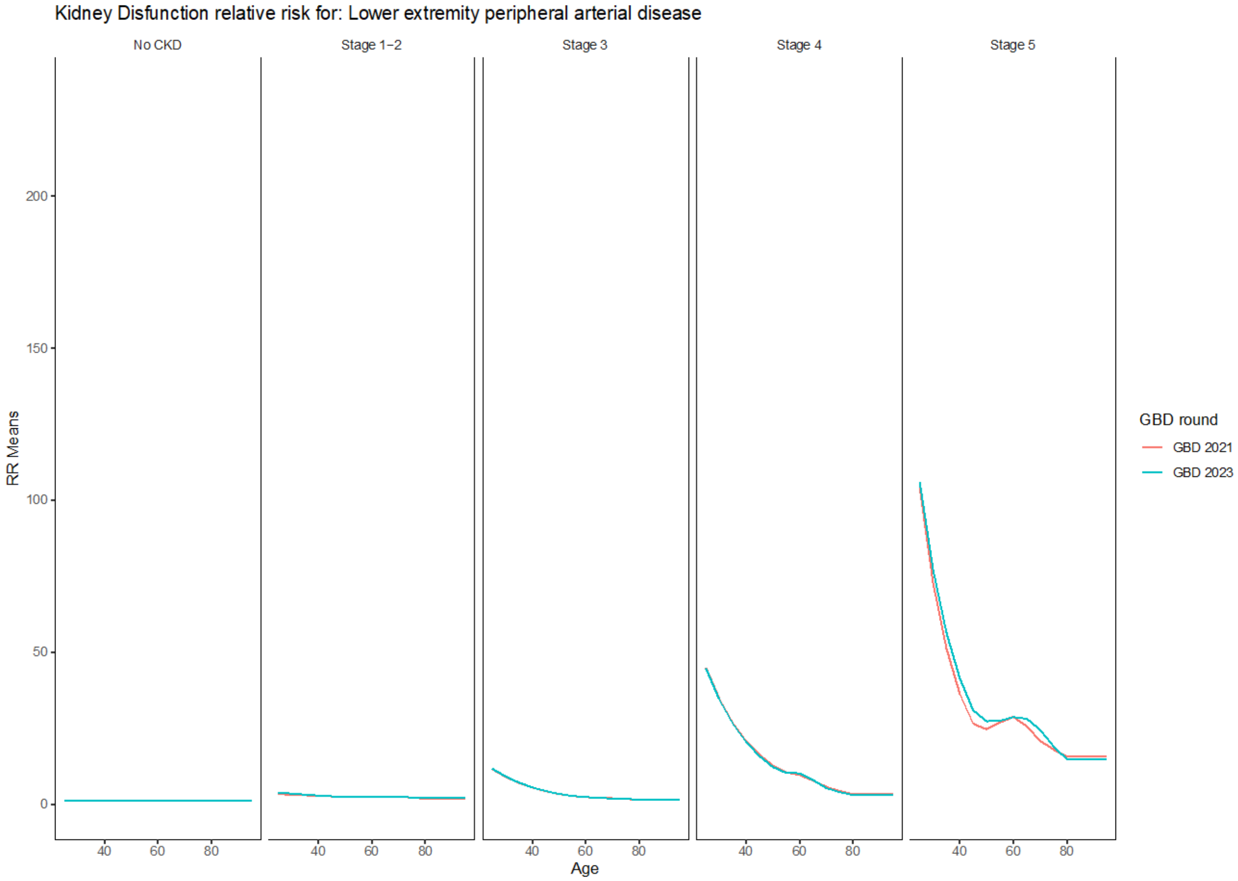

Figure 13: Kidney Dysfunction and stroke Outcomes: Mean Relative Risks Comparison Between GBD 2021 and GBD 2023

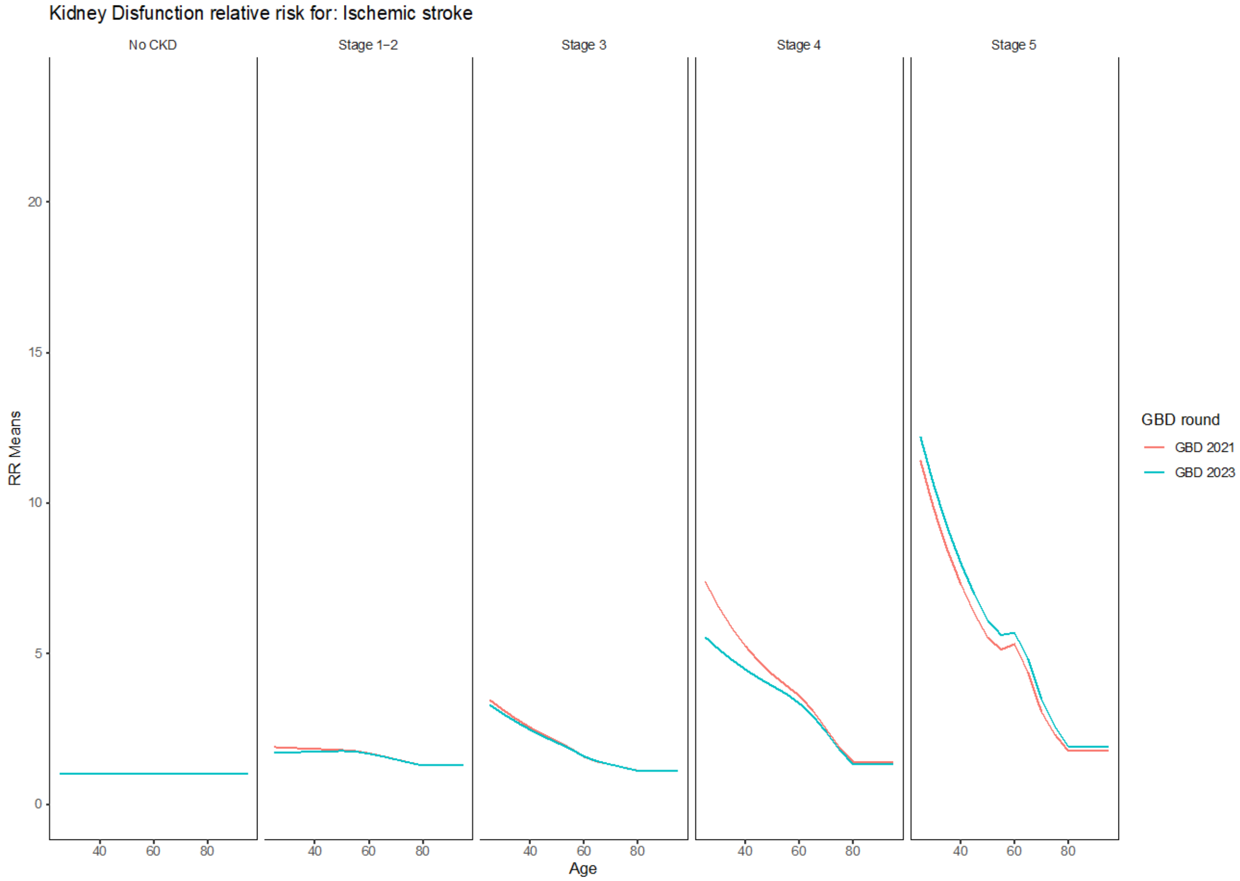

Citations

1. Zheng, P., Barber, R., Sorensen, R. J., Murray, C. J., & Aravkin, A. Y. (2021). Trimmed constrained mixed effects models: formulations and algorithms. *Journal of Computational and Graphical Statistics*, 1-13.
2. Go AS, Chertow GM, Fan D, McCulloch CE, Hsu CY. Chronic kidney disease and the risks of death, cardiovascular events, and hospitalization. N Engl J Med. 2004; 351(13):1296-305.
3. Ninomiya T, Kiyohara Y, Kubo M, Tanizaki Y, Doi Y, Okubo K, et al. Chronic kidney disease and cardiovascular disease in a general Japanese population: the Hisayama Study. Kidney international. 2005; 68(1):228-36.
4. Shara NM, Wang H, Mete M, Al-Balha YR, Azalddin N, Lee ET, et al. Estimated GFR and incident cardiovascular disease events in American Indians: the Strong Heart Study. American journal of kidney diseases: the official journal of the National Kidney Foundation. 2012; 60(5):795-803.
5. Mann JF, Gerstein HC, Pogue J, Bosch J, Yusuf S. Renal insufficiency as a predictor of cardiovascular outcomes and the impact of ramipril: the HOPE randomized trial. Annals of internal medicine. 2001; 134(8):629-36.
6. Chronic Kidney Disease Prognosis C, Matsushita K, van der Velde M, Astor BC, Woodward M, Levey AS, et al. Association of estimated glomerular filtration rate and albuminuria with all-cause and cardiovascular mortality in general population cohorts: a collaborative meta-analysis. Lancet. 2010; 375(9731):2073-81.

7. De Graauw J, Chonchol M, Poppert H, Etgen T, Sander D. Relationship between kidney function and risk of asymptomatic peripheral arterial disease in elderly subjects. Nephrology, dialysis, transplantation: official publication of the European Dialysis and Transplant Association - European Renal Association. 2011;26(3):927-32.
8. Wattanakit K, Folsom AR, Selvin E, Coresh J, Hirsch AT, Weatherley BD. Kidney function and risk of peripheral arterial disease: results from the Atherosclerosis Risk in Communities (ARIC) Study. Journal of the American Society of Nephrology: JASN. 2007; 18(2):629-36.
9. O'Hare AM, Vittinghoff E, Hsia J, Shlipak MG. Renal insufficiency and the risk of lower extremity peripheral arterial disease: results from the Heart and Estrogen/Progestin Replacement Study (HERS). Journal of the American Society of Nephrology: JASN. 2004; 15(4):1046-51.
10. Manjunath G, Tighiouart H, Coresh J, Macleod B, Salem DN, Griffith JL, et al. Level of kidney function as a risk factor for cardiovascular outcomes in the elderly. Kidney international. 2003; 63(3):1121-9.
11. Manjunath G, Tighiouart H, Ibrahim H, MacLeod B, Salem DN, Griffith JL, et al. Level of kidney function as a risk factor for atherosclerotic cardiovascular outcomes in the community. Journal of the American College of Cardiology. 2003; 41(1):47-55.
12. Zheng P, Afshin A, Biryukov S, *et al.* The Burden of Proof studies: assessing the evidence of risk. *Nat Med* 2022; **28**: 2038–44.

Lead exposure

Flowchart

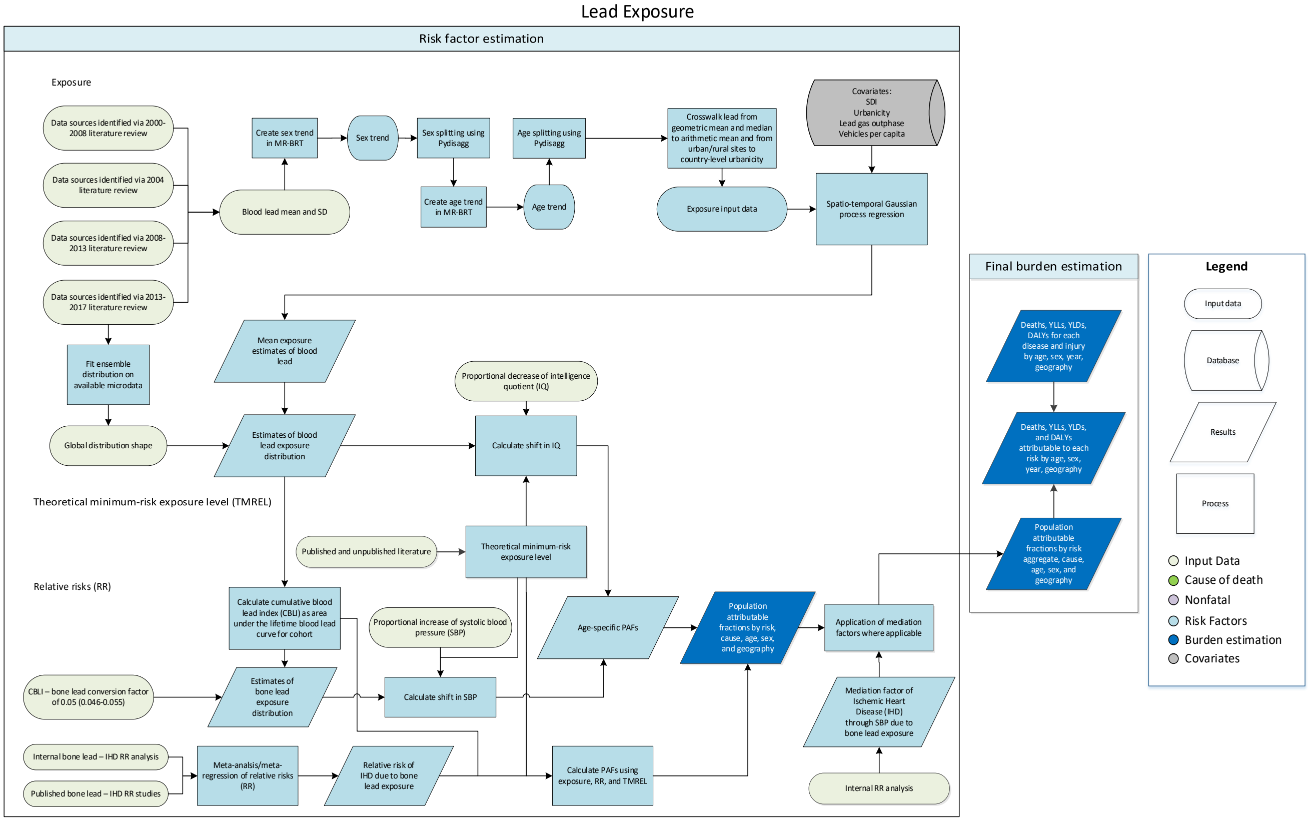

Definitions

Exposure to lead is defined in two different ways according to the currently known pathways of attributable health loss. Acute lead exposure, measured as micrograms of lead per decilitre of blood (µg/dL), is associated with IQ loss in children. Chronic lead exposure, measured as micrograms of lead per gram of bone (µg/g), is associated with cardiovascular diseases (CVD), mediated by the impact of lead on increased systolic blood pressure (SBP). In addition to mediation through SBP, ischaemic heart disease (IHD) is also caused by direct bone lead exposure.

Input data

Exposure

The input data for lead exposure are primarily extracted from literature reports of blood lead levels (BLL), in addition to a few blood lead surveys. Blood lead values are derived from studies that take blood samples and analyse them to determine the level of lead present. The second pathway of burden, bone lead exposure, was estimated by calculating a cumulative blood lead index for cohorts using estimated blood lead exposure over their lifetime. The cumulative blood lead index is then used to estimate bone lead using a scalar defined in literature.[1]

For GBD 2023, we included new sources from a systematic review of blood lead exposure from the scientific literature. The search was an updated search from 2017 and was conducted using the PubMed database using the following search string:

Search String: ("blood lead") AND (("2016/07/01"[Date - Publication] : "2021/12/31"[Date - Publication]))

Inclusion criteria

- Specific to humans
- Study sample is representative of geographical location
- Blood lead was measured using whole blood
- Study reports mean BLL and measure of uncertainty or sample size

Exclusion criteria:

- Not representative of general population
  - Studies specific to workers occupationally exposed to lead or people living close to mines
  - Any case-control study or RCT where the control group was not representative
  - Specifically targeted areas or subgroups of people with previously excessively high lead levels
- No exposure of interest
  - Study reported lead exposure based on hair, teeth, or urine samples
- Duplicate data
  - Studies that have an underlying study population that is the same as a study group we have already included in our exposure model
- Not exposure measure of interest
  - Studies where the limit of detection (LOD) was >3 µg/dl with no report of persons < LOD and studies where % < LOD was larger than 50%
  - Study did not have report measure of uncertainty (eg, CI or SE) or sample size
  - Studies where mean BLL was only reported for a subset of BLL values (eg, mean BLL for people with BLL < 5 µg/dl)
- No study type of interest
  - Systematic reviews/meta-analysis
  - Study does not report original findings / secondary source
  - Study does not provide adequate information regarding sampling, population, or lead testing methodology

The search yielded 1149 results with only 450 passing the initial title-abstract screening and 160 passing the full text screening (Figure 1).

Additionally, five other new data sources were found and added to input data, with three of these sources being collaborator-recommended and the other two identified via the Lead Cooper Project.

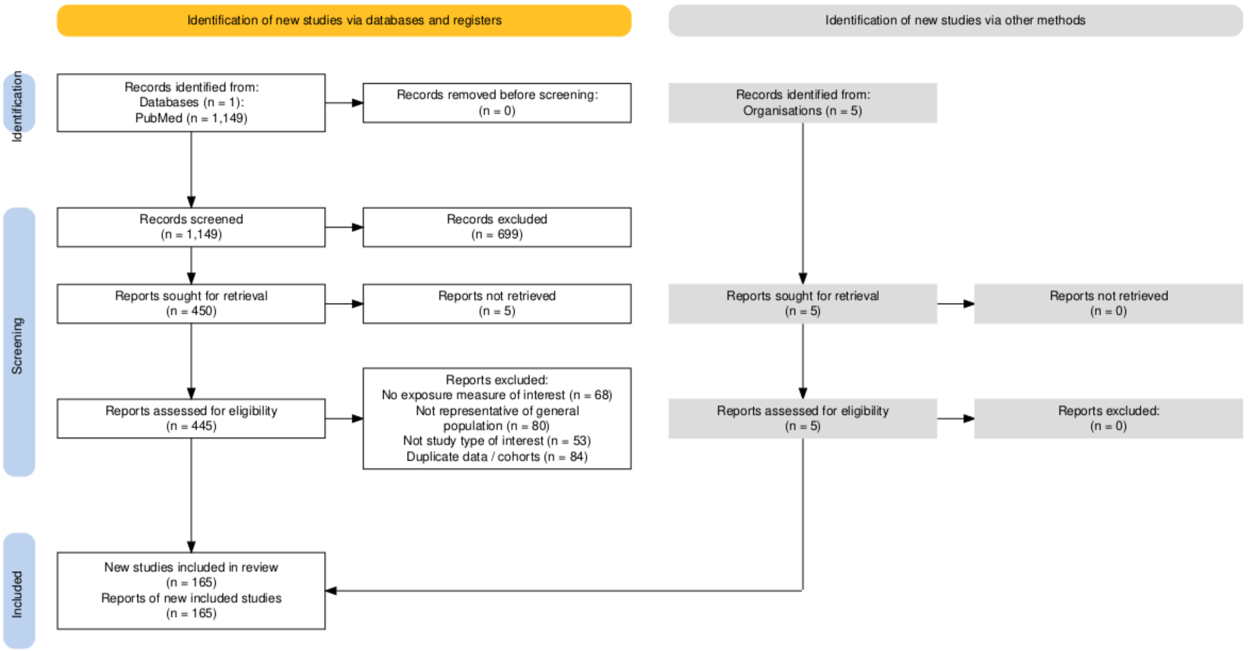

Figure 1: PRISMA diagram for blood lead exposure systematic review

Following extractions, studies were outliered if they had <10 people in their sample size or if their lead analysis was specific to plasma or serum lead.

For GBD 2023, we also extracted three new microdata sources.[2,3,4] These studies (Mexico ENSANUT data) had been previously excluded from our SR due to the reports showing a high percentage of the sampled population being below the limit of detection. We were able to incorporate these data by accessing the microdata for these surveys and setting those non-detect values to $\frac{LOD}{\sqrt{2}}$.

Relative risks

Blood lead

Blood lead relative risks were previously taken from a 2005 pooled analysis that was first incorporated in GBD 2010.[5] Those relative risks were then updated for GBD 2017 using a 2013 re-analysis of the findings of that 2005 paper, providing slightly adjusted relative risk estimates specific to exposure at 24 months of age.[6]

Bone lead

Prior to GBD 2023, all causes associated with bone lead are mediated through high systolic blood pressure (SBP). The shift in SBP due to bone lead exposure was adapted from Navas-Acien, et al. 2008.[7]

For GBD 2023, we re-evaluated our relationship of bone lead exposure to IHD. First, we conducted a survival analysis to calculate hazard ratios between bone lead exposure and CVD, using publicly available data from the National Health and Nutrition Examination Survey (NHANES) with linked mortality data.

These data ranged from 1988 to 2013 within the USA population. We then conducted a systematic review to find the association between blood/bone lead exposure and CVD or IHD.

The systematic review (SR) was submitted to Prospero, and more detailed methodology can be found with Prospero ID: CRD42023453813. Briefly, we used a meta-analysis by Brown et al., 2020 to reduce the year search range to 2018 through August 2023.[8] We searched PubMed, Embase, and Global Medicus Index. Our inclusion criteria were as follows: study design was either cohort or a nested case-control, provided lead levels prior to or concurrently with CVD/IHD diagnosis, provided a relative measure of association along with a measure of uncertainty or provided data to calculate each, measurements must be taken within the same population, and the population must be generalisable (ie, exclude populations that have an elevated risk to CVD). If multiple studies sampled the same cohort, we would include the study with the longest follow-up period. The PubMed search string is below, and the search strings for the other two databases can be found on Prospero's website using the aforementioned Prospero ID.

("Lead Poisoning"[Mesh] OR "Lead"[Mesh] OR Pb[tiab] OR "blood lead level"[tiab] *OR "lead level*"[tiab] OR "blood lead"[tiab] OR "lead blood level"[tiab] *OR "*bone lead"[tiab] *OR "*bone lead level"[tiab]) AND ("Cardiovascular Diseases"[Mesh] OR "Myocardial Ischemia"[Mesh] OR "Myocardial Infarction"[Mesh] OR "Coronary Artery Disease"[Mesh] OR "Angina Pectoris"[Mesh] OR "cardiovascular disease*"[tiab] OR "cvd"[tiab] OR "idh"[tiab] OR "cad"[tiab] OR "chd"[tiab] OR "mi"[tiab] OR "Ischemic heart disease*"[tiab] OR "coronary artery disease*"[tiab] OR "coronary heart disease*"[tiab] OR "ischaemic heart disease*"[tiab] OR "angina"[tiab] OR "angina pectoris"[tiab] OR "myocardial ischemia"[tiab] OR "myocardial ischaemia"[tiab] OR "myocardial infarction"[tiab]) AND (2018[PDAT]:3000[PDAT]) NOT ("Animal Experimentation"[Mesh]) NOT (animals[MeSH] NOT humans[MeSH]) NOT ('animal'/exp NOT human/exp)

Two reviewers performed full-text reviews on all four papers from Brown et al., 2020, but all failed the inclusion criteria.[8] From the systematic review we identified 1460 papers (Figure 2). The first reviewer conducted title/abstract screening on all of the papers, and the second reviewer screened 10% of the excluded papers. This was done to ensure that the second reviewer agreed on the exclusions. Of the 27 papers that passed title/abstract screening, all were full-text screened by both reviewers. If there was disagreement between the two reviewers, a senior expert was consulted. Of the 27 papers, five passed full-text screening (Figure 2). The five papers included both blood and bone lead exposures. All five papers were extracted using a standard template by the first reviewer. The second reviewer reviewed the extractions to check for accuracy and completeness.

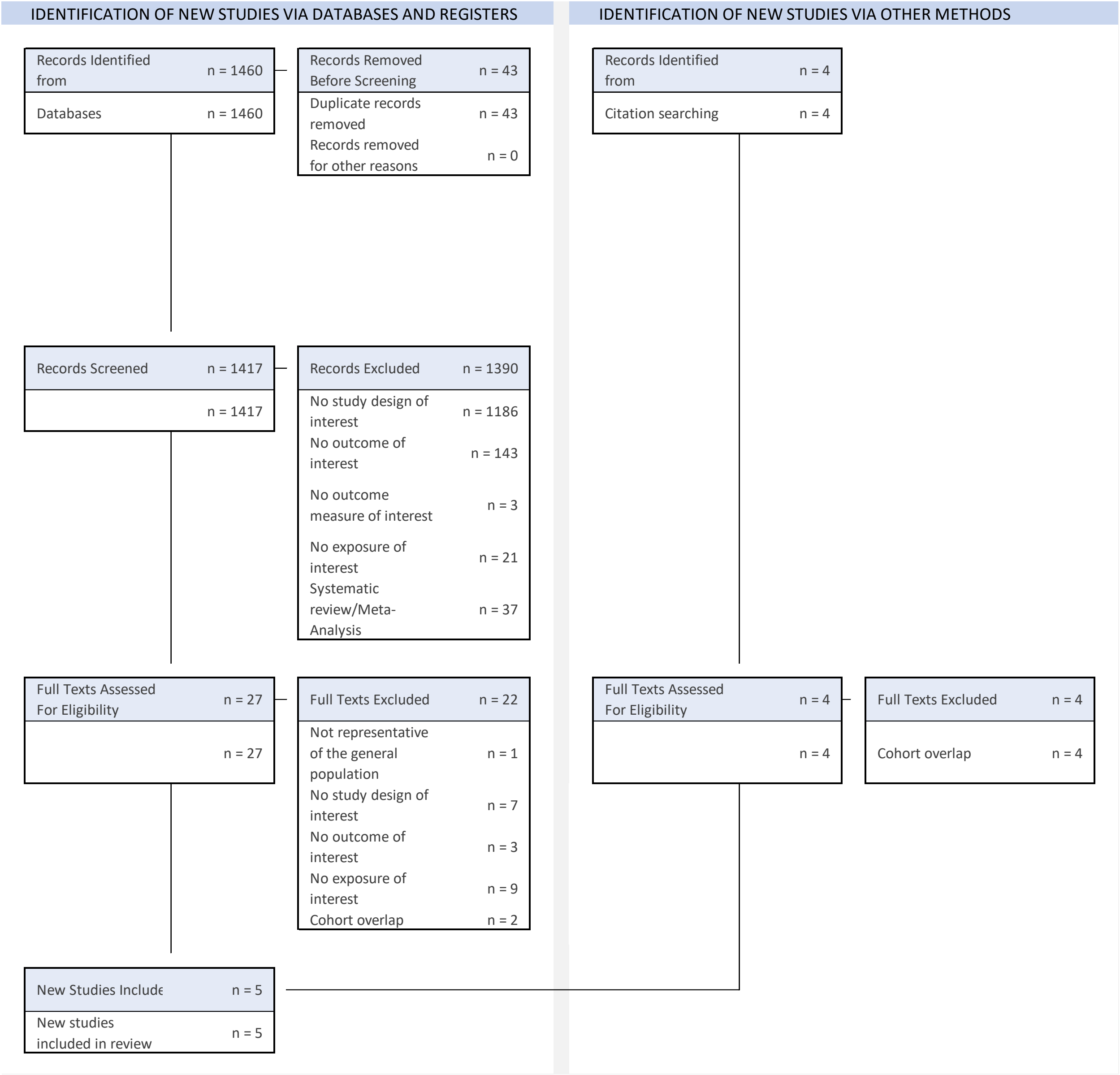

Figure 2: PRISMA diagram for blood/bone lead-CVD systematic review.

Since this particular study is focused on direct CVD/IHD effects of bone lead exposure not mediated by high systolic blood pressure (SBP), we only used one of the five papers in our final analysis which provided bone lead to CVD/IHD measurements of association with adjustment for SBP.[9] In addition to the one paper, we included the NHANES analysis mentioned above in the final analysis which resulted in a total of 20 estimates of relative risk. Please see the *Relative risk* section below for more details on the final analysis.

Data processing

First, we conducted age and sex splitting of exposure input data that did not align with GBD's age and sex groupings. Prior to sex-splitting, we estimated the global sex pattern model, based on exposure data that aligned with GBD's sex groupings for lead (male and female). A tool called meta-regression—

Bayesian, regularised, trimmed (MR-BRT) was used for the sex pattern model and was used in previous rounds.[10] For the sex pattern, we calculated the ratio between females and males, and did not use priors or other covariates in the model. Once a global sex pattern was established, we used a new tool called PyDisagg to conduct sex splitting of all exposure data that were reported as both sexes.[11] PyDisagg disaggregates rate and count observations based on a proportionality assumption. We seek to disaggregate a user-provided datum D into subcomponents D_i, guaranteeing that:

$$sum(D_i) = D$$

$$D_i = g_i p_i$$

where p_i is population weight and g_i a transform of the global rate (f_i).

$$g_i = T^{-1}\big(\beta + T(f_i)\big)$$

The basic use case has $T = ln$, guaranteeing that the recovered rates are proportional to the global. In addition, PyDisagg allows splitting of bounded quantities using the appropriate logit transform for T. PyDisagg version 0.6.0 disaggregates observations of both continuous quantities (eg, age-splitting of age-aggregated observations) as well as discrete quantities (including sex and multiple categories). The uncertainty from the reported data (ie, unsplit datapoint) and uncertainty from the sex pattern were used to estimate the standard errors.

Prior to age-splitting, we estimated the global age pattern model, based on exposure data that aligned with GBD's age groups. In previous GBD cycles, the age pattern was modelled using DisMod ODE.[12] For GBD 2023, we changed the modelling methodology to use MR-BRT to model the global age pattern. The data that are used in the model are post–sex splitting. For the age pattern model, we used no priors and the following covariates: age midpoint and sex. Pydisagg was then used to conduct the age splitting into GBD standard age groups.

Second, we crosswalk the exposure data (post sex and age splitting) using MR-BRT. Blood lead exposure data are reported in the literature as an arithmetic mean, a geometric mean, or a median. To standardise the data, we adjusted all values reported as a geometric mean or median to reflect what they would have been had the study reported the arithmetic mean (see Table 1 for adjustment factors). Additionally, we expected the urbanicity (proportion of the population living in an urban area, for a given location) of a location to affect our estimates. After adjusting to the arithmetic mean, we adjusted our data so that they were equivalent to the average urbanicity of the location from which the data were collected (see Table 2 for adjustment factors). This was only done if the study's reported urbanity did not match the average urbanicity of the location.

Table 1: MR-BRT crosswalk adjustment factors for lead exposure (mean)

Reference or alternative case definition	Gamma	Beta coefficient, log (95% UI)*	Adjustment factor**
Reference (data reported as arithmetic mean)	0.0669	---	---

Alternative (data reported as geometric mean)		–0.295 (–0.479 to –0.111)	0.745 (0.620–0.895)
Alternative (data reported as median)		–0.598 (–0.782 to –0.413)	0.550 (0.458–0.661)

Table 2: MR-BRT crosswalk adjustment factors for lead exposure (urbanicity)

**Reference or alternative case definition**	**Gamma**	**Beta coefficient, log (95% UI)***	**Adjustment factor****
Reference (study urbanicity equals location average urbanicity)	0.033	---	---
Alternative (study urbanicity does not equal location average urbanicity)		–0.149 (–0.264 to –0.034)	0.892 (0.768–0.967)

**MR-BRT crosswalk adjustments can be interpreted as the factor the alternative case definition is adjusted by to reflect what it would have been, had it been measured using the reference case definition.*
***The adjustment factor column is the exponentiated beta coefficient. For log beta coefficients, this is the relative rate between the two case definitions. For logit beta coefficients, this is the relative odds between the two case definitions.*

The average urbanicity of a location is calculated using the one-kilometre resolution GHS-SMOD and world population rasters for the years 1975–2020 at five-year intervals.[13,14] The GHS-SMOD and world population rasters are overlayed such that the locations align. GHS-SMOD assigns categories to each raster cell, ranging from urban center to a water grid cell. We assign the cells that are categorised as urban center and dense urban cluster as 100% of the population lives in an urban area and all other cells as 0%. For each GBD location, we take a population weighted mean of all the cells that reside within the borders of that location. Using the population weighted mean for all locations and available years, we developed a spatiotemporal Gaussian process regression (ST-GPR) model to produce estimates for all locations and years.[15] We included population density over 1000 people per square kilometre as a covariate within the model and is used to predict for location-years without data. The linear regression equation used in the ST-GPR model is shown below:

$$logit(data) \sim pop\ dense\ over\ 1000\ per\ sqkm$$

pop dense over 1000 per sqkm = Proportion of the country with population density over 1000 people per square kilometre

To predict blood lead for all country-year-age-sex groups, we developed a ST-GPR model. ST-GPR was used to produce estimates of mean and standard deviation of blood lead for all age groups, for both sexes, and for all GBD locations and years. For blood lead exposure, the covariates determined to have predictive ability were the lag-distributed income per capita (LDI), urbanicity, the combined number of two- and four-wheeled vehicles per capita, and a covariate indicating whether leaded gasoline had been

phased out in a given country-year (smoothed over the first five years of phase-out to reflect its gradual implementation). LDI is the gross domestic product per capita that has been smoothed over the preceding ten years. In previous GBD cycles, Socio-demographic Index (SDI) was an included covariate instead of LDI. We found, with the addition of new exposure input data, that SDI was leading to unreasonably high exposure estimates in a handful of countries. By replacing SDI with LDI, we were able to mitigate this effect, leading to more reasonable estimates and a more accurate model when compared to the input data. The linear regression equation used in the ST-GPR model is shown below.

$$\log(data) \sim \log(ldi) + urbanicity + (smooth\ leaded\ gas\ outphase * vehicles\ per\ capita) + (1|level_1) + age$$

LDI = lag-distributed income per capita
Urbanicity = proportion of population living in urban areas
Smooth leaded gas outphase = whether a country has banned use of leaded gasoline, smoothed over the first five years of phase-out
Vehicles per capita = number of 2- and 4-wheeled vehicles per capita
$(1|level_1)$ = super-region-level random effects
Age = factor variable of all age groups

In earlier iterations of GBD, the distribution of lead exposure was assumed to be lognormal. Since GBD 2016, ensemble modelling techniques were used to find ensemble weights by fitting a variety of distributions to the available blood lead microdata. This was a common update for all continuous risk factors in the GBD. The ST-GPR mean and standard deviation estimates for blood lead were used with the ensemble weights to determine distributions for blood lead exposure. The distribution ultimately included 11 different probability distributions: exponential, gamma, inverse-gamma, mirrored gamma, log-logistic, Gumbel, mirrored Gumbel, Weibull, lognormal, normal, and beta. The final distribution was log-logistic (35%), inverse-gamma (18%), lognormal (16%), mirrored Gumbel (12%), with the seven other distributions comprising the remaining 19%.

To calculate blood lead over the lifetime of a given cohort, blood lead was assumed to grow linearly from 0.016 µg/dL in 1920 (see section *Theoretical minimum-risk exposure level*) to the estimated value for the cohort in 1980. Using the exposure distributions of blood lead over time and space, cohorts were constructed such that lifetime blood lead could be expressed as a curve over each year of life. The area under this curve was the cumulative blood lead index, which was used to estimate bone lead in a given year with the scalar from Hu et al (2007).[1]

Estimating attributable burden

Assessment of risk–outcome pairs

We included outcomes based on the strength of available evidence supporting a causal relationship. Blood lead level (a measure of acute lead exposure) was paired with idiopathic developmental intellectual disability as modelled through the impact of blood lead levels on IQ in children. Bone lead level (a measure of chronic lead exposure) was paired with systolic blood pressure, and subsequently to all cardiovascular outcomes to which SBP is paired, which include the following: IHD, ischaemic stroke, intracerebral haemorrhage, subarachnoid haemorrhage, atrial fibrillation and flutter, aortic aneurysm, lower extremity peripheral arterial disease, chronic kidney disease due to hypertension, chronic kidney

disease due to diabetes type 2, chronic kidney disease due to glomerulonephritis, hypertensive heart disease, and chronic kidney disease due to other and unspecified causes.

For GBD 2023, we re-evaluated our relationship of bone lead exposure to IHD. As a result, we have updated this relationship to include both mediation through SBP and a direct relationship between bone lead exposure and IHD. See the *Relative risks* section below for details on the direct relationship and the mediation factor for bone lead exposure to IHD.

Theoretical minimum risk exposure level

In previous iterations of GBD, the theoretical minimum risk exposure level (TMREL) was estimated to be 2.0 micrograms of lead per decilitre of blood. In GBD 2021, we re-evaluated our approach and concluded that the blood lead TMREL should be lowered to 0.016 µg/dL, the level experienced by pre-industrial humans, as estimated by Flegal and Smith (1992).[16] Additionally, given that bone lead levels are cumulative, in GBD 2021 we updated our bone lead TMREL to be age-specific (Table 3). To do this, we created synthetic cohorts where every age and sex was exposed to the blood lead TMREL for all years of their lives. Then, we calculated the cumulative blood lead exposure and converted that to bone lead exposure using the scalar from Hu et al (2007).[1] No updates to the TMREL were made for GBD 2023.

Table 3: Bone lead TMRELs

Age group	TMREL in µg/g (95% UI)
25–29	0.022 (0.020–0.024)
30–34	0.026 (0.024–0.028)
35–39	0.030 (0.027–0.033)
40–44	0.034 (0.031–0.037)
45–49	0.038 (0.035–0.041)
50–54	0.042 (0.038–0.046)
55–59	0.046 (0.042–0.050)
60–64	0.050 (0.045–0.054)
65–69	0.054 (0.049–0.059)
70–74	0.058 (0.053–0.063)
75–79	0.062 (0.056–0.067)
80–84	0.066 (0.060–0.072)
85–89	0.070 (0.064–0.076)
90–94	0.074 (0.067–0.080)
95 plus	0.078 (0.071–0.085)

Relative risks

Blood lead

Because the relative risk of IQ loss from lead exposure is specific to children, in early iterations of GBD, no burden of lead via IQ loss was estimated in the population aged 15 and above. To better account for the continued burden of past lead exposure on IQ in older age groups, since GBD 2016 we have constructed cohorts from the entire population. Estimates of a cohort's lead exposure in early childhood

(at 24 months of age) were used to determine past IQ loss and thus calculate burden via the impact on concurrent IQ in the older population (Table 4).

Table 4: Relative risks for exposure to blood lead

Exposure level	IQ shift (95% UI)
0.016 µg/dL	0.0 (0.0–0.0)
2 µg/dL	2.172 (0.813–3.552)
4 µg/dL	3.182 (1.191–5.204)
6 µg/dL	3.847 (1.440–6.291)
8 µg/dL	4.344 (1.626–7.104)
10 µg/dL	4.741 (1.775–7.753)
12 µg/dL	5.071 (1.898–8.293)
15 µg/dL	5.482 (2.052–8.964)
20 µg/dL	6.019 (2.253–9.843)
25 µg/dL	6.442 (2.411–10.534)
30 µg/dL	6.789 (2.542–11.103)
35 µg/dL	7.085 (2.652–11.586)
40 µg/dL	7.342 (2.748–12.006)

Bone lead

In GBD 2010, we modified the results of Navas-Acien, et al. 2008 by re-running the analysis after removing one study (a cross-sectional study of lead workers in Korea).[7] The revised meta-analysis results showed a 0.61 mmHg (95% UI: –0.01 to 1.34) increase in SBP per 10 µg/g bone lead. Because bone lead is associated with increases in SBP, the health loss attributable to bone lead exposure is mediated through SBP (Table 5), except that due to IHD. As such, the relative risks for bone lead exposure are all the same as the relative risks that SBP has for each outcome, with the exception of IHD. Using the internal NHANES analysis mentioned above (see section *Input data–relative risks*), we calculated the mediation factor for IHD by using two models: one that is adjusted for SBP, and another that is not adjusted for SBP. The mediation factor equation is:

$$Mediation\ Factor = \frac{RR\ crude - RR\ adjusted}{RR\ crude - 1}$$

Where *RR crude* is the non-SBP adjusted model, and *RR adjusted* is the SBP adjusted model. All other causes of SBP remained the same (Table 5).

Table 5: SBP mediation factor for bone lead

Cause name	Mediation factor
IHD	0.0586 (0.0519–0.0678)
Ischaemic stroke	1.000 (1.000–1.000)
Intracerebral haemorrhage	1.000 (1.000–1.000)
Subarachnoid haemorrhage	1.000 (1.000–1.000)

Hypertensive heart disease	1.000 (1.000–1.000)
Atrial fibrillation and flutter	1.000 (1.000–1.000)
Aortic aneurysm	1.000 (1.000–1.000)
Lower extremity peripheral arterial disease	1.000 (1.000–1.000)
Chronic kidney disease	1.000 (1.000–1.000)

Estimating the relative risk (RR) of IHD occurring as a function of exposure to bone lead followed the burden of proof approach established by Zheng and colleagues and instantiated in the MR-BRT tool.[10,17] MR-BRT synthesises input data to generate a RR curve by relying on an ensemble spline method to capture the potentially non-linear shape of the risk–outcome relationship; integrating over varying exposure ranges in different comparison groups; trimming potentially distorting outliers; testing, selecting, and adjusting for bias covariates to account for known heterogeneity in input study-design characteristics (eg, confounding, selection bias, exposure measurement); and quantifying remaining between-study heterogeneity (gamma) through random effects modelling and incorporating this value into uncertainty around the mean RR curve.

MR-BRT further evaluates evidence for small-study effects and generates funnel plots that represent potential risk of publication or reporting bias, and generates the burden of proof risk function (BPRF), defined for harmful risks as the 5th and for protective risks as the 95th quantile risk curve – inclusive of between-study heterogeneity/gamma – that is closest to null. The BPRF is transformed into a risk–outcome score (ROS: the signed value of the average log BPRF between the 15th and 85th percentiles of risk exposure levels observed across included studies) and mapped onto a star-rating system from one to five stars. These metrics complement RR estimates by providing an alternative, conservative measure of effect size and evidence strength that formally and systematically incorporates divergence/convergence across input findings, with higher positive ROS values and more stars corresponding to incrementally larger effects and stronger evidence for the risk–outcome relationship.

For this model we imposed a two-degree spline and a right linear tail. The BPRF can be seen in Figure 3. No publication bias was found, and the model returned a risk score of 0.145, which is a three-star rating.

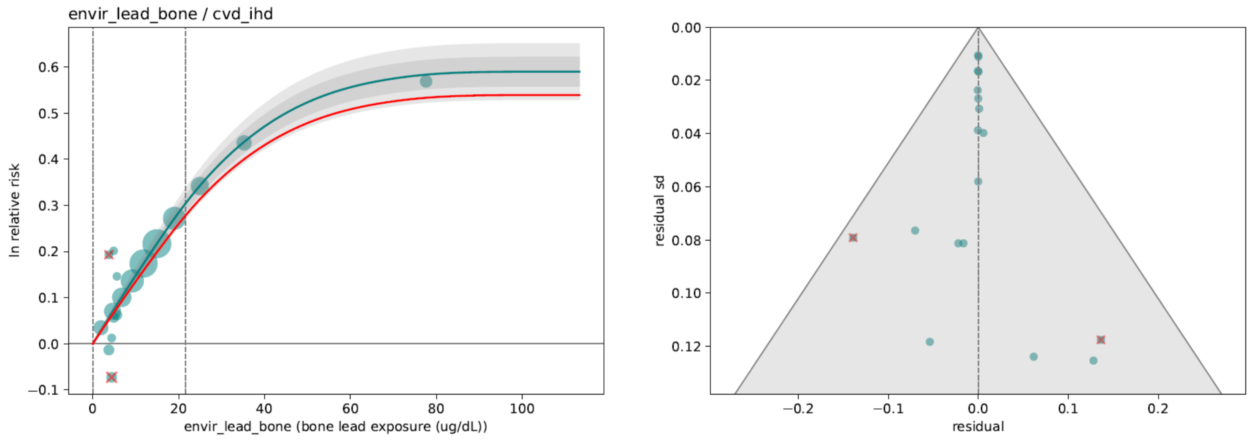

Figure 3: Risk curve (left) and funnel plot (right) showing relative risk of IHD due to bone lead exposure. The final mean log RR curve (thick green line), 95% UIs with between-study heterogeneity (dark grey) and without between-study heterogeneity (light grey), the log BPRF (thick red line), log RRs from included studies, 15th and 85th percentile of exposure observed in the data (dashed lines), and the funnel plot. The size of the log RRs corresponds to the inverse of their standard errors. Trimmed datapoints are marked with a red x.

Population attributable fraction

We used the standard GBD population attributable fraction (PAF) equation to calculate PAFs for bone lead exposure and each of its paired outcomes using exposure estimates and relative risks.[15] We used a similar approach for estimating PAFs for the burden of intellectual disability attributable to blood lead, which uses the estimated distribution of intellectual disability and the modelled shifts in IQ due to blood lead levels to determine the PAF.

In GBD 2023, we changed how the standard deviation was calculated for the estimated distribution of intellectual disability. In previous cycles, we took the overall standard deviation to be the mean standard deviation across all intellectual disability severities. In GBD 2023, we removed any severities with a cumulative YLD rate of zero from the mean standard deviation calculation. This resulted in an overall increase in PAFs for blood lead exposure.

References

1. Hu H, Shih R, Rothenberg S, Schwartz BS. The epidemiology of lead toxicity in adults: measuring dose and consideration of other methodologic issues. *Environ Health Perspect*. 2007;115(3):455-62.
2. Ministry of Health (Mexico), National Institute of Public Health (Mexico). Mexico National Survey of Health and Nutrition (ENSANUT) 2018-2019 100K. Cuernavaca, Mexico: National Institute of Public Health (Mexico).
3. National Institute of Public Health (Mexico), National Institute of Statistics and Geography (INEGI) (Mexico). Mexico National Survey of Health and Nutrition (ENSANUT) 2018-2019. Cuernavaca, Mexico: National Institute of Public Health (Mexico).
4. Ministry of Health (Mexico), National Institute of Public Health (Mexico). Mexico Continuous National Health and Nutrition Survey 2022. Cuernavaca, Mexico: National Institute of Public Health (Mexico), 2023.
5. Lanphear BP, Hornung R, Khoury J, et al. Low-level environmental lead exposure and children's intellectual function: an international pooled analysis. *Environ Health Perspect*. 2005;113(7):894-9.
6. Crump K, Van Landingham C, Bowers T, Cahoy D, Chandalia J. A statistical reevaluation of the data used in the Lanphear et al. (2005) pooled-analysis that related low levels of blood lead to intellectual deficits in children. *Critical Reviews in Toxicology*. 2013;43(9):785-799.
7. Navas-Acien A, Schwartz BS, Rothenberg SJ, Hu H, Silbergeld EK, Guallar E. Bone lead levels and blood pressure endpoints: a meta-analysis. *Epidemiology*. 2008;19(3):496-504.
8. Brown L, Lynch M, Belova A, Klein R, Chiger A. Developing a Health Impact Model for Adult Lead Exposure and Cardiovascular Disease Mortality. *Environ Health Perspect*. 2020 Sep;128(9):97005.
9. Lieberman-Cribbin W, Domingo-Relloso A, Navas-Acien A, Cole S, Haack K, Umans J, Tellez-Plaza M, Colicino E, Baccarelli AA, Gao X, Kupsco A. Epigenetic Biomarkers of Lead Exposure and Cardiovascular Disease: Prospective Evidence in the Strong Heart Study. *J Am Heart Assoc*. 2022 Dec 6;11(23):e026934

10. Zheng P, Barber R, Sorensen RJD, Murray CJL, Aravkin AY. Trimmed constrained mixed effects models: formulations and algorithms. *J Comput Graph Stat* 2021; 30: 544–56.
11. Alexander Hsu, Peng Zheng, Kelsey Maass, Aleksandr Aravkin, & Sameer Ali. (2025). pyDisagg: Dissaggregation under Generalized Proportionality Assumptions (v0.6.0). Zenodo.
12. GBD 2021 Diseases and Injuries Collaborators. Global incidence, prevalence, years lived with disability (YLDs), disability-adjusted life-years (DALYs), and healthy life expectancy (HALE) for 371 diseases and injuries in 204 countries and territories and 811 subnational locations, 1990-2021: a systematic analysis for the Global Burden of Disease Study 2021. *Lancet* 2024; 403: 2133–61.
13. Schiavina M., Melchiorri M., Pesaresi M. (2023): GHS-SMOD R2023A - GHS settlement layers, application of the Degree of Urbanisation methodology (stage I) to GHS-POP R2023A and GHS-BUILT-S R2023A, multitemporal (1975-2030) European Commission, Joint Research Centre (JRC)
14. Geography and Environmental Science, University of Southampton, WorldPop. Age and Sex Structures, Global Per Country 2000-2020. Southampton, United Kingdom: WorldPop, 2018.
15. GBD 2021 Risk Factors Collaborators. Global burden and strength of evidence for 88 risk factors in 204 countries and 811 subnational locations, 1990–2021: a systematic analysis for the Global Burden of Disease Study 2021. *Lancet* 2024; 403: 2162–203.
16. Flegal AR, Smith DR. Lead levels in preindustrial humans. *N Engl J Med*. 1992;326(19):1293-4.
17. Zheng P, Afshin A, Biryukov S, et al. The Burden of Proof studies: assessing the evidence of risk. *Nat Med* 2022; 28: 2038–44.

Low birthweight and short gestation

Flowchart

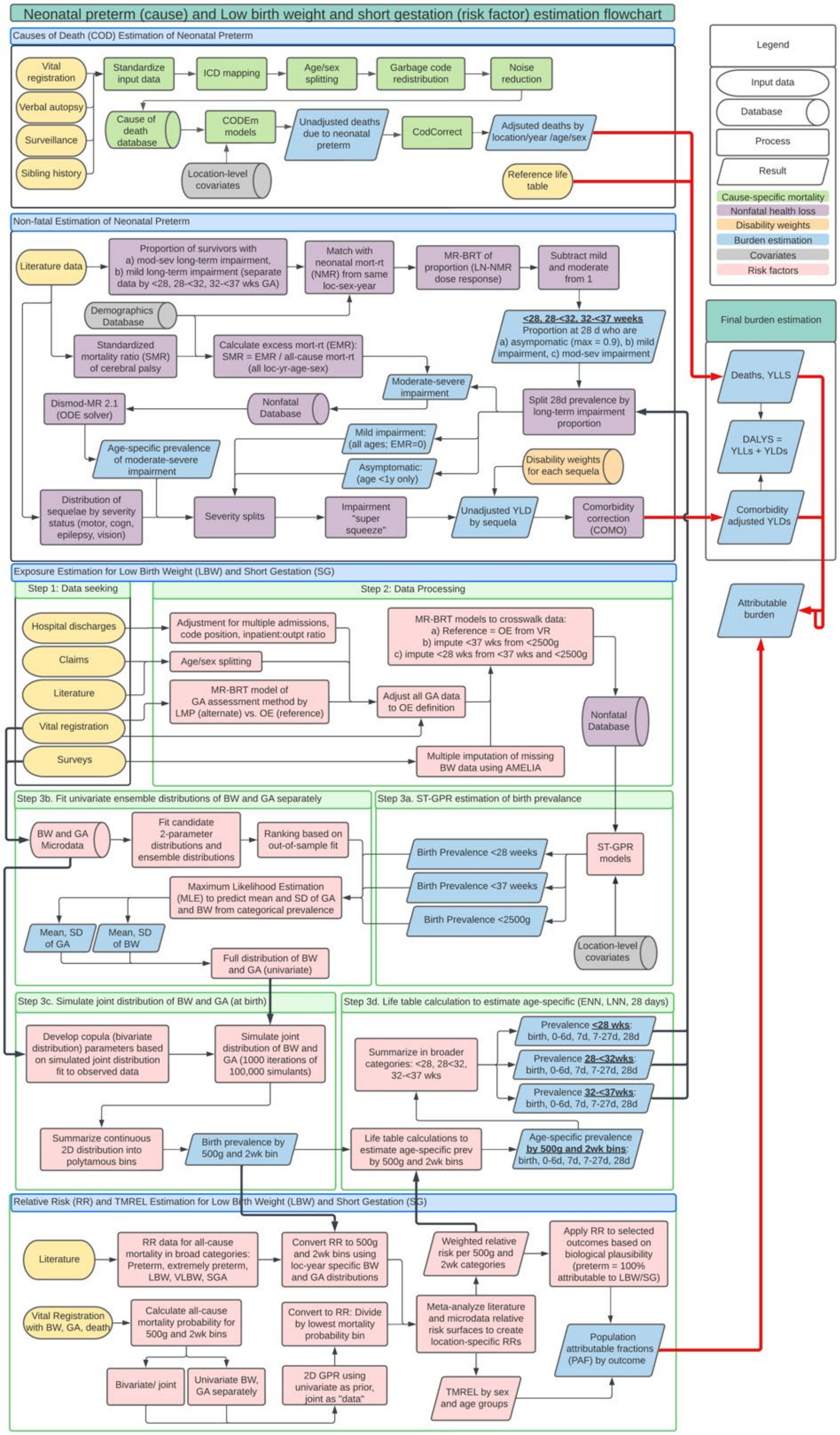

Input data and methodological summary

Low birthweight and preterm birth are highly correlated risk factors associated with poor child health outcomes. The "low birthweight and short gestation" (LBWSG) risk factor quantifies the burden of disease attributable to increased risk of death and disability due to 1) less than ideal birthweight ("low birthweight") and 2) shorter than ideal length of gestation ("short gestation").

Within GBD, attributable burden is generally estimated separately for each individual risk factor, but the combined burden attributable to multiple risk factors is of general interest. In GBD, attributable burden due to multiple risk factors is typically estimated through a "mediation analysis" that is applied after independent estimation of each risk factor's exposure, relative risk, theoretical minimum risk exposure level (TMREL), and population attributable fraction (PAF). In the mediation analysis, a "mediation factor" adjusts the PAF of each risk factor by the amount of attributable burden mediated through the other GBD risk factors. While mediation may be common, direct quantification of the joint exposure, relative risk, and PAF of the combined risk factors is conceptually more straightforward.

In GBD 2016, LBWSG became the first group of GBD risk factors in which combined attributable burden is quantified by direct estimation of the joint exposure, relative risk, TMREL, and PAF of multiple risk factors. After first directly estimating the joint exposure, relative risk, TMREL, and PAF of birthweight and gestational age together, we then separate out the independent PAFs due to birthweight only or gestational age only. Because of this modelling strategy, the joint GBD risk factor quantifying the burden of disease due to both less than ideal birthweight ("low birthweight") and shorter than ideal gestational age ("short gestation") is grouped into a single "parent" risk factor termed "low birthweight and short gestation." LBWSG is disaggregated into two "child" risk factors: "low birthweight" and "short gestation". Low birthweight quantifies the burden of disease attributable to less than ideal birthweight, after adjusting for the influence of gestational age. Likewise, short gestation quantifies the burden of disease attributable to shortened gestational age, after adjusting for the influence of birthweight.

Ideally, the model for joint exposure and joint relative risk would be fully continuous. To simplify the computation for the analysis, a grid of 500-gram and two-week units ("bins") is used as the birthweight and gestational age dimensions and to approximate a fully continuous joint distribution model (see Figure 1).

Figure 1: Fully continuous analysis of joint gestational age and birthweight (left) is approximated with a grid of birthweight and gestational age with 500-gram and two-week "bins" (right)

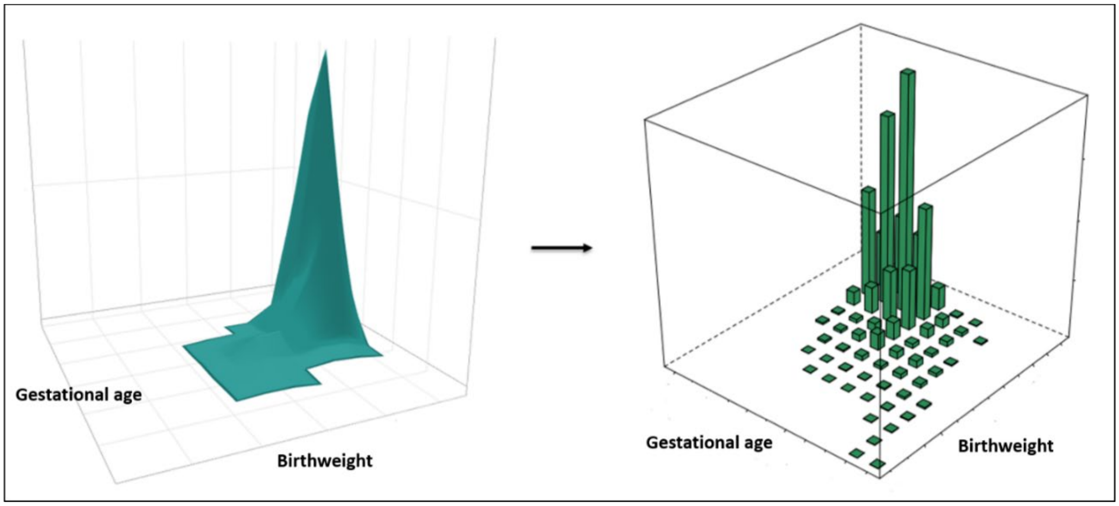
Gestational age
Birthweight
Gestational age
Birthweight

Case definition

"Low birthweight" has historically referred to any birthweight less than 2500 grams, dichotomising birthweight into two categories: "normal" and "low". In the context of the GBD LBWSG risk factor, low birthweight refers to any birthweight less than the birthweight TMREL (the birthweight that minimises risk at the population level). Because LBWSG is estimated in a grid of 500-gram and two-week bins, any 500-gram birthweight unit less than the TMREL, which was determined as [38, 40) weeks and [3500, 4000) g for the LBWSG parent risk factor, is considered "low birthweight". This includes, for example, birthweight of [2500, 3000) grams, which the traditional, dichotomous definition of "low birthweight" would not include.

Like birthweight, gestational age is typically classified into broad categories. "Preterm" is used to describe any newborn baby born less than 37 completed weeks of gestation. In the GBD context, "short gestation" is used to refer to all gestational ages below the gestational age TMREL.

Exposure

In LBWSG, exposure refers to the portion of the joint distribution of gestational age and birthweight less than the TMREL, by location/year/sex (l/y/s), from birth to the end of the neonatal period. Modelling LBWSG exposure can be summarised in three steps:

A. Model univariate gestational age and birthweight distributions at birth, by l/y/s
B. Model joint distributions of gestational age and birthweight at birth, by l/y/s
C. Model joint distributions from birth to the end of the neonatal period, by l/y/s

Table 1: Analytic steps in estimation of YLDs due to preterm birth

	Summary of exposure modelling strategy
**Step A**<br>Model univariate distributions at birth	1. Model mean gestational age, prevalence of gestational age <28 weeks, and prevalence of gestational age <37 weeks, by l/y/s<br>2. Model mean birthweight and prevalence of birthweight <2500 grams, by l/y/s<br>3. Model univariate gestational age and birthweight distributions separately at birth, by l/y/s
**Step B**<br>Model joint distributions at birth	1. Use copulae to model the correlation structure of the joint distribution of gestational age and birthweight, globally<br>2. Model the joint distribution of gestational age and birthweight, by location/year/sex at birth, by applying the globally modelled correlation structure to the location/year/sex-specific univariate models of gestational age and birthweight distributions
**Step C**<br>Model joint distributions from birth to 28 days	1. Model all-cause mortality rates by gestational age and birthweight<br>2. Model gestational age and birthweight distributions of surviving neonates for all l/y/s from birth to end of the neonatal period, using all-cause mortality rates by gestational age and birthweight

Input data and data processing

Input data needed to model univariate gestational age and birthweight distributions at birth (Step A):

- Prevalence of preterm birth (<37 weeks), by l/y/s
- Prevalence of extremely preterm birth (<28 weeks), by l/y/s

- Mean gestational age, by l/y/s
- Gestational age microdata
- Prevalence of low birthweight (<2500 grams), by l/y/s
- Mean birthweight, by l/y/s
- Birthweight microdata

To model joint distributions of gestational age and birthweight (Step B), joint microdata of gestational age and birthweight are also required. Additional inputs to modelling joint distributions from birth to 28 days (Step C) are all-cause mortality by l/y/s and joint birthweight and gestational age microdata linked to mortality outcomes.

Prevalence of extremely preterm birth (<28 weeks) and preterm birth (<37 weeks) were modelled using vital registration, survey, and clinical data. For the preterm models, only inpatient and insurance claims data were included from clinical informatics datasets; outpatient data were excluded because they were more likely to capture repeated visits by the same child rather than unique visits. Prevalence of low birthweight (<2500 grams) was modelled using only vital registration and survey data.

Literature review

Before GBD 2016, available preterm birth data were sourced by a technical working group. In GBD 2016 and GBD 2017, we conducted systematic reviews to identify additional sources beyond the data already used in the models. The PubMed database was searched using the following search string:

> ((("Infant, Premature"[Mesh] OR ("infant"[All Fields] AND "premature"[All Fields]) OR "premature infant"[All Fields] OR ("preterm"[All Fields] AND "infant"[All Fields]) OR "preterm infant"[All Fields] OR ("infant, newborn"[MeSH Terms] OR ("infant"[All Fields] AND "newborn"[All Fields]) OR "newborn infant"[All Fields] OR ("newborn"[All Fields] AND "infant"[All Fields])) AND (premature[All Fields] OR preterm[All Fields]) OR "premature birth"[MeSH Terms] OR ("premature"[All Fields] AND "birth"[All Fields]) OR "premature birth"[All Fields] OR ("preterm"[All Fields] AND "birth"[All Fields]) OR "preterm birth"[All Fields]) ((("Infant, Premature"[Mesh] OR ("infant"[All Fields] AND "premature"[All Fields]) OR "premature infant"[All Fields] OR ("preterm"[All Fields] AND "infant"[All Fields]) OR "preterm infant"[All Fields] OR ("infant, newborn"[MeSH Terms] OR ("infant"[All Fields] AND "newborn"[All Fields]) OR "newborn infant"[All Fields] OR ("newborn"[All Fields] AND "infant"[All Fields])) AND (premature[All Fields] OR preterm[All Fields]) OR "premature birth"[MeSH Terms] OR ("premature"[All Fields] AND "birth"[All Fields]) OR "premature birth"[All Fields] OR ("preterm"[All Fields] AND "birth"[All Fields]) OR "preterm birth"[All Fields]) AND ("1985"[PDAT] : "3000"[PDAT]) AND "humans"[MeSH Terms].

The exclusion criteria were studies that did not provide primary data on epidemiological parameters, non-representative studies (eg, only high-risk pregnancies), and reviews. Table 2 shows the search hits, number of full texts reviewed, and number of extracted sources.

Table 2. LBWSG search hits, full-text review, extracted sources

GBD round	Search date	Hits	Full-text review	Extracted
GBD 2017	6/6/2017	16,174	2200	154

Data processing

Any data that didn't fit a GBD age group was split into GBD age groups using a model that was run using only age-specific data. Starting in GBD 2019, as was the case with all other non-fatal analyses, we applied empirical age and sex ratios from previous models to disaggregate observations that did not

entirely fit in one GBD age category or sex. Ratios were determined by dividing the result for a specific age and sex by the result for the aggregate age and sex specified in a given observation.

Low birthweight (<2500 grams) data were extracted from literature, vital registration systems, and surveys. Survey data (most commonly from DHS and MICS) were observed to have high missingness of birthweight responses. We evaluated the patterns of missingness and found a number of distinct patterns that suggested non-random omission of birthweight observations. We therefore imputed missing birthweight values using the Amelia II (Version 1.7.6) package in R. Birthweight was predicted using the following variables also in the DHS surveys: urbanicity, sex, birthweight recorded on card, birth order, maternal education, paternal education, child age, child weight, child height, mother's age at birth, mother's weight, shared toilet facility, and household water treated.

After imputation, we completed a number of additional steps to standardise the dataset by applying a series of crosswalks. "Crosswalking" is a process of reducing non-random bias by adjusting non-standard data to the likely value had the data been collected using a reference definition, technique, or sample. Three crosswalks were applied for birthweight and gestational age data, all of the statistical models for which were developed using meta-regression—regularised, Bayesian, trimmed (MR-BRT).

First was a crosswalk for method of gestational age assessment that included three separate models. All microdata that reported GA and both obstetric estimate (OE) and last menstrual period were crosswalked to OE using the relationship derived from USA GA microdata (Figure 2). This crosswalk was developed with a spline on LMP in order to reliably match on the data that needed to be crosswalked.

Next, for all data that were only categorical, we adjusted all gestational age data to a reference definition of obstetric estimate (OE), which also included tabulations of the crosswalked microdata above. Two alternate definitions regularly appeared, and both were crosswalked separately. These were last menstrual period (LMP) for each of <37 weeks' and <28 weeks' gestation (Tables 3 and 4) and other measure of gestational age (Table 5 and 6).

The second set of crosswalks adjusted data derived from clinical administrative sources (ie, hospital discharges and insurance claims) to matched vital registration data using OE (Tables 7 and 8).

The third set of crosswalks served to "square the input dataset" to ensure that every location-year with data had an observation for each of <2500g (birthweight), <37 weeks, and <28 weeks. This process utilised relationships between input data types to maximise the volume of data later input to models. Low birthweight data (<2500g) were crosswalked to preterm (<37 weeks) data (Table 9), preterm to extremely preterm (Table 10), and extremely preterm to preterm (Table 11).

Figure 2. MR-BRT OE-LMP crosswalk adjustment factor by LMP-reported gestational age

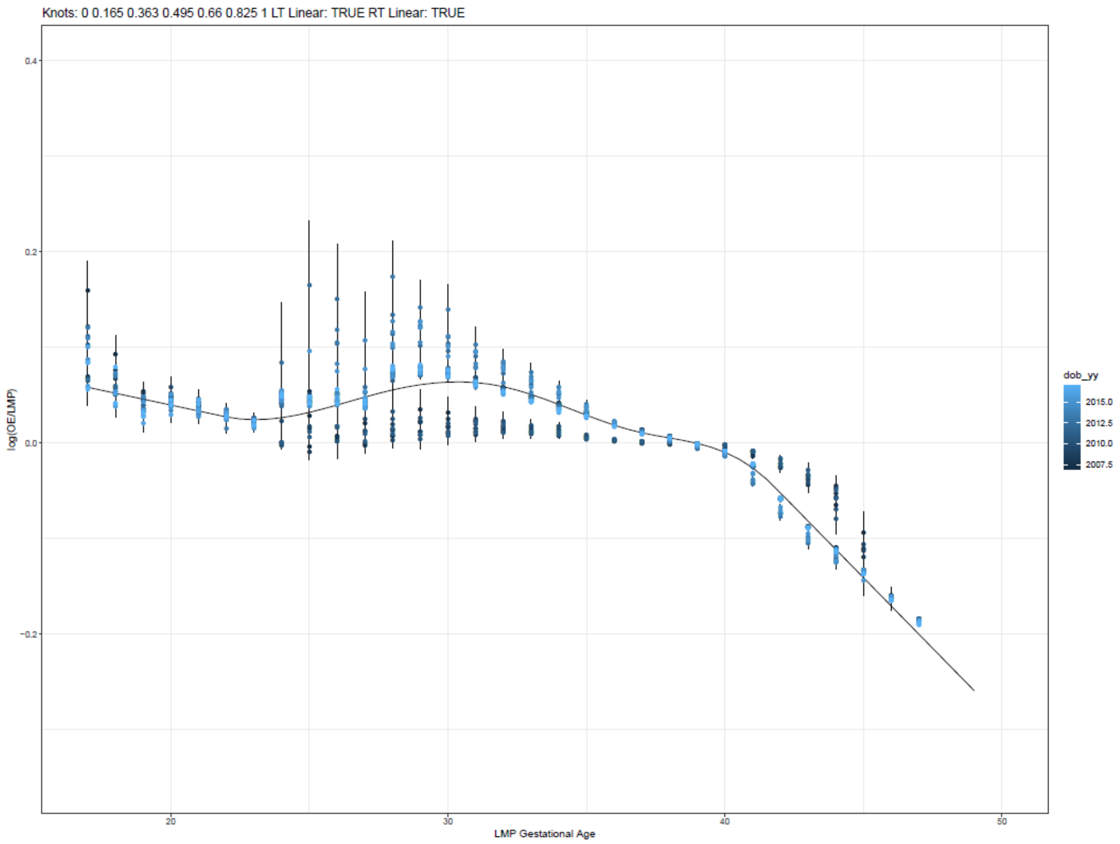

Table 3. MR-BRT OE-LMP crosswalk adjustment factor for preterm birth (<37 weeks' gestation)

Data input	Reference or alternative case definition	Gamma	Beta coefficient, log (95% CI)	Adjustment factor*
Obstetric estimate	Reference	0.01	---	---
Last menstrual period	Alternative		0.187 (0.142,0.231)	1.205 (1.153, 1.260)

Table 4. MR-BRT OE-LMP crosswalk adjustment factor for extremely preterm (<28 weeks' gestation)

Data input	Reference or alternative case definition	Gamma	Beta coefficient, log (95% CI)	Adjustment factor*
Obstetric estimate	Reference	0.00	---	---
Last menstrual period	Alternative		0.0284 (0.268,0.300)	1.328 (1.308, 1.349)

Table 5. MR-BRT OE-other measure crosswalk adjustment factor for preterm birth (<37 weeks' gestation)

Data input	Reference or alternative case definition	Gamma	Beta coefficient, log (95% CI)	Adjustment factor*
Obstetric estimate	Reference	0.10	---	---
Other measurement	Alternative		–0.243 (–0.494, 0.009)	0.785 (0.610, 1.01)

Table 6. MR-BRT OE-other measure crosswalk adjustment factor for extremely preterm birth (<28 weeks' gestation)

Data input	Reference or alternative case definition	Gamma	Beta coefficient, log (95% CI)	Adjustment factor*
Obstetric estimate	Reference	0.37	---	---
Other measurement	Alternative		0.154 (–0.486, 0.793)	1.166 (0.615, 2.210)

Table 7. MR-BRT VR-claims crosswalk adjustment factor for preterm birth (<37 weeks' gestation)

Data input	Reference or alternative case definition	Gamma	Beta coefficient, log (95% CI)	Adjustment factor*
Vital registration	Reference	0.07	---	---
Insurance claims	Alternative		–0.712 (–0.909, –0.515)	0.491 (0.403, 0.597)

Table 8. MR-BRT VR-insurance claims crosswalk adjustment factor for extremely preterm birth (<28 weeks' gestation)

Data input	Reference or alternative case definition	Gamma	Beta coefficient, log (95% CI)	Adjustment factor*
Vital registration	Reference	0.02	---	---
Insurance claims	Alternative		–1.258 (–1.447, –1.07)	0.284 (0.235, 0.344)

Table 9. MR-BRT low birthweight to preterm birth (<37 weeks' gestation)

Data input	Reference or alternative case definition	Gamma	Beta coefficient, log (95% CI)	Adjustment factor*
Preterm birth	Reference	0.08	---	---
Low birthweight	Alternative		–0.479 (–0.518, –0.440)	0.620 (0.596, 0.644)

Table 10. MR-BRT preterm (<37 weeks' gestation) to extremely preterm (<28 weeks' gestation)

Data input	Reference or alternative case definition	Gamma	Beta coefficient, log (95% CI)	Adjustment factor*
28 weeks	Reference	0.06	---	---
37 weeks	Alternative		3.221 (3.161, 3.281)	25.053 (23.600, 26.604)

Table 11. MR-BRT extremely preterm (<28 weeks' gestation) to preterm (<37 weeks' gestation)

Data input	Reference or alternative case definition	Gamma	Beta coefficient, log (95% CI)	Adjustment factor*
37 weeks	Reference	0.05	---	---
28 weeks	Alternative		–3.208 (–3.266, –3.150)	0.0404 (0.0381, 0.0428)

**MR-BRT crosswalk adjustments can be interpreted as the factor the alternative case definition is adjusted by to reflect what it would have been had it been measured using the reference case definition. If the log/logit beta coefficient is negative, then the*

alternative is adjusted up to the reference. If the log/logit beta coefficient is positive, then the alternative is adjusted down to the reference.

***The adjustment factor column is the exponentiated beta coefficient. For log beta coefficients, this is the relative rate between the two case definitions. For logit beta coefficients, this is the relative odds between the two case definitions.*

These data adjustments had the effect of dramatically increasing the size of each of the modelling datasets and are primarily responsible for most changes in preterm estimates between GBD 2019 and subsequent rounds. After all crosswalks, we performed a deduplication step on GA models. Namely, if low birthweight data in countries that were 1) categorised as "data-rich" locations in cause-of-death modelling or had at least ten consecutive years of vital registration data recording gestational age, and 2) had both preterm birth and low birthweight data, then crosswalked low birthweight data were outliered so that the model was informed only by the gestational age data.

Modelling strategy

Step A: Model univariate birthweight and gestational age distributions at birth, by l/y/s

Microdata are the ideal data source for modelling distributions; however, microdata are not widely available for birthweight and are scarcer for gestational age. Categorical prevalence data are more readily available from a wider range of locations and years for low birthweight (<2500 g), extremely preterm (<28 weeks of gestation), and preterm birth (<37 weeks of gestation). Because categorical prevalence has wider availability than microdata, we use prevalence data to assist in modelling birthweight and gestational age ensemble distributions.

Ensemble distribution models can be constructed with three pieces of information: mean of the distribution, variance of the distribution, and the weights of the distributions being used in the ensemble. To model mean and variance for all l/y/s for birthweight and gestational age, we first used spatiotemporal Gaussian process regression (ST-GPR) models to model prevalence of low birthweight, extremely preterm, and preterm birth for all l/y/s at birth. To model mean birthweight for all l/y/s, OLS linear regression was used to regress mean birthweight on log-transformed low birthweight prevalence. This model was then used to predict mean birthweight for all l/y/s, using the prevalence of low birthweight (<2500 grams) modelled for all l/y/s in ST-GPR. Similarly, to model gestational age mean for all l/y/s, OLS linear regression model was used to regress mean gestational age on log-transformed preterm prevalence. Mean gestational age for all l/y/s was predicted using the preterm birth (<37 weeks) estimates modelled in ST-GPR.

Global ensemble weights for gestational age were derived by using all available gestational age and birthweight microdata in Table 12 to select the ensemble weights. The distribution families included in the optimisation process were exponential, gamma, gumbel, Weibull, log-normal, normal, mirrored gamma, and mirrored gumbel. As an advancement starting in GBD 2021, ensemble weights were fit that specifically targeted the fit at 28 weeks and 37 weeks for gestational age and 1500 grams and 2500 grams for low birthweight. In GBD cycles prior to GBD 2021, the fit of these models had been optimised to reduce error across the entire distribution. Additionally, as an improvement starting in GBD 2021, this ensemble weight fitting strategy optimised on all microdata sources simultaneously, as opposed to separately.

For each l/y/s, given the mean and ensemble weights, the variance was optimised to minimise error on the prevalence of preterm birth (<37 weeks) for the gestational age distribution and prevalence of low birthweight (<2500 grams) for the birthweight distribution.

Step B: Model joint birthweight and gestational age distributions at birth, by l/y/s

To model the joint distribution of gestational age and birthweight from separate distributions, information was needed about the correlation between the two distributions. Distributions of gestational age and birthweight are not independent; the Spearman correlation for each country where joint microdata were available (Table 12), pooling across all years of data available, ranged from 0.25 to 0.49. The overall Spearman correlation was 0.38, pooling across all countries in the dataset.

Table 12. Summary of microdata inputs

Location	Years of data	Total births*	Format of data	Spearman correlation	Used in ensemble weight selection	Used in copula parameter selection	Used in relative risk models
BRA	2016	2,854,380	Microdata	0.37	Yes	Yes	No
ECU	2003–2015	2,473,039	Microdata	0.34	Yes	Yes	No
ESP	1990–2014	8,537,220	Microdata	0.42	Yes	Yes	No
JPN	1995–2015	23,644,506	Tabulations	0.41	No	No	Yes
MEX	2008–2012	10,256,117	Microdata	0.35	Yes	Yes	No
NOR	1990–2014	1,489,210	Microdata	0.44	Yes	Yes	Yes
NZL	1990–2016	1,600,501	Microdata	0.25	Yes	Yes	Yes
SGP	1993–2015	972,775	Tabulations	0.41	No	No	Yes
TWN	1998–2002	1,331,760	Tabulations	0.38	No	No	Yes
URY	1996–2014	698,622	Microdata	0.49	Yes	Yes	No
USA	1990–2014	81,929,879	Microdata	0.38	Yes	Yes	Yes

** Pooled across all years and sexes, excluding data missing year of birth, gestational age, or birthweight*

Joint distributions between the birthweight and gestational age marginal distributions were modelled with copulae. The Copula and VineCopula packages in R were used to select the optimal copula family and copula parameters to model the joint distribution, using joint microdata from the country-years in Table 12. The copula family selected from the microdata was "Survival BB8", with theta parameter set to 1.75 and delta parameter set to 1.

The joint distribution of birthweight and gestational age per location-year-sex was modelled using the global copula family and parameters selected and the location-year-sex gestational age and birthweight distributions. The joint distribution was simulated 100 times to capture uncertainty. Each simulation consisted of 10,000 simulated joint birthweight and gestational age datapoints. Each joint distribution was divided into 500-gram by two-week bins to match the categorical bins of the relative risk surface. Birth prevalence was then calculated for each 500-gram by two-week bin.

Step C: Model joint distributions from birth to the end of the neonatal period, by l/y/s

Early neonatal prevalence and late neonatal prevalence were estimated using life table approaches for each 500-gram and two-week bin. Using the all-cause early neonatal mortality rate for each location-year-sex, births per location-year-sex-bin, and the relative risks for each location-year-sex-bin in the early neonatal period, the all-cause early neonatal mortality rate was calculated for each location-year-sex-bin. The early neonatal mortality rate per bin was used to calculate the number of survivors at 7 days of age and prevalence in the early neonatal period. Using the same process, the all-cause late neonatal mortality rate for each location-year-sex was paired with the number of survivors at 7 days and late neonatal relative risks per bin to calculate late neonatal prevalence and survivors at 28 days of age.

Relative risks and theoretical minimum risk exposure level

LBWSG is paired with the outcomes listed in the table below and is only attributed to burden in the early and late neonatal period.

Table 13: Cause list of outcomes for low birthweight and short gestation

Cause name
Diarrhoeal diseases
Lower respiratory infections
Upper respiratory infections
Otitis media
Meningitis
Encephalitis
Neonatal preterm birth complications
Neonatal encephalopathy due to birth asphyxia and trauma
Neonatal sepsis and other neonatal infections
Haemolytic disease and other neonatal jaundice
Other neonatal disorders
Sudden infant death syndrome

Causes

The available data for deriving relative risk was only for all-cause mortality. The exception was the USA linked infant birth-death cohort data, which contained three-digit ICD causes of death, but also had nearly 30% of deaths coded to causes that are ill-defined, or intermediate causes of death, in the GBD cause classification system. We analysed the relative risk of all-cause mortality across all available sources and selected outcomes based on criteria of biological plausibility. Some causes, most notably congenital birth defects, haemoglobinopathies, malaria, and HIV/AIDS, were excluded based on the criteria that reverse causality could not be excluded.

Input data

In the Norway, New Zealand, and USA linked birth/death cohort microdata datasets, livebirths are reported with gestational age, birthweight, and an indicator of death at 7 days and 28 days. For this analysis, gestational age was grouped into two-week categories, and birthweight was grouped into 500-gram categories. The Taiwan, Japan, and Singapore datasets were prepared in tabulations of joint 500-gram and two-week categories. A pooled country analysis of mortality risk in the early neonatal period and late neonatal period by gestational age category in developing countries in Asia and sub-Saharan Africa were also used to inform the relative risk analysis.

Modelling strategy

For each location, data were pooled across years, and the risk of all-cause mortality at the early neonatal period and late neonatal period at joint birthweight and gestational age combinations was calculated. In all datasets except from the USA, sex-specific data were combined to maximise sample size. The USA analyses were sex-specific. To calculate relative risk at each 500-gram and two-week combination, logistic regression was first used to calculate mortality odds for each joint two-week gestational age and

500-gram birthweight category. Mortality odds were smoothed with Gaussian process regression, with the independent distributions of mortality odds by birthweight and mortality odds by gestational age serving as priors in the regression.

A pooled country analysis of mortality risk in the early neonatal period and late neonatal period by gestational age category in developing countries in Asia and sub-Saharan Africa were also converted into 500-gram and two-week bin mortality odds surfaces. The relative risk surfaces produced from microdata and the Asia and Africa surfaces produced from the pooled country analysis were meta-analysed, resulting in a meta-analysed mortality odds surface for each location. The meta-analysed mortality odds surface for each location was smoothed using Gaussian process regression and then converted into mortality risk. To calculate mortality relative risks, the risk of each joint two-week gestational age and 500-gram birthweight category were divided by the risk of mortality in the joint gestational age and birthweight category with the lowest mortality risk.

For each of the country-derived relative risk surfaces, the 500-gram and two-week gestational age joint bin with the lowest risk was identified. This bin differed within each country dataset. To identify the universal 500-gram and two-week gestational age category that would serve as the universal TMREL for our analysis, we chose the bins that were identified to be the TMREL in each country dataset to contribute to the universal TMREL, and these bins were assigned to a relative risk equal to 1.

Population attributable fraction

The total PAF for the low birthweight and short gestation joint risk factor was calculated using the categorical PAF formula, summing the PAF calculated from each 500-gram x two-week category, with the lowest risk category among all the 500-gram x two-week categories serving as the TMREL.

To calculate the PAFs for the univariate risks ("short gestation" and "low birthweight"), relative risks are first weighted by global exposure in the final year in the GBD estimation time series, summed across one of the dimensions (gestational age or birthweight), and then rescaled by the maximum relative risk in the TMREL block (38–42 weeks of gestation and 3500–4500 grams). Any relative risk less than 1 was set to 1. Exposure was also summed across the same dimension, and the univariate PAF equalled the sum of the product of the weighted relative risks and exposures.

Citation

1. Katz J, Lee AC, Kozuki N, Lawn JE, Cousens S, Blencowe H, et al. Mortality risk in preterm and small-for-gestational-age infants in low-income and middle-income countries: a pooled country analysis. *The Lancet*. 2013;382(9890):417–25.

Low bone mineral density

Flowchart

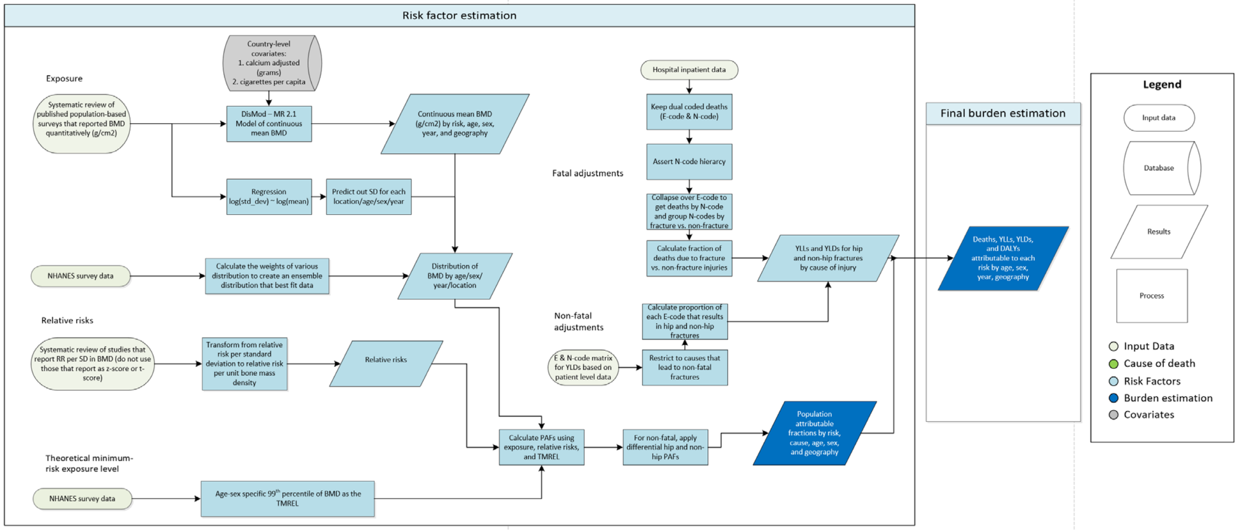

Input data and methodological summary

Definition

Exposure

Bone mineral density (BMD) is a continuous variable measured by dual-X-ray-absorptiometry (DXA) at the femoral neck (FN) and is presented in g/cm^2 after standardising for the brand of densitometer (sBMD). Low BMD is measured in terms of the difference between BMD of a population and the 99th percentile of a reference population at the same age and sex (theoretical minimum risk exposure level, TMREL). The burden attributed to low bone mineral density is estimated for adults 20 years and older.

Input data

Exposure

A systematic review was conducted in GBD 2010 and updated for GBD 2013, 2015, and 2021 using the same search string. It was not updated in 2023. With each successive round of the GBD, new sources suggested by collaborators within the GBD network or identified in the Global Health Data Exchange database by GBD librarians (keywords: bone mineral density or osteoporosis) have been included (GHDX, http://ghdx.healthdata.org/). Inclusion criteria that informed the search are:

- Representative, population-based surveys
- Reporting of quantitative BMD
    - measured by DXA
    - performed at the FN region
    - measured in g/cm^2

Mean BMD was occasionally reported in stratified groups, eg, by fracture status but not for total sample. In these cases, the stratified means were aggregated to obtain a total mean BMD at the population level for an age or sex category.

Table 1. Exposure input data

Input data	Exposure
Source count (total)	168
Number of countries with data	49

Relative risk

To establish the relationship between low BMD and fracture risk, we conducted a systematic review for GBD 2019 to identify studies that reported fracture risk per standard deviation or per unit bone-mass density. This was not updated for 2023. Eligible publication types included peer-reviewed longitudinal cohort studies and meta-analyses published in English that used FNBMD measured by DXA as the exposure variable, and osteoporotic fracture as the outcome of interest. Studies reporting relative risk in an osteoporotic group versus a non-osteoporotic group were excluded. A review of the literature in PubMed from 2010 to 2020 using the following search string yielded 611 results, six of which ultimately met our inclusion criteria:

(((bone mineral density OR bone mineral densities OR bone density) AND

(mean OR average) AND risk) AND fracture).

These six studies were supplemented with six additional studies from a meta-analysis that has been previously used to estimate the relationship between low BMD and fragility fracture.[1] Out of 12 total studies reported in the meta-analysis, six met our inclusion criteria; the remaining six either reported cohorts that used a measure of exposure other than BMD measured at the femoral neck or used mortality as an outcome of interest instead of fracture and were therefore excluded.

Figure 1: PRISMA diagram of BMD RR systematic review from 2019

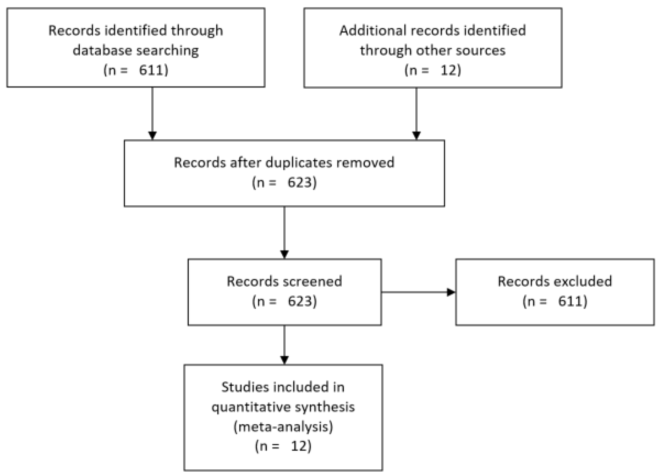

Table 2: Relative risk input data

Input data	Relative risk
Source count (total)	12
Number of countries with data	9

Nine countries were represented among the 12 cohort studies included in the meta-analysis.[2-13]

Modelling strategy

Exposure

We modelled mean BMD in DisMod-MR 2.1 as a single "continuous" parameter model by age and sex, and all GBD locations for years 1990–2019. The model had age mesh points at 0 10 20 25 30 40 50 60 70 80 90 & 100, a time window of ten years for fitting data, and a minimum coefficient of variation of 0.1 for global, 0.06 for super-regions, and 0.08 for the region level. We made no substantive changes in the modelling strategy from GBD 2017.

The country covariates of total physical activity (MET-min/week), tobacco consumption (cigarettes per capita), mean BMI, and unadjusted calcium intake (g) were included in modelling.

Table 3: Covariates included in the BMD DisMod-MR meta-regression model

Covariate	Type	Parameter	Exponentiated beta (95% uncertainty interval)
Total physical activity (MET-min/week), age-standardised	Country-level	Continuous	1.00 (1.00–1.00)
Tobacco consumption (cigarettes per capita)	Country-level	Continuous	0.98 (0.96–0.99)
Mean BMI	Country-level	Continuous	1.01 (1.00–1.01)
Calcium intake (g), unadjusted	Country-level	Continuous	1.00 (1.00–1.01)

Theoretical minimum risk exposure level

The theoretical minimum of risk exposure level or TMREL was chosen as the age-sex specific 99th percentile of BMD from five cycles of NHANES study as the reference population. Below is a descriptive table of the five NHANES cycles used.

Table 4. TMREL summary of NHANES reference population

NHANES cycle	Age range (years)	Number of people tested	BMD range (g/cm^2)
1988	20–90	14,646	0.23–1.84
2005	20–85	3494	0.40–1.50
2007	20–80	4726	0.34–1.46
2009	20–80	5052	0.33–1.63
2013	40–80	3127	0.39–1.36

Figure 2: Plot of 99th percentile of BMD at femoral neck in each cycle of NHANES

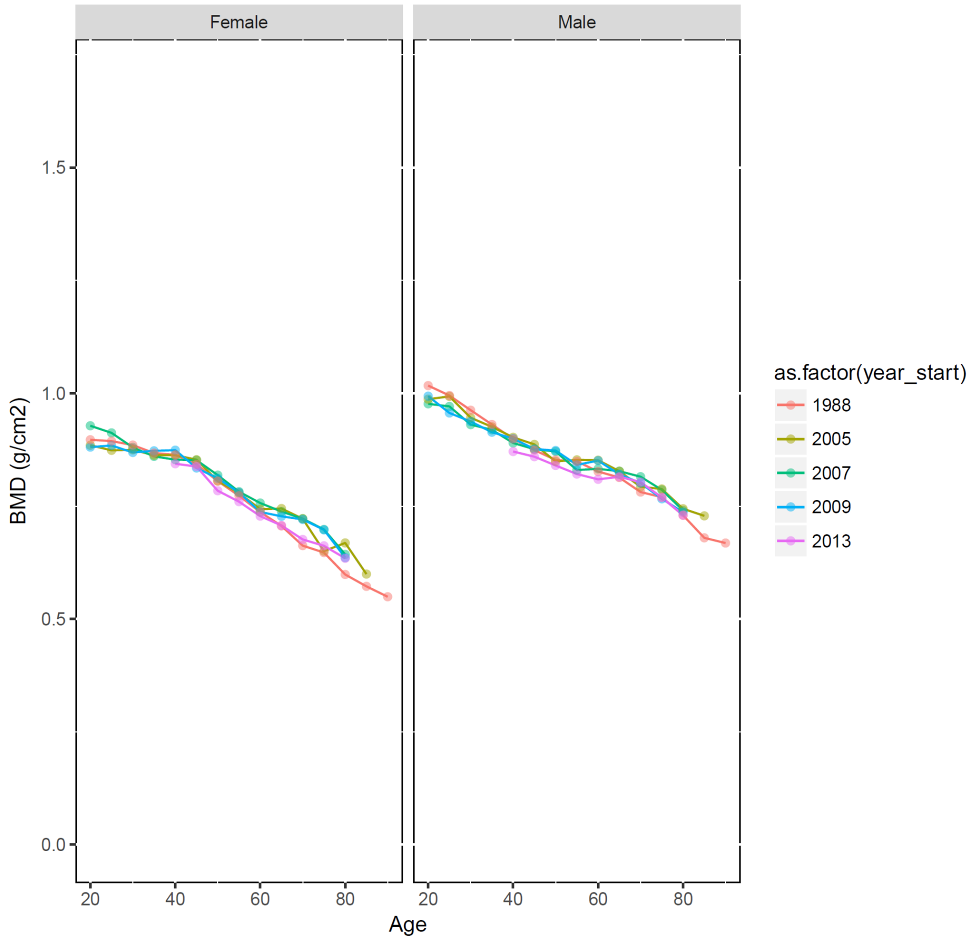

Relative risk

We used study covariates for studies that reported the relative risk of low BMD on hip fracture and non-hip fractures. The mean and standard error for the coefficients were calculated using the MR-BRT crosswalk adjustment method.

We investigated whether adding covariates for the percentage of the cohort that was male (which was always either 1 or 0) or a cubic spline on cohort mean age improved the model fit. An adjustment for percentage male was not included in the final model, as we did not find a significant difference between relative risks for males and females. After testing four iterations with two and three knots placed evenly or by data frequency, it was clear that there was not a reliable relationship between cohort mean age and relative risk. As a result, the age spline was also not included in the final model.

Betas and exponentiated values (which can be interpreted as the relative risks) for the remaining hip and non-hip fracture covariates are shown in the table below:

Table 5. MR-BRT crosswalk results for RR of fracture due to low BMD

Data input	Gamma	Beta coefficient, log (95% CI)	RR per SD unit of BMD
Hip fracture	0.13	0.77 (0.45–1.08)	2.18 (1.66–2.80)
Non-hip fracture		0.57 (0.30–0.84)	1.79 (1.41–2.23)

Attributable burden

The attributable burden of fractures due to low BMD was calculated by comparing the observed distribution of sFNBMD to the counterfactual distribution for each age, sex, year, and cause according to the following formula:

$$PAF_{oasgt} = \frac{\int_{x=l}^{u} RR_o(x)P_{asgt}(x)dx - RR_o(TMREL_{as})}{\int_{x=l}^{u} RR_o(x)P_{asgt}(x)dx}$$

where PAF_{oasgt} represents the population attributable fraction for outcome o (ie, hip or non-hip fracture), age group a, sex s, location g, and year t; RR_o (x) is the relative risk at exposure level x for outcome o with the lowest observed exposure as l and the highest as u; $P_{asgt}(x)$ is the exposure at level x for age group a, sex s, location g, and year t; and $TMREL_{as}$ is the TMREL for age group a and sex s. PAFs were estimated in five-year age groups from 40–44 until 95+.

To obtain the attributable burden of the root causes of fractures due to low BMD, two additional processes are required. Osteoporotic non-hip fractures include fractures of vertebrae, clavicle, scapula, humerus, skull, sternum, face bone, radius or ulna, femur, patella, tibia, fibula, ankle, and pelvis.

First, we calculated the proportion of injury deaths that are due to fractures. We assumed that hip fracture and some non-hip fractures (any fractures apart from those of fingers and toes) are potentially fatal fractures. However, in mortality registration systems, deaths due to injuries are assigned to the cause of injury (eg, falls or road injury) rather than to the nature of injury (eg, fracture or traumatic brain injury). To derive an estimate of the proportion of deaths attributable to the fractures resulting from each of the six categories of injury, inpatient hospital data with detailed diagnostic codes were analysed to estimate the proportion of injury deaths during admission that could be ascribed to fractures. The analysis was restricted to cases that were dual-coded with both the cause of injury ("E-code") and nature of injury ("N-code") that died during the inpatient episode. For those with a code for a fracture, the death was assigned to the fracture in the absence of a more severe injury code that could better explain the death (eg, moderate to severe head trauma, spinal cord lesion, and intra-abdominal or thoracic organ damage). We then collapsed all deaths over E-code to determine the ratio of deaths attributable to fracture versus non-fracture injuries. This ratio was applied to the PAFs to obtain the YLLs from each of the six outcomes attributable to low BMD.

To derive an estimate of the proportion of non-fatal burden attributable to the fractures resulting from each of the six categories of injury, we used the E- to N-code matrix generated from dual-coded (E-code/N-code) patient-level data in the GBD injury analyses to determine the proportion of each E-code that results in a certain N-code. The hip and non-hip fracture population attributable fractions were applied to the appropriate combinations of external cause and fracture estimates of YLD and then summed together to produce a single estimate. Details of the methodology are included below.

Below is the list of injuries for which a PAF was calculated:

- Transport injuries
- Road injuries
- Pedestrian road injuries
- Cyclist road injuries
- Motorcyclist road injuries
- Motor vehicle road injuries
- Other road injuries
- Other transport injuries
- Unintentional injuries
- Falls
- Exposure to mechanical forces
- Other exposure to mechanical forces
- Non-venomous animal contact
- Interpersonal violence
- Assault by other means

References

[1] Johnell O, Kanis JA, Oden A, Johansson H, De Laet C, Delmas P, Eisman JA, Fujiwara S, Kroger H, Mellstrom D, Meunier PJ, Melton LJ, 3rd, O'Neill T, Pols H, Reeve J, Silman A, Tenenhouse A (2005) Predictive value of BMD for hip and other fractures. *J Bone Miner Res* 20 (7):1185-1194

[2] Berger C, Langsetmo L, Joseph L, Hanley DA, Davison KS, Josse RG, Prior JC, Kreiger N, Tenenhouse A, Goltzman D, CaMos Research Group. Association between change in BMD and fragility fracture in women and men. *J Bone Miner Res*. 2009; 24(2): 361-70.

[3]Bow CH, Tsang SW, Loong CH, Soong CS, Yeung SC, Kung AW. Bone mineral density enhances use of clinical risk factors in predicting ten-year risk of osteoporotic fractures in Chinese men: the Hong Kong Osteoporosis Study. *Osteoporosis Int*. 2011; 22(11): 2799-807.

[4] Chalhoub D, Orwoll ES, Cawthon PM, Ensrud KE, Boudreau R, Greenspan S, Newman AB, Zmuda J, Bauer D, Cummings S, Cauley JA, Osteoporotic Fractures in Men (MrOS) Study Research Group. Areal and volumetric bone mineral density and risk of multiple types of fracture in older men. *Bone*. 2016; 92: 100-106.

[5] Crandall CJ, Hovey KM, Andrews CA, Cauley JA, Manson JE, Wactawski-Wende J, Wright NC, Li W, Beavers K, Curtis JR, LeBoff MS. Bone mineral density as a predictor of subsequent wrist fractures: Findings from the Women's Health Initiative Study. *J Clin Endocrinal Metab*. 2015; 100(11): 4315-24.

[6] Dargent-Molina P, Favier F, Grandjean H, Baudoin C, Schott AM, Hausherr E, Meunier PJ, Bréart G. Fall-related factors and risk of hip fracture: the EPIDOS prospective study. *Lancet*. 1996; 348(9021): 145-9.

[7] Fujiwara S, Kasagi F, Masunari N, Naito K, Suzuki G, Fukunaga M. Fracture Prediction From Bone Mineral Density in Japanese Men and Women. *J Bone Miner Res*. 2003; 18(8): 1547-53.

[8] Huopio J, Kröger H, Honkanen R, Saarikoski S, Alhava E. Risk factors for perimenopausal fractures: a prospective study. *Osteoporos Int*. 2000; 11(3): 219-27.

[9] Kwok AW, Gong JS, Wang YX, Leung JC, Kwok T, Griffith JF, Leung PC. Prevalence and risk factors of radiographic vertebral fractures in elderly Chinese men and women: Results of Ms. OS (Hong Kong) and Ms. OS (Hong Kong) studies. *Osteoporosis Int*. 2013; 23(3); 877-85.

[10] Melton LJ, Crowson CS, O'Fallon WM, Wahner HW, Riggs BL. Relative contributions of bone density, bone turnover, and clinical risk factors to long-term fracture prediction. *J Bone Miner Res*. 2003; 18(2): 312–8.

[11] Nguyen TV, Eisman JA, Kelly PJ, Sambrook PN. Risk factors for osteoporotic fractures in elderly men. *Am J Epidemiol*. 1996; 144(3): 255–63.

[12] Sheu Y, Cauley JA, Patrick AL, Wheeler VW, Bunker CH, Zmuda JM. Risk factors for fracture in middle-age and older-age men of African descent. *J Bone Miner Res*. 2014; 29(1): 234-41.

[13] Shin CS, Kim MJ, Shim SM, Kim JT, Yu SH, Koo BK, Cho HY, Choi HJ, Cho SW, Kim SW, Kim SY, Yang SO, Cho NH. The prevalence and risk factors of vertebral fractures in Korea. *J Bone Miner Metab*. 2012; 30(2): 183-192

Low physical activity

Flowchart

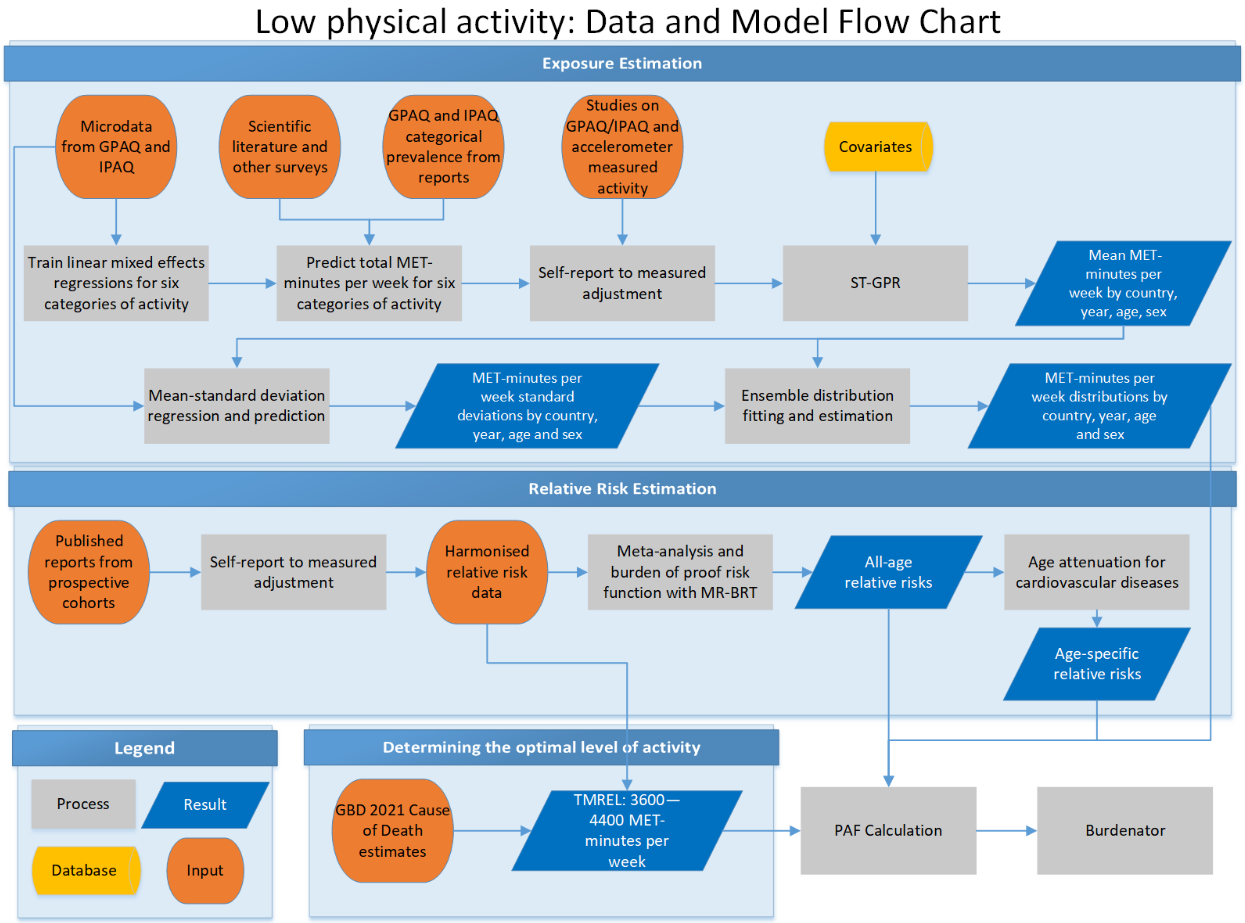

Input data and methodological summary

Definition

Exposure

Low physical activity is defined as objectively measured, total physical activity less than 3600 to 4400 MET-minutes per week. We assess physical activity performed by adults older than 25 years of age, for duration of at least ten minutes at a time, across all domains of life (leisure/recreation, work, household and transport). We use frequency, duration, and intensity of activity to calculate total metabolic equivalent (MET)-minutes per week. MET is the ratio of the working metabolic rate to the resting metabolic rate. One MET is equivalent to 1 kcal/kg/hour and is equal to the energy cost of sitting quietly. A MET is also defined as the oxygen uptake in ml/kg/min with one MET equal to the oxygen cost of sitting quietly, around 3.5 ml/kg/min.

Input data

Exposure

We included surveys of the general adult population that captured self-reported physical activity in all domains of life (leisure/recreation, work/household, and transport), where random sampling was used.

Data were primarily derived from two standardised questionnaires: The Global Physical Activity Questionnaire (GPAQ)[1] and the International Physical Activity Questionnaire (IPAQ),[2] although we included other survey instruments that asked about intensity, frequency, and duration of physical activity performed across all activity domains.

Due to a lack of a consistent relationship on the individual level between activity performed in each domain and total activity, we were not able to use studies that only assessed recreational/leisure activity.

Physical activity level is categorised by total MET-minutes per week using four categories based on rounded values closest to the quartiles of the global distribution of total MET-minutes/week. The lower limit for the Level 1 category (600 MET-min/week) is the recommended minimum amount of physical activity to get any health benefit. We used four categories with higher thresholds rather than the GPAQ and IPAQ recommended three categories to better capture any additional protective effects from higher activity levels.

- Level 0: <600 MET-min/week (inactive)
- Level 1: 600–3999 MET-min/week (low-active)
- Level 2: 4000–7999 MET-min/week (moderately active)
- Level 3: ≥8000 MET-min/week (highly active)

The GHDx was used to locate all surveys that use the GPAQ or IPAQ questionnaire. Although there were many other surveys that focused specifically on leisure activity, we were unable to use these sources because they did not include all three domains (work, transport, and leisure). In addition, we excluded any surveys that did not report frequency, duration, and intensity of activity.

Relative risk

In GBD 2023, we did not conduct an updated systematic review to identify new relative risk data sources. An updated systematic review was conducted in GBD 2021 for studies published before December 31, 2019, evaluating the relationship between physical activity and risk of breast cancer, colon and rectum cancer, diabetes, ischaemic heart disease, and ischaemic stroke. We searched for studies in PubMed using the search strings reported in table 1. We included prospective cohort studies that assessed total physical activity or leisure-time physical activity as the exposure variable and at least one of the five diseases as an outcome. Further, we only included studies that reported risk estimates (relative risk, hazard ratio, or odds ratio) with confidence intervals, standard errors, or enough information to quantify uncertainty. In addition, we only included studies that reported the frequency and duration of activity achieved, excluding studies that reported physical activity using categorical or custom component scores. In future rounds of the GBD, we aim to incorporate new evidence as it becomes available. In addition, we will evaluate the evidence of low physical activity and risk for additional disease endpoints and add these risk–outcome pairs if general GBD inclusion criteria are met.

Table 1: Search strings used to search PubMed database

Outcome	String
Breast cancer	physical activity[Title/Abstract] AND breast cancer [Title/Abstract] AND "humans"[MeSH Terms] AND English[lang] AND ("2014/10/01"[PDAT] : "2019/12/31" [PDAT])
Colon and rectum cancer	physical activity[Title/Abstract] AND colon cancer [Title/Abstract] AND "humans"[MeSH Terms] AND English[lang] AND ("2014/10/01"[PDAT] : "2019/12/31" [PDAT])
Type 2 diabetes	physical activity[Title/Abstract] AND type 2 diabetes[Title/Abstract] AND "humans"[MeSH Terms] AND English[lang] AND ("2014/10/01"[PDAT] : "2019/12/31" [PDAT])
	physical activity[Title/Abstract] AND noninsulin dependent diabetes mellitus [Title/Abstract] AND "humans"[MeSH Terms] AND English[lang] AND ("2014/10/01"[PDAT] : "2019/12/31" [PDAT])
	physical activity[Title/Abstract] AND niddm[Title/Abstract] AND "humans"[MeSH Terms] AND English[lang] AND ("2014/10/01"[PDAT] : "2019/12/31" [PDAT])
Ischaemic heart disease	physical activity[Title/Abstract] AND ischemic heart disease [Title/Abstract] AND "humans"[MeSH Terms] AND English[lang] AND ("2014/10/01"[PDAT] : "2019/12/31" [PDAT])
	physical activity[Title/Abstract] AND ischaemic heart disease [Title/Abstract] AND "humans"[MeSH Terms] AND English[lang] AND ("2014/10/01"[PDAT] : "2019/12/31" [PDAT])
	physical activity[Title/Abstract] AND coronary heart disease [Title/Abstract] AND "humans"[MeSH Terms] AND English[lang] AND ("2014/10/01"[PDAT] : "2019/12/31" [PDAT])
Ischaemic stroke	physical activity[Title/Abstract] AND ischemic stroke [Title/Abstract] AND "humans"[MeSH Terms] AND English[lang] AND ("2014/10/01"[PDAT] : "2019/12/31" [PDAT])
	physical activity[Title/Abstract] AND ischaemic stroke[Title/Abstract] AND "humans"[MeSH Terms] AND English[lang] AND ("2014/10/01"[PDAT] : "2019/12/31" [PDAT])

Data processing

Exposure

Mixed effects modelling

For this round of the GBD, we used six separate linear mixed effects regressions to capture the relationship of reported activity in total MET-minutes per week with the prevalence of the six activity categories (Table 2). We fit the models using tabulated individual level IPAQ and GPAQ data. The general form of the equation was as follows:

$$\ln(MET_i) = \beta_0 + \beta_1 cat_i + \beta_2 age_i + \beta_3 fem_i + \beta_4 IPAQ_i + \beta_5 IPAQ_i * cat_i + \alpha_s + \alpha_r + \alpha_c$$

where MET_i was the reported MET-minutes/week achieved; cat_i was the categorical prevalence of activity (ie, inactive, low/moderately/highly active, low active, moderately/highly active, moderately active, or highly active); age_i was the midpoint of the tabulated age group; fem_i was an indicator for whether the data were for females; $IPAQ_i$ was an indicator for whether the data were from an IPAQ survey; and $\alpha_s, \alpha_r, \alpha_c$ were nested random intercepts at the super-region, region, and country level, respectively. Once these coefficients were estimated, we applied the relationships to the rest of our categorical prevalence data from GPAQ and IPAQ to determine the corresponding levels of MET-minutes per week.

Table 2: Definitions of categorical prevalence of physical activity exposure data

Category	MET-min/week
inactive	<600
low/moderately/highly active	≥600
low active	600–3999
moderately/highly active	>4000
moderately active	4000–7999
highly active	≥8000

Self-report adjustment

Our input data of MET-minutes per week were collected through self-reported questionnaires like GPAQ and IPAQ. Self-reported activity can lead to biased responses and result in exposure measurement error compared to other direct measurement tools (eg, accelerometer and doubly labeled water).[3] To best account for the measurement error, we relied on existing systematic reviews to identify studies that assessed the relationship between self-reported physical activity using GPAQ or IPAQ and accelerometer-measured physical activity.[4,5,6] We extracted 15 studies usable for analysis, collecting information on study name, location, age, sex, type of activity, self-report instrument, self-reported values of activity, accelerometer instrument, and accelerometer-measured values of activity. We used MR-BRT to model the log difference between activity reported through GPAQ or IPAQ questionnaires to activity measured with accelerometers. The results of our crosswalk adjustment are summarised in Table 3. The adjustment factor was then applied to the input data of self-reported total MET-minutes per week to approximate accelerometer-adjusted total MET-minutes per week.

Table 3: MR-BRT crosswalk adjustment factors for low physical activity

Data input	Reference or alternative case definition	Gamma	Beta coefficient, log (95% UI)*	Adjustment factor **
Accelerometer measured	Ref	0.707	---	---
Self-reported with GPAQ or IPAQ	Alt		0.730 (–0.721, 2.181)	2.075

**MR-BRT crosswalk adjustments can be interpreted as the factor the alternative case definition is adjusted by to reflect what it would have been had it been measured using the reference case definition. If the log/logit beta coefficient is negative, then the alternative is adjusted up to the reference. If the log/logit beta coefficient is positive, then the alternative is adjusted down to the reference.*

***The adjustment factor column is the exponentiated beta coefficient. For log beta coefficients, this is the relative rate between the two case definitions. For logit beta coefficients, this is the relative odds between the two case definitions.*

Modelling strategy

Exposure

Accelerometer-adjusted total MET-minutes per week data were then used as inputs into our spatiotemporal Gaussian process regression (ST-GPR) model to generate complete estimates of MET-minutes per week for every country, age group, and sex from 1990 to 2023. Further details on ST-GPR are available in the general methods appendix.

The linear model, which when added to the smoothed residuals forms the mean prior for GPR, is as follows:

$$\log(\mathrm{MET})_{c,a,t} = \beta_0 + \beta_1 \mathrm{agriculture}_{c,t} + \beta_2 \mathrm{vehicles}_{c,t} + \alpha_s + \alpha_r + \alpha_c$$

where agriculture is the proportion of the population working in agriculture and vehicles is the number of four-wheeled vehicles per capita, and α_s, α_r, and α_c are super-region, region, and country nested random intercepts, respectively. Random effects were used in model fitting but were not used in prediction.

Utilising microdata on total MET-minutes/week from individual-level surveys, we characterised the distribution of activity level at the population level. We then used an ensemble approach for distribution fitting, borrowing characteristics from individual distributions to tailor a unique distribution to fit the data using a weighting scheme. We characterised the standard deviation of each population's activity through a linear regression that captured the relationship between standard deviation and mean activity levels in nationally representative IPAQ surveys:

$$ln\,(Standard\ deviation) = \beta_0 + \beta_1 \times ln\,(Mean_i) + \beta_2 \times Age_i + \beta_3 \times SR_i + \beta_4 \times Fem_i$$

Age_i is the youngest age in population *i*'s age group, SR_i is the super-region in which the population lives, and Fem_i is a Boolean value depicting whether the population is female. We then applied the coefficients of this regression to the outputs of our estimate of total MET-minutes per week regression outputs to calculate the standard deviation by country, year, age, and sex.

Relative risk

In GBD 2023, we did not conduct an updated systematic review to identify new relative risk data sources. In GBD 2021, we conducted an updated systematic review and evaluated the relationship between physical activity and risk of the five disease endpoints. We used MR-BRT to estimate the non-linear dose–response relationships between low physical activity and risk for breast cancer, colon cancer, diabetes, ischaemic heart disease, and ischaemic stroke. Specifically, we used the evidence score framework to systematically determine the risk function and evaluate the strength of evidence for each risk–outcome pair. Further details on the evidence score framework are available in the general methods of the Appendix.

The non-linear inverse relationships between physical activity and risk for diseases has been well-defined.[7] We used the MR-BRT tool to estimate the log relative risk associated with each level of total physical activity on a continuous scale. Outcome-specific model characteristics are described in Table 4.

For each risk–outcome pair meta-regression, we considered study-level covariates that could potentially bias the study's reported effect size estimates. These study-level covariates included indication of whether the study used a washout period, whether the study determined outcomes based on administrative records or self-reports, the level of adjustment for relevant confounders like age, sex, smoking, education, and income, and whether the study adjusted for body-mass index (BMI) or another indicator for adiposity. We adjusted for these covariates in our meta-regression if they significantly biased our estimated relative risk function.

When available, we extracted and analysed effect sizes that controlled for BMI or another indicator for adiposity. We evaluated adjustment for BMI as a study-level variable in our covariate selection process described above. We found that this covariate was only significant in our physical activity–breast cancer model, suggesting that our predicted risk functions reflected the protective health effects of total physical activity independent of BMI.

We implemented the Fisher scoring correction to the heterogeneity parameter, which corrects for data-sparse situations. In such cases, the between-study heterogeneity parameter estimate may be zero, simply from lack of data. The Fisher scoring correction uses a quantile of gamma, which is sensitive to the number of studies, study design, and reported uncertainty.

We also added methodology to detect and flag publication bias. The approach is based on the classic Egger's regression strategy, which is applied to the residuals in our model. In the current implementation, we do not correct for publication bias but flag the risk–outcome pairs where the risk for publication bias is significant. We found no evidence of publication bias for the outcomes associated with physical activity.

There is a well-documented attenuation of the risk for cardiovascular disease due to metabolic risks factors throughout one's life.[8] To incorporate this age trend in the relative risks, we first identified the

median age-at-event across all cohorts and considered that as the reference age group. We then assigned our risk curves to this reference age group. Then, we derived attenuation factors for each metabolic mediator (ie, systolic blood pressure and total cholesterol) by taking the ratio of excess risk between each age group and the reference. Finally, we applied 1000 draws of the age-specific attenuation factors averaged across the two metabolic mediators to 1000 draws of the reference age group's risk curve to determine age-specific risk curves that propagated the uncertainty of both the risk function and age pattern. In an update for GBD 2023, age-attenuated risk curves were calculated using age mean relative risk and the associated percentage change:

$$\frac{1}{\left(pct_{s,ag} \cdot \left(\frac{1}{rr_{s,ag}} - 1\right) + 1\right)}$$

Where pct is the age-specific attenuation factor and rr is the relative risk for sex s and age group ag.

Table 4: Model characteristics for outcomes related to high body-mass index in adults

Outcome	Non-linear specifications and constraints	Selected covariates	Mean gamma solution	Publication bias
Breast cancer	*	Adjustment for BMI	0.000	No
Colon and rectum cancer	*		0.000	No
Diabetes mellitus type 2	**		0.105	No
Ischaemic heart disease	**		0.079	No
Ischaemic stroke	*		0.015	No

** Quadratic splines with 4 knots; right linear tail; monotonically decreasing constraint; Gaussian prior (0, 0.01) on max derivative of non-linear intervals*

*** Quadratic splines with 5 knots; right linear tail; monotonically decreasing constraint; Gaussian prior (0, 0.01) on max derivative of non-linear intervals*

Theoretical minimum risk exposure level

The theoretical minimum risk exposure level for physical inactivity is 3600–4400 MET-minutes per week. We calculated this range by taking the weighted average of the 85th percentile of activity achieved across all five risk–outcome pairs. We first determined the 85th percentile of activity for a given study by taking the 85th percentiles of the lower bound and midpoints of the alternative comparison groups. For studies where the reference group was the highest level of activity, we instead took the 85th percentile of the lower bound and midpoint of the reference exposure range. Next, we took the simple average of the 85th percentile ranges across all the studies for a given risk–outcome pair to derive five cause-specific ranges. Finally, using the GBD 2021 Causes of Death analysis, we used the number of global deaths in the year 2021 for adults aged 25+ as weights to estimate our weighted average of the minimum risk level.

Citations

[1] World Health Organization. Global Physical Activity Questionnaire (GPAQ) Analysis Guide. 2011. Geneva, Switzerland: WHO Google Scholar. 2013.

[2] IPAQ Research Committee. Guidelines for data processing and analysis of the International Physical Activity Questionnaire (IPAQ)–short and long forms. Retrieved September. 2005;17:2008.

[3] Shephard RJ. Limits to the measurement of habitual physical activity by questionnaires. *Br J Sports Med* 2003; 37(3): 197–206.

[4] Prince SA, Adamo KB, Hamel ME, Hardt J, Connor Gorber S, Tremblay M. A comparison of direct versus self-report measures for assessing physical activity in adults: a systematic review. *Int J Behav Nutr Phys Act* 2008; 5—56.

[5] Skender S, Ose J, Chang-Claude J, et al. Accelerometry and physical activity questionnaires - a systematic review. *BMC Public Health* 2016; 16—515.

[6] Sember V, Meh K, Sorić M, Starc G, Rocha P, Jurak G. Validity and Reliability of International Physical Activity Questionnaires for Adults across EU Countries: Systematic Review and Meta Analysis. *Int J Environ Res Public Health* 2020; 17(19): 7161.

[7] Kyu HH, Bachman VF, Alexander LT, et al. Physical activity and risk of breast cancer, colon cancer, diabetes, ischemic heart disease, and ischemic stroke events: systematic review and dose-response meta-analysis for the Global Burden of Disease Study 2013. *BMJ* 2016; 354:i3857.

[8] Singh GM, Danaei G, Farzadfar F, et al. The age-specific quantitative effects of metabolic risk factors on cardiovascular diseases and diabetes: a pooled analysis. *PLoS One* 2013; 8(7): e65174.

No access to handwashing facility

Flowchart

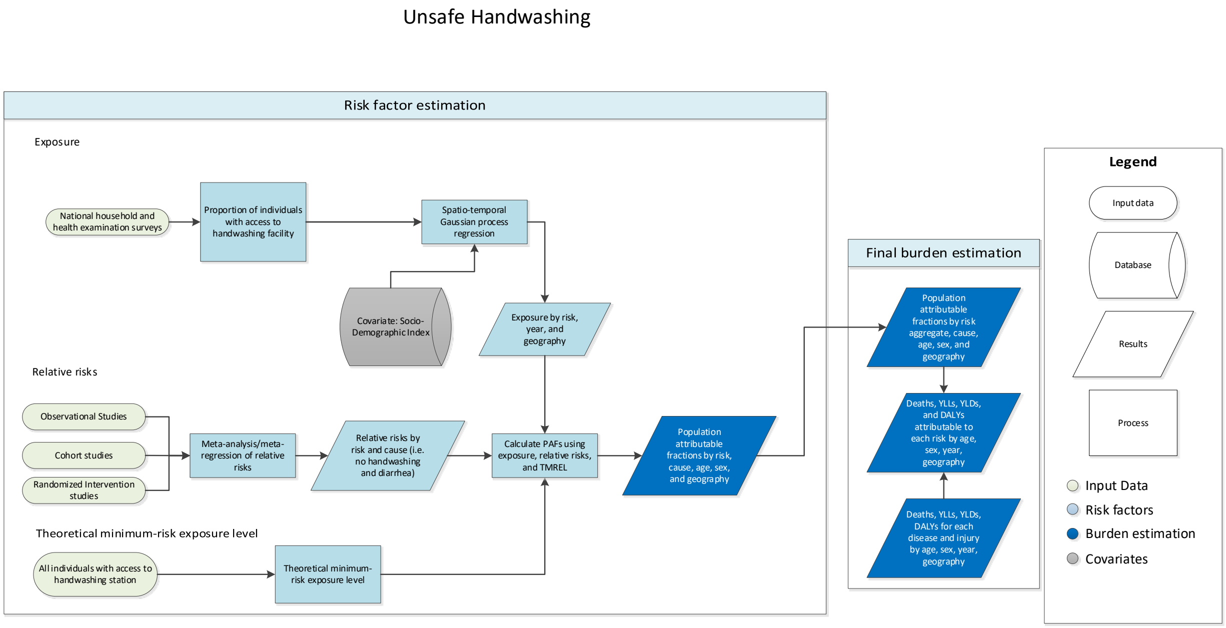

Input data and methodological summary

Definition

Exposure

This risk is defined as the proportion of the population without access to a handwashing facility with soap (bar, liquid, or powder/detergent), water, and wash station (either permanent or mobile).[1] If any criteria are not met, then the individual is counted as not having access.

Input data

Exposure

Input data came primarily from geographically representative household surveys, including the Demographic and Health Surveys (DHS), Multiple Indicator Cluster Surveys (MICS), Performance Monitoring and Accountability 2020 surveys (PMA2020), Performance Monitoring for Action 2021–2022 surveys (PMA2022), along with national surveys in India, the Philippines, and the USA.

Relative risk

Unsafe hygiene is paired with two outcomes: diarrhoeal diseases and lower respiratory infections (LRI). A meta-analysis by Cairncross, et al. 2010 provided relative risk values describing the relationship between lack of facility access and diarrhoeal diseases.[2] A meta-analysis by Rabie & Curtis 2006 provided relative risk evidence for the relationship between lack of facility access and LRI, including the years 1997–2004.[3]

Additionally, a literature review on the relationship between hygiene and LRI was conducted in GBD 2021, which added 15 new studies (Figure 1). A meta-analysis by Jefferson, et al. 2011 was included in the literature review, covering the years 2004–2010. [4] 35 studies were identified from Jefferson, et al 2011 that matched our criteria, of which nine were already extracted in previous rounds.[4] After initial title/abstract screening and full-text review, eight new studies were added. We then searched PubMed for updates to relevant literature published from January 1, 2010, to August 20, 2020, using the search string below:

("Hand Hygiene"[MESH] OR "handwashing"[TiAb] OR "hand hygiene"[TiAb] OR "hand cleansing"[TiAb] OR "hand cleaning"[TiAb]) AND ("lower respiratory tract infection"[TiAb] OR "LTRI"[TiAb] OR "lower respiratory infection"[TiAb] OR "acute respiratory infection" [TiAb] OR "sinusitis"[TiAb] OR "common cold"[TiAb] OR "otitis media"[TiAb] OR "pharyngitis"[TiAb] OR "influenza"[TiAb] OR "coryza"[TiAb] OR "laryngitis"[TiAb] OR "epiglottis"[TiAb] OR "croup"[TiAb] OR "pneumonia"[TiAb] OR "bronchitis"[TiAb] OR "bronchiolitis"[TiAb] OR "pertussis"[TiAb] OR "whooping cough"[TiAb] OR "pneumococcal pneumonia "[TiAb] OR "influenza"[TiAb] OR "respiratory syncytial virus"[TiAb] OR "h influenzae type b"[TiAb] OR "Respiratory Tract Infections"[MESH] OR "Otitis Media"[MESH] OR "Respiratory Syncytial Viruses"[MESH] OR "Croup"[MESH] OR "Haemophilus influenzae type b"[MESH]) AND (2010[PDAT]:3000[PDAT]) NOT(animals[MESH] NOT humans[MESH])

For diarrhoeal diseases, we have a total of 22 studies resulting in 46 relative risk values. For LRI, we have a total of 23 studies resulting in 35 relative risk values.

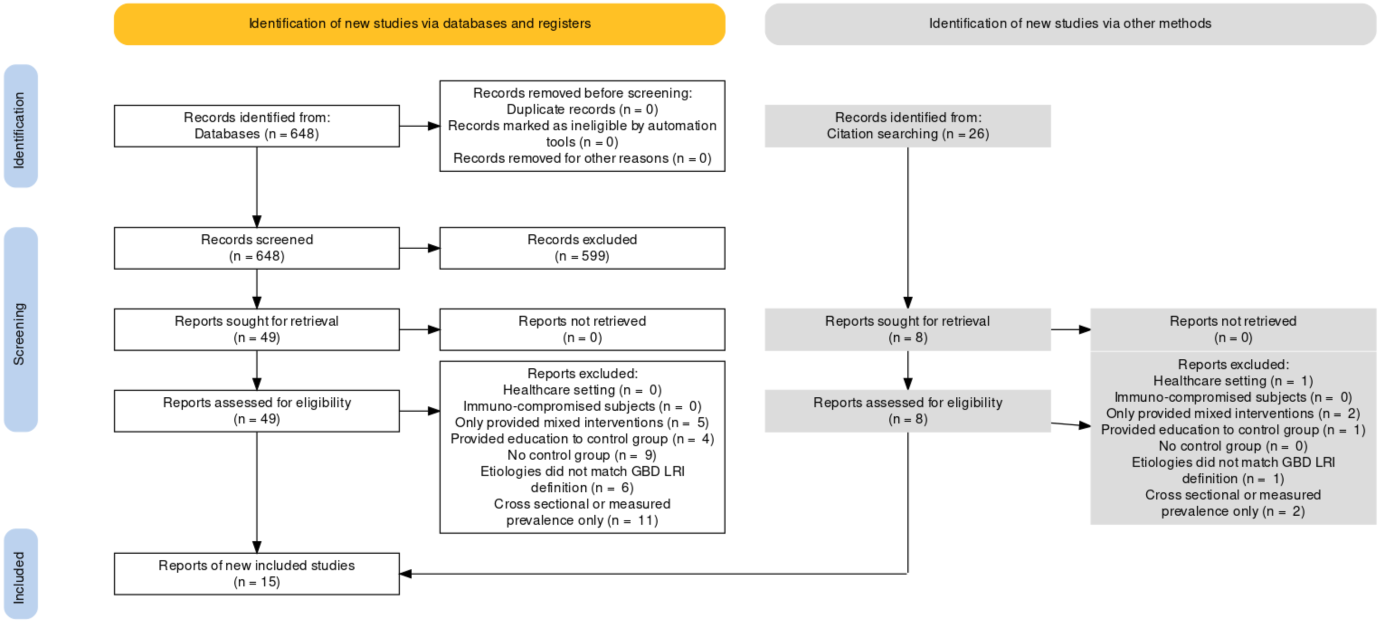

Figure 1: PRISMA diagram for systematic review of hygiene and LRI

Modelling strategy

Exposure

We modelled exposure to this risk using a three-step modelling scheme of mixed effect linear regression followed by spatiotemporal Gaussian process regression (ST-GPR), which outputs full time-series estimates for each GBD 2023 location, for the years 1990-2023. The ST-GPR modelling methodology is detailed in the appendix specific to this analytical technique. Two covariates were used as fixed effects in the linear regression: Socio-demographic Index (SDI), which is a composite measure of development that includes income per capita, education, and fertility, and proportion of individuals with access to piped water. Random effects were set at GBD 2023 region and super-region levels to fit the model but were not used in the predictions. The linear regression equation used in the ST-GPR model is shown below.

$$\text{logit}(data) \sim SDI + piped\ water\ access + (1|level_1|) + (1|level_2|)$$

SDI = Socio-demographic Index

Piped water access = proportion of individuals with access to piped water

(1|$level_1$) = super-region-level random effects

(1|$level_2$) = region-level random effects

Theoretical minimum risk exposure level

The theoretical minimum risk exposure level for unsafe hygiene is defined as having access to a handwashing facility with soap (bar, liquid, or powder/detergent), water, and wash station (either permanent or mobile).

Relative risk

Estimating the relative risk (RR) of diarrhoeal diseases and LRI occurring as a function of exposure to no access to handwashing facilities followed the burden of proof (BoP) approach established by Zheng and colleagues and implemented with the meta-regression—Bayesian, regularised, trimmed (MR-BRT) tool.[5, 6] MR-BRT synthesises input data to generate an RR curve by relying on an ensemble spline method to capture the potentially non-linear shape of the risk–outcome relationship; integrating over varying exposure ranges in different comparison groups; trimming potentially distorting outliers; testing, selecting, and adjusting for bias covariates to account for known heterogeneity in input study-design characteristics (eg, confounding, selection bias, exposure measurement); and quantifying remaining between-study heterogeneity (gamma) through random effects modelling and incorporating this value into uncertainty around the mean RR curve.

MR-BRT further evaluates evidence for small-study effects and generates funnel plots that represent potential risk of publication or reporting bias, and also generates the burden of proof risk function (BPRF), defined for harmful risks as the 5th and for protective risks as the 95th quantile risk curve – inclusive of between-study heterogeneity/gamma – that is closest to null. The BPRF is transformed into a risk–outcome score (ROS: the signed value of the average log BPRF between the 15th and 85th percentiles of risk exposure levels observed across included studies) and mapped onto a star-rating system from one to five stars. These metrics complement RR estimates by providing an alternative, conservative measure of effect size and evidence strength that formally and systematically incorporates divergence/convergence across input findings, with higher positive ROS values and more stars corresponding to incrementally larger effects and stronger evidence for the risk–outcome relationship.

For both the diarrhoeal diseases and LRI models, two study-level covariates were included – whether the study was randomised and whether the percentage of the study population lost to follow-up was greater than 15%. No priors were used.

Figures 2 and 3 show the funnel plots for each MR-BRT analysis, along with the associated "risk–outcome scores," which measure how good the evidence is for that relative risk estimate. The funnel plots (Figures 2-3) below show the protective effects of having access to a handwashing station. Prior to calculating PAFs, we took the inverse of the relative risk to get the relative risk for no access to a handwashing station (Table 1). Before generating a risk–outcome score, we conducted an additional post-analysis step to detect and flag publication bias in the input data. This approach is based on the classic Egger's regression strategy, which is applied to the residuals in our model. In the current implementation, we do not correct for publication bias but flag the risk–outcome pairs where the risk for publication bias is significant.

Table 1: Relative risks (reference: access to handwashing facility)

Outcome	Relative risk (95% CI)
Diarrhoeal diseases	1.49 (1.05–2.12)
Lower respiratory infections	1.37 (0.82–2.3)

For hygiene and diarrhoea, we did not detect evidence of publication bias based on the association between observation residuals and their standard errors (p-value = 0.0265, Egger mean = –0.302, Egger SD = 0.156). The risk–outcome score was 0.054, which is a star rating of two.

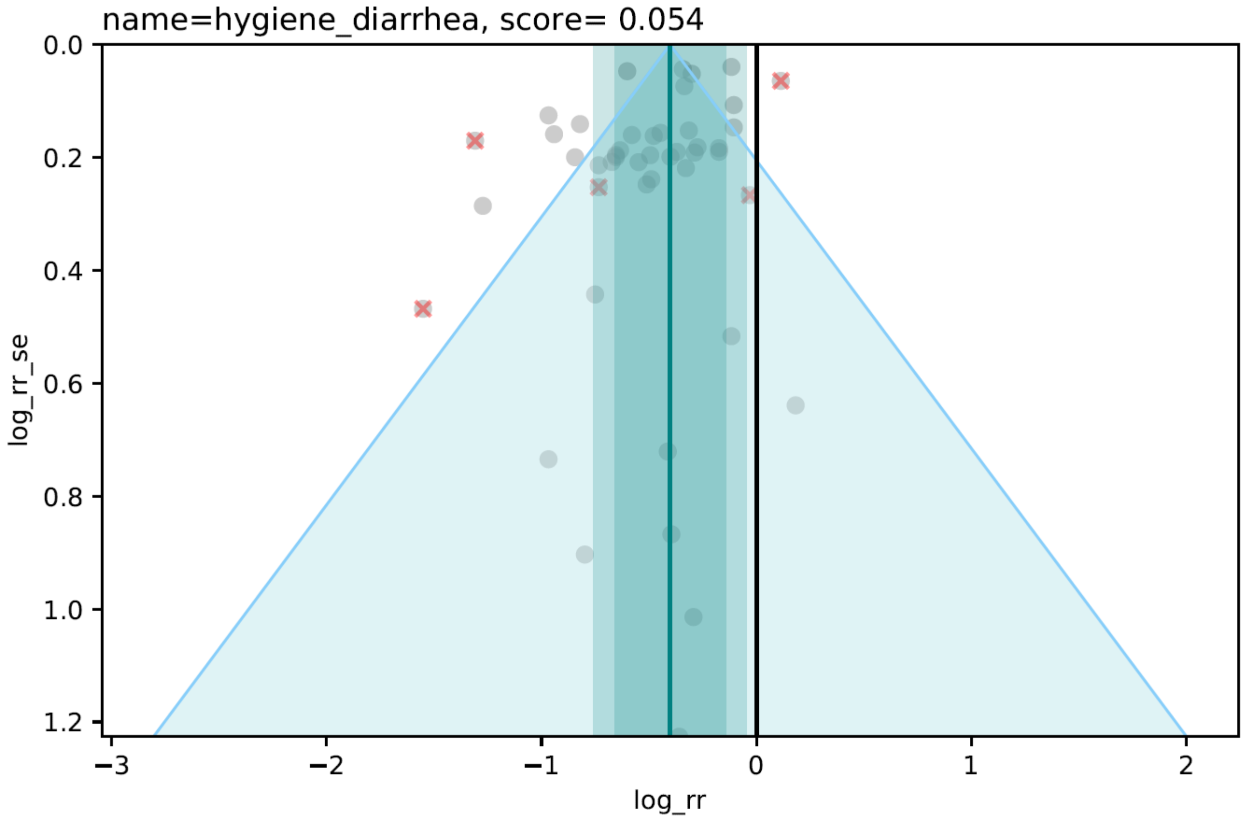

Figure 2: Hygiene–diarrhoea funnel plot and risk–outcome score. The final log RR (thick green line), 95% UIs with between-study heterogeneity (dark green band) and without between-study heterogeneity (light green band), log RRs from included studies, and the funnel. Outliered points are marked with a red x. Prior to PAFs, we take the inverse of the final log RR to get the RR for no access to a handwashing station.

For hygiene and LRI, we did not detect publication bias (p-value = 0.379, Egger mean = –0.0570, Egger SD = 0.186). The risk-outcome score was –0.060, which is a star rating of one.

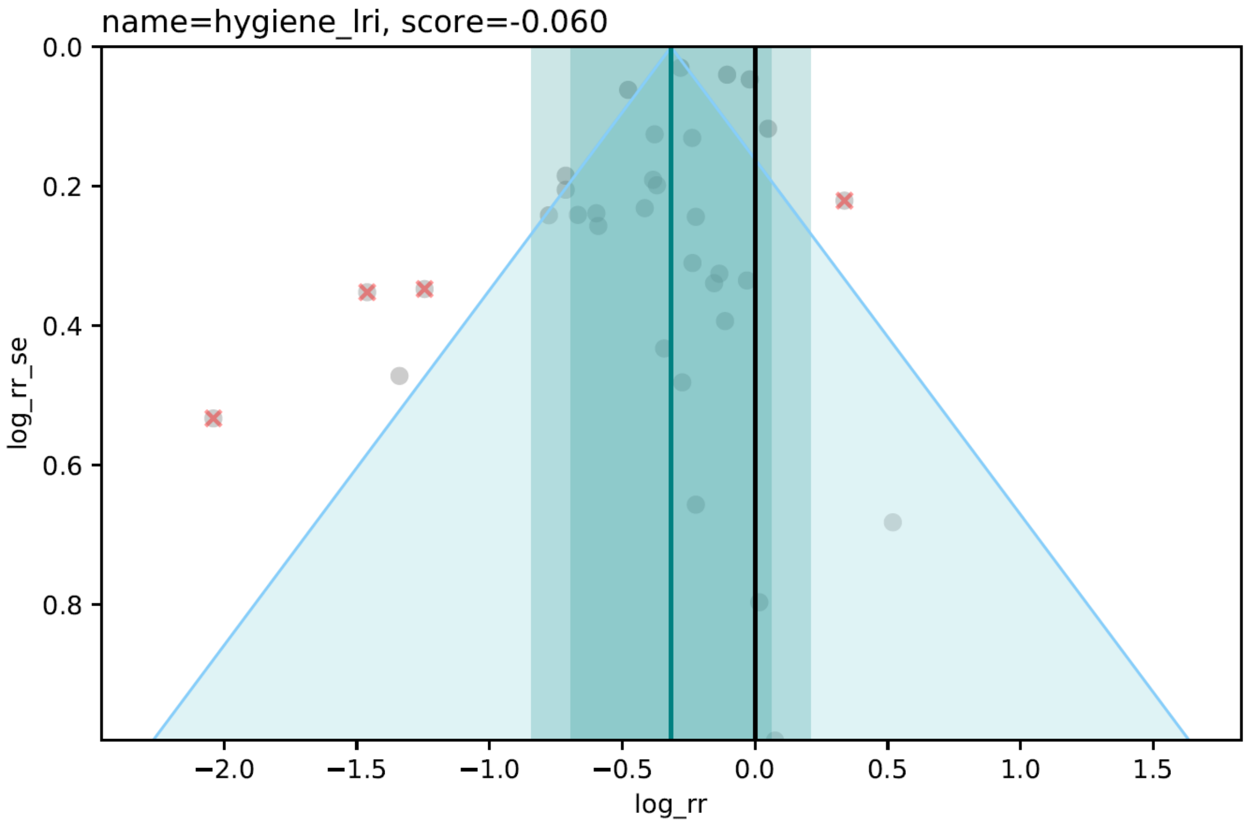

Figure 3: Hygiene–LRI funnel plot and risk–outcome score. The final log RR (thick green line), 95% UIs with between-study heterogeneity (dark green band) and without between-study heterogeneity (light green band), log RRs from included studies, and the funnel. Outliered points are marked with a red x. Prior to PAFs, we take the inverse of the final log RR to get the RR for no access to a handwashing station.

References

1. WHO/UNICEF. JMP Methodology: 2017 update and SDG baselines. 2018.

2. Cairncross S, Hunt C, Boisson S, *et al.* Water, sanitation and hygiene for the prevention of diarrhoea. *International Journal of Epidemiology* 2010; **39**: i193–205.

3. Rabie T, Curtis V. Handwashing and risk of respiratory infections: a quantitative systematic review. *Tropical Medicine and International Health* 2006; **11**: 258–67.

4. Jefferson T, Del Mar CB, Dooley L, Ferroni E, Al-Ansary LA, Bawazeer GA, van Driel ML, Jones MA, Thorning S, Beller EM, Clark J, Hoffmann TC, Glasziou PP, Conly JM. Physical interventions to interrupt or reduce the spread of respiratory viruses. Cochrane Database of Systematic Reviews 2020, Issue 11. Art. No.: CD006207. DOI: 10.1002/14651858.CD006207.pub5. Accessed 13 April 2022.
5. Zheng P, Barber R, Sorensen RJD, Murray CJL, Aravkin AY. Trimmed constrained mixed effects models: formulations and algorithms. *J Comput Graph Stat* 2021; **30**: 544–56.
6. Zheng P, Afshin A, Biryukov S, *et al*. The Burden of Proof studies: assessing the evidence of risk. *Nat Med* 2022; **28**: 2038–44.

Non-optimal temperature

Flowchart

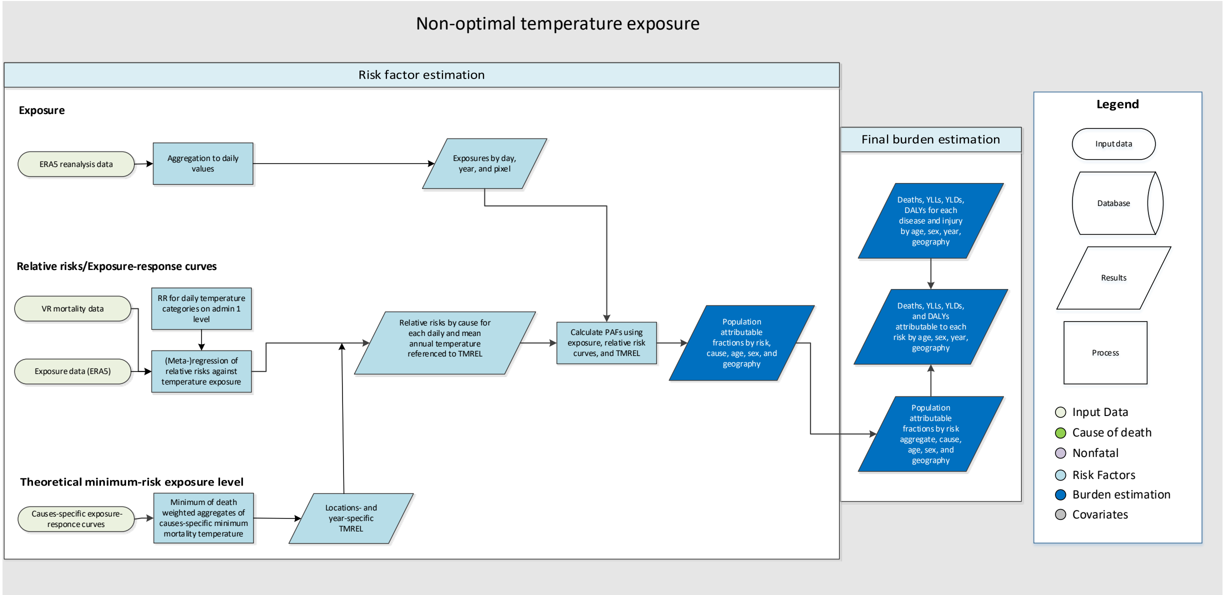

Input data and methodological summary

Definition

The non-optimal temperature risk factor is defined as the same-day exposure to ambient temperature that is either warmer or colder than the temperature of minimum mortality risk. Specifically, we define the theoretical minimum risk exposure level (TMREL) for non-optimal temperature as the temperature that is associated with the lowest overall mortality attributable to this risk in a given location and year. Given varying exposure–response curves for different mean annual temperature zones, as well as spatially and temporally varying cause of death composition, we estimate TMRELs by year and location and not a globally uniform TMREL. High temperature (heat) exposure is defined as exposure to temperatures warmer than the TMREL, and low temperature (cold) is defined as temperatures colder than the TMREL.

Exposure

We assess the daily exposure to non-optimal temperature, which is defined for high temperatures (above the TMREL) and low temperatures (below TMREL). TMRELs for non-optimal temperature exposure vary by year and location and reflect location-specific adaptation to temperature as well as specific composition of causes of death.

Input data

ERA5 data

We derived exposure estimates from the ERA5 reanalysis dataset from the European Centre for Medium-Range Weather Forecasts (ECMWF). ECMWF produced ERA5 estimates using their Integrated Forecast System (IFS). Hourly values of surface temperature are available for a spatial resolution of 0.25° x 0.25°. Uncertainty estimates for these temperature values, ie, the ensemble spread (standard deviation) are available for every three hours (00:00, 03:00, 06:00, 09:00, 12:00, 15:00, 18:00, 21:00) for a spatial resolution of 0.5° x 0.5°. At the time of analysis, data were available from 1979 to December 2023.[1,2] We calculated daily averages of temperature and spread for each pixel and then assigned an uncertainty value to each daily temperature value. Based on the spread, we derived 1000 draws of each daily temperature pixel.

Population data

Population data for calculating population-weighted location means were derived from WorldPop, which is an open-source project initiated in 2013.[3] Multi-temporal, globally consistent, high-resolution human population data at 1 km x 1 km resolution can be downloaded from http://www.worldpop.org.uk/ for 2000, 2005, 2010, 2015, and 2020. For our work, we interpolated between the five-year estimation bins to obtain annual data and extrapolated back to 1990 by using the 2000–2005 growth rate for back-casting. Finally, we rescaled to the pixel-level population estimates so that the sum of all pixels within a GBD location equalled the corresponding GBD population estimate.

Mortality data

Deaths at the individual level that included information on the cause (ie, ICD code), date, and the location at the second administrative level (admin2) or finer were collected from the Global Burden of Disease (GBD) cause of death (CoD) database for vital registration data sources. We adapted the GBD standard procedure for garbage code redistribution to redistribute daily mortality data rather than annual data and mapped ICD causes to Level 3 GBD causes. In total, we analysed 64.9 million deaths from nine different countries and 15,198 administrative units. For Brazil, the data cover a period from 1999 to 2016 for 5570 municipalities and 19.9 million deaths. For Chile, the data cover the period from 1990 to 1996 and 2009 to 2011 for 15 regions and 0.82 million deaths. For Colombia, the data cover a period from 2001 to 2005 for 1125 municipalities and 0.95 million deaths. For Guatemala, the data cover a period from 2009 to 2016 for 333 municipalities and 0.49 million deaths. For Mexico, the data cover a period from 1996 to 2015 for 2438 municipalities and 9.88 million deaths. For New Zealand, the data cover a period from 1988 to 2014 for 20 district health boards and 0.76 million deaths. For South Africa, the data cover the years 1997 to 2016 for one province and 1.8 million deaths. For the USA, the data cover a period from 1980 to 1988 for 3140 municipalities and 18.1 million deaths. For China, the data cover the years 2015 to 2016 for 2556 counties and 12.2 million deaths.

Relative risk modelling strategy

To estimate relative risks for cause-specific mortality, based on average daily temperature and temperature zone (defined by mean annual temperature), we used a robust meta-regression framework, implemented through the MR-BRT (Bayesian, regularised, trimmed) tool. The tool allows three features that are essential to the analysis:[4]

- A meta-analytic framework that can handle heterogeneous data sources.
- A robust approach to outlier detection and removal (trimming).
- Specification of the functional dependence of outcome versus average daily temperature and temperature zone as a two-dimensional surface through a spline interface.

The use of trimming in a vast array of inference and machine learning problems is standard.[5–7] The use of high-dimensional splines has been proposed before,[8] but the methods used for estimation go beyond prior work, and we explain them below.

The functional relationship between any outcome, *y*, and input variables (*t1*, *t2*) models *y* as a linear combination of 2D spline basis elements. Each spline basis element is a product of individual basis elements for 1D splines for *t1* and *t2*. Therefore, the inference problem looks for a combination of simple curvilinear 2D elements that fit the data while preserving smoothness across element boundaries. The MR-BRT tool also allows prior information to influence the shape of the spline, particularly in areas with sparse data.

For modelling the relationship between mortality and mean annual and daily temperature, we imposed monotonicity in the direction of daily temperature. For all J-shaped curves that depicted an increase in mortality above and below a threshold, we forced the curve to monotonically decrease at the lower end of the temperature distribution and to monotonically increase at the upper end. For all external causes that displayed a monotonic increase over the entire temperature range, we imposed monotonicity only in the direction of warmer temperatures. We placed two knots of degree 3 in the direction of mean annual temperature when fitting the surface. In the direction of daily mean temperature, we placed three knots of degree 3 for J-shaped causes and two knots of degree 1 for external causes that monotonically increase over temperature range. Figure 1 shows an example of a relative risk (RR) surface along daily and annual mean temperature for drowning. We extrapolated daily temperature-mortality curves beyond the range of observed temperatures by either linear interpolation, in the case of monotonically increasing response relationships (observed for external causes) or cubic polynomial extrapolation for J- and V-shaped curves (observed for non-external causes).

We estimated uncertainty using a two-step approach. First, we derived the uncertainty of the mean surface from the measurement error using the fit-retrofit error. Second, we added uncertainty from the random effects by sampling it separately from the cold and warm side.

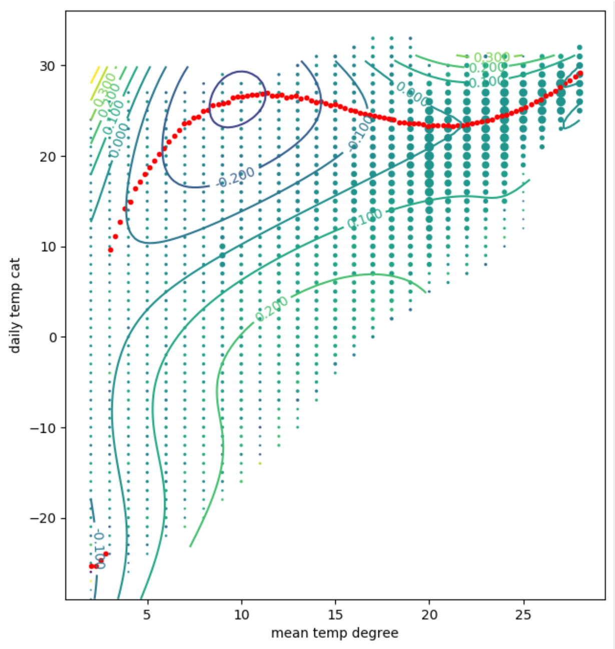

Figure 1: Log relative risk of death from lower respiratory infection along mean annual temperature (mean temp cat) and daily mean temperature (daily temp cat). The red dotted line depicts minimum mortality temperature along mean annual temperature zones. Green and blue lines depict isopleths, ie, lines of equal log RR of mortality

Cause selection

We assessed the effect of temperature on all causes included in Level 3 of the GBD cause hierarchy (N=176). We then reduced the set of potential causes by excluding causes for which no deaths were recorded in our mortality dataset (N=44) or for which classification practices are highly inconsistent across countries, which applied to dementia and protein-energy malnutrition (N=2).[30] We estimated RR surfaces and uncertainties for all remaining (N=130) Level 3 causes. Cause selection or inclusion was based on a risk–outcome score developed for evaluating risk–outcome pairs as part of the Burden of Proof framework. To derive this risk–outcome score, we normalised the risk curve for each cause and each temperature zone so that the RR was equal to 1·0 at the temperature associated with the lowest risk, and log-transformed the resulting normalised curves. The risk-outcome score is based on the area between the lower bound of the 95% UI and the null (ie, log-RR of 0·0). Regions of the risk curve for which the 95% UI includes the null will produce negative scores, and regions that exclude the null will produce positive scores; a score of zero indicates that the lower bound is equal to the null. We then averaged scores across all climate zones for each individual cause and included all causes for which the mean risk–outcome score exceeded zero. A table with average risk–outcome scores and associated star ratings for individual causes is provided below:

Table 1: Average risk–outcome scores across climate zones for individual causes. Causes with an average score above 0 were included into the GBD.

Rank	Cause (level 3 [a])	Parent cause (Level 2 [a])	Cause label (Level 3 [a])	Score average	Star rating
1	Drowning	Unintentional injuries	inj_drowning	0.361087	3
2	Other unintentional injuries	Unintentional injuries	inj_othunintent	0.269039	3
3	Lower respiratory infections	Respiratory infections and tuberculosis	lri	0.118761	2
4	Chronic obstructive pulmonary disease	Chronic respiratory diseases	resp_copd	0.105613	2
5	Animal-related injuries	Unintentional injuries	inj_animal	0.09697	2
6	Ischaemic heart disease	Cardiovascular diseases	cvd_ihd	0.08433	2
7	Stroke	Cardiovascular diseases	cvd_stroke	0.071678	2
8	Suicide	Self−harm and interpersonal violence	inj_suicide	0.070617	2
9	Disaster-related injuries	Unintentional injuries	inj_disaster	0.068935	2
10	Homicide	Self−harm and interpersonal violence	inj_homicide	0.068804	2
11	Mechanical injuries	Unintentional injuries	inj_mech	0.066174	2
12	Other transport-related injuries	Transport injuries	inj_trans_other	0.052465	2
13	Hypertensive heart disease	Cardiovascular diseases	cvd_htn	0.05067	2
14	Transport-related injuries	Transport injuries	inj_trans_road	0.048922	2
15	Diabetes	Diabetes and kidney diseases	diabetes	0.044704	2
16	Cardiomyopathy and myocarditis	Cardiovascular diseases	cvd_cmp	0.044378	2
17	Chronic kidney disease	Diabetes and kidney diseases	ckd	0.032174	2

Theoretical minimum risk exposure level

For the purpose of this analysis, the TMREL was defined as the temperature associated with the lowest mortality for all included causes. We calculated a death-weighted average of the cause-specific exposure–response curves with the minimum of this average curve being the TMREL. This was done for each year and each of the 990 GBD locations using CoD estimates produced for the GBD 2019 study. As climate zones or mean annual temperature can vary within a location, we calculated the TMREL for every mean annual temperature, assuming a consistent cause composition within a location. This approach represents the first use of spatially and temporally varying TMRELs within the GBD study.

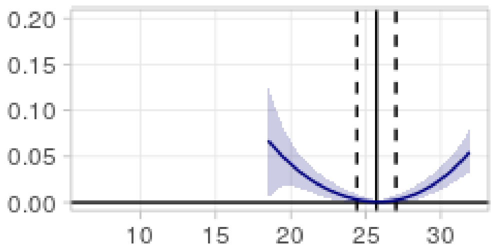

Figure 3: Schematic illustration of the exposure–response relationship between temperature and mortality and associated low temperature (cold) and high temperature effects beyond the theoretical minimum exposure level (TMREL). The blue line depicts the exposure–response curve with blue shaded line showing 95% uncertainty range. The black solid line depicts the TMREL with dashed black lines displaying 95% uncertainty range. Effects left of the TMREL are counted towards cold PAFs and right of the TMREL towards heat PAFs.

Population attributable fractions

The population attributable fraction (PAF) was calculated for each pixel and each day of the year (ie, pixel-day). Subsequently, we population-weighted each pixel using the fraction of the population living in a given pixel relative to the GBD location. Depending on whether the daily mean temperature was below or above the TMREL, the effect was assigned to either low or high temperature. Daily population-weighted high and low temperature PAFs were then aggregated for the location and the year. Temperature effects can be either harmful or protective depending on whether the RR is above or below 1. For harmful temperature effects (ie, effects with a RR above 1), we used the following equation to derive PAFs: PAF=(RR–1)/RR; For temperature effects exhibiting a protective effect, the equation was adapted by implementing the reverse RR: PAF=–((1/RR) –1)/(1/RR). The PAF associated with non-optimal temperature exposure is an aggregate, ie, the sum of heat and cold effects in each location and year. We estimated the temperature-attributable burden as the product of the total burden for that cause and the corresponding PAF for each GBD location, year, age group, and sex.

Citations

1 Hersbach H, Bell B, Berrisford P, *et al.* Global reanalysis : goodbye Global reanalysis : goodbye ERA-Interim, hello. 2019. DOI:10.21957/vf291hehd7.

2 Copernicus Climate Change Service (C3S) (2017): ERA5: Fifth generation of ECMWF atmospheric reanalyses of the global climate. Copernicus Climate Change Service Climate Data Store (CDS), September 2019.

3 Geography and Environmental Science, University of Southampton. Age and Sex Structures, Global Per Country 2000-2020 - WorldPop. Southampton, United Kingdom: Geography and Environmental Science, University of Southampton, 2018.

4 Zheng P, Aravkin AY, Barber R, Sorensen RJD, Murray CJL. Trimmed Constrained Mixed Effects Models: Formulations and Algorithms. 2019; published online Sept 23.

5 Rousseeuw PJ. Least Median of Squares Regression. *Journal of the American Statistical Association* 1984; **79**: 871–80.

6 Aravkin A, Davis D. Trimmed Statistical Estimation via Variance Reduction. *Mathematics of Operations Research* 2019; moor.2019.0992.

7 Yang E, Lozano AC, Aravkin A. A general family of trimmed estimators for robust high-dimensional data analysis. *Electronic Journal of Statistics* 2018; **12**: 3519–53.

8 Pya N, Wood SN. Shape constrained additive models. *Statistics and Computing* 2015; **25**: 543–59.

Occupational risk factors

Flowcharts

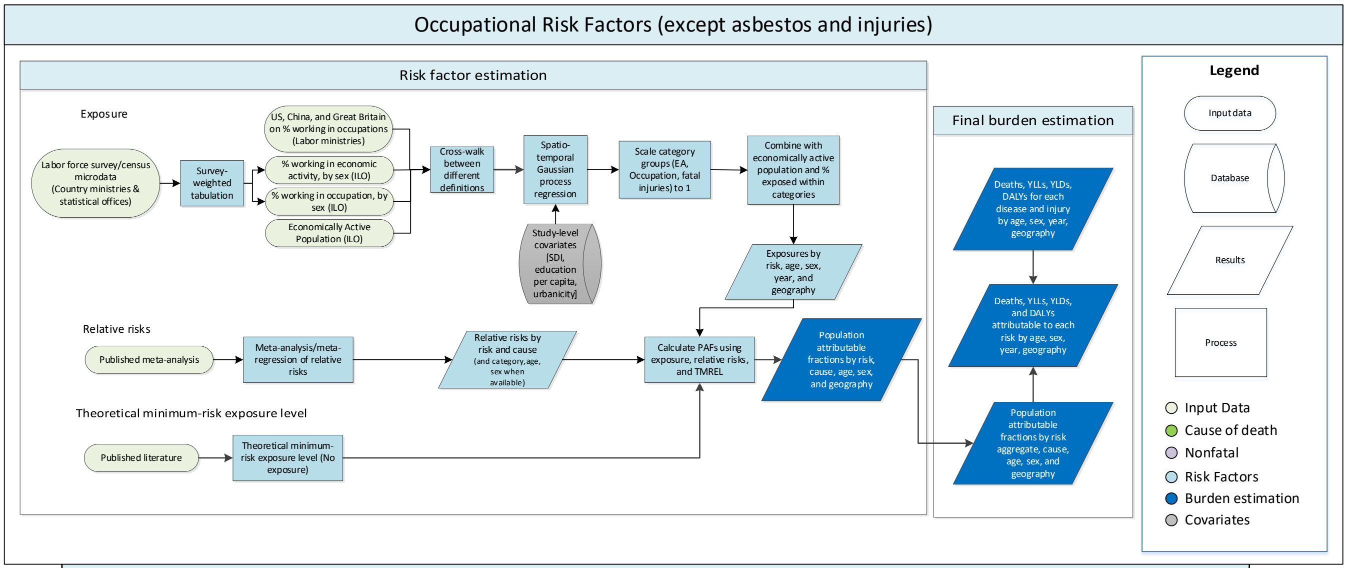

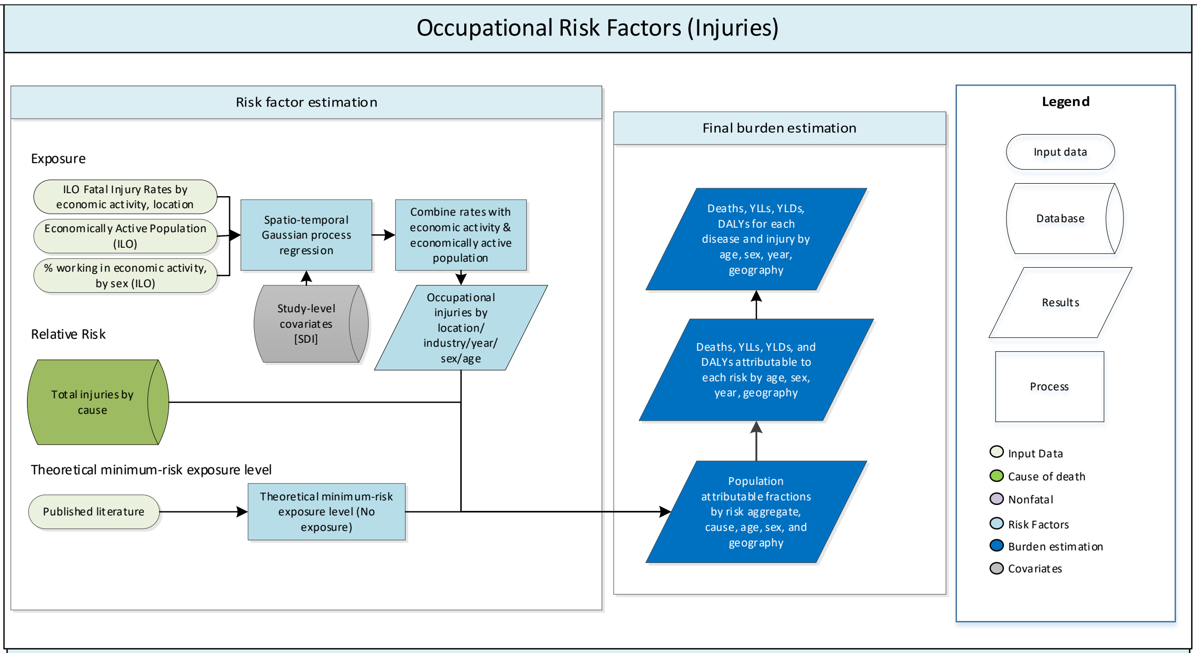

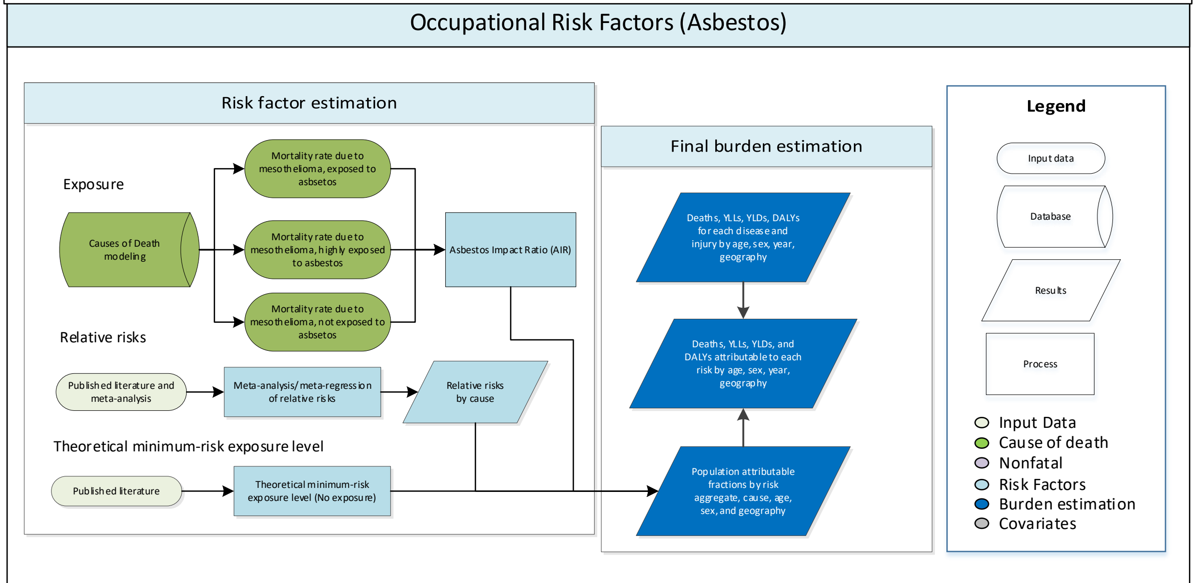

Definitions

Exposure

Table 1 displays the definitions of the occupational exposure risk factors. All exposures were estimated for ages 15 years old and older, unless otherwise noted.

Table 1: Occupational exposure definitions

Occupational exposure	Definition
Occupational asbestos	Proportion of the population occupationally exposed to asbestos, using the mesothelioma death rate as an analogue.
Occupational asthmagens	Proportion of the working population exposed to asthmagens, based on population distributions across nine occupational categories (listed below). Estimated for ages 15–84 years old.
Occupational carcinogens (arsenic, benzene, beryllium, cadmium, chromium, diesel engine exhaust, formaldehyde, nickel, polycyclic aromatic hydrocarbons, silica, sulfuric acid, and trichloroethylene)	Proportion of the population that was ever occupationally exposed to carcinogens at high or low exposure levels, based on population distributions across 17 economic activities (listed below).
Occupational ergonomic factors	Proportion of the working population exposed to work that causes low back pain, based on population distributions across nine occupational categories. Estimated for ages 15–84 years old.
Occupational injuries	Proportion of injuries in the working-age population attributable to occupation, based on fatal injury rates in 17 economic activities.
Occupational noise	Proportion of the population occupationally exposed to 85+ decibels of noise, based on population distributions across 17 economic activities.
Occupational particulates	Proportion of the population occupationally exposed to particles, based on population distributions across 17 economic activities.

Table 2 displays the economic activities and occupation categories for occupational risk factors.

Table 2: Economic activities and occupation categories

Economic activities	Occupations
Agriculture, hunting, forestry	Legislators, senior officials, and managers
Fishing	Professionals
Mining and quarrying	Technicians and associate professionals
Manufacturing	Clerks
Electricity, gas, and water	Service workers and shop/market sales workers
Construction	Skilled agricultural and fishery workers

Wholesale and retail trade/repair	Plant and machine operators and assemblers
Hospitality	Craft and related workers
Transport, storage, and communication	Elementary occupations
Financial intermediation	
Real estate/renting	
Public administration/defense; compulsory social security	
Education	
Health and social work	
Other community/social/personal service activities	
Private households	
Extra-territorial organisations/bodies	

Input data

Exposure

Primary inputs were obtained from the International Labour Organization (ILO).[1-4] These inputs included raw data on economic activity proportions, occupation proportions, fatal injury rates, and employment to population ratio estimates. No data on informal employment were included due to data sparseness. In 2017, a systematic review was conducted to collect the underlying microdata from the ILO's estimates to aid in re-extraction at greater levels of granularity. Where freely available, survey datasets were downloaded from the survey organisations in question. Other datasets were obtained through submission of requests to agencies and through the GBD Collaborator Network. Microdata were tabulated in order to create survey-weighted estimates of economic activities and occupations for the GBD geographies and years. Various classification systems were adjusted to match the ISIC Rev.3 classification (for economic activities) and ISCO 1988 classification (for occupations). In GBD 2021, we updated our ILO data by downloading the data files from their website up to 2019, which was the most recent year at the time. No new ILO data were added for GBD 2023.

For occupational asbestos, primary inputs were obtained through GBD 2023 cause of death estimates and published studies.[5-7]

Uncertainty for inputs where microdata were unavailable was generated by fitting a Loess curve to the data and determining the standard deviation of the data from the fitted curve.

Relative risk

In GBD 2023, we conducted two systematic reviews, one for occupational ergonomics and low back pain, and one for occupational particulate matter and chronic obstructive pulmonary disease (COPD).

Relative risks for all other occupational risk factors were obtained from a systematic review of published meta-analyses, which was last updated in GBD 2016. All relative risks for each occupational risk factor can be found in the relative risk appendix table. For the (new in GBD 2023) occupational ergonomics systematic review, we included papers that provided a measure of association (or values to calculate the association) of low back pain in two or more occupations, with the reference being clerks. We excluded papers that did not report findings for low back pain, papers that only report prevalence, and papers

that only compared two sub-occupations within a given occupation category (Table 2). Only PubMed was searched from 1996 to October 10, 2020. The following search string was used:

("Low Back Pain"[MESH] OR "low back pain"(TiAb) OR "back pain"[TiAb]) AND (occupational[TiAb] OR occupation[TiAb] OR workers[TiAb] or worker[TiAb) AND (1996[PDAT]:3000[PDAT]) NOT (animals[MESH] NOT humans[MESH])

Prior to GBD 2023, a meta-analysis by Driscoll, et al. 2014 was used to estimate occupational ergonomics RR values.[8] We performed full-text reviews on the papers that informed this meta-analysis, and none passed our inclusion criteria. A total of 2682 papers were returned using the search string above. After title/abstract and full-text screening, six papers passed our inclusion criteria. Figure 1 is the PRISMA diagram displaying the number of papers that were included/excluded and their reasonings.

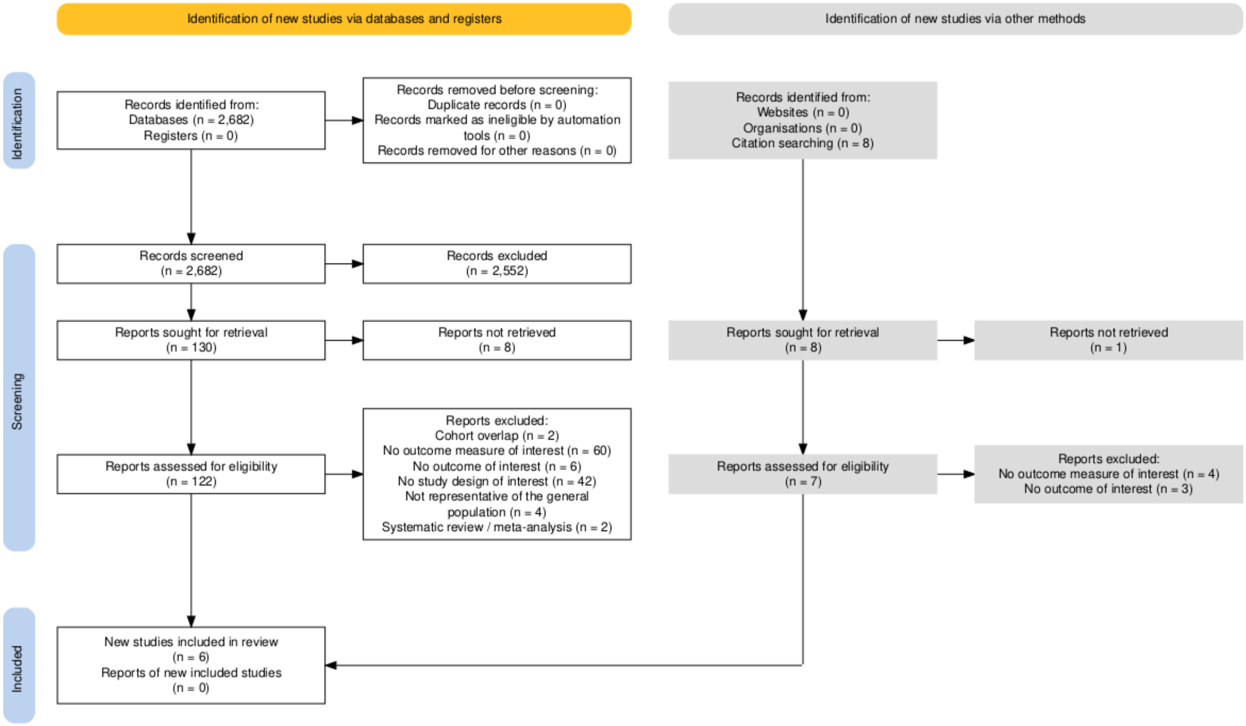

Figure 1: PRISMA diagram for occupational ergonomics – low back pain systematic review. This diagram was made using an online tool.[9]

For the occupational particulate matter systematic review, we included papers that provided a measure of association (or values to calculate the association) of COPD for high or low exposure, with the reference being no exposure. We excluded cross-sectional studies of COPD prevalence, studies that did not report findings for COPD, studies that only reported findings for a sub-cause of COPD via self-reporting, studies on interventions to prevent or heal COPD, and studies that do not stratify exposure into low and high exposure or did not use "no exposure" as the reference. Only PubMed was searched from 2009 to January 25, 2021. The following search string was used:

("occupational"[TiAB] OR "occupation"[TiAB] OR "workers"[TiAB] OR "worker"[TiAB] OR "Occupational Exposure"[MESH]) AND ("dust"[TiAB] OR "gases"[TiAb] OR "fumes"[TiAB] OR "particulates"[TiAB] OR "particles"[TiAB] OR "particulate matter"[TiAb] OR "Particulate Matter"[MESH]) AND ("copd"[TiAB] OR

"Chronic Obstructive Pulmonary Disease"[TiAb] OR "Pulmonary Disease, Chronic Obstructive" [MESH]) AND (2009[PDAT]:3000[PDAT]) NOT (animals[MESH] NOT humans[MESH]).

Prior to GBD 2023, Blanc, et al. 2009 was used to estimate occupational particulate matter RR values, and its publication year (2009) was used as the starting year for our systematic review.[10] Ultimately, this paper did not pass our inclusion criteria and was not included in our final analysis. The search string and year range returned 354 papers. After title/abstract and full-text review, nine papers passed our inclusion criteria. Figure 2 is the PRISMA diagram displaying the number of papers that were included/excluded and their reasonings.

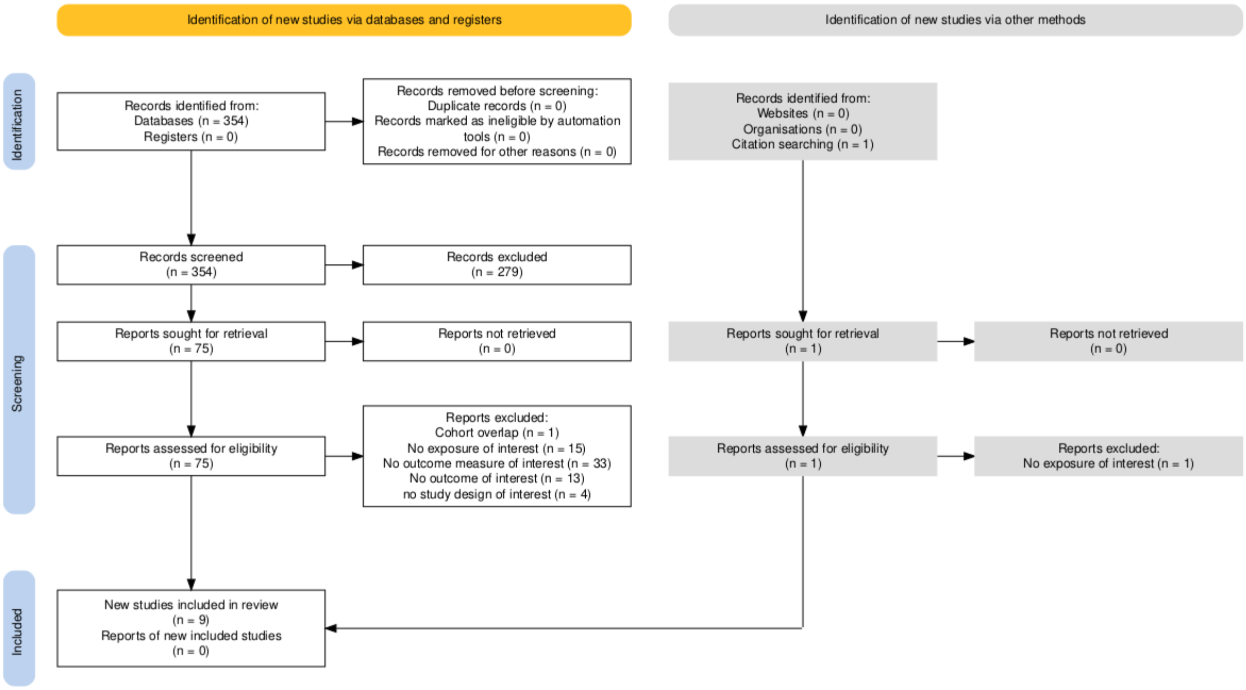

Figure 2: PRISMA diagram for occupational particulate matter – low back pain systematic review. This diagram was made using an online tool.[9]

Data processing

For injuries input data that are reported as both sexes (aggregated), we split these data into male and female data. To do this, we first calculated the female:male ratio for each ISIC code, source, and demographic information for reported sex-specific data. Using this ratio, we ran a meta-regression—Bayesian, regularised, trimmed (MR-BRT) model to determine the relationship between male and female injuries.[11] Using the modelled global ratio, we split all data reported as both-sex into male and female values.

To account for the accuracy of difference data sources, we crosswalked the post-sex-split injuries input data. Occupational injuries exposure data come from a number of different sources: insurance records, labour inspectorate records, establishment surveys, establishment or business registers, labour force surveys, economic or establishment censuses, official estimates, records of employers' organisations, and other administrative records and related sources. We expect insurance records to be the gold-standard source, as people should have more incentive to report injuries when they stand to benefit from their insurance plans. We adjusted all non-insurance record sources as if they reported from

insurance records. To do so, we ran a mixed-effects log-linear regression using a MR-BRT model, with fixed effects on type of data source and random effects on super-region and region. Table 3 shows the beta coefficients from the MR-BRT model, as well as the crosswalk adjustment factors.

Table 3: MR-BRT crosswalk adjustment factors for occupational injuries

Reference or alternative case definition	Gamma	Beta coefficient, log (95% UI)*	Adjustment factor**
Reference (insurance records)	0.29	---	---
Alternative (labour inspectorate records)		–0.22 (–0.24, –0.21)	0.80 (0.79, 0.81)
Alternative (establishment surveys)		–0.46 (–0.47, –0.44)	0.63 (0.62, 0.65)
Alternative (labour force surveys)		0.01 (0.00, 0.02)	1.01 (1.00, 1.02)
Alternative (economic or establishment censuses)		0.17 (0.17, 0.18)	1.19 (1.18, 1.20)
Alternative (official estimates)		–0.54 (–0.56, –0.52)	0.58 (0.57, 0.60)
Alternative (establishment or business registers)		–0.69 (–0.76, –0.62)	0.50 (0.47, 0.54)
Alternative (other administrative records and related sources)		–0.29 (–0.30, –0.28)	0.75 (0.74, 0.75)

**MR-BRT crosswalk adjustments can be interpreted as the factor the alternative case definition is adjusted by to reflect what it would have been had it been measured using the reference case definition. If the beta coefficient is negative, then the alternative is adjusted up to the reference. If the beta coefficient is positive, then the alternative is adjusted down to the reference.*

***The adjustment factor column is the exponentiated beta coefficient. For log beta coefficients, this is the relative rate between the two case definitions.*

Modelling strategy

Exposure

A spatiotemporal Gaussian process regression (ST-GPR) was used to generate estimates for all years and locations for the primary inputs.[12] Space-time parameters were chosen by maximising out-of-sample cross-validation and minimising RMSE. A number of different study-level covariates were used in the linear regression models (Table 5). The linear models for each of the 46 different ST-GPR models used in occupational exposure estimation are listed below (Table 4). Although they may appear to be duplicates, there is a distinction between occupation and economic activity (detailed in the footnotes). For

example, “skilled agriculture/fisheries” involves the proportion of the workforce doing agricultural and fishing work, while “agriculture, hunting, forestry” and “fishing” involve the proportion of the workforce employed in those respective industries (ie, one doesn’t have to be actually doing agricultural or fishing work – someone who transports crops would count as being employed in this industry, but their occupation would fall under “plant and machine operators & assemblers”). Each model included random effects at the region and super-region levels. In addition to these random effects, the employment model included fixed effects on age.

Table 4: Linear regression equations for each ST-GPR model

**ST-GPR model**	**Linear regression equation**
Employment (% of population employed)	$logit(data) = gov\_exp + prop\_muslim + education$
Armed forces*	$logit(data) = sdi + education + urbanicity$
Management*	$logit(data) = sdi + education + urbanicity$
Professional occupations*	$logit(data) = sdi + education + urbanicity$
Scientific/technicians*	$logit(data) = sdi + education + urbanicity$
Clerical work*	$logit(data) = sdi + education + urbanicity$
Service & shop/market sales workers*	$logit(data) = sdi + education + urbanicity$
Skilled agriculture/fisheries*	$logit(data) = sdi + latitude + urbanicity$
Craft and related trades*	$logit(data) = sdi + education + urbanicity$
Plant and machine operators & assemblers*	$logit(data) = sdi + education + urbanicity$
Elementary occupations*	$logit(data) = sdi + education + urbanicity$
Agriculture, hunting, forestry†	$logit(data) = sdi + latitude + urbanicity$
Fishing†	$logit(data) = \log(coastal\_prop + 0.01)$
Mining/quarrying†	$logit(data) = sdi + \log(coastal\_prop + 0.01) + urbanicity + asbestos$
Manufacturing†	$logit(data) = sdi + education + urbanicity$
Electricity/gas/water supply†	$logit(data) = \log(sdi) + urbanicity + temperature$
Construction†	$logit(data) = sdi + urbanicity$
Wholesale and retail trade/repair†	$logit(data) = sdi + education + urbanicity$
Hospitality†	$logit(data) = sdi + urbanicity$
Transport/storage/communications†	$logit(data) = sdi + urbanicity + vehicles\_pc$
Financial intermediation†	$logit(data) = sdi + urbanicity$
Real estate/renting†	$logit(data) = sdi + urbanicity$
Public administration/defence†	$logit(data) = sdi + urbanicity$
Education†	$logit(data) = sdi + education + urbanicity$
Health and social work†	$logit(data) = \log(sdi) + \log(health\_exp)$
Other community/social/personal service activities†	$logit(data) = sdi + urbanicity$
Private households†	$logit(data) = sdi + urbanicity$
Extraterritorial organisations and bodies†	$logit(data) = sdi + urbanicity$
All occupational injuries models‡	$logit(data) = sdi$

**Proportion of workforce working this type of occupation*

†Proportion of workforce employed in this type of economic activity

‡*There are 18 different models, corresponding to one for each type of economic activity and a "total" model*

Table 5: Covariates and their descriptions that are used in the ST-GPR models

Covariate	Description
gov_exp	Total government expenditure
prop_muslim	Proportion of population that is Muslim
education	Age-standardised years of education per capita
sdi	Socio-demographic Index
urbanicity	Proportion of population living in urban areas
latitude	Absolute value of average latitude of country's center point
coastal_prop	Percentage of total country area within 10 km of a coastal zone
asbestos	Asbestos consumption (metric tons per year per capita)
temperature	Population-weighted mean temperature
vehicles_pc	Number of 2- and 4-wheeled vehicles per capita
health_exp	Total health expenditure per capita

The methodology of the urbanicity covariate can be found in the lead risk factor appendix. For economic activity and occupation proportions, estimates from ST-GPR were then re-scaled to sum to 1 across categories by dividing each estimate by the sum of all the estimates.

The following sections describe the modelling approaches for each occupational risk's exposure prevalence. These approaches were developed for GBD 2016 and have not changed substantially since then.

Occupational carcinogens, occupational noise, and occupational particulate matter

Prevalence of exposure to these risks was determined using the following equation:

$$Prevalence\ of\ Exposure_{c,y,s,a,r,l} = \sum_{EA} Proportion_{EA,c,y} * EAP_{c,y,s,a} * Exposure\ rate_{EA,r,l,d}$$

where:

EAP = economically active population	c = country	r = risk
EA = economic activity	d = duration	s = sex
a = age	l = level of exposure	y = year

Exposure rate (proportion of population exposed) was provided by expert group recommendations and literature.[7, 13-15] The CAREX (carcinogen exposure) database was used to quantify the association between exposure by industry/carcinogen to SDI across all the countries in the database.[7] This effect was used to predict exposure in countries that were not included in CAREX. Duration was considered for occupational carcinogens through application of occupational turnover factors and for occupational noise and particulate matter by calculating cumulative exposure as the average exposure over the lifetime (the past 50 years) for each age/sex cohort.[16]

Occupational ergonomic factors and occupational asthmagens

Prevalence of exposure to these risks was determined using the following equation:

$$Prevalence\ of\ Exposure_{c,y,s,a,r} = \sum_{EA} Proportion_{OCC,c,y} * EAP_{c,y,s,a}$$

where:

EAP = economically active population	c = country	r = risk
EA = economic activity	a = age	s = sex
OCC = occupation	y = year	

Occupational injuries

Occupational injury counts were estimated using the following equation:

$$Occupational\ fatal\ injuries_{c,y,a,s} = \sum_{EA} Injury\ rate_{EA,c,y,s} * Population_{c,y,a,s} * EAP_{c,y,s,a} * Proportion_{EA,c,y}$$

where:

EAP = economically active population	c = country	y = year
EA = economic activity	a = age	s = sex

Occupational asbestos

Prevalence of exposure to asbestos was estimated using the asbestos impact ratio (AIR), which is equivalent to the excess deaths due to mesothelioma observed in a population divided by excess deaths due to mesothelioma in a population heavily exposed to asbestos. Formally, this is defined using the following equation:

$$AIR = \frac{Mort_{c,y,s} - N_{c,y,s}}{Mort^{*}_{c,ys,} - N_{c,y,s}}$$

where:

Mort = Mortality rate due to mesothelioma	c = country
$Mort^*$ = Mortality rate due to mesothelioma in population highly exposed to asbestos	y = year
N = Mortality rate due to mesothelioma in population not exposed to asbestos	s = sex

Mortality rate due to mesothelioma was estimated using causes of death results. Mortality rate due to mesothelioma in populations not exposed to asbestos was calculated using the model in Lin and colleagues, while the mortality rate due to high exposure to asbestos was estimated using Goodman and colleagues' model.[5-6] Asbestos exposure prevalence created using the AIR was used to estimate population attributable fractions (PAFs) for all asbestos-associated causes except for mesothelioma. Custom PAFs were calculated for mesothelioma by using the ratio of the excess mortality with respect to an unexposed population (Mort – N) divided by the mortality rate in the population in question (Mort). It is important to note that the AIR does not use the mesothelioma mortality rate specifically for occupational exposures to asbestos; rather it uses the mesothelioma mortality rate in populations highly exposed to asbestos. Although the majority of asbestos exposure is occupational, we note that non-occupational exposure to asbestos may be captured by the AIR. Consequently, as the AIR is used to estimate asbestos exposure, our description of the risk factor "Occupational exposure to asbestos" reflects both occupational and non-occupational exposure.

Theoretical minimum risk exposure level

For all occupational risks, the theoretical minimum risk exposure level was assumed to be no exposure to that risk.

Relative risks

The full table of relative risks (RR) can be found in a separate section of the Appendix. In GBD 2023, we re-evaluated the RR of occupational ergonomics and occupational particulate matter. The re-evaluated RRs from GBD 2023 are compared to the RRs from GBD 2021 in Table 6 for occupational ergonomics and in Table 7 for occupational particulate matter.

Table 6: RR comparisons from GBD 2023 vs GBD 2021 for occupational ergonomics and low back pain.

Labor/ Non-Labor	Occupation (2023)	Occupation (2021)	GBD 2023	GBD 2021
Non-labor	Legislators, senior officials, and managers	Administrative and managerial workers	1.28 (0.91 - 1.74)	1.21 (1.14 - 1.28)
Non-labor	Technicians and associate professionals	Professional, technical and related workers	1.28 (0.91 - 1.74)	1.17 (1.14 - 1.20)
Non-labor	Professionals	Professional, technical and related workers	1.28 (0.91 - 1.74)	1.17 (1.14 - 1.20)
Labor	Elementary occupations	*not estimated prior to GBD 2023*	1.79 (0.53 - 4.50)	*N/A*
Labor	Service workers and shop/market sales workers	Sales workers / Service workers	1.79 (0.53 - 4.50)	1.44 (1.41 - 1.46)
Labor	Skilled agricultural and fishery workers	Agriculture, animal husbandry and forestry workers, fishermen and hunters	1.79 (0.53 - 4.50)	3.78 (3.44 - 4.16)
Labor	Craft and related workers	*not estimated prior to GBD 2023*	1.79 (0.53 - 4.50)	*N/A*
Labor	Plant and machine operators and assemblers	Production and related workers, transport equipment operators and labourers	1.79 (0.53 - 4.50)	1.54 (1.51 - 1.58)
TMREL	Clerical and related workers	Clerical and related workers	1.00 (1.00 - 1.00)	1.00 (1.00 - 1.00)

Table 7: RR comparisons from GBD 2023 vs GBD 2021 for occupational particulate matter and chronic obstructive pulmonary disorder (COPD).

Exposure	GBD 2023	GBD 2021
High	1.58 (0.84 - 2.81)	2.37 (2.19 - 2.57)
Low	1.17 (1.06 - 1.29)	1.46 (1.38 - 1.53)
None (TMREL)	1.00 (1.00 - 1.00)	1.00 (1.00 - 1.00)

Occupational ergonomics – low back pain

Estimating the relative risk (RR) of occupational ergonomics occurring as a function of exposure to low back pain followed the burden of proof approach (BoP) established by Zheng and colleagues and instantiated in the meta-regression—Bayesian, regularised, trimmed (MR-BRT) tool.[11,17] MR-BRT synthesises input data to generate an RR curve by relying on an ensemble spline method to capture the potentially non-linear shape of the risk–outcome relationship; integrating over varying exposure ranges in different comparison groups; trimming potentially distorting outliers; testing, selecting, and adjusting for bias covariates to account for known heterogeneity in input study-design characteristics (eg, confounding, selection bias, exposure measurement); and quantifying remaining between-study heterogeneity (gamma) through random effects modelling and incorporating this value into uncertainty around the mean RR curve.

MR-BRT further evaluates evidence for small-study effects and generates funnel plots that represent potential risk of publication or reporting bias, and also generates the burden of proof risk function (BPRF), defined for harmful risks as the 5th and for protective risks as the 95th quantile risk curve – inclusive of unexplained between-study heterogeneity/gamma – that is closest to null. The BPRF is transformed into a risk–outcome score (ROS: the signed value of the average log BPRF between the 15th and 85th percentiles of risk exposure levels observed across included studies) and mapped onto a star-rating system from one to five stars. These metrics complement RR estimates by providing an alternative, conservative measure of effect size and evidence strength that formally and systematically incorporates divergence/convergence across input findings, with higher positive ROS values and more stars corresponding to incrementally larger effects and stronger evidence for the risk–outcome relationship.

For occupational ergonomics – low back pain, we ran two BoP models, one for labor and another for non-labour occupations. Occupations that fall within labour and non-labour occupations can be seen in Table 8.

Table 8: Occupations that are categorised as labour or non-labour occupations.

Labour occupations	Non-labour occupations
Elementary occupations	Legislators, senior officials, and managers
Service workers and shop/market sales workers	Professionals
Skilled agricultural and fishery workers	Technicians and associate professionals
Plant and machine operators and assemblers	
Craft and related workers	

For the labor BoP dichotomous model, we did not trim any of the data and did not pre-select any of the covariates. Without trimming, there was a total of 14 RR estimates from four studies within the model. No covariates were found to be significant within the model. Publication bias was found, and the model returned a risk score of –0.2269, which is a one star-rating. The modeled RR of lower back pain due to occupational ergonomics in labor occupations is 1.79 (0.53–4.50). The modelled RR and funnel plot can be seen in Figure 3.

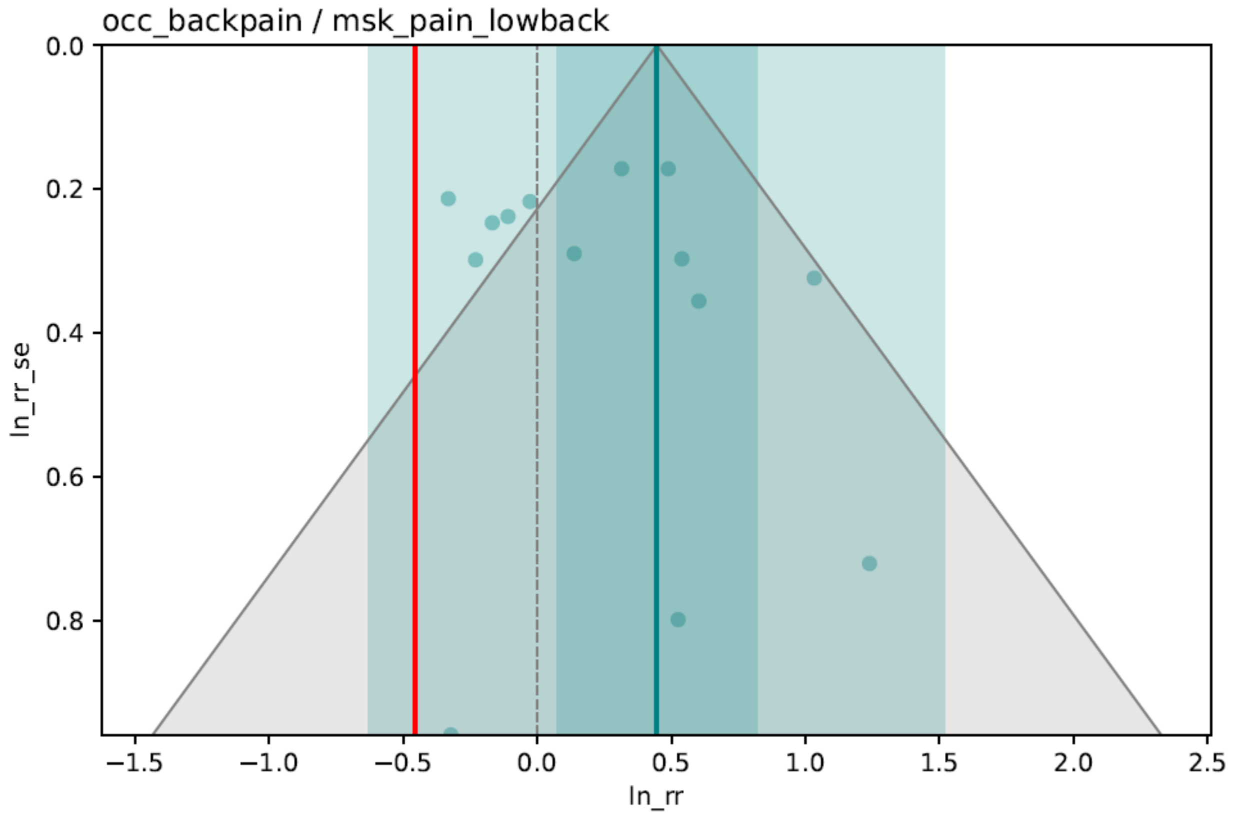

Figure 3: Funnel plot and modelled RR of low back pain due to occupational ergonomics in labour occupations. The final log RR (thick green line), 95% UIs with between-study heterogeneity (dark green band) and without between-study heterogeneity (light green band), the log BPRF (thick red line), log RRs from included studies, and the funnel.

For the non-labour BoP dichotomous model, we did not trim any of the data and did not pre-select any of the covariates. Without trimming, there was a total of 12 RR estimates from six studies within the model. No covariates were found to be significant within the model. Publication bias was not found, and the model returned a risk score of –0.0246, which is a one star-rating. The modeled RR of lower back pain due to occupational ergonomics in labour occupations is 1.28 (0.91–1.74). The modelled RR and funnel plot can be seen in Figure 4.

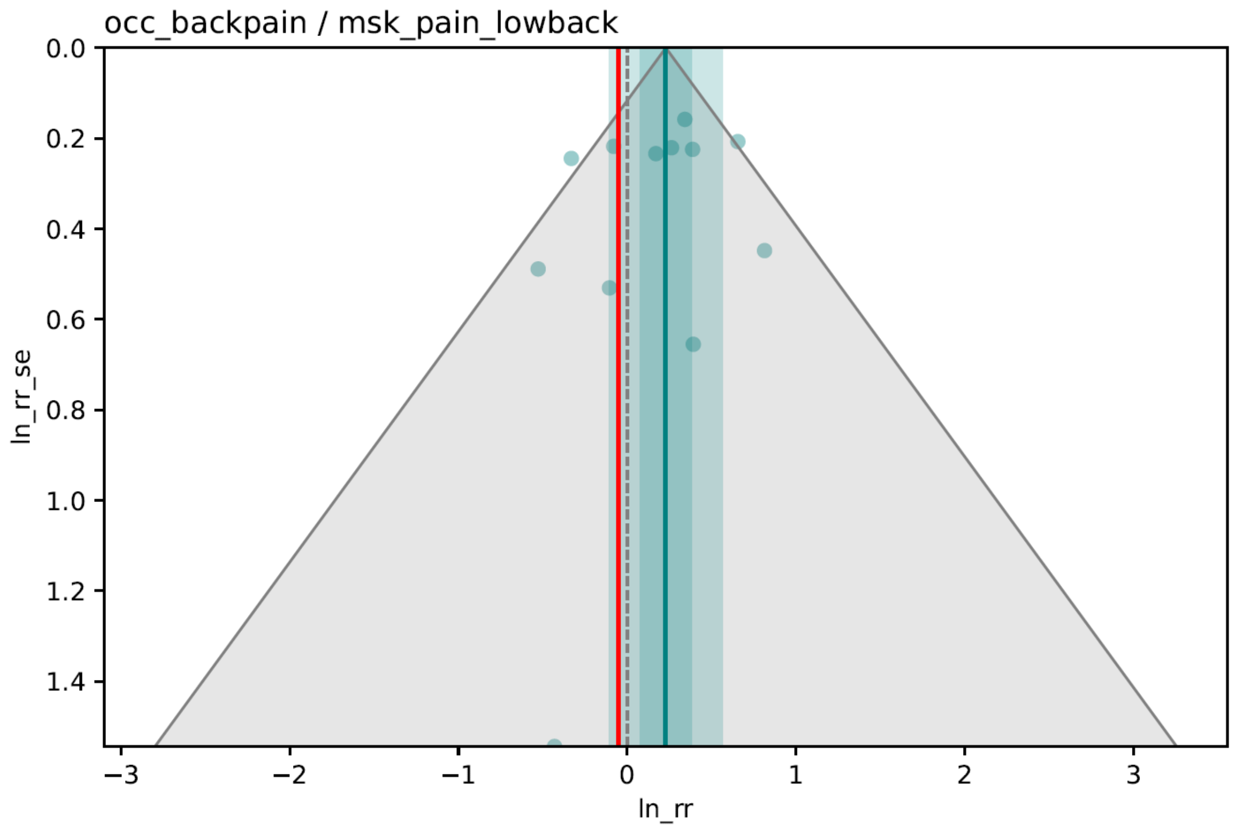

Figure 4: Funnel plot and modelled RR of low back pain due to occupational ergonomics in non-labour occupations. The final log RR (thick green line), 95% UIs with between-study heterogeneity (dark green band) and without between-study heterogeneity (light green band), the log BPRF (thick red line), log RRs from included studies, and the funnel.

Occupational particulate matter – COPD

For occupational particulate matter – COPD, we took a similar approach. We ran two BoP models, one for low and another for high exposure, as defined by each study.

For the high exposure BoP dichotomous model, we trimmed 10% of the data and did not pre-select any of the covariates. Prior to trimming, there was a total of 12 RR estimates from eight studies within the model. Two covariates were found to be significant and were used within the model: whether the study controlled for major confounders, and whether there was greater than 95% follow-up. Publication bias was not found, and the model returned a risk score of –0.0549, which is a one star-rating. The modelled RR of COPD due to occupational particulate matter was 1.58 (0.84–2.81). The modelled RR and funnel plot can be seen in Figure 5.

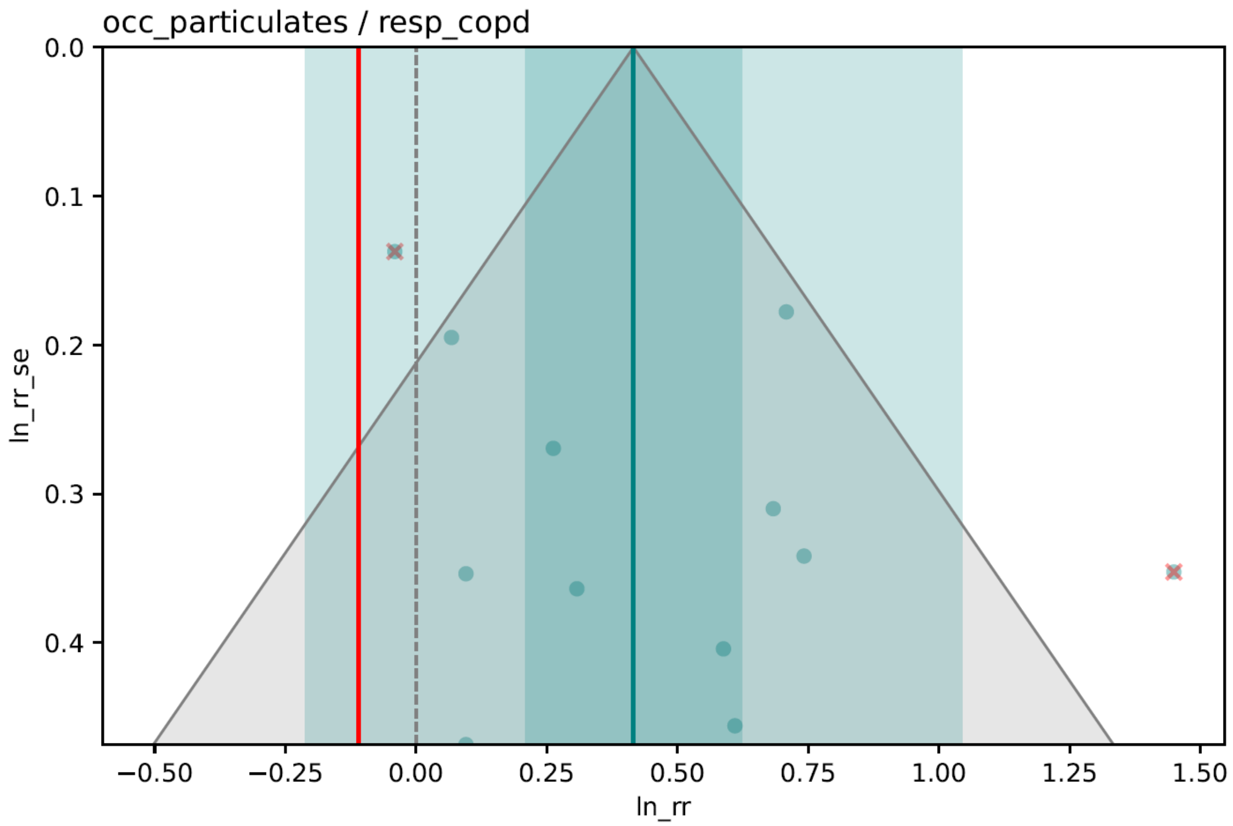

Figure 5: Funnel plot and modelled RR of COPD due to high exposure to occupational particulate matter. The final log RR (thick green line), 95% UIs with between-study heterogeneity (dark green band) and without between-study heterogeneity (light green band), the log BPRF (thick red line), log RRs from included studies, and the funnel. Trimmed datapoints are marked with a red x.

For the low exposure BoP dichotomous model, we trimmed 10% of the data and pre-selected the following covariates: whether the study controlled for major confounders, and whether there was greater than 95% follow-up. The pre-selected covariates were chosen based on the covariates that were found to be significant in the high exposure model. Prior to trimming, there was a total of 12 RR estimates from eight studies within the model. Publication bias was not found, and the model returned a risk score of 0.03612, which is a two star-rating. The modelled RR of COPD due to low exposure to occupational particulate matter is 1.17 (1.06–1.29). The modelled RR and funnel plot can be seen in Figure 6.

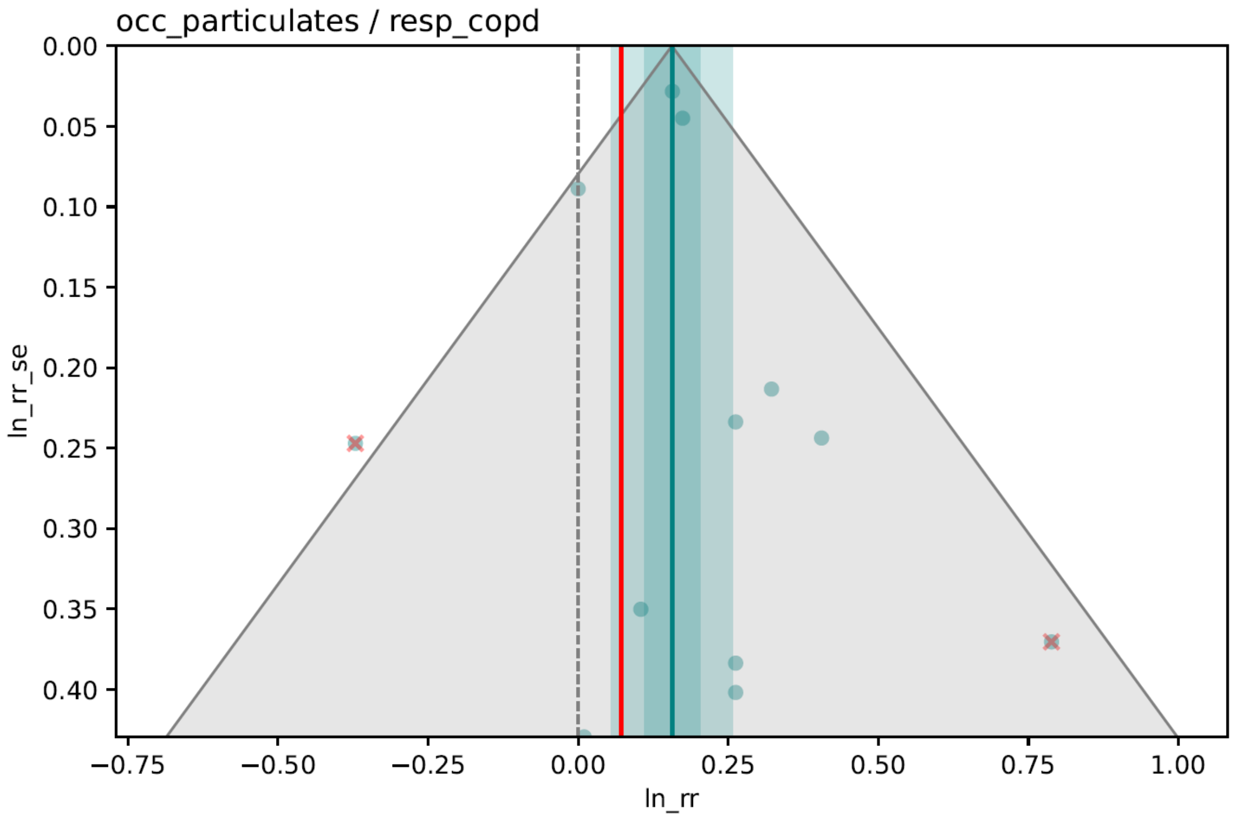

Figure 6: Funnel plot and modelled RR of COPD due to low exposure to occupational particulate matter. The final log RR (thick green line), 95% UIs with between-study heterogeneity (dark green band) and without between-study heterogeneity (light green band), the log BPRF (thick red line), log RRs from included studies, and the funnel. Trimmed datapoints are marked with a red x.

Population attributable fractions (PAFs)

For all occupational risks, with the exception of injuries (outlined below) and asbestos (outlined above), PAFs were calculated using the exposure prevalences estimated above, using the standard GBD PAF formula.[18]

Occupational injuries PAF

The PAFs for occupational injuries were calculated using the following formula:

$$PAF_{c,y,a,s} = \frac{Occupational\ fatal\ injuries_{c,y,a,s} - TMREL}{Fatal\ injuries_{c,y,a,s}}$$

where:

c = country
y = year
a = age
s = sex

Since the TMREL is zero, the occupational injuries PAF is simply the ratio of occupational fatal injuries to total fatal injuries. Fatal injury totals were obtained from GBD 2023 causes of death.

References

1. International Labour Organization (ILO). International Labour Organization Database (ILOSTAT) - Employment by Sex and Economic Activity. International Labour Organization (ILO).
2. International Labour Organization (ILO). International Labour Organization Database (ILOSTAT) - Employment by Sex and Occupation. International Labour Organization (ILO).
3. International Labour Organization (ILO). International Labour Organization Database (ILOSTAT) - Fatal Injuries by Sex and Economic Activity. International Labour Organization (ILO).
4. International Labour Organization (ILO). International Labour Organization LABORSTA Economically Active Population, Estimates and Projections, October 2011. International Labour Organization (ILO), 2011.
5. Lin R-T, Takahashi K, Karjalainen A, et al. Ecological association between asbestos-related diseases and historical asbestos consumption: an international analysis. *Lancet* 2007; 369: 844–9.
6. Goodman M, Morgan RW, Ray R, Malloy CD, Zhao K. Cancer in asbestos-exposed occupational cohorts: a meta-analysis. *Cancer Causes Control* 1999; **10**: 453–65.
7. Kauppinen T, Toikkanen J, Pedersen D, *et al.* Occupational exposure to carcinogens in the European Union. *Occupational and Environmental Medicine* 2000; **57**: 10–8.
8. Driscoll T, Jacklyn G, Orchard J, Passmore E, Vos T, Freedman G, Lim S, Punnett L. The global burden of occupationally related low back pain: estimates from the Global Burden of Disease 2010 study. *Ann Rheum Dis*. 2014; 73(6): 975-81.
9. Haddaway, N. R., Page, M. J., Pritchard, C. C., & McGuinness, L. A. (2022). PRISMA2020: An R package and Shiny app for producing PRISMA 2020-compliant flow diagrams, with interactivity for optimised digital transparency and Open Synthesis Campbell Systematic Reviews, 18, e1230.
10. Blanc PD, Iribarren C, Trupin L, Earnest G, Katz PP, Balmes J, Sidney S, Eisner MD. Occupational exposures and the risk of COPD: dusty trades revisited. *Thorax*. 2009; 64(1): 6-12.
11. Zheng P, Barber R, Sorensen RJD, Murray CJL, Aravkin AY. Trimmed constrained mixed effects models: formulations and algorithms. *J Comput Graph Stat* 2021; **30**: 544–56.
12. GBD 2021 Risk Factors Collaborators. Global burden and strength of evidence for 88 risk factors in 204 countries and 811 subnational locations, 1990–2021: a systematic analysis for the Global Burden of Disease Study 2021. *Lancet* 2024; 403: 2162–203.
13. Wilson DH, Walsh PG, Sanchez L, *et al.* The epidemiology of hearing impairment in an Australian adult population. *Int J Epidemiol* 1999; 28: 247–52
14. Kauppinen T, Toikkanen J, Pederson D, Young R, Kogevinas M, Ahrens W, et al. Occupational Exposure to Carcinogens in the European Union in 1990-93. Helsinki, Finland: Finnish Institute of Occupational Health; 1998.
15. Driscoll T, et al. The global burden of non-malignant respiratory disease due to occupational airborne exposures. *American Journal of Industrial Medicine 2005; 48*(6): 432-445.
16. Nelson, D. I., Concha-Barrientos, M., Driscoll, T., Steenland, K., Fingerhut, M., Punnett, L. & Corvalan, C. (2005). The global burden of selected occupational diseases and injury risks: Methodology and summary. *American journal of industrial medicine*, 48(6), 400-418
17. Zheng P, Afshin A, Biryukov S, *et al.* The Burden of Proof studies: assessing the evidence of risk. *Nat Med* 2022; **28**: 2038–44.
18. GBD 2021 Risk Factors Collaborators. Global burden and strength of evidence for 88 risk factors in 204 countries and 811 subnational locations, 1990-2021: a systematic analysis for the Global Burden of Disease Study 2021. *Lancet* 2024; **403**: 2162–203.

Residential radon pollution

Flowchart

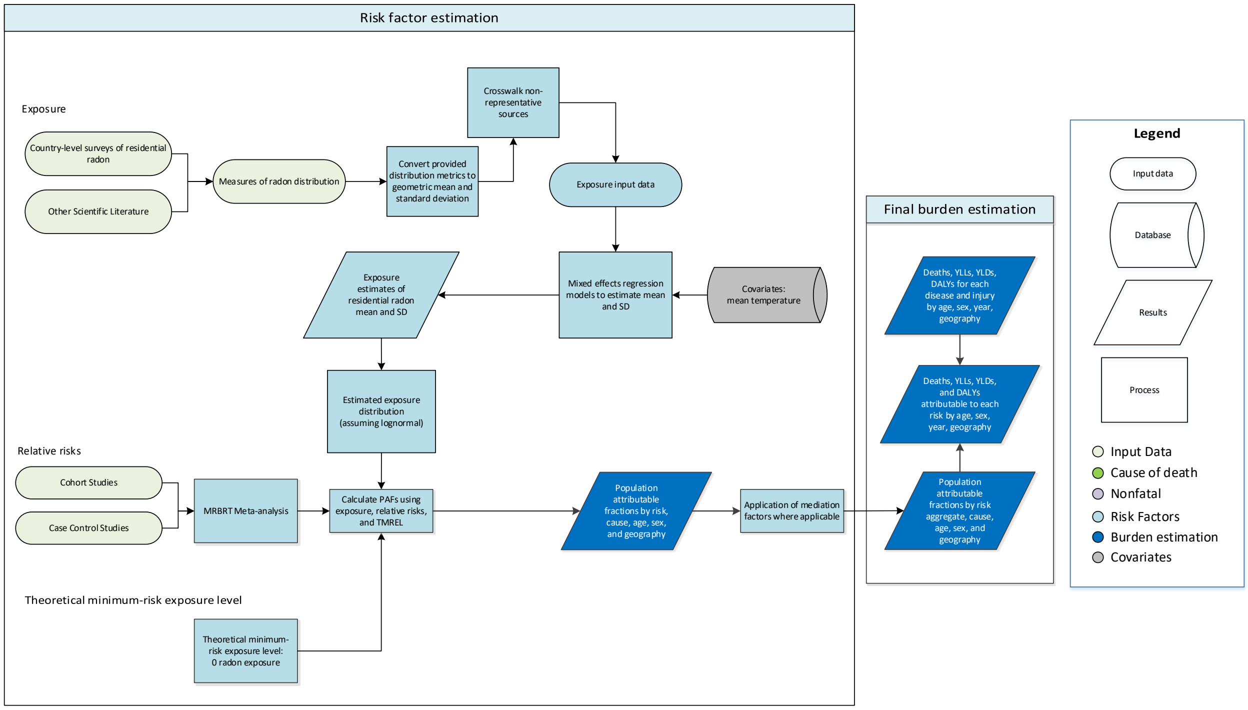

Input data and methodological summary

Exposure

Definition

Radon is a radioactive gas produced as a byproduct of the decay chain of uranium, occurring naturally within the Earth's crust. Some fraction of this natural radon production escapes into the atmosphere, where it is present at low concentrations unless buildup is caused by release into enclosed spaces such as homes, mines, or caves. Radon exposure is expressed as average daily exposure to indoor air radon gas levels measured in Becquerels (disintegrations per second) per cubic metre (Bq/m^3). In the GBD study, we specifically quantify the burden due to indoor radon exposure.

Input data

An expert group curated the original dataset for residential radon exposure. We updated data sources for GBD 2019, and no additional data were included for GBD 2023. Data sources include national surveys, government reports, and scientific literature. We include any sources that report results of residential radon measurement in homes (not schools or workplaces). Due to limited availability of data, we also include sources that are not representative of an entire population, but exclude studies or surveys explicitly conducted in high-radon areas.

From each source, we extracted all available information required to estimate the distribution of radon exposure, including arithmetic mean and standard deviation, geometric mean and standard deviation, median, IQR, range, max, sample size, confidence interval, and/or standard error.

Modelling strategy

Literature suggests that radon exposure follows a lognormal distribution both on the household and national levels.[1] We therefore assume that the distribution of radon exposure is lognormal within any one GBD geography or study. For studies reporting at least one measure of central tendency (arithmetic mean, geometric mean, or median) and a measure of spread (standard deviation [arithmetic or geometric], IQR, confidence interval, or standard error), we are able to directly calculate the geometric mean and geometric standard deviation of the underlying distribution. For those only reporting a measure of central tendency and range, max, or sample size, we estimate the geometric mean and standard deviation based on several assumptions.

- When the range or max is provided, we assume that the range divided by 4 is a reasonable estimate of standard deviation, because 95% of observations occur within 2 standard deviations of the mean. We perform this calculation on log-transformed observations.
- For studies that only provide a measure of central tendency and sample size, we impute standard deviation based on sample size from observed associations between standard deviation and sample size for other available input data.
- If we only have the mean, we impute the median standard deviation of all other studies.

Once we convert all estimates to the mean and standard deviation of a lognormal distribution, we run all analyses on log-transformed data to meet assumptions of normality.

Though we exclude studies intentionally performed in high-exposure areas, we still see a bias in studies that are not representative of their geography. To account for this difference, we perform a crosswalk adjustment using the meta-regression—Bayesian, regularised, trimmed (MR-BRT) tool. We match all locations where we have both representative and non-representative sources. These locations include Canada, Egypt, Gansu, Greece, Hiroshima, Ireland, Jordan, Portugal, Puebla, Querétaro, Romania, San Luis Potosí, Saudi Arabia, Shanghai, Spain, Syria, Taiwan (province of China), Türkiye, Urban Andhra Pradesh, Urban Assam, Urban Gujarat, Urban Haryana, Urban Karnataka, Urban Kerala, Urban Maharashtra, Urban Meghalaya, Urban Punjab, Urban Rajasthan, Urban Tripura, and Urban Uttar Pradesh. We perform the following model on the log difference of the log of the geometric means:

Let $ref = \log(geometric\ mean\ representative)$, and

$alt = \log(geometric\ mean\ non\ representative)$.

$$log\left(\frac{alt}{ref}\right) \sim Beta$$

$$ref \sim e^{-Beta} * alt$$

$$ref \sim (adjustment\ factor) * alt$$

We use the results of this crosswalk to downscale all non-representative input sources and inflate their uncertainty in the model. The effect is equivalent to scaling the log of the geometric mean of non-representative sources by a factor of 0.887.

Table 1: MR-BRT crosswalk adjustment factor for residential radon exposure

Data input	Reference or alternative case definition	Gamma	Beta coefficient, log (95% UI)*	Adjustment factor**
Geographically representative survey or report	Reference	0.25	---	
Estimate not representative of geographical unit	Alternative		0.120 (0.107–0.132)	0.887 (0.876–0.899)

**MR-BRT crosswalk adjustments can be interpreted as the factor the alternative case definition is adjusted by to reflect what it would have been had it been measured using the reference case definition. If the log/logit beta coefficient is negative, then the alternative is adjusted up to the reference. If the log/logit beta coefficient is positive, then the alternative is adjusted down to the reference.*
***The adjustment factor column is the exponentiated negative beta coefficient. For log beta coefficients, this is the relative rate between the two case definitions. For logit beta coefficients, this is the relative odds between the two case definitions.*

After crosswalking non-representative sources, we run a model to estimate the log of the geometric mean residential radon exposure for each GBD most-detailed location. Because radon is naturally occurring and is not considered to have much long-term temporal fluctuation, we used a mixed effects linear model independent of time.[2] The model included nested random effects on super-region, region, and location (most detailed) and one fixed effect covariate: long-term mean temperature (average annual temperature averaged over 1990 to 2020) as a proxy for adequate building ventilation. We weighted the model by inverse standard error. We also tested weighting by inverse variance and sample size, but this resulted in an unstable fit. To predict the log of the geometric mean we used the following model:

$$\log(geometric\ mean) \sim \beta * long\ term\ mean\ temp + (1|super\ region) + (1|region) + (1|location)$$

Table 2: Regression coefficients for predicting mean radon

Input	Coefficient (95% UI)
Intercept	4.008 (3.519 to 4.502)
Long-term mean temperature	–0.0388 (–0.0619 to –0.0145)

We also ran a model to predict the standard deviation (in log space) for each location. We included all studies that were representative of a geography and that included a measure of spread for which we were able to directly calculate the standard deviation. We used a mixed effects linear regression of standard deviation on mean, including random effects on location (most detailed) and region. The model was not stable when including super-region. To predict the log of the geometric standard deviation we used the following model:

$$\log(geometric\ standard\ deviation) \sim \beta * \log(geometric\ mean) + (1|region) + (1|location)$$

Table 3: Regression coefficients for predicting standard deviation of radon

Input	Coefficient (95% UI)
Intercept	0.619 (0.397 to 0.848)
Log(geometric mean)	0.014 (–0.027 to 0.054)

We used the estimated mean and standard deviation for each location to generate an exposure distribution for use in population attributable fraction calculation.

Theoretical minimum risk exposure level

The GBD 2017 study defined the theoretical minimum risk exposure level (TMREL) as a uniform distribution from 7 to 14 Bq/m^3 representing outdoor air. However, in GBD 2019, we updated the radon TMREL to 0. The basis for this decision is that it is theoretically possible with mitigation strategies to reduce all indoor radon exposure to 0. The TMREL of 0 remains unchanged for GBD 2023.

Relative risks

Input data

In GBD 2017, the relative risk (RR) was based on a single meta-analysis (Darby et al., 2005) which reported an RR of 1.16 (1.05–1.31) per 100 Bq/m^3 increase in radon exposure.[3] In GBD 2019, we conducted a systematic review of studies examining residential exposure to radon and lung cancer incidence or mortality. We extracted the component studies from the following meta-analyses: Lubin et al. 2003, Darby et al. 2005, Krewski et al. 2005, Zhang et al. 2012, Torres-Durán et al. 2014, and Dobrzynski et al. 2018.[3,4,5,6,7,8] We excluded studies that were cross-sectional or ecological, studied high-risk populations such as miners, or were not available in English. When multiple studies were published on the same dataset, we retained only the study with the longest follow-up. We also excluded studies that only reported cumulative exposure, because this does not align with our exposure definition.

For GBD 2023, as in GBD 2019, we assumed a log-linear relationship between RR and radon exposure. As before, we converted all reported risks to the RR increase per 100-unit increase in Bq/m^3. Some studies only reported RR between exposure categories. In these instances, we took the mean, median, or midpoint of the exposed and unexposed categories to calculate an "exposure range." We then scaled the reported RR based on that exposure range to estimate the corresponding increase per 100 units. This resulted in a total of 49 estimates from 25 studies in 12 countries including England, the Czech Republic, Finland, France, Germany, Italy, Spain, Sweden, the USA, China, Denmark, and Japan.

For those studies that reported no confidence intervals or standard error, we imputed the standard error based on sample size. To do this we created a model of the following form:

$$se \sim \beta * \frac{1}{\sqrt{n}}$$

Where we predict the standard error, *se*, as a function of some constant, β, times the inverse square root of the sample size, *n*. Here β is an estimate of the population-level standard deviation.

MR-BRT meta-regression

As in GBD 2019, we used the MR-BRT meta-regression tool to estimate a summary effect size from the 49 estimates of the RR increase per 100-unit change in exposure. Since GBD 2021, there were several key updates to the meta-regression process. First, we implemented automated covariate selection to detect significant covariates from those extracted to quantify between-study heterogeneity. The MR-BRT automated covariate selection tool implements a two-step process. First, a series of loosening Lasso penalty parameters are applied to a log-linear meta-regression on all input effect size observations. Then, covariates with a non-zero coefficient are tested for significance using a Gaussian prior (significance threshold = 0.05). No significant covariates were detected for residential radon, so we did not include covariates for selection bias (percentage of cohort retained through follow-up) and quality of exposure measurement (binary indicator for whether or not studies included a full residential history) as we did in GBD 2019. Additionally, we trimmed 10% of input data during model fitting in accordance with GBD protocol across risk factor teams.

We generated 1000 predictions of the effect size for use in calculating burden estimates. These predictions were created using predictions of between-study heterogeneity to characterise the model's uncertainty. We implemented the Fisher scoring correction to the heterogeneity parameter, which corrects for data-sparse situations. In such cases, the between-study heterogeneity parameter estimate may be 0, simply from lack of data. The Fisher scoring correction uses a quantile of gamma, which is sensitive to the number of studies, study design, and reported uncertainty.

The summary effect size for RR of lung cancer per 100 Bq/m^3 of radon exposure is 1.102 (95% UI 0.962–1.266).

Table 4: MR-BRT relative risk model parameters for residential radon pollution

**Covariate**	**Gamma (95% UI)**	**Beta coefficient, log (95% UI)**	**Exponentiated coefficient (95% UI)**
Exposure (per 1 Bq/m^3)	0 (0–0)	9.907e-4 (5.189e-4 to 1.484e-3)	1.00099 (1.00052–1.00149)

Figure 1: Residential radon pollution and lung cancer risk literature funnel plot

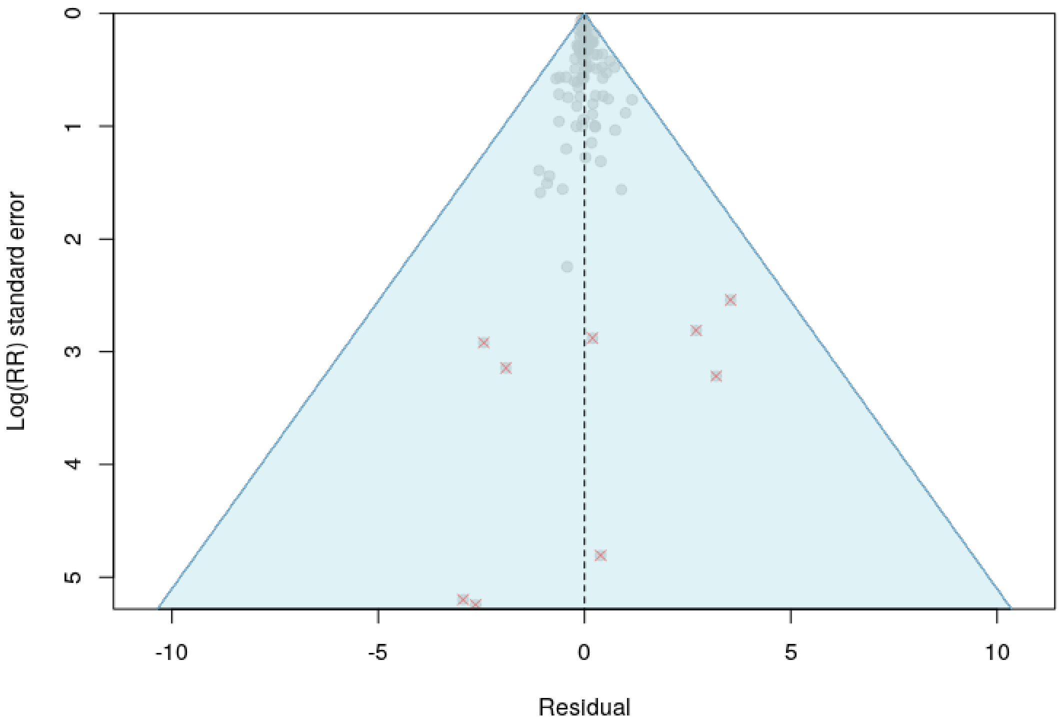

Figure 2: Residential radon pollution and lung cancer log-linear relative risk curve

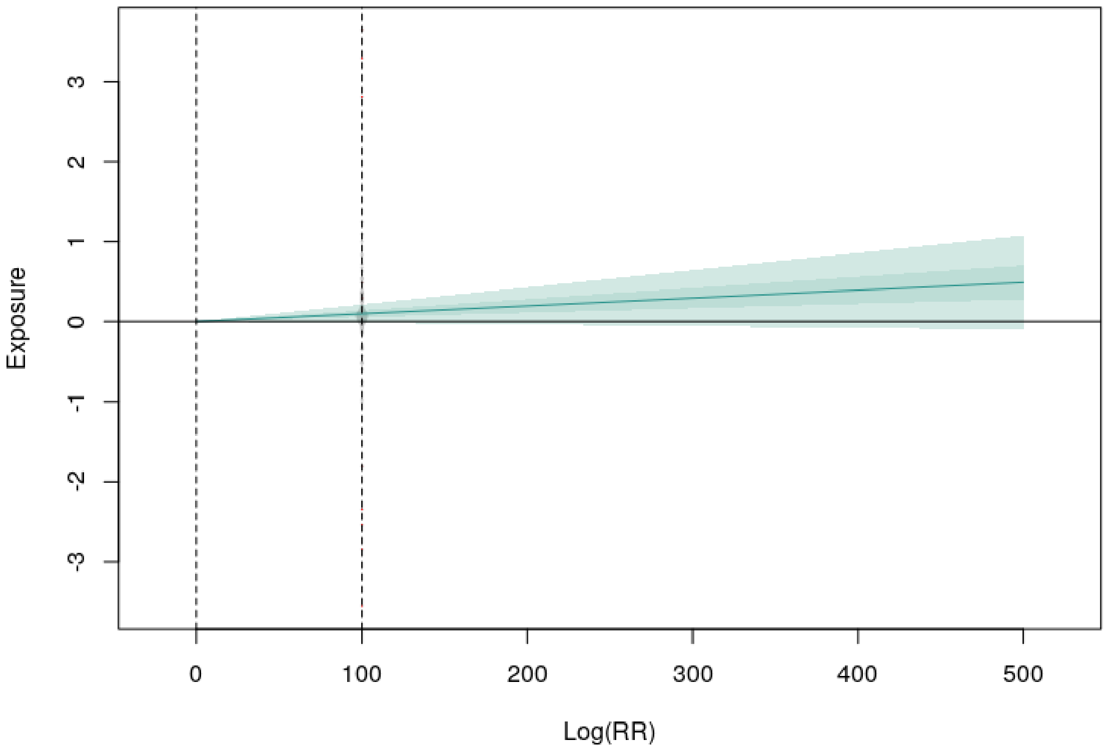

Risk–outcome scoring

Since GBD 2021, we also implemented risk–outcome scoring in the Burden of Proof framework. Risk–outcome scores provide an empirical measure of the strength of evidence for risk–outcome pairs across risk factors in the GBD and are therefore useful for standardised comparison. Risk–outcome scores evaluate the area between the lower bound of the 95% uncertainty interval and the x-axis for harmful risk factors, including residential radon pollution.

Prior to generating a risk–outcome score, we conducted an additional post-analysis step to detect and flag publication bias in the input data. This approach is based on the classic Egger's regression strategy, which is applied to the residuals in our model. In the current implementation, we do not correct for publication bias but flag the risk–outcome pairs where the risk for publication bias is significant. Publication bias was not detected for residential radon pollution.

To calculate the risk–outcome score, we generated an uncertainty interval from 1000 draws of the adjusted summary effect size (retaining uncertainty information from between-study heterogeneity predictions and the Fisher information boost). We then evaluated the risk–outcome score between the 15th and 85th percentiles of the input data exposure distribution (0–100 Bq/m^3). The final risk–outcome score is –0.009, which corresponds to a star rating of 1.

References

1. Daraktchieva Z, Miles JCH, McColl N. Radon, the lognormal distribution and deviation from it. *J Radiol Prot* 2014; 34: 183–190.
2. Steck DJ. Annual average indoor radon variations over two decades. *Health Phys*. 2009;96(1):37-47.
3. Darby S, Hill D, Auvinen A, et al. Radon in homes and risk of lung cancer: collaborative analysis of individual data from 13 European case-control studies. *BMJ*. 2005;330(7485):223
4. Lubin JH. Studies of radon and lung cancer in North America and China. *Radiation Protection Dosimetry*. 2003Jan;104(4):315–9.
5. Krewski D, Lubin JH, Zielinski JM, Alavanja M, Catalan VS, Field RW, et al. Residential Radon and Risk of Lung Cancer. *Epidemiology*. 2005;16(2):137–45.
6. Zhang Z-L, Sun J, Dong J-Y, et al. Residential radon and lung cancer risk: an updated meta- analysis of case-control studies. *Asian Pac J Cancer Prev* 2012; 13: 2459–65.
7. Torres-Durán MCAD, Barros-Dios JM, Fernández-Villar A, Ruano-Ravina A. Residential radon and lung cancer in never smokers. A systematic review. *Cancer Letters*. 2014;345(1):21–6.
8. Dobrzyński L, Fornalski KW, Reszczyńska J. Meta-analysis of thirty-two case–control and two ecological radon studies of lung cancer. *J Radiat Res* 2018; 59: 149–63.

Secondhand smoke

Flowchart

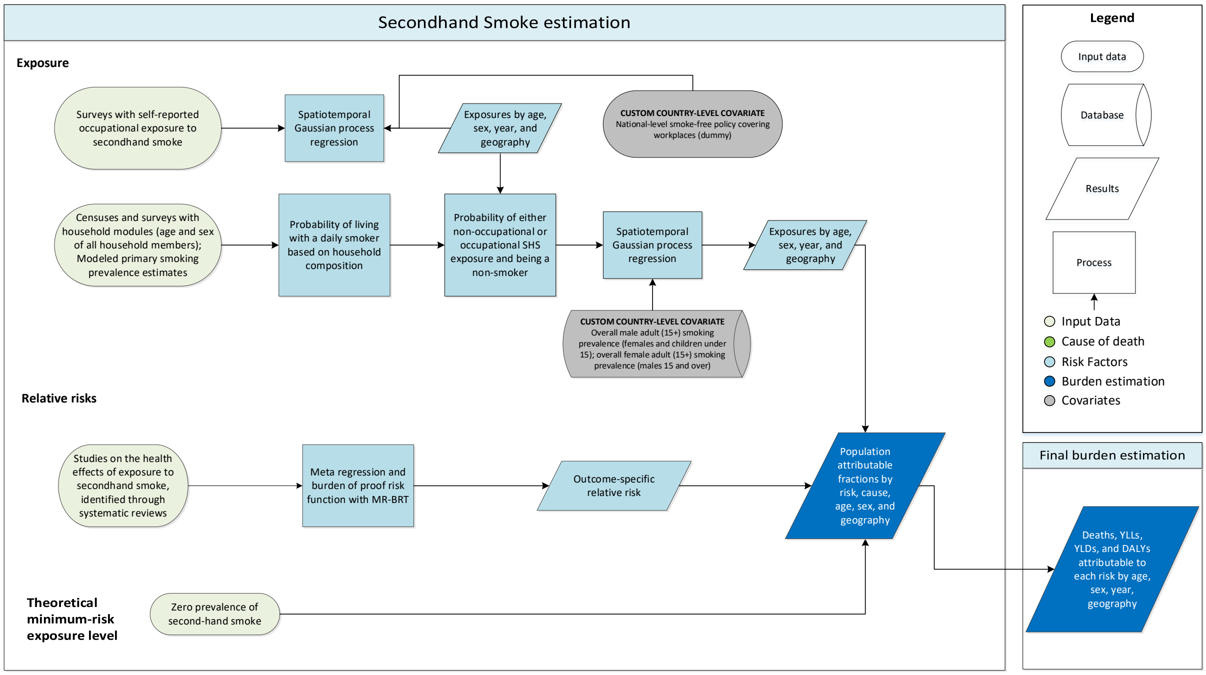

Definition

We define secondhand smoke (SHS) exposure as current exposure to secondhand tobacco smoke at home or work. We use household composition as a proxy for household secondhand smoke exposure and assume that all persons living with a daily smoker are exposed to secondhand smoke. We use surveys to estimate the proportion of the population exposed to secondhand smoke at work. We only consider non-smokers to be exposed to secondhand smoke. Non-smokers are defined as all persons who are not daily smokers. Ex-smokers and occasional smokers are considered non-smokers in this analysis. Exposure is evaluated for both children and adults.

Input data

Exposure

To calculate the proportion of the population exposed to SHS at work, by age and sex, we used cross-sectional surveys that ask respondents about self-reported occupational SHS exposure. When possible, we restricted the exposed population to those exposed in indoor workplace settings. Sources include the Global Adult Tobacco Surveys (GATS), Eurobarometer Surveys, WHO Stepwise Approach to NCD Risk Factor Surveillance (STEPS) Surveys, and other regional and national survey series.

To calculate the proportion of non-smokers who live with at least one daily smoker, two types of data were used: 1) unit record data on household composition, which included the ages and sexes of all persons living in the same household, and 2) GBD daily smoking estimates for each location, year, sex, and age group. Major survey series with a household composition module – including the Demographic Health Surveys (DHS), the Multiple Indicator Cluster Surveys (MICS), and the Living Standards Measurement Surveys (LSMS) – and national and subnational censuses, which included those captured in the Integrated Public Use Microdata Series (IPUMS) project, were used.

To identify new exposure sources for GBD 2023, we searched the Global Health Data Exchange (GHDx) using the keywords *"environmental tobacco smoke"*, for workplace exposure, and *"family composition"*, for identifying household composition modules. We prioritised the extraction of surveys used for estimating workplace exposure and new household modules for filing in location and time gaps. Sources that reported exposure to SHS in a setting other than the workplace were not used. Due to the type of analysis performed, we restricted our data sources to those with available microdata (tabulated data-only sources were excluded). Given the nature of the data used in our models (microdata), no crosswalk for case definition adjustment or age and sex splitting processes were required.

Relative risk

For GBD 2023, we conducted systematic reviews of the literature to identify peer-reviewed studies (including case-control, cohort, case-cohort, or case-crossover studies) reporting on relative measures of association between SHS exposure and nine outcomes of interest: lung and breast cancer, ischaemic heart disease (IHD), stroke, chronic obstructive pulmonary disease (COPD), lower respiratory infections (LRI), type 2 diabetes, otitis media, and asthma. We conducted our searches in two databases: PubMed and Web of Science. We reviewed over 7000 records published between January 1, 1970, and July 31, 2022. Additionally, meta-analyses and systematic reviews identified through our search were reviewed and underlying studies were considered for inclusion if not previously captured by our search strings (Figures 1-9).

Following our pre-defined inclusion and exclusion criteria,[1] we identified publications suitable for our analysis across the nine risk–outcome pairs, all reporting relative risks associated with SHS measured dichotomously, a methodological change from GBD 2021 where the relative risks associated with varying levels of SHS exposure were estimated for lung cancer, IHD, stroke, COPD, LRI, and diabetes. To construct these risk curves in GBD 2021, binary and categorical exposure data from published literature had to be converted into a continuous metric reflecting the number of cigarettes smoked per smoker per day for each location-year using GBD tobacco consumption estimates. The decision to pivot from this methodology stemmed from several considerations: 1) Our systematic review revealed that 90% of the studies identified reported risks associated with binary exposure classifications rather than a continuous spectrum of exposure, facilitating the inclusion of a larger number of studies in our models; 2) Transitioning from a proxy of exposure to a direct measure of SHS exposure simplifies the communication around our results; 3) The new approach eliminates the reliance on cigarette consumption estimates (and its uncertainty), meaning the observed heterogeneity among studies now reflects the input data alone rather than our data manipulation.

Further, we only included studies that reported risk estimates (relative risk, hazard ratio, or odds ratio) with confidence intervals, standard errors, or enough information to quantify uncertainty. In addition, we excluded studies that only reported former exposure to secondhand smoke (eg, child exposure during pregnancy) or only exposure among current smokers.

Our team extracted data from eligible studies using a specifically designed template which captured essential information including study and sample characteristics, definitions of exposure and outcomes, methods for determining these factors, effect sizes with corresponding uncertainties for each model/population, and the covariates factored into the statistical analyses.

Figure 1: PRISMA 2020 flow diagram for secondhand smoke and ischaemic heart disease

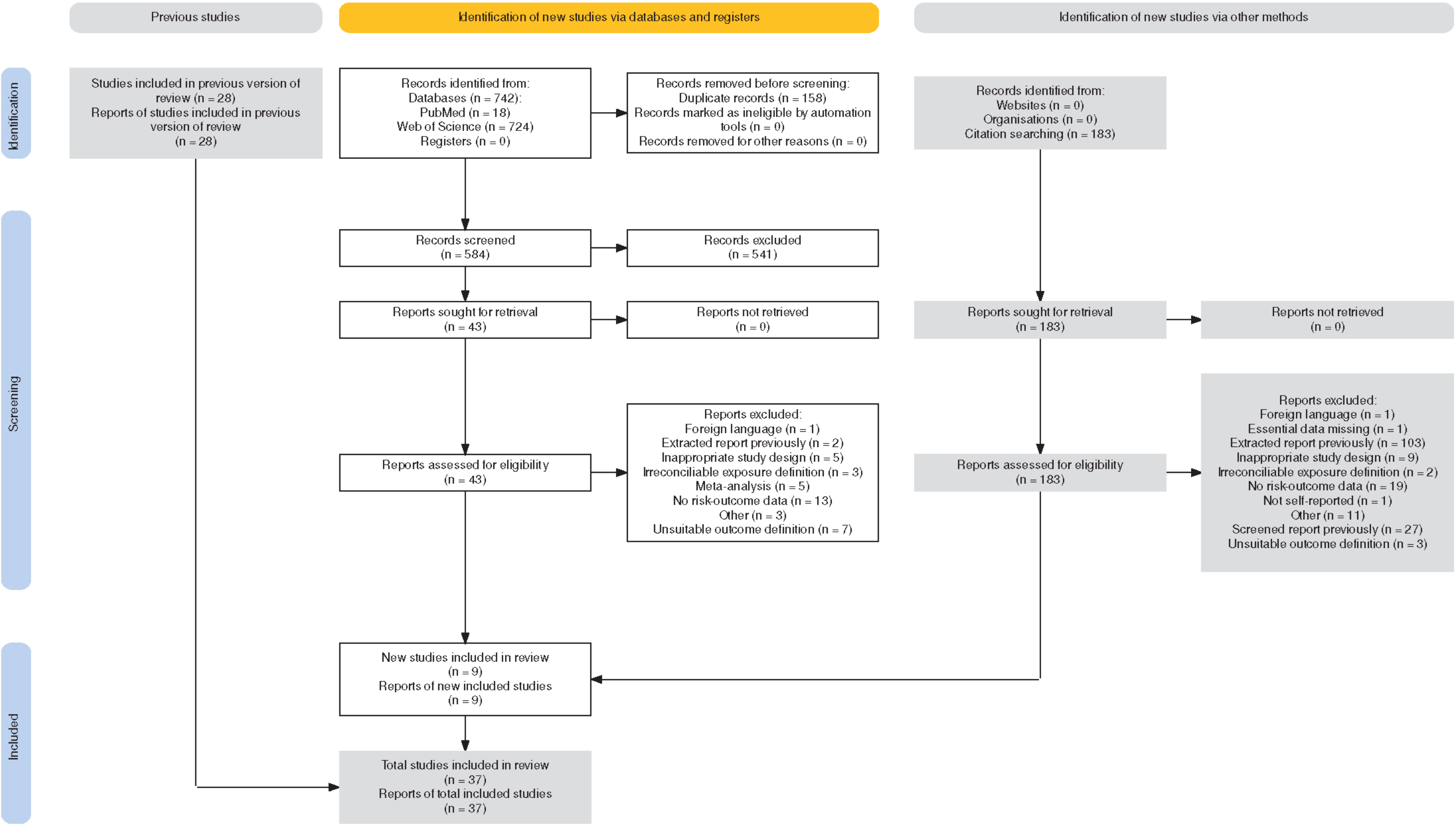

From: Page MJ, McKenzie JE, Bossuyt PM, Boutron I, Hoffmann TC, Mulrow CD, et al. The PRISMA 2020 statement: an updated guideline for reporting systematic reviews. BMJ 2021;372:n71. doi: 10.1136/bmj.n71. For more information, visit: http://www.prisma-statement.org/

Figure 2: PRISMA 2020 flow diagram for secondhand smoke and stroke

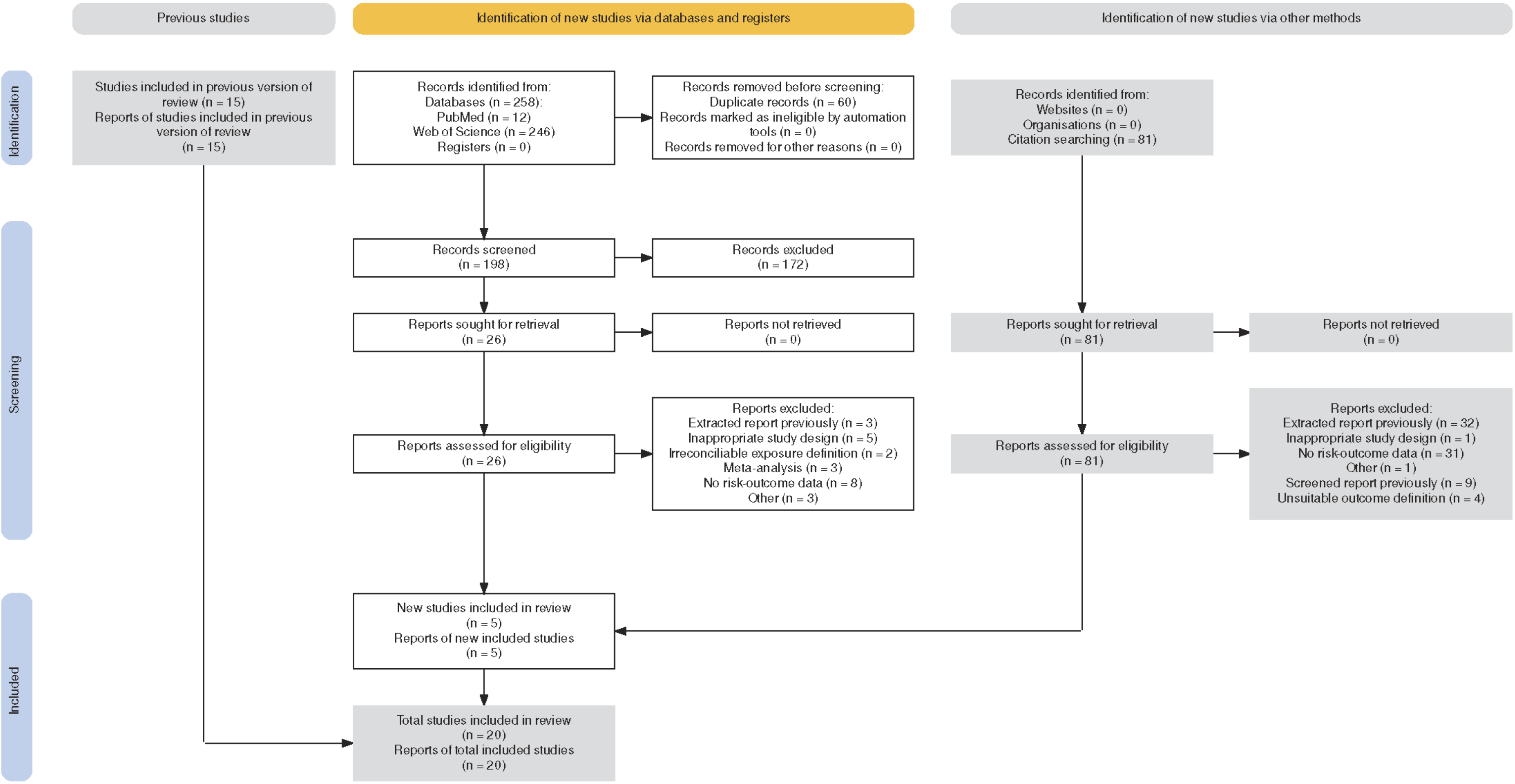

From: Page MJ, McKenzie JE, Bossuyt PM, Boutron I, Hoffmann TC, Mulrow CD, et al. The PRISMA 2020 statement: an updated guideline for reporting systematic reviews. BMJ 2021;372:n71. doi: 10.1136/bmj.n71. For more information, visit: http://www.prisma-statement.org/

Figure 3: PRISMA 2020 flow diagram for secondhand smoke and tracheal, bronchus, and lung cancer

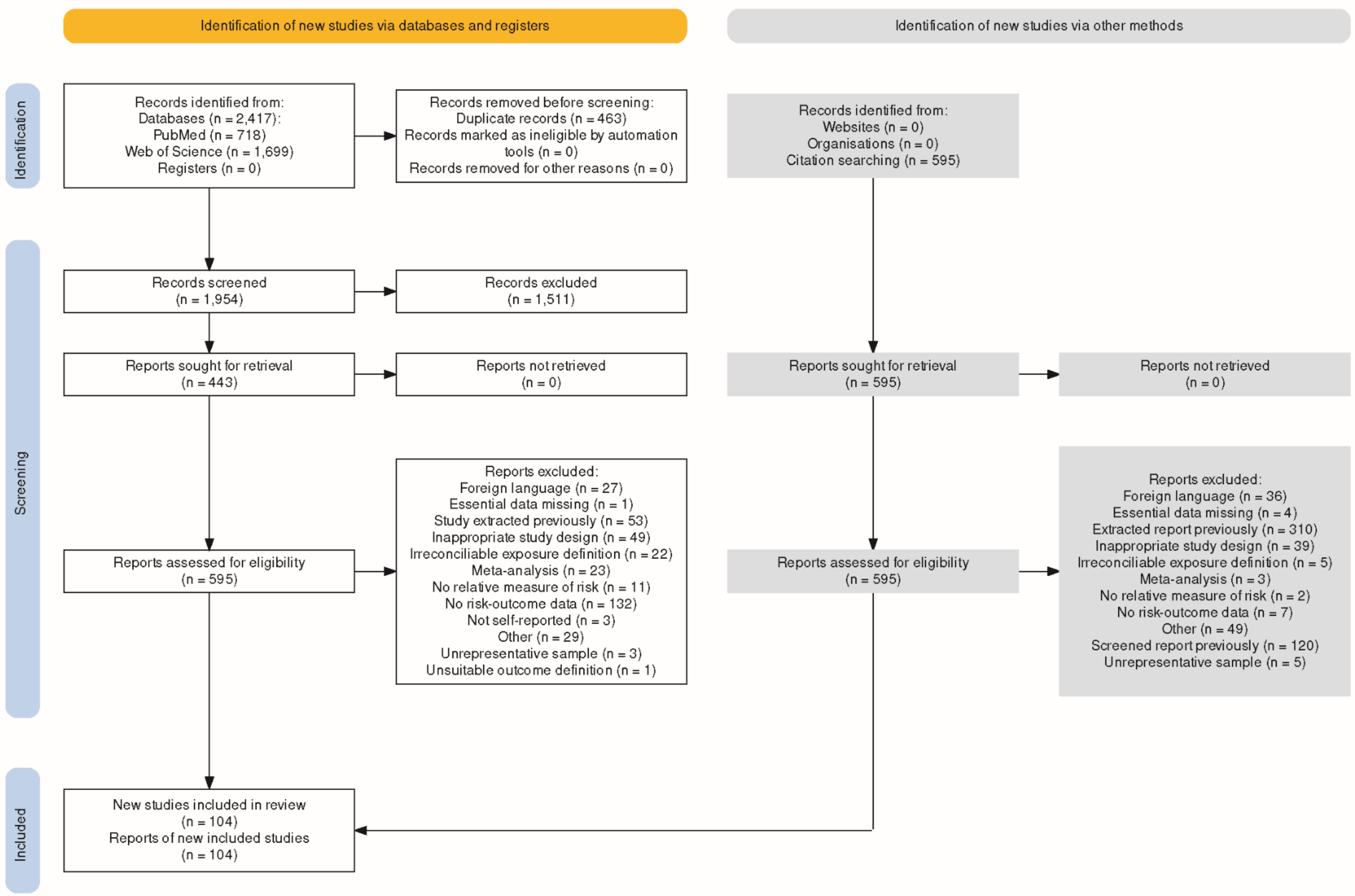

From: Page MJ, McKenzie JE, Bossuyt PM, Boutron I, Hoffmann TC, Mulrow CD, et al. The PRISMA 2020 statement: an updated guideline for reporting systematic reviews. BMJ 2021;372:n71. doi: 10.1136/bmj.n71. For more information, visit: http://www.prisma-statement.org/

Figure 4: PRISMA 2020 flow diagram for secondhand smoke and breast cancer

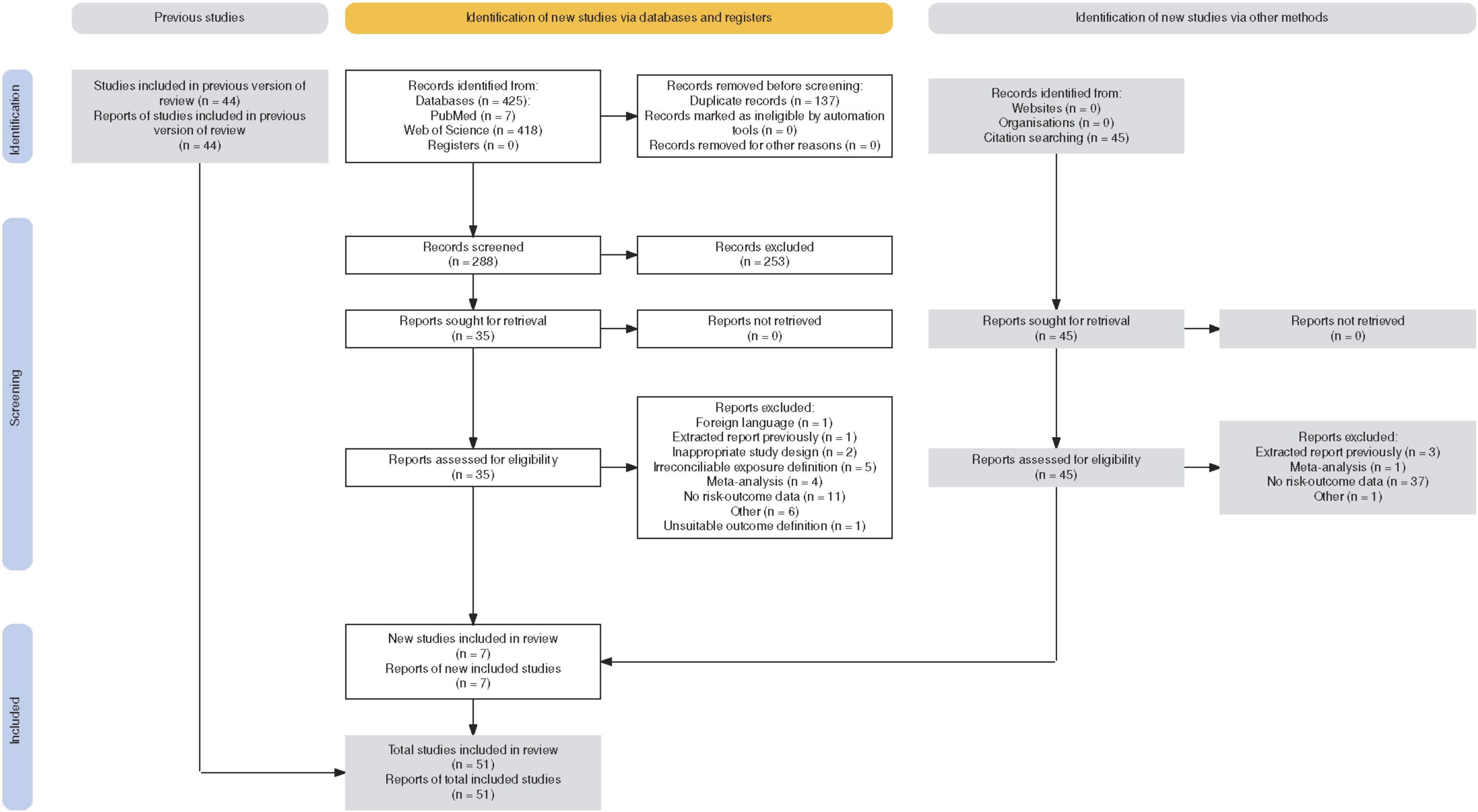

From: Page MJ, McKenzie JE, Bossuyt PM, Boutron I, Hoffmann TC, Mulrow CD, et al. The PRISMA 2020 statement: an updated guideline for reporting systematic reviews. BMJ 2021;372:n71. doi: 10.1136/bmj.n71. For more information, visit: http://www.prisma-statement.org/

Figure 5: PRISMA 2020 flow diagram for secondhand smoke and asthma

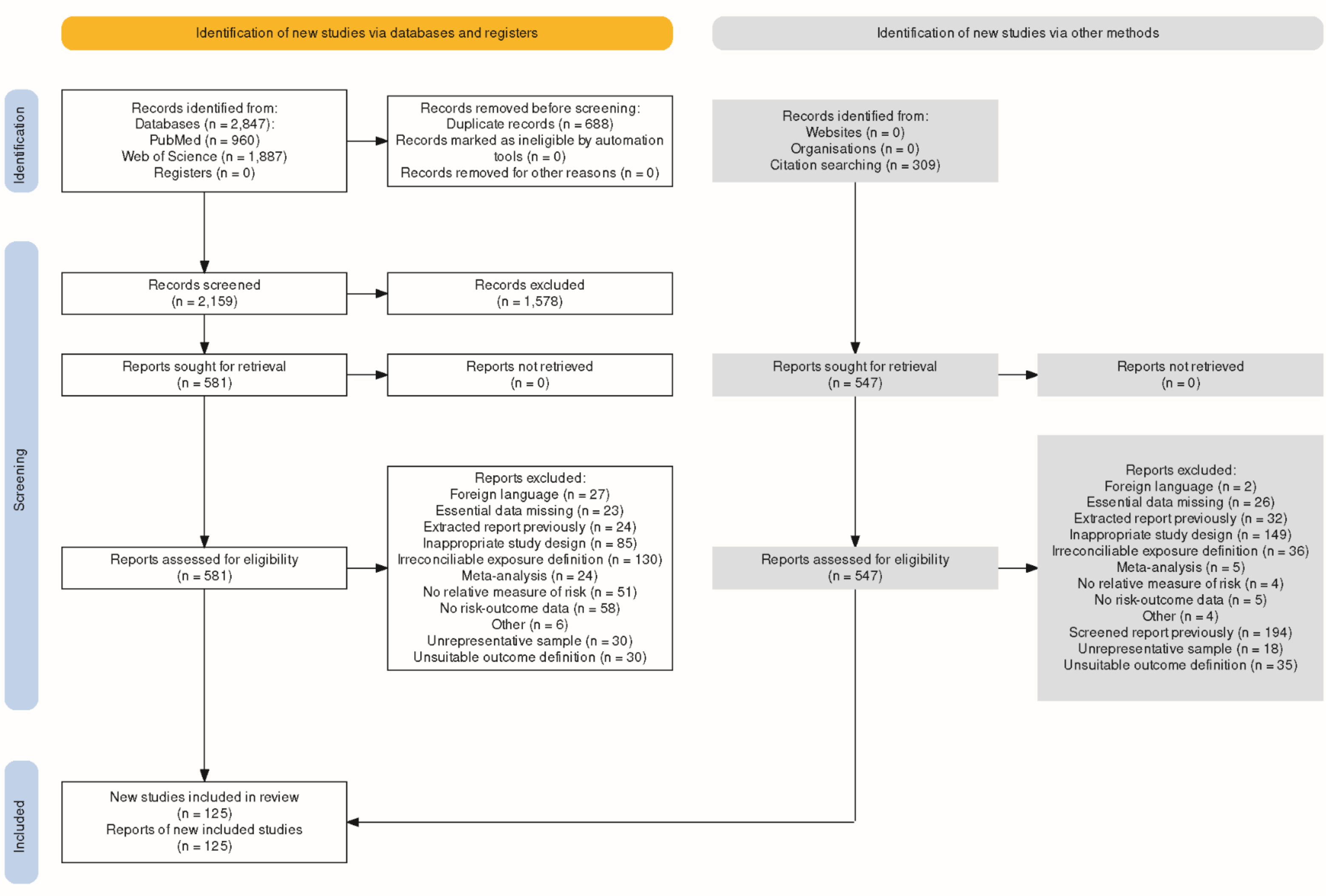

From: Page MJ, McKenzie JE, Bossuyt PM, Boutron I, Hoffmann TC, Mulrow CD, et al. The PRISMA 2020 statement: an updated guideline for reporting systematic reviews. BMJ 2021;372:n71. doi: 10.1136/bmj.n71. For more information, visit: http://www.prisma-statement.org/

Figure 6: PRISMA 2020 flow diagram for secondhand smoke and lower respiratory infections

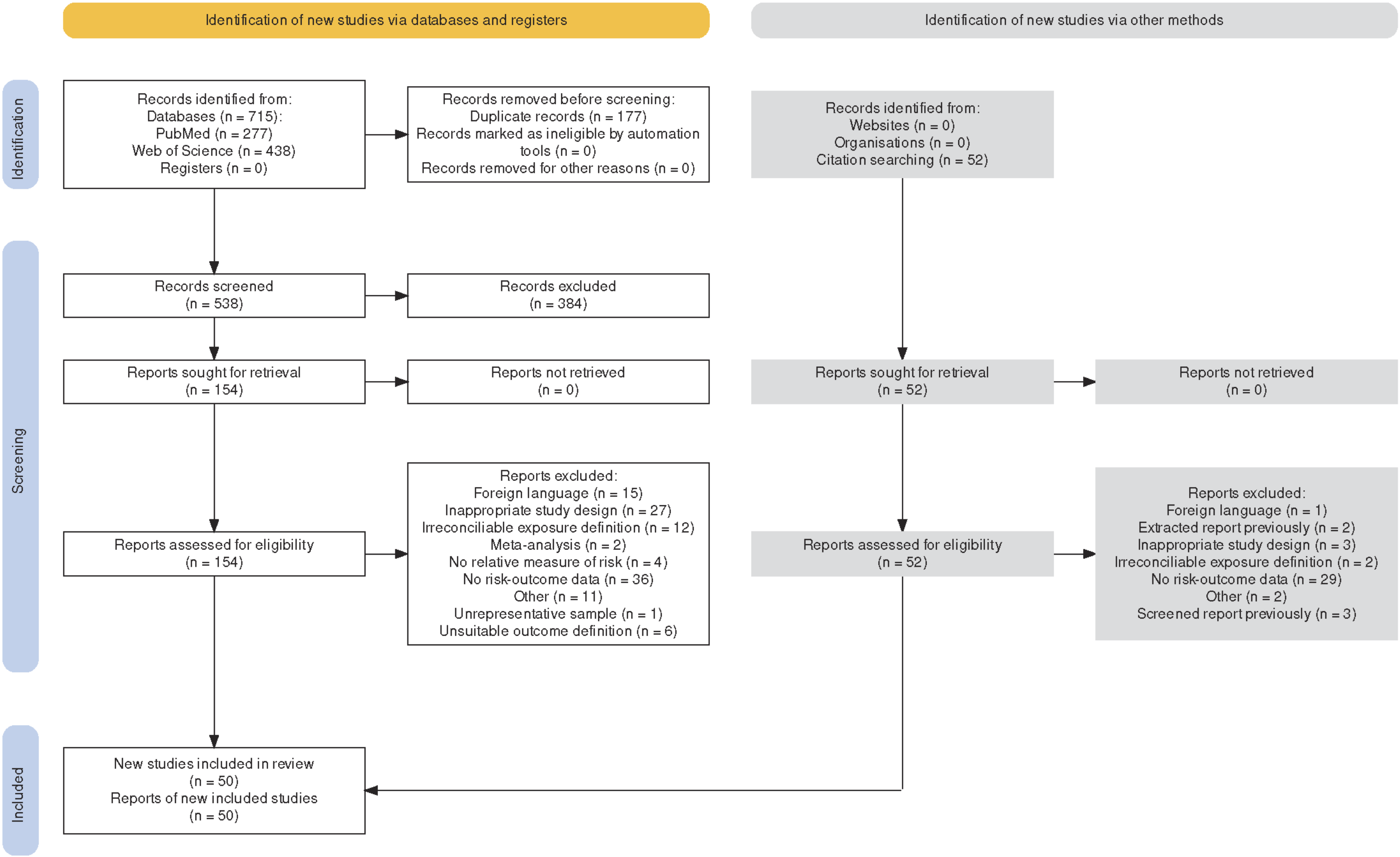

From: Page MJ, McKenzie JE, Bossuyt PM, Boutron I, Hoffmann TC, Mulrow CD, et al. The PRISMA 2020 statement: an updated guideline for reporting systematic reviews. BMJ 2021;372:n71. doi: 10.1136/bmj.n71. For more information, visit: http://www.prisma-statement.org/

Figure 7: PRISMA 2020 flow diagram for secondhand smoke and chronic obstructive pulmonary disease

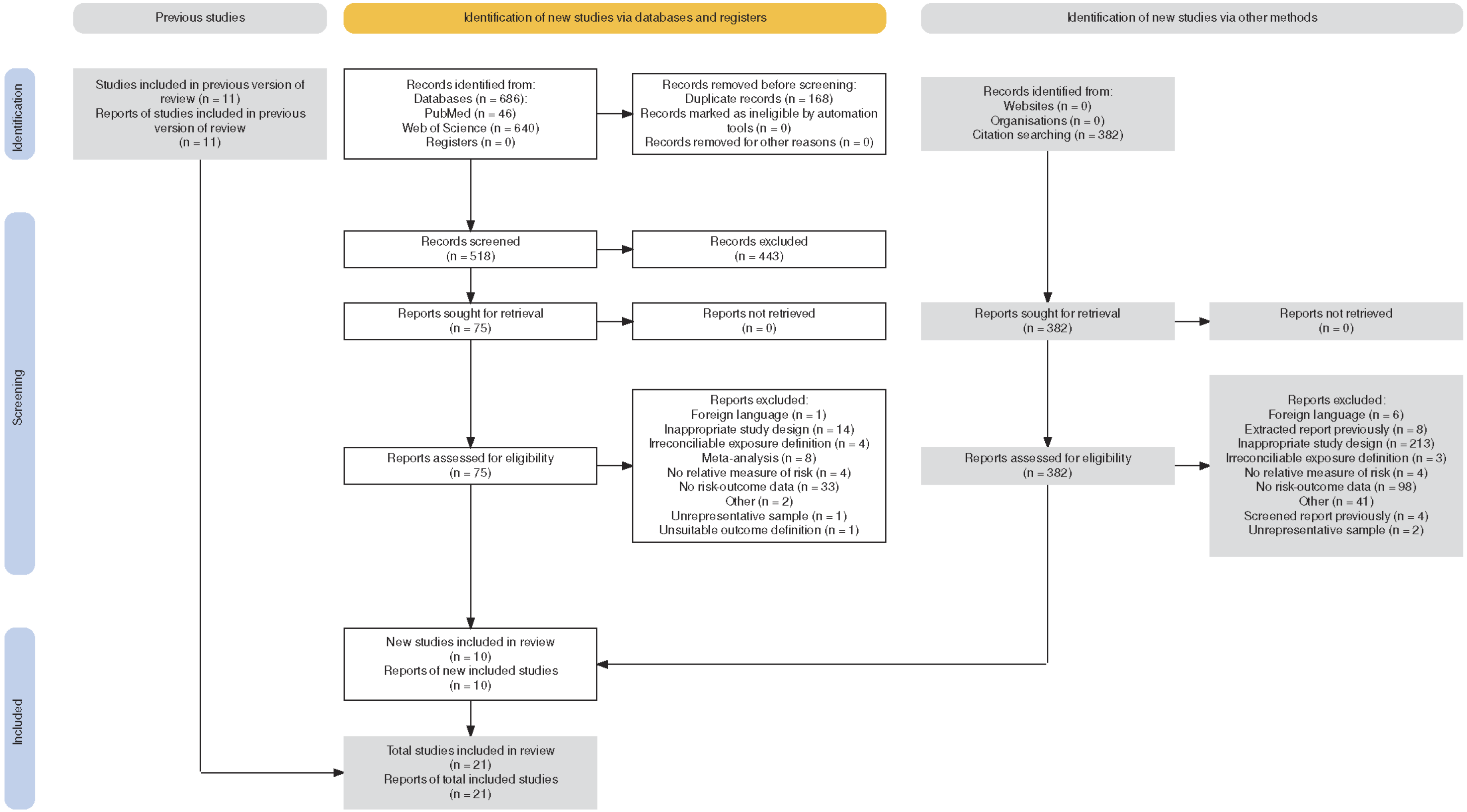

From: Page MJ, McKenzie JE, Bossuyt PM, Boutron I, Hoffmann TC, Mulrow CD, et al. The PRISMA 2020 statement: an updated guideline for reporting systematic reviews. BMJ 2021;372:n71. doi: 10.1136/bmj.n71. For more information, visit: http://www.prisma-statement.org/

Figure 8: PRISMA 2020 flow diagram for secondhand smoke and type 2 diabetes

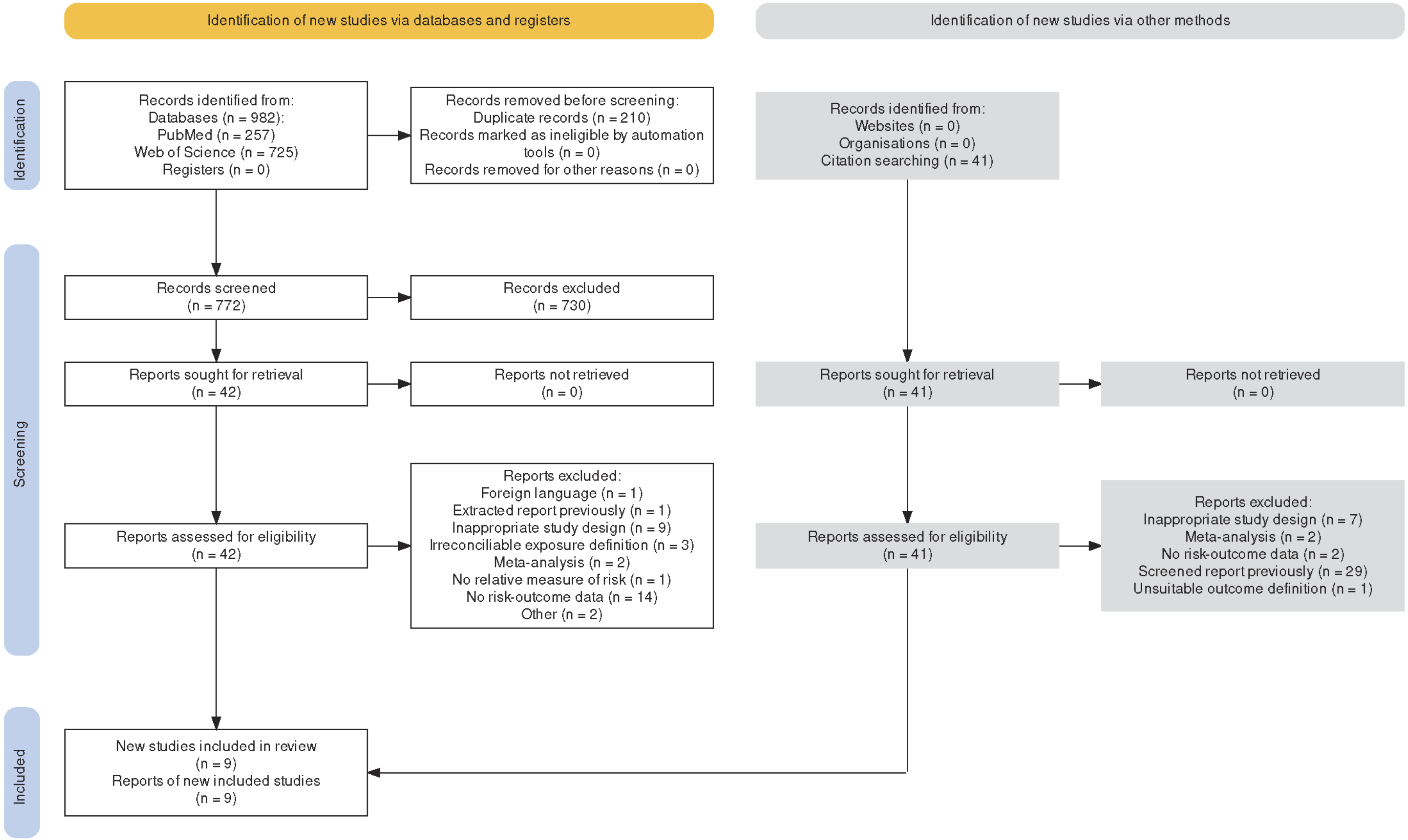

From: Page MJ, McKenzie JE, Bossuyt PM, Boutron I, Hoffmann TC, Mulrow CD, et al. The PRISMA 2020 statement: an updated guideline for reporting systematic reviews. BMJ 2021;372:n71. doi: 10.1136/bmj.n71. For more information, visit: http://www.prisma-statement.org/

Figure 9: PRISMA 2020 flow diagram for secondhand smoke and otitis media

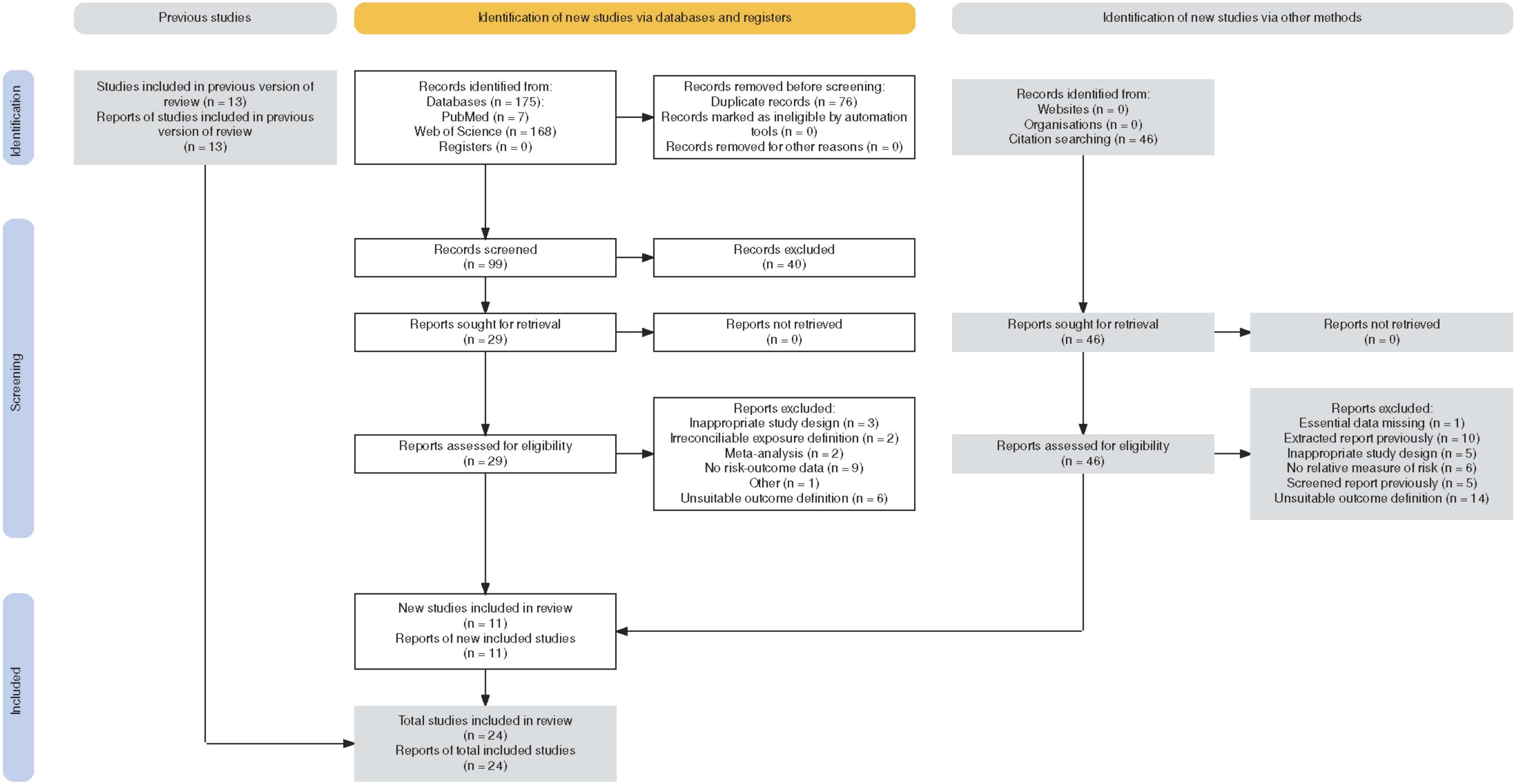

From: Page MJ, McKenzie JE, Bossuyt PM, Boutron I, Hoffmann TC, Mulrow CD, et al. The PRISMA 2020 statement: an updated guideline for reporting systematic reviews. BMJ 2021;372:n71. doi: 10.1136/bmj.n71. For more information, visit: http://www.prisma-statement.org/

Modelling strategy

Exposure

As in GBD 2021, we modelled prevalence of current exposure to secondhand smoke at work, by age, sex, location, and year, in a three-step spatiotemporal Gaussian process regression (ST-GPR), which generates exposure estimates from a mixed-effects hierarchical linear model plus weighted residuals smoothed across time, space, and age. For this, we first processed all data to capture exposure to secondhand smoke at work among anyone working primarily indoors. Using information from survey-specific gateway questions, we considered all those not currently working or not currently working primarily indoors not exposed to secondhand smoke.

The processed microdata were used to generate a complete time series from 1990 to 2023 for the proportion of the population exposed to tobacco smoke at the workplace using the ST-GPR. These estimates were produced for ages 15–79, assuming there would be no workplace SHS exposure outside this age range. The first step of the ST-GPR process is a linear mixed-effects regression of our data on a set of potentially predictive covariates. In addition to the daily smoking prevalence estimates taken from the GBD study covariates database, we updated our dummy covariate to reflect if a national-level smoking ban covering workplaces was in place in each location-year. The data used to create this covariate came mainly from the most recent iteration of the WHO report on the global tobacco epidemic.

Similarly to GBD 2021, we estimated the probability that each person is living with a smoker and is also a non-smoker themselves using set theory. Household composition data were used at the individual level to capture the ages and sexes of each person in the household. In the past, we analysed surveys with both household composition data and tobacco use questions and determined that the distribution of household size, mean age of the household members, and the age distribution were not significantly different between households with and without a self-reported smoker. Since we did not find that household composition varied between smokers and non-smokers, we then used the updated GBD 2023 daily smoking prevalence estimates to calculate the probability that each household member is a daily smoker. Next, we used the probability of the union of sets on each individual household member to calculate the overall probability that at least one of the other household members was a daily smoker.

With the estimated workplace exposure from ST-GPR, in order to avoid double counting, we calculated the probability that an individual is exposed through either household exposure or occupational exposure, given their age, sex, and household composition. Lastly, we multiplied this probability of exposure by the probability that the individual is not a smoker themselves (ie, 1 minus primary daily smoking prevalence for that person's location, year, age, and sex). We then collapsed these individual-level probabilities to produce average probabilities of exposure by location, year, age, and sex.

These final probabilities were modelled in the GBD ST-GPR framework. The linear model formula was fit separately by sex using restricted maximum likelihood in R. We used the sex-specific overall daily smoking prevalence for adults (age 15 and older) as a country-level covariate in the model. The overall male adult daily smoking prevalence was used as the covariate for females of all ages and for males under age 15. The overall female adult daily smoking prevalence was used as the covariate for males aged 15 and older.

All input datapoints from the probability calculation had a measure of uncertainty (variance and sample size) coming from the uncertainty of the primary smoking prevalence model and the sample size from the unit record data going into the modelling process. Geographical random effects were used in model fitting but were not used in prediction.

Theoretical minimum risk exposure level

The theoretical minimum risk exposure level for secondhand smoke is zero exposure among non-smokers, meaning that non-smokers would not live with any daily smokers and would not be exposed to tobacco smoke at their workplace.

Relative risk

We included asthma as a new outcome attributable to SHS in GBD 2023. For children ages 0–14, we estimated the burden of otitis media attributable to secondhand smoke exposure. For all ages, we estimated the burden of LRI and asthma, and for adults greater than or equal to 25 years of age, we estimated the burden of lung cancer, COPD, IHD, ischaemic stroke, breast cancer, and type 2 diabetes.

Estimating the relative risk (RR) of each of these outcomes occurring as a result of SHS exposure followed the burden of proof approach established by Zheng and colleagues[2,3] and instantiated in the meta-regression—Bayesian, regularised, trimmed (MR-BRT) tool. With MR-BRT, we synthesised input data to estimate the risk of developing each outcome for individuals currently exposed to SHS compared to those not exposed by trimming potentially distorting outliers; testing, selecting, and adjusting for bias covariates to account for known heterogeneity in input study-design characteristics (eg, confounding, selection bias, exposure measurement, level of adjustment, and aspects related to SHS exposure, such as source or setting of exposure); and quantifying remaining between-study heterogeneity (gamma) through random effects modelling and incorporating this value into uncertainty around the mean RR value.

MR-BRT further evaluates evidence for small-study effects and generates funnel plots that represent potential risk of publication or reporting bias, and also generates the burden of proof risk function (BPRF), defined for harmful dichotomous risks as the 5th and for protective risks as the 95th quantile of draws closest to the null from the distribution defined by the relative risk UIs inclusive of between-study heterogeneity. The BPRF is transformed into a risk–outcome score (ROS: the signed natural log(BPRF) divided by two for dichotomous risks) and mapped onto a star-rating system from one to five stars. These metrics complement RR estimates by providing an alternative, conservative measure of effect size and evidence strength that formally and systematically incorporates divergence/convergence across input findings, with higher positive ROS values and more stars corresponding to incrementally larger effects and stronger evidence for the risk–outcome relationship.

According to the Burden of Proof approach, all nine outcomes were significantly associated with SHS exposure, including asthma, which had not been included as an SHS-associated outcome in previous GBD cycles. A statistically significant association was identified when the uncertainty interval without between-study heterogeneity did not encompass the null value. The pooled relative risk and uncertainty without between-study heterogeneity informed the calculations of the disease burden attributable to SHS in GBD 2023. No sex-, location-, or age-specific relative risks were produced. Table 1 shows the main outputs from the burden of proof analysis for all nine health outcomes attributable to SHS. Detailed methods and results are published elsewhere.[1]

Table 1: Summary of the Burden of Proof analysis results for the relationship between exposure to secondhand smoke and nine health outcomes

Health outcome	RR (95% UI without between-study heterogeneity)	RR (95% UI with between-study heterogeneity)	BPRF	ROS	Star rating	Publication bias	No. of studies	Selected bias covariates
Ischaemic heart disease	1.26 (1.2–1.32)	1.26 (1.05–1.52)	1.08	0.04	★★	No	37	Baseline exposure assessment; study design (not prospective cohort)
Stroke	1.16 (1.11–1.22)	1.16 (1.03–1.32)	1.05	0.02	★★	No	20	Selection bias; self-reported outcome
Type 2 diabetes	1.16 (1.09–1.24)	1.16 (0.98–1.37)	1.01	0.005	★★	No	9	None
Lung cancer	1.37 (1.3–1.45)	1.37 (0.94–1.99)	1.00	0.001	★★	No	104	Not controlled for smoking
Otitis media	1.12 (1.06–1.18)	1.12 (0.92–1.36)	0.95	–0.03	★	No	24	Study design (not prospective cohort); self-reported outcome
Asthma	1.21 (1.16–1.26)	1.21 (0.88–1.66)	0.93	–0.04	★	No	125	Self-reported outcome; children population
Lower respiratory infections	1.34 (1.23–1.45)	1.34 (0.81–2.19)	0.88	–0.06	★	No	50	Not representative population; ever SHS exposure
Breast cancer	1.22 (1.13–1.31)	1.22 (0.75–1.98)	0.81	–0.11	★	No	51	Study design (not prospective cohort); not controlled for smoking
Chronic obstructive pulmonary disease	1.44 (1.21–1.71)	1.44 (0.67–3.12)	0.75	–0.14	★	No	21	Selection bias; not controlled for smoking

Notably, the SHS relative risks estimated in GBD 2023 are, in general, higher than those utilized in GBD 2021. In Figure 1, we illustrate the continuous risk curve generated for GBD 2021 (depicted in blue) alongside the dichotomous relative risks determined for GBD 2023 (shown in red), which, following the methodological revision detailed in the previous section, do not vary with the level of exposure. Specifically, for COPD, LRI, and lung cancer, the estimated relative risks in GBD 2023 surpass those calculated for GBD 2021. For diabetes, IHD, and stroke, the differences in estimated risks are minimal. Regarding breast cancer and otitis media, conditions for which risks were already modeled dichotomously in GBD 2021, the changes are detailed in Table 2.

Figure 1: Comparison between the relative risk curve produced for GBD 2021 (blue) and the relative risk produced for GBD 2023 (red; same across all ranges of exposure) for selected health outcomes associated with SHS.

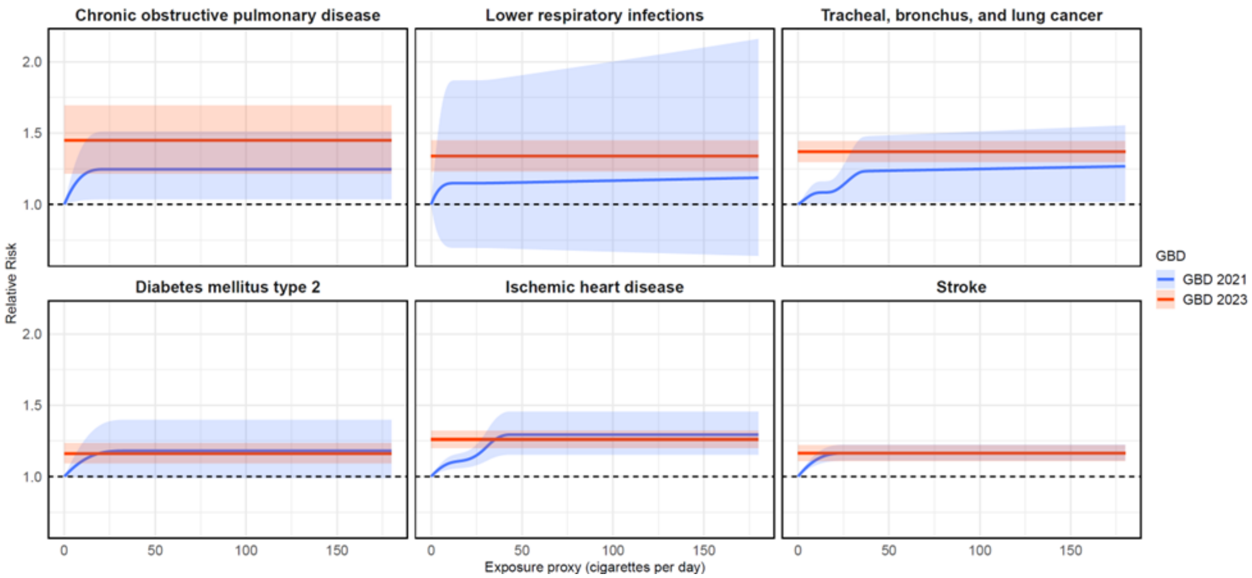

Table 2: Comparison between the SHS relative risks used in GBD 2021 and in GBD 2023 for the associations with breast cancer and otitis media.

	GBD 2021	GBD 2023
Breast cancer	1.04 (1, 1.09)	1.22 (1.13, 1.31)
Otitis	1.23 (1.11, 1.35)	1.12 (1.06, 1.18)

Population attributable fraction

We used the standard GBD population attributable fraction equation for dichotomous risks to estimate burden based on exposure, relative risks, and theoretical minimum risk exposure level.

Citations

[1] Flor LS, Anderson JA, Ahmad N, et al. Health effects associated with exposure to secondhand smoke: a Burden of Proof study. *Nat Med*. 2024 Jan; 30: 149–167.

[2] Zheng P, Barber R, Sorensen RJD, Murray CJL, Aravkin AY. Trimmed constrained mixed effects models: formulations and algorithms. *J Comput Graph Stat* 2021; 30: 544–56.

[3] Zheng P, Afshin A, Biryukov S, et al. The Burden of Proof studies: assessing the evidence of risk. *Nat Med* 2022; 28: 2038–44.

Sexual violence against children

Flowchart

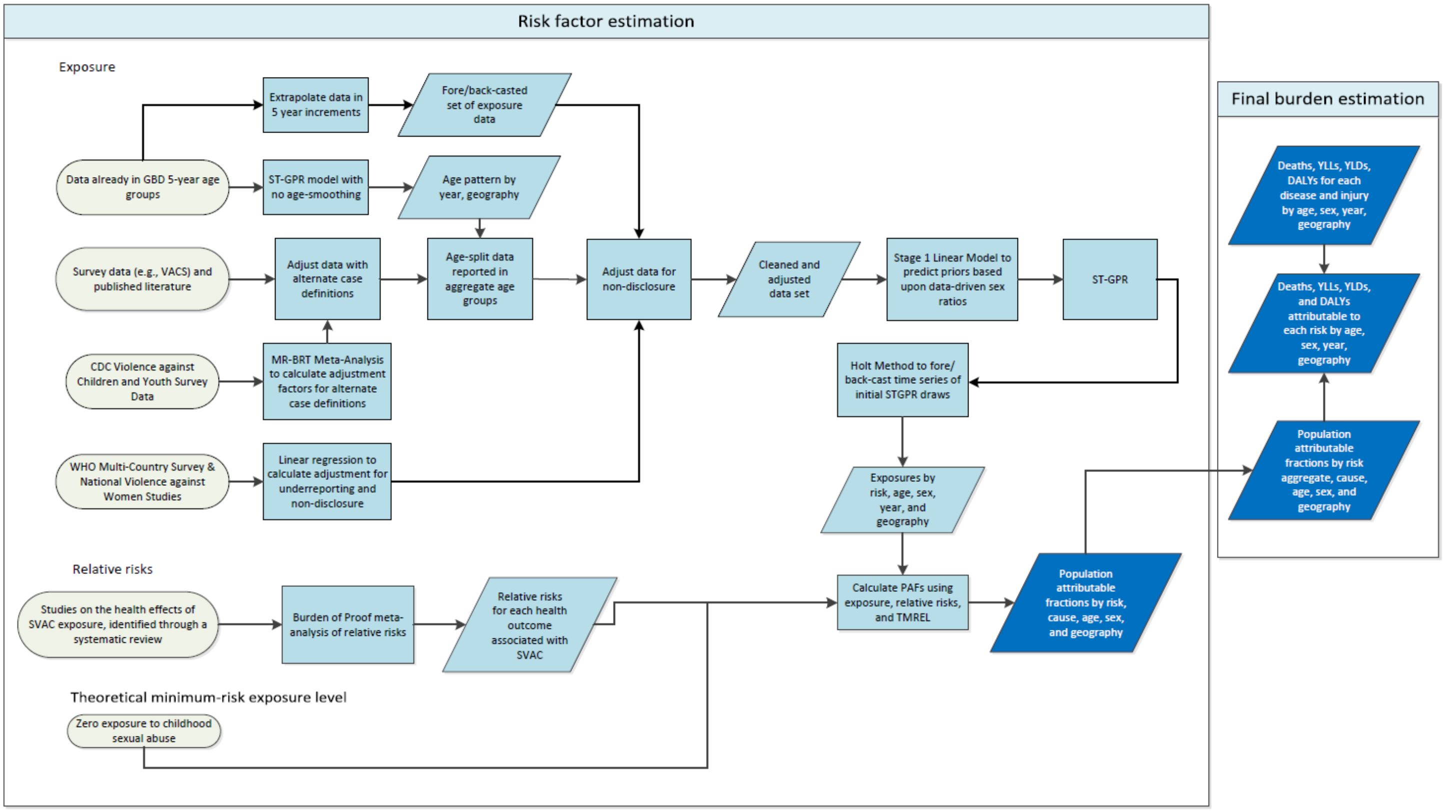

Cumulative risk approach of HIV/AIDS due to SVAC

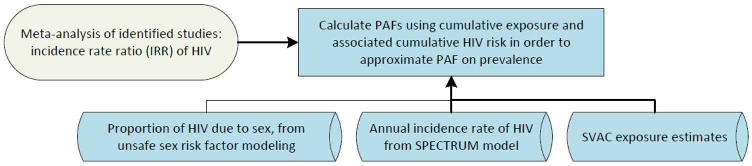

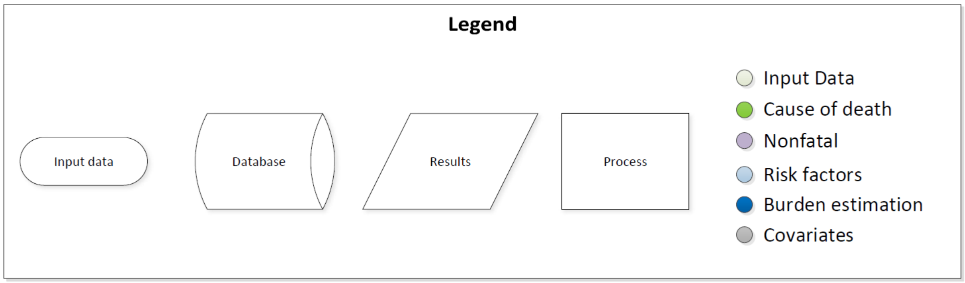

Input data and methodological summary

Definition

Exposure

The case definition for sexual violence against children (SVAC) is ever having experienced intercourse or other contact abuse (ie, fondling and other sexual touching) when under the age of 18 years and in which the contact was unwanted.

Input data

Exposure

Currently, we use self-reported survey data to measure SVAC prevalence, and we do not use data from Child Protection Services (CPS) or other crime data. The reliability and comprehensiveness of CPS and crime statistics vary too much geographically to warrant inclusion (typically identifying only a small proportion of reported cases). In addition, there is a lack of reliable survey data available for children under the age of 10, so our model is restricted to individuals 10 years and older.

For GBD 2023, we incorporated new exposure data sources identified through the Global Health Data Exchange (GHDx), the World Health Organization (WHO) Global Database on the Prevalence of Violence against Women, the United Nations Entity for Gender Equality and the Empowerment of Women (UN Women) Global Database on Violence against Women, and the GBD Collaborator Network. We included all sources that provided population-representative data on the proportion of males or females who experienced sexual violence or abuse before the age of 18. In addition, we accepted sources reporting on the following non-reference cases and populations:

1. Proportion of individuals who experienced intercourse-only SVAC
2. Proportion of individuals who experienced contact or non-contact SVAC
3. Proportion of individuals who experienced SVAC in which the definition of perpetrator is restricted (eg, sexual violence perpetrated by a caregiver)
4. Proportion of individuals whose sexual debut was SVAC (ie, before age 18 and forced, coerced, or unwanted)
5. Proportion of individuals who experienced sexual violence before some age less than 18 (such as before age 12 or 15)
6. Proportion of individuals who experienced SVAC, measured from a student population
7. Subnationally representative populations who experienced SVAC

Relative risk

For GBD 2023, we conducted a new systematic review for SVAC relative risks following a prospectively published protocol.[1] Briefly, we assessed all available literature on the health impacts of sexual violence against children by systematically searching PubMed, Embase/Elsevier, Cumulative Index to Nursing and Allied Health Literature (CINAHL), PsychInfo, Global Index Medicus, Cochrane, and Web of Science. Through this review, we identified peer-reviewed studies published from 1 January 1970 to 31 January 2024 reporting on any health effects associated with any form of violence against children or gender-based violence. The search strings have been published elsewhere[1,2] and do not limit identified studies to any specific health outcome of interest. Instead, the search aims to identify all health outcomes that

have been researched in association with violence against children or gender-based violence, allowing for meta-analyses of those that have been reported on by three or more studies.

The search was first conducted on 30 September 2021, and results up to that moment were published in an October 2024 study in Nature Medicine.[2] The search was updated on 26 February 2024 to include the more recent breadth of literature for the current GBD cycle, which allowed us to analyse one new health outcome in association with SVAC and incorporate newer studies across the previously analysed associations. During both phases of the systematic review, studies were included if they used study designs where temporality between exposure and outcome could be determined (eg, case-control or cohort studies), were conducted among a generalisable study population, and reported an estimate of association between an exposure and outcome of interest or enough data to construct a crude effect size. Studies were only further eligible for inclusion in the analysis of SVAC's health effects if they had data on the association between a health outcome, defined in alignment with GBD standards, and sexual violence against children specifically. Details on the included outcome and exposure definitions can be found elsewhere, as well as a thorough breakdown of the screening process.[1,2]

Figure 1: PRISMA diagram 2020 flow diagram for the health effects of sexual violence against children

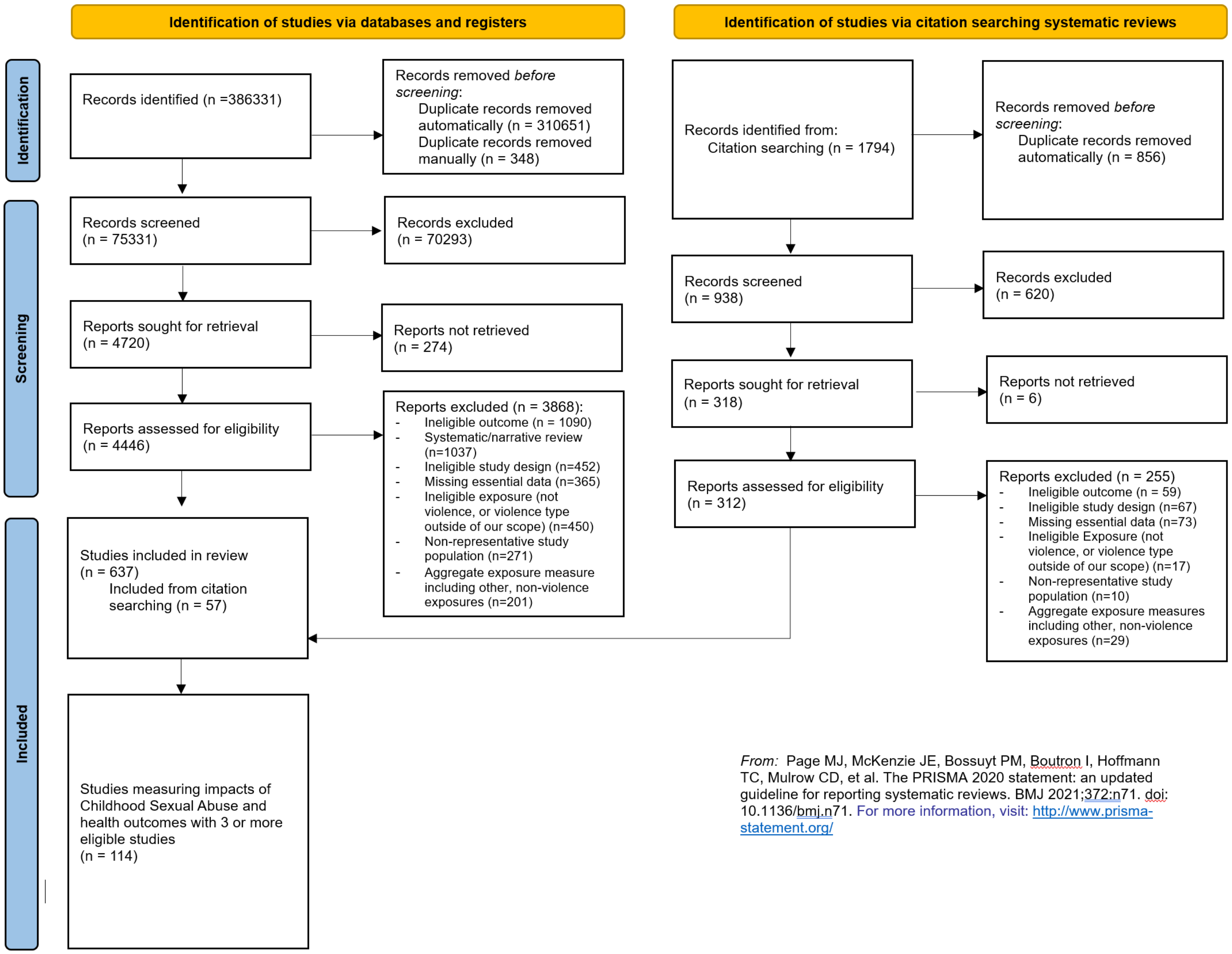

From: Page MJ, McKenzie JE, Bossuyt PM, Boutron I, Hoffmann TC, Mulrow CD, et al. The PRISMA 2020 statement: an updated guideline for reporting systematic reviews. BMJ 2021;372:n71. doi: 10.1136/bmj.n71. For more information, visit: http://www.prisma-statement.org/

Data processing

Crosswalking

For alternate case definitions of SVAC, we used gold-standard data from the CDC Violence Against Children and Youth Surveys (n=15) to run a logit-difference meta-regression with the MR-BRT tool to estimate correction factors. MR-BRT is described in detail in a separate section of this appendix. While we originally intended to fit separate models for each sex, we decided to model one set of crosswalk adjustments as we did not have rich data to inform significant differences between the sexes and observed implausible differences in adjustments made to the same source between the sexes when modelling adjustment factors separately. Our models were fit using 10% trimming and two priors, 1) contact-only (reference) definitions should be less than definitions including contact or non-contact cases, and 2) intercourse-only definitions should be less than contact-only (reference) definitions.

Table 1: MR-BRT crosswalk adjustment factors for SVAC exposure

Data input	Reference or alternative case definition	Gamma	Beta coefficient, logit (SD)*	Adjustment factor**
Contact SVAC	Ref	0.0134	---	---
Contact or non-contact SVAC	Alt		0.4343 (0.0230)	1.5439
Intercourse-only SVAC	Alt		–0.4263 (0.0144)	0.6529
SVAC before 15	Alt		–0.4551 (0.0116)	0.6344
SVAC before 12	Alt		–1.2791 (0.0134)	0.2783

**MR-BRT crosswalk adjustments can be interpreted as the factor the alternative case definition is adjusted by to reflect what it would have been had it been measured using the reference case definition. If the logit beta coefficient is negative, then the alternative is adjusted up to the reference. If the logit beta coefficient is positive, then the alternative is adjusted down to the reference.*

***The adjustment factor column is the exponentiated beta coefficient. For logit beta coefficients, this is the relative odds between the two case definitions.*

Note: Due to insufficient data, over-adjustment issues and other limitations, we decided to not calculate an adjustment factor for data with the restricted perpetrator or sexual debut alternate definitions.

Differential reporting adjustment

We also accounted for differential reporting, recognising that some people might not directly disclose experiences of SVAC to an interviewer. To calculate this adjustment factor, we used data from the WHO Multi-country Study on Women's Health and Domestic Violence against Women and 25 national violence against women surveys, all of which measured female SVAC using face-to-face interviews and anonymous self-report cards. Briefly, respondents were given two opportunities to disclose if someone had ever touched them sexually or made them do something sexual that they did not want to do during childhood. First, respondents verbally replied to the question so that interviewers could mark the response. Later, at the end of the interview, respondents were given another, more private opportunity to reply to the question using a card with pictorial representations of "yes" and "no." Preliminary results from the WHO Multi-country Study and its national adaptations suggest that the private self-report card

was usually able to ascertain more cases of SVAC than the interview alone. Hence, a simple linear regression model was run to estimate the relationship between prevalence estimates derived from each administration method:

$$y_i = mx_i + b$$

where:

- x_i is the prevalence of SVAC derived from the traditional face-to-face interview
- y_i is the prevalence of SVAC derived from private self-report cards
- i is a unique study identifier

We estimated an intercept (b) of 0.058 and a slope (m) of 1.049, and we used these values to predict what prevalence would be in a private, self-report survey given the prevalence from a face-to-face interview. However, our face-to-face input data did not extend beyond 25%, limiting our understanding of reporting patterns in studies already reporting relatively high prevalence estimates. As such, we do not apply the linear shift (intercept) to input values equal to or greater than 25% prevalence. Instead, we only use the modelled slope to relatively increase those values:

$$y_i = \begin{cases} mx_i + b, & x_i < 0.25 \\ mx_i, & x_i \geq 0.25 \end{cases}$$

Age splitting

We split data reported in age groups broader than the standard GBD five-year age groups by adapting the method reported in Ng et al.[3] to split aggregate data using a reference age pattern. We divided the data into two sets: (1) a training dataset, containing data that already fell into GBD five-year age groups, and (2) a split dataset, which contained data reported in aggregate age groups broader than GBD five-year bins. We then used spatiotemporal Gaussian process regression (ST-GPR) to estimate geography-time-specific age patterns using the training dataset. The ST-GPR model used an age-weight parameter value that minimised the effect of any age smoothing within the model. This parameter choice allowed the estimated age pattern to be driven by data rather than enforced by smoothing parameters of the model. Due to data sparsity within the training dataset, estimated geography-time age patterns were aggregated to the GBD region level. For female SVAC, the age pattern from the GBD world region with the most training datapoints (high-income North America) was used to adjust all non-standard age data. Due to extreme data sparsity within the male SVAC model, even the aggregated regional age pattern for the GBD region with the most training datapoints (high-income North America) was unrealistically variable across neighbouring age groups. Therefore, countries within this region were visually examined and the most stable age pattern (Canada) was selected to adjust non-standard age data.

Data extrapolation via cohort imputation

Given the case definition of SVAC, we assume that a population's prevalence of SVAC remains roughly constant after reaching 18 years of age, as any sexual violence experienced after this age would be considered violence during adulthood rather than childhood. Leveraging this assumption that SVAC prevalence should not vary considerably within a birth cohort after they reach age 18, we extrapolated data reported in five-year age groups in five-year increments. For example, the prevalence reported by a given survey for 25–29-year-olds in 2015 can also be considered as an estimate of SVAC prevalence for

20–24-year-olds in 2010 and for 30–34-year-olds in 2020. In recognition of demographic changes over time as well as SVAC's association with adverse health outcomes, both of which might influence prevalence, the uncertainty around extrapolated datapoints were inflated by a factor of 2, which consequently down-weights them in future modelling steps. We did not apply the same assumption or extrapolation to datapoints representative of 10–19-year-olds, as respondents below the age threshold detailed in the SVAC case definition are still at risk of experiencing violence. Thus, their prevalence is subject to change over time and cannot be subject to the same assumptions described above.

Modelling strategy

Exposure

We used ST-GPR to model lifetime SVAC prevalence. Input data were prepared by first adjusting data with alternate case definitions and then splitting data in aggregate age groups by applying modelled reference age patterns, as described above. In previous rounds of the GBD, SVAC exposure was modelled independently for males and females because we observe little correlation between the prevalence of SVAC among females and males. However, previously, in GBD 2021, we found that modelling male and female SVAC entirely independently resulted in non-data-driven sex trends, in which priors from the male model fit in the absence of data were higher than female model fits informed by female-only data. In order to leverage data-driven sex trends, we introduced a stage one linear model that predicted trends using SVAC data from both males and females. This method allowed the global ratio of SVAC exposure between sexes to inform the priors of consequent ST-GPR models in the absence of (super-) regional data. Full details on the ST-GPR method are reported elsewhere in the appendix. Briefly, the mean function input to GPR is a complete time series of estimates generated from the mixed effects hierarchical linear model described above plus weighted residuals smoothed across time, space, and age.

The stage one linear model formula is as follows:

$$logit(p_{g,a,t}) = \beta_0 + \beta_1 S_{A[a],g,t} + \sum_{k=2}^{20} \beta_k I_{A[a]} + \alpha_s + \alpha_r + \alpha_g + \epsilon_{g,a,t}$$

Where $S_{A[a],g,t}$ is the sex of the prevalence point by specific age group A, geography g, and time t, $I_{A[a]}$ is a dummy variable indicating specific age group A that the prevalence point $p_{g,a,t}$ captures, and α_s, α_r, and α_g are super-region, region, and geography random intercepts, respectively. Random effects were used in model fitting and prediction.

Data sparsity within the SVAC models caused poor model fits over time. Thus, we introduced Holt's linear trend method (extended simple exponential smoothing) to fore- and back-cast draws from the initial ST-GPR model. Holt's linear trend method allows forecasting of data with a linear trend using a weighted average of past observations, with weights decaying exponentially as observations get older.[4] We applied this method to location-age-specific draws from our initial ST-GPR model, with the year range of the ST-GPR draws to be used as the initial time series defined based upon location-age data availability. For location-age combinations with available data spanning more than three years, draws were bounded from the minimum year to the maximum year of location-age-specific data. Otherwise, draws were bounded from the minimum year to the maximum year of super-region-age-specific data. For male SVAC, there is one super-region (north Africa and the Middle East) for which we have no data.

In this case, we preserved most of the ST-GPR fit by using the time range of 1990–2019 (ie, forecasting only 2019–2023). To avoid over-forecasting for longer time periods (ie, in locations where only very old data were available), we used a damping parameter (phi=0.9) to enforce a zero-slope linear trend over time. Finally, due to our adjustment to ST-GPR draws, we needed to re-enforce consistency between subnational and national means, so we logit-raked subnational draws to fit national means for countries with subnational estimation.

Theoretical minimum risk exposure level

The theoretical minimum risk exposure level is zero exposure to contact childhood sexual abuse.

Relative risk

We re-examined existing risk–outcome pairs included in the GBD for SVAC and evaluated the strength of evidence and association for new pairs drawing on the updated systematic review described above and the burden of proof risk function (BPRF) methodology developed by Zheng and colleagues.[5,6] Through the systematic review, we identified 16 health outcomes with three or more eligible studies reporting SVAC-related effect sizes (or sufficient data to derive crude effect sizes). The association between SVAC and these health outcomes was consequently assessed through the BPRF methodology, using the meta-regression—Bayesian, regularised, trimmed (MR-BRT) tool to derive a pooled relative risk (RR) and associated measures. With MR-BRT, we synthesised input data to estimate the risk of developing each outcome for individuals who have experienced SVAC compared to those not exposed by trimming potentially distorting outliers; testing, selecting, and adjusting for bias covariates to account for known heterogeneity in input study-design characteristics (eg, confounding, selection bias, sample characteristics, level of adjustment, deviations in outcome definitions, and aspects related to SVAC exposure ascertainment, such as source recall period); and quantifying any remaining between-study heterogeneity (gamma) through random effects modelling. Finally, gamma, and the uncertainty surrounding it, are incorporated into the draws used to derive the 95% uncertainty interval for the mean RR value. More details on the data processing and bias covariates tested can be found in a previously published iteration of these analyses, which reports on findings prior to the most recent systematic review update.[2]

As has been described elsewhere,[5,6] MR-BRT further evaluates evidence for small-study effects and generates funnel plots that represent potential risk of publication or reporting bias. It also produces the burden of proof risk function (BPRF), defined for SVAC and other harmful dichotomous risk factors as the fifth quantile of draws closest to the null when incorporating between-study heterogeneity. The BPRF is transformed into a risk–outcome score (ROS: the signed natural log(BPRF) divided by two for dichotomous risks) and mapped onto a star-rating system from one to five stars. These metrics complement RR estimates by providing an alternative, conservative measure synthesising the effect size and evidence strength by formally and systematically accounting for divergence/convergence across input findings. To this end, positive ROS values and more stars corresponding to incrementally larger effects and stronger evidence for the risk–outcome relationship, measures which can be compared across risk–outcome pairs.

In total, 14 health outcomes met GBD inclusion criteria of a statistically significant association based on conventional RR uncertainty estimates: alcohol use disorders, self-harm, major depressive disorder, anxiety disorders, asthma, bipolar disorder, type 2 diabetes, HIV/AIDS, sexually transmitted infections

excluding HIV, maternal abortion and miscarriage, drug use disorders, conduct disorder, schizophrenia, and bulimia nervosa. Of these, only alcohol use disorder and major depressive disorder had been included as outcomes associated with SVAC in previous GBD cycles. Anorexia nervosa and ischaemic heart disease were tested using the BPRF methodology for potential inclusion with SVAC, but they were not found to have sufficient evidence of a significant association with SVAC to meet the requirements of GBD inclusion. Table 1 shows the main outputs from the burden of proof analysis for all 14 health outcomes now attributable to SVAC within the GBD.

Table 2: Summary of the Burden of Proof analysis results for the relationship between exposure to sexual violence during childhood and associated health outcomes

Health outcome	Pooled mean RR	95% RR UI without between-study heterogeneity	95% RR UI with between-study heterogeneity	BPRF	ROS	Star rating	Publication bias	No. of studies	Selected bias covariates
Self-harm	2.46	2.11–2.86	1.36–4.44	1.49	0.2	★★★	No	22	Risk of reverse causation; unadjusted for potential confounding; unadjusted for sex; effect size for females and males combined
Alcohol use disorder	1.74	1.56–1.94	1.34–2.25	1.4	0.17	★★★	No	12	Unadjusted for potential confounding
Major depressive disorder	1.68	1.51–1.87	1.01–2.80	1.1	0.05	★★	No	33	Unadjusted for sex; includes exposure above 15 years old; effect size for females and males combined; sub-population
Asthma	1.25	1.16–1.35	1.06–1.47	1.09	0.042	★★	No	5	None
Bipolar disorder	3.43	2.03–5.80	0.83–14.16	1.05	0.022	★★	No	3	None
Type 2 diabetes	1.16	1.10–1.23	0.99–1.36	1.02	0.01	★★	No	11	Includes exposure above 15 years old; maximal adjustment; analytical sample includes males; effect size for females and males combined
Drug use disorders	1.87	1.57–2.22	0.81–4.29	0.93	–0.04	★	No	21	Risk of reverse causation; administrative exposure ascertainment; unadjusted for age; maximally adjustment; analytical sample includes males; sub-population
HIV/AIDS	1.34	1.12–1.61	0.87–2.07	0.93	–0.04	★	No	7	None
STIs, excluding HIV/AIDS	1.28	1.04–1.57	0.79–2.08	0.85	–0.081	★	No	4	None
Anxiety disorders	1.85	1.50–2.28	0.72–4.76	0.84	-0.09	★	No	17	Female-only study; risk of selection bias; unadjusted for any potential confounding; unadjusted for age; includes exposure

									above 15 years old; maximal adjustment; analytical sample includes males; PTSD outcome
Maternal abortion and miscarriage	1.35	1.11–1.66	0.75–2.44	0.83	−0.095	★	No	6	None
Conduct disorders	3.42	1.64–7.14	0.45–25.70	0.63	−0.23	★	No	3	None
Schizophrenia	2.89	1.64–5.11	0.38–21.80	0.53	−0.32	★	No	7	Unadjusted for any potential confounding; unadjusted for age
Bulimia nervosa	2.95	1.45–5.97	0.37–23.60	0.51	−0.33	★	No	5	None

Table 3: Changes in relative risks used in GBD 2021 vs. GBD 2023

	Relative Risk		
Outcome	GBD 2021	GBD 2023 (with gamma)	GBD 2023 (without gamma)
Alcohol use disorders	2.21 (1.15, 4.04)	1.74 (1.34, 2.25)	1.74 (1.56, 1.94)
Major depressive disorder	1.56 (1.30, 1.86)	1.68 (1.01, 2.8)	1.68 (1.51, 1.87)

References

[1] Spencer CN, Baeza MJ, Chandan JK, Debure A, Herbert M, Jewell T, et al. Estimating the global health impact of gender-based violence and violence against children: a systematic review and meta-analysis protocol. *BMJ Open*. 2022 Jun;12(6):e061248.

[2] Spencer CN, Khalil M, Herbert M, Aravkin AY, Arrieta A, Baeza MJ, et al. Health effects associated with exposure to intimate partner violence against women and childhood sexual abuse: a Burden of Proof study. *Nat Med*. 2023 Dec;29(12):3243–58.

[3] Ng M, Freeman MK, Fleming TD, Robinson M, Dwyer-Lindgren L, Thomson B, et al. Smoking prevalence and cigarette consumption in 187 countries, 1980-2012. *JAMA*. 2014 Jan 8;311(2):183–92.

[4] Hyndman RJ, Athanasopoulos G. Forecasting: principles and practice. 2nd edition. Lexington, Ky.: Otexts, online, open-access textbook; 2018. 382 p.

[5] Zheng P, Afshin A, Biryukov S, Bisignano C, Brauer M, Bryazka D, et al. The Burden of Proof studies: assessing the evidence of risk. *Nat Med*. 2022 Oct;28(10):2038–44.

[6] Zheng P, Barber R, Sorensen RJD, Murray CJL, Aravkin AY. Trimmed constrained mixed effects models: formulations and algorithms. *J Comput Graph Stat* 2021; 30: 544–56.

Smoking

Flowchart

Smoking

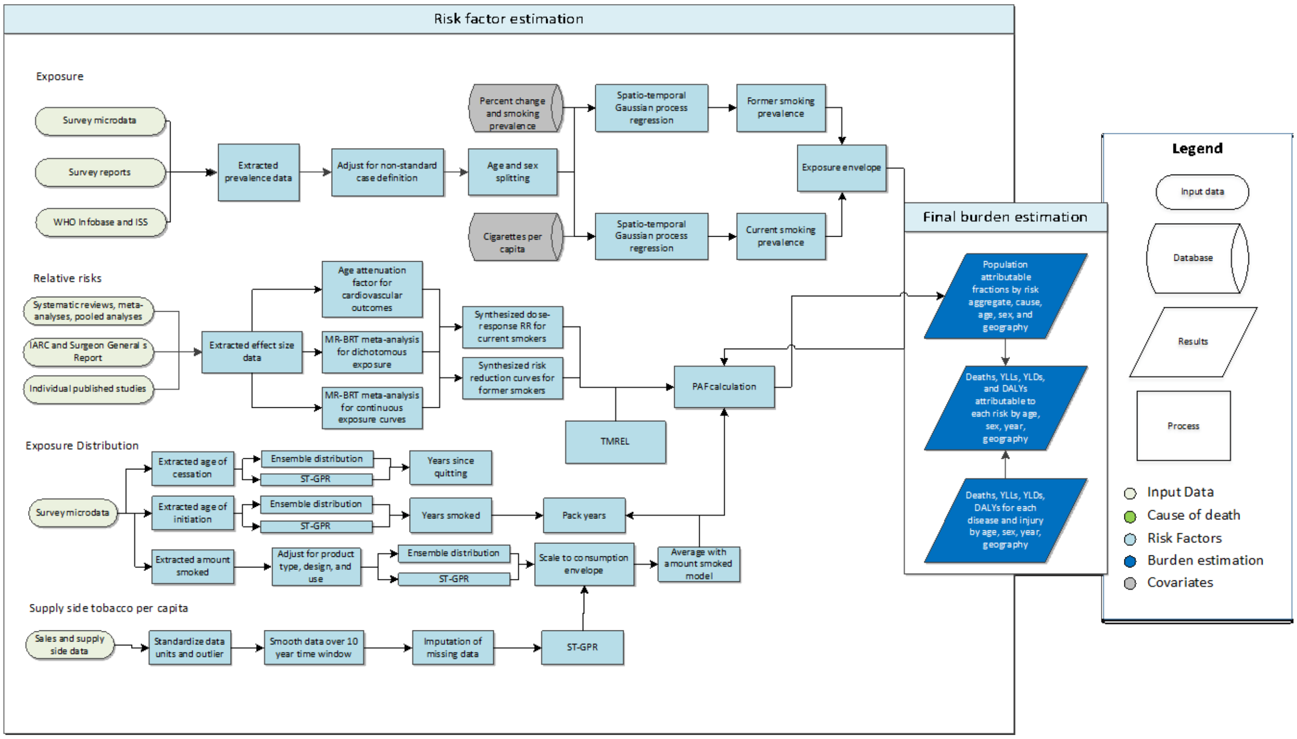

Input data and methodological summary

Definition

Exposure

As in previous GBD cycles, we estimated the prevalence of current smoking and the prevalence of former smoking using data from cross-sectional nationally representative household surveys. We defined current smokers as individuals who currently use any smoked tobacco product on a daily or occasional basis. We defined former smokers as individuals who quit using all smoked tobacco products for at least six months, where possible, or according to the definition used by the given survey.

Input data

Exposure

Our survey data extraction method for smoking exposure has not changed from previous GBD cycles. A systematic review of literature was performed to extract data on our primary exposure indicators. We searched the Global Health Data Exchange (GHDx), a comprehensive online catalog of health-related data created by IHME, for population survey data. We also included surveys that were recommended by our collaborators but were not in the GHDx. Regarding inclusion and exclusion criteria, we only included

surveys that are nationally or subnationally representative (on a state/province level). Surveys conducted among specific populations (eg, pregnant women, physicians) were excluded. Surveys from high-quality standardised series (eg, STEPS NCD, GATS, GYTS) as well as surveys covering data-sparse locations and time periods were prioritized.

We extracted primary data from individual-level microdata and survey report tabulations. Specifically, we extracted data on current, former, and/or ever-smoked tobacco use reported as any combination of frequency of use (daily, occasional, and unspecified, which includes both daily and occasional smokers) and type of smoked tobacco used (all smoked tobacco, cigarettes, hookah, and other smoked tobacco products such as cigars or pipes), resulting in 36 possible combinations. Other variants of tobacco products, for example, hand-rolled cigarettes, were grouped into the four categories listed above based on product similarities.

For microdata, we extracted relevant demographic information, including age, sex, location, and year, as well as survey metadata, including survey weights, primary sampling units, and strata. This information allowed us to tabulate individual-level data into the standard GBD five-year age-sex groups and produce accurate estimates of uncertainty. In GBD 2023, we collapsed individual-level data into detailed older age groups (80–84, 85–89, 90–94, and 95–124 years old). In previous rounds, we had collapsed individual-level data into a combined 80+ age set due to data sparsity. With newly added data, we were able to model detailed age groups directly. For survey report tabulations, we extracted data at the most granular age-sex group provided. After data were extracted, we carefully vetted the extracted data, fixed any extraction errors and cautiously outliered problematic data due to quality concerns based on expert opinion. We documented relevant survey variables from each data source as well as outliered data in spreadsheets. We extracted data using STATA 13.1 and R 4.2.

Table 1: Data inputs for exposure for smoked tobacco

Exposure	Countries with data	New sources	Total sources
Current any smoking	204	364	3145
Current daily smoking	203	338	3008
Former any smoking	187	219	1251
**Total**	**204**	**365**	**3171**

Relative risk

Updates to relative risk estimates were not performed for the GBD 2023 cycle; results from GBD 2021 were used. In GBD 2021, we undertook an effort to improve our relative risk curves by refining our search strings to capture a larger number of studies than was identified by previous search strings. Studies published between 01/01/1970 and 05/31/2022 were reviewed. Of those articles captured in PubMed, prospective cohort and case-control studies were included if they reported the effect sizes (relative risk, hazard ratio, or odds ratio) of an association between a continuous or categorical dose for smoked tobacco consumption and a GBD outcome with uncertainty. Information on study design, confounders controlled for, sample representativeness, and measurement of exposure and outcomes was also extracted. In addition, we adopted a new Burden of Proof approach to re-estimate the relative risks of smoking on 36 health outcomes. More details can be found elsewhere.[3]

In GBD 2021, we also employed a new approach to produce age-specific relative risk (RR) curves for CVD outcomes, which involves estimating an age pattern of excess risk (ie, RR–1) of smoking for CVD outcomes. To estimate the age pattern, we performed a systematic review of literature on risk of smoking for five CVD outcomes, namely, ischaemic heart disease, stroke, atrial fibrillation and flutter, aortic aneurysm, and peripheral arterial disease. We developed a search string to search for articles reporting any association of binary smoking status (ie, current, former, and ever smokers) on the five CVD outcomes from 01/01/1970 to 12/31/2019 and only included studies reporting age-specific risk (RR, OR, HR) of smoking status, which is different from the estimation of dose–response risk of smoking, for which we only included studies reporting dose-specific risk. Information on study design, confounders controlled for, sample representativeness, type of exposure (ie, current, former, and ever smoker), measurement of exposure and outcomes was also extracted for bias adjustment. Table 2 summarises the number of studies included for estimating the dose–response risk curve and the age pattern of risk for the CVD outcomes.

Table 2: Data inputs for relative risks for smoked tobacco use

	Countries with data	New sources	Total sources
Relative risks	55	218	730

Data processing

Crosswalk

Our GBD smoking case definitions were current smoking of any tobacco product and former smoking of any tobacco product. All other definitions were adjusted to be consistent with either of these definitions. Some sources contained information on more than one case definition, and these sources were used to develop the adjustment coefficients to transform alternative case definitions to the GBD case definition. The adjustment coefficients were the beta values derived from linear regression models with one predictor and no intercept. We used the same crosswalk adjustment coefficients as in GBD 2021, and thus we have not included a methods explanation in this appendix, as it has been detailed previously.

Age and sex splitting

As in GBD 2021, data which were reported in broader age groups than the GBD five-year age groups or as both sexes combined were split by adapting the method reported in Ng et al[1] using a sex-geography-time-specific reference age pattern. We separated the data into two sets: a training dataset, with data already falling into GBD sex-specific five-year age groups, and a split dataset, which reported data in aggregated age or sex groups. We then used spatiotemporal Gaussian process regression (ST-GPR) to estimate sex-geography-time-specific age patterns using data in the training dataset. The estimated age patterns were used to split each source in the split dataset.

The ST-GPR model used to estimate the age patterns for age-sex splitting used an age-weight parameter value that minimises the effect of any age smoothing. This parameter choice allowed the estimated age pattern to be driven by data, rather than being enforced by any smoothing parameters of the model.

These age-sex-split datapoints were to be incorporated in the final ST-GPR exposure model; thus, we did not want to doubly enforce a modelled age pattern for a given sex-location-year on a given aggregate datapoint.

As in the data preparation step, for GBD 2023, tabulated data were split into detailed older age sets in order to directly model those groups.

Modelling strategy

Smoking prevalence modelling

We used ST-GPR to model current and former smoking prevalence. The model is identical to that in GBD 2021. Full details on the ST-GPR method are reported elsewhere in the appendix. Briefly, the mean function input to GPR is a complete time series of estimates generated from a mixed-effects hierarchical linear model plus weighted residuals smoothed across time, space, and age. The linear model formula for current smoking, fit separately by sex using restricted maximum likelihood in R, is:

$$logit(p_{g,a,t}) = \beta_0 + \beta_1 CPC_{g,t} + \sum_{k=2}^{19} \beta_k I_{A[a]} + \alpha_s + \alpha_r + \alpha_g + \epsilon_{g,a,t}$$

Where $CPC_{g,t}$ is the tobacco consumption per capita covariate by geography g and time t, described above, $I_{A[a]}$ is a dummy variable indicating specific age group that the prevalence point $p_{g,a,t}$ captures, and a_s, a_r, and a_g are super-region, region, and geography random intercepts, respectively. Random effects were used in model fitting but not in prediction.

The linear model formula for former smoking is:

$$logit(p_{g,a,t}) = \beta_0 + \beta_1 PctChange_{A[a],g,t} + \beta_3 CSP_{A[a],g,t} + \sum_{k=3}^{20} \beta_k I_{A[a]} + \alpha_s + \alpha_r + \alpha_g + \epsilon_{g,a,t}$$

Where $PctChange_{A[a],g,t}$ is the percentage change in current smoking prevalence from the previous year, and $CSP_{A[a],g,t}$ is the current smoking prevalence by specific age group A, geography g, and time t that point $p_{g,a,t}$ captures, both derived from the current smoking ST-GPR model defined above.

Supply-side estimation

The methods for modelling supply-side-level data were consistent with those used in GBD 2021, aside from changes in data source for retail supply. For GBD 2023, we included GlobalData as a new data source for our supply-side estimates. The raw data were domestic supply (USDA Global Surveillance Database and UN FAO) and retail supply (GlobalData) of tobacco. Domestic supply was calculated as production + imports – exports. The data went through three rounds of outliering based on reasonable consumption thresholds of number of cigarettes per smoker per day, distance from the ten-year rolling

mean tobacco per capita, and manual outliering for edge cases. Finally, data smoothing was performed by taking a three-year rolling mean over each location-year.

Next, to impute the missing years for each series and remove compositional bias from our final estimates, we modelled the log ratio of each pair of sources as a function of an intercept and nested random effects on super-region, region, and location. The appropriate predicted ratio was multiplied by each source that we did have, and then the predictions were averaged to get the final imputed value. For some locations where there was limited overlap between series, the predicted ratio did not make sense, and a regional ratio was used.

Finally, variance was calculated both across series (within a location-year) as well as across years (within a location-source). Additionally, if a location-year had one imputed point, the variance was multiplied by 2. If a location-year had two imputed points, the variance was multiplied by 4. The average estimates in each location-year were the input to an ST-GPR model. For this, we used a simple mixed-effects model, which was modelled in log space with nested location random effects. Subnational estimates were then further modelled by splitting the country-level estimates using current smoking prevalence.

Theoretical minimum risk exposure level

The theoretical minimum risk exposure level is zero, or no exposure to smoking.

Exposure among current and former smokers

Identical to GBD 2021, we estimated exposure among current smokers for two continuous indicators: cigarettes per smoker per day and pack-years. Pack-years incorporates aspects of both duration and amount. One pack-year represents the equivalent of smoking one pack of cigarettes (assuming a 20-cigarette pack) per day for one year. Since the pack-years indicator collapses duration and intensity into a single dimension, one pack-year of exposure can reflect smoking 40 cigarettes per day for six months or smoking ten cigarettes per day for two years.

To produce these indicators, we simulated individual smoking histories based on distributions of age of initiation and amount smoked. We informed the simulation with cross-sectional survey data capturing these indicators, modelled at the mean level for all locations, years, ages, and sexes using ST-GPR. We rescaled estimates of cigarettes per smoker per day to an envelope of cigarette consumption based on supply-side data. We estimated pack-years of exposure by summing samples from age- and time-specific distributions of cigarettes per smoker for a birth cohort to capture both age trends and time trends and avoid the common assumption that the amount someone currently smokes is the amount they have smoked since they began smoking. All distributions were age-, sex-, and region-specific ensemble distributions, which were found to outperform any single distribution.

We estimated exposure among former smokers using years since cessation. We used ST-GPR to model mean age of cessation using cross-sectional survey data capturing age of cessation. Using these estimates, we generated ensemble distributions of years since cessation for every location, year, age group, and sex.

Relative risk

The same risk–outcome pairs from GBD 2021 were used for GBD 2023: tuberculosis, lower respiratory tract infections, oesophageal cancer, stomach cancer, bladder cancer, liver cancer, laryngeal cancer, lung cancer, breast cancer, cervical cancer, colorectal cancer, lip and oral cancer, nasopharyngeal cancer, other pharyngeal cancer, pancreatic cancer, kidney cancer, leukaemia, ischaemic heart disease, ischaemic stroke, haemorrhagic stroke, subarachnoid haemorrhage, atrial fibrillation and flutter, aortic aneurysm, peripheral arterial disease, chronic obstructive pulmonary disease, other chronic respiratory diseases, asthma, peptic ulcer disease, gallbladder and biliary tract diseases, Alzheimer's disease and other dementias, Parkinson's disease (protective), multiple sclerosis, type 2 diabetes, rheumatoid arthritis, low back pain, cataracts, macular degeneration, and fracture.

For GBD 2023, the risk of all risk–outcome pairs is evaluated by continuous smoking exposure level (ie, pack-year, cigarettes per smoker per day, and years since cessation), except for fracture, for which risk is evaluated by binary smoking exposure (ie, smoker versus non-smoker/former smoker).

Dose–response risk curves

Dose–response cures were unchanged since GBD 2021. Details on GBD 2021 processes follow.
For GBD 2021, we used the studies identified through the updated systematic review to estimate new dose–response curves using MR-BRT for all outcomes. Importantly, this new method takes into account the risk of biases in the RR estimation by selecting and including important covariates of the risk estimates in the model (eg, measurement of exposure and outcomes, representativeness, and adjustment level of the risk estimates) and incorporates unexplained between-study heterogeneity into the uncertainty of the RR estimates. The results of the meta-regression were used to estimate a non-parametric curve for all doses between zero and 100 pack-years or cigarettes per smoker per day and their corresponding relative risks. For all outcomes, we assumed the relative risk was the same for both sexes, expect for breast cancer, cervical cancer, and prostate cancer, which were assumed to apply only to female or to male.

For data-sparse risk–outcome pairs, we implemented the Fisher scoring correction to the heterogeneity parameter. When data are sparse, the between-study heterogeneity parameter estimate may be zero, simply due to lack of data. The Fisher scoring correction uses a quantile of gamma, which is sensitive to the number of studies, study design, and reported uncertainty.

We have also added methodology that can detect and flag publication bias. The approach is based on the classic Egger's regression strategy, which is applied to the residuals in our model. In the current implementation, we do not correct for publication bias but flag the risk–outcome pairs where the risk for publication bias is significant.

For risk of former smokers, we estimated risk curves of former smokers compared to never smokers taking into account the rate of risk reduction among former smokers seen in the cohort and case-control studies, and the cumulative exposure among former smokers within each age, sex, location, and year group. For GBD 2021, we did not include new data or change the method of estimating the risk curves of former smokers.

In the table below, we list each risk–outcome pair that was updated in GBD 2021 along with several of the key modelling parameters and results. The formulation for MR-BRT is described in detail in the appendix. More details on the modeling strategy, results and the PRIMSA diagrams can be found in Dai et al., 2022.[3]

Table 3: MR-BRT model specifications by risk–outcome pair

Risk–outcome	Type of risk	Spline degree, # interior knots	Priors & constraints
Atrial fibrillation and flutter	Continuous, harmful	Quadratic, 3 I knots	Monotonic increasing, right linear tail, Gaussian max derivative prior on the right tail (0, 0.001)
Alzheimer's and other dementias	Continuous, harmful	Quadratic, 3 I knots	Monotonic increasing, right linear tail, Gaussian max derivative prior on the right tail (0, 0.001)
Aortic aneurism	Continuous, harmful	Quadratic, 3 I knots	Monotonic increasing, right linear tail, Gaussian max derivative prior on the right tail (0, 0.001)
Asthma	Continuous, harmful	Quadratic, 3 I knots	Monotonic increasing, right linear tail, Gaussian max derivative prior on the right tail (0, 0.001)
Bladder cancer	Continuous, harmful	Quadratic, 3 I knots	Monotonic increasing, right linear tail, Gaussian max derivative prior on the right tail (0, 0.001)
Breast cancer	Continuous, harmful	Quadratic, 3 I knots	Monotonic increasing, right linear tail, Gaussian max derivative prior on the right tail (0, 0.001)
Cataracts	Continuous, harmful	Quadratic, 3 I knots	Monotonic increasing, right linear tail, Gaussian max derivative prior on the right tail (0, 0.001)
Cervical cancer	Continuous, harmful	Quadratic, 3 I knots	Monotonic increasing, right linear tail, Gaussian max derivative prior on the right tail (0, 0.001)
Colon and rectum cancer	Continuous, harmful	Quadratic, 3 I knots	Monotonic increasing, right linear tail, Gaussian max derivative prior on the right tail (0, 0.001)
COPD	Continuous, harmful	Quadratic, 3 I knots	Monotonic increasing, right linear tail, Gaussian max derivative prior on the right tail (0, 0.001)
Diabetes	Continuous, harmful	Quadratic, 3 I knots	Monotonic increasing, right linear tail, Gaussian max derivative prior on the right tail (0, 0.001)
Oesophageal cancer	Continuous, harmful	Quadratic, 3 I knots	Monotonic increasing, right linear tail, Gaussian max derivative prior on the right tail (0, 0.001)

Gallbladder diseases	Continuous, harmful	Quadratic, 3 I knots	Monotonic increasing, right linear tail, Gaussian max derivative prior on the right tail (0, 0.001)
Fracture (hip and non-hip)	Dichotomous, harmful	N/A	N/A
Ischaemic heart disease	Continuous, harmful	Quadratic, 3 I knots	Monotonic increasing, right linear tail, Gaussian max derivative prior on the right tail (0, 0.001)
Kidney cancer	Continuous, harmful	Quadratic, 3 I knots	Monotonic increasing, right linear tail, Gaussian max derivative prior on the right tail (0, 0.001)
Laryngeal cancer	Continuous, harmful	Quadratic, 3 I knots	Monotonic increasing, right linear tail, Gaussian max derivative prior on the right tail (0, 0.001)
Lower back pain	Continuous, harmful	Quadratic, 3 I knots	Monotonic increasing, right linear tail, Gaussian max derivative prior on the right tail (0, 0.001)
Leukaemia	Continuous, harmful	Quadratic, 3 I knots	Monotonic increasing, right linear tail, Gaussian max derivative prior on the right tail (0, 0.001)
Lip and oral cavity cancer	Continuous, harmful	Quadratic, 3 I knots	Monotonic increasing, right linear tail, Gaussian max derivative prior on the right tail (0, 0.001)
Liver cancer	Continuous, harmful	Quadratic, 3 I knots	Monotonic increasing, right linear tail, Gaussian max derivative prior on the right tail (0, 0.001)
Lower respiratory infections	Continuous, harmful	Quadratic, 3 I knots	Monotonic increasing, right linear tail, Gaussian max derivative prior on the right tail (0, 0.001)
Lung cancer	Continuous, harmful	Quadratic, 3 I knots	Monotonic increasing, right linear tail, Gaussian max derivative prior on the right tail (0, 0.001)
Macular degeneration	Continuous, harmful	Quadratic, 3 I knots	Monotonic increasing, right linear tail, Gaussian max derivative prior on the right tail (0, 0.001)
Multiple sclerosis	Continuous, harmful	Quadratic, 3 I knots	Monotonic increasing, right linear tail, Gaussian max derivative prior on the right tail (0, 0.001)
Nasopharyngeal cancer	Continuous, harmful	Quadratic, 3 I knots	Monotonic increasing, right linear tail, Gaussian max derivative prior on the right tail (0, 0.001)
Other pharynx cancer	Continuous, harmful	Quadratic, 3 I knots	Monotonic increasing, right linear tail, Gaussian max derivative prior on the right tail (0, 0.001)
Pancreatic cancer	Continuous, Harmful	Quadratic, 3 I knots	Monotonic increasing, right linear tail, Gaussian max derivative prior on the right tail (0, 0.001)

Parkinson's disease	Continuous, protective	Quadratic, 3 I knots	Monotonic decreasing, right linear tail, Gaussian max derivative prior on the right tail (0, 0.001)
Peptic ulcer	Continuous, harmful	Quadratic, 3 I knots	Monotonic increasing, right linear tail, Gaussian max derivative prior on the right tail (0, 0.001)
Peripheral artery disease	Continuous, harmful	Quadratic, 3 I knots	Monotonic increasing, right linear tail, Gaussian max derivative prior on the right tail (0, 0.001)
Prostate cancer	Continuous, harmful	Quadratic, 3 I knots	Monotonic increasing, right linear tail, Gaussian max derivative prior on the right tail (0, 0.001)
Rheumatoid arthritis	Continuous, harmful	Quadratic, 3 I knots	Monotonic increasing, right linear tail, Gaussian max derivative prior on the right tail (0, 0.001)
Stomach cancer	Continuous, harmful	Quadratic, 3 I knots	Monotonic increasing, right linear tail, Gaussian max derivative prior on the right tail (0, 0.001)
Stroke (ischaemic stroke, haemorrhagic stroke, and subarachnoid haemorrhage)	Continuous, harmful	Quadratic, 3 I knots	Monotonic increasing, right linear tail, Gaussian max derivative prior on the right tail (0, 0.001)
Tuberculosis	Continuous, harmful	Quadratic, 3 I knots	Monotonic increasing, right linear tail, Gaussian max derivative prior on the right tail (0, 0.001)

Table 4: MR-BRT estimated parameters and bias covariates by risk–outcome pair

Risk–outcome	Unit of risk	Selected bias covariates	Mean gamma solution	publication bias
Atrial fibrillation and flutter	cigarettes per day	None	0.000	
Alzheimer's and other dementias	cigarettes per day	None	0.054	
Aortic aneurism	cigarettes per day	None	0.000	
Asthma	cigarettes per day	None	1.651	
Bladder cancer	pack-year	None	0.052	
Breast cancer	pack-year	cv_subpopulation	0.000	
Cataracts	cigarettes per day	None	0.000	
Cervical cancer	pack-year	None	0.000	
Colon and rectum cancer	pack-year	None	0.090	
COPD	pack-year	cv_older, cv_adj_L1	0.022	
Diabetes (type 2)	cigarettes per day	cv_subpopulation	0.055	
Oesophageal cancer	pack-year	None	0.106	
Gallbladder diseases	cigarettes per day	cv_adj_L0	0.000	

Fracture (hip and non-hip)	Binary smoking status	cv_subpopulation, cv_risk_measure, cv_adj_L2	0.032	
Ischaemic heart disease	cigarettes per day	cv_adj_L2, cv_subpopulation, cv_older	0.190	
Kidney cancer	pack-year	None	0.078	
Laryngeal cancer	pack-year	None	0.000	
Lower back pain	cigarettes per day	None	0.000	
Leukaemia	pack-year	None	0.000	
Lip and oral cavity cancer	pack-year	cv_adj_L1	0.158	
Liver cancer	pack-year	None	0.429	
Lower respiratory infection	cigarettes per day	None	0.000	
Lung cancer	pack-year	cv_adj_L1, cv_adj_L0, cv_adj_L2	0.063	
Macular degeneration	cigarettes per day	None	0.000	
Multiple sclerosis	cigarettes per day	None	0.000	
Nasopharyngeal cancer	pack-year	cv_adj_L0	0.065	
Other pharynx cancer	pack-year	None	0.000	
Pancreatic cancer	pack-year	None	0.000	
Parkinson's disease	cigarettes per day	cv_adj_L2, cv_outcome_selfreport	0.000	
Peptic ulcer	cigarettes per day	cv_adj_L1, cv_subpopulation	0.000	
Peripheral artery disease	cigarettes per day	cv_subpopulation	0.000	
Prostate cancer	cigarettes per day	None	0.170	
Rheumatoid arthritis	cigarettes per day	None	0.000	
Stomach cancer	pack-year	None	0.000	
Stroke (ischaemic stroke, haemorrhagic stroke, and subarachnoid haemorrhage)	cigarettes per day	None	0.146	
Tuberculosis	cigarettes per day	None	0.038	

† definitions of bias covariates:

cv_subpopulation: 0 for risk estimates are likely generalisable to the general population because the sample was based on the general population with reasonable exclusions for pre-existing disease states; 1 for risk estimates of sub-groups such as high-risk groups.

cv_adj_L0, cv_adj_L1, cv_adj_L2: cascading dummy variables for adjustment level of the risk estimates (ie, how many confounders are adjusted for in the regression model for the risk estimate). There are four adjustment levels, namely, 1. no adjustment, 2. only adjusting for age and sex, 3. adjusting for age and sex and ≤3 other covariates, and 4. adjusting for age and sex and >3 other covariates. If the adjustment level is 1, cv_adj_L0=1, cv_adj_L1=1, cv_adj_L2=1; if the adjustment level is 2, cv_adj_L0=1, cv_adj_L1=1, cv_adj_L2=0; if the adjustment level is 3, then cv_adj_L0=1, cv_adj_L1=0, cv_adj_L2=0; if the adjustment level is 4, then cv_adj_L0=0, cv_adj_L1=0, cv_adj_L2=0.

cv_outcome_selfreport: 0 for measurement of outcome based on assays, tests, or physician observation and 1 for self-report outcome.

cv_older: 0 if the population contains both young and old people; 1 if the population only contains old people.

cv_risk_measure: 0 if the risk is reported as relative risk; 1 if the risk is reported as odds ratio or hazard ratio.

Age-specific dose–response risk curves for CVD outcomes

Age-specific risk curves were not estimated for GBD 2023; results from GBD 2021 were used. Details follow.

For all non-CVD outcomes, we assumed the risk curve to be the same for all ages. However, the risk of smoking on CVD outcomes (ie, stroke, ischaemic heart disease, atrial fibrillation and flutter, aortic aneurysm, and peripheral arterial disease) is well known to attenuate with increasing age, and thus we produced age-specific risk curves for all CVD outcomes. Previously, we used a linear relationship between age and log risk to adjust all RR data to a specific age group (eg, 45–49). Then, we modelled the risk curve for each age group using the adjusted age-group-specific data. This approach often produced curves with different shapes for different age groups and tended to underestimate the risk for older age groups since we set the log RR to be zero for the terminal age group (eg, 95+) in the linear function.

In GBD 2021, we adopted a new approach to produce the age-specific risk curves by producing an age pattern of smoking risk on CVD outcomes and adjusting the risk curve of the reference age group using the age pattern of risk to produce age-group-specific risk curves. Briefly, we first estimated the reference dose–response risk of smoking for each CVD outcome using dose-specific RR data of each outcome regardless of the age group information. This step was the same with other non-CVD outcomes. Once we had the reference curve, we determined the age group of the reference curve by calculating the weighted mean age across all dose-specific RR data (weighted by 1/SE of each datum). For example, if the weighted mean age of all dose-specific RR data was 56.5, we determined the age group of the reference risk curve to be 55–59. For cohort studies, the mean age was calculated as mean age at baseline plus the mean/median years of follow-up (if only maximum years of follow-up is reported, we added half of the maximum years to the mean age at baseline). For the case-control studies, the mean age was just the reported mean age at baseline (in case the mean age is not reported, we used the midpoint of age range as the mean age instead). In the third step, we extracted age-group-specific RR data and relevant bias covariates from literature identified in the systematic review mentioned above, and we used MR-BRT to model the age pattern of excess risk (ie, RR – 1) of smoking on CVD outcomes with age-group-specific excess RR data of all CVD outcomes. In the final model, we included age as spline, random effects of study, and the bias covariates of exposure types (ie, current, former, and ever smokers), which were selected by an algorithm described elsewhere.[2] When predicting the age pattern of the excess risk of smoking on CVD outcomes using the fitted model, we did not include between-study heterogeneity to reduce uncertainty in the prediction. Figure 1 shows the estimated age pattern of excess risk of smoking on CVD along with its 95% uncertainty intervals. In the fourth step, we calculated the age attenuation factors (AF) of excess risk compared with the reference age group for each CVD outcome as ratio of the estimated excess risk of each age group to that of the reference age group. We did the calculation at the draw level to obtain 1000 draws of the AF for each age group. Figure 2 shows the AF for stroke along with its 95% uncertainty intervals. Once we had the AF, in the last step, we adjusted the risk curve of the reference age group from step 1 using equation (1) to produce the age-group-specific risk curves for each CVD outcome.

$$rr_{age_i} = (rr_{ref} - 1) * AF_{age_i} + 1 \qquad (1)$$

We did the age adjustment on draw level so that the uncertainty of the AF can be naturally incorporated in the final adjusted age-specific RR curves. Figure 3 shows the age AF adjusted age-group-specific RR curves for stroke outcome.

Figure 1: Estimated age pattern of excess risk of smoking on CVD outcomes

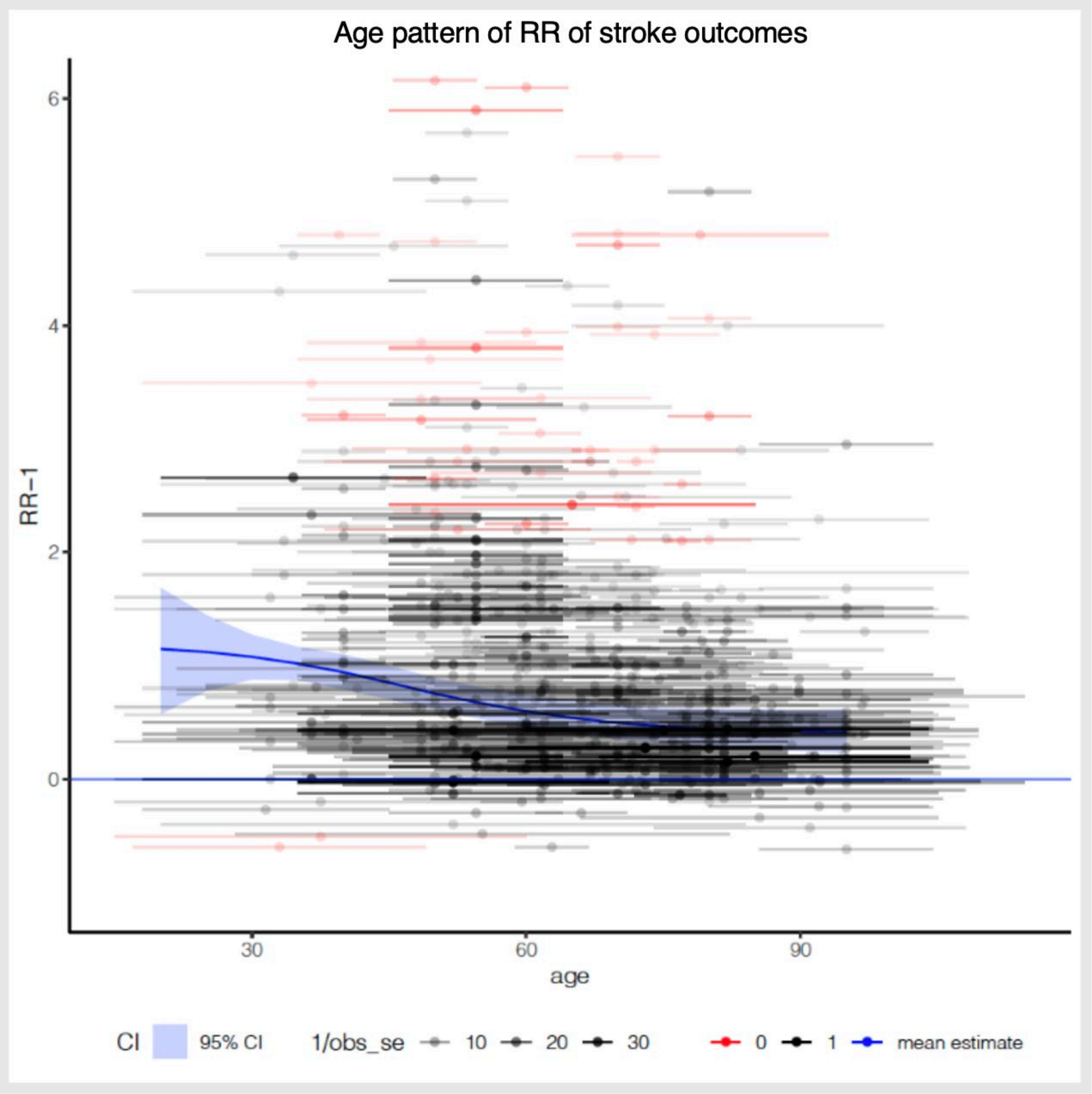

Figure 2: Attenuation factors of excess risk of smoking on stroke compared with the reference age group

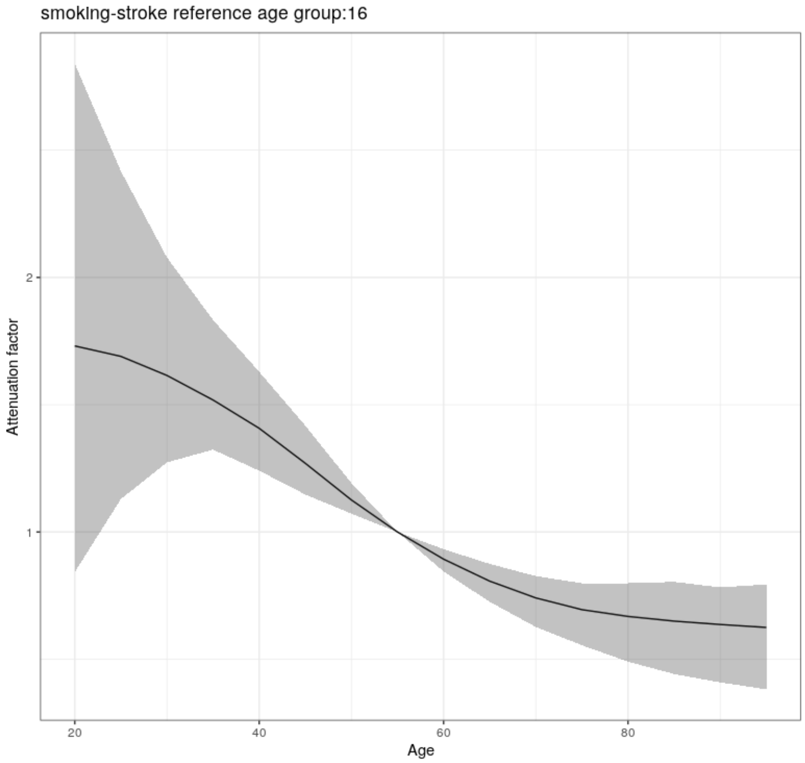

Figure 3: AF adjusted age-group specific RR curves for stroke, reference age group 55–59. The exposure is in units of cigarette-equivalents per smoker per day.

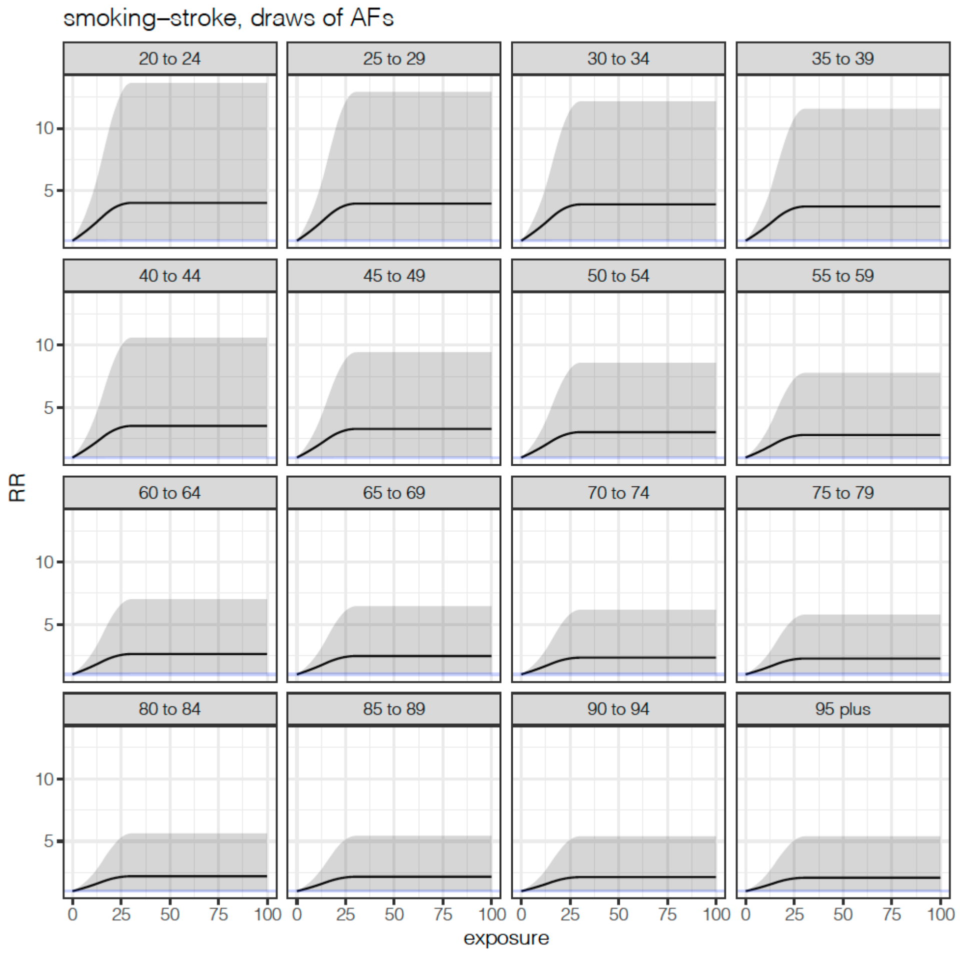

Population attributable fraction (PAF)

As in GBD 2021, we estimated PAFs based on the following equation:

$$PAF = \frac{p(n) + p(f) \int \exp(x) * rr(x) + p(c) \int \exp(y) * rr(y) - 1}{p(n) + p(f) \int \exp(x) * rr(x) + p(c) \int \exp(y) * rr(y)}$$

where $p(n)$ is the prevalence of never smokers, $p(f)$ is the prevalence of former smokers, $p(c)$ is the prevalence of current smokers, $exp(x)$ is a distribution of years since quitting among former smokers, $rr(x)$ is the relative risk for years since quitting, $exp(y)$ is a distribution of cigarettes per smoker per day or pack-years, and $rr(y)$ is the relative risk for cigarettes per smoker per day or pack-years.

We used pack-years as the exposure definition for cancers and chronic respiratory diseases, and cigarettes per smoker per day for cardiovascular diseases and all other health outcomes.

Citations

[1] Ng M, Freeman MK, Fleming TD, Robinson M, Dwyer-Lindgren L, Thomson B, et al. Smoking prevalence and cigarette consumption in 187 countries, 1980-2012. *JAMA*. 2014 Jan 8;311(2):183–92.

[2] Zheng P, Aravkin A, Barber R, Sorensen R, Murray C. Trimmed Constrained Mixed Effects Models: Formulations and Algorithms. bioRxiv. 2020 Jan 29;2020.01.28.923599.

[3] Dai X, Gil GF, Reitsma MB, Ahmad NS, Anderson JA, Bisignano C, Carr S, Feldman R, Hay SI, He J, Iannucci V, Lawlor HR, Malloy MJ, Marczak LB, McLaughlin SA, Morikawa L, Mullany EC, Nicholson SI, O'Connell EM, … Gakidou E. (2022). Health effects associated with smoking: A Burden of Proof study. *Nature Medicine*, 28(10), 2045–2055. https://doi.org/10.1038/s41591-022-01978-x

Suboptimal breastfeeding

Flowchart

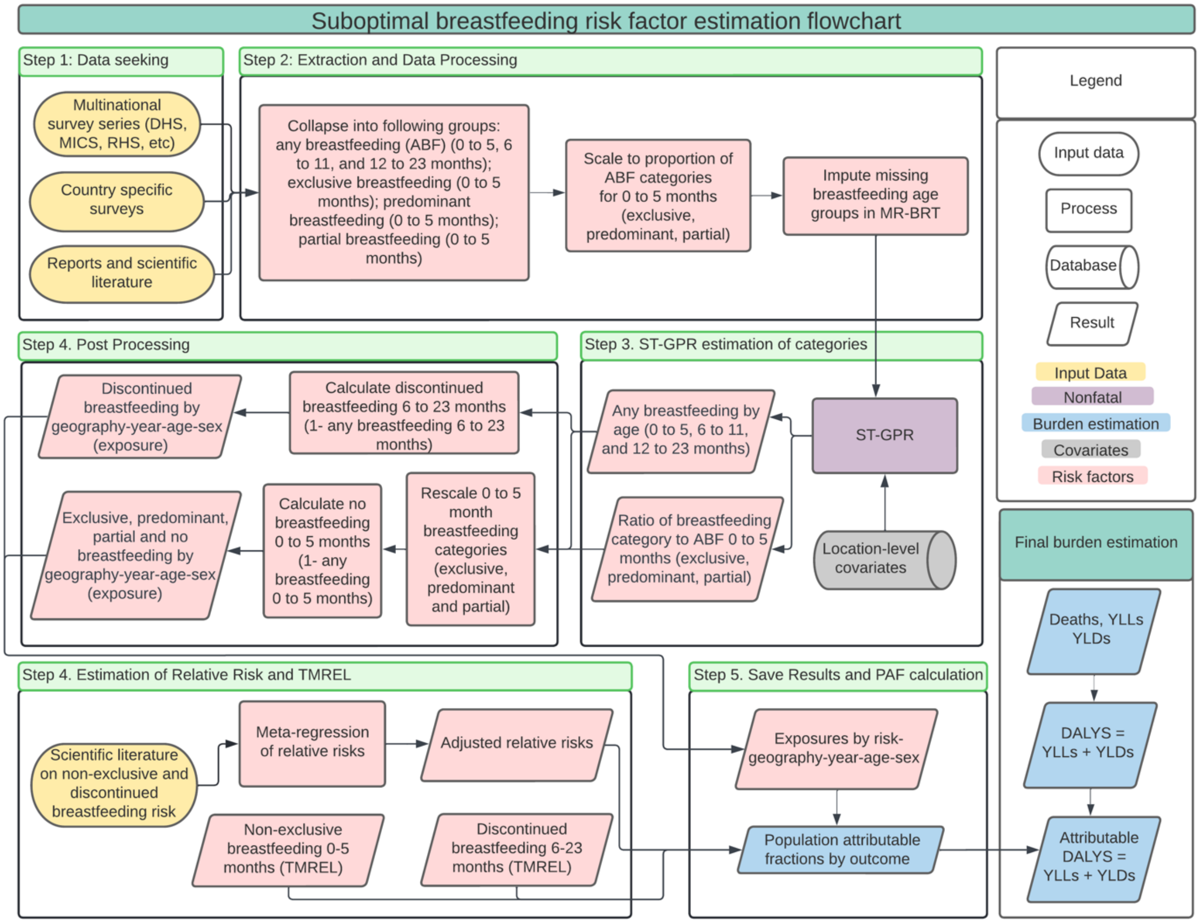

Input data and methodological summary

Definition

Exposure

Exposure to suboptimal breastfeeding is composed of two distinct categories: non-exclusive breastfeeding and discontinued breastfeeding.

Non-exclusive breastfeeding is defined as the proportion of children under 6 months of age who are not exclusively breastfed. We then parse those not exclusively breastfed into three categories – predominant, partial, and no breastfeeding. Exclusive breastfeeding is defined as the proportion of children who receive no other food or drink except breastmilk (allowing for oral rehydration solution, drops, or syrups containing vitamins, minerals, or medicines). Predominant breastfeeding is the proportion of children whose predominant source of nourishment is breastmilk but who also receive other liquids. Partial breastfeeding refers to those infants who receive breastmilk as well as food and

liquids, including non-human milk and formula. No breastfeeding refers to infants who do not receive breastmilk as a source of nourishment.

Discontinued breastfeeding is defined as the proportion of children between 6 and 23 months who receive no breastmilk as a source of nourishment.

Input data

Exposure

The data used in the analysis consist mostly of processed individual-level microdata from surveys; in the cases where microdata were unavailable, we used reported tabulated data from survey reports and scientific literature. Data used to categorise type of non-exclusive breastfeeding (predominant, partial, and none) come from surveys with 24-hour dietary logs based on maternal recall.

We updated our systematic review in GBD 2021 by searching the Global Health Data Exchange (GHDx) using the keyword "breastfeeding." We prioritised extraction of surveys with microdata and new surveys from major survey series such as Demographic and Health Surveys (DHS) and Multiple Indicator Cluster Surveys (MICS).

To better ensure consistency in estimates across age groups, we identified location-years where we had data for "any breastfeeding 6–11 months" but no data for "any breastfeeding 12–23 months." We then imputed data for "any breastfeeding 12–23 months" based on the observed 6–11-month datapoint in that location-year. We estimated the imputation adjustment by meta-analysing proportion ratios of matched pairs by source-location-year for any breastfeeding in these two age groups in meta-regression—Bayesian, regularised, trimmed (MR-BRT),[1] a Bayesian meta-analytic tool.

Table 1. MR-BRT adjustment factor for any breastfeeding 12–23 months imputation

Data input	Reference or alternative definition	Gamma	Beta coefficient, logit (95% UI)*	Adjustment factor**
Any breastfeeding 6–11 months	Ref	0.19	---	---
Any breastfeeding 12–23 months	Alt		−1.54 (−1.58 to −1.50)	0.21 (0.21–0.22)

**MR-BRT crosswalk adjustments can be interpreted as the factor the alternative definition is adjusted by to reflect what it would have been had it been measured using the reference definition.*
***The adjustment factor column is the exponentiated beta coefficient. For logit beta coefficients, this is the relative odds between the two definitions.*

Relative risk

We included outcomes based on the strength of available evidence supporting a causal relationship. Studies evaluating the causal evidence for our risk–outcome pairs came primarily from studies compiled in a published review by the World Health Organization.[2] Non-exclusive breastfeeding was paired with diarrhoea and lower respiratory infection as diseases outcomes. Discontinued breastfeeding was paired with diarrhoea as an outcome.

Modelling strategy

Exposure

Using the processed microdata and tabulated data from reports, we generated a complete time series for 1) any breastfeeding 0–5 months, 6–11 months, and 12–23 months, 2) ratio of exclusive breastfeeding to any breastfeeding 0–5 months, 3) ratio of predominant breastfeeding to any breastfeeding 0–5 months, and 4) ratio of partial breastfeeding to any breastfeeding 0–5 months using a three-step spatiotemporal Gaussian process regression. In previous GBD rounds, "any breastfeeding" was modelled separately for each of the estimated age groups. Starting in GBD 2021, with the addition of new under-5 age groups that aligned with those ages we model, we incorporated the three age groups into a single model of "any breastfeeding." This allowed us to borrow additional strength over space, age, and time by incorporating data from all sources in one model.

The first step of the ST-GPR process is an ensemble linear mixed-effects regression of our data on a set of potentially predictive covariates taken from the GBD study covariates database. We tested every combination of these covariates in individual, sex-specific mixed-effects linear regressions with nested random effects at the super-region, region, and location levels. We then evaluated and ranked each of these sub-models by their out-of-sample root-mean-squared error. Finally, to produce initial estimates for every location, year, age, and sex in the analysis, we averaged the 50 top-performing models where the estimated coefficients were 1) statistically significant at $p < 0.05$ and 2) in the expected direction. We tested the following covariates in the ensemble prior: Socio-demographic Index, SEV unsafe water, total fertility rate, maternal education, antenatal care (4+ visits), HIV mortality in women of reproductive age, high BMI in women of reproductive age, and underweight in women of reproductive age.

The second, spatiotemporal smoothing step of ST-GPR calculates the residual between our stage 1 regression estimate and each of our observed datapoints and then smooths this residual, drawing strength over space, age, and time and producing a revised stage 2 estimate of birth prevalence for every location, year, and sex. The third step of ST-GPR is a Gaussian process regression, using the stage 2 estimates as a prior and the observed datapoints and their variance to 1) further smooth the residual between the stage 2 predictions and observed data and produce a final mean estimate for each location, year, and sex, and 2) estimate uncertainty around this mean estimate, quantified by taking 1000 draws from the posterior Gaussian process. More detailed information on the ST-GPR modelling process can be found in the main text methods appendix.

To generate exposure categories for non-exclusive breastfeeding, we converted the modelled ratios of exclusive, predominant, and partial breastfeeding to the total category prevalence by multiplying each ratio by the estimates of any breastfeeding among infants aged 0–5 months. This ensured that these categories sum correctly to the "any breastfeeding 0–5 months" envelope. We calculated the proportion of infants receiving no breastmilk 0–5 months of age by subtracting the estimates of current breastfeeding from 1. We performed the same operation to estimate discontinued breastfeeding in the 6–11 months and 12–23 months categories.

Theoretical minimum risk exposure level

For non-exclusive breastfeeding, those children who received no source of nourishment other than breastmilk ("exclusively breastfed") were considered to be at the lowest risk of any of the disease outcomes. For discontinued breastfeeding, we assumed that children aged 6–23 months who received any breastmilk as a source of nourishment to be at the lowest risk of disease outcome.

Relative risk

We estimated relative risks for both non-exclusive and discontinued breastfeeding in a meta-analysis using the "metareg" package in Stata. For the 0–5-month age group, we included diarrhoea and lower respiratory infection as outcomes, and for the 6–23-month age group, we included diarrhoea as an outcome. We did not estimate separate relative risks for morbidity and mortality. The estimated relative risks are detailed in Table 2.

Table 2. Suboptimal breastfeeding relative risk estimates

**Exposure category**	**Diarrhoea**		**Lower respiratory infection**	
	**Mortality**	**Morbidity**	**Mortality**	**Morbidity**
0–5 months				
Exclusive breastfeeding	1.00	1.00	1.00	1.00
Predominant breastfeeding	2.35 (1.67–3.23)	2.35 (1.67–3.23)	1.37 (1.06–1.80)	1.37 (1.06–1.80)
Partial breastfeeding	2.63 (1.94–3.48)	2.63 (1.94–3.48)	1.48 (1.21–1.79)	1.48 (1.21–1.79)
No breastfeeding	3.60 (2.72–4.70)	3.60 (2.72–4.70)	1.74 (1.49–2.03)	1.74 (1.49–2.03)
6–23 months				
Any breastfeeding	1.00	1.00	--	--
Discontinued breastfeeding	1.31 (1.11–1.55)	1.31 (1.11–1.55)	--	--

Population attributable fraction

We used the standard GBD population attributable fraction (PAF) equation to calculate PAFs for non-exclusive breastfeeding and discontinued breastfeeding and each of their paired outcomes using exposure estimates, the theoretical minimum risk exposure level, and relative risks.

Citations

1. Vos T, Lim SS, Abbafati C, et al. Global burden of 369 diseases and injuries in 204 countries and territories, 1990–2019: a systematic analysis for the Global Burden of Disease Study 2019. *The Lancet* 2020; 396: 1204–22.
2. Horta, B., Voctora, C. (2013) Short-term effects of breastfeeding: a systematic review on the benefits of breastfeeding on diarrhoea and pneumonia mortality. The World Health Organization.

Unsafe sanitation

Flowchart

Unsafe Sanitation

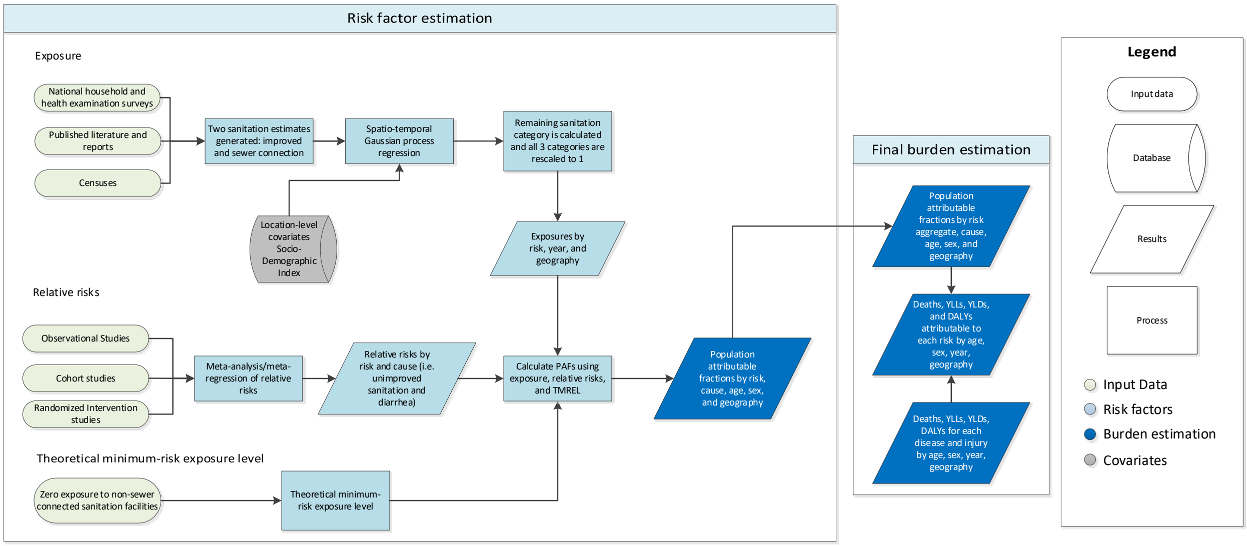

Input data and methodological summary

Definition

Exposure

Exposure to unsafe sanitation is defined based on the primary toilet type used by households. We model three different categories of sanitation: unimproved, improved, and facilities with a sewer connection or septic tank as defined by the WHO/UNICEF Joint Monitoring Programme for Water Supply, Sanitation and Hygiene (JMP).[1] See Table 1 for each category's definition.

Table 1: Exposure categories and definitions

Category	Definition
Unimproved sanitation	Proportion of individuals that use unimproved sanitation facilities, including use of open pit latrines, open defecation, and toilets that flush into creeks or open fields.
Improved sanitation	Proportion of individuals that use improved sanitation facilities, excluding sewer connection or septic tank. Including use of ventilated improved pit latrines, composting toilets, and pit latrines with slabs.
Sanitation facilities with sewer connection or septic tank	Proportion of individuals that use toilet facilities with sewer connection or septic tank.

Input data

Exposure

Input data came primarily from nationally representative surveys, such as the Demographic and Health Survey (DHS), the Multiple Indicator Cluster Surveys (MICS), the World Health Survey (WHS), the DHS AIDS Indicator Survey (AIS), the Malaria Indicator Survey (MIS), the Performance Monitoring and Accountability Survey (PMA), the Performance Monitoring for Action Cross-sectional Household and Female survey (PM-Action), the WHO Study on Global AGEing and Adult Health survey (WHO_SAGE), and numerous national government surveys. Surveys that reported results at the household level were converted to the individual level using household size data, to ensure that our models estimated the proportion of individuals, rather than households, exposed to a given indicator. Surveys and censuses were then tabulated to two sanitation categories, sewer connection and improved sanitation, for each location.

Relative risk

Unsafe sanitation was paired with one outcome, diarrhoeal diseases. Two meta-analyses, by Wolf, et al. 2014 and Wolf, et al. 2018, along with a literature review that used the same search terms as Wolf, et al. 2014, were used to identify relative risk studies.[2,3] We included a total of 16 studies, resulting in 19 relative risk values.

Modelling strategy

Exposure

Sanitation was modelled in an ordinal framework. Two distinct indicators were estimated: (1) the proportion of the total population using sewer connection or septic tank facilities, and (2) the proportion of individuals using improved sanitation within the population not connected to a sewer or septic tank. This ordinal framework allows us to estimate the category with the most data (sewer connection/septic tank prevalence) and leverage that estimate to anchor the estimates for the improved and unimproved sanitation categories.

The two indicators were each modelled using a three-step modelling scheme of mixed effect linear regression followed by spatiotemporal Gaussian process regression (ST-GPR), which produced full time-series estimates for each GBD 2023 location. Socio-demographic Index (SDI), a composite metric combining education per capita, income per capita, and fertility, was set as a fixed effect in the linear regression since it proved to be a significant predictor. Random effects were set at GBD 2023 region and super-region levels to fit the models but were not used in the predictions. The same linear regression equation was used for both ST-GPR models (see below).

$$logit(data) \sim sdi + (1|level\_1) + (1|level\_2)$$

SDI = Socio-demographic Index
(1|level_1) = super-region-level random effects
(2|level_2) = region-level random effects

The process of vetting and evaluating models was largely undertaken for GBD 2021 with additional evaluation of new input data for GBD 2023. Evaluation was accomplished primarily through an examination of ST-GPR scatterplots by location and year. Any poorly fitting datapoints were re-inspected

for error at the level of extraction and survey implementation. If errors in data extraction were found, the study in question was re-extracted. In addition to SDI, a number of different potential fixed effects were considered, including lag-distributed income and urbanicity, but SDI proved to be the strongest predictor of unsafe sanitation in terms of magnitude of the coefficient. Uncertainty in the estimates was initially constructed based on standard deviation around each survey mean, then propagated through ST-GPR modelling by incorporating the variance of each datapoint in the Gaussian process regression step. A datapoint with high variance, for example, would contribute relatively less influence to the model than a datapoint with lower variance.

Full time-series outputs from ST-GPR modelling are then rescaled using the below equations to form three mutually exclusive categories that sum to one for each location-year combination. Rescaling results in the proportion of the population with each category of sanitation (see Table 1 for definitions).

$$\boldsymbol{Sewer} = \frac{\#\ persons\ with\ sewer\ or\ septic\ connection}{\#\ persons\ with\ nonmissing\ response}$$

$$\boldsymbol{Improved} = \left(\frac{\#\ persons\ using\ improved\ facilities}{\#\ persons\ without\ sewer\ or\ septic\ connection}\right) * (1 - \boldsymbol{Sewer})$$

$$\boldsymbol{Unimproved} = 1 - (\boldsymbol{Sewer} + \boldsymbol{Improved})$$

Theoretical minimum risk exposure level

The theoretical minimum risk exposure level for unsafe sanitation was defined as having access to a sanitation facility with sewer connection or septic tank.

Relative risks

Relative risk values were calculated using a network meta-analysis approach with a tool called meta-regression—Bayesian, regularised, trimmed (MR-BRT) in the Burden of Proof (BoP) framework.[4, 5] One study-level covariate was found to be statistically significant – whether the study was generalisable to the general population – and was included in the network meta-analysis. No priors were used. We calculated the risk of developing diarrhoea for those using improved sanitation facilities and sewer or septic facilities, relative to the reference category of those using unimproved facilities (Table 2). Prior to calculating PAFs we took the inverse of the relative risk results such that the reference would be sewer or septic facilities (Table 3).

Table 2: MR-BRT network meta-analysis results (reference: unimproved sanitation)

Intervention	Relative risk (95% CI)
Improved sanitation	0.795 (0.739–0.856)
Sanitation facilities with sewer connection or septic tank	0.310 (0.274–0.352)

Table 3: Relative risks for each exposure category (reference: sewer or septic facilities)

Exposure category	Relative risk (95% CI)
Unimproved sanitation	3.22 (2.74–3.76)

Improved sanitation	2.57 (2.08–3.12)
Sanitation facilities with sewer connection or septic tank	1 (reference)

Figure 1 shows the results of the BoP analysis in graphical form, along with the associated "risk–outcome scores" for each category, which is a measure of how good the evidence is for that relative risk estimate. Prior to generating a risk–outcome score, we conducted an additional post-analysis step to detect and flag publication bias in the input data. This approach is based on the classic Egger's regression strategy, which is applied to the residuals in our model. In the current implementation, we do not correct for publication bias but flag the risk–outcome pairs where the risk for publication bias is significant.

We did not detect publication bias based on the association between observation residuals and their standard errors (p-value = 0.337, Egger mean = –0.102, Egger SD = 0.243). The overall risk–outcome score for unsafe sanitation is 0.518, which is the maximum of the individual category scores. The overall score results in a four-star rating for unsafe sanitation.

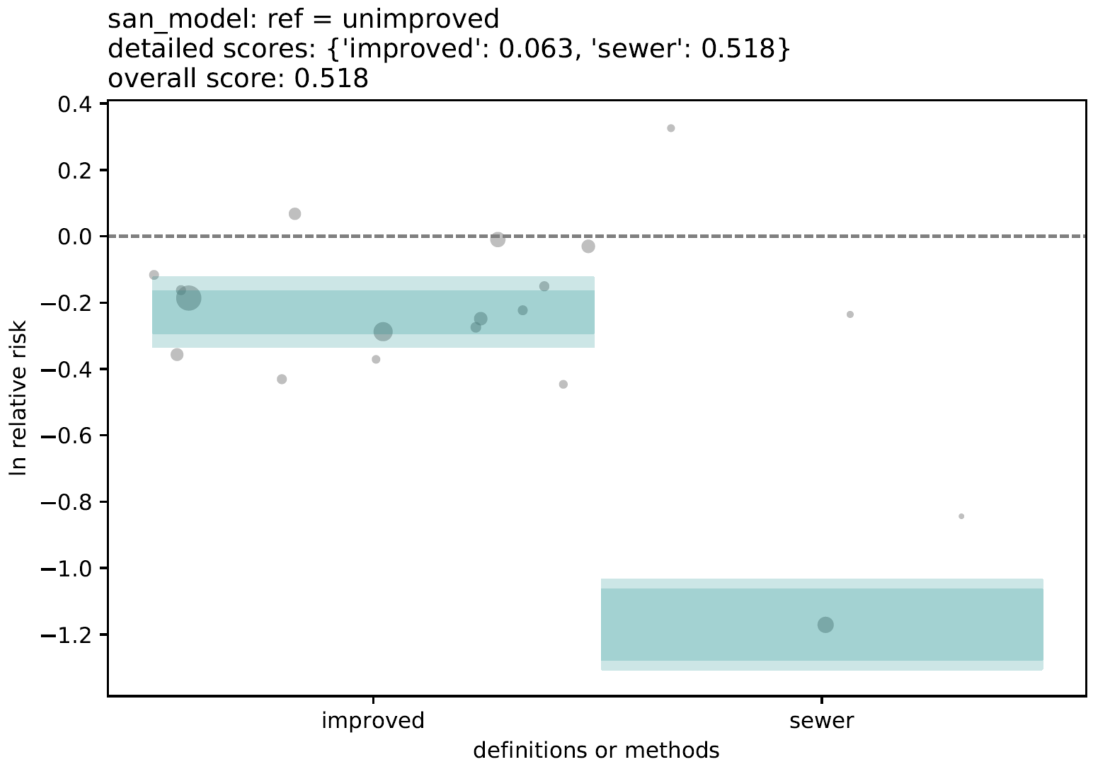

Figure 1: BoP relative risk results and risk–outcome scores

Population attributable fractions (PAFs)

PAFs were calculated using the standard GBD PAF formula.[6]

References

1. WHO/UNICEF Joint Monitoring Programme: Sanitation. https://washdata.org/monitoring/sanitation (accessed Oct 31, 2019).
2. Wolf J, Pruss-Ustun A, Cumming O, *et al.* Assessing the impact of drinking water and sanitation on diarrhoeal disease in low- and middle-income settings: systematic review and meta-regression. *Tropical Medicine and International Health* 2014; **19**: 928–42.
3. Wolf J, Hunter PR, Freeman MC, *et al.* Impact of drinking water, sanitation and handwashing with soap on childhood diarrhoeal disease: updated meta-analysis and meta-regression. *Tropical Medicine and International Health* 2018; **23**: 508–25.
4. Zheng P, Barber R, Sorensen RJD, Murray CJL, Aravkin AY. Trimmed constrained mixed effects models: formulations and algorithms. *J Comput Graph Stat* 2021; **30**: 544–56.
5. Zheng P, Afshin A, Biryukov S, *et al.* The Burden of Proof studies: assessing the evidence of risk. *Nat Med* 2022; **28**: 2038–44.
6. GBD 2021 Risk Factors Collaborators. Global burden and strength of evidence for 88 risk factors in 204 countries and 811 subnational locations, 1990-2021: a systematic analysis for the Global Burden of Disease Study 2021. *Lancet* 2024; **403**: 2162–203.

Unsafe sex

Flowchart

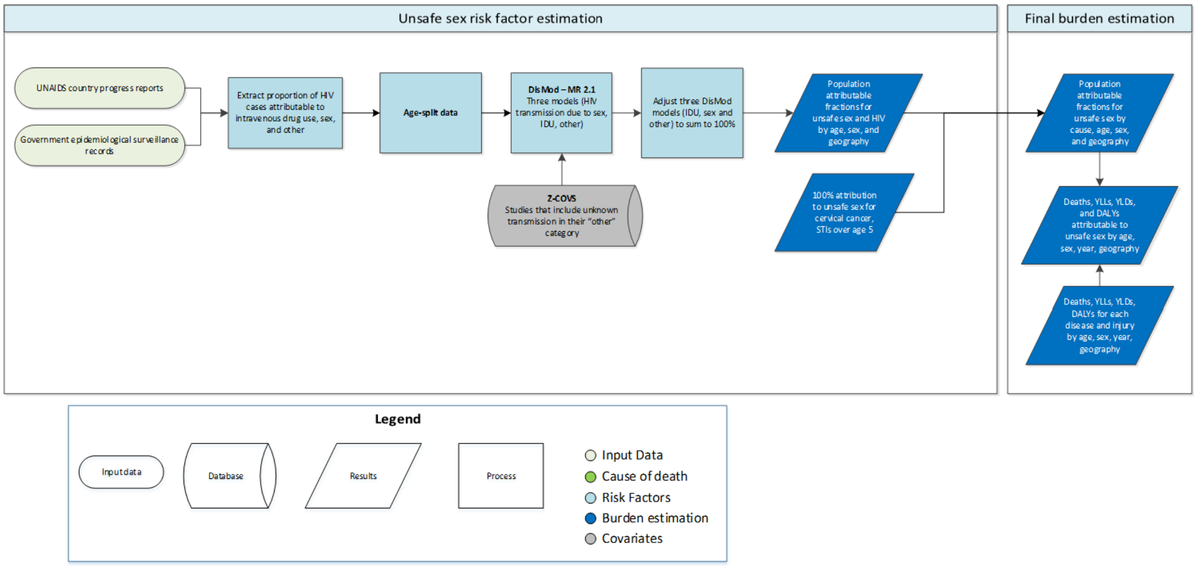

Input data and methodological summary

Definition

Exposure

Unsafe sex is defined as the risk of disease due to sexual transmission. The outcomes associated with unsafe sex that we estimate for the GBD study include HIV, cervical cancer, and all sexually transmitted diseases (STDs) except for those in neonates from vertical transmission, including HIV, *Opthalmia neonatorum*, and neonatal syphilis. We assumed 100% of cervical cancer and STDs were attributable to unsafe sex and modelled the proportion of HIV incidence occurring through sexual transmission to estimate the attributable burden for HIV due to unsafe sex. The theoretical minimum risk exposure level[1] (TMREL, described in Appendix 2 section 2) is defined as the absence of disease transmission due to sexual contact.

Input data

Exposure

To be used in our models, sources must report HIV cases attributable to various modes of transmission. In GBD 2019, we screened UNAIDS country progress reports and searched government epidemiological surveillance records for these data. The primary data sources we used were UNAIDS, the European CDC, and the US CDC.

We excluded all extractions where the "other" category for HIV transmissions accounted for greater than 25% of all cases. We believe that such high proportions raise concerns about the quality of reporting.

Relative risk

We do not estimate relative risks or exposure for unsafe sex due to the inherent challenges in capturing these data. Instead, to determine the proportion of HIV attributable to unsafe sex, we model the proportion of HIV cases due to unsafe sex compared to other transmission modes directly. Additional details are available in the modelling strategy section.

Modelling strategy

We made no substantial changes to the modelling strategy for GBD 2023. We modelled the proportion of HIV cases attributable to unsafe sex. To do this, we collected and processed data, ran three DisMod-MR 2.1 (disease model—Bayesian meta-regression, described in Appendix 1 section 2) models (HIV attributable to sex, HIV attributable to injection drug use, HIV attributable to other routes of transmission), adjusted results of the three DisMod-MR models to sum to one, and then assigned the proportions as direct PAFs (population attributable fractions), described in Appendix 2 section 2.

No country-level covariates were included in the models. We tested an injection drug use (IDU) covariate – an opioid use covariate in the proportion HIV due to drug use model – but found no significant coefficients, so excluded them from the final model.

Since all-age and both-sex datapoints represent the vast majority of the available data, we derived an age-sex pattern for the HIV-IDU transmission model from the age-sex pattern present in the GBD 2017 PAF for hepatitis B attributable to IDU (the model for injecting drug use and hepatitis estimates the cumulative exposure to injecting drug use to capture all infections in people with a history of injecting even if in a more distant past). Assuming the proportion of HIV due to other transmission is constant over age and by sex, the age-sex pattern for the proportion of HIV due to sexual transmission was set to be the complement to 1 of the age-sex pattern for the proportion of HIV due to IDU. The all-age and both-sex data were split according to these age-sex patterns, and the three HIV transmission DisMod-MR models were run on the age- and sex-split data. In previous GBD rounds, only age-splitting had used this approach, while sex-splitting occurred within DisMod-MR. Since most data are for both sexes combined, using the sex ratio – in addition to the age pattern from the IDU-hepatitis B PAF – is much more informative. The impact of this change resulted in general increases in proportion of HIV due to sexual transmission among females, as they generally had lower IDU rates compared to males.

In GBD 2019, we also adjusted the proportion of HIV due to sex DisMod-MR model to operate in complement space, representing (1 - proportion). Since proportions were high in most countries, modelling in complement space resulted in a better model fit. Additional priors were set to inform an age pattern: zero proportion HIV transmission due to IDU before age 15, zero proportion HIV transmission due to sex before age 10 (100 in complement space), and 100% transmission due to other before age 10. The results from these HIV transmission models were adjusted to sum to 100% for a given country-year-age-sex group at each of 1000 draws. This process was continued in GBD 2023.

Squeezed global HIV transmission model, Male

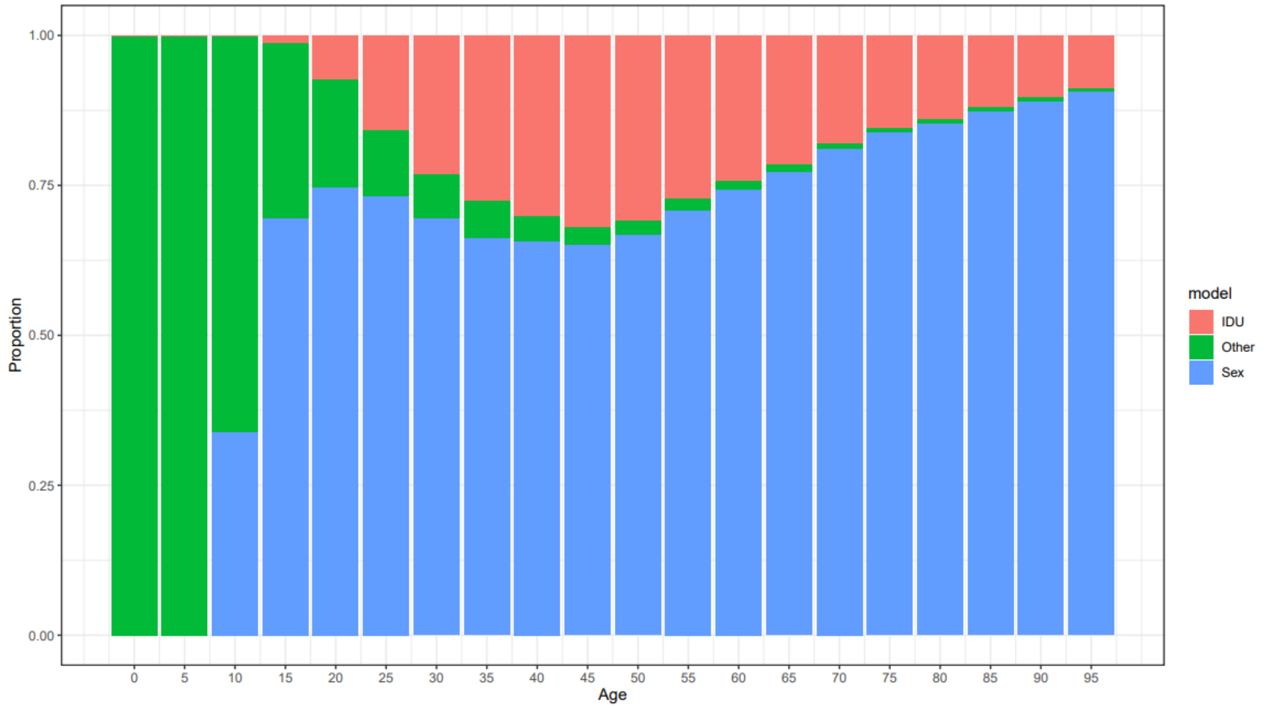

Squeezed global HIV transmission model, Female

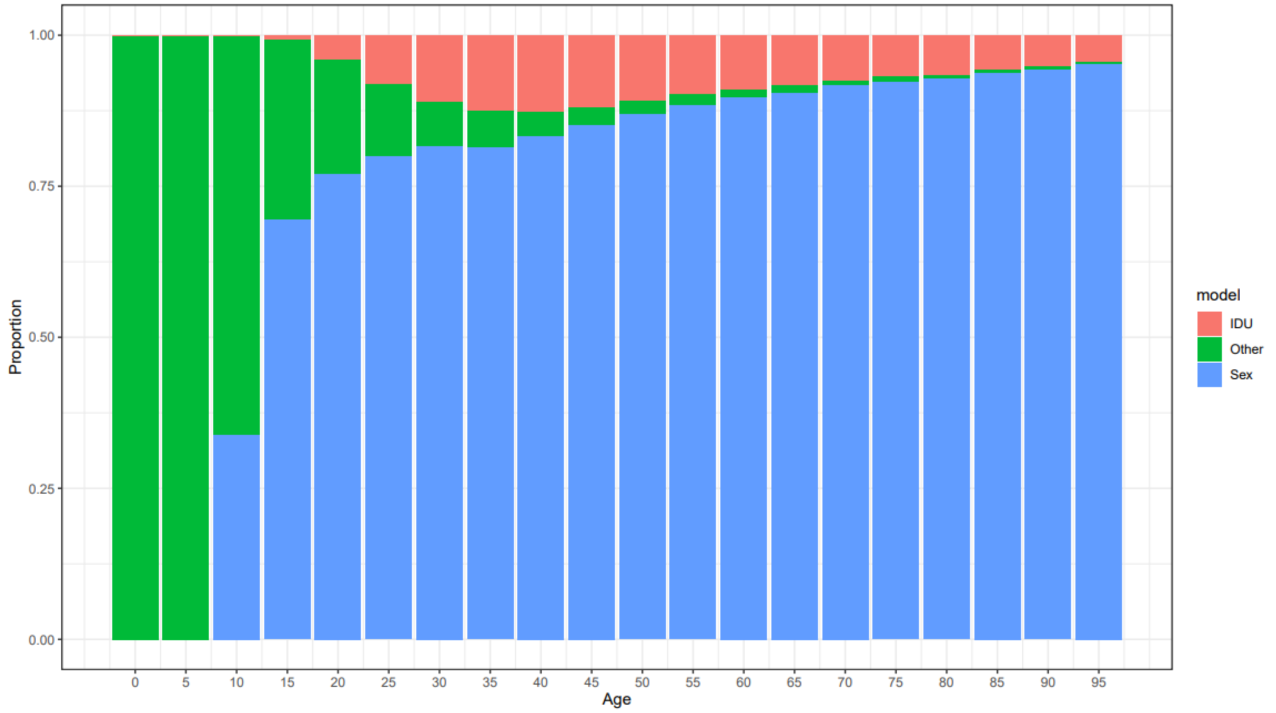

Theoretical minimum risk exposure level

The TMREL used for unsafe sex is the absence of disease transmission due to sexual contact.

Population attributable fraction calculations

Based on evidence in the literature, we attributed 100% of cervical cancer to unsafe sex, as HPV infection is a necessary cause of cervical cancer and is transmitted predominantly through sexual contact.[2] The proportion of STDs attributable to unsafe sex was also 100%.

For HIV, the results from the single parameter proportion DisMod-MR model for HIV transmission due to sex after squeezing were used directly as the population attributable fraction.

References

1. **Brauer M, et al.** Global burden and strength of evidence for 88 risk factors in 204 countries and 811 subnational locations, 1990–2021: a systematic analysis for the Global Burden of Disease Study 2021. *The Lancet* 2024; **403**(10440): 2162–2203. doi:10.1016/S0140-6736(24)00933-4.

2. Burd EM**.** Human papillomavirus and cervical cancer. *Clin Microbiol Rev.* 2003;16(1):1-17. doi:10.1128/CMR.16.1.1-17.2003.

Unsafe water

Flowchart

Unsafe Drinking Water

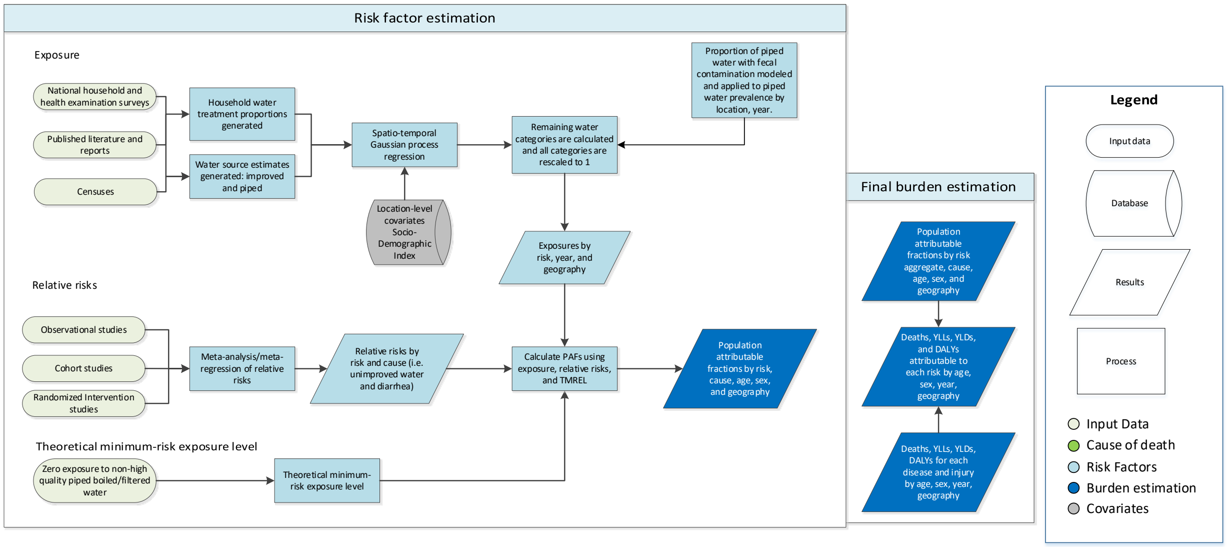

Input data and methodological summary

Exposure

Case definition

Exposure to unsafe water was defined based on (1) reported primary water source used by the household, (2) use of household water treatment (HWT) to improve the quality of drinking water before consumption. Water sources were defined based on the WHO/UNICEF Joint Monitoring Programme for Water Supply, Sanitation and Hygiene (JMP).[1] Examples of "improved" sources include boreholes, tube wells, protected wells, and packaged or delivered water. Piped water is also considered "improved" by the JMP but is placed into its own category for GBD purposes. Thus, our model estimates for improved water source does not include piped water. Examples of "unimproved" sources include unprotected springs, unprotected wells, and surface water. Additionally, four different HWTs were determined to be effective point-of-use treatments based on effect sizes calculated from a network meta-analysis: solar treatment, chlorine treatment, boiling, and filtering. For modelling purposes, we grouped solar and chlorine treatment together, as well as boiling and filtering.

Input data

Exposure

Water source input data came primarily from nationally representative surveys, such as the Demographic and Health Survey (DHS), the Multiple Indicator Cluster Surveys (MICS), the World Health Survey (WHS), the DHS AIDS Indicator Survey (AIS), the WHO Study on Global AGEing and Adult Health (SAGE), the Malaria Indicator Survey (MIS), the Core Welfare Indicators Questionnaire Survey (CWIQ), the Performance Monitoring and Accountability survey (PMA), the Performance Monitoring for Action Cross-sectional Household and Female Survey (PMAction), and numerous national government surveys.

HWT input data were largely limited to the DHS and MICS due to data availability. In GBD 2021, we re-extracted nearly all our sources from 2000 to 2021 in an effort to standardise extraction outputs and fix past extraction errors. New extractions for GBD 2023 were aligned with the GBD 2021 re-extraction approach.

The proportion of piped water with faecal contamination is mostly pulled from Bain et al 2014,[2] along with a few national statistics.

Relative risk

Unsafe water was paired with one outcome – diarrhoeal diseases – given evidence provided by relative risk studies. Two meta-analyses (Wolf et al 2014 and Wolf et al 2018) were used to identify relative risk studies, including years 1970–2016.[3,4] In GBD 2021, a literature review on the relationship between high-quality (HQ) piped water and diarrhoea was conducted, which yielded three studies (Figure 1). We searched PubMed for relevant literature published from January 1, 1970, to July 2, 2020 (date of search), using the search string below:

("Drinking Water"[Mesh] OR "Water Quality"[Mesh] OR "Water Supply"[Mesh] OR "Piped water"[TIAB] OR "Tap water"[TIAB] OR "Potable water"[TIAB]) AND ("Diarrhea"[Mesh] OR Diarrh*[TIAB] OR "Diarrhea incidence"[TIAB] OR "Bacteriological"[TIAB] OR "Microbial water quality"[TIAB]) AND (1970[PDAT] : 3000[PDAT) NOT (animals[MeSH] NOT humans[MeSH])

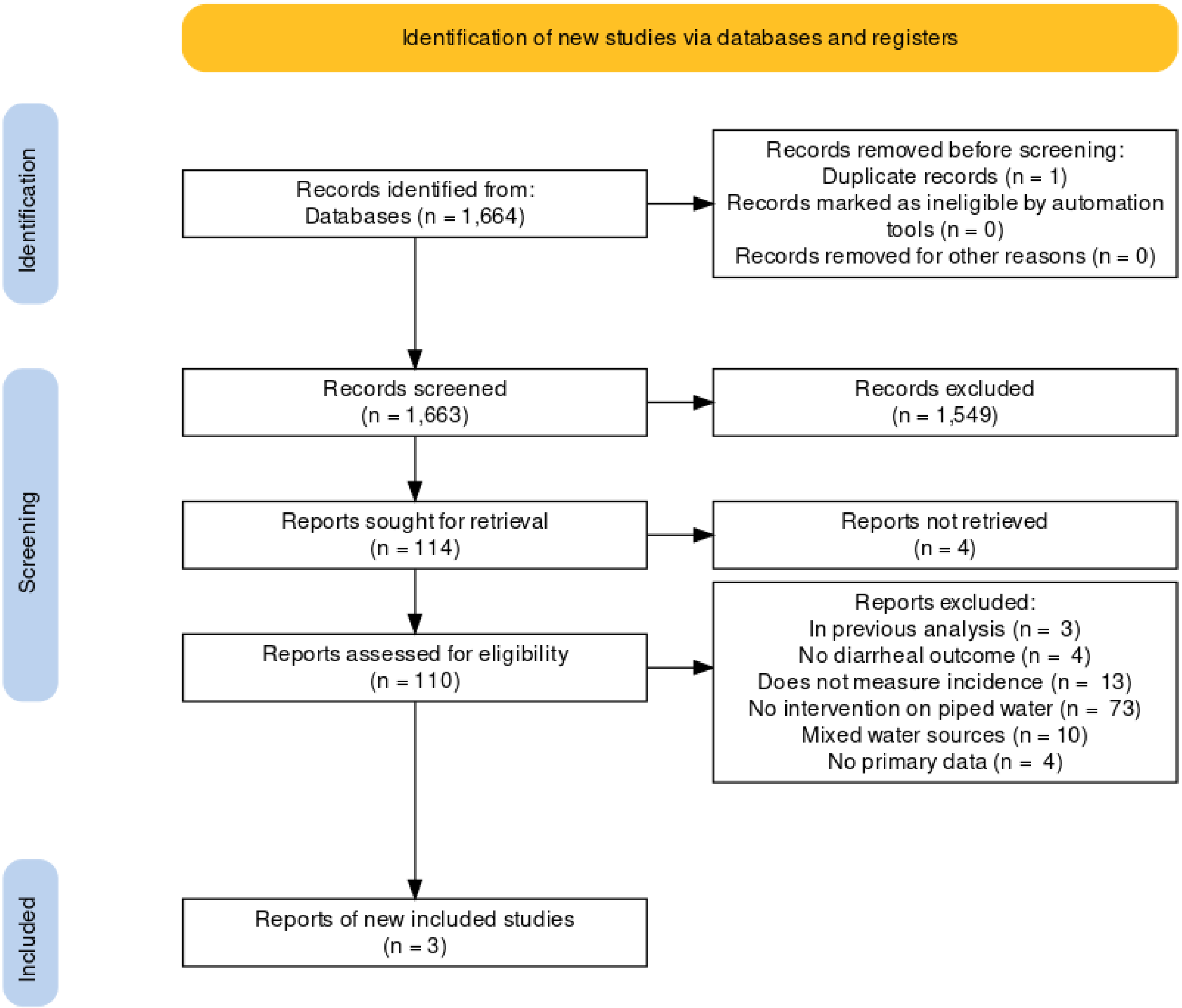

Figure 1: PRISMA diagram for systematic review of HQ piped water and diarrhoeal disease

Data processing

Surveys that reported results at the household level were converted to the individual level using household size data to ensure that our models estimated the proportion of individuals, rather than households, exposed to a given indicator. For water source, we calculated two separate categories, using the available input data: (1) proportion of the total population that uses piped water sources, and (2) proportion of the non-piped population that uses improved water sources. To calculate each category, we used the following equations:

$$\boldsymbol{Piped} = \frac{\#\ persons\ using\ piped\ water}{\#\ persons\ with\ nonmissing\ response}$$

$$\boldsymbol{Improved}_{\ \boldsymbol{non-piped\ population}} = \left(\frac{\#\ persons\ using\ improved\ water}{\#\ persons\ without\ piped\ water}\right)$$

For HWT, we calculated two separate categories, using the available input data: (1) proportion of the total population that does not use any water treatment methods, and (2) proportion of the population

that treat their water that use boiling or filtering, among the population the uses HWT. To calculate each category, we used the following equations:

$$\boldsymbol{No\ HWT} = \frac{\#\ persons\ who\ do\ not\ treat\ water}{\#\ persons\ with\ nonmissing\ response}$$

$$\boldsymbol{Boil\ or\ filter}_{\boldsymbol{HWT\ users}} = \left(\frac{\#\ persons\ who\ treat\ water\ with\ boil\ or\ filter}{\#\ persons\ who\ treat\ water\ with\ any\ method}\right)$$

The proportion of piped water with faecal contamination is already reported as such and does not need to be calculated. Although, the equation below can be used to derive this proportion.

$$\boldsymbol{Faecal\ contamination} = \frac{\#\ contaminated\ piped\ water\ systems}{\#\ piped\ water\ systems\ sampled}$$

Modelling strategy

Exposure

Water source data were modelled using an ordinal framework, with two distinct models: (1) proportion of the total population that uses piped water sources, and (2) proportion of the non-piped population that uses improved water sources. Both models were estimated for all ages and both sexes combined. This ordinal framework allowed estimating the category with the most data (piped water prevalence) and leveraging that estimate to anchor the estimates for the improved and unimproved water categories.

HWT categories were estimated in a similar ordinal framework. The two HWT models were (1) proportion of the total population that does not use any water treatment methods, and (2) proportion of the population that treat their water that use boiling or filtering as their primary HWT. Both HWT models were estimated for all ages and both sexes combined.

We modelled all four of the models using a three-step modelling scheme of mixed-effect linear regression followed by spatiotemporal Gaussian process regression (ST-GPR), which produced full time-series estimates for each location. Covariates are used to predict for location-years that we do not have input data for. Socio-demographic Index (SDI), a composite measure of development combining education per capita, income per capita, and fertility, was set as a fixed effect in the linear regression since it proved to be a significant predictor. Random effects were set on region and super-region levels to fit the models but were not used in the predictions. The linear regression equation for the ST-GPR models was:

$$logit(data) \sim SDI + (1|level\_1) + (1|level\_2)$$

(1|level_1) = super-region-level random effects
(2|level_2) = region-level random effects

ST-GPR was also used to model the proportion of piped water with faecal contamination to produce estimates for location-years we do not have input data for. In addition to SDI, the proportion of the population with piped water access was used as a covariate in the faecal contamination ST-GPR model. The linear regression equation was:

$$logit(data) \sim SDI + piped\ water\ access + (1|level\_1) + (1|level\_2)$$

(1|level_1) = super-region-level random effects
(2|level_2) = region-level random effects

Once all ST-GPR models were run, we used the modelled estimates to calculate all three categories of water source, the three categories of HWT, and split piped water into basic piped and HQ piped. Using the $Piped$ and $Improved_{non-piped\ population}$ modelled estimates, we calculated the proportion of the population with improved (excluding piped) and unimproved water sources for each location-year for all ages and sexes:

$$\boldsymbol{Improved} = \boldsymbol{Improved}_{\boldsymbol{non-piped\ population}} * (1 - \boldsymbol{Piped})$$

$$\boldsymbol{Unimproved} = 1 - (\boldsymbol{Piped} + \boldsymbol{Improved})$$

We define HQ piped water as piped water that enters the household free of faecal contamination and does not receive treatment within the household. Using the $Faecal\ contamination$ and $Piped$ modelled estimates, we calculated the proportion of the population with basic piped and HQ piped for each location-year for all ages and sex:

$$\boldsymbol{Basic\ piped} = \boldsymbol{Piped} * \boldsymbol{Faecal\ contamination}$$

$$\boldsymbol{HQ\ piped} = \boldsymbol{Piped} - \boldsymbol{Basic\ piped}$$

Using the $No\ HWT$ and $Boil\ or\ filter_{HWT\ users}$ modelled estimates, we calculated the proportion of the population that uses boil or filter, or chlorine or solar as HWT for each location-year for all ages and sexes:

$$\boldsymbol{Boil\ or\ filter} = \boldsymbol{Boil\ or\ filter}_{\boldsymbol{HWT\ users}} * (1 - \boldsymbol{No\ HWT})$$

$$\boldsymbol{Chlorine\ or\ solar} = 1 - (\boldsymbol{No\ HWT} + \boldsymbol{Boil\ or\ filter})$$

Once the base categories were calculated for water source and HWT, we combined water source and HWT to estimate ten mutually exclusive categories (Table 1). We then rescaled all ten categories such that they summed to one for each location-year. To do this, we found the total of the combined ten categories, and then divided each category by the total to get a rescaled proportion of the population estimate.

Table 1: Exposure categories, definitions, and formulas

Exposure category	Definition	Formula
Unimproved, no HWT	Proportion of individuals who primarily use unimproved source and *do not* use any HWT to purify their drinking water.	$Unimproved\ * No\ HWT$
Unimproved, chlorine/solar	Proportion of individuals who primarily use unimproved source, and who use solar or chlorine treatment to purify their drinking water.	$Unimproved * Chlorine\ or\ solar$

Unimproved, boil/filter	Proportion of individuals who primarily use unimproved source and who boil or filter to purify their drinking water.	$Unimproved * Boil\ or\ filter$
Improved water except piped, no HWT	Proportion of individuals who primarily use improved sources other than piped water supply and *do not* use any HWT to purify their drinking water.	$Improved * No\ HWT$
Improved water except piped, chlorine/solar	Proportion of individuals who primarily use improved sources other than piped water supply, and who use solar or chlorine treatment to purify their drinking water.	$Improved * Chlorine\ or\ solar$
Improved water except piped, boil/filter	Proportion of individuals who primarily use improved sources other than piped water supply and who boil/filter their drinking water.	$Improved * Boil\ or\ filter$
Basic piped water, no HWT	Proportion of individuals who primarily use basic piped water supply and *do not* use any HWT to purify their drinking water.	$Basic\ piped * No\ HWT$
Basic piped water, chlorine/solar	Proportion of individuals who primarily use basic piped water supply, and who *use* solar or chlorine water treatment, to purify their drinking water.	$Basic\ piped * Chlorine\ or\ solar$
Basic piped water, boil/filter	Proportion of individuals who primarily use basic piped water supply and who boil or filter to purify their drinking water.	$Basic\ piped * Boil\ or\ filter$
High-quality piped water	Proportion of individuals who primarily use high-quality piped water.	$HQ\ piped$

Theoretical minimum risk exposure level

The theoretical minimum risk exposure level for unsafe water is defined as having access to HQ piped water.

Relative risks

In GBD 2021, relative risk values were calculated using a network meta-analysis approach with a tool called meta-regression—Bayesian, regularised, trimmed (MR-BRT) in the Burden of Proof (BoP) framework.[5, 6] One study-level covariate – whether conformity with the study interventions was self-reported or confirmed by the researchers – was included in the network meta-analysis. Several other covariates (whether exposure was captured at the individual level or population level; whether or not the study was randomised; whether or not the study adjusted for all major known confounders; and what the study's follow-up percentage was) were considered but ultimately were not statistically

significant and so were not included in the analysis. No priors were used. The risk of developing diarrhoea relative to using an unimproved water source was calculated for each of the following categories: boil or filter, solar or chlorine, improved, piped, and high-quality piped (Table 2). To match our exposure definitions, we combined the effects of water source and HWT, which were assumed to be multiplicative (Table 3). Additionally, we assumed that the lowest possible risk level is using the best source type (HQ piped water) combined with the best point-of-use treatment (boil/filter). The MR-BRT results were then combined and taken the inverse (1/RR) of to make HQ piped and boil/filter the reference (Table 3). These RR values are then used in our PAF calculations. No updates to relative risks were made for GBD 2023.

Table 2: MR-BRT network meta-analysis results (reference: unimproved water source)

Intervention	Relative risk (95% UI)
Boil/filter water treatment	0.57 (0.37–0.87)
Chlorine/solar water treatment	0.77 (0.51–1.15)
Improved water source	0.84 (0.54–1.28)
Piped water source	0.67 (0.44–1.03)
High-quality piped water source	0.29 (0.12–0.70)

Table 3: Relative risks for each exposure category (reference: high-quality piped water and boil/filter)

Exposure category	Relative risk (95% UI)
Unimproved, no HWT	6.93 (2.20–17.54)
Unimproved, chlorine/solar	5.41 (1.59–14.02)
Unimproved, boil/filter	3.87 (1.42–8.66)
Improved water except piped, no HWT	5.97 (1.77–16.16)
Improved water except piped, chlorine/solar	4.67 (1.25–13.20)
Improved water except piped, boil/filter	3.33 (1.13–8.20)
Basic piped water, no HWT	4.73 (1.33–12.59)
Basic piped water, chlorine/solar	3.69 (1.01–10.14)
Basic piped water, boil/filter	2.64 (0.86–6.34)
High-quality piped water	1 (reference)

Figure 2 shows the results of the BoP analysis in graphical form, along with the associated “risk–outcome score” for each category, which is a measure of how good the evidence is for that particular relative risk estimate. Prior to generating a risk–outcome score, we conducted an additional post-

analysis step to detect and flag publication bias in the input data. This approach is based on the classic Egger's regression strategy, which is applied to the residuals in our model. In the current implementation, we do not correct for publication bias but flag the risk–outcome pairs where the risk for publication bias is significant.

We did not detect publication bias based on the association between observation residuals and their standard errors (p-value = 0.195, Egger mean = –0.101, Egger SD = 0.118). The overall risk–outcome score for this risk factor is 0.231, a 3-star rating, which is the maximum of the individual category scores.

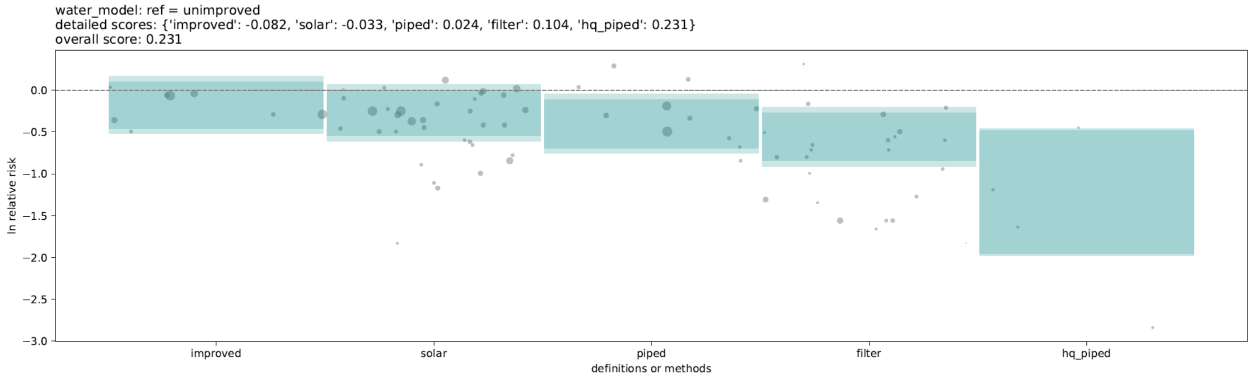

Figure 2: Risk–outcome scores

Population attributable fractions (PAFs)

PAFs were calculated using the standard GBD PAF formula.[7]

References

1. WHO/UNICEF Joint Monitoring Programme: Drinking water. https://washdata.org/monitoring/drinking-water (accessed Oct 31, 2019).
2. Bain R, Cronk R, Wright J, Yang H, Slaymaker T, Bartram J. Fecal Contamination of Drinking-Water in Low- and Middle-Income Countries: A Systematic Review and Meta-Analysis. *PLOS Medicine* 2014; **11**: e1001644.
3. Wolf J, Pruss-Ustun A, Cumming O, *et al.* Assessing the impact of drinking water and sanitation on diarrhoeal disease in low- and middle-income settings: systematic review and meta-regression. *Tropical Medicine and International Health* 2014; **19**: 928–42.
4. Wolf J, Hunter PR, Freeman MC, *et al.* Impact of drinking water, sanitation and handwashing with soap on childhood diarrhoeal disease: updated meta-analysis and meta-regression. *Tropical Medicine and International Health* 2018; **23**: 508–25.
5. Zheng, P., Barber, R., Sorensen, R. J., Murray, C. J., & Aravkin, A. Y. (2021). Trimmed constrained mixed effects models: formulations and algorithms. *Journal of Computational and Graphical Statistics*, 1-13.
6. Zheng P, Afshin A, Biryukov S, *et al.* The Burden of Proof studies: assessing the evidence of risk. *Nat Med* 2022; **28**: 2038–44.
7. GBD 2021 Risk Factors Collaborators. Global burden and strength of evidence for 88 risk factors in 204 countries and 811 subnational locations, 1990-2021: a systematic analysis for the Global Burden of Disease Study 2021. *Lancet* 2024; **403**: 2162–203.

Vitamin A deficiency

Flowchart

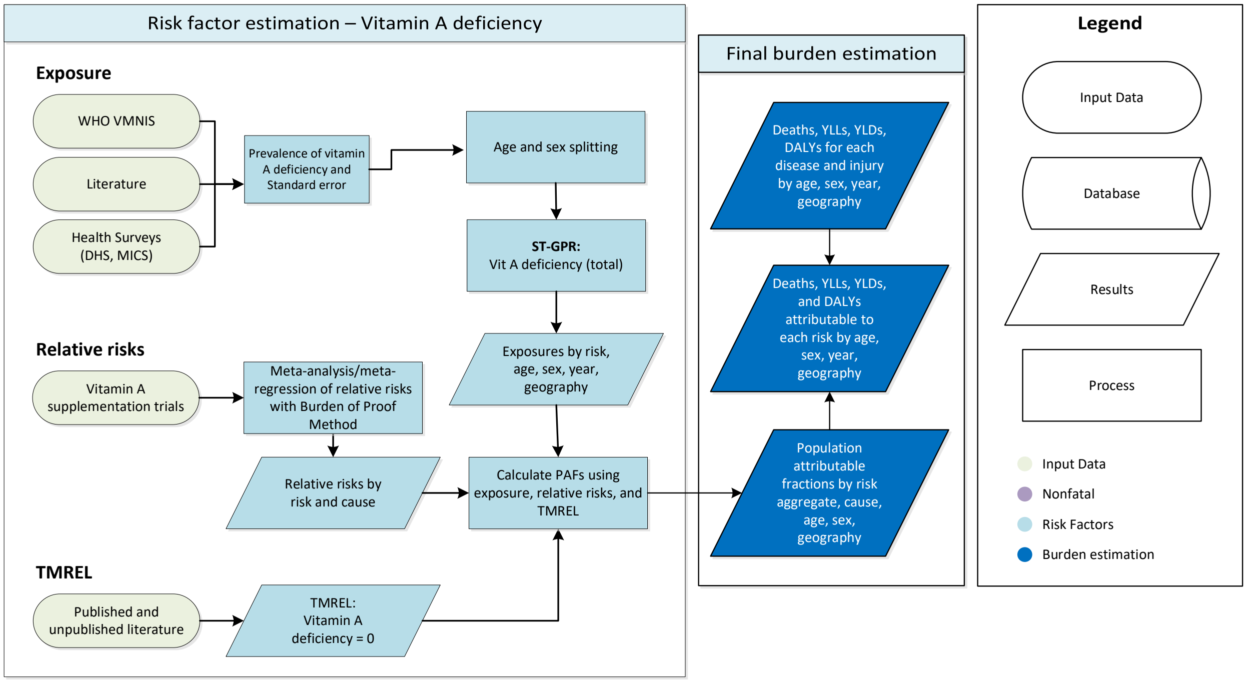

Input data and methodological summary

Definition

Exposure

Vitamin A deficiency is a condition due to low dietary intake or bioavailability of vitamin A that is inadequate to satisfy physiological needs, which is characterised by low serum or/and breastmilk retinol or /and retinol binding concentration, or /and clinical symptoms such as night blindness or xerophthalmia. For GBD 2023, vitamin A deficiency is defined as serum retinol <70 μmol/L. We examined vitamin A deficiency as a risk factor in children aged 6 months to 5 years.

Input data

Exposure

For GBD 2023, we used data from the WHO Vitamin and Mineral Nutrition Information System (WHO VMNIS), health surveys such as DHS and MICS, and studies identified through literature review for the vitamin A deficiency model. We excluded small-scale studies that are not population representative from the vitamin A deficiency model. A systematic review was last conducted for GBD 2013. The PubMed search terms were: ((vitamin A deficiency[Title/Abstract] AND prevalence[Title/Abstract]) AND ("2009"[Date – Publication] : "2013"[Date – Publication])). Exclusion criteria were:

1. Studies that were not population-based, eg, hospital or clinic-based studies

2. Studies that did not provide primary data on epidemiological parameters, eg, commentaries
3. Review articles
4. Case series
5. Self-reported cases

Relative risk

The relative risk data were updated in GBD 2017 to reflect studies included a systematic review by Imdad and colleagues.[1] In GBD 2019, we revisited the underlying studies reported in this analysis and re-analysed and evaluated the evidence on vitamin A deficiency as a risk factor for diarrhoea, measles, and lower respiratory infections using meta-regression—Bayesian, regularised, trimmed (MR-BRT) tool. Lower respiratory infections were removed as an outcome due to insufficient evidence, and the relative risks for diarrhoea and measles were updated. Additionally, in GBD 2019, we found no significant relationship between background vitamin A deficiency prevalence and the magnitude of the relative risk. Thus, starting in GBD 2019, we no longer adjusted relative risks for background vitamin A deficiency prevalence. For GBD 2021, we updated the relative risks using the Burden of Proof methods (BoP), and the relative risk uncertainty estimation included between-study heterogeneity. In both GBD 2019 and GBD 2021, the inclusion and exclusion of a risk–outcome pair were assessed based upon the risk curve without between-study heterogeneity. For GBD 2023, we revisited the Burden of Proof methods analysis of vitamin A deficiency with childhood diarrhoea and measles based on the most updated review by Imdad and colleagues 2022.[2]

Data processing

No major changes to the modelling strategy for vitamin A deficiency were made in GBD 2023 as compared to GBD 2021. We updated a sex-ratio model using MR-BRT based on updated vitamin A deficiency data. The sex ratio model was used to split prevalence data reported for both sexes into male and female prevalences. To derive the age pattern, we utilised a cascading spline model in which country nested within super-region serve as the hierarchical levels of location. In the cascading spline approach, the age pattern estimation for a country is partially informed by the model fitted to data within the super-region and global data, in addition to the data specific to the country. In the absence of data for both a country and a super-region, the age pattern estimation is informed by the global data. The age pattern is used to conduct age splitting for the studies that report vitamin A deficiency for wide non-GBD age groups. We used a new age splitting package called "PyDisagg" to conduct the age splitting. PyDisagg[3] disaggregates proportions, rate, and count observations based on a proportionality assumption. We seek to disaggregate a user-provided datum D into subcomponents D_i, guaranteeing that:

$$sum(D_i) = D$$

$$D_i = g_i p_i$$

where p_i is population weight and g_i a transform of the global rate (f_i).

$$g_i = T^{-1}\big(\beta + T(f_i)\big)$$

The basic use case has T = ln, guaranteeing that the recovered rates are proportional to the global. In addition, PyDisagg allows splitting of bounded quantities using the appropriate logit transform for T. PyDisagg version 0.6.0 disaggregates observations of both continuous quantities (eg, age-splitting of

age-aggregated observations) as well as discrete quantities (including sex and multiple categories). Uncertainty for split datapoints is obtained using asymptotic statistics. Specifically, we use uncertainty of the inputs (ie, uncertainty of the aggregate and uncertainty of the age pattern) together with a generalised delta method that effectively computes a linearised map from the inputs to the outputs and uses that to obtain posterior uncertainty intervals for the split datapoints.

Modelling strategy

Exposure

We applied the spatiotemporal Gaussian process regression (ST-GPR) framework to estimate the mean prevalence of vitamin A deficiency from 1980 to 2023 for national and subnational locations, sexes, and for all GBD age groups. The vitamin A deficiency ST-GPR model used three location-level covariates as a fixed effect: age-standardised summary exposure values for stunting (logit scale), Socio-demographic Index (logit scale), and the availability of retinol activity equivalent (RAE) units in foods (log scale). Since GBD 2021, Vitamin A supplementation was omitted as a covariate in the vitamin A deficiency model due to its lack of statistical significance in the ST-GPR model. We also observed that when the coverage of vitamin A supplementation was included as a covariate in the vitamin A deficiency ST-GPR model, it resulted in an implausible temporal trend. To account for the effect of location levels, the ST-GPR model incorporated these levels (region/super-region/country) as a random effect. Since GBD 2019, we have introduced the assumption that the duration of vitamin A deficiency is one year, which implies that prevalence and incidence are equal. The process of vetting and validating models was accomplished primarily through an examination of ST-GPR scatterplots by GBD 2023 locations from 1990 to 2023. Any poorly fitting datapoints were re-inspected for error at the level of extraction. If errors in data extraction were found, the study in question was re-extracted.

Theoretical minimum risk exposure level

The theoretical minimum risk exposure is a prevalence of vitamin A deficiency of zero.

Relative risk

We applied the Burden of Proof methods[4] to examine the association of vitamin A deficiency with childhood diarrhoea and measles. Studies that evaluated the tolerance or adverse effects of vitamin A supplementation were excluded. We also excluded studies that examined the effect of vitamin A on diarrhoea as a symptom of specific diseases, such as malaria. Bias covariates were extracted for representativeness, exposure measurement, outcome measurement, blinding, confounder adjustment, study design variation, reverse causation, selection bias, and incidence. Most of these other bias covariates did not vary among studies, except for incidence. The incidence bias covariates indicates whether the effect size estimate was for cause-specific mortality or morbidity (incidence). In the updated BoP analysis, we tested additional bias covariates, such as the inclusion of children below 6 months in the study, time interval between vitamin A supplementation and the assessment of the outcome of interest (≤4 months versus >4 months), diarrhoea definitions (applied for diarrhoea only), and the frequency of outcome assessment (endline survey versus continuous surveillance). We also included the background prevalence of vitamin A deficiency (≤50% versus >50%) as a binary bias covariate. The final relative risk used for the population attributable fraction (PAF) calculation incorporated unexplained between-study heterogeneity in the uncertainty estimation.

Table 1: Relative risks for risk–outcome pairs

Cause	GBD 2017 relative risk	GBD 2019 relative risk	GBD 2021 relative risk	GBD 2023 relative risk
Diarrhoea	2.35 (2.17–2.54)	1.14 (1.03–1.26)	1.25 (0.62–2.25)	1.17 (0.72–1.82)
Measles	2.76 (2.01–3.78)	1.39 (1.03–1.90)	1.51 (0.64–3.04)	1.46 (0.60–3.02)

Citations

1 Imdad A, Mayo-Wilson E, Herzer K, Bhutta ZA. Vitamin A supplementation for preventing morbidity and mortality in children from six months to five years of age. *Cochrane Database Syst Rev* 2017; **3**: CD008524.

2 Imdad A, Mayo-Wilson E, Haykal MR, *et al.* Vitamin A supplementation for preventing morbidity and mortality in children from six months to five years of age. *Cochrane Database Syst Rev* 2022; **3**: CD008524.

3 Alexander Hsu, Peng Zheng, Kelsey Maass, Aleksandr Aravkin, Sameer Ali. pyDisagg: Dissaggregation under Generalized Proportionality Assumptions. 2025; published online Jan 13. DOI:10.5281/ZENODO.14641582.

4 Zheng P, Afshin A, Biryukov S, *et al.* The Burden of Proof studies: assessing the evidence of risk. *Nat Med* 2022; **28**: 2038–44.

Zinc deficiency

Flowchart

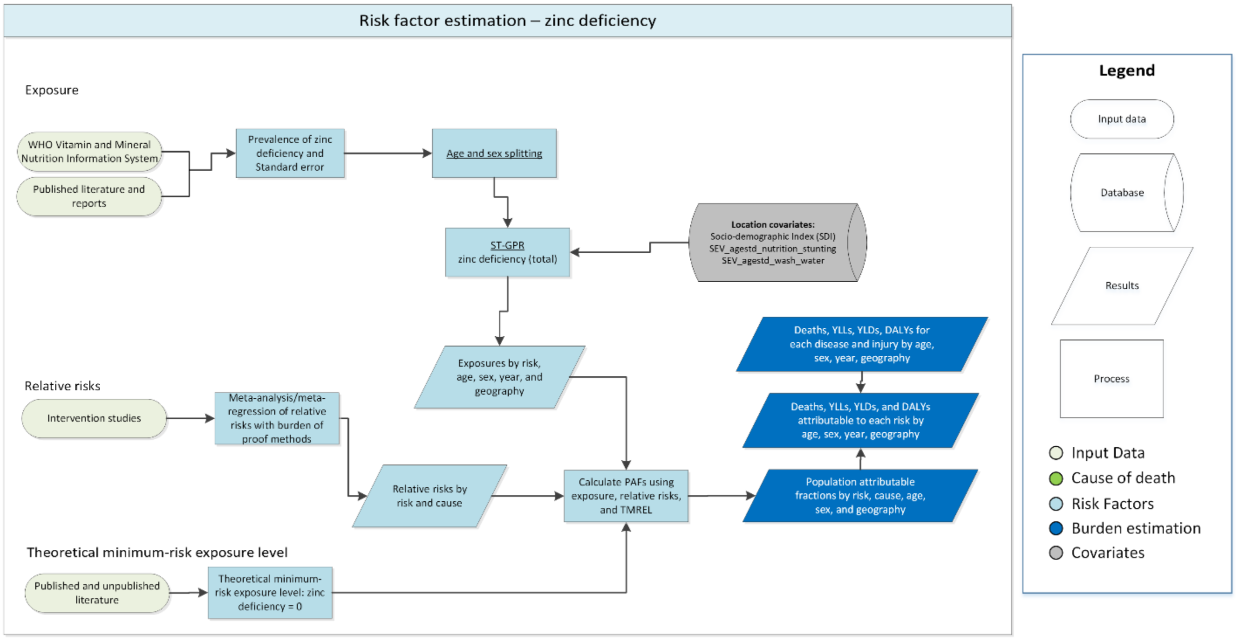

Input data and methodological summary

Definition

Exposure

For GBD 2023, we updated the case definition based on plasma zinc concentrations, and zinc deficiency is now defined as a plasma zinc concentration below 65 µg/dL (morning non-fasting) or 57 µg/dl (afternoon) among children aged 12–59 months.[1,2] In GBD 2021 and prior GBD rounds, zinc deficiency was defined as zinc consumption (in mg/day) less than 2–3 mg from all dietary sources among children aged 12–59 months.

Input data

Exposure

We used nationally representative nutrition surveys that assess zinc status based on plasma zinc concentration. For GBD 2023, most of the data come from the WHO Vitamin and Mineral Nutrition Information System. We also identified nationally representative data sources through literature reviews. The major update in the exposure input data is the use of plasma zinc concentration in GBD 2023, instead of the dietary zinc intake used in GBD 2021 data.

Relative risk

In GBD 2023, no updates were made to the GBD 2021 relative risks. In GBD 2021, a new systematic review was conducted on the effect of zinc supplementation on diarrhoea. In the review, only orally administered zinc supplementation trials were included. Sources were excluded if study designs were not randomised controlled trials (ie, observational, cross-sectional, commentary pieces), included populations with disease of no interest (ie, children less than 6 months of age), had no outcome of interest, or components for analysis were not available (ie, relative risk not measured). Also, dietary zinc and zinc-fortified foods were excluded from the analysis. The details of the systematic review, including PRISMA flow diagram, are described in GBD 2021 appendix.[3]

Data processing

In GBD 2023, the major update in the modelling strategy for zinc deficiency was the use of plasma zinc concentration instead of dietary zinc intake to define zinc deficiency. Based on the available biomarker-based zinc deficiency data, we developed a sex-ratio model using MR-BRT (meta-regression—Bayesian, regularised, trimmed). This sex ratio model was used to split prevalence data reported for both sexes into male and female prevalences. To derive the age pattern, we utilized a cascading spline MR-BRT model in which countries nested within super-regions serve as the hierarchical levels of location. In the cascading spline approach, the age pattern estimation for a country is partially informed by the model fitted to data within the super-region and global data, in addition to the data specific to the country. In the absence of data for both a country and a super-region, the age pattern estimation is informed by the global data. The age pattern is used to conduct age splitting for the studies that report zinc deficiency for wide non-GBD age groups. We used a new age splitting package called "PyDisagg" to conduct the age splitting. PyDisagg[4] disaggregates proportions, rate, and count observations based on a proportionality assumption. We seek to disaggregate a user-provided datum D into subcomponents D_i, guaranteeing that:

$$sum(D_i) = D$$

$$D_i = g_i p_i$$

where p_i is population weight and g_i a transform of the global rate (f_i).

$$g_i = T^{-1}\big(\beta + T(f_i)\big)$$

The basic use case has T = ln, guaranteeing that the recovered rates are proportional to the global. In addition, PyDisagg allows splitting of bounded quantities using the appropriate logit transform for T. PyDisagg version 0.6.0 disaggregates observations of both continuous quantities (eg, age-splitting of age-aggregated observations) as well as discrete quantities (including sex and multiple categories). Uncertainty for split datapoints is obtained using asymptotic statistics. Specifically, we use uncertainty of the inputs (ie, uncertainty of the aggregate and uncertainty of the age pattern) together with a generalised delta method that effectively computes a linearised map from the inputs to the outputs and uses that to obtain posterior uncertainty intervals for the split datapoints.

Modelling strategy

Exposure

We applied the spatiotemporal Gaussian process regression (ST-GPR) framework to estimate the mean prevalence of zinc deficiency from 1980 to 2023 for national and subnational locations, sexes, and age groups between 1 and 5 years. The zinc ST-GPR model used three location-level covariates as a fixed effect: age-standardised summary exposure values for stunting (logit scale), Socio-demographic Index (logit scale), and age-standardised summary exposure value for unsafe water source (logit scale). To account for the effect of location levels, the ST-GPR model incorporated these levels (region/super-region/country) as a random effect. In addition to the three fixed effect covariates used in the final ST-GPR model, additional potential fixed covariates were considered, including calorie availability and national dietary zinc availability. However, both calorie availability and dietary zinc availability were poor predictors.

The process of vetting and validating models was accomplished primarily through an examination of ST-GPR scatterplots by GBD 2023 locations from 1990 to 2023. Any poorly fitting datapoints were re-inspected for error at the level of extraction. If errors in data extraction were found, the study in question was re-extracted.

Theoretical minimum risk exposure level

The theoretical minimum risk exposure is that the prevalence of zinc deficiency is zero.

Relative risk

We made no changes to the GBD 2021 relative risk for GBD 2023. In GBD 2019, we used a published meta-analysis evaluating the effects of zinc supplementation on disease endpoints,[5] and we re-analysed and evaluated the evidence of zinc deficiency as a risk factor for diarrhoea and lower respiratory infections (GBD 2017 outcomes) using MR-BRT. Lower respiratory infections were removed as an outcome due to insufficient evidence, and the relative risks for diarrhoea were updated. Additionally, in GBD 2019 we found no significant relationship between background zinc deficiency prevalence and the magnitude of the relative risk. Thus, starting in GBD 2019, the relative risks were no longer adjusted for background zinc deficiency prevalence. In GBD 2021, we updated the analysis using the Burden of Proof methods.[6] Between GBD 2019 and GBD 2021, the main updates included the addition of new data from the systematic review we conducted, as well as the incorporation of unexplained between-study heterogeneity (gamma) into the uncertainty estimation.

All input data for the relative risk model came from randomised controlled zinc supplementation trials. Bias covariates were extracted for representativeness, exposure measurement, outcome measurement, blinding, confounder adjustment, study design variation, reverse causation, selection bias, and incidence. Most bias covariates did not show variation among studies, and only incidence, which indicates whether the RR estimate was for cause-specific mortality or morbidity, was used in the analysis as a bias covariate.

Table 1: Relative risks for risk–outcome pairs

Cause	GBD 2017 RR mortality incidence	GBD 2019 RR mortality + incidence	GBD 2021 RR mortality + incidence	**GBD 2023 RR** mortality + morbidity
Diarrhoea	1.95 (0.91–3.91) 1.90 (1.52–2.33)	1.14 (1.07–1.21)	1.18 (0.72–1.84)	1.18 (0.72–1.84)

Citations

1 International Zinc Nutrition Consultative Group (IZiNCG), Brown KH, Rivera JA, *et al.* International Zinc Nutrition Consultative Group (IZiNCG) technical document #1. Assessment of the risk of zinc deficiency in populations and options for its control. *Food Nutr Bull* 2004; **25**: S99-203.

2 King JC, Gibson RS, Krebs NF, Lowe NM, Siekmann JH, Raiten DJ. Biomarkers of Nutrition for Development (BOND)—Zinc Review. *The Journal of Nutrition* 2016; **146**: 858S-885S.

3 Brauer M, Roth GA, Aravkin AY, *et al.* Global burden and strength of evidence for 88 risk factors in 204 countries and 811 subnational locations, 1990–2021: a systematic analysis for the Global Burden of Disease Study 2021. *The Lancet* 2024; **403**: 2162–203.

4 Alexander Hsu, Peng Zheng, Kelsey Maass, Aleksandr Aravkin, Sameer Ali. pyDisagg: Dissaggregation under Generalized Proportionality Assumptions. 2025; published online Jan 13. DOI:10.5281/ZENODO.14641582.

5 Mayo-Wilson E, Junior JA, Imdad A, *et al.* Zinc supplementation for preventing mortality, morbidity, and growth failure in children aged 6 months to 12 years of age. *Cochrane Database Syst Rev* 2014; : CD009384.

6 Zheng P, Afshin A, Biryukov S, *et al.* The Burden of Proof studies: assessing the evidence of risk. *Nat Med* 2022; **28**: 2038–44.
