# Supplementary material for: Burden of 375 diseases and injuries, risk-attributable burden of 88 risk factors, and healthy life expectancy in 204 countries and territories, including 660 subnational locations, 1990–2023: a systematic analysis for the Global Burden of Disease Study 2023
Source: Lancet. 2025 Oct 18;406(10513):1873–922. doi: 10.1016/S0140-6736(25)01637-X (PMC12535840; doi:10.1016/S0140-6736(25)01637-X)

THE LANCET

Supplementary appendix 3

This appendix formed part of the original submission and has been peer reviewed. We post it as supplied by the authors.

Supplement to: GBD 2023 Disease and Injury and Risk Factor Collaborators. Burden of 375 diseases and injuries, risk-attributable burden of 88 risk factors, and healthy life expectancy in 204 countries and territories, including 660 subnational locations, 1990–2023: a systematic analysis for the Global Burden of Disease Study 2023. *Lancet* 2025; published online Oct 12. https://doi.org/10.1016/S0140-6736(25)01637-X.

Appendix 3: supplementary results appendix to "Non-fatal burden for 375 diseases and injuries, including risk-attributable burden for 88 risk factors and healthy life expectancy in 204 countries and territories, including 660 subnational locations, 1990–2023: a systematic analysis for the Global Burden of Disease Study 2023"

This supplement provides additional figures containing more detailed results for "Non-fatal burden for 375 diseases and injuries, including risk-attributable burden for 88 risk factors and healthy life expectancy in 204 countries and territories, including 660 subnational locations, 1990–2023: a systematic analysis for the Global Burden of Disease Study 2023".

All supplementary tables can be accessed here https://ghdx.healthdata.org/record/ihme-data/gbd-2023-yld-daly-hale-risk-1990-2023.

List of supplementary results tables and figures

Tables

Table S1. Global age-standardised prevalence rate (per 100·000), by sex in 1990, 2010, 2020, and 2023.

Table S2. Global age-standardised incidence rate (per 100·000), by sex in 1990, 2010, 2020, and 2023.

Table S3. Global all-age DALY counts by cause, by sex in 1990, 2010, 2020, and 2023. DALY=disability-adjusted life-years.

Table S4. Global age-standardised DALY rates (per 100·000), by cause, by sex in 1990, 2010, 2020, and 2023. DALY=disability-adjusted life-years.

Table S5. Global all-age YLD counts by cause, by sex, for 1990, 2010, 2020, and 2023. YLD=years lived with disability.

Table S6. Global age-standardised YLD rates (per 100·000) by cause, by sex in 1990, 2010, 2020, and 2023. YLD=years lived with disability.

Table S7. Global all-age YLL counts by cause, by sex in 1990, 2010, 2020, and 2023. YLL=years of life lost.

Table S8. Global age-standardised YLL rates (per 100·000) by cause, by sex, in1990, 2010, 2020, and 2023. YLL=years of life lost.

Table S9. Healthy life expectancy by location and sex in 1990, 2010, 2020, and 2023.

Table S10. Percentage change between 2010 and 2023 in count and age-standardised rate of DALYs (per 100·000) by cause, global, both sexes. DALY=disability-adjusted life-years.

Table S11. Percentage change between 2010 and 2023 in count and age-standardised rate of YLDs (per 100·000) by cause, global, both sexes. YLD=years lived with disability.

Table S12. Percentage change between 2010 and 2023 in count and age-standardised rate of YLLs (per 100·000) by cause, global, both sexes. YLL=years of life lost.

Table S13a. All-age DALYs, DALY PAFs, age-standardised DALY rate (per 100·000), and percentage change for each risk factor and outcome for both sexes combined in 1990, 2010, and 2023, global. DALYs=disability-adjusted life-years. PAFs=population attributable fractions.

Table S13b. All-age DALYs, DALY PAFs, age-standardised DALY rate (per 100·000), and percentage change for each risk factor and outcome for both sexes combined in 1990, 2010, and 2023, by location. DALYs=disability-adjusted life-years. PAFs=population attributable fractions.

Table S14a. All-age deaths, death PAFs, age-standardised death rate (per 100·000), all-age DALYs, DALY PAFs, age-standardised DALY rate (per 100·000), and percentage change for each risk factor and outcome for both sexes combined in 1990, 2010, and 2023, global. DALYs=disability-adjusted life-years. PAFs=population attributable fractions.

Table S14b. All-age deaths, death PAFs, age-standardised death rate (per 100·000), all-age DALYs, DALY PAFs, age-standardised DALY rate (per 100·000), and percentage change for each risk factor and outcome for both sexes combined in 1990, 2010, and 2023, by location. DALYs=disability-adjusted life-years. PAFs=population attributable fractions.

Table S15a. Age-standardised Summary Exposure Values (SEVs) for most-detailed risk factors and percentage change, by sex, global, in 1990, 2000, 2010 and 2023.

Table S15b. Age-standardised Summary Exposure Values (SEVs) for most-detailed risk factors and percentage change, by sex and by location in 1990, 2000, 2010 and 2023.

Table S16a. All-age PAFs attributable to the joint distribution of all risk factors, both sexes combined for each GBD Level 1, Level 2, and Level 3 cause and all causes in 2023, global. PAFs=population attributable fractions.

Table S16b. All-age PAFs attributable to the joint distribution of all risk factors, both sexes combined for each GBD Level 1, Level 2, and Level 3 cause and all causes in 2023, by location. PAFs=population attributable fractions.

Table S17. Relative risks used by age and sex for each outcome for iron deficiency, high alcohol use, low bone mineral density, occupational risks, non-exclusive breastfeeding, discontinued breastfeeding, intimate partner violence, childhood sexual abuse, chewing tobacco, low birth weight and short gestation, particulate matter pollution, lead exposure in blood. Table only includes relative risks not available in the Burden of Proof visualization tool (https://vizhub.healthdata.org/burden-of-proof/).

Table S18. Risk-outcome scores and star ratings for all scored risk-outcome pairs, 2023.

Figures

Figure S1. Leading cause of age-standardised DALY rates by location, all ages, both sexes in 2023. DALY=disability adjusted life year. 4

Figure S2a. Leading 25 Level 3 risk factors for ages under 5 by attributable DALYs, percentage of total DALYs (2010 and 2023), and percentage change in attributable DALY counts and age-standardised DALY rates from 2010 to 2023. DALY=disability adjusted life year. 5

Figure S2b. Leading 25 Level 3 risk factors for ages 5-14 years by attributable DALYs as percentage of total DALY counts (2010 and 2023), and percentage change in attributable DALY counts and age-standardised DALY rates from 2010 to 2023. DALY=disability adjusted life year. . 6

Figure S2c. Leading 25 Level 3 risk factors for ages 15-49 years by attributable DALYs as percentage of total DALYs (2010 and 2023), and percentage change in attributable DALY counts and age-standardised DALY rates from 2010 to 2023. DALY=disability adjusted life year. 7

Figure S2d. Leading 25 Level 3 risk factors for ages 50-69 years by attributable DALYs as percentage of total DALYs (2010 and 2023), and percentage change in attributable DALY counts and age-standardised DALY rates from 2010 to 2023. DALY = disability-adjusted life-year. 8

Figure S2e. Leading 25 Level 3 risk factors for ages 70+ years by attributable DALYs as percentage of total DALY counts (2010 and 2023), and percentage change in attributable DALY counts and age-standardised DALY rates from 2010 to 2023. DALY=disability adjusted life year. . 9

Figure S2f. Leading 25 Level 3 risk factors for females by attributable DALYs as percentage of total DALY counts (2010 and 2023), and percentage change in attributable DALY counts and age-standardised DALY rates from 2010 to 2023. DALY=disability adjusted life year. 10

Figure S2g. Leading 25 Level 3 risk factors for males, by attributable DALYs as percentage of total DALY counts (2010 and 2023), and percentage change in attributable DALY counts and age-standardised DALY rates from 2010 to 2023. DALY=disability adjusted life year. 11

Figure S3. Age-standardised DALY rate (per 100·000) attributable to all GBD risk factors combined, 2023. DALY=disability adjusted life year. 12

Supplementary Figure 1. Leading cause of age-standardised DALY rates by location, all ages, both sexes in 2023

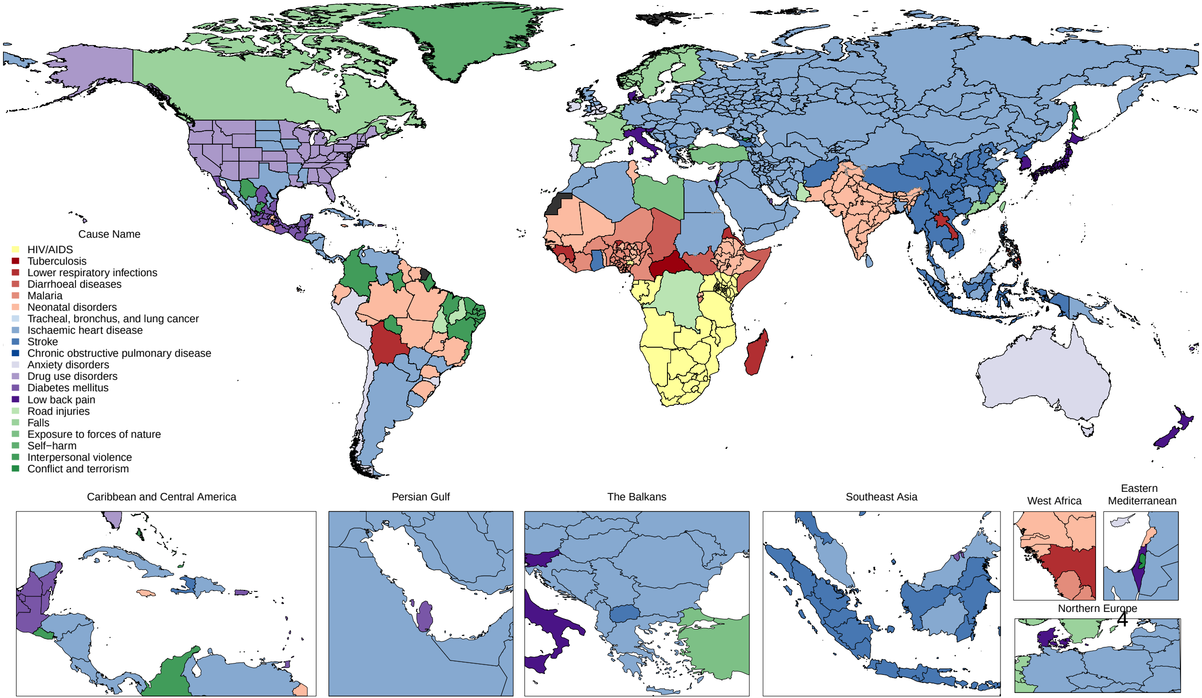

Supplementary Figure 2a. Leading 25* Level 3 risk factors for ages under 5 by attributable DALYs as percentage of total DALY counts (2010 and 2023), and percentage change in attributable DALY counts and age-standardised DALY rates from 2010 to 2023.

Leading risks 2010	Percentage of total DALYs, 2010	Leading risks 2023	Percentage of total DALYs, 2010	Percentage change in number of DALYs, 2010-2023	Percentage change in age-standardised rate of DALYs, 2010-2023
1 Low birth weight and short gestation	29.1 (27.9 to 30.5)	1 Low birth weight and short gestation	30.1 (28.4 to 31.9)	-27.0 (-31.1 to -22.1)	-18.9 (-23.6 to -13.8)
2 Child growth failure	24.2 (16.3 to 29.6)	2 Child growth failure	18.0 (10.5 to 23.7)	-47.3 (-56.1 to -39.9)	-44.3 (-53.1 to -37.0)
3 Particulate matter pollution	13.8 (11.0 to 16.1)	3 Particulate matter pollution	13.7 (11.2 to 15.7)	-30.0 (-35.5 to -23.1)	-24.5 (-28.4 to -20.3)
4 Unsafe water source	7.8 (3.9 to 11.0)	4 Unsafe water source	4.9 (2.4 to 7.5)	-56.2 (-70.7 to -29.4)	-50.1 (-63.0 to -33.5)
5 Unsafe sanitation	6.3 (4.6 to 8.0)	5 Unsafe sanitation	3.7 (2.5 to 5.5)	-58.3 (-72.0 to -33.0)	-54.0 (-64.9 to -38.4)
6 No access to handwashing facility	4.7 (-1.2 to 9.8)	6 No access to handwashing facility	3.4 (-1.1 to 7.9)	-49.2 (-79.4 to -27.9)	-44.9 (-71.7 to -24.7)
7 Suboptimal breastfeeding	3.1 (2.3 to 3.9)	7 Suboptimal breastfeeding	2.2 (1.5 to 2.8)	-50.2 (-62.4 to -33.5)	-46.0 (-59.3 to -28.0)
8 Second-hand smoke	1.4 (1.0 to 1.9)	8 Iron deficiency	1.4 (1.0 to 2.0)	-10.6 (-22.7 to 3.2)	-11.1 (-30.3 to 12.1)
9 High temperature	1.1 (0.6 to 1.6)	9 Second-hand smoke	1.1 (0.7 to 1.5)	-45.0 (-57.4 to -28.2)	-22.4 (-31.2 to -12.8)
10 Iron deficiency	1.1 (0.7 to 1.6)	10 High temperature	0.8 (0.4 to 1.2)	-51.0 (-64.9 to -36.4)	-30.2 (-41.5 to -17.8)
11 Vitamin A deficiency	0.9 (-1.5 to 3.4)	11 Vitamin A deficiency	0.5 (-0.6 to 2.0)	-61.4 (-85.9 to -41.8)	-56.8 (-103.0 to -4.6)
12 Low temperature	0.4 (0.2 to 0.6)	12 Low temperature	0.3 (0.1 to 0.5)	-49.3 (-67.4 to -29.4)	-28.2 (-32.9 to -22.9)
13 Zinc deficiency	0.2 (-0.8 to 1.0)	13 Kidney dysfunction	0.2 (0.1 to 0.2)	15.0 (-24.8 to 79.6)	-6.3 (-12.6 to 0.2)
14 Kidney dysfunction	0.1 (0.1 to 0.1)	14 Zinc deficiency	0.1 (-0.5 to 0.6)	-62.9 (-74.8 to -37.0)	-61.9 (-74.2 to -35.4)
15 High fasting plasma glucose	0.0 (0.0 to 0.1)	15 High fasting plasma glucose	0.1 (0.0 to 0.1)	-12.9 (-44.1 to 35.4)	6.2 (-2.9 to 16.1)
16 Lead exposure	0.0 (0.0 to 0.1)	16 Lead exposure	0.0 (0.0 to 0.1)	-21.6 (-42.9 to -10.1)	-14.0 (-20.2 to -8.0)
17 High body-mass index	0.0 (0.0 to 0.0)	17 High body-mass index	0.0 (0.0 to 0.0)	-0.6 (-17.8 to 20.3)	10.5 (0.5 to 21.1)
18 Ambient nitrogen dioxide pollution	0.0 (0.0 to 0.0)	18 Ambient nitrogen dioxide pollution	0.0 (0.0 to 0.0)	-30.9 (-49.6 to -18.4)	-29.2 (-48.4 to -16.7)
19 High alcohol use	0.0 (0.0 to 0.0)	19 High alcohol use	0.0 (0.0 to 0.0)	-19.9 (-48.8 to -2.0)	-22.6 (-27.0 to -18.2)

Environmental and occupational risks
Behavioural risks
Metabolic risks
*For ages under 5, GBD does not estimate 25 level 3 risk factors.

Supplementary Figure 2b. Leading 25* Level 3 risk factors for ages 5-14 years by attributable DALYs as percentage of total DALY counts (2010 and 2023), and percentage change in attributable DALY counts and age-standardised DALY rates from 2010 to 2023.

Leading risks 2010	Percentage of total DALYs, 2010	Leading risks 2023	Percentage of total DALYs, 2010	Percentage change in number of DALYs, 2010-2023	Percentage change in age-standardised rate of DALYs, 2010-2023
1 Iron deficiency	6.0 (3.8 to 8.6)	1 Iron deficiency	6.5 (4.4 to 9.4)	2.9 (-26.4 to 42.0)	-11.1 (-30.3 to 12.1)
2 Unsafe water source	4.2 (2.1 to 6.1)	2 Unsafe water source	2.8 (1.3 to 4.1)	-36.4 (-52.0 to -16.4)	-50.1 (-63.0 to -33.5)
3 Unsafe sanitation	3.4 (2.3 to 4.8)	3 Low birth weight and short gestation	2.1 (1.7 to 2.6)	16.6 (11.9 to 21.3)	-18.9 (-23.6 to -13.8)
4 No access to handwashing facility	2.0 (-0.4 to 4.0)	4 Unsafe sanitation	2.1 (1.3 to 2.8)	-41.7 (-55.3 to -22.9)	-54.0 (-64.9 to -38.4)
5 Low birth weight and short gestation	1.8 (1.4 to 2.2)	5 No access to handwashing facility	1.5 (-0.4 to 3.3)	-28.9 (-62.3 to 17.4)	-44.9 (-71.7 to -24.7)
6 Particulate matter pollution	1.5 (0.7 to 2.4)	6 Particulate matter pollution	1.5 (0.7 to 2.4)	-7.4 (-33.0 to 32.9)	-24.5 (-28.4 to -20.3)
7 Child growth failure	1.4 (1.2 to 1.7)	7 Child growth failure	0.8 (0.6 to 1.1)	-45.3 (-56.5 to -32.6)	-44.3 (-53.1 to -37.0)
8 High temperature	1.2 (0.7 to 1.7)	8 Bullying victimization	0.7 (0.4 to 1.4)	37.0 (9.0 to 108.1)	18.6 (4.2 to 39.5)
9 Vitamin A deficiency	0.6 (0.3 to 0.9)	9 High temperature	0.6 (0.5 to 0.8)	-48.9 (-63.3 to -22.9)	-30.2 (-41.5 to -17.8)
10 Second-hand smoke	0.6 (0.4 to 0.7)	10 Kidney dysfunction	0.6 (0.5 to 0.9)	14.3 (-16.0 to 53.4)	-6.3 (-12.6 to 0.2)
11 Bullying victimization	0.5 (0.3 to 0.9)	11 Second-hand smoke	0.5 (0.4 to 0.7)	-12.1 (-28.0 to 11.2)	-22.4 (-31.2 to -12.8)
12 Kidney dysfunction	0.5 (0.4 to 0.7)	12 Vitamin A deficiency	0.4 (0.2 to 0.6)	-37.3 (-54.2 to -8.6)	-56.8 (-103.0 to -4.6)
13 Unsafe sex	0.4 (0.4 to 0.5)	13 Lead exposure	0.3 (0.1 to 0.5)	-10.4 (-35.8 to 5.5)	-14.0 (-20.2 to -8.0)
14 Lead exposure	0.3 (0.1 to 0.5)	14 Unsafe sex	0.2 (0.2 to 0.3)	-46.8 (-51.3 to -42.3)	-30.7 (-37.0 to -24.6)
15 High fasting plasma glucose	0.2 (0.1 to 0.3)	15 High fasting plasma glucose	0.2 (0.1 to 0.3)	-8.5 (-33.8 to 28.0)	6.2 (-2.9 to 16.1)
16 High alcohol use	0.1 (0.1 to 0.2)	16 High alcohol use	0.1 (0.1 to 0.2)	-7.2 (-21.3 to 8.6)	-22.6 (-27.0 to -18.2)
17 Ambient nitrogen dioxide pollution	0.1 (-0.1 to 0.4)	17 High body-mass index	0.1 (0.1 to 0.2)	14.0 (-8.2 to 38.8)	10.5 (0.5 to 21.1)
18 High body-mass index	0.1 (0.0 to 0.2)	18 Ambient nitrogen dioxide pollution	0.1 (-0.1 to 0.4)	-18.0 (-39.4 to -4.0)	-29.2 (-48.4 to -16.7)
19 Drug use	0.0 (0.0 to 0.0)	19 Drug use	0.0 (0.0 to 0.0)	-1.9 (-12.5 to 5.1)	8.7 (2.5 to 15.3)
20 Chewing tobacco	0.0 (0.0 to 0.0)	20 Chewing tobacco	0.0 (0.0 to 0.0)	-15.8 (-62.0 to 90.6)	1.6 (-31.3 to 45.9)
21 Low temperature	-0.5 (-0.6 to -0.3)	21 Low temperature	-0.3 (-0.4 to -0.2)	-29.4 (-45.1 to -12.7)	-28.2 (-32.9 to -22.9)

Environmental and occupational risks
Behavioural risks
Metabolic risks
*For ages 5-14 years, GBD does not estimate 25 level 3 risk factors.

Supplementary Figure 2c. Leading 25 Level 3 risk factors for ages 15-49 years by attributable DALYs as percentage of total DALY counts (2010 and 2023), and percentage change in attributable DALY counts and age-standardised DALY rates from 2010 to 2023.

Leading risks 2010	Percentage of total DALYs, 2010	Leading risks 2023	Percentage of total DALYs, 2010	Percentage change in number of DALYs, 2010-2023	Percentage change in age-standardised rate of DALYs, 2010-2023
1 Unsafe sex	5.7 (5.1 to 6.5)	1 Unsafe sex	4.0 (3.5 to 4.8)	-27.6 (-33.6 to -21.8)	-30.7 (-37.0 to -24.6)
2 Occupational injuries	4.8 (4.2 to 5.4)	2 Occupational injuries	3.9 (3.4 to 4.4)	-15.7 (-24.2 to -6.0)	-24.3 (-31.7 to -15.6)
3 Smoking	3.9 (3.1 to 4.7)	3 High body-mass index	3.6 (2.0 to 5.0)	37.5 (22.5 to 52.2)	10.5 (0.5 to 21.1)
4 High alcohol use	3.7 (3.2 to 4.3)	4 High systolic blood pressure	3.2 (2.4 to 4.2)	11.1 (-8.7 to 33.3)	-14.3 (-22.8 to -5.1)
5 Particulate matter pollution	3.0 (2.4 to 3.6)	5 Smoking	3.1 (2.5 to 3.9)	-16.6 (-26.4 to -6.2)	-24.8 (-32.4 to -16.7)
6 High systolic blood pressure	3.0 (2.2 to 3.8)	6 High alcohol use	3.1 (2.7 to 3.6)	-12.4 (-17.7 to -7.0)	-22.6 (-27.0 to -18.2)
7 High body-mass index	2.7 (1.4 to 4.0)	7 High fasting plasma glucose	3.0 (2.5 to 3.4)	29.4 (17.7 to 41.6)	6.2 (-2.9 to 16.1)
8 Sexual violence against children	2.5 (1.3 to 4.0)	8 Particulate matter pollution	2.8 (2.2 to 3.5)	-3.3 (-13.8 to 9.1)	-24.5 (-28.4 to -20.3)
9 Iron deficiency	2.4 (1.8 to 3.1)	9 Sexual violence against children	2.6 (1.4 to 4.1)	5.7 (-21.7 to 40.9)	-3.0 (-29.5 to 30.8)
10 High fasting plasma glucose	2.4 (2.1 to 2.7)	10 Drug use	2.4 (2.1 to 2.7)	22.2 (15.2 to 29.0)	8.7 (2.5 to 15.3)
11 High LDL cholesterol	2.1 (1.5 to 2.6)	11 Iron deficiency	2.2 (1.6 to 3.0)	-4.0 (-25.8 to 22.6)	-11.1 (-30.3 to 12.1)
12 Drug use	2.0 (1.7 to 2.3)	12 High LDL cholesterol	2.2 (1.6 to 2.8)	8.1 (-8.7 to 25.2)	-13.7 (-23.3 to -1.5)
13 Kidney dysfunction	1.7 (1.5 to 2.0)	13 Kidney dysfunction	1.9 (1.6 to 2.3)	16.3 (1.6 to 30.9)	-6.3 (-12.6 to 0.2)
14 Intimate partner violence	1.5 (0.6 to 2.7)	14 Intimate partner violence	1.6 (0.8 to 2.6)	8.2 (-17.7 to 63.3)	0.1 (-23.8 to 44.5)
15 Diet low in fruits	1.3 (0.4 to 2.0)	15 Diet low in fruits	1.4 (0.5 to 2.2)	8.0 (-9.9 to 32.3)	-9.6 (-22.8 to 8.3)
16 Lead exposure	1.1 (0.9 to 1.4)	16 Lead exposure	1.1 (0.8 to 1.3)	-3.2 (-17.1 to 10.7)	-14.0 (-20.2 to -8.0)
17 Unsafe water source	1.0 (0.5 to 1.6)	17 Low birth weight and short gestation	0.9 (0.7 to 1.0)	27.8 (23.3 to 32.1)	-18.9 (-23.6 to -13.8)
18 Unsafe sanitation	0.8 (0.6 to 1.4)	18 Diet low in whole grains	0.8 (0.4 to 1.3)	7.7 (-20.9 to 44.6)	-11.1 (-33.2 to 19.0)
19 Second-hand smoke	0.8 (0.6 to 1.0)	19 Second-hand smoke	0.8 (0.6 to 1.0)	1.5 (-9.9 to 12.9)	-22.4 (-31.2 to -12.8)
20 Diet low in whole grains	0.8 (0.4 to 1.3)	20 Unsafe water source	0.7 (0.4 to 1.1)	-27.1 (-43.4 to -4.3)	-50.1 (-63.0 to -33.5)
21 Occupational ergonomic factors	0.7 (-0.7 to 2.0)	21 Occupational ergonomic factors	0.7 (-0.7 to 2.0)	6.4 (-27.0 to 24.7)	-5.8 (-34.6 to 9.5)
22 Low birth weight and short gestation	0.7 (0.6 to 0.8)	22 Diet low in nuts and seeds	0.7 (0.2 to 1.1)	3.9 (-18.8 to 32.0)	-17.4 (-34.6 to 0.1)
23 High temperature	0.7 (0.5 to 0.9)	23 Diet low in vegetables	0.7 (0.3 to 1.0)	21.6 (-5.7 to 58.6)	-10.1 (-27.5 to 11.0)
24 Diet low in nuts and seeds	0.7 (0.2 to 1.1)	24 High temperature	0.6 (0.4 to 0.7)	-13.8 (-27.8 to 1.8)	-30.2 (-41.5 to -17.8)
25 Diet low in vegetables	0.6 (0.3 to 0.8)	25 Unsafe sanitation	0.5 (0.4 to 0.8)	-34.9 (-49.1 to -13.9)	-54.0 (-64.9 to -38.4)

Environmental and occupational risks
Behavioural risks
Metabolic risks

Supplementary Figure 2d. Leading 25 Level 3 risk factors for ages 50-69 years by attributable DALYs as percentage of total DALY counts (2010 and 2023), and percentage change in attributable DALY counts and age-standardised DALY rates from 2010 to 2023.

Leading risks 2010	Percentage of total DALYs, 2010	Leading risks 2023	Percentage of total DALYs, 2010	Percentage change in number of DALYs, 2010-2023	Percentage change in age-standardised rate of DALYs, 2010-2023
1 High systolic blood pressure	13.6 (11.1 to 16.0)	1 High systolic blood pressure	13.3 (10.7 to 15.8)	27.0 (14.0 to 41.8)	-14.3 (-22.8 to -5.1)
2 Smoking	13.5 (11.3 to 15.9)	2 Smoking	11.2 (9.5 to 13.5)	8.3 (-2.9 to 19.3)	-24.8 (-32.4 to -16.7)
3 Particulate matter pollution	10.9 (9.2 to 12.8)	3 High fasting plasma glucose	9.6 (8.6 to 10.9)	56.2 (41.9 to 70.9)	6.2 (-2.9 to 16.1)
4 High fasting plasma glucose	8.0 (7.2 to 9.2)	4 Particulate matter pollution	9.3 (7.7 to 11.2)	10.3 (3.5 to 17.5)	-24.5 (-28.4 to -20.3)
5 High body-mass index	7.2 (3.7 to 10.5)	5 High body-mass index	8.8 (4.5 to 12.6)	57.6 (43.6 to 72.9)	10.5 (0.5 to 21.1)
6 High LDL cholesterol	5.9 (3.8 to 7.8)	6 High LDL cholesterol	5.8 (3.8 to 7.7)	27.3 (13.6 to 42.4)	-13.7 (-23.3 to -1.5)
7 Kidney dysfunction	4.6 (3.8 to 5.3)	7 Kidney dysfunction	4.8 (4.0 to 5.6)	35.8 (26.9 to 45.1)	-6.3 (-12.6 to 0.2)
8 Lead exposure	4.2 (3.1 to 5.3)	8 Lead exposure	4.1 (3.0 to 5.2)	27.0 (14.6 to 38.9)	-14.0 (-20.2 to -8.0)
9 High alcohol use	3.6 (2.9 to 4.6)	9 Diet low in fruits	3.0 (1.0 to 4.9)	29.0 (9.4 to 56.0)	-9.6 (-22.8 to 8.3)
10 Diet low in fruits	3.0 (1.0 to 4.7)	10 High alcohol use	2.9 (2.3 to 3.8)	6.3 (0.1 to 13.2)	-22.6 (-27.0 to -18.2)
11 Diet high in sodium	2.7 (0.6 to 6.2)	11 Diet high in sodium	2.5 (0.5 to 5.9)	19.9 (-25.8 to 83.0)	-17.2 (-52.3 to 28.0)
12 Second-hand smoke	2.5 (2.1 to 3.0)	12 Second-hand smoke	2.3 (1.9 to 2.8)	18.1 (7.0 to 31.8)	-22.4 (-31.2 to -12.8)
13 Diet low in whole grains	2.0 (0.8 to 3.3)	13 Diet low in whole grains	2.0 (0.9 to 3.3)	29.5 (-4.5 to 74.4)	-11.1 (-33.2 to 19.0)
14 Unsafe sex	1.8 (1.6 to 2.1)	14 Unsafe sex	1.7 (1.5 to 2.0)	23.3 (8.1 to 39.2)	-30.7 (-37.0 to -24.6)
15 Diet low in nuts and seeds	1.5 (0.4 to 2.5)	15 Diet low in nuts and seeds	1.4 (0.4 to 2.4)	20.8 (-4.6 to 46.7)	-17.4 (-34.6 to 0.1)
16 Low temperature	1.5 (1.3 to 1.7)	16 Diet low in vegetables	1.4 (0.7 to 2.1)	26.5 (1.7 to 57.8)	-10.1 (-27.5 to 11.0)
17 Diet low in vegetables	1.4 (0.7 to 2.2)	17 Low bone mineral density	1.2 (1.0 to 1.4)	28.5 (21.8 to 35.3)	-6.2 (-10.6 to -1.6)
18 Occupational injuries	1.3 (1.2 to 1.5)	18 Low temperature	1.2 (1.0 to 1.4)	7.9 (1.3 to 15.0)	-28.2 (-32.9 to -22.9)
19 Low bone mineral density	1.3 (1.0 to 1.5)	19 Sexual violence against children	1.0 (0.5 to 1.8)	39.2 (-0.1 to 91.8)	-3.0 (-29.5 to 30.8)
20 Sexual violence against children	1.0 (0.5 to 1.5)	20 Occupational injuries	1.0 (0.9 to 1.2)	0.4 (-12.4 to 15.8)	-24.3 (-31.7 to -15.6)
21 Diet low in seafood omega-3 fatty acids	1.0 (0.2 to 1.9)	21 Drug use	1.0 (0.8 to 1.1)	44.7 (34.2 to 58.2)	8.7 (2.5 to 15.3)
22 Diet low in omega-6 polyunsaturated fatty acids	1.0 (-2.7 to 3.6)	22 Low physical activity	0.9 (0.4 to 1.3)	46.8 (35.8 to 60.2)	-3.3 (-9.3 to 4.4)
23 Diet low in legumes	0.9 (-0.7 to 2.4)	23 Diet low in omega-6 polyunsaturated fatty acids	0.9 (-2.4 to 3.5)	22.4 (-0.2 to 44.6)	-17.1 (-31.9 to 1.3)
24 Diet high in trans fatty acids	0.9 (0.0 to 1.7)	24 Diet low in legumes	0.9 (-0.7 to 2.2)	24.4 (-3.6 to 58.2)	-15.4 (-33.7 to 7.1)
25 Unsafe water source	0.9 (0.4 to 1.5)	25 Diet low in seafood omega-3 fatty acids	0.8 (0.2 to 1.5)	5.9 (-37.2 to 51.3)	-26.8 (-55.8 to 2.9)
26 Drug use	0.9 (0.7 to 1.0)	28 Diet high in trans fatty acids	0.7 (0.0 to 1.3)	-2.1 (-31.8 to 42.0)	-32.3 (-52.1 to -0.3)
28 Low physical activity	0.8 (0.4 to 1.2)	36 Unsafe water source	0.5 (0.2 to 0.7)	-28.9 (-48.2 to -3.2)	-50.1 (-63.0 to -33.5)

Environmental and occupational risks
Behavioural risks
Metabolic risks

Supplementary Figure 2e. Leading 25 Level 3 risk factors for ages 70+ years by attributable DALYs as percentage of total DALY counts (2010 and 2023), and percentage change in attributable DALY counts and age-standardised DALY rates from 2010 to 2023.

Leading risks 2010	Percentage of total DALYs, 2010	Leading risks 2023	Percentage of total DALYs, 2010	Percentage change in number of DALYs, 2010-2023	Percentage change in age-standardised rate of DALYs, 2010-2023
1 High systolic blood pressure	20.6 (16.9 to 23.4)	1 High systolic blood pressure	18.7 (15.4 to 21.8)	21.1 (10.7 to 33.2)	-14.3 (-22.8 to -5.1)
2 Particulate matter pollution	15.7 (12.9 to 18.4)	2 Particulate matter pollution	12.5 (9.9 to 15.1)	6.4 (-1.3 to 14.5)	-24.5 (-28.4 to -20.3)
3 Smoking	10.5 (8.4 to 12.9)	3 High fasting plasma glucose	11.0 (9.8 to 12.6)	51.5 (38.6 to 66.3)	6.2 (-2.9 to 16.1)
4 High fasting plasma glucose	9.7 (8.7 to 11.2)	4 Smoking	8.8 (7.1 to 11.1)	12.2 (-4.1 to 30.1)	-24.8 (-32.4 to -16.7)
5 Kidney dysfunction	6.4 (5.1 to 7.7)	5 High body-mass index	6.6 (3.1 to 10.0)	52.7 (38.0 to 67.2)	10.5 (0.5 to 21.1)
6 Lead exposure	6.1 (4.5 to 7.7)	6 Kidney dysfunction	6.4 (5.2 to 7.7)	33.7 (27.0 to 40.8)	-6.3 (-12.6 to 0.2)
7 High LDL cholesterol	5.8 (2.9 to 9.0)	7 Lead exposure	5.8 (4.1 to 7.3)	26.6 (17.7 to 35.8)	-14.0 (-20.2 to -8.0)
8 High body-mass index	5.8 (2.7 to 8.8)	8 High LDL cholesterol	5.0 (2.7 to 7.9)	16.6 (1.6 to 33.1)	-13.7 (-23.3 to -1.5)
9 Second-hand smoke	3.0 (2.4 to 3.7)	9 Diet high in sodium	2.7 (0.3 to 6.7)	22.8 (-33.9 to 105.6)	-17.2 (-52.3 to 28.0)
10 Low temperature	2.9 (2.6 to 3.3)	10 Second-hand smoke	2.6 (2.1 to 3.3)	17.5 (4.1 to 31.8)	-22.4 (-31.2 to -12.8)
11 Diet high in sodium	2.9 (0.4 to 7.4)	11 Diet low in fruits	2.4 (1.1 to 3.7)	28.8 (6.6 to 55.6)	-9.6 (-22.8 to 8.3)
12 Diet low in fruits	2.5 (1.1 to 3.8)	12 Low temperature	2.3 (2.0 to 2.6)	5.7 (0.2 to 11.8)	-28.2 (-32.9 to -22.9)
13 Diet low in whole grains	1.8 (0.7 to 3.2)	13 Low bone mineral density	2.0 (1.7 to 2.3)	51.8 (45.9 to 59.1)	-6.2 (-10.6 to -1.6)
14 Low bone mineral density	1.7 (1.5 to 2.1)	14 Diet low in whole grains	1.7 (0.7 to 2.7)	23.7 (-7.6 to 61.4)	-11.1 (-33.2 to 19.0)
15 Diet low in vegetables	1.4 (0.8 to 2.1)	15 Low physical activity	1.4 (0.6 to 2.2)	36.2 (25.0 to 50.7)	-3.3 (-9.3 to 4.4)
16 High alcohol use	1.4 (1.1 to 2.1)	16 Diet low in vegetables	1.3 (0.8 to 2.0)	21.7 (-0.5 to 48.3)	-10.1 (-27.5 to 11.0)
17 Low physical activity	1.4 (0.5 to 2.2)	17 High alcohol use	1.3 (0.9 to 1.8)	17.4 (7.8 to 26.9)	-22.6 (-27.0 to -18.2)
18 Unsafe water source	1.4 (0.7 to 2.2)	18 Diet low in nuts and seeds	1.1 (0.3 to 2.0)	8.7 (-14.0 to 32.0)	-17.4 (-34.6 to 0.1)
19 Diet low in nuts and seeds	1.4 (0.3 to 2.3)	19 Ambient ozone pollution	0.8 (0.2 to 1.4)	31.2 (11.5 to 62.2)	-9.0 (-24.0 to 12.3)
20 Unsafe sanitation	1.1 (0.8 to 1.8)	20 Diet low in legumes	0.8 (-0.6 to 2.2)	15.6 (-10.4 to 41.9)	-15.4 (-33.7 to 7.1)
21 Diet low in legumes	0.9 (-0.7 to 2.6)	21 Unsafe water source	0.8 (0.3 to 1.2)	-25.4 (-44.0 to -1.9)	-50.1 (-63.0 to -33.5)
22 Diet low in seafood omega-3 fatty acids	0.9 (0.2 to 1.8)	22 Diet low in seafood omega-3 fatty acids	0.7 (0.1 to 1.4)	-0.1 (-40.9 to 40.5)	-26.8 (-55.8 to 2.9)
23 Ambient ozone pollution	0.9 (0.2 to 1.5)	23 Diet low in omega-6 polyunsaturated fatty acids	0.6 (-1.5 to 2.6)	5.8 (-15.4 to 25.5)	-17.1 (-31.9 to 1.3)
24 Diet low in omega-6 polyunsaturated fatty acids	0.8 (-2.0 to 3.2)	24 Diet high in red meat	0.6 (0.0 to 1.1)	47.0 (-31.0 to 148.9)	9.9 (-46.9 to 133.0)
25 Diet high in trans fatty acids	0.7 (0.0 to 1.4)	25 High temperature	0.6 (0.4 to 0.9)	36.9 (26.1 to 53.1)	-30.2 (-41.5 to -17.8)
29 High temperature	0.6 (0.4 to 0.9)	29 Unsafe sanitation	0.5 (0.3 to 0.8)	-39.0 (-52.9 to -20.2)	-54.0 (-64.9 to -38.4)
30 Diet high in red meat	0.6 (0.0 to 0.9)	31 Diet high in trans fatty acids	0.5 (0.0 to 0.9)	-10.2 (-36.5 to 31.8)	-32.3 (-52.1 to -0.3)

Environmental and occupational risks
Behavioural risks
Metabolic risks

Supplementary Figure 2f. Leading 25 Level 3 risk factors for females by attributable DALYs as percentage of total DALY counts (2010 and 2023), and percentage change in attributable DALY counts and age-standardised DALY rates from 2010 to 2023.

Leading risks 2010	Percentage of total DALYs, 2010	Leading risks 2023	Percentage of total DALYs, 2010	Percentage change in number of DALYs, 2010-2023	Percentage change in age-standardised rate of DALYs, 2010-2023
1 Particulate matter pollution	8.7 (7.3 to 10.5)	1 High systolic blood pressure	7.9 (6.4 to 9.4)	22.2 (10.1 to 37.5)	-14.5 (-23.2 to -3.9)
2 High systolic blood pressure	6.9 (5.7 to 8.1)	2 Particulate matter pollution	7.7 (6.3 to 9.3)	-5.0 (-11.1 to 1.8)	-23.5 (-27.8 to -18.4)
3 Low birth weight and short gestation	6.5 (5.8 to 7.2)	3 High fasting plasma glucose	5.7 (5.0 to 6.4)	46.9 (32.8 to 63.1)	4.9 (-5.6 to 16.6)
4 Child growth failure	6.3 (4.4 to 8.0)	4 High body-mass index	5.2 (2.5 to 7.6)	51.1 (37.1 to 67.8)	10.6 (0.0 to 23.3)
5 High fasting plasma glucose	4.2 (3.7 to 4.9)	5 Low birth weight and short gestation	4.6 (4.1 to 5.1)	-23.2 (-28.8 to -17.5)	-18.3 (-24.4 to -12.2)
6 High body-mass index	3.7 (1.8 to 5.6)	6 Kidney dysfunction	3.1 (2.6 to 3.6)	32.5 (21.1 to 44.9)	-4.5 (-12.6 to 4.8)
7 Unsafe sex	3.0 (2.7 to 3.4)	7 Child growth failure	3.0 (1.8 to 3.9)	-49.3 (-58.5 to -40.8)	-47.8 (-57.5 to -38.7)
8 Unsafe water source	3.0 (1.6 to 4.2)	8 High LDL cholesterol	2.7 (1.7 to 3.9)	17.0 (-2.2 to 37.9)	-16.2 (-30.0 to 0.2)
9 Kidney dysfunction	2.5 (2.1 to 2.9)	9 Unsafe sex	2.5 (2.1 to 2.9)	-11.6 (-21.1 to -0.6)	-25.9 (-33.8 to -17.0)
10 High LDL cholesterol	2.5 (1.5 to 3.5)	10 Lead exposure	2.2 (1.5 to 2.8)	22.1 (6.5 to 37.0)	-14.2 (-25.0 to -3.7)
11 Unsafe sanitation	2.4 (1.7 to 3.3)	11 Iron deficiency	2.1 (1.5 to 2.9)	-2.1 (-22.6 to 20.8)	-12.6 (-30.6 to 7.6)
12 Iron deficiency	2.3 (1.7 to 3.0)	12 Smoking	1.8 (1.4 to 2.5)	-0.4 (-19.8 to 25.9)	-28.6 (-42.3 to -10.1)
13 Smoking	2.0 (1.4 to 2.6)	13 Second-hand smoke	1.7 (1.4 to 2.1)	0.4 (-10.4 to 12.3)	-24.1 (-32.1 to -15.6)
14 Lead exposure	1.9 (1.4 to 2.4)	14 Unsafe water source	1.5 (0.8 to 2.2)	-46.4 (-62.7 to -22.3)	-50.6 (-66.4 to -27.1)
15 Second-hand smoke	1.8 (1.4 to 2.3)	15 Diet low in fruits	1.4 (0.6 to 2.3)	28.1 (4.7 to 60.3)	-6.4 (-23.6 to 16.7)
16 No access to handwashing facility	1.6 (-0.4 to 3.3)	16 Intimate partner violence	1.4 (0.7 to 2.2)	15.9 (-11.8 to 68.1)	0.5 (-23.4 to 45.3)
17 Intimate partner violence	1.3 (0.5 to 2.2)	17 Sexual violence against children	1.2 (0.6 to 2.0)	13.4 (-11.7 to 48.3)	-3.3 (-24.5 to 25.4)
18 Diet low in fruits	1.2 (0.4 to 1.9)	18 Unsafe sanitation	1.1 (0.8 to 1.6)	-51.6 (-65.4 to -30.6)	-54.9 (-68.4 to -32.8)
19 Sexual violence against children	1.2 (0.6 to 1.9)	19 Low bone mineral density	1.0 (0.8 to 1.2)	36.5 (30.5 to 44.1)	-4.3 (-8.4 to 1.0)
20 Low temperature	0.9 (0.7 to 1.0)	20 Diet low in whole grains	1.0 (0.4 to 1.5)	21.8 (-9.4 to 63.8)	-11.8 (-34.1 to 18.4)
21 Diet high in sodium	0.9 (0.1 to 2.3)	21 Diet high in sodium	0.9 (0.1 to 2.6)	16.3 (-42.7 to 94.8)	-18.5 (-59.3 to 37.3)
22 Diet low in whole grains	0.8 (0.3 to 1.4)	22 No access to handwashing facility	0.8 (-0.3 to 1.9)	-44.8 (-71.4 to -17.5)	-47.0 (-73.4 to -19.2)
23 Low bone mineral density	0.8 (0.6 to 0.9)	23 Low temperature	0.8 (0.7 to 1.0)	-1.0 (-8.3 to 8.2)	-30.8 (-35.7 to -25.2)
24 High temperature	0.8 (0.5 to 1.0)	24 Diet low in vegetables	0.8 (0.4 to 1.2)	31.4 (4.9 to 74.6)	-4.5 (-23.6 to 26.6)
25 High alcohol use	0.7 (0.6 to 0.9)	25 Low physical activity	0.7 (0.3 to 1.1)	36.0 (25.8 to 48.4)	-4.7 (-11.8 to 3.9)
27 Diet low in vegetables	0.6 (0.3 to 1.0)	27 High alcohol use	0.7 (0.6 to 0.8)	-4.6 (-10.5 to 1.6)	-24.4 (-29.1 to -19.7)
30 Low physical activity	0.6 (0.2 to 0.9)	30 High temperature	0.5 (0.4 to 0.7)	-23.7 (-40.2 to -4.7)	-37.1 (-51.6 to -20.9)

Environmental and occupational risks
Behavioural risks
Metabolic risks

Supplementary Figure 2g. Leading 25 Level 3 risk factors for males by attributable DALYs as percentage of total DALY counts (2010 and 2023), and percentage change in attributable DALY counts and age-standardised DALY rates from 2010 to 2023.

Leading risks 2010	Percentage of total DALYs, 2010
1 Particulate matter pollution	9.8 (8.2 to 11.4)
2 Smoking	9.3 (7.8 to 10.9)
3 Low birth weight and short gestation	8.1 (7.6 to 8.8)
4 High systolic blood pressure	7.6 (6.1 to 8.8)
5 Child growth failure	5.7 (3.7 to 7.1)
6 High fasting plasma glucose	4.1 (3.6 to 4.6)
7 High alcohol use	3.5 (2.9 to 4.3)
8 High LDL cholesterol	3.2 (2.1 to 4.4)
9 High body-mass index	3.1 (1.7 to 4.6)
10 Occupational injuries	2.9 (2.6 to 3.2)
11 Kidney dysfunction	2.8 (2.3 to 3.2)
12 Unsafe water source	2.8 (1.4 to 3.9)
13 Lead exposure	2.6 (2.0 to 3.2)
14 Unsafe sanitation	2.2 (1.5 to 3.1)
15 Diet low in fruits	1.7 (0.6 to 2.7)
16 Unsafe sex	1.7 (1.6 to 1.9)
17 Diet high in sodium	1.6 (0.3 to 3.5)
18 No access to handwashing facility	1.5 (-0.4 to 3.2)
19 Second-hand smoke	1.5 (1.2 to 1.9)
20 Drug use	1.1 (1.0 to 1.3)
21 Diet low in whole grains	1.1 (0.5 to 1.8)
22 Iron deficiency	1.0 (0.6 to 1.5)
23 Sexual violence against children	1.0 (0.5 to 1.5)
24 Diet low in nuts and seeds	0.9 (0.3 to 1.5)
25 High temperature	0.8 (0.6 to 1.1)
27 Diet low in vegetables	0.8 (0.4 to 1.2)

Leading risks 2023	Percentage of total DALYs, 2010	Percentage change in number of DALYs, 2010-2023	Percentage change in age-standardised rate of DALYs, 2010-2023
1 Smoking	9.3 (7.9 to 11.1)	5.0 (-4.7 to 15.1)	-24.5 (-31.7 to -17.1)
2 High systolic blood pressure	8.9 (7.2 to 10.5)	22.2 (8.0 to 38.3)	-14.1 (-24.0 to -3.1)
3 Particulate matter pollution	8.7 (7.1 to 10.2)	-8.0 (-14.9 to -1.5)	-25.5 (-30.9 to -20.7)
4 High fasting plasma glucose	5.9 (5.3 to 6.6)	51.0 (37.6 to 64.7)	7.2 (-2.4 to 17.0)
5 Low birth weight and short gestation	5.8 (5.3 to 6.5)	-24.9 (-30.1 to -18.5)	-19.3 (-24.8 to -12.0)
6 High body-mass index	4.5 (2.4 to 6.4)	50.7 (35.5 to 64.9)	10.6 (-0.5 to 20.7)
7 High LDL cholesterol	3.8 (2.5 to 5.1)	21.0 (7.3 to 36.7)	-11.7 (-21.7 to 0.1)
8 Kidney dysfunction	3.4 (2.8 to 4.0)	28.8 (16.5 to 42.1)	-7.8 (-16.2 to 0.9)
9 High alcohol use	3.3 (2.7 to 4.1)	-2.3 (-8.1 to 4.4)	-22.4 (-27.3 to -17.6)
10 Child growth failure	3.1 (1.9 to 4.1)	-43.3 (-54.6 to -30.9)	-41.0 (-52.7 to -27.8)
11 Lead exposure	3.0 (2.3 to 3.8)	21.4 (9.9 to 33.6)	-13.9 (-21.9 to -5.2)
12 Occupational injuries	2.4 (2.0 to 2.7)	-14.9 (-25.1 to -4.3)	-26.2 (-35.0 to -17.1)
13 Diet low in fruits	2.0 (0.7 to 3.1)	19.7 (0.7 to 44.3)	-11.7 (-25.6 to 6.0)
14 Diet high in sodium	1.8 (0.3 to 4.1)	17.7 (-30.1 to 80.4)	-16.9 (-50.9 to 27.6)
15 Second-hand smoke	1.5 (1.2 to 1.9)	4.2 (-8.9 to 19.0)	-20.6 (-30.6 to -9.5)
16 Unsafe water source	1.4 (0.6 to 2.1)	-47.3 (-63.5 to -21.4)	-49.8 (-65.4 to -23.2)
17 Drug use	1.4 (1.2 to 1.5)	25.6 (16.4 to 36.6)	5.6 (-2.3 to 15.0)
18 Diet low in whole grains	1.3 (0.6 to 2.1)	22.5 (-9.8 to 60.9)	-10.4 (-34.0 to 17.9)
19 Unsafe sex	1.2 (1.0 to 1.4)	-26.9 (-34.5 to -19.1)	-38.0 (-44.4 to -31.4)
20 Sexual violence against children	1.1 (0.6 to 1.8)	15.2 (-20.5 to 63.4)	-2.8 (-33.2 to 38.3)
21 Unsafe sanitation	1.0 (0.7 to 1.6)	-51.4 (-67.2 to -28.3)	-53.4 (-69.1 to -28.4)
22 Diet low in nuts and seeds	1.0 (0.3 to 1.6)	13.0 (-13.9 to 43.0)	-16.5 (-35.8 to 5.5)
23 Iron deficiency	1.0 (0.6 to 1.5)	2.3 (-24.1 to 38.7)	-7.7 (-31.7 to 24.0)
24 Diet low in vegetables	0.9 (0.5 to 1.4)	18.3 (-5.0 to 47.2)	-14.2 (-30.7 to 6.3)
25 No access to handwashing facility	0.9 (-0.3 to 2.0)	-41.5 (-67.5 to -9.3)	-43.1 (-68.3 to -11.8)
28 High temperature	0.7 (0.6 to 0.9)	-10.5 (-22.6 to 3.3)	-24.9 (-34.7 to -14.0)

Environmental and occupational risks
Behavioural risks
Metabolic risks

Supplementary Figure 3. Age−standardised DALY rate (per 100·000) attributable to all GBD risk factors combined, 2023

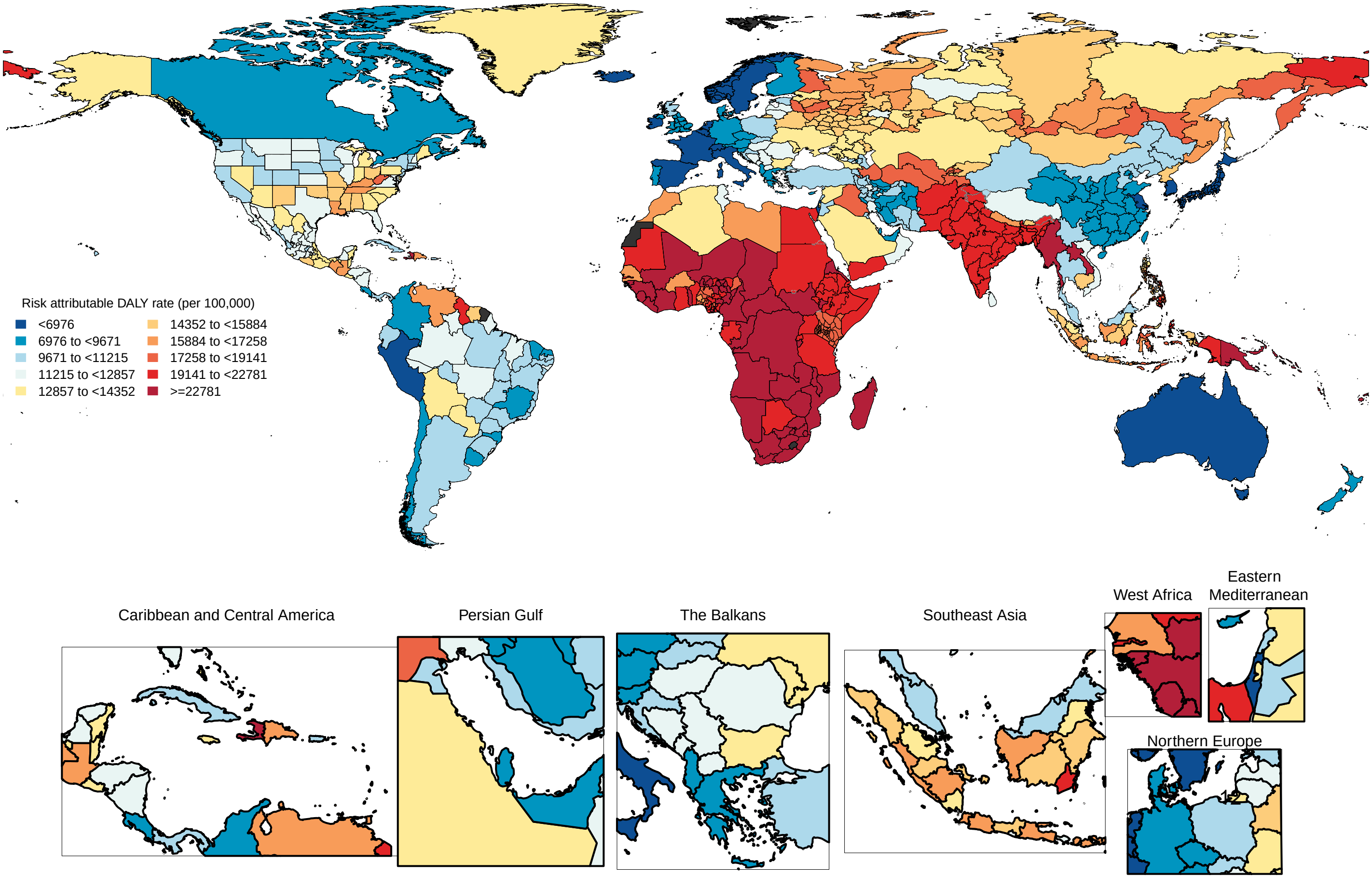

Supplement: Supplementary appendix 3 [file mmc3.pdf]
