# Supplementary material for: Burden of 375 diseases and injuries, risk-attributable burden of 88 risk factors, and healthy life expectancy in 204 countries and territories, including 660 subnational locations, 1990–2023: a systematic analysis for the Global Burden of Disease Study 2023
Source: Lancet. 2025 Oct 18;406(10513):1873–922. doi: 10.1016/S0140-6736(25)01637-X (PMC12535840; doi:10.1016/S0140-6736(25)01637-X)
Supplement: Supplementary appendix 4 [file mmc4.pdf]

THE LANCET

Supplementary appendix 4

This appendix formed part of the original submission and has been peer reviewed. We post it as supplied by the authors.

Supplement to: GBD 2023 Disease and Injury and Risk Factor Collaborators. Burden of 375 diseases and injuries, risk-attributable burden of 88 risk factors, and healthy life expectancy in 204 countries and territories, including 660 subnational locations, 1990–2023: a systematic analysis for the Global Burden of Disease Study 2023. *Lancet* 2025; published online Oct 12. https://doi.org/10.1016/S0140-6736(25)01637-X.

Appendix 4: Authorship appendix to "Burden of 375 diseases and injuries, risk-attributable burden of 88 risk factors, and healthy life expectancy in 204 countries and territories, including 660 subnational locations, 1990–2023: a systematic analysis for the Global Burden of Disease Study 2023"

This appendix provides further authorship detail for "Burden of 375 diseases and injuries, risk-attributable burden of 88 risk factors, and healthy life expectancy in 204 countries and territories, including 660 subnational locations, 1990–2023: a systematic analysis for the Global Burden of Disease Study 2023"

Table of Contents

GBD 2023 Disease and Injury and Risk Factor Collaborators 2

Affiliations 14

Authors' Contributions 74

- Managing the overall research enterprise 74
- Writing the first draft of the manuscript 74
- Primary responsibility for applying analytical methods to produce estimates 74
- Primary responsibility for seeking, cataloguing, extracting, or cleaning data; designing or coding figures and tables 74
- Providing data or critical feedback on data sources 74
- Developing methods or computational machinery 80
- Providing critical feedback on methods or results 80
- Drafting the work or revising it critically for important intellectual content 91
- Managing the estimation or publications process 99

GBD 2023 Disease and Injury and Risk Factor Collaborators

Simon I Hay*, Kanyin Liane Ong*, Damian F Santomauro*, Bhoomadevi A, Mohammad Amin Aalipour, Hasan Aalruz, Hazim S Ababneh, Ukachukwu O Abaraogu, Biruk Beletew Abate, Cristiana Abbafati, Nasir Abbas, Mitra Abbasifard, Mohsen Abbasi-Kangevari, Samar Abd ElHafeez, Ashraf Nabiel Abdalla, Mohammed Altigani Abdalla, Emad M Abdallah, Barkhad Aden Abdeeq, Nadin M I Abdel Razeq, Ahmed Abdelrahman Abdelgalil, Reda Abdel-Hameed, Michael Abdelmasseh, Mahmoud Abdelnabi, Wael M Abdel-Rahman, Sherief Abd-Elsalam, Sepideh Abdi, Mohammad Abdollahi, Meriem Abdoun, Arman Abdous, Jeza Muhamad Abdul Aziz, Deldar Morad Abdulah, Rizwan Suliankatchi Abdulkader, Adam Abdullahi, Auwal Abdullahi, Toufik Abdul-Rahman, Kulmira Abdykerimova, Habtamu Abebe Getahun, Aidin Abedi, Armita Abedi, Asrat Agalu Abejew, Roberto Ariel Abeldaño Zuñiga, E S Abhilash, Shehab Uddin Al Abid, Syed Hani Abidi, Alemwork Abie, Olugbenga Olusola Abiodun, Olumide Abiodun, Richard Gyan Aboagye, Shady Abohashem, Hassan Abolhassani, Ulric Sena Abonie, Nagah M Abourashed, Mohamed Abouzid, Dmitry Abramov, Lucas Guimarães Abreu, Dariush Abtahi, Rana Kamal Abu Farha, Fuad Hamdi A Abuadas, Aminu Kende Abubakar, Bilyaminu Abubakar, Eman Abu-Gharbieh, Sawsan Abuhammad, Ahmad Y Abuhelwa, Hana J Abukhadijah, Niveen ME Abu-Rmeileh, Salahdein Aburuz, Dina Abushanab, Raghu Ram Achar, Anirudh Balakrishna Acharya, Apurba Acharya, Ilana N Ackerman, Juan Manuel Acuna, Ousman Adal, Lisa C Adams, Lawan Hassan Adamu, Mesafint Molla Adane, Zenaw Debasu Addisu, Isaac Yeboah Addo, Oluwafemi Atanda Adeagbo, Tajudeen Adesanmi Adebisi, Isaac Akinkunmi Adedeji, David Adedia, Kamoru Ademola Adedokun, Rufus Adesoji Adedoyin, Oluwatobi E Adegbile, Oyelola A Adegboye, Nurudeen A Adegoke, Olumide Thomas Adeleke, Isaac Ayodeji Adesina, Miracle Ayomikun Adesina, Habeeb Omoponle Adewuyi, Temitayo Esther Adeyeoluwa, Olorunsola Israel Adeyomoye, Kishor Adhikari, Ripon Kumar Adhikary, Usha Adiga, Mohd Adnan, Qorinah Estiningtyas Sakilah Adnani, Prince Owusu Adoma, Leticia Akua Adzigbli, David Adzrago, Giuseppina Affinito, Ahmed M Afifi, Aanuoluwapo Adeyimika Afolabi, Rotimi Felix Afolabi, Saira Afzal, Gizachew Beykaso Agafari, Suneth Buddhika Agampodi, Temesgen Anjulo Ageru, Navidha Aggarwal, Mahdi Aghaalikhani, Sepehr Aghajanian, Seyed Mohammad Kazem Aghamir, César Agostinis Sobrinho, Anurag Agrawal, Williams Agyemang-Duah, Mahsa Ahadi, Bright Opoku Ahinkorah, Aqeel Ahmad, Danish Ahmad, Faisal Ahmad, Khabir Ahmad, Khurshid Ahmad, Muayyad M Ahmad, Noah Ahmad, Rabbiya Ahmad, Sajjad Ahmad, Tauseef Ahmad, Waqas Ahmad, Negar Sadat Ahmadi, Amir Mahmoud Ahmadzade, Mohadese Ahmadzade, Akeem Olayiwola Ahmed, Anisuddin Ahmed, Ayman Ahmed, Gasha Salih Ahmed, Haroon Ahmed, Junaid Ahmed, Luai A Ahmed, Mehrunnisha Sharif Ahmed, Meqdad Saleh Ahmed, Muktar Beshir Ahmed, Mushood Ahmed, Oli Ahmed, Shabbir Ahmed, Sindew Mahmud Ahmed, Gulzhanat Aimagambetova, Ahmed AJ Jabbar, Dolapo Emmanuel Ajala, Marjan Ajami, Azeezat Oluwafunmilayo Ajose, Hossein Akbarialiabad, Saeid Akbarifard, Oluwasefunmi Akeju, Roland Eghoghosoa Akhigbe, Olufemi Ambrose Akinkuotu, Karolina Akinosoglou, Mohammed Ahmed Akkaif, Sreelatha Akkala, Wole Akosile, Hammad Akram, Ashley E Akrami, Ralph Kwame Akyea, Alaa Al Amiry, Salah Al Awaidy, Syed Mahfuz Al Hasan, Omar Al Omari, Mohammad Al Qadire, Omar Al Ta'ani, Wasan A M Al Taie, Yazan Al Thaher, Omar Ali Mohammed Al Zaabi, Mohammad Ahmmad Mahmoud Al Zoubi, Mousa Ali Al-Abbadi, Yazan Al-Ajlouni, Tariq A Alalwan, Ziyad Al-Aly, Khurshid Alam, Manjurul Alam, Mohammad Khursheed Alam, Mostafa Alam, Rasmieh Mustafa Al-Amer, Abebaw Alamrew, Amani Alansari, Turki M Alanzi, Fahmi Y Al-Ashwal, Rahmeh Al-Asmar, Seyed Mohammad Amin Alavi, Mohammed Albashtawy, Astefanos Al-Dalakta, Khalifah A Aldawsari, Wafa A Aldhaleei, Mohammed S Aldossary, Robert W Aldridge, Raouf Alebshehy, Shereen M Aleidi, Bezawit Abeje Alemayehu, Tekletsadik Tekleslassie Alemayehu, Fentahun Alemnew, Melaku Birhanu Alemu, Ayman Al-Eyadhy, Ali M Alfalki, Fahad D

Algahtani, Abdelazeem M Algammal, Mohammed Ridha Algethami, Adel Ali Saeed Al-Gheethi, Khairat Al-Habbal, Khalid F Alhabib, Nma Bida Alhaji, Samar Al-Hajj, Fadwa Naji Alhalaiqa, Mohammed Khaled Al-Hanawi, Aminu Alhassan Ibrahim, Ashraf Alhumaidi, Fahad A Alhumaydhi, Dari Alhuwail, Abid Ali, Haroon Muhammad Ali, Irfan Ali, Maratab Ali, Mohammad Daud Ali, Mohammed Usman Ali, Rafat Ali, Shahid Ali, Syed Shujait Ali, Syed Yusuf Ali, Waad Ali, Akram Al-Ibraheem, Gianfranco Alicandro, Montaha Al-Iede, Sheikh Mohammad Alif, Morteza Alipour, Samah W Al-Jabi, Mohammad A Aljasir, Mohamad Aljofan, Adel Al-Jumaily, Syed Mohamed Aljunid, Ahmad Alkhatib, Mayson H Alkhatib, Mustafa Alkhawam, Atefeh Allahbakhshian, Khaled S Allemailem, Mohammed Z Allouh, Wesam Taher Almagharbeh, Wael Almahmeed, Sabah Al-Marwani, Nihad A Almasri, Joseph Uy Almazan, Hesham M Al-Mekhlafi, Omar Almidani, Amr Almobayed, Khaldoon Aied Alnawafleh, Hasan Yaser Alniss, Margret Beaula Alocious Sukumar, Mahmoud A Alomari, Mohammad R Alosta, Jaber S Alqahtani, Saleh A Alqahtani, Mohammad R Alqudimat, Ahmad Rajeh Al-Qudimat, Ahmad Alrawashdeh, Intima Alrimawi, Sahel Majed Alrousan, Salman Khalifah Al-Sabah, Mohammed A Alsabri, Najim Z Alshahrani, Mansour Abdullah Alshehri, Zaid Altaany, Awais Altaf, Alaa B Al-Tammemi, Jaffar A Al-Tawfiq, Malik A Althobiani, Khalid A Altirkawi, Javier Alvarez-Galvez, Vera L Alves Carneiro, Nelson Alvis-Guzman, Nelson J Alvis-Zakzuk, Hassan Alwafi, Mohammad Al-Wardat, Yaser Mohammed Al-Worafi, Hany Aly, Mohammad Sharif Ibrahim Alyahya, Amal AlZahmi, Hosam Alzahrani, Karem H Alzoubi, Md Akib Al-Zubayer, Uchenna Anderson Amaechi, Ekiyor Joseph Amafah, Joy Amafah, Masoud Aman Mohammadi, Reza Amani-Beni, Adeladza Kofi Amegah, Faten Amer, Bardia Amidi, Amr Amin, Tarek Tawfik Amin, Alireza Amindarolzarbi, Saeed Amini, Ehsan Amini-Salehi, Nafiu Aminu, Majid Aminzare, Sohrab Amiri, Joanne O Amlag, Dickson A Amugsi, Jimoh Amzat, Filippos Anagnostakis, Roshan A Ananda, Robert Ancuceanu, Deanna Anderlini, David B Anderson, Jason A Anderson, Sofia Androudi, Susan C Anenberg, Song Peng Ang, Colin Angus, Nguyen Hoang Anh, Samuel Egyakwa Ankomah, Kabilan Annadurai, Amir Anoushiravani, Iman Ansari, Sumbul Ansari, Umair Ansari, Rahel Mulatie Anteneh, Josep M Antó, Catherine M Antony, Ernoiz Antriyandarti, Boluwatife Stephen Anuoluwa, Saleha Anwar, Sumadi Lukman Anwar, Razique Anwer, Shahnawaz Anwer, Anayochukwu Edward Anyasodor, Geminn Louis Carace Apostol, Juan Pablo Arab, Hossein Arabi, Jalal Arabloo, Mosab Arafat, Aleksandr Y Aravkin, Demelash Areda, Jorge Arias de la Torre, Hany Ariffin, Benedetta Armocida, Johan Ärnlöv, Jesu Arockiaraj, Mahwish Arooj, Anton A Artamonov, Kurnia Dwi Artanti, Raphael Taiwo Aruleba, Deepavalli Arumuganainar, Nurila Aryntayeva, Mahsa Asadi Anar, Muhammad Asaduzzaman, Syed Mohammed Basheeruddin Asdaq, Shewatatek Melaku Asefa, Mulu Tiruneh Asemu, Saeed Asgary, Mohammad Asghari-Jafarabadi, Charlie Ashbaugh, Syed Amir Ashraf, Tahira Ashraf, Mitra Ashrafi, Milad Ashrafizadeh, Bernard Kwadwo Yeboah Asiamah-Asare, Muhammad Shahzad Aslam, Saeed Aslani, Yuni Asri, Batyrbek Assembekov, Thomas Astell-Burt, Mahshid Ataei, Mirbahador Athari, Seyyed Shamsadin Athari, Maha Moh'd Wahbi Atout, Sachin R Atre, Alok Atreya, Julie Alaere Atta, Zeenah A Atwan, Zaure Maratovna Aumoldaeva, Marcel Ausloos, Abolfazl Avan, Núbia Carelli Pereira Avelar, Sana Javaid Awan, Babafela B Awosile, Adedapo Wasiu Awotidebe, Lemessa Assefa A Ayana, Seyyed HamidReza Ayatizadeh, Olatunde O Ayinde, Yusuf Oloruntoyin Ayipo, Seyed Mohammad Ayyoubzadeh, Davood Azadi, Sina Azadnajafabad, Alireza Azarboo, Ali Azargoonjahromi, Masood Azhar, Farya Azimi, Mohd Yusmaidie Aziz, Sadat Abdulla Aziz, Amin Azizan, Ahmed Y Azzam, Domenico Azzolino, Zaharaddeen Shuaibu Babandi, Rasha Babiker, Giridhara Rathnaiah Babu, Israel Tadesse Bacha, Muhammad Badar, Ashish D Badiye, Alaa Aboelnour Badran, Youngoh Bae, Arvind Bagga, Soroush Baghdadi, Nasser Bagheri, Sara Bagheri, Elahe Baghizadeh, Fereshteh Baghizadeh, Sana Baghizadeh, Khlood K Baghlaf, Najmeh Bahmanziari, Mohammad Amin Bahrami, Razieh Bahreini, Ruhai Bai, Atif Amin Baig, Vali Baigi, Shankar M Bakkannavar, Abdulaziz T Bako, Senthilkumar

Balakrishnan, Wondu Feyisa Balcha, Maher Balkis, Jose Balmori-de-la-Miyar, Mohammadreza Balooch Hasankhani, Ovidiu Constantin Baltatu, Shatha Bamashmous, Maciej Banach, Morteza Banakar, Palash Chandra Banik, Rajon Banik, Shirin Barati, Noel C Barengo, Suzanne Lyn Barker-Collo, Hiba Jawdat Barqawi, Ismael A Barreras Beltran, Amadou Barrow, Sandra Barteit, Lingkan Barua, MD Abu Bashar, Zarrin Basharat, Shahid Bashir, Guido Basile, Pritish Baskaran, Rehana Basri, Quique Bassat, Mohammad-Mahdi Bastan, Sanjay Basu, Saurav Basu, Kavita Batra, Bernhard T Baune, Mahdis Bayat, Mohammad Amin Bayat Tork, Mulat Tirfie Bayih, Feyisa Shasho Bayisa, Nebiyou Simegnew Bayleyegn, Thomas Beaney, Neeraj Bedi, Narasimha M Beeraka, Priyamadhaba Behera, Jina Behjati, Babak Behnam, Amir Hossein Behnoush, Bezawit K Bekele, Asnake Gashaw Belayneh, Melesse Belayneh, Abel Cherkos Belete, Gokce Belge Bilgin, Michael Belingheri, Muhammad Bashir Bello, Olorunjuwon Omolaja Bello, Luis Belo, Apostolos Beloukas, Salaheddine Bendak, Riyad Bendardaf, Corina Benjet, Derrick A Bennett, Isabela M Bensenor, Samiun Nazrin Bente Kamal Tune, Habib Benzian, Zombor Berezvai, Maria Bergami, Alemshet Yirga Berhie, Abiye Assefa Berihun, Amiel Nazer C Bermudez, Eduardo Bernabe, Robert S Bernstein, Paulo J G Bettencourt, Ajeet Singh Bhadoria, Akshaya Srikanth Bhagavathula, Neeraj Bhala, Jeetendra Bhandari, Kayleigh Bhangdia, Ravi Bharadwaj, Sonu Bhaskar, Ajay Nagesh Bhat, Anup Bhat, Vivek Bhat, Priyadarshini Bhattacharjee, Shuvarthi Bhattacharjee, Gurjit Kaur Bhatti, Jasvinder Singh Bhatti, Manpreet Singh Bhatti, Rajbir Bhatti, Soumitra S Bhuyan, Sibhatu Kassa Biadgilign, Raluca Bievel-Radulescu, Can Bilgin, Cem Bilgin, Saeed Biroudian, Catherine Bisignano, Atanu Biswas, Bijit Biswas, Raaj Kishore Biswas, Ahmad Naoras Bitar, Molalegne Bitew, Bruno Bizzozero-Peroni, Espen Bjertness, Fiona M Blyth, Trupti Bodhare, Virginia Bodolica, Mahmut Bodur, Lucimere Bohn, Rachael Bokota, Obasanjo Afolabi Bolarinwa, Srinivasa Rao Bolla, Paria Bolourinejad, Aime Bonny, Sri Harsha Boppana, Berrak Bora Basara, Sanaz Bordbar, Hamed Borhany, Alejandro Botero Carvajal, Souad Bouaoud, Soufiane Boufous, Rupert R A Bourne, Christopher Boxe, Marija M Bozic, Jyoti Brahmaiah, Dejana Braithwaite, Nicholas J K Breitborde, Hermann Brenner, Edmond D Brewer, Gabrielle Britton, Julie Brown, Annie J Browne, Traolach Brugha, Claudia Buchweitz, Raffaele Bugiardini, Linh Phuong Bui, Norma B Bulamu, Tsion Samuel Bunare, Danilo Buonsenso, Asmat Burhan, Katrin Burkart, Richard A Burns, Felix Busch, Reinhard Busse, Yasser Bustanji, Zahid A Butt, Channa Buxbaum, Sanjay C J, Jack Cagney, Tianji Cai, Rose Cairns, Mehtap Çakmak Barsbay, Daniela Calina, Luis Alberto Cámera, Luciana Aparecida Campos, Ismael Campos-Nonato, Fan Cao, Yuchen Cao, Angelo Capodici, Rosario Cárdenas, Sinclair Carr, Giulia Carreras, Juan Jesus Carrero, Austin Carter, Andrea Carugno, Andre F Carvalho, Ana Paula Carvalho-e-Silva, Joao Mauricio Castaldelli-Maia, Carlos A Castañeda-Orjuela, Giulio Castelpietra, Alberico L Catapano, Maria Sofia Cattaruzza, Arthur Caye, Christopher R Cederroth, Luca Cegolon, Francieli Cembranel, Muthia Cenderadewi, Kelly M Cercy, Ester Cerin, Sonia Cerrai, Muge Cevik, Madhu Chakkere Shivamadhu, Chiranjib Chakraborty, Promit Ananyo Chakraborty, Sandip Chakraborty, Joht Singh Chandan, Rama Mohan Chandika, Miyuru Chandradasa, Eeshwar K Chandrasekar, Jung-Chen Chang, Vijay Kumar Chattu, Victoria Chatzimavridou-Grigoriadou, Lam Duc Chau, Sirshendu Chaudhuri, Akhilanand Chaurasia, Galmesa Bekana Chemeda, An-Tian Chen, Catherine S Chen, Guangjin Chen, Hana Chen, Haowei Chen, Hui Chen, Junhao Chen, Meng Xuan Chen, Shanquan Chen, Simiao Chen, Xiang Chen, Yifan Chen, Haojin Cheng, Ka Ching Cheung, Nicholas WS Chew, Gerald Chi, Ju-Huei Chien, Odgerel Chimed-Ochir, Patrick R Ching, Jesus Lorenzo Chirinos-Caceres, Clara G Chisari, William C S Cho, Bryan Chong, Yuen Yu Chong, Hou In Chou, Enayet Karim Chowdhury, Mohiuddin Ahsanul Kabir Chowdhury, Hanne Christensen, Steffan Wittrup McPhee Christensen, Dinh-Toi Chu, Isaac Sunday Chukwu, Eric Chung, Erin Chung, Sheng-Chia Chung, Sunghyun Chung, Muhammad Chutiyami, Arrigo Francesco Giuseppe Cicero, Liliana G Ciobanu, Rebecca M Cogen, Aaron J Cohen, Alyssa Columbus, Joao Conde, Stephen E Congly, Nathalie

Conrad, Sara Conti, Mariana Oliveira Corda, Alexandru Corlateanu, Samuele Cortese, Paolo Angelo Cortesi, Claudia Cosma, Ewerton Cousin, Emma Johnson Cowart, Michael H Criqui, Andrew Crist, Jessica A Cruz, Natalia Cruz-Martins, Xiaolin Cui, Garland T Culbreth, Nour Dababo, Ali Dabbagh, Omid Dadras, Tukur Dahiru, Xiaochen Dai, Zhaoli Dai, Mayank Dalakoti, Koustuv Dalal, Gloria Dalla Costa, Giovanni Damiani, Emanuele D'Amico, Yohannes Tefera Damtew, Roy Arokiam Arokiam Daniel, Lucio D'Anna, Pojsakorn Danpanichkul, Samuel Demissie Darcho, Latefa Ali Dardas, Bahar Darouei, Reza Darvishi Cheshmeh Soltani, Anna Dastiridou, Gail Davey, Claudio Alberto Dávila-Cervantes, Nicole Davis Weaver, Dimash Davletov, Kairat Davletov, Elham Davoudi, Fernando Pio De la Hoz, Katie de Luca, Nicole K DeCleene, Edward Christopher Dee, Orla Deegan, Sindhura Deekonda, Amanda Deen, Louisa Degenhardt, Paria Dehesh, Lee Deitesfeld, Tadesse Asmamaw Dejenie, Pouria Delbari, Mohammad Delsoz, Dessalegn Demeke, Andreas K Demetriades, Desalegn Getnet Demsie, Edgar Denova-Gutiérrez, Tadios Niguss Derese, Ismail Dergaa, Hunegnaw Almaw Derseh, Emina Dervišević, Abraham Aregay Desta, Vinoth Gnana Chellaiyan Devanbu, Pradeep Kumar Devarakonda, Syed Masudur Rahman Dewan, Arkadeep Dhali, Kuldeep Dhama, Rajinder K Dhamija, Amol S Dhane, Narender K Dhania, Mandira Lamichhane Dhimal, Meghnath Dhimal, Sameer Dhingra, Bibha Dhungel, Marcello Di Pumpo, Diana Dias da Silva, Daniel Diaz, Luis Antonio Diaz, Kimia Didehvar, Lauren K Dillard, Adriana Dima, Xueting Ding, Temesgien Ergetie Dinkayehu, Huyen Phuc Do, Thao Huynh Phuong Do, Klara Georgieva Dokova, Christiane Dolecek, Regina-Mae Villanueva Dominguez, Francesco Dondi, Mario D'Oria, Fariba Dorostkar, Ojas Prakashbhai Doshi, Paulo Magno Martins Dourado, Robert Kokou Dowou, Menayit Tamrat Dresse, Tim Robert Driscoll, Ashel Chelsea Dsouza, Viola Savy Dsouza, Jiang Du, John Dube, Emeka W Dumbili, Samuel C Dumith, Jennifer Dunne, Andre Rodrigues Duraes, Senbagam Duraisamy, Oyewole Christopher Durojaiye, Ashit Kumar Dutta, Arkadiusz Marian Dziedzic, Abdel Rahman E'mar, Osamudiamen Ebohon, Ejemai Eboreime, Lamiaa Labieb Mahmoud Ebraheim, Alireza Ebrahimi, Mohammad Hossein Ebrahimi, Sara Ebrahimi, Abdelaziz Ed-Dra, Ekaette Godwin Edelduok, Kristina Edvardsson, Ferry Efendi, Behrad Eftekhari, Foolad Eghbali, Fatemeh Ehsani, Ashkan Eighaei Sedeh, Terje Andreas Eikemo, Ebrahim Eini, Michael Ekholuenetale, Temitope Cyrus Ekundayo, Rabie Adel El Arab, Abdelfatteh EL Omri, Maysaa El Sayed Zaki, Mohamed Ahmed Eladl, Reza Elahi, Said El-Ashker, Rana Elbeshbeishy, Noha Mousaad Elemam, Ghada Metwally Tawfik ElGohary, Muhammed Elhadi, Mohamed Elhoumed, Waseem El-Huneidi, Sherif Elkannishy, Omar Abdelsadek Abdou Elmeligy, Rami Elmorsi, Adel B Elmoselhi, Mohamed Hassan Elnaem, Gihan ELNahas, Mohammed Elshaer, Ibrahim Elsohaby, Abdelgawad Salah Abdelgawad Eltahawy, Tadele Emagneneh, Theophilus I Emeto, Victor Oghenekparobo Emojevwe, Destaw Endeshaw, Misganu Endriyas, Holly E Erskine, Christopher Imokhuede Esezobor, Derese Eshetu, Habitu Birhan Eshetu, Gilbert Eshun, Sharareh Eskandarieh, Majid Eslami, Rafaela Cavalheiro do Espírito Santo, Francesco Esposito, Kara Estep, Crystal Amiel M Estrada, Fahima Nasrin Eva, Elochukwu Ezenwankwo, Adewale Oluwaseun Fadaka, Heidar Fadavian, Adeniyi Francis Fagbamigbe, Ayesha Fahim, Ildar Ravisovich Fakhradiyev, Aliasghar Fakhri-Demeshghieh, Qiping Fan, Mohammad Farahmand, Emerito Jose Aquino Faraon, Mohammad Fareed, Zaki Farhana, Carla Sofia e Sá Farinha, MoezAlIslam Ezzat Mahmoud Faris, Andre Faro, Syed Muhammad Yousaf Farooq, Umar Farooque, Hossein Farrokhpour, Fatemeh Farshad, Farima Farsi, Md Omar Faruk, Folorunso Oludayo Fasina, Modupe Margaret Fasina, Emmanuel Toluwani Fasusi, Ali Fatehizadeh, Davood Fathi, Zareen Fatima, Mehdi Fazlzadeh, Li Fei, Valery L Feigin, Alireza Feizkhah, Ginenus Fekadu, Berhanu Elfu Feleke, Dechao Feng, Kaixin Feng, Xiaoqi Feng, Talukdar Raian Ferdous, Seyed-Mohammad Fereshtehnejad, Rodrigo Fernandez-Jimenez, Pietro Ferrara, Alize J Ferrari, André Ferreira, Nuno Ferreira, Natan Feter, Alexander Finnemore, Claudio Fiorilla, Florian Fischer, Ida Fitriana, Luisa S Flor, Federica Fogacci,

Morenike Oluwatoyin Folayan, Marco Fonzo, Lisa M Force, Arianna Fornari, Carla Fornari, Ingeborg Forthun, Daniela Fortuna, Matteo Foschi, Maryam Fotouhi, Kayode Raphael Fowobaje, Juluis Visnel Foyet F, Richard Charles Franklin, Alberto Freitas, Jinming Fu, Nancy Fullman, Blima Fux, Sridevi G, Peter Andras Gaal, Dominic Dormenyo Gadeka, Márió Gajdács, Yaseen Galali, Silvano Gallus, Dhanraj Ganapathy, Shivaprakash Gangachannaiah, Mohd Ashraf Ganie, Dingwei Gao, Xiang Gao, Bashiru Garba, Fernando Barroga Garcia, Vanessa Garcia, Miguel Garcia-Argibay, David Garcia-Azorin, William M Gardner, Jacopo Garlasco, Zisis Gatzioufas, Prem Gautam, Rupesh K Gautam, Federica Gazzelloni, Feven Sahle Gebre, Miglas Welay Gebregergis, Haftay Gebremedhin Gebreslassie, Stefano Gelibter, Nsikakabasi Samuel George, Ali Gerami Matin, Genanew K Getahun, Kalab Yigermal Gete, Delaram J Ghadimi, Keyghobad Ghadiri, Fataneh Ghadirian, Amir Ghaffari Jolfayi, Seyyed-Hadi Ghamari, Arin Ghamkhar, Ali Ghandili, Moein Ghasemi, Mohammad-Reza Ghasemi, Shakiba Ghasemi Assl, Haniyeh Ghasrsaz, Ramy Mohamed Ghazy, Sailaja Ghimire, Nermin Ghith, Nasim Gholizadeh, Elena Ghotbi, Alessandro Gialluisi, Konstantinos Giannakis, Ruth Margaret Gibson, Artyom Urievich Gil, Gabriela Fernanda Gil, Syed Abdullah Gilani, Nora M Gilbertson, Tiffany K Gill, Themba G Ginindza, Bikash Ranjan Giri, Alem Abera Girmay, Alessandro Girombelli, Elena V Gnedovskaya, Laszlo Göbölös, Kimiya Gohari, Mahaveer Golechha, Pouya Goleij, Ali Golestani, Davide Golinelli, Melika Golmohammadi, Wenping Gong, Sameer Vali Gopalani, Yitayal Ayalew Goshu, Alessandra C Goulart, Aman Goyal, Ayman Grada, Simon Matthew Graham, Vittorio Grieco, Michal Grivna, Ashna Grover, Habtamu Alganeh Guadie, Shi-Yang Guan, Zhongyang Guan, Giovanni Guarducci, Mohammed Ibrahim Mohialdeen Gubari, Avirup Guha, Damitha Asanga Gunawardane, Xingzhi Guo, Zhaoyu Guo, Zheng Guo, Zhifeng Guo, Anish Kumar Gupta, Himanshu Gupta, Ishita Gupta, Lalit Gupta, Rajat Das Gupta, Rajeev Gupta, Sapna Gupta, Veer Bala Gupta, Vijai Kumar Gupta, Vipin Gupta, Vivek Kumar Gupta, Yonas Deressa Guracho, Lami Gurmessa, Reyna Alma Gutiérrez, Roberth Steven Gutiérrez-Murillo, Parishma Guttoo, Jose Guzman-Esquivel, Adrina Habibzadeh, Abrham Tesfaye Habteyes, Awoke Derbie Habteyohannes, Tesfahun Simon Hadaro, Najah R Hadi, Zahra Hadian, Abdul Hafiz, Faraidoon Haghdoost, Arian Haghtalab, Hailey Hagins, Demewoz Haile, Haimanot Ewnetu Hailu, Pritam Halder, Aram Halimi, Sebastian Haller, Kosar Hikmat Hama Aziz, Islam M Hamad, Randah R Hamadeh, Nadia M Hamdy, Sajid Hameed, Erin B Hamilton, Ahmad Hammoud, Mohammad Hamza, Umar Sabiu Hamza, Hannah Han, Didem Han Yekdeş, Asif Hanif, Nasrin Hanifi, Graeme J Hankey, Fahad Hanna, Ashanul Haque, Md Aminul Haque, Md Nuruzzaman Haque, Harapan Harapan, Hilda L Harb, Kassandra L Harding, Arief Hargono, Andy Martahan Andreas Hariandja, Josep Maria Haro, Ashley Ann Harris, Eka Mishbahatul Marah Has, Ahmed I Hasaballah, Faizul Hasan, Md Kamrul Hasan, Hamidreza Hasani, Ali Hasanpour- Dehkordi, Arezou Hashem Zadeh, Mohammad Hashem Hashempur, Nada Tawfig Hashim, Ammarah Hasnain, Amr Hassan, Ibrahim Nagmeldin Hassan, Ikrama Hassan, Nageeb Hassan, Mahgol Sadat Hassan Zadeh Tabatabaei, Shokoufeh Hassani, Mohammed Bheser Hassen, Lasanthi Wathsala Hathagoda, Rasmus J Havmoeller, Angie Hawat, Khezar Hayat, Youssef Hbid, Jiawei He, Jue He, Jeffrey J Hebert, Mohammad Heidari, Mehdi Hemmati, Claire A Henson, Molly E Herbert, Claudiu Herteliu, Austin Heuer, Sumudu Avanthi Hewage, Mojtaba Heydari, Zahra Heydarifard, Kamal Hezam, Yuta Hiraike, Ramesh Holla, Julia Hon, Alamgir Hossain, Lubna Hossain, Md Belal Hossain, Md Mahbub Hossain, Md Sabbir Hossain, Mohammad Bellal Hossain, Hassan Hosseinzadeh, Mehdi Hosseinzadeh, Mihaela Hostiuc, Sorin Hostiuc, Jada Averianna Houser, Mila Nu Nu Htay, Chengxi Hu, Yifei Hu, Junjie Huang, Weijun Huang, Yefei Huang, Mega Hasanul Huda, Atanesia Indriyani Human, Kyle Matthew Humphrey, Kiavash Hushmandi, Andreas Kattem Husøy, Javid Hussain, M Azhar Hussain, Salman Hussain, Dursa Hussein, Nawfal R Hussein, Mohamed Ibrahim Husseiny, Hong-Han Huynh, Bing-Fang Hwang, Luigi Francesco Iannone, Ahmed Ibrahim, Khalid S Ibrahim, Ramzi

Ibrahim, Reem Ibrahim, Anel Ibrayeva, Francisco Javier Idalsoaga, Pulwasha Maria Iftikhar, Audrey L Ihler, Nayu Ikeda, Adalia Ikiroma, Jibran Ikram, Olayinka Stephen Ilesanmi, Irena M Ilic, Milena D Ilic, Muhammad Hamza Ilyas, Mohammad Tarique Imam, Masoud Imani, Mustapha Immurana, Lucius Chidiebere Imoh, Leeberk Raja Inbaraj, Arit Inok, Mujahid Iqbal, Lalu Muhammad Irham, Mustafa Alhaji Isa, Dr Md Shahinul Islam, Md Rabiul Islam, Md Shariful Islam, Farhad Islami, Faisal Ismail, Nahlah Elkudssiah Ismail, Yerlan Ismoldayev, Hiroyasu Iso, Gaetano Isola, Mosimah Charles Ituka, Masao Iwagami, Chinwe Juliana Iwu-Jaja, Ihoghosa Osamuyi Iyamu, Mahalaxmi Iyer, Veena J Iyer, Vinothini J, Jalil Jaafari, Louis Jacob, Kathryn H Jacobsen, Ali Jadidi, Farhad Jadidi-Niaragh, Morteza Jafarinia, Shabbar Jaffar, Haitham Jahrami, Ammar Abdulrahman Jairoun, Vikash Jaiswal, Mihajlo Jakovljevic, Ali Jaliliyan, Reza Jalilzadeh Yengejeh, Mohamed Jalloh, Armaan Jamal, Qazi Mohammad Sajid Jamal, Jazlan Jamaluddin, Jerin James, Tyler G James, Hasan Jamil, Safayet Jamil, Roland Dominic G Jamora, Masoud Jamshidi, Shaghayegh JamshidiRastabi, Rajiv Janardhanan, Esmaeil Jarrahi, Syed Sarmad Javaid, Anita Javanmardi, Javad Javidnia, Talha Jawaid, Qassim Jawell Odah Abed, Ruwan Duminda Jayasinghe, Yovanthi Anurangi Jayasinghe, Achala Upendra Jayatilleke, Kimia Jazi, Felix K Jebasingh, Sun Ha Jee, Jayakumar Jeganathan, Tadesse Hailu Jember, Belayneh Hamdela Jena, Diptismita Jena, Seogsong Jeong, Mahsa Jessri, Bijay Mukesh Jeswani, Vivekanand Jha, Zixiang Ji, Min Jiang, Weiqiu Jin, Wenyi Jin, Catherine O Johnson, Emily Katherine Johnson, Mohammad Jokar, Jost B Jonas, Tamas Joo, Abu Jor, Abel Joseph, Alex Joseph, Nitin Joseph, Charity Ehimwenma Joshua, Farahnaz Joukar, George Joy, Jacek Jerzy Jozwiak, Mikk Jürisson, Malik E Juweid, Madhanraj K, Billingsley Kaambwa, Zubair Kabir, Dler H Hussein Kadir, Ethan M Kahn, Ashish Kumar Kakkar, Leila R Kalankesh, Khalil Kalavani, Feroze Kaliyadan, Aidana Kaliyakparova, Sanjay Kalra, Md Moustafa Kamal, Mehnaz Kamal, Sivesh Kathir Kamarajah, Rajesh Kamath, Saltanat Kamenova, Arun Kamireddy, Ramat T Kamorudeen, Oleksandr Kamyshnyi, Haidong Kan, Mona Kanaan, Saddam Fuad Kanaan, Jiseung Kang, Samuel Berchi Kankam, Kehinde Kazeem Kanmodi, Sujitha Kannan, Suthanthira Kannan S, Rami S Kantar, Neeti Kapoor, Sujita Kumar Kar, Paschalis Karakasis, Reema A Karasneh, Hanie Karimi, Arman Karimi Behnagh, Samad Karkhah, Dr Mohmed Isaqali Karobari, Tomasz M Karpiński, Manoj Kumar Kashyap, Abdene Weya Kaso, Hengameh Kasraei, Nigussie Assefa Kassaw, Adarsh Katamreddy, Patrick DMC Katoto, Joonas H Kauppila, Gbenga A Kayode, Nastaran Kazemi Rad, Mohammad-Hossein Keivanlou, Peter Njenga Keiyoro, Chukwudi Keke, John H Kempen, Salima Kerai, Vikash Ranjan Keshri, Kamyab Keshtkar, Emmanuelle Kesse-Guyot, Reza Khademi, Yousef Saleh Khader, Inn Kynn Khaing, Himanshu Khajuria, Sidra Khalid, Sumaira Khalid-Ariturk, Hazim O Khalifa, Anas Husam Khalifeh, Anees Ahmed Khalil, Mariam Khalil, Anita Khalili, Pantea Khalili, Ghazaleh Khalili-Tanha, Mohamed Khalis, Faham Khamesipour, Abdul Arif Khan, Ajmal Khan, Asaduzzaman Khan, Faiz Ullah Khan, Maseer Khan, Md Abdullah Saeed Khan, Mohammad Idreesh Khan, Mohammad Jobair Khan, Muhammad Hamza Khan, Muhammad Mueed Khan, Muhammad Umair Khan, Muhammad Umer Khan, Nusrat Khan, Ruby Khan, Salman Ali Khan, Serab Khan, Sumaiya Khan, Yusuf Saleem Khan, Zahid Khan, Srijana Khanal, Vishnu Khanal, Shaghayegh Khanmohammadi, Sameer Uttamaro Khasbage, Zenith Khashim, Khaled Khatab, Haitham Khatatbeh, Moawiah Mohammad Khatatbeh, Kavin Khatri, Hamid Reza Khayat Kashani, Afshin Khazaei, Peyman Kheirandish Zarandi, Sunil Kumar Khokhar, Mohammad Saeid Khonji, Najmaddin Salih Husen Khoshnaw, Atulya Aman Khosla, Farbod Khosravi, Mahmood Khosrowjerdi, P Ratan Khuman, Helda Khusun, Zemene Demelash Kifle, Hye Jun Kim, Jinho Kim, Min Seo Kim, Sungroul Kim, Ruth W Kimokoti, Yohannes Kinfu, Mary Kirk, Adnan Kisa, Sezer Kisa, Katarzyna Kissimova-Skarbek, Mika Kivimäki, Jessica Klusty, Abdul Basith KM, Shivakumar KM, Ann Kristin Skrindo Knudsen, Nazarii Kobyliak, Jonathan M Kocarnik, Sonali Kochhar, Michail Kokkorakis, Ali-Asghar Kolahi, Diana Gladys Kolieghu Tcheumeni, Farzad Kompani, Aida

Kondybayeva, Anastasios Georgios Panagiotis Konstas, Isaac Koomson, Gerbrand Koren, Tapos Kormoker, Oleksii Korzh, Karel Kostev, Konstantinos Kotsis, Archana Koul, Parvaiz A Koul, Sindhura Lakshmi Koulmane Laxminarayana, Irene Akwo Kretchy, James-Paul Kretchy, Kewal Krishan, Chong-Han Kua, Ananya Kuanar, Barthelemy Kuate Defo, Raja Amir Hassan Kuchay, Burcu Kucuk Bicer, Mohammed Kuddus, Ilari Kuitunen, Omar Kujan, Anit Kujur, Mukhtar Kulimbet, Vishnutheertha Kulkarni, Shweta Kulshreshtha, Dewesh Kumar, Dhasarathi Kumar, Jogender Kumar, Manasi Kumar, Nitesh Kumar, Nithin Kumar, Rakesh Kumar, Sanjay Kirshan Kumar, Tushar Kumar, Vijay Kumar, Subramanian Kumaran, Jibin Kunjavara, Setor K Kunutsor, Almagul Kurmanova, Om P Kurmi, Maria Dyah Kurniasari, Krishna Prasad Kurpad, Pramod Kumar Kushawaha, Asep Kusnali, Christina Yeni Kustanti, Dian Kusuma, Tezer Kutluk, Assylkhan Kuttybayev, Michael Agyemang Kwarteng, Wai Hang Patrick Kwong, Evans F Kyei, Grace Kwakyewaa Kyei, Ville Kytö, Hmwe Hmwe Kyu, Pallavi L C, Adriano La Vecchia, Carlo La Vecchia, Muhammad Awwal Ladan, Lucie Laflamme, Chandrakant Lahariya, Daphne Teck Ching Lai, Anita Lakhani, Dharmesh Kumar Lal, Ratilal Lalloo, Tea Lallukka, Judit Lám, Iván Landires, Berthold Langguth, Ariane Laplante-Lévesque, Savita Lasrado, Kamaluddin Latief, Kenney Ki Lee Lau, Basira Kankia Lawal, Bilkisu Kankia Lawal, Saheed Akinmayowa Lawal, Aliyu Lawan, Harriet L S Lawford, Hilary R Lawlor, Dai Quang Le, Duc Huy Le, Huu-Hoai Le, Long Khanh Dao Le, Minh Huu Nhat Le, Nhi Huu Hanh Le, Thao Thi Thu Le, Trang Diep Thanh Le, Caterina Ledda, Hye Ah Lee, Seung Won Lee, Wei-Chen Lee, Yo Han Lee, James Leigh, Vasileios Leivaditis, Matthew J Lennon, Matilde Leonardi, Elvynna Leong, Janni Leung, Chengcheng Li, Haobo Li, Hui Li, Jianan Li, Jiaying Li, Jie Li, Jinbo Li, Ming-Chieh Li, Peng li, Shaojie Li, Wang-Zhong Li, Wei Li‡, Wei Li§, Weilong Li, Wenjie Li, Xunliang Li, Yichong Li, Yongze Li, Zhengrui Li, Zhihui Li, Yanxue Lian, Xue-Zhen Liang, Stephen S Lim, Jialing Lin, Queran Lin, Ro-Ting Lin, Ya Lin, Daniel Lindholm, Christine Linehan, Yuewei Ling, Gang Liu, Haipeng Liu, Jue Liu, Xianliang Liu, Xiaofeng Liu, Xuefeng Liu, Yubo Liu, Yunfei Liu, Erand Llanaj, Michael J Loftus, Valerie Lohner, José Francisco López-Gil, Platon D Lopukhov, Stefan Lorkowski, Masoud Lotfizadeh, Shanjie Luan, Jailos Lubinda, Taraneh Lucas, Giancarlo Lucchetti, Alessandra Lugo, Raimundas Lunevicius, Peng Luo, Jay B Lusk, Angelina M Lutambi, Ricardo Lutzky Saute, Miltiadis D Lytras, Ellina Lytvyak, Hawraz Ibrahim M Amin, Kevin Sheng-Kai Ma, Zheng Feei Ma, Mahmoud Mabrok, Isis E Machado, Firoozeh Madadi, Seyed Ataollah Madinezad, Christian Madsen, Aurea Marilia Madureira-Carvalho, Mohammed Magdy Abd El Razek, Azzam A Maghazachi, D R Mahadeshwara Prasad, Sasikumar Mahalingam, Mehrdad Mahalleh, Nozad Hussein Mahmood, Alireza Mahmoudi, Farhad Mahmoudi, My Tra Mai, Rituparna Maiti, Azeem Majeed, Konstantinos Christos C Makris, Mohammad-Reza Malekpour, Reza Malekzadeh, Hardeep Singh Malhotra, Ahmad Azam Malik, Farihah Malik, Tabarak Malik, Deborah Carvalho Malta, Abdullah A Mamun, Mustapha Mangdow, Lokesh Manjani, Yosef Manla, Kamaruddeen Mannethodi, Farheen Mansoor, Marjan Mansourian, Mohammad Ali Mansournia, Ana M Mantilla Herrera, Lorenzo Giovanni Mantovani, Changkun Mao, Tahir Maqbool, Sajid Maqsood, Hamid Reza Marateb, Joemer C Maravilla, Konstantinos Margetis, Mirko Marino, Adilson Marques, Randall V Martin, Gabriel Martinez, Bernardo Alfonso Martinez-Guerra, Ramon Martinez-Piedra, Daniela Martini, Francisco Rogerlândio Martins-Melo, Miquel Martorell, Winfried März, Roy Rillera Marzo, Sammer Marzouk, Stefano Masi, Clara N Matei, Yasith Mathangasinghe, Stephanie Mathieson, Alexander G Mathioudakis, Manu Raj Mathur, Medha Mathur, Fernanda Penido Matozinhos, Rita Mattiello, Khurshid A Mattoo, Richard James Maude, Pallab K Maulik, Miranda L May, Mahsa Mayeli, Maryam Mazaheri, Antonio Mazzotti, Chioma Ngozichukwu Pauline Mbachu, Ikechukwu Innocent Mbachu, Martin McKee, Susan A McLaughlin, Steven M McPhail, Enkeleint A Mechili, Rishi P Mediratta, Jitendra Meena, Elahe Meftah, Medhin Mehari, Asim Mehmood, Man Mohan Mehndiratta, Entezar Mehrabi Nasab, Kala M Mehta, Vini Mehta, Subhash Mehto, Toni

Meier, Tesfahun Mekene Meto, Hadush Negash Meles, Endalkachew Belayneh Melese, Satish Melwani, Aishe Memetova, Walter Mendoza, Godfred Antony Menezes, Ritesh G Menezes, Berihun Agegn Mengistie, Emiru Ayalew Mengistie, Sultan Ayoub Meo, Michelangelo Mercogliano, Atte Meretoja, Tuomo J Meretoja, Tomislav Mestrovic, Chamila Dinushi Kukulege Mettananda, Sachith Mettananda, Mohamed M M Metwally, Louise Mewton, Adquate Mhlanga, Andrea Michelerio, Ana Carolina Micheletti Gomide Nogueira de Sá, Hiwot Soboksa Mideksa, Paul Anthony Miller, Ted R Miller, Giuseppe Minervini, Wai-kit Ming, GK Mini, Mojgan Mirghafourvand, Erkin M Mirrakhimov, Seyed Ali Mirshahvalad, Mizan Kiros Mirutse, Yousef Mirzaei, Archana Mishra, Kumar Guru Mishra, Vinaytosh Mishra, Arup Kumar Misra, Philip B Mitchell, Prasanna Mithra, Sayan Mitra, Manasi Murthy Murthy Mittinty, Malihe Moazeni, Mohammadreza Mobayen, Madeline E Moberg, Shivani Modi, Ashraf Mohamadkhani, Jama Mohamed, Mona Gamal Mohamed, Nouh Saad Mohamed, Khabab Abbasher Hussien Mohamed Ahmed, Taj Mohammad, Sakineh Mohammad-Alizadeh-Charandabi, Abdolreza Mohammadi, Mohammad Reza Mohammadi, Seyed Omid Mohammadi, Abdollah Mohammadian-Hafshejani, Ibrahim Mohammadzadeh, Ramin Mohammadzadeh, Ammas Siraj Mohammed, Hussen Mohammed, Omer Mohammed, Shafiu Mohammed, Suleiman Mohammed, Yahaya Mohammed, Syam Mohan, Yugal Kishore Mohanta, Mohammad Mohseni, Amin Mokari-Yamchi, Ali H Mokdad, Alexandr Mokhirev, Peyman Mokhtarzadehazar, Sabrina Molinaro, Amirabbas Mollaei, Shaher Momani, Lorenzo Monasta, Amirabbas Monazzami, Himel Mondal, Stefania Mondello, Ahmed Al Montasir, Catrin E Moore, Yousef Moradi, Maziar Moradi-Lakeh, Paula Moraga, Lidia Morawska, Rafael Silveira Moreira, Brooks W Morgan, Negar Morovatdar, Mahdis Morovvati, Mahmoud M Morsy, Jakub Morze, Reza Mosaddeghi Heris, Jonathan F Mosser, Nogol Motamedgorji, Vincent Mougin, Simin Mouodi, Asma Mousavi, Seyede Zohre Mousavi, Amin Mousavi Khaneghah, Seyed Mohamad Sadegh Mousavi Kiasary, Mohamed Awad Abdalaziz Mousnad, Amanda Movo, Hagar Lotfy Mowafy, Kimia Mozahheb Yousefi, Matías Mrejen, Ahmed Msherghi, Rabia Mubarak, Sumaira Mubarik, Shiv K Mudgal, Syed Aun Muhammad, Muhammad Solihuddin Muhtar, Sukhes Mukherjee, Sumoni Mukherjee, Amartya Mukhopadhyay, Satinath Mukhopadhyay, M A Muktadir, Sileshi Mulatu, Francesk Mulita, Getaneh Baye Mulu, Chalie Mulugeta, Mulyadi Mulyadi, Muneeb Ahmad Muneer, Malaisamy Muniyandi, Kavita Munjal, Yanjinlkham Munkhsaikhan, Javier Muñoz Laguna, Anjana Munshi, Pradeep Manohar Muragundi, Michio Murakami, Yahye Hassan Muse, Ali Mushtaq, Ghulam Mustafa, Sherzad Ibrahim Mustafa, Mubarak Taiwo Mustapha, Sathish Muthu, Saravanan Muthupandian, Claude Mambo Muvunyi, Muhammad Muzaffar, Woojae Myung, Amin Nabavi, Fatemehzahra Naddafi, Ahamarshan Jayaraman Nagarajan, Shankar Prasad Nagaraju, Mohsen Naghavi, Pirouz Naghavi, Ganesh R Naik, Gurudatta Naik, Hiten Naik, Firzan Nainu, Sanjeev Nair, Soroush Najdaghi, Noureddin Nakhostin Ansari, Paul Nam, Vinay Nangia, Jobert Richie Nansseu, Ibrahim A Naqid, Shumaila Nargus, Delaram Narimani Davani, Yvonne Nartey, Bruno Ramos Nascimento, Gustavo G Nascimento, Abdallah Y Naser, Mohammad Naser, Abdulqadir J Nashwan, Hamide Nasiri, Mahmoud Nassar, Zuhair S Natto, Javaid Nauman, Zakira Naureen, Samidi Nirasha Kumari Navaratna, Anum Nawaz, M Omar Nawaz, Biswa Prakash Nayak, Shalini Ganesh Nayak, Javad Nazari, G Takop Nchanji, Rawlance Ndejjo, Anthony Wainaina Ndungu, Amanuel Tebabal Nega, Abigia Ashenafi Negash, Ionut Negoi, Ruxandra Irina Negoi, Alina Gabriela Negru, Jalil Nejati, Chakib Nejjari, Samata Nepal, Olivia D Nesbit, Henok Biresaw Netsere, Charles Richard James Newton, Marie Ng, Georges Nguefack-Tsague, Josephine W Ngunjiri, Anh Thy H Nguyen, Cuong Tat Nguyen, Huong Lan Thi Nguyen, Huong-Dung Thi Nguyen, Nghia Phu Nguyen, Phat Tuan Nguyen, The Phuong Nguyen, Trang Nguyen, Tu Anh Nguyen, Van Thanh Nguyen, Ambe Marius Ngwa, Robina Khan Niazi, Jing Nie, Luciano Nieddu, Yeshambel T Nigatu, Ali Nikoobar, Dina Nur

Anggraini Ningrum, Vikram Niranjan, Abebe Melis Nisro, Jan Rene Nkeck, Princess Afia Nkrumah-Boateng, Chukwudi A Nnaji, Efaq Ali Noman, Shuhei Nomura, Syed Toukir Ahmed Noor, Mohammadamin Noorafrooz, Pardis Noormohammadpour, Mamoona Noreen, Masoud Noroozi, Jean Jacques Noubiap, Taylor Noyes, Valentine C Nriagu, Chisom Adaobi Nri-Ezedi, Jean Claude Nshimiyimana, Fred Nugen, Atoma Negera Nugusa, Mengistu H Nunemo, Aqsha Nur, Dieta Nurrika, Sylvester Dodzi Nyadanu, Felix Kwasi Nyande, Chimezie Igwegbe Nzoputam, Ogochukwu Janet Nzoputam, Bogdan Oancea, George Obaido, Erin M O'Connell, Adashi Margaret Odama, Ramez M Odat, Fabio Massimo Oddi, Ismail A Odetokun, Oluwakemi Ololade Odukoya, Joseph Kojo Oduro, Michael Safo Oduro, Onome Bright Oghenetega, Oluwaseun Adeolu Ogundijo, Abiola Ogunkoya, James Odhiambo Oguta, Doori Oh, Sarah Oh, Edel T O'Hagan, Hassan Okati-Aliabad, Sylvester Reuben Okeke, Deborah Oluwatosin Okeke-Obayemi, Akinkunmi Paul Okekunle, Olalekan John Okesanya, Onyedika A Okoli, Osaretin Christabel Okonji, John Olayemi Okunlola, Oluyemi Adewole Okunlola, Oluwaseyi Isaiah Olabisi, Andrew T Olagunju, Oladotun Victor Olalusi, Matthew Idowu Olatubi, Arão Belitardo Oliveira, Gláucia Maria Moraes Oliveira, Abdulhakeem Abayomi Olorukooba, Erik J Olson, Oluseye Olalekan Oludoye, Ronald Olum, Bolajoko Olubukunola Olusanya, Jacob Olusegun Olusanya, Oluwafemi G Oluwole, Folorunsho Bright Omage, Hany A Omar, Goran Latif Omer, Qi Chwen Ong, Sandersan Onie, Obinna E Onwujekwe, Franklyn Opara, Marcel Opitz, Michal Ordak, Verner N Orish, Raffaele Ornello, Atakan Orscelik, Alberto Ortiz, Esteban Ortiz-Prado, Augustus Osborne, Eric Osei, Samuel M Ostroff, John W Ostrominski, Uchechukwu Levi Osuagwu, Olayinka Osuolale, Godfred Otchere, Elham H Othman, Mostafa Monier Othman, Adrian Otoiu, Oche Joseph Otorkpa, Abdu Oumer, Jerry John Ouner, Amel Ouyahia, Guoqing Ouyang, Mayowa O Owolabi, Irene Amoakoh Owusu, Kolapo Oyebola, Tope Oyelade, Oyetunde T Oyeyemi, Ilker Ozsahin, Mahesh P A, Kevin Pacheco-Barrios, Inderbir Padda, Alicia Padron-Monedero, Jagadish Rao Padubidri, Anton Pak, Pramod Kumar Pal, Tamás Palicz, Raffaele Palladino, Raul Felipe Palma-Alvarez, Tejasri Paluvai, Feng Pan, Hai-Feng Pan, Parsa Panahi, Sujogya Kumar Panda, Songhomitra Panda-Jonas, Deepshikha Pande Katare, Ke Pang, Helena Ullyartha Pangaribuan, Georgios D Panos, Leonidas D Panos, Ioannis Pantazopoulos, Giovanni Paolino, Mario Virgilio Papa, Ilias Papadimopoulos, Paraskevi Papadopoulou, Utsav Parekh, Peyvand Parhizkar Roudsari, Amrita Parida, Chulwoo Park, Eun-Kee Park, Seoyeon Park, Arpit Parmar, Swapnil Parve, Ava Pashaei, Roberto Passera, Bhumi Hemal Patel, Hemal M Patel, Mitesh Patel, Neel Navinkumar Patel, Satyananda Patel, Ashlesh Patil, Shankargouda Patil, Dimitrios Patoulias, Apurba Patra, Mohammad Hridoy Patwary, Hilary Paul, Shrikant Pawar, Shubhadarshini Pawar, Hamidreza Pazoki Toroudi, Amy E Peden, Paolo Pedersini, Jarmila Pekarcikova, Veincent Christian Filipino Pepito, Prince Peprah, João Perdigão, Gavin Pereira, Maria Odete Pereira, Pablo Perez-Lopez, Norberto Perico, Simone Perna, Konrad Pesudovs, Pavlo Petakh, Olumuyiwa James Peter, Fanny Emily Petermann-Rocha, Hoang Nhat Pham, Nhat Truong Pham, Tung Thanh Pham, Anil K Philip, Michael R Phillips, Zayar Phyo, Brandon V Pickering, David M Pigott, Julian David Pillay, Luane Pinheiro Pinheiro Rocha, Zahra Zahid Piracha, Michael A Piradov, Edoardo Pirera, Enrico Pisoni, Dietrich Plass, Evgenii Plotnikov, Indrashis Podder, Dimitri Poddighe, Roman V Polibin, Peter Pollner, Ramesh Poluru, Arjun Pon Avudaiappan, Constance Dimity Pond, Ville T Ponkilainen, Ion Popa, Svetlana Popova, Djordje S Popovic, Maarten J Postma, Sajjad Pourasghary, Reza Pourbabaki, Farzad Pourghazi, Mohsen Poursadeqiyan, Naeimeh Pourtaheri, Attur Ravindra Prabhu, Sergio I Prada, Jalandhar Pradhan, Pranil Man Singh Pradhan, Rifky Octavia Pradipta, Peralam Yegneswaran Prakash, Chandra P Prasad, Akila Prashant, Elton Junio Sady Prates, Tina Priscilla, Natalie Pritchett, Harsh Priya, Hery Purnobasuki, Bharathi M Purohit, Jagadeesh Puvvula, Nameer Hashim Qasim, Xiang Qi, Zhipeng Qi, Jia-Yong Qiu, Zahiruddin Syed Quazi, Shahazad Niwazi Qurashi, Deepthi R,

Navid Rabiee, Basuki Rachmat, Raghu Anekal Radhakrishnan, Venkatraman Radhakrishnan, Maja R Radojčić, Hadi Raeisi Shahraki, Ibrar Rafique, Pankaja Raghav, Pracheth Raghuveer, Leila Rahbarnia, Fakher Rahim, Hawbash Mohammed-Amin Rahim, Sajjad Rahimi, Afarin Rahimi-Movaghar, Vafa Rahimi-Movaghar, Fryad Majeed Rahman, Mahbubur Rahman, Mahfuzur Rahman, Md Mosfequr Rahman, Mohammad Hifz Ur Rahman, Mohammad Meshbahur Rahman, Mosiur Rahman, Saeed Rahmani, Masoud Rahmati, Ghasem Rahmatpour Rokni, Hakim Rahmoune, Pramila Rai, Diego Raimondo, Ivano Raimondo, Sunil Kumar Raina, Jeffrey Pradeep Raj, Adarsh Raja, Sandesh Raja, Sathish Rajaa, Erta Rajabi, Shahryar Rajai Firouzabadi, Gunaseelan Rajendran, Judah Rajendran, Vinoth Rajendran, Shaman Rajindrajith, Mohammad Amin Rajizadeh, Prashant Rajput, Mahmoud Mohammed Ramadan, Majed Ramadan, Kadar Ramadhan, Chitra Ramasamy, Shakthi Kumaran Ramasamy, Sheena Ramazanu, Zahra Ramezani, Marzieh Ramezani Farani, Pramod W Ramteke, Juwel Rana, Shailendra Singh Rana, Chhabi Lal Ranabhat, Nemanja Rancic, Smitha Rani, Fatemeh Ranjbar Noei, Chythra R Rao, Kumuda Rao, Mithun Rao, Sowmya J Rao, Davide Rasella, Vahid Rashedi, Mohammad-Mahdi Rashidi, Mohammad Aziz Rasouli, Ashkan Rasouli-Saravani, Prateek Rastogi, Azad Rasul, Devarajan Rathish, Abdur Rauf, Santosh Kumar Rauniyar, Ilari Rautalin, Ramin Ravangard, Dhwani Ravi, David Laith Rawaf, Salman Rawaf, Reza Rawassizadeh, Ramu Rawat, Ayita Ray, Mohammad Rayati, Iman Razeghian, Bahman Razi, Christian Razo, Filippo Recenti, Murali Mohan Rama Krishna Reddy, Elrashdy Redwan, Sanika Rege, Wajiha Rehman, Lennart Reifels, Giuseppe Remuzzi, Longbing Ren, Andre M N Renzaho, Serge Resnikoff, Luis Felipe Reyes, Mina Rezaei, Nazila Rezaei, Negar Rezaei, Nima Rezaei, Mohsen Rezaeian, Taeho Gregory Rhee, Mavra A Riaz, Antonio Luiz P Ribeiro, Jennifer Rickard, Moattar Raza Rizvi, Hannah Elizabeth Robinson-Oden, Hermano Alexandre Lima Rocha, João Rocha Rocha-Gomes, Mónica Rodrigues, Leonardo Roever, Peter Rohloff, Iftitakhur Rohmah, Susanne Röhr, David Rojas-Rueda, Megan L Rolfzen, Debby Syahru Romadlon, Michele Romoli, Marina Romozzi, Luca Ronfani, Jennifer Jacqueline Rosauer, Amirhossein Roshanshad, Morteza Rostamian, Gregory A Roth, Kunle Rotimi, Himanshu Sekhar Rout, Hanieh Rouzbahani, Reza Rouzbahani, Shiva Rouzbahani, Bedanta Roy, Nitai Roy, Parimal Roy, Poulami Roy, Priyanka Roy, Sharmistha Roy, Shubhanjali Roy, Susovan Roy Chowdhury, Parameswari Royapuram Parthasarathy, Enrico Rubagotti, Guilherme de Andrade Ruela, Susan Fred Rumisha, Michele Russo, Godfrey Mutashambara Rwegerera, Manjula S, Chandan S N, Aly M A Saad, Adnan Saad Eddin, Zahra Saadatian, Maha Mohamed Saber-Ayad, Cameron John Sabet, Siamak Sabour, Kabir P Sadarangani, Seyed Kiarash Sadat Rafiei, Basema Ahmad Saddik, Bashdar Abuzed Sadee, Tarannom Sadegh, Ehsan Sadeghi, Erfan Sadeghi, Fatemeh Sadeghi-Ghyassi, Mohd Saeed, Umar Saeed, Maryam Saeedi, Mehdi Safari, Sare Safi, Sher Zaman Safi, Rajesh Sagar, Mastooreh Sagharichi, Dominic Sagoe, Nondo Saha, Fatemeh Saheb Sharif-Askari, Narjes Saheb Sharif-Askari, Amirhossein Sahebkar, Pragyan Monalisa Sahoo, Kirti Sundar Sahu, Muhammad Soaib Said, Zahra Saif, S Mohammad Sajadi, Md Refat Uz Zaman Sajib, Mirza Rizwan Sajid, Morteza Saki, Nasir Salam, Payman Salamati, Luciane B Salaroli, Mohamed A Saleh, Leili Salehi, Mahdi Salehi, Marwa Rashad Salem, Mohammed Z Y Salem, Dauda Salihu, Sohrab Salimi, Malik Sallam, Giovanni A Salum, Sundeep Santosh Salvi, Hossein Samadi Kafil, Jayami Eshana Samaranayake, Waqas Sami, Yoseph Leonardo Samodra, Vijaya Paul Samuel, Abdallah M Samy, Sandeep G Sangle, Elaheh Sanjari, Sathish Sankar, Francesco Sanmarchi, Francesca Sanna, Lucas H C C Santos, Milena M Santric-Milicevic, Bruno Piassi Sao Jose, Krishna Prasad Sapkota, Sivan Yegnanarayana Iyer Saraswathy, Jacob Owusu Sarfo, Yaser Sarikhani, Hemen Sarma, Mohammad Sarmadi, Gargi Sachin Sarode, Sachin C Sarode, Satish Saroshe, Michele Sassano, Brijesh Sathian, Mukesh Kumar Sathya Narayanan, Paul A Saunders, Mehrdad Savabi Far, Monika Sawhney, Sangeeta Gopal Saxena, Ganesh Kumar Saya, Abu Sayeed, Mete Saylan, Christophe Schinckus, Ione Jayce Ceola Schneider, Rachel D

Schneider, Art Schuermans, Austin E Schumacher, Ghil Schwarz, David C Schwebel, Falk Schwendicke, Catherine Schwinger, Amin Sedigh, Saravanan Sekaran, Mario Šekerija, Muthamizh Selvamani, Vimalraj Selvaraj, Yuliya Semenova, Mohammad H Semreen, Fikadu Waltengus Sendeku, Yigit Can Senol, Subramanian Senthilkumaran, Sadaf G Sepanlou, Andreea Claudia Serban, Edson Serván-Mori, Yashendra Sethi, Christian Sewor, Seyed Mohammad Seyed Alshohadaei, Allen Seylani, Matthew Seymour, Jamileh Shadid, Nilay S Shah, Sweni Shah, Shazlin Shaharudin, Muhammad Shahbaz, Samiah Shahid, Syed Ahsan Shahid, Wajeehah Shahid, Endrit Shahini, Fatemeh Shahrahmani, Hamid R Shahsavari, Moyad Jamal Shahwan, Masood Ali Shaikh, Alireza Shakeri, Ali Shakerimoghaddam, Ali S Shalash, Sunder Sham, Muhammad Aaqib Shamim, Farzane Shams, Mehran Shams-Beyranvand, Mohammad Ali Shamshirgaran, Anas Shamsi, Alfiya Shamsutdinova, Dan Shan, Abhishek Shankar, Mohammed Shannawaz, Xian Shao, Amin Sharifan, Javad Sharifi Rad, Avimanu Sharma, Bhoopesh Kumar Sharma, Bunty Sharma, Kamal Sharma, Manoj Sharma, Sourabh Sharma, Ujjawal Sharma, Vishal Sharma, Rajesh P Shastry, Shamee Shastry, Armin Shavandi, Ramzi Shawahna, Maryam Shayan, Babangida Shehu Bappah, Ali Sheidaei, Suchitra M Shenoy, Samendra P Sherchan, Suraj S Shetty, Fang Shi, Lin-Hong Shi, Mosa Shibani, Belayneh Fentahun Shibesh, Kenji Shibuya, Desalegn Shiferaw, Md Monir Hossain Shimul, Jae Il Shin, Min-Jeong Shin, Rahman Shiri, Reza Shirkoohi, Aminu Shittu, Abdul-karim Olayinka Shitu, Ivy Shiue, Velizar Shivarov, Nathan A Shlobin, Shayan Shojaei, Zahra Shokati Eshkiki, Azad Shokri, Sinegugu Nosipho Shongwe, Sina Shool, Seyed Afshin Shorofi, Gambhir Shrestha, Sunil Shrestha, Kerem Shuval, Nicole Remaliah Samantha Sibuyi, Emmanuel Edwar Siddig, Mohammad Sidiq, Martin Siegel, Diego Augusto Santos Silva, Gustavo Correia Basto da Silva, João Pedro Silva, Juan Carlos Silva, Luís Manuel Lopes Rodrigues Silva, Noah Joseph Bernard Silva de Leonardi, Padam Prasad Simkhada, Abhinav Singh, Akanksha Singh, Ambrish Singh, Baljinder Singh, Bhim Pratap Singh, Harmanjit Singh, Harpreet Singh, Jasbir Singh, Jasvinder A Singh, Kalpana Singh, Lucky Singh, Narinder Pal Singh, Paramdeep Singh, Poornima Suryanath Singh, Prashant Kumar Singh, Puneetpal Singh, Rakesh K Singh, Samer Singh, Satwinder Singh, Surendra Singh, Mukesh Kumar Sinha, Ratnesh Sinha, Robert Sinto, Sarah Brooke Sirota, Søren T Skou, David A Sleet, Erica Leigh N Slepak, Farrukh Sobia, MdSalman Sohel, Somaye Sohrabi, Balamrit Singh Sokhal, Ranjan Solanki, Solikhah Solikhah, Sameh S M Soliman, Weiyi Song, Younseong Song, Aayushi Sood, Prashant Sood, Soroush Soraneh, Reed J D Sorensen, Joan B Soriano, Michele Sorrentino, Fernando Sousa, Marco Aurelio Sousa, Ceren Soylu, Michael Spartalis, Sandra Spearman, Manraj Singh Sra, Chandrashekhar T Sreeramareddy, Bahadar S Srichawla, Suresh Kumar Srinivasamurthy, Shyamkumar Sriram, Lauryn K Stafford, Jeffrey D Stanaway, Antonina V Starodubova, Simona Cătălina Ștefan, Caroline Stein, Dan J Stein, Caitlyn Steiner, Timothy J Steiner, Jaimie D Steinmetz, Paschalis Steiropoulos, Aleksandar Stevanović, Leo Stockfelt, Lars Jacob Stovner, Kurt Straif, Peter Stubbs, Yu Su, Omer Subasi, Narayan Subedi, Claudia Kimie Suemoto, Alisha Suhag, Liang Sui, Thitiporn Sukaew, Surajo Kamilu Sulaiman, Auwal Garba Suleiman, Muritala Suleiman Odidi, Muhammad Suleman, Desy Sulistiyorini, Mark J M Sullman, Anusha Sultan Meo, Haitong Zhe Sun, Jing Sun, Mao-ling Sun, Xiaodong Sun, Zhong Sun, Zhuanlan Sun, Suraj Sundaragiri, Thanigaivel Sundaram, Johan Sundström, David Sunkersing, Sumam Sunny, Vinay Suresh, Chandan Kumar Swain, Vivianne M Swart, Dayinta Annisa Syaiful, Lukasz Szarpak, Mindy D Szeto, Sree Sudha T Y, Payam Tabaee Damavandi, Rafael Tabarés-Seisdedos, Seyed-Amir Tabatabaeizadeh, Shima Tabatabai, Celine Tabche, Ramin Tabibi, Mohammad Tabish, Jyothi Tadakamadla, Santosh Kumar Tadakamadla, Buhari Abdullahi Tafida, Farzad Taghizadeh-Hesary, Yasaman Taheri Abkenar, Moslem Taheri Soodejani, Amir Taherkhani, Jabeen Taiba, Shima Tajabadi, Iman M Talaat, Stella Talic, Byomkesh Talukder, Mircea Tampa, Jacques Lukenze Tamuzi, Jianye Tan, Ker-Kan Tan, Shynar Tanabayeva, Haosu Tang, Ekamol Tantisattamo, Ingan Ukur

Tarigan, Mengistie Kassahun Tariku, Saba Tariq, Md Tariqujjaman, Nathan Y Tat, Razieh Tavakoli Oliaee, Rahele Tavakoly, Seyed Mohammad Tavangar, Mebrahtu G Tedla, Amare Teshome Tefera, Mojtaba Teimoori, Mohamad-Hani Temsah, Corey Teply, Masayuki Teramoto, Amensisa Hailu Tesfaye, Azimeraw Arega Tesfu, Jay Tewari, Alireza Teymouri, Omar Thaher, Pugazhenthan Thangaraju, Kavumpurathu Raman Thankappan, Rekha Thapar, Ismaeel Tharwat, Samar Tharwat, Hadiza Theyra-Enias, Mehakpreet Kaur Thind, Arun James Thirunavukarasu, Muthu Thiruvengadam, Rekha Thiruvengadam, Arulmani Thiyagarajan, Nihal Thomas, Geethika P Thota, Wei Tian, Jansje Henny Vera Ticoalu, Tenaw Yimer Tiruye, Madi Tleshev, Musliu Adetola Tolani, Sojit Tomo, Marcello Tonelli, Roman Topor-Madry, Ali Torkashvand, Mathilde Touvier, Marcos Roberto Tovani-Palone, Khaled Trabelsi, Eugenio Traini, Mai Thi Ngoc Tran, Nghia Minh Tran, Ngoc Ha Tran, Quynh Thuy Huong Tran, Tam Quoc Minh Tran, Thang Huu Tran, Nguyen Tran Minh Duc, Domenico Trico, Indang Trihandini, Samuel Joseph Tromans, Quynh Xuan Nguyen Truong, Thien Tan Tri Tai Truyen, Aristidis Tsatsakis, Gary Tse, Evangelia Eirini Tsermpini, Lorainne Tudor Car, Mike Tuffour Amirikah, Munkhtuya Tumurkhuu, Zhouting Tuo, Sok Cin Tye, Aniefiok John Udoakang, Atta Ullah, Himayat Ullah, Irfan Ullah, Saeed Ullah, Muhammad Umair, Krishna Kishore Umapathi, Lawan Umar, Muhammad Umar¶, Muhammad Umar**, Shehu Salihu Umar, Andrew Underwood-Nakamura, Dinesh Upadhya, Era Upadhyay, Dipan Uppal, Daniele Urso, Jibrin Sammani Usman, Kelechi Julian Uzor, Dilber Uzun Ozsahin, Hande Uzunçıbuk, Pratyusha Vadagam, sara Vahdati, Asokan Govindaraj Vaithinathan, Omid Vakili, Alireza Vakilian, Pascual R Valdez, Mario Valenti, Gelareh Valizadeh, Jef Van den Eynde, Giloume Van Der Walt, Aaron van Donkelaar, Javad Varasteh, Ravi Prasad Varma, Priya Vart, Tommi Juhani Vasankari, Sampara Vasishta, Srivatsa Surya Vasudevan, Prabhakar Veginadu, Ashleigh S Vella, Balachandar Vellingiri, Narayanaswamy Venketasubramanian, Baskar Venkidasamy, Megan Verma, Poonam Verma, Massimiliano Veroux, Georgios-Ioannis Verras, Dominique Vervoort, Simone Vidale, Simone Villa, Jorge Hugo Villafañe, David Villarreal-Zegarra, Francesco S Violante, Sharath Chaitanya Vipparthy, Rachel Visontay, Luciano Magalhães Vitorino, Vasily Vlassov, Martin Vojtek, Stein Emil Vollset, Avina Vongpradith, Mehdi Vosoughi, Elpida Vounzoulaki, Hai Nam Vu, Linh Vu, Yasir Waheed, Mugi Wahidin, Megha Walia, Agnes Wamuyu Wamai, Jin-Yi Wan, Cong Wang, Fang Wang, Lei Wang, Liang Wang, Ruixuan Wang, Shaopan Wang, Shu Wang, Wei Wang, Xing Wang, Xuequan Wang, Yanzhong Wang, Yichen Wang, Yuan-Pang Wang, Zhihua Wang, Tanveer A Wani, Mary Njeri Wanjau, Ahmed Bilal Waqar, Muhammad Waqas, Paul Ward, Toyiba Hiyaru Wassie, Kosala Gayan Weerakoon, Ishanka Weerasekara, Fei-Long Wei, Xueying Wei, Robert G Weintraub, Daniel J Weiss, Eli J Weiss, Yi Feng Wen, Andrea Werdecker, Ronny Westerman, Joanna L Whisnant, Harvey A Whiteford, Taweewat Wiangkham, Yohanes Cakrapradipta Wibowo, Anggi Lukman Wicaksana, Dakshitha Praneeth Wickramasinghe, Nuwan Darshana Wickramasinghe, Samuel Wiebe, Angga Wilandika, Peter Willeit, Shadrach Wilson, Andrew Awuah Wireko, Gemechu Kumera Wirtu, Charles Shey Wiysonge, Abay Tadesse Woday, Marcin W Wojewodzic, Axel Walter Wolf, Tewodros Eshete Wonde, Yohannes Chemere Wondmeneh, Daniel Tarekegn Worede, Minichil Chanie Worku, Nigus Kassie Worku, Ai-Min Wu, Chenkai Wu, Felicia Wu, James Fan Wu, Jiayuan Wu, Jinyi Wu, Shi-Nan Wu, Zenghong Wu, Yihun Miskir Wubie, Yanjie Xia, Zhijia Xia, Guangqin Xiao, Hong Xiao, Na Xiao, Wanqing Xie, Hongquan Xing, Site Xu, Suowen Xu, Wanqing Xu, Xiang Xu, Xiaoyue Xu, Mukesh Kumar Yadav, Vikas Yadav, Mahnaz Yadollahi, Sajad Yaghoubi, Saba Yahoo (Syed), Galal Yahya, Kazumasa Yamagishi, Guangcan Yan, Haibo Yang, Weiguang Yang, Xinxin Yang, Yuichiro Yano, Haiqiang Yao, Laiang Yao, Amir Yarahmadi, Haya Yasin, Mohamed A Yassin, Yuichi Yasufuku, Sanni Yaya, Pengpeng Ye, Meghdad Yeganeh, Ali Cem Yekdeş, Mohammad Hossein YektaKooshali, Kuanysh A Yergaliyev, Renjulal Yesodharan, Subah Abderehim Yesuf, Saber Yezli, Siyan Yi, Muluken Yigezu, Zeamanuel Anteneh Yigzaw, Dehui Yin, Yulai Yin, Paul Yip,

Malede Berihun Yismaw, Yazachew Engida Yismaw, Dong Keon Yon, Naohiro Yonemoto, Mustafa Z Younis, Abdilahi Yousuf, Chuanhua Yu, Jian Yu, Yong Yu, Faith H Yuh, Ghazala Yunus, Umar Yunusa, Aminu Abba Yusuf, Monal Yuwanati, Siddhesh Zadey, Vesna Zadnik, Mubashir Zafar, Manijeh Zaghampour, Emilia Zainal Abidin, Fathiah Zakham, Nazar Zaki, Giulia Zamagni, Burhan Abdullah Zaman, Sojib Bin Zaman, Abu Sarwar Zamani, Nelson Zamora, Aurora Zanghì, Heather J Zar, Kourosh Zarea, Mohammed Zawiah, Mohammed G M Zeariya, Abay Mulu Zenebe, Sebastian Zensen, Eyael M Zeru, Tiansong Zhan, Yongle Zhan, Beijian Zhang, Casper J P Zhang, Haijun Zhang, Jingya Zhang, Liqun Zhang, Meixin Zhang, Xiaoyi Zhang, Yunquan Zhang, Zhiqiang Zhang, Jianhui Zhao, Sheng Zhao, Zhongyi Zhao, Jinxin Zheng, Ming-Hua Zheng, Peng Zheng, Claire Chenwen Zhong, Jiayan Zhou, Juexiao Zhou, Maigeng Zhou, Bin Zhu, Zhengyang Zhu, Abzal Zhumagaliuly, Magdalena Zielińska, Liu Zihao, Ghazal Zoghi, Mohamed Ali Zoromba, Zhiyong Zou, Rafat Mohammad Zrieq, Liesl J Zuhlke, Lilik Zuhriyah, Alimuddin Zumla, Ahed H Zyoud, Sa'ed H Zyoud, Shaher H Zyoud, Michael Brauer†, Theo Vos†, Christopher J L Murray†, and Emmanuela Gakidou†.

* Joint first authors

† Joint senior authors

Affiliations

Institute for Health Metrics and Evaluation (Prof S I Hay FMedSci, K L Ong PhD, N Ahmad BS, R W Aldridge PhD, J O Amlag MPH, J A Anderson BS, C M Antony MA, A Y Aravkin PhD, C Ashbaugh MA, I A Barreras Beltran BA, K Bhangdia MS, C Bisignano MPH, R Bokota MPH, Prof M Brauer DSc, E D Brewer BS, K Burkart PhD, C Buxbaum MPH, J Cagney MSc, A Carter MPH, K M Cercy BS, C S Chen BA, E Chung MD, R M Cogen BA, A J Cohen DSc, E Cousin PhD, E J Cowart BS, A Crist BSc, J A Cruz BS, G T Culbreth PhD, X Dai PhD, N Davis Weaver MPH, N K DeCleene BS, A Deen MPH, Prof L Degenhardt PhD, L Deitesfeld MA, R V Dominguez BS, K Estep MPA, Prof V L Feigin PhD, A J Ferrari PhD, L S Flor MPH, L M Force MD, N Fullman MPH, V Garcia BS, W M Gardner MPH, G F Gil MPH, N M Gilbertson PhD, H Hagins MSPH, D Haile PhD, E B Hamilton MPH, H Han MSc, K L Harding PhD, A A Harris MPH, J He MSc, C A Henson MPH, M E Herbert MSc, A Heuer MSc, J Hon MLS, J A Houser BA, K M Humphrey MS, A L Ihler PSM, C O Johnson PhD, E M Kahn MPH, M Kirk MPH, J Klusty MS, J M Kocarnik PhD, H H Kyu PhD, H R Lawlor BA, Prof S S Lim PhD, M L May MPH, S A McLaughlin PhD, T Mestrovic PhD, P A Miller PhD, M E Moberg MS, Prof A H Mokdad PhD, B W Morgan MSPH, J F Mosser MD, V Mougin BA, A Movo MPH, Prof M Naghavi PhD, P Nam MS, O D Nesbit MA, M Ng PhD, A H Nguyen PhD, T Noyes MPH, E M O'Connell BA, D Oh PhD, E J Olson BS, S M Ostroff PhD, H Paul MS, B V Pickering BS, D M Pigott PhD, N Pritchett DrPH, C Razo PhD, H E Robinson-Oden MLIS, J J Rosauer BA, G A Roth MD, D F Santomauro PhD, R D Schneider MPPM, A E Schumacher PhD, M Seymour MPH, J Shadid BSc, N J B Silva de Leonardi PhD, S B Sirota MA, E N Slepak MLIS, R J D Sorensen PhD, S Spearman MS, L K Stafford MS, J D Stanaway PhD, C Stein PhD, C Steiner MPH, J D Steinmetz PhD, V M Swart MPH, C Teply MS, A Underwood-Nakamura MPH, M Verma MSc, Prof S E Vollset DrPH, A Vongpradith BA, Prof T Vos PhD, H N Vu MPS, E J Weiss BS, J L Whisnant MPH, Prof H A Whiteford PhD, S Wilson BS, F H Yuh MPA, M Zhang MS, P Zheng PhD, Prof C J L Murray DPhil, Prof E Gakidou PhD), Department of Health Metrics Sciences, School of Medicine (Prof S I Hay FMedSci, R W Aldridge PhD, A Y Aravkin PhD, K Burkart PhD, E Cousin PhD, X Dai PhD, L S Flor MPH, L M Force MD, N M Gilbertson PhD, H H Kyu PhD, Prof S S Lim PhD, Prof A H Mokdad PhD, Prof M Naghavi PhD, D M Pigott PhD, G A Roth MD, J D Stanaway PhD, C Stein PhD, Prof S E Vollset DrPH, Prof T Vos PhD, P Zheng PhD, Prof C J L Murray DPhil, Prof E Gakidou PhD), Department of Applied

Mathematics (A Y Aravkin PhD), Department of Pediatrics (E Chung MD), Department Health Metrics Science (M Hassen MSc), Department of Global Health (M Khalil BA, S Kochhar MD, R J D Sorensen PhD), Department of Radiology (F Khosravi MD), Cardiothoracic Imaging Section (F Khosravi MD), Henry M Jackson School of International Studies (S M Ostroff PhD), Division of Cardiology (G A Roth MD), University of Washington, Seattle, WA, USA; Amity Institute of Public Health (Prof B A PhD), Amity University, Uttar Pradesh, India; Shahid Beheshti University of Medical Sciences (M Aalipour MD), Non-communicable Diseases Research Center (M Abbasi-Kangevari MD), Department of Anesthesiology (Prof D Abtahi MD, S Salimi MD, S Seyed Alshohadaei MD, A Shakeri MD), Urology Department (M Ahmadzade MD), National Nutrition and Food Technology Research Institute (M Ajami PhD, Z Hadian PhD), Department of Oral and Maxillofacial Surgery (M Alam MSc), General Surgery Department (I Ansari MD), School of Medicine (M Asadi Anar MD, J Behjati MD, S Ebrahimi MD, M Golmohammadi MD, S Madinezad MD, S Rajai Firouzabadi MD, S Sadat Rafiei MD), Research Institute of Dental Sciences (Prof S Asgary MSc), School of Medical Education and Learning Technologies (S Bagheri PhD, S Sohrabi PhD), Shahid Rajii Hospital (E Baghizadeh MD), Taleghani Anesthesiologist (F Baghizadeh MD), Cancer Research Center (M Bayat MD), Internal Medicine Department (H Borhany MD), Department of Anesthesia, Critical Care and Pain Medicine (Prof A Dabbagh MD), School of Nursing and Midwifery (F Ghadirian PhD), Faculty of Medicine (A Ghaffari Jolfayi MD, M Sagharichi Dipl), Social Determinants of Health Research Center (S Ghamari MD, Prof A Kolahi MD, A Nikoobar BSc, M Rashidi MD), Department of Medical Genetics (M Ghasemi PhD), Center for Comprehensive Genetic Services (M Ghasemi PhD), Obstetrics and Gynecology Department (E Ghotbi MD), Research Center for Social Determinants of Health (A Halimi MSc), Department of Neurosurgery (H Khayat Kashani MD), Injury Prevention and Safety Promotion Research Center (T Lucas PhD), Anesthesiology Research Center (F Madadi MD), Skull Base Research Center (I Mohammadzadeh MD), Department of Audiology, School of Rehabilitation (S Mousavi PhD), Department of Immunology (A Rasouli-Saravani PhD), College of Medicine (I Razeghian MD), Department of Epidemiology (Prof S Sabour PhD), School of Public Health (S Sadat Rafiei MD), Department of Health (M Safari PhD), Ophthalmic Research Center (S Safi PhD), Ophthalmic Research Center (ORC) (M Shayan MD), Department of Medical Education (S Tabatabai PhD), Shahid Beheshti University of Medical Sciences, Tehran, Iran; Department of Nursing (H Aalruz PhD), Al Zaytoonah University of Jordan, Amman, Jordan; Department of Radiation Oncology (H S Ababneh MD), Cardiovascular Research Center (S Abohashem MPH), Department of Orthopaedic Surgery (M Ilyas MBBS), Department of Anesthesia, Critical Care and Pain Medicine (Prof J Kang PhD), Department of Radiology (X Liu PhD), Department of Orthopaedics (O Subasi PhD), Massachusetts General Hospital, Boston, MA, USA (A Eighaei Sedeh MD, M Kim MD); School of Health & Life Sciences (U O Abaraogu PhD), University of the West of Scotland, Paisley, UK; Department of Medical Rehabilitation (U O Abaraogu PhD), Department of Pharmacology and Therapeutics (Prof O E Onwujekwe PhD), University of Nigeria Nsukka, Enugu, Nigeria; Public Health (Prof B B Abate MSc), Curtin School of Population Health (M B Alemu MSc), School of Public Health (E K Chowdhury PhD), School of Population Health (J Dunne PhD, Z Guan MPH, S Nyadanu PhD, Prof G Pereira PhD, D J Weiss PhD), Curtin University, Perth, WA, Australia; Department of Legal and Economic Studies (Prof C Abbafati PhD), Department of Public Health and Infectious Diseases (M S Cattaruzza PhD), La Sapienza University, Rome, Italy; Centre for Regenerative Medicine and Health, Hong Kong Institute of Science and Innovation (N Abbas PhD), Chinese Academy of Sciences, Hong Kong, China; Department of Internal Medicine (M Abbasifard MD), Clinical Research Development Unit (M Abbasifard MD), Department of Epidemiology and Biostatistics (Prof M Rezaeian PhD), Department of Neurology (A Vakilian MD), Non-communicable Diseases

Research Center (A Vakilian MD), Rafsanjan University of Medical Sciences, Rafsanjan, Iran; Department of Epidemiology (S Abd ElHafeez DrPH), Pediatric Dentistry and Dental Public Health Department (Prof O A A Elmeligy PhD), Tropical Health Department (R M Ghazy PhD), Department of Pathology (Prof I M Talaat PhD), Alexandria University, Alexandria, Egypt; College of Pharmacy (Prof A N Abdalla PhD), Umm Al-qura University, Makkah, Saudi Arabia; Hull York Medical School (M A Abdalla PhD), University of Hull, Hull, UK; Department of Biology (Prof E M Abdallah PhD), College of Applied Medical Sciences (Prof F A Alhumaydhi PhD), Department of Medical Laboratories (M A Aljasir PhD, Prof K Allemailem PhD), Department of Health Informatics (Q Jamal PhD), Department of Basic Health Sciences (Prof M I Khan PhD), Qassim University, Buraydah, Saudi Arabia; Department of Health and Nutrition (B A Abdeeq MSc), Save the Children, Hargeisa, Somalia; School of Nursing (Prof N M I Abdel Razeq PhD), The University of Jordan, Amman, Jordan; College of Pharmacy (A Abdelgalil PhD), Pediatric Intensive Care Unit (A Al-Eyadhy MD, Prof M Temsah MD), Department of Cardiac Sciences (Prof K F Alhabib MD), Section of Adult Hematology (Prof G M T ElGohary MD), Department of Physiology (Prof S Meo PhD), University Diabetes Center (A Sultan Meo MPH), Research Chair for Evidence-Based Health Care and Knowledge Translation (Prof M Temsah MD), Department of Pharmaceutical Chemistry College of Pharmacy (Prof T A Wani PhD), King Saud University, Riyadh, Saudi Arabia; Basic Science Department (Prof R Abdel-Hameed PhD), Basic Science Department, Preparatory Year (N M Abourashed PhD), Department of Biology (Prof M Adnan PhD, Prof M Saeed PhD), Department of Public Health (Prof F D Algahtani PhD, M G M Zeariya PhD), College of Applied Medical Science (S Ashraf PhD), Department of Chemistry (A Haque PhD), College of Medicine (Y S Khan MD), Department of Biochemistry (Prof M Kuddus PhD), College of Public Health & Health Informatics (R Kumar PhD), Medical and Diagnostic Research Centre (Prof C T Sreeramareddy MD), Department of Basic Science (G Yunus PhD), Family and Community Medicine Department (M Zafar PhD), Department of Medical-Surgical Nursing (R M Zrieq PhD), University of Hail, Hail, Saudi Arabia; Chemistry Department (Prof R Abdel-Hameed PhD), Department of Zoology and Entomology (A I Hasaballah PhD, M G M Zeariya PhD), Al-Azhar University, Cairo, Egypt; Department of Surgery (M Abdelmasseh MD), College of Health Professions (S Khalid-Ariturk PhD), Marshall University, Huntington, WV, USA; Department of Cardiovascular Medicine (M Abdelnabi MBBCh, R Ibrahim MD), Mayo Clinic, Phoenix, AZ, USA; Department of Medical Laboratory Science (Prof W M Abdel-Rahman PhD), Clinical Sciences Department (Prof E Abu-Gharbieh PhD, H J Barqawi MPhil, Prof A A Maghazachi PhD, Prof M M Saber-Ayad PhD, N Saheb Sharif-Askari PhD, Prof I M Talaat PhD), Department of Nursing (Prof S Abuhammad PhD), Department Pharmacy Practice and Pharmacotherapeutics (A Y Abuhelwa PhD), Department of Restorative Dentistry (A B Acharya PhD), College of Pharmacy (S M Aleidi PhD, H Y Alniss PhD, Prof M H Semreen PhD), College of Medicine (Prof A Amin PhD, Prof M A Saleh PhD), Center of Excellence of Cancer Research (Prof R Bendardaf PhD), Department of Basic Biomedical Sciences (Prof Y Bustanji PhD), Department of Basic Medical Sciences (M A Eladl PhD, Prof W El-Huneidi PhD), Sharjah Institute for Medical Research (N M Elemam PhD), Basic Medical Sciences Department (A B Elmoselhi PhD), Research Institute of Medical & Health Sciences (A B Elmoselhi PhD, Prof M H Semreen PhD), College of Health Sciences (S V Gopalani PhD), Department of Finance and Economics (Prof M Hussain PhD), Department of Pharmacy Practice and Pharmacotherapeutics (Prof H A Omar PhD), Department of Clinical Sciences (Prof M M Ramadan PhD), Sharjah Institute of Medical Sciences (F Saheb Sharif-Askari PhD), Department of Medicinal Chemistry (S S M Soliman PhD), Research Institute for Medical and Health Sciences (Prof D Uzun Ozsahin PhD), University of Sharjah, Sharjah, United Arab Emirates (K A Altirkawi MD); Department of Tropical Medicine and Infectious Diseases (S Abd-Elsalam PhD), Tanta University, Tanta, Egypt; Stanford Cancer Institute (S Abdi MD), Department of Medicine (R M Gibson

PhD), Department of Gastroenterology and Hepatology (A Joseph MD), Management Science and Engineering (Y Ling MS), Division of Pediatric Hospital Medicine (R P Mediratta MD), Department of Biomedical Data Science (S Park MD), Department of Radiology (S Ramasamy MD), School of Medicine (J Zhou PhD), Stanford University, Stanford, CA, USA; The Institute of Pharmaceutical Sciences (TIPS) (Prof M Abdollahi PhD, S Hassani PhD), School of Pharmacy (Prof M Abdollahi PhD), Research Center for Immunodeficiencies (H Abolhassani PhD, Prof N Rezaei PhD), Urology Research Center (Prof S Aghamir PhD, A Mohammadi MD), School of Medicine (N S Ahmadi MD, A Azarboo MD, A Behnoush BS, H Farrokhpour MD, H Karimi MD, N Kazemi Rad MD, S Khanmohammadi MD, M Morovvati MD), Digestive Diseases Research Institute (A Anoushiravani MD, Prof R Malekzadeh MD, A Mohamadkhani PhD, S G Sepanlou MD), Department of Toxicology and Pharmacology (M Ataei PharmD), Department of Health Information Management (S Ayyoubzadeh PhD), Rheumatology Research Center (A Azizan PhD), School of Public Health (N Bahmanziari PhD, A Sheidaei PhD), Sina Trauma and Surgery Research Center (V Baigi PhD, M Hassan Zadeh Tabatabaei MD, M Jalloh MD, Prof V Rahimi-Movaghar MD, Z Ramezani MD, Prof P Salamati MD, S Shool MD), Department of Epidemiology and Biostatistics (V Baigi PhD, M Mansournia PhD, E Sanjari PhD), Dental Research Center (M Banakar PhD), Non-communicable Diseases Research Center (M Bastan MD, S Ghamari MD, M Malekpour MD, M Rashidi MD, N Rezaei MD, N Rezaei PhD), Pastor Institute (M Bayat MD), Department of Public Health (S Bordbar MD), Department of Neurosurgery (P Delbari MD), Department of Radiology (R Elahi MD, A Teymouri MD), Multiple Sclerosis Research Center (S Eskandarieh PhD), Pediatric Infectious Disease Research Center (M Farahmand PhD), Dentistry Research Institute (F Farshad DDS), Obesity and Eating Habits Research Center (F Farsi MD), Department of Environmental Health Engineering (M Fazlzadeh PhD), Neurology Department (M Ghasemi MD), Institute of Radiology and Radiological Sciences (A Hashem Zadeh MD), Iranian Tissue Bank and Research Center (K Jazi MD), Children's Medical Center (Prof F Kompani MD), Department of Ophthalmology (A Mahmoudi MD), Tehran Heart Center, Cardiovascular Diseases Research Institute (E Mehrabi Nasab MD, A Mousavi MD, S Shojaei MD), Urology Department (A Mohammadi MD), Department of Physiotherapy (Prof N Nakhostin Ansari PhD), Research Center for War-affected People (Prof N Nakhostin Ansari PhD), Family Health Research Institute (M Noorafrooz MD), Digestive Diseases Research Center (P Parhizkar Roudsari MD), Cardiac Research Center (P Parhizkar Roudsari MD), Iranian National Center for Addiction Studies (Prof A Rahimi-Movaghar MD), Department of Infectious Diseases and Tropical Medicine (E Rajabi MD), Gene Therapy Center (M Rayati MD), Endocrinology and Metabolism Research Institute (N Rezaei PhD), Epidemiology and Biostatistics (E Sanjari PhD), Department of Medical Education (A Sedigh PhD), Cancer Research Center (R Shirkoohi PhD), Cancer Biology Research Center (R Shirkoohi PhD), Student Scientific Research Center (S Shojaei MD), Department of Pathology (Prof S Tavangar MD), Tehran University of Medical Sciences, Tehran, Iran (A Azizan PhD, M Mahalleh MD, G Valizadeh PhD); Department of Medicine (Prof M Abdoun PhD), University of Setif Algeria, Sétif, Algeria; Department of Health, Sétif, Algeria (Prof M Abdoun PhD); Faculty of Veterinary Medicine (A Abdous MD), Young Researchers and Elite Club (M Jokar DVM), Islamic Azad University, Karaj, Iran; Komar University of Science and Technology, Sulaymaniyah, Iraq (J M Abdul Aziz MSc); Baxshin Hospital (J M Abdul Aziz MSc), Baxshin Research Center, Sulaymaniyah, Iraq; Community and Maternity Nursing Unit (D M Abdulah MPH), Department of Pathology and Microbiology (M S Ahmed PhD), Basic Sciences Department (B A Zaman PhD), University of Duhok, Duhok, Iraq; National Institute of Epidemiology (R Abdulkader PhD), Indian Council of Medical Research, Chennai, India; Department of Population and Global Health (A Abdullahi PhD), Department of Radiology (S Abohashem MPH), Center for Primary Care (S Basu PhD), Department of Epidemiology (S

Carr MS), Division of Cardiovascular Medicine (G Chi MD), Nutrition Department (G Dalla Costa MD), T. H. Chan School of Public Health (S B Kankam MD, M Tuffour Amirikah MD), Department of Ophthalmology (Prof J H Kempen PhD, M Shayan MD), Department of Medicine (M Kokkorakis BSc), Department of Global Health and Population (Z Li PhD), Department of Health Policy and Oral Epidemiology (Z S Natto DrPH), Department of Global Health and Social Medicine (S Onie PhD), Cardiovascular Division (J W Ostrominski MD), Department of Physical Medicine and Rehabilitation (K Pacheco-Barrios MD), T.H. Chan School of Public Health (P M S Pradhan MD), Division of Global Health Equity (P Rohloff MD), Joslin Diabetes Center (S Tye PhD), Department of Social and Behavioral Sciences (W Xu MPH), Harvard University, Boston, MA, USA; Department of Physiotherapy (A Abdullahi PhD, A W Awotidebe PhD, J S Usman PhD), Department of Anatomy (L H Adamu PhD), Department of Clinical Pharmacy and Pharmacy Practice (U Hamza MPH), Department of Nursing Science (M Ladan PhD, U Yunusa PhD), Department of Haematology (A A Yusuf MD), Bayero University Kano, Kano, Nigeria; Department of Physiotherapy (A Abdullahi PhD), Federal University Wukari, Wukari, Nigeria; Department of Research (T Abdul-Rahman MD), Toufik's World Medical Association, Antonova 10, Ukraine; Department of General Surgery and Clinical Anatomy (K Abdykerimova MD), Department of Public Health (N Aryntayeva MSPH, A Kuttybayev MSc), Atchabar Scientific Research Institute (B Assembekov PhD), Atchabarov Scientific-Research Institute of Fundamental and Applied Medicine (D Davletov MD, A Zhumagaliuly MD), Population Health Research Center (Prof K Davletov PhD), Director of the Scientific and Technological Park (I R Fakhradiyev PhD), Science and Technology Park (A Ibrayeva PhD), Department of Urology (Y Ismoldayev PhD), Department of General Medical Practice No. 2 (Prof S Kamenova DMedSc), Scientific and Educational Center for Neurology and Applied Neuroscience (A Kondybayeva PhD), Research and Publication Activity Division (M Kulimbet MSc), Science Department (A Shamsutdinova MD), Department of Medicine (S Tanabayeva PhD), Department of Prosthetic Dentistry (M Tleshev MSc), Kazakh National Medical University, Almaty, Kazakhstan; Department of Epidemiology and Biostatistics (H Abebe Getahun MSc), Department of Health Systems and Policy (M B Alemu MSc), Department of Medical Biochemistry (T A Dejenie MSc), Department of Health Promotion and Health Behavior (H Eshetu MPH), Department of Pharmacology (Z D Kifle MSc), Department of Internal Medicine (E Melese MD), School of Midwifery (B Mengistie MSc), School of Nursing (H B Netsere MSc), Department of Environmental and Occupational Health and Safety (A H Tesfaye MPH), Department of Pharmacy (M Worku MSc), University of Gondar, Gondar, Ethiopia; Department of Neurosurgery (A Abedi MD), Keck School of Medicine (A Abedi MD, M Athari MD), Department of Biological Sciences (N Feter PhD), Department of Radiology (M Fotouhi MD), University of Southern California, Los Angeles, CA, USA; Department of Emergency Medicine (A Abedi MD), Department of Food Safety and Hygiene (M Aminzare PhD), School of Medicine (M Ashrafi MD, H Nasiri MD), Department of Immunology (S Athari PhD), Department of Critical Care and Emergency Nursing (N Hanifi PhD), Zanjan University of Medical Sciences, Zanjan, Iran; School of Pharmacy (A Abejew MSc, T T Alemayehu MSc), Department of Midwifery (A Abie MSc, B A Alemayehu MSc, F Alemnew MSc, W F Balcha MSc, A T Nega MSc, F W Sendeku MSc, A A Tesfu MSc, T H Wassie MSc), Department of Emergency and Critical Care Nursing (O Adal MSc, A G Belayneh MSc, Y M Wubie MPH), College of Medicine and Health Sciences (M M Adane PhD, D G Demsie MSc, K Y Gete MD, Y Guracho PhD, T H Jember PhD, H B Netsere MSc), Department of Clinical Pharmacy (Z Addisu MSc), School of Veterinary Medicine (S M Asefa MSc), Department Nutrition and Dietetics (M T Bayih MSc), Department of Public Health (M Belayneh PhD), Department of Nursing (A Y Berhie MSc), Department of Environmental Health (T S Bunare MPH), Department of Physiology (D Demeke MSc), Department of Nutrition and Dietetics (H A Derseh MPH), Department of Psychiatry (T E

Dinkayehu MSc), Department of Adult Health Nursing (D Endeshaw MSc, E A Mengistie MSc), Department of Epidemiology and Biostatistics (B E Feleke MPH), Department of Health Informatics (H A Guadie MPH), Department of Medical Microbiology (A D Habteyohannes PhD), Department of Pediatrics and Child Health Nursing (S Mulatu MSc), Department of Health Promotion and Behavioral Science (Z A Yigzaw MPH), Department of Pharmacy (M Yismaw MSc), Department of Pharmacology (Y E Yismaw MSc), Bahir Dar University, Bahir Dar, Ethiopia; Postgraduate Department (Prof R Abeldaño Zuñiga PhD), University of Sierra Sur, Miahuatlan de Porfirio Diaz, Mexico; Yhteiskuntadatatieteen keskus (Centre for Social Data Science) (Prof R Abeldaño Zuñiga PhD), Department of Public Health (Prof M Kivimäki PhD, Prof T Lallukka PhD), University of Helsinki, Helsinki, Finland (T J Meretoja MD); Department of Botany (E S Abhilash PhD), Sree Narayana Guru College Chelannur, Kozhikode, India; Nuffield Department of Population Health (S Abid MSc, A O Ajose MBBS, D A Bennett PhD), Oxford Vaccine Group (O Akeju MPH), Nuffield Department of Surgical Sciences (O Almidani MSc), Oxford Centre for Global Health Research (C Dolecek PhD), Nuffield Department of Orthopaedics, Rheumatology, and Musculoskeletal Sciences (S Graham PhD), Big Data Institute (Z Guo PhD), Nuffield Department of Medicine (Prof R J Maude PhD), Department of Psychiatry (Prof C R J Newton MD, V Suresh MBBS), Centre for Global Epilepsy (M Romoli MD), University of Oxford, Oxford, UK; National Heart Foundation Hospital and Research Institute, Dhaka, Bangladesh (S Abid MSc); Department of Biomedical Sciences (S Abidi PhD), Nazarbayev University School of Medicine, Astana, Kazakhstan; Department of Internal Medicine (O O Abiodun FWACP), Federal Medical Centre, Abuja, Nigeria; Department of Community Medicine (Prof O Abiodun MPH), Babcock University, Ilishan-Remo, Nigeria; Department of Family and Community Health (R G Aboagye MPH), School of Basic and Biomedical Sciences (D Adedia PhD), Department of Epidemiology and Biostatistics (L A Adzigbli BSc, R K Dowou MPhil), Institute of Health Research (M Immurana PhD), Department of Nursing (F K Nyande PhD), Department of Microbiology and Immunology (Prof V N Orish PhD), University of Health and Allied Sciences (E Osei PhD), University of Health and Allied Sciences, Ho, Ghana; School of Population Health (R G Aboagye MPH, Z Dai PhD, X Feng PhD, V Keshri PhD, A E Peden PhD, Prof B A Saddik PhD, X Xu PhD), Centre for Social Research in Health (I Y Addo PhD, S R Okeke PhD), St George and Sutherland Clinical School (H Akbarialiabad MD), Transport and Road Safety (TARS) Research Centre (S Boufous PhD), Faculty of Medicine (J Brown PhD), National Drug and Alcohol Research Centre (Prof L Degenhardt PhD), The George Institute for Global health (F Haghdoost PhD), Centre for Healthy Brain Ageing (M J Lennon PhD, L Mewton PhD, S Röhr PhD, A S Vella PhD, R Visontay BA), International Centre for Future Health Systems (J Lin PhD), School of Medicine (Prof P K Maulik PhD), Discipline of Psychiatry and Mental Health (Prof P B Mitchell MD), School of Optometry and Vision Science (Prof K Pesudovs PhD, Prof S Resnikoff MD), Centre for Healthy Brain Ageing (CHeBA) (S Röhr PhD), The George Institute for Global Health (P Ye PhD), University of New South Wales, Sydney, NSW, Australia; Department of Medical Biochemistry and Biophysics (H Abolhassani PhD), Department of Neurobiology, Care Sciences and Society (Prof J Ärnlöv PhD, B Bizzozero-Peroni PhD), Department of Medical Epidemiology and Biostatistics (Prof J J Carrero PhD), Department of Physiology and Pharmacology (C R Cederroth PhD), Department of Neurobiology, Care Sciences, and Society (S Fereshtehnejad PhD), Karolinska Institutet Campus Solna (A Javanmardi MD), Department of Molecular Medicine and Surgery (Prof J H Kauppila MD), Department of Global Public Health (Prof L Laflamme PhD), Karolinska Institute, Stockholm, Sweden; Department of Sport, Exercise and Rehabilitation (U S Abonie PhD), Northumbria University, Newcastle, UK; Zoology Department (N M Abourashed PhD), Benha University, Benha, Egypt; Department of Physical Pharmacy and Pharmacokinetics (M Abouzid PharmD), Chair and Department of Medical Microbiology (Prof T M

Karpiński DDS), Poznan University of Medical Sciences, Poznan, Poland; Department of Cardiovascular Disease (D Abramov MD), Loma Linda University Medical Center, Loma Linda, CA, USA; Department of Pediatric Dentistry (Prof L Abreu PhD), Department of Maternal-Child Nursing and Public Health (Prof D C Malta PhD, Prof F P Matozinhos PhD, Prof A C Micheletti Gomide Nogueira de Sá MSc, E J S Prates BS), Department of Clinical Medicine (Prof B R Nascimento PhD), Clinical Hospital (Prof B R Nascimento PhD), Department of Applied Nursing (Prof M O Pereira PhD), Maternal and Child Nursing Department (L P Pinheiro Rocha MSc), Department of Internal Medicine (Prof A P Ribeiro MD), Centre of Telehealth (Prof A P Ribeiro MD), Department of Infectious Diseases and Tropical Medicine (B P Sao Jose PhD), Faculty of Dentistry (Prof G C B D Silva PhD), Federal University of Minas Gerais, Belo Horizonte, Brazil; Clinical Pharmacy and Therapeutics Department (Prof R K Abu Farha PhD), Applied Science Research Center (A B Al-Tammemi MPH), Department of Clinical Nutrition and Dietetics (Prof M E M Faris PhD), Faculty of Nursing (E H Othman PhD), Applied Science Research Centre (R M Zrieq PhD), Applied Science Private University, Amman, Jordan; Community Health Nursing Department (F H A Abuadas PhD), Preventive Dentistry Department (Prof M K Alam PhD), College of Medicine (R Basri PhD), Jouf University, Sakaka, Saudi Arabia; Graduate School of Public Health (A K Abubakar MPH, H Jamil MD), St. Luke's International University, Tokyo, Japan; Division of Population Data Science (A K Abubakar MPH), Division of Population Data Science, Institute for Cancer Control (H Jamil MD), National Cancer Center, Tokyo, Japan; Department of Pharmacology and Toxicology (B Abubakar PhD), Department of Pharmaceutics and Pharmaceutical Technology (N Aminu PhD), Department of Sociology (Prof J Amzat PhD), Department of Veterinary Microbiology (M B Bello PhD), Department of Veterinary Public Health and Preventive Medicine (B Garba PhD, A Shittu MSc), Medical Microbiology Department (Prof Y Mohammed FWACP), Usmanu Danfodiyo University, Sokoto, Sokoto, Nigeria; Clinical Science Department (Prof M O Folayan PhD), Department of Biochemistry and Nutrition (K Oyebola PhD), Nigerian Institute of Medical Research, Lagos, Nigeria (B Abubakar PhD); Department of Biopharmaceutics and Clinical Pharmacy (Prof E Abu-Gharbieh PhD), College of Pharmacy (Prof S Aburuz PhD), University of Jordan, Amman, Jordan; Maternal and Child Health Nursing (Prof S Abuhammad PhD), Jordan University of Science and Technology, Irbid, Jordan; Medical Research Center (H J Abukhadijah MPH), Department of Pharmacy (D Abushanab MSc), Department of Surgery (A Alansari MD), Surgical Research Section (A R Al-Qudimat MPH, A EL Omri PhD), Nursing & Midwifery Research Department (NMRD) (G Joy MSc, J Kunjavara PhD, A J Nashwan PhD), Corporate Nursing and Midwifery Research Department (K Mannethodi MPH), Department of Geriatric and Long Term Care (B Sathian PhD), Research Department (K Singh PhD), Hematology Section (Prof M A Yassin MD), Hamad Medical Corporation, Doha, Qatar; College of Health Sciences (Prof N M Abu-Rmeileh PhD), College of Nursing (Prof F N Alhalaiqa PhD, W Sami PhD), Rehabilitation Sciences Department (Prof N A Almasri PhD), Department of Pharmaceutical Sciences (Prof K H Alzoubi PhD), Department of Population Medicine (Prof G Babu PhD), Department of Rehabilitation Sciences (O Deegan PhD, S F Kanaan PhD), College of Medicine (Prof Y Kinfu PhD, Prof M A Yassin MD), Qatar University, Doha, Qatar; Birzeit University, Ramallah, Palestine (Prof N M Abu-Rmeileh PhD); Department of Pharmacology and Therapeutics (Prof S Aburuz PhD), Institute of Public Health (Prof L A Ahmed PhD, Prof M Grivna PhD), College of Medicine and Health Sciences (Prof M Z Allouh PhD, J Nauman PhD), Department of Veterinary Medicine (H O Khalifa PhD), Department of Food, Nutrition and Health (Prof S Maqsood PhD), Department of Computer Science and Software Engineering (Prof N Zaki PhD), United Arab Emirates University, Al Ain, United Arab Emirates; Department of Biochemistry (R Achar PhD), Jagadguru Sri Shivarathreeswara University, Mysuru, India; Department of Forensic Medicine and Toxicology (A Acharya MD), Department of General Practice and Emergency

Medicine (J Bhandari MD), Karnali Academy of Health Sciences, Jumla, Nepal; School of Public Health and Preventive Medicine (Prof I N Ackerman PhD, S M Alif PhD, Prof M Asghari-Jafarabadi PhD, P Rai PhD, S Talic PhD), Faculty of Medicine, Nursing, and Health Sciences (S Aslani PhD), Department of Epidemiology and Preventative Medicine (E K Chowdhury PhD), Department of Public Health and Preventive Medicine (H Hailu MPH), Department of Infectious Diseases (M J Loftus MBBS), Department of General Practice (S Melwani PhD), Person-Centered Research (S Shrestha PhD), School of Primary and Allied Health Care (F Sousa PhD), Monash University, Melbourne, VIC, Australia; Department of Clinical Medicine (Prof J M Acuna MD), American University of Antigua, Coolidge, Antigua and Barbuda; FIU Robert Stempel College of Public Health & Social Work (Prof J M Acuna MD), Department of Translational Medicine (N C Barengo PhD), Department of Global Health (B Talukder PhD), Florida International University, Miami, FL, USA; Department of Diagnostic and Interventional Radiology (L C Adams PhD), School of Medicine and Health (F Busch MD), Technical University of Munich, Munich, Germany; Stanford University, Palo Alto, CA, USA (L C Adams PhD); Department of Human Anatomy (L H Adamu PhD), Federal University Dutse, Dutse, Nigeria; School of Medicine (I Y Addo PhD), Translational Research Team (N A Adegoke PhD), Faculty of Medicine and Health (D B Anderson PhD, R Cairns PhD, M M Kamal MPH), Sydney Musculoskeletal Health (D B Anderson PhD, S Mathieson PhD), School of Architecture, Design, and Planning (Prof T Astell-Burt PhD), Charles Perkins Centre (R Biswas PhD), School of Public Health (Prof F M Blyth PhD, Prof T R Driscoll PhD), School of Health Science (A Carvalho-e-Silva PhD), School of Pharmacy and Charles Perkins Centre (Z Dai PhD), Institute for Musculoskeletal Health (M Jamshidi PhD), Asbestos and Dust Diseases Research Institute (J Leigh MD), University of Sydney (S Mathieson PhD), Central Clinical School, Faculty of Medicine and Health (S Mitra PhD), School of Chemical & Biomolecular Engineering (E A Noman PhD), Westmead Applied Research Center (E T O'Hagan PhD), Population Oral Health, Sydney Dental School (P Veginadu PhD), The Matilda Centre for Research in Mental Health and Substance Use (R Visontay BA), University of Sydney, Sydney, NSW, Australia (S R Okeke PhD); Department of Health Promotion, Education and Behavior (O A Adeagbo PhD), Department of Epidemiology and Biostatistics (A M Alfalki MPH, R Gupta MPH), University of South Carolina, Columbia, SC, USA; Department of Public Health (O A Adeagbo PhD), School of Nursing and Public Health (A W Awotidebe PhD), Discipline of Public Health Medicine (T G Ginindza PhD), Department of Public Health Medicine (S N Shongwe MPH), University of KwaZulu-Natal, Durban, South Africa; Department of Microbiology (T A Adebisi BSc), Ladoke Akintola University, Osogbo, Nigeria; NMC Healthcare (T A Adebisi BSc), Independent Consultant, Sharjah, United Arab Emirates; Department of Sociology (I A Adedeji PhD), Olabisi Onabanjo University, Ago-Iwoye, Nigeria; Department of Immunology (K A Adedokun MSc), Roswell Park Comprehensive Cancer Center, Buffalo, NY, USA; Graduate Program Division (K A Adedokun MSc), University at Buffalo, Buffalo, NY, USA; Department of Medical Rehabilitation (Prof R A Adedoyin PhD), Department of Child Dental Health (Prof M O Folayan PhD), Obafemi Awolowo University, Ile-Ife, Nigeria; Department of Pediatrics (O E Adegbile MD), East Tennessee State University, Johnson City, TN, USA; Center for Cardiovascular Risk Research (O E Adegbile MD), Center for Cardiovascular Risk Research, Johnson City, TN, USA; Menzies School of Health Research (Prof O A Adegboye PhD), Charles Darwin University, Darwin, NT, Australia; Melanoma Institute Australia (N A Adegoke PhD), The University of Sydney, Sydney, NSW, Australia; Department of Family Medicine (O T Adeleke MD), Bowen University Hospital (D E Ajala BSc), College of Health Sciences (O I Olabisi PhD), Department of Nursing Science (M I Olatubi PhD), Bowen University, Iwo, Nigeria; Department of Family Medicine (O T Adeleke MD), Bowen University Teaching Hospital, Ogbomoso, Nigeria; Department of Microbiology (I A Adesina PhD, O O Bello PhD, T C Ekundayo PhD), Department

of Pharmacology and Therapeutics (T E Adeyeoluwa PhD), Department of Physiology (O I Adeyomoye PhD, V O Emojevwe PhD), Department of Environmental and Occupational Health (B S Anuoluwa MPH), Mathematical and Computer Sciences (O A Okunlola MSc, O Peter PhD), Department of Biosciences and Biotechnology (O T Oyeyemi PhD, A J Udoakang PhD), University of Medical Sciences, Ondo, Ondo, Nigeria; Slum and Rural Health Initiative Research Academy (M A Adesina BPT), Slum and Rural Health Initiative, Ibadan, Nigeria; Department of Physiotherapy (M A Adesina BPT), Department of Educational Counselling and Developmental Psychology (H O Adewuyi PhD), Department of Veterinary Medicine (T E Adeyeoluwa PhD), Department of Epidemiology and Medical Statistics (R F Afolabi PhD, A F Fagbamigbe PhD), Department of Veterinary Public Health and Preventive Medicine (O A Ogundijo MSc), Department of Health Promotion and Education (A Ogunkoya MPH), Counselling and Human Development Studies (D O Okeke-Obayemi BSc), College of Medicine (A P Okekunle PhD, O I Olabisi PhD), Department of Medicine (O V Olalusi MD, Prof M O Owolabi DrM), University of Ibadan, Ibadan, Nigeria; Department of Educational Psychology (H O Adewuyi PhD), Department of Sociology (Prof J Amzat PhD), Department of Education Leadership and Management (J O Okunlola PhD), University of Johannesburg, Johannesburg, South Africa; Department of Community Medicine (Prof K Adhikari PhD), Tribhuvan University, Bharatpur, Nepal; Public Health Section (Prof K Adhikari PhD), Himalayan Environment and Public Health Network (HEPHN), Chitwan, Nepal; Department of Fisheries and Marine Bioscience (R K Adhikary PhD), Jashore University of Science and Technology, Jashore, Bangladesh; Research School of Population Health (R K Adhikary PhD), School of Medicine and Psychology (D Ahmad PhD), National Centre for Epidemiology and Population Health (R A Burns PhD), Australian National University, Canberra, ACT, Australia; Apollo Institute Of Medical Sciences & Research Chittoor (Prof U Adiga PhD), Apollo Hospital, Chittoor, India; Department of Public Health (Q Adnani PhD), Universitas Padjadjaran (Padjadjaran University), Bandung, Indonesia; Department of Health Administration and Education (P O Adoma PhD), University of Education Winneba, Winneba, Ghana; National Institute on Minority Health and Health Disparities (D Adzrago PhD), Department of Endocrinology, Diabetes and Metabolism (G P Thota MD), National Institutes of Health, Bethesda, MD, USA (A Grover MD); School of Public Health (D Adzrago PhD), University of Texas Health Science Center at Houston, Houston, TX, USA; Department of Public Health and Preventive Medicine (G Affinito PhD), Department of Public Health (C Fiorilla MD, R Palladino MD, M Sorrentino MD), University of Naples "Federico II", Naples, Italy; Department of Surgery (A M Afifi MD), University of Toledo, Toledo, OH, USA; Technical Services Directorate (A A Afolabi MPH), MSI Nigeria Reproductive Choices, Abuja, Nigeria; Department of Community Medicine (Prof S Afzal PhD), King Edward Memorial Hospital, Lahore, Pakistan; Department of Public Health (Prof S Afzal PhD), Public Health Institute, Lahore, Pakistan; Department of Public Health (G B Agafari PhD, M H Nunemo MPH), Department of Epidemiology (B H Jena PhD), Wachemo University, Hossana, Ethiopia; Department of New Initiatives (Prof S B Agampodi MD), International Vaccine Institute, Seoul, South Korea; College of Health Sciences and Medicine (T Ageru PhD), Wolaita Sodo University, Wolaita Sodo, Ethiopia; MM College of Pharmacy (N Aggarwal PhD), Maharishi Markandeshwar (Deemed to be University), Ambala, India; Department of Orthopedic Surgery and Sports Medicine (M Aghaalikhani MD), Boston Children's Hospital, Boston, MA, USA; Department of Neurosurgery (S Aghajanian MD), Research Center for Health, Safety and Environment (Prof L Salehi PhD), School of Medicine (M Shams-Beyranvand MSc), Alborz University of Medical Sciences, Karaj, Iran; Neuroscience Research Center (S Aghajanian MD), Health Management and Economics Research Center (J Arabloo PhD), School of Medicine (M Bastan MD, P Panahi MD), Medical Ethics Department (S Biroudian PhD), Department of Medical Laboratory Sciences (F Dorostkar PhD), Iran University of Medical Sciences (F Eghbali MD), Department of Medicine (M

Fotouhi MD), Department of Cardiology (A Ghaffari Jolfayi MD), Department of Ophthalmology (H Hasani MD), Department of Biostatistics (M Imani MSc), Department of Surgery (A Jaliliyan MD), Endocrine Research Center (A Karimi Behnagh MD), Department of Echocardiography (A Karimi Behnagh MD), Eye Research Center (H Kasraei MD), Department of Obstetrics & Gynecology (P Khalili MD), Bone and Joint Reconstruction Research Center (M Khonji MD), Gastrointestinal and Liver Diseases Research Center (Prof M Moradi-Lakeh MD), Preventive Medicine and Public Health Research Center (Prof M Moradi-Lakeh MD), Antimicrobial Resistance Research Center (K Mozahheb Yousefi MD), Hazrat-e Rasool General Hospital (K Mozahheb Yousefi MD), Physiology Research Center (H Pazoki Toroudi PhD), Department of Physiology (H Pazoki Toroudi PhD), Center for Technology and Innovation in Cardiovascular Informatics (S Shool MD), The Five Senses Health Institute (F Taghizadeh-Hesary MD), Iran University of Medical Sciences, Tehran, Iran; Health Research and Innovation Sciences Center (C Agostinis Sobrinho PhD), Health Research and Innovation Science Centre (R C D Espírito Santo PhD), Klaipeda University, Klaipeda, Lithuania; SPRINT Sport Physical Activity and Health Research & Innovation Center (C Agostinis Sobrinho PhD), Sport Physical Activity and Health Research & Innovation Center (SPRINT) (Prof L M L R Silva PhD), Polytechnic Institute of Guarda, Guarda, Portugal; Trivedi School of Biosciences (Prof A Agrawal PhD), Ashoka University, Sonipat, India; Department of Public Health Sciences (W Agyemang-Duah PhD), Queen's University, Kingston, ON, Canada; Rajaie Trauma Research Center (M Ahadi MD), Health Policy Research Center (B Amidi MD, M Banakar PhD, H Kasraei MD, Y Sarikhani PhD), Trauma Research Center (S Ayatizadeh MD, M Yadollahi MD), Department of Nursing (A Azargoonjahromi BSc), Healthcare Management Department (Prof M Bahrami PhD), Research Center for Traditional Medicine and History of Medicine (Prof M Hashempur PhD), Poostchi Ophthalmology Research Center (M Heydari PhD), Shiraz Neuroscience Research Center (M Jafarinia PhD), Health Policy Research Center (R Khademi MD), Non-communicable Disease Research Center (Prof R Malekzadeh MD), Amir Oncology Hospital (E Meftah MD), Department of Occupational Health and Safety Engineering (R Pourbabaki PhD), Department of Health Services Management (Prof R Ravangard PhD), Department of Biostatistics (E Sadeghi PhD), Basic Sciences in Infectious Diseases Research Center (R Tavakoli Oliaee PhD), Faculty of Medicine (S Vahdati MD), Shiraz University of Medical Sciences, Shiraz, Iran (S Mousavi Kiasary DVM); School of Public Health (B O Ahinkorah MPhil, G K Wirtu PhD), School of Nursing and Midwifery (M Chutiyami PhD), School of Life Sciences (G Liu PhD), Discipline of Physiotherapy (P Stubbs PhD), School of Biomedical Engineering (N Tran MD), Faculty of Health (T E Wonde MPH), University of Technology Sydney, Sydney, NSW, Australia; College of Medicine (A Ahmad PhD, Prof G Mustafa MD, M Tabish MPharm, H Ullah FCPS), Shaqra University, Shaqra, Saudi Arabia; Health Research Institute (D Ahmad PhD, Prof N Bagheri PhD), University of Canberra, Canberra, NSW, Australia; Biological Production Unit National Institute of Health Islamabad Pakistan (F Ahmad PhD), Research and Development Coordination (I Rafique PhD), National Institute of Health, Islamabad, Pakistan; World Health Organisation, Islamabad, Pakistan (F Ahmad PhD); Department of Research (Prof K Ahmad PhD), King Khaled Eye Specialist Hospital & Research Center, Riyadh, Saudi Arabia; Department of Health Informatics (K Ahmad PhD), Qassim University, Buraidha, Saudi Arabia; School of Nursing (Prof M M Ahmad PhD), The University of Jordan School of Medicine (Prof M A Al-Abbadi MD), Faculty of Medicine (R Al-Asmar BSc), School of Pharmacy (S M Aleidi PhD, Prof Y Bustanji PhD), Department of Diagnostic Radiology and Nuclear Medicine (Prof A Al-Ibraheem MD), The School of Medicine (M Al-Iede MD), Department of Radiology and Nuclear Medicine (Prof M E Juweid MD), Department of Mathematics (Prof S Momani PhD), Department of Pathology, Microbiology and Forensic Medicine (M Sallam PhD), Department of Clinical Laboratories and Forensic Medicine (M Sallam PhD), Department of

Movement Sciences and Sports Training (K Trabelsi PhD), The University of Jordan, Amman, Jordan (Prof L A Dardas PhD); Department of Clinical Pharmacy (R Ahmad PhD, M Said PhD), Advanced Medical & Dental Institute (M Aziz PhD), Universiti Sains Malaysia, Penang, Malaysia; Department of Pharmacy Practice (R Ahmad PhD), The Islamia University of Bahawalpur, Bahawalpur, Pakistan; Department of Health and Biological Sciences (S Ahmad PhD), Abasyn University, Peshawar, Pakistan; Department of Natural Sciences (S Ahmad PhD), Gilbert and Rose-Marie Chagoury School of Medicine (Prof L Roever PhD), Lebanese American University, Beirut, Lebanon; School of Public Health (T Ahmad PhD), Department of Epidemiology and Biostatistics (J Zhao MD), Zhejiang University, Hangzhou, China; Department of Community Health Sciences (T Ahmad PhD), Sohail University, Karachi, Pakistan; College of Medicine (W Ahmad PhD), University of Cincinnati, Cincinnati, OH, USA; Department of Neuroscience (A Ahmadzade MD), Faculty of Medicine (R Khademi MD, F Shahrahmani MD), Department of Medical Genetics and Molecular Medicine (G Khalili-Tanha PhD), Clinical Research Development Unit (N Morovatdar MD), Biotechnology Research Center (Prof A Sahebkar PhD), Department of Medicine (A Yarahmadi PhD), Mashhad University of Medical Sciences, Mashhad, Iran; Department of Veterinary Microbiology (A O Ahmed PhD), Department of Veterinary Public Health and Preventive Medicine (I A Odetokun PhD), University of Ilorin, Ilorin, Nigeria; Maternal and Child Health Division (MCHD) (A Ahmed MS, S Ahmed MDS, M Al-Zubayer MSc, R Banik MS, L Hossain MPH, S Noor MS, M Patwary MSc, N Saha MSc, A Sayeed MSc), Nutrition and Clinical Services Division (M Tariqujjaman MSc), International Centre for Diarrhoeal Disease Research, Bangladesh, Dhaka, Bangladesh; Department of Women's and Children's Health (A Ahmed MS), Department of Medical Sciences (D Lindholm MD, Prof J Sundström PhD), Uppsala University, Uppsala, Sweden; Institute of Endemic Diseases (A Ahmed MSc), Department of Oral Rehabilitation (N T Hashim PhD), Faculty of Medicine (K A H Mohamed Ahmed MD), Unit of Basic Medical Sciences (E E Siddig MD), University of Khartoum, Khartoum, Sudan; Swiss Tropical and Public Health Institute (A Ahmed MSc), Department of Ophthalmology (Prof Z Gatzioufas PhD), University of Basel, Basel, Switzerland; Medical Laboratory Science Department (G S Ahmed MSc, H M Rahim MSc), University of Human Development, Sulaymaniyah, Iraq; Department of Biosciences (H Ahmed PhD), COMSATS Institute of Information Technology, Islamabad, Pakistan; Manipal College of Dental Sciences, Mangalore (Prof J Ahmed MDS), Department of General Medicine (A N Bhat MD, J Jeganathan MD), Department of Community Medicine (N Joseph MD, N Kumar MD, P Mithra MD, R Thapar MD), Kasturba Medical College (G A Menezes PhD), Department of Forensic Medicine and Toxicology (Prof J Padubidri MD, Prof P Rastogi MD), Manipal Academy of Higher Education, Mangalore, India; College of Nursing (M S Ahmed MSc), Majmaah University, Al Majmaah, Saudi Arabia; College of Medicine and Public Health (M B Ahmed PhD, B Kaambwa PhD, G R Naik PhD), Flinders Health and Medical Research Institute (N B Bulamu PhD), Health Economics Unit (B Kaambwa PhD), Department of Nursing and Health Sciences (S Shorofi PhD), Flinders University, Adelaide, SA, Australia; Faculty of Public Health (M B Ahmed PhD), Department of Health Behavior and Society (L A A Ayana MPH), Department of Surgery (N S Bayleyegn MD), Institute of Health (Prof T Malik PhD), Department of Epidemiology (D Shiferaw MPH), Jimma University, Jimma, Ethiopia; Department of Medicine (M Ahmed MBBS), Rawalpindi Medical University, Rawalpindi, Pakistan; Department of Psychology (O Ahmed MSc), University of Chittagong, Chattogram, Bangladesh; Department of Public Health Epidemiology (S M Ahmed MSc), Department of Pediatrics and Child Health Nursing (G B Mulu MSc), Debre Berhan University, Debre Berhan, Ethiopia; Menelik II Medical and Health Science College (S M Ahmed MSc), EpiMetrics, Inc., Addis Ababa, Ethiopia; School of Medicine (G Aimagambetova PhD, Y Semenova PhD, K A Yergaliyev DrPH), Department of Biomedical Sciences (M Aljofan PhD, S Bolla PhD), Department of Medicine (J U Almazan PhD), Nazarbayev

University, Astana, Kazakhstan; Clinical Academic Department of Women's Health (G Aimagambetova PhD), NU Medicine, Astana, Kazakhstan; Department of Medical Laboratory Technology (A AJ Jabbar PhD), Erbil Polytechnic University, Erbil, Iraq; Department of Water Engineering (S Akbarifard PhD), Graduate University of Advanced Technology, Kerman, Iran; Department of Physiology (R E Akhigbe PhD), Department of Medicine (A O Shitu MBBS), Ladoke Akintola University, Ogbomoso, Nigeria; School of Veterinary Medicine (O A Akinkuotu PhD, Prof B B Awosile PhD), Texas Tech University, Amarillo, TX, USA; Department of Internal Medicine (K Akinosoglou PhD), Department of Cardiothoracic Surgery (V Leivaditis PhD), University of Patras, Patras, Greece; Department of Internal Medicine and Infectious Diseases (K Akinosoglou PhD), University General Hospital of Patras, Patras, Greece; Department of Cardiology (M Akkaif PhD), School of Public Health (Prof H Kan PhD), Fudan University, Shanghai, China; Department of Management, Policy, and Community Health (S Akkala MPH), School of Public Health (M A M Al Zoubi PhD, H Theyra-Enias MD), Management Policy and Community Health (J A Atta MPH), Department of Epidemiology (L D Chau BA), MD Anderson Cancer Center Department of Plastic Surgery (R Elmorsi MD), University of Texas, Houston, TX, USA; Faculty of Health and Behavioural Sciences (W Akosile PhD), Centre for Sensorimotor Performance (D Anderlini MD), Department of Urology (Prof E Chung MD), School of Public Health (H E Erskine PhD, A J Ferrari PhD, M Islam MSc, A M Mantilla Herrera PhD, J C Maravilla PhD, P A Miller PhD, D F Santomauro PhD, J Shadid BSc, Prof H A Whiteford PhD), School of Health and Rehabilitation Sciences (A Khan PhD, M Rahman MPH), Centre for the Business and Economics of Health (I Koomson PhD, A Pak PhD), School of Dentistry (R Lalloo PhD), UQ Centre for Clinical Research (H L S Lawford PhD), National Centre for Youth Substance Abuse Research (J Leung PhD), Poche Centre for Indigenous Health (Prof A A Mamun PhD), The University of Queensland, Brisbane, QLD, Australia; Department of Infection Prevention & Control (H Akram MD), Baylor Scott & White Health, Frisco, TX, USA; Chicago College of Osteopathic Medicine (A E Akrami BS), Midwestern University, Downers Grove, IL, USA; Feinberg School of Medicine (A E Akrami BS), Department of Microbiology and Immunology (O Ebohon MPH), Medical Scientist Training Program (S Marzouk MA), Department of Medicine (N S Shah MD), Department of Preventive Medicine (M Teramoto MD), Northwestern University, Chicago, IL, USA (M D Szeto MS); Centre for Academic Primary Care (R K Akyea PhD), Institute of Applied Health Research (N Bhala PhD), Division of Ophthalmology & Visual Sciences (Prof G D Panos MD), University of Nottingham, Nottingham, UK; College of Pharmacy and Health Sciences (A Al Amiry MS), Center of Medical and Bio-allied Health Sciences Research (A Al Amiry MS), Nonlinear Dynamics Research Center (NDRC) (Prof S Momani PhD), Center for Medical and Bio-Allied Health Sciences Research (Prof M J Shahwan PhD, A Shamsi PhD), College of pharmacy and Health Science (H Yasin PhD), Ajman University, Ajman, United Arab Emirates (Prof N Hassan PhD); Department of Communicable Diseases (S Al Awaidy MSc), Ministry of Health, Muscat, Oman; Middle East, Eurasia, and Africa Influenza Stakeholders Network, Muscat, Oman (S Al Awaidy MSc); Division of Public Health Sciences (S Al Hasan PhD), Department of Research and Development (Z Al-Aly MD), Department of Surgery (S Azadnajafabad MD, C Wang PhD), School of Public Health (I T Bacha MD), Department of Energy, Environmental, and Chemical Engineering (Prof R V Martin PhD, A van Donkelaar PhD), Brown School (A M Odama MPH), Department of Anesthesiology (S Roy Chowdhury PhD), John T. Milliken Department of Medicine (T Q M Tran MSc), Brown School of Public Health (Y C Wondmeneh MD), Washington University in St. Louis, St. Louis, MO, USA; Fundamentals and Administration Department (Prof O Al Omari PhD), Department of Adult Health and Critical Care (O A M Al Zaabi PhD), Department of Geography (W Ali PhD), Sultan Qaboos University, Muscat, Oman; Department of Community and Mental Health (Prof M Albashtawy PhD), Al Al-Bayt University, Mafraq, Jordan (Prof M Al Qadire PhD);

Department of Internal Medicine (O Al Ta'ani MD), Allegheny Health Network, Pittsburgh, PA, USA; Department of General Education (Prof W A M Al Taie PhD), Hamdan Bin Mohammed Smart University, Dubai, United Arab Emirates; Faculty of Pharmacy (Y Al Thaher PhD), Faculty of Nursing (M M W Atout PhD), Philadelphia University, Amman, Jordan; School of Pharmacy (Y Al Thaher PhD), School of Earth and Environmental Sciences (M Nawaz PhD), Cardiff University, Cardiff, UK; Department of Rehabilitation (Y Al-Ajlouni MD), Montefiore Medical Center, Bronx, NY, USA; Department of Epidemiology (Y Al-Ajlouni MD), Departments of Psychiatry and Epidemiology (Prof M R Phillips MD), Columbia University, New York, NY, USA (D Shan MD); Department of Biology (T A Alalwan PhD), College of Health and Sport Sciences (A G Vaithinathan MSc), University of Bahrain, Zallaq, Bahrain; Clinical Epidemiology Center (Z Al-Aly MD), US Department of Veterans Affairs (VA), St. Louis, MO, USA; Murdoch Business School (K Alam PhD), Murdoch University, Perth, WA, Australia; Department of Bioengineering (M Alam PhD), George Mason University, Fairfax, VA, USA; School of Nursing (R M Al-Amer PhD), Department of Basic Sciences (Z Altaany PhD), Department of Basic Medical Sciences (R A Karasneh PhD, Prof M M Khatatbeh PhD), Faculty of Nursing (H Khatatbeh PhD), Yarmouk University, Irbid, Jordan; School of Nursing and Midwifery (R M Al-Amer PhD), Western Sydney University, Sydney, NSW, Australia; Department of Nursing and Midwifery (A Alamrew MSc), College of Health Science (C Mulugeta MSc), Woldia University, Woldia, Ethiopia; Department of Health Information Management and Technology (Prof T M Alanzi PhD), Deanship of Preparatory Year and Supporting Studies (Prof S El-Ashker PhD), Department of Pathology (Prof R G Menezes MD), Imam Abdulrahman Bin Faisal University, Dammam, Saudi Arabia; Department of Clinical Pharmacy (F Y Al-Ashwal PhD), Al-Ayen Iraqi University, Thi-Qar, Iraq; Department of Clinical Pharmacy and Pharmacy Practice (F Y Al-Ashwal PhD), University of Science and Technology, Sana'a, Yemen; Faculty of Medicine (S Alavi MD), Department of Microbiology (M Saki PhD), Alimentary Tract Research Center (Z Shokati Eshkiki PhD), Nursing Care Research Center in Chronic Diseases (Prof K Zarea PhD), Ahvaz Jundishapur University of Medical Sciences, Ahvaz, Iran; Department of Internal Medicine (A Al-Dalakta MD, A Goyal MD, A Mushtaq MD), Department of Pediatrics (Prof H Aly MD, A E'mar MD), Lerner College of Medicine (M Balkis MD), Department of Cardiovascular Medicine (J Ikram MD, J Rajendran MD), Lerner Research Institute (Prof X Liu PhD), Cleveland Clinic, Cleveland, OH, USA; Division of Pediatric Cardiology (K A Aldawsari MD), University of Colorado, Aurora, CO, USA; Heart Center (K A Aldawsari MD), Liver, Digestive, and Lifestyle Health Research Section (S A Alqahtani MD), Biostatics, Epidemiology, and Science Computing Department (S Yezli PhD), King Faisal Specialist Hospital & Research Center, Riyadh, Saudi Arabia; Division of Gastroenterology and Hepatology (W A Aldhaleei MD), Department of Radiology (G Belge Bilgin MD, C Bilgin MD, F Nugen PhD), Department of Physiology and Biomedical Engineering (Z Khashim PhD, F Pourghazi MD), Department of Cardiovascular Medicine (H Pham MD), Department of Endocrinology (M Salehi MD), Department of Hematology (M S Sra MD), Mayo Clinic, Rochester, MN, USA (D J Ghadimi MD); General Directorate of Research and Studies (M S Aldossary MClinDent), Ministry of Health, Riyadh, Saudi Arabia; Institute of Health Informatics (R W Aldridge PhD), Department of Health Informatics (S Chung PhD), Division of Surgery & Interventional Science (D Feng MD), Institute for Global Health (N Khan PhD), Department of Brain Sciences (Prof M Kivimäki PhD), Division of Medicine (T Oyelade PhD), Division of Psychiatry (L Ren PhD), Department of Population Health Sciences (D Sunkersing PhD), Center for Clinical Microbiology (Prof A Zumla PhD), University College London, London, UK; Department of Health (R Alebshehy PhD), Department for Health (H L Harb MPH), University of Bath, Bath, UK; Department of Bacteriology, Immunology, and Mycology (Prof A M Algammal PhD), Faculty of Veterinary Medicine (M Mabrok PhD), Suez Canal University, Ismailia, Egypt;

Department of Family and Community Medicine (M R Algethami MD, N Z Alshahrani MD), University of Jeddah, Jeddah, Saudi Arabia; Global Centre for Environmental Remediation (A A S Al-Gheethi PhD), University of Newcastle, Newcastle, NSW, Australia; Cooperative Research Centre for Contamination Assessment and Remediation of the Environment, Newcastle, NSW, Australia (A A S Al-Gheethi PhD); College of Medicine and Health Sciences (K Al-Habbal MD), College of Medicine and Health Sciences Academic Programs (Prof W Almahmeed MD), Department of Public Health and Epidemiology (Prof B A Saddik PhD), Khalifa University, Abu Dhabi, United Arab Emirates; Africa Center of Excellence for Mycotoxin and Food Safety, Minna, Nigeria (N B Alhaji PhD); Epidemiology and Population Health Department (Prof S Al-Hajj PhD), American University of Beirut, Beirut, Lebanon; British Columbia Injury Research Prevention Unit (Prof S Al-Hajj PhD), British Columbia Children's Hospital Research Institute, Vancouver, BC, Canada; Department of Health Services and Hospital Administration (M K Al-Hanawi PhD), Health Economics Research Group (M K Al-Hanawi PhD), Department of Respiratory Therapy (M A Althobiani PhD), Respiratory Therapy Unit (M A Althobiani PhD), Pediatric Dentistry Department (K K Baghlaf PhD), Periodontology (S Bamashmous PhD), Rabigh Faculty of Medicine (Prof A Malik PhD), Department of Dental Public Health (Z S Natto DrPH), King Abdulaziz University, Jeddah, Saudi Arabia; Faculty of Applied Health Sciences (Physiotherapy) (A Alhassan Ibrahim PhD), Department of Physiotherapy (S K Sulaiman PhD), Tishk International University, Erbil, Iraq; Faculty of Dentistry (A Alhumaidi DDS), Ibn Al-Nafis University for Medical Sciences, Sana'a, Yemen; Information Science Department (D Alhuwail PhD), Department of Surgery (S K Al-Sabah MD), Kuwait University, Kuwait, Kuwait; Health Informatics Unit and Geohealth Lab (D Alhuwail PhD), Dasman Diabetes Institute, Dasman, Kuwait; Department of Zoology (A Ali PhD), Abdul Wali Khan University Mardan, Mardan, Pakistan; Department of Biotechnology (H M Ali MS), University of Malakand, Chakdara, Pakistan; Department of Statistics and Operations Research (I Ali PhD), Aligarh Muslim University, Aligarh, India; School of Food and Agricultural Sciences (M Ali PhD), Department of Life Sciences (Prof M Umair PhD), University of Management and Technology, Lahore, Pakistan; Department of Pharmacy (M Ali PhD), Mohammed Al-Mana College for Medical Sciences, Dammam, Saudi Arabia; Department of Medical Rehabilitation (Physiotherapy) (M U Ali PhD), Department of Microbiology (M A Isa PhD), University of Maiduguri, Maiduguri, Nigeria; The Nethersole School of Nursing (M U Ali PhD, Y Chong PhD, J Li PhD), Faculty of Medicine (J Huang MD), Department of Orthopaedics and Traumatology (K Lau PhD), The Department of Medicine & Therapeutics (L Shi PhD), School of Public Health and Primary Care (L Yao MSc), Jockey Club School of Public Health and Primary Care (C Zhong PhD), The Chinese University of Hong Kong, Hong Kong, China; Department of Biosciences (R Ali PhD, N Salam PhD), Centre for Interdisciplinary Research in Basic Sciences (CIRBSc) (S Anwar PhD, S Khan MSc, T Mohammad PhD, A Shamsi PhD), Jamia Millia Islamia, New Delhi, India; Centre for Biotechnology and Microbiology (S Ali PhD), University of Swat, Charbagh, Pakistan; Center for Biotechnology and Microbiology (S S Ali PhD, M Suleman PhD), University of Swat, Swat, Pakistan; Biomedical Engineering Department (S Ali MS), Center for Clinical Global Health Education (S R Atre PhD), School of Public Health (A A Berihun MA), Department of Anesthesia and Critical Care Medicine (S Boppana MD), Department of Biostatistics (A Columbus MS), institute of radiology and radiological sciences (A Hashem Zadeh MD), Russell H. Morgan Department of Radiology and Radiological Science (A Kamireddy MD), Department of Public Health (A A Negash MD), Department of Epidemiology (R Olum MD), Department of Health Policy and Management (D Vervoort MD), Department of Psychiatry (E M Zeru MPH), Department of International Health (H Zhang PhD), Johns Hopkins University, Baltimore, MD, USA (E Melese MD); Department of Nuclear Medicine (Prof A Al-Ibraheem MD), King Hussein Cancer Center, Amman, Jordan; Department of

Pathophysiology and Transplantation (G Alicandro PhD), Università degli Studi di Milano (University of Milan), Milan, Italy; Cystic Fibrosis Center (G Alicandro PhD), Pediatric Emergency Department (A La Vecchia MD), Fondazione IRCCS Ospedale Maggiore Policlinico, Milan, Italy; Institute of Health and Wellbeing (S M Alif PhD), Federation University Australia, Melbourne, VIC, Australia; Biomedical Physics Group (M Alipour BSc), University of Hamburg, Hamburg, Germany; Department of Clinical and Community Pharmacy (Prof S W Al-Jabi PhD, Prof S H Zyoud PhD), Department of Pharmacy (F Amer PhD), Department of Physiology, Pharmacology, and Toxicology (Prof R Shawahna PhD), Department of Chemistry (Prof A H Zyoud PhD), An-Najah National University, Nablus, Palestine; School of Physics, Mathematics and Computing (Prof A Al-Jumaily PhD), Centre for Neuromuscular and Neurological Disorders (Perron Institute) (Prof G J Hankey MD), Dental School (O Kujan PhD), School of Biomedical Sciences (Prof L Wang PhD), The University of Western Australia, Perth, WA, Australia; Information and Communication Technology Research Pole (Lab-STICC) (Prof A Al-Jumaily PhD), ENSTA Bretagne, Brest, France; Department of Public Health and Community Medicine (Prof S M Aljunid PhD, Prof C T Sreeramareddy MD), International Medical University, Kuala Lumpur, Malaysia; International Centre for Casemix and Clinical Coding (Prof S M Aljunid PhD), National University of Malaysia, Bandar Tun Razak, Malaysia; College of Life Sciences (Prof A Alkhatib PhD, D Islam PhD), Birmingham City University, Birmingham, UK; Department of Biological Sciences and Chemistry (DBSC) (Prof M H Alkhatib PhD, S A Gilani PhD, Prof J Hussain PhD, Z Naureen PhD), Natural and Medical Sciences Research Center (A Khan PhD, S Shahid MPhil, A Ullah MS), School of Pharmacy (A K Philip PhD), University of Nizwa, Nizwa, Oman; Cardiovascular Division (M Alkhawam MD), University of Alabama, Birmingham, AL, USA; Tabriz University of Medical Sciences (A Allahbakhshian PhD), Department of Immunology (F Jadidi-Niaragh PhD), School of Management and Medical Informatics (L R Kalankesh PhD), Faculty of Nursing and Midwifery (Prof M Mirghafourvand PhD), Social Determinants of Health Research Center (Prof S Mohammad-Alizadeh-Charandabi PhD), Midwifery Department (Prof S Mohammad-Alizadeh-Charandabi PhD), Department of Pharmaceutics (R Mohammadzadeh PharmD), Neurosciences Research Center (NSRC) (R Mosaddeghi Heris MD), Student Research Committee (R Mosaddeghi Heris MD), Department of Geriatric Health (F Naddafi PhD), Infectious and Tropical Diseases Research Center (L Rahbarnia PhD), Iranian Research Center for Evidence-based Medicine (F Sadeghi-Ghyassi PhD), Drug Applied Research Center (H Samadi Kafil PhD), Tabriz University of Medical Sciences, Tabriz, Iran; Faculty of Medicine (Prof M Z Allouh PhD, Prof M S I Alyahya PhD, R M Odat MD), Department of Physical Therapy and Rehabilitation Sciences (Prof M A Alomari PhD), Department of Rehabilitation Sciences and Physical Therapy (Prof M A Alomari PhD), Department of Allied Medical Sciences (A Alrawashdeh PhD), Department of Rehabilitation Sciences (M Al-Wardat PhD), Department of Clinical Pharmacy (Prof K H Alzoubi PhD), Department of Public Health (Prof Y S Khader PhD), Jordan University of Science and Technology, Irbid, Jordan; Faculty of Nursing (W T Almagharbeh PhD), Nursing Faculty (K A Alnawafleh PhD), Prince Fahad bin Sultan Chair for Biomedical Research (S Muthupandian PhD), University of Tabuk, Tabuk, Saudi Arabia (S Muthupandian PhD); Department of Cardiology, Heart, Vascular, and Thoracic Institute (Prof W Almahmeed MD), Department of Urology (O Almidani MSc), Research Department (N Dababo MD), Department of Cardiac Surgery (Prof L Göbölös PhD), Cleveland Clinic Abu Dhabi, Abu Dhabi, United Arab Emirates; Independent Consultant, Amman, Jordan (S Al-Marwani MSc); Department of Parasitology (Prof H M Al-Mekhlafi PhD), Department of Paediatrics (Prof H Ariffin PhD), University of Malaya Medical Centre (Prof H Ariffin PhD), Centre for Population Health (CePH), Department of Social and Preventive Medicine, Faculty of Medicine (M Htay PhD), University of Malaya, Kuala Lumpur, Malaysia; Department of Parasitology (Prof H M Al-Mekhlafi PhD), Sana'a University, Sana'a, Yemen;

Ophthalmology Department (A Almobayed MD), Neurology Department (F Mahmoudi MD), Department of Ophthalmology (S Rouzbahani MD), University of Miami, Miami, FL, USA; School of Public Health (M Alocious Sukumar MPH), SRM Institute of Science and Technology, Chennai, India; Faculty of Nursing (M R Alosta PhD), Department of Nursing (A H Khalifeh PhD), Zarqa University, Zarqa, Jordan; Department of Respiratory Care (J S Alqahtani PhD), Prince Sultan Military College of Health Sciences, Dammam, Saudi Arabia; Division of Gastroenterology and Hepatology (S A Alqahtani MD), Weill Cornell Medicine, New York, NY, USA; American University of the Middle East, Egaila, Kuwait (M R Alqudimat PhD); Department of Nursing (I Alrimawi PhD), Department of Medicine (M Hemmati MD, C J Sabet MA), Georgetown University, Washington, DC, USA; Macro-Fiscal Policy Department (S M Alrousan PhD), Ministry of Finance, Dubai, United Arab Emirates; Jaber Al Ahmad Al Sabah Hospital (S K Al-Sabah MD), Ministry of Health, Kuwait, Kuwait; Department of Emergency Medicine (M A Alsabri MD), Sana'a University, Sanaa, Yemen; Pediatric Emergency Medicine Department (M A Alsabri MD), Department of Biochemistry (M Chakkere Shivamadhu PhD), Drexel Dornsife School of Public Health (E Ezenwankwo MPH), School of Biomedical Engineering, Science and Health Systems (M Noroozi BSc), Drexel University, Philadelphia, PA, USA; Department of Medical Rehabilitation Sciences (M A Alshehri PhD), Department of Clinical Pharmacology and Toxicology (H Alwafi PhD), Department of Microbiology and Parasitology (A Hafiz PhD), Umm Al-Qura University, Makkah, Saudi Arabia; Institute of Molecular Biology and Biotechnology (A Altaf PhD, S J Awan PhD, M Khan PhD, T Maqbool PhD, S Shahid PhD), University College of Medicine & Dentistry (Prof M Arooj PhD), University Institute of Food Science and Technology (S Bashir PhD), University Institute of Radiological Sciences and Medical Imaging Technology (Prof Z Fatima PhD), University Institute of Diet and Nutritional Sciences (A Khalil PhD), University Institute of Public Health (F Malik PhD, S Nargus PhD), Department of Technology (M Muzaffar MBA), Research Centre for Health Sciences (RCHS) (M Muzaffar MBA, S Shahid PhD, M Umar** MBA), Department of Physics (W Shahid PhD), Lahore Business School (M Umar** MBA), Faculty of Sciences (Prof A B Waqar PhD), The University of Lahore, Lahore, Pakistan (M A Riaz Mcom); Faculty of Health Sciences (A Altaf PhD), Equator University of Science and Technology, Uganda, Masaka, Uganda; Research, Policy, and Training Directorate (A B Al-Tammemi MPH), Jordan Center for Disease Control, Amman, Jordan; Department of Specialty Internal Medicine (Prof J A Al-Tawfiq MD), Johns Hopkins Aramco Healthcare, Dhahran, Saudi Arabia; Department of Medicine (Prof J A Al-Tawfiq MD), Indiana University School of Medicine, Indianapolis, IN, USA; Faculty of Health Sciences (J Alvarez-Galvez PhD), University of Cadiz, Cadiz, Spain; School of Sciences (V Alves Carneiro PhD), Life and Health Sciences Research Institute (ICVS) (Prof N Cruz-Martins PhD), University of Minho, Braga, Portugal; Research Group in Health Economics (Prof N Alvis-Guzman PhD), Universidad de Cartagena (University of Cartagena), Cartagena, Colombia; Research Group in Hospital Management and Health Policies (Prof N Alvis-Guzman PhD), Department of Economic Sciences (N J Alvis-Zakzuk MSc), Universidad de la Costa (University of the Coast), Barranquilla, Colombia; National Health Observatory (N J Alvis-Zakzuk MSc), National Institute of Health, Bogota, Colombia; Department of Medical Sciences (Prof Y M Al-Worafi PhD), Azal University for Human Development, Sana'a, Yemen; Department of Clinical Sciences (Prof Y M Al-Worafi PhD), University of Science and Technology of Fujairah, Fujairah, United Arab Emirates; Institute of Public Health (A AlZahmi PhD), United Arab Emirates University, Abu Dhabi, United Arab Emirates; Department of Physiotherapy (H Alzahrani PhD), Taif University, Taif, Saudi Arabia; Evaluation Unit (U A Amaechi BDS), Global Alliance for Vaccines and Immunisations, Geneva, Switzerland; London School of Hygiene and Tropical Medicine (E J Amafah MSc), UCL Institute for Global Health (Prof S Jaffar PhD), University of London, London, UK; Global Health Advocacy Incubator (GHAI) (J Amafah MPH), University of Central

Nicaragua, Washington, DC, USA; Food and Beverages Safety Research Center (M Aman Mohammadi PhD), School of Medicine (A Haghtalab MD, P Mokhtarzadehazar MD, S Pourasghary MD, S Soraneh MD), Maternal and Childhood Obesity Research Center (A Mokari-Yamchi PhD), Urmia University of Medical Sciences, Urmia, Iran; Isfahan Cardiovascular Research Institute, Heart Failure Research Center. (R Amani-Beni MD), Ophthalmology Department (P Bolourinejad MD), Heart Failure Research Center (B Darouei MD, S Najdaghi MD, D Narimani Davani MD), Department of Epidemiology and Biostatistics (Prof M Mansourian PhD), Environment Research Center (M Moazeni PhD), Department of Environmental Health Engineering (M Moazeni PhD), Department of Health Services Management (M Mohseni PhD), Neuroscience Research Center (S Najdaghi MD), Family and Prevention Medicine (R Rouzbahani MD), Department of Clinical Biochemistry (O Vakili PhD), Isfahan University of Medical Sciences, Isfahan, Iran; Department of Biomedical Science (Prof A K Amegah PhD), Department of Management (S E Ankomah PhD, S E Ankomah PhD), Department of Population and Health (J K Oduro PhD), Department of Health, Physical Education and Recreation (J Sarfo PhD), University of Cape Coast, Cape Coast, Ghana; Student Research Committee (B Amidi MD), Lorestan University of Medical Sciences, Khorramabad, Iran; Summer Program (Prof A Amin PhD), Biological Science Division (M Bayat Tork MD), Department of Public Health Sciences (A Jamal BS), Pritzker School of Medicine (Prof H Yao PhD), University of Chicago, Chicago, IL, USA (Prof J Wan PhD); Public Health and Community Medicine Department (Prof T T Amin MD), Department of Neurology (Prof A Hassan MD), Medical Microbiology and Immunology Department (H L Mowafy MD), Cairo University, Cairo, Egypt; Department of Radiology and Radiological Science (A Amindarolzarbi MD), Department of Sociology, Anthropology, and Public Health (C Park DrPH), University of Maryland, Baltimore, MD, USA; Department of Health and Management Sciences (S Amini PhD), Operating Room Department (A Sedigh PhD), Khomein University of Medical Sciences, Khomein, Iran; Gastrointestinal and Liver Diseases Research Center (E Amini-Salehi MD, F Joukar PhD, M Keivanlou MD), Gastrointestinal and Liver Disease Research Center (B Eftekhari MD), Department of Social Medicine and Epidemiology (A Feizkhah MD), Department of Environmental Health Engineering (J Jaafari PhD), Caspian Digestive Disease Research Center (F Joukar PhD), Department of Medical-Surgical Nursing (S Karkhah MSc), Department of Medicine (A Khalili MD), Burn and Regenerative Medicine Research Center (Prof M Mobayen MD), Medical Biotechnology Research Center (M YektaKooshali PhD), Guilan University of Medical Sciences, Rasht, Iran; School of Pharmacy (N Aminu PhD), Department of Internal Medicine (G M Rwegerera MD), University of Botswana, Gaborone, Botswana; Spiritual Health Research Center (S Amiri PhD), Nephrology and Urology Research Center (K Hushmandi PhD), Baqiyatallah University of Medical Sciences, Tehran, Iran; Department of Health and Wellbeing (D A Amugsi PhD), African Population and Health Research Center, Nairobi, Kenya; Center for Biomedical Image Computing & Analytics (F Anagnostakis MD), Penn Medicine (S K Khokhar PhD), Population Studies Center (W Li PhD), Center for Global Health (K Ma DDS), Department of Biostatistics, Epidemiology, and Informatics (J Puvvula PhD), University of Pennsylvania, Philadelphia, PA, USA; Dipartimento di Scienze Mediche e Chirurgiche (M Bergami PhD), Department of Medical and Surgical Sciences (Prof R Bugiardini MD, Prof A F G Cicero PhD, M Sassano MD, Prof F S Violante MD), Dipartimento di Scienze Biomediche e Neuromotorie (DIBINEM) (F Esposito MD), Medical and Surgical Sciences Department (F Fogacci MDc), Department of Biomedical and Neuromotor Sciences (A Mazzotti PhD, F Sanmarchi MD), Department of Medicine and Surgery (I Papadimopoulos MD), University of Bologna, Bologna, Italy (F Anagnostakis MD); Department of General Medicine (R A Ananda MD), Eastern Health, Box Hill, VIC, Australia; Faculty of Pharmacy (Prof R Ancuceanu PhD), Department of Internal Medicine (M Hostiuc PhD), Department of Legal Medicine and Bioethics (Prof S Hostiuc PhD),

Department of Dermatology (C N Matei PhD, M Tampa PhD), Department of General Surgery (I Negoi PhD), Department of Anatomy and Embryology (R I Negoi PhD), Carol Davila University of Medicine and Pharmacy, Bucharest, Romania; Neurology Department (D Anderlini MD), Royal Brisbane and Women's Hospital, Brisbane, QLD, Australia; Department of Medicine (S Androudi PhD), Faculty of Medicine (O J Okesanya MPH), University of Thessaly, Volos, Greece; Department of Environmental and Occupational Health (S C Anenberg PhD), Milken Institute of Public Health (B K Bekele MPH), Department of Global Health (R S Bernstein MD), School of Engineering and Applied Science (A Gerami Matin PhD), George Washington University, Washington, DC, USA; Department of Internal Medicine (S Ang MD), Rutgers University, Toms River, NJ, USA; Sarver Heart Center (S Ang MD), College of medicine (M Asadi Anar MD), Department of Internal Medicine (H Pham MD), University of Arizona, Tucson, AZ, USA; School of Health and Related Research (C Angus MSc), Department of Infection and Tropical Medicine (O C Durojaiye MPH), School of Medicine and Population Health (N S George MPH), Sheffield Centre for Health and Related Research (J O Oguta MSc), University of Sheffield, Sheffield, UK; Department of General Medicine (N Anh MD), Thai Binh University of Medicine and Pharmacy in Vietnam, Thai Binh City, Vietnam; Department of Public Health (K Annadurai PhD), The Apollo University, Chittoor, India; Department of Physiotherapy (S Ansari PhD, M Sidiq PhD), Galgotias Multidisciplinary Research & Development Cell (M Sidiq PhD), Galgotias University, Greater Noida, India; Pharmacy Department, Critical Care (U Ansari PharmD), Cleveland Clinic Abu Dhabi, Abu Dhabi, United Arab Emirates; Department of Public Health (R M Anteneh MPH, M T Asemu MSc), Midwifery Department (Y A Goshu MSc), Debre Tabor University, Debre Tabor, Ethiopia; Environment and Health over the Lifecourse Programme (Prof J M Antó MD), Barcelona Institute for Global Health, Barcelona, Spain (Prof D Rasella PhD); Department of Experimental and Health Sciences (Prof J M Antó MD), Pompeu Fabra University, Barcelona, Spain; Agribusiness Study Program (E Antriyandarti DrAgrSc), Sebelas Maret University, Surakarta, Indonesia; School of Chemical and Life Sciences (SCLS) (S Anwar PhD), Jamia Hamdard, New Delhi, India; Department of Surgery (S Anwar PhD), Department of Pharmacology (I Fitriana PhD), Department of Medical Surgical Nursing (A L Wicaksana PhD), Gadjah Mada University, Yogyakarta, Indonesia; Department of Pathology (R Anwer PhD), Department of Pharmacology (T Jawaid PhD), Imam Mohammad Ibn Saud Islamic University, Riyadh, Saudi Arabia; Department of Rehabilitation Sciences (S Anwer PhD, K Cheung MSc, W P Kwong PhD, J S Usman PhD), Department of Biomedical Engineering (A Jor MSc), Hong Kong Polytechnic University, Hong Kong, China; Rural Health Research Institute (A E Anyasodor PhD, Prof J Sun PhD), Charles Sturt University, Orange, NSW, Australia; School of Medicine and Public Health (G C Apostol MD), Center for Research and Innovation (V F Pepito MSc), Ateneo De Manila University, Pasig City, Philippines; Inter-Agency Committee on Environmental Health (G C Apostol MD), Department of Health Philippines, Manila, Philippines; Division of Gastroenterology, Hepatology, and Nutrition (J Arab MD), Division of Infectious Diseases (P R Ching MD), Virginia Commonwealth University, Richmond, VA, USA; Gastroenterology Department (J Arab MD), Department of Gastroenterology (L Diaz MD), Departamento de Gastroenterologia (Department of Gastroenterology) (F J Idalsoaga MD), Pontifical Catholic University of Chile, Santiago, Chile; Geneva University Hospital (H Arabi PhD), University of Geneva, Geneva, Switzerland; College of Pharmacy (M Arafat PhD), Al Ain University, Abu Dhabi, United Arab Emirates; College of Art and Science (D Areda PhD), Ottawa University, Surprise, AZ, USA; School of Life Sciences (D Areda PhD), Arizona State University, Tempe, AZ, USA; Care in Long Term Conditions Research Division (J Arias de la Torre PhD), Institute of Psychiatry, Psychology & Neuroscience (D Urso MD), School of Life Course and Population Sciences (Prof Y Wang PhD), King's College London, London, UK; CIBER Epidemiology and Public Health (CIBERESP), Madrid,

Spain (J Arias de la Torre PhD); Department of Cardiovascular, Endocrine-Metabolic Diseases and Aging (B Armocida MD), Istituto Superiore di Sanità (ISS), Rome, Italy; School of Health and Social Studies (Prof J Ärnlöv PhD), Dalarna University, Falun, Sweden; Department of Biotechnology (Prof J Arockiaraj PhD, T Sundaram PhD), SRM Medical College Hospital and Research Centre (J James MD), Division of Medical Research (R Janardhanan PhD), Sri Ramaswamy Memorial Institute of Science and Technology, Kattankulathur, India; Institute for Biomedical Problems (A A Artamonov PhD), Russian Academy of Sciences, Moscow, Russia; Division of Epidemiology (K D Artanti DrPH), Advanced Nursing Department (Prof F Efendi PhD), Department of Epidemiology Population Biostatistics and Health Promotion (A Hargono PhD), Department of Advanced Nursing (E M M Has PhD), Department of Fundamental Nursing (R Pradipta MS), Department of Biology (Prof H Purnobasuki PhD), Faculty of Public Health (D A Syaiful MGPH), Universitas Airlangga (Airlangga University), Surabaya, Indonesia; Wake Forest University, Winston Salem, NC, USA (R T Aruleba PhD); Department of Periodontics (D Arumuganainar PhD), Department of Prosthodontics (D Ganapathy PhD), Department of Oral Medicine and Periodontology (Prof R D Jayasinghe MS), Saveetha Medical College and Hospital (Prof M Karobari PhD), Saveetha Dental College and Hospitals (G Minervini PhD, M Selvamani PhD, M Tovani-Palone PhD, M Yuwanati MDS), Department of Biomaterials, Saveetha Dental College and Hospitals, SIMATS (N Rabiee PhD), Department of Biochemistry (P Royapuram Parthasarathy PhD), Center for Global Health Research (Prof A Sahebkar PhD), Department of Microbiology (S Sankar PhD), Department of Pharmacology (S Sekaran PhD), Centre for Global Health Research (R Thiruvengadam PhD), Centre for Biosciences and Biotechnology (B Venkidasamy PhD), Saveetha University, Chennai, India; Department of Clinical Disciplines (N Aryntayeva MSPH), кафедра (Z M Aumoldaeva MSc), Department of Clinical Subjects (A Kurmanova MD), Al Farabi Kazakh National University, Almaty, Kazakhstan; Department of Community Medicine and Global Health (M Asaduzzaman MPH, Prof E Bjertness PhD), University of Oslo, Oslo, Norway; Department of Pharmacy Practice (Prof S Asdaq PhD), College of Medicine (M Fareed PhD), AlMaarefa University, Riyadh, Saudi Arabia (Prof A Dutta PhD); National Agency for Strategic Research in Medical Education (NASRME) (Prof S Asgary MSc), Ministry of Health and Medical Education, Tehran, Iran; Cabrini Research (Prof M Asghari-Jafarabadi PhD), Cabrini Health, Malvern, VIC, Australia; Pioneer Journal of Biostatistics and Medical Research (PJBMR), Pakistan, Pakistan (T Ashraf PhD); Department of Radiation Oncology (M Ashrafizadeh DVM), Shandong University, Shandong, China; School of Health and Social Development (B K Y Asiamah-Asare PhD), School of Exercise and Nutrition Sciences (N Subedi PhD), Deakin University, Melbourne, VIC, Australia; School of Traditional Chinese Medicine (M Aslam PhD), Xiamen University Malaysia, Sepang, Malaysia; Nursing Department (Y Asri PhD), Faculty of Health Science (Y Asri PhD), Institute of Technology and Health Science RS dr Soepraoen, Malang, Indonesia; Birjand University of Medical Sciences, Birjand, Iran (M Ataei PharmD); Hospital and Research Centre (S R Atre PhD), Dr. D. Y. Patil Vidyapeeth Pune (Deemed to be University), Pune, India; Department of Forensic Medicine (A Atreya MD), Department of Community Medicine (S Nepal MD), Lumbini Medical College, Palpa, Nepal; College of Medicine (Prof Z A Atwan PhD), University of Basrah, Basrah, Iraq; School of Business (Prof M Ausloos PhD), Department of Health Sciences (Prof T Brugha MD, S J Tromans PhD), Diabetes Research Centre (E Vounzoulaki PhD), University of Leicester, Leicester, UK; Department of Statistics and Econometrics (Prof M Ausloos PhD, Prof C Herteliu PhD, A Otoiu PhD), Faculty of Management (A Dima PhD), Management Department (Prof I Popa PhD, Prof S Ștefan PhD), Department of Economics and Economic Policies (Prof A Serban PhD), Bucharest University of Economic Studies, Bucharest, Romania; Robarts Research Institute (A Avan MD), School of Physical Therapy (A Lawan PhD), The University of Western Ontario, London, ON, Canada; Department of Physiotherapy (N C P Avelar

DSc), Department of Physiotherapy (I J C Schneider PhD), Federal University of Santa Catarina, Araranguá, Brazil; Department of Public Health (L A A Ayana MPH), Department of Pharmacy (G Fekadu PhD), Department of Nursing (L Gurmessa MSc), Wollega University, Nekemte, Ethiopia; Psychiatry Department (O O Ayinde MD), Pharmacy Department (E T Fasusi MSc), Department of Community Medicine (O S Ilesanmi PhD), Department of Neurology (O V Olalusi MD), Department of Pharmaceutical Services (F Opara BPharm), Department of Medicine (Prof M O Owolabi DrM), University College Hospital, Ibadan, Ibadan, Nigeria; Medicinal Chemistry Unit (Y O Ayipo PhD), Kwara State University, Malete, Ilorin, Nigeria; Centre for Drug Research (Y O Ayipo PhD), Universiti Sains Malaysia, Pinang, Malaysia; Laboratory Sciences Department (D Azadi PhD), Arak University of Medical Sciences, Khomein, Iran; Miami Cardiovascular Institute (M Azhar MD), Baptist Health South Florida, Inc, Miami, FL, USA; Department of Psychiatry (F Azimi MD), Iranian Research Center on Aging (V Rashedi PhD), University of Social Welfare and Rehabilitation Sciences, Tehran, Iran; Department of Anesthesia (S A Aziz PhD), Cihan University -Sulaimaniya, Sulaymaniyah, Iraq; Department of Basic Sciences (S A Aziz PhD), College of Science (Prof K H Hama Aziz PhD, F M Rahman PhD), University of Sulaimani, Sulaymaniyah, Iraq; ASIDE Healthcare, Lewes, DE, USA (A Y Azzam MD); Faculty of Medicine (A Y Azzam MD), October 6 University, 6th of October City, Egypt; Geriatric Unit (D Azzolino PhD), Fondazione IRCCS Ca' Granda Ospedale Maggiore Policlinico, Milan, Italy; Community Medicine Department (Z S Babandi MD), Department of Community Medicine (Prof T Dahiru MA, A A Olorukooba MD, A G Suleiman MPH, S S Umar FWACS), Department of Obstetrics and Gynaecology (B K Lawal MSc), Health Systems and Policy Research Unit (Prof S Mohammed PhD), Department of Surgery (M Tolani FWACS), Ahmadu Bello University, Zaria, Nigeria; Physiology department (R Babiker PhD), RAK Medical and Health Sciences University, Ras Alkhaimah, United Arab Emirates; Directorate of Quality Assurance (M Badar PhD), Gomal University, Dera Ismail Khan, Pakistan; Department of Forensic Science (A D Badiye PhD, N Kapoor PhD), Government Institute of Forensic Science Nagpur, Nagpur, India; Rashtrasant Tukadoji Maharaj Nagpur University, Nagpur, India (A D Badiye PhD); Department of Clinical Pathology (Prof A A Badran MD, Prof M Elshaer PhD), Clinical Pathology Department (Prof M El Sayed Zaki PhD), Department of Anatomy and Embryology (M A Eladl PhD), Department of Toxicology (Prof S Elkannishy PhD), Department of Cardiology (Prof M M Ramadan PhD), Faculty of Pharmacy (Prof M A Saleh PhD), Rheumatology and Immunology Unit (Prof S Tharwat MD), Faculty of Nursing (M Zoromba PhD), Mansoura University, Mansoura, Egypt; Microbiology Department (Prof A A Badran MD), Horus University Egypt, Damietta, Egypt; Department of Precision Medicine (Y Bae MD, Prof S Lee MD), Department of Integrative Biotechnology (N Pham MS), Sungkyunkwan University, Suwon, South Korea; Department of Pediatrics (Prof A Bagga DSc, J Meena DM), Centre for Community Medicine (P Halder MD), Department of Biophysics (T Mohammad PhD), Medical Oncology Lab (C P Prasad PhD), Centre for Dental Education and Research (H Priya MDS, B M Purohit MDS), Department of Psychiatry (Prof R Sagar MD), Department of Radiation Oncology (A Shankar MD), All India Institute of Medical Sciences, New Delhi, India; Department of Orthopedics (S Baghdadi MD), University of California Los Angeles, Los Angeles, CA, USA; Orthopedic Institute for Children, Los Angeles, CA, USA (S Baghdadi MD); Dental Material Research Center (S Baghizadeh DDS), Department of Biology, Science and Research Branch (P Kheirandish Zarandi PhD), Islamic Azad University, Tehran, Iran (M Zaghampour MD); College of Optometry (R Bahreini MS), Pacific University, Forest Grove, OR, USA; Clinical Research Center (Prof R Bai MD), Nanjing Children's Hospital, Nanjing, China; International Medical School (A A Baig PhD), Management and Science University, Alam, Malaysia; Department of Forensic Medicine and Toxicology (S M Bakkannavar MD), Department of Pharmacology (S Gangachannaiah MD, A Parida MD), Kasturba Medical College,

Mangalore (R Holla MD, M Rao MD), Prasanna School of Public Health (R Kamath MHA), Kasturba Medical College, Manipal (P L C MD, J P Raj DM, Prof S Shastry MD), Manipal College of Pharmaceutical Sciences (P M Muragundi PhD), Department of Nephrology (Prof S Nagaraju DM, Prof A Prabhu DM), Manipal College of Nursing (S Nayak PhD), Department of Microbiology (P Y Prakash PhD), Manipal College of Dental Sciences (Prof R A Radhakrishnan PhD), Department of Community Medicine (C R Rao MD), Department of Physiotherapy (M K Sinha PhD), Kasturba Medical College (D Upadhya PhD), Manipal Academy of Higher Education, Manipal, India (Prof V Jha MD); TIRR Memorial Hermann, Houston, TX, USA (A T Bako PhD); Division of Biological Sciences (S Balakrishnan PhD), Tamil Nadu State Council for Science and Technology, Chennai, India; Chen Senior Medical Center, Tamarac, FL, USA (M Balkis MD); Anahuac Business School (J Balmori-de-la-Miyar PhD), Universidad Anahuac Mexico, Mexico City, Mexico; Department of Epidemiology and Biostatistics (M Balooch Hasankhani PhD), Department of Biostatistics and Epidemiology (P Dehesh PhD), Physiology Research Center (M Rajizadeh PhD), Kerman University of Medical Sciences, Kerman, Iran; College of Medicine (Prof O Baltatu PhD), Alfaisal University, Riyadh, Saudi Arabia; Center of Innovation, Technology and Education (CITE) (Prof O Baltatu PhD), Anhembi Morumbi University, São José dos Campos, Brazil; Department of Hypertension (Prof M Banach PhD), Medical University of Lodz, Lodz, Poland; Polish Mothers' Memorial Hospital Research Institute, Lodz, Poland (Prof M Banach PhD); Department of Non-communicable Diseases (P C Banik MPhil, L Barua MPH), Bangladesh University of Health Sciences, Dhaka, Bangladesh; Department of Anatomy (S Barati PhD), Department of Nursing and Midwifery (M Saeedi PhD), Saveh University of Medical Sciences, Saveh, Iran; School of Psychology (Prof S L Barker-Collo PhD), University of Auckland, Auckland, New Zealand; Department of Public and Environmental Health (A Barrow MPH), University of The Gambia, Banjul, The Gambia; Department of Epidemiology (A Barrow MPH, D Braithwaite PhD), Department of Health Services Research, Management and Policy (R Wang PhD), University of Florida, Gainesville, FL, USA; Heidelberg Institute of Global Health (HIGH) (S Barteit PhD, S Chen DSc, Prof S Mohammed PhD), Department of Ophthalmology (S Panda-Jonas MD), Heidelberg University, Heidelberg, Germany; Department of Community and Family Medicine (M Bashar MD, S G MD, V J MD, V Rajendran MD), All India Institute of Medical Sciences, Gorakhpur, India; Alpha Genomics Private Limited, Islamabad, Pakistan (Z Basharat PhD); Department of General Surgery and Medical-Surgical Specialties (Prof G Basile MD, Prof G Isola PhD), Department of Medical and Surgical Sciences and Advanced Technologies "GF Ingrassia" (C G Chisari MD, Prof E D'Amico MD, V Grieco MD, Prof M Veroux PhD), Department of Clinical and Experimental Medicine (Prof C Ledda PhD), University of Catania, Catania, Italy; Department of Community Medicine (P Baskaran MD), Sri Manakula Vinayagar Medical College and Hospital, Puducherry, Puducherry, India; ISGlobal Instituto de Salud Global de Barcelona (Barcelona Institute for Global Health) (Prof Q Bassat MD), Universitat de Barcelona, Barcelona, Spain; Catalan Institution for Research and Advanced Studies (ICREA), Barcelona, Spain (Prof Q Bassat MD); School of Public Health (S Basu PhD, Y Hbid PhD, Q Ong MD), The George Institute for Global Health (T Beaney PhD, Prof S Yaya PhD), Department of Brain Sciences (L D'Anna PhD), UK Dementia Research Institute Care Research & Technology Centre (Y Hbid PhD), WHO Collaborating Centre for Public Health Education and Training (Q Lin MPH, D L Rawaf MD), Department of Primary Care and Public Health (Prof A Majeed MD, R Palladino MD, Prof S Rawaf MD, C Tabche MSc, L Tudor Car PhD), Imperial College London, London, UK; Department of Community Medicine (S Basu MD), Employees State Insurance-Post Graduate Institute of Medical Sciences and Research, Kolkata, India; Department of Medical Education (K Batra PhD), Department of Social and Behavioral Health (Prof M Sharma PhD), University of Nevada Las Vegas, Las Vegas, NV, USA; Department of Psychiatry (Prof B T Baune PhD), University of Münster,

Münster, Germany; Department of Psychiatry (Prof B T Baune PhD), Melbourne Medical School, Melbourne, VIC, Australia; Epidemiology ang Biostatistics (F S Bayisa MPH), Department of Public Health (S D Darcho MPH), School of Pharmacy (A S Mohammed BA), School of Public Health (A Oumer PhD), Haramaya University, Harar, Ethiopia; School of Public Health (Prof N Bedi MD), Dr. D. Y. Patil University, Mumbai, India; Clinical Nutrition Department (R M Chandika PhD), Epidemiology Program (M Khan MD), Department of Prosthetic Dental Sciences (K A Mattoo MD), Department of Public Health (A Mehmood PhD, W Rehman MS, F Sobia PhD), College of Nursing and Health Sciences (S N Qurashi PhD), Jazan University, Jazan, Saudi Arabia (Prof N Bedi MD); Department of Human Anatomy and Histology (Prof N Beeraka PhD), Department of Epidemiology and Evidence-Based Medicine (P D Lopukhov PhD, R V Polibin PhD), I.M. Sechenov First Moscow State Medical University, Moscow, Russia; Department of Community and Family Medicine (P Behera MD), Department of Pharmacology (Prof R Maiti MD, A Mishra DM), Department of Psychiatry (A Parmar DM), All India Institute of Medical Sciences, Bhubaneswar, India; Avicenna Biotech Research, Germantown, MD, USA (B Behnam MD); Department of Regulatory Affairs (B Behnam MD), Amarex Clinical Research, Germantown, MD, USA; Endocrinology and Metabolism Research Institute (H Farrokhpour MD), Quantitative Department (K Gohari MS, A Sheidaei PhD), Department of Epidemiology (S Khanmohammadi MD), Non-Communicable Diseases Research Center (NCDRC), Tehran, Iran (A Behnoush BS, A Golestani MD); Department of Public Health (M Belayneh PhD), Institute for Social and Health Sciences (Prof L Laflamme PhD), University of South Africa, Pretoria, South Africa; Department of Midwifery (A C Belete MSc, T S Hadaro MSc), Department of Public Health (T Mekene Meto MPH), Arba Minch University, Arba Minch, Ethiopia; Department of Medicine and Surgery (M Belingheri MD, A La Vecchia MD), University of Milan Bicocca, Milan, Italy; Direzione Sanitaria (M Belingheri MD), Fondazione IRCCS San Gerardo dei Tintori, Monza, Italy; Infectious Disease Research Department (M B Bello PhD), Medical Genomics Research Department (Prof M Umair PhD), King Abdullah International Medical Research Center, Riyadh, Saudi Arabia; Department of Biological Sciences (Prof L Belo PhD), Research Unit on Applied Molecular Biosciences (UCIBIO) (Prof L Belo PhD, Prof D Dias da Silva PhD, J Silva PhD), Research Centre for Physical Activity, Health, and Leisure (L Bohn PhD), Institute for Research and Innovation in Health (i3S) (Prof N Cruz-Martins PhD), MEDCIDS, Faculty of Medicine of the University of Porto (A Freitas PhD), Faculty of Medicine (J R Rocha-Gomes MD), Faculty of Pharmacy (M Vojtek PhD), University of Porto, Porto, Portugal; Department of Biomedical Sciences (Prof A Beloukas PhD), National AIDS Reference Center of Southern Greece (Prof A Beloukas PhD), University of West Attica, Athens, Greece; Department of Industrial Engineering (Prof S Bendak PhD), School of Business Administration (Prof V Bodolica PhD), American University of Sharjah, Sharjah, United Arab Emirates; Department of Epidemiology and Psychosocial Research (C Benjet PhD), Department of Epidemiology and Psychosocial Research (R A Gutiérrez PhD), Ramón de la Fuente Muñiz National Institute of Psychiatry, Mexico City, Mexico; Department of Internal Medicine (I M Bensenor PhD), Department of Psychiatry (Prof J Castaldelli-Maia PhD, A Caye PhD, Y Wang PhD), Heart Institute (P M Dourado PhD), Center for Clinical and Epidemiological Research (A B Oliveira PhD), University of São Paulo, São Paulo, Brazil (L H C C Santos MSc); BRAC James P Grant School of Public Health (S Bente Kamal Tune MPH), Centre for Noncommunicable Diseases and Nutrition (R Gupta MPH), Pharmacy (M A Haque PhD), School of Pharmacy (M Islam PhD), BRAC University, Dhaka, Bangladesh; Department of Epidemiology and Health Promotion (Prof H Benzian PhD), Department of Child and Adolescent Psychiatry (Prof S Cortese PhD), Institute for Excellence in Health Equity (M Kumar PhD), Rory Meyers College of Nursing (X Qi PhD), New York University, New York, NY, USA; Institute of Marketing and Communication Sciences (Z Berezvai PhD), Corvinus University of Budapest, Budapest, Hungary;

Department of Epidemiology and Biostatistics (A C Bermudez PhD), Department of Environmental and Occupational Health (C M Estrada PhD), Department of Health Policy and Administration (E A Faraon MD, Prof F B Garcia PhD), Department of Neurosciences (Prof R G Jamora PhD), University of the Philippines Manila, Manila, Philippines; Institute of Dentistry (Prof E Bernabe PhD), Barts and the London School of Medicine and Dentistry, London, UK; Hubert Department of Global Health (R S Bernstein MD), Rollins School of Public Health (Prof D A Sleet PhD), Emory University, Atlanta, GA, USA; Faculty of Medicine (P J G Bettencourt PhD), Universidade Católica Portuguesa (Catholic University of Portugal), Sintra, Portugal; Center for Interdisciplinary Research in Health (CIIS) (P J G Bettencourt PhD), Universidade Católica Portuguesa (Catholic University of Portugal), Lisbon, Portugal; Department of Community and Family Medicine (A S Bhadoria MD), All India Institute of Medical Sciences, Rishikesh, India; Community Health Department (A S Bhadoria MD), University of South Wales, South Wales, UK; Department of Public Health (A S Bhagavathula PhD), North Dakota State University, Fargo, ND, USA; Institute of Applied Health Research (N Bhala PhD), Department of Applied Health Sciences (Prof J S Chandan PhD), NIHR Global Health Research Unit on Global Surgery (S K Kamarajah MD), Department of Metabolism and Systems Science (S Tariq PhD), University of Birmingham, Birmingham, UK; Department of Medicine (R Bharadwaj PhD), Department of Neurology (B S Srichawla MD), University of Massachusetts Medical School, Worcester, MA, USA; Global Health Neurology Lab (S Bhaskar MD), NSW Brain Clot Bank, Sydney, NSW, Australia; Division of Cerebrovascular Medicine and Neurology (S Bhaskar MD), National Cerebral and Cardiovascular Center, Suita, Japan; Manipal College of Health Professions (A Bhat MSPT), Kasturba Medical College, Manipal (S Koulmane Laxminarayana MD), Manipal College of Nursing (R Yesodharan MPhil), Manipal Academy of Higher Education, Udupi, India; Department of Medicine (V Bhat MBBS), SUNY Upstate Medical University, Syracuse, NY, USA; Translational and Clinical Research Institute (P Bhattacharjee MD), Newcastle University, Newcastle upon Tyne, UK; School of Sport & Health Sciences (S Bhattacharjee MPH), University of Brighton, Brighton, UK; Department of Public Health Research (S Bhattacharjee MPH), Bengal Rural Welfare Service (BRWS), Kolkata, India; Department of Medical Lab Technology (Prof G K Bhatti PhD), University Centre for Research and Development (S Kalra DM), Chandigarh University, Mohali, India; Laboratory of Translational Medicine and Nanotherapeutics (Prof J S Bhatti PhD), Department of Microbiology (P K Kushawaha PhD, M Yadav PhD), Department of Human Genetics and Molecular Medicine (Prof A Munshi PhD, U Sharma PhD), Department of Biochemistry (B Singh PhD), Department of Computer Science & Engineering (Prof S Singh PhD), Department of Zoology (B Vellingiri PhD), Central University of Punjab, Bathinda, India; Department of Botanical and Environmental Sciences (Prof M S Bhatti PhD), Department of Pharmaceutical Sciences (Prof R Bhatti PhD), Guru Nanak Dev University, Amritsar, India; Department of Health Administration (S S Bhuyan PhD), Institute for Health, Health Care Policy and Aging Research (S Rege PhD), Rutgers University, New Brunswick, NJ, USA; Independent Consultant, Addis Ababa, Ethiopia (S K Biadgilign PhD); Fondazione Banca Degli Occhi Del Veneto (R Bievel-Radulescu MD), Carol Davila University of Medicine and Pharmacy, Venice, Italy; TAF Uludag Winter Training Center (C Bilgin MD), Turkish Ministry of Defence, Bursa, Turkiye; Neurovascular Research Laboratory (C Bilgin MD), Mayo Clinic College of Medicine, Rochester, MN, USA; Department of Neurology (Prof A Biswas DM), Department of Endocrinology & Metabolism (Prof S Mukhopadhyay MD), Institute of Post-Graduate Medical Education and Research and Seth Sukhlal Karnani Memorial Hospital, Kolkata, India; Department of Community and Family Medicine (B Biswas MD), Department of Physiology (H Mondal MD), College of Nursing (S K Mudgal PhD), Department of Pharmacology (S T Y MD), All India Institute of Medical Sciences, Deoghar, India; Clinical Research Centre (R Biswas PhD), Sydney Local Health District,

Sydney, NSW, Australia; Department of Clinical Pharmacy (A Bitar PhD), Universiti Sultan Zainal Abidin, Besut, Malaysia; Health Biotechnology Directorate at Bio and Emerging Technology Institute (M Bitew PhD), School of Public Health (Prof G Davey MD), College of Health Sciences (F S Gebre MD, A M Zenebe MSc), Department of Reproductive, Family and Population Health (N A Kassaw MPH), Addis Ababa University, Addis Ababa, Ethiopia; Department of Physical Education and Health (B Bizzozero-Peroni PhD), Universidad de la República, Rivera, Uruguay; Department of Community and Family Medicine (Prof T Bodhare MD), All India Institute of Medical Sciences, Tamil Nadu, India; Department of Nutrition and Dietetics (M Bodur PhD), Faculty of Health Sciences Healthcare Management Department (M Çakmak Barsbay PhD), Ankara University, Ankara, Turkiye; Faculty of Psychology, Education, and Sport (L Bohn PhD), University Lusofona, Porto, Portugal; Global Healthcare Management (O A Bolarinwa PhD), York University, London, UK; Demography and Population Studies (O A Bolarinwa PhD), University of the Witwatersrand, Johannesburg, South Africa; Faculty of Medicine and Pharmaceutical Sciences (Prof A Bonny MD), University of Douala, Douala, Cameroon; Department of Cardiology (Prof A Bonny MD), Centre Hospitalier Montfermeil (Montfermeil Hospital Center), Montfermeil, France; General Directorate of Health Information Systems (B Bora Basara PhD), Ministry of Health, Ankara, Turkiye; Facultad de Salud (Faculty of Health) (Prof A Botero Carvajal PhD), Universidad Santiago de Cali, Cali, Colombia; Department of Medicine (Prof S Bouaoud DrPH), Faculty of Medicine (Prof A Ouyahia PhD), University Ferhat Abbas of Setif, Setif, Algeria; Department of Epidemiology and Preventive Medicine (Prof S Bouaoud DrPH), University Hospital Saadna Abdenour, Setif, Algeria; Vision and Eye Research Institute (Prof R R A Bourne FRCOphth), Anglia Ruskin University, Cambridge, UK; Department of Earth, Environment, and Equity (C Boxe PhD), Howard University, Washington, DC, USA; Faculty of Medicine (Prof M M Bozic PhD, I M Ilic PhD, Prof M M Santric-Milicevic PhD, A Stevanović MD), School of Public Health and Health Management (Prof M M Santric-Milicevic PhD), University of Belgrade, Belgrade, Serbia; Department Of Pathology (J Brahmaiah MD), Apollo Institute of Medical Sciences & Research Chittoor, Chittoor, India; Cancer Population Sciences Program (D Braithwaite PhD), University of Florida Health Cancer Center, Gainesville, FL, USA; School of Population and Public Health (Prof M Brauer DSc, P A Chakraborty MPH, M Hossain PhD, I O Iyamu MD), Department of Food, Nutrition and Health (M Jessri PhD), Department of Medicine (H Naik MS), School of Nursing (A Pashaei MSc), University of British Columbia, Vancouver, BC, Canada; Department of Psychiatry and Behavioral Health (Prof N J K Breitborde PhD), Department of Psychology (Prof N J K Breitborde PhD), Division of Cardiovascular Medicine (A Guha MD), Center for Tobacco Research (C Keke PhD), Ohio State University, Columbus, OH, USA; Division of Clinical Epidemiology and Aging Research (Prof H Brenner MD), German Cancer Research Center, Heidelberg, Germany; CEVAXIN, Panama City, Panama (G Britton PhD); Institute for Scientific Research and High Technology Services, Panama City, Panama (G Britton PhD); Department of Injury (J Brown PhD), The George Institute for Global Health, Newtown, NSW, Australia; The Malaria Atlas Project (A J Browne DPhil, S F Rumisha PhD), Geospatial Health and Development Team-Child Health Analytics (J Lubinda PhD), Child Health Analytics Research Program (F Sanna PhD, D J Weiss PhD), Telethon Kids Institute, Perth, WA, Australia; Hospital de Clínicas de Porto Alegre (C Buchweitz PhD), Department of Psychiatry (A Caye PhD), School of Medicine (N Feter PhD), Department of Social Medicine (R Mattiello PhD), Department of Psychiatry and Legal Medicine (Prof G A Salum PhD), Federal University of Rio Grande do Sul, Porto Alegre, Brazil; Child & Adolescent Research Program (C Buchweitz PhD), Programa de Depressão na Infância e na Adolescência, Porto Alegre, Brazil; College of Health Sciences (L P Bui PhD, T T Pham PhD, Prof D Poddighe PhD), VinUniversity, Hanoi, Vietnam; Research Advancement Consortium in Health, Hanoi, Vietnam (L P Bui PhD, T T Pham PhD); Department of

Woman and Child Health and Public Health (D Buonsenso MD), Fondazione Policlinico Universitario A. Gemelli IRCCS (Agostino Gemelli University Polyclinic IRCCS), Rome, Italy; Global Health Research Institute (D Buonsenso MD), Università Cattolica del Sacro Cuore (Catholic University of Sacred Heart), Rome, Italy; School of Nursing (A Burhan MSc), Universitas Harapan Bangsa (National Hope University), Banyumas, Indonesia; Department of Radiology (F Busch MD), Department of Public Health and Primary Care (M Dalakoti MPH, Prof P Willeit PhD), University of Cambridge, Cambridge, UK; Department of Health Care Management (Prof R Busse PhD), Technical University of Berlin, Berlin, Germany; School of Public Health Sciences (Z A Butt PhD), University of Waterloo, Waterloo, ON, Canada; Al Shifa School of Public Health (Z A Butt PhD), Al Shifa Trust Eye Hospital, Rawalpindi, Pakistan; JSS Dental College & Hospital (S C J MDS), Department of Respiratory Medicine (Prof M P A DNB), Department of Forensic Medicine and Toxicology (S Rani MD), Department of Oral and Maxillofacial Surgery (M S MDS, C S N PhD), Jagadguru Sri Shivarathreeswara University, Mysore, India; Department of Sociology (Prof T Cai PhD), University of Macau, Macau, China; The Children's Hospital at Westmead (R Cairns PhD), New South Wales Poisons Information Centre, Sydney, NSW, Australia; Department of Clinical Pharmacy (Prof D Calina PhD), University of Medicine and Pharmacy of Craiova, Craiova, Romania; Department of Internal and Geriatric Medicine (Prof L A Cámera MD), Hospital Italiano de Buenos Aires (Italian Hospital of Buenos Aires), Buenos Aires, Argentina; Board of Directors (Prof L A Cámera MD), Argentine Society of Medicine, Buenos Aires, Argentina; Center of Innovation, Technology and Education (CITE) (Prof L A Campos PhD), Anhembi Morumbi University, Sao Jose dos Campos, Brazil; Center for Nutrition and Health Research (I Campos-Nonato PhD), Public Health Intelligence Unit (Prof D Diaz PhD), Center for Health Systems Research (E Serván-Mori PhD), National Institute of Public Health, Cuernavaca, Mexico; Department of Ophthalmology (F Cao MD), Beijing Institute of Ophthalmology, Beijing, China; Department of Surgery (Y Cao MD), Peking Union Medical College Hospital (A Chen PhD), Fuwai Hospital (S Zhao MD), Chinese Academy of Medical Sciences, Beijing, China; Unit of Hygiene and Public Health (A Capodici MD), Romagna Local Health Authority, Forlì-Cesena, Italy; Interdisciplinary Research Center for Health Science (A Capodici MD), Sant'Anna School of Advanced Studies, Pisa, Italy; Department of Health Care (Prof R Cárdenas DSc), Metropolitan Autonomous University, Mexico City, Mexico; Institute for Cancer Research, Prevention and Clinical Network, Florence, Italy (G Carreras PhD); Department of Medicine and Surgery (A Carugno PhD), University of Insubria, Varese, Italy; IMPInstitute for Mental and Physical Health and Clinical Translation (IMPACT) (A F Carvalho MD), School of Medicine (V Gupta PhD), Deakin University, Geelong, VIC, Australia; Education Center of Australia (A Carvalho-e-Silva PhD), Health Science College, Sydney, NSW, Australia; Public Health Department (C A Castañeda-Orjuela PhD), Epidemiology and Public Health Evaluation Group (C A Castañeda-Orjuela PhD), Department of Public Health (Prof F P De la Hoz PhD), National University of Colombia, Bogota, Colombia; Division of Country Health Policies and Systems (CPS) (G Castelpietra PhD), World Health Organisation, Trieste, Italy; Mental Health Flagship (G Castelpietra PhD), World Health Organization (WHO), Copenhagen, Denmark; Department of Pharmacological and Biomolecular Sciences (Prof A L Catapano PhD), IRCCS Istituto Ortopedico Galeazzi (Galeazzi Orthopedic Institute IRCCS) (G Damiani MD), Department of Clinical Sciences and Community Health (Prof C La Vecchia MD), Department of Food, Environmental and Nutritional Sciences (Prof D Martini PhD), University of Milan, Milan, Italy; MultiMedica Sesto San Giovanni IRCCS, Sesto San Giovanni, Italy (Prof A L Catapano PhD); Department of Otolaryngology, Head and Neck Surgery (C R Cederroth PhD), University of Tübingen, Tübingen, Germany; Department of Medical, Surgical, and Health Sciences (Prof L Cegolon PhD, Prof M D'Oria MD), University of Trieste, Trieste, Italy; Public Health Unit (Prof L Cegolon PhD), University Health Agency Giuliano-Isontina

(ASUGI), Trieste, Italy; Department of Nutrition (Prof F Cembranel DSc), Department of Physical Education (Prof D A S Silva PhD), Federal University of Santa Catarina, Florianópolis, Brazil; College of Public Health, Medical, and Veterinary Sciences (M Cenderadewi MPHTM, A E Peden PhD), Department of Public Health and Tropical Medicine (T I Emeto PhD), College of Medicine, Dentistry and Public Health (Prof R C Franklin PhD), James Cook University, Townsville, QLD, Australia; Department of Public Health (M Cenderadewi MPHTM), University of Mataram, Mataram, Indonesia; Mary MacKillop Institute for Health Research (Prof E Cerin PhD), Australian Catholic University, Melbourne, VIC, Australia; School of Public Health (Prof E Cerin PhD, C J P Zhang PhD), Department of Medicine (H Chou MSc), Centre for Suicide Research and Prevention (Prof P Yip PhD), Department of Social Work and Social Administration (Prof P Yip PhD), Department of Surgery (Y Zhan PhD), University of Hong Kong, Hong Kong, China; Institute of Clinical Physiology (S Cerrai MSc), Italian National Council of Research, Pisa, Italy; Infection and Global Health Research (M Cevik MD), University of St Andrews, St Andrews, UK; Regional Infectious Diseases Unit (M Cevik MD), NHS National Services Scotland, Edinburgh, UK; Department of Biotechnology (Prof C Chakraborty PhD), Adamas University, Kolkata, India; Institute for Skeletal Aging & Orthopedic Surgery (Prof C Chakraborty PhD), Hallym University, Chuncheon, South Korea; State Disease Investigation Laboratory (S Chakraborty MVSc), Animal Resources Development Department, Agartala, India; Department of Psychiatry (Prof M Chandradasa MD), Department of Pharmacology (Prof C D K Mettananda PhD), Department of Paediatrics (Prof S Mettananda DPhil), University of Kelaniya, Ragama, Sri Lanka; University Psychiatry Unit (Prof M Chandradasa MD), Clinical Medicine Department (Prof C D K Mettananda PhD), University Paediatrics Unit (Prof S Mettananda DPhil), Colombo North Teaching Hospital, Ragama, Sri Lanka; Department of Anesthesiology and Perioperative Medicine (E K Chandrasekar MD), School of Medicine (Prof S Xu PhD), University of Rochester, Rochester, NY, USA; College of Medicine (J Chang PhD), Institute of Epidemiology and Preventive Medicine (Y L Samodra PhD), National Taiwan University, Taipei, Taiwan; Department of Nursing (J Chang PhD), National Taiwan University Hospital, Taipei, Taiwan; Department of Epidemiology and Biostatistics (V Chattu PhD), Semey Medical University (SMU), Semey, Kazakhstan; Department of Community Medicine (V Chattu PhD), Datta Meghe Institute of Medical Sciences, Sawangi, India; Department of Endocrinology (V Chatzimavridou-Grigoriadou MD), Division of Immunology, Immunity to Infection and Respiratory Medicine (A G Mathioudakis PhD), Division of Psychology and Mental Health (M R Radojčić PhD), University of Manchester, Manchester, UK; Department of Endocrinology (V Chatzimavridou-Grigoriadou MD), Christie Hospital NHS Foundation Trust, Manchester, UK; Department of Public Health (S Chaudhuri MD), Indian Institute of Public Health, Hyderabad, India; Department of Oral Medicine and Radiology (Prof A Chaurasia MD), Department of Psychiatry (S K Kar MD), Department of Neurology (Prof H S Malhotra DM), Internal Medicine Department (J Tewari MBBS), King George's Medical University, Lucknow, India; EPI (G B Chemeda MPH), Oromia Health Bureau, Addis Ababa, Ethiopia; Hospital of Stomatology (G Chen PhD), Sun Yat-sen University, Guangzhou, China; Faculty of Humanities and Health Sciences (H Chen MSc), Curtin University, Miri, Malaysia; Clinical Research Center (H Chen PhD), Zhujiang Hospital of Southern Medical University, Guangzhou, China; Science and Technology Department (H Chen MMed), Northern Jiangsu People's Hospital, Yangzhou, China; Department of Urology (J Chen MSc), Academy of Medical Science, Kunming, China; School of Dentistry (M Chen DDS), Department of Family Medicine (T G James PhD), Management Science and Healthcare Analytics (O A Okoli MS), University of Michigan, Ann Arbor, MI, USA; Faculty of Epidemiology and Population Health (Prof S Chen PhD), Epidemiology Programme (A Hafiz PhD), Department of Non-Communicable Disease Epidemiology (M Iwagami PhD), Department of Health Services Research and Policy (Prof M McKee

DSc), Department of Non-communicable Disease Epidemiology (A Nur MPH), International Centre for Eye Health (A J Thirunavukarasu MA), London School of Hygiene & Tropical Medicine, London, UK; Department of Computer, Electrical and Mathematical Sciences and Engineering (X Chen MSc), Computer, Electrical, and Mathematical Sciences and Engineering Division (P Moraga PhD), King Abdullah University of Science and Technology, Thuwal, Saudi Arabia; Department of Cardiology (Y Chen PhD), Shanghai Jiao Tong University School of Medicine, Shanghai, China; School of Chinese Medicine (H Cheng BSc), Hong Kong Baptist University, Hong Kong, China; Yong Loo Lin School of Medicine (N W Chew MD, M Ng PhD, Prof H Z Sun PhD, Prof N Venketasubramanian MSc), Department of Medicine (B Chong MBBS), Cardiovascular Metabolic Translational Research Program (M Dalakoti MPH), Saw Swee Hock School of Public Health (S Ramazanu PhD, Prof S Yi PhD), Department of Surgery (K Tan PhD), National University of Singapore, Singapore, Singapore; Department of Laboratory Medicine (J Chien PhD), Taichung Tzu-Chi Hospital Buddhist Tzu-Chi Medical Foundation, Tanzih, Taiwan; Department of Medical Laboratory Science and Biotechnology (J Chien PhD), Central Taiwan University of Science and Technology, Taiwan; Department of Public Health and Health Policy (O Chimed-Ochir PhD, I Khaing MPH), Department of Epidemiology (Z Phyo MD), Hiroshima University, Hiroshima, Japan; Department of Public Health, Administration, and Social Sciences (J L Chirinos-Caceres DrPH), Cayetano Heredia University, Lima, Peru; Department of Clinical Oncology (W C S Cho PhD), Queen Elizabeth Hospital, Hong Kong, China; Department of Public Health (M Chowdhury PhD), Asian University for Women, Chittagong, Bangladesh; Bispebjerg Hospital (Prof H Christensen DMSci), Department of Neurology (Prof T J Steiner PhD), University of Copenhagen, Copenhagen, Denmark; Department of Health Science and Technology (S W M Christensen PhD), Aalborg University, Aalborg, Denmark; Department of Physiotherapy (S W M Christensen PhD), University College of Northern Denmark, Aalborg, Denmark; The Interdisciplinary Research Group on Biomedicine and Health (D Chu PhD), VNU International School, Hanoi, Vietnam; Faculty of Applied Sciences (D Chu PhD), VNU International School (VNUIS), Hanoi, Vietnam; Department of Paediatric Surgery (I S Chukwu BMedSc), Federal Medical Centre, Umuahia, Nigeria; Department of AndroUrology (Prof E Chung MD), AndroUrology Centre, Brisbane, QLD, Australia; Health Data Research UK, London, UK (S Chung PhD); Department of Health Behavior (S Chung MPH), Texas A&M University, College Station, TX, USA; Adelaide Medical School (L G Ciobanu PhD, T K Gill PhD), University of Adelaide, Adelaide, SA, Australia; School of Pharmacy and Medical Sciences (L G Ciobanu PhD), The University of Adelaide (Y Damtew PhD), Department of Allied Health and Human Performance (T Y Tiruye PhD), University of South Australia, Adelaide, SA, Australia; Health Effects Institute, Boston, MA, USA (A J Cohen DSc); Nova Medical School (Prof J Conde PhD), National School of Public Health (M O Corda MSc), Nova University of Lisbon, Lisbon, Portugal; Department of Medicine (S E Congly MD), Faculty of Veterinary Medicine (M Jokar DVM), Cumming School of Medicine (M I Olatubi PhD), Department of Medicine (Prof M Tonelli MD), Department of Clinical Neurosciences (Prof S Wiebe MD), Department of Community Health Sciences (Prof S Wiebe MD), University of Calgary, Calgary, AB, Canada; Department of Cardiovascular Sciences (N Conrad PhD, A Schuermans BSc, J Van den Eynde BSc), Faculty of Medicine (A Schuermans BSc), Department of Abdominal Surgery (A Teymouri MD), Katholieke Universiteit Leuven, Leuven, Belgium; General Administration (S Conti PhD), Research Center on Public Health (CESP) (P Cortesi PhD), Center for Public Health Research (P Ferrara PhD), Department of Medicine and Surgery (C Fornari PhD), School of Medicine and Surgery (Prof L G Mantovani DSc), University of Milan Bicocca, Monza, Italy; Egas Moniz Center for Interdisciplinary Research (M O Corda MSc), Egas Moniz School of Health and Science, Almada, Portugal; Department of Respiratory Medicine and Allergology (Prof A Corlateanu PhD), Nicolae Testemitanu State University of Medicine and

Pharmacy, Chisinau, Moldova; School of Psychology (Prof S Cortese PhD), Centre for Innovation in Mental Health (M Garcia-Argibay PhD), Department of Surgery (G Verras MSc), University of Southampton, Southampton, UK; Laboratory of Public Health (P Cortesi PhD), Instituto Auxologico Italiano IRCCS (Italian Auxological Institute), Milan, Italy; Department of Health Sciences (C Cosma MD), Department of Statistics, Computer Science, Applications "G. Parenti" (DiSIA) (A Desta MSc), University of Florence, Florence, Italy; Department of Family Medicine and Public Health (Prof M H Criqui MD), University of California San Diego, La Jolla, CA, USA (L Diaz MD); School of Medicine (X Cui PhD), School of Data Science (J Zhou PhD), The Chinese University of Hong Kong, Shenzhen, Shenzhen, China; Faculty of Medicine (N Dababo MD), University of Aleppo, Aleppo, Syria; Research Center for Child Psychiatry (O Dadras PhD), Heart Center (V Kytö MD), University of Turku, Turku, Finland; Department of Health Statistics and Informatics (O Dadras PhD), Northern Territory Government, Darwin, WA, Australia; Institute for Health Sciences (Prof K Dalal PhD), Mid Sweden University, Sundsvall, Sweden; Department of Dermatology (G Damiani MD), Lerner College of Medicine (Prof L Göbölös PhD), Harrington Heart and Vascular Institute (A Guha MD), Department of Population and Quantitative Sciences (P Guttoo PhD), Department of Quantitative Health Science (Prof X Liu PhD), Department of Endocrinology (A Sood MD), Case Western Reserve University, Cleveland, OH, USA; Department of Community Medicine (R A Daniel MD, S Rajaa MD), Employees' State Insurance Model Hospital, Chennai, India; Department of Internal Medicine (P Danpanichkul MD), Texas Tech University, Lubbock, TX, USA; Department of Environmental Health (R Darvishi Cheshmeh Soltani PhD), Department of Nursing (A Jadidi PhD), Department of Pediatrics (J Nazari MD), Arak University of Medical Sciences, Arak, Iran; 2nd University Ophthalmology Department (A Dastiridou MD), 2nd Department of Cardiology (P Karakasis MD), Department of Ophthalmology (Prof A G P Konstas PhD), First Department of Ophthalmology (Prof G D Panos MD), Second Propedeutic Department of Internal Medicine (Prof D Patoulias PhD), Aristotle University of Thessaloniki, Thessaloniki, Greece; Ophthalmology Department (A Dastiridou MD), University of Thessaly, Greece; Department of Global Health and Infection (Prof G Davey MD), Brighton and Sussex Medical School, Brighton, UK; Department of Population and Development (C A Dávila-Cervantes PhD), Latin American Faculty of Social Sciences Mexico, Mexico City, Mexico; biomedical and nutritional sciences (E Davoudi PharmD), University of Massachusetts Lowell, lowell, MA, USA; School of Health, Medical and Applied Sciences (K de Luca PhD), CQUniversity, Brisbane, QLD, Australia; Memorial Sloan Kettering Cancer Center (E Dee MD), Memorial Sloan Kettering Cancer Center, New York, NY, USA; Department of Pediatrics (S Deekonda MD), Brookdale University Hospital Medical Center, Brooklyn, NY, USA; Ophthalmology Department (M Delsoz MD), Department of Ophthalmology (A Nabavi MD), University of Tennessee, Memphis, TN, USA; Department of Neurosurgery (A K Demetriades MD), College of Medicine and Veterinary Medicine (G Verras MSc), University of Edinburgh, Edinburgh, UK; Department of Neurosurgery (A K Demetriades MD), National Health Service (NHS) Scotland, Edinburgh, UK; Dirección de Nutrición (E Denova-Gutiérrez DSc), Salvador Zubiran National Institute of Medical Sciences and Nutrition, Mexico City, Mexico; Research and Training Directorate (T N Derese MPH), Eka Kotebe General Hospital, Addis Ababa, Ethiopia; Department of Biological Sciences (I Dergaa PhD), University of Manouba, Manouba, Tunisia; Department of Social Sciences (I Dergaa PhD), University of Jendouba, El Kef, Tunisia; Department of Forensic Medicine (E Dervišević PhD), University of Sarajevo, Sarajevo, Bosnia and Herzegovina; Chettinad Hospital & Research Institute (Prof V Devanbu MD), Chettinad Academy of Research and Education, Chennai, India; Department of Cardiology (P Devarakonda MD), Department of Neurosurgery (K Margetis MD), Icahn School of Medicine at Mount Sinai, New York, NY, USA; Department of Pharmacy (S Dewan PhD), United International University,

Dhaka, Bangladesh; Pharmacology Division (S Dewan PhD), Center for Life Sciences Research Bangladesh, Dhaka, Bangladesh; Sheffield Teaching Hospitals NHS Foundation Trust, Sheffield, UK (A Dhali MBBS); Division of Pathology (K Dhama PhD), ICAR-Indian Veterinary Research Institute, Bareilly, India; Neurology Department Institute of Human Behavior and Allied Sciences (Prof R K Dhamija MD), University of Delhi, New Delhi, India; Research and Development Cell (A S Dhane MBA), Department of Oral Pathology and Microbiology (Prof G S Sarode PhD, Prof S C Sarode PhD), Dr. D. Y. Patil Vidyapeeth, Pune (Deemed to be University), Pune, India; Department of Zoology (N K Dhania PhD), University of Delhi, Delhi, India; Research Department (M L Dhimal PhD), Planetary Health Research Centre, Kathmandu, Nepal; Institute of Occupational, Social and Environmental Medicine (M L Dhimal PhD, M Dhimal PhD), Goethe University, Frankfurt am Main, Germany; Research Department (M Dhimal PhD, S Ghimire MPH), Nepal Health Research Council, Kathmandu, Nepal; Department of Pharmacy Practice (S Dhingra PhD), National Institute of Pharmaceutical Education and Research Hajipur, Hajipur, India; Population Interventions Unit (B Dhungel DrPH), Department of Medicine (Prof F K Jebasingh DM), School of Health Sciences (A Meretoja MD), Melbourne School of Population and Global Health (M M Mittinty PhD, L Reifels PhD), University of Melbourne, Melbourne, VIC, Australia; Department of Life Science and Public Health (M Di Pumpo DrPH), Università Cattolica del Sacro Cuore (Catholic University of the Sacred Heart), Rome, Italy; Escola Superior de Saúde (Higher School of Health) (Prof D Dias da Silva PhD), Instituto Politécnico do Porto (Polytechnic Institute of Porto), Porto, Portugal; Department of Anesthesiology (K Didehvar MD), Rutgers University, Newark, NJ, USA; Department of Otolaryngology - Head and Neck Surgery (L K Dillard PhD), Medical University of South Carolina, Charleston, SC, USA; Joe C. Wen School of Population & Public Health (X Ding MA), University of California Irvine, Irvine, CA, USA; College of Health Sciences (H P Do PhD), VinUniversity, Ha Noi, Vietnam; Institute of Health Economics and Technology (iHEAT), Hanoi, Vietnam (H P Do PhD); Department of Medicine (T H P Do MD), Can Tho University of Medicine and Pharmacy, Can Tho, Vietnam; Department of Social Medicine and Health Care Organisation (Prof K G Dokova PhD), Medical University of Varna, Varna, Bulgaria; Mahidol Oxford Tropical Medicine Research Unit (C Dolecek PhD), Mahidol University, Bangkok, Thailand; Nuclear Medicine Department (F Dondi MD), ASST Spedali Civili di Brescia and Università degli Studi di Brescia, Brescia, Italy; Cardio-Thoraco-Vascular Department (Prof M D'Oria MD), Azienda Sanitaria Universitaria Giuliano Isontina, Trieste, Italy; Independent Consultant, Bridgewater, NJ, USA (O P Doshi MS); Clínica Pró Coração, São Paulo, Brazil (P M Dourado PhD); Department of Epidemiology (M Dresse MD), University of Pittsburgh, Pittsburgh, PA, USA; Department of Psychiatry (M Dresse MD), University of Pittsburgh Medical Center, Pittsburgh, PA, USA; Department of Medicine (A C Dsouza MBBS), Bangalore Medical College and Research Institute, Bangalore, India; Faculty of Health, Medicine and Life Sciences (FHML) (V S Dsouza MSc), Care and Public Health Research Institute (CAPHRI) (R Kamath MHA), Maastricht University, Maastricht, Netherlands; Department of Pathology (Prof J Du PhD), China Medical University, Liaoning, China; Office of Institutional Analysis (J Dube MA), University of Windsor, Windsor, ON, Canada; School of Sociology (E W Dumbili PhD), UCD Centre for Disability Studies (C Linehan PhD), University College Dublin, Dublin, Ireland; Postgraduate Program in Health Sciences (S C Dumith PhD), Federal University of Rio Grande do Sul, Rio Grande, Brazil; School of Medicine (Prof A R Duraes PhD), Institute of Collective Health (Prof D Rasella PhD), Federal University of Bahia, Salvador, Brazil; Department of Internal Medicine (Prof A R Duraes PhD), Escola Bahiana de Medicina e Saúde Pública (Bahiana School of Medicine and Public Health), Salvador, Brazil; Faculty of Science and Humanities (S Duraisamy PhD), SRM Institute of Science and Technology, Kattankulathur, India; Department of Conservative Dentistry with Endodontics (A M Dziedzic DSc), Medical University of Silesia, Katowice,

Poland; Department of Biological and Chemical Sciences (O Ebohon MPH), Michael and Cecilia Ibru University, Delta State, Nigeria; Department of Psychiatry (E Eboreime PhD, E Tsermpini PhD), Department of Physics and Atmospheric Science (Prof R V Martin PhD), Dalhousie University, Halifax, NS, Canada; Department of Psychiatry (E Eboreime PhD), Department of Medicine (E Lytvyak MD), Faculty of Nursing (U Yunusa PhD), University of Alberta, Edmonton, AB, Canada; Histology Department (L L M Ebraheim PhD), Department of Animal Medicine (I Elsohaby PhD), Department of Pathology (Prof M M M Metwally PhD), Cardiovascular Department (Prof A M A Saad MD), Department of Microbiology and Immunology (G Yahya PhD), Zagazig University, Zagazig, Egypt (Prof M I Husseiny PhD); Department of Public Health Science (H Xiao PhD), Fred Hutchinson Cancer Research Center, Seattle, WA, USA (A Ebrahimi MD); Environmental and Occupational Health Research Center (M Ebrahimi MD), Shahroud University of Medical Sciences, Shahroud, Iran; Higher School of Technology (Prof A Ed-Dra PhD), Sultan Moulay Slimane University, Beni Mellal, Morocco; Department of Zoology (E G Edelduok PhD), University of Uyo, Ikot Akpaden, Nigeria; School of Nursing and Midwifery (Prof K Edvardsson PhD), School of Nursing and Midwifery (E M M Has PhD), La Trobe University, Bundoora, VIC, Australia; La Trobe University, Melbourne, VIC, Australia (Prof F Efendi PhD); Semnan University of Medical Sciences and Health (F Ehsani PhD), Samara University, Semnan, Iran; Isenberg School of Management (A Eighaei Sedeh MD), University of Massachusetts Amherst, Amherst, MA, USA; Centre for Global Health Inequalities Research (CHAIN) (Prof T Eikemo PhD), Department of Neuromedicine and Movement Science (A K Husøy PhD, Prof T J Steiner PhD, Prof L J Stovner PhD), Department of Circulation and Medical Imaging (J Nauman PhD), Norwegian University of Science and Technology, Trondheim, Norway; Private Orthodontist, Ahvaz, Iran (E Eini MSD); Faculty of Science and Health (M Ekholuenetale PhD), University of Portsmouth, Hampshire, UK; Almoosa College of Health Sciences, Al Ahsa, Saudi Arabia (R A El Arab PhD); College of Medicine (Prof R Elbeshbeishy PhD), Department of Periodontics (N T Hashim PhD), Department of Pharmacology (S Srinivasamurthy MD), RAK Medical and Health Sciences University, Ras Al Khaimah, United Arab Emirates; Faculty of Medicine (Prof R Elbeshbeishy PhD), Department of Internal Medicine (Prof G M T ElGohary MD), Department of Neuropsychiatry (Prof G ELNahas MD), Biochemistry Department (Prof N M Hamdy PhD), Department of Entomology (A M Samy PhD), Medical Ain Shams Research Institute (MASRI) (A M Samy PhD), Neurology Department (Prof A S Shalash PhD), Ain Shams University, Cairo, Egypt; College of Medicine (M Elhadi MD, Prof S Jeong PhD), Department of Medicine (I R Fakhradiyev PhD), School of Health and Environmental Science (Prof J Kang PhD), Department of Health Policy and Management (Prof J Kim PhD), Department of Preventive Medicine (Prof Y Lee PhD), Korea University, Seoul, South Korea (Prof M Shin PhD); Houston Methodist Hospital, Houston, TX, USA (M Elhadi MD); National Institute of Public Health Research (M Elhoumed PhD), Ministry of Health, Nouakchott, Mauritania; Faculty of Applied Health Science (Prof S Elkannishy PhD), Horus University, New Damietta, Egypt; School of Pharmacy and Pharmaceutical Sciences (M Elnaem PhD), Ulster University, Coleraine, UK; Executive Committee (Prof G ELNahas MD), International Association for Women Mental Health, Potomac, MD, USA; Department of Infectious Diseases and Public Health (I Elsohaby PhD, G Fekadu PhD, Prof W Ming MD), Department of Biomedical Sciences (W Jin MD), City University of Hong Kong, Hong Kong, China; Faculty of Veterinary Medicine (Prof A S A Eltahawy PhD), Damanhour University, Damanhur, Egypt; Department of Midwifery (T Emagneneh MSc), Woldia University, Addis Ababa, Ethiopia; Health Research and Technology Transfer Directorate (M Endriyas MSc), South Ethiopia Region Public Health Institute, Jinka, Ethiopia; Department of Public Health (M Endriyas MSc), Department of Pathology (A M Nisro MD), Hawassa University, Hawassa, Ethiopia; Queensland Centre for Mental Health Research, Brisbane, QLD, Australia (H E Erskine PhD);

Department of Paediatrics (C I Esezobor MB), Department of Psychiatry (Prof A T Olagunju PhD), University of Lagos, Lagos, Nigeria; Department of Paediatrics (C I Esezobor MB), Lagos University Teaching Hospital, Lagos, Nigeria; Goba College of Medicine and Health Sciences (D Eshetu MSc), Madda Walabu University, Robe, Ethiopia; Wassa Amenfi East Municipal Health Directorate (G Eshun BSc), Ghana Health Service, Wassa Akropong, Ghana; Department of Bacteriology and Virology (M Eslami PhD), Semnan University of Medical Sciences, Semnan, Iran; Cancer Research Center (M Eslami PhD), Semnan University of Medical Scinces, Semnan, Iran; Department of Public Health (F Eva MPH), North South University, Dhaka, Bangladesh; Department of Anesthesia (A O Fadaka PhD), Cincinnati Children's Hospital Medical Center, Cincinnati, OH, USA; Department of Biotechnology (A O Fadaka PhD), School of Pharmacy (O C Okonji MSc), University of the Western Cape, Cape Town, South Africa; Department of Electrical and Computer Engineering (H Fadavian MSc), Department of Electrical and Computer Engineering (ECE) (Prof D Fathi PhD), Department of Biostatistics (K Gohari MS), Department of Bacteriology (M Mohammadi MSc), Department of Hematology (B Razi PhD), Tarbiat Modares University, Tehran, Iran; Research Centre for Healthcare and Community (A F Fagbamigbe PhD), Faculty of Health and Life Sciences (O P Kurmi PhD), Centre for Intelligent Healthcare (H Liu PhD), Coventry University, Coventry, UK; Department of Oral Biology (A Fahim PhD), Riphah International University, Islamabad, Pakistan (Z Z Piracha PhD); Department of Food Hygiene and Quality Control (A Fakhri-Demeshghieh PhD), School of Biotechnology (M Yeganeh PhD), University of Tehran, Tehran, Iran; Department of Public Health Sciences (Q Fan DrPH), Clemson University, Clemson, SC, USA; Saveetha Medical College and Hospital (M Fareed PhD), Saveetha Institute of Medical and Technical Sciences (SIMATS), Chennai, India; Division of Statistics (Z Farhana MS), Bangladesh Bank, Sylhet, Bangladesh; Environmental Statistics Unit (C S E S Farinha PhD), National Institute of Statistics, Lisbon, Portugal; Ecological Economics and Environmental Management (C S E S Farinha PhD), NOVA University of Lisbon, Lisbon, Portugal; Department of Psychology (A Faro PhD), Federal University of Sergipe, São Cristóvão, Brazil; Department of Radiography and Imaging Technology (S Farooq PhD), Department of Public Health (S Hameed PhD), Green International University, Lahore, Pakistan; Department of Family Medicine (U Farooque MD), Luton & Dunstable University Hospital, Luton, UK; Department of Clinical Psychology (M Faruk MSc), Department of Population Sciences (Prof M B Hossain PhD), University of Dhaka, Dhaka, Bangladesh; Community-based Inclusive Mental Health Department (M Faruk MSc), Centre for Disability in Development (CDD), Dhaka, Bangladesh; Department of Veterinary Tropical Diseases (Prof F O Fasina PhD), University of Pretoria, Pretoria, South Africa; Animal Production and Health Division (EMPRES) (Prof F O Fasina PhD), Food and Agriculture Organization of the United Nations, Rome, Italy; Charité University Berlin (M M Fasina MSc), Institute of Public Health (F Fischer PhD), Charité Medical University Berlin (Charité Universitätsmedizin Berlin), Berlin, Germany; Department of Chemistry and Biochemistry (E T Fasusi MSc), University System of Georgia, Statesboro, GA, USA; School of Engineering (A Fatehizadeh PhD), Edith Cowan University, Joondalup, WA, Australia; Department of Environmental Health Engineering (M Fazlzadeh PhD, M Vosoughi PhD), Department of Occupational Health and Safety Engineering (Prof M Poursadeqiyan PhD), Ardabil University of Medical Science, Ardabil, Iran; Department of Cardiovascular Surgery (Prof L Fei MD), Division of Gastroenterology (Prof Z Wu PhD), Tongji Medical College (G Xiao MD), Huazhong University of Science and Technology, Wuhan, China; National Institute for Stroke and Applied Neurosciences (Prof V L Feigin PhD), The National Institute for Stroke and Applied Neurosciences (I Rautalin PhD), Auckland University of Technology, Auckland, New Zealand; Third Department of Neurology (E V Gnedovskaya PhD), Research Center of Neurology, Moscow, Russia (Prof V L Feigin PhD, Prof M A Piradov DSc); Department of Urology (D Feng MD), The

First Affiliated Hospital of Zhejiang Chinese Medical University, Hangzhou, China; School of Acupuncture-Tuina (K Feng BA), First Clinical Medicine (W Li‡ BSc), School of Acupuncture-Tuina (L Sui MS), Shandong University of Traditional Chinese Medicine, Jinan, China; National Institute of Environmental Health (X Feng PhD), National Center for Chronic and Noncommunicable Disease Control and Prevention (P Ye PhD), Chinese Center for Disease Control and Prevention, Beijing, China; Department of Biomedical Engineering (T Ferdous MSc), Department of Decision and Information Sciences (M Hossain DrPH), Department of Biology and Biochemistry (S Ullah MSc), University of Houston, Houston, TX, USA; Division of Neurology (S Fereshtehnejad PhD), University Health Network (S Mirshahvalad MD), Dalla Lana School of Public Health (S Popova PhD), University of Toronto, Toronto, ON, Canada; Cardiovascular Health and Imaging Laboratory (R Fernandez-Jimenez PhD), Centro Nacional de Investigaciones Cardiovasculares (CNIC) (National Centre for Cardiovascular Disease Research), Madrid, Spain; Department of Cardiology (R Fernandez-Jimenez PhD), Hospital Clinico San Carlos, IdISSC, Madrid, Spain; Laboratory of Public Health (P Ferrara PhD, Prof L G Mantovani DSc), IRCCS Istituto Auxologico Italiano, Milan, Italy; West Moreton Hospital Health Services (A M Mantilla Herrera PhD), Queensland Centre for Mental Health Research, Wacol, QLD, Australia (A J Ferrari PhD, D F Santomauro PhD, J Shadid BSc, Prof H A Whiteford PhD); Ophthalmology (Prof A Ferreira PhD), Hospital Center of Porto, Porto, Portugal; Department of Social Sciences (Prof N Ferreira PhD, Prof M J M Sullman PhD), Department of Life and Health Sciences (Prof M J M Sullman PhD), University of Nicosia, Nicosia, Cyprus; Medical School (A Finnemore Dipl), Universidad de Navarra, Pamplona, Spain; Department of Cardiac, Thoracic, Vascular Sciences and Public Health (M Fonzo MD), University of Padova, Padova, Italy; Division of Pediatric Hematology-Oncology (L M Force MD), St. Jude Children's Research Hospital, Seattle, WA, USA; Department of Neurology, Public Health and Disability (A Fornari PhD), SC Neurologia, Salute Pubblica e Disabilità (Neurology, Public Health, Disability Unit) (M Leonardi MD), Fondazione IRCCS Istituto Neurologico Carlo Besta, Milan, Italy; Department of Disease Burden (I Forthun PhD, C Madsen PhD, C Schwinger PhD), GBD Collaborating Unit (K Giannakis PhD, Prof S E Vollset DrPH), Centre for Disease Burden (A S Knudsen PhD), Norwegian Institute of Public Health, Bergen, Norway; Emilia-Romagna Region - Innovation in Healthcare and Social Services Department, Bologna, Italy (D Fortuna MSc); Department of Neuroscience (M Foschi MD), Multiple Sclerosis Research Center, Ravenna, Italy; Department of Biotechnological and Applied Clinical Sciences (M Foschi MD), University of L’Aquila, L’Aquila, Italy; Clinical Epidemiology Division (KEP) (K R Fowobaje PhD), Karolinska Institute, Stockholm, Sweden; Department of Microbiology and Parasitology (J Foyet F MSc, G Nchanji PhD), University of Buea, Buea, Cameroon; Center for Health Technology and Services Research (CINTESIS), Porto, Portugal (A Freitas PhD); Department of Biostatistics (J Fu MD, Prof X Gao PhD), Key Lab of Environment and Health (Prof X Gao PhD), School of Public Health (Y Huang PhD, Prof J Li PhD, P li PhD, W Li§ PhD, Prof Z Qi PhD, Prof W Song PhD, F Wang PhD, Prof W Wang PhD, X Yang PhD, T Zhan PhD), Department of Epidemiology (D Yin DrPH), Xuzhou Medical University, Xuzhou, China (M Jiang PhD); Department of Pathology (Prof B Fux PhD), Department of Integrated Health Education (Prof L B Salaroli PhD), Federal University of Espirito Santo, Vitória, Brazil; Health Services Management Training Centre (Prof P A Gaal PhD, T Joo PhD, J Lám PhD, T Palicz MD), Institute of Digital Health Sciences (P Pollner PhD), Semmelweis University, Budapest, Hungary; Department of Applied Social Sciences (Prof P A Gaal PhD), Sapientia Hungarian University of Transylvania, Târgu-Mureş, Romania; School of Public Health (D Gadeka PhD), University of Ghana, Legon, Accra, Ghana; Department of Public Health (M Gajdács PhD), University of Szeged, Szeged, Hungary; Department of Food Technology (Y Galali ResM, B A Sadee PhD), Department of Statistics (Prof D H Kadir PhD), Department of Chemistry (H I M Amin PhD), Salahaddin University-

Erbil, Erbil, Iraq; Department of Nutrition and Dietetics (Y Galali ResM, B A Sadee PhD), Department of Medical Biochemical Analysis (A Ghandili PhD, H I M Amin PhD, Y Mirzaei PhD), Department of Business Administrations (Prof D H Kadir PhD), Cihan University-Erbil, Erbil, Iraq; Department of Medical Epidemiology (S Gallus PhD, A Lugo PhD), Mario Negri Institute for Pharmacological Research, Milan, Italy; Department of Endocrinology (Prof M Ganie MD), Department of Internal and Pulmonary Medicine (Prof P A Koul MD), Sheri Kashmir Institute of Medical Sciences, Srinagar, India; Department of Endocrinology and Metabolism (Prof M Ganie MD), All India Institute of Medical Sciences, Delhi, India; Clinical Nuclear Medicine Center (D Gao MS), Shanghai Tenth People's Hospital, Shanghai, China; Department of Nuclear Medicine (D Gao MS), Tongji University Tenth People's Hospital, Shanghai, China; Department of Public Health (B Garba PhD), SIMAD University Mogadishu. Somalia, Mogadishu, Somalia; School of Medicine (M Garcia-Argibay PhD), Orebro University, Orebro, Sweden; Department of Medicine (Prof D Garcia-Azorin MD), University of Valladolid, Valladolid, Spain; Department of Neurology (Prof D Garcia-Azorin MD), Hospital Universitario Rio Hortega, Valladolid, Spain; Infectious Diseases Unit (J Garlasco MD), University of Verona, Verona, Italy; Professional Services Division (P Gautam PhD), Texas State Board of Pharmacy, Austin, TX, USA; Department of Pharmacology (Prof R K Gautam PhD), IES Institute of Pharmacy, Bhopal, India; Independent Consultant, Rome, Italy (F Gazzelloni MSc); Department of Midwifery (M W Gebregergis MSc), School of Public Health (H G Gebreslassie MPH), Department of Epidemiology (M Mehari MPH), Department of Medical Laboratory Sciences (H N Meles MSc), Adigrat University, Adigrat, Ethiopia; Department of Neurosciences, Neurology and Stroke Unit (S Gelibter MD), Stroke Unit and Neurology Unit (G Schwarz MD), ASST Grande Ospedale Metropolitano Niguarda, Milan, Italy; Institute of Public Health (N S George MPH), Department of Health Economics and Social Security (K Kissimova-Skarbek PhD), Jagiellonian University Medical College, Krakow, Poland; Department of Public Health (G K Getahun MPH), Menelik II Medical and Health Science College, Addis Ababa, Ethiopia; Infectious Disease Research Center (Prof K Ghadiri MD), Pediatric Department (Prof K Ghadiri MD), Universal Scientific Education and Research Network (USERN) (P Goleij MSc), Research Center for Environmental Determinants of Health (Prof E Sadeghi PhD), Kermanshah University of Medical Sciences, Kermanshah, Iran; Research Committee of Qom University of Medical Sciences (A Ghamkhar BSc), Qom University of Medical Sciences, Qom, Iran; Department of Global Health Sciences (S Ghasemi Assl MD), Department of Epidemiology and Biostatistics (Prof K M Mehta DSc), Division of Cardiology (J Noubiap MD), Department of Neurosurgery (A Orscelik MD, Y Senol MD), School of Nursing (J Ouner PhD), University of California San Francisco, San Francisco, CA, USA; School of Medicine (H Ghasrsaz MD), Mazandaran University of Medical Sciences, Mazandaran, Iran; Family and Community Medicine Department (R M Ghazy PhD), King Khalid University, Abha, Saudi Arabia; Research Group for Childhood Cancer (N Ghith PhD), Danish Cancer Research Institute, Copenhagen, Denmark; Department of Dermatology (N Gholizadeh MD, G Rahmatpour Rokni MD), Invasive Fungi Research Center (J Javidnia PhD), Department of Medical Mycology (J Javidnia PhD), Department of Medical-Surgical Nursing (S Shorofi PhD), Mazandaran University of Medical Sciences, Sari, Iran; Department of Epidemiology and Prevention (A Gialluisi PhD), IRCCS Neuromed, Pozzilli, Italy; Country Office (A U Gil PhD), World Health Organization (WHO), Astana, Kazakhstan; Department of Zoology (B R Giri PhD), KKS Women's College, Balasore, India; Department of Nursing (A A Girmay MSc), Aksum University, Aksum, Ethiopia; Department of Anesthesiology and Critical Care Medicine (A Girombelli MD), Ospedale SS Annunziata Savigliano, Savigliano, Italy; Department of Health Systems and Policy Research (Prof M Golechha PhD), Indian Institute of Public Health, Gandhinagar, India; Department of Genetics (P Goleij MSc), Sana Institute of Higher Education,

Sari, Iran; Department of Life Sciences, Health and Healthcare Professions (Prof D Golinelli MD), Link Campus University, Rome, Italy; Health Services Research, Evaluation and Policy Unit (Prof D Golinelli MD), AUSL della Romagna, Ravenna, Italy; Research Institute for Endocrine Sciences, Tehran, Iran (M Golmohammadi MD); Senior Department of Tuberculosis (Prof W Gong PhD), The Eighth Medical Center of PLA General Hospital, Beijing, China; Department of Epidemiology (Prof A C Goulart PhD), Universidade de São Paulo (University of São Paulo), São Paulo, Brazil; Department of Dermatology (A Grada MD), Case Western Reserve University, Libertyville, IL, USA; Liverpool Orthopaedic and Trauma Service (S Graham PhD), Institute of Population Health Sciences (M R Mathur PhD), University of Liverpool, Liverpool, UK; Department of Public Health and Preventive Medicine (Prof M Grivna PhD), Charles University, Prague, Czech Republic; Department of Epidemiology and Biostatistics (S Guan MD, Prof H Pan PhD), Department of Urology (C Mao MSc), Anhui Medical University, Hefei, China; Health Direction (G Guarducci MD), Local Health Authority of Ferrara, Ferrara, Italy; Department of Clinical Science (M I M Gubari PhD), University Of Sulaimani, Sulaimani, Iraq; Department of Community Medicine (D A Gunawardane MD, Prof S N K Navaratna MD), University of Peradeniya, Kandy, Sri Lanka; Department of Geriatric Neurology (X Guo PhD), Shaanxi Provincial People’s Hospital, Xi'an, China; Division of Epidemiology (Z Guo PhD), Vanderbilt University Medical Center, Nashville, TN, USA; Nanyang Maternal and Child Health Care Hospital (Z Guo MPH), Nanyang Central Hospital, Nanyang, China; Department of Nephrology (A K Gupta PharmD), Max Super Specialty Hospital, New Delhi, India; Non-communicable Diseases Division (NCD) (A K Gupta PharmD), Indian Council of Medical Research, New Delhi, India (D K Lal MD, L Singh PhD); College of Medicine and Public Health (H Gupta PhD), Flinders University, Adelaide, VIC, Australia; Independent Consultant, Bharatpur, India (I Gupta MD); Independent Consultant, Delhi, India (I Gupta MD); Department of Anaesthesia (Prof L Gupta MD), Maulana Azad Medical College, New Delhi, India; Department of Preventive Cardiology & Medicine (Prof R Gupta MD), Eternal Heart Care Centre & Research Institute, Jaipur, India; Department of Medicine (Prof R Gupta MD), Mahatma Gandhi University Medical Sciences, Jaipur, India; Department of Toxicology (S Gupta PhD), Shriram Institute for Industrial Research, Delhi, India; School of Biotechnology (V Gupta PhD), Dublin City University, Dublin, Ireland; Department of Anthropology (V Gupta PhD), Deemed University, Delhi, India; Faculty of Health, Medicine and Life Sciences (FHML) (Prof V K Gupta PhD), Australian Institute of Health Innovation (P Peprah MSc), Macquarie University, Sydney, NSW, Australia; Doctoral Program in Biomedical Gerontology (R S Gutiérrez-Murillo PhD), Pontifical Catholic University of Rio Grande do Sul, Porto Alegre, Brazil; Research Unit in Epidemiology Clinic (J Guzman-Esquivel DSc), Mexican Institute of Social Segurity, Colima, Mexico; Neurosurgery Department (A Habibzadeh MD), Fasa University of Medical Sciences, Shiraz, Iran; College of Health Science (A Habteyes MPH), Dilla University, Dilla, Ethiopia; Department of Clinical Pharmacology and Medicine (Prof N R Hadi PhD), University of Kufa, Najaf, Iraq; School of Medicine (A Haghtalab MD), Hamedan University of Medical Sciences, Hamedan, Iran; Department of Community Medicine (P Halder MD, A KM MD), Department of Pharmacology (A K Kakkar MD), Department of Pediatrics (J Kumar MD), Post Graduate Institute of Medical Education and Research, Chandigarh, India; Department of Infectious Disease Epidemiology (S Haller MD), Robert Koch Institute, Berlin, Germany; Department of Public Health (S Haller MD), Charité Insitute of Public Health, Berlin, Germany; Department of Pharmacy (Prof I M Hamad PhD), American University of Madaba, Amman, Jordan; Department of Family and Community Medicine (Prof R R Hamadeh PhD), College of Medicine and Health Sciences (H Jahrami PhD), Arabian Gulf University, Manama, Bahrain; Department of Medical and Technical Information Technology (A Hammoud PhD), Bauman Moscow State Technical University, Moscow, Russia; Guthrie Medical Group

(M Hamza MD), Guthrie Medical Group, Cortland, NY, USA; Edirne Public Health Center (D Han Yekdeş MD), Edirne Provincial Health Directorate, Edirne, Turkiye; Sakarya University, Sakarya, Turkiye (A Hanif PhD); Stroke Research Centre (Prof G J Hankey MD), Perron Institute for Neurological and Translational Science, Perth, WA, Australia; Department of Health and Education (F Hanna PhD), Torrens University Australia, Melbourne, VIC, Australia; Department of Population Science and Human Resource Development (Prof M Haque PhD, Prof M Rahman PhD, Prof M Rahman DrPH), Department of Physics (A Hossain PhD), University of Rajshahi, Rajshahi, Bangladesh; Medical Research Unit (H Harapan PhD), Universitas Syiah Kuala (Syiah Kuala University), Banda Aceh, Indonesia; Vital and Health Statistics (H L Harb MPH), Ministry of Health, Beirut, Lebanon; University of Nevada Reno, Reno, NV, USA (K L Harding PhD); Directorate General of Health Human Resources (A M A Hariandja DrPH), Center for Health System and Strategy (A Nur MPH), Ministry of Health, Jakarta, Indonesia; Research Unit (J M Haro MD), Parc Sanitari Sant Joan de Deu, Barcelona, Spain; Department of Mental Health (J M Haro MD), Carlos III Health Institute (Prof R Tabarés-Seisdedos PhD), Biomedical Research Networking Center for Mental Health Network (CiberSAM), Madrid, Spain; Faculty of Nursing (F Hasan PhD, D S Romadlon PhD), Chulalongkorn University, Bangkok, Thailand; Department of Health Research Methods, Evidence, and Impact (M Hasan MPH), Department of Medicine (O P Kurmi PhD), Department of Psychiatry and Behavioural Neurosciences (Prof A T Olagunju PhD), McMaster University, Hamilton, ON, Canada; Department of Biochemistry and Molecular Biology (M Hasan MPH), Tejgaon College, Dhaka, Bangladesh; Department of Medical Surgical (Prof A Hasanpour- Dehkordi PhD), Shahroud University of Medical Sciences, Shahrekord, Iran; Department of Biotechnology (A Hasnain PhD), Lahore University of Biological and Applied Sciences, Lahore, Pakistan; Department of Medicine (I Hassan MD), University of Khartoum Faculty of Medicine, Khartoum, Sudan; Department of Community Medicine (I Hassan PhD), Federal University Teaching Hospital, Lafia, Nigeria; Department of Epidemiology and Community Medicine (I Hassan PhD), Federal University of Lafia, Lafia, Nigeria; National Data Management Center (M Hassen MSc), Ethiopian Public Health Institute, Addis Ababa, Ethiopia; Department of Paediatrics (L W Hathagoda MD, Prof S Rajindrajith PhD), Postgraduate Institute of Medicine (A U Jayatilleke PhD, Prof S N K Navaratna MD, J Samaranayake MBBS), Department of Anatomy, Genetics and Biomedical Informatics (Y Mathangasinghe PhD), Department of Surgery (D P Wickramasinghe MD), University of Colombo, Colombo, Sri Lanka; Skaane University Hospital (R J Havmoeller PhD), Skaane County Council, Malmö, Sweden; Faculty of Medicine (A Hawat MD), Damascus University, Damascus, Syria; Institute of Pharmaceutical Sciences (K Hayat MS), University of Veterinary and Animal Sciences, Lahore, Pakistan; Department of Pharmacy Administration and Clinical Pharmacy (K Hayat MS), Xian Jiaotong University, Xian, China; Department of Neurosurgery (J He MD), Beijing Fengtai Hospital, Beijing, China; Faculty of Kinesiology (Prof J J Hebert PhD), University of New Brunswick, Fredericton, NB, Canada; School of Allied Health (Prof J J Hebert PhD), Murdoch University, Murdoch, WA, Australia; Community-Oriented Nursing Midwifery Research Center (M Heidari PhD), Department of Community Health (M Lotfizadeh PhD), Social Determinants of Health Research Center (M Lotfizadeh PhD), Modeling in Health Research Center (A Mohammadian-Hafshejani PhD), Department of Epidemiology and Biostatistics (H Raeisi Shahraki PhD), Shahrekord University of Medical Sciences, Shahrekord, Iran (S JamshidiRastabi MSc); Department of Medicine (M Hemmati MD), Internal Medicine Department (L Manjani MD), MedStar Health, Washington, DC, USA; Babes-Bolyai University, Cluj-Napoca, Romania (Prof C Herteliu PhD); Australian Centre for Health Service Innovations (S A Hewage MD), Australian Centre for Health Services Innovation (Prof S M McPhail PhD), International Laboratory for Air Quality and Health (Prof L Morawska PhD), Faculty of Science (M Sarmadi MSc), Queensland University of Technology, Brisbane, QLD, Australia;

Department of Virology (Z Heydarifard PhD), Lorestan University of Medical Sciences, Khorramabad, Iran; Department of Microbiology (K Hezam PhD), Faculty of Applied Sciences (E A Noman PhD), Taiz University, Taiz, Yemen; School of Medicine (K Hezam PhD), Nankai University, Tianjin, China; Graduate School of Medicine (Y Hiraike PhD), Department of Global Health Policy (S K Rauniyar PhD), University of Tokyo, Tokyo, Japan; Centre for Advancing Health Outcomes, Vancouver, BC, Canada (M Hossain PhD); Public Health Research Group (M Hossain DrPH), Nature Study Society of Bangladesh, Khulna, Bangladesh; Department of Statistics (M Hossain MSc, S Noor MS), Shahjalal University of Science and Technology, Sylhet, Bangladesh; School of Health and Society (Prof H Hosseinzadeh PhD), Faculty of Science, Medicine and Health (P A Saunders PhD), University of Wollongong, Wollongong, NSW, Australia; School of Engineering and Technology (Prof M Hosseinzadeh PhD), Faculty of Medicine (T Nguyen PhD), Duy Tan University, Da Nang, Vietnam; Jadara Research Center (Prof M Hosseinzadeh PhD), Jadara University, Irbid, Jordan; Department of Clinical Legal Medicine (Prof S Hostiuc PhD), National Institute of Legal Medicine Mina Minovici, Bucharest, Romania; Department of Community Medicine (M Htay PhD), Manipal University College Malaysia, Melaka, Malaysia; Department of Psychological and Cognitive Sciences (C Hu PhD), Tsinghua Vanke School of Public Health (Z Li PhD), Vanke School of Public Health (Y Xia MD), Tsinghua University, Beijing, China; Maternal Care and Child Health Department (Prof Y Hu PhD), Department of Neurosurgery (S Wang MD), Capital Medical University, Beijing, China; Department of Otorhinolaryngology Head and Neck Surgery (W Huang PhD), School of Medicine (Z Li PhD), Shanghai Mental Health Center (Prof M R Phillips MD), Ruijin Hospital (S Xu MPH), School of Global Health (J Zheng PhD), Shanghai Jiao Tong University, Shanghai, China; Pediatric Nursing Department (M H Huda PhD), Faculty of Public Health (D Kusuma DSc, Prof I Trihandini PhD), Centre for Family Welfare (K Latief PhD), University of Indonesia, Depok, Indonesia; East Manggarai Regency Health Office (A I Human BSN), Ministry of Health, Borong, Indonesia; Department of Social Sciences and Business (Prof M Hussain PhD), Roskilde University, Roskilde, Denmark; Czech National Centre for Evidence-Based Healthcare and Knowledge Translation (S Hussain PhD), Institute of Biostatistics and Analyses (S Hussain PhD), Masaryk University, Brno, Czech Republic; Clinical Governance and Quality Improvement Head (D Hussein MPH), Salale University, Gerba Guracha, Ethiopia; Department of Biomolecular Sciences (Prof N R Hussein PhD), Department of Biology (K S Ibrahim PhD), Department of Biomedical Sciences (Prof I A Naqid PhD), University of Zakho, Zakho, Iraq; Artur Riggs Diabetes & Metabolism Research Institute (Prof M I Husseiny PhD), Cancer Prevention and Research Institute, Duarte, CA, USA; International Master Program for Translational Science (H Huynh BS), School of Nursing (M Kurniasari PhD, I Rohmah MSN), Department of Global Health and Health Security (K Latief PhD), International Ph.D. Program in Medicine (M H N Le MD), Research Center for Artificial Intelligence in Medicine (M H N Le MD), International Ph.D. Program in Biotech and Healthcare Management (M Muhtar MBA), Graduate Institute of Biomedical Informatics (D N A Ningrum PhD), Taipei Medical University, Taipei, Taiwan; Department of Occupational Safety and Health (Prof B Hwang PhD), College of Public Health (R Lin PhD), China Medical University, Taiwan, Taichung, Taiwan; Department of Occupational Therapy (Prof B Hwang PhD), Asia University, Taiwan, Taichung, Taiwan; Department of Biomedical, Metabolic, and Neural Science (L F Iannone MD), University of Modena and Reggio Emilia, Modena, Italy; Gastroenterology and Hepatology (A Ibrahim MD), Alexandria University, Charleston, SC, USA; Genetics and Molecular Biology Department (R Ibrahim PhD), Abu Dhabi University, Abu Dhabi, United Arab Emirates; Department of Medicine (F J Idalsoaga MD), Western University, London, ON, Canada; Health Policy and Management Department (P M Iftikhar MD), City University of New York, New York, NY, USA; Center for Nutritional Epidemiology and Policy Research (N Ikeda PhD),

National Institutes of Biomedical Innovation, Health and Nutrition, Settsu, Japan; Collaborative Alliance Research and Education (CARE) Programme (A Ikiroma PhD), Episcope Research Service, Aberdeen, Scotland; West Africa RCC (O S Ilesanmi PhD), Africa Centre for Disease Control and Prevention, Abuja, Nigeria; Faculty of Medical Sciences (Prof M D Ilic PhD), University of Kragujevac, Kragujevac, Serbia; Department of Clinical Pharmacy (M Imam PhD), College of Pharmacy (M Kamal PhD), Department of Computer and Self Development (A S Zamani PhD), College of Nursing (M Zoromba PhD), Prince Sattam bin Abdulaziz University, Al Kharj, Saudi Arabia; Department of Chemical Pathology (L C Imoh MPH), University of Jos, Jos, Nigeria; Department of Chemical Pathology (L C Imoh MPH), Jos University Teaching Hospital, Jos, Nigeria; Department of Health Research (L R Inbaraj MD), ICMR National Institute for Research in Tuberculosis, Chennai, India; Faculty of Health and Life Sciences (A Inok PhD), Living Systems Institute (Y Taheri Abkenar PharmD), University of Exeter, Exeter, UK; Department of Psychology (M Iqbal PhD), Department of Orthopedics (W Jin MD), Department of Epidemiology and Biostatistics (Prof S Mubarik PhD, Prof C Yu PhD), Wuhan University, Wuhan, China; Faculty of Pharmacy (L M Irham PhD), Faculty of Public Health (Prof S Solikhah DrPH), Universitas Ahmad Dahlan, Yogyakarta, Indonesia; Department of Biotechnology (M A Isa PhD), Sharda University, Greater Noida, India; Journal of Biological Sciences and Public Health, Dhaka, Bangladesh (D Islam PhD); Department of Surveillance and Health Equity Science (F Islami PhD), American Cancer Society, Atlanta, GA, USA; Clinical Laboratory Department (F Ismail PhD), Tobruk University, Tobruk, Libya; Department of Blood Transmitted Diseases (F Ismail PhD), National Centre for Disease Control (NCDC), Tobruk, Libya; Department of Clinical Pharmacy & Pharmacy Practice (Prof N E Ismail PhD), Asian Institute of Medicine, Science and Technology, Bedong, Malaysia; Malaysian Academy of Pharmacy, Puchong, Malaysia (Prof N E Ismail PhD); Public Health Department of Social Medicine (Prof H Iso MD), Osaka University, Suita, Japan; Department of Medicine (M C Ituka MD), Department of Internal Medicine and Specialties (J Nkeck MD), University of Yaoundé I, Yaounde, Cameroon; Department of Health Services Research (M Iwagami PhD), Department of Public Health Medicine (Prof K Yamagishi MD), University of Tsukuba, Tsukuba, Japan; Department of Global Health (C J Iwu-Jaja PhD), Cochrane South Africa (Prof C S Wiysonge MD), South African Medical Research Council, Cape Town, South Africa; Department of Global Health (C J Iwu-Jaja PhD, Prof P D Katoto PhD, Prof C S Wiysonge MD), Department of Epidemiology (J L Tamuzi MSc), Stellenbosch University, Cape Town, South Africa; Knowledge Translation Program (I O Iyamu MD), Centre for Health Evaluation and Outcome Sciences, Vancouver, BC, Canada; Department of Biotechnology (M Iyer PhD), Karpagam Academy of Higher Education, Coimbatore, India; Indian Institute of Public Health (V J Iyer MPH), Public Health Foundation of India, Gandhinagar, India; Department of Physical Medicine and Rehabilitation (L Jacob MD), Université Paris Cité, Paris, France; Research and Development Unit (L Jacob MD), Biomedical Research Networking Center for Mental Health Network (CiberSAM), Barcelona, Spain (R F Palma-Alvarez PhD); Department of Health Studies (K H Jacobsen PhD), University of Richmond, Richmond, VA, USA; Government Hospitals, Manama, Bahrain (H Jahrami PhD); Department of Health and Safety (A A Jairoun PhD), Dubai Municipality, Dubai, United Arab Emirates; Department of Research and Academic Affairs (V Jaiswal MD), Larkin Community Hospital, South Miami, FL, USA; Department of Medicine (V Jaiswal MD), AMA School of Medicine, Makati, Philippines; UNESCO-TWAS Section of Economic & Social Sciences, Humanities & Arts (Prof M Jakovljevic PhD), The World Academy of Sciences UNESCO-TWAS, Trieste, Italy; Shaanxi University of Technology, Hanzhong, China (Prof M Jakovljevic PhD); Department of Environmental Engineering (Prof R Jalilzadeh Yengejeh PhD), Islamic Azad University, Ahvaz, Iran; Department of Neurosurgery (M Jalloh MD), Division of Hematology and Oncology (J F Wu MD), Medical College of Wisconsin, Milwaukee, WI, USA;

Department of Primary Care Medicine (J Jamaluddin MMed), Universiti Malaya, Kuala Lumpur, Malaysia; Department of Public Health (S Jamil MPH, M Shimul MPH), Department of Development Studies (M Sohel MPH), Daffodil International University, Dhaka, Bangladesh; Department of Public and Community Health (S Jamil MPH), Frontier University Garowe, Puntland, Somalia; Institute for Neurosciences (Prof R G Jamora PhD), St. Luke's Medical Center, Bonifacio Global City, Philippines; Department of Stem Cells and Developmental Biology (E Jarrahi MSc), Royan Institution, Tehran, Iran; Department of Medicine (S Javaid MD), University of Mississippi Medical Center, Jackson, MS, USA; Department of Medicine (S Javaid MD), Jinnah Sindh Medical University, Karachi, Pakistan; Department of Nursing (Q Jawell Odah Abed PhD), Middle Technical University of Kut Technical Institute, Baghdad, Iraq; Department of Oral Medicine and Periodontology (Prof R D Jayasinghe MS, Y A Jayasinghe BSc), University of Peradeniya, Peradeniya, Sri Lanka; Department of Research (Y A Jayasinghe BSc, Prof K K Kanmodi MPH), University of Puthisastra, Phnom Penh, Cambodia; Faculty of Graduate Studies (A U Jayatilleke PhD), Institute for Violence and Injury Prevention, Colombo, Sri Lanka; Department of Endocrinology, Diabetes and Metabolism (Prof F K Jebasingh DM, Prof N Thomas PhD), Christian Medical College and Hospital (CMC), Vellore, India; Department of Epidemiology and Health Promotion (Prof S Jee PhD), Institute for Global Engagement & Empowerment (Prof S Oh PhD), Department of Pediatrics (Prof J Shin MD), Yonsei University, Seoul, South Korea; Department of Biotechnology (B Sharma PhD), Graphic Era (Deemed to be University), Dehradun, India (D Jena PhD); Department of Internal Medicine (B M Jeswani MBBS), GCS Medical College, Hospital & Research Centre, Ahmedabad, India; Research Division (Prof P K Maulik PhD), The George Institute for Global Health, New Delhi, India (Prof V Jha MD); Department of Public Health (Z Ji MD), School of Medicine (J Yu MD), Tongji University, Shanghai, China; Department of Radiology (W Jin MD), Department of Cardiology (B Zhang PhD), Zhongshan Hospital, Shanghai, China; Danish Center for Health Economics (E K Johnson MSc), Department of Sports Science and Clinical Biomechanics (Prof S T Skou PhD), University of Southern Denmark, Odense, Denmark; Rothschild Foundation Hospital (Prof J B Jonas MD), Institut Français de Myopie, Paris, France;Singapore Eye Research Institute, Singapore, Singapore (Prof J B Jonas MD); Hungarian Health Management Association, Budapest, Hungary (T Joo PhD, T Palicz MD); School of Public Health, (Prof A Joseph PhD), SRM Centre for Clinical Trials and Research (CCTR) (D Kumar PhD), Sri Ramaswamy Memorial Institute of Science and Technology (SRMIST), Chennai, India; Department of Economics (C E Joshua BSc), National Open University, Benin City, Nigeria; Department of Family Medicine and Public Health (J J Jozwiak PhD), University of Opole, Opole, Poland; Institute of Family Medicine and Public Health (M Jürisson PhD), University of Tartu, Tartu, Estonia; School of Public Health (M K MD), National Institute of Epidemiology, Chennai, India; Research Department (Z Kabir PhD), TobaccoFree Research Institute Ireland, Dublin, Ireland; School of Public Health (Z Kabir PhD), University College Cork, Cork, Ireland; Department of Health (K Kalavani PhD), Khoy Medical Sciences, Khoy, Iran; Department of Dermatology (F Kaliyadan MD), King Faisal University, Hofuf, Saudi Arabia; Research Institute of Cardiology and Internal Medicine, Almaty, Almaty, Kazakhstan (A Kaliyakparova MD); Department of Endocrinology (S Kalra DM), Bharti Hospital Karnal, Karnal, India; Canberra Business School (M M Kamal MPH), University of Canberra, Hawker, ACT, Australia; Department of Public Health (R T Kamorudeen MPH), South Wales University, Treforest, UK; Microbiology, Virology and Immunology Department (Prof O Kamyshnyi DSc), I. Horbachevsky Ternopil National Medical University, Ternopil, Ukraine; Department of Health Sciences (Prof M Kanaan PhD), University of York, York, UK; Office of the Executive Director (Prof K K Kanmodi MPH), Cephas Health Research Initiative Inc, Ibadan, Nigeria; MS Ramaiah Memorial Hospital (S Kannan MD), Ramaiah University of Applied Sciences, Banglore, India; Department of Community Medicine (S

Kannan S MD), ESIC Medical College and Hospital Chennai, Chennai, India; The Hansjörg Wyss Department of Plastic and Reconstructive Surgery (R S Kantar MD), NYU Langone Health, New York, NY, USA; Cleft Lip and Palate Surgery Division (R S Kantar MD), Global Smile Foundation, Norwood, MA, USA; Amity Stem Cell Institute (ASCI) (Prof M K Kashyap PhD), Amity University Haryana, Gurugram, India; College of Health Science, Department of Public Health (A Kaso MPH), Arsi University, Asella, Ethiopia; Department of Medicine (A Katamreddy MD), Jacobi Medical Center, New York, NY, USA; Centre for Tropical Diseases and Global Health (Prof P D Katoto PhD), Catholic University of Bukavu, Bukavu, Democratic Republic of the Congo; Surgery Research Unit (Prof J H Kauppila MD), Oulu Business School (I Shiue PhD), Martti Ahtisaari Institute (I Shiue PhD), University of Oulu, Oulu, Finland; International Research Center of Excellence (G A Kayode PhD), Institute of Human Virology Nigeria, Abuja, Nigeria; Julius Centre for Health Sciences and Primary Care (G A Kayode PhD), Copernicus Institute of Sustainable Development (G Koren PhD), Utrecht University, Utrecht, Netherlands; Open, Distance and eLearning Campus (Prof P N Keiyoro PhD), Department of Psychiatry (M Kumar PhD), Department of Management Science and Project Planning (A W Ndungu PhD), Population Studies and Research Institute (A W Wamai MSc), School of Nursing Sciences (M N Wanjau PhD), University of Nairobi, Nairobi, Kenya; Eye Unit (Prof J H Kempen PhD), MyungSung Medical College, Addis Ababa, Ethiopia (A A Negash MD); Center of Global Child Health (S Kerai PhD), Department of Gastroenterology, Hepatology, and Nutrition (M K Thind PhD), The Hospital for Sick Children, Toronto, ON, Canada; Jindal School of Public Health and Human Development (V Keshri PhD), O. P. Jindal Global University, Sonipat, Sonipat, India; Department of Biomedical Informatics (K Keshtkar BSc), Arizona State University, Phoenix, AZ, USA; Department of Human Nutrition of INRAE (E Kesse-Guyot PhD), National Research Institute for Agriculture, Food and Environment, Paris, France; Department of Health, Medicine and Human Biology (M Touvier PhD), Sorbonne Paris Nord University, Bobigny, France (E Kesse-Guyot PhD); Amity Institute of Forensic Sciences (H Khajuria PhD, B P Nayak PhD), Amity Institute of Pharmacy (K Munjal PhD), Amity Institute of Public Health (M Shannawaz PhD), Amity institute of Public Health and Hospital Administration (A Singh PhD), Amity Institute of Public Health and Hospital Administration (P S Singh PhD), Amity University, Noida, India; Lahore Medical Research Center (S Khalid PhD), Lahore Medical Research Center, Lahore, Pakistan; Faculty of Veterinary Medicine (H O Khalifa PhD), Kafrelsheikh University, Kafrelsheikh, Egypt; Department of Public Health (Prof M Khalis PhD), Mohammed VI Center for Research and Innovation, Rabat, Morocco; Higher Institute of Nursing Professions and Health Techniques, Rabat, Morocco (Prof M Khalis PhD); Food and Drug Research Center (F Khamesipour PhD), Iran Food and Drug Administration, Tehran, Iran; NITVAR (A Khan PhD), Indian Council of Medical Research, Pune, India; Academy of Scientific and Innovative Research (AcSIR), India, Ghaziabad, India (A Khan PhD); Department of Pharmacy Administration and Clinical Pharmacy (F U Khan PhD), School of Public Health (S Li MD, H Zhang PhD), Department of Epidemiology and Biostatistics (Prof J Liu PhD), Institute of Child and Adolescent Health (Y Liu MS, Prof Z Zou MD), School of Public Health and China Center for Health Developments (L Ren PhD), Peking University, Beijing, China; Department of Pharmacy Administration and Clinical Pharmacy (F U Khan PhD), The First Affiliated Hospital of Xi'an Jiaotong University, Xi'an, China; Department of Community Medicine (M Khan MPH), Department of Biostatistics (Prof M Rahman MS), National Institute of Preventive and Social Medicine, Dhaka, Bangladesh; BD Statistics Center for Research, Dhaka, Bangladesh (M Khan MPH); Karachi Medical and Dental College, Karachi, Pakistan (M Khan MBBS); Center for Atmospheric Particle Studies (CAPS) (M Khan MSc), Department of Mechanical Engineering (MechE) (M Khan MSc), Carnegie Mellon University, Pittsburgh, PA, USA; Aston Pharmacy School, College of Health and Life Sciences (M Khan PhD), Aston University, Birmingham, UK; Joint

Doctoral School (S A Khan MSc), Silesian University of Technology, Gliwice, Poland (R Khan PhD); Dr. Panjwani Center for Molecular Medicine & Drug Research (S A Khan MSc), International Center for Chemical and Biological Sciences (F Mansoor MS, S Ullah MSc), H.E.J. Research Institute of Chemistry (M Shahbaz MPH), University of Karachi, Karachi, Pakistan; International Center for Chemical and Biological Sciences (S Khan PhD), International Center for Chemical and Biological Sciences, Karachi, Pakistan; Department of Cardiology (Z Khan MD), University of South Wales, Treforest, UK; Department of Cardiology (Z Khan MD), University of Buckingham, Buckingham, UK; Central Department of zoology (S Khanal MSc), Department of Community Medicine and Public Health (P M S Pradhan MD), Tribhuvan University, Kathmandu, Nepal; Department of Health (V Khanal PhD), Nepal Development Society, Chitwan, Nepal; Department of Preventable Non Communicable Disease (V Khanal PhD), Menzies School of Health Research, Alice Springs, NT, Australia; Department of Pharmacology (S U Khasbage MD, P Thangaraju MD), All India Institute of Medical Sciences, Raipur, India; College of Health, Wellbeing and Life Sciences (Prof K Khatab PhD), Sheffield Hallam University, Sheffield, UK; College of Arts and Sciences (Prof K Khatab PhD), Ohio University, Zanesville, OH, USA; Department of Orthopaedics (K Khatri MS), Postgraduate Medical Institute, Sangrur, India; Asadabad School of Medical Sciences, Asadabad, Iran (A Khazaei PhD); University of Sulaimani College of Medicine (N S H Khoshnaw PhD), Sulaimani Polytechnic University, Sulaymaniyah, Iraq; Department of Internal Medicine (A A Khosla MD), Corewell Health East William Beaumont University Hospital, Royal Oak, MI, USA; Department of Medical Oncology (A A Khosla MD), Department of Medical and Surgical Oncology (A Pon Avudaiappan MD), Miami Cancer Institute, Miami, FL, USA; Research Department (M Khosrowjerdi PhD), University of Inland Norway, Elverum, Norway; Ashok & Rita Patel Institute of Physiotherapy (P Khuman PhD), Charotar University of Science and Technology, Changa, Anand, India; Faculty of Health Sciences (H Khusun PhD), University of Muhammadiyah Prof. Dr. Hamka, Jakarta, Indonesia; Program Division (H Khusun PhD), SEAMEO Regional Center for Food and Nutrition, Jakarta, Indonesia; Department of Biomedical Sciences (H Kim MS), Department of Psychiatry (W Myung PhD), Department of Food and Nutrition (A P Okekunle PhD), Seoul National University, Seoul, South Korea; Cardiovascular Disease Initiative (M Kim MD), Broad Institute of MIT and Harvard, Cambridge, MA, USA; Department of Environmental Health Sciences (Prof S Kim PhD), Soonchunhyang University, Asan, South Korea; Health and Healing Research, Education, and Service, Inc., Boston, MA, USA (R W Kimokoti MD); Millennium Prevention, Inc., Westwood, MA, USA (R W Kimokoti MD); The Pacific Community, Noumea, New Caledonia (Prof Y Kinfu PhD); School of Health Sciences (Prof A Kisa PhD), Kristiania University College, Oslo, Norway; Department of International Health and Sustainable Development (Prof A Kisa PhD), Department of Environmental Health Sciences (S P Sherchan PhD), Tulane University, New Orleans, LA, USA (E Lytvyak MD); Department of Nursing and Health Promotion (S Kisa PhD), Faculty of Health Sciences (Prof A W Wolf PhD), Oslo Metropolitan University, Oslo, Norway; Department of Public Health Dentistry (Prof S KM PhD), Krishna Vishwa Vidyapeeth (Deemed to be University), Karad, India; Endocrinology Department (Prof N Kobyliak DSc), Bogomolets National Medical University, Kyiv, Ukraine; Scientific Department (Prof N Kobyliak DSc), Medical Laboratory CSD, Kyiv, Ukraine; Global Healthcare Consulting, New Delhi, India (S Kochhar MD); Mycobacteriology Unit (D Koliegthu Tcheumeni MSc), Center for Health Promotion and Research, Bamenda, Cameroon; Department of Science and Environmental Studies (T Kormoker PhD), The Education University of Hong Kong, Hong Kong, China; Department of General Practice and Family Medicine (Prof O Korzh DSc), Kharkiv National Medical University, Kharkiv, Ukraine; Department of Epidemiology (Prof K Kostev PhD), IQVIA, Frankfurt am Main, Germany; University Hospital Marburg, Marburg, Germany (Prof K Kostev PhD); Department of Psychiatry (Prof K Kotsis PhD), University of

Ioannina, Ioannina, Greece; Amity institute of Public Health and Hospital administration (A Koul MHA), Amity University Noida, Noida, India; School of Pharmacy (Prof I A Kretchy PhD), University of Ghana, Legon, Ghana; Department of Public Health (J Kretchy PhD), Central University, Accra, Ghana (J Kretchy PhD); Department of Anthropology (Prof K Krishan PhD), Institute of Forensic Science & Criminology (V Sharma PhD), Panjab University, Chandigarh, India; School of Applied Science (C Kua PhD), Republic Polytechnic, Singapore, Singapore; Centre for Biotechnology (A Kuanar PhD, S K Panda PhD, S Patel PhD, P Verma PhD), Siksha 'O' Anusandhan Deemed to be University, Bhubaneswar, India; Department of Demography (Prof B Kuate Defo PhD), Department of Social and Preventive Medicine (Prof B Kuate Defo PhD), University of Montreal, Montreal, QC, Canada; Department of Biotechnology (R H Kuchay PhD), Baba Ghulam Shah Badshah University, Jammu and Kashmir, India; Department of Medical Education and Informatics (B Kucuk Bicer PhD), Gazi University Faculty of Medicine, Ankara, Turkiye; Department of Pediatrics (I Kuitunen PhD), Kuopio University Hospital, Kuopio, Finland; Institute of Clinical Medicine (I Kuitunen PhD), University of Eastern Finland, Kuopio, Finland; Rajendra Institute of Medical Sciences (A Kujur PharmD), Ministry of Health and Family Welfare, Ranchi, India; Center of Medicine and Public Health (M Kulimbet MSc), Director of Central Asia Research Collaboration Group (Prof F Rahim PhD), Asfendiyarov Kazakh National Medical University, Almaty, Kazakhstan; Department of Medicine (V Kulkarni MS), Digital Health and Informatics Directorate (Prof S M McPhail PhD), Queensland Health, Brisbane, QLD, Australia; Amity Centre for Water Studies and Research (S Kulshreshtha PhD), Amity Institute of Biotechnology (E Upadhyay PhD), Amity University Rajasthan, Jaipur, India; Department of Community Medicine (D Kumar MD), Department of Anaesthesiology (T Kumar DNB), Rajendra Institute of Medical Sciences, Ranchi, India; Department of Pharmacology and Toxicology (N Kumar PhD), National Institute of Pharmaceutical Education and Research, Hajipur, Hajipur, India; Gastroenterology Department (S Kumar FCPS), Ahalia Hospital, Abu Dhabi, United Arab Emirates; Allied Health Sciences (S Kumar FCPS), Bahria University Medical and Dental College, Karachi, Pakistan; Department of Economics (V Kumar PhD), Manipal University, Jaipur, Jaipur, India; IITM Pravartak Technologies Foundation, Chennai, India (S Kumaran PhD); Section of Cardiology (Prof S K Kunutsor PhD), University of Manitoba, Winnipeg, MB, Canada; Department of Translational Health Sciences (Prof S K Kunutsor PhD), Bristol Medical School (Y Nartey PhD), Integrative Epidemiology Unit (A Suhag PhD), University of Bristol, Bristol, UK; Faculty of Medicine and Health Science (M Kurniasari PhD), Universitas Kristen Satya Wacana (Satya Wacana Christian University), Salatiga, Indonesia; Division of Cardiology (K Kurpad MD), Department of Computer Science (P Naghavi MS), Department of Health and Kinesiology (M Sajib BDS), University of Illinois, Champaign, IL, USA; Research Center for Public Health and Nutrition (B Rachmat MPH), National Research and Innovation Agency, Jakarta, Indonesia (A Kusnali MA, I U Tarigan PhD); Institute for Health Sciences (C Kustanti PhD), STIKES Bethesda Yakkum Yogyakarta Indonesia, Yogyakarta, Indonesia; Department of Public Health and Epidemiology (D Kusuma DSc), Khalifa University of Science and Technology, Abu Dhabi, United Arab Emirates; Department of Pediatric Oncology (Prof T Kutluk MD), Medicana Health International, Istanbul, Turkiye; Department of Pediatric Oncology (Prof T Kutluk MD), Hacettepe University, Ankara, Turkiye; Department of Clinical Surgical Sciences (M A Kwarteng PhD), University of the West Indies, St Augustine, Trinidad and Tobago; Department of Nursing (E F Kyei PhD, G K Kyei BSc), University of Massachusetts Boston, Boston, MA, USA; Clinical Research Center (V Kytö MD), Turku University Hospital, Turku, Finland; Division of Evidence Synthesis (C Lahariya MD), Foundation for People-centric Health Systems, New Delhi, India; Division of Lifestyle Medicine (C Lahariya MD), Centre for Health: The Specialty Practice, New Delhi, India; School of Digital Science (D Lai PhD), Institute of Applied Data Analytics (D Lai PhD), Universiti

Brunei Darussalam (University of Brunei Darussalam), Bandar Seri Begawan, Brunei; Department of Chemistry (Prof A Lakhani PhD), Dayalbagh Educational Institute, Agra, India; NEVES Society for Patient Safety, Budapest, Hungary (J Lám PhD); Unidad de Genética y Salud Pública (Prof I Landires MD), Instituto de Ciencias Médicas, Las Tablas, Panama; Ministry of Health (Prof I Landires MD), Hospital Joaquín Pablo Franco Sayas, Las Tablas, Panama; Department of Psychiatry and Psychotherapy (B Langguth PhD), University of Regensburg, Regensburg, Germany; Department of Behavioural Sciences and Learning (Prof A Laplante-Lévesque PhD), Linköping University, Linköping, Sweden; Department of Otorhinolaryngology (S Lasrado MS), Father Muller Medical College, Mangalore, India; Department of Clinical Pharmacy and Pharmacy Management (B K Lawal PhD), Department of Radiology (H Theyra-Enias MD), Kaduna State University, Kaduna, Nigeria; Department of Obstetrics and Gynaecology (B K Lawal MSc), University of Nottingham, Derby, UK; Health Systems, Administration and Management (S A Lawal PhD), Babcock University, Sagamu, Nigeria; Health Services Management Programme (S A Lawal PhD), Plasma University, Mogadishu, Somalia; Faculty of Medicine, (D Le MD), Nam Can Tho University, Can Tho, Vietnam; PhD Program in Epidemiology (D Le MD), Vanderbilt University, Nashville, TN, USA; Faculty of Medicine (H Le MD, N Le MD), Department of General Medicine (V T Nguyen MD), Department of Internal Medicine (T H Tran MD), University of Medicine and Pharmacy at Ho Chi Minh City, Ho Chi Minh City, Vietnam (T T T Le MD, T D T Le MD); Department of Cardiovascular Research (H Le MD, N Le MD), Methodist Hospital, Merrillville, IN, USA; Health Economics Division (L K D Le PhD), Monash University, Burwood, VIC, Australia; Independent Consultant, Ho Chi Minh City, Vietnam (T D T Le MD); Clinical Trial Center (H Lee PhD), Ewha Womans University, Seoul, South Korea; Department of Family Medicine (W Lee PhD), University of Texas Medical Branch, Galveston, TX, USA; Department of Cardiothoracic and Vascular Surgery (V Leivaditis PhD), Westpfalz Klinikum, Kaiserslautern, Germany; Faculty of Science (E Leong PhD), (Universiti Brunei Darussalam) University of Brunei Darussalam, Bandar Seri Begawan, Brunei; School of Public Health and Management (C Li MD), Guangzhou University of Chinese Medicine, Guangzhou, China; Peking Union Medical College, Beijing, China (H Li MD, X Xu MD); Department of Rheumatology and Immunology (H Li MD), The People’s Hospital of Baoan Shenzhen, Shenzhen, China; Global Health Research Center (Prof J Li PhD), Guangdong Cardiovascular Institute (J Qiu MD), Guangdong Academy of Medical Sciences, Guangzhou, China; Shanxi Medical University, Taiyuan, China (J Li MD); Department of Health Promotion and Health Education (M Li PhD), National Taiwan Normal University, Taipei, Taiwan; The First Affiliated Hospital of Guangzhou Medical University (W Li MD), Guangzhou Medical University, Guangzhou, China; Department of Radiation Oncology (W Li MD), Zhujiang Hospital of Southern Medical University (P Luo MD), Division of Nephrology (X Shao MD), Department of Joint and Orthopedics (J Tan MD), Southern Medical University, Guangzhou, China; Department of Nephrology (X Li MD), the Second Affiliated Hospital of Anhui Medical University, Hefei, China; National Clinical Research Center for Cardiovascular Diseases (Y Li PhD), Chinese Academy of Medical Sciences, Shenzhen, China; Department of Endocrinology and Metabolism (Prof Y Li PhD), The First Hospital of China Medical University, Shenyang, China; Discipline of Physiology (Y Lian MA), National University of Ireland, Galway, Galway, Ireland (D Shan MD); First Clinical Medical College (X Liang DrPH), Shandong University of Chinese Medicine, Jinan, China; Department of Food Science and Human Nutrition (Q Lin MPH), Iowa State University, Ames, IA, USA; Asbestos Diseases Research Institute, Concord, NSW, Australia (R Lin PhD); The First Affiliated Hospital of Wenzhou Medical University (Y Lin MD), Department of Epidemiology and Health Statistics (F Shi PhD), The Second Affiliated Hospital (Prof A Wu MD), Department of Hepatology (Prof M Zheng PhD), Wenzhou Medical University, Wenzhou, China; Department of Medicine (D Lindholm MD), Norrtälje Hospital (Tiohundra),

Norrtälje, Sweden; School of Nursing and Health Sciences (X Liu PhD, S Ramazanu PhD), Hong Kong Metropolitan University, Hong Kong, China; Department of Radiology and Biomedical Imaging (X Liu PhD, M Mayeli MD, S Rahmani MD), Department of Genetics (S Pawar PhD), Department of Psychiatry (T Rhee PhD), Yale University, New Haven, CT, USA; Xiangya Hospital (Y Liu MSc), Anesthesiology Department of The Third Xiangya Hospital (K Pang MS), Central South University, Changsha, China; Department of Molecular Epidemiology (E Llanaj PhD), German Institute of Human Nutrition Potsdam-Rehbrücke, Potsdam, Germany; German Center for Diabetes Research (DZD), München-Neuherberg, Germany (E Llanaj PhD); Department of Infectious Diseases (M J Loftus MBBS), Alfred Health, Melbourne, VIC, Australia; Department of Cardiology (V Lohner PhD), University of Cologne, Cologne, Germany; School of Medicine (J López-Gil PhD), Universidad Espíritu Santo, Samborondón, Ecuador (J Sharifi Rad PhD); Vicerrectoría de Investigación y Postgrado (J López-Gil PhD), Universidad de Los Lagos, Osorno, Chile; Institute of Nutritional Sciences (Prof S Lorkowski PhD), Friedrich Schiller University Jena, Jena, Germany; Competence Cluster for Nutrition and Cardiovascular Health (nutriCARD), Jena, Germany (Prof S Lorkowski PhD); Department of Spine Surgery (S Luan MD), Qingdao Municipal Hospital Group, Qingdao, China; Scientific Research and Surveillance Systems (J Lubinda PhD), Macha Research Trust, Choma, Zambia; School of Medicine (Prof G Lucchetti PhD), Federal University of Juiz de Fora, Juiz de Fora, Brazil; Department of Emergency General and Trauma Surgery (Prof R Lunevicius DSc), NHS University Hospitals of Liverpool Group, Aintree Hospital, Liverpool, UK; Department of Population Health Sciences (J B Lusk MD), Duke Global Health Institute (C Wu PhD), Duke University, Durham, NC, USA; Dodoma Medical Research Centre (A M Lutambi PhD), National Institute for Medical Research in Tanzania, Dodoma, Tanzania; Department of Neurosciences and Behavioral Sciences (R Lutzky Saute MD), University of São Paulo, Ribeirão Preto, Brazil; College of Engineering (Prof M D Lytras PhD), Effat University, Jeddah, Saudi Arabia; Management of Information Systems Department (Prof M D Lytras PhD), The American College of Greece, Aghia Paraskevi, Greece; Centre for Public Health and Wellbeing (Z Ma PhD), University of the West of England, Bristol, UK; Department of Microbiology and Parasitology (M Mabrok PhD), King Salman International University, South of Sinai, Egypt; Department of Family Medicine, Mental and Public Health (Prof I E Machado PhD), Federal University of Minas Gerais, Ouro Preto, Brazil; Associate Laboratory i4HB (A M Madureira-Carvalho PhD), University Institute of Health Sciences (CESPU), Gandra, Portugal; UCIBIO Research Unit on Applied Molecular Biosciences (A M Madureira-Carvalho PhD), University Institute of Health Sciences, Gandra, Portugal; Ophthalmology Department (M Magdy Abd El Razek MSc), Ministry of Health & Population, Aswan, Egypt; Department of Forensic Medicine & Toxicology (D Mahadeshwara Prasad MD), Mysore Medical College & Research Institute, Mysooru, India; Department of Health & Family Welfare (D Mahadeshwara Prasad MD), Government of Karnataka, Bangalore, India; Department of Emergency Medicine (S Mahalingam MD), Sri Lakshmi Narayana Institute of Medical Science, Puducherry, Puducherry, India; Research Center (N H Mahmood PhD), Cihan University-Sulaimaniya, Sulaymaniyah, Iraq; Institute of Health Science (M T Mai MD), Faculty of Medicine (H T Nguyen MD, T T T T Truyen MD), International Medical Faculty (N P Nguyen MD), Nam Can Tho University, Can Tho, Vietnam; Cyprus International Institute for Environmental and Public Health (Prof K C C Makris PhD), Cyprus University of Technology, Limassol, Cyprus; Division of Research and Development (Prof T Malik PhD), Lovely Professional University, Phagwara, India; University of Kansas Medical Center (M Mangdow MSc), A.T. Still University, Kansas City, KS, USA; Internal Medicine Department (Y Manla MD), Eisenhower Health, Palm Desert, CA, USA; Biomedical Engineering Research Center (CREB) (Prof M Mansourian PhD), Automatic Control Department (H Marateb PhD), Universitat Politècnica de Catalunya (Barcelona Tech - UPC), Barcelona,

Spain; Department of Biomedical Engineering (H Marateb PhD, M Noroozi BSc), University of Isfahan, Isfahan, Iran; Far Eastern University, Manila, Philippines (J C Maravilla PhD); Department of Food, Environmental and Nutritional Sciences (M Marino PhD, Prof S Perna PhD), Department of Biomedical Sciences for Health (S Villa MD), University of Milan, Milano, Italy; Faculty of Human Kinetics (Prof A Marques PhD), University of Lisbon, Lisbon, Portugal; Department of Economics (Prof G Martinez PhD), Instituto Tecnologico Autonomo de Mexico (Autonomous Technology Institute of Mexico), Mexico City, Mexico; Department of Infectious Diseases (B A Martinez-Guerra MSc), Instituto Nacional de Nutrición Salvador Zubirán (Salvador Zubiran National Institute of Medical Sciences and Nutrition), Mexico City, Mexico; Department of Non-communicable Diseases and Mental Health (R Martinez-Piedra BSc), Pan American Health Organization, Washington, DC, USA; Campus Fortaleza (F R Martins-Melo PhD), Federal Institute of Education, Science and Technology of Ceará, Fortaleza, Brazil; Department of Nutrition and Dietetics (M Martorell PhD), Centre for Healthy Living (M Martorell PhD), University of Concepción, Concepción, Chile; Clinical Institute of Medical and Chemical Laboratory Diagnostics (Prof W März MD), Medical University of Graz, Graz, Austria; Medical Clinic V (Prof W März MD), Department of Experimental Pharmacology (Y Wibowo MD), Heidelberg University, Mannheim, Germany; Faculty of Humanities and Health Sciences (Prof R R Marzo MD), Curtin University, Sarawak, Malaysia; Jeffrey Cheah School of Medicine and Health Sciences (Prof R R Marzo MD), Monash University, Subang Jaya, Malaysia; Department of Clinical and Experimental Medicine (Prof S Masi PhD, D Trico MD), University of Pisa, Pisa, Italy; Board of Directors (C N Matei PhD), Association of Resident Physicians, Bucharest, Romania; Department of Anatomy and Developmental Biology (Y Mathangasinghe PhD), Monash University, Clayton, VIC, Australia; North West Lung Centre (A G Mathioudakis PhD), Manchester University NHS Foundation Trust, Manchester, UK; Health Policy Research (M R Mathur PhD), Public Health Foundation of India, Gurugram, India; Department of Community Medicine (M Mathur MD), Geetanjali Medical College and Hospital in Udaipur India, Udaipur, India; Department of Epidemiology (Prof R J Maude PhD), Mahidol-Oxford Tropical Medicine Research Unit, Bangkok, Thailand; Department of Social Medicine and Family (M Mazaheri PhD), Dezful University of Medical Sciences, Dezful, Iran; Orthopedic Trauma Pathology Department (A Mazzotti PhD), IRCCS, Bologna, Italy; Department of Paediatrics (C N P Mbachu PhD), Nnamdi Azikiwe University, Nnewi, Nigeria; Department of Obstetrics and Gynaecology (Prof I I Mbachu FWACS), Department of Paediatrics (C A Nri-Ezedi PhD), Nnamdi Azikiwe University, Awka, Nigeria; Department of Healthcare (Prof E A Mechili PhD), University of Vlora, Vlora City, Albania; Clinic of Social and Family Medicine (Prof E A Mechili PhD), Department of Medicine (Prof A Tsatsakis DSc), University of Crete, Heraklion, Greece; Neurology Department (Prof M Mehndiratta MD), Janakpuri Super Specialty Hospital Society, New Delhi, India; Department of Neurology (Prof M Mehndiratta MD), Govind Ballabh Institute of Medical Education and Research, New Delhi, India; Department of Dental Research Cell (Prof V Mehta PhD), Dr. D. Y. Patil University, Pune, India; Department of Biosciences and Bioengineering (S Mehto PhD), Indian Institute of Technology Dharwad, Dharwad, India; Institute for Sustainable Agriculture and Food Economics (INL) (T Meier PhD), Martin Luther University Halle-Wittenberg, Halle, Germany; Office of Innovation (T Meier PhD), Competence Cluster for Nutrition and Cardiovascular Health (nutriCARD), Halle, Germany; Health Care Authority, Olympia, WA, USA (A Memetova MA); Dirección General de Investigación, Desarrollo e Innovación (DGIDI) (W Mendoza MD), Universidad Científica del Sur (University of the South), Lima, Peru; Department of Medical Microbiology and Immunology (G A Menezes PhD), Trinity Medical Sciences University, St. Vincent, Saint Vincent and the Grenadines; Department of Public Health (M Mercogliano MD), University “Federico II” of Naples, Naples, Italy; General Administration Department

(A Meretoja MD), Comprehensive Cancer Center (T J Meretoja MD), Department of Neurosurgery (I Rautalin PhD), Helsinki University Hospital, Helsinki, Finland; University Centre Varazdin (T Mestrovic PhD), University North, Varazdin, Croatia; Stritch School of Medicine (A Mhlanga PhD), Loyola University Chicago, Chicago, IL, USA; Dermatology Unit (A Michelerio PhD), Fondazione IRCCS Policlinico San Matteo, Pavia, Italy; Department of Oncology (H S Mideksa MD), Addis Ababa University, Addis Abeba, Ethiopia; Queensland Centre for Mental Health Research (QCMHR) (P A Miller PhD), The University of Queensland, Wacol, QLD, Australia; College of Human Medicine (T R Miller PhD), Michigan State University, Flint, MI, USA; Multidisciplinary Department of Medical-Surgical and Dental Specialties (G Minervini PhD), University of Campania Luigi Vanvitelli, Naples, Italy; Department of Public Health Dentistry (Prof G Mini PhD), Saveetha Medical College and Hospital (M Tabish MPharm), Saveetha Institute of Medical and Technical Sciences, Chennai, India; Global Institute of Public Health (Prof G Mini PhD), Ananthapuri Hospitals and Research Institute, Trivandrum, India; Internal Medicine Programme (Prof E M Mirrakhimov PhD), Kyrgyz State Medical Academy, Bishkek, Kyrgyzstan; Department of Atherosclerosis and Coronary Heart Disease (Prof E M Mirrakhimov PhD), National Center of Cardiology and Internal Disease, Bishkek, Kyrgyzstan; Department of Radiology (S Mirshahvalad MD), Health Sciences North, Sudbury, ON, Canada; Bergen Center for Ethics and Priority Setting (M K Mirutse PhD), Department of Psychosocial Science (Prof D Sagoe PhD), Center for International Health (C Schwinger PhD), University of Bergen, Bergen, Norway; Department of Community Medicine (K G Mishra MD), Department of Dermatology, Venereology and Leprosy-DVL (Prof T Priscilla MD), Apollo Institute of Medical Sciences and Research, Hyderabad, India; Thumbay College of Management and AI in Healthcare (V Mishra PhD), Gulf Medical University, Ajman, United Arab Emirates; Research and Development Department (V Mishra PhD), Panacea Institute of Interdisciplinary Research and Education, Varanasi, India; Department of Pharmacology (A K Misra MD), All India Institute of Medical Sciences, Mangalagiri, India; Department of Internal Medicine (S Modi MD), Albert Einstein Hospital, Philadelphia, PA, USA; College of Applied and Natural Science (J Mohamed MSc), University of Hargeisa, Hargeisa, Somalia; RAK College of Nursing (M Mohamed PhD), RAK Medical and Health Sciences University, Ras Alkhima, United Arab Emirates; Nursing College (M Mohamed PhD), Sohag University, Sohag, Egypt; Molecular Biology Unit (N S Mohamed MSc), Bio-Statistical and Molecular Biology Department (N S Mohamed MSc), Sirius Training and Research Centre, Khartoum, Sudan; Department of Medicine (S Mohammadi BS), Anne Burnett Marion School of Medicine at Texas Christian University, Fort Worth, TX, USA; Department of Public Health (H Mohammed PhD, A Oumer PhD, N K Worku MPH), Public Health Department (M Yigezu MPH), Dire Dawa University, Dire Dawa, Ethiopia; Department of Medicine (O Mohammed MBBS), Government Medical College Kozhikode, Kozhikode, India; Department of Health Sciences, Azare (S Mohammed MSc), National Institute for Research in Tribal Health, Bauchi, Nigeria; Medical Microbiology Department (Prof Y Mohammed FWACP), Usmanu Danfodiyo University Teaching Hospital, Sokoto, Nigeria; Department of Pharmacology (S Mohan PhD), Dale View College of Pharmacy and Research Centre, Thruvananthapuram, India; School of Health Sciences (S Mohan PhD), University of Petroleum and Energy Studies, Dehradun, India; Department of Applied Biology (Y Mohanta PhD), University of Science and Technology Meghalaya, Ri-Bhoi, India; Department of Health Services Management (M Mohseni PhD), Iran University of Medical Sciences, Iran, Iran; Kazakh-Russian Medical University, Almaty, Kazakhstan (A Mokhirev MSc); Institute of Clinical Physiology (S Molinaro PhD), National Research Council, Pisa, Italy; Department Medical-Surgical Nursing (A Mollaei PhD), Department of Nursing and Midwifery (F Ranjbar Noei MSc), Golestan University of Medical Sciences, Gorgan, Iran; Clinical Epidemiology and Public Health Research Unit (L Monasta DSc, L Ronfani PhD, E

Traini PhD, G Zamagni MSc), Burlo Garofolo Institute for Maternal and Child Health, Trieste, Italy; Department of Sport Physiology (A Monazzami PhD), Razi University, Kermanshah, Iran; Department of Biomedical and Dental Sciences and Morphofunctional Imaging (Prof S Mondello MD), Messina University, Messina, Italy; Department of Medicine (A A Montasir FMD), TMSS Medical College, Bogura, Bangladesh; Department of Medicine (A A Montasir FMD), Sofia Ismail Memorial Medical Centre, Bogura, Bangladesh; Centre for Neonatal and Paediatric Infection (C E Moore PhD), St. George's University of London, London, UK; Department of Epidemiology and Biostatistics (Y Moradi PhD), Epidemiology and Biostatistics (M Rasouli PhD), Social Determinants of Health Research Center (A Shokri PhD), Kurdistan University of Medical Sciences, Sanandaj, Iran; Department of Public Health (Prof R S Moreira PhD), Oswaldo Cruz Foundation, Recife, Brazil; Department of Public Health (Prof R S Moreira PhD), Federal University of Pernambuco, Recife, Brazil; Baan Clinic (M Morovvati MD), Baan Clinic, Tehran, Iran; Faculty of Medicine (M Morsy MD), October 6 University, Giza, Egypt; Department of Biology and Biological Engineering (J Morze PhD), Chalmers University of Technology, Gothenburg, Sweden; College of Medical Sciences (J Morze PhD), SGMK Copernicus University, Warsaw, Poland; NHS National Services Scotland, Belfast, UK (N Motamedgorji MD); Social Determinants of Health Research Center (S Mouodi PhD), School of Medicine (S Soraneh MD), Babol University of Medical Sciences, Babol, Iran; Faculty of Biotechnologies (Prof A Mousavi Khaneghah PhD), ITMO University, Saint Petersburg, Russia; Department of Physical and Environmental Sciences (S Mousavi Kiasary DVM), Texas A&M University, Corpus Christi, TX, USA; School of Pharmacy (M A A Mousnad PhD), Management and Science University (MSU), Shah Alam, Malaysia; International University of Africa (IUA), Khartoum, Sudan (M A A Mousnad PhD); René Rachou Institute (M Mrejen PhD), Oswaldo Cruz Foundation, Belo Horizonte, Brazil; Department of Radiology (A Msherghi MD), University of Tripoli, Tripoli, Libya; PMAS Arid Agriculture University Rawalpindi, Rawalpindi, Pakistan (R Mubarak MSc); Unit of Pharmacotherapy, Epidemiology and Economics (Prof S Mubarik PhD), University of Groningen (Rijksuniversiteit Groningen), Groningen, Netherlands; Institute of Molecular Biology and Biotechnology (S Muhammad PhD), Bahauddin Zakariya University Multan, Multan, Pakistan; Department of Biochemistry (S Mukherjee PhD), Department of Dentistry (Prof A Singh MDS), All India Institute of Medical Sciences, Bhopal, India; Knowledge Management Department (S Mukherjee PhD), Prahlad Omkarwati Foundation (POF), Mumbai, India; Independent Consultant, New Delhi, India (S Mukherjee PhD, P Sood PhD); Department of Medicine (A Mukhopadhyay MD), National University Health System, Singapore, Singapore; Department of Mechanical Engineering (M Muktadir PhD), North Carolina Agricultural and Technical State University, Greensboro, NC, USA; Department of Surgery (F Mulita PhD), General University Hospital of Patras, Patras, Greece; Faculty of Medicine (F Mulita PhD), Department of Emergency Medicine (Prof I Pantazopoulos PhD), University of Thessaly, Larissa, Greece; Department of Nursing (M Mulyadi PhD), Sam Ratulangi University, Manado, Indonesia; Department of Medicine (M Muneer MBBS), Allama Iqbal Medical College, Lahore, Pakistan; Department of Health Economics (M Muniyandi PhD), Department of Epidemiology (M Sathya Narayanan MBBS), National Institute for Research in Tuberculosis, Chennai, India; Department of Community and Global Health (Y Munkhsaikhan MD), The University of Tokyo, Tokyo, Japan; Epidemiology, Biostatistics and Prevention Institute (EBPI) (J Muñoz Laguna MSc), University of Zürich, Zurich, Switzerland; Center for Infectious Disease Education and Research (Prof M Murakami PhD), Department of Biostatistics and Data Science (Y Yasufuku MSc), The University of Osaka, Suita, Japan; School of Postgraduate research and publications (Y H Muse PhD), Amoud University, Hargeisa, Somalia; Department of Pediatrics & Pediatric Pulmonology (Prof G Mustafa MD), Institute of Mother & Child Care, Multan, Pakistan; Department of Pathology and

Microbiology (S I Mustafa PhD), Duhok University, Duhok, Iraq; Operational Research Center in Healthcare (M T Mustapha PhD), Near East University, Nicosia, Cyprus; Department of Research Methods (S Muthu PhD), Orthopaedic Research Group, Coimbatore, India; Central Research Laboratory (S Muthu PhD), Meenakshi Medical College Hospital and Research Institute, Chennai, India; Director General (Prof C M Muvunyi PhD), Rwanda Biomedical Centre, Kigali, Rwanda; College of Medicine and Health Sciences (Prof C M Muvunyi PhD), University of Rwanda, Kigali, Rwanda; Department of Neuropsychiatry (W Myung PhD), Seoul National University Bundang Hospital, Seongnam, South Korea; Department of Health Education & Promotion (F Naddafi PhD), School of Medicine (M Rostamian PhD), Faculty of Medicine (Z Saadatian PhD), Infectious Diseases Research Center (Z Saadatian PhD), Gonabad University of Medical Sciences, Gonabad, Iran; Research and Analytics Department (A J Nagarajan MTech), Initiative for Financing Health and Human Development, Chennai, India; Department of Research and Analytics (A J Nagarajan MTech), Bioinsilico Technologies, Chennai, India; Department of Computer Science and IT (G R Naik PhD), Torrens University, Adelaide, SA, Australia; Department Health Services Research (G Naik MPH), Department of Psychology (D C Schwebel PhD), University of Alabama at Birmingham, Birmingham, AL, USA; Faculty of Pharmacy (Prof F Nainu PhD), Hasanuddin University, Makassar, Indonesia; Department of Pulmonary Medicine (Prof S Nair MD), Government Medical College, Thrissur, Thrissur, India; Health Action by People, Trivandrum, India (Prof S Nair MD); Suraj Eye Institute, Nagpur, India (V Nangia PhD); Department for the Control of Disease, Epidemics, and Pandemics (J Nansseu MD), Ministry of Public Health, Yaoundé, Cameroon; Department of Public Heath (J Nansseu MD), Department of Public Health (G Nguefack-Tsague PhD), University of Yaoundé I, Yaoundé, Cameroon; National Dental Research Institute Singapore (G G Nascimento PhD), Duke-NUS Medical School, Singapore, Singapore; Department of Applied Pharmaceutical Sciences and Clinical Pharmacy (A Y Naser PhD), Isra University, Amman, Jordan; Critical Care Unit (M Naser MD), Sheikh Shakhbout Medical City hospital, Abu Dhabi, United Arab Emirates; Division of Endocrinology and Diabetes (M Nassar PhD), University of Vermont, South Burlington, VT, USA; Department of Internal Medicine (A Nawaz MD), Khyber Medical University, Islamabad, Pakistan; Department of Research (G Nchanji PhD), TroDDIVaT Initiative, Buea, Cameroon; Department of Disease Control and Environmental Health (R Ndejjo PhD), School of Public Health (R Olum MD), Makerere University, Kampala, Uganda; Department of General Surgery (I Negoi PhD), Emergency University Hospital Bucharest, Bucharest, Romania; Department of Cardiology (R I Negoi PhD), Cardio-Aid, Bucharest, Romania; Department of Cardiology (A G Negru PhD), University of Medicine and Pharmacy "Victor Babes", Timisoara, Romania; Rocordis Heart Center (A G Negru PhD), Cardiology and Cardiovascular Surgery Hospital, Timisoara, Romania; Health Promotion Research Center (J Nejati PhD, H Okati-Aliabad PhD), Zahedan University of Medical Sciences, Zahedan, Iran; Euromed Research Center (Prof C Nejjari MD), Euromed University of Fes, Fez, Morocco; Faculty of Medicine, Pharmacy, and Dentistry (Prof C Nejjari MD), University Sidi Mohammed Ben Abdellah, Fez, Morocco; Department of Neurosciences (Prof C R J Newton MD), Kenya Medical Research Institute/Wellcome Trust Research Programme, Kilifi, Kenya; Department of Biological Sciences (J W Ngunjiri PhD), University of Embu, Embu, Kenya; Institute for Global Health Innovations (C T Nguyen MPH, H L T Nguyen MPH), Duy Tan University, Hanoi, Vietnam; Cardiovascular Research Department (N P Nguyen MD), Methodist Hospitals, Merrillville, IN, USA; Department of Surgery (P T Nguyen MD), Danang Family Hospital, Danang, Vietnam; Hitotsubashi Institute for Advanced Study (HIAS) (T Nguyen DrPH), Hitotsubashi University, Tokyo, Japan; Institute for Cancer Control (T Nguyen DrPH), National Cancer Center, Chuo-ku, Japan; Faculty of Public Health (T Nguyen PhD, L Vu PhD), International Institute for Training and Research (INSTAR) (L Vu PhD), VNU University of Medicine and

Pharmacy, Hanoi, Vietnam; Department of Pediatrics (T Nguyen MD), New York Medical College, New York, NY, USA; Tuberculosis Group (V T Nguyen MD), Oxford University Clinical Research Unit, Vietnam, Ho Chi Minh City, Vietnam; Department of Public Health (A M Ngwa MSc), University of Bamenda, Bamenda, Cameroon; International Islamic University Islamabad, Islamabad, Pakistan (R K Niazi PhD); Population Research Institute (J Nie PhD), High-Quality Development Evaluation Research Institute (Z Sun PhD), Nanjing University of Posts and Telecommunications, Nanjing, China; Department of Humanities and Social Science (L Nieddu PhD), University for International Studies in Rome, Rome, Italy; Institute for Mental Health Policy Research (Y T Nigatu PhD, S Popova PhD), Centre for Addiction and Mental Health, Toronto, ON, Canada; Public Health Department (D N A Ningrum PhD), Universitas Negeri Semarang (State University of Semarang), Kota Semarang, Indonesia; School of Medicine (V Niranjan PhD), University of Limerick, Limerick, Ireland; Department of Public Health (V Niranjan PhD), UNICAF, Larnaca, Cyprus; College of Health Sciences (P Nkrumah-Boateng BSc), University of Ghana, Accra, Ghana; Technical Department (C A Nnaji PhD), School of Public Health and Family Medicine (C A Nnaji PhD), Institute of Infectious Disease and Molecular Medicine (O G Oluwole PhD), SAMRC Unit on Risk and Resilience in Mental Disorders (Prof D J Stein FRCPC), Psychiatry and Mental Health (G Van Der Walt BA), Department of Paediatrics and Child Health (Prof H J Zar PhD, Prof L J Zuhlke PhD), Department of Medicine (Prof L J Zuhlke PhD), University of Cape Town, Cape Town, South Africa; Global Research Institute (Prof S Nomura PhD), Keio University, Tokyo, Japan; Canadian Institute for Health Information, Toronto, ON, Canada (P Noormohammadpour MD); Department of Microbiology and Molecular Genetics (M Noreen PhD), The Women University Multan, Multan, Pakistan; Internal Medicine Department (V C Nriagu MD), Maimonides Medical Center, Brooklyn, NY, USA; Global Health Department (J Nshimiyimana MPH), Euclid University, Banqui, Central African Republic; School of Information (F Nugen PhD), College of Computing, Data Science and Society (G Obaido PhD), University of California Berkeley, Berkeley, CA, USA; Department of Public Health (A N Nugusa MPH), Mattu University, Mattu, Ethiopia; Department of Public Health (D Nurrika PhD), Banten School of Health Science, South Tangerang, Indonesia; Ministry of Research, Technology and Higher Education (D Nurrika PhD), Higher Education Service Institutions (LL-DIKTI) Region IV, Bandung, Indonesia; Center of Excellence in Reproductive Health Innovation (CERHI) (C I Nzoputam MPH), University of Benin, Benin City, Nigeria; Department of Physiology (O J Nzoputam PhD), University of Benin, Edo, Nigeria; Department of Physiology (O J Nzoputam PhD), Benson Idahosa University, Benin City, Nigeria; Department of Applied Economics and Quantitative Analysis (Prof B Oancea PhD), University of Bucharest, Bucharest, Romania; Bioinformatics Department (Prof B Oancea PhD), National Institute of Research and Development for Biological Sciences, Bucharest, Romania; Department of Biomedicine and Prevention (F Oddi PhD), University of Rome "Tor Vergata", Rome, Italy; Department of Community Health and Primary Care (Prof O O Odukoya MSc), University of Lagos, Idi Araba, Nigeria; Department of Family and Preventive Medicine (Prof O O Odukoya MSc), Department of Biomedical Informatics (D Villarreal-Zegarra MPH), University of Utah, Salt Lake City, UT, USA; PSSM Data Sciences, Pfizer Research & Development (M Oduro PhD), Pfizer Inc., Groton, CT, USA; Department of Physiology (O B Oghenetega PhD), Adeleke University, Ede, Nigeria; Department of Medical Laboratory Science (O J Okesanya MPH), Federal Neuropsychiatric Hospital, Abeokuta, Nigeria; Associação Brasileira de Cefaleia em Salvas e Enxaqueca (ABRACES), São Paulo, Brazil (A B Oliveira PhD); Cardiology Department (Prof G M M Oliveira PhD), Federal University of Rio de Janeiro, Rio de Janeiro, Brazil; School of Health and Life Sciences (O O Oludoye PhD), Teesside University, Middlesbrough, UK; Research Policy & Administration (J O Olusanya MBA), Centre for Healthy Start Initiative, Lagos, Nigeria (B O Olusanya PhD); Department of

Pharmacology and Therapeutics (O G Oluwole PhD), Olabisi Onabanjo University, Sagamu, Nigeria; Institute of Chemistry (F Omage PhD), Universidade Estadual de Campinas (State University of Campinas), Campinas, Brazil; Department of Computational Biology (F Omage PhD), Brazilian Agricultural Research Institute (EMBRAPA), Campinas, Brazil; Department of Pharmacology and Toxicology (Prof H A Omar PhD), Beni-Suef University, Beni-Suef, Egypt; Surgery Department (G L Omer MD), Sulaimani University, Sulaimani, Iraq; ENT Department (G L Omer MD), Tor Vergata University of Rome, Rome, Italy; Lee Kong Chian School of Medicine (Q Ong MD), Nanyang Technological University, Singapore, Singapore; Wellspring Research (S Onie PhD), Wellspring Center Indonesia, Jakarta, Indonesia; Institute of Diagnostic and Interventional Radiology and Neuroradiology (M Opitz MD), University Hospital Essen, Essen, Germany; Department of Pharmacotherapy and Pharmaceutical Care (M Ordak PhD), Department of Biochemistry and Pharmacogenomics (M Zielińska MPharm), Medical University of Warsaw, Warsaw, Poland; Sickle Cell Unit (Prof V N Orish PhD), Ho Teaching Hospital, Ho, Ghana; Department of Biotechnological and Applied Clinical Sciences (R Ornello PhD), University of L'Aquila, L'Aquila, Italy; Department of Neurology (R Ornello PhD), ASL Avezzano-Sulmona-L'Aquila, L'Aquila, Italy; Department of Nephrology and Hypertension (Prof A Ortiz MD), IIS-Fundacion Jimenez Diaz, Madrid, Spain; Department of Medicine (Prof A Ortiz MD), Faculty of Medicine (P Perez-Lopez MD), Autonomous University of Madrid, Madrid, Spain; One Health Global Research Group (Prof E Ortiz-Prado PhD), Universidad de las Americas (University of the Americas), Quito, Ecuador; Department of Biological Sciences (A Osborne MSc), Njala University, Freetown, Sierra Leone; School of Medicine (U L Osuagwu PhD), Western Sydney University, Bathurst, NSW, Australia; Department of Optometry and Vision Science (U L Osuagwu PhD), University of KwaZulu-Natal, KwaZulu-Natal, South Africa; Department of Biological Sciences (O Osuolale PhD), Elizade University, Ilara-Mokin, Nigeria; Department of Preventive and Social Medicine (G Otchere MSc), University of Otago, Dunedin, New Zealand; Independent Consultant, Gothenburg, Sweden (M Othman MSc); School of Public Health (O J Otorkpa PhD), Texila American University, Georgetown, Guyana; Division of Infectious Diseases (Prof A Ouyahia PhD), University Hospital of Setif, Setif, Algeria; Department of General Surgery (G Ouyang PhD), Central South University, ChangSha, China; West African Center for Cell Biology of Infectious Pathogens (I A Owusu PhD), University of Ghana, Legon-Accra, Ghana; School of Medicine (T Oyelade PhD), Keele University, Keele, UK; Operational Research Center in Healthcare (I Ozsahin PhD, Prof U Saeed PhD), Near East University, Nicosia, Turkiye; Department of Mathematical Sciences (I Ozsahin PhD), Saveetha School of Engineering, SIMATS, Chennai, India; Universidad San Ignacio de Loyola, Lima, Peru (K Pacheco-Barrios MD); Department of Medicine (I Padda MD), Richmond University Medical Center, Staten Island, NY, USA; National School of Public Health (A Padron-Monedero PhD), Institute of Health Carlos III, Madrid, Spain; Department of Neurology (Prof P K Pal DM), National Institute of Mental Health and Neurosciences, Bangalore, India; Department of Mental Health (R F Palma-Alvarez PhD), Hospital Universitari Vall d'Hebron (CIBERSAM), Barcelona, Spain; Primary Health Center (T Paluvai MBBS), Directorate of Public Health and Family Welfare, Eluru District, India; Menzies Institute for Medical Research (F Pan PhD, A Singh PhD), University of Tasmania, Hobart, TAS, Australia; Centre for Medical Biotechnology (Prof D Pande Katare PhD), Amity University Uttar Pradesh, Noida, India; Department of Neurological Sciences (K Pang MS), Department of Anesthesiology (M L Rolfzen MD), Department of Environmental, Agricultural and Occupational Health (J Taiba PhD), University of Nebraska Medical Center, Omaha, NE, USA; National Institute of Health Research and Development (H U Pangaribuan MSc), Ministry of Health Indonesia, Jakarta, Indonesia; Department of Neurology (L D Panos MD), University of Bern, Biel/Biene, Switzerland; Department of Neurology (L D Panos MD),

University of Cyprus, Nicosia, Cyprus; Department of Emergency Medicine (Prof I Pantazopoulos PhD), Division of Neurological Science (F Shams DVM), University of Bern, Bern, Switzerland; Unit of Dermatology (G Paolino PhD), IRCCS Ospedale San Raffaele, Milan, Italy; University of Padua, Padua, Italy (M Papa MD); Medical University of Vienna, Vienna, Austria (I Papadimopoulos MD); Department of Science and Mathematics (Prof P Papadopoulou PhD), Deree-The American College of Greece, Athens, Greece; Department of Biophysics (Prof P Papadopoulou PhD), 3rd Department of Cardiology (M Spartalis PhD), University of Athens, Athens, Greece; Department of Forensic Medicine and Toxicology (U Parekh MD), All India Institute of Medical Sciences, Rajkot, India; Department of Medical Humanities and Social Medicine (Prof E Park PhD), Kosin University, Busan, South Korea; Department of Primary Care and General Practice (S Parve PhD), Kazan State Medical University, Kazan, Russia; Department of Cardiology (S Parve PhD), Parve Nursing Home, Sindkhed Raja, India; Department of Medical Sciences (R Passera PhD), University of Torino, Torino, Italy; Department of Imaging (R Passera PhD), AOU Città della Salute e della Scienza di Torino (AOU City of Health and Science of Turin), Torino, Italy; Institute of Physiotherapy (B H Patel MSPT), Ashok and Rita Patel Institute of Physiotherapy, Anand, India; Department of Physiotherapy (H M Patel PhD), Ashok & Rita Patel Institute of Physiotherapy (S Sunny PhD), Charotar University of Science and Technology, Anand, India; Marwadi University Research and Development Cell (M Patel PhD), Marwadi University, Rajkot, India; Department of Cardiovascular Medicine (N N Patel MD), University of Tennessee, Nashville, TN, USA; Department of Physiology (A Patil MD), All India Institute of Medical Sciences, Nagpur, India; College of Dental Medicine (Prof S Patil PhD), Roseman University of Health Sciences, South Jordan, UT, USA; Department of Human Anatomy (A Patra MD), Department of Dentistry (S S Rana MDS), Department of Radiodiagnosis (P Singh MD), All India Institute of Medical Sciences, Bathinda, India; Department of Interventional Cardiology (S Pawar MD), Cedars Sinai Medical Center, Los Angeles, CA, USA; IRCCS Fondazione Don Carlo Gnocchi, Milan, Italy (P Pedersini MSc); Department of Clinical and Experimental Sciences (P Pedersini MSc), University of Brescia, Brescia, Italy; Department of Public Health (J Pekarcikova PhD), Trnava University, Trnava, Slovakia; Research Institute for Medicines (Prof J Perdigão PhD), Universidade de Lisboa (University of Lisbon), Lisbon, Portugal; Centre for Fertility and Health (Prof G Pereira PhD), Department of Chemical Toxicology (M W Wojewodzic PhD), Norwegian Institute of Public Health, Oslo, Norway; Mario Negri Institute for Pharmacological Research, Bergamo, Italy (N Perico MD, Prof G Remuzzi MD); Department of Biochemistry and Pharmacology (P Petakh PhD), Uzhhorod National University, Uzhhorod, Ukraine; Facultad de Medicina (Faculty of Medicine) (F E Petermann-Rocha PhD), Universidad Diego Portales (Diego Portales University), Santiago, Chile; School of Cardiovascular and Metabolic Health (F E Petermann-Rocha PhD), University of Glasgow, Glasgow, UK; Basic Medical Sciences Department (J D Pillay PhD), Durban University of Technology, Durban, South Africa; International Center of Medical Sciences Research, Islamabad, Pakistan (Z Z Piracha PhD, Prof U Saeed PhD); Department of Promoting Health, Maternal-Infant, Excellence and Internal and Specialized Medicine (PROMISE) G. D'Alessandro (E Pirera MD), University of Palermo, Palermo, Italy; Air and Climate Unit (E Pisoni PhD), European Commission, Ispra, Italy; Department of Environmental Hygiene (D Plass DrPH), German Environment Agency, Berlin, Germany; Mental Health Research Institute (E Plotnikov PhD), Tomsk National Research Medical Center, Tomsk, Russia; Siberian State Medical University, Tomsk, Russia (E Plotnikov PhD); Department of Dermatology (I Podder MD), Sagore Dutta Hospital, Kolkata, India; Data Driven Health Division (P Pollner PhD), Hungarian Healthcare Management Association, Budapest, Hungary; Department of Data Management and Analysis (R Poluru PhD), The INCLEN Trust International, New Delhi, India; Wicking Dementia Research and Teaching Centre (Prof C D Pond PhD), University of

Newcastle, Hobart, TAS, Australia; Department of Orthopedics and Traumatology (V T Ponkilainen PhD), University of Tampere, Tampere, Finland; Academy of Romanian Scientists, Bucharest, Romania (Prof I Popa PhD); Department of Internal Medicine (D S Popovic PhD), University of Novi Sad, Novi Sad, Serbia; Clinic for Endocrinology, Diabetes and Metabolic Disorders (D S Popovic PhD), Clinical Center of Vojvodina, Novi Sad, Serbia; University Medical Center Groningen (Prof M J Postma PhD), Department of Internal Medicine (P Vart PhD), University of Groningen, Groningen, Netherlands; Center of Excellence in Higher Education for Pharmaceutical Care Innovation (Prof M J Postma PhD), Padjadjaran University, Bandung, Indonesia; Non-communicable Diseases Research Center (N Pourtaheri PhD), Bam University of Medical Sciences, Bam, Iran; Centro de Investigaciones Clinicas (Clinical Research Center) (S I Prada PhD), Fundación Valle del Lili (Valle del Lili Foundation), Cali, Colombia; Centro PROESA (S I Prada PhD), Departamento de Ciencias Básicas Médicas (E Rubagotti PhD), Universidad ICESI, Cali, Colombia; Department of Humanities and Social Sciences (Prof J Pradhan PhD), National Institute of Technology Rourkela, Rourkela, India; Research Center in Advancing Community Healthcare, Surabaya, Indonesia (R Pradipta MS); Department of Biochemistry (Prof A Prashant PhD), JSS Academy of Higher Education and Research, Mysuru, India; Department of Medical Instrumentation Techniques Engineering (N H Qasim DSc), Al-Rafidain University College, Baghdad, Iraq; Department of Cybersecurity (N H Qasim DSc), Kyiv National University of Construction and Architecture, Kyiv, Ukraine; Department of Respiratory and Critical Care Medicine (J Qiu MD), Henan University of Science and Technology, Luoyang, China; Global Consortium for Public Health and Research (Prof Z Quazi PhD), Datta Meghe Institute of Higher Education and Research, Wardha, India; Department of Community Medicine (D R MD), Rajiv Gandhi Institute of Medical Sciences, Bengaluru, India; Oman Dental College, Muscat, Oman (Prof R A Radhakrishnan PhD); Department of Medical Oncology (Prof V Radhakrishnan MD), Cancer Institute (W.I.A), Chennai, India; Department of Community and Family Medicine (Prof P Raghav MD), Department of Pharmacology (M Shamim MBBS), Department of Biochemistry (S Tomo MD), All India Institute of Medical Sciences, Jodhpur, India; Department of Epidemiology (P Raghuveer MD), National Institute of Mental Health and Neurosciences, Bengaluru, India; Osh State University, Osh, Kyrgyzstan (Prof F Rahim PhD); Department of Environmental Health Engineering (S Rahimi PhD), Health Science Research Centre (S Rahimi PhD), Health Sciences Research Center (M Sarmadi MSc), Torbat Heydariyeh University of Medical Sciences, Torbat Heydariyeh, Iran; Faculty of Health Sciences (F M Rahman PhD), Qaiwan International University, Sulaymaniyah, Iraq; Department of Epidemiology (M Rahman PhD), Institute of Epidemiology, Disease Control and Research (IEDCR), Dhaka, Bangladesh; Department of Pathobiology and Population Sciences (PPS) (M Rahman PhD), Royal Veterinary College (RVC), London, UK; College of Medicine and Health Sciences (M Rahman PhD), National University of Science and Technology, Sohar, Oman; Health Service Research and Quality of Life Center (CEReSS) (Prof M Rahmati PhD), Aix-Marseille University, Marseille, France; Faculty of Medicine (H Rahmoune PhD), LIRSSEI Research Lab (H Rahmoune PhD), University of Setif Algeria, Setif, Algeria; Division of Gynecology and Human Reproduction Physiopathology (D Raimondo PhD), IRCCS Azienda Ospedaliero-Universitaria di Bologna, Bologna, Italy; Department of Medical, Surgical and Experimental Sciences (I Raimondo MD), University of Sassari, Sassari, Italy; Gynecology and Breast Care Center (I Raimondo MD), Mater Olbia Hospital, Olbia, Italy; Dr. Rajendra Prasad Government Medical College, Tanda, Kangra, India (Prof S K Raina MD); Department of Cardiology (A Raja MD), Department of Medicine (S Raja MD), Dow University of Health Sciences, Karachi, Pakistan; Emergency Medicine Department (G Rajendran MD), Sri Manakula Vinayagar Medical College and Hospital, Puducherry, India; Centre for Chronic Disease Control, New Delhi, India (P Rajput PhD); Department of Population Health (M Ramadan DrPH), King Saud bin

Abdulaziz University for Health Sciences, Jeddah, Saudi Arabia; Department of Midwifery (K Ramadhan MPH), Ministry of Health of the Republic of Indonesia, Palu, Indonesia; Department of Anatomy (C Ramasamy MD), Govt. Siddhartha Medical College, Vijayawada, India; Department of Biological Science and Bioengineering (M Ramezani Farani PhD), Inha University, Incheon, South Korea; Department of Biotechnology (Prof P W Ramteke PhD), Hislop College, Nagpur, India; Department of Molecular Biology & Genetic Engineering (Prof P W Ramteke PhD), RTM Nagpur University, Nagpur, India; South Asian Institute for Social Transformation (SAIST), Dhaka, Bangladesh (J Rana MPH); Department of Epidemiology, Biostatistics and Occupational Health (J Rana MPH), McGill University, Montreal, QC, Canada; Department of Research (C L Ranabhat PhD), Eastern Scientific LLC, Richmond, KY, USA; Planetary Health Research Centre (PHRC), Kathmandu, Nepal (C L Ranabhat PhD); Centre for Clinical Pharmacology (N Rancic PhD), University of Defence in Belgrade, Belgrade, Serbia; Centre for Clinical Pharmacology (N Rancic PhD), Medical College of Georgia at Augusta University, Belgrade, Serbia; Department of Oral Medicine and Radiology (K Rao PhD), KS Hegde Medical Academy (S S Shetty MD), Nitte University, Mangalore, India; Department of Oral Pathology, Microbiology and Forensic Odontology (S J Rao MDS), Sharavathi Dental College and Hospital, Shimogga, India; Department of Geography (A Rasul PhD), Soran University, Soran, Iraq; Department of Family Medicine (Prof D Rathish PhD), Department of Parasitology (Prof K G Weerakoon PhD), Department of Community Medicine (N Wickramasinghe MD), Rajarata University of Sri Lanka, Anuradhapura, Sri Lanka; University of Swabi (A Rauf PhD), University of Swabi, Swabi, Pakistan; Department of Psychiatry (D Ravi MBBS), St. John's National Academy of Health Sciences, Bangalore, India; Inovus Medical, St Helens, UK (D L Rawaf MD); Academic Public Health England (Prof S Rawaf MD), Public Health England, London, UK; Department of Computer Science (R Rawassizadeh PhD), Boston University, Boston, MA, USA; Department of Mathematical Demography & Statistics (R Rawat PhD), International Institute for Population Sciences, Mumbai, India; Interventional Cardiology Department (A Ray MD), Saint Vincent Hospital, Worcester, MA, USA; Department of Hematology (B Razi PhD), North Khorasan University of Medical Sciences, Bojnurd, Iran; Clinical Epidemiology Unit (F Recenti MSc), Lund University, Lund, Sweden; Department of Neurosciences, Rehabilitation, Ophthalmology, Genetics, Maternal and Child Health (F Recenti MSc), University of Genoa, Genoa, Italy; Department of Internal Medicine (M M R K Reddy MD), Northwest Health, Porter, Valparaiso, IN, USA; Department of Biological Sciences (Prof E Redwan PhD), King Abdulaziz University, Jeddah, Egypt; Department of Protein Research (Prof E Redwan PhD), Research and Academic Institution, Alexandria, Egypt; The School of Pharmaceutical Sciences (W Rehman MS), University of Science Malaysia, Penang, Malaysia; School of Medicine (Prof A M N Renzaho PhD), Translational Health Research Institute (Prof A M N Renzaho PhD), Western Sydney University, Campbelltown, NSW, Australia; Brien Holden Vision Institute, Sydney, NSW, Australia (Prof S Resnikoff MD); Unisabana Center for Translational Science (L Reyes PhD), Universidad de La Sabana (Savannah University), Chia, Colombia; Critical Care Department (L Reyes PhD), Clinica Universidad De La Sabana (Savannah University Clinic), Chia, Colombia; School of Environment (M Rezaei PhD), Tehran University, Tehran, Iran; Network of Immunity in Infection, Malignancy and Autoimmunity (NIIMA) (Prof N Rezaei PhD), Universal Scientific Education and Research Network (USERN), Tehran, Iran; Department of Public Health Sciences (T Rhee PhD), University of Connecticut, Farmington, CT, USA; Department of Surgery (J Rickard MD), University of Minnesota, Minneapolis, MN, USA; Department of Surgery (J Rickard MD), University Teaching Hospital of Kigali, Kigali, Rwanda; Department of Physiology and Physiotherapy (Prof M R Rizvi PhD), DIT University, Delhi, India; Community Health Department (Prof H A L Rocha PhD), Federal University of Ceará, Fortaleza, Brazil; Department of Geography and Demography (M Rodrigues

PhD), University of Coimbra, Coimbra, Portugal; Department of Clinical Research (Prof L Roever PhD), University of Sao Paulo, Ribeirão Preto, Brazil; Center for Indigenous Health Research (P Rohloff MD), Wuqu' Kawoq Maya Health Alliance, Tecpan, Guatemala; Department of Environmental and Radiological Health Sciences (Prof D Rojas-Rueda PhD, C Sewor MPH), Colorado State University, Fort Collins, CO, USA; Department of Neurosciences (M Romoli MD), Maurizio Bufalini Hospital, Cesena, Italy; Fondazione Policlinico Universitario A. Gemelli (M Romozzi MD), Cuore Università Cattolica del Sacro Cuore (Catholic University of Sacred Heart), Rome, Italy; Department of Ophthalmology and Visual Sciences (A Roshanshad MD), University of Wisconsin-Madison, Madison, WI, USA; Department of Pharmacy Services (K Rotimi MSc), Alberta Health Services, Edmonton, AB, Canada; West African Postgraduate College of Pharmacists, Lagos, Nigeria (K Rotimi MSc); Department of Analytical and Applied Economics (Prof H Rout PhD, P Sahoo MA, C Swain MPhil), RUSA Centre of Excellence in Public Policy and Governance (Prof H Rout PhD), Utkal University, Bhubaneswar, India; Isfahan University of Medical Sciences (H Rouzbahani MD), Islamic Azad University, Isfahan, Iran; Faculty of Medicine (B Roy PhD), Quest International University Perak, Ipoh, Malaysia; Department of Biochemistry and Food Analysis (N Roy PhD), Department of Post-Harvest Technology and Marketing (A Sayeed MSc), Patuakhali Science and Technology University, Patuakhali, Bangladesh; Department of Veterinary Microbiology (P Roy PhD), College of Veterinary Science and Animal Husbandry, Agartala, India; Department of Medicine (P Roy MBBS), North Bengal Medical College and Hospital, Siliguri, India; Department of Labour (P Roy PhD), Government of West Bengal, Kolkata, India; Department of Public Health (S Roy MD), New Mexico State University, Las Cruces, NM, USA; Research Department (S Roy MSc), Indian Institute of Public Health, Delhi, India; Advanced Campus Governador Valadares (Prof G D A Ruela MSc), Juiz de For a Federal University, Governador Valadares, Brazil; Department of Health Statistics (S F Rumisha PhD), National Institute for Medical Research, Dar es Salaam, Tanzania; Department of Cardiology (M Russo PhD), SS. Annunziata Hospital - ASL2 Abruzzo, Chieti, Italy; Department of Internal Medicine (G M Rwegerera MD), Muhimbili University of Health and Allied Sciences, Dar es Salaam, Tanzania; Department of Critical Care Anesthesiology (A Saad Eddin MD), University of Alabama Birmingham, Birmingham, AL, USA; Department of Medical Pharmacology (Prof M M Saber-Ayad PhD), Public Health and Community Medicine Department (M R Salem MD), Cairo University, Giza, Egypt; Escuela de Kinesiología (Prof K P Sadarangani PhD), Diego Portales University, Santiago de Chile, Chile; Universidad Autónoma de Chile, Santiago de Chile, Chile (Prof K P Sadarangani PhD); Department of Computer (T Sadegh MSc), University of Science and Culture, Tehran, Iran; Faculty of Medicine, Bioscience and Nursing (S Safi PhD), MAHSA University, Selangor, Malaysia; Interdisciplinary Research Centre in Biomedical Materials (IRCBM) (S Safi PhD), COMSATS Institute of Information Technology, Lahore, Pakistan; Canadian Red Cross (K Sahu PhD), Red Cross, Ottawa, ON, Canada; Department of Psychiatry (Z Saif MBA), Ministry of Health, Manama, Bahrain; College of Pharmacy (Prof S Sajadi PhD), Al-Hadba University, Mosul, Iraq; Department of Statistics (M R Sajid PhD), University of Gujrat, Gujrat, Pakistan; Department of Health Education & Promotion (Prof L Salehi PhD), A.C.S. Medical College and Hospital, Karaj, Iran; Student Research Committee (M Salehi MD), Kashan University of Medical Sciences, Kashan, Iran; Technology Management Department (Prof M Z Y Salem PhD), University College of Applied Sciences, Gaza, Palestine; School of Economics and Management (Prof M Z Y Salem PhD), University of Kassel, Kassel, Germany; College of Nursing (D Salihu PhD), Jouf University, Jouf, Saudi Arabia; Department of Global Initiatives (Prof G A Salum PhD), Child Mind Institute, New York, NY, USA; Clinical Research Division (S S Salvi MD), Pulmocare Research and Education (PURE) Foundation, Pune, India; Faculty of Health Sciences (S S Salvi MD), Symbiosis International University, Pune, India; Surgical Department (J

Samaranayake MBBS), North Colombo Teaching Hospital, Ragama, Sri Lanka; Benang Merah Research Center (BMRC), Minahasa Utara, Indonesia (Y L Samodra PhD); Department of Anatomy (Prof V P Samuel PhD), Ras Al Khaimah Medical and Health Sciences University, Ras Al Khaimah, United Arab Emirates; Department of Forensic Biology (S G Sangle PhD), Government Institute of Forensic Science Chhatrapati Sambhajinagar, Chhatrapati Sambhajinagar, India; Primary Healthcare Department (F Sanmarchi MD), Azienda USL di Bologna, Bologna, Italy; Department of Sociology and Gerontology (K P Sapkota MSc), Miami University, Oxford, OH, USA; Independent Consultant, Thiruvananthapuram, India (S Y I Saraswathy PhD); Department of Public Health (Y Sarikhani PhD), Jahrom University of Medical Sciences, Jahrom, Iran; Botany Department (H Sarma PhD), Bodoland University, Kokrajhar, India; Department of Community Medicine (S Saroshe MD), Mahatma Gandhi Memorial Medical College, Indore, India; Faculty of Health & Social Sciences (B Sathian PhD), Bournemouth University, Bournemouth, UK; Precision Medicine Department (M Savabi Far MD, S Tajabadi MSc), Università degli studi della Campania Luigi Vanvitelli (University of Campania Luigi Vanvitelli), Naples, Italy; Department of Public Health Sciences (M Sawhney PhD), University of North Carolina at Charlotte, Charlotte, NC, USA; Department of Public Health Sciences (S G Saxena DrPH), Coastal Carolina University, Conway, SC, USA; Harvard Extension School (S G Saxena DrPH), Department of Orthopaedics (O Subasi PhD), Harvard Kennedy School (K J Uzor MD), Harvard University, Cambridge, MA, USA; Department of Preventive and Social Medicine (G Saya MD), Jawaharlal Institute of Postgraduate Medical Education and Research, Puducherry, India; Psychiatry Clinic (M Saylan MD), Holy Savior Armenian Hospital, Istanbul, Turkiye; Faculty of Business and Computing (Prof C Schinckus PhD), University of the Fraser Valley, Abbotsford, BC, Canada; Graduate School of Business (Prof C Schinckus PhD), ESAN University, Lima, Peru; Clinic for Conservative Dentistry and Periodontology (Prof F Schwendicke PhD), University Hospital of the Ludwig-Maximilians-University Munich, Munich, Germany; Department of Medical Statistics (M Šekerija PhD), University of Zagreb, Zagreb, Croatia; Department of Epidemiology and Prevention of Chronic Noncommunicable Diseases (M Šekerija PhD), Croatian Institute of Public Health, Zagreb, Croatia; Department of Applied Mechanics and Biomedical Engineering (V Selvaraj PhD), Indian Institute of Technology Delhi, Chennai, India; Emergency Department (S Senthilkumaran PhD), Manian Medical Centre, Erode, India; Department of Medicine (Y Sethi MD), Swami Vivekanand Subharti University, Meerut, India; National Heart, Lung, and Blood Institute (A Seylani MD), National Institutes of Health, Rockville, MD, USA; Rita A. Patel Institute of Physiotherapy (S Shah PhD), The Charutar Vidya Mandal (CVM) University, Anand, India; School of Health Sciences (S Shaharudin PhD), Universiti Sains Malaysia, Kota Bharu, Malaysia; Department of Biotechnology (S Shahid MPhil), Quaid-i-Azam University Islamabad, Islamabad, Pakistan; Gastroenterology Unit (E Shahini MD), IRCCS, Bari, Italy; Department of Chemistry (H R Shahsavari PhD), Institute for Advanced Studies in Basic Sciences (IASBS), Zanjan, Iran; Independent Consultant, Karachi, Pakistan (M A Shaikh MD); Noncommunicable Diseases Research Center (A Shakerimoghaddam PhD), Department of Basic Medical Sciences (S Yaghoubi PhD), Neyshabur University of Medical Sciences, Neyshabur, Iran; Department of Pathology and Laboratory Medicine (S Sham MD), Northwell Health, New York, NY, USA; Department of Pathobiology (M Shamshirgaran PhD), Shahid Bahonar University of Kerman, Kerman, Iran; Department for Evidence-based Medicine and Evaluation (A Sharifan PharmD), University for Continuing Education Krems, Krems, Austria; Amity Institute of Biotechnology (A Sharma PhD), Amity University Rajasthan, Rajasthan, India; Department of Forensic Science (Prof B K Sharma PhD, M Walia MPhil), Faculty of Medicine and Health Sciences (Prof N P Singh MD), Shree Guru Gobind Singh Tricentenary University, Gurugram, India; UN Mehta Institute of Cardiology and Research Center (Prof K Sharma MD), B.J. Medical College, Ahmedabad, India;

Department of Cardiology (Prof K Sharma MD), Government Medical College, Ahmedabad, India; Department of Nephrology (S Sharma DNB), Vardhman Mahavir Medical College, New Delhi, India; Yenepoya Research Center (R P Shastry PhD), Yenepoya University, Mangalore, India; Department of Engineering (A Shavandi PhD), Free University of Brussels, Brussels, Belgium; Physiotherapy Department (B Shehu Bappah MSc), Federal University of Health Sciences, Azare, Nigeria; Department of Microbiology (S M Shenoy MD), Kasturba Medical College, Mangalore, India; Department of Biology (S P Sherchan PhD), Morgan State University, Baltimore, MD, USA; General Surgery Department (M Shibani MD), Glasgow Royal Infirmary, Glasgow, UK; Department of HIV/AIDS Prevention and Control (B F Shibesh MPH), Bahir Dar University, Bahir Dar, Ethiopia; Tokyo Foundation for Policy Research, Tokyo, Japan (Prof K Shibuya MD); Department of Public Health (D Shiferaw MPH), Dambi Dollo University, Dembi Dollo, Ethiopia; Finnish Institute of Occupational Health, Helsinki, Finland (R Shiri PhD); Department of Experimental Research (V Shivarov PhD), Medical University Pleven, Pleven, Bulgaria; Department of Genetics (V Shivarov PhD), Sofia University "St. Kliment Ohridiski", Sofia, Bulgaria; Department of Neurosurgery (N A Shlobin MD), Columbia University Medical Center, New York, NY, USA; Department of Community Medicine and Public Health (G Shrestha MD), Institute of Medicine, Kathmandu, Nepal; Department of Research and Academics (S Shrestha PhD), Kathmandu Cancer Center, Bhaktapur, Nepal; Kenneth H. Cooper Institute (Prof K Shuval PhD), Texas Tech University Health Sciences Center, Dallas, TX, USA; Advanced Materials Division (N R S Sibuyi PhD), Mintek, Randburg, South Africa; Department of Biotechnology (N R S Sibuyi PhD), University of the Western Cape, Bellville, South Africa; Department of Medical Microbiology and Infectious Diseases (E E Siddig MD), Erasmus University, Rotterdam, Netherlands; Chair for General Economics, Health Economics and Econometrics (Prof M Siegel PhD), University of Greifswald, Greifswald, Germany; Instituto Salud Publica (J Silva MD), Pontifical Javeriana University, Bogota, Colombia; RISE Health (Prof L M L R Silva PhD), University of Beira Interior, Covilhã, Portugal; School of Human and Health Sciences (Prof P P Simkhada PhD), University of Huddersfield, Huddersfield, UK; Department of Agriculture and Environmental Sceinces (Prof B P Singh PhD), National Institute of Food Technology Entrepreneurship and Management-Kundli (NIFTEM-K), Sonipat, India; Department of Pharmacology (H Singh DM), Government Medical College and Hospital, Chandigarh, India; School of Pharmaceutical Sciences (H Singh PhD), IFTM University, Moradabad, India; Department of Paediatrics (J Singh MD), All India Institute of Medical Sciences, Bilaspur, India; School of Medicine (Prof J A Singh MD), Baylor College of Medicine, Houston, TX, USA; Department of Medicine Service (Prof J A Singh MD), US Department of Veterans Affairs (VA), Houston, TX, USA; National Institute of Cancer Prevention and Research, Noida, India (P Singh PhD); Department of Human Genetics (P Singh PhD), Punjabi University Patiala, Patiala, India; Department of Biochemistry (Prof R K Singh PhD), Institute of Medical Sciences (S Singh PhD), Banaras Hindu University, Varanasi, India; Department of Community Medicine (S Singh MD), Veer Chandra Singh Garhwali Government Institute of Medical Science and Research, Srinagar Garhwal, India; ESIC Medical College and Hospital (R Sinha MD), ESIC Medical College and Hospital, Ranchi, India; Department of Internal Medicine (R Sinto MD), University of Indonesia, Jakarta, Indonesia; Department of Internal Medicine (R Sinto MD), Dr. Cipto Mangunkusumo National Hospital, Jakarta Pusat, Indonesia; Department of Physiotherapy and Occupational Therapy (Prof S T Skou PhD), Næstved-Slagelse-Ringsted Hospitals, Slagelse, Denmark; Division of Injury Prevention (Prof D A Sleet PhD), The Bizzell Group, Atlanta, GA, USA; School of Medicine (B Sokhal MD), Keele University, Stoke-On-Trent, UK; University Hospitals North Midlands, Stoke-on-Trent, UK (B Sokhal MD); Department of Systemic Pathology (R Solanki MD), Touro College of Osteopathic Medicine, Middletown, NY, USA; Department of Pathology (R Solanki MD), American

University of the Caribbean School of Medicine, Cupecoy, Saint Martin; Division of Engineering in Medicine (Y Song PhD), Harvard Medical School, Boston, MA, USA; Hospital Universitario de La Princesa (Prof J B Soriano MD), Universidad Autónoma de Madrid (Autonomous University of Madrid), Madrid, Spain; Centro de Investigación Biomédica en Red Enfermedades Respiratorias (CIBERES) (Center for Biomedical Research in Respiratory Diseases Network), Madrid, Spain (Prof J B Soriano MD); Department of Public Health, Experimental and Forensic Medicine (M Sorrentino MD), University of Pavia, Pavia, Italy; Universidade Federal de Minas Gerais (Federal University of Minas Gerais), Belo Horizonte, Brazil (M A Sousa PhD); Doheny Eye Institute (C Soylu MD), University of California Los Angeles, Pasadena, CA, USA; College of Health and Public Service (S Sriram PhD), University of North Texas, Denton, TX, USA; Nutrition and Dietetics Department (A V Starodubova DSc), Federal Research Institute of Nutrition, Biotechnology and Food Safety, Moscow, Russia; Department of Internal Disease (A V Starodubova DSc), Pirogov Russian National Research Medical University, Moscow, Russia; Department of Medicine (P Steiropoulos MD), Democritus University of Thrace, Alexandroupolis, Greece; Occupational and Environmental Medicine Department (L Stockfelt PhD), Institute of Health and Care Sciences (Prof A W Wolf PhD), University of Gothenburg, Gothenburg, Sweden; Global Observatory on Pollution and Health (Prof K Straif PhD), Boston College, Chestnut Hill, MA, USA; ISGlobal Instituto de Salud Global de Barcelona, Barcelona, Spain (Prof K Straif PhD); Department of Ophthalmology (Y Su PhD), Renmin Hospital of Wuhan University, Wuhan, China; Research Department (N Subedi PhD), Nepal Development Society, Kathmandu, Nepal; Department of Geriatrics (C K Suemoto MD), University of São Paulo, Sao Paulo, Brazil; Praboromarajchanok Institute (T Sukaew PhD), Ministry of Public Health, Nonthaburi, Thailand; Department of Human Anatomy (M Suleiman Odidi PhD), Federal University, Dutse, Dutse, Nigeria; School of Life Sciences (M Suleman PhD), Xiamen University, Xiamen, China; Faculty of Health Science (D Sulistiyorini MSc), Universitas Indonesia Maju, Jakarta, Indonesia; School of Medicine, Medical Sciences and Nutrition (A Sultan Meo MPH), University of Aberdeen, Aberdeen, UK; Yusuf Hamied Department of Chemistry (Prof H Z Sun PhD), University of Cambridge, Cambridgeshire, UK; Institute of Integrated Intelligence and Systems (Prof J Sun PhD), Griffith University, Brisbane, QLD, Australia; The First Hospital of China Medical University (M Sun MM), China Medical University, Shenyang, China; Department of Endocrinology and Metabolism (X Sun PhD), Affiliated Hospital of Shandong Second Medical University, Weifang, China; Department of Biomedical Sciences (Z Sun PhD), Universiti Putra Malaysia, Selangor, Malaysia; Gandhi Medical College (S Sundaragiri MD), Kaloji Narayana Rao University of Health Sciences (KNRUHS), Secunderabad, India; Cardiovascular Program (X Xu PhD), The George Institute for Global Health, Sydney, NSW, Australia (Prof J Sundström PhD); Department of Clinical Research and Development (Prof L Szarpak PhD), LUXMED Group, Warsaw, Poland; Collegium Medicum (Prof L Szarpak PhD), John Paul II Catholic University of Lublin, Lublin, Poland; Department of Neurology (P Tabaee Damavandi MD), Neurocenter of Southern Switzerland (NSI), Lugano, Switzerland; Department of Medicine (Prof R Tabarés-Seisdedos PhD), University of Valencia, Valencia, Spain; Department of Basic Medical Sciences (S Tabatabaeizadeh PhD), Department of Internal Medicine (S Tabatabaeizadeh PhD), Islamic Azad University, Mashhad, Iran; Department of Health, Safety, and Environmental Management (R Tabibi PhD), Abadan School of Medical Sciences, Abadan, Iran; Department of Dentistry and Oral Health (J Tadakamadla PhD), La Trobe University, Bendigo, VIC, Australia; School of Dentistry and Oral Health (S K Tadakamadla PhD), School of Medicine and Dentistry (M T N Tran PhD, M N Wanjau PhD), Griffith University, Gold Coast, QLD, Australia; Department of Physiotherapy (B A Tafida MSc), A.T. Still University, Azare, Nigeria; Department of Biostatistics and Epidemiology (M Taheri Soodejani PhD), Shahid Sadoughi University of Medical

Sciences, Yazd, Iran; Research Center for Molecular Medicine (A Taherkhani PhD), Hamadan University of Medical Sciences, Hamadan, Iran; Department of Dermato-Venereology (M Tampa PhD), Dr. Victor Babes Clinical Hospital of Infectious Diseases and Tropical Diseases, Bucharest, Romania; Department of Medicine (J L Tamuzi MSc), Northlands Medical Group, Omuthiya, Namibia; Department of Orthopaedics (J Tan MD), Western Institute of Digital-Intelligent Medicine (Z Xia MD), Department of Anesthesiology (J Yu MD), Chongqing Medical University, Chongqing, China; State Key Laboratory of Numerical Modeling for Atmospheric Sciences and Geophysical Fluid Dynamics (LASG) (H Tang PhD), Chinese Academy of Sciences, Beijing, China; Department of Medicine (E Tantisattamo MD), University of California Irvine, Orange, CA, USA; Department of Public Health (M K Tariku MPH), Public Health Department (T Y Tiruye PhD), School of Public Health (G K Wirtu PhD), Debre Markos University, Debre Markos, Ethiopia; Department of Pharmacology and Therapeutics (S Tariq PhD), The University of Faisalabad, Faisalabad, Pakistan; Taking Our Best Shot, Houston, TX, USA (N Y Tat MS); Department of Research and Innovation (N Y Tat MS), Enventure Medical Innovation, Houston, TX, USA; Institute of General Practice and Interprofessional Care (R Tavakoly PhD), University Hospital Tübingen, Tübingen, Germany; Robert Bosch Center for Integrative Medicine and Health (R Tavakoly PhD), Bosch Health Campus, Stuttgart, Germany; Indiana University School of Medicine (M G Tedla PhD), University of Missouri, Indianapolis, IN, USA; University of Gondar (A T Tefera MPH), Ethiopian Medical Association, Gondar, Ethiopia; Department of Urology (M Teimoori MD), Sabzevar University of Medical Sciences, Sabzevar, Iran; Department of Surgery (O Thaher PhD), University Hospital Bochum, Herne, Germany; Amrita Vishwa Vidyapeetham (Prof K R Thankappan MD), Amrita Institute of Medical Sciences, Kochi, India; Department of Economics (I Tharwat PhD), The American University in Cairo, Cairo, Egypt; Department of Applied Bioscience (Prof M Thiruvengadam PhD), Konkuk University, Seoul, South Korea; Clinical Epidemiology (A Thiyagarajan MPH), Leibniz Institute for Prevention Research and Epidemiology, Bremen, Germany; School of Public Health (W Tian PhD, G Yan MD), Harbin Medical University, Harbin, China; Faculty of Public Health (J H V Ticoalu MPH), Universitas Sam Ratulangi (Sam Ratulangi University), Manado, Indonesia; Interdisciplinary Health Data Center (R Topor-Madry PhD), Jagiellonian University Medical College, Kraków, Poland; Merilyn and Glick Eye Institute (A Torkashvand MD), University of Indiana, Indianapolis, IN, USA; Nutritional Epidemiology Research Team (EREN) (M Touvier PhD), National Institute for Health and Medical Research (INSERM), Paris, France; High Institute of Sport and Physical Education of Sfax (K Trabelsi PhD), University of Sfax, Sfax, Tunisia; Netherlands Organisation for Applied Scientific Research (TNO) (E Traini PhD), Netherlands Organisation for Applied Scientific Research (TNO), Utrecht, Netherlands; Department of Health (N M Tran MD), Children's Hospital 1, Ho Chi Minh City, Vietnam; Second Department of Internal Medicine (Q T H Tran MD), Kansai Medical University, Hirakata, Japan; Department of Business Analytics (T H Tran MD), University of Massachusetts Dartmouth, Dartmouth, MA, USA; Molecular Neuroscience Research Center (N Tran Minh Duc MD), Shiga University of Medical Science, Shiga, Japan; ALS Vietnam Research and Advocacy Initiative (N Tran Minh Duc MD), ALS Vietnam, Quang Ngai, Vietnam; Adult Learning Disability Service (S J Tromans PhD), Leicestershire Partnership National Health Service Trust, Leicester, UK; College of Health Science (Q X N Truong PhD), Vin University, Hanoi, Vietnam; Department of Cardiology (Prof G Tse PhD), Tianjin Medical University, Tianjin, China; Kent and Medway Medical School (Prof G Tse PhD), Kent and Medway Medical School, Canterbury, UK; Department of Internal Medicine (M Tumurkhuu PhD), Wake Forest University, Winston-Salem, NC, USA; Department of Urology (Z Tuo MS), The Second Hospital of Tianjin Medical University, Tianjin, China; Hayatabad Medical Complex (H Ullah FCPS), Postgraduate Medical Institute, Peshawar, Pakistan; Department of Allied Health Sciences (I Ullah PhD),

Iqra University Chak Shahzad Campus, Islamabad, Pakistan; Department of Pediatric Cardiology (K Umapathi MD), Rush University, Chicago, IL, USA; Department of Physiotherapy (L Umar PhD), Federal Ministry of Health, Azare, Nigeria; Department of Medicine (M Umar¶ MBBS), Khairpur Medical College, Khairpur, Pakistan; Department of Oncology (S S Umar FWACS), Federal Medical Centre, Gusau, Nigeria; Section of Advanced Heart Failure and Transplant (D Uppal MD), North Shore University Hospital, Manhasset, NY, USA; Center for Neurodegenerative Diseases and the Aging Brain (D Urso MD), University of Bari, Tricase, Italy; Operational Research Center in Healthcare (Prof D Uzun Ozsahin PhD), Near East University, Mersin, Turkiye; Department of Orthodontics (H Uzunçıbuk PhD), University of Trakya, Edirne, Turkiye; Johnson & Johnson (P Vadagam MS), Duquesne University, Pittsburgh, PA, USA; Sociedad Argentina de Medicina, Buenos Aires, Argentina (Prof P R Valdez PhD); Hospital Vélez Sarsfield, Buenos Aires, Argentina (Prof P R Valdez PhD); Department of Biomedical Sciences (M Valenti MD), Humanitas University, Milan, Italy; Dermatology Unit (M Valenti MD), IRCCS Humanitas Research Hospital, Milan, Italy; Faculty of Sciences (J Varasteh MSc), University of Guilan, Rasht, Iran; Achutha Menon Centre for Health Science Studies (R P Varma MD), Sree Chitra Tirunal Institute for Medical Sciences and Technology, Thiruvananthapuram, India; UKK Institute, Tampere, Finland (Prof T J Vasankari PhD); Faculty of Medicine and Health Technology (Prof T J Vasankari PhD), Tampere University, Tampere, Finland; Department of Biochemistry (S Vasishta PhD), Apollo Institute of Medical Sciences and Research Chittoor, Chittoor, India; Department of Otolaryngology Head and Neck Surgery (S Vasudevan MS), Louisiana State University Health Sciences Center, Shreveport, LA, USA; School of Dentistry and Medical Sciences (P Veginadu PhD), Charles Sturt University, Wagga Wagga, NSW, Australia; Department of Human Genetics & Molecular Biology (B Vellingiri PhD), Bharathiar University, Coimbatore, India; Raffles Neuroscience Centre (Prof N Venketasubramanian MSc), Raffles Hospital, Singapore, Singapore; Department of Neurology (S Vidale MD), Infermi Hospital, Rimini, Italy; Department of Neurology & Stroke Unit (S Vidale MD), Sant'Anna Hospital, Como, Italy; Department of Physiotherapy (J H Villafañe PhD), Universidad Europea de Madrid (European University of Madrid), Villaviciosa de Odón, Spain; Digital Health Research Center (D Villarreal-Zegarra MPH), Instituto Peruano de Orientación Psicológica, Lima, Peru; Occupational Medicine Unit (Prof F S Violante MD), Sant'Orsola Malpighi Hospital, Bologna, Italy; Cardiac Electrophysiology (S C Vipparthy MD), St Bernard's Medical Center, Jonesboro, AR, USA; Faculty of Medicine of Itajubá, Brazil, Itajubá, Brazil (Prof L M Vitorino PhD); Department of Health Care Administration and Economics (Prof V Vlassov MD), National Research University Higher School of Economics, Moscow, Russia; NUST School of Health Sciences (Prof Y Waheed PhD), National University of Science and Technology (NUST), Islamabad, Pakistan; Széchenyi István University, Gyor, Hungary (Prof Y Waheed PhD); Research Organization for Health (M Wahidin PhD), National Research and Innovation Agency, Bogor, Indonesia; Department of Social Sciences (A W Wamai MSc), Chuka University, Kenya, Nairobi, Kenya; School of Chinese Medicine (Prof J Wan PhD), School of Traditional Chinese Medicine (Prof H Yao PhD), Beijing University of Chinese Medicine, Beijing, China; Brigham and Women's Hospital, Boston, MA, USA (C Wang PhD); Department of Oncology (L Wang MA), Xiang'an Hospital of Xiamen University, Xiamen, China; Department of Laboratory Medicine (Prof L Wang PhD), Guangdong Provincial People's Hospital, Guangzhou, China; Department of Artificial Intelligence (S Wang PhD), Eye Institute of Xiamen University (S Wu MD), Xiamen University Malaysia, Xiamen, China; Department of Neurosurgery (S Wang MD), Beijing Tiantan Hospital, Beijing, China; College of Agriculture (X Wang PhD), Northwest A&F University, Xianyang City, China; Enze Medical Health Academy (X Wang PhD), Taizhou Hospital of Zhejiang Province, Taizhou, China; Division of Gastroenterology and Hepatology (Y Wang MD), Mayo Clinic, Jacksonville, FL, USA; Division of Life

Sciences and Medicine (Prof Z Wang PhD), University of Science and Technology of China, Heifei, China; Key Laboratory of Computer-Aided Drug Design (M Waqas PhD), Guangdong Medical University, Dongguan, China; Department of Biotechnology and Genetic Engineering (M Waqas PhD), Hazara University Mansehra, Mansehra, Pakistan; Centre for Health Policy Research (Prof P Ward PhD), Torrens University Australia, Adelaide, SA, Australia; Institute of Health and Wellbeing (I Weerasekara PhD), Federation University, Melbourne, VIC, Australia; University of Adelaide, North Terrace, NSW, Australia (I Weerasekara PhD); Department of Orthopaedics (F Wei PhD), General Hospital of Central Theater Command, Wuhan, China; Fourth Military Medical University, Xi'an, China (F Wei PhD); Department of Geriatrics (X Wei MS), The Eighth Affiliated Hospital of Sun Yat-sen University, Shenzhen, China; Cardiology Department (Prof R G Weintraub MB), Royal Children's Hospital, Melbourne, VIC, Australia; Department of Critical Care and Neurosciences (Prof R G Weintraub MB), Murdoch Children's Research Institute, Parkville, VIC, Australia; Key Laboratory of Shaanxi Province for Craniofacial Precision Medicine Research (Y Wen PhD), Stomatological Hospital (College) of Xi'an Jiaotong University, Xi'an, China; Demographic Change and Aging Research Area (A Werdecker PhD), Competence Center of Mortality-Follow-Up of the German National Cohort (R Westerman DSc), Federal Institute for Population Research, Wiesbaden, Germany; Department of Physical Therapy (T Wiangkham PhD), Naresuan University, Phitsanulok, Thailand; Department of Nursing (A Wilandika PhD), Universitas Aisyiyah Bandung, Bandung, Indonesia; Institute of Clinical Epidemiology (Prof P Willeit PhD), Medical University Innsbruck, Innsbruck, Austria; Research Organisation (A Wireko MD), Inter-Continental Omni-Research in Medicine Collaborative, Berlin, Germany; Department of Public Health (A T Woday MPH), Samara University, Samara, Ethiopia; Department of Research (M W Wojewodzic PhD), Cancer Registry of Norway, Oslo, Norway; Department of Theory and Empiricism of Healthcare (D T Worede MSc), Universität Kassel, Kassel, Germany; Global Health Research Center (C Wu PhD), Duke Kunshan University, Kunshan, China; Department of Food Science and Human Nutrition (Prof F Wu PhD), Michigan State University, East Lansing, MI, USA; Affiliated Hospital of Guangdong Medical University (J Wu MD), Guangdong Medical University, Zhanjiang, China; Department of Public Health (Prof J Wu MPH), Wuhan Fourth Hospital, Wuhan, China; School of Public Health (H Xiao PhD), Zhejiang University, Zhejiang, China; School of Nursing and Rehabilitation (N Xiao BS), Shandong University, Jinan, China; Department of Intelligent Medical Engineering (Prof W Xie DrPH), Anhui Medical University, Anhui, China; Department of Surgery (Prof W Xie DrPH), The First Affiliated Hospital of Anhui Medical University, Hefei, Anhui, China; Department of Respiratory and Critical Care Medicine (H Xing DrPH), Nanchang University, Jiangxi, China; Department of Endocrinology (Prof S Xu PhD), University of Science and Technology of China, Hefei, China; Department of Nutrition (W Xu MPH), Tufts University, Boston, MA, USA; Department of Environmental Health and Epidemiology (V Yadav MD), National Institute for Research in Environmental Health, Bhopal, India; Department of Community Medicine (S Yahoo (Syed) MD), Apollo Institute of Medical Sciences and Research, Hyderabad, India; Department of Cells and Tissues (G Yahya PhD), Molecular Biology Institute of Barcelona, Barcelona, Spain; Department of Public Health (Prof K Yamagishi MD, Prof N Yonemoto PhD), Faculty of Medicine (Y Yano MD), Juntendo University, Tokyo, Japan; Department of Public Health Administration (H Yang MD), Linyi People's Hospital, Linyi, China; Department of Cardiovascular Surgery (W Yang MD), Tianjin Medical University General Hospital, Tian Jin, China; Department of Medicine (A Yarahmadi PhD), Thomas Jefferson University, Philadelphia, PA, USA; Department of Public Health (A Yekdeş MD), Trakya University, Edirne, Turkiye; Department of Family Medicine (S A Yesuf MSc), St. Paul's Hospital Millennium Medical College, Addis Ababa, Ethiopia; Family Medicine Department (S A Yesuf MSc), St. Peter's Specialized Hospital, Addis Ababa, Ethiopia;

KHANA Center for Population Health Research, Phnom Penh, Cambodia (Prof S Yi PhD); Nankai University (Y Yin PhD), China Population and Development Research Center, Tianjin, China; Pharmacy Department (Y E Yismaw MSc), Alkan Health Science, Business and Technology College, Bahir Dar, Ethiopia; Department of Pediatrics (Prof D Yon MD), Kyung Hee University, Seoul, South Korea; Department of Biostatistics (Prof N Yonemoto PhD), University of Toyama, Toyama, Japan; Department of Health Policy and Management (Prof M Z Younis PhD), Jackson State University, Jackson, MS, USA; School of Business & Economics (Prof M Z Younis PhD), Universiti Putra Malaysia (University of Putra Malaysia), Kuala Lumpur, Malaysia; Department of Public Health (A Yousuf PhD), Jigjiga University, Jigjiga, Ethiopia; School of Public Health (Prof Y Yu MS), Hubei University of Medicine, Shiyan, China; Department of Haematology and Blood Transfusion (A A Yusuf MD), Aminu Kano Teaching Hospital, Kano, Nigeria; Association for Socially Applicable Research (ASAR), Pune, India (S Zadey MS); Department of Emergency Medicine (S Zadey MS), Global Emergency Medicine Innovation and Implementation (GEMINI) Research Center, Durham, NC, USA; Epidemiology and Cancer Registry Sector (Prof V Zadnik PhD), Institute of Oncology Ljubljana, Ljubljana, Slovenia; Department of Environmental and Occupational Health (E Zainal Abidin PhD), Universiti Putra Malaysia, Serdang, Malaysia; Faculty of Medicine and Health Sciences (F Zakham PhD), Hodeidah University, Hodeidah, Yemen; Department of Health Sciences (S Zaman PhD), James Madison University, Harrisonburg, VA, USA; Health Investigation Center (N Zamora MD), Universidad Católica Boliviana San Pablo, Tarija, Bolivia; San Pablo Catholic University Tarija Bolivia, Tarija, Bolivia (N Zamora MD); Sant'Elia Hospital (A Zanghì MD), University of Catania, Caltanissetta, Italy; Unit on Child & Adolescent Health (Prof H J Zar PhD), Medical Research Council South Africa, Cape Town, South Africa; Department of Clinical Practice (M Zawiah PhD), Northern Border University, Rafha, Saudi Arabia; Institute of Diagnostic and Interventional Radiology and Neuroradiology (S Zensen MD), University of Duisburg-Essen, Essen, Germany; School of Public Policy and Administration (J Zhang BA), Xi 'an Jiaotong University, Xi'an, China; Medical Oncology Department of Gastrointestinal Cancer (L Zhang MS), Cancer Hospital of Dalian University of Technology, Shenyang, China; School of Biomedical Engineering (L Zhang MS), Dalian University of Technology, Dalian, China; Department of Internal Medicine (X Zhang MD), Jacobi Medical Center, Bronx, NY, USA; Department of Internal Medicine (X Zhang MD), Albert Einstein College of Medicine, Bronx, NY, USA; School of Public Health (Y Zhang PhD), Hubei Province Key Laboratory of Occupational Hazard Identification and Control (Y Zhang PhD), Wuhan University of Science and Technology, Wuhan, China; Tianjin Medical University General Hospital (Z Zhang MD), Tianjin Centers for Disease Control and Prevention, Tianjin, China; Department of Health Management (Z Zhao PhD), Shengjing Hospital of China Medical University, Shenyang, China; National Institute of Parasitic Diseases (J Zheng PhD), Chinese Center for Disease Control and Prevention, Shanghai, China; National Center for Chronic and Noncommunicable Disease Control China and Prevention, Beijing, China (M Zhou PhD); School of Public Health and Emergency Management (B Zhu PhD), Southern University of Science and Technology, Shenzhen, China; Adama Hospital Medical College (Z Zhu PhD), Adama Hospital Medical College, Adama, Ethiopia; School of Public Health (L Zihao PhD), Bengbu Medical College, Bengbu, China; Endocrinology and Metabolism Research Center (G Zoghi MD), Hormozgan University of Medical Sciences, Bandar Abbas, Iran; Department of Public Health (L Zuhriyah PhD), Universitas Brawijaya, Malang, Indonesia; NIHR-Biomedical Research Centre (NIHR-BRC) (Prof A Zumla PhD), University College London Hospitals, London, UK; Clinical Research Centre (Prof S H Zyoud PhD), An-Najah National University Hospital, Nablus, Palestine; Department of Building Engineering and Environment (S H Zyoud PhD), Civil

Engineering and Sustainable Structures (S H Zyoud PhD), Palestine Technical University (Kadoorie), Tulkarem, Palestine

Authors' Contributions

Managing the overall research enterprise

Robert W Aldridge, Charlie Ashbaugh, Catherine S Chen, Amanda Deen, Kara Estep, Emmanuela Gakidou, Ashley Ann Harris, Simon I Hay, Miranda L May, Ali H Mokdad, Christopher J L Murray, Mohsen Naghavi, Olivia D Nesbit, Erin M O'Connell, Matthew Seymour, Caitlyn Steiner, and Stein Emil Vollset.

Writing the first draft of the manuscript

Simon I Hay, Susan A McLaughlin, and Samuel M Ostroff.

Primary responsibility for applying analytical methods to produce estimates

Michael Brauer, Channa Buxbaum, Jack Cagney, Rebecca M Cogen, Ewerton Cousin, Andrew Crist, Garland T Culbreth, Nicole K DeCleene, Holly E Erskine, Alize J Ferrari, William M Gardner, Gabriela Fernanda Gil, Demewoz Haile, Hannah Han, Kassandra L Harding, Simon I Hay, Claire A Henson, Austin Heuer, Catherine O Johnson, Jonathan M Kocarnik, Madeline E Moberg, Ali H Mokdad, Brooks W Morgan, Mohsen Naghavi, Paul Nam, Taylor Noyes, Kanyin Liane Ong, Natalie Pritchett, Christian Razo, Gregory A Roth, Damian F Santomauro, Sarah Brooke Sirota, Sandra Spearman, Lauryn K Stafford, Jeffrey D Stanaway, Jaimie D Steinmetz, Corey Teply, Andrew Underwood-Nakamura, Avina Vongpradith, Theo Vos, Joanna L Whisnant, and Faith H Yuh.

Primary responsibility for seeking, cataloguing, extracting, or cleaning data; designing or coding figures and tables

Noah Ahmad, Robert W Aldridge, Jason A Anderson, Ismael A Barreras Beltran, Rachael Bokota, Edmond D Brewer, Channa Buxbaum, Jack Cagney, Rebecca M Cogen, Andrew Crist, Nicole K DeCleene, Lee Deitesfeld, Alize J Ferrari, Vanessa Garcia, Kassandra L Harding, Simon I Hay, Claire A Henson, Austin Heuer, Julia Hon, Jada Averianna Houser, Audrey L Ihler, Catherine O Johnson, Mariam Khalil, Jonathan M Kocarnik, Hilary R Lawlor, Ali H Mokdad, Mohsen Naghavi, Paul Nam, Taylor Noyes, Doori Oh, Kanyin Liane Ong, Brandon V Pickering, David M Pigott, Hannah Elizabeth Robinson-Oden, Jennifer Jacqueline Rosauer, Damian F Santomauro, Noah Joseph Bernard Silva de Leonardi, Sarah Brooke Sirota, Erica Leigh N Slepak, Sandra Spearman, Lauryn K Stafford, Jeffrey D Stanaway, Jaimie D Steinmetz, Andrew Underwood-Nakamura, Avina Vongpradith, Hai Nam Vu, Joanna L Whisnant, and Faith H Yuh.

Providing data or critical feedback on data sources

Bhoomadevi A, Ahmed A.J. Jabbar, Mohammad Amin Aalipour, Ukachukwu O Abaraogu, Biruk Beletew Abate, Cristiana Abbafati, Samar Abd ElHafeez, Mohammed Altigani Abdalla, Nadin M. I. Abdel Razeq, Ahmed Abdelrahman Abdelgalil, Wael M Abdel-Rahman, Jeza Muhamad Abdul Aziz, Rizwan Suliankatchi Abdulkader, Adam Abdullahi, Auwal Abdullahi, Toufik Abdul-Rahman, Armita Abedi, Roberto Ariel Abeldaño Zuñiga, E S Abhilash, Olugbenga Olusola Abiodun, Richard Gyan Aboagye, Shady Abohashem, Hassan Abolhassani, Lucas Guimarães Abreu, Sawsan Abuhammad, Hana J Abukhadijah, Niveen ME Abu-Rmeileh, Salahdein Aburuz, Dina Abushanab, Raghu Ram Achar, Anirudh Balakrishna Acharya, Juan Manuel Acuna, Mesafint Molla Adane, Zenaw Debasu Addisu, Isaac Akinkunmi Adedeji, David Adedia, Kamoru Ademola Adedokun, Rufus Adesoji Adedoyin, Olumide Thomas Adeleke, Habeeb Omoponle

Adewuyi, Mohd Adnan, Qorinah Estiningtyas Sakilah Adnani, Leticia Akua Adzigbli, David Adzrago, Saira Afzal, Gizachew Beykaso Agafari, Temesgen Anjulo Ageru, Mahdi Aghaalikhani, César Agostinis Sobrinho, Anurag Agrawal, Williams Agyemang-Duah, Bright Opoku Ahinkorah, Danish Ahmad, Muayyad M Ahmad, Rabbiya Ahmad, Sajjad Ahmad, Tauseef Ahmad, Ayman Ahmed, Gasha Salih Ahmed, Haroon Ahmed, Mehrunnisha Sharif Ahmed, Muktar Beshir Ahmed, Sindew Mahmud Ahmed, Gulzhanat Aimagambetova, Hossein Akbarialiabad, Roland Eghoghosoa Akhigbe, Sreelatha Akkala, Salah Al Awaidy, Omar Al Omari, Mohammad Al Qadire, Omar Al Ta'ani, Wasan A. M. Al Taie, Yazan Al Thaher, Omar Ali Mohammed Al Zaabi, Mohammad Ahmmad Mahmoud Al Zoubi, Mostafa Alam, Rasmieh Mustafa Al-Amer, Abebaw Alamrew, Turki M Alanzi, Mohammed Albashtawy, Robert W Aldridge, Tekletsadik Tekleslassie Alemayehu, Abdelazeem M Algammal, Khalid F Alhabib, Ashraf Alhumaidi, Abid Ali, Shahid Ali, Syed Shujait Ali, Waad Ali, Montaha Al-Iede, Sheikh Mohammad Alif, Morteza Alipour, Mohammad A Aljasir, Mohamad Aljofan, Syed Mohamed Aljunid, Mayson H. Alkhatib, Mustafa Alkhawam, Wesam Taher Almagharbeh, Wael Almahmeed, Sabah Al-Marwani, Joseph Uy Almazan, Hesham M Al-Mekhlafi, Omar Almidani, Amr Almobayed, Khaldoon Aied Alnawafleh, Hasan Yaser Alniss, Jaber S Alqahtani, Saleh A Alqahtani, Intima Alrimawi, Salman Khalifah Al-Sabah, Najim Z. Alshahrani, Mansour Abdullah Alshehri, Awais Altaf, Alaa B Al-Tammemi, Nelson Alvis-Guzman, Nelson J Alvis-Zakzuk, Hassan Alwafi, Mohammad Al-Wardat, Hany Aly, Amal AlZahmi, Reza Amani-Beni, Amr Amin, Tarek Tawfik Amin, Alireza Amindarolzarbi, Saeed Amini, Ehsan Amini-Salehi, Nafiu Aminu, Majid Aminzare, Dickson A Amugsi, Filippos Anagnostakis, Deanna Anderlini, Sofia Androudi, Susan C Anenberg, Nguyen Hoang Anh, Samuel Egyakwa Ankomah, Kabilan Annadurai, Sumbul Ansari, Saleha Anwar, Sumadi Lukman Anwar, Razique Anwer, Geminn Louis Carace Apostol, Jalal Arabloo, Jorge Arias de la Torre, Benedetta Armocida, Jesu Arockiaraj, Mahwish Arooj, Anton A Artamonov, Kurnia Dwi Artanti, Deepavalli Arumuganainar, Nurila Aryntayeva, Mahsa Asadi Anar, Shewatatek Melaku Asefa, Mulu Tiruneh Asemu, Syed Amir Ashraf, Mitra Ashrafi, Muhammad Shahzad Aslam, Yuni Asri, Batyrbek Assembekov, Mirbahador Athari, Seyyed Shamsadin Athari, Maha Moh'd Wahbi Atout, Alok Atreya, Marcel Ausloos, Núbia Carelli Pereira Avelar, Sana Javaid Awan, Olatunde O Ayinde, Davood Azadi, Masood Azhar, Farya Azimi, Mohd Yusmaidie Aziz, Ahmed Y Azzam, Domenico Azzolino, Rasha Babiker, Giridhara Rathnaiah Babu, Ashish D Badiye, Alaa Aboelnour Badran, Youngoh Bae, Arvind Bagga, Soroush Baghdadi, Sana Baghizadeh, Ruhai Bai, Atif Amin Baig, Abdulaziz T Bako, Senthilkumar Balakrishnan, Jose Balmori-de-la-Miyar, Ovidiu Constantin Baltatu, Maciej Banach, Palash Chandra Banik, Rajon Banik, Shirin Barati, Amadou Barrow, Sandra Barteit, MD Abu Bashar, Shahid Bashir, Mohammad-Mahdi Bastan, Sanjay Basu, Mulat Tirfie Bayih, Feyisa Shasho Bayisa, Nebiyou Simegnew Bayleyegn, Narasimha M Beeraka, Jina Behjati, Babak Behnam, Bezawit K Bekele, Melesse Belayneh, Olorunjuwon Omolaja Bello, Apostolos Beloukas, Derrick A Bennett, Samiun Nazrin Bente Kamal Tune, Eduardo Bernabe, Robert S Bernstein, Akshaya Srikanth Bhagavathula, Neeraj Bhala, Jeetendra Bhandari, Kayleigh Bhangdia, Ravi Bharadwaj, Sonu Bhaskar, Ajay Nagesh Bhat, Anup Bhat, Priyadarshini Bhattacharjee, Gurjit Kaur Bhatti, Jasvinder Singh Bhatti, Soumitra S Bhuyan, Sibhatu Kassa Biadgilign, Raluca Bievel-Radulescu, Cem Bilgin, Saeed Biroudian, Bijit Biswas, Ahmad Naoras Bitar, Molalegne Bitew, Lucimere Bohn, Rachael Bokota, Obasanjo Afolabi Bolarinwa, Sri Harsha Boppana, Berrak Bora Basara, Sanaz Bordbar, Hamed Borhany, Souad Bouaoud, Soufiane Boufous, Rupert R A Bourne, Christopher Boxe, Marija M Bozic, Dejana Braithwaite, Michael Brauer, Annie J Browne, Traolach Brugha, Danilo Buonsenso, Asmat Burhan, Katrin Burkart, Felix Busch, Sanjay C J, Jack Cagney, Luciana Aparecida Campos, Yuchen Cao, Juan Jesus Carrero, Joao Mauricio Castaldelli-Maia, Carlos A Castañeda-Orjuela, Christopher R Cederroth, Francieli Cembranel, Madhu Chakkere Shivamadhu, Eeshwar K Chandrasekar, Vijay Kumar Chattu, Victoria

Chatzimavridou-Grigoriadou, Lam Duc Chau, Sirshendu Chaudhuri, Akhilanand Chaurasia, Haowei Chen, Hui Chen, Meng Xuan Chen, Nicholas WS Chew, Clara G Chisari, William C S Cho, Bryan Chong, Dinh-Toi Chu, Eric Chung, Sunghyun Chung, Aaron J Cohen, Alyssa Columbus, Joao Conde, Nathalie Conrad, Sara Conti, Mariana Oliveira Corda, Paolo Angelo Cortesi, Ewerton Cousin, Michael H Criqui, Natalia Cruz-Martins, Xiaochen Dai, Mayank Dalakoti, Gloria Dalla Costa, Giovanni Damiani, Yohannes Tefera Damtew, Lucio D'Anna, Pojsakorn Danpanichkul, Samuel Demissie Darcho, Fernando Pio De la Hoz, Edward Christopher Dee, Sindhura Deekonda, Louisa Degenhardt, Pouria Delbari, Andreas K Demetriades, Tadios Niguss Derese, Ismail Dergaa, Hunegnaw Almaw Derseh, Vinoth Gnana Chellaiyan Devanbu, Pradeep Kumar Devarakonda, Narender K. Dhania, Mandira Lamichhane Dhimal, Meghnath Dhimal, Marcello Di Pumpo, Diana Dias da Silva, Daniel Diaz, Kimia Didehvar, Xueting Ding, Huyen Phuc Do, Thao Huynh Phuong Do, Klara Georgieva Dokova, Christiane Dolecek, Regina-Mae Villanueva Dominguez, Ojas Prakashbhai Doshi, Robert Kokou Dowou, Jiang Du, Jennifer Dunne, Andre Rodrigues Duraes, Senbagam Duraisamy, Lamiaa Labieb Mahmoud Ebraheim, Alireza Ebrahimi, Mohammad Hossein Ebrahimi, Fatemeh Ehsani, Ashkan Eighaei Sedeh, Ebrahim Eini, Michael Ekholuenetale, Temitope Cyrus Ekundayo, Rabie Adel El Arab, Maysaa El Sayed Zaki, Reza Elahi, Rana Elbeshbeishy, Mohammed Elshaer, Abdelgawad Salah Eltahawy, Victor Oghenekparobo Emojevwe, Holly E Erskine, Derese Eshetu, Gilbert Eshun, Majid Eslami, Fahima Nasrin Eva, Adewale Oluwaseun Fadaka, Heidar Fadavian, Adeniyi Francis Fagbamigbe, Ayesha Fahim, Aliasghar Fakhri-Demeshghieh, Qiping Fan, Zaki Farhana, Syed Muhammad Yousaf Farooq, Hossein Farrokhpour, Fatemeh Farshad, Ali Fatehizadeh, Davood Fathi, Valery L Feigin, Alireza Feizkhah, Ginenus Fekadu, Berhanu Elfu Feleke, Dechao Feng, Seyed-Mohammad Fereshtehnejad, Alize J Ferrari, Natan Feter, Alexander Finnemore, Morenike Oluwatoyin Folayan, Lisa M Force, Arianna Fornari, Ingeborg Forthun, Matteo Foschi, Maryam Fotouhi, Richard Charles Franklin, Blima Fux, Sridevi G, Peter Andras Gaal, Yaseen Galali, Silvano Gallus, Dhanraj Ganapathy, Mohd Ashraf Ganie, Bashiru Garba, Fernando Barroga Garcia, William M Gardner, Zisis Gatzioufas, Stefano Gelibter, Nsikakabasi Samuel George, Ali Gerami Matin, Kalab Yigermal Gete, Keyghobad Ghadiri, Arin Ghamkhar, Shakiba Ghasemi Assl, Sailaja Ghimire, Elena Ghotbi, Gabriela Fernanda Gil, Syed Abdullah Gilani, Bikash Ranjan Giri, Alem Abera Girmay, Laszlo Göbölös, Kimiya Gohari, Mahaveer Golechha, Pouya Goleij, Davide Golinelli, Wenping Gong, Yitayal Ayalew Goshu, Aman Goyal, Ayman Grada, Shi-Yang Guan, Avirup Guha, Damitha Asanga Gunawardane, Zheng Guo, Rajat Das Gupta, Rajeev Gupta, Sapna Gupta, Veer Bala Gupta, Vijai Kumar Gupta, Vivek Kumar Gupta, Lami Gurmessa, Adrina Habibzadeh, Awoke Derbie Habteyohannes, Tesfahun Simon Hadaro, Najah R Hadi, Abdul Hafiz, Demewoz Haile, Pritam Halder, Kosar Hikmat Hama Aziz, Islam M Hamad, Nadia M Hamdy, Erin B Hamilton, Mohammad Hamza, Hannah Han, Nasrin Hanifi, Ashanul Haque, Harapan Harapan, Kassandra L Harding, Andy Martahan Andreas Hariandja, Josep Maria Haro, Eka Mishbahatul Marah Has, Faizul Hasan, Ali Hasanpour- Dehkordi, Arezou Hashem Zadeh, Mahgol Sadat Hassan Zadeh Tabatabaei, Mohammed Bheser Hassen, Lasanthi Wathsala Hathagoda, Rasmus J Havmoeller, Simon I Hay, Jeffrey J Hebert, Mehdi Hemmati, Claire A Henson, Claudiu Herteliu, Md Mahbub Hossain, Mohammad Bellal Hossain, Mehdi Hosseinzadeh, Jada Averianna Houser, Weijun Huang, Atanesia Indriyani Human, Andreas Kattem Husøy, Salman Hussain, Dursa Hussein, Nawfal R Hussein, Mohamed Ibrahim Husseiny, Hong-Han Huynh, Luigi Francesco Iannone, Ahmed Ibrahim, Ramzi Ibrahim, Pulwasha Maria Iftikhar, Nayu Ikeda, Jibran Ikram, Olayinka Stephen Ilesanmi, Muhammad Hamza Ilyas, Leeberk Raja Inbaraj, Lalu Muhammad Irham, Md. Shahinul Islam, Nahlah Elkudssiah Ismail, Gaetano Isola, Mahalaxmi Iyer, Kathryn H. Jacobsen, Morteza Jafarinia, Shabbar Jaffar, Haitham Jahrami, Vikash Jaiswal, Mihajlo Jakovljevic, Ali Jaliliyan, Mohamed Jalloh, Armaan Jamal, Jazlan Jamaluddin, Jerin James, Safayet Jamil,

Masoud Jamshidi, Rajiv Janardhanan, Syed Sarmad Javaid, Anita Javanmardi, Talha Jawaid, Qassim Jawell Odah Abed, Yovanthi Anurangi Jayasinghe, Achala Upendra Jayatilleke, Tadesse Hailu Jember, Belayneh Hamdela Jena, Diptismita Jena, Bijay Mukesh Jeswani, Vivekanand Jha, Weiqiu Jin, Wenyi Jin, Catherine O Johnson, Emily Katherine Johnson, Jost B Jonas, Tamas Joo, Abel Joseph, Charity Ehimwenma Joshua, Jacek Jerzy Jozwiak, Billingsley Kaambwa, Zubair Kabir, Dler H. Hussein Kadir, Ethan M Kahn, Ashish Kumar Kakkar, Leila R Kalankesh, Khalil Kalavani, Feroze Kaliyadan, Sivesh Kathir Kamarajah, Saltanat Kamenova, Ramat T. Kamorudeen, Oleksandr Kamyshnyi, Jiseung Kang, Rami S Kantar, Neeti Kapoor, Arman Karimi Behnagh, Mohmed Isaqali Karobari, Tomasz M Karpiński, Manoj Kumar Kashyap, Abdene Weya Kaso, Adarsh Katamreddy, Gbenga A Kayode, Nastaran Kazemi Rad, Mohammad-Hossein Keivanlou, Peter Njenga Keiyoro, Vikash Ranjan Keshri, Emmanuelle Kesse-Guyot, Yousef Saleh Khader, Himanshu Khajuria, Anas Husam Khalifeh, Anees Ahmed Khalil, Mariam Khalil, Pantea Khalili, Faham Khamesipour, Ajmal Khan, Faiz Ullah Khan, Maseer Khan, Mohammad Idreesh Khan, Muhammad Mueed Khan, Muhammad Umer Khan, Ruby Khan, Salman Ali Khan, Zahid Khan, Sameer Uttamaro Khasbage, Zenith Khashim, Khaled Khatab, Atulya Aman Khosla, Farbod Khosravi, Zemene Demelash Kifle, Jinho Kim, Min Seo Kim, Yohannes Kinfu, Mary Kirk, Adnan Kisa, Sezer Kisa, Jessica Klusty, Shivakumar KM, Ann Kristin Skrindo Knudsen, Jonathan M Kocarnik, Michail Kokkorakis, Diana Gladys Kolieghu Tcheumeni, Aida Kondybayeva, Tapos Kormoker, Oleksii Korzh, Archana Koul, Sindhura Lakshmi Koulmane Laxminarayana, Irene Akwo Kretchy, James-Paul Kretchy, Kewal Krishan, Chong-Han Kua, Barthelemy Kuate Defo, Burcu Kucuk Bicer, Shweta Kulshreshtha, Dewesh Kumar, Dhasarathi Kumar, Jogender Kumar, Manasi Kumar, Sanjay Kirshan Kumar, Vijay Kumar, Subramanian Kumaran, Maria Dyah Kurniasari, Asep Kusnali, Dian Kusuma, Assylkhan Kuttybayev, Ville Kytö, Hmwe Hmwe Kyu, Pallavi L C, Muhammad Awwal Ladan, Chandrakant Lahariya, Dharmesh Kumar Lal, Tea Lallukka, Savita Lasrado, Kamaluddin Latief, Saheed Akinmayowa Lawal, Dai Quang Le, Duc Huy Le, Huu-Hoai Le, Long Khanh Dao Le, Minh Huu Nhat Le, Nhi Huu Hanh Le, Thao Thi Thu Le, Trang Diep Thanh Le, Caterina Ledda, Seung Won Lee, Wei-Chen Lee, Yo Han Lee, James Leigh, Vasileios Leivaditis, Janni Leung, Hui Li, Jiaying Li, Weilong Li, Yichong Li, Zhengrui Li, Yanxue Lian, Stephen S Lim, Queran Lin, Gang Liu, Jue Liu, Xuefeng Liu, Erand Llanaj, Michael J Loftus, Stefan Lorkowski, Shanjie Luan, Jailos Lubinda, Jay B Lusk, Angelina M Lutambi, Miltiadis D Lytras, Ellina Lytvyak, Kevin Sheng-Kai Ma, Zheng Feei Ma, Isis E Machado, Seyed Ataollah Madinezad, Christian Madsen, Aurea Marilia Madureira-Carvalho, Mohammed Magdy Abd El Razek, D. R. Mahadeshwara Prasad, Sasikumar Mahalingam, Nozad Hussein Mahmood, My Tra Mai, Rituparna Maiti, Mohammad-Reza Malekpour, Ahmad Azam Malik, Deborah Carvalho Malta, Mustapha Mangdow, Lokesh Manjani, Mohammad Ali Mansournia, Ana M Mantilla Herrera, Lorenzo Giovanni Mantovani, Changkun Mao, Sajid Maqsood, Joemer C Maravilla, Mirko Marino, Randall V Martin, Bernardo Alfonso Martinez-Guerra, Francisco Rogerlândio Martins-Melo, Winfried März, Roy Rillera Marzo, Sammer Marzouk, Stefano Masi, Clara N Matei, Alexander G Mathioudakis, Medha Mathur, Maryam Mazaheri, Chioma Ngozichukwu Pauline Mbachu, Ikechukwu Innocent Mbachu, Steven M McPhail, Enkeleint A Mechili, Medhin Mehari, Asim Mehmood, Entezar Mehrabi Nasab, Vini Mehta, Tesfahun Mekene Meto, Endalkachew Belayneh Melese, Aishe Memetova, Walter Mendoza, Godfred Antony Menezes, Ritesh G Menezes, Berihun Agegn Mengistie, Atte Meretoja, Chamila Dinushi Kukulege Mettananda, Louise Mewton, Andrea Michelerio, Paul Anthony Miller, Ted R Miller, Wai-kit Ming, Erkin M Mirrakhimov, Yousef Mirzaei, Archana Mishra, Kumar Guru Mishra, Arup Kumar Misra, Mohammadreza Mobayen, Mona Gamal Mohamed, Nouh Saad Mohamed, Khabab Abbasher Hussien Mohamed Ahmed, Mohammad Reza Mohammadi, Seyed Omid Mohammadi, Abdollah Mohammadian-Hafshejani, Shafiu Mohammed, Yahaya Mohammed, Yugal Kishore Mohanta,

Ali H Mokdad, Shaher Momani, Lorenzo Monasta, Amirabbas Monazzami, Ahmed Al Montasir, Catrin E Moore, Yousef Moradi, Maziar Moradi-Lakeh, Brooks W Morgan, Mahdis Morovvati, Mahmoud M Morsy, Jakub Morze, Jonathan F Mosser, Nogol Motamedgorji, Vincent Mougin, Seyed Mohamad Sadegh Mousavi Kiasary, Mohamed Awad Abdalaziz Mousnad, Ahmed Msherghi, Rabia Mubarak, Sumaira Mubarik, Shiv K Mudgal, Syed Aun Muhammad, Sukhes Mukherjee, Sumoni Mukherjee, Amartya Mukhopadhyay, Francesk Mulita, Getaneh Baye Mulu, Chalie Mulugeta, Kavita Munjal, Yanjinlkham Munkhsaikhan, Christopher J L Murray, Ali Mushtaq, Ghulam Mustafa, Saravanan Muthupandian, Muhammad Muzaffar, Ahamarshan Jayaraman Nagarajan, Mohsen Naghavi, Ganesh R Naik, Sanjeev Nair, Ibrahim A Naqid, Shumaila Nargus, Yvonne Nartey, Bruno Ramos Nascimento, Abdallah Y Naser, Mohammad Naser, Hamide Nasiri, Zuhair S Natto, Zakira Naureen, Anum Nawaz, M. Omar Nawaz, Biswa Prakash Nayak, Javad Nazari, Ionut Negoi, Ruxandra Irina Negoi, Henok Biresaw Netsere, Josephine W Ngunjiri, Anh Thy H Nguyen, Cuong Tat Nguyen, Huong Lan Thi Nguyen, Nghia Phu Nguyen, Phat Tuan Nguyen, Trang Nguyen, Tu Anh Nguyen, Van Thanh Nguyen, Ambe Marius Ngwa, Robina Khan Niazi, Luciano Nieddu, Yeshambel T Nigatu, Jan Rene Nkeck, Chukwudi A Nnaji, Shuhei Nomura, Pardis Noormohammadpour, Mamoona Noreen, Masoud Noroozi, Jean Jacques Noubiap, Valentine C Nriagu, Fred Nugen, Dieta Nurrika, Chimezie Igwegbe Nzoputam, Ogochukwu Janet Nzoputam, Bogdan Oancea, George Obaido, Ismail A Odetokun, Michael Safo Oduro, Onome Bright Oghenetega, Oluwaseun Adeolu Ogundijo, Abiola Ogunkoya, James Odhiambo Oguta, Doori Oh, Akinkunmi Paul Okekunle, Olalekan John Okesanya, John Olayemi Okunlola, Andrew T Olagunju, Oladotun Victor Olalusi, Matthew Idowu Olatubi, Abdulhakeem Abayomi Olorukooba, Ronald Olum, Bolajoko Olubukunola Olusanya, Jacob Olusegun Olusanya, Folorunsho Bright Omage, Hany A Omar, Kanyin Liane Ong, Sandersan Onie, Obinna E Onwujekwe, Marcel Opitz, Atakan Orscelik, Alberto Ortiz, Samuel M Ostroff, Uchechukwu Levi Osuagwu, Olayinka Osuolale, Godfred Otchere, Mostafa Monier Othman, Adrian Otoiu, Oche Joseph Otorkpa, Abdu Oumer, Jerry John Ouner, Amel Ouyahia, Guoqing Ouyang, Mayowa O Owolabi, Mahesh P A, Kevin Pacheco-Barrios, Inderbir Padda, Jagadish Rao Padubidri, Anton Pak, Raffaele Palladino, Tejasri Paluvai, Sujogya Kumar Panda, Songhomitra Panda-Jonas, Deepshikha Pande Katare, Giovanni Paolino, Mario Virgilio Papa, Paraskevi Papadopoulou, Chulwoo Park, Seoyeon Park, Bhumi Hemal Patel, Hemal M Patel, Mitesh Patel, Neel Navinkumar Patel, Shankargouda Patil, Apurba Patra, Shrikant Pawar, Shubhadarshini Pawar, Paolo Pedersini, Prince Peprah, Gavin Pereira, Maria Odete Pereira, Pablo Perez-Lopez, Simone Perna, Pavlo Petakh, Hoang Nhat Pham, Nhat Truong Pham, David M Pigott, Julian David Pillay, Luane Pinheiro Pinheiro Rocha, Zahra Zahid Piracha, Dietrich Plass, Peter Pollner, Ramesh Poluru, Arjun Pon Avudaiappan, Maarten J Postma, Sajjad Pourasghary, Mohsen Poursadeqiyan, Naeimeh Pourtaheri, Attur Ravindra Prabhu, Sergio I Prada, Jalandhar Pradhan, Elton Junio Sady Prates, Natalie Pritchett, Harsh Priya, Jagadeesh Puvvula, Nameer Hashim Qasim, Xiang Qi, Zahiruddin Syed Quazi, Navid Rabiee, Venkatraman Radhakrishnan, Hadi Raeisi Shahraki, Pankaja Raghav, Pracheth Raghuveer, Leila Rahbarnia, Fakher Rahim, Hawbash Mohammed-Amin Rahim, Sajjad Rahimi, Afarin Rahimi-Movaghar, Vafa Rahimi-Movaghar, Mohammad Meshbahur Rahman, Masoud Rahmati, Ghasem Rahmatpour Rokni, Diego Raimondo, Sunil Kumar Raina, Jeffrey Pradeep Raj, Adarsh Raja, Sandesh Raja, Sathish Rajaa, Erta Rajabi, Judah Rajendran, Mahmoud Mohammed Ramadan, Kadar Ramadhan, Chitra Ramasamy, Shakthi Kumaran Ramasamy, Sheena Ramazanu, Chhabi Lal Ranabhat, Nemanja Rancic, Smitha Rani, Chythra R Rao, Kumuda Rao, Mithun Rao, Sowmya J Rao, Vahid Rashedi, Mohammad Aziz Rasouli, Prateek Rastogi, Santosh Kumar Rauniyar, Ilari Rautalin, David Laith Rawaf, Salman Rawaf, Reza Rawassizadeh, Ramu Rawat, Ayita Ray, Iman Razeghian, Christian Razo, Filippo Recenti, Wajiha Rehman, Andre M N Renzaho, Serge Resnikoff, Mina

Rezaei, Nima Rezaei, Taeho Gregory Rhee, Mavra A Riaz, Antonio Luiz P Ribeiro, Moattar Raza Rizvi, Hermano Alexandre Lima Rocha, João Rocha Rocha-Gomes, Mónica Rodrigues, Leonardo Roever, Peter Rohloff, David Rojas-Rueda, Debby Syahru Romadlon, Michele Romoli, Marina Romozzi, Luca Ronfani, Amirhossein Roshanshad, Kunle Rotimi, Hanieh Rouzbahani, Reza Rouzbahani, Shiva Rouzbahani, Poulami Roy, Priyanka Roy, Sharmistha Roy, Shubhanjali Roy, Parameswari Royapuram Parthasarathy, Enrico Rubagotti, Susan Fred Rumisha, Michele Russo, Godfrey Mutashambara Rwegerera, Aly M A Saad, Cameron John Sabet, Siamak Sabour, Kabir P Sadarangani, Seyed Kiarash Sadat Rafiei, Basema Ahmad Saddik, Bashdar Abuzed Sadee, Tarannom Sadegh, Ehsan Sadeghi, Mohd Saeed, Umar Saeed, Maryam Saeedi, Mehdi Safari, Sher Zaman Safi, Rajesh Sagar, Mastooreh Sagharichi, Fatemeh Saheb Sharif-Askari, Narjes Saheb Sharif-Askari, Pragyan Monalisa Sahoo, Kirti Sundar Sahu, Zahra Saif, S Mohammad Sajadi, Mirza Rizwan Sajid, Morteza Saki, Leili Salehi, Mahdi Salehi, Marwa Rashad Salem, Malik Sallam, Giovanni A Salum, Sundeep Santosh Salvi, Hossein Samadi Kafil, Waqas Sami, Vijaya Paul Samuel, Abdallah M Samy, Elaheh Sanjari, Sathish Sankar, Francesca Sanna, Damian F Santomauro, Milena M Santric-Milicevic, Mohammad Sarmadi, Brijesh Sathian, Paul A Saunders, Monika Sawhney, Mete Saylan, Christophe Schinckus, Ione Jayce Ceola Schneider, David C Schwebel, Falk Schwendicke, Saravanan Sekaran, Muthamizh Selvamani, Vimalraj Selvaraj, Yigit Can Senol, Subramanian Senthilkumaran, Edson Serván-Mori, Yashendra Sethi, Allen Seylani, Jamileh Shadid, Muhammad Shahbaz, Samiah Shahid, Endrit Shahini, Masood Ali Shaikh, Ali Shakerimoghaddam, Sunder Sham, Muhammad Aaqib Shamim, Mehran Shams-Beyranvand, Anas Shamsi, Alfiya Shamsutdinova, Dan Shan, Abhishek Shankar, Mohammed Shannawaz, Xian Shao, Amin Sharifan, Javad Sharifi Rad, Avimanu Sharma, Vishal Sharma, Rajesh P Shastry, Shamee Shastry, Ramzi Shawahna, Maryam Shayan, Ali Sheidaei, Suchitra M Shenoy, Samendra P Sherchan, Lin-Hong Shi, Md Monir Hossain Shimul, Jae Il Shin, Aminu Shittu, Ivy Shiue, Nathan A Shlobin, Shayan Shojaei, Zahra Shokati Eshkiki, Gambhir Shrestha, Sunil Shrestha, Mohammad Sidiq, Juan Carlos Silva, Luís Manuel Lopes Rodrigues Silva, Noah Joseph Bernard Silva de Leonardi, Padam Prasad Simkhada, Abhinav Singh, Ambrish Singh, Baljinder Singh, Bhim Pratap Singh, Harmanjit Singh, Harpreet Singh, Jasvinder A Singh, Kalpana Singh, Lucky Singh, Narinder Pal Singh, Paramdeep Singh, Prashant Kumar Singh, Rakesh K Singh, Samer Singh, Surendra Singh, Mukesh Kumar Sinha, Ratnesh Sinha, David A Sleet, Md.Salman Sohel, Balamrit Singh Sokhal, Solikhah Solikhah, Younseong Song, Aayushi Sood, Soroush Soraneh, Reed J D Sorensen, Fernando Sousa, Michael Spartalis, Sandra Spearman, Chandrashekhar T Sreeramareddy, Shyamkumar Sriram, Lauryn K Stafford, Jeffrey D Stanaway, Antonina V Starodubova, Caroline Stein, Timothy J Steiner, Jaimie D Steinmetz, Leo Stockfelt, Lars Jacob Stovner, Muhammad Suleman, Anusha Sultan Meo, Haitong Zhe Sun, Xiaodong Sun, Zhuanlan Sun, Suraj Sundaragiri, Thanigaivel Sundaram, Johan Sundström, David Sunkersing, Chandan Kumar Swain, Dayinta Annisa Syaiful, Lukasz Szarpak, Mindy D Szeto, Sree Sudha T Y, Rafael Tabarés-Seisdedos, Shima Tabatabai, Celine Tabche, Ramin Tabibi, Yasaman Taheri Abkenar, Amir Taherkhani, Jabeen Taiba, Stella Talic, Mircea Tampa, Jianye Tan, Ker-Kan Tan, Shynar Tanabayeva, Haosu Tang, Jay Tewari, Pugazhenthan Thangaraju, Nihal Thomas, Geethika P Thota, Madi Tleshev, Musliu Adetola Tolani, Roman Topor-Madry, Mathilde Touvier, Marcos Roberto Tovani-Palone, Mai Thi Ngoc Tran, Quynh Thuy Huong Tran, Tam Quoc Minh Tran, Nguyen Tran Minh Duc, Domenico Trico, Indang Trihandini, Quynh Xuan Nguyen Truong, Gary Tse, Munkhtuya Tumurkhuu, Sok Cin Tye, Himayat Ullah, Irfan Ullah, Muhammad Umair, Muhammad Umar¶, Dinesh Upadhya, Era Upadhyay, Dilber Uzun Ozsahin, sara Vahdati, Jef Van den Eynde, Aaron van Donkelaar, Tommi Juhani Vasankari, Sampara Vasishta, Srivatsa Surya Vasudevan, Ashleigh S Vella, Balachandar Vellingiri, Narayanaswamy Venketasubramanian, Georgios-Ioannis Verras, David Villarreal-Zegarra, Sharath Chaitanya Vipparthy,

Luciano Magalhães Vitorino, Vasily Vlassov, Martin Vojtek, Theo Vos, Mehdi Vosoughi, Linh Vu, Yasir Waheed, Megha Walia, Agnes Wamuyu Wamai, Jin-Yi Wan, Liang Wang, Shu Wang, Xing Wang, Yichen Wang, Tanveer A. Wani, Daniel J Weiss, Andrea Werdecker, Ronny Westerman, Joanna L Whisnant, Harvey A Whiteford, Taweewat Wiangkham, Angga Wilandika, Peter Willeit, Andrew Awuah Wireko, Gemechu Kumera Wirtu, Charles Shey Wiysonge, Axel Walter Wolf, Tewodros Eshete Wonde, Yohannes Chemere Wondmeneh, Daniel Tarekegn Worede, Minichil Chanie Worku, Nigus Kassie Worku, Ai-Min Wu, Felicia Wu, Jiayuan Wu, Zenghong Wu, Hong Xiao, Na Xiao, Hongquan Xing, Site Xu, Suowen Xu, Wanqing Xu, Sajad Yaghoubi, Kazumasa Yamagishi, Weiguang Yang, Haiqiang Yao, Laiang Yao, Amir Yarahmadi, Haya Yasin, Sanni Yaya, Pengpeng Ye, Mohammad Hossein YektaKooshali, Subah Abderehim Yesuf, Siyan Yi, Muluken Yigezu, Yulai Yin, Malede Berihun Yismaw, Naohiro Yonemoto, Mustafa Z Younis, Abdilahi Yousuf, Chuanhua Yu, Yong Yu, Siddhesh Zadey, Vesna Zadnik, Mubashir Zafar, Emilia Zainal Abidin, Burhan Abdullah Zaman, Kourosh Zarea, Sebastian Zensen, Beijian Zhang, Jingya Zhang, Xiaoyi Zhang, Jianhui Zhao, Zhongyi Zhao, Claire Chenwen Zhong, Juexiao Zhou, Maigeng Zhou, Abzal Zhumagaliuly, Magdalena Zielińska, Liu Zihao, Liesl J Zuhlke, Alimuddin Zumla, and Ahed H Zyoud.

Developing methods or computational machinery

Robert W Aldridge, Aleksandr Y Aravkin, Michael Brauer, Jack Cagney, Austin Carter, Kelly M Cercy, Catherine S Chen, Emma Johnson Cowart, Jessica A Cruz, Garland T Culbreth, Xiaochen Dai, Nicole K DeCleene, Lisa M Force, William M Gardner, Nora M Gilbertson, Demewoz Haile, Erin B Hamilton, Hannah Han, Kassandra L Harding, Simon I Hay, Jiawei He, Austin Heuer, Kyle Matthew Humphrey, Catherine O Johnson, Jonathan M Kocarnik, Madeline E Moberg, Ali H Mokdad, Jonathan F Mosser, Vincent Mougin, Christopher J L Murray, Mohsen Naghavi, Paul Nam, Doori Oh, Erik J Olson, Kanyin Liane Ong, Christian Razo, Gregory A Roth, Austin E Schumacher, Reed J D Sorensen, Sandra Spearman, Lauryn K Stafford, Jeffrey D Stanaway, Caitlyn Steiner, Jaimie D Steinmetz, Vivianne M Swart, Corey Teply, Megan Verma, Theo Vos, Eli J Weiss, Joanna L Whisnant, Shadrach Wilson, Faith H Yuh, Meixin Zhang, and Peng Zheng.

Providing critical feedback on methods or results

Bhoomadevi A, Ahmed A.J. Jabbar, Mohammad Amin Aalipour, Hazim S Ababneh, Ukachukwu O Abaraogu, Biruk Beletew Abate, Cristiana Abbafati, Nasir Abbas, Mitra Abbasifard, Samar Abd ElHafeez, Mohammed Altigani Abdalla, Emad M. Abdallah, Barkhad Aden Abdeeq, Nadin M. I. Abdel Razeq, Reda Abdel-Hameed, Michael Abdelmasseh, Mahmoud Abdelnabi, Wael M Abdel-Rahman, Sherief Abd-Elsalam, Sepideh Abdi, Meriem Abdoun, Arman Abdous, Jeza Muhamad Abdul Aziz, Deldar Morad Abdulah, Rizwan Suliankatchi Abdulkader, Adam Abdullahi, Auwal Abdullahi, Toufik Abdul-Rahman, Kulmira Abdykerimova, Habtamu Abebe Getahun, Armita Abedi, Asrat Agalu Abejew, Roberto Ariel Abeldaño Zuñiga, E S Abhilash, Syed Hani Abidi, Alemwork Abie, Olugbenga Olusola Abiodun, Richard Gyan Aboagye, Shady Abohashem, Hassan Abolhassani, Ulric Sena Abonie, Nagah M. Abourashed, Mohamed Abouzid, Lucas Guimarães Abreu, Dariush Abtahi, Rana Kamal Abu Farha, Fuad Hamdi A. Abuadas, Bilyaminu Abubakar, Eman Abu-Gharbieh, Sawsan Abuhammad, Ahmad Y Abuhelwa, Hana J Abukhadijah, Niveen ME Abu-Rmeileh, Salahdein Aburuz, Dina Abushanab, Raghu Ram Achar, Anirudh Balakrishna Acharya, Apurba Acharya, Ilana N Ackerman, Juan Manuel Acuna, Ousman Adal, Lisa C Adams, Lawan Hassan Adamu, Mesafint Molla Adane, Zenaw Debasu Addisu, Isaac Yeboah Addo, Oluwafemi Atanda Adeagbo, Tajudeen Adesanmi Adebisi, Isaac Akinkunmi Adedeji, David Adedia, Kamoru Ademola Adedokun, Rufus Adesoji Adedoyin, Oluwatobi E Adegbile, Oyelola A Adegboye, Nurudeen A Adegoke, Olumide Thomas Adeleke, Isaac Ayodeji Adesina, Miracle Ayomikun Adesina,

Habeeb Omoponle Adewuyi, Temitayo Esther Adeyeoluwa, Kishor Adhikari, Ripon Kumar Adhikary, Mohd Adnan, Qorinah Estiningtyas Sakilah Adnani, Leticia Akua Adzigbli, David Adzrago, Giuseppina Affinito, Ahmed M Afifi, Aanuoluwapo Adeyimika Afolabi, Rotimi Felix Afolabi, Saira Afzal, Gizachew Beykaso Agafari, Suneth Buddhika Agampodi, Temesgen Anjulo Ageru, Navidha Aggarwal, Mahdi Aghaalikhani, Sepehr Aghajanian, Seyed Mohammad Kazem Aghamir, César Agostinis Sobrinho, Anurag Agrawal, Williams Agyemang-Duah, Mahsa Ahadi, Bright Opoku Ahinkorah, Aqeel Ahmad, Danish Ahmad, Faisal Ahmad, Khabir Ahmad, Khurshid Ahmad, Muayyad M Ahmad, Rabbiya Ahmad, Sajjad Ahmad, Tauseef Ahmad, Waqas Ahmad, Negar Sadat Ahmadi, Amir Mahmoud Ahmadzade, Mohadese Ahmadzade, Akeem Olayiwola Ahmed, Anisuddin Ahmed, Ayman Ahmed, Gasha Salih Ahmed, Haroon Ahmed, Junaid Ahmed, Luai A Ahmed, Mehrunnisha Sharif Ahmed, Muktar Beshir Ahmed, Mushood Ahmed, Oli Ahmed, Shabbir Ahmed, Sindew Mahmud Ahmed, Dolapo Emmanuel Ajala, Hossein Akbarialiabad, Saeid Akbarifard, Oluwasefunmi Akeju, Roland Eghoghosoa Akhigbe, Olufemi Ambrose Akinkuotu, Karolina Akinosoglou, Sreelatha Akkala, Wole Akosile, Hammad Akram, Ashley E Akrami, Ralph Kwame Akyea, Alaa Al Amiry, Salah Al Awaidy, Syed Mahfuz Al Hasan, Omar Al Omari, Mohammad Al Qadire, Omar Al Ta'ani, Wasan A. M. Al Taie, Yazan Al Thaher, Omar Ali Mohammed Al Zaabi, Mousa Ali Al-Abbadi, Yazan Al-Ajlouni, Ziyad Al-Aly, Khurshid Alam, Manjurul Alam, Mohammad Khursheed Alam, Mostafa Alam, Rasmieh Mustafa Al-Amer, Abebaw Alamrew, Amani Alansari, Turki M Alanzi, Fahmi Y Al-Ashwal, Rahmeh Al-Asmar, Seyed Mohammad Amin Alavi, Mohammed Albashtawy, Astefanos Al-Dalakta, Khalifah A Aldawsari, Robert W Aldridge, Shereen M Aleidi, Bezawit Abeje Alemayehu, Tekletsadik Tekleslassie Alemayehu, Fentahun Alemnew, Melaku Birhanu Alemu, Ali M Alfalki, Fahad D Algahtani, Abdelazeem M Algammal, Mohammed Ridha Algethami, Adel Ali Saeed Al-Gheethi, Khairat Al-Habbal, Khalid F Alhabib, Nma Bida Alhaji, Mohammed Khaled Al-Hanawi, Aminu Alhassan Ibrahim, Ashraf Alhumaidi, Fahad A. Alhumaydhi, Dari Alhuwail, Abid Ali, Haroon Muhammad Ali, Irfan Ali, Maratab Ali, Mohammad Daud Ali, Mohammed Usman Ali, Rafat Ali, Shahid Ali, Syed Shujait Ali, Syed Yusuf Ali, Waad Ali, Akram Al-Ibraheem, Gianfranco Alicandro, Montaha Al-Iede, Sheikh Mohammad Alif, Morteza Alipour, Samah W Al-Jabi, Mohammad A Aljasir, Mohamad Aljofan, Adel Al-Jumaily, Syed Mohamed Aljunid, Mayson H. Alkhatib, Mustafa Alkhawam, Atefeh Allahbakhshian, Khaled S. Allemailem, Mohammed Z. Allouh, Wesam Taher Almagharbeh, Wael Almahmeed, Sabah Al-Marwani, Joseph Uy Almazan, Hesham M Al-Mekhlafi, Omar Almidani, Amr Almobayed, Khaldoon Aied Alnawafleh, Hasan Yaser Alniss, Margret Beaula Alocious Sukumar, Mahmoud A Alomari, Mohammad R Alosta, Jaber S Alqahtani, Saleh A Alqahtani, Mohammad R Alqudimat, Ahmad Alrawashdeh, Intima Alrimawi, Salman Khalifah Al-Sabah, Mohammed A Alsabri, Najim Z. Alshahrani, Mansour Abdullah Alshehri, Zaid Altaany, Awais Altaf, Alaa B Al-Tammemi, Jaffar A Al-Tawfiq, Malik A Althobiani, Khalid A Altirkawi, Javier Alvarez-Galvez, Nelson Alvis-Guzman, Nelson J Alvis-Zakzuk, Hassan Alwafi, Mohammad Al-Wardat, Yaser Mohammed Al-Worafi, Hany Aly, Mohammad Sharif Ibrahim Alyahya, Amal AlZahmi, Hosam Alzahrani, Karem H Alzoubi, Md. Akib Al-Zubayer, Ekiyor Joseph Amafah, Joy Amafah, Masoud Aman Mohammadi, Reza Amani-Beni, Adeladza Kofi Amegah, Faten Amer, Amr Amin, Tarek Tawfik Amin, Alireza Amindarolzarbi, Saeed Amini, Ehsan Amini-Salehi, Nafiu Aminu, Majid Aminzare, Sohrab Amiri, Dickson A Amugsi, Jimoh Amzat, Filippos Anagnostakis, Roshan A Ananda, Robert Ancuceanu, Deanna Anderlini, David B Anderson, Susan C Anenberg, Song Peng Ang, Colin Angus, Nguyen Hoang Anh, Samuel Egyakwa Ankomah, Kabilan Annadurai, Amir Anoushiravani, Sumbul Ansari, Umair Ansari, Rahel Mulatie Anteneh, Josep M Antó, Ernoiz Antriyandarti, Boluwatife Stephen Anuoluwa, Saleha Anwar, Sumadi Lukman Anwar, Razique Anwer, Shahnawaz Anwer, Anayochukwu Edward Anyasodor, Geminn Louis Carace Apostol, Juan Pablo Arab, Jalal Arabloo, Mosab Arafat, Demelash Areda, Jorge

Arias de la Torre, Hany Ariffin, Benedetta Armocida, Jesu Arockiaraj, Mahwish Arooj, Anton A Artamonov, Raphael Taiwo Aruleba, Deepavalli Arumuganainar, Mahsa Asadi Anar, Muhammad Asaduzzaman, Syed Mohammed Basheeruddin Asdaq, Shewatatek Melaku Asefa, Mulu Tiruneh Asemu, Saeed Asgary, Mohammad Asghari-Jafarabadi, Charlie Ashbaugh, Syed Amir Ashraf, Tahira Ashraf, Mitra Ashrafi, Milad Ashrafizadeh, Bernard Kwadwo Yeboah Asiamah-Asare, Muhammad Shahzad Aslam, Saeed Aslani, Yuni Asri, Batyrbek Assembekov, Thomas Astell-Burt, Mahshid Ataei, Mirbahador Athari, Seyyed Shamsadin Athari, Maha Moh'd Wahbi Atout, Sachin R Atre, Alok Atreya, Julie Alaere Atta, Zaure Maratovna Aumoldaeva, Marcel Ausloos, Abolfazl Avan, Núbia Carelli Pereira Avelar, Sana Javaid Awan, Babafela B Awosile, Adedapo Wasiu Awotidebe, Lemessa Assefa A Ayana, Seyyed HamidReza Ayatizadeh, Davood Azadi, Sina Azadnajafabad, Alireza Azarboo, Ali Azargoonjahromi, Masood Azhar, Farya Azimi, Mohd Yusmaidie Aziz, Sadat Abdulla Aziz, Amin Azizan, Ahmed Y Azzam, Domenico Azzolino, Zaharaddeen Shuaibu Babandi, Rasha Babiker, Giridhara Rathnaiah Babu, Israel Tadesse Bacha, Muhammad Badar, Alaa Aboelnour Badran, Youngoh Bae, Arvind Bagga, Nasser Bagheri, Sara Bagheri, Elahe Baghizadeh, Fereshteh Baghizadeh, Sana Baghizadeh, Khlood K Baghlaf, Najmeh Bahmanziari, Mohammad Amin Bahrami, Razieh Bahreini, Ruhai Bai, Atif Amin Baig, Abdulaziz T Bako, Senthilkumar Balakrishnan, Wondu Feyisa Balcha, Maher Balkis, Jose Balmori-de-la-Miyar, Mohammadreza Balooch Hasankhani, Ovidiu Constantin Baltatu, Shatha Bamashmous, Maciej Banach, Morteza Banakar, Palash Chandra Banik, Rajon Banik, Noel C Barengo, Hiba Jawdat Barqawi, Amadou Barrow, Sandra Barteit, Lingkan Barua, MD Abu Bashar, Zarrin Basharat, Shahid Bashir, Guido Basile, Pritish Baskaran, Rehana Basri, Quique Bassat, Mohammad-Mahdi Bastan, Sanjay Basu, Saurav Basu, Kavita Batra, Bernhard T Baune, Mahdis Bayat, Mohammad Amin Bayat Tork, Mulat Tirfie Bayih, Feyisa Shasho Bayisa, Nebiyou Simegnew Bayleyegn, Thomas Beaney, Narasimha M Beeraka, Priyamadhaba Behera, Jina Behjati, Babak Behnam, Amir Hossein Behnoush, Bezawit K Bekele, Asnake Gashaw Belayneh, Melesse Belayneh, Abel Cherkos Belete, Gokce Belge Bilgin, Michael Belingheri, Olorunjuwon Omolaja Bello, Apostolos Beloukas, Salaheddine Bendak, Derrick A Bennett, Isabela M Bensenor, Samiun Nazrin Bente Kamal Tune, Habib Benzian, Zombor Berezvai, Maria Bergami, Alemshet Yirga Berhie, Abiye Assefa Berihun, Amiel Nazer C Bermudez, Eduardo Bernabe, Robert S Bernstein, Paulo J G Bettencourt, Ajeet Singh Bhadoria, Akshaya Srikanth Bhagavathula, Neeraj Bhala, Jeetendra Bhandari, Kayleigh Bhangdia, Ravi Bharadwaj, Sonu Bhaskar, Ajay Nagesh Bhat, Anup Bhat, Vivek Bhat, Priyadarshini Bhattacharjee, Shuvarthi Bhattacharjee, Gurjit Kaur Bhatti, Jasvinder Singh Bhatti, Manpreet Singh Bhatti, Rajbir Bhatti, Soumitra S Bhuyan, Sibhatu Kassa Biadgilign, Raluca Bievel-Radulescu, Can Bilgin, Cem Bilgin, Saeed Biroudian, Bijit Biswas, Raaj Kishore Biswas, Ahmad Naoras Bitar, Molalegne Bitew, Bruno Bizzozero-Peroni, Espen Bjertness, Trupti Bodhare, Virginia Bodolica, Lucimere Bohn, Obasanjo Afolabi Bolarinwa, Paria Bolourinejad, Aime Bonny, Sri Harsha Boppana, Sanaz Bordbar, Hamed Borhany, Alejandro Botero Carvajal, Souad Bouaoud, Rupert R A Bourne, Christopher Boxe, Marija M Bozic, Jyoti Brahmaiah, Dejana Braithwaite, Michael Brauer, Nicholas J K Breitborde, Hermann Brenner, Gabrielle Britton, Julie Brown, Annie J Browne, Traolach Brugha, Claudia Buchweitz, Raffaele Bugiardini, Linh Phuong Bui, Norma B Bulamu, Tsion Samuel Bunare, Danilo Buonsenso, Asmat Burhan, Katrin Burkart, Richard A Burns, Felix Busch, Yasser Bustanji, Zahid A Butt, Channa Buxbaum, Sanjay C J, Jack Cagney, Tianji Cai, Rose Cairns, Mehtap Çakmak Barsbay, Luis Alberto Cámera, Luciana Aparecida Campos, Ismael Campos-Nonato, Fan Cao, Yuchen Cao, Angelo Capodici, Rosario Cárdenas, Sinclair Carr, Juan Jesus Carrero, Andre F Carvalho, Ana Paula Carvalho-e-Silva, Joao Mauricio Castaldelli-Maia, Giulio Castelpietra, Christopher R Cederroth, Luca Cegolon, Francieli Cembranel, Muthia Cenderadewi, Ester Cerin, Sonia Cerrai, Muge Cevik, Madhu Chakkere Shivamadhu, Chiranjib Chakraborty, Promit Ananyo Chakraborty, Joht Singh Chandan, Rama

Mohan Chandika, Eeshwar K Chandrasekar, Jung-Chen Chang, Vijay Kumar Chattu, Victoria Chatzimavridou-Grigoriadou, Lam Duc Chau, Sirshendu Chaudhuri, Akhilanand Chaurasia, Galmesa Bekana Chemeda, An-Tian Chen, Guangjin Chen, Hana Chen, Haowei Chen, Hui Chen, Meng Xuan Chen, Shanquan Chen, Xiang Chen, Yifan Chen, Haojin Cheng, Nicholas WS Chew, Gerald Chi, Odgerel Chimed-Ochir, Jesus Lorenzo Chirinos-Caceres, William C S Cho, Bryan Chong, Yuen Yu Chong, Hou In Chou, Enayet Karim Chowdhury, Mohiuddin Ahsanul Kabir Chowdhury, Hanne Christensen, Dinh-Toi Chu, Isaac Sunday Chukwu, Eric Chung, Erin Chung, Sheng-Chia Chung, Sunghyun Chung, Muhammad Chutiyami, Arrigo Francesco Giuseppe Cicero, Liliana G Ciobanu, Aaron J Cohen, Alyssa Columbus, Joao Conde, Nathalie Conrad, Sara Conti, Mariana Oliveira Corda, Alexandru Corlateanu, Samuele Cortese, Paolo Angelo Cortesi, Ewerton Cousin, Michael H Criqui, Natalia Cruz-Martins, Xiaolin Cui, Garland T Culbreth, Nour Dababo, Ali Dabbagh, Omid Dadras, Tukur Dahiru, Xiaochen Dai, Zhaoli Dai, Mayank Dalakoti, Koustuv Dalal, Gloria Dalla Costa, Giovanni Damiani, Yohannes Tefera Damtew, Roy Arokiam Daniel, Lucio D'Anna, Pojsakorn Danpanichkul, Samuel Demissie Darcho, Latefa Ali Dardas, Bahar Darouei, Reza Darvishi Cheshmeh Soltani, Anna Dastiridou, Gail Davey, Dimash Davletov, Kairat Davletov, Elham Davoudi, Fernando Pio De la Hoz, Edward Christopher Dee, Orla Deegan, Sindhura Deekonda, Louisa Degenhardt, Paria Dehesh, Tadesse Asmamaw Dejenie, Pouria Delbari, Mohammad Delsoz, Dessalegn Demeke, Andreas K Demetriades, Desalegn Getnet Demsie, Tadios Niguss Derese, Ismail Dergaa, Hunegnaw Almaw Derseh, Emina Dervišević, Abraham Aregay Desta, Vinoth Gnana Chellaiyan Devanbu, Pradeep Kumar Devarakonda, Syed Masudur Rahman Dewan, Arkadeep Dhali, Kuldeep Dhama, Rajinder K Dhamija, Amol S Dhane, Narender K. Dhania, Mandira Lamichhane Dhimal, Meghnath Dhimal, Bibha Dhungel, Marcello Di Pumpo, Diana Dias da Silva, Daniel Diaz, Kimia Didehvar, Lauren K Dillard, Adriana Dima, Xueting Ding, Temesgien Ergetie Dinkayehu, Huyen Phuc Do, Thao Huynh Phuong Do, Klara Georgieva Dokova, Christiane Dolecek, Mario D'Oria, Fariba Dorostkar, Ojas Prakashbhai Doshi, Robert Kokou Dowou, Tim Robert Driscoll, Viola Savy Dsouza, Jiang Du, Emeka W Dumbili, Jennifer Dunne, Senbagam Duraisamy, Oyewole Christopher Durojaiye, Ashit Kumar Dutta, Arkadiusz Marian Dziedzic, Abdel Rahman E’mar, Osamudiamen Ebohon, Ejemai Eboreime, Lamiaa Labieb Mahmoud Ebraheim, Alireza Ebrahimi, Mohammad Hossein Ebrahimi, Sara Ebrahimi, Abdelaziz Ed-Dra, Kristina Edvardsson, Ferry Efendi, Foolad Eghbali, Fatemeh Ehsani, Ashkan Eighaei Sedeh, Terje Andreas Eikemo, Ebrahim Eini, Michael Ekholuenetale, Temitope Cyrus Ekundayo, Rabie Adel El Arab, Abdelfatteh EL Omri, Maysaa El Sayed Zaki, Mohamed Ahmed Eladl, Reza Elahi, Said El-Ashker, Rana Elbeshbeishy, Noha Mousaad Elemam, Muhammed Elhadi, Mohamed Elhoumed, Waseem El-Huneidi, Sherif Elkannishy, Omar Abdelsadek Abdou Elmeligy, Rami Elmorsi, Adel B Elmoselhi, Mohamed Hassan Elnaem, Gihan ELNahas, Mohammed Elshaer, Ibrahim Elsohaby, Abdelgawad Salah Eltahawy, Tadele Emagneneh, Theophilus I Emeto, Destaw Endeshaw, Misganu Endriyas, Holly E Erskine, Derese Eshetu, Habitu Birhan Eshetu, Gilbert Eshun, Sharareh Eskandarieh, Majid Eslami, Rafaela Cavalheiro do Espírito Santo, Crystal Amiel M Estrada, Fahima Nasrin Eva, Elochukwu Ezenwankwo, Adewale Oluwaseun Fadaka, Heidar Fadavian, Adeniyi Francis Fagbamigbe, Ayesha Fahim, Ildar Ravisovich Fakhradiyev, Aliasghar Fakhri-Demeshghieh, Qiping Fan, Mohammad Farahmand, Emerito Jose Aquino Faraon, Mohammad Fareed, Zaki Farhana, Carla Sofia e Sá Farinha, Andre Faro, Syed Muhammad Yousaf Farooq, Umar Farooque, Hossein Farrokhpour, Fatemeh Farshad, Farima Farsi, Md. Omar Faruk, Emmanuel Toluwani Fasusi, Ali Fatehizadeh, Davood Fathi, Zareen Fatima, Mehdi Fazlzadeh, Li Fei, Valery L Feigin, Alireza Feizkhah, Ginenus Fekadu, Berhanu Elfu Feleke, Dechao Feng, Xiaoqi Feng, Talukdar Raian Ferdous, Seyed-Mohammad Fereshtehnejad, Rodrigo Fernandez-Jimenez, Pietro Ferrara, Alize J Ferrari, André Ferreira, Natan Feter, Alexander Finnemore, Florian Fischer, Ida Fitriana, Luisa S Flor, Federica Fogacci, Morenike

Oluwatoyin Folayan, Lisa M Force, Arianna Fornari, Ingeborg Forthun, Matteo Foschi, Maryam Fotouhi, Kayode Raphael Fowobaje, Juluis Visnel Foyet F, Richard Charles Franklin, Alberto Freitas, Nancy Fullman, Blima Fux, Sridevi G, Peter Andras Gaal, Márió Gajdács, Yaseen Galali, Dhanraj Ganapathy, Mohd Ashraf Ganie, Dingwei Gao, Xiang Gao, Bashiru Garba, Fernando Barroga Garcia, Miguel Garcia-Argibay, David Garcia-Azorin, William M Gardner, Jacopo Garlasco, Zisis Gatzioufas, Rupesh K Gautam, Federica Gazzelloni, Feven Sahle Gebre, Miglas Welay Gebregergis, Haftay Gebremedhin Gebreslassie, Stefano Gelibter, Nsikakabasi Samuel George, Ali Gerami Matin, Genanew K Getahun, Kalab Yigermal Gete, Delaram J Ghadimi, Amir Ghaffari Jolfayi, Arin Ghamkhar, Ali Ghandili, Moein Ghasemi, Mohammad-Reza Ghasemi, Shakiba Ghasemi Assl, Haniyeh Ghasrsaz, Ramy Mohamed Ghazy, Sailaja Ghimire, Nermin Ghith, Elena Ghotbi, Alessandro Gialluisi, Konstantinos Giannakis, Ruth Margaret Gibson, Gabriela Fernanda Gil, Syed Abdullah Gilani, Tiffany K Gill, Themba G Ginindza, Bikash Ranjan Giri, Alem Abera Girmay, Alessandro Girombelli, Elena V Gnedovskaya, Laszlo Göbölös, Kimiya Gohari, Mahaveer Golechha, Ali Golestani, Melika Golmohammadi, Wenping Gong, Yitayal Ayalew Goshu, Alessandra C Goulart, Aman Goyal, Ayman Grada, Simon Matthew Graham, Vittorio Grieco, Michal Grivna, Ashna Grover, Habtamu Alganeh Guadie, Shi-Yang Guan, Zhongyang Guan, Mohammed Ibrahim Mohialdeen Gubari, Avirup Guha, Damitha Asanga Gunawardane, Xingzhi Guo, Zhaoyu Guo, Zheng Guo, Zhifeng Guo, Anish Kumar Gupta, Himanshu Gupta, Ishita Gupta, Lalit Gupta, Rajat Das Gupta, Rajeev Gupta, Sapna Gupta, Veer Bala Gupta, Vipin Gupta, Vivek Kumar Gupta, Yonas Deressa Guracho, Lami Gurmessa, Reyna Alma Gutiérrez, Roberth Steven Gutiérrez-Murillo, Parishma Guttoo, Jose Guzman-Esquivel, Adrina Habibzadeh, Abrham Tesfaye Habteyes, Awoke Derbie Habteyohannes, Tesfahun Simon Hadaro, Najah R Hadi, Zahra Hadian, Abdul Hafiz, Arian Haghtalab, Demewoz Haile, Haimanot Ewnetu Hailu, Pritam Halder, Aram Halimi, Sebastian Haller, Kosar Hikmat Hama Aziz, Islam M Hamad, Randah R Hamadeh, Nadia M Hamdy, Ahmad Hammoud, Mohammad Hamza, Umar Sabiu Hamza, Hannah Han, Didem Han Yekdeş, Asif Hanif, Nasrin Hanifi, Fahad Hanna, Ashanul Haque, Md Nuruzzaman Haque, Md. Aminul Haque, Harapan Harapan, Hilda L Harb, Kassandra L Harding, Arief Hargono, Eka Mishbahatul Marah Has, Ahmed I Hasaballah, Faizul Hasan, Md Kamrul Hasan, Hamidreza Hasani, Ali Hasanpour-Dehkordi, Arezou Hashem Zadeh, Mohammad Hashem Hashempur, Ammarah Hasnain, Ibrahim Nagmeldin Hassan, Ikrama Hassan, Mahgol Sadat Hassan Zadeh Tabatabaei, Shokoufeh Hassani, Mohammed Bheser Hassen, Lasanthi Wathsala Hathagoda, Rasmus J Havmoeller, Angie Hawat, Simon I Hay, Khezar Hayat, Youssef Hbid, Jue He, Jeffrey J Hebert, Mohammad Heidari, Mehdi Hemmati, Claudiu Herteliu, Austin Heuer, Sumudu Avanthi Hewage, Mojtaba Heydari, Zahra Heydarifard, Kamal Hezam, Yuta Hiraike, Ramesh Holla, Alamgir Hossain, Lubna Hossain, Md Belal Hossain, Md Mahbub Hossain, Md Sabbir Hossain, Mohammad Bellal Hossain, Hassan Hosseinzadeh, Mehdi Hosseinzadeh, Mihaela Hostiuc, Chengxi Hu, Yifei Hu, Weijun Huang, Yefei Huang, Mega Hasanul Huda, Atanesia Indriyani Human, Kyle Matthew Humphrey, Kiavash Hushmandi, Andreas Kattem Husøy, Javid Hussain, M Azhar Hussain, Salman Hussain, Dursa Hussein, Nawfal R Hussein, Mohamed Ibrahim Husseiny, Hong-Han Huynh, Bing-Fang Hwang, Luigi Francesco Iannone, Ahmed Ibrahim, Khalid S Ibrahim, Ramzi Ibrahim, Francisco Javier Idalsoaga, Jibran Ikram, Olayinka Stephen Ilesanmi, Irena M Ilic, Milena D Ilic, Muhammad Hamza Ilyas, Mohammad Tarique Imam, Masoud Imani, Mustapha Immurana, Lucius Chidiebere Imoh, Leeberk Raja Inbaraj, Arit Inok, Mujahid Iqbal, Lalu Muhammad Irham, Mustafa Alhaji Isa, Md Rabiul Islam, Md Shariful Islam, Md. Shahinul Islam, Farhad Islami, Nahlah Elkudssiah Ismail, Gaetano Isola, Mosimah Charles Ituka, Masao Iwagami, Chinwe Juliana Iwu-Jaja, Ihoghosa Osamuyi Iyamu, Mahalaxmi Iyer, Veena J Iyer, Vinothini J, Jalil Jaafari, Louis Jacob, Kathryn H. Jacobsen, Ali Jadidi, Farhad Jadidi-Niaragh, Morteza Jafarinia, Shabbar Jaffar, Haitham Jahrami, Ammar Abdulrahman

Jairoun, Vikash Jaiswal, Mihajlo Jakovljevic, Ali Jaliliyan, Reza Jalilzadeh Yengejeh, Mohamed Jalloh, Armaan Jamal, Jerin James, Tyler G. James, Hasan Jamil, Safayet Jamil, Roland Dominic G Jamora, Masoud Jamshidi, Shaghayegh JamshidiRastabi, Rajiv Janardhanan, Esmaeil Jarrahi, Syed Sarmad Javaid, Anita Javanmardi, Javad Javidnia, Qassim Jawell Odah Abed, Ruwan Duminda Jayasinghe, Yovanthi Anurangi Jayasinghe, Achala Upendra Jayatilleke, Kimia Jazi, Felix K Jebasingh, Sun Ha Jee, Jayakumar Jeganathan, Tadesse Hailu Jember, Belayneh Hamdela Jena, Diptismita Jena, Seogsong Jeong, Bijay Mukesh Jeswani, Zixiang Ji, Min Jiang, Weiqiu Jin, Wenyi Jin, Catherine O Johnson, Emily Katherine Johnson, Mohammad Jokar, Jost B Jonas, Tamas Joo, Abu Jor, Abel Joseph, Alex Joseph, Nitin Joseph, Charity Ehimwenma Joshua, Farahnaz Joukar, George Joy, Jacek Jerzy Jozwiak, Mikk Jürisson, Malik E Juweid, Madhanraj K, Billingsley Kaambwa, Zubair Kabir, Dler H. Hussein Kadir, Ethan M Kahn, Ashish Kumar Kakkar, Leila R Kalankesh, Khalil Kalavani, Feroze Kaliyadan, Aidana Kaliyakparova, Md Moustafa Kamal, Mehnaz Kamal, Sivesh Kathir Kamarajah, Rajesh Kamath, Saltanat Kamenova, Ramat T. Kamorudeen, Oleksandr Kamyshnyi, Haidong Kan, Mona Kanaan, Jiseung Kang, Samuel Berchi Kankam, Kehinde Kazeem Kanmodi, Suthanthira Kannan S, Rami S Kantar, Sujita Kumar Kar, Paschalis Karakasis, Hanie Karimi, Arman Karimi Behnagh, Samad Karkhah, Mohmed Isaqali Karobari, Tomasz M Karpiński, Manoj Kumar Kashyap, Abdene Weya Kaso, Nigussie Assefa Kassaw, Adarsh Katamreddy, Patrick DMC Katoto, Gbenga A Kayode, Nastaran Kazemi Rad, Mohammad-Hossein Keivanlou, Peter Njenga Keiyoro, Chukwudi Keke, John H Kempen, Vikash Ranjan Keshri, Kamyab Keshtkar, Emmanuelle Kesse-Guyot, Reza Khademi, Yousef Saleh Khader, Inn Kynn Khaing, Himanshu Khajuria, Sidra Khalid, Sumaira Khalid-Ariturk, Hazim O. Khalifa, Anas Husam Khalifeh, Anees Ahmed Khalil, Pantea Khalili, Ghazaleh Khalili-Tanha, Mohamed Khalis, Faham Khamesipour, Abdul Arif Khan, Ajmal Khan, Asaduzzaman Khan, Faiz Ullah Khan, Maseer Khan, Md Abdullah Saeed Khan, Mohammad Jobair Khan, Muhammad Hamza Khan, Muhammad Mueed Khan, Muhammad Umair Khan, Muhammad Umer Khan, Nusrat Khan, Ruby Khan, Salman Ali Khan, Serab Khan, Sumaiya Khan, Yusuf Saleem Khan, Zahid Khan, Srijana Khanal, Vishnu Khanal, Shaghayegh Khanmohammadi, Sameer Uttamaro Khasbage, Zenith Khashim, Khaled Khatab, Haitham Khatatbeh, Moawiah Mohammad Khatatbeh, Kavin Khatri, Afshin Khazaei, Peyman Kheirandish Zarandi, Sunil Kumar Khokhar, Mohammad Saeid Khonji, Najmaddin Salih Husen Khoshnaw, Atulya Aman Khosla, Farbod Khosravi, Mahmood Khosrowjerdi, P Ratan Khuman, Helda Khusun, Zemene Demelash Kifle, Hye Jun Kim, Jinho Kim, Min Seo Kim, Sungroul Kim, Ruth W Kimokoti, Yohannes Kinfu, Adnan Kisa, Sezer Kisa, Katarzyna Kissimova-Skarbek, Mika Kivimäki, Jessica Klusty, Abdul Basith KM, Shivakumar KM, Ann Kristin Skrindo Knudsen, Jonathan M Kocarnik, Sonali Kochhar, Michail Kokkorakis, Ali-Asghar Kolahi, Diana Gladys Kolieghu Tcheumeni, Farzad Kompani, Aida Kondybayeva, Anastasios Georgios Panagiotis Konstas, Isaac Koomson, Gerbrand Koren, Tapos Kormoker, Oleksii Korzh, Karel Kostev, Konstantinos Kotsis, Archana Koul, Sindhura Lakshmi Koulmane Laxminarayana, Irene Akwo Kretchy, James-Paul Kretchy, Kewal Krishan, Chong-Han Kua, Barthelemy Kuate Defo, Burcu Kucuk Bicer, Mohammed Kuddus, Ilari Kuitunen, Omar Kujan, Anit Kujur, Shweta Kulshreshtha, Dewesh Kumar, Dhasarathi Kumar, Jogender Kumar, Nitesh Kumar, Nithin Kumar, Tushar Kumar, Vijay Kumar, Subramanian Kumaran, Jibin Kunjavara, Setor K Kunutsor, Om P Kurmi, Maria Dyah Kurniasari, Krishna Prasad Kurpad, Pramod Kumar Kushawaha, Asep Kusnali, Christina Yeni Kustanti, Dian Kusuma, Tezer Kutluk, Michael Agyemang Kwarteng, Wai Hang Patrick Kwong, Evans F Kyei, Grace Kwakyewaa Kyei, Ville Kytö, Hmwe Hmwe Kyu, Pallavi L C, Adriano La Vecchia, Carlo La Vecchia, Muhammad Awwal Ladan, Chandrakant Lahariya, Daphne Teck Ching Lai, Anita Lakhani, Dharmesh Kumar Lal, Tea Lallukka, Judit Lám, Iván Landires, Ariane Laplante-Lévesque, Savita Lasrado, Kamaluddin Latief, Basira Kankia Lawal, Bilkisu Kankia Lawal, Saheed Akinmayowa Lawal, Aliyu Lawan, Harriet L S Lawford, Dai Quang Le,

Duc Huy Le, Huu-Hoai Le, Long Khanh Dao Le, Minh Huu Nhat Le, Nhi Huu Hanh Le, Thao Thi Thu Le, Trang Diep Thanh Le, Caterina Ledda, Seung Won Lee, Wei-Chen Lee, Yo Han Lee, James Leigh, Vasileios Leivaditis, Matthew J Lennon, Elvynna Leong, Chengcheng Li, Haobo Li, Hui Li, Jianan Li, Jiaying Li, Jie Li, Jinbo Li, Ming-Chieh Li, Peng li, Wang-Zhong Li, Wei Li‡, Wei Li§, Weilong Li, Wenjie Li, Xunliang Li, Yongze Li, Zhengrui Li, Zhihui Li, Yanxue Lian, Xue-Zhen Liang, Stephen S Lim, Jialing Lin, Queran Lin, Ro-Ting Lin, Ya Lin, Daniel Lindholm, Yuewei Ling, Gang Liu, Haipeng Liu, Jue Liu, Xianliang Liu, Xiaofeng Liu, Xuefeng Liu, Yubo Liu, Yunfei Liu, Erand Llanaj, Valerie Lohner, José Francisco López-Gil, Stefan Lorkowski, Masoud Lotfizadeh, Shanjie Luan, Jailos Lubinda, Taraneh Lucas, Giancarlo Lucchetti, Peng Luo, Jay B Lusk, Angelina M Lutambi, Miltiadis D Lytras, Ellina Lytvyak, Hawraz Ibrahim M. Amin, Kevin Sheng-Kai Ma, Zheng Feei Ma, Mahmoud Mabrok, Firoozeh Madadi, Seyed Ataollah Madinezad, Christian Madsen, Aurea Marilia Madureira-Carvalho, Mohammed Magdy Abd El Razek, Azzam A Maghazachi, Sasikumar Mahalingam, Nozad Hussein Mahmood, Alireza Mahmoudi, Farhad Mahmoudi, My Tra Mai, Rituparna Maiti, Azeem Majeed, Konstantinos Christos C. Makris, Reza Malekzadeh, Hardeep Singh Malhotra, Ahmad Azam Malik, Farihah Malik, Tabarak Malik, Deborah Carvalho Malta, Abdullah A Mamun, Mustapha Mangdow, Lokesh Manjani, Yosef Manla, Kamaruddeen Mannethodi, Farheen Mansoor, Marjan Mansourian, Mohammad Ali Mansournia, Lorenzo Giovanni Mantovani, Changkun Mao, Tahir Maqbool, Sajid Maqsood, Hamid Reza Marateb, Joemer C Maravilla, Konstantinos Margetis, Mirko Marino, Adilson Marques, Randall V Martin, Bernardo Alfonso Martinez-Guerra, Ramon Martinez-Piedra, Daniela Martini, Francisco Rogerlândio Martins-Melo, Miquel Martorell, Winfried März, Roy Rillera Marzo, Sammer Marzouk, Stefano Masi, Clara N Matei, Yasith Mathangasinghe, Stephanie Mathieson, Alexander G Mathioudakis, Manu Raj Mathur, Medha Mathur, Fernanda Penido Matozinhos, Rita Mattiello, Khurshid A Mattoo, Richard James Maude, Mahsa Mayeli, Chioma Ngozichukwu Pauline Mbachu, Ikechukwu Innocent Mbachu, Martin McKee, Steven M McPhail, Enkeleint A Mechili, Rishi P Mediratta, Jitendra Meena, Elahe Meftah, Medhin Mehari, Asim Mehmood, Entezar Mehrabi Nasab, Kala M Mehta, Vini Mehta, Subhash Mehto, Toni Meier, Tesfahun Mekene Meto, Hadush Negash Meles, Endalkachew Belayneh Melese, Satish Melwani, Walter Mendoza, Godfred Antony Menezes, Ritesh G Menezes, Berihun Agegn Mengistie, Emiru Ayalew Mengistie, Sultan Ayoub Meo, Michelangelo Mercogliano, Atte Meretoja, Tuomo J Meretoja, Tomislav Mestrovic, Chamila Dinushi Kukulege Mettananda, Sachith Mettananda, Mohamed M. M. Metwally, Louise Mewton, Adquate Mhlanga, Andrea Michelerio, Ana Carolina Micheletti Gomide Nogueira de Sá, Hiwot Soboksa Mideksa, Paul Anthony Miller, Ted R Miller, Giuseppe Minervini, Wai-kit Ming, GK Mini, Erkin M Mirrakhimov, Seyed Ali Mirshahvalad, Mizan Kiros Mirutse, Yousef Mirzaei, Archana Mishra, Kumar Guru Mishra, Vinaytosh Mishra, Prasanna Mithra, Sayan Mitra, Manasi Murthy Murthy Mittinty, Mohammadreza Mobayen, Shivani Modi, Ashraf Mohamadkhani, Jama Mohamed, Mona Gamal Mohamed, Nouh Saad Mohamed, Khabab Abbasher Hussien Mohamed Ahmed, Taj Mohammad, Sakineh Mohammad-Alizadeh-Charandabi, Abdolreza Mohammadi, Mohammad Reza Mohammadi, Seyed Omid Mohammadi, Abdollah Mohammadian-Hafshejani, Ibrahim Mohammadzadeh, Ramin Mohammadzadeh, Ammas Siraj Mohammed, Omer Mohammed, Shafiu Mohammed, Suleiman Mohammed, Yahaya Mohammed, Syam Mohan, Yugal Kishore Mohanta, Mohammad Mohseni, Ali H Mokdad, Peyman Mokhtarzadehazar, Sabrina Molinaro, Amirabbas Mollaei, Shaher Momani, Lorenzo Monasta, Amirabbas Monazzami, Himel Mondal, Stefania Mondello, Ahmed Al Montasir, Catrin E Moore, Yousef Moradi, Maziar Moradi-Lakeh, Paula Moraga, Lidia Morawska, Rafael Silveira Moreira, Negar Morovatdar, Mahdis Morovvati, Mahmoud M Morsy, Reza Mosaddeghi Heris, Jonathan F Mosser, Nogol Motamedgorji, Simin Mouodi, Asma Mousavi, Seyede Zohre Mousavi, Amin Mousavi Khaneghah, Seyed Mohamad Sadegh Mousavi

Kiasary, Mohamed Awad Abdalaziz Mousnad, Hagar Lotfy Mowafy, Kimia Mozahheb Yousefi, Ahmed Msherghi, Rabia Mubarak, Sumaira Mubarik, Shiv K Mudgal, Syed Aun Muhammad, Muhammad Solihuddin Muhtar, Sukhes Mukherjee, Sumoni Mukherjee, Amartya Mukhopadhyay, Satinath Mukhopadhyay, M A Muktadir, Sileshi Mulatu, Francesk Mulita, Getaneh Baye Mulu, Chalie Mulugeta, Mulyadi Mulyadi, Muneeb Ahmad Muneer, Malaisamy Muniyandi, Kavita Munjal, Yanjinlkham Munkhsaikhan, Javier Muñoz Laguna, Anjana Munshi, Pradeep Manohar Muragundi, Michio Murakami, Christopher J L Murray, Yahye Hassan Muse, Ali Mushtaq, Ghulam Mustafa, Sherzad Ibrahim Mustafa, Mubarak Taiwo Mustapha, Sathish Muthu, Saravanan Muthupandian, Claude Mambo Muvunyi, Muhammad Muzaffar, Woojae Myung, Amin Nabavi, Fatemehzahra Naddafi, Ahamarshan Jayaraman Nagarajan, Shankar Prasad Nagaraju, Mohsen Naghavi, Pirouz Naghavi, Ganesh R Naik, Gurudatta Naik, Hiten Naik, Firzan Nainu, Soroush Najdaghi, Paul Nam, Vinay Nangia, Jobert Richie Nansseu, Ibrahim A Naqid, Shumaila Nargus, Delaram Narimani Davani, Yvonne Nartey, Bruno Ramos Nascimento, Abdallah Y Naser, Mohammad Naser, Abdulqadir J Nashwan, Hamide Nasiri, Mahmoud Nassar, Zuhair S Natto, Javaid Nauman, Zakira Naureen, Samidi Nirasha Kumari Navaratna, Anum Nawaz, M. Omar Nawaz, Biswa Prakash Nayak, Shalini Ganesh Nayak, Javad Nazari, G. Takop Nchanji, Rawlance Ndejjo, Anthony Wainaina Ndungu, Amanuel Tebabal Nega, Abigia Ashenafi Negash, Ionut Negoi, Ruxandra Irina Negoi, Jalil Nejati, Chakib Nejjari, Henok Biresaw Netsere, Charles Richard James Newton, Marie Ng, Georges Nguefack-Tsague, Josephine W Ngunjiri, Cuong Tat Nguyen, Huong Lan Thi Nguyen, Nghia Phu Nguyen, Phat Tuan Nguyen, The Phuong Nguyen, Trang Nguyen, Tu Anh Nguyen, Van Thanh Nguyen, Ambe Marius Ngwa, Robina Khan Niazi, Jing Nie, Luciano Nieddu, Yeshambel T Nigatu, Ali Nikoobar, Dina Nur Anggraini Ningrum, Vikram Niranjan, Abebe Melis Nisro, Princess Afia Nkrumah-Boateng, Chukwudi A Nnaji, Efaq Ali Noman, Shuhei Nomura, Syed Toukir Ahmed Noor, Pardis Noormohammadpour, Mamoona Noreen, Masoud Noroozi, Jean Jacques Noubiap, Taylor Noyes, Valentine C Nriagu, Chisom Adaobi Nri-Ezedi, Jean Claude Nshimiyimana, Fred Nugen, Atoma Negera Nugusa, Mengistu H Nunemo, Aqsha Nur, Dieta Nurrika, Sylvester Dodzi Nyadanu, Felix Kwasi Nyande, Chimezie Igwegbe Nzoputam, Ogochukwu Janet Nzoputam, Bogdan Oancea, George Obaido, Adashi Margaret Odama, Ramez M Odat, Fabio Massimo Oddi, Ismail A Odetokun, Joseph Kojo Oduro, Michael Safo Oduro, Onome Bright Oghenetega, Oluwaseun Adeolu Ogundijo, Abiola Ogunkoya, James Odhiambo Oguta, Sarah Oh, Hassan Okati-Aliabad, Akinkunmi Paul Okekunle, Olalekan John Okesanya, Osaretin Christabel Okonji, John Olayemi Okunlola, Oluyemi Adewole Okunlola, Oluwaseyi Isaiah Olabisi, Andrew T Olagunju, Oladotun Victor Olalusi, Matthew Idowu Olatubi, Gláucia Maria Moraes Oliveira, Abdulhakeem Abayomi Olorukooba, Oluseye Olalekan Oludoye, Ronald Olum, Bolajoko Olubukunola Olusanya, Jacob Olusegun Olusanya, Oluwafemi G. Oluwole, Folorunsho Bright Omage, Hany A Omar, Goran Latif Omer, Kanyin Liane Ong, Qi Chwen Ong, Obinna E Onwujekwe, Marcel Opitz, Michal Ordak, Atakan Orscelik, Samuel M Ostroff, John W Ostrominski, Uchechukwu Levi Osuagwu, Olayinka Osuolale, Godfred Otchere, Elham H Othman, Mostafa Monier Othman, Adrian Otoiu, Oche Joseph Otorkpa, Abdu Oumer, Jerry John Ouner, Amel Ouyahia, Mayowa O Owolabi, Irene Amoakoh Owusu, Kolapo Oyebola, Tope Oyelade, Oyetunde T Oyeyemi, Ilker Ozsahin, Mahesh P A, Kevin Pacheco-Barrios, Inderbir Padda, Alicia Padron-Monedero, Jagadish Rao Padubidri, Anton Pak, Pramod Kumar Pal, Tamás Palicz, Tejasri Paluvai, Feng Pan, Hai-Feng Pan, Parsa Panahi, Sujogya Kumar Panda, Songhomitra Panda-Jonas, Deepshikha Pande Katare, Ke Pang, Georgios D Panos, Leonidas D Panos, Ioannis Pantazopoulos, Giovanni Paolino, Mario Virgilio Papa, Ilias Papadimopoulos, Paraskevi Papadopoulou, Utsav Parekh, Peyvand Parhizkar Roudsari, Amrita Parida, Chulwoo Park, Eun-Kee Park, Seoyeon Park, Swapnil Parve, Ava Pashaei, Roberto Passera, Bhumi Hemal Patel, Hemal M Patel, Mitesh Patel, Neel Navinkumar Patel, Satyananda Patel, Ashlesh Patil,

Shankargouda Patil, Dimitrios Patoulias, Apurba Patra, Mohammad Hridoy Patwary, Shrikant Pawar, Shubhadarshini Pawar, Amy E Peden, Paolo Pedersini, Jarmila Pekarcikova, Prince Peprah, Gavin Pereira, Maria Odete Pereira, Pablo Perez-Lopez, Simone Perna, Konrad Pesudovs, Pavlo Petakh, Olumuyiwa James Peter, Fanny Emily Petermann-Rocha, Hoang Nhat Pham, Nhat Truong Pham, Tung Thanh Pham, Anil K Philip, Michael R Phillips, Zayar Phyo, David M Pigott, Julian David Pillay, Luane Pinheiro Pinheiro Rocha, Zahra Zahid Piracha, Michael A Piradov, Enrico Pisoni, Dietrich Plass, Evgenii Plotnikov, Roman V Polibin, Peter Pollner, Ramesh Poluru, Arjun Pon Avudaiappan, Constance Dimity Pond, Ville T Ponkilainen, Ion Popa, Svetlana Popova, Maarten J Postma, Sajjad Pourasghary, Reza Pourbabaki, Farzad Pourghazi, Mohsen Poursadeqiyan, Naeimeh Pourtaheri, Attur Ravindra Prabhu, Sergio I Prada, Jalandhar Pradhan, Pranil Man Singh Pradhan, Rifky Octavia Pradipta, Akila Prashant, Elton Junio Sady Prates, Tina Priscilla, Natalie Pritchett, Harsh Priya, Hery Purnobasuki, Bharathi M Purohit, Jagadeesh Puvvula, Nameer Hashim Qasim, Xiang Qi, Zhipeng Qi, Jia-Yong Qiu, Zahiruddin Syed Quazi, Shahazad Niwazi Qurashi, Deepthi R, Navid Rabiee, Basuki Rachmat, Raghu Anekal Radhakrishnan, Venkatraman Radhakrishnan, Maja R Radojčić, Hadi Raeisi Shahraki, Pankaja Raghav, Pracheth Raghuveer, Leila Rahbarnia, Fakher Rahim, Hawbash Mohammed-Amin Rahim, Sajjad Rahimi, Afarin Rahimi-Movaghar, Vafa Rahimi-Movaghar, Fryad Majeed Rahman, Mahbubur Rahman, Mahfuzur Rahman, Md. Mosfequr Rahman, Mohammad Meshbahur Rahman, Mosiur Rahman, Saeed Rahmani, Masoud Rahmati, Ghasem Rahmatpour Rokni, Hakim Rahmoune, Pramila Rai, Diego Raimondo, Sunil Kumar Raina, Jeffrey Pradeep Raj, Adarsh Raja, Sandesh Raja, Sathish Rajaa, Erta Rajabi, Gunaseelan Rajendran, Judah Rajendran, Vinoth Rajendran, Shaman Rajindrajith, Mohammad Amin Rajizadeh, Prashant Rajput, Mahmoud Mohammed Ramadan, Majed Ramadan, Kadar Ramadhan, Chitra Ramasamy, Shakthi Kumaran Ramasamy, Zahra Ramezani, Marzieh Ramezani Farani, Juwel Rana, Shailendra Singh Rana, Chhabi Lal Ranabhat, Nemanja Rancic, Smitha Rani, Chythra R Rao, Kumuda Rao, Mithun Rao, Sowmya J Rao, Vahid Rashedi, Mohammad-Mahdi Rashidi, Mohammad Aziz Rasouli, Ashkan Rasouli-Saravani, Prateek Rastogi, Azad Rasul, Devarajan Rathish, Abdur Rauf, Santosh Kumar Rauniyar, Ilari Rautalin, Ramin Ravangard, Dhwani Ravi, David Laith Rawaf, Salman Rawaf, Reza Rawassizadeh, Ramu Rawat, Ayita Ray, Mohammad Rayati, Iman Razeghian, Bahman Razi, Christian Razo, Filippo Recenti, Murali Mohan Rama Krishna Reddy, Elrashdy Redwan, Sanika Rege, Wajiha Rehman, Lennart Reifels, Longbing Ren, Andre M N Renzaho, Serge Resnikoff, Luis Felipe Reyes, Mina Rezaei, Nazila Rezaei, Negar Rezaei, Nima Rezaei, Mohsen Rezaeian, Taeho Gregory Rhee, Antonio Luiz P Ribeiro, Jennifer Rickard, Moattar Raza Rizvi, Hermano Alexandre Lima Rocha, João Rocha Rocha-Gomes, Mónica Rodrigues, Leonardo Roever, Peter Rohloff, Iftitakhur Rohmah, Susanne Röhr, Megan L Rolfzen, Debby Syahru Romadlon, Marina Romozzi, Amirhossein Roshanshad, Gregory A Roth, Kunle Rotimi, Himanshu Sekhar Rout, Hanieh Rouzbahani, Reza Rouzbahani, Shiva Rouzbahani, Parimal Roy, Poulami Roy, Priyanka Roy, Sharmistha Roy, Shubhanjali Roy, Susovan Roy Chowdhury, Parameswari Royapuram Parthasarathy, Enrico Rubagotti, Michele Russo, Godfrey Mutashambara Rwegerera, Aly M A Saad, Adnan Saad Eddin, Zahra Saadatian, Maha Mohamed Saber-Ayad, Cameron John Sabet, Kabir P Sadarangani, Seyed Kiarash Sadat Rafiei, Basema Ahmad Saddik, Bashdar Abuzed Sadee, Tarannom Sadegh, Ehsan Sadeghi, Erfan Sadeghi, Fatemeh Sadeghi-Ghyassi, Mohd Saeed, Umar Saeed, Maryam Saeedi, Mehdi Safari, Sare Safi, Sher Zaman Safi, Rajesh Sagar, Mastooreh Sagharichi, Dominic Sagoe, Nondo Saha, Fatemeh Saheb Sharif-Askari, Narjes Saheb Sharif-Askari, Pragyan Monalisa Sahoo, Kirti Sundar Sahu, Muhammad Soaib Said, Zahra Saif, S Mohammad Sajadi, Md Refat Uz Zaman Sajib, Mirza Rizwan Sajid, Morteza Saki, Payman Salamati, Mohamed A Saleh, Leili Salehi, Mahdi Salehi, Marwa Rashad Salem, Sohrab Salimi, Malik Sallam, Jayami Eshana Samaranayake, Waqas Sami, Yoseph Leonardo Samodra, Vijaya Paul Samuel,

Abdallah M Samy, Sandeep G Sangle, Elaheh Sanjari, Sathish Sankar, Damian F Santomauro, Lucas H C C Santos, Milena M Santric-Milicevic, Krishna Prasad Sapkota, Sivan Yegnanarayana Iyer Saraswathy, Jacob Owusu Sarfo, Yaser Sarikhani, Hemen Sarma, Mohammad Sarmadi, Gargi Sachin Sarode, Sachin C Sarode, Satish Saroshe, Michele Sassano, Brijesh Sathian, Paul A Saunders, Mehrdad Savabi Far, Monika Sawhney, Sangeeta Gopal Saxena, Ganesh Kumar Saya, Abu Sayeed, Mete Saylan, Christophe Schinckus, Ione Jayce Ceola Schneider, Art Schuermans, Austin E Schumacher, Ghil Schwarz, David C Schwebel, Falk Schwendicke, Catherine Schwinger, Amin Sedigh, Saravanan Sekaran, Mario Šekerija, Muthamizh Selvamani, Vimalraj Selvaraj, Yuliya Semenova, Mohammad H Semreen, Fikadu Waltengus Sendeku, Yigit Can Senol, Subramanian Senthilkumaran, Sadaf G Sepanlou, Andreea Claudia Serban, Edson Serván-Mori, Yashendra Sethi, Seyed Mohammad Seyed Alshohadaei, Nilay S Shah, Sweni Shah, Shazlin Shaharudin, Muhammad Shahbaz, Samiah Shahid, Syed Ahsan Shahid, Wajeehah Shahid, Endrit Shahini, Fatemeh Shahrahmani, Hamid R Shahsavari, Masood Ali Shaikh, Ali Shakerimoghaddam, Ali S Shalash, Muhammad Aaqib Shamim, Farzane Shams, Mehran Shams-Beyranvand, Mohammad Ali Shamshirgaran, Anas Shamsi, Dan Shan, Abhishek Shankar, Mohammed Shannawaz, Xian Shao, Amin Sharifan, Javad Sharifi Rad, Avimanu Sharma, Bhoopesh Kumar Sharma, Bunty Sharma, Kamal Sharma, Sourabh Sharma, Ujjawal Sharma, Vishal Sharma, Rajesh P Shastry, Shamee Shastry, Armin Shavandi, Ramzi Shawahna, Maryam Shayan, Babangida Shehu Bappah, Ali Sheidaei, Suchitra M Shenoy, Samendra P Sherchan, Fang Shi, Lin-Hong Shi, Mosa Shibani, Belayneh Fentahun Shibesh, Kenji Shibuya, Desalegn Shiferaw, Md Monir Hossain Shimul, Min-Jeong Shin, Rahman Shiri, Aminu Shittu, Ivy Shiue, Nathan A Shlobin, Shayan Shojaei, Zahra Shokati Eshkiki, Sina Shool, Seyed Afshin Shorofi, Gambhir Shrestha, Sunil Shrestha, Kerem Shuval, Nicole Remaliah Samantha Sibuyi, Emmanuel Edwar Siddig, Mohammad Sidiq, Martin Siegel, Diego Augusto Santos Silva, Gustavo Correia Basto da Silva, Luís Manuel Lopes Rodrigues Silva, Padam Prasad Simkhada, Abhinav Singh, Ambrish Singh, Baljinder Singh, Bhim Pratap Singh, Harmanjit Singh, Harpreet Singh, Jasbir Singh, Jasvinder A Singh, Kalpana Singh, Lucky Singh, Narinder Pal Singh, Paramdeep Singh, Prashant Kumar Singh, Puneetpal Singh, Rakesh K Singh, Samer Singh, Satwinder Singh, Surendra Singh, Mukesh Kumar Sinha, Ratnesh Sinha, Søren T Skou, David A Sleet, Farrukh Sobia, Md.Salman Sohel, Balamrit Singh Sokhal, Solikhah Solikhah, Sameh S M Soliman, Weiyi Song, Younseong Song, Aayushi Sood, Prashant Sood, Soroush Soraneh, Reed J D Sorensen, Joan B Soriano, Michele Sorrentino, Fernando Sousa, Marco Aurelio Sousa, Ceren Soylu, Michael Spartalis, Manraj Singh Sra, Chandrashekhar T Sreeramareddy, Suresh Kumar Srinivasamurthy, Shyamkumar Sriram, Lauryn K Stafford, Jeffrey D Stanaway, Antonina V Starodubova, Simona Cătălina Ștefan, Caroline Stein, Timothy J Steiner, Jaimie D Steinmetz, Aleksandar Stevanović, Kurt Straif, Peter Stubbs, Omer Subasi, Claudia Kimie Suemoto, Alisha Suhag, Liang Sui, Thitiporn Sukaew, Muritala Suleiman Odidi, Muhammad Suleman, Desy Sulistiyorini, Mark J M Sullman, Anusha Sultan Meo, Haitong Zhe Sun, Jing Sun, Mao-ling Sun, Xiaodong Sun, Zhong Sun, Zhuanlan Sun, Suraj Sundaragiri, Thanigaivel Sundaram, Johan Sundström, David Sunkersing, Sumam Sunny, Vinay Suresh, Chandan Kumar Swain, Dayinta Annisa Syaiful, Lukasz Szarpak, Mindy D Szeto, Sree Sudha T Y, Payam Tabaee Damavandi, Rafael Tabarés-Seisdedos, Shima Tabatabai, Celine Tabche, Ramin Tabibi, Mohammad Tabish, Jyothi Tadakamadla, Santosh Kumar Tadakamadla, Buhari Abdullahi Tafida, Farzad Taghizadeh-Hesary, Yasaman Taheri Abkenar, Moslem Taheri Soodejani, Jabeen Taiba, Shima Tajabadi, Iman M Talaat, Stella Talic, Byomkesh Talukder, Mircea Tampa, Jacques Lukenze Tamuzi, Jianye Tan, Ker-Kan Tan, Shynar Tanabayeva, Haosu Tang, Ekamol Tantisattamo, Ingan Ukur Tarigan, Mengistie Kassahun Tariku, Saba Tariq, Md. Tariqujjaman, Razieh Tavakoli Oliaee, Rahele Tavakoly, Seyed Mohammad Tavangar, Mebrahtu G. Tedla, Amare Teshome Tefera, Mojtaba Teimoori, Mohamad-Hani Temsah, Corey Teply,

Masayuki Teramoto, Amensisa Hailu Tesfaye, Azimeraw Arega Tesfu, Jay Tewari, Alireza Teymouri, Pugazhenthan Thangaraju, Kavumpurathu Raman Thankappan, Rekha Thapar, Ismaeel Tharwat, Hadiza Theyra-Enias, Mehakpreet Kaur Thind, Arun James Thirunavukarasu, Muthu Thiruvengadam, Rekha Thiruvengadam, Arulmani Thiyagarajan, Nihal Thomas, Geethika P Thota, Wei Tian, Jansje Henny Vera Ticoalu, Tenaw Yimer Tiruye, Madi Tleshev, Marcello Tonelli, Roman Topor-Madry, Ali Torkashvand, Mathilde Touvier, Marcos Roberto Tovani-Palone, Khaled Trabelsi, Eugenio Traini, Mai Thi Ngoc Tran, Nghia Minh Tran, Ngoc Ha Tran, Quynh Thuy Huong Tran, Tam Quoc Minh Tran, Nguyen Tran Minh Duc, Domenico Trico, Indang Trihandini, Samuel Joseph Tromans, Quynh Xuan Nguyen Truong, Gary Tse, Evangelia Eirini Tsermpini, Lorainne Tudor Car, Mike Tuffour Amirikah, Munkhtuya Tumurkhuu, Zhouting Tuo, Sok Cin Tye, Aniefiok John Udoakang, Atta Ullah, Himayat Ullah, Irfan Ullah, Saeed Ullah, Muhammad Umair, Krishna Kishore Umapathi, Muhammad Umar¶, Muhammad Umar**, Shehu Salihu Umar, Dinesh Upadhya, Era Upadhyay, Dipan Uppal, Jibrin Sammani Usman, Kelechi Julian Uzor, Dilber Uzun Ozsahin, Hande Uzunçıbuk, sara Vahdati, Omid Vakili, Pascual R Valdez, Mario Valenti, Gelareh Valizadeh, Jef Van den Eynde, Priya Vart, Sampara Vasishta, Srivatsa Surya Vasudevan, Prabhakar Veginadu, Ashleigh S Vella, Balachandar Vellingiri, Narayanaswamy Venketasubramanian, Baskar Venkidasamy, Megan Verma, Massimiliano Veroux, Georgios-Ioannis Verras, Dominique Vervoort, Simone Villa, Jorge Hugo Villafañe, David Villarreal-Zegarra, Francesco S Violante, Sharath Chaitanya Vipparthy, Rachel Visontay, Luciano Magalhães Vitorino, Martin Vojtek, Stein Emil Vollset, Theo Vos, Mehdi Vosoughi, Elpida Vounzoulaki, Linh Vu, Yasir Waheed, Mugi Wahidin, Megha Walia, Jin-Yi Wan, Cong Wang, Fang Wang, Liang Wang, Ruixuan Wang, Shaopan Wang, Shu Wang, Wei Wang, Xing Wang, Xuequan Wang, Yanzhong Wang, Yichen Wang, Yuan-Pang Wang, Zhihua Wang, Tanveer A. Wani, Mary Njeri Wanjau, Ahmed Bilal Waqar, Muhammad Waqas, Paul Ward, Toyiba Hiyaru Wassie, Kosala Gayan Weerakoon, Ishanka Weerasekara, Fei-Long Wei, Yi Feng Wen, Andrea Werdecker, Ronny Westerman, Joanna L Whisnant, Harvey A Whiteford, Taweewat Wiangkham, Yohanes Cakrapradipta Wibowo, Anggi Lukman Wicaksana, Dakshitha Praneeth Wickramasinghe, Nuwan Darshana Wickramasinghe, Angga Wilandika, Peter Willeit, Andrew Awuah Wireko, Gemechu Kumera Wirtu, Charles Shey Wiysonge, Abay Tadesse Woday, Marcin W Wojewodzic, Axel Walter Wolf, Tewodros Eshete Wonde, Yohannes Chemere Wondmeneh, Daniel Tarekegn Worede, Minichil Chanie Worku, Ai-Min Wu, Chenkai Wu, Felicia Wu, James Fan Wu, Jiayuan Wu, Jinyi Wu, Yihun Miskir Wubie, Zhijia Xia, Guangqin Xiao, Hong Xiao, Na Xiao, Wanqing Xie, Hongquan Xing, Site Xu, Suowen Xu, Wanqing Xu, Xiang Xu, Xiaoyue Xu, Mukesh Kumar Yadav, Vikas Yadav, Mahnaz Yadollahi, Saba Yahoo (Syed), Galal Yahya, Guangcan Yan, Haibo Yang, Weiguang Yang, Xinxin Yang, Yuichiro Yano, Haiqiang Yao, Laiang Yao, Amir Yarahmadi, Haya Yasin, Mohamed A Yassin, Sanni Yaya, Pengpeng Ye, Meghdad Yeganeh, Ali Cem Yekdeş, Mohammad Hossein YektaKooshali, Kuanysh A. Yergaliyev, Renjulal Yesodharan, Subah Abderehim Yesuf, Saber Yezli, Siyan Yi, Muluken Yigezu, Zeamanuel Anteneh Yigzaw, Dehui Yin, Yulai Yin, Paul Yip, Malede Berihun Yismaw, Yazachew Engida Yismaw, Dong Keon Yon, Naohiro Yonemoto, Mustafa Z Younis, Abdilahi Yousuf, Chuanhua Yu, Jian Yu, Yong Yu, Ghazala Yunus, Umar Yunusa, Aminu Abba Yusuf, Monal Yuwanati, Siddhesh Zadey, Vesna Zadnik, Mubashir Zafar, Manijeh Zaghampour, Emilia Zainal Abidin, Fathiah Zakham, Nazar Zaki, Giulia Zamagni, Burhan Abdullah Zaman, Sojib Bin Zaman, Abu Sarwar Zamani, Heather J Zar, Kourosh Zarea, Mohammed Zawiah, Mohammed G M Zeariya, Sebastian Zensen, Eyael M Zeru, Tiansong Zhan, Yongle Zhan, Beijian Zhang, Casper J P Zhang, Haijun Zhang, Jingya Zhang, Liqun Zhang, Xiaoyi Zhang, Yunquan Zhang, Zhiqiang Zhang, Jianhui Zhao, Zhongyi Zhao, Jinxin Zheng, Ming-Hua Zheng, Claire Chenwen Zhong, Jiayan Zhou, Juexiao Zhou, Maigeng Zhou, Bin Zhu, Zhengyang Zhu,

Magdalena Zielińska, Liu Zihao, Mohamed Ali Zoromba, Zhiyong Zou, Rafat Mohammad Zrieq, Liesl J Zuhlke, Lilik Zuhriyah, Alimuddin Zumla, Ahed H Zyoud, Sa'ed H Zyoud, and Shaher H Zyoud.

Drafting the work or revising it critically for important intellectual content

Bhoomadevi A, Ahmed A.J. Jabbar, Mohammad Amin Aalipour, Hasan Aalruz, Hazim S Ababneh, Ukachukwu O Abaraogu, Biruk Beletew Abate, Cristiana Abbafati, Mohsen Abbasi-Kangevari, Samar Abd ElHafeez, Ashraf Nabiel Abdalla, Emad M. Abdallah, Nadin M. I. Abdel Razeq, Reda Abdel-Hameed, Michael Abdelmasseh, Sherief Abd-Elsalam, Mohammad Abdollahi, Arman Abdous, Jeza Muhamad Abdul Aziz, Auwal Abdullahi, Toufik Abdul-Rahman, Aidin Abedi, Armita Abedi, Roberto Ariel Abeldaño Zuñiga, Shehab Uddin Al Abid, Olugbenga Olusola Abiodun, Olumide Abiodun, Shady Abohashem, Hassan Abolhassani, Ulric Sena Abonie, Nagah M. Abourashed, Mohamed Abouzid, Dmitry Abramov, Lucas Guimarães Abreu, Rana Kamal Abu Farha, Fuad Hamdi A. Abuadas, Aminu Kende Abubakar, Bilyaminu Abubakar, Eman Abu-Gharbieh, Sawsan Abuhammad, Ahmad Y Abuhelwa, Hana J Abukhadijah, Niveen ME Abu-Rmeileh, Salahdein Aburuz, Dina Abushanab, Raghu Ram Achar, Anirudh Balakrishna Acharya, Apurba Acharya, Ilana N Ackerman, Juan Manuel Acuna, Lawan Hassan Adamu, Mesafint Molla Adane, Zenaw Debasu Addisu, Isaac Yeboah Addo, Oluwafemi Atanda Adeagbo, Tajudeen Adesanmi Adebisi, Isaac Akinkunmi Adedeji, Kamoru Ademola Adedokun, Rufus Adesoji Adedoyin, Oluwatobi E Adegbile, Nurudeen A Adegoke, Olumide Thomas Adeleke, Isaac Ayodeji Adesina, Habeeb Omoponle Adewuyi, Olorunsola Israel Adeyomoye, Usha Adiga, Mohd Adnan, Qorinah Estiningtyas Sakilah Adnani, Prince Owusu Adoma, David Adzrago, Ahmed M Afifi, Aanuoluwapo Adeyimika Afolabi, Rotimi Felix Afolabi, Saira Afzal, Gizachew Beykaso Agafari, Suneth Buddhika Agampodi, Mahdi Aghaalikhani, César Agostinis Sobrinho, Mahsa Ahadi, Bright Opoku Ahinkorah, Danish Ahmad, Khabir Ahmad, Muayyad M Ahmad, Rabbiya Ahmad, Tauseef Ahmad, Waqas Ahmad, Negar Sadat Ahmadi, Mohadese Ahmadzade, Akeem Olayiwola Ahmed, Ayman Ahmed, Gasha Salih Ahmed, Haroon Ahmed, Junaid Ahmed, Luai A Ahmed, Mehrunnisha Sharif Ahmed, Meqdad Saleh Ahmed, Muktar Beshir Ahmed, Mushood Ahmed, Oli Ahmed, Shabbir Ahmed, Sindew Mahmud Ahmed, Gulzhanat Aimagambetova, Dolapo Emmanuel Ajala, Marjan Ajami, Azeezat Oluwafunmilayo Ajose, Hossein Akbarialiabad, Roland Eghoghosoa Akhigbe, Mohammed Ahmed Akkaif, Wole Akosile, Hammad Akram, Ashley E Akrami, Alaa Al Amiry, Salah Al Awaidy, Omar Al Omari, Omar Al Ta'ani, Wasan A. M. Al Taie, Yazan Al Thaher, Omar Ali Mohammed Al Zaabi, Mohammad Ahmmad Mahmoud Al Zoubi, Yazan Al-Ajlouni, Tariq A Alalwan, Khurshid Alam, Mostafa Alam, Rasmieh Mustafa Al-Amer, Abebaw Alamrew, Amani Alansari, Fahmi Y Al-Ashwal, Seyed Mohammad Amin Alavi, Mohammed Albashtawy, Astefanos Al-Dalakta, Khalifah A Aldawsari, Wafa A Aldhaleei, Mohammed S Aldossary, Robert W Aldridge, Raouf Alebshehy, Tekletsadik Tekleslassie Alemayehu, Fentahun Alemnew, Ayman Al-Eyadhy, Fahad D Algahtani, Abdelazeem M Algammal, Khalid F Alhabib, Nma Bida Alhaji, Samar Al-Hajj, Fadwa Naji Alhalaiqa, Mohammed Khaled Al-Hanawi, Aminu Alhassan Ibrahim, Ashraf Alhumaidi, Fahad A. Alhumaydhi, Dari Alhuwail, Abid Ali, Mohammed Usman Ali, Shahid Ali, Syed Shujait Ali, Waad Ali, Akram Al-Ibraheem, Gianfranco Alicandro, Montaha Al-Iede, Morteza Alipour, Samah W Al-Jabi, Mohammad A Aljasir, Ahmad Alkhatib, Mayson H. Alkhatib, Mustafa Alkhawam, Atefeh Allahbakhshian, Khaled S. Allemailem, Mohammed Z. Allouh, Nihad A Almasri, Hesham M Al-Mekhlafi, Omar Almidani, Amr Almobayed, Khaldoon Aied Alnawafleh, Hasan Yaser Alniss, Mahmoud A Alomari, Mohammad R Alosta, Jaber S Alqahtani, Saleh A Alqahtani, Ahmad Rajeh Al-Qudimat, Ahmad Alrawashdeh, Intima Alrimawi, Sahel Majed Alrousan, Najim Z. Alshahrani, Mansour Abdullah Alshehri, Zaid Altaany, Awais Altaf, Alaa B Al-Tammemi, Jaffar A Al-Tawfiq, Malik A Althobiani, Vera L Alves Carneiro, Nelson Alvis-Guzman, Nelson J Alvis-Zakzuk, Hassan Alwafi, Mohammad Al-Wardat, Yaser Mohammed Al-Worafi,

Hany Aly, Mohammad Sharif Ibrahim Alyahya, Amal AlZahmi, Hosam Alzahrani, Karem H Alzoubi, Uchenna Anderson Amaechi, Reza Amani-Beni, Adeladza Kofi Amegah, Faten Amer, Bardia Amidi, Amr Amin, Tarek Tawfik Amin, Alireza Amindarolzarbi, Saeed Amini, Ehsan Amini-Salehi, Nafiu Aminu, Majid Aminzare, Sohrab Amiri, Dickson A Amugsi, Jimoh Amzat, Filippos Anagnostakis, Roshan A Ananda, Robert Ancuceanu, Deanna Anderlini, David B Anderson, Song Peng Ang, Colin Angus, Nguyen Hoang Anh, Samuel Egyakwa Ankomah, Kabilan Annadurai, Amir Anoushiravani, Iman Ansari, Sumbul Ansari, Umair Ansari, Rahel Mulatie Anteneh, Josep M Antó, Ernoiz Antriyandarti, Boluwatife Stephen Anuoluwa, Saleha Anwar, Shahnawaz Anwer, Anayochukwu Edward Anyasodor, Geminn Louis Carace Apostol, Juan Pablo Arab, Hossein Arabi, Jalal Arabloo, Jorge Arias de la Torre, Hany Ariffin, Benedetta Armocida, Johan Ärnlöv, Jesu Arockiaraj, Mahwish Arooj, Kurnia Dwi Artanti, Raphael Taiwo Aruleba, Deepavalli Arumuganainar, Mahsa Asadi Anar, Muhammad Asaduzzaman, Syed Mohammed Basheeruddin Asdaq, Shewatatek Melaku Asefa, Mulu Tiruneh Asemu, Saeed Asgary, Mitra Ashrafi, Bernard Kwadwo Yeboah Asiamah-Asare, Muhammad Shahzad Aslam, Yuni Asri, Seyyed Shamsadin Athari, Maha Moh'd Wahbi Atout, Alok Atreya, Zeenah A Atwan, Marcel Ausloos, Abolfazl Avan, Núbia Carelli Pereira Avelar, Adedapo Wasiu Awotidebe, Seyyed HamidReza Ayatizadeh, Olatunde O Ayinde, Yusuf Oloruntoyin Ayipo, Seyed Mohammad Ayyoubzadeh, Davood Azadi, Sina Azadnajafabad, Alireza Azarboo, Farya Azimi, Sadat Abdulla Aziz, Ahmed Y Azzam, Domenico Azzolino, Giridhara Rathnaiah Babu, Israel Tadesse Bacha, Muhammad Badar, Ashish D Badiye, Alaa Aboelnour Badran, Youngoh Bae, Soroush Baghdadi, Elahe Baghizadeh, Fereshteh Baghizadeh, Khlood K Baghlaf, Atif Amin Baig, Vali Baigi, Shankar M Bakkannavar, Abdulaziz T Bako, Senthilkumar Balakrishnan, Mohammadreza Balooch Hasankhani, Ovidiu Constantin Baltatu, Shatha Bamashmous, Maciej Banach, Morteza Banakar, Palash Chandra Banik, Noel C Barengo, Suzanne Lyn Barker-Collo, Hiba Jawdat Barqawi, Amadou Barrow, Sandra Barteit, MD Abu Bashar, Shahid Bashir, Guido Basile, Pritish Baskaran, Quique Bassat, Mohammad-Mahdi Bastan, Sanjay Basu, Bernhard T Baune, Mahdis Bayat, Mulat Tirfie Bayih, Feyisa Shasho Bayisa, Thomas Beaney, Neeraj Bedi, Priyamadhaba Behera, Jina Behjati, Babak Behnam, Amir Hossein Behnoush, Bezawit K Bekele, Melesse Belayneh, Muhammad Bashir Bello, Olorunjuwon Omolaja Bello, Luis Belo, Apostolos Beloukas, Salaheddine Bendak, Riyad Bendardaf, Corina Benjet, Isabela M Bensenor, Samiun Nazrin Bente Kamal Tune, Habib Benzian, Maria Bergami, Paulo J G Bettencourt, Ajeet Singh Bhadoria, Akshaya Srikanth Bhagavathula, Neeraj Bhala, Jeetendra Bhandari, Ravi Bharadwaj, Sonu Bhaskar, Anup Bhat, Vivek Bhat, Priyadarshini Bhattacharjee, Shuvarthi Bhattacharjee, Gurjit Kaur Bhatti, Jasvinder Singh Bhatti, Raluca Bievel-Radulescu, Saeed Biroudian, Catherine Bisignano, Atanu Biswas, Bijit Biswas, Bruno Bizzozero-Peroni, Espen Bjertness, Fiona M Blyth, Virginia Bodolica, Mahmut Bodur, Lucimere Bohn, Rachael Bokota, Obasanjo Afolabi Bolarinwa, Srinivasa Rao Bolla, Paria Bolourinejad, Sri Harsha Boppana, Sanaz Bordbar, Hamed Borhany, Alejandro Botero Carvajal, Souad Bouaoud, Soufiane Boufous, Christopher Boxe, Marija M Bozic, Dejana Braithwaite, Michael Brauer, Nicholas J K Breitborde, Hermann Brenner, Gabrielle Britton, Julie Brown, Traolach Brugha, Raffaele Bugiardini, Norma B Bulamu, Danilo Buonsenso, Felix Busch, Reinhard Busse, Yasser Bustanji, Sanjay C J, Jack Cagney, Rose Cairns, Mehtap Çakmak Barsbay, Daniela Calina, Luciana Aparecida Campos, Ismael Campos-Nonato, Yuchen Cao, Angelo Capodici, Giulia Carreras, Juan Jesus Carrero, Andrea Carugno, Andre F Carvalho, Ana Paula Carvalho-e-Silva, Joao Mauricio Castaldelli-Maia, Carlos A Castañeda-Orjuela, Giulio Castelpietra, Alberico L Catapano, Maria Sofia Cattaruzza, Arthur Caye, Christopher R Cederroth, Luca Cegolon, Francieli Cembranel, Muthia Cenderadewi, Ester Cerin, Sonia Cerrai, Muge Cevik, Madhu Chakkere Shivamadhu, Chiranjib Chakraborty, Sandip Chakraborty, Joht Singh Chandan, Rama Mohan Chandika, Miyuru Chandradasa, Eeshwar K Chandrasekar, Vijay

Kumar Chattu, Victoria Chatzimavridou-Grigoriadou, Lam Duc Chau, Sirshendu Chaudhuri, Akhilanand Chaurasia, An-Tian Chen, Guangjin Chen, Hana Chen, Haowei Chen, Junhao Chen, Meng Xuan Chen, Simiao Chen, Haojin Cheng, Ka Ching Cheung, Nicholas WS Chew, Ju-Huei Chien, Patrick R Ching, Jesus Lorenzo Chirinos-Caceres, William C S Cho, Bryan Chong, Yuen Yu Chong, Hou In Chou, Hanne Christensen, Steffan Wittrup McPhee Christensen, Sunghyun Chung, Muhammad Chutiyami, Arrigo Francesco Giuseppe Cicero, Liliana G Ciobanu, Alyssa Columbus, Joao Conde, Stephen E Congly, Nathalie Conrad, Sara Conti, Mariana Oliveira Corda, Samuele Cortese, Paolo Angelo Cortesi, Claudia Cosma, Michael H Criqui, Natalia Cruz-Martins, Xiaolin Cui, Garland T Culbreth, Xiaochen Dai, Zhaoli Dai, Mayank Dalakoti, Koustuv Dalal, Gloria Dalla Costa, Emanuele D'Amico, Roy Arokiam Daniel, Lucio D'Anna, Samuel Demissie Darcho, Latefa Ali Dardas, Anna Dastiridou, Gail Davey, Claudio Alberto Dávila-Cervantes, Nicole Davis Weaver, Dimash Davletov, Katie de Luca, Edward Christopher Dee, Orla Deegan, Sindhura Deekonda, Louisa Degenhardt, Paria Dehesh, Andreas K Demetriades, Edgar Denova-Gutiérrez, Tadios Niguss Derese, Ismail Dergaa, Emina Dervišević, Abraham Aregay Desta, Pradeep Kumar Devarakonda, Syed Masudur Rahman Dewan, Arkadeep Dhali, Amol S Dhane, Narender K. Dhania, Mandira Lamichhane Dhimal, Meghnath Dhimal, Sameer Dhingra, Marcello Di Pumpo, Diana Dias da Silva, Daniel Diaz, Luis Antonio Diaz, Kimia Didehvar, Lauren K Dillard, Adriana Dima, Xueting Ding, Huyen Phuc Do, Klara Georgieva Dokova, Christiane Dolecek, Francesco Dondi, Mario D'Oria, Ojas Prakashbhai Doshi, Paulo Magno Martins Dourado, Menayit Tamrat Dresse, Tim Robert Driscoll, Ashel Chelsea Dsouza, Viola Savy Dsouza, Jiang Du, John Dube, Emeka W Dumbili, Samuel C Dumith, Jennifer Dunne, Senbagam Duraisamy, Oyewole Christopher Durojaiye, Arkadiusz Marian Dziedzic, Abdel Rahman E’mar, Osamudiamen Ebohon, Lamiaa Labieb Mahmoud Ebraheim, Mohammad Hossein Ebrahimi, Sara Ebrahimi, Ekaette Godwin Edelduok, Ferry Efendi, Behrad Eftekhari, Foolad Eghbali, Fatemeh Ehsani, Ashkan Eighaei Sedeh, Ebrahim Eini, Michael Ekholuenetale, Rabie Adel El Arab, Abdelfatteh EL Omri, Maysaa El Sayed Zaki, Rana Elbeshbeishy, Noha Mousaad Elemam, Ghada Metwally Tawfik ElGohary, Muhammed Elhadi, Mohamed Elhoumed, Sherif Elkannishy, Omar Abdelsadek Abdou Elmeligy, Rami Elmorsi, Adel B Elmoselhi, Mohamed Hassan Elnaem, Mohammed Elshaer, Abdelgawad Salah Eltahawy, Theophilus I Emeto, Victor Oghenekparobo Emojevwe, Destaw Endeshaw, Holly E Erskine, Christopher Imokhuede Esezobor, Derese Eshetu, Sharareh Eskandarieh, Majid Eslami, Rafaela Cavalheiro do Espírito Santo, Francesco Esposito, Fahima Nasrin Eva, Elochukwu Ezenwankwo, Adewale Oluwaseun Fadaka, Heidar Fadavian, Adeniyi Francis Fagbamigbe, Ayesha Fahim, Ildar Ravisovich Fakhradiyev, Aliasghar Fakhri-Demeshghieh, Emerito Jose Aquino Faraon, Mohammad Fareed, Zaki Farhana, MoezAlIslam Ezzat Mahmoud Faris, Andre Faro, Hossein Farrokhpour, Fatemeh Farshad, Farima Farsi, Folorunso Oludayo Fasina, Modupe Margaret Fasina, Ali Fatehizadeh, Davood Fathi, Zareen Fatima, Li Fei, Berhanu Elfu Feleke, Kaixin Feng, Talukdar Raian Ferdous, Seyed-Mohammad Fereshtehnejad, Rodrigo Fernandez-Jimenez, Pietro Ferrara, Alize J Ferrari, André Ferreira, Nuno Ferreira, Natan Feter, Alexander Finnemore, Claudio Fiorilla, Florian Fischer, Ida Fitriana, Luisa S Flor, Federica Fogacci, Marco Fonzo, Lisa M Force, Carla Fornari, Ingeborg Forthun, Daniela Fortuna, Matteo Foschi, Maryam Fotouhi, Juluis Visnel Foyet F, Alberto Freitas, Jinming Fu, Blima Fux, Peter Andras Gaal, Dominic Dormenyo Gadeka, Márió Gajdács, Yaseen Galali, Silvano Gallus, Shivaprakash Gangachannaiah, Mohd Ashraf Ganie, Xiang Gao, Bashiru Garba, Fernando Barroga Garcia, Miguel Garcia-Argibay, David Garcia-Azorin, Jacopo Garlasco, Zisis Gatzioufas, Prem Gautam, Rupesh K Gautam, Miglas Welay Gebregergis, Haftay Gebremedhin Gebreslassie, Stefano Gelibter, Ali Gerami Matin, Kalab Yigermal Gete, Delaram J Ghadimi, Fataneh Ghadirian, Seyyed-Hadi Ghamari, Arin Ghamkhar, Moein Ghasemi, Mohammad-Reza Ghasemi, Shakiba Ghasemi Assl, Haniyeh Ghasrsaz, Ramy Mohamed Ghazy,

Sailaja Ghimire, Nermin Ghith, Nasim Gholizadeh, Elena Ghotbi, Alessandro Gialluisi, Ruth Margaret Gibson, Artyom Urievich Gil, Gabriela Fernanda Gil, Syed Abdullah Gilani, Tiffany K Gill, Alem Abera Girmay, Alessandro Girombelli, Elena V Gnedovskaya, Laszlo Göbölös, Ali Golestani, Davide Golinelli, Melika Golmohammadi, Wenping Gong, Sameer Vali Gopalani, Yitayal Ayalew Goshu, Alessandra C Goulart, Aman Goyal, Ayman Grada, Vittorio Grieco, Michal Grivna, Ashna Grover, Habtamu Alganeh Guadie, Shi-Yang Guan, Giovanni Guarducci, Avirup Guha, Damitha Asanga Gunawardane, Zhaoyu Guo, Zheng Guo, Zhifeng Guo, Himanshu Gupta, Ishita Gupta, Lalit Gupta, Rajat Das Gupta, Rajeev Gupta, Sapna Gupta, Veer Bala Gupta, Vivek Kumar Gupta, Lami Gurmessa, Reyna Alma Gutiérrez, Roberth Steven Gutiérrez-Murillo, Adrina Habibzadeh, Awoke Derbie Habteyohannes, Tesfahun Simon Hadaro, Najah R Hadi, Zahra Hadian, Abdul Hafiz, Faraidoon Haghdoost, Arian Haghtalab, Haimanot Ewnetu Hailu, Pritam Halder, Aram Halimi, Kosar Hikmat Hama Aziz, Islam M Hamad, Randah R Hamadeh, Sajid Hameed, Erin B Hamilton, Ahmad Hammoud, Mohammad Hamza, Umar Sabiu Hamza, Didem Han Yekdeş, Nasrin Hanifi, Graeme J Hankey, Fahad Hanna, Md. Aminul Haque, Harapan Harapan, Kassandra L Harding, Josep Maria Haro, Eka Mishbahatul Marah Has, Ahmed I Hasaballah, Faizul Hasan, Md Kamrul Hasan, Ali Hasanpour- Dehkordi, Arezou Hashem Zadeh, Mohammad Hashem Hashempur, Nada Tawfig Hashim, Amr Hassan, Ibrahim Nagmeldin Hassan, Nageeb Hassan, Mahgol Sadat Hassan Zadeh Tabatabaei, Lasanthi Wathsala Hathagoda, Rasmus J Havmoeller, Angie Hawat, Simon I Hay, Jue He, Jeffrey J Hebert, Mehdi Hemmati, Claudiu Herteliu, Austin Heuer, Sumudu Avanthi Hewage, Kamal Hezam, Yuta Hiraike, Ramesh Holla, Alamgir Hossain, Lubna Hossain, Md Mahbub Hossain, Md Sabbir Hossain, Mohammad Bellal Hossain, Mehdi Hosseinzadeh, Sorin Hostiuc, Mila Nu Nu Htay, Chengxi Hu, Junjie Huang, Weijun Huang, Yefei Huang, Atanesia Indriyani Human, M Azhar Hussain, Salman Hussain, Dursa Hussein, Nawfal R Hussein, Mohamed Ibrahim Husseiny, Hong-Han Huynh, Luigi Francesco Iannone, Ahmed Ibrahim, Ramzi Ibrahim, Reem Ibrahim, Anel Ibrayeva, Francisco Javier Idalsoaga, Pulwasha Maria Iftikhar, Adalia Ikiroma, Jibran Ikram, Olayinka Stephen Ilesanmi, Irena M Ilic, Milena D Ilic, Muhammad Hamza Ilyas, Masoud Imani, Mustapha Immurana, Lucius Chidiebere Imoh, Arit Inok, Mujahid Iqbal, Lalu Muhammad Irham, Mustafa Alhaji Isa, Md Rabiul Islam, Farhad Islami, Faisal Ismail, Nahlah Elkudssiah Ismail, Yerlan Ismoldayev, Hiroyasu Iso, Gaetano Isola, Mosimah Charles Ituka, Chinwe Juliana Iwu-Jaja, Ihoghosa Osamuyi Iyamu, Mahalaxmi Iyer, Jalil Jaafari, Louis Jacob, Kathryn H. Jacobsen, Morteza Jafarinia, Shabbar Jaffar, Haitham Jahrami, Vikash Jaiswal, Mihajlo Jakovljevic, Ali Jaliliyan, Mohamed Jalloh, Armaan Jamal, Qazi Mohammad Sajid Jamal, Jazlan Jamaluddin, Jerin James, Tyler G. James, Hasan Jamil, Safayet Jamil, Masoud Jamshidi, Shaghayegh JamshidiRastabi, Rajiv Janardhanan, Esmaeil Jarrahi, Syed Sarmad Javaid, Anita Javanmardi, Ruwan Duminda Jayasinghe, Yovanthi Anurangi Jayasinghe, Kimia Jazi, Felix K Jebasingh, Tadesse Hailu Jember, Belayneh Hamdela Jena, Seogsong Jeong, Mahsa Jessri, Bijay Mukesh Jeswani, Vivekanand Jha, Weiqiu Jin, Wenyi Jin, Jost B Jonas, Tamas Joo, Abel Joseph, Nitin Joseph, Charity Ehimwenma Joshua, George Joy, Jacek Jerzy Jozwiak, Mikk Jürisson, Malik E Juweid, Madhanraj K, Ashish Kumar Kakkar, Leila R Kalankesh, Khalil Kalavani, Feroze Kaliyadan, Sanjay Kalra, Md Moustafa Kamal, Sivesh Kathir Kamarajah, Rajesh Kamath, Saltanat Kamenova, Arun Kamireddy, Ramat T. Kamorudeen, Oleksandr Kamyshnyi, Mona Kanaan, Saddam Fuad Kanaan, Jiseung Kang, Samuel Berchi Kankam, Kehinde Kazeem Kanmodi, Sujitha Kannan, Suthanthira Kannan S, Rami S Kantar, Neeti Kapoor, Paschalis Karakasis, Reema A Karasneh, Samad Karkhah, Mohmed Isaqali Karobari, Tomasz M Karpiński, Manoj Kumar Kashyap, Abdene Weya Kaso, Hengameh Kasraei, Adarsh Katamreddy, Patrick DMC Katoto, Joonas H Kauppila, Gbenga A Kayode, Mohammad-Hossein Keivanlou, Peter Njenga Keiyoro, Chukwudi Keke, John H Kempen, Salima Kerai, Vikash Ranjan Keshri, Emmanuelle Kesse-Guyot, Yousef Saleh Khader, Himanshu Khajuria, Hazim O.

Khalifa, Anas Husam Khalifeh, Anees Ahmed Khalil, Anita Khalili, Pantea Khalili, Ghazaleh Khalili-Tanha, Ajmal Khan, Faiz Ullah Khan, Maseer Khan, Md Abdullah Saeed Khan, Mohammad Jobair Khan, Muhammad Hamza Khan, Muhammad Mueed Khan, Muhammad Umair Khan, Muhammad Umer Khan, Nusrat Khan, Ruby Khan, Salman Ali Khan, Sumaiya Khan, Yusuf Saleem Khan, Zahid Khan, Vishnu Khanal, Shaghayegh Khanmohammadi, Sameer Uttamaro Khasbage, Zenith Khashim, Khaled Khatab, Haitham Khatatbeh, Moawiah Mohammad Khatatbeh, Hamid Reza Khayat Kashani, Afshin Khazaei, Sunil Kumar Khokhar, Atulya Aman Khosla, Mahmood Khosrowjerdi, Hye Jun Kim, Min Seo Kim, Adnan Kisa, Sezer Kisa, Katarzyna Kissimova-Skarbek, Mika Kivimäki, Jessica Klusty, Abdul Basith KM, Shivakumar KM, Ann Kristin Skrindo Knudsen, Nazarii Kobyliak, Jonathan M Kocarnik, Sonali Kochhar, Michail Kokkorakis, Diana Gladys Kolieghu Tcheumeni, Aida Kondybayeva, Oleksii Korzh, Karel Kostev, Konstantinos Kotsis, Parvaiz A Koul, Sindhura Lakshmi Koulmane Laxminarayana, Irene Akwo Kretchy, James-Paul Kretchy, Kewal Krishan, Chong-Han Kua, Ananya Kuanar, Barthelemy Kuate Defo, Raja Amir Hassan Kuchay, Burcu Kucuk Bicer, Mohammed Kuddus, Ilari Kuitunen, Mukhtar Kulimbet, Vishnutheertha Kulkarni, Shweta Kulshreshtha, Dewesh Kumar, Jogender Kumar, Rakesh Kumar, Tushar Kumar, Setor K Kunutsor, Almagul Kurmanova, Om P Kurmi, Maria Dyah Kurniasari, Krishna Prasad Kurpad, Asep Kusnali, Christina Yeni Kustanti, Dian Kusuma, Tezer Kutluk, Ville Kytö, Pallavi L C, Adriano La Vecchia, Carlo La Vecchia, Muhammad Awwal Ladan, Lucie Laflamme, Chandrakant Lahariya, Daphne Teck Ching Lai, Anita Lakhani, Ratilal Lalloo, Tea Lallukka, Judit Lám, Iván Landires, Berthold Langguth, Ariane Laplante-Lévesque, Savita Lasrado, Kamaluddin Latief, Kenney Ki Lee Lau, Basira Kankia Lawal, Saheed Akinmayowa Lawal, Aliyu Lawan, Harriet L S Lawford, Dai Quang Le, Huu-Hoai Le, Minh Huu Nhat Le, Nhi Huu Hanh Le, Thao Thi Thu Le, Caterina Ledda, Hye Ah Lee, James Leigh, Vasileios Leivaditis, Matthew J Lennon, Matilde Leonardi, Elvynna Leong, Janni Leung, Chengcheng Li, Hui Li, Jiaying Li, Jie Li, Shaojie Li, Wei Li§, Zhengrui Li, Zhihui Li, Yanxue Lian, Queran Lin, Ya Lin, Daniel Lindholm, Christine Linehan, Yuewei Ling, Jue Liu, Xianliang Liu, Yubo Liu, Erand Llanaj, Michael J Loftus, Valerie Lohner, José Francisco López-Gil, Platon D Lopukhov, Stefan Lorkowski, Masoud Lotfizadeh, Giancarlo Lucchetti, Alessandra Lugo, Raimundas Lunevicius, Jay B Lusk, Angelina M Lutambi, Ricardo Lutzky Saute, Miltiadis D Lytras, Ellina Lytvyak, Kevin Sheng-Kai Ma, Zheng Feei Ma, Mahmoud Mabrok, Isis E Machado, Seyed Ataollah Madinezad, Christian Madsen, Aurea Marilia Madureira-Carvalho, Mohammed Magdy Abd El Razek, Sasikumar Mahalingam, Mehrdad Mahalleh, Nozad Hussein Mahmood, Farhad Mahmoudi, My Tra Mai, Rituparna Maiti, Mohammad-Reza Malekpour, Reza Malekzadeh, Hardeep Singh Malhotra, Ahmad Azam Malik, Farihah Malik, Tabarak Malik, Deborah Carvalho Malta, Abdullah A Mamun, Mustapha Mangdow, Lokesh Manjani, Kamaruddeen Mannethodi, Marjan Mansourian, Lorenzo Giovanni Mantovani, Changkun Mao, Tahir Maqbool, Sajid Maqsood, Hamid Reza Marateb, Konstantinos Margetis, Adilson Marques, Randall V Martin, Gabriel Martinez, Bernardo Alfonso Martinez-Guerra, Ramon Martinez-Piedra, Daniela Martini, Francisco Rogerlândio Martins-Melo, Miquel Martorell, Winfried März, Roy Rillera Marzo, Sammer Marzouk, Stefano Masi, Clara N Matei, Yasith Mathangasinghe, Stephanie Mathieson, Alexander G Mathioudakis, Medha Mathur, Fernanda Penido Matozinhos, Rita Mattiello, Khurshid A Mattoo, Pallab K Maulik, Mahsa Mayeli, Antonio Mazzotti, Chioma Ngozichukwu Pauline Mbachu, Ikechukwu Innocent Mbachu, Susan A McLaughlin, Steven M McPhail, Enkeleint A Mechili, Rishi P Mediratta, Elahe Meftah, Medhin Mehari, Asim Mehmood, Man Mohan Mehndiratta, Entezar Mehrabi Nasab, Kala M Mehta, Vini Mehta, Toni Meier, Hadush Negash Meles, Endalkachew Belayneh Melese, Satish Melwani, Walter Mendoza, Godfred Antony Menezes, Ritesh G Menezes, Sultan Ayoub Meo, Michelangelo Mercogliano, Atte Meretoja, Tuomo J Meretoja, Tomislav Mestrovic, Chamila Dinushi Kukulege Mettananda, Sachith Mettananda, Mohamed M. M.

Metwally, Louise Mewton, Andrea Michelerio, Ana Carolina Micheletti Gomide Nogueira de Sá, Ted R Miller, Wai-kit Ming, Mojgan Mirghafourvand, Seyed Ali Mirshahvalad, Yousef Mirzaei, Archana Mishra, Philip B Mitchell, Prasanna Mithra, Malihe Moazeni, Mona Gamal Mohamed, Nouh Saad Mohamed, Khabab Abbasher Hussien Mohamed Ahmed, Taj Mohammad, Sakineh Mohammad-Alizadeh-Charandabi, Abdollah Mohammadian-Hafshejani, Ramin Mohammadzadeh, Ammas Siraj Mohammed, Hussen Mohammed, Omer Mohammed, Shafiu Mohammed, Suleiman Mohammed, Yahaya Mohammed, Yugal Kishore Mohanta, Amin Mokari-Yamchi, Ali H Mokdad, Alexandr Mokhirev, Sabrina Molinaro, Amirabbas Mollaei, Lorenzo Monasta, Amirabbas Monazzami, Himel Mondal, Stefania Mondello, Ahmed Al Montasir, Catrin E Moore, Maziar Moradi-Lakeh, Paula Moraga, Rafael Silveira Moreira, Mahdis Morovvati, Mahmoud M Morsy, Jakub Morze, Reza Mosaddeghi Heris, Nogol Motamedgorji, Simin Mouodi, Asma Mousavi, Seyede Zohre Mousavi, Amin Mousavi Khaneghah, Mohamed Awad Abdalaziz Mousnad, Hagar Lotfy Mowafy, Kimia Mozahheb Yousefi, Matías Mrejen, Ahmed Msherghi, Shiv K Mudgal, Syed Aun Muhammad, Sukhes Mukherjee, Amartya Mukhopadhyay, Francesk Mulita, Getaneh Baye Mulu, Chalie Mulugeta, Muneeb Ahmad Muneer, Malaisamy Muniyandi, Yanjinlkham Munkhsaikhan, Javier Muñoz Laguna, Michio Murakami, Ali Mushtaq, Ghulam Mustafa, Sathish Muthu, Claude Mambo Muvunyi, Muhammad Muzaffar, Amin Nabavi, Ahamarshan Jayaraman Nagarajan, Shankar Prasad Nagaraju, Mohsen Naghavi, Hiten Naik, Sanjeev Nair, Soroush Najdaghi, Noureddin Nakhostin Ansari, Paul Nam, Jobert Richie Nansseu, Ibrahim A Naqid, Shumaila Nargus, Bruno Ramos Nascimento, Gustavo G Nascimento, Abdallah Y Naser, Mohammad Naser, Abdulqadir J Nashwan, Hamide Nasiri, Mahmoud Nassar, Zuhair S Natto, Javaid Nauman, Samidi Nirasha Kumari Navaratna, Biswa Prakash Nayak, Shalini Ganesh Nayak, Javad Nazari, G. Takop Nchanji, Anthony Wainaina Ndungu, Ionut Negoi, Ruxandra Irina Negoi, Alina Gabriela Negru, Jalil Nejati, Samata Nepal, Charles Richard James Newton, Georges Nguefack-Tsague, Josephine W Ngunjiri, Cuong Tat Nguyen, Huong Lan Thi Nguyen, Huong-Dung Thi Nguyen, Nghia Phu Nguyen, Phat Tuan Nguyen, Trang Nguyen, Tu Anh Nguyen, Van Thanh Nguyen, Ambe Marius Ngwa, Robina Khan Niazi, Luciano Nieddu, Yeshambel T Nigatu, Dina Nur Anggraini Ningrum, Vikram Niranjan, Abebe Melis Nisro, Jan Rene Nkeck, Princess Afia Nkrumah-Boateng, Mohammadamin Noorafrooz, Pardis Noormohammadpour, Mamoona Noreen, Masoud Noroozi, Jean Jacques Noubiap, Valentine C Nriagu, Chisom Adaobi Nri-Ezedi, Jean Claude Nshimiyimana, Fred Nugen, Sylvester Dodzi Nyadanu, Chimezie Igwegbe Nzoputam, Ogochukwu Janet Nzoputam, Bogdan Oancea, Ramez M Odat, Ismail A Odetokun, Oluwakemi Ololade Odukoya, Onome Bright Oghenetega, Oluwaseun Adeolu Ogundijo, Abiola Ogunkoya, James Odhiambo Oguta, Sarah Oh, Edel T O'Hagan, Sylvester Reuben Okeke, Deborah Oluwatosin Okeke-Obayemi, Olalekan John Okesanya, Onyedika A Okoli, Osaretin Christabel Okonji, John Olayemi Okunlola, Oluyemi Adewole Okunlola, Andrew T Olagunju, Oladotun Victor Olalusi, Matthew Idowu Olatubi, Arão Belitardo Oliveira, Abdulhakeem Abayomi Olorukooba, Oluseye Olalekan Oludoye, Bolajoko Olubukunola Olusanya, Jacob Olusegun Olusanya, Folorunsho Bright Omage, Hany A Omar, Kanyin Liane Ong, Qi Chwen Ong, Sandersan Onie, Obinna E Onwujekwe, Franklyn Opara, Marcel Opitz, Michal Ordak, Verner N Orish, Raffaele Ornello, Atakan Orscelik, Alberto Ortiz, Esteban Ortiz-Prado, Augustus Osborne, Eric Osei, Samuel M Ostroff, John W Ostrominski, Uchechukwu Levi Osuagwu, Godfred Otchere, Elham H Othman, Mostafa Monier Othman, Adrian Otoiu, Oche Joseph Otorkpa, Jerry John Ouner, Amel Ouyahia, Guoqing Ouyang, Mayowa O Owolabi, Irene Amoakoh Owusu, Kolapo Oyebola, Tope Oyelade, Ilker Ozsahin, Mahesh P A, Kevin Pacheco-Barrios, Alicia Padron-Monedero, Jagadish Rao Padubidri, Anton Pak, Tamás Palicz, Raffaele Palladino, Raul Felipe Palma-Alvarez, Tejasri Paluvai, Feng Pan, Sujogya Kumar Panda, Songhomitra Panda-Jonas, Deepshikha Pande Katare, Helena Ullyartha Pangaribuan, Leonidas D Panos,

Ioannis Pantazopoulos, Giovanni Paolino, Mario Virgilio Papa, Ilias Papadimopoulos, Paraskevi Papadopoulou, Amrita Parida, Arpit Parmar, Swapnil Parve, Roberto Passera, Mitesh Patel, Neel Navinkumar Patel, Satyananda Patel, Ashlesh Patil, Shankargouda Patil, Dimitrios Patoulias, Apurba Patra, Hilary Paul, Shrikant Pawar, Shubhadarshini Pawar, Hamidreza Pazoki Toroudi, Amy E Peden, Paolo Pedersini, Veincent Christian Filipino Pepito, João Perdigão, Gavin Pereira, Norberto Perico, Simone Perna, Konrad Pesudovs, Pavlo Petakh, Fanny Emily Petermann-Rocha, Hoang Nhat Pham, Nhat Truong Pham, Michael R Phillips, David M Pigott, Julian David Pillay, Luane Pinheiro Pinheiro Rocha, Zahra Zahid Piracha, Michael A Piradov, Edoardo Pirera, Dietrich Plass, Indrashis Podder, Dimitri Poddighe, Peter Pollner, Ville T Ponkilainen, Ion Popa, Svetlana Popova, Djordje S Popovic, Maarten J Postma, Sajjad Pourasghary, Reza Pourbabaki, Sergio I Prada, Jalandhar Pradhan, Pranil Man Singh Pradhan, Peralam Yegneswaran Prakash, Chandra P Prasad, Akila Prashant, Elton Junio Sady Prates, Tina Priscilla, Harsh Priya, Jagadeesh Puvvula, Xiang Qi, Zhipeng Qi, Jia-Yong Qiu, Zahiruddin Syed Quazi, Shahazad Niwazi Qurashi, Deepthi R, Navid Rabiee, Raghu Anekal Radhakrishnan, Venkatraman Radhakrishnan, Maja R Radojčić, Hadi Raeisi Shahraki, Ibrar Rafique, Pankaja Raghav, Hawbash Mohammed-Amin Rahim, Sajjad Rahimi, Afarin Rahimi-Movaghar, Vafa Rahimi-Movaghar, Mahbubur Rahman, Md. Mosfequr Rahman, Mohammad Hifz Ur Rahman, Mohammad Meshbahur Rahman, Masoud Rahmati, Ghasem Rahmatpour Rokni, Hakim Rahmoune, Diego Raimondo, Ivano Raimondo, Sunil Kumar Raina, Jeffrey Pradeep Raj, Sandesh Raja, Sathish Rajaa, Shahryar Rajai Firouzabadi, Gunaseelan Rajendran, Judah Rajendran, Vinoth Rajendran, Shaman Rajindrajith, Prashant Rajput, Mahmoud Mohammed Ramadan, Kadar Ramadhan, Chitra Ramasamy, Shakthi Kumaran Ramasamy, Zahra Ramezani, Pramod W Ramteke, Shailendra Singh Rana, Chhabi Lal Ranabhat, Nemanja Rancic, Smitha Rani, Fatemeh Ranjbar Noei, Chythra R Rao, Kumuda Rao, Mithun Rao, Sowmya J Rao, Davide Rasella, Vahid Rashedi, Mohammad-Mahdi Rashidi, Mohammad Aziz Rasouli, Ashkan Rasouli-Saravani, Devarajan Rathish, Ilari Rautalin, Ramin Ravangard, Dhwani Ravi, David Laith Rawaf, Salman Rawaf, Reza Rawassizadeh, Ayita Ray, Iman Razeghian, Bahman Razi, Filippo Recenti, Elrashdy Redwan, Sanika Rege, Wajiha Rehman, Giuseppe Remuzzi, Longbing Ren, Andre M N Renzaho, Luis Felipe Reyes, Mina Rezaei, Nima Rezaei, Mavra A Riaz, Antonio Luiz P Ribeiro, Jennifer Rickard, Moattar Raza Rizvi, Hermano Alexandre Lima Rocha, João Rocha Rocha-Gomes, Mónica Rodrigues, Leonardo Roever, Peter Rohloff, Susanne Röhr, David Rojas-Rueda, Megan L Rolfzen, Debby Syahru Romadlon, Michele Romoli, Marina Romozzi, Luca Ronfani, Amirhossein Roshanshad, Morteza Rostamian, Gregory A Roth, Kunle Rotimi, Hanieh Rouzbahani, Reza Rouzbahani, Shiva Rouzbahani, Bedanta Roy, Nitai Roy, Poulami Roy, Priyanka Roy, Sharmistha Roy, Shubhanjali Roy, Susovan Roy Chowdhury, Enrico Rubagotti, Guilherme de Andrade Ruela, Susan Fred Rumisha, Michele Russo, Godfrey Mutashambara Rwegerera, Manjula S, Chandan S N, Aly M A Saad, Adnan Saad Eddin, Maha Mohamed Saber-Ayad, Cameron John Sabet, Siamak Sabour, Kabir P Sadarangani, Seyed Kiarash Sadat Rafiei, Basema Ahmad Saddik, Bashdar Abuzed Sadee, Tarannom Sadegh, Umar Saeed, Maryam Saeedi, Mehdi Safari, Rajesh Sagar, Mastooreh Sagharichi, Dominic Sagoe, Fatemeh Saheb Sharif-Askari, Narjes Saheb Sharif-Askari, Amirhossein Sahebkar, Kirti Sundar Sahu, Zahra Saif, Md Refat Uz Zaman Sajib, Mirza Rizwan Sajid, Morteza Saki, Nasir Salam, Luciane B Salaroli, Leili Salehi, Mahdi Salehi, Marwa Rashad Salem, Mohammed Z Y Salem, Dauda Salihu, Malik Sallam, Giovanni A Salum, Sundeep Santosh Salvi, Waqas Sami, Vijaya Paul Samuel, Abdallah M Samy, Sandeep G Sangle, Elaheh Sanjari, Sathish Sankar, Francesco Sanmarchi, Damian F Santomauro, Lucas H C C Santos, Milena M Santric-Milicevic, Bruno Piassi Sao Jose, Jacob Owusu Sarfo, Yaser Sarikhani, Mohammad Sarmadi, Gargi Sachin Sarode, Sachin C Sarode, Michele Sassano, Mukesh Kumar Sathya Narayanan, Sangeeta Gopal Saxena, Ganesh Kumar Saya, Abu Sayeed, Mete Saylan, Ione

Jayce Ceola Schneider, Art Schuermans, Ghil Schwarz, David C Schwebel, Catherine Schwinger, Mario Šekerija, Muthamizh Selvamani, Vimalraj Selvaraj, Yuliya Semenova, Mohammad H Semreen, Yigit Can Senol, Sadaf G Sepanlou, Edson Serván-Mori, Yashendra Sethi, Christian Sewor, Seyed Mohammad Seyed Alshohadaei, Allen Seylani, Nilay S Shah, Sweni Shah, Shazlin Shaharudin, Muhammad Shahbaz, Samiah Shahid, Endrit Shahini, Fatemeh Shahrahmani, Moyad Jamal Shahwan, Alireza Shakeri, Ali Shakerimoghaddam, Ali S Shalash, Muhammad Aaqib Shamim, Farzane Shams, Mehran Shams-Beyranvand, Anas Shamsi, Alfiya Shamsutdinova, Dan Shan, Abhishek Shankar, Mohammed Shannawaz, Amin Sharifan, Javad Sharifi Rad, Avimanu Sharma, Bunty Sharma, Kamal Sharma, Manoj Sharma, Sourabh Sharma, Ujjawal Sharma, Vishal Sharma, Rajesh P Shastry, Shamee Shastry, Ramzi Shawahna, Babangida Shehu Bappah, Samendra P Sherchan, Suraj S Shetty, Fang Shi, Mosa Shibani, Kenji Shibuya, Md Monir Hossain Shimul, Reza Shirkoohi, Aminu Shittu, Abdul-karim Olayinka Shitu, Velizar Shivarov, Nathan A Shlobin, Azad Shokri, Sinegugu Nosipho Shongwe, Sina Shool, Seyed Afshin Shorofi, Gambhir Shrestha, Sunil Shrestha, Kerem Shuval, Emmanuel Edwar Siddig, Mohammad Sidiq, Martin Siegel, Diego Augusto Santos Silva, Gustavo Correia Basto da Silva, João Pedro Silva, Luís Manuel Lopes Rodrigues Silva, Padam Prasad Simkhada, Abhinav Singh, Akanksha Singh, Ambrish Singh, Harmanjit Singh, Jasbir Singh, Jasvinder A Singh, Kalpana Singh, Lucky Singh, Narinder Pal Singh, Paramdeep Singh, Poornima Suryanath Singh, Puneetpal Singh, Rakesh K Singh, Samer Singh, Satwinder Singh, Surendra Singh, Mukesh Kumar Sinha, Ratnesh Sinha, Robert Sinto, Søren T Skou, Farrukh Sobia, Somaye Sohrabi, Balamrit Singh Sokhal, Ranjan Solanki, Solikhah Solikhah, Prashant Sood, Soroush Soraneh, Joan B Soriano, Michele Sorrentino, Fernando Sousa, Marco Aurelio Sousa, Ceren Soylu, Michael Spartalis, Manraj Singh Sra, Chandrashekhar T Sreeramareddy, Bahadar S Srichawla, Shyamkumar Sriram, Antonina V Starodubova, Simona Cătălina Ștefan, Dan J Stein, Timothy J Steiner, Paschalis Steiropoulos, Aleksandar Stevanović, Leo Stockfelt, Peter Stubbs, Yu Su, Omer Subasi, Narayan Subedi, Claudia Kimie Suemoto, Alisha Suhag, Thitiporn Sukaew, Surajo Kamilu Sulaiman, Auwal Garba Suleiman, Muritala Suleiman Odidi, Muhammad Suleman, Mark J M Sullman, Anusha Sultan Meo, Haitong Zhe Sun, Mao-ling Sun, Xiaodong Sun, Zhuanlan Sun, Suraj Sundaragiri, Thanigaivel Sundaram, Johan Sundström, David Sunkersing, Chandan Kumar Swain, Lukasz Szarpak, Sree Sudha T Y, Payam Tabaee Damavandi, Rafael Tabarés-Seisdedos, Seyed-Amir Tabatabaeizadeh, Shima Tabatabai, Celine Tabche, Jyothi Tadakamadla, Santosh Kumar Tadakamadla, Buhari Abdullahi Tafida, Farzad Taghizadeh-Hesary, Yasaman Taheri Abkenar, Iman M Talaat, Stella Talic, Mircea Tampa, Jacques Lukenze Tamuzi, Jianye Tan, Ker-Kan Tan, Saba Tariq, Nathan Y Tat, Razieh Tavakoli Oliaee, Rahele Tavakoly, Seyed Mohammad Tavangar, Mojtaba Teimoori, Mohamad-Hani Temsah, Amensisa Hailu Tesfaye, Azimeraw Arega Tesfu, Alireza Teymouri, Omar Thaher, Pugazhenthan Thangaraju, Ismaeel Tharwat, Samar Tharwat, Mehakpreet Kaur Thind, Arun James Thirunavukarasu, Arulmani Thiyagarajan, Nihal Thomas, Tenaw Yimer Tiruye, Musliu Adetola Tolani, Sojit Tomo, Marcello Tonelli, Roman Topor-Madry, Ali Torkashvand, Mathilde Touvier, Marcos Roberto Tovani-Palone, Khaled Trabelsi, Mai Thi Ngoc Tran, Nghia Minh Tran, Ngoc Ha Tran, Tam Quoc Minh Tran, Thang Huu Tran, Nguyen Tran Minh Duc, Domenico Trico, Samuel Joseph Tromans, Thien Tan Tri Tai Truyen, Aristidis Tsatsakis, Gary Tse, Evangelia Eirini Tsermpini, Lorainne Tudor Car, Mike Tuffour Amirikah, Sok Cin Tye, Aniefiok John Udoakang, Himayat Ullah, Irfan Ullah, Muhammad Umair, Krishna Kishore Umapathi, Lawan Umar, Shehu Salihu Umar, Era Upadhyay, Dipan Uppal, Daniele Urso, Jibrin Sammani Usman, Dilber Uzun Ozsahin, Pratyusha Vadagam, sara Vahdati, Asokan Govindaraj Vaithinathan, Alireza Vakilian, Pascual R Valdez, Mario Valenti, Jef Van den Eynde, Giloume Van Der Walt, Javad Varasteh, Ravi Prasad Varma, Priya Vart, Tommi Juhani Vasankari, Sampara Vasishta, Srivatsa Surya Vasudevan, Ashleigh S Vella, Balachandar Vellingiri, Narayanaswamy

Venketasubramanian, Poonam Verma, Massimiliano Veroux, Georgios-Ioannis Verras, Dominique Vervoort, Simone Vidale, Simone Villa, Jorge Hugo Villafañe, David Villarreal-Zegarra, Sharath Chaitanya Vipparthy, Rachel Visontay, Luciano Magalhães Vitorino, Vasily Vlassov, Martin Vojtek, Stein Emil Vollset, Theo Vos, Mehdi Vosoughi, Linh Vu, Yasir Waheed, Mugi Wahidin, Fang Wang, Lei Wang, Liang Wang, Shaopan Wang, Shu Wang, Wei Wang, Xing Wang, Xuequan Wang, Yanzhong Wang, Yichen Wang, Yuan-Pang Wang, Tanveer A. Wani, Ahmed Bilal Waqar, Paul Ward, Kosala Gayan Weerakoon, Ishanka Weerasekara, Fei-Long Wei, Xueying Wei, Robert G Weintraub, Andrea Werdecker, Ronny Westerman, Taweewat Wiangkham, Dakshitha Praneeth Wickramasinghe, Nuwan Darshana Wickramasinghe, Samuel Wiebe, Peter Willeit, Andrew Awuah Wireko, Gemechu Kumera Wirtu, Charles Shey Wiysonge, Abay Tadesse Woday, Marcin W Wojewodzic, Axel Walter Wolf, Tewodros Eshete Wonde, Yohannes Chemere Wondmeneh, Daniel Tarekegn Worede, Minichil Chanie Worku, Ai-Min Wu, James Fan Wu, Jinyi Wu, Shi-Nan Wu, Yanjie Xia, Na Xiao, Site Xu, Suowen Xu, Wanqing Xu, Mukesh Kumar Yadav, Vikas Yadav, Mahnaz Yadollahi, Saba Yahoo (Syed), Galal Yahya, Kazumasa Yamagishi, Haibo Yang, Weiguang Yang, Xinxin Yang, Laiang Yao, Amir Yarahmadi, Haya Yasin, Yuichi Yasufuku, Sanni Yaya, Pengpeng Ye, Meghdad Yeganeh, Ali Cem Yekdeş, Mohammad Hossein YektaKooshali, Kuanysh A. Yergaliyev, Saber Yezli, Muluken Yigezu, Yulai Yin, Malede Berihun Yismaw, Dong Keon Yon, Naohiro Yonemoto, Jian Yu, Yong Yu, Faith H Yuh, Ghazala Yunus, Umar Yunusa, Aminu Abba Yusuf, Monal Yuwanati, Mubashir Zafar, Manijeh Zaghampour, Emilia Zainal Abidin, Fathiah Zakham, Burhan Abdullah Zaman, Sojib Bin Zaman, Nelson Zamora, Aurora Zanghì, Heather J Zar, Kourosh Zarea, Mohammed Zawiah, Mohammed G M Zeariya, Abay Mulu Zenebe, Sebastian Zensen, Beijian Zhang, Casper J P Zhang, Haijun Zhang, Xiaoyi Zhang, Zhiqiang Zhang, Jianhui Zhao, Sheng Zhao, Zhongyi Zhao, Jiayan Zhou, Bin Zhu, Zhengyang Zhu, Abzal Zhumagaliuly, Magdalena Zielińska, Liu Zihao, Ghazal Zoghi, Mohamed Ali Zoromba, Rafat Mohammad Zrieq, Liesl J Zuhlke, Alimuddin Zumla, Ahed H Zyoud, and Sa'ed H Zyoud

Managing the estimation or publications process

Joanne O Amlag, Catherine M Antony, Charlie Ashbaugh, Catherine S Chen, Amanda Deen, Lisa M Force, Hailey Hagins, Erin B Hamilton, Ashley Ann Harris, Simon I Hay, Catherine O Johnson, Jonathan M Kocarnik, Hmwe Hmwe Kyu, Miranda L May, Madeline E Moberg, Ali H Mokdad, Amanda Movo, Mohsen Naghavi, Olivia D Nesbit, Erin M O'Connell, Erik J Olson, Samuel M Ostroff, Rachel D Schneider, Matthew Seymour, Jeffrey D Stanaway, Caitlyn Steiner, and Faith H Yuh.
